# Fungal communities decline with urbanization—more in air than in soil

**DOI:** 10.1038/s41396-020-0732-1

**Published:** 2020-08-05

**Authors:** Nerea Abrego, Brittni Crosier, Panu Somervuo, Natalia Ivanova, Arusyak Abrahamyan, Amir Abdi, Karoliina Hämäläinen, Kaisa Junninen, Minna Maunula, Jenna Purhonen, Otso Ovaskainen

**Affiliations:** 1 Department of Agricultural Sciences, University of Helsinki, P.O. Box 27, FI-00014 Helsinki, Finland; 2 Organismal and Evolutionary Biology Research Programme, University of Helsinki, P.O. Box 65, FI-00014 Helsinki, Finland; 3 Centre for Biodiversity Genomics, Biodiversity Institute of Ontario, University of Guelph, 50 Stone Road East, Guelph, ON Canada; 4 Department of Integrative Biology, College of Biological Sciences, University of Guelph, 50 Stone Road East, Guelph, ON Canada; 5 Trycksbackantie 20, 10360 Mustio, Finland; 6 School of Forest Sciences, University of Eastern Finland, P.O. Box 111, FI-80101 Joensuu, Finland; 7 Department of Microbiology, University of Helsinki, P.O. Box 56, FI-00014 Helsinki, Finland; 8 Department of Biological and Environmental Science, University of Jyväskylä, P.O. Box 35, FI-40014 Jyväskylä, Finland; 9 School of Resource Wisdom, University of Jyväskylä, P.O. Box 35, FI-40014 Jyväskylä, Finland; 10 Department of Music, Art and Culture Studies, University of Jyväskylä, P.O. Box 35, FI-40014 Jyväskylä, Finland; 11 Centre for Biodiversity Dynamics, Department of Biology, Norwegian University of Science and Technology, N-7491 Trondheim, Norway

**Keywords:** Community ecology, Fungal ecology

## Abstract

Increasing evidence suggests that degradation of biodiversity in human populated areas is a threat for the ecosystem processes that are relevant for human well-being. Fungi are a megadiverse kingdom that plays a key role in ecosystem processes and affects human well-being. How urbanization influences fungi has remained poorly understood, partially due to the methodological difficulties in comprehensively surveying fungi. Here we show that both aerial and soil fungal communities are greatly poorer in urban than in natural areas. Strikingly, a fivefold reduction in fungal DNA abundance took place in both air and soil samples already at 1 km scale when crossing the edge from natural to urban habitats. Furthermore, in the air, fungal diversity decreased with urbanization even more than in the soil. This result is counterintuitive as fungal spores are known to disperse over large distances. A large proportion of the fungi detectable in the air are specialized to natural habitats, whereas soil fungal communities comprise a large proportion of habitat generalists. The sensitivity of the aerial fungal community to anthropogenic disturbance makes this method a reliable and efficient bioindicator of ecosystem health in urban areas.

## Introduction

Biodiversity in urban areas contributes to many kinds of ecosystem processes important for human well-being, including amelioration of climate, soil erosion control, water quality and flow regulation, noise abatement, air pollution control, and pest control [1, 2]. Environmental degradation in urban areas threatens biodiversity [3], which in turn has negative impacts on humans due to the degradation of the ecosystem services [4–6]. Furthermore, there is increasing evidence that a lack of contact with natural biodiversity impacts human health through negative effects in the microbiome and immune system [7, 8]. Given that urban areas are projected to increase in both in their extent and in their human density [9], gaining a predictive understanding on how urbanization affects biodiversity is a key priority for sustainable development.

Fungi are a megadiverse kingdom providing an array of ecosystem services, and many fungal species have been harnessed as tools for evaluating environmental quality. In terms of their ecological functions, lichens fix atmospheric nitrogen into nitrates that plants use as nutrients [10]. Mycorrhizal fungi improve plant primary production by facilitating their nutrient and water uptake [11], and endophytic fungi act as biocontrols protecting plants from pathogen infections [12]. Wood-decaying fungi are primary agents of deadwood decomposition, and thus they play a pivotal role in the biogeochemical cycle of carbon and nitrogen [13]. As tools, lichenized fungi are well-known indicators of air quality [14], mycorrhizal fungi are highly sensitive to increased levels of nutrients and pollutants in soil [15], and wood-decaying fungi are used as indicators of forest naturalness [16]. Fungal diversity is particularly high in the air [17], the main dispersal mean for fungi [18]. While air is routinely biomonitored to inform the public about the presence of allergy- and disease-causing fungi [19–21], little is known about the fungal composition in the air; yet, many of the unmonitored species of fungi can have direct consequences to ecosystem services and human well-being.

**Fig. 1:**
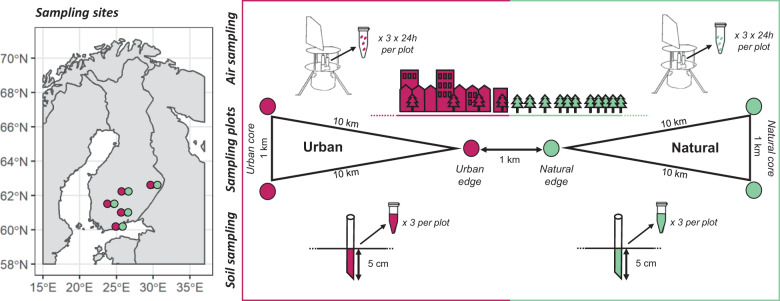
Study design. The location of the five Finnish cities are shown in the left-hand panel and the sampling sceme within each of the cities is shown in the right-hand panel, fuksia (respectively green) color illustrating the sampling carried out in urban areas (respectively natural areas). Within each site, three plots were located in natural and other three plots in urban areas, representing the core and edges of both area types. Fungal communities were sampled from the air (24 h sample taken by the cyclone sampler) and from the soil (a mixture of three soil samples). From each plot, three replicate air and three replicate soil samples were taken.

Currently, we lack information on how urbanization influences fungal diversity, largely due to methodological challenges in comprehensively surveying this megadiverse kingdom. A recent methodological breakthrough shows that, together with molecular species identification, sampling fungal spores directly from the air with a cyclone sampler is a highly efficient way of surveying fungal diversity, revealing much higher diversity than substrate-specific DNA sampling [17]. In this study, we systematically apply both aerial and soil sampling in urban and surrounding natural forest areas in five Finnish cities to assess the impact of urbanization on fungal communities (Fig. 1). In particular, we ask (1) whether fungal communities differ between urban and natural areas in terms of species composition, richness and abundance, (2) which is the spatial scale at which fungal communities change in the environmental gradient from natural to urban areas, and (3) which of the fungal survey methods (i.e., soil sampling or air sampling) best captures the fungal community changes between urban and natural areas. Given the lack of host resources (e.g., deadwood and symbiotic plants) and increased levels of pollution in urban areas compared to natural areas, we expected to find poorer fungal diversity in urban areas. As airborne spores have a much greater dispersal potential than mycelia in the soil, we further hypothesized that only the fungal communities in the soil would be dissimilar between urban and natural environments.

Our results provide the first quantitative results of the biological impacts of urbanization on the fungal diversity: the species richness drops to almost one half and the fungal DNA abundance to one fifth already at 1 km scale when crossing the edge from natural to urban habitats. Contrary to our expectation, fungal diversity decreased with urbanization more in the air than in the soil, demonstrating the feasibility of air sampling for fungal biomonitoring in urban habitats.

## Materials and methods

### Study design and sampling protocol

The sampling scheme consisted of five sites representing Finnish cities, the distances among which ranged from ca. 100 to ca. 500 km (Fig. 1). In each site, six sampling plots were established (Fig. 1). These consisted of three natural and three urban plots, where the urban plots were located within settlement areas (noncultivated lawns, road sides, and backyards) and the natural plots were located in the surrounding forests (Supplementary Fig. 1). Two of the urban plots were located in the core urban area, and the distance between them was ca. 1 km. Two of the natural plots were located in the core natural area, and the distance between them was ca. 1 km. One urban plot and one natural plot were located at the edge between the urban and natural area, and the distance between them was ca. 1 km. The distance between the edge and the core plots was ca. 10 km. The motivation for this study design was to include pairs of plots that were either urban–urban, natural–natural, or urban–natural, and the distances between which were ca. 1, 10, 100, or 500 km, thus covering a wide range of distances at a logarithmic scale. The list and description of the samples is given in Supplementary Table 1.

From each plot, we acquired both soil and air samples. To acquire soil samples, leaf litter was first removed from the surface. Then soil cores were taken with a 2.5 cm diameter cylinder, to 5 cm depth, or until reaching rock. For each soil sample, three soil cores were collected within a 1 m square, which were pooled and mixed and then 2 mL were taken for the final sample tube. The sampling was conducted during the short period of 8 days in 16th to 23rd August 2019, and it was synchronized across the sites to minimize variation seasonality and weather conditions. The soil samples were kept in cool storage until processing in the lab. The air samples were acquired by the cyclone sampler, which collects particles greater than 1 µm directly to an Eppendorf vial [17]. We followed the protocol of the Global Spore Sampling Project [22] and thus let each air sample accumulate for 24 h. From each plot, we collected three replicate samples of both soil and air. As there were 30 plots but we had only 15 cyclone samplers, we alternated the sampling between the natural and urban locations. The sampling was synchronized so that it took place at the same times across the five study sites (see Supplementary Information for the sampling dates). As we acquired three replicates of two sample types from six plots in five sites, the total number of samples was 180, out of which 90 were soil and 90 air samples.

The samples were preprocessed at the University of Helsinki, Finland. All soil samples were freeze dried for 48 h at 0.57 mbar vacuum and −80 °C. Before drying, air samples were cleaned of larger items, such as arthropods. To clean, sterile water was added to the sample tube and then the sample was vortexed for 10 s to release any spores that may have been attached to larger items. After vortexing, all visible contaminants were removed from the tube using sterile tweezers. Spore samples were then covered with parafilm and freeze dried for 24 h at 0.57 mbar vacuum and −80 °C temperature. Dry samples were stored in −20 °C freezers until being shipped for DNA analysis. All samples were sent to the University of Guelph, Canada, for molecular analyses: DNA extraction, PCR, and indexed library preparation were done at the Center for Biodiversity Genomics; Illumina MiSeq runs were processed at the AAC Genomics Facility.

### DNA extraction, sequencing, and bioinformatics analyses

As our aim was to analyze the samples for their fungal communities, we targeted the DNA work to the ITS region which is the universal molecular barcode of fungi [23]. Concerning the air samples, we followed the DNA extraction and sequencing protocol of Ovaskainen et al. [22]. This includes adding synthetic DNA as a positive control (also called spiking) which allows translating the raw sequence counts into quantitative estimates of DNA amount. This was achieved by adding to each sample nine positive control plasmids that were prepared from synthetic sequences that are generally consistent with fungal ITS sequences, yet different from all known natural sequences [24]. In this experiment we used 0.1 µl of 0.01 ng/µl spike per each 12.5 µl round one PCR reaction. The soil samples were processed with the following modifications: one 3/8′ sterile stainless-steel bead and 3–5 ml of ILB+1% PVPP buffer with 25 µl of Proteinase K (20 mg/ml) per each 1 ml of buffer were added to each sample [25]. Soil samples were homogenized on Genie 2 vortex on max speed for 5 min. Tubes were incubated at 56 °C for 2 h on the orbital shaker, followed by 2 h incubation at 65 °C. Tubes were centrifuged for 5 min at 2000 × *g* and a volume of 200 µl of each lysate was transferred to a 500 µl Eppendorf deep-well plate using Biomek NX; 100 µl of lysate from each replicate was mixed with 200 µl of 5M GuSCN Plant Binding buffer and applied to a 96-well Acroprep plate with 1 µm Glass Fiber membrane (PALL) for DNA binding. The washes were conducted at 5000 × *g* as described in Ivanova et al. [25] with the following modifications: 1st wash—300 µl of 5M GuSCN buffer; 2nd wash—300 µl of plant PWB; 3rd and 4th washes—600 µl of WB. After the last wash plate was incubated at 56 °C to dry the membrane; DNA was eluted in 80 µl of 10 mM Tris-HCL pH 8.0. DNA from some soil sampled was not completely purified from humic acids, resulting in dark brown DNA extracts, therefore 70 µl of each DNA extract was transferred to a 500 µl Eppendorf deep-well plate containing 70 µl of 1% ILB+PVPP and 280 µl of 5M GuSCN binding buffer; DNA was bound to 1 µm glass fiber membrane as described above; 1st wash consisted of 150 µl of 5M GuSCN binding buffer, followed by two washes with 600 µl of WB. After the last wash plate was incubated at 56 °C to dry the membrane; DNA was eluted in 70 µl of 10 mM Tris-HCL pH 8.0.

All samples were sequenced on Illumina MiSeq following the procedure described in Ovaskainen et al. [22] with minor modifications: to increase complexity of amplicon libraries 6Ns were added before Illumina “mis” adapter to ITS3, ITS_S2F and ITS4 primers [26, 27] for the first round of PCR.

ITS_S2F-misN6 TCGTCGGCAGCGTCAGATGTGTATAAGAGACAGNNNNNNATGCGATACTTGGTGTGAAT

ITS3-misN6 TCGTCGGCAGCGTCAGATGTGTATAAGAGACAGNNNNNNGCATCGATGAAGAACGCAGC

ITS4-misN6 GTCTCGTGGGCTCGGAGATGTGTATAAGAGACAGNNNNNNTCCTCCGCTTATTGATATGC

Raw Illumina data were paired using Geneious Prime 2019.0.4, short sequences (<100 bp) were discarded and 5′-end and 3′-end were trimmed by quality (QV20) using BBDuk. The following bioinformatics workflow was used to process paired-end data: Cutadapt (v1.8.1) was used to trim primer sequences; Sickle (v1.33) was used for filtering (<200 bp) and Uclust (v1.2.22q) was used to cluster that sequences with 99.5% similarity threshold. We note that this initial clustering was conducted to reduce the computational cost of analysing the data rather than to identify ecologically meaningful taxonomical units. Clusters that corresponded to the spikes were identified using Ublast with 95% similarity threshold.

### OTU clustering and taxonomic placement

We denote for each of the *i* = 1, …, 180 samples the total number of sequences by *n*_*i*_, the number of spike sequences by *s*_*i*_, the number of sequences that were estimated to represent plant (or other non-fungal) DNA by *p*_*i*_, and and by *f*_*i*_ = *n*_*i*_ − *s*_*i*_ − *p*_*i*_ the number of sequences representing fungal DNA. We used the proportion *w*_*i*_ = *f*_*i*_/*s*_*i*_ as an estimate of the total amount of fungal DNA in the sample, which measure has been shown to agree well with qPCR-based estimates of absolute DNA amount [22]. We applied probabilistic taxonomic placement with the software PROTAX [28], following the specific implementation to fungi [29]. This yields for each sequence the most likely taxonomic identity at the levels of phylum, class, order, family, genus, and species, and the uncertainty in these assignments as measured by probability of correct placement. We note that the uncertainty estimates of PROTAX account for the possibility that the species might be unknown to science (i.e., not included in the taxonomy database), or known to science but lacking reference sequences [28, 29]. We followed Somervuo et al. [28] by considering an identification reliable if the probability of taxonomic placement was >90%. We excluded potentially non-fungal sequences by including in our analyses only those sequences that were reliably classified to belong to a known fungal phylum.

We considered the reliably classified sequences (at the levels of class, order, family, genus, and species) as a backbone against which the non-reliably classified sequences were clustered. To do so, we applied constrained clustering to these sequences in a hierarchical manner, starting from the class level and finishing with the species levels. Sequences that were reliably classified by PROTAX were first clustered within their taxon to yield representative sequences of the taxa. All reliably classified sequences under the current node of the hierarchical clustering were then mapped against the representative sequences to obtain distributions of sequence similarities both within and among taxa. These distributions were used to define the optimal sequence similarity threshold, defined by the criteria that the amounts of false positives and false negatives were equal. The optimal sequence similarity thresholds were thus optimized separately for each taxonomic level. Sequences that were not reliably classified by PROTAX were mapped against the representative sequences. If the best sequence similarity of these mappings exceeded the optimal threshold, the sequence was classified to the corresponding taxon. Sequences for which the best similarity did not exceed the threshold were clustered denovo assuming the optimal similarity threshold. In these analyses, we used LAST for mapping [30] and UCLUST for clustering [31]. For more technical details, including the scripts used in the analyses, see Supplementary Information.

### Statistical analyses

We excluded from all analyses those six samples for which the sequencing failed technically, producing less than 10,000 sequences. These included five soil samples from Urban sites (JOE-U1-210819-S, JOE-U1-200819-S, TAM-U3-160819-S, JOE-U3-200819-S, and JYV-U1-200819-S) and one soil sample from a natural site (TAM-N1-220819-S). The remaining 174 samples contained on average 300,000 sequences, with a minimum of 17,000 and a maximum of 510,000 sequences. We measured the abundance of each OTU by multiplying the estimate of total fungal DNA amount (obtained by the spiking approach, see above) by relative OTU abundance, defined as the fraction of focal sequences out of all fungal sequences. As the unit of DNA amount is not comparable between air and soil samples, we normalized the data obtained these two sampling methods separately so that the total DNA amount of both sampling methods summed to one. The normalization was performed by dividing the amount of DNA estimated for each sample by the total amount of DNA estimated for all samples sampled by the same method. Thus, when evaluating whether the taxa were predominantly found from air or soil (see below), we assumed to have equally as much DNA from both sample types.

We classified the sequences placed at each taxonomical level (from phylum to species) as predominantly air detectable if their abundance was at least ten times higher in the air than in the soil. Similarly, we classified the taxa as predominantly soil detectable if their abundance was at least ten times higher in the soil than in the air. We further classified the taxa as natural or urban specialists, based on whether they were at least ten times more abundant in one of these two habitat types compared to the other. We excluded from both kinds of classifications all taxa that were present in less than five samples, as their classifications would not be reliable.

**Fig. 2:**
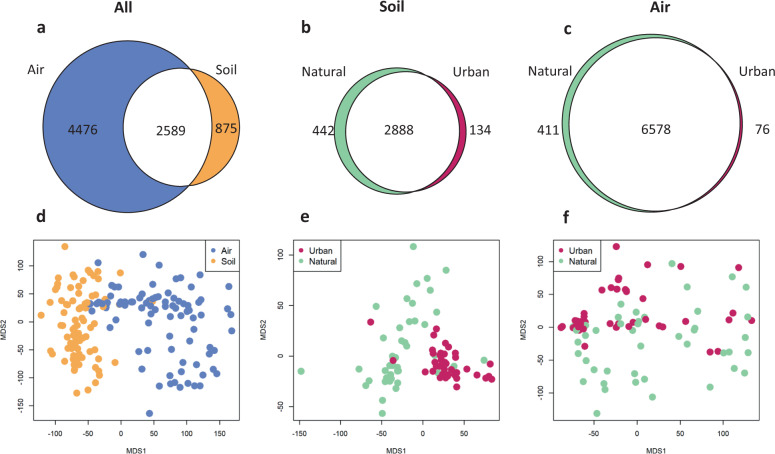
Fungal communities in air and soil of urban and natural sites. The upper row of panels: Euler diagrams for numbers of species shared or not shared between (**a**) air and soil, (**b**) urban and natural soil, (**c**) urban and natural air. The lower row of panels: fungal community composition for (**d**) all data, (**e**) soil data and (**f**) air data in the ordination space. The ordinations plots are based on Euclidian distance applied to log-transformed DNA abundance data. Only OTUs present in at least five samples were included in these analyses.

To visualize the community composition of the samples (Fig. 2), we first log(*x* + 10^−6^) transformed the DNA amount data, then computed sample distances using the dist function, and performed ordination with the sammon function in R software [32]. We tested for the significance of the different factors influencing community composition by applying PERMANOVA [33] with the adonis function of the R-package vegan [34]. In analyses restricted to air or soil, we explained variation in the community distance matrix with the habitat type (natural or urban), the site, and the plot. In the analysis of all data, we additionally included sample type (air or soil) and its interaction with the above-mentioned factors. To avoid spurious inference with noisy data, we excluded from these analyses those OTUs that were present in less than in five samples.

We examined how the DNA amount and OTU richness varied among the two sampling methods (air/soil) and the habitat types (modeled either with the four types of combining natural/urban with core/edge; or with the two types of natural/urban) with generalized linear mixed models. We log-transformed the DNA amount and assumed a linear mixed model, whereas for OTU richness we fitted a Poisson mixed model with the log link function. All models included the log-transformed sequencing depth to control for the effect of sample size, and the nested random effects of plot and site to control for the nature of the study design. The full model consisted of the main effects of sampling method and the habitat type, and the interaction between these. Reduced models consisted of those without the interaction, as well as those without the main effects of the sampling method and the habitat. As an exception, for the DNA abundance model we kept the effect of the sampling method without testing for it, for the reason that DNA abundance was measured in different units for soil and air and thus a direct comparison is not meaningful. We selected the best supported model with AIC.

To examine in more detail the responses of the most prevalent species to the environmental and spatial predictors, we fitted the joint species distribution model of Hierarchical Modeling of Species Communities (HMSC, [35]) separately to the air (model HMSC-air) and to the soil (model HMSC-soil) data with the R-package Hmsc [36]. For these analyses, we selected species which occurred in at least 20 samples of each sample type. To account for the zero-inflated nature of the data, we applied a hurdle-approach, first fitting probit models to presence–absence data, and then log-linear models to abundance conditional on presence. We included habitat (natural versus urban) and log-transformed sequencing depth as fixed effects, and the nested structure of plot within sites as random effects. We assumed the default prior distribution of the R-package Hmsc [36]. We fitted the models with two Markov Chain Monte Carlo (MCMC) chains, each of which consisted of 150,000 iterations, out of which we discarded the first 50,000 as burn-in and thinned the remaining by 100 to yield in total 2000 posterior samples. We assessed the convergence of the MCMC chains by examining the distribution of the potential scale reduction factor over the parameters that measure the responses of the OTUs to the fixed effects included in the model.

## Results

The data consist of 52 million ITS2 sequences. The combination of probabilistic taxonomic placement and clustering resulted in 79,155 operational taxonomical units (OTUs) representing the species level. Out of the 7940 OTUs present in at least 5 samples, 33% were found from both the air and the soil samples, 56% exclusively from the air samples, and 11% exclusively from the soil samples (Fig. 2). The distinct nature of air and soil communities is illustrated by their full separation in the ordination space (Fig. 2; PERMANOVA *p* < 0.001, Supplementary Table 2). When considering the air and the soil samples separately, the natural and urban habitats separated in the ordination space (Fig. 2; PERMANOVA *p* < 0.001 for soil and *p* = 0.002 for air, Supplementary Table 2); yet, almost all OTUs were present in both the natural and the urban habitats (Fig. 2; Euler diagrams). Thus, the difference between these two habitat types is not in the total species richness, but in the species abundance, richness, and composition per sample.

**Fig. 3:**
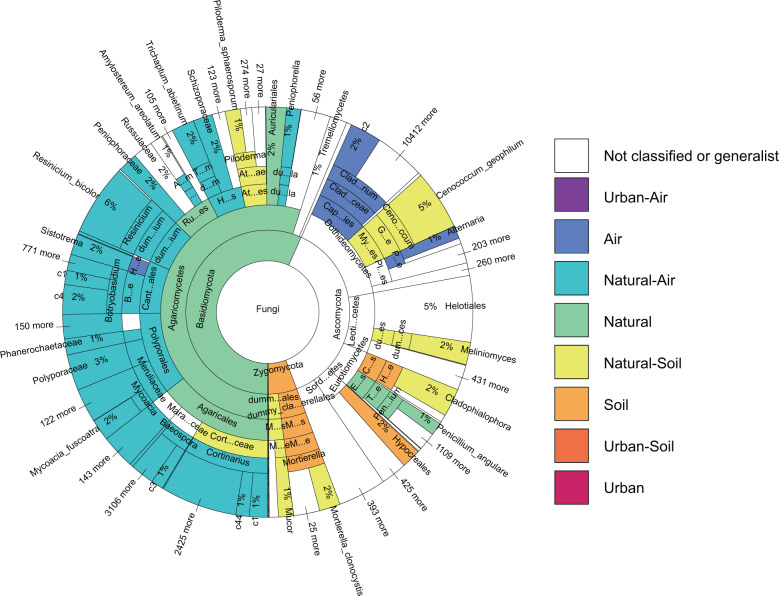
Fungal taxonomic composition in air and soil of urban and natural sites. The Krona wheel showing the taxonomic levels to which each of the 79,155 identified fungal species (OTUs) belongs. The coloring shows the taxa that are predominantly (with >10 times higher DNA abundance) found from air or from soil, from natural or from urban habitats, and the combination of these two classifications (see “Methods” for criteria used when performing the classifications). Sector areas are proportional to DNA abundance, the percentage numbers showing the percentage of total DNA abundance belonging to the taxa contained in the sector. This figure shows pooled data from all sample types, whereas sample-type specific versions are shown in Figure. For an interactive version of the Krona wheel that allows detailed examination of each taxonomic level, as well as for the same information as numerical table format, see Supplementary Information.

We found a large proportion of all taxa to be specialized to natural areas, such as the entire phylum Basidiomycota, whereas almost no taxa were specialized to urban areas (Fig. 3). A large proportion of the taxa were found predominantly in air samples from natural areas: the orders Polyporales and Hymenochaetales which comprise wood-decaying fungi, Cantharellales which comprises mostly ectomycorrhizal fungi but also saprotrophic taxa, and many genera of the order Agaricales which comprises a high diversity of saprotrophic and ectomycorrhizal taxa. Also Lecanorales which comprises lichens and Pucciniales which comprises plant pathogenic fungi were predominantly found from the air. Considerably fewer taxa were found predominantly from the soil. These included the phylum Chytridiomycota which comprises saprotrophic fungi and animal parasites inhabiting water environments, and the phylum Glomeromycotina which comprises endomycorrhizal fungi not developing aboveground structures. The species from the order Atheliales, which are decomposers of the litter material as well as ectomycorrhizal, were found mostly in soil samples from natural areas.

**Fig. 4:**
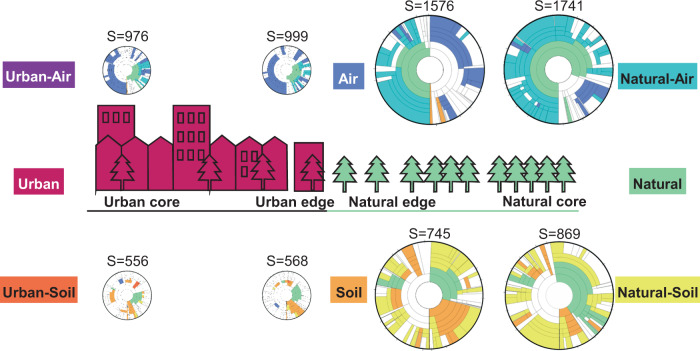
Taxonomic composition and DNA abundance of fungi in air and soil along the gradient from urban core areas to natural core areas. The Krona wheels show the taxonomic composition of the OTUs found from each sample type. The predicted species richness per sample is shown by S, and the predicted amount of DNA per sample is represented by the relative sizes of the Krona wheels. The coloring follows the classification in Fig. 3. As DNA abundance was measured in different units for the soil and the air, the sizes for the Krona wheels are not comparable between these two sample types. For interactive versions of the eight Krona wheels shown in this figure that allow detailed examination of each taxonomic level, see Supplementary Information.

Fungal abundance and diversity were notably higher in samples from natural than urban habitats, both in the air and in the soil (Fig. 4, Supplementary Table 3). Quite strikingly, fungal abundance and diversity dropped abruptly at the 1 km scale when crossing the edge from natural to urban habitats (Fig. 4). This result is in stark contrast with our hypothesis of air showing only a minor difference between natural and urban areas. In quantitative terms, the amount of fungal DNA was five times greater at the natural than at the urban edge, both in the air and in the soil.

The joint species distribution models were fitted to those 928 species that occurred in at least 20 air samples and to those 166 species that occurred in at least 20 soil samples. In HMSC-air, 95% of the species showed higher prevalence and 100% of the species showed higher DNA abundance in natural than in urban habitats (Table 1). Therefore, natural and urban air communities have a nested composition, with the urban air communities being a subsample of the natural air communities. The results were much less clear in the soil than in the air: in HMSC-soil, 6% of the species showed higher prevalence and 14% higher DNA abundance in natural than in urban habitats. While in the air none of the species was more prevalent nor abundant in urban habitats, in the soil 1% of the species (*Knufia peltigerae* and a species from the Herpotrichiellaceae family) were more prevalent in urban habitats (Table 1). Hence, as compared to the air, in the soil a large proportion of the most common species are generalists found from both the natural and the urban areas. In both HMSC-air and HMSC-soil, unexplained variation among plots within sites was higher than unexplained variation among sites (Table 1), suggesting that the results were consistent among the five cities in which the research was conducted.

**Table 1:** Results of joint species distribution models HMSC-soil and HMSC-air.

Substrate	Model	Explanatory power	Proportion of explained variance				Proportion of responding species	
			Habitat	Sequencing depth	Site	Plot	Natural habitat	Urban habitat
Soil	Presence–absence	0.84	0.11	0.10	0.11	0.67	0.06	0.01
	Abundance	0.24	0.26	0.25	0.23	0.26	0.14	0.00
Air	Presence–absence	0.77	0.25	0.08	0.13	0.54	0.95	0.00
	Abundance	0.39	0.35	0.05	0.16	0.44	1.00	0.00

For presence–absence models, explanatory power is measured by AUC, for abundance models conditional on presence, it is measured by *R*^2^. The following columns show the average proportions of variance attributed to the fixed and random effects in the model. The last two columns indicate the proportion of species that showed with at least 95% posterior probability a positive response to natural sites or to urban sites, respectively. The models were fitted to those species that occur in at least 20 (out of the 90) sampling units, totaling 166 species for the soil analyses and 928 species for the air analyses.

## Discussion

The rate at which community similarity decays with increasing distance has been studied extensively across ecosystems and organism groups [37, 38]. Compared to terrestrial environments, the atmosphere (i.e., the air environment) has been considered much more homogeneous. In particular, microorganisms were previously considered essentially not limited by dispersal [39]. A large body of more recent literature has shown that microbial communities are also spatially structured [40–42], even within the air environment [17, 43, 44]. Our results bring strong evidence for dispersal limitation of fungal spores, showing that aerial fungal communities vary greatly already at small spatial scales.

Anthropogenic disturbances, such as urbanization, have been found to increase similarity among ecological communities, by the so-called biotic homogenization process [3, 45]. While a large body of literature has assessed how communities in urban environments differ from those in nonurban environments [46–49], the influence of urbanization on microbial communities has remained little studied. A recent study in this area [50] showed that urbanization causes homogenization in soil microbial communities, and more so for fungi than for bacteria. Our results support this finding by showing that a large proportion of fungi are specialized to natural areas. As a new and unexpected finding, we furthermore show that the pattern of urbanization is more marked in the air than in the soil.

Air sampling is efficient for capturing fungal diversity changes of not only soil-inhabiting fungi, such as ectomycorrhizal and saprotrophic fungi, but also wood-decaying fungi, lichens, endophytes, and plant pathogens. We conclude that urbanization can result in marked variation in fungal community composition already at the local scale, and that such variation is more efficiently captured by air sampling than by soil sampling. Aerial fungal sampling can be applied globally with standardized methods [22], providing an exciting avenue of research for the global impacts of urbanization on the fungal kingdom. Our results suggest that the aerial fungal communities to be highly sensitive to anthropogenic disturbance and thus be reliable bioindicator of ecosystem health. Identifying the particular stressors causing the marked variation in aerial fungal communities is an important avenue for future research.

## Supplementary information

Supplemental information

Supplemental data

Bioinformatic scripts

## Data Availability

Raw sequence data have been deposited into ENA, accession number PRJEB37919.
