# Supplementary material for: Fungal communities decline with urbanization—more in air than in soil
Source: ISME J. 2020 Aug 5;14(11):2806–15. doi: 10.1038/s41396-020-0732-1 (PMC7784924; doi:10.1038/s41396-020-0732-1)
Supplement: Supplementary file 1 — Supplemental information [file 41396_2020_732_MOESM1_ESM.pdf]

Fungal communities decline with urbanization – more in air than in soil

Abrego, N., Crosier, B. Somervuo, P., Ivanova, N., Abrahamyan, A., Abdi, A., Hämäläinen, K., Junninen, K., Maunula, M., Purhonen, J. & Ovaskainen, O.

SUPPLEMENTARY TABLES

Supporting Table 1. Description of the samples. The Sample ID includes information about location, date and type of the sample, so that e.g. HEL-N1-170819-A is the air (A) sample from Helsinki (HEL), natural plot 1 (N1), sampled 17th August 2019 (170819).

Sample ID	Site	Plot	Type	Latitude	Longitude	Habitat	RefinedHabitat
HEL-N1-170819-A	Helsinki	HEL-N1	Air	60.29107	24.45114	Natural	NaturalCore
HEL-N1-170819-S	Helsinki	HEL-N1	Soil	60.29107	24.45114	Natural	NaturalCore
HEL-N1-180819-A	Helsinki	HEL-N1	Air	60.29107	24.45114	Natural	NaturalCore
HEL-N1-180819-S	Helsinki	HEL-N1	Soil	60.29107	24.45114	Natural	NaturalCore
HEL-N1-220819-A	Helsinki	HEL-N1	Air	60.29107	24.45114	Natural	NaturalCore
HEL-N1-220819-S	Helsinki	HEL-N1	Soil	60.29107	24.45114	Natural	NaturalCore
HEL-N2-170819-A	Helsinki	HEL-N2	Air	60.2943	24.51658	Natural	NaturalCore
HEL-N2-170819-S	Helsinki	HEL-N2	Soil	60.2943	24.51658	Natural	NaturalCore
HEL-N2-180819-A	Helsinki	HEL-N2	Air	60.2943	24.51658	Natural	NaturalCore
HEL-N2-180819-S	Helsinki	HEL-N2	Soil	60.2943	24.51658	Natural	NaturalCore
HEL-N2-220819-A	Helsinki	HEL-N2	Air	60.2943	24.51658	Natural	NaturalCore
HEL-N2-220819-S	Helsinki	HEL-N2	Soil	60.2943	24.51658	Natural	NaturalCore
HEL-N3-170819-A	Helsinki	HEL-N3	Air	60.23775	24.66243	Natural	NaturalEdge
HEL-N3-170819-S	Helsinki	HEL-N3	Soil	60.23775	24.66243	Natural	NaturalEdge
HEL-N3-180819-A	Helsinki	HEL-N3	Air	60.23775	24.66243	Natural	NaturalEdge
HEL-N3-180819-S	Helsinki	HEL-N3	Soil	60.23775	24.66243	Natural	NaturalEdge
HEL-N3-220819-A	Helsinki	HEL-N3	Air	60.23775	24.66243	Natural	NaturalEdge
HEL-N3-220819-S	Helsinki	HEL-N3	Soil	60.23775	24.66243	Natural	NaturalEdge
HEL-U1-160819-A	Helsinki	HEL-U1	Air	60.15399	24.9422	Urban	UrbanCore
HEL-U1-160819-S	Helsinki	HEL-U1	Soil	60.15399	24.9422	Urban	UrbanCore
HEL-U1-200819-A	Helsinki	HEL-U1	Air	60.15399	24.9422	Urban	UrbanCore
HEL-U1-200819-S	Helsinki	HEL-U1	Soil	60.15399	24.9422	Urban	UrbanCore
HEL-U1-210819-A	Helsinki	HEL-U1	Air	60.15399	24.9422	Urban	UrbanCore
HEL-U1-210819-S	Helsinki	HEL-U1	Soil	60.15399	24.9422	Urban	UrbanCore
HEL-U2-160819-A	Helsinki	HEL-U2	Air	60.1724	24.95002	Urban	UrbanCore
HEL-U2-160819-S	Helsinki	HEL-U2	Soil	60.1724	24.95002	Urban	UrbanCore
HEL-U2-200819-A	Helsinki	HEL-U2	Air	60.1724	24.95002	Urban	UrbanCore
HEL-U2-200819-S	Helsinki	HEL-U2	Soil	60.1724	24.95002	Urban	UrbanCore
HEL-U2-210819-A	Helsinki	HEL-U2	Air	60.1724	24.95002	Urban	UrbanCore
HEL-U2-210819-S	Helsinki	HEL-U2	Soil	60.1724	24.95002	Urban	UrbanCore
HEL-U3-160819-A	Helsinki	HEL-U3	Air	60.22722	25.01652	Urban	UrbanEdge

HEL-U3-160819-S	Helsinki	HEL-U3	Soil	60.22722	25.01652	Urban	UrbanEdge
HEL-U3-200819-A	Helsinki	HEL-U3	Air	60.22722	25.01652	Urban	UrbanEdge
HEL-U3-200819-S	Helsinki	HEL-U3	Soil	60.22722	25.01652	Urban	UrbanEdge
HEL-U3-210819-A	Helsinki	HEL-U3	Air	60.22722	25.01652	Urban	UrbanEdge
HEL-U3-210819-S	Helsinki	HEL-U3	Soil	60.22722	25.01652	Urban	UrbanEdge
JOE-N1-170819-A	Joensuu	JOE-N1	Air	62.61314	30.1733	Natural	NaturalCore
JOE-N1-170819-S	Joensuu	JOE-N1	Soil	62.61314	30.1733	Natural	NaturalCore
JOE-N1-180819-A	Joensuu	JOE-N1	Air	62.61314	30.1733	Natural	NaturalCore
JOE-N1-180819-S	Joensuu	JOE-N1	Soil	62.61314	30.1733	Natural	NaturalCore
JOE-N1-220819-A	Joensuu	JOE-N1	Air	62.61314	30.1733	Natural	NaturalCore
JOE-N1-220819-S	Joensuu	JOE-N1	Soil	62.61314	30.1733	Natural	NaturalCore
JOE-N2-170819-A	Joensuu	JOE-N2	Air	62.618	30.16288	Natural	NaturalCore
JOE-N2-170819-S	Joensuu	JOE-N2	Soil	62.618	30.16288	Natural	NaturalCore
JOE-N2-180819-A	Joensuu	JOE-N2	Air	62.618	30.16288	Natural	NaturalCore
JOE-N2-180819-S	Joensuu	JOE-N2	Soil	62.618	30.16288	Natural	NaturalCore
JOE-N2-230819-A	Joensuu	JOE-N2	Air	62.618	30.16288	Natural	NaturalCore
JOE-N2-230819-S	Joensuu	JOE-N2	Soil	62.618	30.16288	Natural	NaturalCore
JOE-N3-170819-A	Joensuu	JOE-N3	Air	62.62875	30.10211	Natural	NaturalEdge
JOE-N3-170819-S	Joensuu	JOE-N3	Soil	62.62875	30.10211	Natural	NaturalEdge
JOE-N3-180819-A	Joensuu	JOE-N3	Air	62.62875	30.10211	Natural	NaturalEdge
JOE-N3-180819-S	Joensuu	JOE-N3	Soil	62.62875	30.10211	Natural	NaturalEdge
JOE-N3-220819-A	Joensuu	JOE-N3	Air	62.62875	30.10211	Natural	NaturalEdge
JOE-N3-220819-S	Joensuu	JOE-N3	Soil	62.62875	30.10211	Natural	NaturalEdge
JOE-U1-160819-A	Joensuu	JOE-U1	Air	62.60496	29.74068	Urban	UrbanCore
JOE-U1-160819-S	Joensuu	JOE-U1	Soil	62.60496	29.74068	Urban	UrbanCore
JOE-U1-200819-A	Joensuu	JOE-U1	Air	62.60496	29.74068	Urban	UrbanCore
JOE-U1-200819-S	Joensuu	JOE-U1	Soil	62.60496	29.74068	Urban	UrbanCore
JOE-U1-210819-A	Joensuu	JOE-U1	Air	62.60496	29.74068	Urban	UrbanCore
JOE-U1-210819-S	Joensuu	JOE-U1	Soil	62.60496	29.74068	Urban	UrbanCore
JOE-U2-160819-A	Joensuu	JOE-U2	Air	62.61026	29.78303	Urban	UrbanCore
JOE-U2-160819-S	Joensuu	JOE-U2	Soil	62.61026	29.78303	Urban	UrbanCore
JOE-U2-200819-A	Joensuu	JOE-U2	Air	62.61026	29.78303	Urban	UrbanCore
JOE-U2-200819-S	Joensuu	JOE-U2	Soil	62.61026	29.78303	Urban	UrbanCore
JOE-U2-210819-A	Joensuu	JOE-U2	Air	62.61026	29.78303	Urban	UrbanCore
JOE-U2-210819-S	Joensuu	JOE-U2	Soil	62.61026	29.78303	Urban	UrbanCore
JOE-U3-160819-A	Joensuu	JOE-U3	Air	62.63427	29.89303	Urban	UrbanEdge
JOE-U3-160819-S	Joensuu	JOE-U3	Soil	62.63427	29.89303	Urban	UrbanEdge
JOE-U3-200819-A	Joensuu	JOE-U3	Air	62.63427	29.89303	Urban	UrbanEdge
JOE-U3-200819-S	Joensuu	JOE-U3	Soil	62.63427	29.89303	Urban	UrbanEdge
JOE-U3-210819-A	Joensuu	JOE-U3	Air	62.63427	29.89303	Urban	UrbanEdge
JOE-U3-210819-S	Joensuu	JOE-U3	Soil	62.63427	29.89303	Urban	UrbanEdge
JYV-N1-170819-A	Jyväskylä	JYV-N1	Air	62.14443	25.73463	Natural	NaturalEdge
JYV-N1-170819-S	Jyväskylä	JYV-N1	Soil	62.14443	25.73463	Natural	NaturalEdge
JYV-N1-180819-A	Jyväskylä	JYV-N1	Air	62.14443	25.73463	Natural	NaturalEdge
JYV-N1-180819-S	Jyväskylä	JYV-N1	Soil	62.14443	25.73463	Natural	NaturalEdge
JYV-N1-220819-A	Jyväskylä	JYV-N1	Air	62.14443	25.73463	Natural	NaturalEdge

JYV-N1-220819-S	Jyväskylä	JYV-N1	Soil	62.14443	25.73463	Natural	NaturalEdge
JYV-N2-170819-A	Jyväskylä	JYV-N2	Air	62.21892	25.59721	Natural	NaturalCore
JYV-N2-170819-S	Jyväskylä	JYV-N2	Soil	62.21892	25.59721	Natural	NaturalCore
JYV-N2-180819-A	Jyväskylä	JYV-N2	Air	62.21892	25.59721	Natural	NaturalCore
JYV-N2-180819-S	Jyväskylä	JYV-N2	Soil	62.21892	25.59721	Natural	NaturalCore
JYV-N2-220819-A	Jyväskylä	JYV-N2	Air	62.21892	25.59721	Natural	NaturalCore
JYV-N2-220819-S	Jyväskylä	JYV-N2	Soil	62.21892	25.59721	Natural	NaturalCore
JYV-N3-170819-A	Jyväskylä	JYV-N3	Air	62.20428	25.60934	Natural	NaturalCore
JYV-N3-170819-S	Jyväskylä	JYV-N3	Soil	62.20428	25.60934	Natural	NaturalCore
JYV-N3-180819-A	Jyväskylä	JYV-N3	Air	62.20428	25.60934	Natural	NaturalCore
JYV-N3-180819-S	Jyväskylä	JYV-N3	Soil	62.20428	25.60934	Natural	NaturalCore
JYV-N3-220819-A	Jyväskylä	JYV-N3	Air	62.20428	25.60934	Natural	NaturalCore
JYV-N3-220819-S	Jyväskylä	JYV-N3	Soil	62.20428	25.60934	Natural	NaturalCore
JYV-U1-160819-A	Jyväskylä	JYV-U1	Air	62.23031	25.74405	Urban	UrbanCore
JYV-U1-160819-S	Jyväskylä	JYV-U1	Soil	62.23031	25.74405	Urban	UrbanCore
JYV-U1-200819-A	Jyväskylä	JYV-U1	Air	62.23031	25.74405	Urban	UrbanCore
JYV-U1-200819-S	Jyväskylä	JYV-U1	Soil	62.23031	25.74405	Urban	UrbanCore
JYV-U1-210819-A	Jyväskylä	JYV-U1	Air	62.23031	25.74405	Urban	UrbanCore
JYV-U1-210819-S	Jyväskylä	JYV-U1	Soil	62.23031	25.74405	Urban	UrbanCore
JYV-U2-160819-A	Jyväskylä	JYV-U2	Air	62.22907	25.71911	Urban	UrbanCore
JYV-U2-160819-S	Jyväskylä	JYV-U2	Soil	62.22907	25.71911	Urban	UrbanCore
JYV-U2-200819-A	Jyväskylä	JYV-U2	Air	62.22907	25.71911	Urban	UrbanCore
JYV-U2-200819-S	Jyväskylä	JYV-U2	Soil	62.22907	25.71911	Urban	UrbanCore
JYV-U2-210819-A	Jyväskylä	JYV-U2	Air	62.22907	25.71911	Urban	UrbanCore
JYV-U2-210819-S	Jyväskylä	JYV-U2	Soil	62.22907	25.71911	Urban	UrbanCore
JYV-U3-160819-A	Jyväskylä	JYV-U3	Air	62.15381	25.75899	Urban	UrbanEdge
JYV-U3-160819-S	Jyväskylä	JYV-U3	Soil	62.15381	25.75899	Urban	UrbanEdge
JYV-U3-200819-A	Jyväskylä	JYV-U3	Air	62.15381	25.75899	Urban	UrbanEdge
JYV-U3-200819-S	Jyväskylä	JYV-U3	Soil	62.15381	25.75899	Urban	UrbanEdge
JYV-U3-210819-A	Jyväskylä	JYV-U3	Air	62.15381	25.75899	Urban	UrbanEdge
JYV-U3-210819-S	Jyväskylä	JYV-U3	Soil	62.15381	25.75899	Urban	UrbanEdge
LAH-N1-170819-A	Lahti	LAH-N1	Air	61.10316	25.7946	Natural	NaturalCore
LAH-N1-170819-S	Lahti	LAH-N1	Soil	61.10316	25.7946	Natural	NaturalCore
LAH-N1-180819-A	Lahti	LAH-N1	Air	61.10316	25.7946	Natural	NaturalCore
LAH-N1-180819-S	Lahti	LAH-N1	Soil	61.10316	25.7946	Natural	NaturalCore
LAH-N1-220819-A	Lahti	LAH-N1	Air	61.10316	25.7946	Natural	NaturalCore
LAH-N1-220819-S	Lahti	LAH-N1	Soil	61.10316	25.7946	Natural	NaturalCore
LAH-N2-170819-A	Lahti	LAH-N2	Air	61.09949	25.80648	Natural	NaturalCore
LAH-N2-170819-S	Lahti	LAH-N2	Soil	61.09949	25.80648	Natural	NaturalCore
LAH-N2-180819-A	Lahti	LAH-N2	Air	61.09949	25.80648	Natural	NaturalCore
LAH-N2-180819-S	Lahti	LAH-N2	Soil	61.09949	25.80648	Natural	NaturalCore
LAH-N2-220819-A	Lahti	LAH-N2	Air	61.09949	25.80648	Natural	NaturalCore
LAH-N2-220819-S	Lahti	LAH-N2	Soil	61.09949	25.80648	Natural	NaturalCore
LAH-N3-170819-A	Lahti	LAH-N3	Air	61.02676	25.79969	Natural	NaturalEdge
LAH-N3-170819-S	Lahti	LAH-N3	Soil	61.02676	25.79969	Natural	NaturalEdge
LAH-N3-180819-A	Lahti	LAH-N3	Air	61.02676	25.79969	Natural	NaturalEdge

LAH-N3-180819-S	Lahti	LAH-N3	Soil	61.02676	25.79969	Natural	NaturalEdge
LAH-N3-220819-A	Lahti	LAH-N3	Air	61.02676	25.79969	Natural	NaturalEdge
LAH-N3-220819-S	Lahti	LAH-N3	Soil	61.02676	25.79969	Natural	NaturalEdge
LAH-U1-160819-A	Lahti	LAH-U1	Air	60.96621	25.6525	Urban	UrbanCore
LAH-U1-160819-S	Lahti	LAH-U1	Soil	60.96621	25.6525	Urban	UrbanCore
LAH-U1-200819-A	Lahti	LAH-U1	Air	60.96621	25.6525	Urban	UrbanCore
LAH-U1-200819-S	Lahti	LAH-U1	Soil	60.96621	25.6525	Urban	UrbanCore
LAH-U1-210819-A	Lahti	LAH-U1	Air	60.96621	25.6525	Urban	UrbanCore
LAH-U1-210819-S	Lahti	LAH-U1	Soil	60.96621	25.6525	Urban	UrbanCore
LAH-U2-160819-A	Lahti	LAH-U2	Air	61.00863	25.66531	Urban	UrbanCore
LAH-U2-160819-S	Lahti	LAH-U2	Soil	61.00863	25.66531	Urban	UrbanCore
LAH-U2-200819-A	Lahti	LAH-U2	Air	61.00863	25.66531	Urban	UrbanCore
LAH-U2-200819-S	Lahti	LAH-U2	Soil	61.00863	25.66531	Urban	UrbanCore
LAH-U2-210819-A	Lahti	LAH-U2	Air	61.00863	25.66531	Urban	UrbanCore
LAH-U2-210819-S	Lahti	LAH-U2	Soil	61.00863	25.66531	Urban	UrbanCore
LAH-U3-160819-A	Lahti	LAH-U3	Air	61.01642	25.80325	Urban	UrbanEdge
LAH-U3-160819-S	Lahti	LAH-U3	Soil	61.01642	25.80325	Urban	UrbanEdge
LAH-U3-200819-A	Lahti	LAH-U3	Air	61.01642	25.80325	Urban	UrbanEdge
LAH-U3-200819-S	Lahti	LAH-U3	Soil	61.01642	25.80325	Urban	UrbanEdge
LAH-U3-210819-A	Lahti	LAH-U3	Air	61.01642	25.80325	Urban	UrbanEdge
LAH-U3-210819-S	Lahti	LAH-U3	Soil	61.01642	25.80325	Urban	UrbanEdge
TAM-N1-170819-A	Tampere	TAM-N1	Air	61.50889	23.98333	Natural	NaturalCore
TAM-N1-170819-S	Tampere	TAM-N1	Soil	61.50889	23.98333	Natural	NaturalCore
TAM-N1-180819-A	Tampere	TAM-N1	Air	61.50889	23.98333	Natural	NaturalCore
TAM-N1-180819-S	Tampere	TAM-N1	Soil	61.50889	23.98333	Natural	NaturalCore
TAM-N1-220819-A	Tampere	TAM-N1	Air	61.50889	23.98333	Natural	NaturalCore
TAM-N1-220819-S	Tampere	TAM-N1	Soil	61.50889	23.98333	Natural	NaturalCore
TAM-N2-170819-A	Tampere	TAM-N2	Air	61.5378	24.02935	Natural	NaturalCore
TAM-N2-170819-S	Tampere	TAM-N2	Soil	61.5378	24.02935	Natural	NaturalCore
TAM-N2-180819-A	Tampere	TAM-N2	Air	61.5378	24.02935	Natural	NaturalCore
TAM-N2-180819-S	Tampere	TAM-N2	Soil	61.5378	24.02935	Natural	NaturalCore
TAM-N2-220819-A	Tampere	TAM-N2	Air	61.5378	24.02935	Natural	NaturalCore
TAM-N2-220819-S	Tampere	TAM-N2	Soil	61.5378	24.02935	Natural	NaturalCore
TAM-N3-170819-A	Tampere	TAM-N3	Air	61.59444	24.11595	Natural	NaturalEdge
TAM-N3-170819-S	Tampere	TAM-N3	Soil	61.59444	24.11595	Natural	NaturalEdge
TAM-N3-180819-A	Tampere	TAM-N3	Air	61.59444	24.11595	Natural	NaturalEdge
TAM-N3-180819-S	Tampere	TAM-N3	Soil	61.59444	24.11595	Natural	NaturalEdge
TAM-N3-220819-A	Tampere	TAM-N3	Air	61.59444	24.11595	Natural	NaturalEdge
TAM-N3-220819-S	Tampere	TAM-N3	Soil	61.59444	24.11595	Natural	NaturalEdge
TAM-U1-160819-A	Tampere	TAM-U1	Air	61.49972	23.76167	Urban	UrbanCore
TAM-U1-160819-S	Tampere	TAM-U1	Soil	61.49972	23.76167	Urban	UrbanCore
TAM-U1-200819-A	Tampere	TAM-U1	Air	61.49972	23.76167	Urban	UrbanCore
TAM-U1-200819-S	Tampere	TAM-U1	Soil	61.49972	23.76167	Urban	UrbanCore
TAM-U1-210819-A	Tampere	TAM-U1	Air	61.49972	23.76167	Urban	UrbanCore
TAM-U1-210819-S	Tampere	TAM-U1	Soil	61.49972	23.76167	Urban	UrbanCore
TAM-U2-160819-A	Tampere	TAM-U2	Air	61.50356	23.7489	Urban	UrbanCore

TAM-U2-160819-S	Tampere	TAM-U2	Soil	61.50356	23.7489	Urban	UrbanCore
TAM-U2-200819-A	Tampere	TAM-U2	Air	61.50356	23.7489	Urban	UrbanCore
TAM-U2-200819-S	Tampere	TAM-U2	Soil	61.50356	23.7489	Urban	UrbanCore
TAM-U2-210819-A	Tampere	TAM-U2	Air	61.50356	23.7489	Urban	UrbanCore
TAM-U2-210819-S	Tampere	TAM-U2	Soil	61.50356	23.7489	Urban	UrbanCore
TAM-U3-160819-A	Tampere	TAM-U3	Air	61.44282	23.86826	Urban	UrbanEdge
TAM-U3-160819-S	Tampere	TAM-U3	Soil	61.44282	23.86826	Urban	UrbanEdge
TAM-U3-200819-A	Tampere	TAM-U3	Air	61.44282	23.86826	Urban	UrbanEdge
TAM-U3-200819-S	Tampere	TAM-U3	Soil	61.44282	23.86826	Urban	UrbanEdge
TAM-U3-210819-A	Tampere	TAM-U3	Air	61.44282	23.86826	Urban	UrbanEdge
TAM-U3-210819-S	Tampere	TAM-U3	Soil	61.44282	23.86826	Urban	UrbanEdge

Supporting Table 2. Outputs from the PERMANOVA analyses. Outputs from the analyses applied to all data (A), or air (B) and soil (C) separately. These analyses correspond respectively to Figure 2a, Figure 2b, and Figure 2c from the main paper.

A. All data

Variable	Degrees of freedom	Sums of squares	Mean squares	F- statistic	R^2	p (F-based)
**Sample type**	1	208412	208412	28.7002	0.12534	0.001
**Habitat**	1	38593	38593	5.3146	0.02321	0.001
**Site**	4	62858	15715	2.1640	0.03780	0.001
**Plot**	24	224339	9347	1.2872	0.13492	0.002
**Sample type x Habitat**	1	30365	30365	4.1816	0.01826	0.001
**Sample type x Site**	4	59363	14841	2.0437	0.03570	0.002
**Sample type x Plot**	24	210975	8791	1.2105	0.12688	0.004
**Residuals**	114	827833	7262		0.49787	
**Total**	173	1662738			1.00000	

B. Air

Variable	Degrees of freedom	Sums of squares	Mean squares	F- statistic	R^2	p (F-based)
**Habitat**	1	26916	26915.6	2.7292	0.02796	0.002
**Site**	4	79243	19810.6	2.0088	0.08233	0.001
**Plot**	24	264605	11025.2	1.1180	0.27492	0.032
**Residuals**	60	591716	9861.9		0.61478	
**Total**	89	962479			1.00000	

C. Soil

Variable	Degrees of freedom	Sums of squares	Mean squares	F- statistic	R^2	p (F-based)

**Habitat**	1	42667	42667	9.7580	0.08675	0.001
**Site**	4	42892	10723	2.4524	0.08721	0.001
**Plot**	24	170171	7090	1.6216	0.34598	0.001
**Residuals**	54	236117	4373		0.48006	
**Total**	83	491847			1.00000	

Supporting Table 3. Outputs from the generalized linear mixed models fitted to the OTU richness data (A) and DNA abundance data (B). The models with lowest AIC values are indicated with an asterisk (*).

A. Outputs from the models applied to the OTU richness data.

Fixed effects included in the model	Degrees of freedom	AIC
Sample type + habitat type (including edge/core categories) + interaction between sample type and habitat type + sequencing depth	11	95356.48*
Sample type + habitat type (not including edge/core categories) + interaction between sample type and habitat type + sequencing depth	7	95365.32
Sample type + habitat type (including edge/core categories) + sequencing depth	8	95584.15
Sample type + habitat type (not including edge/core categories) + sequencing depth	6	95580.44
Habitat type (including edge/core categories) + sequencing depth	7	113453.66
Habitat type (not including edge/core categories) + sequencing depth	5	113450.02
Sample type + sequencing depth	5	95587.84
Sequencing depth	4	113457.13

B. Outputs from the models applied to the DNA abundance data.

Fixed effects included in the model	Degrees of freedom	AIC
Sample type + habitat type (including edge/core categories) + interaction between sample type and habitat type + sequencing depth	12	782.9658
Sample type + habitat type (not including edge/core categories) + interaction between sample type and habitat type + sequencing depth	8	780.0033
Sample type + habitat type (including edge/core categories) + sequencing depth	9	781.7218
Sample type + habitat type (not including edge/core categories) + sequencing depth	7	779.0088*
Sample type + sequencing depth	6	796.8022

SUPPLEMENTARY FIGURES

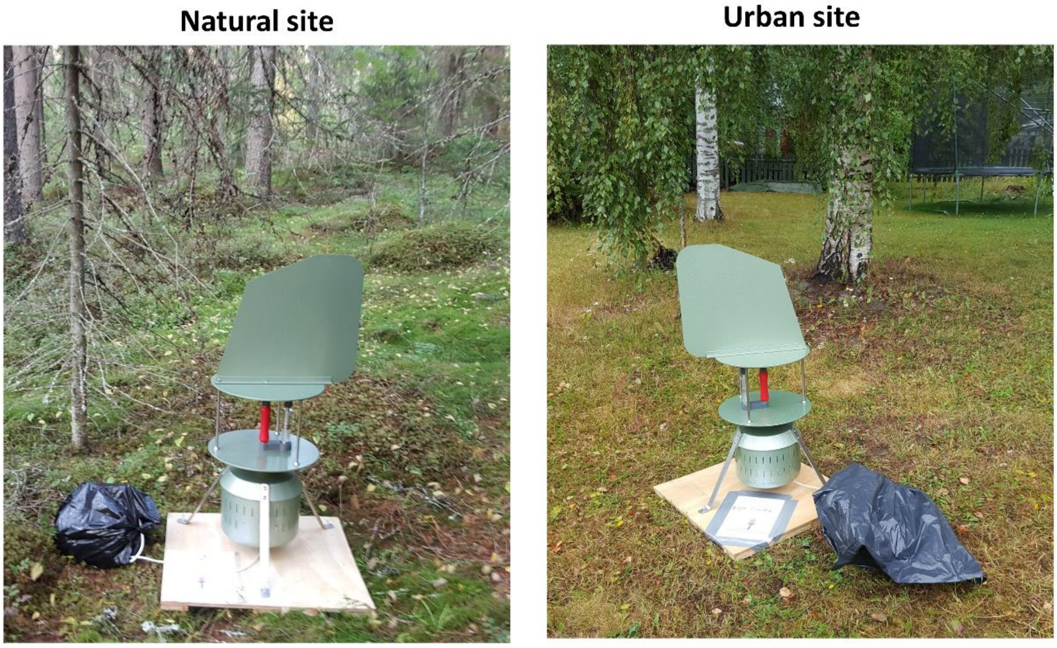

Supporting Figure 1. Pictures showing typical habitat types in the natural and urban sampling plots. The pictures were taken in Jyväskylä by Jenna Purhonen.

SUPPLEMENTARY NOTES

Technical details of the constrained clustering method

Sequences were considered to be reliable fungi sequences if their PROTAX classification probability (based on the method of Abarenkov et al. 2018) was 0.9 or higher to a known fungi phylum. Here we describe how we applied constrained clustering to these sequences, hierarchically at the taxonomic levels of class, order, family, genus, and species.

In the beginning, the set of parent nodes were known fungi phyla and the clustering started at the class level. At each level, the process consisted of three steps:

1) Sequences that were reliably classified by PROTAX (probability 0.9 or higher) into a known taxon of the focal taxonomy level were clustered within their taxon to get representative sequences of the taxa. All reliably classified sequences in the focal level were mapped against the representative sequences in order to define the optimal similarity that delineates whether the sequence belongs to an already existing cluster. As optimality criteria, we required that the amounts of false positives and false negatives were equal. For each taxa, two means were calculated, one from the similarities corresponding to correct and the other from the incorrect classifications. These taxon-specific values were used to obtain a pooled estimate of the threshold, i.e. a shared value among all taxa for each focal level.

2) Sequences that were not reliably classified into a known taxon were mapped against the representative s equences. If the best similarity exceeded the threshold, the sequence was classified to the corresponding ta xon.

3) Sequences that were not reliably classified by PROTAX nor mapped to the known class with the required s imilarity were clustered denovo, with the same similarity threshold as was used for mapping in step 2.

In steps 1 and 2 we used LAST for mapping and in steps 1 and 3 we used uclust for clustering. In step 1, clust ering threshold was 80%, otherwise the optimal mapping and clustering thresholds were found to be 93%, 9 3%, 93%, 95%, and 97% for the levels of class, order, family, genus, and species.

The scripts that were used to implement the above described steps are found from the file scripts.zip.

MCMC convergence of the HMSC models

To evaluate the convergence of the MCMC chains of the HMSC models, we computed the potential scale re duction factors for the parameters measuring the species responses to the fixed effects. The median and (0. 025,0.975) quantiles of these were 1.00 (1.00,1.02) for the HMSC-soil, presence-absence; 1.00 (1.00…1.02) f or the HMSC-soil, abundance conditional on presence; 1.00 (1.00…1.02) for the HMSC-air, presence-absenc e; 1.00 (1.00…1.02) for the HMSC-air, abundance conditional on presence. As the potential scale reduction f actors are close to one in all cases, the MCMC convergence can be considered satisfactory.

CONTENTS OF THE PROVIDED DATA FILES

Interactive version of the Krona wheel shown in Figure 3. The Krona wheel of Fig. 3 is given as an interactive html-page (Krona_PooledData.html). Select “Color by TaxonIndex” to show the classification with the following colors:

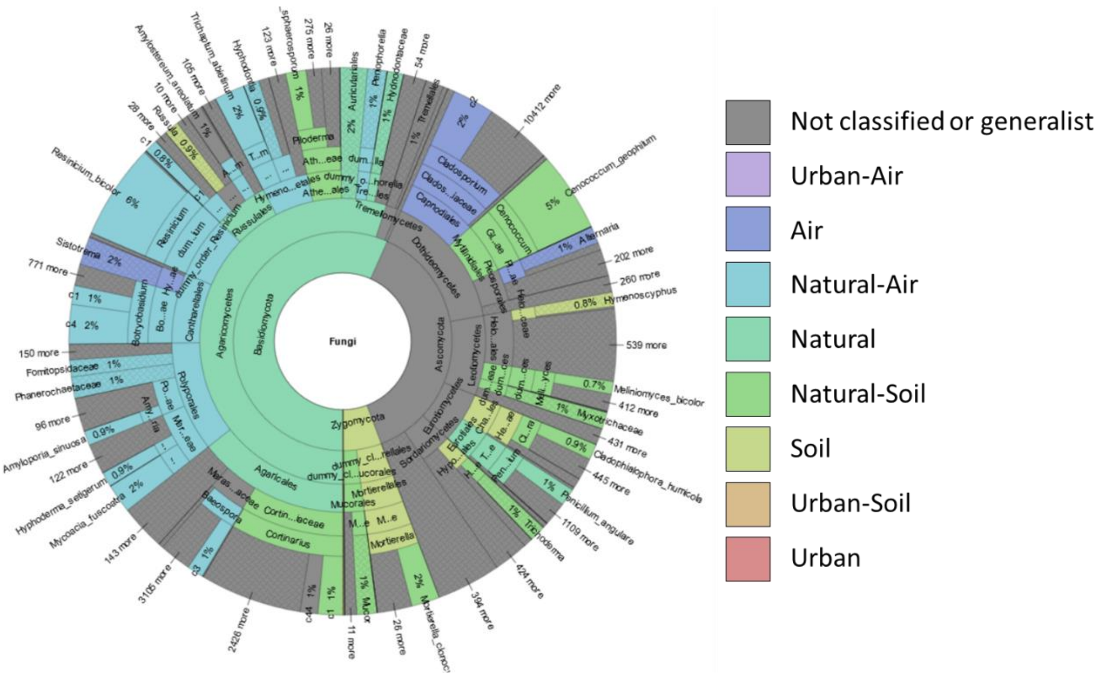

Interactive version of the Krona wheels shown in Figure 4. The eight Krona wheels of Fig. 4 is given as an interactive html-pages (e.g. Krona_AirNaturalCore.html). Select "Color by TaxonIndex" to show the classification with colors used in the interactive version of the Krona wheel shown in Fig. 3.

Data Table 1. The contents of the Krona wheel shown in Figure 3 as numerical data for the species level. The file contains the four columns of TAXON, OCCURRENCES, ABUNDANCE and CLASSIFICATION. TAXON contains the well identified levels with Latin names, and de novo clustered levels (see Methods) with cluster IDs. OCCURRENCES shows the number of samples (out of the 174 samples for which at least 10,000 sequences were obtained) from which the taxon is found. ABUNDANCE gives the relative DNA abundance of each taxon, the numbers summing to one over all taxa. CLASSIFICATION corresponds to the classification of nine types illustrated by the colors of the Krona wheel.

Data Table 2. The contents of the Krona wheel shown in Figure 3 as numerical data for the genus level. The file contains the four columns of TAXON, OCCURRENCES, ABUNDANCE and CLASSIFICATION. TAXON contains the well identified levels with Latin names, and de novo clustered levels (see Methods) with cluster IDs. OCCURRENCES shows the number of samples (out of the 174 samples for which at least 10,000 sequences were obtained) from which the taxon is found. ABUNDANCE gives the relative DNA abundance of each taxon, the numbers summing to one over all taxa. CLASSIFICATION corresponds to the classification of nine types illustrated by the colors of the Krona wheel.

Data Table 3. The contents of the Krona wheel shown in Figure 3 as numerical data for the family level. The file contains the four columns of TAXON, OCCURRENCES, ABUNDANCE and CLASSIFICATION. TAXON contains the well identified levels with Latin names, and de novo clustered levels (see Methods) with cluster IDs. OCCURRENCES shows the number of samples (out of the 174 samples for which at least 10,000 sequences were obtained) from which the taxon is found. ABUNDANCE gives the relative DNA abundance of each taxon, the numbers summing to one over all taxa. CLASSIFICATION corresponds to the classification of nine types illustrated by the colors of the Krona wheel.

Data Table 4. The contents of the Krona wheel shown in Figure 3 as numerical data for the order level. The file contains the four columns of TAXON, OCCURRENCES, ABUNDANCE and CLASSIFICATION. TAXON contains the well identified levels with Latin names, and de novo clustered levels (see Methods) with cluster IDs. OCCURRENCES shows the number of samples (out of the 174 samples for which at least 10,000 sequences were obtained) from which the taxon is found. ABUNDANCE gives the relative DNA abundance of each taxon, the numbers summing to one over all taxa. CLASSIFICATION corresponds to the classification of nine types illustrated by the colors of the Krona wheel.

Data Table 5. The contents of the Krona wheel shown in Figure 3 as numerical data for the class level. The file contains the four columns of TAXON, OCCURRENCES, ABUNDANCE and CLASSIFICATION. TAXON contains the well identified levels with Latin names, and de novo clustered levels (see Methods) with cluster IDs. OCCURRENCES shows the number of samples (out of the 174 samples for which at least 10,000 sequences were obtained) from which the taxon is found. ABUNDANCE gives the relative DNA abundance of each taxon, the numbers summing to one over all taxa. CLASSIFICATION corresponds to the classification of nine types illustrated by the colors of the Krona wheel.

Data Table 6. The contents of the Krona wheel shown in Figure 3 as numerical data for the phylum level. The file contains the four columns of TAXON, OCCURRENCES, ABUNDANCE and CLASSIFICATION. TAXON contains the well identified levels with Latin names, and de novo clustered levels (see Methods) with cluster IDs. OCCURRENCES shows the number of samples (out of the 174 samples for which at least 10,000 sequences were obtained) from which the taxon is found. ABUNDANCE gives the relative DNA abundance of each taxon, the numbers summing to one over all taxa. CLASSIFICATION corresponds to the classification of nine types illustrated by the colors of the Krona wheel.
