# Supplementary material for: Fungal communities decline with urbanization—more in air than in soil
Source: ISME J. 2020 Aug 5;14(11):2806–15. doi: 10.1038/s41396-020-0732-1 (PMC7784924; doi:10.1038/s41396-020-0732-1)
Supplement: Supplementary file 2 — Supplemental data [file 41396_2020_732_MOESM2_ESM.zip › Krona_AirNaturalCore.html]

Javascript must be enabled to view this page.

num
probth


437924

84485.1

0.569407
3

0.569407
3

0.569407
3

0.569407
3

0.569407
3

0

0

0

0

0

0

0

0

0

0

0

0

0

0

0

0

0

0

0

0

0

0

0

0

0

0

0

0

0

0

0

0

0

0

0

0

0
4

0
4

0

0

0

0
4

0

0

0
4

0
4

0

0

0

0
4

0
4

0
4

0
4

0

0

0

0

0

0

0
4

0
4

0
4

0
4

0.281441

0.281441

0.281441

0.281441

0.281441

0
4

0
4

0
4

0

0

0

0

0
4

0
4

0
4

0
4

57560.4

94.5618

74.7223

58.3456

0
3

0
2

0

0.136044

0.764607

0.271308

0

0

0

0

0

0

0

0

0

0

0.327165

0

0

0

0.0404642

0

0

0
3

0.0599054

0.0404642

0

0.0404642

0.0404642

0

0

0

0

0

0.657404

0

0.0599054

0

0

0

0

0.0168673

0

0

0

0.291999

0

0

0

0.0599054

0.357411

0

0

0

0

0

2.54598

0

0

0

0.211081

0.0599054

0

0.0125159

0

0

0

4.57028
7

0

0

0

0

0

0

0

0

0

0.465099

0.272793
7

0.343945

0

0

0

0.00458879

0

0

0

0

0

0.299527
7

0

0

0

0

0.20597

0

0.0404642

0

0.062322

0.00500636

0

0

0

0

0.0809283

0.310066

0.0595195

0.195564

0

0

0

0

0.0469069

7.86305

0

0

0

0

0

0

0

0

0

0.54759

0.00690984

0

0

0

0.0340111

0

0

0.0506019

0

0.0809283

0.351025
6

0

0

0

0.0703603

0

0

0

0.0316877

0

0

0

0

0

0.0469069

0.934504
6

3.45846
7

0

0.431342

1.70996

0
1

0

0
2

0

0.920309

3.37563

0.236099

0

4.3004

0.539429

0.0815314

0.141625
3

0

3.30271

0

0

0

0.320407

0

0.242785

0

0

0
2

0

0.0809283

0

0.078714

0.135654

0.0703603

0

0

0

0

0
2

0

0.258752

0

0.0898581

0

0

0

0

0

0.281543

1.61454
7

0.0224897

0

0.700552

0.545039

0.566498

0.926608

0.0367103

0.703603

0.182089

0

4.17166
6

0

0

0

0.0572897

0.0557601

0

0

0

0.21317

1.15152

3.03401
6

0.294223

0

0

0

0

0.649597

0

0

0.389385

0

2.55906407176099e-14

0
4

0.536117
3

0.536117
3

0

0

0

0
4

0.0351807

0.0351807

0
4

0.807802

0.807802

0
4

0

0

0
4

2.91674

2.91674

0
4

0.134278

0.0938138

0.0404642

0
4

0.0469069

0

0.0469069

0
4

0.423168

0.0387582

0.384409

0
4

0.0599054

0.0599054

0
4

0.0775164

0.0775164

0
4

0

0

0
4

3.03327
6

0.408428

0

2.62484

0

4.44089209850063e-16
6

0
4

0

0

0
4

0

0

0

0
4

0

0

0

0
4

1.26681

1.09253

0.174282

0
4

0.0768796

0.0768796

0
4

0.071891

0

0.071891

0
4

0.0168673

0.0168673

0

0
4

0

0

0
4

0

0

0
4

0

0

0
4

2.56494
2

2.56494

0

0

0

0
4

0

0

0
4

0

0

0
4

0.027535

0.027535

0
4

0

0

0
4

0.0775164

0.0775164

0
4

0.536117

0.536117

0
4

0

0

0
4

0

0

0
4

0

0

0
4

0

0

0
4

0.0404642
6

0.0404642

0

0
4

0

0

0
4

0

0

0
4

0

0

0
4

0

0

0
4

0.290687

0.290687

0
4

0

0

0
4

0

0

0
4

0.0404642

0.0404642

0
4

0

0

0
4

0

0

0
4

0.130322

0.130322

0

0
4

0

0

0
4

0.0125159

0.0125159

0
4

0.0703603

0.0703603

0
4

0

0

0
4

0

0

0
4

0.0599054

0.0599054

0
4

0.119811

0.119811

0
4

0

0

0
4

0

0

0
4

0

0

0
4

0.043779

0.043779

0
4

0.536117

0.536117

0
4

0

0

0
4

0

0

0
4

2.28237
7

0.47645

1.80592

0
4

0.0404642

0.0404642

0
4

0

0

0

0
4

2.97262214843386e-14

0
4

4.30627
6

4.30627
6

4.30627
6

0
4

0
4

0

0

0

0
4

0

0

0
4

0
4

1.90517

1.90517

1.90517

0
4

0
4

0

0

0

0

0
4

0
4

0

0

0

0
4

0
4

1.70996

1.70996

1.70996

0
4

0
4

0

0

0

0
4

0
4

0.0112449

0.0112449

0.0112449

0
4

0
4

0

0

0

0
4

0
4

0

0

0

0
4

0
4

2.56494

2.56494

2.56494

0
4

0
4

0.910405
6

0.829477
6

0.829477
6

0
4

0.0809283

0.0809283

0
4

0

0

0
4

0
4

0

0

0

0
4

0
4

0

0

0

0
4

0
4

0.0255083

0.0255083

0.0255083

0
4

0
4

0

0

0

0
4

0
4

5.36117

5.36117

5.36117

0
4

0
4

0

0

0

0
4

0
4

0

0

0

0
4

0
4

0

0

0

0
4

0
4

0

0

0

0
4

0
4

0.714822

0.714822

0.714822

0

0
4

0
4

0

0

0

0

0

0
4

0

0

0
4

0
4

1.07223

1.07223

1.07223

0
4

0
4

0

0

0

0
4

0
4

0

0

0

0
4

0
4

1.07562

1.07562

1.07562

0
4

0
4

0.182089

0.182089

0.182089

0
4

0
4

0
4

42724.2
7

0

0

0

0

0

0

0
4

0

0

0

0
4

0

0

0

0
4

0

0

0

0
4

0

0

0
4

0
4

41397.7
7

41397.3
7

3962.97
7

79.4411
7

363.54
6

1.11748

0.0112377

0

0

0.0809283

0

0

0

0.0606962

0.007332

0

0.109713
7

0.0404642

0

0

0

0.0549439

0

0

0

0

0

0.103117
7

0

0

0.010998

0

0.0469069

0

0

0

0

0

0.0170056

0

0.0469069

0

0

0

0

0.021996

0

0.0404642

0.0316877

0.661862
7

0

0.179716

0

0

0

0.0633753

0.117267

0

0

0

0.515976

0

0

0

0

0

0

0

0

0

0

0.753125
7

0.01833

0

0

0

1.70996

0

0

0

0.0170056

0

0
8

0

0

0

0.0633753

0.007332

0.0404642

0

0

0

0

0.165655

0

0

0

0.0697535

0.136044

0

0

0

0

0

6.16881

0.0255083

0

0

0

0.0255083

0.0606962

0

0.0475315

0

0

1.86185
7

0.753581
7

0

0

0

0.0599054

0.0469069

0

0

0

0.0172746

0

0.0843364

0

0

0

0.0170056

0

0

0

0

0

0.0852543

2.35441
6

0

0

0

0

0

0

0.299527

0.121392

0

0

0.265398
7

0.0404642

0.0170056

0

0.0232512

0.0809283

0.0340111

0

0

0

0

0.360995
7

0

0.0469069

0.552681

0

0

0

0.0170056

0

0

0.0404642

0.0112449
8

0

0

0

0

0

0

0.0234908

0

0

0

0.182519
8

0

0

0.00939633

0

0

0.0404642

0.032994

0

0

0

0.950256
7

0.0170056

0.0938138

0

0.0425139

0

0.0255083

0

0

0

0

0.661748
7

0

0.221814

0

0

0

0.0244735

0.714822

0.0103648

0.0387519

0

7.08031
6

0

0

0

0.0310016

0

0

0.00835115

0

0

0

18.5908
6

0.007332
1

0.0155008

0

0

0.0938138

0

0.0809283

0

0

0.0850278

0

0.737404
7

0

0

0.0425139

0.119811

0

0

0.0599054

0.0155008

0

0

1.03672
7

0

0.029229

0.0898581

0

0

0

0

0

2.56494

0

0.007332
1

0.0170056

0.0404642

0.331672

0

1.70996

0

0.0155008

0

0

0

0
7

0

0.0122835

0.21317

0.170509

0

0

0.10116

0

0

0

1.90234
6

0.0255083

0

0

0

0

0

0

0

0.141625

0.0112377

0.0837993
8

0.0387582

0

0

0.00525911

0.0469069

0

0

0.60979

0.0599054

0.00525911

0.276796
7

0

0

0.357411

0.0112449

0

0

0

0.119811

0

0

0.130507

0

0

0.014664

0.0155008

0

0.0281121

0.0387582

0

0

0

2.08828
6

0

0

0

0.357411

0.0255083

0

0

0.00525911

0.0599054

0

1.42973
7

0.172855

0

0

0

0

0

0.536117

0.0387582

0.0469069

0

0

0.767318
6

0

0

1.25094

0

0

0

0

0

0

0.0170056

0.110907
1

0.00939633

0

0

0

0.0300732

0

0

0

0

2.0273

0.041084
7

0

0

1.25094

0

0.0316877

0

0.135654

0.0155008

0

0

0.0859213
7

0

0.0549439

0

0

0

0

0.0851081

0

0.0469069

0.0792192

1.0958
7

0

0.0112377

0

0.119039

0

0

0

0.0425139

0

0

0.906196
7

0

0.0170056

0

0.0316877

0

0

0.0387582

0

0

2.14447

0.19346
5

0.0310943

0

2.14447

0

0.0809283

0

0

0

0.0599054

0

0.829506
7

0.007332

0

0

0

0

0

0

0.891439

0.0599054

0.536117

0.740023
6

0

0

0.681285

0

0

0

0

0.0155008

0

0

9.09283
7

0.15991
7

0

0.007332

0

0.0387582

0

0

0.007332

0

0

0.0968956

0.431793
7

0.0404642

0.0224897

0

0.140721
7

0.456824
7

0
8

0.0155008
7

0.615837
6

0.761669
6

0.399787
7

0.473894

14.8803
7

0.322007
7

0.629549
7

0.41195
7

0.161857
7

0.223203

0.19243

0.261674

0.297597
7

1.197
7

0.0112449

3.35488

0.221333
7

0.240144

0.13296
7

0.083374

0.0224897
7

0.0942448
7

0.137176
7

2.96952
3

0.158474

0.22265
6

4.65379
7

0.166772
7

0.0170056
7

0.117682

0.256627
7

0.0748928

0.313478
7

0
7

0.0654693

396.548
6

0.728091
6

5.73833

0.0405173
8

4.56541
6

0.0599054
7

1.74013
6

0.277015

0.166718

0.82262

0.272156

0.0809283
7

0.88737

4.66558
7

0.243466
7

0.0721518
7

0.0818709

0.0487595
7

13.1042
6

0.210399
7

0.313163
2

0.308706

0.0971901

0.0823808
8

15.2223

4.22734
7

2.9586
6

48.55
6

0.0280942
8

0.962963
7

0.304462
7

0.491476
7

0.559911
8

143.708
6

0.01833

0.497654
6

2.18188

0.147276

0.685191

0.405005
6

0.128616
8

42.7489
6

0.142251
7

0.0850278
7

1.31952
6

0.007332
8

1.52427
7

5.62509
7

0.27283
7

0.0610836
8

0.31106

1.8436
6

0.241143
7

0.0497562

0.204021
7

0.368203
7

0.11949
2

0.226658

1.70499
7

0.0823951

0.670652

0.226297
6

0.470216
7

0.209669
8

0.0255083
7

0.0155008

0.139055

0.23566
7

0.785162
7

1.28291

0.0404642
1

0.0677918
8

0.512978
7

3.65008

0.893685
7

3.62105
7

1.93776
6

0.942221
7

0.120157
7

0.219901
7

9.50353
7

0.246562
7

0.266032

0.127806
7

2.18323
6

0.144955
7

2.51949
6

0.0112449

0.632842

0.147785
7

0.0930047
7

2.84033
7

0.41309
6

0.0940339
7

0.150812
7

0.145609
7

1.08717

0.270409

1.89712
6

0.0155008
8

15.9668
6

0.155033
7

8.9771
7

0.781649
7

0.0637338
7

0.0626892
7

0.364149
7

0.469622
6

0.114499
7

0.468111
7

0.0328872
7

0.601609
7

0
7

1.9845
7

0.267218

3.62233
6

0.304063
7

0.66339
6

0.0316877
7

5.21529
6

1.16891
7

13.8625
6

0.0423649
7

0.112267
7

5.36472
7

0.580456
7

0.240528
7

0.570162
7

0.304581

0.506699
7

0.893528
6

0.992945
7

0.102033

0.0599054
7

0.120908
7

35.374
7

1.80487
7

1.27464
6

0.226013

0.226525
7

5.38195
6

0.277991
7

0.195396

0.197734
6

0.0704459
7

0.0609939
5

1.34455
7

11.505
7

0.0316877
7

0.0340111
7

0.193692
7

0.0898581
8

0.058656
8

1.73321
7

0.0112377

0.1525
7

0.0622615
1

1.27254

4.03172
7

0.508841
7

0.206396
7

1.01148
6

0.0558848
7

2.00109
7

0.276119
5

0.0425139

0.254776
7

2.79769
6

0.689043
6

13.4126
7

0.432785
7

0.0711503
8

1.76948
6

0.275641

0.0387582
1

0.0469069
7

1.46442
7

0.199268
7

0.0802426

1.49511
7

4.63882
7

0.0703603

3.45311
6

0.0775164
7

0.473749
7

0.0112449

0.372912
7

0.814742
7

0.28069

2.61184
6

0.376808
7

2.55897
7

0.155033
7

0.0323573

0.909029
6

0.133813
7

0.576581
7

0.445894
7

0.0497562
7

0.0338308
7

0.303537
7

35.6518
6

1.8111
7

0.116029

0.131057
7

0.0185769
7

0.260199
7

0.190961
7

2.18196
6

1.46243
7

0
7

0.0803331
2

0.0155008

3.27951
7

0.157731
1

0.30442
7

0.0170056
7

0

0.007332

1.67637
7

0.350244
7

0.180965
7

0.0255083

0.039357
7

1.65054
7

0.179716

0.600196
7

2.39381
6

0
7

0.133458
5

0.837348
7

0.127416
7

1.38358
7

0.0112449
2

0.794042

3.40598
7

0.142644
7

0.654903
7

0.356544
7

0.176606
5

0.254499
6

0.364743
7

0.0750046
7

0.132713

0.0874452
8

0.214464

36.3391
7

7.83931
7

0.214177
7

0.286674
7

0.186664
7

0.196563
6

0.182581
7

0.236561
7

0
7

0.480692
7

0.218253
7

0.330556
7

1.19906

0.0727693
7

0.0404642
7

0.0316877
7

0.128724
7

0.075019
8

0.095063

0.713411
7

0.524753
7

0.276864
7

0.0938138

1.34596
7

1.60928
7

0.15898

0.16293
7

0.262879

1.32806
6

0
2

0.71008
7

0.0103648

0.226637
7

0.0729705

1.41762

0
7

0.244746
7

4.33559
6

0.165552
6

0.698443
6

36.3615
7

0.209669

0.188423

0.0487595
7

0.256489
7

3.40033

0.159114

0.574869
7

0.309312
7

0.192092
7

0.210652
5

0.164174
7

0.88036
7

0.0404642
2

1.53702
7

0.339171
7

10.6442
7

3.46963
6

0.136044

0.0726311
7

5.68479
6

0.398214
7

0.0728249
6

0.454307

1.85452
7

0.411652
7

1.24942

3.20212
7

0.380902
7

0.058656

0.226249

0.208499
2

0.149764
7

0.512859
7

0.396096

0.207395

6.84009
6

0.22996

1.00464
7

0.170732
7

0.353985
7

0.167744
5

0.306466
7

1.36923
6

0.159879
7

0.0862046

0.283923
7

0.152365
7

0.118428
7

1.36715
7

0.242602
4

0.910534
6

0.353015
7

1.78534
7

0.203128

0.0932828
2

0.325208
7

0.00690984

0
7

0.197111
7

50.5656
6

2.00481
6

0.983386

0.104651
7

3.1396
6

0.0514621
7

0.076911
7

0.202932
7

0.48736
7

0
7

0.0112377
7

34.2089
7

2.355

3.78815
6

0.0986636
7

1.88305
7

0.126751

0.567118

0.279845
7

46.6213
7

0

0.225439
7

0.108364
5

2.90401
7

0.832775
6

0.135654

0.0620094

0.33133
6

0.189515
7

0.105889
6

0.0310016
2

0.938655
7

0
7

0

1.24058
7

0.0809283
4

0.251899
8

0.0478669

0.233105

0.0358731
7

6.80837
6

0.0680222
7

0.339362
7

0.0255083
8

0.18169
7

1.67841
7

0.369373
7

0.01833
7

0
1

0.0387582
7

0.145935

0.954862
6

1.30677
6

0.032994
7

0.0854939
7

0.0461953
8

2.07675
7

0

0.434184
7

0.161553
7

0.289695

0.101709

0.195868
7

0.179683
7

1.85357
6

1.32438
6

0.0224754

3.53084
7

0
8

0.0938138
8

2.36173
6

0.324054
7

0.0750202
7

0.0316877
7

2.45609
7

5.21964
6

0.437836
6

0.0939165
7

1.07101

0.455453

0.540597

0.0775164

0
7

1.76986
7

0.10037
7

0.218427
7

0.0228328
7

0.0387582
7

0.128616
7

1.23334
7

0.0375802

0.282927

0.119811
7

0.259237
7

1.4106
6

1.64004
8

0.308003

0.364321
7

0.608973
6

0.233978

2.71299

0.0922735
7

0.266605
7

0.561216
7

0.0475315

2.02213
7

7.83204
6

0.00939633
7

0.243285
7

0.346235
7

0.331852

5.87967
7

0.0947232
6

0.122895
7

0.550302
6

0.778232

0
7

0.0693018
1

0.044301
7

0.278938
6

0.414638
7

0.141627
6

17.0105
7

0.892168
7

0.572777
7

0.069994
8

0.232549
7

3.10052
6

1.17905
7

0.189907
7

0.166863
7

0.0274961
7

0.0168673

1.13175
6

7.53257
7

0.182089

0.0112449
7

0.357411
7

0.0523289
7

0.396919
7

0.229946
7

0.24169
7

0

0.0439702
7

0

1.56763
7

0.140517
6

1.31338
6

0.444777

0.173832
7

0.200043
7

0.347091

1.77607
6

0.473686
7

0.718276

0.981501

5.91048
7

0.0155008
7

0.0809283
7

0
8

0.0477962
7

0

0.460935
6

0.0606962
7

1.70111
5

0.010998
7

0.0425139
6

4.5054
7

1.28013
7

1.48636
6

1.16837
7

0.191485
7

0.0280942
7

0.0785331
7

0.496536
6

0.293182
5

0.0112449
8

0.0404642
7

15.1486
7

0.169081
6

0.00350607
8

0.0340111
7

0.267556
7

1.18723
6

0.0300375
7

2.60306
6

0.271045
7

0.126408

0
7

3.78476
7

0.117059

0.10699
7

0.0606962

0.215191
7

0
7

0.0423649
7

0.390312
6

0.26501
7

0.0566661
7

3.41991
7

1.53018
7

0

0.0234908
7

0.0449723

0.182125
7

0.0475315

0.32779
6

0.710198

0.0465023

0.798679

0.00939633
5

3.81239

0

1.35325
6

0.382751
6

0
1

0.194079
6

0.412355
7

0
8

0.119811

0

0.0155008

1.39384
7

0.131087
2

0.470641

0.120303

12.6537

0.444999
7

0.304895
7

0.105669
7

0.0387582
7

0.581623
7

0.105622
6

13.2327

10.3335
7

0.359974
7

0.0232512

0.536117

2.36193
6

0.155033
7

0.188003
6

0.0717522
6

0.273351
7

0.223708

0.0224897
7

3.39606

0.353321
7

0.433372
7

1.83936

0.0599054
7

0

0.0310016
7

0.0282504
7

0.0100127
8

0.107843

0.0232512

5.22434
7

2.59044
6

0.0621233
7

1.72096
7

0.214163

0.0809984
5

0.104457
7

0.664045
7

0.95944
6

0.452819
6

0
2

5.83353
7

0.0680282
7

0.0316877

0.028189
1

0.158549
6

0.0552368
7

1.03425
6

0.0606962
7

0.185568
2

0.021996

0.149378
7

0.954272
7

0.136765
7

0.534264
7

0
8

0
7

0.143882
7

0.0310016
7

1.72696
6

0.111247
7

0
1

0.157096

1.77282
7

0.00939633
7

0.179522
7

0.130907

0

1.38959
6

0.173033
7

0.583648

0.0750046
7

0.0651112
7

0.110536
7

3.14752
7

0.110564
6

0.199167
6

55.5736
6

0.220779

0.0316877
7

0.379003
7

0.120704

0.274779

0
1

0
7

1640.21
7

0.0316877

25.4366
6

0.00500636
8

1.29798

0.0850278
8

0.0792192
1

0.0337346

0.355782
6

0.140448
7

0.259599
7

7.94611
6

0.126279
7

0.257988

0.20558
7

0

0.0250645
7

0
7

0.323463

0

0.377968

0.0709034
7

5.40775
7

0.0469069
7

0.106437
7

0.010998
7

0
7

2.52901
6

0.109888
8

0
8

0.0232512

0.914864
7

89.6455
6

47.9183
7

1.10924

0.0944093

0.0606962
1

0.181837

0.0981762
7

0.0856651
8

0.611137
5

0.290557
7

0.501281
6

0.199094
7

0.536117

1.5739

0.0437512
7

0.37927
6

0.076911
7

0.0943837
7

0.00939633
7

0.200311
7

0.0454705

2.56494
7

0.0471885
7

0.290638

0.248528
7

0.0387582
5

0.112
7

0.122674
7

0.0986386

0.141625
7

0.0624152
7

0.0328662
7

0.0595195
7

5.72369
6

0.24759
6

3.63639
7

0.438653

0.0534515
7

0.0999836
7

0
7

0.0340111
2

0.413581
6

0.136291
7

38.4909
6

0.014664
7

0.0469069
8

1.03949
7

0.466902
7

0.0316877
7

0.021996
7

0.138403
7

0.0475315

0.167072
8

0.0538343
4

0.0420524

0.166271
7

0.111449
8

5.63399
7

0.549688
7

0.43628
6

0.0259088
7

1.33532

0.11075
6

0.007332

4.46467
6

0.249217
7

0.165363

0.0469069
7

8.90104

0.137422

0.447269
7

0.191418
7

0.774342

0

0
7

0.0316877
7

0.0792192
7

0.255895
7

0.0898581

6.69637

0.118166
7

0.0404642
7

0.40266
6

0.0404642
7

0.0255083
7

0.0478741
7

0.220124

0
7

0.090264
7

0.0900182
7

5.73702
6

0.0809283
5

0.188921
6

0.0465023
6

0.234534
6

0
5

0.43405
7

3.28603
6

0

0.173421
7

0.0334734

4.08663
6

0.129402
2

0.559827
6

0.00939633
8

0.0155008
7

0.051709
7

4.93559
6

0.0469069
7

0.260147
7

0.0869128

0.536117

24.8272
7

26.7701
7

1.00176
6

0.0898581

2.60745
6

0.459459

0.128616
6

0.0323681

0.142595

0
8

0.0155008
8

0.237658
7

0.676318
7

0.139919

0.0637153

0.0387582
7

0.897741
6

0.0387582
7

0.0606962
6

0.633243
7

0.414473
6

0.1602
7

0
6

2.85495
7

0
8

0
6

0.205476
7

0.0968956
7

0.234534
7

0
7

0.534922
6

0.0224897

0.316384
6

2.39681
6

2.57414

0
1

0.0887613
5

0.691731
6

0.0167023
7

0.729486
6

0.176971
6

0.0573497

0.161066

0.756761
6

0.033713
2

7.84086

0.766809
6

0
8

0.239622

0.0112449
7

0.0112449
7

0.0393319
7

0.132372
7

0.167215
7

0.310701
6

0.0659725
7

70.8525
6

0
7

2.59044
6

0.095063
7

0
8

0.421948
7

0.524129
6

0.701474
5

0.172314
7

0.850464
5

0.0996978
7

0.759413
7

0
7

0.18627
7

0.0656988

0.120246
7

0.129592
7

0
7

0
5

0.0581373
7

0

0.166031

36.4408
6

0.0957434
7

0.0170056

0.102048
6

7.88008
6

0.0385975

0
8

0.0112377

0.235174

0
8

0.0719339
7

1.91109
7

0.120602
7

0.102048
6

0.134903
6

0.39016
6

41.928
6

0.0112449
6

0.107094
7

0.0280942
7

0.0581373
8

0.22386

0.667875
4

0.12988
7

0.0224456
5

0
7

0.289836

0.0103648
1

0.403831
6

0.813303
6

0.163972

0.774195
7

0.606477

28.5531
7

6.4201

1.79982
7

6.58508
6

0.191906
7

2.09062
6

0.128533
6

0.0703603
7

0.419303
7

0.0316877

0.669095
6

0.0549439

1.02428
7

0

0.45185
6

0.179716

0.194773
7

0.311672
7

0.357411
7

0.0216649
7

0.0753602
8

0.118562
7

5.58311
6

0.564902
7

0
8

0.0373457
7

0.014664
8

10.4231
6

0.326275
6

0

0.140437
6

0.0469069
1

7.37595
7

0.63825
7

0.715973
7

1.76809
6

0
7

0.179716
7

0.0812875
7

0.554224
6

0.10116

0.411664
6

0
7

0.118028
7

0.158774
8

1436.56
7

0
8

0.0112449
7

0.0721518
7

0.0786981
7

0.249417
6

3.12015
6

0.0475315
8

13.634
6

0.182089
6

0
1

2.77229
7

0.230141

1.05434

0.039357
7

0.141625
7

5.34561
6

0.0954804
7

0.141062
7

0.095063
7

0.209669

0.0524776
7

4.55
6

0.552984
7

0.0747907
7

0.141105
7

0.0282575
7

0.0338621

0.730323
7

0.0316877

0.0544164
7

0
6

0.0560252
1

2.55976
7

0.0337346
7

0
7

0
7

0
8

0.561456
7

0.121457

0.52851
6

0.0595195
7

0.552984
7

0.271515
7

4.90735
6

0.214773
7

0.189913
7

0

0.10702
7

0.222636
7

0
8

0.179716
7

0.0633753
7

0.266222
7

0.644622
7

4.6118
5

0
7

0
7

0.00614177
2

0.0255083
7

0.6793
6

0.329287
7

0.119811
7

1.80685
6

0.481396
7

0

16553.7
7

50.8221
7

3.21236
6

1.70996
7

0.101895
7

0.135654
7

1.44089
7

30.9053
6

0.374268
7

0
7

0.0703603
7

0.0703603
7

1.79331
6

14.6427
8

2.20301
6

1.23767
6

0.144111
7

0.310066
7

0.365366

0.0170056

0.113763
7

0.090464
5

0.110536

0.367519
7

0.402747

0.0557638
7

0.299784
7

0.358729
6

0.0770934
8

0.0375853
7

0.274938
7

0
4

0.714822
7

1.8094
6

0.741585
7

0.245184
7

0.20078
7

0.183059
7

0.0170056
1

0.01833
7

0.630513
6

0.487522
6

0.0655658
7

0.172418
6

0
7

0

3.98116
7

0

0.0859984
6

0
8

0.007332
7

4.13474
6

0.0938138

0.0232512
7

0.280171

0.0255083
7

0.0475315
8

7.57927
7

0.0595195

0.120952
7

0
7

0.316877

0.0316877

0
8

0.357411

0.0541774
6

0.10116

0.261813

0.887567
7

0.0730916
7

0.197111

0.125378
7

0.0366292
7

0.105208
5

0.550359
6

0.0155008
7

2.67385
6

0.0170056

0.0469069
7

3.33004

0
7

0.0797567

0.164174
7

0

0.010998

0.104623
7

0.0938138
7

0.144547
6

0
7

0.0711503
7

0.87939

0.198685
6

0

0.375068
6

0.195441
2

0.0471885
7

0
8

1.42964
6

0.328348
1

0.0366292
6

0.19258
6

0.953685
7

0
7

0.357411

0.0168673
8

0.584568
7

0.135654
7

1.75749
6

0.0898581
7

0

0.613371
7

0

7.4536
7

3.97156

0.0879957
7

2.09934
6

0

0.067884
7

0
8

0.0893278
7

5.03207
7

0.348045
7

2.44298
6

0.10247
7

0.605147

0

0.295083

0.0703603
6

0
7

0.110907
7

0.0703603
7

10.3688
6

0.0898581

0.258766
7

0.0155008

0.676855
7

0
8

0
7

0.361167
7

0.0986015
6

0.582776
6

0.110907

0.0542527

1.95375
6

0.0680222
7

0.121392
7

2.87941
7

0.160218

0

0.273472
5

0.0968956

0.0456681
7

0.356289

0.0366292
7

0.385523
6

0.0425139

0.021996
7

1.67898
7

0.039357
2

0.179716
7

0.533625

0.116275

0

0.007332
7

0
7

0.776749

0.010998
7

0.134421
7

4.9171
7

0.0475315
7

0.0809283
7

0.0645371
7

0.010998
1

0.536117
7

9.07106

0.209261

0.272652

0

0.0606962
5

0.737513
7

0.0387582
7

0.0599054
7

0
1

0
6

0.0599054
7

0
4

0.0232512

0
8

1.07223
7

0.205724
7

11.0596
7

0.0168673

0.0316877
7

0.110023
6

0.286153

0.110907
7

0.110536

0.0816823
6

0.0255083
2

0.0898581
6

0
8

1.29953
7

0

0.352749
7

2.67385
6

0
1

0.238078

0.0581446
7

0.0676061
7

0.0680222
7

0.357411

0.080918
8

0.84697

0.0112377
7

0.116156
7

0
8

0.0718323
7

0

0
8

0.0905962
7

0.363848

0.0606962

0.0168673
8

9.60541
7

0.663564

0.0346894

0.00939633
8

0.0404642
7

0.179716
6

0
8

0.0255083
8

1.07724
6

0.00500636
8

0.131793

1.73867
6

3.75851

0

0
7

1.44217

0.0850278

0.128724
7

1.80349
6

0.206809
7

0.0753542

0.0305205
6

0.0776923

3.95397
7

0.0358731

0.0170056
6

0.0475315
7

0.141625
7

0.772959
6

0.0305147

0.105059
7

0.161415
7

0.128691
8

0.0255083
7

5.47712
7

0.132848
7

1.26218

0.161857

0.0571779
7

0.119811
6

0.520814
6

9.12132
6

0

1.81112
6

0.0812721
5

3.14413
6

0.536117

0.0706404

0.0739432

0

0.10116
7

0.0785946

0.0310016

0
2

0.0387582
1

0

0.547376
7

0.237831
7

0.0475315
7

0.253414
6

0.129646

0
7

0.220907

0

0.0719411
7

3.41991
6

0.0232512
7

7.51072
7

0.769761

0

0

0.0112449

0.149764
7

0.142305
6

0.080918
7

0
7

0.10116

0

1.72387

0.119811

0.0232512
7

1.70996

0.0340111

0.0510167
7

0.014664
7

0
7

2.70656
6

0
7

4.0788
6

4.90455
7

0.243658
5

0
8

0.0140572

0.221706

0.287605

0.0316877
7

0

0.0112449

0.0170056

0.158022

4.63273
7

0.359055
6

0.0633753
7

0.0264019
5

0
7

1.76063
6

0.209669
6

0.107331
7

0.0735155
7

0.0809283
7

1.70996
6

21.0513
7

3.10199
7

0.0167023
7

0.146901
7

0

0.0775164

0.102033
7

0.036694
7

0.179735
5

0.0565099
7

0
7

0.528892
7

1.93072

0

0.0170056
8

1.45359

0.0475315
7

0.127825

0.0648061
7

0.178559
7

2.58044
6

0

0.0255083
7

1.1137

1.85068
6

0

0.0500031
7

0.0599054
8

0
7

0

0.143042
7

0.0542527

0.0168673
7

0.0703603

0.838106

0.422162
6

0
7

0.0772702
6

0.209388

0

2.62116

0
1

0.536117

0.292625

0
7

1.53674
7

0

1.01744
6

0
8

2.56494
6

0.00500636
6

0.190724
7

0.852543

0.00507012

0.0400509
8

0.0637742
6

2.37872
7

0.0316877
1

0.222917
7

0.0465023
1

0.0387519

0.195564

0.0337238
8

0.0112377
7

0.0938138
7

3.3125
6

0
7

0.18873
7

0.0968956
7

0.239622

0.141625
7

0.30298
6

0.0703603
7

0

0.0112449
7

1.90106
6

0.0255083

0.56276
6

8.89407
6

0.537128
6

0.007332
4

0

0.551618
6

0.443136
7

2.57559
6

0.117267

0.0701581
7

0
7

0.0168673
8

4.42928
6

0.661215

0.222553
7

0.0155008

0

0.034496
6

0.131935
7

0
1

0.0255083
7

0.20597

48.6112
6

46.16
6

0
7

1.70996

0.336783
6

0.0324182
7

0.307554
7

0.855567
7

0
7

0.199987
7

0.0170056
8

0.010998
7

20.5054
7

0.426353

0.007332

0

0.0906715
6

3.41991
6

0.229879

0.278302
6

0.0316877
1

0

0

0.0457822
7

0.933503
7

0

0.570128
6

0.0746145

0.117267
7

0.0155008

0.007332
7

0.0702702
8

0.112405

0.0404642
6

0.357411

0.76755
7

0
7

0.132768
6

2.36554

0.007332
7

0.47911
7

0.164247
7

4.27489
6

0
6

0
7

0.164174

5.5316
7

0

0

0.0599054

0

0.0366292

0.193544

0

0.536117

0.821541

0.0599054

1.01327
7

0.0469069

0.0387582

0.0475315

0.0620094

0.181952

0

0

0

0

0

0.332299
7

0.0856182

0.0880176

0

0.0816052

0.684973

0.0387582

0.118759

0

0.0340111

0.0232512

0.325671

0.116275

0.101204

0.0170056

0.0599054

2.10648

0

0

0.0387582

0.116275

0.0310016

4.28691
6

2.87947

0.125389

0

0.0425139

0.428812

0.124899

0.0267456

0.239622

0.10037

0.0338621

2.56142

0.117267

0.0340111

0.402882

0

0.0475315

0.0606962

0.0387519

0.0281121

0

0.0155008

0.478028
7

0.140471

0

0.357411

0.0606962

0.0899589

16.6601

0

0.0510167

0.0843364

0

9.00234
7

63.7881
6

0.0137834

0.0340111

0.190632

0

0.0727693

0

16.2622

0.121392

0

0

4.63663
7

0.0404642

0.215787

3.41991

0.0155008

0.0413431

0.054259

0

0.269345

0.333267

0

0.314128
7

0.0599054

0

0.0481546

0

1.85068

0.128265

0

0.121392

0

0

2.76048
7

0

0.0050122

0.0599054

5.91998

0

0.0340111

0.119996

0.0387582

0

0.0672374

0.443028
7

0.134083

0.0404642

0

0

0.0968956

0.714822

0.19667

0.095063

0.237658

0.0103648

0.466444

0.283249

0.0898581

0.151966

0.10116

4.27489

0.273489

0.0460256

0.0155008

0

0

4.41971
7

0.0938138

0.0475315

0.357411

0.249349

0.188423

0.0469069

0.0537588

0.458166

0.161857

0.0643988

0.943123
7

0.0187927

0.0425139

0.121392

0.0510167

0.0899589

0

0.0337346

0

0.0581373

0.10321

0.585466
7

0

0.0168673

0.867283

0.0595195

0

0

1.80539

0.025662

0.0387582

0

6.519
6

0.0224754

5.2064

0

1.70996

0.0387582

0.0606962

0.357411

0

0.0475315

0

15.5311
7

17.5569
6

0.360574

0.0170056

0

0.140721

0.0681501

0.0748011

0.271301

1.70996

0

0.0620031

2.67103
7

0.0155008

0.0337346

0.161387

0

0

0.0153857

0

0.0255083

3.41991

0.380662

0.763332
7

0.359606

0.0471094

0.917588

0.190795

0.051709

0

0

0

0.0475315

5.42615

2.25255
7

0

0.0581373

0

0.132758

1.8545

0.187628

0.0915931

0.0228328

0

0.0465023

0.661423

0

0.0316877

0.0898581

0

0.116166

0.0680222

0.121392

0.263378

0.774728

0.0387582

1.59441
7

0.110907

0.237658

0.0879996

0.0404642

0

0.291388

0.496122

0.140471

0.510702

0

1.54905
7

0.0387582

0.126487

0

0.0170056

0.130322

0.0170056

0

0.0112449

0.0626892

0.121884

3.73926
7

0.0633753

0.0557638

0.0935306

0.010998

0.0772328

0

0.0112449

0.501399

0.602066

0.152394

0.912094

0.302997

0

0

0.357411

0.120153

0

0.0167023

0.568892

0.0112449

0.141625

0.609718
7

0

0.119425

0.0944384

0.151665

0.0775164

0

0.0898581

0.0316877

0

0

21.2007
7

17.9235

0.0581373

2.63449

0.0112449

0

0

0.0308409

0.0595195

0.437467

0.0170056

0.536117

2.84506
7

0.0757748

0

0.118028

0.357411

1.78032

1.99966

0

0

0.0224897

0.115669

5.08279
6

0.0968956

0.135654

0.0404642

0

0

0.00690984

0.0581373

0.0606962

0.0469069

0

0.633034

0.887919

0.0809283

0.0387582

0

0.0525768

0.0255083

0.0170056

0.0155008

0.0542527

0.135654

0.878525

0

0.0633753

1.13561

0.120609

0.232549

0.0905763

0

2.66

0.0809283

1.38874

7.827

0.0633753

0.0775164

0.0622001

0.010998

0.039357

0.010998

3.17247

0.357411

0

0.0168673

0.469014
7

0.039357

0.0624077

0.357411

0.0599054

0

0.0661887

0.0112449

0

0

0.0404642

0.653185
7

0.095063

0.0112449

0.0366292

0.0775164

0.0340111

0

0.0574697

0.117558

0

0.010998

4.91872
7

0.0387582

0

0

0

0.365469

0

0.202321

0.0366292

0

0.033713

12.3172
6

0

0

0.847126

0.0404642

0.124586

0.536117

0.0123384

0.0856651

0.216314

0

4.36706
7

4.76322
7

0.0519133

0.32806

0

0.0872276

0.0140945

0.119811

0.113393

0

0.0387582

1.78235

4.76361
7

0.181298

0.536117

0.0557638

0.0168673

0.0898581

0.147712

0.179716

0.406171

0

0.109204

7.43938
7

0.99154

0.126669

0

0.0155008

0

0.172409

0

0

0.0404642

0.0170056

1.85935

0.0450091

0.0944308

0

0.0693903

0.0654693

0.271308

0.0387582

0.0595195

0.0168673

0.164174

1.04642
7

0

0.0938138

0

0.010998

1.76986

0.0599054

0.106812

0.11193

0

0

0.894527

4.27489

0.0542389

0

0.160171

0.135527

0.0606962

0.075789

0.0112377

0.069273

0.536117

0.377277

0.0155008

0.0469069

0

3.52128

0.563499

0.0708849

0.357411

0.027232

0.0404642

0.198887

0.828074
7

0.0404642

0

1.70996

0.20597

0.0170056

0

3.87974

0

0.496746

0

0.870589
7

0.139308

0.267943

0

0

0.0439702

0.0310016

0.222553

0.290687

0.0807958

0

0.866955
7

0.0873659

0.0243376

0.0125267

0.269345

0.120602

0.178168

0.113018

0

0.490023

0

31.5932
7

4.49719
7

0.268721

0.0680222

0.21756

0.064528

5.15798

0

0.194615

0.0599054

0.331044

0.007332

3.62213
6

0.357411

0.0606962

0.259791

0.17133

0.119811

0.0123384

0.357411

0

0.0968956

0.301033

12.0465
7

0.0316877

0.893528

0.149764

0.130266

0.112616

0.145484

0

0.0232512

0.0316877

0.0915643

0.53213

0

0.357411

0.283985

0

0.638945

0.0595195

0.0711503

0

0

0.0581373

8.34854
7

0

0.014664

0

4.33688

0.0387582

1.96286

0.0599054

0.0404642

0.536117

0.105059

0.690264

0

0.0155008

0.0103648

0.0606962

0

0

0.0255083

0.0775164

0

0.0935306

4.89707
7

1.70996

0

0

0.0606962

0.0168673

0.893528

0.662914

0.0809283

0

0.21317

3.44747

0

0.245779

0

0.110536

0.209669

0.0225286

0.0337346

0

0.714822

0.00500636

17.3373
7

0

0.0904569

0.0404642

0

0.0898581

0.10037

0.221814

0.379901

0.191019

0

0.289158
7

0

0

0

0.010998

0

0.0387582

0

0

0

0.321056

758.384

8.99964
7

0.600348
7

0.076744

0

0.108478

1.36257

0.233571

0.0510167

0.007332

0

0

0.0680222

0.732447
7

0

0.0366292

0.714822

0.0316877

0.0112449

0.0704459

0

0.144387

0.039357

0.357411

1.33663
6

0.0316877

0.0898581

0

0.761882

0

0

0

1.70996

0

0.0404642

1.05098
7

0.893528

0.249646

0.0404642

0

0.239622

0.0968956

0

0.0469069

0.0898581

0

0.899075
7

0

0

0.10116

0.0434074

53.4955

1.82977

0

0.158438

0

0.0255083

1.13319
7

1.70996

0

0.0404642

0.239622

0.0170056

0.0230103

0.00500636

0.076376

0.138776

0.0542389

0.650465
7

0.0316877

3.41991

16.2763

0.0404642

0.714822

0.0898581

0.0809283

1.783

0

0.0437646

0.385662
7

0

0.0387582

0.10116

10.9465

0.00939633

0.0475315

0.276514

0.0140945

0

0

0.635696

0.0170056

0

0.0387582

0.112348

0.032994

0.149764

0.234534

0

0.362166

0

3.50201
7

0

0

0.0170056

0.0112449

0.0112449

4.27489

0.0170056

0

0.010998

0

12.5345
7

3.77806
7

0

0.0325063

0

0.0404642

0.0630323

0.0599054

0

0

0

0

28.6617
6

0

0.040326

0.0404642

0.0425139

0

0

0

2.50188

0

1.72696

3.16069
7

0.0606962

0

0.0469069

0.0234908

0

0.280592

0.0606962

0.106812

0

0

1.82061
7

0

0.135231

0

0.0366292

0

0

0.007332

0.0549439

0

0

2.62936
7

0.0338621

0.014664

0.0542527

0.01833

0.014664

0.0112377

0.0404642

0

0

3.08513

1.81593
7

0.0366292

0

0.0170056

0.050355

0.014664

0.0542527

3.41991

0.00668293

0.095063

0

1.30635
7

0

0

0.119811

0.0232512

0

0.0168565

0

0.0741987

0.007332

0

2.56857
7

0

0.0606962

0.186754

0.0469069

0.119811

0.039357

0

8.54979

0

0

0.561525
7

0

0.0170056

0

0.168094

0

0.0775164

0.102033

0.0703603

0.199301

0.0475315

0.144606
7

2.56494

0

0.0775164

0

0.0112377

0.41167

0.241203

0

0

0.0387582

6.42193

1.22473

0.893528

0

0.0155008

0

6.52747

3.22681

0.0155008

0

0.193791

0

0.529926
7

0

2.80997

0

0.0255083

0

0.0938138

0.0155008

0.164174

0.143444

0.0475315

0.922543
7

0.0549388

0.0404642

0.076911

0.0404642

0.029328

0

0.232549

0.0809283

0

0.00939633

4.51409
7

0.458501

0.076911

0

0

0

0

0

0.0168673

0

0.0410091

0.240792

0

0.714822

0.218749

0

0.0310016

0.00500636

0

0.792192

0.0475315

0.0703603

0.453534

0

0.0792192

3.41991

0

0.551618

0.0162512

0.193791

2.56494

0.256627

1.42964

0.879582
7

0.0549439

0.0835361

0.043992

0

0.0404642

0

0.095063

0.0900454

0.561625

0.0753542

1.75065
7

0.0465023

0.0232512

0.0224897

0.0703603

0.014664

0

0.0078762

0.0039381

0.0112377

0.0510167

0.214989
4

0.0599054

0

0.0510167

0

0

0

0.0224897

0

0.0816052

0.0404642

0.904472
7

0.536117

0.0170056

0

0.221814

0.0387582

0.0957428

0.0683169

0.127835

0.0809283

0.0429325

278.359
6

1.11965
7

0.110907

0.219315

0.0733881

0.748818

0.0887904

0.0475315

0

0

0.497256

0.09472

0.534893
5

0.0469069

0

0.174282

0.00835115

0.0155008

0.00835115

0.0792192

0

0.0809283

0

9.87244
7

0

0.213561

0

0.0112449

0

0.00939633

0

0

0

0.0404642

2.42072
7

0

0.0387582

0

0

0

0.104812

0.0938138

0

1.07223

0.0310016

2.16107
7

0.357411

0.0465023

0.0469069

0

0.0792192

0

0

2.59662

0.0466059

0.0599054

0.311434

0

0

0.0599054

0.143998

0

0.0168565

0.11067

0.0471885

0.917143

83.732

0.914962
7

0.0404642

0.511314

0.0599054

0.0500031

0.0703603

0.0170056

0.653384

0

0.0337346

0

1.58385
7

0.0581373

0

0

0

0

0.250294

0.0170056

0.0711431

0.0140945

0.381961

2.16926
7

0.0475315

0

0.0330803

0

0.0938138

0

0

0

0.146891

0.222553

0.179407
7

0.19673

0

2.56494

0.014664

0

0

0.0404642

0.0779085

0.536117

0.11323

8.61573

1.73946
7

0

0

0.182089

0.0429325

0

0.00690984

0.0620031

0.103414

0.428315

0

5.71077
7

1.42996

0.00835115

0.148053

0

0

0.0387582

0.0155008

0.158492

0.0469069

0

1.36731
7

0

0.0510167

0

0.00835115

0.0310016

0

0.00350607

0.0170056

1.93909

0.0112449

66.6076
6

0

0

0.0599054

0.010998

0.119811

0

0.007332

0

0.0633753

0.144427

126.372
7

0.357411

0.119811

0.0255083

0

0.359446

0.0387582

0.119811

1.16209

0

0.0340111

14.4676
6

0.0310016

0

0

0.158438

0

0.0155008

15.7643

0.0633753

0

0.222553

6.49519
7

0.007332

0.205537

0.0280942

0.0425139

0

0.0973865

0.0948645

0.357411

0.0968956

4.05745

0.880761
7

0.117267

0.00939633

0

1.29479

0.0721518

0

0.0581373

0

0.014664

0.0633753

0.781377
7

0

0

0.357411

0.112547

0.0633753

0

0.0316877

0

0.116275

0.164174

0.235789
7

0

0

3.41991

0.0505696

1.78648

0

0.0528527

0.0469069

0

0

12.4279
7

2.09891

0.0281121

0

0.0367532

0

0.539149

0.0281121

0.126751

0

0.0112449

0.271243

1.82216
7

0.187048

0.372912

0.0599054

0

0.102033

0.0449507

0.0469069

0

0.010998

0.130236

6.08861
5

0.107437

0

0.116275

0

0.0465023

0.0469069

0

0.010998

0

0

4.45557
6

0.722374

0

0

0

0

0

0.0404642

0

0.232549

0.0387582

0.943737
7

0.0469069

0.0887613

0.116275

0.174412

0.271308

0

0

0.0316877

0.119811

0.0316877

0.754355
7

0

0.106812

0

0

8.38834

5.12987

0.0599054

0

0

0

8.99113
7

0.0282504

0

1.0723

0.218288

0

0.0809283

0.0469069

0

0.00500636

0

0.699607

0.192782

0

0

0.037586

0

0.057196

0.0935344

0

0.151278

0.0469069

2.05759
7

0

0.0475315

0

0

0.095063

0

0

0.72582

0.819178

0.149764

0.940882

0

0.00939633

0.283249

0.0775164

0.0792224

0.161553

0.0316877

0.029328

0.00939633

0.0703603

8.5737
7

1.52555
7

0

0

0.0510167

0.0112377

0

0.021996

0.0680222

0

0

0

1.01766
7

0

0.0255083

0

0

0.0224897

0.0595195

0

0.110557

0.0187927

0

0.253313
2

0.110907

0

0.406391

0.021996

0.0324182

0

0

0.0906195

0

0

0.94175

0.0425139

0.0680222

0.0170056

0.0170056

0

0

0.249328

0.0170056

0.0387582

0

3.726
7

0

0.0829781

0.0112377

0.351936

0

1.43465

0

0.0968956

0

6.96952

0.883279
7

0

0.123444

0

0

0.290687

0

0.120962

1.8911

1.07223

0

2.25212
7

0

0.0968956

0

0.176509

0.0255083

0

0.714049

0

0.007332

0.014664

12.6337
7

0

0.187741

0

0

0.0732585

0.0510167

0

0.0255083

0

0.0101402

0.371689

0

0.0103648

0.0703603

0.0112377

11.6856

0.0112449

0.0429254

0.187628

0.105059

0.11323

4.27953

0.0168673

0.0469069

0.32948

0.398189

0.0527845

0

1.07223

0

0.0775164

0.0606962

12.7629
7

5.1464
7

0

0.0469069

0.0581373

0

0

0.0510167

0.00690984

0.0728013

0.0829781

0

7.22857
7

0.128616

0

0

0

0.0548363

0

0.0799309

0.0310016

0.0243376

0.0255083

0.370198

0

0.0366292

0

0.161553

0.0170056

0

1.8774

0

0

0.193811

1.56017
7

0

7.16506

0.0170056

0

0.714822

0

0.251928

0.014664

0.0599054

0.0316877

0.793452
7

0

0.0316877

0.0170056

0.00500636

0.0255083

0.0891389

0.15644

0.0703603

0

0.010998

1.14138
7

0.0889467

0

0

0.0581373

0.0337346

0.119401

1.70951

0.260572

0

0

5.52714
5

0

0

0.0682566

0

0.357411

0.0950228

0.26784

0.0387582

1.70996

0.0581373

3.17491
6

0

0.0232512

0.357411

0

1.03425

0.0792192

0

0

0

0.0316877

8.99401
7

0

0.0232512

0.236142

0.0404642

0

0.0340111

0.00500636

0

0.007332

0.00690984

4.01844
7

0

0

0.0599054

0.141625

0.0968956

0.0255083

0.0316877

0.01833

0.179716

4.27489

28.7966
7

6.23273
6

0.0170056

0.0469069

0.0112377

0

0.0112377

0

0.0549439

0.0316877

0.1719

0.0340111

5026.56
6

0

0.0599054

0

0.0599054

0

0.0170056

0.0542527

0.603113

0.0785946

0

0.653396
7

0

0

0

0

0.0606962

0

0

0.0170056

0

0

5.01884
7

0

0

0

0

0

0

0

0

0

0.0549439

0.170809

0

0

0

0

0

0.357411

0

0

0.140721

1.70996

1.36491

0

0.0316877

0.0898581

0.123614

0.0581373

0

0

0.0387582

0.0478669

0

17.5325
6

0

0

0

0.0469069

0.0168565

0.101139

0.110536

0.0475315

0.0599054

0.021996

0.593059
7

0

0

0

1.19613

512.987

0.0581373

0.0404642

0

0.0255083

0

0.384982
7

0

0.0170056

0.374417

0

0.523236

0

0.0775164

0.0232512

0.0152104

0

0.405045

0

0

0

0

0.177841

0.00500636

0

0

0.010998

0

56.7849
7

0.939425
7

0

0

0

0.0606962

0.0155008

0

0.0680222

0

0

0

2.33345
7

0

0.0500031

0

0

0

0

0

0

0

0.0968956

1.29907

0

0

0.00350607

0.0316877

0.161553

0

0.0425139

0

0.0935306

0.143756

0.956912
7

0.0599054

0.209388

0

0

0

0.77897

0.0775164

0.0316877

0

0

0.0843208

1.07223

0

0

0

0.46353

0

0.0599054

1.02562

0.0510167

0

0.829651
7

0

0

0

0

0.03666

0.0170056

0

0

0

0

2.47656

0

0

0

0

0

0.0337346

0.179716

0.0606962

0

0.0542527

0.119228
8

0

0.0112449

0.106826

0

0.775519

0

0.0316877

0.039357

0

0

1.53952
7

0

0.161857

0

0

0

0

0

0

0.0404642

0

14.2156
6

0.357411

0

0.0264988

0.0323573

0

0

0

0

0.0790087

0.0323086

907.494

3.13472

0.475124
7

0

0

0

0

0

0.135654

0.0595195

0.0404642

0.0599054

0.0316877

3.237
7

0

0

0

0

0.0425139

0.040326

0.357411

1.70996

4.12547

0.243547

5.34641

0.0281121

0.0979339

0.0809283

0.0898581

0.00939633

0

0

0

0.0170056

0.10116

1.03072

0

0.362418

0

0.0599054

0

0.0140945

0.0316877

0

0.0224897

0.0170056

0.607113

0

0.0469069

0

0.0170056

0.0792192

0.116275

0

0

0.0316877

0

191.895
6

0.0112377

0.0255083

0

0.0938138

0

0.135654

0

0.146183

0

0

0.971111
7

0.0581373

0

0.0725533

0

0.0809283

0

0

0

0

0.0155008

0.133466

0.0599054

0

0

0

0.110825

0.0574697

0

0.667659

0

0

0.40528
7

0

0

0

0.0581373

0.0775164

0

0

0

0

0

18.1533

0.0581373

0.0658126

0.0170056

0

0

0.0387582

4.11023

0

7.22285

0

6.41743
7

34.1607
6

0.898581

0

0

0.498132

0

0.357411

0

0

0.0581373

0.0404642

2.67141
7

0.007332

0

0.330781

0

0.121392

0

0

0

0.0255083

0

0.626484

0

0.0469069

0

0.0387582

0.0599054

0.0775164

0

0

0.00939633

0

0.949606
7

0.0620031

0.0140945

0

0

0.0170056

0.164174

0

0

0.0775164

0

0.202133
7

0

0.121392

0.0387582

0

0

0

0.00939633

0.0792192

0.0599054

0

0.144084
7

0

0.0168673

0

0

0.0340111

0

0

0

0

0

0.292128
7

0

0.0224754

0.0979339

0

0.007332

2.86773

0.0170056

0

0.0581373

0.0579265

6.15343
6

0.0898581

0

0.303481

0

0

0.0316877

0.0140945

0

0.149764

0.0112449

1.12163
7

0.0387519

0

0

0.0316877

0

0

0.0366292

0

0.0187927

0.174282

15.092
6

0

1.76986

0

0

0.0310016

0

0.007332

0

0.368656

0.0542527

4.44704

1.23702

1.70996

0.0255083

0

0

0.0898581

0

0

0

0.021996

0

2.06675
7

0

0.0425139

0

0.0375477

0

0

0

0

0

0

0.565978

0.357411

0

0.076986

0.0425139

0

0

0

0

0

0

0.372647
7

0

0.142595

0.0168673

0.202407

0

0

0.0581446

0

0

0.0809283

0.202578

0.100752

0.058656

0

0.0469069

0

0.112398

0

0.0316877

0.0140945

0

2.52981
7

0

0.0599054

0

0

0.167133

0.104533

0.0915931

0

0.0404642

0

1.02317
7

0.0168673

0

0

0

0

0

0

0

0

0.304895

0.185387

0

0.0469069

0.0387582

0

0.0792192

0

0.0768595

0.020347

0.0721518

0

5.5757
6

0

0.121392

0.0606962

0

0

0

0.0775164

0.00835115

0.419338

0.0170056

0.53236
7

0

0

0.0948645

0

0.0234908

0

0

0

0.0469069

0.021996

19.5918
7

0.735121
7

0

0.0404642

0.0310016

0.174282

0.0316877

1.02611

0

0.0393391

0.0968956

0

4.41105
7

0.181353

0.0703603

0

0

0

0

0.0207295

7.70863

0

0.536117

0.551579

0.00939633

0.0170056

1.10825

0.179716

0

0.242775

0

0.0310016

0.357411

0

0.333179
1

0

0

0.0316877

0.0393319

0

0.304895

0

0

0

0.0730286

0.444301

0.0606962

0

0

0.10116

0.242785

0

0

0.0792192

0

0.0469069

0.762871
7

0.10814

0.0316877

0

0.014664

0.0475315

0

1.00771

0

0

0.0703603

0.784651
7

0

0.0255083

0.007332

0

0.0350445

0

0.109888

0

0

0.0170056

0.534243

0

0

0.953434

0.0387582

0

0.26427

0.095063

0.357411

0

0.0170056

1.10173
7

0

0.299527

0.106826

0.014664

0

3.75282

0

0.0232512

0

0.0255083

2.24224
7

0

0

0

0

0.0469069

0

0.0366292

0

1.08348

0

37.7983
7

0.378518
7

0.0155008

0.0599054

0

0.795751

0.357411

0

0

0

0.0155008

0.0168673

0.758463
7

0.112616

0.0168673

0

0

0

0

0.117267

0.10116

0

0.0155008

1.03867
7

0.39056

0.0469816

0.0680222

0.0170056

0

0

0.0898581

0

0.0680222

0.164174

0.746716
7

0

0.465338

0

0.0662348

0

0

0

0

0.007332

0

2.15229
7

0.186009

0.0974578

0

0

0

0

0

0

0

0.040326

12.4464
6

0

0.01833

0

0

0

0

0

0.01833

0.36562

0.076525

5.20563
6

0.121392

0

0.0469069

0

0

0

0.0898581

0.212599

0.357411

0

0.288471

0

0

0

0

0

0.0587336

0.159462

0

0.0316877

0

0.44152

0

0.130322

0

0.0425139

0.0267385

0

0.0581373

0.0316877

0

0.146609

2.50522
7

0.140721

0.201207

3.48564

0

0

0

3.41991

0.109888

0

0.0724152

12.436
7

0.285046
7

0

0

0.0112449

0.0255083

0.0316877

0

0.0898581

0

0

0.0404642

1.53383
7

0

0

0

0.007332

0

0

0

1.70996

0

0.0792192

1.14761
7

0.0155008

0.007332

0.0168673

0

0

0.130322

0.0854138

0

4.6675

0.0651645

1.65999
7

0

0.112741

0.119811

0.0255083

0.0387519

0

0

0

0.0218018

0

0.337632

0

0

0.014664

0

0.0155008

0.0112449

0

0

0.0542527

94.0477

0.399318
7

0.0465023

0.0170056

0.0606962

0.0255083

0.218718

0

0

0.509196

0.0850278

0

1.80391

0

0.0599054

0.0316877

0

0.177948

0.0898581

0

0

0.0813885

0.120962

0.285888
7

0.0255083

0

0

0

0

0

0.0581373

0

0

0

0.059553
7

0

0

0.007332

0.0155008

0.587293

0

0

0

0

0.007332

2.57185

0.0387582

0.359433

0.15305

0

0

0

0

0

0.0425139

0

19.5598
7

0.471211
7

0.0328403

0

0

0

0

0

0

0

0

0.375014

0.213693

0.00500636

0

0

0.00350607

0

0.554534

1.96576

0.10116

0.0782354

0.0197611

2.73625
7

0

0.0340111

0

0

0

0.0454705

0.357411

0

0.0316877

0

0.611615
7

0

0.0606962

0

0

0

0

0

0.0680222

0

0.0404642

0.48147
7

0

0

0.38292

0.0775164

0

0

0.0475315

0

0.0168673

0

0.781842
7

0

0

0

0

0.0703603

0.384409

0

0

0

1.70996

0.513202

0

0.0732585

0

0.0267456

0.0935306

0

0.007332

0.0112377

0.0475315

0.007332

1.75211

0.148692

0.0255083

0

0.0112449

0

0

10.7223

0.0827043

0.0469069

0.0510167

89.6459
6

0

0.014664

0.0938138

0

0.0170056

0.0938138

0.0633753

0.007332

1.98944

0.633243

0.838269
7

0.138739

0.0387582

0

0.0224897

0

0.119811

0

0.00939633

0

1.24652

4.78459

0.624785

0

0

0.0938138

0.0224897

0.958487

0

0

0.0606962

0

0.357411

0.185018

0

0

0

0

0.0633753

0.0168673

0

0.0112449

0.0340111

0

0.810641
7

0.094835

0

0

0.0112449

0.539149

0

0.0155008

0

0

0.121392

0.922181
7

0

0

0

0

0

0.36562

0.0155008

0.127542

0

0.0316877

0.378517
7

0.0703603

0

0

0.0112449

0.0809283

0

0

0.0155008

0

0

0.368201

14.5469

0

0.0170056

0

0

0

0

0.179716

0.00525911

0

3.68791
7

0.276425

0

0.117267

0

0

0

0

0

0.0938138

0.010998

0.49434
7

0.255763

0

0

0

0

0

0

0

0.310066

0

0.596155

0.0387582

0.0465023

0

0.0168673

0.257988

0.0732585

0

0.253501

0

0

0.526685

0.119811

0

0.00690984

0

0

0

0

0

0.0483689

0

13.7711
7

0.318925
7

0

2.56494

0.357411

0

0

0

0

0.007332

0.416636

0

0.822141
7

0

5.12987

0.0475315

0

0

0

0.101915

0

0

0

4.26085
7

0

0

0

0.0899589

1.70996

0

0.0599054

0.0234908

0.0620031

0

2.24344

0

0

0.0155008

0

0.357411

0

0.209669

0.357411

0.0390197

1.70996

0.238643

0.00350607

0.010998

0.155033

0

0.0898581

0

0.0155008

0.0387582

0

0.0155008

0.731124
7

0.0606962

0.094522

0.376462

0.193791

0

0.0316877

0.100005

0

0.0387519

0

0.264628

0.0310016

0

0.375255

0.15305

0

0.0938138

1.47216

0.281441

0

0

1.52887
5

0

0.0606962

0

0.170056

0.117971

0.101401

0.010998

0.0633753

0.406691

0.0606962

0.34483
7

0.161553

0.0374968

0

0

0

0.0112377

0

0.552984

0

0.0112377

4.42931
7

0.0316877

0

0.0316877

0

0.0598804

0

0

0

0

0

7.57348
7

0.493826
7

0

0.347978

0.025662

0

0.0606962

0.0935306

0.0425139

0.748834

0

0

1.32988
7

0

0.0581373

0.0170056

0.0606962

0.0809283

0.0809283

0

0

0.014664

0

0.109345
7

0.0659725

0

0

0.010998

0

0

0.0911696

0

0.0809283

0

0.152561
7

0.359433

0

0.0404642

0.10116

0

0.119811

0

0.0606962

0.0170056

0.155033

6.33702
6

0.0168565

0

0

0.084567

0

0

0

0.0170056

0.0599054

0

0.227595
7

0.536117

0

0

0

0

0.0112449

0.0937482

0.775519

0.0316877

0

0.398557
7

0

0

0.0316877

0

0

0

0.0898581

0.0404642

0

0.0224897

3.81775
6

0.0935306

0

0.0914808

0.0792192

0.0475315

0

0

0.0387582

0

0.915731

0.0486751

0

0

0

0

0

0

0

0.0422835

0.0255083

0.0170056

3.84521
7

0

0.211378

0

0

0.0898581

0

0.0170056

0

0.0187927

0.0310016

359.594

1.63792
7

1.61039
7

0

0.0155008

0

0.0232512

0

0

0.10116

0

0.0168673

0.0542527

2.09343
7

0

0

0

0.190126

0.0255083

0.0337346

0.0170056

0

0

0

0.215372
8

0.164832

0

0

0

0

0

0

0.0168673

0

0.0915731

1.28569
6

0

0.0792671

0.0316877

0

0

0.893528

0.0475315

0.0167023

0.0615709

0

0.685425
6

0

0

0

0

0

0.0581373

0

0

0.0340111

0.102033

0.516475
7

2.56494

0.00500636

0

0

0.007332

0.204067

0

3.41991

0.0316877

0.0599054

0.198149

0.033713

0

0

0

0

1.70996

0.139722

0

0

0

0.84524

0

0.0495119

0

0.0741987

0

0

0.0633753

0.0340111

0

0

0.155916
7

0.0469069

0.00876519

0

0.235177

0.0170056

0

0.0310016

0

0.0103648

0

3.70767
7

0.104228

0

0.0282432

0

0

0

0

0

0.0932828

0.357411

18.2528
7

2.07019
7

0

0.007332

0.536117

0.14516

0.0968956

0.0387582

0.0633753

0.187628

0

0.117267

0.182057
7

0.161857

0.0316877

0.0449794

0.174282

0

0.0809283

0.0366292

0

0

0.170914

0.11225
7

0

0

0.0356217

0.0599054

0.0633753

0.0387582

0

0.0170056

0.102048

0

0.141625

0.007332

0

0.0244735

0

0.0923839

0.0599054

0

0

0

0.0316877

0.963567
7

0.110907

0.281441

0

0

0

0.0469069

0

4.31488

0.0125267

0.0387582

0.663325
7

0

0.0170056

0

0

0.174412

0.021996

0.0210901

0

0

0

3.1269

0.0255083

0

0

0.0404642

0

0

0

0.310039

0

0.0112377

0.167752

0

0

0

0

0.0255083

3.41991

0

0.0809283

3.41991

0.007332

11.2328

0

0.364177

0

0

0

0

0

0

5.98485

0

4.38348
6

0

0.0606962

0.0112377

0

0.722154

0

0.0633753

0.0806519

0.0170056

0

3.89219

0.822064
7

0

0

0

0.0155008

0.0425139

0.0340111

0.951665

2.96532

0

0.0633753

6.88369
7

0.357411

0.0375853

0.0581518

0

0.0599054

0

0.0469069

0

0

0

0.901061
7

0.283249

0

0.107603

0

0.0112449

0.0112449

0

0

0.116275

0.00939633

3.13624
7

0

0

0

0.108505

0.0425139

0

0.0727693

0

0

0.0281121

0.980189
7

0.167835

0

0.0255083

0

0

0.0792161

0

0.0469069

0.0170056

0.0809283

0.400535

0.893528

0

0.0668641

0

0

0.0404642

0

0.713036

0

0

0.887155
7

0.0316877

0

0

0

0

0

0.0112449

0.0469069

0.48789

0

0.332304

0.007332

0

0.0898581

0

0.536117

0.076525

0.0792192

0

0

0

1.76557
7

0

0

0.014664

0

0

0.0103648

0

0.357411

0.0170056

0.0599054

0.391382
7

3.45365

0.0112377

0

0.11239

0

0

0.057196

0.0606962

0.0255083

0.0168565

5.26315

2.0161

0.0387582

0

0.32948

0.0316877

0.0170056

0.0170056

0

0

0

0

0.230627

0

0.0281121

0

0

0

0.0112449

0.0599054

0

0.110907

0.117996

0.157878
7

0

0.0792192

0.0112449

0.0475315

0

0

0.0510167

0.0168673

0.0606962

0

0.236305
7

0.0387582

0

0.26594

0

0.0255083

0

0.0155008

0

0

0.0380041

22.0841
7

0.0170056

0.0545227

0

0

0

0

0.0224897

0

0.469069

0.0630615

0.417735

0.00939633

0.117267

0.633243

0

0.186616

0.239622

0.0475315

0.121392

0

0.0792192

0.240686

0.179716

0

0.269574

0

0.00334147

0

0

1.0085

0.0656988

0

0.77181
7

0.329445

0.00939633

0

0

0

0.0168673

0

0.0170056

0.0581373

0.0469069

2.12886

0.117267

2.56494

0.0475315

0.175295

0.0387582

0

0

0

0

0

2.15742
7

0.0170056

0

0

0.0170056

0.149764

0

0

0

0

0

19.1868
7

2.4596
7

0.357411

0.010998

0

0

0

0

0

0

0

0.0112377

0.413436
7

0.007332

0.0170056

0

0

0

0

0.0404642

0

0

0

147.135
6

0

0

0

0

0

0.0610761

0.893528

0

0

0.021996

0.54947
7

0

0.0809283

0.0366292

0

0

0

0

0.0674261

0

0

8.82519
7

0

0

0

0

0

0.0316877

0

0.0168673

0

0.119039

31.5823
6

0.0968956

0

0.0404642

0.0606962

0

0

0.0170056

0

0

0

1.21636
6

0.0703603

0.007332

0.0775164

0

0

0.0170056

0

0

0

0.0170056

0.468391
5

0

0.0366292

0.128202

0

0.0404642

0

0

0

0.010998

0

0.8171
7

0

0.465338

0.007332

0

0.0255083

0

0

0

0

0

0.223263
7

0.007332

0

0.0224754

0

0

0.121392

0

0

0.025662

0

12.288
7

2.24069
7

0

0.0469069

0

0.117267

0

0

0.00690984

0

0

0

0.0990147
7

0

0

0.0633753

0.095063

0

0

0

0

0.0155008

0

0.698385
7

0

0.0475315

0

0

0

0

0

0

0

0.348824

0.246819
8

0

0.00939633

0

0

0

0

0

0.0404642

0

0.078714

0.351212
7

0

0.0599054

0

0.039357

0

0

0

0

0.0775164

0

0.391472
7

0

0.0899014

0

0

0

0

0.0404642

0.0387582

0

0

0.0436672
7

0

0

0

0

0.0112449

0

0

0

0.238078

0.0340111

0.365758
2

0

0

0

0

0.0680222

0

0

0

0

0

1.97818

1.70996

0

0

0

0.007332

0

0.209669

0

0

0.121392

0.682062
7

0

0.0469069

0.0562243

0

0

0

0.0340111

0

0.0703603

0.714822

5.58482
7

0.007332
7

0.536117

0

0.0103648

0

0

0

0

0.025662

0

0

0.320614

0

0

0

0.561183

4.27489

0

1.58749

0

0

0.0475315

0.917213
7

0

0

0

0

0

0

0

0

0.0138197

0

0.155477
7

0

0

0.102033

0.0505696

0

0.280592

0.0581373

0

0.0459313

0

0.378107
7

0

0

0

0

0.0599054

0

0

0

0

0

3.76332
6

0

0

0

0

0

0

0

0.102033

0

0

0.360605
7

0

0

0

0.0340111

0

0

0.0366292

0

0

0

0.465005

0

0.029328

0

0

0

0

0

0

0

0

0.618126
7

0

0.0404642

0

0

0

0

0

0

0

0.0232512

3.50148
6

0.0366292

0

0

0

0

0

0.0316877

0

0

0

5.85766
7

1.16794
7

0

0

0.0112377

0.359433

0.0170056

0

0

0.00350607

0

1.70996

0.258445
7

0

0

0

0

0.00750954

0

0

0

0.0469069

0

0.196068
6

0

0

0.179716

0

0

0

0

0

0.187628

0

0.179396

0

0

0.0732585

0.0316877

0

0.10116

0

0

0

0.0449507

12.4824
6

0.0469069

0

0.0606962

0.714822

0

0

0.0404642

5.98485

0.0112449

0.141625

121.362
6

0

0.116275

0

0

0.357411

0

0

0

0.0697535

0

3.56205
7

0

0

0.0465023

0

0.0633753

0

0

0.357411

0

0

0.199373
8

0

0.0404642

0

0.0809283

0.0469069

0

0

0

0

0

0.600449
7

1.43405

0

0

0.0562243

0.025662

0

0

0

0

0

0.842647

0

0

0

2.56494

0

0

0

0.237658

0

0.0112449

4.67074

0.430302
7

0

0.566498

0.0606962

0

0

0.010998

0

0

0.0633753

0

2.10648

0.10116

0

0

0.0387582

0.121392

0.121392

1.2329

0.00500636

0

0

0.0426675

0

0.257988

0

0

0

0

0

0

0

0

10.2178
6

0

0.076525

0.182089

0.0606962

0

0.0112449

0

0

0.209669

0

0.0874515
6

0

0

0.0112377

0.0366292

0

0

0.00701215

0

0

0

7.74989
6

0

0

0.0938138

0

0

0.0850278

0

0

0

0.00525911

27.055
6

0

0

0

0.0155008

0

0

0

2.56494

0

0

0.494153

0

0

0

0

0

0

0

5.12987

0

0

0.10814
8

0

0

0

0

0

0

0

0.0232512

0

0

0.280387
7

0.0469069

0

0

0

0

0

0

0

0

0.0404642

3.26993

5.91947
6

0

0

0

0.0510167

0

0

0

0

0

0.0281121

0.111368
7

0

0

0

0.0898581

0

0

0

0

0.950908

0.0599054

1.35603
7

0

0

0

0.0404642

0

0

0

0

0.0170056

0.0404642

0.475236
6

0.0387582

0.29218

0

0

0

0.0680222

0

0.0469069

0

0

0.296602
7

0

0.141625

0

0

0

0

0.0170056

0

0

0.149764

1.94366
7

0

0

0.0599054

0

0

0

0.0340111

0

0

0.357411

0.472988
7

0

0

0.0898581

0

0

0

0

0.0155008

0

0

0.352791
7

3.86982

0

0.237658

0

0.0337346

0.42634

0

2.56494

0

0

0.622828

0

0.142595

0

0

0

0.182089

0.0599054

0.119811

0

0.0255083

75.1001
6

0

0

0

0

0

0.0792192

0.0404642

0

0

0

918.154
7

2.99187

0.132536
7

0

0

0.144547

0

3.41991

0

0.00350607

0

0

0

0.333372
7

0

0

0

0

0

0

0.0606962

0

0

0

0.330716
6

0.110907

0

0

0

0.0404642

0

0

0

0.357411

0.234534

0.616443

0

0

0

0

0

0

0

0.10116

0

0

1.36383
6

0.0281121

0.0915731

0

5.98485

0

0.357411

0

0

0.357411

0.161857

0.276953
7

0

0.0387582

0

0

0

0

0

0

0

0

0.687456
7

0

0

0

0

0.00939633

0.0469069

0

0

0

0

4.50482
8

0

0.0255083

0

0

0

0

0.0168565

0

0

4.27489

0.460967
7

0

0

0

0

0

0

0

0

0

0

0.393207
7

0

0.00939633

0.0634879

0

0

0

0

0

0.0606962

0.893528

11.0884

0.77678
5

0.0703603

0

0

0

0

0.0595195

0

0.117267

0.0112377

0

0.0984674
7

0

0.00350607

0

0

1.70996

0

0

0

0

0

0.501643
7

0

0

0.0366292

0

0.123614

0.0898581

0

0

0

0

0.216083
7

0

0

0.010998

0

0

0

0

0

0.110536

0

1.8485
7

0

0

0

0

0

0.0475315

0

0

0

0

114.33
6

0

0

0

0

0

0

0

0.0792192

0

0

1.85782

0.0581373

0

0.0938138

0.0606962

0.119811

0

0

0

0

0

1.67375
7

1.70996

0

0

0

0

0

0.0898581

0

0

0

1.4031
7

0.0387582

0.0340111

0

0

0

0

0

0.0606962

0

0

0.234142

0

0

1.70996

0

0

0

0

0.0105182

0.0599054

0

4.07621
7

17.5327
6

0

0

0

0

0

0.0680222

0.234534

0

0

0

1.73144
7

0

0.007332

0.0316877

0

0

0

0

0.0138197

0

0.581373

3.83939
7

0

0

0

0

0.007332

0.0599054

0.0170056

0

0.00350607

0

0.388726
7

0

0.095063

0.170056

0.0703603

0

0.0581373

0

0

0

0

1.77719
7

0.149764

0

0.076525

0

0

0

0

0

0.127542

0

0.320959
7

0

0.0606962

0.0422835

0

0

0

0.0465023

0

0

0.0168673

0.63478
7

0

0

0

0.0792192

0

0

0

0

0

0

6.6066
7

0

0

0.0581373

0

0

0

0.368203

0.0425139

0

0

0.123636

0

0

0.0549439

0.0606962

0

0.0112377

0

0.0599054

0

0

1.40242

0

0

0

0

0.0316877

0

1.70996

0

0

0

5.79862
7

8.85509
6

0

0.0606962

0.0387582

0.0316877

0.0316877

0

0

0

0

0

0.0536025
8

0

0

0

0.123614

0

0.374122

0.0168565

0

0.0155008

0

0.517973

0

0

0

0

0

0

0

0

0

0

0.426003
6

0

2.85929

0

0.10116

0.0232512

0

0.142595

0.0387519

0

0.0340111

0.358043
6

0.536117

0.0968956

0

0.0112377

0

0.0404642

0

0.0170056

0

0

0.245178

0

0.0425139

0

0.0155008

0.0387582

0

0.10116

0

0

0

0.459766

0.0898581

0

0.0732585

0

0.357411

0

0.0599054

0.0168673

0.121392

0.0792192

0.4523
7

0.155033

0.0510167

0

0.164174

0.0898581

0

0

0

0.0697535

0.283249

1.09644
7

0

0

0

1.70996

0

0

0

0

0

0

19.9432
6

0.174412

0

0.00690984

0

0

0.00525911

0

0

0

0

5.18556

0.525854
7

0.0404642

0

0

0.0340111

0.0606962

0.0255083

0

0

0.536117

0.424874

0.462455

0

0.0224754

0

0

0.01833

0

0

0

0

0

0.288727
7

0.0581373

0

0

0.0510167

0.0316877

0

0.0938138

0.0155008

0

0.0599054

0.76001
7

0

0.0340111

0

0.0469069

0

0

0

0

1.42964

0.0281121

0.394147
7

0.0469069

0

0

0

0.010998

0

0

0.0599054

0

0

0.0639125
7

0

0.010998

0

0

0.007332

0

0

0

0.0170056

0.0469069

0.326435
7

0.0168673

0

0

0

0.00690984

0.0232512

0.0599054

0.285189

0.116275

0

1.18404
7

0

0

0.0606962

0

0

0.0387519

0

0

0

0.0170056

0.304129
7

0

0

0

0.0404642

0

0

0

0

0

0

0.388742
7

0

0.0703603

0.0404642

0

0

0

0

0

0

0

4.93864
7

1.35781
7

0

0

0.0316877

0.0316877

0

0

0.0581373

0

0

0

2.09608
7

0

0.0633753

0.0703603

0

0.007332

0

0

0.0103648

0.0809283

0

2.48783
6

0

0

0.0469069

0

0

1.70996

0

0.222553

0.0340111

0

0.79048

0

0

0

0

0

0.0703603

0

0

0.100755

0

1.59489
7

0

0

0

0

0

0

0

0

0

0

0.172511
7

0.142595

0.095063

0

0.0775164

0.492522

0

0

0.182089

0.0475315

0.389385

0.242602

0

0

0

0

0

0.357411

0

0

0

0.0703603

1.92223
7

0

0

0.01833

0

0

0

0.007332

0

0

0.389385

0.288883
7

0.0170056

0

0

0

0.0606962

0

0

0

0

0.119811

0.144719

0.164174

0

0

0

0

0

0

0.0172746

0

0

26.7439
7

10.4826
7

0

0

0

0

0

0

0

0

0

0

1.01247
7

0

0

0

0

0

0

0

0.0898581

0

0.014664

0.351697
7

0

0

0

0

0

0.714822

0

0.0599054

0

0

0.157145
1

1.70996

0.0599054

0

0

67.5433

0

0

0

0

0.0935306

0.79155
6

0

0

0

0.007332

0

4.27489

0

0

0

0

0.215137
7

0

0

0.0703603

0.029328

0

0

0

0.0404642

0

0

1.18035
6

0

2.56494

0

0.285189

0

0

0

0

0

0.007332

0.282776
7

0.0599054

0

0

0.0703603

0.118071

0

0

0.0112377

0.0898581

0

28.5652
6

0

0

0.00690984

0.290687

0

0.0469069

0

4.27489

0.0387582

0.0599054

1.08195
7

0

0.0510167

0

0.110907

0

0

0

0

0

0

17.5401
7

0.310996
7

0.0112377

0

0

0

0

0

0.0112449

0

0

0.0316877

0.864655

0

0.0938138

1.70996

0

0

0

0

0

0

0

4.39117
7

1.70996

0.357411

0

0

0.164174

0

0

0

0

0

0.170904
7

0

0.0316877

0

0

0

0.257988

0

0

0

0

1.21151
7

0

0

0

0.119811

0

1.70996

0

0.536117

0.301033

0

0.764941
7

0

0

3.41991

0

0

0.0404642

0

0

0

0

0.780521
6

0

0

0

0

0

0

0

0.0387519

0.0938138

0

0.611619
7

0

0.014664

0

0.0599054

0

0

0.0809283

0.0898581

1.70996

0

0.331246
7

0

0

0

0

0

0

1.70996

0.03666

0

1.70996

3.87545

0.0792192

0

0

0.0469069

0

0

0

0

0

0.007332

4.25755
7

2.68575
6

0

0

0

0

0

0.00939633

0

0

2.36626

0

0.171969

0.893528

0

0.0404642

0

0.328348

0.299527

0

0

0

0

1.523

0

0

0

0.239622

0.076525

0

0.0599054

0

0

0

0.260602
7

0

0

0

0.028189

0

0

0

0.0170056

0

0

0.406205
7

0

0

0.0112377

0

0.0449794

0

0

0

0.0606962

0

0.0968956

0

0

0

0

0

0

0.0469069

0

0

0

0.0931996

0.0680222

0

0.0404642

0

0.0938138

0

0.0703603

0

0

0

0.45862
7

0.0606962

0

0

0

0.010998

0.118071

0.10116

0

0

0

0.434907
6

0.0809283

0

0.040326

0

0

0

0

0

0

0

2.07247
7

0

0.0469069

0

0.117267

0

0

0

0

0

0

2.64272

0.177169
7

0.135654

0

0

0

0

0

0.372019

0

0.0039381

1.70996

0.138793

0

3.038

0

0.542615

0.0100127

0

0

0

0

0

0.0449507

0.536117

0

0.10116

0

1.70996

0

0.0404642

0

0.0155008

0.029328

0.211453
7

0

0

0

0

0.119039

0

0

0.0316877

0

0

0.586239
7

0.025662

0

0

0

0

0

0

0

0

0

0.891019
7

0.0898581

0

0

0

0

0.0168673

0.357411

0

0.0606962

0

0.118438
8

0

0.095063

0

0

0

0.0968956

0

0

0

0

0.222885
7

0

0.0404642

0

0

0

0

0

0

0

0

3.24268
7

0

0

0

0

0

0

0

0

0.0340111

0

0.202321

0.366292

0

0.0168565

0

0

0

0.007332

0.00350607

0

0

160.614

6.31734
7

0.942241
7

0

0

1.96576

0.0170056

0.0599054

0

0.00939633

0

0.0469069

1.78706

1.76933
7

0

0

0

0

0.0449507

0.095063

0

0

0

0

0.533647
7

0.0232512

0

0

0.0255083

0

0.0581373

0

0

0

0

0.236034
7

0

0

0.0170056

0

0

0.0387582

0

0

0

0

0.391331
7

0

0

0

0.10116

0

0.0387582

0.0393319

0

0

0

0.896966

0.0170056

0

0.119811

0

0

0.0224754

0

0

1.70996

0

0.320063
7

0.536117

0

0

0

0

0.0387582

0

0

0

0

0.0425139

0

0.539429

0

0

0

0

0

0.0469069

0

0.0938138

0.444234
7

0.0310016

0.0316877

0.179716

0

0

0.03666

0

0

0.0606962

0

0.0168673
1

0

0

0.0112377

0

0

0

0.0599054

0

0.010998

0

62.4186
6

1.10392

0

0

0.357411

0

0

0

0

0.141625

0

0

1.22449

0

0.0340111

0

0

0

0

0

0

0.10116

0

0.216965

0

0

0

0

0

0

0

0

0.0112377

0

0.309914
7

0

0

0

0

0.0633753

0.357411

0

0

0

0

0.187372

0

0

0

0

0

0

0

0

0

0

0.540841
7

0.193791

0

0.629007

0

0

0

0

0

0

0

0.215114

0.0375853

0

0

0

0

0

0

0

0

0

0.292455
7

0

0.007332

0

0

0

0

0.357411

0

0

0

2.75465
7

0

0

0

0

0

0

0.257988

0.00690984

0

0

2.74914
5

0

0.0425139

0

0

0

0

0

0

0.00305919

0.0549439

58.0885
7

0.0894349

0.0112449

0.0599054

0.893528

0

0.310066

0.179716

0

0

0

0

1.9544
7

0

0

0

0.0170056

0

0

0.0112377

0.179716

0

0

0.06883
7

0

0.0633753

0

0

0.0316877

0

0.025662

0

0

0

0.303613
2

0.257988

0

0.00500636

0

0.0475315

0

0.010998

0.714822

0

3.57411

0.113407

0.0809283

0

0

0

0

0.0404642

0

0

0

0

1.80008
7

0

0

0.0168565

0

0

0

0

0

0

0.110536

2.95941
6

0

0.0255083

0.0316877

0

0

0

0

0

0

0.0703603

0.266449

0

0

0

0.0168565

0.161857

0

0.0340111

0

0

0

3.68408
6

0

0.0387582

0

0

0

0

0

0

0

0.158438

0.714822
7

0

0.0112377

0

0.0316877

0.0255083

0

0

0.0206752

0

0.0606962

3.74853
7

0.0966785

0

0.007332

0

0.0510167

0.0898581

0

0.0425139

0

0

0

0.229515

0

0

0

0

0

0

0.0337346

0

0

0

4.75334
7

0.00500636

0

0

0

0.0680222

0

0

0.0155008

0.109888

0

0.511127
7

0

0

0

0

0

0

0.102033

0

0

0.007332

0.387554
7

0.010998

0.0404642

0.0170056

0.0469069

0

0

0

0

0

0

0.157592
7

0

0

0

0.021996

0.141625

0

0

0.0775164

0

0.0140945

4.76409
6

0

0.007332

0.0168673

0.007332

0

0

0

0

0

0

0.560945
7

0.239622

0

0.0775164

0

0.0935306

0

0

0

0

0

0.129312

0

0

0

0

0

0

0

0

0

0

0.309064

0

0.0599054

0

0

0

0

0

0

0

0

9.45842
7

0.18472

0

0

0.029328

0

0

0

0

0

0

0

1.13007
7

0.0170056

0

0

0

0.0606962

0.0404642

0

0

0.126751

0.0316877

1.62251
6

0

0

0.010998

0.00690984

0

0.0599054

0

0

0

0.0340111

0.215765

0.0469069

0

0

0.0404642

0

0

0

0

0

0.007332

7.8683
6

0

0

0

0.0170056

0.0606962

0

0

0

0.0469069

0

0.296545
7

0

0

0

0

0

0

0

0

0

0.0595195

0.250223
7

0

0.178558

0.0316877

3.038

0.10116

0

1.78706

0

0

0.0175223

0.069705
7

0

0

0.0469069

0

0

0

0

0

0.0170056

0.0170056

0.106408
7

0.0703603

0

0

0

0

0

0.0599054

0

0

0

0.27394
7

0

0.0599054

0.119039

0

0

0.0316877

0

0

0

0

10.3425
6

0.271893

0.0232512

0.00500636

0

0.0425139

0.222553

0.0168673

0.359433

0

0

0

21.8169
6

0

0

0

0

0.0955203

0

0.0316877

0

0

0

2.01226
6

0

0

0

0.0168673

0

0

0

0

0.0112377

0

0.25001
5

0

0.00939633

0.0404642

0

0

0.0633753

0.149764

0

0

0

0.327613

0

0.202321

0

0

0

0

0.328348

0

0

0

0.116928
7

0

0

0

0

0.0255083

0

0

0.0170056

0

0

0.138901

0

0.536117

0.357411

0

0

0.183146

0

0

0.0250534

0

1.29729
6

0.264254

0.007332

0

0

0

0

0

0

0

0

0.140127

0.0125267

0.0809283

0.0387582

0

0

0

0

0

0

0

0.23205
7

0

0

0.0898581

0

1.70996

0.687891

0

0

0

0

32.0641
6

0.40376
7

0

0

0

0

1.25094

0

0

0

0

0

0.322075
7

0

0

0.239622

0

0

0

0

0

0

0

0.790426
7

0.0475315

0

0

0.0366292

0.0168673

0

0

0

0.0938138

0.0606962

1.30597
6

0.0599054

0

0.0599054

0

0

0

0

0

0

0

3.97262
6

0.0232512

0

0

0

0

0

0

0

0.0404642

0

0.164008
7

0

0

0

0

0

0.174412

0.032994

0.021996

0.0140945

0

0.306177

0.0187927

0

0

0

0.0809283

0

0

0

0

0

1.16206
7

0

0

0

0.357411

0

0

0

0

0

0

0.503491
7

0

0

0.647427

0

0

0.0387582

0

0.0475315

2.56494

0

0.0401723
8

0.0606962

0.0475315

0

0

0.00939633

0.110907

0

0

0

0

11.3771
7

0.841241
7

0

0

0.0703603

0

0

0

0

0

0.404642

0

0.230274
7

0

0.121392

0

0.0775164

0.0599054

0.0581373

0.10116

0

0

0

0.588262
7

0

0.264254

0

0

0

0.0549439

0

0

0

0

2.24712
6

0

0

0

0

0

0.0599054

0

0.0224897

0

0

1.00472
7

0.0449794

0

0

0

0.714822

0

0.0316877

0.0152104

0

0.357411

0.462463
7

0

0

0.0775164

0.179716

0

0

0

0

0

0

0.222148
7

0

0

0.0633753

0

0.121392

0

0

0

0

0.0168565

0.806496
7

0.0112377

0.0475315

0

0

0

0

0

0.095063

0.010998

0

0.269574
7

0.0510167

0.299527

0

0.134852

0

0

0

0

0

0

0.185329
7

0

0

0

0

0.140721

0

0

0

0

0

2.42474
7

0.0112449
7

0.0232512

0

0.149764

0

0.303481

0.0404642

0

0

0

0.0180541

0.307495
7

0

0

0.014664

0

0

0

0

0

0

0

0.0927926
7

0

0.0425139

0

0

0.0475315

0

0

0

0.0387582

0

13.1944
7

0

0.0255083

0

0.0316877

0

0.010998

0

0

0

0

0.202321
7

0

0.201461

0

0.195564

0

0

0

0

0

0.0775164

1.38955
7

0

0

2.56494

0

0.10116

0

0

0

0.0935306

1.42964

16.3835
6

0

0

0

0

0

0

1.07223

0

0

0

0.10261
7

0

0

0

0

0

0

0

0

0

0.119811

0.620585
7

0.00939633

1.70996

0

0

0

0

0

0

0

0.00590715

0.195506
7

0.0340111

0.0475315

0

0

0

0

0.007332

0

0

0.0599054

7.03417
7

0.4779
7

0

0

0

0.0340111

0

0

0

0

0

0

4.51643
6

0

0

0.0510167

0

0

0

0

30.5985

0

0

0.0761566
2

0

0

0.0469069

0

0

0

0

0

0

0.0155008

0.320153
6

0

0.00939633

0

0

0

0

0

0

0

0

0.510257
7

0.110536

0

0.357411

0

0.239622

2.36715

0.110907

0

0

0

0.67936
7

0

0

0

0

0.0581373

0

0.0366292

0

0

0.0281121

0.196675
8

0

0

0

0

0

0

0

0

0

0

0.273933
7

0

0

0

0

0

0

0

0

0.0112449

0

0.386025

0

0.0170056

0

0

0

0

0

0.00305919

0

0.357411

0.18203
7

0

0

0

0.0792192

0.0170056

0

0.116275

0

0

0

51.5004
7

3.87527
7

0.0865512
7

0.0618072

0

0.0387582

0.0112449

0.0792192

0.222553

0

0

0

0

0.47837
7

0

0

0

0

0

0

0

0.333606

0

0

0.478739
7

0

0

0

0

0.119811

0

0.0898581

0

0.142595

0

0.33642

0

0

0

0.007332

0.0599054

0

0

0.539149

0

0

0.428249

0

0.0232512

0.0404642

0

0.0103648

0

0

0

4.72284

0.0168673

0.182966

0

0

0

0

0.00690984

0

0

0.117267

0

1.70996

0.239622
7

0.0898581

0

0.0581373

0

0

0.007332

0

0

0.158438

0

0.289318

0

0.0170056

0

0

0

0

0.007332

0

0

0

9.80126
6

0.0561884

0.280592

0

0

0

0.076525

0

0

0.0334046

0.283249

0.142595

0

0.00525911

0

0

0

0.0469069

0

0

0

0.714822

4.4905
7

0.984011
7

0

0

0

0.0316877

0

0

0

0

0

0

0.285865
7

0

0

0

0

0.0255083

0.00690984

0.0469069

0

0

0

0.343947
7

0

0

0

0

0

0.0316877

0.010998

0.076525

0

0.0599054

0.480442
5

0.357411

0.469069

0.10116

0.0100127

0

0

0

0

0

0

0.549271

0

0

0

0.0170056

0.00939633

0

0

0

0

0

0.0168673
7

0.126751

0

0

0.0281121

0

0

0.00750954

0.0775164

0

0

0.116275

0

0

0.0340111

0.0316877

0

0.0170056

0.0425139

0.0469069

0

0.029328

0.114314
7

0

0

0

0.0898581

0

0

0

0

9.40477

0

0.531092
7

0

0

0

0

0.0633753

0

0.0112377

0.0170056

0

0

1.05223
7

0.01833

0.00701215

0.00939633

0.209669

0

0

0.0337346

0

0

0

10.9107
7

0.531492
7

1.52759

0.127542

0

0

0.357411

0

0

0.0599054

0

0.007332

0.368203
7

0

0.0404642

0.0606962

0

0

0

0

0.0155008

0

0

0.435334
7

0

0

0.158438

0.010998

0

0

0

0.0232512

0

0.0425139

0.313722
7

0

0.116275

0

0

0

0.0155008

0

2.10723

0

0

0.0877699

0

0.0469069

0.00690984

0

0.0387582

0

0

0

0

0

0.254462
6

0.0633753

0

0

0

0

0.0170056

0

0

0.179716

0.142595

1.3775
7

0

0.21317

0.010998

0

2.56494

0

0

0

0.0404642

0.0404642

0.177331

0.119811

0

0.0483689

0.443627

0.809283

0

0

0.0316877

0.0599054

0

0.324931
7

0

0

0

0

0

0

0

0.0599054

0.0255083

0.0469069

2.84658
7

0

0

0

0

0

0

0

0.0465023

0

0

9.50905
7

0.736845
7

0.0606962

0.0387582

0

0.714822

0

0.040326

0.0316877

0

0

0

0.0936396

0

0

0.893528

0

0

1.07223

0

0

0.211081

0

3.56109
6

0

0.0170056

2.42617

0

0

0

0

1.70996

0.357411

0

0.0816282
7

0.0703603

0

0.0168673

0

0

0

0

0.0224897

0

0

12.2449
7

0

0.375255

0

0.0216596

0

0.10116

0.144547

0

0

0

0.499458
7

0

0

0

0

0

0

0.007332

0

0.0170056

0

0.0631797
4

0.0549439

0

0

0.01833

0.269345

0

0

0

0.0599054

5.12987

0.441359

0

0

1.6388

0

0

0

0

0.357411

0

0

0.183516
7

0.290687

0

0.0599054

0.0506019

0.00760519

0

0

0.0234908

0

0

0.252918
7

0

0

0

0.0224897

0

0

0

0

0

0

13.3549
6

1.02852
7

0

1.70996

0

0

0

0.10116

0.0232512

0

0

0

4.29794
7

0

0

0

0

0.112756

0

0.0170056

0.007332

0

0

0.309139
7

0

0

0

0

0

0

0.536117

5.98485

0.0475315

0

0.334685
7

0

0.140721

0

0

0

0

0

0.0732585

0

0.242785

0.595412
7

0

0

0

0.0340111

0

0

0

0

0.0680222

0

1.89082
7

0

0

0

0.0310016

0

0

0

0

0

0

0.0167023
7

0

0.00525911

0

0

1.70996

0.0809283

0

0

0

0

0.31556

0

0

0

0.0170056

0

0.0112449

0

0

0

0

6.6346
7

0.0469069

0

0.0475315

0.007332

0.0703603

0

0

0

0

0

0.553626
7

0

0

0.174282

0

0

0.0599054

0

0

0

0.0469069

9.11238
7

1.88495
6

0.0255083

0

0.950908

0

0

0.0469069

0.61791

0.242785

0

0.0510167

0.365113

0

0

0

0

0

0.010998

0

0

0

0

0.417943
7

0.0404642

0

0

0

0

207.363

0.0809283

0

0.0103648

0

0.487592
7

0

0

0

0

0

0.263586

0

0.0167023

0

0

0.0504769
7

0.0155008

0

0

0.232549

0

0

0.0425139

0

0

0

1.90369
7

0.0155008

0

0

0

0.155008

0

0

0

0.0387582

0

0.17817

0.357411

0

0

0

0

0

0

0.00939633

0.119811

0.0792192

0.292358
7

0

0

0

0

0

0.0112377

0.0581373

0

0.0915731

0.0255083

0.293435
6

0

0.0232512

0

0.0581373

0.0387582

0

0

0.0599054

0

0

0.286509
7

0

0

0

0

0

0

0.0581373

0

0.0280942

0.714822

7.52982
7

0

0

0

0

0

0

0

0.0935306

0

0.0404642

0

0.0473868
7

0

0

0.0170056

0.0170056

0.0112449

0

0

0

0.161857

0

0.0224897

0

0.102033

0

0

0

0.251928

0.007332

0.021996

0

0.0469069

0.221649
6

0.0280942

0.0232512

0.0606962

0.00690984

0

0

0

0

0

0

0.37566
7

0.0606962

0

0

0

0

0

4.27489

0

0

0

0.257428

0

0

0

0

0

0

0

0

0

0

0.1385
7

0.007332

0

0

0

0.119811

0

0

0

0.0404642

0.007332

0.204839
7

0

0

0

0

0

0.0475315

0.095063

0

0

0

0.483061

0

0

0

0

0

0

0

0.0475315

0

0

0.536117

0

0

0.0155008

0

0

0

0

0

0

0

5.86117
7

0.190307

0

0.0155008

0

0.0599054

0

0

0

0

0

0

3.7505
7

0

0

0

0

0

0.116275

0.0595195

0

0

0.0387582

0.311859
7

0

0.357411

0

0

0

0

0

0

0.149764

0

4.3858
6

0

0

0

2.56494

0

0

0.0112449

0

0.00835115

0.728355

2.20809
6

0.359433

0.0549439

0

0.0168673

0.0703603

0.0232512

0

0

0

0

0.67083
5

0

0

0.161857

0

1.70996

0

0.0898581

0

0

0

0.707478
6

0

0

0.0475315

0

0.0168565

0

0

0

0

0.0475315

0.893528
7

0.0281121

0

0

0

0

0

0.0599054

0

0

0

0.158844

0

0.285189

0

0

0.0595195

0.219775

0.140721

0.179716

0

0.0469069

0.683184

0.00939633

0

0

0

0

0

0

0

0.0898581

0

7.56033
7

0.430181
7

0.714822

0

0

1.70996

0

0

0.536117

0

0

0

0.473783
7

0

0.032994

0

0

0

0.0562243

0

0

0

0

0.794688
7

0.357411

0

0

0.0316877

0

0

0.0387519

0

1.70996

0

0.0243376
7

0.179716

0

0

0

0

0

0

0.0469069

0

0

0.681571
7

0

0.174412

0

0

0.0898581

0

0

0

0

0

0.327006
7

0.0155008

0.0469069

0

0.109888

0

0

0

0

0.0155008

0

0.561014

0

0

0.142595

0

0

0

0.0170056

0

0

0

0.794572
7

0

0

0

0

0

0

0

0

0

0

0.275617
7

0

0

0

0

0

0

0

0

0

0

0.400843

0.0404642

0

0

0

0

0.0898581

0.0510167

0

0.021996

0

1.64191
7

0.135775

0.0599054

0

0.007332

0

0

0

0

0

0

0

0.18169
2

1.70996

0.007332

0

0

0.0103648

0

0

0

0.00690984

0

0.718589
7

0

0

0

0.0393319

0

0.007332

0

0

0

0.014664

0.0337346
7

0

0.0938138

0

0.069654

0

0

0.0404642

0

0

0

0.0700926
6

0.0316877

0

0

0

0

0.0775164

0

1.62403

0

0

2.66875

0

0

0

0

0.0792192

0

0

0.0633753

0

0

0.0228328
7

0.280592

2.56494

0.00835115

0.03666

0.0898581

0

0

0.0404642

0.0170056

4.3499

0.112616
7

0.357411

0

0.0581373

0.536117

0

0.0112449

0.389385

0

0.0168673

0

0.904541

0

0.0170056

0.0606962

0.140721

0

0.0316877

0

0

0.00750954

0.0252757

0.0224754
7

0

0

0

0

0

0.0366292

0.0310943

0

0

0

292.754
7

6.31016
7

0.14003

0

0

0

0

0

0.0633753

0

0

0.0475315

0

0.118435

0

0.546266

0.0404642

0.0775164

0.0112449

0.0469069

3.41991

0

0

0.142595

0.276217
6

0.007332

0

0

0.082918

0

0

0

0.0232512

0

0

0.363808
7

0

5.18246

0

0

0.0387582

0.0898581

0.0168565

0

0

0

3.95953
7

0

0

0

0.00350607

0

0.893528

46.1689

0

0.714822

0.0680222

0.182089
7

0

0

0

0

0.0387582

0

0.0549439

0.0387582

0

0

0.0703603

0

0

0

0

0.0469069

0

0

0

0.0404642

0

10.3306
6

0

7.69481

0

0

0

11.1147

0

0.00690984

0

0.0316877

0.134605
8

0

0

0

0

0.0387582

0

0

0

0

0

0.0145041
7

0

0

0

0

0

0

0.0340111

0.161857

0

0

1.67247

0.704486

0

0

0

0.149764

0.116275

0

0

0

0

0

0.262647
7

0

0.283249

0.357411

1.03855

0.0387519

0.0112449

0

0

0

0

0.736849
7

0.161857

0

0

0.0170056

0

0.025662

0.0469069

0.141625

0.0155008

0.0730916

1.31181
7

0.140721

0

0

0

0

0

0

0

0

0

0.155033
7

0

0.0843179

0

0.0732585

0

0

0

0

0

0

9.8283
6

0

0

0.0112449

0

0.0168673

0.0340111

0

0

0

0

0.108958
7

0.662441

0

0

0

0.0606962

0

0

0

0

0

0.399874
7

0

0

0.0425139

0.014664

0

0.0599054

0.191041

0

0

0

3.38936
6

0

0

0.404642

0

0

0

0

0

0.116275

0

0.370644
7

0

0

0

0

0

0

0

0

0

0

2.61006
7

0.0167283
7

0

0

0.0316877

0

0.0599054

0.0809283

0

0.0316877

0.0697535

0

0.10116

0

0

0

0.0387582

0.536117

61.0436

0

0.0170056

0.0170056

0

0.090464
7

0

0

0

0

0.0703603

0

0

0

0.119039

0.135654

0.872626
7

0.0703603

0.007332

0

0.121392

0.0255083

0.007332

0.0170056

0

0

0

0.0449794

0

0

0

0

0

0.797417

0

0

0

0.0469069

0.989526
7

0

0

0.0968956

0

0.0255083

0

0.00939633

0

0

0

0.247269
7

0

0

0

0

0

0.0606962

0.0112449

0

0.0606962

0

0.217259
7

0.0155008

0

0.029328

0

0.0232512

0

0

0

0.303481

0

30.6032
6

0

0

0.0112377

0.0599054

0

0

0

0.0469069

0

0.0170056

0.184752
7

0

0.10116

0.00500636

0

0

0

0.357411

0.380252

0

0.281441

10.5431
7

5.24621
7

0.10116

0

0

0.0340111

0.0549439

0

0.301033

0.0510167

0

0

0.263374
7

0

0

0

0

0

0

0.0387582

0

0

0

1.64513
7

0

0.014664

0

0.00939633

0

0

0

0

0

0

0.178588

0

0

0

0

0

0

0.0475315

0

0.141625

0

1.25533
7

0

23.0844

0

0

0

0

0.0465023

0.141625

0

0

175.454
6

0

0.0393319

0

0.119811

0.0170056

0.0809283

0

0

0.0168565

0

0.401488
7

0.007332

0

0.141625

0

0.0387582

0

0

0

0.141625

0

0.145059
2

0

0

0

0

0.0170056

0

0.536117

0

0.0387582

0

0.269429
7

0

0

0.0387582

0

0.0280942

0.170509

0

0

0

0.0581373

0.0399578

0.0606962

0.0112377

0

0

0.140721

0.0155008

2.56494

0

0

0

3.87471
7

1.14999
7

0.014664

0

0

0

0.0599054

0

0

0

0

0

0.190257
7

0

0

0.0387582

0

0

0

1.70996

0

0

0

0.119811
7

0

0.00500636

0

0

0

0

0

0

0

0.0542527

0.102872
8

0.0633753

0

1.90462

0.0316877

0

0.0387582

0

0

0

0

0.427287

0

0

0.357411

0

0.00750954

16.977

0

0

0

0

0.007332

0

0

0

0

0.0155008

0

0

0.0165402

0

0.357411

0.258129

0

0

0.010998

0

0.0606962

0.0316877

1.70996

0

0.0465023

0

1.01961
7

0.357411

0.10116

0

0.0316877

0

0.0168565

0

0

0

0

2.77221
7

0

0

0

0

0

0

0

0

0

0

0.163762
7

0.0898581

0

0

0

0

0

0

0

0

0

6.8911
7

0.0267385
7

0.0475315

0

0

0

0

0

0.0898581

0

0

0

1.41448
6

0

0.0366292

0

0

0

0

0

0

0

0

0.39011
7

0.0310016

0

0.0581373

0

0

0

0.0703603

0.0232512

2.56494

0

0.866071
7

0

0

0

0.0510167

0

0

0

0

0

0

0.081598

0

0

0

0

0

0.0155008

0

0

0

0

1.47655
7

0

0.0170056

0

0.0599054

0

0

0

0

0

0.449291

0.267284
7

0

0.0316877

0.0469069

0

0

0

0

0.0224897

0.0157524

0

0.317275
6

0.0366292

0

0.748818

0

0

0

0

0.0581373

0

0

0.575229
7

0.0170056

0

0

0

0.314856

0

0

0.0387582

0.0581373

0.120922

1.15165
7

0

0

0.0469069

0

0

0

0.0316877

0.0475315

0

0

3.3483
7

1.22587
7

0

0

0

0.0809283

0

0

0

0.0633753

0

0

0.0938138

0

0

0

0

0

0.0387582

0

0

0

0

0.148067

0

0

0.328348

0

0.0703603

0

0

0

0

0

1.15045
6

0

0

0

0.0340111

0.0232512

0

0

0

0

0

3.51154
7

0

0

0.0404642

0

0

0.0404642

0

0

0.195564

0

0.241699

0

0

0.357411

0

0.0475315

0

0.158438

0.0404642

0

0

0.136765
7

0

0

0

0

0

0

0.0581373

0

0

0

0.136816

3.2167

0

0

0

0

0

0

0

0.0170056

0

0.159168
7

0

0

0.010998

0

0.0898581

0.0316877

0.0953159

0.179716

0.0465023

0

0.304015
7

0

0.357411

0.0898581

0.0938138

0

0

0

0.0280942

0

0.0606962

2.05657
7

0.767489
7

0.0422835

0

0

0.0170056

0.0155008

0

0.0775164

0

0.357411

0

0.294675
7

0

0

0

0

0.222553

0

0.007332

0.0633753

0

0

0.468034
7

0

0

0

0

0

0

0

0.0620031

0

0

0.928816
7

0

0

0.0255083

0

0

0

0

0

0

0.0581373

0.942973
7

0

0

0

0.014664

0

0

0

0

0

0

1.09408
8

0

0

0.119811

0.014664

0

0.136044

0

0

0.0703603

0

0.117474
7

0.0425139

0.0404642

0

0

0

0.0112449

0

0

0

0

0.19151
7

0

0

0.0404642

0

0.222553

0

0

0

0

0.48557

2.84776
6

0

0.007332

0

0

0

0

0.0387582

0

0

0

0.58973
7

0

0.00525911

0

0

0

0

0.0112377

0

0

0

16.5143
7

0
8

0.0224897

0.0469069

0

0.129233

0

0

0

0.0155008

0.010998

0

0.491406
7

0

0.0850278

0

0.065988

0.0697535

0

0

0

0.00690984

0

0.578579
7

0

0.0112377

0

0.0674261

0.714822

0

0.0155008

0.0340111

0.0510167

0

0.282136

0.467653

0.0898581

0

0

0.0170056

0

0

0

0.135654

0

3.67865
6

0

0.191163

0

0

0

0.0112449

0

0

0.0599054

0

0.596813
7

0

0

0.136247

0

0.209669

0.0898581

0

0

0

0

0.222185
5

0

0

0.0387582

0

0

0

0

0

0

0.01833

0
1

0.0387582

0.0633753

0

0

0

0

0.0469069

0

0

0

0.135654
7

0.0168673

0.0316877

0.007332

0

0.00690984

0

0

0

0.0224897

0

0.286048
6

0

0

0

0

0

0.0475315

0

0

0.0809283

0.0703603

10.8159
7

1.19772
7

0

0.222553

0

0

0

0.142595

0

0

0

0

0.617268
5

0

0

0

0

0.0809283

0.095063

0

0.0750954

0

1.70996

0.0704319
7

0.0425139

0

0

0

0

0

0.0155008

0.0915731

0

0

0.16088
7

0.00939633

0

0.0112449

0

0

0

0.0387582

0

0.014664

0.121392

0.302475
8

0.033713

0

0.076525

0.0703603

0

0

0.0168673

0

0

0

0.124072

0

0

0

0.0255083

0

0.0101402

0.0168673

0

0

0

0.389099
7

5.12987

0.0168565

0

0.0112449

0.893528

0

0.0475315

0

0

0

2.45647
7

0

0

0.357411

0

0

0.0155008

0.0938138

0

0

0

3.70874
6

11.6159

0

0

0.0112377

0

0

0

0.0387582

0

0

0.20605
7

0

0

0

0.0103648

0

0.0232512

0

0.0387582

0

0.0224897

8.17903630240258e-10
7

0
4

0.167684
5

0.167684

0

0

0
4

0.0282504
7

0.0282504
7

0
4

0.141417
7

0.0170056

0.124412

0
4

0

0

0
4

0

0

0
4

0

0

0

0
4

0

0

0
4

0.0606962

0.0606962

0
4

0

0

0
4

4.98452668029614e-12
7

0
4

4.17348
7

4.16597
7

0.86913
7

2.57251
7

0.724335
7

0

0
4

0.00750954

0.00750954

0
4

0
4

850.982

91.1532
7

46.1751
7

0.043744
7

0

0

0

0

0

0

0

0.0103648

0

0.00690984

0.2699
7

2.56494

0

0

0.0404642

0

0.0155008

0

0.0809283

0

0

2.09023
6

0

0

0.357411

0

0.328348

0

0.0112377

0

0

0

0.0505696

0.0542527

0.0316877

0.0404642

0

0.0112377

0.0310016

0

0

0.0469069

0

0.0683169
7

3.14447
6

0.287406

1.28343
7

0.417317
6

0.033713

5.39277
7

0.0168565
7

0

2.34679
7

0

0.125383

0.460194

0.647427

0.117996

0.0600865

0.0138197

3.6421
7

0.0915731

0.340988

0.0484629

0.323419

0

0

0.0659725

0.116256

0.0207295

0.381936

2.79711
6

0.0469069

0.071061

0

0.0112377

0.0316877

1.70996

0

0

0.100755

0.0753806

3.01583
6

0

0.0155008

0

0.0207295

0.0255083

0.0112377

0

0

0.0404642

0

0.444042
7

0.0620031

0

0.374417

0.262574

0.100755

0.0155008

0.0112377

0

0

0

0.596714
7

0.0232512

0

0

0

0.0404642

0.0155008

0.0469069

0

0

0

3.8618
6

0

0.357411

0.0155008

0

0

0

0

0

0

0

4.82316
6

0.255083

0.0606962

0

0

0

0.0404642

0.0469069

0

0.0168673

0

1.16726073251527e-13
7

0
4

9.471

1.28888

1.70779

6.43387

0

0.0404642

0
4

12.6493

12.6493

0
4

94.3345

89.3484

3.42492

1.04517
7

0.515976

0
4

0.563703

0.271412

0

0.283939

0

0.00835115

0

2.77555756156289e-17

0
4

70.4023
7

35.7091
7

0.161857

1.13293
6

4.31707
6

0.420658

0.173911

0.00305919
5

0

0.379396

0.0183781

0.265849

5.44954

0

0.0809283

1.43904

0.343945

0.0127378

0

0.222553

0.140721

0.0965753

0.10037

7.66789
6

0.0809283

0.0170056

0.196767

0.131057

0.0469069

0.121392

0.117892

0.112348

0

0.0599054

0.740323
7

0.0469069

0.0599054

0.00835115

0.00334147

0.189755

0.0835361

0.0404642

0

0.0475315

0

0.699068
7

0.0116951

0

0

0.0340111

0

0

0

0.0168673

0

0.0112377

2.7381
7

0

0

0

0

0.00334147

0

0

0

0

0

3.94541
6

0.0224897

0

0

0.0898581

0

0

0

0.0506019

0

0

1.77019
7

0

0.242785

0

0.0404642

0

0

0.0469069

0

0.438549

4.61297666731753e-14
7

0
4

15.7063
6

4.74121
6

0

0.00750954

0

0

0

0

1.70996

0

0.0469069

0

0.33295

0

7.15816
6

0

0.184921

1.52471

0

0

0

1.11022302462516e-15
6

0
4

548.691
6

10.5896
6

2.15754
7

0.076525

0.0809283

0.0404642

0.0721518

0.0606962

0

0.107199

0.0606962

0

0.00525911

1.36021
6

0

0.0404642

0.831759

1.70996

0.0597998

1.81199

0.0112377

0

0.0620031

0

20.4191

0.0404642

0.048555

0

0

0.0168673

0.0155008

0.0337346

0.0387582

0

0

13.0443
7

0.0366292

0.0404642

0

0

0

0

0.0366292

0.0366292

0.0232512

0

5.21776
6

0.455417

0

0.0404642

0

0

0.0232512

0

0

0

0.0404642

11.4975

0.0404642

0.0155008

0

0

0.0112377

0

0.0316877

0.0404642

0.0155008

0

1.74367
7

0.0599054

0

0

0.0599054

0.147257

0.0155008

0.0366292

0.0599054

0.0404642

0

10.5128
6

0

0.0224754

0.0170056

0.00525911

0.45853

0.714822

0

0.0366292

0

0

4.10401
6

0.0168673

0.0112449

0

0

0.0168565

0.0232512

0.0155008

0.0310016

0.0449794

0

4.25874
7

0

0

0.10116

0.0404642

0

0.0633753

0

0

0.0606962

0.0449794

67.319
6

0.84613
6

0.110907

0

0.0404642

0.0155008

0.0449794

0

0.302265

0.0366292

0.357411

0

2.15468

0

0

0.0112377

0

0.0112449

0.0155008

0

0

0.0469069

0

0.519261
6

0

0.357411

0

0.0112449

0.0404642

0

0

0.0224754

0.0606962

0

0.344338
7

0

0

0.0404642

0

0.0404642

0.0112377

0.0168673

0.0404642

0

0.0633753

0.741365
6

0

0.0281121

0.0404642

0.0255083

0.161857

0.0155008

0.570514
7

0.733655
6

0.794619
6

0.553501
7

3.85049
6

24.853
6

0.904985
6

0.271937
6

0.285202

1.87489
6

0.717153

0.3313
6

2.64244

0.104179
6

123.558
6

0.300882
7

45.7175
6

0.106774

4.86781
7

1.12422
6

0.0665316
6

0.147877
7

1.65217
6

1.07223

0.114663

0.0809283

0.0338621

3.90224

2.504

0.0404642

0.0898581
7

0.10116

0.136044

0.138591
6

0.168766

1.82722

0.209786

0.124

76.8165
6

0.0141315

0.696651

6.98929

0.0232512

0.326849

0.0315547

0.311349

0.202321

0

0.452355

17.601
6

0.0170056

0

8.47051

1.88575

0

1.72696

0.194691

0.806934

1.83135

0

6.87242
7

0.0606962

0

0.0591046

0.252848

0.0232512

0.0404642

0.165809

0.43834

0

0

27.4048
7

0.0316877

0.694555

0.0105182

0.0563032

0

0.0155008

0

0

0

0.536117

0
4

0.971378

0.932781

0

0.0316877
5

0

0

0.00690984

0

0
4

0.086373

0.086373

0
4

0

0

0

0

0
4

0.127832

0.127832

0
4

0.0469069

0.0469069

0
4

0.0809283

0.0404642

0.0404642

0
4

0

0

0
4

0.117267

0.117267

0
4

0

0

0
4

0

0

0
4

0

0

0
4

1.1996

1.1996

0

0
4

0

0

0
4

0

0

0
4

0.0469069

0.0469069

0
4

0

0

0
4

0

0

0
4

0.0469069

0.0469069

0
4

0

0

0
4

0

0

0

0

0
4

0.039357

0.039357

0
4

0

0

0

0

0

0

0
4

0

0

0

0

0
4

0

0

0
4

0.0155008

0.0155008

0

0
4

5.23103

5.23103

0
4

0
4

247.719

7.57448
7

7.57448
7

0
4

34.859
5

32.1691
5

1.86133
5

0.230718

0.126126

0.00750954

0.357411

0

0.0469069

0.0599054

0
4

0.51609

0.483584

0.0325063

0

0

0

0

6.93889390390723e-18

0
4

16.8316
7

16.7248
7

0.106812
7

0

1.55431223447522e-15
7

0
4

0

0

0

0

0

0

0

0

0
4

6.08296
5

6.05127
5

0.0316877

0

0
4

1.796
7

1.73786
7

0.0581373

0

0
4

0.336161
5

0.0809283
5

0.127604

0

0.0606962

0.0469069

0.0200254

0

5.89805981832114e-17
5

0
4

2.88887
6

2.75659
6

0.0982777

0.0340111

6.93889390390723e-17
6

0
4

1.106

1.06553

0

0.0404642

0
4

0.14206
4

0.0919641
4

0.0125159

0

0

0.0208779

0.0167023

0

6.93889390390723e-18
4

0
4

0

0

0

0

0

0

0

0
4

41.7845

27.9397
5

0

0

0

0

0

11.874
6

0.103629

0.0316877

1.75686

0

0.0469069

0.0316877

0

1.66533453693773e-15

0
4

0.027535
3

0.0225286
4

0.00500636

0
4

0

0

0

0
4

0.0387519

0.0387519

0

0

0
4

0.524986
7

0.195559

0.329427

0
4

0.127662
5

0.0100127

0.00500636

0.00750954

0

0.00500636

0

0.00500636

0.0951208

0
4

0.579692

0.579692

0
4

0.0170056

0

0

0

0

0.0170056

0

0
4

2.23228
7

2.23228
7

0
4

1.18719
6

1.18719
6

0

0
4

0.104377

0.104377

0
4

9.42172
5

6.59762
5

0

0

0

0

0.0170056

0

0

0.0599054

1.70996

0

0.454307
5

0

0.0404642

0.121392

0.271308

0

0.0898581

0

0.0599054

0

0
4

0
4

0

0

0
4

0.989753

0.0792192

0.0170056

0.536117

0

0.357411

0
4

0.0404642
7

0

0

0

0.0404642

0
4

0

0

0

0
4

0

0

0

0
4

0.0809283

0.0809283

0
4

0

0

0

0
4

0.868629

0.77877

0.0898581

0

1.38777878078145e-17

0
4

0

0

0
4

0.0736276

0.0736276

0

0
4

14.792
7

5.93165
7

0

0

0.237241
7

7.90296
7

0.228486
7

0.444782
6

0.0469069
7

0

0

0

0
4

0

0

0
4

0.0465023

0.0155008

0.0310016

3.46944695195361e-18

0
4

0.0620031

0.0620031

0
4

0.0100127

0.0100127

0
4

0.00500636

0.00500636

0

0

0
4

0

0

0
4

0.304895

0.304895

0
4

0

0

0

0

0
4

0.0469069

0.0469069

0

0
4

0

0

0

0

0
4

3.20865
5

2.75527
5

0.0884071
3

0.200797
5

0.164174

0

0

0

2.22044604925031e-16
5

0
4

0.269574

0

0.269574

0
4

0.351802

0.351802

0
4

0

0

0

0
4

0.236086

0.236086

0
4

0.0316877

0.0316877

0

0
4

0

0

0
4

0.357411

0.357411

0
4

0.0599054

0

0.0599054

0
4

0.00750954

0.00750954

0
4

0.0375477

0.0375477

0

0
4

16.3345

13.1995

2.29154
6

0.0475315

0.095063

0.0599054

0.357411

0.257988

0.0255083

0
4

0

0

0
4

0

0

0
4

0

0

0
4

0.0469069

0.0469069

0
4

0

0

0
4

0

0

0
4

0.0340111

0.0340111

0
4

0

0

0
4

0

0

0
4

0

0

0
4

67.1475

67.0264

0

0.0425139

0.0469069

0

0

0.0316877

0

5.21804821573824e-15

0
4

0

0

0
4

0.00350607

0.00350607

0
4

0

0

0
4

0

0

0
4

0.0170056

0.0170056

0
4

0.00827008

0.00827008

0
4

0

0

0
4

0

0

0
4

0

0

0
4

0.0898581

0.0898581

0
4

0.526034

0.121392

0

0

0.364177

0

0

0

0.0404642

0

0

0

2.77555756156289e-17

0
4

0.0469069

0.0469069

0
4

0

0

0
4

0

0

0
4

0

0

0
4

0

0

0
4

0.0387582

0.0387582

0
4

0

0

0
4

0

0

0
4

0

0

0
4

0

0

0
4

10.801
5

9.71794

0.725614

0.357411

0

0

7.21644966006352e-16
5

0
4

0

0

0
4

0

0

0
4

2.56494

2.56494

0
4

0
4

8.21855

8.21855

1.21934

6.99085

0

0

0

0.00835115

0
4

0
4

0

0

0

0
4

0
4

0

0

0

0
4

0
4

0

0

0

0

0
4

0
4

0.00500636

0.00500636

0

0

0.00500636

0
4

0
4

0.290687

0.290687

0.290687

0
4

0
4

0

0

0

0

0
4

0
4

0

0

0

0

0
4

0
4

0.0703603

0.0703603

0.0703603

0
4

0
4

0

0

0

0
4

0
4

23.0844

23.0844

0

23.0844

0
4

0
4

0.217432
3

0.217432
3

0.0170056

0

0

0

0

0

0

0.007332

0

0.0599054

0.0316877

0.0732585

0

0

0.00334147

0

0

0.00334147

0

0

0

0

0.0107277
5

0

0

0

0

0

0
3

0
3

0.0108324

0

0

0

0
4

0

0

0
4

0
4

0.147688

0.147688

0

0.147688

0
4

0
4

0

0

0

0
4

0

0

0
4

0
4

0

0

0

0
4

0

0

0
4

0
4

0

0

0

0
4

0
4

0

0

0

0
4

0
4

0.0170056

0.0170056

0.0170056

0
4

0
4

0

0

0

0
4

0
4

0

0

0

0
4

0
4

0

0

0

0
4

0
4

0

0

0

0
4

0
4

0.0240116
3

0.0240116
3

0.0240116

0

0

0

0
4

0

0

0
4

0

0

0
4

0
4

0.00701215

0.00701215

0.00701215

0
4

0
4

0

0

0

0
4

0
4

0

0

0

0
4

0
4

0

0

0

0
4

0
4

0

0

0

0
4

0
4

0

0

0

0
4

0
4

0.0310016

0.0310016

0.0310016

0
4

0
4

0

0

0

0
4

0
4

0

0

0

0
4

0
4

0

0

0

0
4

0
4

0.198775
6

0.198775
6

0.176286
6

0

0

0.0224897

0

3.46944695195361e-18
6

0
4

0
4

0.0606962

0.0606962

0.0606962

0
4

0
4

0

0

0

0
4

0
4

0

0

0

0
4

0
4

0.136044

0.136044

0.136044

0
4

0
4

0.389385

0.389385

0.389385

0
4

0
4

0

0

0

0
4

0
4

3.41991

3.41991

3.41991

0
4

0
4

0

0

0

0
4

0
4

0

0

0

0

0

0

0
4

0
4

0.00350607
5

0.00350607

0.00350607

0
4

0

0

0
4

0

0

0
4

0
4

0

0

0

0

0
4

0
4

0

0

0

0

0
4

0
4

24.4245

18.4278

18.0815

0.0898581

0.0310016

0

0.0898581

0

0.135654

0

0
4

4.17094
6

4.03244
6

0

0

0

0.0809283

0.00500636

0.0525668

0

2.0122792321331e-16
6

0
4

0

0

0
4

0.119811

0.119811

0
4

0.290687

0.290687

0
4

0.411952
5

0.119811

0.0337346

0.0469069

0.185235

0.0150191

0

0.0112449

0

0

0
4

0.327995
7

0.250729
6

0.0737606

0.00350607

3.46944695195361e-18
7

0
4

0.350725
7

0.319037
7

0.0316877

4.85722573273506e-17
7

0
4

0.0125159

0.0125159

0

0
4

0.0938138

0.0938138

0
4

0.177726

0.177726

0
4

0.0404642

0.0404642

0
4

0

0

0
4

0
4

39.3307

21.3977

1.88004

0.10116
5

0.491747
7

0.134938

2.2165

3.03014
5

0

0

0
5

0

0

1.20014

0.00750954

0.0112449

0

0.00750954

0.0620256

0

1.18924

0

0

0

2.12913
6

0

0

0

0.00500636

0.112405

0.007332

0

0.209669

0.419338

0

2.03678
5

0.0693966

0.346099

0

0

0

0.0124051

0.00500636

0

0

0.0599054

3.29745
6

0

0

0

0

0.0125159

0.0599054

0.0606962

0.232549

0

0.0599054

0

0.007332

0

0.007332

0

0

0

0

0

0

0

0

0

0.00500636

0

0.0155008

0.0703603

0

0

0.0281121

0.0404642

0.0232512

1.14526
7

0

0.0703603

0

0.0606962

0.00500636

0

0.0599054

0.140721

0.250744

0
4

3.47686

3.47686
7

0

0
4

0

0

0

0
4

0.0599054

0.0599054

0
4

0.10116

0.10116

0
4

0.0404642

0.0404642

0
4

0

0

0
4

0.0425139

0.0425139

0
4

0.239622

0.239622

0
4

0

0

0
4

0.91409
5

0.852164
6

0.0100127

0.0469069

0.00500636

0
4

1.19538
6

0.231489

0.821293

0.10384

0.0387582

9.0205620750794e-17
6

0
4

10.6964
5

10.6964
5

0
4

0.211404
7

0.0915931
7

0.0599054

0.0599054

0
4

0.456075
7

0.456075
7

0
4

0

0

0

0
4

0.204096

0.0633753

0.140721

2.77555756156289e-17

0
4

0.295011

0.295011

0
4

3.3584246494911e-14

0
4

11.2557

10.6118

2.83244
5

0

0

0.789051

0.0809283

0

0

0

0.0155008

0

0

5.05575
5

0

0

0

0

0

0

0

0

0

0

0.818213

0

0

0

0.90756
6

0

0

0

0

0.112348

2.91433543964104e-16

0
4

0

0

0
4

0.597033

0.597033

0
4

0

0

0
4

0.0469069

0.0469069

0
4

3.95516952522712e-16

0
4

25.2987
5

25.1725
5

2.76477
5

0.104233
5

0.0538741
5

0.0267385
6

0.105059
5

0.219938

0.150554

0.0443583

0.13148

0.0275327

0.140721

6.31912

0.0137664

0

0.00458879

0

0

0.0393319

0.0465825

0.0292811

0

0.0278609

1.50102
6

0.536117

0.624031

0

0.0183552

0

0.0122368

0.0224754

0.00305919

0

0.317391

0.197497
5

0

0.0112377

0

0

0

0.00305919

0

0

0.0404642

0.0122368

1.56864
5

0

0

0

0.0606962

0

0.0140945

0

0

0

0.0404642

2.70982
5

0.0107072

0

0.00458879

0

0.0809283

0

0.0404642

0.0100127

0

0

2.4486
6

0

0

0.00764798

0.0155008

0

0

0.27353
4

4.33788
6

0
4

0.104733

0.0572012

0.0475315

0
4

0

0

0

0
4

0.00458879

0.00458879

0
4

0.0168565

0.0168565

0
4

0
4

48.0367

40.7089

23.6942

11.2892
5

0.445616

0

0

0.0404642

0.0125159

0

0

0.239622

0.357411

0

1.68936
5

0

0

0.076525

0.656697

0

0

0

0

0

0

1.91507

0

0.0469069

0

0.0506019

0

0.106059
7

0.0887272

0

2.5951463200613e-15

0
4

3.79697
5

3.79697
5

0
4

2.00623

2.00623

0
4

0.00750954

0

0.00750954

0
4

0

0

0

0

0
4

0.112396

0.112396

0
4

0

0

0
4

0.0425139

0.0425139

0
4

0

0

0

0
4

0

0

0
4

0

0

0
4

0.0112449

0.0112449

0
4

0

0

0

0
4

0

0

0
4

0

0

0
4

0

0

0
4

0

0

0
4

0

0

0
4

0.0337346

0.0337346

0
4

0.00500636

0.00500636

0
4

0.0938138

0.0938138

0
4

0.357411

0.357411

0
4

0.0703603

0.0703603

0
4

0.32948

0

0.239622

0.0898581

0
4

0

0

0
4

0

0

0
4

0

0

0
4

0

0

0
4

0.119811

0.119811

0
4

0

0

0
4

0

0

0
4

0.0140243

0.00350607

0.00701215

0

0.00350607

0
4

0

0

0

0

0

0
4

0.327356

0.327356

0
4

0

0

0
4

0

0

0
4

0

0

0

0
4

1.12132525487141e-14

0
4

12.699
5

10.7272
7

9.69343
7

1.03379
7

0

0
4

0.0599054
4

0.0599054
4

0

0

0
4

0

0

0
4

0

0

0
4

0

0

0
4

0.095063

0.095063

0
4

0

0

0

0
4

0.0599054
4

0.0599054
4

0

0

0
4

1.75686

1.70996

0

0.0469069

0
4

0

0

0

0

0
4

0

0

0
4

0

0

0
4

0

0

0
4

0

0

0
4

2.66453525910038e-15
5

0
4

24.3142

24.3142

0

3.69477
5

0

2.21548

0.140721

0.0469069

0.328348

0.0938138

0

0

0.0469069

6.73003

0

0.140721

0.0581373

0

0

0

0

0

0

0.117267

0.146331

0.0938138

0.0469069

0

0.0938138

0.0387582

0

0.0316877

0

0.211081

0.0316877

1.34735
7

0

4.52267
7

0.500559

0.917583

0.0622912

2.65659

0
4

0
4

0.129659
4

0

0

0

0

0

0
4

0

0

0

0

0
4

0.129659

0.129659

0
4

0

0

0
4

0

0

0
4

0

0

0
4

0
4

1.87828
4

1.37815
4

0.934886
5

0

0

0

0.149764

0

0

0.0898581

0.0469069

0

0

0.0475315

0.109204

0

0

0

0

1.52655665885959e-16
4

0
4

0.500125

0.500125

0
4

0

0

0
4

0

0

0
4

0
4

0
4

1504.58

762.522

18.3497

13.727
5

1.00354

0.707914

0

2.62563

0

0.191753

0.0938138

0

0

0

1.88737914186277e-15

0
4

301.043

296.327

0.346704
7

0.10116

0

0

0

0

0

0

0.0469069

0

0

3.07332
7

0

0.0703603

0.844652
7

0.202321

0.0255135

0

0

0

0.00500636

1.42819783777171e-14

0
4

187.677

151.337
7

0.164174
7

0

0

0

0

0

0.0404642

0

0

0

0

0.914406
7

0

0

0.00305919

0

0

0.0316877

0

0.0898581

0

0

0.826103
7

0

0

0

0

0

0

0.0316877

0

0.0039381

0

0.0387582
8

0

0.0404642

0

0

0.121392

0

0

0

0

0

0.10116
7

0

0

0

0

0

0

0

1.70996

0

0

0.44429
7

0

0

0

0

0

0

0.0599054

0

0

0

0
1

0

0.0366292

0

0

0

0

0

0

0

0

0

0

0.222553

0

0

0

0

0.0633753

0

0

0

0
8

0

0

0

0

0

0

0

0

0

0

0.0316877

0

0

0.0155008

0

0

0

0

0

0

0

0.948171

0

0

0

0

0

0

0

0

0

0

0

0
8

0

0

0

0

0

0

0

4.27489

0

0

0
7

0

0

0

0

0.0599054

0

0

0

0

0.00500636

0.0366292
7

0

0

0

0.0732585

0

0

0

0

0

0

0.334625
7

0

0

0

0.0606962

0

0

0.0310016

0

0

0.149764
7

0.0606962

0

6.31587
7

0
7

0

0.0316877

0.00500636

0.14483

0

0

0

0

0

13.4358

0

0.141625

0

0

0.0170056

0

0.142595

0

0

0

2.32121
7

0

0

0.00917758

0

0

0

0.00500636

0.0316877

0

0

0.172914
1

0.00500636

0

0

0

0

0.0599054

0.0316877

0.0107072

0

0

1.10203
1

0.0581373

0

0

0.299527

0.0599054

0

0

0

0

0

0.563613
8

0.0703603

0

0

0

0

0.0449794

0

0.126751

0

0

0
8

0

0.0599054

0

0

0

0

0

0.0475315

0

0

0
4

22.2152
7

19.979
7

2.23623

0
4

0.363339
7

0.132032
7

0

0.0559649

0.0155008

0.0599054

0.0155008

0

0.0404642

0.0404642

0.00350607

0
4

16.423
6

10.1208
6

6.24025
6

0

0.0620031

0

0
4

2.28455
7

0.293232
7

1.78918
6

0.0155008

0.186636

2.77555756156289e-16
7

0
4

0.122938
7

0

0

0.0599054

0

0.0475315

0

0

0

0

0.0155008

0
4

0.0404642

0

0

0

0.0404642

0

0
4

0
1

0
1

0
4

0.288628
6

0.188022
6

0.0456618

0.0549439

2.08166817117217e-17
6

0
4

0

0

0

0

0
4

0

0

0

0
4

63.1623
5

41.6075
7

5.21702

0.2107
6

0.127511

0.11564

0.188686

0.417317

0.0542527

0.1065

0.0404642

0.0232512

7.21536

0

0.0475315

0.0366292

0.0155008

0.0404642

0.119811

0

0.0310016

0

0.0366292

3.68089
6

0

0.141625

0.877634

0.937922
6

0.279696
5

0.978664
6

0.49687
6

0.117267
6

0
4

0.232637
6

0.232637
6

0
4

0

0

0

0
4

0

0

0

0
4

0

0

0

0
4

0

0

0

0
4

0.0387519

0

0.0387519

0
4

0.0598804

0.0232512

0.0366292

0
4

0.0599054

0.0599054

0
4

0

0

0
4

0

0

0
4

80.0884

0.00500636
1

0.0404642

0

0

0.0732121

0.232042

0.0604476

0.0146653

0.0366292
7

0.0559649
2

0.0100127
8

79.5599
6

1.4210854715202e-14

0
4

0

0

0
4

0.10116

0.10116

0
4

0

0

0
4

0

0

0
4

0

0

0
4

0

0

0
4

0

0

0
4

0

0

0
4

0

0

0
4

0

0

0
4

41.0824

10.8901
5

0

28.7344

0.462644
7

0.569721
7

0.161857

0.0559649

0

0.126751

0.0809283

4.38538094726937e-15

0
4

0

0

0
4

0

0

0
4

0

0

0
4

0

0

0
4

0.0404642

0.0404642

0
4

0

0

0
4

0.0387582

0.0387582

0
4

0.0404642

0.0404642

0
4

0

0

0
4

0

0

0
4

2.32739

0.117581
1

0.0105687
1

0.078195

0

0.0469069

0.364177

1.70996

0

0
4

0

0

0
4

0.273801
7

0.273801
7

0
8

0

0

0

0

0
4

24.677
6

24.5771
6

0.0190069
7

0.0104159

0

0.0549439

0.0155008

0

6.08887940067859e-16
6

0
4

0.472609
8

0.44542
8

0
8

0

0.0039381

0.0232512

0

0

0
4

1.01795

0.937018

0.0404642

0

0

0.0404642

0

0
4

9.34363697524532e-13

0
4

190.134

189.25

185.869

0

0

0

0.121392

0

0

0

0

0

0

0

2.2834

0

0

0.0606962

0

0.21317

0

0.501057

0.0599054

0.140834

0

0

0

0

0
4

0.00350607
8

0
8

0

0

0

0

0

0

0

0.00350607

0

0

0
8

0

0

0

0

0

0

0

0

0

0

0
8

0

0

0

0

0

0

0

0

0
4

0.486149

0.486149

0
4

0

0

0
4

0.0770934

0.0770934

0
4

0

0

0
4

0

0

0
4

0.242785

0.242785

0
4

0.0168673

0.0168673

0
4

0.0581373

0.0581373

0
4

0

0

0
4

0

0

0
4

4.01831345975268e-14

0
4

270.853

228.59

94.6668

0.223763
5

0.0168673

0

0.0404642

0

0

0

0

0

0.121392

0

0.445106

0

0

0.0404642

0

0

0.0599054

0

0.0404642

0

0.0404642

3.26039
6

0

0

0.0404642

0.0469069

0.0112377

0

0

0

0

0

0.599911
7

0

0.0404642

0

0

0

0

0

0.0469069

0

0

0.492378

0

0.00611838

0

0.0112377

0.0404642

0

0

0.357411

0.0633753

0.0606962

0.380252

0

0

0.200045
6

0.125908
7

0.00611838

0.139125
6

19.6581

0.243055
7

0.095063

0.0855171

0.0606962
7

0.0822784

0.149764

0

0.119811

0.0267596

0.20597

76.935
7

0.142595

1.70996

0.158492

0.289468

0

0.0404642

0.0450529

0

0.0143041

0

14.9738

0

0

2.81501

0.0606962

0.0404642

0.00458879

0.263017

0

0.0112449

0.0599054

2.08532
6

0.0404642

0.0898581

0

0.0404642

0.0404642

0.0387582

0.0404642

0.10037

0.0404642

0

1.79149
7

0

0.00305919

0.00458879

0.0599054

0

0.0633753

0

0

0.209669

0.0785946

0.0761492
1

0.119811

0

0.0112449

0

0

0

0.0404642

0

0

0

1.26551

0.0809283

0

0

0.0155008

0.0581373

0.0809283

0

0

0

1.70996

0.271792
7

0.0606962

0

0

0

0

0.0898581

0.00939633

0.0155008

0.0404642

0

0
4

0.224136

0.0938138

0.0404642

0.0898581

1.38777878078145e-17

0
4

0

0

0

0

0
4

0.301769

0.263017

0.0387519

0
4

0

0

0
4

0.0404642

0.0404642

0
4

0

0

0
4

0.0809283

0.0809283

0
4

0

0

0
4

33.6729

5.27096

1.70996

1.31237

0.499036

0.10116

0.856447

0.0435233

0.0404642

0.182089

0

0.0606962

4.10711
6

0.121392

0.0938138

0.58673

0.00305919

0

0

0.0316877

0.0469069

0

0

5.77338
6

0.0316877

0

0.0606962

0.202321

0.0469069

0.0404642

0.0404642

0.0404642

0

0.242785

4.50569
6

0.0404642

0.0606962

0.141625

0

0.536117

0.0404642

0

0

0.0606962

0

1.51741

0.0404642

0.0404642

1.25094

3.86074

0.0305919

0

0

0
4

2.26054
5

2.02289
6

0

0.0404642

0.0168673

0.0112449

0.0898581

0.0792192

0

0

0

0
4

0.407722

0.317616
6

0

0.00305919

0.00305919

0.0404642

0

0.0404642

0.00305919

0

0

0

0
4

1.36671
7

1.36671
7

0

0

0

0
4

3.03283
6

0.0587764

0.0404642

0.368656

2.56494

0

0
4

0.161857
7

0.0404642

0.121392

0

0

0
4

0.531575

0.303483

0.0873711

0.0469069

0.0938138

0
4

0.182089

0.10116

0

0

0.0404642

0.0404642

1.38777878078145e-17

0
4

1.36918254511897e-13

0
4

0.0680222

0.0680222

0.0680222

0
4

0

0

0
4

0
4

0.0475315

0.0475315

0.0475315

0
4

0

0

0
4

0
4

0

0

0

0

0
4

0
4

0

0

0

0

0

0
4

0
4

0.075211

0.075211

0.0721518

0.00305919

4.33680868994202e-18

0
4

0
4

0

0

0

0

0
4

0
4

0

0

0

0

0
4

0
4

0

0

0

0

0
4

0

0

0
4

0
4

0.0112377

0.0112377

0.0112377

0
4

0
4

0.0170056

0.0170056

0.0170056

0
4

0
4

98.9514
7

6.35562
7

0.276756
7

0

0.0703603

0

0

0

0

3.46085
7

2.47197
7

0.0255489
7

0.0112377

0.00939633

0

0.0107072

0.0187927

0
4

87.2068
6

84.5925
6

0.00458879
7

0.0168256

0.0137664

2.42495

0.0668146

0.0404642

0.0469069

2.07125983031631e-14
6

0
4

0.124642

0.059357

0.065285

0
4

0.657192

0.657192

0

0
4

4.59592
6

0.30402

0.0170056

4.27489

8.88178419700125e-16
6

0
4

0.0112449

0

0.0112449

0
4

0

0

0
4

0

0

0
4

0

0

0
4

5.05446379195362e-14
7

0
4

0

0

0

0
4

0

0

0
4

0
4

0

0

0

0
4

0

0

0
4

0
4

0.0606962

0.0606962

0.0606962

0

0
4

0
4

0.135654

0.135654

0.135654

0
4

0
4

1.25094

1.25094

1.25094

0
4

0
4

0

0

0

0
4

0
4

0

0

0

0
4

0
4

0.0170056

0.0170056

0.0170056

0
4

0
4

0.164174

0.164174

0.164174

0
4

0
4

0.164436

0.164436

0.164436

0
4

0
4

4.41803
7

2.52197
7

0.488104
7

1.81033

0.110922

0.0809283

0.0316877

0
4

1.02981
7

0.298739
7

0.27633

0.0606962

0

0.357411

0.0366292

5.55111512312578e-17
7

0
4

0.825792
6

0.567805

0

0.257988

0
4

0.0404642

0.0404642

0
4

3.05311331771918e-16
7

0
4

0

0

0

0
4

0
4

0

0

0

0
4

0
4

0

0

0

0
4

0
4

0

0

0

0
4

0
4

0

0

0

0
4

0
4

0

0

0

0
4

0
4

6.08164
6

4.61108
6

2.816
6

0.0633753

1.15323

0.0404642

0.234534

0.303481

0
4

0.179896
7

0.0900378
7

0.0898581

0

0
4

0.918347
7

0.918347
6

0

0
4

0.0898581

0

0.0898581

0

0
4

0.222553

0.222553

0
4

0.0599054

0.0599054

0
4

3.33066907387547e-16
6

0
4

8.96665
6

8.96665
6

7.84483
6

0.706944

0.357411

0.0170056

0.0404642

0
4

0
4

0
2

0
2

0
2

0

0

0

0

0

0

0

0
4

0
4

1.25894
6

0.629931
6

0.536117

0.0938138

0

0

0

0
4

0.629007

0.629007

0

0
4

0
4

0.714822

0.714822

0.714822

0
4

0
4

0.221814

0.221814

0

0.110907

0.110907

0

0

0
4

0
4

158.45

158.41

61.5111

0.0947074
7

0.151289
7

0.0914808

0

0.0574697
7

0.0387519

0.0404642

0.0809283

0.10116

0.383619

17.6116
7

0.116256

0.19376

0

0.120602

0

0

0.141625

0.357411

0

0.0469069

72.1259

0.0340111

0.0809283

0

0

0.0595195

0

0

0

0

0

0

0.0680222

0.0606962

0.0968956

0.718733

0.121392

0.121392

0

0

0

0

2.70516
7

0

0

0

0.0404642

0

0.10116

0.0404642

0

0

0

0

0

0

0

0.0340111

0

0

0

0

0.0387582

0

0
8

0

0

0

0.0404642

0

0

0

0

0

0.606962

0

0

0

0

0

0

0

0

0

0

0

0.0744753

0.0606962

0.0404642

0
4

0

0

0
4

0

0

0
4

0.0404642

0.0404642

0
4

0
4

5.08748598804232e-12

0
4

78.737

0.148629
7

0.0465023

0.0465023

0

0
4

0

0

0

0
4

0

0

0
4

0

0

0
4

0.0542527

0.0542527

0
4

0

0

0
4

0.0366292

0.0366292

0
4

0.0112449

0.0112449

0
4

0
4

8.77701
7

8.77701
7

1.51396

0.479243

0

0

0

0.167341

1.83853

0.451225

0.230692

0.285189

0

0

0.0606962

0

0.351294

0

0

0.0224897

0.160485

0.222553

0.0606962

0

0

0.304895

0

0.0606962

0

0.0938138

0

0

0

0.0469069

0.222553

0

0.124377

0.0792192

0.0155008

0.0606962

0.095063

0

0.0703603

0.0168673

0

0

0.156952

0.0581373

0

0.039357

0.0387582

0.629007

0.0112449

0

0

0

0.0168673

0.0967377

0

0

0.164174

0

0

0.0168673

0.0112449

0.0606962

0

0.0316877

0

0

0

0.0112449

0

0.0606962

0.229528

0.10846

5.27355936696949e-15
7

0
4

0

0

0
4

0
4

2.96148
6

2.96148
6

2.92102
6

0.0404642

0
4

0
4

0

0

0

0
4

0
4

0

0

0

0
4

0
4

6.16803
7

6.16803
7

6.16803
7

0
4

0
4

0.345292

0.345292

0.345292

0
4

0
4

0.0606962

0.0606962

0.0606962

0
4

0
4

0

0

0

0
4

0
4

0.095063

0.095063

0.095063

0
4

0
4

0

0

0

0
4

0
4

0.182089

0.182089

0.182089

0
4

0
4

0

0

0

0
4

0
4

59.9987

56.5311

22.131

11.9594
7

4.27489

0

0.0112449

0

0.0105182

0

0

0

0.0337346

0

7.03237
6

0

0.149764

0

0

0.117267

0

1.70996

0

0.0350607

0.0606962

0.761275

0

0.0809283

0.0214143

0.881089
7

0.151806

5.25541
7

0.264254

1.46133

0.127632

1.21014309684142e-14

0
4

0.676701

0.66443

0.00876519

0.00350607

0
4

0.714822

0.714822

0
4

1.20616

1.20616

0
4

0.769694

0.769694

0
4

0.0157773

0.00525911

0.0105182

0
4

0

0

0
4

0.0809283

0.0809283

0
4

0.00350607

0.00350607

0
4

4.33333924299006e-15

0
4

0
4

19.5523
4

15.2347
4

13.2761
4

1.65376
4

4.11317

0

0

0

0.0140243

0.0425139

0.141625

0.0255083

0.140721

0.129316

0

4.75607

0

0

0.676838

0

0.10116

0

0

0.0387582

0

0

0.453622
7

0

0

0

0

0

0.0170056

0

0

0.00525911

0

0.0809283

0

0

0

0

0

0

0

0

0

0

0.00350607

0.0112449

0

0

0

0.00305919

0

0

0

0

0.868043
6

0

0

0

0
4

0

0

0
4

0.0968956

0.0968956

0
4

1.70996

1.70996

0
4

0

0

0
4

0.10116

0.10116

0
4

0

0

0
4

0.0112449

0.0112449

0
4

0

0

0
4

0

0

0
4

0.0168565

0.0168565

0
4

0

0

0
4

0

0

0
4

0.0224754

0.0224754

0
4

0
4

0

0

0

0
4

0
4

0

0

0

0

0
4

0
4

0

0

0

0
4

0
4

0

0

0

0
4

0
4

0

0

0

0
4

0
4

0

0

0

0
4

0
4

0

0

0

0
4

0
4

0

0

0

0
4

0
4

0

0

0

0
4

0
4

0

0

0

0
4

0
4

0

0

0

0
4

0
4

0.716264

0.716264

0.464073

0.252192

0
4

0
4

0.0170056

0.0170056

0.0170056

0
4

0
4

0.0599054

0.0599054

0.0599054

0
4

0
4

0.0168565

0.0168565

0.0168565

0
4

0
4

0

0

0

0
4

0
4

0.0168565

0.0168565

0.0168565

0
4

0
4

0

0

0

0
4

0
4

0

0

0

0
4

0
4

0.0595195

0.0595195

0.0595195

0
4

0
4

0

0

0

0

0
4

0
4

0

0

0

0
4

0
4

3.41991

3.41991

3.41991

0

0
4

0
4

0

0

0

0
4

0
4

0.0112377

0.0112377

0.0112377

0
4

0
4

0
4

12420.1

0.484478
1

0

0

0

0

0

0

0
4

0

0

0
4

0

0

0
4

0

0

0
4

0

0

0
4

0

0

0
4

0

0

0
4

0

0

0
4

0.484478

0.484478

0
4

0

0

0
4

0
4

26.1625

20.5715

1.55127

0
2

0
3

0

0

0

0

0

0

1.70996

0

5.24962

0.0606962

0

0

0.119811

0

0

0

0

0

0

0
4

0

0

0.0475315

0

0

0

0

0

0

0

0.548359

0

0

0

0

0

0

0.0155008

8.54979

0

0.0898581

0

0

0

0

0

0.0155008

0

0

0

0

0

0
4

0

0

0

0.364177

0

0.357411

0

0

0

0.0404642

0
3

0

1.70996

0

0

0

0

0

0

0

0

0

0

0.141625

0

0

0

0

0

0

0

0

0
3

0

0

0

0

0

0

0

0

0

0
4

0
3

0
3

0

0

0

0

0
4

0

0

0
4

0

0

0
4

0

0

0
4

0.0475315

0.0475315

0
4

0.0404642

0.0404642

0
4

0

0

0
4

0

0

0
4

1.70996

1.70996

0
4

1.70996

1.70996

0
4

0

0

0
4

0
3

0
3

0
4

0

0

0

0
4

0.0599054

0

0.0599054

0
4

2.02321

2.02321

0
4

0

0

0

0
4

0

0

0
4

0

0

0
4

0

0

0
4

3.99680288865056e-15

0
4

15.5376
1

3.45317
1

1.16616
1

0

0

0.406961

0

0.356518

0

0

0

0

0

1.12825
1

0

0

0.317766

0

0

0

0

0

0

0

0.0775164

0

0

0

0

0

0

0

0

0

0

0

0

0

0

0

0

0

0

0

0

0

0

0

0

0

0

0

0

0

0

0

0

0

0

0

0

0

0

0

0

0

0

0

0

0

1.52655665885959e-16
1

0
4

4.31497
2

3.95756
2

0.357411

0
4

3.8093

3.8093

0

0

0

0
4

0

0

0
4

0

0

0
4

0

0

0
4

0.0155008

0.0155008

0
4

0

0

0
4

0

0

0
4

0

0

0
4

0

0

0
4

0

0

0
4

0

0

0
4

3.92911

3.92911

0

0

0
4

0

0

0
4

0

0

0
4

0

0

0
4

0

0

0

0
4

0

0

0
4

0

0

0
4

0.0155008

0.0155008

0
4

0

0

0
4

1.28889954265077e-15
1

0
4

3309.56

26.352

0
2

0

0

1.76019

1.70996

0

0

0

0

0.0809283

0

10.27
6

0

0

0

0

0

0

0

0

0

0

0.652392
3

0

0

0

0

0

0.0404642

0

0

0

0

0.0968956
2

0

0

0.0404642

0

0

0

0

0

0

0

0.966751
3

0

0.0469069

0.768819

0

0

0

0

0

0

0

9.91828
6

0

0

0

0

0

0

0

0
2

0

0
2

0
4

3159.88

3062.83

1.99789
7

0.362464

0.0168565

0

0.0404642

0.0170056

0.357411

0.0606962

0.0703603

0.116256

0

0

0.73205
7

0.0232512

0

0

0

0

0.0396852

0

0.0170056

0.0779085

0.179918

0.23497

0

0.0155008

0

0.0646343

0.0620031

0

0.0471885

0.0606962

0

0

0.737708
7

0

0

0.0404642

0

0

0

0.0112377

0.10116

0.0232512

0

0.873227

0

0

0

0

0

0

0.029328

0

0

0

0.471168
7

0

0.0155008

0

0

0

0

0

0.0155008

0

0

0.413545
6

0.0404642

0.0387519

0

0

0.0310016

0

0.0387519

0

0

0

0.0937197
7

0

0

0

0

0

0

0

0

0

0

0.482121
7

0

0

0

0

0

0

0

0

0

0.0449794

0.449502
7

0

0

0

0

0.0310016

0

0

0

0

0.0340111

25.6142
7

1.75783
7

0

0

0.00690984

0

0

0

0

0

0

0

0.152394
7

0

0

0

0

0

0

0

0

0.0404642

0

0.704293
7

0

0.121392

0

0

0.0404642

0.0155008

0

0.0469069

0

0

0.969385
7

0

0.142595

0

0.0170056

0.257988

0

0

0

0

0

10.0098
6

0

0

0

0

0

0.0506019

0

0.0155008

0

0

0.144644
2

0.0232512

0

0.0469069

0

0.0155008

0

0

0

0

0

0.279355
7

0

0

0

0

0.0469069

0

0

0.0112377

0.0404642

0

0.166659

0

0.0404642

0

0

0.0703603

0

0

0.0775039

0

0

0.170913
7

0.0232512

0.0366292

0

0

0

0

0

0

0

0

0.139705
6

0.0469069

0

0

0

0

0.00939633

0

0.0809283

0.0404642

0.0404642

3.81252
7

0.139943
6

0

0.128202

0

0

0

0.0155008

0

0.0404642

0.0316877

0

0.243884
7

0

0

0.0225286

0.0155008

0.0549439

0

0

0.0155008

0.0138197

0.00690984

1.2907

0

0

0

0

0.0112377

0

0

0

0

0

0.291999
7

0

0

0

0

0

0.0224897

0

0.0112377

0

0

0.121546
7

0

0.0155008

0.0775039

0

0.0606962

0

0

0.0232512

0.0606962

0

0.0777018
8

0

0.0112377

0.222553

0.0404642

0

0.0155008

0

0.0542527

0

0.0469069

0.0325063
1

0

0

0

0

0

0

0.00690984

0.0404642

0

0.0232512

0.0328401
7

0

0.0255083

0

0

0.0404642

0

0.00690984

0.161857

0

0.0404642

0.106025

0

0.0155008

0.0112377

0

0

0

0

0

0.121392

0.0103648

0.292913
7

0

0.0310016

0

0

0

0

0

0

3.6585
7

0.313845

0.0379114
7

1.47694

0.0170056
7

0.0714657
7

0.305359
6

0.0155008
8

0.214104

0.209669

0.0794396

4.79256
7

0
7

0
7

0.0241844

10.4986
6

0.158438

0

0.0898581

0.0873711

0.0404642

0

5.63349
7

0.0155008

0.124899

0.114704

0

0.116256

0.0112377

0.0282504

0.0809283

0.0267385

0

4.12864
7

0

0.0620031

0.0620031

0.0112377

0.0549439

0

0.0809283

0.0620094

0.497256

0

3.03335

0

0.0232512

0

0

0

0

0

0

0

0.154977

2.05697

0.0792671

0.0606962

0

0

0.295325

0

0

0

0

0.0232512

2.20511109372268e-12

0
4

55.2333
7

55.2333
7

0

0

0
4

55.5051

3.48086

21.5927

0

0

0

0

0

0

12.8247

0.0637153

0.852543

0

0.258362
6

0.286561

0

0

0.738719

0.0404642
4

0

3.37876

0.536117

0.00690984

0.0404642

0.0809283

0

0.0618072

0

0

0
2

0

0

0

0

0

0.0155008

0

0

0.0155008

0

0.0973313
3

0

0

0

0.0255083

0

0

0

0

0

0

9.42556
6

0

0

0

0

0

0

0

0

0.141625

0.0232512

0.0241844
1

0.039357

0

0.0340111

0

0

0

0

0

0

0.0809283

0.388505

0

0

0

0

0

0

0.714822

0

0.0155008

0

0
3

0.0255083

0

0

0

0.0112377

0

0

0

0

0

0.095063
4

0

0

0

0

0

0

0.0599054

0.0170056

0

0.0112449

0
4

9.61116

9.33177

0.256141
7

0.0232512

0
4

0.028105

0.028105

0
4

0

0

0

0
4

0

0

0
4

0

0

0
4

0.121392

0.121392

0
4

0

0

0
4

0

0

0
4

0

0

0
4

0

0

0
4

0

0

0
4

0.194446

0.0170056
1

0

0.0404642

0

0.0595195

0

0

0.0449507

0

0.0170056

0.0155008

0

5.20417042793042e-18

0
4

0

0

0
4

0

0

0
4

0

0

0
4

0

0

0
4

2.28915
7

1.70996
7

0.313503

0.204997

0.0606962

0

0

1.80411241501588e-16
7

0
4

0.0328401

0.0103648
1

0

0

0

0

0.0224754

0

0
4

0.0654904

0.0654904

0

0

0

0

0
4

0.15305

0

0

0.15305

0

0
4

0
1

0

0

0

0
4

0

0

0

0

0

0
4

0.0938138

0.0938138

0

0

0
4

1.32088784354778e-12

0
4

213.908

209.518

208.57

0.0510167

0.357411

0

0

0.539429

0

3.77475828372553e-14

0
4

0
1

0
2

0
1

0

0

0
4

0

0

0
4

0

0

0
4

0

0

0
4

0.121392

0.0809283

0.0404642

0
4

0

0

0

0
4

0

0

0
4

0

0

0

0
4

0

0

0
4

0

0

0
4

0.0898581

0.0898581

0
4

0

0

0

0
4

0.0155008

0

0.0155008

0
4

0

0

0

0
4

0

0

0
4

0.142595

0.142595

0
4

0

0

0
4

0.0170056

0.0170056

0
4

0

0

0
4

0.0404642

0.0404642

0
4

0

0

0
4

0

0

0
4

0.119811
2

0.119811
2

0

0
4

0

0

0
4

0

0

0
4

0

0

0
4

0

0

0
4

0

0

0
4

1.70996

1.70996

0
4

0

0

0
4

0

0

0
4

0

0

0
4

0

0

0
4

0
2

0
2

0
4

0

0

0
4

0

0

0
4

0

0

0
4

0

0

0
4

0.0155008

0.0155008

0
4

0

0

0
4

0

0

0
4

0

0

0
4

2.11866

2.11866

0
4

0

0

0

0
4

0

0

0
4

0

0

0

0
4

0
4

14.4222
3

14.3967

14.3579

0

0

0.0387582

6.86950496486816e-16

0
4

0

0

0

0

0
4

0

0

0
4

0

0

0

0
4

0

0

0

0

0
4

0.0255083

0.0255083

0
4

0

0

0

0
4

0

0

0
4

0

0

0
4

0

0

0
4

0

0

0
4

5.10008701937181e-16
3

0
4

30.1467

18.799

0
8

0

0

0

0

0

0

0

0

0

0

0

0

0

0

0

0

0

0.0387582

0

0.0316877

0

0

0.100755

0

0

0

0.0792192

1.70996

0

0

0.434022

0

0.290687

1.62785

0.382828

0

0

0

0

0

2.56494

3.81588

0

0

0

0

0

0

0

0

0.0475315

0

0

0.271264

0

0

0

0.443524

0

0

0

0

0

0

0.0792192

0

0.714822

0

0

0

0

0

0.0633753

0

0

0.536117

0.0404642

0

0

0

0

0

0

0

0.0316877

0.0316877

1.12399

0

0

0

0.095063

0.536117

0

0

0

0

0

0

0

0

0

0

0

0.0316877

0

0

0

0

3.63717

0

0

0

0

0

0

0

0

0

0.0387582

0
4

6.00481

4.07187

0.982318

0.396096

0.0792192

0.126751

0.0316877

0.316877

7.21644966006352e-16

0
4

0

0

0
4

0.887255

0.649597

0.0475315

0.0316877

0.158438

0
4

0.142595

0.142595

0
4

0.269345

0.237658

0.0316877

0
4

0

0

0
4

0.110907

0.0792192

0

0.0316877

6.93889390390723e-18

0
4

0

0

0

0

0
4

0.142595

0.095063

0.0475315

0

0
4

1.61857

1.61857

0

0
4

0

0

0
4

0

0

0

0

0

0
4

0

0

0

0

0
4

0

0

0
4

0.237658

0.237658

0
4

0.0316877

0.0316877

0

0

0
4

0

0

0

0

0
4

0

0

0
4

0

0

0
4

0

0

0

0
4

0.174282

0.174282

0
4

0

0

0

0
4

0

0

0

0
4

0.0404642

0.0404642

0
4

0.158438

0.158438

0
4

0

0

0
4

0

0

0
4

0.0599054

0.0599054

0
4

0

0

0
4

0

0

0
4

0.0316877

0.0316877

0
4

0

0

0
4

0.0316877

0.0316877

0
4

0.536117

0.536117

0

0

0

0

0

0
4

0

0

0
4

0

0

0
4

0

0

0
4

0

0

0
4

0

0

0
4

0

0

0
4

0

0

0
4

0

0

0
4

0

0

0
4

0.0316877

0.0316877

0
4

0.727057
7

0.727057
7

0

0
4

0

0

0
4

0

0

0
4

0

0

0
4

0

0

0
4

0

0

0
4

0

0

0
4

0.0316877

0.0316877

0
4

0

0

0
4

0

0

0
4

0

0

0

0

0

0

0
4

0

0

0
4

0

0

0

0

0
4

0.0792192

0.0792192

0

0
4

5.9535709695524e-15

0
4

1.64056

1.49079

0.782598

0.161553

0

0

0

0

0

0

0

0

0

0

0.141652

0

0

0

0

0

0

0

0.00690984

0

0

0.170056

0

0

0

0

0.0241844

0.20384

0

2.22044604925031e-16

0
4

0.0898581

0

0.0898581

0

0

0

0
4

0

0

0
4

0

0

0

0
4

0

0

0
4

0

0

0
4

0

0

0
4

0.0599054

0.0599054

0
4

0
4

6.86609

0

0

0

0
4

0.0599054

0

0

0

0

0

0

0

0.0599054

0

0
4

0

0

0
4

1.45343

1.45343

0

0
4

0

0

0
4

0

0

0
4

0

0

0

0
4

0.136044

0.136044

0
4

0

0

0

0
4

0

0

0
4

0.0599054

0.0599054

0
4

0

0

0
4

0

0

0

0

0

0
4

0

0

0
4

0.536117

0.536117

0
4

0

0

0
4

0.170056

0.170056

0
4

0

0

0
4

1.25094

1.25094

0
4

0.0599054

0.0599054

0
4

0.357411

0.357411

0
4

0.0898581

0

0.0898581

0

0

0
4

0.930197

0.930197

0

0
4

1.07223

1.07223

0

0
4

0.241956

0.212628

0.029328

0
4

0.157443

0.110536

0.0469069

0
4

0
2

0
2

0

0

0
4

0.290687

0.251928

0.0387582

2.08166817117217e-17

0
4

0
4

113.603

113.603

111.765

0

0

0.0935306

0

0

0.0340111

1.70996

0

1.24344978758018e-14

0
4

0

0

0

0
4

0

0

0

0
4

0

0

0
4

0

0

0
4

0

0

0
4

0
4

31.8222

0

0

0

0

0

0

0

0

0

0

0

0
4

31.8222

31.8222

0
4

0

0

0

0
4

0
4

416.608

6.72239
7

0

0

1.70996

0.0606962

0

0.0809283

0

0

0.0404642

0

0

0.464107

0

0.0404642

0

0.121392

0.0606962

0

0.142595

0.0475315

0.364177

0.0606962

0.627194

0.0404642

0.0404642

0

0

0.364177

0.0792192

0

1.66235

0.714822

0
4

3.59595

1.14026
7

0

0

0.0404642

0

0

0

0

0

0

0

1.99004
7

0

0

0

0.425196
7

0

0

0

0

0

0

0
4

181.161

111.178
7

28.4318
7

0.144225

0.12831

0.140542

0.187628

0

0.0898581

0

0.007332

0.0898581

0.0168673

36.328
7

0.032994

0.141625

0.0323681

0.140721

0

0

0

0

0

0

0.635024
6

0.0469069

0.0938138

0

0.211081

0

0.010998

0

0.211081

0

0.007332

0

0

0

0.117267

0

0.0404642

0.010998

0.007332

0.007332

0.117267

0

0.271264

0

0

0.0155008

0

0

0.0404642

0.0404642

0

0

0.0938138

0.386528

0.0155008

0.010998

0

0.182089

0

0

0.0703603

0.0606962

0

0.007332

0.0542527

0

0.0404642

0.515958

0.748193

0
4

95.4027

37.5261

15.4249
7

0.310288

0

0

0

0

0

0

0

0

0

0.0170056

1.58102
7

0

0

0.00350607

0.0404642

0.0262956

0.0549439

0

0.311349

0.0316877

0

1.14402
7

0.014664

0.0232512

0.0542527

0.121392

0

0

0.007332

0

0

0

0.480541
7

0.0155008

0

0

0.0316877

0.0366292

0

0

0.01833

0

0.0155008

4.1736
6

0.0155008

0.00350607

0

0

0

0.00525911

0.155033

0

0

0

1.39125

0.0549439

0

0

0

0

0

0.014664

0

0.0366292

0

0.182089

0

0

0

0

0

0

0

0

0.424874
7

0.357411

0.0549439

6.75059
7

0.087353

0.243767

0.127774

0.0606962

0.0170056

0.0366292

0

4.30394

0

0.0620031

1.39715

0

0.368656

4.3218

0.076197

0

0.13153

0

1.11276

0

0

3.48615
5

0.014664

0

0.0112449

0

0

0.384409

0.117454

0

0

0

1.48192
7

0

0

0

0.0989819

0

0.010998

0

0.0366292

0.0770934

0.0208779

0.345339
7

0.109888

0.109888

0

0.0366292

0

0

0.0112377

0.0843179

0

0.0843364

0.0953159

0

0

0

0

0

0.01833

0

0

0

0

2.95277
7

0

2.14447

0.010998

0

0.007332

0.010998

0

0

0

0

0.357411

0

0

0

0.007332

0

0

0.0175304

0

0

0

8.3544282603043e-15

0
4

5.50281
2

0
2

0

0

0

0

0

2.56494

0.0938138

0

0

0

2.56494
2

0

0

0

0

0

0

0

0

0.117267

0

0

0

0

0

0

0

0

0

0

0

0

0
2

0

0

0

0

0

0

0

0

0.161857

0

0

0

0

0

0

0

0

0

0

0

0

0

0

0

0

0

0

0

0

0

0

0

0

0

0
4

4.60196

4.60196

0
4

16.4041
7

16.3724
7

0

0.0316877

0
4

8.34274

3.51641

0

0

0

0.0549439

0.0366292

0.128202

0.0366292

0.0366292

0.0404642

4.26731

0.0606962

0

0

0

0.0915731

0.0732585

0
4

4.97481

4.47638

0

0

0

0

0

0

0.139507

0

0.00827008

0

0.127797

0.0232512

0.0542527

0

0.0248102

0.007332

0

0.043992

0.00350607

0.0657026

3.19189119579733e-16

0
4

0.00350607
3

0

0.00350607

0

0

0
4

1.9231
6

1.91186
6

0.0112449

0
4

0.105391

0.0888507

0.0165402

0

0

0
4

0.357411

0.357411

0

0

0

0

0
4

4.04929

3.51318

0.536117

0
4

0

0

0

0
4

0.0792192

0.0792192

0

0

0
4

0.124817

0.00500636

0

0.119811

0
4

0.007332

0

0

0.007332

0
4

0

0

0
4

30.2585

9.12316

0.00827008

0.007332

0.109888

0.00350607

0.10116

17.4698
7

0

0

0.0404642

3.3544

0

0

0.0404642

0
4

0.110907

0.110907

0
4

0.172791

0.132327

0.0404642

0
4

0

0

0
4

0.475315

0.475315

0
4

0.714822

0.714822

0

0

0
4

0.007332

0.007332

0

0

0
4

1.70996

1.70996

0

0
4

0.0112449

0.0112449

0

0
4

0

0

0
4

0.222553

0.121392

0.10116

0
4

0.461575
7

0.35918
7

0.095063
7

0

0.007332

0

0

0

0
4

1.43698

0.007332

1.42964

0
4

0.025662

0.01833

0.007332

1.73472347597681e-18

0
4

0

0

0

0
4

0.544786

0.544786

0
4

0

0

0

0
4

0.0654693

0.007332

0.0581373

0
4

0.0633753

0.0633753

0
4

0

0

0
4

0

0

0
4

0.00701215

0.00701215

0
4

28.8258
7

28.6493
7

0.176509

0
4

0

0

0
4

0.010998

0.010998

0
4

0.142974

0.142974

0
4

0.222553

0.222553

0
4

0.146517

0.146517

0
4

0

0

0
4

0.505802

0.505802

0
4

0.0276393

0.0276393

0
4

0

0

0
4

0

0

0
4

11.5754
7

9.21175

1.70996

0.278544

0.37515

0
4

0

0

0
4

0

0

0
4

0.0387582

0.0387582

0
4

0

0

0
4

0

0

0
4

0

0

0
4

0.0850278

0.0850278

0
4

0

0

0
4

0.0809283

0.0809283

0
4

0

0

0
4

0.544377
7

0.544377
7

0
4

0.010998

0.010998

0
4

0.0170056

0.0170056

0
4

0.0633753

0.0633753

0
4

0.007332

0.007332

0
4

0

0

0
4

0

0

0
4

0

0

0
4

2.43314
7

2.38212

0

0.0510167

3.46944695195361e-17
7

0
4

1.73562
5

0

1.73562

0

0

0
4

0.486362
7

0.486362
7

0

0

0
4

2.71782596428238e-13

0
4

14.7496

14.7496

14.7496

0

0

0

0
4

0

0

0

0

0

0

0

0
4

0

0

0
4

0

0

0
4

0

0

0
4

0

0

0
4

0

0

0
4

0

0

0
4

0

0

0

0

0

0

0

0

0
4

0

0

0

0

0

0

0

0
4

0

0

0

0

0

0
4

0

0

0

0
4

0

0

0

0
4

0

0

0

0
4

0

0

0

0
4

0

0

0
4

0
4

7556.69
7

5203.82
7

964.86
7

87.507
7

0.189729
7

0.479243

0

0

0.0599054

0.043992

0

0

0.0112449

0.0581446

0

0.870741
7

0.161857

0.10247

0.0469069

0

0.242785

0.182089

0

0

0.0112449

0

1.05225
7

0

0

0.0898581

0

0.0404642

0.0732585

0.00500636

0

0.0469069

0

0.288991
7

0.0606962

0

0.0809283

0

0

0

0.0703603

0

0.0100127

0.0155008

1.91242
7

0

0.0387519

0

0.536117

0

0

0.536117

0

0

0

0.142527

0

0

1.07223

0

0.0599054

0

0

0.10037

0

0.0155008

0.140721
7

0

0

0.0928501

0.0500031

0.0155008

0

0

0.0232512

0.0633753

0

2.20385
7

0.0469069

0.0823951

0

0.0599054

0

0.0155008

0

0

0

0

0.769075
7

0.0280942

0

0

0

0

0.0599054

0

0.0155008

0.714822

0

0.0890175

0

0

0

0.178259

0

0.0337346

0.0404642

0.0366292

0.0469069

0

4.51248
7

0.0559649
8

0

0.0387582

0

0.00939633

0

0.0633753

0

0

0

0.00500636

0.112348
7

0.0599054

0

0

0

0

0

0.0404642

0

0

0

1.02153
8

0

0

0

0

0

0

0

0.0633753

0

0

1.4756
7

0.0599054

0

0

0.0581373

0

0.0404642

0.32948

0

0.0155008

0.0469069

0.101103
7

0.0661607

0

0.0150191

0

0.0606962

0.255763

0

0

0

0

2.08986
7

0

0

0.0469069

0

0

0.0469069

0.164174

0

0

0

4.24879
7

0.0155008

0.0404642

0

0

0.0469069

0.110907

0.0155008

0

0

0

1.19438
7

0

0

0

0.0112449

0

0

0

0

0.0404642

0

1.31814
7

0.0938138

0.0599054

0.0469069

0

0.0809283

0

0.0155008

0

0

0.140721

0.756622
7

0.0599054

0

0

0

0

0

0

0

0

0

25.9886
7

0.0492354
7

0

0

0

0

0.0112449

0.357411

0.0809283

0.0606962

0

0.0469069

7.46001
7

0.0155008

0.0404642

0

0

1.70996

0

0

0.0703603

0

0

0.107279
7

0.0469069

0

0.357411

0.137664

0.187061

0

0

0

0

0.00690984

3.12039
7

0

0

0.0938138

0

0

0

0

0

0

0.0155008

0.209045
7

0

0.0168673

0

0

0

0.0703603

0

0

0

0

0.188815
6

0

0

0.357411

0

0

0.0606962

0

0.00350607

1.70996

0

0.730958
7

0.0599054

0

0.0606962

0

0

0.0155008

0

0.007332

0.0404642

0

0.617207
7

0.0404642

0

0.0469069

0

0.0100127

0

0.0155008

0.0404642

0

0

0.154968
7

0

0

0.599054

0

0

0.110907

0

0

0

0.187628

0
7

0

0

0.10116

0

0

0

0.161857

0.0112449

0

0

114.503
7

0.294034
7

0.0316877

0.0599054

0

0

0.0112449

0

0

0

0

0.0232512

0.180679
7

0

0.117267

0

0

0.140721

0

0

0

0

0

0.546482
7

0

0

0.0387519

0.0809283

0

0

0

0.0112449

0.0938138

0

0
7

0.0224897

0.0703603

0

0

0

0.0938138

0.0112449

0

0

0.0404642

37.3393
6

0

0

0

0

0

0.0469069

0.0404642

0.0112449

0

0.0599054

0.0599054
7

0

0.0599054

0

0

0

0

0

0

0

0.0316877

24.8832

0

0

0

0

1.70996

0

0

0

0

0.0404642

0.312411
7

0

0

0.0599054

0

0

0.0938138

0

0

0

0

3.79129
7

0.0387582

0.0387582

0.0599054

0

0.0469069

0

0.0404642

0

0

0

0.179473
7

0

0

0.0155008

0

0

0

0

0

0

0

28.7493
7

1.26708
7

0

0.0469069

0

0

0

0

0

0.0703603

0

0

0.153719
7

0.0469069

0

0

0

0

0.119811

0.0599054

0

0

0

0.0599054
8

0

0.0606962

0

0

0.0168673

0.164174

0.0703603

0

0.0968956

0

0.151488
7

0.536117

0

0.953298

0.0400509

0.0475315

0

0.0112449

0

0.714822

0

2.05301
6

0

0.0112449

0

0

0

0

0

0

0

0

0.00939633
6

0

0

0.0404642

0

0.0155008

0.0898581

0.0316877

0

0

0.0732585

0.667173

0

0

0.0898581

0

0

0

0.119811

0

0

0.0809283

0.207973
7

0.116275

0

0.0898581

0

0.0465023

0.849747

0

0

0

0

0.112405
8

0.0469069

0

0

0

0.0599054

0.0469069

0

0

0.102033

0

0.286529

0

0

0

0

0

0

0

0

0.0404642

0.0155008

9.5184
7

0.187548
7

0.0155008

0

0.140721

0.0599054

0

0

0

0.868044

0

0

0.980899
7

0.0606962

0

0

0.0404642

0

0.0404642

0

0

0

0

2.318
7

0

0

0

0

0

0.00827008

0

0

0.0469069

0.0103648

0.211081
7

0

0.140721

0

0

0

0

0.0155008

1.25094

0

0.007332

0.220971
7

0

0.714822

0

0

0

0

0

0

0.117996

0

0.228898
7

0

0.0809283

0

0

0.0469069

0

0

0

0.0469069

0

2.15784
7

0

0

0

0.0606962

0.0599054

0.239622

0

0

0

0

0.0672777
7

0

0.0469069

0

0

0.0155008

0

0

0

0.0155008

0.0899589

2.6972
6

0

0.0599054

0

0.0599054

0

0.0366292

0

0

0

0

0.684957
7

0

0

0.357411

0

0.0387582

0.310016

0

0

0

0.0506019

12.4133
7

0.145826
7

0

0

0

0.0792192

0

0.0703603

0.117267

0

0.0703603

0

0.109112
7

0

0

0.0469069

0

0

0.0599054

0

0

0.0938138

0

0.346535
6

0.0281121

0.0404642

0

0.357411

0.00690984

0.539429

0

0

0

0.0599054

1.22334
6

0

0

0

0

0.116275

0.00690984

0.599054

0

0

0

0.0809283

0

0.0404642

0

0.0898581

0.0703603

0

0.0404642

0.0310016

0.0606962

0

0
7

0.00500636

0

0

0.0469069

0

0.0562243

0

0.0938138

0

0

0.0366292

0

0

0

0

0.0404642

0.0606962

0

0.0938138

0.182089

0

0.417317
7

0

0

0

0.0599054

0

0.0404642

0

0

0

0

0.233523
7

0

0

0.0938138

0.0578906

0

0

0

0

0

0

0.460593
7

0

0

0

0

0.0599054

0

0.0404642

0

0.00750954

0

5.83241
7

0.964493
6

0.0404642

0

0

0

0

0

0

0

0

0

0
8

0

0

0.0168673

0.0155008

0

0

0

0

0

0

0.266342
7

0

0

0

0

0

0

0

0.357411

0

0

0.161756
7

0.0232512

0.0599054

0

0

0.0112449

0

0

0

0

0

3.71557
6

0

0

0

0.0898581

0

0.0404642

0

0

0.0469069

0

0.131057
7

0

0

0

0

0.0469069

0

0

0

0

0

0.561728
7

0.0475315

0

0

0

0

0

0

0

0

0

0.0947472
7

0.0155008

0

0

0

0.095063

0

0

0

0

0

0.13791
7

0

0

0

0

0

0

0

0

0.135654

0

0.0155008
8

0

0.0469069

0

0.0599054

0

0

0

1.70996

0.0469069

0

17.959
7

0.477222
7

0.0112449

0

0

0.0170056

0.0224897

0

0.00500636

0

0

0

0.0921732
7

0

0

0.0542527

0.0404642

0.0469069

0.0125159

0

0.0112449

0

0

0.126034
8

0

0

0.0155008

0

0

0

0

0.007332

0

0

0.269815
7

0

0

0

0

0

0

0

0

0

0.0898581

0.103117
7

0

1.70996

0.0469069

0

0

0.0542527

0

0

0.0469069

0

0.283249
7

0

0.0404642

0

0.0898581

0

0

0

1.70996

0

0

7.26148
6

0

0.0469069

0.0310016

0

0

0

0

0

0.0469069

0.0703603

0.339214
7

0

0

0

0

0

0

0

0.121392

0

0

1.44669
7

0

0

0

0

0

0

0

0.357411

0

0

0.0471885

0

0

0.10116

0

0

0

0.0112449

0

0

0.0599054

8.38618
7

0.665017
6

0

0.0809283

0.0112449

0

0

0.0703603

0

0.0475315

0

0

0.0703603
7

0

0

0.161857

0

0

0

0

0

0

0

0.17736
6

0

0.021996

0

0

0

0

0

0

0

0.0703603

0.00690984
7

0

0

0

0

0.0703603

0

0

0.00750954

0

0.0366292

0.3315
7

0

0

0.0469069

0

0

0

0

0.891231

0

0

0.43834
7

0

0

0

0.162758

0

0

0

0

0

0

0

0

0

0

0

0

0.0112449

0.0224897

0.0703603

0

0.0549439

0.0938138
7

0

0.0898581

0

0

0

0

0

0.0938138

0.10116

0

0.231426
7

0.164174

0

0.0599054

0

0

0

0

0

0

0

0.449291
7

0

0

0

0.0404642

0.0404642

0.0224897

0

0

0

0.10116

618.181
7

25.9952
7

1.8702
7

0

0

0.007332

1.70996

0

0.0703603

0

0

0.0155008

0

29.0646
6

0

0

0

0

0

0.0404642

0.0150191

0

0

0

0.0633753

0

0

0.0170056

0

0

0.149764

0.0449794

0.0232512

0

0.117267

1.19842
7

0

0.0168673

0

0.0310016

0

0

0

0

0

1.24303

1.07451
7

0

0

0

3.41991

0.224897

0

0

0

0

0

0.234
7

0.0469069

0

0.0599054

0

0

0

0

0.0310016

0

0

3.94027
7

0

0.0155008

0

0.0606962

0

0

0

0

0

0

3.05383
8

0

0

0

0.0155008

0

0

0.0599054

0

0

0

0.0657026
7

0

0

0

0.0475315

0

0.0469069

0.0316877

0

0

0

0.149764
7

0.0316877

0

0

0

0

0

0.0852543

0

0

0

3.21437
7

0.0856651
7

0

0

0

0

0

0

0

0

0

0

0.596316
7

0

0

0.0404642

0

0

0

0

0.0404642

0.0404642

0

0.0559649
7

0

0.0232512

0.0606962

0

0.0469069

0

0

0

0

0

0.0475315
7

0

0

0

0

0

0

0.0404642

0.0281121

0

0.0155008

0.130322
7

0.0316877

0

0

0.0404642

0.0703603

0

0

0

0.0599054

0

0.187628
6

0.0316877

1.96576

0.0155008

0

0

0

0

0

0

0

2.61184
6

0.0606962

0

0.0606962

0

0.0469069

0

1.42964

0

0.0703603

0

5.41635
6

0

0.0606962

0

0.0599054

0.0316877

0.0232512

0.140721

0

0.0404642

0.0938138

0.694555

0

0

0

0

0

0

0

0.357411

0

0

0.0404642
7

0.0599054

0.095063

0

0

0.0606962

0

0

0

0

0.0232512

0.348558
7

1.97293
7

0

0.0599054

0

0

0.48557

0.0366292

0

0

0.119811

0

0.357411
7

0

0

0.0404642

0

0.0155008

0

0

0

0

0

1.79598
6

0

0

0

0

0

0

0

0

0.0599054

0.00750954

0.117267
7

0

0.0465023

0

0.0316877

0

0

0

0.0599054

0

0

0
7

0

0.0175223

0

0.0112449

0

0

0.0606962

0.0599054

0

0.0469069

0.0719411
7

0

0.0465023

0.0898581

0

0

0

0

0

0

0

0.0581446
7

0

0.0599054

0

0.121392

0.0938138

0.0606962

0

0.0281121

0

0.0469069

0.347267
7

0

0.0155008

0

0

0.0606962

0.0599054

0

0

0

0

0.205674
7

0

0

0.0404642

0.00500636

0

0

0

0

0

0

0.0112449
8

0.0232512

0.00500636

0.0404642

0

0

0

0.536117

0

0

0.144547

35.0112
7

1.7212
6

0

0.0620031

0.893528

0.0404642

0

0

0

0.0898581

0.357411

0

0.0112449

0

2.56494

0

0

0.149764

0

0

0.0898581

0.0366292

0

0.975153
8

0

0

0

0

0

0

0.0316877

0

0.257988

0

0
7

0

0

0

0.182089

0

0

0.0599054

0

0

0

0.0155008
8

0.0599054

0.0898581

0

0.0404642

0.0155008

0.0599054

0

0

0

0

0.0783774

0

0

0

0

0.0599054

0

0

0.0404642

0

0.0112449

4.00684
6

0.0112449

0.10116

0

0

0.0112449

0.179716

0.0809283

0

0

0

0
7

0

0.0599054

0

0

0.0938138

0.0469069

0

0

0

0

0.34389
7

0.0366292

0

0

0

0

0

0.0465023

0

0

0

1.71747
6

0

0

0

0

0

0

0.0703603

0

0.0155008

0.0469069

19.2755
7

0.318398

0

0.0112449

0

0.0170056

0.0140945

0

1.70996

0

0.00690984

0

0.257223
7

0

0

0

0

0

0

0.161857

0

0

0

0.716624
6

0

0.0232512

0

0.0469069

0

0.0404642

0

0.0606962

0.0155008

0.00690984

0.0351937
7

0.00525911

0

0

0.0703603

0.0168673

0

0.00690984

0

0

0.0232512

0.141908
7

0

0.00500636

0.0599054

0

0

0

0

0

0

0

0

0

1.42964

0

0.0232512

0

0

0

0.0404642

0

0

2.30335
7

0

0.0703603

0

0

0

0

0

0

0.0469069

0.0404642

0.0809283
7

0

0

0.0703603

0

0

0

0.0618467

0

0

0.0599054

0.073248
8

0

0.0775039

0

0

0.0703603

0

0

0.0387582

0

0.0366292

0.582282
7

0

0.0232512

0

0

0

0

0

0

0

0.0599054

23.7866
7

0.0477962

0

0.0469069

0.164174

0.0469069

0

0

0

0

0

0

0
7

0

0

0.0454854

0

0.0404642

0

0.0469069

0

3.41991

0

0.0205071
7

0.0775164

0

0.0316877

0.0469069

0.928534

0

0

0.0599054

0

0

0.313407

0.0809283

0.0703603

0

0

0

1.70996

0

0

0

0

0.444782
7

0

0

0

0

0.0404642

0

0

0.0469069

0

0

0.0404642

0.0155008

0.0224897

0

0.0938138

0

0.0155008

0

0.0599054

0

0.116275

1.90432
6

0

0

0

0

0

0

0

0.0852543

0

0

0.0112449
7

0

0.121392

0

0

0.0366292

0

0.0387519

0

0

0

0.422162

0.0404642

0

0

0

0

0

0

0.0155008

0

0

0.693149
6

0

0.0606962

0.141625

0

0.0122368

0

0

0

0

0

10.2073
7

0.10116

2.14447

0

0.357411

0.0387519

0.0475315

0

0

0

0

0

0.147995
7

0

0.0469069

0

0.0225286

0

0

0

0.0469069

0

0

0
8

0.0599054

0.117267

0.164174

0

0.257988

0

1.70996

0

0

0.0475315

0.770008
7

0

0.0387519

0

0

0.0775164

0.0469069

0.0155008

0

0

0

0.0112449
7

0.0155008

0

0

0.0112449

0.0366292

0

0

0

0.182089

0

5.87281
6

0

0.015296

0

0.0809283

0

0.0968956

0.0633753

0

0

0.0404642

0.0155008
7

0

0

0.128202

0.0232512

1.78706

0

0

0

0

0

0.219775
7

0

0.0599054

0.00500636

0

0

0

0

0

0

0.0938138

0

0

0.00500636

0

0

0

0

0

0.0581373

0

0

0.00500636

0

0

0

0

0

0.0168673

0.357411

0

0

0

7.39011

8.06365
7

0

0

0

0

0

0.00500636

0

0

0

0

0
7

0.23809

0

0

0

0

0.357411

0

0.0168673

0

0

0.132161
7

0

0

0

0

0

0

0

0

0

0

0.346434
6

1.52759

0

0

0.0232512

0

0

0

0

0

0

0.606477

0

0

0.357411

0

0

0

0.0155008

0.0792192

0

0

0.313851
7

0.357411

0

0

0

0.0112449

0

0

0.0280942

0

0

0

0

0

0

0.893528

0

0

0

0.0316877

0

0

0.0599054

0

0.0599054

0.0898581

0

0

0.0404642

0.117267

1.70996

0

0

0.0898581

0.142595

0

0.0232512

0

0

0.187628

0

0

0

0

0

0

0

0.0103648

0.00500636

0.0404642

0

0.0469069

0.0475315

0.0232512

0

6.21109
7

0

0.00500636
7

0

0
7

0.0471885
7

0.132057
7

0.194974
7

0.0404642
8

0.162018

0.753581
7

14.7079
7

0.0792224
7

0.141221
6

0.0124051
7

0.150812

0.130266
7

0.452474
7

0.0950228
7

1.19677
6

0.307551
6

0.106812
8

489.428
7

788.886
6

0.0898581

0.007332

0.155033

2.59662
6

0
7

0.0469069

0.586336

0.10116

0
8

0.536117

12.863
7

0.251715

0.099241
7

0

0

0.248324
7

0.0232512

0

0.181658
8

2.79739
7

0.417317
7

0.453172
7

0.581491
6

0.114063
6

0

0

0.979389
6

0
7

0.222553
7

0.0469069

0

0
8

0.445478
7

0
7

4.3218
6

0.0754062
7

0.0599054
7

0.106812
7

17.2951

0.501565

0.0469069

0.00500636

0.0383336

0.752388
7

0.00750954
8

0.150554
7

9.30368
6

0

0.0469069

0

0

0

0

0.0112449
7

0.381599
7

0

0

0.588432
6

0.0404642

0

0.182225

0.048555

0

0

0.143022

8.18447
7

0.117611
6

6.04276

0.373354
7

0
7

0.0869245

0.147662

0

0.0112377

0.134938

0

139.886
5

0.122859

0

0.428699

0.0732585

1.07886

0

0.0465023

0

0.0599054

0.00690984

13.7182
7

0.121392

0

0

0

0.396163

0.0469069

0

0.146556

0.209669

0.039357

37.5429
7

0

0.0469069

0.0695127

0

7.77574

0.282345

0

5.07149

0.0404642

0.0938138

528.931
7

3.57312
7

0.0898581

0.119279

0

0.0155008

0.0938138

0

0

0.119811

0.0232512

0.0112449

23.7201
7

3.35332

0.0469069

0

0

0

0.0232512

0.0404642

0

0.0873711

0.149764

1.57467
7

0.127406

0.0840898

0.10037

0

0.0809283

0.536117

0.236067

0.189313

0

0.357411

4.69828
7

0.0155008

0.0630425

0.430432

0.0898581

0.149764

0.0112449

0.0649118

0

0

0.0155008

47.2069
7

0.0606962

0

0

0

0.0457641

0

0.0404642

0.0168673

0.204707

0

0.148192
7

0

0

0.625975

0

0

0

5.00376

0.0112449

0.438495

0.0155008

9.30709
6

0.357411

0.695758

0

0.057196

0

0

0.0155008

7.32693

0

0.0316877

0.451407
7

0

0.0404642

0.00827008

0.283249

0

0.343445

0.248699

0.147257

0

0.146339

3.13492
7

0

0.181298

0

0.0454705

0.0469069

0

0.357411

0

0.10116

0.0968956

0.274433
7

0

0.0387519

0

0

0.0703603

0.755287

0.0898581

0

0.0649118

0

13.3972
7

0.77707
7

0.0873711

0.0469069

0.0150191

0

0.0190069

0

0.142595

0.0858611

0.0155008

0.0469069

68.1838
7

0.00305919

0.106812

0

0.105359

0.0775039

0.179716

0.0469816

0

0

1.84022

1.71009
7

0.299527

0

0

0

0

0.0856651

0

0.0599054

0

0

0.259972
7

0.0469069

0.0155008

0.0775164

0.0387519

0.0703603

1.42964

0.0469069

0.0599054

0

0

1.82418
7

0.13739

0.0599054

0.0112449

0

0

0.0366292

0.0316877

0.914685

0.596701

0.0639125

0.791738
7

0

0.0922289

2.80545

7.69481

0.563381

0

0.269574

0.0733881

0

0

2.34689
7

0

0.773308

0

0

0.0248102

0.0469069

0.0112449

0.340035

0.0155008

0.0316877

3.00151
7

0.357411

0.357411

0.40912

0.0207295

0

1.70996

0

0

0.0112449

0

1.80141
7

0

3.06618

0.110907

0.0703603

0.236341

0.357411

0.10116

0.164174

0.164174

0.0155008

1.19343
7

0

0.0276393

0

0

0.0112449

0

0

0

0.404642

0.0140945

16.9783
7

2.40318
7

0

0.0809283

0.0938138

0

0

0.00690984

0.106725

0.0168673

0

0

9.60638
7

0.0404642

0

0.0155008

0

0.522847

0.0938138

1.76986

0

0.0898581

0

3.78893
7

0.0404642

0.0548327

0.0232512

0.134278

0

0

0

0.0155008

0

0.160275

5.84853
7

0

0.117267

0.0155008

0.0310016

0

0

0.835644

0.211251

0

0

0.436739
7

0.0404642

0

0.0404642

0.0915731

0

0.0404642

0

0

0.0469069

0

0.206782
7

0

0.182089

0

0.0404642

0

0

0.0170056

0.00690984

0

0

0.268536

0.0606962

0

0.0103648

0.0469069

0

0

0

0

0

0.242599

2.19317
7

0.0569196

0

0

0

0.0595195

0

0

0.0404642

0.0404642

0

0.135312
8

0

0.357411

0

0.0168673

0

0

0.0599054

0.422162

0

0

0.223732

0.351802

0.357411

0.0232512

0

0

0.00350607

0

0.0858611

0.0469069

0

11.4162
7

0.27015

0

0

0.136678

0.0232512

0

0.0155008

0

0.0581373

0

0.00750954

0.299424

0.0968956

0

0.0938138

0

0

0.0873711

0.0606962

0.0898581

0

0

4.30284
7

0.10116

0.0475315

0.0404642

0

0

0.0125159

0

0.234534

0.0232512

0.0898581

0.0469218
7

0

0.0599054

0.0168673

0

0.0542527

0

0

0.0280942

0

0

0.341787
7

0.0112449

0

24.7944

0.536117

0.309982

0.217011

0

0.0469069

0.0986636

0

2.24892
7

0.364408

0.0469069

0

0

0.0168673

0.062249

0.143062

0.16447

0

0.0262639

0.444904
7

0

0

0.404318

0

0

0

0

0

0.187628

0

1.03135
7

0.0915731

0

0.0620031

0

0.117267

0.357411

0.0316877

0

0

0.124817

0.291053
7

0.0714657

0

0.106812

0.139507

0

0.0103648

0.00701215

0.095063

0

0.105059

0.155162

0

0

0

0.135654

0.0112449

0.00500636

0.160275

0

0

0.0599054

22.6922
7

10.8803
7

0

0

0.141625

0

0.0255083

0

0.155033

0

0.0404642

0

0.111414
7

0

0.0316877

0

0.0310016

0.136765

0

0

0.0599054

0.0232512

0.00827008

0.25432
7

0.0938138

0.0809283

0

0.0599054

0

0

0

0

0

0

0.187628
7

0.0935306

0

0

0.117267

0

0.0316877

0

0.140721

0

0.0138197

14.1517
6

0

0.0155008

0.0469069

0.0112449

0.0809283

0.83209

0

0

0

0

0.592646
8

0

0.0404642

0

0.0309618

0.0703603

0

0

0.0232512

0

0.0668153

1.11551
7

0.209669

0

0.0366292

0

0

0

0.0387519

0.0155008

0

0.0703603

0.736386
7

0

0.241994

0.130322

0

1.78706

0

0

0.357411

0

0

0.632958
7

6.4334

0.0170056

0.119811

0

0.0703603

0

0

0.351599

0

0

0.0967726
7

0.0404642

0.164832

0.0170056

0.00500636

0.536117

0

0

0.0404642

0

0.417317

57.2257
7

0.493307

0.357411

0

0

0

0

0

0.182561

0.0155008

0

0.397875

0.0253797
7

0

0

0.0599054

0.0404642

0

0.0674692

0

0.0224897

0

0.0711503

0.177079
8

0.161857

0.00750954

0.583024

77.0221

0.0898581

0.161857

0

0

0

0

0.136063
7

0.0404642

0

0

0.0107072

0

0.0224897

0

0

0.19667

0

0.795085
7

0.0155008

0

0.0155008

0.0112449

0

0.0938138

0

0.0471885

0.893528

0.583024

1.44553
7

0.0809283

0.357411

0.160275

145.466

0

0.0469069

0.0775039

0

0

0.140834

8.74253
6

1.69495

0

0

0.140721

0.0475315

0

0

0.445106

0

0.914685

2.69622
6

0

0

0

0

29.9243

0

0.0112449

0

0.157726

0.0944384

3.43598
7

0.0606962

0

0

0

1.82089

0.0232512

0

0.415411

0

0.0150191

0.635025
7

0

0

0.161857

3.41991

0.0938138

0

0.0633753

0.140834

0

0

1.921474090949e-11
7

0
4

167.407
6

167.407
6

0
4

160.176

18.9083
7

60.5421
5

1.16287

0.223531

0

1.75707

1.04938

0.116799

0.129864

0.397875

0.04839

0.0404642

42.553
5

0.0918626

0.0400388

0

0.714822

0.0404642

1.75749

0

0.0475315

0

0.00835115

2.94043
6

1.25094

0

0

0.0633753

0

0.0250534

0.00835115

0

0

0

0

0

0

0

0

0

0

0.0599054

0

0.190126

1.70996

20.1931
5

0.0316877

0.0208779

0.00835115

0

0.0387582

0

0.82388

0.0316877

0.0125267

0.00835115

0

0

0

0.0316877

0

0.0250534

0

0

0

2.01587
6

0

1.05538
6

0
4

0

0

0

0

0

0

0
4

2.86042
7

0

0

0

0

0

0

0

0

0

2.56494

0

0

0

0.0599054

0

0

0

0

0

0

0.039357

0.196223

0

0

0

0

1.94289029309402e-16
7

0
4

1385.18
7

98.7357
7

24.4859
7

8.63824
7

10.6476
6

1242.07
7

0.0469069

0.0138197

0.0404642

0.158569

0.0404642

0.161857

0.0316877

0.110907

0

0

0

2.21711538017644e-13
7

0
4

500.276
7

497.636
7

2.45725
7

0.142595

0.0404642

0

0

0
4

86.4419
7

7.33585

0.957717
7

0

0

0.0170056

0

0.0155008

0.0328872

0.0155008

0

0

0

0.848449
6

0.357411

0

0

0

0

1.70996

0.0232512

0

0.0469069

0

0.418521

0.0465023

0

0

0

0

0.0404642

0

0.0404642

0

0.110907

0.164876
7

0

0.0232512

0

0.0703603

0.0155008

0

0

0

0

0.0581518
7

0.148067

0.0689921

11.1472
6

0

2.56494
7

5.78784
6

0

0.0852543

0

0.801015

0.326883

0

0.940435

1.36185

0.10831

1.72546

3.43518
6

0

0

0

0

0

1.31164

0.0155008

0

0

0.0155008

29.2499
6

0

0

0

0.0545587

0.417317

0.010998

0

0

0

0.0155008

0.761729

0

0.139507

0.0232512

0.0228328

1.70996

0.232512

0.0232512

0

0

0.357411

8.46603
7

0.0599054

0.0469069

0

0

0

0.0404642

0.0599054

0

0

0

0.0469069
7

0

0

0

0

0

0

0

0.357411

0.0316877

0

1.7836
7

0

0

0

0

0

0

0.211081

0

0.0469069

0.0155008

0

0

0

0

0

0

0

0

0

0.0633753

0

6.86117829218347e-14
7

0
4

15.1538
7

9.3688
7

0.429609
7

0.140067
7

0.085465
7

0

0

0

5.12987

0

8.88178419700125e-16
7

0
4

0.664231
7

0.664231
6

0

0
4

1.75014
7

0.161857

1.58829

0
4

0.16837
7

0.112405

0.0155008

0

0.0404642

0
4

0.0274961
7

0.00500636

0

0.0224897

3.46944695195361e-18
7

0
4

0

0

0

0
4

6.88607

0.0358731

6.83983

0.0103648

0

0

2.9316826744008e-16

0
4

0

0

0

0

0
4

0

0

0

0

0
4

0

0

0
4

0.272089

0.272089

0
4

7.76144

7.53363

0.10116

0.0809283

0

0.0404642

0

0.00525911

0

0

5.3776427755281e-16

0
4

0

0

0

0
4

0.0873711

0.0873711

0
4

0.112616

0.0721518

0.0404642

0
4

0

0

0
4

0

0

0
4

0.0404642

0.0404642

0
4

0.0469816

0.0469816

0
4

0.0232512

0.0232512

0
4

0.110907

0

0.110907

0
4

0.134278

0.134278

0
4

1.70996
3

1.70996
3

0

0

0

0

0

0

0

0

0
4

0.953434

0.893528

0.0599054

0
4

0.0469069

0.0469069

0

0
4

0

0

0

0
4

0.0599054

0.0599054

0
4

0

0

0
4

0

0

0
4

0

0

0
4

0.0170056

0.0170056

0
4

0

0

0
4

0

0

0
4

1.26694
7

1.15617
7

0.100755

0

0.00500636

0.00500636

0

0

2.60208521396521e-16
7

0
4

0

0

0
4

0.0170056

0.0170056

0
4

0

0

0
4

0.0155008

0.0155008

0
4

0

0

0
4

0

0

0
4

0

0

0
4

0

0

0
4

0

0

0
4

0.0387582

0.0387582

0
4

7.64647
7

7.39855
7

0.0821109

0

0.141625

0.0241844

0
4

0

0

0
4

0.0316877

0.0316877

0
4

0

0

0
4

0

0

0
4

0

0

0
4

0.007332

0.007332

0
4

0

0

0
4

0

0

0
4

0

0

0
4

0.0404642

0.0404642

0
4

2.41796
7

2.25611
7

0.161857

0

0

1.38777878078145e-16
7

0
4

0.0404642

0.0404642

0
4

0.358541
7

0.108505
7

0.234534
7

0.0155008

0
4

0.902363
7

0.902363
7

0

0
4

1.70996
3

1.70996
3

0

0
4

1.49200651833326e-11
7

0
4

112.772

0
2

0
2

0

0

0

0

0

0

0

0

0

0

0

0
4

112.772

112.772

0

0

0

0
4

0
2

0
2

0
4

0

0

0

0
4

0

0

0
4

0

0

0
4

0
4

23.014

9.3472

0.187628
3

0

0

0

0

0

0

0

0

0

0

0
2

0

0

0

0

0

0

0.492522

0

0

0

0
2

0

0

0

0

0

0

0

0

0

0

0
2

0

0

3.41991

0

3.53718

1.70996

0
2

0
4

0.0469069

0.0469069

0

0
4

0

0

0

0
4

0

0

0

0
4

0

0

0

0
4

0

0

0
4

0

0

0
4

0.0703603
3

0
3

0

0

0

0

0

0

0.0703603

0

0

0

0

0

0

0

0

0

0

0

0

0

0

0

0

0

0

0
4

10.8095

10.0008

0

0

0

0
2

0.808723
3

0

0

0

0

0

0

1.11022302462516e-15

0
4

2.65479

0
3

0

0

0.0898581

2.56494

0

0

0

0

0

0

0
4

0

0

0

0

0
4

0
2

0

0

0

0

0

0
4

0
2

0

0

0

0

0

0
4

0

0

0

0
4

0.0852543

0.0852543

0
4

0
4

0.234534
2

0.234534
2

0.234534

0

0

0

0

0

0

0

0

0

0
4

0

0

0

0

0

0

0

0
4

0

0

0

0

0

0

0

0

0

0

0
4

0

0

0

0
4

0

0

0

0

0
4

0

0

0

0
4

0
4

0

0

0

0
4

0

0

0
4

0
4

0

0

0

0

0
4

0
4

0.0732585

0.0366292

0.0366292

0
4

0.0366292

0.0366292

0
4

0
4

0.424874

0.424874

0.424874

0
4

0
4

0

0

0

0
4

0

0

0
4

0
4

0

0

0

0
4

0

0

0
4

0
4

0.202321

0.202321

0.202321

0
4

0
4

0

0

0

0
4

0
4

0

0

0

0
4

0
4

0.359433

0.359433

0.359433

0
4

0
4

12.6199

10.2597

10.2597

0

0

0

0

0

0

0
4

0
3

0
2

0

0

0

0

0

0

0

0

0

0

0
4

2.3602

0

2.3602

0

0

0

0

0
4

0

0

0

0

0

0
4

0

0

0
4

0

0

0
4

0

0

0
4

0

0

0
4

0

0

0
4

0
4

0.00701215

0.00701215

0.00350607

0.00350607

0
4

0
4

0

0

0

0
4

0
4

0.933992

0.893528

0.893528

0
4

0.0404642

0.0404642

0
4

0
4

0

0

0

0
4

0
4

0

0

0

0

0
4

0
4

0.10116

0.10116

0.10116

0
4

0
4

0

0

0

0
4

0
4

0

0

0

0
4

0

0

0
4

0
4

0

0

0

0
4

0

0

0
4

0
4

0

0

0

0

0
4

0
4

94.4661

24.9015

23.9004

0.237078

0.377138

0.267031

0.119811

0

0

0

9.0205620750794e-16

0
4

19.8756
7

0.0745694

4.38087

15.3872

0.007332

0.010998

0.007332

0.007332

5.82867087928207e-16
7

0
4

49.6291

0

0.007332

49.5888

0

0

0

0.032994

0
4

0

0

0

0

0

0
4

0.0599054

0

0.0599054

0
4

0

0

0
4

0
4

0

0

0

0
4

0
4

0.00525911

0.00525911

0.00525911

0
4

0
4

0

0

0

0
4

0
4

0

0

0

0
4

0
4

0

0

0

0
4

0
4

0

0

0

0
4

0
4

0

0

0

0
4

0
4

0.0316877

0.0316877

0.0316877

0
4

0
4

0

0

0

0
4

0
4

0

0

0

0
4

0
4

0.714822
2

0
1

0

0

0

0

0

0

0

0
4

0.714822

0

0

0

0

0.357411

0

0.357411

0

0

0

0
4

0

0

0

0

0

0

0
4

0

0

0

0

0

0
4

0

0

0

0
4

0

0

0

0
4

0

0

0
4

0
4

0

0

0

0
4

0
4

0.0316877

0.0316877

0.0316877

0
4

0
4

0

0

0

0
4

0
4

0

0

0

0
4

0
4

0.0170056

0.0170056

0.0170056

0
4

0
4

0

0

0

0
4

0
4

0

0

0

0
4

0
4

0

0

0

0
4

0
4

0.0155008

0.0155008

0.0155008

0
4

0
4

0.10116

0.10116

0.10116

0
4

0
4

23.6959

15.2617

0.727057
3

2.56494

0

11.9697

0
4

0

0

0

0

0

0

0

0
4

0.304895

0.304895

0

0
4

0

0

0

0
4

8.12932

8.12932

0
4

0

0

0
4

0
4

0.0606962

0.0606962

0.0606962

0
4

0
4

0

0

0

0
4

0
4

0

0

0

0
4

0
4

0

0

0

0
4

0
4

0

0

0

0
4

0
4

0.0155008

0.0155008

0.0155008

0
4

0
4

0

0

0

0
4

0
4

0

0

0

0
4

0
4

0

0

0

0
4

0
4

0

0

0

0
4

0
4

5.71399
7

5.22028
7

3.0016
7

0.257988

0.109112

1.70996

0

0.0809283

0.0606962

5.68989300120393e-16
7

0
4

0.251715
7

0.251715
7

0
4

0.241994

0.0599054

0.182089

0
4

0
4

0

0

0

0
4

0
4

0

0

0

0
4

0
4

0

0

0

0
4

0
4

0

0

0

0
4

0
4

0

0

0

0
4

0
4

0

0

0

0
4

0
4

0.0232512

0.0232512

0.0232512

0
4

0
4

0

0

0

0
4

0
4

0

0

0

0
4

0
4

0.0175304

0.0175304

0.0175304

0
4

0
4

0
3

0
3

0
3

0

0

0

0

0

0

0
4

0
4

0.0516798

0.0516798

0.0516798

0
4

0
4

0.0606962

0.0606962

0.0606962

0
4

0
4

0

0

0

0
4

0
4

0

0

0

0
4

0
4

1.70996

1.70996

1.70996

0
4

0
4

0.0168565

0.0168565

0.0168565

0
4

0
4

0

0

0

0
4

0
4

0

0

0

0
4

0
4

0.119811

0.119811

0.119811

0
4

0
4

0

0

0

0
4

0
4

6.7656

2.36368
6

1.04848

0.209261

0.950908

0.155033

2.77555756156289e-16
6

0
4

0

0

0
4

0.010998

0.010998

0
4

0

0

0
4

0.718206
7

0.718206

0

0
4

3.46038

0

0

0.0404642

0

3.41991

0
4

0.0123384

0

0.007332

0.00500636

0
4

0.156012

0.13035

0.025662

1.38777878078145e-17

0
4

0.014664

0.007332

0.007332

0
4

0

0

0
4

0.029328

0.007332

0.021996

0
4

0

0

0
4

5.72458747072346e-16

0
4

0

0

0

0
4

0
4

0

0

0

0
4

0
4

0

0

0

0
4

0
4

0

0

0

0
4

0
4

0

0

0

0
4

0
4

0.141625

0.141625

0.141625

0
4

0
4

0

0

0

0
4

0
4

0

0

0

0
4

0
4

0

0

0

0
4

0
4

0

0

0

0
4

0
4

0.314189
7

0.132443
7

0
7

0.100755

0

0

0.0316877

0

6.93889390390723e-18
7

0
4

0.0155008
7

0.0155008
6

0

0

0
4

0.166245

0.111993

0.0542527

0
4

0

0

0

0
4

0

0

0
4

0
4

0

0

0

0
4

0
4

0

0

0

0
4

0
4

0.0366292

0.0366292

0.0366292

0
4

0
4

0

0

0

0
4

0
4

0

0

0

0
4

0
4

0

0

0

0

0

0

0

0

0

0

0
4

0

0

0
4

0

0

0
4

0
4

94.4387

93.2848

60.819

1.60749

0.754439

0.122852

0.153972

0

0

0

0

0

0.029328

2.47169

0

0

0

0

0

0

0

0

0.007332

0

11.5078

0.0898581

0

0

0

0.03666

0.357411

0

0

0.119811

0

0.568492

0.0599054

0

0.007332

0

0

0

0.0599054

0.149764

0

0

0.167469

0

0

0

0

0

0

0.209669

0.007332

0

0

1.14162

0

0.010998

0

12.8247

5.32907051820075e-15

0
4

0.776272

0.249995

0.15218

0.0898581

0.269574

0

0.014664

2.42861286636753e-17

0
4

0.221165

0.117267

0.01833

0

0.0599054

0.025662

0

0

0

0

0

0

0
4

0.156471

0.141807

0.007332

0

0.007332

0
4

0

0

0
4

5.4678483962789e-15

0
4

0

0

0

0

0

0

0

0

0

0
4

0

0

0
4

0
4

0

0

0

0

0
4

0
4

0
4

0
4

0

0

0

0

0

0
4

0
4

0

0

0

0
4

0

0

0

0

0

0
4

0
4

0

0

0

0

0

0

0

0

0
4

0
4

0

0

0

0

0

0

0
4

0

0

0

0

0
4

0

0

0

0

0
4

0

0

0
4

0
4

0

0

0

0

0

0

0
4

0

0

0
4

0
4

0
3

0
3

0
3

0

0

0

0

0
4

0

0

0

0
4

0

0

0
4

0
4

0
8

0
8

0

0

0

0

0

0
4

0

0

0
4

0
4

0
3

0
3

0
3

0
4

0
4

0
3

0
2

0
2

0

0
4

0
3

0

0

0

0
4

0

0

0
4

0
4

2.29558
1

0
1

0

0

0

0

0

0

0

0

0

0

0

0
2

0

0

0

0

0

0

0

0

0
4

0

0

0
4

0

0

0
4

0
1

0
2

0

0

0

0

0

0

0

0

0

0

0
1

0

0

0

0

0

0

0

0

0

0

0
4

0.829397
1

0

0

0.0879839

0

0.205296

0

0

0

0

0.536117

0
4

0

0

0

0

0
4

1.46619

0.597558

0.868629

0
4

0

0

0

0
4

0

0

0

0
4

0

0

0

0
4

0

0

0

0
4

0
4

0
3

0
3

0
3

0

0

0

0

0
4

0

0

0

0

0

0
4

0
4

0

0

0

0

0

0

0
4

0

0

0

0

0

0

0
4

0

0

0
4

0

0

0

0
4

0

0

0

0
4

0

0

0
4

0
4

0

0

0

0

0

0

0
4

0

0

0

0

0

0

0
4

0
4

0.209669

0.209669

0.209669

0

0

0

0
4

0
4

0.328348
2

0.328348

0

0.328348

0

0

0

0
4

0

0

0
4

0

0

0
4

0
4

0

0

0

0

0
4

0

0

0

0
4

0

0

0

0
4

0
4

6.83983

6.83983

0
2

6.83983

0

0
4

0

0

0
4

0

0

0
4

0
4

2.86422
7

0.110825

0

0.110825

0
4

0.0404642

0

0.0404642

0

0
4

2.71293

0.147995

2.56494

0
4

0

0

0
4

0
4

0
2

0

0

0

0

0
4

0
3

0

0

0

0

0
4

0

0

0
4

0
4

0.403914

0

0

0

0

0

0
4

0.403914

0.403914

0

0
4

0
4

25.6761

16.2301
7

14.4047
7

0

0

0

0.0843179

0.0332337

0.00500636

0.161857

0

0.647427

0.893528

0

0
4

0

0

0
4

0.182089

0

0.182089

0
4

0.222553

0.182089

0.0404642

0
4

0

0

0
4

1.67705

0.431899

0.424874

0.152623

0.58673

0

0

0.0404642

0.0404642

0
4

0.154248
1

0

0.07332

0.0809283

0

0

0

0

0

1.38777878078145e-17
1

0
4

0.873134
6

0.197929

0.007332

0

0.667873

0

0

0
4

0.988852
6

0.959524
6

0.029328

4.85722573273506e-17
6

0
4

2.50465

0.263017

1.95194

0.202321

0.0469069

0.0404642

0
4

2.78749
7

0

2.56494

0

0.222553

1.66533453693773e-16
7

0
4

0.0559649

0

0

0

0.0404642

0.0155008

0
4

0

0

0

0

0

0
4

0
4

0
8

0
8

0
8

0
4

0

0

0
4

0

0

0
4

0
4

0
3

0

0

0

0
4

0

0

0
4

0

0

0
4

0

0

0
4

0

0

0
4

0
4

0.428384
7

0.428384
7

0.428384
7

0

0
4

0
4

0.158894
7

0.158894
7

0.0703603

0.0885339

0

0
4

0

0

0
4

0
4

0.040326

0.040326

0.032994

0

0

0.007332

0

0
4

0

0

0
4

0

0

0
4

0
4

0
2

0
2

0

0

0

0

0
4

0

0

0
4

0

0

0
4

0
4

0
4

0

0

0

0

0

0

0
4

0

0

0
4

0
4

0

0

0

0

0

0

0
4

0
4

0.128302
7

0.128302
7

0.128302
7

0

0

0
4

0
4

0
4

0
4

0

0

0
4

0

0

0
4

0

0

0
4

0

0

0
4

0
4

22.9626
7

5.41002
7

3.57511
7

1.49971
6

0.0431401
8

0.127527

0.0316877

0.10116

0.0316877

0

0

6.24500451351651e-17
7

0
4

16.8851
7

15.7909
7

1.09416

0

0
4

0.31351
7

0.0112449
7

0

0

0.302265

0

0
4

0.271313
7

0.0379905
8

0.161857

0.0310016

0

0

0

0.0404642

0
4

0.0112449

0

0

0.0112449

0
4

0.0714657

0.0310016

0.0404642

0

0
4

0

0

0

0
4

0

0

0
4

8.04911692853238e-16
7

0
4

0

0

0

0

0

0

0
4

0

0

0

0

0
4

0

0

0
4

0
4

1.62312

1.62312

1.087

0.536117

0
4

0
4

0.119039

0.119039

0.119039

0
4

0
4

0.536117

0.536117

0.536117

0

0

0

0
4

0
4

0
3

0
3

0

0

0

0
4

0

0

0
4

0
4

0

0

0

0

0
4

0

0

0
4

0

0

0
4

0
4

1.0017

1.0017

0.761325

0.164174

0.0155008

0.0606962

0
4

0
4

0

0

0

0

0
4

0

0

0

0
4

0
4

0

0

0

0

0
4

0

0

0

0
4

0
4

0

0

0

0

0

0
4

0

0

0
4

0
4

0
3

0
3

0
3

0

0

0

0

0
3

0

0

0

0

0

0

0

0
4

0

0

0

0
4

0

0

0
4

0
4

0.0581373

0

0

0

0

0

0

0
4

0.0581373

0.0581373

0
4

0
4

0

0

0

0

0

0
4

0
4

0.0689029

0

0

0

0
4

0.0542389

0.0469069

0.007332

0
4

0.014664

0.014664

0
4

3.46944695195361e-18

0
4

0

0

0

0

0
4

0

0

0
4

0

0

0

0
4

0
4

0

0

0

0

0

0

0
4

0
4

0.128117
8

0.0155008

0.0155008

0

0
4

0.0316877

0.0316877

0
4

0.0809283

0.0809283

0
4

0
4

0

0

0

0
4

0

0

0
4

0

0

0
4

0
4

0.0469069

0.0469069

0.0469069

0

0

0
4

0

0

0
4

0
4

0

0

0

0

0
4

0
4

0

0

0

0

0
4

0

0

0

0
4

0

0

0
4

0
4

12.8211

11.1579

8.70005

0

0

0

0

1.81112

0.0232512

0.536117

0

0

0.0873659

0

0

0
4

0.362934

0.149764

0

0

0

0.21317

0

0

0

0

0

0
4

1.22404

1.22404

0
4

0

0

0
4

0.0606962

0.0606962

0
4

0.0155008

0.0155008

0
4

0

0

0
4

0

0

0
4

5.8113236445223e-16

0
4

3.88248

3.85969

3.85969

0
4

0.0227895

0.0227895

0
4

1.14491749414469e-16

0
4

0

0

0

0
4

0

0

0
4

0
4

0

0

0

0

0
4

0
4

15.3896

13.6797

13.6797

0
4

1.70996

1.70996

0

0
4

0

0

0
4

0
4

0

0

0

0
4

0

0

0

0
4

0
4

0.0123384
7

0.00500636

0.00500636

0
4

0.007332

0.007332

0

0
4

0
4

0

0

0

0
4

0
4

0.076197

0.0606962

0.0606962

0
4

0.0155008

0.0155008

0
4

0
4

0.0325413

0.0325413

0.0225286

0.0100127

0
4

0
4

0

0

0

0
4

0
4

34.4541

31.5504

22.3531

0

0.0155008

7.70605

0

0.164174

0

0.0606962

0

1.25094

0

0
4

0

0

0
4

0

0

0
4

0.207631
7

0.160724
7

0

0.0469069

0

6.93889390390723e-18
7

0
4

2.328
7

0

2.08521

0.0404642

0.161857

0.0404642

8.32667268468867e-17
7

0
4

0

0

0

0

0

0
4

0.31314

0.31314

0

0
4

0.0232512

0.0232512

0
4

0

0

0
4

0.0316877

0.0316877

0
4

0

0

0
4

0
4

0

0

0

0
4

0
4

0

0

0

0

0

0
4

0
4

0

0

0

0
4

0
4

0

0

0

0

0
4

0
4

0

0

0

0

0
4

0

0

0
4

0
4

0

0

0

0

0
4

0

0

0
4

0
4

0

0

0

0

0
4

0

0

0
4

0
4

0.0404642

0.0404642

0.0404642

0

0
4

0
4

0

0

0

0

0

0
4

0
4

0

0

0

0
4

0
4

0

0

0

0

0

0

0

0

0

0

0

0

0

0

0

0

0

0

0

0

0

0

0

0

0

0

0

0

0

0
4

0

0

0

0
4

0
4

0.537128

0.537128

0.537128

0
4

0
4

0.343945

0.343945

0.161857

0.0404642

0.141625

0
4

0
4

0.0873711

0.0469069

0

0.0469069

0
4

0.0404642

0.0404642

0
4

6.93889390390723e-18

0
4

0

0

0

0

0
4

0

0

0
4

0
4

0

0

0

0

0

0
4

0
4

0

0

0

0

0
4

0
4

0.0155008

0.0155008

0.0155008

0

0
4

0
4

0.162758

0.162758

0.162758

0
4

0

0

0
4

0
4

0.182089

0.182089

0.182089

0
4

0

0

0
4

0

0

0
4

0
4

0.0404642

0.0404642

0

0.0404642

0
4

0

0

0
4

0
4

0
3

0
3

0

0

0

0

0

0

0
4

0
4

14.8055
3

14.7886
3

3.44914
3

11.3395

0

0
4

0

0

0
4

0

0

0
4

0.0168565

0.0168565

0
4

0

0

0
4

0

0

0
4

1.15879528195251e-15
3

0
4

139.671

139.671

16.996
7

2.77505
7

0

0

0

0

0.182089

0

0

0

0.0469069

0

0.0155008
8

0.0542527

0.0469069

0.0595195

0

0

0.0703603

0

0.0404642

0

1.43648

0
3

0.0404642

0

0

0.0155008

0.0703603

0

0

0

0

0

0.0935306

0

0

0.0938138

0

0

0.0469069

0

0

0

0

0.0170056

0

0

0

0

0.0469069

0

0.0469069

0

0.0155008

0

0.377394
7

0

0

0.0469069

0.0155008

0.0469069

0

0

0

0

0

13.8781

0

0.0469069

0

0

0.0469069

0.140721

0

0

0

0

0.10727
7

0

0

0.0255083

0

0

0

0

0

0

0

0.60587

0.0310016

0

0.0469069

0

0

0

0.076525

0

0.0232512

0

1.72696

0

0.0469069

0

0.0112377

0

0

0
3

0.0155008
7

0.0340111

0.0935306

6.72718
6

0

0.0703603
7

0.0325063
7

1.18709

0

0.730323

33.909
7

5.12987

0.132566
7

0.0112377

0

0

4.2424

0

0

0

0

9.8467
7

2.11081

0.117267

0.0310016

0.0775039

0

2.67482

6.67274

0

0

0.0703603

6.44383
6

0

0

0

0.273489

0.0469069

0.0703603

0

0.582619

0

0

0
3

5.12987

0.251545

1.17346

0.181185

0

0

0

0

0.0155008

0.117267

9.81592

0.187628

0.219775

0

0

0

0

0

0

0

0.0732585

0
3

0

0

0.0155008

0

0.109315

0

0

0

0

0.0703603

0.629206

0.0469069

0

0.0155008

0

0.0938138

0.68015

0

0

0

0

0
4

0

0

0

0

0

0
4

0

0

0

0
4

0

0

0
4

0

0

0
4

0
4

0.144304
3

0.0809283
4

0.0809283

0

0
4

0

0

0
4

0.0316877

0.0316877

0
4

0

0

0
4

0

0

0
4

0

0

0
4

0.0316877

0.0316877

0
4

0
4

0
4

143.058
3

140.517
3

9.25144

9.07289

0

0.0750954

0

0

0.0125159

0

0

0.0404642

0

0

0

0

0

0

0

0

0

0.00500636

0

0.0454705

0

0
4

45.2541
5

42.553
5

0.931796
6

0

0.00500636

0.0404642

0.0404642

0.112405

0.859653

0.100634

0.182089

0

0.0991568

0.32948

0

0
4

84.3389
3

6.55894

0.664697
3

22.8286

0
4

0.0469069

0

0

0.01833

0

0

0

0.069654

0

0

0
4

0

0.0599054

0

0

0

0

0

0

0

0

0.113646
3

0

0

0

0

0

0

0

0

0

0

0.0172746
3

0

0

0.021996

0

0

0

0

0

0

0.0599054

0.0843179
4

0

0

0.043992

0

0

0

0

0

0.212569

0

0.820082

0

0

0

0.007332

0

0

0

0

0

0

0.0898581
3

0

0

0.299527

0.153972

0.007332

0

0

0.119811

0

0.014664

0.893528

0

0

0

0

0

0

0

0

0

0

0
4

0

0

0

0

0

0

0

1.70996

0

0

0
4

0

0

0

0

0.043992

0

0

0.021996

0

0.007332

0

0
4

0

0.014664

0

0

0.0469069

0

0

0

0.007332

0

0.00690984
3

0

0

0

0

0

0

0

0

0

0.357411

0
4

0

0

0

0

0

0

0.021996

0

0

0

0

0

0

0

0

0

0

0

0.0703603

0

0

8.1156

0

0

0.00500636

0

0

0

0.007332

1.70996

0

0

0
3

0

0

0

0

0

0

0.174412

0

0

0

0.0712022

0

0

0

0.357411

0.007332

1.50134
5

1.47047
6

0.014664

3.36706

0
3

0

0

0

0.208207
3

0

0

0.0138197
4

0

0

4.85918

4.38485
6

0
4

0

0

0
4

0.0404642

0

0

0

0

0
4

0.0599054

0

0

0

0

0

0.010998

0

0

8.54979

0
3

0

0.0606962

0.05499

0

0

0.0521608

0

0

0

0

5.12987

0.0599054

0

0

0

0

0

0

0

0

0

0.65416
3

0

0

0

0

0

0

0

0

0

0

6.4344

0

0

0

1.42964

0

0

0

0

0.01833

0

0
4

0.107603

0.0606962

0.0469069

0
4

0

0

0
4

0.247815

0.247815

0
4

0.10116

0.10116

0
4

0.0404642

0.0404642

0
4

0.244927

0.149864

0.095063

0
4

0.239622

0.149764

0.0898581

1.38777878078145e-17

0
4

0.200626

0.200626

0
4

0.0809283

0.0809283

0
4

0

0

0
4

0.0469069

0.0469069

0
4

0.357411

0.357411

0
4

0.00500636

0.00500636

0
4

0
4

0
3

0
3

0
3

0

0

0

0

0

0

0

0

0

0

0

0

0

0

0

0

0

0

0

0
4

0
3

0

0

0

0

0

0
4

0
3

0

0
2

0

0

0

0

0

0

0

0
4

0
4

0

0

0

0

0

0

0

0

0

0

0

0

0

0

0

0

0
4

0

0

0
4

0
4

0
4

0
4

0
4

0
4

0

0

0
4

0
4

0

0

0

0

0

0

0

0
4

0

0

0
4

0
4

0.111503

0.111503

0.111503

0
4

0
4

0

0

0

0

0

0
4

0
4

0

0

0

0

0
4

0

0

0
4

0

0

0
4

0
4

0

0

0

0

0

0

0
4

0

0

0

0
4

0
4

0.107102

0.107102

0.107102

0

0
4

0
4

0

0

0

0

0

0
4

0

0

0
4

0

0

0
4

0
4

0

0

0

0
4

0

0

0
4

0
4

0

0

0

0

0
4

0
4

0
3

0
3

0
3

0

0

0

0

0

0

0

0
4

0

0

0
4

0

0

0
4

0

0

0

0

0

0

0

0
4

0
2

0

0

0

0

0
4

0
3

0

0

0

0
4

0

0

0
4

0

0

0
4

0

0

0
4

0

0

0
4

0

0

0
4

0
4

0.0606962

0.0606962

0.0606962

0
4

0
4

0.10116

0.10116

0.10116

0
4

0
4

0

0

0

0
4

0

0

0
4

0
4

0.743697

0.743697

0.743697

0
4

0
4

0.0475315

0.0475315

0.0475315

0
4

0

0

0
4

0
4

0

0

0

0

0
4

0

0

0
4

0
4

0

0

0

0
4

0

0

0
4

0
4

0

0

0

0
4

0
4

0

0

0

0
4

0

0

0
4

0

0

0
4

0
4

0.594246

0.594246

0.580426

0.0138197

0
4

0
4

0
3

0
3

0
3

0

0

0

0

0

0
3

0
4

0

0

0

0

0

0

0
4

0

0

0
4

0

0

0
4

0

0

0

0
4

0
3

0

0

0

0

0

0

0
4

0

0

0
4

0

0

0

0
4

0

0

0

0
4

0

0

0

0
4

0

0

0
4

0

0

0
4

0
4

0

0

0

0
4

0

0

0
4

0

0

0
4

0
4

0.0949499

0.0949499

0.0949499

0
4

0
4

0.357411

0.357411

0.357411

0
4

0
4

0

0

0

0
4

0
4

0.207597

0.207597

0.182089

0.0255083

1.04083408558608e-17

0
4

0
4

0

0

0

0

0
4

0
4

0

0

0

0
4

0
4

0.00750954

0.00750954

0.00750954

0
4

0
4

0

0

0

0
4

0
4

0

0

0

0
4

0
4

0.0475315
4

0.0475315
4

0
4

0.0475315
4

0

0

0

0

0

0

0
4

0
4

0
4

0

0

0

0

0

0
4

0

0

0

0

0
4

0

0

0

0
4

0
4

0

0

0

0
4

0
4

0.0599054

0.0599054

0.0599054

0
4

0
4

0

0

0

0
4

0
4

0

0

0

0
4

0
4

0

0

0

0
4

0
4

0

0

0

0
4

0
4

0

0

0

0
4

0
4

0

0

0

0

0

0

0

0

0

0

0

0

0

0

0

0

0
4

0

0

0

0

0

0

0

0

0

0

0

0

0

0

0
4

0

0

0

0

0

0
4

0

0

0

0
4

0
4

0

0

0

0

0

0

0

0

0

0
4

0

0

0

0

0

0

0

0

0
4

0

0

0

0

0

0

0
4

0
4

0
4

0

0

0

0

0

0

0

0

0

0

0
4

0

0

0

0

0

0
4

0
4

0

0

0

0

0

0
4

0
4

0
4

0
4

0

0

0

0

0

0
4

0

0

0

0
4

0

0

0

0
4

0

0

0

0
4

0

0

0
4

0
4

1.04777297948999e-14
3

0
4

0

0

0

0

0

0

0

0

0

0

0

0

0

0

0
4

0

0

0

0

0

0
4

0

0

0

0

0

0
4

0

0

0

0
4

0
4

0
4

0.199782
7

0.199782
7

0.199782
7

0.199782
7

0

0

0
4

0
4

0
4

0.0281014

0.0281014

0.0281014

0.0281014

0
4

0
4

0
4

0

0

0

0

0

0
4

0
4

0
4

0

0

0

0

0

0
4

0
4

0
4

0

0

0

0

0
4

0
4

0
4

0

0

0

0

0
4

0
4

0
4

0

0

0

0

0
4

0
4

0
4

0

0

0

0

0
4

0
4

0
4

0.117267

0.117267

0.117267

0.117267

0
4

0
4

0
4

0.891439

0.891439

0.891439

0.891439

0
4

0
4

0
4

0.283249

0.283249

0.283249

0.283249

0

0
4

0
4

0
4

1.41029
7

1.41029
7

1.41029
7

1.22266
7

0.0469069

0.140721

0
4

0
4

0
4

0

0

0

0

0

0
4

0
4

0
4

0.943078

0.943078

0.943078

0.943078

0
4

0
4

0
4

0

0

0

0

0
4

0
4

0
4

0.0465023

0.0465023

0.0465023

0.0465023

0
4

0
4

0
4

0.00350607

0.00350607

0.00350607

0.00350607

0
4

0
4

0
4

1.44703

1.44703

1.44703

1.44703

0
4

0
4

0
4

0.369287

0.369287

0.369287

0.369287

0
4

0
4

0
4

0.234534

0.234534

0.0938138

0.0938138

0
4

0.140721

0.140721

0
4

0
4

0
4

0

0

0

0

0
4

0
4

0
4

0.0680282

0.0680282

0.0680282

0.0680282

0
4

0
4

0
4

6.49499
7

6.49499
7

6.35427
7

6.35427
7

0
4

0.140721

0.140721

0
4

0
4

0
4

0

0

0

0

0
4

0

0

0
4

0
4

0
4

0

0

0

0

0
4

0
4

0
4

0

0

0

0

0
4

0

0

0
4

0
4

0
4

0

0

0

0

0
4

0

0

0
4

0
4

0
4

1.38936

1.38936

1.38936

1.38936

0
4

0
4

0
4

0.0394338

0.0394338

0.0394338

0.0112449

0.028189

0
4

0
4

0
4

1.40778

1.40778

1.40778

1.40778

0
4

0
4

0
4

0

0

0

0

0
4

0
4

0
4

0.303481

0.303481

0.303481

0.303481

0
4

0
4

0
4

0

0

0

0

0
4

0
4

0
4

0

0

0

0

0

0

0
4

0

0

0

0
4

0
4

0

0

0

0
4

0

0

0
4

0
4

0
4

0

0

0

0

0

0
4

0
4

0
4

0

0

0

0

0

0
4

0
4

0
4

0.536117

0.536117

0.536117

0.536117

0
4

0
4

0
4

0

0

0

0

0
4

0

0

0
4

0
4

0
4

0

0

0

0

0
4

0

0

0
4

0
4

0
4

0

0

0

0

0
4

0
4

0
4

0

0

0

0

0
4

0
4

0
4

0

0

0

0

0
4

0
4

0
4

0

0

0

0

0
4

0
4

0
4

0

0

0

0

0
4

0
4

0
4

0

0

0

0

0

0

0

0

0

0

0
4

0

0

0

0

0

0
4

0

0

0
4

0
4

0
4

0.526034

0.526034

0.526034

0.526034

0
4

0
4

0
4

0

0

0

0

0
4

0
4

0
4

0

0

0

0

0
4

0
4

0
4

0.0170056

0.0170056

0.0170056

0.0170056

0
4

0
4

0
4

0

0

0

0

0
4

0
4

0
4

0.357411

0.357411

0.357411

0.357411

0
4

0
4

0
4

0

0

0

0

0
4

0
4

0
4

0

0

0

0

0
4

0
4

0
4

0

0

0

0

0
4

0
4

0
4

0.269574

0.269574

0.269574

0.269574

0
4

0
4

0
4

3.32393
7

1.52887
7

1.36199

1.36199

0

0
4

0.166877

0.0633753

0.0565951

0.0469069

0
4

0

0

0
4

0

0

0
4

8.32667268468867e-17
7

0
4

1.79506
6

1.37019

1.05509

0

0.315097

0
4

0.384409

0.384409

0
4

0.0404642

0.0404642

0
4

2.77555756156289e-17
6

0
4

0
4

0.0475315

0.0475315

0.0475315

0.0475315

0
4

0
4

0
4

0.0510167

0.0510167

0.0510167

0.0510167

0
4

0
4

0
4

0.102033

0.102033

0.102033

0.102033

0
4

0
4

0
4

0

0

0

0

0
4

0
4

0
4

0

0

0

0

0
4

0
4

0
4

0.400509

0.400509

0.400509

0.400509

0
4

0
4

0
4

0

0

0

0

0
4

0
4

0
4

0

0

0

0

0
4

0
4

0
4

0.0232512

0.0232512

0.0232512

0.0232512

0
4

0
4

0
4

0

0

0

0

0
4

0
4

0
4

2.46863
3

2.46863
3

2.46863
3

2.46863
3

0

0
4

0

0

0
4

0
4

0
4

0

0

0

0

0
4

0
4

0
4

0

0

0

0

0
4

0
4

0
4

0

0

0

0

0
4

0
4

0
4

0

0

0

0

0
4

0
4

0
4

0

0

0

0

0
4

0
4

0
4

0.125159

0.125159

0.125159

0.125159

0
4

0
4

0
4

0

0

0

0

0
4

0
4

0
4

0.202321

0.202321

0.202321

0.202321

0
4

0
4

0
4

0.00500636

0.00500636

0.00500636

0.00500636

0
4

0
4

0
4

0

0

0

0

0
4

0
4

0
4

15.0445
5

15.0445
5

13.3176
5

10.1356
5

3.18198

0

4.44089209850063e-16
5

0
4

1.70996

1.70996

0
4

0.0170056

0.0170056

0
4

0
4

0
4

0

0

0

0

0
4

0
4

0
4

0

0

0

0

0
4

0
4

0
4

0.0404642

0.0404642

0.0404642

0.0404642

0
4

0
4

0
4

0

0

0

0

0
4

0
4

0
4

0

0

0

0

0
4

0
4

0
4

0

0

0

0

0
4

0
4

0
4

0

0

0

0

0
4

0
4

0
4

0

0

0

0

0
4

0
4

0
4

0.076525

0.076525

0.076525

0.076525

0
4

0
4

0
4

0

0

0

0

0
4

0
4

0
4

0

0

0

0

0

0

0

0
4

0

0

0

0
4

0
4

0
4

0

0

0

0

0
4

0
4

0
4

0.0809283

0.0809283

0.0809283

0.0809283

0
4

0
4

0
4

0

0

0

0

0
4

0
4

0
4

0

0

0

0

0
4

0
4

0
4

0.0599054

0.0599054

0.0599054

0.0599054

0
4

0
4

0
4

0

0

0

0

0
4

0
4

0
4

0.0152104

0.0152104

0.0152104

0.0152104

0
4

0
4

0
4

0.121392

0.121392

0.121392

0.121392

0
4

0
4

0
4

0

0

0

0

0
4

0
4

0
4

0.117267

0.117267

0.117267

0.117267

0
4

0
4

0
4

0

0

0

0

0

0

0

0
4

0

0

0
4

0
4

0
4

0

0

0

0

0
4

0
4

0
4

0

0

0

0

0
4

0
4

0
4

0.357411

0.357411

0.357411

0.357411

0
4

0
4

0
4

0.0170056

0.0170056

0.0170056

0.0170056

0
4

0
4

0
4

0.0703603

0.0703603

0.0703603

0.0703603

0
4

0
4

0
4

0

0

0

0

0
4

0
4

0
4

0

0

0

0

0
4

0
4

0
4

0

0

0

0

0
4

0
4

0
4

0

0

0

0

0
4

0
4

0
4

0.0112449

0.0112449

0.0112449

0.0112449

0
4

0
4

0
4

0

0

0

0

0

0

0

0

0

0

0
4

0

0

0

0

0

0
4

0

0

0

0

0
4

0

0

0
4

0
4

0
4

3.69557
7

3.69557
7

3.69557
7

3.69557
7

0
4

0
4

0
4

0.119811

0.119811

0.119811

0.119811

0
4

0
4

0
4

0

0

0

0

0
4

0
4

0
4

0

0

0

0

0
4

0
4

0
4

0.0404642

0.0404642

0.0404642

0.0404642

0
4

0
4

0
4

0

0

0

0

0
4

0
4

0
4

0

0

0

0

0
4

0
4

0
4

0.0599054

0.0599054

0.0599054

0.0599054

0
4

0
4

0
4

0

0

0

0

0
4

0
4

0
4

0

0

0

0

0
4

0
4

0
4

0

0

0

0

0
4

0
4

0
4

0

0

0

0

0

0

0
4

0
4

0
4

0.193791

0.193791

0.193791

0.193791

0
4

0
4

0
4

0

0

0

0

0
4

0
4

0
4

0

0

0

0

0
4

0
4

0
4

0

0

0

0

0
4

0
4

0
4

0.0404642

0.0404642

0.0404642

0.0404642

0
4

0
4

0
4

0.0505696

0.0505696

0.0505696

0.0505696

0
4

0
4

0
4

0

0

0

0

0
4

0
4

0
4

0

0

0

0

0
4

0
4

0
4

0.141625

0.141625

0.141625

0.141625

0
4

0
4

0
4

0

0

0

0

0
4

0
4

0
4

0

0

0

0

0

0
4

0
4

0
4

0.0898581

0.0898581

0.0898581

0.0898581

0
4

0
4

0
4

0

0

0

0

0
4

0
4

0
4

0

0

0

0

0
4

0
4

0
4

0

0

0

0

0
4

0
4

0
4

0.0898581

0.0898581

0.0898581

0.0898581

0
4

0
4

0
4

0

0

0

0

0
4

0
4

0
4

0.0316877

0.0316877

0.0316877

0.0316877

0
4

0
4

0
4

0

0

0

0

0
4

0
4

0
4

0

0

0

0

0
4

0
4

0
4

0

0

0

0

0
4

0
4

0
4

3.89122
7

0.860949

0.860949

0.402377

0.397875

0.0606962

0
4

0
4

3.03027

0.465338

0.465338

0
4

2.56494

2.56494

0
4

0
4

0
4

0

0

0

0

0
4

0
4

0
4

0

0

0

0

0
4

0
4

0
4

0

0

0

0

0
4

0
4

0
4

1.25094

1.25094

1.25094

1.25094

0
4

0
4

0
4

0

0

0

0

0
4

0
4

0
4

0

0

0

0

0
4

0
4

0
4

0

0

0

0

0
4

0
4

0
4

0

0

0

0

0
4

0
4

0
4

0

0

0

0

0
4

0
4

0
4

0.0898581

0.0898581

0.0898581

0.0898581

0
4

0
4

0
4

12.3141
6

12.3141
6

12.3141
6

12.3141
6

0
4

0
4

0
4

0

0

0

0

0
4

0
4

0
4

0

0

0

0

0
4

0
4

0
4

0

0

0

0

0
4

0
4

0
4

0

0

0

0

0
4

0
4

0
4

0.106826

0.106826

0.106826

0.106826

0
4

0
4

0
4

0

0

0

0

0
4

0
4

0
4

0

0

0

0

0

0

0
4

0
4

0

0

0

0

0
4

0
4

0
4

0
3

0
3

0
3

0

0

0

0

0
4

0
4

0
4

17.3963
6

17.3963
6

17.3963
6

0

4.08705

13.3093

0
4

0
4

0
4

0
4

0
4

0
4

0
4

0
4

0

0

0
4

0
4

0
4

0.783667
7

0.783667
7

0.783667
7

0.783667
7

0
4

0
4

0
4

0.317344
1

0.317344
1

0.317344
1

0.303524
1

0

0.00690984

0.00690984

0

4.5102810375397e-17
1

0
4

0

0

0
4

0
4

0
4

1.30938
7

1.30938
7

1.30938
7

1.30938
7

0
4

0
4

0
4

6.32046
6

6.32046
6

6.32046
6

6.32046
6

0

0
4

0
4

0
4

0

0

0

0

0

0

0

0
4

0
4

0
4

1.01803

1.01803

1.01803
7

1.01803
7

0

0
4

0

0

0
4

0
4

0
4

0

0

0

0

0

0

0
4

0

0

0

0

0
4

0
4

0

0

0

0
4

0

0

0
4

0
4

0
4

0.830723
7

0.830723
7

0.830723
7

0.825717
7

0.00500636

0
4

0
4

0
4

1.79429

1.79429

1.79429

0.0843364

1.70996

0
4

0
4

0
4

0

0

0

0

0

0
4

0

0

0

0
4

0
4

0
4

0.794544

0.794544

0.794544

0.620132

0.0775164

0.0968956

8.32667268468867e-17

0
4

0
4

0
4

0

0

0

0

0

0
4

0

0

0

0
4

0

0

0
4

0

0

0
4

0
4

0
4

3.43199
6

3.43199
6

3.43199
6

3.30444
6

0.127542

0
4

0
4

0
4

0.179716
7

0.179716
7

0.179716
7

0

0.179716

0

0

0
4

0
4

0
4

0.629798
7

0.629798
7

0.629798
7

0.629798
7

0

0
4

0
4

0
4

0.855407

0.855407

0.579964

0.182089

0.397875

0
4

0.275443

0.275443

0
4

1.11022302462516e-16

0
4

0
4

0.15765

0.15765

0.15765

0.117185

0.0404642

1.38777878078145e-17

0
4

0
4

0
4

0.0606962

0.0606962

0

0

0

0

0
4

0.0606962

0.0606962

0
4

0

0

0
4

0
4

0
4

0.0143885

0.0143885

0.0143885

0.00611838

0

0.00827008

0

0
4

0
4

0
4

0

0

0

0

0
4

0
4

0
4

0.529841

0.529841

0.529841

0.529841

0

0
4

0
4

0
4

1.40321
7

1.40321
7

1.40321
7

1.40321
7

0
4

0
4

0
4

0

0

0

0

0

0

0

0
4

0
4

0
4

8.31729
5

8.31729
5

3.66798
5

3.61221
5

0.0557638

0
4

2.92235

2.92235

0
4

1.70996

0

1.70996

0
4

0.0170056

0.0170056

0
4

1.59594559789866e-16
5

0
4

0

0

0

0

0

0
4

0
4

0
4

1.42964

1.42964

1.42964

0

1.42964

0

0

0
4

0
4

0
4

4.08485
6

4.08485
6

4.08485
6

4.08485
6

0
4

0
4

0
4

0

0

0

0

0

0
4

0

0

0
4

0
4

0
4

0

0

0

0

0

0

0
4

0
4

0
4

0

0

0

0

0
4

0

0

0

0
4

0
4

0
4

0.304895

0.304895

0.304895

0.304895

0

0
4

0

0

0

0
4

0
4

0
4

0

0

0

0

0

0

0

0
4

0
4

0
4

0

0

0

0

0

0
4

0

0

0
4

0
4

0
4

0

0

0

0

0
4

0

0

0
4

0

0

0
4

0

0

0
4

0
4

0
4

0.182089

0.182089

0.182089

0

0.182089

0

0
4

0
4

0
4

0.510841
7

0.510841
7

0.510841
7

0.479153
7

0.0316877

0

0
4

0
4

0
4

0

0

0

0

0

0

0
4

0
4

0
4

0

0

0

0

0

0
4

0
4

0
4

0
3

0
3

0

0

0
4

0

0

0
4

0
4

0
4

0

0

0

0

0
4

0
4

0
4

1.42021

1.42021

1.42021

1.42021

0
4

0
4

0
4

0

0

0

0

0
4

0
4

0
4

0.00500636

0.00500636

0.00500636

0

0.00500636

0
4

0
4

0
4

0.373843

0.373843

0.373843

0.373843

0
4

0
4

0
4

0.234133

0.234133

0.234133

0.226623

0.00750954

0
4

0
4

0
4

0

0

0

0

0

0
4

0
4

0
4

0

0

0

0

0

0

0

0

0

0

0

0

0
4

0

0

0

0

0

0

0
4

0

0

0

0
4

0

0

0

0
4

0
4

0
4

2.09589

2.09589

2.09589

0.929845

0.0938138

1.07223

0
4

0
4

0
4

0

0

0

0

0

0
4

0
4

0
4

0

0

0

0

0
4

0
4

0
4

0

0

0

0

0

0

0
4

0
4

0
4

0

0

0

0

0
4

0
4

0
4

0

0

0

0

0
4

0
4

0
4

0

0

0

0

0

0
4

0

0

0

0
4

0
4

0
4

0.2025

0.2025

0.2025

0.2025

0
4

0

0

0
4

0
4

0

0

0

0
4

0
4

0
4

1.09607

1.09607

1.09607

1.09607

0

0
4

0
4

0
4

5.12987

5.12987

5.12987

5.12987

0
4

0
4

0
4

1.80721
7

1.80721
7

1.80721
7

1.31489
7

0.0393319

0.357411

0.0843364

0.0112377

0
4

0
4

0
4

0

0

0

0

0

0
4

0

0

0
4

0
4

0
4

0.894539

0.894539

0.894539

0.894539

0
4

0
4

0
4

2.20248

2.20248

2.20248

2.20248

0
4

0
4

0
4

0

0

0

0

0
4

0
4

0
4

0.0656434

0.0656434

0.0656434

0.0656434

0

0
4

0
4

0
4

1.60835

1.60835

1.60835

1.60835

0
4

0
4

0
4

0.898357

0.898357

0.898357

0.888344

0.0100127

0
4

0
4

0
4

0

0

0

0

0

0

0
4

0
4

0
4

0.516667

0.516667

0.491159

0.0792192

0.41194

0
4

0.0255083

0.0255083

0
4

1.04083408558608e-17

0
4

0
4

0

0

0

0

0
4

0

0

0
4

0
4

0
4

7.63574
6

7.63574
6

4.04911

1.58042

2.46869

0

0

0

8.88178419700125e-16

0
4

0.166718

0

0.0469069

0

0.119811

0
4

3.41991

3.41991

0
4

0
4

0
4

0.447269

0.447269

0.447269

0.447269

0
4

0
4

0
4

0.175586

0.175586

0.175586

0.175586

0
4

0
4

0
4

0

0

0

0

0
4

0

0

0
4

0
4

0
4

0

0

0

0

0

0

0
4

0
4

0
4

0

0

0

0

0
4

0
4

0

0

0

0
4

0
4

0
4

1.09475

1.09475

1.09475

1.09475

0
4

0
4

0
4

0

0

0

0

0

0
4

0

0

0
4

0
4

0
4

0.177224

0.177224

0.177224

0.177224

0
4

0
4

0
4

0.749287

0.749287

0.749287

0.21317

0.536117

0
4

0
4

0
4

0.164832

0.164832

0.164832

0.164832

0
4

0
4

0
4

0.432726
1

0

0

0

0
4

0
4

0

0

0

0
4

0
4

0

0

0

0
4

0
4

0.032994

0.032994

0.025662

0.007332

0
4

0
4

0

0

0

0
4

0
4

0

0

0

0
4

0
4

0

0

0

0
4

0
4

0.359268

0

0

0
4

0.359268

0.359268

0
4

0
4

0

0

0

0
4

0
4

0

0

0

0

0
4

0
4

0

0

0

0
4

0
4

0.0404642
1

0.0404642
2

0

0

0

0

0

0

0

0.0404642

0

0
4

0

0

0
4

0
4

0
4

142.509
7

0

0

0

0

0
4

0
4

0

0

0

0
4

0
4

0

0

0

0
4

0
4

0

0

0

0
4

0
4

0

0

0

0
4

0
4

0

0

0

0
4

0
4

0

0

0

0
4

0
4

142.509
7

139.816
7

139.816
7

0

0
4

2.26587
7

2.26587
7

0
4

0

0

0
4

0.0775039

0.0775039

0
4

0.0549439

0.0549439

0
4

0.0366292

0.0366292

0
4

0.219775

0.219775

0
4

0.0155008

0.0155008

0
4

0.0232512

0.0232512

0
4

0
4

0
4

8.75491
3

6.39181
3

6.22764
3

6.04555

0

0

0

0

0

0

0

0

0

0

0.141625
3

0

0

0

0

0

0

0

0

0

0

0
2

0

0

0

0

0.0404642
3

0
2

0
3

0
2

0
3

0
2

2.4980018054066e-16
3

0
4

0.164174

0.164174

0
4

0

0

0
4

0
4

2.05944

2.05944

1.70996

0.349484

0

1.11022302462516e-16

0
4

0
4

0

0

0

0

0
4

0
4

0

0

0

0
4

0
4

0.127662

0.127662

0.127662

0
4

0
4

0

0

0

0

0
4

0
4

0.095063

0

0

0
4

0.095063

0.095063

0
4

0
4

0.0809283

0.0809283

0.0809283

0
4

0

0

0
4

0
4

0

0

0

0
4

0
4

0

0

0

0
4

0
4

0
4

60.7167
7

0

0

0

0
4

0
4

0

0

0

0
4

0
4

0

0

0

0
4

0
4

0

0

0

0
4

0
4

0

0

0

0
4

0
4

0

0

0

0
4

0
4

0.0404642

0.0404642

0.0404642

0
4

0
4

0.0809283

0.0809283

0.0809283

0
4

0
4

0.0606962

0.0606962

0.0606962

0
4

0
4

0.0404642

0.0404642

0.0404642

0
4

0
4

0.0404642

0.0404642

0.0404642

0
4

0
4

60.4537
7

59.2689
7

46.3427
7

5.70262
5

0.0281121

0.0581373

0

0.0510167

0

0.211081

0

0

0

0

0.190228
7

0.359433

0

0.0404642

0.0674692

0.10116

0.0599054

0

0

0.0112449

0

3.62518
6

0.0852543

0

0

0.82388

0.357411

0

0

0.0581373

0.179716

0.0599054

0

0.0732585

0

0.290597
7

0

0.229483

0.138793

0.123693

4.16333634234434e-15
7

0
4

1.14439

1.14439

0
4

0

0

0
4

0.0404642

0.0404642

0
4

0
4

0
4

29.2218

10.1981
5

10.1577
5

2.76022

0

0.0404642

0.00458879

2.87458
5

4.39118
6

0.0774698

0

0

0.00611838

0.00305919

0

0
4

0.0404642

0.0404642

0
4

0
4

16.6646
5

16.6616
5

16.5818
5

0

0.0404642

0.00764798

0.0316877

9.64506252643105e-16
5

0
4

0.00305919

0.00305919

0
4

0
4

0

0

0

0
4

0
4

0

0

0

0
4

0
4

0.449291

0.449291

0.449291

0
4

0
4

1.90976

1.90976
7

0

0

0

0

0

0

0

0

0

0.106826

0

0

0

0

0

0

0

0

1.70996

0

0.0674692

0.0255083

2.42861286636753e-17
7

0
4

0

0

0

0

0
4

0

0

0
4

0

0

0
4

0

0

0
4

0
4

1.77635683940025e-15

0
4

38.6188

0.434886

0.424874

0

0.161857

0

0

0.263017

0

0

0

0

0
4

0.0100127

0.00500636

0.00500636

0
4

0

0

0
4

0
4

8.41201
5

8.41201
5

7.11868
5

0.0898581

0

0.0898581

0.609957
7

0.162946

0

0

0

0.0112377

0

0.32948

1.94289029309402e-15
5

0
4

0
4

0.0469069

0.0469069

0.0469069

0

0
4

0

0

0
4

0
4

0.357411

0.357411

0.357411

0
4

0
4

0.174412

0.174412

0.174412

0
4

0
4

0

0

0

0
4

0
4

0.445106

0.445106

0.445106

0
4

0
4

5.98485

5.98485

5.98485

0
4

0
4

0

0

0

0

0
4

0
4

0.211081

0.211081

0

0.211081

0
4

0
4

0

0

0

0
4

0
4

0.0581373

0.0581373

0.0581373

0
4

0
4

15.0193
5

13.2689
5

12.9978
5

0

0.0703603

0

0.119811

0.0809283

0

0
4

0.0404642

0.0404642

0
4

1.70996

1.70996

0
4

0
4

0

0

0

0
4

0
4

0

0

0

0
4

0
4

0

0

0

0
4

0
4

0

0

0

0
4

0
4

1.70996

1.70996

1.70996

0
4

0
4

0

0

0

0
4

0
4

0

0

0

0
4

0
4

0

0

0

0
4

0
4

0.0599054

0.0599054

0.0599054

0
4

0
4

0.290687

0.290687

0.290687

0
4

0
4

0.951472
7

0.951472
7

0.480142
7

0

0.0404642
7

0.117701
7

0

0

0.304895

0

0.00827008

0
4

0
4

0.619358
5

0.535022
5

0.368656

0.0600763

0

0.0200254

0

0

0.0469069

0.039357

1.04083408558608e-16
5

0
4

0.0843364

0

0

0.0843364

0

0
4

0
4

2.20463
5

2.19462
5

2.12453

0

0

0

0

0.070089

0
4

0.0100127

0.00500636

0.00500636

0
4

3.29597460435593e-16
5

0
4

0.357411

0.357411

0.357411

0
4

0

0

0
4

0
4

1.28132

1.28132

1.28132

0

0

0
4

0

0

0
4

0
4

0
7

0
7

0

0

0

0

0

0
4

0
4

0

0

0

0
4

0

0

0
4

0
4

1.37667655053519e-14

0
4

56.8461

0

0

0

0

0

0

0

0

0

0

0
4

0

0

0
4

0
4

0.0103648

0.0103648

0.0103648

0

0

0
4

0
4

0

0

0

0

0
4

0
4

0

0

0

0

0
4

0

0

0
4

0
4

0

0

0

0

0
4

0
4

0.0103648

0

0

0
4

0.0103648

0.0103648

0
4

0
4

0

0

0

0
4

0
4

0

0

0

0
4

0
4

0

0

0

0
4

0
4

0

0

0

0
4

0

0

0
4

0
4

0

0

0

0
4

0
4

0.54664

0.54664

0.337231

0.168945

0.0404642

0
4

0
4

0.0316877

0.0316877

0.0316877

0
4

0
4

0

0

0

0
4

0
4

0

0

0

0
4

0
4

0

0

0

0
4

0
4

0.174282

0.174282

0.174282

0
4

0
4

0.0103648

0.0103648

0.0103648

0
4

0
4

0.00690984

0.00690984

0.00690984

0
4

0
4

0

0

0

0
4

0
4

0

0

0

0
4

0
4

0

0

0

0
4

0
4

0

0

0

0

0
4

0

0

0

0
4

0
4

0

0

0

0
4

0
4

0

0

0

0
4

0
4

0

0

0

0
4

0
4

0

0

0

0
4

0
4

2.56494

2.56494

2.56494

0
4

0
4

1.70996

1.70996

1.70996

0
4

0
4

0

0

0

0

0
4

0
4

0

0

0

0

0

0
4

0

0

0
4

0
4

0.0404642

0

0

0

0
4

0

0

0
4

0.0404642

0.0404642

0
4

0
4

0.0170056

0

0

0
4

0.0170056

0.0170056

0

0
4

0
4

0

0

0

0

0
4

0
4

0

0

0

0

0
4

0

0

0

0
4

0
4

51.7231

49.519

46.5001

1.2984

0

0.00500636

0

0

0

0

0

0

1.61857

0

0

0

0

0

0
3

0

0.0968956

0

0

0

0

0
4

0.0760082

0.0449139

0.0207295

0

0

0

0.0103648

8.67361737988404e-18

0
4

0

0

0
4

0

0

0
4

0

0

0
4

0.0208779

0.0208779

0
4

0

0

0
4

0

0

0
4

0.0340111

0.0340111

0
4

2.05597

2.04344

0

0

0

0.0125267

5.72458747072346e-17

0
4

0

0

0
4

0.0103648

0.0103648

0
4

0

0

0

0
4

0

0

0
4

0

0

0
4

0

0

0
4

0.00690984

0.00690984

0
4

7.9207473913101e-15

0
4

0
4

7.16662
5

0.443704
6

0.42634

0.193791

0

0

0.232549

0

0

0

0

0
4

0.00611838

0.00611838

0

0
4

0

0

0

0
4

0.0112449

0.0112449

0
4

0

0

0
4

0
4

3.71689

2.82261
5

2.74766
6

0.0198848

0.0520063

0

0.00305919

0

0

0

1.56992474575901e-16
5

0
4

0.889689

0.889689

0

0
4

0.00458879

0.00458879

0
4

5.24753851482984e-16

0
4

0

0

0

0
4

0
4

0

0

0

0
4

0
4

0

0

0

0
4

0
4

0.00917758

0.00917758

0.00917758

0
4

0
4

0.00305919

0.00305919

0.00305919

0
4

0
4

2.08686
5

2.08686
5

1.77958
6

0.104958

0.141625

0

0

0.0606962

2.35922392732846e-16
5

0
4

0
4

0.179803

0.179803

0.0449507

0.0280942

0.0280942

0

0.0786638

0
4

0
4

0.0306229
5

0.0137664

0.00458879

0

0.00611838

0.00305919

0
4

0.0168565

0.0168565

0
4

0

0

0
4

0
4

0.214163

0.214163

0.214163

0
4

0
4

0.10116

0.10116

0.0404642

0.0606962

0
4

0
4

0.323713

0.323713

0.323713

0
4

0
4

0.0404642

0.0404642

0.0404642

0
4

0
4

0.0170056

0.0170056

0.0170056

0
4

0
4

2.6853519408121e-15
5

0
4

0.287398
3

0

0

0

0

0
4

0
4

0.049085

0.049085

0.049085

0
4

0
4

0.238313
3

0.0809283

0.0809283

0
4

0

0

0

0

0
4

0.116921

0.116921

0
4

0

0

0
4

0.0404642

0.0404642

0
4

2.77555756156289e-17
3

0
4

0
4

56.2287

11.0309

11.0309

10.9504
7

0

0

0

0

0

0

0.0549439

0

0

0.0255083
8

0
8

0
1

0

0

0

0

0

0
4

0

0

0

0

0
4

0

0

0

0
4

0
4

0

0

0

0

0
4

0
4

0

0

0

0

0

0
4

0

0

0
4

0
4

0

0

0

0

0
4

0

0

0

0
4

0
4

4.37605

4.37605

4.37605

0
4

0
4

0

0

0

0
4

0
4

0

0

0

0

0
4

0

0

0
4

0
4

0

0

0

0
4

0
4

0

0

0

0

0
4

0
4

0

0

0

0
4

0
4

0.697129

0

0

0
4

0.697129

0.697129

0
4

0
4

14.3172

13.6797
7

0

0

0

0

0

0

0

13.6797

0

0

0

0
4

0.637582
1

0.637582
8

0

0

0
4

0

0

0
4

0

0

0
4

0
4

0

0

0

0
4

0
4

0

0

0

0

0
4

0
4

0

0

0

0
4

0
4

0

0

0

0
4

0
4

0

0

0

0
4

0
4

0

0

0

0
4

0
4

0

0

0

0
4

0
4

0

0

0

0
4

0
4

0

0

0

0
4

0
4

0.0809283

0.0809283

0.0809283

0
4

0
4

0.0915731

0

0

0

0

0

0
4

0

0

0

0
4

0.0915731

0.0915731

0
4

0

0

0
4

0
4

0

0

0

0
4

0
4

0.402922

0.402922

0.402922

0
4

0
4

0

0

0

0
4

0
4

0

0

0

0
4

0
4

0

0

0

0
4

0
4

0.0809283

0.0809283

0.0809283

0
4

0
4

0

0

0

0
4

0
4

0

0

0

0
4

0
4

0

0

0

0
4

0
4

1.70996

1.70996

1.70996

0
4

0
4

0

0

0

0

0

0

0
4

0

0

0

0
4

0
4

4.31536

0

0

0

0
4

4.31536

4.31536

0

0

0

0
4

0

0

0

0
4

0
4

0
3

0
3

0

0

0

0

0

0

0

0

0
4

0

0

0
4

0

0

0
4

0
4

0
3

0
3

0

0

0

0

0

0
4

0
4

2.19083
6

0.242785

0.202321

0

0.0404642

0
4

1.70996

1.70996

0
4

0.23809

0.23809

0
4

0
4

0

0

0

0

0

0
4

0
4

16.9349

15.4516

1.44182
7

1.70996

0

0.065988

0.0775164

0.0514621

2.56494

0

0

0

0

0

0

0

0

0

0

0.00750954

0

0.014664

0

0

0.0775039

0

0

0.315276

0

0

0

0

0

0.0366292

0

0.480196

0

0.00701215

0

0.0404642

0

0

0

0

0

0

0.901988
7

0.0404642

0

0

0

0.0404642

0.0232512

0

0

0

0.010998

0

0

1.70996

0

0

0

0

0

0

0

0.0606962

2.92437

0

0.131757

2.71675

0
4

0

0

0

0

0
4

0

0

0
4

0

0

0
4

0

0

0

0
4

0

0

0

0
4

0

0

0
4

0.00750954

0.00750954

0
4

0

0

0
4

0

0

0
4

0

0

0
4

0

0

0
4

1.32165
6

1.3104
6

0.0112449

0

0
4

0

0

0
4

0

0

0
4

0

0

0
4

0

0

0
4

0

0

0
4

0.138619

0.0224897

0.0599054

0

0.0562243

6.93889390390723e-18

0
4

0

0

0

0

0
4

0
3

0

0

0

0
4

0.0155008

0

0.0155008

0
4

0

0

0

0
4

0

0

0

0
4

0

0

0
4

0
4

1.4210854715202e-14

0
4

30.7149
7

0.718624
6

0.718624
6

0.361213
6

0.357411

0
4

0
4

1.12166
6

1.12166
6

1.12166
6

0
4

0
4

0

0

0

0
4

0
4

0

0

0

0
4

0
4

0

0

0

0
4

0
4

0

0

0

0
4

0
4

28.8746
7

28.8746
7

28.8746
7

0
4

0
4

7.105427357601e-15
7

0
4

0
4

5765.6

0

0

0

0

0

0

0
4

0
4

0
4

738.35
3

10.2686

10.2686

10.0209

0

0

0

0

0

0

0

0

0.10116

0.0366292
2

0

0

0

0

0.109888

0

0

0
4

0

0

0
4

0

0

0
4

0
4

627.82
3

198.85

0

38.9182

1.47576

0.359433

0.109888

0

0.0404642

0.375255

0.0599054

0.351802

0

0.0469069

0

5.31265

0

0

0

0

0

0

0.0335696

0

2.56494

0.0404642

12.1699

0.117267

0

0.0155008

0

0.303481

0

0

0

0

0.357411

7.56984

0.0252756

0

0

0.00827008

0

0

0

0

0

0

4.81291

0

0.0606962

0.136765

0

0

0

0.119811

0

0

0

9.16647
6

0

0

0.0475315

0.209669

0

0.479243

0

0

0.0599054

0

0.837784
7

0.0606962

0

0

0

0

0

0

0

0

0

0.360223

0

0

0

0

0

0

0

0.0366292

0

0.0599054

0.587382

0

0

0.0599054

0.0599054

0

0

0

0

0

0

0.0599054

0.0633753

0

0

0

0

0

0.0599054

0

0

0

9.42298

0.170574
5

0

0

0

0

0

0

0.0112449

0

0

0

0

0

0

0

0

1.60835

2.56494

0

0

2.56494

0

0
2

0

0

0

1.70996

0

0

0

0

0.0469069

0.0316877

0

0

0

0

0.0915731

0.0599054

0

0

0

0

0.0599054

1.25094

0.0475315

0

0

0.0404642

0

0.00827008

0

0

0.0366292

0

0.622704

0

0

0

0

0

0

0.0404642

0.0404642

0.0404642

0

3.66474
6

0

0

0.00835115

0

0

0

0.0898581

0

0.0898581

0

0.647454

0

0

0

0

0

0

0.161857

0

0

0

0.807206

0.0155008

0

0

0

0.0599054

0

0

0.0703603

0

0

0

0

0

0

0.0775164

0

0

0

0

0

0

10.8023

0.666383

0

0

0

0.209669

0

0.108505

0

0.121392

0

0.119811

0

0

0

0

0

0

0.0404642

0

0

0

0

0
2

0

0

0.0898581

0

0

0

0.0469069

0

0

0

0

0

0

0

0

0

0

0

0

0.0599054

0

0.357411

0.0469069

0

0.0599054

0

0

0

0

0.0549439

0

0

0

0

0

0

0.0703603

0

0

0

0

0

0.239622

0

0

0

0

0

0

0

0.0599054

0.149764

0

0

0.370677

0

0

0

0

0

25.7897

0.133618

0.140518

2.60408
6

0

0

0
2

0

0

0.175069
7

0

9.20025

0.0404642

0.318577

0

0

0.243128

0

0

0

0

0.179716

5.81899

0

0

0.0898581

0.485199

0

0

0.259959

0

0

0.509196

11.6222

0

0

0

0

0

0

0

0

0

0

10.2344

0

0

0.00827008

0

0.202321

0

0

0

0.940435

0

1.22455

1.04834

0

0.0475315

0.0404642

0

0

0

0

0

0.0599054

0
4

231.355
4

5.08155
3

36.1686
4

174.995
3

1.77192
6

0
4

0

0

0

0

0

0

0

0

0

0

0
4

0

0

0

0

0

0

0

0

0

0

0
4

0

0

0

0

0.0170056

0

0

0

1.13293

0

0
4

0

0

0

0

0.35329

0.940758

0

0

0

0

0.0633753
4

0

0.386842

0

0

0

0

0

0

0

0

0
2

0

0

0

0

0

0

0

0.984397

0

0

0
3

0

0

0

0

0

0.0674692

0

0.110441

0

0

0
4

0

0

0

0

0

0

0

0.0345492

0

0

0

0.0606962

0

0

0

0

0

0

0

0

0

0
2

0

0

0

0

0

0

0

0.0850278

0

0

0
3

0
4

0

0

0

0

0

0

0

0

0

0

0

0

0

0

0

0.00690984

0

0

0

0

0

0

0

0

0

0

0

0.119811

0

0

0

0

0
4

0

0

0

0

0

0

0

0

0

0

0

0

0

0

0

0

0

0

0

0

0

0
3

0

0

0

0

0

0

0

0

0

0

0

0

0

0

0

0

0

0

0

0

0

2.5005

0

0

0

0

0

0

0

0

0

0

0
4

0

0

0

0

0

0

0

0

0

0

0.269345
4

0

0

0

0

0

0

0

0

0

0

0
4

0

0

0

0

0

0

0

0

0

0

0

0

0

0

0

0

0

0

0

0.0112449

0

0

0

0

0

0

0

0

0

0

0

0

0

0
4

0

0

0

0

0

0

0

0

0

0

0
3

0

0

0.0404642

0

0

0

0

0

0

0

0

0

0

0

0

0

0

0

0

0

0

0

0

0

0

0

0

0

0

0

0

0

0
4

0

0

0

0

0

0.0633753

0

0

0

0

0.662612

0

0

0

0

0

0

0

0

0

0

0.0674692

0

0

0

0

0

0

0

0

0

0

0
4

0

0

0

0

0

0

0.121392

0

0

0

0

0

0

0

0.0103648

0

0

0

0

0

0

0

0

0

0

0

0

0

0

0

0

0.0105182

0

0
2

0

0

0

0

0

0

0

0

0

0

0

0

0

0

0
4

0

0

0

0
3

0
4

0.89461

0

0

0
4

0

0
4

0

0

0

0

0
4

0

0

0

0

0

0

0

0

0

0

0
4

0

1.8684

0.0782354

0

0.100231

0

0

2.27543

0

0

0
3

0

0

0

0

0

0

0

0

0

0

0
3

0

0

0

0

0

0

0

0

0

0

0
4

4.62234
3

0
3

0.140721
3

1.39703
3

0.357411
3

0.321618
2

1.42128
3

0
2

0.17728
1

0

0

0

0

0

0

0

0

0

0

0.783151

0

0.00835115

0

0

0

0

0

0

0

0

0

0

0

0.0155008

0

0

0
8

0

0

0

0

0

5.39499001028787e-16
3

0
4

103.103

18.1687
7

2.55775
6

0

0

0

0

0

0.0229265

0

0

0

0

1.05122
7

0.0170056

0

0

0

0

0

0

0

0

0

0.0404642
7

0

0

0

0

0

0

0

0

0.00750954

0

0.897308
5

0

0.893528

0

0

0

0

0

0

0.0730916

0.0387582

3.43413
6

0

0

0.121392

0

0

0

0

0

0

0

0.0425139
7

0

0

0.0703603

0.357411

0.0112377

0

0

0

2.56494

0.00690984

0.01833

0

0

0

0

0

0

0.00750954

0

0

0

0

0

0

0

0

0

0

0

0

43.7247

0
8

0.0425139

0.210088

1.37152

0.0404642

0.141625

0

0.188776

0

0

7.81988

0

0

0

0

0.597033

0

0

4.4117

0

0

0.288904
3

0

1.25094

0

0.00500636

0.0200254

0

0

0

0

0

6.70648
7

0.0316877

0

0

0

0

0

0.0573163

0

0

0

2.46513
3

0

0

0.0843364

0

0

0

0.095063

0.0404642

0.140721

0

1.1452
7

0.00701215

0

0

0

0.0155008

0

0

0.0310943

0

0

0.010998
1

1.70996

0

0

0

0

0

0

0

0

0

0

0

0.00500636

0

0.0404642

0

0

0

0

0

0

6.65301147506625e-14

0
4

6.28626
2

0

0

0

0

0

0

0

0

0.323713

0

0

0

1.64112

0

0

0

1.7902

0

0

0

0

0

0

0

0

0

0

1.82459

0.237658

0

0

0

0

0.126751

0

0

0

0

0

0

0

0

0

0

0

0

0.263017

0

0

0

0

0

0

0

0

0

0

0

0

0

0

0

0

0

0

0

0.0792192

0

0

0

0

0

0

0

0

0

0

0

0

0

0

0

0

0

0

0

7.91033905045424e-16
2

0
4

23.0914
3

21.8214
3

0

0.0933642

0.0105182

0

0.021996

0.00350607

0.0599054

0

0.00525911

0

0.510154
4

0

0

0

0

0

0

0

0.0404642

0

0.0792192

0.295942
4

0

0

0

0

0

0.0122713

0.0316877
4

0.0669176
3

0.0126753
4

0.00350607
3

0.00525911
4

0.0173414

0
4

0.644749
3

0.557752

0.0210364

0

0

0.00701215
4

0.0262956

0.0155008

0

0

0.0171524

0

0

7.28583859910259e-17
3

0
4

0

0

0
4

0

0

0
4

0

0

0
4

0

0

0
4

0

0

0

0
4

0

0

0
4

0.00500636

0.00500636

0

0
4

0

0

0
4

0

0

0
4

0

0

0

0
4

0.18534
3

0.0181234
4

0.0175304
3

0.0629237
3

0.029139
3

0.0155883

0.0243535

0.00500636

0.0126753

1.04083408558608e-17
3

0
4

0

0

0
4

0

0

0

0
4

0

0

0
4

0.0599054

0.0599054

0

0
4

0

0

0
4

0

0

0
4

0

0

0

0
4

0

0

0
4

0

0

0
4

1.70996

1.70996

0
4

0
3

0
3

0

0

0

0

0

0

0

0
4

0

0

0
4

0

0

0
4

0

0

0
4

0

0

0
4

0

0

0
4

0

0

0
4

0

0

0
4

0

0

0
4

0

0

0
4

0

0

0
4

0
2

0
2

0

0

0

0

0

0

0
4

0.00876519

0.00876519

0
4

0

0

0
4

0

0

0
4

0.0599054

0.0599054

0
4

0.119811

0.119811

0
4

0

0

0
4

0

0

0
4

0

0

0
4

0

0

0
4

0

0

0
4

0

0

0

0

0

0

0

0

0
4

0

0

0
4

0

0

0
4

0

0

0
4

0

0

0
4

0

0

0
4

0

0

0
4

0

0

0
4

0

0

0
4

0

0

0
4

0

0

0
4

0.826455
4

0.826455
4

0

0

0

0
4

0

0

0
4

0

0

0
4

0

0

0
4

0

0

0
4

0

0

0
4

0

0

0
4

0

0

0
4

0

0

0
4

0

0

0
4

0

0

0
4

0

0

0

0

0

0
4

0.00690984

0.00690984

0
4

0

0

0
4

0

0

0
4

0

0

0
4

0

0

0
4

0

0

0
4

0

0

0
4

0

0

0
4

0

0

0
4

0

0

0
4

0
3

0
3

0

0

0

0
4

0

0

0
4

0

0

0
4

0

0

0
4

0

0

0
4

1.70996

1.70996

0
4

0

0

0
4

0

0

0
4

0.0599054

0.0599054

0
4

0

0

0
4

0

0

0
4

0
4

0
4

0

0

0

0
4

0

0

0
4

0

0

0
4

0

0

0
4

0

0

0
4

0

0

0
4

0

0

0
4

0

0

0
4

0.095063

0.095063

0
4

0

0

0
4

0

0

0
4

0

0

0

0

0

0

0
4

0

0

0
4

0.0157773

0.0157773

0
4

0.0475315

0.0475315

0
4

0

0

0
4

0

0

0
4

0

0

0
4

0

0

0
4

0

0

0
4

0.0404642

0.0404642

0
4

0

0

0
4

0.504979
3

0.262475
3

0.0192585

0.0208475

0

0

0

0

0

0.00350607

0

0.00525911

0.0389155
4

0.00525911

0

0

0.00507012

0.00701215

0

0.0039381

0.00701215
4

0.0250223
4

0
3

0.050413

0.0105182

0.0125159

0.0279558

0
4

0

0

0

0

0

0
4

0.0229265

0.0229265

0
4

0

0

0
4

0

0

0
4

0.116275

0.116275

0
4

0

0

0
4

0.174295
3

0.174295
3

0

0

0
4

0.31161
4

0

0.296946

0

0.007332

0.007332

2.42861286636753e-17
4

0
4

0
2

0

0

0

0

0

0

0

0
4

0

0

0

0

0

0

0

0

0

0
4

0.584944

0.584944

0

0
4

0
3

0

0

0

0

0

0

0
4

0
3

0

0

0

0

0

0

0

0
4

0

0

0

0
4

0
2

0

0

0

0

0

0
4

32.807
3

29.0519
3

0

0.0599054

0

0.00350607

0

0

0

0

0

0

0.197606
4

0

0

0.110441
4

0.0120823

0.0122713

0

3.30845

0.0508381

5.93969318174459e-15
3

0
4

0
3

0

0

0

0

0

0
4

0.00350607
4

0

0

0

0.00350607

0

0
4

0

0

0
4

0
4

0
4

0

0

0

0
4

0

0

0

0
4

0

0

0

0

0
4

0
4

0
4

0

0
4

0.631013
5

0.625943
5

0.00507012

8.93382590128056e-17
5

0
4

0
2

0

0

0

0

0

0
4

0
4

0
4

0
4

3.61423
5

3.07218
5

0.119811

0.0170056

0

0

0

0

0

0

0

0

0

0

0

0

0

0

0

0.170056

0.115366

0.119811

0

0

0

0

2.91433543964104e-16
5

0
4

0.270546
4

0.082918

0.187628

0
4

0.0227895

0.0105182

0.0122713

0

0
4

0.665441

0.665441

0
4

0

0

0

0

0
4

0.033055
4

0.033055

0

0
4

0

0

0

0

0

0
4

0

0

0

0
4

0

0

0

0

0

0
4

0

0

0
4

0

0

0
4

0.213727
6

0.0170056

0.0599054

0

0

0

0

0

0

0

0

0

0.0170056

0

0

0

0

0

0

0

0

0

0

0

0

0

0

0

0

0

0.119811

0

0

1.38777878078145e-17
6

0
4

0
2

0

0

0
4

0

0

0

0

0
4

0

0

0

0
4

0

0

0

0

0
4

0.44559

0.174282

0

0.271308

0
4

0

0

0

0

0
4

0

0

0

0
4

0

0

0
4

0

0

0

0
4

0.179716

0

0

0.179716

0
4

1.80762
2

1.68781
2

0.119811
2

0

0

0

0

0

0

6.93889390390723e-17
2

0
4

0

0

0
4

0

0

0

0
4

0.261405

0.261405

0
4

0

0

0

0
4

0.947122

0.947122

0
4

0.420129

0.420129

0
4

0.780352

0.780352

0
4

0

0

0

0

0

0
4

0

0

0

0
4

0.0489653

0.0489653

0
4

0
3

0
4

0

0

0

0

0

0

0

0

0

0

0

0

0

0

0

0

0

0

0

0

0

0

0

0

0

0

0

0

0
4

0

0

0
4

0.0469069

0.0469069

0
4

0.216294

0.021996

0.194298

0
4

0

0

0

0
4

0

0

0

0
4

0.0316877

0.0316877

0

0
4

0

0

0

0
4

0

0

0

0
4

1.37578

1.33532

0.0404642

0
4

0

0

0

0
4

6.75917
6

6.19122
6

0

0

0

0.178558

0.0898581

0

0.299527

0

0

0

0

0
4

0

0

0

0
4

0.21317

0.174412

0.0387582

0
4

0.0599054

0.0599054

0
4

0

0

0

0
4

0

0

0
4

0

0

0
4

0

0

0

0
4

0

0

0

0
4

0

0

0
4

0

0

0
4

0.602888
4

0.532567
4

0.0192834
4

0.0475315
4

0

0

0.00350607

0

0

0

8.67361737988404e-17
4

0
4

0

0

0
4

0

0

0
4

0

0

0
4

0

0

0
4

0

0

0

0
4

0.74466

0.712973

0.0316877

4.85722573273506e-17

0
4

0

0

0
4

0

0

0

0
4

0

0

0
4

0

0

0

0
4

9.97313343020778e-13
3

0
4

0
2

0
2

0
2

0

0

0

0

0

0

0

0

0

0

0

0

0

0

0

0

0

0

0

0

0

0
2

0

0

0

0

0

0

0

0

0

0

0

0

0

0

0

0

0

0

0

0

0

0

0

0

0

0

0

0

0

0

0

0

0

0

0

0

0

0

0

0

0

0

0

0

0

0

0

0

0

0

0

0

0
4

0
2

0

0

0

0

0

0

0

0

0

0

0

0
4

0

0

0

0

0

0

0

0

0
4

0

0

0

0

0

0
4

0

0

0
4

0
4

0.387679

0.371435

0.234255
7

0.13718

0

0

0

0

0
4

0.0112377

0.0112377

0
4

0.00500636

0.00500636

0
4

0
4

0

0

0

0
4

0

0

0
4

0
4

0

0

0

0
4

0

0

0
4

0
4

0

0

0

0
4

0

0

0
4

0
4

0

0

0

0

0
4

0
4

0

0

0

0
4

0

0

0
4

0
4

0

0

0

0
4

0
4

0

0

0

0
4

0
4

0

0

0

0
4

0
4

0

0

0

0
4

0

0

0
4

0
4

0

0

0

0
4

0
4

1.96576

1.96576

1.96576

0

0

0

0

0

0
4

0
4

0

0

0

0

0
4

0
4

0

0

0

0
4

0

0

0
4

0
4

0

0

0

0

0
4

0
4

0.142595

0.142595

0.142595

0
4

0
4

0

0

0

0

0
4

0
4

0

0

0

0
4

0
4

0

0

0

0
4

0
4

0

0

0

0
4

0
4

0

0

0

0
4

0
4

0

0

0

0

0
4

0
4

7.91206
7

7.91206
7

7.91206

0

0

0

0

0

0
4

0
4

0

0

0

0

0
4

0
4

0.00690984

0.00690984

0.00690984

0
4

0
4

0.0938138

0.0938138

0.0938138

0
4

0
4

0

0

0

0
4

0
4

0

0

0

0
4

0
4

0

0

0

0
4

0
4

0.0404642

0.0404642

0.0404642

0
4

0
4

0

0

0

0
4

0
4

0

0

0

0
4

0
4

0

0

0

0
4

0
4

0
2

0
2

0
2

0

0

0

0

0

0

0
4

0
4

0

0

0

0
4

0
4

0

0

0

0
4

0
4

0

0

0

0
4

0
4

0

0

0

0
4

0
4

0.140721

0.140721

0.140721

0
4

0
4

0

0

0

0
4

0
4

0.117267

0.117267

0.117267

0
4

0
4

0

0

0

0
4

0
4

0

0

0

0
4

0
4

0

0

0

0
4

0
4

2.61187

0.820981
5

0.786381

0.0103648

0

0.00690984

0.00690984

0.00690984

0.00350607

0
4

1.79089

0

1.75042

0

0

0.0404642

0
4

0

0

0
4

4.44089209850063e-16

0
4

0.00525911

0.00525911

0.00525911

0
4

0
4

0.0316877

0.0316877

0.0316877

0
4

0
4

0.0316877

0.0316877

0.0316877

0
4

0
4

0

0

0

0
4

0
4

0.117267

0.117267

0.117267

0
4

0
4

0

0

0

0
4

0
4

0

0

0

0
4

0
4

0

0

0

0
4

0
4

0

0

0

0
4

0
4

0

0

0

0
4

0
4

0

0

0

0

0

0

0

0

0
4

0
4

0

0

0

0
4

0
4

0

0

0

0
4

0
4

0.357411

0.357411

0.357411

0
4

0
4

0.00876519

0.00876519

0.00876519

0
4

0
4

0

0

0

0
4

0
4

0.0475315

0.0475315

0.0475315

0
4

0
4

0

0

0

0
4

0
4

0

0

0

0
4

0
4

0

0

0

0
4

0
4

0

0

0

0
4

0
4

0

0

0

0

0

0

0

0
4

0
4

0

0

0

0
4

0
4

0

0

0

0
4

0
4

0

0

0

0
4

0
4

0

0

0

0
4

0
4

0.00701215

0.00701215

0.00701215

0
4

0
4

0

0

0

0
4

0
4

0

0

0

0
4

0
4

0.0112377

0.0112377

0.0112377

0
4

0
4

0

0

0

0
4

0
4

0

0

0

0
4

0
4

0

0

0

0

0

0

0

0

0

0

0

0

0
4

0
4

0

0

0

0
4

0
4

0.0168673

0.0168673

0.0168673

0
4

0
4

0.0316877

0.0316877

0.0316877

0
4

0
4

0

0

0

0
4

0
4

0

0

0

0
4

0
4

0

0

0

0
4

0
4

0

0

0

0
4

0
4

0

0

0

0
4

0
4

0.0469069

0.0469069

0.0469069

0
4

0
4

0

0

0

0
4

0
4

0

0

0

0

0

0

0
4

0

0

0
4

0
4

0

0

0

0
4

0
4

0

0

0

0
4

0
4

0.299527

0.299527

0.299527

0
4

0
4

0

0

0

0
4

0
4

0.00690984

0.00690984

0.00690984

0
4

0
4

0

0

0

0
4

0
4

0

0

0

0
4

0
4

0

0

0

0
4

0
4

0

0

0

0
4

0
4

0

0

0

0
4

0
4

3.84191
6

3.84191
6

3.26808
6

0.459802

0.0306347

0.0140945

0.0599054

0.00939633

2.8275992658422e-16
6

0
4

0
4

0

0

0

0
4

0
4

0

0

0

0
4

0
4

0

0

0

0
4

0
4

0
2

0
2

0
2

0

0

0

0

0

0

0

0

0

0

0
2

0

0

0

0

0

0

0

0

0

0

0

0

0

0

0

0

0

0

0

0

0

0
2

0

0

0

0

0

0

0

0

0

0

0

0

0

0

0

0

0

0

0

0

0

0

0

0

0

0

0

0

0

0

0

0

0

0

0

0

0

0

0

0

0

0

0

0
2

0

0

0

0

0

0

0

0

0

0

0
4

0
2

0
2

0

0

0

0

0

0

0

0

0

0
4

0
2

0

0
2

0

0

0

0
4

0

0

0

0

0

0
4

0

0

0

0

0
4

0

0

0

0

0
4

0

0

0

0
4

0
4

0
2

0
2

0
2

0

0

0

0
4

0

0

0

0
4

0

0

0
4

0
4

0

0

0

0

0

0

0
4

0
4

0.528587
5

0.528587
5

0.509303
5

0.0157773

0.00350607

2.08166817117217e-17
5

0
4

0
4

0

0

0

0

0

0

0

0

0

0
4

0

0

0

0
4

0

0

0
4

0
4

0

0

0

0

0

0

0

0
4

0

0

0
4

0
4

0

0

0

0

0

0

0
4

0

0

0

0
4

0

0

0
4

0

0

0
4

0
4

0

0

0

0

0

0

0
4

0

0

0
4

0

0

0
4

0

0

0
4

0
4

1.79906
5

1.79906
5

1.74165

0

0.0155008

0

0

0.0168565

0.0138197

0.0112377

8.67361737988404e-17
5

0
4

0
4

0.136004
4

0.0656434

0.0656434

0

0

0
4

0

0

0
4

0.0703603

0.0703603

0
4

0
4

1.02906
7

1.02906
7

0.855758
7

0.168299

0.00500636

8.15320033709099e-17
7

0
4

0
4

0.0618467
2

0.0618467
2

0.0618467
2

0

0

0

0

0

0

0

0

0
2

0

0

0

0

0

0

0

0
4

0
2

0
2

0

0

0

0
4

0

0

0

0

0

0

0

0

0

0

0
4

0

0

0

0
4

0
4

0

0

0

0

0

0

0
4

0
4

12.6172
6

12.6172
6

12.6172
6

0
4

0
4

0
2

0

0

0

0

0

0
4

0

0

0

0
4

0

0

0
4

0
4

9.97943
7

9.97943
7

9.97943
7

0

0
4

0
4

2.14884
7

2.14884
7

1.70996

0.438884

1.11022302462516e-16
7

0
4

0
4

2.32317

2.32317

2.32317

0

0

0
4

0

0

0
4

0
4

0

0

0

0

0

0

0

0
4

0

0

0
4

0

0

0
4

0
4

14.9928
6

14.9928
6

14.8107
6

0.182089

0
4

0
4

0

0

0

0

0
4

0
4

0.0775039

0.0775039

0.0775039

0
4

0

0

0
4

0

0

0
4

0
4

0
2

0
2

0
2

0

0

0

0

0

0

0

0

0

0

0

0

0

0

0

0

0

0

0

0

0

0

0
4

0

0

0
4

0
2

0

0

0

0

0

0

0
4

0

0

0

0

0

0
4

0

0

0

0

0
4

0

0

0
4

0

0

0

0
4

0

0

0

0
4

0

0

0
4

0

0

0
4

0
4

0.257421

0.257421

0.206405

0.0510167

3.46944695195361e-17

0
4

0

0

0

0
4

0
4

0

0

0

0

0
4

0

0

0
4

0
4

0

0

0

0

0

0

0

0
4

0

0

0

0
4

0
4

4.13474
6

4.13474
6

4.13474
6

0
4

0
4

1.87443

1.87443

1.87443

0
4

0
4

0

0

0

0

0
4

0
4

0

0

0

0

0
4

0

0

0

0
4

0
4

0

0

0

0

0

0
4

0
4

0.344719

0.286581

0.194875

0.0229265

0.0229265

0.045853

2.08166817117217e-17

0
4

0.0581373

0.0581373

0
4

0
4

0

0

0

0
4

0
4

0

0

0

0

0

0

0

0

0

0

0

0

0

0

0

0

0

0

0
4

0

0

0
4

0

0

0

0
4

0
4

0

0

0

0

0

0
4

0

0

0
4

0

0

0
4

0
4

0

0

0

0
4

0
4

0

0

0

0

0
4

0
4

0.161857

0

0

0
4

0.161857

0.161857

0
4

0
4

0

0

0

0

0
4

0

0

0
4

0
4

0

0

0

0

0
4

0
4

0

0

0

0

0

0
4

0

0

0
4

0

0

0
4

0
4

0

0

0

0

0
4

0

0

0
4

0

0

0
4

0

0

0
4

0
4

1.70996

1.70996

0

1.70996

0
4

0
4

0

0

0

0
4

0
4

0
2

0
2

0
2

0
2

0

0

0

0

0

0

0

0
4

0

0

0

0
4

0
4

0

0

0

0

0
4

0

0

0
4

0

0

0
4

0

0

0
4

0
4

0.09589
7

0.0923839

0.0923839

0

0
4

0.00350607

0.00350607

0
4

3.46944695195361e-18
7

0
4

0

0

0

0
4

0
4

0

0

0

0
4

0

0

0

0
4

0

0

0
4

0
4

0

0

0

0

0
4

0
4

0

0

0

0

0
4

0

0

0
4

0
4

0.0581373

0.0581373

0.0581373

0

0

0
4

0
4

0

0

0

0
4

0

0

0
4

0
4

0.0202805

0.0202805

0.0202805

0
4

0
4

0.323713

0.323713

0.283249

0.0404642

0
4

0
4

0
2

0
2

0
2

0

0

0

0

0

0
4

0
2

0

0

0

0

0

0

0

0
4

0
2

0

0

0

0

0

0
4

0

0

0

0
4

0

0

0

0
4

0

0

0
4

0

0

0
4

0

0

0
4

0

0

0
4

0
4

0.0633753

0.0633753

0.0633753

0
4

0
4

0.0775039

0.0775039

0.0775039

0
4

0

0

0
4

0
4

0

0

0

0

0

0
4

0
4

0

0

0

0
4

0
4

0

0

0

0

0

0
4

0
4

0.0486477

0.0486477

0.0401353

0.00500636

0.00350607

1.73472347597681e-18

0
4

0
4

0

0

0

0
4

0

0

0
4

0
4

2.06529

1.79089

1.70996

0.0809283

0
4

0.274404

0.274404

0
4

5.55111512312578e-17

0
4

0.0404642

0.0404642

0.0404642

0
4

0
4

0.222553

0

0

0
4

0.222553

0.222553

0
4

0
4

0

0

0

0

0

0

0

0

0

0

0
4

0
4

0.296984

0.296984

0.296984

0
4

0
4

0

0

0

0
4

0
4

0

0

0

0

0

0
4

0
4

0.141625

0.141625

0.141625

0

0
4

0
4

0.326936

0.326936

0.326936

0
4

0
4

0

0

0

0

0
4

0
4

0.276796

0.0340111

0.0340111

0
4

0.242785

0.242785

0
4

2.77555756156289e-17

0
4

0.0112377

0.0112377

0.0112377

0

0
4

0
4

0

0

0

0
4

0
4

0

0

0

0
4

0

0

0
4

0
4

0

0

0

0

0

0

0

0

0

0

0
4

0

0

0
4

0

0

0

0
4

0
4

0

0

0

0
4

0

0

0
4

0
4

5.48728

5.48728

5.48728

0
4

0
4

0

0

0

0

0
4

0
4

0

0

0

0

0
4

0
4

0.095063

0.095063

0.095063

0
4

0
4

1.77065

1.70996

1.70996

0
4

0.0606962

0.0606962

0
4

0
4

0

0

0

0
4

0

0

0
4

0
4

0.141625

0.141625

0.141625

0

0
4

0
4

0

0

0

0

0
4

0
4

0

0

0

0
4

0

0

0
4

0
4

16.2394

13.534

13.534

0

0

0

0

0

0
4

0
1

0
1

0
4

0

0

0
4

0

0

0

0
4

0

0

0

0
4

0.140471

0.112377

0.0280942

0
4

2.56494

2.56494

0
4

0
4

1.84741111297626e-13
3

0
4

1.11072
4

1.0597
4

0.484478

0.484478

0

0
4

0

0

0
4

0.575226

0.575226

0
4

0

0

0
4

0
4

0

0

0

0
4

0
4

0

0

0

0

0
4

0
4

0.0340111

0.0340111

0.0340111

0
4

0
4

0.0170056

0.0170056

0.0170056

0
4

0
4

3.46944695195361e-17
4

0
4

4896.11
5

0.0613563
4

0.0613563
4

0
4

0

0

0

0

0

0

0

0

0

0

0

0

0

0

0

0

0

0

0

0

0

0

0

0

0

0

0

0

0

0

0

0

0

0

0

0

0

0

0

0

0

0

0

0

0

0

0
4

0

0

0

0
4

0.00876519

0

0

0

0

0

0

0

0

0

0

0
4

0

0

0

0

0

0

0

0

0

0

0
4

0

0

0

0

0

0

0

0

0

0

0.0175304
4

0

0

0

0

0

0

0

0

0

0

0

0

0

0

0

0

0

0

0

0

0

0
4

0

0

0

0

0

0

0

0

0

0

0
4

0

0

0

0

0

0

0

0

0

0

0.0350607
4

0

0

0

0

0

0

0

0

0

0

0
4

0
4

4895.69
5

24.2189

5.52742

0.174412
2

4.3818

0
4

0

0
4

0.0562243

0

0.133075

0

0.0250534

0

1.14124

1.28335
7

0

0

0

0.21317

0

0.174282

0.110907

0

0

0

0
3

0

0.0229265

0.0475315

0

0

0

0

0

0

0.0229265

0
4

0

0.0475315

0.0343898

0

0

0

0.0633753

0

0.0469069

0.310066

1.76534

0

0

0.0475315

0

0.137559

0

0.158438

0

0.0343898

0

5.96972

0

0.0316877

0

0.045853

0

0

0.513944

0

0

0

1.19218

0.0316877

0.0316877

0

0

0

0.0343898

0

0

0

0

0

0.0792192

0

0.0343898

0.0316877

0

0

0.239622

0

0

0

0

0

0.0229265

0

0
4

0

0

0

0

0

0
4

0

0

0
4

4855
5

1599.52
5

0.160486
3

244.093
3

5.90017
4

671.117
5

0
4

30.8247

0.389751

0

0.0229265

0

0

0.366824

0.126096

0

0

0

0

0.928524

0.045853

0

0

0

0

0

0.0343898

0.0343898

0.49292

0

1.40998

0

0.126096

0

0

0

0

0.0343898

0.0229265

0

0.447067

0.355361

0

0.0229265

0

0.252192

0.309508

0.0343898

0.045853

0

0

0

0

0.045853

0

0.114633

0

0.0343898

0

0.0229265

0.0802428

0.0229265

0

0
4

0

0

0

0

0.389751

0.0573163

0

0.0343898

0.0229265

0.0802428

0

0

0

0.045853

0

0

0.252192

0

0.091706

0.0343898

0

1.19218

0.0229265

0.0229265

0.0229265

0.045853

0

0

0.0229265

0

0.045853

0

0

0.114633

0.0229265

0.126096

0

0.137559

0

0

0.160486

0

0

0

0.045853

0.126096

0

0.0573163

0.0802428

0

0.091706

0.0229265

0

0

0.275118
4

0.974377

0.0229265

0.149022

0

0.0229265

0

0

0
3

0

0.0573163
4

0.722185

0
4

0

0.859744

0
4

0.045853

0.114633
4

0.619016

0

0
4

0

0

0.49292

0

0
4

0

0.95145

26.996

0

0

0

0.217802

0

2.20095

0

0.0229265

0

0

14.4896

0.194875

0

0.515847

0.997303

0.447067

0.183412

0.149022

0.504383

0.584626

0

61.936

0

0

0

0

0.320971

0.0687795

0

0

0

0

0
4

0.641942

0

0.309508

0

0

0

0.641942

0.378287

0.733648

0

0
3

0.0229265

0

0

1.54754

0

0.0343898

0.298045

0

0

0.905597

0
4

0

0

0.217802

0

0

1.23803

0.126096

0

0.206339

0

0
4

0

1.08901

0.194875

0

0

0

0.699259

0.596089

0.859744

0

0.0802428
4

14.9595

0.435161

0

0

0.149022

0

0

0

0.298045

0

1.74241

0.091706
4

0

0.355361

0.481457

0

0

0.42414

0

0

1.02104

0.447067

8.91841

0

0

0.298045

0.504383

0

0.722185

0.091706

0.905597

0

0.802428

0
4

0

0

1.58193

0.0802428

0.229265

0.756575

0.332434

0.309508

0.286581

0

9.04451

0.091706

1.73095

0.928524

0

0.286581

0

0

0.0802428

0.0573163

0.0687795

0
4

0.653406

0.194875

0.240728

0.813891

0

0.378287

0

0.298045

0.140721

0

0
4

0.045853

0

0

0.194875

0.538773

0.98584

0

0.103169

0.217802

0

0.0229265
4

0

0

0

0.045853

0.0573163

0

0.0802428

0.378287

0.0343898

0.114633

0
4

0

0.252192

0.091706

1.27242

0

0.234534

0

0

0.928524

0

0
4

0.756575

0

0.126096

0.194875

0

0

0.5617

0.103169

0.275118

0.137559

37.0951

0
4

0.0802428

0

0

0.229265

0.240728

0.091706

0

0.0687795

0.045853

0

0
4

0

0

0.0573163

0.0802428

0.183412

0.52731

0.5617

0

0

0

7.56575

0.137559

0

0

0.45853

0

0.275118

0.114633

0.126096

0

0

5.2731

0

0

0.0687795

0.194875

0.0802428

0.905597

0

0.217802

0

0

0
4

0.194875

0.149022

0.745112

0

0

0

0.091706

0

0.160486

0

6.00675

0.103169

0

0

0.0687795

0.0687795

0

0.103169

0.0573163

0

0

2.44167

0

0

0

0.366824

0

0

0.091706

0

0.045853

0.0687795

0
4

0

0

0.355361

0.0573163

0.0229265

0

0.103169

0

0

0

2.87728

0

0

0.0802428

0.653406

0.676332

0.126096

0

0

0.504383

0.045853

4.63116

1.39852

0.91706

0

0

0

0

0.263655

0

0

0

1707.21
6

0
3

0

0

0

0.0343898

0

0

0.0802428

0.0573163

0

0

3.10654

0.0229265

0.045853

0.0802428

0.171949

0

0

0

0.103169

0.091706

0.0343898

0
3

0

0

0.114633

0

0.0229265

0.0573163

0

0

0.0229265

0.0229265

0.0229265
4

0.0343898

0

0

0

0.0343898

0.114633

0.0229265

0

0

0.366824

3.95482

0.0343898

0

0.0229265

0.0573163

0.045853

0

0.0573163

0.0229265

0.091706

0.217802

0
4

0

0.045853

0

0.0802428

0

0

0.0229265

0

0.0687795

0

2.07485

0

0.0343898

0

0

0

0

0

0

0

0.0343898

0
4

0.149022

0

0.0573163

0

0.0687795

0.103169

0.103169

0

0

0.045853

0
4

0

0.149022

0.0687795

0.0229265

0

0.309508

0.137559

0.0343898

0

0

2.71679

0.343898

0

0

0

0

0.0229265

0

0

0

0

82.2832

5.0553

0.0229265

0

0.045853

0.0343898

0.0687795

0.0343898

0

0

0

0.0687795

17.149

0

0

0

0.733648

0

0.103169

0

0.103169

0.0229265

0

0
4

0

0

0.0802428

0.160486

0.091706

0.045853

0

0

0

0

0
4

0.0229265

0.263655

0

0.401214

0

0.114633

0

0

0.149022

0.045853

1.29535

0.091706

0.0343898

0

0.401214

0.0573163

0.252192

0.209669

0

0

0.0229265

0
4

0.871207

0

0

0.0343898

0.0229265

0

0.607553

0.206339

0.0573163

0

0
3

0.0343898

0

0

0

0

0.0343898

0.045853

0.0343898

0

0

0
3

0.045853

0

0

0

0.0229265

0.045853

0

0

0

0

6.90088

0

0

0.045853

0

0.0687795

0.0802428

0.0343898

0

0.332434

0.0687795

0
4

0

0.0229265

0.137559

0

0

0.0343898

0

0

0.0343898

0

0.0469069
4

1.7768

0.263655

0.0229265

0.137559

0

0.0687795

0

0.091706

0

0

0.126096

0

0.0343898

0

0

0

0

0

0.045853

0

0.0343898

0

0.756575

0

0

1.02023

0.091706

0.0343898

0.0229265

0

0.0687795

0

0

0
4

0

0.0343898

0

0.0229265

0

0

0.206339

0

0.160486

0.0573163

0
4

0.0343898

0

0.091706

0.42414

0.171949

0.229265

0

0

0.0229265

0

1.05462

0.0343898

0

0

0.309508

0

0.0573163

0

0.0229265

0

0

1.15779

0

0.412677

0.045853

0

0

0.0687795

0

0

0

0

0
4

0.103169

0.0343898

0

0.0229265

0.0229265

0

0.0229265

0.0343898

0

0

0.515847

0

0

0

0.298045

0.0343898

0

0

0.0343898

0

0

0.481457

0.103169

0

0.160486

0

0.0687795

0

0

0.0229265

0.389751

0.469993

0
4

0
4

0

0

0

0

0.0229265

0.160486

1.42144

0.194875

0.045853

0.0229265

0.768038

0

0

0.0229265

0.0229265

0.0343898

0.0573163

0.0343898

0

0

0

0
4

0

0.045853

0.263655

0

0

0

0.126096

0

0

0.149022

4.68847

0

0

0.0573163

0

0.103169

0

0

0.126096

0

0.0343898

1.54754

0

0

0

0.0229265

0

0.149022

0.813891

0.240728

0.206339

0.0229265

0
3

0

0

0.045853

0.0229265

0

0

0.779501

0.0229265

0

0.0229265

1.23803

0.0573163

0.573163

0

0.0343898

0.114633

0.0229265

0.091706

0

0

0.309508

0
4

0

0

0

0.0802428

0

0

0.0687795

0.0343898

0

0.137559

0.939987

0.0343898

0

0.126096

0

0.206339

0.0802428

0

0.0687795

0.00305919

0

0
4

0.0802428

0

0

0.0802428

0.229265

0.263655

0.0343898

0

0.045853

0.0229265

0
3

17.7415

0

0

0.091706

0.0229265

0

0.0229265

0.045853

0.263655

0

0.126096

0

0.0687795

0.0229265

0.0343898

0.0573163

0.0229265

0

0

0.0229265

0.217802

0.137559

0.332434

0

0

0.0229265

0.0229265

0.52731

0

0

0

0.126096

0

1.40998

0.045853

0

0.114633

0.0802428

0.0229265

0

0

0.607553

0.045853

1.02023

6.82064

0

0

0

0

0.0687795

0

0.160486

0.45853

0.045853

0

0

0.0229265

0

0

0.045853

0

0

0

0

0.126096

0

1.87997

0.149022

0.160486

0.0343898

0.103169

0.309508

0.0229265

0

0.0573163

0.0229265

0.045853

0.0316877
4

0.091706

0

0

0

0

0.045853

0.0343898

0

0.114633

0

1.20364

0

0

0.206339

0

0

0

0

0

0

0.0229265

0.229265

0.103169

0.206339

0.0229265

0.137559

0.0687795

0.0802428

0

0

0.0229265

0.045853

89.8719

3.55361

10.5118

0.481457

0.0229265

1.63925

0.504383

0.0573163

0.0229265

0

0.045853

0.538773

0

0

0.0229265

0

0.126096

0.0229265

0

0

0.515847

0

0

0
4

0.117267

0

0

0.217802

0

0.0573163

0

0.0229265

0

0.0802428

0

0

0

0

0

0

0

0.0573163

0

0.0229265

0

0
4

0.733648

0

0.0573163

0.0229265

0

0.275118

0.0229265

0

0

0

3.17532

0.298045

0.0343898

0.0343898

0.103169

0

0

0.0343898

0

0.320971

0

2.49899

0.0229265

0

0.298045

0.0343898

0.0343898

0.0343898

0

0.194875

0

0

0.905597

0.045853

0

0.0229265

0

0

0

0

0

0

0

1.36413

0.103169

0.0802428

0.309508

2.44167

0.0343898

0

0.0229265

0

0.00458879

0.0229265

0.217802

0

0

0.171949

0.0687795

0

0.045853

0

0.275118

0.00305919

0

9.52952040195187e-12
5

0
4

0

0

0

0
4

1.22184
4

1.22184

0
4

0

0

0

0
4

14.693

14.3646
5

0

0

0

0

0

0

0

0

0

0

0

0.281441

0

0.0469069

5.62050406216485e-16

0
4

0

0

0
4

0

0

0
4

0.263017

0.263017

0
4

0

0

0
4

0

0

0
4

0

0

0
4

0.283249

0.283249

0
4

0.00590715

0.00590715

0
4

0
4

0

0

0

0
4

0
4

0

0

0

0
4

0
4

0

0

0

0
4

0
4

0

0

0

0
4

0
4

0.357411
4

0.357411
4

0

0

0.357411
4

0
4

0

0

0

0
4

0

0

0
4

0

0

0
4

0
4

0
4

0
3

0

0

0

0

0

0

0
4

0
4

0

0

0

0
4

0
4

0

0

0

0
4

0
4

0

0

0

0

0
4

0
4

0

0

0

0

0
4

0
4

0

0

0

0
4

0
4

0

0

0

0
4

0
4

0

0

0

0
4

0
4

0

0

0

0
4

0
4

0

0

0

0
4

0
4

0

0

0

0
4

0
4

0
4

6.14847

6.14847

6.14847

0.0425139

0.0850278

0.0595195

0

0

0.119811

0

0

0.0850278

0

0

0.27882
1

0

0

0.536117

0

0

0

0

0

0

0

1.40778

0

0

0.714822

0

0

0

0.239622

0

0.179716

0

0.175292

0

0

0

0

0

0

0

0.402252

1.76721

0.0549439

0

0

1.49880108324396e-15

0
4

0
4

0

0

0

0
4

0
4

0
4

17.0135

17.0135

15.4919

0.903127
7

0

0

0

2.68822

0

1.94891

0

0

0

0

1.78275
6

0.121392

0

0

0

0

0

0

0

0

0.141625

0

0

0

0

0

0

0

0

0

0

0

0

0

0

0

0

0

0

0

0.0809283

0.0404642

0

5.9143
6

0

0

0

0

0

0

0

0

1.87023

0

0

0
4

0

0

0

0

0

0

0
4

1.16209

1.16209

0
4

0

0

0

0
4

0

0

0

0
4

0

0

0
4

0.359433

0.359433

0
4

2.94209101525666e-15

0
4

0

0

0

0
4

0
4

0

0

0

0
4

0
4

0

0

0

0
4

0
4

0
4

25.6343
7

25.6343
7

22.9115
7

5.94102
7

0

0

0

0

0

0

0

0

0

0

10.9978
6

2.79247
7

0

0.399925

2.72928

0.0340111

0.0170056

0

0
4

2.71347
6

2.14447

0.536117

0.0328872

6.24500451351651e-17
6

0
4

0.00939633

0.00939633

0

0
4

0
4

0
4

0.890211
7

0.890211
7

0.890211
7

0.829515

0.0606962

0

0

0

0
4

0
4

0
4

0

0

0

0

0
4

0
4

0
4

0

0

0

0

0
4

0
4

0
4

0

0

0

0

0
4

0
4

0
4

0

0

0

0

0
4

0
4

0
4

0

0

0

0

0
4

0
4

0
4

0

0

0

0

0
4

0
4

0
4

0.161857

0.161857

0.161857

0.161857

0
4

0
4

0
4

0

0

0

0

0
4

0
4

0
4

0

0

0

0

0
4

0
4

0
4

0

0

0

0

0
4

0
4

0
4

0.0487709
7

0.0487709
7

0.0487709
7

0.0100127
7

0.0387582
7

0

0
4

0

0

0
4

0
4

0
4

1.70996

1.70996

1.70996

1.70996

0
4

0
4

0
4

2.56494

2.56494

2.56494

2.56494

0
4

0
4

0
4

0

0

0

0

0
4

0
4

0
4

0.007332

0.007332

0.007332

0.007332

0
4

0
4

0
4

0

0

0

0

0
4

0
4

0
4

0

0

0

0

0
4

0
4

0
4

0

0

0

0

0
4

0
4

0
4

1.70996

1.70996

1.70996

1.70996

0
4

0
4

0
4

0

0

0

0

0
4

0
4

0
4

0

0

0

0

0
4

0
4

0
4

0

0

0

0

0

0

0
4

0

0

0
4

0
4

0
4

0

0

0

0

0
4

0
4

0
4

0

0

0

0

0
4

0
4

0
4

0.16497

0.16497

0.16497

0.16497

0
4

0
4

0
4

0

0

0

0

0
4

0
4

0
4

1.70996

1.70996

1.70996

1.70996

0
4

0
4

0
4

0.0168565

0.0168565

0.0168565

0.0168565

0
4

0
4

0
4

0

0

0

0

0
4

0
4

0
4

0

0

0

0

0
4

0
4

0
4

0

0

0

0

0
4

0
4

0
4

0

0

0

0

0
4

0
4

0
4

0.151696
7

0.151696
7

0.127511
7

0.0676061

0.0599054

0

1.38777878078145e-17
7

0
4

0.0241844

0.0103648

0.00690984

0.00690984

0
4

0
4

0
4

0

0

0

0

0
4

0
4

0
4

0

0

0

0

0
4

0
4

0
4

0.00500636

0.00500636

0.00500636

0.00500636

0
4

0
4

0
4

3.41991

3.41991

3.41991

3.41991

0
4

0
4

0
4

0

0

0

0

0
4

0
4

0
4

0

0

0

0

0
4

0
4

0
4

0

0

0

0

0
4

0
4

0
4

0

0

0

0

0
4

0
4

0
4

0

0

0

0

0
4

0
4

0
4

0

0

0

0

0
4

0
4

0
4

0.147653

0.147653

0.100121

0.036746

0

0

0.0633753

0
4

0.0475315

0

0.0475315

0
4

1.38777878078145e-17

0
4

0
4

0

0

0

0

0
4

0
4

0
4

0

0

0

0

0
4

0
4

0
4

0

0

0

0

0
4

0
4

0
4

0.0549439

0.0549439

0.0549439

0.0549439

0
4

0
4

0
4

0.00690984

0.00690984

0.00690984

0.00690984

0
4

0
4

0
4

0

0

0

0

0
4

0
4

0
4

0.0425139

0.0425139

0.0425139

0.0425139

0
4

0
4

0
4

0

0

0

0

0
4

0
4

0
4

0.914685

0.914685

0.914685

0.914685

0
4

0
4

0
4

0.0229265

0.0229265

0.0229265

0.0229265

0
4

0
4

0
4

0

0

0

0

0

0

0
4

0
4

0
4

0

0

0

0

0
4

0
4

0
4

0.536117

0.536117

0.536117

0.536117

0
4

0
4

0
4

0

0

0

0

0
4

0
4

0
4

0

0

0

0

0
4

0
4

0
4

0

0

0

0

0
4

0
4

0
4

0

0

0

0

0
4

0
4

0
4

0

0

0

0

0
4

0
4

0
4

0

0

0

0

0
4

0
4

0
4

0

0

0

0

0
4

0
4

0
4

0.357411

0.357411

0.357411

0.357411

0
4

0
4

0
4

0.0599054

0.0599054

0.0599054

0

0

0.0599054

0

0
4

0
4

0
4

0.00690984

0.00690984

0.00690984

0.00690984

0
4

0
4

0
4

0

0

0

0

0
4

0
4

0
4

0.256576
7

0.256576
7

0.256576
7

0.256576

0

0

0
4

0
4

0
4

0

0

0

0

0

0

0
4

0

0

0
4

0
4

0
4

0.0229265

0.0229265

0

0

0
4

0

0

0

0

0
4

0.0229265

0.0229265

0
4

0
4

0
4

36.3573
7

36.3573
7

36.3573
7

35.6425
7

0.357411

0.357411

0

2.99760216648792e-15
7

0
4

0
4

0

0

0

0
4

0
4

0
4

0

0

0

0

0

0

0
4

0
4

0
4

3.77733

3.77733

3.77733

3.77733

0

0
4

0
4

0
4

0.536117

0.536117

0.536117

0.536117

0
4

0
4

0
4

0.164295

0.164295

0.164295

0.164295

0
4

0
4

0
4

2.02297

0.117449

0.094522

0.094522

0
4

0.0229265

0.0229265

0
4

0
4

1.90552

0.195564

0.195564

0
4

1.70996

1.70996

0
4

0
4

0
4

0

0

0

0

0

0
4

0
4

0
4

0.0255083

0.0255083

0.0255083

0.0255083

0

0
4

0
4

0
4

0

0

0

0

0

0
4

0
4

0
4

0

0

0

0

0
4

0
4

0
4

0

0

0

0

0
4

0
4

0
4

0
2

0
2

0
2

0
2

0

0

0

0

0
4

0
2

0

0

0

0

0

0

0
4

0

0

0
4

0

0

0
4

0
4

0
4

0

0

0

0

0

0

0
4

0
4

0
4

0.378997

0.378997

0.378997

0.378997

0
4

0
4

0
4

0

0

0

0

0
4

0

0

0
4

0

0

0
4

0
4

0
4

0

0

0

0

0

0
4

0
4

0
4

0

0

0

0

0

0

0

0
4

0
4

0
4

0

0

0

0

0
4

0
4

0
4

0.0573163

0.0573163

0.0573163

0.0573163

0

0
4

0

0

0
4

0
4

0

0

0

0
4

0
4

0
4

0

0

0

0

0

0
4

0

0

0
4

0
4

0
4

0

0

0

0

0
4

0
4

0
4

0

0

0

0

0

0

0
4

0
4

0
4

1.70996

1.70996

1.70996

0

0

1.70996

0

0

0

0
4

0
4

0
4

0

0

0

0

0

0
4

0

0

0
4

0
4

0
4

0.241994

0.241994

0.241994

0.241994

0
4

0
4

0
4

0

0

0

0

0
4

0
4

0
4

0

0

0

0

0
4

0

0

0
4

0
4

0
4

0

0

0

0

0

0
4

0

0

0
4

0
4

0
4

0.479243

0.479243

0.479243

0.479243

0

0
4

0
4

0
4

0

0

0

0

0

0
4

0
4

0
4

0

0

0

0

0
4

0
4

0
4

0.435161

0.435161

0.435161

0.0599054

0.375255

0
4

0
4

0
4

0

0

0

0

0

0
4

0

0

0
4

0
4

0
4

0.780144
4

0.780144
4

0.780144
4

0.460835

0.282551

0.0316877

0.00507012

0

0

2.68882138776405e-17
4

0
4

0

0

0
4

0
4

0
4

0

0

0

0

0
4

0
4

0

0

0

0
4

0
4

0
4

0

0

0

0

0
4

0

0

0
4

0

0

0
4

0
4

0
4

0.0606962

0.0606962

0.0606962

0.0606962

0

0
4

0
4

0
4

0.00851243

0.00851243

0.00851243

0.00500636

0.00350607

0
4

0
4

0
4

0.00701215

0.00701215

0.00701215

0.00350607

0.00350607

0
4

0
4

0
4

0

0

0

0

0
4

0

0

0
4

0
4

0
4

0

0

0

0

0
4

0

0

0
4

0
4

0
4

0

0

0

0

0
4

0
4

0
4

0

0

0

0

0
4

0

0

0
4

0
4

0
4

1.96576

1.96576

1.96576

1.96576

0
4

0

0

0
4

0
4

0
4

0.448467
7

0.448467
7

0.371556
7

0

0.0898581

0

0.131935

0.149764

0

0

0

0
4

0.076911

0.0599054

0.0170056

0
4

5.55111512312578e-17
7

0
4

0
4

0

0

0

0

0
4

0
4

0
4

0

0

0

0

0
4

0
4

0
4

0

0

0

0

0

0
4

0
4

0
4

0

0

0

0

0
4

0

0

0
4

0
4

0
4

0.357411

0.357411

0.357411

0.357411

0
4

0
4

0
4

0

0

0

0

0
4

0

0

0
4

0
4

0
4

0

0

0

0

0
4

0

0

0
4

0
4

0
4

0

0

0

0

0

0
4

0
4

0
4

0

0

0

0

0
4

0

0

0
4

0
4

0
4

0.357411

0.357411

0.357411

0.357411

0

0
4

0
4

0
4

0.0555929

0.0555929

0.0555929

0.048555

0

0.00703794

0

0

0

1.73472347597681e-18

0
4

0

0

0
4

0
4

0

0

0

0

0
4

0
4

0
4

0

0

0

0

0
4

0
4

0
4

0

0

0

0

0
4

0

0

0
4

0
4

0
4

0

0

0

0

0
4

0
4

0
4

0

0

0

0

0

0
4

0
4

0
4

0

0

0

0

0
4

0
4

0
4

1.70996

1.70996

0

0

0
4

1.70996

1.70996

0
4

0
4

0
4

0

0

0

0

0
4

0
4

0
4

0

0

0

0

0
4

0

0

0
4

0
4

0
4

0

0

0

0

0
4

0

0

0
4

0
4

0
4

0

0

0

0

0

0
4

0
4

0
4

0.298627
5

0.298627
5

0.270855
5

0.262342
5

0.00500636

0.00350607

1.73472347597681e-18
5

0
4

0.027772

0.00701215

0.0155008

0.00525911

0

1.73472347597681e-18

0
4

3.81639164714898e-17
5

0
4

0
4

0

0

0

0

0

0
4

0
4

0
4

0

0

0

0

0

0
4

0
4

0
4

0

0

0

0

0

0
4

0
4

0
4

0

0

0

0

0
4

0
4

0
4

0

0

0

0

0
4

0

0

0
4

0
4

0
4

0.63118

0.63118

0.63118

0.63118

0
4

0
4

0
4

0

0

0

0

0
4

0
4

0
4

0.106314

0.106314

0.106314

0.106314

0
4

0
4

0
4

0

0

0

0

0
4

0

0

0
4

0
4

0
4

0.0282575

0.0282575

0.0282575

0.0282575

0
4

0
4

0
4

2.1519
7

2.14839
7

1.54464
7

0.991516
7

0.553122

0
4

0.0538193

0.0368138

0.0170056

0
4

0.549937

0.549937

0
4

2.22044604925031e-16
7

0
4

0.00350607

0.00350607

0.00350607

0
4

0
4

1.14491749414469e-16
7

0
4

0

0

0

0

0

0
4

0
4

0
4

0.0316877

0.0316877

0.0316877

0.0316877

0
4

0
4

0
4

0

0

0

0

0
4

0
4

0
4

0.459471

0.459471

0.459471

0.459471

0
4

0
4

0
4

0.536117

0.536117

0.536117

0.536117

0
4

0
4

0
4

0

0

0

0

0
4

0
4

0
4

0

0

0

0

0
4

0
4

0
4

0

0

0

0

0
4

0
4

0
4

0

0

0

0

0
4

0
4

0
4

0

0

0

0

0
4

0
4

0
4

10.0614

9.44116

7.74531

0.729842

0

0

0

0

0.933992

0.464867

0

0.0155008

0

0.0404642

0.571636

0

0

0

0

0.0340111

0.20597

0.596022

0.418107

0

0

0.0316877
4

0.00750954

0.0404642

0

0

0

0

0

0

0

0

0

1.25094

0.014664

0

0

0.536117

0

0.0469069

0

0

0

0

0.303481

0

0.007332

0

0.0316877

0.01833

0

0.357411

0.025662

0

0

0

0

0

0

0

0

0

0

0

0

0

0.27495

0

0

0

0

0

0

0.0112449

0.0506019

0

0.0792192

0

0

0

0.424874

0

0

0.221814

0

0

1.66533453693773e-16

0
4

0.802979

0.721056

0.0345492

0.0404642

0.00690984

0
4

0.0475315

0.0475315

0
4

0

0

0
4

0.119811

0.119811

0
4

0.0380041

0.0380041

0
4

0.00690984

0.00690984

0
4

0

0

0

0

0
4

0

0

0

0
4

0

0

0

0

0
4

0.466414

0.445684

0.0138197

0.00690984

0
4

0.214205

0.214205

0

0
4

0

0

0
4

0

0

0
4

0

0

0
4

0
4

0.568387

0.568387

0.43532

0.0276393

0.095063

0.0103648

0
4

0
4

0.0518238

0.0380041

0.0207295

0.00690984

0.0103648

3.46944695195361e-18

0
4

0.0138197

0.0138197

0
4

1.73472347597681e-18

0
4

0

0

0

0

0
4

0
4

0

0

0

0
4

0
4

0

0

0

0
4

0
4

1.36002320516582e-15

0
4

0

0

0

0

0
4

0
4

0
4

0
4

6.93901
3

6.25443
3

5.98485
3

5.98485

0

0

0

0

0

5.98485

0

0

0

0

0

0
4

0
3

0
3

0

0

0
4

0

0

0
4

0

0

0

0

0

0

0

0

0

0
4

0

0

0

0

0

0
4

0

0

0
4

0

0

0
4

0

0

0
4

0

0

0
4

0

0

0

0
4

0

0

0
4

0

0

0

0
4

0

0

0

0
4

0

0

0
4

0

0

0

0
4

0

0

0
4

0

0

0
4

0
4

0

0

0

0

0

0

0

0

0
4

0
4

0

0

0

0

0
4

0
4

0

0

0

0
4

0
4

0.269574

0.269574

0.269574

0
4

0
4

3.33066907387547e-16
3

0
4

0.29392

0.29392

0.29392

0.282675

0.0112449

0

0
4

0
4

0
4

0

0

0

0

0
4

0
4

0
4

0

0

0

0

0
4

0
4

0
4

0

0

0

0

0
4

0
4

0
4

0

0

0

0

0
4

0
4

0
4

0

0

0

0

0
4

0
4

0
4

0

0

0

0

0
4

0
4

0
4

0

0

0

0

0
4

0
4

0
4

0

0

0

0

0
4

0
4

0
4

0.0112449

0.0112449

0.0112449

0.0112449

0
4

0
4

0
4

0

0

0

0

0

0
4

0
4

0
4

0.0220119

0.0220119

0.0220119

0.0170056

0.00500636

0
4

0
4

0
4

0

0

0

0

0
4

0
4

0
4

0.357411

0.357411

0.357411

0.357411

0
4

0
4

0
4

0

0

0

0

0
4

0
4

0
4

0

0

0

0

0
4

0
4

0
4

0

0

0

0

0
4

0
4

0
4

5.55111512312578e-17
3

0
4

2583.77

1.12476
4

0

0

0

0

0

0

0

0

0

0

0

0

0

0

0

0

0

0

0

0

0

0

0

0

0

0

0

0

0

0

0

0

0

0

0

0
4

0

0

0

0

0
4

0

0

0

0
4

0

0

0

0
4

0
4

0
4

0

0

0

0

0

0
4

0

0

0

0

0
4

0

0

0
4

0

0

0
4

0
4

0

0

0

0

0

0
4

0

0

0

0
4

0

0

0
4

0
4

0.205401
6

0.205401
6

0.205401
6

0
4

0
4

0

0

0

0
4

0

0

0
4

0
4

0

0

0

0

0
4

0

0

0

0
4

0

0

0
4

0
4

0

0

0

0

0
4

0
4

0

0

0

0

0

0
4

0
4

0

0

0

0

0
4

0

0

0
4

0
4

0.0336511

0.0336511

0.0336511

0
4

0
4

0

0

0

0
4

0

0

0
4

0
4

0
4

0
4

0

0

0

0

0

0

0

0

0

0

0

0

0

0

0

0

0

0
4

0

0

0

0

0

0

0
4

0

0

0

0

0

0

0

0
4

0
4

0

0

0

0
4

0
4

0

0

0

0
4

0
4

0

0

0

0
4

0
4

0

0

0

0
4

0
4

0

0

0

0
4

0
4

0

0

0

0
4

0
4

0

0

0

0
4

0
4

0
4

0
4

0

0

0

0

0

0

0

0

0

0

0
4

0

0

0
4

0

0

0

0
4

0

0

0
4

0
4

0
4

0
4

0

0

0

0

0
4

0

0

0

0

0
4

0

0

0
4

0

0

0
4

0
4

0
4

0
4

0

0
4

0

0

0

0

0

0

0

0
4

0
4

0

0

0

0

0

0

0

0

0
4

0

0

0

0

0
4

0

0

0

0

0

0
4

0

0

0
4

0

0

0
4

0
4

0.885708
6

0.885708
6

0.885708
6

0

0

0
4

0
4

0

0

0

0

0

0

0

0

0

0

0
4

0

0

0

0
4

0

0

0

0

0
4

0

0

0
4

0
4

0

0

0

0

0

0

0

0
4

0

0

0
4

0
4

0
4

6.42898
5

6.42898
5

0.135172

0.135172

0
4

6.29381
5

5.14688
5

0

0

0

0.450942

0

0

0

0.695993

0

0

0

8.88178419700125e-16
5

0
4

0
4

0
4

2471.42
7

2.68449
6

2.67948
6

1.55303

1.12646

0
4

0.00500636

0.00500636

0
4

0
4

88.202

14.4699

4.0013

0
1

0

0.161857

0.0262956

0.049085

0.00876519

0.0771336

0

0.240166

0.0105182

1.53566

0.152514

0.0280486

0.00701215

0.045579

0.0403199

0.0140243

0.108688

0.00701215

0.00525911

0.00525911

0.918175

0.0122713

0

0

0.00525911

0.0245425

0.0280486

0.00525911

0.0298016

0.00876519

0.00350607

5.08984

0.00350607

0.0403199

0.00350607

0.0105182

0

0.00350607

0.00350607

0

0.0631093

0.0404642

0.361126

0.0140243

0.00350607

0.211081

0.00350607

0.0262956

0.0262956

0.0210364

0.0105182

0.00876519

0.00350607

0.0403199

0.0157773

0

0.164174

0

0.00350607

0.0438259

0.00350607

0

0.00701215

0.440012

0.124466

0.119207

5.6621374255883e-15

0
4

1.37578

0.546266

0

0

0.708123

0

0.0809283

0

0

0

0.0404642

0
4

24.1146

6.22605

0

0

0

0

0.128202

0

0.0404642

0

0

0.0112449

0.0112449

0.141625

0

0

0

0

0

0

2.1446

0

0.0404642

0

0

0

0

0

0.0224897

0

0

0

0.0404642

0

0

0

0

0

0

0

0

0

0.141625

0

0

0.202321

0

0

0

0

0

0

0

0

0

0

0

0

0

0

0.182089

0

0

0

0

0

0

0

0

0

0

0

0

0

5.31115
6

0

0

0

0

0

0

0

0

0

0

0.120285
7

0

0

0

0.0404642

0

0.0404642

0

0

0

0

0.0809283
8

0

0

0

0

0.0549439

2.56494

0

0

0.357411

0

0.986641

1.73996

0

0

0

0

0

0

0

0

0

0.183146

0.0112449

0

0

0.141625

0

0

0

0

0

0

0.151289

0

0

0.0281121

0

0.0809283

0.0404642

0

0

0

0

0.0629539
7

0

0

0

0

0

0

0

0.303611

0

0

0.161857

0.0938138

2.22553

0

0

0

0

0

0

0

0

0
4

1.96777
6

0.00525911

0

1.96251

0

0

0

0
4

0

0

0
4

0.00750954
7

0

0.00750954

0
4

0.141625

0.0809283

0.0606962

0

1.38777878078145e-17

0
4

0

0

0

0

0
4

0.027535

0

0

0.027535

0
4

0.977839

0.977839

0

0
4

0

0

0
4

0.0469069

0.0469069

0

0
4

0.0150191

0.0100127

0.00500636

0
4

33.4954

30.9306

0

0.0809283

0.456555

0

0.222553

1.80479

0

0

0

0

0
4

0.0454705

0.0454705

0
4

0

0

0

0
4

0.0809283

0.0809283

0
4

1.75042

1.70996

0.0404642

0
4

0

0

0
4

0

0

0
4

0.0112449

0.0112449

0
4

0.00500636

0.00500636

0
4

0

0

0
4

0

0

0
4

0.800413

0.0861874

0.553964

0.123021

0.0337346

0

0.00350607

0

0
4

0

0

0
4

0

0

0
4

0.0112449

0.0112449

0
4

0

0

0
4

0

0

0
4

0

0

0
4

0

0

0
4

0.0404642

0.0404642

0
4

0.10116

0.10116

0
4

0.0404642

0.0404642

0
4

1.58573

0.188358
7

0.00750954

0

0.571345

0.242785
7

0.505642

0.0125159

0.0525668

0

0.00500636

0

3.38271077815477e-16

0
4

0.00750954

0.00750954

0
4

0.182089

0.182089

0
4

0

0

0
4

0

0

0
4

0.546266

0.546266

0
4

0.357411

0.357411

0
4

0

0

0
4

0

0

0
4

0.357411

0.357411

0
4

0.00500636

0.00500636

0
4

1.43653

0.50806

0

0

0

0.10116

0

0.576581
6

0.117558

0

0.133171

0

0

0

0

0
4

0

0

0
4

0

0

0
4

0

0

0
4

0.602348

0.215273

0.0666674

0.0125159

0.0826049

0.182732

0

0

0.0300382

0.0125159

0
4

1.5142

1.40603
7

0

0.10116

0

0

0.00701215

3.46944695195361e-18

0
4

1.81067

0.0504769

0

1.76019

0

0

0
4

0.270113

0.222553

0.00750954

0.0125159

0.0150191

0

0.00500636

0.00750954

0
4

0
4

2356.57
7

119.832
6

26.8976
6

7.94925
7

3.74041

2.08002

0.739831

0.0893428

0.397297

0.466245

0.599159

0.642286

3.72411

0.345236

9.23672
6

1.77455

0

1.77772

0.239622

0.337391

0.0122713

0.19667

2.60589

0.0150191

0.149764

0.17881

0.0915931

0.0982093

0.0894049

0.375255

0.149764

3.45507

0.119811

0.0599054

0.0898581

0.209669

2.49791

0

0.0898581

0.119811

0.411964

0

0

0.119811

0.239622

0.0469069

0.0599054

1.08519

0.0404642

0.0599054

0.0599054

0.0968956

0.0599054

0.0250534

0.599054

0.0898581

0

0.119811

2.32264
5

0.0599054

0

0.0599054

0.0606962

0.0469069

0.0469069

0.00701215

0.0599054

0.0316877

0

6.72376
6

0.00525911

0.0599054

0.00350607

0.0703603

0.00350607

0.0599054

0.0475315

0.0475315

0.269574

34.5719
6

0.518456
6

1.7874590696465e-14
6

0
4

5.27661
7

5.17924
7

0.00750954

0

0.0898581

0
4

1826.88
7

1518.7
7

305.206
7

0.155008

0.01833

0.0404642

0.082434

0.0898581

0.198185

0

0

0.536117

0

0

0.0809283

0

0.257988

0

0

0

0

0

0

0.00500636

0

0

0

0.01833

0

0.0703603

0.0483689

0

0

0

0

0.0469069

0.104073

0.0404642

0

0.357411

0.453446

0

0.186009

0.120216

0.0562243

4.27921587053959e-14
7

0
4

1.90224
7

1.85513

0

0.0387519

0.00835115

0

0
4

0.985301

0.938394

0

0

0.0469069

0
4

33.8062
7

31.1356
7

0.0792192

0.0873711

0.0404642

0.00500636

0

0.0771336

0

0.0112377

0

0

0

0.0826049
7

0

0.0404642

0

0.00827008

0.0316877

0.0155008

0.00500636

0

0

0.00500636

1.48835
6

0.0454854

0

0.0316877

0

0

0

0.0385975

0.0112449

0.0606962

0.0404642

0.202158

0.263017

1.18793863634892e-14
7

0
4

197.033
6

191.123
6

5.56557

0.183059

0.161857

0
4

85.6801
6

85.476
6

0.140721

0

0.0469069

0.0165402

0

3.94476118437126e-15
6

0
4

44.6483
6

2.05966
7

0.539643
6

0.0743248
7

0.569102

0.0599054

0.617299

0.310193

0

0.0316877

1.23582

0.0125159

0.0124051

0.247014

0

0.0112449

0

0

0.0112449

0.0404642

0

0.0103648

0

0

0.311785
7

0.00750954

0

0.0316877

0

0

0

0

0

26.5844
6

0.13541
7

6.8642
6

1.03145
7

0.116011
7

3.72294
6

1.99840144432528e-14
6

0
4

1.09843
7

1.08376
7

0

0

0

0

0

0.014664

0

0
4

2.84834

0.61791

0.0898581

0.0475315

0.966474

0.0152104

0.110907

0.539149

0.00507012

0.0633753

0.32948

0.0633753

0
4

6.8823
6

6.8823
6

0

0
4

14.0022
6

13.243
6

0.00500636

0.00500636

0.00827008

0.740941

1.33226762955019e-15
6

0
4

0

0

0

0
4

0.629007

0.629007

0
4

0.251545

0.0404642

0.211081

0
4

0

0

0
4

1.64004

1.64004

0
4

0.0126753

0.0126753

0
4

0

0

0
4

0.484478

0.484478

0
4

0.10116

0.10116

0
4

0.00458879

0.00458879

0
4

1.57643
7

1.32511
7

0.150958

0.0599054

0

0.0404642

0
4

0.00350607

0.00350607

0
4

0.656697

0.656697

0
4

0

0

0
4

0

0

0
4

0.0250318

0.0250318

0
4

0.0404642

0.0404642

0
4

0

0

0
4

0

0

0
4

0

0

0
4

0

0

0
4

3.16353
7

3.11183
7

0.0517018

2.98372437868011e-16
7

0
4

0

0

0
4

0.0599054

0.0599054

0
4

0

0

0
4

0

0

0
4

0

0

0
4

0

0

0
4

0.808723

0.808723

0
4

0.049085

0.049085

0
4

0.0316877

0.0316877

0
4

0

0

0
4

0.0595195

0.0595195

0

0

0

0
4

0

0

0
4

0

0

0
4

0.00500636

0.00500636

0
4

0

0

0
4

0.0316877

0.0316877

0
4

1.61603
7

1.61603
7

0
4

0

0

0
4

3.83949

3.83949

0
4

0.396895

0.396895

0

0
4

0.21317

0.21317

0
4

1.14480647184223e-12
7

0
4

3.54453
7

3.54453
6

1.41798

2.00674
6

0.119811

0

0

0
4

0

0

0

0
4

0
4

2.12115
7

1.72474

1.72474

0
4

0.00350607
8

0.00350607
8

0

0
4

0.0633753

0.0316877

0.0316877

0
4

0.007332

0.007332

0
4

0

0

0
4

0.0404642

0.0404642

0
4

0.0227895

0.0227895

0
4

0

0

0
4

0.195564

0.195564

0
4

0.0633753

0.0633753

0
4

0
4

8.23559

2.35222

1.75039

0.601832

4.44089209850063e-16

0
4

0.385786

0.076197

0.161857

0.147733

0

0

0
4

0

0

0
4

0

0

0
4

0

0

0
4

0.0112449

0.0112449

0
4

4.23978
5

4.17641
5

0.0633753

0
4

0.364177
7

0.222553

0.141625

0
4

0.00525911

0.00525911

0
4

0

0

0

0
4

0.233226

0.20027

0.0329558

0
4

0

0

0

0
4

0.633753

0.633753

0
4

0.0101402

0.0101402

0
4

0
4

9.41366
7

9.41366
7

6.4334

0

0

2.94857
6

0.0316877

0
4

0
4

0.510674

0.510674

0.510674

0
4

0
4

0

0

0

0
4

0
4

0

0

0

0
4

0
4

0.0562243

0.0562243

0.0562243

0
4

0
4

0

0

0

0

0

0
4

0
4

0

0

0

0
4

0
4

0.0404642

0.0404642

0.0404642

0

0
4

0
4

0

0

0

0
4

0
4

0.0387582

0.0387582

0.0387582

0
4

0
4

0

0

0

0
4

0
4

0

0

0

0
4

0
4

0

0

0

0
4

0
4

0
4

12.6372

11.8174

7.35849
7

1.45355

0

0

0

0

0.0469069

0

0.0898581

0.0599054

0.281441

0

4.66869
6

0

0

0.0599054

0

0

0.0469069

0.346434

0

0.304895

0

0

1.4432899320127e-15
7

0
4

1.25094

1.25094

0
4

2.95602

0.173826
7

0

1.07223

1.70996

0
4

0

0

0

0
4

0

0

0
4

0

0

0
4

0

0

0
4

0.251928

0.251928

0
4

0
4

0.281063

0.281063

0.0333077

0.175881

0.0420729

0.0105182

0.0157773

0.00350607

1.38777878078145e-17

0
4

0
4

0.0475315

0.0475315

0.0475315

0
4

0
4

0.0475315

0.0475315

0.0475315

0
4

0
4

0.0387582

0.0387582

0.0387582

0
4

0
4

0

0

0

0

0
4

0
4

0.357411

0

0

0
4

0.357411

0.357411

0
4

0
4

0

0

0

0
4

0
4

0

0

0

0
4

0

0

0
4

0
4

0

0

0

0
4

0
4

0.0475315

0.0475315

0.0475315

0
4

0
4

0

0

0

0
4

0
4

0

0

0

0
4

0
4

9.0205620750794e-16

0
4

6.089

5.80222

5.73623

1.63192
6

0.389385

0.0633753

0.0404642

0

0.0599054

0

0

0

0

0

2.09139
6

0

0.00305919

0

0.0404642

0

0.0404642

0

0

0.296135

0.380252

0.65896

0.0404642

0

0

3.7470027081099e-15

0
4

0

0

0
4

0.0107072

0.0107072

0
4

0.0552787

0.0552787

0
4

0

0

0
4

0
4

0

0

0

0

0
4

0
4

0

0

0

0
4

0
4

0

0

0

0
4

0
4

0

0

0

0
4

0
4

0.176201

0.176201

0.176201

0
4

0
4

0

0

0

0
4

0
4

0

0

0

0
4

0
4

0.0207295

0.0207295

0.0207295

0
4

0
4

0.0898581

0.0898581

0.0898581

0
4

0
4

0
4

6.53652

0.142595

0.142595

0.110907

0.0316877

0
4

0
4

1.40421

0

0

0
4

0.392606
1

0

0.301033

0

0.0915731

0
4

1.0116

1.0116

0

0

0
4

0

0

0
4

2.22044604925031e-16

0
4

4.98972

0

0

0

0
4

4.98972

0
1

4.98972

0

0
4

0
4

0
4

46.6849
5

46.4939
5

28.5537

4.91863

22.8554
7

0

0.0915731

0

0

0.00305919

0.137675

0.437102

0

0.0851081

0

0.0125217

0.00500636

0

0.00764798

0
4

4.53611
6

1.8418
6

0.313383
7

1.53149
6

0.0519133

0.718865

0.00750954

0.0599054

0.0112449

0
4

0

0

0
4

0

0

0
4

0

0

0
4

0

0

0
4

0

0

0
4

0.301033

0.301033

0
4

12.548
6

12.548
6

0

0
4

0

0

0

0

0

0

0
4

0

0

0

0

0
4

0

0

0

0

0
4

0

0

0

0
4

0

0

0
4

0.555114

0.555114

0
4

0

0

0
4

0
4

0

0

0

0

0
4

0
4

0.0262639

0.0262639

0.0262639

0

0
4

0
4

0.0633753

0.0633753

0.0633753

0
4

0
4

0.0475315

0.0475315

0.0475315

0
4

0
4

0.0469069

0.0469069

0.0469069

0
4

0
4

0

0

0

0
4

0
4

0.00690984

0.00690984

0.00690984

0
4

0
4

8.30065183254902e-15
5

0
4

1.95383

1.95383

1.30712

1.21246

0.0333077

0.0122713

0.0122713

0.0210364

0.00701215

0.00350607

0.00525911

0
4

0.147255

0.0508381

0.0227895

0.0683684

0.00525911

2.42861286636753e-17

0
4

0.0403199

0.0227895

0.00525911

0.00525911

0.00701215

0
4

0.0140243

0.0140243

0
4

0.445106

0.445106

0
4

0
4

0
4

0
3

0
3

0
3

0
3

0

0
4

0
4

0
4

1.04824
6

1.04824
6

0.977881
6

0.977881
6

0

0
4

0.0703603

0.0703603

0
4

2.77555756156289e-17
6

0
4

0
4

3.67572

3.67572

3.67572

3.67572

0

0

0

0
4

0

0

0

0
4

0
4

0
4

3.35713

3.35713

3.35713

3.24622

0.0792192

0.0316877

2.84494650060196e-16

0
4

0
4

0
4

0
4

0
4

0
4

0
4

0

0

0
4

0

0

0
4

0
4

0
4

0.450703

0.450703

0.450703

0.380342

0

0.0703603

0
4

0
4

0
4

0.595801

0.595801

0.595801

0.588891

0.00690984

0
4

0
4

0
4

0.0404642

0.0404642

0.0404642

0.0404642

0

0

0

0
4

0
4

0
4

0.0387582

0.0387582

0.0387582

0

0.0387582

0
4

0
4

0
4

1.30116

1.30116

1.30116

1.30116

0
4

0
4

0
4

0.184736
6

0.184736
6

0.184736
6

0

0

0.117267

0.0674692

0

0

0

0
4

0
4

0
4

7.38713
6

7.38713
6

7.38713

7.38713

0
4

0

0

0
4

0
4

0
4

0

0

0

0

0

0
4

0
4

0
4

0.283249

0.283249

0.283249

0.283249

0
4

0
4

0
4

0

0

0

0

0

0
4

0

0

0
4

0
4

0
4

0

0

0

0

0

0
4

0
4

0
4

0.0449139

0.0449139

0.0449139

0.0172746

0.00690984

0.0207295

0
4

0
4

0
4

0

0

0

0

0

0
4

0
4

0
4

0.282117

0.282117

0.282117

0.282117

0
4

0
4

0
4

0.323713

0.323713

0.202321

0.202321

0
4

0.121392

0.121392

0
4

0
4

0
4

0

0

0

0

0

0

0
4

0
4

0
4

0
4

0
4

0
4

0

0

0

0

0

0

0

0
4

0
4

0

0

0

0
4

0
4

0
4

0

0

0

0

0

0
4

0
4

0
4

0.121392

0.121392

0.0809283

0.0404642

0.0404642

0
4

0.0404642

0.0404642

0
4

0
4

0
4

2.00214

2.00214

2.00214

2.00214

0
4

0

0

0
4

0
4

0
4

0.123385

0.123385

0.123385

0.123385

0
4

0
4

0
4

1.70996

1.70996

1.70996

0

1.70996

0
4

0
4

0
4

0.334486

0.334486

0.334486

0.334486

0
4

0
4

0
4

0

0

0

0

0

0
4

0

0

0
4

0
4

0
4

0.569959

0.569959

0.569959

0.569959

0
4

0
4

0
4

0

0

0

0

0
4

0
4

0
4

0

0

0

0

0
4

0
4

0
4

0
4

0
4

0
4

0

0

0

0

0

0

0

0

0
4

0

0

0

0

0

0
4

0

0

0
4

0
4

0
4

0.849201

0.849201

0.849201

0.849201

0
4

0
4

0
4

0

0

0

0

0
4

0
4

0
4

0

0

0

0

0
4

0
4

0
4

0

0

0

0

0

0
4

0
4

0
4

0.0714657

0.0714657

0.0714657

0.0714657

0
4

0
4

0
4

0

0

0

0

0
4

0

0

0
4

0
4

0
4

0

0

0

0

0
4

0
4

0
4

0.0469069

0.0469069

0.0469069

0.0469069

0
4

0
4

0
4

0

0

0

0

0
4

0
4

0
4

0

0

0

0

0
4

0
4

0
4

0
4

0
4

0
4

0
4

0

0

0

0

0
4

0

0

0
4

0

0

0

0
4

0

0

0
4

0
4

0
4

0

0

0

0

0
4

0
4

0
4

0.0137664

0.0137664

0.0137664

0.0137664

0
4

0
4

0
4

0.0229265

0.0229265

0.0229265

0.0229265

0
4

0
4

0
4

0

0

0

0

0
4

0
4

0
4

0

0

0

0

0
4

0
4

0
4

0

0

0

0

0
4

0
4

0
4

0

0

0

0

0
4

0
4

0
4

0

0

0

0

0
4

0
4

0
4

0

0

0

0

0
4

0
4

0
4

0.00458879

0.00458879

0.00458879

0.00458879

0
4

0
4

0
4

2.4356

2.4356

1.56401

1.54473

0.00350607

0.00350607

0.00525911

0.00701215

0
4

0.0262956

0.00525911

0.00701215

0.00876519

0.00525911

0
4

0.829515

0.829515

0
4

0.0122713

0.00525911

0.00701215

0
4

0.00350607

0.00350607

0
4

0
4

0
4

0

0

0

0

0
4

0
4

0
4

0.0606962

0.0606962

0.0606962

0.0606962

0
4

0
4

0
4

0.0229265

0.0229265

0.0229265

0.0229265

0
4

0
4

0
4

0

0

0

0

0
4

0
4

0
4

0

0

0

0

0
4

0
4

0
4

0

0

0

0

0
4

0
4

0
4

0

0

0

0

0
4

0
4

0
4

0.00750954

0.00750954

0.00750954

0.00750954

0
4

0
4

0
4

0.0606962

0.0606962

0.0606962

0.0606962

0
4

0
4

0
4

1.70996

1.70996

1.70996

1.70996

0
4

0
4

0
4

0.416469
7

0.416469
7

0.416469
7

0.359148
7

0.0573207

0

0

0

0

0

0
4

0
4

0
4

0

0

0

0

0
4

0
4

0
4

0

0

0

0

0
4

0
4

0
4

0.536117

0.536117

0.536117

0.536117

0
4

0
4

0
4

0

0

0

0

0
4

0
4

0
4

0.0172746

0.0172746

0.0172746

0.0172746

0
4

0
4

0
4

0

0

0

0

0
4

0
4

0
4

0

0

0

0

0
4

0
4

0
4

0.0112377

0.0112377

0.0112377

0.0112377

0
4

0
4

0
4

0.0105182

0.0105182

0.0105182

0.0105182

0
4

0
4

0
4

0

0

0

0

0
4

0
4

0
4

0.0469069
7

0.0469069
7

0.0469069
7

0.0469069
7

0

0

0
4

0
4

0
4

0

0

0

0

0
4

0
4

0
4

0

0

0

0

0
4

0
4

0
4

0

0

0

0

0
4

0
4

0
4

0

0

0

0

0
4

0
4

0
4

0

0

0

0

0
4

0
4

0
4

0.0404642

0.0404642

0.0404642

0.0404642

0
4

0
4

0
4

0

0

0

0

0
4

0
4

0
4

0

0

0

0

0
4

0
4

0
4

0

0

0

0

0
4

0
4

0
4

0.536117

0.536117

0.536117

0.536117

0
4

0
4

0
4

0

0

0

0

0

0

0

0

0
4

0
4

0
4

0.0561884

0.0561884

0.0561884

0.0561884

0
4

0
4

0
4

0

0

0

0

0
4

0
4

0
4

0

0

0

0

0
4

0
4

0
4

0

0

0

0

0
4

0
4

0
4

0.0460296

0.0460296

0.0460296

0.0460296
8

0

0
4

0

0

0
4

0
4

0

0

0

0
4

0
4

0

0

0

0
4

0
4

0
4

0
4

8726.31

1059.85
7

1059.77
7

338.931
7

338.931
7

0

0
4

267.231
7

18.2104
7

38.9959
7

0
7

0.0100127

0

0.0310016

0.0475315

0

0

0

0

0

0

0.346688
7

0

0

0.0506019

0

0.0449794

0.276842

0

0

0.0598804

0.00750954

0.172253
7

0.149764

0

0

0

0

0.0581373

0.369927

0

0

0

4.48056
7

0.0606962

0.0401185

0

0.0170056

0

0

0.0387519

0.0542527

0

0

0.72628
7

0.0926176

0.00500636

0

0

0.0310016

0.0581373

0

0

0

0

6.497
6

0.0155008

0

0

0

0.0599054

0

0

0

0

0.0155008

0.315851
6

0.0103648

0

0

0

0

0

0

0

0

0.0387519

0.246642
7

0

0

0

0.0155008

0

0.0138197

0

0

0.00500636

0

0.357675
7

0

0.0232512

0

0

0.00500636

0

0.0232512

0

0.10116

0.357411

5.22789
7

0.0310016

0.0366292

0

0

0.10116

0

0

1.70996

0

0.0935306

14.6127
7

2.91392
6

0

0

0

0.0155008

1.07223

0.0155008

0

0.0366292

0

0

0.0772174
7

0

0

0.0469069

0

0.00750954

0

0.0155008

0

0

0.0168673

0.13382
7

0

0.0599054

0

0

0

0

0.00500636

0

0

0

0.156221
7

0

0.536117

0

0

0.161857

0

0

0

0.0599054

0.0100127

16.4439
7

0.0366292

0

0

0

0

0

0

0.0155008

0

0

0.069375
7

0

0.0155008

0

0

0

0

0

0.00500636

0

0

0.364603
7

0

0

0

0

0.0155008

0.0112449

0.0155008

0.146517

0

0

12.248
6

0.00500636

0

0

0.0155008

0

0

0

0

0

0

0.435589
7

0

0
7

67.9588
7

2.58819
6

3.63266
6

2.19278
7

0.0532893
7

0

0.0823951
7

0
8

0.00750954
7

0.0212576
7

0.0162512
7

21.939
7

0.184578

0.206637

0.0898581
7

0

0.0125159
7

0.465338

0.0549439

0.132448

0.0986324

0
8

11.1562
6

0.0915731

0.445106

0.0155008

0

0

0.0529801

0

0.0175223

0.195833

0

0

0

0.0495119

0.0232512

0.0529801

0.0599054

0.167306

0

0

0.0549439

0.0119162

6.07639
6

3.64596

0.041459

0

0.0597998

0.0387519

0.033023

0.118071

0.455875

0.0112449

0.534777

3.28355
6

0

0.0150191

0

6.64208

0.121884

0

0

0

0

0.16066

3.6498
7

0.0930047

0

0

0.0250318

0.317766

0.0255083

0.0230103

0.192634

0

0

0
4

111.651
7

0

5.86152
7

1.41761
7

26.5698
7

31.5795
7

46.2222
7

0

0

0

0

0

0

0

2.1316282072803e-14
7

0
4

23.8496
7

19.5747
7

4.27489

0

8.88178419700125e-16
7

0
4

9.89595
7

8.1642
7

0

0

0

0

0.384607

0.0930047

0.0542527

0.0387519

0.0310016

0.927924

0.0704446

0.108505

0.0232512

1.13797860024079e-15
7

0
4

6.16988

2.83893

3.27025

0.0606962

1.80411241501588e-16

0
4

299.124
7

65.0517
7

233.338
7

0

0.0155008

0.0542527

0.0155008

0.237658

0.0387519

0.0387519

0.0775039

0.0465023

0.108505

0

0.0549439

0.0465023

2.80817036291126e-14
7

0
4

0.560412
7

0.52941
7

0

0.0310016

2.42861286636753e-17
7

0
4

2.29788
8

2.14822
8

0.110907

0.0387519

3.05311331771918e-16
8

0
4

0.0633753

0.0633753

0
4

0

0

0
4

0

0

0
4

0
4

0

0

0

0
4

0
4

0

0

0

0

0
4

0
4

0

0

0

0
4

0
4

0

0

0

0
4

0
4

0.0599054

0.0599054

0.0599054

0
4

0
4

0.0155008

0.0155008

0.0155008

0
4

0
4

0

0

0

0
4

0
4

0

0

0

0
4

0
4

0
4

6284.56

36.0739

1.97372

1.26563

0.0469069

0.404318

0.0637742

0.0955813

0

0

0.0506019

0.0469069

0

0
4

0

0

0
4

2.95513

2.95513

0
4

0

0

0
4

0

0

0
4

0

0

0
4

0

0

0
4

0

0

0
4

1.70996

1.70996

0
4

0

0

0
4

0

0

0
4

8.76704

4.49215

0

0

0

4.27489

0

0

0
4

0

0

0
4

1.70996

1.70996

0
4

0

0

0
4

0.871411

0.697129

0.174282

0

0

0

0

0
4

14.9343

1.53882

12.3657

1.02985

0
4

0.68505

0.656938

0.0281121

0
4

2.43916

1.99354

0.281441

0.0938138

0.0703603

8.32667268468867e-17

0
4

0

0

0
4

0

0

0

0
4

0.0281121

0.0281121

0
4

0
4

0.233802

0.233802

0.233802

0
4

0
4

105.444

0
3

0

0

0

0

0

0

0
4

0.0232512
3

0

0

0

0

0.0232512

0

0

0

0

0

0

0

0

0

0

0

0

0

0

0

0

0

0

0

0

0

0

0

0

0

0
4

0

0

0
4

3.84928
3

3.81827
3

0

0

0

0

0

0.0310016

0

0

0

0

3.57353036051222e-16
3

0
4

17.4517

9.38051
5

2.60349

0.151839

0.0724152

0

0

0.357411

0

0.0581373

0.00703794

0

1.35774

0

0.045853

0

0

0

1.28404

0.951279
5

0

0.091706

0.776749

0.31352

0

0
4

0

0

0

0

0

0

0
4

0

0

0
4

0

0

0

0

0

0
4

0

0

0

0

0

0

0

0
4

0

0

0

0

0

0
4

0

0

0

0

0

0
4

0.0138197

0.00690984

0.00690984

0

0

0
4

0

0

0

0
4

1.70996

0

1.70996

0

0
4

0

0

0

0

0
4

41.0946
5

38.1894
5

0

0

0.560198

1.84022

0.0775164

0.0469069

0

0.357411

0.0229265

0

7.04644675941779e-15
5

0
4

3.48832

3.41991

0.0340111

0.0343898

2.77555756156289e-17

0
4

0

0

0
4

0

0

0

0

0
4

0.0103648

0

0

0.0103648

0
4

0

0

0

0

0
4

0

0

0

0
4

1.70996

0

0

1.70996

0
4

0

0

0

0
4

0

0

0

0

0
4

0

0

0
4

21.6571
5

20.711
5

0.451933

0.404318

0

0.0898581

0
4

0

0

0

0
4

0

0

0
4

0

0

0
4

0

0

0

0
4

2.56494

2.56494

0
4

0

0

0
4

0

0

0
4

0

0

0
4

0.0138197

0.0138197

0
4

0.0229265

0.0229265

0
4

0
3

0

0

0

0

0

0

0

0
4

0

0

0

0
4

0

0

0

0
4

0

0

0

0
4

0

0

0

0
4

0

0

0

0
4

0.131287

0.131287

0
4

0

0

0

0
4

0

0

0
4

0

0

0

0
4

0

0

0
4

0.0775003
5

0

0.0175948

0

0

0

0.0599054

0

6.93889390390723e-18
5

0
4

0.0170056

0.0170056

0

0
4

0

0

0

0
4

0.0281121

0.0281121

0
4

0

0

0
4

0

0

0
4

0.0255083

0.0255083

0
4

0

0

0
4

0

0

0
4

0

0

0
4

0

0

0
4

0

0

0

0

0

0

0

0

0
4

0

0

0
4

0.357411

0.357411

0
4

0.0170056

0.0170056

0
4

0

0

0
4

0

0

0
4

0

0

0
4

0.0103648

0.0103648

0
4

0

0

0
4

0

0

0
4

0

0

0
4

10.3141
5

10.3141
5

0

0
4

0.0387582

0.0387582

0
4

0

0

0
4

0

0

0
4

0

0

0
4

0

0

0
4

0

0

0
4

0

0

0
4

0

0

0
4

0

0

0
4

0

0

0
4

0.459798

0.362902

0

0

0.0968956

0

0

4.16333634234434e-17

0
4

0

0

0
4

0

0

0
4

0

0

0
4

0

0

0
4

0

0

0
4

0

0

0
4

0

0

0
4

0

0

0
4

0

0

0
4

0

0

0
4

0

0

0

0

0

0

0
4

0.357411

0.357411

0
4

0
4

316.532
3

68.1504

0.218412

0.0606962
3

0

0

0

0

0

0

0

0.169482

0

0.404642

0.20151

0

0

0

1.70996

7.2394
5

0

0

0.141625

0.116863

0

0

0

0

0

0

0.170509

0

0.0465023

0

0

0

0

0.0606962

0.161857

0

0

0
3

0

0

0

0

0

0

0

0

2.56494

0

0.760341

0

0

0

0

0.0606962

0

0

0

0

0

0
3

0

0

0

0

0.0404642

0

0

0.0387582

0

0

27.4806
6

0

0

0.182089

0

0

0

0

0

0

0

6.54538

0

0

0

0.357411

0

0

0

0

0

0

17.4851

0.121392

0

0

0

0

0.10116

0

1.70996

0

0

9.54791801177635e-15

0
4

66.9634

34.3183
6

0.271284

0.0510167

0

1.85071

2.92235

0

0

0.0542527

0

0.124006

0.709193

1.70996

0.285189

0.0510167

0

0

0

0

2.92235

0

0.119811

5.51011

0

0.299527

0.0935306

0.090521

0

0.0775039

0.0155008

0.179716

0

0.126751

0.188887
7

0

0

0

0.0155008

0

0

0

0

0

0

1.88967

0

0

0

0.0155008

0

11.9697

0

0

0.0465023

0

0.420535
6

0.174282

0

0

0.0103648

0

0.0340111

0

0

0.095063

0

0.10116
7

0

0

0

0

0

0

0.0155008

0

0

0.0340111

0.0897686

0

0.0633753

0

0

0

0

0

0

0.0170056

0

0
3

0

0
4

65.1605

0

63.5799

0

0

0.0316877

0

0

0

0

0

0.0172746

0

0

0

0

0

0

0

0

0

0.102033

0

0

0.893528

0.536117

8.99280649946377e-15

0
4

92.7934
3

0.0899589
4

0.0224897
4

0

0

0

0

0

0

0

0

0

0

0

0
4

0

0

0

0

0

0

0

0

0

0

0
4

0

0

0

0

0

0

0

0

0

0

0

0

0

0

0

0

0

0

0

0

0

0
4

0

0

0

0

0

0

0

0

0

0

0

0.439551

0

0

0

0

0

0

0

0

0

0

0

0

0

0

0

0

0

0

0

0

0

0

0

0

0

0

0

0

0

0

0

0

0

0

0

0

0

0

0

0

0

0

0

0

0

0

0

0

0

0

0

0

0

0

0

0

0

0

0

0

0

0

0

0

0

0

0

0

0

0

0

0

0

0

0

0

1.6662

0

0

0

0

0

0

0

0

0

0

0

0

0

0

0

0

0

0

0

0

0

0

0

0

0

0

0

0

0

0

0

0

0

0

0

0

0

0

0

0

0

0

0

0

0

0

0

0

0

0

0

0

0

0

8.54979

0

0

0

0

0

0

0

0

0

0

0

0

0

0

0

0

0

0

0

0

0

0
4

0

0

0

0

0

0

0

0

0

0

0

0

0

0

0

0

0

0

0

0

0

0

0

0

0

0

0

0

0

0

0

0

0

0
4

0

0

0

0

0

0

0

0

0

0

0

0

0

0

0

0

0

0

0

0

0

0

0

0

0

0

0

0

0

0

0

0

0

0

0

0

0

0

0

0

0

0

0

0

0

0

0

0

0

0

0

0

0

0

0

0

0

0

0

0

0

0

0

0

0

0

0

0

0

0

0

0

0

0

0

0

0

0

0

0

0

0

0

0

0

0

0

0

0
4

0

0

0

0

0

0

0

0

0

0

0
4

0

0

0

0

0

0

0

0

0

0

0

0

0

0

0

0

0

0

0

0

0

0

0

0

0

0

0

0

0

0

0

0

0

0

0

0

0

0

0

0

0

0

0

0

0

0

0

0

0

0

0

0

0

0

0

0

0

0

0

0

0

0

0

0

0

0

0

0

0

0

0

0

0

0

0

0

0

0

0

0

0

0

0

0

0

0

0

0

0

0

0

0

0

0

0

0

0

0

0

0

0

0

0

0

0

0

0

0

0

0

0
4

0

0

0

0

0

0

0

0

0

0

0
4

0

0

0

0

0

0

0

0

0

0

0

0

0

0

0

0

0

0

0

0

0

0

0

0

0

0

0

0

0

0

0

0

0

0

0

0

0

0

0

0

0

0

0

0

0

0

0

0

0

0

0

0

0

0

4.62287

0

1.70996

0

0

0

0

0

0

0

0

0

0

0

0

0

0

0

0

0

0

0

0

0

0

0

0

0

0

0

0

0

0

0

0

0

0

0

0

0

0

0

0

0

2.56494

0

0

0

0

0.536117

0

0

0

0

0

0

0.0898279
7

0

0

0

0

0

0

0

0

0

0

0

0

0

0

0

0

0

0

0

0

0

0

0

0

0

0

0

0

0

0

0

0

0

0

0

0

0

0

0

0

0

0

0

0

0

0

0

0

0

0

0

0

0

0

0

0

0

0

0

0

0

0

0

0

0

0

0

0

0

5.12987

0

0

0

0

0

0
3

0

0

0

0

0

0

1.70996

0

0

0

0

0

0

0

0

0

0

0

0

0

0

0

0

0

0

0

0

0

0

0

0

0

0

0

0

0

0

0

0

0

0

0

0

0

0

0

0

0

0

0

2.14447

0

0

0

0

0
4

0

0

0

0

0

0

0

0

0

0

0
4

0

0

0

0

0

0

0

0

0

0

0
3

0

0

0

0

0

0

0

0

0

0

0

0

0

0

0

0

0

0

0

0

0

9.12445

0

0

0

0

0

0

0

0

0

0

0
4

0

0

0

0

0

0

0

0

0

0

0
4

0

0

0

0

0

0

0

0

0

0

0.123693
4

0
4

0

0

0

0

0

0

0

0

0

0

0
4

0

0

0

0

0

0

0

0

0

0

0

0

0

0

0

0

0

0

0

0

0

0

0

0

0

0

0

0

0

0

0

0

0

0

0

0

0

0

0

0

0

0

0.00701215

0
4

0

0

0

0

0

0

0

0

0

0

0
4

0

0

0

0

0

0

0

0

0

0

0
4

0

0

0

0

0

0

0

0

0

0

0
4

0

0

0

0

0

0

0

0

0

0

0
3

0

0

0

0

0

0

0

0

0

0

0
4

0

0

0

0

0

0

0

0

0

0

0

0
4

0

0

0

0

0

0

0

0

0

0

0
4

0

0

0

0

0

0

0

0

0

0

0

0

0

0

0

0

0

0

0

0

0

0
4

0

0

0

0

0

0

0

0

0

0

0

0

0

0

0

0

0

0

0

0

0

1.1655
3

0

0

0

0

0

0

5.48728

0

0

0

0
4

0

0

0

0

0

0

0

0

0

0

0

0

0

0

0

0

0

0

0

0

0

0

0

0

0

0

0

0

0

0

0

0

0

0
4

0

7.69481

0

0

0

0

0

0

0

0

0
4

0

0

0

0

0

0

0

1.70996

0

0

0

0

0

0

0

0

0

0

0

0

0

0
4

0

0

0

0

0

0

0

0

0

0

0

0

0

0

0

0

0

0

0

0

0

0

0

0

0

0

0

0

0

0

3.41991

0

0

0

0

0

0

0

0

0

0

0

0

0
4

0

0

0

0

0

0

0

0

0

0

0.936619
7

0

0

0

0.0469069

0

0

0

2.85929

0

0

16.3217

0

0

0

0

0

0

0

0

0

0

0
4

0
4

0

0

0

0

0

0

0

0

0

0

0
4

0

0

0

0

0

0

0

0

0

0

0
4

0

0

0

0

0

0

0

0

0

0

0
4

0

0

0

0

0

0

0

0

0

0

0
4

0

0

0

0

0

0

0

0

0

0

0
4

0

1.70996

0

0

0

0

0

0

0

0

0

0

0

0

0

0

0

0

0

0

0

0
4

0

0

0

0

0

0

0

0

0

0

0
4

0

0

0

0

0

0

0

0

0

0

0

0

0

0

0

0

0

0

0

0

0

0

0
4

0

0

0

0

0

0

0

0

0

0

0
4

0

0

0

0

0

0

0

0

0

0

0

0

0

0

0

0

0

0

0

0

0

0

0

0

0

0

0

0

0

0

0

0

0

0

0

0

0

0

0

0

0

0

0

0

0

0

0

0

0

0

0

0

0

0

0

0

0

0

0

0

0

0.0469069

0

0

0

0
4

0

0

0

0

0

0

0

0

0

0

0

0

0

0

0

0

0

0

0

0

0

0

0

0

0

0

0

0

0

0

0

0

0

0
4

0

0

0

0

0

0

0

0

0

0

0

0

0

0

0

0

0

0

0

0

0

5.12987

0

0

0

0

0

0

0

0

0

0

0
4

0

0

0

0

0

0

0

0

0

0

0
4

0

0

0

0

0

0

0

0

0

0

0
4

0

0

0

0

0

0

0

0

0

0

0

0

0

0

0

0

0

0.0387582

0

0

0

0

0

0

0

0

0

0

0

0

0

0

0
3

0

0

0

0

0

0

0

0

0

0

0
4

0

0

0

0

0

0

0

0

0

0

0

0

0

0

0

0

0

0

0

0

0

0

0
4

0

0

0

0

0

0

0

0

0

0

0

0

0

0

0

0

0

0

0

0

0

0
4

0

0

0

0

0

0

0

0

0

0

0
4

0

0

0

0

0

0

0

0

0

0

0
4

0

0

0

0

0

0

0

0

0

0

0
4

0

0

0

0

0

0

0

0

0

0

0

0

0

0

0

0

0

0

0

0

0

0

0

0

0

0

0

0

0

0

0

0

7.69481

0

0

0

0

0

0

0

0

0

0

3.5527136788005e-15
3

0
4

18.844

14.4397

0.366005

0

0

0

0.0595195

0.396096

0.162758

1.70996

0

1.70996

0

0

1.77635683940025e-15

0
4

0.218408
4

0.0633753
4

0
4

0
4

0

0

0.155033

0
4

1.12275

1.12275

0
4

0
4

0

0

0

0

0

0
4

0

0

0

0

0
4

0

0

0

0

0

0
4

0

0

0

0
4

0

0

0

0

0

0

0

0
4

0

0

0

0

0

0
4

0

0

0
4

0

0

0

0
4

0

0

0

0

0
4

0.0387582
3

0
3

0

0

0

0

0

0

0

0

0

0

0

0

0

0

0

0.0387582

0
4

0

0

0
4

0.0753812

0.0366292

0.0232512

0.0155008

0
4

0

0

0
4

0

0

0
4

0.104614

0.0984725

0.00614177

8.67361737988404e-19

0
4

0

0

0

0

0
4

0

0

0

0
4

0

0

0

0

0
4

0

0

0

0
4

0

0

0
4

0.644707
3

0.095063
4

0

0

0

0

0.549644

0

0

0

0

0

0

0

0
4

0

0

0

0
4

0

0

0
4

0

0

0
4

0

0

0

0
4

0

0

0

0
4

0.109105

0.0774173

0.0316877

6.93889390390723e-18

0
4

0

0

0
4

0

0

0
4

0

0

0
4

0

0

0

0
4

0
3

0

0

0

0

0

0

0

0

0

0

0

0

0

0

0

0
4

0

0

0
4

0

0

0
4

0

0

0
4

0

0

0

0
4

0

0

0

0
4

0

0

0

0
4

0

0

0

0
4

0

0

0

0
4

0

0

0

0
4

0

0

0
4

0
3

0

0

0

0

0

0

0
4

0

0

0
4

0

0

0

0
4

0

0

0
4

0

0

0
4

0

0

0
4

0

0

0
4

0

0

0
4

0

0

0
4

0

0

0
4

0

0

0
4

0
4

0
4

0

0

0

0

0

0

0

0

0
4

0

0

0
4

0

0

0
4

0

0

0
4

0

0

0
4

0

0

0
4

0

0

0
4

0

0

0
4

0

0

0
4

0

0

0
4

0

0

0
4

0.209991

0.192985

0

0.0170056

0

0
4

0.00350607

0.00350607

0
4

0

0

0
4

0

0

0
4

0

0

0
4

0

0

0
4

0.0155008

0.0155008

0
4

0.281441

0.281441

0
4

0

0

0
4

0

0

0
4

0.0125267

0.0125267

0
4

1.73671

0

0

1.70996

0

0.0232512

0

0.00350607

0
4

0

0

0
4

0

0

0
4

0

0

0
4

0

0

0
4

0

0

0
4

0

0

0
4

0

0

0
4

0

0

0
4

0

0

0
4

0

0

0
4

0

0

0

0

0

0

0

0

0
4

0

0

0
4

0

0

0
4

0

0

0
4

0

0

0
4

0.0469069

0.0469069

0
4

0

0

0
4

0

0

0
4

0

0

0
4

0

0

0
4

0
4

2.06737
4

0
4

0
4

0

0

0

0

0

0

0

0

0

0

0

0

0

0

0

0

0

0

0

0

0

0

0

0

0

0

0

0

0

0

0

0

0

0

0

0

0

0

0

0

0

0

0

0

0
4

2.06737
4

2.06737
4

0

0
4

0

0

0
4

0

0

0
4

0

0

0
4

0
4

270.621

86.3675

26.5364
7

1.64189
7

0

0

0

0

0

0

0

0

0.357411

0.0469069

0
8

0.536117

0

0

0

0

0

0

0

0.007332

0

0.893528
7

0

0

1.70996

0

0.0469069

0.00917758

0

0

0

0.299527

1.83269

0

0

0

0

0

0

0

0.0510167

0

0

0.0606962
7

0.357411

0

0

0.0229439

0

0.010998

0.0387582

1.22025
7

1.8684

0
8

0.187628
3

0.007332
7

3.49929

0

0

0.106812
7

0.00500636
8

0

0

1.55995
7

0.151777

0

0.127344

8.71026
7

0.536117

2.82396

0

0.0300382

1.96346
7

0

0.00500636

0

3.41991

2.60349

4.53872

0.202407

0

0.0510167

0

0.0703603

0

0.893528

0

0.0935306

0

0.131723
8

0

0

0

0

0

1.73726

0.0850278

0

0

0

0
3

0

0

0

0

0

0.0255083

0

0.0170056

0.0633753

0.007332

0.0316877
8

0

1.70996

0

0

0

0

0

0

0.0449794

3.77733

9.19977
7

0

0

0

0

0

0

0

0

0

0

0.328533
7

0

0

0.0112449

0

0

0

0.0340111

0.00458879

0

0.0229265

2.42930675575792e-14

0
4

0

0

0

0
4

0
2

0

0

0

0

0

0

0

0

0

0

0
4

1.84062
4

1.70996

0
4

0

0

0

0

0

0

0

0.0138197

0

0

0.00690984

0

0

0.0253567

0

0

0
4

0

0

0

0

0

0

0

0

0

0

0
4

0

0

0

0.0207295

0

0

0

0

0

0.0599054

0
3

0

0

0

0

0

0

0

0

0

0

0
4

0

0

0

0

0

0

0

0

0

0

0

0

0

0

0

0

0

0

0

0

0

0

0

0

0

0

0

0

0

0

0

0

0.0039381
8

0

0

0

0

0

0

0

0

0

0

0

0

0

0

0

0

0

0

0

0

0

6.76542155630955e-17
4

0
4

3.48192
5

0

0

0

0

1.70996

0

1.70996

0

0

0.0620031

0

0

0

0

0

0

0

0

0
4

95.2937
6

0

0

0

0

0

0.00350607

0.0668153

0

0

0

0

39.676

0

0

0.0281121

0

0

0

0

0

0

0

0.100724

0

0

0.00690984

0

0

0

0.0103648

0

0

0

45.2018

0

2.56494

0

0.0137664

0.00350607

0

0

0

0

0.00690984

0

0

0

0

0

0

0.0207295

0

3.85442

2.9609

0.0595195

0.714822

2.56461518688411e-14
6

0
4

13.9413
5

3.77733
5

0

0

0

0

0

0

0

0

0

0

2.00779

0

0

0.149764

0

0

0

0

0

0

0

0

0

0

0

0

0

0.0316877

0

0

0

0

7.73336
5

0

0

0

0

0

0

0

0

0

0

0

0

0

0.241357

0

5.27355936696949e-16
5

0
4

0

0

0

0

0

0

0

0

0

0

0

0

0

0

0

0

0

0

0

0

0
4

0

0

0

0

0

0

0

0

0
4

0

0

0
4

0

0

0
4

0

0

0
4

0

0

0
4

0.0898581

0.0898581

0
4

0

0

0
4

0

0

0
4

0

0

0
4

0.536117

0.536117

0
4

0

0

0
4

0.0577309
7

0.0518238
7

0.00590715

0

0

0

2.60208521396521e-18
7

0
4

0

0

0
4

0

0

0
4

0

0

0
4

0

0

0
4

0

0

0
4

0.536117

0.536117

0
4

0

0

0
4

0

0

0
4

0

0

0
4

0

0

0
4

0
4

0

0

0

0
4

0

0

0
4

0

0

0
4

0

0

0
4

0

0

0
4

0

0

0
4

0

0

0
4

0

0

0
4

0

0

0
4

0

0

0
4

0

0

0
4

0

0

0

0

0

0

0

0

0
4

0

0

0
4

0

0

0
4

0

0

0
4

0

0

0
4

0

0

0
4

0

0

0
4

0

0

0
4

0.0629317
7

0.0224754

0.0404563

0

0

0
4

0
4

0

0

0

0

0

0
4

0
4

0

0

0

0

0
4

0.0978942

0.079539

0.0183552

0
4

0.106812
7

0.106812
7

0
4

1.30093
7

1.30093
7

0
4

0
4

0
4

0
4

0
4

0

0

0

0
4

0

0

0

0
4

0.0968956
8

0

0.0968956

0
4

0.0330188
6

0.0221708

0.0108479

0

0
4

0

0

0

0

0

0
4

0

0

0
4

0

0

0

0

0
4

0

0

0
4

0

0

0

0

0

0
4

0

0

0

0

0
4

0

0

0

0
4

50.7179
7

50.3605
7

0

0.357411

0
4

0

0

0
4

0

0

0

0

0
4

2.56494

2.56494

0

0
4

0

0

0
4

0.0775164

0

0.0775164

0
4

2.56494

2.56494

0

0
4

0.597033

0.597033

0
4

0

0

0
4

0

0

0
4

0

0

0

0

0
4

0.024589
7

0.00590715

0.00350607

0

0

0.0039381

0

0.0112377

0
4

0

0

0

0

0
4

0

0

0
4

0

0

0

0
4

0

0

0

0
4

0

0

0

0
4

0.111661

0.104013

0.00305919

0.00458879

1.73472347597681e-18

0
4

0

0

0

0
4

4.27489

4.27489

0
4

0

0

0

0
4

0

0

0

0
4

0

0

0

0

0

0

0

0

0

0

0

0

0
4

0.00703794

0.00703794

0

0
4

0.0138197

0

0.0138197

0
4

0

0

0
4

0.0599054

0.0599054

0

0
4

3.85442

3.85442

0
4

0

0

0
4

0.125492

0.0404642

0.0850278

0
4

0

0

0

0
4

0.0138197

0

0.0138197

0
4

0

0

0
4

1.10595
7

0.731534
7

0.0170056

0

0.357411

0
4

0

0

0

0
4

0

0

0
4

0

0

0

0
4

0

0

0
4

0

0

0
4

0

0

0
4

0

0

0

0
4

0

0

0

0
4

0

0

0
4

0

0

0
4

0
3

0

0

0

0
4

0

0

0
4

0

0

0
4

0

0

0
4

0

0

0
4

0

0

0
4

0

0

0
4

0

0

0
4

0

0

0
4

0

0

0
4

0

0

0
4

0

0

0

0

0
4

0

0

0
4

0.0118143

0.0118143

0
4

0

0

0
4

0

0

0
4

0

0

0
4

0

0

0
4

0

0

0
4

0

0

0
4

0.253009

0.253009

0
4

0.0103648

0.0103648

0
4

0.340111
7

0.340111
7

0

0
4

0

0

0
4

0

0

0
4

0.0316877

0.0316877

0
4

0

0

0
4

0

0

0
4

0

0

0
4

0

0

0
4

0

0

0
4

0

0

0
4

0.0170056

0.0170056

0
4

0
4

576.327

5.65388
7

1.93716
7

2.00676

0

0

0

0

1.70996

0

0

0
4

468.454

68.8564
7

11.4578
7

0

0
3

0.357411
6

1.93103

0

0

0

0.0606962

0

0

3.58828
3

0.0387582

0.0890208

0

0.0510167

0.306175

0

0.0167023

0

0

0

4.54844

0

0

0

0

0

0

0.0345492

0

0.00690984

0

1.11794
7

0

0.10384

0.0676061

0

0

0

0

0.714822

0.0167023

0

39.0964
7

0.357411

0.0281121

0

0

0

0

0.0517018

0

0

0

12.0134
6

0.357411

0

0

0

0

0

0

0

0

0

0

0

0

0

0

0.0691495

0.0469069

0.357411

0

0.0112449

0

9.07865

0

0

0

0

0

0.010998

0

0

0

0.536117

13.7776

0

1.70996

0

0

0

0

0.0039381

0.158438

0

1.08924

0.128189

0

0

0

0

0

0

0

0

0

0

0
3

1.07016
7

0.202321

0

0

0

0

0

1.70996

0.00690984

0

0

1.88124
7

0

0

0

0

0

0

0

0

0

0.0103648

0
2

0.00690984

0

0

0

0

0

0

0

0

0

0
3

0

0

0

0

0.144547

0

0.0443759

0

0

0

2.22935
3

0.0760082

0

0

0.0606962

0

0.00690984

0.357411

0

0

0

0.58137
7

0

0

0

0

0.0680222

0

0.0255083

0

0

0.0801017

1.20905
7

0

1.74872

0

0

0

0

0

0

0

0

0
3

0.0340111

0

0

0

0

0

0

0

0.337176

0

3.45846
7

0

0

0

0

0

0

0

0

0

0.0170056

0.0510167
2

0.00835115

0

0

1.70996

0

0

0

0

0.00690984

0

87.7911
7

4.17266

0

0

0

0

0

0.0170056

0

0

0.00690984

0

2.86734
6

0

0

0

0

0

0

0

0

0

0

0
2

0.202321

0

0

0

0

0

0

0.00305919

0

0.357411

0
3

0

0

0

0

0

0

0

4.27489

0

0.00525911

1.37195
7

0

0

0

0

0.0425139

0

0

0

0

0

0
2

0

0.00458879

0

0

0

0

0

0

0

0.0170056

4.42202

0

0

0

0

0

0

0

0

1.42964

0

1.71733
5

0

0

0

0

0

0.714822

0

0

0

0

0.214143
5

0

0

0

0

0

0

0

0

0.0404642

0

0
3

0

0.0125267

0

0

0

0.00690984

0

0

0

0

0
4

5.9843
6

0.0898581

0

0

0

0

0

0

0

0

0

0
3

0

0

0

0

0.0112449

0

0

0.0599054

0

0

0.0404642

0

0

0.00690984

0.00458879

0

0

0

0

0.010998

0

0
3

0

0

0

0

0

0

0

0

0

0.0680222

0.216982
7

0.0633753

0

0

0.0170056

0

0

0

0

0

0

0
3

0

0

0

0

0

6.83983

0

0

0

0

0.714822
3

0

0

0

0

0

0

0.007332

0

0

0

0.848103
7

0

0

0

0

0

0

0

0

0.164174

0

0
3

0

0

0.0280942

0

0

0

0

0

0

0.00690984

1.70996
7

0

0

0

0

0

0.0103648

0

0

0

0.0170056

8.1974
7

0
2

0

1.70996

0

0

0

0

0

0

0

0

1.92819

0

0

0

0

0

0

0

0

0

0.0850278

0.283794

0

0.0103648

0.00690984

0

0

0

0.714822

0

0

0

0

0

0

0

0.00525911

0

0

0

0.0103648

0

0

0.357411

0.00458879

0

0.536117

0

0

0

0

0

0

0

16.6829

0

0

0

0

0.0124051

0

0

0.0140759

0

0

0
3

0

0.0725533

0

0

0

0

0

0

0

0

0.0316877

0.0112377

0

0

0

0

0

0

0

0

0

0.24503
5

0.0316877

0.116275

0

0

0

0

0

0

0

0

0
3

0

0

0

0

0

0

0

0

1.70996

0

18.5511
7

0
3

3.41991

0

0

0

0

0

0

0

0

0

0
3

0

0

0

0.357411

0

0

0

0

0.0469069

0.00835115

0
3

0

0

0

0.357411

0

0

0

0

0

0

3.08912
6

0

0

0

0

0

0

0

0

0

0

0.0968961
7

0

0

0

0

0

0

0

0

0

0.0340111

0.0323573
7

0

0

0

0

0

0

0

0

0

0

0.380342
7

0

0.00305919

0.357411

0

0

0

0

0

0

0

1.42964
7

0

0.141652

0

0

0

0

0

0

0

0.0581373

0
2

0

0.00835115

0

0

0.00835115

0

0

0

0

0

0
3

0

0

0

0

0

0

0

0.0595195

0

0

57.0621

0.0316877
2

0

0

0.0387582

0

0

0.0935306

0

0

0

0

0
3

0

0

0

0

0

0

0

0

0

0.179716

0
3

0

0

0

0

0

0

0

0

0

0

1.28772

0

0

0

0

0

0

0

0

0

0

0

0

0

0

0

0

0

0

0.0510167

0.893528

0

0.714822
6

0

0

0

0

0

0

0

0.00690984

0

0

0
3

0

0

0

0

0

0

0

0

0

0

0
2

0

0.0224897

0.00827008

0

0

0

0

0

0

0

0.108674
7

0

0

0

0

0.00835115

0.0112449

0

0

0

0

0.476542

0

0

0

0

0

0

0

0

0

0

0
3

1.73802

0

0

0

0

0

0.00690984

0

0

0

0

2.92235
7

0

0

0

0

0

0

0.0506019

0

0

0

0

0

0

0

0

0.0103648

0

0.0898581

0

0

0

0

0

0.0276393

0

0

0

0.357411

0

0

0

4.63253
6

0

0.0170056
7

0

0
7

0.0606962

9.5954

0.0239154

0
3

0.057196

0.0419207

0.714822

0

9.39555
6

0.0290443
7

0.468318

0

2.44582132324922e-13

0
4

0

0

0

0

0

0

0

0

0

0

0

0

0

0

0

0

0
4

91.5111

77.7032

0.494028
7

0
4

0

0

0

0

0

0

0

0.0168673

0

0
4

0

1.25801

0

0

0

0

0

0

0

0

9.12781
7

0

0

0

0

0

0

0

0

0

0

0.822583
7

0

0

0

0

0

0

0

0

0

0

1.70996
4

0

0

0

0

0

0

0

0.0170056

0

0

0.159781
7

0.0103648

0

0

0

0

0

0

0

0

0

0
4

0

0

0

0

0.0167023

0

0

0

0

0

0.0340111

0

0

0.140721
7

1.85962356624714e-15

0
4

8.63503
7

2.55203
7

0

0

0.0425139

0.119811

0.0898581

0

0

0.0207295

0

0

3.80011
6

0

0

0

0

0

0

0.297686
7

0.00690984

0.876558
6

0.0581373
7

0.739857
7

0.0138197

0.0170056

1.31838984174237e-16
7

0
4

0
4

0

0

0

0

0
4

0

0

0
4

0

0

0
4

0

0

0
4

0

0

0
4

0.174412

0.174412

0
4

0

0

0
4

0

0

0
4

0

0

0
4

0

0

0
4

0

0

0
4

0
4

0
4

0

0

0
4

0.0125267

0.0125267

0
4

0

0

0
4

0

0

0
4

0

0

0
4

0

0

0
4

0

0

0
4

0

0

0
4

0

0

0
4

0

0

0
4

0

0

0
4

0.0207295
7

0.0207295
7

0

0
4

0

0

0
4

0

0

0
4

0

0

0
4

0

0

0
4

0

0

0
4

0.0170056

0.0170056

0
4

0

0

0
4

0

0

0
4

0

0

0
4

0

0

0
4

0

0

0

0

0

0
4

0

0

0
4

0

0

0
4

0

0

0
4

0

0

0
4

0.0170056

0.0170056

0
4

0

0

0
4

0

0

0
4

0

0

0
4

0

0

0
4

0

0

0
4

0
3

0

0

0

0
4

0

0

0
4

0

0

0
4

0

0

0
4

0

0

0
4

0

0

0
4

0

0

0
4

0

0

0
4

0

0

0
4

0

0

0
4

0

0

0
4

0
2

0

0

0

0

0
4

0

0

0

0

0

0

0
4

0.0704459
8

0.0704459

0

0
4

0

0

0

0

0

0
4

0.0112449
7

0.0112449
7

0
4

0
4

0
4

0

0

0

0

0

0

0

0

0

0

0
4

0

0

0

0

0

0

0

0

0

0

0
4

0

0
4

0
4

0
4

0

0

0

0
4

0

0

0
4

0.0194366

0.0125267

0.00690984

0
4

0

0

0

0

0
4

0

0

0

0
4

1.34157

1.34157

0
4

0

0

0

0
4

0

0

0

0
4

0

0

0

0

0
4

0

0

0
4

0

0

0

0

0
4

0
4

0
4

0
4

0

0

0

0

0

0

0
4

0

0

0

0

0
4

0

0

0

0
4

0

0

0
4

0

0

0

0

0
4

0.017152

0.00590715

0.0112449

1.73472347597681e-18

0
4

0

0

0

0

0
4

0

0

0

0

0
4

0

0

0
4

0

0

0

0
4

0

0

0

0
4

0.160275
5

0
7

0.0425139
5

0

0

0.0170056

0

0.100755

0

0
4

0

0

0

0
4

0.0170056

0.0170056

0

0
4

0

0

0

0
4

0

0

0

0
4

0

0

0
4

0

0

0
4

0

0

0

0
4

0

0

0

0
4

0

0

0

0
4

0

0

0

0
4

0
3

0
3

0

0

0

0

0

0

0
4

0.029328

0.010998

0.01833

0
4

0

0

0
4

0

0

0
4

0.0156239

0.0156239

0
4

0

0

0
4

0

0

0

0
4

0

0

0

0
4

0

0

0

0
4

0

0

0
4

0

0

0
4

0.0469069
3

0
3

0

0.0469069

0

0
4

0

0

0
4

0.0475315

0.0475315

0
4

0

0

0
4

0

0

0
4

0

0

0
4

0

0

0
4

0

0

0
4

0

0

0
4

0

0

0
4

0

0

0
4

0
4

0
4

0

0
4

0

0

0
4

0

0

0
4

0

0

0
4

0

0

0
4

0

0

0
4

0

0

0
4

0

0

0
4

0.007332

0.007332

0
4

0

0

0
4

0

0

0
4

0
3

0
4

0

0

0

0
4

0

0

0
4

0

0

0
4

0

0

0
4

0

0

0
4

0

0

0
4

0.0469069

0.0469069

0
4

0

0

0
4

0

0

0
4

0

0

0
4

0

0

0
4

0
4

0

0

0

0

0

0
4

0

0

0
4

0

0

0
4

0

0

0
4

0

0

0
4

0

0

0
4

0

0

0
4

0

0

0
4

0

0

0
4

0

0

0
4

0

0

0
4

5.81430736890098e-13

0
4

3.17516
3

2.03831
3

2.03831
3

0

0

0

0

0

0

0

0

0

0

0

0

0

0

0

0

0

0

0

0

0

0
4

0
4

0
4

0

0

0
4

0

0

0
4

0

0

0
4

0

0

0

0
4

0

0

0

0
4

0

0

0

0
4

0

0

0
4

0

0

0
4

0

0

0
4

0

0

0
4

0

0

0
4

0

0

0

0
4

0

0

0
4

0

0

0
4

0

0

0
4

0

0

0
4

0

0

0
4

0

0

0
4

0.0175304

0.0175304

0
4

1.07886

1.07886

0
4

0

0

0
4

0

0

0
4

0

0

0
4

0

0

0
4

0

0

0
4

0

0

0
4

0

0

0
4

0

0

0
4

0.0404642

0.0404642

0
4

0

0

0
4

0

0

0
4

0

0

0
4

0

0

0
4

0

0

0

0

0
4

0

0

0
4

0

0

0
4

0

0

0
4

0

0

0
4

0

0

0

0

0
4

0

0

0
4

0

0

0
4

0

0

0
4

0

0

0

0
4

0
4

10.6552
2

10.6552
2

0.427784
2

0.0475315
2

0

0

0

0

0

0

0

0

0

0

0

0

0

0

0

0

0

0

0

0

0

0

0

0

0

0

0

0

0

0

0

0

0

0

0

0

0

0

0

0

0

0

0

0

0

0

0

4.27489

0

0

0

0

0

5.12987

0

0

0

0

0

0

0

0

0

0

0

0

0.135654

0

0

0

0

0

0

0

0.0549439

0

0

0

0

0

0

0

0

0

0

0

0

0

0

0

0

0

0.507003
2

0

0

0

0

0

0

0

0

0

0

0

0

0

0

0

0

0.0775164

0

0

0

0

0
4

0

0

0
4

0

0

0
4

0
4

1696.74

1669.76
7

511.043
7

7.5403
7

0.109888

0.0806519

0.0170056

0.0387582

0.029229

0.0325063

0.0155008

0.0282504

0

0.0966996

2.15194

0.058656

0

0.0255083

0

1.78177

4.45804

0

0.0425139

0.879102

0

0.506292

0.0366292

0

0.021996

0.567753

0

0.128202

0.0915731

0.0366292

0

0

6.23432
6

0

0

0.110233

0.982287

0.164832

0.0719494

0

0

0.055258

0.0866208

0.427894

0.211081

0

0

0.0366292

0.0724152

0.399925

0.054259

1.02091

0.0366292

0.731828

0.982448
7

0.0167023

0.0680222

0.0216649

0.00835115

0.0449794

0.0125267

0.025662

0.128202

0.0366292

0

7.61411
6

0

0.114849

2.72888

0

0.155008

0.0366292

0.0250534

0.0112449

0.0599054

0.0915731

0.381929
7

0.00835115

0

0.219775

0.0366292

0

0.0923175

0

0.219775

0

0.0621376

0.176449
7

0.0170056

0.388999

0.0366292

0.0366292

0.0125267

0.0170056

0.106812

0.0170056

0

0.179641

0.315276
7

0.102033

0

0

0.0125267

0.0484126

0.179716

0

0.0375802

0

0

844.189
7

4.93107
6

0.0478741

0

0.0549439

0.0255083

0

0

0.0510167

0.00835115

0.0367532

0

0.39571
7

0.0469069

0.0775164

0

0

0.0915731

0.0703603

0.0340111

11.9867

0

0

0.647498
7

0

0.0599054

0.0598804

0.0510167

0.0366292

0.0208779

0.0170056

0.0170056

0

0

1.5718
7

3.41991

0

0.00835115

0

0

0.0170056

0

0

0

0

0.0845033
7

0.0310016

0.135642

0

0.0549439

0

0.0170056

0.183146

0

0

0

7.13547
6

0.0340111

0

0

0

0

0

0.0549439

0.0915731

0

0

3.21328
7

0

0.943808

0.007332

0

0.0366292

0

0.0155008

0.0732585

0

0

0.877642
7

0.00835115

0.0232512

0.0167023

0

0

0

0

0.0170056

0

0.00835115

2.80981
6

0

0

0.0167023

0

0

0.0255083

0

0

0

0.0112377

15.6074
7

0.0170056

0

0

0

0

0

0

0.0366292

0

0

48.2325
7

0.225829
5

0.0340111

0

0.0775039

0.174412

0

0

0.0898581

0

0

0.0549439

0.37751
7

0

0

0

0

0

0.007332

0.0155008

0.109888

0.269574

0

0.0999242
7

0

0.0387582

0

0

0

0.100755

0.0366292

0.0425139

0

0

0.27145
7

0.0449794

0.00835115

0

0

0

0.299527

0

0

0

0

1.52241
7

0.0387582

0

0

1.70996

0

0

0

0

0.0170056

1.70996

0.342753
7

0

0.133618

0

0

0

0

0

0

0.0469069

0

4.27489

0

0

0

0.0898581

0

0

0.0170056

0

0

0

0.215848
7

0.0255083

0.0170056

0

0.357411

0.0510167

0.357411

0

0.183146

0

0.0170056

5.5599
6

0.0170056

0

0.0170056

0.0366292

0.0366292

0.0366292

0.0366292

0

0

0

2.15927

0.0898581

0

0

0

0

0

0

0.293034

0.0843364

0.0170056

45.0267
6

0.391544
6

0

0.0599054

0.0112377

0

0.0366292

0.0366292

0.0387582

0

0.007332

0

0.415549

0.0366292

2.89094

0

0.0366292

0

0

0.0732585

0.0250534

0

0

0.187419
7

0

0.0387582

0

0

0

0.0366292

0

0

1.60835

0

0.723435
7

0

0

0

1.70996

0

0

0

0

0

0.641012

3.24425

0

0.007332

3.41991

0.0549439

0

0

0.010998

0

0.0155008

0

0.342811
6

0

0

1.70996

0

0

0

0

0

0

0

0.502481
6

0

0.0125267

0

0.0155008

0.0340111

0

0.128202

0

0.0366292

1.70996

0.640892
7

0

0.183146

0

0

0

0

0

0.0310016

0

0

0.168098
7

0

0

0

0

0

0.0968956

0.0170056

0

0

0

2.33344
7

0

0

0

0

0

0.209669

0

0.544178

0

0.0170056

4.22996
7

0

0

0

0

0

0.0433298

0

0.0170056

0

0

0

4.37354

0

0.0599054

0.0255083

0

0.0549439

0.0549439

0

0

0

0

0.0712583
7

0

0

0.0366292

0.256405

0.00835115

0

0

0

0

0

0.719776
6

0.0155008

0

0.0366292

0

0

0.014664

0

0.0366292

0.0599054

0

0.521742
7

0.0732585

0.0366292

0.0549439

0

0

0

0

0

0.0170056

0

0.389748
7

0.007332

0

0.162847

0

0

0

0

0.0168673

0

0.0170056

0.438228
6

0.00835115

0

0

0.0170056

0

0

0

0

0.0915731

0.0732585

0

0

0

0

0

0.0125267

0

0

0

0.0250534

0.0732585

0.188108
7

0.0465023

0

0.0224754

0

0

0

0

0.0255083

0

0.0155008

0.0983119
7

0.0167023

0.732585

0.149764

0

0

0

0

0

0

0.0366292

3.25041
7

0.403196
7

0

0

4.27489

0

0

0.0170056

0.0170056

0

0.0599054

0.0340111

0.488707
7

0

0.155033

0.0387582

0

0

0.032994

0

0.0469069

0.00835115

0

0.748815
7

0

0

0

0

0.0732585

0

0.0549439

0.0599054

0

0.0366292

0.591061
6

0

0

0

0

0

0.0125267

0

0

0.0255083

0

0.162325

7.22922

0.069654

8.94164
6

0.732585

0.0536348
8

2.01177
5

0.0253567
7

0.0112377

8.56103
6

0.0366292

0

9.53823

0.029229

0
7

0.161

0.0366292

7.1706
7

0.0549439

0.0170056
7

0

0.0224897

0.0933789

0.0170056
7

0.128202

0

0.224737

0.108579

0.404315
7

0.15305

0.765839

0.0898581

0.535902

0

2.50527

0.0595195

1.24539

0.0232512

0.0232512

1.63599689351202e-12
7

0
4

20.7475
7

20.7475
7

0

0
4

4.29021
4

1.00778
4

0.674544

0.025662

0.007332

0.014664

0.007332

0

0

0

0.382625

2.02363

0.01833

0

0

0

0.12831

2.77555756156289e-17
4

0
4

0

0

0
4

0

0

0
4

0

0

0
4

0

0

0
4

0

0

0
4

0

0

0
4

0.0549439

0.0549439

0
4

0

0

0
4

0

0

0
4

0

0

0
4

0

0

0
4

1.07223

0

1.07223

0
4

0.0155008

0.0155008

0
4

0

0

0
4

0

0

0
4

0.076911

0.076911

0
4

0

0

0
4

0

0

0

0
4

0

0

0
4

0

0

0
4

0

0

0
4

0.714822

0.714822

0
4

0
4

3.07492
4

2.8353
4

0
4

1.19001
5

0

0

0

0

0

0

0

0

0

0

0
3

0

0

0

0

0

0

0

0

0

0

0
4

0

0

0

0

0

0

0

0

0

0

0
4

0

0

0

0

0

0

0

0

0

0

0

0

0

0

0

0

0

0

0

0

0

0

0

0

0

0

0

0

0

0

0

0

0
4

0

0

0

0

0

0

0

0

0

0

0.208304
5

0

0

0

0

0

0

0

0

0

0

0

0

0

0

0

0

0

0

0

0

0

0

0

0

0

0

0

0

0

0

0

0

0

0
4

0

0

0

0

0

0

0

0

0

0

0

0

0

0

0

0

0

0

0

0

0

0

0

0

0

0

0

0

0

0

0

0

0

0

0

0

0

0

0

0

0

0

0

0

0

0

0

0

0

0

0

0

0

0

0

0

0

0

0

0

0

0

0

0

0

0

0

0

0

0

0

0

0

0

0

0

0

0

0

0

0

0

0

0

0

0

0

0

0

0

0

0

0

0

0

0.332434

0

0

0
4

0

0

0

0

0

0

0

0

0

0

0

0

0

0

0

0

0.00750954

0

0

0

0

0

0

0

0

0

0

0

0

0

0

0

0

0

0

0

0

0

0

0

0

0

0

0

0

0

0

0

0

0

0

0

0

0

0

0

0

0

0

0

0

0

0

0

0

0

0
3

0

0

0

0

0

0

0

0

0

0

0

0

0

0

0

0

0

0

0

0

0

0

0

0

0

0

0

0

0

0

0

0

0

0

0

0

0

0

0

0

0

0

0

0

0

0

0

0

0

0

0

0

0

0

0

0

0

0

0

0

0

0

0

0.0599054

0

0

0.00750954

0

0

0

0

0

0

0

0

0

0

0

0

0

0

0

0

0

0

0

0

0

0

0

0

0

0

0

0

0

0

0

0

0

0

0

0

0

0

0

0

0

0

0

0

0

0

0

0

0

0

0

0

0

0

0
4

0

0

0

0

0

0

0

0

0

0

0

0

0

0

0

0

0

0

0

0

0

0

0

0

0

0
4

0
4

0

0

0

0

0

0

0.0300382

0

0

0

0
4

0

0

0.0599054

0

0

0

0

0

0

0

0
3

0

0

0

0

0

0

0

0

0

0

0

0

0

0

0

0

0

0

0

0

0

0.917153
5

0

0

0

0

0.0225286

0

0

0

0

0

2.77555756156289e-16
4

0
4

0.239622
4

0.239622
4

0

0

0

0

0

0

0

0

0

0

0

0

0

0

0

0

0

0

0
4

0

0

0

0

0
4

0

0

0

0
4

0

0

0

0
4

0

0

0

0

0

0
4

0

0

0

0
4

0

0

0
4

0

0

0

0
4

0

0

0

0

0
4

0

0

0

0
4

0

0

0

0

0
4

0
4

0
4

0

0

0
4

0
4

0

0

0

0

0

0

0
4

0

0

0

0
4

0

0

0
4

0

0

0

0
4

0

0

0
4

0

0

0
4

0

0

0

0
4

0

0

0
4

0

0

0

0
4

0

0

0

0
4

0

0

0
4

0
3

0
3

0

0

0

0

0

0
4

0

0

0
4

0

0

0
4

0

0

0
4

0

0

0
4

0

0

0
4

0

0

0
4

0

0

0
4

0

0

0
4

0

0

0
4

0

0

0
4

0
3

0
3

0
4

0

0

0
4

0

0

0
4

0

0

0
4

0

0

0
4

0

0

0
4

0

0

0
4

0

0

0
4

0

0

0
4

0

0

0
4

0

0

0
4

0

0

0

0

0
4

0

0

0

0

0
4

0

0

0

0

0
4

0

0

0

0

0

0
4

0

0

0

0

0

0
4

2.77555756156289e-17
4

0
4

340.724
4

175.422
5

27.5509

79.9472
5

0.00984525
4

0

0

0

0.161857

0

0

0

0

0

0

2.51738
5

0

0

0

0

0

0

0

0

0

0

0.761729
5

0

0

0

0

0

0

0

0

0

0

1.07223

0

0

0

0.00703794

0

1.70996

0

0

0

4.88154
5

0

1.70996

2.06737

0

0.0823951

0

0

0

0

0

23.8868
5

0.0343898

0

0

0

0.0039381

0

0

0

0

0

26.9487
6

0

0

0

0

0

0

0

0

0

0

0.357411
4

0

0

0

0

0

0

0

0

0

0

0.429556
5

0

0

0

0

0

0

0

0

0

0

0.0523816

0.0170056

0

0

0

0

0

0

0

0

0.0316877

1.08589
5

0

0

0.0581373

0

0

0.0229265

0

0

0

0

0.0137834

0

0

0

0

0

0

0

0

0

0

0
4

157.067
4

42.0556
6

0.505802
3

0.492127
4

12.1519
5

35.9793
5

42.536
5

1.29984
4

0
4

1.70996
4

0
4

0

0

0

0

0

0

0

0

0

0

0
4

0

0

0

0

0

0

0

0

0

0

0

0

0

0

0

0

0

0

0

0

0

0
4

0

0

0

0

0

0

0.42634

0

0

0

0

0

0

0

0

0

0

0

0

0

0

0

0

0

0

0

0

0

0

0

0

0

0
4

0

0

0

0

0

0

0

0

0

0

0

0

0

0

0

0

0

0

0

0

0

0

0

0

0

0

0

0

0

0

0

0

0

0

0

0

0

0

0

0

0

0

0

0
4

6.6261
7

0

0

0

0

0

0

0

0

0

0

0

0

0

0

0

0

0

0

0

0

0

0

0

0

0

0

0

0

0

0

0

0

0

0

0

0

0

0

0

0

0

0

0

0

0

0

0

0

0

0

0

0

0

0

0
4

0

0

0

0

0

0

0

0

0

0

0

0

0

0

0

0

0

0

0

0

0

0

0

0

0

0

0

0

0

0

0

0

0

0

7.69481
4

0

0

0

0

0

0

0

0

0

0

0
3

0

0

0

0

0

0

0

0

0

0

5.12987

0

0

0

0

0.135654

0

0

0

0

0

0
4

0

0

0

0

0

0

0

0

0

0

0
4

0

0

0.323713

0

0

0

0

0

0

0

0
4

0

0

0

0

0

0

0

0

0

0

0
3

0

0

0

0

0

0

0

0

0

0

0
4

7.8515

0
8

0.0255083

0

0

0

0

0

0

0

0.0469069

0

0
8

0

0

0

0

0

0

0

0

0

0.0316877

0

0

0

0

0

0

0

0

0

0

2.32317

0

0

0

0

0

5.42423

0

1.77635683940025e-15

0
4

0

0

0

0

0

0

0

0

0
4

0

0

0
4

0

0

0
4

0

0

0
4

0

0

0
4

0

0

0
4

0

0

0
4

0

0

0
4

0

0

0
4

0

0

0
4

0

0

0
4

0.0404642
4

0.0404642
4

0

0

0

0
4

0

0

0
4

0.0581373

0.0581373

0
4

0

0

0
4

0

0

0
4

0.207597
4

0.207597
4

0

0

0
4

0
4

0

0

0

0

0
4

0

0

0

0

0
4

0

0

0
4

0

0

0
4

0

0

0
4

0.0775164

0.0775164

0
4

2.04489203348146e-13
4

0
4

0.907468
3

0
4

0
4

0

0

0

0

0

0

0

0

0

0

0
4

0.357411

0

0

0

0.357411

0
4

0

0

0
4

0

0

0
4

0

0

0
4

0

0

0
4

0

0

0
4

0.268464
3

0.116275
4

0

0

0

0

0

0

0

0
4

0.0914932

0.0606962
4

0

0

0

0
4

0.0751706
3

0.0610761
3

0.0140945
4

0
4

1.73472347597681e-18
3

0
4

0
4

0
4

0

0

0

0

0

0

0
4

0.146517
3

0

0

0

0

0

0

0.146517

0
4

0
4

0
4

0

0

0

0
4

0
4

0

0

0

0

0

0
4

0.0599054

0

0.0599054

0

0
4

0

0

0

0

0

0
4

0
4

9.02849

8.92651

0.813825

0.0333077

0

7.06356
7

0

0.300998

0

0

0

0.714822

0

0
4

0

0

0

0

0

0
4

0.0350607

0.00350607

0.00350607

0.0105182

0.0105182

0.00701215

0
4

0.0669176

0.00701215

0.0599054

0
4

0

0

0
4

4.9960036108132e-16

0
4

0
4

0
4

0

0

0

0

0
4

0

0

0

0
4

0
4

0

0

0

0

0

0

0
4

0

0

0

0
4

0
4

0.484658

0.484658

0

0.484658

0
4

0

0

0

0
4

0

0

0
4

0
4

0

0

0

0

0

0
4

0
4

0
3

0
3

0
3

0

0
4

0

0

0
4

0
4

0
4

0

0

0

0
4

0

0

0
4

0

0

0
4

0
4

0.0935306

0.076525

0.0425139

0

0

0.0340111

0
4

0.0170056

0.0170056

0
4

0
4

0

0

0

0

0
4

0

0

0

0

0
4

0

0

0
4

0
4

14.1495
6

14.1495
6

14.1495
6

0
4

0
4

3.34758

3.34758

3.34758

0
4

0
4

0
3

0
3

0
3

0

0

0
3

0

0

0

0

0

0

0

0
4

0

0

0

0

0

0
4

0

0

0

0

0
4

0
4

0

0

0

0

0

0
4

0

0

0
4

0
4

0
4

0

0

0

0

0
4

0

0

0
4

0

0

0
4

0
4

0.0387582

0.0387582

0.0387582

0

0
4

0
4

0
3

0

0

0
4

0

0

0

0
4

0

0

0
4

0

0

0
4

0

0

0
4

0

0

0
4

0
4

0.645834

0.645834

0.301889

0.343945

0
4

0
4

0

0

0

0

0
4

0

0

0

0

0
4

0

0

0
4

0

0

0
4

0
4

0

0

0

0

0

0

0
4

0
4

0.762053

0.762053

0.579964

0

0.182089

0
4

0

0

0

0

0
4

0
4

0.0898581

0.0898581

0.0898581

0

0
4

0
4

0

0

0

0

0
4

0
4

0.476725
3

0.476725
3

0.283249
3

0

0.00690984
4

0.186566

0

0

0

0

0

0

5.55111512312578e-17
3

0
4

0

0

0

0

0
4

0

0

0

0
4

0

0

0

0
4

0

0

0

0
4

0
4

0

0

0

0

0
4

0

0

0

0
4

0

0

0
4

0
4

0

0

0

0

0

0

0

0

0
4

0
4

0
4

0
4

0

0

0

0
4

0

0

0
4

0

0

0
4

0
4

0

0

0

0

0

0
4

0

0

0

0
4

0
4

0.0207295

0.0207295

0.00690984

0.0138197

0
4

0

0

0

0
4

0

0

0
4

0
4

0

0

0

0

0

0

0
4

0

0

0
4

0
4

0

0

0

0

0

0
4

0

0

0
4

0

0

0
4

0

0

0
4

0
4

1.70996
4

0
4

0
4

0
4

1.70996

1.70996

0
4

0

0

0
4

0
4

0
4

0
4

0

0

0

0
4

0
4

0

0

0

0

0
4

0

0

0

0
4

0
4

4.26047
3

3.98045
3

3.87709
3

0.10336
3

0

0

0

0

0

0

0

0
4

0

0

0
4

0

0

0
4

0

0

0
4

0

0

0
4

0

0

0
4

0
4

0
4

0

0

0

0
4

0.26594
4

0.110907

0.155033

0

0
4

0

0

0

0
4

0.0140759

0.00703794

0.00703794

0
4

0

0

0
4

0

0

0
4

0

0

0
4

0

0

0
4

1.83880688453542e-16
3

0
4

5.63664
6

5.63664
6

5.63664
6

0
4

0
4

0

0

0

0
4

0

0

0
4

0

0

0

0
4

0

0

0
4

0
4

0

0

0

0

0
4

0
4

0
3

0
3

0

0

0

0
4

0

0

0
4

0
4

0

0

0

0

0

0
4

0

0

0
4

0
4

0.357411

0.357411

0.357411

0
4

0

0

0
4

0

0

0
4

0

0

0
4

0
4

0

0

0

0

0

0
4

0
4

0.714822

0.714822

0

0

0

0.714822

0
4

0

0

0
4

0
4

0

0

0

0
4

0

0

0
4

0

0

0
4

0
4

0.388634

0.388634

0.357411

0.0172746

0.00690984

0.00703794

0
4

0
4

2.89569
5

2.89569
5

1.46598
5

0

0.0170056

0.0170056

0

0.0170056

0.0425139

0

0.0170056

0.0112449

0

0.730752
5

0.0469069

0.269308
4

0.0316877

0.174282

0.0449794

0.0100127

0

0

2.61943244872498e-16
5

0
4

0
4

0

0

0

0

0

0

0

0
4

0
4

0

0

0

0

0

0

0
4

0

0

0
4

0
4

0

0

0

0

0
4

0
4

0

0

0

0

0

0

0
4

0

0

0
4

0

0

0
4

0
4

0

0

0

0

0
4

0

0

0

0
4

0

0

0
4

0
4

0

0

0

0

0
4

0

0

0
4

0
4

0

0

0

0

0

0
4

0
4

0

0

0

0

0
4

0

0

0
4

0
4

0.194012

0.102419

0.0599054

0.0425139

0

6.93889390390723e-18

0
4

0.0915931

0.0915931

0
4

0
4

0

0

0

0

0

0
4

0
4

4.67691
5

4.67691
5

4.15775
5

0.0724152

0.179716

0.0703603

0.0469069

0.149764

0

0

0

2.4980018054066e-16
5

0
4

0
4

0

0

0

0

0
4

0

0

0
4

0

0

0
4

0

0

0
4

0
4

0

0

0

0

0

0
4

0

0

0

0
4

0
4

0

0

0

0

0

0
4

0

0

0

0
4

0
4

0

0

0

0

0
4

0

0

0
4

0

0

0
4

0
4

0

0

0

0

0
4

0

0

0
4

0

0

0
4

0

0

0
4

0
4

0.93988

0.93988

0.430684

0.509196

0
4

0
4

0
4

0

0

0

0
4

0

0

0
4

0
4

5.34713

5.34713

5.34713

0
4

0
4

0

0

0

0

0

0
4

0

0

0
4

0
4

0

0

0

0

0

0
4

0

0

0
4

0

0

0
4

0
4

0.269574
4

0.269574
4

0.269574
4

0

0

0

0

0

0

0

0

0

0

0

0
4

0

0

0
4

0

0

0

0
4

0

0

0
4

0

0

0
4

0

0

0
4

0

0

0
4

0

0

0
4

0

0

0
4

0

0

0
4

0
4

0
4

0

0

0
4

0

0

0
4

0
4

0

0

0

0

0
4

0

0

0
4

0
4

0.0518238

0

0

0

0
4

0.0518238

0.0518238

0

0
4

0
4

0

0

0

0

0
4

0

0

0
4

0
4

0

0

0

0

0

0
4

0

0

0

0
4

0
4

0.0822895

0.0822895

0.0822895

0
4

0
4

0

0

0

0

0

0
4

0

0

0

0
4

0
4

2.05402

1.89946

1.85361

0.045853

0
4

0.0399321

0.0229265

0.0170056

3.46944695195361e-18

0
4

0.114633

0.114633

0
4

0
4

0.126751

0.0475315

0

0.0475315

0

0
4

0.0792192

0.0792192

0

0
4

0
4

0

0

0

0

0

0
4

0

0

0
4

0

0

0
4

0
4

2.28609

0.516808

0.509898
6

0.00690984

0

0

0

0

0

0
4

1.76928

1.76237
6

0

0

0

0.00690984

0

0

0

7.80625564189563e-17

0
4

0

0

0

0
4

0

0

0

0
4

2.22044604925031e-16

0
4

0

0

0

0

0

0

0
4

0

0

0
4

0
4

0

0

0

0

0

0
4

0

0

0
4

0

0

0
4

0
4

0

0

0

0

0

0
4

0

0

0
4

0
4

0

0

0

0
4

0
4

0

0

0

0

0
4

0

0

0
4

0

0

0
4

0
4

0

0

0

0

0
4

0

0

0

0
4

0

0

0
4

0
4

0

0

0

0
4

0
4

0

0

0

0

0

0
4

0
4

0

0

0

0

0
4

0
4

0

0

0

0

0

0

0
4

0
4

43.8069

43.2233
6

42.0701
6

0.714822

0.0809283

0.357411

0

0

0

0

7.105427357601e-15
6

0
4

0
2

0

0

0

0

0

0

0
4

0
2

0

0

0

0

0
4

0.0475315

0

0

0.0475315

0
4

0

0

0

0
4

0.536117

0

0.536117

0
4

0

0

0
4

0
4

0

0

0

0

0

0

0
4

0
4

0

0

0

0

0
4

0

0

0

0
4

0
4

0

0

0

0
4

0
4

0

0

0

0

0
4

0

0

0
4

0
4

0

0

0

0

0
4

0

0

0
4

0
4

0

0

0

0

0
4

0
4

0

0

0

0

0

0
4

0
4

0

0

0

0

0
4

0

0

0
4

0
4

0

0

0

0
4

0

0

0

0
4

0
4

0

0

0

0
4

0

0

0
4

0
4

21.353
5

21.348
5

21.1387
5

0.149764

0.0595195

0
4

0.00500636

0.00500636

0
4

0

0

0
4

0
4

0

0

0

0

0

0
4

0

0

0
4

0
4

0

0

0

0

0
4

0
4

0

0

0

0
4

0
4

0

0

0

0

0
4

0

0

0
4

0
4

0.0275625

0

0

0
4

0.0275625

0.0105569

0.0170056

0
4

0
4

0

0

0

0
4

0

0

0
4

0

0

0
4

0
4

0

0

0

0

0

0
4

0
4

0

0

0

0

0
4

0

0

0
4

0
4

0

0

0

0

0

0
4

0

0

0
4

0
4

0

0

0

0

0

0

0
4

0
4

0

0

0

0

0

0

0

0

0

0

0

0

0

0

0

0

0

0

0

0

0

0

0

0

0

0

0

0

0

0

0

0

0

0

0

0

0

0
4

0

0

0

0

0

0

0

0

0

0

0
4

0
4

0

0

0

0

0

0

0

0

0

0

0

0

0

0

0

0

0
4

0
4

0

0

0

0
4

0

0

0
4

0

0

0
4

0
4

0

0

0

0

0
4

0
4

0

0

0

0

0
4

0
4

0

0

0

0
4

0

0

0
4

0

0

0
4

0
4

0

0

0

0
4

0

0

0
4

0
4

0

0

0

0
4

0

0

0
4

0
4

0

0

0

0

0
4

0
4

0

0

0

0

0
4

0

0

0
4

0
4

0

0

0

0

0
4

0

0

0
4

0
4

0

0

0

0

0
4

0

0

0
4

0
4

0

0

0

0

0

0

0

0

0

0

0

0

0

0

0

0

0

0

0

0

0

0
4

0

0

0

0

0

0

0

0
4

0

0

0

0
4

0
4

0

0

0

0

0
4

0
4

0.600363

0.600363

0.600363

0
4

0
4

0

0

0

0

0
4

0
4

0

0

0

0
4

0

0

0
4

0
4

0

0

0

0

0
4

0

0

0
4

0
4

0

0

0

0

0

0
4

0
4

3.77733

2.06737

2.06737

0
4

1.70996

1.70996

0
4

0
4

0

0

0

0
4

0

0

0
4

0
4

0

0

0

0

0
4

0

0

0
4

0
4

0

0

0

0

0
4

0
4

0
3

0
3

0
3

0

0

0

0

0

0

0

0

0
4

0

0

0

0
4

0

0

0
4

0
4

0

0

0

0
4

0

0

0
4

0
4

0

0

0

0

0
4

0
4

0

0

0

0
4

0

0

0
4

0

0

0
4

0
4

0

0

0

0

0
4

0

0

0
4

0
4

0

0

0

0

0
4

0

0

0
4

0
4

0

0

0

0

0
4

0

0

0
4

0
4

0

0

0

0

0
4

0
4

142.75

142.75

142.75

0
4

0
4

0

0

0

0

0
4

0
4

0.0281121

0.0281121

0

0.0281121

0
4

0

0

0
4

0
4

0
4

0
4

0

0

0

0

0

0

0

0

0
4

0
4

0
4

0

0

0

0

0
4

0

0

0

0

0

0
4

0

0

0

0
4

0

0

0
4

0
4

0

0

0

0
4

0

0

0
4

0
4

0

0

0

0
4

0

0

0
4

0

0

0
4

0
4

0

0

0

0

0
4

0
4

0

0

0

0

0
4

0
4

0

0

0

0
4

0
4

0

0

0

0
4

0

0

0
4

0
4

0

0

0

0
4

0
4

0.126751

0.126751

0

0.126751

0
4

0
4

0

0

0

0

0

0
4

0
4

0

0

0

0

0
4

0
4

0.263017

0
5

0

0

0

0

0

0

0

0

0
4

0.263017

0.222553

0

0.0404642

2.77555756156289e-17

0
4

0

0

0

0
4

0

0

0
4

0
4

0

0

0

0

0
4

0
4

0

0

0

0
4

0

0

0
4

0
4

0

0

0

0
4

0
4

0

0

0

0
4

0
4

0

0

0

0
4

0
4

0

0

0

0

0
4

0
4

0

0

0

0
4

0

0

0
4

0
4

0

0

0

0

0
4

0
4

0

0

0

0

0
4

0
4

0

0

0

0
4

0

0

0
4

0
4

0
2

0
2

0

0

0

0

0
2

0

0

0

0

0

0

0

0
4

0

0

0

0
4

0
4

0

0

0

0
4

0
4

0

0

0

0

0
4

0
4

0

0

0

0
4

0
4

0

0

0

0

0
4

0
4

0

0

0

0

0
4

0
4

0

0

0

0

0
4

0
4

0.0168673

0.0168673

0

0.0168673

0
4

0
4

0

0

0

0
4

0

0

0
4

0
4

0

0

0

0

0
4

0
4

0

0

0

0
4

0

0

0
4

0
4

0
3

0
3

0
3

0

0

0

0

0

0

0

0
4

0
3

0

0

0

0

0

0

0

0
4

0
4

0

0

0

0
4

0
4

0

0

0

0
4

0

0

0
4

0
4

0

0

0

0
4

0

0

0
4

0
4

0

0

0

0
4

0

0

0
4

0
4

0

0

0

0

0
4

0
4

0

0

0

0
4

0

0

0
4

0
4

0

0

0

0
4

0
4

0

0

0

0

0
4

0
4

0

0

0

0

0
4

0
4

0

0

0

0
4

0

0

0
4

0
4

0.121392
4

0.121392
4

0.121392
4

0

0

0

0

0

0

0

0
4

0

0

0

0

0

0

0
4

0

0

0

0

0
4

0

0

0

0
4

0

0

0

0
4

0

0

0

0
4

0

0

0
4

0
4

0

0

0

0
4

0

0

0
4

0
4

0

0

0

0

0
4

0
4

0

0

0

0

0
4

0
4

0

0

0

0
4

0

0

0
4

0
4

0

0

0

0
4

0

0

0
4

0
4

0

0

0

0
4

0

0

0
4

0
4

0

0

0

0

0
4

0
4

0

0

0

0

0
4

0
4

0

0

0

0
4

0

0

0
4

0
4

0

0

0

0
4

0
4

2.7664
3

0
3

0
3

0
2

0

0

0

0

0
4

0
3

0
3

0

0

0

0
4

2.7664

2.7664

0

0
4

0

0

0

0

0

0
4

0

0

0
4

0
4

0

0

0

0

0
4

0
4

0

0

0

0
4

0
4

0

0

0

0

0
4

0
4

0

0

0

0
4

0

0

0
4

0
4

0

0

0

0

0
4

0
4

0

0

0

0

0
4

0
4

0

0

0

0
4

0
4

3.93152

3.93152

1.96576

1.96576

0
4

0
4

0.0387409

0.0387409

0.0337346

0.00500636

0
4

0
4

0

0

0

0

0
4

0
4

3.72287
4

3.51149
4

3.51149
4

0

0

0

0

0

0

0

0
4

0.211384

0.211384

0
4

0

0

0
4

1.11022302462516e-16
4

0
4

0

0

0

0
4

0
4

0.0475315

0.0475315

0.0475315

0
4

0

0

0
4

0
4

0.045853

0.045853

0.0229265

0.0229265

0
4

0
4

2.06737

2.06737

2.06737

0
4

0
4

0

0

0

0
4

0

0

0
4

0
4

0

0

0

0

0
4

0
4

0.0170056

0.0170056

0.0170056

0
4

0

0

0
4

0
4

0

0

0

0

0
4

0
4

0

0

0

0

0
4

0
4

0

0

0

0
4

0

0

0
4

0
4

0
2

0
2

0
2

0

0

0

0

0

0
3

0

0

0

0

0

0

0
4

0
2

0
2

0

0

0

0

0

0

0

0

0

0

0

0
2

0
3

0

0

0

0

0

0
4

0

0

0

0
4

0

0

0

0

0

0
4

0

0

0
4

0

0

0
4

0
4

34.4883

34.4883

34.1309

0

0

0.357411

0
4

0

0

0
4

0

0

0
4

0
4

0

0

0

0
4

0

0

0
4

0
4

0

0

0

0
4

0
4

0

0

0

0
4

0

0

0
4

0
4

0

0

0

0
4

0
4

0

0

0

0
4

0

0

0
4

0
4

0

0

0

0
4

0
4

0

0

0

0
4

0

0

0
4

0
4

0

0

0

0
4

0
4

0

0

0

0
4

0
4

0.0255083

0.0255083

0.0255083

0
4

0
4

0.982465
7

0.912019
7

0.126751
7

0
8

0.0316877

0

0

0

0.714822

0.0387582

0

2.08166817117217e-17
7

0
4

0.0387582

0.0387582

0

0

0

0
4

0.0316877

0.0316877

0

0
4

0
4

0

0

0

0
4

0
4

0

0

0

0

0
4

0
4

0

0

0

0
4

0
4

0

0

0

0
4

0
4

0

0

0

0
4

0
4

0

0

0

0
4

0
4

0

0

0

0
4

0
4

0

0

0

0
4

0
4

0

0

0

0
4

0
4

0

0

0

0
4

0
4

0
2

0
2

0
2

0
2

0

0

0

0
4

0

0

0

0
4

0

0

0
4

0

0

0
4

0
4

0

0

0

0
4

0
4

0

0

0

0
4

0
4

0.269574

0.269574

0.269574

0
4

0
4

0

0

0

0
4

0
4

0

0

0

0
4

0
4

0

0

0

0
4

0
4

0.0170056

0.0170056

0.0170056

0
4

0
4

0

0

0

0
4

0
4

0

0

0

0
4

0
4

0

0

0

0
4

0
4

0
4

0
4

0
4

0

0

0

0

0

0

0

0
4

0
4

0

0

0

0
4

0
4

0

0

0

0
4

0
4

0

0

0

0
4

0
4

0

0

0

0
4

0
4

0

0

0

0
4

0
4

0

0

0

0
4

0
4

0.00350607

0.00350607

0.00350607

0
4

0
4

0

0

0

0
4

0
4

0

0

0

0
4

0
4

0

0

0

0
4

0
4

0

0

0

0

0

0

0

0

0

0
4

0

0

0
4

0
4

0.00690984

0.00690984

0.00690984

0
4

0
4

0

0

0

0
4

0
4

0

0

0

0
4

0
4

0

0

0

0
4

0
4

0

0

0

0
4

0
4

0

0

0

0
4

0
4

0

0

0

0
4

0
4

0

0

0

0
4

0
4

0

0

0

0
4

0
4

0

0

0

0
4

0
4

0
3

0
3

0
3

0
3

0

0

0
4

0

0

0

0

0
4

0
4

0

0

0

0
4

0
4

0

0

0

0
4

0
4

0

0

0

0
4

0
4

0

0

0

0
4

0
4

0.047658

0.047658

0.047658

0
4

0
4

0

0

0

0
4

0
4

0

0

0

0
4

0
4

0

0

0

0
4

0
4

0.0475315

0.0475315

0.0475315

0
4

0
4

0

0

0

0
4

0
4

0
4

0
4

0

0

0

0

0

0

0

0

0

0
4

0

0

0

0

0

0

0
4

0

0

0
4

0
4

0.491159

0.491159

0.491159

0
4

0
4

0

0

0

0
4

0
4

0

0

0

0
4

0
4

0

0

0

0
4

0
4

0

0

0

0
4

0
4

0

0

0

0
4

0
4

0.0469069

0.0469069

0.0469069

0
4

0
4

0.0599054

0.0599054

0.0599054

0
4

0
4

0

0

0

0
4

0
4

0

0

0

0
4

0
4

0
3

0
3

0
3

0

0

0

0

0
4

0

0

0
4

0

0

0
4

0
4

0.357411

0.357411

0.357411

0
4

0
4

0

0

0

0
4

0
4

0.0295358

0.0295358

0.0295358

0
4

0
4

0

0

0

0
4

0
4

0

0

0

0
4

0
4

0

0

0

0
4

0
4

0

0

0

0
4

0
4

0

0

0

0
4

0
4

0

0

0

0
4

0
4

0

0

0

0
4

0
4

0

0

0

0

0

0

0

0
4

0

0

0
4

0
4

0

0

0

0
4

0
4

0

0

0

0
4

0
4

0.357411

0.357411

0.357411

0
4

0
4

0.10116

0.10116

0.10116

0
4

0
4

0

0

0

0
4

0
4

0

0

0

0
4

0
4

0

0

0

0
4

0
4

0

0

0

0
4

0
4

0

0

0

0
4

0
4

0

0

0

0
4

0
4

0

0

0

0

0

0

0

0

0
4

0

0

0

0

0
4

0

0

0

0
4

0

0

0

0
4

0

0

0
4

0
4

0

0

0

0
4

0
4

0

0

0

0
4

0
4

0

0

0

0
4

0
4

0

0

0

0
4

0
4

0

0

0

0
4

0
4

0

0

0

0
4

0
4

0

0

0

0
4

0
4

0

0

0

0
4

0
4

0.0140759

0.0140759

0.0140759

0
4

0
4

0.00500636

0.00500636

0.00500636

0
4

0
4

0.223514
3

0.0792192
3

0.0792192
5

0

0

0

0

0

0

0

0

0

0

0

0

0

0

0

0

0

0

0
4

0

0

0

0
4

0

0

0

0

0
4

0.0668604

0

0.0668604

0

0

0
4

0.0175948

0.00703794

0.0105569

0

0
4

0

0

0

0
4

0

0

0

0
4

0

0

0

0
4

0

0

0

0
4

0

0

0

0
4

0

0

0
4

0

0

0

0

0

0

0

0

0

0

0

0
4

0

0

0
4

0

0

0
4

0
3

0

0

0

0

0

0

0

0

0

0

0

0

0
4

0
3

0

0

0

0

0

0

0
4

0
4

0

0

0

0

0

0

0

0

0

0
4

0

0

0

0

0

0
4

0.0528015
5

0.0316877

0

0

0

0.0211138

0
4

0.00703794
4

0

0

0.00703794

0

0

0
4

0

0

0

0

0

0
4

5.72458747072346e-17
3

0
4

0
3

0
3

0
3

0

0

0

0

0

0

0
4

0

0

0

0

0
4

0

0

0

0
4

0
4

0

0

0

0
4

0
4

0.0155008

0.0155008

0.0155008

0
4

0
4

0

0

0

0
4

0
4

0.091706

0.091706

0.091706

0
4

0
4

0

0

0

0
4

0
4

0

0

0

0
4

0
4

0

0

0

0
4

0
4

0

0

0

0
4

0
4

0

0

0

0
4

0
4

0

0

0

0
4

0
4

0
4

0
4

0
4

0

0

0

0
4

0

0

0

0

0

0
4

0
4

0

0

0

0
4

0
4

0

0

0

0
4

0
4

0

0

0

0
4

0
4

0

0

0

0
4

0
4

0

0

0

0
4

0
4

0

0

0

0
4

0
4

0

0

0

0
4

0
4

0

0

0

0
4

0
4

0

0

0

0
4

0
4

0

0

0

0
4

0
4

0

0

0

0

0

0

0

0

0
4

0

0

0
4

0
4

0

0

0

0
4

0
4

0

0

0

0
4

0
4

0

0

0

0
4

0
4

0

0

0

0
4

0
4

0

0

0

0
4

0
4

0

0

0

0
4

0
4

0

0

0

0
4

0
4

0

0

0

0
4

0
4

0

0

0

0
4

0
4

0

0

0

0
4

0
4

0
4

0

0

0

0

0

0

0

0

0
4

0

0

0

0

0

0
4

0

0

0

0
4

0

0

0
4

0
4

0

0

0

0
4

0
4

0.00350607

0.00350607

0.00350607

0
4

0
4

0.00690984

0.00690984

0.00690984

0
4

0
4

0

0

0

0
4

0
4

0

0

0

0
4

0
4

0

0

0

0
4

0
4

0.0599054

0.0599054

0.0599054

0
4

0
4

0.0599054

0.0599054

0.0599054

0
4

0
4

0

0

0

0
4

0
4

0

0

0

0
4

0
4

0.298357
3

0.298357
3

0.197197

0

0

0

0.10116

0

0

0
4

0

0

0

0

0
4

0

0

0
4

0
4

0

0

0

0
4

0
4

0

0

0

0
4

0
4

0

0

0

0
4

0
4

0

0

0

0
4

0
4

0

0

0

0
4

0
4

0

0

0

0
4

0
4

0

0

0

0
4

0
4

0

0

0

0
4

0
4

0

0

0

0
4

0
4

0

0

0

0
4

0
4

7.12286
7

7.12286
7

7.04865
7

0.0742095
7

0
4

0
4

0

0

0

0
4

0
4

0

0

0

0
4

0
4

0

0

0

0
4

0
4

0

0

0

0
4

0
4

0.0316877

0.0316877

0.0316877

0
4

0
4

0.0155008

0.0155008

0.0155008

0
4

0
4

0

0

0

0
4

0
4

0

0

0

0
4

0
4

0

0

0

0
4

0
4

0

0

0

0
4

0
4

0
4

0
4

0
4

0

0

0

0

0

0

0
4

0

0

0

0

0
4

0

0

0

0
4

0
4

0

0

0

0
4

0
4

0

0

0

0
4

0
4

0

0

0

0
4

0
4

0.00350607

0.00350607

0.00350607

0
4

0
4

0

0

0

0
4

0
4

0

0

0

0
4

0
4

0.0276393

0.0276393

0.0276393

0
4

0
4

0

0

0

0
4

0
4

0

0

0

0
4

0
4

0

0

0

0
4

0
4

0
4

0
4

0
4

0

0

0

0

0

0
4

0
4

0

0

0

0
4

0

0

0
4

0

0

0
4

0

0

0
4

0
4

0

0

0

0
4

0
4

0

0

0

0
4

0
4

0

0

0

0
4

0
4

0

0

0

0
4

0
4

0

0

0

0
4

0
4

0

0

0

0
4

0
4

0

0

0

0
4

0
4

0

0

0

0
4

0
4

0

0

0

0
4

0
4

0

0

0

0
4

0
4

0

0

0

0

0

0

0

0

0

0

0
4

0

0

0

0
4

0

0

0
4

0

0

0
4

0

0

0
4

0
4

0.0100127

0.0100127

0.0100127

0
4

0
4

0

0

0

0
4

0
4

0

0

0

0
4

0
4

0

0

0

0
4

0
4

0

0

0

0
4

0
4

0

0

0

0
4

0
4

0

0

0

0
4

0
4

0

0

0

0
4

0
4

0

0

0

0
4

0
4

0

0

0

0
4

0
4

0.358029

0.342528

0.294521

0.0170056
7

0.0155008

0.0155008

1.73472347597681e-17

0
4

0.0155008

0

0.0155008

0
4

0

0

0
4

1.21430643318376e-17

0
4

0

0

0

0
4

0
4

0

0

0

0
4

0
4

0

0

0

0
4

0
4

0

0

0

0
4

0
4

0.0792192

0.0792192

0.0792192

0
4

0
4

0

0

0

0
4

0
4

0

0

0

0
4

0
4

0

0

0

0
4

0
4

0

0

0

0
4

0
4

0

0

0

0
4

0
4

0
4

0
4

0

0

0

0

0

0

0

0

0

0

0

0

0

0

0

0

0

0

0

0

0

0

0

0

0

0

0

0

0

0

0

0

0

0

0

0

0

0

0

0

0

0

0

0

0

0

0

0

0

0

0

0

0

0

0

0
4

0

0

0
4

0

0

0

0

0

0

0
4

0

0

0

0

0
4

0

0

0

0
4

0

0

0

0

0
4

0

0

0
4

0

0

0

0
4

0

0

0
4

0

0

0
4

0
4

0
4

0
4

0

0

0

0

0

0

0
4

0

0

0

0

0
4

0

0

0

0
4

0

0

0

0
4

0
4

0

0

0

0
4

0
4

0

0

0

0
4

0
4

0

0

0

0
4

0
4

0

0

0

0
4

0
4

0

0

0

0
4

0
4

0

0

0

0
4

0
4

0

0

0

0
4

0
4

0

0

0

0
4

0
4

0

0

0

0
4

0
4

0

0

0

0
4

0
4

0
4

0
4

0

0

0

0

0

0
4

0
3

0

0

0

0

0
4

0

0

0
4

0

0

0
4

0
4

0

0

0

0
4

0
4

0

0

0

0
4

0
4

0

0

0

0

0

0

0
4

0

0

0

0
4

0

0

0
4

0
4

0
4

0
4

0
4

0

0

0
4

0

0

0

0

0

0

0
4

0

0

0

0

0

0

0
4

0

0

0
4

0
4

0
4

0
4

0

0

0

0

0

0

0

0

0
4

0

0

0

0

0
4

0

0

0
4

0
4

0.51598
3

0.396169

0.396169

0

0
4

0.119811

0.119811

0

0

0

0

0
4

0

0

0
4

0

0

0
4

0

0

0
4

1.38777878078145e-17
3

0
4

1.70996
2

1.70996
2

1.70996
2

0

0

0
4

0
4

0
3

0

0

0

0

0

0

0
4

0

0

0

0

0

0
4

0

0

0

0

0
4

0

0

0
4

0

0

0
4

0

0

0
4

0
4

0

0

0

0

0

0

0

0

0
4

0

0

0

0

0

0

0
4

0
4

0
3

0
4

0
4

0

0

0

0

0

0
4

0

0

0

0

0
4

0

0

0
4

0
4

1.90964
3

0.141633
3

0.127557
4

0

0

0.0140759

0

0

0

0

0

0

0

0

0
4

0

0

0
4

0

0

0
4

0

0

0
4

0.664298
3

0.632627
5

0

0

0

0

0

0

0

0

0

0.0316707

0

0
4

0.0140945
4

0.0140945
4

0

0

0

0

0

0

0
4

0.374796
3

0.374796
7

0

0

0

0

0

0

0
4

0.714822
5

0

0

0

0.714822

0

0

0

0
4

0

0

0

0

0

0

0
4

0

0

0

0
4

0

0

0

0
4

0

0

0
4

2.22044604925031e-16
3

0
4

0

0

0

0

0

0
4

0

0

0

0

0

0
4

0

0

0
4

0

0

0
4

0

0

0
4

0
4

0

0

0

0

0

0

0

0

0

0
4

0

0

0
4

0
4

0

0

0

0

0

0

0

0

0

0
4

0

0

0

0

0

0
4

0
4

0
4

0
4

0

0

0

0

0

0
4

0
4

0

0

0

0

0

0

0

0

0
4

0

0

0
4

0
4

0.861656
7

0.28123
7

0.204198
7

0.0560972

0.0140243

0.00690984

5.55111512312578e-17
7

0
4

0.566607

0.566607

0
4

0.0138197

0.0138197

0
4

0

0

0
4

0

0

0
4

0

0

0
4

0

0

0
4

8.50014503228635e-17
7

0
4

0
4

0
4

0

0

0

0

0

0

0

0
4

0

0

0

0
4

0
4

2.30857
3

2.30857
3

2.30857
3

0
4

0

0

0
4

0
4

0
4

0

0

0

0

0

0
4

0

0

0

0

0
4

0

0

0

0

0
4

0

0

0
4

0
4

0

0

0

0

0

0

0

0

0
4

0

0

0

0
4

0
4

2.08434
7

2.08434
7

0.749776
7

0

0.0138197

0

0

0.00690984

0.357411

0

0.894083
7

0

0.0103648
7

0.015261
7

0

0.0125267

0.0241844

0

5.20417042793042e-17
7

0
4

0
7

0

0

0

0

0
4

0
4

0
4

0

0

0

0

0

0

0

0
4

0

0

0

0

0
4

0

0

0
4

0
4

0

0

0

0
4

0

0

0

0
4

0

0

0

0

0
4

0

0

0

0
4

0
4

0

0

0

0

0

0
4

0
4

0.602237
4

0.602237
4

0.602237
4

0
4

0
4

0
4

0

0

0

0

0

0
4

0

0

0
4

0

0

0
4

0

0

0

0
4

0

0

0
4

0

0

0
4

0
4

0
2

0
2

0

0

0

0

0
4

0

0

0

0

0
4

0

0

0

0
4

0

0

0
4

0
4

0

0

0

0

0

0

0
4

0

0

0
4

0
4

1.59215
7

0.843331
7

0.48592
7

0.357411

0
4

0.748818

0.748818

0

0
4

0
4

0

0

0

0

0
4

0

0

0

0
4

0

0

0
4

0
4

0
4

0

0

0

0

0

0

0
4

0

0

0

0
4

0
4

4.42141
3

1.70996
3

0
2

0
3

0
2

1.70996

0

0

0

0

0
4

0

0

0

0

0

0

0
4

2.67482

2.56494

0

0.109888

0

0
4

0

0

0

0

0
4

0

0

0
4

0.0366292

0.0366292

0
4

0

0

0
4

0
4

0
4

0

0

0

0

0
4

0

0

0

0

0
4

0

0

0
4

0

0

0
4

0

0

0
4

0
4

0.0316877
4

0

0

0

0

0

0

0
4

0

0

0

0

0

0
4

0

0

0
4

0

0

0
4

0.0316877

0.0316877

0
4

0
4

0

0

0

0

0

0

0

0

0

0
4

0

0

0

0

0
4

0
4

0
3

0
3

0
3

0

0

0

0
4

0

0

0

0
4

0

0

0
4

0

0

0
4

0
4

0

0

0

0

0

0

0

0
4

0

0

0

0
4

0
4

0
1

0

0

0

0

0

0

0
4

0

0

0
4

0

0

0
4

0

0

0
4

0

0

0
4

0

0

0
4

0
4

0
3

0

0

0

0

0

0

0
4

0
4

0

0

0

0

0

0
4

0
4

0.292913

0.292913

0.292913

0

0
4

0

0

0

0
4

0

0

0

0

0
4

0
4

0

0

0

0

0

0

0

0

0
4

0
4

0.313292

0.0522545

0

0.0229265

0.029328

0

0

3.46944695195361e-18

0
4

0.140863

0.140863

0
4

0.0972483

0.0802428

0.0170056

0
4

0

0

0
4

0

0

0
4

0.0229265

0.0229265

0
4

2.42861286636753e-17

0
4

0
3

0
4

0
4

0

0

0

0

0

0

0

0

0

0

0

0

0

0

0
4

0
3

0
4

0

0

0

0

0

0
3

0

0

0

0

0

0

0

0
4

0
3

0

0

0
4

0

0

0
4

0
4

0
2

0
2

0
2

0

0

0

0
4

0
4

0.0170056
7

0

0

0

0

0

0
4

0

0

0

0

0

0
4

0.0170056

0.0170056

0

0
4

0
4

0.161857
4

0.161857
4

0.161857

0

0

0

0
4

0

0

0

0

0
4

0

0

0
4

0
4

0.11296
7

0.11296
7

0.0316877

0.0812721

0

0
4

0
4

0
4

0
4

0

0

0

0

0
4

0

0

0
4

0
4

0
4

0

0

0

0

0

0
4

0

0

0

0

0
4

0

0

0
4

0

0

0
4

0
4

0.565214
3

0.207802
3

0.0968956

0.110907

0

1.38777878078145e-17
3

0
4

0.357411

0.357411

0
4

0

0

0
4

0
4

0
2

0
2

0

0

0

0

0
4

0

0

0
4

0
4

0
3

0
3

0
3

0

0
4

0

0

0

0

0
4

0
4

0
4

0
4

0
4

0

0

0
4

0
4

16.0351

1.74571

1.70525

0.0404642

0
4

14.2313
6

13.6952

0.536117

0
4

0

0

0

0

0
4

0.0581373

0.0581373

0
4

0

0

0
4

0

0

0
4

0
4

132.829
3

129.024
3

2.50188
3

30.7514

35.5342
3

0.327074

4.39917
7

0.0168565

0

0.0599054

0

2.33008
7

0

0.0561884

0

0.0232512

0

1.70996

0

0

10.964
6

0

0

0.0170056

0.0170056

0

0

0.0387519

0

0

0.0170056

35.7697
7

0

0.343945

0

0

0

0

0.0138197

0

0

0.0112377

0.535256
7

0

0

0.179716

0

0

0.0198848

0

0

0

0

0.187537
7

0

0

0

0

0.00835115

0

0

0

0

0

0.882389
7

0

0

0

0

0

0

0

0

0

0

0

0

0

0

0

0

0

0

0

0.0170056

0

2.06737
7

0

0

0

0

0.178259

0

0

0

0

0

0.0387519
2

0

0

0.007332

0

0

0

0

0

0

0

0
4

1.29576

0.178467
5

0.0170056

0.930994
7

0.0425139
7

0

0.0850278

0

0

0.0417557

0

0
4

0

0

0
4

0

0

0
4

0

0

0
4

0

0

0
4

0

0

0
4

0

0

0
4

0

0

0
4

0.187628

0.187628

0
4

0

0

0
4

0

0

0
4

0.189415
4

0.0914808
4

0.0979339
4

0

0

0

0
4

0

0

0
4

0

0

0
4

0

0

0
4

0

0

0
4

0

0

0
4

0

0

0
4

0

0

0
4

0

0

0
4

2.0549
7

1.98129
7

0.0736082

0

3.19189119579733e-16
7

0
4

0.076911
7

0

0

0.0170056

0

0

0.0599054

0
4

0

0

0
4

0

0

0

0

0
4

0

0

0
4

0

0

0
4

0

0

0

0
4

0
4

92.1556

76.4516

0
3

0

0

0
4

0

8.35534
6

0

0

6.87838

0

0

16.7896

0

0.304288

0

0

5.12987

0

2.56494

0

0

0

11.3195

0

0

0

0.164174

0.0155008

0

0

0

0.0703603

0

2.6822

0

1.70996

1.70996

0

0

0

0

0

0

0

0

0

0

0

0

0

0

0

0

0

0

0
4

0

0.357411

0

0

0.0155008

0

0

0

0

0

18.3377

0

0

0

0

0

0

0

0

0

0

0

0.0469069

0

0

0

0

0

0

0

0
4

0
3

0
3

0
2

0

0

0

0

0

0

0
4

0

0

0

0
4

0

0

0

0
4

0

0

0

0
4

0

0

0
4

0

0

0

0
4

0

0

0

0
4

0

0

0

0
4

0

0

0
4

0

0

0

0
4

0

0

0
4

11.1616

11.1147

0.0469069

6.17561557447743e-16

0
4

0

0

0
4

0

0

0
4

0

0

0
4

0

0

0
4

0

0

0
4

0.391128

0.391128

0
4

0

0

0
4

0.314603

0.314603

0
4

1.70996

1.70996

0
4

0

0

0
4

0

0

0

0

0
4

0

0

0
4

0

0

0
4

0.0469069

0.0469069

0
4

0

0

0
4

0

0

0
4

0

0

0
4

0
4

0

0

0

0

0
4

0.24188
7

0.24188
7

0
4

1.70996

1.70996

0

0

0
4

0

0

0

0

0

0
4

0.0655176

0.0404642

0.0167023

0.00835115

0
4

0.0624077

0.0624077

0
4

1.17614251671228e-14

0
4

447.136

446.993

120.389
6

0
4

0

0

0

0

0

0

0

0.095063

0.0105182

0

0.0775164
3

0

0.0387582

0

0.228201

0

0

0

0

0.0475315

0

2.55329
7

0

0.0255083

0

0

0

0

0

0.104103

1.10461

0

0.443564
6

0

0.0404529

0

0.341716

0

0.0935306

0

0.117331

0

0

0
2

0.357411

0

0

0

0

0.53869

0

0

0

0

0
4

0

0

0

0

0.0785946

0

0

0

0

0

0.647427
3

0

0.0633753

0

0

0

0.0316877

0.0192834

0

0

0

0
4

1.70996

0

0.357411

0

0.00350607

0

0

0

0.0703603

0

0

0

0

0

0

0

0.10116

0.536117

0

0.0649954

0

0.10116
3

0

2.06737

0.0656988

0.364408

0

0

0

0

0

0

185.203
6

0
3

0

0

0

0

0.0351937

0

0

0.0175304

0.357411

0

0.0850278

0

0

0

0.108228

0

0

0

0

0

0.0205116

0
4

0.00350607

0.0440173

0

0

0

0

0

0

0

0

3.17057
6

0.0985691

0

0

0.00876519

0

0

0.164785

0

0

0

0
4

0

0.0475315

0

0.0255083

0

0

0

0

0

0.00350607

0.625574
5

0.00525911

0

0

0

0

0

0

0

0

0

4.27489
7

0

2.42411

0

0

0

0

0

0.00350607

0

0

0.183521

0

0

0.0387582

0

0

0.00350607

1.70996

0

0.00350607

2.56494

0
4

0

0.0192834

0.0170056

0.010998

0

0

0.0316877

0

0

0

0.191236
7

0

0.0316877

0.0792192

0

0

0

0

0

0.00525911

0

0
4

1.0723
3

0

0

0

0

0.007332

0

0

0

0

0

0

0.00525911

0

0

0

0

0

0

0

0

0

0
4

0

0

0

0

0

0.00350607

0

0.110907

1.70996

0

0.412136
6

0

0

0

0

0

0

0

0.140721

0

0

0
4

0

0

0

0

0

0

0

0

0.0170056

0

0

0

0.0140243

0

0

0

0

0.0316877

0

0

0

0
4

0

0

0

0.0316877

0.0255083

0

0

0

0

0.0792192

0

0.00525911

0

0

0.0316877

0

0

0.0898581

0

0

0

0

0

0

0

0

0

0

0.0633753

0

0.0316877

0

0

0

0.00525911

0

0

0.076525

0

0

0.0475315

0

0

41.3326
7

0
3

0

0.357411

0

0

0

0

0

0

0

0

0
3

0

0.0633753

0.00350607

0

0

0

0

0

0

0.0316877

0.152427

0

0

0

0

0

0.0316877

0

0

0

0

0
3

0.00350607

0.0404642

0.0280942

0

0

0

0.0315547

0

0.00350607

0

8.08738

0.014664

0

0.759065

0

0

0

0

0

0

0.0316877

0
4

0

0.00350607

0

0

0.00525911

0

0.0475315

0.0316877

0

0

1.70996
7

0.00350607

0

0

0.00350607

0

0

0

0

0.00525911

0

0.0925192

0.00350607

0

0.0316877

2.56494

0.0316877

0

0

0

0

0.33483

0.068568

0.0809283

0

0

0

0

0.00350607

0

0

0.190126

0

0
4

0

0.00350607

0

0

0

0

0

0

0

0

0.0387582
4

0.58799

0

0

0

0

0

0

0.00525911

0

0

0

7.91206

0.0155008

0

0

0

0

0.0599054

0

0.0404642

0

0

0
2

0.174282

0.0475315

0

0

0

0

0

0.00525911

0

0

0.833302

0

0

0

0

0

0

0

0

0

0

0

0.00350607

0

0

0

0

0

0

0

0.0475315

0

0

0

0

0

0

0

0

0

0

0

0

0.179716

0

0

0

0

0

0

0

0

0.357411

0

0
3

0

0

0

0

0

0

0

0.0606962

0.0469069

0

0

0

0.00350607

0

0.357411

0

0

0

0

0

0.0255083

0.13231
6

0.00350607

0

0

0

0

0

0.0469069

0.0316877

0

0

0.0915731

0

0

0.0316877

0

0

0.00701215

0

0

0

0

0

0.364726

0

0

0

0

0

0

0

0

0.00350607

0

0

0.142595

0

0

0

0

0

0

0

0

0

0
4

0

0

0

0

0

0.00350607

0

0

0.0387582

0

0
4

0

0.0316877

0

0

0

0

0

0

0.0465023

0

0

0

0.00525911

0

0.00876519

0

0

0

0.357411

0

0.0316877

0
4

0

0

0

0

0

0

0

0.0170056

0

0

0

0

0

0.0170056

0.0404642

0

0

0

0

0

0.0633753

0

0

0

0

0

0

0

0

0

0.536117

0.00350607

0

0

0

0

0

0

0

0.00350607

0

0.0633753

0

1.69526
6

0

0

0.0105182

0.0340111

0.0475315

0

0

0

0

0

0

5.33913

0

0

0

0.110907

0.357411

0

0

0.0387582

0.0792192

0

0

0

0

0

0

0

0

0.0898581

0

0

0

0.893528

0

0

0

0

1.70996

0.0475315

0.0475315

0.0599054

0.161857

0

0.0913574

0

0.0316877

0.00350607

0

0.0170056

0.187628

0.0170056

0.544517

0

0

0

6.67251
6

0

2.03944

0

0.13019

0

0

0.0862313

5.46278

0

0

11.9158
6

0.049218

0

0

0.15039

0

1.23792

0

0

0

0

0
4

0

0

0

0
4

0

0

0
4

0

0

0

0
4

0

0

0
4

0

0

0
4

0.0792192

0.0792192

0
4

0.0633753

0.0633753

0
4

0

0

0
4

0

0

0
4

0
4

10.6811

1.25043

0.438329
7

0

0

0

0

0.0595195

0

0

0

0

0

0.705955
7

0

0

0

0

0

0

0

0

0

0

0.0466222

0

0

0

3.19189119579733e-16

0
4

9.23715
7

5.51593
7

0

0

0.357411

0.427772
6

0.269832
6

0.897607

1.70996

0.0246328

0

0

0.0340111

0
4

0

0

0
4

0.0209857
7

0.0209857

0

0
4

0.109168

0.0915731

0.0175948

6.93889390390723e-18

0
4

0.0281518

0.0211138

0.00703794

0

1.73472347597681e-18

0
4

0

0

0
4

0

0

0
4

0.0246328

0.0246328

0
4

0

0

0
4

0.0105569

0.0105569

0
4

1.40512601554121e-16

0
4

102.553

2.39654
3

2.33852
3

0

0

0

0

0

0

0

0

0

0

0

0

0

0

0

0

0

0

0.014664

0

0

0

0

0

0.0103648

0

0

0

0

0

0

0.032994

0

0

0

0

0

0

0

0

0
4

29.0629
7

27.3004
7

0.029328

0.0232512

0

0

0

1.70996

0
4

0
3

0

0

0

0

0
4

0

0

0
4

0

0

0
4

0.116256

0.116256

0
4

0

0

0
4

0

0

0
4

0

0

0
4

0

0

0
4

1.74397
7

1.74397
6

0

0
4

0
3

0

0

0

0

0

0
4

0

0

0

0

0

0
4

0

0

0

0
4

0.933216
7

0.933216
7

0
4

0

0

0

0

0
4

0

0

0
4

0.0255083
7

0.0255083

0

0
4

0

0

0

0
4

37.1709
6

36.6479
6

0.0837039

0.0651753

0.0492656

0

0

0.277999

0.0469069

0
4

6.83983

0

6.83983

0
4

0

0

0

0
4

0

0

0

0

0

0
4

0

0

0
4

0.0508363

0.0170056

0.0155008

0.01833

0

0
4

0

0

0

0
4

1.20302

1.07223

0.130787

0
4

0.029328

0

0.029328

0
4

0.093011

0.093011

0
4

0

0

0

0

0
4

12.7928
7

11.1009
7

0

0

0

1.20493
7

0

0

0.095063

0.0775039

0.314398

0

0

3.33066907387547e-16
7

0
4

0.10998

0.10998

0
4

0

0

0

0
4

0.0635079

0.0170056

0.0155008

0.0310016

3.46944695195361e-18

0
4

4.51255

0.237658

4.27489

0
4

0

0

0

0
4

0.629647

0.629647

0
4

0

0

0
4

0

0

0
4

0

0

0
4

0

0

0

0
4

0
8

0
8

0

0

0

0

0

0

0

0

0
4

0.10037

0.10037

0
4

0

0

0

0
4

0.0425139

0

0.0425139

0
4

0

0

0

0
4

0

0

0

0
4

0

0

0
4

0

0

0
4

0

0

0
4

0

0

0

0
4

0

0

0
4

0.0170056
8

0

0

0.0170056

0

0

0

0

0

0
4

0

0

0
4

0

0

0
4

0

0

0

0
4

0

0

0
4

0

0

0
4

0

0

0

0
4

0

0

0

0
4

0

0

0
4

0

0

0
4

0

0

0
4

0
3

0

0

0

0

0

0

0
4

0

0

0
4

0

0

0
4

0

0

0
4

0

0

0
4

0

0

0
4

0

0

0
4

0

0

0
4

3.41991

3.41991

0
4

0

0

0
4

0.0105569

0.0105569

0
4

0

0

0

0

0
4

0.357411

0.357411

0
4

0

0

0
4

0

0

0
4

0

0

0
4

0

0

0
4

0

0

0
4

0.00690984

0.00690984

0
4

0.0606962

0.0606962

0
4

0

0

0
4

0

0

0
4

0

0

0

0

0

0
4

0.0316877

0.0316877

0
4

0

0

0
4

0

0

0
4

0

0

0
4

0

0

0
4

0.357411

0.357411

0
4

0

0

0
4

0

0

0
4

0

0

0
4

0

0

0
4

0

0

0

0

0

0
4

0

0

0
4

0

0

0
4

0

0

0
4

0.0170056

0.0170056

0
4

0

0

0
4

0

0

0
4

0.357411

0.357411

0
4

0

0

0
4

0

0

0
4

0

0

0
4

4.86832796298131e-14

0
4

35.931
3

35.5735
3

35.1247
3

0
2

0
2

0

0

0

0

0

0

0

0

0

0

0
3

0

0

0

0

0

0

0

0

0

0

0
3

0

0

0

0

0

0

0

0

0

0

0
2

0

0

0

0

0

0

0

0

0

0

0

0

0

0

0

0

0

0

0

0

0

0
2

0

0

0

0

0

0

0

0

0

0.357411

0

0

0

0

0

0

0

0

0

0

0

0

0

0

0

0

0

0

0

0

0

0

0
2

0

0

0

0

0

0

0

0

0

0

0

0

0

0

0

0

0

0

0

0

0

0

0

0

0.00690984

0

0

0

0

0

0

0.0316877

0

0

0

0

0

0

0

0

0

0

0

0

0

0

0

0

0

0

0

0.0340111

0

0

0

0

0

0

0

0

0

0

0

0

0

0

0

0

0

0

0

0

0

0

0

0

0

0

0

0.00690984

0

0

0

0

0

0.00500636

0

0

0

0

0

0
2

0

0

0

0

0

0

0

0

0

0

0

0

0

0

0

0

0

0

0.00690984

0

0

0
3

0

0

0

0

0

0

0

0

0

0

0

0

0

0

0

0

0

0

0

0

0

0

0

0

0

0

0

0

0

0

0

0

0
3

0

0

0

0

0

0

0

0

0

0

0

0

0

0

0

0

0

0

0

0

0

1.30399163689177e-14
3

0
4

0

0

0

0

0

0
4

0

0

0

0

0
4

0

0

0
4

0

0

0

0

0
4

0

0

0

0

0
4

0

0

0

0
4

0

0

0
4

0

0

0

0
4

0

0

0

0
4

0

0

0

0
4

0

0

0
4

0
3

0

0

0

0
4

0

0

0

0
4

0

0

0
4

0

0

0

0
4

0

0

0
4

0

0

0

0
4

0

0

0
4

0

0

0
4

0.357411

0.357411

0
4

0

0

0
4

0

0

0
4

0
2

0

0

0

0
4

0

0

0
4

0

0

0
4

0

0

0
4

0

0

0
4

0

0

0
4

0

0

0
4

0

0

0
4

0

0

0
4

0

0

0
4

0

0

0
4

0

0

0
4

0

0

0
4

0

0

0
4

0

0

0
4

0

0

0
4

0

0

0
4

0

0

0
4

0

0

0
4

0

0

0
4

0

0

0
4

0

0

0
4

0

0

0

0
4

0

0

0
4

0

0

0
4

0

0

0
4

0

0

0
4

0

0

0
4

0

0

0
4

0

0

0
4

0

0

0
4

0

0

0
4

0

0

0
4

0

0

0
4

0

0

0
4

0

0

0
4

0

0

0

0

0
4

0

0

0

0

0
4

0

0

0

0

0
4

0
4

0.640725
3

0.0518238
3

0
2

0

0

0

0

0

0

0

0

0

0

0
2

0

0

0

0

0.0138197

0

0

0

0

0

0
3

0

0

0.0380041

0

0

0

0

0

0

0

0

0

0

0

0

0

0

0

0

0

0

0

0

0
4

0

0

0

0

0

0

0
4

0

0

0

0
4

0

0

0

0

0
4

0.0527845

0.0211138

0.0316707

0
4

0

0

0
4

0

0

0
4

0.536117

0.536117

0
4

0

0

0
4

0

0

0
4

0
4

27.7334
5

27.0116
5

18.7848
5

0.505292
7

0.0316877

0.114633

0.0366292

0

0.0316877

0

0.0229265

0

0.045853

0

0.841426

0

0

0

0.0229265

0.0138197

0

0.0343898

0.0316877

0.0809283

0

6.03371

0.119811

0.103169

0

0.091706

0

0.064528

0
4

0

0

0
4

0

0

0
4

0

0

0
4

0

0

0
4

0

0

0
4

0.714822

0.714822

0
4

0.00690984

0.00690984

0
4

8.55218673656566e-16
5

0
4

223.289
4

86.4158
4

70.5782
4

3.96931
7

0.23052

0

0.0449139

0

0.0103648

0

0

0

0

0

0

0.110907

0

0

0

0

0

0

0

0

0

0

0

0.00500636

0

0

0.0337346

0

0

0.00458879

0

0

0
4

0.0310943

0.0112449

0

0

0

0

0.119811

0

0.0404642

0

0.995147
6

0

0

0.0112449

0

0

0.0100127

0

0

0

0.0151575

1.22312
7

0

0

0.0229265

0

0.0697535

0

0

0

0

0

0.278053
7

0

0

0

0.0155008

0

0.0562243

0

0.0229265

0

0

4.74872
7

0

0.0103648

0

0.0366292

0

0

0

0.00701215

0

0

2.8983
7

0

0

0.00458879

0

0

0

0

0

0

0

0.723397
5

0.00690984

0.0224897

0

0

0

0.0112449

0

0

0

0

0.00611838
6

0

0

0

0

0

0.0229265

0

0

0.00690984

0

6.65838911784178e-14
4

0
4

16.2112
6

10.8419
6

0.0753806

0.0886887

0.0483689

0.041459

0.0173769

0.0138197

0.0207295

0.0172746

0.0690984

0.0207295

1.07145
6

0.0138197

0.0227895

0.0103648

0.0103648

0.00690984

0.0103648

0.00690984

0.00350607

0.0103648

0.00690984

1.24701
6

0.0103648

0.00690984

0.00350607

0.0138197

0.00350607

0.0241844

0.0103648

0.00690984

0.00876519

0.00876519

0.680926

0.00690984

0.00350607

0.0207295

0.0103648

0.00690984

0.00690984

0.00690984

0.0138197

0.0276393

0.00525911

0.133296

0.0105182

0.00690984

0.0172746

0.0595195

0.00690984

0.0103648

0.00690984

0.00350607

0.0380041

0.00690984

0.186566

0.00690984

0.0241844

0.0138197

0.0207295

0.0633753

0.0276393

0.00690984

0.00525911

0.00690984

0.00690984

0.256073

0.00690984

0.0172746

0.0921436

0.606882

0
4

0.0469069
3

0

0

0

0

0

0

0

0.0469069

0

0

0
4

0

0

0
4

0

0

0

0
4

0

0

0
4

0

0

0

0
4

0

0

0

0
4

0

0

0
4

0

0

0

0
4

0

0

0

0
4

0

0

0

0
4

0

0

0
4

0
4

0
4

0
4

0

0

0

0

0

0
4

1.70996

1.70996

0

0
4

0

0

0

0
4

0.078762

0.078762

0
4

0

0

0
4

0

0

0

0
4

0

0

0

0
4

0

0

0

0
4

0

0

0

0
4

2.02567

2.02567

0
4

0.0241844

0.0138197

0.0103648

0
4

0.0775164
2

0.0775164

0
3

0

0

0

0

0

0

0
4

0

0

0
4

0.0469069

0

0.0469069

0
4

0

0

0
4

0.0711503

0.0711503

0
4

0.183672

0.0898581

0.0938138

1.38777878078145e-17

0
4

0

0

0

0
4

0

0

0

0
4

0

0

0

0
4

0

0

0
4

0.0207295

0.0207295

0
4

1.63212

0.27299

0.107205

0.0573387

0.335913

0.0276393

0.41459

0.00876519

0.041459

0.366221

1.11022302462516e-16

0
4

0

0

0

0
4

0.013922

0.00690984

0.00701215

0
4

0

0

0

0
4

0.357411

0.357411

0
4

0

0

0
4

0

0

0
4

0

0

0
4

0

0

0
4

0

0

0
4

0

0

0
4

5.62015
5

5.28481
5

0.00525911

0.211081

0.0427829

0.0103648

0.0103648

0.0103648

0.0241844

0.00690984

0.0140243

0
4

0.0039381

0.0039381

0
4

0

0

0
4

0

0

0
4

0

0

0
4

0

0

0
4

0

0

0
4

0.00350607

0.00350607

0
4

0.124006

0.124006

0
4

0

0

0
4

0.0599054

0.0599054

0
4

0.239622

0.239622

0

0

0
4

1.70996

1.70996

0
4

0

0

0
4

0

0

0
4

0

0

0
4

0

0

0
4

0

0

0
4

0

0

0
4

0

0

0
4

0

0

0
4

0.0103648

0.0103648

0
4

0.606514

0.522812

0.0511953

0.0177215

0.00690984

0.0078762

0
4

0

0

0
4

0.00350607

0.00350607

0
4

0

0

0
4

0

0

0
4

0

0

0
4

0.0792192

0.0792192

0
4

0

0

0
4

0.0138197

0.0138197

0
4

0

0

0
4

0

0

0
4

0.623573

0.503762

0.119811

0

0

0
4

0

0

0
4

0.0232512

0.0232512

0
4

0

0

0
4

0

0

0
4

0.0316877

0.0316877

0
4

0

0

0
4

0

0

0
4

0

0

0
4

0

0

0
4

0

0

0
4

0.454574
5

0.454574
5

0

0
4

0

0

0
4

0

0

0
4

0.0599054

0.0599054

0
4

0

0

0
4

0

0

0
4

0

0

0
4

0

0

0
4

0

0

0
4

0

0

0
4

0.174412

0.174412

0
4

0.260588

0.200683
5

0

0

0

0.0599054

0

2.77555756156289e-17

0
4

0

0

0
4

0

0

0
4

0

0

0
4

0

0

0
4

0.0316707

0.0316707

0
4

0

0

0
4

0.0465023

0.0465023

0
4

0

0

0
4

0

0

0
4

0

0

0
4

0
4

0
4

0

0

0

0

0

0

0

0

0

0

0
4

0

0

0

0

0

0

0

0

0

0

0

0

0

0

0

0

0
4

5.02827

4.67085

0.357411

0
4

0

0

0
4

0

0

0
4

0

0

0
4

0

0

0
4

0.0703603

0.0703603

0
4

0

0

0
4

0

0

0
4

0

0

0
4

0

0

0
4

0

0

0
4

16.3544
6

15.9768

0.0780682

0.0416125

0.0639927

0.0676523

0.0710049

0.0241844

0.0310943

2.60902410786912e-15
6

0
4

0

0

0
4

0

0

0
4

0.357411

0.357411

0
4

0

0

0
4

0

0

0
4

0.161857

0.161857

0
4

0

0

0
4

0

0

0
4

0

0

0
4

0

0

0
4

0.0703603
3

0.0703603

0

0

0

0

0

0
4

0

0

0
4

0

0

0
4

0

0

0
4

0

0

0
4

0

0

0
4

0.00703794

0.00703794

0
4

0

0

0
4

0.00690984

0.00690984

0
4

0

0

0
4

0

0

0
4

0
4

0

0

0

0

0

0

0

0

0
4

0

0

0
4

0

0

0
4

0

0

0
4

0

0

0
4

0

0

0
4

0.0255083

0.0255083

0
4

0.0633753

0.0633753

0
4

0

0

0
4

0

0

0
4

0

0

0
4

0
3

0

0

0

0

0

0
4

0

0

0
4

0.0775164

0.0775164

0
4

0

0

0
4

0

0

0
4

0

0

0
4

0.357411

0.357411

0
4

0.00350607

0.00350607

0
4

0.240728

0.240728

0
4

0

0

0
4

0
4

0
4

0

0

0

0
4

0
2

0

0

0

0

0

0

0
4

0.186566

0.0967377

0

0.0310943

0

0.0587336

0
4

0
4

0

0

0

0

0

0

0
4

0

0

0

0

0
4

0
4

0
4

0

0

0

0

0

0

0

0

0

0

0
4

0

0

0

0

0

0
3

0
4

0

0

0

0

0

0
4

0
4

0
4

0

0

0

0
4

0.0793456
4

0.040326

0

0.007332

0

0.0316877

0

0
4

0

0

0

0

0

0

0

0
4

0

0

0

0

0

0

0

0

0
4

0.061117
7

0.0342788
7

0.0268383

0

6.93889390390723e-18
7

0
4

0.221814
4

0.221814
4

0

0
4

0.345885

0.345885

0

0

0
4

0.363491
7

0.363491
7

0
4

0
4

0

0

0

0
4

0.317106

0.197295

0.0599054

0.0599054

0

0
4

0
4

0
4

0

0

0

0

0
4

0
4

0

0

0

0

0

0

0
4

0.153719

0.0938138

0.0599054

0
4

0

0

0

0

0
4

2.33321
6

2.33321
6

0
4

1.17839
5

1.17839
5

0
4

0

0

0

0
4

0

0

0

0
4

0.986475

0.986475

0
4

0

0

0

0

0

0
4

0.082918

0

0.0656434

0.0172746

0

0
4

0.0766449

0.0588871

0

0.0039381

0.0138197

0
4

53.6741

24.7574

0

26.3132

0

0

0

0.536117

0

1.70996

0.357411

2.83106871279415e-15

0
4

0

0

0

0

0
4

0.18038

0.18038

0

0
4

0

0

0

0

0

0
4

0.0105569

0

0

0.0105569

0
4

0.00939633

0.00939633

0

0

0
4

0.179716

0.179716

0
4

1.84696

1.48955

0.357411

0
4

5.70454

5.70454

0
4

1.58336

1.42964

0.153719

5.55111512312578e-17

0
4

0.369226

0.369226

0

0
4

3.36201

0.243393
3

0

0.196267
2

0

2.92235

0

0

0

0

0

0
4

0

0

0

0

0
4

0

0

0

0
4

0.766536

0.766536

0
4

0

0

0

0

0

0
4

0.882665

0.882665

0

0
4

0

0

0

0

0
4

0

0

0

0
4

0

0

0
4

0

0

0

0
4

0

0

0
4

0
4

0
4

0
4

0

0

0

0
4

0

0

0
4

0

0

0

0

0
4

0.394125

0.00703794

0.00703794

0.380049

5.55111512312578e-17

0
4

0

0

0

0
4

0.200626

0.200626

0
4

0

0

0

0
4

0

0

0

0
4

0.082918

0.082918

0
4

0.121392

0.121392

0

0
4

0

0

0

0

0
4

2.18725
4

0
4

2.02185

0

0

0

0

0.1654

0

1.66533453693773e-16
4

0
4

0

0

0

0

0
4

0.0296993

0.00690984

0.00350607

0.0192834

0
4

0

0

0

0
4

0

0

0

0

0
4

0

0

0
4

0

0

0

0
4

0

0

0

0
4

0.553122

0.536117

0.0170056

4.85722573273506e-17

0
4

0

0

0
4

0

0

0

0
4

2.24957
6

2.24957
6

0
4

0

0

0

0
4

0

0

0
4

0

0

0
4

0.0554322

0.0554322

0
4

0.109204

0.0775164

0.0316877

6.93889390390723e-18

0
4

0.00701215

0.00350607

0.00350607

0
4

0.0633753

0.0633753

0
4

0.357411

0

0.357411

0
4

0

0

0

0
4

0

0

0
4

0
4

34.6484

30.3278

27.4199

2.67592
6

0

0

0

0

0

0.135105

0.0968956

0

0

0

0

0

3.20576898360514e-15

0
4

4.28897
6

0.0140759

4.27489

0
4

0

0

0
4

0

0

0
4

0

0

0
4

0.0316877

0.0316877

0
4

3.49026363366534e-15

0
4

26.4291
3

6.84829
3

6.73738
3

0
4

0

0

0

0

0

0.0633753

0

0

0

0

0
4

0

0

0

0

0

0

0

0

0

0

0

0

0

0

0

0

0

0

0

0

0

0.0475315
4

0

0

0

0

0

0

0

0

0

0

0

0

0

0

2.77555756156289e-16
3

0
4

11.0213
6

6.83298
6

0.751452

0.0170056

3.41991

0
4

0

0

0
4

0

0

0
4

0

0

0
4

0

0

0
4

0.0938138
4

0.0938138
4

0
4

0

0

0

0
4

8.35514

8.26947

0.0856651

0
4

0.110536

0.110536

0
4

0

0

0
4

0

0

0

0
4

0

0

0
4

0

0

0
4

2.73392419813945e-15
3

0
4

227.902

210.607

108.218

16.4952
7

0.0806349
6

0

0

0

0

0.0340111

0

0

0

0

0.00350607

1.07223

0

0.00835115

0

0

0

0

0

0

0

0

0.190081
7

0

0

0

0

0

0

0

0

0

0.0340111

0.0232512

0.110907

0

0

0

0

0

0

0

0

4.27489

7.70605

0

0.140721

0

0

0

0

0

0.32948

0

0

0

0

0

0

0

0

0.119811

0

0

0

0

0.700948
5

0

0

0

0

0

0

0

0

0.155008

0.10116

1.63203
7

0.380662
7

2.91232
7

0.0599054

9.38026

0.631622

1.07223

0

1.75042

0.269345

0.155014

0

0.116275

14.5612
6

10.0951

0.0224754

2.9609

0.0239154

4.11023

0.0599054

0

0

0

0

1.4121
7

0

0

0

0.536117

0

0

0.0425139

0.0039381

0.0112449

0

0

0

0.0250534

0.0542527

0

0

0.192243

0

0

0.155033

0.0606962

0.73132
5

0

0

0.00350607

0

0

0

0

0

0

0

3.65425
7

0

0

0.0599054

0.0387519

0

0

0

0

2.56494

0

10.2726

0

0

0.0170056

0

0

0

0

0

0

0

0.690324

0

0

0

0

0

0.0898581

0

0

0

0

0
4

1.40285
7

0.524921
7

0.00703794

0

0

0

0.00690984

0

0

0.0366292

0

0.241067
7

0
8

0.544826
7

0

0

0.0310943

0

0.0103648

2.75821032680312e-16
7

0
4

0.357411

0.357411

0
4

0

0

0
4

0

0

0
4

0

0

0
4

0

0

0
4

0

0

0
4

14.8088

14.8088

0

0

0
4

0.33972

0.305708

0

0

0

0.0170056

0

0.0170056

0

0
4

0.0542527
4

0
4

0

0.0542527

0

0
4

0.195564

0.0595195

0.119039

0.0170056

0
4

0.0938138

0.0938138

0

0
4

0

0

0
4

0.0425139

0.0425139

0
4

0

0

0
4

4.31391034005912e-14

0
4

244.733

183.601

7.23619

0.136583

0

0

0

0

0

0

0

0

0

0

5.98632
6

0

0

0

0

0

0

0

0

0

0

0.117267
7

0

0

0

0

0

0

0

0

0

0

0

0

0

0

0

0

0

0.0200254

0

0

0

0.149764

0

0

0.00500636

0

0

0.0298016

0

0

0

0

4.60523
7

0

0

0

0

0

0

0

0

0

0

0.0552787

0.0108324

0

0.00690984

0.00350607

0

0

0.00690984

0

0

0

0

0

0

0

0.0170056

0

0

0

0

0

0

0.378141

0

0

0

0

0

0

0

0

0

0

0.33496
6

0.0404642

0

0

0

2.31116
7

0.0557638

0.0340111
7

0

0

0.520599
7

0.103762
6

0

0.117817
7

0.0210364

11.3268
6

11.3734

0.668371

0.0842788

0.0773331

2.56494

0.0340111

0

0.438549

0

0

0

1.1777

0

0.0255083

0

0.0368138

0

0.0316877

0.0251374

0

0

0

1.4718
7

0

0

0.242304

0

0

0.0105182

0.10384

0

0.00701215

0.0122713

6.65352
7

0

0

0.105382

0

0

0.0475315

0

0

0

0

63.3904
7

0

0

0

0.0938138

0

0

0

1.71687

0

0

19.4948
7

0.0425139

0

0

0

0

0

0

0.0168673

0

0

39.4212
6

0

0

0

0

0.00750954

0

0.536117

0.0475315

0

0.0100127

1.88096066500165e-14

0
4

46.327
6

46.327
6

0
4

14.433

14.433

0
4

0

0

0
4

0

0

0
4

0.0150191

0.0150191

0
4

0

0

0
4

0.357411

0.357411

0
4

0

0

0
4

3.10862446895044e-15

0
4

139.978
3

138.829
3

137.059
3

1.76986
3

0

0

0

0

0
4

1.0073

0.970675

0.0366292

1.11022302462516e-16

0
4

0

0

0
4

0

0

0
4

0.141625

0.141625

0
4

0

0

0
4

0
4

95.222

95.2074

45.9226

0.0626892
7

0

0

0

0.0255083

0

0.0366292

0

0.0134738

0

0

13.7729
6

0

0

0

0

0.0465023

0

0

0

0

0

0
3

0

0

0.372019

0

0

0

0

0

0.00350607

0

0
3

0

0

0

0.0140243

0.0425139

0

0

0

0

0.0633753

0.0232512
3

0.0648624

0

0

0

1.56558

0

0.00701215

0.0155008

0

0

0.141346
7

0.0340111

0

0

0

0

0.0316877

0

0

0

0.0140243

0.0140243
7

0.0581373

0

0

0.174412

0

0.00703794

0

0

0

0.00350607

0.0775039
7

0

0

0

0

0.0930047

0.0310016

0.0155008

0

0.076525

0

0
3

0.007332

0.00350607

0

0.0366292

0

0

0

0

0

0.076525

1.74898
6

0

0

0.0255083

0.0316877

0

0

0

0

0

0

19.2112
7

0.00350607

0

0

0.0155008

0

0

0

0

0

0.0316877

0

0
3

0

0.0938138

0

0

0

0.00525911

0

0

0

0.0316877

0.118419

0.0633753

0

0

0

0

0

0

0

0

0

0
3

0

0

0

0

0

0

0

0

0

0

0

0.00350607

0

0.0170056

0

0

0

0

0

0

0.0465023

0.029328
7

0

0

0

0

0

0

0

0

0

0

0.0536733
7

0

0

0.007332

0

0

0

0

0

0

0

0

0

0

0

0.170056

0

0

0

0.0155008

0.357411

0.0155008

0.0450998
4

0

0.0155008

0

0

0

0

0

0

0

0

0.0852543

0

0

0

0

0

0

0

0

0

0

4.82572
7

0.0542527
7

0

0

0.0170056

0

0

0.0469069

0

0

0.0155008

0.0157773

0
3

0

0

0

0

0

0.0155008

0

0

0

0

0.0968956

0

0

0

0

0

0

0

0.0465023

0

0

0.0486932
7

0

0.0510167

0.007332

0

0

0

0

0

0

0

0
3

0

0

0.714822

0.065988

0

0

0

0

0

0

0

0

0

0

0

0

0

0

0

0.0155008

0

0.0542527

0.0232512

0

0

0

0.714822

0.00701215

0

0

0.0170056

0

0

0

0.10116

0

0

0.126751

0.0310016

0

0

0.00703794

0

0.0797611

0

0

0

0

0

0

0

0.0155008

0

0

0

0.0255083

0

0

0

0

0.0112377

0.0310016

0

0

0

0
4

0.0155008

0

0.0387582

0

0

0

0.0155008

0

0

0

0.0595195

0.126408

0

0

0

0

0.0122713

0

0

0

0

0

0.0780393

0

0

0

0

0

0

0

0

0

0.007332

0.0155008

0

0

0

0

0.343886
7

0

0

0.0255083

0

0

0

0.00525911

0.007332

0

0

0.562862
7

0.0255083

0.0697535

0.010998

0

0

0.0316877

0

0

0

0

0.007332
3

0

0

0

0

0.0105569

0.0465023

0

0.00703794

0

0

0.119762
7

0

0.302265

0

0.256405

0

0

0

0

0

0

0.426081
7

0.0175304

0

0

0.010998

0

0

0

0

0

0.0785946

0
4

0

0

0
4

0

0

0
4

0

0

0
4

0.014664

0.014664

0
4

0

0

0
4

0

0

0
4

0

0

0
4

0

0

0
4

6.5746019739521e-15

0
4

714.191

65.3211
3

2.91595
6

0.878845
7

0.0232512

0.091706

0

0

0.0775407

0

0

0.0809283

0.0687795

0.0621885

8.29962
6

0

0

0

0.0802428

0.0207295

0

0

0

0

0.0573163

0
3

0

0

0

0.0574697

0.121733

0

0.0802428

0.103169

0

0

0.643178
6

0

0.0241844

0

0.184067

0

0

0.126096

0.027844

0

0.0316695

0.0625795
2

0.0898581

0

0

0

0.095063

0.0294435

0

0

0.275118

0

0

0.0138197

0

0.0229265

0

0

0

0

0.0599054

0.893528

0

0
4

0

0.095063

0.0316877

0.0310016

0.0792192

0.00690984

0

0

0.0846112

0.0229265

0

0

0

0

0

0.103169

0.126751

0.357411

0

0.0633753

0

1.05146

0.0633753

0.0316877

0

0

0

0.0229265

0.045853

0.0721643

0

0.0138197

0.721537
7

0

0

0

0

0.0316877

0

0

0.0229265

0.0633753

0

0.042554
2

0

0

0

0

0

0

0

0

0

0

0

0.913671
6

0

0.0390197

0.0167023

0.045853

0

0

0

0.0633753

0

0

3.78432
6

0

0

0.0229265

0.0616847

0

0

0

0

0

0

0.337678
6

0

0.0316877

0.0316877

0

0.536117

0.0475315

0.0229265

0

0

0.0475315

0.467034

0

0

0

0.0633753

0

0.00690984

0

0

0

0.0316877

4.85797
6

0

0.0229265

0.0112449

0

0.0316877

0

0.00690984

0

0

0

0

0

0.0404642

0

0

0.00690984

0.045853

0.01833

0.0229265

0

0

0
2

0

0.00703794

0

0.0229265

0.0316877

0

0

0

0

0

0.508786
6

0

0

0

0

0

0

0

0

0.00500636

0

0.413977
7

0

0.0229265

0

0

0

0.0316877

0.0573163

0

0

0

2.68407
7

0

0

0.0343898

0

0

0

0

0

0

0.00690984

0.0316877

0
4

0.0229265

0

0.0404642

0

0

0.007332

0

0.0475315

0

0.0229265

0.156963
7

0.0229265

0.0316877

0

0.0316877

0

0.0316877

0

0

0.0343898

0

0.373025
5

0.0633753

0

0

0.0229265

0.00703794

0.00690984

0.00690984

0

0

0.00690984

0.263655

0.0229265

0.0475315

0

0

0

0

0.00690984

0

0

0

0.20751
7

0.0229265

0

0

0.126096

0.0112449

0

0

0

0

0.0343898

0

0.0316877

0

0

0

0.00690984

0

0

0.0229265

0

0.0229265

0.0469069

0.0224897

0

0

0

0.0316877

0

0.00350607

0

0

0.0606962

0.197716

0.00690984

0

0

0.00690984

0.00703794

0

0

0.00690984

0

0.0229265

0

0

0

0.0343898

0

0

0

0

0

0

0.01833

0

0.131473
7

0

1.70996

0

0

0.357411

0

0

0

0

0

0.137559

0

0.0229265

0.0316877

0.0316877

0

0

0

0.0229265

0

0

0.864697

0

0

0.0140759

0

0.0229265

0

0

0

0

0

0

0.0229265

0

0

0.0316877

0.0606962

0

0

0

0.0404642

0.045853

0.0604795

0

0.0112449

0.00690984

0

0

0

0.0229265

0.0316877

0.00690984

0

0.275118

0.00690984

0.0343898

0.0633753

0.0229265

0

0.0316877

0

0

0

0

0

0

0

0

0

0

0

0.0343898

0.0475315

0

0.0404642

3.99923

0

0

0

0.0316877

0.0340111

0

0.0229265

0

0.0229265

0.0475315

0

0.0316877

0

0.357411

0.0229265

0.0229265

0

0

0.0316877

0

0

0

0.01833

0.0475315

0

0.0229265

0

0.0229265

0

0.0170056

0.0343898

0

0.65431
4

0.178701
5

0

0.0229265

0.00703794

0.0229265

0.387582

0

0.0229265

0.0633753

0

0

0

0

0

0

0

0.0229265

0.0316877

0.095063

0

0.0316877

0

0.0874019

0.121392

0

0.0229265

0

0

0.045853

0.0469069

0.091706

0.0229265

0

0.0792192

0.357411

0.10116

0

0

0.0229265

0.0343898

0

0.0599054

0

0

0

0

0.00750954

0

0.0229265

0

0.007332

0

0.0343898

0

0

0

0

0.0170056

0

0.0229265

0.0229265

0

0.0340111

0

0

0.0229265

0

0

0

0

0

0.045853

0.0475315

0

0

0.0229265

0

0.149927

0

0.357411

0.045853

0.0168673

0.0492656

0

0.0809283

0.0687795

0.0316877

0

0

0.0316877

0

0.045853

0.0229265

0

0

0

0

0.0229265

0

0.0952454

0.045853

0

0.045853

0.0316877

0.0792192

0

0.0229265

0

0

0

1.99738
7

0

0.0157773

0

0

0

0

0.0475315

0.00690984

0.0316877

0

0

0.0928813
7

0

0

0

0

0

0.007332

0.0229265

0.0404642

0.0573163

0.0382631
7

0

0.0138197

0.0696078

0
4

0

5.84626

0
4

1.04069
5

0.133821

0.0721518

0.165509

0.0573163

0.193791

0.0170056

0.0225849

0.0856651

0.105889

0

0.00500636
2

0.0585295

0

0.104728

0

0.137559

0

0.140892

0

0

0

2.68929
6

0.095063

0.114633

0

0

0

0

0.134158

0.0792192

0

1.84548

0
4

102.711

60.8232

0.435604

0.110907

0.0316877

0.045853

0.0229265

0.0343898

0.0229265

0.0229265

0.0343898

0.0229265

0

0.328192
6

0.0343898

0.0573163

0.0687795

0.0802428

0.045853

0

0.0343898

0

0.0343898

0

0.045853
7

0.0229265

0.045853

0.0229265

0.0229265

0.0343898

0

0.0343898

0.0343898

0

0.0343898

1.53417

0.0229265

0

0

0

0

0

0.0229265

0

0.320971

0.010998

5.01202

0.0573163

0

0.0229265

0.0687795

0.0343898

0.0229265

0.0316877

0.007332

0.0343898

0

0.236597

0.091706

0.0229265

0

0.0792192

0

0.0343898

0.0229265

0.0573163

0.0316877

0

0.137568
7

0.0229265

0

0.045853

0.010998

0

0

0.103169

0.045853

0.0343898

0.014664

1.42144

0.0343898

0.0229265

0.0229265

0.0687795

0.250183

0.0460902
7

12.94
7

0.474541

1.36143

0.343898

0.160486

0.252192

0.774721
6

0.103169

0.0316877

0.240728

0.0229265

2.08428
7

0.366824

0.142565

0.160486

0.184233

0.045853

0.183412

0.137559

0.0680222

0.183412

0.0343898

1.04316

0.0206752

0

0.435604

0.142595

0.137559

0.114633

0.192549

0.0513953

0

0.0687795

0.852649
5

0.149022

0.0875748

0.103169

0.0802428

0.114633

0.045853

0.0613538

0.091706

0.0343898

0.0573163

2.33612
7

0

0

0

0.045853

0.0343898

0.0340111

0.0475315

0.0343898

0.0340111

0

0

0.0255083

0

0.045853

0.0343898

0.045853

0.007332

0

0

0.0573163

0.0687795

2.60216

0.0229265

0.0138197

0

0.0229265

0

0.0687795

0.0316877

0.0229265

0

0

0.10116

0

0.0343898

0

0.0229265

0

0.00690984

0.0175223

0.0229265

0

0.0343898

0
4

0
2

0
2

0

0

0

0
4

0.0775164

0.0775164

0

0
4

0.0486932

0.0170056

0.0316877

0
4

0.114633

0.114633

0
4

0.0475315

0.0475315

0

0
4

0.374417

0.374417

0
4

1.11614

1.11614

0
4

0

0

0

0
4

0

0

0

0
4

0

0

0

0
4

0.194875

0.194875

0
4

0
4

0
4

0

0

0

0

0

0

0

0

0

0
4

0

0

0
4

0

0

0

0
4

0

0

0

0
4

0.126096

0.126096

0
4

0

0

0

0
4

0

0

0
4

0

0

0
4

0.0809283

0.0809283

0
4

0

0

0
4

0.0469069

0.0469069

0
4

6.94396
6

3.23578
6

3.20971

0.217802

0.0229265

0.103169

0.0229265

0.0170056

0.045853

0.0687795

1.20736753927986e-15
6

0
4

0

0

0
4

0.110907

0.110907

0
4

0

0

0
4

0

0

0
4

0

0

0
4

0

0

0
4

0.0316877

0.0316877

0
4

0

0

0
4

0

0

0
4

0

0

0
4

0
3

0
3

0

0

0

0

0

0

0

0

0

0
4

0

0

0
4

0

0

0
4

0

0

0
4

0

0

0
4

0

0

0
4

0

0

0
4

0

0

0
4

0

0

0
4

0

0

0
4

0

0

0
4

6.20027
6

5.35199
6

0.0687795

0.045853

0

0.0687795

0.664869

4.44089209850063e-16
6

0
4

0

0

0
4

0

0

0
4

0

0

0
4

0

0

0
4

0

0

0
4

0.0366292

0.0366292

0
4

0.0599054

0.0599054

0
4

0

0

0
4

0

0

0
4

0.0170056

0.0170056

0
4

0.0138197
3

0
3

0

0

0

0

0.0138197

0
4

0

0

0
4

0.0469069

0.0469069

0
4

0

0

0
4

0

0

0
4

0.00690984

0.00690984

0
4

0

0

0
4

0

0

0
4

0.0404642

0.0404642

0
4

0

0

0
4

0

0

0
4

0

0

0

0

0
4

0

0

0
4

0

0

0
4

0.0792192

0.0792192

0
4

0

0

0
4

0.536117

0.536117

0
4

0

0

0
4

0

0

0
4

0.0229265

0.0229265

0
4

0

0

0
4

0

0

0
4

0.117267
3

0.117267
3

0

0

0

0

0

0

0

0

0

0
4

0

0

0
4

0

0

0
4

0

0

0
4

0

0

0
4

0

0

0
4

0.021996

0.021996

0
4

0

0

0
4

0.0606962

0.0606962

0
4

0

0

0
4

0

0

0
4

0.427784

0

0.285189

0

0

0

0.0792192

0.0633753

0
4

0

0

0
4

0.00350607

0.00350607

0
4

0

0

0
4

0

0

0
4

0.0475315

0.0475315

0
4

0

0

0
4

0

0

0
4

0

0

0
4

0

0

0
4

1.10047

1.10047

0
4

0.522544
5

0.203612

0.0255083

0.0510167

0.0802428

0.091706

0.0475315

0

0

0.0229265

0
4

0

0

0
4

0.0172746

0.0172746

0
4

0

0

0
4

0

0

0
4

0

0

0
4

0.536117

0.536117

0
4

0

0

0
4

0.091706

0.091706

0
4

0.0232512

0.0232512

0
4

0

0

0
4

292.719

287.064

0.286581

0

0

0

0

0

0

0.194875

1.98314

0

0.143441
7

0

0.0573163

0.0229265

0.286581

0.0475315

0.653406

1.70996

0.0803809

0

0.0883669

0.0687795

0.0316877

2.83731371730767e-14

0
4

0.242785
4

0.242785
4

0

0

0

0

0

0
4

0

0

0
4

0.298045

0.298045

0
4

0.0229265

0.0229265

0
4

0

0

0
4

0

0

0
4

0

0

0
4

0

0

0
4

0.00690984

0.00690984

0
4

0.0633753

0.0633753

0
4

0.457865

0.457865

0
4

0

0

0

0

0

0

0
4

0.0155008

0.0155008

0
4

0

0

0
4

0

0

0
4

0

0

0
4

0

0

0
4

0

0

0
4

0.624841
6

0.0802428

0.308466

0.126096

0.01833

0.091706

0
4

0
4

0

0

0

0

0

0

0
4

0
4

0

0

0

0

0

0
4

0.921752

0

0.773753

0.0792192

0.0343898

0.0343898

0

9.71445146547012e-17

0
4

0
4

0

0

0

0

0
4

0

0

0

0

0

0

0
4

0.236373

0.236373

0

0

0
4

0.182423
6

0.0588871

0.0725533

0.0439702

0.00701215

3.46944695195361e-18
6

0
4

0
4

0
4

0

0

0

0

0

0

0

0

0

0

0

0

0

0

0

0

0

0

0

0

0

0

0

0

0

0

0

0

0

0

0

0

0

0

0

0

0

0

0

0

0

0

0

0

0

0

0

0

0

0

0

0

0

0

0

0

0

0

0

0

0

0

0

0
4

3.86887
6

3.86887
6

0
4

1.19811
7

1.19811

0

0
4

0.836215

0

0.121392

0

0.714822

0
4

0

0

0

0

0
4

0

0

0

0

0
4

0

0

0

0

0

0
4

0

0

0

0
4

0

0

0

0
4

0.599054

0.449291

0.149764

0
4

0

0

0

0

0
4

12.3647

5.01002
7

0.127832

0.0577387

0

0.047374

0.00690984

0

0

0

0.323713

0

1.07646
6

0.0103648

0.0809283

0.00690984

0.0404642

0.0404642

0.357411

1.60835

0.0809283

0.0450572

0.141625

0.548777

0

0.439191

1.94444

0.0697535
2

0.198861

0.10116

0

0
4

0

0

0

0

0
4

0

0

0

0

0
4

0.0585295

0.0585295
7

0

0
4

0.459471

0.158438

0.221814

0.0316877

0.0475315

6.24500451351651e-17

0
4

0.0498905
7

0

0.0343898

0

0.0155008

0
4

0

0

0

0

0
4

0

0

0

0

0
4

0

0

0

0

0

0
4

0.0345492

0.0276393

0

0.00690984

1.73472347597681e-18

0
4

0

0

0

0
4

113.84
6

111.807
6

0.0527198

0.687795

0.160486

0

0.00827008

0

0.0229265

1.10047

5.10702591327572e-15
6

0
4

1.12178

0.714822

0.406961

0
4

0

0

0

0
4

0.00722163

0.00722163

0

0
4

0

0

0

0

0
4

13.0419

12.6845

0.357411

0
4

7.37595

0.536117

6.83983

0

0
4

0

0

0

0
4

0

0

0

0
4

0.368656

0.357411

0.0112449

0

0
4

0

0

0

0

0
4

8.2926

5.76121
6

0.712973

0.0243376

0.0170056

0.0316877

0

0.301033

0

0

0.0475315

0

0.333882

0

0.0792192

0.095063

0.127542

0.0316877

0.095063

0

0.0316877

0.0792192

0.140892

0

0.143756

0.0792192

0

0.126751

0.0328403

0
4

0

0

0

0

0
4

0

0

0
4

0

0

0
4

0

0

0

0

0
4

0

0

0

0

0
4

0.420787

0

0.0633753

0.357411

0
4

0.357411

0

0

0.357411

0
4

0

0

0

0
4

0

0

0
4

3.1948

0.0404642

3.15434

0

0
4

33.7247
6

28.4755
6

0.0155008

0

0.103648

0.536117

0.61073
7

0

3.20443

0.00835115

0.770447

0

0

0

0
4

0

0

0

0

0
4

0

0

0

0
4

0

0

0
4

0.82388

0.82388

0
4

0

0

0

0

0
4

0

0

0

0
4

0.161539

0.0573163

0.0469069

0.0573163

0
4

0.095063

0.095063

0
4

0

0

0
4

5.12987

5.12987

0

0
4

23.2172

19.8693

3.04965

0.23148

0

0.0599054

0.00690984

0

1.63237479089418e-15

0
4

0

0

0

0

0
4

0.144918

0.144918

0
4

0.0734094

0.0734094

0
4

0

0

0

0
4

0

0

0

0
4

0

0

0

0
4

0

0

0

0
4

0

0

0

0
4

0

0

0

0
4

0

0

0
4

0
3

0
3

0

0

0

0

0

0

0

0

0

0
4

0

0

0

0
4

0

0

0

0
4

0

0

0

0
4

0

0

0

0
4

0.123693

0.123693

0
4

0

0

0

0
4

0

0

0

0
4

0

0

0
4

0

0

0

0
4

0.0387256

0.0316877

0.00703794

1.73472347597681e-18

0
4

7.14955872282985e-13

0
4

1.81898940354586e-12

0
4

99.9551
4

98.6748
4

98.4769
4

35.6465
4

0

0.893528

0

0

0

0.0404642

0

0

0

0

0.174282

1.703

0

0.0387582

0

0

0.0809283

0

0

0

0

0.0404642

0.310991

0

0

0

0

0.0606962

0

0

0

0.0387582

0

0

0

0

0

0

0

0.0809283

0

0.0404642

0

0

0
4

0

0.00350607

0

0

0

0.0809283

0.0404642

0

0.0404642

0

0.00350607
4

0

0

0.343945

0.0404642

0.0725922

0

0.0140243

0

0

0

0.0105182

0

0

0

0

0

0

0

0

0.0404642

0

0
4

0

0.0105182

0

0

0.0112377

0.0404642

0.0606962

0.00750954

0.00500636

0

0

0

0

0.0404642

0

0

0

0

0.0100127

0

0

0.00350607
4

0.0404642

0

0

0.0404642

0

0

0

0

51.3814
5

0

0

0.263017

0.0606962

0

0.108463

0

0.384409

0.174282

0

2.71193

0.401932

0.0290144

0

0.121392

0

0.0884379

0

0

0.182089

0.10116

0.788224

0

0

0

0

0

0.0474763

0.00525911

0

0

0

0.542071
6

0.00500636

0

0

0

0.0657026

0

0

0

0

0.343945

0

0.242785

0.0125159

0

0.00525911

0.121392

0

0

0

0

0

0

0

0.0112449

0

0

0

0

0.0404642

0

0

0

0
4

0

0.0809283

0

0

0

0

0

0

0

0

0
4

0

0

0

0.0404642

0

0

0.00500636

0

0

0

0
4

0.137176
4

0.0599054

0.0703603

0

0

0.00690984

0

0

0

0
4

0

0

0
4

0

0

0

0

0

0
4

0

0

0

0

0

0
4

0

0

0

0
4

0.0606962

0

0.0606962

0
4

0

0

0

0
4

0

0

0
4

0

0

0
4

0

0

0
4

1.42247325030098e-14
4

0
4

0

0

0

0

0

0

0

0
4

0
4

0

0

0

0

0
4

0
4

0

0

0

0

0
4

0

0

0
4

0
4

0

0

0

0

0
4

0
4

0

0

0

0

0
4

0
4

0

0

0

0
4

0
4

0.140721

0.140721

0.140721

0
4

0
4

0

0

0

0
4

0
4

0

0

0

0
4

0

0

0
4

0

0

0
4

0
4

0

0

0

0
4

0

0

0
4

0
4

0.0316877

0

0

0
4

0.0316877

0.0316877

0
4

0
4

0
4

0
4

0

0

0

0

0

0

0
4

0
4

0

0

0

0
4

0
4

0

0

0

0
4

0

0

0
4

0
4

0

0

0

0
4

0
4

0.13093

0.13093

0.13093

0
4

0
4

0.147912

0.147912

0.0968956

0.0510167

0
4

0
4

0

0

0

0

0
4

0
4

0

0

0

0
4

0
4

0.0168673

0.0168673

0.0168673

0
4

0
4

0.014664

0.014664

0.014664

0
4

0
4

0

0

0

0
4

0
4

0

0

0

0

0

0

0
4

0

0

0
4

0

0

0
4

0

0

0
4

0
4

0

0

0

0
4

0
4

0

0

0

0
4

0
4

0

0

0

0
4

0
4

0

0

0

0
4

0
4

0

0

0

0
4

0
4

0

0

0

0
4

0
4

0

0

0

0
4

0
4

0

0

0

0
4

0
4

0.0170056

0.0170056

0.0170056

0
4

0
4

0

0

0

0
4

0
4

0.237341
6

0.237341
6

0.123578

0.0750046

0.0387582

0
4

0
4

0

0

0

0
4

0
4

0.536117

0.536117

0.536117

0
4

0
4

0

0

0

0
4

0
4

0

0

0

0
4

0
4

0

0

0

0
4

0
4

0

0

0

0
4

0
4

0

0

0

0
4

0
4

0

0

0

0
4

0
4

0

0

0

0
4

0
4

0

0

0

0
4

0
4

0

0

0

0

0

0

0
4

0
4

0

0

0

0
4

0
4

0

0

0

0
4

0
4

0

0

0

0
4

0
4

0

0

0

0
4

0
4

0

0

0

0
4

0
4

0.00703794

0.00703794

0.00703794

0
4

0
4

0

0

0

0
4

0
4

0

0

0

0
4

0
4

0

0

0

0

0

0
4

0
4

0

0

0

0
4

0
4

0

0

0

0

0
4

0

0

0
4

0
4

0
4

595.549

42.9278
5

41.0563
5

19.7921
6

21.2642
5

3.5527136788005e-15
5

0
4

0

0

0

0

0

0

0
4

0

0

0
4

0

0

0
4

0

0

0
4

0

0

0
4

0

0

0
4

0

0

0
4

0.161553

0.161553

0
4

0

0

0
4

0

0

0
4

0

0

0
4

1.70996
4

0

0

0

1.70996

0

0

0
4

0

0

0
4

0

0

0
4

0

0

0
4

0

0

0

0
4

0

0

0

0
4

0

0

0

0
4

0

0

0
4

0

0

0

0
4

0

0

0
4

0

0

0
4

1.77635683940025e-15
5

0
4

548.06

36.8502

2.77289
7

0

0

0

0

0

0

0.0224897

0

0

0

0

0.226471

0

0

0

0

0

0

0

0.0340111

0

0.0599054

4.32496
7

0.0112449

0

0

0.119039

0

0

0

0

0

0

0
7

0

0

0

0

0

0

0

0.0112449

0

0

0.140721
7

1.70996

0

0

0

0

0

0

0

0

0

0.15305

0

0

0.149764

0

0

0

0

0

0

0

0.0500031
7

0.599054

0

0

0

0.039357

0.0170056

0

0

0

0

0.0938138

0

0

0

0.727292
6

0.854186
6

0

8.51745

0

0

0

1.4346
7

1.70996
7

0.398191

0

0.0170056
8

0

0

0

0.0168673

0.127825

0.600752

5.57714

0.119039

0

0.0340111

0.0938138
7

0

1.20828

0

0.0112449

0.0469069

0

0

0.357411

0

0

1.06468
7

0.00350607

0

0

0

0

0

0

0

0.0170056

0.0775164

0

0.0255083

0.0112449

0

0

0.0703603

0

0

0

0

0

0

0

0

0.0599054

0

0.0449794

0.0425139

0

0

0

0

0

0

0

0

0

0

0.357411

0

0.536117

0

0

1.91472
7

0

0

0

0

0.0281121

0

0

0.209669

0

0

2.56461518688411e-14

0
4

12.0519

2.5348
7

0

0

0

0.357411

0

0

0.0425139

0

0

0

0
7

0

0

0

0

0

0

0

0

0.119811

0

2.80356
7

0

0.00827008

0

0

0

0

0

0

0

0

6.15156
6

0

0

0

0

0

0.0340111

3.19189119579733e-16

0
4

37.0043

9.45892
7

0.246967

0.357411

3.49644
7

0.119425
7

8.54327

0.357411

2.94915

0.536117

0

0

1.47834
7

0

0

0

0.0703603

0

0

0.714822

0.0680222

0.0633753

0.0316877

0.16842
8

0

0

0

0

0

0.0170056

0

0

0

0

0.0510167
8

0.0170056

0.357411

0

0

0.102033

0

0

0

0.0469069

0

3.01848
7

0

0

0

0

0

0

0.0581373
7

1.12266
7

0.584238
7

2.96925
7

6.21724893790088e-15

0
4

0.357411

0

0

0

0

0

0

0

0

0

0

0

0

0

0

0.357411

0

0

0

0

0
4

448.826

0.487155
7

409.088

36.6897

0.297878

0.209669

0

0.10116

0

0.357411

0

0.479243

0

0

0.161857

0.893528

0

0.0599054

0

8.11017919488677e-14

0
4

0

0

0
4

12.343
7

12.1205
7

0.222553

0

0

0
4

0

0

0

0

0

0

0

0

0
4

0.507175

0.357411

0.0898581

0.0599054

0

0

1.38777878078145e-17

0
4

0

0

0
4

0

0

0
4

0

0

0
4

0.119811

0.119811

0
4

0

0

0
4

0

0

0
4

0

0

0
4

1.11868847518792e-13

0
4

0.419338

0.149764

0

0

0

0

0.149764

0

0

0

0
4

0.269574

0.209669

0

0.0599054

0
4

0
4

0.212569

0.212569

0.212569

0

0
4

0

0

0
4

0
4

0.288704

0.288704

0.288704

0
4

0
4

0

0

0

0

0
4

0

0

0
4

0
4

0

0

0

0

0

0
4

0
4

0

0

0

0
4

0

0

0
4

0
4

0

0

0

0
4

0

0

0
4

0
4

0.116275

0.116275

0.116275

0
4

0
4

0

0

0

0

0
4

0
4

1.97688

1.97688

1.97688

0
4

0
4

0.0469069

0

0

0
4

0.0469069

0.0469069

0
4

0
4

0.16824
7

0.16133
7

0.0122368

0.0519133
7

0.0734195

0.00500636

0.00750954

0.0112449

0
4

0.00690984

0.00690984

0
4

0
4

0.0168673

0.0168673

0.0168673

0
4

0
4

0.155033

0.155033

0.155033

0
4

0
4

0

0

0

0
4

0
4

0

0

0

0
4

0
4

0

0

0

0
4

0
4

0

0

0

0
4

0
4

0

0

0

0
4

0
4

0

0

0

0
4

0
4

0

0

0

0
4

0
4

0

0

0

0
4

0
4

0

0

0

0

0
4

0

0

0
4

0
4

0

0

0

0
4

0
4

0

0

0

0
4

0
4

0

0

0

0

0

0
4

0

0

0
4

0
4

0

0

0

0
4

0
4

0.258939
6

0.00305919

0.00305919

0

0

0
4

0.00305919

0.00305919

0
4

0.252821

0.252821

0
4

0
4

0

0

0

0

0

0
4

0

0

0

0
4

0
4

0.0599054

0.0599054

0.0599054

0

0
4

0
4

0.842118

0.842118

0.842118

0

0
4

0
4

1.06248343456627e-13

0
4

6.03788

5.59715

5.55505

1.94228

0.0606962

0

0

0

0

0.0316877

0

0

0.0404642

0.0404642

0.0404642
1

0.546266

2.85272

0

0

0

0

0

4.44089209850063e-16

0
4

0

0

0

0
4

0.0316877

0.0316877

0

0
4

0.00350607

0.00350607

0
4

0

0

0
4

0

0

0
4

0.00690984

0.00690984

0
4

0
4

0.377338

0.377338

0.377338

0
4

0
4

0

0

0

0
4

0
4

0

0

0

0

0
4

0
4

0

0

0

0
4

0
4

0

0

0

0
4

0
4

0

0

0

0
4

0
4

0.0229265

0.0229265

0.0229265

0
4

0
4

0

0

0

0
4

0
4

0.0404642

0.0404642

0.0404642

0
4

0
4

0

0

0

0
4

0
4

8.60422844084496e-16

0
4

10.5542
5

10.5542
5

10.0411
5

5.70592
5

3.22543

0.617411

0

0.229575

0.136044

0.0316877

0.0316877

0.0633753

0
4

0

0

0

0

0
4

0.190126

0.190126

0

0

0
4

0.257988

0.257988

0
4

0.0599381

0.0282504

0

0.0316877

0
4

0

0

0
4

0.00500636

0.00500636

0
4

1.12236608895699e-15
5

0
4

0
4

0
4

0
4

0
4

0
4

0

0

0

0

0
4

0

0

0

0

0

0

0

0
4

0
4

0
4

0.222553

0.222553

0.182089

0.141625

0.0404642

0
4

0.0404642

0.0404642

0
4

0
4

0
4

0.0599054

0.0599054

0.0599054

0

0.0599054

0
4

0
4

0
4

0.01833

0.01833

0.01833

0.01833

0

0
4

0

0

0

0
4

0
4

0
4

0

0

0

0

0

0
4

0
4

0
4

0.0659725

0.0659725

0.0659725

0.0255083

0

0.0404642

0
4

0
4

0
4

0

0

0

0

0
4

0
4

0
4

1.97997

1.97997

1.97997

1.9749

0.00507012

8.93382590128056e-17

0
4

0
4

0
4

0

0

0

0

0
4

0
4

0
4

0.208293

0.208293

0.0656988

0.0340111

0.0316877

6.93889390390723e-18

0
4

0.142595

0.142595

0
4

0
4

0
4

0.100755

0.100755

0.100755

0.100755

0
4

0
4

0
4

0.893528
2

0
2

0
2

0

0

0

0

0

0

0

0

0

0
4

0

0

0
4

0
4

0.893528

0.893528

0.893528

0

0

0

0

0
4

0

0

0

0
4

0
4

0
4

0.110922

0.110922

0.0599054

0.0599054

0
4

0.0510167

0.0510167

0
4

6.93889390390723e-18

0
4

0
4

0

0

0

0

0

0

0
4

0
4

0
4

0

0

0

0

0
4

0

0

0
4

0
4

0
4

0

0

0

0

0

0
4

0
4

0

0

0

0
4

0
4

0
4

0.268503

0.268503

0.268503

0.268503

0
4

0
4

0
4

0.237658

0.237658

0.237658

0.237658

0
4

0

0

0
4

0
4

0
4

2.92235

2.92235

2.92235

2.92235

0
4

0
4

0
4

0

0

0

0

0

0
4

0

0

0
4

0
4

0
4

0

0

0

0

0
4

0

0

0
4

0
4

0
4

0

0

0

0

0

0

0
4

0
4

0
4

2.84801
7

2.84801
7

1.11195
7

0.99931
7

0.0988208
7

0.0138197

4.33680868994202e-17
7

0
4

1.6139
7

1.59689
7

0.0170056

0

0
4

0.111798

0.0730398

0

0.0387582

6.93889390390723e-18

0
4

0.0103648

0.0103648

0

0
4

0
4

0
4

0

0

0

0

0

0
4

0
4

0
4

0.0360266

0.0360266

0.00701215

0.00701215

0
4

0.00350607

0.00350607

0
4

0.0255083

0.0255083

0
4

3.46944695195361e-18

0
4

0
4

0

0

0

0

0
4

0

0

0
4

0
4

0
4

0

0

0

0

0

0
4

0
4

0
4

0

0

0

0

0

0
4

0
4

0
4

2.42478

2.42478

2.42478

2.42478

0
4

0
4

0
4

0

0

0

0

0
4

0
4

0
4

0

0

0

0

0
4

0
4

0
4

0

0

0

0

0

0
4

0
4

0
4

0.714822

0.714822

0.714822

0.714822

0
4

0

0

0
4

0
4

0
4

22.3467

22.3467

22.3467

18.5559

0

3.76664

0.0241844

0

0

0

0

0

0

1.37737043992558e-15

0
4

0

0

0
4

0

0

0

0
4

0
4

0

0

0

0

0
4

0
4

0
4

0

0

0

0

0

0
4

0
4

0
4

0

0

0

0

0
4

0

0

0
4

0
4

0
4

0

0

0

0

0

0
4

0
4

0
4

0

0

0

0

0
4

0
4

0
4

0

0

0

0

0

0
4

0

0

0
4

0
4

0
4

0.0549439

0.0549439

0.0549439

0.0549439

0
4

0

0

0
4

0
4

0
4

0

0

0

0

0
4

0
4

0
4

0

0

0

0

0

0
4

0
4

0

0

0

0
4

0
4

0
4

1.98118

1.98118

1.98118

1.98118

0
4

0

0

0
4

0
4

0
4

0

0

0

0

0
4

0
4

0
4

0
2

0
2

0
2

0

0

0

0

0

0

0
4

0

0

0

0

0

0

0
4

0

0

0

0

0

0
4

0
4

0
4

0

0

0

0

0
4

0
4

0
4

0

0

0

0

0
4

0

0

0
4

0

0

0
4

0
4

0
4

0

0

0

0

0
4

0
4

0
4

0

0

0

0

0

0
4

0
4

0
4

0

0

0

0

0

0

0
4

0
4

0
4

0

0

0

0

0

0
4

0
4

0
4

0

0

0

0

0

0
4

0
4

0
4

0.149764

0.149764

0.149764

0.149764

0
4

0
4

0
4

0

0

0

0

0
4

0

0

0
4

0
4

0
4

0

0

0

0

0
4

0
4

0
4

8.73242

8.73242

3.03122

2.20945
6

0.655156
7

0.0579945

0.0809283

0.0241844

0.00350607

4.78783679369599e-16

0
4

5.69429
6

5.60646
6

0.0809283

0

0.00690984

0
4

0

0

0

0
4

0

0

0
4

0.00690984

0.00690984

0
4

0

0

0
4

1.41033018596914e-15

0
4

0
4

0

0

0

0

0
4

0

0

0
4

0
4

0
4

0

0

0

0

0

0
4

0
4

0
4

0

0

0

0

0
4

0

0

0
4

0
4

0
4

0

0

0

0

0

0
4

0
4

0
4

0

0

0

0

0

0
4

0
4

0
4

0.0599054

0.0599054

0.0599054

0.0599054

0

0
4

0
4

0
4

0

0

0

0

0
4

0

0

0
4

0
4

0
4

0

0

0

0

0
4

0

0

0
4

0
4

0
4

0.0316877

0.0316877

0.0316877

0.0316877

0
4

0

0

0
4

0
4

0
4

0

0

0

0

0
4

0
4

0
4

0.851704
7

0.851704
7

0.757712
7

0.721083
7

0.0366292

0

0

0

0
4

0.0939915

0.076986

0.0170056

0

0

0

6.93889390390723e-18

0
4

1.38777878078145e-16
7

0
4

0
4

0.182089

0.182089

0

0

0
4

0.182089

0.182089

0
4

0
4

0
4

0.149764

0.149764

0

0

0
4

0.149764

0.149764

0
4

0
4

0
4

0

0

0

0

0

0
4

0
4

0
4

0.160486

0.160486

0.160486

0.045853

0.114633

0
4

0
4

0
4

0

0

0

0

0

0
4

0
4

0
4

0

0

0

0

0
4

0
4

0
4

0

0

0

0

0
4

0
4

0
4

0

0

0

0

0
4

0

0

0
4

0
4

0
4

0

0

0

0

0
4

0

0

0
4

0
4

0
4

2.06737

2.06737

2.06737

2.06737

0
4

0
4

0
4

6.32234
7

6.32234
7

5.51246
7

3.18641
7

1.70996

0.272089

0.283303

0

0.0606962

0
4

0.809885

0.809885

0

0

0
4

0

0

0
4

1.11022302462516e-16
7

0
4

0
4

0.0112449

0.0112449

0

0

0
4

0.0112449

0.0112449

0
4

0
4

0
4

0

0

0

0

0
4

0
4

0
4

0

0

0

0

0
4

0

0

0
4

0
4

0
4

0

0

0

0

0

0
4

0
4

0
4

0

0

0

0

0
4

0
4

0
4

0

0

0

0

0

0
4

0
4

0
4

0

0

0

0

0

0
4

0
4

0
4

0

0

0

0

0

0
4

0
4

0
4

0

0

0

0

0

0
4

0
4

0
4

0

0

0

0

0
4

0

0

0
4

0
4

0
4

2.05063
7

2.05063
7

2.05063
7

1.00207
7

0.155033

0.893528

1.11022302462516e-16
7

0
4

0

0

0
4

0

0

0
4

0
4

0
4

0

0

0

0

0

0
4

0
4

0
4

0.0938138

0.0938138

0.0938138

0.0938138

0
4

0
4

0
4

0

0

0

0

0
4

0
4

0
4

0

0

0

0

0
4

0

0

0
4

0
4

0
4

0.045853

0.045853

0.045853

0.045853

0
4

0
4

0
4

0

0

0

0

0

0
4

0
4

0
4

0

0

0

0

0
4

0

0

0
4

0
4

0
4

0

0

0

0

0
4

0
4

0
4

0

0

0

0

0
4

0
4

0
4

0

0

0

0

0
4

0
4

0
4

0.587135
7

0.587135
7

0.400865
7

0.0597819
7

0.244187
7

0.0968956

0

0

1.38777878078145e-17
7

0
4

0.18627
7

0.138739
7

0

0.0475315

1.38777878078145e-17
7

0
4

0
4

0
4

1.70996

1.70996

0

0

0
4

1.70996

1.70996

0
4

0
4

0
4

0

0

0

0

0

0
4

0
4

0
4

0.095063

0.095063

0.095063

0.095063

0
4

0
4

0
4

0

0

0

0

0
4

0

0

0
4

0
4

0
4

0

0

0

0

0
4

0
4

0
4

0

0

0

0

0

0
4

0
4

0
4

0

0

0

0

0
4

0

0

0
4

0
4

0
4

0

0

0

0

0
4

0
4

0
4

0

0

0

0

0
4

0

0

0
4

0
4

0
4

0

0

0

0

0
4

0

0

0
4

0
4

0
4

0.0340111
4

0.0340111
4

0

0

0

0

0

0

0

0

0

0

0

0

0

0

0

0

0

0

0
4

0.0170056
4

0

0

0

0

0

0

0

0.0170056

0

0

0

0

0

0

0

0
4

0.0170056

0.0170056

0

0

0

0

0

0
4

0

0

0

0
4

0
4

0

0

0

0
4

0
4

0
4

0

0

0

0

0

0

0

0

0

0

0
4

0

0

0

0

0
4

0
4

0
4

0

0

0

0

0
4

0
4

0
4

0

0

0

0

0

0
4

0
4

0
4

0

0

0

0

0
4

0
4

0
4

0

0

0

0

0

0
4

0
4

0
4

0

0

0

0

0

0
4

0
4

0
4

0

0

0

0

0
4

0
4

0
4

0

0

0

0

0
4

0

0

0
4

0
4

0
4

0.299527

0.299527

0.299527

0.299527

0
4

0
4

0
4

0

0

0

0

0

0
4

0
4

0
4

0

0

0

0

0

0
4

0
4

0
4

2.27329
7

2.27329
7

2.27329
7

2.17219
7

0.0898581
7

0.0112449

0
4

0

0

0

0
4

0
4

0

0

0

0
4

0
4

0
4

0.0469069

0.0469069

0.0469069

0.0469069

0
4

0
4

0
4

0.0898581

0.0898581

0.0898581

0.0898581

0
4

0
4

0
4

0

0

0

0

0
4

0
4

0
4

0

0

0

0

0
4

0
4

0
4

0.0316877

0.0316877

0.0316877

0.0316877

0
4

0
4

0
4

0.0316877

0.0316877

0.0316877

0.0316877

0
4

0
4

0
4

0

0

0

0

0
4

0
4

0
4

0

0

0

0

0
4

0
4

0
4

0.00690984

0.00690984

0.00690984

0.00690984

0
4

0
4

0
4

0

0

0

0

0
4

0
4

0
4

3.94812

3.94812

2.61796

2.09472
7

0.523236

0

0
4

1.25094

1.25094

0
4

0.0316877

0.0316877

0
4

0.0475315

0.0475315

0
4

0
4

0
4

0.0207295

0.0207295

0.0207295

0.0207295

0
4

0
4

0
4

0

0

0

0

0
4

0
4

0
4

0

0

0

0

0
4

0
4

0
4

0

0

0

0

0
4

0
4

0
4

0.0340111

0.0340111

0.0340111

0.0340111

0
4

0
4

0
4

1.70996

1.70996

1.70996

1.70996

0
4

0
4

0
4

0

0

0

0

0
4

0
4

0
4

0

0

0

0

0
4

0
4

0
4

0

0

0

0

0
4

0
4

0
4

0

0

0

0

0
4

0
4

0
4

1.07223
2

1.07223
2

1.07223
2

1.07223
2

0

0

0

0

0

0
4

0
2

0

0

0

0
4

0

0

0
4

0
4

0
4

0

0

0

0

0
4

0
4

0
4

0.0316877

0.0316877

0.0316877

0.0316877

0
4

0
4

0
4

0

0

0

0

0
4

0
4

0
4

0

0

0

0

0
4

0
4

0
4

0

0

0

0

0
4

0
4

0
4

0.0316877

0.0316877

0.0316877

0.0316877

0
4

0
4

0
4

0

0

0

0

0
4

0
4

0
4

0

0

0

0

0
4

0
4

0
4

0.449291

0.449291

0.449291

0.449291

0
4

0
4

0
4

0

0

0

0

0
4

0
4

0
4

0
4

0
4

0
4

0

0

0

0

0

0

0

0

0

0
4

0
4

0
4

0
4

0
4

0

0

0

0
4

0
4

0
4

0

0

0

0

0
4

0
4

0
4

0

0

0

0

0
4

0
4

0
4

0

0

0

0

0
4

0
4

0
4

0

0

0

0

0
4

0
4

0
4

0

0

0

0

0
4

0
4

0
4

0

0

0

0

0
4

0
4

0
4

0.00690984

0.00690984

0.00690984

0.00690984

0
4

0
4

0
4

0.0316877

0.0316877

0.0316877

0.0316877

0
4

0
4

0
4

0

0

0

0

0
4

0
4

0
4

0

0

0

0

0
4

0
4

0
4

10.9285
5

10.9285
5

10.9285
5

10.9285
5

0

0

0
4

0
4

0
4

0

0

0

0

0
4

0
4

0
4

0

0

0

0

0
4

0
4

0
4

0

0

0

0

0
4

0
4

0
4

0

0

0

0

0
4

0
4

0
4

0

0

0

0

0
4

0
4

0
4

0

0

0

0

0
4

0
4

0
4

0.0809283

0.0809283

0.0809283

0.0809283

0
4

0
4

0
4

0

0

0

0

0
4

0
4

0
4

0

0

0

0

0
4

0
4

0
4

0

0

0

0

0
4

0
4

0
4

1.04708
7

1.04708
7

0.332255
7

0.0312224
7

0.301033

0
4

0.714822

0.714822

0
4

1.11022302462516e-16
7

0
4

0

0

0

0

0

0
4

0
4

0
4

0

0

0

0

0
4

0
4

0
4

0

0

0

0

0
4

0
4

0
4

0

0

0

0

0
4

0
4

0
4

0

0

0

0

0
4

0
4

0
4

0

0

0

0

0
4

0
4

0
4

0

0

0

0

0
4

0
4

0
4

0

0

0

0

0
4

0
4

0
4

0

0

0

0

0
4

0
4

0
4

0

0

0

0

0
4

0
4

0
4

0

0

0

0

0
4

0
4

0
4

0

0

0

0

0

0

0

0

0

0

0
4

0
4

0
4

0

0

0

0

0
4

0
4

0
4

0

0

0

0

0
4

0
4

0
4

0

0

0

0

0
4

0
4

0
4

0

0

0

0

0
4

0
4

0
4

0.00500636

0.00500636

0.00500636

0.00500636

0
4

0
4

0
4

0

0

0

0

0
4

0
4

0
4

1.70996

1.70996

1.70996

1.70996

0
4

0
4

0
4

0.0475315

0.0475315

0.0475315

0.0475315

0
4

0
4

0
4

0

0

0

0

0
4

0
4

0
4

0

0

0

0

0
4

0
4

0
4

0

0

0

0

0

0

0

0

0
4

0
4

0
4

0

0

0

0

0
4

0
4

0
4

0

0

0

0

0
4

0
4

0
4

0

0

0

0

0
4

0
4

0
4

0

0

0

0

0
4

0
4

0
4

0

0

0

0

0
4

0
4

0
4

0

0

0

0

0
4

0
4

0
4

0

0

0

0

0
4

0
4

0
4

0

0

0

0

0
4

0
4

0
4

0

0

0

0

0
4

0
4

0
4

0

0

0

0

0
4

0
4

0
4

0
4

0
4

0
4

0
4

0

0

0

0
4

0
4

0
4

0

0

0

0

0
4

0
4

0
4

0

0

0

0

0
4

0
4

0
4

0

0

0

0

0
4

0
4

0
4

0

0

0

0

0
4

0
4

0
4

0.0103648

0.0103648

0.0103648

0.0103648

0
4

0
4

0
4

0

0

0

0

0
4

0
4

0
4

0

0

0

0

0
4

0
4

0
4

0

0

0

0

0
4

0
4

0
4

0

0

0

0

0
4

0
4

0
4

0

0

0

0

0
4

0
4

0
4

0
4

0
4

0
4

0
4

0

0

0

0

0

0

0

0

0

0

0
4

0

0

0
4

0

0

0

0

0

0

0
4

0
4

0

0

0

0

0

0

0
4

0

0

0

0
4

0

0

0
4

0
4

0
4

3.37949
5

3.37949
5

3.28963
5

3.10859
5

0.0469069

0

0.0168673

0.0469069

0

0.0703603

3.05311331771918e-16
5

0
4

0.0898581

0.0898581

0
4

1.2490009027033e-16
5

0
4

0
4

0

0

0

0

0
4

0
4

0
4

0

0

0

0

0
4

0
4

0
4

0

0

0

0

0
4

0
4

0
4

0

0

0

0

0
4

0
4

0
4

0

0

0

0

0
4

0
4

0
4

0

0

0

0

0
4

0
4

0
4

0

0

0

0

0
4

0
4

0
4

0

0

0

0

0
4

0
4

0
4

0

0

0

0

0
4

0
4

0
4

0

0

0

0

0
4

0
4

0
4

0.0469069
4

0.0469069
4

0.0469069
4

0

0

0.0469069

0

0

0

0

0
4

0
4

0
4

0

0

0

0

0
4

0
4

0
4

0

0

0

0

0
4

0
4

0
4

0

0

0

0

0
4

0
4

0
4

0.0475315

0.0475315

0.0475315

0.0475315

0
4

0
4

0
4

0

0

0

0

0
4

0
4

0
4

0

0

0

0

0
4

0
4

0
4

0

0

0

0

0
4

0
4

0
4

0

0

0

0

0
4

0
4

0
4

0

0

0

0

0
4

0
4

0
4

0

0

0

0

0
4

0
4

0
4

2.36562

2.36562

0.10236
6

0.0416636

0

0.0606962

6.93889390390723e-18
6

0
4

1.78706

0

0

1.78706

0
4

0.124377

0.124377

0
4

0.283249

0.0606962

0.222553

0
4

0.0404642

0.0404642

0
4

0.0281121

0.0281121

0
4

0
4

0
4

0

0

0

0

0
4

0
4

0
4

0

0

0

0

0
4

0
4

0
4

0

0

0

0

0
4

0
4

0
4

0

0

0

0

0
4

0
4

0
4

0.202321

0.202321

0.202321

0.202321

0
4

0
4

0
4

0

0

0

0

0
4

0
4

0
4

0

0

0

0

0
4

0
4

0
4

0

0

0

0

0
4

0
4

0
4

0

0

0

0

0
4

0
4

0
4

0

0

0

0

0
4

0
4

0
4

11.075

11.075

11.075

9.11737
7

1.95763

0

0

8.88178419700125e-16

0
4

0
4

0
4

0

0

0

0

0
4

0
4

0
4

0

0

0

0

0
4

0
4

0
4

0

0

0

0

0
4

0
4

0
4

0

0

0

0

0
4

0
4

0
4

0

0

0

0

0
4

0
4

0
4

0

0

0

0

0
4

0
4

0
4

0

0

0

0

0
4

0
4

0
4

0

0

0

0

0
4

0
4

0
4

0

0

0

0

0
4

0
4

0
4

0

0

0

0

0
4

0
4

0
4

3.59433
3

3.59433
3

1.88437
4

1.88437

0

0

0

0
4

1.70996

1.70996

0
4

0

0

0
4

0

0

0

0
4

0
4

0
4

0.269574

0.269574

0.269574

0.269574

0
4

0
4

0
4

1.70996

1.70996

1.70996

1.70996

0
4

0
4

0
4

0

0

0

0

0
4

0
4

0
4

0

0

0

0

0
4

0
4

0
4

0

0

0

0

0
4

0
4

0
4

0

0

0

0

0
4

0
4

0
4

0

0

0

0

0
4

0
4

0
4

0

0

0

0

0
4

0
4

0
4

0.025662

0.025662

0.025662

0.025662

0
4

0
4

0
4

0

0

0

0

0
4

0
4

0
4

0.458572
7

0.458572
7

0.418107
7

0

0.0606962

0.357411

0
4

0.0404642

0.0404642

0

0

0

0
4

0

0

0
4

0
4

0
4

0

0

0

0

0
4

0
4

0
4

0

0

0

0

0
4

0
4

0
4

0.0475315

0.0475315

0.0475315

0.0475315

0
4

0
4

0
4

0

0

0

0

0
4

0
4

0
4

0

0

0

0

0
4

0
4

0
4

0

0

0

0

0
4

0
4

0
4

0

0

0

0

0
4

0
4

0
4

0

0

0

0

0
4

0
4

0
4

0.0792192

0.0792192

0.0792192

0.0792192

0
4

0
4

0
4

0

0

0

0

0
4

0
4

0
4

0.222553

0.222553

0.222553

0.222553

0

0

0
4

0

0

0

0
4

0
4

0

0

0

0

0
4

0

0

0
4

0
4

0
4

0

0

0

0

0
4

0
4

0
4

0

0

0

0

0
4

0
4

0
4

0

0

0

0

0
4

0
4

0
4

0.182089

0.182089

0.182089

0.182089

0
4

0
4

0
4

0.182089

0.182089

0.182089

0.182089

0
4

0
4

0
4

0.00939633

0.00939633

0.00939633

0.00939633

0
4

0
4

0
4

0

0

0

0

0
4

0
4

0
4

0.0404642

0.0404642

0.0404642

0.0404642

0
4

0
4

0
4

0.0170056

0.0170056

0.0170056

0.0170056

0
4

0
4

0
4

0.0112377

0.0112377

0.0112377

0.0112377

0
4

0
4

0
4

0.5675
5

0.5675
5

0.5675
5

0.5675
5

0

0
4

0
4

0
4

0

0

0

0

0
4

0
4

0
4

0

0

0

0

0
4

0
4

0
4

0

0

0

0

0
4

0
4

0
4

0

0

0

0

0
4

0
4

0
4

0

0

0

0

0
4

0
4

0
4

0

0

0

0

0
4

0
4

0
4

0

0

0

0

0
4

0
4

0
4

0.0404642

0.0404642

0.0404642

0.0404642

0
4

0
4

0
4

0

0

0

0

0
4

0
4

0
4

0

0

0

0

0
4

0
4

0
4

0.726818
7

0.726818
7

0.16692

0.152256

0.014664

0
4

0.559897

0.559897

0

0
4

0
4

0
4

0

0

0

0

0
4

0
4

0
4

0.357411

0.357411

0.357411

0.357411

0
4

0
4

0
4

1.70996

1.70996

1.70996

1.70996

0
4

0
4

0
4

0

0

0

0

0
4

0
4

0
4

0

0

0

0

0
4

0
4

0
4

0

0

0

0

0
4

0
4

0
4

0.110907

0.110907

0.110907

0.110907

0
4

0
4

0
4

0

0

0

0

0
4

0
4

0
4

0

0

0

0

0
4

0
4

0
4

0

0

0

0

0
4

0
4

0
4

0
4

0
4

0
4

0

0

0

0
4

0
4

0
4

0
4

0

0

0
4

0

0

0
4

0
4

0
4

0

0

0

0

0
4

0
4

0
4

0

0

0

0

0
4

0
4

0
4

0

0

0

0

0
4

0
4

0
4

0

0

0

0

0
4

0
4

0
4

0

0

0

0

0
4

0
4

0
4

0

0

0

0

0
4

0
4

0
4

0

0

0

0

0
4

0
4

0
4

0

0

0

0

0
4

0
4

0
4

0

0

0

0

0
4

0
4

0
4

0.095063

0.095063

0.095063

0.095063

0
4

0
4

0
4

8.86205
7

8.86205
7

5.9685
7

5.36412
7

0

0.0578963
6

0.546482

0

0

9.99200722162641e-16
7

0
4

2.89356
7

0.203352
7

0.0324182
7

2.59282
7

0.0579265
7

0.00703794

0

0

0
4

0
4

0
4

0

0

0

0

0

0

0

0
4

0

0

0

0
4

0
4

0
4

0.0138197

0.0138197

0.0138197

0.0138197

0
4

0
4

0
4

0

0

0

0

0
4

0
4

0
4

1.92318

1.92318

1.92318

1.92318

0
4

0
4

0
4

0

0

0

0

0
4

0
4

0
4

0

0

0

0

0
4

0
4

0
4

0

0

0

0

0
4

0
4

0
4

0

0

0

0

0
4

0
4

0
4

0

0

0

0

0
4

0
4

0
4

0.110907

0.110907

0.110907

0.110907

0
4

0
4

0
4

0

0

0

0

0
4

0
4

0
4

2.56494
7

2.56494
7

2.56494
7

2.56494
6

0

0

0
4

0

0

0
4

0

0

0
4

0
4

0
4

0

0

0

0

0
4

0
4

0
4

0.0170056

0.0170056

0.0170056

0.0170056

0
4

0
4

0
4

0

0

0

0

0
4

0
4

0
4

0

0

0

0

0
4

0
4

0
4

0

0

0

0

0
4

0
4

0
4

0

0

0

0

0
4

0
4

0
4

0

0

0

0

0
4

0
4

0
4

0

0

0

0

0
4

0
4

0
4

0

0

0

0

0
4

0
4

0
4

0.893528

0.893528

0.893528

0.893528

0
4

0
4

0
4

0.564706
7

0.564706
7

0.564706

0.207295

0

0.357411

0
4

0

0

0

0

0
4

0
4

0
4

0

0

0

0

0
4

0
4

0
4

0

0

0

0

0
4

0
4

0
4

0

0

0

0

0
4

0
4

0
4

0

0

0

0

0
4

0
4

0
4

5.06879
6

5.06879
6

5.06879
6

3.3113
6

1.70996

0.0475315

8.46545056276682e-16
6

0
4

0
4

0
4

0

0

0

0

0

0

0

0
4

0

0

0

0
4

0

0

0
4

0

0

0

0
4

0
4

0

0

0

0
4

0
4

0
4

1.05774
7

1.05774
7

1.05774
7

0.997837
7

0.0599054

5.55111512312578e-17
7

0
4

0

0

0
4

0

0

0
4

0
4

0
4

1.67279
7

1.67279
7

1.67279
7

1.67279
7

0
4

0
4

0
4

0

0

0

0

0

0

0

0

0
4

0

0

0
4

0
4

0
4

0
4

0
4

0

0

0

0

0
4

0

0

0
4

0
4

0
4

0

0

0

0

0

0
4

0
4

0
4

10.09
5

10.09
5

7.90557
6

4.86048
6

0.00690984

0

0.0103648

0.0449139

0.00350607

0.0587336

0.0932828

0

0.194992

0.0103648

0

0.0190788

0.0207295

2.56494

0.0103648

0.00690984

3.71230823859037e-16
6

0
4

2.14447
5

0
7

2.14447
6

0

0

0
4

0.0399618

0.0172746

0.00690984

0.0157773

3.46944695195361e-18

0
4

1.31145094783847e-15
5

0
4

0
4

0

0

0

0

0

0
4

0

0

0

0
4

0
4

0
4

0
4

0
4

0
4

0

0

0

0

0
4

0

0

0

0
4

0
4

0
4

0

0

0

0

0

0

0

0
4

0
4

0
4

0.089895
7

0.089895
7

0.089895
7

0.0868359

0.00305919

4.33680868994202e-18
7

0
4

0
4

0
4

1.88852

1.88852

1.70996

0

0

1.70996

0
4

0.178558

0.178558

0
4

0
4

0
4

0.0138197

0.0138197

0.0138197

0

0.00690984

0.00690984

0

0

0
4

0

0

0
4

0
4

0
4

0.0968956

0.0968956

0.0968956

0

0.0968956

0

0

0
4

0
4

0
4

0

0

0

0

0

0

0
4

0
4

0
4

0

0

0

0

0
4

0
4

0
4

0.0465023

0.0465023

0.0465023

0.0465023

0

0
4

0
4

0
4

25.7026

25.7026

25.1875
5

22.4
6

0.007332

0

0.183581

0

0

0.0316877

0

2.56494

0

0
4

0.515119
7

0.478366
7

0.0112449

0.0255083

0
4

0

0

0

0

0

0
4

0

0

0

0
4

0
4

0

0

0

0
4

0
4

0
4

0

0

0

0

0

0

0
4

0

0

0

0
4

0
4

0

0

0

0
4

0
4

0
4

0
4

0
4

0
4

0
4

0

0
4

0
4

0
4

0.82229
7

0.82229
7

0.82229
7

0.807626
7

0.014664

1.35308431126191e-16
7

0
4

0
4

0
4

0.161857

0.161857

0.161857

0.161857

0

0
4

0

0

0
4

0
4

0
4

0

0

0

0

0

0
4

0

0

0
4

0

0

0

0
4

0

0

0
4

0
4

0
4

0

0

0

0

0

0
4

0
4

0
4

0
4

0
4

0

0

0

0
4

0

0

0

0
4

0

0

0
4

0
4

0
4

0

0

0

0

0

0

0
4

0
4

0
4

0
4

0
4

0

0

0
4

0

0

0
4

0

0

0
4

0
4

0
4

2.10312
5

2.10312
5

2.10312
5

2.10312
5

0
4

0
4

0
4

34.1806

34.1806

33.2263

33.2194

0

0.00690984

1.41033018596914e-15

0
4

0

0

0
4

0

0

0
4

0.0680222
4

0.0680222
4

0

0

0

0

0
4

0

0

0

0

0
4

0.690324

0.690324

0
4

0.0103648

0.0103648

0
4

0.18554

0.18554

0
4

0

0

0
4

0

0

0
4

0

0

0
4

5.19029264012261e-15

0
4

0
4

0

0

0

0

0

0
4

0
4

0
4

0

0

0

0

0

0

0
4

0

0

0

0
4

0
4

0
4

0.309982

0.309982

0.309982

0.309982

0
4

0
4

0
4

0

0

0

0

0

0
4

0

0

0

0
4

0
4

0
4

0.0211138

0.0211138

0.0211138

0.0140759

0.00703794

0
4

0
4

0
4

0

0

0

0

0

0

0

0

0
4

0

0

0
4

0
4

0
4

0.0823951

0.0823951

0.0823951

0.0599054

0.0224897

0

3.46944695195361e-18

0
4

0
4

0
4

0

0

0

0

0
4

0

0

0

0
4

0
4

0
4

0.0255083
7

0.0255083
7

0.0255083

0.0255083

0
4

0

0

0
4

0
4

0
4

0

0

0

0

0

0
4

0

0

0

0
4

0
4

0
4

0.0404642

0.0404642

0.0404642

0

0

0

0

0

0

0

0

0.0404642

0

0

0
4

0

0

0

0

0

0

0

0

0
4

0

0

0

0

0

0

0

0

0

0
4

0
4

0
4

0

0

0

0

0

0

0
4

0
4

0
4

2.18527
5

2.18527
5

2.18527
5

2.18527

0

0
4

0
4

0
4

0

0

0

0

0
4

0
4

0
4

1.95398

1.95398

1.95398

1.95398

0
4

0
4

0
4

0.388254

0.388254

0.388254

0.388254

0

0
4

0
4

0
4

0

0

0

0

0
4

0
4

0
4

0

0

0

0

0

0
4

0

0

0
4

0
4

0
4

0

0

0

0

0

0

0
4

0
4

0
4

0

0

0

0

0

0
4

0
4

0
4

0

0

0

0

0

0
4

0

0

0

0
4

0
4

0
4

1.49476
7

1.49476
7

1.48439
7

0.505498
7

0.275285
7

0.304274
7

0

0.0483689

0.0170056

0.121392

0.212569

0

8.32667268468867e-17
7

0
4

0.0103648

0

0

0.0103648

0
4

0

0

0
4

7.11236625150491e-17
7

0
4

0
4

0

0

0

0

0

0

0
4

0
4

0
4

0

0

0

0

0

0

0
4

0
4

0
4

0

0

0

0

0

0
4

0
4

0
4

0

0

0

0

0
4

0
4

0
4

0

0

0

0

0

0
4

0
4

0
4

0.0968956

0.0968956

0.0968956

0.0968956

0
4

0

0

0
4

0
4

0
4

0.357411

0.357411

0.357411

0.357411

0
4

0
4

0
4

0.104013

0.104013

0.104013

0

0.104013

0
4

0
4

0
4

0

0

0

0

0

0
4

0

0

0
4

0
4

0

0

0

0
4

0
4

0
4

0

0

0

0

0

0

0
4

0
4

0
4

13.6802

0
4

0
4

0

0

0

0
4

0
4

0.0599054

0.0599054

0.0599054

0
4

0
4

0

0

0

0
4

0
4

0

0

0

0
4

0
4

0.427305

0.427305

0.427305

0
4

0
4

0

0

0

0
4

0
4

0

0

0

0
4

0
4

0

0

0

0
4

0
4

0

0

0

0
4

0
4

9.40477

9.40477

9.40477

0
4

0
4

0

0

0

0
4

0
4

0

0

0

0

0
4

0
4

0.0599054

0.0599054

0.0599054

0
4

0
4

0

0

0

0
4

0
4

0

0

0

0
4

0
4

0

0

0

0
4

0
4

0

0

0

0
4

0
4

0

0

0

0
4

0
4

0

0

0

0
4

0
4

0

0

0

0
4

0
4

0.0792192

0.0792192

0.0792192

0
4

0
4

0

0

0

0
4

0
4

0.646242

0.646242

0.646242

0
4

0
4

0

0

0

0
4

0
4

0

0

0

0

0

0
4

0
4

0

0

0

0

0
4

0
4

0

0

0

0
4

0
4

0

0

0

0
4

0

0

0
4

0
4

0

0

0

0
4

0
4

0

0

0

0
4

0
4

3.0029
3

1.75686
2

0
3

0
2

0

0

0

0

0

0

0

0

0

0

0
2

0

0

0

0

0

0

0

0

0

0

0

0

0

0

0

1.70996

0

0

0

0

0

0

0

0

0.0469069

0
4

0.665978

0.515976

0.0561884

0.0938138

0

0

0

0

0

0

0
4

0

0

0
4

0

0

0

0
4

0

0

0
4

0

0

0

0
4

0

0

0
4

0

0

0
4

0.0316877

0.0316877

0
4

0

0

0
4

0

0

0
4

0

0

0
4

0.0112377

0.0112377

0

0

0

0

0

0
4

0

0

0
4

0

0

0
4

0

0

0
4

0

0

0
4

0

0

0
4

0

0

0
4

0

0

0
4

0

0

0
4

0

0

0
4

0.357411

0.357411

0
4

0

0

0

0

0
4

0

0

0
4

0

0

0
4

0

0

0
4

0.179716

0.0898581

0.0898581

0
4

0

0

0

0
4

0

0

0

0

0
4

0

0

0

0

0
4

0

0

0
4

0

0

0

0
4

0
4

0
4

38.4785
3

0

0

0

0

0

0

0

0

0
4

0

0

0

0

0
4

0

0

0
4

0
4

3.05953
6

3.05953
6

3.05953
6

0
4

0
4

0

0

0

0
4

0
4

0

0

0

0
4

0
4

0

0

0

0
4

0
4

0

0

0

0
4

0
4

0

0

0

0
4

0
4

0

0

0

0
4

0
4

0

0

0

0
4

0
4

0.149764

0.149764

0.149764

0
4

0
4

0.0155008

0.0155008

0.0155008

0
4

0
4

0

0

0

0
4

0
4

0

0

0

0

0

0

0

0
4

0

0

0

0
4

0

0

0
4

0
4

0

0

0

0
4

0
4

0

0

0

0
4

0
4

1.70996

1.70996

1.70996

0
4

0
4

0

0

0

0
4

0
4

0

0

0

0
4

0
4

0

0

0

0
4

0
4

0

0

0

0
4

0
4

0

0

0

0
4

0
4

0

0

0

0
4

0
4

0

0

0

0
4

0
4

0

0

0

0

0
4

0

0

0

0
4

0
4

0

0

0

0
4

0
4

0

0

0

0
4

0
4

0.121392

0.121392

0.121392

0
4

0
4

0.714822

0.714822

0.714822

0
4

0
4

0

0

0

0
4

0
4

0

0

0

0
4

0
4

0

0

0

0
4

0
4

0

0

0

0
4

0
4

0

0

0

0
4

0
4

0

0

0

0
4

0
4

0

0

0

0

0

0

0

0
4

0

0

0
4

0
4

0

0

0

0
4

0
4

0

0

0

0
4

0
4

0

0

0

0
4

0
4

0.0170056

0.0170056

0.0170056

0
4

0
4

0

0

0

0
4

0
4

0

0

0

0
4

0
4

0

0

0

0

0

0
4

0

0

0

0
4

0

0

0
4

0
4

0
3

0

0

0

0

0

0
4

0

0

0

0
4

0

0

0
4

0

0

0
4

0

0

0
4

0
4

0
2

0

0

0

0

0
4

0

0

0
4

0

0

0

0
4

0

0

0
4

0

0

0
4

0
4

0

0

0

0

0

0

0
4

0
4

0.431889

0.431889

0.329756

0.102134

0
4

0
4

14.9091
6

14.9091
6

14.9091
6

0
4

0
4

0
4

0
4

0
4

0
4

0

0

0
4

0

0

0
4

0

0

0
4

0
4

0
3

0

0

0

0

0

0
4

0

0

0
4

0

0

0
4

0
4

0
3

0

0

0

0

0
4

0

0

0
4

0

0

0
4

0

0

0
4

0

0

0
4

0
4

0

0

0

0

0
4

0

0

0
4

0
4

1.70996

1.70996

1.70996

0

0
4

0
4

0

0

0

0

0
4

0
4

0

0

0

0
4

0

0

0
4

0
4

0.10116

0.10116

0.10116

0
4

0

0

0

0
4

0
4

0

0

0

0

0

0
4

0

0

0
4

0
4

0
4

0
4

0

0

0

0

0

0

0
4

0
4

0
4

0

0

0

0

0

0
4

0

0

0

0
4

0
4

0.161857
3

0.161857
3

0.161857

0

0

0

0
4

0

0

0

0

0
4

0

0

0
4

0
4

0

0

0

0

0
4

0

0

0
4

0
4

0

0

0

0

0

0

0
4

0
4

6.75054
6

6.75054
6

6.75054
6

0
4

0
4

0

0

0

0

0
4

0

0

0

0
4

0
4

0

0

0

0

0
4

0

0

0
4

0
4

0.0387582

0.0387582

0.0387582

0

0
4

0
4

0

0

0

0

0

0
4

0

0

0
4

0
4

0

0

0

0

0

0
4

0
4

0

0

0

0

0
4

0

0

0
4

0
4

0

0

0

0

0
4

0
4

0
2

0

0

0

0

0
4

0

0

0

0

0
4

0

0

0
4

0

0

0
4

0

0

0
4

0
4

0

0

0

0

0
4

0

0

0
4

0
4

0

0

0

0
4

0
4

0.0581373

0.0581373

0.0581373

0
4

0
4

0.389745

0.389745

0.389745

0
4

0
4

0

0

0

0
4

0
4

0

0

0

0

0
4

0
4

0

0

0

0
4

0

0

0
4

0
4

0

0

0

0

0
4

0

0

0
4

0
4

0

0

0

0
4

0
4

0.327539

0.327539

0.327539

0
4

0
4

0

0

0

0

0

0

0

0
4

0
4

0.0138197

0.0138197

0.0138197

0
4

0

0

0
4

0
4

0

0

0

0

0
4

0

0

0
4

0
4

0

0

0

0

0
4

0
4

0

0

0

0

0
4

0
4

0

0

0

0

0
4

0
4

0

0

0

0
4

0

0

0
4

0
4

0

0

0

0

0
4

0
4

0

0

0

0
4

0

0

0
4

0
4

0

0

0

0
4

0

0

0
4

0
4

0

0

0

0
4

0
4

0
4

0

0

0

0

0

0
4

0

0

0
4

0

0

0
4

0

0

0
4

0
4

0

0

0

0
4

0
4

0

0

0

0

0
4

0
4

0

0

0

0
4

0
4

0.0809283

0.0809283

0.0404642

0.0404642

0
4

0
4

0

0

0

0
4

0

0

0
4

0
4

0.0316877

0

0

0
4

0.0316877

0.0316877

0
4

0
4

0

0

0

0
4

0

0

0
4

0
4

0

0

0

0
4

0
4

0

0

0

0
4

0
4

0

0

0

0

0
4

0
4

0

0

0

0

0
4

0

0

0

0

0
4

0

0

0
4

0
4

0

0

0

0

0
4

0
4

0

0

0

0
4

0

0

0
4

0
4

0

0

0

0
4

0

0

0
4

0
4

0

0

0

0

0
4

0
4

0.110536

0.110536

0.110536

0
4

0
4

0

0

0

0
4

0
4

0

0

0

0

0
4

0
4

0

0

0

0
4

0
4

0

0

0

0
4

0
4

0

0

0

0
4

0
4

0

0

0

0

0

0
4

0

0

0
4

0

0

0
4

0

0

0

0
4

0
4

0.0168673

0.0168673

0.0168673

0
4

0
4

0

0

0

0
4

0
4

0

0

0

0
4

0
4

0

0

0

0
4

0
4

0

0

0

0
4

0
4

0

0

0

0
4

0
4

0.0732585

0.0732585

0.0732585

0
4

0
4

0

0

0

0
4

0
4

0

0

0

0
4

0
4

0

0

0

0
4

0
4

0.114392
3

0.114392

0.0633753

0.0510167

0
4

0

0

0

0

0
4

0

0

0
4

0

0

0
4

0
4

0

0

0

0
4

0
4

0

0

0

0
4

0
4

0

0

0

0
4

0
4

0

0

0

0
4

0
4

0

0

0

0
4

0
4

0

0

0

0
4

0
4

1.07223

1.07223

1.07223

0
4

0
4

0

0

0

0
4

0
4

0.311349

0.311349

0.311349

0
4

0
4

0.357411

0.357411

0.357411

0
4

0
4

5.62941
3

3.45618
3

1.68552
3

0
3

0

0

0

0

0

0

0

0

0

1.70996

0

0

0

0

0

0

0

0

0

0

0

0

0

0

0

0

0

0

0

0

0

0

0

0

0

0

0

0

0

0

0

0

0

0

0

0

0.0606962

6.24500451351651e-16
3

0
4

1.67355
7

1.67355
7

0
4

0

0

0
4

0

0

0
4

0

0

0
4

0.484478

0.484478

0
4

0

0

0
4

0.0152104

0.0152104

0
4

0

0

0
4

0

0

0
4

0

0

0
4

0

0

0
4

0

0

0
4

0

0

0
4

0

0

0
4

0

0

0
4

0

0

0

0
4

0

0

0
4

0

0

0
4

0

0

0
4

0

0

0
4

0
4

0
4

336.249
4

3.262
7

3.262
7

3.0771
7

0.126126

0

0.0112449

0.0475315

0

0

0
4

0
4

4.04347
7

4.04347
7

4.04347
7

0
4

0

0

0
4

0
4

0

0

0

0

0
4

0
4

0

0

0

0

0
4

0
4

0.0633753

0.0633753

0.0316877

0.0316877

0
4

0
4

0

0

0

0

0
4

0
4

0

0

0

0

0
4

0
4

1.70996

1.70996

1.70996

0
4

0
4

0

0

0

0

0
4

0
4

0

0

0

0

0
4

0
4

0

0

0

0
4

0

0

0
4

0
4

0

0

0

0
4

0
4

0
4

0
4

0

0

0

0

0

0

0

0

0
4

0

0

0

0
4

0

0

0

0
4

0

0

0
4

0

0

0
4

0
4

0.0316877

0.0316877

0.0316877

0
4

0

0

0
4

0
4

0

0

0

0
4

0

0

0
4

0
4

0

0

0

0
4

0

0

0
4

0
4

0

0

0

0
4

0

0

0
4

0
4

0

0

0

0

0
4

0
4

0

0

0

0
4

0

0

0
4

0
4

0

0

0

0
4

0
4

0

0

0

0

0
4

0
4

0

0

0

0
4

0

0

0
4

0
4

0.0282504

0.0282504

0.0170056

0.0112449

1.73472347597681e-18

0
4

0
4

5.64651
7

5.59074
7

5.50549
7

0.0852543

0

0
4

0.0557638

0.0170056

0.0387582

0
4

0

0

0
4

0
4

0

0

0

0
4

0

0

0
4

0
4

0

0

0

0
4

0
4

0

0

0

0
4

0
4

0

0

0

0
4

0

0

0
4

0
4

0

0

0

0
4

0
4

0

0

0

0
4

0

0

0
4

0
4

0

0

0

0
4

0

0

0
4

0
4

0

0

0

0
4

0
4

0

0

0

0

0
4

0
4

0

0

0

0
4

0

0

0
4

0
4

0.0829781
4

0.0829781
4

0.0425139

0

0

0.0404642

0

6.93889390390723e-18
4

0
4

0

0

0

0
4

0

0

0

0
4

0
4

0

0

0

0
4

0
4

0

0

0

0
4

0
4

0

0

0

0
4

0

0

0
4

0
4

0

0

0

0
4

0
4

0

0

0

0
4

0
4

0.051324

0.051324

0.051324

0
4

0
4

0

0

0

0
4

0

0

0
4

0
4

0

0

0

0
4

0

0

0
4

0
4

0

0

0

0
4

0

0

0
4

0
4

0

0

0

0
4

0

0

0
4

0
4

3.38287
7

3.38287
7

3.37452
7

0.00835115

2.77555756156289e-17
7

0
4

0

0

0
4

0
4

0

0

0

0
4

0

0

0
4

0
4

0

0

0

0
4

0
4

0.0255083

0.0255083

0.0255083

0
4

0

0

0
4

0
4

0

0

0

0
4

0

0

0
4

0
4

0

0

0

0
4

0
4

0

0

0

0
4

0
4

0.0633753

0.0633753

0.0633753

0
4

0
4

0

0

0

0
4

0
4

0

0

0

0
4

0
4

0.10116

0.10116

0.10116

0
4

0
4

0.0595195
7

0.0595195
6

0

0

0

0.0595195

0

0

0
4

0

0

0
4

0

0

0
4

0

0

0
4

0
4

0

0

0

0
4

0
4

0

0

0

0
4

0
4

0

0

0

0
4

0
4

0

0

0

0
4

0
4

0

0

0

0
4

0
4

0

0

0

0
4

0
4

0

0

0

0
4

0
4

0

0

0

0
4

0
4

0

0

0

0
4

0
4

0

0

0

0
4

0
4

0

0

0

0

0

0

0

0
4

0

0

0

0

0

0
4

0

0

0

0

0
4

0
4

0

0

0

0
4

0
4

0

0

0

0
4

0
4

0

0

0

0
4

0
4

0

0

0

0
4

0
4

0

0

0

0
4

0
4

0

0

0

0
4

0
4

0

0

0

0
4

0
4

0

0

0

0
4

0
4

0

0

0

0
4

0
4

0

0

0

0
4

0
4

0.547311
5

0

0

0

0

0
4

0.450416

0.411664

0.0387519

0
4

0.0968956

0.0968956

0
4

0

0

0
4

0

0

0
4

0

0

0
4

0
4

0

0

0

0
4

0
4

3.93152

3.93152

3.93152

0
4

0
4

0

0

0

0
4

0
4

0

0

0

0
4

0
4

0

0

0

0
4

0
4

0

0

0

0
4

0
4

0

0

0

0
4

0
4

0

0

0

0
4

0
4

0

0

0

0
4

0
4

0

0

0

0
4

0
4

1.03717
4

1.03717
4

0.989634
6

0

0.0475315

0

0
4

0

0

0

0

0
4

0
4

0

0

0

0
4

0
4

0

0

0

0
4

0
4

0

0

0

0
4

0
4

0.0112449

0.0112449

0.0112449

0
4

0
4

0

0

0

0
4

0
4

0

0

0

0
4

0
4

0

0

0

0
4

0
4

0.0316877

0.0316877

0.0316877

0
4

0
4

0

0

0

0
4

0
4

0

0

0

0
4

0
4

0
4

0
4

0

0

0

0

0

0

0
4

0

0

0

0
4

0
4

0

0

0

0
4

0
4

0

0

0

0
4

0
4

0

0

0

0
4

0
4

0

0

0

0
4

0
4

0

0

0

0
4

0
4

0.0122713

0.0122713

0.0122713

0
4

0
4

0

0

0

0
4

0
4

0

0

0

0
4

0
4

0

0

0

0
4

0
4

0

0

0

0
4

0
4

0
4

0
4

0
4

0

0

0

0

0
4

0

0

0

0

0
4

0

0

0

0
4

0

0

0

0
4

0

0

0

0
4

0

0

0
4

0

0

0
4

0
4

0.15305
4

0.15305
4

0.15305
4

0

0

0
4

0

0

0

0
4

0

0

0
4

0
4

0.010998

0.010998

0.010998

0
4

0
4

0

0

0

0
4

0
4

0

0

0

0
4

0
4

0

0

0

0
4

0
4

0

0

0

0
4

0
4

0

0

0

0
4

0
4

0

0

0

0
4

0
4

0.00701215

0.00701215

0.00701215

0
4

0
4

0

0

0

0
4

0
4

0

0

0

0
4

0
4

0

0

0

0

0

0
4

0

0

0

0

0
4

0

0

0

0
4

0
4

0

0

0

0
4

0
4

0

0

0

0
4

0
4

0

0

0

0
4

0
4

0.536117

0.536117

0.536117

0
4

0
4

0

0

0

0
4

0
4

0

0

0

0
4

0
4

0

0

0

0
4

0
4

0

0

0

0
4

0
4

0

0

0

0
4

0
4

0

0

0

0
4

0
4

0

0

0

0

0

0

0

0

0
4

0

0

0

0

0
4

0
4

0

0

0

0
4

0
4

0

0

0

0
4

0
4

0

0

0

0
4

0
4

0

0

0

0
4

0
4

0

0

0

0
4

0
4

0

0

0

0
4

0
4

0

0

0

0
4

0
4

0.0155008

0.0155008

0.0155008

0
4

0
4

0

0

0

0
4

0
4

0

0

0

0
4

0
4

0
4

0

0

0

0

0

0
4

0

0

0

0

0
4

0

0

0
4

0

0

0
4

0
4

0

0

0

0
4

0
4

0.0404642

0.0404642

0.0404642

0
4

0
4

1.70996

1.70996

1.70996

0
4

0
4

0.0170056

0.0170056

0.0170056

0
4

0
4

0

0

0

0
4

0
4

0

0

0

0
4

0
4

0

0

0

0
4

0
4

0

0

0

0
4

0
4

0

0

0

0
4

0
4

0

0

0

0
4

0
4

0
4

0

0

0

0

0
4

0

0

0

0
4

0

0

0
4

0

0

0
4

0
4

0

0

0

0
4

0
4

0

0

0

0
4

0
4

0.357411

0.357411

0.357411

0
4

0
4

0

0

0

0
4

0
4

0

0

0

0
4

0
4

0

0

0

0
4

0
4

0.007332

0.007332

0.007332

0
4

0
4

0

0

0

0
4

0
4

0

0

0

0
4

0
4

0

0

0

0
4

0
4

0
4

0
4

0
4

0

0
4

0

0

0
4

0

0

0
4

0
4

0

0

0

0
4

0
4

0

0

0

0
4

0
4

0.893528

0.893528

0.893528

0
4

0
4

0

0

0

0
4

0
4

0

0

0

0
4

0
4

0

0

0

0
4

0
4

0

0

0

0
4

0
4

0

0

0

0
4

0
4

0.714822

0.714822

0.714822

0
4

0
4

0

0

0

0
4

0
4

0

0

0

0

0
4

0

0

0

0
4

0

0

0
4

0

0

0
4

0
4

0

0

0

0
4

0
4

0

0

0

0
4

0
4

0

0

0

0
4

0
4

0.0469069

0.0469069

0.0469069

0
4

0
4

0

0

0

0
4

0
4

0

0

0

0

0

0
4

0

0

0
4

0

0

0
4

0
4

0
4

0

0

0

0
4

0

0

0

0
4

0

0

0
4

0

0

0
4

0

0

0
4

0
4

0

0

0

0

0

0

0

0

0
4

0

0

0
4

0

0

0
4

0

0

0
4

0
4

0.178558
4

0.178558
4

0.178558
4

0

0

0

0

0

0

0
4

0

0

0

0
4

0
4

0

0

0

0
4

0

0

0

0

0
4

0

0

0

0
4

0

0

0
4

0
4

0
3

0
3

0

0

0

0

0

0
4

0
4

0
3

0

0

0

0

0
4

0

0

0

0
4

0

0

0
4

0
4

0.357411

0

0

0

0
4

0

0

0

0
4

0

0

0
4

0

0

0
4

0

0

0
4

0.357411

0.357411

0
4

0
4

0
4

0
4

0

0

0

0

0

0
4

0
4

0

0

0

0

0

0

0

0
4

0

0

0
4

0

0

0
4

0
4

0
3

0

0

0

0
4

0

0

0

0
4

0

0

0
4

0

0

0
4

0
4

0

0

0

0

0

0
4

0
4

0

0

0

0
4

0

0

0
4

0

0

0
4

0

0

0
4

0

0

0
4

0

0

0
4

0
4

0.181614
6

0.134083

0.134083

0
4

0.0475315

0.0475315

0
4

1.38777878078145e-17
6

0
4

2.88
7

2.88
7

0.299934
7

0.8531
6

0

1.70996

0

0

0.0170056

1.59594559789866e-16
7

0
4

0
4

0

0

0

0

0

0
4

0

0

0
4

0

0

0
4

0
4

0.131287

0.131287

0.117467

0.00690984

0.00690984

3.46944695195361e-18

0
4

0
4

0
3

0
3

0
3

0
4

0

0

0
4

0

0

0
4

0
4

0.0463996

0.0338728

0

0.0170056

0.0168673

0
4

0.0125267

0

0.0125267

0

0
4

0

0

0
4

1.73472347597681e-18

0
4

2.33328
5

1.82086

1.82086

0
4

0.512419

0.512419

0
4

0

0

0
4

4.44089209850063e-16
5

0
4

2.85929

2.85929

0

2.85929

0
4

0
4

0

0

0

0

0
4

0

0

0

0

0
4

0
4

0

0

0

0

0
4

0

0

0
4

0

0

0
4

0
4

0
4

0

0

0
4

0

0

0
4

0

0

0
4

0

0

0
4

0

0

0
4

0
4

0
4

0

0

0

0
4

0

0

0
4

0

0

0
4

0

0

0
4

0
4

0
4

0
4

0

0

0

0

0

0

0

0

0
4

0
4

0

0

0

0

0

0
4

0

0

0

0
4

0

0

0

0

0
4

0

0

0
4

0

0

0

0
4

0

0

0
4

0

0

0
4

0

0

0
4

0
4

0

0

0

0

0
4

0
4

0.0469069

0

0

0
4

0

0

0
4

0

0

0
4

0

0

0
4

0.0469069

0.0469069

0
4

0
4

0
4

0

0

0

0
4

0

0

0
4

0
4

0.0316877

0

0

0

0
4

0

0

0
4

0.0316877

0.0316877

0
4

0

0

0
4

0
4

0

0

0

0

0
4

0

0

0
4

0

0

0
4

0

0

0
4

0
4

0

0

0

0

0
4

0
4

0

0

0

0

0

0
4

0

0

0
4

0
4

0

0

0

0

0
4

0

0

0
4

0
4

1.70996

1.70996

0

0

0

1.70996

0
4

0

0

0
4

0
4

0.785183

0

0

0

0
4

0.785183

0.785183

0
4

0
4

0
4

0
4

0
4

0

0

0

0

0
4

0

0

0

0

0
4

0
4

0

0

0

0

0
4

0

0

0

0
4

0

0

0
4

0
4

0

0

0

0

0
4

0

0

0
4

0

0

0
4

0
4

0.0469069

0

0

0

0
4

0.0469069

0.0469069

0
4

0

0

0
4

0
4

0

0

0

0

0
4

0

0

0

0
4

0
4

0

0

0

0

0
4

0

0

0
4

0

0

0
4

0
4

0.135654

0

0

0
4

0.135654

0.135654

0
4

0

0

0
4

0
4

0

0

0

0
4

0

0

0
4

0

0

0
4

0
4

1.87092

1.87092

0.262574

1.60835

0
4

0
4

0

0

0

0
4

0

0

0
4

0
4

0.193791

0

0

0
4

0.193791

0.193791

0
4

0

0

0
4

0

0

0
4

0
4

16.9267
6

1.48955
7

1.48955
7

0

0

0

0
4

15.4371
6

15.3896
6

0

0.0475315

0

4.02455846426619e-16
6

0
4

0
4

0

0

0

0
4

0

0

0
4

0

0

0
4

0
4

0

0

0

0
4

0
4

0

0

0

0
4

0

0

0
4

0

0

0
4

0
4

0

0

0

0
4

0

0

0
4

0
4

0.761729

0.404318

0.0469069

0.357411

0
4

0.357411

0.357411

0
4

5.55111512312578e-17

0
4

0

0

0

0

0
4

0

0

0
4

0
4

0

0

0

0
4

0
4

0

0

0

0
4

0

0

0
4

0

0

0
4

0
4

0

0

0

0

0
4

0

0

0
4

0
4

0

0

0

0

0
4

0
4

1.9841
7

1.97719
7

1.51862
7

0

0.10116

0.357411

0
4

0.00690984

0.00690984

0
4

7.80625564189563e-17
7

0
4

0

0

0

0
4

0
4

0

0

0

0

0
4

0
4

0

0

0

0
4

0

0

0
4

0
4

0

0

0

0
4

0

0

0
4

0
4

0

0

0

0
4

0

0

0
4

0

0

0
4

0
4

0

0

0

0

0
4

0

0

0
4

0
4

0

0

0

0

0
4

0
4

0

0

0

0
4

0

0

0
4

0
4

0

0

0

0

0
4

0

0

0
4

0
4

1.50001

1.14259

0.0703603

1.07223

0
4

0.357411

0.357411

0
4

0
4

0
4

0

0

0

0

0
4

0

0

0

0

0
4

0

0

0

0
4

0

0

0

0
4

0

0

0
4

0

0

0
4

0

0

0
4

0
4

0

0

0

0

0
4

0

0

0
4

0
4

0.0633391

0.029328

0.029328

0
4

0.0340111

0.0340111

0
4

6.93889390390723e-18

0
4

0

0

0

0
4

0
4

0

0

0

0

0
4

0

0

0
4

0
4

0

0

0

0
4

0
4

0

0

0

0

0

0
4

0
4

0

0

0

0
4

0

0

0
4

0

0

0
4

0
4

0

0

0

0
4

0

0

0
4

0

0

0
4

0
4

0

0

0

0

0
4

0

0

0
4

0
4

0

0

0

0

0
4

0
4

272.59
4

267.282
4

94.0386
4

16.2699
4

3.45463
4

49.3829
7

0

0

0.0787115

0.0334046

0

0.0654693

0

0

0.102619

0.0469069

0

0

0

0

0

0.0039381

0

0

0

0

0

0

0.230144
7

0

0

0

0

0

0.0337079

0

0

0

0

1.88656
6

0.119322

0

0

0

0.237658

0

0

0

0

0

0.88342
7

0

0

0.0170056

0

0

0

0

0.0112449

0

0

0.132521
7

0

0

0.00590715

0

0

0

0

0

0

0

5.11558
7

0

0

5.12987

0.0224897

0

0

0

0

0

0.00690984

0

0

0

0

0

0

0

0

0

0

0

0.938176
6

0

0

0

0

0

0

0

0

0

0

0.102133
7

0

0

0

0

0

0

0

0

0

0

71.3534
7

0
4

0

0

0

0

0

0

0

0

0

0.00590715

0
7

0

0

0

0

0

0

0

0

0

0.0170056

1.78276
6

0

0

0

0

0

0

0

0

0

0

3.63705
7

0.0475315

0

0

0

0.0039381

0

0

0

0

0

0.0208054
7

0.0078762

0

0

0

0

0

0.00590715

0

0

0

0
3

0

0

0

0

0.0170056

0

0.00590715

0

0

0

0.41191
6

0.0165402

0

0

0

0

0

0

0

0

0

0

0

0

0

0

0

0

0

0

0

0

0.237078
6

0.0167023

0

0

0

0

0.0039381

0.0232512

0.0316877

0

0

0.762398
6

0

0

0

0

0

0

0

0

0

0

2.86189
7

1.06087

0

0.007332

0

0.0255083

0

0

0

0.0112449

0.0898581

0

0.14703
6

0

0

0

0

0

0

0

0.0316877

0

0

0

0

0

0

0

0

0

0.0112449

0.0703603

0.0168673

0

0
3

0

0

0

0

0

0.00703794

0

0

0

0

0.351802
4

0.0168673

0

0

0

0

0

0

0

0

0

0

0

0

0

0.0475315

0

0

0

0

0

0

0.0168673
6

0

0

0.00835115

0

0

0

0.0599054

0

0.00590715

0

0.0293996
7

0

0

0

0

0

0.0581373

0

0

0

0

0
7

0

0

0.0167023

0

0

0

0

0

0

0

0

0.0599054

0.00703794

0.0168673

0

0

0

0

0

0

0

0
4

0.00690984
7

0

0

0

0.007332

0

0

0

0

0

0.0387519

0.0820978

0

0

0

0.0599054

0

0

0

0.140703

1.0994
7

0.0469069

0.0599054

0

0.0167023

0

0.0366292

0

0

0

0

0
3

0

0

0

2.12264

0.149764

0.00590715

0

0

0

0

0.636226
7

0

0

0.00835115

0.095063

0

0

0

0

0

0.03666

0.617802
7

0

0

0

0

0

0

0

0

0.0404642

0

0
4

0

0

0

0.0680222

0

0

0.0170056

0.0938138

0

0

0
4

0.281815
7

0.195611
7

0.0255083

0

0.0606962

0
4

0

0

0

0

0
4

0

0

0
4

0

0

0
4

0

0

0
4

0

0

0
4

0

0

0
4

0.110907

0

0.0792192

0.0316877

0

6.93889390390723e-18

0
4

0

0

0

0
4

0

0

0

0
4

0

0

0

0
4

0

0

0

0

0
4

0

0

0

0
4

0

0

0
4

0

0

0

0
4

0

0

0
4

0

0

0

0

0

0
4

0

0

0

0
4

0.0224897

0.0224897

0
4

0

0

0
4

0

0

0

0
4

0

0

0

0
4

0

0

0
4

0

0

0

0
4

0

0

0

0
4

0.5395

0.182089

0.357411

0
4

0

0

0

0
4

0.156025
7

0.0387582

0.117267

0

1.38777878078145e-17
7

0
4

0

0

0

0
4

0

0

0
4

0

0

0

0
4

0

0

0
4

0.0366292

0.0366292

0
4

0

0

0
4

0

0

0
4

0

0

0
4

0

0

0
4

0

0

0
4

2.13396
6

2.13396
6

0
4

0

0

0
4

0.0155008

0.0155008

0
4

0

0

0
4

0.0599054

0.0599054

0
4

0

0

0
4

0

0

0
4

0

0

0
4

0

0

0
4

0.00690984

0.00690984

0
4

0

0

0
4

0.205956
6

0.205956
6

0
4

0

0

0
4

0

0

0
4

0

0

0
4

0

0

0
4

0

0

0
4

0

0

0
4

0

0

0
4

0

0

0
4

0

0

0
4

0

0

0
4

0
4

0
4

0
4

0

0

0
4

0

0

0
4

0

0

0
4

0

0

0
4

0

0

0
4

0

0

0
4

0

0

0
4

0

0

0
4

0

0

0
4

1.70996

1.70996

0
4

0

0

0

0

0
4

0

0

0
4

0

0

0
4

0

0

0
4

0

0

0
4

0

0

0
4

0

0

0
4

0

0

0
4

0

0

0
4

0

0

0
4

0

0

0
4

0

0

0

0

0
4

0

0

0
4

0

0

0
4

0.0155008

0.0155008

0
4

0

0

0
4

0

0

0
4

0.0138197

0.0138197

0
4

0

0

0
4

0

0

0
4

0

0

0
4

0

0

0
4

0

0

0

0
4

0

0

0
4

0

0

0
4

0

0

0
4

0

0

0
4

0

0

0
4

0

0

0
4

0

0

0
4

0

0

0
4

0

0

0
4

0

0

0
4

4.05647737622417e-14
4

0
4

0
4

34.7531
4

29.407
4

0

0

0

0

0

0
4

16.8186
4

0
4

0.292526
4

2.60691
3

3.12212
4

0
4

0.463896
4

0.89581
4

9.43737
4

0
3

0

0

0

0

0

0

0

0

0

0

0

0

1.77635683940025e-15
4

0
4

4.92371
3

3.67564
3

1.09175
3

0.0366292
3

0.0387582
4

0
4

0

0
4

0

0

0

0

0

0

0

0
4

0

0

0

0

0

0.0809283

0

0

0

0

0
3

0

0

0

0

0

0

0

0

0

0

0
3

0

0

0

0

0

0

0

0

0

0

0

0

0

0

0

0

0

0

0

0

0

0
3

0

0

0

0

0

0

0

0

0

0

0
4

0

0

0

0

0

0

0

0

0

0

0
4

0
4

7.49400541621981e-16
3

0
4

7.02516
5

7.02516
5

0
4

0

0

0
4

0

0

0
4

0

0

0
4

0

0

0
4

0

0

0
4

0

0

0
4

0

0

0
4

0

0

0
4

0

0

0
4

0

0

0
4

0.359433

0

0.359433

0
4

0

0

0
4

0

0

0
4

0

0

0
4

0

0

0
4

0

0

0
4

0

0

0
4

0

0

0
4

0

0

0
4

0

0

0
4

0.209669

0.209669

0

0
4

0

0

0
4

0.0703603

0.0703603

0
4

0

0

0
4

0

0

0
4

0

0

0
4

0

0

0
4

0
4

0
4

0
4

0

0

0

0

0

0

0

0

0
4

0

0

0

0
4

0

0

0
4

0
4

0
4

0

0

0

0

0
4

0

0

0
4

0
4

0
4

0

0

0
4

0

0

0
4

0

0

0
4

0

0

0
4

0
4

0

0

0

0
4

0

0

0

0
4

0

0

0
4

0
4

0

0

0

0
4

0

0

0
4

0
4

0

0

0

0

0

0

0
4

0
4

0

0

0

0
4

0

0

0
4

0

0

0
4

0
4

0

0

0

0
4

0

0

0
4

0

0

0
4

0
4

0

0

0

0

0
4

0
4

0

0

0

0

0

0
4

0
4

0

0

0

0
4

0
4

0
4

0
4

0
4

0

0
4

0

0

0

0
4

0

0

0
4

0
4

0

0

0

0
4

0

0

0
4

0

0

0
4

0
4

0

0

0

0
4

0

0

0
4

0

0

0
4

0
4

0

0

0

0

0
4

0
4

0

0

0

0

0
4

0

0

0
4

0
4

0

0

0

0

0
4

0
4

0

0

0

0

0
4

0

0

0
4

0
4

0

0

0

0
4

0
4

0

0

0

0
4

0
4

0

0

0

0

0
4

0
4

0

0

0

0
4

0
4

0
4

0
4

0
4

0

0

0
4

0

0

0

0
4

0

0

0
4

0
4

0

0

0

0
4

0
4

0.0404642

0.0404642

0.0404642

0
4

0
4

0

0

0

0
4

0
4

0

0

0

0

0
4

0
4

0

0

0

0
4

0
4

0

0

0

0
4

0

0

0
4

0
4

0.0898581

0.0898581

0.0898581

0

0
4

0
4

0

0

0

0

0
4

0
4

0

0

0

0
4

0

0

0
4

0
4

0.0337346

0.0337346

0.0337346

0
4

0
4

2.35395
5

2.26829

0.558329

1.70996

0
4

0.0856651

0.0387582

0

0.0469069

0

0
4

0
4

0

0

0

0
4

0
4

0

0

0

0
4

0
4

0

0

0

0
4

0
4

0.00701215

0.00701215

0.00701215

0
4

0
4

0

0

0

0
4

0
4

0

0

0

0
4

0
4

0

0

0

0
4

0
4

0

0

0

0
4

0
4

0

0

0

0
4

0
4

0

0

0

0
4

0
4

0
4

0
4

0
4

0

0

0
4

0

0

0
4

0

0

0
4

0
4

0

0

0

0
4

0
4

0

0

0

0
4

0
4

0

0

0

0
4

0
4

0

0

0

0
4

0
4

0

0

0

0
4

0
4

0

0

0

0
4

0
4

0

0

0

0
4

0
4

0

0

0

0
4

0
4

0

0

0

0
4

0
4

0

0

0

0
4

0
4

0
4

0
4

0

0

0

0

0
4

0
4

0

0

0

0
4

0
4

0

0

0

0
4

0
4

0

0

0

0
4

0
4

0

0

0

0
4

0
4

0

0

0

0
4

0
4

0

0

0

0
4

0
4

2.56494

2.56494

2.56494

0
4

0
4

0.179716

0.179716

0.179716

0
4

0
4

0

0

0

0
4

0
4

0

0

0

0
4

0
4

0

0

0

0

0
4

0

0

0
4

0

0

0
4

0

0

0
4

0
4

0

0

0

0
4

0
4

0

0

0

0
4

0
4

0

0

0

0
4

0
4

0

0

0

0
4

0
4

0

0

0

0
4

0
4

0

0

0

0
4

0
4

0

0

0

0
4

0
4

0

0

0

0
4

0
4

0

0

0

0
4

0
4

0

0

0

0
4

0
4

0

0

0

0

0

0
4

0

0

0
4

0
4

0

0

0

0
4

0
4

0

0

0

0
4

0
4

0

0

0

0
4

0
4

0

0

0

0
4

0
4

0

0

0

0
4

0
4

0.0316877

0.0316877

0.0316877

0
4

0
4

0

0

0

0
4

0
4

0

0

0

0
4

0
4

0

0

0

0
4

0
4

0

0

0

0
4

0
4

0
4

0
4

0
4

0

0
4

0
4

0.0103648

0.0103648

0.0103648

0
4

0
4

0

0

0

0
4

0
4

0

0

0

0
4

0
4

0

0

0

0
4

0
4

0

0

0

0
4

0
4

0

0

0

0
4

0
4

0.0343898

0.0343898

0.0343898

0
4

0
4

0

0

0

0
4

0
4

0

0

0

0
4

0
4

0
4

29.2902
5

0.47645

0.47645

0.47645

0

0

0
4

0

0

0
4

0
4

0

0

0

0
4

0
4

0.269574

0.269574

0.269574

0
4

0
4

0

0

0

0
4

0
4

0

0

0

0
4

0
4

0

0

0

0
4

0
4

0

0

0

0
4

0
4

0

0

0

0
4

0
4

0.351802

0.351802

0.351802

0
4

0
4

0

0

0

0
4

0
4

0

0

0

0
4

0
4

0
4

0
4

0

0

0

0

0
4

0

0

0

0
4

0

0

0
4

0
4

0

0

0

0
4

0
4

0.0599054

0.0599054

0.0599054

0
4

0
4

0

0

0

0
4

0
4

7.15348
6

7.15348
6

7.15348

0

0

0
4

0
4

1.98205
7

1.98205
7

1.98205
7

0
4

0
4

12.2351
6

12.2351
6

12.1876
6

0.0475315

4.02455846426619e-16
6

0
4

0
4

0

0

0

0
4

0

0

0
4

0
4

1.02957

1.02957

1.02957

0
4

0
4

0

0

0

0

0
4

0
4

0

0

0

0
4

0
4

5.73231

1.80377
5

0

1.70996

0

0

0

0.0938138

0

0
4

1.70996
7

1.70996

0

0
4

0

0

0
4

1.70996

1.70996

0
4

0.0613563

0.0613563

0
4

0

0

0
4

0.0898581

0.0898581

0
4

0.357411

0

0.357411

0
4

0

0

0

0
4

0

0

0
4

0

0

0
4

0

0

0
4

0

0

0
4

0

0

0
4

0

0

0
4

3.33066907387547e-16

0
4

8.88178419700125e-16
5

0
4

3.42765815730672e-11

0
4

23.0337

23.0337

19.9277

1.70996
3

1.70996
3

0

0

0

0

0

0
4

0
3

0
3

0

0

0

0

0

0

0

0

0
2

0
3

0

0

0

0

0

0

0
4

0

0

0

0

0
4

18.1248
7

6.0234

0

0

0.0404642

0.357411

0.0404642

0

0

0.0792192

0

3.19192
6

1.88967

2.9609

1.70996

1.70996

0.0809283

0

0.0404642

0
4

0.0930047

0.0930047

0
4

0

0

0
4

0

0

0
4

0
4

0.667659
3

0

0

0

0
4

0.667659

0

0.667659

0

0

0
4

0

0

0

0

0
4

0

0

0
4

0
4

0

0

0

0
4

0
4

0

0

0

0
4

0
4

0

0

0

0
4

0
4

0

0

0

0
4

0

0

0
4

0
4

0.728355
3

0.728355
3

0

0

0.728355

0
4

0

0

0

0
4

0

0

0
4

0
4

0

0

0

0

0
4

0
4

0
2

0
2

0
2

0

0
4

0
4

0

0

0

0

0
4

0
4

0

0

0

0
4

0
4

1.70996

1.70996

1.70996

0
4

0
4

0

0

0

0
4

0
4

0
4

0
2

0
2

0
2

0

0

0

0

0

0
4

0
3

0

0

0

0

0

0
4

0

0

0
4

0
4

0
4

0

0

0

0

0
4

0
4

0
4

0

0

0

0

0

0
4

0
4

0
4

0

0

0

0

0

0

0
4

0
4

0
4

0

0

0

0

0
4

0
4

0
4

0

0

0

0

0
4

0
4

0
4

0
4

4167.14

4166.81

0.815863
3

0.815863
3

0

0

0

0

0

0

0

0

0

0

0

0
3

0.0100127

0

0

0

0

0.260331

0

0

0.158438

0

0
3

0

0

0

0.126751

0.00500636

0

0

0

0

0

0
3

0

0

0

0

0

0.00500636

0

0

0

0

0

0

0.00750954

0

0

0

0.00750954

0

0

0

0

0
4

0

0

0.235299

0
4

0

0
4

0
4

4.63473
3

4.63473
3

0.16305
1

0

0

0

0

0

0

0

0

0

0

0

0

0

0

0

0

0

0

0

0

0

0
4

0

0

0

0

0

0

0

0

1.70996
4

0

2.76172

0

0

0

0
4

0
4

2038.89
6

2038.89
6

884.823
6

0

0.357411

5.71858

20.3793

12.3657

0.231522
7

6.24066

1.07223

0.536117

0

18.656
5

0

0

0

1.78706

0

0

0

1.70996

0

1.70996

25.2472
5

0

0

0

1.70996

0

0.0316877

0.714822

0

0

0

1.482
6

0.357411

0

0

7.69481

1.70996

1040.84
6

2.68098
6

0.164882
7

0.673157
6

0
4

0

0

0
4

0
4

0.131398
7

0.131398
7

0.0486932
7

0.0827043
7

0
4

0
4

13.3936
3

13.3936
3

0

0

0

0

0

0

0.100953

0

0.0618072

0.0122368

0

0

0

4.31344

0

0

0

0

0.0542527

0

0.0168256

0

0

0

0

0.1499

0

0

6.91693

0

0

0

0

0

0

0

0

0

0

0

0

0

0

0

0

0

0

0

0

0

0

1.70996

0

0

0

0

0

0

0

0.0542527

0

0

0

0

0

0

0

0

0

0

0

0

0

0

0

0

0

0

0

0

0

0

0

0

0

0

0

0.00305919

0

5.38631639290799e-16
3

0
4

0

0

0

0

0

0
4

0

0

0
4

0

0

0
4

0

0

0

0

0
4

0

0

0
4

0

0

0

0
4

0

0

0

0
4

0

0

0
4

0

0

0
4

0

0

0
4

0

0

0
4

0
4

29.1109
3

0
3

0

0

0
4

1.70996
3

0
4

0

0

0

0

0

0

0

0

0

0

0
2

0

0

0

0

0

0

0

0

0

0

0

0

0

0

0

0

0

0

0

0

0

0
4

0

0

0

0

0

0

0

0

0

0

1.70996

0

0

0

0

0

0

0

0

0

0

0
4

0

0

0

0

0

0

0

0

0

0
4

0

0

0

0
4

0

0

0
4

0.348824

0.348824

0
4

0

0

0

0

0
4

0

0

0

0

0

0

0

0

0

0

0

0

0
4

0

0

0

0

0
4

27.0521

25.3421

1.70996

0

0

0

0

0

0

0

0

0

0

0

0

0

1.33226762955019e-15

0
4

0

0

0
4

0

0

0
4

0
4

2079.67
5

199.838
6

199.838
6

0
4

1790.71
5

1790.71
5

0
4

89.1197
7

6.97927

0

0.378287

0.435604

0.206339

0.217802

0.102033

0.091706

0.178558

0.171949

0.0687795

23.9697

0.240728

0.275118

0.0802428

0.045853

0.0573163

0

0.355361

0.114633

0.0229265

0.045853

44.1916

0.0229265

0.0229265

0.045853

0.0573163

0.0573163

0.0229265

0

0.0343898

0.0687795

0.045853

0.974377

0.0229265

0

0.0343898

0.0229265

0.160486

0.0343898

0.045853

0.0343898

0.0229265

0.0229265

0.848281

0.0229265

0.0229265

0.0343898

0

0.0229265

0.103169

0.0229265

1.04316

0

0.045853

0.905597

0

0.0687795

0

0.0229265

0

0.206339

0.469993

0.0687795

0.160486

0.0229265

0.710722

0.0229265

0.0343898

0.0229265

0.0573163

0.091706

0.0229265

0.0229265

0.0229265

1.49022

0.641942

1.9029

0
4

0

0

0

0
4

0

0

0
4

0

0

0
4

0

0

0
4

8.5265128291212e-14
5

0
4

0
3

0
3

0
4

0

0

0

0

0

0

0

0

0

0
4

0
4

0
3

0
3

0
3

0

0

0

0

0

0
3

0

0

0

0

0

0

0

0
4

0

0

0
4

0

0

0
4

0
4

0

0

0

0
4

0
4

0

0

0

0
4

0
4

0

0

0

0
4

0
4

0

0

0

0
4

0
4

0

0

0

0
4

0
4

0

0

0

0
4

0
4

0

0

0

0
4

0
4

0

0

0

0
4

0
4

0

0

0

0
4

0
4

0

0

0

0
4

0
4

0
3

0
3

0
3

0
4

0
4

0

0

0

0

0

0

0

0
4

0
4

0

0

0

0

0
4

0
4

0

0

0

0

0
4

0

0

0
4

0
4

0

0

0

0
4

0

0

0
4

0
4

0.0510167

0.0510167

0.0510167

0
4

0
4

0

0

0

0

0
4

0
4

0.117267

0

0

0
4

0.117267

0.117267

0
4

0
4

5.86489190546047e-13

0
4

0
3

0
3

0
3

0
3

0
3

0

0

0

0

0
4

0

0

0
4

0

0

0

0
4

0
4

0
4

0

0

0

0

0

0

0
4

0

0

0
4

0
4

0
4

0.0703603

0.0703603

0.0703603

0.0703603

0

0
4

0
4

0
4

0.0938138

0.0938138

0.0938138

0.0938138

0

0
4

0
4

0
4

0

0

0

0

0

0
4

0
4

0
4

0

0

0

0

0
4

0

0

0
4

0
4

0
4

0

0

0

0

0
4

0
4

0
4

0

0

0

0

0

0
4

0
4

0
4

0

0

0

0

0
4

0
4

0
4

0

0

0

0

0
4

0
4

0
4

0.0802428

0.0802428

0.0802428

0.0802428

0
4

0
4

0
4

0
3

0
3

0
3

0
3

0

0

0

0
4

0
4

0
4

0

0

0

0

0
4

0
4

0
4

0

0

0

0

0
4

0
4

0
4

0

0

0

0

0
4

0
4

0
4

0

0

0

0

0
4

0
4

0
4

0

0

0

0

0
4

0
4

0
4

0

0

0

0

0
4

0
4

0
4

0

0

0

0

0
4

0
4

0
4

0

0

0

0

0
4

0
4

0
4

0

0

0

0

0
4

0
4

0
4

0

0

0

0

0
4

0
4

0
4

0

0

0

0

0

0

0

0

0
4

0

0

0
4

0
4

0
4

0

0

0

0

0
4

0
4

0
4

0

0

0

0

0
4

0
4

0
4

0.0340111

0.0340111

0.0340111

0.0340111

0
4

0
4

0
4

0

0

0

0

0

0
4

0
4

0
4

0.0469069

0.0469069

0

0

0

0

0
4

0

0

0
4

0.0469069

0.0469069

0
4

0
4

0
4

0

0

0

0

0

0
4

0
4

0
4

0

0

0

0

0

0

0

0
4

0

0

0
4

0
4

0
4

0

0

0

0

0

0

0
4

0
4

0
4

0

0

0

0

0

0

0
4

0

0

0
4

0
4

0
4

0
4

8.89721
3

8.89721
3

0
4

0
4

0
4

0
4

0
4

0.696057

0

0

0

0

0

0

0

0

0

0

0

0

0

0

0

0

0

0

0
4

0.594024

0.134874

0.136044

0.0255083

0.0170056

0.0170056

0.0595195

0.0340111

0.0595195

0.0425139

0.0170056

0.0170056

0.0170056

0.0170056

2.77555756156289e-17

0
4

0.102033

0.0680222

0.0340111

0
4

0
4

0

0

0

0
4

0
4

0.264254

0

0

0
4

0.264254

0.264254

0

0
4

0
4

1.81193

0
1

0
1

0
4

1.74434

1.74434

0
4

0

0

0
4

0.0675858
4

0

0
4

0.0675858

0

0

0

0
4

0

0

0
4

0

0

0
4

0

0

0
4

0

0

0
4

0
4

0.386964
7

0.386964
7

0.386964
7

0
4

0
4

2.13308
7

2.13308
7

2.13308
7

0

0

0

0
4

0
4

0
4

0
4

0
4

0

0

0

0

0

0

0

0
4

0

0

0
4

0

0

0
4

0
4

0
4

0
4

0

0

0

0
4

0
4

0

0

0

0
4

0
4

0

0

0

0
4

0
4

1.56336
3

0.977139
3

0

0
3

0.496893

0
3

0

0

0

0

0

0

0

0

0

0

0

0

0

0

0

0

0

0

0

0

0

0

0

0

0

0

0

0

0

0

0

0

0

0

0

0

0.480246

0
4

0.586222

0.586222

0
4

0
4

1.88833
4

1.88833
4

1.88833
4

0
4

0
4

0
3

0

0

0

0

0

0
4

0
4

0.153233

0.153233

0.153233

0

0
4

0
4

0
4

0

0

0

0

0
4

0
4

0
4

0

0

0

0

0
4

0
4

0
4

0
4

2647.86

129.571

67.087

67.087

67.087

0
4

0
4

16.4312

8.21186
3

2.55526
3

0

0

0

0

0.0170056

0

1.07223

0

0

0

3.5888
3

0

0

0

0

0

0

0

0

0.0680222
3

0

0

0.893528

0

0

0.0170056

6.03683769639929e-16
3

0
4

0.0996883

0.0996883

0
4

8.11968

8.11968

0

0

0

0

0
4

0
4

46.0525

39.2092

16.4587

22.6406

0.0465023

0

0

0

0

0

0

0

0

0

0

0

0

0.0633753

0

0

0

0

0

0
4

6.84329

3.62986

0.606559

0.43834

1.70996

0.458572

0
4

1.33226762955019e-14

0
4

0

0

0

0
4

0
4

0

0

0

0
4

0
4

0
4

0.284297
1

0.0229265

0.0229265

0

0

0

0

0.0229265

0

0

0

0

0

0

0

0
4

0

0

0

0

0

0

0
4

0

0

0
4

0

0

0
4

0
4

0

0

0

0
4

0
4

0

0

0

0
4

0
4

0.0229265

0.0229265

0

0.0229265

0

0

0

0

0
4

0

0

0
4

0

0

0
4

0
4

0.0404642
8

0.0404642
8

0

0.0404642

0

0
4

0

0

0
4

0
4

0.0126753

0.0126753

0.0126753

0

0

0
4

0

0

0
4

0
4

0.109888

0

0

0
4

0.109888

0.109888

0
4

0
4

0

0

0

0

0

0
4

0

0

0
4

0
4

0.0278857

0.0278857

0.0278857

0

0

0
4

0
4

0.0475315

0

0

0
4

0.0475315

0.0475315

0
4

0
4

0

0

0

0
4

0
4

2.08166817117217e-17
1

0
4

3.69908
3

3.69908
3

2.00854
3

0

0

0

0

0

0

0.108195

0

0

0

0

0.110536

0

0

0

0

0

0

0

0.179883

0

0.0170056

0

0

0

0.0170056

0

0

0

0.0510167

0

0

0

0

0.117267

0

0.140721

0

0

0

0

0

0.620703

0.0170056

0.433642

0

0

0

0

0.0595195

0

0

0

0.136044

0

0

0

0
4

0
4

0
4

0
4

0

0

0

0

0

0

0

0

0

0

0
4

0

0

0

0

0

0

0

0

0

0

0
4

0

0

0

0

0

0

0

0

0

0

0
4

0

0

0

0

0

0

0

0

0

0

0

0

0

0

0

0

0

0

0

0

0

0
4

0

0

0

0

0

0

0

0

0

0

0
4

0

0

0

0

0

0

0

0

0

0

0
4

0

0

0

0

0

0

0

0

0

0

0
4

0

0

0

0

0

0

0

0

0

0

0
4

0

0

0

0

0

0

0

0

0

0

0
4

0
4

0

0

0

0

0

0

0

0

0

0

0
3

0
4

0
4

0
3

0

0
4

0

0

0
4

0

0
3

0

0

0

0

0

0

0

0

0
4

0

0

0

0

0

0

0

0

0

0

0
4

0

0

0

0

0

0

0

0

0

0

0
4

0

0

0

0

0

0

0

0

0

0

0
4

0

0

0

0

0

0

0

0

0

0

0
4

0

0

0

0

0

0

0

0

0

0

0
4

0

0

0

0

0

0

0

0

0

0

0
4

0
2

0
2

0

0

0
4

0.0850278
2

0

0

0.0850278

0

0

0

0

0

0

0

0

0

0

0

0

0

0

0

0

0

0

0

0

0

0

0

0

0

0

0

0

0

0

0

0

0

0

0

0

0

0

0

0

0

0

0
4

0

0

0

0

0
4

0

0

0

0

0

0

0

0

0

0

0

0
4

0.114633

0.114633

0
4

0

0

0
4

0

0

0
4

0

0

0
4

0

0

0

0

0

0

0

0
4

0

0

0

0
4

0

0

0

0
4

1.49088

1.36413

0.126751

0

0
4

0

0

0

0
4

0

0

0
4

0

0

0
4

0

0

0
4

2.22044604925031e-16
3

0
4

0

0

0

0
4

0

0

0
4

0
4

0

0

0

0

0
4

0
4

0

0

0

0
4

0
4

0

0

0

0
4

0
4

0
4

12.1112
3

8.33957
3

1.99731
3

0
2

0.162137

0

0

0

0

0.0581373

0

0

0

0

0

0.193791

0
3

0.0387582

0

0

0

0

0.444628

0

0

0.0387582

0

0

0.0310016

0

0

0.00764798

0

0

0

0

0

0

0.0775164

0

0

0

0

0

0

0

0

0

0

0

0.0170056

0

0

0

0

0

0

0

0

0

0

0

0

0

0

0

0

0

0

0

0.00305919

0.331608

0.0792192

0.0310016

0

0

0

0

0

0

0

0

0.0581373
4

0

0

0.0387582

0

0

0.206495

0

0

0

0

0.116275

0

0

0

0

0.0633753

0

0

0

0

0

1.38777878078145e-16
3

0
4

0
4

0

0
4

0

0
4

0

0

0
4

0

0

0
4

0

0

0
4

0

0

0

0
4

0
4

0
4

0
4

6.34226

6.34226

0
4

0

0

0

0
4

0

0

0
4

0

0

0
4

0

0

0
4

0

0

0
4

0
4

0.283303

0.283303

0.283303

0

0

0

0
4

0

0

0
4

0
4

3.41991

3.41991

3.41991

0
4

0

0

0
4

0
4

0

0

0

0

0

0
4

0
4

0

0

0

0
4

0
4

0

0

0

0
4

0
4

0

0

0

0
4

0
4

0

0

0

0
4

0
4

0

0

0

0
4

0
4

0

0

0

0
4

0
4

0.0683684

0.0683684

0.0683684

0
4

0
4

0

0

0

0
4

0
4

0

0

0

0

0
4

0

0

0
4

0
4

0

0

0

0
4

0
4

0

0

0

0
4

0
4

0

0

0

0
4

0
4

0

0

0

0
4

0
4

0

0

0

0
4

0
4

0

0

0

0
4

0
4

0

0

0

0
4

0
4

0

0

0

0
4

0
4

0

0

0

0
4

0
4

0

0

0

0
4

0
4

0

0

0

0

0

0

0
4

0

0

0
4

0
4

0

0

0

0
4

0

0

0

0

0
4

0
4

0

0

0

0

0
4

0
4

0

0

0

0

0

0
4

0
4

0

0

0

0

0
4

0
4

0

0

0

0
4

0
4

0

0

0

0
4

0
4

7.91033905045424e-16
3

0
4

261.835

0.373731
4

0.373731
5

0

0.178259

0.0404642

0.155008

0

0

0

0

0

0

0

0
4

0

0

0

0
4

0

0

0
4

0
4

214.966
7

0.102033
8

0.076525

0.0255083
8

0
4

0.0734847
1

0.0734847
1

0

0

0

0
4

0
1

0

0

0

0

0

0

0

0

0

0

0

0

0

0
4

0.357411

0.357411

0
4

207.222
7

2.86161
7

155.319
7

13.7171
7

0

0.0243376

0

0.01833

0

0

0.0138197

0

0.014664

0.357411

5.13574
7

0

0.007332

0

0

0

0

0

0

0

0.007332

29.0346
7

0.069654

0

0

0

0.414339
7

0.127874
7

0.0213627

0.0606962

0.0172746

0

0
4

5.8546

5.32324

0.173954
7

0

0

0

0

0

0

0

0

0

0.357411

0

0
4

1.07223

1.07223

0

0
4

0.284009

0.269345

0.014664

2.42861286636753e-17

0
4

0

0

0
4

0

0

0
4

0
4

6.9222
7

2.4632
7

2.33878
7

0.0469069

0.0775164

0
4

4.459
7

4.459
6

0

0
4

0
4

34.6134

0.311349
1

0

0

0

0

0

0

0

0

0

0

0

0

0

0

0

0

0

0

0

0

0

0

0.0732585

0

0

0

0

0

0

0

0

0

0

0.0366292

0

0

0

0

0

0

0

0

0

0

0

0

0

0

0

0

0

0

0

0

0.201461

0

0

0

0
4

2.19973

0

2.0209

0

0.0443759

0.0579265

0.0425139

0

0

0.0340111

0

0

0
4

32.1023

4.14463

1.28704

1.64717

0

0.536117

0

0.0170056

0

4.69058

5.31303
7

0.289045

11.2098
5

0.28598

1.78918

0.892792

0

0
4

0

0

0
4

0

0

0
4

0
4

0.490058

0.490058

0.320003

0.136044

0.0340111

0

1.38777878078145e-17

0
4

0

0

0
4

0
4

0.0581373

0.0581373

0.0581373

0
4

0
4

0

0

0

0
4

0
4

0

0

0

0
4

0
4

0

0

0

0
4

0
4

0.526772

0.406961

0.406961

0

0
4

0.119811

0.119811

0
4

0
4

0.378287

0.378287

0.275118

0.0229265

0.0802428

0
4

0
4

0

0

0

0

0
4

0

0

0
4

0
4

0

0

0

0
4

0
4

0

0

0

0
4

0
4

0

0

0

0

0
4

0
4

0

0

0

0
4

0
4

0

0

0

0
4

0
4

3.506
7

3.506
7

3.506
7

0
4

0
4

0
4

695.496
3

2.50838
3

0

0

0
4

0.0366292
3

0
3

0

0

0.0366292

0
4

1.20037
2

0.119811
2

0
2

0
2

0.860787

0.183146

0

0

0.0366292

0
4

0

0

0

0
4

1.19045

0

1.19045

0
4

0

0

0

0
4

0.0809283

0.0809283

0
4

0

0

0
4

0

0

0
4

0

0

0
4

1.66533453693773e-16
3

0
[truncated: 353,560 more chars]
