# Supplementary material for: Fungal communities decline with urbanization—more in air than in soil
Source: ISME J. 2020 Aug 5;14(11):2806–15. doi: 10.1038/s41396-020-0732-1 (PMC7784924; doi:10.1038/s41396-020-0732-1)
Supplement: Supplementary file 2 — Supplemental data [file 41396_2020_732_MOESM2_ESM.zip › Krona_AirNaturalEdge.html]

Javascript must be enabled to view this page.

num
probth


16410

8059.31

0.855149
3

0.855149
3

0.855149
3

0.855149
3

0.855149
3

0

0

0

0

0

0

0

0

0

0

0

0

0

0

0

0

0

0

0

0

0

0

0

0

0

0

0

0

0

0

0

0

0

0

0

0

0
4

0
4

0

0

0

0
4

0

0

0
4

0
4

0

0

0

0
4

0
4

0
4

0
4

0

0

0

0

0

0

0
4

0
4

0
4

0
4

0

0

0

0

0

0
4

0
4

0
4

0

0

0

0

0
4

0
4

0
4

0
4

6174.54

18.4907

13.3035

11.5185

0
3

0
2

0

0

0

0

0

0.0347507

0

0

0

0

0.0756917

0

0

0

0

0

0

0

0

0

0

0
3

0

0

0

0

0

0

0.065547

0

0

0

0.466306

0

0

0

0

0

0

0

0.0163868

0

0

0

0.0409669

0

0

0

0

0

0

0

0.0163868

0

0.0843952

0

0

0

0

0

0

0

0

0

0

0.83999
7

0

0

0.0173753

0

0.0434384

0

0

0

0

0

0.119382
7

0

0.0163868

0

0

0

0.0245801

0

0.0163868

0

0

0.58321
7

0

0.0163868

0

0

0

0

0

0.00997446

0

0

0

0

0

0.0163868

0

0

0

0

0

0

0.0347507

0

0.131094

0

0

0

0

0

0

0

0

0

0.0163868

0.536237

0

0

0

0

0

0

0

0

0

0

0.0524242
6

0

0

0

0

0.0327735

0.0163868

0

0

0

0

0

0.0163868

0.00710613

0

0.0245801
6

0.0423545
7

0

0

0

0.526002
1

0

0
2

0

0

0

0

0

0

0

0.0235571

0
3

2.64646

0.116855

0.168658

0

0

0.683922

0.213028

0

0

0

0
2

0

0

0.122901

0.0129839

0

0

0

0.245801

0

0

0
2

0

0

0.0213864

0

0

0

0

0

0

0

0.14038
7

0.0163868

0

0

0

0

0

0

1.50758

0

0

0.22736
6

0

0

0.114707

0

0

0

0

0.0491603

0

0

1.03237
6

0

0

0

0

0

0.234567

0

0

0

0

0
4

0
3

0
3

0

0

0

0
4

0

0

0
4

0

0

0
4

0.682776

0.682776

0
4

0

0

0
4

0

0

0

0
4

0

0

0

0
4

0

0

0

0
4

0

0

0
4

0

0

0
4

0.204834

0.204834

0
4

0
6

0

0

0

0

0
4

0

0

0
4

0

0

0

0
4

0

0

0

0
4

0

0

0

0
4

0

0

0
4

0

0

0

0
4

0

0

0

0
4

0

0

0
4

0

0

0
4

0

0

0
4

0
2

0

0

0

0

0
4

0.0368421

0.0368421

0
4

0

0

0
4

0

0

0
4

0

0

0
4

0

0

0
4

0

0

0
4

0

0

0
4

0

0

0
4

0

0

0
4

0

0

0
4

0.132156
6

0.115769

0.0163868

1.73472347597681e-17
6

0
4

0

0

0
4

0

0

0
4

0

0

0
4

0

0

0
4

0

0

0
4

0.00498723

0.00498723

0
4

0

0

0
4

0

0

0
4

0

0

0
4

0

0

0
4

0.131736

0.0955644

0.0361715

0
4

0

0

0
4

0

0

0
4

0

0

0
4

0

0

0
4

0

0

0
4

0

0

0
4

0

0

0
4

0

0

0
4

0

0

0
4

0

0

0
4

0.409669

0.409669

0
4

0

0

0
4

0

0

0
4

0

0

0
4

0
7

0

0

0
4

0.182034

0.182034

0
4

0

0

0

0
4

0
4

0.080909
6

0.080909
6

0.080909
6

0
4

0
4

0

0

0

0
4

0

0

0
4

0
4

0

0

0

0
4

0
4

0

0

0

0

0
4

0
4

0

0

0

0
4

0
4

0

0

0

0
4

0
4

0.0259677

0.0259677

0.0259677

0
4

0
4

0

0

0

0
4

0
4

0

0

0

0
4

0
4

0

0

0

0
4

0
4

0

0

0

0
4

0
4

1.15444
6

1.14945
6

1.14945
6

0
4

0

0

0
4

0.00498723

0.00498723

0
4

1.30104260698261e-17
6

0
4

0

0

0

0
4

0
4

0

0

0

0
4

0
4

0

0

0

0
4

0
4

3.22

3.22

3.22

0
4

0
4

0

0

0

0
4

0
4

0.00498723

0.00498723

0.00498723

0
4

0
4

0.0245801

0.0245801

0.0245801

0
4

0
4

0

0

0

0
4

0
4

0

0

0

0
4

0
4

0.128319

0.128319

0.106932

0.0213864

0
4

0
4

0

0

0

0

0

0
4

0

0

0
4

0
4

0.19723

0.19723

0.19723

0
4

0
4

0

0

0

0
4

0
4

0.254349

0.254349

0.254349

0
4

0
4

0

0

0

0
4

0
4

0.0964573

0.0964573

0.0964573

0
4

0
4

0
4

3986.28
7

0

0

0

0

0

0

0
4

0

0

0

0
4

0

0

0

0
4

0

0

0

0
4

0

0

0
4

0
4

3850.25
7

3849.97
7

340.522
7

6.24903
7

0.496389
6

0

0

0

0

0

0

0

0

0

0

0.00736842

0.0552932
7

0

0

0.0173753

0.00950508

0

0

0

0.00748085

0

0.0199489

0.102104
7

0

0

0

0

0

0

0

0

0.243255

0.0695014

0.0790499

0.065547

0

0

0

0.0104881

0

0

0

0

0

0.0409669
7

0

0

0

0

0

0

0

0

0

0

0.238291

0

0

0

0

0.0160762

0.0163868

0.0347507

0

0

0

0.0870338
7

0

0

0

0.0327735

0

0.00736842

0

0.0189229

0

0

0.305146
8

0.0839044

0.416305

0

0

0

0

0

0.0324597

0.0189229

0

0.0409669

0.0262201

0

0

0

0

0.0142123

0.00748085

0

0.0163868

0

0.12913

0

0.0163868

0.0173753

0.00475254

0

0

0.156378

0

0

0

0.811772
7

0.032061
7

0.0163868

0

0

0

0

0

0

0

0

0

0.202344

0.0163868

0

0

0

0.0409669

0

0

0

0

0

0.0862552
6

0

0.0434384

0.0110526

0

0

0

0

0

0

0.125263

0.111304
7

0

0

0

0

0

0

0

0

0

0

0.0491603
7

0

0

0

0

0

0

0

0

0

0

0.0763603
8

0

0

0

0

0

0

0

0

0

0

0.0570244
8

0

0

0

0.0434384

0

0

0

0

0

0.026063

0.194224
7

0

0

0

0

0.0104881

0

0

0

0

0.0129839

0.172221
7

0

0

0

0.0901271

0.0378459

0

0

0

0

0

0.0283844
6

0

0

0.0245801

0

0

0.0189229

0

0

0.0173753

0.00997446

1.10395
6

0.037564
1

0

0.0080381

0.00498723

0

0

0

0

0.0173753

0

0

0.336947
7

0

0

0

0

0

0.0283844

0

0

0

0

0.19201
7

0

0

0

0.0163868

0

0

0

0

0

0

0.285071
1

0

0

0

0

0

0.0194758

0

0.0160762

0.634527

0

0.509506
7

0.0194758

0

0

0

0

0.0173753

0

0.0174553

0

0.0173753

0.0623613
6

0

0.382258

0.605949

0.131094

0

0.00950508

0

0

0

0

0.0378459
8

0

0

0

0

0

0

0.0173753

0

0

0

0.752172
7

0

0.00498723

0

0

0.0573536

0.0129839

0

0

0

0

0.0641219

0

0

0

0

0

0

0

0.00710613

0

0

0.107502
6

0

0

0

0

0

0

0

0

0

0

0.390232
7

0.0383987

0

0

0

0

0.0189229

0

0

0

0

0

0.0986765
6

0

0.0245801

0

0

0

0

0.0157321

0

0.0149617

0

0.0344652
1

0

0

0

0

0

0

0

0.00710613

0.0104881

0

0.351498
7

0.0409669

0.0327735

0

0

0

0.0129839

0

0

0

0

0.178
7

0

0

0

0.0173753

0

0

0

0

0

0

0
7

0

0

0

0

0

0

0

0

0.0245801

0

0.148436
7

0

0

0

0

0

0

0

0

0

0

0.2379
5

0

0.0129839

0

0

0

0

0.0100064

0.026063

0

0.0189229

0
7

0

0.0347507

0

0.0491603

0

0.256957

0.0892155

0

0

0

0
6

0

0

0

0

0

0.0245801

0.00748085

0

0

0

0.59079
7

0.046632
7

0.00498723

0

0

0

0.065547

0

0

0

0

0

0.0324648
7

0

0

0

0.0173753
7

0.0996004
7

0.0571984
8

0.0588746
7

10.5496
6

0.0616311
6

0.0646803
7

0.00498723

0.745809
7

0.0275324
7

0.026063
7

0.0315876
7

0.0868767
7

0.01814

0.0409669

0.0999679

0.172061
7

0.281437
7

0.0384265

0.333427

0
7

0.114564

0.142872
7

0.0829224

0.121251
7

1.55269
7

0.021374
7

0
3

0.118525

0.13752
6

1.45259
7

0.139189
7

0.130942
7

0.166866

0.026063
7

0.00498723

0.135048
7

0.126544
7

0.133156

167.125
6

0.178814
6

0.65721

0.0665969
8

0.0408535
6

0.0730057
7

23.8197
6

0.212743

0.0163868

0.0221279

0.0284245

0.0275686
7

0.177362

1.33493
7

0.130273
7

0.0080381
7

0.0289293

0.230896
7

0.00736842
6

0.026063
7

0
2

0.00475254

0.0189229

0.00615279
8

9.53285

0.92033
7

0.0785401
6

0.196641
6

0
8

0.0104881
7

0.105427
7

0.0189229
7

0.569222
8

0.0851532
6

0.38604

0.7727
6

0.611213

0.0481909

0.123889

0.0259633
6

0.0150095
8

0.0389516
6

0.0130253
7

0.0418285
7

0.0387924
6

0.0593308
8

0.148873
7

1.16408
7

0.0539508
7

0.0245801
8

0.0672569

0.0104881
6

0.104473
7

0.0118814

0.0446684
7

0.0916293
7

0.0457194
2

0.0241147

0.335617
7

0.0129839

0

0.167452
6

0.0177653
7

0.172299
8

0.150941
7

0.0542326

0.0326333

0.0786637
7

0
7

0.884007

0
1

0
8

0.189047
7

0.105929

0.0163868
7

0.052126
7

0.0452387
6

0.0327735
7

0.232075
7

0.0910777
7

0.59815
7

0.151566
7

0.0323729

0.0514549
7

0.0503139
6

0.366586
7

4.13941
6

0.136923

0.0347507

0.0484256
7

0.00997446
7

0.566966
7

0.112777
6

0.0737404
7

0.143121
7

0.0152406
7

0.030955

0

0.173753
6

0
8

0.131094
6

0.232267
7

2.13779
7

0.170306
7

0.149266
7

0.0830627
7

0.0355681
7

0.233398
6

0.0218877
7

0.026063
7

0.0195035
7

0.336996
7

0.206666
7

1.74451
7

0.127152

0.0804148
6

0.0378459
7

0.17919
6

0.0956478
7

0.0298298
6

0
7

0.0263931
6

0.0520308
7

0.221202
7

0.508233
7

0.0249362
7

0.42053
7

0
7

0.135369

0
7

32.9972
6

0.0501961
7

0.0737404

0.103393
7

0
7

2.08475
7

0.354492
7

0.22132
6

0.184172

0
7

0
6

0.0278634
7

0.217231

0.0426742
6

0.0260291
7

0.142991
5

0.0836375
7

0.287883
7

0.208504
7

0.0589358
7

0.065547
7

0
8

0.026063
8

0.027055
7

0.0668175

0.0644598
7

0.0794758
1

0.0259677

0.477215
7

0.11389
7

0.242914
7

0.00710613
6

0.0254135
7

0.101781
7

0.0385311
5

0.097959

0.029411
7

0
6

0
6

0.439973
7

0.0901271
7

0.180254
8

0.0184584
6

0.0163868

0.00498723
1

0.0800313
7

0.38792
7

0.0378459
7

0.0142123

0.196349
7

0.194916
7

0.0156464

0.148123
6

0.0858881
7

0.456257
7

0.0826044

0.0245801
7

0.0439388
7

0.0485359

0.363415
6

0.0409669
7

1.16195
7

0.1058
7

0.284031

0.0670215
6

0.0231273
7

0.0149617
7

0.00498723
7

0.0224425
7

0.0444939
7

0.132969
7

0.0173753
6

0.379699
7

0.145124

0.0482459
7

0.0703132
7

0.0933277
7

0.223345
7

0.0983205
6

0.0189229
7

0.124715
7

0.0337621
2

0.170125

1.41348
7

0.151435
1

0.130315
7

0.131804
7

0.0480521

0.0818513

0
7

0.0324597
7

0.108295
7

0.0848043

0.278013
7

0.998375
7

0.0378459

0.0403122
7

0.377646
6

0.0471474
7

0.0708021
5

0
7

0.0911157
7

0.0955644
7

0.146132
2

0.0567688

0.872077
7

0.145561
7

0.052126
7

0.0269567
7

0.15313
5

0.124682
6

0.0513353
7

0.114707
7

0.00748085

0.0335099
8

0

8.17215
7

0.336686
7

0.0516261
7

1.68071
7

0.0598898
7

0.0163868
6

0.0411076
7

0.0603213
7

0.0189229
7

0.106052
7

0.833722
7

0.0423915
7

0.889498

0.0901404
7

0.0368789
7

0.168316
7

0.0163868
7

0.0853938
8

0.122999

0
7

0.0449956
7

0.178487
7

0.0189648

0.465067
7

0.0955644
7

0.00475254

0.157167
7

0.218959

0.0404655
6

0.15692
2

0.0946147
7

0.188425

0.140484
7

0.356195

0.251017

0.0643486
7

0.0106592
7

0.065547
6

0.137864
6

0.0341011
6

17.4191
7

0.03396

0.114707

0.0798014
7

0.0544071
7

0.40815

0.00748085

0.0651099
7

0.00748085
7

0.0908871
7

0.0914238
5

0.0283844
7

0.0106592
7

0.13363
2

0
7

0
7

0.523658
7

0.104252
6

0.0173753

0.00475254
7

0.0189229
6

0.0163868
7

0.0822705
6

0

0.267759
7

0.0245801
7

0.0378459

0.407749
7

0.121627
7

0

0.065547

0
2

0.0585022
7

0.0714113
7

0.0245801

0

0.208039
6

0.026063

1.22073
7

0.0194758
7

0
7

0.0539128
5

0.0559316
7

0.0157321
6

0.0568786
7

0

0.114706
7

0.0491603
7

0.108651
7

0.378734
7

0.065547
4

0.0573536
6

0.0378459
7

0.0850228
7

0.0259677

0.0177653
2

0.0703366
7

0.0129839

0.102671
7

0.0943576
7

0.475664
6

0.0852334
6

0.0473073

0.032417
7

0.0173753
6

0.0173753
7

0.00498723
7

0.0669346
7

0.286496
7

0.0683691
7

0.117531
7

4.70977
7

0.897123

0.0437019
6

0.0768572
7

0.0571928
7

0.0129839

0.0347507

0.0819338
7

0
7

0.0364225

0
7

0.223408
5

0.194383
7

0.0745341
6

0.138421

0.026063

0.0288548
6

0.392232
7

0.968236
6

0.0283844
2

0
7

0.7281
7

0.0327735

2.30851
7

0.0921043
4

0.0149617
8

0.0310502

0.0901271

0.0534128
7

0
6

0.00498723
7

0.026063
7

0.0106592
8

0.132482
7

0.846183
7

0.0254135
7

0.520901
7

0.0327735
1

0.171804
7

0.0262201

0.0263612
6

0.0327735
6

0.0347507
7

0.062266
7

0.0491603
8

0.808033
7

0.219197

0.0505445
7

0.117673
7

0.141839

0.00475254

0.052126
7

0
7

0.102873
6

0.333667
6

0.0241143

0.708512
7

0
8

0
8

0.0995074
6

0.204122
7

0.138062
7

0.440921
7

0.0195035
7

0
6

0.0872378
6

0.113538
7

0.111121

0.0245801

0

0.0173753

0.561111
7

0.0163868
7

0.066806
7

0.0259677
7

0.0918384
7

0.0958014
7

0.0857804
7

1.05887
7

0.0716028

0.0177653

0.0760362
7

0.0395658
7

0.0173753
6

0.0365511
8

0.0695014

0.0819338
7

0.00950508
6

0.0573536

0.718079

0.0627712
7

0.213921
7

0.0124681
7

0.0857459

0
7

0.0890227
6

0.199229
7

2.81917
7

0.106514
7

0.0864302

6.09212
7

0.0408473
6

0.153907
7

0.0388725
6

0.0624516

0.064877
7

0
1

0.104421
7

0.0383589
6

0.208749
7

0.085738
6

3.50507
7

0.15609
7

0.0689854
7

0.0209761
8

0.0284245
7

0.0209761
6

0.0209761
7

0
7

0.235726
7

0.139165
7

0.0452387

0.108355
6

0.523578
7

0

0.074729
7

0.444345
7

0.046746
7

0
7

0.00736842
7

0.0573536
7

0.11294

0.0482488
7

0.0129839

0.163476
7

0.0941462
6

0.0473073
6

0.0983205

0.0189229
7

0
7

0.0199489

0
6

0.00748085
7

0

0

0.14076
7

0.0611579
7

0.097379
7

0
8

0.0124681
7

0.286149

0.901104
6

0.0383987
7

0.0327735
5

0.113315
7

0.690486
6

0.308063
7

0.0157321
7

0
6

0.0719713
7

0.0347507
7

0.245616
7

0.00710613
7

0.147848
6

0
5

0.0129839
8

0.0677098
7

0.767164
7

0.0666992
6

0.00997446
8

0.170806
7

0.0901271
7

0
6

0.0367082
7

0.109991
6

0
7

0.0473073

0.0499306
7

0.538316
7

0

0.0173753
7

0.104385

0.0401495
7

0.0953592
7

0
7

0.0485051
6

0.0245801
7

0.0485056
7

0.20414
7

15.437
7

0.0402079

0.305877
7

0.126855

0.100857
7

0.0993091

0.131094
6

0.0457574

0.0189229

0.128965

0.14404
5

0.198332

0.0129839

0.00710613
6

0.156378
6

0.0173753
1

0.229852
6

0.0509839
7

0.0511374
8

0.187735

0.0935872

0.283685

1.99018
7

0.0177653
2

0.0496108

0.221221

0.0983205

0.243255
7

0
7

0
7

0.0944018
7

0.0569172
7

0.0645207
6

1.5209

0.881764
7

0.0279934
7

0.106573

0.0852133

0
6

0.0434384
7

0.0173753
6

3.39751
6

0
7

0.0998034

0.0347507
7

1.11823

0.0519355
7

0.17203
7

0

0.00475254
7

0.111586

0.0839109
7

0.227136
7

0
8

0

0.0367082

0.286293
7

0.399501
6

0.229822
7

0.0787854
7

0.0434384

0.113538
5

0.0278634
7

0
7

0.00710613
6

0.0491603
6

0.0889772
2

0.153677
7

0.0104881
7

0.0514549

0.0270459
1

0.0434384
6

0.0579665
7

0.100298
6

0.280502
7

0
2

0.0659083

0.0129839
7

0.417252
7

0.0637309
7

0.0434327
7

0.140722
8

0.0466827
7

0.0104881
7

0.0925181
7

0.0163868
6

0.0189229
7

0.00498723
1

0.0365511

0.68228
7

0.0983205
7

0
7

0.047107

0.0603249

0.065547
6

0.0249362
7

0

0.0608137
7

0.0123056
7

0.00997446
7

0.364546
7

0.0509413
6

0.133869
6

0.0607184
6

0

0.0362983
7

0.0419522
7

0

0.173753

0
1

0.176757
7

20.5501
7

0.0958786

0
6

0.0783297
8

0.625512

0
8

0
1

0.0442105

0.0245801
6

0.0358028
7

0.00748085
7

5.84369
6

0.0163868
7

0.0173753

0.0104881
7

0.104252

0.0673281
7

0.0129839
7

0

0.0695014

0.0454436

0.0189229
7

0.582281
7

0.0242186
7

0.155674
7

0.0390469
7

0.0955644
7

0.547242
6

0.222693
8

0.0129839
8

0

0.00710613
7

0.0314642
6

1.29866
7

0.450384

0.0573536

0
1

0.0163868

0.0149617
7

0.109974
8

0
5

0.310075
7

0
6

0.362149
7

0.0299234

1.29797

0.0993091
7

0
6

0.0129839
7

0
7

0.0812791
7

0.270116
7

0.0647216

0.0242283
7

0.0379178
7

0.0819338

0.482099
7

0.0266175
5

0
7

0
7

0.07461

0.175554
7

0.0163868
7

0.021374
7

0.665475
7

0
6

0.097112
6

0.710472
7

0

0.0505445
7

0.667962
7

0.198124
7

0.0104881
2

0.0440559
6

0.0283844
7

0.00710613
6

0.0506471
7

0
8

0.323829
7

0.00997446
7

0.142793
7

0.127973
7

0
7

0.0641219

0.0173753
8

0.0819828
4

0.0491603

0.0104881
7

0.0104881
8

0.541492
7

0.0200127
7

0.107347
6

0.0163868
7

0.012116

0.0348459
6

0.0211393

0.0299234
6

0.0378459
7

0.0567688

0.0785309
7

0.287757

0.0409669

0.0403122
7

0.0245801
7

0.344122

0.130738

0.0471197
7

0.0173753
7

0.0505696
7

0
7

0.225067

0.448881

0.0118587
7

0.0403122
7

0.0364558
6

0.0819338
7

0.0410184
7

0.0347507
7

0.158146

0.0971767
7

0
7

0.0491603
7

0.394311
6

0.101771
5

0.0491603
6

0.0445869
6

0.123575
6

0.0767029
5

0.0497429
7

0.0766075
6

0.0475806

0.0828318
7

0.026063

43.8584
6

0.122901
2

0.0173429
6

0.0163868
8

0.00475254
7

0.0295821
7

0.0150095
6

0.113063
7

0.0209761
7

0.0163868

0.0189229

2.48612
7

0.927288
7

0
6

0.00498723

0.128338
6

0.00498723

0.131455
6

0.0245801

0.0584274

0.0209761
8

0.026063
8

0
7

0.609877
7

0.052126

0.0983205

0.0244815
7

0
6

0.00997446
7

3.11167
6

0
7

0.0104881
6

0.0409669
7

0.341138
6

0.192432
7

0.0409669
8

2.17979
6

0.0434327
7

0
7

0.0374653
7

0.151494
7

0.0104881
6

0

0.237951
6

0.163342
6

0.316763

0
1

0.0189229
5

0.0401419
6

0.1442
7

0.0120572
6

0.0608137
6

0

0

0
6

0.12457
2

15.5518

0.278575
6

0.0435183
8

0

0.0503971
7

0.0448851
7

0.0160762
7

0.0473073
7

0.0177653
7

0.00498723
6

0.0245801
7

0.449445
6

0.0580101
7

0
6

0.0163868
7

0.0104881
8

0
7

0.0189229
6

0.026063
5

0.196932
7

0.0118814
5

0
7

0.301313
7

0.209791
7

0.0397379
7

0.0283844

0
7

0.026063
7

0.118169
7

0.193391
5

0.0189229
7

0

0

0.310136
6

0
7

0

0.0998034
6

0
6

0.0409669

0
8

0.032417

0

0
8

0
7

0.261163
7

0.155674
7

0.10898
6

0.204834
6

0.0347507
6

0.052126
6

9.75514
6

0.0245801
7

0.241001
7

0
8

0

0.171263
4

0.0236431
7

0.0173753
5

0.262849
7

0.0129839

0
1

0.0283844
6

0.037564
6

0.00710613

0.211488
7

0.0283844

10.745
7

0.951115

0.00498723
7

0.0422786
6

0
7

0.0173753
6

0.0874886
6

0.182346
7

0.0901271
7

0.0737404

0.0409669
6

0.0762766

0.24101
7

0.0781891

0
6

0

0.0672104
7

0
7

0
7

0.0303592
7

0.0129839
8

0.114911
7

0.00710613
6

0.397837
7

0.0347507
8

0.00498723
7

0.00712881
8

0.0347507
6

0.00997446
6

0.0163868

0.032061
6

0
1

0.125362
7

0.0142123
7

0.248911
7

0.0409669
6

0.0577531
7

0.0573536
7

0.0209761
7

0.0331369
6

0.00498723

0.0149617
6

0.0635891
7

0
7

0
8

135.669
7

0.0194758
8

0.0573536
7

0.173753
7

0.0695014
7

0.026063
6

0.0377607
6

0.026063
8

0.163868
6

0.161522
6

0.0378459
1

0.343645
7

0

0.0911157

0.0768294
7

0.321973
7

0.466928
6

0.0209761
7

0
7

0.0245801
7

0

0.0163868
7

0.308409
6

0.0899719
7

0.184228
7

0
7

0.0194758
7

0.0283844

0.0983205
7

0.0491603

0
7

1.01258
6

0
1

0.598058
7

0.102671
7

0.0621062
7

0.0434384
7

0
8

0.0520308
7

0.0534601

0.143024
6

6.37316
7

0.026063
7

0.0434384
7

0.279845
6

0.165066
7

0
7

0

1.24976
7

0
7

0.0199489
8

0.0536736
7

0
7

0.140666
7

0.026063
7

0.289325
5

0.101126
7

0.139287
7

0.0142123
2

0.0422315
7

0
6

0
7

0.0173753
7

0.128297
6

0.0106592
7

0

1304.47
7

12.0367
7

0.418181
6

0.0177653
7

0.0389356
7

0.0246112
7

0
7

0.0362983
6

0.0163868
7

0.0426368
7

0
7

0.0173753
7

0.0516261
6

1.65792
8

0
6

0.0819338
6

0.069235
7

0.00475254
7

0

0.114383

0.0983205
7

0.0199489
5

0.0171352

0.155674
7

0.131329

0.0395032
7

0.0823695
7

0.0163868
6

0.026063
8

0.0716018
7

0.281041
7

0
4

0.00950508
7

0
6

0.100298
7

0.332701
7

0.037662
7

0
7

0.0409669
1

0.0327735
7

0.0598898
6

0.253995
6

0.0104881
7

0.0437042
6

0.042778
7

0.0505445

0.463281
7

0

0.344122
6

0.0157321
8

0.0447712
7

0.0529645
6

0.195815

0.0173753
7

0

0.0491603
7

0.0104881
8

0.513576
7

0.026063

0.0104881
7

0.0756917
7

0.188448

0.0106592

0
8

0.0148493

0.0481909
6

0

0

0.17662
7

0.0491603
7

0.0173753

0.0491603
7

0.102529
7

0.0324597
5

0.0163868
6

0.0317089
7

0
6

0.172061

0.0191367
7

0.398055

0.0613552
7

0.00710613

0
7

0.0383266

0

0
7

0.026063
7

0.0906486
6

0.0110526
7

0.0209761
7

0.373866

0.0163868
6

0.0888342

0.0129839
6

0
2

0.0129839
7

0
8

0.0173753
6

0
1

0.134243
6

0.0389516
6

0.296734
7

0.0670299
7

0.0189229

0.0234929
8

0
7

0.0557184
7

0.0163868
6

0.00710613
7

0.0124681

0.00748085
7

0.00710613

2.3656
7

0.212234

0
7

0.179475
6

0.0177653

0.0335439
7

0
8

0.0179711
7

0
7

0.478899
7

0.00498723
6

0.0231273
7

0.202768

0

0

0.429462
6

0.0166339
7

0
7

0.0341011
7

0.0491603
6

0

0.0327735
7

0

0.292395
7

0
8

0.00710613
7

0.0695014
7

0.0303592
6

0.0409669
6

0.254843

0.147481

0.130315
6

0.0259677
7

0.0383987
7

0.259285
7

0

0.125437

0
5

0.097608

0.0303592
7

0

0.00498723
7

0.0662303
6

0

0.00748085
7

0.303814
7

0.0149617
2

0
7

0.0173753

0.0347507

0

0.0173753
7

0.0381961
7

0.0221053

0.399633
7

0
7

0.317669
7

0.0612015
7

0.00710613
7

0
7

0.00712881
1

0.0313484
7

0

0.0940815

0.0118814

0.0122334

0.0540064
5

0.265594
7

0.0852717
7

0.0316863
7

0.0173753
1

8.07514
6

0.0149617
7

0
4

0.0491603

0
8

0.00997446
7

0
7

0.781215
7

0

0.155674
7

0.0152406
6

0

0.0259677
7

0.152271

0.0511374
6

0.0327735
2

0.0605727
6

0.0173753
8

0.0787276
7

0.0525115

0
7

0.00498723
6

0.0173753
1

0.0573536

0.0327735
7

0.272919
7

0.16743
7

0.0785309

0.0142576
8

0.38509

0
7

0.052126
7

0
8

0.0173753
7

0.0256687

0
8

0.0209761
7

0.00710613

0.0129839

0.00712881
8

4.77487
7

1.17198

0.00498723

0
8

0.0163868
7

0.038593
6

0.0129839
8

0.052126
8

0.0271961
6

0
8

0

0
6

0.426534

0.09161

0.0245801
7

0.133071

0.0209761

0.00475254
7

0.0299639
6

0.026063
7

0

0.0695014
6

0.159362

0.319214
7

0.0737404

0.116306
6

0.0629283
7

0.00475254
7

0.00712881
6

0.0262201

0.0129839
7

0
7

0
8

0.0347507
7

0.135619
7

0
7

0.0163868

0.0629283

0.0491603
7

1.30052
6

0
6

1.17322
6

0.0565287

0.0163868
6

0.0104881
5

148.173
6

0.153326

0.0150095

0

0.282801

0.131094
7

0

0.0819338

0.0080381
2

0
1

0.0737869

0.522401
7

0
7

0
7

0
6

0

0.711882
7

0.0583422

0.0223626

0.00950508
7

0.02009
6

0.052126
7

0.170138
7

0

0.0157321

0

0

0
7

0.00615279
6

0.0245801
7

0.218467
7

0.0378459

0.5361

0.612124

0.0491603

0.0299234
7

0

0.0163868

0.0345865
7

0.00498723
7

0.139003
7

0.15539
6

0.163049
7

0.00498723
6

0.79213
7

0
5

0.00710613
8

0

0.0316863

0

0
7

0

0.0163868

0

0.0189229

6.01275
7

0.0239102
6

0.0254135
7

0.166701
5

0.106514
7

0.180254
6

0.0734266
6

0.0378373
7

0
7

0.0163868
7

0.0347507
6

4.4538
7

0.228043
7

0
7

0.199758
7

0.122544

0

0
7

0
7

0.211698
5

0.0194758
7

0.0080381
7

0.0189229
7

0.223188

0.0868767

0.0215348
8

0

0.0473073
7

0.052126

0.0868767
7

0.0894995
7

0.15057
6

0.201771

0.00748085
7

0.191738

0.0434384
6

0.0542326

0.0378459
7

0
8

0.0737404
7

0.188978

0
7

0.26931

0.0367222
7

0.0434384

0.556989

0.0514197
6

0.251943
7

0.0424498
6

0

0.109827

0

0
1

0.0425984

0.0245801

0.145325
7

24.8743
7

0.0551791

0.0347507
6

0
8

0.086921
6

0.861637
6

0.0104881
7

0.529948

0

0
8

0.0608784
6

0.625116
7

0.0118814
1

0
7

0
1

0.0635064

0.0216211

0.00498723
8

0.032061
7

0.0224425
7

0
6

0.0173753
7

0.420204
7

0
7

0.0779032

0.00997446
7

0.0423915
6

0.0491603
7

0.0324597

0.0585227
7

0
6

0.0157321

0.180958
6

0.674569
6

0.0142123
6

0
4

0

0.0129839
6

0
7

0
6

0.0695014

0.0110526
7

0.0373629
7

0
8

0.511492
6

0

0.0194758
7

0.184519

0.0695014

0.13693
6

0
7

0.00615279
1

0.11294
7

0

0.0367082
6

3.52591
6

0.0544474
7

0.0491603

0.026063
6

0
7

0.00736842
7

0.0498723
7

0.0570113
7

0
7

0.0173753
8

0.0602912
7

3.01754
7

0.515861

0.0487588

0.00710613

0.052126
6

0.0129839
6

0.107274

0.0950508
6

0.026063
1

0.0955644

0.0224425

0.0567688
7

1.52623
7

0.0194758

0
6

0

0.231951
7

0.0552212

0.0726531
7

0
8

0

0.102143
6

0

0.334418
7

0.0949148
7

0.0293327
6

0.0256159

0.037564
7

0.0245801
7

0.00498723
7

0.0179689
6

0.268353
6

0.0124681
7

0.00710613

0.412649
7

0.0163868

0.106514

0.0142349

0.0363357

0

0.0473073

0.340587

0.117513

0

0.152271

0.552525
7

0

0.0173753

0

0

0

0.0695014

0.0473417

0.254983

0

0.0347507

0.166924
7

0

0.426056

0.052126

0

0.026063

0

0.00710613

0.0793151

2.38252

0.062426

0.207226

0.0608137

0.026063

0.00748085

0.0144745

0

0.0100064

0.376625

0.0245801

0.101286

0

0.132444
6

0.501217

0

0.0173753

0

0.0189229

0

0.0184584

0.0154117

0.0695014

0

0.540537

0.00922919

0

0

0.0149617

0.0583422

0.0209761

0.052126

0.00710613

0.00475254

0.0189229

0.405509
7

0

0.0357639

0.0951995

0.0130253

0

0.0541739

0.0737404

0.0694817

0.0524724

0.0321188

2.15931
7

0.224676
6

0

0

0.00710613

0.0260517

0.22768

0.0163868

0.0104881

0.0129839

0

0.0558062

0.145973
7

0.0163868

0.0886437

0

0.061756

0.0124681

0.0511374

0.122901

0.0200953

0

0.0568124

0.590055
7

0.0422315

0.0555443

0

0.0104881

0

0.0173753

0

0

0.106514

0

0.0863854
7

0.0245801

0.0378459

0.00710613

0.0868767

0.14055

0

0.0319776

0.0234719

0

0

0.139668
7

0.0118814

0.0163868

0.047414

0.0120572

0.0314642

0

0

0

0.0157321

0.0756917

0.494892

0.0173753

0

0.0163868

0.0327735

0.0177653

0.819338

0.0173753

0

0.0652263

0.052126

0.139525
7

0.0258918

0

0.0303592

0

0.0177653

0.217818

0

0.0388725

0

0.122901

0.208138
7

0

0.133071

1.03438

0.0173753

0

0.154419

0

0

0.026063

0.0894995

0.244161
7

0.0173753

0.00997446

0.0983205

0.0420354

0

0

0

0

0.0866863

0

0.0700237
6

0.00475254

0.116903

0.0491603

0.0378459

0

0

0

0

0

0.245801

1.36491
7

11.5381
6

0

0.00475254

0.393302

0.0292553

0

0

0.0149617

0.0608137

0.0390436

0

0.664097
7

0

0

0.0157321

0

0.0918133

0

0.032061

0.026063

0

0.516183

0.241838
7

0.0308043

0

0

0.0819338

0

0

0.0149617

0

0.0173753

0.026063

0.259395
7

0.175711

0.172061

0

0

0

0

0.0508297

0.00498723

0

0.179669

0.271105

0.0157321

0.0634902

0.159134

0.0434384

0.368702

0

0.0209761

0

0

0.0199808

0.415091
7

0.0695014

0.0163353

0

0.0104881

0.014587

0

0.065547

0

0.065547

0.0347507

0.14423
7

0.0447712

0.0166408

0

0.0658009

0.00736842

0

0

0

0.0245801

0

0.169248
7

0.0378459

0.0337621

0.0235156

0.0697527

0

0.0173753

0.0353097

0.0124681

0.00712881

0.0173753

0.352602

0.0447712

0.0904775

0.0461898

0.0142123

0.0245801

0.242918

0.00748085

0

0

0.00736842

0.489202
7

0.026063

0

0.116684

0.0189229

0.0173753

0.0505479

0

0.0714113

0.0372411

0.117673

2.74896
7

4.59348

0.0319068

0.0195035

0

0.0150095

0.0104881

0

0.00748085

0

0

0.0234929

1.7377
7

0

0.0409669

0

0.146031

0

0

0.248594

0.14769

0.0245801

0.0491603

0.267909
6

0

0

0.0367082

0.0213184

0

0.0150095

0.0307203

0.00498723

0.0610853

0.073022

0.815788

0

0.072075

0.0273498

0.0149617

0.0124681

0.0106592

0.0593276

0.00475254

0.142759

0

1.02767

0

0.0433431

1.45397

0

0.0577954

0

0.026063

0.0288974

0.0389516

0.0623409

0.150112

0

0.172278

0

0.0259677

0.0249362

0

0.0195035

0

0

0.0100064

0.332041
7

0.0347507

0.0262201

0

0.028457

0.0349106

0

0.425696

0.0209761

0.0321188

2.40885

0.417919
7

0.0163868

0

0

0.065547

0

0.102398

0.0932162

0

0.00498723

0.0157321

1.1028
7

0.0634147

0.0245801

0.0819338

0.0641213

0

0

0.110142

0.121672

0.0389516

0.324696

0.0944964
6

0.00997446

0.0142123

0.356195

0.0695014

0.0571133

0.0597173

0

0

0

0.0324597

1.65144
7

0.29327
7

0.225746

0

0

0

0

0.026063

0.0283844

0.055216

0.00475254

0.00498723

0.0652337
7

0.0450286

0

0.00498723

0.0327735

0.0327735

0

0.1748

0

0

0.0166339

0.322396
7

0.0473073

0

1.15347

0.0529645

0.238312

0.0118814

0.00748085

0.0695014

0

0

0.154951

0

0.0473073

0.287757

0.0100064

0.0314642

0.0129839

0

0

0.0955644

0.0837148

0.309969
7

0.0283844

1.09791

0

0

0.0163868

0.0353097

0.00997446

0.0473073

0.0173753

0

0.557641

0

0.00997446

0.0173753

0

0.0781891

0.00712881

0

0.0100064

0

0

0.161035

0.136316

0.864678

0.00475254

0

0

0

0.0236431

0.0129839

0.0762119

0

0.423018
7

0.0283844

0

0

0

0

0.0284439

0.00710613

0.0901271

0.0213184

0.0163868

0.866688
7

0.14769

0

0.164856

0.0224425

0.0327735

0

0

0.0602912

0.142123

0.0303592

0.189761
7

0.0149617

0

0.0118814

0

0.00997446

0.0283844

0.00498723

0.0491603

0

0.0327735

1.33948
7

0.547214
7

0.163043

0.03396

0

0

0.0362983

0.131094

0.00710613

0.0104881

0

0.0157321

0.225227
6

0.0435955

0.0245801

0

0

0

0

0

0.0378459

0.0419522

0

1.39069
7

0.106514

0.0245801

0.0104881

0.0118814

0

0

0.0223626

0.00498723

0

0.00748085

0.176

0.0245801

0.0327735

0.00748085

0.11294

0.0163868

0.0946147

0.425696

0.00748085

0.0901271

0.0197621

0.289272
7

0.052126

0.147481

0.364882

3.39215

0.0851532

0

0

0

0.0129839

0.0157321

0.202097

0.0163868

0.0368512

0.0409669

0.0405263

0.153537

0

0.0163868

0.0434384

0.0522493

0

0.559532
7

0.0189229

0.0189229

0.065547

0.0662303

0

0.0695014

0.0283844

0

0.0695014

0

1.97626

0

0

0

0

0.0367082

0

0.0435031

0

0

0

0.19902
7

0

0

0

0

0.0189229

0.052126

0

0

0.104252

0.0784929

0.0236755
7

0.0163868

0.0189229

0.0189229

0.00498723

0.0398978

0

0.0150095

0

0

0

107.934

4.4496
7

0.410136
7

0

0.0596483

0

0.00748085

0

0

0.0289293

0

0.00710613

0.0389516

0.219196
7

0

0.139287

0.00498723

0.0163868

0

0

0

0.0173753

0.0245042

0.0347507

0.978555
6

0

0

0.0106592

1.60874

0

0.0150095

0

0.0245801

0.0473073

0

0.37715
7

0.0129839

0.155806

0

0

0

0

0.139287

0.0142123

0

0.120076

0.145807
7

0.0104881

0.0245801

0.00475254

0

17.097

0.0160762

0.0189229

0.0367082

0.143055

0.0163868

0.185895
7

0

0

0

0

0.0129839

0

0

0

0

0.0245801

0.151337
7

0

0.0983205

0

0.026063

0

0

0

0

0.0963718

0

0.135622
7

0.0199489

0.0104881

0

0

0

0.0189229

0

0.0224425

0.0409669

0.0337621

0.0708355

0

0

0

0

0

0

0.0400254

0.0157321

0

0

0.238161
7

0

0

0.0473073

0

0

0

0

0.086921

0

0.213028

4.53084
7

0.123219
7

0.0173753

0

0.106514

0.0209761

0

0

0.0283844

0.0262914

0.0259677

0.0173753

0.700062
6

0.00748085

0

0

0.558227

0.106514

0.0129839

0.0841904

0.0150095

0

0.0163868

0.479072
7

0

0.441452

0.0409669

0.0519355

0.0163868

0.0955644

0.0260291

0.0163868

0.0160762

0.0100064

1.48543
7

0.0608137

0

0

0

0

0

0.0327735

0

0.026063

0

2.19792
7

0

0.0361715

0.0149617

0

0.0245801

0

0

0.0901271

0.0363657

0.026063

0.14729
7

0

0.0695014

0

0

0

0.0150095

0.245801

0.0434384

0.0409669

0

0.283592
7

0.0331074

0.00475254

0

0

0

0

0.0110526

0

0

0.0851532

0.21483
7

0

0.0245801

0

0

0.0200953

0

0

0

0

0.0308156

0.388171
7

0.0435955

0

0.0608137

0.0189229

0

0.0263737

0.00498723

0

0

0

0.446806
7

0

0.0378459

0.0245801

0

0.0106592

0

0

0.0163868

0.0157321

0.0347507

3.49283

0.493657

0.00710613

0.0104881

0.0224425

0

0

0.0163868

0

0.0419522

0

0.0129839

0.529675
7

0.141922

0

0.0315662

0

0.0163868

0

0

0

0

0

0.116351
7

0

0.026063

0.00748085

0.00736842

0

0.0104881

0.0245801

0

0.052126

0

0.245147
7

0.0901271

0

0.0209761

0.0367082

0.0129839

0.565343

0

0.0189229

0.0259677

0

0.421978

0

0.119338

0

0.0345546

0

0.0080381

0.0163868

0.0163868

0

0

0.150839

0.0274298

0.0327735

0

0

0.0189229

0

0

0.0608137

0.00615279

0

0.112152
7

0

0.0163868

0.0100064

0.0733364

0.0129839

0.00615279

0

0

0

0.0177653

0.139349
7

0.0129839

0.0394806

0

0.0831763

0

0

0.646304

0.11294

0

0.0434384

0.324789
4

0

0.0491603

0

0

0.425654

0.114707

0

0.0163868

0

0

0.304354
7

0

0.0695014

0

0.0491603

0

0.0189229

0.0573536

0

0.0157321

0

1.54383
6

0.162504
7

0

0

0

0

0

0.0189229

0

0.026063

0

0

0.333551
5

0

0.0608137

0.0236755

0.0737404

0

0.0173753

0

0

0

0.0106592

0.482808
7

0.0983205

0

0

0.104661

0.00748085

0

0.0983205

1.6872

0.0283844

0.052126

0.117647
7

0.0394247

0

0.0166339

0.163868

0

0

0.0163868

0.00997446

0.0118814

0.0983205

0.0398558
7

0

0

0

0

0.0567688

0.0781891

0.0224425

0

0

0

0.0648616

0.00615279

0.0417951

0.0327735

0

0

0

0

0

0

0.188448

0.590489
7

0.0268748

0.0573536

0.0537496

0.0173753

0.0781891

0

0

0.0106592

0.0491603

0.071049

0.311881
7

0.0177653

0.132636

0.0830902

0

0.018421

0

0.0080381

0

0.0189229

0

0.211336
7

0

0.00950508

0.0104881

0.0882071

0

0.0173753

0

0.0457194

0

0.00475254

0.584502
7

0.026063

0

0

0

0.052126

0.0451491

0

0

0

0

1.4042

0.0799894
7

0.0245801

0.0327735

0.0507191

0.0173753

0.00712881

0.0173753

0.0142576

0

0

0.163868

0.0662303
7

0.11294

0.0473073

0.026063

0.147481

0.0256159

0

0

0

0.0245801

0

0.292414
7

0

0.0106592

0

0.0378459

0

0

0.0142123

0.0199489

0.0695014

0.0844001

0.0345865
6

0.0194758

0.286768

0

0

0

0.0327735

0

0

0

0

0.313588
7

0.00475254

0.00748085

0.0142576

0

0

0

0.180254

0

0.00475254

0.0461898

0.19403
6

0.0110526

0.065547

0.00710613

0.0163868

0.204834

0

0.0416889

0

0.00748085

0

0.0923829
7

0.0750477

0

0

0.0118814

0.026063

0

0

0.0491603

0.0189229

0.0509192

0.0906199
7

0.29891

0.0104881

0.065547

0

0.156378

0

0

0.0163868

0

0

0.115253
7

0.0189229

0.206736

0

0

0.0106592

0.221221

0.0163868

0.0163868

0

0.0163868

0.936896
7

0.0378459

0.00748085

0

0

0

0.0163868

0.0106592

0

0.0292553

0

2.41937
7

0.431539

0.0434384

0

0

0.0424498

0

0

0.0824488

0.0347507

0

0

0.338795
7

0

0

0.00498723

0.0946147

0.0519174

0.00922919

0.0283844

0.039007

0

0.00498723

0.0469036
5

0.0129839

0.0440836

0

0.0698421

0

0.0234719

0

0.0189229

0.0378459

0.0651099

0.0797978
6

0.157321

0.0350682

0.10695

0

0.0378459

0.00498723

0.115696

0.111773

0.00710613

0

0.397774
7

0.0189229

0

0.0245801

0.0608137

0

0

0.0209761

0.121627

1.17165

0

0.0727137
7

0.0334442

0

0.0173753

0

0

0.039204

0

0.720605

0.0163868

0.0163868

0.165767
7

0

0.0163868

0

0.0737404

0

0.110318

0

0.052126

0

0

0.130858

0

0.113995

0.0283844

0

0.0163868

0

0

0

0.331089

0

0.261986
7

0

0.00710613

0.0973648

0

0.00748085

0.0419555

0.0209761

0

0

0.482631

0.200148

0.0347507

0

0.0245801

0.00475254

0

0.0259677

0

0

0

0.0104881

3.64601
7

0.257804
7

0

0.00712881

0.106514

0

0.0124681

0.0851532

0.0104881

0.0129839

0

0

0.503064
7

0.0491603

0.131736

0.0118814

0.00498723

0.0118814

0.0173753

0.22588

0.00710613

0.0457574

0.0273498

0.495507
2

0

0

0.0347507

0

0

0.0608137

0.0324597

0.0419522

0.0129839

0.0781674

0.165472

0

0.0756917

0.0695014

0.065547

0.00748085

0.00997446

0.0245801

0.0104881

0

0.0432616

0.383744
7

0.0157321

0

0

0

0

0

0.0250159

0.0245801

0.180254

0

0.0682834
7

0.0189229

0

0.0146097

0.0163868

0.0324597

0.0321188

0

0.00997446

0.0675082

0

0.220862
7

0.00498723

0

0.00498723

0.0819338

0

0.0173753

0

0

0

0.0975176

0.186595
7

0.0124681

0

0.124895

0.0157321

0.0454436

0.00950508

0

0

0.0173753

0.0106592

0.14748

0.0163868

0

0

0.00498723

0

0

0

0

0

0.0150095

0.493315

0

0

0.0573536

0

0.0163868

0.0221053

0

0

0.052126

0

2.47008
7

0.0587412
7

0.51985

0

0.0163868

0

0.13231

0

0.0516964

0.0173753

0

0

0.216074
7

0

0

0.0209761

0

0.0177653

0.0129839

0

0.0080381

0

0

0.196482

0

0

0

0.0181588

0

0.0283844

0

0

0.065547

0.0409669

0.22203
7

0.0163868

0.0124681

0

0.390259

0

0.0259677

0

0.0901271

0.0189229

0

0.156061
7

0

0.0347507

0

0.0327735

0.0409669

0

0

0

0.0327735

0.0129839

0.306597
7

0

0.266846

0.0174553

0.0259677

0.0434384

0

0

0.0129839

0.0409669

0

0.259573
5

0.0544474

0

0

1.16151

0

0

0.0662303

0.026063

0.0866863

0.0409669

0.0412855
6

0.0104881

0

0.176838

0

0

0

0

0

0

0

0.322782
7

0.0106592

0

0.0189229

0.00922919

0

0.0189229

0

0

0

0

0.257517
7

0.0409669

0

0.0173753

0

0

0

0

0.0409669

0.052126

0

1.5873
7

0.229074
6

0

0.0245801

0

0

0.026063

0

0.0194758

0.0163868

0

0.0283844

0.219841
6

0.0129839

0.039007

0

0.00498723

0.052126

0

0

0

0

0

0.195399
7

0.0262201

0.0409669

0

0.139003

0.312756

0

0.230196

0.38792

0

0

0.0855947
7

0.283051

0.0283844

0

0.0104881

0.0299234

0.0347507

0

0.0163868

0

0.0163868

0.0961767

0

0.0174553

0.0327735

0

0

0

0

0

0

0

0.173443

0

0.0723148

0

0.0327735

0

0.00997446

0

0

0

0

0.0817667
6

0.0173753

0.0398978

0

0

0.052126

0

0

0.0142123

0.106592

0

0.19263
7

0

0

0.0434384

0

0

0

0

0.0189229

0

0.139003

0.734563
7

0

0.00498723

0

0

0

0

0

0

0

0.0173753

0.108579

0.0756917

0.0292553

0

0.0129839

0

0

0.0347507

0.0491603

0

0

1.97634
7

0.257502
7

0

0

0.196641

0

0

0.00712881

0

0

0

0.0173753

0.050015
7

0.0189229

0

2.20667

0.742194

0.00748085

0.0163868

0

0

3.64266

0

0.0406178

0.0737404

0

0

0

0.0378459

0

0

0.0173753

0.132461

0

0.183465
7

0

0

0.0106592

0

0.00712881

0

0

0

0.0163868

0.00475254

0.28337

0.0104881

0

0

0

0

0

0

0.0194758

0

0

0
7

0.0589936

0.0100064

0.0163868

0

0.0491603

0

0.0163868

0.0299234

0.0163868

0.0901271

0.223309

0

0.11294

0.0228382

0.0737404

0.162298

0

0.180254

0.0908871

0

0

0.0409669
8

0

0

0.188266

0.0901271

0

0.0163868

0

0.0608137

0

0.0163868

0.332498
7

0.00710613

0.0781891

0

0

0

0.00748085

0.0142123

0.0106592

0

0

0.971972
6

0

0.0118814

0

0

0

0

0.0293706

0.00997446

0

0

334.475

2.69508

0.0690718
7

0.0173753

0

0

0

0

0

0.0901271

0.0124681

0.0104881

0

0.152816
7

0.0573536

0.00498723

0

0.0283844

0

0.104252

0.0149617

0.0283844

0.0245801

0

0.403928

0

0

0

0.0163868

0

0

0.251943

0

0

0.0142123

0.186222

0.0695014

0

0.0149617

0

0

0.0173753

0.0434384

0

0

0

0.154127

0.0378459

0.0209761

0.0142123

0

0.0347507

0

0.0608137

0.0442096

0

0.0283844

0.136701
6

0.0199489

0

0.0173753

0

0

0

0.0245801

0.245801

0

0

0.0735125
7

0

0

0

0

0

0

0

0

0

0.0327735

0.106192

0

0

0.0173753

0

0

0

0.0110526

0

0.0416954

0

0.290852
7

0.0129839

0.053578

0

0

0

0.0768895

0

0.0473073

0

0.0353097

0.489622

0.00748085

0

0

0.262188

0

0.0245801

0

0

0

0.0530046

1.42325
7

0.820376
6

0.22588

0.0585106

0.0409669

0

0

0.0327735

0

0

0

0

0.341863
7

0

0

0

0.0104881

0.0434384

0.0713018

0.0147368

0.25421

0

0

0.151319

0

0

0.0129839

0

0

0.0245801

0

0

0

0

0.224681
7

0

0.00710613

0.0245801

0

0

0.0080381

0.0163868

0

0

0.0123056

0.222111
7

0

0

0

0.00498723

0.0157321

0

0

0

0

0

0.102637
7

0

0

0

0.0221053

0

0.0173753

0

0

0.0150095

0.00475254

0.13319
7

0

0.00710613

0

0

0.065547

0

0

0.0173753

0

0

0.319157
6

0

0.0409669

0

0.267445

0

0

0.0189229

0.0224425

0

0

0.130266
7

0

0.0194758

0

0.0491603

0

0

0.0327735

0

0.0327735

0

0.0351222
6

0

0

0.0283844

0.114393

0

0.0911157

0

0

0

0

1.8643

0.303764

0.00710613

0

0.0283844

0

0

0.180254

0

0.0194758

0

0

0.0149617
7

0

0

0

0.335928

0

0

0

0

0

3.83174

0.459892

0.0129839

0.0104881

0.0779032

0.0163868

0.0157321

0

0.0189229

0.026063

0.0501488

0.130315

0.119052
7

0

0

0

0.0983205

0.0166339

0.0118814

0

0

0

0.0573536

0.101986

0

0.0173753

0

0

0.0104881

0

0.127888

0

0

0

0.031862
7

0.0173753

0.0194758

0

0.0473073

0

0

0

0.0471962

0.00710613

0

0.326158
7

0.0189229

0.0955644

0.0262201

0.00712881

0.0245801

0

0.122901

0.0104881

0

0.0347507

3.10795

0

0.0163868

0.0573536

0.0245801

0

0.0173753

0

0

0

0

0.125968
6

0.0110526

0.0173753

0

0

0

0

0.00639934

0.0262201

0.0106592

0

0.134619
7

0

0

0

0.0245801

0

0.0173753

0.0471962

0.0491603

0

0

1.29399
7

0.0970596
7

0.0194758

0

0.00710613

0

0

0.0173753

0

0

0

0

0.317138
7

0

0

0

0.00475254

0.0327735

0.0400254

0

0

0

0.0194758

0.291978

0.0259677

0

0

0.0129839

0.104732

0

0

0.114707

0.0163868

0

0.681868
1

0.0163868

0

0.0142123

0.0245801

0

0.0897701

0

0.163868

0

0

0.22581

0

0

0.0327735

0

0

0

0.0347507

0

0

0

0.290787
7

0

0

0

0

0

0

0.0142123

0.0573536

0

0

0.395341
7

0.065547

0.0367082

0.0324597

0.00498723

0

0.0505445

0

0.00615279

0

0

0.311131

0.0245801

0.0163868

0

0.00712881

0.00710613

0

0

0

0

0.0245801

0.59758
7

0

0.0163868

0

0

0.00950508

0.0129839

0.196641

0

0.00475254

0.0080381

0.128027
7

0

0.0189229

0

0

0

0.182441

0

0

0

0.0604021

1.92516
7

0.237293
7

0.0195035

0

0.00736842

0

0

0

0.026063

0.0819338

0

0.00997446

0.243081
7

0

0

0.0314642

0.0262201

0.122901

1.93014

0

0

0

0

0.131452
7

0

0

0.026063

0.0378459

0

0

0

0.0157321

0.0955644

0

0.488845
7

0

0

0

0

0

0.0163868

0

0.0104881

0

0

0.191005
7

0.00498723

0

0.0189229

0

0.0293706

0.0163868

0

0.0100064

0

0

0.235998
6

0

0

0

0

0.114707

0

0.065547

0.0194758

0

0

0.0608137
6

0

0

0

0

0.0163868

0

0.00498723

0

0

0.0567688

0.15779

0

0

0

0

0

0

0

0.00498723

0

0

0.187836

0.0378459

0

0.0819338

0.0245801

0

0.0189229

0

0

0.00997446

0

0.161215
7

0.0262201

0

0

0

0

0.0662303

0

0

0.0347507

0

1.51682
7

0.123956
7

0.074729

0

0

0

0.0173753

0.0519355

0

0.00710613

0

0.0245801

0.0798898
7

0

0

0

0

0.0409669

0

0

0

0

0

0.365225
7

0

0

0

0

0

0

0

0

0

0

0.344279
7

0

0

0.0163868

0

0

0

0.0163868

0.0104881

0

0.00498723

0.626107

0

0

0

0.0458033

0

0

0

0.0497429

0.0163868

0

1.11597
7

0

0

0

0

0

0

0.0149617

0.026063

0

0.0245801

0.375763

0.0194758

0

0

0.00710613

0

0

0.0173753

0

0

0

0.461173
7

0

0

0

0

0.0908871

0

0

0.104252

0.721017

0.00710613

0.13863
7

0.0327735

0.0542326

0.026063

0.0901271

0

0.00475254

0.0189229

0

0

0

0.276076

0

0

0

0

0.0173753

0

0.14769

0

0

0

1.33019
7

0.119677
7

0

0

0

0.171747

0.0234929

0.25046

0

0.0327735

0.0942393

0

0.43626

0

0

0

0.0409669

0.0839044

0.0327735

0.0129839

0.1741

0

0

0.33544
7

0

0.179768

0

0.0225405

0

0

0

0

0

0.0194758

0.110146
7

0

0

0

0

0.00498723

0.0160762

0.0173753

0

0

0

0.263556
7

0

0.0163868

0

0

0

0

0

0.00615279

0

0.0104881

0.100632
7

0.0245801

0

0.0100064

0.0189229

0

0

0.00498723

0

0

0.0283844

0.139287

0

0

0

0

0

0.0163868

0

0

0.0695014

0

0.0661451

0

0

0.0819338

0.0120572

0

0.0163868

0.00498723

0

0.0259677

0

0.319335
6

0.0163868

0

0.213028

0.0327735

0.0080381

0

0

0

0

0.199273

0.199234
7

0

0

0.0104881

0.052126

0.0157321

0

0.0327735

0.0173753

0.595551

0

3.22315

0.170207

0.029411

0.00498723

0

0.00498723

0.00710613

0.0118814

0

0

0.0213184

0.0189229

0.138127

0

0

0

0.0189229

0.0189229

0

0

0

0

0

0.415817
7

0

0.0688569

0.0173753

0

0.0163868

0

0

0.0245801

0.0189229

0

0.187164
7

0.0891981

0.0173753

0.0118814

0

0.00498723

0.0473073

0.026063

0.269318

0.0652332

0

0.0459541
7

0

0

0

0.0173753

0.156028

0.237608

0.0106592

0

0.0786604

0

0.0836746

0

0

0.0623404

0

0

0

0

0

0

0.0567688

0.0543521
7

0

0

0

0

0

0

0

0.0209761

0

0

0.0868767
7

0

0

0.0514765

0

0.0245801

0.0149617

0

0

0

0

0.587494

0

0.0409669

0.0851532

0.0327735

0

0

15.0248

0.052126

0.0868767

0.0434384

0.11065

0.12119

0.067336

0

0.0925986

0

0

0.0491603

0

0.0163868

0.0163868

2.03038
7

0.0321188
7

0.0327735

0.0283844

0

0.0217385

0

0.0327735

0.270381

0

0

0.0104881

0.0439033
7

0

0

0

0

0

0.0173753

0

0.0542921

0

0

0.473201
7

0.0434384

0.0331691

0.032417

0

0.0194758

0.0347507

0

0

0.0104881

1.41064

0.161398

0.0608137

0

0.0129839

0

0

0.052126

0.0434384

0.0901271

0

0

0.052277

0.0189229

0.0157321

0.0209761

0.0389516

0

0

0

0

0.0283844

0.0080381

0.052126
7

0

0

0

0.0714113

0

0

0

0.434249

0.00712881

0

0.0347507

0.00498723

0

0

0

0.00498723

0

0

0

0.0163868

0.0209761

2.96121
5

0

0

0.0409669

0

0

0

0.0245801

0

0

0

0.208471
7

0

0

0.0270459

0

0

0.0189229

0

0

0

0

0.195222
7

0

0.0327735

1.12277

0.0129839

0

0

0.0104881

0

0

0.0106592

1.87603
7

0.825818
7

0.0194758

0

0

0.0584274

0

0

0.00736842

0

0.0324597

0.0310502

0.304764
7

0.0157321

0

0

0.026063

0

0.0163868

0.0758775

0

0

0.0149617

0.0919802
7

0

0

0

0

0.0163868

0

0

0.0106592

0

0

0.143001
7

0

0

0

0.0195035

0.0163868

0

0

0.0129839

0

0

0.118161
6

0

0

0.0340092

0

0

0.245801

0.0616311

0

0

0.026063

0.309291
7

0

0.130315

0.131094

0.0118814

0.0470391

0.00498723

0

0

0

0.0292553

0.0235156
7

0

0

0

0

0.00498723

0.00498723

0

0

0

0

0.225176
6

0

0

0

0

0

0.903518

0.0245801

0.0173753

0

0.00498723

0.0631092

0

0.026063

0

0.0607184

0.0403122

0

0

0

0.165066

0.0173753

0.299284
7

0.0819338

0

0.052126

0.0567688

0.0157321

0

0

0

0.0104881

0

36.6687

1.77034
7

0.17982
7

0

0

0.157697

0

0

0.0347507

0.0378459

0

0

0.00615279

0.287011
7

0

0.0978838

0

0.0147368

0

0

0.0149617

0

0.0573536

0

0.224539
8

0

0.0347507

0.0223626

0

0

0

0.0100064

0

0.0983205

0

0.126667
6

0.105801

0

0

0.0409669

0.0209761

0

0

0

0

0

0.910646
6

0

0.0173753

0

0.0471962

0.0163868

0.106514

0.0173753

0.0163868

0

0

0.0405054
7

0

0

0.026063

0

0

0.00748085

0

0

0

0

0.20605

0

0

0.0409669

0

0.0209761

0

0

0.00748085

0.0104881

0.0757176

0.264981

0

0

0

0

0

0.0189229

0

0

0.0173753

0.00498723

0.189826
7

0

0

0

0

0.0129839

0.0142123

0

0.00710613

0

0.00748085

0.116082
7

0

0

0

0.0189229

0.00615279

0.0312928

0.0634094

0.0362983

0

0

2.08099
7

0.0713315
7

0.0150095

0.0209761

0

0

0.00748085

0

0

0.0163868

0

0

0.0163868
7

0

0.0283844

0

0

0

0.0104881

0

0

0

0

0.0401506
7

0.0519355

0.0157321

0

0.0737404

0

0

0

0.0367082

0

0.483409

0.0744547

0

0

0

0.0414026

0

0

0.0573536

0

0

0.0129839

0.246396
7

0.0129839

0

0

0

0.0213864

0.0194758

0.0194758

0.286693

0

0.0283844

0.22222
7

0.0419522

0.00710613

0

0.0726758

0.00748085

0.0347507

0

0.121627

0.0157321

0

0.0852952

0

0.021374

0.00712881

0

0.0540976

0.0157321

0.0819338

0

0

0.00748085

0.152064

0.451759

0

0

0.0150095

0

0

0.0110526

0

0

0

0.465978

0.0245801

0

0.0142576

0

0.0434384

0.0389516

0

0

0

0.0327735

0.470433
6

0.57715

0

0

0.173753

0

0.00736842

0.00475254

0.00997446

0.139003

0

2.87582

0.575811
7

0

0.0434384

0

0.0189229

0

0.00748085

0

0.0163868

0.149315

0

1.50483
7

0

0

0

0.0983205

0

0.0163868

0

0

0.0471962

0.0394771

0.174099
7

0.00710613

0.065547

0

0.026063

0

0

0

0.0409669

0

0

0.199147
7

0

0

0.0457574

0

0

0

0

0.0100064

0.0491603

0

0.0189229
7

0

0.0194758

0

1.52034

0

0

0

0

0

0.00475254

0.230442

0.0194758

0

0

0

0.0218849

0

0

0

0.0104881

0

0.0177364
7

0

0.0378459

0

0

0.00498723

0

0

0

0

0

0.288302

0.0163868

0.0324624

0

0

0.0283844

0

0

0.00997446

0

0

0.0982034
7

0

0

0

0.0245801

0

0

0.0567688

0

0.0173753

0.00748085

0.0884289
7

0

0

1.4765

0

0

0.0347507

0

0.0573536

0

0

3.19083

0.231474

0

0.0209761

0.286693

0

0

0

0

0.0573536

0.026063

0

0.1074

0.449474

0

0

0.00997446

0

0

0

0

0.0209761

0

0.0244815
7

0

0

0

0

0

0

0

0

0

0

0.475426
7

0

0

0

0

0

0

0

0

0.0293706

0

47.6502
7

0

0

0

0.00475254

0.150883

0.0779032

0.0573536

0.00475254

0.026063

0

0.83566

0.0434384

0.00748085

0

0

0

0

0

0

0.0737404

0.0245801

0.617302

0

0.0949176

0.0608137

0

0

0

0

0

0

0

0.239585
7

0.0573536

0.0756917

0

0

0.0163868

0

0.0245801

0.0118814

0

0

0.16153

0

0.0142576

0

0

0

0

0.0473073

0

0

0

0.433039
7

0

0.026063

0.0245801

0

0

0.172061

0

0.0491603

0

0.0259677

2.21295
7

0.205626
7

0

0

0

0

0

0.155674

0

0

0

0

0.057255
7

0

0

0

0

0

0

0

0

0

0

0.0805104
6

0.00710613

0

0

0

0.0608137

0

0

0

0

0

0.12169
7

0

0

0

0

0.0173753

0.0124681

0.026063

0

0.0327735

0

0.135533
7

0

0

0

0

0

0

0

0

0

0

0.440783
6

0

0

0

0

0

0

0

0.0245801

0.0819338

0

0.484923
6

0

0

0

0.0245801

0

0

0

0

0

0

0.142625
5

0.0245801

0

0

0.639083

0

0

7.84226

0

0

0

0.0189229
7

0

0

0

0

0

0

0

0

0

0

0.109433
7

0

0

0

0.0562667

0

0

0.0163868

0

0

0

1.62549
7

0.879583
7

0

0

0

0

0.00498723

0.0284245

0

0.0157321

0

0

0.0612305
7

0.0409669

0

0

0

0

0.0473073

0

0

0

0

0.0484903
7

0.55585

0

0

0.0819338

0

0.0129839

0

0.85531

0.0163868

0

0.121361
8

0

0

0.00736842

0

0.0608137

0.0124681

0.00736842

0

0

0

0.13363
7

0

0

0

0

0.026063

0

0

0.251943

0

0

0.175996
7

0

0

0

0

0

0.00950508

0

0

0

0.0600381

0.108104
7

0.0150095

0

0

0

0

0

0

0

0

0

0.026063
2

0

0

0

0

0

0

0.00498723

0

0

0.0173753

0.0573536

0

0.00736842

0

0.0104881

0

0.0173753

0

0

0

0

0.0397207
7

0

0

0

0.0245801

0

0

0

0.0200127

0

0

1.55242
7

0.422901
7

0

0

0

0.0080381

0.0274298

0.00615279

0.0283844

0

0.173753

0

0.0279456

0.0163868

0.0473073

0

0

0

0

0

0

0.00498723

0

0.0149617
7

0

0.052126

0

0.0245801

0.00710613

0

0.0189229

0

0

0

0.0703375
7

0

0

0

0

0

0

0

0

0

0

0.260086
7

0

0

0

0

0

0

0

0.14769

0

0

0.0730968
6

0.0737404

0

0

0

0

0.0450286

0

0

0.026063

0.0129839

0.0428331
7

0.0602858

0

0

0

0.130315

0.0737404

0

0

0

0

0.288998

0

0

0

0

0.00498723

0

0

0

0

0.00748085

0.241257
7

0

0

0

0

0

0

0.026063

0

0

0

0.0629142
6

0

0.0245801

0

0.141922

0

0

0

0

0

0

1.14949
7

0.327124
7

0

0

0

0

0

0

0

0

0

0

0.140276
7

0

0.00498723

0

0

0

0

0

0.0409669

0

0

23.9006
6

0.0106592

0

0

0.0245801

0.0245801

0

0

0

0

0

2.38662

0

0.0150095

0

0

0

0

0

0

0

0

0.154743
6

0

0

0

0

0

0.0189229

0

0

0

0

0.129241
6

0.0120572

0

0.0368421

0

0

0.00498723

0.140667

0

0

0

0.102244
7

0

0

0

0

0

0

0

0

0.0173753

0

0.00498723
8

0

0

0

0

0

0

0.0249362

0.0189229

0

0

0.390939
7

0

0

0

0

0

0

0

0

0

0

0.534104

0.16879

0

0

0

0

0

0

0

0

0

0.562582

0.0685877
7

0

0

0

0

0

0

0

0

0

0

0.0365511

0

0

0.0129839

0

0

0

0

0

0

0

0.139287

0

0

0

0

0

0

0.0327735

0.0245801

0

0.0473787

0.303155
6

0.0174553

0

0

0

0.122901

0

0

0.0163868

0

0.00712881

0.619564
6

0

0.0194758

0

0

0

0

0

0

0

0

0.155514
6

0

0.0786604

0

0

0.0259677

0

0

0.0695014

0

0

0.143322
6

0.0409669

0

0.018421

0

0.0190102

0

0.0573536

0

0.00615279

0

0.0602032

0.0163868

0

0

0.00748085

0

0.0100064

0.0756917

0

0

0.0409669

0.102191
8

0

0

0.00950508

0

0

0.00710613

0

0

0

0

0.305189
7

0

0

0

0

0

0.0104881

0.0338404

0

0.026139

0

1.92862

0.0228382
6

0.0189229

0

0

0

0

0

0

0

0

0

0.221099
7

0

0

0

0

0.0213184

0

0.0955644

0

0

0

0.231723
7

0

0

0.0327735

0

0

0.0189229

0

0

0

0

0.34278
6

0

0

0.0245801

0

0

0

0

0

0.0259677

0

0.26978
7

0

0

0

0.065547

0.0189229

0.0104881

0

0

0

0

0.00950508
7

0

0

0

0.00997446

0

0

0

0

0.026063

0

0.0551361
7

1.22053

0.0163868

0

0

0.0324597

0

0

0

0

0.0163868

0.0530043
7

0

0

0

0.0327735

0

0

0

0

0

0.0868767

0.579877

0

0

0

0.0150095

0

0

0

0

0

0

0.171461
6

0.0189229

0

0

0.262188

0

0

0

0

0

0

24.1482
7

1.16469

0.198834
7

0

0.0491603

0

0

0

0

0

0.0367082

0

0.00748085

0.0567688
7

0

0.00498723

0.030764

0.0163868

0

0.0173753

0

0

0.0955644

0

0.150153
6

0

0

0

0

0

0.0347507

0

0

0

0

0.430815

0

0

0

0

0.0163868

0.00498723

0

0

0

0

0.13245
6

0

0

0.0427729

0

0

0

0

0.0189229

0

0

0.133565
7

0

0

0

0

0

0

0.0104881

0

0.0195035

0

0.150742
7

0

0

0.00498723

0

0

0

0

0

0

0

0.0955644
8

0

0

0.0245801

0

0.0662303

0

0

0

0

0

0.118642
7

0.0173753

0

0.0189229

0.0118814

0

0

0.00498723

0.0434384

0

0

0.391779
7

0

0

0

0

0.00498723

0

0.0194758

0

0

0

1.96631

0.247262
5

0

0.00498723

0

0.065547

0.00498723

0

0

0

0

0.0781891

0.062098
7

0

0

0.163868

0

0

0

0

0.0173753

0

0

0.100106
7

0.0519355

0.0157321

0

0

0

0

0

0.0163868

0

0

0.0936161
7

0

0.122901

0

0.0189229

0

0

0

0

0

0

0.143766
7

0

0

0

0

0.0347507

0

0

0.0080381

0

0

0.104252
6

0.0321524

0

0

0.0173753

0

0

0

0

0

0.0781891

0.261964

0

0.00498723

0

0

0

0

0

0.0283844

0.00498723

0

0.0770204
7

0

0

0

0

0.0194758

0

0

0.155674

0

0

0.151217
7

0

0

0

0

0

0

0.0104881

0

0

0.0173753

0.106077

0

0

0

0

0

0

0

0

0

0

1.02811
7

0.110894
6

0

0

0

0

0

0

0

0

0

0

0.0819338
7

0.065547

0

0

0

0

0

0

0

0

0

0
7

0

0.052126

0

0

0

0

0

0

0

0

0.115512
7

0.0327735

0

0

0

0

0

0

0.0409669

0

0.0283844

0.536492
7

0

0

0

0

0

0

0

0

0

0.026063

0.153027
7

0

0

0

0

0

0

0

0

0.0189229

0

0.18303
7

0

0

0

0

0

0.0245801

0

0

0.0781891

0

0.0669665
7

0.0245801

0

0

0

0.0347507

0.0299234

0

0

0

0.0409669

0.322352

0

0.052126

0

0

0.0434384

0

0

0

0

0

0.148549

0

0

0.0129839

0.0173753

0

0

0

0.0695014

0

0.0409669

5.38406
7

0.272812
6

0

0

0

0

0

0

0

0

0

0.0129839

0.0362167
8

0

0

0.0104881

0

0.0199489

0

0

0

0

0

0

0

0

0

0

0

0

0

0

0

0.0314642

0.126689
6

0.0194758

0

0

0

0

0

0

0

0.0213864

0

40.9611
6

0

0

0

0

0

0

0.139287

0

0

0

0.102663

0

0

0

0

0

0

0

0.0327735

0

0

0.0679198

0

0

0

0

0

0

0

0

0

0

0.109974
7

0

0

0.026063

0

0

0

0.00498723

0

0

0

0.0342624
7

0

0

0

0

0

0

0

0.0292553

0.00475254

0

0.175607
6

0

0

0

0.0259677

0

0

0.0434384

0

0

0

1.75965

0.768886
7

0

0

0.0262201

0

0

0

0.052126

0

0

0

0.136432

0

0

0

0.175532

0

0

0.0189229

0.0157321

0.0189229

0

0.0157321
7

0

0

0.0163868

0

0

0.00748085

0

0

0.0473073

0

0.185152
7

0.131101

0

0

0

0.0491603

0

0

0

0

0

0.107092
7

0

0

0

0

0

0

0

0

0.00950508

0

0.0728289
7

0

0

0

0.0173753

0

0

0

0

0

0

0.147481
7

0

0

0

0

0

0

0

0

0

0.00498723

0.103111
7

0

0.0173753

0

0

0

0

0.00997446

0.0573536

0

0

0.258584
7

0

0

0.0983205

0

0

0.0314642

0

0.0209761

0

0

0.204834
7

0

0

0

0

0

0

0

0.11294

0

0

1.22564
7

0.32399
7

0

0

0

0

0

0

0

0

0

0

0.481687
7

0.25421

0

0

0

0

0

0

0

0

0

0.329309
6

0

0

0

0.0163868

0

0

0

0

0

0

0.500319

0

0.189229

0.0104881

0

0

0

0.00498723

0

0

0.0157321

0.173863
7

0

0

0

0.0245801

0

0.0695014

0

0

0

0.0189229

0.213371
7

0

0

0

0

0

0

0.0173753

0

0

0

0.0705342

0

0

0

0.0327735

0

0

0

0

0

0

1.51382
7

0

0

0

0

0

0.0173753

0

0

0.0163868

0

0.142822
7

0

0

0

0

0

0

0

0

0.0189229

0

0.0499306

0

0

0.00498723

0.179768

0

0

0

0

0

0.0482286

1.4981
7

0.122901
7

0.0324597

0.0163868

0

0.0327735

0

0.0124681

0.0390837

0

0

0.0173753

0.320805
7

0

0

0

0

0

0

0

0

0

0

0.0785298
7

0

0

0

0

0

0

0

0

0

0.188448

0.0247003
1

0

0

0

0

0

0

0

0.0347507

0.0129839

0

0.167994
6

0

0

0

0

0

0

0

0

0

0

0.0589448
7

0

0

0

0

0

0

0

0

0

0.00615279

0.316009
6

0

0

0

0

0.0259677

0

0.0434384

0

0

0

0.164519
7

0

0

0

0

0

0.00498723

0

0

0

0.0124681

0.0355306
6

0

0

0

0

0.0173753

0

0

0

0

0

0.0924382
7

0.390837

0

0

0

0

0.0157321

0

0

0

0

1.53659
7

0.0649015
7

0

0.026063

0.0163868

0

0

0

0

0

0

0

0.0667563

0

0

0

0.0573536

0.0409669

0

0

0.0194758

0

0.0327735

0.322562
7

0

0

0.0781891

0.052126

0

0

0.694637

0

0

0

0.118544
7

0

0

0

0

0

0

0.039007

0

0

0

0.0616255
7

0

0

0

0

0

0

0

0

0

0

0.114302
7

0

0

0

0

0

0

0

0

0

0.0573536

0.936122
6

0

0

0

0

0

0

0

0

0

0

0.0695014
7

0

0

0.0163868

0

0

0

0

0

0

0

0.0450286
7

0

0

0.0129839

0

0.0283844

0.204517

0

0

0

0

0.0730857

0

0

0.00710613

0

0.0283844

0

0.00498723

0.265168

0

0

3.05006
7

0.0297766
6

0.0173753

0

0

0.0150095

0

0

0

0.0177653

0

0

0.306903

0

0

0

0.139287

0

0

0

0

0

0

0.14717

0.0245801

0

0

0

0

0

0

0

0

0.0104881

0.0756857
7

0.114707

0.00922919

0

0

0

0

0.0106592

0

0

0

0.00710613
7

0

0

0

0.0189229

0

0

0

0.00475254

0

0

0.113592

0

0

0

0

0

0

0

0.0173753

0

0

0.0174553

0

0

0

0

0

0.0189229

0

0

0.0283844

0

0.0492295
7

0

0

0

0

0

0

0

0

0.243255

0.0249362

0.0831763
6

0

0.00997446

0

0.0675082

0

0

0

0.0163868

0

0.0209761

0.0150095
7

0

0

0

0

0

0

0

0

0

0.0259677

1.56145

0.317171
7

0

0

0

0

0

0.0173753

0

0.0129839

0

0

0.0573536

0

0

0

0

0

0.0163868

0

0

0.0149617

0.0163868

0.0728783

0

0

0

0

0

0

0

0

0

0

0.0173753
7

0

0.00748085

0.00710613

0.00498723

0

0

0.00712881

0

0

0.0283844

0.0921843
7

0

0

0.0173753

0.0819338

0

0

0

0

0

0

0.228327
7

0

0

0

0

0

0

0

0

0

0

0.0352393
8

0.0419522

0

0.106932

0

0

0

0.0104881

0.00615279

0

0

0.576284
7

0

0

0.0104881

0.0283844

0

0

0

0

0

0.0163868

0.0675279
7

0

0

0.0819338

0

0.0189229

0

0

0

0

0

0.209257

0

0.0819338

0

0

0.130315

0.0868767

0

0

0.00615279

0

19.5888

0.824103
7

0.199386
7

0

0

0

0

0

0.0819338

0

0

0

0

0.0452143
7

0.0174553

0

0.00712881

0

0

0

0

0

0

0.0150095

0.624392
7

0

0

0

0

0

0

0

0

0.0163868

0

0.168239
7

0

0.0142576

0

0

0

0

0

1.35681

0

0

0.0402543
7

0.00710613

0.00475254

0.026063

0

0

0

0

0.0434384

0

0

0.119627

0

0

0

0

0.278575

0

0.0173753

0

0

0

0.0080381
7

0

0

0

0

0

0

0

0.0173753

0

0

0.11167

0.0173753

0

0

0.0327735

0

0

0.00748085

0

0

0

0.143242
7

0

0

0

0

0.0245801

0

0.0471962

0

0

0.0163868

0.0163868
1

0

0

0

0

0

0

0

0.0868767

0

0

1.03477
6

0.0189229

0

0

0

0.0409669

0

0.0106592

0

0

0.0245801

0

1.06995

0

0

0

0.0195035

0

0

0

0

0

0.0491603

0.109683

0.0245801

0

0

0.026063

0

0

0

0

0

0

0.14055
7

0.0104881

0

0

0.0819338

0

0

0.0163868

0.0104881

0.0189229

0

0

0

0

0

0

0.0163868

0.0245801

0

0.00475254

0

0

0.0955644
7

0

0.0283844

0

0

0

0

0

0

0

0

0.033701

0

0

0

0

0

0

0.0189229

0.0163868

0

0

0.0286281
7

0

0

0.0946147

0

0

0

0

0.0163868

0

0.00748085

0.134249
7

0

0

0.0327735

0.121627

0.052126

0

0

0

0

0.0157321

1.18152
5

0.00475254

0

0.0347507

0.0409669

0

0

0.0324597

0.0189229

0

0

6.41579
7

0.0868767

0

0

0

0.0104881

0

0

0.147481

0.0434384

0

0

0.065547
7

0

0.0104881

0

0

0.0426368

0

0

0

0

0.00710613

0.0640233
7

0

0

0.0124681

0.0173753

0

0.0163868

0

0

0

0

0.0478482
2

0

0

0

0

0

0

0

0

0.026063

0

0.0358652

0

0.00710613

0

0

0

0

0

0.22588

0

0.0608137

0.0638238
7

0

0.0851532

0

0

0.0283844

0.0389516

0

0

0.264921

0

0.119115
6

0

0

0

0

0

0

0.217192

0

0.00498723

0

0.0425967

0.00950508

0

0

0

0

0.0319776

0

0

0.0714113

0.0781891

0.149791
6

0.0104881

0

0

0

0.0608137

0

0

0

0

0

0.157416
7

0

0

0.052126

0

0

0.0173753

0

0

0

0

1.16435
7

0.142808

0

0

0

0

0

0

0

0

0

0.0106592

0.110645

0

0

0

0

0

0

0

0.0189229

0

0.00748085

0.242179
7

0

0.052126

0

0

0

0

0.385089

0

0

0.026063

0
7

0

0

0.0819338

0

0.0129839

0.0189229

0

0.0104881

0

0

0.191731
7

0

0

0

0

0

0.142823

0

0

0.0378459

0.0166339

0.0941748
7

0

0.0173753

0.0104881

0

0

0.0378459

0

0

0.0403966

0

0.0938789
6

0.0173753

0

0

0

0

0

0.0491603

0.0163868

0.00498723

0

0.0686361
7

0

0.0409669

0

0

0

0

0

0

0

0

0.0615762

0

0

0

0.0409669

0

0

0

0

0

0

0.0195035

0.0173753

0

0

0

0

0

0

0.495197

0.0491603

0

0.901468
7

0.087712

0

0

0

0

0

0

0.0173753

0

0

0

0.0536634
7

0

0

0

0

0

0

0

0.0327735

0

0

0.295666
6

0

0

0

0

0

0

0

0

0

0

0.128901

0

0.0173753

0

0

0

0.0209761

0

0

0.026063

0

0.236276
6

0

0.0608137

0

0

0

0.0100064

0.0163868

0.0129839

0

0

0.189486
7

0

0

0.00736842

0

0.104252

0

0

0

0.0245801

0

0.12599
7

0

0

0

0

0

0

0

0.0120572

0.188785

0

0.264954
7

0

0

0

0

0

0

0.0245801

0

0

0

0.159199
7

0

0

0

0.0174553

0

0

0

0

0

0.0157321

0.170644
7

0.0245801

0

0

0

0.0245801

0

0

0

0

0

0.723648
6

0.00997446

0

0

0

0

0

0

0

0

0.0327735

0.0324597

0.0977872
6

0

0.0523659

0

0

0

0

0

0.0129839

0

0.0189229

0.417008
6

0

0

0

0

0.0173753

0

0

0

0

0

0.610098
5

0.114707

0

0

0

0

0

0

0

0.00498723

0

0.117019

0

0

0

0

0.0608137

0

0

0.0173753

0

0.00736842

0.502174
7

0

0.0142123

0.196641

0.0189229

0

0

0

0

0

0.0104881

0.192679

0

0

0

0

0

0

0

0.00736842

0

0

0.0884529
6

0

0

0.0163868

0.147481

0

0.0454436

0

0

0

0

0.0224425

0

0

0

0

0

0

0

0

0

0

0.275172
7

0

0.0163868

0

0

0

0

0

0

0

0.0237627

3.77846
6

1.74182
7

0.0259677

0.00475254

0.0163868

0

0

0

0

0

0

0

0.165066
7

0

0

0

0.0843952

0

0

0

0.0163868

0.0473073

0

0.3146
7

0

0

0

0

0

0.0163868

0

0

0

0

0.060383
6

0

0

0

0

0

0

0

0.0695014

0

0

0.0979294
6

0

0

0

0

0

0

0

0.385089

0

0

0.0695014
7

0.0573536

0

0

0

0

0

0

0

0

0.026063

0.0432836

0

0.00748085

0

0

0

0.0262201

0

0

0

0

0.0142123
7

0

0

0

0

0

0

0

0

0

0

0.0295951
7

0

0

0

0

0

0

0.0100064

0

0

0

0
8

0

0

0

0

0

0

0

0.0327735

0

0

1.31149
7

0.0395231
7

0

0.00475254

0

0.616825

0

0

0

0

0

0.104252

0.0613701
7

0.0173753

0

0.0200127

0

0

0

0

0

0

0.0104881

0.210066
7

0.0129839

0

0.204834

0

0

0

0

0.0389516

0

0.0681724

0.148841
6

0.00710613

0

0

0

0.0104881

0

0.0584274

0

0

0

0.1859
7

0

0

0

0

0

0.0174553

0

0

0

0

0.0864889
7

0.0100064

0.052126

0

0

0.0434384

0

0.00710613

0

0

0

0.0608137
7

0

0.0194758

0

0

0

0.0163868

0

0

0

0

0.136559
7

0

0

0.0327735

0

0.0409669

0

0

0

0

0

0.318268
7

0

0

0

0

0

0

0.0189229

0

0

0

0.208745
7

0.0573536

0

0

0.0129839

0

0

0

0

0.0163868

0

1.25782
7

0.0142123
7

0

0

0

0.0129839

0

0

0.0908871

0

0

0

0.0979421
7

0

0

0

0.0327735

0

0

0

0

0.0163868

0

0.19802
7

0

0

0

0

0

0.00710613

0

0.0142123

0

0

0.237608
7

0.0434384

0

0

0

0

0

0

0

0

0

0.306868
7

0

0

0

0

0

0.0189229

0

0

0.0327735

0

0.0801606
7

0

0

0

0

0

0

0.0851532

0

0

0

0.0626141
6

0

0

0

0

0

0

0

0.00498723

0

0

0.06736
7

0

0.0245801

0

0.0173753

0.104252

0.0389516

0.0080381

0

0

0

0.0410726
7

0

0

0

0

0

0.0163868

0.0786604

0

0

0

0.174691
7

0

0

0.0262201

0

0

0

0

0

0.0173753

0

1.42494
7

0.324884
7

0

0.0491603

0.0080381

0

0

0

0.00498723

0

0

0.0409669

0.100774
6

0

0

0

0

0

0

0

0

0

0.132461

0.11327
2

0

0.0129839

0

0

0.065547

0

0.0519355

0

0

0

0.532135
6

0

0

0

0

0

0

0

0.0104881

0

0

0.106514
7

0

0

0

0

0

0

0

0

0

0

0.0173753
7

0

0

0

0

0

0

0

0.00712881

0.0781891

0

0.0644598
8

0

0

0

0

0.00748085

0.0147368

0.065547

0

0

0

0.0104881
7

0

0.026063

0.0129839

0

0.0106592

0.0473073

0.0163868

0

0

0

0.086921

0.372326

0

0.00922919

0.0080381

0.0194758

0

0.0173753

0

0

0

0.181279
7

0

0

0

0

0

0

0

0

0.026063

0

16.1727
7

0.699481
7

0.150971
7

0

0

0

0

0

0

0.0283844

0

0.163868

0

0.188343
7

0

0

0

0

0.0347507

0

0

0

0

0

0.575488
7

0.0209761

0

0

0

0

0

0

0

0

0

0.246188

0

0

0

0

0

0

0

0

0

0.0756917

0.222533

0

0

0

0

0

0

0

0.0434384

0

0

0.142114

0

0

0

0

0

0

0.0163868

0

0.00498723

0

0.116938
7

0

0

0

0

0.234567

0

0.00710613

0.0124681

0

0

0.0163868

0

0

0

0

0

0.0189229

0

0.0423915

0.0199489

0

0.177682
6

0

0

0

0.026063

0

0

0.443071

0

0

0

0.0142123

0

0

0

0

0

0

0

0

0.122901

0

1.04517
7

0.15422
7

0.230737

0.0491603

0.0737404

0

0

0

0

0

0

0

0.0514549
7

0

0

0

0

0

0

0

0.0142123

0

0.00710613

0.052359
7

0

0

0

0

0.842071

0

0

0

0

0

0.0569347
5

0

0

0

0

0.00475254

0.00748085

0

0.0173753

0.629718

0.566355

0.454944

0

0

0

0

0

0.0173753

0

0.0868767

0

0

0.551492
7

0

0

0

0

0

0.0473073

0

0

0.0124681

0.0157321

0.322522

0.0160762

0.0163868

0

0

0.0361715

0

0

0

0

0

0.0573536
7

0.755828

0.0147368

0

0

0.0983205

0.0104881

0

0

0

0

0.0923515
7

0.00475254

0

0

0.00498723

0

0.0124681

0

0

0

0

0
7

0

0

0

0

0

0

0

0

0

0

1.32435
7

0.106514
7

0

0

0

0

0

0

0

0

0

0

0.206474
7

0.0608137

0

0

0

0

0

0

0

0

0

0.229392
7

0.0327735

0.0129839

0

0

0.180254

0.0608137

0

0

0.0324597

0

0.0409669
7

0.0491603

0

0

0

0

0

0.0262201

0

0

0.0283844

0.0836877

0

0

0

0

0

0

0

0

0

0.065547

8.4237
6

0

0

0.556011

0

0.0189229

0

0

0

0

0

0.374492
7

0.170306

0

0

0

0

0

0

0.052126

0

0

0.0712134

0

0

0

0

0

0

0.065547

0

0

0.0118814

0.0275686
7

0

0

0

0

0.0209761

0.0714113

0.0283844

0

0

0

0.0830602
7

0.0378459

0.0327735

0.052126

0

0

0

0

0

0.00498723

0

1.84278
7

0.182315
7

0

0

0

0

0

0

0

0

0

0

0.140474

0

0

0

0.0163868

0

0

0.0163868

0

0

0

0.327407
6

0.0189229

0

0

0

0

0

0.0901271

0

0

0

0.0360726
7

0

0

0

0

0.0473073

0

0

0

0

0

0.0767527
7

0

0

0

0

0

0

0

0.0129839

0

0.0163868

0.310079
7

0

0

0.0157321

0.0129839

0.11294

0

0

0

0

0

0.130315
4

0

0

0.117251

0

0

0

0.0129839

0

0

0

0.0129839

0

0

0

0.0173753

0

0.0737404

0

0

0

0

0.112854
7

0

0

0

0

0

0

0

0

0.0150095

0

0.0468256
7

0

0

0

0

0

0

0

0

0

0

1.66981
6

0.0603455
7

0

0

0.0163868

0.0163868

0

0

0

0

0

0

0.270259
7

0

0

0

0.215005

0

0

0

0

0

0

0.289008
7

0

0.460447

0

0.00498723

0

0.0473073

0

0

0

0

0.151923
7

0

0

0

0

0.0157321

0

0

0

0

0

0.0104881
7

0.0434384

0

0

0

0.0163868

0

0

0.0104881

0

0.0163868

0.00475254
7

0.0189229

0

0

0

0

0

0

0

0.0163868

0.00712881

0.0612536
7

0

0

0.0173753

0.026063

0

0

0

0

0.0177653

0

0.207211

0

0

0

0

0

0

0

0

0

0.0173753

0.0837275
7

0

0

0

0

0

0

0.065547

0.0100064

0.0104881

0

0.319471
7

0

0.0378459

0

0

0

0

0.00498723

0.026063

0

0

0.640403
7

0.222507
6

0

0

0

0.182441

0

0

0

0

0.0163868

0

0.097112

0

0.00475254

0

0

0

0

0

0

0.0149617

0.00710613

0.00498723
7

0

0

0.0189229

0.0347507

0

0

0

0

0

0.00748085

0.148066
7

0

0

0.0409669

0

0

0

0.0427729

0

0.0173753

0.026063

0.177014
7

0

0

0

0

0.385829

0.00475254

0

0.0434384

0

0.0194758

0.0166339
7

0

0

0

0

0

0.0955644

0

0

0

0

0.149662

0

0.0283844

0

0

0

0

0.00615279

0

0

0

0.174729
7

0

0

0.0327735

0

0

0

0

0.0327735

0

0

0.156507
6

0.00498723

0

0

0

0

0

0.0491603

0

0

0

0.25965
7

0.0157321

0

0

0

0

0.00748085

0

0

0

0

1.12915
7

0.320211

0.0245801

0

0

0

0.0573536

0

0

0.0173753

0

0

0.00712881
7

0

0

0

0

0

0

0

0

0

0

0.149386

0.0819338

0

0

0.0080381

0.208504

0

0

0

0

0

1.0385
6

0

0

0

0

0

0.0173753

0

0

0

0

2.35775
7

0

0.0195035

0

0

0

0.0434384

0

0

0

0.122901

0.0262201

0

0

0.0209761

0

0

0

0

0

0.139287

0.097379

0.114609
7

0

0

0

0

0

0

0

0

0

0

0.0439034
7

0

0

0.0943925

0

0

0

0

0

0

0

0.240677

0

0.0104881

0

0.0245801

0.00498723

0

0

0

0

0

0.169058

0

0

0

0.00950508

0

0

0

0

0

0.0434384

1.68816
7

0.0602129

0

0

0.0245801

0

0

0

0

0

0

0

0.0658474
7

0

0.0163868

0

0

0

0

0

0.0189229

0.0163868

0

0.0106592
7

0

0

0

0.0262201

0

0

0

0

0

0

0.45383
6

0.026063

0.0189229

0

0

0

0

0

0

0

0

0.0534594
6

0

0

0

0

0

0

0

0

0

0

0.143646
5

0

0

0

0

0

0

0

0

0

0.0173753

0.0390436
6

0.495197

0

0

0

0

0.0347507

0

0

0.00950508

0

0.0795585
7

0

0

0

0.0173753

0

0.0104881

0

0

0

0

0.0174553

0

0

0

0

0

0

0

0

0

0

0.246886

0

0

0

0.0104881

0

0.0409669

0.00710613

0

0

0

2.19808
7

0.0483339
7

0

0

0

0

0

0

0

0.00712881

0.0338404

0.0129839

0.327735
7

0

0

0

0

0

0

0

0

0

0

0.150587
7

0

0.00498723

0

0

0

0

0

0.0189229

0

0

0.212929
7

0

0

0

0

0.122999

0

0.00710613

0

0

0

0.0110526
7

0

0

0.00997446

0.0737404

0

0.0491603

0

0.0491603

0.0189229

0

0.0839416
7

0

0

0.0173753

0

0

0

0

0

0

0

0

0.104881

0

0

0

0.0491603

0

0

0

0.0695014

0

0.20579
7

0

0

0.0573536

0

0

0

0

0

0

0

0.0729996
7

0.0548595

0.0104881

0

0

0

0

0.0124681

0.052126

0

0

0.0262201

0

0

0

0

0

0

0

0

0

0

1.2427
7

0.0924218

0

0

0

0.0367082

0

0

0.0194758

0

0.0163868

0.0163868

0.0819624
2

0

0

0.0189229

0.213028

0

0

0.0189229

0.0189229

0

0

0.0289293
7

0.0104881

0.0662303

0

0

0.0106592

0

0

0.0173753

0

0

0.0243494
7

0

0

0.199816

0

0.00748085

0

0

0

0

0

9.47483
6

0

0

0

0.00997446

0

0

0

0

0.00736842

0

0.113538

0

0.0163868

0.0471962

0.00710613

0

0

0

0

0

0

0.095403
7

0

0

0

0

0

0

0

0

0

0

0.144882
7

0

0.0104881

0

0

0

0

0

0.0080381

0

0

0.564699

0.0491603

0

0

0

0

0

0.00736842

0

0

0

0.130282
7

0

0.052126

0

0

0

0

0

0

0

0

8.49738
7

0.926024
7

0.118985

0

0.155674

0

0

0.0448851

0

0

0

0

0.00615279

0.335747

0

0

0

0

0

0

0

0

0

0

0.400104
6

0

0

0

0

0.0129839

0

0.00710613

0

0

0

0.0924218
7

0

0

0.0901271

0.00748085

0

0

0

0.0080381

0

0

0.0426069
7

0

0

0.974531

0

0

0

0

0.026063

0

0

0.253717
7

0

0.0245801

0.00475254

0

0

0.052126

0

0

0

0

0.0827825

0

0

0

0.0194758

0

0

0

0.0347507

0

0

12.8223
6

0

0

0.0163868

0.026063

0

0

0.00475254

0

0

0

0.0327735
8

0.0104881

0

0

0

0

0.0104881

0

0

0

0

0.0765237
7

0.0157321

0.0695014

0.052126

0

0.00615279

0

0

0

0.0473073

0.00498723

0.85926

0.0209761

0

0

0

0

0

0

0.0129839

0.0283844

0

0

0.0273498
7

0.131094

0

0

0

0

0

0

0

0.0173753

0.0245801

0.0195035
7

0

0.0189229

0

0

0

0

0

0

0

0

0.101488
7

0

0

0.0173753

0.00950508

0

0

0

0

0.0163868

0

0.247901
7

0

0

0.065547

0

0

0

0

0.0374042

0

0

0.0283844
6

0.0100064

0

0

0.0129839

0

0

0

0.0173753

0.0283844

0

0.0960267
7

0

0

0

0

0

0

0

0

0

0.0189229

0.0173753
7

0.0129839

0.026063

0

0

0

0

0

0

0

0

0.14399
6

0

0

0

0.0245801

0.0378459

0.0194758

0

0

0

0

0.192426
7

0

0.0491603

0

0

0

0.0245801

0.0573536

0

0.00498723

0.0327735

0.542997
7

0.206319
7

0

0

0

0.0245801

0

0

0

0

0

0

0.635085

0

0

0.0283844

0

0

0

0

0

0

0

0.122901
7

0

0

0

0

0

0

0.0157321

0

0

0

0.1621
7

0

0

0

0

0

0

0

0

0

0

0.338183

0

0.229415

0.0189229

0.0378459

0

0

0

0

0

0

0.183011
7

0.0173753

0

0

0

0

0

0

0

0

0.0166339

0.102769
7

0

0

0

0

0

0

0

0.0409669

0

0

0.052882
7

0

0

0

0

0

0

0.0157321

0.0524403

0

0

0.0314642
6

0

0.0259677

0

0

0

0

0

0

0

0

0.364313
7

0.0274298

0

0

0

0

0.00748085

0

0

0.0209761

0

1.14865
7

0.267419
7

0

0.0106592

0

0

0

0.0347507

0

0

0

0.026063

0.0621465
7

0

0

0.00475254

0

0.00615279

0

0

0

0

0.026063

0.377632
7

0.270381

0

0.065547

0

0

0

0.0194758

0.0150095

0

0

0.0593308

0

0.0174553

0.0245801

0.0283844

0.0361715

0

0

0

0

0

0.0293706
7

0.0259677

0

0

0

0

0.0434384

0

0

0

0

0.276947
6

0.052126

0

0

0

0

0

0

0

0

0

0.032417
7

0

0

0

0

0

0

0

0.0649194

0

0

0.0478602
2

0

0

0.0314642

0

0

0

0

0

0

0

0.0104881
7

0.00615279

0.0327735

0

0

0

0

0

0.0519355

0.0327735

0

0.0514549

0

0

0.0471962

0

0

0

0

0.0434384

0

0

2.06319
7

0.0383515
7

0

0

0.00710613

0.0129839

0

0.0163868

0

0

0.00710613

0

0.0770237
7

0

0.0194758

0

0

0

0

0

0

0

0

0.122252
7

0

0

0

0.00475254

0

0

0.131094

0

0

0

0.0927001
8

0

0

0

0

0

0

0

0.0409669

0

0.327735

0.0932162

0

0.0163868

0

0.0573536

0

0

0

0

0

0.0409669

0.0508921

0

0

0.0149617

0

0

0.0283844

0

0

0

0

0.0513826

0

0.0173753

0

0.0434384

0

0

0

0

0

0

0.276028
7

0

0

0.00997446

0

0.00498723

0

0

0

0

0

0.0283844
7

0

0

0.0163868

0.0177653

0

0

0

0

0

0.0491603

0
7

0

0

0

0

0

0

0

0

0

0.052126

1.42511
7

0.0632132
7

0

0

0

0

0

0.00748085

0

0.0163868

0.0177653

0

0.0778179
6

0

0

0

0

0

0

0.0104881

0.596915

0

0.0104881

0.00710613
7

0

0

0

0.00498723

0

0.0327735

0

0

0

0.0189229

0.250289
7

0

0

0

0

0

0

0.0104881

0

0

0

0.0418568

0

0

0

0.0163868

0

0

0

0.0983205

0

0

0.151169
7

0

0

0.0106592

0

0

0

0

0

0

0

0.0245801
7

0

0

0

0

0

0.0100064

0

0

0

0

0.0671768
6

0

0

0

0

0

0

0.085054

0

0

0.00498723

0.0433862
7

0

0

0

0

0

0

0.052126

0

0

0

0.00498723
7

0.0129839

0

0

0

0

0.00710613

0

0

0

0

0.433731
7

0.0819018
7

0.0522477

0

0

0

0

0

0.0209761

0

0

0.0173753

0.10981

0

0

0.0378459

0

0

0

0

0

0

0.00498723

0.172061

0

0

0

0.0209761

0

0.0157321

0.0129839

0

0.0868767

0

0.153652
6

0

0

0.0491603

0

0

0.0194758

0

0.0104881

0

0

0.250306
7

0.0695014

0.0157321

0

0

0

0

0.0173753

0

0

0

0.0327735

0

0.030764

0

0.0163868

0

0.0983205

0

0

0

0

0.290068
7

0

0.0189229

0.0367082

0.0756917

0

0

0

0

0

0

0.13835

0

0

0

0

0

0

0.0173753

0.026063

0

0

0.104499
7

0

0.0189229

0

0

0

0

0

0

0

0

0.125366
7

0.065547

0

0

0

0

0.0163868

0

0

0.0209761

0

0.654729
7

0.104252
7

0

0.0163868

0

0

0

0

0

0.0173753

0

0

0.0682946
7

0

0

0

0

0

0

0

0

0.0142123

0

0.173563
7

0.0100064

0.0124681

0.0434384

0

0

0

0.0851532

0

0

0.0173753

0.122999
7

0

0

0

0

0

0.191129

0

0

0

0

0.0295674
7

0

0

0

0

0

0

0.0347507

0

0

0.0378459

0.120772
8

0.0314642

0

0

0

0

0

0

0

0

0

0.0934926
7

0

0

0.0245801

0

0

0

0

0

0

0

0
7

0.0868767

0

0

0.0773684

0

0

0.0163868

0

0.0409669

0

0.0327735
6

0

0

0

0.00475254

0

0.0173753

0

0

0

0

0.0805087
7

0

0

0.00498723

0

0

0

0

0.0662303

0

0

1.96152
7

0.0378459
8

0

0

0

0

0.00498723

0

0

0

0

0

0.426056
7

0

0

0

0

0

0.0104881

0.0189229

0

0

0

0.0378459
7

0

0

0

0

0

0

0

0

0

0.0262201

0.0848995

0

0

0.0423915

0

0

0.0901271

0

0

0

0

0.0122334
6

0

0

0.0163868

0

0

0

0.0163868

0

0

0.0199489

0.00748085
7

0

0.0283844

0

0.0104881

0

0

0

0

0

0.00498723

0.534564
5

0.0868767

0

0

0

0.0189229

0

0

0

0.065547

0

0.0912399
1

0

0

0

0

0

0

0

0.121627

0

0.143684

0.0458345
7

0

0

0

0

0

0

0

0

0

0

0.716284
6

0

0.0173753

0.00997446

0

0.0573536

0

0

0

0

0

1.39394
7

0.0351407
7

0.0177653

0

0

0

0

0

0

0

0

0

0.114547
5

0

0

0

0

0

0

0.00710613

0

0

0

0.0271961
7

0

0.0173753

0

0

0

0

0

0

0

0

0.101861
7

0

0

0

0

0.0810526

0.0104881

0

0

0

0

0.0194758
8

0

0

0

0

0

0

0

0.0189229

0

0

0.0583894

0.0378459

0.0157321

0

0

0

0

0

0

0

0

0.0147368
7

0

0

0

0

0

0

0

0.0974271

0

0

0.314857
7

0

0

0

0

0

0

0

0

0

0

0.0245801
6

0

0

0.110125

0

0

0.0104881

0

0

0

0

0.226317
7

0.0327735

0

0

0

0.382258

0

0

0

0.0209761

0

1.31427597971356e-10
7

0
4

0.0199489
5

0.0149617

0

0.00498723

8.67361737988404e-19
5

0
4

0
7

0
7

0
4

0.0104881
7

0.0104881

0

0
4

0.101761

0.101761

0
4

0.136345

0.136345

0
4

0

0

0

0
4

0

0

0
4

0

0

0
4

0.0163868

0.0163868

0
4

9.22362880517724e-13
7

0
4

3.75529
7

3.75529
7

2.764
7

0.220898
7

0.770389
7

0

1.11022302462516e-16
7

0
4

0

0

0
4

0
4

62.3693

7.02241
7

3.10859
7

0.0213184
7

0

0.00712881

0

0.0080381

0

0

0

0

0

0

0
7

0

0

0

0

0

0

0.0173753

0

0

0.0100064

0
6

0

0.0104881

0

0.0163868

0

0

0

0.00710613

0.0184584

0

0

0

0

0

0

0

0

0

0

0

0.00712881

0.00710613
7

0
6

0.00950508

0.039007
7

0.0485289
6

0

0.892149
7

0.0104881
7

0

0
7

0.0292553

0

0

0.0461898

0

0.0746143

0.0249626

0.209293
7

0.0120572

0.0123056

0.0228382

0

0.052126

0

0

0

0.0301627

0

0.275873
6

0

0.0080381

0.00950508

0.0438143

0.0245801

0

0.026063

0.0861391

0

0.0104881

0.338717
6

0.0570305

0.0163868

0

0.0576843

0.0129839

0

0

0.0299443

0

0

0.0886247
7

0

0.0288669

0

0.0522477

0.00710613

0

0

0

0

0.0104881

0.210343
7

0.0177653

0

0

0.0129839

0

0

0

0.00475254

0

0

0.0280345
6

0

0

0

0

0

0.00736842

0

0.0157321

0

0.0129839

0.757488
6

0

0

0.00710613

0

0

0

0

0.0106592

0

0

4.90926743701436e-16
7

0
4

0.0129839

0

0

0

0.0129839

0

0
4

1.63713

1.63713

0
4

7.7494

7.7494

0

0
7

0

0
4

0

0

0

0

0

0

0

0
4

8.66886
7

4.24088
7

0.145676

0.0762766
6

0.0163868
6

0.0409669

0

0.0692293
5

0

0.0225395

0.0601801

0

1.95354

0.0234719

0.0324597

0.0184584

0

0.00922919

0

0.188312

0

0

0.0129839

0.196507
6

0

0.00922919

0.0129839

0

0

0

0

0

0.0573536

0

0.240335
7

0.0163868

0

0.0104881

0.00615279

0

0

0

0.0163868

0

0.0245801

0.191203
7

0

0.00615279

0.0104881

0

0.0262201

0

0.0163868

0

0

0

0.0753804
7

0

0.0194758

0.32513

0

0

0.0471962

0

0

0

0

0.167802
6

0

0

0

0

0

0

0

0

0

0.0142123

0.00615279
7

0

0

0.0245801

0

0.00922919

0.135361

0

0.0194758

0.0734164

0
4

2.19891
6

0.548956
6

0.234567

0

0

0.0163868

0

0

0

0.0163868

0

0

0.0573536

0.0163868

0.786564
6

0.103039

0.00922919

0.0163868

0.191129

0.1741

0.0284245

1.38777878078145e-16
6

0
4

32.7978
6

0.543496
6

1.05409
7

0.220726

0

0.0213184

0

0.0195035

0

0

0.0157321

0

0

0.445898
6

0

0.0157321

0

0

0

0

0

0.0372934

0

0.0259677

2.75139

0

0

0

0

0

0

0

0

0

0.00710613

0.0746143
7

0

0

0

0.0100064

0

0.0104881

0

0

0

0

1.19419
6

0

0

0

0.0194758

0.0104881

0

0.0129839

0.00710613

0

0

1.43774

0

0

0.0195035

0.0585106

0

0

0

0

0

0.0129839

0
7

0

0

0.0106592

0

0

0

0

0

0

0

0.24008
6

0

0

0

0

0

0

0

0

0.0104881

0

0.114557
6

0

0

0

0

0

0

0

0

0

0

0.16578
7

0

0

0

0

0

0

0.00710613

0.0104881

0

0

3.60356
6

0.0935783
6

0

0

0

0

0

0.0142123

0

0

0

0.0080381

0

0

0

0

0.0110526

0

0

0

0

0

0

0.0822947
6

0

0

0

0

0

0.0160762

0.00710613

0

0

0.0487588

0.00710613
7

0

0

0

0.0378459

0

0

0

0

0

0

0.176432
6

0.0106592

0

0

0

0

0

0.0662303
7

0.280958
6

0.00710613
6

0.15952
7

0.319587
6

0.528923
6

0.128651
6

0.0195035
6

0

0.0284245
6

0

0.0595018
6

0.0945541

0.0874661
6

3.32535
6

0
7

4.51046
6

0.129839

0
7

0.00615279
6

0.114582
6

0.136216
7

0.335213
6

0.195124

0.0303592

0

0

1.01802

0

0

0.256957
7

0.0142123

0

0.0157321
6

2.18152

0

0.00922919

0

1.11009
6

0.209761

0

0

0

0.00710613

0.0321524

0

0.097379

0.0341485

0.0473073

0.707944
6

0.0213184

0.0389516

0

0

0.12791

0.0195035

0

0

0

0.0294325

0.053296
7

0

0.28197

0

0

0.0195035

0

0

0

0.0408219

0.0110526

2.90603
7

0

0

0

0

0.11007

0

0.0387414

0.0195035

0.025041

0.0471962

0
4

0.716487

0.519018

0.11619

0.0345645
5

0.00736842

0.0319776

0

0.00736842

1.04083408558608e-16

0
4

0

0

0
4

0

0

0

0

0
4

0

0

0
4

0

0

0
4

0

0

0

0
4

0.052126

0.052126

0
4

0

0

0
4

0

0

0
4

0

0

0
4

0.0194758

0.0194758

0
4

0.860665

0.853297

0.00736842

6.93889390390723e-18

0
4

0

0

0
4

0

0

0
4

0

0

0
4

0

0

0
4

0

0

0
4

0

0

0
4

0.145676

0.145676

0
4

0

0

0

0

0
4

0.429078

0.429078

0
4

0

0

0

0

0

0

0
4

0.0478107

0.0259677

0.0147368

0.00710613

0
4

0

0

0
4

0.0104881

0.0104881

0

0
4

0

0

0
4

1.20840837336544e-14

0
4

16.1279

0.0524403
7

0.0524403
7

0
4

0.527946
5

0.470593
5

0.0163868
5

0.0409669

0

0

0

0

0

0

4.16333634234434e-17
5

0
4

0.205786

0.177362

0.0106592

0.0177653

0

0

0

0
4

1.62261
7

1.5657
7

0.0569139
7

0

2.15105711021124e-16
7

0
4

0.357067

0.32465

0.0274298

0

0

0

0.00498723

0

0
4

0.28383
5

0.28383
5

0

0

0
4

0
7

0
7

0

0

0
4

0.0622265
5

0.0163868
5

0.0174553

0

0.0283844

0

0

0

6.93889390390723e-18
5

0
4

0
6

0
6

0

0

0
4

0.946425

0.930038

0.0163868

0

4.5102810375397e-17

0
4

0.0174553
4

0.00498723
4

0

0

0.0124681

0

0

0

0
4

0

0

0

0

0

0

0

0
4

1.29827

1.05367
5

0.0215348

0

0

0

0

0.0692484
6

0.0338404

0.0615279

0

0.00615279

0.0461459

0

0.00615279

9.28077059647592e-17

0
4

0.0245801
3

0.0245801
4

0

0
4

0

0

0

0
4

0.251713

0.225493

0.0262201

0

0
4

0.429078
7

0.429078

0

0
4

0.00498723
5

0.00498723

0

0

0

0

0

0

0

0
4

0.236385

0.236385

0
4

0

0

0

0

0

0

0

0
4

0
7

0
7

0
4

0.536347
6

0.536347
6

0

0
4

0.0129839

0.0129839

0
4

2.00633
5

0.968173
5

0.026063

0.00710613

0.00710613

0

0

0

0

0

0

0

0.417008
5

0

0

0.00710613

0

0.0262201

0.0104881

0.451759

0.00710613

0.0781891

0
4

0.026063
4

0.026063

0

0
4

0

0

0

0

0

0

0
4

0.0334974
7

0.0106592

0.00710613

0.0157321

0

0
4

0

0

0

0
4

0

0

0

0
4

0

0

0
4

0

0

0

0
4

0

0

0

0

0
4

0

0

0
4

0.00615279

0

0.00615279

0
4

1.49378
7

1.05492
7

0

0.0367082

0.0604052
7

0
7

0.0439344
7

0.0494331
6

0.209687
7

0.00748085

0.0262201

0.00498723

2.87096735274162e-16
7

0
4

0

0

0
4

0

0

0

0
4

0.32513

0.32513

0
4

0.00498723

0.00498723

0
4

0

0

0

0

0
4

0

0

0
4

0

0

0
4

0.00498723

0.00498723

0

0

0
4

0

0

0

0
4

0.0338404

0.0215348

0.00615279

0.00615279

0
4

0.184761
5

0.184761
5

0
3

0
5

0

0

0

0

0
4

0.00922919

0.00922919

0

0
4

0

0

0
4

0.0173753

0.0173753

0

0
4

0

0

0
4

0

0

0

0
4

0

0

0
4

0.0487588

0.0487588

0
4

0

0

0

0
4

0

0

0
4

0

0

0

0
4

0.249035

0.241554

0
6

0

0.00748085

0

0

0

0

0
4

0

0

0
4

0

0

0
4

0

0

0
4

0

0

0
4

0

0

0
4

0

0

0
4

0

0

0
4

0

0

0
4

0

0

0
4

0.0163868

0.0163868

0
4

1.6947

1.68855

0

0

0

0

0.00615279

0

0

7.89299181569447e-17

0
4

0

0

0
4

0

0

0
4

0

0

0
4

0

0

0
4

0

0

0
4

0

0

0
4

0

0

0
4

0

0

0
4

0

0

0
4

0

0

0
4

0.755385

0.443591

0

0

0.0923796

0.0262201

0.122988

0.0333263

0.0157321

0.0106592

0

0.0104881

0
4

0

0

0
4

0

0

0
4

0

0

0
4

0

0

0
4

0

0

0
4

0

0

0
4

0

0

0
4

0.0157321

0.0157321

0
4

0

0

0
4

0

0

0
4

2.31218
5

1.64481

0

0

0.660263

0.00710613

0
4

0

0

0
4

0.0195035

0.0195035

0
4

0

0

0
4

0
4

0.413051

0.413051

0.413051

0

0

0

0

0

0
4

0
4

0

0

0

0
4

0
4

0

0

0

0
4

0
4

0.0118814

0.0118814

0.0118814

0

0
4

0
4

0

0

0

0

0

0
4

0
4

0

0

0

0
4

0
4

0.0349106

0.0349106

0.0349106

0

0
4

0
4

0

0

0

0

0
4

0
4

0

0

0

0
4

0
4

0.124357

0.124357

0.124357

0
4

0
4

0

0

0

0

0
4

0
4

0.141233
3

0.141233
3

0.0395418

0

0

0

0

0.0157321

0

0

0

0

0

0

0

0

0

0

0

0

0

0

0

0

0.0631214
5

0

0

0

0

0

0
3

0
3

0

0

0.00710613

0.0157321

2.77555756156289e-17
3

0
4

0

0

0
4

0
4

0.0163868

0.0163868

0.0163868

0

0
4

0
4

0

0

0

0
4

0

0

0
4

0
4

0

0

0

0
4

0

0

0
4

0
4

0

0

0

0
4

0
4

0.0292553

0.0292553

0.0292553

0
4

0
4

0

0

0

0
4

0
4

0

0

0

0
4

0
4

0

0

0

0
4

0
4

0

0

0

0
4

0
4

0

0

0

0
4

0
4

0.00570748
3

0.00570748
3

0

0

0.00570748

0

0
4

0

0

0
4

0

0

0
4

0
4

0

0

0

0
4

0
4

0.0292553

0.0292553

0.0292553

0
4

0
4

0

0

0

0
4

0
4

0.039007

0.039007

0.039007

0
4

0
4

0

0

0

0
4

0
4

0

0

0

0
4

0
4

0

0

0

0
4

0
4

0

0

0

0
4

0
4

0.0245801

0.0245801

0.0245801

0
4

0
4

0.0487588

0.0487588

0.0487588

0
4

0
4

0.111304
6

0.111304
6

0.0163868
6

0.0129839

0.065547

0

0.0163868

3.46944695195361e-18
6

0
4

0
4

0

0

0

0
4

0
4

0.00498723

0.00498723

0.00498723

0
4

0
4

0

0

0

0
4

0
4

0

0

0

0
4

0
4

0

0

0

0
4

0
4

0

0

0

0
4

0
4

0

0

0

0
4

0
4

0.448891

0.448891

0.448891

0
4

0
4

0

0

0

0

0

0

0
4

0
4

0.00950508
5

0

0

0
4

0

0

0
4

0.00950508

0.00950508

0
4

0
4

0.0817205

0.0817205

0.0817205

0

0
4

0
4

0

0

0

0

0
4

0
4

8.87863

5.0156

4.96045

0

0

0.0356441

0

0.0195035

0

0

0
4

2.36628
6

2.01046
6

0.0440559

0.0830627

0.125437

0.0868767

0

0

0.0163868

1.73472347597681e-17
6

0
4

0.0434384

0.0434384

0
4

0

0

0
4

0

0

0
4

0.627631
5

0

0.0292553

0.039007

0.477836

0

0

0.0327735

0.0487588

0

4.85722573273506e-17
5

0
4

0.148039
7

0.137551
6

0.0104881

0

3.46944695195361e-18
7

0
4

0
7

0
7

0

0
4

0

0

0

0
4

0.6342

0.6342

0
4

0

0

0
4

0

0

0
4

0.0434384

0.0434384

0
4

3.88578058618805e-16

0
4

1.39168

1.05094

0.0358626

0.0419555
5

0
7

0.0245801

0

0
5

0

0

0.0273817
5

0.0419522

0

0.0491603

0.149509

0.052126

0

0.0189229

0

0

0

0

0.0868767

0

0.146778
6

0

0

0

0

0

0

0.0100064

0

0

0.0222131

0
5

0

0

0

0

0.0292553

0

0

0.00475254

0

0

0.280233
6

0

0

0

0.0163868

0

0

0

0

0

0

0

0

0

0

0

0

0

0

0

0

0

0

0.0129839

0

0

0

0

0

0

0

0

0

0
7

0

0

0

0

0

0

0

0

0

2.1163626406917e-16

0
4

0.117482

0.117482
7

0

0
4

0

0

0

0
4

0

0

0
4

0

0

0
4

0

0

0
4

0.0996365

0.0996365

0
4

0

0

0
4

0

0

0
4

0

0

0
4

0.0724681
5

0.0724681
6

0

0

0

0
4

0.0316863
6

0.0245801

0.00710613

0

0

0
4

0
5

0
5

0
4

0
7

0
7

0

0

0
4

0.0194758
7

0.0194758
7

0
4

0

0

0

0
4

0

0

0

0
4

0

0

0
4

0
4

4.64645

4.6172

1.44215
5

0.0347507

0

0

0

0

0.00710613

0.0434384

0

0

0

1.73589
5

0.0195035

0

0

0.0173753

0.0173753

0

0.0695014

0.188448

0

0.0173753

0

0

0.0173753

0

0.121627
6

0

0.780141

0.0887464

0

0.0163868

0
4

0

0

0
4

0

0

0
4

0.0292553

0.0292553

0
4

0

0

0
4

2.77555756156289e-16

0
4

12.5062
5

12.2613
5

0.255278
5

0.0585106
5

0.00748085
5

0.40183
6

0.914395
5

0

0.117021

0

0

0

0

5.83041

0

0.0573536

0.0195035

0.0245801

0.0327735

0

0

0

0

0

0.53199
6

0

0

0

0

0

0.039007

0.0245801

0.0292553

0.0194758

0

0.659234
5

0.0173753

0

1.26997

0.0276876

0

0

0.253546

0

0

0

0.110363
5

0.0245801

0

0.00615279

0

0

0

0

0.0409669

0.0173753

0

0.395116
5

0

0.0163868

0

0.0163868

0

0.0195035

0

0

0

0.00615279

0.0723147
6

0.0245801

0

0

0

0

0.0292553

0.0819338
4

0.808978
6

0
4

0.208152

0.208152

0

0
4

0.0292553

0.0292553

0

0
4

0.00748085

0.00748085

0
4

0

0

0
4

5.20417042793042e-17
5

0
4

2.70953

1.76433

0.743048

0
5

0

0

0

0

0

0

0

0

0

0

0
5

0

0

0

0

0

0

0

0.039007

0

0

0.289905

0.0195035

0

0.0292553

0.614361

0

0.0292553
7

0

0

5.55111512312578e-17

0
4

0
5

0
5

0
4

0.0163868

0.0163868

0
4

0.126773

0.126773

0

0
4

0

0

0

0

0
4

0

0

0
4

0

0

0
4

0

0

0
4

0

0

0

0
4

0

0

0
4

0.00615279

0.00615279

0
4

0

0

0
4

0

0

0

0
4

0.0195035

0.0195035

0
4

0.0163868

0.0163868

0
4

0

0

0
4

0

0

0
4

0

0

0
4

0

0

0
4

0

0

0
4

0

0

0
4

0

0

0
4

0

0

0
4

0.104252

0.0868767

0.0173753

0

0
4

0

0

0
4

0

0

0
4

0

0

0
4

0

0

0
4

0

0

0
4

0.318105

0.318105

0
4

0

0

0
4

0

0

0

0

0

0
4

0

0

0

0

0

0
4

0.16879

0.16879

0
4

0.039007

0.039007

0
4

0.129839

0.129839

0
4

0

0

0

0
4

0
4

20.2035
5

19.8652
7

17.3246
7

2.54062
7

0

1.33226762955019e-15
7

0
4

0
4

0
4

0

0

0
4

0

0

0
4

0

0

0
4

0

0

0
4

0

0

0
4

0

0

0

0
4

0
4

0
4

0

0

0
4

0.0147368

0.0147368

0

0

0
4

0

0

0

0

0
4

0.302767

0.302767

0
4

0

0

0
4

0.0207193

0.0207193

0
4

0

0

0
4

0
4

1.29019

1.29019

0.122901

0.0608137
5

0.172061

0

0

0

0

0

0

0.0100064

0

0.439499

0

0

0

0

0.0163868

0

0.0163868

0

0

0

0.0324597

0

0

0

0

0

0

0

0.0150095

0

0

0.0329966
7

0.0150095

0.159028
7

0.0737404

0.106514

0.0173753

0

2.46330733588707e-16

0
4

0
4

0
4

0

0

0

0

0

0
4

0

0

0

0

0
4

0

0

0
4

0

0

0
4

0

0

0
4

0

0

0
4

0
4

0.571383
4

0.571383
4

0
5

0

0.284245

0

0

0.155674

0

0

0

0

0

0.131463

0

0

0

0

0

0
4

0

0

0
4

0

0

0
4

0

0

0
4

0
4

9.26614340812648e-12
7

0
4

160.778

74.1117

0.745253

0.515287
5

0

0

0.0511374

0.129668

0

0

0.0327735

0

0

0.0163868

0
4

39.5392

37.4408

0.508148
7

0

0.00710613

0

0.0104881

0

0.0367082

0.0163868

0

0.0319776

0

1.07263
7

0

0

0.346057
7

0

0

0.0419522

0.0104881

0.0163868

0

0
4

20.1296

14.6175
7

0.026063
7

0.0434384

0.0104881

0.0142123

0

0

0

0

0

0

0

0.0106592
7

0

0

0

0

0.0163868

0

0.0209761

0

0.0245801

0.0142123

0
7

0

0

0

0

0

0

0

0

0

0

0.0157321
8

0

0

0.00736842

0.0104881

0

0

0

0

0

0

0.00710613
7

0.0104881

0

0

0

0

0.0104881

0

0

0

0

0.0178565
7

0

0

0

0

0

0

0

0

0

0

0
1

0

0

0

0

0

0

0

0

0

0

0

0

0

0

0

0

0

0

0

0

0.0104881

0.0104881
8

0

0

0

0

0

0

0

0

0

0

0.260537

0

0

0

0

0.00736842

0

0

0

0

0

0.274976

0.0524403

0

0

0

0

0

0

0

0

0

0

0.0104881
8

0

0

0

0

0

0

0.015382

0

0.0157321

0

0.0327735
7

0

0.0104881

0

0

0

0

0

0

0

0

0.073981
7

0

0

0

0

0

0

0

0

0.00712881

0

0.0209761
7

0

0

0

0

0

0

0

0.0209761

0

0
7

0

0.0147368

0.272494
7

0.0166408
7

0

0

0

0

0.165066

0

0

0

0.00736842

2.55082

0.0157321

0

0

0.0257895

0

0

0.0473073

0

0

0

0.534323
7

0.0451491

0

0

0

0

0

0

0

0.0104881

0.0104881

0.10128
1

0

0

0

0

0

0

0

0

0

0.0157321

0.178158
1

0

0.0147368

0

0

0

0.0104881

0

0

0

0

0.22415
8

0

0

0

0

0.0245801

0

0

0

0.0104881

0

0
8

0

0.0104881

0

0

0.110125

0

0

0

0.0106592

0

0
4

0.660448
7

0.660448
7

0

0
4

0.0539207
7

0.0106592
7

0

0.0104881

0.0163868

0

0.0163868

0

0

0

0

0
4

0.317498
6

0.0144745
6

0.00710613
6

0.0173429

0

0.278575

0
4

0.0447712
7

0
7

0.0283844
6

0.0163868

0

0
4

0.0209761
7

0.0209761

0

0

0

0

0

0

0

0

0

0
4

0

0

0

0

0

0

0
4

0.0209761
1

0.0209761
1

0
4

0.0327735
6

0.0327735
6

0

0

0
4

0

0

0

0

0
4

0

0

0

0
4

4.83291
5

3.41738
7

0.0426368

0
6

0.0149617

0.129037

0

0

0.0104881

0

0

0

0.446761

0

0

0

0

0

0

0.00475254

0

0.0327735

0

0.239125
6

0

0

0.109944

0.095606
6

0.0216211
5

0.0421284
6

0.131531
6

0.0941559
6

4.16333634234434e-16
5

0
4

0.0245801
6

0.0245801
6

0
4

0

0

0

0
4

0

0

0

0
4

0

0

0

0
4

0.0262201

0.0157321

0.0104881

0
4

0

0

0

0
4

0

0

0

0
4

0

0

0
4

0

0

0
4

0

0

0
4

0.417629

0.115196
1

0.0471962

0.00475254

0

0.0518623

0.0624725

0.0480618

0.0267847

0
7

0.0106592
2

0
8

0.0506431
6

1.38777878078145e-17

0
4

0

0

0
4

0

0

0
4

0.0104881

0.0104881

0
4

0

0

0
4

0

0

0
4

0

0

0
4

0

0

0
4

0

0

0
4

0.0104881

0.0104881

0
4

0

0

0
4

4.62023

2.18299
5

0.00710613

1.24552

0.280808
7

0.855775
7

0.0106592

0

0.0209761

0.0163868

0

0
4

0

0

0
4

0.0347507

0.0347507

0
4

0

0

0
4

0

0

0
4

0

0

0
4

0

0

0
4

0

0

0
4

0

0

0
4

0

0

0
4

0

0

0
4

0.105105

0.0530048
1

0.0520997
1

0

0

0

0

0

0

0
4

0

0

0
4

0.188778
7

0.151415
7

0
8

0.0163868

0.0209761

0

0

0
4

0.996982
6

0.948252
6

0.0258918
7

0

0.0157321

0

0

0.00710613

0
4

1.08093
8

1.08093
8

0
8

0

0

0

0

0

0
4

0.197192

0.165728

0.0104881

0

0.0209761

0

0

0
4

2.80331313717852e-15

0
4

36.6473

35.4155

35.073

0

0.037564

0

0

0

0

0

0.221221

0

0

0

0

0

0

0

0

0

0.00498723

0.0573536

0.00498723

0.0163868

0

0

0

0

0
4

1.16478
8

0.215199
8

0

0

0

0

0

0

0

0

0

0

0.651489
8

0

0

0

0

0

0.0173753

0

0

0

0.0106592

0.163441
8

0

0

0

0.0106592

0.0106592

0

0.0852952

0

0
4

0

0

0
4

0

0

0
4

0

0

0
4

0

0

0
4

0

0

0
4

0.026063

0.026063

0
4

0

0

0
4

0

0

0
4

0

0

0
4

0.0409669

0.0409669

0
4

0
4

17.8312

16.9802

4.57423

0.982986
5

0

0

0

0

0.0173753

0

0.0173753

0

0

0

0.0245801

0.0434384

0

0

0

0.0194758

0

0

0

0.0163868

0

0.0409669
6

0

0.0173753

0

0

0

0.0123056

0.0327735

0.0819338

0

0.0347507

0.211963
7

0.0163868

0

0

0

0.0163868

0

0

0

0

0

0

0

0

0

0

0

0

0

0

0

0

0

0.0100064

0

0.0184584
6

0.0238676
7

0

0.0778457
6

1.4357

0
7

0.11294

0

0.0337621
7

0.00615279

0

0

0

0.065547

0

3.04019
7

0.0665323

0

0

0

0

0

0.0245801

0.0163868

0

0.0598251

3.72078

0

0

0

0.0110526

0.032417

0

0

0.0273817

0.0573532

0

1.42707
6

0

0

0.0367082

0.00498723

0

0

0.0173753

0

0.0173753

0

0.0448895
7

0

0.0245801

0.0080381

0

0

0

0

0

0

0

0.0829521
1

0

0

0

0

0

0

0

0.00498723

0

0

0.103594

0

0

0

0

0

0

0

0

0

0

0.206138
7

0

0

0

0

0

0

0

0

0

0

0
4

0.0123056

0.0123056

0

0

0
4

0.0189229

0

0.0189229

0

0
4

0

0

0

0
4

0

0

0
4

0

0

0
4

0

0

0
4

0

0

0
4

0

0

0
4

0.325867

0.09599

0

0

0

0.00615279

0

0

0

0

0.0129839

0

0.0629283
6

0

0

0

0

0.0173753

0

0

0

0

0

0
6

0

0

0

0

0

0

0

0

0

0

0.0142123
6

0

0

0

0

0

0

0

0.0461898

0

0

0.0367082

0

0

0

0

0

0.0333263

0

4.16333634234434e-17

0
4

0.220798
5

0.170809
6

0

0.0172206

0.0172928

0

0

0

0.0104881

0.00498723

0

3.38271077815477e-17
5

0
4

0.0409669

0
6

0.0409669

0

0

0

0

0

0

0

0

0

0
4

0.0389516
7

0.0389516
7

0

0

0

0
4

0.098872
6

0.0347507

0.0347507

0

0.0163868

0.0129839

1.04083408558608e-17
6

0
4

0.0943925
7

0

0

0

0.0943925

0
4

0

0

0

0

0

0
4

0

0

0

0

0

0

0
4

0
4

0.305121

0.305121

0.305121

0
4

0

0

0
4

0
4

0.032337

0.0149617

0.0149617

0
4

0.0173753

0.0173753

0
4

3.46944695195361e-18

0
4

0

0

0

0

0
4

0
4

0

0

0

0

0

0
4

0
4

0

0

0

0

0
4

0
4

0

0

0

0

0
4

0
4

0

0

0

0

0
4

0
4

0

0

0

0

0
4

0

0

0
4

0
4

0.0163868

0.0163868

0.0163868

0
4

0
4

0

0

0

0
4

0
4

2.88681
7

1.5287
7

0.0633708
7

0

0

0

0

0

0

0.00498723
7

1.35368
7

0.0245801
7

0.0310502

0

0.0100064

0.0335439

0.00748085

2.04697370165263e-16
7

0
4

0.0141909
6

0.0141909
6

0
7

0

0

0

0

0

0

0
4

0.00615279

0.00615279

0

0
4

1.26178

0

1.26178

0
4

0.052126
6

0

0.052126

0

0
4

0.00748085

0.00748085

0

0
4

0

0

0
4

0

0

0
4

0.0163868

0.0163868

0
4

2.63677968348475e-16
7

0
4

0

0

0

0
4

0

0

0
4

0
4

0.00498723

0.00498723

0.00498723

0
4

0

0

0
4

0
4

0.0163868

0.0163868

0

0.0163868

0
4

0
4

0

0

0

0
4

0
4

0.471962

0.471962

0.471962

0
4

0
4

0.00736842

0.00736842

0.00736842

0
4

0
4

0

0

0

0
4

0
4

0

0

0

0
4

0
4

0

0

0

0
4

0
4

0

0

0

0
4

0
4

0.137792
7

0.0444905
7

0.0271151
7

0.0173753

0

0

0

0
4

0.0933011
7

0.0759257
7

0

0

0.0173753

0

0

0
4

0
6

0

0

0

0
4

0

0

0
4

1.38777878078145e-17
7

0
4

0

0

0

0
4

0
4

0

0

0

0
4

0
4

0

0

0

0
4

0
4

0

0

0

0
4

0
4

0

0

0

0
4

0
4

0

0

0

0
4

0
4

6.93898
6

6.80835
6

0.764206
6

6.04415

0

0

0

0

0
4

0.074729
7

0.0573536
7

0

0.0173753

3.46944695195361e-18
7

0
4

0.0497429
7

0.0497429
6

0

0
4

0.00615279

0

0

0.00615279

0
4

0

0

0
4

0

0

0
4

0
4

0.162298
6

0.162298
6

0.162298
6

0

0

0

0

0
4

0
4

0
2

0
2

0
2

0

0

0

0

0

0

0

0
4

0
4

0.0314642
6

0
6

0

0

0

0

0

0
4

0.0314642

0

0.0314642

0
4

0
4

0.40379

0.40379

0.40379

0
4

0
4

0

0

0

0

0

0

0

0
4

0
4

20.7721

20.7311

8.28894

0.0327735
7

0.0293706
7

0.00498723

0

0.207742
7

0

0.0488464

0.0785309

0.0293706

0.0163868

0.692525
7

0

0

0

0

0

0

0

0.0378459

0.0194758

0.0324597

10.0111

0

0

0

0

0

0

0.037564

0.0163868

0.253186

0

0

0.0173753

0.0163868

0

0

0

0.0163868

0

0

0

0

0.473929
7

0

0

0

0

0

0

0

0

0.0163868

0.0163868

0

0

0

0

0

0

0

0.0245801

0

0

0

0
8

0

0.0129839

0.0163868

0

0

0.0129839

0

0

0.0163868

0

0

0

0

0

0

0.0163868

0

0

0.0163868

0

0

0.220726

0

0

3.38618022510673e-15

0
4

0

0

0
4

0.0409669

0.0409669

0
4

0

0

0
4

0
4

2.41584530158434e-13

0
4

13.1602

0.601089
7

0

0

0

0
4

0.200127

0.120076

0.0800508

0
4

0.0779032

0.0779032

0
4

0.0779032

0.0779032

0
4

0

0

0
4

0.245156

0.245156

0
4

0

0

0
4

0

0

0
4

8.32667268468867e-17
7

0
4

3.60503
7

3.60503
7

0

0

0

0

0.352315

0

0

0

0

0

0

0.676214

0.0157321

0

0

0

0

0

0

0

0

0

0.399633

0

0.0327735

0

0

0

0

0.0189229

0

0

0

0.477822

0

0

0

0

0

0.0434384

0

0

0.0163868

0

0

0

0

0

0

0

0

0

0

0.0283844

0

0

0

0.0283844

0

0.052126

0.136331

0

0

0

0

0

1.2736

0

0.0245801

0

0.0283844

0

0

0

8.60422844084496e-16
7

0
4

0

0

0
4

0
4

0.743863
6

0.743863
6

0.743863
6

0

0
4

0
4

0.0843952

0.0843952

0.0843952

0
4

0
4

0

0

0

0
4

0
4

0.0819338
7

0.0819338
7

0.0819338
7

0
4

0
4

0

0

0

0
4

0
4

0

0

0

0
4

0
4

0

0

0

0
4

0
4

0

0

0

0
4

0
4

0

0

0

0
4

0
4

0

0

0

0
4

0
4

0

0

0

0
4

0
4

8.04386

7.86834

2.85026

1.54049
7

0

0

0.026063

0

0

0.0584274

0

0

0

0.0737404

0.906868
6

0

0

0

0

0

0

0

0

0

0

0.725153

0.0327735

0

0

0
7

0.0781891

1.23826
7

0.165066

0.155674

0.0173753

5.3776427755281e-16

0
4

0

0

0

0

0
4

0.175521

0.175521

0
4

0

0

0
4

0

0

0
4

0

0

0

0
4

0

0

0
4

0

0

0
4

0

0

0
4

2.4980018054066e-16

0
4

0
4

2.39647
4

2.24602
4

2.24602
4

1.53963
4

0.0983205

0

0

0

0

0

0

0

0

0

0

0.106514

0

0.237608

0

0

0

0

0

0

0.0737404

0

0
7

0

0

0

0

0

0

0

0

0

0

0

0

0

0

0

0

0.00748085

0

0.139287

0

0

0

0

0

0

0

0

0

0.0434384

0

0

0
6

0

0

0

0
4

0

0

0
4

0

0

0
4

0

0

0
4

0

0

0
4

0

0

0
4

0

0

0
4

0

0

0
4

0

0

0
4

0

0

0
4

0

0

0
4

0

0

0
4

0

0

0
4

0

0

0
4

0
4

0

0

0

0
4

0
4

0.0173753

0.0173753

0

0.0173753

0
4

0
4

0

0

0

0
4

0
4

0

0

0

0
4

0
4

0.0327735

0.0327735

0.0327735

0
4

0
4

0.0491603

0.0491603

0.0491603

0
4

0
4

0

0

0

0
4

0
4

0

0

0

0
4

0
4

0

0

0

0
4

0
4

0

0

0

0
4

0
4

0

0

0

0
4

0
4

0

0

0

0

0
4

0
4

0

0

0

0
4

0
4

0

0

0

0
4

0
4

0

0

0

0
4

0
4

0

0

0

0
4

0
4

0

0

0

0
4

0
4

0

0

0

0
4

0
4

0

0

0

0
4

0
4

0

0

0

0
4

0
4

0

0

0

0

0
4

0
4

0

0

0

0
4

0
4

0.0163868

0.0163868

0

0.0163868

0
4

0
4

0

0

0

0
4

0
4

0.0347507

0.0347507

0.0347507

0
4

0
4

0
4

1921.65

0.217192
1

0.0868767

0.0868767

0

0

0

0

0
4

0

0

0
4

0

0

0
4

0.130315

0.130315

0
4

0

0

0
4

0

0

0
4

0

0

0
4

0

0

0
4

0

0

0
4

0

0

0
4

0
4

0.901599

0.901599

0.147481

0
2

0
3

0.0163868

0

0

0

0

0

0.0852735

0.0461898

0.204834

0

0

0

0

0

0

0

0

0

0

0
4

0.243255

0

0

0

0

0

0

0

0

0

0

0

0

0

0

0

0

0

0

0

0

0

0

0

0

0

0

0.0104881

0

0

0

0

0
4

0

0

0

0

0

0

0

0

0

0

0
3

0

0

0

0

0

0

0

0

0

0

0

0

0

0

0

0

0

0.14769

0

0

0

0
3

0

0

0

0

0

0

0

0

0

0
4

0
3

0
3

0

0

0

0

0
4

0

0

0
4

0

0

0
4

0

0

0
4

0

0

0
4

0

0

0
4

0

0

0
4

0

0

0
4

0

0

0
4

0

0

0
4

0

0

0
4

0
3

0
3

0
4

0

0

0

0
4

0

0

0

0
4

0

0

0
4

0

0

0

0
4

0

0

0
4

0

0

0
4

0

0

0
4

0
4

0
1

0
1

0
1

0

0

0

0

0

0

0

0

0

0

0
1

0

0

0

0

0

0

0

0

0

0

0

0

0

0

0

0

0

0

0

0

0

0

0

0

0

0

0

0

0

0

0

0

0

0

0

0

0

0

0

0

0

0

0

0

0

0

0

0

0

0

0

0

0

0

0

0

0
4

0
2

0
2

0

0
4

0

0

0

0

0

0
4

0

0

0
4

0

0

0
4

0

0

0
4

0

0

0
4

0

0

0
4

0

0

0
4

0

0

0
4

0

0

0
4

0

0

0
4

0

0

0
4

0

0

0

0

0
4

0

0

0
4

0

0

0
4

0

0

0
4

0

0

0

0
4

0

0

0
4

0

0

0
4

0

0

0
4

0

0

0
4

0
4

572

7.05172

0
2

0.00475254

0

0.23238

0

0

0.00475254

0

0

0.0608137

0.00475254

2.90502
6

0

0

0.00475254

0

0

0.0285152

0.0337621

0

0

0.269318

0
3

0

0.00498723

0

0.026063

0.052126

0

0

0

0

0

0
2

0

0

0

0

0

0

0

0

0

0

0
3

0

0

0

0.0163868

0

0

0.0608137

0

0.0245801

0

2.99298
6

0

0

0

0

0.0943925

0.122901

0.0173753

0
2

0.0902983

0
2

3.19189119579733e-16

0
4

544.409

518.346

1.20763
7

0.168558

0.460927

0.0194758

0.0129839

0.0104881

0

0

0.0194758

0

0

0

0.313731
7

0.00710613

0.0478845

0

0.0604021

0.0147368

0

0

0

0

0

0.29005

0

0

0

0

0

0.0142123

0

0.0245801

0.0367082

0.0347507

0.18481
7

0.0163868

0.0262201

0

0

0

0

0

0

0

0.0173753

0.367307

0

0

0

0.00736842

0

0

0

0.0163868

0

0.0173753

0.068356
7

0

0

0

0

0

0

0

0

0.0129839

0

0.177339
6

0

0

0.0209761

0

0

0

0

0.0163868

0

0

0.14853
7

0

0.0120572

0

0

0

0

0

0

0

0.104252

0.210188
7

0

0

0

0.0327735

0

0.00498723

0.0262201

0

0

0

0.216215
7

0

0

0.0245801

0

0

0

0

0

0

0

2.0761
7

0.0451027
7

0

0.00736842

0

0

0

0.00475254

0

0.0163868

0

0

0.115124
7

0.0163868

0

0

0

0

0.0259677

0

0

0

0

0.115928
7

0

0

0.0104881

0

0

0

0

0

0.0129839

0.0163868

0.00710613
7

0.0157321

0

0

0

0

0

0

0

0

0

0.0976314
6

0

0.0163868

0

0.0327735

0

0

0

0

0.0163868

0

0.0416998
2

0

0

0

0

0

0

0

0

0.0110526

0

0.0454468
7

0

0.00710613

0

0

0

0.00710613

0.0163868

0

0

0.589923

0

0.018421

0

0

0

0

0

0

0

0

0

0.0270459
7

0

0

0.0104881

0.0129839

0

0

0.0163868

0

0

0

0.0707809
6

0

0

0

0

0

0

0

0

0

0

1.22959
7

0.0838153
6

0

0

0

0

0

0

0

0

0

0

0.12159
7

0

0

0

0

0

0.0491603

0.00748085

0

0

0

0.00498723

0

0

0

0

0

0.0209761

0

0

0

0

0.0299639
7

0.0163868

0.0173753

0.00498723

0

0.0106592

0

0

0

0

0.0080381

0.0120934
7

0

0

0

0.0173753

0

0.0737404

0

0

0

0

0.0434384
8

0

0

0

0

0

0

0

0

0

0

0
1

0

0

0.0173753

0

0

0.00710613

0

0

0.0163868

0

0.0248714
7

0.0314642

0

0.0779032

0

0

0

0

0

0.00710613

0

0

0.0129839

0

0

0

0

0

0.0106592

0

0

0

0.0964567
7

0

0

0

0

0

0

0

0

0.835147
7

0.0839044

0.0305008
7

0

0.0163868
7

0
7

0.141922
6

0.0157321
8

0

0.0104881

0.0104881

0.765179
7

0.0245801
7

0.22123
7

0.0565067

0.00498723
6

0

0.0173753

0.0491603

0.0245801

0

0.121627

11.9195
7

0.0157321

0.0715066

0

0.0211393

0.0106592

0

0.026961

0

0.0200953

0.0174553

0.363908
7

0.0163868

0.0080381

0

0

0.00710613

0

0.0100064

0

0

0.0110526

0.470332

0

0.00710613

0.026063

0

0

0.0104881

0

0

0

0

0.485524

0

0

0

0

0

0.0314642

0.0163868

0

0

0.0157321

0
4

10.6733
7

10.6733
7

0

0

0
4

7.9131

2.53329

0.111103

0

0

0.0163868

0

0.00736842

0.0163868

1.42458

0.065547

0

0

0.647277
6

0

0.0327735

0.0901271

0

0.0207193
4

0

0

0

0.068827

0.0819338

0

0.0529645

0

0

0

0
2

0.0189229

0

0.0194758

0.0104881

0

0

0

0

0.0163868

0

0.0327735
3

0

0.0409669

0

0

0

0

0

0

0.0245801

0

2.26957
6

0.0163868

0

0.0409669

0

0

0

0.0163868

0

0

0

0.0163868
1

0

0

0

0

0

0

0

0

0

0

0.021374

0

0

0

0

0

0

0

0

0

0

0.0194758
3

0

0

0

0

0

0

0.0104881

0

0.00498723

0

0.0740711
4

0

0

0

0

0.0245801

0.065547

0

0

0

0

0
4

1.04669

0.763409

0.283278
7

0

5.55111512312578e-17

0
4

0.0173753

0.0173753

0
4

0.0163868

0.0163868

0

0
4

0

0

0
4

0

0

0
4

0

0

0
4

0

0

0
4

0

0

0
4

0.0173753

0.0173753

0
4

0

0

0
4

0

0

0
4

0.108491

0
1

0

0

0

0.0347507

0

0

0

0.0737404

0

0

0

0
4

0.0163868

0.0163868

0
4

0

0

0
4

0

0

0
4

0

0

0
4

0.271238
7

0
7

0.0163868

0.254852

0

0

0

0
4

0

0
1

0

0

0

0

0

0

0
4

0.245801

0.0245801

0.0901271

0.0163868

0.114707

0

0
4

0.180254

0.180254

0

0

0

0
4

0.00710613
1

0

0

0.00710613

0
4

0

0

0

0

0

0
4

0.026063

0

0

0.026063

0
4

2.38756930892592e-13

0
4

8.86699

8.73079

8.73079

0

0

0

0

0

0

0
4

0.0522987
1

0.0246112
2

0.00615279
1

0.0215348

0

0
4

0

0

0
4

0

0

0
4

0

0

0
4

0

0

0

0
4

0

0

0

0
4

0

0

0
4

0

0

0

0
4

0

0

0
4

0

0

0
4

0

0

0
4

0

0

0

0
4

0

0

0

0
4

0

0

0

0
4

0

0

0
4

0

0

0
4

0.0104881

0.0104881

0
4

0

0

0
4

0

0

0
4

0

0

0
4

0

0

0
4

0

0

0
4

0
2

0
2

0

0
4

0

0

0
4

0.0734164

0.0734164

0
4

0

0

0
4

0

0

0
4

0

0

0
4

0

0

0
4

0

0

0
4

0

0

0
4

0

0

0
4

0

0

0
4

0
2

0
2

0
4

0

0

0
4

0

0

0
4

0

0

0
4

0

0

0
4

0

0

0
4

0

0

0
4

0

0

0
4

0

0

0
4

0

0

0
4

0

0

0

0
4

0

0

0
4

0

0

0

0
4

0
4

0.24889
3

0.24889

0.24889

0

0

0

0
4

0

0

0

0

0
4

0

0

0
4

0

0

0

0
4

0

0

0

0

0
4

0

0

0
4

0

0

0

0
4

0

0

0
4

0

0

0
4

0

0

0
4

0

0

0
4

0
4

2.27263

0.633826

0
8

0

0.0104881

0

0

0

0

0.00736842

0

0

0

0

0.245801

0

0

0.00736842

0

0.0157321

0

0

0

0

0

0

0

0

0

0.131101

0

0

0

0

0

0

0.0737404

0

0

0

0

0

0.0573536

0

0

0

0

0

0

0.0147368

0

0

0

0.0104881

0

0

0

0

0

0

0

0

0

0

0

0

0

0

0

0

0

0

0

0

0.0245801

0

0

0

0

0

0

0

0

0

0

0

0

0

0

0.0245801

0

0

0

0

0

0

0

0

0

0.0104881

0

0

0

0

0

0

0

0

0

0

0

0

0

0

0

0

0

0

0

0

0

0

0
4

0.844288

0.0943925

0.739408

0.0104881

0

0

0

0

0
4

0.246469

0.246469

0
4

0

0

0

0

0

0
4

0.0104881

0.0104881

0
4

0

0

0

0
4

0

0

0
4

0.0367082

0

0.0367082

0

0
4

0

0

0

0

0
4

0.0209761

0.0104881

0

0.0104881

0
4

0

0

0

0
4

0.0221053

0.0221053

0
4

0

0

0

0

0

0
4

0

0

0

0

0
4

0

0

0
4

0.0314642

0.0314642

0
4

0.0314642

0

0.0209761

0.0104881

0
4

0

0

0

0

0
4

0.245801

0.245801

0
4

0

0

0
4

0

0

0

0
4

0

0

0
4

0

0

0

0
4

0

0

0

0
4

0

0

0
4

0

0

0
4

0.0901271

0.0901271

0
4

0

0

0
4

0

0

0
4

0

0

0
4

0.00498723

0.00498723

0
4

0

0

0
4

0

0

0
4

0

0

0
4

0

0

0

0

0

0

0

0
4

0

0

0
4

0

0

0
4

0

0

0
4

0

0

0
4

0.0173753

0.0173753

0
4

0

0

0
4

0

0

0
4

0

0

0
4

0

0

0
4

0

0

0
4

0
7

0
7

0

0
4

0

0

0
4

0

0

0
4

0

0

0
4

0

0

0
4

0.026063

0.026063

0
4

0

0

0
4

0

0

0
4

0.0104881

0.0104881

0
4

0

0

0
4

0

0

0

0

0

0

0
4

0

0

0
4

0

0

0

0

0
4

0

0

0

0
4

7.52869988573934e-16

0
4

0.509235

0.509235

0.441554

0

0

0

0

0

0

0

0

0

0

0

0

0

0

0

0

0

0

0

0

0

0

0

0

0

0.0676807

0

0

0

0

0
4

0

0

0

0

0

0

0
4

0

0

0
4

0

0

0

0
4

0

0

0
4

0

0

0
4

0

0

0
4

0

0

0
4

0
4

0.306299

0

0

0

0
4

0

0

0

0

0

0

0

0

0

0

0
4

0

0

0
4

0.0943925

0.0839044

0.0104881

3.46944695195361e-18

0
4

0

0

0
4

0

0

0
4

0

0

0

0
4

0

0

0
4

0

0

0

0
4

0

0

0
4

0

0

0
4

0

0

0
4

0

0

0

0

0

0
4

0.0163868

0.0163868

0
4

0

0

0
4

0

0

0
4

0

0

0
4

0

0

0
4

0

0

0
4

0

0

0
4

0

0

0
4

0

0

0

0

0

0
4

0.0681724

0.0681724

0

0
4

0

0

0

0
4

0

0

0

0
4

0.11686

0.11686

0

0
4

0
2

0
2

0

0

0
4

0.0104881

0.0104881

0

0
4

3.46944695195361e-18

0
4

3.29961

3.28322

3.28322

0

0

0

0

0

0

0

0

0
4

0

0

0

0
4

0

0

0

0
4

0

0

0
4

0.0163868

0.0163868

0
4

0

0

0
4

0
4

1.26546

0.356594

0

0.0104881

0

0.141589

0

0.204517

0

0

0

0

2.77555756156289e-17

0
4

0.908871

0.908871

0
4

0

0

0

0
4

1.11022302462516e-16

0
4

28.3434

0
7

0

0

0

0

0

0

0

0

0

0

0

0

0

0

0

0

0

0

0

0

0

0

0

0

0

0

0

0

0

0

0

0

0
4

0.589739

0.0209761
7

0

0

0

0.00736842

0

0

0

0

0

0

0.561395
7

0

0

0

0
7

0

0

0

0

0

0

0
4

15.4165

11.7949
7

1.20297
7

0

0

0.0106592

0

0

0.00736842

0.0177653

0.00710613

0.239474

0.0245801

1.61558
7

0

0

0

0

0

0.0294737

0.00736842

0

0

0

0.110214
6

0

0

0

0

0

0

0.0283844

0

0

0

0.195263

0

0.0213184

0

0.0378459

0

0

0

0

0

0

0

0

0

0

0.00710613

0

0

0

0

0.0110526

0

0.0355306

0

0

0

0

0.00498723

0

0

0

0

0

0

0

0

0

0.00748085

5.8113236445223e-17

0
4

4.50778

2.17692

0.938467
7

0

0

0

0

0

0

0.0294737

0

0

0

0

0.0124681
7

0

0

0

0

0

0

0

0

0

0

0.204929
7

0

0

0

0

0.00498723

0

0

0

0

0

0.032417
7

0

0

0.0104881

0

0

0.00736842

0.0173753

0

0

0

0.0248562
6

0

0

0

0

0

0

0

0

0

0

0

0

0.0157321

0

0

0

0

0

0

0

0.00710613

0.0629283

0

0

0.00736842

0

0

0

0

0.0104881

0.0104881
7

0

0.0355306

0.07
7

0

0

0

0

0

0

0.0473814

0

0

0.0200953

0.0773302

0.0284245

0

0.0209761

0

0

0

0.019538

0

0

0

0.0888482
5

0

0.0434384

0

0.0548595

0

0

0

0

0

0

0.0962487
7

0

0

0

0

0.0120572

0

0

0

0

0

0.0891485
7

0

0

0

0

0

0

0

0.162105

0.00736842

0

0

0

0

0.0120572

0

0

0

0

0

0

0

0
7

0

0

0

0

0

0

0

0

0.0209761

0

0.0170444

0

0

0.0104881

0

0

0

0

0

0

0

0
4

0
2

0
2

0

0

0

0

0

0

0

0

0

0

0
2

0

0

0

0

0

0

0

0

0

0

0

0

0

0

0

0

0

0

0

0

0

0
2

0

0

0

0

0

0

0

0

0

0

0

0

0

0

0

0

0

0

0

0

0

0

0

0

0

0

0

0

0

0

0

0

0

0

0
4

1.14852

1.14852

0
4

2.19603
7

2.14359
7

0.0524403

0

0
4

0.554654

0.00748085

0

0

0

0

0

0

0

0

0.139287

0

0.188448

0.097251

0.122187

0

0

0

4.16333634234434e-17

0
4

0.532143

0.110125

0.131101

0

0.0129839

0.0419522

0

0

0

0

0

0

0

0

0

0

0

0.125857

0.0157321

0.0943925

0

0

6.93889390390723e-17

0
4

0
3

0

0

0

0

0
4

0
6

0
6

0

0
4

0.0157321

0

0

0.0157321

0

0
4

0.0194758

0

0

0

0

0.0194758

0
4

0

0

0

0
4

0

0

0

0
4

0

0

0

0

0
4

0.0209761

0

0.0209761

0

0
4

0.0839044

0.0839044

0

0

0
4

0

0

0
4

1.32329

1.13795

0

0

0

0

0

0.159112
7

0.0104881

0

0

0

0

0.0157321

0

2.77555756156289e-17

0
4

0

0

0
4

0

0

0

0
4

0

0

0
4

0

0

0
4

0

0

0

0

0
4

0

0

0

0

0
4

0

0

0

0
4

0

0

0

0
4

0

0

0
4

0

0

0

0
4

0.226017
7

0.195418
7

0.0163868
7

0

0.0142123

0

0

0

0
4

0

0

0

0
4

0

0

0

0
4

0

0

0

0
4

0

0

0
4

0.0174553

0

0.0174553

0
4

0

0

0

0
4

0

0

0
4

0

0

0
4

0

0

0
4

0

0

0
4

0.662492
7

0.662492
7

0

0
4

0

0

0
4

0

0

0
4

0

0

0
4

0

0

0
4

0

0

0
4

0

0

0
4

0

0

0
4

0

0

0
4

0

0

0
4

0

0

0
4

0.0847368
7

0.0847368

0

0

0

0
4

0

0

0
4

0

0

0
4

0

0

0
4

0

0

0
4

0

0

0
4

0

0

0
4

0

0

0
4

0

0

0
4

0

0

0
4

0.0124681

0.0124681

0
4

0.327895
7

0.327895
7

0
4

0

0

0
4

0

0

0
4

0

0

0
4

0

0

0
4

0

0

0
4

0

0

0
4

0

0

0
4

0
7

0

0

0

0
4

0.35135
5

0.35135

0

0

0

0
4

0.252268
7

0.252268
7

0

0

0
4

0
4

4.71921

4.71921

4.71921

0

0

0

0
4

0

0

0

0

0

0

0

0
4

0

0

0
4

0

0

0
4

0

0

0
4

0

0

0
4

0

0

0
4

0

0

0
4

0

0

0

0

0

0

0

0

0
4

0

0

0

0

0

0

0

0
4

0

0

0

0

0

0
4

0

0

0

0
4

0

0

0

0
4

0

0

0

0
4

0

0

0

0
4

0

0

0
4

0
4

1265.04
7

754.019
7

114.253
7

7.77484
7

0.109814
7

0

0

0.0223626

0

0

0.0327735

0.0173753

0.0423915

0

0.0173753

0.0930797
7

0

0

0.0173753

0.0104881

0.256957

0

0.0608137

0.0737404

0

0

0.0501488
7

0

0.0163868

0.0174553

2.74891

0

0

0

0

0

0.247582

0.0811957
7

0

0.196641

0

0.0163868

0.0104881

0

0

0

0

0.0327735

0.146416
7

0

0.00922919

0

0.314642

0.0110526

0

0

0.049903

0

0

0.0283844

0.0695014

0.0173753

0

0.0163868

0.0245801

0

0.052126

0

0

0

0.217623
7

0.104881

0.0163868

0

0

0

0.0262201

0.0124681

0

0

0

0.199145
7

0

0

0.00615279

0

0.0491603

0

0.0163868

0.00498723

0

0

0.00712881
7

0

0.0245801

0

0

0.0173753

0

0

0

0

0

0.0438597

0

0

0

0

0.0157321

0

0

0

0

0.00498723

0.779595
7

0.0945641
8

0

0

0

0

0

0

0.0163868

0.0149617

0

0

0.434933
7

0

0

0

0

0.0189229

0

0

0.0163868

0.132461

0.0163868

0.893078
8

0.0283844

0

0

0

0

0

0

0

0

0.0163868

0.292793
7

0

0.00748085

0

0

0.0245801

0

0

0

0

0

0.0911736
7

0

0.026063

0

0

0

0

0.00615279

0.0851532

0.0378459

0.0299234

0.0209761
7

0

0

0

0

0

0

0

0.0327735

0.0173753

0

0.0772005
7

0

0

0

0.00498723

0

0

0

0.0173753

0.0327735

0.00498723

0.0378459
7

0

0

0

0

0.0209761

0.477206

0

0

0

0

0.441094
7

0

0

0

0.0124681

0

0

0

0.026063

0.0173753

0

0.323199
7

0

0

0

0.0173753

0.0163868

0

0

0

0.052126

0

4.67942
7

0.0245801
7

0

0

0

0.065547

0

0

0

0

0.0434384

0

0.173186
7

0

0

0

0

0

0

0.0209761

0

0.00748085

0

0.0772005
7

0

0.026063

0

0

0

0.026063

0

0.0245801

0.0104881

0

0.0459167
7

0.0189229

0

0

0.0955644

0

0

0.0163868

0

0

0

0.0523246
7

0

0

0

0

0.0163868

0

0

0.0434384

0

0

0.286333
6

0.0409669

0.104252

0

0

0

0

0.0245801

0

0

0.0283844

0.088006
7

0

0

0

0

0.0245801

0

0

0

0

0.0173753

0.120135
7

0

0.00498723

0

0

0

0.0245801

0

0

0.00498723

0

0.055216
7

0.0124681

0.104252

0

0

0

0

0.00615279

0

0.0349106

0

0.148723
7

0

0

0

0.0104881

0

0

0

0

0.0983205

0

11.2011
7

0.213028
7

0

0

0

0

0

0.0189229

0

0.0409669

0

0

0.0486661
7

0

0

0.0163868

0

0

0.0491603

0.00498723

0.0104881

0.0283844

0

0.0974955
7

0

0

0

0

0

0.0189229

0

0

0

0.0163868

0.213714
7

0

0

0.0245801

0

0.0314642

0

0

0.026063

0.0173753

0

0.357268
6

0.00498723

0

0

0.026063

0.0259677

0

0

0

0

0

0.0688775
7

0.0173753

0

0

0.0327735

0

0

0.0104881

0

0

0

0.135086

0.352315

0.0173753

0.0163868

0

0

0

0.0104881

0

0

0

0.0639845
7

0.0434384

0.026063

0

0.0163868

0

0

0.0737404

0.0573536

0.0104881

0

0.0506431
7

0

0

0

0

0

0

0

0

0

0

0.0497584
7

0.0173753

0

0

0

0.0173753

0.0104881

0.251713

0

0.0163868

0

3.44007
7

0.120639
7

0

0

0

0

0

0

0

0

0

0

0.085217
7

0

0.00498723

0.0163868

0

0.00710613

0

0

0

0

0.0173753

0.021374
8

0.00736842

0

0

0.0245801

0

0

0

0

0

0

0.104661
7

0

0.0163868

0

0

0

0

0

0.00748085

0

0

0.125866
6

0.0189229

0

0

0

0

0.0173753

0

0

0

0

2.15306
6

0.0163868

0

0

0.0104881

0

0

0

0

0.0157321

0

0.507989

0

0

0

0

0

0

0

0

0.0409669

0

0.0234929
7

0

0

0

0

0

0

0.00498723

0.0189229

0

0.0163868

0.15864
8

0

0

0.0245801

0.0573536

0

0

0

0.0245801

0

0

0

0

0

0

0

0.0163868

0

0

0

0

0

4.48463
7

0.0409669
7

0

0

0

0

0

0.0163868

0

0

0

0

0.0658009
7

0

0.0367082

0.0173753

0

0

0

0.0163868

0

0

0.0104881

0.187651
7

0

0

0

0

0.0173753

0

0

0.0142123

0

0

0.0834745
7

0.876773

0

0

0

0

0

0

0

0.0163868

0

0.0452416
7

0.0241143

0

0

0.0173753

0

0

0.026063

0.0756917

0

0.0163868

0.186843
7

0

0

0.0106592

0

0

0

0.0975176

0

0

0

0.0163868
7

0

0

0

0

0

0

0.0189229

0.0189229

0.0955644

0.0189229

0.111951
7

0

0

0

0.0157321

0

0.0173753

0.0163868

0

0

0

0.247243
6

0

0

0.0104881

0

0

0

0

0.00922919

0

0

0.122901
7

0

0

0

0.0283844

0

0

0.0163868

0

0.0189229

0

1.02912
7

0.131094
7

0

0

0.0327735

0

0

0

0

0.00498723

0

0

0
7

0

0.0173753

0

0

0.00748085

0

0.0327735

0.0209761

0

0

0.347745
6

0

0

0

0

0

0

0.0104881

0.0409669

0

0

0.104252
6

0

0

0

0

0

0

0

0.0209761

0.0327735

0

0.00498723

0.0157321

0

0.0819338

0

0

0

0

0

0

0

0.0853906
7

0

0

0.0104881

0

0.0163868

0

0.0245801

0

0.0189229

0

0.0682822

0.0245801

0.0283844

0

0

0

0

0.00498723

0

0

0.0249362

0.0715228
7

0

0

0

0

0

0

0.0163868

0.106514

0.0163868

0

0.110786
7

0.0189229

0.0946147

0

0

0

0.0100064

0.00997446

0

0

0.0163868

0.0721136
7

0.0245801

0.0104881

0.0245801

0

0

0

0

0.0163868

0

0.0163868

1.97193
7

0.137552
6

0

0.106514

0

0.0347507

0

0

0.0124681

0

0.00615279

0

0.0327735
8

0.0378459

0

0

0

0.0163868

0

0

0

0.0868767

0

0.376943
7

0

0.0104881

0.0173753

0

0

0.0104881

0.00498723

0

0.0245801

0

0.0720511
7

0

0

0.0104881

0.0473073

0

0.0327735

0.0347507

0.393282

0

0

0
6

0.0173753

0

0

0

0

0

0.0189229

0

0

0

0.0295674
7

0.163868

0

0

0.0163868

0

0

0

0

0.0163868

0.0491603

0.0542326
7

0

0

0

0

0

0

0.0163868

0

0.0189229

0

0.0661342
7

0

0.00748085

0

0

0

0

0

0.0173753

0.0104881

0

0.0488037
7

0

0.00498723

0.0173753

0.0104881

0.0104881

0.0434384

0

0

0

0

0.0596868
8

0

0

0

0

0

0

0.0245801

0

0

0.00748085

1.714
7

0.0780122
7

0

0.026063

0

0

0

0.0327735

0

0.0173753

0.0367082

0.199816

0.00748085
7

0.0104881

0

0

0

0

0

0

0

0

0

0.0894995
8

0

0.0491603

0

0

0

0.0173753

0

0

0

0

0.129327
7

0.0104881

0.0245801

0.0163868

0

0

0

0.0245801

0

0.0245801

0

0.0931507
7

0.0163868

0

0

0

0.0573536

0

0

0

0

0.0173753

0.0906346
7

0

0

0

0

0

0

0.0173753

0

0.0245801

0.0080381

0.053349
6

0

0

0

0.0283844

0

0

0.0163868

0.00997446

0

0

0.140364
7

0.0104881

0

0.065547

0

0.155674

0.0173753

0

0

0.0163868

0

0
7

0

0

0.0249362

0

0.0283844

0.0163868

0

0

0

0

0.0245801

0.0163868

0.0173753

0

0.052126

0.00498723

0

0

0

0.00475254

0

0.781505
7

0.0327735
6

0.0163868

0

0

0

0.00950508

0

0

0

0

0

0.189436
7

0.162565

0

0

0

0

0

0

0

0

0

0.0998034
6

0

0

0

0.0245801

0

0

0

0

0.360509

0

1.85765
7

0

0.052126

0

0

0

0.0173753

0.00498723

0

0

0

0.149432
7

0.0163868

0.0245801

0

0

0.0347507

0

0

0

0.0173753

0

0.0648345
7

0

0.00498723

0

0

0

0.0347507

0.0104881

0.0163868

0

0.0409669

0

0

0.00748085

0.0262201

0.0378459

0

0

0

0

0.026063

0

0.397871
7

0

0

0.0473073

0.026063

0

0

0.0189229

0

0

0

0.199816
7

0

0.0163868

0

0.00498723

0

0.00498723

0.0839044

0.00498723

0.0245801

0

0.170636
7

0.0163868

0

0.0409669

0

0

0

0

0

0

0

89.8288
7

2.86588
7

0
7

0.0262201

0

0

0

0.0163868

0

0

0

0

0

0.0397379
6

0.0173753

0.00498723

0.0245801

0

0

0

0

0

0

0.0163868

0.0573536

0.0173753

0

0

0

0

0

0

0

0.00498723

0

0
7

0

0

0.0163868

0

0.0163868

0

0

0

0

0

0
7

0.0173753

0

0

0

0

0.0283844

0

0.0163868

0.0737404

0.0209761

0.0434384
7

0

0.00615279

0

0

0

0

0.0209761

0

0

0.0100064

0
7

0

0

0

0

0

0

0

0

0.0173753

0

0.00498723
8

0

0.0695014

0.0673276

0

0.0189229

0.0523659

0

0

0.052126

0

0.0839109
7

0

0.0573536

0

0

0.126773

0

0

0.0173753

0.0189229

0

0.0573536
7

0

0

0.0245801

0.0173753

0.0163868

0

0

0.393282

0.0149617

0

0.431912
7

0.073677
7

0

0.0163868

0.00498723

0.0471962

0

0

0

0.0409669

0

0

0.052278
7

0.0163868

0

0

0.0299234

0

0

0

0

0

0

0.0173753
7

0.0173753

0

0

0

0

0.00498723

0.0378459

0.0104881

0.0104881

0.0434384

0.1764
7

0

0

0.0173753

0.026063

0.0104881

0

0

0

0

0

0.100102
7

0

0

0

0

0

0.0378459

0

0

0

0.0173753

0.0921043
6

0

0

0

0.0378459

0.0163868

0.00498723

0

0.0245801

0.283177

0

0.0254497
6

0

0.0173753

0

0

0

0

0

0.0786604

0

0

0
6

0

0

0.026063

0

0

0

0

0.026063

0

0

0.0189229

0

0.026063

0

0.0283844

0

0

0

0

0

0

0.398499
7

0

0

0

0

0

0

0.0245801

0.0104881

0

0

0.335364
7

0.0209761
7

0.0327735

0

0.0104881

0

0

0

0.0955644

0.0173753

0

0

0.074729
7

0.00498723

0

0

0

0

0.0245801

0.0173753

0.00498723

0

0

0.449576
6

0

0

0.0157321

0.026063

0.0163868

0.0104881

0

0.106514

0

0

0.0583422
7

0

0

0.0245801

0

0

0

0

0

0

0.026063

0.185733
7

0

0

0.0245801

0

0

0.0573536

0

0

0

0

0.0593308
7

0

0

0

0

0

0.00710613

0

0.00615279

0.0163868

0.0245801

0.00710613
7

0

0

0

0

0

0

0

0

0

0

0.00498723
7

0.0173753

0

0.0245801

0

0

0

0.0314642

0

0.0248714

0.0519355

0.101604
7

0

0.00710613

0

0

0.00615279

0.0245801

0

0

0

0.0173753

0.026063
8

0

0

0

0.0104881

0

0

0

0.0262201

0

0

4.68971
7

0.0378459
6

0

0

0

0

0.0173753

0.0104881

0

0

0

0.00736842

0.0573536

0.0245801

0

0.0434384

0

0

0.0163868

0.0173753

0

0

0.00498723

0.150084
8

0.0327735

0

0.0409669

0.0327735

0

0.0756917

0

0.0173753

0

0.0819338

0.145847
7

0

0

0

0

0.0163868

0.0163868

0

0

0

0.0314642

0.0307329
8

0

0

0

0

0

0

0

0.0245801

0.065547

0.052126

0.00710613

0.0163868

0.0163868

0.00498723

0

0

0

0

0

0.0163868

0

0.0173753
6

0

0

0

0

0

0

0

0.0123056

0.0378459

0

0.407006
7

0

0

0

0

0

0

0

0

0.0314642

0.0173753

0.0337621
7

0

0.0245801

0.0245801

0.0173753

0.0104881

0

0

0

0

0

0.0263612
6

0

0

0.00475254

0

0

0.0173753

0

0

0

0

3.43343
7

0

0

0

0.0163868

0

0

0

0

0

0

0

0.00922919
7

0.032417

0.0189229

0

0

0.0327735

0

0

0

0

0.0173753

0.0173429
6

0.0163868

0

0.0249362

0

0.0209761

0

0

0

0

0

0.208152
7

0

0.0163868

0

0

0

0.0104881

0

0.0695014

0.0163868

0

0.00615279
7

0.0573536

0

0

0.0163868

0.0163868

0

0.0163868

0.0245801

0

0

0

0.0163868

0

0

0

0

0

0

0

0.0157321

0

0.00475254
7

0

0

0.0245801

0.0173753

0

0

0.00498723

0.0163868

0

0

0.0104881
7

0.00615279

0

0

0

0

0.0409669

0

0.0163868

0

0

0.0797957
8

0.0163868

0

0.0163868

0.0163868

0

0.0378459

0.0189229

0

0

0

0.0328506
7

0.00997446

0

0.0434384

0

0

0.0245801

0.0245801

0

0.0173753

0

1.19433
7

0

0.0378459

0

0

0

0

0

0.0327735

0.0314642

0

0

0.187107
7

0

0.0491603

0

0

0

0

0

0

0

0

0.0317687
7

0

0

0

0

0

0.026063

0

0

0

0.0245801

0.199113

0

0

0

0

0

0

0.0983205

0

0.106514

0

0
7

0

0

0.0100064

0.0173753

0

0

0

0

0

0

0.253995

0

0

0.0314642

0

0

0

0.0173753

0

0

0

0.0280345
6

0

0

0

0

0.0173753

0

0.0104881

0

0

0

0.0419555
7

0.0173753

0

0

0.0123056

0

0

0

0.0163868

0

0.0819338

0

0

0.0163868

0

0

0

0

0

0

0.14769

0

0.0470391
6

0

0

0

0

0

0

0

0

0.0157321

0.00997446

0.696855
7

0.0662303

0

0

0

0

0

0

0

0.0245801

0

0

0.0238676
7

0

0

0

0

0

0.0163868

0.340613

0

0

0

0.0295674
8

0

0

0

0.0080381

0

0.0573536

0

0

0.026063

0

0.0848995
7

0

0

0.0245801

0

0

0

0

0.0955644

0.00710613

0

2.25011
7

0

0.0157321

0.0157321

0

0

0.0163868

0.0163868

0

0

0.0245801

0.122999
6

0

0

0.0367082

0

0.0623404

0

0

0

0.0173753

0

0.0330276
7

0

0.0163868

0

0

0

0.0224425

0.0737404

0

0.0567688

0.00498723

8.42704
7

0

0

0

0

0.0209761

0.00748085

0.0327735

0

0

0

0.272689

0

0

0

0

0

0

0

0

0

0

0.0983205

0

0.0163868

0

0

0.0189229

0

0

0.0163868

0.0104881

0

0.297393

0.0567688
7

0

0

0

0

0

0

0

0.0173753

0

0

0.0583422
7

0

0.0173753

0

0.0163868

0.0163868

0

0.0163868

0

0

0.0327735

0.0245801
7

0

0

0

0

0

0

0

0.0189229

0.0347507

0.0327735

0.0272673
6

0

0

0.0163868

0

0.0163868

0.0163868

0.026063

0

0.0104881

0

0.205823

0

0

0

0

0

0

0

0

0

0

0.0189229
7

0

0

0

0.0737404

0

0.0189229

0

0

0.0163868

0

0.0423915

0

0.0245801

0

0

0.0245801

0

0

0

0.0189229

0

0.0857061

0.0327735

0

0

0.0163868

0.0868767

0

0

0

0.0189229

0.0473073

0

0

0.0163868

0

0

0.0173753

0

0

0.00475254

0.0173753

0.0163868

0.0224425

0

0.052126

0

0

0

0

0

0

0

0

1.52184
7

0.0189229

0.0173753
7

0.0163868

0.128163
7

0.0491603
7

0.0409669
7

0.114707
7

0
8

0

0.0173753
7

26.8377
7

0.0327735
7

0.0398978
6

0.0788127
7

0.0367082

0.0767029
7

0.0283844
7

0.0175942
7

0
6

0
6

0
8

133.722
7

4.20514
6

0

0.0321188

0

0.0473073
6

0.10905
7

0.0327735

0

0.0695014

0.00736842
8

0

0.535457
7

0

0.0409669
7

0.100792

0.0863509

0.0901271
7

0

0.021374

0.0156464
8

0
7

0
7

0.374273
7

0.0337621
6

0.0851675
6

0.136345

0.00973977

0.0245801
6

0.0573536
7

0.00498723
7

0

0.0378459

0.00748085
8

0.108228
7

0.0970916
7

0.021374
6

0.0176169
7

0.131806
7

0.0176169
7

0

0

0.0149617

0.0983205

0.0378459

0.572718
7

0.00710613
8

0.0163868
7

0.0106592
6

0.0573536

0.0582788

0.0321188

0.0283844

0.149648

0.021374

0.0104881
7

0.518958
7

0.0894146

0.0199489

0.00997446
6

0.00710613

0.0394806

0

0.0378459

0.270381

0.151258

0

0.643368
7

0.0978912
6

0

0
7

0.038186
7

0

0.071049

0.0783297

0.0512157

0

0.0221279

0.69097
5

0

0

0

0.0737404

0.0347507

0

0.0583422

0.0639089

0.0163868

0.065547

1.16832
7

0.104252

0.0149617

0.0397379

0.0644598

0

0.0921043

0.00475254

0

0.0245801

0.0163868

3.56358
7

0.021374

0.0501488

0

0.0417951

8.82755

0.0163868

0.0901271

0

0

0.0434384

99.5896
7

0.166956
7

0.0104881

0

0.0106592

0

0.0173753

0.164223

0.0596483

0.0173753

0

0.0608137

1.30799
7

0

0.0347507

0.031862

0.0529645

0.155674

0

0.0347507

0.0737404

0.0621005

0.0173753

0.442258
7

0

0.0157321

0.00498723

0.0268748

0.139003

0.0360375

0

0

0.065547

0.0435955

0.464003
7

0

0

0.026063

0.25546

0.026063

0.026063

0.0189229

0

0.0223626

0.0367082

2.63367
7

0.00710613

0

0.021374

0.0224425

0

11.4062

0.0347507

0.021374

0.0209761

0.0173753

0.521826
7

0.163868

0.335618

0.0409669

0.0248562

0.0432616

0.0662303

0.0173753

0.026063

0

0.0466306

0.57658
6

0.279563

0.0173753

0.0163868

0.0737404

0.422815

0

0.0163868

0.0245801

0

0.0157321

0.376925
7

0

0

8.01386

0.0262201

0.0474183

0

0

0.0245801

0.0327735

0

0.349952
7

0.0359962

0.0573536

0.0157321

0

0

0.00498723

0.0409669

0.00615279

0.0080381

0.062426

0.522656
7

0.0347507

0.0104881

0.0473073

0

0

0.0955644

10.8483

0.00712881

0

0

2.93152
7

0.21046
7

0.0173753

0.106514

0

0.0173753

0

0.0355306

0.359536

0

0.0270459

0.0104881

11.6987
7

0

0.0209761

0

0.0283844

0

0.0349106

0.0106592

0.0670877

0.032337

0

0.247932
7

0

0.0274298

0.0104881

0.00498723

0.296743

0.026063

0.153412

0

0.0173753

0.104252

0.28141
7

0.106514

0

0.381174

0

0

0

0

0.00498723

0.0432616

0

1.04799
7

0

0.065547

0.0163868

0

0.286693

0.0174553

0

1.22053

0.0245801

0

0.293442
7

0.0163868

0

0

0.0955644

0.0163868

0.0765736

0.0104881

0.350074

0.0347507

0

2.27981
7

0.0491603

0.0184584

0.0511374

0.0781891

0.0213184

0.128672

0

0

0.0434384

0.0163868

1.47032
7

0.701175

0.0362983

0

0.00615279

0.00997446

0

0.065547

0

0.0104881

0.0278634

0.285873
7

0.0337621

0

0.0163868

0.00736842

0.0662303

0.0173753

0.0106592

0

0.0163868

0

0.0753004
7

0.0471197

0

0.0353442

0.0174553

0.660263

0

0.0506431

0.0573536

0

0.00498723

1.36562
7

0.583409
7

0.278575

0.0347507

0.0163868

0.0245801

0.0573536

0.0322158

0

0.0163868

0

0.0173753

0.0717764
7

0.00748085

0

0

0.16538

0.0611579

0.052126

0.0163868

0.0163868

0.0491603

0.00498723

2.51952
7

0.0163868

0

0.0157321

0.0157321

0.0163868

0.0245801

0.00498723

0.0327735

0.0124681

0

0.561638
7

0.0868767

0

0.0680185

0

0.065547

0.00748085

0

0.0163868

0

0

0.0835937
7

0.115369

0.0573536

0.0245801

0.0245801

0.0567688

0

0.0163868

0

0

0.0321188

0.129606
7

0.104495

0.0189229

0

0.0434384

0.065547

0.0347507

0.0163868

0

0.0173753

0.0238676

0.0764513

0.0327735

0.0321188

0.0104881

0.0104881

0.0274298

0.0245801

0.0471962

0.127844

0

0

0.387763
7

0

0.0163868

0

0.147481

0

0

0.0570305

0.0173753

0

0.0163868

0.147798
8

0

0

0

0

0.0990038

0.0452387

0

0

0.00997446

0

0.268769

0.0163868

0

0

0.00498723

0

0.0173753

0

0

0

0

3.10452
7

0.155525

0.0189229

0

0

0

0

0

0.0819338

0.0283844

0.00498723

0

0.107998

0

0.122901

0.0163868

0.656459

0.106514

0

0.0409669

0.0149617

0.0106592

0.0491603

0.598676
7

0

0.147481

0

0.0245801

0

0.0163868

0.0347507

0

0

0

0.29109
7

0.213028

0.0173753

0

0.0163868

0

0.0179689

0

0

0.0163868

0.304226

0.248757
7

0.0163868

0.00498723

0

0

0

0

0

0

0

0.0163868

0.811898
7

0

0

0

0

0

0

0

0

0.0629283

0

0.331628
7

0.0868767

0

0

0.0327735

0

0.304163

0.0473073

0.0299234

0

0.0409669

0.0923804
7

0

0

0

0.026063

0.0157321

0.141922

0.00748085

0

0.00748085

0

0.409669
7

0

0

0

0

0.0327735

0.0327735

0

0

0.00615279

0

0.0824754

0.0337621

0

0

0

0

0

0

0

0.0491603

0

2.29504
7

0.918784
7

0.0727989

0

0

0.0104881

0

0.0163868

0

0

0.0737404

0

0.138607
7

0

0

0.0491603

0

0

0

0.0163868

0.0347507

0.0283844

0.0347507

0.414228
7

0.0163868

0

0

0

0.0347507

0.0409669

0.0213184

0

0.00498723

0

0.269779
7

0

0

0

0.0573536

0.163868

0.0189229

0

0

0

0

2.65642
6

0.196641

0

0

0.00710613

0

0

0

0.0347507

0.0583422

0

0.149952
8

0

0.0163868

0.141922

0

0.00615279

0.0104881

0.0367082

0

0.052126

0

0.231427
7

0

0.0163868

0.0245801

0.0409669

0

0.0189229

0

0

0

0

0.167546
7

0

0

0

0.0426069

0

0.00710613

0.0937437

0

0.0737404

0.0163868

0.546916
7

0

0.0104881

0

0

0

0.0163868

0.0207193

0

0

0

0.149644
7

0

0.0245801

0

0.0163868

0.0224425

0.00498723

0.0173753

0

0

0

6.53022
7

0.0962332

0.00498723

0

0

0

0.00475254

0.0283844

0

0.0245801

0.139003

0

0.108701
7

0.0173753

0

0.969065

0.0163868

0.0920785

0.0173753

0.00997446

0.179768

0.0174553

0

0.143753
8

0

0.026063

0

0

0

0

0.0163868

0.0295674

0.0173753

0.0104881

0.365119
7

0.00498723

0.408094

0

0

0.0104881

0.0163868

0

0.360509

0

0

0.171024
7

0.0491603

0.131094

0.0473073

0.00498723

0

0

0

0

0

0

0.00997446
7

0.0283844

0

0

0

0.0327735

0.0163868

0

0.0567688

0.0501488

0

0.535302
6

0

0.00748085

0

0

0.0245801

0.021374

0

0.0104881

0

0

0.87768
6

0.0163868

0.0283844

0.0163868

0.0245801

0.0163868

0.0163868

0.0327735

0.0173753

0

0

0.018421
7

0

0.032417

0.0173753

0

0.0819338

0

0.00748085

0

0.0173753

45.8255

1.4751
7

0.0104881

0

0

0

0

0.0347507

0

0

0.0173753

0.00997446

0
4

55.528
6

55.528
6

0
4

1.35168

0.00498723
7

0.463037
5

0.0174553

0.00498723

0.039491

0

0

0

0

0

0

0

0.443647
5

0

0

0

0.0173753

0.0174553

0

0

0

0

0

0.0967816
6

0

0

0.026063

0

0

0

0

0

0

0

0

0

0.00498723

0

0

0

0

0

0

0

0

0.0922638
5

0

0

0

0.00748085

0

0.00498723

0

0

0

0

0

0

0

0

0

0

0

0

0

0.0609736
6

0

0.0497124
6

2.63677968348475e-16

0
4

4.65839

4.61315

0.0104881

0

0.0173753

0.0173753

0
4

4.62704
7

1.6059

0.0163868

0.0245801

0.0163868

0

0.0245801

0.0173753

0.243255

0.0163868

0

0.0163868

2.06473

0.0163868

0

0

0.0409669

0

0.0157321

0.0491603

0.360509

0

0

0.0491603

0.0245801

0.0245801

0

6.93889390390723e-16
7

0
4

146.279
7

9.77163
7

2.10311
7

0.0410247
7

1.30601
6

132.278
7

0.695782

0

0

0.0327735

0.0173753

0

0

0

0

0.0163868

0.0173753

2.1468937738689e-14
7

0
4

279.117
7

276.271
7

1.7429
7

0.0629283

0

1.04056

0

0
4

11.201
7

1.36461

0.22588
7

0

0.0157321

0

0.0173753

0

0

0

0.0173753

0.0173753

0

0.270974
6

0

0

0.0163868

0

0

0

0

0

0

0

0.376895

0

0

0

0.0100064

0.026063

0

0

0

0.00615279

0

0.097608
7

0

0

0.0173753

0

0

0.0163868

0

0

0

0.555963
7

0.1131

0.0174553

0.0150095
6

0.121627

0.0901271
7

0.445307
6

0.151383

0.0537748

0

0.00997446

0

1.11807

0.0245801

0.0473073

0

0

1.33048
6

0

0

0

0.103393

0.739875

0.0374042

0.0367222

0

0

0.0245801

1.74604
6

0.0299234

0

0

0

0

0

0

0.0149617

0

0.151383

0.114556

0.0283844

0.0163868

0.0189229

0

0.0100064

0.141922

0.0573536

0.0608137

0.00498723

0.0163868

0.106743
7

0

0

0.0163868

0

0

0

0

0

0

0

0.222469
7

0

0

0.052126

0.0189229

0

0.0104881

0

0

0

0.0173753

0.0500318
7

0.0347507

0

0

0.0434384

0

0

0

0.417862

0

0

0.0868767

0.0189229

0

0.0983205

0

0

0.0209761

0

0

0

0.0409669

0
4

5.84848
7

4.93689
7

0.278557
7

0.322356
7

0.17951
7

0.126181

0

0

0

0.00498723

0
4

0.13685
7

0.13685
6

0

0
4

0
7

0

0

0
4

0
7

0

0

0

0

0
4

0.0397379
7

0.0347507

0.00498723

0

0
4

0.0665356

0.061783

0.00475254

4.33680868994202e-18

0
4

0

0

0

0

0

0

0
4

0

0

0

0

0
4

0

0

0

0

0
4

0

0

0
4

0

0

0
4

0

0

0

0

0

0

0

0

0

0

0
4

0.0163868

0.0163868

0

0
4

0.026063

0.026063

0
4

0

0

0

0
4

0

0

0
4

0

0

0
4

0

0

0
4

0.0110526

0.0110526

0
4

0

0

0
4

0.00498723

0.00498723

0

0
4

0

0

0
4

0.191129
3

0.14769
3

0.0434384

0

0

0

0

0

0

0

0
4

0

0

0

0
4

0.0173753

0

0.0173753

0
4

0.0195035

0

0.0195035

0
4

0

0

0
4

0

0

0
4

0

0

0
4

0

0

0
4

0

0

0
4

0.0245801

0.0245801

0
4

0

0

0
4

0.325516
7

0.309129
7

0.0163868

0

0

0

0

0

0
4

0

0

0
4

0

0

0
4

0

0

0
4

0

0

0
4

0

0

0
4

0

0

0
4

0

0

0
4

0

0

0
4

0

0

0
4

0

0

0
4

0
7

0
7

0

0

0

0

0
4

0

0

0
4

0

0

0
4

0.15921

0.15921

0
4

0

0

0
4

0.0209761

0.0209761

0
4

0

0

0
4

0

0

0
4

0

0

0
4

0

0

0
4

0

0

0
4

0.288415
7

0.222868
7

0

0.0245801

0.0409669

6.93889390390723e-18
7

0
4

0

0

0
4

0.564912
7

0.291999
7

0.272912
7

0

0
4

0.235469
7

0.235469
7

0

0
4

0.259147
3

0.234567
3

0.0245801

0
4

1.71399006099193e-11
7

0
4

0

0
2

0
2

0

0

0

0

0

0

0

0

0

0

0

0
4

0

0

0

0

0

0
4

0
2

0
2

0
4

0

0

0

0
4

0

0

0
4

0

0

0
4

0
4

0

0

0
3

0

0

0

0

0

0

0

0

0

0

0
2

0

0

0

0

0

0

0

0

0

0

0
2

0

0

0

0

0

0

0

0

0

0

0
2

0

0

0

0

0

0

0
2

0
4

0

0

0

0
4

0

0

0

0
4

0

0

0

0
4

0

0

0

0
4

0

0

0
4

0

0

0
4

0
3

0
3

0

0

0

0

0

0

0

0

0

0

0

0

0

0

0

0

0

0

0

0

0

0

0

0

0

0
4

0

0

0

0

0

0
2

0
3

0

0

0

0

0

0

0
4

0

0
3

0

0

0

0

0

0

0

0

0

0

0
4

0

0

0

0

0
4

0
2

0

0

0

0

0

0
4

0
2

0

0

0

0

0

0
4

0

0

0

0
4

0

0

0
4

0
4

0
2

0
2

0

0

0

0

0

0

0

0

0

0

0
4

0

0

0

0

0

0

0

0
4

0

0

0

0

0

0

0

0

0

0

0
4

0

0

0

0
4

0

0

0

0

0
4

0

0

0

0
4

0
4

0.0399931

0.0399931

0.0399931

0
4

0

0

0
4

0
4

0

0

0

0

0
4

0
4

0

0

0

0
4

0

0

0
4

0
4

0

0

0

0
4

0
4

0.0177653

0

0

0
4

0.0177653

0.0177653

0
4

0
4

0

0

0

0
4

0

0

0
4

0
4

0

0

0

0
4

0
4

0

0

0

0
4

0
4

0

0

0

0
4

0
4

0

0

0

0
4

0
4

0

0

0

0

0

0

0

0

0

0
4

0
3

0
2

0

0

0

0

0

0

0

0

0

0

0
4

0

0

0

0

0

0

0

0
4

0

0

0

0

0

0
4

0

0

0
4

0

0

0
4

0

0

0
4

0

0

0
4

0

0

0
4

0
4

0

0

0

0

0
4

0
4

0

0

0

0
4

0
4

0

0

0

0
4

0

0

0
4

0
4

0

0

0

0
4

0
4

2.45801

2.45801

0

2.45801

0
4

0
4

0

0

0

0
4

0
4

0

0

0

0
4

0
4

0

0

0

0
4

0

0

0
4

0
4

0

0

0

0
4

0

0

0
4

0
4

0.0209761

0.0209761

0.0104881

0.0104881

0
4

0
4

0

0

0

0

0

0

0

0

0

0

0
4

0
7

0

0

0

0

0

0

0

0
4

0

0

0

0

0

0

0

0

0
4

0

0

0

0

0

0
4

0

0

0

0
4

0

0

0
4

0
4

0

0

0

0
4

0
4

0

0

0

0
4

0
4

0

0

0

0
4

0
4

0

0

0

0
4

0
4

0

0

0

0
4

0
4

0

0

0

0
4

0
4

0.0524403

0.0524403

0.0524403

0
4

0
4

0

0

0

0
4

0
4

0

0

0

0
4

0
4

0

0

0

0
4

0
4

0
2

0
1

0

0

0

0

0

0

0

0
4

0

0

0

0

0

0

0

0

0

0

0

0
4

0

0

0

0

0

0

0
4

0

0

0

0

0

0
4

0

0

0

0
4

0

0

0

0
4

0

0

0
4

0
4

0

0

0

0
4

0
4

0

0

0

0
4

0
4

0

0

0

0
4

0
4

0.0434384

0.0434384

0.0434384

0
4

0
4

0

0

0

0
4

0
4

0

0

0

0
4

0
4

0.0355306

0.0355306

0.0355306

0
4

0
4

0

0

0

0
4

0
4

0

0

0

0
4

0
4

0

0

0

0
4

0
4

0

0

0
3

0

0

0

0
4

0

0

0

0

0

0

0

0
4

0

0

0

0
4

0

0

0

0
4

0

0

0
4

0

0

0
4

0
4

0

0

0

0
4

0
4

0

0

0

0
4

0
4

0

0

0

0
4

0
4

0

0

0

0
4

0
4

0

0

0

0
4

0
4

0

0

0

0
4

0
4

0

0

0

0
4

0
4

0

0

0

0
4

0
4

0

0

0

0
4

0
4

0

0

0

0
4

0
4

0.605112
7

0.449438
7

0.375552
7

0.0475254

0

0.00997446

0.0163868

0

0

0
4

0.155674
7

0.155674
7

0
4

0

0

0

0
4

5.55111512312578e-17
7

0
4

0

0

0

0
4

0
4

0

0

0

0
4

0
4

0

0

0

0
4

0
4

0

0

0

0
4

0
4

0

0

0

0
4

0
4

0

0

0

0
4

0
4

0

0

0

0
4

0
4

0

0

0

0
4

0
4

0

0

0

0
4

0
4

0

0

0

0
4

0
4

0
3

0
3

0
3

0

0

0

0

0

0

0
4

0
4

0

0

0

0
4

0
4

0

0

0

0
4

0
4

0

0

0

0
4

0
4

0.0129839

0.0129839

0.0129839

0
4

0
4

0

0

0

0
4

0
4

0

0

0

0
4

0
4

0

0

0

0
4

0
4

0

0

0

0
4

0
4

0

0

0

0
4

0
4

0

0

0

0
4

0
4

1.90936

0.475216
6

0

0.475216

0

0

0
4

0

0

0
4

0

0

0
4

0

0

0
4

0.0259677
7

0

0.0259677

0
4

0

0

0

0

0

0

0
4

0

0

0

0

0
4

0

0

0

0
4

0

0

0

0
4

1.1296

1.1296

0
4

0

0

0

0
4

0.278575

0.278575

0
4

0
4

0

0

0

0
4

0
4

0

0

0

0
4

0
4

0

0

0

0
4

0
4

0

0

0

0
4

0
4

0

0

0

0
4

0
4

0

0

0

0
4

0
4

0.0245801

0.0245801

0.0245801

0
4

0
4

0

0

0

0
4

0
4

0

0

0

0
4

0
4

0

0

0

0
4

0
4

0.31863
7

0.0459541
7

0.021374
7

0

0

0.0245801

0

0

0
4

0.190742
7

0.166162
6

0.0245801

0

6.93889390390723e-18
7

0
4

0.065547

0.0327735

0.0327735

0
4

0

0

0

0
4

0.0163868

0.0163868

0
4

0
4

0

0

0

0
4

0
4

0

0

0

0
4

0
4

0

0

0

0
4

0
4

0

0

0

0
4

0
4

0

0

0

0
4

0
4

0

0

0

0

0

0

0

0

0

0

0
4

0

0

0
4

0

0

0
4

0
4

0

0

0

0

0

0

0

0

0

0

0

0

0

0

0

0

0

0

0

0

0

0

0

0

0

0

0

0

0

0

0

0

0

0

0

0

0

0

0

0

0

0

0

0

0

0

0

0

0

0

0

0

0

0

0

0

0

0

0

0

0

0

0
4

0

0

0

0

0

0

0

0
4

0

0

0

0

0

0

0

0

0

0

0

0

0
4

0

0

0

0

0

0
4

0

0

0
4

0
4

0

0

0

0

0

0

0

0

0

0
4

0

0

0
4

0
4

0

0

0

0

0
4

0
4

0
4

0
4

0

0

0

0

0

0
4

0
4

0

0

0

0
4

0

0

0

0

0

0
4

0
4

0

0

0

0

0

0

0

0

0
4

0
4

0

0

0

0

0

0

0
4

0

0

0

0

0
4

0

0

0

0

0
4

0

0

0
4

0
4

0

0

0

0

0

0

0
4

0

0

0
4

0
4

0
3

0
3

0
3

0

0

0

0

0
4

0

0

0

0
4

0

0

0
4

0
4

0
8

0
8

0

0

0

0

0

0
4

0

0

0
4

0
4

0
3

0
3

0
3

0
4

0
4

0.17822
3

0
2

0
2

0

0
4

0.087922
3

0

0

0.087922

0
4

0.0902983

0.0902983

0
4

1.38777878078145e-17
3

0
4

0
1

0
1

0

0

0

0

0

0

0

0

0

0

0

0
2

0

0

0

0

0

0

0

0

0
4

0

0

0
4

0

0

0
4

0
1

0
2

0

0

0

0

0

0

0

0

0

0

0
1

0

0

0

0

0

0

0

0

0

0

0
4

0
1

0

0

0

0

0

0

0

0

0

0

0
4

0

0

0

0

0
4

0

0

0

0
4

0

0

0

0
4

0

0

0

0
4

0

0

0

0
4

0

0

0

0
4

0
4

0
3

0
3

0
3

0

0

0

0

0
4

0

0

0

0

0

0
4

0
4

0

0

0

0

0

0

0
4

0

0

0

0

0

0

0
4

0

0

0
4

0

0

0

0
4

0

0

0

0
4

0

0

0
4

0
4

0

0

0

0

0

0

0
4

0

0

0

0

0

0

0
4

0
4

0

0

0

0

0

0

0
4

0
4

0
2

0

0

0

0

0

0

0
4

0

0

0
4

0

0

0
4

0
4

0

0

0

0

0
4

0

0

0

0
4

0

0

0

0
4

0
4

0.0104881

0.0104881

0.0104881
2

0

0

0
4

0

0

0
4

0

0

0
4

0
4

0.0500877
7

0.0163868

0.0163868

0

0
4

0.0262201

0

0

0.0262201

0
4

0.00748085

0.00748085

0

0
4

0

0

0
4

0
4

0
2

0

0

0

0

0
4

0
3

0

0

0

0

0
4

0

0

0
4

0
4

0

0

0

0

0

0

0
4

0

0

0

0
4

0
4

2.11597

0.2229
7

0.0932307
7

0

0.00997446

0

0

0

0

0

0.114707

0

0

0.00498723

2.68882138776405e-17
7

0
4

0.221221

0.221221

0
4

0

0

0

0
4

0

0

0

0
4

0.0194758

0.0194758

0
4

0.103871

0

0

0.103871

0

0

0

0

0

0
4

0.268366
1

0

0.229415

0.0389516

0

0

0

0

0

1.38777878078145e-17
1

0
4

0.188266
6

0.188266

0

0

0

0

0

0
4

0.0389516
6

0.0389516
6

0

0
4

0

0

0

0

0

0

0
4

0.0129839
7

0

0.0129839

0

0

0
4

0.0364558

0.0259677

0.0104881

0

0

0

0
4

1.00347

0.0327735

0.954315

0.0163868

0

4.5102810375397e-17

0
4

4.44089209850063e-16

0
4

0
8

0
8

0
8

0
4

0

0

0
4

0

0

0
4

0
4

0
3

0

0

0

0
4

0

0

0
4

0

0

0
4

0

0

0
4

0

0

0
4

0
4

0.124232
7

0.124232
7

0.113744
7

0.0104881

0
4

0
4

0
7

0
7

0

0

0

0
4

0

0

0
4

0
4

0

0

0

0

0

0

0

0
4

0

0

0
4

0

0

0
4

0
4

0
2

0
2

0

0

0

0

0
4

0

0

0
4

0

0

0
4

0
4

0
4

0

0

0

0

0

0

0
4

0

0

0
4

0
4

0

0

0

0

0

0

0
4

0
4

0.0259633
7

0.0259633
7

0.0209761
7

0

0.00498723

0
4

0
4

0
4

0
4

0

0

0
4

0

0

0
4

0

0

0
4

0

0

0
4

0
4

3.32987
7

1.81642
7

0.308104
7

0.95115
6

0
8

0

0.331151

0.122901

0.0129839

0

0.0901271

5.55111512312578e-17
7

0
4

0.792481
7

0.534924
7

0.237608

0.0199489

0
4

0.144809
7

0.032065
7

0.0831763

0.0245801

0

0.00498723

1.99493199737333e-17
7

0
4

0.335928
7

0
8

0

0.335928

0

0

0

0

0
4

0.205165

0.205165

0

0

0
4

0

0

0

0

0
4

0.0350682

0.0104881

0.0245801

0
4

0

0

0
4

0
4

0

0

0

0

0

0

0
4

0

0

0

0

0
4

0

0

0
4

0
4

0

0

0

0

0
4

0
4

0

0

0

0
4

0
4

0

0

0

0

0

0

0
4

0
4

0
3

0
3

0

0

0

0
4

0

0

0
4

0
4

0

0

0

0

0
4

0

0

0
4

0

0

0
4

0
4

0

0

0

0

0

0

0
4

0
4

0

0

0

0

0
4

0

0

0

0
4

0
4

0

0

0

0

0
4

0

0

0

0
4

0
4

0

0

0

0

0

0
4

0

0

0
4

0
4

0
3

0
3

0
3

0

0

0

0

0
3

0

0

0

0

0

0

0

0
4

0

0

0

0
4

0

0

0
4

0
4

0

0

0

0

0

0

0

0
4

0

0

0
4

0
4

0

0

0

0

0

0
4

0
4

0

0

0

0

0
4

0

0

0

0
4

0

0

0
4

0
4

0

0

0

0

0
4

0

0

0
4

0

0

0

0
4

0
4

0

0

0

0

0

0

0
4

0
4

0
8

0

0

0

0
4

0

0

0
4

0

0

0
4

0
4

0

0

0

0
4

0

0

0
4

0

0

0
4

0
4

0

0

0

0

0

0
4

0

0

0
4

0
4

0

0

0

0

0
4

0
4

0

0

0

0

0
4

0

0

0

0
4

0

0

0
4

0
4

0.0491603

0.0163868

0

0

0

0

0

0

0.0163868

0

0

0

0

0

0

0
4

0.0163868

0

0

0

0

0

0

0.0163868

0

0

0

0
4

0

0

0
4

0

0

0
4

0

0

0
4

0

0

0
4

0.0163868

0.0163868

0
4

0

0

0
4

0
4

0

0

0

0
4

0

0

0
4

0
4

0.208084

0.052278

0.052278

0
4

0.155806

0.155806

0
4

0
4

0

0

0

0

0
4

0
4

0

0

0

0
4

0

0

0

0
4

0

0

0
4

0
4

0

0

0

0
4

0

0

0

0
4

0
4

0.0173753
7

0

0

0
4

0.0173753

0

0.0173753

0
4

0
4

0

0

0

0
4

0
4

0.139287

0.139287

0.139287

0
4

0

0

0
4

0
4

0

0

0

0

0
4

0
4

0

0

0

0
4

0
4

6.42557

0.457092

0.02009

0

0

0.149942

0

0.262188

0

0

0

0

0.0248714

0
4

0

0

0
4

0

0

0
4

5.91029
7

0.224088
7

5.6862

0

0

0
4

0.0304689
7

0.0147368

0.0157321

0

0

0

3.46944695195361e-18
7

0
4

0

0

0

0

0

0
4

0.0147368

0

0.0147368

0
4

0

0

0
4

0.0129839

0.0129839

0
4

0

0

0
4

0

0

0
4

0
4

0

0

0

0
4

0
4

0

0

0

0

0

0
4

0
4

0

0

0

0
4

0
4

0

0

0

0

0
4

0
4

0

0

0

0

0
4

0

0

0
4

0
4

0

0

0

0

0
4

0

0

0
4

0
4

0

0

0

0

0
4

0

0

0
4

0
4

0

0

0

0

0
4

0
4

0

0

0

0

0

0
4

0
4

0.142823

0.142823

0.142823

0
4

0
4

0

0

0

0

0

0

0

0

0

0

0

0

0

0

0

0

0

0

0

0

0

0

0

0

0

0

0

0

0

0
4

0

0

0

0
4

0
4

0

0

0

0
4

0
4

0

0

0

0

0

0
4

0
4

0

0

0

0

0
4

0

0

0
4

0
4

0

0

0

0

0
4

0

0

0
4

0
4

0

0

0

0

0

0
4

0
4

0.0409669

0.0409669

0.0245801

0.0163868

0
4

0
4

0.0363357

0.0363357

0.0163868

0.0199489

0
4

0
4

0

0

0

0
4

0

0

0
4

0
4

0

0

0

0
4

0

0

0
4

0

0

0
4

0
4

0.0173753

0

0

0

0
4

0.0173753

0.0173753

0
4

0
4

0
3

0
3

0

0

0

0

0

0

0
4

0
4

0
3

0
3

0
3

0

0

0
4

0

0

0
4

0

0

0
4

0

0

0
4

0

0

0
4

0

0

0
4

0
4

15.0155

15.0155

3.37744
7

0.180254
7

0

0

0.0173753

0

0

0

0

0

0

0.0163868

0
8

0

0

0

0

0

0

0

0

0

0

0
3

0

0

0.026063

0

0

0

0

0.0163868

0

0

0.0409669

0

0

0

0

0

0

0

0

0

0

0.0163868

0

0

0

0.0173753

0

0

0

0

0

0

0.0983205
7

0

0

0

0

0

0

0

0

0.0163868

0

0.68005

0

0

0

0

0

0

0

0

0

0

0.0633725
7

0

0

0

0

0

0

0

0

0

0

0

0

0.0245801

0

0

0.0163868

0

0

0

0

0.0327735

0.0491603

0

0

0

0

0

0

0
3

0
7

0.0491603

0

1.5504
6

0

0
7

0
7

0

0

0

4.71007
7

0.443071

0.0173753
7

0.0901271

0

0

0

0

0

0

0

0.221221
7

0

0.0245801

0

0

0

0.213028

0

0.0608137

0

0.0245801

1.32719
6

0

0.0163868

0

0

0

0

0

0

0

0

0
3

0.564699

0

0

0

0

0

0

0

0

0

0.540763

0

0

0

0

0

0.026063

0

0

0

0.180254

0
3

0

0

0

0

0

0

0

0

0

0

0.253995

0

0

0

0

0

0

0

0

0

0.0120572

3.11382863937837e-15

0
4

0

0

0

0

0

0
4

0

0

0

0
4

0

0

0
4

0

0

0
4

0
4

0.194854
3

0.190102
4

0.190102

0

0
4

0

0

0
4

0

0

0
4

0.00475254

0.00475254

0
4

0

0

0
4

0

0

0
4

0

0

0
4

4.33680868994202e-18
3

0
4

0
4

8.44403
3

7.98651
3

1.56571

1.54932

0

0.0163868

0

0

0

0

0

0

0

0

0

0

0

0

0

0

0

0

0

0

0

4.5102810375397e-17

0
4

1.73816
5

1.61137
5

0.0124681
6

0

0

0

0

0.0199489

0.0259677

0

0.00997446

0

0

0

0.0584274

9.71445146547012e-17
5

0
4

4.66768
3

0.450636

0.158005
3

1.10045

0
4

0

0

0

0

0

0.147481

0

0

0

0

0
4

0

0

0

0

0

0

0

0

0

0

0.45415
3

0

0.026063

0

0

0

0

0

0

0

0

0
3

0

0

0

0

0

0

0

0

0

0

0
4

0

0

0

0

0

0

0

0

0

0

0

0

0.0129839

0

0

0

0

0

0

0

0

0.0683222
3

0

0

0

0

0

0

0

0

0

0

0.139287

0

0

0

0

0

0

0

0

0

0

0
4

0.0189229

0.0245801

0

0

0

0

0

0

0

0

0
4

0

0.0200127

0

0

0

0

0

0

0

0

0

0
4

0

0

0

0.0300191

0

0

0

0

0

0

0
3

0.245801

0

0

0

0

0

0

0

0.0378459

0

0.208152
4

0

0

0

0

0

0

0

0

0

0

0

0

0

0

0

0

0.0163868

0

0

0

0

0

0

0

0

0

0

0

0

0

0

0

0
3

0

0

0

0

0

0

0

0

0

0

0.0649194

0

0.0662303

0

0

0

0.319542
5

0.0434384
6

0

0

0.0756917
3

0

0

0

0
3

0

0

0
4

0

0

0.264011

0.121627
6

0
4

0

0

0.0163868
4

0

0

0

0

0

0
4

0.0259677

0

0

0.0129839

0

0

0

0

0

0

0
3

0

0

0

0

0

0

0

0

0

0

0

0

0

0

0

0.0150095

0

0

0

0

0

0
3

0

0

0

0

0

0

0

0

0

0

0.472774

0

0

0

0

0.0100064

0

0

0

0

0

7.73686670285656e-16
3

0
4

0.0149617

0.0149617

0

0
4

0

0

0
4

0

0

0
4

0

0

0
4

0

0

0
4

0

0

0

0
4

0

0

0

0
4

0

0

0
4

0

0

0
4

0

0

0
4

0

0

0
4

0

0

0
4

0

0

0
4

0
4

0.0473073
3

0.0473073
3

0.0473073
3

0

0

0

0

0

0

0

0

0

0

0

0

0

0

0

0

0

0

0

0
4

0
3

0

0

0

0

0

0
4

0
3

0

0
2

0

0

0

0

0

0

0

0
4

0
4

0

0

0

0

0

0

0

0

0

0

0

0

0

0

0

0

0
4

0

0

0
4

0
4

0.0195035
4

0.0195035
4

0.0195035
4

0
4

0

0

0
4

0
4

0

0

0

0

0

0

0

0
4

0

0

0
4

0
4

0.107269

0.107269

0.107269

0
4

0
4

0

0

0

0

0

0
4

0
4

0

0

0

0

0
4

0

0

0
4

0

0

0
4

0
4

0

0

0

0

0

0

0
4

0

0

0

0
4

0
4

0

0

0

0

0
4

0
4

0

0

0

0

0

0
4

0

0

0
4

0

0

0
4

0
4

0

0

0

0
4

0

0

0
4

0
4

0

0

0

0

0
4

0
4

0.113538
3

0.113538
3

0.113538
3

0

0

0

0

0

0

0

0
4

0

0

0
4

0

0

0
4

0

0

0

0

0

0

0

0
4

0
2

0

0

0

0

0
4

0
3

0

0

0

0
4

0

0

0
4

0

0

0
4

0

0

0
4

0

0

0
4

0

0

0
4

0
4

0.0522493

0.0522493

0.0522493

0
4

0
4

0.0539508

0.0539508

0.0539508

0
4

0
4

0

0

0

0
4

0

0

0
4

0
4

0

0

0

0
4

0
4

0

0

0

0
4

0

0

0
4

0
4

0

0

0

0

0
4

0

0

0
4

0
4

0

0

0

0
4

0

0

0
4

0
4

0

0

0

0
4

0
4

0

0

0

0
4

0

0

0
4

0

0

0
4

0
4

0

0

0

0

0
4

0
4

0.0473073
3

0.0473073
3

0.0189229
3

0

0

0

0

0

0
3

0
4

0

0

0

0

0.0283844

0

0
4

0

0

0
4

0

0

0
4

0

0

0

0
4

0
3

0

0

0

0

0

0

0
4

0

0

0
4

0

0

0

0
4

0

0

0

0
4

0

0

0

0
4

0

0

0
4

0

0

0
4

0
4

0

0

0

0
4

0

0

0
4

0

0

0
4

0
4

0

0

0

0
4

0
4

0

0

0

0
4

0
4

0

0

0

0
4

0
4

0

0

0

0

0
4

0
4

0

0

0

0

0
4

0
4

0

0

0

0
4

0
4

0

0

0

0
4

0
4

0

0

0

0
4

0
4

0.0163868

0.0163868

0.0163868

0
4

0
4

0
4

0
4

0
4

0
4

0

0

0

0

0

0

0
4

0
4

0
4

0

0

0

0

0

0
4

0

0

0

0

0
4

0

0

0

0
4

0
4

0

0

0

0
4

0
4

0

0

0

0
4

0
4

0

0

0

0
4

0
4

0

0

0

0
4

0
4

0

0

0

0
4

0
4

0

0

0

0
4

0
4

0

0

0

0
4

0
4

0

0

0

0

0

0

0

0

0

0

0

0

0

0

0

0

0
4

0

0

0

0

0

0

0

0

0

0

0

0

0

0

0
4

0

0

0

0

0

0
4

0

0

0

0
4

0
4

0

0

0

0

0

0

0

0

0

0
4

0

0

0

0

0

0

0

0

0
4

0

0

0

0

0

0

0
4

0
4

0
4

0

0

0

0

0

0

0

0

0

0

0
4

0

0

0

0

0

0
4

0
4

0

0

0

0

0

0
4

0
4

0
4

0
4

0

0

0

0

0

0
4

0

0

0

0
4

0

0

0

0
4

0

0

0

0
4

0

0

0
4

0
4

0
4

0

0

0

0

0

0

0

0

0

0

0

0

0

0

0
4

0

0

0

0

0

0
4

0

0

0

0

0

0
4

0

0

0

0
4

0
4

0
4

0.620844
7

0.620844
7

0.620844
7

0.547103
7

0.0737404

0

2.77555756156289e-17
7

0
4

0
4

0
4

0

0

0

0

0
4

0
4

0
4

0

0

0

0

0

0
4

0
4

0
4

0

0

0

0

0

0
4

0
4

0
4

0

0

0

0

0
4

0
4

0
4

0

0

0

0

0
4

0
4

0
4

0.0629283

0.0629283

0.0629283

0.0629283

0
4

0
4

0
4

0

0

0

0

0
4

0
4

0
4

0

0

0

0

0
4

0
4

0
4

0

0

0

0

0
4

0
4

0
4

0

0

0

0

0

0
4

0
4

0
4

0.289015
7

0.289015
7

0.289015
7

0.289015
7

0

0

0
4

0
4

0
4

0

0

0

0

0

0
4

0
4

0
4

0

0

0

0

0
4

0
4

0
4

0

0

0

0

0
4

0
4

0
4

0

0

0

0

0
4

0
4

0
4

0.00498723

0.00498723

0.00498723

0.00498723

0
4

0
4

0
4

0

0

0

0

0
4

0
4

0
4

0

0

0

0

0
4

0
4

0
4

0

0

0

0

0
4

0

0

0
4

0
4

0
4

0

0

0

0

0
4

0
4

0
4

0

0

0

0

0
4

0
4

0
4

0.782043
7

0.782043
7

0.678172
7

0.678172
7

0
4

0.103871

0.103871

0
4

0
4

0
4

0.0215407

0.0215407

0.0104881

0.0104881

0
4

0.0110526

0.0110526

0
4

0
4

0
4

0

0

0

0

0
4

0
4

0
4

0

0

0

0

0
4

0

0

0
4

0
4

0
4

0

0

0

0

0
4

0

0

0
4

0
4

0
4

0

0

0

0

0
4

0
4

0
4

0

0

0

0

0

0
4

0
4

0
4

0

0

0

0

0
4

0
4

0
4

0

0

0

0

0
4

0
4

0
4

0.487588

0.487588

0.487588

0.487588

0
4

0
4

0
4

0

0

0

0

0
4

0
4

0
4

0

0

0

0

0

0

0
4

0

0

0

0
4

0
4

0

0

0

0
4

0

0

0
4

0
4

0
4

0

0

0

0

0

0
4

0
4

0
4

0

0

0

0

0

0
4

0
4

0
4

0

0

0

0

0
4

0
4

0
4

0

0

0

0

0
4

0

0

0
4

0
4

0
4

0

0

0

0

0
4

0

0

0
4

0
4

0
4

0

0

0

0

0
4

0
4

0
4

0

0

0

0

0
4

0
4

0
4

0

0

0

0

0
4

0
4

0
4

0

0

0

0

0
4

0
4

0
4

0

0

0

0

0
4

0
4

0
4

0

0

0

0

0

0

0

0

0

0

0
4

0

0

0

0

0

0
4

0

0

0
4

0
4

0
4

0

0

0

0

0
4

0
4

0
4

0.0157321

0.0157321

0.0157321

0.0157321

0
4

0
4

0
4

0

0

0

0

0
4

0
4

0
4

0

0

0

0

0
4

0
4

0
4

0

0

0

0

0
4

0
4

0
4

0

0

0

0

0
4

0
4

0
4

0

0

0

0

0
4

0
4

0
4

0

0

0

0

0
4

0
4

0
4

0

0

0

0

0
4

0
4

0
4

0

0

0

0

0
4

0
4

0
4

0.052126
7

0
7

0

0

0

0
4

0

0

0

0

0
4

0

0

0
4

0

0

0
4

0
4

0.052126
6

0.052126

0.0347507

0.0173753

0

3.46944695195361e-18

0
4

0

0

0
4

0

0

0
4

0
4

0
4

0

0

0

0

0
4

0
4

0
4

0

0

0

0

0
4

0
4

0
4

0

0

0

0

0
4

0
4

0
4

0

0

0

0

0
4

0
4

0
4

0.0163868

0.0163868

0.0163868

0.0163868

0
4

0
4

0
4

0

0

0

0

0
4

0
4

0
4

0

0

0

0

0
4

0
4

0
4

0

0

0

0

0
4

0
4

0
4

0

0

0

0

0
4

0
4

0
4

0

0

0

0

0
4

0
4

0
4

0
3

0
3

0
3

0
3

0

0
4

0

0

0
4

0
4

0
4

0

0

0

0

0
4

0
4

0
4

0

0

0

0

0
4

0
4

0
4

0

0

0

0

0
4

0
4

0
4

0

0

0

0

0
4

0
4

0
4

0

0

0

0

0
4

0
4

0
4

0

0

0

0

0
4

0
4

0
4

0

0

0

0

0
4

0
4

0
4

0

0

0

0

0
4

0
4

0
4

0

0

0

0

0
4

0
4

0
4

0

0

0

0

0
4

0
4

0
4

0
5

0
5

0
5

0
5

0

0

0
4

0

0

0
4

0

0

0
4

0
4

0
4

0

0

0

0

0
4

0
4

0
4

0

0

0

0

0
4

0
4

0
4

0

0

0

0

0
4

0
4

0
4

0

0

0

0

0
4

0
4

0
4

0

0

0

0

0
4

0
4

0
4

0

0

0

0

0
4

0
4

0
4

0

0

0

0

0
4

0
4

0
4

0

0

0

0

0
4

0
4

0
4

0

0

0

0

0
4

0
4

0
4

0.0409669

0.0409669

0.0409669

0.0409669

0
4

0
4

0
4

0

0

0

0

0

0

0

0
4

0

0

0

0
4

0
4

0
4

0

0

0

0

0
4

0
4

0
4

0

0

0

0

0
4

0
4

0
4

0

0

0

0

0
4

0
4

0
4

0

0

0

0

0
4

0
4

0
4

0

0

0

0

0
4

0
4

0
4

0

0

0

0

0
4

0
4

0
4

0

0

0

0

0
4

0
4

0
4

0

0

0

0

0
4

0
4

0
4

0.0737404

0.0737404

0.0737404

0.0737404

0
4

0
4

0
4

0

0

0

0

0
4

0
4

0
4

0

0

0

0

0

0

0

0
4

0

0

0
4

0
4

0
4

0

0

0

0

0
4

0
4

0
4

0

0

0

0

0
4

0
4

0
4

0

0

0

0

0
4

0
4

0
4

0

0

0

0

0
4

0
4

0
4

0

0

0

0

0
4

0
4

0
4

0

0

0

0

0
4

0
4

0
4

0

0

0

0

0
4

0
4

0
4

0

0

0

0

0
4

0
4

0
4

0

0

0

0

0
4

0
4

0
4

0

0

0

0

0
4

0
4

0
4

0

0

0

0

0

0

0

0

0

0

0
4

0

0

0

0

0

0
4

0

0

0

0

0
4

0

0

0
4

0
4

0
4

1.39287
7

1.39287
7

1.39287
7

1.39287
7

0
4

0
4

0
4

0

0

0

0

0
4

0
4

0
4

0.00922919

0.00922919

0.00922919

0.00922919

0
4

0
4

0
4

0.106514

0.106514

0.106514

0.106514

0
4

0
4

0
4

0

0

0

0

0
4

0
4

0
4

0

0

0

0

0
4

0
4

0
4

0

0

0

0

0
4

0
4

0
4

0

0

0

0

0
4

0
4

0
4

0

0

0

0

0
4

0
4

0
4

0

0

0

0

0
4

0
4

0
4

0

0

0

0

0
4

0
4

0
4

0

0

0

0

0

0

0
4

0
4

0
4

0

0

0

0

0
4

0
4

0
4

0

0

0

0

0
4

0
4

0
4

0

0

0

0

0
4

0
4

0
4

0

0

0

0

0
4

0
4

0
4

0

0

0

0

0
4

0
4

0
4

0

0

0

0

0
4

0
4

0
4

0

0

0

0

0
4

0
4

0
4

0

0

0

0

0
4

0
4

0
4

0

0

0

0

0
4

0
4

0
4

0

0

0

0

0
4

0
4

0
4

0

0

0

0

0

0
4

0
4

0
4

0

0

0

0

0
4

0
4

0
4

0

0

0

0

0
4

0
4

0
4

0

0

0

0

0
4

0
4

0
4

0

0

0

0

0
4

0
4

0
4

0

0

0

0

0
4

0
4

0
4

0.0984447

0.0984447

0.0984447

0.0984447

0
4

0
4

0
4

0

0

0

0

0
4

0
4

0
4

0

0

0

0

0
4

0
4

0
4

0

0

0

0

0
4

0
4

0
4

0

0

0

0

0
4

0
4

0
4

0
7

0

0

0

0

0

0
4

0
4

0

0

0

0
4

0

0

0
4

0
4

0
4

0

0

0

0

0
4

0
4

0
4

0

0

0

0

0
4

0
4

0
4

0.0110526

0.0110526

0.0110526

0.0110526

0
4

0
4

0
4

0

0

0

0

0
4

0
4

0
4

0

0

0

0

0
4

0
4

0
4

0

0

0

0

0
4

0
4

0
4

0

0

0

0

0
4

0
4

0
4

0

0

0

0

0
4

0
4

0
4

0.143684

0.143684

0.143684

0.143684

0
4

0
4

0
4

0

0

0

0

0
4

0
4

0
4

0.44016
6

0.44016
6

0.44016
6

0.44016
6

0
4

0
4

0
4

0

0

0

0

0
4

0
4

0
4

0

0

0

0

0
4

0
4

0
4

0.0100064

0.0100064

0.0100064

0.0100064

0
4

0
4

0
4

0

0

0

0

0
4

0
4

0
4

0

0

0

0

0
4

0
4

0
4

0

0

0

0

0
4

0
4

0
4

0

0

0

0

0

0

0
4

0
4

0

0

0

0

0
4

0
4

0
4

0.0491603
3

0.0491603
3

0.0491603
3

0

0

0.0491603

0

0
4

0
4

0
4

0
6

0
6

0
6

0

0

0

0
4

0
4

0
4

0
4

0
4

0
4

0
4

0
4

0

0

0
4

0
4

0
4

0.0173753
7

0.0173753
7

0.0173753
7

0.0173753
7

0
4

0
4

0
4

0.0576843
1

0.0576843
1

0.0471962
1

0.0471962
1

0

0

0

0

0
4

0.0104881

0.0104881

0
4

0
4

0
4

1.20611
7

1.20611
7

1.20611
7

1.20611
7

0
4

0
4

0
4

0.052126
6

0.052126
6

0.052126
6

0.052126
6

0

0
4

0
4

0
4

0

0

0

0

0

0

0

0
4

0
4

0
4

0.103039

0.103039

0.103039
7

0.103039
7

0

0
4

0

0

0
4

0
4

0
4

0

0

0

0

0

0

0
4

0

0

0

0

0
4

0
4

0

0

0

0
4

0

0

0
4

0
4

0
4

0.0675082
7

0.0675082
7

0.0675082
7

0.0675082
7

0

0
4

0
4

0
4

0.356195

0.356195

0.356195

0

0.356195

0
4

0
4

0
4

0

0

0

0

0

0
4

0

0

0

0
4

0
4

0
4

0

0

0

0

0

0

0
4

0
4

0
4

0

0

0

0

0

0
4

0

0

0

0
4

0

0

0
4

0

0

0
4

0
4

0
4

0.46231
6

0.46231
6

0.46231
6

0.46231
6

0

0
4

0
4

0
4

0.0215348
7

0.0215348
7

0.0215348
7

0

0.0215348

0

0

0
4

0
4

0
4

1.16862
7

1.16862
7

1.16862
7

1.08669
7

0.0819338

0
4

0
4

0
4

0.0573536

0.0573536

0.0573536

0.0573536

0

0
4

0

0

0
4

0
4

0
4

0

0

0

0

0

0
4

0
4

0
4

0

0

0

0

0

0

0
4

0

0

0
4

0

0

0
4

0
4

0
4

0.0194758

0.0194758

0.0194758

0

0.0194758

0

0

0
4

0
4

0
4

0

0

0

0

0
4

0
4

0
4

0

0

0

0

0

0
4

0
4

0
4

0.406745
7

0.406745
7

0.406745
7

0.406745
7

0
4

0
4

0
4

0

0

0

0

0

0

0

0
4

0
4

0
4

0
5

0
5

0
5

0
5

0

0
4

0

0

0
4

0

0

0

0
4

0

0

0
4

0
4

0

0

0

0

0

0
4

0
4

0
4

0

0

0

0

0

0

0

0
4

0
4

0
4

0.393701
6

0.393701
6

0.393701
6

0.393701
6

0
4

0
4

0
4

0

0

0

0

0

0
4

0

0

0
4

0
4

0
4

0

0

0

0

0

0

0
4

0
4

0
4

0.22721

0.22721

0.157799

0.157799

0
4

0.0694108

0.0120572

0.0573536

0
4

1.38777878078145e-17

0
4

0
4

0.0274298

0.0274298

0.0174553

0

0.0174553

0
4

0.00997446

0.00498723

0.00498723

0
4

0
4

0
4

0

0

0

0

0

0

0

0
4

0
4

0
4

0.0292553

0.0292553

0.0292553

0.0292553

0

0
4

0

0

0
4

0
4

0
4

0

0

0

0

0
4

0

0

0
4

0

0

0
4

0

0

0
4

0
4

0
4

0.0199489

0.0199489

0.0199489

0.00997446

0.00498723

0.00498723

0
4

0
4

0
4

0.691546
7

0.691546
7

0.691546
7

0.684065
7

0

0.00748085

2.42861286636753e-17
7

0
4

0
4

0
4

0

0

0

0

0

0

0
4

0
4

0
4

0

0

0

0

0

0
4

0
4

0
4

0
3

0
3

0

0

0
4

0

0

0
4

0
4

0
4

0

0

0

0

0
4

0
4

0
4

0

0

0

0

0
4

0
4

0
4

0

0

0

0

0
4

0
4

0
4

0.0897257

0.0897257

0.0897257

0.0733389

0.0163868

0
4

0
4

0
4

0.0259677

0.0259677

0.0259677

0.0259677

0
4

0
4

0
4

0

0

0

0

0

0
4

0
4

0
4

0

0

0

0

0

0
4

0
4

0
4

0

0

0

0

0

0

0

0

0

0

0

0

0
4

0

0

0

0

0

0

0
4

0

0

0

0
4

0

0

0

0
4

0
4

0
4

0

0

0

0

0

0

0
4

0
4

0
4

0.125263

0.125263

0.125263

0.125263

0

0
4

0
4

0
4

0

0

0

0

0
4

0
4

0
4

0.0515789

0.0515789

0.0515789

0

0

0.0515789

0
4

0
4

0
4

0

0

0

0

0
4

0
4

0
4

0

0

0

0

0
4

0
4

0
4

0

0

0

0

0

0
4

0

0

0

0
4

0
4

0
4

0

0

0

0

0
4

0

0

0
4

0
4

0

0

0

0
4

0
4

0
4

0.131094

0.131094

0.131094

0

0.131094

0
4

0
4

0
4

0.163868

0.163868

0.163868

0.163868

0
4

0
4

0
4

0.966818
7

0.966818
7

0.966818
7

0.909465
7

0.0245801

0.0327735

0

0

0
4

0
4

0
4

0

0

0

0

0

0
4

0

0

0
4

0
4

0
4

0

0

0

0

0
4

0
4

0
4

0

0

0

0

0
4

0
4

0
4

0

0

0

0

0
4

0
4

0
4

0

0

0

0

0

0
4

0
4

0
4

0

0

0

0

0
4

0
4

0
4

0

0

0

0

0

0
4

0
4

0
4

0.0199489

0.0199489

0.0199489

0.00498723

0.00997446

0.00498723

8.67361737988404e-19

0
4

0
4

0
4

0

0

0

0

0

0
4

0

0

0
4

0
4

0
4

0

0

0

0

0
4

0

0

0
4

0
4

0
4

0
6

0
6

0

0

0

0

0

0

0
4

0

0

0

0

0

0
4

0

0

0
4

0
4

0
4

0

0

0

0

0
4

0
4

0
4

0

0

0

0

0
4

0
4

0
4

0

0

0

0

0
4

0

0

0
4

0
4

0
4

0

0

0

0

0

0

0
4

0
4

0
4

0

0

0

0

0
4

0
4

0

0

0

0
4

0
4

0
4

0

0

0

0

0
4

0
4

0
4

0

0

0

0

0

0
4

0

0

0
4

0
4

0
4

0

0

0

0

0
4

0
4

0
4

0

0

0

0

0

0
4

0
4

0
4

0

0

0

0

0
4

0
4

0
4

0.290495
1

0

0

0

0
4

0
4

0

0

0

0
4

0
4

0

0

0

0
4

0
4

0

0

0

0

0
4

0
4

0.205786

0.205786

0.205786

0
4

0
4

0

0

0

0
4

0
4

0

0

0

0
4

0
4

0

0

0

0
4

0

0

0
4

0
4

0

0

0

0
4

0
4

0.084709

0.084709

0.0327735

0.0519355

0
4

0
4

0

0

0

0
4

0
4

0
1

0
2

0

0

0

0

0

0

0

0

0

0
4

0

0

0
4

0
4

1.38777878078145e-17
1

0
4

7.33155
7

0

0

0

0

0
4

0
4

0

0

0

0
4

0
4

0

0

0

0
4

0
4

0

0

0

0
4

0
4

0.00475254

0.00475254

0.00475254

0
4

0
4

0

0

0

0
4

0
4

0

0

0

0
4

0
4

7.3268
7

7.27764
7

7.27764
7

0

0
4

0.0491603
7

0.0491603
7

0
4

0

0

0
4

0

0

0
4

0

0

0
4

0

0

0
4

0

0

0
4

0

0

0
4

0

0

0
4

0
4

0
4

0.747017
3

0.73063
3

0.73063
3

0.239711

0

0

0

0

0

0

0

0

0.141589

0

0.320615
3

0

0

0

0

0

0

0

0

0

0

0.028716
2

0

0

0

0

0
3

0
2

0
3

0
2

0
3

0
2

7.97972798949331e-17
3

0
4

0

0

0
4

0

0

0
4

0
4

0

0

0

0

0

0
4

0
4

0

0

0

0

0
4

0
4

0

0

0

0
4

0
4

0

0

0

0
4

0
4

0

0

0

0

0
4

0
4

0

0

0

0
4

0

0

0
4

0
4

0.0163868

0

0

0
4

0.0163868

0.0163868

0
4

0
4

0

0

0

0
4

0
4

0

0

0

0
4

0
4

4.5102810375397e-17
3

0
4

10.3833
7

0.0767062

0.0767062

0.0767062

0
4

0
4

0.0409669

0.0409669

0.0409669

0
4

0
4

0

0

0

0
4

0
4

0

0

0

0
4

0
4

0

0

0

0
4

0
4

0

0

0

0
4

0
4

0

0

0

0
4

0
4

0

0

0

0
4

0
4

0

0

0

0
4

0
4

0

0

0

0
4

0
4

0

0

0

0
4

0
4

10.2657
7

10.2657
7

7.65837
7

0.513337
5

0

0

0.0573536

0.0274298

0.65547

0

0

0

0

0.0419522

0.22714
7

0

0.0327735

0

0

0

0

0

0.119694

0

0

0.0409669
6

0

0

0

0

0

0

0.139003

0

0

0

0.115882

0

0

0.339071
7

0.201649

0.0955644

0

0

1.19348975147204e-15
7

0
4

0

0

0
4

0

0

0
4

0

0

0
4

0
4

0
4

6.85436

2.61546
5

2.54719
5

1.80111

0.0194758

0

0

0
5

0.685568
6

0.0215348

0.0195035

0

0

0

0

3.2612801348364e-16
5

0
4

0.0682623

0.0682623

0
4

0
4

4.07183
5

4.07183
5

4.06568
5

0.00615279

0

0

0

0
4

0

0

0
4

0
4

0

0

0

0
4

0
4

0

0

0

0
4

0
4

0

0

0

0
4

0
4

0.167072

0.167072
7

0

0.00748085

0

0

0

0

0

0

0

0

0

0

0

0

0

0

0

0

0

0.159591

0

0

0
4

0

0

0

0

0
4

0

0

0
4

0

0

0
4

0

0

0
4

0
4

1.66533453693773e-16

0
4

9.9389

0.870441

0.870441

0.263966

0

0.0679262

0.410876

0

0.0177653

0.0380203

0.0473073

0.0245801

0
4

0

0

0

0
4

0

0

0
4

0
4

0.342841
5

0.342841
5

0.316448
5

0

0

0

0.0163868
7

0

0

0

0.0100064

0

0

0

0
4

0
4

0.0897701

0.0847829

0.0548595

0.0299234

0
4

0.00498723

0.00498723

0
4

0
4

0.189035

0.189035

0.189035

0
4

0
4

0

0

0

0
4

0
4

0

0

0

0
4

0
4

0

0

0

0
4

0
4

0

0

0

0
4

0
4

0

0

0

0

0
4

0
4

0.00748085

0.00748085

0.00748085

0

0
4

0
4

0

0

0

0
4

0
4

0

0

0

0
4

0
4

0.536089
5

0.536089
5

0.536089
5

0

0

0

0

0

0

0
4

0

0

0
4

0

0

0
4

0
4

0

0

0

0
4

0
4

0

0

0

0
4

0
4

0

0

0

0
4

0
4

0

0

0

0
4

0
4

0

0

0

0
4

0
4

0

0

0

0
4

0
4

0.0213184

0.0213184

0.0213184

0
4

0
4

0

0

0

0
4

0
4

0

0

0

0
4

0
4

0

0

0

0
4

0
4

0
7

0
7

0
7

0

0
7

0
7

0

0

0

0

0

0
4

0
4

1.24435
5

1.07857
5

0.0983205

0

0.941246

0.039007

0

0

0

0

0
4

0.16578

0.126773

0.039007

0

0

1.38777878078145e-17

0
4

0
4

6.41157
5

6.41157
5

6.36281

0.0292553

0.0195035

0

0

0

4.64905891561784e-16
5

0
4

0

0

0

0
4

0
4

0.17536

0.170373

0.170373

0
4

0.00498723

0.00498723

0
4

1.30104260698261e-17

0
4

0

0

0

0

0

0
4

0

0

0
4

0
4

0.0506431
7

0.0506431
7

0.0506431

0

0

0

0

0
4

0
4

0

0

0

0
4

0

0

0
4

0
4

0
4

2.98594

0

0

0

0

0

0

0

0

0

0

0
4

0

0

0
4

0
4

0.0215348

0.0215348

0

0

0.0215348

0
4

0
4

0

0

0

0

0
4

0
4

0

0

0

0

0
4

0

0

0
4

0
4

0

0

0

0

0
4

0
4

0

0

0

0
4

0

0

0
4

0
4

0

0

0

0
4

0
4

0

0

0

0
4

0
4

0

0

0

0
4

0
4

0

0

0

0
4

0

0

0
4

0
4

0

0

0

0
4

0
4

0.0173753

0.0173753

0.0173753

0

0

0
4

0
4

0

0

0

0
4

0
4

0

0

0

0
4

0
4

0

0

0

0
4

0
4

0

0

0

0
4

0
4

0

0

0

0
4

0
4

0

0

0

0
4

0
4

0

0

0

0
4

0
4

0

0

0

0
4

0
4

0

0

0

0
4

0
4

0.0104881

0.0104881

0.0104881

0
4

0
4

0

0

0

0

0
4

0

0

0

0
4

0
4

0

0

0

0
4

0
4

0

0

0

0
4

0
4

0

0

0

0
4

0
4

0.125857

0.125857

0.125857

0
4

0
4

0

0

0

0
4

0
4

0

0

0

0
4

0
4

0

0

0

0

0
4

0
4

0

0

0

0

0

0
4

0

0

0
4

0
4

0.121627

0.121627

0.121627

0

0
4

0

0

0
4

0

0

0
4

0
4

0

0

0

0
4

0

0

0

0
4

0
4

0

0

0

0

0
4

0
4

0

0

0

0

0
4

0

0

0

0
4

0
4

2.68906

2.63676

2.57184

0

0.0649194

0

0

0

0

0

0

0

0

0

0

0

0

0

0
3

0

0

0

0

0

0

0
4

0.0399931

0.0215348

0.00922919

0

0.00922919

0

0

3.46944695195361e-18

0
4

0

0

0
4

0

0

0
4

0

0

0
4

0

0

0
4

0

0

0
4

0

0

0
4

0

0

0
4

0

0

0

0

0

0

0
4

0

0

0
4

0.00615279

0.00615279

0
4

0

0

0

0
4

0

0

0
4

0

0

0
4

0.00615279

0.00615279

0
4

0

0

0
4

0
4

4.44089209850063e-16

0
4

4.54861
5

0.56707
6

0.562082

0.177653

0.114706

0.0174553

0.195418

0.00710613

0

0.0497429

0

0
4

0.00498723

0

0.00498723

0
4

0

0

0

0
4

0

0

0
4

0

0

0
4

1.30104260698261e-17
6

0
4

3.3527

2.2684
5

1.99043
6

0.0349106

0.138484

0.0292553

0.00748085

0.0327735

0.0104881

0.0245801

2.60208521396521e-16
5

0
4

1.0843

1.01603

0.0682623

8.32667268468867e-17

0
4

0

0

0
4

0
4

0

0

0

0
4

0
4

0

0

0

0
4

0
4

0.0177653

0.0177653

0.0177653

0
4

0
4

0

0

0

0
4

0
4

0

0

0

0
4

0
4

0.408321
5

0.408321
5

0.0955644
6

0.278006

0.0347507

0

0

0

2.08166817117217e-17
5

0
4

0
4

0.158763

0.158763

0.0604427

0

0.0983205

0

0

0
4

0
4

0.0439943
5

0.039007

0.0195035

0.0195035

0

0

0
4

0

0

0
4

0.00498723

0.00498723

0
4

0
4

0

0

0

0
4

0
4

0

0

0

0

0
4

0
4

0

0

0

0
4

0
4

0

0

0

0
4

0
4

0

0

0

0
4

0
4

7.21644966006352e-16
5

0
4

0
3

0

0

0

0

0
4

0
4

0

0

0

0
4

0
4

0
3

0

0

0
4

0

0

0

0

0
4

0

0

0
4

0

0

0
4

0

0

0
4

0
4

0
4

5.37617

0.165066

0.165066

0.130315
7

0

0

0

0

0

0.0173753

0

0

0

0.0173753
8

0
8

0
1

0

0

0

0

0

2.08166817117217e-17

0
4

0

0

0

0

0
4

0

0

0

0
4

0
4

0

0

0

0

0
4

0
4

0.491603

0.491603

0.491603

0

0

0
4

0

0

0
4

0
4

0

0

0

0

0
4

0

0

0

0
4

0
4

0

0

0

0
4

0
4

0

0

0

0
4

0
4

0

0

0

0

0
4

0

0

0
4

0
4

0.0807932

0.0807932

0.0807932

0
4

0
4

0

0

0

0

0
4

0
4

0

0

0

0
4

0
4

0

0

0

0
4

0

0

0
4

0
4

0.208504

0
7

0

0

0

0

0

0

0

0

0

0

0

0
4

0.208504
1

0.208504
8

0

0

0
4

0

0

0
4

0

0

0
4

0
4

0

0

0

0
4

0
4

0

0

0

0

0
4

0
4

0

0

0

0
4

0
4

0

0

0

0
4

0
4

0

0

0

0
4

0
4

0.0843952

0.0843952

0.0843952

0
4

0
4

0

0

0

0
4

0
4

0.0215348

0.0215348

0.0215348

0
4

0
4

0.0327735

0.0327735

0.0327735

0
4

0
4

0

0

0

0
4

0
4

0

0

0

0

0

0

0
4

0

0

0

0
4

0

0

0
4

0

0

0
4

0
4

0

0

0

0
4

0
4

0

0

0

0
4

0
4

0

0

0

0
4

0
4

0

0

0

0
4

0
4

0

0

0

0
4

0
4

0

0

0

0
4

0
4

0

0

0

0
4

0
4

0

0

0

0
4

0
4

0

0

0

0
4

0
4

0

0

0

0
4

0
4

0.245801

0.065547

0.0491603

0

0

0.0163868

3.46944695195361e-18

0
4

0.180254

0.0327735

0.147481

0
4

0
4

0.319542

0

0

0

0
4

0

0

0

0

0

0
4

0.319542

0.319542

0

0
4

0
4

0
3

0
3

0

0

0

0

0

0

0

0

0
4

0

0

0
4

0

0

0
4

0
4

0.0819338
3

0.0819338
3

0

0.0245801

0.0409669

0.0163868

0

1.04083408558608e-17
3

0
4

0
4

0
6

0

0

0

0

0
4

0

0

0
4

0

0

0
4

0
4

0

0

0

0

0

0
4

0
4

3.64423

2.29312

1.11202
7

0.052126

0.0695014

0.0259677

0

0.0259677

0.20125

0

0

0

0

0

0

0

0

0

0

0

0

0

0

0

0.052126

0

0

0

0

0

0

0

0

0

0

0.0259677

0.0259677

0

0

0

0.0245801

0

0

0

0

0

0
7

0

0

0

0

0

0

0

0

0

0

0

0

0

0

0

0

0

0.0173753

0

0

0

0.512573

0.0173753

0.130315

0

0
4

0

0

0

0

0
4

0.131094

0.131094

0
4

0

0

0
4

0

0

0

0
4

0.0819338

0

0.0819338

0
4

0

0

0
4

0

0

0
4

0

0

0
4

0

0

0
4

0

0

0
4

0

0

0
4

0.999082
6

0.981707
6

0

0.0173753

0
4

0.0173753

0.0173753

0
4

0

0

0
4

0

0

0
4

0

0

0
4

0

0

0
4

0.104252

0.0434384

0.0434384

0.0173753

0

0
4

0

0

0

0

0
4

0
3

0

0

0

0
4

0.0173753

0

0.0173753

0
4

0

0

0

0
4

0

0

0

0
4

0

0

0
4

2.04697370165263e-16

0
4

0
4

3.19753
7

0.199974
6

0.199974
6

0.199974
6

0

0
4

0
4

0.0734164
6

0.0734164
6

0.0734164
6

0
4

0
4

0

0

0

0
4

0
4

0.0173753

0.0173753

0.0173753

0
4

0
4

0.0110526

0.0110526

0.0110526

0
4

0
4

0.0284245

0.0284245

0.0284245

0
4

0
4

2.86728
7

2.86728
7

2.86728
7

0
4

0
4

0
4

0
4

293.144

0

0

0

0

0

0

0
4

0
4

0
4

229.319
3

1.05528

1.05528

0.933653

0

0

0

0

0

0

0

0

0

0
2

0

0

0

0

0

0

0.121627

5.55111512312578e-17

0
4

0

0

0
4

0

0

0
4

0
4

224.306
3

14.6213

0.0173753

1.73329

0

0

0

0

0

0

0

0

0

0

0

0.421835

0.00615279

0

0

0

0

0

0

0

0

0

1.10383

0.0123056

0

0

0

0

0

0

0

0

0

0

0

0

0

0

0

0

0

0

0

0

0.789893

0

0

0

0

0

0

0

0

0

0

0.0454436
6

0

0

0

0

0

0

0

0

0

0

0.732496
7

0

0

0

0

0

0

0

0

0

0

0.312056

0

0.0195035

0

0

0.00615279

0

0

0

0

0

0.0780141

0

0

0

0

0.0245801

0

0

0

0

0

0

0

0

0

0

0.0173753

0.0163868

0

0.0173753

0

0

0.618293

0.0669665
5

0

0.0434384

0

0

0

0

0

0

0

0

0

0

0.00615279

0

0

0

0

0

0

0

0.0184584

0
2

0

0.0369167

0

0

0

0

0

0

0

0

0.0173753

0

0

0

0

0

0

0

0

0

0

0

0

0

0

0

0.0585106

0

0

0

0

0

0.065578

0

0

0

0

0

0

0

0

0

0

0.030764
6

0

0

0

0

0

0

0

0

0

0

0

0

0

0

0

0

0

0

0

0

0

0.0163868

0

0

0

0

0

0

0

0

0

0.0195035

0

0

0

0

0

0

0

0

0

0

0

0.870585

0

0

0

0

0

0

0

0

0

0.039007

0

0

0.015382

0

0.0163868

0

0

0

0

0.0245801

0

0

0
2

0

0

0

0

0

0

0

0

0

0.0184584

0

0

0

0

0

0.00748085

0

0

0

0

0.0195035

0

0

0

0

0

0

0

0

0

0

0

0

0

0

0

0

0

0

0

0

0

0

0

0

0

0

0

0

0

0

0

0

0

0.039007

0

0

0

0

0

0.232205

0.125245

0

0
6

0

0.362412

0
2

0

0

0.00615279
7

0.0245801

1.53691

0

0.827531

0

0

0

0

0

0

0

0

0

0

0

0

0

0

0

0

0

0

0

0.0868767

0

0

0

0

0

0

0

0

0

0

3.35777

0

0

0.00615279

0

0

0.039007

0.204283

0

0

0

0.39203

0

0

0

0.0173753

0

0

0

0

0

0

0
4

60.4686
4

0.435254
3

10.0593
4

20.5115
3

22.7599
6

0
4

0

0

0.130315

0

0

0

0

0

0

0

0
4

0

0

0

0

0

0

0.0104881

0

0

0

0
4

0.14769

0.286693

0

0

0.0173753

0

0

0

0

0

0
4

0

0

0

0

0

0

0

0

0

0

0.0314642
4

0

0

0

0

0

0

0

0

0

0

0
2

0

0

0

0.0608137

0

0

0

0

0

0.121627

0
3

0

0

0

0

0

0

0

0

0

0

0
4

0

0

0

0

0

0

0.052126

0

0

0.0157321

0.556011

0

0

0

0

0

0

0

0

0

0

0
2

0

0

0

0

0

0

0

0

0

0

0.398546
3

0
4

0

0

0

0

0

0

0

0.0695014

0

0

0

0

0.026063

0

0

0

0

0

0

0

0

0

0

0

0

0

0

0

0.0173753

0

0

0

0
4

0

0

0

0

0

0

0

0

0

0

0

0

0.0173753

0

0

0

0

0

0

0

0

0
3

0

0

0

0

0

0

0

0

0

0

0

0

0

0

0

0

0

0

0

0.0104881

0

0

0

0

0

0

0

0

0

0

0

0

0.0262201
4

0

0.110125

0

0

0.026063

0

0

0

0

0

0
4

0

0

0

0

0.495197

0

0

0

0

0

1.02259
4

0.40379

0.0173753

0

0

0

0

0

0

0

0

0

0

0

0.0173753

0

0

0.199816

0

0

0

0

0

0

0.0104881

0

0

0

0

0

0

0

0

0

0
4

0

0

0

0

0

0

0

0

0

0

0
3

0

0

0

0

0

0

0

0

0.0173753

0

0

0

0

0

0

0.0347507

0

0.0104881

0

0

0

0.6342

0

0

0

0

0

0

0

0

0

0

0
4

0

0

0

0

0

0

0

0

0

0

0

0

0

0

0

0

0

0

0

0

0

0

0.026063

0

0

0

0

0

0

0

0

0.188785

0
4

0

0

0

0

0.0695014

0

0

0

0

0

0

0

0

0

0

0

0

0

0.0173753

0

0

0

0

0

0

0

0

0

0

0

0

0

0

0
2

0.0434384

0

0

0

0

0

0

0

0

0

0

0

0

0

0
4

0

0.269318

0

0
3

0
4

0.390945

0

0

0
4

0

0
4

0.208504

0.11294

0

0

0
4

0

0

0

0

0

0

0

0

0

0

0
4

0

0.0327735

0

0

0

0

0

0

0

0

0
3

0

0.0868767

0

0.139003

0

0.121627

0

0

0

0

0
3

0

0

0

0

0

0

0

0

0

0

7.82707232360735e-15
4

0
4

0.198421
3

0
3

0
3

0
3

0
3

0.0163868
2

0
3

0.182034
2

0
1

0

0

0

0

0

0

0

0

0

0

0

0

0

0

0

0

0

0

0

0

0

0

0

0

0

0

0

0
8

0

0

0

0

0

0
4

2.82054

0.537163
7

0
6

0.0100064

0

0

0

0

0

0

0

0

0

0.139287
7

0

0

0

0

0

0

0

0

0

0

0
7

0

0.00475254

0

0

0

0

0

0

0

0

0
5

0

0

0

0.0163868

0

0

0

0

0

0

0
6

0

0

0

0

0

0.0080381

0

0.0237627

0

0

0
7

0

0

0

0

0

0

0

0

0

0

0.0794349

0

0

0

0

0

0

0

0.00615279

0

0

0

0

0

0

0.0080381

0

0

0

0

1.13067

0
8

0

0

0

0

0.0204104

0

0

0.00922919

0

0.395219

0

0

0

0

0

0

0.00475254

0

0.026063

0

0.080381
3

0

0

0

0

0

0

0.00710613

0

0.0250159

0

0
7

0.00736842

0

0

0.0361715

0

0

0

0

0.0177653

0

0.152724
3

0

0.0281334

0

0

0

0

0

0

0

0

0.0184584
7

0

0

0

0

0

0

0

0

0

0

0.0180341
1

0

0

0

0.0100064

0

0

0

0

0

0

0

0

0

0

0

0

0

0

0

0

0

0
4

0
2

0

0

0

0

0

0

0

0

0

0

0

0

0

0

0

0

0

0

0

0

0

0

0

0

0

0

0

0

0

0

0

0

0

0

0

0

0

0

0

0

0

0

0

0

0

0

0

0

0

0

0

0

0

0

0

0

0

0

0

0

0

0

0

0

0

0

0

0

0

0

0

0

0

0

0

0

0

0

0

0

0

0

0

0

0

0

0
4

0.548307
3

0.50423
3

0

0

0

0

0

0

0.0104881

0

0

0

0.0104881
4

0

0

0

0

0

0

0

0

0

0

0
4

0

0

0

0

0

0

0
4

0
3

0
4

0.0157321
3

0
4

0.00736842

0
4

0
3

0

0

0

0

0
4

0

0

0

0

0

0

0

0
4

0

0

0
4

0

0

0
4

0

0

0
4

0.234567

0.234567

0
4

0

0

0

0
4

0.00475254

0.00475254

0
4

0

0

0

0
4

0

0

0
4

0

0

0
4

0

0

0

0
4

0
3

0
4

0
3

0
3

0
3

0

0

0

0

0
4

0

0

0
4

0

0

0

0
4

0

0

0
4

0

0

0

0
4

0

0

0
4

0

0

0
4

0

0

0

0
4

0

0

0
4

0

0

0
4

0

0

0
4

0.330374
3

0
3

0

0

0

0

0

0.330374

0

0
4

0

0

0
4

0

0

0
4

0

0

0
4

0

0

0
4

0

0

0
4

0

0

0
4

0

0

0
4

0

0

0
4

0

0

0
4

0

0

0
4

0
2

0
2

0

0

0

0

0

0

0
4

0

0

0
4

0

0

0
4

0

0

0
4

0

0

0
4

0

0

0
4

0

0

0
4

0

0

0
4

0

0

0
4

0

0

0
4

0

0

0
4

0

0

0

0

0

0

0

0

0
4

0

0

0
4

0

0

0
4

0

0

0
4

0

0

0
4

0

0

0
4

0

0

0
4

0

0

0
4

0.0173753

0.0173753

0
4

0

0

0
4

0

0

0
4

0
4

0
4

0

0

0

0
4

0

0

0
4

0

0

0
4

0

0

0
4

0

0

0
4

0

0

0
4

0

0

0
4

0

0

0
4

0

0

0
4

0

0

0
4

0

0

0
4

0

0

0

0

0

0
4

0

0

0
4

0

0

0
4

0

0

0
4

0

0

0
4

0

0

0
4

0

0

0
4

0

0

0
4

0

0

0
4

0

0

0
4

0

0

0
4

0
3

0
3

0

0

0

0
4

0

0

0
4

0

0

0
4

0

0

0
4

0

0

0
4

0

0

0
4

0.173753

0.173753

0
4

0

0

0
4

0

0

0
4

0

0

0
4

0

0

0
4

0.0576843
4

0
4

0.0576843

0

0

0
4

0

0

0
4

0

0

0
4

0

0

0
4

0

0

0
4

0

0

0
4

0

0

0
4

0

0

0
4

0

0

0
4

0

0

0
4

0.0390837

0.0390837

0
4

0

0

0

0

0

0

0
4

0

0

0
4

0

0

0
4

0

0

0
4

0

0

0
4

0

0

0
4

0.0157321

0.0157321

0
4

0

0

0
4

0

0

0
4

0

0

0
4

0

0

0
4

0.0836077
3

0.0178565
3

0

0

0

0

0

0

0

0

0

0

0.00736842
4

0

0

0

0

0

0

0

0.0478947
4

0.0104881
4

0
3

0

0

0

0

1.04083408558608e-17
3

0
4

0

0

0

0

0

0
4

0

0

0
4

0

0

0
4

0.0173753

0.0173753

0
4

0

0

0
4

0

0

0
4

0.0292553
3

0.0292553
3

0

0

0
4

0
4

0

0

0

0

0

0
4

0
2

0

0

0

0

0

0

0

0
4

0

0

0

0

0

0

0

0

0

0
4

0.0955644

0.0347507

0.0608137

0
4

0
3

0

0

0

0

0

0

0
4

0
3

0

0

0

0

0

0

0

0
4

0

0

0

0
4

0
2

0

0

0

0

0

0
4

0.717513
3

0.707024
3

0

0

0

0

0

0

0

0

0

0

0
4

0

0

0
4

0

0.0104881

0

0

0

0
4

0
3

0

0

0

0

0

0
4

0
4

0

0

0

0

0

0
4

0

0

0
4

0
4

0
4

0

0

0

0
4

0

0

0

0
4

0

0

0

0

0
4

0.312056
4

0.312056
4

0

0
4

0
5

0
5

0

0
4

0
2

0

0

0

0

0

0
4

0
4

0
4

0
4

6.75919
5

1.75244
5

0.026063

0.026063

0.0434384

0.0434384

0.0173753

0.139003

0.0173753

0.0173753

0.495197

0.0347507

1.43982

0

0.173753

0.026063

0

0.0173753

0.0173753

1.3342

0.312756

0.347507

0.130315

0.121627

0.11294

0.11294

0
4

0
4

0

0

0
4

0

0

0

0

0
4

0

0

0
4

0

0

0

0

0
4

0
4

0

0

0
4

0

0

0

0

0

0
4

0

0

0

0
4

0

0

0

0

0

0
4

0

0

0
4

0

0

0
4

46.5833
6

43.4297

0.0173753

0.0347507

0.0434384

0.0608137

0.0434384

0.0173753

0.0173753

0.0173753

0.0173753

0.0173753

0.356195

0.0434384

0.0173753

0.026063

0.026063

0.0173753

0.0173753

0.0608137

0.026063

0.0347507

0.0173753

0.104252

0.0347507

0.0173753

0.243255

0.26063

0.495197

0.0695014

0.0434384

0.920893

0.0347507

0
4

0.0215407
2

0.0215407

0

0
4

0

0

0

0

0
4

0.0157321

0

0.0157321

0
4

0

0

0

0

0
4

0

0

0

0

0
4

0

0

0

0

0
4

0

0

0

0
4

0.0104881

0.0104881

0
4

0.0450286

0.0450286

0

0
4

0

0

0

0

0
4

0
2

0
2

0
2

0

0

0

0

0

0

0
4

0

0

0
4

0

0

0

0
4

0

0

0
4

0

0

0

0
4

0

0

0
4

0

0

0
4

0

0

0
4

0

0

0

0

0

0
4

0.0195035

0.0195035

0

0
4

0

0

0
4

0.131101
3

0.0262201
4

0

0

0

0

0

0

0

0

0

0

0

0

0

0

0

0

0

0

0

0

0

0

0.104881

0

0

0

0

0

0
4

0

0

0
4

0.11294

0.11294

0
4

0

0

0

0
4

0

0

0

0
4

0.0173753

0.0173753

0

0
4

0

0

0

0
4

0

0

0

0
4

0

0

0

0
4

0

0

0

0
4

0

0

0

0
4

89.7694
6

87.7141
6

0.0347507

0

0.0173753

1.24732

0.477822

0.173753

0

0.0173753

0.052126

0.0173753

0.0173753

0
4

0

0

0

0
4

0

0

0

0
4

0

0

0
4

0

0

0

0
4

0

0

0
4

0

0

0
4

0

0

0

0
4

0.0347507

0.0173753

0.0173753

0
4

0

0

0
4

0

0

0
4

0
4

0
4

0
4

0
4

0

0

0

0

0

0

0
4

0

0

0
4

0

0

0
4

0

0

0
4

0

0

0
4

0

0

0

0
4

0

0

0

0
4

0

0

0
4

0

0

0

0
4

0

0

0
4

0

0

0

0
4

0
4

0
2

0
2

0
2

0

0

0

0

0

0

0

0

0

0

0

0

0

0

0

0

0

0

0

0

0

0
2

0

0

0

0

0

0

0

0

0

0

0

0

0

0

0

0

0

0

0

0

0

0

0

0

0

0

0

0

0

0

0

0

0

0

0

0

0

0

0

0

0

0

0

0

0

0

0

0

0

0

0

0

0
4

0
2

0

0

0

0

0

0

0

0

0

0

0

0
4

0

0

0

0

0

0

0

0

0
4

0

0

0

0

0

0
4

0

0

0
4

0
4

0.281337

0.281337

0.144145
7

0

0.0914344

0.0327735

0

0.0129839

2.77555756156289e-17

0
4

0

0

0
4

0

0

0
4

0
4

0

0

0

0
4

0

0

0
4

0
4

0

0

0

0
4

0

0

0
4

0
4

0

0

0

0
4

0

0

0
4

0
4

0

0

0

0

0
4

0
4

0

0

0

0
4

0

0

0
4

0
4

0

0

0

0
4

0
4

0

0

0

0
4

0
4

0

0

0

0
4

0
4

0

0

0

0
4

0

0

0
4

0
4

0

0

0

0
4

0
4

0.120258

0.120258

0.09429

0.0129839

0

0

0

0.0129839

0
4

0
4

0

0

0

0

0
4

0
4

0

0

0

0
4

0

0

0
4

0
4

0

0

0

0

0
4

0
4

0

0

0

0
4

0
4

0

0

0

0

0
4

0
4

0

0

0

0
4

0
4

0

0

0

0
4

0
4

0

0

0

0
4

0
4

0

0

0

0
4

0
4

0

0

0

0

0
4

0
4

0
7

0
7

0

0

0

0

0

0

0
4

0
4

0

0

0

0

0
4

0
4

0

0

0

0
4

0
4

0

0

0

0
4

0
4

0

0

0

0
4

0
4

0

0

0

0
4

0
4

0

0

0

0
4

0
4

0

0

0

0
4

0
4

0

0

0

0
4

0
4

0

0

0

0
4

0
4

0

0

0

0
4

0
4

0
2

0
2

0
2

0

0

0

0

0

0

0
4

0
4

0

0

0

0
4

0
4

0

0

0

0
4

0
4

0

0

0

0
4

0
4

0

0

0

0
4

0
4

0

0

0

0
4

0
4

0

0

0

0
4

0
4

0

0

0

0
4

0
4

0

0

0

0
4

0
4

0

0

0

0
4

0
4

0

0

0

0
4

0
4

0.0292553

0
5

0

0

0

0

0

0

0

0
4

0.0292553

0.0292553

0

0

0

0

0
4

0

0

0
4

0
4

0

0

0

0
4

0
4

0

0

0

0
4

0
4

0

0

0

0
4

0
4

0

0

0

0
4

0
4

0

0

0

0
4

0
4

0

0

0

0
4

0
4

0

0

0

0
4

0
4

0

0

0

0
4

0
4

0

0

0

0
4

0
4

0

0

0

0
4

0
4

0

0

0

0

0

0

0

0

0
4

0
4

0

0

0

0
4

0
4

0

0

0

0
4

0
4

0

0

0

0
4

0
4

0

0

0

0
4

0
4

0

0

0

0
4

0
4

0

0

0

0
4

0
4

0

0

0

0
4

0
4

0

0

0

0
4

0
4

0

0

0

0
4

0
4

0

0

0

0
4

0
4

0

0

0

0

0

0

0

0
4

0
4

0

0

0

0
4

0
4

0

0

0

0
4

0
4

0

0

0

0
4

0
4

0

0

0

0
4

0
4

0

0

0

0
4

0
4

0

0

0

0
4

0
4

0

0

0

0
4

0
4

0

0

0

0
4

0
4

0

0

0

0
4

0
4

0

0

0

0
4

0
4

0

0

0

0

0

0

0

0

0

0

0

0

0
4

0
4

0

0

0

0
4

0
4

0

0

0

0
4

0
4

0

0

0

0
4

0
4

0

0

0

0
4

0
4

0

0

0

0
4

0
4

0

0

0

0
4

0
4

0

0

0

0
4

0
4

0.0851532

0.0851532

0.0851532

0
4

0
4

0

0

0

0
4

0
4

0

0

0

0
4

0
4

0

0

0

0

0

0

0
4

0

0

0
4

0
4

0

0

0

0
4

0
4

0

0

0

0
4

0
4

0

0

0

0
4

0
4

0

0

0

0
4

0
4

0

0

0

0
4

0
4

0

0

0

0
4

0
4

0

0

0

0
4

0
4

0

0

0

0
4

0
4

0

0

0

0
4

0
4

0

0

0

0
4

0
4

0
6

0
6

0
6

0

0

0

0

0

0
4

0
4

0

0

0

0
4

0
4

0

0

0

0
4

0
4

0

0

0

0
4

0
4

0.0473073
2

0.0473073
2

0.0283844
2

0

0

0

0

0

0

0

0

0

0

0
2

0

0

0

0

0

0

0

0

0

0

0

0

0

0

0

0

0

0

0

0

0

0
2

0

0

0

0

0

0

0

0

0

0

0

0

0

0

0

0

0

0

0

0

0

0

0

0

0

0

0

0

0

0

0

0

0

0

0

0

0

0

0

0

0

0

0

0
2

0

0

0

0

0

0.0189229

0

0

0

0

0
4

0
2

0
2

0

0

0

0

0

0

0

0

0

0
4

0
2

0

0
2

0

0

0

0
4

0

0

0

0

0

0
4

0

0

0

0

0
4

0

0

0

0

0
4

0

0

0

0
4

0
4

0
2

0
2

0
2

0

0

0

0
4

0

0

0

0
4

0

0

0
4

0
4

0

0

0

0

0

0

0
4

0
4

0
5

0
5

0
5

0

0

0
4

0
4

0.582074

0.547323

0.0695014

0.0434384

0.139003

0.130315

0.052126

0

0.11294

0
4

0.0347507

0.0347507

0

0
4

0

0

0
4

2.08166817117217e-17

0
4

0

0

0

0

0

0

0

0
4

0

0

0
4

0
4

0

0

0

0

0

0

0
4

0

0

0

0
4

0

0

0
4

0

0

0
4

0
4

0

0

0

0

0

0

0
4

0

0

0
4

0

0

0
4

0

0

0
4

0
4

0
5

0
5

0

0

0

0

0

0

0

0

0
4

0
4

0
4

0

0

0

0

0
4

0

0

0
4

0

0

0
4

0
4

0.350565
7

0.350565
7

0.350565
7

0

0

0
4

0
4

0
2

0
2

0
2

0

0

0

0

0

0

0

0

0
2

0

0

0

0

0

0

0

0
4

0
2

0
2

0

0

0

0
4

0

0

0

0

0

0

0

0

0

0

0
4

0

0

0

0
4

0
4

0

0

0

0

0

0

0
4

0
4

0.0649194
6

0.0649194
6

0.0649194
6

0
4

0
4

0
2

0

0

0

0

0

0
4

0

0

0

0
4

0

0

0
4

0
4

0
7

0
7

0
7

0

0
4

0
4

0
7

0
7

0

0

0
4

0
4

0.164314

0.164314

0.164314

0

0

0
4

0

0

0
4

0
4

0

0

0

0

0

0

0

0
4

0

0

0
4

0

0

0
4

0
4

0
6

0
6

0
6

0

0
4

0
4

0

0

0

0

0
4

0
4

0

0

0

0
4

0

0

0
4

0

0

0
4

0
4

0
2

0
2

0
2

0

0

0

0

0

0

0

0

0

0

0

0

0

0

0

0

0

0

0

0

0

0

0
4

0

0

0
4

0
2

0

0

0

0

0

0

0
4

0

0

0

0

0

0
4

0

0

0

0

0
4

0

0

0
4

0

0

0

0
4

0

0

0

0
4

0

0

0
4

0

0

0
4

0
4

0

0

0

0

0
4

0

0

0

0
4

0
4

0

0

0

0

0
4

0

0

0
4

0
4

0

0

0

0

0

0

0

0
4

0

0

0

0
4

0
4

0
6

0
6

0
6

0
4

0
4

0

0

0

0
4

0
4

0.344647

0.344647

0.333988

0.0106592

0
4

0
4

0

0

0

0

0
4

0

0

0

0
4

0
4

0.11294

0.11294

0.0434384

0.052126

0.0173753

0
4

0
4

0

0

0

0

0

0

0
4

0

0

0
4

0
4

0

0

0

0
4

0
4

0

0

0

0

0

0

0

0

0

0

0

0

0

0

0

0

0

0

0
4

0

0

0
4

0

0

0

0
4

0
4

0

0

0

0

0

0
4

0

0

0
4

0

0

0
4

0
4

0

0

0

0
4

0
4

0

0

0

0

0
4

0
4

0.120613

0.120613

0.120613

0
4

0

0

0
4

0
4

0

0

0

0

0
4

0

0

0
4

0
4

0

0

0

0

0
4

0
4

0

0

0

0

0

0
4

0

0

0
4

0

0

0
4

0
4

0

0

0

0

0
4

0

0

0
4

0

0

0
4

0

0

0
4

0
4

0

0

0

0

0
4

0
4

0

0

0

0
4

0
4

0
2

0
2

0
2

0
2

0

0

0

0

0

0

0

0
4

0

0

0

0
4

0
4

0

0

0

0

0
4

0

0

0
4

0

0

0
4

0

0

0
4

0
4

0
7

0

0

0

0
4

0

0

0
4

0
4

0

0

0

0
4

0
4

0

0

0

0
4

0

0

0

0
4

0

0

0
4

0
4

0

0

0

0

0
4

0
4

0

0

0

0

0
4

0

0

0
4

0
4

0

0

0

0

0

0
4

0
4

0

0

0

0
4

0

0

0
4

0
4

0

0

0

0
4

0
4

0

0

0

0

0
4

0
4

0
2

0
2

0
2

0

0

0

0

0

0
4

0
2

0

0

0

0

0

0

0

0
4

0
2

0

0

0

0

0

0
4

0

0

0

0
4

0

0

0

0
4

0

0

0
4

0

0

0
4

0

0

0
4

0

0

0
4

0
4

0.231322

0.231322

0.231322

0
4

0
4

0

0

0

0
4

0

0

0
4

0
4

0

0

0

0

0

0
4

0
4

0

0

0

0
4

0
4

0

0

0

0

0

0
4

0
4

0

0

0

0

0

0
4

0
4

0

0

0

0
4

0

0

0
4

0
4

0

0

0

0

0
4

0

0

0
4

0
4

0

0

0

0
4

0
4

0.167809

0.167809

0.167809

0
4

0

0

0
4

0
4

0

0

0

0

0

0

0

0

0

0

0
4

0
4

0

0

0

0
4

0
4

0

0

0

0
4

0
4

0

0

0

0

0

0
4

0
4

0.0195035

0.0195035

0

0.0195035

0
4

0
4

0

0

0

0
4

0
4

0

0

0

0

0
4

0
4

0

0

0

0
4

0

0

0
4

0
4

0

0

0

0

0
4

0
4

0

0

0

0
4

0
4

0

0

0

0
4

0

0

0
4

0
4

0

0

0

0

0

0

0

0

0

0

0
4

0

0

0
4

0

0

0

0
4

0
4

0

0

0

0
4

0

0

0
4

0
4

0

0

0

0
4

0
4

0

0

0

0

0
4

0
4

0

0

0

0

0
4

0
4

0.236893

0.236893

0.236893

0
4

0
4

0

0

0

0
4

0

0

0
4

0
4

0

0

0

0
4

0

0

0
4

0
4

0.0292553

0.0292553

0

0.0292553

0
4

0
4

0

0

0

0

0
4

0
4

0

0

0

0
4

0

0

0
4

0
4

0.969366

0.810945

0.746262

0.0646827

0

0

0

0

8.32667268468867e-17

0
4

0
1

0
1

0
4

0.158421

0.158421

0
4

0

0

0

0
4

0

0

0

0
4

0

0

0

0
4

0

0

0
4

0
4

0
4

0
4

0
4

0

0

0

0
4

0

0

0
4

0

0

0
4

0

0

0
4

0
4

0

0

0

0
4

0
4

0

0

0

0

0
4

0
4

0

0

0

0
4

0
4

0

0

0

0
4

0
4

0
4

60.2202
5

0.246694
4

0.246694
4

0.0389516
4

0.0129839

0

0

0

0

0

0

0

0

0

0

0

0

0

0

0

0

0

0

0

0

0

0.0454436

0

0

0

0

0

0

0

0.0129839

0

0

0

0

0

0

0

0

0

0

0

0

0

0

0

0
4

0.0129839

0

0

0
4

0

0

0

0

0

0

0

0

0

0

0

0
4

0

0

0

0

0

0

0

0

0

0

0.123347
4

0

0

0

0

0

0

0

0

0

0

0
4

0

0

0

0

0

0

0

0

0

0

0

0

0

0

0

0

0

0

0

0

0

0
4

0

0

0

0

0

0

0

0

0

0

0
4

0

0

0

0

0

0

0

0

0

0

0
4

0

0

0

0

0

0

0

0

0

0

0
4

0
4

59.8112
5

0.991854

0.668332

0.175148
2

0.0173753

0
4

0

0
4

0.0080381

0

0

0

0

0

0

0
7

0

0

0

0

0

0

0

0

0

0

0
3

0

0

0

0

0

0

0

0

0

0

0
4

0

0

0

0

0

0

0

0

0

0

0

0

0

0

0

0

0

0

0

0

0.0283844

0.0347507

0

0

0

0

0

0

0

0

0

0

0

0

0

0

0

0

0

0

0

0

0

0.0424498

0

0

0

0

0

0

0

0

0

0

0

0

0

0.0173753

1.42247325030098e-16

0
4

0

0

0

0

0

0
4

0

0

0
4

58.2383
5

14.3543
5

0
3

31.8644
3

3.03665
4

8.28586
5

0
4

0

0

0

0

0

0

0

0

0

0

0

0

0

0

0

0

0

0

0

0

0

0

0

0

0

0

0

0

0

0

0

0

0

0

0

0.0283844

0

0

0

0

0

0

0

0

0

0

0

0

0

0

0

0

0

0

0

0

0
4

0

0

0

0

0

0

0

0

0

0

0

0

0

0

0

0

0

0

0

0

0

0

0

0

0

0

0

0

0

0

0

0

0

0

0

0

0

0

0

0

0

0

0

0

0

0

0

0

0

0

0

0

0

0

0
4

0

0

0

0

0

0

0

0
3

0

0
4

0

0
4

0

0

0
4

0

0
4

0

0

0
4

0

0

0

0.245998

0
4

0

0

0

0

0

0

0

0

0

0

0

0

0

0

0

0

0

0

0

0

0

0

0

0

0

0

0

0

0

0

0

0

0

0

0

0
4

0

0

0

0

0

0

0

0

0

0

0
3

0

0

0

0

0

0

0

0

0

0

0
4

0

0

0

0

0

0

0

0

0

0

0
4

0

0

0

0

0

0

0

0

0

0

0.0177653
4

0

0

0

0

0

0

0

0

0

0

0

0
4

0

0

0

0

0

0

0

0

0

0

0

0

0

0

0

0

0

0

0

0

0

0
4

0

0

0

0

0

0

0

0

0

0

0

0

0

0

0

0

0

0

0

0

0

0
4

0

0

0

0

0

0

0

0

0

0

0
4

0

0

0

0

0

0

0

0

0

0

0
4

0

0

0

0

0

0

0

0

0

0

0
4

0

0

0

0

0

0

0.0519355

0

0

0

0
4

0

0

0

0

0

0

0

0

0

0

0

0.0194758
4

0

0

0

0

0

0

0

0

0

0

0
4

0

0

0

0

0

0

0

0

0

0

0

0

0

0

0

0

0

0

0

0

0

0

0

0

0

0

0

0

0

0

0

0

0
4

0

0

0

0.0129839

0

0

0

0

0

0

0

0

0

0

0

0

0

0

0

0

0

0

0

0

0

0

0

0

0

0

0

0

0
4

0

0

0

0

0

0

0

0

0

0

0

0

0

0

0

0

0

0

0

0

0

0

0

0

0

0

0

0

0

0

0

0

0.0779032
6

0
3

0

0

0

0

0

0

0

0

0

0

0

0

0

0

0

0

0

0

0

0

0

0
3

0

0

0

0

0

0

0

0

0

0

0
4

0

0

0

0

0

0

0

0

0

0

0

0

0

0

0

0

0

0

0

0

0

0
4

0

0

0

0

0

0

0

0

0

0

0

0

0

0

0

0

0

0

0

0

0

0
4

0

0

0

0

0

0

0

0

0

0

0
4

0

0

0

0

0

0

0

0

0

0

0

0

0

0

0

0

0

0

0

0

0

0

0

0

0

0

0

0

0

0

0

0

0

0

0

0

0

0

0

0

0

0

0

0

0.0409669
4

0

0

0

0

0

0

0

0

0

0

0
4

0

0

0

0

0

0

0

0

0

0

0

0

0

0

0

0

0

0

0

0

0

0
4

0

0

0

0

0

0

0

0

0

0

0.0189229
3

0

0

0

0

0

0

0

0

0

0

0
3

0

0

0

0

0

0

0

0

0

0

0

0

0

0

0

0

0

0

0

0

0

0
4

0

0

0

0

0

0

0

0

0

0

0
4

0

0

0

0

0

0

0

0

0

0

0

0

0

0

0

0

0

0

0

0

0

0

0

0

0

0

0

0

0

0

0

0

0

0
4

0

0

0

0

0

0

0

0

0

0

0
4

0

0

0

0

0

0

0

0

0

0

0

0

0

0

0

0

0

0

0

0

0

0

0

0

0

0

0

0

0

0

0

0

0
4

0

0

0

0

0

0

0

0

0

0

0

0

0

0

0

0

0

0

0

0

0

0

0

0

0

0

0

0

0

0

0

0

0
4

0
4

0

0

0

0

0

0

0

0

0

0

0

0

0

0

0

0

0

0

0

0

0

0
4

0

0

0

0

0

0

0

0

0

0

0

0

0

0

0

0

0

0

0

0

0

0

0

0

0

0

0

0

0

0

0

0

0
3

0

0

0

0

0

0

0

0

0

0

0

0

0

0

0

0

0

0

0

0

0

0
4

0

0

0

0

0

0

0

0

0

0

0

0

0

0

0

0

0

0

0

0

0

0
4

0

0

0

0

0

0

0

0

0

0

0
3

0

0

0

0

0

0

0

0

0

0

0

0

0

0

0

0

0

0

0

0

0

0

0

0

0

0

0

0

0

0

0

0

0

0

0

0

0

0

0

0

0

0

0

0

0

0

0

0

0

0

0

0

0

0

0

0

0

0

0

0

0

0

0

0.0189229

0

0

0

0

0

0

0

0

0

0

0

0

0

0.0737404
4

0

0

0

0

0

0

0

0

0

0

0

0

0

0

0

0

0

0

0

0

0

0

0

0

0

0

0

0

0

0

0

0

0

0

0

0

0

0

0

0

0

0

0

0

0

0

0

0

0

0

0

0

0

0

0

0.0901271
4

0

0

0

0

0

0

0

0

0

0

0

0

0

0

0

0

0

0

0

0

0

0
4

0

0

0

0

0

0

0

0

0

0

0

0

0

0

0

0

0

0

0

0

0

0

0

0

0

0

0

0

0

0

0

0

0

0

0

0

0

0

0

0

0

0

0

0

0

0

0

0

0

0

0

0

0

0

0

0

0

0

0

0

0

0

0

0

0

0
4

0

0

0

0
4

0
4

0

0
4

0

0

0

0
4

0.512863

0
5

0.110363

0.103871

0

0

0

0

0

0

0

0.0259677

0.0584274

0.214234

0

0

0
4

0

0

0
4

0

0

0
4

0.0681724

0.0681724

0
4

0

0

0
4

0

0

0
4

0

0

0
4

0

0

0
4

0

0

0
4

0
4

0

0

0

0
4

0
4

0

0

0

0
4

0
4

0

0

0

0
4

0
4

0

0

0

0
4

0
4

0.162298
4

0.162298
4

0

0.162298

0
4

0
4

0

0

0

0
4

0

0

0
4

0

0

0
4

0
4

0
4

0
3

0

0

0

0

0

0

0
4

0
4

0

0

0

0
4

0
4

0

0

0

0
4

0
4

0

0

0

0

0
4

0
4

0

0

0

0

0
4

0
4

0

0

0

0
4

0
4

0

0

0

0
4

0
4

0

0

0

0
4

0
4

0

0

0

0
4

0
4

0

0

0

0
4

0
4

0

0

0

0
4

0
4

0
4

0.222386

0.222386

0.222386

0.0573536

0

0.0426368

0

0

0

0

0

0

0

0

0
1

0

0

0

0

0

0

0

0

0

0

0

0

0

0

0

0

0

0

0

0

0

0.065547

0

0

0

0

0

0

0

0.056849

0

0

0

0

1.38777878078145e-17

0
4

0
4

0

0

0

0
4

0
4

0
4

0.125857

0.125857

0.125857

0.104881
7

0

0

0

0

0

0

0

0

0

0

0.0209761
6

0

0

0

0

0

0

0

0

0

0

0

0

0

0

0

0

0

0

0

0

0

0

0

0

0

0

0

0

0

0

0

0

0
6

0

0

0

0

0

0

0

0

0

0

0

0
4

0

0

0

0

0

0

0
4

0

0

0
4

0

0

0

0
4

0

0

0

0
4

0

0

0
4

0

0

0
4

0
4

0

0

0

0
4

0
4

0

0

0

0
4

0
4

0

0

0

0
4

0
4

0
4

0.206755
7

0.206755
7

0.107269
7

0.107269
7

0

0

0

0

0

0

0

0

0

0

0
6

0
7

0

0

0

0

0

0

0
4

0.0177653
6

0

0.0177653

0

0
4

0.0817205

0.0746143

0.00710613

4.33680868994202e-18

0
4

0
4

0
4

0.343092
7

0.343092
7

0.343092
7

0.30807

0

0

0.0250159

0.0100064

2.08166817117217e-17
7

0
4

0
4

0
4

0

0

0

0

0
4

0
4

0
4

0

0

0

0

0
4

0
4

0
4

0

0

0

0

0
4

0
4

0
4

0

0

0

0

0
4

0
4

0
4

0

0

0

0

0
4

0
4

0
4

0.0163868

0.0163868

0.0163868

0.0163868

0
4

0
4

0
4

0

0

0

0

0
4

0
4

0
4

0

0

0

0

0
4

0
4

0
4

0

0

0

0

0
4

0
4

0
4

0

0

0

0

0
4

0
4

0
4

0.0755788
7

0.0755788
7

0.0705916
7

0.0224425
7

0.032417
7

0.0157321

1.04083408558608e-17
7

0
4

0.00498723

0.00498723

0
4

0
4

0
4

0

0

0

0

0
4

0
4

0
4

0

0

0

0

0
4

0
4

0
4

0

0

0

0

0
4

0
4

0
4

0

0

0

0

0
4

0
4

0
4

0

0

0

0

0
4

0
4

0
4

0

0

0

0

0
4

0
4

0
4

0

0

0

0

0
4

0
4

0
4

0

0

0

0

0
4

0
4

0
4

0

0

0

0

0
4

0
4

0
4

0

0

0

0

0
4

0
4

0
4

0.097379

0.097379

0.097379

0.097379

0

0

0
4

0

0

0
4

0
4

0
4

0

0

0

0

0
4

0
4

0
4

0

0

0

0

0
4

0
4

0
4

0

0

0

0

0
4

0
4

0
4

0

0

0

0

0
4

0
4

0
4

0

0

0

0

0
4

0
4

0
4

0

0

0

0

0
4

0
4

0
4

0

0

0

0

0
4

0
4

0
4

0

0

0

0

0
4

0
4

0
4

0

0

0

0

0
4

0
4

0
4

0

0

0

0

0
4

0
4

0
4

0.0163868
7

0.0163868
7

0.0163868
7

0

0.0163868

0

0
4

0

0

0

0

0
4

0
4

0
4

0

0

0

0

0
4

0
4

0
4

0

0

0

0

0
4

0
4

0
4

0

0

0

0

0
4

0
4

0
4

0

0

0

0

0
4

0
4

0
4

0

0

0

0

0
4

0
4

0
4

0

0

0

0

0
4

0
4

0
4

0

0

0

0

0
4

0
4

0
4

0

0

0

0

0
4

0
4

0
4

0

0

0

0

0
4

0
4

0
4

0

0

0

0

0
4

0
4

0
4

0.0248714

0.0248714

0

0

0

0

0

0
4

0.0248714

0.0248714

0

0
4

0
4

0
4

0

0

0

0

0
4

0
4

0
4

0

0

0

0

0
4

0
4

0
4

0

0

0

0

0
4

0
4

0
4

0

0

0

0

0
4

0
4

0
4

0

0

0

0

0
4

0
4

0
4

0.0163868

0.0163868

0.0163868

0.0163868

0
4

0
4

0
4

0

0

0

0

0
4

0
4

0
4

0

0

0

0

0
4

0
4

0
4

0

0

0

0

0
4

0
4

0
4

0

0

0

0

0
4

0
4

0
4

0

0

0

0

0

0

0
4

0
4

0
4

0

0

0

0

0
4

0
4

0
4

0

0

0

0

0
4

0
4

0
4

0

0

0

0

0
4

0
4

0
4

0

0

0

0

0
4

0
4

0
4

0.0163868

0.0163868

0.0163868

0.0163868

0
4

0
4

0
4

0

0

0

0

0
4

0
4

0
4

0

0

0

0

0
4

0
4

0
4

0.0163868

0.0163868

0.0163868

0.0163868

0
4

0
4

0
4

0

0

0

0

0
4

0
4

0
4

0

0

0

0

0
4

0
4

0
4

0

0

0

0

0

0

0

0
4

0
4

0
4

0

0

0

0

0
4

0
4

0
4

0

0

0

0

0
4

0
4

0
4

0.158763
7

0.158763
7

0.158763
7

0

0.139287

0.0194758

6.93889390390723e-18
7

0
4

0
4

0
4

0

0

0

0

0

0

0
4

0

0

0
4

0
4

0
4

0

0

0

0

0
4

0

0

0

0

0
4

0

0

0
4

0
4

0
4

0.296325
7

0.296325
7

0.296325
7

0.296325
7

0

0

0

0
4

0
4

0

0

0

0
4

0
4

0
4

0

0

0

0

0

0

0
4

0
4

0
4

0

0

0

0

0

0
4

0
4

0
4

0.162298

0.162298

0.162298

0.162298

0
4

0
4

0
4

0

0

0

0

0
4

0
4

0
4

0

0

0

0

0
4

0

0

0
4

0
4

0

0

0

0
4

0

0

0
4

0
4

0
4

0

0

0

0

0

0
4

0
4

0
4

0.0585227

0.0585227

0.0585227

0.0390469

0.0194758

0
4

0
4

0
4

0

0

0

0

0

0
4

0
4

0
4

0

0

0

0

0
4

0
4

0
4

0

0

0

0

0
4

0
4

0
4

0
2

0
2

0
2

0
2

0

0

0

0

0
4

0
2

0

0

0

0

0

0

0
4

0

0

0
4

0

0

0
4

0
4

0
4

0

0

0

0

0

0

0
4

0
4

0
4

0.0163868

0.0163868

0.0163868

0.0163868

0
4

0
4

0
4

0

0

0

0

0
4

0

0

0
4

0

0

0
4

0
4

0
4

0

0

0

0

0

0
4

0
4

0
4

0

0

0

0

0

0

0

0
4

0
4

0
4

0

0

0

0

0
4

0
4

0
4

0

0

0

0

0

0
4

0

0

0
4

0
4

0

0

0

0
4

0
4

0
4

0

0

0

0

0

0
4

0

0

0
4

0
4

0
4

0

0

0

0

0
4

0
4

0
4

0

0

0

0

0

0

0
4

0
4

0
4

0

0

0

0

0

0

0

0

0

0
4

0
4

0
4

0

0

0

0

0

0
4

0

0

0
4

0
4

0
4

0

0

0

0

0
4

0
4

0
4

0

0

0

0

0
4

0
4

0
4

0

0

0

0

0
4

0

0

0
4

0
4

0
4

0

0

0

0

0

0
4

0

0

0
4

0
4

0
4

0

0

0

0

0

0
4

0
4

0
4

0

0

0

0

0

0
4

0
4

0
4

0.0466306

0.0466306

0.0466306

0.0466306

0
4

0
4

0
4

0.0519355

0.0519355

0.0519355

0.0519355

0

0
4

0
4

0
4

0

0

0

0

0

0
4

0

0

0
4

0
4

0
4

0
4

0
4

0
4

0

0

0

0

0

0

0
4

0

0

0
4

0
4

0
4

0

0

0

0

0
4

0
4

0

0

0

0
4

0
4

0
4

0

0

0

0

0
4

0

0

0
4

0

0

0
4

0
4

0
4

0

0

0

0

0

0
4

0
4

0
4

0

0

0

0

0

0
4

0
4

0
4

0

0

0

0

0

0
4

0
4

0
4

0

0

0

0

0
4

0

0

0
4

0
4

0
4

0

0

0

0

0
4

0

0

0
4

0
4

0
4

0

0

0

0

0
4

0
4

0
4

0

0

0

0

0
4

0

0

0
4

0
4

0
4

0

0

0

0

0
4

0

0

0
4

0
4

0
4

0.122618
7

0.122618
7

0.103142
7

0.0311169

0

0

0

0.0194758

0.02009

0.0324597

0

6.93889390390723e-18
7

0
4

0.0194758

0.0194758

0

0
4

6.93889390390723e-18
7

0
4

0
4

0

0

0

0

0
4

0
4

0
4

0

0

0

0

0
4

0
4

0
4

0

0

0

0

0

0
4

0
4

0
4

0

0

0

0

0
4

0

0

0
4

0
4

0
4

0

0

0

0

0
4

0
4

0
4

0

0

0

0

0
4

0

0

0
4

0
4

0
4

0

0

0

0

0
4

0

0

0
4

0
4

0
4

0

0

0

0

0

0
4

0
4

0
4

0

0

0

0

0
4

0

0

0
4

0
4

0
4

0

0

0

0

0

0
4

0
4

0
4

0.0546248

0.0372495

0.0372495

0.0181588

0.0080381

0.0110526

0

0

0

0
4

0

0

0
4

0
4

0.0173753

0.0173753

0

0.0173753

0
4

0
4

0
4

0

0

0

0

0
4

0
4

0
4

0

0

0

0

0
4

0

0

0
4

0
4

0
4

0

0

0

0

0
4

0
4

0
4

0

0

0

0

0

0
4

0
4

0
4

0

0

0

0

0
4

0
4

0
4

0

0

0

0

0
4

0

0

0
4

0
4

0
4

0

0

0

0

0
4

0
4

0
4

0.0262201

0.0262201

0.0262201

0.0262201

0
4

0

0

0
4

0
4

0
4

0

0

0

0

0
4

0

0

0
4

0
4

0
4

0.185118

0.185118

0.185118

0

0.185118

0
4

0
4

0
4

0
5

0
5

0
5

0
5

0

0

0
4

0

0

0

0

0

0
4

0
4

0
4

0

0

0

0

0

0
4

0
4

0
4

0

0

0

0

0

0
4

0
4

0
4

0

0

0

0

0

0
4

0
4

0
4

0

0

0

0

0
4

0
4

0
4

0

0

0

0

0
4

0

0

0
4

0
4

0
4

0

0

0

0

0
4

0
4

0
4

0

0

0

0

0
4

0
4

0
4

0

0

0

0

0
4

0
4

0
4

0

0

0

0

0
4

0

0

0
4

0
4

0
4

0

0

0

0

0
4

0
4

0
4

0
7

0
7

0
7

0
7

0

0
4

0

0

0

0
4

0

0

0
4

0
4

0

0

0

0
4

0
4

0
4

0

0

0

0

0

0
4

0
4

0
4

0

0

0

0

0
4

0
4

0
4

0

0

0

0

0
4

0
4

0
4

0

0

0

0

0
4

0
4

0
4

0

0

0

0

0
4

0
4

0
4

0

0

0

0

0
4

0
4

0
4

0

0

0

0

0
4

0
4

0
4

0

0

0

0

0
4

0
4

0
4

0

0

0

0

0
4

0
4

0
4

0

0

0

0

0
4

0
4

0
4

1.24785

1.22548

1.17577

0.0423545

0.109669

0.0523659

0

0

0.173053

0

0

0.186879

0

0.0129839

0.239128

0

0

0

0

0

0

0

0

0

0

0
4

0

0

0

0.0293706

0

0

0

0

0

0

0

0

0

0.0173753

0

0

0

0

0

0

0

0.182441

0

0

0

0

0

0

0

0

0

0

0

0

0

0

0

0

0.0649194

0

0.0327735

0

0

0

0

0

0

0

0

0.0324597

0

0

0

0

0

0

0

0

0

0

0

0

0

0

0
4

0

0

0

0

0

0
4

0

0

0
4

0

0

0
4

0

0

0
4

0

0

0
4

0

0

0
4

0

0

0

0

0
4

0

0

0

0
4

0

0

0

0

0
4

0

0

0

0

0
4

0.0347507

0

0.0347507

0
4

0

0

0
4

0

0

0
4

0.0149617

0.0149617

0
4

0
4

0

0

0

0

0

0

0
4

0
4

0

0

0

0

0

0
4

0

0

0
4

0
4

0

0

0

0

0
4

0
4

0.0173753

0.0173753

0.0173753

0
4

0
4

0.00498723

0.00498723

0.00498723

0
4

0
4

0
4

0

0

0

0

0
4

0
4

0
4

6.23945339839338e-14

0
4

0.201871
3

0.122076
3

0.00736842
3

0

0

0

0

0

0

0

0

0

0

0

0

0
4

0
3

0
3

0

0

0
4

0.00736842

0.00736842

0
4

0

0

0

0

0

0

0

0

0

0
4

0

0

0

0

0

0
4

0

0

0
4

0

0

0
4

0

0

0
4

0

0

0
4

0

0

0

0
4

0

0

0
4

0

0

0

0
4

0

0

0

0
4

0

0

0
4

0

0

0

0
4

0

0

0
4

0

0

0
4

0
4

0

0

0

0

0

0

0

0

0
4

0
4

0

0

0

0

0
4

0
4

0.114707

0.114707

0.114707

0
4

0
4

0

0

0

0
4

0
4

0
4

0

0

0

0

0

0

0
4

0
4

0
4

0

0

0

0

0
4

0
4

0
4

0

0

0

0

0
4

0
4

0
4

0

0

0

0

0
4

0
4

0
4

0

0

0

0

0
4

0
4

0
4

0

0

0

0

0
4

0
4

0
4

0

0

0

0

0
4

0
4

0
4

0

0

0

0

0
4

0
4

0
4

0

0

0

0

0
4

0
4

0
4

0.0797957

0.0797957

0.0797957

0.0797957

0
4

0
4

0
4

0

0

0

0

0

0
4

0
4

0
4

0

0

0

0

0

0
4

0
4

0
4

0

0

0

0

0
4

0
4

0
4

0

0

0

0

0
4

0
4

0
4

0

0

0

0

0
4

0
4

0
4

0

0

0

0

0
4

0
4

0
4

0

0

0

0

0
4

0
4

0
4

0
4

619.848

1.54092
4

0

0

0

0

0

0

0

0

0

0

0

0

0

0

0

0

0

0

0

0

0

0

0

0

0

0

0

0

0

0

0

0

0

0

0

0
4

0

0

0

0

0
4

0

0

0

0
4

0

0

0

0
4

0
4

0
4

0

0

0

0

0

0
4

0

0

0

0

0
4

0

0

0
4

0

0

0
4

0
4

0

0

0

0

0

0
4

0

0

0

0
4

0

0

0
4

0
4

0.649479
6

0.649479
6

0.649479
6

0
4

0
4

0.263298

0.263298

0.263298

0
4

0

0

0
4

0
4

0

0

0

0

0
4

0

0

0

0
4

0

0

0
4

0
4

0

0

0

0

0
4

0
4

0

0

0

0

0

0
4

0
4

0

0

0

0

0
4

0

0

0
4

0
4

0

0

0

0
4

0
4

0

0

0

0
4

0

0

0
4

0
4

0
4

0
4

0

0

0

0

0

0

0

0

0

0

0

0

0

0

0

0

0

0
4

0

0

0

0

0

0

0
4

0

0

0

0

0

0

0

0
4

0
4

0

0

0

0
4

0
4

0

0

0

0
4

0
4

0.0573536

0.0573536

0.0573536

0
4

0
4

0.0104881

0.0104881

0.0104881

0
4

0
4

0

0

0

0
4

0
4

0

0

0

0
4

0
4

0.0491603

0.0491603

0.0491603

0
4

0
4

0
4

0
4

0

0

0

0

0

0

0

0

0

0

0
4

0

0

0
4

0

0

0

0
4

0

0

0
4

0
4

0
4

0
4

0

0

0

0

0
4

0

0

0

0

0
4

0

0

0
4

0

0

0
4

0
4

0
4

0
4

0

0
4

0

0

0

0

0

0

0

0
4

0
4

0

0

0

0

0

0

0

0

0
4

0

0

0

0

0
4

0

0

0

0

0

0
4

0

0

0
4

0

0

0
4

0
4

0.511146
6

0.511146
6

0.46707
6

0.0367082

0.00736842

0
4

0
4

0

0

0

0

0

0

0

0

0

0

0
4

0

0

0

0
4

0

0

0

0

0
4

0

0

0
4

0
4

0

0

0

0

0

0

0

0
4

0

0

0
4

0
4

0
4

1.01121
5

1.01121
5

0

0

0
4

1.01121
5

1.01121
5

0

0

0

0

0

0

0

0

0

0

0

0
4

0
4

0
4

594.637
7

3.17084
6

3.17084
6

2.82671

0.344122

5.55111512312578e-17
6

0
4

0

0

0
4

0
4

14.9583

1.6864

0.953381

0
1

0.0110526

0.0819338

0

0

0

0

0.00736842

0

0

0

0

0

0

0

0

0

0

0

0

0

0.221221

0

0

0

0

0

0

0

0

0

0

0.368702

0

0

0

0

0.00710613

0

0

0

0

0

0

0

0

0

0

0

0

0

0

0

0

0

0

0.0245801

0

0

0

0

0

0.0110526

0

0

0

0

0
4

0

0

0

0

0

0

0

0

0

0

0

0
4

4.65945

0.192314

0.0368421

0.00736842

0

0

0

0

0

0

0

0

0

0.0248714

0.00498723

0.101521

0

0

0.0104881

0

0

0

0

0

0

0

0.0589474

0

0

0

0.0515789

0

0

0

0

0.0157321

0.00710613

0

0

0

0

0.0327735

0

0

0.0150095

0

0.0104881

0

0.0110526

0

0.00748085

0.0405263

0

0

0.00748085

0.0184584

0

0

0.0262201

0

0

0

0

0

0

0

0.0147368

0

0

0.519474

0

0

0.00710613

0

0.761092
6

0

0

0.0163868

0

0

0.0319776

0

0

0.0173753

0

0.110164
7

0.00748085

0

0.251713

0

0

0

0

0

0

0

0.0471962
8

0

0.00736842

0

0

0

0

0.0294737

0

0

0

0.169474

0

0

0

0

0

0

0.0104881

0

0

0.0104881

0.776787

0

0

0

0

0.00710613

0

0.0491603

0.0598468

0

0

0.0110526

0

0.0434384

0

0

0

0

0.00736842

0

0

0

0.125857
7

0

0

0

0

0

0.0409669

0

0

0.0173753

0.0639551

0.0221053

0

0

0

0.718432

0.00498723

0

0

0

0

0.0177653

1.42594269725294e-15

0
4

0.0129839
6

0.0129839

0

0

0

0

0

0
4

0.0629283

0.0629283

0
4

0.117251
7

0.117251

0

0
4

0.0231005

0.0231005

0

0

0
4

0.00736842

0

0.00736842

0

0
4

0

0

0

0

0
4

0

0

0

0
4

0

0

0
4

0

0

0

0
4

0

0

0

0
4

6.69714

6.60526

0.0104881

0

0.0147368

0.0104881

0.0104881

0.0142123

0

0.0104881

0

0.0209761

2.81025203108243e-16

0
4

0

0

0
4

0.0163868

0.0163868

0

0
4

0

0

0
4

0

0

0

0
4

0

0

0
4

0.0129839

0.0129839

0
4

0

0

0
4

0

0

0
4

0.0129839

0.0129839

0
4

0

0

0
4

0.382264

0.245934

0.0194758

0.103871

0

0

0

0.0129839

0
4

0

0

0
4

0

0

0
4

0

0

0
4

0

0

0
4

0

0

0
4

0

0

0
4

0

0

0
4

0

0

0
4

0

0

0
4

0

0

0
4

0.020365

0.00710613
7

0

0

0.00710613

0.00615279
7

0

0

0

0

0

0

0
4

0

0

0
4

0

0

0
4

0

0

0
4

0

0

0
4

0

0

0
4

0

0

0
4

0.0245801

0.0245801

0
4

0

0

0
4

0

0

0
4

0

0

0
4

0.748521

0.225997

0

0.0104881

0

0

0

0.0355306
6

0.0348778

0.383943

0.0157321

0.0209761

0.0209761

0

0

9.71445146547012e-17

0
4

0

0

0
4

0

0

0
4

0

0

0
4

0.163441

0.124357

0

0

0

0

0.0390837

0

0

0

1.38777878078145e-17

0
4

0.27656

0.27656
7

0

0

0

0

0

0
4

0.0265279

0.0104881

0.00498723

0

0

0.0110526

0
4

0.00710613

0

0.00710613

0

0

0

0

0

0
4

0
4

569.921
7

3.56428
6

1.86164
6

0.183973
7

0.444855

0

0

0.13406

0.0194758

0

0

0

0.156784

0

0.20456
6

0

0

0

0

0.0163868

0

0

0

0

0

0

0

0

0

0.0163868

0

0

0

0

0

0

0.0324597

0

0

0

0

0.0163868

0.00997446

0

0

0

0

0.00997446

0

0

0

0

0

0

0

0

0.0129839

0

0
5

0

0

0

0

0

0

0

0

0

0.0159983

0.0194758
6

0

0

0

0

0

0

0

0

0

0.383024
6

0.0258751
6

7.91033905045424e-16
6

0
4

0.0248714
7

0.0248714
7

0

0

0

0
4

519.994
7

396.971
7

122.812
7

0

0

0

0

0

0

0

0

0

0.0173753

0.0839044

0.0338421

0.0194758

0

0

0

0

0

0.00498723

0

0

0

0

0.0245801

0

0.0163868

0

0

0

0

0

0

0

0

0

0

0

0

0

0

0.00997446

0

4.20757179098175e-14
7

0
4

0.0270925
7

0.0270925

0

0

0

0

0
4

2.0996

2.0996

0

0

0

0
4

25.5559
7

23.7548
7

0.118047

0

0.139287

0.0248714

0.0843952

0

0.113796

0.00710613

0

0.0106592

0

0.141173
7

0.00710613

0

0

0

0

0

0

0.056849

0.0104881

0

0.868709
6

0

0

0

0

0

0

0

0.0106592

0.0314642

0.0142123

0.0106592

0.151644

0
4

1.53357
6

1.52111
6

0

0.0124681

0

6.93889390390723e-18
6

0
4

5.24449
6

5.22351
6

0

0.0209761

0

0

0

1.73472347597681e-16
6

0
4

6.02347
6

0.716485
7

2.52961
6

0.0408219
7

0.0142123

0.0163868

0

0

0.0195035

0.00710613

0

0

0

1.42573

0.00615279

0

0

0

0

0

0.0319776

0

0

0.0173753

0.299945
7

0

0

0

0.0173753

0

0.0245801

0

0

0.0577572
6

0.262775
7

0.181456
6

0.0142123
7

0.0266097
7

0.313396
6

0
4

0.312896
7

0.224069
7

0

0

0

0.0888266

0

0

0

1.38777878078145e-17
7

0
4

0

0

0

0

0

0

0

0

0

0

0

0

0
4

4.26722
6

4.23954
6

0.0276876

1.56125112837913e-16
6

0
4

0.779419
6

0.738452
6

0.0409669

0

0

0

5.55111512312578e-17
6

0
4

0

0

0

0
4

0

0

0
4

0

0

0

0
4

0

0

0
4

0

0

0
4

0

0

0
4

0

0

0
4

0

0

0
4

0

0

0
4

0

0

0
4

0.0426368
7

0.0426368
7

0

0

0

0

0
4

0

0

0
4

0

0

0
4

0.206119

0.206119

0
4

0

0

0
4

0

0

0
4

0

0

0
4

0

0

0
4

0

0

0
4

0.0129839

0.0129839

0
4

0

0

0
4

0
7

0
7

0

0
4

0.00710613

0.00710613

0
4

0

0

0
4

0

0

0
4

0

0

0
4

0.0245801

0.0245801

0
4

0

0

0
4

0

0

0
4

0

0

0
4

0

0

0
4

0

0

0
4

0

0

0

0

0

0
4

0

0

0
4

0

0

0
4

0

0

0
4

0.0681724

0.0681724

0
4

0

0

0
4

0.0817205
7

0.0817205
7

0
4

0

0

0
4

0

0

0
4

0.0511374

0.0347507

0.0163868

0
4

0

0

0
4

1.24733556816636e-13
7

0
4

0
7

0
6

0

0
6

0

0

0

0
4

0

0

0

0
4

0
4

0
7

0

0

0
4

0
8

0
8

0

0
4

0

0

0

0
4

0

0

0
4

0

0

0
4

0

0

0
4

0

0

0
4

0

0

0
4

0

0

0
4

0

0

0
4

0
4

4.1279

3.73314

2.0373

1.69583

4.44089209850063e-16

0
4

0.037564

0

0.0245801

0

0

0.0129839

0
4

0

0

0
4

0

0

0
4

0

0

0
4

0

0

0
4

0
5

0
5

0

0
4

0.160465
7

0.160465

0

0
4

0

0

0
4

0.122999

0.122999

0

0
4

0

0

0

0
4

0.0737404

0.0737404

0

0
4

0

0

0
4

0

0

0
4

1.52655665885959e-16

0
4

2.37285
7

2.37285
7

0.194758

0

0

2.17809
6

0

0
4

0
4

0

0

0

0
4

0
4

0.0157321

0.0157321

0.0157321

0
4

0
4

0.0106592

0.0106592

0.0106592

0
4

0
4

0

0

0

0
4

0
4

0

0

0

0

0

0
4

0
4

0

0

0

0
4

0
4

0

0

0

0

0
4

0
4

0

0

0

0
4

0
4

0

0

0

0
4

0
4

0.0245801

0.0245801

0.0245801

0
4

0
4

0.0347507

0.0347507

0.0347507

0
4

0
4

0

0

0

0
4

0
4

3.36258798583344e-13
7

0
4

1.17639

0.975474

0.146276
7

0

0

0

0.0877659

0

0

0

0

0

0

0

0
6

0

0

0

0

0.0585106

0

0

0

0

0

0

6.93889390390723e-18
7

0
4

0

0

0
4

0.0283844

0.0283844
7

0

0

0

0
4

0.0797957

0.0748085

0.00498723

0
4

0.0163868

0.0163868

0
4

0

0

0
4

0.70463

0.70463

0
4

0

0

0
4

0
4

0

0

0

0

0

0

0

0

0
4

0
4

0

0

0

0
4

0
4

0

0

0

0
4

0
4

0

0

0

0
4

0
4

0

0

0

0

0
4

0
4

0.0398978

0.0398978

0.0398978

0
4

0

0

0
4

0
4

0

0

0

0
4

0
4

0

0

0

0
4

0

0

0
4

0
4

0

0

0

0
4

0
4

0

0

0

0
4

0
4

0.14463

0.14463

0.14463

0
4

0
4

0.0163868

0.0163868

0.0163868

0
4

0
4

4.5102810375397e-17

0
4

6.13745

6.06008

6.06008

2.7811
6

0

0

0

0

0

0

0

0

0.0163868

0.0173753

3.1672
6

0.0327735

0

0

0

0

0

0.0173753

0

0

0.0278634

0

0

0

0

0
4

0

0

0
4

0

0

0
4

0

0

0
4

0

0

0
4

0
4

0.0773684

0.0773684

0.0663158

0.0110526

0
4

0
4

0

0

0

0
4

0
4

0

0

0

0
4

0
4

0

0

0

0
4

0
4

0

0

0

0
4

0
4

0

0

0

0
4

0
4

0

0

0

0
4

0
4

0

0

0

0
4

0
4

0

0

0

0
4

0
4

1.38777878078145e-17

0
4

0.110125

0

0

0

0

0
4

0
4

0.110125

0

0

0
4

0.110125
1

0

0

0

0.110125

0
4

0

0

0

0

0
4

0

0

0
4

0
4

0

0

0

0

0
4

0

0
1

0

0

0
4

0
4

0
4

8.72939
5

5.96525
5

2.93697

2.56285

0.026063
7

0.00498723

0

0.0157321

0

0

0

0.0249362

0

0.232924

0.0224425

0

0.026063

0.0209761

0

8.32667268468867e-17

0
4

0.382487
6

0.314645
6

0.0163868
7

0.0514549
6

0

0

0

0

0

0
4

0

0

0
4

0

0

0
4

0

0

0
4

0

0

0
4

0

0

0
4

0

0

0
4

2.5236
6

2.50722
6

0.0163868

0
4

0

0

0

0

0

0

0
4

0.122187

0.0922638

0.0249362

0.00498723

2.60208521396521e-18

0
4

0

0

0

0

0
4

0

0

0

0
4

0

0

0
4

0

0

0
4

0

0

0
4

1.77635683940025e-15
5

0
4

0.608442

0.608442

0.59348

0.0149617

0
4

0
4

2.14572

2.14572

0.434249

1.71147

0
4

0
4

0

0

0

0
4

0
4

0

0

0

0
4

0
4

0

0

0

0
4

0
4

0.00997446

0.00997446

0.00997446

0
4

0
4

0

0

0

0
4

0
4

0
4

0

0

0

0

0

0

0

0

0

0

0

0
4

0

0

0

0

0

0
4

0

0

0

0

0

0
4

0

0

0
4

0

0

0
4

0
4

0
4

0
3

0
3

0
3

0
3

0

0
4

0
4

0
4

0.491603
6

0.491603
6

0.491603
6

0.475216
6

0.0163868

0
4

0

0

0
4

0
4

0
4

0

0

0

0

0

0

0

0
4

0

0

0

0
4

0
4

0
4

0.0245801

0.0245801

0.0245801

0.0245801

0

0

0
4

0
4

0
4

0
4

0
4

0
4

0
4

0

0

0
4

0

0

0
4

0
4

0
4

0.139003

0.139003

0.139003

0.139003

0

0

0
4

0
4

0
4

0

0

0

0

0

0
4

0
4

0
4

0.026063

0.026063

0.026063

0

0

0

0.026063

0
4

0
4

0
4

0.147481

0.147481

0.147481

0.147481

0

0
4

0
4

0
4

0

0

0

0

0
4

0
4

0
4

2.78305
6

2.78305
6

2.78305
6

0.361574

0.827008

1.55964

0.00498723

0.00498723

0.0173753

0.00748085

6.93889390390723e-18
6

0
4

0
4

0
4

0.00498723
6

0.00498723
6

0

0

0
4

0.00498723

0.00498723

0
4

0
4

0
4

0

0

0

0

0

0
4

0
4

0
4

0

0

0

0

0
4

0
4

0
4

0

0

0

0

0

0
4

0

0

0
4

0
4

0
4

0

0

0

0

0

0
4

0
4

0
4

0

0

0

0

0

0

0
4

0
4

0
4

0

0

0

0

0

0
4

0
4

0
4

0.224891

0.224891

0.224891

0.224891

0
4

0
4

0
4

0

0

0

0

0
4

0

0

0
4

0
4

0
4

0

0

0

0

0

0

0
4

0
4

0
4

0
4

0
4

0
4

0

0

0

0

0

0

0

0
4

0
4

0

0

0

0
4

0
4

0
4

0

0

0

0

0

0
4

0
4

0
4

0

0

0

0

0

0
4

0

0

0
4

0
4

0
4

0.582074

0.582074

0

0

0
4

0.582074

0.582074

0
4

0
4

0
4

0

0

0

0

0
4

0
4

0
4

0

0

0

0

0

0
4

0
4

0
4

0.204834

0.204834

0.204834

0.204834

0
4

0
4

0
4

0

0

0

0

0

0
4

0

0

0
4

0
4

0
4

0

0

0

0

0
4

0
4

0
4

0

0

0

0

0
4

0
4

0
4

0.267445

0.267445

0.267445

0.267445

0
4

0
4

0
4

0
4

0
4

0
4

0

0

0

0

0

0

0

0

0
4

0

0

0

0

0

0
4

0

0

0
4

0
4

0
4

0

0

0

0

0
4

0
4

0
4

0

0

0

0

0
4

0
4

0
4

0

0

0

0

0
4

0
4

0
4

0

0

0

0

0

0
4

0
4

0
4

0

0

0

0

0
4

0
4

0
4

0

0

0

0

0
4

0

0

0
4

0
4

0
4

0

0

0

0

0
4

0
4

0
4

0

0

0

0

0
4

0
4

0
4

0

0

0

0

0
4

0
4

0
4

0

0

0

0

0
4

0
4

0
4

0
4

0
4

0
4

0
4

0

0

0

0

0
4

0

0

0
4

0

0

0

0
4

0

0

0
4

0
4

0
4

0

0

0

0

0
4

0
4

0
4

0

0

0

0

0
4

0
4

0
4

0

0

0

0

0
4

0
4

0
4

0.0943925

0.0943925

0.0943925

0.0943925

0
4

0
4

0
4

0

0

0

0

0
4

0
4

0
4

0.00498723

0.00498723

0.00498723

0.00498723

0
4

0
4

0
4

0

0

0

0

0
4

0
4

0
4

0.0327735

0.0327735

0.0327735

0.0327735

0
4

0
4

0
4

0.00736842

0.00736842

0.00736842

0.00736842

0
4

0
4

0
4

0

0

0

0

0
4

0
4

0
4

0

0

0

0

0

0

0

0

0
4

0

0

0

0

0

0
4

0

0

0
4

0

0

0

0
4

0

0

0
4

0
4

0
4

0

0

0

0

0
4

0
4

0
4

0

0

0

0

0
4

0
4

0
4

0

0

0

0

0
4

0
4

0
4

0

0

0

0

0
4

0
4

0
4

0

0

0

0

0
4

0
4

0
4

0

0

0

0

0
4

0
4

0
4

0

0

0

0

0
4

0
4

0
4

0

0

0

0

0
4

0
4

0
4

0

0

0

0

0
4

0
4

0
4

0

0

0

0

0
4

0
4

0
4

0.248661
7

0.248661
7

0.248661
7

0.0901271
7

0

0.131094

0

0.0110526

0

0.0163868

0
4

0
4

0
4

0

0

0

0

0
4

0
4

0
4

0

0

0

0

0
4

0
4

0
4

0

0

0

0

0
4

0
4

0
4

0.0327735

0.0327735

0.0327735

0.0327735

0
4

0
4

0
4

0

0

0

0

0
4

0
4

0
4

0

0

0

0

0
4

0
4

0
4

0

0

0

0

0
4

0
4

0
4

0

0

0

0

0
4

0
4

0
4

0

0

0

0

0
4

0
4

0
4

0

0

0

0

0
4

0
4

0
4

0.29686
7

0.29686
7

0.29686
7

0.223119
7

0.0737404

0

0
4

0
4

0
4

0

0

0

0

0
4

0
4

0
4

0

0

0

0

0
4

0
4

0
4

0

0

0

0

0
4

0
4

0
4

0.141589

0.141589

0.141589

0.141589

0
4

0
4

0
4

0.40379

0.40379

0.40379

0.40379

0
4

0
4

0
4

0

0

0

0

0
4

0
4

0
4

0.321808

0.321808

0.321808

0.321808

0
4

0
4

0
4

0

0

0

0

0
4

0
4

0
4

0

0

0

0

0
4

0
4

0
4

0

0

0

0

0
4

0
4

0
4

0

0

0

0

0

0

0

0

0
4

0
4

0
4

0

0

0

0

0
4

0
4

0
4

0

0

0

0

0
4

0
4

0
4

0

0

0

0

0
4

0
4

0
4

0

0

0

0

0
4

0
4

0
4

0.0245801

0.0245801

0.0245801

0.0245801
8

0

0
4

0

0

0
4

0
4

0

0

0

0
4

0
4

0

0

0

0
4

0
4

0
4

0
4

620.446

130.448
7

130.435
7

28.1237
7

28.1237
7

0

0
4

62.0292
7

20.1932
7

11.7538
7

0.639551
7

0

0.0766075

0

0

0.0179689

0.026063

0.0939211

0

0.0912097

0.00710613

0.281787
7

0

0.0248714

0

0

0

0

0.0209761

0

0

0.0106592

0.0860288
7

0

0

0

0.0142123

0.0104881

0.0104881

0

0

0

0.0209761

0
7

0

0

0

0.0142123

0.0319776

0

0

0

0

0.00736842

0
7

0

0

0.0157321

0

0

0

0

0.0163868

0

0

0.194677
6

0

0

0.0104881

0.00710613

0

0

0

0.0224425

0

0

0.222288
6

0

0

0.0104881

0.0104881

0.0573536

0

0.0262201

0

0.0104881

0

0
7

0.00710613

0

0

0

0

0

0

0

0

0

0.265819
7

0

0

0.0173753

0.0157321

0

0

0

0

0

0

1.65217
7

0

0

0

0

0

0

0.0106592

0

0.00736842

0

0.203091
7

1.30184
6

0

0

0

0

0

0

0

0

0

0.00710613

0.0696585
7

0.0434384

0.00710613

0

0

0

0

0

0.00710613

0

0

0
7

0

0

0

0.0419522

0

0.0104881

0

0.0213184

0.0173753

0

0.0347507
7

0

0

0.00710613

0

0

0

0.0491603

0

0

0

0.611014
7

0

0.0209761

0.0104881

0

0

0.0173753

0

0

0.026063

0.0104881

0.0234929
7

0.00710613

0

0

0.0106592

0

0

0

0

0

0

0.0582489
7

0

0

0

0.00710613

0

0

0

0

0

0.0157321

0.0378459
6

0

0.00710613

0

0

0

0

0.330374

0.00710613

0.0157321

0

0.00736842
7

0

0.0488395
7

16.2692
7

0.174382
6

0.0983205
6

0.0157321
7

0.0173753
7

0

0.0149617
7

0
8

0.0349695
7

0.0318064
7

0.0209761
7

1.5091
7

0

0

0.0262201
7

0

0.0157321
7

0

0.0213184

0

0

0
8

0
6

0.00710613

0

0

0

0.0106592

0.0742721

0.053296

0.0157321

0

0

0

0

0.412631

0.0209761

0

0.0507521

0.0213184

0.0996365

0

0.0142123

0.0104881

0.491874
6

0

0.807087

0.0284245

0

0.0173753

0

0

0

0.0262201

0

1.73614
6

0

0.0104881

0.026063

0

0

0

0

0

0.0524543

0.0955644

0.457809
7

0

0.0390837

0

0.0331691

0

0

0

0

0

0.0576843

4.10990685928425e-14
7

0
4

20.7099
7

0

4.37371
7

0.0347507
7

0
7

14.347
7

1.92962
7

0

0

0

0

0.0248714

0

0

0
4

3.57844
7

3.57844
7

0

0

0
4

0.122901
7

0
7

0

0.122901

0

0

0

0

0

0

0

0

0

0

0

0
4

0

0

0

0

0
4

15.8337
7

1.32062
7

14.5131
7

0

0

0

0

0

0

0

0

0

0

0

0

0

0
4

0.0245801
7

0.0245801
7

0

0

0
4

0
8

0
8

0

0

0
4

0

0

0
4

0.0129839

0.0129839

0
4

0

0

0
4

6.40112962635442e-14
7

0
4

0

0

0

0
4

0
4

0.0129839

0.0129839

0.0129839

0

0
4

0
4

0

0

0

0
4

0
4

0

0

0

0
4

0
4

0

0

0

0
4

0
4

0

0

0

0
4

0
4

0

0

0

0
4

0
4

0

0

0

0
4

0
4

2.11497486191092e-14
7

0
4

380.209

8.26587

0.690683

0.578568

0

0

0.0157321

0

0.0649194

0.0209761

0

0

0.0104881

0
4

0

0

0
4

0

0

0
4

0.0194758

0.0194758

0
4

0

0

0
4

0

0

0
4

0

0

0
4

0

0

0
4

0

0

0
4

0.0129839

0.0129839

0
4

0.0519355

0.0519355

0
4

1.55157

0.558307

0.292137

0.584274

0.0454436

0.0324597

0.0129839

0.0259677

1.83880688453542e-16

0
4

0

0

0
4

0

0

0
4

0.0314642

0.0314642

0
4

1.60904

1.35549

0.0195035

0.0195035

0.0195035

0.16578

0.0292553

0
4

3.52438

1.74633

0.0129839

1.76507

0
4

0.744371

0.744371

0

0
4

0

0

0

0

0

0
4

0

0

0
4

0.0299639

0.0104881

0.0194758

0
4

0

0

0
4

4.54497550705923e-16

0
4

0

0

0

0
4

0
4

2.92664

0.136345
3

0

0.136345

0

0

0

0

0
4

0.0189229
3

0

0

0

0

0

0

0

0

0

0

0

0

0

0

0

0

0

0

0

0.0189229

0

0

0

0

0

0

0

0

0

0

0
4

0.0327735

0.0327735

0
4

0.37757
3

0.37757
3

0

0

0

0

0

0

0

0

0

0

0
4

0.816792

0.576311
5

0

0

0

0.0160762

0.0118814

0

0

0

0

0

0

0

0

0

0.0129839

0

0.0843952

0.0389516
5

0

0.0177653

0

0.0584274

0

0
4

0

0

0

0

0

0

0
4

0

0

0
4

0

0

0

0

0

0
4

0

0

0

0

0

0

0

0
4

0

0

0

0

0

0
4

0

0

0

0

0

0
4

0

0

0

0

0

0
4

0

0

0

0
4

0

0

0

0

0
4

0

0

0

0

0
4

0.686103
5

0.686103
5

0

0

0

0

0

0

0

0

0

0

0
4

0

0

0

0

0
4

0

0

0
4

0

0

0

0

0
4

0

0

0

0

0
4

0

0

0

0

0
4

0

0

0

0
4

0

0

0

0

0
4

0

0

0

0
4

0

0

0

0

0
4

0

0

0
4

0.241989
5

0.229005
5

0

0

0.0129839

0

0
4

0

0

0

0
4

0

0

0
4

0

0

0
4

0

0

0

0
4

0

0

0
4

0

0

0
4

0

0

0
4

0

0

0
4

0

0

0
4

0

0

0
4

0
3

0

0

0

0

0

0

0

0
4

0

0

0

0
4

0

0

0

0
4

0

0

0

0
4

0

0

0

0
4

0

0

0

0
4

0

0

0
4

0

0

0

0
4

0

0

0
4

0

0

0

0
4

0

0

0
4

0.0166112
5

0.00950508

0

0

0

0.00710613

0

0

8.67361737988404e-19
5

0
4

0.0200953

0

0.0200953

0
4

0

0

0

0
4

0

0

0
4

0

0

0
4

0

0

0
4

0

0

0
4

0

0

0
4

0

0

0
4

0

0

0
4

0

0

0
4

0

0

0

0

0

0

0

0

0
4

0

0

0
4

0

0

0
4

0

0

0
4

0

0

0
4

0.0129839

0.0129839

0
4

0

0

0
4

0

0

0
4

0

0

0
4

0

0

0
4

0

0

0
4

0.548718
5

0.54068
5

0.0080381

2.94902990916057e-17
5

0
4

0

0

0
4

0

0

0
4

0.0129839

0.0129839

0
4

0

0

0
4

0

0

0
4

0

0

0
4

0

0

0
4

0

0

0
4

0

0

0
4

0

0

0
4

0

0

0

0

0

0

0

0
4

0

0

0
4

0

0

0
4

0

0

0
4

0

0

0
4

0

0

0
4

0

0

0
4

0

0

0
4

0.00475254

0.00475254

0
4

0

0

0
4

0

0

0
4

0

0

0

0

0

0

0
4

0

0

0
4

0
4

9.83182
3

4.59076

0.209447

0.0173753
3

0

0

0

0

0

0

0

0

0

0.0608137

0

0.095739

0

0

0.0901271

0.304423
5

0

0

0

0

0

0

0.0331074

0

0

0.0173753

0

0

0

0

0

0

0

0.0347507

0

0

0

0
3

0

0

0

0.0221053

0

0

0.0209761

0

0

0.0110526

0.657134

0

0

0

0

0

0

0.0173753

0

0

0

0.106514
3

0

0

0

0.026063

0

0.0173753

0

0

0.321444

0.0173753

0
6

0

0

0

0

0.0434384

0

0

0

0.0347507

0

2.23186

0.0173753

0

0

0

0

0.0419522

0

0.0104881

0

0.0608137

0.0695014

0

0

0

0

0

0

0

0

0

0

0
4

1.72721

0.205417
6

0

0

0

0

0

0

0

0

0

0

0.0539508

0

0

0

0

0

0

0

0

0

0

0.995765

0

0

0

0

0

0

0

0

0

0

0.305121
7

0

0

0

0

0

0

0

0

0

0

0

0

0

0

0

0

0

0

0

0

0

0.147481
6

0

0

0

0

0

0

0

0

0

0

0
7

0

0

0

0

0

0

0

0

0

0

0.0194758

0

0

0

0

0

0

0

0

0

0

0
3

0

0
4

0.108514

0

0.108514

0

0

0

0

0

0

0

0

0

0

0

0

0

0

0

0

0

0

0

0

0

0

0

0
4

1.99651
3

0.540135
4

0
4

0

0

0

0

0

0

0

0

0

0

0

0
4

0

0

0

0

0

0

0

0

0

0

0
4

0

0

0

0

0

0

0

0

0

0

0

0

0

0

0

0

0

0

0

0

0

0
4

0

0

0

0

0

0

0

0

0

0

0

0

0

0

0

0

0

0

0

0

0

0

0

0

0

0

0

0

0

0

0

0

0

0

0

0

0

0

0

0

0

0

0

0

0

0

0

0

0

0

0

0

0

0

0

0

0

0

0

0

0

0

0

0

0

0

0

0

0

0

0

0

0

0

0

0

0

0

0

0

0

0

0

0

0

0

0

0

0

0

0

0

0

0

0

0

0

0

0

0

0

0

0

0

0

0

0

0

0

0

0

0

0

0

0

0

0

0

0

0

0

0

0

0

0

0

0

0

0

0

0

0

0

0

0

0

0

0

0

0

0

0

0

0

0

0

0

0

0

0

0

0

0

0

0

0

0

0

0

0

0

0

0

0

0

0
4

0

0

0

0

0

0

0

0

0

0

0

0

0

0

0

0

0

0

0

0

0

0

0

0

0

0

0

0

0

0

0

0

0

0
4

0

0

0

0

0

0

0

0

0

0

0

0

0

0

0

0

0

0

0

0

0

0

0

0

0

0

0

0

0

0

0

0

0

0

0

0

0

0

0

0

0

0

0

0

0

0

0

0

0

0

0

0

0

0

0

0

0

0

0

0

0

0

0

0

0

0

0

0

0

0

0

0

0

0

0

0

0

0

0

0

0

0

0

0

0

0

0

0

0
4

0

0

0

0

0

0

0

0

0

0

0
4

0

0

0

0

0

0

0

0

0

0

0

0

0

0

0

0

0

0

0

0

0

0

0

0

0

0

0

0

0

0

0

0

0

0

0

0

0

0

0

0

0

0

0

0

0

0

0

0

0

0

0

0

0

0

0

0

0

0

0

0

0

0

0

0

0

0

0

0

0

0

0

0

0

0

0

0

0

0

0

0

0

0

0

0

0

0

0

0

0

0

0

0

0

0

0

0

0

0

0

0

0

0

0

0

0

0

0

0

0

0

0
4

0

0

0

0

0

0

0

0

0

0

0
4

0

0

0

0

0

0

0

0

0

0

0

0

0

0

0

0

0

0

0

0

0

0

0

0

0

0

0

0

0

0

0

0

0

0

0

0

0

0

0

0

0

0

0

0

0

0

0

0

0

0

0

0

0

0

0

0

0

0

0

0

0

0

0

0

0

0

0

0

0

0

0

0

0

0

0

0

0

0

0

0

0

0

0

0

0

0

0

0

0

0

0

0

0

0

0

0

0

0

0

0

0

0

0

0

0

0

0

0

0

0

0.0717251
7

0

0

0

0

0

0

0

0

0.0209761

0

0

0

0

0

0

0

0

0

0

0

0

0

0

0

0

0

0

0

0

0

0

0

0

0

0

0

0

0

0

0

0

0

0

0

0

0

0

0

0

0

0

0

0

0

0

0

0

0

0

0

0

0

0

0

0

0

0

0

0

0

0

0

0

0

0

0
3

0

0

0

0

0

0

0

0

0

0

0

0

0

0

0

0

0

0

0

0

0

0

0

0

0

0

0

0

0

0

0

0

0

0

0

0

0

0

0

0

0

0

0

0

0

0.0524403

0

0

0

0

0

0

0

0.0104881

0.0681724

0
4

0

0

0

0

0

0

0

0

0

0

0
4

0

0

0

0

0

0

0

0

0

0

0
3

0

0

0

0

0

0

0

0

0

0

0

0

0

0

0

0

0

0

0

0

0

0

0

0

0

0

0

0

0

0

0

0

0
4

0

0

0

0

0

0

0

0

0

0

0
4

0

0

0

0

0

0

0

0

0

0

0
4

0
4

0

0

0

0

0

0

0

0

0

0

0
4

0

0

0

0

0

0

0

0

0

0

0

0

0

0

0

0

0

0

0

0

0

0

0

0

0

0

0

0

0

0

0

0

0

0

0

0

0

0

0

0

0

0

0

0
4

0

0

0

0

0

0

0

0

0

0

0
4

0

0

0

0

0

0

0

0

0

0

0
4

0

0

0

0

0

0

0

0

0

0

0
4

0

0

0

0

0

0

0

0.0157321

0

0

0
3

0

0

0

0

0

0

0

0

0

0

0
4

0

0

0

0

0

0

0

0

0

0

0

0
4

0

0

0

0

0

0

0

0

0

0

0
4

0

0

0

0

0

0

0

0

0

0

0

0

0

0

0

0

0

0

0

0

0

0
4

0

0

0

0

0

0

0

0

0

0

0

0

0

0

0

0

0

0

0

0

0

1.02782
3

0

0

0

0

0

0

0

0

0

0

0
4

0

0

0

0

0

0

0

0

0

0

0

0

0

0

0

0

0

0

0

0

0

0

0

0

0

0

0

0

0

0

0

0

0

0
4

0

0

0

0

0

0

0

0

0

0

0
4

0

0

0

0

0

0

0

0

0

0

0

0

0

0

0

0

0

0

0

0

0

0
4

0

0

0

0

0

0.0104881

0

0

0

0

0

0

0

0

0

0

0

0

0

0

0

0

0

0

0

0

0

0

0

0

0

0

0

0

0

0

0

0

0

0

0

0

0

0
4

0

0

0

0

0

0

0

0

0

0

0.110363
7

0

0

0

0

0

0

0

0

0

0

0

0

0

0

0

0

0

0

0

0

0

0.0104881
4

0
4

0

0

0

0

0

0

0

0

0

0

0
4

0

0

0

0

0

0

0

0

0

0

0
4

0

0

0

0

0

0

0

0

0

0

0
4

0

0

0

0

0

0

0

0

0

0

0
4

0

0

0

0

0

0

0

0

0

0

0
4

0

0

0

0

0

0

0

0

0

0

0

0

0

0

0

0

0

0

0

0

0

0
4

0

0

0

0

0

0

0

0

0

0

0
4

0

0

0

0

0

0

0

0

0

0

0

0

0

0

0

0

0

0

0

0

0

0

0
4

0

0

0

0

0

0

0

0

0

0

0
4

0

0

0

0

0

0

0

0

0

0

0

0

0

0

0

0

0

0

0

0

0

0

0

0

0

0

0

0

0

0

0

0

0

0

0

0

0

0

0

0

0

0

0

0

0

0

0

0

0

0

0

0

0

0

0

0

0

0

0

0

0

0

0

0

0

0
4

0

0

0

0

0

0

0

0

0

0

0

0

0

0

0

0

0

0

0

0

0

0

0

0

0

0

0

0

0

0

0

0

0

0
4

0

0

0

0

0

0

0

0

0

0

0

0

0

0

0

0

0

0

0

0

0

0

0

0

0

0

0

0

0

0

0

0

0
4

0

0

0

0

0

0

0

0

0

0

0.0576843
4

0

0

0

0

0

0

0

0

0

0

0
4

0

0

0

0

0

0

0

0

0

0

0

0

0

0

0

0

0

0

0

0

0

0

0

0

0

0

0

0

0

0

0

0

0
3

0

0

0

0

0

0

0

0

0

0

0
4

0

0

0

0

0

0

0

0

0

0

0

0

0

0

0

0

0

0

0

0

0

0

0
4

0

0

0

0

0

0

0

0

0

0

0

0

0

0

0

0

0

0

0

0

0

0
4

0

0

0

0

0

0

0

0

0

0

0
4

0

0

0

0

0

0

0

0

0

0

0
4

0

0

0

0

0

0

0

0

0

0

0
4

0

0

0

0

0

0

0

0

0

0

0

0

0

0

0

0

0

0

0

0

0

0

0

0

0

0

0

0

0

0

0

0

0

0

0

0

0

0

0

0

0

0

0

0
4

0.397819

0.370858

0

0

0

0

0

0.0080381

0

0

0

0

0

0.0189229

3.12250225675825e-17

0
4

0.227075
4

0
4

0
4

0
4

0

0.227075

0

0
4

0

0

0
4

0
4

0

0

0

0

0

0
4

0

0

0

0

0
4

0

0

0

0

0

0
4

0

0

0

0
4

0

0

0

0

0

0

0

0
4

0

0

0

0

0

0
4

0

0

0
4

0

0

0

0
4

0

0

0

0

0
4

0
3

0
3

0

0

0

0

0

0

0

0

0

0

0

0

0

0

0

0

0
4

0

0

0
4

0

0

0

0

0
4

0

0

0
4

0.179768

0.179768

0
4

0

0

0

0
4

0

0

0

0

0
4

0

0

0

0
4

0

0

0

0

0
4

0

0

0

0
4

0

0

0
4

0.388088
3

0
4

0

0

0

0

0.132629

0

0.25546

0

0

0

0

0

0
4

0

0

0

0
4

0

0

0
4

0

0

0
4

0

0

0

0
4

0

0

0

0
4

0

0

0

0
4

0

0

0
4

0

0

0
4

0

0

0
4

0

0

0

0
4

0
3

0

0

0

0

0

0

0

0

0

0

0

0

0

0

0

0
4

0

0

0
4

0

0

0
4

0

0

0
4

0

0

0

0
4

0.0283844

0.0283844

0

0
4

0

0

0

0
4

0

0

0

0
4

0

0

0

0
4

0

0

0

0
4

0

0

0
4

0.0189229
3

0

0

0

0

0

0.0189229

0
4

0

0

0
4

0

0

0

0
4

0

0

0
4

0

0

0
4

0.0195035

0.0195035

0
4

0

0

0
4

0

0

0
4

0

0

0
4

0

0

0
4

0

0

0
4

0
4

0
4

0

0

0

0

0

0

0

0

0
4

0

0

0
4

0

0

0
4

0

0

0
4

0

0

0
4

0

0

0
4

0

0

0
4

0

0

0
4

0

0

0
4

0

0

0
4

0

0

0
4

0.13288

0.104495

0.0283844

0

0

0
4

0

0

0
4

0

0

0
4

0

0

0
4

0

0

0
4

0

0

0
4

0

0

0
4

0

0

0
4

0

0

0
4

0

0

0
4

0

0

0
4

0.0163868

0

0

0

0

0

0.0163868

0

0
4

0

0

0
4

0

0

0
4

0

0

0
4

0

0

0
4

0

0

0
4

0

0

0
4

0

0

0
4

0

0

0
4

0

0

0
4

0

0

0
4

0

0

0

0

0

0

0

0

0
4

0

0

0
4

0

0

0
4

0

0

0
4

0

0

0
4

0

0

0
4

0

0

0
4

0

0

0
4

0

0

0
4

0

0

0
4

7.11236625150491e-16
3

0
4

0
4

0
4

0
4

0

0

0

0

0

0

0

0

0

0

0

0

0

0

0

0

0

0

0

0

0

0

0

0

0

0

0

0

0

0

0

0

0

0

0

0

0

0

0

0

0

0

0

0

0
4

0
4

0
4

0

0
4

0

0

0
4

0

0

0
4

0

0

0
4

0
4

9.16241

4.60789

0.592529
7

0.120572
7

0

0.00475254

0.00475254

0

0

0

0.00712881

0.0194758

0

0

0.0327735
8

0

0

0

0

0

0

0

0

0

0

0
7

0.00475254

0

0

0

0

0

0

0

0

0

0

0

0

0

0

0

0

0

0

0

0

0.214548
7

0

0.065547

0

0

0

0

0

0.16879
7

0

0.0221279
8

0
3

0.0293706
7

0.543852

0.0378459

0

0.117021
7

0
8

0.0120572

0

0.142823
7

0.0259677

0.0779032

0

0.321444
7

0.061783

0

0

0

0
7

0

0

0.00950508

0.065547

0

0.89397

0.0129839

0.0129839

0

0

0.052126

0

0

0

0

0

0.0643048
8

0

0

0

0

0.0080381

0

0

0

0

0

0
3

0

0

0

0

0.0695014

0

0.0327735

0

0

0

0
8

0

0

0

0

0

0

0

0

0

0

0.499264
7

0

0

0

0

0

0

0

0

0

0

0.146132
7

0

0.11294

0

0

0

0

0

0

0

0

0
4

0

0

0

0
4

0
2

0

0

0

0

0

0

0

0

0

0

0
4

0.281551
4

0

0
4

0

0

0

0

0

0

0

0.0194758

0

0

0.0259677

0

0

0

0

0

0
4

0

0

0

0

0

0

0.188266

0

0

0

0
4

0

0

0

0.015382

0

0

0

0

0

0.0324597

0
3

0

0

0

0

0

0

0

0

0

0

0
4

0

0

0

0

0

0

0

0

0

0

0

0

0

0

0

0

0

0

0

0

0

0

0

0

0

0

0

0

0

0

0

0

0
8

0

0

0

0

0

0

0

0

0

0

0

0

0

0

0

0

0

0

0

0

0

0
4

0.0378459
5

0

0

0

0

0

0

0

0

0

0

0

0

0

0

0

0

0

0.0378459

0
4

0.0943696
6

0

0

0

0

0

0

0

0

0

0

0

0

0

0

0

0

0

0

0

0

0

0

0.00498723

0

0

0

0

0

0

0

0

0

0

0

0

0

0

0

0

0

0.00498723

0

0

0

0

0

0.0843952

0

0

0

0

0

0

0

0

0

0
4

0.385089
5

0.155674
5

0

0

0

0

0

0

0

0

0

0

0

0

0

0

0

0

0

0

0

0

0

0

0

0

0

0

0

0

0

0

0

0

0.213028
5

0

0

0

0

0

0

0

0

0

0

0.0163868

0

0

0

0

1.73472347597681e-17
5

0
4

0

0

0

0

0

0

0

0

0

0

0

0

0

0

0

0

0

0

0

0

0
4

0

0

0

0

0

0

0

0

0
4

0

0

0
4

0

0

0
4

0

0

0
4

0

0

0
4

0

0

0
4

0

0

0
4

0

0

0
4

0

0

0
4

0

0

0
4

0

0

0
4

0.115928
7

0.0732908
7

0

0

0.0426368

0

0
4

0

0

0
4

0

0

0
4

0

0

0
4

0

0

0
4

0.00498723

0.00498723

0
4

0

0

0
4

0

0

0
4

0

0

0
4

0

0

0
4

0

0

0
4

0
4

0

0

0

0
4

0

0

0
4

0

0

0
4

0

0

0
4

0

0

0
4

0

0

0
4

0

0

0
4

0

0

0
4

0

0

0
4

0

0

0
4

0

0

0
4

0

0

0

0

0

0

0

0

0
4

0.00475254

0.00475254

0
4

0

0

0
4

0

0

0
4

0

0

0
4

0

0

0
4

0

0

0
4

0

0

0
4

0.171188
7

0.171188

0

0

0

0
4

0
4

0

0

0

0

0

0
4

0
4

0

0

0

0

0
4

0

0

0

0
4

0.0170806
7

0.0170806
7

0
4

0.767675
7

0.767675
7

0
4

0
4

0
4

0
4

0
4

0

0

0

0
4

0

0

0

0
4

0
8

0

0

0
4

0.00615279
6

0

0

0.00615279

0
4

0

0

0

0

0

0
4

0

0

0
4

0

0

0

0

0
4

0

0

0
4

0.0389516

0.0129839

0

0.0259677

0

0
4

0

0

0

0

0
4

0

0

0

0
4

1.13788
7

1.13788
7

0

0

0
4

0

0

0
4

0

0

0

0

0
4

0

0

0

0
4

0

0

0
4

0

0

0

0
4

0

0

0

0
4

0

0

0
4

0

0

0
4

0

0

0
4

0

0

0

0

0
4

0.00736842
7

0

0

0

0.00736842

0

0

0

0
4

0

0

0

0

0
4

0

0

0
4

0.0263786

0.00736842

0.0190102

0
4

0

0

0

0
4

0

0

0

0
4

0

0

0

0

0
4

0

0

0

0
4

0.114707

0.114707

0
4

0

0

0

0
4

0

0

0

0
4

0

0

0

0

0

0

0

0

0

0

0

0

0
4

0.156743

0

0.156743

0
4

0

0

0

0
4

0.0636941

0.0636941

0
4

0.0195035

0

0.0195035

0
4

0

0

0
4

0

0

0
4

0

0

0

0
4

0

0

0

0
4

0.0129839

0.0129839

0

0
4

0

0

0
4

0.50378
7

0.50378
7

0

0

0

0
4

0

0

0

0
4

0

0

0
4

0

0

0

0
4

0

0

0
4

0.026063

0.026063

0
4

0

0

0
4

0

0

0

0
4

0

0

0

0
4

0

0

0
4

0

0

0
4

0
3

0

0

0

0
4

0

0

0
4

0

0

0
4

0

0

0
4

0

0

0
4

0

0

0
4

0

0

0
4

0.0129839

0.0129839

0
4

0.0194758

0.0194758

0
4

0

0

0
4

0

0

0
4

0

0

0

0

0
4

0

0

0
4

0

0

0
4

0

0

0
4

0

0

0
4

0

0

0
4

0

0

0
4

0

0

0
4

0

0

0
4

0

0

0
4

0

0

0
4

0.37991
7

0.37991
7

0

0
4

0

0

0
4

0

0

0
4

0

0

0
4

0

0

0
4

0

0

0
4

0

0

0
4

0

0

0
4

0

0

0
4

0.147481

0.147481

0
4

0

0

0
4

0
4

43.5867

0
7

0
7

0

0

0

0

0

0

0

0

0
4

22.0705

3.09443
7

0.569533
7

0

0
3

0.0331352
6

0

0

0

0

0.0106592

0

0

0.0819338
3

0

0

0

0.0359741

0

0

0

0

0

0

1.32375

0

0

0

0

0

0

0

0

0.0194758

0

0.475351
7

0

0

0

0

0

0

0

0

0

0

1.3455
7

0.0491603

0.00997446

0

0

0

0

0.00475254

0

0

0

0.253281
6

0.0409669

0.056327

0

0

0

0

0

0

0

0

0.175532

0

0

0

0

0

0

0.0292553

0

0

0

0.723029

0

0

0.0194758

0

0

0

0

0

0

0

0.155806

0

0

0

0

0

0

0

0

0

0

0.159395

0

0

0

0

0

0

0

0

0

0

0
3

0.0877659
7

0.0189229

0

0

0

0

0

0

0.0195035

0.0129839

0

0.310325
7

0

0

0

0

0

0

0

0

0

0

0
2

0

0

0

0

0

0

0

0

0

0

0
3

0

0

0

0

0

0

0

0

0

0.0585106

0
3

0

0

0

0

0

0

0

0.149315

0

0

0.065547
7

0

0

0

0

0

0

0

0

0

0.0173753

0.71208
7

0

0

0

0

0

0.0324597

0.0562667

0

0

0

0
3

0

0

0

0

0

0

0

0

0

0

0.208679
7

0

0.0173753

0

0

0

0

0

0

0

0

0.0194758
2

0

0

0

0

0

0

0

0

0

0

4.83015
7

0.443071

0

0

0

0

0

0

0

0

0

0.0200953

0
6

0

0

0

0

0

0

0

0

0

0

0
2

0

0

0

0

0

0

0.0294737

0

0

0

0
3

0

0

0

0

0

0

0

0

0

0

0.618296
7

0

0

0

0

0

0

0

0

0

0

0
2

0

0

0

0

0

0

0

0

0

0

0

0

0

0

0

0

0

0

0

0

0

0.368997
5

0

0

0

0

0.0129839

0

0.00736842

0

0

0

0.198922
5

0

0

0

0

0.0189229

0

0

0

0

0.0129839

0
3

0

0

0

0

0

0

0.0173753

0

0

0

0
4

0
6

0

0

0

0

0

0

0

0

0

0

0
3

0

0

0.0142123

0

0

0

0

0

0

0

0

0

0

0

0

0

0

0

0

0

0

0
3

0

0

0

0

0

0

0

0

0

0

0.49777
7

0

0.0324597

0

0

0

0

0

0

0

0

0
3

0

0

0

0

0

0

0

0

0

0

0
3

0

0

0

0

0

0

0

0

0

0

0
7

0

0

0

0

0

0.0129839

0

0

0

0.039007

0
3

0

0

0

0

0

0

0

0

0

0

0.204787
7

0

0

0

0

0

0

0

0

0

0

0.174126
7

0
2

0

0

0

0

0

0

0

0

0.0259677

0

0

0

0

0

0

0

0

0

0

0

0

0

0

0

0

0

0

0

0

0

0

0

0

0

0

0

0

0

0

0

0

0

0

0

0

0

0

0.00748085

0.00748085

0

0

0

0

0

0

0

0

0

0

0

0

0

0

0

0

0
3

0

0

0

0

0

0

0

0

0.0200127

0

0

0

0

0.0259677

0

0

0

0

0

0.0149617

0

0.0129839
5

0

0

0

0

0

0

0

0

0

0

0
3

0

0

0

0

0

0.0129839

0

0

0

0

1.16721
7

0
3

0

0

0

0

0

0

0

0

0

0

0
3

0

0

0.0194758

0

0

0

0

0

0

0

0
3

0

0

0

0

0

0

0

0

0

0

0
6

0

0

0

0

0

0

0

0

0

0

0
7

0

0

0

0

0

0

0

0

0

0

0.0256563
7

0

0

0

0.0129839

0

0

0

0

0

0

0.0173753
7

0.0080381

0

0

0

0

0

0

0

0

0

0.357691
7

0

0

0.0189229

0.00710613

0

0

0.0189229

0

0

0

0
2

0

0

0

0

0

0

0

0

0

0

0
3

0

0

0

0

0

0

0

0

0

0

0.804669

0
2

0

0

0

0

0

0

0

0

0

0

0
3

0

0

0

0

0

0.0283844

0

0

0

0

0
3

0

0

0

0

0

0

0

0

0

0

0.207742

0.0194758

0

0

0

0

0

0

0

0

0

0

0

0

0

0

0

0.00710613

0

0

0

0

0.0336881
6

0

0

0

0

0

0

0

0

0.0245801

0

0
3

0

0

0

0.0361715

0

0

0

0

0

0

0
2

0.00475254

0

0

0

0

0

0

0

0

0

0.107269
7

0.0129839

0

0

0

0

0

0

0

0

0

0

0

0

0

0

0

0

0

0

0

0

0
3

0

0

0

0

0

0

0

0

0

0

0

0.292553
7

0

0

0

0

0

0

0

0

0

0

0.0189229

0

0

0

0

0

0

0

0

0

0

0

0

0

0

0

0.136525

0

0

0

0

0
6

0

0.0129839
7

0

0.0455665
7

0

0.43965

0

0
3

0.039007

0.00997446

0

0

0
6

0
7

0

0

0
4

0

0

0

0

0

0

0

0

0

0

0

0

0

0

0

0

0
4

17.3567

11.2937

1.97387
7

0
4

0.00615279

0.285645

0

0

0

0

0

0

0

0
4

2.06444

0

0

0

0

0

0

0

0

0.0129839

0.123347
7

0

0

0

0

0

0

0

0

0

0

0.0812759
7

0

0

0

0

0

0

0

0

0

0

0.227075
4

0

0

0

0

0

0

0

0

0

0

0.131103
7

0

0

0

0

0

0

0

0

0

0

0
4

0

0

0

0

0

0

0

0.0129839

0

0

0

0

0

1.14413
7

0
4

2.04005
7

1.29916
7

0

0.0129839

0.0189229

0

0

0.00922919

0

0

0

0

0.06318
6

0

0

0

0

0.0129839

0.0454436

0.171528
7

0

0.0955644
6

0.0643559
7

0
7

0.162298

0.0843952

1.2490009027033e-16
7

0
4

0
4

0

0

0

0

0
4

0

0

0
4

0

0

0
4

0

0

0
4

0.0189229

0.0189229

0
4

0

0

0
4

0

0

0
4

0

0

0
4

0

0

0
4

0

0

0
4

0

0

0
4

0
4

0
4

0

0

0
4

0

0

0
4

0

0

0
4

0

0

0
4

0

0

0
4

0

0

0
4

0

0

0
4

0

0

0
4

0

0

0
4

0

0

0
4

0.0173753

0.0173753

0
4

0.0300283
7

0.0300283
7

0

0
4

0

0

0
4

0

0

0
4

0

0

0
4

0.0300191

0.0300191

0
4

0

0

0
4

0

0

0
4

0

0

0
4

0.14769

0.14769

0
4

0

0

0
4

0

0

0
4

0

0

0

0

0

0
4

0

0

0
4

0

0

0
4

0

0

0
4

0

0

0
4

0

0

0
4

0

0

0
4

0

0

0
4

0

0

0
4

0

0

0
4

0

0

0
4

0
3

0

0

0

0
4

0

0

0
4

0

0

0
4

0

0

0
4

0

0

0
4

0

0

0
4

0

0

0
4

0

0

0
4

0

0

0
4

0

0

0
4

0

0

0
4

0
2

0

0

0

0

0
4

0

0

0

0

0

0

0
4

0
8

0

0

0
4

0

0

0

0

0

0
4

0.00748085
7

0.00748085
7

0
4

0
4

0
4

0

0

0

0

0

0

0

0

0

0

0
4

0

0

0

0

0

0

0

0

0

0

0
4

0

0
4

0
4

0
4

0

0

0

0
4

0.0195035

0.0195035

0
4

0

0

0

0
4

0

0

0

0

0
4

0

0

0

0
4

0

0

0
4

0

0

0

0
4

0

0

0

0
4

0

0

0

0

0
4

1.24823

1.24823

0
4

0

0

0

0

0
4

0
4

0
4

0
4

0

0

0

0

0

0

0
4

0

0

0

0

0
4

0

0

0

0
4

0

0

0
4

0

0

0

0

0
4

0.0714113

0.0714113

0

0
4

0

0

0

0

0
4

0

0

0

0

0
4

0

0

0
4

0

0

0

0
4

0

0

0

0
4

0.521659
5

0.385089
7

0.11199
5

0

0

0

0

0

0.0245801

6.24500451351651e-17
5

0
4

0

0

0

0
4

0

0

0

0
4

0

0

0

0
4

0

0

0

0
4

0

0

0
4

0

0

0
4

0

0

0

0
4

0

0

0

0
4

0

0

0

0
4

0

0

0

0
4

0
3

0
3

0

0

0

0

0

0

0
4

0

0

0

0
4

0

0

0
4

0

0

0
4

0

0

0
4

0

0

0
4

0

0

0

0
4

0

0

0

0
4

0

0

0

0
4

0

0

0
4

0

0

0
4

0
3

0
3

0

0

0

0
4

0

0

0
4

0

0

0
4

0

0

0
4

0

0

0
4

0

0

0
4

0

0

0
4

0

0

0
4

0

0

0
4

0

0

0
4

0

0

0
4

0
4

0
4

0

0
4

0

0

0
4

0

0

0
4

0

0

0
4

0

0

0
4

0

0

0
4

0

0

0
4

0

0

0
4

0

0

0
4

0

0

0
4

0

0

0
4

0
3

0
4

0

0

0

0
4

0

0

0
4

0

0

0
4

0

0

0
4

0

0

0
4

0

0

0
4

0

0

0
4

0

0

0
4

0

0

0
4

0

0

0
4

0

0

0
4

0
4

0

0

0

0

0

0
4

0

0

0
4

0

0

0
4

0

0

0
4

0.00710613

0.00710613

0
4

0

0

0
4

0

0

0
4

0

0

0
4

0

0

0
4

0

0

0
4

0

0

0
4

0
4

1.00154
3

0.630975
3

0.612052
3

0

0

0.0189229

0

0

0

0

0

0

0

0

0

0

0

0

0

0

0

0

0

0

0
4

0
4

0
4

0

0

0
4

0

0

0
4

0

0

0
4

0

0

0

0
4

0

0

0

0
4

0

0

0

0
4

0

0

0
4

0

0

0
4

0

0

0
4

0

0

0
4

0

0

0
4

0.370567

0.370567

0

0
4

0

0

0
4

0

0

0
4

0

0

0
4

0

0

0
4

0

0

0
4

0

0

0
4

0

0

0
4

0

0

0
4

0

0

0
4

0

0

0
4

0

0

0
4

0

0

0
4

0

0

0
4

0

0

0
4

0

0

0
4

0

0

0
4

0

0

0
4

0

0

0
4

0

0

0
4

0

0

0
4

0

0

0
4

0

0

0

0

0
4

0

0

0
4

0

0

0
4

0

0

0
4

0

0

0
4

0

0

0

0

0
4

0

0

0
4

0

0

0
4

0

0

0
4

0

0

0

0
4

1.11022302462516e-16
3

0
4

0.428468
2

0.408992
2

0
2

0
2

0

0

0

0

0

0

0

0

0

0

0

0

0

0

0

0

0

0

0.181774

0

0

0

0

0

0

0

0

0

0

0

0

0

0

0

0

0.227218

0

0

0

0

0

0

0

0

0

0

0

0

0

0

0

0

0

0

0

0

0

0

0

0

0

0

0

0

0

0

0

0

0

0

0

0

0

0

0

0

0

0

0

0

0

0

0

0

0

0

0

0

0

0

0

0

0
2

0

0

0

0

0

0

0

0

0

0

0

0

0

0

0

0

0

0

0

0

0

2.77555756156289e-17
2

0
4

0

0

0
4

0.0194758

0.0194758

0
4

0
4

112.586

100.265
7

25.7041
7

0.229997
7

0.0259677

0

0.0584274

0

0.0324597

0

0

0.0173753

0

0.266784

0.331089

0

0.0661477

0

0.0358626

0

0.0129839

0

0.0194758

0

0

0.0749257

0.0519355

0.0500318

0

0.0293706

0.0497429

0

0.0163868

0.0604427

0.0776666

0

0.519545
6

0.0389516

0

0

0

0.097379

0

0.0194758

0.0163868

0

0

0.107481

0.0389516

0

0.0194758

0

0

0.0324597

0

0

0

0.00710613

0.292137
7

0.131094

0

0

0.0129839

0.0324597

0.0259677

0

0

0

0

0.302703
6

0.0129839

0

0

0

0

0.0177653

0

0.0389516

0.0129839

0.0100064

0.169418
7

0

0.0129839

0

0

0

0

0.0129839

0

0.0539508

0

0.142823
7

0

0

0

0.311613

0

0.0409669

0

0.0129839

0.0761927

0

0.0893235
7

0

0

0.0173753

0

0

0

0

0

0.0100064

0

51.0105
7

0.370222
6

0

0.0194758

0

0

0.055035

0

0

0

0

0.0259677

0.138042
7

0

0.0129839

0

0

0

0

0.0434384

0

0

0.0129839

0.0519355
7

0.0454436

0.0129839

0

0

0

0

0

0

0.0129839

0.0173753

0.17515
7

0

0

0.052126

0

0

0

0.0163868

0

0

0.0259677

0.0522493
7

0

0

0

0

0

0

0

0

0.0163868

0

0.14699
6

0

0

0.0129839

0.0349106

0

0.0129839

0

0

0

0

0.272197
7

0.0129839

0

0

0.0173753

0

0

0

0

0

0

0.163679
7

0

0

0

0

0.0519355

0

0.0194758

0

0

0

0.0259677
6

0

0

0

0

0

0

0

0

0.0129839

0

0.331089
7

0

0.0714113

0.0454436

0

0.0194758

0

0

0

0

0

3.19119
7

0.0293706
5

0

0.0163868

0

0

0.0129839

0

0

0

0

0

0.266665
7

0.0173753

0

0.0194758

0

0.0129839

0

0

0

0

0.0129839

0.0359741
7

0.0129839

0

0.0163868

0.0259677

0

0

0

0

0

0

0.031533
7

0

0

0

0.0194758

0.0454436

0

0

0.0163868

0

0.0129839

0.056327
7

0

0.0173753

0

0

0

0

0

0

0

0

0.159837
7

0.0189229

0

0

0.0160762

0

0

0

0

0

0.0129839

0

0

0

0.0129839

0

0.0163868

0

0

0

0

0

0.0163868
7

0

0

0

0

0

0

0

0

0.0163868

0

0.0265819
6

0

0

0

0

0

0

0

0

0

0

0.0129839

0

0

0

0.0714113

0.0189229

0

0

0

0

0

1.27315
6

1.32034
6

0

0

0

0.0189229

0

0

0

0

0

0

0

0

0

0

0

0

0.0129839

0

0

0

0

0.4025
7

0

0

0

0

0.0245801

0

0

0.0129839

0

0.0129839

0.0737976
7

0.0129839

0

0.0868767

0

0

0

0

0

0

0

0

0

0

0

0

0

0

0

0.00712881

0

0

0.0649194
6

0

0

0

0

0.0390837

0

0

0.0129839

0

0

0.0194758
6

0

0

0

0

0

0

0

0.0245801

0

0

0.0259677
7

0

0

0

0

0.0129839

0

0.0259677

0

0

0.20125

0.0983205
7

0.0173753

0

0

0.0194758

0.0129839

0

0

0

0

0

0.0142123
7

0

0

0

0

0

0

0

0

0.0129839

0

0.434811
7

0.199816

0

0

0

0

0

0

0

0.0409669

0.0163868

0

0.57129

0.0129839

0

0

0

0

0

0.0129839

0

0

0

0.0992423
7

0.0163868

0

0

0

0

0

0

0.0409669

0

0.0129839

0.0129839
6

0

0

0

0.0843952

0

0

0

0

0

0

0.0142123
7

0

0

0

0

0

0

0.0259677

0

0

0

0.0491603
7

0

0.0129839

0

0.0100064

0

0

0

0

0

0

0.0737404
6

0

0.0163868

0

0

0

0.0194758

0

0

0

0

0

0.0129839

0

0

0

0

0

0

0.0194758

0

0

0
7

0

0

0

0.0129839

0

0

0.0194758

0

0.0129839

0

0.0368512
7

0

0

0

0

0.0106592

0

0

0

0.0120572

0

1.57469
7

0.181014
7

0

0

0

0

0.0450286

0

0

0

0

0

0.0454436
7

0

0

0

0

0

0

0.00710613

0

0

0

0
7

0

0

0

0

0

0.0129839

0

0

0

0

0.0621441
6

0

0

0

0.0200127

0

0

0.0194758

0

0

0.0259677

0.0327735

0

0.0194758

0.0448907
6

0

0.0129839
8

0.213613
5

0.180254
7

0

0.0129839
6

0

0.0819338

0

0

0.0693108
7

0

0.0129839

2.78993
7

0

0.0549393
7

0.0434384

0.0522493

0.0163868

0.0150095
7

0.0194758

0

0

0.0194758

0.110179
7

0

0

0

0.817984

0.208504

0.0259677

0

0

0

0

3.23751442321552e-13
7

0
4

11.8253
7

11.8253
7

0

0
4

0.103871
4

0.0259677
4

0.0779032

0

0

0

0

0

0

0

0

0

0

0

0

0

0

0
4

0.0259677

0.0259677

0
4

0.0324597

0.0324597

0
4

0.0259677

0.0259677

0
4

0

0

0
4

0

0

0
4

0

0

0
4

0

0

0
4

0.0129839

0.0129839

0
4

0.0129839

0.0129839

0
4

0

0

0
4

0

0

0
4

0.206222

0.206222

0

0
4

0

0

0
4

0

0

0
4

0

0

0
4

0.0129839

0.0129839

0
4

0

0

0
4

0

0

0

0
4

0

0

0
4

0.0454436

0.0454436

0
4

0.0173753

0.0173753

0
4

0

0

0
4

1.24168036963468e-13

0
4

1.8924
4

1.71843
4

0.469134
4

0
5

0

0

0

0

0

0

0

0

0

0

0
3

0

0

0

0

0

0

0

0

0

0

0
4

0

0

0

0

0

0

0

0

0

0

0.0173753
4

0

0

0

0

0

0

0

0

0

0.167809

0

0

0

0

0

0

0

0

0

0

0

0

0

0

0

0

0

0

0

0

0

0

0
4

0

0

0

0

0

0

0

0

0

0

0
5

0

0

0

0

0

0

0

0

0

0

0

0

0

0

0

0

0

0

0

0

0

0

0

0

0

0

0

0

0

0.251713

0

0

0

0
4

0

0

0

0

0

0

0

0

0

0

0.0173753

0

0

0

0

0

0

0

0

0

0

0

0

0

0

0

0

0

0

0

0

0

0

0

0

0

0

0

0

0

0

0

0

0

0

0

0

0

0

0

0

0

0

0

0

0

0

0

0

0

0

0

0

0

0

0

0

0

0

0

0

0

0

0

0

0

0

0

0

0

0

0

0

0

0

0

0

0

0

0

0

0.0129839

0

0

0

0

0

0.0104881

0
4

0

0

0

0

0

0

0

0

0

0

0

0

0

0

0

0

0

0

0

0

0

0

0

0

0

0

0

0

0

0

0

0

0

0

0

0

0

0

0

0

0

0

0

0

0

0

0

0

0

0

0

0

0

0

0

0

0

0

0

0

0

0

0

0

0

0

0
3

0

0

0

0

0

0

0

0

0

0

0

0

0

0

0

0

0

0

0

0

0

0

0

0

0

0

0

0

0.0104881

0

0

0

0

0

0

0

0

0

0

0

0

0

0

0

0

0

0

0

0

0

0

0

0

0.0157321

0

0.0891485

0

0

0

0

0

0

0

0

0

0

0

0

0

0

0

0

0

0

0

0

0

0

0

0

0

0

0

0

0

0

0

0

0

0

0

0

0

0

0

0

0

0

0

0.0524403

0

0

0

0

0

0

0

0

0

0.0129839

0

0

0

0

0

0

0

0

0

0

0

0
4

0

0

0

0

0

0

0

0

0

0

0

0

0

0

0

0

0

0

0

0

0

0

0

0

0

0
4

0
4

0

0

0

0

0

0

0

0

0

0

0.590762
4

0

0

0

0

0

0

0

0

0

0

0
3

0

0

0

0

0

0

0

0

0

0

0

0

0

0

0

0

0

0

0

0

0

0
5

0

0

0

0

0

0

0

0

0

0

2.22044604925031e-16
4

0
4

0
4

0
4

0

0

0

0

0

0

0

0

0

0

0

0

0

0

0

0

0

0

0
4

0

0

0

0

0
4

0

0

0

0
4

0

0

0

0
4

0

0

0

0

0

0
4

0

0

0

0
4

0.0314642

0.0314642

0
4

0

0

0

0
4

0

0

0

0

0
4

0

0

0

0
4

0

0

0

0

0
4

0
4

0
4

0

0

0
4

0
4

0

0

0

0

0

0

0
4

0

0

0

0
4

0

0

0
4

0

0

0

0
4

0

0

0
4

0

0

0
4

0

0

0

0
4

0

0

0
4

0

0

0

0
4

0

0

0

0
4

0

0

0
4

0
3

0
3

0

0

0

0

0

0
4

0.0314642

0.0314642

0
4

0

0

0
4

0

0

0
4

0

0

0
4

0

0

0
4

0

0

0
4

0

0

0
4

0

0

0
4

0

0

0
4

0

0

0
4

0.104881
3

0.104881
3

0
4

0.00615279

0.00615279

0
4

0

0

0
4

0

0

0
4

0

0

0
4

0

0

0
4

0

0

0
4

0

0

0
4

0

0

0
4

0

0

0
4

0

0

0
4

0

0

0

0

0
4

0

0

0

0

0
4

0

0

0

0

0
4

0

0

0

0

0

0
4

0

0

0

0

0

0
4

1.62196645003831e-16
4

0
4

3.8334
4

1.04255
5

0.196013

0.470651
5

0.00710613
4

0

0

0

0

0

0

0

0

0

0

0.11294
5

0

0

0

0

0

0

0

0

0

0

0
5

0

0

0

0

0

0

0

0

0

0

0

0

0

0

0

0

0

0

0

0

0
5

0

0

0

0

0

0

0

0

0

0

0.163853
5

0

0

0

0

0

0

0

0

0

0

0.0604021
6

0

0

0

0

0

0

0

0

0

0

0.0173753
4

0

0

0

0

0

0

0

0

0

0

0
5

0

0

0

0

0

0

0

0

0

0

0

0

0

0

0

0

0

0

0

0

0

0
5

0

0

0

0

0

0

0

0

0.00710613

0

0

0

0

0

0

0

0.00710613

0

0

0

0

0
4

2.77446
4

0.124357
6

0
3

0
4

1.97551
5

0
5

0.0129839
5

0
4

0
4

0
4

0
4

0

0

0

0

0

0

0

0

0

0

0
4

0

0

0

0

0

0

0

0

0

0

0

0

0

0

0

0

0

0

0

0

0

0
4

0

0

0

0

0

0

0

0

0

0

0

0

0

0

0

0

0

0

0

0

0

0

0

0

0

0

0

0

0

0

0

0

0
4

0

0

0

0

0

0

0

0

0

0

0

0

0

0

0

0

0

0

0

0

0

0

0

0

0

0

0

0

0

0

0

0

0

0

0

0

0

0

0

0

0

0

0

0
4

0.448581
7

0

0

0

0

0

0

0

0

0

0

0

0

0.196641

0

0

0

0

0

0

0

0

0

0

0

0

0

0

0

0

0

0

0

0

0

0

0

0

0

0

0

0

0

0

0

0

0

0

0

0

0

0

0

0

0

0
4

0

0

0

0

0

0

0

0

0

0

0

0

0

0

0

0

0

0

0

0

0.0163868

0

0

0

0

0

0

0

0

0

0

0

0

0

0
4

0

0

0

0

0

0

0

0

0

0

0
3

0

0

0

0

0

0

0

0

0

0

0

0

0

0

0

0

0

0

0

0

0

0
4

0

0

0

0

0

0

0

0

0

0

0
4

0

0

0

0

0

0

0

0

0

0

0
4

0

0

0

0

0

0

0

0

0

0

0
3

0

0

0

0

0

0

0

0

0

0

1.83880688453542e-16
4

0
4

0.0163868

0
8

0

0

0

0

0

0

0.0163868

0

0

0

0
8

0

0

0

0

0

0

0

0

0

0

0

0

0

0

0

0

0

0

0

0

0

0

0

0

0

0

0

0

0
4

0

0

0

0

0

0

0

0

0
4

0

0

0
4

0

0

0
4

0

0

0
4

0

0

0
4

0

0

0
4

0

0

0
4

0

0

0
4

0

0

0
4

0

0

0
4

0

0

0
4

0
4

0
4

0

0

0

0
4

0

0

0
4

0

0

0
4

0

0

0
4

0

0

0
4

0
4

0
4

0

0

0
4

0
4

0

0

0

0

0
4

0

0

0

0

0
4

0

0

0
4

0

0

0
4

0

0

0
4

0

0

0
4

0
4

0.196545
3

0
4

0
4

0

0

0

0

0

0

0

0

0

0

0
4

0

0

0

0

0

0
4

0

0

0
4

0

0

0
4

0

0

0
4

0

0

0
4

0

0

0
4

0.130315
3

0
4

0

0

0

0

0

0

0

0
4

0.130315

0
4

0

0

0

0
4

0.0662303
3

0
3

0.0662303
4

0
4

0
4

0
4

0
4

0

0

0

0

0

0

0
4

0
3

0

0

0

0

0

0

0

0
4

0
4

0
4

0

0

0

0
4

0
4

0

0

0

0

0

0
4

0

0

0

0

0
4

0

0

0

0

0

0
4

0
4

0.40524

0.40524

0.0993841

0

0

0.225534
7

0

0.0104881

0.0129839

0.056849

0

0

0

0
4

0

0

0

0

0

0
4

0

0

0

0

0

0

0
4

0

0

0

0
4

0

0

0
4

0
4

0
4

0
4

0

0

0

0

0
4

0

0

0

0
4

0
4

0

0

0

0

0

0

0
4

0

0

0

0
4

0
4

0

0

0

0

0
4

0

0

0

0
4

0

0

0
4

0
4

0

0

0

0

0

0
4

0
4

0
3

0
3

0
3

0

0
4

0

0

0
4

0
4

0
4

0

0

0

0
4

0

0

0
4

0

0

0
4

0
4

0

0

0

0

0

0

0
4

0

0

0
4

0
4

0

0

0

0

0
4

0

0

0

0

0
4

0

0

0
4

0
4

0.0695014
6

0.0695014
6

0.0695014
6

0
4

0
4

0

0

0

0
4

0
4

0
3

0
3

0
3

0

0

0
3

0

0

0

0

0

0

0

0
4

0

0

0

0

0

0
4

0

0

0

0

0
4

0
4

0

0

0

0

0

0
4

0

0

0
4

0
4

0
4

0

0

0

0

0
4

0

0

0
4

0

0

0
4

0
4

0

0

0

0

0
4

0
4

0
3

0

0

0
4

0

0

0

0
4

0

0

0
4

0

0

0
4

0

0

0
4

0

0

0
4

0
4

0

0

0

0

0
4

0
4

0

0

0

0

0
4

0

0

0

0

0
4

0

0

0
4

0

0

0
4

0
4

0

0

0

0

0

0

0
4

0
4

0

0

0

0

0

0
4

0

0

0

0

0
4

0
4

0

0

0

0

0
4

0
4

0

0

0

0

0
4

0
4

1.2259
3

1.16747
3

0.0573536
3

0

0.902379
4

0

0.162298

0

0

0

0.0324597

0.0129839

0
4

0.0584274

0.0324597

0.0259677

0

3.46944695195361e-18

0
4

0

0

0

0
4

0

0

0

0
4

0

0

0

0
4

0
4

0

0

0

0

0
4

0

0

0

0
4

0

0

0
4

0
4

0

0

0

0

0

0

0

0

0
4

0
4

0
4

0
4

0

0

0

0
4

0

0

0
4

0

0

0
4

0
4

0

0

0

0

0

0
4

0

0

0

0
4

0
4

0

0

0

0

0
4

0

0

0

0
4

0

0

0
4

0
4

0

0

0

0

0

0

0
4

0

0

0
4

0
4

0

0

0

0

0

0
4

0

0

0
4

0

0

0
4

0

0

0
4

0
4

0.0189229
4

0
4

0
4

0
4

0

0

0
4

0.0189229

0.0189229

0
4

0
4

0
4

0
4

0

0

0

0
4

0
4

0

0

0

0

0
4

0

0

0

0
4

0
4

0.626565
3

0.626565
3

0.626565
3

0
3

0

0

0

0

0

0

0

0
4

0

0

0
4

0

0

0
4

0

0

0
4

0

0

0
4

0

0

0
4

0
4

0
4

0

0

0

0
4

0
4

0

0

0

0
4

0

0

0

0
4

0

0

0

0
4

0

0

0
4

0

0

0
4

0

0

0
4

0

0

0
4

0
4

0.0487588
6

0.0487588
6

0.0487588
6

0
4

0
4

0

0

0

0
4

0

0

0
4

0

0

0

0
4

0

0

0
4

0
4

0

0

0

0

0
4

0
4

0
3

0
3

0

0

0

0
4

0

0

0
4

0
4

0

0

0

0

0

0
4

0

0

0
4

0
4

0

0

0

0
4

0

0

0
4

0

0

0
4

0

0

0
4

0
4

0

0

0

0

0

0
4

0
4

0

0

0

0

0

0

0
4

0

0

0
4

0
4

0

0

0

0
4

0

0

0
4

0

0

0
4

0
4

0

0

0

0

0

0

0
4

0
4

0.199389
5

0.199389
5

0.120858
5

0

0

0

0

0

0

0

0

0

0

0.0785309
5

0

0
4

0

0

0

0

0

0

1.38777878078145e-17
5

0
4

0
4

0.0209761

0.0209761

0

0.0104881

0.0104881

0

0

0
4

0
4

0

0

0

0

0

0

0
4

0

0

0
4

0
4

0

0

0

0

0
4

0
4

0

0

0

0

0

0

0
4

0

0

0
4

0

0

0
4

0
4

0

0

0

0

0
4

0

0

0

0
4

0

0

0
4

0
4

0

0

0

0

0
4

0

0

0
4

0
4

0

0

0

0

0

0
4

0
4

0

0

0

0

0
4

0

0

0
4

0
4

0

0

0

0

0

0
4

0

0

0
4

0
4

0

0

0

0

0

0
4

0
4

1.53391
5

1.53391
5

1.1518
5

0.33722

0.0259677

0

0

0

0

0.0189229

0

1.38777878078145e-17
5

0
4

0
4

0

0

0

0

0
4

0

0

0
4

0

0

0
4

0

0

0
4

0
4

0

0

0

0

0

0
4

0

0

0

0
4

0
4

0

0

0

0

0

0
4

0

0

0

0
4

0
4

0

0

0

0

0
4

0

0

0
4

0

0

0
4

0
4

0

0

0

0

0
4

0

0

0
4

0

0

0
4

0

0

0
4

0
4

0.184528

0.184528

0.184528

0

0
4

0
4

0
4

0

0

0

0
4

0

0

0
4

0
4

0

0

0

0
4

0
4

0

0

0

0

0

0
4

0

0

0
4

0
4

0

0

0

0

0

0
4

0

0

0
4

0

0

0
4

0
4

0
4

0
4

0
4

0

0

0

0

0

0

0

0

0

0

0

0
4

0

0

0
4

0

0

0

0
4

0

0

0
4

0

0

0
4

0

0

0
4

0

0

0
4

0

0

0
4

0

0

0
4

0

0

0
4

0
4

0
4

0

0

0
4

0

0

0
4

0
4

0

0

0

0

0
4

0

0

0
4

0
4

0.0173753

0

0

0

0
4

0.0173753

0

0.0173753

0
4

0
4

0

0

0

0

0
4

0

0

0
4

0
4

0

0

0

0

0

0
4

0

0

0

0
4

0
4

0.0473073

0.0473073

0.0473073

0
4

0
4

0

0

0

0

0

0
4

0

0

0

0
4

0
4

0

0

0

0

0
4

0

0

0

0
4

0

0

0
4

0
4

0

0

0

0

0

0
4

0

0

0

0
4

0
4

0

0

0

0

0

0
4

0

0

0
4

0

0

0
4

0
4

0.330446

0.260224

0.0737361
6

0.129134

0.0573536

0

0

0

0

0
4

0.0702222

0.0292553
6

0.0245801

0.0163868

0

0

0

0

0

0
4

0

0

0

0
4

0

0

0

0
4

0
4

0

0

0

0

0

0

0
4

0

0

0
4

0
4

0

0

0

0

0

0
4

0

0

0
4

0

0

0
4

0
4

0

0

0

0

0

0
4

0

0

0
4

0
4

0

0

0

0
4

0
4

0

0

0

0

0
4

0

0

0
4

0

0

0
4

0
4

0

0

0

0

0
4

0

0

0

0
4

0

0

0
4

0
4

0.0487588

0.0487588

0.0487588

0
4

0
4

0

0

0

0

0

0
4

0
4

0

0

0

0

0
4

0
4

0

0

0

0

0

0

0
4

0
4

0.295134

0.288005
6

0.263777
6

0.0194758

0

0

0

0

0.00475254

0

1.12757025938492e-17
6

0
4

0.00712881
2

0

0

0.00712881

0

0

0

0
4

0
2

0

0

0

0

0
4

0

0

0

0

0
4

0

0

0

0
4

0

0

0

0
4

0

0

0
4

0
4

0

0

0

0

0

0

0
4

0
4

0

0

0

0

0
4

0

0

0

0
4

0
4

0

0

0

0
4

0
4

0

0

0

0

0
4

0

0

0
4

0
4

0.0129839

0

0

0

0
4

0.0129839

0.0129839

0
4

0
4

0

0

0

0

0
4

0
4

0

0

0

0

0

0
4

0
4

0

0

0

0

0
4

0

0

0
4

0
4

0

0

0

0
4

0

0

0

0
4

0
4

0

0

0

0
4

0

0

0
4

0
4

1.86074
5

1.86074
5

1.86074
5

0

0

0
4

0

0

0
4

0

0

0
4

0
4

0

0

0

0

0

0
4

0

0

0
4

0
4

0

0

0

0

0
4

0
4

0

0

0

0
4

0
4

0

0

0

0

0
4

0

0

0
4

0
4

0

0

0

0
4

0

0

0

0
4

0
4

0

0

0

0
4

0

0

0
4

0

0

0
4

0
4

0

0

0

0

0

0
4

0
4

0

0

0

0

0
4

0

0

0
4

0
4

0

0

0

0

0

0
4

0

0

0
4

0
4

0

0

0

0

0

0

0
4

0
4

0

0

0

0

0

0

0

0

0

0

0

0

0

0

0

0

0

0

0

0

0

0

0

0

0

0

0

0

0

0

0

0

0

0

0

0

0

0
4

0

0

0

0

0

0

0

0

0

0

0
4

0
4

0

0

0

0

0

0

0

0

0

0

0

0

0

0

0

0

0
4

0
4

0

0

0

0
4

0

0

0
4

0

0

0
4

0
4

0

0

0

0

0
4

0
4

0

0

0

0

0
4

0
4

0

0

0

0
4

0

0

0
4

0

0

0
4

0
4

0

0

0

0
4

0

0

0
4

0
4

0

0

0

0
4

0

0

0
4

0
4

0

0

0

0

0
4

0
4

0

0

0

0

0
4

0

0

0
4

0
4

0

0

0

0

0
4

0

0

0
4

0
4

0

0

0

0

0
4

0

0

0
4

0
4

0

0

0

0

0

0

0

0

0

0

0

0

0

0

0

0

0

0

0

0

0

0
4

0

0

0

0

0

0

0

0
4

0

0

0

0
4

0
4

0

0

0

0

0
4

0
4

0

0

0

0
4

0
4

0

0

0

0

0
4

0
4

0

0

0

0
4

0

0

0
4

0
4

0

0

0

0

0
4

0

0

0
4

0
4

0

0

0

0

0

0
4

0
4

0

0

0

0
4

0

0

0
4

0
4

0

0

0

0
4

0

0

0
4

0
4

0.155674

0.155674

0.155674

0

0
4

0

0

0
4

0
4

0

0

0

0

0
4

0
4

0
3

0
3

0
3

0

0

0

0

0

0

0

0

0
4

0

0

0

0
4

0

0

0
4

0
4

0

0

0

0
4

0

0

0
4

0
4

0

0

0

0

0
4

0
4

0

0

0

0
4

0

0

0
4

0

0

0
4

0
4

0

0

0

0

0
4

0

0

0
4

0
4

0

0

0

0

0
4

0

0

0
4

0
4

0

0

0

0

0
4

0

0

0
4

0
4

0

0

0

0

0
4

0
4

0

0

0

0
4

0
4

0

0

0

0

0
4

0
4

0

0

0

0

0
4

0

0

0
4

0
4

0.141922
4

0
4

0

0

0

0

0

0

0

0

0
4

0.141922
4

0.141922
4

0

0

0

0

0
4

0

0

0

0

0

0
4

0

0

0

0
4

0

0

0
4

0
4

0

0

0

0
4

0

0

0
4

0
4

0

0

0

0
4

0

0

0
4

0

0

0
4

0
4

0

0

0

0

0
4

0
4

0

0

0

0

0
4

0
4

0

0

0

0
4

0
4

0

0

0

0
4

0

0

0
4

0
4

0

0

0

0
4

0
4

0

0

0

0

0
4

0
4

0

0

0

0

0

0
4

0
4

0

0

0

0

0
4

0
4

1.15921

1.01762
5

0

0.731499

0.0576843

0.0819338

0.0491603

0.0573536

0.0184584

0.0215348

0
4

0.141589

0.141589

0

0

0
4

0

0

0

0
4

0

0

0
4

0
4

0

0

0

0

0
4

0
4

0

0

0

0
4

0

0

0
4

0
4

0

0

0

0
4

0
4

0

0

0

0
4

0
4

0

0

0

0
4

0
4

0

0

0

0

0
4

0
4

0

0

0

0
4

0

0

0
4

0
4

0

0

0

0

0
4

0
4

0

0

0

0

0
4

0
4

0

0

0

0
4

0

0

0
4

0
4

0
2

0
2

0

0

0

0

0
2

0

0

0

0

0

0

0

0
4

0

0

0

0
4

0
4

0

0

0

0
4

0
4

0

0

0

0

0
4

0
4

0

0

0

0
4

0
4

0

0

0

0

0
4

0
4

0

0

0

0

0
4

0
4

0

0

0

0

0
4

0
4

0.0173753

0.0173753

0.0173753

0

0
4

0
4

0

0

0

0
4

0

0

0
4

0
4

0.0819338

0.0819338

0.0163868

0.065547

0
4

0
4

0

0

0

0
4

0

0

0
4

0
4

0
3

0
3

0
3

0

0

0

0

0

0

0

0
4

0
3

0

0

0

0

0

0

0

0
4

0
4

0

0

0

0
4

0
4

0

0

0

0
4

0

0

0
4

0
4

0

0

0

0
4

0

0

0
4

0
4

0

0

0

0
4

0

0

0
4

0
4

0

0

0

0

0
4

0
4

0

0

0

0
4

0

0

0
4

0
4

0

0

0

0
4

0
4

0

0

0

0

0
4

0
4

0

0

0

0

0
4

0
4

0

0

0

0
4

0

0

0
4

0
4

0
4

0
4

0
4

0

0

0

0

0

0

0

0
4

0

0

0

0

0

0

0
4

0

0

0

0

0
4

0

0

0

0
4

0

0

0

0
4

0

0

0

0
4

0

0

0
4

0
4

0

0

0

0
4

0

0

0
4

0
4

0

0

0

0

0
4

0
4

0

0

0

0

0
4

0
4

0

0

0

0
4

0

0

0
4

0
4

0

0

0

0
4

0

0

0
4

0
4

0

0

0

0
4

0

0

0
4

0
4

0

0

0

0

0
4

0
4

0

0

0

0

0
4

0
4

0.0662303

0.0662303

0.0662303

0
4

0

0

0
4

0
4

0

0

0

0
4

0
4

0.0756917
3

0.0756917
3

0.0756917
3

0
2

0

0

0

0

0
4

0
3

0
3

0

0

0

0
4

0

0

0

0
4

0

0

0

0

0

0
4

0

0

0
4

0
4

0

0

0

0

0
4

0
4

0

0

0

0
4

0
4

0

0

0

0

0
4

0
4

0

0

0

0
4

0

0

0
4

0
4

0

0

0

0

0
4

0
4

0

0

0

0

0
4

0
4

0

0

0

0
4

0
4

0

0

0

0

0
4

0
4

0

0

0

0

0
4

0
4

0

0

0

0

0
4

0
4

0
4

0
4

0
4

0

0

0

0

0

0

0

0
4

0

0

0
4

0

0

0
4

0
4

0

0

0

0
4

0
4

0

0

0

0
4

0

0

0
4

0
4

0

0

0

0

0
4

0
4

0

0

0

0
4

0
4

0

0

0

0
4

0

0

0
4

0
4

0

0

0

0

0
4

0
4

0

0

0

0
4

0

0

0
4

0
4

0

0

0

0

0
4

0
4

0

0

0

0

0
4

0
4

0

0

0

0
4

0

0

0
4

0
4

0
2

0
2

0
2

0

0

0

0

0

0
3

0

0

0

0

0

0

0
4

0
2

0
2

0

0

0

0

0

0

0

0

0

0

0

0
2

0
3

0

0

0

0

0

0
4

0

0

0

0
4

0

0

0

0

0

0
4

0

0

0
4

0

0

0
4

0
4

0.46296

0.46296

0.46296

0

0

0

0
4

0

0

0
4

0

0

0
4

0
4

0

0

0

0
4

0

0

0
4

0
4

0

0

0

0
4

0
4

0

0

0

0
4

0

0

0
4

0
4

0

0

0

0
4

0
4

0

0

0

0
4

0

0

0
4

0
4

0

0

0

0
4

0
4

0

0

0

0
4

0

0

0
4

0
4

0

0

0

0
4

0
4

0

0

0

0
4

0
4

0

0

0

0
4

0
4

0.0434384
7

0
7

0
7

0
8

0

0

0

0

0

0

0

0
4

0.0434384

0

0

0

0.0434384

0
4

0

0

0

0
4

0
4

0

0

0

0
4

0
4

0.00710613

0.00710613

0

0.00710613

0
4

0
4

0

0

0

0
4

0
4

0

0

0

0
4

0
4

0

0

0

0
4

0
4

0

0

0

0
4

0
4

0

0

0

0
4

0
4

0

0

0

0
4

0
4

0

0

0

0
4

0
4

0

0

0

0
4

0
4

0
2

0
2

0
2

0
2

0

0

0

0
4

0

0

0

0
4

0

0

0
4

0

0

0
4

0
4

0

0

0

0
4

0
4

0

0

0

0
4

0
4

0

0

0

0
4

0
4

0

0

0

0
4

0
4

0

0

0

0
4

0
4

0

0

0

0
4

0
4

0

0

0

0
4

0
4

0

0

0

0
4

0
4

0

0

0

0
4

0
4

0

0

0

0
4

0
4

0
4

0
4

0
4

0

0

0

0

0

0

0

0
4

0
4

0

0

0

0
4

0
4

0

0

0

0
4

0
4

0

0

0

0
4

0
4

0

0

0

0
4

0
4

0

0

0

0
4

0
4

0

0

0

0
4

0
4

0

0

0

0
4

0
4

0

0

0

0
4

0
4

0.0163868

0.0163868

0.0163868

0
4

0
4

0

0

0

0
4

0
4

0

0

0

0

0

0

0

0

0

0
4

0

0

0
4

0
4

0

0

0

0
4

0
4

0

0

0

0
4

0
4

0

0

0

0
4

0
4

0

0

0

0
4

0
4

0

0

0

0
4

0
4

0

0

0

0
4

0
4

0

0

0

0
4

0
4

0.0104881

0.0104881

0.0104881

0
4

0
4

0

0

0

0
4

0
4

0

0

0

0
4

0
4

0
3

0
3

0
3

0
3

0

0

0
4

0

0

0

0

0
4

0
4

0

0

0

0
4

0
4

0

0

0

0
4

0
4

0

0

0

0
4

0
4

0

0

0

0
4

0
4

0

0

0

0
4

0
4

0

0

0

0
4

0
4

0

0

0

0
4

0
4

0

0

0

0
4

0
4

0

0

0

0
4

0
4

0

0

0

0
4

0
4

0
4

0
4

0

0

0

0

0

0

0

0

0

0
4

0

0

0

0

0

0

0
4

0

0

0
4

0
4

0

0

0

0
4

0
4

0

0

0

0
4

0
4

0

0

0

0
4

0
4

0.0129839

0.0129839

0.0129839

0
4

0
4

0

0

0

0
4

0
4

0

0

0

0
4

0
4

0

0

0

0
4

0
4

0

0

0

0
4

0
4

0

0

0

0
4

0
4

0

0

0

0
4

0
4

0
3

0
3

0
3

0

0

0

0

0
4

0

0

0
4

0

0

0
4

0
4

0

0

0

0
4

0
4

0

0

0

0
4

0
4

0

0

0

0
4

0
4

0

0

0

0
4

0
4

0.0283844

0.0283844

0.0283844

0
4

0
4

0

0

0

0
4

0
4

0

0

0

0
4

0
4

0.0104881

0.0104881

0.0104881

0
4

0
4

0

0

0

0
4

0
4

0

0

0

0
4

0
4

0

0

0

0

0

0

0

0
4

0

0

0
4

0
4

0

0

0

0
4

0
4

0

0

0

0
4

0
4

0

0

0

0
4

0
4

0

0

0

0
4

0
4

0

0

0

0
4

0
4

0

0

0

0
4

0
4

0

0

0

0
4

0
4

0

0

0

0
4

0
4

0

0

0

0
4

0
4

0

0

0

0
4

0
4

0

0

0

0

0

0

0

0

0
4

0

0

0

0

0
4

0

0

0

0
4

0

0

0

0
4

0

0

0
4

0
4

0

0

0

0
4

0
4

0

0

0

0
4

0
4

0

0

0

0
4

0
4

0

0

0

0
4

0
4

0

0

0

0
4

0
4

0

0

0

0
4

0
4

0

0

0

0
4

0
4

0

0

0

0
4

0
4

0

0

0

0
4

0
4

0

0

0

0
4

0
4

0.056327
3

0.0129839
3

0.0129839
5

0

0

0

0

0

0

0

0

0

0

0

0

0

0

0

0

0

0

0
4

0

0

0

0
4

0

0

0

0

0
4

0

0

0

0

0

0
4

0

0

0

0

0
4

0

0

0

0
4

0

0

0

0
4

0

0

0

0
4

0

0

0

0
4

0

0

0

0
4

0

0

0
4

0

0

0

0

0

0

0

0

0

0

0

0
4

0

0

0
4

0

0

0
4

0
3

0

0

0

0

0

0

0

0

0

0

0

0

0
4

0
3

0

0

0

0

0

0

0
4

0
4

0

0

0

0

0

0

0

0

0

0
4

0

0

0

0

0

0
4

0.0259677
5

0.0259677

0

0

0

0

0
4

0.0173753
4

0

0.0173753

0

0

0

0
4

0

0

0

0

0

0
4

0
4

0
3

0
3

0
3

0

0

0

0

0

0

0
4

0

0

0

0

0
4

0

0

0

0
4

0
4

0

0

0

0
4

0
4

0

0

0

0
4

0
4

0

0

0

0
4

0
4

0

0

0

0
4

0
4

0

0

0

0
4

0
4

0

0

0

0
4

0
4

0

0

0

0
4

0
4

0

0

0

0
4

0
4

0

0

0

0
4

0
4

0

0

0

0
4

0
4

0
4

0
4

0
4

0

0

0

0
4

0

0

0

0

0

0
4

0
4

0

0

0

0
4

0
4

0

0

0

0
4

0
4

0

0

0

0
4

0
4

0

0

0

0
4

0
4

0

0

0

0
4

0
4

0

0

0

0
4

0
4

0

0

0

0
4

0
4

0

0

0

0
4

0
4

0

0

0

0
4

0
4

0

0

0

0
4

0
4

0

0

0

0

0

0

0

0

0
4

0

0

0
4

0
4

0

0

0

0
4

0
4

0

0

0

0
4

0
4

0

0

0

0
4

0
4

0

0

0

0
4

0
4

0

0

0

0
4

0
4

0

0

0

0
4

0
4

0

0

0

0
4

0
4

0

0

0

0
4

0
4

0

0

0

0
4

0
4

0

0

0

0
4

0
4

0
4

0

0

0

0

0

0

0

0

0
4

0

0

0

0

0

0
4

0

0

0

0
4

0

0

0
4

0
4

0

0

0

0
4

0
4

0

0

0

0
4

0
4

0

0

0

0
4

0
4

0

0

0

0
4

0
4

0

0

0

0
4

0
4

0

0

0

0
4

0
4

0

0

0

0
4

0
4

0

0

0

0
4

0
4

0

0

0

0
4

0
4

0

0

0

0
4

0
4

0
3

0
3

0

0

0

0

0

0

0

0
4

0

0

0

0

0
4

0

0

0
4

0
4

0

0

0

0
4

0
4

0

0

0

0
4

0
4

0

0

0

0
4

0
4

0

0

0

0
4

0
4

0

0

0

0
4

0
4

0

0

0

0
4

0
4

0

0

0

0
4

0
4

0.0080381

0.0080381

0.0080381

0
4

0
4

0

0

0

0
4

0
4

0

0

0

0
4

0
4

7.50046
7

7.50046
7

7.44833
7

0.052126
7

0
4

0
4

0

0

0

0
4

0
4

0

0

0

0
4

0
4

0

0

0

0
4

0
4

0

0

0

0
4

0
4

0

0

0

0
4

0
4

0

0

0

0
4

0
4

0

0

0

0
4

0
4

0

0

0

0
4

0
4

0

0

0

0
4

0
4

0

0

0

0
4

0
4

0
4

0
4

0
4

0

0

0

0

0

0

0
4

0

0

0

0

0
4

0

0

0

0
4

0
4

0

0

0

0
4

0
4

0

0

0

0
4

0
4

0

0

0

0
4

0
4

0

0

0

0
4

0
4

0

0

0

0
4

0
4

0

0

0

0
4

0
4

0

0

0

0
4

0
4

0

0

0

0
4

0
4

0

0

0

0
4

0
4

0

0

0

0
4

0
4

0
4

0
4

0
4

0

0

0

0

0

0
4

0
4

0

0

0

0
4

0

0

0
4

0

0

0
4

0

0

0
4

0
4

0

0

0

0
4

0
4

0

0

0

0
4

0
4

0.0104881

0.0104881

0.0104881

0
4

0
4

0

0

0

0
4

0
4

0

0

0

0
4

0
4

0

0

0

0
4

0
4

0

0

0

0
4

0
4

0

0

0

0
4

0
4

0

0

0

0
4

0
4

0

0

0

0
4

0
4

0

0

0

0

0

0

0

0

0

0

0
4

0

0

0

0
4

0

0

0
4

0

0

0
4

0

0

0
4

0
4

0

0

0

0
4

0
4

0

0

0

0
4

0
4

0

0

0

0
4

0
4

0

0

0

0
4

0
4

0

0

0

0
4

0
4

0

0

0

0
4

0
4

0

0

0

0
4

0
4

0

0

0

0
4

0
4

0

0

0

0
4

0
4

0

0

0

0
4

0
4

0.0516964

0.0516964

0.0327735

0.0189229
7

0

0

3.46944695195361e-18

0
4

0

0

0

0
4

0

0

0
4

0
4

0

0

0

0
4

0
4

0

0

0

0
4

0
4

0

0

0

0
4

0
4

0.0157321

0.0157321

0.0157321

0
4

0
4

0

0

0

0
4

0
4

0

0

0

0
4

0
4

0

0

0

0
4

0
4

0

0

0

0
4

0
4

0

0

0

0
4

0
4

0

0

0

0
4

0
4

0
4

0
4

0

0

0

0

0

0

0

0

0

0

0

0

0

0

0

0

0

0

0

0

0

0

0

0

0

0

0

0

0

0

0

0

0

0

0

0

0

0

0

0

0

0

0

0

0

0

0

0

0

0

0

0

0

0

0

0
4

0

0

0
4

0

0

0

0

0

0

0
4

0

0

0

0

0
4

0

0

0

0
4

0

0

0

0

0
4

0

0

0
4

0

0

0

0
4

0

0

0
4

0

0

0
4

0
4

0
4

0
4

0

0

0

0

0

0

0
4

0

0

0

0

0
4

0

0

0

0
4

0

0

0

0
4

0
4

0

0

0

0
4

0
4

0

0

0

0
4

0
4

0

0

0

0
4

0
4

0

0

0

0
4

0
4

0

0

0

0
4

0
4

0

0

0

0
4

0
4

0

0

0

0
4

0
4

0

0

0

0
4

0
4

0

0

0

0
4

0
4

0

0

0

0
4

0
4

0
4

0
4

0

0

0

0

0

0
4

0
3

0

0

0

0

0
4

0

0

0
4

0

0

0
4

0
4

0

0

0

0
4

0
4

0

0

0

0
4

0
4

0

0

0

0

0

0

0
4

0

0

0

0
4

0

0

0
4

0
4

0
4

0
4

0
4

0

0

0
4

0

0

0

0

0

0

0
4

0

0

0

0

0

0

0
4

0

0

0
4

0
4

0
4

0
4

0

0

0

0

0

0

0

0

0
4

0

0

0

0

0
4

0

0

0
4

0
4

0.122901
3

0.0409669

0

0.0409669

0
4

0.0491603

0

0

0

0.0491603

0

0
4

0.0327735

0.0327735

0
4

0

0

0
4

0

0

0
4

0
4

0
2

0
2

0
2

0

0

0
4

0
4

0
3

0

0

0

0

0

0

0
4

0

0

0

0

0

0
4

0

0

0

0

0
4

0

0

0
4

0

0

0
4

0

0

0
4

0
4

0

0

0

0

0

0

0

0

0
4

0

0

0

0

0

0

0
4

0
4

0
3

0
4

0
4

0

0

0

0

0

0
4

0

0

0

0

0
4

0

0

0
4

0
4

0.470422
3

0.101771
3

0.0887866
4

0

0

0

0

0

0.0129839

0

0

0

0

0

0
4

0

0

0
4

0

0

0
4

0

0

0
4

0.153706
3

0.153706
5

0

0

0

0

0

0

0

0

0

0

0

0
4

0
4

0
4

0

0

0

0

0

0

0
4

0.0824853
3

0.0824853
7

0

0

0

0

0

0

0
4

0
5

0

0

0

0

0

0

0

0
4

0

0

0

0

0

0

0
4

0.132461

0.132461

0

0
4

0

0

0

0
4

0

0

0
4

0
4

0

0

0

0

0

0
4

0

0

0

0

0

0
4

0

0

0
4

0

0

0
4

0

0

0
4

0
4

0

0

0

0

0

0

0

0

0

0
4

0

0

0
4

0
4

0

0

0

0

0

0

0

0

0

0
4

0

0

0

0

0

0
4

0
4

0
4

0
4

0

0

0

0

0

0
4

0
4

0

0

0

0

0

0

0

0

0
4

0

0

0
4

0
4

0.125884
7

0.0347507
7

0.0347507
7

0

0

0

0
4

0

0

0
4

0

0

0
4

0

0

0
4

0.039007

0.039007

0
4

0.052126

0.052126

0
4

0

0

0
4

0
4

0
4

0
4

0

0

0

0

0

0

0

0
4

0

0

0

0
4

0
4

1.43364
3

1.41417
3

1.41417
3

0
4

0.0194758

0.0194758

0
4

0
4

0
4

0

0

0

0

0

0
4

0

0

0

0

0
4

0

0

0

0

0
4

0

0

0
4

0
4

0

0

0

0

0

0

0

0

0
4

0

0

0

0
4

0
4

1.10751
7

1.09046
7

0.470333
7

0

0

0

0

0

0

0

0.0194758
7

0.376532

0.0179711
7

0.0649194
7

0.0525549

0

0.0756917

0.0129839

1.38777878078145e-17
7

0
4

0.0170444
7

0.0120572

0

0

0.00498723

8.67361737988404e-19
7

0
4

0
4

0
4

0

0

0

0

0

0

0

0
4

0

0

0

0

0
4

0

0

0
4

0
4

0

0

0

0
4

0

0

0

0
4

0

0

0

0

0
4

0

0

0

0
4

0
4

0

0

0

0

0

0
4

0
4

0
4

0
4

0
4

0
4

0
4

0
4

0

0

0

0

0

0
4

0

0

0
4

0

0

0
4

0

0

0

0
4

0

0

0
4

0

0

0
4

0
4

0
2

0
2

0

0

0

0

0
4

0

0

0

0

0
4

0

0

0

0
4

0

0

0
4

0
4

0

0

0

0

0

0

0
4

0

0

0
4

0
4

0.129942
7

0.107499
7

0.107499
7

0

0
4

0.0224425

0.00748085

0.0149617

1.73472347597681e-18

0
4

0
4

0

0

0

0

0
4

0

0

0

0
4

0

0

0
4

0
4

0
4

0

0

0

0

0

0

0
4

0

0

0

0
4

0
4

0.0567688
3

0.0567688
3

0.0567688
2

0
3

0
2

0

0

0

0

0

0
4

0

0

0

0

0

0

0
4

0

0

0

0

0

0
4

0

0

0

0

0
4

0

0

0
4

0

0

0
4

0

0

0
4

0
4

0
4

0

0

0

0

0
4

0

0

0

0

0
4

0

0

0
4

0

0

0
4

0

0

0
4

0
4

0
4

0

0

0

0

0

0

0
4

0

0

0

0

0

0
4

0

0

0
4

0

0

0
4

0

0

0
4

0
4

0

0

0

0

0

0

0

0

0

0
4

0

0

0

0

0
4

0
4

0.00475254
3

0.00475254
3

0.00475254
3

0

0

0

0
4

0

0

0

0
4

0

0

0
4

0

0

0
4

0
4

0

0

0

0

0

0

0

0
4

0

0

0

0
4

0
4

0
1

0

0

0

0

0

0

0
4

0

0

0
4

0

0

0
4

0

0

0
4

0

0

0
4

0

0

0
4

0
4

0
3

0

0

0

0

0

0

0
4

0
4

0

0

0

0

0

0
4

0
4

0.327295

0

0

0

0
4

0.316807

0.316807

0

0
4

0.0104881

0.0104881

0

0

0
4

0
4

0

0

0

0

0

0

0

0

0
4

0
4

0

0

0

0

0

0

0

0
4

0

0

0
4

0

0

0

0
4

0

0

0
4

0

0

0
4

0

0

0
4

0
4

0
3

0
4

0
4

0

0

0

0

0

0

0

0

0

0

0

0

0

0

0
4

0
3

0
4

0

0

0

0

0

0
3

0

0

0

0

0

0

0

0
4

0
3

0

0

0
4

0

0

0
4

0
4

0
2

0
2

0
2

0

0

0

0
4

0
4

0.0189875
7

0

0

0

0

0

0
4

0.0189875

0

0.00475254

0.00710613

0.00712881

0
4

0

0

0

0
4

0
4

0
4

0
4

0

0

0

0

0
4

0

0

0

0

0
4

0

0

0
4

0
4

0
7

0
7

0

0

0

0
4

0
4

0
4

0
4

0

0

0

0

0
4

0

0

0
4

0
4

0
4

0

0

0

0

0

0
4

0

0

0

0

0
4

0

0

0
4

0

0

0
4

0
4

0.0641593
3

0.0641593
3

0.0641593

0

0

0
4

0

0

0
4

0

0

0
4

0
4

0
2

0
2

0

0

0

0

0
4

0

0

0
4

0
4

0
3

0
3

0
3

0

0
4

0

0

0

0

0
4

0
4

0
4

0
4

0
4

0

0

0
4

0
4

1.17016

1.08328

1.08328

0

0
4

0
6

0

0

0
4

0

0

0

0

0
4

0.0868767

0.0868767

0
4

0

0

0
4

0

0

0
4

1.11022302462516e-16

0
4

8.06699
3

7.08452
3

0.151383
3

2.32463

3.18785
3

0.84343

0.218226
7

0

0

0

0

0
7

0

0

0

0.0194758

0

0.0129839

0

0

0.136952
6

0

0.0248714

0

0.00922919

0

0

0

0

0

0

0
7

0

0

0

0

0

0

0

0

0

0

0
7

0

0

0

0

0

0

0

0

0

0

0
7

0.0737404

0

0

0

0

0.0284245

0

0

0

0

0
7

0

0

0

0

0

0

0

0

0

0

0

0

0

0

0

0

0

0

0

0

0

0
7

0.00712881

0

0

0

0

0

0

0

0

0

0.0461898
2

0

0

0

0

0

0

0

0

0

0

0
4

0.928994

0.83222
5

0

0.00615279
7

0.0236431
7

0.0215348

0

0

0.0324597

0

0.0129839

1.73472347597681e-16

0
4

0

0

0
4

0

0

0
4

0

0

0
4

0

0

0
4

0

0

0
4

0

0

0
4

0

0

0
4

0

0

0
4

0

0

0
4

0

0

0
4

0
4

0
4

0
4

0

0

0

0
4

0

0

0
4

0

0

0
4

0

0

0
4

0

0

0
4

0

0

0
4

0

0

0
4

0

0

0
4

0

0

0
4

0.0275139
7

0.0194758
7

0

0.0080381

1.73472347597681e-18
7

0
4

0.0259677
7

0

0.0129839

0

0

0.0129839

0

0
4

0

0

0
4

0

0

0

0

0
4

0

0

0
4

0

0

0
4

0

0

0

0
4

0
4

0

0

0
3

0

0

0
4

0

0
6

0

0

0

0

0

0

0

0

0

0

0

0

0

0

0

0

0

0

0

0

0

0

0

0

0

0

0

0

0

0

0

0

0

0

0

0

0

0

0

0

0

0

0

0

0

0

0

0

0

0
4

0

0

0

0

0

0

0

0

0

0

0

0

0

0

0

0

0

0

0

0

0

0

0

0

0

0

0

0

0

0

0
4

0
3

0
3

0
2

0

0

0

0

0

0

0
4

0

0

0

0
4

0

0

0

0
4

0

0

0

0
4

0

0

0
4

0

0

0

0
4

0

0

0

0
4

0

0

0

0
4

0

0

0
4

0

0

0

0
4

0

0

0
4

0

0

0

0
4

0

0

0
4

0

0

0
4

0

0

0
4

0

0

0
4

0

0

0
4

0

0

0
4

0

0

0
4

0

0

0
4

0

0

0
4

0

0

0
4

0

0

0

0

0
4

0

0

0
4

0

0

0
4

0

0

0
4

0

0

0
4

0

0

0
4

0

0

0
4

0
4

0

0

0

0

0
4

0
7

0
7

0
4

0

0

0

0

0
4

0

0

0

0

0

0
4

0

0

0

0

0
4

0

0

0
4

0
4

16.9265

16.9265

2.30129
6

0.331151
4

0

0

0

0

0.014727

0

0

0

0

0

0
3

0

0

0

0

0

0

0

0

0.00498723

0

0.0741044
7

0

0.104252

0

0

0

0

0

0

0

0

0.0363357
6

0

0

0

0

0

0

0

0

0.172061

0

0
2

0

0

0.262188

0

0

0

0

0

0

0

0
4

0

0

0

0

0

0

0

0

0

0

0
3

0

0.0163868

0

0

0

0

0

0

0

0

0.0851532
4

0

0

0

0

0.0189229

0

0

0

0

0

0

0

0

0

0

0

0

0

0

0

0

0
3

0

0

0

0

0

0

0

0

0

0

6.76404
6

0
3

0

0

0

0

0

0

0

0

0.0173753

0

0

0

1.68784

0

0

0

0

0

0

0

0

0
4

0

0

0

0

0

0

0

0

0

0

1.08972
6

0

0

0

0

0

0

0

0

0

0

0
4

0

0

0

0

0

0

0

0

0

0

0
5

0

0

0

0

0

0

0

0

0

0

0
7

0

0

0

0

0

0

0

0

0

0

0.0448851

0

0

0

0.0157321

0

0

0

0

0

0

0
4

0.0378459

0

0

0

0

0

0

0

0

0

0.0988345
7

0

0

0

0

0

0.194029

0

0

0

0

0.132461
4

0
3

0

0

0

0

0

0

0

0

0

0

0

0

0

0

0.0104881

0

0

0

0

0

0

0
4

0

0

0

0

0

0

0

0

0

0

0.0690718
6

0

0

0

0

0

0.0163868

0

0

0

0

0
4

0

0

0

0

0

0

0

0.00748085

0

0.0163868

0

0

0

0

0

0

0

0

0

0

0

0
4

0

0

0

0

0

0

0

0

0

0

0

0

0

0.00498723

0

0

0

0

0

0

0

0

0

0

0

0

0

0

0

0

0

0

0.934045

0

0

0

0

0

0

0

0

0

0

0
7

0
3

0

0

0

0

0

0

0

0

0

0

0
3

0

0

0

0

0

0

0.0327735

0.0104881

0

0

0.0129839

0

0

0.174553

0

0

0

0.0163868

0.00498723

0

0

0
3

0

0

0

0

0

0

0

0

0

0

0

0

0

0

0

0

0

0

0

0

0

0
4

0

0

0

0

0

0

0

0

0

0

0
7

0

0

0

0

0

0

0.00748085

0

0

0

0

0

0

0

0

0

0

0

0

0

0

0

0

0

0

0

0

0

0

0

0

0

0
4

0

0

0

0

0

0

0

0

0

0

0
4

0.0129839

0

0

0

0.0163868

0

0

0

0

0

0

0

0

0

0

0

0

0

0

0

0

0

0
2

0

0

0

0

0

0

0

0

0

0

0

0

0

0

0

0

0

0

0

0.0245801

0

0.393282

0

0

0

0

0.0259677

0

0

0

0

0

0

0

0

0

0

0

0

0

0

0

0

0

0.0163868

0

0

0

0

0

0

0

0

0

0
3

0

0

0

0

0

0

0

0

0

0

0

0

0

0

0

0.0163868

0

0

0

0

0

0.0324597
6

0

0

0

0

0

0

0

0

0

0

0.0608137

0

0

0

0

0

0

0

0

0

0

0

0

0

0

0

0

0

0

0

0

0

0

0

0

0

0

0

0

0

0

0

0

0

0
4

0

0

0

0

0

0

0

0

0

0

0
4

0

0

0

0

0

0

0

0

0

0

0

0

0

0

0

0.00997446

0

0

0

0

0

0
4

0

0

0

0

0

0

0

0

0

0

0

0

0

0

0

0.00748085

0.0106592

0

0

0

0

0

0

0

0

0

0.0173753

0

0

0

0

0

0

0

0

0.0174553

0

0

0

0

0

0

0

0.212858
6

0

0

0

0

0

0

0

0

0

0

0

0

0

0

0

0

0

0

0

0

0

0

0

0

0

0

0

0

0

0

0

0

0

0

0

0

0

0.0104881

0

0

0

0

0

0

0.00498723

0

0

0

0

0

0

0

0

0

0

0

0.625575
6

0

0.00498723

0

0

0

0

0

0

0

0

0.487858
6

0.0676841

0

0

0.00710613

0

0.0199489

0

0

0

0

0
4

0

0

0

0
4

0

0

0
4

0

0

0

0
4

0

0

0
4

0

0

0
4

0

0

0
4

0

0

0
4

0

0

0
4

0

0

0
4

0
4

2.99585

1.27497

0.199258
7

0

0

0

0

0

0

0

0.00498723

0

0.00712881

1.06359
7

0

0

0

0

0

0

0

0

0

0

0

0

0

0

0
4

1.44784
7

0.923117
7

0.00475254

0.0080381

0

0.073722
6

0.182925
6

0

0.206917

0

0.0199489

0.0284245

0

0
4

0.263298

0.263298

0
4

0.00973977
7

0.00973977

0

0
4

0

0

0

0
4

0

0

0

0

0
4

0

0

0
4

0

0

0
4

0

0

0
4

0

0

0
4

0

0

0
4

5.44703171456717e-16

0
4

2.53755

0.0485359
3

0.0404978
3

0

0

0

0

0

0

0

0

0

0

0

0

0

0

0

0

0

0

0

0

0

0

0

0

0

0

0

0

0

0

0

0

0

0

0.0080381

0

0

0

0

0

0
4

0.310541
7

0.294809
7

0

0

0

0

0.0157321

0

2.42861286636753e-17
7

0
4

0
3

0

0

0

0

0
4

0

0

0
4

0.0324597

0.0324597

0
4

0

0

0
4

0

0

0
4

0

0

0
4

0

0

0
4

0

0

0
4

0.092904
7

0.092904
6

0

0
4

0
3

0

0

0

0

0

0
4

0

0

0

0

0

0
4

0

0

0

0
4

0
7

0
7

0
4

0

0

0

0

0
4

0

0

0
4

0.116855
7

0.116855

0

0
4

0

0

0

0
4

0.639671
6

0.60476
6

0

0

0

0

0.0349106

0

0

2.77555756156289e-17
6

0
4

0

0

0

0
4

0

0

0

0
4

0

0

0

0

0

0
4

0

0

0
4

0

0

0

0

0

0
4

0

0

0

0
4

0

0

0

0
4

0.0389516

0.0389516

0

0
4

0.132629

0.132629

0
4

0

0

0

0

0
4

0.68336
7

0.613858
7

0

0

0

0
7

0.0695014

0

0

0

0

0

0

4.16333634234434e-17
7

0
4

0.389516

0.389516

0
4

0

0

0

0
4

0

0

0

0

0
4

0

0

0

0
4

0

0

0

0
4

0

0

0
4

0

0

0
4

0

0

0
4

0

0

0
4

0

0

0

0
4

0.052126
8

0.052126
8

0

0

0

0

0

0

0

0

0
4

0

0

0
4

0

0

0

0
4

0

0

0

0
4

0

0

0

0
4

0

0

0

0
4

0

0

0
4

0

0

0
4

0

0

0
4

0

0

0

0
4

0

0

0
4

0
8

0

0

0

0

0

0

0

0

0
4

0

0

0
4

0

0

0
4

0

0

0

0
4

0

0

0
4

0

0

0
4

0

0

0

0
4

0

0

0

0
4

0

0

0
4

0

0

0
4

0

0

0
4

0
3

0

0

0

0

0

0

0
4

0

0

0
4

0

0

0
4

0

0

0
4

0

0

0
4

0

0

0
4

0

0

0
4

0

0

0
4

0

0

0
4

0

0

0
4

0

0

0
4

0

0

0

0

0
4

0

0

0
4

0

0

0
4

0

0

0
4

0

0

0
4

0

0

0
4

0

0

0
4

0

0

0
4

0

0

0
4

0

0

0
4

0

0

0
4

0

0

0

0

0

0
4

0

0

0
4

0

0

0
4

0

0

0
4

0

0

0
4

0

0

0
4

0

0

0
4

0

0

0
4

0

0

0
4

0

0

0
4

0

0

0
4

0

0

0

0

0

0
4

0

0

0
4

0

0

0
4

0

0

0
4

0

0

0
4

0

0

0
4

0

0

0
4

0

0

0
4

0

0

0
4

0

0

0
4

0

0

0
4

4.16333634234434e-16

0
4

1.97502
3

1.97502
3

1.92586
3

0
2

0
2

0

0

0

0

0

0

0

0

0

0

0
3

0

0

0

0

0

0

0

0

0

0

0
3

0

0

0

0

0

0

0

0

0

0

0
2

0

0

0

0

0

0

0

0

0

0

0

0

0

0

0

0

0

0

0

0

0

0
2

0

0

0

0

0

0

0

0

0

0

0

0

0

0

0

0

0

0

0

0

0

0

0

0

0

0

0

0

0

0

0

0

0
2

0

0

0

0

0

0

0

0

0

0

0

0

0

0

0

0

0

0

0

0

0

0

0

0

0

0

0

0

0

0

0

0

0

0

0

0

0

0

0

0

0

0

0

0.0491603

0

0

0

0

0

0

0

0

0

0

0

0

0

0

0

0

0

0

0

0

0

0

0

0

0

0

0

0

0

0

0

0

0

0

0

0

0

0

0

0

0

0

0

0

0

0

0

0
2

0

0

0

0

0

0

0

0

0

0

0

0

0

0

0

0

0

0

0

0

0

0
3

0

0

0

0

0

0

0

0

0

0

0

0

0

0

0

0

0

0

0

0

0

0

0

0

0

0

0

0

0

0

0

0

0
3

0

0

0

0

0

0

0

0

0

0

0

0

0

0

0

0

0

0

0

0

0

1.38777878078145e-17
3

0
4

0

0

0

0

0

0
4

0

0

0

0

0
4

0

0

0
4

0

0

0

0

0
4

0

0

0

0

0
4

0

0

0

0
4

0

0

0
4

0

0

0

0
4

0

0

0

0
4

0

0

0

0
4

0

0

0
4

0
3

0

0

0

0
4

0

0

0

0
4

0

0

0
4

0

0

0

0
4

0

0

0
4

0

0

0

0
4

0

0

0
4

0

0

0
4

0

0

0
4

0

0

0
4

0

0

0
4

0
2

0

0

0

0
4

0

0

0
4

0

0

0
4

0

0

0
4

0

0

0
4

0

0

0
4

0

0

0
4

0

0

0
4

0

0

0
4

0

0

0
4

0

0

0
4

0

0

0
4

0

0

0
4

0

0

0
4

0

0

0
4

0

0

0
4

0

0

0
4

0

0

0
4

0

0

0
4

0

0

0
4

0

0

0
4

0

0

0
4

0

0

0

0
4

0

0

0
4

0

0

0
4

0

0

0
4

0

0

0
4

0

0

0
4

0

0

0
4

0

0

0
4

0

0

0
4

0

0

0
4

0

0

0
4

0

0

0
4

0

0

0
4

0

0

0
4

0

0

0

0

0
4

0

0

0

0

0
4

0

0

0

0

0
4

0
4

0.182034
3

0.1172
3

0
2

0

0

0

0.0124681

0

0.0224425

0

0

0.0174553

0

0
2

0

0.00748085

0.00498723

0

0

0.00748085

0

0.0124681

0

0

0
3

0

0

0

0

0

0

0.0124681

0.00498723

0.00997446

0

0

0.00498723

0

0

0

0

0

0

0

0

0

0

0

0
4

0

0

0

0

0

0

0
4

0

0

0

0
4

0

0

0

0

0
4

0

0

0

0
4

0.064834

0.064834

0
4

0

0

0
4

0

0

0
4

0

0

0
4

0

0

0
4

0
4

3.86249
5

3.84151
5

3.67342
5

0.0574008
7

0

0

0

0

0

0.0163868

0

0

0

0

0

0

0

0.0221053

0

0

0

0

0

0

0

0

0.064834

0

0

0

0.00736842

0

1.45716771982052e-16
5

0
4

0

0

0
4

0.0104881

0.0104881

0
4

0

0

0
4

0.0104881

0.0104881

0
4

0

0

0
4

0

0

0
4

0

0

0
4

1.73472347597681e-16
5

0
4

77.8329
4

71.9549
4

64.3673
4

4.62918
7

0.0370482

0

0

0

0

0

0

0

0

0

0

0

0.0163868

0.0423915

0.0163868

0.0149617

0

0

0

0

0

0

0

0

0

0

0.0491603

0

0.0179711

0

0

0.021374

0.20697
4

0

0

0

0

0

0.0295674

0.0149617

0

0.0157321

0

0.768122
6

0

0

0.0194758

0

0

0

0

0

0

0

0.0571932
7

0.0508297

0

0

0

0

0.026063

0

0

0

0

0.234357
7

0

0

0

0

0.00498723

0

0

0

0

0

0.473235
7

0

0

0

0

0

0

0

0

0

0

0.0559286
7

0

0.0163868

0

0

0

0

0.00498723

0

0

0.151383

0.0997446
5

0

0

0.0434384

0

0

0

0.00498723

0

0

0

0.464438
6

0

0

0

0

0

0

0

0

0

0

0
4

0.200922
6

0.0786604
6

0

0

0

0

0

0.0209761

0

0

0

0

0
6

0

0

0

0

0

0

0

0

0

0

0.0209761
6

0

0

0

0

0

0

0

0

0

0

0.0698212

0

0

0

0

0

0

0

0

0

0

0

0

0

0

0

0

0

0

0

0

0

0

0

0

0

0

0

0

0

0

0

0

0

0

0

0.0104881

0

0
4

0
3

0

0

0

0

0

0

0

0

0

0

0
4

0

0

0
4

0

0

0

0
4

0

0

0
4

0

0

0

0
4

0.00498723

0.00498723

0

0
4

0

0

0
4

0

0

0

0
4

0

0

0

0
4

0

0

0

0
4

0

0

0
4

0
4

0
4

0
4

0

0

0

0

0

0
4

0

0

0

0
4

0

0

0

0
4

0

0

0
4

0

0

0
4

0

0

0

0
4

0

0

0

0
4

0

0

0

0
4

0

0

0

0
4

0

0

0
4

0

0

0

0
4

0
2

0

0
3

0

0

0

0

0

0

0
4

0

0

0
4

0.0519355

0.0519355

0

0
4

0

0

0
4

0

0

0
4

0

0

0

0
4

0

0

0

0
4

0

0

0

0
4

0

0

0

0
4

0

0

0
4

0

0

0
4

0

0

0

0

0

0

0

0

0

0

0
4

0

0

0

0
4

0

0

0

0
4

0

0

0

0
4

0

0

0
4

0

0

0
4

0

0

0
4

0

0

0
4

0

0

0
4

0

0

0
4

0

0

0
4

0.0922638
5

0.0922638
5

0

0

0

0

0

0

0

0

0

0
4

0

0

0
4

0

0

0
4

0

0

0
4

0

0

0
4

0

0

0
4

0

0

0
4

0

0

0
4

0

0

0
4

0

0

0
4

0

0

0
4

0

0

0

0

0
4

0

0

0
4

0

0

0
4

0

0

0
4

0

0

0
4

0

0

0
4

0

0

0
4

0

0

0
4

0

0

0
4

0

0

0
4

0

0

0
4

0

0

0

0

0

0

0
4

0

0

0
4

0

0

0
4

0

0

0
4

0

0

0
4

0

0

0
4

0

0

0
4

0

0

0
4

0

0

0
4

0

0

0
4

0.00748085

0.00748085

0
4

0

0

0

0

0

0
4

0

0

0
4

0

0

0
4

0

0

0
4

0

0

0
4

0

0

0
4

0

0

0
4

0

0

0
4

0

0

0
4

0

0

0
4

0

0

0
4

0.129839
5

0.129839
5

0

0
4

0

0

0
4

0

0

0
4

0

0

0
4

0.0129839

0.0129839

0
4

0

0

0
4

0

0

0
4

0

0

0
4

0

0

0
4

0

0

0
4

0

0

0
4

0.0433431

0.0129839
5

0

0.0129839

0

0

0.0173753

0
4

0

0

0
4

0.0189229

0.0189229

0
4

0

0

0
4

0

0

0
4

0

0

0
4

0

0

0
4

0

0

0
4

0

0

0
4

0

0

0
4

0.0129839

0.0129839

0
4

0
4

0
4

0

0

0

0

0

0

0

0

0

0

0
4

0

0

0

0

0

0

0

0

0

0

0

0

0

0

0

0

0
4

0

0

0

0
4

0

0

0
4

0

0

0
4

0

0

0
4

0.0104881

0.0104881

0
4

0

0

0
4

0

0

0
4

0

0

0
4

0

0

0
4

0

0

0
4

0

0

0
4

0
6

0

0

0

0

0

0

0

0

0
4

0

0

0
4

0

0

0
4

0

0

0
4

0

0

0
4

0

0

0
4

0

0

0
4

0

0

0
4

0

0

0
4

0

0

0
4

0

0

0
4

0
3

0

0

0

0

0

0

0
4

0

0

0
4

0

0

0
4

0

0

0
4

0

0

0
4

0

0

0
4

0

0

0
4

0.0080381

0.0080381

0
4

0

0

0
4

0

0

0
4

0

0

0
4

0.0224425
4

0

0

0

0.00997446

0

0.0124681

0

0

1.73472347597681e-18
4

0
4

0

0

0
4

0

0

0
4

0

0

0
4

0

0

0
4

0

0

0
4

0

0

0
4

0

0

0
4

0

0

0
4

0

0

0
4

0

0

0
4

0
3

0

0

0

0

0

0
4

0.131101

0.131101

0
4

0

0

0
4

0

0

0
4

0

0

0
4

0

0

0
4

0

0

0
4

0

0

0
4

0

0

0
4

0

0

0
4

0
4

0
4

0

0

0

0
4

0
2

0

0

0

0

0

0

0
4

0

0

0

0

0

0

0
4

0
4

0

0

0

0

0

0

0
4

0

0

0

0

0
4

0
4

0
4

0

0

0

0

0

0

0

0

0

0

0
4

0

0

0

0

0

0
3

0
4

0

0

0

0

0

0
4

0
4

0
4

0

0

0

0
4

0
4

0

0

0

0

0

0

0
4

0

0

0

0

0

0

0

0
4

0

0

0

0

0

0

0

0

0
4

0.0584274
7

0
7

0.0194758

0.0389516

0
4

0
4

0
4

0

0
4

0.0129839

0

0.0129839

0

0
4

0
7

0
7

0
4

0
4

0

0

0

0
4

0

0

0

0

0

0
4

0
4

0
4

0

0

0

0

0
4

0
4

0

0

0

0

0

0

0
4

0

0

0

0
4

0

0

0

0

0
4

1.99023
6

1.99023
6

0
4

0
5

0
5

0
4

0

0

0

0
4

0

0

0

0
4

0

0

0
4

0

0

0

0

0

0
4

0

0

0

0

0

0
4

0

0

0

0

0

0
4

0.705383

0.582968

0

0.0380203

0

0.0843952

0

0

0

0

0

0
4

0

0

0

0

0
4

0

0

0

0
4

0

0

0

0

0

0
4

0.0330791

0.025041

0.0080381

0

1.73472347597681e-18

0
4

0

0

0

0

0
4

0

0

0
4

0

0

0

0
4

0

0

0
4

0

0

0

0
4

0

0

0

0
4

0.0979191

0.0487588
3

0

0.0491603
2

0

0

0

0

0

0

0

6.93889390390723e-18

0
4

0

0

0

0

0
4

0

0

0

0
4

0

0

0
4

0

0

0

0

0

0
4

0

0

0

0
4

0

0

0

0

0
4

0

0

0

0
4

0.175282

0.175282

0
4

0

0

0

0
4

0

0

0
4

0
4

0
4

0
4

0

0

0

0
4

0

0

0
4

0.0534128

0.0434384

0.00997446

0

1.73472347597681e-18

0
4

0

0

0

0

0
4

0

0

0

0
4

0

0

0
4

0

0

0

0
4

0

0

0

0
4

0

0

0
4

0.0224425

0.00498723

0.0174553

0
4

0

0

0

0

0
4

0.209761
4

0
4

0.209761

0

0

0

0

0

0

0
4

0

0

0

0

0
4

0

0

0

0

0
4

0

0

0

0
4

0

0

0

0

0
4

0

0

0
4

0

0

0

0
4

0

0

0

0
4

0

0

0

0
4

0

0

0
4

0

0

0

0
4

1.68041
6

1.68041
6

0
4

0

0

0

0
4

0

0

0
4

0

0

0
4

0

0

0
4

0

0

0

0
4

0

0

0

0
4

0

0

0
4

0

0

0

0
4

0.0908871

0.0389516

0.0519355

0
4

0

0

0
4

9.29811783123569e-15
4

0
4

0.330105

0.283309

0.270325

0
6

0

0

0

0

0

0

0

0.0129839

0

0

0

0

5.55111512312578e-17

0
4

0.0467966
6

0.0272931

0.0195035

0
4

0

0

0
4

0

0

0
4

0

0

0
4

0

0

0
4

2.77555756156289e-17

0
4

0.232051
3

0.121688
3

0.0649194
3

0
4

0

0

0

0

0

0

0

0

0.0567688

0

0
4

0

0

0

0

0

0

0

0

0

0

0

0

0

0

0

0

0

0

0

0

0

0
4

0

0

0

0

0

0

0

0

0

0

0

0

0

0

0
4

0
6

0
6

0

0

0

0
4

0

0

0
4

0

0

0
4

0

0

0
4

0

0

0
4

0.097379
4

0.097379
4

0
4

0

0

0

0
4

0

0

0

0
4

0

0

0
4

0

0

0
4

0

0

0

0
4

0

0

0
4

0.0129839

0.0129839

0
4

0
4

18.0006

8.90743

4.34107

1.96099
7

0.101313
6

0

0.0129839

0

0

0

0

0

0

0

0

0

0.0248714

0

0

0

0

0

0

0

0

0

0.155674
7

0

0

0

0

0

0

0

0

0.0080381

0

0

0

0

0

0

0.0319776

0.0149617

0

0

0

0

0

0

0

0.0129839

0

0

0

0

0

0

0

0

0.0173753

0

0

0

0

0

0

0

0

0

0
5

0

0

0

0

0

0

0

0

0

0

0.273296
7

0.052126
7

1.17022
7

0.0737404

0

0

0

0

0

0

0

0

0.0229902

0.155806
6

0

0

0

0

0

0

0

0

0

0.0194758

0.134625
7

0.052126

0

0

0

0

0

0.00498723

0

0

0

0

0

0

0

0.123347

0

0

0

0

0

0

0.0964676
5

0

0

0

0

0

0

0

0.0129839

0

0

0
7

0.0129839

0

0

0

0

0

0

0

0

0

0

0

0

0

0

0

0

0

0

0

0

0

0

0

0.0200127

0

0

0

0

0

0

0

0
4

0.344744
7

0.0129839
7

0

0

0.0259677

0

0

0.0129839

0

0

0.0129839

0.0653925
7

0.0899604
8

0.111488
7

0.0129839

0

0

0

0

0
4

0

0

0
4

0

0

0
4

0

0

0
4

0

0

0
4

0

0

0
4

0

0

0
4

8.4022

8.37361

0.0224425

0.00615279

0
4

0.0718875

0.0718875

0

0

0

0

0

0

0

0
4

0.274383
4

0.274383
4

0

0

0

0
4

0

0

0

0

0
4

0

0

0

0
4

0

0

0
4

0

0

0
4

0

0

0
4

0
4

8.30916

7.85621

0.518309

0.0194758

0

0

0

0

0

0

0

0

0.0104881

0

0.214234
6

0.204787

0

0

0

0

0

0

0

0.141922

0.0245801

0.15725
7

0

0

0

0

0

0

0

0

0

0

0

0

0

0

0

0

0

0

0

0

0

0.131101

0

0

0

0

0

0

0

0

0

0

0.194758
7

0

0.146276

0

0.00498723

0

0

0

0

0

0

0

0

0

0

0

0

0

0

0

0

0

0

0

0

0

0

0

0

0

0

0

0

0.0194758

0

0

0

0

0

0

0

0

0

0

0.157157
6

0

0

0.00748085

0

1.17186
7

0

0.0292553
7

0

0.561111

0.555747
7

0.00498723
6

0.0629283

0.0367082
7

0.0179689

0.294247
6

1.96392

0

0.00498723

0.0163868

0

0

0

0.026063

0

0

0

0.235154

0.156028

0

0

0.00498723

0

0

0

0

0

0

0.129839
7

0

0

0

0

0

0

0

0

0.00498723

0.00748085

0.0163868
7

0

0

0

0

0

0

0

0

0.0781891

0

0.279153
7

0

0

0

0

0

0

0

0

0

0

0.0245801
7

0

0

0

0

0.0157321

0.0104881

0

0

0

0

0.194758
6

0

0

0

0

0

0

0

0

0

0

6.66133814775094e-16

0
4

0.452949
6

0.452949
6

0
4

0

0

0
4

0

0

0
4

0

0

0
4

0

0

0
4

0

0

0
4

0

0

0
4

0

0

0
4

0
4

0.122901
3

0.122901
3

0.122901
3

0
3

0

0

0

0

0
4

0

0

0

0
4

0

0

0
4

0

0

0
4

0

0

0
4

0

0

0
4

0
4

7.78424

7.74529

4.00965

0.0663752
7

0

0.00736842

0

0.0163868

0

0

0

0

0

0

0
6

0

0.0080381

0

0

0

0

0

0

0

0

0
3

0.0120572

0

0

0

0.00736842

0.0434384

0

0

0

0

0
3

0

0

0

0

0

0

0

0

0

0

0
3

0

0

0

0

0

0

0

0

0

0

0.0118814
7

0

0

0

0

0

0

0

0

0

0

0
7

0

0

0.0173753

0

0

0

0

0

0

0

0.0173753
7

0

0

0

0

0

0

0

0

0

0

0
3

0

0

0

0

0

0

0

0.0104881

0

0

0.0294325
6

0

0

0

0

0.0294737

0.00475254

0.2532

0.0190102

0

0

1.59427
7

0

0

0

0

0

0

0

0

0

0

0

0.00736842
3

0

0

0

0

0.0129839

0

0

0

0

0

0

0

0

0

0

0

0.0080381

0

0.0760407

0

0

0
3

0

0

0

0

0

0

0

0

0.0347507

0

0

0

0

0

0.018421

0

0

0

0

0

0

0.0342404
7

0

0

0

0

0

0

0

0

0

0

0
7

0.0292553

0

0

0

0

0

0

0

0

0

0.00736842

0

0.0163868

0

0

0

0

0

0

0

0

0.0257895
4

0

0

0

0

0

0

0

0

0

0

0.0213864

0

0

0

0

0

0

0.0442096

0

0.00736842

0

0.607199
7

0
7

0

0

0

0

0

0

0

0

0

0

0
3

0

0

0

0

0

0

0

0

0

0

0.0983205

0

0

0

0

0

0

0

0

0

0

0.0868767
7

0

0

0

0

0

0

0

0

0

0

0
3

0

0

0

0

0

0

0

0

0

0

0

0

0

0

0

0

0.0104881

0

0

0

0

0

0

0

0

0

0

0

0

0

0

0

0.0409669

0.0080381

0

0

0

0

0

0

0

0

0

0

0

0

0

0

0

0

0

0

0

0

0

0

0.0173753

0

0

0

0

0

0

0

0

0
4

0

0

0

0

0

0

0

0.0195035

0

0

0

0.097379

0

0

0

0.00475254

0

0

0

0

0

0

0.00712881

0

0

0

0

0

0.0120572

0

0

0.0262201

0

0

0

0

0

0

0
7

0

0.0163868

0

0

0

0

0

0

0

0

0.0436178
7

0

0

0

0.0491603

0

0

0

0.0218656

0

0

0.00712881
3

0.0200953

0

0

0

0

0

0

0

0

0.0237627

0.0173753
7

0

0

0

0

0

0

0

0

0

0.0080381

0
7

0

0

0

0

0

0

0

0

0

0

8.13585310233123e-16

0
4

0

0

0
4

0

0

0
4

0

0

0
4

0

0

0
4

0

0

0
4

0

0

0
4

0

0

0
4

0.0389516

0.0389516

0
4

2.91433543964104e-16

0
4

15.4264

2.53703
3

0.293715
6

0.0295099
7

0

0

0

0

0

0

0

0

0

0

0.0372155
6

0

0

0

0

0.0487588

0

0

0

0

0

0
3

0

0

0

0

0

0

0

0

0

0

0.156615
6

0

0

0.0129839

0

0

0

0

0

0

0

0
2

0

0.0129839

0

0

0

0.0195035

0

0

0

0

0

0

0

0

0

0

0

0

0

0

0.0248714

0
4

0

0

0.0400254

0

0

0.0195035

0

0

0

0.0259677

0

0

0

0

0

0

0

0

0

0

0

0.0292553

0

0.00475254

0

0.0473073

0

0

0

0

0

0

0.0266097
7

0

0

0

0

0.00712881

0

0

0.0129839

0

0

0.0901271
2

0

0

0

0

0

0

0

0

0

0

0

0.0514765
6

0

0

0

0

0

0

0

0

0

0

0
6

0.00710613

0

0.0173753

0

0

0

0.00498723

0

0

0

0.0533696
6

0

0

0

0

0

0

0

0

0

0

0.0129839

0

0

0

0

0

0

0

0

0

0

0
6

0.00475254

0

0

0

0

0

0

0

0

0

0

0

0

0

0

0

0

0

0

0

0

0.0147368
2

0

0

0

0

0

0

0

0

0

0

0.039007
6

0

0.0129839

0

0

0

0

0

0

0

0

0
7

0

0

0

0

0.0195035

0

0

0

0

0

0.0163868
7

0

0

0

0

0

0

0

0

0

0

0

0
4

0

0

0

0

0

0

0

0

0

0

0.0350222
7

0

0

0

0

0

0

0

0

0

0

0
5

0

0

0

0

0

0

0

0

0

0

0

0

0

0

0

0.0195035

0

0

0

0

0

0
7

0

0

0

0

0

0

0

0

0

0

0

0

0

0

0

0

0

0

0

0

0

0

0

0

0

0

0

0

0

0

0

0

0

0

0

0

0

0

0

0

0

0.0262201

0

0

0

0

0

0

0

0

0

0.731382

0

0

0

0.00498723
7

0

0

0

0

0

0

0

0

0

0

0.0195035

0

0

0

0

0

0

0.0174553

0

0

0

0

0

0

0

0

0

0

0

0

0

0.00475254

0

0

0

0

0

0

0

0.00736842

0

0

0

0.00498723

0

0

0

0

0

0

0

0

0

0

0

0

0

0

0

0

0

0

0

0

0

0

0

0

0

0

0

0

0

0

0

0

0

0

0

0

0

0

0

0

0

0

0

0

0

0

0

0

0

0

0

0

0

0

0

0

0

0

0

0

0

0

0

0

0

0
4

0
5

0

0

0

0

0

0

0

0

0

0

0

0

0

0

0

0

0

0

0

0

0

0

0

0

0

0

0

0

0

0

0

0

0.0242561

0

0

0

0

0

0

0

0

0

0

0

0

0

0

0

0

0

0

0

0

0

0

0

0

0

0

0

0

0

0

0

0

0

0

0

0

0

0

0

0

0

0

0

0

0

0

0

0

0

0.00498723

0

0

0

0

0

0

0

0

0

0

0

0

0

0

0

0

0

0

0

0

0

0

0

0.0195035

0

0

0.0200127
7

0

0

0

0

0

0

0

0

0

0

0

0
7

0

0.0163868

0

0

0

0

0

0

0

0
7

0

0.0292553

0.0672388

0
4

0.0491603

0

0
4

0.0194758
5

0

0.0771878

0

0

0

0

0.0877659

0

0

0

0
2

0

0

0.0129839

0

0

0

0

0

0

0

0.0236431
6

0.0195035

0

0

0

0

0

0

0

0

0

1.28369537222284e-15
3

0
4

1.92467

1.20243

0

0

0

0

0

0

0

0

0

0

0.026063

0
6

0

0

0

0

0

0

0

0.0259677

0

0

0.269181
7

0

0

0

0

0

0

0

0

0

0

0

0

0

0

0

0

0

0

0

0

0

0

0

0

0

0

0

0

0

0

0

0.11007

0

0

0

0

0

0

0

0

0

0

0

0
7

0

0

0

0

0

0

0

0

0

0

0

0

0

0

0

0

0.0194758
7

0.101879
7

0

0.0163868

0

0.0195035

0

0
6

0

0

0

0.0163868

0.0300191
7

0

0

0

0

0

0

0

0

0

0.0195035

0

0

0

0

0

0

0

0

0

0

0

0
5

0

0

0

0

0

0

0

0

0

0

0.0350222
7

0

0

0

0

0

0

0

0

0

0

0

0

0

0

0

0

0

0

0

0

0

0

0

0

0

0

0

0

0

0

0

0

0

0.0327735

0

0

0

0

0

0

0

0

0

0
4

0
2

0
2

0

0

0

0
4

0

0

0

0
4

0

0

0

0
4

0

0

0
4

0.0213864

0

0.0213864

0
4

0

0

0
4

0

0

0
4

0

0

0

0
4

0

0

0

0
4

0

0

0

0
4

0

0

0
4

0
4

0
4

0

0

0

0

0

0

0

0

0

0
4

0

0

0
4

0

0

0

0
4

0

0

0

0
4

0

0

0
4

0

0

0

0
4

0

0

0
4

0

0

0
4

0

0

0
4

0

0

0
4

0

0

0
4

0.0163868
6

0.0163868
6

0

0

0

0

0

0

0

0

0
4

0

0

0
4

0

0

0
4

0

0

0
4

0

0

0
4

0

0

0
4

0

0

0
4

0

0

0
4

0

0

0
4

0

0

0
4

0

0

0
4

0
3

0
3

0

0

0

0

0

0

0

0

0

0
4

0

0

0
4

0

0

0
4

0

0

0
4

0

0

0
4

0

0

0
4

0

0

0
4

0

0

0
4

0

0

0
4

0

0

0
4

0.0189229

0.0189229

0
4

0.286773
6

0.286773
6

0

0

0

0

0

0
4

0

0

0
4

0

0

0
4

0

0

0
4

0

0

0
4

0

0

0
4

0

0

0
4

0

0

0
4

0

0

0
4

0

0

0
4

0

0

0
4

0
3

0
3

0

0

0

0

0

0
4

0

0

0
4

0

0

0
4

0

0

0
4

0

0

0
4

0

0

0
4

0

0

0
4

0

0

0
4

0

0

0
4

0

0

0
4

0.00498723

0.00498723

0
4

0

0

0

0

0
4

0

0

0
4

0

0

0
4

0

0

0
4

0

0

0
4

0

0

0
4

0

0

0
4

0.0106592

0.0106592

0
4

0

0

0
4

0

0

0
4

0

0

0
4

0
3

0
3

0

0

0

0

0

0

0

0

0

0
4

0

0

0
4

0

0

0
4

0

0

0
4

0

0

0
4

0

0

0
4

0

0

0
4

0

0

0
4

0

0

0
4

0

0

0
4

0.039007

0.039007

0
4

0

0

0

0

0

0

0

0

0
4

0

0

0
4

0

0

0
4

0

0

0
4

0

0

0
4

0

0

0
4

0

0

0
4

0

0

0
4

0

0

0
4

0.00475254

0.00475254

0
4

0

0

0
4

0.0519355
5

0

0.0519355

0

0

0

0

0

0

0

0
4

0

0

0
4

0

0

0
4

0

0

0
4

0

0

0
4

0

0

0
4

0

0

0
4

0

0

0
4

0

0

0
4

0

0

0
4

0

0

0
4

4.72793

4.48775

0

0

0

0

0

0

0

0

0

0.217192

0.0129839
7

0

0

0

0

0

0.0100064

0

0

0

0

0

0

7.97972798949331e-17

0
4

0
4

0
4

0

0

0

0

0

0
4

0

0

0
4

0

0

0
4

0

0

0
4

0

0

0
4

0

0

0
4

0

0

0
4

0

0

0
4

0

0

0
4

0

0

0
4

0

0

0
4

0

0

0

0

0

0

0
4

0

0

0
4

0

0

0
4

0

0

0
4

0

0

0
4

0

0

0
4

0

0

0
4

0.0195035
6

0.0195035

0

0

0

0

0
4

0
4

0

0

0

0

0

0

0
4

0
4

0

0

0

0

0

0
4

0

0

0

0

0

0

0

0
4

0
4

0

0

0

0

0
4

0

0

0

0

0

0

0
4

0.0163868

0

0

0.0163868

0
4

0.0292553
6

0.0292553

0

0

0

0
4

0
4

0
4

0

0

0

0

0

0

0

0

0

0

0

0

0

0

0

0

0

0

0

0

0

0

0

0

0

0

0

0

0

0

0

0

0

0

0

0

0

0

0

0

0

0

0

0

0

0

0

0

0

0

0

0

0

0

0

0

0

0

0

0

0

0

0

0
4

0.0296178
6

0.0296178
6

0
4

0.0283844
7

0.0283844

0

0
4

0

0

0

0

0

0
4

0

0

0

0

0
4

0

0

0

0

0
4

0

0

0

0

0

0
4

0

0

0

0
4

0

0

0

0
4

0.0324597

0.0324597

0

0
4

0

0

0

0

0
4

0.728057

0
7

0.0349106

0

0

0

0.0487588

0

0.0292553

0.0157321

0

0.039007

0.00498723
6

0

0

0

0

0

0

0

0

0

0

0.0434384

0

0.0129839

0.157321

0.294466
2

0

0.0471962

0

2.28983498828939e-16

0
4

0

0

0

0

0
4

0

0

0

0

0
4

0.039007

0.039007
7

0

0
4

0

0

0

0

0

0
4

0
7

0

0

0

0

0
4

0

0

0

0

0
4

0

0

0

0

0
4

0

0

0

0

0

0
4

0

0

0

0

0
4

0

0

0

0
4

1.5587
6

1.5587
6

0

0

0

0

0

0

0

0

0
4

0

0

0

0
4

0

0

0

0
4

0

0

0

0
4

0

0

0

0

0
4

0

0

0

0
4

0

0

0

0

0
4

0

0

0

0
4

0

0

0

0
4

0.00615279

0.00615279

0

0

0
4

0

0

0

0

0
4

0.273418

0.168984
6

0

0

0

0

0

0

0

0

0

0

0.0361715

0

0

0

0

0

0

0

0

0

0.0682623

0

0

0

0

0

0

2.77555756156289e-17

0
4

0

0

0

0

0
4

0

0

0
4

0

0

0
4

0

0

0

0

0
4

0

0

0

0

0
4

0.0852735

0.0852735

0

0

0
4

0.056849

0.0213184

0.0355306

0

0
4

0

0

0

0
4

0

0

0
4

0

0

0

0

0
4

1.55458
6

0.591113
6

0

0

0

0

0
7

0.0955644

0

0

0.867907

0

0

0

0
4

0.162565

0

0

0.162565

0
4

0

0

0

0
4

0

0

0
4

0

0

0
4

0.113538

0

0

0.113538

0
4

0

0

0

0
4

0

0

0

0

0
4

0.00475254

0.00475254

0
4

0

0

0
4

0

0

0

0
4

1.0107

0.845622

0.0157321

0

0.0195035

0

0

0.129839

1.66533453693773e-16

0
4

0

0

0

0

0
4

0

0

0
4

0

0

0
4

0

0

0

0
4

0

0

0

0
4

0

0

0

0
4

0

0

0

0
4

0

0

0

0
4

0

0

0

0
4

0

0

0
4

0
3

0
3

0

0

0

0

0

0

0

0

0

0
4

0

0

0

0
4

0

0

0

0
4

0

0

0

0
4

0

0

0

0
4

0

0

0
4

0.0327735

0

0.0327735

0
4

0

0

0

0
4

0

0

0
4

0

0

0

0
4

0

0

0

0
4

0
4

0
4

8.93446
4

8.22209
4

7.35161
4

1.42016
4

0

0

0

0

0

0

0

0

0

0

0

0

0

0

0

0

0

0

0

0

0.0434384

0

0.192896

0

0

0

0

0

0.0163868

0

0.660747

0

0

0.11294

0

0.0129839

0

0

0

0

0

0

0

0

0
4

0

0

0

0

0

0

0

0

0

0

0
4

0

0.0157321

0

0

0

0

0

0

0

0

0.0129839

0

0

0

0

0

0

0

0

0

0

0
4

0

0

0

0

0

0

0

0

0

0

0

0

0

0

0

0

0

0

0

0

0

0
4

0

0

0

0

0

0

0

0

3.47114
5

0

0.0584274

0.0347507

0.130315

0

0

0

0.0173753

0

0

0.751369

0

0

0

0

0

0

0

0

0

0.0173753

0.128338

0

0

0

0

0

0

0

0

0

0

0.224891
6

0

0

0

0

0

0

0

0

0

0

0

0

0

0

0

0

0

0

0

0

0

0.0129839

0

0

0

0

0.0163868

0

0

0

0

0

0
4

0

0

0

0

0

0

0

0

0

0

0
4

0

0

0

0

0

0

0

0

0

0

0
4

0.0245801
4

0.0245801

0

0

0

0

0

0

0

0
4

0

0

0
4

0

0

0

0

0

0
4

0.0163868

0

0.0163868

0

0

0
4

0

0

0

0
4

0.794757

0.794757

0

0
4

0.0347507

0.0347507

0

0
4

0

0

0
4

0

0

0
4

0

0

0
4

2.42861286636753e-16
4

0
4

0

0

0

0

0

0

0

0
4

0
4

0

0

0

0

0
4

0
4

0

0

0

0

0
4

0

0

0
4

0
4

0

0

0

0

0
4

0
4

0

0

0

0

0
4

0
4

0

0

0

0
4

0
4

0

0

0

0
4

0
4

0

0

0

0
4

0
4

0

0

0

0
4

0

0

0
4

0

0

0
4

0
4

0

0

0

0
4

0

0

0
4

0
4

0

0

0

0
4

0

0

0
4

0
4

0
4

0
4

0

0

0

0

0

0

0
4

0
4

0.498183

0.498183

0.498183

0
4

0
4

0

0

0

0
4

0

0

0
4

0
4

0.0368789

0.0368789

0.0368789

0
4

0
4

0

0

0

0
4

0
4

0

0

0

0

0
4

0
4

0

0

0

0

0
4

0
4

0

0

0

0
4

0
4

0

0

0

0
4

0
4

0

0

0

0
4

0
4

0

0

0

0
4

0
4

0

0

0

0

0

0

0
4

0

0

0
4

0

0

0
4

0

0

0
4

0
4

0

0

0

0
4

0
4

0

0

0

0
4

0
4

0

0

0

0
4

0
4

0.136345

0.136345

0.136345

0
4

0
4

0

0

0

0
4

0
4

0

0

0

0
4

0
4

0

0

0

0
4

0
4

0

0

0

0
4

0
4

0

0

0

0
4

0
4

0

0

0

0
4

0
4

0.0409669
6

0.0409669
6

0

0

0.0409669

0
4

0
4

0

0

0

0
4

0
4

0

0

0

0
4

0
4

0

0

0

0
4

0
4

0

0

0

0
4

0
4

0

0

0

0
4

0
4

0

0

0

0
4

0
4

0

0

0

0
4

0
4

0

0

0

0
4

0
4

0

0

0

0
4

0
4

0

0

0

0
4

0
4

0

0

0

0

0

0

0
4

0
4

0

0

0

0
4

0
4

0

0

0

0
4

0
4

0

0

0

0
4

0
4

0

0

0

0
4

0
4

0

0

0

0
4

0
4

0

0

0

0
4

0
4

0

0

0

0
4

0
4

0

0

0

0
4

0
4

0

0

0

0

0

0
4

0
4

0

0

0

0
4

0
4

0

0

0

0

0
4

0

0

0
4

0
4

0
4

39.9753

2.59273
5

0
5

0
6

0
5

0
4

1.0446

0.888266

0.103039

0.0213184

0.0177653

0.0142123

0
4

0.0106592

0.0106592

0
4

0

0

0
4

0

0

0
4

0

0

0
4

0.0194758

0.0194758

0
4

0

0

0
4

0

0

0
4

0

0

0
4

0.103871

0.103871

0
4

0

0

0
4

0
4

0

0

0

0

0

0

0
4

0.00710613

0.00710613

0
4

0

0

0
4

0

0

0
4

0

0

0

0
4

1.35016

1.3395

0.0106592

8.50014503228635e-17

0
4

0

0

0

0
4

0

0

0
4

0.056849

0.0461898

0.0106592

1.73472347597681e-18

0
4

0

0

0
4

0

0

0
4

0
4

27.402

9.65812

0.0327735
7

0.662178

0

0

0.0324597

0

0

0

0

0

0

0

0.913834

0

0.0194758

0

0

0

0

0

0

0.0129839

0

0
7

0

0.0194758

0.0173753

0

0

0

0

0

0

0

0.166745
7

0

0

0.0324597

0

0

0

0.0189229

0

0

0

1.08489
7

0

0

0

0

0

0

0

0

0

0.0194758

0

0.0292553

0

0

0

0

0.0129839

0

0

0

0

0.103871
7

0

0

0.0129839

0

0

0

0.0259677

0.0584274

0

0.0324597

0.365839

0.0347507

1.0452

0

1.31945
6

0.0652609
6

0.292518

0

0

0

0

0.23371
7

0
7

0

0

0.118662
8

0

0

0.2339

0.218625

0

0

0

0

0.162298

0

0.996218
7

0.0584274

0

0.0649194

0.0520308

0

0

0

0

0

0

0.181774
7

0

0

0

0

0

0

0

0

0

0

0

0

0.0975176

0

0

0.0245801

0

0

0.549424

0.0194758

0

0

0.0567688

0.0473073

0

0

0

0

0

0.0129839

0

0

0

0

0

0

0

0

0

0

0

0

0.0129839

0
7

0

0

0

0

0

0

0.026063

0

0.0129839

0.0454436

1.18655085756814e-15

0
4

0.640903

0
7

0

0

0

0.0195035

0

0

0

0

0

0

0.06835
7

0

0

0.00710613

0

0

0

0

0.0129839

0

0.00710613

0
7

0.0177653

0

0

0

0

0

0.00710613

0

0.00710613

0

0
6

0

0.241608

0

0

0.252268

0

5.55111512312578e-17

0
4

0.396248

0
7

0.278575

0

0
7

0.0347507
7

0

0

0

0

0

0

0.0173753
7

0

0

0

0

0

0

0

0

0

0

0
8

0

0

0

0

0

0

0

0

0

0

0
8

0

0

0

0

0

0

0

0

0

0

0
7

0

0

0

0

0

0

0.065547
7

0
7

0
7

0
7

5.55111512312578e-17

0
4

0.720605

0.20125

0

0

0

0

0

0

0

0

0

0

0.23371

0

0.123347

0

0

0

0

0.162298

0
4

12.2085

0.113698
7

3.57998

8.38495

0

0

0

0

0

0

0

0

0.129839

0

0

0

0

0

0

1.88737914186277e-15

0
4

2.44372

2.44372

0
4

1.31751
7

1.28826
7

0

0

0.0292553

5.55111512312578e-17
7

0
4

0

0

0

0

0

0

0

0

0
4

0

0

0

0

0

0

0
4

0

0

0
4

0

0

0
4

0

0

0
4

0

0

0
4

0

0

0
4

0.0163868

0.0163868

0
4

0

0

0
4

0
4

0

0

0

0

0

0

0

0

0

0

0
4

0

0

0

0

0
4

0
4

0.0259677

0.0259677

0

0.0259677

0
4

0

0

0
4

0
4

0

0

0

0
4

0
4

0

0

0

0

0
4

0

0

0
4

0
4

0

0

0

0

0

0
4

0
4

0

0

0

0
4

0

0

0
4

0
4

0

0

0

0
4

0

0

0
4

0
4

0.026063

0.026063

0.026063

0
4

0
4

0.0355306

0.0355306

0.0106592

0.0248714

0
4

0
4

0

0

0

0
4

0
4

0

0

0

0
4

0

0

0
4

0
4

0.112293
7

0.112293
7

0.0491603

0.0293706
7

0.0337621

0

0

0

0
4

0

0

0
4

0
4

0

0

0

0
4

0
4

0

0

0

0
4

0
4

0

0

0

0
4

0
4

0.00710613

0.00710613

0.00710613

0
4

0
4

0

0

0

0
4

0
4

0

0

0

0
4

0
4

0.0149617

0.0149617

0.0149617

0
4

0
4

0

0

0

0
4

0
4

0

0

0

0
4

0
4

0.0194758

0.0194758

0.0194758

0
4

0
4

0

0

0

0

0
4

0

0

0
4

0
4

0

0

0

0
4

0
4

0

0

0

0
4

0
4

0.0157321

0

0

0

0

0
4

0.0157321

0.0157321

0
4

0
4

0

0

0

0
4

0
4

0.0245801
6

0.0245801

0.0245801

0

0

0
4

0

0

0
4

0

0

0
4

0
4

0

0

0

0

0

0
4

0

0

0

0
4

0
4

0

0

0

0

0
4

0
4

9.6989

9.6989

9.68643

0.0124681

0
4

0
4

3.5527136788005e-15

0
4

0.529948

0.529948

0.529948

0.217192

0

0

0

0.0173753

0

0

0

0

0

0

0
1

0

0

0

0

0.26063

0.0347507

0

2.08166817117217e-17

0
4

0

0

0

0
4

0

0

0

0
4

0

0

0
4

0

0

0
4

0

0

0
4

0

0

0
4

0
4

0

0

0

0
4

0
4

0

0

0

0
4

0
4

0

0

0

0

0
4

0
4

0

0

0

0
4

0
4

0

0

0

0
4

0
4

0

0

0

0
4

0
4

0

0

0

0
4

0
4

0

0

0

0
4

0
4

0

0

0

0
4

0
4

0

0

0

0
4

0
4

0
4

1.67817
5

1.67817
5

1.25104
5

0.978354
5

0

0.272689

0

0

0

0

0

0

0
4

0.274383

0.274383

0

0

0
4

0.126521

0.113538

0

0.0129839

1.38777878078145e-17

0
4

0

0

0
4

0.0262201

0

0.0262201

0

0
4

0

0

0
4

0

0

0
4

1.66533453693773e-16
5

0
4

0
4

0
4

0
4

0
4

0
4

0

0

0

0

0
4

0

0

0

0

0

0

0

0
4

0
4

0
4

0

0

0

0

0

0
4

0

0

0
4

0
4

0
4

0.0737404

0.0737404

0.0737404

0.0737404

0

0
4

0
4

0
4

0

0

0

0

0

0
4

0

0

0

0
4

0
4

0
4

0

0

0

0

0

0
4

0
4

0
4

0

0

0

0

0

0

0
4

0
4

0
4

0

0

0

0

0
4

0
4

0
4

0

0

0

0

0

0
4

0
4

0
4

0

0

0

0

0
4

0
4

0
4

0

0

0

0

0

0
4

0

0

0
4

0
4

0
4

0

0

0

0

0
4

0
4

0
4

0
2

0
2

0
2

0

0

0

0

0

0

0

0

0

0
4

0

0

0
4

0
4

0

0

0

0

0

0

0

0
4

0

0

0

0
4

0
4

0
4

0

0

0

0

0
4

0

0

0
4

0
4

0
4

0

0

0

0

0

0

0
4

0
4

0
4

0

0

0

0

0
4

0

0

0
4

0
4

0
4

0

0

0

0

0

0
4

0
4

0

0

0

0
4

0
4

0
4

0

0

0

0

0
4

0
4

0
4

0

0

0

0

0
4

0

0

0
4

0
4

0
4

0

0

0

0

0
4

0
4

0
4

0

0

0

0

0

0
4

0

0

0
4

0
4

0
4

0

0

0

0

0
4

0

0

0
4

0
4

0
4

0

0

0

0

0

0

0
4

0
4

0
4

0.221925
7

0.221925
7

0.143736
7

0.143736
7

0
7

0

0
4

0.0781891
7

0.0781891
7

0

0

0
4

0

0

0

0

0
4

0

0

0

0
4

2.77555756156289e-17
7

0
4

0
4

0

0

0

0

0

0
4

0
4

0
4

0

0

0

0

0
4

0

0

0
4

0

0

0
4

0
4

0
4

0

0

0

0

0
4

0

0

0
4

0
4

0
4

0.0276005

0.0276005

0.0276005

0.0171125

0.0104881

0
4

0
4

0
4

0

0

0

0

0

0
4

0
4

0
4

0

0

0

0

0
4

0
4

0
4

0

0

0

0

0
4

0
4

0
4

0

0

0

0

0
4

0
4

0
4

0

0

0

0

0

0
4

0
4

0
4

0

0

0

0

0
4

0

0

0
4

0
4

0
4

1.56802

1.56802

1.56802

1.40952

0.0163868

0.101143

0.0163868

0

0

0

0.0245801

0

0

1.90819582357449e-16

0
4

0

0

0
4

0

0

0

0
4

0
4

0

0

0

0

0
4

0
4

0
4

0

0

0

0

0

0
4

0
4

0
4

0

0

0

0

0
4

0

0

0
4

0
4

0
4

0

0

0

0

0

0
4

0
4

0
4

0

0

0

0

0
4

0
4

0
4

0

0

0

0

0

0
4

0

0

0
4

0
4

0
4

0.0245801

0.0245801

0.0245801

0.0245801

0
4

0

0

0
4

0
4

0
4

0

0

0

0

0
4

0
4

0
4

0

0

0

0

0

0
4

0
4

0

0

0

0
4

0
4

0
4

0

0

0

0

0
4

0

0

0
4

0
4

0
4

0

0

0

0

0
4

0
4

0
4

0
2

0
2

0
2

0

0

0

0

0

0

0
4

0

0

0

0

0

0

0
4

0

0

0

0

0

0
4

0
4

0
4

0

0

0

0

0
4

0
4

0
4

0

0

0

0

0
4

0

0

0
4

0

0

0
4

0
4

0
4

0

0

0

0

0
4

0
4

0
4

0.0163868

0.0163868

0.0163868

0

0.0163868

0
4

0
4

0
4

0

0

0

0

0

0

0
4

0
4

0
4

0

0

0

0

0

0
4

0
4

0
4

0

0

0

0

0

0
4

0
4

0
4

0

0

0

0

0
4

0
4

0
4

0

0

0

0

0
4

0

0

0
4

0
4

0
4

0

0

0

0

0
4

0
4

0
4

0

0

0

0
6

0
7

0

0

0

0

0
4

0
6

0
6

0

0

0

0
4

0

0

0

0
4

0

0

0
4

0

0

0
4

0

0

0
4

0
4

0
4

0

0

0

0

0
4

0

0

0
4

0
4

0
4

0

0

0

0

0

0
4

0
4

0
4

0

0

0

0

0
4

0

0

0
4

0
4

0
4

0

0

0

0

0

0
4

0
4

0
4

0.0173753

0.0173753

0.0173753

0.0173753

0

0
4

0
4

0
4

0

0

0

0

0

0
4

0
4

0
4

0

0

0

0

0
4

0

0

0
4

0
4

0
4

0

0

0

0

0
4

0

0

0
4

0
4

0
4

0

0

0

0

0
4

0

0

0
4

0
4

0
4

0

0

0

0

0
4

0
4

0
4

0
7

0
7

0
7

0
7

0

0

0

0

0
4

0

0

0

0

0

0

0
4

0
4

0
4

0

0

0

0

0
4

0

0

0
4

0
4

0
4

0

0

0

0

0
4

0

0

0
4

0
4

0
4

0

0

0

0

0

0
4

0
4

0
4

0

0

0

0

0

0
4

0
4

0
4

0

0

0

0

0

0
4

0
4

0
4

0.104252

0.104252

0.104252

0.104252

0
4

0
4

0
4

0

0

0

0

0
4

0
4

0
4

0

0

0

0

0
4

0

0

0
4

0
4

0
4

0

0

0

0

0
4

0

0

0
4

0
4

0
4

0

0

0

0

0
4

0
4

0
4

0.723796
7

0.723796
7

0.718808
7

0.130315
7

0.588493

0

0

0

0

0
4

0

0

0

0

0
4

0.00498723

0.00498723

0
4

1.30104260698261e-17
7

0
4

0
4

0.0163868

0.0163868

0.0163868

0.0163868

0
4

0

0

0
4

0
4

0
4

0

0

0

0

0
4

0
4

0
4

0

0

0

0

0
4

0

0

0
4

0
4

0
4

0

0

0

0

0

0
4

0
4

0
4

0

0

0

0

0
4

0
4

0
4

0

0

0

0

0

0
4

0
4

0
4

0

0

0

0

0

0
4

0
4

0
4

0

0

0

0

0

0
4

0
4

0
4

0

0

0

0

0

0
4

0
4

0
4

0

0

0

0

0
4

0

0

0
4

0
4

0
4

0.295381
7

0.295381
7

0.139003
7

0.139003
7

0

0

0
4

0.156378

0.156378

0
4

0

0

0
4

0
4

0
4

0

0

0

0

0

0
4

0
4

0
4

0

0

0

0

0
4

0
4

0
4

0

0

0

0

0
4

0
4

0
4

0

0

0

0

0
4

0

0

0
4

0
4

0
4

0

0

0

0

0
4

0
4

0
4

0

0

0

0

0

0
4

0
4

0
4

0

0

0

0

0
4

0

0

0
4

0
4

0
4

0

0

0

0

0
4

0
4

0
4

0

0

0

0

0
4

0
4

0
4

0

0

0

0

0
4

0
4

0
4

0
7

0
7

0
7

0
7

0
7

0

0

0

0
4

0
7

0
7

0

0

0
4

0
4

0
4

0

0

0

0

0
4

0

0

0
4

0
4

0
4

0

0

0

0

0

0
4

0
4

0
4

0

0

0

0

0
4

0
4

0
4

0.196641

0.196641

0

0

0
4

0.196641

0.196641

0
4

0
4

0
4

0

0

0

0

0
4

0
4

0
4

0

0

0

0

0

0
4

0
4

0
4

0

0

0

0

0
4

0

0

0
4

0
4

0
4

0

0

0

0

0
4

0
4

0
4

0

0

0

0

0
4

0

0

0
4

0
4

0
4

0

0

0

0

0
4

0

0

0
4

0
4

0
4

0
4

0
4

0

0

0

0

0

0

0

0

0

0

0

0

0

0

0

0

0

0

0
4

0
4

0

0

0

0

0

0

0

0

0

0

0

0

0

0

0

0
4

0

0

0

0

0

0

0

0
4

0

0

0

0
4

0
4

0

0

0

0
4

0
4

0
4

0

0

0

0

0

0

0

0

0

0

0
4

0

0

0

0

0
4

0
4

0
4

0

0

0

0

0
4

0
4

0
4

0

0

0

0

0

0
4

0
4

0
4

0

0

0

0

0
4

0
4

0
4

0

0

0

0

0

0
4

0
4

0
4

0

0

0

0

0

0
4

0
4

0
4

0

0

0

0

0
4

0
4

0
4

0

0

0

0

0
4

0

0

0
4

0
4

0
4

0

0

0

0

0
4

0
4

0
4

0

0

0

0

0

0
4

0
4

0
4

0

0

0

0

0

0
4

0
4

0
4

2.44392
7

2.44392
7

2.44392
7

2.21199
7

0.231926
7

0

0
4

0

0

0

0
4

0
4

0

0

0

0
4

0
4

0
4

0

0

0

0

0
4

0
4

0
4

0

0

0

0

0
4

0
4

0
4

0

0

0

0

0
4

0
4

0
4

0

0

0

0

0
4

0
4

0
4

0

0

0

0

0
4

0
4

0
4

0

0

0

0

0
4

0
4

0
4

0

0

0

0

0
4

0
4

0
4

0

0

0

0

0
4

0
4

0
4

0

0

0

0

0
4

0
4

0
4

0

0

0

0

0
4

0
4

0
4

0.0435901

0.0435901

0.0241143

0.0241143
7

0

0

0
4

0

0

0
4

0.0194758

0.0194758

0
4

0

0

0
4

3.46944695195361e-18

0
4

0
4

0

0

0

0

0
4

0
4

0
4

0

0

0

0

0
4

0
4

0
4

0

0

0

0

0
4

0
4

0
4

0

0

0

0

0
4

0
4

0
4

0

0

0

0

0
4

0
4

0
4

0

0

0

0

0
4

0
4

0
4

0

0

0

0

0
4

0
4

0
4

0

0

0

0

0
4

0
4

0
4

0

0

0

0

0
4

0
4

0
4

0

0

0

0

0
4

0
4

0
4

0
2

0
2

0
2

0
2

0

0

0

0

0

0
4

0
2

0

0

0

0
4

0

0

0
4

0
4

0
4

0

0

0

0

0
4

0
4

0
4

0

0

0

0

0
4

0
4

0
4

0

0

0

0

0
4

0
4

0
4

0

0

0

0

0
4

0
4

0
4

0

0

0

0

0
4

0
4

0
4

0

0

0

0

0
4

0
4

0
4

0

0

0

0

0
4

0
4

0
4

0

0

0

0

0
4

0
4

0
4

0

0

0

0

0
4

0
4

0
4

0

0

0

0

0
4

0
4

0
4

0
4

0
4

0
4

0

0

0

0

0

0

0

0

0

0
4

0
4

0
4

0
4

0
4

0

0

0

0
4

0
4

0
4

0

0

0

0

0
4

0
4

0
4

0

0

0

0

0
4

0
4

0
4

0

0

0

0

0
4

0
4

0
4

0

0

0

0

0
4

0
4

0
4

0

0

0

0

0
4

0
4

0
4

0

0

0

0

0
4

0
4

0
4

0

0

0

0

0
4

0
4

0
4

0

0

0

0

0
4

0
4

0
4

0.00498723

0.00498723

0.00498723

0.00498723

0
4

0
4

0
4

0

0

0

0

0
4

0
4

0
4

2.26152
5

2.26152
5

2.26152
5

2.23555
5

0.0259677

0

0
4

0
4

0
4

0

0

0

0

0
4

0
4

0
4

0

0

0

0

0
4

0
4

0
4

0

0

0

0

0
4

0
4

0
4

0

0

0

0

0
4

0
4

0
4

0

0

0

0

0
4

0
4

0
4

0

0

0

0

0
4

0
4

0
4

0

0

0

0

0
4

0
4

0
4

0

0

0

0

0
4

0
4

0
4

0

0

0

0

0
4

0
4

0
4

0

0

0

0

0
4

0
4

0
4

0
7

0
7

0
7

0
7

0

0
4

0

0

0
4

0
4

0

0

0

0

0

0
4

0
4

0
4

0.0567688

0.0567688

0.0567688

0.0567688

0
4

0
4

0
4

0

0

0

0

0
4

0
4

0
4

0

0

0

0

0
4

0
4

0
4

0

0

0

0

0
4

0
4

0
4

0

0

0

0

0
4

0
4

0
4

0

0

0

0

0
4

0
4

0
4

0

0

0

0

0
4

0
4

0
4

0

0

0

0

0
4

0
4

0
4

0

0

0

0

0
4

0
4

0
4

0

0

0

0

0
4

0
4

0
4

0

0

0

0

0

0

0

0

0

0

0
4

0
4

0
4

0

0

0

0

0
4

0
4

0
4

0

0

0

0

0
4

0
4

0
4

0

0

0

0

0
4

0
4

0
4

0

0

0

0

0
4

0
4

0
4

0

0

0

0

0
4

0
4

0
4

0

0

0

0

0
4

0
4

0
4

0

0

0

0

0
4

0
4

0
4

0

0

0

0

0
4

0
4

0
4

0

0

0

0

0
4

0
4

0
4

0

0

0

0

0
4

0
4

0
4

0

0

0

0

0

0

0

0

0
4

0
4

0
4

0

0

0

0

0
4

0
4

0
4

0

0

0

0

0
4

0
4

0
4

0

0

0

0

0
4

0
4

0
4

0

0

0

0

0
4

0
4

0
4

0

0

0

0

0
4

0
4

0
4

0

0

0

0

0
4

0
4

0
4

0

0

0

0

0
4

0
4

0
4

0.0259677

0.0259677

0.0259677

0.0259677

0
4

0
4

0
4

0

0

0

0

0
4

0
4

0
4

0

0

0

0

0
4

0
4

0
4

0
4

0
4

0
4

0
4

0

0

0

0
4

0
4

0
4

0

0

0

0

0
4

0
4

0
4

0

0

0

0

0
4

0
4

0
4

0

0

0

0

0
4

0
4

0
4

0.0737404

0.0737404

0.0737404

0.0737404

0
4

0
4

0
4

0

0

0

0

0
4

0
4

0
4

0

0

0

0

0
4

0
4

0
4

0

0

0

0

0
4

0
4

0
4

0

0

0

0

0
4

0
4

0
4

0

0

0

0

0
4

0
4

0
4

0

0

0

0

0
4

0
4

0
4

0
4

0
4

0
4

0
4

0

0

0

0

0

0

0

0

0

0

0
4

0

0

0
4

0

0

0

0

0

0

0
4

0
4

0

0

0

0

0

0

0
4

0

0

0

0
4

0

0

0
4

0
4

0
4

0.271208
5

0.271208
5

0.271208
5

0.115749
5

0

0.122999

0

0

0.0324597

0

0
4

0

0

0
4

0
4

0
4

0

0

0

0

0
4

0
4

0
4

0

0

0

0

0
4

0
4

0
4

0

0

0

0

0
4

0
4

0
4

0

0

0

0

0
4

0
4

0
4

0

0

0

0

0
4

0
4

0
4

0

0

0

0

0
4

0
4

0
4

0

0

0

0

0
4

0
4

0
4

0

0

0

0

0
4

0
4

0
4

0

0

0

0

0
4

0
4

0
4

0

0

0

0

0
4

0
4

0
4

0
4

0
4

0
4

0

0

0

0

0

0

0

0
4

0
4

0
4

0

0

0

0

0
4

0
4

0
4

0

0

0

0

0
4

0
4

0
4

0

0

0

0

0
4

0
4

0
4

0

0

0

0

0
4

0
4

0
4

0

0

0

0

0
4

0
4

0
4

0

0

0

0

0
4

0
4

0
4

0

0

0

0

0
4

0
4

0
4

0.00736842

0.00736842

0.00736842

0.00736842

0
4

0
4

0
4

0

0

0

0

0
4

0
4

0
4

0

0

0

0

0
4

0
4

0
4

0.46427

0.46427

0.146166
6

0

0.146166

0

0
4

0.318105

0.318105

0

0

0
4

0

0

0
4

0

0

0

0
4

0

0

0
4

0

0

0
4

0
4

0
4

0

0

0

0

0
4

0
4

0
4

0.0080381

0.0080381

0.0080381

0.0080381

0
4

0
4

0
4

0

0

0

0

0
4

0
4

0
4

0

0

0

0

0
4

0
4

0
4

0

0

0

0

0
4

0
4

0
4

0

0

0

0

0
4

0
4

0
4

0.141922

0.141922

0.141922

0.141922

0
4

0
4

0
4

0

0

0

0

0
4

0
4

0
4

0.00736842

0.00736842

0.00736842

0.00736842

0
4

0
4

0
4

0

0

0

0

0
4

0
4

0
4

0

0

0

0
7

0

0

0

0
4

0
4

0
4

0

0

0

0

0
4

0
4

0
4

0

0

0

0

0
4

0
4

0
4

0

0

0

0

0
4

0
4

0
4

0

0

0

0

0
4

0
4

0
4

0

0

0

0

0
4

0
4

0
4

0

0

0

0

0
4

0
4

0
4

0

0

0

0

0
4

0
4

0
4

0

0

0

0

0
4

0
4

0
4

0

0

0

0

0
4

0
4

0
4

0

0

0

0

0
4

0
4

0
4

0.00710613
3

0.00710613
3

0
4

0

0

0

0

0
4

0

0

0
4

0

0

0
4

0.00710613

0.00710613

0

0
4

0
4

0
4

0

0

0

0

0
4

0
4

0
4

0

0

0

0

0
4

0
4

0
4

0

0

0

0

0
4

0
4

0
4

0

0

0

0

0
4

0
4

0
4

0.0157321

0.0157321

0.0157321

0.0157321

0
4

0
4

0
4

0

0

0

0

0
4

0
4

0
4

0

0

0

0

0
4

0
4

0
4

0

0

0

0

0
4

0
4

0
4

0

0

0

0

0
4

0
4

0
4

0

0

0

0

0
4

0
4

0
4

0.00475254
7

0.00475254
7

0.00475254
7

0.00475254

0

0

0
4

0

0

0

0

0

0
4

0

0

0
4

0
4

0
4

0

0

0

0

0
4

0
4

0
4

0.00498723

0.00498723

0.00498723

0.00498723

0
4

0
4

0
4

0

0

0

0

0
4

0
4

0
4

0

0

0

0

0
4

0
4

0
4

0

0

0

0

0
4

0
4

0
4

0

0

0

0

0
4

0
4

0
4

0

0

0

0

0
4

0
4

0
4

0

0

0

0

0
4

0
4

0
4

0

0

0

0

0
4

0
4

0
4

0

0

0

0

0
4

0
4

0
4

0

0

0

0

0

0

0
4

0

0

0

0
4

0
4

0

0

0

0

0
4

0

0

0
4

0
4

0
4

0

0

0

0

0
4

0
4

0
4

0

0

0

0

0
4

0
4

0
4

0

0

0

0

0
4

0
4

0
4

0

0

0

0

0
4

0
4

0
4

0

0

0

0

0
4

0
4

0
4

0

0

0

0

0
4

0
4

0
4

0

0

0

0

0
4

0
4

0
4

0

0

0

0

0
4

0
4

0
4

0

0

0

0

0
4

0
4

0
4

0

0

0

0

0
4

0
4

0
4

0
5

0
5

0
5

0
5

0

0
4

0
4

0
4

0

0

0

0

0
4

0
4

0
4

0

0

0

0

0
4

0
4

0
4

0

0

0

0

0
4

0
4

0
4

0

0

0

0

0
4

0
4

0
4

0

0

0

0

0
4

0
4

0
4

0

0

0

0

0
4

0
4

0
4

0

0

0

0

0
4

0
4

0
4

0

0

0

0

0
4

0
4

0
4

0

0

0

0

0
4

0
4

0
4

0

0

0

0

0
4

0
4

0
4

0.195035
7

0.195035
7

0.195035

0.195035

0

0
4

0

0

0

0
4

0
4

0
4

0

0

0

0

0
4

0
4

0
4

0

0

0

0

0
4

0
4

0
4

0

0

0

0

0
4

0
4

0
4

0

0

0

0

0
4

0
4

0
4

0

0

0

0

0
4

0
4

0
4

0

0

0

0

0
4

0
4

0
4

0

0

0

0

0
4

0
4

0
4

0

0

0

0

0
4

0
4

0
4

0

0

0

0

0
4

0
4

0
4

0

0

0

0

0
4

0
4

0
4

0
4

0
4

0
4

0

0

0

0
4

0
4

0
4

0
4

0

0

0
4

0

0

0
4

0
4

0
4

0

0

0

0

0
4

0
4

0
4

0

0

0

0

0
4

0
4

0
4

0

0

0

0

0
4

0
4

0
4

0

0

0

0

0
4

0
4

0
4

0

0

0

0

0
4

0
4

0
4

0

0

0

0

0
4

0
4

0
4

0

0

0

0

0
4

0
4

0
4

0

0

0

0

0
4

0
4

0
4

0

0

0

0

0
4

0
4

0
4

0

0

0

0

0
4

0
4

0
4

1.38735
7

1.38735
7

0.978225
7

0.501045
7

0.124681

0.3525
6

0

0

0

5.55111512312578e-17
7

0
4

0.409121
7

0.0604052
7

0.0699066
7

0.127078
7

0.097379
7

0.0283844

0.0129839

0.0129839

0
4

2.77555756156289e-16
7

0
4

0
4

0

0

0

0

0

0

0

0
4

0

0

0

0
4

0
4

0
4

0

0

0

0

0
4

0
4

0
4

0

0

0

0

0
4

0
4

0
4

0

0

0

0

0
4

0
4

0
4

0

0

0

0

0
4

0
4

0
4

0

0

0

0

0
4

0
4

0
4

0

0

0

0

0
4

0
4

0
4

0

0

0

0

0
4

0
4

0
4

0

0

0

0

0
4

0
4

0
4

0

0

0

0

0
4

0
4

0
4

0

0

0

0

0
4

0
4

0
4

0.328718
7

0.328718
7

0.302655
7

0.233153
6

0

0.0695014

0
4

0

0

0
4

0.026063

0.026063

0
4

0
4

0
4

0

0

0

0

0
4

0
4

0
4

0

0

0

0

0
4

0
4

0
4

0

0

0

0

0
4

0
4

0
4

0.00615279

0.00615279

0.00615279

0.00615279

0
4

0
4

0
4

0

0

0

0

0
4

0
4

0
4

0

0

0

0

0
4

0
4

0
4

0

0

0

0

0
4

0
4

0
4

0

0

0

0

0
4

0
4

0
4

0

0

0

0

0
4

0
4

0
4

0

0

0

0

0
4

0
4

0
4

0.0249362
7

0.0249362
7

0

0

0

0

0
4

0.0249362

0

0.00748085

0.0174553

0
4

0
4

0
4

0

0

0

0

0
4

0
4

0
4

0

0

0

0

0
4

0
4

0
4

0

0

0

0

0
4

0
4

0
4

0

0

0

0

0
4

0
4

0
4

0.052126
6

0.052126
6

0.052126
6

0.052126
6

0

0

0
4

0
4

0
4

0

0

0

0

0

0

0

0
4

0

0

0

0
4

0

0

0
4

0

0

0

0
4

0
4

0

0

0

0
4

0
4

0
4

0
7

0
7

0
7

0
7

0

0
4

0

0

0
4

0

0

0
4

0
4

0
4

0.538831
7

0.538831
7

0.538831
7

0.538831
7

0
4

0
4

0
4

0

0

0

0

0

0

0

0

0
4

0

0

0
4

0
4

0
4

0
4

0
4

0

0

0

0

0
4

0

0

0
4

0
4

0
4

0

0

0

0

0

0
4

0
4

0
4

1.55534
5

1.55534
5

1.32685
6

1.13335
6

0

0.039007

0

0

0

0

0

0

0

0.0763004

0.0781891

0

0

0

0

0

1.38777878078145e-16
6

0
4

0.228489
5

0.102283
7

0.126205
6

0

0

2.77555756156289e-17
5

0
4

0

0

0

0

0
4

0
4

0
4

0

0

0

0

0

0
4

0

0

0

0
4

0
4

0
4

0
4

0
4

0
4

0

0

0

0

0
4

0

0

0

0
4

0
4

0
4

0

0

0

0

0

0

0

0
4

0
4

0
4

0.024463
7

0.024463
7

0.024463
7

0.0194758

0.00498723

0
4

0
4

0
4

0.558227

0.558227

0.558227

0.104076

0.45415

0

5.55111512312578e-17

0
4

0

0

0
4

0
4

0
4

0.0129839

0.0129839

0.0129839

0.0129839

0

0

0

0

0
4

0

0

0
4

0
4

0
4

0

0

0

0

0

0

0

0
4

0
4

0
4

0

0

0

0

0

0

0
4

0
4

0
4

0.0608137

0.0608137

0.0608137

0.0608137

0
4

0
4

0
4

0.100476

0.100476

0.100476

0.0200953

0.080381

0
4

0
4

0
4

0.0120572

0.0120572

0
5

0
6

0

0

0

0

0

0

0

0

0

0
4

0.0120572
7

0.0120572
7

0

0

0
4

0

0

0

0

0

0
4

0

0

0

0
4

0
4

0

0

0

0
4

0
4

0
4

0

0

0

0

0

0

0
4

0

0

0

0
4

0
4

0

0

0

0
4

0
4

0
4

0
4

0
4

0
4

0
4

0

0
4

0
4

0
4

0.380319
7

0.380319
7

0.380319
7

0.380319
7

0

0
4

0
4

0
4

0

0

0

0

0

0
4

0

0

0
4

0
4

0
4

0

0

0

0

0

0
4

0

0

0
4

0

0

0

0
4

0

0

0
4

0
4

0
4

0

0

0

0

0

0
4

0
4

0
4

0
4

0
4

0

0

0

0
4

0

0

0

0
4

0

0

0
4

0
4

0
4

0.389004

0.389004

0.389004

0.239387

0.00498723

0.14463

2.77555756156289e-17

0
4

0
4

0
4

0
4

0
4

0

0

0
4

0

0

0
4

0

0

0
4

0
4

0
4

0
5

0
5

0
5

0
5

0
4

0
4

0
4

2.17721

2.17721

2.17721

2.17721

0

0

0
4

0

0

0
4

0

0

0
4

0
4

0
4

0

0

0

0

0
4

0

0

0

0

0
4

0

0

0
4

0

0

0
4

0

0

0
4

0

0

0
4

0

0

0
4

0

0

0
4

0
4

0
4

0

0

0

0

0

0
4

0
4

0
4

0

0

0

0

0

0

0
4

0

0

0

0
4

0
4

0
4

0

0

0

0

0
4

0
4

0
4

0

0

0

0

0

0
4

0

0

0

0
4

0
4

0
4

0

0

0

0

0

0
4

0
4

0
4

0

0

0

0

0

0

0

0

0
4

0

0

0
4

0
4

0
4

0.0195035

0.0195035

0.0195035

0

0

0.0195035

0
4

0
4

0
4

0

0

0

0

0
4

0

0

0

0
4

0
4

0
4

0.0473787
7

0.0473787
7

0.0473787

0.0473787

0
4

0

0

0
4

0
4

0
4

0

0

0

0

0

0
4

0

0

0

0
4

0
4

0
4

0

0

0

0

0

0

0

0

0

0

0

0

0

0

0
4

0

0

0

0

0

0

0

0

0
4

0

0

0

0

0

0

0

0

0

0
4

0
4

0
4

0.0943925

0.0943925

0.0943925

0.0681724

0.0157321

0.0104881

0
4

0
4

0
4

0
5

0
5

0
5

0

0

0
4

0
4

0
4

0

0

0

0

0
4

0
4

0
4

0

0

0

0

0
4

0
4

0
4

0

0

0

0

0

0
4

0
4

0
4

0

0

0

0

0
4

0
4

0
4

0

0

0

0

0

0
4

0

0

0
4

0
4

0
4

0

0

0

0

0

0

0
4

0
4

0
4

0

0

0

0

0

0
4

0
4

0
4

0

0

0

0

0

0
4

0

0

0

0
4

0
4

0
4

0.146564
7

0.146564
7

0.121627
7

0.121627
7

0
7

0
7

0

0

0

0

0

0

0
4

0.0249362

0

0.0249362

0

0
4

0

0

0
4

3.46944695195361e-18
7

0
4

0
4

0

0

0

0

0

0

0
4

0
4

0
4

0

0

0

0

0

0

0
4

0
4

0
4

0

0

0

0

0

0
4

0
4

0
4

0

0

0

0

0
4

0
4

0
4

0

0

0

0

0

0
4

0
4

0
4

0

0

0

0

0
4

0

0

0
4

0
4

0
4

0

0

0

0

0
4

0
4

0
4

0.114707

0.114707

0.114707

0

0.114707

0
4

0
4

0
4

0

0

0

0

0

0
4

0

0

0
4

0
4

0

0

0

0
4

0
4

0
4

0.0149617

0.0149617

0.0149617

0.0149617

0

0

0
4

0
4

0
4

0.736133

0
4

0
4

0

0

0

0
4

0
4

0

0

0

0
4

0
4

0

0

0

0
4

0
4

0

0

0

0
4

0
4

0

0

0

0
4

0
4

0

0

0

0
4

0
4

0

0

0

0
4

0
4

0

0

0

0
4

0
4

0

0

0

0
4

0
4

0

0

0

0
4

0
4

0

0

0

0
4

0
4

0

0

0

0

0
4

0
4

0

0

0

0
4

0
4

0

0

0

0
4

0
4

0.0347507

0.0347507

0.0347507

0
4

0
4

0

0

0

0
4

0
4

0

0

0

0
4

0
4

0

0

0

0
4

0
4

0

0

0

0
4

0
4

0

0

0

0
4

0
4

0

0

0

0
4

0
4

0

0

0

0
4

0
4

0

0

0

0
4

0
4

0

0

0

0
4

0
4

0.316689

0.316689

0.00498723

0.29674

0.0149617

0
4

0
4

0

0

0

0

0
4

0
4

0

0

0

0
4

0
4

0

0

0

0
4

0

0

0
4

0
4

0

0

0

0
4

0
4

0

0

0

0
4

0
4

0.384693
3

0.0327735
2

0
3

0
2

0

0

0

0

0

0

0.0327735

0

0

0

0
2

0

0

0

0

0

0

0

0

0

0

0

0

0

0

0

0

0

0

0

0

0

0

0

0

0

0
4

0.17954

0.0349106

0.0448851

0.0274298

0.0149617

0.0249362

0.0149617

0.00748085

0.00498723

0.00498723

2.94902990916057e-17

0
4

0

0

0
4

0.0245801

0

0.0245801

0
4

0

0

0
4

0.00997446

0.00498723

0.00498723

0
4

0

0

0
4

0

0

0
4

0

0

0
4

0

0

0
4

0

0

0
4

0

0

0
4

0.0598468

0.00498723

0.00748085

0

0.0149617

0.0274298

0.00498723

6.07153216591882e-18

0
4

0

0

0
4

0

0

0
4

0

0

0
4

0

0

0
4

0

0

0
4

0

0

0
4

0.0129839

0.0129839

0
4

0

0

0
4

0

0

0
4

0

0

0
4

0

0

0

0

0
4

0

0

0
4

0

0

0
4

0.0245801

0.0245801

0
4

0.0129839

0

0.0129839

0
4

0

0

0

0
4

0

0

0

0

0
4

0

0

0

0

0
4

0

0

0
4

0.0274298

0.0174553

0.00997446

1.73472347597681e-18

0
4

0
4

0
4

1.04688
3

0

0

0

0

0

0

0

0

0
4

0

0

0

0

0
4

0

0

0
4

0
4

0.119279
6

0.119279
6

0.119279
6

0
4

0
4

0

0

0

0
4

0
4

0

0

0

0
4

0
4

0

0

0

0
4

0
4

0

0

0

0
4

0
4

0

0

0

0
4

0
4

0

0

0

0
4

0
4

0

0

0

0
4

0
4

0

0

0

0
4

0
4

0

0

0

0
4

0
4

0

0

0

0
4

0
4

0

0

0

0

0

0

0

0
4

0

0

0

0
4

0

0

0
4

0
4

0

0

0

0
4

0
4

0

0

0

0
4

0
4

0

0

0

0
4

0
4

0

0

0

0
4

0
4

0

0

0

0
4

0
4

0

0

0

0
4

0
4

0

0

0

0
4

0
4

0

0

0

0
4

0
4

0

0

0

0
4

0
4

0

0

0

0
4

0
4

0

0

0

0

0
4

0

0

0

0
4

0
4

0

0

0

0
4

0
4

0

0

0

0
4

0
4

0

0

0

0
4

0
4

0

0

0

0
4

0
4

0

0

0

0
4

0
4

0

0

0

0
4

0
4

0.331151

0.331151

0.331151

0
4

0
4

0

0

0

0
4

0
4

0

0

0

0
4

0
4

0

0

0

0
4

0
4

0

0

0

0

0

0

0

0
4

0

0

0
4

0
4

0

0

0

0
4

0
4

0

0

0

0
4

0
4

0

0

0

0
4

0
4

0

0

0

0
4

0
4

0

0

0

0
4

0
4

0

0

0

0
4

0
4

0

0

0

0

0

0
4

0

0

0

0
4

0

0

0
4

0
4

0
3

0

0

0

0

0

0
4

0

0

0

0
4

0

0

0
4

0

0

0
4

0

0

0
4

0
4

0
2

0

0

0

0

0
4

0

0

0
4

0

0

0

0
4

0

0

0
4

0

0

0
4

0
4

0

0

0

0

0

0

0
4

0
4

0.172061

0.172061

0.172061

0

0
4

0
4

0
6

0
6

0
6

0
4

0
4

0
4

0
4

0
4

0
4

0

0

0
4

0

0

0
4

0

0

0
4

0
4

0
3

0

0

0

0

0

0
4

0

0

0
4

0

0

0
4

0
4

0
3

0

0

0

0

0
4

0

0

0
4

0

0

0
4

0

0

0
4

0

0

0
4

0
4

0

0

0

0

0
4

0

0

0
4

0
4

0

0

0

0

0
4

0
4

0

0

0

0

0
4

0
4

0

0

0

0
4

0

0

0
4

0
4

0

0

0

0
4

0

0

0

0
4

0
4

0

0

0

0

0

0
4

0

0

0
4

0
4

0
4

0
4

0

0

0

0

0

0

0
4

0
4

0
4

0

0

0

0

0

0
4

0

0

0

0
4

0
4

0
3

0
3

0

0

0

0

0
4

0

0

0

0

0
4

0

0

0
4

0
4

0

0

0

0

0
4

0

0

0
4

0
4

0

0

0

0

0

0

0
4

0
4

0.0781891
6

0.0781891
6

0.0781891
6

0
4

0
4

0

0

0

0

0
4

0

0

0

0
4

0
4

0

0

0

0

0
4

0

0

0
4

0
4

0

0

0

0

0
4

0
4

0

0

0

0

0

0
4

0

0

0
4

0
4

0

0

0

0

0

0
4

0
4

0

0

0

0

0
4

0

0

0
4

0
4

0

0

0

0

0
4

0
4

0
2

0

0

0

0

0
4

0

0

0

0

0
4

0

0

0
4

0

0

0
4

0

0

0
4

0
4

0

0

0

0

0
4

0

0

0
4

0
4

0

0

0

0
4

0
4

0

0

0

0
4

0
4

0

0

0

0
4

0
4

0.0080381

0.0080381

0.0080381

0
4

0
4

0

0

0

0

0
4

0
4

0

0

0

0
4

0

0

0
4

0
4

0

0

0

0

0
4

0

0

0
4

0
4

0

0

0

0
4

0
4

0

0

0

0
4

0
4

0

0

0

0

0

0

0

0
4

0
4

0

0

0

0
4

0

0

0
4

0
4

0

0

0

0

0
4

0

0

0
4

0
4

0

0

0

0

0
4

0
4

0

0

0

0

0
4

0
4

0

0

0

0

0
4

0
4

0

0

0

0
4

0

0

0
4

0
4

0

0

0

0

0
4

0
4

0

0

0

0
4

0

0

0
4

0
4

0

0

0

0
4

0

0

0
4

0
4

0

0

0

0
4

0
4

0
4

0

0

0

0

0

0
4

0

0

0
4

0

0

0
4

0

0

0
4

0
4

0

0

0

0
4

0
4

0

0

0

0

0
4

0
4

0

0

0

0
4

0
4

0

0

0

0

0
4

0
4

0

0

0

0
4

0

0

0
4

0
4

0

0

0

0
4

0

0

0
4

0
4

0

0

0

0
4

0

0

0
4

0
4

0

0

0

0
4

0
4

0

0

0

0
4

0
4

0

0

0

0

0
4

0
4

0

0

0

0

0
4

0

0

0

0

0
4

0

0

0
4

0
4

0

0

0

0

0
4

0
4

0

0

0

0
4

0

0

0
4

0
4

0

0

0

0
4

0

0

0
4

0
4

0

0

0

0

0
4

0
4

0

0

0

0
4

0
4

0

0

0

0
4

0
4

0

0

0

0

0
4

0
4

0

0

0

0
4

0
4

0

0

0

0
4

0
4

0

0

0

0
4

0
4

0

0

0

0

0

0
4

0

0

0
4

0

0

0
4

0

0

0

0
4

0
4

0

0

0

0
4

0
4

0

0

0

0
4

0
4

0

0

0

0
4

0
4

0

0

0

0
4

0
4

0

0

0

0
4

0
4

0

0

0

0
4

0
4

0

0

0

0
4

0
4

0

0

0

0
4

0
4

0

0

0

0
4

0
4

0

0

0

0
4

0
4

0.0843952
3

0.0843952

0

0.0843952

0
4

0

0

0

0

0
4

0

0

0
4

0

0

0
4

0
4

0

0

0

0
4

0
4

0

0

0

0
4

0
4

0

0

0

0
4

0
4

0

0

0

0
4

0
4

0.00736842

0.00736842

0.00736842

0
4

0
4

0

0

0

0
4

0
4

0

0

0

0
4

0
4

0

0

0

0
4

0
4

0

0

0

0
4

0
4

0

0

0

0
4

0
4

0.246399
3

0.0283844
3

0
3

0
3

0

0

0

0

0

0

0

0

0

0

0

0

0

0

0

0

0

0

0

0

0

0

0

0

0

0

0

0

0

0

0

0

0

0

0

0

0

0

0

0

0.0283844

0

0

0

0

0

0

0
4

0.213028
7

0.213028
7

0
4

0

0

0
4

0

0

0
4

0

0

0
4

0

0

0
4

0.00498723

0.00498723

0
4

0

0

0
4

0

0

0
4

0

0

0
4

0

0

0
4

0

0

0
4

0

0

0
4

0

0

0
4

0

0

0
4

0

0

0
4

0

0

0

0
4

0

0

0
4

0

0

0
4

0

0

0
4

0

0

0
4

1.30104260698261e-17
3

0
4

0
4

37.7002
4

0.245998
7

0.245998
7

0.198691
7

0.0473073

0

0

0

0

0

0
4

0
4

0.274383
7

0.274383
7

0.274383
7

0
4

0

0

0
4

0
4

0

0

0

0

0
4

0
4

0

0

0

0

0
4

0
4

0

0

0

0

0
4

0
4

0

0

0

0

0
4

0
4

0

0

0

0

0
4

0
4

0

0

0

0
4

0
4

0

0

0

0

0
4

0
4

0

0

0

0

0
4

0
4

0

0

0

0
4

0

0

0
4

0
4

0

0

0

0
4

0
4

0
4

0
4

0

0

0

0

0

0

0

0

0
4

0

0

0

0
4

0

0

0

0
4

0

0

0
4

0

0

0
4

0
4

0

0

0

0
4

0

0

0
4

0
4

0

0

0

0
4

0

0

0
4

0
4

0

0

0

0
4

0

0

0
4

0
4

0

0

0

0
4

0

0

0
4

0
4

0

0

0

0

0
4

0
4

0

0

0

0
4

0

0

0
4

0
4

0

0

0

0
4

0
4

0

0

0

0

0
4

0
4

0

0

0

0
4

0

0

0
4

0
4

0

0

0

0

0
4

0
4

0
7

0
7

0
7

0

0

0
4

0

0

0

0
4

0

0

0
4

0
4

0

0

0

0
4

0

0

0
4

0
4

0

0

0

0
4

0
4

0

0

0

0
4

0
4

0

0

0

0
4

0

0

0
4

0
4

0

0

0

0
4

0
4

0

0

0

0
4

0

0

0
4

0
4

0

0

0

0
4

0

0

0
4

0
4

0.015382

0.015382

0.015382

0
4

0
4

0

0

0

0

0
4

0
4

0

0

0

0
4

0

0

0
4

0
4

0
4

0
4

0

0

0

0

0

0
4

0

0

0

0
4

0

0

0

0
4

0
4

0

0

0

0
4

0
4

0

0

0

0
4

0
4

0

0

0

0
4

0

0

0
4

0
4

0

0

0

0
4

0
4

0

0

0

0
4

0
4

0

0

0

0
4

0
4

0

0

0

0
4

0

0

0
4

0
4

0

0

0

0
4

0

0

0
4

0
4

0

0

0

0
4

0

0

0
4

0
4

0

0

0

0
4

0

0

0
4

0
4

0.552434
7

0.552434
7

0.552434
7

0

0
4

0

0

0
4

0
4

0

0

0

0
4

0

0

0
4

0
4

0

0

0

0
4

0
4

0

0

0

0
4

0

0

0
4

0
4

0

0

0

0
4

0

0

0
4

0
4

0

0

0

0
4

0
4

0

0

0

0
4

0
4

0

0

0

0
4

0
4

0

0

0

0
4

0
4

0

0

0

0
4

0
4

0

0

0

0
4

0
4

0.887295
7

0.861327
6

0.720605

0.114754

0.0259677

0

0

0

1.04083408558608e-17
6

0
4

0

0

0
4

0.0259677

0.0259677

0
4

0

0

0
4

1.04083408558608e-17
7

0
4

0

0

0

0
4

0
4

0

0

0

0
4

0
4

0

0

0

0
4

0
4

0

0

0

0
4

0
4

0

0

0

0
4

0
4

0

0

0

0
4

0
4

0.0129839

0.0129839

0.0129839

0
4

0
4

0

0

0

0
4

0
4

0

0

0

0
4

0
4

0

0

0

0
4

0
4

0

0

0

0

0

0

0

0
4

0

0

0

0

0

0
4

0

0

0

0

0
4

0
4

0

0

0

0
4

0
4

0

0

0

0
4

0
4

0

0

0

0
4

0
4

0

0

0

0
4

0
4

0

0

0

0
4

0
4

0

0

0

0
4

0
4

0

0

0

0
4

0
4

0

0

0

0
4

0
4

0

0

0

0
4

0
4

0

0

0

0
4

0
4

0
5

0

0

0

0

0
4

0

0

0

0
4

0

0

0
4

0

0

0
4

0

0

0
4

0

0

0
4

0
4

0

0

0

0
4

0
4

0

0

0

0
4

0
4

0

0

0

0
4

0
4

0

0

0

0
4

0
4

0

0

0

0
4

0
4

0

0

0

0
4

0
4

0

0

0

0
4

0
4

0

0

0

0
4

0
4

0

0

0

0
4

0
4

0

0

0

0
4

0
4

0.0129839
4

0.0129839
4

0.0129839
6

0

0

0

0
4

0

0

0

0

0
4

0
4

0

0

0

0
4

0
4

0

0

0

0
4

0
4

0

0

0

0
4

0
4

0

0

0

0
4

0
4

0

0

0

0
4

0
4

0

0

0

0
4

0
4

0

0

0

0
4

0
4

0

0

0

0
4

0
4

0

0

0

0
4

0
4

0

0

0

0
4

0
4

0
4

0
4

0

0

0

0

0

0

0
4

0

0

0

0
4

0
4

0

0

0

0
4

0
4

0

0

0

0
4

0
4

0

0

0

0
4

0
4

0

0

0

0
4

0
4

0

0

0

0
4

0
4

0

0

0

0
4

0
4

0

0

0

0
4

0
4

0

0

0

0
4

0
4

0

0

0

0
4

0
4

0

0

0

0
4

0
4

0
4

0
4

0
4

0

0

0

0

0
4

0

0

0

0

0
4

0

0

0

0
4

0

0

0

0
4

0

0

0

0
4

0

0

0
4

0

0

0
4

0
4

0
4

0
4

0
4

0

0

0
4

0

0

0

0
4

0

0

0
4

0
4

0

0

0

0
4

0
4

0.0194758

0.0194758

0.0194758

0
4

0
4

0

0

0

0
4

0
4

0

0

0

0
4

0
4

0

0

0

0
4

0
4

0.0129839

0.0129839

0.0129839

0
4

0
4

0

0

0

0
4

0
4

0

0

0

0
4

0
4

0

0

0

0
4

0
4

0

0

0

0
4

0
4

0

0

0

0

0

0
4

0

0

0

0

0
4

0

0

0

0
4

0
4

0

0

0

0
4

0
4

0.0173753

0.0173753

0.0173753

0
4

0
4

0

0

0

0
4

0
4

0

0

0

0
4

0
4

0

0

0

0
4

0
4

0

0

0

0
4

0
4

0

0

0

0
4

0
4

0

0

0

0
4

0
4

0

0

0

0
4

0
4

0

0

0

0
4

0
4

0

0

0

0

0

0

0

0

0
4

0

0

0

0

0
4

0
4

0

0

0

0
4

0
4

0

0

0

0
4

0
4

0

0

0

0
4

0
4

0

0

0

0
4

0
4

0

0

0

0
4

0
4

0.245801

0.245801

0.245801

0
4

0
4

0

0

0

0
4

0
4

0

0

0

0
4

0
4

0

0

0

0
4

0
4

0

0

0

0
4

0
4

0
4

0

0

0

0

0

0
4

0

0

0

0

0
4

0

0

0
4

0

0

0
4

0
4

0

0

0

0
4

0
4

0

0

0

0
4

0
4

0

0

0

0
4

0
4

0

0

0

0
4

0
4

0

0

0

0
4

0
4

0.0245801

0.0245801

0.0245801

0
4

0
4

0

0

0

0
4

0
4

0

0

0

0
4

0
4

0

0

0

0
4

0
4

0

0

0

0
4

0
4

0
4

0

0

0

0

0
4

0

0

0

0
4

0

0

0
4

0

0

0
4

0
4

0

0

0

0
4

0
4

0

0

0

0
4

0
4

0

0

0

0
4

0
4

0

0

0

0
4

0
4

0

0

0

0
4

0
4

0

0

0

0
4

0
4

0

0

0

0
4

0
4

0

0

0

0
4

0
4

0

0

0

0
4

0
4

0

0

0

0
4

0
4

0
4

0
4

0
4

0

0
4

0

0

0
4

0

0

0
4

0
4

0

0

0

0
4

0
4

0.0173753

0.0173753

0.0173753

0
4

0
4

0

0

0

0
4

0
4

0

0

0

0
4

0
4

0

0

0

0
4

0
4

0

0

0

0
4

0
4

0

0

0

0
4

0
4

0

0

0

0
4

0
4

0

0

0

0
4

0
4

0

0

0

0
4

0
4

0

0

0

0

0
4

0

0

0

0
4

0

0

0
4

0

0

0
4

0
4

0

0

0

0
4

0
4

0

0

0

0
4

0
4

0

0

0

0
4

0
4

0

0

0

0
4

0
4

0

0

0

0
4

0
4

0

0

0

0

0

0
4

0

0

0
4

0

0

0
4

0
4

0
4

0

0

0

0
4

0

0

0

0
4

0

0

0
4

0

0

0
4

0

0

0
4

0
4

0

0

0

0

0

0

0

0

0
4

0

0

0
4

0

0

0
4

0

0

0
4

0
4

0
4

0
4

0
4

0

0

0

0

0

0

0
4

0

0

0

0
4

0
4

0

0

0

0
4

0

0

0

0

0
4

0

0

0

0
4

0

0

0
4

0
4

0
3

0
3

0

0

0

0

0

0
4

0
4

0
3

0

0

0

0

0
4

0

0

0

0
4

0

0

0
4

0
4

0

0

0

0

0
4

0

0

0

0
4

0

0

0
4

0

0

0
4

0

0

0
4

0

0

0
4

0
4

0
4

0
4

0

0

0

0

0

0
4

0
4

0

0

0

0

0

0

0

0
4

0

0

0
4

0

0

0
4

0
4

0
3

0

0

0

0
4

0

0

0

0
4

0

0

0
4

0

0

0
4

0
4

0

0

0

0

0

0
4

0
4

0

0

0

0
4

0

0

0
4

0

0

0
4

0

0

0
4

0

0

0
4

0

0

0
4

0
4

0.0163868
6

0

0

0
4

0.0163868

0.0163868

0
4

0
4

0.452353
7

0.452353
7

0.289849
7

0.134119
6

0

0

0.0283844

0

0

0
4

0
4

0

0

0

0

0

0
4

0

0

0
4

0

0

0
4

0
4

0

0

0

0

0

0
4

0
4

0
3

0
3

0
3

0
4

0

0

0
4

0

0

0
4

0
4

0

0

0

0

0

0
4

0

0

0

0

0
4

0

0

0
4

0
4

0
5

0

0

0
4

0

0

0
4

0

0

0
4

0
4

0

0

0

0

0
4

0
4

0

0

0

0

0
4

0

0

0

0

0
4

0
4

0

0

0

0

0
4

0

0

0
4

0

0

0
4

0
4

0
4

0

0

0
4

0

0

0
4

0

0

0
4

0

0

0
4

0

0

0
4

0
4

0
4

0

0

0

0
4

0

0

0
4

0

0

0
4

0

0

0
4

0
4

0
4

0
4

0

0

0

0

0

0

0

0

0
4

0
4

0

0

0

0

0

0
4

0

0

0

0
4

0

0

0

0

0
4

0

0

0
4

0

0

0

0
4

0

0

0
4

0

0

0
4

0

0

0
4

0
4

0

0

0

0

0
4

0
4

0

0

0

0
4

0

0

0
4

0

0

0
4

0

0

0
4

0

0

0
4

0
4

0
4

0

0

0

0
4

0

0

0
4

0
4

0

0

0

0

0
4

0

0

0
4

0

0

0
4

0

0

0
4

0
4

0

0

0

0

0
4

0

0

0
4

0

0

0
4

0

0

0
4

0
4

0

0

0

0

0
4

0
4

0

0

0

0

0

0
4

0

0

0
4

0
4

0

0

0

0

0
4

0

0

0
4

0
4

0

0

0

0

0

0

0
4

0

0

0
4

0
4

0

0

0

0

0
4

0

0

0
4

0
4

0
4

0
4

0
4

0

0

0

0

0
4

0

0

0

0

0
4

0
4

0

0

0

0

0
4

0

0

0

0
4

0

0

0
4

0
4

0

0

0

0

0
4

0

0

0
4

0

0

0
4

0
4

0.00615279

0.00615279

0

0.00615279

0
4

0

0

0
4

0

0

0
4

0
4

0

0

0

0

0
4

0

0

0

0
4

0
4

0

0

0

0

0
4

0

0

0
4

0

0

0
4

0
4

0.0283844

0

0

0
4

0

0

0
4

0.0283844

0.0283844

0
4

0
4

0

0

0

0
4

0

0

0
4

0

0

0
4

0
4

0

0

0

0

0
4

0
4

0

0

0

0
4

0

0

0
4

0
4

0.0283844

0.0283844

0.0283844

0
4

0

0

0
4

0

0

0
4

0

0

0
4

0
4

0.135086
6

0
7

0
7

0

0

0

0
4

0.135086
6

0.135086
6

0

0

0

0
4

0
4

0

0

0

0
4

0

0

0
4

0

0

0
4

0
4

0.204834

0.204834

0.204834

0
4

0
4

0

0

0

0
4

0

0

0
4

0

0

0
4

0
4

0

0

0

0
4

0

0

0
4

0
4

0

0

0

0

0
4

0

0

0
4

0
4

0

0

0

0

0
4

0

0

0
4

0
4

0

0

0

0
4

0
4

0

0

0

0
4

0

0

0
4

0

0

0
4

0
4

0

0

0

0

0
4

0

0

0
4

0
4

0

0

0

0

0
4

0
4

0
7

0
7

0
7

0

0

0

0
4

0

0

0
4

0
4

0

0

0

0
4

0
4

0

0

0

0

0
4

0
4

0

0

0

0
4

0

0

0
4

0
4

0

0

0

0
4

0

0

0
4

0
4

0

0

0

0
4

0

0

0
4

0

0

0
4

0
4

0.141922

0

0

0

0
4

0.141922

0.141922

0
4

0
4

0

0

0

0

0
4

0
4

0

0

0

0
4

0

0

0
4

0
4

0.0173753

0

0

0

0
4

0.0173753

0.0173753

0
4

0
4

0

0

0

0

0
4

0

0

0
4

0
4

0
4

0

0

0

0

0
4

0

0

0

0

0
4

0

0

0

0
4

0

0

0

0
4

0

0

0
4

0

0

0
4

0

0

0
4

0
4

0

0

0

0

0
4

0

0

0
4

0
4

0

0

0

0
4

0

0

0
4

0
4

0

0

0

0
4

0
4

0

0

0

0

0
4

0

0

0
4

0
4

0.0324597

0.0324597

0.0324597

0
4

0
4

0

0

0

0

0

0
4

0
4

0

0

0

0
4

0

0

0
4

0

0

0
4

0
4

0

0

0

0
4

0

0

0
4

0

0

0
4

0
4

0

0

0

0

0
4

0

0

0
4

0
4

0

0

0

0

0
4

0
4

34.2978
4

34.1447
4

5.42482
4

0.0892155
4

0.887133
4

9.22498
7

0

0

0

0

0

0

0

0

0

0

0

0

0

0

0

0

0

0

0

0

0

0

0.103687
7

0

0

0

0.0203523

0

0

0

0

0

0

0
6

0

0

0

0

0

0

0

0

0

0

0.191756
7

0

0

0

0

0

0

0

0

0

0

0
7

0

0

0

0

0

0

0

0

0

0.0194758

0.116855
7

0

0

0

0

0

0

0

0

0

0

0

0

0

0

0

0.0100064

0

0.00615279

0

0

0

0.0259677
6

0

0

0

0

0

0

0

0

0

0

0.253186
7

0

0.0473073

0

0

0.0173753

0

0.0129839

0

0

0

14.3326
7

0
4

0

0

0

0

0

0

0

0

0

0

0.090563
7

0.0194758

0

0.0189229

0

0

0

0

0

0

0

0
6

0

0

0

0

0

0

0

0

0

0

0.179768
7

0

0

0

0

0

0

0

0

0

0

0.0256563
7

0

0

0

0

0

0

0

0

0

0

0
3

0

0

0

0

0

0

0

0

0

0

0.0191367
6

0

0

0

0

0

0

0

0

0

0

0

0

0

0

0.0080381

0

0

0

0

0

0

0.0792057
6

0

0

0

0

0

0

0

0

0

0

0
6

0

0

0

0

0

0

0

0

0

0

1.03263
7

0

0

0

0

0

0

0

0

0

0

0

0.207079
6

0

0

0

0

0.0194758

0

0

0

0

0

0

0

0.0442105

0

0

0

0

0

0

0

0.0123056

0
3

0

0

0

0

0

0

0

0.00498723

0.0129839

0

0
4

0

0

0

0

0

0

0

0

0

0

0

0

0

0

0

0

0

0

0

0

0

0.0380596
6

0

0

0

0

0

0

0

0

0

0

0.00615279
7

0

0.0584274

0

0

0

0

0.00615279

0

0

0

0.0360734
7

0

0

0

0

0

0.00922919

0.0324597

0

0

0

0

0

0

0

0

0

0

0

0

0

0.0129839

0
4

0.0386125
7

0

0

0

0

0

0

0

0

0

0

0

0

0

0

0

0

0

0

0.0129839

0.129852
7

0.0851532

0.0129839

0

0.0194758

0

0

0.355695

0

0

0

0
3

0

0

0

0

0.0584274

0

0

0

0

0

0.139187
7

0

0

0.149315

0

0

0

0

0

0

0

0.30413
7

0

0

0

0.0489815

0

0

0.00615279

0

0

0

0
4

0

0

0

0

0.0129839

0

0.0129839

0

0

0

0
4

0
7

0
7

0

0

0

0
4

0

0

0

0

0
4

0

0

0
4

0.0189229

0.0189229

0
4

0

0

0
4

0

0

0
4

0

0

0
4

0

0

0

0

0

0
4

0

0

0

0
4

0

0

0

0
4

0

0

0

0
4

0

0

0

0

0
4

0

0

0

0
4

0

0

0
4

0

0

0

0
4

0

0

0
4

0

0

0

0

0

0
4

0

0

0

0
4

0

0

0
4

0

0

0
4

0

0

0

0
4

0

0

0

0
4

0

0

0
4

0

0

0

0
4

0

0

0

0
4

0

0

0

0
4

0

0

0

0
4

0.0584274
7

0

0.0584274

0

0
4

0

0

0

0
4

0

0

0
4

0

0

0

0
4

0

0

0
4

0

0

0
4

0

0

0
4

0

0

0
4

0

0

0
4

0

0

0
4

0

0

0
4

0
6

0
6

0
4

0

0

0
4

0

0

0
4

0

0

0
4

0

0

0
4

0

0

0
4

0

0

0
4

0

0

0
4

0

0

0
4

0

0

0
4

0

0

0
4

0.0378459
6

0.0378459
6

0
4

0

0

0
4

0

0

0
4

0

0

0
4

0

0

0
4

0

0

0
4

0

0

0
4

0

0

0
4

0.0378459

0.0378459

0
4

0

0

0
4

0

0

0
4

0
4

0
4

0
4

0

0

0
4

0

0

0
4

0

0

0
4

0

0

0
4

0

0

0
4

0

0

0
4

0

0

0
4

0

0

0
4

0

0

0
4

0

0

0
4

0

0

0

0

0
4

0

0

0
4

0

0

0
4

0

0

0
4

0

0

0
4

0

0

0
4

0

0

0
4

0

0

0
4

0

0

0
4

0

0

0
4

0

0

0
4

0

0

0

0

0
4

0

0

0
4

0

0

0
4

0

0

0
4

0

0

0
4

0

0

0
4

0

0

0
4

0

0

0
4

0

0

0
4

0

0

0
4

0

0

0
4

0

0

0

0
4

0

0

0
4

0

0

0
4

0

0

0
4

0

0

0
4

0

0

0
4

0

0

0
4

0

0

0
4

0

0

0
4

0

0

0
4

0

0

0
4

0
4

0
4

0.921129
4

0.714525
4

0

0

0

0

0

0
4

0.464428
4

0
4

0.0946147
4

0.0519355
3

0
4

0
4

0.188266
4

0
4

0.05094
4

0
3

0

0

0

0

0.0213184

0

0

0.0573536

0

0

0

0

0
4

0
3

0
3

0
3

0
3

0
4

0
4

0

0
4

0

0

0

0

0

0

0

0
4

0

0

0

0

0

0

0

0

0

0

0
3

0

0

0

0

0

0

0

0

0

0

0
3

0

0

0

0

0

0

0

0

0

0

0

0

0

0

0

0

0

0

0

0

0

0
3

0

0

0

0

0

0

0

0

0

0

0
4

0

0

0

0

0

0

0

0

0

0

0
4

0
4

0
4

0
5

0
5

0
4

0

0

0
4

0

0

0
4

0

0

0
4

0

0

0
4

0

0

0
4

0

0

0
4

0.20125

0.20125

0
4

0

0

0
4

0

0

0
4

0

0

0
4

0.0324597

0

0.0324597

0
4

0

0

0
4

0

0

0
4

0

0

0
4

0

0

0
4

0

0

0
4

0

0

0
4

0

0

0
4

0

0

0
4

0

0

0
4

0

0

0

0
4

0

0

0
4

0

0

0
4

0.0163868

0.0163868

0
4

0

0

0
4

0

0

0
4

0

0

0
4

5.20417042793042e-17
4

0
4

0
4

0
4

0

0

0

0

0

0

0

0

0
4

0

0

0

0
4

0

0

0
4

0
4

0
4

0

0

0

0

0
4

0

0

0
4

0
4

0
4

0

0

0
4

0

0

0
4

0

0

0
4

0

0

0
4

0
4

0

0

0

0
4

0

0

0

0
4

0

0

0
4

0
4

0

0

0

0
4

0

0

0
4

0
4

0

0

0

0

0

0

0
4

0
4

0

0

0

0
4

0

0

0
4

0

0

0
4

0
4

0

0

0

0
4

0

0

0
4

0

0

0
4

0
4

0

0

0

0

0
4

0
4

0

0

0

0

0

0
4

0
4

0

0

0

0
4

0
4

0
4

0
4

0
4

0

0
4

0

0

0

0
4

0

0

0
4

0
4

0

0

0

0
4

0

0

0
4

0

0

0
4

0
4

0

0

0

0
4

0

0

0
4

0

0

0
4

0
4

0

0

0

0

0
4

0
4

0

0

0

0

0
4

0

0

0
4

0
4

0

0

0

0

0
4

0
4

0

0

0

0

0
4

0

0

0
4

0
4

0

0

0

0
4

0
4

0

0

0

0
4

0
4

0

0

0

0

0
4

0
4

0

0

0

0
4

0
4

0
4

0
4

0
4

0

0

0
4

0

0

0

0
4

0

0

0
4

0
4

0

0

0

0
4

0
4

0.0173753

0.0173753

0.0173753

0
4

0
4

0

0

0

0
4

0
4

0

0

0

0

0
4

0
4

0

0

0

0
4

0
4

0

0

0

0
4

0

0

0
4

0
4

0.170306

0.170306

0

0.170306

0
4

0
4

0

0

0

0

0
4

0
4

0

0

0

0
4

0

0

0
4

0
4

0

0

0

0
4

0
4

0
5

0

0

0

0
4

0

0

0

0

0

0
4

0
4

0

0

0

0
4

0
4

0

0

0

0
4

0
4

0

0

0

0
4

0
4

0

0

0

0
4

0
4

0

0

0

0
4

0
4

0

0

0

0
4

0
4

0.0189229

0.0189229

0.0189229

0
4

0
4

0

0

0

0
4

0
4

0

0

0

0
4

0
4

0

0

0

0
4

0
4

0
4

0
4

0
4

0

0

0
4

0

0

0
4

0

0

0
4

0
4

0

0

0

0
4

0
4

0

0

0

0
4

0
4

0

0

0

0
4

0
4

0

0

0

0
4

0
4

0

0

0

0
4

0
4

0

0

0

0
4

0
4

0

0

0

0
4

0
4

0

0

0

0
4

0
4

0

0

0

0
4

0
4

0

0

0

0
4

0
4

0
4

0
4

0

0

0

0

0
4

0
4

0

0

0

0
4

0
4

0

0

0

0
4

0
4

0

0

0

0
4

0
4

0

0

0

0
4

0
4

0

0

0

0
4

0
4

0

0

0

0
4

0
4

0

0

0

0
4

0
4

0

0

0

0
4

0
4

0

0

0

0
4

0
4

0

0

0

0
4

0
4

0

0

0

0

0
4

0

0

0
4

0

0

0
4

0

0

0
4

0
4

0

0

0

0
4

0
4

0

0

0

0
4

0
4

0

0

0

0
4

0
4

0

0

0

0
4

0
4

0

0

0

0
4

0
4

0

0

0

0
4

0
4

0

0

0

0
4

0
4

0

0

0

0
4

0
4

0

0

0

0
4

0
4

0

0

0

0
4

0
4

0

0

0

0

0

0
4

0

0

0
4

0
4

0

0

0

0
4

0
4

0

0

0

0
4

0
4

0

0

0

0
4

0
4

0

0

0

0
4

0
4

0

0

0

0
4

0
4

0

0

0

0
4

0
4

0

0

0

0
4

0
4

0

0

0

0
4

0
4

0

0

0

0
4

0
4

0

0

0

0
4

0
4

0
4

0
4

0
4

0

0
4

0
4

0

0

0

0
4

0
4

0

0

0

0
4

0
4

0

0

0

0
4

0
4

0

0

0

0
4

0
4

0

0

0

0
4

0
4

0

0

0

0
4

0
4

0

0

0

0
4

0
4

0

0

0

0
4

0
4

0

0

0

0
4

0
4

0
4

0.895836
5

0

0

0

0

0

0
4

0

0

0
4

0
4

0

0

0

0
4

0
4

0

0

0

0
4

0
4

0

0

0

0
4

0
4

0.0519355

0.0519355

0.0519355

0
4

0
4

0.00710613

0.00710613

0.00710613

0
4

0
4

0

0

0

0
4

0
4

0

0

0

0
4

0
4

0

0

0

0
4

0
4

0

0

0

0
4

0
4

0.0129839

0.0129839

0.0129839

0
4

0
4

0
4

0
4

0

0

0

0

0
4

0

0

0

0
4

0

0

0
4

0
4

0.286768

0.286768

0.286768

0
4

0
4

0

0

0

0
4

0
4

0

0

0

0
4

0
4

0
6

0
6

0

0

0

0
4

0
4

0
7

0
7

0
7

0
4

0
4

0
6

0
6

0
6

0

0
4

0
4

0

0

0

0
4

0

0

0
4

0
4

0

0

0

0
4

0
4

0.283062

0.283062

0.266676

0.0163868

0
4

0
4

0

0

0

0
4

0
4

0.25398

0.20308
5

0.0516964

0

0

0

0.151383

0

0

0
4

0
7

0

0

0
4

0

0

0
4

0

0

0
4

0

0

0
4

0

0

0
4

0

0

0
4

0

0

0

0
4

0

0

0

0
4

0

0

0
4

0.0319776

0.0319776

0
4

0

0

0
4

0

0

0
4

0

0

0
4

0.0189229

0.0189229

0
4

0
4

0
4

2.07633910065397e-12

0
4

0.0327735

0.0327735

0.0327735

0
3

0
3

0

0

0

0

0

0
4

0
3

0
3

0

0

0

0

0

0

0

0

0
2

0
3

0

0

0

0

0

0

0
4

0

0

0

0

0
4

0
7

0

0

0

0

0

0

0

0

0

0

0
6

0

0

0

0

0

0

0

0
4

0

0

0
4

0

0

0
4

0.0327735

0.0327735

0
4

0
4

0
3

0

0

0

0
4

0

0

0

0

0

0
4

0

0

0

0

0
4

0

0

0
4

0
4

0

0

0

0
4

0
4

0

0

0

0
4

0
4

0

0

0

0
4

0
4

0

0

0

0
4

0

0

0
4

0
4

0
3

0
3

0

0

0

0
4

0

0

0

0
4

0

0

0
4

0
4

0

0

0

0

0
4

0
4

0
2

0
2

0
2

0

0
4

0
4

0

0

0

0

0
4

0
4

0

0

0

0
4

0
4

0

0

0

0
4

0
4

0

0

0

0
4

0
4

0
4

0
2

0
2

0
2

0

0

0

0

0

0
4

0
3

0

0

0

0

0

0
4

0

0

0
4

0
4

0
4

0

0

0

0

0
4

0
4

0
4

0

0

0

0

0

0
4

0
4

0
4

0

0

0

0

0

0

0
4

0
4

0
4

0

0

0

0

0
4

0
4

0
4

0

0

0

0

0
4

0
4

0
4

0
4

38.3904

38.3904

0
3

0
3

0

0

0

0

0

0

0

0

0

0

0

0
3

0

0

0

0

0

0

0

0

0

0

0
3

0

0

0

0

0

0

0

0

0

0

0
3

0

0

0

0

0

0

0

0

0

0

0

0

0

0

0

0

0

0

0

0

0

0
4

0

0

0

0
4

0

0
4

0
4

0.61468
3

0.61468
3

0.284245
1

0

0

0

0

0

0

0

0

0

0

0

0

0

0

0

0

0

0

0

0

0

0
4

0

0

0

0

0.00710613

0

0

0

0
4

0

0.309117

0

0.0142123

0

2.08166817117217e-17
3

0
4

0
4

0.20125
6

0.20125
6

0
6

0

0

0

0

0

0
7

0

0

0

0

0.0908871
5

0

0

0

0

0

0

0

0

0

0

0.110363
5

0

0

0

0

0

0

0

0

0

0

0
6

0

0

0

0

0

0
6

0
6

0
7

0
6

0
4

0

0

0
4

0
4

0
7

0
7

0
7

0
7

0
4

0
4

0.210133
3

0
3

0

0

0

0

0

0

0

0

0

0

0

0

0

0

0

0

0

0

0

0

0

0

0

0

0

0

0

0

0

0

0

0

0

0

0

0

0

0

0

0

0

0

0

0

0

0

0

0

0

0

0

0

0

0

0

0

0

0

0

0

0

0

0

0

0

0

0

0

0

0

0

0

0

0

0

0

0

0

0

0

0

0

0

0

0

0

0

0

0

0
4

0

0

0

0

0

0
4

0.0950604

0.0950604

0
4

0.115073

0.115073

0
4

0

0

0

0

0
4

0

0

0
4

0

0

0

0
4

0

0

0

0
4

0

0

0
4

0

0

0
4

0

0

0
4

0

0

0
4

0
4

0
3

0
3

0

0

0
4

0
3

0
4

0

0

0

0

0

0

0

0

0

0

0
2

0

0

0

0

0

0

0

0

0

0

0

0

0

0

0

0

0

0

0

0

0

0
4

0

0

0

0

0

0

0

0

0

0

0

0

0

0

0

0

0

0

0

0

0

0
4

0

0

0

0

0

0

0

0

0

0
4

0

0

0

0
4

0

0

0
4

0

0

0
4

0

0

0

0

0
4

0

0

0

0

0

0

0

0

0

0

0

0

0
4

0

0

0

0

0
4

0

0

0

0

0

0

0

0

0

0

0

0

0

0

0

0

0
4

0

0

0
4

0

0

0
4

0
4

37.3643
5

0.37004
6

0.37004
6

0
4

36.9943
5

36.9943
5

0
4

0
7

0

0

0

0

0

0

0

0

0

0

0

0

0

0

0

0

0

0

0

0

0

0

0

0

0

0

0

0

0

0

0

0

0

0

0

0

0

0

0

0

0

0

0

0

0

0

0

0

0

0

0

0

0

0

0

0

0

0

0

0

0

0

0

0

0

0

0

0

0

0

0

0

0

0

0

0

0

0

0
4

0

0

0

0
4

0

0

0
4

0

0

0
4

0

0

0
4

7.105427357601e-15
5

0
4

0
3

0
3

0
4

0

0

0

0

0

0

0

0

0

0
4

0
4

0
3

0
3

0
3

0

0

0

0

0

0
3

0

0

0

0

0

0

0

0
4

0

0

0
4

0

0

0
4

0
4

0

0

0

0
4

0
4

0

0

0

0
4

0
4

0

0

0

0
4

0
4

0

0

0

0
4

0
4

0

0

0

0
4

0
4

0

0

0

0
4

0
4

0

0

0

0
4

0
4

0

0

0

0
4

0
4

0

0

0

0
4

0
4

0

0

0

0
4

0
4

0
3

0
3

0
3

0
4

0
4

0

0

0

0

0

0

0

0
4

0
4

0

0

0

0

0
4

0
4

0

0

0

0

0
4

0

0

0
4

0
4

0

0

0

0
4

0

0

0
4

0
4

0

0

0

0
4

0
4

0

0

0

0

0
4

0
4

0

0

0

0
4

0

0

0
4

0
4

0
4

0
3

0
3

0
3

0
3

0
3

0

0

0

0

0
4

0

0

0
4

0

0

0

0
4

0
4

0
4

0

0

0

0

0

0

0
4

0

0

0
4

0
4

0
4

0

0

0

0

0

0
4

0
4

0
4

0

0

0

0

0

0
4

0
4

0
4

0

0

0

0

0

0
4

0
4

0
4

0

0

0

0

0
4

0

0

0
4

0
4

0
4

0

0

0

0

0
4

0
4

0
4

0

0

0

0

0

0
4

0
4

0
4

0

0

0

0

0
4

0
4

0
4

0

0

0

0

0
4

0
4

0
4

0

0

0

0

0
4

0
4

0
4

0
3

0
3

0
3

0
3

0

0

0

0
4

0
4

0
4

0

0

0

0

0
4

0
4

0
4

0

0

0

0

0
4

0
4

0
4

0

0

0

0

0
4

0
4

0
4

0

0

0

0

0
4

0
4

0
4

0

0

0

0

0
4

0
4

0
4

0

0

0

0

0
4

0
4

0
4

0

0

0

0

0
4

0
4

0
4

0

0

0

0

0
4

0
4

0
4

0

0

0

0

0
4

0
4

0
4

0

0

0

0

0
4

0
4

0
4

0

0

0

0

0

0

0

0

0
4

0

0

0
4

0
4

0
4

0

0

0

0

0
4

0
4

0
4

0

0

0

0

0
4

0
4

0
4

0

0

0

0

0
4

0
4

0
4

0

0

0

0

0

0
4

0
4

0
4

0

0

0

0

0

0

0
4

0

0

0
4

0

0

0
4

0
4

0
4

0

0

0

0

0

0
4

0
4

0
4

0

0

0

0

0

0

0

0
4

0

0

0
4

0
4

0
4

0

0

0

0

0

0

0
4

0
4

0
4

0

0

0

0

0

0

0
4

0

0

0
4

0
4

0
4

0
4

1.92738
3

1.92738
3

0
4

0
4

0
4

0
4

0
4

0

0

0

0

0

0

0

0

0

0

0

0

0

0

0

0

0

0

0

0
4

0

0

0

0

0

0

0

0

0

0

0

0

0

0

0
4

0

0

0

0
4

0
4

0

0

0

0
4

0
4

0

0

0

0
4

0

0

0

0
4

0
4

0.167809

0
1

0
1

0
4

0

0

0
4

0

0

0
4

0
4

0

0
4

0

0

0

0

0
4

0

0

0
4

0

0

0
4

0.0629283

0.0629283

0
4

0.104881

0.104881

0
4

1.38777878078145e-17

0
4

0.104252
7

0.104252
7

0.104252
7

0
4

0
4

0.319749
7

0.319749
7

0.319749
7

0

0

0

0
4

0
4

0
4

0
4

0
4

0

0

0

0

0

0

0

0
4

0

0

0
4

0

0

0
4

0
4

0
4

0
4

0

0

0

0
4

0
4

0

0

0

0
4

0
4

0

0

0

0
4

0
4

1.04881
3

1.04881
3

0

0
3

0

0
3

0

0.146833

0

0

0

0

0

0.157321

0

0

0

0

0

0.718432

0

0

0

0

0.0104881

0

0

0

0.0157321

0

0

0

0

0

0

0

0

0

0

0

0

0

0

1.66533453693773e-16
3

0
4

0

0

0
4

0
4

0.286768
4

0.286768
4

0.286768
4

0
4

0
4

0
3

0

0

0

0

0

0
4

0
4

0

0

0

0

0
4

0
4

5.55111512312578e-17
3

0
4

0

0

0

0

0
4

0
4

0
4

0

0

0

0

0
4

0
4

0
4

0
4

156.606

8.60269

0

0

0

0
4

0
4

0

0
3

0
3

0

0

0

0

0

0

0

0

0

0

0
3

0

0

0

0

0

0

0

0

0
3

0

0

0

0

0

0

0
4

0

0

0
4

0

0

0

0

0

0

0
4

0
4

8.60269

7.65208

0

4.12645

0

0.0328506

0.0209761

0.0262201

0

0.0209761

0

0

0.0104881

0.0157321

0.625912

0

0.349379

0.0627712

2.05502

0

0

0.17346

0.131854

2.4980018054066e-16

0
4

0.950609

0.950609

0

0

0

0

0
4

2.22044604925031e-16

0
4

0

0

0

0
4

0
4

0

0

0

0
4

0
4

0
4

0.0104881
1

0

0

0

0

0

0

0

0

0

0

0

0

0

0

0
4

0

0

0

0

0

0

0
4

0

0

0
4

0

0

0
4

0
4

0.0104881

0.0104881

0.0104881

0
4

0
4

0

0

0

0
4

0
4

0

0

0

0

0

0

0

0

0
4

0

0

0
4

0

0

0
4

0
4

0
8

0
8

0

0

0

0
4

0

0

0
4

0
4

0

0

0

0

0

0
4

0

0

0
4

0
4

0

0

0

0
4

0

0

0
4

0
4

0

0

0

0

0

0
4

0

0

0
4

0
4

0

0

0

0

0

0
4

0
4

0

0

0

0
4

0

0

0
4

0
4

0

0

0

0
4

0
4

0
4

0.100064
3

0.100064
3

0
3

0

0

0

0

0

0

0

0

0

0

0

0

0

0

0

0

0

0

0

0

0

0

0

0

0

0

0

0

0

0

0

0

0

0

0

0

0

0

0

0

0

0

0

0

0

0

0

0

0

0

0

0

0

0

0

0

0

0
4

0
4

0
4

0
4

0

0

0

0

0

0

0

0

0

0

0
4

0

0

0

0

0

0

0

0

0

0

0
4

0

0

0

0

0

0

0

0

0

0

0
4

0

0

0

0

0

0

0

0

0

0

0

0

0

0

0

0

0

0

0

0

0

0
4

0

0

0

0

0

0

0

0

0

0

0
4

0

0

0

0

0

0

0

0

0

0

0
4

0

0

0

0

0

0

0

0

0

0

0
4

0

0

0

0

0

0

0

0

0

0

0
4

0

0

0

0

0

0

0

0

0

0

0
4

0
4

0

0

0

0

0

0

0

0

0

0

0
3

0
4

0
4

0
3

0

0
4

0

0

0
4

0

0
3

0

0

0

0

0

0

0

0

0
4

0

0

0

0

0

0

0

0

0

0

0
4

0

0

0

0

0

0

0

0

0

0

0
4

0

0

0

0

0

0

0

0

0

0

0
4

0

0

0

0

0

0

0

0

0

0

0
4

0

0

0

0

0

0

0

0

0

0

0
4

0

0

0

0

0

0

0

0

0

0

0
4

0
2

0
2

0

0

0
4

0
2

0

0

0

0

0

0

0

0

0

0

0

0

0

0

0

0

0

0

0

0

0

0

0

0

0

0

0

0

0

0

0

0

0

0

0

0

0

0

0

0

0

0

0

0

0

0
4

0

0

0

0

0
4

0

0

0

0

0

0

0

0

0

0

0

0
4

0

0

0
4

0

0

0
4

0

0

0
4

0

0

0
4

0

0

0

0

0

0

0

0
4

0

0

0

0
4

0

0

0

0
4

0

0

0

0

0
4

0

0

0

0
4

0

0

0
4

0.100064

0.100064

0
4

0

0

0
4

0
4

0

0

0

0
4

0

0

0
4

0
4

0

0

0

0

0
4

0
4

0

0

0

0
4

0
4

0

0

0

0
4

0
4

0
4

0.294962
3

0.294962
3

0.294962
3

0
2

0

0

0

0

0

0

0

0

0

0

0

0

0
3

0

0

0

0

0

0

0

0

0

0

0

0

0

0

0

0

0

0

0

0

0

0

0

0

0

0

0

0

0

0

0

0

0.065547

0

0

0.0491603

0

0

0

0

0

0

0

0

0

0

0

0

0

0

0

0

0

0

0

0

0

0

0

0

0

0

0

0

0

0
4

0

0

0

0

0

0

0

0

0

0

0.180254

0

0

0

0

0

0

0

0

0

0

0
4

0
4

0

0
4

0

0
4

0

0

0
4

0

0

0
4

0

0

0
4

0

0

0

0
4

0
4

0
4

0
4

0

0

0
4

0

0

0

0
4

0

0

0
4

0

0

0
4

0

0

0
4

0

0

0
4

0
4

0

0

0

0

0

0

0
4

0

0

0
4

0
4

0

0

0

0
4

0

0

0
4

0
4

0

0

0

0

0

0
4

0
4

0

0

0

0
4

0
4

0

0

0

0
4

0
4

0

0

0

0
4

0
4

0

0

0

0
4

0
4

0

0

0

0
4

0
4

0

0

0

0
4

0
4

0

0

0

0
4

0
4

0

0

0

0
4

0
4

0

0

0

0

0
4

0

0

0
4

0
4

0

0

0

0
4

0
4

0

0

0

0
4

0
4

0

0

0

0
4

0
4

0

0

0

0
4

0
4

0

0

0

0
4

0
4

0

0

0

0
4

0
4

0

0

0

0
4

0
4

0

0

0

0
4

0
4

0

0

0

0
4

0
4

0

0

0

0
4

0
4

0

0

0

0

0

0

0
4

0

0

0
4

0
4

0

0

0

0
4

0

0

0

0

0
4

0
4

0

0

0

0

0
4

0
4

0

0

0

0

0

0
4

0
4

0

0

0

0

0
4

0
4

0

0

0

0
4

0
4

0

0

0

0
4

0
4

0
4

19.4032

0.0950508
4

0.0950508
5

0

0

0

0

0.0427729

0

0

0

0

0

0.052278

0
4

0

0

0

0
4

0

0

0
4

0
4

2.43921
7

0
8

0

0
8

0
4

0.0487588
1

0.0487588
1

0

0

0

0
4

0
1

0

0

0

0

0

0

0

0

0

0

0

0

0

0
4

0

0

0
4

2.39045
7

0.893842
7

0.682076
7

0.177838
7

0

0

0

0

0

0

0

0

0

0

0.039007
7

0

0

0

0

0

0

0

0

0

0

0.578209
7

0

0

0

0

0
7

0
7

0

0

0

0.0194758

3.67761376907083e-16
7

0
4

0

0

0
7

0

0

0

0

0

0

0

0

0

0

0

0
4

0

0

0

0
4

0

0

0

0
4

0

0

0
4

0

0

0
4

0
4

0
7

0
7

0
7

0

0

0
4

0
7

0
6

0

0
4

0
4

16.752

0.0355306
1

0

0.0248714

0

0

0

0

0

0

0

0

0

0

0

0

0

0

0

0

0

0

0

0

0

0

0

0

0

0

0

0

0

0

0

0

0

0

0

0

0

0

0

0

0

0

0

0

0

0

0

0

0

0

0

0

0

0

0.0106592

0

1.73472347597681e-18
1

0
4

7.42232

0

6.74714

0.0195035

0.53865

0

0

0.0292553

0.0195035

0

0.0487588

0.0195035

7.91033905045424e-16

0
4

9.29411

0.0877659

0.117021

0

0.0292553

0

0

0

0.0195035

8.50511

0.146276
7

0.202659

0
5

0.052126

0

0.0856377

0.0487588

3.25434124093249e-15

0
4

0

0

0
4

0

0

0
4

0
4

0.0780141

0.0780141

0.0292553

0

0.0292553

0.0195035

0
4

0

0

0
4

0
4

0

0

0

0
4

0
4

0

0

0

0
4

0
4

0

0

0

0
4

0
4

0.0195035

0.0195035

0.0195035

0
4

0
4

0.0195035

0.0195035

0.0195035

0

0
4

0

0

0
4

0
4

0

0

0

0

0

0
4

0
4

0

0

0

0

0
4

0

0

0
4

0
4

0

0

0

0
4

0
4

0

0

0

0
4

0
4

0

0

0

0

0
4

0
4

0

0

0

0
4

0
4

0

0

0

0
4

0
4

0
7

0
7

0
7

0
4

0
4

0
4

52.1603
3

0.0639551
3

0

0

0
4

0
3

0
3

0

0

0

0
4

0.0639551
2

0.0639551
2

0
2

0
2

0

0

0

0

0

0
4

0

0

0

0
4

0

0

0

0
4

0

0

0

0
4

0

0

0
4

0

0

0
4

0

0

0
4

0

0

0
4

0
4

0.485181
3

0.417008
7

0.417008
6

0

0
4

0
3

0
3

0
4

0
3

0
3

0
2

0
4

0
3

0

0
3

0

0

0

0

0

0

0

0

0

0

0

0

0

0

0

0

0

0

0

0

0
4

0.0681724
3

0

0

0

0

0

0

0

0

0

0

0

0

0

0.0367082
7

0.0314642
7

0

0

0

0

0

0
4

0

0

0

0
4

0

0

0
4

0

0

0
4

0

0

0
4

0

0

0
4

0
4

3.23038
3

1.46881

1.46881

0

0

0

0

0

0
4

0
5

0

0

0

0

0

0

0

0

0

0
4

1.27903

1.00878

0.107074
6

0

0

0.150193
6

0.0129839

0

0

0

0

0

0

1.11022302462516e-16

0
4

0.482535
3

0
3

0.482535
4

0
4

0

0

0

0

0

0
4

0

0

0

0

0

0

0

0
4

0

0

0

0
4

0

0

0

0
4

0

0

0
4

0

0

0
4

0

0

0
4

0

0

0
4

0

0

0

0

0
4

0

0

0

0
4

0

0

0
4

0

0

0
4

0

0

0
4

0

0

0
4

0

0

0
4

0

0

0
4

0
4

0.776352
4

0

0

0
4

0

0

0

0
4

0.40927
4

0.324216
4

0.085054
4

0
4

0

0

0

0

0

0

0

0

0

0

0
4

0

0

0

0

0

0

0

0

0

0

0
4

0

0

0

0

0

0

0

0

0

0

0
4

0

0

0

0

0

0

0

0

0
4

0

0

0
4

0

0

0
4

0

0

0

0

0

0
4

0
4

0

0

0

0
4

0

0

0

0

0

0

0

0

0

0

0
4

0

0

0

0

0

0

0

0

0

0

0
4

0

0

0

0

0

0

0

0

0

0

0
4

0

0

0

0

0

0

0

0

0

0

0
3

0

0

0

0

0

0

0

0

0

0

0

0

0

0

0

0

0

0

0

0

0

0
4

0

0

0

0

0

0

0

0

0

0

0
4

0

0

0
4

0

0

0
4

0

0

0
4

0

0

0
4

0

0

0
4

0

0

0
4

0

0

0
4

0

0

0
4

0

0

0
4

0

0

0
4

0

0

0
4

0

0

0

0

0
4

0.367082

0.367082

0
4

0

0

0
4

0

0

0
4

0

0

0
4

0

0

0
4

0

0

0
4

0

0

0
4

0

0

0
4

0

0

0
4

0

0

0
4

0

0

0

0
4

0

0

0
4

0

0

0
4

0

0

0
4

0

0

0
4

0

0

0

0
4

0

0

0
4

0

0

0
4

0

0

0
4

5.55111512312578e-17
4

0
4

36.0235

3.76899
6

1.11685
6

2.37982
6

0.128765

0.117482
6

0.026063

0

0

0

2.08166817117217e-16
6

0
4

0
3

0

0

0

0

0

0

0

0

0

0

0

0

0

0

0

0

0

0

0

0

0

0

0

0

0

0

0

0

0

0

0

0
4

0
3

0
3

0
2

0

0

0

0
2

0

0

0

0

0

0

0

0
4

0
2

0
2

0

0

0

0

0

0

0
2

0

0

0

0

0

0

0

0
4

9.72925

0.778975
3

0.703579
7

0
3

0

8.16497

0.0817205
2

0

0

0

0

0

0

0

0

0

0

0

0

0

0

0

0

0

0

0

0

0

0

0

0

0

0

1.26287869051112e-15

0
4

11.8471

11.8471

0

0

0

0

0

0

0

0
4

10.6782
6

4.11524
6

0.107269

0

0

0

0.0195035

0

4.82712

0

0

0

0.136525

0

0

0.039007

0

0

0

0

0

0.0195035

0

0.0195035

0.672872

0

0

0

0

0

0

0

0

0.0975176

0.136525

0

0

0

0

0

0.0195035

0

0

0

0

0

0

0

0

0.0585106

0

0

0

0

0

0

0

0

0

0

0

0

0.292553

0

0

0

0

0.0780141

0

0

0

0

0

0

0

0

0

0

0
6

0

0

0

0

0

0

0

0

0

0

0.0195035

0.0195035

6.10622663543836e-16
6

0
4

0
3

0
3

0
2

0

0

0

0

0

0

0

0

0

0

0
3

0

0

0

0

0

0

0

0

0

0

0
2

0

0

0

0

0

0

0

0

0

0

0

0

0

0

0

0

0

0

0

0

0

0

0

0

0

0

0

0

0

0

0

0

0

0

0

0

0

0

0

0

0

0

0

0

0

0

0

0

0
2

0

0
4

0
3

0

0

0
4

0

0

0

0

0

0

0

0

0

0

0
4

0

0

0

0

0

0

0
4

0

0

0

0

0

0

0
4

0

0

0

0
4

0

0

0
4

0

0

0
4

0

0

0
4

0

0

0
4

0

0

0
4

0

0

0
4

0

0

0
4

0

0

0
4

0

0

0
4

0

0

0
4

0

0

0
4

0

0

0
4

0

0

0
4

0

0

0
4

0

0

0
4

0

0

0
4

0

0

0

0
4

0

0

0
4

0

0

0
4

0

0

0
4

0

0

0
4

0
4

0.0173753
3

0

0

0
4

0
3

0
2

0

0

0

0

0
4

0
3

0
3

0

0

0

0

0

0

0

0

0

0
4

0

0

0
4

0

0

0
4

0

0

0
4

0

0

0
4

0

0

0
4

0.0173753

0

0.0173753

0
4

0

0

0
4

0

0

0
4

0

0

0
4

0

0

0
4

0

0

0
4

0
4

0.00710613

0

0

0

0
4

0.00710613
2

0

0.00710613

0

0
4

0

0

0
4

0

0

0
4

0

0

0
4

0
4

0.664364
6

0.399697
6

0.338883
6

0.0608137

0

0

6.93889390390723e-18
6

0
4

0.171175
7

0.0813615
6

0.0604427
7

0.0163868

0

0

0.0129839

0

0

0

1.04083408558608e-17
7

0
4

0.0934919
6

0

0.0457574

0.0129839

0

0

0

0

0.0347507

0
4

1.66533453693773e-16
6

0
4

0

0

0

0
4

0
4

0

0

0

0
4

0

0

0
4

0
4

0

0

0

0

0

0
4

0

0

0
4

0
4

0

0

0

0

0

0
4

0

0

0
4

0

0

0
4

0
4

0

0

0

0

0
4

0

0

0
4

0

0

0
4

0
4

0

0

0

0

0
4

0

0

0
4

0

0

0
4

0
4

0

0

0

0

0

0
4

0
4

0

0

0

0

0
4

0
4

0

0

0

0

0
4

0
4

0

0

0

0

0
4

0
4

0
4

0
4

0
4

0

0

0
4

0

0

0
4

0
4

0

0

0

0

0
4

0
4

0

0

0

0
4

0
4

0

0

0

0

0
4

0
4

0

0

0

0
4

0
4

0

0

0

0

0
4

0
4

0

0

0

0
4

0
4

0

0

0

0
4

0
4

0

0

0

0

0
4

0
4

0

0

0

0

0
4

0
4

0

0

0

0
4

0
4

0
3

0
3

0
3

0
4

0

0

0

0

0
4

0

0

0

0
4

0

0

0
4

0
4

0

0

0

0
4

0
4

0

0

0

0
4

0
4

0

0

0

0
4

0
4

0

0

0

0
4

0
4

0

0

0

0
4

0
4

0

0

0

0
4

0
4

0

0

0

0
4

0
4

0

0

0

0
4

0
4

0

0

0

0
4

0
4

0

0

0

0
4

0
4

0
3

0
3

0
3

0

0

0

0
4

0
4

0

0

0

0
4

0
4

0

0

0

0
4

0
4

0

0

0

0
4

0
4

0

0

0

0
4

0
4

0

0

0

0
4

0
4

0

0

0

0
4

0
4

0

0

0

0
4

0
4

0

0

0

0
4

0
4

0

0

0

0
4

0
4

0

0

0

0
4

0
4

0

0

0

0

0
4

0
4

0

0

0

0
4

0
4

0

0

0

0
4

0
4

0

0

0

0
4

0
4

0

0

0

0
4

0
4

0

0

0

0
4

0
4

0

0

0

0
4

0
4

0

0

0

0
4

0
4

0

0

0

0
4

0
4

0

0

0

0
4

0
4

0

0

0

0
4

0
4

0

0

0

0

0

0

0
4

0
4

0

0

0

0
4

0
4

0.0419555
7

0.0419555
7

0

0.0419555

0
4

0
4

0

0

0

0
4

0
4

0
2

0
2

0

0

0
4

0
4

1.49978

1.39674

0
2

0
2

0.0627852

0

0.0106592
3

0
6

0

0.080381

0

0.00710613
2

0

0.0390837
7

0.0284245

0.177544
7

0

0

0

0.0199931

0

0

0.0110526

0

0

0

0.424949
7

0

0

0

0

0

0

0

0

0

0.021022

0.200374

0

0

0

0

0

0

0

0

0

0

0.111229
7

0

0

0

0

0

0

0

0

0

0

0

0

0

0

0

0

0

0

0

0

0

0
2

0

0

0

0.104751

0.0973823
5

0
4

0.0959327
2

0.0959327
2

0

0

0

0

0

0
4

0

0

0

0
4

0

0

0
4

0

0

0
4

0
2

0
2

0

0

0
4

0

0

0

0

0
4

0.00710613
2

0

0.00710613

0

0
4

0

0

0
4

0

0

0
4

0

0

0

0
4

0

0

0
4

0

0

0

0
4

0
4

0.274589
4

0
4

0
4

0

0
4

0.248621
7

0.248621
7

0
7

0

0

0

0

0

0
4

0

0

0
4

0.0259677

0

0.0259677

0
4

0

0

0
4

0

0

0
4

0

0

0
4

0

0

0
4

0

0

0
4

0

0

0
4

0

0

0
4

1.04083408558608e-17
4

0
4

0.0434384

0

0

0

0

0

0

0

0

0

0

0

0

0

0
4

0.0434384
3

0.0173753
3

0.026063

0
4

0

0

0
4

0
4

0.188448

0.114707

0.114707

0
4

0.0245801

0.0245801

0
4

0

0

0
4

0

0

0
4

0.0491603

0.0491603

0
4

0

0

0
4

0

0

0
4

0
4

0.230146

0.230146

0

0

0

0

0

0

0

0.100064

0

0.130083

0

0

0

0

0

0
4

0

0

0
4

0

0

0
4

0

0

0
4

0

0

0
4

0
4

0
3

0
3

0
3

0
4

0
3

0

0
3

0
4

0

0

0

0

0

0
3

0

0

0

0

0

0

0

0

0

0

0
3

0

0

0

0

0

0

0

0

0

0

0
3

0

0

0

0

0

0

0

0
3

0
3

0
3

0
3

0

0
4

0
2

0
2

0

0

0

0

0
4

0

0

0
4

0

0

0
4

0

0

0
4

0

0

0
4

0
2

0

0

0

0

0

0

0
4

0

0

0

0
4

0

0

0
4

0

0

0
4

0

0

0

0
4

0

0

0

0
4

0

0

0

0
4

0

0

0
4

0
4

0
3

0
3

0
2

0

0

0

0

0

0

0

0

0

0

0
2

0

0

0

0

0

0

0

0

0

0

0
2

0

0
2

0

0

0

0
4

0

0

0

0
4

0

0

0
4

0

0

0
4

0

0

0
4

0

0

0
4

0

0

0
4

0

0

0
4

0

0

0
4

0

0

0
4

0
4

8.20176

8.20176

0.00498723
2

8.19678

0

0
4

0

0

0

0

0

0
4

0

0

0

0

0
4

0

0

0

0
4

0

0

0
4

0

0

0
4

0

0

0
4

0
4

0.411968

0.357876

0.357876

0
4

0.0327735

0.0327735

0
4

0.0213184

0.0213184

0
4

4.16333634234434e-17

0
4

0
4

0
2

0
2

0

0

0

0

0

0

0
4

0
2

0
2

0
4

0

0

0
4

0

0

0
4

0

0

0
4

0
4

0

0

0

0
4

0
4

0
4

13.6369
4

0

0

0

0

0
4

0
4

0.139003
2

0
2

0
2

0

0
2

0

0

0

0

0

0
4

0.139003

0.139003

0
4

0

0

0

0

0
4

0

0

0

0
4

0
4

0.397501
4

0.377406
4

0.369367
7

0

0

0

0
7

0

0

0

0

0.0080381

0

0
4

0

0

0

0

0

0

0

0

0

0

0

0

0

0

0

0
4

0

0

0

0

0
4

0.0200953

0.0080381

0

0.0120572

0
4

0

0

0

0
4

0

0

0
4

0

0

0
4

0
4

0.0519355
7

0.0519355
7

0.0519355

0

0

0

0
4

0

0

0

0
4

0
4

0.0129839

0.0129839

0.0129839

0
4

0
4

0

0

0

0
4

0
4

0

0

0

0
4

0
4

0

0

0

0
4

0
4

0

0

0

0
4

0
4

0

0

0

0
4

0
4

0

0

0

0
4

0
4

0

0

0

0
4

0
4

0

0

0

0
4

0
4

0.0389516

0.0389516

0.0389516

0
4

0
4

0.106288
5

0.106288
7

0.106288
7

0
4

0

0

0
4

0

0

0
4

0
4

0

0

0

0
4

0
4

0

0

0

0
4

0
4

0

0

0

0
4

0
4

0

0

0

0
4

0
4

0.0129839

0.0129839

0.0129839

0
4

0
4

0

0

0

0
4

0
4

0

0

0

0
4

0
4

0

0

0

0
4

0
4

0

0

0

0
4

0
4

0

0

0

0
4

0
4

0.166937
7

0.166937
7

0.142823
7

0

0.0241143

0

0

0

0

0

1.04083408558608e-17
7

0
4

0
4

0.0851532

0.0851532

0.0851532

0
4

0
4

0

0

0

0
4

0
4

0

0

0

0
4

0
4

0

0

0

0
4

0
4

0

0

0

0
4

0
4

0

0

0

0
4

0
4

0

0

0

0
4

0
4

0

0

0

0
4

0
4

0

0

0

0
4

0
4

0

0

0

0
4

0
4

0.0567692

0.0194758
7

0

0.0194758

0

0

0
4

0.0292553

0

0

0.0292553

0
4

0.0080381

0.0080381

0
4

0

0

0
4

1.73472347597681e-18

0
4

0

0

0

0
4

0
4

0

0

0

0
4

0
4

0

0

0

0
4

0
4

0

0

0

0
4

0
4

0

0

0

0
4

0
4

0

0

0

0
4

0
4

0

0

0

0
4

0
4

0

0

0

0
4

0
4

0

0

0

0
4

0
4

0

0

0

0
4

0
4

0.00498723

0

0

0

0

0

0
4

0

0

0

0
4

0

0

0

0
4

0

0

0
4

0.00498723

0.00498723

0
4

0

0

0
4

0

0

0
4

0
4

0

0

0

0
4

0
4

0

0

0

0
4

0
4

0

0

0

0
4

0
4

0

0

0

0
4

0
4

0

0

0

0
4

0
4

0.0274298

0.0274298

0.0274298

0
4

0
4

0

0

0

0
4

0
4

0

0

0

0
4

0
4

0

0

0

0
4

0
4

0

0

0

0
4

0
4

0

0

0

0

0

0
4

0
4

0

0

0

0
4

0
4

0

0

0

0
4

0
4

0.0649194
7

0.0649194
7

0.0649194
7

0

0
4

0

0

0
4

0

0

0
4

0
4

0.0223626
5

0.00498723
7

0

0

0

0.00498723

0
4

0

0

0
4

0.0173753

0.0173753

0
4

0
4

0

0

0

0

0
4

0

0

0

0
4

0

0

0

0
4

0

0

0
4

0
4

0

0

0

0

0

0

0
4

0

0

0

0
4

0
4

0

0

0

0

0

0

0

0

0

0

0

0

0

0

0

0

0

0

0

0

0
4

0

0

0

0

0

0

0
4

0
4

0

0

0

0

0
4

0
4

0.0104881

0

0

0

0

0

0
4

0.0104881

0.0104881

0
4

0
4

0
4

0

0

0

0
4

0

0

0
4

0

0

0
4

0
4

0

0

0

0

0

0

0
4

0

0

0

0
4

0
4

0
3

0
3

0
3

0

0
4

0

0

0
4

0
4

0

0

0

0
4

0
4

0
2

0

0

0
4

0

0

0
4

0

0

0
4

0
4

0

0

0

0

0
4

0

0

0
4

0
4

0
6

0

0

0

0

0
4

0

0

0

0
4

0
4

0

0

0

0

0

0
4

0
4

0
4

0
4

0
4

0

0

0

0
4

0

0

0

0

0

0

0

0
4

0

0

0
4

0
4

0

0

0

0

0
4

0
4

0

0

0

0

0

0
4

0

0

0

0
4

0
4

0.33156
5

0.33156
5

0.33156
5

0
4

0
4

0

0

0

0
4

0

0

0
4

0

0

0
4

0

0

0
4

0

0

0
4

0
4

0
2

0

0

0

0

0
4

0

0

0
4

0

0

0
4

0
4

0

0

0

0
4

0
4

0

0

0

0

0
4

0

0

0
4

0

0

0
4

0

0

0
4

0
4

0

0

0

0

0
4

0
4

0.16879

0.16879

0.16879

0

0

0
4

0
4

0

0

0

0
4

0
4

0.471207
4

0

0

0

0

0

0

0

0

0

0
4

0.471207

0.471207

0

0

0

0
4

0

0

0

0

0
4

0

0

0
4

0

0

0
4

0
4

0

0

0

0
4

0
4

0

0

0

0

0
4

0

0

0
4

0
4

0.103039

0.103039

0.0959327

0.00710613

4.33680868994202e-18

0
4

0
4

0

0

0

0

0
4

0
4

0

0

0

0

0
4

0

0

0

0
4

0
4

0

0

0

0

0

0
4

0
4

0.0104881

0.0104881

0.0104881

0

0
4

0

0

0
4

0
4

0

0

0

0
4

0
4

0

0

0

0
4

0

0

0

0
4

0
4

0

0

0

0

0
4

0
4

0.297372
6

0.297372
6

0.297372
6

0

0

0
4

0
4

0

0

0

0

0
4

0
4

0.165066

0.165066

0.165066

0
4

0
4

0

0

0

0
4

0

0

0
4

0
4

0

0

0

0
4

0
4

0

0

0

0
4

0
4

0.0163868

0.0163868

0.0163868

0

0

0
4

0
4

0

0

0

0
4

0

0

0
4

0
4

0

0

0

0

0
4

0

0

0
4

0
4

0

0

0

0

0
4

0
4

0

0

0

0
4

0

0

0
4

0

0

0
4

0
4

0.0655581
3

0.0477016

0.029674

0.00736842
3

0.0106592

0

0
4

0.0178565

0.0178565

0

0
4

0

0

0
4

0

0

0
4

1.73472347597681e-17
3

0
4

0

0

0

0

0
4

0

0

0
4

0
4

0.103871

0.103871

0.103871

0
4

0
4

0

0

0

0

0
4

0

0

0
4

0
4

0

0

0

0
4

0
4

0

0

0

0
4

0

0

0
4

0
4

0

0

0

0
4

0
4

0

0

0

0
4

0

0

0
4

0
4

0

0

0

0
4

0

0

0
4

0
4

0

0

0

0
4

0

0

0
4

0
4

0

0

0

0
4

0
4

0.524958
6

0.524958
6

0.524958
6

0

0
4

0
4

0

0

0

0
4

0
4

0

0

0

0

0
4

0
4

0

0

0

0
4

0

0

0
4

0
4

0

0

0

0

0
4

0
4

0

0

0

0
4

0
4

0

0

0

0
4

0

0

0
4

0
4

0

0

0

0
4

0

0

0
4

0
4

0.0347507

0.0347507

0.0347507

0

0
4

0
4

0

0

0

0
4

0
4

0

0

0

0
4

0
4

0.0471078
2

0.0471078
2

0.0257895

0
2

0

0

0

0.0213184

0

3.46944695195361e-18
2

0
4

0

0

0
4

0
4

0

0

0

0

0
4

0
4

0

0

0

0

0
4

0
4

0

0

0

0
4

0
4

0

0

0

0
4

0
4

0

0

0

0
4

0
4

0

0

0

0
4

0
4

0

0

0

0
4

0
4

0

0

0

0
4

0
4

0.120804

0.120804

0.120804

0
4

0
4

0

0

0

0
4

0
4

0.0714259

0.0609378

0.0209761
6

0.0294737

0

0

0.0104881

6.93889390390723e-18

0
4

0

0

0

0

0
4

0

0

0
4

0.0104881

0.0104881

0
4

3.46944695195361e-18

0
4

0

0

0

0
4

0
4

0

0

0

0
4

0
4

0

0

0

0
4

0
4

0

0

0

0
4

0
4

0

0

0

0
4

0
4

0

0

0

0
4

0
4

0

0

0

0
4

0
4

0

0

0

0
4

0
4

0

0

0

0
4

0
4

0

0

0

0
4

0
4

9.90583

9.63597

4.15923

4.29424

0.708065
2

0
3

0
3

0

0.110363

0.0434384

0

0

0

0

0.00498723

0

0.0118814

0
3

0

0

0

0

0

0

0

0

0

0

0

0

0

0

0.0080381

0

0

0

0

0

0

0.204834

0

0

0

0

0

0

0

0

0

0

0.0908871

0

0

0

0

0

0

6.38378239159465e-16

0
4

0

0

0

0
4

0

0

0
4

0

0

0

0
4

0

0

0
4

0

0

0
4

0

0

0

0
4

0

0

0
4

0

0

0
4

0

0

0
4

0.0157321

0

0.0157321

0
4

0

0

0
4

0

0

0
4

0

0

0
4

0

0

0
4

0

0

0
4

0

0

0
4

0

0

0
4

0

0

0
4

0

0

0
4

0

0

0
4

0

0

0
4

0

0

0
4

0

0

0

0

0

0

0
4

0

0

0
4

0.0173753

0.0173753

0
4

0

0

0
4

0

0

0
4

0

0

0
4

0.0124681

0.0124681

0
4

0

0

0
4

0

0

0
4

0

0

0
4

0

0

0
4

0
3

0
3

0
4

0

0

0
4

0

0

0
4

0

0

0
4

0

0

0
4

0

0

0
4

0

0

0
4

0

0

0
4

0

0

0

0
4

0.224291

0.224291

0
4

0

0

0

0
4

0

0

0

0
4

0

0

0

0

0
4

9.15933995315754e-16

0
4

1.77635683940025e-15
4

0
4

0.0189229

0.0189229

0.0189229

0.0189229

0

0

0
4

0

0

0
4

0
4

0
4

1.63292
3

0.141922
3

0
3

0

0

0

0

0

0

0

0

0

0

0

0

0

0

0

0
4

0
3

0
3

0
3

0

0

0
3

0

0

0
3

0

0

0

0
3

0

0
2

0

0

0

0

0

0

0

0

0
3

0

0

0

0

0

0

0

0

0

0

0
3

0

0

0

0

0

0

0

0

0

0

0
3

0

0

0

0

0

0

0

0

0

0

0
3

0

0

0

0

0

0

0

0

0

0

0
3

0

0

0

0

0

0

0

0

0

0

0
3

0

0

0

0

0

0

0

0

0

0

0
2

0

0

0

0

0

0

0

0
4

0
3

0
3

0

0

0

0

0

0

0

0

0

0

0
3

0

0

0

0

0

0

0

0

0

0
3

0
3

0
2

0

0

0

0

0
4

0
2

0
3

0

0

0

0

0

0

0
4

0

0

0

0
4

0

0

0
4

0

0

0
4

0

0

0
4

0

0

0

0
4

0

0

0
4

0

0

0
4

0

0

0
4

0

0

0
4

0

0

0
4

0
2

0
3

0

0

0

0

0

0
4

0

0

0
4

0

0

0
4

0

0

0
4

0

0

0
4

0

0

0
4

0

0

0
4

0

0

0
4

0

0

0
4

0

0

0
4

0

0

0
4

0

0

0
4

0

0

0
4

0

0

0
4

0

0

0
4

0

0

0
4

0

0

0
4

0

0

0
4

0

0

0
4

0

0

0

0
4

0

0

0

0
4

0

0

0

0

0

0

0

0

0
4

0

0

0

0
4

0.141922

0.0851532

0.0567688

1.38777878078145e-17

0
4

0

0

0

0

0
4

0
4

1.491
3

0.14769
3

0.14769
3

0

0

0

0

0

0

0

0

0
4

0
2

0

0

0

0

0

0

0

0

0

0

0
4

0

0

0

0

0
4

0

0

0

0
4

0.237608

0.237608

0

0
4

0.455289
2

0.410261
3

0
2

0
3

0

0

0

0

0

0

0

0

0

0

0

0

0

0

0

0

0

0

0

0

0

0
2

0

0

0

0.0100064

0

0

0

0

0

0

0
2

0

0

0

0

0

0

0

0

0

0

0
3

0

0

0

0

0

0

0

0

0

0

0

0

0

0

0

0

0

0

0

0

0

0
2

0

0

0

0

0

0

0

0

0

0

0

0

0

0

0

0

0

0

0

0

0

0
2

0

0

0

0

0

0.0200127

0

0

0

0

0

0

0

0

0

0

0

0

0

0.0150095

0

0
3

0

0

0

0

0

0

0

0

0

0

0

0
2

0

0

0

0

0

0
2

0
2

0

0

0

0

0

0

0

0

0
2

0

0

0

0

0
2

0

0

0

0

0

0

0

0

0

0

0
3

0

0

0

0

0

0

0

0

0

0

0
2

0

0

0

0

0

0

0

0

0

0

0
3

0

0

0

0

0

0

0

0

0

0

0
2

0

0

0

0

0

0

0

0

0

0

0

0

0

0

0

0

0

0

0

0

0

0
4

0
3

0
3

0

0

0

0

0
4

0
3

0

0

0

0

0

0

0

0

0

0

0

0

0

0
4

0.0600381

0

0.0300191

0.0300191

0
4

0

0

0
4

0

0

0
4

0

0

0
4

0.590375

0

0.590375

0
4

0

0

0
4

0

0

0
4

0

0

0
4

0

0

0
4

0

0

0
4

0

0

0
4

0

0

0
4

0
4

0

0

0

0

0

0

0

0

0

0

0
4

0

0

0

0

0

0

0

0

0

0

0

0

0

0

0

0

0

0

0

0

0

0

0

0

0

0

0

0

0

0

0

0

0

0

0

0

0

0

0

0

0

0

0

0

0

0

0

0

0

0

0

0

0

0

0

0

0

0

0

0

0

0

0

0

0

0

0

0

0

0

0

0

0

0

0

0

0

0

0

0

0

0

0

0

0

0
4

0

0
7

0

0

0
4

0

0

0
4

0

0

0
4

0

0

0
4

0

0

0
4

0

0

0
4

0

0

0
4

0

0

0
4

0

0

0
4

0

0

0
4

0

0

0
4

0

0

0
4

0

0

0

0

0
4

0
7

0
7

0
4

0

0

0

0
4

0

0

0
4

0

0

0

0

0
4

0

0

0
4

0

0

0
4

0
4

0
3

0
3

0
3

0

0
3

0
2

0
3

0

0

0

0

0

0
4

0
3

0
3

0

0
2

0

0

0

0

0

0

0

0
4

0

0

0

0

0
4

0
3

0

0

0

0

0

0

0

0
4

0

0

0

0

0

0
4

0

0

0

0

0
4

0
2

0

0

0

0

0
4

0

0

0

0
4

0

0

0

0
4

0
4

0

0

0

0
4

0

0

0

0
4

0
4

0

0

0

0

0
4

0

0

0
4

0
4

0

0

0

0
4

0
4

0

0

0

0
4

0
4

0

0

0

0

0
4

0
4

0

0

0

0
4

0
4

0

0

0

0
4

0
4

0

0

0

0
4

0
4

0

0

0

0

0
4

0
4

0

0

0

0
4

0
4

0

0

0

0

0

0

0

0

0

0

0

0

0

0

0

0

0

0

0

0

0

0

0

0

0
4

0

0

0

0

0
4

0

0

0
4

0
4

0

0

0

0
4

0
4

0

0

0

0
4

0
4

0

0

0

0
4

0
4

0

0

0

0
4

0
4

0

0

0

0
4

0
4

0

0

0

0
4

0
4

0

0

0

0
4

0
4

0

0

0

0
4

0
4

0

0

0

0
4

0
4

0

0

0

0
4

0
4

0

0

0

0

0

0

0

0

0

0

0

0
4

0

0

0

0

0

0
4

0
4

0

0

0

0
4

0
4

0

0

0

0
4

0
4

0

0

0

0
4

0
4

0

0

0

0
4

0
4

0
3

0
3

0
3

0

0

0

0

0

0

0

0

0
4

0
3

0
3

0

0
4

0

0

0
4

0

0

0
4

0

0

0
4

0
4

0
3

0
3

0

0

0

0

0

0

0

0
4

0
3

0

0

0

0

0
4

0

0

0
4

0

0

0

0
4

0
4

0
2

0
2

0

0

0

0

0

0

0
4

0

0

0

0

0
4

0

0

0

0
4

0

0

0
4

0

0

0
4

0

0

0
4

0
4

0

0

0

0

0

0

0

0
4

0

0

0

0

0

0
4

0

0

0
4

0

0

0
4

0
4

0

0

0

0

0
4

0

0

0

0
4

0

0

0
4

0
4

0

0

0

0
4

0

0

0
4

0
4

0

0

0

0
4

0
4

0
4

10.7559

0

0

0

0

0

0
4

0

0

0
4

0

0

0
4

0

0

0
4

0
4

0.29891

0

0

0
4

0

0

0
4

0.29891

0.120613

0.146833

0.0104881

0.0104881

0.0104881

0
4

0

0

0

0

0

0

0

0

0
4

0
4

0

0

0

0

0

0

0

0
4

0

0

0

0

0
4

0

0

0

0
4

0

0

0
4

0

0

0
4

0
4

0.513698
5

0.513698
5

0.33393
5

0.179768

0
4

0
4

1.38867

0

0

0

0
4

1.1312
7

1.1312
7

0
4

0.181774
7

0.181774
7

0
4

0
2

0
2

0

0

0

0

0

0

0

0

0

0

0

0

0

0

0
4

0

0

0

0

0

0
4

0

0

0
4

0

0

0
4

0

0

0

0

0
4

0

0

0
4

0

0

0

0

0
4

0

0

0

0

0
4

0

0

0
4

0

0

0

0
4

0

0

0
4

0
2

0
2

0

0

0

0

0

0

0
4

0

0

0

0
4

0

0

0
4

0

0

0

0
4

0

0

0
4

0

0

0
4

0

0

0

0
4

0

0

0

0
4

0

0

0

0
4

0

0

0
4

0

0

0
4

0
2

0
2

0

0

0
4

0

0

0
4

0

0

0
4

0

0

0
4

0

0

0
4

0

0

0
4

0

0

0
4

0

0

0
4

0.0567688

0.0567688

0
4

0

0

0
4

0

0

0
4

0
2

0

0

0

0

0

0

0

0

0
4

0

0

0
4

0.0189229

0.0189229

0
4

0

0

0
4

0

0

0
4

0

0

0
4

0

0

0
4

0

0

0
4

0

0

0
4

0

0

0
4

0

0

0
4

0
2

0

0

0

0

0

0

0
4

0

0

0

0
4

0

0

0

0

0
4

0
2

0
2

0
4

0

0

0

0

0

0

0
4

8.67361737988404e-17

0
4

8.44453
6

8.43404
6

8.43404
6

0
4

0.0104881

0.0104881

0
4

9.74914593498966e-16
6

0
4

0

0

0

0
4

0
4

0.110125

0.110125

0.110125

0
4

0
4

0

0

0

0
4

0
4

0

0

0

0
4

0
4

0
7

0
7

0
7

0
4

0
4

0

0

0

0
4

0
4

0

0

0

0

0

0
4

0
4

0

0

0

0

0
4

0

0

0
4

0
4

0

0

0

0
4

0
4

0

0

0

0

0
4

0
4

0

0

0

0
4

0
4

0

0

0

0
4

0
4

0
4

5.61587
6

5.61587
6

4.41522
7

2.60836
7

0
6

0

0

0

0.039007

0

0.0358903
6

0
6

0.061715
7

0

0.0142123
7

0

0
6

0

0

1.13559
6

0.0129839

0

0

0.0129839

0

0.0292553

0

0

0

0

0.0160762
7

0

0

0.0946147

0

0.0195035

0

0

0

0

0

0.00710613
6

0

0

0

0

0

0

0

0

0

0

0
6

0

0

0

0

0

0.0585106

0

0

0

0

0.0843952
6

0

0

0

0

0

0

0

0

0

0

0
6

0

0

0

0

0

0

0

0

0

0

0
7

0.126773

0

0

0

0

0

0

0

0

0

0.0482286
6

0

0

0

0

0

0

0

0.0100064

0

0

2.74086309204336e-16
7

0
4

0.777528
6

0.777528
6

0

0

0

0

0

0

0

0
4

0.0200127
6

0.0200127

0

0

0

0

0

0

0

0
4

0.0723156
7

0
7

0.0642775

0

0

0.0080381

0

0

1.73472347597681e-18
7

0
4

0.217029
6

0.217029

0

0

0

0

0

0
4

0.097379

0.0714113

0.0259677

0

0
4

0.0163868

0

0.0163868

0

0

0
4

0

0

0

0

0
4

0

0

0
4

4.05925293378573e-16
6

0
4

0
4

0.335012

0.312907

0.0976748

0

0

0

0

0.0819428
5

0.0157321

0

0

0

0

0

0

0
4

0
2

0
2

0

0
4

0.166072

0.139287

0

0.0267847

0

0

0
4

0.0491603

0

0

0.0491603

0
4

0
4

0.0221053
7

0.00736842
7

0

0.00736842

0
4

0.0147368

0.0147368

0

0
4

0
4

0

0

0

0

0
4

0

0

0
4

0
4

0

0

0

0

0

0

0
4

0
4

0
4

0

0

0

0

0
4

0

0

0
4

0
4

0
4

0

0

0

0

0
4

0
4

0
4

0.0163868

0.0163868

0

0

0
4

0.0163868

0.0163868

0
4

0
4

0
4

0

0

0

0

0
4

0

0

0
4

0

0

0
4

0
4

0

0

0

0
4

0
4

0
4

0.0283844

0.0283844

0

0

0
4

0.0283844

0.0283844

0

0
4

0
4

0
4

0

0

0

0

0

0
4

0

0

0
4

0
4

0
4

0

0

0

0

0

0

0
4

0

0

0
4

0
4

0
4

0

0

0

0

0
4

0

0

0
4

0
4

0

0

0

0
4

0
4

0
4

0

0

0

0

0

0

0
4

0

0

0
4

0
4

0
4

0

0

0

0

0
4

0
4

0
4

0.564932
6

0.564932
6

0.510324
6

0.500318
6

0.0100064

5.20417042793042e-17
6

0
4

0.0425512
6

0
7

0.0195035

0

0

0.0080381

0

0.0150095

1.73472347597681e-18
6

0
4

0
6

0
7

0

0

0
4

0.0120572

0.0120572

0
4

0

0

0
4

0
4

0
4

0

0

0

0

0

0
4

0
4

0
4

0

0

0

0

0
4

0
4

0
4

0

0

0

0

0
4

0
4

0
4

0

0

0

0

0
4

0

0

0
4

0
4

0
4

0

0

0

0

0

0
4

0

0

0
4

0
4

0
4

0

0

0

0

0
4

0
4

0
4

0

0

0

0

0
4

0

0

0
4

0
4

0

0

0

0
4

0
4

0
4

0

0

0

0

0

0

0
4

0
4

0
4

0

0

0

0

0
4

0

0

0
4

0
4

0
4

0

0

0

0

0

0
4

0
4

0
4

0
3

0
3

0
4

0
3

0

0

0

0

0

0

0

0

0

0

0
4

0
3

0

0

0

0

0
4

0

0

0
4

0
4

0
4

0

0

0

0

0
4

0
4

0
4

0

0

0

0

0
4

0
4

0
4

0.108548

0.108548

0

0

0
4

0.0129839

0.0129839

0
4

0.0955644

0.0955644

0
4

0
4

0
4

0

0

0

0

0
4

0

0

0
4

0
4

0
4

0

0

0

0

0

0
4

0
4

0
4

0

0

0

0

0
4

0

0

0
4

0
4

0
4

0.0409669

0.0409669

0.0409669

0.0409669

0
4

0
4

0
4

0

0

0

0

0
4

0
4

0
4

0

0

0

0

0
4

0

0

0
4

0
4

0
4

0

0

0

0

0
4

0
4

0
4

0.188582

0.122476

0.122476

0.0389793
7

0.0389516

0

0

0

0.0120572

0.0195035

0

0.0129839

0
4

0

0

0

0

0
4

0

0

0

0

0
4

0

0

0
4

0

0

0
4

0
4

0.0661064

0

0

0

0

0

0

0

0

0

0
4

0

0

0

0

0
4

0.0292553

0.0292553

0

0
4

0.0173753

0.0173753

0
4

0.0194758

0.0194758

0
4

3.46944695195361e-18

0
4

0

0

0

0
4

0

0

0
4

0
4

0

0

0

0
4

0
4

1.38777878078145e-17

0
4

0

0

0

0

0

0

0
4

0
4

0
4

0

0

0

0

0

0
4

0
4

0
4

0.244374

0.244374

0.244374

0.244374

0
4

0
4

0
4

0

0

0

0

0
4

0

0

0
4

0
4

0
4

0

0

0

0

0
4

0
4

0
4

0

0

0

0

0
4

0
4

0
4

0

0

0

0

0
4

0

0

0
4

0
4

0

0

0

0
4

0
4

0
4

0

0

0

0

0
4

0
4

0
4

0

0

0

0

0
4

0

0

0
4

0
4

0

0

0

0
4

0
4

0
4

0

0

0

0

0

0
4

0
4

0
4

0.621134

0.621134

0.416497

0.384037

0

0

0.0324597

0

0
4

0.204637
5

0.146359
5

0.052126

0.00615279

0
4

0

0

0

0
4

0
4

0
4

0

0

0

0

0

0
4

0

0

0
4

0
4

0
4

0

0

0

0

0
4

0
4

0
4

0

0

0

0

0
4

0

0

0
4

0
4

0
4

0

0

0

0

0

0

0
4

0
4

0
4

0

0

0

0

0
4

0
4

0
4

0

0

0

0

0
4

0

0

0
4

0
4

0
4

0.0293706

0.0293706

0.0293706

0.0293706

0

0
4

0
4

0
4

0

0

0

0

0
4

0

0

0
4

0
4

0

0

0

0
4

0
4

0
4

0

0

0

0

0
4

0
4

0
4

0

0

0

0

0
4

0
4

0
4

0.477266

0.477266

0.385906

0.359938

0.0129839

0

0.0129839

0

0
4

0.0913602

0.0783763

0.0129839

0

0

0
4

5.55111512312578e-17

0
4

0
4

0

0

0

0

0

0
4

0

0

0
4

0
4

0
4

0

0

0

0

0
4

0

0

0
4

0
4

0

0

0

0
4

0
4

0
4

0

0

0

0

0
4

0

0

0
4

0
4

0
4

0

0

0

0

0
4

0

0

0
4

0
4

0
4

0

0

0

0

0

0
4

0
4

0
4

0

0

0

0

0

0
4

0
4

0
4

0

0

0

0

0

0
4

0
4

0
4

0

0

0

0

0
4

0
4

0
4

0

0

0

0

0
4

0

0

0
4

0
4

0
4

0

0

0

0

0
4

0
4

0
4

0.11405
6

0.11405
6

0.11405
6

0.0151442
6

0

0

0

0

0.0195035
7

0
6

0.0163868

0

0

0

0.0500318

0.0129839

6.93889390390723e-18
6

0
4

0

0

0
4

0
4

0
4

0

0

0

0

0
4

0

0

0
4

0
4

0
4

0

0

0

0

0
4

0

0

0
4

0
4

0
4

0

0

0

0

0
4

0
4

0
4

0

0

0

0

0
4

0
4

0
4

0

0

0

0

0
4

0

0

0
4

0
4

0
4

0.151053

0.151053

0.151053

0.0147368

0.136316

0
4

0
4

0
4

0

0

0

0

0

0
4

0
4

0
4

0

0

0

0

0
4

0
4

0
4

0

0

0

0

0
4

0
4

0
4

0

0

0

0

0
4

0

0

0
4

0
4

0
4

0.589047
7

0.563079
7

0.563079
7

0.550095
7

0.0129839

0

0
4

0
4

0.0259677

0.0259677

0.0259677

0
4

0

0

0
4

0
4

1.04083408558608e-17
7

0
4

0

0

0

0

0
4

0

0

0
4

0
4

0
4

0

0

0

0

0

0
4

0
4

0
4

0

0

0

0

0
4

0
4

0
4

0

0

0

0

0
4

0

0

0
4

0
4

0
4

0

0

0

0

0
4

0
4

0
4

0

0

0

0

0
4

0
4

0
4

0

0

0

0

0
4

0

0

0
4

0
4

0
4

0

0

0

0

0
4

0

0

0
4

0
4

0
4

0

0

0

0

0

0
4

0
4

0
4

0

0

0

0

0

0
4

0
4

0
4

0

0

0

0

0

0

0

0

0

0

0
4

0

0

0
4

0
4

0
4

0

0

0

0

0

0
4

0
4

0
4

0

0

0

0

0
4

0
4

0
4

0

0

0

0

0
4

0

0

0
4

0
4

0
4

0

0

0

0

0
4

0

0

0
4

0
4

0
4

0

0

0

0

0
4

0

0

0
4

0
4

0
4

0

0

0

0

0
4

0

0

0
4

0
4

0
4

0

0

0

0

0
4

0

0

0
4

0
4

0
4

0

0

0

0

0

0
4

0
4

0
4

0

0

0

0

0
4

0
4

0
4

0

0

0

0

0

0
4

0
4

0
4

0.0942292

0.0942292

0.0195035
5

0.0195035

0

0

0

0

0

0
4

0

0

0

0

0
4

0.0747257
3

0.0419522

0.0327735

0
4

0

0

0
4

0

0

0
4

1.38777878078145e-17

0
4

0

0

0

0

0

0
4

0

0

0

0
4

0
4

0
4

0

0

0

0

0
4

0
4

0
4

0

0

0

0

0

0
4

0
4

0
4

0

0

0

0

0
4

0

0

0
4

0
4

0
4

0.0224425

0.0224425

0.00748085

0.00748085

0
4

0.0149617

0.0149617

0
4

1.73472347597681e-18

0
4

0
4

0

0

0

0

0
4

0
4

0
4

0

0

0

0

0
4

0
4

0
4

0

0

0

0

0
4

0
4

0
4

0

0

0

0

0
4

0

0

0
4

0
4

0
4

0

0

0

0

0
4

0
4

0
4

0

0

0

0

0
4

0
4

0
4

3.2846

3.2846

3.1154

1.98335

0.0147368

0

0

0

0.0104881

0

0

0

0.0209761

0

0.831458

0

0

0.0104881

0.00712881

0.0142123

0.0106592

0

0

0

0

0
3

0

0

0

0

0

0

0

0

0

0.077036
3

0.0106592
3

0.0442105
3

0.0622354

0

0.0177653

5.23886489744996e-16

0
4

0.144328

0.0292553

0.115073

1.38777878078145e-17

0
4

0.00710613

0

0

0.00710613

0

0
4

0.0106592

0.0106592

0
4

0.00710613

0.00710613

0

0
4

0

0

0
4

0

0

0
4

0

0

0
4

0
4

0
4

0.240202
7

0.240202
7

0.097379
7

0.097379
7

0
7

0

0

0

0

0
4

0.142823

0.142823

0
4

0

0

0

0
4

0

0

0
4

2.77555756156289e-17
7

0
4

0
4

0.0908871

0.0908871

0.0908871

0.0908871

0
4

0
4

0
4

0

0

0

0

0
4

0

0

0
4

0
4

0
4

0

0

0

0

0
4

0
4

0
4

0

0

0

0

0
4

0
4

0
4

0

0

0

0

0

0
4

0
4

0
4

0

0

0

0

0
4

0
4

0
4

0.121627

0.121627

0.121627

0.121627

0
4

0
4

0
4

0

0

0

0

0
4

0
4

0
4

0

0

0

0

0
4

0
4

0
4

0

0

0

0

0
4

0
4

0
4

0.155806

0.155806

0
2

0
2

0

0

0
4

0.155806
5

0.155806
5

0

0
4

0

0

0

0

0
4

0
4

0

0

0

0
4

0
4

0
4

0

0

0

0

0

0
4

0
4

0
4

0

0

0

0

0
4

0
4

0
4

0

0

0

0

0
4

0

0

0
4

0
4

0
4

0

0

0

0

0

0
4

0
4

0
4

0

0

0

0

0
4

0
4

0
4

0

0

0

0

0
4

0
4

0
4

0.285645

0.285645

0.285645

0.285645

0
4

0
4

0
4

0

0

0

0

0
4

0
4

0
4

0

0

0

0

0
4

0
4

0
4

0

0

0

0

0
4

0
4

0
4

0

0

0

0

0

0

0

0

0

0

0
4

0
4

0
4

0

0

0

0

0
4

0
4

0
4

0

0

0

0

0
4

0
4

0
4

0

0

0

0

0
4

0
4

0
4

0

0

0

0

0
4

0
4

0
4

0

0

0

0

0
4

0
4

0
4

0

0

0

0

0
4

0
4

0
4

0

0

0

0

0
4

0
4

0
4

0

0

0

0

0
4

0
4

0
4

0

0

0

0

0
4

0
4

0
4

0

0

0

0

0
4

0
4

0
4

0.136174
6

0.136174
6

0.0684932
6

0.0684932
6

0

0
4

0.0676807
6

0.0676807

0

0

0
4

0

0

0

0
4

0

0

0
4

0
4

0
4

0

0

0

0

0
4

0
4

0
4

0

0

0

0

0
4

0
4

0
4

0

0

0

0

0
4

0
4

0
4

0

0

0

0

0
4

0
4

0
4

0.0129839

0.0129839

0.0129839

0.0129839

0
4

0
4

0
4

0

0

0

0

0
4

0
4

0
4

0.00498723

0.00498723

0.00498723

0.00498723

0
4

0
4

0
4

0

0

0

0

0
4

0
4

0
4

0

0

0

0

0
4

0
4

0
4

0

0

0

0

0
4

0
4

0
4

0

0

0

0

0

0

0

0
4

0

0

0
4

0
4

0

0

0

0

0
4

0

0

0
4

0
4

0
4

0

0

0

0

0
4

0
4

0
4

0

0

0

0

0
4

0
4

0
4

0

0

0

0

0
4

0
4

0
4

0

0

0

0

0
4

0
4

0
4

0

0

0

0

0
4

0
4

0
4

0

0

0

0

0
4

0
4

0
4

0

0

0

0

0
4

0
4

0
4

0

0

0

0

0
4

0
4

0
4

0

0

0

0

0
4

0
4

0
4

0

0

0

0

0
4

0
4

0
4

0.0215407

0.0215407

0
2

0
2

0

0

0

0
4

0.0215407

0.0215407

0
4

0

0

0

0

0
4

0

0

0
4

0

0

0
4

0
4

0
4

0

0

0

0

0
4

0
4

0
4

0

0

0

0

0
4

0
4

0
4

0

0

0

0

0
4

0
4

0
4

0

0

0

0

0
4

0
4

0
4

0

0

0

0

0
4

0
4

0
4

0

0

0

0

0
4

0
4

0
4

0

0

0

0

0
4

0
4

0
4

0

0

0

0

0
4

0
4

0
4

0

0

0

0

0
4

0
4

0
4

0

0

0

0

0
4

0
4

0
4

0.538636
7

0.538636
7

0.538636
7

0.538636
7

0
4

0

0

0
4

0
4

0
4

0

0

0

0

0
4

0
4

0
4

0

0

0

0

0
4

0
4

0
4

0

0

0

0

0
4

0
4

0
4

0

0

0

0

0
4

0
4

0
4

0

0

0

0

0
4

0
4

0
4

0

0

0

0

0
4

0
4

0
4

0

0

0

0

0
4

0
4

0
4

0

0

0

0

0
4

0
4

0
4

0

0

0

0

0
4

0
4

0
4

0

0

0

0

0
4

0
4

0
4

0
7

0
7

0
7

0

0

0

0
4

0

0

0

0

0
4

0

0

0
4

0
4

0
4

0

0

0

0

0
4

0
4

0
4

0.0245801

0.0245801

0.0245801

0.0245801

0
4

0
4

0
4

0

0

0

0

0
4

0
4

0
4

0

0

0

0

0
4

0
4

0
4

0

0

0

0

0
4

0
4

0
4

0.00997446

0.00997446

0.00997446

0.00997446

0
4

0
4

0
4

0

0

0

0

0
4

0
4

0
4

0

0

0

0

0
4

0
4

0
4

0

0

0

0

0
4

0
4

0
4

0

0

0

0

0
4

0
4

0
4

0.0983205

0

0

0

0

0
4

0

0

0

0
4

0

0

0
4

0
4

0.0983205

0.0983205

0

0

0

0

0.0983205

0
4

0
4

0

0

0

0

0
4

0
4

0
4

0

0

0

0

0
4

0
4

0
4

0

0

0

0

0
4

0
4

0
4

0

0

0

0

0
4

0
4

0
4

0

0

0

0

0
4

0
4

0
4

0.0129839

0.0129839

0.0129839

0.0129839

0
4

0
4

0
4

0

0

0

0

0
4

0
4

0
4

0

0

0

0

0
4

0
4

0
4

0

0

0

0

0
4

0
4

0
4

0

0

0

0

0
4

0
4

0
4

0.0104881

0.0104881

0.0104881

0.0104881

0
4

0
4

0
4

0.0409669

0.0409669

0.0409669

0.0409669

0

0

0

0
4

0
4

0
4

0

0

0

0

0
4

0
4

0
4

0

0

0

0

0
4

0
4

0
4

0

0

0

0

0
4

0
4

0
4

0

0

0

0

0
4

0
4

0
4

0

0

0

0

0
4

0
4

0
4

0

0

0

0

0
4

0
4

0
4

0

0

0

0

0
4

0
4

0
4

0

0

0

0

0
4

0
4

0
4

0

0

0

0

0
4

0
4

0
4

0

0

0

0

0
4

0
4

0
4

3.29453
7

3.21421
7

3.15392
7

1.83688
7

0

0

0

0

0

0

0.00712881

0

0

0

0.751668
7

0

0.0213864

0

0

0

0.0080381

0

0

0

0

0.178429
6

0

0

0

0

0

0

0

0

0

0

0.0374087
7

0

0

0

0

0

0

0

0

0

0

0

0

0.00712881

0.185283

0.120572

0

0
4

0.0602858
6

0

0.0241143

0

0.0361715

0

0

0

0
4

0
4

0.0803195
6

0.0803195
6

0

0.0292553

0.0120572

0

0

0.039007

0
4

0

0

0

0

0
4

0

0

0
4

0
4

5.55111512312578e-17
7

0
4

0.0360889
4

0.0360889
4

0.0360889

0

0

0

0.0200127

0

0.0160762

0

3.46944695195361e-18

0
4

0

0

0

0

0
4

0

0

0

0
4

0

0

0
4

0

0

0
4

0
4

0
4

0

0

0

0

0
4

0
4

0
4

0

0

0

0

0
4

0
4

0
4

0

0

0

0

0
4

0
4

0
4

0

0

0

0

0
4

0
4

0
4

0

0

0

0

0
4

0
4

0
4

0

0

0

0

0
4

0
4

0
4

0

0

0

0

0
4

0
4

0
4

0

0

0

0

0
4

0
4

0
4

0

0

0

0

0
4

0
4

0
4

0

0

0

0

0
4

0
4

0
4

0
4

0
4

0
4

0

0

0

0

0

0

0
4

0
4

0

0

0

0

0

0
4

0

0

0
4

0
4

0
4

0

0

0

0

0
4

0
4

0
4

0

0

0

0

0
4

0
4

0
4

0

0

0

0

0
4

0
4

0
4

0

0

0

0

0
4

0
4

0
4

0

0

0

0

0
4

0
4

0
4

0.0662303

0.0662303

0.0662303

0.0662303

0
4

0
4

0
4

0

0

0

0

0
4

0
4

0
4

0

0

0

0

0
4

0
4

0
4

0

0

0

0

0
4

0
4

0
4

0

0

0

0

0
4

0
4

0
4

0
6

0
7

0

0

0

0

0
4

0

0

0

0

0
4

0

0

0

0
4

0
4

0

0

0

0

0
4

0

0

0
4

0
4

0
4

0

0

0

0

0
4

0
4

0
4

0

0

0

0

0
4

0
4

0
4

0

0

0

0

0
4

0
4

0
4

0

0

0

0

0
4

0
4

0
4

0

0

0

0

0
4

0
4

0
4

0

0

0

0

0
4

0
4

0
4

0

0

0

0

0
4

0
4

0
4

0

0

0

0

0
4

0
4

0
4

0

0

0

0

0
4

0
4

0
4

0

0

0

0

0
4

0
4

0
4

0.0737404

0.0737404

0.0737404
6

0.0737404
6

0

0
4

0

0

0

0
4

0

0

0
4

0
4

0
4

0

0

0

0

0
4

0
4

0
4

0

0

0

0

0
4

0
4

0
4

0

0

0

0

0
4

0
4

0
4

0

0

0

0

0
4

0
4

0
4

0

0

0

0

0
4

0
4

0
4

0

0

0

0

0
4

0
4

0
4

0

0

0

0

0
4

0
4

0
4

0

0

0

0

0
4

0
4

0
4

0

0

0

0

0
4

0
4

0
4

0

0

0

0

0
4

0
4

0
4

0.0975176

0.0975176

0.0975176
5

0.0195035

0.0487588

0

0.0292553

0
4

0

0

0

0

0
4

0

0

0

0

0
4

0

0

0
4

0

0

0
4

0
4

0
4

0

0

0

0

0
4

0
4

0
4

0

0

0

0

0
4

0
4

0
4

0

0

0

0

0
4

0
4

0
4

0.0262201

0.0262201

0.0262201

0.0262201

0
4

0
4

0
4

0

0

0

0

0
4

0
4

0
4

0

0

0

0

0
4

0
4

0
4

0

0

0

0

0
4

0
4

0
4

0

0

0

0

0
4

0
4

0
4

0

0

0

0

0
4

0
4

0
4

0

0

0

0

0
4

0
4

0
4

0
4

0
4

0
4

0

0

0

0

0
4

0

0

0

0

0

0
4

0
4

0
4

0

0

0

0

0
4

0
4

0
4

0

0

0

0

0
4

0
4

0
4

0

0

0

0

0
4

0
4

0
4

0

0

0

0

0
4

0
4

0
4

0

0

0

0

0
4

0
4

0
4

0

0

0

0

0
4

0
4

0
4

0

0

0

0

0
4

0
4

0
4

0

0

0

0

0
4

0
4

0
4

0

0

0

0

0
4

0
4

0
4

0

0

0

0

0
4

0
4

0
4

0

0

0

0

0

0

0

0

0

0
4

0

0

0

0

0
4

0

0

0

0
4

0

0

0
4

0

0

0
4

0
4

0

0

0

0
4

0
4

0

0

0

0
4

0
4

0
4

0

0

0

0

0
4

0
4

0
4

0

0

0

0

0
4

0
4

0
4

0.0142123

0.0142123

0.0142123

0.0142123

0
4

0
4

0
4

0.00710613

0.00710613

0.00710613

0.00710613

0
4

0
4

0
4

0

0

0

0

0
4

0
4

0
4

0

0

0

0

0
4

0
4

0
4

0

0

0

0

0
4

0
4

0
4

0

0

0

0

0
4

0
4

0
4

0

0

0

0

0
4

0
4

0
4

0

0

0

0

0
4

0
4

0
4

0

0

0

0

0

0
4

0

0

0

0

0

0

0
4

0
4

0
4

0

0

0

0

0
4

0
4

0
4

0

0

0

0

0
4

0
4

0
4

0

0

0

0

0
4

0
4

0
4

0

0

0

0

0
4

0
4

0
4

0

0

0

0

0
4

0
4

0
4

0

0

0

0

0
4

0
4

0
4

0

0

0

0

0
4

0
4

0
4

0

0

0

0

0
4

0
4

0
4

0

0

0

0

0
4

0
4

0
4

0

0

0

0

0
4

0
4

0
4

0.593488

0.593488

0.593488

0.593488

0

0
4

0

0

0
4

0
4

0
4

0

0

0

0

0
4

0
4

0
4

0

0

0

0

0
4

0
4

0
4

0

0

0

0

0
4

0
4

0
4

0

0

0

0

0
4

0
4

0
4

0

0

0

0

0
4

0
4

0
4

0

0

0

0

0
4

0
4

0
4

0

0

0

0

0
4

0
4

0
4

0

0

0

0

0
4

0
4

0
4

0

0

0

0

0
4

0
4

0
4

0

0

0

0

0
4

0
4

0
4

0

0

0

0

0

0

0

0

0

0
4

0

0

0

0
4

0
4

0
4

0.0245801

0.0245801

0.0245801

0.0245801

0
4

0
4

0
4

0

0

0

0

0
4

0
4

0
4

0

0

0

0

0
4

0
4

0
4

0

0

0

0

0
4

0
4

0
4

0

0

0

0

0
4

0
4

0
4

0

0

0

0

0
4

0
4

0
4

0

0

0

0

0
4

0
4

0
4

0

0

0

0

0
4

0
4

0
4

0

0

0

0

0
4

0
4

0
4

0

0

0

0

0
4

0
4

0
4

0

0

0

0

0

0

0

0

0

0

0

0

0

0

0

0

0

0

0

0

0

0

0

0

0

0

0

0

0

0

0
4

0

0

0

0

0

0

0
4

0

0

0

0

0

0

0
4

0

0

0

0

0

0

0

0
4

0

0

0

0
4

0

0

0
4

0
4

0

0

0

0

0

0

0

0

0

0

0

0

0

0

0

0

0

0

0

0

0
4

0

0

0

0

0

0

0

0

0

0
4

0

0

0

0

0

0

0
4

0

0

0

0

0
4

0

0

0
4

0

0

0
4

0
4

0

0

0

0

0

0
4

0
4

0

0

0

0
4

0

0

0
4

0
4

0
4

0.0163868
6

0.0163868
6

0.0163868
6

0

0.0163868

0

0
4

0

0

0
4

0
4

0
4

0

0

0

0

0
4

0
4

0
4

0

0

0

0

0
4

0
4

0
4

0

0

0

0

0
4

0
4

0
4

0

0

0

0

0
4

0
4

0
4

0

0

0

0

0

0

0
4

0

0

0
4

0
4

0

0

0

0
4

0
4

0
4

0
6

0
6

0
6

0
6

0
4

0
4

0
4

0.039007

0.039007

0.039007

0

0.039007

0

0
4

0

0

0

0
4

0

0

0

0

0
4

0
4

0
4

0
7

0
7

0
7

0

0

0

0

0
4

0
4

0
4

0
4

0
4

0

0

0

0

0
4

0

0

0

0

0
4

0

0

0
4

0

0

0
4

0
4

0

0

0

0

0
4

0

0

0
4

0

0

0
4

0
4

0
4

0

0

0

0

0

0
4

0

0

0
4

0
4

0

0

0

0
4

0
4

0
4

0.0129839
3

0.0129839
3

0.0129839
3

0.0129839

0

0

0
4

0

0

0

0

0

0
4

0
4

0
4

0

0

0

0

0

0

0

0

0
4

0

0

0
4

0
4

0
4

0
3

0
3

0
3

0

0

0

0

0

0
4

0
4

0
4

5.30289
7

5.30289
7

5.24411
7

5.24411
7

0

0

0

0

0

0

0

0

0

0

0

0
4

0

0

0
4

0

0

0
4

0.024264

0.00475254

0.0100064
6

0

0.00950508

0

0

0
4

0.0150095
6

0.0150095
6

0

0
4

0

0

0
4

0

0

0
4

0

0

0
4

0.0195035

0.0195035

0
4

0

0

0
4

0

0

0
4

0
4

0
4

0

0

0

0

0

0
4

0
4

0
4

0.0283844
4

0.0283844
4

0.0283844

0

0.0283844

0

0

0
4

0

0

0

0

0

0
4

0
4

0
4

0

0

0

0

0

0

0

0

0
4

0
4

0
4

0
4

0
4

0
4

0

0

0
4

0

0

0

0
4

0

0

0
4

0

0

0
4

0
4

0
4

0

0

0

0

0

0

0
4

0

0

0
4

0
4

0
4

0
4

0
4

0
4

0

0

0

0
4

0
4

0

0

0

0
4

0
4

0
4

0

0

0

0

0

0

0

0

0
4

0
4

0
4

0

0

0

0

0

0

0

0

0
4

0

0

0
4

0
4

0
4

0

0

0

0

0

0
4

0

0

0
4

0
4

0
4

0

0

0

0

0

0

0

0
4

0
4

0
4

0.735364
5

0.69545
5

0.630732
6

0.283785
6

0

0

0.0173753

0

0

0

0

0

0.235247

0

0

0.039007

0

0.0292553

0.026063

0

0
4

0.0647174

0.0377607

0

0.00748085

0

0

0

0

0

0.0194758

0

3.46944695195361e-18

0
4

0
4

0

0

0

0

0

0

0

0

0
4

0

0

0

0

0

0

0
4

0

0

0

0

0
4

0

0

0
4

0

0

0
4

0

0

0
4

1.52655665885959e-16
5

0
4

0.0399145
6

0.0292553

0

0

0.0292553

0

0
4

0

0

0

0

0

0
4

0.0106592

0.0106592

0

0

0

0
4

0

0

0
4

1.73472347597681e-18
6

0
4

0

0

0

0
4

0

0

0
4

0
4

0
4

0

0

0

0

0

0

0
4

0
4

0
4

0

0

0

0

0

0
4

0

0

0
4

0

0

0
4

0
4

0
4

0.0129839
6

0.0129839
6

0

0

0

0
4

0.0129839

0.0129839

0

0
4

0
4

0
4

0

0

0

0

0

0
4

0
4

0

0

0

0
4

0

0

0
4

0
4

0
4

0.055035

0.055035

0.055035

0.055035

0

0
4

0
4

0

0

0

0
4

0
4

0

0

0

0
4

0
4

0
4

0

0

0

0

0

0

0

0
4

0
4

0
4

0

0

0

0

0

0
4

0

0

0
4

0
4

0
4

0

0

0

0

0

0
4

0
4

0
4

0
3

0
3

0
3

0

0

0
4

0

0

0
4

0
4

0
4

0

0

0

0

0

0

0
4

0

0

0
4

0
4

0
4

4.59867

4.59867

4.30801

3.99142

0.278575

0

0.012121
3

0

0.00475254
3

0
3

0.00475254

0

0.0163868

0

5.34294830600857e-16

0
4

0.175532

0.175532

0
4

0.0118587
2

0.00710613

0

0

0.00475254

0
4

0

0

0

0

0
4

0.0259677

0.0259677

0
4

0.0773021

0.0773021

0
4

0

0

0

0
4

0

0

0

0
4

0

0

0
4

0

0

0
4

0
4

0
4

0

0

0

0

0

0
4

0

0

0
4

0

0

0

0
4

0
4

0
4

0

0

0

0

0

0

0
4

0

0

0
4

0

0

0
4

0
4

0
4

0.20513

0.20513

0.20513

0.20513

0

0
4

0

0

0
4

0
4

0

0

0

0
4

0
4

0
4

0.0080381
6

0.0080381

0

0

0

0
4

0

0

0
4

0.0080381

0.0080381

0
4

0
4

0

0

0

0
4

0
4

0
4

0
6

0
6

0
7

0
7

0
4

0

0

0
4

0
4

0
4

0

0

0

0

0

0

0
4

0
4

0
4

0

0

0

0

0

0

0
4

0

0

0
4

0
4

0

0

0

0

0
4

0
4

0
4

0.0195035
7

0.0195035
7

0

0

0

0
4

0.0195035

0.0195035

0
4

0
4

0
4

0

0

0

0

0

0
4

0
4

0

0

0

0
4

0

0

0
4

0
4

0
4

0.141922

0.141922

0

0

0
4

0.141922

0.141922

0
4

0
4

0
4

0.331814
7

0.321808
6

0.282801
7

0.282801
6

0
7

0

0

0

0

0

0
4

0.039007
6

0
6

0

0

0

0.039007

0
4

0

0

0

0
4

0
4

0.0100064
7

0.0100064
7

0

0

0

0

0

0

0

0

0

0.0100064

0

0
4

0

0

0

0

0
4

0

0

0

0
4

0

0

0
4

0
4

0
4

0.0355306

0.0355306

0.0355306

0.0355306

0
4

0
4

0
4

0

0

0

0

0

0
4

0

0

0

0
4

0

0

0
4

0
4

0
4

0.0972791

0.0972791

0.0972791

0.0922919

0.00498723

0
4

0
4

0
4

0

0

0

0

0

0
4

0

0

0
4

0
4

0
4

0

0

0

0

0
4

0

0

0
4

0
4

0
4

0

0

0

0

0

0
4

0

0

0
4

0
4

0

0

0

0
4

0
4

0

0

0

0
4

0
4

0
4

0

0

0

0

0
4

0

0

0
4

0
4

0
4

0

0

0

0

0

0

0
4

0

0

0
4

0
4

0
4

0.0454436

0.0454436

0

0

0
4

0.0454436

0.0454436

0
4

0

0

0
4

0
4

0
4

0

0

0

0

0

0

0
4

0
4

0
4

0
4

0
4

0
4

0
4

0

0

0

0

0

0

0
4

0

0

0
4

0
4

0
4

0

0

0

0

0

0

0

0

0

0
4

0

0

0

0

0
4

0

0

0

0

0

0
4

0

0

0
4

0
4

0

0

0

0

0

0
4

0

0

0
4

0

0

0
4

0
4

0

0

0

0
4

0

0

0
4

0
4

0
4

0
6

0
6

0
6

0
6

0
4

0
4

0
4

0

0

0

0

0
4

0
4

0
4

0

0

0

0

0
4

0
4

0
4

0.0189229

0.0189229

0

0

0
4

0.0189229

0.0189229

0
4

0
4

0
4

0

0

0

0

0

0
4

0
4

0
4

0

0

0

0

0

0
4

0

0

0

0
4

0
4

0
4

0

0

0

0

0
4

0
4

0
4

0

0

0

0

0

0
4

0
4

0
4

0

0

0

0

0

0

0
4

0
4

0
4

0

0

0

0

0
4

0
4

0
4

0
3

0
3

0
3

0

0

0

0

0

0
3

0
3

0

0

0

0

0

0

0
4

0

0

0
4

0

0

0
4

0

0

0
4

0
4

0

0

0

0

0

0
4

0
4

0

0

0

0
4

0
4

0
4

0.0758859
3

0

0

0

0
4

0
4

0

0

0

0

0
4

0
4

0

0

0

0
4

0
4

0

0

0

0
4

0
4

0

0

0

0
4

0
4

0.0758859
3

0
3

0

0

0

0

0

0

0

0

0

0

0

0

0

0

0

0

0

0

0

0

0

0

0

0

0

0

0

0

0

0

0

0
3

0

0

0

0

0
4

0.0758859

0.0195035

0.039007

0.0173753

3.46944695195361e-18

0
4

0

0

0
4

0

0

0
4

0

0

0
4

0
4

0
4

0.20125
3

0

0

0

0
4

0
4

0.20125
3

0.20125
3

0.20125

0

0

0

0

0

0
4

0

0

0
4

0

0

0
4

0
4

0
4

0
4

0
4

0
4

0

0

0

0

0

0

0

0
4

0

0

0

0
4

0
4

0

0

0
4

0
3

0

0

0

0

0

0

0

0

0

0

0

0

0

0

0

0

0
4

0

0

0
4

0

0

0
4

0

0

0
4

0
4

0

0

0

0

0
4

0
4

0

0

0

0
4

0
4

0

0

0

0
4

0
4

0

0

0

0
4

0
4

0

0

0

0
4

0
4

0

0

0

0
4

0
4

0

0

0

0
4

0
4

0
4

0

0

0

0

0

0
4

0
4

0

0

0

0

0
4

0
4

0

0

0

0
4

0
4

0

0

0

0

0
4

0
4

0

0

0

0
4

0
4

0

0

0

0

0

0

0

0

0

0

0

0

0

0

0

0

0

0

0

0

0

0

0

0

0

0

0

0
4

0

0

0
4

0

0

0
4

0

0

0
4

0
4

0
4

0.135016
3

0.135016
3

0.135016
2

0.135016
2

0

0

0
4

0
3

0

0

0

0

0

0

0

0

0

0

0

0

0

0

0

0

0
4

0

0

0
4

0

0

0
4

0
4

0

0

0

0

0
4

0
4

0

0

0

0
4

0
4

0

0

0

0
4

0
4

0

0

0

0
4

0
4

0

0

0

0
4

0
4

0
4

2.4936

0

0

0

0
4

0
4

2.4936

2.4936

2.26652

0
3

0.227075

0

0

0

0

0

0

0

0
4

0

0

0
4

0
4

0
4

3.23205
7

3.23205
7

3.23205
7

3.23205
7

0
4

0
4

0
4

13.5238

13.5238

9.96942

9.96942

0

0

0
4

0

0

0
4

3.2256
3

0.180385
3

2.91771
5

0.127506
3

1.66533453693773e-16
3

0
4

0.328794
3

0.0819338

0.24686
3

0
4

0
4

0

0

0

0

0

0

0
4

0

0

0
4

0

0

0

0
4

0

0

0
4

0

0

0
4

0

0

0
4

2.77555756156289e-16

0
4

0

0

0

0

0
4

0
4

0

0

0

0
4

0
4

0

0

0

0
4

0
4

0

0

0

0
4

0
4

0

0

0

0
4

0
4

0
4

0
2

0
2

0
2

0

0

0

0

0

0
4

0
4

0
4

0.0573536

0.0409669

0.0409669

0.0409669

0
4

0
4

0

0

0

0

0
4

0
4

0

0

0

0
4

0

0

0
4

0
4

0

0

0

0

0
4

0
4

0

0

0

0
4

0
4

0

0

0

0
4

0
4

0.0163868

0

0

0

0
4

0.0163868

0.0163868

0
4

0

0

0
4

0

0

0
4

0

0

0
4

0

0

0
4

0

0

0
4

0

0

0
4

0
4

3.46944695195361e-18

0
4

6.02712324493382e-14

0
4

33.9931
7

33.9931
7

33.9931
7

1.35756
6

0.0163868

0.117908
7

0

0

0

0.0383515

0

0

0

0

0

0.026063

0

0.0522832

0

0

0

0

0

0

0

0

0

0.852817
7

0

0

0

0

0.110278
6

0.0953683
6

0.0173753
6

0.0307329
6

0

0

3.7470027081099e-16
6

0
4

0.199816
7

0.0955644
7

0.104252
7

0

0
4

32.4357
7

2.75242

0.58173
7

11.7132
7

3.56342
7

0.174511
6

12.6872
7

0

0.758405
6

0.0901271

0.114707

0

0

0

1.2642664692919e-14
7

0
4

0
4

0
4

0
4

0

0

0

0

0

0

0

0

0

0

0

0

0

0

0

0

0

0

0

0

0

0

0

0

0

0

0

0

0

0

0

0

0

0

0

0

0

0

0

0

0

0

0

0

0

0

0

0

0

0

0

0

0

0

0

0

0

0

0

0

0

0

0

0

0

0

0

0

0

0

0

0

0

0

0

0

0

0

0

0

0

0

0

0

0

0

0

0

0

0

0

0

0

0

0

0

0

0

0

0

0

0

0

0

0

0

0

0

0

0

0

0

0

0

0

0

0

0

0

0

0

0

0

0

0

0

0

0

0

0

0

0

0

0

0

0

0

0

0

0

0

0

0

0
4

0

0

0

0

0

0

0

0

0

0

0

0

0

0

0

0

0

0

0

0
4

0

0

0

0

0

0

0

0

0
4

0
4

0
4

0
4

6.5298
7

6.5298
7

6.5298
7

6.49697
7

6.45848
7

0.0173753

0.01114

0.00997446

0
4

0.0328373

0.01671

0.01114

0.00498723

0

8.67361737988404e-19

0
4

2.08166817117217e-17
7

0
4

0
4

0
4

0

0

0

0

0

0
4

0

0

0
4

0

0

0
4

0
4

0
4

0
4

0

0

0

0

0

0

0
4

0

0

0
4

0

0

0
4

0
4

0
4

0
4

0

0

0

0

0

0

0
4

0
4

0
4

0
4

0

0

0

0

0

0

0
4

0

0

0
4

0
4

0
4

0
4

0
3

0
3

0
3

0
3

0

0

0
4

0
4

0
4

0
4

0

0

0

0

0

0

0
4

0
4

0
4

0
4

0

0

0

0

0

0

0
4

0
4

0
4

0
4

0

0

0

0

0

0

0
4

0
4

0
4

0
4

0

0

0

0

0

0
4

0
4

0
4

0
4

0

0

0

0

0

0
4

0

0

0
4

0

0

0
4

0
4

0
4

0
4

0.0378459
3

0.0378459
3

0.0378459
3

0.0378459
3

0.0378459
3

0

0

0

0

0

0
4

0

0

0

0
4

0

0

0

0
4

0

0

0

0
4

0

0

0
4

0
4

0
4

0
4

0

0

0

0

0

0

0
4

0
4

0
4

0
4

0

0

0

0

0

0

0
4

0
4

0
4

0
4

0

0

0

0

0

0
4

0
4

0
4

0
4

0

0

0

0

0

0

0
4

0
4

0
4

0
4

0.188266

0.188266

0.188266

0.188266

0.16879

0.0194758

6.93889390390723e-18

0
4

0
4

0
4

0
4

0

0

0

0

0

0
4

0
4

0
4

0
4

0

0

0

0

0

0
4

0
4

0
4

0
4

0

0

0

0

0

0
4

0

0

0
4

0

0

0
4

0

0

0
4

0
4

0
4

0
4

0

0

0

0

0

0
4

0
4

0
4

0
4

0

0

0

0

0

0

0
4

0
4

0
4

0
4

0
4

0
4

0
4

0
4

0
4

0

0

0

0
4

0
4

0
4

0
4

0
4

0
4

0
4

0

0

0
4

0

0

0

0

0

0

0
4

0
4

0
4

0
4

0

0

0

0

0

0
4

0
4

0
4

0
4

0

0

0

0

0

0

0
4

0

0

0
4

0
4

0
4

0
4

0

0

0

0

0

0

0
4

0
4

0
4

0
4

0

0

0

0

0

0

0
4

0
4

0
4

0
4

0

0

0

0

0

0
4

0

0

0
4

0
4

0
4

0
4

0

0

0

0

0

0
4

0
4

0
4

0
4

0

0

0

0

0

0

0
4

0
4

0
4

0
4

0

0

0

0

0

0
4

0
4

0
4

0
4

0.0822893

0.0822893

0.0822893

0.0822893

0.0822893

0
4

0
4

0
4

0
4

0

0

0

0

0

0

0
4

0
4

0
4

0
4

2.85568
6

2.85568
6

2.85568
6

2.84071
6

2.54647
6

0.0548595

0.109719

0.0249362

0.0872765

0.00498723

0.00498723

0.00748085

1.06685493772574e-16
6

0
4

0.0149617

0.00498723

0.00997446

1.73472347597681e-18

0
4

4.85722573273506e-17
6

0
4

0
4

0
4

0

0

0

0

0

0

0
4

0

0

0
4

0
4

0
4

0
4

0

0

0

0

0

0

0

0
4

0

0

0
4

0
4

0
4

0
4

0

0

0

0

0

0

0
4

0
4

0
4

0
4

0

0

0

0

0

0

0

0
4

0

0

0
4

0
4

0
4

0
4

0

0

0

0

0

0
4

0
4

0
4

0
4

0

0

0

0

0

0
4

0
4

0
4

0
4

0

0

0

0

0

0

0
4

0
4

0
4

0
4

0

0

0

0

0

0

0

0
4

0
4

0
4

0
4

0.00950508

0.00950508

0.00950508

0.00950508

0

0.00950508

0
4

0

0

0
4

0
4

0
4

0
4

0

0

0

0

0

0
4

0
4

0
4

0
4

0

0

0

0

0

0

0

0

0

0

0

0

0
4

0

0

0

0

0

0

0

0
4

0

0

0

0

0
4

0
4

0
4

0

0

0

0

0

0

0
4

0

0

0

0
4

0
4

0
4

0

0

0

0

0
4

0
4

0
4

0
4

0

0

0

0

0

0
4

0

0

0
4

0

0

0
4

0
4

0
4

0
4

0

0

0

0

0

0

0
4

0
4

0
4

0

0

0

0

0
4

0
4

0
4

0
4

0

0

0

0

0

0
4

0

0

0
4

0
4

0
4

0
4

0

0

0

0

0

0

0
4

0
4

0
4

0
4

0

0

0

0

0

0

0
4

0
4

0
4

0
4

0

0

0

0

0

0
4

0

0

0
4

0
4

0
4

0
4

0.0374042

0.0374042

0.0374042

0.0374042

0.0374042

0
4

0
4

0
4

0
4

0

0

0

0

0

0
4

0
4

0
4

0
4

0

0

0

0

0

0
4

0
4

0
4

0
4

0

0

0

0

0

0
4

0
4

0
4

0
4

0
3

0
3

0
3

0
3

0
3

0
3

0

0

0

0

0

0

0
4

0

0

0

0
4

0
4

0
4

0

0

0

0

0
4

0
4

0
4

0
4

0

0

0

0

0

0

0
4

0

0

0
4

0
4

0
4

0
4

1.36343

1.36343

1.36343

1.36343

1.36343

0
4

0
4

0
4

0
4

0

0

0

0

0

0

0
4

0

0

0
4

0
4

0
4

0
4

0

0

0

0

0

0
4

0
4

0
4

0
4

0

0

0

0

0

0

0
4

0
4

0
4

0
4

0.0149617

0.0149617

0.0149617

0.0149617

0.0149617

0

0
4

0
4

0
4

0
4

0

0

0

0

0

0
4

0
4

0
4

0
4

0

0

0

0

0

0

0
4

0
4

0
4

0
4

0

0

0

0

0

0
4

0
4

0
4

0
4

0

0

0

0

0

0
4

0
4

0
4

0
4

0

0

0

0

0

0

0

0
4

0

0

0

0
4

0

0

0

0
4

0

0

0
4

0
4

0
4

0

0

0

0

0
4

0

0

0
4

0
4

0
4

0

0

0

0

0
4

0
4

0
4

0
4

0

0

0

0

0

0
4

0
4

0
4

0
4

0.0245801

0.0245801

0.0245801

0.0245801

0.0245801

0
4

0
4

0
4

0
4

0

0

0

0

0

0
4

0
4

0
4

0
4

0

0

0

0

0

0
4

0
4

0
4

0
4

0

0

0

0

0

0

0
4

0
4

0
4

0
4

0.0163868

0.0163868

0.0163868

0.0163868

0.0163868

0

0
4

0
4

0
4

0
4

0

0

0

0

0

0

0
4

0
4

0
4

0
4

0

0

0

0

0

0
4

0
4

0
4

0
4

0

0

0

0

0

0
4

0
4

0
4

0
4

0

0

0

0

0

0
4

0
4

0
4

0
4

0.0573536
7

0.0573536
7

0.0573536
7

0.0573536
7

0
7

0.0573536
7

0

0

0

0
4

0

0

0

0
4

0
4

0
4

0
4

0

0

0

0

0

0
4

0
4

0
4

0
4

0

0

0

0

0

0
4

0
4

0
4

0
4

0

0

0

0

0

0
4

0
4

0
4

0
4

3.37715

3.37715

3.37715

3.37715

3.37715

0
4

0
4

0
4

0
4

0

0

0

0

0

0
4

0
4

0
4

0
4

0

0

0

0

0

0
4

0
4

0
4

0
4

0

0

0

0

0

0
4

0
4

0
4

0
4

0

0

0

0

0

0

0
4

0
4

0
4

0
4

0.00748085

0.00748085

0.00748085

0.00748085

0.00748085

0
4

0
4

0
4

0
4

0

0

0

0

0

0
4

0
4

0
4

0
4

0
4

0
4

0
4

0
4

0
4

0

0

0

0

0

0

0
4

0
4

0

0

0

0
4

0
4

0
4

0
4

0

0

0

0

0

0
4

0
4

0
4

0
4

0

0

0

0

0

0

0
4

0
4

0
4

0
4

0

0

0

0

0

0
4

0
4

0
4

0
4

0

0

0

0

0

0
4

0
4

0
4

0
4

0

0

0

0

0

0

0
4

0
4

0
4

0
4

0

0

0

0

0

0

0
4

0
4

0
4

0
4

0

0

0

0

0

0
4

0

0

0
4

0
4

0
4

0
4

0

0

0

0

0

0
4

0
4

0
4

0
4

0

0

0

0

0

0
4

0
4

0
4

0
4

0

0

0

0

0

0
4

0
4

0
4

0
4

0.0973892

0.092402

0.092402

0.0766699

0.0231005
1

0.0430814

0.0104881

0

0

3.46944695195361e-18

0
4

0

0

0
4

0.0157321

0.0157321

0
4

0
4

0
4

0.00498723

0.00498723

0.00498723

0.00498723

0
4

0
4

0
4

0
4

0

0

0

0

0

0
4

0
4

0
4

0
4

0

0

0

0

0

0

0
4

0
4

0
4

0
4

0

0

0

0

0

0

0
4

0
4

0
4

0
4

0.0491603

0.0491603

0.0491603

0.0491603

0.0491603

0
4

0
4

0
4

0
4

0

0

0

0

0

0
4

0
4

0
4

0
4

0

0

0

0

0

0

0
4

0
4

0
4

0
4

0

0

0

0

0

0
4

0

0

0
4

0
4

0
4

0
4

0

0

0

0

0

0

0
4

0
4

0
4

0
4

0

0

0

0

0

0

0
4

0
4

0
4

0
4

0

0

0

0

0

0
4

0
4

0
4

0
4

10.4123
3

10.4123
3

10.4123
3

10.4123
3

8.34461
3

0.0355306
4

0.0293706
5

0.0104881
4

0
2

0
4

0

0

0

0

0

0.134793
3

0.0194758

0

0.129839

0

0

0

0.00710613

0

0.170547

0

0.217827
3

0.613167
3

0.167353

0.165066
3

0.0565662
3

0.119498

0.191069
3

0
4

0
4

0
4

0
4

0
4

0
4

0
4

0
3

0
3

0
4

0
3

0
4

0

0
4

0
4

0

0

0

0
4

0

0

0

0
4

0

0

0
4

0

0

0
4

0

0

0

0
4

0
4

0
4

0
4

0

0

0

0

0

0
4

0
4

0
4

0
4

0

0

0

0

0

0
4

0
4

0
4

0
4

0

0

0

0

0

0

0
4

0
4

0
4

0
4

0

0

0

0

0

0
4

0
4

0
4

0
4

0.188448

0.188448

0.188448

0.188448

0.188448

0
4

0
4

0
4

0
4

0

0

0

0

0

0
4

0
4

0
4

0
4

0

0

0

0

0

0
4

0
4

0
4

0
4

0

0

0

0

0

0
4

0
4

0
4

0
4

0

0

0

0

0

0
4

0
4

0
4

0
4

0

0

0

0

0

0
4

0

0

0
4

0
4

0
4

0
4

0

0

0

0

0

0

0

0

0

0

0

0
4

0

0

0
4

0
4

0
4

0
4

0

0

0

0

0

0

0
4

0
4

0
4

0
4

0

0

0

0

0

0
4

0
4

0
4

0
4

0.185283

0.185283

0.185283

0.185283

0.185283

0
4

0
4

0
4

0
4

0

0

0

0

0

0

0
4

0
4

0
4

0
4

0

0

0

0

0

0
4

0
4

0
4

0
4

0

0

0

0

0

0
4

0
4

0
4

0
4

0

0

0

0

0

0
4

0
4

0
4

0
4

0

0

0

0

0

0

0
4

0
4

0
4

0
4

0

0

0

0

0

0
4

0

0

0
4

0
4

0
4

0
4

0

0

0

0

0

0
4

0

0

0
4

0
4

0
4

0
4

0
4

0
4

0
4

0
4

0
4

0

0

0

0

0

0

0

0
4

0
4

0
4

0
4

0

0

0

0

0

0
4

0
4

0
4

0
4

0

0

0

0

0

0

0
4

0
4

0
4

0
4

0.209631

0.209631

0.209631

0.209631

0.209631

0
4

0
4

0
4

0
4

0

0

0

0

0

0
4

0
4

0
4

0
4

0

0

0

0

0

0
4

0
4

0
4

0
4

0.00498723

0.00498723

0.00498723

0.00498723

0.00498723

0
4

0
4

0
4

0
4

0

0

0

0

0

0
4

0
4

0
4

0
4

0

0

0

0

0

0
4

0
4

0
4

0
4

0

0

0

0

0

0
4

0
4

0
4

0
4

0

0

0

0

0

0
4

0
4

0
4

0
4

0

0

0

0

0

0

0

0

0

0

0

0
4

0

0

0

0

0
4

0
4

0
4

0
4

0

0

0

0

0

0
4

0
4

0
4

0
4

0

0

0

0

0

0
4

0
4

0
4

0
4

0

0

0

0

0

0
4

0
4

0
4

0
4

0

0

0

0

0

0
4

0
4

0
4

0
4

0

0

0

0

0

0
4

0
4

0
4

0
4

0

0

0

0

0

0
4

0
4

0
4

0
4

0

0

0

0

0

0
4

0
4

0
4

0
4

0

0

0

0

0

0
4

0
4

0
4

0
4

0

0

0

0

0

0
4

0
4

0
4

0
4

0

0

0

0

0

0
4

0
4

0
4

0
4

0.282245
7

0.282245
7

0.282245
7

0.282245
7

0.282245
7

0
7

0

0
4

0
4

0
4

0
4

0

0

0

0

0

0
4

0
4

0
4

0
4

0

0

0

0

0

0
4

0
4

0
4

0
4

0

0

0

0

0

0
4

0
4

0
4

0
4

0

0

0

0

0

0
4

0
4

0
4

0
4

0

0

0

0

0

0
4

0
4

0
4

0
4

0

0

0

0

0

0
4

0
4

0
4

0
4

0

0

0

0

0

0
4

0
4

0
4

0
4

0

0

0

0

0

0
4

0
4

0
4

0
4

0

0

0

0

0

0
4

0
4

0
4

0
4

0

0

0

0

0

0
4

0
4

0
4

0
4

0

0

0

0

0

0

0

0

0

0

0
4

0

0

0

0

0

0

0
4

0
4

0
4

0

0

0

0

0

0

0

0
4

0

0

0
4

0

0

0
4

0
4

0
4

0
4

0

0

0

0

0

0
4

0
4

0
4

0
4

0

0

0

0

0

0
4

0
4

0
4

0
4

0

0

0

0

0

0
4

0
4

0
4

0
4

0

0

0

0

0

0
4

0
4

0
4

0
4

0

0

0

0

0

0
4

0
4

0
4

0
4

0

0

0

0

0

0
4

0
4

0
4

0
4

0

0

0

0

0

0
4

0
4

0
4

0
4

0

0

0

0

0

0
4

0
4

0
4

0
4

0

0

0

0

0

0
4

0
4

0
4

0
4

0

0

0

0

0

0
4

0
4

0
4

0
4

0.141432
3

0.141432
3

0.141432
3

0.026063
4

0.026063
4

0
3

0

0
4

0.115369

0.115369

0

0
4

1.38777878078145e-17
3

0
4

0
4

0
4

0

0

0

0

0

0
4

0
4

0
4

0
4

0

0

0

0

0

0
4

0
4

0
4

0
4

0

0

0

0

0

0
4

0
4

0
4

0
4

0

0

0

0

0

0
4

0
4

0
4

0
4

0

0

0

0

0

0
4

0
4

0
4

0
4

0

0

0

0

0

0
4

0
4

0
4

0
4

0.00498723

0.00498723

0.00498723

0.00498723

0.00498723

0
4

0
4

0
4

0
4

0

0

0

0

0

0
4

0
4

0
4

0
4

0

0

0

0

0

0
4

0
4

0
4

0
4

0

0

0

0

0

0
4

0
4

0
4

0
4

0.104881

0.104881

0.104881

0.104881

0.0734164

0

0

0

0

0.0314642

0

0
4

0
4

0
4

0
4

0

0

0

0

0

0
4

0
4

0
4

0
4

0

0

0

0

0

0
4

0
4

0
4

0
4

0

0
[truncated: 224,860 more chars]
