# Supplementary material for: Fungal communities decline with urbanization—more in air than in soil
Source: ISME J. 2020 Aug 5;14(11):2806–15. doi: 10.1038/s41396-020-0732-1 (PMC7784924; doi:10.1038/s41396-020-0732-1)
Supplement: Supplementary file 2 — Supplemental data [file 41396_2020_732_MOESM2_ESM.zip › Krona_AirUrbanCore.html]

Javascript must be enabled to view this page.

num
probth


41427

27375.4

4.44515
3

4.44515
3

4.44515
3

4.44515
3

4.44515
3

0

0

0

0

0

0

0

0

0

0

0

0

0

0

0

0

0

0

0

0

0

0

0

0

0

0

0

0

0

0

0

0

0

0

0

0

0
4

0
4

0

0

0

0
4

0

0

0
4

0
4

0

0

0

0
4

0
4

0
4

0
4

1.87455

1.87455

1.87455

1.87455

0.793077

1.08147

0
4

0
4

0
4

0
4

0

0

0

0

0

0
4

0
4

0
4

0

0

0

0

0
4

0
4

0
4

0
4

22757.9

19.991

19.0262

15.9998

0.0311165
3

0.0938984
2

0.265631

0

0

0

0

0

0

0

0

0

0.509301

0

0

0

0

0

0

0

0

0

0

0.101129
3

0

0

0

0

0

0.0129576

0

0

0.144196

0.216294

0

0

0

0

0.0887784

0

0

0

0

0.20715

0

0.4276

0

0.00897447

0.0129576

0

0

0

0

0

0

0.325521

0.0710561

0

0

0

0

0

0

0

0

0

0.565392

1.87865
7

0

0.0129576

0

0

0

0

0

0.347468

0.0712667

0

0.216294
7

0

0

0

0

0

0

0.00408015

0

0

0

0.826438
7

0

0

0.0887784

0

0

0

0

0

0

0

0

0

0

0

0

0

0

0

0

0

0

0

0

0

0.15413

0.0103722

0.0156497

0.0518304

0

0.00458025

0

0

0

0

0

0

0.00673085

0

0

0

0

0

0

0

0
6

0

0

0

0

0

0

0

0

0

0

0.282642

0

0

0

0.0595432
6

0.144196
7

0

0

0

4.57138
1

0

0.36049
2

0.0285235

0

0

0

0

0.123097

0

0

0
3

0

0.17468

0

0

0

0.288392

0

0.144196

0

0

0.288392
2

0

0

1.31789

0

0.109545

0

0

0

0

0

0
2

0

0

0.15413

0.0259152

0.0207444

0

0

0

0

0

0.80918
7

0

0

0

0

0

0

0

0

0

0

0.07993
6

0

0

0

0

0

0

0

0

0.245736

0

0
6

0

0

0

0

0

0

0

0

0

0

2.22044604925031e-15

0
4

0.0156497
3

0.0156497
3

0

0

0

0
4

0

0

0
4

0

0

0
4

0

0

0
4

0

0

0
4

0

0

0

0
4

0

0

0

0
4

0

0

0

0
4

0.216294

0.216294

0
4

0.103624

0.103624

0
4

0

0

0
4

0.136055
6

0.0129576

0.0388728

0

0.0842243

0
4

0

0

0
4

0

0

0

0
4

0

0

0

0
4

0

0

0

0
4

0

0

0
4

0

0

0

0
4

0.0134617

0

0.0134617

0
4

0

0

0
4

0

0

0
4

0

0

0
4

0.399068
2

0

0.399068

0

0

0
4

0

0

0
4

0

0

0
4

0

0

0
4

0.00897447

0.00897447

0
4

0

0

0
4

0

0

0
4

0.00777913

0.00777913

0
4

0

0

0
4

0

0

0
4

0

0

0
4

0
6

0

0

0
4

0.0591856

0.0591856

0
4

0.0770648

0.0770648

0
4

0

0

0
4

0

0

0
4

0

0

0
4

0

0

0
4

0

0

0
4

0

0

0
4

0.20715

0.20715

0
4

0

0

0
4

0

0

0

0
4

0

0

0
4

0

0

0
4

0

0

0
4

0

0

0
4

0

0

0
4

0

0

0
4

0

0

0
4

0.00518609

0.00518609

0
4

0.269727

0.269727

0
4

0

0

0
4

0

0

0
4

0

0

0
4

0.732115

0.732115

0
4

0.00411208

0.00411208

0
4

0.770977
7

0.770977

0

0
4

0

0

0
4

0

0

0

0
4

0
4

0.269727
6

0.269727
6

0.269727
6

0
4

0
4

0

0

0

0
4

0

0

0
4

0
4

0

0

0

0
4

0
4

0

0

0

0

0
4

0
4

0.0194364

0.0194364

0.0194364

0
4

0
4

0

0

0

0
4

0
4

0

0

0

0
4

0
4

0

0

0

0
4

0
4

0

0

0

0
4

0
4

0

0

0

0
4

0
4

0

0

0

0
4

0
4

0.144196
6

0.144196
6

0.144196
6

0
4

0

0

0
4

0

0

0
4

0
4

0

0

0

0
4

0
4

0.0259152

0.0259152

0.0259152

0
4

0
4

0

0

0

0
4

0
4

0

0

0

0
4

0
4

0

0

0

0
4

0
4

0

0

0

0
4

0
4

0

0

0

0
4

0
4

0

0

0

0
4

0
4

0

0

0

0
4

0
4

0.15413

0.15413

0.15413

0

0
4

0
4

0

0

0

0

0

0
4

0

0

0
4

0
4

0.20715

0.20715

0.20715

0
4

0
4

0

0

0

0
4

0
4

0

0

0

0
4

0
4

0

0

0

0
4

0
4

0.144196

0.144196

0.144196

0
4

0
4

0
4

13008.7
7

0.179888

0.0696445

0.0161178

0.0183607

0.0322355

0.0029305

0
4

0.0991524

0.0991524

0

0
4

0.00816031

0.00816031

0

0
4

0.0029305

0.0029305

0

0
4

0

0

0
4

4.77048955893622e-18

0
4

12814
7

12813.4
7

1315.87
7

34.292
7

1.62652
6

0.013839

0

0

0.0156497

0

0.0129576

0

0.0156497

0

0

0

0.0244187
7

0

0

0

0

0

0

0

0

0.216294

0

0.0567664
7

0.0107775

0

0

0.0591856

0

0

0

0.00615463

0

0

0.234686

0

0

0

0.0770648

0

0.266335

0

0.0769775

0

0

0.156505
7

0.0091605

0

0.288392

0.216294

0.464213

0

0

0.0100616

0.0352157

0

0.477939

0

0.216294

0.00661392

0.0201232

0

0

0

0

0.236743

0.00458025

2.13068
7

0

0

0

0

0

0

0.0236854

0

0

0.118371

10.3815
8

0

0

0.15413

0

0

0

0

0

0

0

0.445545

0

0.00987078

0

0

0

0

0

0.0107775

0

0

0.130952

0

0

0

0

0

0

0

0

0

0

1.42988
7

0.273306
7

0

0.0156497

0

0

0

0

0

0.017764

0

0

0.0242866

0

1.15357

0

0

0

0

0

0

0.00888202

0

0.0107775
6

0.00518609

0

0

0.144196

0

0

0

0

0

0

0.825102
7

0

0

0.0148034

0

0

0

0.0129576

0

0.118371

0.36049

1.31921
7

0.0029305

0

0

0

0

0

0

0

0

0

1.29438
8

0.0591856

0

0

0.0100616

0.0591856

0.0770648

0

0.00394831

0.0286603

0

5.32619
8

0

0

0

0

0.00822417

0

0

0.062599

0.0156497

0

0.657969
7

0

0

0

0

0

0

0.00923194

0

0.00612023

0

0.148359
7

0.0234746

0

0.0118427

0

0

0

0

0

0

0

0.156421
6

0.0156497

0.0391243

0

0

0.0591856

0

0

0

0

0

1.1369
6

0.0592918
1

0

0

0

0

0

0

0

0

0

0

0.924105
7

0.36049

0

0

0

0.295928

0

0

0

0

0.00592247

0.239768
7

0

0

0

0

0

0

0

0

0

0.147964

4.31994
1

0

0

0

0.0391243

0

0

0

0

0

0

0.991851
7

0

0

0

0

0

0

0

0

0

0

0.139273
6

0

0

0

0

0

0

0.0150924

0.115597

0

0

7.3948
8

0

0

0

0

0

0

0

0

0

0

1.35403
7

0.0156497

0

0

0

0

0

0.0234746

0

0.539453

0.00615463

0.0450468

0

0.0201232

0

0

0

0

0

0

0

0

0.0904828
6

0.0118449

0

0

0

0

0

0

0

0

0

2.26138
7

0.163057

0

0

0

0.0161662

0

0

0

0

0.00408015

1.80245

0.0230192
6

0

0

0

0.144196

0.0887784

0.0591856

0

0

0

0

1.23806
1

0

0

0.00458025

0

0

0.0114506

0.288392

0

0

0

0.0150924
7

0

0

0

0

0

0

0

0

0.00458025

0.0352743

0.23475
7

0

0

0.00777913

0

0.00408015

0

0

0

0

0

0.154973
7

0

0

0.0110232

0

0

0.0114506

0

0

0

0

0.15413
7

0

0

0

0

0.00967569

0.118371

0

0.00518609

0.0129576

0

0.0263585
5

0

0

0

0

0

0.00458025

0

0

0

0

0.259886
7

0

0

0

0

0.216294

0

0

0

0

0

0.0150924
6

0

0.00967569

0

0.330419

0.0389321

0

0

0

0

0

4.41205
7

0.046467
7

0

0

0.00777913

0

0

0.0712667

0

0

0

0

0.554676
7

0

0

0

0.384267
7

0.0743356
7

1.4152
8

0.159846
7

0.959074
6

0.0372208
6

0.0337096
7

0.549167

22.0202
7

0.0418882
7

0.362369
7

0.36049
7

0.516948
7

0.360878

0.189414

0.996423

0.00624502
7

0.36049
7

0.0632658

0.620321

0.328961
7

0.049186

0.102097
7

0.0259152

0.173838
7

0.44884
7

0
7

0
3

0.0949466

0.0156497
6

3.43855
7

0.526258
7

0.0118427
7

0.157591

0.00934944
7

0.049186

1.63193
7

0.521631
7

0.212583

6.8488
6

0
6

1.18967

1.19616
8

0.0259152
6

0.0105016
7

1.1492
6

0.219096

0.0598761

0.0118449

0

0.029112
7

0.0176371

1.90209
7

0.373313
7

0.0848966
7

0.303778

0.057242
7

0.0770648
6

0.0783346
7

0.0800101
2

0.0290271

0

1.19357
8

16.04

1.19261
7

0.00777913
6

3.55405
6

0.82978
8

1.15357
7

0
7

0.0556354
7

36.3915
8

0.0889846
6

0.626825

0.0770648
6

1.90375

0.0629144

0

0
6

5.07109
8

1.16754
6

0.646055
7

0.485138
7

0
6

1.03956
8

0.168385
7

1.12875
7

0.663639
7

2.57606
8

0.060927

0.0129652
6

0.216281
7

0.0538742

0.00448724
7

0.564671
7

0.00987078
2

0.20715

3.6237
7

0.0469492

0.0436087

0.0194364
6

2.81182
7

1.22567
8

0.0187684
7

0.396922

0.0105027

1.01816
7

0.082829
7

1.92322

1.27054
1

0.0955962
8

0.031904
7

0.0290271

0.0264272
7

0.597627
7

0.0402465
6

0.0490788
7

2.81182
7

1.57829
7

4.03284
7

0.032394
7

0.0301849

0
7

0.0889075
6

0.0737611
7

0.144196
6

0.00458025

0.0149524

0.0256351
7

0
7

2.01383
7

0
6

0.270557
7

0.100733
7

0.0591856
7

0.36049

0.16745

0.00592134
6

1.73595
8

0.147964
6

1.69328
7

2.8328
7

0.144196
7

0.157853
7

0.229931
7

0.0620416
7

0.00615463
6

0.362211
7

0.345773
7

1.01395
7

0.0150636
7

0.068772
7

1.17465
7

0.133438

0.118371
6

1.87455
7

0
6

0.0343557
7

0.0378456
6

0.664531
7

0.255533
6

0.102659
7

0.0538875
7

4.07734
7

0.0422797
7

0.0647912
7

0.576113
7

0.158026

0.754392
7

0.203171
6

0.144196
7

1.81217

0.408136
7

0.0118427
7

4.85109
7

5.43817
7

0
6

0.0152477

0.0111074
7

0.0194364
6

0.0445273
7

0.236087

0.0162093
6

0.244743
7

0.00888202
5

0.216294
7

0.932317
7

0.0351952
7

0.00394831
7

0.0356026
7

1.10716
8

1.83794
8

0.300448
7

0.0928586

0.368816
7

0.0396914
1

0.216294

0.702702
7

0.292555
7

0.308622
7

0.0144709
6

0.022497
7

0.18414
7

0
5

0.02023

0.157153
7

0.00967569
6

0.0257114
6

2.11675
7

0.937273
7

5.40735
8

0.0553802
6

0.104271

0.101213
1

0.18003
7

0.262236
7

0.0294089
7

0.039932

0.266647
7

1.55556
7

0.160179

0.0150924
6

0.377105
7

0
7

0.131941

0.105807
7

0.186824
7

0.04973

0.00458025
6

0.648882
7

1.72108
7

0.309947
7

0.0632658

0.0464915
6

0.0547741
7

0.738809
7

0.12378
7

0.374131
7

0.347418
7

0.0118427
7

0.818175
6

0.565412
7

0.0207247

0.37614
7

0.0286073
7

0.153667
7

0.0378272
7

0.0352704
6

1.74981
7

0.0296067
7

0.00592134
2

0.0150924

13.8766
7

0.532093
1

0.0442647
7

0.72098
7

0.0100616

0.0145717

1.54633
7

0.065357
7

0.648882
7

0.384887

0.0281216
7

3.35045
7

0.0357627

0.201719
7

0.148673
6

0.153872
7

0.022013
5

0.0811449
7

0.0149732
7

0.143943
7

0.0388948
2

0.138101

5.30138
7

0.144196
7

0.139072
7

0
7

0.0156497
5

0.0236407
6

0.144196
7

0.728289
7

0.153887

2.16743
8

0

31.5732
7

2.07517
7

0.648882
7

1.24577
7

0.00624502
7

0.0082435
6

0.36049
7

0.242176
7

0.0889051
7

0.589741
7

0.144594
7

1.09114
7

0.326616

0.260489
7

0.148776
7

0.626954
7

0.188464
7

1.45647
8

0.0467021

0.19589
7

0.032394
7

0.325521
7

0.0143997

1.29595
7

0.00411208
7

0.291657

0
7

0.169984

0.0355557
6

0.0103722
2

0.304511
7

0.05964

0.527097
7

0.0301849

0.842725

0.188318
7

0.0153866
7

0.296926
6

0
6

0.0524008
6

44.2468
7

0.0375445

0.215272

0.00888202
7

0.402099
7

0.881123

0.025154

0.0887784
7

0.0704238
7

0.150316
7

0
5

0.0591856
7

0.222708
7

0.00615463
2

0.118045
7

0.0156497
7

3.79895
7

0.019828
6

0.278186

0.0900454
7

0.0249074
6

0.057548
7

0
6

0.5291

0.0842243
7

0.00661392
7

0

2.04754
7

1.00922
7

0.218642

0.0208358

0.0517979
2

0.0632658
7

0.220115
7

0.0132278

0.0592476

0.0123996
6

0.00967569

0.3293
7

0.0111086
7

0.0507155
7

0
5

0.216294
7

0.00458025
6

0.325521
7

0.111514

0.0469492
7

0
7

1.53883
7

0.394214
7

0.0314655
4

0.0262692
6

0.0165977
7

0.373455
7

0.149382

0.0414274
2

0.432588
7

0.0156497

0.00777913
7

0.371922
7

3.9775
6

0.0259152
6

0.119709

0.0284045
7

0.0374197
6

0.0208358
7

0.0102348
7

0.16413
7

0.096339
7

0.266201
7

0.240691
7

16.8894
7

0.582237

0.00458025
6

0.134949
7

0.681649
7

0.0581965

0

0
7

20.9659
7

0.221261

0
7

0.0181437
5

4.19733
7

0.0230192
6

0

0.126375

0
6

0.15447
7

0.0274229
6

0.0121052
2

1.22567
7

1.95538
7

0.0358797

2.75169
7

0.00440928
4

3.82638
8

0.00543325

0.432588

0.0933587
7

0.00394831
6

0.0162162
7

0.270415
7

1.03123
8

1.00937
7

1.31379
7

0.0129576
7

0.439202
7

0.301349
1

0.0588542
7

0.00592134

0
6

0.0532921
6

0.287094
7

0.504686
7

1.35361
8

4.62711
7

0.0393351

0.0113407
7

0.0455714
7

0.0469492

0.0186989

0.0862815
7

0.627092
7

0.115597
6

0
6

0.0427377

1.23321
7

0.0933168
8

0.961248
8

0.00532728
6

0.676097
7

1.96974
7

0.266335
7

0.216294
7

0.017764
6

0.0274229
6

0.0150924
7

0.0975914

0

0.0465234

0.475528

0.784748
7

0.203472
7

0.0933587
7

0.506469
7

0
7

0.118371
7

0.00661392
7

1.60247
7

0

0.0100616

0.144196
7

0.60426
7

0.0107775
6

56.5122
8

0.159237

0.0548458
7

0.00612023
6

0.581985

2.20479

0
7

0.108215
7

0.0422358
7

0

0.193403
7

0.237319
6

0.370551
7

3.35675
7

0.0907031
7

0.624502

46.3549
7

0
6

0.941354
7

0
6

0.00881856

0.171047
7

0.661839
1

0.0100616
7

0.00888202
6

0.23801
7

0.00458025
6

9.49836
7

3.93592
7

0.20715
7

4.19002
8

0.855676
7

0.0220866
6

0
7

0.20715
7

0.0770648
7

0.00799092
7

0.0158303

0.0172682
6

3.32746
7

0.288392

0.0591856
7

0.0469492
7

0.144196
7

0.0925453
7

1.97013
7

0.0391243
7

0.0991506

0.0100616
7

0.0810131

1.59893
7

0
6

0.0404067
6

0

0.154973
7

0.0150022
7

0.0666168

0
6

0
7

0.335427

0.0487592

0.634251
7

0.0927268
7

0.00687037
7

0.153872
8

0.00592134
7

0.00624502

0.0201232
6

0.575109
7

0
5

0.0709939
7

0.0452773
6

1.52282
7

0.865175
7

0.0106546
6

2.31751
7

0.00448724
7

0.836577
7

0.0831093
7

0.0313712
6

0.00615463
5

0.446918
8

0.0104084
7

1.82417
7

0.00458025
6

0.0869671
8

0.0402465
7

0.548057
7

0.0258273
6

0.316767
7

0
6

0.72098
7

0.504686

0.101687
7

0.77332
7

0.0398577

0.437705
7

0.0573576

0
7

0.144196
7

0.0322932
7

0.00923194
6

0.0374171
7

0.0553389
7

0.643246
7

3.25251
7

0.098531

0.0305316
7

0.0391243

0.0234746
7

0.0275548

0.00661392
6

0

0.017764

0

0.0456376
5

1.0579

0.0911186

0.00881856
6

0.00661392
6

0.0514981
1

0
6

0.504686
7

3.40477
8

0.0153866

0.134623

0.157451

1.11976
7

0.15653
2

0

0.334784

0

3.30569
7

0.682509
7

0.756195
7

0.0929572
7

0.0553916
7

0.00881856
6

1.49006

1.02284
7

0.0259152
7

0.169464

0.144196

0.0322055
6

1.48968
7

0.0100616
6

0
6

0.060336
7

0.00394831

0.0799482
7

0.870678

0.0880207
7

0
7

0.115597

0.169052
7

0.029498

0.160334
7

0.00687037
7

1.26223
8

0.0156497

0.0312995

0.597872
7

0
6

0.0452773
7

0.231194
7

0

0.0265826
5

0
7

0.47586
7

0.0904851
6

0
6

0.0783581
2

0.682593
7

0.576784
7

0.216294

0.445553
1

0.00592134
6

0.00592134
7

0.0112181
6

0.564967
7

0
2

0.544932

0.216294
7

1.36808
7

0.0234746
7

0.144196
7

2.04852
8

0.0129576
7

0.15081
7

0.031768
7

0
6

0.00661392
7

0.327516
1

0.0156497

0.97593
7

0.0406932
7

0.177557
7

0

0

0.0266235
6

0.236743
7

0.598791

0.297622
7

0.0485621
7

0.129576
7

0.992156
7

0
6

0
6

0.0234746
6

0

0.168203
7

0.545512
7

0.05964

0.0123093

0.221718
1

0.103725
7

272.664
7

0.00592134

0.0259152
6

1.37954
8

0

1.75698
8

0.0234746
1

0.176447

0
6

0.216294
7

0.0572757
7

0.167943
6

0.0235724
7

0.376955

0.0153577
7

0.276759

0.0633021
7

0.0614332
7

0.0176371

0.0156497

0.240013

0.00777913
7

0.949304
7

0.216294
7

0.141844
7

0.148308
7

0.650395
7

0.0633883
6

7.20972
8

0.112665
8

0.0119425

1.2322
7

0.0947013
6

4.39662
7

0.233451

0.0148034

0.0255517
1

0.0655711

0.144196
7

0
8

0.00408015
5

5.29882
7

0.0234746
6

0.120006
7

0

0.925124

0.0295385
7

0.0193514
6

0.0100616
7

0.0312995
7

0.0162896
7

0
7

0.0156497

0.660322
7

0.0670823
7

0.0123093

1.45121
7

0.0100847
5

0.0352157
7

0.444664
7

0

0.103988
7

0.0237351
7

0.36049
7

0.144196
7

0.0995559
6

0.00661392
6

2.76796
7

0.144196

0.117373
7

0.153078
7

0.231339
7

0.0821649
2

0
6

0.29216
7

0.247593
6

0.0165286
7

0.657341
8

1.07873
7

0.557114
7

0.0309062
7

0
7

0.0372909
7

0.0591856

8.08385
8

0
4

0.0984541

0.0843397
7

0.00411208
8

0.699841
7

0.151975
7

0
6

0.0503081
7

0.432588

0
6

0.0234746

0.0154959
6

0.118371
7

0.00777913

0.708067
7

0.818615

0

0.148359
7

0.0591856
7

0

0.216294

0.00592134
7

0.292014
7

0.0118427
7

0.0469492
7

0.369786

1.92199

0.36049
7

0.00592134
7

0.0107775
6

0.00923194
7

0.013624
7

0.0399574
7

0

0.0637659
7

0.159846
7

0.0246798
7

0.0571039
6

0.00440928
5

0
6

0
6

0.0192045
6

0.0106419
5

2.95478
7

0.15413
6

0.0111074

0
7

0.0122405

0.151617
6

0
2

0.00987078
6

0.504686
8

0.127532
7

0.284389
7

0
6

0.0591856
7

0.0153866
7

0.00483473

0.0234746

2.73329
7

4.61779
7

0.00615463
6

0

0.0391243
6

0.20715

0
6

0.0237351

0.0591856

2.89633
8

2.49926
8

0.318897
7

1.29835
7

0

0.222414

0.00458025
7

0.0362217
6

0.42607
7

0.0166534
6

0.442408
7

0.016563
6

0.025506
7

0.0162162
6

0.633414
7

1.01065
8

0.064779
6

0.118371
7

0.154973
7

0.288392
7

0.0132278
7

0
6

0.0234746

0
6

0.00616813
6

1.28948

0.400434
1

0.0239808
5

0
6

0.648882
7

0.0127685
6

0.0157524
6

0.0207247

0.025154

0.0150924
6

0.0091605
2

0.338837

0
6

0
8

0.00661392

0.177557
7

0.0382486
7

0.0936841
7

0.216294
7

0.0284107
7

0
6

0.00440928
7

0.285255
6

0.261488
7

0.0591856
6

0.0770648
7

0.358378
8

0.0122405
7

0.0435978
6

0.00543325
5

0.195404
7

0
5

0.0457383
7

0.56404
7

0.0368221
7

0
7

0.355114

0.823156
7

0.231194
7

0.148144
7

0
5

0.0250623
7

0.0129576

0.124293

1.51293
6

0.0356729
7

0.116618

0.00416335
6

0.319266
6

0.00532728

11.3816
8

0.0195715

0.72098

1.00887
8

0.0386233
7

1.40363
7

1.01553
7

0.00458025
6

0
6

0.0156497
6

0.00789662
6

0.347577
6

0
7

0.00967569
7

10.5208
8

0.109548

0.847894
4

0.0293174
7

0.00874359
5

1.18834
7

0

0.0129576
1

0.012076
6

0
6

0

0
7

0

9.45284
7

0.303373

0.383964
7

0.0100616
6

0.00458025
7

0
6

0
6

0.00394831
7

0
7

0

0
6

0

2.78635
7

0.390985

0.0111086
6

0.0157932

0
7

0.0198131
7

0.354759
7

0.0323325
7

1.03895
8

0.00789662
7

0.415264
6

0.966819
7

0.72098
8

0.0714027
7

0.348376
8

0.0117274
6

0.00687037
6

0.0100616

0
6

0.750843
1

1.51406
7

0.0861294
7

2.65817
7

0
6

0.0553389
7

0.0145135
7

0.0161662
7

0
6

0.216294

0.0249632
6

0.00967569
7

0.0242034
7

3.10909
8

1196.56
7

0.239984
8

0.38065
7

0.731757
7

0.0301849
7

0.00592247
6

0.0312995
6

0.730655
8

0.138854
6

0
6

0.080687
1

2.75587
7

0.020059

0.0325674

0.0262279
7

0.0547741
7

0
6

0.159846
7

0.0104086
7

0.116501
7

7.22065

0.144196
7

0.254117
6

0
7

0.36049
7

0.504686
7

0.0296139
7

0.0151034

0.550679
7

0.167671

0.395469
7

0.0403861
6

0.490419
1

2.21663
7

0.0436087
7

0.178448
7

0.287644
7

0.0989745
8

0.0938984
7

0

0
6

2.18422
7

0.292788
7

0.576784
7

0.274786
6

1.22567
7

0.427869
7

0.344298

0.51084
7

0
7

0.0438197
8

0
7

0.0547833
7

0.0978565
7

0.883639
7

0.27182
5

0.021812
7

1.28418
7

0.0268449
2

0.203382
7

0.0625788
6

0.0553389
7

0.441748
7

0
6

0
7

1.02502

4479.32
7

9.02228
7

0.213199
6

0.508454
7

0.00799092
7

0.0135128
7

0.00416335
7

0
6

0.519778
7

0.0690564
7

0.231386
7

0.0305148
7

0
6

245.052
8

0.0129576
6

0
6

1.58615
7

0.00616813
7

0.00322316

0.708067

0.0257114
7

0.00592247
5

0.0148618

0.0391243
7

0.127388

0.00923194
7

0.0804854
7

0.0206111
6

1.72372
8

0.00661392
7

0.177557
7

0.287363
4

0.668695
7

0.0301849
6

0.144196
7

0.612663
7

0.865175
7

0.0241892
7

0.462389
1

0.160936
7

0.0177674
6

0
6

0.0091605
7

0
6

0.216294
7

0.36049

1.33816
7

0.903726

0.0173914
6

0.227317
8

0
7

0.0100616
6

0

0.0156497
7

0.0150924

0.00967569
7

0.653462
8

1.43002
7

0.00394831

0.00518609
7

0.0324592
7

0.0091605

0

0.0400119
8

0

0.00967569
6

0.301947

0.032394

0.205941
7

0.996918
7

0

0.0232675
7

0.617145
7

0.00518609
5

0.00458025
6

0.231386
7

0.021555
6

0.144196

0.00661392
7

1.40664

0.399614
7

0.00616813

0.288392
7

0.134021

0.0591856

0
7

0.128241
7

0.0137407
6

0.0982642
7

0
7

0.859521

0.0201232
6

0

0.00661392
6

0
2

0.0591856
7

0.160159
8

0.0460479
6

0.0591856
1

0
6

0
6

0.51134
7

0.00948324
7

0

0.600895
8

0.220406
7

0.36049
7

0.144196
6

0.0387028
7

0.00592134

0
7

0.0651081

10.213
7

0.45342

0.206374
7

0
6

0.00518609

0.288392
7

0.466131
8

0
7

0.725389
7

0.192662
7

0.0390034
6

0.144196
7

0.399547

0.0215711

0.062599

0.0107775
6

0.0301986
7

0.0483591
7

0.00416335
7

0
6

0.0518304

0.0770648
7

0.0832194

1.88653
7

0.569099
8

0.0341096
7

0.144196
7

0.00888202
6

0
6

0

0

0
6

0
7

0.0603697
7

0.776445
7

0

0.817294

0.0301633
5

0

0.0591856
7

0.0312995

0.0369127
7

0.00923194
6

0.026521

0.00923194
7

0.133315
7

0.00881856
2

0.873336
7

0

0.288392

0.014166

0.0091605
7

0
7

0

0.0887784
7

0.0171286
7

1.71929
7

0
7

0.00394831
7

0.0591856
7

0.144196
1

0.0769921
7

0

0

0.109548

0.00416335

0
5

0.530295
7

0.159582
7

0.185548
7

0.118371
1

0.0657704
6

0.508798
7

0
4

0.0709886

0.0991234
8

0.124616
7

0.0352157
7

1.24264
7

0.0377212

0.221356
7

0
6

0.00416335

0.295006
7

0

0
6

0
2

0
6

0.830623
8

0.695702
7

0

0.0123093
7

0.0453516
6

0.153078
1

0.236743

0.648882
7

0.423376
7

0.437555
7

0.0100616

5.23313
8

1.8004

0.0328662
7

0.0137407
7

0.0340104
8

0
7

0.648882

0.465949
8

0.0748354
7

0

0.118371

0.276729
8

15.1909
7

0.771382

0

0.0948801
8

0.146505
7

0
6

0.170652
8

1.12774
8

0.0712667
6

0.0104097
8

0.0100616

0.00518609
6

1.93948

0.576784

0.225037
7

0

0.0123093

0.01249
7

0.00615463
6

0.510608
7

9.08434

0.00843555
6

0

0.0881919
7

0

0
6

0
7

0.141644
7

0
6

0.216294

0.0169059
7

0.0205604
7

1.28285
8

0.0870247
7

0.448044
7

0.0210138
7

0

0.0927268

0.144196
7

0.00458025
6

0.0234746
6

0.36049
6

0

0.00362217
6

0
5

0.615812
6

0.0503081

0

0

0.0591856

0.147964
7

0

0

0.0457027
2

0.532671
1

0.0118427

1.4613
7

0.0921377
7

0.144196
7

0
6

0.0290271

0.0841657
7

0

0.0100616

0.0194364
7

0.0437046
6

0.0525861
7

0.278617
7

0.216294

0.576784

0.146979

0

0.30546
7

0
6

0.648882
7

0.0234746
7

0.144196

0

0.156796

0.0100616

0.11356
7

0

0.0118427

0.152544
7

0
7

0.0378111
7

0.0269437
6

0.235677
7

0
6

2.98583
7

0
5

0.108846
8

0.00518609

0

0

0.0285623
7

0.305216

0.0156497

0.360441

0.144196

3.7082
7

0
6

0.00923194
7

0
5

0.0547741
7

0
6

0.0091605
6

0
7

0.0252668
7

0.0738555
7

0.112253
6

14.3765
7

0.905083
7

0.0808274
7

0
7

0

0.253167

0.0268311
7

0.167881
7

0
5

0.0234746
7

0.0100616
7

0.453954
7

0.29399

0

0.822198
8

0.0207444

0.0156497
7

0.453211

0.024865
7

0.144196
7

0
6

0

0.0239744
7

0.558016

0.00408015
6

0.0770648

0.0266461
7

0.567848
8

0.297624
7

0

0.0107775
7

0.72098

0.16494
7

0.0319637

0.275456

0.00987078
6

0.111592
7

0.00440928
6

0.0296067

0.0145135

0.0237912

0.275443
1

0

0.0107775

0.165153
7

2.42349
7

0.0591856

0
6

0.582938
8

0.0289079
6

0.0103722
6

0.118371
7

0.0150924

0.0298032

0.382031
8

0
6

0.327065
7

0.898808
1

0.0469492
7

0.370551
1

0

0

0
8

0.00592247
7

0.0383944
7

0.108702
6

0.0208391
7

1.40555
7

0.0391243
7

1.58615

0.0532921
7

0
6

0
7

0

0.432588
7

0.0100616
6

0

0
6

0.525408
6

0
6

0.00458025
4

0.813689

0.00967569
6

0.197357
7

0.115597
6

0.0452773

0.0194364
7

0.00458025
7

0.172803
8

0.16308
6

0

0.0309999
7

0.0270617

0

0
6

0.0544202
7

0.00518609
1

0.144196
7

0.059052

0.457688
6

1.28106
6

0.147964
7

0.0234746

0
6

0.150118
7

0.0770648
7

0.00923194
7

0.0215412
7

0.0234746
7

0.767253
8

0.00661392
7

11.1589
7

1.49226

0.0547741

0

0
6

0.0391243
6

0

0
6

0.00458025
1

0.00416335

0.0164229

0.00789662
7

1.92561
7

0.117373

0.0103722
6

0

0.0350248
7

0.0129576

0.0391243
7

0.737317
8

0

0
6

0.0911119

0.399002
7

0.0887784
7

0.00888202
6

0

0.0156497
7

0.144196
7

0.20715
7

0.0678996
6

0.0172087
6

0.0269437
7

0.0301849

2.06256
7

0.167097

0.0518912

0

0.0254941

0.0169135

0.231194

0.0388728

0

0.0670724

0.0100616

12.0922
7

0.216294

0.0100616

0.0347229

0

0

0.0342521

0.0887784

0.144196

0.391781

0

0.811682
7

0

0

0.00408015

0.0562528

0

0

0

0.00615463

0

0

0.53582

0.0855237

0.0114506

0.0224408

0

0

0.0075591

1.80245

0

0

0.0195958

0.214738
6

0

0

0.0234746

0

0.36049

0

0

0

0

0

0.432518

0.00897447

0

0.0100616

0.164319

0.0591856

0.216294

0.0384887

0

2.02574

0.0194364

1.54257
7

0.0151418

0

0.00624502

0

0

0

0.0091605

0

0

10.5054

7.97487
7

1.05773
6

0.021555

3.5328

0

0.00592134

0

0.00416335

0.0450296

0

0.0129652

0

0.513846
7

0.0100616

0

0.035773

0

0

0

0.164487

0.239768

0.0364856

0

1.0873
7

0

0

0.00615463

0.0770648

0.72098

0.00967569

0.0887784

0.225018

0.0301849

0.0162542

1.2844
7

0.0342521

0.59217

0.0118427

0

0

88.8113

0

0

0.144196

0.0736992

1.76704
7

0.216294

0.0176371

0

0.0503081

0

0.183217

0.0148034

0.00923194

0

0.0770648

0.38177

0.0595022

0.0240166

0

0.0150924

0.192662

0.709344

0.0330696

0.00724433

0

0.155861

0.580233
7

0.0129576

0.865175

0.0129576

0

0

0.00440928

0.0987192

0

0.00615463

0

4.26313
7

0

0

0.0483785

0.0234746

0.0105295

0

0

51.9826

0.0234746

0

0.294613
7

0.00411208

0

0.0156497

0

0.386582

0.349104

0.049186

0.0373581

0.144196

0.130096

0.292381
6

0.102219

0.0259152

0

0.0440039

0.0452773

0

0.0774055

0.0129576

0.937273

0.701684

13.3506
7

0.483107
6

2.31722

0.0156497

0

0

0.0117274

0.00440928

0.0301849

0

0

0.0452773

0.315245
7

0.37228

0.0796255

0

0.266602

0.00592247

0.0301849

0.023674

0.0100616

0

0.00923194

0.747996
7

0

0.0129576

0.0148034

0

0

5.63841

0.364899

0.0444424

0.144196

0.0156497

1.29377
7

0.23573

0.00411208

0.168701

0.144196

0.0150924

0.0622331

0

0.0107775

0.226355

0.0591856

0.197357

0.223164

0

0

0.0452773

0

0.504686

0.0654307

0

0

0

0.569439
7

0.126918

0.0156497

0.216294

0

0.00362217

0.432588

0.00687037

0.00362217

0

0.0234746

0.448401
7

0.0156497

0

0.0530582

0

0

0.00888202

0.0397029

0.0194364

0.00967569

0.0469492

1.06546
7

0.0118427

0

0.0591856

0.147964

0

0.148359

0

0.0193514

0

0

0.297481

0

0.144196

0.00673085

0.0156497

0

0.189473

1.44591

0.0193514

0.032394

0.00789662

1.14765
7

0.0187351

0.119709

0

0

0

0.0236854

0.0123093

0

0.00732626

0

19.2479
7

6.56212

0

0

0.00923194

0

0

0.039379

0.144196

0.0408038

0

0

0.404167
7

0

0.020059

0.0129576

0

0.0106941

7.76369

0.00624502

0.154258

0.00411208

0

0.0681577
6

0.655036

0.597873

0

1.59231

0.439202

0.0129576

0.0100616

0.00458025

0.0156497

0

1.35446

0.00448724

1.00937

0

0.0435978

0

0.0156497

0

0.0194364

0

0.00362217

0.140086

1.74244

0.00394831

0

0.447159

0

0.00888202

0.0190407

0

0.0534571

0.72098

0.960023

0.00592247

0

0

0.0505647

0.0918063

0.00881856

0

0.203382

1.88791

0

1.58649
7

0.0224752

0

0.15081

0.0730438

0.00592134

0.00592134

0.760151

0.00408015

0.0275867

0.222448

2.49007
7

0.165843

0.0100616

0.0234746

0.0242511

0.0887784

0

0

0.280752

0.295331

1.66237

0.638576
7

0

0.012736

0

0

0.00440928

0.0156497

0

0

0.0938338

0.00408015

0.24622
6

0.22597

0.0194364

0.0301849

0.0186455

0

0.0770648

0

0.325521

0

0.0226904

7.02786
7

0.458749
7

0.0591856

0.00624502

2.11074

0.02805

0.0304531

0.0223806

0

0

0

0

2.35784
7

0.00448724

0.025506

0.0100616

0.379926

0.429239

0

0

0

0.00977679

0

1.54058
7

0

0

1.50008

0.216294

0.0301849

0

0.0233956

0.0198131

0.432588

0.00592134

1.00896

0.00822417

0.0547741

0.0150924

0.0312995

0

0.00612023

0.0928905

0.288392

2.95602

0

0.712364
7

0.15317

0.0181513

0.865175

0.343166

0.00518609

0

0.115597

0

0.00518609

0.239521

3.36127

0.0260594

0

0.0430109

0

0

0.576784

0

0

0.00687037

0.029498

0.295149

0.00923194

0.00816031

0.0354628

0.216294

0.00615463

0

0

0

0

0

0.104981
7

0

0.150351

0.0280549

0.0156497

0.0156497

0

0.0107775

0.746691

0

1.1052

3.21969
7

0.00416335

0.0100616

0.0100616

0.123296

0

0.0114506

0.27249

0

0

0.00592134

0.348595
7

0

1.08147

0

0.0161662

0.00394831

0.144196

0

0.00986966

0.021555

0.625683

15.1548
7

0.122138
7

0

0

0.0259152

0

0

0.21173

0

0.0431422

6.41672

0

0.0110232
6

0.0156497

0

0.00592134

0.503078

0.0173669

0.0129652

0

0

0.144196

0.0132278

3.71487
7

0.00592247

0.0241892

0

0

0.0107775

0

0.0208391

0.00448724

0.0156497

0

0.0674434

0.0118449

0.0161662

0.0150924

0

0

0

0.0129576

0.0264272

0

0

1.14162
7

0.065107

0.0257114

0.0361062

0.0469492

0.0389071

0.00440928

0.222216

0.00888202

0.025154

0

3.0087

0.39434

0

0.0091605

0.021555

0.0829774

0.0469492

0.00394831

0

0

0.00615463

33.1783
7

0.0591856

0.0100616

0.378264

0.118371

0.00458025

0.0207247

0

0.0312995

0

0.0639346

0.273047

0

0

0.288392

0

0

0.0286073

0

0.0360161

0.00888202

0.00967569

8.93634
7

0.0156497

0

0.157153

0.0254716

0.144196

0

0

0.00881856

0

0.0181513

1.39906
7

0

0.144196

0

0.36049

0.583398

0

0.0137878

0

0.44443

0.0103722

271.646

14.5724
7

0.636221
7

0

0

0

0.0301849

0

0.0377212

0

0.00394831

0.0583091

0

2.29708
7

0

0

0

0

0.0236854

0.00532728

0.017764

0

0

0

0.185878
6

0.0374633

0.148776

0.0111074

0

0.0453285

0.367104

0.00416335

0.216294

0.282502

0.0091605

0.705647
7

0

0

0.0528706

0.0306972

0.0770648

0

0.214627

0.0201232

0.346792

0.216294

0.489241
7

0.72098

0.288392

0

0.0176371

0

0

0.288392

0

0

0

2.75425
7

0.216294

0.0103252

0.231944

0

0.00789662

0

0.0156497

0.0107775

0

0

0.318673
7

0.101088

0

0.00592247

0

0

0.0118427

0.0591856

0

0

0

0.544375
7

0

0.00518609

0.148605

0

1.15972

0

0

0.288392

0.147964

0

0.466944

0.0312995

0.00458025

0.298453

0.0156497

0

1.08147

0.00394831

0.0107775

0.0301849

0.00967569

1.39888
7

0.0194364

0.0150924

2.59786

0.00777913

0.0362609

0.144196

0.0107775

0.0150924

0.23246

0.0176371

5.61537
7

0.448939
7

0.0526906

0.0104084

0

0.00888202

0

0.0129576

0.0140341

0.0107775

0.793077

0.00789662

0.917688
6

0.15764

0.0804929

0.0234746

0.00416335

0.231944

0.197756

0

0

0.00394831

0

0.495023
7

0.0530301

0.0100616

0

0.0224408

0.937273

0.00881856

0

0

0.272109

0

2.44866
7

0.0118427

0

0.0902369

0

0.0126545

0.0254941

0.0234746

0

0.177557

0.144624

0.654608
7

0.0887784

0.00518609

0

0.0100616

0.0312995

0.0150924

0.0123093

0

0

0.0129576

1.33261
7

0.0538875

0.0345941

0.147964

0.118371

0.0469492

4.14299

0.0770648

0.144196

0

0.0638868

0.2732
7

0.0103722

0.263561

0.0201232

0.208984

0.0154959

0.147964

0.00592134

0

0.0100616

0.0201232

0.361902
7

0.262567

0.0161662

0.00661392

0

0.0444101

0.144196

0.174162

0.00923194

0

0.0156497

0.659254
7

0

0

0

0

0.062599

0

0.00532728

0.00822417

0

0

0.497541
7

0.00612023

0

0

0.161977

0

0

0

0.0178408

0

0.0391243

4.41422

1.18193

0.00458025

0.0266729

0.0402465

0.00857263

0

0

0.438509

0

0.226165

0.01249

0.984495
7

0

0.0162027

0

0.0591856

0.0103722

0.0321507

0.018321

0.573685

0.0469492

0.0208358

0.91646
7

0

0

0

0

0.177604

0

0

0.00448724

0.00661392

0

1.2841
7

0

0.144196

0

0.20715

0.0193514

0.00448724

0.0887784

0

0.0193514

1.29776

0.527629

0.144196

0

0

0.0552764

0.0257114

0

0.158999

0

0

0.0453516

1.54432

0.0238024

0

0.0224843

0.0404928

0

0

0.00448724

0

0

2.97802

1.84064
7

0.0756009

0

0.144196

0.0887784

0

0.414299

0.00615463

0.0770648

0

0

2.14639
7

0

0

0.0201926

0

0.0162896

0.0128589

0

0

0.344294

0

0.517476
4

0.0234746

0.514747

0.216294

0.16197

0.0259152

0.0269351

1.09117

0.00687037

0.216294

0.0250483

0.364585
7

0.150351

0

0.216294

0.0591856

0

0

0

0.0234746

0.0770648

0

7.20651
6

0.537583
7

0.436536

0

0

0.0648357

0

0

0.078622

0.00967569

0.00362217

0

0.0545578
5

0.0591856

0

0

0.00822417

0.00923194

0

0.0100616

0.0215711

0.288392

0.216294

1.0024
7

0.0654005

0.36049

0.144196

0

0

0.00881856

0.00687037

0.0100616

0

0

0.35584
7

0

0.937273

0.00592134

0

1.58615

0

0.144196

0

0

0

1.89894
7

0.061388

0

0.216294

2.97437

0

0.110826

0

0

0

0.0402465

0.302485

0.144196

0

0

0

0.0931877

0

0.0887784

0

0

0

0.118646
7

0

0.216294

0

0

0

0.00661392

0

0

0.00439576

0

0.866753
7

0

0

0

0.947376

0.0234746

0

0.118371

0

0

0.00440928

0.43238
7

0.0156497

0.00881856

0.00967569

0

0

0.0150924

0.0206582

0

0.0091605

0

1.37393
7

0

0.00592134

0.00518609

0.00394831

0

0.049237

0.163547

0

0.0201306

0.0091605

6.3963

2.13975
7

0.118371

0

0

0

0

0

0

0

0

0.00687037

1.88762
7

0

0

0

0.255167

0.0754621

0.0184639

0.0141629

0.36049

0

0.00458025

0.866447
7

0.0526729

0.118371

0.0110232

0

0

0.0843397

0.0312995

0

0

0.144196

0.600953
6

0.504686

0.0860736

0.00408015

0.0312995

1.15357

0

1.07233

0.00448724

0

0

5.08507
7

0

0.432588

0

0.0100616

0

0.0125042

0.0754621

0.149012

0.0129576

0

0.851438
6

0

0

0.0310743

0.288392

0.0503081

0

0

0.0201232

0.00661392

0

0.153093
7

0

0

0.0234746

0.01249

0.0166988

1.2429

0.0469492

0

0.0591856

0

0.472262
7

0

0.147964

0

0

0

0.0728644

2.39236

0.0100858

0.0129652

0.0958858

0.525003
7

0.00458025

0.414176

0.284939

0

0.216294

0

0.00416335

0

0

0

0.577092
7

0.153078

0.0161662

0.00532728

0

0

0.236743

0

0.0257114

0.0605221

0.00518609

9.82104
7

0.234948

0.00592134

0.0591856

0

0

0

0

0

0.0388728

0.144196

0

0.516417
7

0.00394831

0

0

0

0

0

0.00967569

0

0.00518609

0

1.77132
5

0

0

0.0234746

0

0

0

0.267945

0.00777913

0.0953924

0.618334

0.128912
6

0

0

1.94664

0

0

0

0

0.021555

0

1.33925

1.36662
7

0.0129576

0

0.025154

0.00888202

0.0156497

0.126006

0.0591856

0.432588

0

0.216294

0.913235
7

0

0

0.0154325

0.0301849

0

0

0.0186455

0.216294

0.0887784

0

0.88803
7

0

0.00777913

0.0969388

0

0.187965

0

0.100841

0

0.148276

0.865175

0.18784

0

0

0.230624

0

0.144196

0.0156497

0.0118427

0.0391243

0

0.00592134

2.06151
7

0

0.0887784

0

0.342515

0.00448724

0

0.0100616

0

0

0.0166534

0.99041

0.0129576

0.281695

0

0

0

0.0118427

0.36049

0.00440928

0.0887784

0

6.05451
7

0.597113
7

0.325521

0.0194364

0

0.144196

0.0100616

0.062599

0.00687037

0.01249

2.67355

0.0321298

0.027638
7

0

0

0.0245318

0.0163206

0

0.0100616

0

0

0

0

0.259793
2

0.0349527

0.00408015

0

0.00394831

0.00440928

0.0145105

0

0

0.0270617

0

0.156708

0.0591856

0

0

0

0

0

0

0

0.0654005

0.0156497

0.910979
7

0

0.0234746

0.0118593

1.77181

0.139543

0.0129576

0

0.00458025

0

0.0770648

1.21819
7

0.0156497

0

0

0.156039

0

0.0770648

0.216294

0

0.0187351

0.0603697

1.63005
7

0.00943936

0

0.0391243

0

0.00440928

0

0.115597

0

0

0.0215412

2.35425
7

24.2427

0

0

0.062599

0

0

0.017764

0.0156497

0

0

1.16385

0.00362217

0.0181437

0.0469492

0.00448724

0.369176

0.00592134

0.0110232

0.144196

2.57458

0

0.269482

0

0.00411208

0

0.0352157

0

0

0.0150924

0.606422

0.0770648

0

9.63902
7

0.583637
7

0.0704313

0.0167577

0

0

0.0104084

0.226355

0

0

0

0.118371

0.574233
7

0.091605

0.240912

0

0.0156497

0

1.00937

0

0

0.0156497

0.36049

1.10771

0.0195981

0

0.0848439

0

0.00440928

0.0195327

2.88392

0

0

0

0.159412
7

0.0538875

0

0.021555

0

7.15212

0.00777913

0.025154

0

0

0.0129576

0.180284
7

0

0

0.144196

0.177557

0

0.12933

0.062599

0

0.144196

0

0.151834
7

0

0

0.118371

0.101723

0

0

0.0091605

0

0.0259304

0.164322

0.36676
5

0

0.220242

0

0.0234746

0

0

0

0

0

0.0201232

0.00777913
6

0.0091605

0.0452773

0

0.36049

0.865175

0.115234

0

1.31714

0

0

1.15437
7

0.0156497

0.0887784

0

0

0.0147258

0.0156497

0.294546

0.368925

0.00394831

0.00408015

1.9635
7

0.00881856

0.0572147

0

0.156497

0

0

0

0

0

0.00687037

5.53392
7

0.274855
6

0

0

0.00592134

0.00592134

0

0

0

0

0

0

0.41084
6

0

0

0.0770648

0

0

0

0

0

0

0.263243

2.67424
7

0.00923194

0.42573

0.0374541

0

0

0.177757

0

0

0.0139473

0.288392

1.29537
7

0

0

0.0123996

0

0

0

0.0201232

0

0.02023

0

0.529305

0.288392

0

0

0

0

0.0391243

0.0156497

0.0163066

0.00661392

0.0352743

0.993164

0.0591856

0

0.504686

0

0

0.0150924

0.576784

0

0

0

1.39831
6

0.0234746

0.00592247

0.00832669

0

0

17.4937

0.118371

0

0

0

1.74761
7

0.0241892

0.0388948

0

0

0

0.0274815

0.00592247

0.0150924

0

0

2.83952
7

0.0194364

0

0

1.40848

0

0.148359

0

0.0887784

0.288392

0.00592134

0.170846

0

0

0.36049

0.153438

0

0.00923194

0

0

2.59553

0.0887784

10.6861
7

0.0297343
7

0.222799

0

0

0.025664

0

0

0.0156497

0.469922

0.36049

0.00416335

0.677652
7

0

0

0.0156497

0

0.0201232

0

0

0

0.0887784

0

0.892862

0

0.144196

0.037509

0

0

38.0174

0.0102004

0.0107775

0

0

1.08876
7

0

0

0.0377212

0.0156497

0.00408015

0

0.118371

0

0.00394831

0

0.284261

0

0.865175

0.0804929

0.369291

0

0.0234746

0.144196

0

0

1.34126

0.99305
7

0

0

0

0.0113407

0

0

0

0.123097

0.0296067

0

7.32475

0.0156497

0.00440928

0

0.216294

0.00394831

0.0194364

0

0

0.159846

0.025154

1.55495
8

0

0.0100616

0

0

0

0.216294

0.0100616

0

0.0234746

0.0269437

0.292722
7

0.0547741

0

0.00592134

0.0591856

0.027638

0

0

0

0

0.147489

0.444035
6

0

0

0

0

0.0826602

0.0133182

0

3.66951

0

0

1169.2

3.10201

0.647051
7

0.00624502

0.216325

0.0228984

0.00518609

0.0887784

0

0

0

0

0

0.426128
7

0

0.00518609

0.148276

0

0

0

0

0

0

0

1.05586

0

0

0

0

0

0.0161662

0.36049

0.0547741

0.0591856

0

0.999415

0.135402

0

0

0

0.509872

0

0

0.0100616

0

0

0.473674

0.0194364

0

0.021555

1.15357

0

0

0.0153866

0

0.00416335

0

0.298117
6

0

0.0100616

0.0107775

0

0

0

0.0887784

0

0.793077

0

0.217036
7

0

0.0154325

0

0.0273676

0

0

0

0.0161662

0.0887784

0

0.494957

0.00518609

0

0

0.0513281

0

0

0.0391243

0

0

0.275479

1.31295
7

0.115597

0

0.00789662

0.00411208

0

0

0.0100616

0.00518609

0.177557

0

0.803741

0

0

0.00448724

0.115597

0

0

0

0.144196

2.8705

0

2.52627
7

1.87822
6

0

0

0

0

0

0

0.00518609

0

0

0

1.95589
7

0.0155583

0.0542797

0

0.00967569

0

0

0

0

0.165798

0.00973656

0.691687

0.00615463

0

0

0.0754621

0.310982

0

0.00615463

0

0

0.157153

0.537892
7

0

0

0.00888202

0

0

0

0.00592247

0.00673085

0

0

0.0949379
7

0

0

0

0.147964

0

0.260992

0

0.00518609

0

0.0591856

1.6048
7

0.0255526

0.0314107

0.00448724

0

0

0

0.0188064

0.177557

0.36049

2.01874

0.48163
7

0

0

0

0

0

0

0

0.0241892

0.0301849

0

0.124199
6

0

0

0.0100616

0.00416335

0

0

0

0.0154325

0

0.0091605

0.50794
7

0.288392

0.0100616

0.0103722

0

0.144196

0.0239316

0

0.504686

0

0

0.366257
6

0

0

0.0246185

0

0

0

0.0234746

0.00394831

0

0.144196

6.15679

2.02805

0

0

0.11014

0.509898

0.648882

0.0184639

0.299876

0

0

0.674797

1.73331
7

0.0150924

0.0887784

0.0107775

0

0.0591856

2.37923

0.288392

0.0228984

0

0

0.0388189

0

0.0156497

0

0

0

0.0513198

0.346792

0.00416335

0

0.0520418

0.164068
7

0.36049

0.01249

0

0

0.00789662

0.0129576

0

0.106721

0

0

0.262082

0

0

0.0127406

0.0161662

0

0

0

0.00416335

0

0.0150924

0.326238
7

0.118371

0

0.0264272

0

0

0

0

0

0

0

0.683428
7

0

0

0.0150924

0

0

0.118371

0.432588

0

0.0591856

0

0.502669

0.0770648

0

0

0

0.00822417

0

0

0

0

0.72098

0.0391243
6

0

0

0.145893

0.0176371

0

0

0

0

0

0

4.38794
7

0.288392

0.0391243

0

0

0.216294

0

0

0

0.144196

0.00592134

9.84462
7

0.300054
7

0

0

0

1.55452

0

0

0.0591856

0

0.0100616

0

0.415658
7

0

0.147964

0.00592134

0

0

0

0

0

0

0

1.13112

0

0.144196

0.216294

0

0.00458025

0

0

0

0

0.105961

8.29561
1

0

0.0782487

0

0

0

0

0.0181437

0

0.02023

0

0.424813

0.0156497

0

0.0145135

0

0.172147

0.00518609

0

0

0.00518609

0.0301849

2.83016
7

0

0

0.0269437

0

0.144196

0.275479

0

0

0.169276

0.00967569

2.02832
7

0.36049

0

0

0.0887784

0.00881856

0

0.144196

0.00394831

0.0129576

0

0.148108

0

0.0242511

0

0

1.22567

0

0.0129576

0.00615463

0.0194364

0

0.658519
7

0.221261

0

0

0.0107775

0.0160309

0

0

0

0

0

0.274335
7

0.36049

0

0.508766

0.00518609

0

0

0

0.0591856

0

0.0259152

2.86049
7

0.106356
7

0

0

0.0822434

0

0

0

0

3.24441

0.36049

0

0.360944
7

0

0.216294

0

0

0

0

0

0

0.00532728

0

0.501978
7

0

0.0107775

0

0

0.698923

0.0312995

0

0.0100616

0

0

0.912284
7

0

0

0.062599

0

0

0

1.51406

0

2.92997

0.00416335

0.352085
7

0

0

0

0.00661392

0

0

0.0416782

0

0.0234746

0

0.860791
6

0.0245325

0

0.0518609

0.00923194

0.36049

0

0

0

0

0.00408015

0.0587044
6

0

0

0

0

0

0

0

0

0

0

0.416131

0.265765

0

0.492031

0.00440928

0.0234746

0.0591856

0

0.00458025

0

0.144196

0.540555

0.288392

0

0.00416335

0

0

0.017764

0

0

0

0

0.947604
7

0

0

0.0782487

0.36049

0.288392

0

0

0

0.0388957

0

6.85628
7

0.621954
7

0

0

0

0

0

0

0

0

0

0

8.81627
7

0

1.22567

0.0259152

0

0.00458025

0

0.72098

0

0.0770648

0

0.0366474
7

0

0.199436

0

0.00416335

0.00923194

0

0

0

0

0

0.328447
7

0.15413

0

0

0.0770648

0.518304

0

0

0

0

0.0312995

0.317334

0

0

0

0

0

0

0.0148034

0.0312995

0

0.0150924

0.28959
7

0

0

0.0391243

0.0770648

0

0.0114506

0.00832669

0

0

0.0161662

0.570226

0

0.144196

0

0

0

0

0

0.0678494

0

0

0.128163
7

0.00408015

0.0162946

0.153356

0.00394831

0

0.025154

0

0

0.00923194

0.288392

0.700379
7

0.00967569

0

0

0

0

0

0

0.0100616

0.0848439

0

0.077688

0.00987078

0.0782487

0

0.0150924

0.00431055

0

3.6049

0.238851

0.00416335

0.00416335

8.44485
7

0.174253
7

0

0

0.288392

0

0

0

0.0933587

0.0591856

0

0

0.0368762

0

0.334665

0.288392

0

0

0

0

0

0

0

1.05682
7

1.51406

0

0

0

0.0799382

0

0

0.0257114

0

0

0.163547
7

0

0.0391243

0.664651

0.216294

0

0

0

0.0156497

0.00624502

0

0.584632
7

0.0453516

0.0194364

0

0.0156497

0

0.0161662

0

0.00905542

0.177557

0

0.893029
7

0

0.0137407

0.0156497

0

0.00440928

0

0

0.177557

0

0

0.140739

0.0400051

0.00967569

0.119761

0

0

0

0

0.288392

0

0.0369278

0.131701

0

0

0

0

0.216294

0

0

0

0

0.138596

0.313394
6

0.0107775

0.0591856

0

0.00518609

0

0

0.0154325

0.00615463

0

0

0.743259
7

0

0.937273

0.00416335

0

0.0163206

0.0518304

0

0

0

0

4.45172

0.208417

0

0

0

0

0

0

0.00458025

0.00416335

0.00612023

0

0.114059

0

0.216294

0.288392

0.144196

0

0

0

0

0.0201232

0

0.322405
7

0.062599

0

0

0.865175

0

0.0337548

0.144196

0

0.295928

0.144196

0.853886
7

0

0.00440928

0.0270617

0.216294

0.177557

0

0

0

0

0.00448724

0.643635
7

0

0.0458346

0.0107775

0

0

0.00416335

0

0

0.0704238

0.74959

0.0772877

0

29.2822

0

0

0

0.0355307

0.154258

0.0241892

0

0

0.121341
7

0

0

0.0156497

0.0103722

0.106909

2.45133

0.0473708

0

0

0.0241892

0.87476
7

0

0

0

0.0312995

0.00789662

0.0161662

0.144196

0

0

0.00661392

0.0181043

0.0159818

0

0.0156497

0

0.00661392

0

0

0

0.0591856

0

0.450186

0

0

0.00881856

0

0

0.0129652

0

0.0938984

0

0

7.49187
7

0.251363
7

0.00615463

0

0

0

0.00416335

0.288392

0

0

0

0.0100616

0.332489
7

0.0620416

0.30517

0.432588

0.09831

0.680635

0

0

0

0.0241892

0

0.0978601
7

0

0

0

0

0

0.00448724

0

0.00532728

0

0

0.131303

0.025154

0

0

0.0194364

0.00458025

0.0312995

0

0

0

0.0591856

0.741107

0

0

0

0

0

0.0887784

0

0.0269437

0.0887784

0

0.328982
7

0

0

0.00592134

0

0.0156497

0

0

0.0107775

0

0

0.139385

0

0

0.00440928

0.133023

0

0.0132278

0

0

0

0.0156497

0.24853
5

0.0156497

0.0161662

0.0194364

0

0

0

0

0.0233374

0

0.0242511

3.39212
7

0.0591856

0

0

0.725143

0

0

0

0

0

0

1.51336
7

0.0194364

0

0

0

0

0.0639491

0

0

0.00926624

0.147964

87.1802
7

0.169257
7

0

0.00408015

0.0114948

0

0

0

0

0

0

0

0.504303
7

0

0.0654005

0.0591856

0

0

0

0

0.288392

0

0

0.589741
7

0

0

0

0.0469492

0

0.0129576

0

0.0150924

0.00615463

0.00592134

0.235199
7

0.00518609

0.0196727

0.288392

0

0.432588

0.0591856

0

0

0

0

0.0234746
6

0

0.0150924

0

0.0503827

0.167671

0

0

0.00448724

0

0.00592134

0.446935
7

0.0123093

0

0.00777913

0

0

0

0

0

0

0.0887784

0.165035
7

0.093926

0

0.00483191

0.00518609

0

0

0

1.77557

0.157153

0

0.118243
6

0.0591856

0.242209

0

0.147964

0.0194364

0.268536

0

0

0.0100616

0

0.126885

0.288392

0

0.508198

0

0

0.0107349

0

0

0

0

0.589996
7

0

0

0

0.0704238

0

0.0100616

0.00518609

0.0100616

0

0

55.4882

15.0019
7

0.0704238
7

0

0

0

0

0.00518609

0

0

0.04311

8.34108

0

3.49656
7

0.0580508

0

0.0100616

0

0.00777913

0

0

0

0

0.0561436

21.5272
8

0

0.0110232

0

0.304042

0.0201232

0

0

0

0

0

0.0731885
6

0

0

0.0162997

0

0.0177674

0

0

0.030865

0

0.00458025

0.0841401
6

0

0.00458025

0

0

0.144196

0

0.36049

0

0.00615463

0

0.639154
7

0

1.44196

0.0091605

0

0.0469492

0

0

0.0312995

0.0704238

0.0887784

1.49425

0

0.0100616

0

0.101736

0.00789662

0

0

0

0.0129576

0

0.0647465

0.504686

0

0

0

0.731041

0

0.00543325

0

0.0583091

0.0100616

0.558348
7

0

0

0.0161662

0

0

0

0.150924

0.025154

0.137431

0.0100616

1.16398
7

0

0.36049

0

0.432588

0.0107775

0

0

0

0.0156497

0

11.4862
7

1.43949
7

0.0118427

0

0

0

0

0

0.0234746

0

0

0

0.312374
7

0.0234746

0

0.021555

0.0137407

0.0218007

0

6.515

0.0102348

0.0796974

0

0.12022
7

0

0.0161662

0

0

0

0.00789662

0

0

0

0

0.273968

0.00592134

0

0

0

0

0

0

0.0201232

0.0163206

0

0.837487
7

0

0.0129576

0.00394831

0.0770648

0

0

0

0

0.0156497

0

0.183408
7

0

0

0.136835

0

0

0

0

0.0325674

0.0325674

0.72098

0.224721

0

0

0

0.144196

0

0.00408015

0

0

0.00615463

0

0.218577

0

0.00440928

0.0156497

0.216294

0.0355348

0.0312995

0.00408015

0

0

0

0.608137

0.00411208

0.115597

0

0.172147

0

0.116618

0

0.0118593

0.00440928

0.017764

0.127133
6

0

0.0234746

0

0

0

0

0

0

0

0.124526

3.34813

0.600599
7

0

0

0.304784

0

0

0

0

0

0.0234746

0

0.778414
7

0

0

0

0

0.00615463

0.0259152

0.0234746

0.0156497

0

0

0.553665
7

0

0

0

0

0.0181513

0.0181513

0.0107775

0.0091605

0

0

0.330714
7

0.00888202

0.0148034

0

0.125198

0.177557

0

0

0

0.0150924

0.503078

1.15071
7

0

0

0

0

0.0617713

0

0

0.576784

0.00661392

0

0.0154959

0

0.165843

0

0.0107775

0

0.216294

0.00615463

0.00448724

0

0.0145135

0.103925
7

0.36049

0

0.220874

0

0

0.0388728

0

0.144196

0

0

0.888535

0

0

0

0

0

1.98707

0

0.0887784

0

0.0748354

0.520024
7

0.72098

0.0194364

0.0580542

0.651042

0

0

0

0.00440928

0

0

0.266949
7

0

0

0

0

0

0.00624502

0

0

0.0129576

0.0241892

3.95075

0.2462

0.0104084

0.0234746

0

0

0

0.269727

0.0293552

0.0187351

0

0.0466748

0.220242

0

0

0

0

0

0

0

0.0440549

0

0.144196

0.107005
7

0

0

0

0.0129576

0

0.0129576

0

0

0

0.0176371

0.755289
7

0.00624502

0

0

0.0252928

0

0.246274

0.00592134

0.00592247

0

0.0100616

11.6944
7

0

0

0.0201232

0

0

0

0

0.00440928

0

0

0.0318013

0

0

0.0156497

0.288392

0

0.0129576

0.0107775

0.0312995

0

0

0.096189

0

0

0

0

0

0.0145135

0.0312995

0

0

0.216294

0.0692103
7

0

0

0.182899

0.0591856

0

0

0.0978386

0

0.0591856

0

0.164322

0.0161662

0

0.0100616

0

0

0.0201232

0

0.0296123

0.00448724

0

2.98732
7

0

0

0

0

0

0

0

0

0

0

3.08316
7

0.154258
7

0

0

0

0

0

0

0.0106546

0

0.432588

0

0.0356592
7

0

0

0

0.0201232

0

0.865175

0

0.177557

0.504686

0.0129576

1.2993
6

0

0.00458025

0.00458025

0

0

0

0

0

0.00967569

0

0.143602
7

0

0

0

0

0

0

0

0

0

0

0.402055
7

0

0.0887784

0.0129576

0

0.00518609

0

0.0207444

0

0.00687037

0

0.292818
6

0

0.0110232

0

0

0

0

0

0

0

0.0132278

0.044031
6

0

0

0

0

0.0126776

0

0

0.288392

0

0

0.118594
5

0

0

0

0

0

0.0704238

0

0

0

0

0.504353
7

0.648882

0

0

0

0

0

0.432588

0

0

0

0.201709
7

0

0

0

0

0

0

0

0.793077

0

0.00777913

4.53399
7

3.13145
7

0

0

0.0156497

0

0

0

0

0

0

0

0.203492
7

0

0

0

0

0

0

0

0.20715

0

0

0.0857859
7

0

0

0.0100616

0

0.0591856

0

0

0

0

0

6.87316
8

0

0

0

0.0129576

0

0

0

0

0.00458025

0

0.0620193
7

0

0

0.00518609

0

0

0.00967569

0

0

0

0

0.0718602
7

0.212865

0

0

0.00416335

0

0

0

0

0

0

0.403328
7

0

0

0.00448724

0.0452773

0

0.0469492

0

0.00661392

0

0

1.1116
2

0

0.00518609

0

0.0154325

0

0.144196

0

0

0.144196

0

0.336137

0

0

0.00440928

0

0

0

0

0.147964

0

0

1.08295
7

0

0

0

0

0

0

0

0

0

0

7.35353
7

1.19994
7

0

0.147964

0

0

0

0

0

0

0

0

0.202835

0

0

0

0

0

0

0

0.00661392

0

0

2.75001
7

0.0234746

0

0.00661392

0

0

0.144196

0

0

0

0

0.616191
7

0

0

0

0

0.0118427

0

0

0.115597

0

0.0138191

0.377959
7

0

0

0

0

0

0.0591856

0

0

0

0.18614

0.201549
6

0

0.0118427

0.144196

0.865175

0

0

0.147964

0

0

0

1.09114
7

0

0

0.0201232

0

0

0

0

0.0107775

0

0

0.898163

0.00416335

0

0

0

0

0.0156497

0.00408015

0.0150924

0

0

0.226447
7

0.00888202

0

0

0

0

0.00881856

0

0

0.0123093

0

0.062599
6

0

0

0.144196

0

0.0402465

0

0

0.00923194

0

0

6.82027
7

0.296528
7

0

0

0

0

0

0.144196

0.0782487

0

0.144196

0

0.275925
7

0.0201232

0

0

0

0

0

0

0

0

0

1.33748
6

0

0

0

0

0

0.00416335

0

0

0

0

1.58862

0

0

0

0

0.00532728

0

0.0100616

0.0110232

0.0150924

0

0.602495
6

0

0

0

0

0

0

0

0

0

0

0.550295
6

0

0

0

0.0156497

0

0

0

0.0129576

0

0

0.420383
7

0.0547741

0

0

0

0

0.026521

0

0

0

0.00518609

60.3173
8

0.00777913

0

0.0234746

0

0

0

0

0

0

0

0.325444
7

0

0.00612023

0.00612023

0

0

0.00888202

0

0

0.0312995

0

0.405527

0

0

0.216294

0

0.177557

0

0

0

0

0

1.63479

1.2219
7

0.032394

0

0

0.0143923

0.216294

0

0

0.0150924

0

0.00322316

0.0193467

0

0.504686

0

0

0

0

0

0

0

0.0201232

0.455866

0

0

0.00448724

0.00612023

0

0

0

0

0.0194364

0

0.473638
6

0

0

0

0

0

0

0.0156497

0

0

0

0.046812
6

0.0770648

0

0

0

0

0.144196

0

0.00518609

0.0156497

0

0.298345
6

0

0

0

0

0

0

0.288392

0

0.295928

0

0.0125716
6

0

0.0156497

0

0

0

0.00408015

0

0

0

0.0154325

1.28884

0

0.0129576

0

0

0

0

0

0

0

0

3.27754
8

0

0.00673085

0

1.51406

0.00458025

0

0

0

0

0.00408015

0.172803
7

0

0

0

0

0.00592134

0

0

0

0

0

3.82497

0.299729
6

0

0

0.0100616

0

0

0

0.0591856

0.648882

0.00592134

0

0.172056
7

0.025154

0

0.0156497

0

0

0.20715

0

0.00440928

0

0

0.145685
7

0

0

0

0

0

0

0

0.144196

0

0

0.032862
6

0

0

0

0

0.0217157

0

0

0

0

0

0.690002
7

0.0583091

0

0

0

0

0

0

0

0.00394831

0

0.56975
7

0.0234746

0

0

0

0.576784

0

0

0.0104084

0

0

0.187683
7

0

0

0

0.0150924

0

0.0102004

0.288392

0

0.00532728

0

2.05161
7

0

0.0137407

0

0

0

0

0

0

0

0

0.102085

0.216294

0

0

0

0.0770648

0

0

0

0.0947996

0

1.10555
6

0

0.0118427

0.00448724

0

0.00416335

0

0

0.00592134

0

0.00592134

487.571
7

2.98339

0.00416335
7

0

0

0

0.0132278

0

0.00394831

0

0

0

0

0.746883
7

0.00518609

0

0

0

0.0391243

0

0

0

0

0

0.0187167
6

0

0.0402465

0

0

0

0

0.0301849

0

0

0

0.0576021

0

0

0

16.2941

0

0

0

0

0.0102004

0

0.0170299
6

0

0

0

0

0

0

0.0110232

0

0

0

0.373789
7

0

0

0.0156497

0

0.00592134

0

0

0

0

0.0194364

0.313717
7

0

0.0156497

0

0.0547741

0

0

0

0

0.937273

0

97.3401
8

0.00777913

0

0

0

0

0

0

0.0391243

0.115597

0

0.448199
7

0

0.504686

0

0

0.00518609

0

0

0

0

0.140863

0.254343
7

0.0591856

0

0

0.00394831

0

0.0118427

0

0

0

0

2.82045

0.0112797
5

0

0

0

0

0

0

0.0311165

0

0

0

0.874336
7

0

0

0

0.0323325

0

0.00458025

0.0103722

0

0.0156497

0.0100616

0.049186
7

0

0

0

0

0

0

0.0118449

0

0

0

0.799228
7

0.216294

0

0

0

0

0

0

0

0

0

0.734566
7

0

0

0

0

0

0

0.00624502

0

0

0

0.0141455
6

0

0

0

0

0

0

0.00724433

0

0

0

0.0524869

0

0

0

0

0

0

0.532671

0

0

0

3.56613
7

0

0.0770648

0

0

0

0

0

0

2.09084

0.318687

0.0326351
7

0

0

0.00592134

0

0

0.0229012

0

0

0.00458025

0

0.61276

0

0.0860736

0

0

0.0201232

0

0

0

0

0

3.78375
7

0.172721
6

0.00615463

0.00518609

0.924777

0

0

0

0

0.115597

0

0.0145135

0.277346
7

0

0

0

0.0591856

0.0091605

0

0.36642

0

0

0

0.9802
7

0

0

0

0

0

0

0

0.0129576

0

0

0.131558
7

0

0

0

0

0

0

0.288392

0

0

0

0.366707
7

0

0.0312995

0

0

0.118371

0.00458025

0.0129652

1.00937

0

0

0.0657996
7

0

0

0

0

0

0

0

0.432588

0

0

0.639726
7

0

0

0

0

0.0591622

0

0

0.00612023

0

0.00411208

1.79309
7

0

0.01249

0

0

0

0

0

0

0

0

0.263061

0

0

0

0

0

0

0

0

0

0.0129576

2.0168

0

0

0

0

0

0

0

0

0.0100616

0

6.99943
7

0.36049
6

0

0

0

0

0

0

0.216294

0.0591856

0

0

1.65794
8

0.00612023

0

0

0

0

0

0

0.0469492

0

0

0.164131

0

0

0

0

0

0

0

0.00923194

0.0156497

0

0.0171569
6

0

0

0

0

0

0.0547741

0

0

0

0

0.239715
6

0

0

0.0503081

0

0

0

0

0

0.288392

0

0.0132407

0

0

0.0338649

0

0

0

0

0

0.0107775

0

0.900702

0

0

0

0.0156497

0

0

0

0

0

0

0.298453
7

0

0

0

0

0

0

0

0

0

0

1.45593
7

0.216294

0.144196

0

0

0

0

0

0

0

0

0.029352
6

0

0

0

0

0

0

0

0.0388728

0

0.0236854

2.31782

0.116211
7

0

0.032394

0

0

0

0

0

0

0

0

0.0197373

0

0

0

0

0

0

0

0

0

0

0.495854
7

0

0

0

0

0

0

0

0

0

0

0.695296
7

0

0

0

0

0

0.00615463

0.00624502

0

0

0

0.017764
7

0

0

0

0.0727532

0

0.00440928

0

0

0

0.0156497

0.564966
7

0

0

0.0770648

0

0

0

0

0

0

0

0.189076
7

0

0.865175

0

0

0

0

0

0

0

0

0.21462
7

0.0591856

0

0

0

0.288392

0

0

0

0.00888202

0

0.950759
7

0.00777913

0

0

0

0

0

0

0

0

0

0.455605
7

0

0

0

0.0104084

0

0.00518609

0

0

0

0

16.121
7

0.798797
7

0.0150924

0

0

0

0

0.0157053

0

0

0

0

0.168876
7

0

0

0

0.0887784

0

0

0.0091605

0

0

0

0.029396
6

0.0220464

0.288392

0

0

0.00592134

0

0.288392

0

0

0

0.183479

0.0782487

0

0

0

0

0

0

0

0

0

0.682887
7

0

0

0

0

0.00411208

0

0.144196

0

0.0591856

0

0.51067
7

0

0

0

0

0

0

0

0

0

0

0.305762

0.0855237

0

0

0

0.00987078

0

0

0

0

0

0.467004
7

0.0311165

0

0

0.793077

0

0

0

4.32588

0

0

1.0407
7

0

0

0

0

0

0.0296123

0.00967569

0.0591856

0

0

0.255985

0

0

0

0

0.00518609

0.0112181

0.144196

0

0.504686

0

6.19874
7

2.31329
7

0

0

0.216294

0

0.0114506

0

0

0.00458025

0

0

0.296991
7

0.0194364

0

0

0.00777913

0.00518609

0.144196

0

0

0

0

1.00492
7

0

0.00967569

0

0.0770648

0.00816031

0

0

0

0.0571222

0

0.601547
1

0

0

0

0.062599

0

0.0234746

0.0591856

0

0

0

0.0156497
6

0

0

0.0259152

0

0

0

0.177557

0

0

0.144196

0.232806
7

0

0.00440928

0

0

0.937273

0.36049

0.0269437

0

0

0

0.0478746
6

0

0

0

0

0

0

0

0.147964

0

0

0.0274947
7

0

0

0

0

0

0

0

0

0

0

0.857572
6

0.00440928

0

0

0

0

0

0.0129652

0

0

0

0.11968
7

0

0

0.0100616

0

0

0

0

0.147964

0.0100616

0

1.16505
7

0.383534
7

0

0

0

0.144196

0.0887784

0.0591856

0

0.0156497

0

0

0.0593543

2.59152

0

0

0

0

0.115597

0.0201232

0

0

0

0.519135
7

0

0

0

0

0

0.118371

0

0

0

0

0.879817
7

0

0

0.00923194

0

0

0

0

0

0

0.216294

0.474288
7

0.266335

0.288392

0.00661392

0

0

0

0

0

0

0.00592134

0.0239187
7

0.00394831

0.0388728

0

0.0104084

0.0738555

0

0.648882

0.0100616

0

0

0.0765784
6

0.288392

0

0.288392

0

0

0.0887784

0

0

0

0.0129576

1.49239
7

0.0102004

0

0

0

0

0

0

0

0

0.00777913

2.01409
7

0

0

0

0

0

0

0

0

0

0

0.0326351

0

0.0770648

0

0

0

0

0

0

0.337064

0

2.48534
7

0.084206
6

0

0.0150924

0

0

0

0

0.00832669

0

0

0

0.587561

0

0.0156497

0

0

0

0

0.00799092

0.0134617

0

0.0161662

0.746114

0

0

0.36049

0

0

0

0

0

0.0129576

0

0.0985631
7

0

0

0.00440928

0

0.00967569

0

0

0

0

0.00394831

0.210805
7

0

0

0

0

0

0.166017

0.00416335

0

0

0

0.025594

0

0.00440928

0

0.00440928

0.0391243

0

0

0

0.887784

0.0591856

0.0164213

0

0

0

0

0

0

0

0

0

0

2.47662
7

0

0

0

0.0591856

0

0

0

0

0

0

0.00976634
6

0

0

0

0

0

0

0

0

0

0

1.98091
7

0

0

0.00416335

0

0.216294

0

0

0

0

0

2.74589

0.741938
7

0

0.00458025

0

0.504686

0

0

0

0

0

0

0.0965819

0.0161662

0

0

0

0

0

0.414299

0

0

0

0.356709

0

0

0

0.770648

0

0.0150924

0

0.00408015

0

0

0.187234
7

0

0

0

0

0

0

0

0

0

0

0.131083
7

0

0

0

0

0.00615463

0

0

0.0153866

0.062599

0.00518609

1.15798
7

0

0

0

0.144196

0.0150924

0

0

0.00592247

0

0

2.65648
8

0

0

0

0.0452773

0

0

0

0

0.118371

0.432588

0.725241
7

0

0

0

0

0.0234746

0.0301849

0.147964

0

0

0

1.59134
7

0.216294

0.0156497

0

0.00612023

0

0

0

0

0

0.00615463

0.935008

0

0

0

0.0591856

0

0

0

0

0

0.00394831

27.4233

4.49723
7

0.00458025
7

0

0

0

0

0

0

0

0.00888202

0

0

0.727342
7

0

0.00881856

0

0

0

0

0.00440928

0.0123093

0

0

0.164348
7

0

0.00440928

0.0234746

0

0

0

0

0.00832669

0

0.00592134

0.021555
7

0

0

0

0

0

0

0

0

0

0

0.238953
7

0

0

0

0

0

0

0

0

0.216294

0

0.904089

0

0.0148034

0

0.0591856

0

0

0

1.15357

0

0.0887784

0.118161
7

0

0.0887784

0.00687037

0.0150924

0

0

0.144196

0

0

0

0.0310754

0

0

0.0547741

0

0

0.00744712

0

0

150.252

0

0.152856
7

0

0

0

0.062599

0

0

0

0

0

0

0.606799
1

0

0.0391243

0

0

0

0.00518609

0

0

0

0

4.53887
6

0.105168

0

0

0

0

0.72098

0

0.00394831

0

0

0.0469492

0.0352157

0.0201232

0

0.0110232

0

0

0

0

0

0

0

0.0997456

0

0

0.0591856

0

0.0430824

0

0

0

0

0

0.174638
7

0

0.0100616

0.216294

0

0

0

0

0

0

0

0.47446

0

0

0

0

0

0

0.216294

0

0

0

0.0864135
7

0

0

0

0.680635

0.00518609

0.144196

0.0150924

0.0110232

0.00518609

0

0.504686

0

0.0100616

0

0.00518609

0.00687037

0

0

0

0.00967569

0

0.98935
7

0.00592134

0

0

0

0.00440928

0

0

0

0

0

0.359319
7

0.0145717

0

0

0

0

0.110678

0

0

0

0

0.216294
5

0

0

0

0

0

0

0

0

0

0

8.5976
7

0.0443137

0

0

0

0

0

0

0

0

0

0

2.22009
7

0

0

1.08147

0

0

0.0145135

0

0

0

0

0.050967
7

0.885422

0

0

0

0

0

0

0

0

0.00888202

0.0205894
2

0

0.452655

0

0

0

0

0

0

0

0

0.064288

0

0

0

0

0.0803844

0

0.216294

0

0

0

0.233534
7

0.00394831

0

0

0.00615463

0

0

0.0129576

0

0

0

0.0547741
6

0.0100616

0

0

0

0.0129576

0.0591856

0

0.00923194

0

0

0.552327

0

0

0.0352157

0

0

0

0

0

0

0

0.0293072
6

0

0

0.147964

0

0

0

0

0.0100616

0.00416335

0

0.0511706
7

0

0

0

0

0

0

0.00518609

0

0

0

7.41214
7

1.06342

0.00411208

0

0.0156497

0

0

0.144196

0

0

0

0

0.0887784

0

0.00543325

0

0.784852

0

0.144196

0

0

0

0

0.752929
7

0

0

0.576784

0

0

0.00615463

0

0

0

0

0.956946
7

0

0

0

0.0150924

0

0

0

0

0.0326412

0

0.693266
7

0

0

0

0

0

0

0

0

0

0

0.344233
7

0.00888202

0

0

0

0

0

0

0

0

0

0.0379865
6

0

0

0

0

0.00624502

0.0123363

0

0

0

0.0591856

0.0705452
7

0

0

0

0

0

0.216294

0

0.288392

0.236743

0

0.26705

0.0156497

0

0

0

0

0.0269437

0

0

0

0

1.76462

0

0

0.144196

0.0822434

0

0.0153866

0.28952

0

0

0

4.08416
7

0.321605

0.00923194

0.144196

0

0

0

0

0

0.00616813

0.144196

0

0.521755
7

0

0

0

0

0

0

0

0

0

0

0.129468
6

0

0.177557

0

0

0.0770648

0

0

0.00881856

0.0301849

0

0.323964

0

0

0.118371

0

0

0

0

0

0

0

0.113981
6

0.0100616

0

0

0

0

0

0

0

0

0

4.08356
7

0.00518609

0.00518609

0

0

0

0

0

2.59087

0

0

0.13534
7

0

0

0

0

0

0

0

0

0

0

0.75403
7

0

0.118371

0

0

0.0234746

0.00687037

0

0.0591856

0

0

0.238216
7

0

0

0.0312995

0

0

0.00416335

0

0.0770648

0.0118427

0

0.122416
7

0

0

0

0

0

0

0.216294

0

0.0234746

0

0.842386
6

0.0100616

0

0

0

0

0

0

0

0

0

0

0.144196
6

0.288392

0

0.00832669

0

0

0

0

0

0

0

0.177265
6

0.00592134

0.00624502

0.0160309

0

0

2.95602

0

0.0469492

0

0.0201232

0.37194
5

0

0

0

0.00458025

0

0

0

0

0

0

0.0163206

0

0

0.00897447

0.0704238

0

0.00518609

0

0

0

0

0.242338
7

0.0145042

0

0

0

0

0

0

0

0.0100616

0

0.29038

0

0

0

0.00362217

0

0

0

0

0

0

0.0296292
6

0

0

0

0

0

0

0

0

0.576784

0.00888202

0.050503

0

0

0

0

0

0.0402465

0

0.00888202

0

0

0
7

0

0

0

0.36049

0

0

0

0

0

0

2.65687
6

0.576784
7

0

0

0

0

0

0.00592134

0

0

0.00816031

0

0.374389
7

0

0

0

0

0.0312995

0

0

0

0

0

1.27261
7

0

0.025664

0.0129576

0

0

0

0

0

0

0

0
6

0

0.0107775

0

0

0

0.0153866

0.0301849

0

0

0

0.250132
6

0

0

0

0.0100616

0

0.288392

0

0

0

0.0550821

0.257321
7

0

0

0

0.144196

0

0

0

0

0

0

0.0357088

0

0

0

0

0

0

0.0150924

0.00923194

0.0100616

0

0.547786
7

0

0.0156497

0

0

0

0

0.00394831

0

0

0.00777913

0.842269
7

0.144196

0.0312995

0

0.0123093

0

0

0

0

0

0.177557

1.02165
8

0

0

0

1.22567

0

0

0

0

0

0

1.52779
7

0.0697848
7

0

0

0

0

0.00881856

0.109548

0.0138191

0.105647

0

0

1.73035
7

0

0

0

0

0

0

0

0.0770648

0.0156497

0

0.334181
7

0

0

0

0.432588

0.0312995

0

0.0156497

0

0

0

0.0415649
6

0

0.01249

0.144196

0

0

0

0

0

0

0

0.158026
7

0

0.025154

0

0.0259152

0

0

0

0

0.216294

0

0.437555
7

0

0

0

0

0

0

0

0

0

0.0615768

0.380562
7

0.118371

0

0

0

0

0

0.288392

0.0129576

0

0

2.93723
7

0

0

0

0.0507067

0

0

0

0

0

0.0100616

2.57255
7

0

0

0

0

0

0

0

0

0

0

0.468105
7

0

0.0156497

0

0

0

0.00411208

0

0.0100616

0

0.577986

0.343032
7

0.127808
7

0

0

0

0

0

0

0

0.0156497

0.0156497

0

2.71783
7

0

0

0

0

0

0

0.0201232

0.00394831

0

0.0107775

0.348273
7

0

0

0

0

0

0

0

0

0

0

2.08695
7

0

0

0

0

0.00967569

0

0

0

0.0100616

0

0.429723
7

0.0770648

0

0.0503081

0

0

0

0.00816031

0

0

0

0.919204
7

0

0.0129576

0

0

0

0

0

0.00518609

0

0

0.0448914
6

0.00394831

0.0138191

0.0128851

0

0.432588

0

0

0

0

0.00532728

1.40916
7

0

0

0.0391243

0

0

0

0

0.0156497

0

0

0.0648357
7

0

0

0.00687037

0.144196

0

0

0

0.00967569

0.00615463

0

0.0207444
7

0

0

0

0

0

0

0

0.0483785

0

0

8.26337
7

2.05232
7

0.0234746

0

0

0

0

0

0

0

0.00394831

0

0.179113
6

0.00362217

0

0

0.192662

0.0150924

0.0150924

0

0

0

0

0.370551
2

0.443892

0

0

0

0

0.021555

0

0.144196

0

0

0.0100015
6

0

0

0

0.144196

0.147964

0.0234746

0

0

0.447037

0.0269234

0.409426
7

0

0.0156497

0

0

0

0

0

0

0

0.216294

0.708067
7

0.0259152

0.00624502

0

0.0207247

0

0.0150924

0

0

0

0

0
8

0.0452773

0.0129576

0.0150924

0

0

0

0

0

0

0.0107775

0.486995
7

0.0150924

0

0

0

0

0

0

0

0

0.105823

0.37217

0

0

0

0

0

0

0

0

0.0391243

0

0.113458
7

0

0

0.00923194

0

0

0.0201232

0

0

0

0.00440928

29.3935
7

8.8088
7

0.0496223
7

0

0

0

0

0

0

0

0

0

0

0.0665345
7

0

0.021555

0

0.0156497

0

0.0156497

0.00615463

0

0

0.0148034

0.648882
7

0

0

0.00687037

0.0100616

0

0

0

0.231194

0

0

0.0635949

0

0

0.0887784

0

0

0.0103722

0.144196

0

0

0

0.170475

0.00416335

0

0

0.0129652

0

0

0.0156497

0

0

0

0.0171286

0

0.00624502

0.172147

0

0

0

0

0

0

0

0.148907
7

0

0.504686

0

0

0

0

0

0

0

0

0.357176

0

0

0

0.025154

0.0133182

0

0

0

0

0

0.0431422
6

0

0

0

0

0

0

0

0

0

0

0.383858

0

0

0.147964

0.0591856

0.281725

0

0.0312995

0

0

0

4.94324
7

0.679125
7

0

0

0

0

0

0.504686

0.00411208

0.0547741

0.0100616

0.00458025

0.0332869
7

0.288392

0.00394831

0.0234746

0.00967569

0

0

0

0

0.0155583

0

0.0454374
7

0.0259152

0.0156497

0.0091605

0

0

0

0

0

0.0100616

0

0.015127
5

0

0

0

0

0

0

0

0

0

0

0.443989

0.147964

0

0.0156497

0

0

0

0.0855237

0

0.0150924

0

0.268661
7

0

0.177557

0

0

0

0

0

0

0

0

0.00592247

0

0

0

0

0

0

0

0

0

0

0.313589
7

0

0

0

0

0

0

0

0

0

0

0.413263
7

0

0.00777913

0

0

0

0

0

0

0

0

0.23584
7

0

0

0

0

0.00408015

0.00615463

0

0

0.0150924

0.0591856

11.0008
7

0.0507031
7

0

0

0.0234746

0.15413

0

0

0.0150924

0

0.937273

0

1.6283
7

0

0

0

0.00440928

0.0234746

0

0.20715

0

0

0

1.18326
7

0

0

0

0

0

0

0

0

0

0

0.027613
7

0

0

0

0

0.36049

0

0

0

0

0

0.176704

0

0

0

0

0

0

0

0

0.00592134

0

0.0401746
6

0

0.216294

0

0.00777913

0

0

0

0.00967569

0

0

0.261741
7

0

0

0

0

0

0.0728586

0

0

0

0

0.00458025

0

0.0770648

0

0

0

0

0

0

0

0

0.282973
7

0

0

0.0123093

0.0478785

0

0

0

0

0

0

0.461634
7

0

0

0

0.0236899

0.00888202

0

0

0

0

0.156125

4.97727
7

3.60873
7

0

0

0.144196

0

0.00789662

0

0

0

0.0156497

0

0.0193836

0.0523143

0.00394831

0

0

0.0680955

0

0

0

0

0.144196

0.0166573
6

0

0

0

0

0

0.236743

0

0

0

0

0.255678
7

0

0.591856

0

0.00408015

0

0

0

0

0

0.140863

1.389
7

0

0

0

0

0

0

0

0

0.0591856

0

0.28698
7

0

0.216294

0

0

0

0.0091605

0

0

0

0

0.0234746
4

0

0

0

0

0

0.0312995

0

0.0156497

0

0

0.0129576

0.00673085

0

0

0

0.144196

0

0.00440928

0

0

0

0.132009
7

0

0.0145717

0

0

0

0

0

0

0

0

0.0250336
7

0.00967569

0.15413

0.432588

0

0.118371

0

0.216294

0

0.0103722

0

0.706794
6

1.34637
7

0

0

0

0

0

0

0

0.00888202

0.0123093

0

1.99038
7

0

0.0266461

0

0

0

0.00458025

0

0

0.0338649

0.288392

0.00987078
7

0

0

0.0887784

0

0.00905542

0

0

0

0

0.504686

0.0748354
7

0

0

0

0

0

0

0

0

0

0

0.429425
7

0

0.0100616

0

0

0

0.0160309

0

0

0

0

0.428679
7

0

0

0.144196

0

0

0.0100616

0.432588

0.0312995

0

0

0.375295
7

0.00458025

0

0

0

0

0

0.216294

0

0

0

0.364258

0

0

0

0

0

0

0

0

0

0

2.86839
7

0

0.0770648

0

0

0

0

0

0

0

0.032394

1.30567
7

0

0

0

0

0

0

0

0

0

0

5.69071
7

0.158805
6

0

0.0129576

0

0

0

0

0

0

0

0

0.14991

0.00416335

0

0

0.0100616

0.132209

0

0

0.00789662

0

0

0.0105134
7

0

0.288392

0

0

0.0301849

0

0

0.0887784

0

0

0.803855
7

0.00923194

0.00592247

0

0.00394831

0.0591856

0

0

0

0

0

1.14049
7

0

0

0

0

0

0

0

0

0.144196

0

0.421418
7

0

0.0153866

0

0

0

0

0.0217157

0.216294

0

0

0.118371

0

0

0

0.00905542

0.00687037

0

0

0

0

0

1.07227
7

0

0.308259

0

0.00518609

0.00394831

0

0

0

0

0

0
6

0

0

0

0

0

0

0

0

0

0.144196

0.0082435
7

0

0

0

0

0.0770648

0

0

0

0

0

5.31485
7

2.23531

0

0

0.0483191

0

0

0

0

0

0

0.15413

0.432588
7

0.0107775

0

0

0

0

0

0

0

0

0

0.0407353

0

0

0

0

0

0

0

0

0.216294

0

0.0278114
6

0

0

0

0

0.0194364

0

0

0.0234746

0

0

0.32177
7

0

0

0

0.0100616

0

0

0

0

0.00616813

0

0.281082

0.00615463

0.118371

0

0.147964

0

0.0391243

0.0887784

0.0325674

0

0

0.847852
7

0

0

0

0

0

0.0102802

0.00789662

0

0

0

0.0207444
7

0

0

0

0

0

0

0

0

0.0107775

0.0887784

0.00615463

0.0770648

0

0.0107775

0

0

0

0

0

0.216294

0.00832669

0

0

0.00673085

0

0

0.0103722

0

0

0

0

0

3.69203
7

0.114583

0

0

0

0

0

0

0

0.0156497

1.69647

0.576784

0.591279
7

0.144196

0

0.106604

0.0132278

0

0

0

0

0

0

0.220637
7

0.0440928

0

0

0

0.0887784

0.0259152

0.025154

0

0

0

0.0307136
6

0

0

0

0

0

0

0

0.0444101

0

0

0.0206111
6

0

0

0.06173

0

0

0

0.00687037

0.115597

0

0.0301849

0.01249
5

0

0

0

0

0

0

0

0.36049

0

0

0.0410832
6

0

0

0

0.0225489

0

0

4.32588

0

0

0

0.0154325
7

0

0

0

0

0.887784

0

0

0.144196

0

0.0390648

0.262709

0

0

0

0.355114

0

0

0

0

0

0

0.0329328

0

0

0.0808312

0

0

0

0

0.144196

0

0

13.5456
7

0.219916
7

0

0.0553916

0.0213091

0

0.0153866

0.0916087

0

0

0

0

1.53883
7

0

0

0.0220464

0.00394831

0.00362217

0

0

0.576784

0

0

1.14954
7

0

0

0.0103722

0

0.0591856

0

0

0

0

0

0.504686
7

0

0

0.36049

0.00592134

0

0.144196

0

0

0.0388728

0

0.118371
7

0.0114506

0

0

0

0

0

0

0

0

0

0.00923194
7

0

0

0

0

0.00592134

0.00661392

0.00448724

0

0

0.745061

0.157494

0

0

0

0

0

0

0

0.0388728

0

0.144196

0.227113
7

0.0469492

0.144196

0

0

0

0

0

0.0129652

0.0480926

0

0.0982845
7

0

0

0.00592134

0

0

0

0

0

0.0156497

0

0.35619

0

0

0

0.0217157

0

0

0

0.0391243

0

0.0107775

0.905405
7

0.0248102

0

0.216294

0

0

0.216294

0

0

0

0

0

0.126701
2

0

0

0

0

0

0.144196

0

0

0

0.00661392

0.0770648
7

0

0

0.144196

0

0

0

0.109548

0

0.0533022

0

0.29234
7

0.00416335

0

0

0

0

0

0

0

0.288392

0.0156497

0.0417203
6

0

0

0.0201232

0

0.0591856

0

0

0

0

0

0.00394831

0.0177674

0

0

0

0

0.0205604

0

0

0

0

0.0790783
7

0

0

0

0

0

0

0.00592134

0

0

0

0.0343099
7

0

0

0

0

0

0

0

0

0

0.0206111

0.313281

0

0

0

0

0

0

0

0.00416335

0

0

0.128433
7

0.00789662

0

0

0.0104084

0

0

0

0.15413

0.0145135

0.0156497

35.7738
7

0.713249
7

1.40463

0.485142

0

0.0132278

0

0

0

0

0.0770648

0

0

0.0645483

0

0

0

0

0

0

0

0.0224408

0.0938984

0

0.0614628
6

0

0

0

0

0

0

0

0

0

0

0.00777913
7

0

0

0

0

0

0

0

0

0

0

0.877373
7

0.216294

0

0

0

0

0

0

0

0

0

0.0947009
7

0

0

0

0.72098

0

0

0

0

0.00661392

0

0.166917

0

0.00416335

0.937273

0

0

0.00661392

0

0

0

0

0.0391243
6

0

0

0

0

0

0

0

0

0.0100579

0

11.0217
8

0

0

0

0

0

0

0.462389

0.0887784

0.00518609

0

0.685792
7

0

0

0

0

0

0

0

0

0

0

3.80643

0.100539

0.0683439

0

0.00592247

0

0

0

0

0

0

0

0.418659
7

0

0

0

0

0

0

0.0887784

0

0

0

1.18101
7

0

0

0.144196

0

0.432588

0

0

0

0

0

0.00408015
7

0

0

0

0

0.0933496

0.0377212

0

0.00592134

0

0

0.456656
7

0.0352157

0

0

0

0

0

0

0

0

0

0.250432
6

0

0

0

0

0

0

0.0148034

0

0

0.00832669

0
7

0

0.00440928

0

0

0

0.0259304

0

0.15413

0.36049

0

0.025154
7

0

0

0

0

0

0

0

0

0

0

0.0362133
6

0.216294

0.0591856

0

0

0

0

0

0.144196

0

0.0201232

0.314991
7

0.0110232

0

0.216294

0

0

0

0

0

0

0

1.89063
7

0.00986966
7

0.00661392

0

0

0

0

0

0

0

0

0

0.115161

0

0

0

0

0

0

0

0

0

0

0.865669
7

0

0

0.0887784

0

0

0

0

0

0

0

0.648882
7

0

0

0

0

0

0

0

0

0.0461597

0.062892

0.385397

0

0

0

0

0.0100616

0

0

0

0

0

0.0291024
7

0

0.0166534

0

0

0

0

0

0

0

0

0.178683
7

0

0.144196

0

0

0.0259152

0

0

0

0

0

0.341473
7

0

0

0

0

0

0.00408015

0

0

0

0.0107775

1.75117
6

0

0

0

0

0.0591856

0.0887784

0.288392

0

0

0

0.77139
7

0

0

0

0

0.00440928

0

0

0

0

0

2.94461
7

3.57809
7

0

0

0.288392

0

0

0

0

0

0.177557

0

0.00440928
7

0

0.36049

0

0.0391243

0

0

0

0.00394831

0

0

0.698501
7

0

0

0

0

0

0.0547741

0

0

0.00411208

0

0.27559

0

0

0

0

0

0

0

0.0107775

0

0

0.562928
7

0

0

0

0

0

0

0

0

0

0.00518609

0.170111
6

0

0

0

0

0

0

0.0156497

0

0

0

0.364977
7

0

0.00592134

0

0.00394831

0

0.0312995

0.0091605

0

0

0

0.0526251
2

0.177557

0.0259152

0

0.00483473

0

0.00394831

0

0.0201232

0

1.08147

0.206217
7

0

0

0

0.00518609

0

0

0.053042

0

0

0

0.0393848

0

0

0

0

0

0

0

0

0

0.00673085

7.80067
7

0.905987
7

0

0

0

0

0

0

0

0

0

0.288392

1.10864
7

0

0

0

0

0

0

0

0

0.0194364

0

0.0480971
7

0

0

0.00673085

0

0

0

0

0

0

0

0.952923
8

0

0

0

0

0

0

0.0198418

0

0.00881856

0

0.281821

0.0148034

0

0

0

0

0

0.00799092

0

0

0

0.0871475

0.469492

0.288392

0

0.0129576

0

0

0.0129576

0

0.0615463

0

0.0283442

0

0

0

0

0

0

0

0.216294

0

0.0312995

1.91647
7

0

0

0

0

0

0

0.00967569

0

0

0.0234746

0.519899
7

0.0336543

0

0

0

0

0

0

0.0645319

0

0

0.173592
7

0

0.0107775

0

0

0

0.0154325

0.0201232

0

0.00416335

0

2.12503
7

0.667789
7

0

0.216294

0

0

0

0

0

0

0

0

0.0514978
6

0

0

0

0.120737

0.36049

0

0

0

0

0

0.124327
7

0

0

0

0

0.0156497

0

0

0

0

0

0.293578
7

0

0

0

0

0

0

0

0

0.0391243

0

0.129025

0.0591856

0

0.00661392

0

0.500921

0

0

0

0

0.00592134

3.27599
7

0.0603697

0

0

0

0.769413

0

0.032394

0

0.00322316

0

1.69748
7

0.0156497

0

0

0

0

0

0

0

0

0.288392

0.0145489
6

0

0.0129576

0

0

0.00518609

0

0

0

0

0

0
7

0

0.0177674

0

0.0391243

0

0.00416335

0

0

0

0

0.0786493
7

0

0.0234746

0

0.0301849

0

0

0

0

0.00967569

0

9.26097
7

0.280251
7

0

0

0.00416335

0

0

0

0

0

0.0156497

0

0

0

0.00592247

0

0.0591856

0.290992

0

0

0

0.00592247

0

0.0353709

0.147964

0

0

0

0

0

0

0.144196

0

0

0
6

0.0100616

0

0

0

0

0

0

0

0

0.0194364

0.364899
7

0

0

0

0.0201232

0.216294

0

0

0.0156497

0

0

0.172803

0

0

0

0

0

0

0

0

0.00881856

0.0591856

0.592459
7

0

0

0

0

0.0887784

0.0156497

0

0.432588

0

0

0.180045

0

0.236743

0

0

0.00394831

0

0

0

0

0

0.166104
7

0

0

0

0.0107775

0

0

0

0

0

0.00777913

1.31555
7

0

0

0

0

0

0

0

0

0

0

18.4542
7

0.118574
7

0

0

0.144196

0

0

0.0107775

0

0

0

0.288392

0.231501
7

0

0.937273

0

0.2138

0

0.091605

0

0

0

0.00416335

2.78816
7

0

0

0

0

0.282642

0

0

0

0.00967569

0

0.248932
7

0.00832669

0

0

0

0

0

0.216294

0.00661392

0.00416335

0

0.419675
7

0.123941

0.00440928

0

0

0

0

0

0.216294

0

0

14.3212
8

0

0

0

0

0

0

0

0

0

0.0157053

0.119783
7

0

0

0

0

0.0234746

0

0.00394831

0

0

0

0.0215711
7

0

0

0

0

0

0.0923652

0

0.0234746

0

0

0.0103722
6

0.462389

0

0.0154325

0

0.0311165

0

0

0.00789662

0

0

0.435373
7

0

0

0

0

0.0100616

0.0402465

0

0

0

0.00440928

11.5822
7

0.447032
8

0

0

0

0

0

0.00440928

0

0

0

0

0.370085
7

0

0

0.0129576

0

0

0

0

0

0

0.0469492

0.733835
7

0

0

0.177557

0

0

0

0

0

0

0

0.165843

0

0

0

0.0770648

0

0

0.144196

0

0

0.00799092

0
6

0.0591856

0

0

0

0.00615463

0

0

0

0

0

0.246972
7

0

0

0

0

0

0

0.432588

0.00518609

0.216294

0

0.00458025
5

0

0.00687037

0

0

0

0.793077

0.0234746

0.0887784

0

0

0.261603
1

0

0

0

0

0

0.576784

0

0

0

0

0.371052
7

0

0

0

0

0

0.025154

0

0

0

0

0.0262279
6

0.00394831

0

0

0.216294

0

0

0.0132278

0.00518609

0

0

3.04274
7

0.207164
7

0

0

0.288392

0

0

0

0.0150924

0.00458025

0

0.0091605

0.0156497
5

0.00322316

0

0.00518609

0

0

0

0

0

0

0

0.0202785
7

0

0

0

0

0

0

0

0

0

0.00789662

0.309653
7

0

0.00789662

0

0.648882

0

0

0

0.00616813

0

0

11.8521
8

0

0.00518609

0

0

0.504686

0

0

0

0.144196

0.118371

0.399835

0

0

0.140848

0

3.89329

0

0

0

0.0234746

0

0.867946
7

0

0

0.00615463

0

0

0.00394831

0

0

0

0

0.180358
7

0

0.00888202

0

0.00789662

0.134243

0

0

0

0.021555

0

0.0383126
6

0

0.144196

0

0

0.144196

0

0.0107775

0

0

0

0.371774
7

0

0.00440928

0

0

0

0

0.00724433

0

0

0

0
4

0.032394
5

0

0.032394

0

0
4

0.0242529
7

0.0242529
7

0
4

0.144196
7

0.144196

0

0
4

0.0312995

0.0312995

0
4

0

0

0
4

0.288392

0.144196

0.144196

0
4

0

0

0
4

0

0

0
4

0

0

0
4

0
4

2.33924
7

2.32628
7

1.22754
7

0.15413
7

0.939425
7

0.00518609

0
4

0.0129576

0.0129576

0
4

2.96637714392034e-16
7

0
4

90.8118

13.8124
7

6.54872
7

0.158838
7

0

0

0.00408015

0

0.0114506

0.0156497

0.00411208

0

0.00458025

0

0.439743
7

0

0.00458025

0.0306012

0

0

0

0

0

0.00592134

0

0.115597
6

0

0

0

0

0

0.00888202

0

0

0

0.0156497

0

0

0

0

0.00458025

0

0

0.0704238

0.0435406

0

0

0.158838
7

0.0914161
6

0.018321

0.216294
7

0
6

0

1.41165
7

0.225841
7

0

1.18532
7

0.116618

0.00458025

0

0.0107775

0.0770648

0

0

1.24208
7

0.00458025

0

0

0

0

0.0268084

0.144196

0.0161662

0

0

0.027297
6

0.0230192

0

0.00458025

0

0.0325674

0

0

0

0.0469492

0

0.0687037
6

0

0

0

0

0

0.0029305

0.00869233

0

0

0

0.161262
7

0

0

0

0

0

0.0343519

0

0

0.0860736

0

0.359944
7

0

0.0417956

0.00458025

0

0

0

0

0

0.0122405

0.00458025

0.0201232
6

0.00411208

0

0

0

0

0

0.15413

0

0.00967569

0

0.231194
6

0

0

0

0.0251914

0.0029305

0

0

0

0

0.00799092

0
4

0

0

0

0

0

0

0
4

5.5464

5.5464

0
4

18.0529

15.3059

0.174927

2.57208
7

0

0
4

0

0

0

0

0

0

0

0
4

14.8718
7

7.22823
7

0

0.0156497
6

0.0226333
6

0

0

0
5

0.0712667

0

0

0.0388728

1.85784

0.0323166

0.0388728

0.0712667

0

0.0129576

0.125198

0.156497

0.062599

0

0

0.600668
6

0.136055

0.0129576

0

0

0.0591856

0.0259152

0

0

0.0194364

0.596049

0.148246
7

0

0.0777455

0

0

0

0

0.0129576

0.0712667

0

0

0.138927
7

0

0

0

0

0

0

0

0

0.0388728

0

1.42988
7

0.36049

0

0

0.0129576

0

0

0.0259152

0

0.0426287

0.0453516

0.0234289
6

0

0

0.00777913

0

0.0156497

0.00518609

0.0259152

0

0.0194364

0

1.05613
7

0.0129576

0

0

0

0

0

0

0

0.115597

3.95516952522712e-15
7

0
4

0.702314
6

0.0770648
6

0

0

0.144196

0

0

0

0

0

0

0

0

0

0.192662
6

0

0

0

0

0.144196

0.144196

5.55111512312578e-17
6

0
4

34.7142
6

0.266428
6

1.6033
7

0

0.00411208

0

0

0

0.00416335

0

0

0.0603697

0.00411208

0.0248102
6

0

0

0

0.432588

0

0

0.00458025

0

0

0.0100616

4.75294

0.0156497

0

0.0100334

0.00532728

0

0

0

0

0

0

5.41706
7

0

0

0.115597

0

0.0194364

0

0

0

0

0.00408015

0.281695
6

0

0.00408015

0

0

0

0

0

0

0.00411208

0

0.652087

0

0

0

0

0

0

0

0

0

0

0.183739
7

0

0.00816031

0

0

0

0

0

0

0

0

0.0192221
6

0

0

0

0

0

0

0

0

0

0

0
6

0

0

0.0164483

0.0156497

0

0

0

0

0

0.0159818

0.456743
7

0.144196

0.00799092

0

0

0.103661

0

0

0

0

0

3.3696
6

0.0156497
6

0

0.423856

0

0

0

0

0

0

0

0

0

0.0591856

0.0822417

0

0

0

0

0

0.00616813

0

0.0156497

0.0181513
6

0.00532728

0

0.0091605

0

0

0

0

0

0

0

0.535762
7

0.0133182

0.00687037

0

0

0

0

0

0

0.00687037

0

0.0770648
6

0

0

0

0

0

0

0.335197
7

0
6

0.0541905
6

0.203382
7

0.144196
6

1.5952
6

0.00458025
6

0.00687037
6

1.73035

0
6

0.187687

0
6

0

0.00458025
6

0.652223
6

0.0589374
7

0.740139
6

0.0201232

0.504686
7

0.00411208
6

0
6

0.148308
7

0
6

0

0

0.423778

0.0320617

0.398991

0

0.269727

0.328051
7

0.018321

0.36049

0
6

0

0.288392

0

0.00416335

0.697475
6

0.0274815

0.305072

0

0.120946

0.0091605

0

0

0

0

0

0.174727
6

0

0.0100616

0

0

0

0

0.025154

0

0

0

2.23596
7

0.0137407

0

0

0.0137407

0

0.0326412

0

0

0

0.0234746

2.92568
7

0.168449

0

0

0

0

0.0887784

0

0.00967569

0

0

7.67615138119737e-15
6

0
4

0.237412

0.19101

0.0289471

0.00967569
5

0.00777913

0

0

0

0
4

0.0129576

0.0129576

0
4

0.0269234

0.00673085

0.0157053

0.00448724

2.60208521396521e-18

0
4

0.155491

0.155491

0
4

0.0971819

0.0971819

0
4

0

0

0

0
4

0

0

0
4

0

0

0
4

0.00448724

0.00448724

0
4

0.00532728

0.00532728

0
4

0

0

0
4

0.596943

0.596943

0

0
4

0.0647879

0.0647879

0
4

0

0

0
4

0

0

0
4

0

0

0
4

0

0

0
4

0

0

0
4

0

0

0
4

0.619239

0.585584

0.029167

0.00448724

0
4

0.524782

0.524782

0
4

0.756099

0.73815

0.00448724

0.00448724

0.00448724

0.00448724

0
4

0

0

0

0

0
4

0

0

0
4

0

0

0

0
4

0.0100616

0.0100616

0
4

1.41830991395864e-14

0
4

44.6327

1.09972
7

1.09972
7

0
4

2.14991
5

2.124
5

0.0129576
5

0

0

0.0129576

0

0

0

0

1.49186218934005e-16
5

0
4

0.73185

0.624601

0

0

0.0150924

0.0770648

0.0150924

0
4

7.69354
7

7.59181
7

0.0782487
7

0.0234746

1.56125112837913e-16
7

0
4

0.168616

0.1249

0

0.0312251

0.00416335

0.00416335

0

0.00416335

1.21430643318376e-17

0
4

0.221272
5

0.221272
5

0

0

0
4

0.399076
7

0.375552
7

0.0167925

0.00673085

0
4

0.032394
5

0.0194364
5

0

0

0

0

0

0.0129576

1.73472347597681e-18
5

0
4

0.155491
6

0.142533
6

0.0129576

0

0
4

0.894412

0.884736

0.00967569

0

0
4

0.0583091
4

0.0453516
4

0

0.0129576

0

0

0

0

0
4

0

0

0

0

0

0

0

0
4

2.02574

0.830357
5

0

0.0887784

0.325521

0.00923194

0

0.33457
6

0.141354

0

0

0

0

0.0887784

0.20715

1.66533453693773e-16

0
4

0
3

0
4

0

0
4

0

0

0

0
4

0

0

0

0

0
4

0.129576
7

0.129576

0

0
4

0
5

0

0

0

0

0

0

0

0

0
4

0.92399

0.92399

0
4

0.0686952

0.0541235

0

0.0104084

0.00416335

0

0

2.60208521396521e-18

0
4

0.440479
7

0.440479
7

0
4

0.129576
6

0
6

0.129576

0
4

0

0

0
4

1.21826
5

0.727408
5

0

0

0

0.0234746

0

0

0

0

0

0

0.10689
5

0

0

0.36049

0

0

0

0

0

0

0
4

0.147799
4

0.00416335

0.143635

0
4

0.0770648

0

0

0

0.0770648

0

0
4

0.158709
7

0.144196

0.0145135

0

0

0
4

0.0416335

0.0416335

0

0
4

0.00416335

0.00416335

0

0
4

0.063349

0.063349

0
4

0

0

0

0
4

0.0259152

0.0129576

0

0.0129576

0
4

0.0907031

0.0907031

0
4

0

0

0

0
4

6.97304
7

1.33671
7

0.0156497

0

0.413668
7

4.81714
7

0.0312995
7

0.0156497
6

0.272501
7

0.0234746

0.0469492

0

6.59194920871187e-16
7

0
4

0

0

0
4

0

0

0

0
4

0

0

0
4

0

0

0
4

0

0

0

0

0
4

0

0

0
4

0.292868

0.292868

0
4

0.0104084

0

0.00416335

0.00624502

8.67361737988404e-19

0
4

0.0129576

0.0129576

0

0
4

0

0

0

0

0
4

0.246701
5

0.246701
5

0
3

0
5

0

0

0

0

0
4

0

0

0

0
4

0.168449

0.168449

0
4

0

0

0

0
4

0

0

0
4

0

0

0

0
4

0

0

0
4

0

0

0
4

0.144196

0.144196

0

0
4

0.032394

0.032394

0
4

0

0

0

0
4

6.20626

5.87801

0.00411208
6

0.313807

0.00416335

0.00616813

0

0

0

0
4

0.00832669

0.00832669

0
4

0.0194364

0.0194364

0
4

0

0

0
4

0

0

0
4

0

0

0
4

0

0

0
4

0

0

0
4

0

0

0
4

0

0

0
4

0

0

0
4

8.62416

8.53127

0.00411208

0

0

0

0

0

0.0887784

6.24500451351651e-16

0
4

0

0

0
4

0

0

0
4

0

0

0
4

0.0156497

0.0156497

0
4

0

0

0
4

0

0

0
4

0

0

0
4

0.0129576

0.0129576

0
4

0.0100616

0.0100616

0
4

0

0

0
4

0.615226

0.256406

0.0887784

0

0

0.147964

0.0591856

0

0

0

0.062892

0

2.77555756156289e-17

0
4

0

0

0
4

0.00967569

0.00967569

0
4

0

0

0
4

0

0

0
4

0

0

0
4

0

0

0
4

0

0

0
4

0

0

0
4

0.36049

0.36049

0
4

0.0887784

0.0887784

0
4

1.63039
5

1.61374

0

0.0166534

0

0

0
4

0

0

0
4

0

0

0
4

0

0

0
4

2.48689957516035e-14

0
4

5.51977

5.51977

5.49629

0

0

0.0234746

0

0

2.39391839684799e-16

0
4

0
4

0.00745395

0.00745395

0.00745395

0
4

0
4

0

0

0

0
4

0
4

0

0

0

0

0
4

0
4

0.0175378

0.0175378

0.00458025

0.0129576

0

1.73472347597681e-18

0
4

0
4

0

0

0

0
4

0
4

0

0

0

0

0
4

0
4

0.0186455

0.0186455

0

0.0186455

0
4

0
4

0

0

0

0
4

0
4

0.440558

0.440558

0.440558

0
4

0
4

0

0

0

0

0
4

0
4

0.0707038
3

0.0707038
3

0.0134623

0

0.00733715

0

0.0147258

0.00408015

0

0

0

0

0

0

0.00888202

0

0

0

0

0

0

0

0

0

0.0103571
5

0

0.00408015

0

0

0

0
3

0.00777913
3

0

0

0

0

0
4

0

0

0
4

0
4

0

0

0

0

0
4

0
4

0

0

0

0
4

0

0

0
4

0
4

0.0583091

0.0583091

0.0583091

0
4

0

0

0
4

0
4

0

0

0

0
4

0
4

0

0

0

0
4

0
4

0

0

0

0
4

0
4

0

0

0

0
4

0
4

0.421122

0.421122

0.421122

0
4

0
4

0

0

0

0
4

0
4

0

0

0

0
4

0
4

0.025685
3

0.025685
3

0.0127999

0

0.0128851

0

0
4

0

0

0
4

0

0

0
4

0
4

0

0

0

0
4

0
4

0

0

0

0
4

0
4

0

0

0

0
4

0
4

0

0

0

0
4

0
4

0

0

0

0
4

0
4

0.0312995

0.0312995

0.0312995

0
4

0
4

0

0

0

0
4

0
4

0

0

0

0
4

0
4

0

0

0

0
4

0
4

0

0

0

0
4

0
4

0.00673085
6

0.00673085
6

0.00673085
6

0

0

0

0

0
4

0
4

0

0

0

0
4

0
4

0

0

0

0
4

0
4

0.504686

0.504686

0.504686

0
4

0
4

0

0

0

0
4

0
4

0

0

0

0
4

0
4

0

0

0

0
4

0
4

0

0

0

0
4

0
4

0.179024

0.179024

0.179024

0
4

0
4

0

0

0

0

0

0

0
4

0
4

0.0157126
5

0

0

0
4

0.0157126

0.0157126

0
4

0

0

0
4

0
4

0.155491

0.155491

0.0971819

0.0583091

6.93889390390723e-18

0
4

0
4

0.549726

0.549726

0.541946

0.00777913

0
4

0
4

4.56036

4.09912

4.09912

0

0

0

0

0

0

0

0
4

0.216482
6

0.203524
6

0.0129576

0

0

0

0

0

0

0
4

0

0

0
4

0

0

0
4

0

0

0
4

0
5

0

0

0

0

0

0

0

0

0

0
4

0.0921572
7

0.0150924
6

0.0770648

0

0
4

0.152595
7

0.152595
7

0

0
4

0

0

0

0
4

0

0

0
4

0

0

0
4

0

0

0
4

0

0

0
4

9.15933995315754e-16

0
4

8.42725

6.28717

0.556908

0.032394
5

0.59976
7

0.144196

0.0453516

0.0445904
5

0

0.0259152

0.0234746
5

0

0.105647

0.500915

0

0.308515

0.113944

0

0.00586101

0.032394

0.025154

1.08159

0

0.0234789

0
6

0

0.0194947

0

0.432588

0

0

0

0

0

0

0.0129576
5

0

0

0

0.0100616

0

0

0

0

0.0100616

0

0
6

0.00408015

0

0.0100616

0

0

0

0

0

0

0

0.191171

0

0.0150924

0

0.144196

0

0.00439576

0.00586972

0.0259152

0.0156497

0

0.677523

0

0

0

0

0

0.0100616

0.0150924

0

0

0

0.997162
7

0.0156497

0

0

0

0

0

0

0

0

3.81639164714898e-16

0
4

1.59991

1.59991
7

0

0
4

0.0129576

0

0.0129576

0
4

0

0

0
4

0

0

0
4

0

0

0
4

0

0

0
4

0

0

0
4

0

0

0
4

0.0129576

0.0129576

0
4

0
5

0
6

0

0

0

0
4

0.0194364
6

0.0194364

0

0

0

0
4

0.0842243
5

0.0842243
5

0
4

0.0330808
7

0.0330808
7

0

0

0
4

0
7

0
7

0
4

0.140765

0.053042

0.0877233

0
4

0.236743

0.236743

0

0
4

0

0

0
4

0
4

1.11977

1.07167

0
5

0

0

0

0

0

0

0

0

0.72098

0

0
5

0

0

0.00777913

0

0

0

0

0

0.0388728

0

0

0.288392

0

0

0
6

0

0

0.0156497

0

0

0
4

0.0480926

0.0480926

0
4

0

0

0
4

0

0

0
4

0

0

0
4

0
4

4.07055
5

4.07055
5

0.288968
5

0.0129576
5

0
5

0
6

0
5

0

0.0259152

0

0

0

0

2.26792

0

0

0

0

0.0770648

0

0

0

0.0777455

0

0.149012
6

0

0

0.129576

0

0.0907031

0

0

0

0.0129576

0

0
5

0

0

0

0

0.0129576

0

0

0

0

0

0
5

0

0

0

0

0.0129576

0

0

0

0

0

0
5

0

0

0

0

0

0

0

0

0

0

0.2138
6

0

0.0129576

0

0

0.0129576

0

0.25745
4

0.414643
6

5.55111512312578e-16
5

0
4

0

0

0

0
4

0

0

0

0
4

0

0

0
4

0

0

0
4

0
4

9.67771

7.92035

5.03036

0.216294
5

0

0

0.464433

0.325521

0.0129576

0.0129576

0.0129576

0

0

0

0
5

0.118371

0

0

0

0

0

0.116618

0

0

0.0129576

0

0

0

0.0712667

1.05604

0.391866

0.0777455
7

0

0

0
4

0.168449
5

0.168449
5

0
4

0

0

0
4

0.0129576

0.0129576

0

0
4

0.0328967

0.00411208

0.00822417

0.0205604

0
4

0

0

0
4

0

0

0
4

0

0

0
4

0.648882

0.36049

0.288392

0
4

0.00411208

0.00411208

0
4

0

0

0
4

0

0

0
4

0.492213

0.492213

0

0
4

0

0

0
4

0

0

0
4

0

0

0
4

0

0

0
4

0.0102004

0.0102004

0
4

0

0

0
4

0

0

0
4

0

0

0
4

0

0

0
4

0

0

0
4

0.144196

0.144196

0

0

0
4

0.0156497

0.0156497

0
4

0

0

0
4

0.0185044

0.0185044

0
4

0.00822417

0.00822417

0
4

0

0

0
4

0

0

0
4

0.0575692

0.0575692

0
4

0.0100616

0

0

0.0100616

0

0
4

0.0267285

0.0102802

0.00822417

0.00411208

0.00411208

3.46944695195361e-18

0
4

0

0

0
4

0.0388728

0.0388728

0
4

0

0

0
4

0.0678494

0.0370088

0.0308406

6.93889390390723e-18

0
4

6.66133814775094e-16

0
4

13.0173
5

7.98735
7

5.41537
7

2.21149
7

0.36049

0
4

0
4

0
4

0

0

0
4

0.0129576

0.0129576

0
4

0.00408015

0.00408015

0
4

0

0

0
4

0

0

0
4

0

0

0

0
4

0
4

0
4

0

0

0
4

0

0

0

0

0
4

0

0

0

0

0
4

0.025154

0.025154

0
4

4.97476

4.97476

0
4

0

0

0
4

0.0129576

0.0129576

0
4

1.62890534394222e-15
5

0
4

6.57931

6.57931

0.187618

0
5

0.527463

0

0

0.110232

0

0.216294

0.36049

0.144196

0.00440928

2.40103

0

0

0

0

0

0.648882

0

0.00440928

0

0

0.239768

0

0

0.144196

0

0

0.00440928

0

0

0

0

0.144196
7

0

1.22567
7

0.0440928

0.171962

0

0

5.55111512312578e-17

0
4

0
4

1.14041
4

1.06418

1.06418

0

0

0

0
4

0

0

0

0

0
4

0.00408015

0.00408015

0
4

0

0

0
4

0.0591856

0.0591856

0
4

0.0129576

0.0129576

0
4

1.71737624121704e-16
4

0
4

0.194364
4

0.0971819
4

0
5

0

0.0453516

0

0

0

0

0

0

0

0

0.0194364

0.032394

0

0

0

0

0
4

0.0712667

0.0712667

0
4

0

0

0
4

0.0259152

0.0259152

0
4

0
4

0
4

1559.23

609.558

2.18526

0.415725
5

0.663986

0

0

0

0.129576

0

0

0.975974

0

0

0
4

39.4059

37.7966

0.235929
7

0

0

0

0

0.0156497

0

0

0

0

0.144196

0.791286
7

0

0

0.422258
7

0

0

0

0

0

0

0
4

495.65

381.568
7

0.667876
7

0

0.0102004

0

0.132278

0.443892

0

0.00612023

0.0103722

0.288392

0.00612023

0.957768
7

0.24673

0.0591856

0

0.0102004

0

0

0

0

0

0

0.385497
7

0.00408015

0.0591856

0

0.00408015

0.0122405

0.0224408

0

0.147964

0

0.0369278

0.619974
8

0.0156497

0

0

0

0

0

0

0.0693626

0.0156497

0.00416335

0.735578
7

0

0.118371

0

0.144196

0.0246798

0

0.00408015

0

0.0156497

0

0.146333
7

0.00816031

1.18371

0.0770648

0.00612023

0.0887784

0.00624502

0

0.0591856

0.029305

0.0244809

0.233681
1

0.288392

0

0.0234746

0.00416335

0

0.00624502

0.00615463

0.00416335

0.0403851

0.0591856

0.303394

0.00518609

0

0.0122405

0.0591856

2.80103

0

0

0

0.00661392

0

0.704568
8

0.0591856

0.00448724

0.00416335

0.00612023

0

0.00879151

0.00987078

0.0591856

0.00967569

0

0.236743

0.0887784

0

0

0

0

0

0.144196

0

0.00816031

0

5.94167

0.212039

0

0

0

0.00408015

0.118371

0.00408015

0.0102004

0.0591856

0.144196

0.0591856

0.254419
8

0.00624502

0.0591856

0.144196

0.0591856

0.0887784

0.0312995

0

0

0

0.266335

0.147341
7

0.00408015

0

0.0187351

0.00612023

0

0.0591856

0.00832669

0.0306012

0.00408015

0

0.0948987
7

0

0.0887784

0

0

0.144196

0.00416335

0.00408015

0.00416335

0

0.0591856

0.0748354
7

0.00416335

0

0.00394831

0

0

0.0156497

0

0.0673459

0.144892

0.66933
7

0.440748

1.81966

12.1959
7

0.0916878
7

0.488346

0.275504

0.0902219

0

0.0469218

0.160517

0.186348

0.00874359

0.042106

35.5005

0.09831

0.156846

0.505923

0.0632658

0.156846

0.00816031

0

0.0510294

0.237055

0.128572

3.78036
7

0.970876

0.610828

0.113759

0.97116

0.443892

0.164009

0.215968

1.38371

0.0251392

0.0100015

3.31837
1

0.0929418

0.103225

0.0142805

0.00816031

0.227517

0.288392

0.00458025

0.0458025

0.387806

0.0156497

11.0373
1

0.00816031

0.0591856

0

0

0.118371

0.0591856

0.0591856

0.118371

0.0770648

0.22609

11.9738
8

0.504686

0.0102004

0.246049

0.275479

0

0.00408015

0.0122405

0.00923194

0

0.00440928

1.22599
8

0

0

0.0714261

0.275479

0.00624502

0.153885

0.0887784

0.0591856

0.144196

0.0770648

0
4

2.15781
7

2.15781
7

0

0
4

0.134021
7

0.0591856
7

0.0156497

0

0

0

0

0.0591856

0

0

0

2.08166817117217e-17
7

0
4

0.0704238
6

0.0469492
6

0
6

0.0234746

0

0

0
4

0.236743
7

0.236743
7

0
6

0

0

0
4

0.933578
7

0.36661

0

0.147964

0.144196

0

0.0591856

0.0591856

0.144196

0.0122405

0

0
4

0.384565

0.0224362

0.285846

0.0695522

0

0.00673085

2.60208521396521e-17

0
4

0.158121
1

0.158121
1

0
4

0
6

0
6

0

0

0
4

0

0

0

0

0
4

0.053042

0.0367214

0.0163206

0
4

6.00568
5

4.25828
7

0

0
6

0.0293

0

0.032394

0

0

0

0

0

1.19942

0

0

0

0

0

0

0

0

0

0

0.227873
6

0

0

0.234939

0
6

0
5

0.0234746
6

0
6

0
6

1.40512601554121e-15
5

0
4

0
6

0
6

0
4

0.122859

0.118371

0.00448724

6.07153216591882e-18

0
4

0.325521

0.266335

0.0591856

0
4

0

0

0

0
4

0

0

0

0
4

0.118371

0.118371

0

0
4

0

0

0

0
4

0

0

0
4

0.528903

0.528903

0
4

1.87455

1.87455

0
4

20.193

0.676043
1

2.54314

0.0133182

0.0591856

15.394

0.358201

0.247228

0.235358

0.0838906
7

0.0326412
2

0.310885
8

0.239103
6

0
4

0

0

0
4

0

0

0
4

0

0

0
4

0.0591856

0.0591856

0
4

0.00888202

0.00888202

0
4

0.0887784

0.0887784

0
4

0

0

0
4

0

0

0
4

0

0

0
4

0.144196

0.144196

0
4

7.82851

1.39086
5

0

5.63873

0.582626
7

0.216294
7

0

0

0

0

0

1.33226762955019e-15

0
4

0

0

0
4

0

0

0
4

0.0129576

0.0129576

0
4

0

0

0
4

0

0

0
4

0.144196

0.144196

0
4

0

0

0
4

0

0

0
4

0.00816031

0.00816031

0
4

0.0591856

0.0591856

0
4

1.75966

1.15168
1

0.282841
1

0.00532728

0.0938435

0.216294

0

0

0.00967569

3.48679418671338e-16

0
4

0.0112181

0.0112181

0
4

3.59149
7

2.56462
7

0.393099
8

0.189881

0.147964

0.0591856

0.236743

7.7715611723761e-16
7

0
4

0.830757
6

0.825429
6

0
7

0

0.00532728

0

0

0

4.77048955893622e-17
6

0
4

23.8592
8

23.5429
8

0.0598579
8

0.0244809

0.0156497

0

0.216294

0

1.19348975147204e-15
8

0
4

0.613895

0.555295

0

0.0586001

0

0

0

2.08166817117217e-17

0
4

0
4

822.132

720.646

720.15

0

0

0

0

0

0

0.00416335

0

0.144196

0.144196

0

0

0

0

0

0

0

0

0.144196

0.0591856

0

0

0

0

0

0
4

96.2731
8

5.76664
8

0.321753

0.355114

0.216294

0.20715

0.0591856

0.0591856

0.0887784

0

0.0591856

0.216294

48.2268
8

5.23793

0.0591856

1.03575

0.118371

0.00518609

0

0

0.0591856

0.144196

0

10.7717
8

0.0887784

0.0887784

16.0344

1.605

3.87666

1.33866

0

0.232974

1.03805852802452e-14
8

0
4

5.13466

5.13466

0
4

0

0

0
4

0

0

0
4

0

0

0
4

0.0782487

0.0782487

0
4

0

0

0
4

0

0

0
4

0

0

0
4

0

0

0
4

0

0

0
4

0
4

65.8635

64.2105

8.66358

0.0156497
5

0

0.00411208

0

0.00458025

0

0.00799092

0

0.00888202

0

0

0.199504

0

0.0129576

0

0.00532728

0

0

0

0

0

0

0.00777913
6

0

0

0

0

0

0

0

0

0

0

0.655051
7

0

0

0

0.0259152

0

0

0.0143923

0

0.00687037

0.0129576

0

0

0

0.0133182

0

0

0.269727

0.269727

0

0

0

0.269727

0

0.018321

0.00777913
6

0.15413
7

0.0253246

0
6

26.8427

0.389904
7

0.0251914

0.924777

0.0229012
7

0.00987078

0.179321

0

0.136227

0

1.27157

5.66578
7

0

0

0.0194364

0

0.298764

0

0

0.0591856

0.00458025

0

11.6382

0.0129576

0.23417

0

2.16294

0.144196

0

0

0

0

0

0.0391243
6

0

0.115597

0

0

0

0.0155583

0

0

0

0.0570164

0.937638
7

0.0216737

0

0

0

0.0219522

0

0.0170423

0.115597

0.0103722

0

0.527266
1

0.00592134

0.0102004

0

0.0156497

0.288392

0.00518609

0

0

0.00416335

0.00518609

0.828176

0

0.0887784

0.00458025

0

0

0

0.00458025

0.0887784

0

0

0.225431
7

0

0

0.0266461

0

0.0137407

0

0

0

0

0

3.3362201889986e-14

0
4

0

0

0

0

0
4

0.015791

0.0118427

0

0.00394831

0
4

0

0

0

0
4

0

0

0
4

0

0

0
4

0.144196

0.144196

0
4

0

0

0
4

0.00408015

0.00408015

0
4

0.871161

0.366126

0.318182

0

0

0.00518609

0.0194364

0

0

0.0207444

0

0

0.0285969
6

0

0

0

0

0

0.0107775

0

0

0

0.00967569

0
6

0

0

0

0

0

0

0

0

0

0

0
6

0

0

0

0

0

0

0.00799092

0

0

0.0103722

0

0

0

0

0.0194364

0.0233374

0.0312995

0

0
4

0.231772
5

0.20715
6

0

0

0

0

0.00518609

0

0

0

0.0194364

3.46944695195361e-18
5

0
4

0.0493075

0.0091344
6

0

0

0

0.0129576

0.00777913

0

0

0.0194364

0

0

3.46944695195361e-18

0
4

0.198387
7

0.15413
7

0

0.0312995

0.0129576

5.20417042793042e-18
7

0
4

0.00518609
6

0

0.00518609

0

0

0

0
4

0.133135
7

0.0175378

0

0

0.115597

0
4

0

0

0

0

0

0
4

0

0

0

0

0

0

0
4

9.85322934354826e-15

0
4

0.216294

0.216294

0.216294

0
4

0

0

0
4

0
4

0.0156497

0.0156497

0.0156497

0
4

0

0

0
4

0
4

0.00518609

0.00518609

0.00518609

0

0
4

0
4

0

0

0

0

0

0
4

0
4

0

0

0

0

0
4

0
4

0

0

0

0

0
4

0
4

0

0

0

0

0
4

0
4

0

0

0

0

0
4

0

0

0
4

0
4

0

0

0

0
4

0
4

0

0

0

0
4

0
4

29.9578
7

29.8818
7

0.0368733
7

0.00592134

0

0.0259152

0.00592134

0.00822417

0

29.2648
7

0.48259
7

0.0273343
7

0.0114506

0.00362217

0.0091605

0

0

2.24993634834192e-15
7

0
4

0.0277102
6

0.00673085
6

0.0091344
7

0.00789662

0.00394831

0

0

0

0

0
4

0.00518609

0

0.00518609

0
4

0.0224408

0.0224408

0

0
4

0
6

0

0

0

0
4

0.00687037

0.00687037

0

0
4

0.00687037

0.00687037

0
4

0.00687037

0.00687037

0
4

0

0

0
4

2.02962646689286e-16
7

0
4

0.0887784

0

0

0
4

0.0887784

0.0887784

0
4

0
4

0.15413

0

0

0
4

0.15413

0.15413

0
4

0
4

0

0

0

0

0
4

0
4

0

0

0

0
4

0
4

0

0

0

0
4

0
4

0

0

0

0
4

0
4

0

0

0

0
4

0
4

0

0

0

0
4

0
4

0

0

0

0
4

0
4

0

0

0

0
4

0
4

1.21544
7

0.226737
7

0.222657
7

0

0.00408015

0

0

2.16840434497101e-17
7

0
4

0.975734
7

0.916548
7

0

0.0591856

0

0

0

0
4

0.0129652
6

0.0129652

0

0

0
4

0

0

0
4

9.19403442267708e-17
7

0
4

0

0

0

0
4

0
4

0

0

0

0
4

0
4

0.0388728

0.0388728

0.0388728

0
4

0
4

0

0

0

0
4

0
4

0

0

0

0
4

0
4

0

0

0

0
4

0
4

0.946189
6

0.0358985
6

0.0183607
6

0

0.0175378

0

0

0

0
4

0.871097
7

0.871097
7

0

0

0
4

0.0235441
7

0.0235441
6

0

0
4

0.0156497

0.0156497

0

0

0
4

0

0

0
4

0

0

0
4

0
4

0.306997
6

0.306997
6

0.281082
6

0.0259152

0

0

0

0
4

0
4

0.258221
2

0.258221
2

0.258221
2

0

0

0

0

0

0

0

0
4

0
4

0.00967569
6

0.00967569
6

0

0

0.00967569

0

0

0
4

0

0

0

0
4

0
4

0

0

0

0
4

0
4

0

0

0

0

0

0

0

0
4

0
4

28.4674

28.1564

8.87177

0
7

0.15413
7

0

0.183642

0.144196
7

0.171366

0.00322316

0

0.0194364

0

1.57983
7

0.0367214

0.477378

0.120365

0

0.0163206

0.224954

0

0

0

0

9.97244

0.0770648

0

0.0109505

0.0122405

0

0.0183607

0

0

0.855201

0.0086604

1.54748

0

0

0.00322316

0

0

0

0.0086604

0.0114506

0.00816031

0

0.370978
7

0.0163206

0

0.00408015

0

0.0163206

0

0

0.00408015

0

0

0.220929

0.00408015

0

0

0

0.0122405

0

0

0

0

0

2.42359
8

0.00408015

0

0

0

0.00408015

0

0.00408015

0.00408015

0

0

0.0693626

0

0.00408015

0.103661

0.168449

0

0

0.0489618

0

0.00612023

0

0.129576

0

0

0
4

0.310982

0.310982

0
4

0

0

0
4

0

0

0
4

1.22124532708767e-15

0
4

0
4

21.9985

0.095757
7

0.0395518

0.0333068

0.00624502

0
4

0

0

0

0
4

0

0

0
4

0

0

0
4

0.0562052

0.0562052

0
4

0

0

0
4

0

0

0
4

0

0

0
4

0
4

1.65472
7

1.65472
7

0

0

0.0150924

0

0

0

0

0

0

0

0.062599

0

0

0.0553389

0

0.0907031

0.192662

0

0

0

0

0.123097

0

0

0

0

0

0

0.269727

0

0

0

0

0.201121

0

0

0

0

0

0

0

0

0

0.0770648

0

0

0.0483785

0

0

0

0

0

0.0704238

0

0

0

0.216294

0

0

0

0

0

0

0

0.232217

0

0

0

0

0

0

0

0

0

1.38777878078145e-16
7

0
4

0

0

0
4

0
4

0.148535
6

0.148535
6

0.148535
6

0

0
4

0
4

0

0

0

0
4

0
4

0.693231

0.693231

0.693231

0
4

0
4

4.4566
7

4.4566
7

4.4566
7

0
4

0
4

0

0

0

0
4

0
4

0

0

0

0
4

0
4

0

0

0

0
4

0
4

0

0

0

0
4

0
4

0

0

0

0
4

0
4

0

0

0

0
4

0
4

0.115597

0.115597

0.115597

0
4

0
4

14.834

12.2871

3.71822

4.66875
7

0.025154

0.222445

0

0

0

0

0.0355281

0.0887784

0

0

0.691448
6

0

0

0

0.0583091

0

0

0

0.0770648

0

0

1.09379

0

0

0

0.200843
7

0.118371

1.19958
7

0.0887784

0

0

6.93889390390723e-16

0
4

1.98981

1.98981

0

0

0
4

0

0

0
4

0

0

0
4

0

0

0
4

0

0

0

0
4

0.557176

0.557176

0
4

0

0

0
4

0

0

0
4

1.88737914186277e-15

0
4

0
4

11.4253
4

10.0092
4

9.91914
4

0.0445268
4

3.46163

0.401685

0

0

0

0

0

0

0

0.149012

0.115709

4.75399

0

0

0

0.032394

0

0

0.0155583

0

0

0

0.437614
7

0

0

0

0

0

0

0

0.0100616

0

0

0

0

0.0259152

0

0

0

0

0.0301849

0

0.0100616

0.216294

0.169222

0

0

0

0

0

0

0

0

0

0
6

0

0

0.0452773

1.08940634291343e-15
4

0
4

0.0647879

0.0647879

0
4

0

0

0
4

0

0

0
4

0

0

0
4

0

0

0
4

0

0

0
4

0

0

0
4

0

0

0
4

0

0

0
4

0

0

0
4

0.00518609

0.00518609

0
4

0.0201232

0.0201232

0
4

0

0

0
4

0
4

0

0

0

0
4

0
4

0

0

0

0

0
4

0
4

0.58957

0.58957

0.58957

0
4

0
4

0

0

0

0
4

0
4

0

0

0

0
4

0
4

0

0

0

0
4

0
4

0

0

0

0
4

0
4

0.192662

0.192662

0.192662

0
4

0
4

0

0

0

0
4

0
4

0.134838

0.134838

0.134838

0
4

0
4

0.00777913

0.00777913

0.00777913

0
4

0
4

0

0

0

0

0
4

0
4

0

0

0

0
4

0
4

0

0

0

0
4

0
4

0

0

0

0
4

0
4

0

0

0

0
4

0
4

0

0

0

0
4

0
4

0.231194

0.231194

0.231194

0
4

0
4

0

0

0

0
4

0
4

0.144196

0.144196

0.144196

0
4

0
4

0.0907031

0.0907031

0.0647879

0.0259152

0
4

0
4

0.025154

0.025154

0.025154

0
4

0
4

0

0

0

0

0
4

0
4

0

0

0

0
4

0
4

0

0

0

0
4

0
4

7.8756445809347e-16
4

0
4

7882.35

1.6572
1

0

0

0

0

0

0

0
4

0

0

0
4

0.710228

0.710228

0
4

0

0

0
4

0

0

0
4

0.94697

0.94697

0
4

0

0

0
4

0

0

0
4

0

0

0
4

0

0

0
4

1.11022302462516e-16
1

0
4

7.10766

5.17704

1.26349

0
2

0
3

0

0.00673085

0

0

0

0

0

0

2.58653

0

0

0

0.0534571

0

0

0

0

0

0

0.144196
4

0

0

0

0

0

0

0

0

0

0.00592247

0.466876

0

0

0

0

0

0

0

0

0

0

0

0

0

0

0

0

0

0

0

0

0

0
4

0

0

0

0

0

0

0

0

0.0143923

0

0.00673085
3

0

0

0.00411208

0

0

0.00362217

0

0

0

0.00448724

0

0

0

0

0

0

0.00897447

0

0

0

0

0.607515
3

0

0

0

0

0

0

0

0

0

0
4

0
3

0
3

0

0

0

0

0
4

0

0

0
4

0

0

0
4

0

0

0
4

0

0

0
4

0

0

0
4

0

0

0
4

0

0

0
4

0

0

0
4

0

0

0
4

0

0

0
4

0
3

0
3

0
4

0

0

0

0
4

0.0822417

0.0102802

0.0719615

0
4

0

0

0
4

1.84221

1.82165

0.0205604

1.00613961606655e-16

0
4

0

0

0
4

0

0

0
4

0.00616813

0.00616813

0
4

0
4

33.8462
1

32.9294
1

13.9852
1

0.252673

0.032394

0.0259152

0.0583091

0

0.187885

0.0518304

0.621449

0.0518304

0.0453516

15.1281
1

0.0647879

0.032394

0

0.032394

0.0583091

0.0129576

0.0777455

0.0388728

0.116618

0.187885

0.349855

0.0259152

0.00518609

0.0129576

0.0453516

0.129576

0.0129576

0.0129576

0.651042

0.0129576

0

0.0583091

0.0259152

0.0129576

0.0129576

0.00777913

0

0

0.0103722

0.0259152

0.0129576

0.0194364

0.0453516

0

0

0

0

0.0259152

0.0129576

0

0.0194364

0.0129576

0

0

0.0129576

0.0194364

0.0129576

0.0194364

0.0388728

0.0259152

0.0259152

0.0129576

0.032394

0.0647879

0

0.0388728

9.86016823745217e-15
1

0
4

0.730199
2

0.344875
2

0.385324

0
4

0

0

0

0

0

0
4

0

0

0
4

0

0

0
4

0

0

0
4

0

0

0
4

0

0

0
4

0

0

0
4

0

0

0
4

0

0

0
4

0

0

0
4

0.149012

0.149012

0
4

0

0

0

0

0
4

0

0

0
4

0

0

0
4

0

0

0
4

0

0

0

0
4

0

0

0
4

0.00518609

0.00518609

0
4

0

0

0
4

0.032394

0.032394

0
4

0
4

2010.39

0.706473

0
2

0

0

0

0

0

0

0

0.0579547

0

0

0
6

0

0

0

0

0.00615463

0

0

0

0.00362217

0

0.0430824
3

0

0

0

0

0

0

0

0

0.0887784

0

0.0677009
2

0

0

0

0

0

0

0

0

0

0.288392

0.144634
3

0

0

0

0

0

0

0

0

0

0

0
6

0

0

0

0.00615463

0

0

0

0
2

0

0
2

3.29597460435593e-17

0
4

1992.53

1928.34

6.97618
7

0.672373

0

0.144196

0

0

0

0.0100616

0

0

0

0.0129576

1.57647
7

0

0

0.0748354

0

0.0103722

0

0

0.0259304

0

0

0.567722

0

0.0156497

0.0938984

0

0.0107775

0.0156497

0

0

0.0201232

0

0.973272
7

0.00615463

0

0.0129576

0.2374

0

0.0114506

0

0

0

0

0.568695

0

0

0

0

0.0312995

0

0

0

0

0

0.516231
7

0.0307731

0

0

0

0.0148034

0

0

0

0

0.0887784

0.00518609
6

0

0

0

0.00592134

0

0

0

0

0.0580542

1.15357

0.246588
7

0

0

0.00518609

0

0.0919191

0.0100616

0.0194364

0

0

0

0.930651
7

0.0145135

0.0100616

0

0

0.0518304

0

0

0

0.00518609

0

1.36472
7

0.621449

0

0

0

0

0.00518609

0

0

0.0193514

0

3.61825
7

0.392657
7

0

0

0

0.0484987

0

0

0

0

0

0

0.132004
7

0

0

0

0.327003

0.0156497

0

0

0

0

0

0.305222
7

0.0234746

0

0

0.0591856

0

0

0

0

0

0

0.268384
7

0

0

0.0103722

0

0

0

0

0

0.00888202

0

0.389413
6

0

0

0

0

0

0

0

0

0

0

0.0680466
2

0

0

0

0

0

0.0887784

0.00615463

0

0

0

0
7

0

0

0

0.0145135

0

0

0

0

0

0

0.0302001

0

0

0.216294

0.00967569

0

0.0860736

0.00319998

0

0

0

0.485794
7

0

0

0

0

0.0129576

0

0

0

0.0591856

0.0595432

0.0135722
6

0

0

0

0.0591856

0

0

0.0495996

0

0

0

1.98861
7

0.0129576
6

0

0

0

0

0.0377212

0

0

0

0

0

0.591856
7

0.0100616

0

0

0

0

0

0

0

0

0

0.0156497

0.0452773

0

0

0.0100616

0

0

0.0156497

0

0

0

0.0984541
7

0

0

0

0

0

0

0

0

0.00615463

0

0.238694
7

0

0

0

0

0

0

0.0391243

0

0

0

1.80196
8

0

0

0

0

0

0

0.0241892

0

0.0435406

0

0.0343327
1

0.0777455

0.0156497

0

0.00777913

0

0

0

0

0

0

0.150909
7

0

0

0

0.00518609

0

0.0352157

0

0

0

0

0.0226333

0

0

0

0.00777913

0.00532728

0

0

0.00967569

0

0

0.0984541
7

0

0

0

0

0.00967569

0.00777913

0.0156497

0.0100616

3.46133
7

0.0129576

0
7

0

0.120638
7

0.435871
7

0.0229012
6

0.39364
8

0

0.00408015

0.101723

2.55939
7

0.0517492
7

0
7

0.00440928

0
6

0.00362217

2.01647

1.84215

0

0.0777455

0.0129652

3.77122
7

0.0688613

0

0

0.123557

0.669828

0.238083

0

0.0201232

0

0.144196

6.30407
7

0.236743

0

0

0

0.0194364

0

0.144196

0.0161662

0

0.0129576

1.22161

0.0287819

0.0234746

0.0129652

2.48698

0

0.0365573

0.00362217

0

0.0991506

0

7.22001

0

0.0100616

0.118371

0.184442

0

0.00362217

0.0985547

0.0285235

0.032394

0

0
4

0.799233
7

0.799233
7

0

0

0
4

3.5439

1.13559

0.159297

0.00777913

0

0

0.00673085

0

0

0.344294

0.0591856

0.15413

0

0.00394831
6

0.291546

0.0156497

0.0194364

0

0.172358
4

0

0

0.00777913

0

0

0

0

0

0

0.0670833

0.15492
2

0

0

0

0

0

0.118371

0.00448724

0.0156497

0

0

0.277687
3

0.0181513

0

0.0181513

0

0

0

0

0

0

0.0751983

0.0161576
6

0

0

0

0

0

0

0

0

0

0

0.239932
1

0

0

0

0

0

0

0

0

0

0

0.0153577

0.00897447

0

0.00967569

0.00479996

0

0

0

0.0091605

0

0

0.0547857
3

0

0

0

0

0

0.0207444

0

0.00319998

0

0

0
4

0

0

0.0259152

0

0

0

0

0

0.00777913

0

1.46931078415236e-15

0
4

9.56568

8.20887

1.35681
7

0

0
4

0

0

0
4

0.144196

0

0.144196

0
4

0

0

0
4

0

0

0
4

0

0

0
4

0.00543325

0.00543325

0
4

0.144196

0.144196

0
4

0

0

0
4

0.0591856

0.0591856

0
4

0

0

0
4

0.154186

0.0591856
1

0.0194364

0

0

0.0129652

0

0

0

0.0469492

0

0

0.0156497

0
4

0

0

0
4

0

0

0
4

0

0

0
4

0

0

0
4

0.613706
7

0.321546
7

0.144196

0

0.0887784

0.0591856

0

4.16333634234434e-17
7

0
4

0.22148

0.22148
1

0

0

0

0

0

0

0
4

0.504686

0.504686

0

0

0

0

0
4

1.31458

0

1.31458

0

0

0
4

0.0170377
1

0.0129576

0.00408015

0

0
4

0.00777913

0.00777913

0

0

0

0
4

0.0591856

0

0.0591856

0

0
4

0
4

143.892

140.955

140.523

0

0.432588

0

0

0

0

0
4

0.306765
1

0.0873904
2

0.188258
1

0.0311165

0

0
4

0

0

0
4

0

0

0
4

0.306012

0.306012

0
4

0

0

0

0
4

0

0

0

0
4

0

0

0
4

0.20715

0.20715

0

0
4

0

0

0
4

0.148673

0.148673

0
4

0.388728

0.388728

0
4

0

0

0

0
4

0.00518609

0.00518609

0

0
4

0.017764

0.0118427

0.00592134

0
4

0.435219

0.435219

0
4

0

0

0
4

0

0

0
4

0

0

0
4

0

0

0
4

0

0

0
4

0

0

0
4

0

0

0
4

0.0296067
2

0.0296067
2

0

0
4

0

0

0
4

0

0

0
4

0

0

0
4

0

0

0
4

0

0

0
4

0

0

0
4

0.346792

0.346792

0
4

0

0

0
4

0.216294

0.216294

0
4

0

0

0
4

0
2

0
2

0
4

0.187797

0.187797

0
4

0.144196

0.144196

0
4

0.15413

0.15413

0
4

0

0

0
4

0

0

0
4

0

0

0
4

0

0

0
4

0.0346813

0.0346813

0
4

0

0

0
4

0

0

0

0
4

0

0

0
4

0.00777913

0

0.00777913

0
4

4.90354284954364e-14

0
4

1.82619
3

1.62105

1.60677

0

0.0142805

0

0
4

0

0

0

0

0
4

0.00612023

0.00612023

0
4

0.129652

0.129652

0

0
4

0.0652825

0.00612023

0.026521

0.0326412

6.93889390390723e-18

0
4

0

0

0
4

0

0

0

0
4

0

0

0
4

0

0

0
4

0.00408015

0.00408015

0
4

0

0

0
4

4.9439619065339e-17
3

0
4

8.03744

4.39385

2.41911
8

0

0

0.0153866

0.00411208

0

0

0

0

0

0

0

0.308259

0

0.00616813

0

0

0

0

0

0

0

0

0

0.0156497

0

0.24942

0

0

0

0

0.435233

0

0

0

0

0

0

0

0

0

0

0

0

0

0

0.0149524

0

0

0.0155583

0

0

0

0

0.303394

0

0

0

0

0

0.0297196

0.0380138

0

0

0

0.331716

0

0.144196

0.015477

0

0

0

0

0

0.0164483

0.00416335

0

0

0

0

0.0123363

0

0

0

0

0

0

0

0

0

0

0

0

0

0

0

0

0

0

0

0

0

0

0

0

0.00416335

0

0

0

0

0

0

0

0

0

0.00518609

0

0

0.00518609

0

9.8879238130678e-16

0
4

0

0

0

0

0

0

0

0

0
4

0

0

0
4

0

0

0

0

0

0
4

0

0

0
4

0

0

0

0
4

0.534311

0.534311

0
4

0

0

0

0

0
4

0.0583091

0.032394

0.0129576

0.0129576

0
4

0

0

0

0

0
4

0

0

0

0
4

0.144196

0.144196

0
4

0.0863538

0.0616813

0.0143923

0.00411208

0.00616813

0
4

0

0

0

0

0
4

0

0

0
4

0

0

0
4

0

0

0

0

0
4

0

0

0

0

0
4

0.385324

0.385324

0
4

0

0

0
4

0.0123996

0.00615463

0.00624502

0
4

0

0

0
4

0

0

0

0
4

0

0

0

0
4

0

0

0
4

0

0

0
4

0

0

0
4

0

0

0
4

0

0

0
4

0

0

0
4

0

0

0
4

0

0

0
4

0

0

0
4

0

0

0
4

0

0

0

0

0

0

0

0
4

0.0143923

0.0143923

0
4

0.144196

0.144196

0
4

0

0

0
4

0.0194364

0.0194364

0
4

0

0

0
4

0

0

0
4

0

0

0
4

0

0

0
4

0.00411208

0.00411208

0
4

0

0

0
4

0.432588
7

0.144196
7

0.288392

0
4

0

0

0
4

0.118371

0.118371

0
4

0

0

0
4

0

0

0
4

0

0

0
4

0

0

0
4

0

0

0
4

0

0

0
4

0.00416335

0.00416335

0
4

0.0884098

0.0328967

0.0123363

0.0164483

0.0123363

0.0143923

3.46944695195361e-18

0
4

0.423089

0.423089

0
4

0.71116

0.672036

0.0234746

0.0156497

6.59194920871187e-17

0
4

0.462786

0.444635

0.0181513

0
4

1.49880108324396e-15

0
4

6.43363

6.24726

4.921

0

0

0

0

0

0

0

0

0.115597

0.266335

0.231194

0.0971819

0

0

0

0.115597

0

0.0591856

0

0

0

0

0

0.144196

0

0

0.147964

0

0.149012

0

0
4

0

0

0

0

0

0

0
4

0.0322355

0.0322355

0
4

0

0

0

0
4

0

0

0
4

0.15413

0.15413

0
4

0

0

0
4

0

0

0
4

2.4980018054066e-16

0
4

7.65769

0

0

0

0
4

1.73884

0.654005

0.0100616

0.865175

0.025154

0

0.0100616

0.144196

0

0.0301849

0
4

0

0

0
4

0

0

0

0
4

0

0

0
4

0

0

0
4

0

0

0

0
4

0

0

0
4

1.29776

1.15357

0.144196

0
4

0

0

0
4

0

0

0
4

0

0

0
4

0

0

0

0

0

0
4

0

0

0
4

0

0

0
4

0.216294

0.216294

0
4

0

0

0
4

0.0100616

0.0100616

0
4

0

0

0
4

0

0

0
4

0

0

0
4

0.789837

0.467865

0.291787

0.0201232

0.0100616

0
4

3.10021

0

3.10021

0
4

0.504686

0.504686

0

0
4

0

0

0

0
4

0

0

0

0
4

0
2

0
2

0

0

0
4

0

0

0

0
4

0
4

28.7028

28.7028

28.6872

0

0

0

0

0.0156497

0

0

0

0
4

0

0

0

0
4

0

0

0

0
4

0

0

0
4

0

0

0
4

0

0

0
4

0
4

2.81397

1.08362

0.106963

0

0.308259

0.115597

0.423856

0

0.123081

0.0029305

0

0.0029305

6.07153216591882e-18

0
4

1.44196

1.44196

0
4

0.288392

0.288392

0

0
4

3.88578058618805e-16

0
4

159.461

3.08898
7

0.633758

0.0483785

0

0

0

0

0.80918

0.062892

0

0.686974

0.00967569

0

0.0290271

0

0.00967569

0

0

0.144196

0

0

0

0

0

0

0

0.00967569

0.616518

0

0

0.0290271

0

0

2.74086309204336e-16
7

0
4

1.9287

0.481674
7

0

0

0

0

0

0.00518609

0

0

0

0

1.39846
7

0

0

0.0233374

0.0200479
7

0

0

0

0

0

0

0
4

44.5098

25.2369
7

5.85207
7

0

0

0

0

2.95602

0

0.144196

0

0

0

7.4678
7

0

0.00923194

0

0

0

0

0

0

0.144196

0.144196

0
6

0

0

0

0

0.00394831

0

0

0

0

0

1.00937

0

0

0

0

0

0

0

0

0

0

0

0

0.36049

0

0

0.36049

0

0

0

0

0

0

0

0

0.0907031

0

0

0.504686

0

0

0

0

0

0

0

0.216294

0.00923194

6.5294991635767e-15

0
4

64.5321

29.5539

4.59253
7

0

0

0.0100616

0

0

0.00967569

0

0

0.0194364

0

0

0.331852
7

0.0194364

0.00777913

0

0

0

0

0.0156497

0

0

0

0.234746
7

0

0

0

0

0

0

0

0

0.0156497

0

0
7

0

0

0

0

0

0

0

0

0

0

0.144196
6

0

0

0

0

0.269727

0

0

0.00518609

0.144196

0

0.0770648

0

0

0

0

0

0

0

0

0

0

0.30221

0

0.115597

0

0.0129576

0

0.00923194

0.144196

0

0.48553
7

0.233438

0.0234746

7.12606
7

0.577986

0

0

8.51133

0.292555

0

0.101723

0

0

0

1.08618

0.0591856

0

0

0.252673

2.37923

0.192662

0.0156497

0

0.0156497

0.159846

0.0469492
5

0

0.0241892

0

0.0312995

0.36049

0

0.0770648

0.00967569

0

2.77433

0.554604
7

0

0.153428

0.865175

0

0

0.0184639

0

0

0

0

0
7

0

0

0.36049

0

0

0.270911

0

0

0.0153866

0

0.341144

0

0.00615463

0

0

0.00615463

0

0

0.0100616

0.115597

0.346792

0.385324
7

0

0

0

0.00416335

0

0

0

0.00923194

0

0

0.0300678

0.0591856

0.0855237

0

0

0.0156497

0

0

0.00615463

0

0

2.07733136248223e-14

0
4

0.645008
2

0
2

0.295607

0

0

0

0

0

0

0

0

0

0.15413
2

0

0

0

0

0

0

0

0

0

0

0.0726053

0

0

0

0

0

0

0

0

0

0

0.0770648
2

0

0

0

0.0337096

0.00777913

0

0

0

0

0

0

0

0

0

0

0

0

0

0

0

0

0

0

0.00411208

0

0

0

0

0

0

0

0

0

0

1.30104260698261e-17
2

0
4

2.10356

2.10356

0
4

2.01874
7

2.01874
7

0

0

0
4

0.868219

0

0.432588

0.00518609

0

0

0

0

0

0

0.127059

0

0.158176

0

0

0.145211

0

0

0
4

2.28272

1.43501

0.269727

0

0

0

0

0

0

0

0

0

0.231194

0

0

0

0.192662

0

0

0

0

0.15413

0
4

0
3

0

0

0

0

0
4

0
6

0
6

0

0
4

0.259793

0.144196

0

0

0.115597

2.77555756156289e-17

0
4

0.250929

0.218288

0.0183607

0.00408015

0.0102004

0

2.25514051876985e-17

0
4

0

0

0

0
4

0.0801856

0.0637373

0.0164483

3.46944695195361e-18

0
4

0.539453

0

0.423856

0.115597

0
4

0

0

0

0

0
4

0

0

0

0

0
4

0

0

0
4

5.50203

0.648882

0

0

0

0

0

4.47007
7

0

0

0.0770648

0

0.306012

0

0

2.22044604925031e-16

0
4

0

0

0
4

0

0

0

0
4

0.510522

0.510522

0
4

0

0

0
4

0.0142805

0

0.00408015

0.0102004

0
4

0.36049

0

0.36049

0

0
4

0.504686

0

0.504686

0
4

0

0

0

0
4

0

0

0
4

0

0

0

0
4

1.93226
7

1.0495
7

0.721994
7

0.145211

0

0.00518609

0.00518609

0.00518609

0
4

0

0

0

0
4

0

0

0

0
4

0.025154

0.0100616

0.0150924

0
4

0

0

0
4

0.770648

0.770648

0

0
4

0

0

0

0
4

0

0

0
4

0.00416335

0.00416335

0
4

0

0

0
4

0

0

0
4

10.6321
7

10.6321
7

0

0
4

0.00777913

0.00777913

0
4

0

0

0
4

0

0

0
4

0

0

0
4

0

0

0
4

3.24441

3.24441

0
4

0

0

0
4

0

0

0
4

0

0

0
4

0.216294

0.216294

0
4

7.37751
7

1.3913

1.16967

4.81655

0

0
4

0

0

0
4

0.144196

0.144196

0
4

0

0

0
4

0.00624502

0.00624502

0
4

1.29776

1.29776

0
4

0

0

0
4

0

0

0
4

0.09869

0.09869

0
4

0

0

0
4

0

0

0
4

0.38506
7

0.38506
7

0
4

0

0

0
4

0

0

0
4

0

0

0
4

0

0

0
4

0.00518609

0.00518609

0
4

0.0468881

0.0468881

0
4

0.144196

0.144196

0
4

0.775295
7

0.577986

0.197309

0

2.77555756156289e-17
7

0
4

0.0770648
5

0

0.0770648

0

0

0
4

2.27002
7

2.26484
7

0.00518609

0

7.11236625150491e-17
7

0
4

7.061018436616e-14

0
4

9.65159

8.36862

8.36862

0

0

0

0
4

0.477002

0.127475

0.0842977

0.0226165

0

0.236445

0.00616813

6.41847686111419e-17

0
4

0

0

0
4

0.0123363

0.0123363

0
4

0

0

0
4

0.00616813

0.00616813

0
4

0

0

0
4

0

0

0
4

0

0

0

0

0

0

0

0

0
4

0

0

0

0

0

0

0

0
4

0.0164483

0.00411208

0.00411208

0.00411208

0.00411208

0
4

0

0

0

0
4

0.756624

0.748399

0.00822417

0
4

0.00822417

0.00411208

0.00411208

0
4

0

0

0

0
4

0.00616813

0.00616813

0
4

2.42514341941558e-15

0
4

5247.31
7

3885.1
7

458.036
7

76.0593
7

1.09158
7

0.115597

0.216294

0

0

0.0887784

0

0.00673085

0

0

0

0.20715
7

0

0

0

0

0

0

0

0.00362217

0.144196

0.00908513

0.564464
7

0.460699

0.0591856

0

0

0.20715

0.0591856

0.144196

0.0591856

0.0591856

0

0.741519
7

0.00362217

0.0159818

0

0.0591856

0.144196

0.275479

0.384707

0.0591856

0.00624502

0

0.715604
7

0.203382

0

0

0

0.0129576

0.147964

0.0591856

0

0.769413

0.118371

0.037939

0

0

0.00673085

0.118371

0

0.114694

0

0

0.147964

0.0547741

0.440914
7

0

0.414299

0

0

0.0887784

0.00440928

0

0.00661392

0.0770648

0.0635949

0.372763
7

0.0591856

0

0.00416335

0.0591856

0

0

0

0

17.6669

0.0591856

2.26988
7

0

0

0.0591856

0.0591856

0

0

0.147964

0

0

0

0.0586584

0.00532728

0.0591856

0.0488992

0

0

0

0

0

0

0

5.08644
7

2.27486
8

0

0

0

0

0

0

0

0

0.0591856

0

0.220242
7

0

0.0129576

0.144196

0.0129576

0

0.0547741

0

0

0

0

62.3654
8

0

0.144196

0.144196

0.0887784

0.0118427

0.144196

0.0591856

0

0

0

0.583228
7

0

0

0.0591856

0

0

0

0

0.0591856

0

0

1.17244
7

0

0

0

0.865175

0

0

0

0

0

0

1.14506
7

0.144196

0.0887784

0

0.144196

0.0591856

0

0

0

0

0.432588

0.482629
7

0

0

0.144196

0

0

0

0

0

0

0

2.59036
7

0.144196

0.144196

0.0145717

0

0

0

0.288392

0.432588

0

0.0591856

1.34942
7

0

0

0

0

0

0.00416335

0

0

0

0

0.225614
7

0

0.36049

0

0

0

0.0591856

0.0591856

0.00499292

0

0.651042

18.2079
7

0.516035
7

0.00615463

0.0234746

0.0591856

0

0

0

0

0

0

0

1.33001
7

0

0

0.0591856

0.216294

0

0

0

0

0

0.0156497

0.514627
7

0

0

0

0

0

0

0

0

0

0

1.23493
7

0

0.118371

0

0

0.288392

0.00448724

0

0.0591856

0.36049

0

0.931897
7

0.0591856

0

0.0887784

0

0

0

0.118371

0

0

0

0.00448724
6

0

0

0

0.144196

0

0

0

0

0

0

0.707101
7

0

0.144196

0

0.0591856

0

0

0.0591856

0

0

0

0.516479
7

0

0

0

0.0887784

0

0

0

0

0

0.144196

1.46416
7

0

0

0

0.0887784

0.147964

0

0

0.216294

0

0

0.476739
7

0.144196

0.144196

0

0

0.144196

0.0887784

0

0

0

0

423.122
7

0.288392
7

0

0

0.00448724

0.0129576

0

0

0.144196

0

0.0591856

0

0.682243
7

0.0104084

0

0

0.0156497

0

0

0

0

0

0.0887784

0.127647
7

0.0591856

0.118371

0

0

0.144196

0

0.00440928

0

0

0

0.671372
7

0

0

0

0.0100616

0

0

0

0

0

0

0
6

0

0.144196

0.0194364

0

0

0

0

0

0.0887784

0

0.626825
7

0

0

0.0591856

0

0.144196

0.144196

0

0.00624502

0.0104084

0

0

0

0

0

0.144196

0

0.0529114

0

0.177557

1.44196

0

0.191652
7

0

0

0

0

0.216294

0

0

0

0

0.0591856

1.43862
7

0

0

0

0.355114

0

0.216294

0

0.216294

0.144196

0.144196

0.635454
7

0

0.0591856

0

0.118371

0

0

0

0.0591856

0

0.0591856

20.2867
7

0.287434
7

0.0591856

0

0.0591856

0.00416335

0.0591856

0

0.144196

0

0.144196

0

1.12779
7

0

0

0

0.0591856

0

0

0

0.00615463

0.0591856

0

5.1043
8

0

0

0.0591856

0

0

0

0

0.36049

0

0.295928

1.28545
7

0

0

0

0

0

0.144196

0

0

0

0

0.0201232
6

0

0

0.0388728

0.00897447

0.0591856

0

0

0.0591856

0.0591856

0

0.02987
6

0

0.0591856

0

0

0

0

0

0.36049

0

0

0

0.443892

0

0

0.0887784

0.0591856

0.00362217

0

0.0129576

0

0

0.436751
7

0

0.0591856

0

0.20715

0

0

0

0

0.0591856

0

9.1318
8

0

0.00615463

0

0

0

0

0.0591856

0

0

0.144196

0.918985

0.0591856

0.144196

0.00416335

0.0770648

0

0.0591856

0

0.0107775

0

0

8.30075
7

0.890811
7

0

0

0

0

0.288392

0

0

0

0.288392

0.00440928

0.597278
7

0

0

0

0

0.0591856

0

0

0.00458025

0.0591856

0

0.32406
7

0.0388728

0

0.144196

0.0591856

0

0

0.0591856

0

0

0

0.288482
7

0

0

0.0591856

0.00881856

0.0591856

0.118371

0

0

0

0

0.639783
7

0

0

0

0

0.0591856

0.0591856

0

0

0

0

0.728911
7

0.288392

0

0

15.2995

0

0.0591856

0

0.0156497

0

0.144196

0.332518
7

0.0029305

0.00416335

0.0887784

0

0

0

0

0

0

0

0.36301
7

0.118371

0

0.00416335

0

0

0

0

0.0591856

0

0

0.0591856
6

0.00897447

0

0

0

0.0156497

0

0.216294

0

0.0591856

0.216294

0.104428
7

0.216294

0.216294

0

0

0

0

0

4.49811

0

0

6.33153
7

0.530478
7

0

0.032394

0

0

0.0591856

0

0

0

0

0

0.976568
7

0.144196

0

0

0.976563

0

0

0

0

0

0.144196

0
6

0

0

0.216294

0

0

0

0

0

0.0591856

0

0.078622
6

0.0591856

0.0591856

0.00897447

0

0

0

0

0

0

0.36049

0.410531

0

0

0

0

0

0.144196

0

0

0

0.0156497

0.569834
7

0

0.144196

0

0

0

0

0

0

0

0

0.241153

0

0

0.144196

0.177557

0

0

0

0

0

0

0.800614
7

0.0259152

0.0591856

0.00592134

0

0.01249

0

0

0

0

0.216294

1.51124
7

0

0

0

0

0.236743

0

0

0.0591856

0.0129576

0

0.887784
7

0

0

0

0.0591856

0

0.0176371

0

0

0

0

3.85374
7

0.0623288
6

0

0

0.118371

0

0.0122405

0.00416335

0

0.144196

0

0.144196

1.10333
8

0

0.0887784

0

0

0

0.144196

0.00448724

0.288392

0

0.0591856

0.266731
7

0.144196

0

0

0.0887784

0.0887784

0

0

0

0

0.216294

0.285772
7

0

0

0

0

0

0

0

0

0.147964

0.0591856

0.125576
6

0

0.0204008

0.288392

0

0.0591856

0

0

0.216294

0

0.562264

0.616743
7

0

0.144196

0.0887784

0

0

0.0591856

0.0129576

0.793077

0

0

0.607787
7

0

0.0129576

0.36049

0.0591856

0.504686

0.00440928

0

1.00937

0

0

0.220457
7

0

0

0.0887784

3.82119

0

0.216294

0.144196

0

0

0.00362217

0.148359
7

0.0194364

0

0

0

0

0

0.00592247

0.0887784

0

0.00448724

0.879207
8

0.473485

0

0.0591856

0

0.147964

0.0591856

0

0

0

0

5.45114
7

0.550959
7

0

0

0.288392

0

0

0

0

0

0

0

0.74505
7

0

0.0591856

0

0

0

0

0.144196

0

0.266335

0.288392

6.23933
8

0.0129576

0

0

0.0591856

0.144196

0

0.144196

0

0.00832669

0.0582868

0.482008
7

0

0

0

0.00416335

0.144196

0.144196

0

0

0

0

0.108215
7

0

0

0

0.0591856

0

0

0.00416335

0

0

0

1.12356
7

0.00897447

0

0

0

0.01249

0.0591856

0

0

0

0

0.0183607
6

0.288392

0

0

0

0.00592134

0.144196

0

0

0

0

0.144196
7

0

0.288392

0

0.0591856

0

0

0.36049

0

0

0.0712667

0.955123
7

0.147964

0.216294

0

0.147964

0

0

0.216294

0

0.0134617

0.0259152

0.414299

0

0

0

0

0

0.288392

0

0.0591856

0

0

3.21304
7

0.00458025
6

0

0

0

0.00832669

0

0

0.216294

0

0.0887784

0.15413

0.351346
7

0

0.0887784

0

0.0276958

0

0.00440928

0.144196

0.0591856

0.00923194

0.0591856

0.0156497
6

0.0503081

0

0.177557

0

0.144196

0.118371

0.144196

0.0503081

0

0

0.609629
7

0.0591856

0

0.216294

0.0591856

0

0

0

0

0.0547741

0

0.0591856
7

0

0

0

0.0259152

0

0.0591856

0.0591856

0

0

0.216294

0.482485
7

0.0591856

0

0.0887784

0

0.144196

0

0

0

0.00987078

0

0

0.288392

0

0

0

0.115597

0

0

0

0

0

1.55872
7

0.0591856

0

0

0

0

0.00543325

0

0

0

0.144196

0.576784
7

0

0

0

0

0.216294

0

0

0

0

0.0887784

0.251902
7

0

0.0770648

0

0

0

0

0.0591856

0.144196

0.00416335

0

407.933
7

12.2893
7

0.937273
7

0

0

0

0

0

0

0.216294

0.00687037

0

0.0591856

1.03809
6

0

0

0

0.144196

0.00832669

0

0

0.192662

0.0591856

0

0.113556

0

0.0591856

0

0.144196

0.0591856

0

0

0

0

0

0.19363
7

0.473485

0

0

0

0

0.144196

0.00416335

0.0403851

0.125198

0

0.503078
7

0

0.147964

0.288392

0

0

0

0.0591856

0

0

0

0.0545225
7

0

0

0

0.118371

0.144196

0.00923194

0

0

0.144196

0

0.72098
7

0.00592134

0

0.0161662

0

0.0887784

0.0194364

0.20715

0.118371

0

0

46.0714
8

0.0591856

0

0

0

0

0

0

0

0

0.0887784

0.0887784
7

0.030865

0

0.144196

0

0

0

0

0

0

0

0.302048
7

0

0.0591856

0

0

0

0.0156497

0

0

0

0.144196

2.95775
7

0.351346
7

0.00408015

0

0

0

0

0.0591856

0.00624502

0

0.0591856

0.0887784

1.04489
7

0

0.0887784

0

0

0.0194364

0.0591856

0.144196

0

0

0.0591856

0.132851
7

0

0

0

0.20715

0

0

0

0

0

0

0.53802
7

0.144196

0.432588

0

0

0

0.0591856

0

0

0.0104084

0

1.35695
7

0

0.144196

0.36049

0

0

0

0.144196

0.0591856

0

0

0.0129576
6

0

0

0

0

0

0

0.504686

0

0

0.0547741

0.0224362
6

0

0

0

0.0591856

0

0.0887784

0

0

0

0.0591856

0.236353
6

0.0591856

0

0

0

0

0

0

0

0

0

0.118371

0.144196

0

0.144196

0

0.504686

0.00362217

0.144196

0

0.00408015

0.144196

0.393851
7

0

0

0.0129576

0.0591856

0

0.0591856

0

0

0.00416335

0

4.46864
7

0.305072
7

0

0

0

0.0484987

0

0

0

0

0

0.0591856

0.203382
7

0

0.236743

0

0.144196

0

0

0

0

0.216294

0.36049

0
6

0.144196

0.0591856

0

0

0

0

0.144196

0

0

0

0.232974
7

0

0

0

0

0.0591856

0.00615463

0.0107775

0

0.0591856

0

0.436356
7

0.0591856

0

0

0

0.216294

0

0

0

0.00624502

0

0.177557
7

0.266335

0

0

0.144196

0.00967569

0

0.118371

0

0

0

0.449268
7

0.0029305

0

0

0

0

0

0.288392

0

0.144196

0

0.236743
7

0

0

0

0.0887784

0

0

0

0.0183607

0

0

0.266335
7

0.937273

0

0

0

0

0

0.00967569

0.473485

0.503078

0

0.59622
8

0

0

0

0

0.00416335

0.144196

0

0

0.144196

0

12.3772
7

0.0675123
6

0.00673085

0

0

0

0

0

0.118371

0

0

0

0.292555

0

0

0

0.20715

0

0

0

0

0

0

35.0396
8

0

0.0129576

0

0

0.144196

0

0

0

0

0

1.34677
7

0.0887784

0.0591856

0.021555

0

0

0

0

0.144196

0.144196

0

0.47964
8

0

0

0.00416335

0

0

0

0

0

0

0

0

0

0

0

0.0184639

0

0.147964

0.032394

0

0

0

0.144196
6

0

0

0.0402465

0.266335

0

0

0

0

0

0

0.745196
7

0

0

0.0591856

0.144196

0

0

0.216294

0.00923194

0

0

0.144196
7

0

0

0

0

0

0.216294

0

0.00416335

0.144196

0.0107775

0.0988401
6

0.144196

0.0591856

0

0

0.0938984

0

0

0.118371

0

0

25.1099
7

0

0.00416335

0

0

0

0

0.118371

0

0.576784

0

0

0.0704238
7

0

0

0.144196

0.216294

0

0

0

0.216294

0.118371

0

0.0171209
6

0

0

0

0

0

0

0.0591856

0

0

0

0.377977
7

0

0

0.384707

0

0

0

0

0

0

0

1.00471
7

0

0

0

0

0

0.216294

0

0

0.144196

0.00416335

0

0

0

0.00732626

0

0

0

0.0647879

0

0

0.144196

1.26239
7

0.0887784

0

0

0

0.0887784

0.144196

0

0

0

0

0.154084
7

0

0.118371

0

0.144196

0.288392

0

0

0

0.0591856

0

8.34305
8

0

0

0

0

0

0

0

0

0.216294

0

0.0875011
7

0

0

0

4.32588

0.0591856

0

0

0.0591856

0

0

10.0707
7

0.211313

0

0

0

0

0.025154

0.0591856

0

0

0.144196

0.0591856

0.0129819
7

0.00416335

0

0

0.0591856

0

0.216294

0

0.144196

0

0.0887784

0.120078
7

0

0.00615463

0

0

0

0

0.0100616

0

0.0591856

0

0

0

0

0.00448724

0.0887784

0.0887784

0

0

0.0887784

0

0.144196

3.3574
7

0.0259152

0

0

0

0

0.144196

0.118371

0

0.20715

0.00624502

0

0

0

0

0

0.0887784

0

0

0

0

0

0.0179489
6

0.144196

0.216294

1.22567

0.00897447

0

0.0591856

0

0

0.144196

0

0.360535
7

0

0

0.0194364

0

0

0.0591856

0

0

0.00416335

0

1.52536

0

0

0.0591856

0.01249

0.504686

0

0.36049

0

0

0.144196

0
6

0.144196

0

0

0.144196

0

0.0591856

0.144196

0.144196

0

0

4.40247
7

7.70534

0

0.0887784

0

0

0

0

0.0964756

0

0.216294

0.0591856

0.220374
7

0.0129576

0

0.021555

0

0.216294

0

0

0

0.00592134

0.144196

265.396
8

0

0

0

0

0

0

0

0.00615463

0

0

1.00616
7

0.0398438

0

0

0.118371

0

0

0

0

0

0.144196

0.705456
7

0

0

0

0

0

0

0

0.032394

0

0

0
6

0.0234746

0

0

0

0

0

0

0.00661392

0

0

0.432588
7

0.0110232

0

0

0

0

0

0

0

0

0

0.656418
7

0.144196

0

0

0.216294

0

0

0

0.0249801

0.0591856

0

0

0.00687037

0

0.0312995

0.0591856

0.0887784

0.216294

0.0591856

0

0.865175

0.118371

0.177557

0

0

0.0591856

0.0137407

0

0

0

0

0

0.144196

3.0357

1.65825
7

0

0

0

0.216294

0.20715

0

0.144196

0

0.288392

0.00673085

0.0651081
7

0

0

0.118371

0

0

0

0

0

0.062599

0

0.216339
7

0.00543325

0.144196

0.144196

0.118371

0.0591856

0.144196

0.00673085

0

0

0

0
6

0

0.0129576

0

0

0

0

0

0.144196

0

0.144196

0

0

0.0591856

0

0.144196

0.0591856

0.0129576

0

0

0.591856

0.00923194

0.203382
7

0

0.00362217

0.147964

0

0

0

0.144196

0

0

0.144196

0.190514

0.00408015

0

0

0

0

0.0591856

0.648882

0

0

0.0615794

0.295928

0

0

0

0

0

0

0

0

0

0

1.26737

0

0

0

0.00416335

0

0

0.0259152

0

0

0

0.0591856

0.144196

0

0

0

0

0.0591856

0

0

0

1.44196

2.98957
7

0.0323325

0.0872356
7

0.556434

0.449268
7

0.297624
7

0.0591856
7

0.321753
7

0.3493
8

3.10021

0.193115
7

4.60572
7

0.0170377
7

0.0129576
6

0.0905847
7

0.147964

0.275479
7

0.879008
7

0.00897447
7

0.0228984
6

0.0128303
6

4.4045
8

695.62
7

5.12585
6

0.626825

0.147964

0

0.0690564
6

0.09831
7

0.118371

0

0.232974

0.228536
8

0.456625

2.30314
7

0.384707

0.0628078
7

0.177557

0.216294

0.623057
7

0.124616

0.366644

2.59553
8

0.720021
7

0.216339
7

1.43975
7

0
6

0.00799092
6

0

0.288392

0.0100616
6

0.134634
7

0.282004
7

0.236743

0.562264

0.106546
8

2.24976
7

0.131329
7

0.0591856
6

0.432588
7

0.229522
7

0.0887784
7

0

0.0129576

0.443892

0

0.0591856

2.7224
7

145.809
8

0.203382
7

0.373447
6

0.377762

0.295928

0.806035

0.206124

0.288392

49.7476

0.171041
7

1.55203
7

0

0.293993

0.00673085
6

0.183479

0.865175

0.118371

0.0887784

0

0.36049

0

1.41043
7

0
6

0.20715

0.234295
7

0.0887784
7

0.177557

0

0.0239728

0.147964

0.177557

0.15081

4.16098
5

0.144196

0

0.0887784

7.75332

0.325521

0.0591856

0

0

0.163632

0

3.43898
7

0.288392

0.648882

0.00897447

0.0887784

0.118371

0

0.0163206

0.00777913

0.13598

0.393851

22.5141
7

0.0149408

0

0.144196

0.063349

0.359944

0.0591856

0.0129576

1.84697

0.207791

0.37717

439.991
7

10.2613
7

0.449268

0

0.872712

0.08099

0.0163297

0.160362

0.648882

0.118371

1.44743

0.36049

8.25133
7

0.0215412

0.183802

0.0887784

0.288392

0.144196

0.20715

0.147964

0.351658

0

0.00416335

1.98466
7

0.118371

0.0591856

0.216294

0.165843

0.144196

0.0591856

0.36049

0.00416335

0.0153866

0

1.12511
7

0.550959

0.00624502

0.216294

0

0.128433

0.00673085

0.0591856

0.198817

0.268124

0.148359

0.625103
7

0.203382

0.183479

0.0933587

0.292555

0

0.144196

0.374465

0.144196

0

0.144196

0.220033
7

0.185669

0.29216

0

0.232974

0.177557

0.20715

6.25011

0.504686

0.36049

0.0591856

0.616135
6

0.288392

0

0.20715

0.144196

0

0.34082

0.150351

0.122535

0.0191042

0.144196

2.18526
7

0.076283

0.301349

0.00923194

0.00448724

0

0

0.0591856

0.00362217

0.216294

0

2.57613
7

0

0

0.373447

0.0591856

0.275479

0.432588

0

0.150351

0.288392

0

3.04423
7

0.321753

0.144196

0.144196

0.0449363

0.370423

0

0

0.0688613

0.0887784

0.348349

9.28665
7

1.48272
7

0

0

0.0259152

0.0591856

0.00615463

0.186726

0.140165

0.0591856

0

0.288392

6.13895
7

0.150351

0

0.562264

0

0.215463

0

0.0129576

0.00673085

0.144196

0

1.45655
7

0.236743

0.0591856

0.00416335

2.67179

0.0166534

0

0

0.288392

0.159628

0.211098

1.44437
7

0.0270617

0.118371

0

0.203382

0.203382

0.0591856

0.0915181

0.144196

0.216294

0.443892

5.70043
7

0

0.0887784

0.0887784

0.185804

0.182044

0.00416335

0.118371

0

0

0.032394

4.04948
7

3.95248

0

0.36049

0.216294

0

0

0.00416335

0

0.0106792

0.177557

3.29555
7

0.177557

0

0.144196

0

0.144196

0

0.266335

0

0.00362217

0.0591856

6.10202
7

0

0

0

0

0.0591856

1.51783

0.364899

0.366411

0.0887784

0.00673085

1.6528
7

0

12.3288

0.0919191

0.216294

0

0.00624502

0.266335

0

0.36049

0.0400195

1.59778
7

0

0

0

0.0129576

0.00615463

0.147964

0.0194364

0.155219

0

0.0201926

7.31339
7

3.49584
7

0

0.0129576

0.0100616

0.295928

0

0

0.20715

0.0842243

0.212356

0.0397109

1.46914
7

0.144196

0

0.648882

0.144196

0

0.36049

0

1.1372

0

0

2.80364
7

0.0333068

0

0.0323325

0

0.0932657

0.491773

0.148144

0

0.0129576

0

2.12896
7

0.216294

0.118371

0

0

0

0.382267

0.288392

0

0.0678362

0

0.829512
7

0

0.0591856

0.0259152

0.288392

0

0

0.232974

0.443892

2.67139

0.00440928

0.0711509
7

0

0.00888202

0.504686

0

0.0156497

0

0

0.144196

0.0887784

0

0.301499

0

0

0

0

0.144196

0.0591856

0.144196

0

0.0391243

0

5.78055
7

0

0.36049

1.15357

0

0

0.828599

0.0591856

0

0.00518609

0.216294

6.09921
8

0.20715

0

0.380938

0.01249

0

0

0.144196

0.0887784

0

0.0887784

0.20253

0

0.0591856

0

0.216294

0.0591856

0

0.203382

0

0.144196

13.507

10.9191
7

0.215996

0.147964

0.0110232

0

0.72098

0.288392

0.0123093

0

0

0.144196

0.621449

0.203596

0.0591856

0

0

0

0

0

0

0

0.0887784

0

1.03327
7

0

0

0.144196

0.36049

0.232974

0

0.216294

0.144196

0.0591856

0.144196

0.151621
7

0

0

0.0887784

0.20715

0

0

0.0946998

0.0591856

0

0

0.824831
7

0

0.144196

2.10109

0.00408015

0

0.00416335

0.0887784

0.118371

0

5.55154

0.432588
7

0.00416335

0.00624502

0.160922

0.288392

0.443892

0

0

0

0

0

3.45156
7

0.118371

0.732115

0

0

0.491773

0.00448724

0.0129576

0

0.216294

0.00832669

2.59152
7

0.00362217

0.247593

0

0.00448724

0

0

0

0.797487

0

0

0.418463
7

0

0.0253577

0

0.0234746

0

0

0.00532728

0.0887784

0.0887784

0

0.0725084

0

0

0.157153

0.216294

0.0591856

0.36049

0

0.0190778

0

0

10.4129
7

1.12599
7

0

0.144196

0

0.0591856

0.144196

0.144196

0

0.288392

0

0.0169321

0.219313
7

0.20715

0.0591856

0

0

0

0.144196

0.0591856

0

0

0

0.481742
7

0

0.144196

0.72098

0.236743

0.0887784

0.147964

0.00448724

0.0314107

0.432588

1.08147

0.783919
7

0

0.118371

0.0591856

0

0

0

0.0357867

0

0.0887784

0

1.05357
6

0

0

0.00905542

0

0

0

0

0.0591856

0

0.0832194

8.78066
8

0.385108

0

0

0

0

0.0887784

0.0107775

0.288392

0

0

1.9965
7

0.504686

0.0887784

0

0.0194364

0.0161662

0.118371

0

0.118371

0.0518304

0.0107775

1.13905
7

0.13625

0

0

0

2.73972

0.00673085

0

0.36049

0.0591856

0.00881856

0.280907
7

0.144196

0

0.562264

0.0169312

0.0234746

0.00458025

0

0

0.0277631

0.708067

0.0890482
7

0.118371

0

0

0

0

0.00448724

0.118371

0.144196

0.0591856

0

13.3975
7

0.273017

0

0.118371

0.266335

0.295928

0.264372

0.0887784

0

0

0

0

0.281393
7

0.0887784

0.410531

0

0

0

0

0.00440928

0

0

0

3.85359
8

0.00673085

0

0

0.15413

0.0591856

0.00967569

0.20715

0

0.0887784

0.0591856

0.769433
7

0

0

0.032394

0.144196

0.144196

0

0.0453516

0

0

0.604479

0.754341
7

0

0

0

0

0.118371

0.144196

0.288392

0

0

0

0.585356
7

0

0.144196

0

0.576784

0.0887784

0

0.144196

0.0591856

0

0

0.266731
6

0

0.72098

0.0452819

0.0591856

0

0

0.150316

0

0.231194

0.216294

0.0156497
6

0.0161662

0.118371

0.0591856

0

0

0.0591856

0

0.00416335

0

0

4.15823
7

0.288392

0.032394

0.118371

0.295928

0

0.0369278

0.0887784

0

0.0129576

0

1.38721
7

0.0887784

0.436751

0

0.36049

0.0234746

0.00615463

0

0

0.0887784

0

0
4

12.784
6

12.784
6

0
4

37.7632

36.2923
7

0.423856
5

0.0544539

0

0.00518609

0

0

0

0

0

0

0.00518609

0.539453
5

0

0

0.0388957

0

0

0

0

0

0.00518609

0

0.114438
6

0

0

0

0

0

0

0

0.0887784

0.00881856

0.00518609

0

0

0

0.00777913

0.0233374

0

0.00518609

0

0

0

0

0
5

0

0

0

0

0

0

0

0

0

0

0

0

0.00518609

0

0.0544539

0

0.0129652

0.00777913

0.0103722

0.0259304
6

0

0.0285235
6

0
4

0.0591856

0

0

0.0591856

0

0

0
4

0.613913
7

0

0

0

0

0.0591856

0

0

0

0

0

0

0

0

0

0.144196

0

0

0

0

0

0.266335

0

0

0

0

0.144196

0
4

1064.35
7

42.4555
7

20.0843
7

8.83525
7

0.432059
6

992.495
7

0.0333068

0

0

0

0

0

0

0

0.00967569

0

0

0
4

205.206
7

203.674
7

1.5264
7

0

0

0

0.00518609

0
4

15.4517
7

1.7177

0.154119
7

0.00816031

0

0

0

0

0

0

0

0

0.00394831

0
6

0

0

0

0.00408015

0

0

0

0.00615463

0

0.0244809

0.147964

0

0.144196

0

0

0

0

0

0

0

0

0.1674
7

0.00448724

0

0

0

0

0

0.0693626

0.0591856

0.00448724

1.34065
7

0

0.648882

0.156039
6

0

0.41118
7

0.159901
6

0.748147

0

0.835256

0

0.132943

0.383534

0.144196

0.20715

0.0591856

0

0.223895
6

0.0592762

0.680635

0.0122405

0

0

0

0

0

0.0699631

0

0.155299
6

0.288392

0.266335

0.186789

0

0

0.0591856

0.184288

0

0.08622

0

0.371708

0.0591856

0

0

0

0

0

0

0.216294

0.00897447

0

2.2452
7

0

0

0

0.94842

0.0887784

0

0

0.00408015

0

0.00408015

0.236695
7

0.00448724

0.0591856

0

0

0.0100616

0

0.0323325

0

0

0

0.3919
7

0

0.216294

0

0

0.0107775

0.0129576

0

0

0

0

0.324372

0

0

0

0.0955854

0

0

0.00624502

0.0887784

0

0

5.44009282066327e-15
7

0
4

10.4004
7

8.49434
7

0.797807
7

0.661589
7

0.261781
7

0

0.157196

0.0277096

0

0

0
4

0.0655516
7

0.0547741
6

0.0107775

0
4

0.704653
7

0.576784

0.127869

2.77555756156289e-17
7

0
4

0.406763
7

0.0591856

0.203382

0.144196

0

0
4

0.0283442
7

0.0153866

0.0129576

0

0
4

0

0

0

0
4

0.00881856

0

0

0

0.00440928

0.00440928

0
4

0

0

0

0

0
4

0.0285235

0.00777913

0.00777913

0.0129652

0
4

0

0

0
4

0.0100616

0.0100616

0
4

2.34847

2.13964

0.192662

0

0

0

0

0

0

0.0161662

1.31838984174237e-16

0
4

0.00362217

0

0.00362217

0
4

0

0

0
4

0

0

0

0
4

0

0

0
4

0.319618

0.319618

0
4

0.0181513

0.0181513

0
4

0

0

0
4

0.0123093

0.0123093

0
4

0

0

0

0
4

0

0

0
4

0.0714027
3

0.0673225
3

0

0.00408015

0

0

0

0

0

0

0
4

0

0

0

0
4

0

0

0

0
4

0.0156497

0.0156497

0

0
4

0

0

0
4

0.00586101

0.00586101

0
4

0.0177674

0.0177674

0
4

0

0

0
4

0

0

0
4

0

0

0
4

0.0234746

0.0234746

0
4

4.67977
7

4.26547
7

0

0.266335

0

0

0.0591856

0.0887784

0
4

0.0029305

0.0029305

0
4

0

0

0
4

0.20715

0.20715

0
4

0

0

0
4

0

0

0
4

0

0

0
4

0.00905542

0.00905542

0
4

0.147964

0.147964

0
4

0

0

0
4

0

0

0
4

0.545024
7

0.545024
7

0

0

0

0

0
4

0.0591856

0.0591856

0
4

0

0

0
4

0

0

0
4

0

0

0
4

0

0

0
4

0

0

0
4

0.0103722

0.0103722

0
4

0

0

0
4

0

0

0
4

0

0

0
4

3.44604
7

3.44604
7

0

0

0

0
4

0

0

0
4

0.465756
7

0.459835
7

0.00592134
7

0

0
4

1.82832
7

1.70995
7

0.118371

0
4

0.0979237
3

0.0979237
3

0

0
4

2.30753749441703e-11
7

0
4

0.929259

0.325521
2

0.325521
2

0

0

0

0

0

0

0

0

0

0

0

0
4

0.603738

0.603738

0

0

0

0
4

0
2

0
2

0
4

0

0

0

0
4

0

0

0
4

0

0

0
4

0
4

0.114094

0.114094

0
3

0

0

0

0

0.0648261

0

0

0

0

0

0.0207444
2

0

0

0

0

0

0

0

0

0

0

0.00777913
2

0

0

0

0.00518609

0

0.00518609

0

0

0

0

0.00518609
2

0

0

0

0

0

0

0.00518609
2

6.93889390390723e-18

0
4

0

0

0

0
4

0

0

0

0
4

0

0

0

0
4

0

0

0

0
4

0

0

0
4

0

0

0
4

0
3

0
3

0

0

0

0

0

0

0

0

0

0

0

0

0

0

0

0

0

0

0

0

0

0

0

0

0

0
4

0

0

0

0

0

0
2

0
3

0

0

0

0

0

0

0
4

0

0
3

0

0

0

0

0

0

0

0

0

0

0
4

0

0

0

0

0
4

0
2

0

0

0

0

0

0
4

0
2

0

0

0

0

0

0
4

0

0

0

0
4

0

0

0
4

0
4

0
2

0
2

0

0

0

0

0

0

0

0

0

0

0
4

0

0

0

0

0

0

0

0
4

0

0

0

0

0

0

0

0

0

0

0
4

0

0

0

0
4

0

0

0

0

0
4

0

0

0

0
4

0
4

0.0712667

0

0

0
4

0.0712667

0.0712667

0
4

0
4

0

0

0

0

0
4

0
4

0

0

0

0
4

0

0

0
4

0
4

0

0

0

0
4

0
4

0.0156497

0.0156497

0.0156497

0
4

0

0

0
4

0
4

0.0143923

0.00616813

0.00616813

0
4

0.00822417

0.00822417

0
4

0
4

0

0

0

0
4

0
4

0

0

0

0
4

0
4

0

0

0

0
4

0
4

0.804929

0.804929

0.804929

0
4

0
4

0.00518609

0

0

0

0

0

0

0

0

0
4

0.00518609
3

0
2

0

0

0

0.00518609

0

0

0

0

0

0

0
4

0

0

0

0

0

0

0

0
4

0

0

0

0

0

0
4

0

0

0
4

0

0

0
4

0

0

0
4

0

0

0
4

0

0

0
4

0
4

0

0

0

0

0
4

0
4

0

0

0

0
4

0
4

0

0

0

0
4

0

0

0
4

0
4

0

0

0

0
4

0
4

0.0887784

0.0887784

0.0887784

0

0
4

0
4

0

0

0

0
4

0
4

2.45133

2.45133

2.45133

0
4

0
4

0

0

0

0
4

0

0

0
4

0
4

0

0

0

0
4

0

0

0
4

0
4

0

0

0

0

0
4

0
4

29.2119

16.2472

16.2472

0

0

0

0

0

0

0

0
4

12.9055
7

0

12.9055

0

0

0

0

0

0
4

0

0

0

0

0

0

0

0

0
4

0

0

0

0

0

0
4

0

0

0

0
4

0.0591856

0.0591856

0
4

0
4

0

0

0

0
4

0
4

0

0

0

0
4

0
4

0

0

0

0
4

0
4

0

0

0

0
4

0
4

0.0091605

0.0091605

0.0091605

0
4

0
4

0

0

0

0
4

0
4

0

0

0

0
4

0
4

0

0

0

0
4

0
4

0

0

0

0
4

0
4

0

0

0

0
4

0
4

2.89633
2

2.60417
1

0

0.325521

2.27865

0

0

0

0

0
4

0.147964

0

0

0

0

0

0

0

0

0

0.147964

0
4

0

0

0

0

0

0

0
4

0.144196

0.144196

0

0

0

0
4

0

0

0

0
4

0

0

0

0
4

0

0

0
4

4.71844785465692e-16
2

0
4

0

0

0

0
4

0
4

0

0

0

0
4

0
4

0

0

0

0
4

0
4

0

0

0

0
4

0
4

0

0

0

0
4

0
4

0

0

0

0
4

0
4

0

0

0

0
4

0
4

0

0

0

0
4

0
4

0

0

0

0
4

0
4

0

0

0

0
4

0
4

0.140024

0.140024

0.140024
3

0

0

0

0
4

0

0

0

0

0

0

0

0
4

0

0

0

0
4

0

0

0

0
4

0

0

0
4

0

0

0
4

0
4

0

0

0

0
4

0
4

0

0

0

0
4

0
4

0

0

0

0
4

0
4

0.155491

0.155491

0.155491

0
4

0
4

0

0

0

0
4

0
4

0

0

0

0
4

0
4

0

0

0

0
4

0
4

0

0

0

0
4

0
4

0.0518304

0.0518304

0.0518304

0
4

0
4

0

0

0

0
4

0
4

0.646148
7

0.486765
7

0.48052
7

0.00624502

0

0

0

0

0

3.46944695195361e-17
7

0
4

0.159383
7

0.159383
7

0
4

0

0

0

0
4

0
4

0.0108665

0.0108665

0.0108665

0
4

0
4

0

0

0

0
4

0
4

0

0

0

0
4

0
4

0

0

0

0
4

0
4

0

0

0

0
4

0
4

0

0

0

0
4

0
4

0

0

0

0
4

0
4

0.0201232

0.0201232

0.0201232

0
4

0
4

0

0

0

0
4

0
4

0

0

0

0
4

0
4

0
3

0
3

0
3

0

0

0

0

0

0

0
4

0
4

0

0

0

0
4

0
4

0

0

0

0
4

0
4

0

0

0

0
4

0
4

0

0

0

0
4

0
4

0

0

0

0
4

0
4

0

0

0

0
4

0
4

0.0591856

0.0591856

0.0591856

0
4

0
4

0

0

0

0
4

0
4

0

0

0

0
4

0
4

0.0194364

0.0194364

0.0194364

0
4

0
4

2.37923

0
6

0

0

0

0

0
4

0

0

0
4

0

0

0
4

0.288392

0.288392

0
4

1.08147
7

1.08147

0

0
4

0

0

0

0

0

0

0
4

1.00937

1.00937

0

0

0
4

0

0

0

0
4

0

0

0

0
4

0

0

0
4

0

0

0

0
4

0

0

0
4

0
4

0

0

0

0
4

0
4

0

0

0

0
4

0
4

0.0389321

0.0389321

0.0389321

0
4

0
4

0

0

0

0
4

0
4

0

0

0

0
4

0
4

0

0

0

0
4

0
4

0

0

0

0
4

0
4

2.23504

2.23504

2.23504

0
4

0
4

0

0

0

0
4

0
4

0.0129576

0.0129576

0.0129576

0
4

0
4

1.16574
7

0.553719
7

0.0148034
7

0

0.538916

0

0

0

0
4

0.0185577
7

0.00967569
6

0

0.00888202

0
4

0.0887784

0.0887784

0

0
4

0.504686

0

0.504686

0
4

0

0

0
4

2.22044604925031e-16
7

0
4

0

0

0

0
4

0
4

0.0704238

0.0704238

0.0704238

0
4

0
4

0

0

0

0
4

0
4

0

0

0

0
4

0
4

0

0

0

0
4

0
4

0

0

0

0

0

0

0

0

0

0

0
4

0

0

0
4

0

0

0
4

0
4

6.54308

6.31114

4.88425

0

0

0

0

0

0

0

0.0156497

0

0

0.0391243

0

0

0

0

0

0

0

0.562264

0

0

0

0

0

0

0

0

0

0

0

0

0

0.0156497

0

0

0

0

0

0

0

0

0

0.28952

0

0

0

0

0

0

0

0

0

0

0

0

0

0

0

0.504686

4.44089209850063e-16

0
4

0.216294

0

0

0

0.216294

0

0

0
4

0.0156497

0

0

0

0

0

0

0

0

0.0156497

0

0

0
4

0

0

0

0

0

0
4

0

0

0
4

0
4

0

0

0

0

0

0

0

0

0

0
4

0

0

0
4

0
4

0

0

0

0

0
4

0
4

0
4

0
4

0

0

0

0

0

0
4

0
4

0

0

0

0
4

0

0

0

0

0

0
4

0
4

0.299687

0.299687

0.116618

0.0388728

0

0.144196

0

0

2.77555756156289e-17

0
4

0
4

0

0

0

0

0

0

0
4

0

0

0

0

0
4

0

0

0

0

0
4

0

0

0
4

0
4

0

0

0

0

0

0

0
4

0

0

0
4

0
4

0
3

0
3

0
3

0

0

0

0

0
4

0

0

0

0
4

0

0

0
4

0
4

0.366031
8

0.357704
8

0.189007

0.100109

0.0479529

0.0144813

0.00615463

0
4

0.00832669

0.00832669

0
4

8.67361737988404e-18
8

0
4

0
3

0
3

0
3

0
4

0
4

0
3

0
2

0
2

0

0
4

0
3

0

0

0

0
4

0

0

0
4

0
4

22.2773
1

6.08798
1

2.01874

0

0.144196

0

0

0

0

0

0

0

0

2.73972
2

0

0.288392

0.680635

0

0

0.216294

0

0

0
4

0.216294

0.216294

0
4

0

0

0
4

13.321
1

6.32312
2

0

0.0591856

0.118371

0

0

0

1.00937

0

0

0

5.21748
1

0

0

0

0.305072

0.288392

0

0

0

0

0

0
4

2.24152
1

1.83475

0

0

0

0

0.0591856

0

0.288392

0.0591856

0

0
4

0.177557

0

0

0.177557

0
4

0

0

0

0
4

0.0887784

0

0.0887784

0
4

0.144196

0.144196

0

0
4

0

0

0

0
4

0

0

0

0
4

2.4980018054066e-15
1

0
4

0
3

0
3

0
3

0

0

0

0

0
4

0

0

0

0

0

0
4

0
4

0

0

0

0

0

0

0
4

0

0

0

0

0

0

0
4

0

0

0
4

0

0

0

0
4

0

0

0

0
4

0

0

0
4

0
4

0

0

0

0

0

0

0
4

0

0

0

0

0

0

0
4

0
4

1.11811

1.11811

1.01246

0.0804929

0.0100616

0.0150924

9.36750677027476e-17

0
4

0
4

0
2

0

0

0

0

0

0

0
4

0

0

0
4

0

0

0
4

0
4

0

0

0

0

0
4

0

0

0

0
4

0

0

0

0
4

0
4

0

0

0
2

0

0

0
4

0

0

0
4

0

0

0
4

0
4

1.81214
7

1.65825

1.65825

0

0
4

0.153885

0.153885

0

0

0
4

0

0

0

0
4

0

0

0
4

5.55111512312578e-17
7

0
4

0
2

0

0

0

0

0
4

0
3

0

0

0

0

0
4

0

0

0
4

0
4

0

0

0

0

0

0

0
4

0

0

0

0
4

0
4

8.08188

6.62632
7

6.33792
7

0

0

0

0.288392

0

0

0

0

0

0

0

0
4

0

0

0
4

0

0

0

0
4

0

0

0

0
4

0

0

0
4

0

0

0

0

0

0

0

0

0

0
4

0.216294
1

0

0

0.216294

0

0

0

0

0

0
4

0.026521
6

0

0

0.0122405

0

0.00816031

0.00612023

0
4

0
6

0
6

0

0
4

0

0

0

0

0

0

0
4

1.06856
7

0.432588

0

0.203382

0.432588

2.22044604925031e-16
7

0
4

0

0

0

0

0

0

0
4

0.144196

0

0

0

0.144196

0
4

1.38777878078145e-16

0
4

2.34578
8

2.32623
8

2.32623
8

0
4

0.0133953

0.0133953

0
4

0.00615463

0.00615463

0
4

0
4

0
3

0

0

0

0
4

0

0

0
4

0

0

0
4

0

0

0
4

0

0

0
4

0
4

0.909832
7

0.909832
7

0.909832
7

0

0
4

0
4

0.267484
7

0.267484
7

0.257422

0

0.0100616

2.77555756156289e-17
7

0
4

0

0

0
4

0
4

0

0

0

0

0

0

0

0
4

0

0

0
4

0

0

0
4

0
4

0
2

0
2

0

0

0

0

0
4

0

0

0
4

0

0

0
4

0
4

0
4

0

0

0

0

0

0

0
4

0

0

0
4

0
4

0

0

0

0

0

0

0
4

0
4

0.314307
7

0.314307
7

0.170111
7

0.144196

0

0
4

0
4

0
4

0
4

0

0

0
4

0

0

0
4

0

0

0
4

0

0

0
4

0
4

30.8939
7

12.9574
7

0.897996
7

0.0533174
6

10.0462
8

1.92353

0

0

0

0.0363026

0

0
4

15.5032
7

15.5032
7

0

0

0
4

0.510081
7

0.311402
7

0.159807

0.0388728

0

0

0
4

1.78412
7

1.04871
8

0.36049

0.0156497

0.300092

0

0.0591856

0

4.16333634234434e-17
7

0
4

0

0

0

0

0
4

0.0591856

0

0

0.0591856

0
4

0

0

0

0
4

0.07993

0.07993

0
4

1.04360964314765e-14
7

0
4

0

0

0

0

0

0

0
4

0

0

0

0

0
4

0

0

0
4

0
4

0

0

0

0

0
4

0
4

0.11014

0.11014

0.11014

0
4

0
4

0

0

0

0

0

0

0
4

0
4

0.185329
3

0.18092
3

0.18092

0

0

0
4

0.00440928

0.00440928

0
4

0
4

0.118371

0.0591856

0

0.0591856

0
4

0.0591856

0.0591856

0
4

0

0

0
4

0
4

0

0

0

0

0

0

0
4

0
4

0

0

0

0

0
4

0

0

0

0
4

0
4

0

0

0

0

0
4

0

0

0

0
4

0
4

0

0

0

0

0

0
4

0

0

0
4

0
4

0
3

0
3

0
3

0

0

0

0

0
3

0

0

0

0

0

0

0

0
4

0

0

0

0
4

0

0

0
4

0
4

0.608083

0.608083

0.144196

0.144196

0.0312995

0.144196

0.144196

5.55111512312578e-17

0
4

0

0

0
4

0
4

0

0

0

0

0

0
4

0
4

0.216294

0.216294

0.216294

0

0
4

0

0

0

0
4

0

0

0
4

0
4

0

0

0

0

0
4

0

0

0
4

0

0

0

0
4

0
4

0.0916087

0.0916087

0.0916087

0

0

0

0
4

0
4

1.22567
8

0.288392

0.288392

0

0
4

0.937273

0.937273

0
4

0

0

0
4

0
4

0

0

0

0
4

0

0

0
4

0

0

0
4

0
4

0.0518304

0.0518304

0.0194364

0.0129576

0.0194364

0
4

0

0

0
4

0
4

0

0

0

0

0
4

0
4

0

0

0

0

0
4

0

0

0

0
4

0

0

0
4

0
4

3.38639

0.698755

0.61711

0.00458025

0

0

0

0

0

0

0.0770648

0

0

0

0

0
4

2.68764

1.75648

0

0.532091

0.359944

0

0

0

0.0156497

0

0.0234746

1.76941794549634e-16

0
4

0

0

0
4

0

0

0
4

0

0

0
4

0

0

0
4

0

0

0
4

0

0

0
4

0
4

0

0

0

0
4

0

0

0
4

0
4

0.288392

0

0

0
4

0.288392

0.288392

0
4

0
4

2.37923

2.37923

2.23504

0.144196

0
4

0
4

0.232974

0.144196

0.144196

0
4

0.0887784

0

0.0887784

0
4

0

0

0
4

0
4

0.36049

0

0

0
4

0.36049

0

0.36049

0
4

0
4

0.144196
7

0.144196

0.144196

0
4

0

0

0

0
4

0
4

0

0

0

0
4

0
4

0.0391243

0.0391243

0.0391243

0
4

0

0

0
4

0
4

0.155491

0.155491

0.155491

0

0
4

0
4

0

0

0

0
4

0
4

6.93185

1.76167

1.39819

0

0

0.062599

0

0.288392

0

0

0.01249

0

0

0
4

0

0

0
4

0

0

0
4

3.57589
7

1.85187
7

1.71365

0.00518609

0.00518609

0
4

1.53808
7

1.53009

0

0.00799092

0

0

0
4

0

0

0

0

0

0
4

0

0

0

0
4

0.0453516

0.0453516

0
4

0

0

0
4

0

0

0
4

0.0108665

0.0108665

0
4

9.4542429440736e-16

0
4

0

0

0

0
4

0
4

0

0

0

0

0

0
4

0
4

0

0

0

0
4

0
4

0.399614

0.399614

0.36049

0.0391243

1.38777878078145e-17

0
4

0
4

0

0

0

0

0
4

0

0

0
4

0
4

0

0

0

0

0
4

0

0

0
4

0
4

0

0

0

0

0
4

0

0

0
4

0
4

0

0

0

0

0
4

0
4

0

0

0

0

0

0
4

0
4

0.144196

0.144196

0.144196

0
4

0
4

0

0

0

0

0

0

0

0

0

0

0

0

0

0

0

0

0

0

0

0

0

0

0

0

0

0

0

0

0

0
4

0

0

0

0
4

0
4

0

0

0

0
4

0
4

0

0

0

0

0

0
4

0
4

0

0

0

0

0
4

0

0

0
4

0
4

0.0103306

0.00440928

0.00440928

0

0
4

0.00592134

0.00592134

0
4

0
4

0.236026

0.236026

0.0236899

0.20715

0.00518609

1.56125112837913e-17

0
4

0
4

0

0

0

0

0
4

0
4

0

0

0

0

0
4

0
4

0

0

0

0
4

0

0

0
4

0
4

0

0

0

0
4

0

0

0
4

0

0

0
4

0
4

0.0129576

0.0129576

0.0129576

0

0
4

0

0

0
4

0
4

0.266046
3

0.266046
3

0

0.0156497

0.250396

0

0

0

0
4

0
4

1.85109
3

1.85109
3

0.695115
3

1.15597

0

0
4

0

0

0
4

0

0

0
4

0

0

0
4

0

0

0
4

0

0

0
4

0
4

75.1273

75.1221

18.1324
7

3.70887
7

0.0770648

0

0

0

0

0.00612023

0

0.00592134

0

0

1.21958
8

0

0

0

0.0469492

0

0

0.00592134

0

0.00416335

0

0
3

0

0.288392

0

0

0

0.0156497

0.0236854

0

0.0201232

0

0.207247

0.00592134

0.0234746

0

0.0156497

0.0166534

0

0.0562528

0.00592134

0.00592134

0.00592134

0.213168

0.0201232

0

0.144196

0

0

0

0

0.0118427

0

0

0.0760926
7

0.0201232

0.0249801

0

0

0

0.0207247

0.937273

0.0118427

0

0

1.29776

0.144196

0

0

0.0156497

0

0

1.22567

0.0770648

0

0.00592134

0.530698
7

0.00592134

0.0100616

0

0

0

0.017764

0.0148034

0.0156497

0.00888202

0

0.102558

0

0

0

0

0

0

0

0

0

0

0.0621741

0.0296067

0

0.0770648

0

0.00592134

0.115597

0
3

0.113098
7

0.161664

1.44185

0.291787
6

0.181567

0.0820921
7

0.0693114
7

0

0

0.0918681

26.5155
7

0

0.0812013
7

0

0.937273

0.0584393

0.648882

0

0.0260907

0.0299304

0.516513

4.50299
7

0

0

0.0208391

0.00592134

0.0307863

0

0.504686

0.164191

0

0.0156497

0.261602
6

0.247308

0.0198131

0.03111

0

0.0236854

0.0156497

0.36049

0

0.113336

0

0
3

0

0

0

0

0.0361754

0.0829861

0.204772

0.0248881

0.00592134

0

0.937273

0

0.0161662

0.0810131

0.0148034

4.18168

0.0156497

0.169779

0.01249

0

0

0
3

0

0.017764

0.0452773

0.36049

0

0.504686

0

0.0148034

0.00592134

0

0.577331

0

0.937273

0

0

0

0

0.00592134

0.0592134

0.103624

0

1.9595436384634e-14

0
4

0

0

0

0

0

0
4

0

0

0

0
4

0

0

0
4

0.00518609

0.00518609

0
4

0
4

0.144196
3

0
4

0

0

0
4

0

0

0
4

0

0

0
4

0

0

0
4

0.144196

0.144196

0
4

0

0

0
4

0

0

0
4

0
4

6.41321717953502e-11

0
4

8.91008
3

6.68159
3

1.16028

1.16028

0

0

0

0

0

0

0

0

0

0

0

0

0

0

0

0

0

0

0

0

0

0
4

1.21712
5

1.21712
5

0
6

0

0

0

0

0

0

0

0

0

0

0

0

0
4

4.30418
3

0.625208

0.113932
3

1.14953

0
4

0.0591856

0

0

0

0

0

0

0

0

0

0
4

0

0

0

0

0

0

0

0

0

0

0
3

0

0

0

0

0

0

0

0

0

0

0
3

0

0

0

0

0

0

0

0

0

0

0
4

0

0

0

0

0

0

0

0

0

0

0.0770648

0

0

0

0

0

0

0

0

0

0

0
3

0

0

0

0

0

0

0

0

0

0

0.118371

0

0.0156497

0

0

0

0

0.414299

0

0

0

0
4

0

0

0

0

0

0

0

0

0

0

0
4

0

0

0

0

0

0

0

0

0

0

0

0
4

0

0

0

0

0

0

0

0

0

0

0
3

0

0

0

0

0

0

0

0

0

0

0
4

0

0

0

0

0

0

0

0

0

0

0

0

0

0

0

0

0

0.0887784

0

0

0

0

0

0

0

0

0

0

0

0

0

0.0312995

0
3

0

0

0

0

0.0391243

0

0

0

0

0

0.0156497

0

0

0

0

0

0.231194
5

0
6

0.0770648

0

0
3

0

0

0

0
3

0

0

0
4

0

0

0.265217

0
6

0
4

0

0

0
4

0

0

0

0

0

0
4

0

0

0

0

0

0

0

0

0

0

0
3

0

0

0.15413

0

0

0

0

0

0

0

0

0

0

0

0

0.0692472

0

0

0

0

0

0
3

0

0

0

0

0

0

0.062599

0

0

0

0.661423

0

0

0

0

0.0352157

0

0

0

0

0

0
4

0

0

0

0
4

0

0

0
4

0

0

0
4

0

0

0
4

0

0

0
4

0

0

0

0
4

0

0

0

0
4

0

0

0
4

0

0

0
4

0

0

0
4

0

0

0
4

0

0

0
4

0

0

0
4

8.88178419700125e-16
3

0
4

0.748946
3

0.748946
3

0.628148
3

0

0

0

0

0.00483191

0

0

0

0

0

0

0

0

0

0

0.00322127

0

0

0

0
4

0
3

0.0241595

0

0.088585

0

0

0
4

0
3

0

0
2

0

0

0

0

0

0

0

0
4

0
4

0

0

0

0

0

0

0

0

0

0

0

0

0

0

0

0

0
4

0

0

0
4

0
4

0.0144957
4

0.0144957
4

0.0144957
4

0
4

0

0

0
4

0
4

0

0

0

0

0

0

0

0
4

0

0

0
4

0
4

0

0

0

0
4

0
4

0

0

0

0

0

0
4

0
4

0

0

0

0

0
4

0

0

0
4

0

0

0
4

0
4

0

0

0

0

0

0

0
4

0

0

0

0
4

0
4

0

0

0

0

0
4

0
4

0

0

0

0

0

0
4

0

0

0
4

0

0

0
4

0
4

0

0

0

0
4

0

0

0
4

0
4

0

0

0

0

0
4

0
4

1.08718
3

0.998595
3

0.998595
3

0

0

0

0

0

0

0

0
4

0

0

0
4

0

0

0
4

0.0595935

0.00322127

0.0161064

0

0.0144957

0.00644255

0.0193276

0
4

0.00644255
2

0

0

0

0.00644255

0
4

0.00805318
3

0.00805318

0

0

0
4

0

0

0
4

0.0144957

0.0144957

0
4

0

0

0
4

0

0

0
4

0

0

0
4

3.33066907387547e-16
3

0
4

0.0129576

0.0129576

0.0129576

0
4

0
4

0

0

0

0
4

0
4

0

0

0

0
4

0

0

0
4

0
4

0

0

0

0
4

0
4

0.00322127

0

0

0
4

0.00322127

0.00322127

0
4

0
4

0.00483191

0.00483191

0.00483191

0

0
4

0

0

0
4

0
4

0

0

0

0
4

0

0

0
4

0
4

0

0

0

0
4

0
4

0

0

0

0
4

0

0

0
4

0

0

0
4

0
4

0

0

0

0

0
4

0
4

0.238374
3

0.0193276
3

0.0193276
3

0

0

0

0

0

0
3

0
4

0

0

0

0

0

0

0
4

0.00805318

0.00805318

0
4

0.0112745

0.0112745

0
4

0.154621

0.149789

0.00483191

1.21430643318376e-17

0
4

0
3

0

0

0

0

0

0

0
4

0.0193276

0.0193276

0
4

0.0128851

0.0128851

0

0
4

0

0

0

0
4

0.00644255

0

0.00644255

0
4

0

0

0
4

0.00644255

0.00644255

0
4

0
4

0

0

0

0
4

0

0

0
4

0

0

0
4

0
4

0

0

0

0
4

0
4

0

0

0

0
4

0
4

0

0

0

0
4

0
4

0

0

0

0

0
4

0
4

0

0

0

0

0
4

0
4

0

0

0

0
4

0
4

0

0

0

0
4

0
4

0

0

0

0
4

0
4

0

0

0

0
4

0
4

0
4

0
4

0
4

0
4

0

0

0

0

0

0

0
4

0
4

0
4

0

0

0

0

0

0
4

0

0

0

0

0
4

0

0

0

0
4

0
4

0

0

0

0
4

0
4

0

0

0

0
4

0
4

0

0

0

0
4

0
4

0

0

0

0
4

0
4

0.0156497

0.0156497

0.0156497

0
4

0
4

0.0770648

0.0770648

0.0770648

0
4

0
4

0.00966382

0.00966382

0.00966382

0
4

0
4

0

0

0

0

0

0

0

0

0

0

0

0

0

0

0

0

0
4

0

0

0

0

0

0

0

0

0

0

0

0

0

0

0
4

0

0

0

0

0

0
4

0

0

0

0
4

0
4

0

0

0

0

0

0

0

0

0

0
4

0

0

0

0

0

0

0

0

0
4

0

0

0

0

0

0

0
4

0
4

0
4

0

0

0

0

0

0

0

0

0

0

0
4

0

0

0

0

0

0
4

0
4

0

0

0

0

0

0
4

0
4

0.0161064
4

0.0161064
4

0.0161064

0

0

0

0

0
4

0

0

0

0
4

0

0

0

0
4

0

0

0

0
4

0

0

0
4

0
4

0
4

0

0

0

0

0

0

0

0

0

0

0

0

0

0

0
4

0

0

0

0

0

0
4

0

0

0

0

0

0
4

0

0

0

0
4

0
4

0
4

0.115221
7

0.115221
7

0.115221
7

0.0957847
7

0

0.0194364

3.46944695195361e-18
7

0
4

0
4

0
4

0

0

0

0

0
4

0
4

0
4

0

0

0

0

0

0
4

0
4

0
4

0

0

0

0

0

0
4

0
4

0
4

0.625281

0.625281

0.625281

0.625281

0
4

0
4

0
4

0

0

0

0

0
4

0
4

0
4

0

0

0

0

0
4

0
4

0
4

0

0

0

0

0
4

0
4

0
4

0

0

0

0

0
4

0
4

0
4

0

0

0

0

0
4

0
4

0
4

0

0

0

0

0

0
4

0
4

0
4

3.06148
7

3.06148
7

3.06148
7

2.84518
7

0.216294

0

0
4

0
4

0
4

0

0

0

0

0

0
4

0
4

0
4

0

0

0

0

0
4

0
4

0
4

0

0

0

0

0
4

0
4

0
4

0.0201232

0.0201232

0.0201232

0.0201232

0
4

0
4

0
4

0

0

0

0

0
4

0
4

0
4

0

0

0

0

0
4

0
4

0
4

0

0

0

0

0
4

0
4

0
4

0

0

0

0

0
4

0

0

0
4

0
4

0
4

0.0547741

0.0547741

0.0547741

0.0547741

0
4

0
4

0
4

0

0

0

0

0
4

0
4

0
4

0.720476
7

0.720476
7

0.720476
7

0.720476
7

0
4

0

0

0
4

0
4

0
4

0

0

0

0

0
4

0

0

0
4

0
4

0
4

0

0

0

0

0
4

0
4

0
4

0.00592134

0.00592134

0

0

0
4

0.00592134

0.00592134

0
4

0
4

0
4

0

0

0

0

0
4

0

0

0
4

0
4

0
4

0

0

0

0

0
4

0
4

0
4

0

0

0

0

0

0
4

0
4

0
4

0

0

0

0

0
4

0
4

0
4

0

0

0

0

0
4

0
4

0
4

0

0

0

0

0
4

0
4

0
4

0.0313917

0.0313917

0.0313917

0.0313917

0
4

0
4

0
4

0

0

0

0

0

0

0
4

0

0

0

0
4

0
4

0

0

0

0
4

0

0

0
4

0
4

0
4

0

0

0

0

0

0
4

0
4

0
4

0.72098

0.72098

0.72098

0.144196

0.576784

0
4

0
4

0
4

0

0

0

0

0
4

0
4

0
4

0

0

0

0

0
4

0

0

0
4

0
4

0
4

0

0

0

0

0
4

0

0

0
4

0
4

0
4

0

0

0

0

0
4

0
4

0
4

0

0

0

0

0
4

0
4

0
4

0

0

0

0

0
4

0
4

0
4

0

0

0

0

0
4

0
4

0
4

0

0

0

0

0
4

0
4

0
4

0

0

0

0

0

0

0

0

0

0

0
4

0

0

0

0

0

0
4

0

0

0
4

0
4

0
4

0

0

0

0

0
4

0
4

0
4

0

0

0

0

0
4

0
4

0
4

0.00777913

0.00777913

0.00777913

0.00777913

0
4

0
4

0
4

0

0

0

0

0
4

0
4

0
4

0.0770648

0.0770648

0.0770648

0.0770648

0
4

0
4

0
4

0

0

0

0

0
4

0
4

0
4

0

0

0

0

0
4

0
4

0
4

0

0

0

0

0
4

0
4

0
4

0

0

0

0

0
4

0
4

0
4

0

0

0

0

0
4

0
4

0
4

0.56398
7

0.558793
7

0.0532921

0

0.0532921

0
4

0

0

0

0

0
4

0.00458025

0.00458025

0
4

0.500921

0.500921

0
4

0
4

0.00518609
6

0

0

0

0

0
4

0.00518609

0.00518609

0
4

0

0

0
4

0
4

0
4

0

0

0

0

0
4

0
4

0
4

0

0

0

0

0
4

0
4

0
4

0

0

0

0

0
4

0
4

0
4

0

0

0

0

0
4

0
4

0
4

0

0

0

0

0
4

0
4

0
4

0

0

0

0

0
4

0
4

0
4

0

0

0

0

0
4

0
4

0
4

0

0

0

0

0
4

0
4

0
4

0

0

0

0

0
4

0
4

0
4

0

0

0

0

0
4

0
4

0
4

0.0156497
3

0.0156497
3

0.0156497
3

0.0156497
3

0

0
4

0

0

0
4

0
4

0
4

0

0

0

0

0
4

0
4

0
4

0

0

0

0

0
4

0
4

0
4

0

0

0

0

0
4

0
4

0
4

0

0

0

0

0
4

0
4

0
4

0

0

0

0

0
4

0
4

0
4

0

0

0

0

0
4

0
4

0
4

0

0

0

0

0
4

0
4

0
4

0

0

0

0

0
4

0
4

0
4

0

0

0

0

0
4

0
4

0
4

0

0

0

0

0
4

0
4

0
4

0.937273
5

0.937273
5

0.937273
5

0.576784
5

0.36049

0

0
4

0

0

0
4

0

0

0
4

0
4

0
4

0

0

0

0

0
4

0
4

0
4

0

0

0

0

0
4

0
4

0
4

0

0

0

0

0
4

0
4

0
4

0

0

0

0

0
4

0
4

0
4

0

0

0

0

0
4

0
4

0
4

0

0

0

0

0
4

0
4

0
4

0

0

0

0

0
4

0
4

0
4

0

0

0

0

0
4

0
4

0
4

0

0

0

0

0
4

0
4

0
4

0

0

0

0

0
4

0
4

0
4

0

0

0

0

0

0

0

0
4

0

0

0

0
4

0
4

0
4

0

0

0

0

0
4

0
4

0
4

0

0

0

0

0
4

0
4

0
4

0

0

0

0

0
4

0
4

0
4

0.36049

0.36049

0.36049

0.36049

0
4

0
4

0
4

0

0

0

0

0
4

0
4

0
4

0

0

0

0

0
4

0
4

0
4

0

0

0

0

0
4

0
4

0
4

0

0

0

0

0
4

0
4

0
4

0

0

0

0

0
4

0
4

0
4

0

0

0

0

0
4

0
4

0
4

0

0

0

0

0

0

0

0
4

0

0

0
4

0
4

0
4

0

0

0

0

0
4

0
4

0
4

0

0

0

0

0
4

0
4

0
4

0

0

0

0

0
4

0
4

0
4

0

0

0

0

0
4

0
4

0
4

0

0

0

0

0
4

0
4

0
4

0

0

0

0

0
4

0
4

0
4

0.0712667

0.0712667

0.0712667

0.0712667

0
4

0
4

0
4

0

0

0

0

0
4

0
4

0
4

0

0

0

0

0
4

0
4

0
4

0

0

0

0

0
4

0
4

0
4

0

0

0

0

0

0

0

0

0

0

0
4

0

0

0

0

0

0
4

0

0

0

0

0
4

0

0

0
4

0
4

0
4

0.880276
7

0.880276
7

0.880276
7

0.880276
7

0
4

0
4

0
4

0

0

0

0

0
4

0
4

0
4

0

0

0

0

0
4

0
4

0
4

0

0

0

0

0
4

0
4

0
4

0

0

0

0

0
4

0
4

0
4

0.144196

0.144196

0.144196

0.144196

0
4

0
4

0
4

0

0

0

0

0
4

0
4

0
4

0

0

0

0

0
4

0
4

0
4

0

0

0

0

0
4

0
4

0
4

0.00448724

0.00448724

0.00448724

0.00448724

0
4

0
4

0
4

0

0

0

0

0
4

0
4

0
4

0

0

0

0

0

0

0
4

0
4

0
4

0

0

0

0

0
4

0
4

0
4

0

0

0

0

0
4

0
4

0
4

0.0842243

0.0842243

0.0842243

0.0842243

0
4

0
4

0
4

0

0

0

0

0
4

0
4

0
4

0

0

0

0

0
4

0
4

0
4

0

0

0

0

0
4

0
4

0
4

0.216294

0.216294

0.216294

0.216294

0
4

0
4

0
4

0

0

0

0

0
4

0
4

0
4

0

0

0

0

0
4

0
4

0
4

0

0

0

0

0
4

0
4

0
4

0

0

0

0

0

0
4

0
4

0
4

0

0

0

0

0
4

0
4

0
4

0

0

0

0

0
4

0
4

0
4

0.15413

0.15413

0.15413

0.15413

0
4

0
4

0
4

0

0

0

0

0
4

0
4

0
4

0

0

0

0

0
4

0
4

0
4

0

0

0

0

0
4

0
4

0
4

0

0

0

0

0
4

0
4

0
4

0

0

0

0

0
4

0
4

0
4

0

0

0

0

0
4

0
4

0
4

0

0

0

0

0
4

0
4

0
4

1.80245
7

1.80245

1.80245

1.80245

0

0

0
4

0
4

0

0

0

0
4

0

0

0
4

0
4

0
4

0.144196

0.144196

0.144196

0.144196

0
4

0
4

0
4

0

0

0

0

0
4

0
4

0
4

0

0

0

0

0
4

0
4

0
4

0

0

0

0

0
4

0
4

0
4

0

0

0

0

0
4

0
4

0
4

0

0

0

0

0
4

0
4

0
4

0

0

0

0

0
4

0
4

0
4

0.473485

0.473485

0.473485

0.473485

0
4

0
4

0
4

0

0

0

0

0
4

0
4

0
4

0

0

0

0

0
4

0
4

0
4

0.635486
6

0.635486
6

0.635486
6

0.635486
6

0
4

0
4

0
4

0

0

0

0

0
4

0
4

0
4

0

0

0

0

0
4

0
4

0
4

0

0

0

0

0
4

0
4

0
4

0

0

0

0

0
4

0
4

0
4

0

0

0

0

0
4

0
4

0
4

0.0971819

0.0971819

0.0971819

0.0971819

0
4

0
4

0
4

0

0

0

0

0

0

0
4

0
4

0

0

0

0

0
4

0
4

0
4

0
3

0
3

0
3

0

0

0

0

0
4

0
4

0
4

1.00739
6

1.00739
6

1.00739
6

0.718995

0.288392

0

0
4

0
4

0
4

0
4

0
4

0
4

0
4

0
4

0

0

0
4

0
4

0
4

0.108453
7

0.108453
7

0.108453
7

0.108453
7

0
4

0
4

0
4

0.602528
1

0.602528
1

0.602528
1

0.58957
1

0

0.0129576

0

0

0
4

0

0

0
4

0
4

0
4

1.58371
7

1.58371
7

1.58371
7

1.58371
7

0
4

0
4

0
4

0.0259152
6

0.0259152
6

0.0259152
6

0.0259152
6

0

0
4

0
4

0
4

0

0

0

0

0

0

0

0
4

0
4

0
4

0.913871

0.913871

0.913871
7

0.803732
7

0.11014

9.71445146547012e-17
7

0
4

0

0

0
4

0
4

0
4

0

0

0

0

0

0

0
4

0

0

0

0

0
4

0
4

0

0

0

0
4

0

0

0
4

0
4

0
4

0.463329
7

0.463329
7

0.463329
7

0.463329
7

0

0
4

0
4

0
4

0.0712667

0.0712667

0.0712667

0.0712667

0

0
4

0
4

0
4

0

0

0

0

0

0
4

0

0

0

0
4

0
4

0
4

0

0

0

0

0

0

0
4

0
4

0
4

0

0

0

0

0

0
4

0

0

0

0
4

0

0

0
4

0

0

0
4

0
4

0
4

0.334838
6

0.334838
6

0.334838
6

0.334838
6

0

0
4

0
4

0
4

1.08109
7

1.08109
7

1.08109
7

1.06511

0

0.0159818

0

0
4

0
4

0
4

1.00023
7

1.00023
7

1.00023
7

1.00023
7

0

0
4

0
4

0
4

0

0

0

0

0

0
4

0

0

0
4

0
4

0
4

0

0

0

0

0

0
4

0
4

0
4

0.0423984

0.0423984

0.0275951

0.0127917

0.00592134

0.00888202

0
4

0

0

0
4

0.0148034

0.0148034

0
4

0
4

0
4

0

0

0

0

0

0

0

0
4

0
4

0
4

0

0

0

0

0
4

0
4

0
4

0.673795

0.673795

0.673795

0.673795

0

0
4

0
4

0
4

0.346792
7

0.346792
7

0.346792
7

0.346792
7

0
4

0
4

0
4

0

0

0

0

0

0

0

0
4

0
4

0
4

0.576784
5

0.576784
5

0.432588
5

0.432588
5

0

0
4

0.144196

0.144196

0
4

0

0

0

0
4

0

0

0
4

2.77555756156289e-17
5

0
4

0

0

0

0

0

0
4

0
4

0
4

0.15413

0.15413

0.15413

0.15413

0

0

0

0
4

0
4

0
4

0
6

0
6

0
6

0
6

0
4

0
4

0
4

0

0

0

0

0

0
4

0

0

0
4

0
4

0
4

0

0

0

0

0

0

0
4

0
4

0
4

0

0

0

0

0
4

0

0

0

0
4

0
4

0
4

0

0

0

0

0

0
4

0

0

0

0
4

0
4

0
4

0

0

0

0

0

0

0

0
4

0
4

0
4

0.0161064

0.0161064

0.0161064

0.0161064

0

0
4

0

0

0
4

0
4

0
4

0.0428416

0.0428416

0

0

0
4

0.0428416

0.0428416

0
4

0

0

0
4

0

0

0
4

0
4

0
4

0

0

0

0

0

0

0
4

0
4

0
4

1.15853
7

1.15853
7

1.15853
7

1.15853
7

0

0

0
4

0
4

0
4

0

0

0

0

0

0

0
4

0
4

0
4

0.220279

0.220279

0.220279

0.207321

0.0129576

0
4

0
4

0
4

0
3

0
3

0

0

0
4

0

0

0
4

0
4

0
4

0

0

0

0

0
4

0
4

0
4

0.0770648

0.0770648

0.0770648

0.0770648

0
4

0
4

0
4

0

0

0

0

0
4

0
4

0
4

0

0

0

0

0

0
4

0
4

0
4

0.0148034

0.0148034

0.0148034

0.0148034

0
4

0
4

0
4

0

0

0

0

0

0
4

0
4

0
4

0

0

0

0

0

0
4

0
4

0
4

0

0

0

0

0

0

0

0

0

0

0

0

0
4

0

0

0

0

0

0

0
4

0

0

0

0
4

0

0

0

0
4

0
4

0
4

0

0

0

0

0

0

0
4

0
4

0
4

0.234389

0.234389

0.234389

0

0.234389

0
4

0
4

0
4

3.82119

3.82119

3.82119

3.82119

0
4

0
4

0
4

0.0320617

0.0320617

0.0320617

0.0274815

0.00458025

0

0
4

0
4

0
4

0

0

0

0

0
4

0
4

0
4

0.0370088

0.0370088

0.0370088

0.0370088

0
4

0
4

0
4

0

0

0

0

0

0
4

0

0

0

0
4

0
4

0
4

0

0

0

0

0
4

0

0

0
4

0
4

0

0

0

0
4

0
4

0
4

0

0

0

0

0

0
4

0
4

0
4

0.736881

0.736881

0.736881

0.736881

0
4

0
4

0
4

0.44126
7

0.44126
7

0.44126
7

0.436773
7

0.00448724

0

0

0

1.99493199737333e-17
7

0
4

0
4

0
4

0

0

0

0

0

0
4

0

0

0
4

0
4

0
4

0.20715

0.20715

0.20715

0.20715

0
4

0
4

0
4

0

0

0

0

0
4

0
4

0
4

0

0

0

0

0
4

0
4

0
4

0

0

0

0

0

0
4

0
4

0
4

0

0

0

0

0
4

0
4

0
4

0

0

0

0

0

0
4

0
4

0
4

0

0

0

0

0

0

0
4

0
4

0
4

0

0

0

0

0

0
4

0

0

0
4

0
4

0
4

0

0

0

0

0
4

0

0

0
4

0
4

0
4

0.429904
6

0.429904
6

0.314307

0

0

0

0.0259152

0.288392

0
4

0.115597

0

0

0.115597

0

0
4

0

0

0
4

0
4

0
4

0.20715

0.20715

0.20715

0.20715

0
4

0
4

0
4

0

0

0

0

0
4

0
4

0
4

0

0

0

0

0
4

0

0

0
4

0
4

0
4

0

0

0

0

0

0

0
4

0
4

0
4

0

0

0

0

0
4

0
4

0

0

0

0
4

0
4

0
4

0

0

0

0

0
4

0
4

0
4

0

0

0

0

0

0
4

0

0

0
4

0
4

0
4

0

0

0

0

0
4

0
4

0
4

0

0

0

0

0

0
4

0
4

0
4

0.0770648

0.0770648

0.0770648

0.0770648

0
4

0
4

0
4

2.83454
1

0

0

0

0
4

0
4

1.29776

1.29776

1.29776

0
4

0
4

0.385324

0.385324

0.385324

0
4

0
4

0

0

0

0

0
4

0
4

0.104044

0.104044

0.104044

0
4

0
4

0

0

0

0
4

0
4

0

0

0

0
4

0
4

0

0

0

0
4

0

0

0
4

0
4

0.72098

0.72098

0.72098

0
4

0
4

0

0

0

0

0
4

0
4

0

0

0

0
4

0
4

0.326433
1

0.216294
2

0

0

0

0

0

0.216294

0

0

0

0
4

0.11014

0.11014

0
4

0
4

0
4

81.055
7

0

0

0

0

0
4

0
4

0.129531

0.129531

0.129531

0
4

0
4

0

0

0

0
4

0
4

0.00822417

0.00822417

0.00822417

0
4

0
4

0

0

0

0
4

0
4

0.0770648

0.0770648

0.0770648

0
4

0
4

0

0

0

0
4

0
4

80.8402
7

78.4222
7

78.4222
7

0

0
4

1.697
7

1.697
7

0
4

0

0

0
4

0.72098

0.72098

0
4

0

0

0
4

0

0

0
4

0

0

0
4

0

0

0
4

0

0

0
4

0
4

0
4

1.60832
3

0.857465
3

0.740847
3

0.216294

0

0

0

0

0

0

0

0

0.308259

0

0.216294
3

0

0

0

0

0

0

0

0

0

0

0
2

0

0

0

0

0
3

0
2

0
3

0
2

0
3

0
2

0
4

0

0

0
4

0.116618

0.116618

0
4

0
4

0.123097

0.123097

0.0712667

0.0388728

0.0129576

0
4

0
4

0

0

0

0

0
4

0
4

0.0907031

0.0907031

0.0907031

0
4

0
4

0.103661

0.103661

0.103661

0
4

0
4

0

0

0

0

0
4

0
4

0.356334

0.356334

0.356334

0
4

0

0

0
4

0
4

0

0

0

0
4

0

0

0
4

0
4

0

0

0

0
4

0
4

0.0770648

0.0770648

0.0770648

0
4

0
4

0
4

31.9235
7

0

0

0

0
4

0
4

0

0

0

0
4

0
4

0.181406

0.181406

0.181406

0
4

0
4

0.0129576

0.0129576

0.0129576

0
4

0
4

0.0259152

0.0259152

0.0259152

0
4

0
4

0.00448724

0.00448724

0.00448724

0
4

0
4

0

0

0

0
4

0
4

0

0

0

0
4

0
4

0

0

0

0
4

0
4

0

0

0

0
4

0
4

0

0

0

0
4

0
4

31.6987
7

31.352
7

27.6276
7

0.548296
5

0.0553389

0

0.0453516

0

0

0

0

0

0.0259152

0

0.144196
7

0

0

0

0

0

0

0

0

0

0.269727

0.0194364
6

0

0.0714027

0.0571222

0

0

0

0

0

0

0

0.964331

0

0

0.242209
7

1.08027

0

0

0.200843

1.88737914186277e-15
7

0
4

0.288392

0.288392

0
4

0.0583091

0.0583091

0
4

0

0

0
4

0
4

0
4

8.97163

0.724042
5

0.724042
5

0.691648

0

0

0

0.0194364
5

0
6

0

0

0

0

0

0.0129576

6.41847686111419e-17
5

0
4

0

0

0
4

0
4

1.69735
5

1.69735
5

1.69735
5

0

0

0

0

0
4

0

0

0
4

0
4

0.329317

0.329317

0.329317

0
4

0
4

0

0

0

0
4

0
4

0

0

0

0
4

0
4

6.22092

6.14464
7

0.2109

0

0.0233374

0.00448724

0.00518609

0.00518609

0.0426287

0.346792

0.00673085

0

0.00777913

1.34218

0.00673085

0.00448724

0.0134617

0.271478

2.77467

0.385324

0.693278

0

0

0

1.33226762955019e-15
7

0
4

0.0605777

0.047116

0.00448724

0.00897447

0
4

0.00448724

0.00448724

0
4

0

0

0
4

0.0112181

0.0112181

0
4

6.33174068731535e-16

0
4

0
4

7.38125

0.576784

0.576784

0.288392

0

0

0.288392

0

0

0

0

0

0
4

0

0

0

0
4

0

0

0
4

0
4

0.44906
5

0.44906
5

0.0156497
5

0

0

0

0.433411
7

0

0

0

0

0

0

0

0
4

0
4

0

0

0

0

0
4

0

0

0
4

0
4

0.15413

0.15413

0.15413

0
4

0
4

0

0

0

0
4

0
4

0.149012

0.149012

0.149012

0
4

0
4

0.0770648

0.0770648

0.0770648

0
4

0
4

0.15413

0.15413

0.15413

0
4

0
4

0.220279

0.220279

0.207321

0.0129576

0
4

0
4

0

0

0

0

0
4

0
4

0

0

0

0
4

0
4

0

0

0

0
4

0
4

1.00917
5

0.989048
5

0.948244
5

0.025154

0

0

0

0

0.0156497

1.00613961606655e-16
5

0
4

0.0201232

0.0201232

0
4

0

0

0
4

0
4

0.0107775

0.0107775

0.0107775

0
4

0
4

0.0622331

0.0622331

0.0622331

0
4

0
4

0.0591856

0.0591856

0.0591856

0
4

0
4

0.0100616

0.0100616

0.0100616

0
4

0
4

0

0

0

0
4

0
4

0.308259

0.308259

0.308259

0
4

0
4

0

0

0

0
4

0
4

0.010272

0.010272

0.010272

0
4

0
4

0

0

0

0
4

0
4

0

0

0

0
4

0
4

0.975604
7

0.975604
7

0.281294
7

0.167721

0.249341
7

0.157449
7

0.0220942

0.0791236

0.0029305

0.0156497

0

9.36750677027476e-17
7

0
4

0
4

0
5

0
5

0

0

0

0

0

0

0

0

0
4

0

0

0

0

0

0
4

0
4

1.51406
5

1.51406
5

1.51406

0

0

0

0

0

0
4

0

0

0

0
4

0
4

0.0770648

0.0770648

0.0770648

0
4

0

0

0
4

0
4

1.50406

1.49967

1.48208

0.0102568

0.00732626

0
4

0.00439576

0.00439576

0
4

9.54097911787244e-18

0
4

0.0600453
7

0.0600453
7

0

0.0388957

0.0107775

0.0103722

0

0
4

0
4

0

0

0

0
4

0

0

0
4

0
4

0
4

41.0335

0

0

0

0

0

0

0

0

0

0

0
4

0

0

0
4

0
4

0

0

0

0

0

0
4

0
4

0

0

0

0

0
4

0
4

0

0

0

0

0
4

0

0

0
4

0
4

0

0

0

0

0
4

0
4

0

0

0

0
4

0

0

0
4

0
4

0

0

0

0
4

0
4

0

0

0

0
4

0
4

0.00362217

0.00362217

0.00362217

0
4

0
4

0.0518304

0.0518304

0.0518304

0
4

0

0

0
4

0
4

0.32394

0.32394

0.32394

0
4

0
4

0.796892

0.796892

0

0.796892

0

0
4

0
4

0

0

0

0
4

0
4

0

0

0

0
4

0
4

0

0

0

0
4

0
4

0

0

0

0
4

0
4

0

0

0

0
4

0
4

0

0

0

0
4

0
4

0

0

0

0
4

0
4

0

0

0

0
4

0
4

0

0

0

0
4

0
4

0

0

0

0
4

0
4

0

0

0

0

0
4

0

0

0

0
4

0
4

0.115597

0.115597

0.115597

0
4

0
4

0

0

0

0
4

0
4

0

0

0

0
4

0
4

0

0

0

0
4

0
4

0

0

0

0
4

0
4

0

0

0

0
4

0
4

0.272109

0.272109

0.226758

0.0453516

0
4

0
4

0

0

0

0

0

0
4

0

0

0
4

0
4

0.174927

0.155491

0.0129576

0.142533

0
4

0.0194364

0.0194364

0
4

0

0

0
4

0
4

0.0344106

0.0289773

0.0289773

0
4

0.00543325

0

0.00543325

0
4

0
4

0

0

0

0

0
4

0
4

0

0

0

0

0
4

0

0

0

0
4

0
4

39.2602

38.1069

37.9122

0.0824445

0

0

0

0.00687037

0

0

0

0

0

0

0

0

0

0

0.105346
3

0

0

0

0

0

0

0
4

0.0251914

0.00687037

0

0.0114506

0

0.00687037

0

8.67361737988404e-19

0
4

0.00362217

0.00362217

0
4

0.432588

0.432588

0
4

0.432588

0.432588

0
4

0

0

0
4

0.032394

0.032394

0
4

0

0

0
4

0

0

0
4

0.0647879

0.0388728

0.0129576

0

0.0129576

0

3.46944695195361e-18

0
4

0

0

0
4

0

0

0
4

0.0453516

0.032394

0.0129576

0
4

0

0

0
4

0

0

0
4

0.116796

0.116796

0
4

0

0

0
4

5.21804821573824e-15

0
4

0
4

1.50751
5

0.0583341
6

0.0426287

0

0

0

0

0

0.00448724

0

0.0381415

0
4

0

0

0

0
4

0.0112181

0.00673085

0.00448724

8.67361737988404e-19

0
4

0

0

0
4

0.00448724

0.00448724

0
4

0
4

1.35848

0.269727
5

0.269727
6

0

0

0

0

0

0

0

0
4

1.08875

1.08875

0

0
4

0

0

0
4

0
4

0.0907031

0.0907031

0.0907031

0
4

0
4

0

0

0

0
4

0
4

0

0

0

0
4

0
4

0

0

0

0
4

0
4

0

0

0

0
4

0
4

0
5

0
5

0
6

0

0

0

0

0

0
4

0
4

0

0

0

0

0

0

0

0
4

0
4

0
5

0

0

0

0

0

0
4

0

0

0
4

0

0

0
4

0
4

0

0

0

0
4

0
4

0

0

0

0

0
4

0
4

0

0

0

0
4

0
4

0

0

0

0
4

0
4

0

0

0

0
4

0
4

0
4

0
3

0

0

0

0

0
4

0
4

0

0

0

0
4

0
4

0
3

0

0

0
4

0

0

0

0

0
4

0

0

0
4

0

0

0
4

0

0

0
4

0
4

0
4

37.1543

13.3685

13.2724

10.0214
7

0.0201232

0.0452773

0.0100616

0.00394831

0.0402465

0

0

0.0201232

0.0100616

0.718482
8

1.65563
8

0.512326
1

0.109793

0.0452773

0.0138191

0.0317773

0.0140099

4.2708891978549e-15

0
4

0.064318

0.0503081

0.0100616

0.00394831

0
4

0.0317773

0.0217157

0.0100616

3.46944695195361e-18

0
4

0
4

0.484436

0.484436

0.484436

0

0
4

0
4

0

0

0

0

0

0
4

0

0

0
4

0
4

0

0

0

0

0
4

0

0

0

0
4

0
4

0.432588

0.432588

0.432588

0
4

0
4

0

0

0

0
4

0
4

0

0

0

0

0
4

0

0

0
4

0
4

0

0

0

0
4

0
4

0

0

0

0

0
4

0
4

0.0259152

0.0259152

0.0259152

0
4

0
4

0.00543325

0.00543325

0.00543325

0
4

0

0

0
4

0
4

13.8805

2.19665
7

0.0508181

0.140863

0.00592247

1.37437

0.0942105

0.0140099

0.0240715

0.492388

0

0

0

5.55111512312578e-17
7

0
4

11.6799
1

11.6447
8

0.0100616

0.025154

2.46330733588707e-16
1

0
4

0

0

0
4

0.00394831

0.00394831

0
4

0
4

0.0801856

0.0801856

0.0801856

0
4

0
4

0

0

0

0

0
4

0
4

0.0388728

0.0388728

0.0388728

0
4

0
4

0.0185044

0.0185044

0.0185044

0
4

0
4

0

0

0

0
4

0
4

0

0

0

0
4

0
4

0.0129576

0.0129576

0.0129576

0
4

0
4

0

0

0

0
4

0
4

0

0

0

0
4

0
4

0

0

0

0
4

0
4

1.00952

0.972508

0.347471

0.608589

0.0123363

0.00411208

0
4

0.0328967

0.0287846

0.00411208

8.67361737988404e-19

0
4

0

0

0
4

0.00411208

0.00411208

0
4

0
4

0.0887784

0.0887784

0.0887784

0
4

0
4

0

0

0

0
4

0
4

0.0129576

0.0129576

0.0129576

0
4

0
4

0

0

0

0
4

0
4

0

0

0

0
4

0
4

0

0

0

0
4

0
4

0

0

0

0
4

0
4

0.0129576

0.0129576

0.0129576

0
4

0
4

0

0

0

0
4

0
4

0

0

0

0
4

0
4

0.353161

0.353161

0.345917

0.00362217

0.00362217

0

2.60208521396521e-18

0
4

0

0

0

0
4

0
4

0.37273

0

0

0

0
4

0.36049

0

0.144196

0

0.216294

2.77555756156289e-17

0
4

0.0122405

0

0.0122405

0
4

0
4

0
3

0
3

0

0

0

0

0

0

0

0

0
4

0

0

0
4

0

0

0
4

0
4

0
3

0
3

0

0

0

0

0

0
4

0
4

0
6

0

0

0

0

0
4

0

0

0
4

0

0

0
4

0
4

0

0

0

0

0

0
4

0
4

6.95635

4.95966

1.58615
7

0

0.0199219

0

0.20715

0

0

0

0.0100616

0

0

0

0.20715

0.288392

0

0

0

0

0.239715

0

0

0.144196

0.11772

0

0

0

0.0102802

0.129576

0.0156497

0

0

0

0

0

0

0

0.0123363

0

0

0.0469492

0

0.0591856

0

0

1.0796
7

0

0

0

0.0259152

0

0

0

0

0

0

0

0

0

0

0

0

0

0

0.36049

0

0

0.216294

0

0.182919

0

0
4

0

0

0

0

0
4

0

0

0
4

0

0

0
4

0

0

0

0
4

0

0

0

0
4

0

0

0
4

0

0

0
4

0

0

0
4

0

0

0
4

0

0

0
4

0

0

0
4

0.216294
6

0.216294
6

0

0

0
4

0

0

0
4

0

0

0
4

0.0259152

0.0259152

0
4

0

0

0
4

0

0

0
4

0.504686

0

0

0.504686

0

0
4

0.576784

0.432588

0

0.144196

2.77555756156289e-17

0
4

0
3

0

0

0

0
4

0.0253552

0.0253552

0

0
4

0

0

0

0
4

0

0

0

0
4

0.647653

0.647653

0
4

2.22044604925031e-15

0
4

8.88178419700125e-16

0
4

3.05023
7

0
6

0
6

0
6

0

0
4

0
4

0
6

0
6

0
6

0
4

0
4

0.157086

0.157086

0.157086

0
4

0
4

0

0

0

0
4

0
4

0

0

0

0
4

0
4

0

0

0

0
4

0
4

2.89315
7

2.89315
7

2.89315
7

0
4

0
4

4.44089209850063e-16
7

0
4

2.67674327147915e-10

0
4

292.219

0

0

0

0

0

0

0
4

0
4

0
4

208.248
3

0.582785

0.582785

0.569828

0

0

0

0

0

0.0129576

0

0

0

0
2

0

0

0

0

0

0

0

0
4

0

0

0
4

0

0

0
4

0
4

183.27
3

66.9518

0

10.8546

1.30224

0

0

0

0

0.0583091

0

0.00458025

0

0.0887784

0

1.04834

0

0

0.0259152

0

0

0

0

0

0.149012

0.0194364

4.72785

0

0

0.0194364

0

0

0

0

1.03013

0

0

0.436829

0

0

0.178464

0.032394

0

0.0583091

0

0

0

0

0.712667

0

0.0712667

0

0

0

0.0259152

0

0

0.0518304

0

0
6

0

0.032394

0

0

0

0

0.0129576

0

0

0

0.753132
7

0

0

0

0.0156497

0

0

0

0

0

0

0.397046

0.0194364

0

0.0453516

0

0

0

0

0

0.20715

0

0

0.032394

0

0

0

0

0

0.0129576

0

0.0453516

0

3.02811

0

0

0.0129576

0.0129576

0

0

0

0

0

0.0129576

1.09666

0.0583091
5

0.0129576

0

0

0.0259152

0.0591622

0

0

0

0

0.0129576

0

0

0

0.207321

0.0391243

0

0

0.0453516

0

0

0

0
2

0.0129576

0

0

0

0

0

0.0194364

0

0

0

0.310982

0

0.0259152

0

0

0

0.0777455

0.0129576

0

0.0674192

0

5.64951

0

0

0.00799092

0

0

0

0.0129576

0

0

0.0129576

0.118371

0.00777913

0.0129576

0.0591856

0

0.0129576

0

0

0

0

0

0.0129576
6

0.0156497

0

0

0

0

0

0

0

0

0.278588

0.146568

0

0.0259152

0

0

0

0.0129576

0

0.0129576

0.0129576

0

0.13625

0

0

0.147964

0

0

0

0

0

0

0

0

0.0194364

0.0129576

0.0591856

0

0

0.0194364

0.147964

0

0.0259152

0.237064

4.11418

0.218136

0

0.0194364

0

0

0

0

0

0

0

0

0

0

0.0129576

0

0.0129576

0.0129576

0

0.0129576

0

0.144196

0.0194364

0
2

0

0.0129576

0

0

0

0

0

0.0129576

0.0129576

0

0

0

0.0259152

0

0.0777455

0

0.0907031

0

0.032394

0

0

0.139623

0

0

0

0

0

0

0

0

0

0.0091605

0

0.207321

0

0

0

0

0.032394

0

0.0194364

0

0

0

0

0

0.0129576

0

0

0

0

0

0.0129576

0.0907031

0.0129576

0

0.0129576

0

0

0

7.14958

0.479431

0.0777455

0
6

0

0.0938951

0
2

0

0.17668

0.147964
7

0.0841393

2.42707

0

0

0

0

0.58891

0

0

0.2138

0

0.0129576

0.321883

0.103661

0

3.4607

0.18321

0

0.142533

0.00297885

0.0453516

1.01717

0

3.28261

0

0

0

0.0372909

0

0

0

0

0.0480437

0

5.28607

0

0

0.0129576

0

0.155491

0.0518304

0

0

0.0194364

0

0.984948

0.0591856

0

0

0

0

0

0

0

0

0.0591856

9.51183576347603e-14

0
4

75.0944
4

0
3

6.93439
4

65.1848
3

1.41901
6

0.187797
4

0

0

0

0

0

0

0

0

0

0

0
4

0

0

0

0

0

0

0

0

0

0

0
4

0

0

0

0

0

0

0

0

0

0.298328

0
4

0

0

0

0

0

0

0

0

0

0

0
4

0

0

0

0

0

0

0

0

0

0

0
2

0

0

0

0

0

0

0

0

0.0649673

0

0
3

0

0

0

0

0

0

0

0.032394

0

0

0
4

0

0

0

0

0

0

0

0

0

0

0.0454056

0

0

0

0

0

0

0

0

0

0

0
2

0

0

0

0

0

0

0

0

0

0

0
3

0
4

0

0

0

0

0

0

0

0

0

0

0

0

0

0

0

0

0

0

0

0

0

0

0

0

0

0

0

0

0

0

0

0

0
4

0

0

0

0

0

0

0

0

0

0.0129576

0

0

0

0

0

0

0

0

0

0

0

0
3

0

0

0

0

0

0

0

0

0

0

0

0

0

0

0

0

0

0.00518609

0

0

0

0

0

0

0

0

0

0

0

0

0.00394831

0

0
4

0

0

0

0

0

0

0

0

0

0

0
4

0

0

0

0

0

0

0

0

0

0

0
4

0

0

0

0

0

0

0

0

0

0

0

0

0

0

0

0

0

0

0

0

0

0

0

0

0.00592247

0

0

0

0

0

0

0

0

0
4

0

0

0

0

0

0

0

0

0

0

0
3

0

0

0

0

0

0

0

0

0

0

0

0

0

0

0

0

0

0

0

0

0

0

0

0

0

0

0

0

0

0

0

0

0
4

0

0

0

0

0

0

0

0

0

0

0

0

0

0

0

0

0

0

0

0

0

0

0

0

0

0

0

0

0

0

0

0

0
4

0

0

0

0

0

0

0

0

0

0

0

0

0

0

0

0

0

0

0

0

0

0

0

0

0

0

0.00319998

0

0

0

0

0

0

0
2

0

0

0.00987078

0

0

0

0

0

0

0

0

0

0

0

0
4

0

0

0

0
3

0
4

0

0

0

0
4

0

0
4

0

0

0

0

0
4

0

0

0

0

0

0

0

0

0

0

0
4

0

0.886245

0

0

0

0

0

0

0

0

0
3

0

0

0

0

0

0

0

0

0

0

0
3

0

0

0

0

0

0

0

0

0

0

5.55111512312578e-16
4

0
4

10.3083
3

0
3

0.109523
3

0.00322127
3

0
3

0.15413
2

0.666791
3

0.064665
2

3.22438
1

0

0

0.115597

0

0

0

0

0

0.288392

0

3.95033

0.00408015

0

0

0

0

0

0

0

0

0

0.569998

0.144196

0

0

0

0

0.950379
8

0

0

0

0

0.062599

2.3037127760972e-15
3

0
4

9.24415

1.16473
7

0
6

0

0

0

0

0

0

0.192662

0

0.0532163

0.15413

1.02983
7

0

0

0

0

0

0

0

0.0129576

0.00799092

0

0.0348438
7

0

0

0.0591856

0

0

0

0

0

0

0

0
5

0

0

0

0

0

0

0

0

0

0

0
6

0

0

0

0

0

0

0

0

0

0

0.171576
7

0

0

0

0

0

0

0

0

0

0

0

0

0

0

0

0

0

0

0

0

0

0

0

0.0133182

0

0

0

0

0.0770648

0

3.18582

0.0633123
8

0

0

0

0.0259152

0

0.336897

0

0

0.00967569

1.0855

0

0.00532728

0

0

0

0.0106546

0

0

0.00408015

0.15413

0
3

0

0

0.0346273

0

0

0.0159818

0

0

0

0

1.18189
7

0

0

0.0239728

0.0091605

0

0

0

0

0

0

0
3

0

0

0

0

0

0

0

0.0106546

0

0

0.0533863
7

0

0

0.00687037

0

0

0

0

0

0

0

0.0506741
1

0

0

0

0

0

0

0

0.00411208

0

0

0

0

0

0

0

0

0

0

0

0

0

0
4

1.09824
2

0

0

0

0

0

0.032394

0

0

0.144196

0

0

0

0

0

0

0

0

0

0

0

0

0

0

0

0

0

0

0

0.285067

0.0842243

0

0

0

0.317461

0

0

0

0

0

0

0

0

0

0

0

0

0

0

0

0

0

0

0

0

0

0

0

0

0

0

0

0.0907031

0

0

0

0

0

0.144196

0

0

0

0

0

0

0

0

0

0

0

0

0

0

0

0

0

0

0
4

4.97066
3

4.4326
3

0.0194364

0

0

0

0.0259152

0

0

0.0591856

0.00592134

0

0.0647879
4

0

0

0.0971819

0

0

0

0.0129576

0

0

0

0.0194364
4

0.032394

0

0

0

0

0

0.0194364
4

0.0194364
3

0.0712667
4

0.0907031
3

0
4

0

0
4

0.0259152
3

0.0259152

0

0

0

0
4

0

0

0

0

0

0

0

0
4

0

0

0
4

0

0

0
4

0

0

0
4

0

0

0
4

0

0

0

0
4

0

0

0
4

0

0

0

0
4

0.0744307

0.0744307

0
4

0

0

0
4

0

0

0

0
4

0.0129576
3

0
4

0.0129576
3

0
3

0
3

0

0

0

0

0
4

0

0

0
4

0

0

0

0
4

0

0

0
4

0

0

0

0
4

0

0

0
4

0

0

0
4

0

0

0

0
4

0.0029305

0.0029305

0
4

0

0

0
4

0

0

0
4

0
3

0
3

0

0

0

0

0

0

0

0
4

0.447037

0.447037

0
4

0

0

0
4

0

0

0
4

0

0

0
4

0

0

0
4

0

0

0
4

0

0

0
4

0

0

0
4

0

0

0
4

0

0

0
4

0
2

0
2

0

0

0

0

0

0

0
4

0

0

0
4

0

0

0
4

0

0

0
4

0

0

0
4

0

0

0
4

0.0129576

0.0129576

0
4

0

0

0
4

0

0

0
4

0.00732626

0.00732626

0
4

0

0

0
4

0

0

0

0

0

0

0

0

0
4

0

0

0
4

0

0

0
4

0

0

0
4

0

0

0
4

0

0

0
4

0

0

0
4

0

0

0
4

0

0

0
4

0.00319998

0.00319998

0
4

0

0

0
4

0
4

0
4

0

0

0

0
4

0

0

0
4

0

0

0
4

0

0

0
4

0

0

0
4

0

0

0
4

0

0

0
4

0

0

0
4

0

0

0
4

0

0

0
4

0

0

0
4

0.568052

0

0.336858

0

0.231194

0
4

0

0

0
4

0

0

0
4

0

0

0
4

0

0

0
4

0

0

0
4

0

0

0
4

0

0

0
4

0

0

0
4

0

0

0
4

0

0

0
4

0
3

0
3

0

0

0

0
4

0

0

0
4

0

0

0
4

0

0

0
4

0

0

0
4

0

0

0
4

0

0

0
4

0

0

0
4

0

0

0
4

0

0

0
4

0

0

0
4

0
4

0
4

0

0

0

0
4

0

0

0
4

0

0

0
4

0

0

0
4

0

0

0
4

0

0

0
4

0

0

0
4

0

0

0
4

0

0

0
4

0

0

0
4

0

0

0
4

0

0

0

0

0

0

0
4

0

0

0
4

0

0

0
4

0

0

0
4

0

0

0
4

0

0

0
4

0

0

0
4

0.0770648

0.0770648

0
4

0

0

0
4

0

0

0
4

0

0

0
4

0.512291
3

0.065107
3

0

0

0

0

0

0

0

0

0

0

0.349683
4

0

0

0

0

0

0

0

0.0780646
4

0
4

0.0194364
3

0

0

0

0

0
4

0

0

0

0

0

0
4

0

0

0
4

0

0

0
4

0

0

0
4

0

0

0
4

0

0

0
4

0.0547741
3

0.0547741
3

0

0

0
4

0
4

0

0

0

0

0

0
4

0
2

0

0

0

0

0

0

0

0
4

0

0

0

0

0

0

0

0

0

0
4

0

0

0

0
4

0
3

0

0

0

0

0

0

0
4

0
3

0

0

0

0

0

0

0

0
4

0

0

0

0
4

0
2

0

0

0

0

0

0
4

6.02095
3

5.92376
3

0

0

0

0

0

0

0

0

0

0

0.0842243
4

0

0

0
4

0.0129576

0

0

0

0

4.21537804662364e-16
3

0
4

0
3

0

0

0

0

0

0
4

0
4

0

0

0

0

0

0
4

0

0

0
4

0
4

0
4

0

0

0

0
4

0

0

0

0
4

0

0

0

0

0
4

0.144196
4

0.144196
4

0

0
4

0
5

0
5

0

0
4

0
2

0

0

0

0

0

0
4

0.500921
4

0.500921
4

0
4

0.538988
5

0.349387
5

0

0

0

0.144196

0

0

0

0

0

0

0.0157932

0.0177674

0

0

0.00394831

0

0

0.00789662

0

0

0

0

0

0

0
4

0
4

0

0

0
4

0

0

0

0

0
4

0

0

0
4

0

0

0

0

0
4

0
4

0

0

0
4

0

0

0

0

0

0
4

0

0

0

0
4

0

0

0

0

0

0
4

0

0

0
4

0

0

0
4

0.100623
6

0

0

0

0

0

0

0

0

0

0

0

0.00789662

0

0

0

0

0

0

0

0

0

0

0

0

0

0

0.0927268

0

0

0

0

0

0
4

0
2

0

0

0
4

0

0

0

0

0
4

0

0

0

0
4

0

0

0

0

0
4

0

0

0

0

0
4

0

0

0

0

0
4

0.078622

0.078622

0

0
4

0

0

0
4

0.0984541

0.0984541

0

0
4

0

0

0

0

0
4

3.16222
2

3.16222
2

0
2

0

0

0

0

0

0

0
4

0

0

0
4

0

0

0

0
4

0

0

0
4

0.032394

0.032394

0

0
4

0

0

0
4

0.200843

0.200843

0
4

0.291546

0.291546

0
4

0

0

0

0

0

0
4

0

0

0

0
4

0

0

0
4

0
3

0
4

0

0

0

0

0

0

0

0

0

0

0

0

0

0

0

0

0

0

0

0

0

0

0

0

0

0

0

0

0
4

0

0

0
4

0

0

0
4

0

0

0

0
4

0

0

0

0
4

0

0

0

0
4

0

0

0

0
4

0

0

0

0
4

0

0

0

0
4

0

0

0

0
4

0

0

0

0
4

2.62124
6

2.62124
6

0

0

0

0

0

0

0

0

0

0

0

0
4

0

0

0

0
4

0

0

0

0
4

0

0

0
4

0

0

0

0
4

0

0

0
4

0

0

0
4

0

0

0

0
4

0

0

0

0
4

0

0

0
4

0

0

0
4

0
4

0
4

0
4

0
4

0

0

0

0

0

0

0
4

0

0

0
4

0

0

0
4

0

0

0
4

0

0

0
4

0

0

0

0
4

0

0

0

0
4

0

0

0
4

0

0

0

0
4

0

0

0
4

0.0129576

0.0129576

0

0
4

5.62518781554999e-14
3

0
4

0.144196
2

0.144196
2

0.144196
2

0

0

0

0

0

0

0

0

0

0

0

0

0

0

0

0

0

0

0

0

0

0
2

0

0

0

0

0

0

0

0

0

0

0

0

0

0

0

0

0

0

0

0

0

0

0

0

0

0

0

0

0

0

0

0

0

0

0

0

0

0

0

0

0

0

0

0

0

0

0

0

0

0

0

0

0
4

0
2

0

0

0

0

0

0

0

0

0

0

0

0
4

0

0

0

0

0

0

0

0

0
4

0

0

0

0

0

0
4

0

0

0
4

0
4

0.294519

0.284844

0.214606
7

0.0145135

0

0.0453516

0.0103722

0

3.98986399474666e-17

0
4

0.00967569

0.00967569

0
4

0

0

0
4

1.56125112837913e-17

0
4

0

0

0

0
4

0

0

0
4

0
4

0

0

0

0
4

0

0

0
4

0
4

0

0

0

0
4

0

0

0
4

0
4

0.0518304

0.0518304

0.0388728

0.0129576

0
4

0
4

0

0

0

0
4

0

0

0
4

0
4

0

0

0

0
4

0
4

0

0

0

0
4

0
4

0

0

0

0
4

0
4

0

0

0

0
4

0

0

0
4

0
4

0

0

0

0
4

0
4

1.01069

1.01069

0.881116

0.0518304

0.0388728

0.0129576

0.0259152

0

0
4

0
4

0

0

0

0

0
4

0
4

0

0

0

0
4

0

0

0
4

0
4

0.0156497

0.0156497

0.0156497

0

0
4

0
4

0

0

0

0
4

0
4

0

0

0

0

0
4

0
4

0

0

0

0
4

0
4

0

0

0

0
4

0
4

0

0

0

0
4

0
4

0

0

0

0
4

0
4

0

0

0

0

0
4

0
4

1.1793
7

1.1793
7

0.988727

0.0674801

0.0583091

0.0129576

0.0129576

0.0388728

0
4

0
4

0

0

0

0

0
4

0
4

0

0

0

0
4

0
4

0

0

0

0
4

0
4

0

0

0

0
4

0
4

0

0

0

0
4

0
4

0

0

0

0
4

0
4

0

0

0

0
4

0
4

0

0

0

0
4

0
4

0

0

0

0
4

0
4

0

0

0

0
4

0
4

0.0127999
2

0.0127999
2

0.00319998
2

0

0.00959993

0

0

0

0

0
4

0
4

0

0

0

0
4

0
4

0.15413

0.15413

0.15413

0
4

0
4

0.192662

0.192662

0.192662

0
4

0
4

0

0

0

0
4

0
4

0

0

0

0
4

0
4

0

0

0

0
4

0
4

0

0

0

0
4

0
4

0

0

0

0
4

0
4

0

0

0

0
4

0
4

0.0156497

0.0156497

0.0156497

0
4

0
4

0.170423

0
5

0

0

0

0

0

0

0

0
4

0.170423

0.0887784

0

0.0770648

0.00458025

0

1.73472347597681e-17

0
4

0

0

0
4

0
4

0

0

0

0
4

0
4

0

0

0

0
4

0
4

0

0

0

0
4

0
4

0.00687037

0.00687037

0.00687037

0
4

0
4

0

0

0

0
4

0
4

0

0

0

0
4

0
4

0

0

0

0
4

0
4

0

0

0

0
4

0
4

0

0

0

0
4

0
4

0

0

0

0
4

0
4

0

0

0

0

0

0

0

0

0
4

0
4

0.0770648

0.0770648

0.0770648

0
4

0
4

0

0

0

0
4

0
4

0

0

0

0
4

0
4

0

0

0

0
4

0
4

0

0

0

0
4

0
4

0

0

0

0
4

0
4

0

0

0

0
4

0
4

0

0

0

0
4

0
4

0

0

0

0
4

0
4

0

0

0

0
4

0
4

0.37506

0.37506

0.264387

0.0209561

0.0426182

0.0289471

0.0181513

3.46944695195361e-17

0
4

0
4

0.00458025

0.00458025

0.00458025

0
4

0
4

0

0

0

0
4

0
4

0

0

0

0
4

0
4

0

0

0

0
4

0
4

0

0

0

0
4

0
4

0

0

0

0
4

0
4

0

0

0

0
4

0
4

0

0

0

0
4

0
4

0

0

0

0
4

0
4

0

0

0

0
4

0
4

0

0

0

0

0

0

0

0

0

0

0

0

0
4

0
4

0

0

0

0
4

0
4

0

0

0

0
4

0
4

0

0

0

0
4

0
4

0

0

0

0
4

0
4

0

0

0

0
4

0
4

0

0

0

0
4

0
4

0

0

0

0
4

0
4

0

0

0

0
4

0
4

0

0

0

0
4

0
4

0

0

0

0
4

0
4

0.144196

0.144196

0.144196

0

0

0

0
4

0

0

0
4

0
4

0

0

0

0
4

0
4

0

0

0

0
4

0
4

0

0

0

0
4

0
4

0.171759

0.171759

0.171759

0
4

0
4

0

0

0

0
4

0
4

0.118371

0.118371

0.118371

0
4

0
4

0

0

0

0
4

0
4

0

0

0

0
4

0
4

0

0

0

0
4

0
4

0

0

0

0
4

0
4

0
6

0
6

0
6

0

0

0

0

0

0
4

0
4

0

0

0

0
4

0
4

0

0

0

0
4

0
4

0

0

0

0
4

0
4

0
2

0
2

0
2

0

0

0

0

0

0

0

0

0

0

0
2

0

0

0

0

0

0

0

0

0

0

0

0

0

0

0

0

0

0

0

0

0

0
2

0

0

0

0

0

0

0

0

0

0

0

0

0

0

0

0

0

0

0

0

0

0

0

0

0

0

0

0

0

0

0

0

0

0

0

0

0

0

0

0

0

0

0

0
2

0

0

0

0

0

0

0

0

0

0

0
4

0
2

0
2

0

0

0

0

0

0

0

0

0

0
4

0
2

0

0
2

0

0

0

0
4

0

0

0

0

0

0
4

0

0

0

0

0
4

0

0

0

0

0
4

0

0

0

0
4

0
4

0
2

0
2

0
2

0

0

0

0
4

0

0

0

0
4

0

0

0
4

0
4

0.397083

0.397083

0.35166

0.0263745

0.0161178

0.0029305

0
4

0
4

0
5

0
5

0
5

0

0

0
4

0
4

0.0592247

0.0414573

0.00987078

0.00394831

0.00592247

0.00592247

0.00592247

0.00987078

0

0
4

0.0138191

0.00592247

0.00789662

0
4

0.00394831

0.00394831

0
4

2.60208521396521e-18

0
4

0

0

0

0

0

0

0

0
4

0

0

0
4

0
4

0

0

0

0

0

0

0
4

0

0

0

0
4

0

0

0
4

0

0

0
4

0
4

0

0

0

0

0

0

0
4

0

0

0
4

0

0

0
4

0

0

0
4

0
4

0.153872
5

0.153872
5

0.144196

0

0.00967569

0

0

0

0

0

0
4

0
4

0
4

0

0

0

0

0
4

0

0

0
4

0

0

0
4

0
4

0.0591856
7

0.0591856
7

0.0591856
7

0

0

0
4

0
4

0
2

0
2

0
2

0

0

0

0

0

0

0

0

0
2

0

0

0

0

0

0

0

0
4

0
2

0
2

0

0

0

0
4

0

0

0

0

0

0

0

0

0

0

0
4

0

0

0

0
4

0
4

0.0518304

0.0518304

0

0.0388728

0

0.0129576

0
4

0
4

0
6

0
6

0
6

0
4

0
4

0.153599
2

0

0

0

0

0

0
4

0.153599

0.148799

0.00479996

0
4

0

0

0
4

0
4

1.12082
7

1.12082
7

1.06899
7

0.0518304

0
4

0
4

4.39269
7

4.39269
7

3.27525

1.11744

0
4

0
4

1.08196

1.05604

1.03013

0.0129576

0.0129576

1.49186218934005e-16

0
4

0.0259152

0.0259152

0
4

2.77555756156289e-17

0
4

0.443971

0.0205135

0.00732626

0.00439576

0.0029305

0.0029305

0.0029305

2.60208521396521e-18

0
4

0.417597

0.417597

0
4

0.00586101

0.00586101

0
4

6.07153216591882e-18

0
4

0
6

0
6

0
6

0

0
4

0
4

0

0

0

0

0
4

0
4

0.216294

0.216294

0.216294

0
4

0

0

0
4

0

0

0
4

0
4

0.288392
2

0.288392
2

0.288392
2

0

0

0

0

0

0

0

0

0

0

0

0

0

0

0

0

0

0

0

0

0

0

0
4

0

0

0
4

0
2

0

0

0

0

0

0

0
4

0

0

0

0

0

0
4

0

0

0

0

0
4

0

0

0
4

0

0

0

0
4

0

0

0

0
4

0

0

0
4

0

0

0
4

0
4

0

0

0

0

0
4

0

0

0

0
4

0
4

0

0

0

0

0
4

0

0

0
4

0
4

0

0

0

0

0

0

0

0
4

0

0

0

0
4

0
4

0.252605
6

0.252605
6

0.252605
6

0
4

0
4

0.693583

0.693583

0.693583

0
4

0
4

0

0

0

0

0
4

0
4

0.719146

0.693231

0.492388

0.200843

0
4

0.0259152

0.0129576

0.0129576

0
4

0
4

0.00789662

0.00789662

0.00789662

0

0

0
4

0
4

0

0

0

0

0

0

0
4

0

0

0
4

0
4

0

0

0

0
4

0
4

0

0

0

0

0

0

0

0

0

0

0

0

0

0

0

0

0

0

0
4

0

0

0
4

0

0

0

0
4

0
4

0

0

0

0

0

0
4

0

0

0
4

0

0

0
4

0
4

0

0

0

0
4

0
4

0.144196

0.144196

0.144196

0

0
4

0
4

0

0

0

0
4

0

0

0
4

0
4

0

0

0

0

0
4

0

0

0
4

0
4

0

0

0

0

0
4

0
4

0

0

0

0

0

0
4

0

0

0
4

0

0

0
4

0
4

0

0

0

0

0
4

0

0

0
4

0

0

0
4

0

0

0
4

0
4

0.191171

0.191171

0.0755741

0.115597

1.38777878078145e-17

0
4

0
4

0

0

0

0
4

0
4

0.143999
2

0.0879993
2

0
2

0.0879993
2

0

0

0

0

0

0

0

0
4

0.0559996

0.0383997

0.0175999

3.46944695195361e-18

0
4

1.38777878078145e-17
2

0
4

0

0

0

0

0
4

0

0

0
4

0

0

0
4

0

0

0
4

0
4

0.0280549
7

0.0280549

0.0234746

0.00458025

0
4

0

0

0
4

0
4

0

0

0

0
4

0
4

0

0

0

0
4

0

0

0

0
4

0

0

0
4

0
4

0.151148

0.151148

0.141988

0.0091605

6.93889390390723e-18

0
4

0
4

0

0

0

0

0
4

0

0

0
4

0
4

0.153006

0.153006

0.153006

0

0

0
4

0
4

0

0

0

0
4

0

0

0
4

0
4

0

0

0

0
4

0
4

0

0

0

0

0
4

0
4

0
2

0
2

0
2

0

0

0

0

0

0
4

0
2

0

0

0

0

0

0

0

0
4

0
2

0

0

0

0

0

0
4

0

0

0

0
4

0

0

0

0
4

0

0

0
4

0

0

0
4

0

0

0
4

0

0

0
4

0
4

0

0

0

0
4

0
4

0.216294

0.216294

0.216294

0
4

0

0

0
4

0
4

0

0

0

0

0

0
4

0
4

0

0

0

0
4

0
4

0.00448724

0.00448724

0

0.00448724

0

0
4

0
4

0

0

0

0

0

0
4

0
4

0

0

0

0
4

0

0

0
4

0
4

0

0

0

0

0
4

0

0

0
4

0
4

0.0583091

0.0583091

0.0583091

0
4

0
4

0

0

0

0
4

0

0

0
4

0
4

0

0

0

0

0

0

0

0

0

0

0
4

0
4

0.144196

0.144196

0.144196

0
4

0
4

0

0

0

0
4

0
4

0.00532728

0.00532728

0

0.00532728

0

0
4

0
4

0

0

0

0

0
4

0
4

0.216294

0.216294

0.216294

0
4

0
4

0

0

0

0

0
4

0
4

0

0

0

0
4

0

0

0
4

0
4

0.00458025

0.00458025

0.00458025

0

0
4

0
4

0

0

0

0
4

0
4

0

0

0

0
4

0

0

0
4

0
4

1.82702

1.47717

1.24393

0.0647879

0.0583091

0.0388728

0.0129576

0.0259152

0.0194364

0.0129576

2.51534904016637e-16

0
4

0.32394

0.32394

0
4

0.0259152

0.0129576

0.0129576

0
4

1.49186218934005e-16

0
4

0

0

0

0
4

0

0

0
4

0
4

0.20715

0.20715

0.20715

0
4

0
4

0

0

0

0

0
4

0
4

0

0

0

0

0
4

0
4

0

0

0

0
4

0
4

0

0

0

0
4

0

0

0
4

0
4

0

0

0

0
4

0

0

0
4

0
4

0

0

0

0

0
4

0
4

0

0

0

0

0
4

0
4

0

0

0

0
4

0

0

0
4

0
4

6.66767

4.03276

4.02757

0

0

0

0.00518609

0

5.15212872365112e-16

0
4

0.738565
1

0.738565
1

0
4

1.8181

1.8181

0
4

0

0

0

0
4

0

0

0

0
4

0.0782487

0.0782487

0

0
4

0

0

0
4

5.82867087928207e-16

0
4

1.69642078162724e-13
3

0
4

0
4

0
4

0

0

0

0
4

0

0

0
4

0

0

0
4

0

0

0
4

0
4

0

0

0

0
4

0
4

0

0

0

0

0
4

0
4

0

0

0

0
4

0
4

0

0

0

0
4

0
4

0
4

51.5603
5

0.504686
4

0.504686
4

0.216294
4

0

0

0

0

0

0

0

0

0

0

0

0

0

0

0

0

0

0

0

0

0

0

0

0

0

0

0

0

0

0

0

0

0

0

0

0

0

0

0

0

0

0

0

0

0

0

0
4

0

0

0

0
4

0

0

0

0

0

0

0

0

0

0

0

0
4

0

0

0

0

0

0

0

0

0

0

0
4

0

0

0

0

0

0

0.288392

0

0

0

0
4

0

0

0

0

0

0

0

0

0

0

0

0

0

0

0

0

0

0

0

0

0

0
4

0

0

0

0

0

0

0

0

0

0

0
4

0

0

0

0

0

0

0

0

0

0

0
4

0

0

0

0

0

0

0

0

0

0

0
4

0
4

50.6066
5

5.26708

1.13506

0.00518609
2

1.25166

0
4

0

0
4

0.0493593

0

0

0

0

0

0

0.357526
7

0

0

0

0

0

0

0

0

0

0

0
3

0

0

0

0

0

0

0

0

0

0

0
4

0.00448724

0

0

0

0

0.144196

0

0

0

0

0

0

0

0

0

0

0

0

0

0

0

1.79578

0.0207444

0

0

0

0

0.177557

0

0

0.266335

0

0

0

0

0

0

0

0

0

0

0.0591856

0

0

0

0

0

0

0

0

0

0

0

0

0

0

0

0

2.63677968348475e-16

0
4

0

0

0

0

0

0
4

0

0

0
4

44.9562
5

3.05806
5

0
3

23.8097
3

2.68902
4

14.3426
5

0.026521
4

0

0

0

0

0

0

0

0

0

0

0

0

0

0

0

0

0

0

0

0

0

0

0

0

0

0

0

0

0

0

0

0

0

0

0

0

0

0

0

0

0

0

0

0

0

0

0

0

0

0

0

0

0

0

0

0

0
4

0

0

0

0

0

0

0

0

0

0

0

0

0

0

0

0

0

0

0

0

0

0

0

0

0

0

0

0

0

0

0

0

0

0

0

0

0

0

0

0

0

0

0

0

0

0

0

0

0

0

0

0

0

0

0.00408015
4

0

0

0

0

0

0

0

0
3

0

0
4

0

0
4

0

0

0
4

0

0.144196
4

0

0

0
4

0

0

0

0

0
4

0

0

0

0

0

0

0

0

0

0

0

0

0

0

0

0

0

0

0

0

0

0

0

0

0

0

0

0

0

0

0

0

0

0

0

0
4

0

0

0

0

0

0

0

0

0

0

0
3

0

0

0

0

0

0

0

0

0

0

0
4

0

0

0

0

0

0

0

0

0

0

0
4

0

0

0

0

0

0

0

0

0

0

0.00816031
4

0

0

0

0

0

0

0

0

0

0

0

0
4

0

0

0

0

0

0

0

0

0

0

0

0

0

0

0

0

0

0

0

0

0

0
4

0

0

0

0

0

0

0

0

0

0

0

0

0

0

0

0

0

0

0

0

0

0
4

0

0

0

0

0

0

0

0

0

0

0
4

0

0

0

0

0

0

0

0

0

0

0
4

0

0

0

0

0

0

0

0

0

0

0
4

0

0

0

0

0

0

0

0

0

0

0
4

0

0

0

0

0

0

0

0

0

0

0

0
4

0

0

0

0

0

0

0

0

0

0

0
4

0

0

0

0

0

0

0

0

0

0

0

0

0

0

0

0

0

0

0

0

0

0

0

0

0

0

0

0

0

0

0

0

0
4

0

0

0

0

0

0

0

0

0

0

0

0

0

0

0

0

0

0

0

0

0

0

0.115597

0

0

0

0

0

0

0

0

0

0
4

0

0

0

0

0

0

0

0

0

0

0

0

0

0

0

0

0

0

0

0

0

0

0

0

0

0

0

0

0

0

0

0

0.648882
6

0
3

0

0

0

0

0

0

0

0

0

0

0

0

0

0

0

0

0

0

0

0

0

0
3

0

0

0

0

0

0

0

0

0

0

0
4

0

0

0

0

0

0

0

0

0

0

0

0

0

0

0

0

0

0

0

0

0

0
4

0

0

0

0

0

0

0

0

0

0

0

0

0

0

0

0

0

0

0

0

0

0
4

0

0

0

0

0

0

0

0

0

0

0
4

0

0

0

0

0

0

0

0

0

0

0

0

0

0

0

0

0

0

0

0

0

0

0

0

0

0

0

0

0

0

0

0

0

0

0

0

0

0

0

0

0

0

0

0

0
4

0

0

0

0

0

0

0

0

0

0

0
4

0

0

0

0

0

0

0

0

0

0

0

0

0

0

0

0

0

0

0

0

0

0
4

0

0

0

0

0

0

0

0

0

0

0
3

0

0

0

0

0

0

0

0

0

0

0
3

0

0

0

0

0

0

0

0

0

0

0

0

0

0

0

0

0

0

0

0

0

0
4

0

0

0

0

0

0

0

0

0

0

0
4

0

0

0

0

0

0

0

0

0

0

0

0

0

0

0

0

0

0

0

0

0

0

0

0

0

0

0

0

0

0

0

0

0

0
4

0

0

0

0

0

0

0

0

0

0

0
4

0

0

0

0

0

0

0

0

0

0

0

0

0

0

0

0

0

0

0

0

0

0

0

0

0

0

0

0

0

0

0

0

0
4

0

0

0

0

0

0

0

0

0

0

0

0

0

0

0

0

0

0

0

0

0

0

0

0

0

0

0

0

0

0

0

0

0
4

0
4

0

0

0

0

0

0

0

0

0

0

0

0

0

0

0

0

0

0

0

0

0

0
4

0

0

0

0

0

0

0

0

0

0

0

0

0

0

0

0

0

0

0

0

0

0

0

0

0

0

0

0

0

0

0

0

0
3

0

0

0

0

0

0

0

0

0

0

0

0

0

0

0

0

0

0

0

0

0

0
4

0

0

0

0

0

0

0

0

0

0

0

0

0

0

0

0

0

0

0

0

0

0
4

0

0

0

0

0

0

0

0

0

0

0.00612023
3

0.099172

0

0

0

0

0

0

0

0

0

0

0

0

0

0

0

0

0

0

0

0

0

0

0

0

0

0

0

0

0

0

0

0

0

0

0

0

0

0

0

0

0

0

0

0

0

0

0

0

0

0

0

0

0

0

0

0

0

0

0

0

0

0

0

0

0

0

0

0

0

0

0

0

0

0

0

0

0
4

0

0

0

0

0

0

0

0

0

0

0

0

0

0

0

0

0

0

0

0

0

0

0

0

0

0

0

0

0

0

0

0

0

0

0

0

0

0

0

0

0

0

0

0

0

0

0

0

0

0

0

0

0

0.00408015

0

0
4

0

0

0

0

0

0

0

0

0

0

0

0

0

0

0

0

0

0

0

0

0

0
4

0

0

0

0

0

0

0

0

0

0

0

0

0

0

0

0

0

0

0

0

0

0

0

0

0

0

0

0

0

0

0

0

0

0

0

0

0

0

0

0

0

0

0

0

0

0

0

0

0

0

0

0

0

0

0

0

0

0

0

0

0

0

0

0

0

0
4

0

0

0

0
4

0.231194
4

0.231194

0
4

0

0

0

0
4

0.152147

0.152147
5

0

0

0

0

0

0

0

0

0

0

0

0

0

0

0
4

0

0

0
4

0

0

0
4

0

0

0
4

0

0

0
4

0

0

0
4

0

0

0
4

0

0

0
4

0

0

0
4

0
4

0

0

0

0
4

0
4

0.025154

0.025154

0.025154

0
4

0
4

0

0

0

0
4

0
4

0

0

0

0
4

0
4

0.423856
4

0.423856
4

0.192662

0.231194

0
4

0
4

0

0

0

0
4

0

0

0
4

0

0

0
4

0
4

0
4

0.308259
3

0.0770648

0.0770648

0

0.0770648

0

0

0
4

0
4

0

0

0

0
4

0
4

0

0

0

0
4

0
4

0.231194

0.231194

0.231194

0

0
4

0
4

0

0

0

0

0
4

0
4

0

0

0

0
4

0
4

0

0

0

0
4

0
4

0

0

0

0
4

0
4

0

0

0

0
4

0
4

0

0

0

0
4

0
4

0

0

0

0
4

0
4

0
4

12.4173

12.0156

12.0156

0.755716

0

0.20715

1.41238

1.18939

0.0453516

0.0485602

0.0259152

0

0.266335

0.0770648

0.600486
1

0.0259152

0.462389

0

0

0.0194364

0.0129576

0.207321

0

0.0129576

0.0129576

0.0518304

0.355114

0

0

0

0.0129576

0.0129576

0

0

0

0.0129576

1.80952

0.0129576

0.0194364

0.032394

0.0388728

0.616518

0.0129576

0.0129576

1.21429

1.61426

0.32394

0.0194364

0.459994

0
4

0
4

0.401685

0.401685

0.401685

0
4

0
4

0
4

0

0

0

0
7

0

0

0

0

0

0

0

0

0

0

0
6

0

0

0

0

0

0

0

0

0

0

0

0

0

0

0

0

0

0

0

0

0

0

0

0

0

0

0

0

0

0

0

0

0
6

0

0

0

0

0

0

0

0

0

0

0

0
4

0

0

0

0

0

0

0
4

0

0

0
4

0

0

0

0
4

0

0

0

0
4

0

0

0
4

0

0

0
4

0
4

0

0

0

0
4

0
4

0

0

0

0
4

0
4

0

0

0

0
4

0
4

0
4

3.92967
7

3.92967
7

3.8526
7

2.31711
7

0

0.15413

0.0114506

0

0

0

0.00362217

0.00518609

0

0.192662

0.382335
6

0.308259
7

0.365457

0.00411208

0

0.0770648

0.0270968

0.00411208

4.48426018540005e-16
7

0
4

0.0770648
6

0

0

0.0770648

0
4

0

0

0

0
4

0
4

0
4

0.269727
7

0.269727
7

0.269727
7

0.269727

0

0

0

0

0
4

0
4

0
4

0

0

0

0

0
4

0
4

0
4

0.00408015

0.00408015

0.00408015

0.00408015

0
4

0
4

0
4

0

0

0

0

0
4

0
4

0
4

0

0

0

0

0
4

0
4

0
4

0

0

0

0

0
4

0
4

0
4

0

0

0

0

0
4

0
4

0
4

0

0

0

0

0
4

0
4

0
4

0

0

0

0

0
4

0
4

0
4

0.0770648

0.0770648

0.0770648

0.0770648

0
4

0
4

0
4

0

0

0

0

0
4

0
4

0
4

0.85273
7

0.85273
7

0.85273
7

0.138015
7

0.625936
7

0.0887784

1.38777878078145e-16
7

0
4

0

0

0
4

0
4

0
4

0

0

0

0

0
4

0
4

0
4

0

0

0

0

0
4

0
4

0
4

0

0

0

0

0
4

0
4

0
4

0

0

0

0

0
4

0
4

0
4

0

0

0

0

0
4

0
4

0
4

0

0

0

0

0
4

0
4

0
4

0

0

0

0

0
4

0
4

0
4

0

0

0

0

0
4

0
4

0
4

0

0

0

0

0
4

0
4

0
4

0

0

0

0

0
4

0
4

0
4

0.318327

0.318327

0.302296

0.277105

0.00687037

0.018321

2.77555756156289e-17

0
4

0.0160309

0.0160309

0
4

0
4

0
4

0

0

0

0

0
4

0
4

0
4

0

0

0

0

0
4

0
4

0
4

0

0

0

0

0
4

0
4

0
4

0.0259304

0.0259304

0.0259304

0.0259304

0
4

0
4

0
4

0

0

0

0

0
4

0
4

0
4

0

0

0

0

0
4

0
4

0
4

0.054963

0.054963

0.054963

0.054963

0
4

0
4

0
4

0

0

0

0

0
4

0
4

0
4

0

0

0

0

0
4

0
4

0
4

0

0

0

0

0
4

0
4

0
4

0.144196
7

0.144196
7

0.144196
7

0.144196

0

0

0
4

0

0

0

0

0
4

0
4

0
4

0

0

0

0

0
4

0
4

0
4

0

0

0

0

0
4

0
4

0
4

0

0

0

0

0
4

0
4

0
4

0

0

0

0

0
4

0
4

0
4

0

0

0

0

0
4

0
4

0
4

0

0

0

0

0
4

0
4

0
4

0.0770648

0.0770648

0.0770648

0.0770648

0
4

0
4

0
4

0

0

0

0

0
4

0
4

0
4

0

0

0

0

0
4

0
4

0
4

0

0

0

0

0
4

0
4

0
4

0.153962

0.153962

0.153962

0.148776

0

0.00518609

0

0
4

0

0

0

0
4

0
4

0
4

0

0

0

0

0
4

0
4

0
4

0

0

0

0

0
4

0
4

0
4

0

0

0

0

0
4

0
4

0
4

0

0

0

0

0
4

0
4

0
4

0

0

0

0

0
4

0
4

0
4

0

0

0

0

0
4

0
4

0
4

0

0

0

0

0
4

0
4

0
4

0

0

0

0

0
4

0
4

0
4

0

0

0

0

0
4

0
4

0
4

0

0

0

0

0
4

0
4

0
4

0

0

0

0

0

0

0
4

0
4

0
4

0.72098

0.72098

0.72098

0.72098

0
4

0
4

0
4

0

0

0

0

0
4

0
4

0
4

0

0

0

0

0
4

0
4

0
4

0

0

0

0

0
4

0
4

0
4

0

0

0

0

0
4

0
4

0
4

0

0

0

0

0
4

0
4

0
4

0

0

0

0

0
4

0
4

0
4

0

0

0

0

0
4

0
4

0
4

0

0

0

0

0
4

0
4

0
4

0

0

0

0

0
4

0
4

0
4

0

0

0

0

0

0

0

0
4

0
4

0
4

0

0

0

0

0
4

0
4

0
4

0

0

0

0

0
4

0
4

0
4

0.726627
7

0.726627
7

0.726627
7

0.726627

0

0

0
4

0
4

0
4

0

0

0

0

0

0

0
4

0

0

0
4

0
4

0
4

0.0129576

0.0129576

0

0

0
4

0.0129576

0.0129576

0

0

0
4

0

0

0
4

0
4

0
4

5.30892
7

5.30892
7

5.30892
7

5.30892
7

0

0

0

0
4

0
4

0

0

0

0
4

0
4

0
4

0

0

0

0

0

0

0
4

0
4

0
4

0

0

0

0

0

0
4

0
4

0
4

0.371

0.371

0.371

0.371

0
4

0
4

0
4

0.0156497

0.0156497

0.0156497

0.0156497

0
4

0
4

0
4

0

0

0

0

0
4

0

0

0
4

0
4

0

0

0

0
4

0

0

0
4

0
4

0
4

0

0

0

0

0

0
4

0
4

0
4

0

0

0

0

0

0
4

0
4

0
4

0.0712667

0.0712667

0.0712667

0.0583091

0.0129576

0
4

0
4

0
4

0

0

0

0

0
4

0
4

0
4

0

0

0

0

0
4

0
4

0
4

0
2

0
2

0
2

0
2

0

0

0

0

0
4

0
2

0

0

0

0

0

0

0
4

0

0

0
4

0

0

0
4

0
4

0
4

0.285067

0.285067

0.285067

0.239715

0.032394

0.0129576

0
4

0
4

0
4

0

0

0

0

0
4

0
4

0
4

0

0

0

0

0
4

0

0

0
4

0

0

0
4

0
4

0
4

0.221356

0.221356

0.221356

0.206263

0.0150924

0
4

0
4

0
4

0.00881856

0.00881856

0.00881856

0

0.00881856

0

0

0
4

0
4

0
4

0.0946041

0.0946041

0.0946041

0.0946041

0
4

0
4

0
4

0

0

0

0

0

0
4

0

0

0
4

0
4

0

0

0

0
4

0
4

0
4

0.00518609

0.00518609

0

0

0

0
4

0.00518609

0.00518609

0
4

0
4

0
4

0

0

0

0

0
4

0
4

0
4

0

0

0

0

0

0

0
4

0
4

0
4

0

0

0

0

0

0

0

0

0

0
4

0
4

0
4

0.0234746

0.0234746

0.0234746

0

0.0234746

0
4

0

0

0
4

0
4

0
4

0

0

0

0

0
4

0
4

0
4

0.0435124

0.0435124

0.0435124

0.0435124

0
4

0
4

0
4

0

0

0

0

0
4

0

0

0
4

0
4

0
4

0

0

0

0

0

0
4

0

0

0
4

0
4

0
4

0.15413

0.15413

0.15413

0.15413

0

0
4

0
4

0
4

0.0770648

0.0770648

0.0770648

0.0770648

0

0
4

0
4

0
4

0

0

0

0

0
4

0
4

0
4

0

0

0

0

0

0
4

0
4

0
4

0

0

0

0

0

0
4

0

0

0
4

0
4

0
4

0
4

0
4

0
4

0

0

0

0

0

0

0
4

0

0

0
4

0
4

0
4

0

0

0

0

0
4

0
4

0

0

0

0
4

0
4

0
4

0

0

0

0

0
4

0

0

0
4

0

0

0
4

0
4

0
4

0

0

0

0

0

0
4

0
4

0
4

0

0

0

0

0

0
4

0
4

0
4

0

0

0

0

0

0
4

0
4

0
4

0

0

0

0

0
4

0

0

0
4

0
4

0
4

0

0

0

0

0
4

0

0

0
4

0
4

0
4

0

0

0

0

0
4

0
4

0
4

0.0160309

0.0160309

0.00687037

0.00687037

0
4

0.0091605

0.0091605

0
4

0
4

0
4

0.0129576

0.0129576

0

0

0
4

0.0129576

0.0129576

0
4

0
4

0
4

0.359051
7

0.359051
7

0.359051
7

0.00777913

0.0312995

0.0887784

0

0.231194

0

0

0

2.77555756156289e-17
7

0
4

0

0

0

0
4

0
4

0
4

0

0

0

0

0
4

0
4

0
4

0

0

0

0

0
4

0
4

0
4

0

0

0

0

0

0
4

0
4

0
4

0.472952

0.472952

0.459994

0.459994

0
4

0.0129576

0.0129576

0
4

1.90819582357449e-17

0
4

0
4

0.192662

0.192662

0.192662

0.192662

0
4

0
4

0
4

0

0

0

0

0
4

0

0

0
4

0
4

0
4

0

0

0

0

0
4

0

0

0
4

0
4

0
4

0

0

0

0

0

0
4

0
4

0
4

0

0

0

0

0
4

0

0

0
4

0
4

0
4

0.937273

0.937273

0.937273

0

0.937273

0
4

0
4

0
4

0.131247

0.131247

0.115597

0

0.115597

0

0

0

0

0
4

0.0156497

0.0156497

0
4

0
4

0

0

0

0

0
4

0
4

0
4

0

0

0

0

0
4

0
4

0
4

0.0520819

0.0520819

0.0391243

0.0391243

0
4

0.0129576

0.0129576

0
4

0
4

0
4

0

0

0

0

0
4

0
4

0
4

0

0

0

0

0

0
4

0
4

0
4

0

0

0

0

0
4

0
4

0
4

0

0

0

0

0
4

0

0

0
4

0
4

0
4

0

0

0

0

0
4

0
4

0
4

0

0

0

0

0
4

0

0

0
4

0
4

0
4

0

0

0

0

0
4

0

0

0
4

0
4

0
4

0

0

0

0

0

0
4

0
4

0
4

0
5

0
5

0
5

0
5

0

0

0
4

0

0

0

0

0

0
4

0
4

0
4

0

0

0

0

0

0
4

0
4

0
4

0

0

0

0

0

0
4

0
4

0
4

0

0

0

0

0

0
4

0
4

0
4

0

0

0

0

0
4

0
4

0
4

0

0

0

0

0
4

0

0

0
4

0
4

0
4

0

0

0

0

0
4

0
4

0
4

0.455867

0.455867

0.455867

0.455867

0
4

0
4

0
4

0

0

0

0

0
4

0
4

0
4

0

0

0

0

0
4

0

0

0
4

0
4

0
4

0

0

0

0

0
4

0
4

0
4

0.632168
7

0.632168
7

0.632168
7

0.632168
7

0

0
4

0

0

0

0
4

0

0

0
4

0
4

0

0

0

0
4

0
4

0
4

0

0

0

0

0

0
4

0
4

0
4

0

0

0

0

0
4

0
4

0
4

0

0

0

0

0
4

0
4

0
4

0

0

0

0

0
4

0
4

0
4

0

0

0

0

0
4

0
4

0
4

0

0

0

0

0
4

0
4

0
4

0

0

0

0

0
4

0
4

0
4

0.0583091

0.0583091

0.0583091

0.0583091

0
4

0
4

0
4

0

0

0

0

0
4

0
4

0
4

0

0

0

0

0
4

0
4

0
4

2.31645

2.31645

2.31645

0.0887784

0

0

0.285067

0

0

0

0

0

0

0

0

0

0

0

0

0

0

0

0

0

0

0.103661
4

0

0.0194364

0

0

0

0.165843

0

0.42663

0

0

0.155491

0

0

0

0

0

0

0

0

0

0

0

0

0

0

0

0

0

0

0

0

0

0

0

0.0129576

0

0

0.00319998

0

0

0

0

0

0

0

0

0

0.0194364

0

0

0

0

0

0

0.459994

0

0.504686

0

0

0

0

0

0

0.0712667

0
4

0

0

0

0

0

0
4

0

0

0
4

0

0

0
4

0

0

0
4

0

0

0
4

0

0

0
4

0

0

0

0

0
4

0

0

0

0
4

0

0

0

0

0
4

0

0

0

0

0
4

0

0

0

0
4

0

0

0
4

0

0

0
4

0

0

0
4

0
4

0

0

0

0

0

0

0
4

0
4

0

0

0

0

0

0
4

0

0

0
4

0
4

0

0

0

0

0
4

0
4

0

0

0

0
4

0
4

0

0

0

0
4

0
4

0
4

0

0

0

0

0
4

0
4

0
4

0
4

0.492677
3

0.492677
3

0.492677
3

0

0

0

0

0

0

0

0

0

0

0

0

0
4

0.0388728
3

0
3

0

0.0388728

0
4

0

0

0
4

0

0

0

0

0

0

0

0

0

0
4

0

0

0

0

0

0
4

0.00416335

0.00416335

0
4

0

0

0
4

0

0

0
4

0

0

0
4

0.195677

0.191514

0.00416335

4.33680868994202e-18

0
4

0

0

0
4

0

0

0

0
4

0

0

0

0
4

0

0

0
4

0.253964

0.00416335

0.249801

0
4

0

0

0
4

0

0

0
4

0
4

0

0

0

0

0

0

0

0

0
4

0
4

0

0

0

0

0
4

0
4

0

0

0

0
4

0
4

0

0

0

0
4

0
4

0
4

0

0

0

0

0

0

0
4

0
4

0
4

0

0

0

0

0
4

0
4

0
4

0

0

0

0

0
4

0
4

0
4

0

0

0

0

0
4

0
4

0
4

0

0

0

0

0
4

0
4

0
4

0

0

0

0

0
4

0
4

0
4

0

0

0

0

0
4

0
4

0
4

0

0

0

0

0
4

0
4

0
4

0

0

0

0

0
4

0
4

0
4

0

0

0

0

0
4

0
4

0
4

0

0

0

0

0

0
4

0
4

0
4

0

0

0

0

0

0
4

0
4

0
4

0

0

0

0

0
4

0
4

0
4

0

0

0

0

0
4

0
4

0
4

0

0

0

0

0
4

0
4

0
4

0

0

0

0

0
4

0
4

0
4

0

0

0

0

0
4

0
4

0
4

0
4

619.862

0
4

0

0

0

0

0

0

0

0

0

0

0

0

0

0

0

0

0

0

0

0

0

0

0

0

0

0

0

0

0

0

0

0

0

0

0

0
4

0

0

0

0

0
4

0

0

0

0
4

0

0

0

0
4

0
4

0
4

0

0

0

0

0

0
4

0

0

0

0

0
4

0

0

0
4

0

0

0
4

0
4

0

0

0

0

0

0
4

0

0

0

0
4

0

0

0
4

0
4

0
6

0
6

0
6

0
4

0
4

0

0

0

0
4

0

0

0
4

0
4

0

0

0

0

0
4

0

0

0

0
4

0

0

0
4

0
4

0

0

0

0

0
4

0
4

0

0

0

0

0

0
4

0
4

0

0

0

0

0
4

0

0

0
4

0
4

0

0

0

0
4

0
4

0

0

0

0
4

0

0

0
4

0
4

0
4

0
4

0

0

0

0

0

0

0

0

0

0

0

0

0

0

0

0

0

0
4

0

0

0

0

0

0

0
4

0

0

0

0

0

0

0

0
4

0
4

0

0

0

0
4

0
4

0

0

0

0
4

0
4

0

0

0

0
4

0
4

0

0

0

0
4

0
4

0

0

0

0
4

0
4

0

0

0

0
4

0
4

0

0

0

0
4

0
4

0
4

0
4

0

0

0

0

0

0

0

0

0

0

0
4

0

0

0
4

0

0

0

0
4

0

0

0
4

0
4

0
4

0
4

0

0

0

0

0
4

0

0

0

0

0
4

0

0

0
4

0

0

0
4

0
4

0
4

0
4

0

0
4

0

0

0

0

0

0

0

0
4

0
4

0

0

0

0

0

0

0

0

0
4

0

0

0

0

0
4

0

0

0

0

0

0
4

0

0

0
4

0

0

0
4

0
4

0
6

0
6

0
6

0

0

0
4

0
4

0

0

0

0

0

0

0

0

0

0

0
4

0

0

0

0
4

0

0

0

0

0
4

0

0

0
4

0
4

0

0

0

0

0

0

0

0
4

0

0

0
4

0
4

0
4

3.65608
5

3.65608
5

0

0

0
4

3.65608
5

1.92212
5

0

0

0

0

1.3101

0

0.115597

0

0

0.308259

0

0
4

0
4

0
4

597.716
7

0
6

0
6

0

0

0
4

0

0

0
4

0
4

36.203

1.06208

0.942263

0.0080604
1

0.00411208

0

0

0

0.0129576

0

0

0

0

0

0

0

0

0

0

0

0

0

0

0

0.0647879

0

0

0.0239728

0

0

0

0

0

0

0

0

0

0

0

0

0

0

0

0.00592247

0

0

0

0

0

0

0

0

0

0

0

0

0

0

0

0

0

0

0

0

0

0

0

0

0

0

2.60208521396521e-17

0
4

0

0

0

0

0

0

0

0

0

0

0

0
4

14.3808

1.15193

0.17668

0

0.0770648

0.125643

0

0.146088

0

0

0.266335

0

0

0.0453516

0

0

0.0246725

0

0

1.29776

0

0.00592247

0

0

0.0516032

0

0

0

0

0

0

0

0

0.0194364

0.00458025

0.00987078

0

0.00448724

0

0.0215412

0.0137407

0

0

0

0

0

0.00927559

0.0887784

0

0

0

0

0.577986

0.00799092

0

0

0.0138191

0.180072

0

0

0

0

0.00592247

0.0458025

0

0.385324

0

0.115597

0

0

0

0.0444196

0.00394831

0.167087

0.241168
6

0

0.308259

0.0352157

0.0767946

0.0358979

0.591856

0.00411208

0.00448724

0.0194364

0.00448724

0.737886
7

0

0

0

0

0

0

0.0246185

0.144196

0.327003

0.00394831

1.32742
8

0.025154

0

0

0.032394

0

0

0

0.0100616

0

0.0887784

0.899235

0

1.61836

0.917377

0

0

0.00532728

0

0

0.0381415

0

0.0903737

0

0.479431

0

0

0

0.0259152

0

0

0.0134617

0.0129576

0.365457

0

0

0

0

0

0

0

0.00394831

0.0336543

0

0.123494
7

0

0.0194364

0

0.236743

0.00448724

0

0.0770648

0

0

0

0

0

0

0

0

0

0

0

0.122527

0.16154

0

8.60422844084496e-16

0
4

0.0129576
6

0

0.0129576

0

0

0

0

0
4

0

0

0
4

0.540895
7

0.540895

0

0
4

0.00448724

0

0

0.00448724

0
4

0.00458025

0

0

0.00458025

0
4

0.144196

0

0.144196

0

0
4

0.0156497

0

0.0156497

0
4

0.062599

0.062599

0
4

0

0

0

0
4

0

0

0

0
4

17.1541

17.1446

0

0

0

0.00943936

0

0

0

0

0

0

0
4

0

0

0
4

0.0770648

0

0.0770648

0
4

0

0

0
4

0

0

0

0
4

0.0387615

0.0387615

0
4

0

0

0
4

0

0

0
4

0

0

0
4

0

0

0
4

0

0

0
4

0.248733

0.104537

0.144196

0

0

0

0

0

0
4

0.0129576

0.0129576

0
4

0.00394831

0.00394831

0
4

0

0

0
4

0.053042

0.053042

0
4

0.0842243

0.0842243

0
4

0.00411208

0.00411208

0
4

0

0

0
4

0

0

0
4

0

0

0
4

0

0

0
4

0.168279

0.00411208
7

0

0

0.00411208

0.0941384
7

0.0591856

0

0

0.00673085

0

0

1.21430643318376e-17

0
4

0

0

0
4

0

0

0
4

0

0

0
4

0.00448724

0.00448724

0
4

0

0

0
4

0

0

0
4

0

0

0
4

0.00394831

0.00394831

0
4

0

0

0
4

0

0

0
4

0.194653

0.123097

0.00615463

0

0.0129576

0

0

0
6

0

0.0524433

0

0

0

0

0

0
4

0.346792

0.346792

0
4

0.174927

0.174927

0
4

0.109548

0.109548

0
4

0.912104

0.912104

0

0

0

0

0

0

0

0

0
4

0.216294

0.216294
7

0

0

0

0

0

0
4

0.0762553

0.0632977

0.0129576

0

0

0

0
4

0.0904659

0.0904659

0

0

0

0

0

0

0
4

0
4

554.441
7

4.74768
6

0.156031
6

1.43557
7

0

0

0

0

0

0

0

0

0

0

0.504869
6

0

0.136055

0

0

0

0

0

0

0

0

0

0

0

0

0

0

0

0

0

0

0

0

0.00967569

0

0

0

0

0

0

0

0

0

0

0

0

0

0

0

0

0

0

0

0

0
5

0

0

0

0

0

0

0

0

0

0

0
6

0

0

0

0

0

0

0

0

0

2.50548
6

0
6

0
4

0.386405
7

0.242209
7

0

0.144196

0

0
4

510.789
7

428.053
7

81.5785
7

0

0

0.232217

0

0.0259152

0

0

0.0259152

0

0

0

0

0

0

0

0

0

0.0547741

0

0.31191

0

0.291731

0

0

0

0

0

0

0

0

0.0156497

0.032394

0

0.144196

0

0.0224362

0

0

0

0

0

0

2.78804757058992e-14
7

0
4

0.469218
7

0

0.440657

0

0

0.0285611

3.46944695195361e-18
7

0
4

1.44335

1.44335

0

0

0

0
4

28.8312
7

28.1593
7

0

0

0

0

0

0

0

0

0.00362217

0

0

0.193917
7

0

0

0

0

0

0

0

0

0

0

0
6

0

0

0

0

0.0156497

0

0.423856

0.00362217

0.0312995

0

0

0

2.26207941267376e-15
7

0
4

0.423856
6

0.423856
6

0

0

0

0
4

1.07234
6

0.918209
6

0

0

0

0

0.15413

0
4

2.16458
6

0.032394
7

0.129718
6

0
7

0

0.00532728

0

0

0

0

0

0

0

0.408344

0

0

0

0

0

0

0

0

0.0156497

0

0.0133182
7

0

0.00362217

0

0

0

0

0.144196

0

0.0110232
6

0.717269
7

0.00408015
6

0.660289
7

0.0193514
7

0
6

8.46545056276682e-16
6

0
4

0.433325
7

0.0388728
7

0.240729

0.134645

0.0129576

0

0.00612023

0

0

5.20417042793042e-18
7

0
4

0

0

0

0

0

0

0

0

0

0

0

0

0
4

0
6

0
6

0

0
4

0.591856
6

0.591856
6

0

0

0

0

0
4

0

0

0

0
4

0

0

0
4

0

0

0

0
4

0

0

0
4

0

0

0
4

0

0

0
4

0

0

0
4

0

0

0
4

0

0

0
4

0

0

0
4

0.216294
7

0
7

0.216294

0

0

0

0
4

0

0

0
4

0

0

0
4

0

0

0
4

0

0

0
4

0

0

0
4

0

0

0
4

0

0

0
4

0

0

0
4

0

0

0
4

0.0102802

0.0102802

0
4

0.96319
7

0.923638
7

0.0395518

4.16333634234434e-17
7

0
4

0

0

0
4

0

0

0
4

0.00408015

0.00408015

0
4

0.0129576

0.0129576

0
4

0

0

0
4

0.504686

0.504686

0
4

0

0

0
4

0

0

0
4

0

0

0
4

0

0

0
4

0.42663

0

0.0591856

0.308259

0.0591856

0
4

0.00408015

0.00408015

0
4

0.0770648

0.0770648

0
4

0

0

0
4

0

0

0
4

0

0

0
4

0.72098
7

0.72098
7

0
4

0

0

0
4

0.147799

0.147799

0
4

0

0

0

0
4

0

0

0
4

0
4

0.415377
7

0.175662
6

0.0773557

0.0870252
6

0

0.00805789

0.00322316

2.25514051876985e-17
6

0
4

0.239715

0.116618

0.123097

1.38777878078145e-17

0
4

0
4

1.31072
7

0

0

0
4

1.29776
8

1.29776
8

0

0
4

0

0

0

0
4

0

0

0
4

0

0

0
4

0

0

0
4

0

0

0
4

0.0129576

0.0129576

0
4

0

0

0
4

0

0

0
4

7.45931094670027e-17
7

0
4

3.71922

3.13046

2.35765

0.77281

0
4

0.117495

0.117495

0

0

0

0

0
4

0.00408015

0.00408015

0
4

0

0

0
4

0.200843

0.200843

0
4

0

0

0
4

0
5

0
5

0

0
4

0.266335
7

0

0.266335

0
4

0

0

0
4

0

0

0

0
4

0

0

0

0
4

0

0

0

0
4

0

0

0
4

0

0

0
4

5.55111512312578e-17

0
4

1.60074
7

1.60074
7

0

1.28328

0.317461

0
6

0

1.11022302462516e-16
7

0
4

0
4

0

0

0

0
4

0
4

0

0

0

0
4

0
4

0

0

0

0
4

0
4

0

0

0

0
4

0
4

0

0

0

0

0

0
4

0
4

0

0

0

0
4

0
4

0.0129576

0.0129576

0

0.0129576

0
4

0
4

0

0

0

0
4

0
4

0

0

0

0
4

0
4

0

0

0

0
4

0
4

0

0

0

0
4

0
4

0.0129576

0.0129576

0.0129576

0
4

0
4

0
4

0.506967

0.385022

0.00888202
7

0

0

0

0

0

0

0.00888202

0

0

0

0

0
6

0

0

0

0

0

0

0

0

0

0

0

0
4

0.36049

0.36049

0
4

0.0156497

0.0156497
7

0

0

0

0
4

0

0

0

0
4

0

0

0
4

0

0

0
4

0

0

0
4

0

0

0
4

0
4

0.0129576

0.0129576

0

0.0129576

0

0

0

0

0
4

0
4

0

0

0

0
4

0
4

0

0

0

0
4

0
4

0

0

0

0
4

0
4

0

0

0

0

0
4

0
4

0

0

0

0
4

0

0

0
4

0
4

0

0

0

0
4

0
4

0.108988

0.00532728

0.00532728

0
4

0.103661

0.103661

0
4

0
4

0

0

0

0
4

0
4

0

0

0

0
4

0
4

0

0

0

0
4

0
4

0

0

0

0
4

0
4

0
4

0.595443

0.595443

0.595443

0.162725
6

0

0

0

0

0

0.0583091

0

0

0

0

0.219939
6

0

0

0.115597

0

0

0

0

0

0

0

0

0

0

0.0388728

0
4

0

0

0
4

0

0

0
4

0

0

0
4

0

0

0
4

0
4

0

0

0

0

0
4

0
4

0

0

0

0
4

0
4

0

0

0

0
4

0
4

0

0

0

0
4

0
4

0

0

0

0
4

0
4

0

0

0

0
4

0
4

0

0

0

0
4

0
4

0

0

0

0
4

0
4

0

0

0

0
4

0
4

0
4

3.68079

0

0

0

0

0
4

0
4

2.00963

1.07717

1.07717

0
4

0.564692
1

0

0

0.564692

0

0
4

0.367764

0.253258

0.0526729

0.0618334

0
4

0

0

0
4

2.77555756156289e-16

0
4

1.67116

0.288392

0.144196

0.144196

0
4

1.38277

1.38277
1

0

0

0
4

0
4

0
4

4.23463
5

4.23463
5

3.56798

1.2659

2.30208
7

0

0

0

0

0

0

0

0

0

0

0

0

0

0

0
4

0.103661
6

0.0583091
6

0.0129576
7

0.032394
6

0

0

0

0

0

6.93889390390723e-18
6

0
4

0

0

0
4

0

0

0
4

0

0

0
4

0

0

0
4

0

0

0
4

0

0

0
4

0.562995
6

0.562995
6

0

0
4

0

0

0

0

0

0

0
4

0

0

0

0

0
4

0

0

0

0

0
4

0

0

0

0
4

0

0

0
4

0

0

0
4

0

0

0
4

0
4

0

0

0

0

0
4

0
4

0

0

0

0

0
4

0
4

0

0

0

0
4

0
4

0

0

0

0
4

0
4

0

0

0

0
4

0
4

0

0

0

0
4

0
4

0

0

0

0
4

0
4

0
4

0.432588

0.432588

0.432588

0.144196

0.288392

0

0

0

0

0

0

0
4

0

0

0

0

0

0
4

0

0

0

0

0

0
4

0

0

0
4

0

0

0
4

0
4

0
4

0
3

0
3

0
3

0
3

0

0
4

0
4

0
4

0
6

0
6

0
6

0
6

0

0
4

0

0

0
4

0
4

0
4

0.252673

0.252673

0.252673

0

0.032394

0.207321

0.0129576

1.90819582357449e-17

0
4

0

0

0

0
4

0
4

0
4

0

0

0

0

0

0

0
4

0
4

0
4

0
4

0
4

0
4

0
4

0

0

0
4

0

0

0
4

0
4

0
4

0

0

0

0

0

0

0
4

0
4

0
4

0

0

0

0

0

0
4

0
4

0
4

0

0

0

0

0

0

0

0
4

0
4

0
4

0

0

0

0

0

0
4

0
4

0
4

0

0

0

0

0
4

0
4

0
4

0.192662
6

0.192662
6

0.192662
6

0

0

0.192662

0

0

0

0

0
4

0
4

0
4

0
6

0
6

0

0

0
4

0

0

0
4

0
4

0
4

0

0

0

0

0

0
4

0
4

0
4

0

0

0

0

0
4

0
4

0
4

1.14268

1.14268

0.277506

0.269727

0.00777913

0
4

0.865175

0.865175

0
4

0
4

0
4

0

0

0

0

0

0
4

0
4

0
4

0

0

0

0

0

0

0
4

0
4

0
4

0

0

0

0

0

0
4

0
4

0
4

0

0

0

0

0
4

0
4

0
4

0

0

0

0

0
4

0

0

0
4

0
4

0
4

0

0

0

0

0

0

0
4

0
4

0
4

0
4

0
4

0
4

0

0

0

0

0

0

0

0
4

0
4

0

0

0

0
4

0
4

0
4

0

0

0

0

0

0
4

0
4

0
4

0

0

0

0

0

0
4

0

0

0
4

0
4

0
4

0

0

0

0

0
4

0

0

0
4

0
4

0
4

0

0

0

0

0
4

0
4

0
4

0.032394

0.032394

0.032394

0.032394

0

0
4

0
4

0
4

0

0

0

0

0
4

0
4

0
4

0

0

0

0

0

0
4

0

0

0
4

0
4

0
4

0

0

0

0

0
4

0
4

0
4

0

0

0

0

0
4

0
4

0
4

0

0

0

0

0
4

0
4

0
4

0
4

0
4

0
4

0

0

0

0

0

0

0

0

0
4

0

0

0

0

0

0
4

0

0

0
4

0
4

0
4

0

0

0

0

0
4

0
4

0
4

0

0

0

0

0
4

0
4

0
4

0

0

0

0

0
4

0
4

0
4

0

0

0

0

0

0
4

0
4

0
4

0

0

0

0

0
4

0
4

0
4

0.0156497

0.0156497

0

0

0
4

0.0156497

0.0156497

0
4

0
4

0
4

0

0

0

0

0
4

0
4

0
4

0

0

0

0

0
4

0
4

0
4

0.0194364

0.0194364

0.0194364

0.0194364

0
4

0
4

0
4

0

0

0

0

0
4

0
4

0
4

0
4

0
4

0
4

0
4

0

0

0

0

0
4

0

0

0
4

0

0

0

0
4

0

0

0
4

0
4

0
4

0.0194364

0.0194364

0.0194364

0.0194364

0
4

0
4

0
4

0

0

0

0

0
4

0
4

0
4

0

0

0

0

0
4

0
4

0
4

0

0

0

0

0
4

0
4

0
4

0

0

0

0

0
4

0
4

0
4

0

0

0

0

0
4

0
4

0
4

0.308259

0.308259

0.308259

0.308259

0
4

0
4

0
4

0

0

0

0

0
4

0
4

0
4

0

0

0

0

0
4

0
4

0
4

0

0

0

0

0
4

0
4

0
4

0.504686

0.504686

0.504686

0.504686

0

0

0

0

0
4

0

0

0

0

0

0
4

0

0

0
4

0

0

0

0
4

0

0

0
4

0
4

0
4

0.0388728

0.0388728

0.0388728

0.0388728

0
4

0
4

0
4

0

0

0

0

0
4

0
4

0
4

0

0

0

0

0
4

0
4

0
4

0

0

0

0

0
4

0
4

0
4

0

0

0

0

0
4

0
4

0
4

0

0

0

0

0
4

0
4

0
4

0.00448724

0.00448724

0.00448724

0.00448724

0
4

0
4

0
4

0

0

0

0

0
4

0
4

0
4

0

0

0

0

0
4

0
4

0
4

0

0

0

0

0
4

0
4

0
4

0.705134
7

0.705134
7

0.705134
7

0.676221
7

0.0206373

0

0.00416335

0

0.00411208

0

0
4

0
4

0
4

0

0

0

0

0
4

0
4

0
4

0

0

0

0

0
4

0
4

0
4

0

0

0

0

0
4

0
4

0
4

0

0

0

0

0
4

0
4

0
4

0

0

0

0

0
4

0
4

0
4

0

0

0

0

0
4

0
4

0
4

0

0

0

0

0
4

0
4

0
4

0

0

0

0

0
4

0
4

0
4

0

0

0

0

0
4

0
4

0
4

0

0

0

0

0
4

0
4

0
4

0.642546
7

0.642546
7

0.642546
7

0.580649
7

0.0541177

0.00777913

0
4

0
4

0
4

0

0

0

0

0
4

0
4

0
4

0

0

0

0

0
4

0
4

0
4

0.0118449

0.0118449

0.0118449

0.0118449

0
4

0
4

0
4

0

0

0

0

0
4

0
4

0
4

0

0

0

0

0
4

0
4

0
4

0

0

0

0

0
4

0
4

0
4

0

0

0

0

0
4

0
4

0
4

0.0129652

0.0129652

0.0129652

0.0129652

0
4

0
4

0
4

1.44196

1.44196

1.44196

1.44196

0
4

0
4

0
4

0

0

0

0

0
4

0
4

0
4

0

0

0

0

0

0

0

0

0
4

0
4

0
4

0

0

0

0

0
4

0
4

0
4

0.0518304

0.0518304

0.0518304

0.0518304

0
4

0
4

0
4

0.0860736

0.0860736

0.0860736

0.0860736

0
4

0
4

0
4

0.0453516

0.0453516

0.0453516

0.0453516

0
4

0
4

0
4

3.51063

3.45232

3.45232

3.45232
8

0

0
4

0

0

0
4

0
4

0

0

0

0
4

0
4

0.0583091

0.0583091

0.0583091

0
4

0
4

0
4

0
4

2510.41

1162.18
7

1162.06
7

766.823
7

766.793
7

0.0307731

0
4

203.378
7

24.1907
7

6.30578
7

0.73777
7

0.00408015

0

0.00448724

0.0259152

0

0.0100616

0

0.014451

0

0

0.214096
7

0.00687037

0

0

0.116938

0.0179489

0

0.0107775

0.0156497

0

0

1.10524
7

0.0156497

0.269727

0.0118427

0.00394831

0.0156497

0

0

0.0161662

0.0408038

0.0296067

1.29527
7

0.117373

0

0.0430824

0

0

1.96515

0

0

0.15413

0

0.160448
7

0

0

0

0.0160309

0

0

0.393896

0

0.00615463

0.0430824

0.0183924
6

0

0.0770648

0

0

0

0.0100616

0.0770648

0

0.00615463

0

0
6

0

0.00615463

0

0

0

0.0091605

0

0.00592134

0

0

0.0805605
7

0

0.0107775

0.0112181

0

0.00888202

0

0

0.0156497

0

0.0107775

2.17057
7

0.00458025

0

0

0

0

0.0107775

0

0.0100616

0

0

3.09549
7

0

0

0.0201232

0.847713

0

0.00888202

0

0

0

0

3.72045
7

0.203382
6

0.0461597

0.017764

0.0770648

0

0

0

0.00394831

0

0.269727

0

0.214681
7

0

0

0

8.28446

0

0.0307731

0

0

0.0118427

0

0.066408
7

0.0184639

0

0.00458025

0

0.0770648

0

0

0

0

0.021555

0.279865
7

0.00458025

0

0

0.0156497

0

0.00615463

0

0.0118427

0

0

6.86616
7

0

0

0

0.0100616

0.00615463

0

0.00448724

0

0

0

0.137501
7

0

0

0.0523143

0

0.0887784

0.00458025

0.00615463

0

0.0091605

0.0269437

0.0815565
7

0.00592134

0.0184639

0

0

0

0

0

0

0.115597

0

0.307731
6

0

0

0.00687037

0

0.0156497

0.0123093

0

0

0

0.15413

0.196669
7

3.00553

0.0320434
7

13.2705
7

0.0161662
6

0
6

0.650451
7

0.0823653
7

2.14958

0.0518592
7

0.0419072
8

0.0301185
7

0.0292751
7

0.0385808
7

3.92035
7

0.0324538

1.08147

0.0235692
7

0.13027

0.0325892
7

2.96699

0

0.00592134

0.021555

0.802001
8

0.769329
6

0.241448

0

0.0519997

49.2482

0.375594

0

0

0.0153151

0.0164229

1.11744

49.5713

0.0769328

0.0091605

0.0269437

0.032394

0

0.00394831

0.0674696

0.339491

0.0452773

0.0161662

0.296325
6

0.288392

0.0312995

0.144196

0

0.00592134

0.00592134

0

0

0

0

0.27449
6

0.346792

0.00615463

0.0134623

0

0

0.587015

0.031524

4.00737

0

0

0.47966
7

0

0

0.166017

0

0.0251914

0.0371476

0

0

0.770648

0.0114506

1.73402958658642e-14
7

0
4

73.1221
7

0.648261

16.3742
7

0.744881
7

23.5989
7

22.7083
7

9.04746
7

0

0

0

0

0

0

0

2.1316282072803e-14
7

0
4

10.7318
7

10.3802
7

0

0.3516

0
4

2.88416
7

2.69044
7

0.00592134

0

0.00458025

0.00458025

0

0.17863

0

0

0

0

0

0

0

1.11022302462516e-16
7

0
4

0

0

0

0

0
4

80.9413
7

23.8216
7

56.9499
7

0.15413

0

0

0

0

0

0

0

0

0

0.0156497

0

0

0
4

0.548514
7

0.541643
7

0.00687037

0

1.47451495458029e-17
7

0
4

23.6237
8

23.6237
8

0

0

0
4

0

0

0
4

0

0

0
4

0.0091605

0.0091605

0
4

1.38528771786994e-12
7

0
4

0.0172426

0.0172426

0.0172426

0
4

0
4

0.0102802

0.0102802

0

0.0102802

0
4

0
4

0.00518609

0.00518609

0.00518609

0
4

0
4

0.0591856

0.0591856

0.0591856

0
4

0
4

0

0

0

0
4

0
4

0

0

0

0
4

0
4

0.00458025

0.00458025

0.00458025

0
4

0
4

0.0234746

0.0234746

0.0234746

0
4

0
4

0
4

985.019

1.87198

0.36049

0.36049

0

0

0

0

0

0

0

0

0

0
4

0

0

0
4

0

0

0
4

0

0

0
4

0

0

0
4

0

0

0
4

0

0

0
4

0

0

0
4

0

0

0
4

0

0

0
4

0

0

0
4

0.500921

0.500921

0

0

0

0

0

0

0
4

0

0

0
4

0

0

0
4

0

0

0
4

0

0

0

0

0

0

0

0
4

0.577986

0

0.577986

0

0
4

0.432588

0.432588

0

0
4

0

0

0

0

0

0
4

0

0

0
4

0

0

0

0
4

0

0

0
4

2.22044604925031e-16

0
4

0

0

0

0
4

0
4

9.99896

0
3

0

0

0

0

0

0

0
4

0.802031
3

0

0

0.0156497

0

0

0

0

0

0

0

0

0

0

0

0

0

0

0

0

0

0

0

0.357142

0

0

0

0

0.429239

0

0

0
4

0

0

0
4

0.362812
3

0.362812
3

0

0

0

0

0

0

0

0

0

0

0
4

2.56963

0.147964
5

0.308259

0.0259152

0

0.115597

0.20715

0

0

0

0

0

1.23041

0

0

0.0194364

0

0

0.302121

0.0259152
5

0.115597

0

0

0

0.0712667

1.38777878078145e-16

0
4

0

0

0

0

0

0

0
4

0

0

0
4

0

0

0

0

0

0
4

0

0

0

0

0

0

0

0
4

0

0

0

0

0

0
4

0

0

0

0

0

0
4

0

0

0

0

0

0
4

0.162307

0.162307

0

0
4

0.192662

0.0770648

0

0.115597

1.38777878078145e-17

0
4

0

0

0

0

0
4

3.82101
5

3.32009
5

0

0

0.500921

0

0

0

0

0

0

0

0
4

0

0

0

0

0
4

0

0

0
4

0

0

0

0

0
4

0

0

0

0

0
4

0

0

0

0

0
4

0

0

0

0
4

0

0

0

0

0
4

0

0

0

0
4

0

0

0

0

0
4

0

0

0
4

1.25779
5

1.24483
5

0.0129576

0

0

0

7.45931094670027e-17
5

0
4

0

0

0

0
4

0

0

0
4

0.0297716

0.0297716

0
4

0

0

0

0
4

0

0

0
4

0

0

0
4

0

0

0
4

0

0

0
4

0

0

0
4

0

0

0
4

0
3

0

0

0

0

0

0

0

0
4

0

0

0

0
4

0

0

0

0
4

0

0

0

0
4

0

0

0

0
4

0

0

0

0
4

0

0

0
4

0

0

0

0
4

0

0

0
4

0

0

0

0
4

0

0

0
4

0.0194364
5

0

0.0194364

0

0

0

0

0

0
4

0

0

0

0
4

0

0

0

0
4

0

0

0
4

0

0

0
4

0

0

0
4

0

0

0
4

0

0

0
4

0

0

0
4

0

0

0
4

0

0

0
4

0

0

0

0

0

0

0

0

0
4

0.0129576

0.0129576

0
4

0

0

0
4

0

0

0
4

0

0

0
4

0

0

0
4

0

0

0
4

0

0

0
4

0.144196

0.144196

0
4

0

0

0
4

0

0

0
4

0
5

0
5

0

0
4

0

0

0
4

0

0

0
4

0

0

0
4

0

0

0
4

0

0

0
4

0

0

0
4

0

0

0
4

0

0

0
4

0.0129576

0.0129576

0
4

0

0

0
4

0.462389

0.346792

0.115597

0

0

0

0

0
4

0

0

0
4

0

0

0
4

0

0

0
4

0

0

0
4

0

0

0
4

0

0

0
4

0

0

0
4

0

0

0
4

0

0

0
4

0.149012

0.149012

0
4

0

0

0

0

0

0

0
4

0

0

0
4

1.66533453693773e-16

0
4

51.3086
3

5.5134

0.359482

0
3

0

0

0.115597

0

0

0.00799092

0

0

0

0.0799092

0.144196

0

0

0

0.144196

0.0338649
5

0.0770648

0.00532728

0

0

0.00532728

0

0

0.504686

0

0

0.144196

0

0

0

0

0

0.346792

0

0

0

0

0
3

0

0

0

0

0

0

0

0

0

0

0.710987

0

0.00532728

0

0

0

0

0

0

0

0

0.288392
3

0

0

0

0

0

0

0

0

0

0

0.271691
6

0.15413

0

0

0

0

0

0

0

0

0.00532728

1.53213

0

0

0

0

0

0

0

0

0

0

0

0

0

0

0

0

0

0.576784

0

0

0

0
4

14.135

2.69903
6

0

0

0.0614843

0

0

0

0.231194

0.168758

0.790312

0

1.05454

1.94839

0.266335

0

0.0358797

0.0782487

0

0.0156497

0

0.36049

0.693583

1.63141

0.0469492

0.0469492

0

0

0

0.355114

0.226921

0.924777

0

0.144196

0.577986
7

0.0470882

0

0.0391243

0

0

0

0

0.291546

0

0

0.0312995

0.0129576

0.0156497

0.0156497

0

0

0

0.0469492

0.0547741

0

0

0.0156497
6

0

0

0

0

0

0

0

0.0156497

0

0

0.722182
7

0

0.250396

0

0.0234746

0.0770648

0.0156497

0

0

0.0156497

0

0

0.0234746

0

0

0.0156497

0

0

0

0

0

0.0156497

0.0312995
3

0

2.28983498828939e-15

0
4

6.46208

0

5.87114

0

0

0

0

0

0.0129576

0.500921

0

0

0

0

0

0

0

0

0

0

0

0.0770648

0

0

0

0

0
4

17.9756
3

0
4

0
4

0

0

0

0

0

0

0

0

0

0

0

0
4

0

0

0

0

0

0

0

0

0

0

0
4

0

0

0

0

0

0

0

0

0

0

0

0

0

0

0

0

0

0

0

0

0

0
4

0

0

0

0

0

0

0.0647879

0

0

0

0

0

0

0

0

0

0

0

0

0

0

0

0

0

0

0

0

0

0

0

0

0

0

0

0

0

0

0

0

0

0

0

0

0

0

0

0

0

0

0

0

0

0

0

0

0

0

0

0

0

0

0

0

0

0

0

0

0

0

0.0129576

0

0

0

0

0

0

0

0

0

0

0

0

0.0453516

0

0

0

0

0

0.508616

0

0

0

0

0

0

0

0

0

0

0

0

0

0

0

0

0

0

0

0

0

0

0

0

0

0

0

0

0

0

0

0.0145135

0

0

0

0

0

0

0

0

0

0

0

0

0

0

0

0

0

0

0

0

0

0

0.0583091

0

0

0

0

0

0

0

0

0

0

0

0

0

0

0

0

0

0

0

0

0

0
4

0

0

0.0129576

0

0

0

0

0

0

0

0

0

0.0194364

0

0

0.0453516

0

0

0.0453516

0.0453516

0

0.0129576

0

0

0

0

0

0

0

0

0

0

0

0
4

0

0

0

0

0

0

0

0

0

0

0

0

0

0

0

0.0194364

0

0

0

0

0

0

0.0129576

0

0

0

0

0

0

0

0

0

0

0

0

0

0

0

0

0

0

0

0

0

0

0

0

0

0

0

0

0

0

0

0

0

0

0

0

0

0

0

0

0

0

0

0

0

0

0

0

0

0

0

0

0

0

0

0

0.0129576

0

0

0

0

0

0

0

0

0
4

0

0.0971819

0

0

0

0

0

0

0

0

0
4

0

0.123097

0

0

0

0

0

0

0

0

0

0

0

0

0

0

0

0

0

0

0

0

0.0129576

0

0

0

0

0

0

0

0

0

0

0

0

0

0

0

0

0

0

0

0

0

0

0

0

0

0

0

0

0

0

0

0

0

0

0

0

0

0

0

0

0

0

0

0

0

0

0

0

0

0

0

0

0

0.0129576

0

0

0

0

0

0

0

0

0

0

0

0

0

0

0

0

0

0

0

0

0

0

0

0

0

0

0

0

0

0

0

0

0

0
4

0

0

0

0

0

0

0

0

0

0

0
4

0

0

0

0

0

0

0

0

0

0

0

0

0

0

0

0

0

0

0

0

0

0

0

0.0194364

0

0

0

0

0

0

0

0

0

0

0

0

0

0

0

0

0

0

0

0

0

0.0129576

0

0

0

0

0.0156497

0

0

0

0

0

0

0

0

0

0

0

0.0129576

0

0

0

0.032394

0

0

0

0

0

0

0

0

0

0

0

0

0

0

0

0

0

0

0

0

0

0

0

0

0

0

0

0

0

0

0.330419

0

0

0

0

0

0

0

0

0

0

0

0

0.15413
7

0

0

0

0

0

0

0

0

0

0

0

0

0

0

0

0

0

0

0

0

0

0

0

0

0

0

0

0

0

0

0

0

0

0

0

0

0

0

0

0

0

0

0

0

0

0

0

0

0

0

0

0.0971819

0

0

0

0

0

0

0.395206

0

0

0

0

0

0

0

0

0

0

0.187885

0

0

2.54617

0

0

0
3

0

0

0

0

0

0

0.421122

0

0

0

0

0

0

0

0

0

0

0

0

0

0

0

0

0

0

0

0

0

0

0

0

0

0

0

0.00967569

0

0

0

0

0

0

0

0

0

0

0

0

0

0.0388728

0

0

0

0

0

0

0
4

0

0

0

0

0

0

0

0

0

0

0
4

0

0

0

0

0

0

0

0

0

0

0
3

0

0

0

0

0

0

0

0

0

0

0

0

0

0

0

0

0

0

0

0

0

2.03434

0

0

0

0

0

0

0

0

0

0

0
4

0

0

0

0

0

0

0.0453516

0

0

0

0
4

0

0

0

0

0

0.0388728

0

0

0.174927

0

0
4

0
4

0

0

0

0

0

0

0

0

0

0

0
4

0

0

0

0

0

0

0

0

0

0

0.181406

0

0

0

0

0

0

0

0

0

2.28054

0

0

0

0

0

0

0

0

0

0

0

0

0

0

0

0

0

0

0

0

0

0

0
4

0

0

0

0

0

0

0

0

0

0

0
4

0

0

0

0

0

0

0

0

0

0

0
4

0

0

0

0

0

0

0

0

0

0

0
4

0

0

0

0

0

0

0

0

0

0

0
3

0

0

0

0

0

0

0

0

0

0

0
4

0

0

0

0

0

0

0

0

0

0

0

0
4

0

0

0

0

0

0

0

0

0

0

0
4

0

0

0

0

0

0

0

0

0

0

0.0153866

0

0

0

0

0

0

0

0

0

0

0
4

0

0

0

0

0

0

0

0

0

0

0

0

0

0

0

0

0

0

0

0

0

0.424027
3

0

0

0

0

0

0

0

0

0

0

0
4

0

0

0

0

0

0

0

0

0

0

0

0

0

0

0

0

0

0

0

0

0

0

0

0

0

0

0

0

0

0

0

0

0

0
4

0

0.252673

0

0

0

0

0.187885

0

0

0

0
4

0

0

0

0

0

0.0194364

0

0

0

0

0

0

0

0

0

0

0

0

0

0

0

0
4

0

0

0

0

0

0

0

0

0

0

0

0

0

0

0

0

0

0

0

0

0

0

0

0

0

0

0

0

0

0

0

0

0

0

0

0

0

0

0

0

0

0

0

0
4

0

0

0

0

0

0

0

0

0

0

0.370981
7

0

0

0

0

0

0

0

0

0

0

2.92842

0

0

0

0

0

0

0

0

0

0

0
4

0
4

0

0

0

0

0

0

0

0

0

0

0
4

0

0

0

0

0

0

0

0

0

0

0
4

0

0.11014

0

0

0

0

0

0

0

0

0
4

0

0

0

0

0

0

0

0

0.0194364

0

0
4

0

0

0.0129576

0

0

0

0

0.0518304

0

0

0
4

0

0

0

0

0

0

0

0

0

0

0

0

0

0

0

0

0

0

0

0

0

0
4

0

0

0

0

0

0

0

0

0

0

0
4

0

0

0

0

0

0

0

0

0

0

0

0

0

0

0

0

0

0

0

0

0

0

0
4

0.032394

0.0194364

0

0

0

0

0

0

0

0

0
4

0

0

0

0

0

0

0

0

0

0

0

0

0

0

0

0

0

0

0

0

0

0

0

0

0

0

0

0

0

0

0

0

0

0

0

0

0.0129576

0

0

0

0

0

0

0

0

0

0

0

0

0

0

0

0

0

0

0

0

0

0

0.0129576

0

0

0

0

0

0
4

0

0

0

0

0

0

0

0

0

0

0

0

0

0

0

0

0

0

0

0

0

0

0

0

0

0

0

0

0

0

0

0

0

0
4

0

0

0

0

0

0

0

0

0

0

0

0

0

0

0

0

0

0

0

0.103661

0

0.0453516

0

0

0

0

0

0

0

0

0

0

0
4

0

0

0

0

0

0

0

0

0

0

0
4

0

0

0

0

0

0

0

0

0

0

0
4

0

0

0

0

0

0

0

0

0

0

0

0

0.0194364

0

0

0

0

0

0

0

0

0

0

0

0

0

0

0

0

0

0

0

0
3

0

0

0.032394

0

0

0

0

0

0

0

0
4

0

0

0

0

0

0

0.0194364

0

0

0

0

0

0

0

0

0

0

0

0

0

0

0

0
4

0.0129576

0

0

0

0

0

0

0

0

0

0

0

0

0

0.829286

0

0

0

0

0

0

0
4

0

0

0

0

0

0

0

0

0

0

0
4

0

0.0194364

0

0

0

0

0

0

0.0129576

0

0
4

0

0

0

0

0

0

0

0

0

0

0
4

0

0

0

0

0

0

0

0

0

0

0

0

0

0

0

0

0

0

0

0

0

1.91772

0

0

0

0

0

0

0

0.0129576

0

0.0129576

0.259152

0

0

0

0

0

0

0

0

0

0

0
4

6.17339

6.08665

0.0770648

0

0

0

0

0.00967569

0

0

0

0

0

0

0
4

0
4

0
4

0
4

0
4

0

0

0

0
4

0.655051

0.655051

0
4

0
4

0

0

0

0

0

0
4

0

0

0

0

0
4

0

0

0

0

0

0
4

0

0

0

0
4

0

0

0

0

0

0

0

0
4

0

0

0

0

0

0
4

0

0

0
4

0.0156497

0

0.0156497

0
4

0

0

0

0

0
4

0
3

0
3

0

0

0

0

0

0

0

0

0

0

0

0

0

0

0

0

0
4

0

0

0
4

0.144196

0.144196

0

0

0
4

0

0

0
4

0

0

0
4

0.144196

0.144196

0

0
4

0

0

0

0

0
4

0

0

0

0
4

0

0

0

0

0
4

0

0

0

0
4

0

0

0
4

0
3

0
4

0

0

0

0

0

0

0

0

0

0

0

0

0
4

0

0

0

0
4

0

0

0
4

0

0

0
4

0

0

0

0
4

0

0

0

0
4

0

0

0

0
4

0

0

0
4

0

0

0
4

0

0

0
4

0

0

0

0
4

0
3

0

0

0

0

0

0

0

0

0

0

0

0

0

0

0

0
4

0

0

0
4

0

0

0
4

0

0

0
4

0

0

0

0
4

0.0129576

0

0.0129576

0
4

0

0

0

0
4

0

0

0

0
4

0

0

0

0
4

0

0

0

0
4

0

0

0
4

0
3

0

0

0

0

0

0

0
4

0

0

0
4

0

0

0

0
4

0

0

0
4

0

0

0
4

0

0

0
4

0

0

0
4

0

0

0
4

0

0

0
4

0

0

0
4

0

0

0
4

0
4

0
4

0

0

0

0

0

0

0

0

0
4

0

0

0
4

0

0

0
4

0

0

0
4

0

0

0
4

0

0

0
4

0

0

0
4

0

0

0
4

0

0

0
4

0

0

0
4

0

0

0
4

0

0

0

0

0

0
4

0

0

0
4

0

0

0
4

0

0

0
4

0

0

0
4

0

0

0
4

0

0

0
4

0

0

0
4

0

0

0
4

0

0

0
4

0

0

0
4

0

0

0

0

0

0

0

0

0
4

0

0

0
4

0

0

0
4

0

0

0
4

0

0

0
4

0

0

0
4

0

0

0
4

0

0

0
4

0.0770648

0.0770648

0
4

0

0

0
4

0

0

0
4

0

0

0

0

0

0

0

0

0
4

0

0

0
4

0

0

0
4

0

0

0
4

0

0

0
4

0

0

0
4

0

0

0
4

0

0

0
4

0

0

0
4

0

0

0
4

0
4

1.80758
4

0.466473
4

0
4

0

0.0907031

0.149012

0

0

0

0.0907031

0

0.0194364

0

0

0

0

0

0

0.0129576

0.0129576

0

0

0

0

0

0

0

0

0.0259152

0

0.0129576

0

0

0

0

0

0

0.0194364

0.0129576

0.0194364

0

0

0

0

0

0

0

1.04083408558608e-17
4

0
4

1.32815
4

1.32815
4

0

0
4

0

0

0
4

0.0129576

0.0129576

0
4

0

0

0
4

7.45931094670027e-17
4

0
4

65.4594

43.7112

5.01233
7

5.08943
7

0

0

0

0

0.00777913

0.453516

0

0

0

0

0.769966
8

0

0

0.00967569

0

0.0770648

0.00518609

0

0.0156497

0

0

0.571315
7

0

0

0

0.0156497

0

0

0

0

0.0234746

0

0

0

0.0887784

0.062599

0.00777913

0.0233003

0

0.0100616

0

0

0.00518609

0.572803
7

0

0

0.0156497

0

1.07891

0

0

0.576784
7

0.218835

0.64223
8

0
3

0.0466748
7

4.65565

1.35641

1.29683

0.038551
7

0.151233
8

0.162347

0

0.648882
7

0

0.346792

0

2.80531
7

0.0804929

0

0.558649

0.192805

0.441089
7

0.395206

0.0228112

0.00518609

0

0

7.15525

0

0.0770648

0

0.0256199

0.0234746

0

0.0156497

0.272501

0.0402465

0.367536

2.0382
8

0

0

0.0186455

0

0.00518609

0.0156497

0

0.115597

0

0

0
3

0

0

0.0267285

0.0211679

0

0

0

0.0770648

0.187797

0.0391243

1.03967
8

0.311799

0.15413

0

0

0.00777913

0.15413

0.00777913

0

0

0

2.39006
7

0

0.0129576

0

0.0156497

0.0129576

0.00518609

0.0181513

0

0

0

0.478856
7

0

0

0

0

0.00518609

0.0155583

0

0

0

0

0
4

0

0

0

0
4

0
2

0

0

0

0

0

0

0

0

0

0

0
4

0.305513
4

0

0
4

0

0

0

0

0

0.0164483

0

0.00687037

0

0

0.00518609

0

0

0.0114506

0

0

0
4

0

0

0

0

0

0

0.036642

0

0

0

0
4

0

0

0

0

0

0

0

0

0

0

0
3

0

0

0

0

0

0.00440928

0

0

0

0

0
4

0.0100616

0

0

0

0

0.00967569

0

0

0

0

0

0

0

0

0

0

0

0

0

0

0

0

0

0

0

0

0

0

0

0

0

0

0.165644
8

0

0

0.0391243

0

0

0

0

0

0

0

0

0

0

0

0

0

0

0

0

0

0

0
4

0
5

0

0

0

0

0

0

0

0

0

0

0

0

0

0

0

0

0

0

0
4

0.608001
6

0

0

0

0.00518609

0.169222

0

0

0.151975

0.0129576

0.0770648

0

0

0.00616813

0

0

0

0.00998585

0

0

0

0

0

0.0267285

0.00518609

0

0

0

0

0.0100616

0

0.0129576

0.0241892

0.0855705

0

0.00458025

0

0

0

0

0

0

0

0

0

0

0

0

0

0

0

0

0

0

0

0.00616813

0

9.54097911787244e-17
6

0
4

2.05395
5

0.333743
5

0.0129576

0

0.103661

0

0

0

0

0

0

0

1.13379

0

0

0

0

0

0

0

0

0

0

0.0777455

0

0

0

0

0

0

0

0.0259152

0

0

0.346701
5

0

0

0

0

0

0

0

0

0

0

0

0

0

0.0194364

0

4.5102810375397e-17
5

0
4

0

0

0

0

0

0

0

0

0

0

0

0

0

0

0

0

0

0

0

0

0
4

0

0

0

0

0

0

0

0

0
4

0.0312995

0.0312995

0
4

0

0

0
4

0

0

0
4

0

0

0
4

0

0

0
4

0

0

0
4

0

0

0
4

0

0

0
4

0

0

0
4

0.00518609

0.00518609

0
4

0.0201566
7

0.00905542
7

0

0.00448724

0

0.00661392

1.73472347597681e-18
7

0
4

0

0

0
4

0

0

0
4

0

0

0
4

0

0

0
4

0

0

0
4

0

0

0
4

0.0770648

0.0770648

0
4

0

0

0
4

0

0

0
4

0.0156497

0.0156497

0
4

0
4

0

0

0

0
4

0

0

0
4

0

0

0
4

0

0

0
4

0

0

0
4

0

0

0
4

0

0

0
4

0

0

0
4

0

0

0
4

0

0

0
4

0

0

0
4

0

0

0

0

0

0

0

0

0
4

0

0

0
4

0

0

0
4

0

0

0
4

0

0

0
4

0.0129576

0.0129576

0
4

0

0

0
4

0

0

0
4

0.0557938
7

0.00897447

0

0.0160309

0.0307884

3.46944695195361e-18
7

0
4

0
4

0

0

0

0

0

0
4

0.00789662
4

0.00789662

0

0

0

0
4

0

0

0

0
4

0.216294
7

0.216294
7

0
4

0.309131
7

0.309131
7

0
4

0
4

0
4

0
4

0
4

0

0

0

0
4

0

0

0

0
4

2.40725
8

0.675758

1.73149

0
4

0.00362217
6

0.00362217

0

0

0
4

0

0

0

0

0

0
4

0

0

0
4

0

0

0

0

0
4

0

0

0
4

0.0156497

0

0.0156497

0

0

0
4

0.325521

0.177557

0.0887784

0.0591856

1.38777878078145e-17

0
4

0

0

0

0
4

10.7167
7

10.7022
7

0.0145135

0

5.8113236445223e-16
7

0
4

0

0

0
4

0.00592247

0.00592247

0

0

0
4

0

0

0

0
4

0

0

0
4

1.03575

1.03575

0

0
4

0

0

0

0
4

0.15413

0.15413

0
4

0

0

0
4

0

0

0
4

0

0

0

0

0
4

0.0387013
7

0.0119798

0.00362217

0.0105027

0.00448724

0.00448724

0.00362217

0

3.90312782094782e-18
7

0
4

0

0

0

0

0
4

0.032394

0.032394

0
4

0

0

0

0
4

0

0

0

0
4

0

0

0

0
4

0

0

0

0

0
4

0.0208358

0.0156497

0.00518609

1.73472347597681e-18

0
4

0

0

0
4

0

0

0

0
4

0

0

0

0
4

0

0

0

0

0

0

0

0

0

0

0

0

0
4

0

0

0

0
4

0

0

0

0
4

0

0

0
4

0

0

0

0
4

0

0

0
4

0

0

0
4

0

0

0

0
4

0

0

0

0
4

0

0

0

0
4

0

0

0
4

2.39134
7

2.19868
7

0

0.192662

0

0
4

0

0

0

0
4

0.00411208

0.00411208

0
4

0.125273

0.115597

0.00967569

0
4

0

0

0
4

0

0

0
4

0

0

0
4

0

0

0

0
4

0

0

0

0
4

0

0

0
4

0

0

0
4

0
3

0

0

0

0
4

0

0

0
4

0.0234746

0.0234746

0
4

0.00822417

0.00822417

0
4

0

0

0
4

0

0

0
4

0

0

0
4

0

0

0
4

0

0

0
4

0

0

0
4

0

0

0
4

0

0

0

0

0
4

0

0

0
4

0

0

0
4

0

0

0
4

0

0

0
4

0

0

0
4

0

0

0
4

0

0

0
4

0

0

0
4

0

0

0
4

0

0

0
4

0.649389
7

0.639017
7

0.0103722

1.21430643318376e-17
7

0
4

0

0

0
4

0

0

0
4

0

0

0
4

0

0

0
4

0.0709939

0.0709939

0
4

0

0

0
4

0

0

0
4

0

0

0
4

0

0

0
4

0

0

0
4

3.08503222967715e-14

0
4

143.862

0.969756
7

0.683649
7

0

0.0748354

0.0312995

0

0.179972

0

0

0

5.55111512312578e-17
7

0
4

79.3694

6.87201
7

2.00305
7

0

0
3

0.0105027
6

0

0

0

0

0.0483785

0

0

3.14558
3

0.0207162

0

0

0.0770648

0

0

0.0188218

0

0

0

1.49714

0

0

0.101723

0

0

0

0

0

0

0

1.66017
7

0

0.144196

0

0.101723

0

0

0

0.409808

0

0.158685

5.23639
7

0.118371

0.0770648

0

0

0

0

0

0

0

0

1.16019
6

0.0591856

0.0533022

0

0

0.0134617

0

0

0

0

0.0241892

0

0.00543325

0

0

0

0

0.0391243

0

0.0317961

0.00687037

0

0.793845

0

0

0.0770648

0

0

0.288392

0

0

0

0

0.841572

0

0.00661392

0

0

0

0

0.00592247

0

0

0

0.0615935

0

0

0

0

0

0

0.00998585

0

0

0.0437921

0
3

0.746259
7

0

0

0

0.0100616

0

0

0

0

0

0

0.452455
7

0

0

0

0

0

0

0

0.00967569

0

0

0
2

0

0

0

0

0

0

0

0

0

0

0
3

0

0

0

0

0

0

0

0.147964

0

0

0
3

0.0297716

0

0

0.0100616

0

0

0.0770648

0

0.062599

0

2.11448
7

0

0

0

0

0

0

0.00408015

0

0

0

2.91042
7

0

0

0

0

0

0

0.025154

0.00332862

0

0

0
3

0.115597

0

0

0.00673085

0

0

0

0

0

0

1.76143
7

0

0

0

0

0

0

0

0.00615463

0

0

0
2

0

0

0

0

0

0.216294

0

0

0

0

13.7358
7

2.74129

0

0.135832

0.0770648

0

0

0

0.00532728

0

0

0

0.147964
6

0

0

0

0

0.0156497

0.0156497

0.00458025

0.0770648

0

0

0
2

0

0

0

0

0

0

0

0

0

0

0
3

0

0

0

0

0.539453

0

0

0

0

0

1.94666
7

0

0

0

0

0

0

0

0

0

0

0
2

0.00411208

0

0

0

0

0

0

0

0

0

0.363433

0

0

0.00724433

0

0

0

0

0

0

0

0.819567
5

0.00532728

0

0.00394831

0.0325995

0

0

0

0

0

0

0
5

0

0.0156497

0

0

0

0

0

0.0100616

0

0

0
3

0

0

0

0

0

0

0

0.0770648

0

0.0108665

0
4

0.221261
6

0

0

0

0

0

0

0.00881856

0

0

0

0.00440928
3

0.0234746

0

0

0

0

0

0

0

0

0

0

0

0

0

0

0

0.025154

0

0

0

0

0
3

0

0

0

0

0

0

0

0

0.0156497

0

0.0626555
7

0

0

0

0

0

0

0

0

0

0

0
3

0.00448724

0

0

0

0

0

0.0145135

0

0.0091605

0

0
3

0

0

0

0

0

0

0

0

0

0

0.220057
7

0

0

0

0

0

0

0

0

0

0

0
3

0

0

0

0

0

0.0156497

0

0

0

0

0.165978
7

0

0

0

0

0

0

0

0

0

0

4.27967
7

0
2

0

0

0.177557

0

0

0.00518609

0.00394831

0.00799092

0

0

0

0.0133182

0

0

0

0

0

0

0

0

0

0.383419

0

0

0

0

0

0

0

0

0

0.00616813

0

0

0

0

0

0

0

0

0

0

0

0

0

0

0

0

0

0

0

0

0

0

0.462028

0

0.00411208

0

0

0

0

0

0

0

0

0
3

0

0

0

0

0

0

0

0

0

0.0312995

0.336469

0

0.00616813

0

0

0

0

0

0

0

0

0.0156497
5

0

0

0

0

0

0

0

0

0

0

0
3

0

0

0

0.0234746

0

0

0

0

0

0

4.34608
7

0
3

0

0

0

0

0.0234746

0

0

0

0

0

0
3

0

0

0

0

0

0

0

0.00777913

0

0

0
3

0

0

0.0161662

0

0.0234746

0

0

0

0.00411208

0

0.0102802
6

0

0

0

0

0

0.0259304

0

0

0

0

0.112107
7

0.00592247

0.0391243

0

0

0

0

0

0

0

0

0.00543325
7

0.0129652

0

0

0

0

0

0

0

0.00665723

0

0.268743
7

0

0

0

0

0

0

0

0

0

0

0.81996
7

0

0

0

0

0

0

0

0

0

0

0.00661392
2

0

0

0.0233374

0

0

0

0

0

0

0

0
3

0

0

0

0.0220464

0

0.00518609

0

0

0

0

6.67513

0
2

0.0156497

0

0

0

0

0

0

0

0

0.00411208

0
3

0

0

0

0

0

0

0.0860736

0

0.00362217

0

0
3

0

0

0

0

0

0

0

0

0

0.0129652

0.0390648

0

0

0

0

0

0

0

0

0

0

0

0

0

0

0

0

0

0.00881856

0

0

0

0.00592247
6

0

0

0

0.00332862

0

0.00799092

0

0

0

0.00458025

0
3

0

0

0

0

0.0198418

0

0

0

0

0

0
2

0

0

0

0.00532728

0.00408015

0

0

0

0

0

0.250166
7

0

0

0

0

0

0

0

0

0

0

0.461667

0

0

0

0

0

0

0

0

0

0

0.346792
3

0.00967569

0.00518609

0

0

0

0.1465

0

0

0

0

0

0.223304
7

0

0

0

0

0

0

0

0

0

0

0

0.0156497

0

0

0

0

0

0

0

0

0

0

0

0

0.0156497

0

0

0

0

0

0

0.298325
6

0

0.0862253
7

0

0.093514
7

0.171251

1.34857

0

0
3

0.0234746

0

0.275246

0

0.777504
6

0.192662
7

0.672939

0

4.89608353859694e-14

0
4

0

0

0

0

0

0

0

0

0

0

0

0

0

0

0

0

0
4

53.9668

49.8009

1.56425
7

0
4

0.0811769

0.0155583

0

0.701764

0

0

0

0.0770648

0

0
4

0

0

0

0

0

0

0

0

0

0.00518609

0.0890088
7

0

0

0

0

0

0

0

0

0

0

0.0673886
7

0

0

0.00458025

0

0

0

0

0.00518609

0

0

0.181095
4

0

0.0234746

0

0

0

0

0

0

0

0

0.0770648
7

0

0

0

0.0201232

0

0

0.0129576

0

0

0

0
4

0

0

0.0770648

0

0

0

0

0

0

0

0.123821

0

0

1.03913
7

0
4

6.78772
7

2.80116
7

0

0

0

0.00458025

0

0

0

0

0

0

0.172494
6

0

0

0.0312995

0

0

0

1.09952
7

0.239887

0.0103722
6

0.973682
7

1.45473
7

0

0

1.11022302462516e-15
7

0
4

0
4

0

0

0

0

0
4

0

0

0
4

0

0

0
4

0

0

0
4

0

0

0
4

0

0

0
4

0

0

0
4

0

0

0
4

0

0

0
4

0

0

0
4

0

0

0
4

0
4

0
4

0

0

0
4

0

0

0
4

0

0

0
4

0

0

0
4

0

0

0
4

0

0

0
4

0

0

0
4

0.0100616

0.0100616

0
4

0

0

0
4

0

0

0
4

0

0

0
4

0.100539
7

0.0770648
7

0.0234746

3.46944695195361e-18
7

0
4

0

0

0
4

0

0

0
4

0

0

0
4

0

0

0
4

0

0

0
4

0

0

0
4

0

0

0
4

0

0

0
4

0

0

0
4

0

0

0
4

0

0

0

0

0

0
4

0

0

0
4

0

0

0
4

0

0

0
4

0

0

0
4

0

0

0
4

0

0

0
4

0

0

0
4

0

0

0
4

0

0

0
4

0

0

0
4

0
3

0

0

0

0
4

0

0

0
4

0

0

0
4

0

0

0
4

0.288392

0.288392

0
4

0

0

0
4

0

0

0
4

0

0

0
4

0

0

0
4

0

0

0
4

0

0

0
4

0
2

0

0

0

0

0
4

0

0

0

0

0

0

0
4

0.90972
8

0.886245

0.0234746

1.73472347597681e-17
8

0
4

0

0

0

0

0

0
4

0.144196
7

0.144196
7

0
4

0
4

0
4

0

0

0

0

0

0

0

0

0

0

0
4

0

0

0

0

0

0

0

0

0

0

0
4

0

0
4

0
4

0
4

0

0

0

0
4

0.269727

0.269727

0
4

0.0156497

0.0156497

0

0
4

0

0

0

0

0
4

0

0

0

0
4

0

0

0
4

0

0

0

0
4

0

0

0

0
4

0

0

0

0

0
4

0.158019

0.158019

0
4

0

0

0

0

0
4

0
4

0
4

0
4

0

0

0

0

0

0

0
4

0.0276382

0.0276382

0

0

0
4

0

0

0

0
4

0

0

0
4

0.00394831

0

0

0.00394831

0
4

0

0

0

0
4

0

0

0

0

0
4

0

0

0

0

0
4

0.146897

0.146897

0
4

0

0

0

0
4

0

0

0

0
4

0.335952
5

0.200992
7

0
5

0.0842243

0.0194364

0

0.0312995

0

0

0
4

0

0

0

0
4

0

0

0

0
4

0.0388728

0.0388728

0

0
4

0

0

0

0
4

0.0194364

0.0194364

0
4

0

0

0
4

0

0

0

0
4

0

0

0

0
4

0

0

0

0
4

0

0

0

0
4

0
3

0
3

0

0

0

0

0

0

0
4

0

0

0

0
4

0

0

0
4

0

0

0
4

0

0

0
4

0

0

0
4

0

0

0

0
4

0

0

0

0
4

0

0

0

0
4

0

0

0
4

0

0

0
4

0
3

0
3

0

0

0

0
4

0.00665723

0.00665723

0
4

0

0

0
4

0

0

0
4

0

0

0
4

0

0

0
4

0

0

0
4

0

0

0
4

0

0

0
4

0

0

0
4

0

0

0
4

0
4

0
4

0

0
4

0

0

0
4

0

0

0
4

0

0

0
4

0

0

0
4

0

0

0
4

0

0

0
4

0

0

0
4

0

0

0
4

0.0391243

0.0391243

0
4

0.0156497

0.0156497

0
4

0.00592247
3

0.00592247
4

0

0

0

0
4

0

0

0
4

0

0

0
4

0

0

0
4

0

0

0
4

0

0

0
4

0

0

0
4

0

0

0
4

0

0

0
4

0

0

0
4

0

0

0
4

0
4

0

0

0

0

0

0
4

0

0

0
4

0

0

0
4

0

0

0
4

0

0

0
4

0

0

0
4

0

0

0
4

0.0156497

0.0156497

0
4

0

0

0
4

0.216294

0.216294

0
4

0

0

0
4

2.40696351738734e-13

0
4

4.88629
3

4.85499
3

4.84203
3

0

0

0

0

0

0

0

0

0

0

0

0

0

0

0

0

0.0129576

0

0

0

0

7.40726924242097e-16
3

0
4

0
4

0
4

0

0

0
4

0

0

0
4

0

0

0
4

0

0

0

0
4

0

0

0

0
4

0

0

0

0
4

0

0

0
4

0

0

0
4

0

0

0
4

0

0

0
4

0

0

0
4

0

0

0

0
4

0

0

0
4

0

0

0
4

0

0

0
4

0

0

0
4

0

0

0
4

0

0

0
4

0

0

0
4

0

0

0
4

0

0

0
4

0

0

0
4

0

0

0
4

0

0

0
4

0

0

0
4

0

0

0
4

0

0

0
4

0

0

0
4

0

0

0
4

0.0156497

0.0156497

0
4

0

0

0
4

0

0

0
4

0

0

0
4

0

0

0

0

0
4

0

0

0
4

0

0

0
4

0

0

0
4

0

0

0
4

0

0

0

0

0
4

0

0

0
4

0

0

0
4

0.0156497

0.0156497

0
4

0

0

0

0
4

0
4

0.0091605
2

0.0091605
2

0
2

0
2

0

0

0

0

0

0

0

0

0

0

0

0

0

0

0

0

0

0

0

0

0

0

0

0

0

0

0

0

0

0

0

0

0

0

0

0

0

0

0

0

0

0

0

0

0

0

0

0

0

0

0

0

0

0

0

0

0

0

0

0

0

0

0

0

0

0

0

0

0

0

0

0

0

0

0

0

0

0

0

0

0

0

0

0

0

0

0

0

0

0

0

0

0
2

0

0

0

0

0

0

0

0.00458025

0

0

0

0

0

0

0

0.00458025

0

0

0

0

0

0
4

0

0

0
4

0

0

0
4

0
4

304.593

268.936
7

67.3476
7

2.74273
7

0

0

0.0118427

0.0145135

0.0100616

0.36049

0.0668185

0.00394831

0.516937

0

0

0

0

0

0

0

0

0

0.00394831

0.693583

0.0281396

0.0906012

0.115597

0.00458025

0

0

0.0091605

0

0.0269437

0

0

0

0.288392
6

0.0897827

0

0

0

0

0.0100616

0.203382

0.0150924

0

0

0.167456

0

0.169019

0.0100616

0.0308921

0.0145135

0

0.00967569

0

0

0

0.507668
7

0

0.0402465

0.00458025

0

0

0

0.00592134

0

0

0.0582868

0.264437
6

0.0100616

0

12.7613

0.0384887

0

0

0.0138191

0

0

0

0.125682
7

0

0.107775

0

0.0384887

0

0

0

0

0

0

0.0961339
7

0.0100616

0

0.0343519

0

1.48522

0

0

0

0

0

0.327563
7

0

0.00923194

0.0236854

0.0100616

0

0

0.109491

0

0

0.414299

124.126
7

0.179444
6

0

0

0.00615463

0.36049

0.00394831

0.159846

0.432588

0

0

0

0.472643
7

0.0296067

0

0.0409438

0

0

0.432588

0

0

0.00687037

0

0.0752577
7

0.0100616

0

0

0.0591856

0

0

0.144196

0.0100616

0.00967569

0.0177674

2.62408
7

0

0

0

0

0.00592134

0.0887784

0

0

0.0870812

0

0.0722383
7

0

0

0.0298032

0

0

0

0

0

0

0.00888202

0
6

0

0.00394831

0

0

0

0

0

0

0.0290271

0.00624502

1.89062
7

0

0

0

0

0

0

0

0

0.147964

0.0100616

0.240354
7

0

0

0

0

0

0

0

0

0

0

0.161128
6

0.0809404

0.00518609

0

0.0206111

0

0

0

0.00416335

0

0

7.00649
7

0

0

0

0

0

0.0201232

0.0100616

0

0.0100616

0.0100616

5.17651
7

0.0549943
5

0

0

0

0

0

0

0

0.0118427

0

0

0.571082
7

0

0

0

0.0377212

0

0

0

0

0

0

0.0842411
7

0

0

0

0

0.0104084

0

0

0

0

0

0.306066
7

0

0

0.00592247

0

0

0

0

0

0

0

0.304185
7

0

0

0

0

0

0

0

0.00592134

0

0

0.673919
7

0

0

0

0

0.00592134

0

0.0100616

0.0207247

0

0

0

0.0100616

0.025154

0

0

0

0

0

0

0.0100616

0

0.139016
7

0

0

0.0241892

0

0

0

0.0118427

0

0

0

0
6

0

0

0

0

0

0

0

0

0.0091605

0

0

0

0.144196

0

0

0

0

0.00615463

0

0

0

1.8974
6

0.0100616
6

0.021555

0

0

0

0

0

0

0.0100616

0

0

0

0

0

0.140298

0

0

0

0

0

0

0.0160309

0.884913
7

0.00592247

0

0

0

0

0

0.0770648

0

0

0

0.237226
7

0

0.00394831

0

0

0

0.423856

0

0

0.0704313

0

0

0

0

0

0

0.0145135

0

0

0

0

0

0.0316133
6

0.0100616

0.0118427

0

0

0

0.00458025

0.00687037

0

0

0

0.0350024
6

0

0

0

0

0

0

0

0

0

0

0.0929762
7

0

0

0

0.118371

0

0.00592247

0

0

0.0145135

0

0.491773
7

0

0.177557

0

0

0

0

0

0

0

0.0100616

0.380847
7

0

0.0241892

0.0201232

0

0.216294

0

0.0100616

0

0

0

9.93024
7

0

0

0.621449

0

0.216294

0

0

0

0

0

0.00687037

0.00888202

0

0

0

0.00888202

0

0

0

0

0

0.00394831

0.0118427
7

0

0

0

0

0

0.0100616

0.0100616

0

0.0145135

0

0.00967569
6

0

0

0

0

0.0591856

0

0.0091605

0

0

0

0.504686
7

0

0

0

0.144196

0

0

0

0

0

0

0.597799
7

0

0

0

0

0.00394831

0.0107775

0

0

0.167051

0

0.0107775
6

0

0

0.0118427

0

0

0

0

0.0513281

0

0

0.312572

0

0.118371

0.00592247

0.0236854

0

0.00394831

0

0

0

0

0.0215206
7

0

0

0

0

0

0.0100616

0

0

0

0

0.025154
7

0

0

0

0.0887784

0

0.144196

0.0100616

0.0100616

0

0

2.44903
7

0.216294
7

0

0

0

0.00394831

0

0

0

0.0156497

0

0

0.0452773
7

0

0

0

0.144196

0

0

0

0

0

0.00394831

0.129643
7

0

0.0591856

0.0107775

0.144196

0

0

0

0

0.00394831

0

0.0201232
6

0

0.0100616

0

0

0

0

0

0.0395518

0

0

0.0591856

0.231194

0

0
6

0.0100616

7.32731
8

0.105834
5

0.519778
7

0

0.00967569
6

0.0549636

0.0100616

0.269727

0.0679061

0.0108253
7

0

0.0716802

0.917056
7

0.0562306

0
7

0

0

0

0.0337386
7

0.0107775

0

0.00615463

0.301849

0.470097
7

0

0.0150924

0

0

0

0

0.0100616

0.0150924

0.00923194

0.00967569

0
4

34.6042
7

34.6042
7

0

0
4

0.0870247
4

0.0709939
4

0

0

0

0

0

0

0

0

0

0

0

0.0160309

0

0

0

0
4

0.00976634

0.00976634

0
4

0

0

0
4

0

0

0
4

0

0

0
4

0

0

0
4

0

0

0
4

0

0

0
4

0

0

0
4

0

0

0
4

0

0

0
4

0

0

0
4

0

0

0

0
4

0

0

0
4

0.504686

0.504686

0
4

0

0

0
4

0

0

0
4

0.0181513

0.0181513

0
4

0.432588

0.432588

0

0
4

0

0

0
4

0

0

0
4

0

0

0
4

0

0

0
4

0
4

1.78856
4

1.69786
4

0
4

0.892984
5

0

0

0

0

0

0

0

0

0

0

0
3

0

0

0

0

0

0

0

0

0

0

0
4

0

0

0

0

0

0

0

0

0

0

0
4

0

0

0

0

0

0

0

0

0

0

0

0

0

0

0

0

0

0

0

0

0

0

0

0

0

0

0

0

0

0

0

0

0
4

0

0

0

0

0

0

0

0

0

0.0234746

0.156605
5

0

0

0

0

0

0

0

0

0

0

0

0

0

0

0

0

0

0

0

0

0

0

0

0

0

0

0

0

0

0

0

0

0

0
4

0

0

0

0

0

0

0

0

0

0

0

0

0

0.0194364

0

0

0

0

0

0

0

0

0

0

0

0

0

0

0

0

0

0

0

0

0

0

0

0

0

0.0129576

0

0

0

0

0

0

0

0

0

0

0

0

0

0

0

0

0

0

0

0

0

0

0

0

0

0

0

0

0

0

0

0

0

0

0

0

0

0

0

0

0

0

0

0

0

0

0

0

0

0

0

0

0

0

0

0

0

0

0
4

0

0

0

0

0

0

0

0

0

0

0

0

0

0

0

0

0

0

0

0

0

0

0

0

0

0

0

0

0

0

0

0

0

0

0

0

0

0

0

0

0

0

0

0

0

0

0

0

0

0

0

0

0

0

0

0

0

0

0

0

0

0

0

0

0

0

0
3

0

0

0

0

0

0

0

0

0

0

0

0

0

0

0

0

0

0

0

0

0

0

0

0

0

0

0

0

0

0

0

0

0

0

0

0

0

0

0

0

0

0

0

0

0

0

0

0

0

0

0

0

0

0

0

0

0

0

0

0

0

0

0

0

0

0

0.0683708

0

0

0

0

0

0

0

0

0

0

0

0

0

0

0

0

0

0

0

0

0

0

0

0

0

0

0

0

0

0

0

0

0

0

0

0

0

0

0

0

0.0129576

0

0

0

0

0

0

0

0

0

0

0

0

0

0
4

0

0

0

0

0

0

0

0

0

0

0

0

0

0

0

0

0.0518304

0

0

0

0

0

0

0

0

0
4

0
4

0

0

0

0

0

0

0.0770648

0

0

0

0
4

0

0

0

0

0

0

0

0

0

0

0
3

0

0

0

0

0

0

0

0

0

0

0

0

0

0

0

0

0

0

0

0

0

0.382175
5

0

0

0

0

0

0

0

0

0

0

0
4

0
4

0
4

0

0

0

0

0

0

0

0

0

0

0

0

0

0

0

0

0

0

0
4

0

0

0

0

0
4

0

0

0

0
4

0

0

0

0
4

0

0

0

0

0

0
4

0

0

0

0
4

0

0

0
4

0

0

0

0
4

0

0

0

0

0
4

0

0

0

0
4

0

0

0

0

0
4

0
4

0
4

0

0

0
4

0
4

0

0

0

0

0

0

0
4

0

0

0

0
4

0

0

0
4

0

0

0

0
4

0

0

0
4

0

0

0
4

0

0

0

0
4

0

0

0
4

0

0

0

0
4

0

0

0

0
4

0.0907031

0.0907031

0
4

0
3

0
3

0

0

0

0

0

0
4

0

0

0
4

0

0

0
4

0

0

0
4

0

0

0
4

0

0

0
4

0

0

0
4

0

0

0
4

0

0

0
4

0

0

0
4

0

0

0
4

0
3

0
3

0
4

0

0

0
4

0

0

0
4

0

0

0
4

0

0

0
4

0

0

0
4

0

0

0
4

0

0

0
4

0

0

0
4

0

0

0
4

0

0

0
4

0

0

0

0

0
4

0

0

0

0

0
4

0

0

0

0

0
4

0

0

0

0

0

0
4

0

0

0

0

0

0
4

0
4

22.7911
4

11.6729
5

1.67799

5.6822
5

0
4

0

0

0

0

0

0

0

0

0

0

0.181406
5

0

0

0

0

0

0

0.15413

0

0

0.0388728

0.104984
5

0

0

0

0

0

0

0

0

0

0

0.0453516

0

0

0

0.00777913

0

0

0.0194364

0

0

0.593584
5

0

0.0129576

0

0

0

0

0

0

0.0129576

0

1.96494
5

0

0.11014

0

0.136055

0

0

0

0

0

0

0.418799
6

0

0

0

0

0

0

0

0

0

0

0.0770648
4

0.123097

0

0

0

0

0

0

0

0

0

0.10636
5

0

0

0

0.0712667

0

0.0155583

0

0

0

0

0.0738903

0

0.0388728

0.00518609

0

0

0

0

0

0

0

0
5

0

0

0

0

0

0

0

0

0

0

0

0

0

0

0

0

0

0

0

0

0

0
4

5.94893
4

1.64217
6

0
3

0
4

0
5

0.620904
5

0.64006
5

0.986035
4

0
4

0.142533
4

0
4

0

0

0

0

0

0

0

0

0.0129576

0

0
4

0

0

0

0

0

0

0

0

0

0

0

0

0

0

0

0

0

0

0

0

0

0
4

0

0

0

0

0

0

0

0

0

0

0

0

0

0

0

0

0

0

0

0

0

0

0

0

0

0

0

0

0

0

0

0

0
4

0

0

0

0

0

0

0

0

0

0

0

0

0

0

0

0

0

0

0

0

0

0

0

0

0

0

0

0

0

0

0

0

0

0

0

0

0

0

0

0

0

0

0

0
4

0.732115
7

0

0

0

0

0

0

0

0

0

0

0

0

0

0

0

0

0

0

0

0

0

0

0

0

0

0

0

0

0

0

0

0

0

0

0

0

0

0

0

0

0

0

0

0

0

0

0

0

0

0

0

0

0

0

0
4

0

0

0

0

0

0

0.0129576

0

0

0

0

0

0

0

0

0

0

0

0

0

0

0

0

0

0

0

0

0

0

0

0

0.0583091

0

0

0.511144
4

0

0

0

0

0

0

0.576784

0

0

0

0
3

0

0

0

0

0

0

0

0

0

0

0

0

0

0

0

0

0

0

0

0

0

0
4

0

0

0

0

0

0

0

0

0

0

0
4

0

0

0

0

0

0

0

0

0

0

0
4

0

0

0

0

0

0

0

0

0

0

0.0129576
3

0

0

0

0

0

0

0

0

0

0

8.51749226704612e-16
4

0
4

5.10446

1.56509
8

0

0.0228984

0.0234746

0.504686

0.00416335

0

0

0

0

0

1.91912
8

0

0

0

0

0

0

0.0156497

0

0

0

0.0399847

0

0

0.00592247

0

0

0

0

0

0

0

0.339559

0

0.576784

0

0.0871264

0

0

3.19189119579733e-16

0
4

0

0

0

0

0

0

0

0

0
4

0

0

0
4

0

0

0
4

0

0

0
4

0

0

0
4

0

0

0
4

0

0

0
4

0

0

0
4

0

0

0
4

0

0

0
4

0

0

0
4

0
4

0
4

0

0

0

0
4

0

0

0
4

0

0

0
4

0

0

0
4

0.0647879

0.0647879

0
4

0
4

0
4

0

0

0
4

0
4

0

0

0

0

0
4

0

0

0

0

0
4

0

0

0
4

0

0

0
4

0

0

0
4

0

0

0
4

3.46944695195361e-16
4

0
4

0.231587
3

0
4

0
4

0

0

0

0

0

0

0

0

0

0

0
4

0

0

0

0

0

0
4

0

0

0
4

0

0

0
4

0

0

0
4

0

0

0
4

0

0

0
4

0.221716
3

0.0592247
4

0

0.0138191

0

0

0

0.148673

0

0
4

0

0
4

0

0

0

0
4

0
3

0
3

0
4

0
4

0
4

0
4

0
4

0

0

0

0

0

0

0
4

0
3

0

0

0

0

0

0

0

0
4

0
4

0
4

0

0

0

0
4

0.00987078
4

0

0

0.00987078

0

0

0
4

0

0

0

0

0
4

0

0

0

0

0

0
4

1.90819582357449e-17
3

0
4

1.77939

1.67885

0.555103

0

0

1.04668
7

0

0

0

0.0770648

0

0

0

0
4

0.0770648

0

0

0

0.0770648

0
4

0.0234746

0.0234746

0

0

0

0

0
4

0

0

0

0
4

0

0

0
4

3.12250225675825e-17

0
4

0
4

0
4

0

0

0

0

0
4

0

0

0

0
4

0
4

0

0

0

0

0

0

0
4

0

0

0

0
4

0
4

0.868158

0.848722

0.848722

0

0
4

0.0194364

0.0194364

0

0
4

0

0

0
4

0
4

0

0

0

0

0

0
4

0
4

0
3

0
3

0
3

0

0
4

0

0

0
4

0
4

0
4

0

0

0

0
4

0

0

0
4

0

0

0
4

0
4

0

0

0

0

0

0

0
4

0

0

0
4

0
4

0

0

0

0

0
4

0

0

0

0

0
4

0

0

0
4

0
4

0.144196
6

0.144196
6

0.144196
6

0
4

0
4

1.47069

1.47069

1.47069

0
4

0
4

0
3

0
3

0
3

0

0

0
3

0

0

0

0

0

0

0

0
4

0

0

0

0

0

0
4

0

0

0

0

0
4

0
4

0

0

0

0

0

0
4

0

0

0
4

0
4

0
4

0

0

0

0

0
4

0

0

0
4

0

0

0
4

0
4

0.0106546

0.0106546

0.0106546

0

0
4

0
4

0
3

0

0

0
4

0

0

0

0
4

0

0

0
4

0

0

0
4

0

0

0
4

0

0

0
4

0
4

0

0

0

0

0
4

0
4

0

0

0

0

0
4

0

0

0

0

0
4

0

0

0
4

0

0

0
4

0
4

0

0

0

0

0

0

0
4

0
4

0.508454

0.508454

0.36049

0.147964

0

5.55111512312578e-17

0
4

0

0

0

0

0
4

0
4

0.0591856

0.0591856

0.0591856

0

0
4

0
4

0

0

0

0

0
4

0
4

0
3

0
3

0
3

0

0
4

0

0

0

0

0

0

0

0
4

0

0

0

0

0
4

0

0

0

0
4

0

0

0

0
4

0

0

0

0
4

0
4

0

0

0

0

0
4

0

0

0

0
4

0

0

0
4

0
4

0

0

0

0

0

0

0

0

0
4

0
4

0
4

0
4

0

0

0

0
4

0

0

0
4

0

0

0
4

0
4

0

0

0

0

0

0
4

0

0

0

0
4

0
4

0.0178209

0.0137407

0.0091605

0.00458025

0
4

0

0

0

0
4

0.00408015

0.00408015

0
4

8.67361737988404e-19

0
4

0

0

0

0

0

0

0
4

0

0

0
4

0
4

0

0

0

0

0

0
4

0

0

0
4

0

0

0
4

0

0

0
4

0
4

0
4

0
4

0
4

0
4

0

0

0
4

0

0

0
4

0
4

0
4

0
4

0

0

0

0
4

0
4

0

0

0

0

0
4

0

0

0

0
4

0
4

1.0303
3

0.485432
3

0.485432
3

0
3

0

0

0

0

0

0

0

0
4

0

0

0
4

0

0

0
4

0

0

0
4

0

0

0
4

0

0

0
4

0.505384
4

0.491565
4

0.00789662

0

0.00592247

0
4

0.0394831
4

0

0.0394831

0

0
4

0

0

0

0
4

0

0

0

0
4

0

0

0
4

0

0

0
4

0

0

0
4

0

0

0
4

0
4

0.346792
6

0.346792
6

0.346792
6

0
4

0
4

0

0

0

0
4

0

0

0
4

0

0

0

0
4

0

0

0
4

0
4

0

0

0

0

0
4

0
4

0
3

0
3

0

0

0

0
4

0

0

0
4

0
4

0

0

0

0

0

0
4

0

0

0
4

0
4

0.691248

0.691248

0.691248

0
4

0

0

0
4

0

0

0
4

0

0

0
4

0
4

0.375912

0.375912

0.305858

0.0161662

0.0538875

0
4

0
4

0.019841

0.0144077

0.00543325

0.00448724

0.00448724

0

0
4

0.00543325

0.00543325

0
4

0
4

0

0

0

0
4

0

0

0
4

0

0

0
4

0
4

0.0770648

0.0770648

0.0770648

0

0

0

0
4

0
4

0.163632
5

0.163632
5

0.0194364
5

0

0

0

0.144196

0

0

0

0

0

0

0
5

0

0
4

0

0

0

0

0

0

0
4

0
4

0

0

0

0

0

0

0

0
4

0
4

0

0

0

0

0

0

0
4

0

0

0
4

0
4

0

0

0

0

0
4

0
4

0

0

0

0

0

0

0
4

0

0

0
4

0

0

0
4

0
4

0

0

0

0

0
4

0

0

0

0
4

0

0

0
4

0
4

0

0

0

0

0
4

0

0

0
4

0
4

0

0

0

0

0

0
4

0
4

0

0

0

0

0
4

0

0

0
4

0
4

0

0

0

0

0

0
4

0

0

0
4

0
4

0

0

0

0

0

0
4

0
4

0.840535
5

0.840535
5

0.250748
5

0.314307

0.0591856

0

0.216294

0

0

0

0

1.11022302462516e-16
5

0
4

0
4

0

0

0

0

0
4

0

0

0
4

0

0

0
4

0

0

0
4

0
4

0

0

0

0

0

0
4

0

0

0

0
4

0
4

0

0

0

0

0

0
4

0

0

0

0
4

0
4

0

0

0

0

0
4

0

0

0
4

0

0

0
4

0
4

0

0

0

0

0
4

0

0

0
4

0

0

0
4

0

0

0
4

0
4

0

0

0

0

0
4

0
4

0
4

0

0

0

0
4

0

0

0
4

0
4

0.0156497

0.0156497

0.0156497

0
4

0
4

0

0

0

0

0

0
4

0

0

0
4

0
4

0

0

0

0

0

0
4

0

0

0
4

0

0

0
4

0
4

0
4

0
4

0
4

0

0

0

0

0

0

0

0

0

0

0

0
4

0

0

0
4

0

0

0

0
4

0

0

0
4

0

0

0
4

0

0

0
4

0

0

0
4

0

0

0
4

0

0

0
4

0

0

0
4

0
4

0
4

0

0

0
4

0

0

0
4

0
4

0

0

0

0

0
4

0

0

0
4

0
4

0.0150924

0.0150924

0

0.0150924

0
4

0

0

0

0
4

0
4

0

0

0

0

0
4

0

0

0
4

0
4

0

0

0

0

0

0
4

0

0

0

0
4

0
4

0

0

0

0
4

0
4

0

0

0

0

0

0
4

0

0

0

0
4

0
4

0

0

0

0

0
4

0

0

0

0
4

0

0

0
4

0
4

0

0

0

0

0

0
4

0

0

0

0
4

0
4

0

0

0

0

0

0
4

0

0

0
4

0

0

0
4

0
4

0.0259152

0.0259152

0
6

0

0.0259152

0

0

0

0

0
4

0

0
6

0

0

0

0

0

0

0

0
4

0

0

0

0
4

0

0

0

0
4

0
4

0

0

0

0

0

0

0
4

0

0

0
4

0
4

0

0

0

0

0

0
4

0

0

0
4

0

0

0
4

0
4

0.00616813

0.00616813

0.00616813

0

0

0
4

0

0

0
4

0
4

0

0

0

0
4

0
4

0

0

0

0

0
4

0

0

0
4

0

0

0
4

0
4

0

0

0

0

0
4

0

0

0

0
4

0

0

0
4

0
4

0.027638

0.027638

0.027638

0
4

0
4

0

0

0

0

0

0
4

0
4

0

0

0

0

0
4

0
4

0

0

0

0

0

0

0
4

0
4

0.289163

0
6

0
6

0

0

0

0

0

0

0

0
4

0
2

0

0

0

0

0

0

0
4

0.0194364
2

0

0.0194364

0

0

0
4

0

0

0

0

0
4

0

0

0

0
4

0.269727

0.269727

0

0
4

0

0

0
4

0
4

0

0

0

0

0

0

0
4

0
4

0

0

0

0

0
4

0

0

0

0
4

0
4

0

0

0

0
4

0
4

0

0

0

0

0
4

0

0

0
4

0
4

0

0

0

0

0
4

0

0

0
4

0
4

0.195622

0.195622

0.109548

0.0860736

0
4

0
4

0

0

0

0

0

0
4

0
4

0

0

0

0

0
4

0

0

0
4

0
4

0

0

0

0
4

0

0

0

0
4

0
4

0

0

0

0
4

0

0

0
4

0
4

1.75137
5

1.75137
5

1.75137
5

0

0

0
4

0

0

0
4

0

0

0
4

0
4

0

0

0

0

0

0
4

0

0

0
4

0
4

0

0

0

0

0
4

0
4

0

0

0

0
4

0
4

0

0

0

0

0
4

0

0

0
4

0
4

0.0084121

0.0084121

0.0084121

0
4

0

0

0

0
4

0
4

0

0

0

0
4

0

0

0
4

0

0

0
4

0
4

0

0

0

0

0

0
4

0
4

0

0

0

0

0
4

0

0

0
4

0
4

0

0

0

0

0

0
4

0

0

0
4

0
4

0

0

0

0

0

0

0
4

0
4

0

0

0

0

0

0

0

0

0

0

0

0

0

0

0

0

0

0

0

0

0

0

0

0

0

0

0

0

0

0

0

0

0

0

0

0

0

0
4

0

0

0

0

0

0

0

0

0

0

0
4

0
4

0.315243

0.315243

0

0

0

0

0

0

0.144196

0

0

0

0

0

0.171047

0

0
4

0
4

0

0

0

0
4

0

0

0
4

0

0

0
4

0
4

0

0

0

0

0
4

0
4

0

0

0

0

0
4

0
4

0

0

0

0
4

0

0

0
4

0

0

0
4

0
4

0

0

0

0
4

0

0

0
4

0
4

0

0

0

0
4

0

0

0
4

0
4

0

0

0

0

0
4

0
4

0

0

0

0

0
4

0

0

0
4

0
4

0

0

0

0

0
4

0

0

0
4

0
4

0

0

0

0

0
4

0

0

0
4

0
4

0

0

0

0

0

0

0

0

0

0

0

0

0

0

0

0

0

0

0

0

0

0
4

0

0

0

0

0

0

0

0
4

0

0

0

0
4

0
4

0

0

0

0

0
4

0
4

0.614406

0.614406

0.614406

0
4

0
4

0

0

0

0

0
4

0
4

0

0

0

0
4

0

0

0
4

0
4

0

0

0

0

0
4

0

0

0
4

0
4

0

0

0

0

0

0
4

0
4

0

0

0

0
4

0

0

0
4

0
4

0

0

0

0
4

0

0

0
4

0
4

0

0

0

0

0
4

0

0

0
4

0
4

0

0

0

0

0
4

0
4

0
3

0
3

0
3

0

0

0

0

0

0

0

0

0
4

0

0

0

0
4

0

0

0
4

0
4

0

0

0

0
4

0

0

0
4

0
4

0

0

0

0

0
4

0
4

0

0

0

0
4

0

0

0
4

0

0

0
4

0
4

0

0

0

0

0
4

0

0

0
4

0
4

0

0

0

0

0
4

0

0

0
4

0
4

0

0

0

0

0
4

0

0

0
4

0
4

0

0

0

0

0
4

0
4

0

0

0

0
4

0
4

0.116687

0.116687

0.111501

0.00518609

1.73472347597681e-18

0
4

0
4

0.194433

0.0777455

0.0777455

0

0
4

0.116687

0.116687

0
4

0
4

0.0236899
4

0
4

0

0

0

0

0

0

0

0

0
4

0
4

0
4

0

0

0

0

0
4

0.00987078

0

0.00394831

0.00592247

0

8.67361737988404e-19

0
4

0.0138191

0.00789662

0.00592247

0
4

0

0

0
4

0
4

0.0107644

0.00660102

0.00660102

0
4

0.00416335

0.00416335

0
4

8.67361737988404e-19

0
4

0

0

0

0
4

0

0

0
4

0

0

0
4

0
4

0

0

0

0

0
4

0
4

0

0

0

0

0
4

0
4

0

0

0

0
4

0
4

0

0

0

0
4

0

0

0
4

0
4

0

0

0

0
4

0
4

0.203382

0.203382

0.203382

0

0
4

0
4

0

0

0

0

0

0
4

0
4

0

0

0

0

0
4

0
4

1.29576

0.0583091
5

0

0

0.0583091

0

0

0

0

0

0
4

1.19858

1.19858

0

0

0
4

0.0388728

0.0194364

0.0194364

0
4

0

0

0
4

2.0122792321331e-16

0
4

0

0

0

0

0
4

0
4

0

0

0

0
4

0

0

0
4

0
4

0

0

0

0
4

0
4

0

0

0

0
4

0
4

0

0

0

0
4

0
4

0

0

0

0

0
4

0
4

0

0

0

0
4

0

0

0
4

0
4

0

0

0

0

0
4

0
4

0

0

0

0

0
4

0
4

0

0

0

0
4

0

0

0
4

0
4

0
2

0
2

0

0

0

0

0
2

0

0

0

0

0

0

0

0
4

0

0

0

0
4

0
4

0

0

0

0
4

0
4

0

0

0

0

0
4

0
4

0

0

0

0
4

0
4

0

0

0

0

0
4

0
4

0

0

0

0

0
4

0
4

0

0

0

0

0
4

0
4

0

0

0

0

0
4

0
4

0

0

0

0
4

0

0

0
4

0
4

0

0

0

0

0
4

0
4

0

0

0

0
4

0

0

0
4

0
4

0.217157
3

0.189519
3

0.1757
3

0.0138191

0

0

0

0

0

0

1.21430643318376e-17
3

0
4

0.0276382
3

0.0118449

0.0118449

0.00394831

0

0

0

0

0
4

0
4

0

0

0

0
4

0
4

0

0

0

0
4

0

0

0
4

0
4

0.0129652

0.00518609

0.00518609

0
4

0.00777913

0.00777913

0
4

0
4

0

0

0

0
4

0

0

0
4

0
4

0

0

0

0

0
4

0
4

0

0

0

0
4

0

0

0
4

0
4

0

0

0

0
4

0
4

0

0

0

0

0
4

0
4

0

0

0

0

0
4

0
4

0

0

0

0
4

0

0

0
4

0
4

0
4

0
4

0
4

0

0

0

0

0

0

0

0
4

0

0

0

0

0

0

0
4

0

0

0

0

0
4

0

0

0

0
4

0

0

0

0
4

0

0

0

0
4

0

0

0
4

0
4

0

0

0

0
4

0

0

0
4

0
4

0

0

0

0

0
4

0
4

0

0

0

0

0
4

0
4

0

0

0

0
4

0

0

0
4

0
4

0

0

0

0
4

0

0

0
4

0
4

0

0

0

0
4

0

0

0
4

0
4

0

0

0

0

0
4

0
4

0

0

0

0

0
4

0
4

0

0

0

0
4

0

0

0
4

0
4

0

0

0

0
4

0
4

0.654433
3

0.20715
3

0
3

0
2

0.20715

0

0

0

0
4

0
3

0
3

0

0

0

0
4

0.447284

0.447284

0

0
4

0

0

0

0

0

0
4

0

0

0
4

0
4

0

0

0

0

0
4

0
4

0

0

0

0
4

0
4

0

0

0

0

0
4

0
4

0.362812

0

0

0
4

0.362812

0.362812

0
4

0
4

0

0

0

0

0
4

0
4

0

0

0

0

0
4

0
4

0.032394

0.032394

0.032394

0
4

0
4

0

0

0

0

0
4

0
4

0

0

0

0

0
4

0
4

0

0

0

0

0
4

0
4

0.0129576
4

0.0129576
4

0
4

0

0

0

0.0129576

0

0

0

0
4

0

0

0
4

0

0

0
4

0
4

0

0

0

0
4

0
4

0

0

0

0
4

0

0

0
4

0
4

0

0

0

0

0
4

0
4

0

0

0

0
4

0
4

0

0

0

0
4

0

0

0
4

0
4

0

0

0

0

0
4

0
4

0

0

0

0
4

0

0

0
4

0
4

0

0

0

0

0
4

0
4

0

0

0

0

0
4

0
4

0.00518609

0

0

0
4

0.00518609

0.00518609

0
4

0
4

0
2

0
2

0
2

0

0

0

0

0

0
3

0

0

0

0

0

0

0
4

0
2

0
2

0

0

0

0

0

0

0

0

0

0

0

0
2

0
3

0

0

0

0

0

0
4

0

0

0

0
4

0

0

0

0

0

0
4

0

0

0
4

0

0

0
4

0
4

2.60133

2.60133

2.60133

0

0

0

0
4

0

0

0
4

0

0

0
4

0
4

0

0

0

0
4

0

0

0
4

0
4

0

0

0

0
4

0
4

0

0

0

0
4

0

0

0
4

0
4

0.0297989

0.0297989

0.0297989

0
4

0
4

0

0

0

0
4

0

0

0
4

0
4

0

0

0

0
4

0
4

0

0

0

0
4

0

0

0
4

0
4

0

0

0

0
4

0
4

0

0

0

0
4

0
4

0.0160309

0.0160309

0.0160309

0
4

0
4

1.66399
7

0.472879
7

0.135585
7

0.251162
8

0

0

0.00448724

0.00458025

0

0

0.0770648

5.55111512312578e-17
7

0
4

1.17663

0.945431

0.15413

0.0770648

0

1.38777878078145e-17

0
4

0.0144887

0.0144887

0

0
4

0
4

0

0

0

0
4

0
4

0

0

0

0

0
4

0
4

0

0

0

0
4

0
4

0

0

0

0
4

0
4

0

0

0

0
4

0
4

0

0

0

0
4

0
4

0

0

0

0
4

0
4

0

0

0

0
4

0
4

0

0

0

0
4

0
4

0.0129576

0.0129576

0.0129576

0
4

0
4

0.0591856
2

0
2

0
2

0
2

0

0

0

0
4

0

0

0

0
4

0.0591856

0.0591856

0
4

0

0

0
4

0
4

0

0

0

0
4

0
4

0

0

0

0
4

0
4

0

0

0

0
4

0
4

0

0

0

0
4

0
4

0

0

0

0
4

0
4

0.36049

0.36049

0.36049

0
4

0
4

0

0

0

0
4

0
4

0

0

0

0
4

0
4

0

0

0

0
4

0
4

0

0

0

0
4

0
4

0
4

0
4

0
4

0

0

0

0

0

0

0

0
4

0
4

0.0285235

0.0285235

0.0285235

0
4

0
4

0.0107775

0.0107775

0.0107775

0
4

0
4

0

0

0

0
4

0
4

0

0

0

0
4

0
4

0

0

0

0
4

0
4

0

0

0

0
4

0
4

0

0

0

0
4

0
4

0

0

0

0
4

0
4

0

0

0

0
4

0
4

0.0770648

0.0770648

0.0770648

0
4

0
4

0

0

0

0

0

0

0

0

0

0
4

0

0

0
4

0
4

0

0

0

0
4

0
4

0.0199717

0.0199717

0.0199717

0
4

0
4

0

0

0

0
4

0
4

0

0

0

0
4

0
4

0

0

0

0
4

0
4

0

0

0

0
4

0
4

0

0

0

0
4

0
4

0

0

0

0
4

0
4

0

0

0

0
4

0
4

0

0

0

0
4

0
4

0
3

0
3

0
3

0
3

0

0

0
4

0

0

0

0

0
4

0
4

0

0

0

0
4

0
4

0

0

0

0
4

0
4

0

0

0

0
4

0
4

0

0

0

0
4

0
4

0

0

0

0
4

0
4

0

0

0

0
4

0
4

0

0

0

0
4

0
4

0.0156497

0.0156497

0.0156497

0
4

0
4

0

0

0

0
4

0
4

0

0

0

0
4

0
4

0
4

0
4

0

0

0

0

0

0

0

0

0

0
4

0

0

0

0

0

0

0
4

0

0

0
4

0
4

0

0

0

0
4

0
4

0

0

0

0
4

0
4

0

0

0

0
4

0
4

0

0

0

0
4

0
4

0

0

0

0
4

0
4

0

0

0

0
4

0
4

0

0

0

0
4

0
4

0

0

0

0
4

0
4

0

0

0

0
4

0
4

0

0

0

0
4

0
4

0.144196
3

0.144196
3

0
3

0

0.144196

0

0

0
4

0

0

0
4

0

0

0
4

0
4

0

0

0

0
4

0
4

0

0

0

0
4

0
4

0

0

0

0
4

0
4

0

0

0

0
4

0
4

0

0

0

0
4

0
4

0

0

0

0
4

0
4

0

0

0

0
4

0
4

0

0

0

0
4

0
4

0

0

0

0
4

0
4

0

0

0

0
4

0
4

0

0

0

0

0

0

0

0
4

0

0

0
4

0
4

0.288392

0.288392

0.288392

0
4

0
4

0

0

0

0
4

0
4

0

0

0

0
4

0
4

0

0

0

0
4

0
4

0

0

0

0
4

0
4

0

0

0

0
4

0
4

0.0233374

0.0233374

0.0233374

0
4

0
4

0

0

0

0
4

0
4

0.216294

0.216294

0.216294

0
4

0
4

0

0

0

0
4

0
4

0

0

0

0

0

0

0

0

0
4

0

0

0

0

0
4

0

0

0

0
4

0

0

0

0
4

0

0

0
4

0
4

0

0

0

0
4

0
4

0

0

0

0
4

0
4

0

0

0

0
4

0
4

0

0

0

0
4

0
4

0

0

0

0
4

0
4

0

0

0

0
4

0
4

0

0

0

0
4

0
4

0.0091605

0.0091605

0.0091605

0
4

0
4

0

0

0

0
4

0
4

0

0

0

0
4

0
4

0.103913
3

0.0312995
3

0
5

0

0

0

0

0

0

0

0

0

0

0

0

0

0

0.0312995

0

0

0

0
4

0

0

0

0
4

0.00987078

0.00394831

0.00592247

0

8.67361737988404e-19

0
4

0.00592247

0

0

0.00592247

0

0
4

0

0

0

0

0
4

0

0

0

0
4

0

0

0

0
4

0

0

0

0
4

0

0

0

0
4

0

0

0

0
4

0

0

0
4

0

0

0

0

0

0

0

0

0

0

0

0
4

0

0

0
4

0

0

0
4

0.0156497
3

0.0156497

0

0

0

0

0

0

0

0

0

0

0

0
4

0.00394831
3

0

0

0

0.00394831

0

0

0
4

0.00592247
4

0

0

0

0

0

0

0.00592247

0

0

0
4

0

0

0

0

0

0
4

0
5

0

0

0

0

0

0
4

0
4

0

0

0

0

0

0
4

0.0312995

0.0312995

0

0

0

0
4

6.93889390390723e-18
3

0
4

0
3

0
3

0
3

0

0

0

0

0

0

0
4

0

0

0

0

0
4

0

0

0

0
4

0
4

0

0

0

0
4

0
4

0

0

0

0
4

0
4

0

0

0

0
4

0
4

0

0

0

0
4

0
4

0

0

0

0
4

0
4

0

0

0

0
4

0
4

0

0

0

0
4

0
4

0

0

0

0
4

0
4

0

0

0

0
4

0
4

0

0

0

0
4

0
4

0.362812
4

0.362812
4

0.362812
4

0

0

0

0
4

0

0

0

0

0

0
4

0
4

0

0

0

0
4

0
4

0

0

0

0
4

0
4

0

0

0

0
4

0
4

0

0

0

0
4

0
4

0

0

0

0
4

0
4

0

0

0

0
4

0
4

0

0

0

0
4

0
4

0

0

0

0
4

0
4

0

0

0

0
4

0
4

0.00411208

0.00411208

0.00411208

0
4

0
4

0

0

0

0

0

0

0

0

0
4

0

0

0
4

0
4

0

0

0

0
4

0
4

0

0

0

0
4

0
4

0

0

0

0
4

0
4

0

0

0

0
4

0
4

0

0

0

0
4

0
4

0

0

0

0
4

0
4

0.0129576

0.0129576

0.0129576

0
4

0
4

0

0

0

0
4

0
4

0

0

0

0
4

0
4

0

0

0

0
4

0
4

0
4

0

0

0

0

0

0

0

0

0
4

0

0

0

0

0

0
4

0

0

0

0
4

0

0

0
4

0
4

0

0

0

0
4

0
4

0

0

0

0
4

0
4

0

0

0

0
4

0
4

0

0

0

0
4

0
4

0

0

0

0
4

0
4

0

0

0

0
4

0
4

0

0

0

0
4

0
4

0

0

0

0
4

0
4

0.018321

0.018321

0.018321

0
4

0
4

0

0

0

0
4

0
4

0
3

0
3

0

0

0

0

0

0

0

0
4

0

0

0

0

0
4

0

0

0
4

0
4

0

0

0

0
4

0
4

0

0

0

0
4

0
4

0

0

0

0
4

0
4

0

0

0

0
4

0
4

0

0

0

0
4

0
4

0

0

0

0
4

0
4

0

0

0

0
4

0
4

0

0

0

0
4

0
4

0.00532728

0.00532728

0.00532728

0
4

0
4

0

0

0

0
4

0
4

5.0795
7

5.0795
7

5.00693
7

0.0725677
7

1.38777878078145e-16
7

0
4

0
4

0

0

0

0
4

0
4

0.0100616

0.0100616

0.0100616

0
4

0
4

0

0

0

0
4

0
4

0

0

0

0
4

0
4

0

0

0

0
4

0
4

0

0

0

0
4

0
4

0

0

0

0
4

0
4

0

0

0

0
4

0
4

0

0

0

0
4

0
4

0

0

0

0
4

0
4

0
4

0
4

0
4

0

0

0

0

0

0

0
4

0

0

0

0

0
4

0

0

0

0
4

0
4

0

0

0

0
4

0
4

0

0

0

0
4

0
4

0

0

0

0
4

0
4

0

0

0

0
4

0
4

0

0

0

0
4

0
4

0

0

0

0
4

0
4

0

0

0

0
4

0
4

0

0

0

0
4

0
4

0

0

0

0
4

0
4

0.0194364

0.0194364

0.0194364

0
4

0
4

0
4

0
4

0
4

0

0

0

0

0

0
4

0
4

0

0

0

0
4

0

0

0
4

0

0

0
4

0

0

0
4

0
4

0

0

0

0
4

0
4

0

0

0

0
4

0
4

0

0

0

0
4

0
4

0

0

0

0
4

0
4

0

0

0

0
4

0
4

0

0

0

0
4

0
4

0.00448724

0.00448724

0.00448724

0
4

0
4

0

0

0

0
4

0
4

0

0

0

0
4

0
4

0

0

0

0
4

0
4

0

0

0

0

0

0

0

0

0

0

0
4

0

0

0

0
4

0

0

0
4

0

0

0
4

0

0

0
4

0
4

0

0

0

0
4

0
4

0.0129576

0.0129576

0.0129576

0
4

0
4

0

0

0

0
4

0
4

0

0

0

0
4

0
4

0

0

0

0
4

0
4

0

0

0

0
4

0
4

0

0

0

0
4

0
4

0

0

0

0
4

0
4

0.192662

0.192662

0.192662

0
4

0
4

0

0

0

0
4

0
4

0.251865

0.223081

0.16208

0.0610013
7

0

0

0
4

0.0246725

0.0185044

0.00616813

0
4

0.00411208

0.00411208

0
4

2.16840434497101e-17

0
4

0

0

0

0
4

0
4

0

0

0

0
4

0
4

0

0

0

0
4

0
4

0

0

0

0
4

0
4

0

0

0

0
4

0
4

0

0

0

0
4

0
4

0

0

0

0
4

0
4

0.144196

0.144196

0.144196

0
4

0
4

0

0

0

0
4

0
4

0.288392

0.288392

0.288392

0
4

0
4

0
4

0
4

0

0

0

0

0

0

0

0

0

0

0

0

0

0

0

0

0

0

0

0

0

0

0

0

0

0

0

0

0

0

0

0

0

0

0

0

0

0

0

0

0

0

0

0

0

0

0

0

0

0

0

0

0

0

0

0
4

0

0

0
4

0

0

0

0

0

0

0
4

0

0

0

0

0
4

0

0

0

0
4

0

0

0

0

0
4

0

0

0
4

0

0

0

0
4

0

0

0
4

0

0

0
4

0
4

0
4

0
4

0

0

0

0

0

0

0
4

0

0

0

0

0
4

0

0

0

0
4

0

0

0

0
4

0
4

0

0

0

0
4

0
4

0

0

0

0
4

0
4

0.0234746

0.0234746

0.0234746

0
4

0
4

0

0

0

0
4

0
4

0.00394831

0.00394831

0.00394831

0
4

0
4

0

0

0

0
4

0
4

0

0

0

0
4

0
4

0.0155583

0.0155583

0.0155583

0
4

0
4

0

0

0

0
4

0
4

0

0

0

0
4

0
4

0
4

0
4

0

0

0

0

0

0
4

0
3

0

0

0

0

0
4

0

0

0
4

0

0

0
4

0
4

0

0

0

0
4

0
4

0

0

0

0
4

0
4

0

0

0

0

0

0

0
4

0

0

0

0
4

0

0

0
4

0
4

0
4

0
4

0
4

0

0

0
4

0

0

0

0

0

0

0
4

0

0

0

0

0

0

0
4

0

0

0
4

0
4

0.0129576
4

0.0129576
4

0

0

0

0

0

0

0.0129576

0

0
4

0

0

0

0

0
4

0

0

0
4

0
4

0.24686
3

0.24686

0.083722

0.163138

0
4

0

0

0

0

0

0

0
4

0

0

0
4

0

0

0
4

0

0

0
4

0
4

0
2

0
2

0
2

0

0

0
4

0
4

0
3

0

0

0

0

0

0

0
4

0

0

0

0

0

0
4

0

0

0

0

0
4

0

0

0
4

0

0

0
4

0

0

0
4

0
4

0

0

0

0

0

0

0

0

0
4

0

0

0

0

0

0

0
4

0
4

0.025664
3

0.025664
4

0.025664
4

0

0

0

0

0

0
4

0

0

0

0

0
4

0

0

0
4

0
4

0.177557
3

0
3

0
4

0

0

0

0

0

0

0

0

0

0

0

0
4

0

0

0
4

0

0

0
4

0

0

0
4

0
3

0
5

0

0

0

0

0

0

0

0

0

0

0

0
4

0
4

0
4

0

0

0

0

0

0

0
4

0.177557
3

0.177557
7

0

0

0

0

0

0

0
4

0
5

0

0

0

0

0

0

0

0
4

0

0

0

0

0

0

0
4

0

0

0

0
4

0

0

0

0
4

0

0

0
4

0
4

0

0

0

0

0

0
4

0

0

0

0

0

0
4

0

0

0
4

0

0

0
4

0

0

0
4

0
4

0

0

0

0

0

0

0

0

0

0
4

0

0

0
4

0
4

0

0

0

0

0

0

0

0

0

0
4

0

0

0

0

0

0
4

0
4

0
4

0
4

0

0

0

0

0

0
4

0
4

0

0

0

0

0

0

0

0

0
4

0

0

0
4

0
4

0.564789
7

0.447923
7

0.447923
7

0

0

0

0
4

0.0330696

0.0330696

0
4

0

0

0
4

0.0687037

0.0687037

0
4

0

0

0
4

0

0

0
4

0.0150924

0.0150924

0
4

3.81639164714898e-17
7

0
4

0
4

0
4

0

0

0

0

0

0

0

0
4

0

0

0

0
4

0
4

0
3

0
3

0
3

0
4

0

0

0
4

0
4

0
4

0

0

0

0

0

0
4

0

0

0

0

0
4

0

0

0

0

0
4

0

0

0
4

0
4

0

0

0

0

0

0

0

0

0
4

0

0

0

0
4

0
4

1.15344
7

1.06632
7

0.447408
7

0

0

0

0

0

0

0

0.188881
7

0.0363026

0.0160309
7

0.227744
7

0

0.0343519

0.115597

0

0
4

0.0871264
7

0

0.0871264

0

0

0
4

0
4

0
4

0

0

0

0

0

0

0

0
4

0

0

0

0

0
4

0

0

0
4

0
4

0

0

0

0
4

0

0

0

0
4

0

0

0

0

0
4

0

0

0

0
4

0
4

0

0

0

0

0

0
4

0
4

0.192662
4

0.192662
4

0.192662
4

0
4

0
4

0
4

0

0

0

0

0

0
4

0

0

0
4

0

0

0
4

0

0

0

0
4

0

0

0
4

0

0

0
4

0
4

0
2

0
2

0

0

0

0

0
4

0

0

0

0

0
4

0

0

0

0
4

0

0

0
4

0
4

0.0809404

0.0809404

0.0809404

0

0

0

0
4

0

0

0
4

0
4

0.231194
7

0.231194
7

0.231194
7

0

0
4

0

0

0

0
4

0
4

0

0

0

0

0
4

0

0

0

0
4

0

0

0
4

0
4

0
4

0

0

0

0

0

0

0
4

0

0

0

0
4

0
4

0.272501
3

0
3

0
2

0
3

0
2

0

0

0

0

0

0
4

0

0

0

0

0

0

0
4

0.272501

0.118371

0

0

0.15413

2.77555756156289e-17

0
4

0

0

0

0

0
4

0

0

0
4

0

0

0
4

0

0

0
4

0
4

0
4

0

0

0

0

0
4

0

0

0

0

0
4

0

0

0
4

0

0

0
4

0

0

0
4

0
4

0
4

0

0

0

0

0

0

0
4

0

0

0

0

0

0
4

0

0

0
4

0

0

0
4

0

0

0
4

0
4

0

0

0

0

0

0

0

0

0

0
4

0

0

0

0

0
4

0
4

0
3

0
3

0
3

0

0

0

0
4

0

0

0

0
4

0

0

0
4

0

0

0
4

0
4

0

0

0

0

0

0

0

0
4

0

0

0

0
4

0
4

0.277167
1

0.00332862

0.00332862

0

0

0

0

0
4

0.269727

0.269727

0
4

0.00411208

0.00411208

0
4

0

0

0
4

0

0

0
4

0

0

0
4

0
4

0
3

0

0

0

0

0

0

0
4

0
4

0

0

0

0

0

0
4

0
4

1.03661

0.932946

0.932946

0

0
4

0

0

0

0
4

0.103661

0

0.0842243

0.0194364

3.46944695195361e-18

0
4

0
4

0.0770648

0.0770648

0

0

0

0

0.0770648

0

0
4

0
4

0.0615366

0.0615366

0.00967569

0

0

0.0388957

0.0129652

1.73472347597681e-18

0
4

0

0

0
4

0

0

0

0
4

0

0

0
4

0

0

0
4

0

0

0
4

0
4

0
3

0
4

0
4

0

0

0

0

0

0

0

0

0

0

0

0

0

0

0
4

0
3

0
4

0

0

0

0

0

0
3

0

0

0

0

0

0

0

0
4

0
3

0

0

0
4

0

0

0
4

0
4

0
2

0
2

0
2

0

0

0

0
4

0
4

0.0239728
7

0.0159818

0.00532728

0

0.00532728

0.00532728

1.73472347597681e-18

0
4

0

0

0

0

0

0
4

0.00799092

0

0.00799092

0
4

0
4

0
4

0
4

0

0

0

0

0
4

0

0

0

0

0
4

0

0

0
4

0
4

0.303132
7

0.303132
7

0.270062

0

0.0330696

0
4

0
4

0.0453516
4

0.0453516
4

0.0259152

0.0194364

0

0

3.46944695195361e-18
4

0
4

0

0

0
4

0
4

0
4

0

0

0

0

0

0
4

0

0

0

0

0
4

0

0

0
4

0

0

0
4

0
4

0
3

0
3

0

0

0

0
4

0

0

0
4

0

0

0
4

0
4

0
2

0
2

0

0

0

0

0
4

0

0

0
4

0
4

0.215183
3

0.207286
3

0.207286
3

0

0
4

0.00789662

0.00394831

0.00394831

0

0
4

5.20417042793042e-18
3

0
4

0
4

0
4

0
4

0

0

0
4

0
4

0.832812

0

0

0

0
4

0.216294
6

0.216294

0

0
4

0

0

0

0

0
4

0

0

0
4

0.0770648

0.0770648

0
4

0.539453

0.539453

0
4

0
4

29.5469
3

27.9356
3

0.655643
3

5.43591

2.39751
3

0.751245

1.00246
7

0

0

0

0

0.0156497
7

0

0.118371

0

0.144196

0

0

0

0

0.222996
6

0

0

0.0129576

0

0

0.0143923

0

0

0

0

11.1674
7

0.131247

0

0

0.0123594

0

0

0.00458025

0

0

0

2.71125
7

0

0

0

0

0

0

0

0

0

0

1.32664
7

0

0

0

0

0

0

0

0

0

0.0782487

0.143258
7

0

0

0

0

0

0

0

0

0

0

0

0

0.216294

0

0

0.0770648

0.0156497

0

0

0

0

0.268226
7

0

0

0

0

0

0

0

0

0

0

0.865175
2

0

0

0

0

0

0

0.115597

0

0.0312995

0

0
4

0.693967

0.172467
5

0

0.0091605
7

0.285968
7

0.197848

0

0.0285235

0

0

0

2.77555756156289e-17

0
4

0.0591856

0.0591856

0
4

0

0

0
4

0

0

0
4

0

0

0
4

0

0

0
4

0

0

0
4

0

0

0
4

0

0

0
4

0

0

0
4

0

0

0
4

0
4

0
4

0
4

0

0

0

0
4

0

0

0
4

0.0916087

0.0916087

0
4

0

0

0
4

0

0

0
4

0

0

0
4

0.0312995

0.0312995

0
4

0.00777913

0.00777913

0
4

0

0

0
4

0.722289
7

0.698952
7

0.0233374

0

0
4

0.00518609
7

0.00518609

0

0

0

0

0

0
4

0

0

0
4

0

0

0

0

0
4

0

0

0
4

0

0

0
4

0

0

0

0
4

9.59302082215174e-16
3

0
4

2.35643

1.75242

0
3

0

0

0
4

0

0.0870247
6

0

0

0.0503827

0

0

0

0

0.177557

0

0

0

0

0

0

0

0

0.347383

0

0

0

0.347126

0

0

0

0.0201232

0

0

0

0

0

0

0

0

0

0

0.00458025

0

0

0

0.115597

0

0

0

0

0

0

0

0

0

0
4

0

0

0

0

0

0

0

0

0

0

0.54787

0

0

0

0

0

0.0547741

0

0

0

0

0

0

0

0

0

0

0

0

0

3.40005801291454e-16

0
4

0
3

0
3

0
2

0

0

0

0

0

0

0
4

0

0

0

0
4

0

0

0

0
4

0

0

0

0
4

0

0

0
4

0

0

0

0
4

0

0

0

0
4

0

0

0

0
4

0

0

0
4

0

0

0

0
4

0

0

0
4

0

0

0

0
4

0

0

0
4

0.36049

0.36049

0
4

0

0

0
4

0

0

0
4

0

0

0
4

0

0

0
4

0

0

0
4

0

0

0
4

0

0

0
4

0

0

0
4

0

0

0

0

0
4

0

0

0
4

0.0100616

0.0100616

0
4

0

0

0
4

0

0

0
4

0

0

0
4

0

0

0
4

0
4

0

0

0

0

0
4

0.210563
7

0.210563
7

0
4

0

0

0

0

0
4

0

0

0

0

0

0
4

0

0

0

0

0
4

0.0229012

0.0229012

0
4

0
4

21.2579

21.2579

1.88243
6

0
4

0

0

0

0

0

0

0

0

0

0

0
3

0

0

0

0

0

0

0

0

0

0

0.481924
7

0

0.0312995

0

0

0

0

0

0

0

0

0
6

0

0

0

0

0.135374

0

0

0

0

0

0
2

0.432588

0

0

0

0

0.648882

0

0

0

0.0971819

0
4

0

0

0

0

0

0

0

0

0

0

0
3

0.0965012

0

0

0

0

0

0

0

0

0

0.0246798
4

0.115597

0.0129576

0.144196

0

0

0

0

0.0129576

0

0

0

0.0259152

0

0

0.221261

0.11014

0

0.0518304

0

0

0

0
3

0

0

0

0

0

0.539453

0

0

0

0

4.47788
6

0
3

0.142533

0

0

0

0

0

0

0

0

0

1.30323

0

0

0

0

0

0

0.0133182

0

0

0

0
4

0

0

0

0

0.0194364

0.173566

0

0

0

0

0
6

0

0

0

0

0

0

0

0

0

0

0
4

0

0

0

0

0

0

0

0

0.0129576

0

0
5

0

0

0

0

0

0

0

0.0591856

0

0.0134617

0.986766
7

0

0

0

0

0

0

0

0

0

0

0

0

0

0

0

0

0

0

0

0

0

0
4

0

0

0

0

0

0

0

0

0

0

0
7

0

0

0

0

0

0

0

0.2138

0

0

0.0852575
4

0
3

0

0

0

0

0

0

0

0

0

0

0

0

0

0

0

0

0

0

0

0

0

0
4

0

0

0

0

0

0

0

0

0

0

0
6

0

0

0

0

0

0

0.0129576

0

0

0

0
4

0

0

0

0

0

0

0

0

0

0

0

0

0

0

0

0

0

0

0

0

0.0388728

0
4

0

0

0

0

0

0

0

0

0

0

0

0

0

0

0

0

0

0

0

0

0

0

0

0.0129576

0.00458025

0

0

0

0

0

0

0

0

0

0

0

0

0

0

0

0

0

0

6.64449
7

0
3

0

0

0

0

0

0

0

0

0

0

0
3

0

0

0

0

0

0

0

0

0

0

0

0

0

0

0

0

0

0

0

0

0

0.00448724
3

0

0

0

0.0134617

0

0

0

0

0

0

0

0

0

0

0

0

0

0

0.0194364

0

0

0
4

0

0

0

0

0

0

0

0

0

0

0.481054
7

0

0

0

0

0

0

0

0

0

0.0129576

0

0

0

0

0

0

0

0

0

0

0

0

0

0

0

0

0

0

0

0

0

0

0
4

0

0

0

0

0

0

0

0

0

0

0
4

0

0

0

0

0

0

0

0

0

0.0129576

0

0

0

0

0

0.0194364

0

0

0

0

0

0

0
2

0

0

0

0

0

0

0

0

0

0

0

0

0

0

0

0

0

0

0

0

0

0

0

0

0.0388728

0

0

0

0

0.204169

0

0.0129576

0

0

0

0

0

0

0

0

0

0

0

0

0

0

0

0

0

0.144196

0

0

0

0

0
3

0

0

0

0

0

0

0

0

0

0

0

0

0

0

0

0

0

0

0

0

0

0
6

0

0

0

0

0

0

0

0

0

0

0

0

0

0

0

0

0

0

0

0

0

0

0

0

0.0129576

0

0

0

0

0

0

0

0

0

0

0

0.0129576

0

0

0

0

0

0

0

0.0650649
4

0

0

0

0

0

0

0

0

0

0

0
4

0

0

0

0

0

0

0

0

0

0

0

0

0

0

0

0

0

0

0

0

0

0
4

0

0

0

0

0

0

0

0

0

0

0

0

0

0

0

0

0

0

0

0

0

0

0

0

0

0

0

0

0.0591856

0

0

0

0

0

0

0

0

0

0

0

0

0

0

0
6

0

0

0

0

0

0

0

0

0

0

0

0

0

0

0

0

0

0

0

0

0

0

0

0

0

0

0

0

0

0

0

0

0

0

0

0

0

0

0

0

0

0

0

0

0

0

0

0

0

0

0

0

0

0

0

0

0
6

0

0

0

0

0

0

0

0

0

0

0.727287
6

0

0

0

0

0

0

0

0

0.136055

0

0
4

0

0

0

0
4

0

0

0
4

0

0

0

0
4

0

0

0
4

0

0

0
4

0

0

0
4

0

0

0
4

0

0

0
4

0

0

0
4

0
4

6.82734

3.95709

0.196774
7

0

0

0

0.00615463

0

0.00624502

0

0

0.0184639

0

3.64203
7

0

0

0

0

0

0

0

0.0874303

0

0

0

0

0

0

1.94289029309402e-16

0
4

2.71612
7

2.67198
7

0

0

0

0.00616813
6

0
6

0.0102802

0.0276958

0

0

0

0

0
4

0

0

0
4

0
7

0

0

0
4

0

0

0

0
4

0.15413

0

0

0.15413

0
4

0

0

0
4

0

0

0
4

0

0

0
4

0

0

0
4

0

0

0
4

0
4

35.4538

1.02319
3

0.905247
3

0

0.0236854

0.0413017

0

0

0

0

0

0

0

0

0

0

0

0

0

0

0

0

0

0

0

0

0

0

0.00411208

0

0

0

0

0.032394

0

0

0

0

0

0

0.0164483

0

0

4.5102810375397e-17
3

0
4

8.86928
7

6.94266
7

0.269727

0

0

1.65689

0

0

1.77635683940025e-15
7

0
4

0.144196
3

0

0.144196

0

0

0
4

0

0

0
4

0

0

0
4

0

0

0
4

0

0

0
4

0.0129576

0.0129576

0
4

0

0

0
4

0

0

0
4

0.0532163
7

0.0532163
6

0

0
4

0
3

0

0

0

0

0

0
4

0.0102348

0.00615463

0

0

0.00408015

0
4

0

0

0

0
4

0.405733
7

0.405733
7

0
4

0

0

0

0

0
4

0.274603

0.274603

0
4

0.0268678
7

0.0268678

0

0
4

0.594625

0.219031

0.375594

0
4

2.01868
6

1.93446
6

0.032394

0

0

0.0518304

0

0

0

0
4

0

0

0

0
4

1.29103

0.674515

0.616518

0
4

0

0

0

0

0

0
4

0

0

0
4

0.0100616

0

0

0

0.0100616

0
4

0.109548

0

0.109548

0
4

0.15413

0.15413

0

0
4

0.693583

0.693583

0

0
4

0.0955854

0.0955854

0
4

0.00411208

0

0

0.00411208

0
4

15.2005
7

13.5295
7

0

0.0770648

0.0156497

0.793627
7

0.131247

0.648882

0

0

0

0.00458025

0

0
4

0

0

0
4

0

0

0

0
4

0

0

0

0

0
4

0.144196

0.144196

0

0
4

0

0

0

0
4

0

0

0
4

0.15413

0.15413

0
4

0

0

0
4

0

0

0
4

0

0

0

0
4

3.60416
8

3.17704
8

0.0828289

0.062599

0.140848

0.0156497

0.0234746

0.0234746

0.062599

0.0156497

4.09394740330526e-16
8

0
4

0

0

0
4

0

0

0

0
4

0.15413

0.15413

0

0
4

0.126776

0.123154

0.00362217

1.30104260698261e-18

0
4

0.0234746

0

0.0234746

0
4

0

0

0
4

0

0

0
4

0

0

0
4

0

0

0

0
4

0

0

0
4

0.16141
8

0.0145135

0.0312995

0.115597

0

0

0

0

0

1.38777878078145e-17
8

0
4

0

0

0
4

0

0

0
4

0

0

0

0
4

0

0

0
4

0

0

0
4

0

0

0

0
4

0

0

0

0
4

0

0

0
4

0

0

0
4

0.0156497

0.0156497

0
4

0
3

0

0

0

0

0

0

0
4

0.0144887

0.0144887

0
4

0

0

0
4

0

0

0
4

0

0

0
4

0

0

0
4

0

0

0
4

0

0

0
4

0

0

0
4

0

0

0
4

0

0

0
4

0

0

0

0

0
4

0

0

0
4

0

0

0
4

0

0

0
4

0

0

0
4

0

0

0
4

0

0

0
4

0

0

0
4

0

0

0
4

0

0

0
4

0.0234746

0.0234746

0
4

0

0

0

0

0

0
4

0

0

0
4

0

0

0
4

0

0

0
4

0

0

0
4

0.00332862

0.00332862

0
4

0

0

0
4

0.0129576

0.0129576

0
4

0.0234746

0.0234746

0
4

0

0

0
4

0

0

0
4

0

0

0

0

0

0
4

0

0

0
4

0

0

0
4

0

0

0
4

0

0

0
4

0

0

0
4

0

0

0
4

0

0

0
4

0

0

0
4

0

0

0
4

0

0

0
4

5.79050696281058e-15

0
4

5.10149
3

4.77591
3

4.29506
3

0.0591856
2

0
2

0

0

0

0

0

0

0

0

0.144196

0

0
3

0.0770648

0

0

0

0

0

0

0

0

0

0
3

0

0

0

0

0

0

0

0

0

0

0
2

0

0

0

0.0102802

0

0

0

0

0

0

0

0

0

0

0

0

0

0

0

0

0

0
2

0

0

0

0

0

0

0

0

0

0

0

0

0

0

0

0

0

0

0

0

0

0

0

0

0

0

0

0

0

0

0

0

0
2

0

0

0

0

0

0

0

0

0

0

0

0

0

0

0

0

0

0

0

0

0.00616813

0

0

0

0

0

0

0

0

0

0

0

0

0

0

0

0

0

0

0

0

0

0

0

0

0

0

0

0

0

0

0

0

0

0

0

0

0

0

0

0

0

0

0

0

0

0

0

0

0

0

0

0

0

0

0

0

0

0

0

0

0

0.00362217

0

0

0

0

0

0

0

0

0
2

0

0

0

0

0

0

0

0

0

0

0

0

0

0

0

0

0

0

0

0

0

0
3

0

0

0

0

0

0

0

0

0

0

0

0

0

0

0

0

0

0

0.174162

0

0

0

0

0

0

0

0

0

0

0

0

0

0
3

0

0

0

0

0

0

0

0

0

0

0

0

0

0

0.00616813

0

0

0

0

0

0

9.19403442267708e-17
3

0
4

0

0

0

0

0

0
4

0

0

0

0

0
4

0

0

0
4

0

0

0

0

0
4

0

0

0

0

0
4

0

0

0

0
4

0

0

0
4

0

0

0

0
4

0

0

0

0
4

0

0

0

0
4

0

0

0
4

0
3

0

0

0

0
4

0

0

0

0
4

0.155491

0.155491

0
4

0

0

0

0
4

0

0

0
4

0

0

0

0
4

0

0

0
4

0

0

0
4

0

0

0
4

0

0

0
4

0.00362217

0.00362217

0
4

0
2

0

0

0

0
4

0

0

0
4

0

0

0
4

0

0

0
4

0

0

0
4

0

0

0
4

0

0

0
4

0

0

0
4

0

0

0
4

0

0

0
4

0

0

0
4

0

0

0
4

0

0

0
4

0

0

0
4

0

0

0
4

0

0

0
4

0

0

0
4

0

0

0
4

0

0

0
4

0

0

0
4

0.00411208

0.00411208

0
4

0.15413

0.15413

0
4

0

0

0

0
4

0

0

0
4

0

0

0
4

0

0

0
4

0

0

0
4

0

0

0
4

0

0

0
4

0

0

0
4

0

0

0
4

0.00822417

0.00822417

0
4

0

0

0
4

0

0

0
4

0

0

0
4

0

0

0
4

0

0

0

0

0
4

0

0

0

0

0
4

0

0

0

0

0
4

5.84601811404184e-16
3

0
4

0
3

0
3

0
2

0

0

0

0

0

0

0

0

0

0

0
2

0

0

0

0

0

0

0

0

0

0

0
3

0

0

0

0

0

0

0

0

0

0

0

0

0

0

0

0

0

0

0

0

0

0

0

0
4

0

0

0

0

0

0

0
4

0

0

0

0
4

0

0

0

0

0
4

0

0

0

0
4

0

0

0
4

0

0

0
4

0

0

0
4

0

0

0
4

0

0

0
4

0
4

1.84513
5

1.83994
5

1.4496
5

0.143303
7

0

0

0.00624502

0

0.00458025

0

0

0

0

0

0

0

0

0

0

0

0

0

0

0

0.00458025

0.0103722

0

0

0.221261

0

0

0

1.38777878078145e-16
5

0
4

0

0

0
4

0

0

0
4

0.00518609

0.00518609

0
4

0

0

0
4

0

0

0
4

0

0

0
4

0

0

0
4

7.11236625150491e-17
5

0
4

39.0783
4

30.6071
4

21.5037
4

2.80182
7

0

0.00624502

0

0

0

0.00458025

0

0

0

0

0

0

0

0

0

0

0

0

0

0

0.00967569

0

0

0

0

0

0

0

0

0.00448724

0

0.144196

0.216294
4

0

0.144196

0

0

0

0

0

0

0

0

0.144196
6

0

0

0

0

0

0

0

0

0

0

0.534891
7

0

0

0

0

0

0

0

0

0

0

0.514361
7

0

0

0

0

0

0

0

0

0

0

4.1603
7

0

0

0

0

0

0

0

0

0

0

0.374684
7

0.0389321

0

0

0

0

0

0

0

0

0

0
5

0

0

0

0

0

0

0

0

0

0

0.00448724
6

0

0

0

0

0

0

0

0

0

0

0
4

0.908573
6

0.704268
6

0

0

0

0

0

0

0

0

0

0

0.0253552
6

0.0543325

0

0

0

0

0

0

0

0

0

0.117373
6

0

0

0

0

0

0

0

0

0

0

0.00724433

0

0

0

0

0

0

0

0

0

0

0

0

0

0

0

0

0

0

0

0

0

0

0

0

0

0

0

0

0

0

0

0

0

0

0

0

0

8.5868812060852e-17
6

0
4

0
3

0

0

0

0

0

0

0

0

0

0

0
4

0.591856

0.591856

0
4

0

0

0

0
4

0

0

0
4

0

0

0

0
4

0.00624502

0

0.00624502

0
4

0

0

0
4

0

0

0

0
4

0

0

0

0
4

0

0

0

0
4

0

0

0
4

0
4

0
4

0
4

0

0

0

0

0

0
4

0

0

0

0
4

0

0

0

0
4

0

0

0
4

0

0

0
4

0

0

0

0
4

0

0

0

0
4

0

0

0

0
4

0

0

0

0
4

0

0

0
4

0

0

0

0
4

0
2

0

0
3

0

0

0

0

0

0

0
4

0

0

0
4

0

0

0

0
4

0

0

0
4

0

0

0
4

0

0

0

0
4

0

0

0

0
4

0

0

0

0
4

0

0

0

0
4

0

0

0
4

0.00362217

0.00362217

0
4

0.0271662

0.0199219

0.00724433

0

0

0

0

0

0

0

0
4

0.192662

0.115597

0.0770648

1.38777878078145e-17

0
4

0

0

0

0
4

0

0

0

0
4

0

0

0
4

0

0

0
4

0

0

0
4

0

0

0
4

0

0

0
4

0

0

0
4

0

0

0
4

0.610633
5

0.547741
5

0

0.062892

0

0

0

0

0

0

0

4.16333634234434e-17
5

0
4

0

0

0
4

0

0

0
4

0

0

0
4

0

0

0
4

0

0

0
4

0

0

0
4

0

0

0
4

0

0

0
4

0

0

0
4

0

0

0
4

0.653668

0.642218

0.00687037

0.00458025

0
4

0

0

0
4

0

0

0
4

0

0

0
4

0

0

0
4

0

0

0
4

0

0

0
4

0

0

0
4

0

0

0
4

0

0

0
4

0

0

0
4

0.144196

0.144196

0

0

0

0

0
4

0

0

0
4

0

0

0
4

0

0

0
4

0.0453516

0.0453516

0
4

0

0

0
4

0

0

0
4

0

0

0
4

0

0

0
4

0.00687037

0.00687037

0
4

0

0

0
4

0

0

0

0

0

0
4

0

0

0
4

0

0

0
4

0

0

0
4

0.0137407

0.0137407

0
4

0

0

0
4

0

0

0
4

0.0290271

0.0290271

0
4

0

0

0
4

0

0

0
4

0

0

0
4

0.0647879
5

0
5

0.0647879

0
4

0

0

0
4

0

0

0
4

0

0

0
4

0

0

0
4

0

0

0
4

0

0

0
4

0

0

0
4

0.00458025

0.00458025

0
4

0

0

0
4

0

0

0
4

0.447102

0.105115
5

0.264923

0

0.0770648

0

0

0
4

0

0

0
4

0

0

0
4

0

0

0
4

0

0

0
4

0

0

0
4

0

0

0
4

0

0

0
4

0.0129576

0.0129576

0
4

0

0

0
4

0

0

0
4

0
4

0
4

0

0

0

0

0

0

0

0

0

0

0
4

0

0

0

0

0

0

0

0

0

0

0

0

0

0

0

0

0
4

0

0

0

0
4

0

0

0
4

0.00458025

0.00458025

0
4

0

0

0
4

0

0

0
4

0

0

0
4

0

0

0
4

0

0

0
4

0

0

0
4

0

0

0
4

0

0

0
4

0.36575
6

0.358505

0.00724433

0

0

0

0

0

0

2.60208521396521e-18
6

0
4

0

0

0
4

0

0

0
4

0

0

0
4

0

0

0
4

0

0

0
4

0

0

0
4

0

0

0
4

0

0

0
4

0

0

0
4

0

0

0
4

0
3

0

0

0

0

0

0

0
4

0

0

0
4

0

0

0
4

0

0

0
4

0

0

0
4

0

0

0
4

0

0

0
4

0

0

0
4

0

0

0
4

0

0

0
4

0

0

0
4

0
4

0

0

0

0

0

0

0

0

0
4

0.00458025

0.00458025

0
4

0

0

0
4

0

0

0
4

0.308259

0.308259

0
4

0

0

0
4

0

0

0
4

0

0

0
4

0

0

0
4

0

0

0
4

0

0

0
4

0
3

0

0

0

0

0

0
4

0

0

0
4

0

0

0
4

0

0

0
4

0

0

0
4

0

0

0
4

0

0

0
4

0

0

0
4

0

0

0
4

0

0

0
4

0
4

0
4

0

0

0

0
4

0
2

0

0

0

0

0

0

0
4

0.0253552

0

0.021733

0

0.00362217

0

1.30104260698261e-18

0
4

0
4

0

0

0

0

0

0

0
4

0

0

0

0

0
4

0
4

0
4

0

0

0

0

0

0

0

0

0

0

0
4

0

0

0

0

0

0
3

0
4

0

0

0

0

0

0
4

0
4

0
4

0

0

0

0
4

0.00458025
4

0

0

0

0.00458025

0

0

0
4

0

0

0

0

0

0

0

0
4

0

0

0

0

0

0

0

0

0
4

0.00673085
7

0.00673085
7

0

0

0
4

0
4

0
4

0

0
4

0.255418

0.239768

0

0.0156497

2.08166817117217e-17

0
4

0.783406
7

0.783406
7

0
4

0
4

0

0

0

0
4

0

0

0

0

0

0
4

0
4

0
4

0

0

0

0

0
4

0
4

0

0

0

0

0

0

0
4

0

0

0

0
4

0.118912

0.076283

0.00448724

0.0381415

0
4

0.192662
6

0.192662
6

0
4

0
5

0
5

0
4

0

0

0

0
4

0

0

0

0
4

0

0

0
4

0

0

0

0

0

0
4

0.0156497

0

0

0

0.0156497

0
4

0

0

0

0

0

0
4

0.159846

0.0156497

0

0

0

0.144196

0

0

0

0

0

0
4

0

0

0

0

0
4

0

0

0

0
4

0

0

0

0

0

0
4

0

0

0

0

0
4

0.00458025

0.00458025

0

0

0
4

0.222847

0.222847

0
4

0

0

0

0
4

0

0

0
4

0.282529

0.282529

0

0
4

0.259793

0.144196

0.115597

2.77555756156289e-17

0
4

0.00458025

0
3

0.00458025

0
2

0

0

0

0

0

0

0

0
4

0

0

0

0

0
4

0

0

0

0
4

0

0

0
4

0

0

0

0

0

0
4

0.170515

0

0.170515

0
4

0

0

0

0

0
4

0

0

0

0
4

0.15413

0.15413

0
4

0

0

0

0
4

0

0

0
4

0
4

0
4

0
4

0

0

0

0
4

0

0

0
4

0.36049

0

0

0.36049

0
4

0

0

0

0

0
4

0

0

0

0
4

0

0

0
4

0

0

0

0
4

0

0

0

0
4

0.0591856

0.0591856

0
4

0

0

0

0
4

0

0

0

0

0
4

0.0156497
4

0
4

0.0156497

0

0

0

0

0

0

0
4

0

0

0

0

0
4

0

0

0

0

0
4

0

0

0

0
4

0

0

0

0

0
4

0

0

0
4

0

0

0

0
4

0

0

0

0
4

0

0

0

0
4

0

0

0
4

0

0

0

0
4

0.15413
6

0.15413
6

0
4

0

0

0

0
4

0

0

0
4

0

0

0
4

0

0

0
4

0

0

0

0
4

0

0

0

0
4

0

0

0
4

0.00458025

0.00458025

0

0
4

0

0

0

0
4

0.769413

0.769413

0
4

0
4

2.80403

2.67032

2.65075

0
6

0

0

0

0

0

0

0

0

0

0.00499292

0

0.0145717

2.96637714392034e-16

0
4

0
6

0

0

0
4

0

0

0
4

0.0181108

0.0181108

0
4

0.115597

0.115597

0
4

0

0

0
4

0
4

1.93068
3

1.01434
3

0.937273
3

0
4

0

0

0

0

0

0

0

0

0

0

0
4

0

0

0

0

0

0

0

0

0

0

0

0

0

0

0.0770648

0

0

0

0

0

0

0
4

0

0

0

0

0

0

0

0

0

0

0

0

0

0

1.38777878078145e-17
3

0
4

0.15413
6

0.15413
6

0

0

0

0
4

0

0

0
4

0

0

0
4

0

0

0
4

0

0

0
4

0.576784
4

0.576784
4

0
4

0

0

0

0
4

0

0

0

0
4

0.185429

0.185429

0
4

0

0

0
4

0

0

0

0
4

0

0

0
4

0

0

0
4

0
4

53.4553

41.7157

29.6711

2.31777
7

0.00458025
6

0

0

0

0

0

0

0

0.0107775

0

0

0

0

0

0

0

0

0.0156497

0

0

0

0.0194364

0.470139
7

0

0

0

0.269727

0

0

0

0

0

0

0

0

0

0

0

0

0

0.00332862

0.0469492

0

0

0.982511

0

0

0

0

0

0.00458025

0

0

0

0

0

0

0.0234746

0

0.00518609

0

0

0.00458025

0.0234746

0

0

0.0874806
5

0

0

0.00518609

0

0.0156497

0.0469492

0

0

0

0.10818

0.308259
7

0.0744407
7

1.42508
7

0.0312995

0.0312995

0

0.500792

0.0453516

0.159846

0

0.73982

0.0904659

0

0.638494
6

0.0770648

0

0

0

0

0

0

0.0770648

0

1.3101

0.208561
7

0

0

0

0.234746

0

0.0211818

0

0

0.0156497

0.331349

0

0

0

0

0.0591856

0

0

0

0

0

0

0.0241892
5

0

0

0

0.00411208

0.0234746

0

0

0

0

0

0.777925
7

0

0

0

0

0.0156497

0

0.00362217

0.0156497

0

0

0.062599

0

0

0

0.0770648

0

0

0

0

0

0

0.139811

0.0156497

0

0

0.0234746

0

0

0.0156497

0

0

0

0
4

6.05466
7

0.0507359
7

0

0.0156497

0

0.0778642

0

0

0.231194

0

0

0.00458025
7

0.880894
8

4.13075
7

0.423856

0.239144

0

0

0

4.71844785465692e-16
7

0
4

0

0

0
4

0

0

0
4

0

0

0
4

0

0

0
4

0.0194364

0.0194364

0
4

0

0

0
4

4.97764

4.83457

0.143079

0

8.60422844084496e-16

0
4

0.263984

0.169849

0.0941345

0

0

0

0

0

0

1.38777878078145e-17

0
4

0.346854
4

0.346854
4

0

0

0

0
4

0.0770648

0

0.0770648

0

0
4

0

0

0

0
4

0

0

0
4

0

0

0
4

0

0

0
4

0
4

45.5755

43.5387

2.73289

0.813786

0

0

0.0842243

0

0

0

0

0.0156497

0

0

0.335536
6

0

0

0

0

0.00616813

0.576784

0.0129576

0.0129576

0

0

0.298508
7

0

0

0

0

0.0194364

0

0.216294

0

0

0.00543325

0.126776

0

0.0129576

0.00362217

0.0770648

0

0

0

0

0

0

0.036295

0.0259152

0.0453516

0

0

0

0

0.0129576

0.0647879

0.00458025

0

1.46541
7

0

0

0.00332862

0

0

0

0.00332862

0.00905542

0

0

0.281995

0

0

0

0

0.115597

0.00362217

0

0.0129576

0.0129576

0

0

0

0.190164

0.00362217

0

0

0

0

0.0770648

0.285067

0

0.203447

0.0312995

0.0194364

0.0312995

0

0

0.0129576

0.115597

0.0860736

0

0.0194364

0
6

0

0.0129576

0

0

0.845951
7

0

0.278922
7

0

0.481262

0.615485
7

0
6

0.0721432

0.149382
7

0

0.0591856
6

6.90013

3.14279

0

0

0.0208358

0.123097

0

0

0.408037

0

0

0.926657

0

0.118371

0.194364

0

0.101723

0.032394

0

0

0.137642

0

3.0776
7

0.246194

0.0271662

0.220279

0

0

0

0

0.00362217

0

0

1.75759
7

0

0.0176371

0

0.00724433

0.0437075

0.0129576

0

0

0

0.032394

9.41646
7

0

0

0.167087

0.00967569

0.0971819

0

0

0

0

0.0770648

2.91441
7

0

0

0.129576

0

0

0

0.231194

0

0.0194364

0.0129576

1.75514
6

0.0770648

0

0

0.0391243

0

0

0

0

0

0

0
4

2.03672
6

2.03672
6

0
4

0

0

0
4

0

0

0
4

0

0

0
4

0

0

0
4

0

0

0
4

0

0

0
4

0

0

0
4

0
4

8.73364
3

8.73364
3

8.71912
3

0.0145135
3

0

0

0

0

0
4

0

0

0

0
4

0

0

0
4

0

0

0
4

0

0

0
4

0

0

0
4

0
4

26.566

26.566

13.5817

0.0983739
7

0

0.0145135

0.0469492

0

0

0

0.00532728

0

0

0

0.437735
6

0

0

0

0

0

0

0

0

0

0

0.0156497
3

0

0.0150924

0

0.0156497

0

0

0

0

0

0

0
3

0

0.15413

0

0

0

0

0.0603697

0

0

0

0.00440928
3

0

0.0183607

0

0

0

0.00687037

0

0

0

0

0.0550138
7

0

0

0.00532728

0

0.0132278

0

0.0518304

0

0

0

0.0448122
7

0

0

0

0

0

0

0

0

0

0

0.177467
7

0.00483473

0

0

0

0

0

0

0

0

0.0153866

0
3

0

0

0

0

0

0.0586001

0.0100616

0

0

0

0
6

0

0

0

0

0

0

0

0

0

0

4.65555
7

0.00973656

0

0

0

0.00518609

0

0

0

0

0

0.00458025

0.0133182
3

0

0

0

0

0

0

0.00458025

0

0

0

0.655051

0

0.0193514

0.00532728

0.00532728

0

0

0

0

0.288392

0

0
3

0.0156497

0

0

0

0.0100616

0

0

0

0

0

0

0

0

0

0

0

0

0.288392

0

0

0

0.00940743
7

0

0

0.0129576

0.00458025

0.0266364

0

0

0

0

0

0.0110023
7

0

0

0

0

0

0

0

0

0

0.00518609

0.0133182

0

0

0.00799092

0

0

0

0

0

0

0

0
4

0.00881856

0

0

0

0

0

0

0

0

0

0.0129576

0

0

0

0

0

0.0312995

0

0

0

0.00532728

2.24101
7

0.00518609
7

0

0

0

0.0156497

0

0

0

0

0

0

0
3

0

0

0

0

0

0

0.0156497

0.242571

0.00483473

0.00532728

0

0

0

0

0.0770648

0

0

0

0

0

0.0639273

0.231194
7

0.0106546

0

0

0

0.0156497

0.0234746

0

0

0

0

0
3

0

0.0692546

0

0

0

0

0.500921

0

0

0.0100616

0.117373

0

0.00458025

0

0

0

0

0

0

0

0

0

0

0

0.00518609

0

0

0

0

0

0

0

0.0770648

0

0

0

0.00799092

0

0

0

0

0

0

0.00479996

0

0.0559364

0

0

0

0

0

0

0

0.0639273

0.0984135

0

0

0

0

0

0

0

0

0.00458025

0.0213091

0
4

0.217816

0

0

0.0234746

0

0

0

0

0

0

0

0.00967569

0

0

0

0

0

0

0

0

0

0

0.00440928

0

0

0.0129652

0.00532728

0

0

0

0

0.0125712

0.00408015

0.0210848

0

0

0

0

0.137561
7

0.00799092

0.0248857

0

0

0

0

0

0.120739

0

0

0.167251
7

0.032394

0

0.0150924

0.00518609

0

0

0

0

0

0

0.0178984
3

0

0

0.148667

0.0547741

0.0133182

0

0

0.00644631

0

0

0.265952
7

0

0.00518609

0

0

0

0

0

0

0.0346273

0

0.142652
7

0

0

0.0186455

0

0

0

0

0

0

0

0
4

0

0

0
4

0

0

0
4

0

0

0
4

0

0

0
4

0

0

0
4

0

0

0
4

0

0

0
4

0

0

0
4

0
4

63.1054

5.31408
3

0.25639
6

0.141329
7

0

0.0122405

0

0

0

0

0.0843091

0.365923

0

0

0.307081
6

0

0

0

0

0

0

0

0

0.00394831

0.0194364

0
3

0

0

0

0.00777913

0

0

0

0

0

0.00518609

0
6

0.144196

0

0.117311

0

0

0

0

0

0

0.00518609

0.295544
2

0.0200479

0

0

0

0

0

0

0

0

0

0

0

0

0

0

0.15413

0

0

0

0

0

0
4

0

0

0

0

0

0

0

0

0

0

0.00966382

0

0

0

0

0

0

0.00543325

0

0

0

0

0

0

0

0

0

0

0

0

0

0

0.321978
7

0

0

0

0

0

0

0.00518609

0

0

0

0.160142
2

0

0

0

0

0

0

0

0

0

0

0

0.0807313
6

0

0

0

0

0.00411208

0

0

0

0

0

0.0188362
6

0.00411208

0

0

0

0

0

0

0

0

0

0.00967569
6

0

0

0

0

0

0

0

0

0

0

0

0.00408015

0

0

0

0

0

0

0

0

0

0.144196
6

0

0

0

0

0

0

0

0

0

0

0

0

0

0

0

0

0

0

0

0.0091605

0

0.00777913
2

0

0

0

0

0

0

0

0

0

0

0.0483785
6

0

0

0

0

0

0.00777913

0

0

0

0

0.0914571
7

0

0

0.00967569

0

0

0

0

0

0

0

0.233286
7

0

0

0

0

0

0

0

0

0

0

0

0
4

0

0

0

0

0

0

0

0

0.00458025

0

0.15413
7

0

0

0

0

0

0

0

0

0

0

0.0303119
5

0

0

0

0

0

0

0

0

0

0

0.0134617

0

0

0

0

0

0.0103722

0

0

0.00967569

0.00967569

0.149382
7

0

0

0

0

0

0

0

0

0.00458025

0

0

0

0

0

0

0

0

0.00394831

0

0

0

0.0798074

0

0.00518609

0

0

0

0

0

0

0.0129576

0

0

0

0

0

0

0

0

0

0

0

0

0

0

0.0234746

0

0

0

0

0

0

0

0

0

0.0156497
7

0

0

0

0

0

0

0

0.00967569

0

0

0.00458025

0

0

0

0

0.00518609

0

0

0

0.00687037

0

0

0

0

0

0

0

0

0

0

0.00458025

0

0

0

0

0.0234746

0

0

0

0

0

0

0

0

0

0

0

0

0

0.0157053

0

0

0

0

0

0

0

0

0

0

0

0

0

0

0

0

0

0

0

0

0

0

0

0

0

0

0.0770648

0

0

0

0

0

0

0

0

0

0

0

0

0

0

0

0

0

0

0

0

0

0

0

0

0

0

0

0

0

0

0

0

0.0112181
4

0.00458025
5

0

0

0

0

0

0

0

0

0

0

0

0

0

0

0

0

0

0

0

0

0

0

0

0

0

0

0

0

0

0

0

0

0

0

0

0

0

0

0

0.00518609

0

0.0137407

0

0

0

0

0

0

0

0

0

0

0

0

0

0

0

0

0

0

0

0

0

0

0

0

0

0

0

0

0

0

0

0.0770648

0

0

0.00518609

0

0

0

0

0

0

0

0

0

0

0

0

0.0770648

0

0

0

0

0

0

0

0

0.00673085

0

0

0

0

0

0

0

0

0

0.00458025

0.391993
7

0

0

0

0.00777913

0

0

0

0

0

0

0

0.0556495
7

0.0129576

0

0

0

0

0

0

0

0

0.014264
7

0

0.0391243

0

0
4

0.0129576

0

0
4

0.0166367
5

0.00518609

0

0

0.00673085

0.423856

0.0163206

0

0

0

0

0.0725511
2

0.00518609

0

0

0

0

0

0

0

0

0

0.00518609
6

0

0.0196996

0

0

0

0.140751

0.062892

0.00897447

0

0

0
4

19.2661

14.8318

0.00408015

0

0

0

0

0

0

0

0

0

0

0.0286073
6

0

0

0

0

0

0

0

0

0

0.0100616

0.0757727
7

0

0

0

0

0

0

0

0

0

0

0

0

0

0

0.0150924

0

0

0

0

0

0

0

0

0.00518609

0

0

0

0

0

0

0

0

0.0239316

0

0

0.00518609

0

0.0770648

0

0

0

0

0

0.0590174
7

0

0

0

0

0

0.115597

0

0

0

0

0

0

0

0

0

0.00518609

0.125242
7

2.7802
7

0.00518609

0

0

0

0

0.0100616
6

0.100616

0

0

0.0148618

0.0155583
7

0

0.0112181

0

0

0

0

0

0.00777913

0

0

0

0

0

0

0

0

0.00967569

0

0

0

0.0654005

0
5

0

0

0

0

0

0

0

0

0

0

0.323449
7

0

0.0103722

0.00777913

0

0

0

0

0

0

0

0

0

0

0

0

0

0

0.0129652

0

0

0

0

0

0

0.00518609

0

0.00448724

0

0

0

0.100616

0.346792

0.0370088

0

0

0

0

0

0

0

0

0.0150924

0

5.9015292652731e-15

0
4

0.0322127
2

0.0322127
2

0

0

0

0
4

0

0

0

0
4

0

0

0

0
4

0

0

0
4

0

0

0

0
4

0

0

0
4

0

0

0
4

0

0

0

0
4

0

0

0

0
4

0

0

0

0
4

0

0

0
4

0.288392
4

0.288392
4

0

0

0

0

0

0

0

0

0

0
4

0.0312995

0.0312995

0
4

0

0

0

0
4

0

0

0

0
4

0

0

0
4

0

0

0

0
4

0

0

0
4

0

0

0
4

0

0

0
4

0

0

0
4

0

0

0
4

0.288392
6

0.288392
6

0

0

0

0

0

0

0

0

0
4

0

0

0
4

0

0

0
4

0

0

0
4

0.00789662

0.00789662

0
4

0.0453516

0.0453516

0
4

0

0

0
4

0

0

0
4

0

0

0
4

0

0

0
4

0

0

0
4

0
3

0
3

0

0

0

0

0

0

0

0

0

0
4

0.0129576

0.0129576

0
4

0

0

0
4

0

0

0
4

0

0

0
4

0.0770648

0.0770648

0
4

0

0

0
4

0

0

0
4

0

0

0
4

0

0

0
4

0

0

0
4

0.316562
6

0.316562
6

0

0

0

0

0

0
4

0

0

0
4

0

0

0
4

0.15413

0.15413

0
4

0

0

0
4

0

0

0
4

0

0

0
4

0

0

0
4

0

0

0
4

0

0

0
4

0

0

0
4

0.732115
3

0.732115
3

0

0

0

0

0

0
4

0

0

0
4

0

0

0
4

0

0

0
4

0

0

0
4

0

0

0
4

0

0

0
4

0

0

0
4

0

0

0
4

0

0

0
4

0

0

0
4

0

0

0

0

0
4

0

0

0
4

0

0

0
4

0

0

0
4

0

0

0
4

0

0

0
4

0

0

0
4

0

0

0
4

0

0

0
4

0

0

0
4

0

0

0
4

0
3

0
3

0

0

0

0

0

0

0

0

0

0
4

0

0

0
4

0

0

0
4

0

0

0
4

0

0

0
4

0

0

0
4

0

0

0
4

0

0

0
4

0

0

0
4

0

0

0
4

0

0

0
4

0

0

0

0

0

0

0

0

0
4

0

0

0
4

0

0

0
4

0

0

0
4

0.00518609

0.00518609

0
4

0

0

0
4

0

0

0
4

0

0

0
4

0

0

0
4

0

0

0
4

0

0

0
4

0
5

0

0

0

0

0

0

0

0

0

0
4

0.00518609

0.00518609

0
4

0

0

0
4

0.00408015

0.00408015

0
4

0

0

0
4

0

0

0
4

0

0

0
4

0

0

0
4

0

0

0
4

0

0

0
4

0

0

0
4

26.7304

26.6945

0

0

0

0

0

0

0

0

0

0

0.0261982
7

0

0

0

0

0

0

0

0

0.00967569

0

0

0

1.42420797377696e-15

0
4

0
4

0
4

0

0

0

0

0

0
4

0

0

0
4

0

0

0
4

0

0

0
4

0

0

0
4

0

0

0
4

0.00440928

0.00440928

0
4

0

0

0
4

0

0

0
4

0

0

0
4

0

0

0
4

0

0

0

0

0

0

0
4

0

0

0
4

0

0

0
4

0

0

0
4

0.00448724

0.00448724

0
4

0

0

0
4

0

0

0
4

0.029373
6

0.00967333

0

0.0196996

0

0

0
4

0
4

0

0

0

0

0

0

0
4

0
4

0

0

0

0

0

0
4

0.286012

0.0777913

0.192662

0

0

0

0.0155583

0
4

0
4

0

0

0

0

0
4

0

0

0

0

0

0

0
4

0

0

0

0

0
4

0.00440928
6

0

0.00440928

0

0

0
4

0
4

0
4

0

0

0

0

0

0

0

0

0

0

0

0

0

0

0

0

0

0

0

0

0

0

0

0

0

0

0

0

0

0

0

0

0

0

0

0

0

0

0

0

0

0

0

0

0

0

0

0

0

0

0

0

0

0

0

0

0

0

0

0

0

0

0

0
4

0
6

0
6

0
4

0.0173059
7

0.00724433

0.0100616

0
4

0.0492679

0

0.0492679

0

0

0
4

0

0

0

0

0
4

0

0

0

0

0
4

0

0

0

0

0

0
4

0

0

0

0
4

0

0

0

0
4

0.00543325

0.00543325

0

0
4

0

0

0

0

0
4

1.9995

1.70502
7

0

0

0.0183607

0

0

0.00440928

0

0

0

0

0
6

0

0

0

0

0

0

0

0

0

0

0.241371

0.00440928

0

0

0
2

0

0

0.0259304

0
4

0

0

0

0

0
4

0

0

0

0

0
4

0.15413

0.15413
7

0

0
4

0

0

0

0

0

0
4

0.060525
7

0.0553389

0

0.00518609

0

1.73472347597681e-18
7

0
4

0

0

0

0

0
4

0

0

0

0

0
4

0

0

0

0

0

0
4

0.00440928

0

0.00440928

0

0
4

0

0

0

0
4

1.60808
6

1.5604
6

0.0163206

0

0

0.00687037

0

0.0244809

0

0

0
4

0.462389

0

0.462389

0
4

0

0

0

0
4

0

0

0

0
4

0

0

0

0

0
4

0

0

0

0
4

0.0160309

0.0160309

0

0

0
4

0

0

0

0
4

0

0

0

0
4

0

0

0

0

0
4

0

0

0

0

0
4

0.56187

0.543719
6

0

0

0

0

0

0

0

0

0

0.00777913

0

0

0

0

0

0

0

0

0

0

0

0

0.0103722

0

0

0

0

3.46944695195361e-18

0
4

0

0

0

0

0
4

0

0

0
4

0

0

0
4

0

0

0

0

0
4

0

0

0

0

0
4

0

0

0

0

0
4

0

0

0

0

0
4

0

0

0

0
4

0

0

0
4

0.00458025

0

0

0.00458025

0
4

1.64377
6

1.44029
6

0

0

0

0

0.0947198
7

0.0544668

0

0.035253

0

0.00897447

0.0100616

0

0
4

0

0

0

0

0
4

0.00394831

0

0.00394831

0
4

0

0

0
4

0

0

0
4

0

0

0

0

0
4

0

0

0

0
4

0

0

0

0

0
4

0

0

0
4

0

0

0
4

0

0

0

0
4

2.52727

2.52727

0

0

0

0

0

0

0
4

0

0

0

0

0
4

0

0

0
4

0

0

0
4

0

0

0

0
4

0

0

0

0
4

0

0

0

0
4

0

0

0

0
4

0

0

0

0
4

0

0

0

0
4

0

0

0
4

0.346792
3

0
3

0

0

0

0.346792

0

0

0

0

0

0
4

0

0

0

0
4

0

0

0

0
4

0

0

0

0
4

0

0

0

0
4

0

0

0
4

0

0

0

0
4

0

0

0

0
4

0

0

0
4

0

0

0

0
4

0

0

0

0
4

2.42028619368284e-14

0
4

0
4

7.78031
4

6.36666
4

6.32779
4

0.561567
4

0

0

0

0

0

0

0

0

0

0

0

0

0

0

0

0

0

0

0

0

0

0

0

0

0

0

0

0

0

0

0

0

0

0

0

0

0

0

0

0

0

0

0

0

0.00411208
4

0

0

0

0

0

0

0

0

0

0

0
4

0

0

0

0

0

0

0

0

0

0

0

0

0

0

0

0

0

0

0

0

0

0
4

0

0

0

0

0

0

0

0

0

0

0

0

0

0

0

0

0

0

0

0

0

0
4

0

0

0

0

0

0

0

0

2.4037
5

0

0.0259152

0

0.158242

0

0

0

0

0

0

1.7748

0.144196

0

0

0

0

0

0

0

0.144196

0.0267285

0.161302

0

0

0

0

0

0

0

0

0

0

0
6

0

0

0

0

0

0

0

0

0

0

0

0

0

0

0

0

0

0

0

0

0

0

0

0

0

0.00518609

0

0

0

0

0

0.0526729

0.865175
4

0

0

0

0

0

0

0

0

0

0

0
4

0

0

0

0

0

0

0

0

0

0

1.99840144432528e-15
4

0
4

0.0388728
4

0

0.0388728

0

0

0

0

0

0

0
4

0

0

0
4

0

0

0

0

0

0
4

0

0

0

0

0

0
4

0

0

0

0
4

0

0

0

0
4

0

0

0

0
4

0

0

0
4

0

0

0
4

0

0

0
4

5.48172618408671e-16
4

0
4

0

0

0

0

0

0

0

0
4

0
4

0

0

0

0

0
4

0
4

0.0352157

0.0352157

0

0.0352157

0
4

0

0

0
4

0
4

0

0

0

0

0
4

0
4

0.0860393

0.0860393

0.00897447

0.0770648

1.38777878078145e-17

0
4

0
4

0.0583091

0.0583091

0.0583091

0
4

0
4

0

0

0

0
4

0
4

0

0

0

0
4

0
4

0

0

0

0
4

0

0

0
4

0

0

0
4

0
4

0

0

0

0
4

0

0

0
4

0
4

0.0591856

0.0591856

0.0591856

0
4

0

0

0
4

0
4

0
4

0
4

0

0

0

0

0

0

0
4

0
4

0

0

0

0
4

0
4

0

0

0

0
4

0

0

0
4

0
4

0

0

0

0
4

0
4

0

0

0

0
4

0
4

0

0

0

0

0
4

0
4

0

0

0

0

0
4

0
4

0

0

0

0
4

0
4

0

0

0

0
4

0
4

0

0

0

0
4

0
4

0

0

0

0
4

0
4

0

0

0

0

0

0

0
4

0

0

0
4

0

0

0
4

0

0

0
4

0
4

0

0

0

0
4

0
4

0.0194364

0.0194364

0.0194364

0
4

0
4

0

0

0

0
4

0
4

0

0

0

0
4

0
4

0

0

0

0
4

0
4

0

0

0

0
4

0
4

0.144196

0.144196

0.144196

0
4

0
4

0

0

0

0
4

0
4

0

0

0

0
4

0
4

0

0

0

0
4

0
4

0.00673085
6

0.00673085
6

0

0.00673085

0

0
4

0
4

0.0770648

0.0770648

0.0770648

0
4

0
4

0

0

0

0
4

0
4

0

0

0

0
4

0
4

0

0

0

0
4

0
4

0

0

0

0
4

0
4

0

0

0

0
4

0
4

0.0194364

0.0194364

0.0194364

0
4

0
4

0

0

0

0
4

0
4

0

0

0

0
4

0
4

0.648882

0.648882

0.648882

0
4

0
4

0.226758

0.226758

0.155491

0.0388728

0.0129576

0.0194364

0
4

0
4

0

0

0

0
4

0
4

0.032394

0.032394

0.032394

0
4

0
4

0

0

0

0
4

0
4

0

0

0

0
4

0
4

0

0

0

0
4

0
4

0

0

0

0
4

0
4

0

0

0

0
4

0
4

0

0

0

0
4

0
4

0

0

0

0

0

0
4

0
4

0

0

0

0
4

0
4

0

0

0

0

0
4

0

0

0
4

0
4

0
4

170.492

3.06433
5

1.87455
5

0.144196
6

1.73035
5

0
4

0

0

0

0

0

0

0
4

0

0

0
4

0.0770648

0.0770648

0
4

0

0

0
4

0

0

0
4

0

0

0
4

0.144196

0.144196

0
4

0

0

0
4

0.00518609

0.00518609

0
4

0

0

0
4

0

0

0
4

0
4

0

0

0

0

0

0

0
4

0

0

0
4

0.0726053

0.0726053

0
4

0.231194

0.231194

0
4

0

0

0

0
4

0

0

0

0
4

0

0

0

0
4

0.539453

0.539453

0
4

0

0

0

0
4

0.115597

0.115597

0
4

0.00448724

0.00448724

0
4

0
4

163.278

24.4122

7.82545
7

0

0

0

0

0.00440928

0

0

0

0.00661392

0

0

0.937273

0

0

0

0

0

0.0770648

0

0

0

0

0.852107
7

0

0

0

0

0.018321

0

0

0

0

0

0.0770648
7

0.36049

0.192662

0

0

0

0

0

0

0.0770648

0.00458025

0.115597
7

0

0

0

0.00881856

0

0

0

0

0.115597

0

0.354495

0

0.00440928

0

0.15413

0

0

0

0

0

0

0.732115
7

0

0.00440928

0

0

0

0

0

0

0

0

0

0

0

0.298325

0.144196
6

0
6

0.144196

0

0

0.284819

0

0.504686
7

0.13199
7

0.0770648

0

4.55184
8

0.0770648

0

0.216294

0.15413

0

2.86525

0

0.00458025

0

0.423856

0.0770648
7

0

0

0

0

0.115597

0

0

0

0.15413

0

0.36049
7

0.0770648

0

0.00458025

0

0.081645

0

0.00687037

0

0

0

0

0

0

0

0

0

0.0091605

0

0

0

0

0.144196

0

0

0.144196

0.081645

0

0.924777

0

0

0

0

0

0.0312995

0

0

0.115597

0

0

0.00458025

0

0

0

0.10604
7

0

0.0181513

0

0.0770648

0

0.0770648

0

0

0

0

0
4

12.7491

3.55064
7

0.385324

1.37126

0

0.00518609

0.0822509

0.770648

0

0.0770648

0.00518609

0

0.0907486
7

0

0.0770648

0

0.00777913

0.115597

0.144196

0

0

0

0

4.92963
7

0

0

0.115597

0.00518609

0

0

0

0.500921

0

0.00518609

0.365457
6

0

0

0

0

0

0.144196

0
4

30.235

14.7722
7

0.886245

0.0414887

0.601618
7

0.115597
7

0

0

0

0.192662

0

0.0770648

7.46191
7

0.588047

0.770648

0.15413

0.115597

0.115597

0.0091605

0

0

0

0

0.212785
8

0

0.0770648

0

0.0770648

0

0

0.0770648

0.00518609

0

0

1.17313
8

0

0

0.0100616

0

0

0

0

0.15413

0

0

0.323574
7

0

0

0

0

0.0770648

0.0770648

0.231194
7

0.32658
7

1.0101
7

0.500921
7

0
4

2.07714

0.555042

0.00777913

0.00322316

0

0.00322316

0.0591856

0

0

0.0193389

0.00805789

0.00483473

0.72843

0.0360492

0

0.462389

0

0.0354547

0.15413

0

0
4

63.7521

0.781127
7

42.0466

19.4531

0

0.093231

0

0

0.0100616

0

0

0.759292

0.224142

0.0129652

0.192662

0

0

0

0.17892

4.57966997657877e-15

0
4

11.2595

11.2595

0
4

15.1161
7

11.786
7

3.3301

0

0

1.33226762955019e-15
7

0
4

1.61795

1.5246

0.0492679

0.0155583

0.00518609

0.0103722

0.00518609

0.00777913

7.45931094670027e-17

0
4

0.167051

0.134719

0

0

0.0161662

0.0161662

0
4

0

0

0
4

0

0

0
4

0

0

0
4

0

0

0
4

0.0770648

0.0770648

0
4

0

0

0
4

1.81513

1.81513

0
4

2.5157653738006e-13

0
4

0

0

0

0

0

0

0

0

0

0

0
4

0

0

0

0

0
4

0
4

0.0499908

0.0391243

0.0391243

0

0
4

0.0108665

0.0108665

0
4

1.73472347597681e-18

0
4

0

0

0

0
4

0
4

0.0492679

0.0440818

0.0129652

0.0311165

0
4

0.00518609

0.00518609

0
4

1.73472347597681e-18

0
4

0

0

0

0

0

0
4

0
4

0

0

0

0
4

0

0

0
4

0
4

0.0712667

0.0518304

0.0518304

0
4

0.0194364

0.0194364

0
4

0
4

0

0

0

0
4

0
4

0

0

0

0

0
4

0
4

0

0

0

0
4

0
4

0.0129576

0.0129576

0.0129576

0
4

0

0

0
4

0
4

0.0757369
7

0.0757369
7

0.0515476

0.00967569
7

0

0.0145135

0

0

0
4

0

0

0
4

0
4

0

0

0

0
4

0
4

0

0

0

0
4

0
4

0.0129576

0.0129576

0.0129576

0
4

0
4

0

0

0

0
4

0
4

0

0

0

0
4

0
4

0

0

0

0
4

0
4

0

0

0

0
4

0
4

0

0

0

0
4

0
4

0

0

0

0
4

0
4

0

0

0

0
4

0
4

0

0

0

0

0
4

0

0

0
4

0
4

0

0

0

0
4

0
4

0

0

0

0
4

0
4

0

0

0

0

0

0
4

0

0

0
4

0
4

3.87306

3.87306

3.87306

0
4

0
4

0.00448724
6

0.00448724

0

0

0.00448724

0
4

0

0

0
4

0

0

0
4

0
4

0

0

0

0

0

0
4

0

0

0

0
4

0
4

0

0

0

0

0
4

0
4

0

0

0

0

0
4

0
4

0
4

6.48733

6.48733

6.26773

2.64682

0.00518609

0

0

0

0

0

0

0

0

0

0.381234
1

0.0181513

3.21115

0.00518609

0

0

0

0

7.11236625150491e-17

0
4

0

0

0

0
4

0.0770648

0

0.0770648

0
4

0

0

0
4

0

0

0
4

0.142533

0.142533

0
4

0

0

0
4

0
4

0

0

0

0
4

0
4

0

0

0

0
4

0
4

0

0

0

0

0
4

0
4

0

0

0

0
4

0
4

0

0

0

0
4

0
4

0

0

0

0
4

0
4

0

0

0

0
4

0
4

0

0

0

0
4

0
4

0

0

0

0
4

0
4

0

0

0

0
4

0
4

0
4

1.43512
5

1.43512
5

1.21882
5

0.358827
5

0.859996

0

0

0

0

0

0

0

0
4

0

0

0

0

0
4

0

0

0

0

0
4

0.216294

0.216294

0
4

0

0

0

0

0
4

0

0

0
4

0

0

0
4

0
4

0
4

0
4

0
4

0
4

0
4

0

0

0

0

0
4

0

0

0

0

0

0

0

0
4

0
4

0
4

0

0

0

0

0

0
4

0

0

0
4

0
4

0
4

0.0695522

0.0695522

0.0695522

0.0695522

0

0
4

0
4

0
4

0.062599

0.062599

0.0156497

0

0.0156497

0
4

0.0469492

0.0312995

0.0156497

0
4

0
4

0
4

0

0

0

0

0

0
4

0
4

0
4

0.0156497

0.0156497

0.0156497

0.0156497

0

0

0
4

0
4

0
4

0.304503

0.304503

0.304503

0.304503

0
4

0
4

0
4

0

0

0

0

0

0
4

0
4

0
4

0

0

0

0

0
4

0
4

0
4

0

0

0

0

0

0
4

0

0

0
4

0
4

0
4

0

0

0

0

0
4

0
4

0
4

0
2

0
2

0
2

0

0

0

0

0

0

0

0

0

0
4

0

0

0
4

0
4

0

0

0

0

0

0

0

0
4

0

0

0

0
4

0
4

0
4

0.0100616

0.0100616

0.0100616

0.0100616

0
4

0

0

0
4

0
4

0
4

0

0

0

0

0

0

0
4

0
4

0
4

0.856444

0.856444

0.432588

0.432588

0
4

0.423856

0.423856

0
4

0
4

0
4

0

0

0

0

0

0
4

0
4

0

0

0

0
4

0
4

0
4

0

0

0

0

0
4

0
4

0
4

0

0

0

0

0
4

0

0

0
4

0
4

0
4

0

0

0

0

0
4

0
4

0
4

0.0770648

0.0770648

0.0770648

0.0770648

0

0
4

0

0

0
4

0
4

0
4

0

0

0

0

0
4

0

0

0
4

0
4

0
4

0

0

0

0

0

0

0
4

0
4

0
4

2.49214
7

2.49214
7

1.29183
7

0.498748
7

0.793077
7

0

1.11022302462516e-16
7

0
4

0.891468
7

0.216294
7

0.598109

0.0770648

1.38777878078145e-17
7

0
4

0.269727

0

0.269727

0

0
4

0.0391243

0

0.0391243

0
4

6.93889390390723e-17
7

0
4

0
4

0

0

0

0

0

0
4

0
4

0
4

0

0

0

0

0
4

0

0

0
4

0

0

0
4

0
4

0
4

0

0

0

0

0
4

0

0

0
4

0
4

0
4

0

0

0

0

0

0
4

0
4

0
4

0

0

0

0

0

0
4

0
4

0
4

0

0

0

0

0
4

0
4

0
4

0

0

0

0

0
4

0
4

0
4

0.123097

0.123097

0.123097

0.123097

0
4

0
4

0
4

0

0

0

0

0

0
4

0
4

0
4

0

0

0

0

0
4

0

0

0
4

0
4

0
4

0.18278

0.142533

0.142533

0.142533

0

0

0

0

0

0

0

0

0

0
4

0

0

0
4

0

0

0

0
4

0
4

0.0402465

0.0402465

0.0402465

0

0
4

0
4

0
4

0

0

0

0

0

0
4

0
4

0
4

0

0

0

0

0
4

0

0

0
4

0
4

0
4

0

0

0

0

0

0
4

0
4

0
4

0

0

0

0

0
4

0
4

0
4

0

0

0

0

0

0
4

0

0

0
4

0
4

0
4

0.96331

0.96331

0

0

0
4

0.96331

0.96331

0
4

0
4

0
4

0

0

0

0

0
4

0
4

0
4

0

0

0

0

0

0
4

0
4

0

0

0

0
4

0
4

0
4

0

0

0

0

0
4

0

0

0
4

0
4

0
4

0.140848

0.140848

0.140848

0.140848

0
4

0
4

0
4

0
2

0
2

0
2

0

0

0

0

0

0

0
4

0

0

0

0

0

0

0
4

0

0

0

0

0

0
4

0
4

0
4

0

0

0

0

0
4

0
4

0
4

0

0

0

0

0
4

0

0

0
4

0

0

0
4

0
4

0
4

0

0

0

0

0
4

0
4

0
4

0

0

0

0

0

0
4

0
4

0
4

0

0

0

0

0

0

0
4

0
4

0
4

0

0

0

0

0

0
4

0
4

0
4

0

0

0

0

0

0
4

0
4

0
4

0.385324

0.385324

0.385324

0.385324

0
4

0
4

0
4

0

0

0

0

0
4

0

0

0
4

0
4

0
4

0

0

0

0

0
4

0
4

0
4

1.7697

1.7697

1.47036

0
6

1.47036
7

0

0

0

0

0
4

0.268042
6

0.268042
6

0

0

0

0
4

0.0312995

0.0156497

0.0156497

0
4

0

0

0
4

0

0

0
4

0

0

0
4

0
4

0
4

0.365457

0.365457

0.288392

0.288392

0
4

0.0770648

0.0770648

0
4

1.38777878078145e-17

0
4

0
4

0

0

0

0

0

0
4

0
4

0
4

0

0

0

0

0
4

0

0

0
4

0
4

0
4

0.00362217

0.00362217

0.00362217

0.00362217

0

0
4

0
4

0
4

0

0

0

0

0

0
4

0
4

0
4

0

0

0

0

0

0
4

0
4

0
4

0

0

0

0

0
4

0

0

0
4

0
4

0
4

0

0

0

0

0
4

0

0

0
4

0
4

0
4

0

0

0

0

0
4

0

0

0
4

0
4

0
4

0

0

0

0

0
4

0
4

0
4

0.794905
7

0.794905
7

0.56212
7

0.514139
7

0.0241892

0.00440928

0.00615463

0.0132278

0
4

0.232785

0.226171

0.00661392

0

0

0

7.80625564189563e-18

0
4

2.77555756156289e-17
7

0
4

0
4

0

0

0

0

0
4

0

0

0
4

0
4

0
4

0

0

0

0

0
4

0

0

0
4

0
4

0
4

0

0

0

0

0

0
4

0
4

0
4

0

0

0

0

0

0
4

0
4

0
4

0

0

0

0

0

0
4

0
4

0
4

0

0

0

0

0
4

0
4

0
4

0

0

0

0

0
4

0
4

0
4

0

0

0

0

0
4

0

0

0
4

0
4

0
4

0.0259152

0.0259152

0.0129576

0.0129576

0
4

0.0129576

0.0129576

0
4

0
4

0
4

0

0

0

0

0
4

0
4

0
4

4.57926
7

4.57926
7

4.10977
7

3.8662
7

0.118371

0

0

0.125198

0

5.55111512312578e-17
7

0
4

0.469492

0

0.30517

0.164322

2.77555756156289e-17

0
4

0

0

0
4

0
4

0
4

0

0

0

0

0
4

0

0

0
4

0
4

0
4

0

0

0

0

0
4

0
4

0
4

0

0

0

0

0
4

0

0

0
4

0
4

0
4

0

0

0

0

0

0
4

0
4

0
4

0

0

0

0

0
4

0
4

0
4

0

0

0

0

0

0
4

0
4

0
4

0

0

0

0

0

0
4

0
4

0
4

0

0

0

0

0

0
4

0
4

0
4

0

0

0

0

0

0
4

0
4

0
4

0.0122664

0.0122664

0.00448724

0.00448724

0
4

0.00777913

0.00777913

0
4

8.67361737988404e-19

0
4

0
4

5.78765
7

5.78765
7

5.40969
7

3.02068
7

1.69543

0.693583

0
4

0.377954

0.377954

0
4

0

0

0
4

0
4

0
4

0

0

0

0

0

0
4

0
4

0
4

0

0

0

0

0
4

0
4

0
4

0

0

0

0

0
4

0
4

0
4

0

0

0

0

0
4

0

0

0
4

0
4

0
4

0

0

0

0

0
4

0
4

0
4

0

0

0

0

0

0
4

0
4

0
4

0.308259

0.308259

0.308259

0.308259

0
4

0

0

0
4

0
4

0
4

0.166017

0.166017

0.166017

0.166017

0
4

0
4

0
4

0.355114

0.355114

0.355114

0.355114

0
4

0
4

0
4

0.0259152

0.0259152

0.0259152

0.0259152

0
4

0
4

0
4

2.02347
7

2.02347
7

1.75681
7

0.188819
7

0.219356
7

0.886245

0.462389

0

0
4

0.266663
7

0.259793
7

0.00687037

0

0
4

0
4

0
4

0.0583091

0.0583091

0.0583091

0.0583091

0
4

0

0

0
4

0
4

0
4

0

0

0

0

0

0
4

0
4

0
4

0

0

0

0

0
4

0
4

0
4

0

0

0

0

0
4

0

0

0
4

0
4

0
4

0

0

0

0

0
4

0
4

0
4

0

0

0

0

0

0
4

0
4

0
4

0

0

0

0

0
4

0

0

0
4

0
4

0
4

0

0

0

0

0
4

0
4

0
4

0.0129576

0.0129576

0.0129576

0.0129576

0
4

0

0

0
4

0
4

0
4

0

0

0

0

0
4

0

0

0
4

0
4

0
4

0.732371
4

0.732371
4

0

0

0

0

0

0

0

0

0

0

0

0

0

0

0

0

0

0

0
4

0.712248
4

0.712248

0

0

0

0

0

0

0

0

0

0

0

0

0

0

0
4

0.0201232

0

0

0.0201232

0

0

0

0
4

0

0

0

0
4

0
4

0

0

0

0
4

0
4

0
4

3.03925

3.03925

2.74437

0.786933

0.0959427

1.68921

0.0129652

0.0770648

0.00518609

0.0770648

6.93889390390723e-17

0
4

0.294881

0.263764

0.00518609

0.0259304

0
4

8.32667268468867e-16

0
4

0
4

0

0

0

0

0
4

0
4

0
4

0

0

0

0

0

0
4

0
4

0
4

0

0

0

0

0
4

0
4

0
4

0

0

0

0

0

0
4

0
4

0
4

0

0

0

0

0

0
4

0
4

0
4

0

0

0

0

0
4

0
4

0
4

0.0388957

0.0388957

0.0181513

0.0181513

0
4

0.0207444

0.0207444

0
4

0
4

0
4

0

0

0

0

0
4

0
4

0
4

0

0

0

0

0

0
4

0
4

0
4

0

0

0

0

0

0
4

0
4

0
4

3.6371
7

3.36738
7

2.67379
7

2.67379
7

0
7

0

0
4

0.693583

0

0.693583

0
4

4.44089209850063e-16
7

0
4

0.269727

0.269727

0.269727

0
4

0
4

0
4

0

0

0

0

0
4

0
4

0
4

0

0

0

0

0
4

0
4

0
4

0

0

0

0

0
4

0
4

0
4

0.0129576

0.0129576

0.0129576

0.0129576

0
4

0
4

0
4

0

0

0

0

0
4

0
4

0
4

0

0

0

0

0
4

0
4

0
4

0

0

0

0

0
4

0
4

0
4

0

0

0

0

0
4

0
4

0
4

0

0

0

0

0
4

0
4

0
4

0

0

0

0

0
4

0
4

0
4

2.26404

2.26404

1.99432

1.06954
7

0.924777

0

3.33066907387547e-16

0
4

0.269727

0.269727

0
4

0

0

0
4

0

0

0
4

0
4

0
4

0

0

0

0

0
4

0
4

0
4

0

0

0

0

0
4

0
4

0
4

0

0

0

0

0
4

0
4

0
4

0

0

0

0

0
4

0
4

0
4

0

0

0

0

0
4

0
4

0
4

0

0

0

0

0
4

0
4

0
4

0

0

0

0

0
4

0
4

0
4

0

0

0

0

0
4

0
4

0
4

0

0

0

0

0
4

0
4

0
4

0

0

0

0

0
4

0
4

0
4

0
2

0
2

0
2

0
2

0

0

0

0

0

0
4

0
2

0

0

0

0
4

0

0

0
4

0
4

0
4

0

0

0

0

0
4

0
4

0
4

0

0

0

0

0
4

0
4

0
4

0

0

0

0

0
4

0
4

0
4

0.115597

0.115597

0.115597

0.115597

0
4

0
4

0
4

0

0

0

0

0
4

0
4

0
4

0

0

0

0

0
4

0
4

0
4

0

0

0

0

0
4

0
4

0
4

0

0

0

0

0
4

0
4

0
4

0

0

0

0

0
4

0
4

0
4

0

0

0

0

0
4

0
4

0
4

0.0129576
4

0
4

0
4

0

0

0

0

0

0

0

0

0

0
4

0
4

0
4

0
4

0
4

0.0129576

0.0129576

0.0129576

0
4

0
4

0
4

0

0

0

0

0
4

0
4

0
4

0

0

0

0

0
4

0
4

0
4

0

0

0

0

0
4

0
4

0
4

0

0

0

0

0
4

0
4

0
4

0

0

0

0

0
4

0
4

0
4

0

0

0

0

0
4

0
4

0
4

0

0

0

0

0
4

0
4

0
4

0

0

0

0

0
4

0
4

0
4

0

0

0

0

0
4

0
4

0
4

0.0129576

0.0129576

0.0129576

0.0129576

0
4

0
4

0
4

0.950674
5

0.950674
5

0.950674
5

0.426552
5

0.504686

0.0194364

0
4

0
4

0
4

0

0

0

0

0
4

0
4

0
4

0

0

0

0

0
4

0
4

0
4

0

0

0

0

0
4

0
4

0
4

0

0

0

0

0
4

0
4

0
4

0

0

0

0

0
4

0
4

0
4

0

0

0

0

0
4

0
4

0
4

0

0

0

0

0
4

0
4

0
4

0

0

0

0

0
4

0
4

0
4

0

0

0

0

0
4

0
4

0
4

0

0

0

0

0
4

0
4

0
4

0.342744
7

0.31974
7

0.073291
7

0.073291
7

0

0
4

0.246449

0.246449

0
4

2.77555756156289e-17
7

0
4

0.0230036

0.0230036

0.0148942

0.00448724

0.00362217

2.16840434497101e-18

0
4

0
4

0
4

0

0

0

0

0
4

0
4

0
4

0

0

0

0

0
4

0
4

0
4

0

0

0

0

0
4

0
4

0
4

0

0

0

0

0
4

0
4

0
4

0

0

0

0

0
4

0
4

0
4

0.0770648

0.0770648

0.0770648

0.0770648

0
4

0
4

0
4

0

0

0

0

0
4

0
4

0
4

0.0712667

0.0712667

0.0712667

0.0712667

0
4

0
4

0
4

0

0

0

0

0
4

0
4

0
4

0

0

0

0

0
4

0
4

0
4

0

0

0

0

0

0

0

0

0

0

0
4

0
4

0
4

0

0

0

0

0
4

0
4

0
4

0

0

0

0

0
4

0
4

0
4

0

0

0

0

0
4

0
4

0
4

0

0

0

0

0
4

0
4

0
4

0

0

0

0

0
4

0
4

0
4

0

0

0

0

0
4

0
4

0
4

0

0

0

0

0
4

0
4

0
4

0

0

0

0

0
4

0
4

0
4

0

0

0

0

0
4

0
4

0
4

0

0

0

0

0
4

0
4

0
4

0

0

0

0

0

0

0

0

0
4

0
4

0
4

0.0156497

0.0156497

0.0156497

0.0156497

0
4

0
4

0
4

0

0

0

0

0
4

0
4

0
4

0

0

0

0

0
4

0
4

0
4

0

0

0

0

0
4

0
4

0
4

0

0

0

0

0
4

0
4

0
4

0

0

0

0

0
4

0
4

0
4

0

0

0

0

0
4

0
4

0
4

0

0

0

0

0
4

0
4

0
4

0

0

0

0

0
4

0
4

0
4

0

0

0

0

0
4

0
4

0
4

0
4

0
4

0
4

0
4

0

0

0

0
4

0
4

0
4

0

0

0

0

0
4

0
4

0
4

0

0

0

0

0
4

0
4

0
4

0

0

0

0

0
4

0
4

0
4

0

0

0

0

0
4

0
4

0
4

0

0

0

0

0
4

0
4

0
4

0

0

0

0

0
4

0
4

0
4

0

0

0

0

0
4

0
4

0
4

0

0

0

0

0
4

0
4

0
4

0

0

0

0

0
4

0
4

0
4

0

0

0

0

0
4

0
4

0
4

0
4

0
4

0
4

0
4

0

0

0

0

0

0

0

0

0

0

0
4

0

0

0
4

0

0

0

0

0

0

0
4

0
4

0

0

0

0

0

0

0
4

0

0

0

0
4

0

0

0
4

0
4

0
4

0.0194364
5

0.0194364
5

0.0194364
5

0
5

0.0194364

0

0

0

0

0

0
4

0

0

0
4

0
4

0
4

0

0

0

0

0
4

0
4

0
4

0

0

0

0

0
4

0
4

0
4

0.103661

0.103661

0.103661

0.103661

0
4

0
4

0
4

0

0

0

0

0
4

0
4

0
4

0

0

0

0

0
4

0
4

0
4

0

0

0

0

0
4

0
4

0
4

0

0

0

0

0
4

0
4

0
4

0

0

0

0

0
4

0
4

0
4

0.00616813

0.00616813

0.00616813

0.00616813

0
4

0
4

0
4

0

0

0

0

0
4

0
4

0
4

0.12577
4

0.12577
4

0.12577
4

0

0

0.12577

0

0

0

0

0
4

0
4

0
4

0

0

0

0

0
4

0
4

0
4

0

0

0

0

0
4

0
4

0
4

0

0

0

0

0
4

0
4

0
4

0

0

0

0

0
4

0
4

0
4

0

0

0

0

0
4

0
4

0
4

0

0

0

0

0
4

0
4

0
4

0

0

0

0

0
4

0
4

0
4

0

0

0

0

0
4

0
4

0
4

0

0

0

0

0
4

0
4

0
4

0

0

0

0

0
4

0
4

0
4

0.963633

0.963633

0
6

0

0

0

0
4

0.963633

0.963633

0

0

0
4

0

0

0
4

0

0

0

0
4

0

0

0
4

0

0

0
4

0
4

0
4

0.00967569

0.00967569

0.00967569

0.00967569

0
4

0
4

0
4

0

0

0

0

0
4

0
4

0
4

0

0

0

0

0
4

0
4

0
4

0

0

0

0

0
4

0
4

0
4

0

0

0

0

0
4

0
4

0
4

0.0770648

0.0770648

0.0770648

0.0770648

0
4

0
4

0
4

0

0

0

0

0
4

0
4

0
4

0

0

0

0

0
4

0
4

0
4

0

0

0

0

0
4

0
4

0
4

0

0

0

0

0
4

0
4

0
4

3.18714

3.18714

3.18714

3.18714
7

0

0

0

0
4

0
4

0
4

0

0

0

0

0
4

0
4

0
4

0

0

0

0

0
4

0
4

0
4

0.0770648

0.0770648

0.0770648

0.0770648

0
4

0
4

0
4

0

0

0

0

0
4

0
4

0
4

0

0

0

0

0
4

0
4

0
4

0.0234746

0.0234746

0.0234746

0.0234746

0
4

0
4

0
4

0

0

0

0

0
4

0
4

0
4

0

0

0

0

0
4

0
4

0
4

0.00408015

0.00408015

0.00408015

0.00408015

0
4

0
4

0
4

0

0

0

0

0
4

0
4

0
4

0.255418
3

0.255418
3

0.216294
4

0.216294

0

0

0

0
4

0

0

0
4

0

0

0
4

0.0391243

0

0.0391243

0
4

1.38777878078145e-17
3

0
4

0
4

0

0

0

0

0
4

0
4

0
4

0

0

0

0

0
4

0
4

0
4

0

0

0

0

0
4

0
4

0
4

0

0

0

0

0
4

0
4

0
4

0

0

0

0

0
4

0
4

0
4

0

0

0

0

0
4

0
4

0
4

0.144196

0.144196

0.144196

0.144196

0
4

0
4

0
4

0

0

0

0

0
4

0
4

0
4

0

0

0

0

0
4

0
4

0
4

0

0

0

0

0
4

0
4

0
4

0.130588
7

0.130588
7

0.0453516
7

0

0

0.0453516

0
4

0.0719183

0

0

0.0612637

0.0106546

0
4

0.0133182

0.0133182

0
4

8.67361737988404e-18
7

0
4

0
4

0

0

0

0

0
4

0
4

0
4

0

0

0

0

0
4

0
4

0
4

0

0

0

0

0
4

0
4

0
4

0.032394

0.032394

0.032394

0.032394

0
4

0
4

0
4

0

0

0

0

0
4

0
4

0
4

0.0100616

0.0100616

0.0100616

0.0100616

0
4

0
4

0
4

0

0

0

0

0
4

0
4

0
4

0

0

0

0

0
4

0
4

0
4

0

0

0

0

0
4

0
4

0
4

0

0

0

0

0
4

0
4

0
4

9.0278

8.67795

8.65203

8.53541

0.032394

0.0842243

0
4

0.0259152

0.0129576

0.0129576

0
4

1.48145384848419e-15

0
4

0.349855

0.259152

0.246194

0.0129576

1.90819582357449e-17

0
4

0.0907031

0.0907031

0
4

0
4

9.43689570931383e-16

0
4

0

0

0

0

0
4

0
4

0
4

0

0

0

0

0
4

0
4

0
4

0

0

0

0

0
4

0
4

0
4

0

0

0

0

0
4

0
4

0
4

0

0

0

0

0
4

0
4

0
4

0

0

0

0

0
4

0
4

0
4

0

0

0

0

0
4

0
4

0
4

0

0

0

0

0
4

0
4

0
4

0

0

0

0

0
4

0
4

0
4

0

0

0

0

0
4

0
4

0
4

0
5

0
5

0
5

0
5

0

0
4

0
4

0
4

0

0

0

0

0
4

0
4

0
4

0.0103722

0.0103722

0.0103722

0.0103722

0
4

0
4

0
4

0

0

0

0

0
4

0
4

0
4

0.0647879

0.0647879

0.0647879

0.0647879

0
4

0
4

0
4

0

0

0

0

0
4

0
4

0
4

0

0

0

0

0
4

0
4

0
4

0

0

0

0

0
4

0
4

0
4

0

0

0

0

0
4

0
4

0
4

0

0

0

0

0
4

0
4

0
4

0

0

0

0

0
4

0
4

0
4

0.0967569
7

0.0967569
7

0

0

0

0
4

0.0967569

0.0870812

0.00967569

1.73472347597681e-18

0
4

0
4

0
4

0

0

0

0

0
4

0
4

0
4

0

0

0

0

0
4

0
4

0
4

0

0

0

0

0
4

0
4

0
4

0

0

0

0

0
4

0
4

0
4

0

0

0

0

0
4

0
4

0
4

0

0

0

0

0
4

0
4

0
4

0

0

0

0

0
4

0
4

0
4

0

0

0

0

0
4

0
4

0
4

0

0

0

0

0
4

0
4

0
4

0

0

0

0

0
4

0
4

0
4

0.032394
4

0.032394
4

0
4

0

0

0

0
4

0
4

0
4

0
4

0

0

0
4

0.032394

0.032394

0
4

0
4

0
4

0

0

0

0

0
4

0
4

0
4

0

0

0

0

0
4

0
4

0
4

0

0

0

0

0
4

0
4

0
4

0

0

0

0

0
4

0
4

0
4

0.0391243

0.0391243

0.0391243

0.0391243

0
4

0
4

0
4

0.346792

0.346792

0.346792

0.346792

0
4

0
4

0
4

0

0

0

0

0
4

0
4

0
4

0

0

0

0

0
4

0
4

0
4

0

0

0

0

0
4

0
4

0
4

0

0

0

0

0
4

0
4

0
4

5.39566
7

5.39566
7

4.84296
7

3.04585
7

1.76276

0.0206111
6

0

0.0137407

0

0
4

0.552699
7

0.0297716
7

0.105925
7

0.100616
7

0.190727
7

0.0100616

0.115597

0

0
4

3.33066907387547e-16
7

0
4

0
4

0

0

0

0

0

0

0

0
4

0

0

0

0
4

0
4

0
4

0

0

0

0

0
4

0
4

0
4

0.0156497

0.0156497

0.0156497

0.0156497

0
4

0
4

0
4

0

0

0

0

0
4

0
4

0
4

0

0

0

0

0
4

0
4

0
4

0

0

0

0

0
4

0
4

0
4

0

0

0

0

0
4

0
4

0
4

0.00518609

0.00518609

0.00518609

0.00518609

0
4

0
4

0
4

0

0

0

0

0
4

0
4

0
4

0

0

0

0

0
4

0
4

0
4

0

0

0

0

0
4

0
4

0
4

0.252329
7

0.252329
7

0.236679
7

0.206458
6

0.0302214

0

0
4

0.0156497

0.0156497

0
4

0

0

0
4

0
4

0
4

0.0156497

0.0156497

0.0156497

0.0156497

0
4

0
4

0
4

0

0

0

0

0
4

0
4

0
4

0

0

0

0

0
4

0
4

0
4

0

0

0

0

0
4

0
4

0
4

0

0

0

0

0
4

0
4

0
4

0

0

0

0

0
4

0
4

0
4

0

0

0

0

0
4

0
4

0
4

0

0

0

0

0
4

0
4

0
4

0

0

0

0

0
4

0
4

0
4

0

0

0

0

0
4

0
4

0
4

0.0234746
7

0.0234746
7

0.0234746

0

0.0234746

0

0
4

0

0

0

0

0
4

0
4

0
4

0

0

0

0

0
4

0
4

0
4

0

0

0

0

0
4

0
4

0
4

0

0

0

0

0
4

0
4

0
4

0

0

0

0

0
4

0
4

0
4

0
6

0
6

0
6

0
6

0

0

0
4

0
4

0
4

0

0

0

0

0

0

0

0
4

0

0

0

0
4

0

0

0
4

0

0

0

0
4

0
4

0

0

0

0
4

0
4

0
4

1.09001
7

1.09001
7

1.05114
7

1.05114
7

0

0
4

0.0388728

0.0388728

0
4

0

0

0
4

1.04083408558608e-16
7

0
4

0
4

2.47944
7

2.47944
7

2.47944
7

2.47944
7

0
4

0
4

0
4

0

0

0

0

0

0

0

0

0
4

0

0

0
4

0
4

0
4

0
4

0
4

0

0

0

0

0
4

0

0

0
4

0
4

0
4

0

0

0

0

0

0
4

0
4

0
4

0.130521
5

0.130521
5

0.0606548
6

0.0156497
6

0

0

0

0

0

0

0

0.00440928

0

0.0338649

0

0

0.00673085

0

0

0

0
4

0.0451862
5

0.0155583
7

0.0134617
6

0

0.0161662

0
4

0.0246798

0.0246798

0

0

0
4

0
4

0
4

0

0

0

0

0

0
4

0

0

0

0
4

0
4

0
4

0.0712667
4

0.0712667
4

0.0712667
4

0.0712667

0

0

0

0
4

0

0

0

0
4

0
4

0
4

0

0

0

0

0

0

0

0
4

0
4

0
4

0.269727
7

0.269727
7

0.269727
7

0

0.269727

0
4

0
4

0
4

0

0

0

0

0

0

0
4

0

0

0
4

0
4

0
4

0.0435124

0.0435124

0.0389321

0.00458025

0.0160309

0

0.0091605

0.0091605

0
4

0.00458025

0.00458025

0
4

3.46944695195361e-18

0
4

0
4

0

0

0

0

0

0

0

0
4

0
4

0
4

0.312547

0.312547

0.312547

0.118552

0.0323325

0.161662

0
4

0
4

0
4

0

0

0

0

0
4

0
4

0
4

0.0704313

0.0704313

0.0704313

0.0704313

0

0
4

0
4

0
4

1.92433

1.92433

1.3862
5

0.6653
6

0

0.693583

0.0176371

0

0

0.00967569

0

0

0

1.57859836313889e-16
5

0
4

0.538133
7

0.456561
7

0.0815717

0

0
4

0

0

0

0

0

0
4

0

0

0

0
4

0
4

0

0

0

0
4

0
4

0
4

0

0

0

0

0

0

0
4

0

0

0

0
4

0
4

0

0

0

0
4

0
4

0
4

0
4

0
4

0
4

0
4

0

0
4

0
4

0
4

0.179
7

0.179
7

0.179
7

0.179
7

0

0
4

0
4

0
4

0

0

0

0

0

0
4

0

0

0
4

0
4

0
4

0.600397

0.600397

0

0

0

0
4

0.587079

0.587079

0
4

0.00532728

0.00532728

0

0
4

0.00799092

0.00799092

0
4

8.67361737988404e-18

0
4

0
4

0

0

0

0

0

0
4

0
4

0
4

0
4

0
4

0

0

0

0
4

0

0

0

0
4

0

0

0
4

0
4

0
4

0

0

0

0

0

0

0
4

0
4

0
4

0
4

0
4

0

0

0
4

0

0

0
4

0

0

0
4

0
4

0
4

0
5

0
5

0
5

0
5

0
4

0
4

0
4

1.53244

1.53244

0.2138

0.2138

0

0

0
4

0.0129576

0.0129576

0
4

0

0

0
4

1.30568
4

1.30568
4

0

0

0

0

0
4

0

0

0

0

0
4

0

0

0
4

0

0

0
4

0

0

0
4

0

0

0
4

0

0

0
4

0

0

0
4

0
4

0
4

0

0

0

0

0

0
4

0
4

0
4

0

0

0

0

0

0

0
4

0

0

0

0
4

0
4

0
4

0

0

0

0

0
4

0
4

0
4

0

0

0

0

0

0
4

0

0

0

0
4

0
4

0
4

0.00411208

0.00411208

0.00411208

0.00411208

0

0
4

0
4

0
4

0

0

0

0

0

0

0

0

0
4

0

0

0
4

0
4

0
4

0.0103722

0.0103722

0.0103722

0.00518609

0.00518609

0

0
4

0
4

0
4

0.958862

0.958862

0.907031

0.907031

0
4

0.0518304

0.0259152

0.0259152

0
4

5.55111512312578e-17

0
4

0
4

0.148509
7

0.148509
7

0

0

0
4

0.148509

0.148509

0
4

0
4

0
4

0

0

0

0

0

0
4

0

0

0

0
4

0
4

0
4

5.63291

5.63291

3.80589

0.168449

0.0129576

0.0129576

0.080588

0.116618

3.24588

0.0388728

0.103661

0

0.0129576

0.0129576

0
4

1.10787

0.732104

0.0971819

0.103661

0.0453516

0.0453516

0.0583091

0.0259152

0
4

0.719146

0.0453516

0.349855

0.0388728

0.16197

0.0518304

0.0129576

0.0129576

0.0453516

0
4

6.66133814775094e-16

0
4

0
4

0

0

0

0

0

0

0
4

0
4

0
4

0
5

0
5

0
5

0

0

0
4

0
4

0
4

2.59339

2.59339

2.59339

2.59339

0
4

0
4

0
4

0

0

0

0

0
4

0
4

0
4

0.306997

0.306997

0.306997

0.216294

0.0907031

0
4

0
4

0
4

0

0

0

0

0
4

0
4

0
4

0

0

0

0

0

0
4

0

0

0
4

0
4

0
4

0.0194364

0.0194364

0.0194364

0.0194364

0

0

0
4

0
4

0
4

1.03661

1.03661

1.03661

1.01717

0.0194364

1.00613961606655e-16

0
4

0
4

0
4

0.0712667

0.0712667

0.0712667

0.0583091

0.0129576

0
4

0

0

0

0
4

0
4

0
4

2.14415
7

2.14415
7

2.04242
7

0.756644
7

0.774662
7

0.135524
7

0.226921

0.0938984

0

0

0

0.0547741

2.42861286636753e-16
7

0
4

0.0860736

0.0860736

0

0

0
4

0.0156497

0.0156497

0
4

1.59594559789866e-16
7

0
4

0
4

0

0

0

0

0

0

0
4

0
4

0
4

0

0

0

0

0

0

0
4

0
4

0
4

0

0

0

0

0

0
4

0
4

0
4

0

0

0

0

0
4

0
4

0
4

0

0

0

0

0

0
4

0
4

0
4

0

0

0

0

0
4

0

0

0
4

0
4

0
4

0

0

0

0

0
4

0
4

0
4

0

0

0

0

0

0
4

0
4

0
4

0

0

0

0

0

0
4

0

0

0
4

0
4

0

0

0

0
4

0
4

0
4

0.0363026

0.0363026

0.0363026

0.00518609

0.00518609

0.0259304

0
4

0
4

0
4

2.1339

0
4

0
4

0

0

0

0
4

0
4

0

0

0

0
4

0
4

0

0

0

0
4

0
4

0

0

0

0
4

0
4

0

0

0

0
4

0
4

0

0

0

0
4

0
4

0

0

0

0
4

0
4

0

0

0

0
4

0
4

0

0

0

0
4

0
4

0

0

0

0
4

0
4

0.0583091

0.0583091

0.0583091

0
4

0
4

0

0

0

0

0
4

0
4

0

0

0

0
4

0
4

0

0

0

0
4

0
4

0

0

0

0
4

0
4

0

0

0

0
4

0
4

0

0

0

0
4

0
4

0

0

0

0
4

0
4

0

0

0

0
4

0
4

0

0

0

0
4

0
4

0

0

0

0
4

0
4

0

0

0

0
4

0
4

0

0

0

0
4

0
4

0

0

0

0
4

0
4

0

0

0

0

0

0
4

0
4

0

0

0

0

0
4

0
4

0

0

0

0
4

0
4

0

0

0

0
4

0

0

0
4

0
4

0

0

0

0
4

0
4

0.032394

0.032394

0.032394

0
4

0
4

2.04319
3

1.09186
2

0.0129576
3

0
2

0

0

0

0

0

0

0

0

0.231194

0

0
2

0

0

0

0

0

0

0

0

0

0

0

0

0

0

0

0

0

0

0

0

0

0

0

0

0.847713

0
4

0

0

0

0

0

0

0

0

0

0

0
4

0

0

0
4

0.0100616

0.0100616

0

0
4

0

0

0
4

0

0

0

0
4

0.0129576

0.0129576

0
4

0

0

0
4

0

0

0
4

0

0

0
4

0

0

0
4

0

0

0
4

0.0259152

0.0129576

0

0.0129576

0

0

0

0
4

0.0129576

0.0129576

0
4

0

0

0
4

0

0

0
4

0

0

0
4

0

0

0
4

0

0

0
4

0

0

0
4

0

0

0
4

0

0

0
4

0

0

0
4

0.343376

0.259152

0.0259152

0.0583091

0
4

0.0234746

0.0234746

0
4

0

0

0
4

0

0

0
4

0.224561

0.209468

0.0150924

0
4

0.298025

0.278588

0.0194364

0
4

0

0

0

0

0
4

0

0

0

0

0
4

0

0

0
4

0

0

0

0
4

0
4

0
4

6.65602
3

0

0

0

0

0

0

0

0

0
4

0

0

0

0

0
4

0

0

0
4

0
4

0.192662
6

0.192662
6

0.192662
6

0
4

0
4

0

0

0

0
4

0
4

0

0

0

0
4

0
4

0

0

0

0
4

0
4

0

0

0

0
4

0
4

0

0

0

0
4

0
4

0

0

0

0
4

0
4

0.118371

0.118371

0.118371

0
4

0
4

0

0

0

0
4

0
4

0

0

0

0
4

0
4

0

0

0

0
4

0
4

0

0

0

0

0

0

0

0
4

0

0

0

0
4

0

0

0
4

0
4

0

0

0

0
4

0
4

0

0

0

0
4

0
4

0

0

0

0
4

0
4

0

0

0

0
4

0
4

0

0

0

0
4

0
4

0

0

0

0
4

0
4

0

0

0

0
4

0
4

0

0

0

0
4

0
4

0

0

0

0
4

0
4

0

0

0

0
4

0
4

0

0

0

0

0
4

0

0

0

0
4

0
4

0

0

0

0
4

0
4

0

0

0

0
4

0
4

0

0

0

0
4

0
4

0

0

0

0
4

0
4

0

0

0

0
4

0
4

0

0

0

0
4

0
4

0

0

0

0
4

0
4

0

0

0

0
4

0
4

0

0

0

0
4

0
4

0

0

0

0
4

0
4

0

0

0

0

0

0

0

0
4

0

0

0
4

0
4

0

0

0

0
4

0
4

0

0

0

0
4

0
4

0

0

0

0
4

0
4

0

0

0

0
4

0
4

0

0

0

0
4

0
4

0

0

0

0
4

0
4

0

0

0

0

0

0
4

0

0

0

0
4

0

0

0
4

0
4

0
3

0

0

0

0

0

0
4

0

0

0

0
4

0

0

0
4

0

0

0
4

0

0

0
4

0
4

0
2

0

0

0

0

0
4

0

0

0
4

0

0

0

0
4

0

0

0
4

0

0

0
4

0
4

0

0

0

0

0

0

0
4

0
4

0

0

0

0

0
4

0
4

0.0547011
6

0.0547011
6

0.0547011
6

0
4

0
4

0
4

0
4

0
4

0
4

0

0

0
4

0

0

0
4

0

0

0
4

0
4

0
3

0

0

0

0

0

0
4

0

0

0
4

0

0

0
4

0
4

0
3

0

0

0

0

0
4

0

0

0
4

0

0

0
4

0

0

0
4

0

0

0
4

0
4

0

0

0

0

0
4

0

0

0
4

0
4

0.142617

0.142617

0.132245

0.0103722

1.21430643318376e-17

0
4

0
4

0

0

0

0

0
4

0
4

0

0

0

0
4

0

0

0
4

0
4

0.495005

0.486781

0.486781

0
4

0.00822417

0.00411208

0.00411208

0
4

0
4

0

0

0

0

0

0
4

0

0

0
4

0
4

0
4

0
4

0

0

0

0

0

0

0
4

0
4

0
4

0

0

0

0

0

0
4

0

0

0

0
4

0
4

0
3

0
3

0

0

0

0

0
4

0

0

0

0

0
4

0

0

0
4

0
4

0

0

0

0

0
4

0

0

0
4

0
4

0

0

0

0

0

0

0
4

0
4

0
6

0
6

0
6

0
4

0
4

0

0

0

0

0
4

0

0

0

0
4

0
4

0

0

0

0

0
4

0

0

0
4

0
4

0.38229

0.38229

0.288392

0.0938984

1.38777878078145e-17

0
4

0
4

0

0

0

0

0

0
4

0

0

0
4

0
4

0

0

0

0

0

0
4

0
4

0

0

0

0

0
4

0

0

0
4

0
4

0

0

0

0

0
4

0
4

0
2

0

0

0

0

0
4

0

0

0

0

0
4

0

0

0
4

0

0

0
4

0

0

0
4

0
4

0

0

0

0

0
4

0

0

0
4

0
4

0

0

0

0
4

0
4

0.485688

0.485688

0.485688

0
4

0
4

0.231194

0.231194

0.231194

0
4

0
4

0

0

0

0
4

0
4

0

0

0

0

0
4

0
4

0

0

0

0
4

0

0

0
4

0
4

0

0

0

0

0
4

0

0

0
4

0
4

0

0

0

0
4

0
4

0

0

0

0
4

0
4

2.37124

2.37124

2.14448

0.0453516

0.0518304

0.0971819

0.032394

2.28983498828939e-16

0
4

0
4

0

0

0

0
4

0

0

0
4

0
4

0

0

0

0

0
4

0

0

0
4

0
4

0

0

0

0

0
4

0
4

0

0

0

0

0
4

0
4

0

0

0

0

0
4

0
4

0

0

0

0
4

0

0

0
4

0
4

0

0

0

0

0
4

0
4

0

0

0

0
4

0

0

0
4

0
4

0

0

0

0
4

0

0

0
4

0
4

0

0

0

0
4

0
4

0
4

0

0

0

0

0

0
4

0

0

0
4

0

0

0
4

0

0

0
4

0
4

0

0

0

0
4

0
4

0

0

0

0

0
4

0
4

0

0

0

0
4

0
4

0

0

0

0

0
4

0
4

0

0

0

0
4

0

0

0
4

0
4

0

0

0

0
4

0

0

0
4

0
4

0

0

0

0
4

0

0

0
4

0
4

0.0156497

0.0156497

0.0156497

0
4

0
4

0.0488992

0.0488992

0.0488992

0
4

0
4

0

0

0

0

0
4

0
4

0

0

0

0

0
4

0

0

0

0

0
4

0

0

0
4

0
4

0

0

0

0

0
4

0
4

0

0

0

0
4

0

0

0
4

0
4

0

0

0

0
4

0

0

0
4

0
4

0

0

0

0

0
4

0
4

0

0

0

0
4

0
4

0

0

0

0
4

0
4

0

0

0

0

0
4

0
4

0

0

0

0
4

0
4

0

0

0

0
4

0
4

0

0

0

0
4

0
4

0.531023

0.147296

0.139072

0.00411208

0.00411208

1.73472347597681e-18

0
4

0.209835

0.209835

0
4

0.0197618

0.0197618

0
4

0.15413

0.0770648

0.0770648

0
4

2.77555756156289e-17

0
4

0

0

0

0
4

0
4

0

0

0

0
4

0
4

0

0

0

0
4

0
4

0

0

0

0
4

0
4

0

0

0

0
4

0
4

0

0

0

0
4

0
4

0

0

0

0
4

0
4

0

0

0

0
4

0
4

0

0

0

0
4

0
4

0.158176

0.158176

0.158176

0
4

0
4

0
3

0

0

0

0
4

0

0

0

0

0
4

0

0

0
4

0

0

0
4

0
4

0

0

0

0
4

0
4

0

0

0

0
4

0
4

0

0

0

0
4

0
4

0

0

0

0
4

0
4

0

0

0

0
4

0
4

0

0

0

0
4

0
4

0

0

0

0
4

0
4

0

0

0

0
4

0
4

0

0

0

0
4

0
4

0

0

0

0
4

0
4

1.4285
3

1.03022
3

0.718707
3

0
3

0

0

0

0

0

0

0

0

0

0

0

0

0.144196

0

0

0

0

0

0

0

0

0

0

0

0

0

0

0

0

0

0.147964

0

0

0

0

0

0

0

0

0

0

0

0

0

0

0

0.0193514

0
4

0.385324
7

0.385324
7

0
4

0

0

0
4

0.0129576

0.0129576

0
4

0

0

0
4

0

0

0
4

0

0

0
4

0

0

0
4

0

0

0
4

0

0

0
4

0

0

0
4

0

0

0
4

0

0

0
4

0

0

0
4

0

0

0
4

0

0

0
4

0

0

0

0
4

0

0

0
4

0

0

0
4

0

0

0
4

0

0

0
4

0
4

0
4

84.2563
4

1.25232
7

1.25232
7

1.20242
7

0.0129576

0.0129576

0

0

0.0110232

0.0129576

0
4

0
4

1.82306
7

1.81644
7

1.81644
7

0
4

0.00661392

0.00661392

0
4

0
4

0

0

0

0

0
4

0
4

0

0

0

0

0
4

0
4

0

0

0

0

0
4

0
4

0

0

0

0

0
4

0
4

0

0

0

0

0
4

0
4

0.385324

0.385324

0.385324

0
4

0
4

0

0

0

0

0
4

0
4

0

0

0

0

0
4

0
4

0

0

0

0
4

0

0

0
4

0
4

0.15413

0.15413

0.15413

0
4

0
4

0
4

0
4

0

0

0

0

0

0

0

0

0
4

0

0

0

0
4

0

0

0

0
4

0

0

0
4

0

0

0
4

0
4

0.0416549

0

0

0
4

0.0416549

0.0416549

0
4

0
4

0

0

0

0
4

0

0

0
4

0
4

0

0

0

0
4

0

0

0
4

0
4

0

0

0

0
4

0

0

0
4

0
4

0

0

0

0

0
4

0
4

0

0

0

0
4

0

0

0
4

0
4

0

0

0

0
4

0
4

0

0

0

0

0
4

0
4

0

0

0

0
4

0

0

0
4

0
4

0

0

0

0

0
4

0
4

1.20455
7

1.20455
7

1.08091
7

0.11906

0.00458025

1.00613961606655e-16
7

0
4

0

0

0

0
4

0

0

0
4

0
4

0

0

0

0
4

0

0

0
4

0
4

0

0

0

0
4

0
4

0

0

0

0
4

0
4

0

0

0

0
4

0

0

0
4

0
4

0

0

0

0
4

0
4

0

0

0

0
4

0

0

0
4

0
4

0

0

0

0
4

0

0

0
4

0
4

0

0

0

0
4

0
4

0

0

0

0

0
4

0
4

0

0

0

0
4

0

0

0
4

0
4

0
4

0
4

0

0

0

0

0

0
4

0

0

0

0
4

0

0

0

0
4

0
4

0

0

0

0
4

0
4

0

0

0

0
4

0
4

0

0

0

0
4

0

0

0
4

0
4

0.0091605

0.0091605

0.0091605

0
4

0
4

0

0

0

0
4

0
4

0.0770648

0.0770648

0.0770648

0
4

0
4

0

0

0

0
4

0

0

0
4

0
4

0

0

0

0
4

0

0

0
4

0
4

0

0

0

0
4

0

0

0
4

0
4

0

0

0

0
4

0

0

0
4

0
4

2.41099
7

2.40217
7

2.40217
7

0

0
4

0.00881856

0.00881856

0
4

0
4

0

0

0

0
4

0

0

0
4

0
4

0

0

0

0
4

0
4

0.0777455

0

0

0
4

0.0777455

0.0777455

0
4

0
4

0

0

0

0
4

0

0

0
4

0
4

0

0

0

0
4

0
4

0

0

0

0
4

0
4

0

0

0

0
4

0
4

0

0

0

0
4

0
4

0

0

0

0
4

0
4

0

0

0

0
4

0
4

0.0248391
7

0.0133885
6

0.00518609

0

0

0

0.00458025

0.00362217

0
4

0.0114506

0.0114506

0
4

0

0

0
4

0

0

0
4

0
4

0

0

0

0
4

0
4

0

0

0

0
4

0
4

0

0

0

0
4

0
4

0

0

0

0
4

0
4

0

0

0

0
4

0
4

0

0

0

0
4

0
4

0

0

0

0
4

0
4

0

0

0

0
4

0
4

0

0

0

0
4

0
4

0

0

0

0
4

0
4

0

0

0

0

0

0

0

0
4

0

0

0

0

0

0
4

0

0

0

0

0
4

0
4

0

0

0

0
4

0
4

0

0

0

0
4

0
4

0

0

0

0
4

0
4

0

0

0

0
4

0
4

0

0

0

0
4

0
4

0

0

0

0
4

0
4

0

0

0

0
4

0
4

0.0591856

0.0591856

0.0591856

0
4

0
4

0

0

0

0
4

0
4

0.216294

0.216294

0.216294

0
4

0
4

0.0890823
5

0

0

0

0

0
4

0

0

0

0
4

0.0307731

0.0307731

0
4

0

0

0
4

0

0

0
4

0.0583091

0.0583091

0
4

6.93889390390723e-18
5

0
4

0

0

0

0
4

0
4

0

0

0

0
4

0
4

0.0201232

0.0201232

0.0201232

0
4

0
4

0

0

0

0
4

0
4

0

0

0

0
4

0
4

0

0

0

0
4

0
4

0

0

0

0
4

0
4

0

0

0

0
4

0
4

0

0

0

0
4

0
4

0

0

0

0
4

0
4

0
4

0
4

0
6

0

0

0

0
4

0

0

0

0

0
4

0
4

0

0

0

0
4

0
4

0.144196

0.144196

0.144196

0
4

0
4

0.0103722

0.0103722

0.0103722

0
4

0
4

0

0

0

0
4

0
4

0

0

0

0
4

0
4

0

0

0

0
4

0
4

0

0

0

0
4

0
4

0

0

0

0
4

0
4

0.231194

0.231194

0.231194

0
4

0
4

0

0

0

0
4

0
4

0
4

0
4

0

0

0

0

0

0

0
4

0

0

0

0
4

0
4

0

0

0

0
4

0
4

0

0

0

0
4

0
4

0.231194

0.231194

0.231194

0
4

0
4

0

0

0

0
4

0
4

0

0

0

0
4

0
4

0

0

0

0
4

0
4

0

0

0

0
4

0
4

0

0

0

0
4

0
4

0

0

0

0
4

0
4

0

0

0

0
4

0
4

0
4

0
4

0
4

0

0

0

0

0
4

0

0

0

0

0
4

0

0

0

0
4

0

0

0

0
4

0

0

0

0
4

0

0

0
4

0

0

0
4

0
4

0
4

0
4

0
4

0

0

0
4

0

0

0

0
4

0

0

0
4

0
4

0

0

0

0
4

0
4

0

0

0

0
4

0
4

0

0

0

0
4

0
4

0

0

0

0
4

0
4

0

0

0

0
4

0
4

0

0

0

0
4

0
4

0

0

0

0
4

0
4

0

0

0

0
4

0
4

0

0

0

0
4

0
4

0

0

0

0
4

0
4

0

0

0

0

0

0
4

0

0

0

0

0
4

0

0

0

0
4

0
4

0

0

0

0
4

0
4

0

0

0

0
4

0
4

0

0

0

0
4

0
4

0

0

0

0
4

0
4

0

0

0

0
4

0
4

0

0

0

0
4

0
4

0

0

0

0
4

0
4

0

0

0

0
4

0
4

0

0

0

0
4

0
4

0.148673

0.148673

0.148673

0
4

0
4

0

0

0

0

0

0

0

0

0
4

0

0

0

0

0
4

0
4

0

0

0

0
4

0
4

0

0

0

0
4

0
4

0

0

0

0
4

0
4

0

0

0

0
4

0
4

0

0

0

0
4

0
4

0

0

0

0
4

0
4

0

0

0

0
4

0
4

0

0

0

0
4

0
4

0

0

0

0
4

0
4

0

0

0

0
4

0
4

0
4

0

0

0

0

0

0
4

0

0

0

0

0
4

0

0

0
4

0

0

0
4

0
4

0

0

0

0
4

0
4

0

0

0

0
4

0
4

0

0

0

0
4

0
4

0

0

0

0
4

0
4

0

0

0

0
4

0
4

0

0

0

0
4

0
4

0

0

0

0
4

0
4

0

0

0

0
4

0
4

0

0

0

0
4

0
4

0

0

0

0
4

0
4

0
4

0

0

0

0

0
4

0

0

0

0
4

0

0

0
4

0

0

0
4

0
4

0

0

0

0
4

0
4

0

0

0

0
4

0
4

0

0

0

0
4

0
4

0

0

0

0
4

0
4

0

0

0

0
4

0
4

0

0

0

0
4

0
4

0

0

0

0
4

0
4

0

0

0

0
4

0
4

0

0

0

0
4

0
4

0

0

0

0
4

0
4

0
4

0
4

0
4

0

0
4

0

0

0
4

0

0

0
4

0
4

0

0

0

0
4

0
4

0

0

0

0
4

0
4

0

0

0

0
4

0
4

0

0

0

0
4

0
4

0

0

0

0
4

0
4

0

0

0

0
4

0
4

0

0

0

0
4

0
4

0

0

0

0
4

0
4

0

0

0

0
4

0
4

0

0

0

0
4

0
4

0

0

0

0

0
4

0

0

0

0
4

0

0

0
4

0

0

0
4

0
4

0

0

0

0
4

0
4

0

0

0

0
4

0
4

0

0

0

0
4

0
4

0

0

0

0
4

0
4

0

0

0

0
4

0
4

0

0

0

0

0

0
4

0

0

0
4

0

0

0
4

0
4

0
4

0

0

0

0
4

0

0

0

0
4

0

0

0
4

0

0

0
4

0

0

0
4

0
4

0

0

0

0

0

0

0

0

0
4

0

0

0
4

0

0

0
4

0

0

0
4

0
4

0
4

0
4

0
4

0

0

0

0

0

0

0
4

0

0

0

0
4

0
4

0

0

0

0
4

0

0

0

0

0
4

0

0

0

0
4

0

0

0
4

0
4

0
3

0
3

0

0

0

0

0

0
4

0
4

0
3

0

0

0

0

0
4

0

0

0

0
4

0

0

0
4

0
4

0

0

0

0

0
4

0

0

0

0
4

0

0

0
4

0

0

0
4

0

0

0
4

0

0

0
4

0
4

0
4

0
4

0

0

0

0

0

0
4

0
4

0

0

0

0

0

0

0

0
4

0

0

0
4

0

0

0
4

0
4

0
3

0

0

0

0
4

0

0

0

0
4

0

0

0
4

0

0

0
4

0
4

0

0

0

0

0

0
4

0
4

0

0

0

0
4

0

0

0
4

0

0

0
4

0

0

0
4

0

0

0
4

0

0

0
4

0
4

0
6

0

0

0
4

0

0

0
4

0
4

1.38697
7

1.38697
7

1.36155
7

0
6

0.0121884

0.00661392

0

0.00661392

0

0
4

0
4

0

0

0

0

0

0
4

0

0

0
4

0

0

0
4

0
4

0.584154

0.584154

0.584154

0

0

0
4

0
4

0
3

0
3

0
3

0
4

0

0

0
4

0

0

0
4

0
4

0.1519

0

0

0

0

0
4

0.0363026

0

0

0.0363026

0
4

0.115597

0.115597

0
4

0
4

0.0523143
5

0.0523143

0.0523143

0
4

0

0

0
4

0

0

0
4

0
4

0

0

0

0

0
4

0
4

0

0

0

0

0
4

0

0

0

0

0
4

0
4

0

0

0

0

0
4

0

0

0
4

0

0

0
4

0
4

0
4

0

0

0
4

0

0

0
4

0

0

0
4

0

0

0
4

0

0

0
4

0
4

0
4

0

0

0

0
4

0

0

0
4

0

0

0
4

0

0

0
4

0
4

0
4

0
4

0

0

0

0

0

0

0

0

0
4

0
4

0

0

0

0

0

0
4

0

0

0

0
4

0

0

0

0

0
4

0

0

0
4

0

0

0

0
4

0

0

0
4

0

0

0
4

0

0

0
4

0
4

0

0

0

0

0
4

0
4

0

0

0

0
4

0

0

0
4

0

0

0
4

0

0

0
4

0

0

0
4

0
4

0
4

0

0

0

0
4

0

0

0
4

0
4

0

0

0

0

0
4

0

0

0
4

0

0

0
4

0

0

0
4

0
4

0.0591856

0.0591856

0

0.0591856

0
4

0

0

0
4

0

0

0
4

0

0

0
4

0
4

0

0

0

0

0
4

0
4

0

0

0

0

0

0
4

0

0

0
4

0
4

0

0

0

0

0
4

0

0

0
4

0
4

0.0194364

0.0194364

0.0194364

0

0

0

0
4

0

0

0
4

0
4

0

0

0

0

0
4

0

0

0
4

0
4

0
4

0
4

0
4

0

0

0

0

0
4

0

0

0

0

0
4

0
4

0

0

0

0

0
4

0

0

0

0
4

0

0

0
4

0
4

0

0

0

0

0
4

0

0

0
4

0

0

0
4

0
4

0.0770648

0

0

0

0
4

0

0

0
4

0.0770648

0.0770648

0
4

0
4

0

0

0

0

0
4

0

0

0

0
4

0
4

0

0

0

0

0
4

0

0

0
4

0

0

0
4

0
4

0

0

0

0
4

0

0

0
4

0

0

0
4

0
4

0

0

0

0
4

0

0

0
4

0

0

0
4

0
4

0.375594

0.375594

0.375594

0

0
4

0
4

0

0

0

0
4

0

0

0
4

0
4

0

0

0

0
4

0

0

0
4

0

0

0
4

0

0

0
4

0
4

1.00504
6

0.838261
7

0.576495
7

0.0839352

0.17325

0.00458025

3.46944695195361e-18
7

0
4

0.166778
6

0.161592
6

0

0

0.00518609

0
4

5.55111512312578e-17
6

0
4

0

0

0

0
4

0

0

0
4

0

0

0
4

0
4

0

0

0

0
4

0
4

0

0

0

0
4

0

0

0
4

0

0

0
4

0
4

1.09712

1.08147

1.08147

0
4

0.0156497

0.0156497

0
4

0
4

0.0129576

0.0129576

0.0129576

0

0
4

0

0

0
4

0
4

0

0

0

0

0
4

0

0

0
4

0
4

0

0

0

0
4

0
4

0

0

0

0
4

0

0

0
4

0

0

0
4

0
4

0

0

0

0

0
4

0

0

0
4

0
4

0

0

0

0

0
4

0
4

0.337306
7

0.337306
7

0.260241
7

0.0770648

0

0

1.38777878078145e-17
7

0
4

0

0

0
4

0
4

0

0

0

0
4

0
4

0

0

0

0

0
4

0
4

0

0

0

0
4

0

0

0
4

0
4

0.0184639

0

0

0
4

0.0184639

0.0184639

0
4

0
4

0

0

0

0
4

0

0

0
4

0

0

0
4

0
4

0

0

0

0

0
4

0

0

0
4

0
4

0.0712667

0.0712667

0.0583091

0.0129576

0
4

0
4

0

0

0

0
4

0

0

0
4

0
4

0

0

0

0

0
4

0

0

0
4

0
4

0

0

0

0

0
4

0

0

0
4

0
4

0
4

0

0

0

0

0
4

0

0

0

0

0
4

0

0

0

0
4

0

0

0

0
4

0

0

0
4

0

0

0
4

0

0

0
4

0
4

0

0

0

0

0
4

0

0

0
4

0
4

0

0

0

0
4

0

0

0
4

0
4

0

0

0

0
4

0
4

0

0

0

0

0
4

0

0

0
4

0
4

0.00518609

0.00518609

0.00518609

0
4

0
4

0

0

0

0

0

0
4

0
4

0

0

0

0
4

0

0

0
4

0

0

0
4

0
4

0

0

0

0
4

0

0

0
4

0

0

0
4

0
4

0

0

0

0

0
4

0

0

0
4

0
4

0

0

0

0

0
4

0
4

70.3912
4

69.6538
4

25.9237
4

2.20228
4

0
4

12.7166
7

0

0

0

0

0

0

0

0

0

0

0

0

0

0

0

0.00687037

0

0

0

0

0

0

0.155458
7

0

0

0

0

0

0

0

0

0.100539

0

0.0770648
6

0

0

0

0

0.216294

0

0.0271662

0

0

0

0.156873
7

0

0

0

0

0

0

0

0

0

0

0.143594
7

0

0

0

0.0156497

0

0

0

0

0

0

1.77207
7

0

0.0770648

0

0

0

0

0

0

0

0

0

0

0

0

0

0

0

0

0.0091605

0.0129576

0

0.00687037
6

0

0

0

0

0

0

0

0

0

0

0.0156497
7

0

0

0

0

0

0

0

0

0

0

19.4317
7

0
4

0

0

0

0

0.00458025

0

0

0

0

0

0.313095
7

0

0

0

0

0

0

0

0

0

0

0.0338468
6

0.00458025

0

0

0

0.00440928

0.00440928

0

0

0

0

1.12675
7

0

0

0.00615463

0

0

0

0

0.0100616

0.00687037

0

0.01815
7

0

0

0

0

0

0

0

0

0

0

0
3

0

0

0

0

0

0

0

0

0

0

0
6

0

0

0

0

0

0

0

0.115597

0

0

0

0

0

0

0

0

0

0

0

0

0

0
6

0

0

0

0

0.00724433

0

0

0

0

0

0.00777913
6

0

0

0.0770648

0

0

0

0

0.00881856

0

0.00440928

0.624455
7

0.462389

0.0110232

0

0

0

0

0

0

0

0

0

0.0160309
6

0

0

0

0

0

0

0

0

0

0.15413

0

0

0

0

0

0

0

0

0

0

0

0
3

0

0.00673085

0

0

0

0

0

0

0

0

0.352119
4

0

0

0

0

0

0.0770648

0

0

0.00448724

0

0

0.0301849

0

0

0

0

0.00687037

0

0

0

0

0.00458025
6

0.115597

0.0887784

0

0.0220464

0

0

0

0

0

0

0.00543325
7

0

0

0.00687037

0

0

0

0

0

0

0

0.00458025
7

0

0

0

0

0

0

0

0

0.0770648

0

0

0

0

0

0

0

0

0

0

0

0

0
4

0.00820242
7

0

0

0

0

0

0

0

0

0

0

0

0

0

0

0.00458025

0

0

0

0

0.593906
7

0.0770648

0.00458025

0

1.34863

0

0.0091605

0

0

0

0

0
3

0

0

0

0

0.00543325

0.0112797

0

0

0

0

0.522619
7

0

0

0

0

0

0

0

0

0

0

0.0824599
7

0

0

0

0

0

0

0.00967569

0

0.0129576

0

0
4

0

0

0.0114506

0.0156497

0.00458025

0

0

0.0100616

0.0296007

0

4.38780956013574e-14
4

0
4

0.298325
7

0.221261
7

0

0.0770648

0

1.38777878078145e-17
7

0
4

0

0

0

0

0
4

0

0

0
4

0

0

0
4

0

0

0
4

0

0

0
4

0

0

0
4

0.0518304

0

0

0

0.0518304

0
4

0

0

0

0
4

0

0

0

0
4

0

0

0

0
4

0

0

0

0

0
4

0

0

0

0
4

0.0114506

0.0114506

0
4

0

0

0

0
4

0

0

0
4

0

0

0

0

0

0
4

0

0

0

0
4

0

0

0
4

0

0

0
4

0

0

0

0
4

0

0

0

0
4

0.0547741

0.0547741

0
4

0

0

0

0
4

0

0

0

0
4

0

0

0

0
4

0

0

0

0
4

0.269727
7

0

0.115597

0.15413

0
4

0

0

0

0
4

0

0

0
4

0

0

0

0
4

0

0

0
4

0

0

0
4

0

0

0
4

0

0

0
4

0

0

0
4

0

0

0
4

0

0

0
4

0
6

0
6

0
4

0

0

0
4

0

0

0
4

0

0

0
4

0

0

0
4

0.0156497

0.0156497

0
4

0

0

0
4

0

0

0
4

0

0

0
4

0

0

0
4

0

0

0
4

0.0181513
6

0.0181513
6

0
4

0

0

0
4

0

0

0
4

0

0

0
4

0

0

0
4

0

0

0
4

0

0

0
4

0

0

0
4

0

0

0
4

0

0

0
4

0

0

0
4

0
4

0
4

0
4

0

0

0
4

0

0

0
4

0

0

0
4

0

0

0
4

0

0

0
4

0

0

0
4

0

0

0
4

0

0

0
4

0

0

0
4

0

0

0
4

0

0

0

0

0
4

0

0

0
4

0

0

0
4

0

0

0
4

0

0

0
4

0

0

0
4

0.00458025

0.00458025

0
4

0

0

0
4

0

0

0
4

0

0

0
4

0

0

0
4

0

0

0

0

0
4

0

0

0
4

0

0

0
4

0

0

0
4

0

0

0
4

0.0129652

0.0129652

0
4

0

0

0
4

0

0

0
4

0

0

0
4

0

0

0
4

0

0

0
4

0

0

0

0
4

0

0

0
4

0

0

0
4

0

0

0
4

0

0

0
4

0

0

0
4

0

0

0
4

0

0

0
4

0

0

0
4

0

0

0
4

0

0

0
4

8.70657712592759e-15
4

0
4

0
4

4.05143
4

3.51666
4

0

0

0

0

0

0
4

3.10383
4

0
4

0
4

0.0259152
3

1.40711
4

0
4

0.26026
4

0.359798
4

1.05075
4

0
3

0

0

0

0

0

0

0

0

0

0

0

0

1.11022302462516e-15
4

0
4

0.29615
3

0.0129576
3

0.231194
3

0
3

0
4

0
4

0

0
4

0

0

0

0

0

0

0

0
4

0

0

0

0

0

0

0

0

0

0

0
3

0

0.0285235

0

0

0

0

0

0

0

0

0
3

0

0

0

0

0

0

0

0

0

0

0

0

0

0

0

0

0

0

0

0

0

0
3

0

0.0234746

0

0

0

0

0

0

0

0

0
4

0

0

0

0

0

0

0

0

0

0

0
4

0
4

1.73472347597681e-17
3

0
4

0
5

0
5

0
4

0

0

0
4

0

0

0
4

0

0

0
4

0

0

0
4

0

0

0
4

0

0

0
4

0

0

0
4

0

0

0
4

0

0

0
4

0.00518609

0.00518609

0
4

0

0

0

0
4

0

0

0
4

0.0129576

0.0129576

0
4

0

0

0
4

0

0

0
4

0.0829774

0.0829774

0
4

0

0

0
4

0

0

0
4

0

0

0
4

0

0

0
4

0

0

0

0
4

0

0

0
4

0

0

0
4

0

0

0
4

0.0155583

0.0155583

0
4

0

0

0
4

0

0

0
4

0
4

0
4

0
4

0

0

0

0

0

0

0

0

0
4

0

0

0

0
4

0

0

0
4

0
4

0
4

0

0

0

0

0
4

0

0

0
4

0
4

0
4

0

0

0
4

0

0

0
4

0

0

0
4

0

0

0
4

0
4

0

0

0

0
4

0

0

0

0
4

0

0

0
4

0
4

0

0

0

0
4

0

0

0
4

0
4

0

0

0

0

0

0

0
4

0
4

0.0129576

0

0

0
4

0.0129576

0.0129576

0
4

0

0

0
4

0
4

0

0

0

0
4

0

0

0
4

0

0

0
4

0
4

0

0

0

0

0
4

0
4

0

0

0

0

0

0
4

0
4

0.239715

0.239715

0.239715

0
4

0
4

0
4

0
4

0
4

0

0
4

0

0

0

0
4

0

0

0
4

0
4

0

0

0

0
4

0

0

0
4

0

0

0
4

0
4

0

0

0

0
4

0

0

0
4

0

0

0
4

0
4

0.129652

0.129652

0.0103722

0.11928

1.38777878078145e-17

0
4

0
4

0

0

0

0

0
4

0

0

0
4

0
4

0

0

0

0

0
4

0
4

0

0

0

0

0
4

0

0

0
4

0
4

0

0

0

0
4

0
4

0

0

0

0
4

0
4

0

0

0

0

0
4

0
4

0

0

0

0
4

0
4

0
4

0
4

0
4

0

0

0
4

0

0

0

0
4

0

0

0
4

0
4

0

0

0

0
4

0
4

0

0

0

0
4

0
4

0

0

0

0
4

0
4

0

0

0

0

0
4

0
4

0

0

0

0
4

0
4

0

0

0

0
4

0

0

0
4

0
4

0

0

0

0

0
4

0
4

0

0

0

0

0
4

0
4

0

0

0

0
4

0

0

0
4

0
4

0

0

0

0
4

0
4

0
5

0

0

0

0
4

0

0

0

0

0

0
4

0
4

0

0

0

0
4

0
4

0

0

0

0
4

0
4

0

0

0

0
4

0
4

0

0

0

0
4

0
4

0

0

0

0
4

0
4

0

0

0

0
4

0
4

0

0

0

0
4

0
4

0.00411208

0.00411208

0.00411208

0
4

0
4

0

0

0

0
4

0
4

0

0

0

0
4

0
4

0
4

0
4

0
4

0

0

0
4

0

0

0
4

0

0

0
4

0
4

0

0

0

0
4

0
4

0

0

0

0
4

0
4

0

0

0

0
4

0
4

0.0129652

0.0129652

0.0129652

0
4

0
4

0.0129576

0.0129576

0.0129576

0
4

0
4

0

0

0

0
4

0
4

0

0

0

0
4

0
4

0

0

0

0
4

0
4

0

0

0

0
4

0
4

0.0770648

0.0770648

0.0770648

0
4

0
4

0
4

0
4

0

0

0

0

0
4

0
4

0

0

0

0
4

0
4

0

0

0

0
4

0
4

0

0

0

0
4

0
4

0

0

0

0
4

0
4

0

0

0

0
4

0
4

0

0

0

0
4

0
4

0

0

0

0
4

0
4

0

0

0

0
4

0
4

0

0

0

0
4

0
4

0

0

0

0
4

0
4

0

0

0

0

0
4

0

0

0
4

0

0

0
4

0

0

0
4

0
4

0

0

0

0
4

0
4

0

0

0

0
4

0
4

0

0

0

0
4

0
4

0

0

0

0
4

0
4

0

0

0

0
4

0
4

0

0

0

0
4

0
4

0

0

0

0
4

0
4

0

0

0

0
4

0
4

0

0

0

0
4

0
4

0

0

0

0
4

0
4

0

0

0

0

0

0
4

0

0

0
4

0
4

0

0

0

0
4

0
4

0

0

0

0
4

0
4

0

0

0

0
4

0
4

0

0

0

0
4

0
4

0

0

0

0
4

0
4

0

0

0

0
4

0
4

0.0453516

0.0453516

0.0453516

0
4

0
4

0

0

0

0
4

0
4

0

0

0

0
4

0
4

0

0

0

0
4

0
4

0
4

0
4

0
4

0

0
4

0
4

0

0

0

0
4

0
4

0

0

0

0
4

0
4

0

0

0

0
4

0
4

0

0

0

0
4

0
4

0

0

0

0
4

0
4

0

0

0

0
4

0
4

0

0

0

0
4

0
4

0

0

0

0
4

0
4

0

0

0

0
4

0
4

0
4

4.45618
5

0

0

0

0

0

0
4

0

0

0
4

0
4

0.00518609

0.00518609

0.00518609

0
4

0
4

0

0

0

0
4

0
4

0

0

0

0
4

0
4

0

0

0

0
4

0
4

0

0

0

0
4

0
4

0

0

0

0
4

0
4

0

0

0

0
4

0
4

0

0

0

0
4

0
4

0

0

0

0
4

0
4

0

0

0

0
4

0
4

0.865175
4

0
4

0

0

0

0

0
4

0.865175

0.865175

0

0
4

0

0

0
4

0
4

0

0

0

0
4

0
4

0

0

0

0
4

0
4

0.216294

0.216294

0.216294

0
4

0
4

0
6

0
6

0

0

0

0
4

0
4

1.21435
7

1.21435
7

1.21435
7

0
4

0
4

0
6

0
6

0
6

0

0
4

0
4

1.29776

0

0

0
4

1.29776

1.29776

0
4

0
4

0

0

0

0
4

0
4

0

0

0

0

0
4

0
4

0

0

0

0
4

0
4

0.857413

0
5

0

0

0

0

0

0

0

0
4

0.275443
7

0.259793

0.0156497

0
4

0

0

0
4

0

0

0
4

0

0

0
4

0

0

0
4

0

0

0
4

0

0

0

0
4

0

0

0

0
4

0.36049

0.36049

0
4

0

0

0
4

0.00518609

0.00518609

0
4

0

0

0
4

0.216294

0.216294

0
4

0

0

0
4

2.77555756156289e-17

0
4

0
4

9.36140054363932e-13

0
4

6.75788

6.75788

6.6283

0.66327
3

0.487191
3

0

0

0.120739

0.0553389

0

0
4

0.522059
3

0
3

0

0

0.11014

0.0388728

0

0

0.115597

0

0
2

0
3

0

0

0

0

0.25745

0

0
4

0.55842

0

0.548358

0.0100616

0
4

3.65889
7

0.790413

0.187885

0.220279

0

0

0

0.0388728

0.36049

0

0.0194364

0.192662
6

0.15413

1.03013

0.0777455

0.252673

0.192662

0.0259152

0.115597

5.27355936696949e-16
7

0
4

1.22567

1.22567

0
4

0

0

0
4

0

0

0
4

1.77635683940025e-15

0
4

0
3

0

0

0

0
4

0

0

0

0

0

0
4

0

0

0

0

0
4

0

0

0
4

0
4

0

0

0

0
4

0
4

0.129576

0.129576

0.129576

0
4

0
4

0

0

0

0
4

0
4

0

0

0

0
4

0

0

0
4

0
4

0
3

0
3

0

0

0

0
4

0

0

0

0
4

0

0

0
4

0
4

0

0

0

0

0
4

0
4

0
2

0
2

0
2

0

0
4

0
4

0

0

0

0

0
4

0
4

0

0

0

0
4

0
4

0

0

0

0
4

0
4

0

0

0

0
4

0
4

0
4

0
2

0
2

0
2

0

0

0

0

0

0
4

0
3

0

0

0

0

0

0
4

0

0

0
4

0
4

0
4

0

0

0

0

0
4

0
4

0
4

0

0

0

0

0

0
4

0
4

0
4

0

0

0

0

0

0

0
4

0
4

0
4

0

0

0

0

0
4

0
4

0
4

0

0

0

0

0
4

0
4

0
4

0
4

28.2227

27.9964

0
3

0
3

0

0

0

0

0

0

0

0

0

0

0

0
3

0

0

0

0

0

0

0

0

0

0

0
3

0

0

0

0

0

0

0

0

0

0

0
3

0

0

0

0

0

0

0

0

0

0

0

0

0

0

0

0

0

0

0

0

0

0
4

0

0

0

0
4

0

0
4

0
4

0.411595
3

0.411595
3

0.147964
1

0

0

0

0

0

0

0

0

0

0

0

0

0

0

0

0

0

0

0

0

0

0
4

0.0156497

0

0

0

0

0

0

0

0.0547741
4

0

0.177557

0.0156497

0

0

0
4

0
4

1.55845
6

1.51932
6

0.101723
6

0

0.115597

0

0

0

0.231194
7

0

0.259793

0

0

0
5

0

0

0

0

0

0

0

0

0

0

0
5

0

0

0.0156497

0

0

0

0

0

0

0

0.119814
6

0

0

0

0

0

0.333667
6

0.172105
6

0.15413
7

0.0156497
6

0
4

0.0391243

0.0391243

0
4

0
4

0.28547
7

0.28547
7

0.130467
7

0.155004
7

0
4

0
4

0.954991
3

0.0107775
3

0

0

0

0

0

0

0

0

0

0

0

0

0

0

0

0

0

0

0

0

0

0

0

0

0

0

0

0

0

0

0

0

0

0

0

0

0

0

0

0

0

0

0

0

0

0

0

0

0

0

0

0

0.0107775

0

0

0

0

0

0

0

0

0

0

0

0

0

0

0

0

0

0

0

0

0

0

0

0

0

0

0

0

0

0

0

0

0

0

0

0

0
4

0

0

0

0

0

0
4

0

0

0
4

0

0

0
4

0

0

0

0

0
4

0

0

0
4

0.0153866

0.00615463

0.00923194

0
4

0.021555

0.0107775

0.0107775

0
4

0.538875

0.538875

0
4

0.362242

0.362242

0
4

0

0

0
4

0.00615463

0.00615463

0
4

0
4

1.3377
3

0
3

0

0

0
4

0.352537
3

0
4

0

0

0

0

0

0

0

0

0

0

0.0312995
2

0

0

0

0

0

0

0

0

0

0

0

0

0

0

0

0

0

0

0

0

0

0
4

0

0

0

0

0

0

0

0

0

0

0.164322

0

0

0

0

0

0

0

0

0.156916

0

0
4

0

0

0

0

0

0

0

0

0

2.77555756156289e-17
3

0
4

0

0

0

0
4

0

0

0
4

0

0

0
4

0

0

0

0

0
4

0

0

0

0

0

0

0

0

0

0

0

0

0
4

0

0

0

0

0
4

0.985162

0.369009

0.372144

0.00616813

0

0

0

0

0.139152

0

0

0

0.00822417

0.0904659

0

0

0
4

0

0

0
4

0

0

0
4

1.11022302462516e-16
3

0
4

23.3308
5

4.69146
6

4.69146
6

0
4

16.9457
5

16.9457
5

0
4

1.69363
7

0.216294

0

0

0

0

0

0.187885

0

0.330419

0

0

0.0712667

0

0

0

0

0

0.0259152

0

0

0

0

0.607515

0

0

0

0

0

0

0.032394

0

0

0

0.157153

0

0

0

0

0

0

0

0

0

0

0

0

0

0

0

0

0

0

0

0

0

0

0.0129576

0

0

0

0

0

0

0

0

0

0.0518304

0

0

0

0

0

0

0

0

0

0

0

0
4

0

0

0

0
4

0

0

0
4

0

0

0
4

0

0

0
4

0
4

0
3

0
3

0
4

0

0

0

0

0

0

0

0

0

0
4

0
4

0
3

0
3

0
3

0

0

0

0

0

0
3

0

0

0

0

0

0

0

0
4

0

0

0
4

0

0

0
4

0
4

0

0

0

0
4

0
4

0

0

0

0
4

0
4

0

0

0

0
4

0
4

0

0

0

0
4

0
4

0

0

0

0
4

0
4

0

0

0

0
4

0
4

0

0

0

0
4

0
4

0

0

0

0
4

0
4

0

0

0

0
4

0
4

0

0

0

0
4

0
4

0
3

0
3

0
3

0
4

0
4

0

0

0

0

0

0

0

0
4

0
4

0.117373

0.117373

0.117373

0

0
4

0
4

0

0

0

0

0
4

0

0

0
4

0
4

0

0

0

0
4

0

0

0
4

0
4

0

0

0

0
4

0
4

0

0

0

0

0
4

0
4

0

0

0

0
4

0

0

0
4

0
4

2.09554595897998e-15

0
4

0
3

0
3

0
3

0
3

0
3

0

0

0

0

0
4

0

0

0
4

0

0

0

0
4

0
4

0
4

0

0

0

0

0

0

0
4

0

0

0
4

0
4

0
4

0

0

0

0

0

0
4

0
4

0
4

0

0

0

0

0

0
4

0
4

0
4

0.0323325

0.0323325

0.0323325

0.0161662

0.0161662

0
4

0
4

0
4

0

0

0

0

0
4

0

0

0
4

0
4

0
4

0

0

0

0

0
4

0
4

0
4

0

0

0

0

0

0
4

0
4

0
4

0

0

0

0

0
4

0
4

0
4

0

0

0

0

0
4

0
4

0
4

0

0

0

0

0
4

0
4

0
4

0
3

0
3

0
3

0
3

0

0

0

0
4

0
4

0
4

0

0

0

0

0
4

0
4

0
4

0

0

0

0

0
4

0
4

0
4

0

0

0

0

0
4

0
4

0
4

0

0

0

0

0
4

0
4

0
4

0

0

0

0

0
4

0
4

0
4

0

0

0

0

0
4

0
4

0
4

0

0

0

0

0
4

0
4

0
4

0.187797

0.187797

0.187797

0.187797

0
4

0
4

0
4

0

0

0

0

0
4

0
4

0
4

0

0

0

0

0
4

0
4

0
4

0

0

0

0

0

0

0

0

0
4

0

0

0
4

0
4

0
4

0

0

0

0

0
4

0
4

0
4

0.00615463

0.00615463

0.00615463

0.00615463

0
4

0
4

0
4

0

0

0

0

0
4

0
4

0
4

0

0

0

0

0

0
4

0
4

0
4

0

0

0

0

0

0

0
4

0

0

0
4

0

0

0
4

0
4

0
4

0

0

0

0

0

0
4

0
4

0
4

0

0

0

0

0

0

0

0
4

0

0

0
4

0
4

0
4

0

0

0

0

0

0

0
4

0
4

0
4

0

0

0

0

0

0

0
4

0

0

0
4

0
4

0
4

2.75300615637519e-15

0
4

17.5629
3

17.5629
3

0
4

0
4

0
4

0
4

0
4

1.02186

1.02186

0

0

0

0.216294

0

0

0.216294

0

0.576784

0

0.00624502

0

0

0

0

0.00624502

0

0
4

0

0

0

0

0

0

0

0

0

0

0

0

0

0

0
4

0

0

0

0
4

0
4

0

0

0

0
4

0
4

0.0887784

0.0887784

0.0887784

0
4

0

0

0

0
4

0
4

1.89755

1.5449
1

1.5449
1

0
4

0

0

0
4

0.123649

0.123649

0
4

0
4

0

0
4

0

0

0

0

0
4

0.163206

0.163206

0
4

0.0657934

0.0657934

0
4

0

0

0
4

0

0

0
4

0
4

2.57762
7

2.57762
7

2.57762
7

0
4

0
4

3.37681
7

3.37681
7

3.04816
7

0.179972

0.125198

0.0234746

0
4

0
4

0
4

0
4

0
4

0

0

0

0

0

0

0

0
4

0

0

0
4

0

0

0
4

0
4

0
4

0
4

0

0

0

0
4

0
4

0

0

0

0
4

0
4

0

0

0

0
4

0
4

8.60024
3

8.60024
3

0.199664

0.805962
3

0

0
3

0.219096

0

0

0.0391243

0.0547741

0

0

0

0.0547741

0

2.69175

0

0

0

0

0

0.0234746

0

0

0

0.0156497

0.341508

0

0

0

0

0

0

0

0

0

0.226921

0.532091

3.0204

0

0.375044

0

7.7715611723761e-16
3

0
4

0

0

0
4

0
4

0
4

0
4

0
4

0
4

0
4

0
3

0

0

0

0

0

0
4

0
4

0

0

0

0

0
4

0
4

3.5527136788005e-15
3

0
4

0

0

0

0

0
4

0
4

0
4

0

0

0

0

0
4

0
4

0
4

0
4

618.384

14.2747

0

0

0

0
4

0
4

2.34778

1.72633
3

0.429175
3

0

0

0

0

0

0.0391243

0.181406

0

0

0.0129576

0.680413
3

0

0

0

0

0

0

0

0

0.0388728
3

0.0129576

0

0.16197

0

0.156497

0.0129576

0
4

0.621449

0.621449

0
4

0

0

0

0

0

0

0
4

0
4

11.9269

9.14774

7.69972

0.346708

0

0

0

0

0

0

0

0

0

0

0

0

0.00687037

0

1.08147

0.0129652

0

0

0

4.25007251614318e-16

0
4

2.77916

2.73661

0.0425476

0

0

0

0
4

0
4

0

0

0

0
4

0
4

0

0

0

0
4

0
4

0
4

13.5657
1

5.61064

5.44219

3.70587

0.0194364

0.032394

0.0129576

0.181406

0.233237

0.272109

0.0971819

0.466473

0.317461

0.0129576

0.0907031

0
4

0.136055

0.0518304

0.032394

0.0259152

0.0129576

0.0129576

0
4

0.0194364

0.0194364

0
4

0.0129576

0.0129576

0
4

2.58473797920544e-16

0
4

0

0

0

0
4

0
4

0

0

0

0
4

0
4

5.33853

5.26726

1.91124

2.67574

0.382249

0.0907031

0.187885

0.0194364

5.16947595841089e-16

0
4

0.0194364

0.0194364

0
4

0.0518304

0.0518304

0
4

7.14706072102445e-16

0
4

0.700686
8

0.645912
8

0.583313

0.0469492

0.0156497

8.32667268468867e-17
8

0
4

0.0547741

0.0547741

0
4

6.93889390390723e-18
8

0
4

0.388728

0.336897

0.298025

0.0259152

0.0129576

4.68375338513738e-17

0
4

0.0518304

0.0518304

0
4

6.93889390390723e-18

0
4

0.115597

0

0

0
4

0.115597

0.115597

0
4

0
4

0.919989

0.919989

0.887595

0.0129576

0.0194364

0
4

0

0

0
4

0
4

0.421122

0.421122

0.388728

0.0129576

0.0194364

1.04083408558608e-17

0
4

0
4

0.0704238

0.0704238

0.0704238

0
4

0

0

0
4

0
4

0

0

0

0
4

0
4

9.99200722162641e-16
1

0
4

1.07891
3

1.07891
3

0
3

0

0

0

0

0

0

0

0

0

0

0

0

0

0

0

0

0

0

0

0

0

0

0

0

0

0

0

0

0

0

0

0

0

0

0

0

0

0

0

0

0

0

0

0

0

0

0

0

0

0

0

0

0

0

0

0

0

0
4

0
4

0
4

0
4

0

0

0

0

0

0

0

0

0

0

0
4

0

0

0

0

0

0

0

0

0

0

0
4

0

0

0

0

0

0

0

0

0

0

0
4

0

0

0

0

0

0

0

0

0

0

0

0

0

0

0

0

0

0

0

0

0

0
4

0

0

0

0

0

0

0

0

0

0

0
4

0

0

0

0

0

0

0

0

0

0

0
4

0

0

0

0

0

0

0

0

0

0

0
4

0

0

0

0

0

0

0

0

0

0

0
4

0

0

0

0

0

0

0

0

0

0

0
4

0
4

0

0

0

0

0

0

0

0

0

0

0
3

0
4

0
4

0
3

0

0
4

0

0

0
4

0

0
3

0

0

0

0

0

0

0

0

0
4

0

0

0

0

0

0

0

0

0

0

0
4

0

0

0

0

0

0

0

0

0

0

0
4

0

0

0

0

0

0

0

0

0

0

0
4

0

0

0

0

0

0

0

0

0

0

0
4

0

0

0

0

0

0

0

0

0

0

0
4

0

0

0

0

0

0

0

0

0

0

0
4

0
2

0
2

0

0

0
4

0
2

0

0

0

0

0

0

0

0

0

0

0

0

0

0

0

0

0

0

0

0

0

0

0

0

0

0

0

0

0

0

0

0

0

0

0

0

0

0

0

0

0

0

0

0

0

0
4

0.96331

0.423856

0

0.539453

0
4

0

0

0

0

0

0

0

0

0

0

0

0
4

0

0

0
4

0

0

0
4

0

0

0
4

0

0

0
4

0

0

0

0

0

0

0

0
4

0

0

0

0
4

0

0

0

0
4

0.115597

0.115597

0

0

0
4

0

0

0

0
4

0

0

0
4

0

0

0
4

0

0

0
4

0
4

0

0

0

0
4

0

0

0
4

0
4

0

0

0

0

0
4

0
4

0

0

0

0
4

0
4

0

0

0

0
4

0
4

0
4

3.16543
3

2.67304
3

2.40006
3

0
2

0

0

0

0

0

0

0.144196

0

0

0

0

0

0
3

0

0

0

0

0

0

0

0

0

0

0

0

0

0

0

0

0

0

0.032394

0

0

0.224087

0

0

0

0.00416335

0

0.00624502

0

0

0

0

0

0

0

0

0

0

0.00416335

0

0

0

0

0

0

0

0

0

0

0

0

0

0

0

0.648882

0

0

0

0

0

0

0

0

0

0

0
4

0

0

0

0

0.0208167

0

0

0

0

0

1.29383

0

0

0.0129576

0

0

0

0.00832669

0

0

0

0
4

0
4

0

0
4

0

0
4

0.0591856

0.0591856

0
4

0

0

0
4

0

0

0
4

0

0

0

0
4

0
4

0
4

0
4

0.2138

0.2138

0
4

0

0

0

0
4

0

0

0
4

0

0

0
4

0

0

0
4

0

0

0
4

0
4

0

0

0

0

0

0

0
4

0

0

0
4

0
4

0.0647879

0.0518304

0.0518304

0
4

0.0129576

0.0129576

0
4

0
4

0

0

0

0

0

0
4

0
4

0

0

0

0
4

0
4

0.395206

0.395206

0.395206

0
4

0
4

0

0

0

0
4

0
4

0

0

0

0
4

0
4

0

0

0

0
4

0
4

0

0

0

0
4

0
4

0

0

0

0
4

0
4

0

0

0

0
4

0
4

0

0

0

0

0
4

0

0

0
4

0
4

0

0

0

0
4

0
4

0

0

0

0
4

0
4

0

0

0

0
4

0
4

0

0

0

0
4

0
4

0

0

0

0
4

0
4

0

0

0

0
4

0
4

0

0

0

0
4

0
4

0

0

0

0
4

0
4

0

0

0

0
4

0
4

0

0

0

0
4

0
4

0

0

0

0

0

0

0
4

0

0

0
4

0
4

0

0

0

0
4

0

0

0

0

0
4

0
4

0

0

0

0

0
4

0
4

0

0

0

0

0

0
4

0
4

0

0

0

0

0
4

0
4

0

0

0

0
4

0
4

0.032394

0.032394

0.032394

0
4

0
4

0
4

120.268

0
4

0
5

0

0

0

0

0

0

0

0

0

0

0

0
4

0

0

0

0
4

0

0

0
4

0
4

90.2509
7

14.1231
8

0.938476

13.1847
8

1.77635683940025e-15
8

0
4

1.39976
1

1.37643
1

0.0129576

0.0103722

0

1.97758476261356e-16
1

0
4

1.54853
1

0.349863

0

0.0129576

0.0129576

0.0129576

0.80731

0.168449

0.0829622

0

0

0.0311013

0.00518609

0.0647879

0
4

7.37911

7.37911

0
4

62.6669
7

16.6227
7

23.1587
7

3.36564
7

0.0336943

0

0.177557

0

0

0.00967569

0

0

0

0

1.59145
7

0.115597

0

0.0591856

0.00592134

0.0207444

0.0129576

0

0.144196

0.00518609

0

15.5431
7

0

0.00543325

0.0259152

0.0103722

0.0415649
7

0.136977
7

0.285285

0.975751

0.3063

0.0129576

4.18241830058008e-15
7

0
4

1.42992

0.72098

0.0482619
7

0

0.0129576

0.0129576

0.37717

0.144196

0

0.0887784

0

0.00518609

0

0.0194364

3.46944695195361e-18

0
4

1.70354

1.27095

0.432588

1.11022302462516e-16

0
4

0

0

0

0
4

0

0

0
4

0

0

0
4

6.66133814775094e-15
7

0
4

0.924777
7

0.385324
7

0.385324
7

0

0

0
4

0.539453
7

0.423856
6

0.115597

0
4

0
4

28.1146

23.4215
1

6.24643

0.304503

0.0777455

0.0453516

0.220279

0.181406

0.0518304

0.032394

0.11014

0.0712667

0

0.200843

0.0156497

0.0259152

0.0453516

0

0.0777455

0.0842243

0.0194364

0.0583091

0.0129576

0.0129576

0.971819

0.0103722

0.0129576

0.032394

2.94137

0.032394

0.0129576

0.0129576

0.0194364

0.0453516

0.0129576

2.20174

0.0129576

0.0259152

0.0194364

0.0388728

0.0647879

0

0.0129576

0.0129576

0.0129576

0.123097

0.220279

0.0259152

0.0129576

0.0777455

0.0129576

0.0388728

0.0129576

0.0129576

0.0194364

0.0259152

8.19638

0

0.116618

0.123097

2.06779038336435e-15
1

0
4

1.09951

0.653493

0.406893

0

0.0391243

0

0

0

0

0

0

0

0
4

3.59363

1.19522

0.412575

0

0

0

0

0

0

0.61034

1.19763
7

0.0234746

0.0777455
5

0.0156497

0.0453516

0

0.0156497

5.82867087928207e-16

0
4

0

0

0
4

0

0

0
4

1.77635683940025e-15

0
4

0.0156497

0.0156497

0.0156497

0

0

0

0
4

0

0

0
4

0
4

0

0

0

0
4

0
4

0

0

0

0
4

0
4

0

0

0

0
4

0
4

0

0

0

0
4

0
4

0.220498

0.220498

0.0684176

0.152081

0
4

0

0

0
4

0
4

0

0

0

0

0

0
4

0
4

0

0

0

0

0
4

0

0

0
4

0
4

0

0

0

0
4

0
4

0

0

0

0
4

0
4

0

0

0

0

0
4

0
4

0

0

0

0
4

0
4

0

0

0

0
4

0
4

0.741237
7

0.741237
7

0.741237
7

0
4

0
4

5.42899059041702e-14

0
4

285.692
3

0.953376
3

0

0

0
4

0
3

0
3

0

0

0

0
4

0.953376
2

0.837779
2

0.115597
2

0
2

0

0

0

0

0

0
4

0

0

0

0
4

0

0

0

0
4

0

0

0

0
4

0

0

0
4

0

0

0
4

0

0

0
4

0

0

0
4

0
4

4.0013
3

2.65937
7

2.65937
6

0

0
4

0
3

0
3

0
4

0.609647
3

0.532582
3

0.0770648
2

1.38777878078145e-17
3

0
4

0.288392
3

0

0.288392
3

0

0

0

0

0

0

0

0

0

0

0

0

0

0

0

0

0

0

0

0

0
4

0.443892
3

0

0

0

0

0

0

0

0

0

0

0

0

0

0.20715
7

0.236743
7

0

0

0

0

0

2.77555756156289e-17
3

0
4

0

0

0

0
4

0

0

0
4

0

0

0
4

0

0

0
4

0

0

0
4

0
4

7.92003
3

2.25681

2.25681

0

0

0

0

0

0
4

1.14657
5

0.275757

0.609569

0.179

0

0.0725677

0

0

0.00967569

0

1.56125112837913e-17
5

0
4

0.225737

0.0770648

0.0704238
6

0

0

0.0782487
6

0

0

0

0

0

0

0

2.77555756156289e-17

0
4

4.29091
3

0.288392
3

4.00252
4

0
4

0

0

0

0

0

0
4

0

0

0

0

0

0

0

8.88178419700125e-16
3

0
4

0

0

0

0
4

0

0

0

0
4

0

0

0
4

0

0

0
4

0

0

0
4

0

0

0
4

0

0

0

0

0
4

0

0

0

0
4

0

0

0
4

0

0

0
4

0

0

0
4

0

0

0
4

0

0

0
4

0

0

0
4

0
4

0.96696
4

0

0

0
4

0.568052

0.568052

0

0
4

0.398908
4

0.295928
4

0
4

0
4

0

0

0

0

0

0

0

0

0

0

0
4

0

0

0

0

0

0

0

0

0

0

0
4

0

0

0

0

0

0

0

0

0

0

0
4

0

0

0

0

0

0

0

0

0
4

0

0

0
4

0

0

0
4

0

0

0

0

0

0
4

0
4

0

0

0

0
4

0

0

0

0

0

0

0

0

0

0

0
4

0

0

0

0.0770648

0

0

0

0

0

0

0
4

0

0

0

0

0

0

0

0

0

0

0
4

0

0

0

0

0

0

0

0

0

0

0
3

0

0

0

0

0

0

0

0

0

0

0

0

0

0

0

0

0

0

0

0

0.0259152

0
4

0

0

0

0

0

0

0

0

0

0

0
4

0

0

0
4

0

0

0
4

0

0

0
4

0

0

0
4

0

0

0
4

0

0

0
4

0

0

0
4

0

0

0
4

0

0

0
4

0

0

0
4

0

0

0
4

0

0

0

0

0
4

0

0

0
4

0

0

0
4

0

0

0
4

0

0

0
4

0

0

0
4

0

0

0
4

0

0

0
4

0

0

0
4

0

0

0
4

0

0

0
4

0

0

0

0
4

0

0

0
4

0

0

0
4

0

0

0
4

0

0

0
4

0

0

0

0
4

0

0

0
4

0

0

0
4

0

0

0
4

5.55111512312578e-17
4

0
4

203.385

3.52806
6

0.982858
6

2.52955
6

0

0
6

0

0.0156497

0

0

1.59594559789866e-16
6

0
4

1.20843
3

0

0

0

0.648882

0

0

0

0

0

0

0

0

0

0

0

0

0

0

0

0

0

0

0.221567

0

0

0

0

0

0

0.337977

0

0
4

0.305624
3

0
3

0.20715
2

0

0

0

0
2

0

0

0

0

0

0

0.098474

1.38777878078145e-17
3

0
4

0.0518304
2

0
2

0

0

0

0

0

0

0
2

0

0

0

0

0

0.0518304

0

0
4

144.908

0.764498
3

83.5796
7

0
3

0

60.1073

0.0194364
2

0.288392

0

0.0129576

0

0

0

0

0

0

0

0

0

0

0

0

0

0

0

0

0.11014

0

0

0

0.00624502

0

0.0194364

0
4

51.1812

51.1812

0

0

0

0

0

0

0

0
4

0.746596
6

0.276441
6

0

0

0

0

0

0

0.173734

0

0

0

0

0.0591856

0.00777913

0

0

0

0

0

0.0337096

0

0

0

0

0

0

0

0

0

0

0

0

0.00518609

0

0

0.0259304

0

0

0

0

0

0

0

0

0

0

0

0

0

0

0

0

0

0

0

0

0

0

0

0

0

0

0

0

0

0

0

0

0

0

0

0

0

0

0

0

0

0.154258
6

0

0

0

0

0

0

0

0

0

0.0103722

0

0

6.76542155630955e-17
6

0
4

0.731261
3

0
3

0
2

0

0

0

0

0

0

0

0.15413

0

0

0
3

0

0

0

0

0

0.0194364

0

0

0

0

0.236743
2

0

0

0

0

0

0

0

0

0

0

0

0

0

0

0

0

0

0

0

0

0

0

0

0

0

0

0.0770648

0

0

0

0

0

0

0

0

0.153866

0

0

0

0

0

0.0129576

0.0770648

0

0

0

0

0

0
2

0

5.55111512312578e-17
3

0
4

0
3

0

0

0
4

0

0

0

0

0

0

0

0

0

0

0
4

0

0

0

0

0

0

0
4

0

0

0

0

0

0

0
4

0

0

0

0
4

0

0

0
4

0

0

0
4

0

0

0
4

0

0

0
4

0

0

0
4

0

0

0
4

0.0751983

0.0751983

0
4

0

0

0
4

0

0

0
4

0

0

0
4

0

0

0
4

0.36049

0.36049

0
4

0.144196

0.144196

0
4

0

0

0
4

0

0

0
4

0

0

0
4

0

0

0

0
4

0.144196

0.144196

0
4

0

0

0
4

0

0

0
4

0

0

0
4

8.61533067109121e-14

0
4

0.515981
3

0

0

0
4

0
3

0
2

0

0

0

0

0
4

0.155491
3

0.155491
3

0

0

0

0

0

0

0

0

0

0
4

0

0

0
4

0

0

0
4

0.144196

0.144196

0
4

0

0

0
4

0

0

0
4

0

0

0

0
4

0

0

0
4

0.216294

0.216294

0
4

0

0

0
4

0

0

0
4

0

0

0
4

0
4

0.144196

0.144196

0

0.144196

0
4

0
2

0

0

0

0
4

0

0

0
4

0

0

0
4

0

0

0
4

0
4

0.546093
6

0.0229939
6

0
6

0.00967569

0.0133182

0

0
4

0.423394
7

0
6

0.144196
7

0.020977

0.258221

0

0

0

0

0

0
4

0.0997057
6

0.0770648

0

0.00967569

0

0

0.0129652

0

0

0
4

6.93889390390723e-17
6

0
4

0

0

0

0
4

0
4

0

0

0

0
4

0

0

0
4

0
4

0

0

0

0

0

0
4

0

0

0
4

0
4

0

0

0

0

0

0
4

0

0

0
4

0

0

0
4

0
4

0

0

0

0

0
4

0

0

0
4

0

0

0
4

0
4

0

0

0

0

0
4

0

0

0
4

0

0

0
4

0
4

0

0

0

0

0

0
4

0
4

0

0

0

0

0
4

0
4

0.174417

0.174417

0.174417

0

0
4

0
4

0

0

0

0

0
4

0
4

0.362866
4

0.123097
4

0.123097
4

0

0

0
4

0.239768

0.239768

0
4

0
4

0

0

0

0

0
4

0
4

0

0

0

0
4

0
4

0

0

0

0

0
4

0
4

0.0518304

0.0518304

0.0518304

0
4

0
4

0

0

0

0

0
4

0
4

0

0

0

0
4

0
4

0

0

0

0
4

0
4

0

0

0

0

0
4

0
4

0.288392

0.288392

0.144196

0.144196

0
4

0
4

0

0

0

0
4

0
4

0.0259152
3

0.0259152
3

0.0259152
3

0
4

0

0

0

0

0
4

0

0

0

0
4

0

0

0
4

0
4

0

0

0

0
4

0
4

0

0

0

0
4

0
4

0

0

0

0
4

0
4

0.0129576

0.0129576

0.0129576

0
4

0
4

0

0

0

0
4

0
4

0

0

0

0
4

0
4

0

0

0

0
4

0
4

0

0

0

0
4

0
4

0

0

0

0
4

0
4

0.0259152

0.0259152

0.0259152

0
4

0
4

0.429
3

0.429
3

0.181406
3

0.247593

0

0

2.77555756156289e-17
3

0
4

0
4

0

0

0

0
4

0
4

0

0

0

0
4

0
4

0

0

0

0
4

0
4

0

0

0

0
4

0
4

0

0

0

0
4

0
4

0

0

0

0
4

0
4

0

0

0

0
4

0
4

0

0

0

0
4

0
4

0

0

0

0
4

0
4

0.0259152

0.0259152

0.0259152

0
4

0
4

0

0

0

0

0
4

0
4

0.0129576

0.0129576

0.0129576

0
4

0
4

0

0

0

0
4

0
4

0

0

0

0
4

0
4

0

0

0

0
4

0
4

0

0

0

0
4

0
4

0

0

0

0
4

0
4

0

0

0

0
4

0
4

0

0

0

0
4

0
4

0

0

0

0
4

0
4

0.0770648

0.0770648

0.0770648

0
4

0
4

0

0

0

0

0

0

0
4

0
4

0

0

0

0
4

0
4

0.144196
7

0.144196
7

0.144196

0

0
4

0
4

0.11144

0.11144

0.11144

0
4

0
4

0
2

0
2

0

0

0
4

0
4

4.92525

3.77921

0
2

0.115597
2

0

0

0
3

0.00416335
6

0

0.0770648

0

0
2

0

0.144196
7

0

0.520565
7

0

0

0.00518609

0

0

0

0

0

0

0

0.897284
7

0

0

0

0

0

0.0770648

0.216294

0

0

0

0.115597

1.08147

0

0

0

0

0

0

0

0

0

0.0363601
7

0

0

0

0

0

0.144196

0

0

0

0

0

0

0.00448724

0

0

0

0

0

0

0

0

0.147804
2

0

0

0

0.191885

0
5

0
4

1.14604
2

1.14604
2

0

0

0

0

0

0
4

0

0

0

0
4

0

0

0
4

0

0

0
4

0
2

0
2

0

0

0
4

0

0

0

0

0
4

0
2

0

0

0

0
4

0

0

0
4

0

0

0
4

0

0

0

0
4

0

0

0
4

0

0

0

0
4

1.33226762955019e-15

0
4

0.694678
4

0
4

0
4

0

0
4

0.576306
7

0.429954
7

0.0692876
7

0

0.0770648

0

0

0

0
4

0.0591856

0.0591856

0
4

0.0591856

0.0591856

0

0
4

0

0

0
4

0

0

0
4

0

0

0
4

0

0

0
4

0

0

0
4

0

0

0
4

0

0

0
4

0
4

2.68368

2.61889

0.695951

0

0.0156497

0

0.346792

1.23659

0.0770648

0

0.0156497

0

0.231194

0

2.77555756156289e-16

0
4

0.0647879
3

0.0647879
3

0

0
4

0

0

0
4

0
4

1.95856

1.9456

1.9456

0
4

0

0

0
4

0.0129576

0.0129576

0
4

0

0

0
4

0

0

0
4

0

0

0
4

0

0

0
4

7.45931094670027e-17

0
4

0.144196

0.144196

0

0.144196

0

0

0

0

0

0

0

0

0

0

0

0

0

0
4

0

0

0
4

0

0

0
4

0

0

0
4

0

0

0
4

0
4

0
3

0
3

0
3

0
4

0
3

0

0
3

0
4

0

0

0

0

0

0
3

0

0

0

0

0

0

0

0

0

0

0
3

0

0

0

0

0

0

0

0

0

0

0
3

0

0

0

0

0

0

0

0
3

0
3

0
3

0
3

0

0
4

0
2

0
2

0

0

0

0

0
4

0

0

0
4

0

0

0
4

0

0

0
4

0

0

0
4

0
2

0

0

0

0

0

0

0
4

0

0

0

0
4

0

0

0
4

0

0

0
4

0

0

0

0
4

0

0

0

0
4

0

0

0

0
4

0

0

0
4

0
4

0.794599
3

0.289913
3

0.0414887
2

0

0.062599

0

0.00518609

0

0

0

0

0

0

0
2

0

0

0

0

0

0

0.0323325

0

0

0

0
2

0.0547741

0.0622331
2

0.0312995

0

0

0
4

0.504686

0.504686

0

0
4

0

0

0
4

0

0

0
4

0

0

0
4

0

0

0
4

0

0

0
4

0

0

0
4

0

0

0
4

0

0

0
4

0
4

53.5509

53.4687

0.365457
2

53.1032

0

0
4

0

0

0

0

0

0
4

0

0

0

0

0
4

0.00518609

0

0.00518609

0
4

0.0770648

0.0770648

0
4

0

0

0
4

0

0

0
4

0
4

0.764479

0.432588

0.432588

0
4

0.331891

0.331891

0
4

0

0

0
4

0
4

1.71862524211974e-13
3

0
4

0.576784
2

0.576784
2

0.576784

0

0

0

0.288392

0.288392

0
4

0
2

0
2

0
4

0

0

0
4

0

0

0
4

0

0

0
4

0
4

0

0

0

0
4

0
4

0
4

47.1919
4

0

0

0

0

0
4

0
4

0.15413
2

0.15413
2

0
2

0

0.15413
2

0

0

0

0

0

0
4

0

0

0
4

0

0

0

0

0
4

0

0

0

0
4

0
4

8.10792
4

7.98195
4

7.45999
7

0

0.0156497

0

0.375622
7

0

0.0150924

0

0.115597

0

0

1.41553435639707e-15
4

0
4

0

0

0

0

0

0

0

0

0

0

0

0

0

0

0

0
4

0

0

0

0

0
4

0.0103722

0

0.0103722

0

0
4

0

0

0

0
4

0

0

0
4

0.115597

0.115597

0
4

1.38777878078145e-17
4

0
4

3.15279
7

0.528879
7

0.490006

0

0.0129576

0.0259152

0
4

2.62391

0.0518304

2.57208

0
4

0
4

0

0

0

0
4

0
4

0.266335

0.266335

0.266335

0
4

0
4

0

0

0

0
4

0
4

0

0

0

0
4

0
4

0

0

0

0
4

0
4

0

0

0

0
4

0
4

0

0

0

0
4

0
4

0

0

0

0
4

0
4

0

0

0

0
4

0
4

0

0

0

0
4

0
4

0.0352157
5

0.0352157
7

0.0352157
7

0
4

0

0

0
4

0

0

0
4

0
4

0

0

0

0
4

0
4

0

0

0

0
4

0
4

0

0

0

0
4

0
4

0

0

0

0
4

0
4

0

0

0

0
4

0
4

0.00458025

0.00458025

0.00458025

0
4

0
4

0

0

0

0
4

0
4

0

0

0

0
4

0
4

0.118371

0.118371

0.118371

0
4

0
4

0

0

0

0
4

0
4

2.58494
7

2.58494
7

2.3621
7

0

0

0

0.0201232

0.0100616

0.0770648

0.115597

0
4

0
4

0

0

0

0
4

0
4

0

0

0

0
4

0
4

0

0

0

0
4

0
4

0.0591856

0.0591856

0.0591856

0
4

0
4

0

0

0

0
4

0
4

0

0

0

0
4

0
4

0

0

0

0
4

0
4

0

0

0

0
4

0
4

0

0

0

0
4

0
4

0

0

0

0
4

0
4

0

0
7

0

0

0

0

0
4

0

0

0

0

0
4

0

0

0
4

0

0

0
4

0
4

0

0

0

0
4

0
4

0

0

0

0
4

0
4

0

0

0

0
4

0
4

0

0

0

0
4

0
4

0

0

0

0
4

0
4

0

0

0

0
4

0
4

0

0

0

0
4

0
4

0

0

0

0
4

0
4

0

0

0

0
4

0
4

0

0

0

0
4

0
4

0.00448724

0

0

0

0

0

0
4

0.00448724

0.00448724

0

0
4

0

0

0

0
4

0

0

0
4

0

0

0
4

0

0

0
4

0

0

0
4

0
4

0

0

0

0
4

0
4

0

0

0

0
4

0
4

0

0

0

0
4

0
4

0

0

0

0
4

0
4

0

0

0

0
4

0
4

0

0

0

0
4

0
4

0

0

0

0
4

0
4

0

0

0

0
4

0
4

0

0

0

0
4

0
4

0

0

0

0
4

0
4

1.29576

1.29576

0.194364

0.706189

0.395206

0
4

0
4

0

0

0

0
4

0
4

0

0

0

0
4

0
4

1.04002
7

0.696646
7

0.683689
7

0.0129576

0
4

0.103661

0.103661

0
4

0.239715

0.239715

0
4

8.32667268468867e-17
7

0
4

0
5

0
7

0

0

0

0

0
4

0

0

0
4

0

0

0
4

0
4

0

0

0

0

0
4

0

0

0

0
4

0

0

0

0
4

0

0

0
4

0
4

0

0

0

0

0

0

0
4

0

0

0

0
4

0
4

0

0

0

0

0

0

0

0

0

0

0

0

0

0

0

0

0

0

0

0

0
4

0

0

0

0

0

0

0
4

0
4

0

0

0

0

0
4

0
4

0.148359

0.148359

0.148359

0

0

0

0
4

0

0

0
4

0
4

0
4

0

0

0

0
4

0

0

0
4

0

0

0
4

0
4

0

0

0

0

0

0

0
4

0

0

0

0
4

0
4

0
3

0
3

0
3

0

0
4

0

0

0
4

0
4

0

0

0

0
4

0
4

0.0156497
2

0

0

0
4

0.0156497

0.0156497

0
4

0

0

0
4

0
4

0

0

0

0

0
4

0

0

0
4

0
4

0
6

0

0

0

0

0
4

0

0

0

0
4

0
4

0.278588

0.278588

0.233237

0.032394

0.0129576

0
4

0
4

0.0704238
4

0.0704238
4

0
4

0

0

0

0
4

0

0

0.0704238

0

0

0

0

0
4

0

0

0
4

0
4

0.388728

0.388728

0.2138

0.174927

2.77555756156289e-17

0
4

0
4

0

0

0

0

0

0
4

0

0

0

0
4

0
4

0.216294
5

0.216294
5

0.216294
5

0
4

0
4

0

0

0

0
4

0

0

0
4

0

0

0
4

0

0

0
4

0

0

0
4

0
4

0.123376
2

0.00777913

0

0

0.00777913

0
4

0.115597

0.115597

0
4

0

0

0
4

0
4

0

0

0

0
4

0
4

0.0289915

0

0

0

0
4

0

0

0
4

0

0

0
4

0.0289915

0.0289915

0
4

0
4

0

0

0

0

0
4

0
4

0.837762

0.837762

0.837762

0

0

0
4

0
4

0

0

0

0
4

0
4

6.71267
4

0

0

0

0

0

0

0

0

0

0
4

6.71267

6.71267

0

0

0

0
4

0

0

0

0

0
4

0

0

0
4

0

0

0
4

0
4

0

0

0

0
4

0
4

0

0

0

0

0
4

0

0

0
4

0
4

0

0

0

0

0
4

0
4

0.308259

0.308259

0.308259

0

0
4

0
4

0

0

0

0

0
4

0

0

0

0
4

0
4

0

0

0

0

0

0
4

0
4

0.221261

0.144196

0

0.144196

0
4

0.0770648

0.0770648

0
4

0
4

0

0

0

0
4

0
4

0.0351065

0.01249

0.01249

0
4

0.0226165

0.0226165

0

0
4

0
4

0

0

0

0

0
4

0
4

0.955115
6

0.955115
6

0.895929
6

0

0.0591856

0
4

0
4

0

0

0

0

0
4

0
4

0.149012

0.149012

0.149012

0
4

0
4

0

0

0

0
4

0

0

0
4

0
4

0

0

0

0
4

0
4

0

0

0

0
4

0
4

0.0129652

0.0129652

0

0.00518609

0.00777913

0
4

0
4

0

0

0

0
4

0

0

0
4

0
4

0.487988

0

0

0

0
4

0.487988

0.487988

0
4

0
4

0.0770648

0.0770648

0

0.0770648

0
4

0
4

0.0984754

0

0

0
4

0.0984754

0.0984754

0
4

0

0

0
4

0
4

0.147417
3

0.147417

0.147417

0
3

0

0

0
4

0

0

0

0
4

0

0

0
4

0

0

0
4

0
4

0.00362217

0

0

0

0
4

0.00362217

0.00362217

0
4

0
4

0

0

0

0
4

0
4

0

0

0

0

0
4

0

0

0
4

0
4

0.462389

0.462389

0.462389

0
4

0
4

0

0

0

0
4

0

0

0
4

0
4

0.330419

0.330419

0.330419

0
4

0
4

0

0

0

0
4

0

0

0
4

0
4

0.673795

0

0

0
4

0.673795

0.673795

0
4

0
4

0

0

0

0
4

0

0

0
4

0
4

0

0

0

0
4

0
4

1.55583
6

1.55583
6

1.54018
6

0.0156497

0
4

0
4

0.0215412

0.0215412

0.0215412

0
4

0
4

0

0

0

0

0
4

0
4

0

0

0

0
4

0

0

0
4

0
4

0

0

0

0

0
4

0
4

0

0

0

0
4

0
4

0.0388728

0.0129576

0.0129576

0
4

0.0259152

0.0259152

0
4

0
4

0.216294

0

0

0
4

0.216294

0.216294

0
4

0
4

0

0

0

0

0
4

0
4

0

0

0

0
4

0
4

0.310982

0.310982

0.310982

0
4

0
4

0.00483191
2

0.00483191
2

0

0
2

0.00483191

0

0

0

0

0
4

0

0

0
4

0
4

0

0

0

0

0
4

0
4

0

0

0

0

0
4

0
4

0

0

0

0
4

0
4

0

0

0

0
4

0
4

0

0

0

0
4

0
4

0

0

0

0
4

0
4

0.0312995

0.0312995

0.0312995

0
4

0
4

0

0

0

0
4

0
4

0

0

0

0
4

0
4

0

0

0

0
4

0
4

0.148359

0.148359

0
6

0

0.148359

0

0

0
4

0

0

0

0

0
4

0

0

0
4

0

0

0
4

0
4

0

0

0

0
4

0
4

0

0

0

0
4

0
4

0

0

0

0
4

0
4

0

0

0

0
4

0
4

0

0

0

0
4

0
4

0

0

0

0
4

0
4

0

0

0

0
4

0
4

0

0

0

0
4

0
4

0.00612023

0.00612023

0.00612023

0
4

0
4

0

0

0

0
4

0
4

15.2503

13.798

1.67953

4.1481

0.731959
2

0
3

0
3

0

0

0

0.0234746

0

0.0938984

0

0

0

0

0
3

0

0

0

0

0

0

0

0

0.0156497

0

0

0

0

0

0

0

0

0

0.115709

0.226758

0.0129576

0

0

0

4.47007

0

0

0

0

0

0

1.42217

0

0

0.36049

0.497211

0

0

0

3.60822483003176e-15

0
4

0

0

0

0
4

0

0

0
4

0

0

0

0
4

0

0

0
4

0

0

0
4

0

0

0

0
4

0

0

0
4

0

0

0
4

0

0

0
4

0

0

0

0
4

0

0

0
4

0.423856

0.423856

0
4

0

0

0
4

0.0091605

0.0091605

0
4

0

0

0
4

0.0194364

0.0194364

0
4

0.15413

0.15413

0
4

0

0

0
4

0

0

0
4

0

0

0
4

0

0

0
4

0

0

0
4

0

0

0

0

0

0

0
4

0.216294

0.216294

0
4

0

0

0
4

0.0129576

0.0129576

0
4

0

0

0
4

0

0

0
4

0

0

0
4

0

0

0
4

0

0

0
4

0

0

0
4

0

0

0
4

0
3

0
3

0
4

0

0

0
4

0

0

0
4

0

0

0
4

0

0

0
4

0

0

0
4

0

0

0
4

0

0

0
4

0.462389

0.462389

0

0
4

0.0770648

0.0770648

0
4

0.0770648

0.0770648

0

0
4

0

0

0

0
4

0

0

0

0

0
4

0
4

0
4

0

0

0

0

0

0

0
4

0

0

0
4

0
4

0
4

13.8818
3

0.290703
3

0
3

0

0

0

0

0

0

0

0

0

0

0

0

0

0

0

0
4

0.0391243
3

0
3

0
3

0

0

0
3

0

0

0
3

0

0

0

0
3

0

0
2

0

0

0

0

0

0

0

0

0
3

0

0

0

0

0

0

0

0

0

0

0
3

0

0

0

0

0

0

0

0

0

0

0
3

0

0

0

0

0

0

0

0

0

0

0
3

0

0

0

0

0

0

0

0

0

0

0
3

0

0

0

0

0

0

0.0391243

0

0

0

0
3

0

0

0

0

0

0

0

0

0

0

0
2

0

0

0

0

0

0

0

0
4

0
3

0
3

0

0

0

0

0

0

0

0

0

0

0
3

0

0

0

0

0

0

0

0

0

0
3

0
3

0
2

0

0

0

0

0
4

0
2

0
3

0

0

0

0

0

0

0
4

0

0

0

0
4

0

0

0
4

0

0

0
4

0

0

0
4

0

0

0

0
4

0

0

0
4

0

0

0
4

0

0

0
4

0

0

0
4

0.0312995

0.0312995

0
4

0
2

0
3

0

0

0

0

0

0
4

0

0

0
4

0

0

0
4

0.0129576

0.0129576

0
4

0.032394

0.032394

0
4

0

0

0
4

0

0

0
4

0.142533

0.142533

0
4

0

0

0
4

0

0

0
4

0.032394

0.032394

0
4

0

0

0
4

0

0

0
4

0

0

0
4

0

0

0
4

0

0

0
4

0

0

0
4

0

0

0
4

0

0

0
4

0

0

0

0
4

0

0

0

0
4

0

0

0

0

0

0

0

0

0
4

0

0

0

0
4

0

0

0

0
4

0

0

0

0

0
4

0
4

0.811259
3

0.67993
3

0.67993
3

0

0

0

0

0

0

0

0

0
4

0.0129576
2

0

0

0

0

0

0

0

0.0129576

0

0

0
4

0

0

0

0

0
4

0

0

0

0
4

0

0

0

0
4

0.118371
2

0
3

0
2

0
3

0

0

0

0

0

0

0

0

0

0

0

0

0

0

0

0

0

0

0

0

0

0
2

0

0

0

0

0

0

0

0

0

0

0
2

0

0

0

0

0

0

0

0

0

0

0
3

0

0

0

0

0

0

0

0

0

0

0

0

0

0

0

0

0

0

0

0

0

0
2

0

0

0

0

0

0

0

0

0

0

0

0

0

0

0

0

0

0

0

0

0

0.0591856
2

0

0

0

0

0

0

0

0

0

0

0

0

0

0

0

0

0

0

0

0

0

0.0591856
3

0

0

0

0

0

0

0

0

0

0

0

0
2

0

0

0

0

0

0
2

0
2

0

0

0

0

0

0

0

0

0
2

0

0

0

0

0
2

0

0

0

0

0

0

0

0

0

0

0
3

0

0

0

0

0

0

0

0

0

0

0
2

0

0

0

0

0

0

0

0

0

0

0
3

0

0

0

0

0

0

0

0

0

0

0
2

0

0

0

0

0

0

0

0

0

0

0

0

0

0

0

0

0

0

0

0

0

0
4

0
3

0
3

0

0

0

0

0
4

0
3

0

0

0

0

0

0

0

0

0

0

0

0

0

0
4

0

0

0

0

0
4

0

0

0
4

0

0

0
4

0

0

0
4

0

0

0

0
4

0

0

0
4

0

0

0
4

0

0

0
4

0

0

0
4

0

0

0
4

0

0

0
4

0

0

0
4

4.16333634234434e-17
3

0
4

12.6502

9.73604

4.46852

0

0.0591856

4.942

0.0591856

0.0591856

0.0887784

0.0591856

1.23512311489549e-15

0
4

0.651042

0.236743

0

0

0

0

0

0

0

0

0

0

0

0

0

0

0

0

0

0

0

0

0

0

0

0

0

0

0

0

0

0

0

0

0.414299

0

0

0

0

0

0

0

0

0

0

0

0

0

0

0

0

0

0

0

0

0

0

0

0

0

0

0

0

0

0

0

0

0

0

0

0

0

0

0

0

0

0

0

0

0

0

0

0

0

0

0
4

0.575109

0.575109
7

0

0

0
4

0

0

0
4

0

0

0
4

0

0

0
4

0

0

0
4

0

0

0
4

0

0

0
4

0

0

0
4

0

0

0
4

0.0860736

0.0860736

0
4

0.0770648

0.0770648

0
4

0

0

0
4

0

0

0

0

0
4

0.694157
7

0.694157
7

0
4

0

0

0

0
4

0.83075

0.83075

0
4

0

0

0

0

0
4

0

0

0
4

0

0

0
4

0
4

0
3

0
3

0
3

0

0
3

0
2

0
3

0

0

0

0

0

0
4

0
3

0
3

0

0
2

0

0

0

0

0

0

0

0
4

0

0

0

0

0
4

0
3

0

0

0

0

0

0

0

0
4

0

0

0

0

0

0
4

0

0

0

0

0
4

0
2

0

0

0

0

0
4

0

0

0

0
4

0

0

0

0
4

0
4

0

0

0

0
4

0

0

0

0
4

0
4

0

0

0

0

0
4

0

0

0
4

0
4

0

0

0

0
4

0
4

0

0

0

0
4

0
4

0

0

0

0

0
4

0
4

0

0

0

0
4

0
4

0

0

0

0
4

0
4

0

0

0

0
4

0
4

0

0

0

0

0
4

0
4

0

0

0

0
4

0
4

0

0

0

0

0

0

0

0

0

0

0

0

0

0

0

0

0

0

0

0

0

0

0

0

0
4

0

0

0

0

0
4

0

0

0
4

0
4

0.129576

0.129576

0.129576

0
4

0
4

0

0

0

0
4

0
4

0

0

0

0
4

0
4

0

0

0

0
4

0
4

0

0

0

0
4

0
4

0

0

0

0
4

0
4

0

0

0

0
4

0
4

0

0

0

0
4

0
4

0

0

0

0
4

0
4

0

0

0

0
4

0
4

0

0

0

0

0

0

0

0

0

0

0

0
4

0

0

0

0

0

0
4

0
4

0

0

0

0
4

0
4

0

0

0

0
4

0
4

0

0

0

0
4

0
4

0

0

0

0
4

0
4

0
3

0
3

0
3

0

0

0

0

0

0

0

0

0
4

0
3

0
3

0

0
4

0

0

0
4

0

0

0
4

0

0

0
4

0
4

0
3

0
3

0

0

0

0

0

0

0

0
4

0
3

0

0

0

0

0
4

0

0

0
4

0

0

0

0
4

0
4

0
2

0
2

0

0

0

0

0

0

0
4

0

0

0

0

0
4

0

0

0

0
4

0

0

0
4

0

0

0
4

0

0

0
4

0
4

0

0

0

0

0

0

0

0
4

0

0

0

0

0

0
4

0

0

0
4

0

0

0
4

0
4

0

0

0

0

0
4

0

0

0

0
4

0

0

0
4

0
4

0

0

0

0
4

0

0

0
4

0
4

0

0

0

0
4

0
4

3.05311331771918e-16
3

0
4

27.4804

0

0

0

0

0

0
4

0

0

0
4

0

0

0
4

0

0

0
4

0
4

3.05799

0

0

0
4

0

0

0
4

0

0

0

0

0

0

0
4

3.05799

0.738583

0.609007

1.58083

0.0388728

0.0194364

0.0583091

0.0129576

0
4

0
4

0

0

0

0

0

0

0

0
4

0

0

0

0

0
4

0

0

0

0
4

0

0

0
4

0

0

0
4

0
4

0.748347
5

0.748347
5

0.748347
5

0

0
4

0
4

20.7821

1.73899

1.73899

0

0
4

3.92405
7

3.92405
7

0
4

14.3981
7

14.3981
7

0
4

0
2

0
2

0

0

0

0

0

0

0

0

0

0

0

0

0

0

0
4

0

0

0

0

0

0
4

0

0

0
4

0

0

0
4

0

0

0

0

0
4

0

0

0
4

0

0

0

0

0
4

0

0

0

0

0
4

0

0

0
4

0

0

0

0
4

0

0

0
4

0
2

0
2

0

0

0

0

0

0

0
4

0

0

0

0
4

0

0

0
4

0

0

0

0
4

0.36049

0.36049

0
4

0

0

0
4

0

0

0

0
4

0

0

0

0
4

0

0

0

0
4

0

0

0
4

0

0

0
4

0
2

0
2

0

0

0
4

0

0

0
4

0

0

0
4

0

0

0
4

0

0

0
4

0

0

0
4

0

0

0
4

0

0

0
4

0

0

0
4

0

0

0
4

0

0

0
4

0
2

0

0

0

0

0

0

0

0

0
4

0

0

0
4

0

0

0
4

0

0

0
4

0

0

0
4

0

0

0
4

0

0

0
4

0

0

0
4

0

0

0
4

0

0

0
4

0

0

0
4

0
2

0

0

0

0

0

0

0
4

0

0

0

0
4

0.36049

0.36049

0

0

0
4

0
2

0
2

0
4

0

0

0

0

0

0

0
4

0
4

0.333432
6

0.333432
6

0.333432
6

0
4

0

0

0
4

0
4

0.00362217

0.00362217

0.00362217

0
4

0
4

0

0

0

0
4

0
4

0.0194364

0.0194364

0.0194364

0
4

0
4

0.115597

0.115597

0.115597

0
4

0
4

0.689483
7

0.689483
7

0.689483
7

0
4

0
4

1.15357

1.15357

1.15357

0
4

0
4

0

0

0

0

0

0
4

0
4

0.576784

0.288392

0.288392

0

0
4

0.288392

0.288392

0
4

0
4

0

0

0

0
4

0
4

0

0

0

0

0
4

0
4

0

0

0

0
4

0
4

0

0

0

0
4

0
4

1.4432899320127e-15

0
4

20.0683
6

20.0683
6

18.8212
7

13.6801
7

0
6

0.0201926

0

0

0

0

0.0234746
6

0.00967569
6

0.226527
7

0

0.00448724
7

0

0.00724433
6

0

0.00615463

2.84778
6

0.0338649

0

0

0

0.0547741

0.0577035

0

0.00967333

0

0.0145135

0.223635
7

0.00518609

0.0123093

0

0

0.00518609

0.00644631

0

0

0

0.00362217

0.13625
6

0

0

0

0

0

0

0

0.00724433

0.00615463

0

0.134673
6

0

0.0225621

0.0887784

0

0

0

0

0

0

0

0.432588
6

0

0

0

0

0

0

0

0

0

0

0.202331
6

0

0

0

0

0

0.0396835

0

0

0

0

0.0531208
7

0

0

0.0314107

0

0

0

0

0

0

0.0677298

0.00448724
6

0

0.00543325

0.0770648

0

0.15413

0

0.0156497

0

0.0123093

0.0770648

0
4

0.953615
6

0.682036
6

0.271579

0

0

0

0

0

0

0
4

0.0177425
6

0

0

0

0.00543325

0

0

0

0.0123093

0
4

0.212881
7

0.153695
7

0

0

0.0591856

0

0

0

0
4

0
6

0

0

0

0

0

0

0
4

0.0538468

0.0381415

0.00448724

0.0112181

1.73472347597681e-18

0
4

0

0

0

0

0

0
4

0.00897447

0

0

0.00897447

0
4

0

0

0
4

3.64291929955129e-15
6

0
4

0
4

3.31249

2.64871

0.393053

0.210037

0

0

0

0.0622331
5

0.120783

0

0

0

0

0

0

1.38777878078145e-17

0
4

0.144196
2

0.144196
2

0

0
4

0.0927145

0.0927145

0

0

0

0

0
4

2.01874

0

2.01874

0

0
4

0
4

0.216294
7

0.216294
7

0.216294

0

0
4

0

0

0

0
4

0
4

0.0770648

0

0

0

0
4

0.0770648

0.0770648

0
4

0
4

0.370423

0.370423

0.15413

0.216294

0

0

0
4

0
4

2.22044604925031e-16

0
4

0.237005

0.237005

0.237005

0.237005

0
4

0

0

0
4

0
4

0
4

0.0285611

0.0285611

0.0285611

0.0285611

0
4

0
4

0
4

0

0

0

0

0
4

0

0

0
4

0
4

0
4

0

0

0

0

0
4

0

0

0
4

0

0

0
4

0
4

0

0

0

0
4

0
4

0
4

0.2138

0.2138

0.2138

0.2138

0
4

0

0

0

0
4

0
4

0
4

0.0770648

0.0770648

0

0

0

0
4

0.0770648

0.0770648

0
4

0
4

0
4

0

0

0

0

0

0

0
4

0

0

0
4

0
4

0
4

0

0

0

0

0
4

0

0

0
4

0
4

0

0

0

0
4

0
4

0
4

0

0

0

0

0

0

0
4

0

0

0
4

0
4

0
4

0

0

0

0

0
4

0
4

0
4

2.4533
6

2.4533
6

2.40485
6

2.40485
6

0

0
4

0.0394812
6

0.00967569
7

0.00673085

0

0.0230747

0

0

0

0
4

0.00448724
6

0.00448724
7

0

0

0
4

0

0

0
4

0.00448724

0.00448724

0
4

3.10515502199848e-16
6

0
4

0
4

0

0

0

0

0

0
4

0
4

0
4

0

0

0

0

0
4

0
4

0
4

0

0

0

0

0
4

0
4

0
4

0

0

0

0

0
4

0

0

0
4

0
4

0
4

0

0

0

0

0

0
4

0

0

0
4

0
4

0
4

0.262454

0.262454

0.262454

0.262454

0
4

0
4

0
4

0

0

0

0

0
4

0

0

0
4

0
4

0

0

0

0
4

0
4

0
4

0.142533

0.142533

0.142533

0.103661

0.0129576

0.0259152

0
4

0
4

0
4

0.269727

0.269727

0.192662

0.192662

0
4

0.0770648

0.0770648

0
4

1.38777878078145e-17

0
4

0
4

0

0

0

0

0

0
4

0
4

0
4

0
3

0
3

0
4

0
3

0

0

0

0

0

0

0

0

0

0

0
4

0
3

0

0

0

0

0
4

0

0

0
4

0
4

0
4

0.770648

0.770648

0.770648

0.770648

0
4

0
4

0
4

0.539453

0.539453

0.539453

0.539453

0
4

0
4

0
4

0

0

0

0

0
4

0

0

0
4

0

0

0
4

0
4

0
4

0

0

0

0

0
4

0

0

0
4

0
4

0
4

0

0

0

0

0

0
4

0
4

0
4

0

0

0

0

0
4

0

0

0
4

0
4

0
4

0.115597

0.115597

0.115597

0.115597

0
4

0
4

0
4

0

0

0

0

0
4

0
4

0
4

0

0

0

0

0
4

0

0

0
4

0
4

0
4

0.0777455

0.0777455

0.0777455

0.0777455

0
4

0
4

0
4

0.158855

0.122468

0.122468

0.122468
7

0

0

0

0

0

0

0

0

0
4

0

0

0

0

0
4

0

0

0

0

0
4

0

0

0
4

0

0

0
4

0
4

0.0363878

0.0295175

0.0132278

0

0

0.00661392

0

0.00967569

0

0

1.73472347597681e-18

0
4

0.00687037

0

0.00687037

0

0
4

0

0

0

0
4

0

0

0
4

0

0

0
4

0
4

0

0

0

0
4

0

0

0
4

0
4

0

0

0

0
4

0
4

0
4

0

0

0

0

0

0

0
4

0
4

0
4

0

0

0

0

0

0
4

0
4

0
4

0.772727

0.772727

0.772727

0.772727

0
4

0
4

0
4

0

0

0

0

0
4

0

0

0
4

0
4

0
4

0

0

0

0

0
4

0
4

0
4

0

0

0

0

0
4

0
4

0
4

0

0

0

0

0
4

0

0

0
4

0
4

0

0

0

0
4

0
4

0
4

0

0

0

0

0
4

0
4

0
4

0

0

0

0

0
4

0

0

0
4

0
4

0

0

0

0
4

0
4

0
4

0.0129652

0.0129652

0.0129652

0.0129652

0

0
4

0
4

0
4

0.351686

0.351686

0.325771

0.302296

0

0.0234746

0

0

1.73472347597681e-17

0
4

0.0259152
5

0.0259152
5

0

0

0
4

0

0

0

0
4

0
4

0
4

0

0

0

0

0

0
4

0

0

0
4

0
4

0
4

0

0

0

0

0
4

0
4

0
4

0

0

0

0

0
4

0

0

0
4

0
4

0
4

0

0

0

0

0

0

0
4

0
4

0
4

0

0

0

0

0
4

0
4

0
4

0

0

0

0

0
4

0

0

0
4

0
4

0
4

0.0091605

0.0091605

0.0091605

0

0.0091605

0
4

0
4

0
4

0

0

0

0

0
4

0

0

0
4

0
4

0

0

0

0
4

0
4

0
4

0

0

0

0

0
4

0
4

0
4

0

0

0

0

0
4

0
4

0
4

1.37564

1.37564

0.837088

0.837088

0

0

0

0

0
4

0.538551

0.538551

0

0

0

0
4

0
4

0
4

0

0

0

0

0

0
4

0

0

0
4

0
4

0
4

0

0

0

0

0
4

0

0

0
4

0
4

0

0

0

0
4

0
4

0
4

0

0

0

0

0
4

0

0

0
4

0
4

0
4

0

0

0

0

0
4

0

0

0
4

0
4

0
4

0

0

0

0

0

0
4

0
4

0
4

0

0

0

0

0

0
4

0
4

0
4

0

0

0

0

0

0
4

0
4

0
4

0

0

0

0

0
4

0
4

0
4

0

0

0

0

0
4

0

0

0
4

0
4

0
4

0

0

0

0

0
4

0
4

0
4

0.591746
6

0.591746
6

0.44755
6

0.15413
6

0

0

0.00923194

0.00967569

0.138528
7

0.0221244
6

0

0.113861

0

0

0

0

0
4

0.144196

0.144196

0
4

8.32667268468867e-17
6

0
4

0
4

0

0

0

0

0
4

0

0

0
4

0
4

0
4

0

0

0

0

0
4

0

0

0
4

0
4

0
4

0

0

0

0

0
4

0
4

0
4

0.147964

0.147964

0.147964

0.147964

0
4

0
4

0
4

0

0

0

0

0
4

0

0

0
4

0
4

0
4

0

0

0

0

0

0
4

0
4

0
4

0

0

0

0

0

0
4

0
4

0
4

0.032394

0.032394

0.032394

0.032394

0
4

0
4

0
4

0

0

0

0

0
4

0
4

0
4

0

0

0

0

0
4

0

0

0
4

0
4

0
4

1.89128
7

1.89128
7

1.89128
7

1.88212
7

0

0.0091605

0
4

0
4

0

0

0

0
4

0

0

0
4

0
4

0
4

0

0

0

0

0
4

0

0

0
4

0
4

0
4

0

0

0

0

0

0
4

0
4

0
4

0

0

0

0

0
4

0
4

0
4

0.0770648

0.0770648

0.0770648

0.0770648

0
4

0

0

0
4

0
4

0
4

0.00967569

0.00967569

0.00967569

0.00967569

0
4

0
4

0
4

0

0

0

0

0
4

0
4

0
4

0

0

0

0

0
4

0

0

0
4

0
4

0
4

0

0

0

0

0
4

0

0

0
4

0
4

0
4

0

0

0

0

0

0
4

0
4

0
4

0

0

0

0

0

0
4

0
4

0
4

2.6887

2.6887

2.61743

2.18335

0.103661

0.0453516

0.0777455

0.168449

0.0129576

0.0259152

0
4

0.0712667

0.0712667

0
4

0
4

0
4

0

0

0

0

0

0
4

0
4

0
4

0

0

0

0

0
4

0
4

0
4

0

0

0

0

0
4

0

0

0
4

0
4

0
4

0

0

0

0

0
4

0

0

0
4

0
4

0
4

0

0

0

0

0
4

0

0

0
4

0
4

0
4

0

0

0

0

0
4

0

0

0
4

0
4

0
4

0

0

0

0

0
4

0

0

0
4

0
4

0
4

0.032394

0.032394

0.032394

0.0194364

0.0129576

1.73472347597681e-18

0
4

0
4

0
4

0.0647879

0.0647879

0.0647879

0.0647879

0
4

0
4

0
4

0.0129576

0.0129576

0.0129576

0

0.0129576

0
4

0
4

0
4

0.236734

0.232154

0
5

0

0

0

0

0

0

0
4

0.232154

0.220703

0

0.0114506

0
4

0
3

0

0

0
4

0

0

0
4

0

0

0
4

0
4

0.00458025

0

0

0

0

0
4

0.00458025

0

0.00458025

0
4

0
4

3.46944695195361e-18

0
4

0

0

0

0

0
4

0
4

0
4

0

0

0

0

0

0
4

0
4

0
4

0

0

0

0

0
4

0

0

0
4

0
4

0
4

0

0

0

0

0
4

0

0

0
4

0
4

0
4

0

0

0

0

0
4

0
4

0
4

0

0

0

0

0
4

0
4

0
4

0

0

0

0
[truncated: 266,331 more chars]
