# Supplementary material for: Fungal communities decline with urbanization—more in air than in soil
Source: ISME J. 2020 Aug 5;14(11):2806–15. doi: 10.1038/s41396-020-0732-1 (PMC7784924; doi:10.1038/s41396-020-0732-1)
Supplement: Supplementary file 2 — Supplemental data [file 41396_2020_732_MOESM2_ESM.zip › Krona_AirUrbanEdge.html]

Javascript must be enabled to view this page.

num
probth


4239

2820.17

0.252259
3

0.252259
3

0.252259
3

0.252259
3

0.252259
3

0

0

0

0

0

0

0

0

0

0

0

0

0

0

0

0

0

0

0

0

0

0

0

0

0

0

0

0

0

0

0

0

0

0

0

0

0
4

0
4

0

0

0

0
4

0

0

0
4

0
4

0

0

0

0
4

0
4

0
4

0
4

0

0

0

0

0

0

0
4

0
4

0
4

0
4

0

0

0

0

0

0
4

0
4

0
4

0

0

0

0

0
4

0
4

0
4

0
4

2219.39

2.52309

2.48482

2.03749

0
3

0
2

0

0

0

0

0

0

0

0

0

0

0

0

0

0.0849438

0

0.40817

0

0

0

0

0

0
3

0

0

0

0

0

0

0

0

0

0

0

0.00364411

0

0

0

0

0

0

0

0

0

0.288667

0

0

0

0

0

0

0

0

0

0

0

0

0.00655526

0

0

0

0

0

0

0

0

0.145989
7

0

0

0

0

0

0

0

0

0

0

0
7

0

0

0

0

0

0

0

0

0.00780182

0

0
7

0

0

0

0

0

0

0

0

0

0

0

0

0

0

0

0

0

0

0.0446436

0

0

0

0.0923949

0

0

0

0

0

0

0

0.0127553

0

0

0

0

0

0

0.013003

0

0

0

0

0

0

0
6

0

0

0

0

0

0

0

0

0

0

0

0

0

0

0.00755116
6

0.232628
7

0

0

0.10842

0.213659
1

0

0
2

0

0

0

0

0

0.00520121

0

0

0
3

0

0

0

0

0

0

0

0

0

0

0
2

0

0

0.173714

0

0

0

0

0

0

0

0
2

0

0

0

0.00780182

0

0

0

0

0

0

0.0907113
7

0

0

0

0

0

0

0

0

0

0

0
6

0

0

0

0

0

0

0.0182206

0

0

0

0.0710112
6

0

0

0

0

0

0

0

0

0

0

3.33066907387547e-16

0
4

0
3

0
3

0

0

0

0
4

0

0

0
4

0

0

0
4

0

0

0
4

0

0

0
4

0

0

0

0
4

0.00780182

0.00780182

0

0
4

0

0

0

0
4

0

0

0
4

0

0

0
4

0

0

0
4

0.0104024
6

0

0.0104024

0

0

0
4

0

0

0
4

0.00894146

0.00894146

0

0
4

0

0

0

0
4

0

0

0

0
4

0

0

0
4

0

0

0

0
4

0

0

0

0
4

0

0

0
4

0

0

0
4

0.019133

0.019133

0
4

0
2

0

0

0

0

0
4

0

0

0
4

0

0

0
4

0

0

0
4

0

0

0
4

0

0

0
4

0

0

0
4

0

0

0
4

0

0

0
4

0

0

0
4

0

0

0
4

0
6

0

0

0
4

0

0

0
4

0

0

0
4

0

0

0
4

0

0

0
4

0

0

0
4

0

0

0
4

0

0

0
4

0

0

0
4

0

0

0
4

0

0

0
4

0

0

0

0
4

0

0

0
4

0

0

0
4

0

0

0
4

0

0

0
4

0.0273308

0.0273308

0
4

0

0

0
4

0

0

0
4

0

0

0
4

0

0

0
4

0.00443786

0.00443786

0
4

0

0

0
4

0

0

0
4

0

0

0
4

0

0

0
4

0.369286
7

0.369286

0

0
4

0

0

0
4

0

0

0

0
4

0
4

0
6

0
6

0
6

0
4

0
4

0

0

0

0
4

0

0

0
4

0
4

0

0

0

0
4

0
4

0

0

0

0

0
4

0
4

0

0

0

0
4

0
4

0

0

0

0
4

0
4

0

0

0

0
4

0
4

0

0

0

0
4

0
4

0

0

0

0
4

0
4

0

0

0

0
4

0
4

0

0

0

0
4

0
4

0
6

0
6

0
6

0
4

0

0

0
4

0

0

0
4

0
4

0

0

0

0
4

0
4

0

0

0

0
4

0
4

0

0

0

0
4

0
4

0

0

0

0
4

0
4

0

0

0

0
4

0
4

0

0

0

0
4

0
4

0

0

0

0
4

0
4

0

0

0

0
4

0
4

0

0

0

0
4

0
4

0.019133

0.019133

0.019133

0

0
4

0
4

0

0

0

0

0

0
4

0

0

0
4

0
4

0.019133

0.019133

0.019133

0
4

0
4

0

0

0

0
4

0
4

0

0

0

0
4

0
4

0

0

0

0
4

0
4

0

0

0

0
4

0
4

3.12250225675825e-16

0
4

1736.16
7

0

0

0

0

0

0

0
4

0

0

0

0
4

0

0

0

0
4

0

0

0

0
4

0

0

0
4

0
4

1723.22
7

1723.21
7

172.216
7

2.8913
7

0.271819
6

0

0

0

0

0

0

0.00780182

0

0

0

0

0.011858
7

0

0.0352388

0

0

0

0.00665679

0.00887572

0

0

0

0.00520121
7

0

0.0133136

0

0

0

0.019133

0.121175

0

0

0

0.00503411

0

0

0.00447073

0

0

0

0

0

0

0

0.0760447
7

0

0

0

0

0

0

0

0

0

0.0163169

0.0689777

0.00527298

0

0

0

0

0

0

0.00503411

0

0

0.0573989
7

0

0.00520121

0.00665679

0

0

0

0

0

0

0

0.140486
8

0

0

0

0

0

0

0.0049252

0

0

0.0255106

0.038356

0

0

0.00351532

0

0

0

0

0

0

0

0

0

0

0

0

0

0

0

0

0.00665242

0

0.37886
7

0.155421
7

0

0

0.114798

0

0

0.00332561

0

0

0

0

0.0280925

0

0

0.00447073

0

0

0.00443786

0.00443786

0.10842

0

0

0.00443786
6

0

0

0

0

0.019133

0.127553

0

0

0.0382659

0

0.0163169
7

0

0

0

0

0

0

0

0.00443786

0

0

0
7

0

0

0

0

0

0.226535

0

0.0125853

0

0

0.0212976
8

0

0.195045

0.00443786

0

0

0

0

0

0

0

0.0176194
8

0

0.019133

0

0

0

0

0

0

0

0

0.0255106
7

0

0

0

0

0

0

0

0.00703064

0

0

0
7

0

0

0

0

0

0

0

0

0

0.0208048

0
6

0

0

0

0

0

0

0

0.00670609

0

0

0.137691
6

0.0888713
1

0

0

0

0

0.160604

0

0.031065

0

0

0.00443786

0.0655526
7

0

0.0318883

0

0

0

0

0

0

0.0302047

0

0.017456
7

0

0

0

0

0.0624145

0

0.0382659

0.0377558

0

0

0.522967
1

0

0

0

0

0

0

0

0

0

0.363526

0.00983289
7

0

0

0

0

0.0255106

0

0

0

0.0855799

0

0
6

0

0

0

0

0.00755116

0

0

0

0

0

0.00987683
8

0

0.0156036

0.0598139

0

0

0.00887572

0

0

0

0

0.158513
7

0

0

0

0

0

0

0

0

0

0

0.058823

0.0379465

0

0

0

0.0110946

0

0

0

0.00351532

0.0276876

0.0726678
6

0

0

0.0251705

0

0

0

0.00703064

0

0

0.00780182

0.422573
7

0.0268773

0.00503411

0.024626

0

0

0

0

0

0

0

0

0.0243342
6

0.0127553

0

0

0

0

0

0

0.0221893

0

0

0.019133
1

0

0.0201364

0

0.0510212

0

0

0

0

0

0

0.0503828
7

0

0

0

0

0

0

0

0

0

0

0.0402729
7

0.0637765

0

0

0

0

0

0

0

0

0

0.112891
7

0.00997863

0

0

0

0.00738779

0

0.102042

0

0

0.0255106

9.90315
7

0.00443786

0

0.013003

0

0

0

0

0

0

0

0.0127553
5

0

0

0

0.00447073

0

0

0

0

0

0

0.153717
7

0

0

0

0

0

0

0

0

0

0

0.0151023
6

0.0266271

0

0

0

0

0

0

0

0.0110946

0

0.0659692
7

0.0829095
7

0

0

0

0

0

0

0

0.0151023

0.024626

0

0.00543897
7

0

0

0.0104024

0.0755116
7

0.00985039
7

0.119066
8

0.00871653
7

0.00527298
6

0
6

0.0212668
7

0

5.77956
7

0.0412444
7

0.0302047
7

0.00755116
7

0.0562166
7

0

0.248728

0.0217559

0.129988
7

0.00780182
7

0.0500224

0.131345

0.0255106
7

0.0956648

0
7

0

0.00985039
7

0.00665679
7

0.0683191
7

0
3

0

0.00939592
6

0.288155
7

0
7

0.0127524
7

0.00520121

0.0701542
7

0.0160791

0.00503411
7

0.0417864
7

0.0318883

11.2691
6

0.0501233
6

0.272021

0.0104024
8

0.0127553
6

0.0699423
7

0.0127553
6

0.578364

0.0338707

0

0.0446436

0.0646566
7

0.0829095

0.177849
7

0.019133
7

0.0517076
7

0

0
7

0.0764519
6

0.0238327
7

0.162914
2

0.00665679

0

0.0266841
8

7.06393

0.272611
7

0.0701542
6

0.0155654
6

0.0135974
8

0.013003
7

0.04296
7

0.0049252
7

0
8

0.0356689
6

0.0709963

0.00887572
6

0.305064

0.00665242

0.0652088

0.0127553
6

0.00887572
8

0.403714
6

0.00351532
7

0.00503411
7

0
6

0.0523589
8

0.00443786
7

0.546451
7

0.0111768
7

0.0188965
8

0.0244059

0.0847185
6

0.00520121
7

0.0317677

0.0203871
7

0.293515
7

0.00520121
2

0.139868

0.0637765
7

3.29561

0.0493934

0
6

0.221028
7

2.44829
8

0.0628744
7

0.0255106

0.0688106

0.00527298
7

0.0221634
7

1.13189

0
1

3.22793
8

0.12014
7

1.32051

0.0686369
7

0
7

0.126921
6

0.118302
7

0.0318883
7

0.0100682
7

0.61412
7

0.0330144
7

0.0218323

0.0274256
7

0.00351532
6

0
7

0
6

0.059743

0.100593

0.00665242
7

0.20574
7

0.467412
7

0
6

0.0253406
7

0.0133136
7

0.0247813
7

0.179691

0.0140613

0
6

0.248154
8

0.0344366
6

0.649581
7

0.0634173
7

0.130139
7

0
7

0.125799
7

0
7

0.00655526
6

0.0149934
7

0.019133
7

0.0280965
7

0.113372
7

0.0176786
7

0.266194
7

0

0
6

0.0958424
7

0.0571908
6

0.0284962
7

0.0255106
6

0.293429
7

0.0151023
6

0.308795
7

0.119547
7

0.265384
7

0.0829095
7

0.423194
7

0.0049252
7

0

0.41291
7

0.0176194
6

0
7

0.0637765

0.00543897
7

0.208353
7

0.843889
7

0.38342
7

0.0544864
6

0.019133

0.0685963
7

0.146686
6

0.019133
7

0.031065

0
6

0
7

0
5

0
7

0.256862
7

0.0613284
7

0.028772
7

0.0115894
7

0.198711
8

0.00983289
8

0
7

0.0402729

0.0104024
7

2.44894
1

0.0049252

0.435881
7

0
7

0
7

0.0726522
6

0.0127553
7

0.0190364
7

0.0184133
5

0.0318883

0
7

0.0329554
6

0
6

0.100699
7

0.0226535
7

0.0829442
8

0.0784514
6

0.0255106

0.0194121
1

0
7

0.00755116
7

0.0831886
7

0.0177514

0
7

0.0955063
7

0.0199186

0.00443786
6

0.0373272
7

1.62809
7

0.00447073

0.0762863
7

0
7

0.010349

0.0387614
6

0.00703064
7

0.116576
7

0.1074
7

0

0
6

0.0271242
7

0.00665242
7

0.00527298
7

0.02465
7

0.00351532
7

0.119019
7

0
6

0.0977233
7

0.0382659

0.00887572
7

0.0221893
7

0.00543897
7

0.0607426
7

0.0701542
6

0
7

0.0805322
7

0.0276876
2

0.0510212

0.582788
7

0
1

0.0970889
7

0.244065
7

0.0929312

0.0299485

0.125258
7

0.0318883
7

0.0573989
7

0.0131105

0.0587278
7

0.300348
7

0.155295

0
7

0
6

0.0404944
7

0
5

0.020507
7

0.733838
7

0.0570588
7

0.019133
2

0

0.211997
7

0.00443786
7

0.101104
7

0.132412
7

0
5

0
6

0.0194077
7

0
7

0.0122725

0
8

0.0305447

2.05936
7

0.297595
7

0.0271094
7

0
7

0.0679075
7

0.0106402
6

0
7

0.00887572
7

0.038181
7

0
7

0.0510212
7

0.0184291
7

0.168422

0.0266841
7

0.0360161
7

0.0393203
7

0.00755116
7

0.0100682
8

0.0446436

0.0176194
7

0.0288461
7

0.00665679
7

0.0246072

0.0385078
7

0.0446436
7

0

0.0343863
7

0.127383

0.0133136
6

0.0245719
2

0.0382659
7

0.353139

0.0637765
7

0.0139098

0.116982

0.0305447
7

0.0171932
7

0
6

0.0186315
6

0.00738779
6

0.0123036
7

0.00503411

0.013212

0.00564832
7

0.0127553
7

0.124471

0.00755116

0.447269
7

0.199714
7

0.0139098
7

0.0104024
5

0.0812174
7

0.100682
7

0.0122397
2

0.0332839
7

0.0300622
7

0.138382
7

0.0110946
6

0.013212

0.0049252
7

0.0125853
6

0.00543897
7

0.0104024
6

0

0.161361
7

0.045307
7

0.172197

0.376656
7

0.0302047
7

0

0

0.0293425
2

0.0115776
7

0.0176194
7

0.0271671

0.0133136

0.019133
6

0.019133

0.27531
7

0.0151023
7

0.10873
7

0.0125853
5

0.0510212
7

0.012313
6

0.0226535
7

0.00503411

0.0156475
7

0.95027
7

0.0829095
7

0.217465
7

0
4

0.038545
6

0.0723312
7

0.0199704
7

0

0.0127553
2

0.00670609
7

0.0553752

0.0900826
7

0.0701542
7

0.269466
6

0.00854943
6

0.0149038

0.0151023
7

0.0127553
6

0.0173471
7

0.0406818
7

0.0368134
7

0
7

0.00364411
7

0.100693
7

3.21887
7

0.330953

0.0104024
6

0.0176499
7

0
7

0.0255754

0.0210166

0.0417147
7

0
7

0.0321674

0.0636799
7

0.013003
5

0.169149
7

0.00670609
6

0.0427899

0.0701542

0.0110946
6

0
7

0
6

0.0087883
2

0
7

0.00703064
7

0.146686

0.298821
7

0.0448212
4

0
8

0.0941512

0.0939812

0.0503411
7

0.133107
6

0.0336719
7

0.0176194
7

0
8

0.0382659
7

0.30698
7

0.10943
7

0.0309595
7

0.0573989
1

0.0125853
7

0.00520121

0.0318883
6

0
6

0.0127553
7

0.010546
7

0.0829095
8

0.167835
7

0.0472795

1.08847
7

0.0120914
7

0.0276876

0.0343781

0.0450808
7

0.0251705
7

0
6

0.10723
6

0.0108779

0.139299
7

0.03627
8

0.0746782
8

0.019133
6

0.00527298
7

0
7

0.013003
7

0.0307895
7

0.0783126
6

0.00755116
6

0.113772
7

0.343944

0.0163169

0.0127553

0.0354894

0.0317182
7

0.040083
7

0.013003
7

0.0127553
7

0.0307932
7

0
7

0.0665905
7

0.221447
7

0.0125853

0.0344742

0.0373625
7

0
7

0
6

0
8

0

0.031065
7

0.0172556
6

0.036939

0.233338

0.0419052
7

0.0402729
7

0.0637765
7

0.05571

0.0164269
7

0.0655526
6

0
7

0
7

0.0650151
7

0.0615181

0.519784
7

0.00985039
6

0.0216576
7

0.0382659
6

0.0260061

0.0248511
7

0.0228235
1

0.0897164
7

0
6

0
7

0.0127553
6

1.02949
7

0.191157
7

0
7

0
8

0.0233343
7

0.0271522
6

0.0598969
7

0.0664811
7

0.0149934
7

0.0629264
7

0.0136874

0.0701542
6

0.244476
7

0.0151023

0.0967085
7

0.0876035
7

0
7

0.0812657
7

0.0241915
7

0.0125853
7

0.453987

0
7

0.0273064

0.0551529
7

0.0127524
6

0.0255106
6

0.387991

0
7

0.0214971
7

0.0177514

0.157922
6

0.483015
7

0.0049252

0.012727

0.0965022
7

0.242271
7

0.211196
7

1.83608
8

0.0148845
7

0

0
6

0.0701542
7

0.00887572
5

0.00527298
7

0.0127553
6

0.159378
7

0.00520121
7

0.0292011
6

0
7

0.0503411
7

0.0483341
7

0.0382659
7

0
6

0.00503411
5

0.0279265
8

0.0100682
7

0.186275
7

0
6

0.0423019
8

0.0609142
7

0.0427899
7

0.00543897
6

0.0510212
7

0
6

0.00546617
7

0.0363885

0.00351532
7

0.350193
7

0.0125853

0.00780182
7

0.0163169

0.0892871
7

0.0222604
7

0.0229906
7

0.00543897
6

0.0190364
7

0.00527298
7

0.0255106
7

0.163445
7

0.0260061

0.0510212
7

0.0260061

0.0904606
7

0.0604093

0.0125853
6

0.12557

0.0235708

0.00894146

0
5

0.293933

0.0127553

0.0573989
6

0.0131105
6

0.0201388
1

0.0127553
6

0.0510212
7

0.00887572
8

0.0276876

0

0

0.244711
7

0.0208048
2

0

0

0.0956648

0
7

0.094322
7

0.00447073
7

0
7

0.019133
7

0
6

0.697329

0.100103
7

0.012313
7

0

0.00503411

0.192925
6

0.0527275
7

0.0049252
6

0
6

0
7

0.00780182

0.0892871
7

0.646576

0
7

0.0876265
7

0.00985039

0.0269348
7

0.00655526

0
7

0.37685
7

0.729851
8

0.00665679

0.00520121

0.414719
7

0.21684
6

0
7

0.00703064
7

0.00527298

0
5

0.0255106
7

0.019133
7

0
6

0.0235784
6

0.0736695
2

0.158782
7

0.0127553
7

0.00780182

0.0127553
1

0.00887572
6

0.0230837
7

0.00364411
6

0
7

0.00947197
2

0.0399407

0.0255106
7

0.301633
7

0
7

0.00503411
7

0.155954
8

0.0129901
7

0.0176194
7

0
7

0.121631
6

0.0162959
7

0.0124568
1

0

0.226398
7

0.019133
7

0.00755116
7

0.00351532

0.0177514

0
6

0.15531
7

0.0327217

0
7

0
7

0.0430236
7

0.158102
7

0.00987683
6

0.00520121
6

0.0597459
6

0.0655273

0
7

0.127828
7

0.00997863

0.0192781

0.0939759
1

0.0829095
7

2.24794
7

0.0049252

0
6

0.0110946
8

0

0
8

0.0903997
1

0.0765318

0.00503411
6

0.00443786
7

0.00527298
7

0.21869
6

0
7

0.114798

0.0767094
7

0.00755116

0.00815845
7

0.0177514
7

0.0100682

0.11471

0.00527298

0.0561159
7

0.368853
7

0.0458171
7

0
7

0.0171932
7

0.0204714
7

0.019133
6

0.0318883
8

0.0133136
8

0.0900507

0
7

0
6

0.844119
7

0.160045

0

0.0210733
1

0.0234054

0.0382659
7

23.4144
8

0.0363261
5

0
7

0.0170548
6

0.0573989
7

0.0308524

0.446563

0
7

0.00808197
6

0.0363261
7

0.0467116
7

0.0161288
7

0.7079
7

0

0.0200525
7

0.00887572
7

0.0245719

0.381724
7

0
5

0.040764
7

0.00780182
7

0

0.0302047
7

0.00665242
7

0
7

2.01645
7

0.0510354
6

0.0100682
6

0.0730595
7

0.0765318

0
7

0.0127553
7

0.0295354
7

0.0226535
2

0.00543897
6

0
7

0.0179565
6

0.0127553
7

0.00987683
8

0.0691646
7

0.00755116
7

0
7

0.05747
7

0.0151023
7

0.0221893

0
8

0
4

0

0.00847249
7

1.36242
8

0.227977
7

0
7

0.0326338
6

0.0363261
7

0.0151023

0.00780182
6

0.0510212

0.00665242
6

0.0049252
7

0

0.0318883
7

0.306127

0.0455459

0
7

0.0151023
7

0.102042

0.0382659

0.0670987
7

0.0132408
7

0.00780182
7

0.414547
7

0

0.791705

0
7

0.0171932
7

0
6

0.0192134
7

0
7

0.0446436
7

0

0
7

0.0318883
7

0.121175
7

0.101861
6

0.0100682
5

0.0127553
6

0.00755116
6

0
6

0
5

0
7

0.0112966
6

0

0.0353533
7

0.00443786

0.38534
6

0.0156036
2

0.00780182
6

0.0369224
8

0.0456537
7

0
7

0.17747
6

0.00997863
7

0.0309595
7

0.00815845

0.0382659

0.552918
7

0.101438
7

0.019133
6

0.0315653

0
6

0

0.00443786
6

0

0.0385136

0.112137
8

0.00443786
8

0.133938
7

0.165964
7

0.0682144

0

0.0141487
7

0.0340463
6

0
7

0.00503411
6

0.0783126
7

0.00443786
6

0
7

0.0108779
6

0.118008
7

0
8

0.0104024
6

0
7

0.0170592
7

0
7

0.182468
7

0
6

0.0232295

0
6

0
6

0.0424701

0.0626001
1

0
5

0
6

0
7

0.00755116
6

0
6

0

0.0100682

0.0266271
6

0
2

0.166935

0.075041
6

1.71614
8

0.181197

0.00520121
7

0
7

0.133931
7

0.0328917
7

0.012313
7

0.0276876
6

0.012589
7

0.234328
6

0.0255106
7

0.0927598
6

0.0255823
7

0.29108
8

0.0394394
7

0
6

0.0127553
5

0.0765318
7

0.0327217
5

0.00665242
7

0.107497
7

0.0377558
7

0.0477029
7

0.00503411

0
7

0.0226535
7

0
7

0.019111
5

0.0106402
7

0.0305447

0

0.188971
6

0
7

0.0446436

0
6

0
6

0

0.0197008
8

0.019133

0

0.093725
8

0.0255106
7

0.121362
7

0.00543897
7

0
6

0.0251705
6

0
6

0.0151023
6

0.00503411
6

0.0385407
7

0.0562965
7

0.0573989
8

0.140308

0.0717963
4

0
7

0
5

0
7

0

0.0983472
1

0.00780182
6

0.00543897
6

0.0382659

0.00947197
7

0.0103071

1.39112
7

0.12782

0
7

0
6

0.0866
7

0.0193106
6

0.00670609
6

0.0282128
7

0.162218
7

0.0243447

0.00543897
6

0.0256882

0.0684118
7

0

0.00520121
6

0.0901387

1.97517
7

0.0138009
7

0.00503411
7

0.0127553
7

0.00887572
8

0.0251705
7

0
6

0.128196
7

0.0182749
8

0.167253
7

0.0956648
8

0.00755116
6

0
6

0.0211629

0.00808197
6

0.0127553
1

0.0637765
7

0
7

0.128742
7

0.126074
6

0.0764828
7

0.0367118
7

0.0302047
7

0.019133
6

0.0139177

0
6

0.0321674
7

0.0492145
7

0.00351532
8

52.6789
7

0.0500925
8

0.00503411
7

0
7

0.062433
7

0.00755116
6

0
6

0.0360991
8

0
6

0.013003
6

0.071935
1

0.0176805
7

0.0104024

0

0.0104024
7

0.0701542
7

0
6

0
7

0.054365
7

0.0201364
7

0.0227339

0.0127553
7

0.0376388
6

0.0829095
7

0.0127553
7

0
7

0
7

0

0
7

0

0
7

0.00665679
6

0
1

10.2695
7

0.0364085
7

0.00520121
7

0.109434
7

0.0303747
8

0
7

0.0255106

0.00503411
6

0.00815845
7

0.00887572
7

0.024626
7

0.038545
6

0
7

0.00780182
7

0.0462312

0
7

0.199751
7

0.29046
8

0.81809
7

0.0655573
7

0
7

0
7

0.0980216
5

0
7

0.00351532
7

0.0226535
2

0
7

0
6

0.0175484
7

0.0123036
7

0.0829095
6

0.115077
7

0.0490814

631.304
7

0.595681
7

0.0738263
6

0.00963907
7

0.0133136
7

0.0177471
7

1.33629
7

0
6

0.0223536
7

0.0512689
7

0.0761918
7

0.00738779
7

0
6

0.226135
8

0
6

0.00503411
6

0.0276876
7

0.0536588
7

0.210462

0

0.0151023
7

0.00443786
5

0

0.327228
7

0.139989

0.00543897
7

0
7

0
6

0.0318883
8

0.014867
7

0
7

0.0226535
4

0.00670609
7

0.0249352
6

0
7

0.551545
7

0.0201364
7

0.0308814
7

0.00520121
1

0.00655526
7

0
6

0
6

0.019133
7

0
6

0.0151023
7

0.00527298

0.208008
7

0.100529

0
6

0.017456
8

0.0201431
7

0
6

0

0.0253406
7

0.00443786

0.0148109
7

0.00503411
8

0.105393
7

0.0100682

0.0829095
7

0.0806188
7

0

0.0760801

0.125791
8

0.12837

0
6

0

0

0.0614334
7

0.166652
7

0

0
7

0.0125853
7

0
5

0
6

0
7

0
6

0

0.00443786
7

0.108728

0.0133048
7

0

0.272885
7

0

0.0114459

0.0390329
7

0
7

0
6

0.00520121
7

0.0179565
7

0.770376

0
6

0.0218787

0
6

0.00689936
2

0.00665242
7

0.0104731
8

0.019133
6

3.09584
1

0.00527298
6

0.00755116
6

0.0371592
7

0.019133
7

0.0474208

0
8

0.0255106
7

0.0197008
7

0.00738779
6

0.0493376
7

0.0286431

0.102042
7

0.031065

0.935995
7

0.209375

0
7

0.00703064
6

0.0796279

0
7

0.0135974
8

0.0163169
7

0.0352388
7

0
7

0
6

0
7

0.141012

0.0354036

0.00503411

0
6

0.0131105
7

0.0330618
7

0.019133
7

0.00503411
6

0

0
7

0.00887572

0.484727
7

0.0401068
8

0.00755116
7

0.121175
7

0
6

0.0395797
6

0.00755116

0.00755116

0
6

0.0523799
7

0.00443786
7

0.163403
7

0.0446436

0

0
5

0.00520121

0
7

0

0.0956648
7

0.019133
6

0.0203065

0.0236704
7

0.202613
7

0
2

0.0360812
7

0.0176194

0.0576922

0.0127553

0.081733
7

0.072171
7

0.0765318

0.0469906
7

0
7

0.108081
7

0.0127553
7

0.0410248
7

0.0203065
7

0.0446436
1

0.0637765
7

0

0.00780182

0

0.00655526

0
5

0.0821134
7

0
7

0
7

0.0410012
1

0.00351532
6

0.0127553
7

0.0248014
4

0

0.0343863
8

0.0100682
7

0.0255106
7

0.91185
7

0.0252427

0.0347944
7

0
6

0

0.0446436
7

0.047824

0
6

0.0104731
2

0.0108779
6

0.00543897
8

0.105426
7

0.0382659

0.0573989
7

0
6

0.00815845
1

0.00527298

0.0133136
7

0
7

0
7

0

0
8

0.627596

0.0564701
7

0.00443786
7

0.0327213
8

0.124691
7

0.00983289

0.064742
8

0
7

0.0197008

0.0049252

0.00543897
8

1.29581
7

0.317777

0.0201364

0
8

0
7

0.0130748
6

0.0124219
8

0.0140613
8

0
6

0.223126
8

0

0.0780102
6

0

0.173594

0.00447073
7

0

0.0318883

0.00443786
7

0
6

0
7

0.00738779

0
6

0

0.234884
7

0.0553752

0
6

0.0280829
7

0.0135974
7

0.0151023
6

0

0
7

0.27284
7

0
8

0.019322
7

5.11164
7

0.0127553
7

0.0255106

0

0.0104024
7

0.0125963
6

0.019133
6

0
6

0.00665679

0
6

0.0510212
5

0.136414
6

0

0.0110946

0

0.113137

0.0755931
7

0.02821

0.0253406

0
2

0.0099593
1

0

0.0814954
7

0
7

0.0316518
7

0.0164269
6

0

0.0765318
7

0

0.00755116

0.0221893
7

0
6

0.0177514
7

4.02037
7

0

0.00520121

0.0108779

0.0798823

0.0457118
7

0
6

0.00887572
7

0.0864402
7

0.00447073

0.0382659

0.172369

0.00503411

0.0377218
7

0.0318883

0

0
7

0.0916198
7

0.0318883
7

0
6

0.019133
7

0.0175766
6

0.0452381
7

0.0443111
5

0.0266271
8

0.0176194

0

0.00887572

0.0517452
7

0

0.00655526

0.167652

0

0.0486683
7

0
6

0.00815845
7

0
5

0.075467
7

0
6

0.0127553
6

0.0201364
7

0.00887572
7

0.0289172
7

0.0266271
6

1.46488
7

0.155995
7

0.045307
7

0.0637765
7

0.0269348

0

0.01393
7

0.00520121
7

0
5

0.0127553
7

0.0349712
7

0
7

0.106824

0.0304434

0.00364411
8

0

0.0162706
7

0

0
7

0
7

0.00665242
6

0.0382659

0.0104024
7

0.0686051

0.0127553
6

0

0.0127553
7

0.0546653
8

0.123016
7

0.0110946

0.0331085
7

0.00503411

0
7

0.00703064

0.13838

0.0299143
6

0.0116865
7

0
6

0.0255106

0

0

0.0134122
1

0.114798

0

0.0446436
7

0.134397
7

0.00780182

0.0312073
6

0.0183477
8

0
6

0.00364411
6

0.0255106
7

0

0.0260061

0.0218084
8

0
6

1.38699
7

0
1

0.0715316
7

0.00665242
1

0.0510212

0.00443786

0.814354
8

0.0131105
7

0.0125853
7

0
6

0.0404281
7

0.14127
7

0.0996636
7

0.00520121

0.0200744
7

0
6

0.0280829
7

0.371469

0.00503411
7

0
6

0.121863

0
6

0.296361
6

0.0240581
6

0.00755116
4

0.0175766

0.0226535
6

0.00503411
7

0.0682768
6

0.0127553

0.0302047
7

0.040514
7

0.00738779
8

0.15431
6

0.0210919

0.00351532
7

0

0.0104024

0
6

0.0104024
7

0.152646
1

0.0488266
7

0

0.0318883
6

0.412678
6

0.0124219
7

0.00780182

0
6

0.0553752
7

0.00655526
7

2.8674
7

0.00503411
7

0.0382659
7

0.0127553
8

0.0110946
7

1.29346
7

0.194812

0.00503411

0.0324465

0.00703064
6

0.00665679
6

0

0.00815845
6

0.0427899
1

0.127553

0.00503411

0.0135974
7

0.252368
7

0.0241671

0.0148845
6

0.0382659

0
7

0

0.00520121
7

0
8

0.0952224

0.00503411
6

0.00985039

0.454891
7

0.0344987
7

0
6

0

0.00520121
7

0.00447073
7

0.0127553
7

0
6

0
6

0.038742
7

0.00887572

0.386357
7

0.00665679

0

0

0

0.00780182

0

0

0

0.0260061

0

0.0582363
7

0.0158414

0.00443786

0.00655526

0.0201364

0.0324465

0

0

0.0318883

0

0.0829095

0.0856502
7

0.00780182

0

0.0104024

0

0.00665242

0.093855

0

0.0125853

0.0110946

0.00983289

0.053072

0.019133

0.00443786

0.0110946

0.0100682

0

0.00670609

0.0395777

0.0142707

0.00780182

0

0.019133
6

57.4818

0

0.0499165

0.0510212

0

0.0127553

0.0127553

0

0.0286067

0.0311361

0.0562942

0.0135974

0.0105003

0

0

0

0

0.0208048

0.0100682

0

0.0355029

0.304084
7

0.00755116

0.0327353

0

0.00520121

0.0585154

0

0.0424342

0.0100682

0

0

0.954102
7

0.277542
6

0.00520121

0.0127553

0.0125853

0.00815845

0

0.017226

0

0.534993

0.0255106

0

0.119236
7

0.00503411

0

0

0.0127553

0.0318883

0

0

0

0.00738779

0.0087883

0.691284
7

0.00543897

0

0.0100682

0.0513496

0

0

0.0176805

0

0.0640556

0.0108779

0.0816514
7

0

0

0.00503411

0

0.0127553

0.0382659

0.0151023

0.00985039

0.0255077

0

0.179612
7

0

0

0.00887572

0.101025

0.0127553

0.0144586

0

0.0342353

0.00443786

0

0.144746

0

0.0637765

0.0127553

0.0176194

0

0

0

0.0433

0.0123036

0.0182042

0.0318883
7

0

0.0956648

0

0.455587

0.0151023

0

0.0510212

0

0.0321255

0.0131105

0.0720828
7

0.0252639

0.121175

0

0

0.0155325

0.0179565

0.0122397

0.030933

0.00543897

0

0.0499188
7

0.0139098

0.0233343

0

0

0.00351532

0.0127553

0

0.0510212

0

0

0.0510212
6

0

0

0.0598131

0

0.0125853

0.0277391

0.00755116

0.0127553

0.0151023

0

0.592271
7

0.133532
6

1.52282

0.00503411

0.0307118

0.0318883

0

0

0

0.338015

0.011989

0.00520121

0.25544
7

0.0196547

0

0

0.035764

0

0.0288461

0

0.0104024

0.0892871

0

0.411361
7

0

0.0104024

0

0

0.0905051

0

0.00503411

0.164201

0.00503411

0.0110946

0.0433901
7

0

0

0.0213499

0

0

0.0148152

0

0

0.165819

0

0.0127553

0

0

0

0.00665679

0.00520121

0.0255106

0

0.0133136

4.47573

0.010546

0.0321701
7

0

0

0.0966713

0.0124764

0.00520121

0.00443786

0

0

0.0151023

0.00503411

3.5058
7

0

0

0

0.0135974

0.00665679

0.00443786

0.00520121

0.0110946

0

0.0127553

0.227438
7

0.0100682

0

0

0

0

0.00520121

0.0260061

0

0.0200032

0

0.161583

0

0.00443786

0.13033

0.0049252

0.0177894

0

0

0

0.104716

0.0100682

0.474097
7

0.00520121

0

0

0

0.0133136

0.0234054

0.00689936

0.0211154

0.0503411

0.0194121

2.06154
7

1.4216

0

0

0.0158414

0.0281729

0.0560179

0

0.00503411

0

0.0255106

0.0127553

0.065983
7

0.010546

0.0255106

0.0382659

0

0

0

0

0.00665679

0.00755116

0

0.136058
6

0.013003

0

0

0

0

0

0

0.019133

0

0.0236513

0.127119

0.0416097

0

0.0049252

0.0442103

0

0.019133

0

0

0

0.0520247

0.0856983

0.00443786

0

0

0

0

0.0100682

0.00755116

0

0.0276876

0

0.122995

0.0276876

0.0309595

0.0300386

0

0.0176194

0.131991

0

0.010349

0.00780182

0.00703064

0.215062
7

0

0

0.00443786

0

0.0255106

0.00543897

0

0.0235708

0

0

0.129949
7

0

0.0100682

0.0195402

0

0.0194121

0.00780182

0

0

0

0

0.0602597
7

0

0.00546617

0

0

0.00364411

0.0277333

0

0

0.013003

0

0.0610039
6

0.00665242

0.00947197

0

0.0701542

0

0

0.0127553

0.00780182

0.0151023

0.0637765

0.90297
7

0.16355
7

0

0

0.00543897

0

0.0127553

0

0

0.0120958

0

0

0.127213
7

0

0.0104024

0

0

0

0

0

0.00665242

0.0892871

0.0163169

0.0682144
7

0

0.00503411

0.0573989

0

0.0427899

0

0.00520121

0.0155325

0.0155325

0.0310606

0.169187

0.0355029

0

0.0255106

0

0.019133

0

0.00520121

0.00983289

0.32526

0.0318883

0.0613026
7

0.0108779

0.0251705

0

0

0

0.306245

0

0.00755116

0.014208

0

0.261484

0

0.00443786

0

0.0255106

0

0

0.0886535

0.0266841

0

0.0147756

0.254285

0.0177514

0

0.0201364

0

0

0.00351532

0.0125853

0.00503411

0

0

0.0781335
7

0.0336691

0.0670609

0.00520121

0.020045

0.0102353

0.0317182

0

0

0

0

0.114346
7

0

0

0.0510212

0.00728822

0.019133

0.0151023

0

0.00728822

0.00780182

0.0382632

0.0456762
7

0

0

0.0156036

0.0199704

0

0

0

0.0200426

0

0

0.47177
7

0.139247
7

0

0

0.0299143

0

0

0.0318883

0

0

0.0255106

0.0427662

0.0468703
6

0

0.0148403

0.00364411

0.0255106

0.00443786

0.00985039

0.0231577

0

0.0127553

0.00332561

0.0939304
7

0

0.0318883

0.0305447

0.0318883

0.00503411

0.0382659

0

0

0.0513003

0.00780182

0.0883214

0.00716994

0.0829095

0

0.0701542

0.00443786

0.0133136

0

0.0100682

0.013003

0.0221634

0.251133
7

0.0255106

0

0.0100682

0.0112966

0

0.0123036

0.00443786

0.00503411

0.00520121

0.0127553

0.223254

0.019133

0.019133

0.0382659

0.0446436

0.0104024

0.310531

0

0

0.00520121

0.0110946

0.224781
7

0

0.00503411

0

0

0.0087883

0

0

0

0.00755116

0.0798814

0.0335232

0.0960007

0.0104024

0.0182042

0.0510212

0

0

0

0.00983289

0.00503411

0.0127553

0.375664
7

0.0171932

0

0

0

0

0

0.0449227

0

0

0

0
7

0.023881

0.0100682

0.0266271

0

0

0.0148394

0

0.0259306

0

0

43.4258

0.274088
7

0.439129
7

0

0.0255106

0.0155325

0

0

7.97844

0

0.133931

0.0255106

0

0.00755116
7

0.102976

0

0.0255106

0.00543897

0.00443786

0

0.019133

0

0

0.019133

0.0220878
6

0

0

0

0.0108779

0

0

0.108162

0

0

0.0255106

0.0648588
7

0

0

0

0

0.00983289

0.00443786

0

0

0.00738779

0.0127553

0.651365
7

0.00364411

0.00364411

0.00815845

0

0.0127553

0

0.0155325

0.0446436

0.00443786

0.00503411

0.986435
7

0.0125853

0.0780287

0

0.0204706

0

0.00665242

0.0578923

0

0

0

0.0525322
7

0

0

0

0.00447073

0.498023

0.00503411

0

0.0573989

0

0.00443786

0.093068
7

0.081544

0

0

0.019133

0

0

0

0

0.0510212

0.00503411

0.0547492

0.0255106

0.0318883

0

0

0

0.0765318

0

0

0

0.0670661

0.0426118
7

0.0240581

0.0127553

0

0.00815845

0

0.127553

0.00543897

0

0

0

1.05161
7

0.0273925
7

0

0

0.0370895

0

0.0049252

0.0127553

0

0

0.0276876

0.0446436

1.00171
6

0

0

0.00985039

0

0

0

0

0.00703064

0.0715783

0

0.0179913
7

0

0.0156036

0.00983289

0

0.0127553

0

0

0

0.00443786

0

0.237601
7

0.0338079

0

0

0.00755116

0.0318883

0

0

0.0222604

0

0

0.270608
7

0

0

0.0573989

0.00503411

0

0.00780182

0.121175

0.0296084

0

0

0.0640043
7

0.00738779

0

0.482845

0

0.00527298

0

0

0

0.0125853

0.00527298

0.0266701
7

0

0

0.0156036

0

0.0151023

0.00985039

0.0100682

0.00520121

0.0163169

0.0127553

0.207436
7

0

0

0

0

0

0

0

0

0.0380959

0

0.274154
7

0

0.0701542

0.0244082

0

0.0261636

0

0

0

0

0.0690518

0.26572
7

0

0.00503411

0

0

0

0

0.0573989

0

0.165819

0

2.19664

0.0354854

0

0

0

0.00665679

0.0318883

0

0

0

0

0.00527298

0
7

0

0

0

0.00665679

0.0176194

0

0

0

0

0

0.0511019
7

0

0

0

0.0135974

0

0.0280087

0

0

0

0.0452018

0.243317
7

0

0

0.00887572

0.00815845

0.00503411

0.0127553

0.525872

0.00665679

0.0127553

0

0.125613

0.00503411

0

0

0

0

0.0151023

0

0.0155325

0.0103642

0

0.160886

0

0.00755116

0

0.00755116

0

0.0127553

0

0.0133136

0

0

0.106869
7

0

0

0

0

0.103113

0.00443786

0.0100682

0

0.0127553

0

0.0830072
7

0.0151023

0

0.00543897

0

0

0.223218

0

0.019133

0.019133

0.00443786

0.312135
4

0.00520121

0

0

0.0241671

0

0

0

0.00520121

0

0

0.0303571
7

0

0

0.019133

0

0.0180242

0

0

0

0

0

0.7647
6

0.0769327
7

0

0.0829095

0.00543897

0

0

0.0510212

0.0151023

0.00983289

0

0.0704775

0.0882339
5

0

0.0125853

0

0

0.0765318

0.0127553

0

0.00665679

0.00543897

0.00443786

0.0691753
7

0

0

0.00443786

0

0.160399

0.00520121

0.0104024

0

0.0344366

0.0390091

0.179345
7

0.00847249

0

0.0637765

0.00543897

0.0486844

0.00503411

0

0.183608

0

0

0.220303
7

0

0

0.0312073

0

0.0133136

0

0.0437049

0.0573989

0.00443786

0.0318883

0.0424753

0.00351532

0

0

0

0

0.0225882

0

0

0.031065

0

0.0880497
7

0

0

0

0

0.00665679

0

0.0155325

0.0586689

0

0.019133

0.142031
7

0.106625

0

0

0

0.0127553

0.0127553

0

0

0.00985039

0

0.0170989
7

0

0.0318883

0

0

0.0203442

0

0

0.0382659

0

0

0.0638288
7

0

0.025839

0.00351532

0

0

0.00443786

0

0.00351532

0

0

0.904603

0.0935932
7

0.0956648

0

0

0

0.00503411

0.0100682

0

0.00703064

0

0.0155325

0.0358865
7

0

0.00887572

0

0

0

0.0676157

0

0

0.0172382

0.00665242

0.162746
7

0.0161288

0

0.00520121

0.00815845

0.0446436

0.0158189

0

0.00780182

0

0

0.234349
6

0.00447073

0.00665679

0.00887572

0.00543897

0.0110946

0

0

0.0125853

0.0317182

0.0049252

34.6135
7

0.0133048

0

0.0312073

0.0333124

0

0

0

0

0

0.0255106

0.067484
6

0.00703064

0.0254093

0

0

0.00364411

0.0347366

0

0.0127553

0.00443786

0

0.506136
7

0

0

0.0244082

0

0

0

0

0.0049252

0

0

0.286533
7

0.0573989

0

0.013003

0.00543897

0

0

0

0

0.00351532

0

0.0130748
7

0.0244754

0

0.0629264

0

0

0.0129901

0

0.00947197

0.0538505

0.00447073

0.138435
7

0

0.0226535

0.00755116

0.00755116

0.00364411

0.019133

0

0

0

0.0468206

0.916834
7

0.151796

0

0.00527298

0.00351532

0.0255106

0.0770996

0.0355788

0

0.0127553

0

0

0.1858
7

0

0.0182042

0.0365817

0

0

0.0318883

0

0.00520121

0.00520121

0

0.110958
5

0

0.0573989

0.0110946

0

0.00543897

0

29.8793

0

0

0

0.0230584
6

0.00983289

0.00443786

0

0.0883575

0.0110946

0.67127

0

0

0.0127553

0

0.0612273
7

0

0.0536591

0

0

0.00887572

0.013003

0

0

0.0488164

0.0127553

0.0388242
7

0.019133

0.0087883

0.00443786

0.019133

0.00887572

0

0.0226535

0

0.00543897

0.0174016

0.0177894
7

0.0110946

0.00997863

0

0

0.309749

0

0

0.154237

0

0.0197691

0.0445791

0

0

0

0

0.00543897

0

0

0.0206981

0

0.0127553

0.46595
7

0.103717

0

0.0049252

0.00543897

0

0

0.0156036

0

0.00351532

0

0.0961849

0.00447073

0.0131105

0.00527298

0

0.019133

0

0.00503411

0

0.0503411

0

1.32399
7

0.012391
7

0.00351532

0.0176194

0.00755116

0.019133

0

0

0

0.00780182

0.0087883

0.0446436

0.136855
7

0.00447073

0

0

0.00503411

0

0

0.00780182

0

0

0.0125853

0.113455
2

0

0.0211615

0

0

0

0

0

0

0.0228496

0.112858

0.0382659

0

0

0.449905

0

0

0.0396901

0

0

0.019133

0

0.119242
7

0

0

0

0

0

0

0.0261827

0

0.0110946

0.0127553

0.215692
7

0.00443786

0.0127553

0.0108779

0

0.00332561

0

0

0.0382659

0

0.280617

0.0327263
7

0

0

0.0049252

0

0.0318883

0.0140769

0

0.113454

0.00520121

0

0.0484597
7

0

0

0

0

0.0135974

0.0318883

0.0713044

0.0127553

0.0185148

0.0111768

0.0624215

0.019133

0

0.0127553

0

0

0.019133

0

0

0

0

0.67981

4.68489

0.0127553

0.00815845

0

0.0125853

0.215922

0.0382659

0

0

0.013003

0.424238
7

0.0226321
7

0

0

0.00887572

0.0452018

0

0

0

0

0.00520121

0.0523227

0.18949
7

0

0

0.00520121

0.0446436

0

0

0.0156036

0.00503411

0

0

0.0339039

0.0125853

0.0382659

0.00728822

0

0.0318883

0

0

0.0595204

0.019133

0

0.211498
7

0

0

0.00887572

0.0276876

0

0

0.0829095

0.0255106

0.0260061

0.00520121

0.0826904
7

0.0612565

0.0255106

0.0382659

0

0

0

0

0

0

0

0.250957
7

0

0

0.00443786

0

0.0127553

0

0

0

0

0.00755116

0.0750875
5

0

0.0049252

0.00443786

0

0.589277

0.0255106

0.0255106

0.00520121

0

0

0.0754674
6

0.131088

0.00738779

0.0416097

0.0133136

0

0

0.00447073

0

0.0165072

0.0100682

0.0259099
7

0.00351532

0.00665679

0

0

0.00887572

0

0

0

0.00503411

0

0.0705119
7

0

0

0

0

0.00503411

0.00755116

0.019133

0

0

0

1.21245
7

0.113693
6

0.0163169

0

0

0.0553752

0

0

0

0

0

0

0.061053
6

0.00738779

0

0

0

0.00351532

0.0127553

0.0127553

0

0

0

0.0444735
7

0

0

0

0.00443786

0

0

0

0

0

0.00351532

0.018759
7

0

0.00503411

0

0

0

0.0637765

0.0127553

0.00443786

0

0

0.131764

0.00365461

0.0255106

0.00527298

0

0.0578923

0

0.0087883

0

0

0

0

0.0701542

0

0

0

0

0

0.0049252

0.0573989

0

0.00665679

0.0863924
6

0

0

0.0151023

0.0127553

0

0

0

0

0

0.00520121

0.113411
7

0.0100682

0

0

0

0

0

0

0

0.0327217

0

0.0311361
7

0.0446436

0

0

0

0.0255106

0

0.0382659

0

0

0

0.0888356

0.0199704

0.00755116

0.00503411

0

0

0

0.00543897

0.00665679

0

0.00780182

1.44337
7

0.13507
7

0

0.0732203

0.00443786

0

0.00755116

0

0

0

0

0

0.115173
7

0.0127553

0

0

0

0

0.0049252

0.0257897

0.0201364

0

0.00665679

0.391542

0.019133

0

0

0.00503411

0

0

0

0

0

0

0.117251
7

0.0127553

0

0

0.00543897

0

0

0

0

0

0.00503411

0.0575886

0

0.0765318

0.0386685

0.0127553

0

0.00985039

0

0

0.0151023

0

0.0453187
7

0

0.00503411

0.0573989

0

0

0

0.00655526

0

0

0.0251705

0.0892871

0

0

0

0

0

0

0

0

0

0

0.0785785
8

0

0

0

0.00503411

0

0

0

0

0.00351532

0

0.225928
7

0

0

0.0402729

0.00503411

0

0

0

0

0.0127553

0

0.0934374
6

0.0104024

0.00527298

0

0

0

0

0

0

0

0.0127553

80.2887

1.64348

0.0261408
7

0

0.0255106

0.00351532

0

0

0

0

0

0

0.0624145

0.0968383
7

0

0

0

0.00887572

0.452813

0

0

0

0

0

0.0190364

0

0

0.00443786

0

0.0127553

0.0182042

0

0.0104024

0

0

0.0535987

0

0

0.00520121

0.019133

0

0

0

0

0.00520121

0

0.057678

0

0

0

0

0

0

0

0.0127553

0

0.00755116

0.0349665
6

0

0

0

0.0338079

0.0763814

0

0

0

0

0.0460677

0.129011
7

0.00364411

0

0.0104024

0

0

0.0302047

0.0322575

0.0302047

0

0

0.306806

0

0.0269348

0.0276876

0.0127553

0

0

0

0.0956648

0

0

0.0719415
7

0

0

0.00780182

0

0.00443786

0

0

0

0.0318883

0

0.0679221

0

0

0

0

0.0232383

0

0.0318883

0.161238

0

0

1.11324
7

0.210122
6

0

0.019133

0

0

0.128727

0

0.00520121

0.0491903

0.0182042

0

0.0295512
7

0

0

0

0

0

0

0.019133

0.00503411

0

0

0.048983

0.00665679

0.019133

0

0

0

0

0.00665679

0.0257897

0.019133

0

0.0584094
7

0.0573989

0

0

0.0251926

0.00738779

0

0

0.0110946

0.0206981

0.00543897

0.0497669
7

0.00967194

0.00755116

0.0125853

0

0.0266271

0

0.00443786

0

0

0

0.0545324
7

0

0

0.0199704

0.0147756

0.0049252

0.00887572

0

0

0

0

0.157804
7

0.127832

0

0

0

0

0

0.0255106

0

0

0

0.0576736
6

0.0382659

0.00503411

0

0

0.00527298

0.00520121

0

0

0.00703064

0

0.273194
7

0

0

0.0654434

0

0.0163169

0

0

0.0108779

0

0.00520121

0.136278
6

0.0354894

0

0

0

0

0

0

0.00443786

0

0

1.59534

0.206153

0

0.0177514

0

0.0104024

0

0

0

0.0155325

0.0133136

0

0.139493
7

0.0442103

0

0.00665679

0

0.00689936

0.00443786

0.00364411

1.49237

0.0203065

0

0.00665242

0

0

0

0

0.0127553

0

0

0

0

0

0.038622
7

0

0

0.0127553

0

0

0

0

0

0.0254341

0

0.0297101

0

0

0

0

0.0255106

0

0

0

0.00503411

0.0489507

0.800499
7

0

0

0

0

0

0

0

0.0765318

0

0.00755116

0
7

0

0

0

0

0

0.0109323

0

0.0163169

0

0

0.0377218

0.00527298

0

0

0.00443786

0

0.00520121

0

0

0

0

0.0958842
6

0.019133

0

0

0.019133

0.0102353

0.274239

0

0

0

0.00443786

0.0946496
7

0.013003

0.0510212

0

0.0172382

0

0.0100682

0.00520121

0.00738779

0

0

1.3932
7

0.0637765
7

0.0510212

0

0

0

0.00443786

0

0

0

0

0.00447073

0.0447691
7

0

0

0.0318883

0.0255106

0.0884206

0.264291

0.0892871

0

0

0

0.173055

0

0

0

0

0

0

0.0127553

0

0

0

0.148923
1

0.0127553

0

0

0

0.0182042

0

0

0.0255106

0

0

0.0631524

0

0.0127524

0

0

0

0.019133

0.00447073

0

0.0104024

0

0.0359778
7

0

0.00780182

0

0.121175

0

0

0

0.00689936

0

0

0.0895914
7

0

0

0

0

0

0

0

0

0.00503411

0.0510212

0.0226535

0.00997863

0

0

0

0

0

0

0

0

0

0.104665
7

0

0

0.00738779

0

0

0

0.0127553

0.00689936

0.00351532

0

0.0534692
7

0.0127553

0.0318883

0

0

0.0127553

0.0208048

0

0.00520121

0

0

0.971345
7

0.135044
7

0

0.0226535

0

0

0.0255106

0.0619191

0.0446436

0

0

0

0.0717308
7

0

0

0

0.013003

0.00755116

0.0210919

0.0049252

0

0.00503411

0.00985039

0.203236
7

0

0

0

0

0

0.00815845

0.00780182

0

0

0.00755116

0.109163
7

0.0127553

0.0318883

0.019133

0

0

0.00665679

0

0.0382659

0

0.0147756

0.0255106
7

0

0

0.00527298

0.0234054

0

0.0125853

0

0.00443786

0.153064

0

0.0503892
6

0

0.0510212

0

0.00520121

0

0.0110946

0

0

0

0

0.096072
6

0.00983289

0.0318883

0.0049252

0.0645197

0.0127553

0.0228235

0

0

0.0176194

0

0.201216

0

0.0498448

0

0.00738779

0.0302047

0

0

0

0.0127553

0.00447073

0.143449

0

0

0

0

0

0

0.0255106

0.121175

0.0104024

0

0.0898075
7

0

0

0

0.00447073

0.0532194

0

0

0

0

0

0.451557
7

0.0721913
7

0

0.0179086

0

0.0255106

0

0.0382659

0

0.00443786

0.00503411

0

0.0398412
7

0.133931

0

0.00364411

0.0510212

0

0.0754122

0.00520121

0

0.0276876

0.0201364

0.139267
7

0.0892871

0

0.0318883

0.204085

0

0

0

0

0

0

0.490557
7

0.00520121

0.00887572

0

0

0

0.0829095

0.00655526

0.00503411

0

0

0.0307102

0.0553752

0.00755116

0

0

0

0.102042

0.121175

0

0

0

0.108179
7

0

0.00443786

0

0

0

0.0156036

0

0

0

0

0.171854

0.0637765

0

0

0.0127553

0

0.0177514

0.00443786

0.0127553

0

0

0.35669
7

0

0

0

0.0338079

0.019133

0.127553

0.0127553

0.00520121

0

0

0.141465
7

0

0

0

0

0

0.0295512

0.0127553

0.0446436

0

0.010349

0.12602

0

0

0.0087883

0.00520121

0

0.0218787

0

0

0

0.0510212

1.19
7

0.149346
7

0

0.019133

0.0127553

0

0

0

0

0

0

0

0.231307

0.0049252

0

0.00689936

0

0.0255106

0

0

0

0

0

0.120629
7

0.0318883

0

0.00520121

0

0.0125853

0

0.0302047

0

0

0.0637765

0.0352775
7

0.133931

0

0

0

0.00543897

0.00755116

0.0049252

0

0.0320138

0.0104024

0.0100682
7

0.0255106

0

0

0

0

0.0276876

0.00443786

0

0

0

0.138067
7

0.00894146

0.0255106

0

0

0

0.0127553

0

0.00755116

0.0160198

0

0.0609194

0

0

0

0

0.0127553

0.0176194

0.00520121

0

0

0

0.150738

0

0.0127553

0.0446436

0

0.00443786

0.00503411

0

0

0

0

0.0268322
6

0

0

0

0

0

0.133931

0

0

0

0

0.110083
7

0

0

0

0

0

0

0.0255106

0

0

0

2.73448

0.153064

0

0.0156036

0.0255106

0

0

0.147586

0.0488164

0

0

0

0.0486322

0.00780182

0.00983289

0

0

0

0.0127553

0.0346655

0.0829095

0

0.0257897

0
7

0

0

0.0255106

0

0

0

0

0

0

0

0.166261
7

0

0

0

0.0353533

0

0

0

0

0

0

0.0883484
7

0.019133

0

0

0

0

0

0.0382659

0.00520121

0

0.0155325

0.0104024

0

0

0

0.0637765

0.0347366

0

0

0

0

0.0104024

0.0861415
7

0

0.0337367

0

0.0127553

0

0

0

0.00520121

0.0318883

0

0.0886855
7

0.00755116

0.011582

0

0.00665679

0

0

0.00780182

0.10842

2.06409

0.00520121

0.149976

0

0

0

0

0

0.0125853

0

0

0

0.019133

0.0543976

0

0

0

0

0.0127553

0

0

0.0780182

0

0

0.599026
7

0.085909
7

0

0

0.00520121

0

0.0573989

0

0

0.00503411

0

0

0.0495532
7

0

0

0

0

0

0.0127553

0.0544875

0

0.0871792

0.0349446

0.262623
7

0.0255106

0

0.019133

0

0

0

0.00351532

0

0

0.00665679

0.0872635

0

0.0639246

0

0.00520121

0

0

0

0

0

0

0.00364411

0

0

0

0.0127553

0.00665679

0.0049252

0.0087883

0

0

0

0.390327
7

0.0176194

0

0

0

0

0.0382659

0

0

0

0.0255106

0.0943263

0

0.0255106

0

0

0.0151023

0

0

0

0.0176194

0

0.0890729
5

0

0

0

0

0

0

0

0

0

0

0.122822
7

0

0

0

0

0.00985039

0

0

0

0.0177894

0.00503411

0.00780182
7

0

0.00703064

0

0.00527298

0

0

0.00443786

0.00665679

0

0

0.62169
7

0.0128359
7

0.00351532

0

0

0.00655526

0

0.0049252

0

0

0.00520121

0

0.128026
7

0.0251705

0

0

0

0.00665679

0

0

0.00443786

0.00527298

0

0.0679119
7

0

0

0.643363

0

0.21684

0.00520121

0

0

0

0.0226535

0.0777952
7

0

0

0

0

0

0

0

0

0.0127553

0.00755116

0.00351532
6

0

0.0182042

0

0

0

0.0226535

0

0

0.0687868

0

0.0489716
7

0

0

0

0

0

0

0

0

0.0127553

0

0.134548
7

0

0

0

0.00443786

0.0100682

0.00503411

0

0

0

0.0134122

0.00443786
6

0

0

0

0

0

0

0

0

0

0

0.120247

0.019133

0.0133048

0

0

0

0

0.019133

0.00503411

0

0

0.386172
7

0.0049252

0

0.0127553

0

0

0.019133

0

0.00738779

0

0.0516702

52.1513

0.488565
7

0.0613166
7

0.0087883

0.0108779

0

0.0049252

0.0110946

0.047824

0

0.0302047

0

0

0.134172
7

0

0

0.0100682

0

0

0

0

0.00351532

0

0.400163

0.114781
8

0

0

0

0

0.0364085

0.0253406

0.0260061

0.0573989

0

0

0.00755116
6

0

0

0

0

0

0.00447073

0

0

0

0

0.0616464
6

0.0209138

0

0.0734261

0

0

0

0

0.00780182

0

0.0125853

0.0953247
7

0.00665679

0

0

0.0637765

0

0

0.0892871

0

0

0

0.134424

0.00887572

0.00503411

0

0

0

0.0318883

0

0.00503411

0

0

0.105802

0

0

0.00947197

0

0

0.019133

0

0.0151023

0

0

0.101683
7

0.0701542

0.10842

0.0176194

0

0

0

0

0

0

0

0.914034
7

0

0.0573989

0

0

0

0

0

0

0

0.00780182

0.960115
7

0.0355029
7

0

0

0

0

0

0.0100682

0

0

0.0176805

0

0.0231489
7

0

0

0

0

0

0

0

0

0

0

0.0343863
7

0.0151023

0

0

0

0

0

0.162858

0

0

0.00689936

0.0648334

0

0.0203065

0

0

0

0.0177514

0

0.10842

0.00755116

0

0.0377558
7

0

0

0.0318883

0.121175

0.00780182

0

0.00913654

0

0

0

0.03221
7

0

0

0

0

0

0

0

0

0

0.00665679

0.163151

0.0156036

0

0.019133

0

0

0

0.0255106

0

0

0

0

0.0352388

0.0226535

0.00983289

0

0

0

0

0.00503411

0

0

0

0

0

0.0637765

0.0108779

0

0

0.0765318

0

0

0

0.00997863
6

0.0573989

0

0.0327217

0.0163169

0

0.00755116

0

0

0

0

2.17237

0.00527298
7

0.0252433

0.00665679

0

0

0.0127553

0

0

0

0

0.0100682

0.0537643
7

0.00443786

0.0049252

0

0.0125853

0

0

0

0.0226535

0.0156475

0

0.00815845
7

0

0.019133

0

0.00364411

0

0

0.00780182

0

0

0.0177514

0.0637765
7

0.0110946

0.0125853

0

0

0

0.00871653

0

0.00520121

0

0

0.023978
7

0

0.0201183

0

0

0

0

0

0

0

0

0.195238

0

0

0

0.0049252

0

0

1.44949

0

0.0637765

0.012313

0.195353
7

0

0.0127553

0

0.00815845

0.0208048

0.00780182

0

0

0

0.0179565

0

0

0

0

0.0201364

0

0

0.00520121

0

0.0318883

0

0.0226535
7

0

0.56382

0

0

0.00887572

0.00520121

0

0

0

0

0.0487174
7

0

0.0049252

0

0

0.0959439

0

0

0

0

0

2.55006

0.059119

0

0

0

0

0.00755116

0

0

0

0.0312073

0.0206981

0.0102353

0

0

0

0.175098

0.0226535

0.00665679

0.0201364

0

0

0

0.0557959
7

0.0446436

0.00520121

0.0108779

0

0

0.00703064

0.0104024

0

0.0127553

0.0111768

0.0200919
7

0

0.00351532

0

0

0.0127553

0

0

0.00543897

0

0

6.75879
7

0.00755116

0

0.00780182

0.00780182

0

0.165585

0

0

0

0

0.0393348

0

0

0

0.0382659

0

0

0

0

0

0

0.0281935

0.00503411

0

0

0.0127553

0.0104024

0.00443786

0.013003

0.00780182

0

0.00755116

0.0384514
7

0

0

0

0.0133048

0.0382659

0

0

0

0

0

0.120325

0

0

0

0

0.0382659

0

0

0

0

0.00443786

0.0612271
7

0

0

0

0

0

0

0

0

0

0

0.640972
7

0.0882183
7

0

0

0.0456992

0.00755116

0.0353533

0

0

0.00351532

0

0

0.0822277
7

0

0

0.00520121

0

0.00443786

0

0

0

0

0

0.138455
6

0

0

0

0.00738779

0

0

0

0.00665679

0

0

0.0663274
7

0.00351532

0

0

0.00894146

0

0

0

0

0

0.0049252

0.440058
7

0.00543897

0

0

0.00443786

0

0

0

0

0

0

0.0190364
6

0

0

0

0

0

0.00443786

0

0

0

0

0.0701542
6

0

0

0

0

0

0

0.0829095

0

0.0156036

0

0.00887572
5

0

0

0

0

0

0

0

0

0

0.019133

0.216547
7

0

0

0

0.0276876

0

0.0127553

0

0

0.0127553

0

0.0766254
7

0

0.0156036

0

0

0.00351532

0

0

0

0

0

0.594752
7

0.00670609
7

0.00503411

0

0

0

0

0

0

0

0.00503411

0.00665242

0.0508293
7

0

0.0637765

0

0

0

0

0.019133

0

0

0.00503411

0.0155281
7

0

0

0

0

0

0

0.00665679

0

0

0

0.0363474
8

0

0

0

0

0

0

0

0

0

0

0.0469906
7

0.0151023

0

0

0

0

0

0.00887572

0

0

0.00351532

0.170622
7

0

0

0.019133

0

0.0127553

0

0

0

0

0

0.045307
7

0

0

0

0

0

0

0

0

0

0

0.0477111
2

0

0

0.00665242

0

0

0

0

0.0299143

0

0

0.0323286

0

0

0

0

0

0

0

0

0.00780182

0

0.265744
7

0.159441

0

0

0

0.00815845

0.0127553

0

0

0

0

0.665154
7

0.28022
7

0

0

0

0

0

0

0

0

0

0.905626

0.135581

0

0

0.0182042

0

0

0.0416097

0

0

0

0

2.33049
7

0

0

0

0

0

0

0

0.00503411

0

0.00503411

0.0467285
7

0.0255106

0.0201364

0

0

0

0

0

0

0

0

0.11246
7

0

0.0468109

0.0100682

0.0049252

0

0

0.019133

0

0.0255106

0

0.15437
6

0

0

0

0

0

0

0

0

0

0

0.0601202
7

0

0.0049252

0

0

0

0

0

0

0.0127553

0.0190364

0.00543897

0

0

0.00755116

0.0147756

0

0

0

0

0

0

0.0624772
7

0

0

0.0255106

0.0163169

0.00351532

0

0

0.00755116

0

0

0.133931
6

0

0

0

0

0

0

0

0

0

0.0109638

0.384459
7

0.00887572
7

0

0.00527298

0

0

0

0

0

0

0

0

0.0343311
7

0

0

0.00443786

0

0

0.00332561

0.00689936

0

0

0.00815845

0.157088
6

0

0.0127553

0

0

0

0

0.0318883

0.0049252

0

0.047824

0.0386211

0.0604093

0

0

0

0

0

0

0

0

0

0.133892
6

0

0.0133136

0

0

0.0573989

0

0

0

0

0

7.50386
6

0

0

0

0

0

0

0

0

0

0.019133

0.0454857
7

0

0.0127553

0

0

0

0

0

0

0

0

0.0987667
8

0

0

0

0

0

0

0

0

0.0087883

0

0.0296806
7

0

0

0

0

0

0

0

0.0276876

0

0.0125853

0.117978

0

0.0156036

0

0

0

0.00738779

0.0377558

0

0

0

0.26317

0.0443035
7

0

0

0

0

0

0

0.0104024

0

0

0

0.0903118

0

0

0

0

0

0

0

0

0.0255106

0

0.108349

0.0510212

0

0

0

0.00503411

0.0566397

0

0

0

0

0.0483018
6

0

0

0

0

0

0

0

0

0

0

0.00780182
6

0

0

0

0

0.0049252

0

0

0

0

0.00443786

0.00780182
6

0.0318883

0

0

0.248377

0

0

0

0

0

0

0.13051
6

0

0

0

0

0

0

0

0

0

0

0.0592445

0

0

0.0286067

0

0.10842

0

0

0

0.00443786

0

0.239297
8

0.0255106

0

0

0

0

0

0.0125853

0

0.00665679

0

0.112849
7

0

0.0637765

0.00887572

0.031065

0

0

0

0.00503411

0

0

1.33778

0.0337429
6

0

0.00887572

0

0

0

0

0

0

0

0

0.0263166
7

0

0.0765318

0

0

0

0

0

0

0

0

0.45588
7

0

0.0443268

0

0

0.0127553

0

0.0251705

0

0

0

0.0152694
6

0

0

0

0

0

0

0.00703064

0

0

0

0.0275697
7

0

0

0

0

0

0

0

0.00665679

0

0

0.129063
7

0

0.0255106

0

0

0

0.00443786

0

0

0

0

0.0618991
7

0

0

0

0

0

0

0

0

0

0

0.0856737
7

0

0

0

0

0

0

0.0100682

0

0.0127553

0

0.0473687

0

0

0

0

0

0

0

0

0

0

0.0103071
6

0

0

0

0

0

0

0

0

0.0446436

0

27.4557
7

0.369559

0.143112
7

0

0

0

0

0

0

0

0

0

0

0.085426
7

0

0

0

0

0

0

0

0.0352388

0

0.00543897

0.0101721
6

0

0

0.0298802

0.0127553

0

0

0

0.0469426

0

0

0.0145818

0.0701542

0.00503411

0.0377558

0

0

0

0.0127553

0

0

0.0127553

0.057678
6

0

0

0

0

0.0318883

0

0

0

0

0

0
7

0.0125853

0

0

0.0255106

0

0.0049252

0

0.0446436

0

0

0.178959
7

0.0104024

0

0

0

0

0

0.0127553

0

0

0.0104024

0.0356087
8

0

0

0

2.58933

0

0.0110946

0

0

0

0

0
7

0

0

0

0

0

0.961275

0

0

0

0

0.025839
7

0

0

0

0

0

0

0

0

0

0

0.875599

0.0860369
5

0

0

0.0163881

0

0

0

0

0

0

0

0.0819425
7

0.00443786

0

0

0

0

0

0

0

0

0

0.011989
7

0

0

0

0

0

0

0

0

0.0100682

0.00503411

0.195676
7

0

0

0

0

0.0451866

0

0.00665679

0

0

0.0127553

0.0201364
7

0.00503411

0.0197008

0.121175

0

0

0

0

0

0.0151023

0.00665242

0.107318
6

0

0.031065

0.00351532

0

0

0

0

0

0

0

0.00887572

0

0

0

0

0

0.00520121

0

0

0

0

0.0634365
7

0

0

0.00503411

0.00498842

0

0.00503411

0

0

0

0

0.154289
7

0

0

0

0.00887572

0.00503411

0

0

0

0

0

0.011858

0.00755116

0

0

0.00503411

0

0.0127553

0.0701542

0

0

0

0.305124
7

0.0582971
6

0

0

0

0.0151023

0.102042

0

0

0

0.0390091

0

0.0758327
7

0

0

0

0

0

0.0127553

0

0

0.561233

0

0
7

0

0

0

0.0352388

0

0

0

0

0

0.0276876

0.0201364
7

0

0

0

0

0.00503411

0

0

0

0.00527298

0

0.019133
7

0

0

0

0.00665679

0

0

0

0

0

0

0.00665679
7

0.00780182

0

0

0

0

0

0

0

0

0

0.0199573
7

0.0151023

0.00665679

0.00365461

0

0

0

0.00780182

0

0

0

0.0133048
7

0

0

0

0.00520121

0

0

0

0

0.0465975

0

0.0795786

0

0

0

0

0

0

0.0255106

0

0.00503411

0

0.0829095

0.0104024

0.00665679

0

0

0

0.0133048

0

0

0

0

0.49926
7

0.0327264
6

0.0255106

0

0

0

0

0

0

0

0.0327217

0

0.160423
8

0

0.00520121

0

0

0

0

0

0

0

0.00520121

0.0103071

0

0.0318883

0.0163881

0.146353

0

0.0127553

0

0

0

0

0.0235708
6

0

0

0

0

0

0

0

0

0

0

0.0458918
6

0

0

0

0

0.0829095

0

0

0

0

0.00364411

0.0508582

0.0176194

0

0

0

0

0.00443786

0

0

0

0.00503411

0

0

0.00543897

0

0

0

0.00443786

0

0

0

0

0.0388808
7

0

0

0

0

0

0.0127553

0

0

0

0

0.010546
7

0

0

0.0221893

0

0.0255106

0.00520121

0.00665679

0

0

0.0318883

0.0505695
6

0

0.0087883

0

0

0.361685

0

0

0

0

0

1.84164

0.0427899
7

0

0

0

0

0

0

0

0.121175

0

0

0.0222273

0.0382659

0

0.121175

0

0

0.00815845

0

0

0

0.0251705

0.0282004
7

0

0.0318883

0

0

0

0

0

0

0

0

0.0637765
7

0

0

0

0

0

0

0

0.00503411

0

0

0.149874
7

0

0.0127553

0.0382659

0

0

0

0.0049252

0

0

0

0.0115894
7

0

0

0

0

0

0.00665679

0.0127553

0.00780182

0

0

0.091916
7

0

0

0

0.0344764

0

0

0

0

0

0

0.218535
7

0

0

0

0.0255106

0

0

0

0

0

0

0.00887572
7

0

0.00887572

0

0

0.00443786

0

0.0199704

0

0.0110946

0.013003

0.00755116
7

0

0

0

0

0.0312073

0

0.00351532

0

0.0353533

0.00503411

0.858512
7

0.0443256
7

0

0.0127553

0

0

0

0

0

0.0892871

0.0127553

0.0598139

0.0958869
7

0

0

0

0

0

0.00815845

0

0

0

0.00443786

0
6

0

0

0

0

0

0

0

0

0

0.0351532

0.118485

0

0

0

0.0100682

0.0382659

0

0

0.0100682

0

0

0.0201364
7

0.0145764

0.0127553

0

0

0

0

0

0

0

0

0.0127553
7

0

0

0.0446436

0

0

0.00503411

0

0

0

0

0.0104024

0

0.00351532

0.0127553

0

0

0

0.0382659

0

0

0

0.0135974
7

0

0.0199704

0

0

0.0226535

0

0

0

0

0

0.04296
7

0

0.00443786

0.0226535

0

0

0

0

0

0

0

0.0161288

0

0

0

0

0

0

0

0

0

0

0.48997
7

0.0985131
7

0

0

0

0

0

0

0

0

0

0

0.0909112
7

0

0

0

0

0

0

0.00755116

0

0.019133

0

0.011989
7

0.00738779

0

0.0155325

0

0

0

0

0

0

0

0.093309
1

0

0

0.402729

0

0

0

0

0

0

0

0.0365633
6

0.0087883

0.00738779

0

0

0.0182042

0

0

0.00815845

0.00364411

0

0.0442845
7

0

0

0

0

0

0

0

0

0.00498842

0

0.0253406
6

0

0

0

0

0

0.0147756

0

0

0.153064

0

0.0606634
7

0

0.0312073

0

0

0

0

0.0100682

0

0

0

0.109742
6

0

0.0206981

0

0

0

0

0

0

0

0

0.160686
7

0

0

0

0

0.0127553

0

0.0110946

0

0

0.0503411

0.831155
7

0.0251705
7

0

0

0

0

0

0

0

0

0.019133

0

0.056724

0

0

0

0

0

0

0

0

0.00503411

0

0.325654
7

0

0

0

0

0

0

0

0.00655526

0.00503411

0.00665242

0.0360566
7

0.045307

0

0

0.0382659

0.00520121

0

0

0

0.0127553

0

0.0535353
7

0

0

0

0

0.0446436

0

0.0155325

0

0

0

0.0646139
7

0

0

0

0

0

0

0

0

0.00665679

0

0.0536966
6

0

0

0

0.0133136

0

0

0.0125853

0

0

0

0.101036
7

0

0

0

0

0.00655526

0.019133

0

0

0

0

0.0176194
7

0.0123036

0

0

0

0

0

0

0

0.00546617

0

0.0140613

0

0

0

0

0

0.019133

0

0

0

0

0.35129
7

0.041815
6

0

0

0.0133136

0

0.0871792

0

0

0

0

0.00543897

0.00443786

0

0

0

0

0

0

0

0

0.0127553

0

0.0127553

0

0.0127553

0

0

0

0.0251705

0

0.00780182

0

0

0.0521502
7

0

0

0

0

0

0.00887572

0

0

0.0125853

0

0.014522
7

0

0.0399145

0

0

0

0

0

0

0

0

0.0468568

0.0446436

0

0.019133

0

0

0

0

0

0

0

0.0381209

0

0

0

0.00665679

0

0

0

0.0446436

0

0

0.073914
7

0

0.00983289

0.0151023

0

0

0

0

0.0637765

0

0

0.0154407
6

0

0

0

0

0.0156036

0

0

0

0.0151023

0

0.0569499
7

0.0251705

0

0

0

0

0.0864815

0.0125853

0.0156036

0.00520121

0

1.30361

0.0362457
7

0

0

0.0255106

0

0.00703064

0

0

0

0

0

0.0399412

0

0

0

0

0

0

0

0.00364411

0

0

0.0343863

0

0.0110946

0

0

0

0

0

0

0

0

0.0276876
7

0

0

0

0

0

0.0127553

0

0

0

0

0.0133136
7

0

0.209162

0

0

0

0

0

0

0

0

0.0536552
7

0

0

0.00543897

0

0

0

0

0

0

0.0318883

0.0255106
8

0

0

0

0

0.00503411

0

0

0

0

0

0.268297
7

0.0127553

0

0

0

0

0

0

0.0701542

0

0

0.0436447
7

0

0

0

0

0

0.0510212

0

0.019133

0

0

0.0345789

0

0

0

0

0

0

0

0

0

0

15.4105

0.427127
7

0.0988934
7

0.0176194

0.0110946

0

0

0

0

0

0

0

0

0.0174409
7

0

0

0

0

0

0

0

0

0

0

0.0941512
7

0

0

0

0

0.0177514

0

0.267861

0

0

0

0.031103
7

0.00755116

0

0

0

0.0578923

0

0.0127553

0

0.019133

0.00527298

0.0260061
7

0

0

0

0

0.00520121

0

0

0

0

0.0674161

0.00665679

0

0

0

0

0

0

0

0

0

0

0.0437128
7

0

0

0

0

0.0234054

0

0

0

0.00503411

0.00443786

0.0544939

0

0

0

0

0.0255106

0

0

0

0

0

0.0359078
7

0

0

0

0

0

0

0

0.0131105

0

0

0.0871532
1

0.0127553

0

0

0.0100682

0.483727

0

0

0

0

0.00520121

0.530674
6

0.109594

0

0

0

0

0

0

0

0

0

0

0.0803411

0

0

0

0

0.369904

0.0201004

0

0.00703064

0

0

0.00780182

0

0.00543897

0

0

0

0.00665679

0.00527298

0.00520121

0

0.0133136

0.0178881
7

0

0

0

0

0

0

0

0

0

0.00351532

0.0658639

0.0127553

0

0

0

0

0

0

0

0

0.00503411

0.387626
7

0

0

0

0

0

0

0

0

0

0.045307

0.0601519

0

0

0

0

0

0.00520121

0

0

0

0.0382659

0.0590919
7

0

0

0

0.0127553

0

0.00503411

0

0

0.00780182

0

0.0446436
7

0

0

0

0

0

0

0

0

0

0

0.0449227
5

0

0

0

0

0

0.0578923

0

0

0

0

0.435077
7

0.0833126

0

0

0

0

0

0

0

0

0.00655526

0.00503411

0.0242762
7

0.00665679

0

0

0

0

0

0

0

0.00443786

0

0.040484
7

0

0

0

0

0

0

0

0.0127553

0

0

0.113263
2

0

0

0

0

0

0.127553

0

0

0

0

0.0701295

0

0

0.0127553

0.0108779

0

0

0

0

0.0156036

0

0.081457
7

0

0

0

0

0

0

0

0

0

0

0.073739
6

0

0

0

0

0

0

0

0

0

0

0.0110946

0

0

0

0

0

0

0

0.0137987

0

0

0.00755116
6

0

0

0

0

0

0.0127553

0

0

0

0

0.155138
7

0.0127553

0

0

0

0

0

0

0

0.0255106

0

0.508811
7

0.0124219

0

0

0

0

0

0

0

0.0226535

0.00543897

0

0.0251463

0.0140613

0

0

0

0

0

0

0

0.019133

0

0.641878
7

0

0

0

0.0111768

0

0

0

0

0

0

0.00503411
7

0

0.0176194

0

0

0

0

0

0

0

0

0.0170342
7

0

0

0

0

0.00755116

0

0.0137987

0.00815845

0

0

0.00520121
7

0

0

0

0

0

0

0.00520121

0

0

0

0.0246072
6

0

0

0

0

0

0

0

0

0

0

0.0255106
7

0

0

0

0.0127553

0

0

0.00443786

0

0

0.00503411

0.0118246

0

0.00655526

0.019133

0

0.00443786

0

0.0255106

0.0271948

0

0

0.0434073

0

0

0

0

0.00503411

0

0

0

0

0

0.426567
7

0.0168635

0

0

0

0.00443786

0.0125853

0.0134122

0

0

0

0.019133

0.0271948
7

0

0

0.00780182

0.00447073

0

0

0.0364085

0

0

0

0.0498448
6

0.0140613

0

0

0

0

0

0.00689936

0

0

0

0.0255106

0

0

0

0

0.0125853

0

0.00351532

0.0276876

0

0

0.0834029
6

0

0

0.00703064

0

0

0

0

0

0

0.00351532

0.0177865
7

0

0

0

0

0

0.0127553

0.00665679

0

0

0

0.0151023
7

0.0382659

0

0

0

0

0.00503411

0

0

0

0

0.078606
7

0.0956648

0

0

0.0166311

0

0

0

0

0

0

0.0919133
7

0

0.019133

0

0

0.0446436

0

0

0

0

0

0.0607409
7

0

0

0

0.0377218

0

0

0

0.0327217

0

0.00443786

0.169417
6

0.0457952

0

0

0.0251705

0

0

0

0

0.012313

0

0

0.0454857
6

0

0

0

0.00755116

0

0.0701542

0

0

0

0

0.0206981
6

0

0

0

0

0

0

0.0286067

0

0

0

0.040545
5

0

0

0

0

0.0465975

0

0

0.0177514

0

0.0140613

0.14017

0.00503411

0

0

0

0

0

0

0

0.00655526

0

0.00503411
7

0

0

0

0

0

0.0255106

0.0255106

0

0

0

0.0241671

0.00503411

0

0

0

0.0176194

0

0.0140613

0

0

0.013003

0.0171932
6

0

0

0

0

0.019133

0

0

0.00351532

0

0

0.053097

0

0

0

0

0.0125853

0

0.0123036

0

0.00780182

0.0288461

0.144166
7

0.019133

0

0

0

0

0

0.019133

0.00665679

0.00503411

0

0.320542
6

0.0380728
7

0

0

0

0

0

0

0

0.00443786

0

0

0.0653319
7

0.00503411

0.0100682

0

0

0

0

0.00780182

0

0

0.0197008

0.0547118
7

0

0

0

0

0

0

0.153064

0.0127553

0

0

0.0752439
6

0

0

0

0.0100682

0.0221634

0

0

0

0.0382659

0.0446436

0.0148845
6

0

0.0125853

0.0244082

0

0.0176194

0

0.0210919

0

0

0

0.0730229
7

0

0.27942

0.00503411

0

0.0133136

0

0

0

0

0

0.00503411

0

0

0

0.0543897

0

0

0

0

0

0.0133136

0.0127553
7

0.00503411

0

0.0637765

0

0.0302047

0.0104024

0

0

0

0

0.0701542
7

0

0

0

0

0.0382659

0

0

0

0

0

0.0396766
8

0

0

0.0829095

0

0

0

0.00780182

0

0.00447073

0.0255106

0.548961
7

0.112296
7

0.0382659

0

0

0

0

0

0

0

0

0

0.0697025
7

0

0

0

0

0

0

0

0

0

0

0.0421597
7

0

0

0

0

0

0

0

0

0.0127553

0

0.0157751
6

0

0

0

0.00443786

0

0

0

0

0.0127553

0.00670609

0.00997863
7

0

0

0.0765318

0

0

0

0

0

0

0

0.0502934
7

0

0

0

0

0

0.0837283

0

0.0151023

0.00503411

0

0.00689936
7

0

0

0

0.121175

0

0

0

0

0.0446436

0

0.0558853
7

0

0

0

0

0

0.0049252

0

0

0

0

0.199382
7

0

0

0.00443786

0

0.0377218

0.0158189

0

0.00503411

0

0.00443786

0.0720208
7

0

0

0.00887572

0

0

0

0.0255106

0

0

0

0.755851
7

0.104338
7

0

0.00503411

0

0

0

0

0

0

0

0

0.0790032
7

0.019133

0.00520121

0

0

0.0251705

0.00527298

0

0

0

0

0.0905852
7

0

0

0.0255106

0.0049252

0

0

0.0255106

0

0

0

0.017428
7

0

0

0.00443786

0

0

0

0.0446436

0.0615649

0

0.101606

0.448934
7

0

0

0

0

0

0

0

0.00543897

0

0

0.0127553
7

0.0110946

0

0

0

0

0.0127553

0

0

0

0

0.0279213
6

0

0

0

0

0

0.00665679

0

0

0.0251705

0

0.0127553
7

0

0

0

0

0

0

0

0

0.00351532

0

0.120807
7

0

0

0

0

0.0338079

0

0

0

0

0

0.0166161
7

0

0

0

0.0127553

0.0244754

0.159441

0

0

0

0

1.29493
7

0.0133136
7

0

0

0

0

0.0226535

0

0

0.00665679

0

0

0.0465632
6

0

0.0318883

0

0

0

0

0.0127553

0

0.0327217

0

0.0148403
2

0

0

0

0.00755116

0

0

0

0

0.0127553

0

0.00351532
6

0.00443786

0

0.00780182

0

0

0

0.0049252

0

0

0

0.0390809
7

0

0

0

0.0498932

0

0

0

0.010546

0.0177514

0

0.0321674
7

0

0

0.00755116

0

0

0

0

0

0

0

34.536
8

0

0

0

0.00543897

0

0

0

0.0125853

0.0100682

0

0.0179338
7

0

0

0

0.00503411

0

0

0

0.019133

0

0

0.219851

0

0

0

0

0

0.00755116

0

0

0

0

0.342011
7

0.0133048

0.00520121

0

0

0

0

0

0.0327217

0

0

4.30595
7

0.394906
7

0.019133
7

0

0.0510212

0

0

0

0

0

0.0573989

0

0.00527298

0.0661954
7

0.0049252

0

0.0829095

0

0

0

0

0

0

0

0.373914
7

0

0.0127553

0

0

0

0.0127553

0

0

0

0

0.0178007

0.00543897

0.0049252

0

0

0

0

0

0

0.00520121

0

0.00351532

0

0

0

0

0

0.00527298

0

0

0

0

0.0594288

0

0

0

0

0

0.0251705

0

0

0

0

0.198372
7

0

0

0

0.00738779

0

0

0

0

0

0.331638

0.075893

0.00503411

0

0.0127553

0

0

0

0

0

0

0.00520121

0.0135974
6

0

0

0.0049252

0

0

0

0

0.00351532

0

0

0.0349826

0.0125853

0

0

0

0

0

0

0.0637765

0

0

0.247021
7

0.0276876
7

0

0

0

0

0

0

0

0

0

0

0.0381694
7

0

0

0

0

0

0

0

0

0

0

0.0176231
7

0

0

0

0.00351532

0

0

0

0

0

0

0.0125853
5

0

0

0

0

0

0

0

0

0

0

0.0108779

0

0.00443786

0

0

0

0

0

0

0

0

0.0127553
7

0

0

0.00443786

0

0.019133

0

0

0

0

0

0.0087883

0

0

0

0

0

0

0

0

0.0140613

0

0.0171932
7

0

0

0

0

0

0

0.00755116

0.0352388

0

0.0100682

0.0255106
7

0

0

0.0127553

0

0

0

0

0

0.019133

0

0.103205
7

0

0

0

0

0

0

0

0.0318883

0

0

0.225234
7

0.0601289
7

0

0

0

0

0

0.0382659

0

0

0

0

0.0815046
7

0

0

0

0

0

0.312505

0

0

0.00443786

0.019133

0.0882843
7

0

0

0

0

0

0

0

0

0

0

0.115513
7

0

0

0.0147756

0.0049252

0

0

0

0

0.0510212

0

0.0181446

0

0

0

0.0108779

0

0.019133

0.0221893

0

0

0

0.00443786
6

0

0

0

0

0

0

0.0127553

0

0

0

0.00738779
7

0

0

0

0.00520121

0

0

0

0

0

0

0.167438

0

0

0

0

0

0.0049252

0

0

0

0

0.0244082
7

0.00503411

0

0

0

0

0

0

0

0

0

0
7

0

0

0

0

0

0

0.0100682

0

0

0

0.575928
7

0.0449227
7

0

0

0

0

0

0

0

0.00670609

0

0.00443786

0.0189439

0

0

0

0

0

0

0

0.0226535

0

0

0.0528581
6

0

0

0

0.00503411

0.019133

0

0

0

0

0.0182909

0.163033
7

0

0

0

0

0

0.00520121

0.00543897

0

0

0

0.00520121
7

0.0288461

0

0

0

0.0127553

0

0

0

0

0

0.036359
7

0.0127553

0

0

0

0

0

0

0.00503411

0

0.21684

0.119122
4

0

0.00665242

0

0

0

0

0

0

0

0

0.0568735

0

0

0

0

0

0

0

0

0.00755116

0

0.159441
7

0

0

0

0

0

0.00527298

0.0510212

0

0

0.0364085

0.0111231
7

0

0

0

0

0

0.00503411

0

0.00546617

0

0.0352388

0.0778244
6

0.0401068
7

0.0151023

0

0

0

0.00543897

0

0

0

0

0.0087883

0
7

0.00520121

0

0.00543897

0

0

0

0

0

0

0

0.0446436
7

0.00443786

0

0

0

0

0

0

0

0

0

0.10631
7

0.0190364

0

0

0.0956648

0

0

0.0573989

0

0.0125853

0

0.0370943
7

0

0

0

0

0

0

0.0701542

0

0

0

0.0618991
7

0

0.00443786

0

0

0.00520121

0

0

0

0

0

0.033329
7

0

0

0

0

0

0

0

0.0049252

0

0.0127553

0

0.0208048

0.0226535

0.0177514

0

0

0

0.00985039

0

0.0127553

0

0.0255106
7

0

0

0

0

0

0.00520121

0

0

0

0

0.0364085
7

0.00815845

0

0

0

0

0

0

0

0.0175766

0

0.27827
7

0.0125853
6

0

0

0

0

0.0147756

0

0

0

0

0

0.0226483

0

0

0.0125853

0

0

0

0

0

0

0

0.0476536
7

0

0

0

0

0

0

0

0

0

0

0.116717
7

0

0

0

0

0

0

0

0

0

0

0.234221
7

0

0.00503411

0

0

0

0

0

0

0

0

0.00703064
7

0

0

0

0.0163169

0

0

0

0

0

0

0.146686

0

0

0.293372

0

0

0

0

0

0

0

0
7

0.019133

0

0

0

0

0

0

0

0

0

0.00503411
6

0

0

0.0210919

0

0

0.0110946

0

0

0

0

0.0688077
7

0

0.338015

0

0.121272

0

0

0

0.0765318

0

0

0.384086
7

0.0338796

0

0.0127553

0

0

0

0.0318883

0

0

0

0

0.0559902
7

0

0.0133136

0

0

0

0.019133

0.00503411

0.0127553

0

0

0.0422274

0

0

0.019133

0

0

0

0

0

0

0

0.0495076
6

0

0

0

0

0

0

0.0127553

0

0

0

0
7

0

0

0.00503411

0

0.047824

0

0

0.00503411

0

0

0.0607114

0

0

0

0

0.0382659

0

0

0

0

0

0.102961
7

0

0.203881

0.0765318

0.0127553

0

0

0

0.00351532

0

0

0.0578649
7

0.019133

0

0

0.0176194

0.00738779

0

0

0

0

0

0.014208

0

0

0

0

0

0.00780182

0.0637765

0

0

0

0.0701542

0.438342

0

0

0

0

0

0.0226535

0.031065

0.131105

0

0.471575
7

0.00503411

0.0729946

0

0

0

0

0.0494115

0.0127553

0

0

0

0.0133136
7

0

0

0

0

0.0510212

0

0

0

0

0

0.630964
7

0

0

0.00527298

0

0

0

0

0

0

0.0446436

0.0276223
6

0

0

0.0127553

0

0

0.19133

0

0

0

0

0.115155
6

0

0

0

0

0

0

0

0

0.031065

0

0.0327217
5

0.00755116

0.0127553

0

0

0

0.0221893

0

0

0.00443786

0

0.00351532
6

0

0.00351532

0

0

0

0

0

0.0182042

0

0

0.118149
7

0

0.00665242

0.00755116

0

0

0

0

0

0.019133

0

0.0200275

0.0125853

0

0.0251705

0

0

0

0

0

0.0155325

0

0.159441

0

0.00738779

0

0

0.101606

0

0

0

0

0.00670609

14.8583
7

0.102322
7

0

0

0

0

0

0

0

0

0

0

0.2384
7

0.019133

0

0

0

0

0

0.0251705

0

0.0221893

0.0151023

0.0699841
7

0

0

0

0

0

0

0

0

0

0

0.0437136
7

0

0

0

0

0

0

0

0

0

0.00351532

0.10869
7

0

0

0

0

0

0

0

0

0

0.00520121

0.117074
7

0

0

0

0

0

0

0

0.019133

0

0

0.0369109

0

0

0

0.0573989

0

0

0

0

0

0

0.00987683
7

0

0

0

0

0.0255106

0.00983289

0

0

0

0.0255106

0.142931
7

0

0

0

0.0255106

0

0.0382659

0

0

0

0

0

0

0.0382659

0

0

0.0578923

0

0

0

0

0

0.252849
7

0.0293059

0

0

0

0

0

0.00755116

0

0.0166311

0

0

0.0150167
2

0

0

0

0

0

0

0

0

0

0

0.0446436
7

0

0

0

0

0

0

0

0

0

0

0.147101
7

0

0

0

0

0

0.0135974

0

0.00665679

0

0

0.0182042
6

0

0.0637765

0

0

0

0

0.00755116

0

0

0.00520121

0.0556602

0

0

0

0

0

0

0.0510212

0

0.0201364

0

0.585639
7

0

0

0

0

0

0.0127553

0

0

0

0

0.0144494
7

0

0

0

0

0.0127553

0

0

0

0

0

0.0799569

0

0

0

0

0.0510212

0

0

0

0

0

0.00503411
7

0

0

0.00780182

0

0

0

0

0

0

0

3.4591
7

0.271859
7

0.0466592

0

0

0

0

0

0

0

0

0

0

0.0446436

0.00520121

0

0

0

0

0

0

0

0

0

0
6

0

0.0127553

0.010546

0

0

0

0

0

0.00670609

0.0087883

0.114728
7

0.00520121

0

0

0

0

0

0

0

0.0510212

0.0255106

0
7

0

0

0

0

0.133931

0

0

0

0

0

0.916871
7

0.0127553

0

0

0

0

0

0

0

0

0

0.0446436

0.0489507

0

0

0

0

0

0.0637765

0

0

0.00520121

0
6

0

0

0

0

0.0127553

0

0

0

0

0

0.0971074
8

0

0.00755116

0.0255106

0.0127553

0

0

0

0

0

0.019133

0.0288461
7

0

0

0

0.10842

0

0

0

0

0

0

0.339765

0

0

0

0

0

0

0.0127553

0

0

0.00447073

0

0.0763998
7

0

0

0

0

0

0

0

0.00527298

0

0

0.113114
7

0

0

0

0

0

0

0

0

0

0

0.145532
7

0

0.019133

0

0

0

0

0.0127553

0

0

0.0510212

0.0205542
7

0

0

0

0

0.00520121

0.00351532

0.00815845

0

0.0127553

0

0.00665242
6

0

0.00351532

0

0

0

0

0

0

0

0

0.0887951
7

0

0

0.0125853

0.00520121

0

0

0.0049252

0

0

0

0.0382659
7

0

0

0

0

0

0

0

0

0.00665679

0

0.0492287
6

0

0

0

0

0

0

0.0100682

0

0

0

0.00780182
7

0

0

0

0.019133

0

0

0

0.00887572

0

0

0.424621
7

0.0241671
7

0

0

0

0

0

0

0.00503411

0

0

0.00780182

0.105643

0.0123036

0.010546

0

0

0

0

0.00665679

0

0

0.00447073

0.183567
7

0.00755116

0.00503411

0

0.0104024

0

0

0

0.00503411

0

0

0.0127553
7

0

0

0

0

0

0

0

0

0

0

0.00443786

0.0637765

0

0

0

0

0

0.00520121

0.0127553

0.0178829

0

0.147429
7

0

0

0

0.00503411

0

0.00665679

0

0.0100682

0.0260061

0

0.10726
7

0

0

0.00503411

0

0

0

0

0

0

0.00503411

0.0600772
7

0

0.0127553

0

0.00815845

0

0

0

0

0

0

0.0176194
6

0.013003

0

0

0

0

0

0

0

0

0

0
7

0

0

0

0

0

0

0

0

0

0

0.213054
7

0
7

0

0

0

0

0

0

0

0

0

0

0.0611376
7

0.019133

0

0

0

0

0

0

0

0.00351532

0

0
7

0

0

0

0

0

0

0

0

0

0.0182731

0.0446436

0

0

0

0

0

0.00503411

0

0

0

0.0327217

0.00665679
7

0

0

0

0.0140613

0.0127553

0

0

0

0.00503411

0

0.783379
6

0

0

0.019133

0

0

0

0

0.0127553

0

0.00755116

0.0765318
7

0

0

0

0

0

0

0

0

0

0

0.0337545
2

0

0

0

0

0

0

0

0

0

0

0.0127553
7

0

0

0

0

0

0

0

0

0

0

0.0214306

0

0

0

0

0

0

0

0

0.0318883

0

0.118747
7

0.00443786
7

0

0.0166311

0

0

0

0

0.00503411

0.013003

0

0

0.0557181
7

0

0

0

0

0

0.0338079

0

0.0338079

0

0.0255106

0.102248
7

0.0127553

0

0

0

0

0.0127553

0

0.0049252

0.0755116

0

1.74121
8

0

0.00520121

0

0

0.0127553

0

0

0

0

0

0.107244

0

0

0

0

0

0

0

0

0.0200426

0

0.161015

0

0

0

0

0

0

0

0

0

0

0.0099097

0.631387

0

0

0

0

0

0

0

0

0

0.19133
7

0

0

0

0

0

0

0

0.0151023

0.00755116

0

0.181722
7

0

0

0

0

0

0.00503411

0.0127553

0

0.00503411

0

0.0255794
7

0

0

0.00351532

0

0

0

0

0.0318883

0

0

0.167056
7

0.0155325
7

0

0

0

0.502691

0

0

0

0

0

0.0049252

0.019133
6

0.0127553

0

0.0276876

0

0

0

0

0

0.0127553

0

0.00443786
7

0

0.140308

0

0

0

0

0

0

0

0

0.0692155
7

0

0.0125853

0.00543897

0

0.140308

0.00520121

0

0.0176194

0

0.00887572

0.0307118

0

0.0318883

0

0

0

0

0.0498932

0

0.0127553

0

0.0249527
7

0

0

0

0

0

0.0100682

0

0.00738779

0

0

0.0100682
7

0

0

0

0.0049252

0

0

0.00670609

0

0

0

0.00503411
6

0

0

0

0.0127553

0

0.00443786

0

0

0.0701542

0

1.4061
7

0

0

0.0573989

0

0

0

0

0

0

0

7.32792
7

0

0

0

0

0.00520121

0

0

0

0

0.019133

0.484512
7

0
7

0

0.00443786

0

0

0

0

0

0

0

0

0.201025

0.00447073

0

0

0

0

0

0.0151023

0.0255106

0

0

0.0751504

0

0.0255106

0

0

0

0

0

0

0

0.010546

0.0208647
6

0

0.0125853

0

0

0

0

0.00527298

0

0.0318883

0

0.047979
7

0

0

0

0

0

0

0

0

0

0.00443786

0.00503411

0

0

0

0

0

0

0

0

0

0

0.114878
7

0

0

0

0

0

0

0

0

0.00997683

0.0127553

0.071935

0

0

0.00527298

0.0156036

0

0.0234054

0

0

0

0.0127553

0.016102
7

0.0255106

0

0

0

0

0

0

0

0

0

0.0523376
7

0

0

0

0

0.0318883

0

0.0127553

0

0

0

0.387756
7

0.212469
7

0

0

0

0

0

0

0

0

0

0

0.0395105
7

0.0255106

0

0.00520121

0

0

0

0

0

0

0

0.04313
7

0

0

0

0

0

0.00755116

0

0

0

0

0.0352388
7

0

0.0199704

0

0.0892871

0.00738779

0

0

0

0

0

0
7

0

0

0.0503411

0

0.00755116

0.00738779

0

0

0.00351532

0

0
8

0

0.0127553

0

0

0.0276876

0

0.00520121

0.0049252

0

0

0.0510212
7

0

0

0

0.00543897

0

0

0

0.0151023

0.047824

0.00447073

0.0380959
7

0

0.00780182

0

0

0

0

0

0

0

0

0.0235847
6

0

0

0

0

0

0

0

0

0

0.0127553

0.0266271
7

0.0765318

0

0

0.00755116

0

0

0

0

0.0226535

0

0.214479
7

0.00854943
8

0

0

0.303993

0

0

0

0

0

0

0

1.04576
7

0.013003

0

0

0

0

0

0

0.00985039

0

0

0.0244754
7

0.013003

0

0

0

0

0.00527298

0

0

0

0

0.104676

0

0

0

0

0

0

0

0.0125853

0

0

0.0581207
6

0

0

0

0.00443786

0

0

0

0.0234054

0

0

0.126198
7

0

0

0

0

0

0

0

0

0

0

0.00665679
5

0

0

0

0.00503411

0

0

0

0

0

0

0.505592
1

0

0

0

0.00665679

0.00887572

0

0

0

0.0163169

0

0.0328917
7

0

0

0

0

0

0

0.0637765

0.00503411

0

0

0.036359
6

0

0

0

0

0

0

0

0

0

0

0.931483
7

0.0104024
7

0

0

0

0.00997863

0.0271948

0

0

0

0.0125853

0

0
5

0

0.0127553

0

0.0127553

0

0

0

0

0.0382659

0

0.0406556
7

0

0

0.019133

0.00503411

0.0127553

0.0255106

0

0

0.0127553

0

0.0210919
7

0

0

0

0

0

0

0

0

0

0

0.114798
8

0

0

0

0

0

0.0100682

0

0

0

0

0.00527298

0

0

0

0

0

0

0

0

0

0

0.0602443
7

0

0

0

0

0

0

0

0

0.0446436

0.00443786

0.0318883
7

0.00447073

0

0

0

0

0

0

0.00443786

0

0.0104024

0.00351532
6

0

0

0

0

0

0

0

0

0.0765318

0.0318883

0
7

0

0

0.0510354

0

0

0

0

0

0

0

0
4

0
5

0

0

0

0
4

0.0110946
7

0.0110946
7

0
4

0
7

0

0

0
4

0

0

0
4

0

0

0
4

0

0

0

0
4

0

0

0
4

0

0

0
4

0

0

0
4

0
4

0.582534
7

0.582534
7

0.0765318
7

0.506002
7

0
7

0

0
4

0

0

0
4

0
4

7.08778

1.96536
7

0.665329
7

0.0540775
7

0

0

0

0

0

0

0

0

0

0

0
7

0

0

0

0

0.00665679

0

0

0

0

0

0.0199704
6

0.00665679

0

0

0

0

0

0

0

0

0

0.0599111

0

0

0

0

0

0

0

0

0

0

0.0535193
7

0
6

0

0
7

0
6

0.0399407

0.259028
7

0
7

0.263452

0.127553
7

0

0

0

0

0.00665679

0

0

0.10842
7

0

0

0

0

0.0171932

0

0

0

0

0

0.0127553
6

0

0

0.00443786

0

0

0

0.0238499

0

0

0.0049252

0
6

0

0

0.0221893

0

0

0

0

0

0.0255106

0.135355

0
7

0

0

0

0

0

0

0.00443786

0.0110946

0

0.00665242

0
7

0

0

0

0

0

0

0

0

0

0

0
6

0

0

0

0.019133

0.00665679

0

0

0

0

0

0
6

0

0

0

0

0

0

0

0

0

0

0
4

0

0

0

0

0

0

0
4

0

0

0
4

0.223218

0.223218

0

0
7

0

0
4

0

0

0

0

0

0

0

0
4

0.886493
7

0.465568
7

0

0.0127553
6

0
6

0

0

0
5

0

0

0

0

0.274239

0

0

0

0

0

0

0

0

0

0

0.102042
6

0

0

0

0

0

0

0

0

0

0

0
7

0

0

0

0

0

0

0

0

0

0

0
7

0

0

0

0

0

0

0

0

0

0

0
7

0

0

0

0

0

0

0

0.0318883

0

0

0
6

0

0

0

0

0

0

0

0

0

0

0
7

0

0

0

0

0

0

0

0

0

0
4

0.567611
6

0.0892871
6

0

0

0

0

0

0

0

0

0

0

0.172197

0

0.306127
6

0

0

0

0

0

0

5.55111512312578e-17
6

0
4

3.21064
6

0.00665242
6

0.00997863
7

0

0

0

0

0

0.00447073

0

0

0.0318883

0

0
6

0.0100682

0

0

0

0

0

0

0

0

0

0.133931

0

0

0

0

0

0

0

0

0.0127553

0

0.296455
7

0

0

0

0

0

0

0

0

0

0

0.172197
6

0

0

0

0

0

0

0

0

0

0

0.321214

0

0

0

0

0

0.00665679

0

0

0

0

0.019133
7

0

0

0

0

0

0

0

0

0

0.00503411

0.019133
6

0.00447073

0

0

0

0

0

0.00503411

0

0

0.00997863

0
6

0

0

0

0

0

0

0

0

0

0

0.0975057
7

0

0

0

0

0

0

0

0

0

0

0.81049
6

0.019133
6

0

0

0

0

0

0

0

0

0

0

0

0

0

0

0

0

0

0.0127553

0

0

0

0
6

0

0

0

0

0

0

0

0

0

0

0
7

0

0

0

0

0

0

0

0

0

0

0
6

0

0

0

0

0

0

0.0421937
7

0.0318883
6

0.0201183
6

0
7

0
6

0.0510212
6

0
6

0
6

0

0
6

0

0
6

0

0
6

0
6

0.0446436
7

0.0266097
6

0

0
7

0
6

0
6

0
7

0.0255106
6

0.019133

0

0

0

0.0717321

0

0.0510212

0
7

0

0

0
6

0

0

0

0

0.225965
6

0

0

0

0.0926328

0

0

0

0.0318883

0

0

0.10842
6

0

0

0

0

0

0

0

0

0

0.00665242

0.178702
7

0

0

0

0

0.00503411

0

0

0

0

0

0.168538
7

0

0

0

0

0

0

0

0

0

0

2.22044604925031e-16
6

0
4

0.0956648

0.0829095

0

0
5

0.0127553

0

0

0

5.20417042793042e-18

0
4

0

0

0
4

0

0

0

0

0
4

0

0

0
4

0

0

0
4

0

0

0

0
4

0.00564832

0.00564832

0
4

0

0

0
4

0

0

0
4

0

0

0
4

0

0

0
4

0.0956648

0.0956648

0

0
4

0

0

0
4

0

0

0
4

0

0

0
4

0

0

0
4

0.00447073

0.00447073

0
4

0

0

0
4

0

0

0
4

0

0

0

0

0
4

0

0

0
4

0

0

0

0

0

0

0
4

0

0

0

0

0
4

0

0

0
4

0.0133136

0.00887572

0.00443786

0
4

0.0197008

0.0197008

0
4

2.69576028166796e-15

0
4

1.0418

0.221634
7

0.221634
7

0
4

0
5

0
5

0
5

0

0

0

0

0

0

0

0
4

0

0

0

0

0

0

0

0
4

0
7

0
7

0
7

0

0
4

0

0

0

0

0

0

0

0

0
4

0
5

0
5

0

0

0
4

0
7

0
7

0

0

0
4

0
5

0
5

0

0

0

0

0

0

0
4

0
6

0
6

0

0

0
4

0.144774

0.144774

0

0

0
4

0
4

0
4

0

0

0

0

0

0

0
4

0

0

0

0

0

0

0

0
4

0.0692381

0.0692381
5

0

0

0

0

0

0
6

0

0

0

0

0

0

0

0
4

0
3

0
4

0

0
4

0

0

0

0
4

0

0

0

0

0
4

0
7

0

0

0
4

0
5

0

0

0

0

0

0

0

0

0
4

0

0

0
4

0

0

0

0

0

0

0

0
4

0
7

0
7

0
4

0
6

0
6

0

0
4

0

0

0
4

0.165819
5

0.165819
5

0

0

0

0

0

0

0

0

0

0

0
5

0

0

0

0

0

0

0

0

0

0
4

0
4

0

0

0
4

0

0

0

0

0

0

0
4

0
7

0

0

0

0

0
4

0

0

0

0
4

0

0

0

0
4

0.00364411

0.00364411

0
4

0

0

0

0
4

0

0

0

0

0
4

0

0

0
4

0.0049252

0.0049252

0

0
4

0
7

0
7

0

0

0
7

0
7

0
7

0
6

0
7

0

0

0

0
4

0

0

0
4

0

0

0

0
4

0

0

0
4

0

0

0
4

0

0

0

0

0
4

0

0

0
4

0

0

0
4

0

0

0

0

0
4

0

0

0

0
4

0

0

0

0

0
4

0
5

0
5

0
3

0
5

0

0

0

0

0
4

0

0

0

0
4

0

0

0
4

0

0

0

0
4

0

0

0
4

0

0

0

0
4

0

0

0
4

0

0

0
4

0

0

0

0
4

0

0

0
4

0

0

0

0
4

0.0127544

0.0127544

0
6

0

0

0

0

0

0

0
4

0

0

0
4

0

0

0
4

0

0

0
4

0

0

0
4

0.00443786

0.00443786

0
4

0

0

0
4

0

0

0
4

0

0

0
4

0

0

0
4

0

0

0
4

0.102035

0.102035

0

0

0

0

0

0

0

0
4

0

0

0
4

0

0

0
4

0

0

0
4

0

0

0
4

0

0

0
4

0

0

0
4

0

0

0
4

0

0

0
4

0

0

0
4

0

0

0
4

0.114826

0.0776625

0

0.00443786

0

0.0155325

0

0.00443786

0

0.0127553

0

0

0
4

0

0

0
4

0

0

0
4

0

0

0
4

0

0

0
4

0

0

0
4

0

0

0
4

0

0

0
4

0

0

0
4

0

0

0
4

0

0

0
4

0.197707
5

0.197707

0

0

0

0

0
4

0

0

0
4

0

0

0
4

0

0

0
4

5.55111512312578e-17

0
4

0.725843

0.725843

0.725843

0

0

0

0

0

0
4

0
4

0.00364411

0.00364411

0.00364411

0
4

0
4

0

0

0

0
4

0
4

0

0

0

0

0
4

0
4

0

0

0

0

0

0
4

0
4

0

0

0

0
4

0
4

0

0

0

0

0
4

0
4

0

0

0

0

0
4

0
4

0

0

0

0
4

0
4

0

0

0

0
4

0
4

0

0

0

0

0
4

0
4

0.0438633
3

0.0438633
3

0

0

0

0

0

0

0

0

0

0

0

0.0127553

0

0

0

0

0.00443786

0

0

0

0

0

0.00351532
5

0

0

0

0

0

0
3

0
3

0

0.0231548

0

0

0
4

0

0

0
4

0
4

0

0

0

0

0
4

0
4

0

0

0

0
4

0

0

0
4

0
4

0

0

0

0
4

0

0

0
4

0
4

0

0

0

0
4

0
4

0

0

0

0
4

0
4

0

0

0

0
4

0
4

0

0

0

0
4

0
4

0

0

0

0
4

0
4

0

0

0

0
4

0
4

0

0

0

0
4

0
4

0.0157018
3

0.00738779
3

0

0

0

0.00738779

0
4

0.00831403

0.00831403

0
4

0

0

0
4

1.73472347597681e-18
3

0
4

0

0

0

0
4

0
4

0

0

0

0
4

0
4

0.0714153

0.0714153

0.0714153

0
4

0
4

0

0

0

0
4

0
4

0

0

0

0
4

0
4

0

0

0

0
4

0
4

0

0

0

0
4

0
4

0.195266

0.195266

0.195266

0
4

0
4

0

0

0

0
4

0
4

0

0

0

0
4

0
4

0.0110946
6

0.0110946
6

0.0110946
6

0

0

0

0

0
4

0
4

0

0

0

0
4

0
4

0

0

0

0
4

0
4

0

0

0

0
4

0
4

0

0

0

0
4

0
4

0

0

0

0
4

0
4

0

0

0

0
4

0
4

0

0

0

0
4

0
4

0

0

0

0
4

0
4

0

0

0

0

0

0

0
4

0
4

0
5

0

0

0
4

0

0

0
4

0

0

0
4

0
4

0

0

0

0

0
4

0
4

0

0

0

0

0
4

0
4

0.390311

0.390311

0.390311

0

0

0

0

0

0

0

0
4

0
6

0
6

0

0

0

0

0

0

0

0
4

0

0

0
4

0

0

0
4

0

0

0
4

0
5

0

0

0

0

0

0

0

0

0

0
4

0
7

0
6

0

0

0
4

0
7

0
7

0

0
4

0

0

0

0
4

0

0

0
4

0

0

0
4

0

0

0
4

0

0

0
4

0
4

0.936142

0.738626

0

0.0226535
5

0
7

0

0

0
5

0.176289

0

0
5

0.0702163

0

0.115785

0

0

0

0

0

0

0

0

0

0

0.106625
6

0

0

0

0

0

0.0104024

0.00520121

0

0

0

0
5

0

0

0

0

0

0

0

0

0

0

0.231454
6

0

0

0

0

0

0

0

0

0

0

0

0

0

0

0

0

0

0

0

0

0

0

0

0

0

0

0

0

0

0

0

0

0
7

0

0

0

0

0

0

0

0

0

0
4

0.0765318

0.0765318
7

0

0
4

0

0

0

0
4

0

0

0
4

0

0

0
4

0

0

0
4

0

0

0
4

0

0

0
4

0

0

0
4

0

0

0
4

0
5

0
6

0

0

0

0
4

0
6

0

0

0

0

0
4

0
5

0
5

0
4

0.0255106
7

0.0255106
7

0

0

0
4

0.0954735
7

0.0954735
7

0
4

0

0

0

0
4

0

0

0

0
4

0

0

0
4

2.77555756156289e-17

0
4

0

0

0
5

0

0

0

0

0

0

0

0

0

0

0
5

0

0

0

0

0

0

0

0

0

0

0

0

0

0

0
6

0

0

0

0

0

0
4

0

0

0
4

0

0

0
4

0

0

0
4

0

0

0
4

0
4

0.024626
5

0.024626
5

0.024626
5

0
5

0
5

0
6

0
5

0

0

0

0

0

0

0

0

0

0

0

0

0

0

0

0

0

0
6

0

0

0

0

0

0

0

0

0

0

0
5

0

0

0

0

0

0

0

0

0

0

0
5

0

0

0

0

0

0

0

0

0

0

0
5

0

0

0

0

0

0

0

0

0

0

0
6

0

0

0

0

0

0

0
4

0
6

0
4

0

0

0

0
4

0

0

0

0
4

0

0

0
4

0

0

0
4

0
4

0.183438

0.183438

0.132417

0.0510212
5

0

0

0

0

0

0

0

0

0

0

0
5

0

0

0

0

0

0

0

0

0

0

0

0

0

0

0

0

0
7

0

0

0
4

0
5

0
5

0
4

0

0

0
4

0

0

0

0
4

0

0

0

0

0
4

0

0

0
4

0

0

0
4

0

0

0
4

0

0

0

0
4

0

0

0
4

0

0

0
4

0

0

0
4

0

0

0

0
4

0

0

0
4

0

0

0
4

0

0

0
4

0

0

0
4

0

0

0
4

0

0

0
4

0

0

0
4

0

0

0
4

0

0

0
4

0

0

0
4

0

0

0

0

0
4

0

0

0
4

0

0

0
4

0

0

0
4

0

0

0
4

0

0

0
4

0

0

0
4

0

0

0
4

0

0

0

0

0

0
4

0

0

0

0

0

0
4

0

0

0
4

0

0

0
4

0

0

0
4

0

0

0

0
4

0
4

1.6263
5

1.52426
7

0.535723
7

0.988536
7

0

1.11022302462516e-16
7

0
4

0
4

0
4

0

0

0
4

0

0

0
4

0

0

0
4

0

0

0
4

0

0

0
4

0

0

0

0
4

0
4

0
4

0

0

0
4

0.102042

0.0765318

0.0255106

0

0
4

0

0

0

0

0
4

0

0

0
4

0

0

0
4

0

0

0
4

0

0

0
4

1.11022302462516e-16
5

0
4

0

0

0

0
5

0

0

0

0

0

0

0

0

0

0

0

0

0

0

0

0

0

0

0

0

0

0

0

0

0

0

0

0

0

0

0

0
7

0

0
7

0

0

0

0

0
4

0
4

0.00670609
4

0.00670609

0

0

0

0.00670609

0
4

0

0

0

0

0
4

0

0

0
4

0

0

0
4

0

0

0
4

0

0

0
4

0
4

0
4

0
4

0
5

0

0

0

0

0

0

0

0

0

0

0

0

0

0

0

0

0
4

0

0

0
4

0

0

0
4

0

0

0
4

0
4

0
4

60.9068

31.5197

0.0510212

0.0510212
5

0

0

0

0

0

0

0

0

0

0

0
4

18.0183

18.0042

0
7

0

0

0

0

0

0

0

0

0

0

0
7

0

0

0
7

0.0140446

0

0

0

0

0

0
4

9.02882

6.3587
7

0.0354363
7

0

0

0.0223536

0

0

0

0

0

0

0

0.0251705
7

0

0

0

0

0

0

0

0

0

0

0.311744
7

0

0

0.00520121

0

0

0

0

0

0

0

0.0127553
8

0

0

0

0

0

0.090614

0.00447073

0

0

0

0.0111768
7

0

0

0.00447073

0

0

0

0

0

0

0

0.0049252
7

0

0

0

0

0

0

0

0

0

0

0.176599
1

0

0

0

0

0.00447073

0

0

0

0

0

0.0111768

0

0

0

0

0

0.00351532

0

0.00447073

0

0

0
8

0

0

0

0

0.019133

0

0

0

0

0

0

0

0.00689936

0

0.00755116

0

0.0049252

0

0

0

0

0.149458

0

0

0.00527298

0.00447073

0

0

0

0

0

0

0

0.0049252
8

0

0

0

0

0

0

0

0

0

0

0.0100682
7

0

0

0

0

0

0

0

0

0

0

0.00815845
7

0.019133

0

0

0

0

0

0

0

0

0

0.00503411
7

0

0.0151023

0

0

0

0

0

0.00689936

0

0.0557094
7

0

0

0.179635
7

0
7

0

0

0

0.0232608

0

0

0

0

0

0.66773

0

0

0

0

0

0.00503411

0.0855799

0

0

0

0.0926173
7

0

0

0

0

0

0

0

0

0

0

0.0631289
1

0

0

0

0

0

0

0

0

0

0

0.0869615
1

0

0

0.0087883

0.00670609

0

0

0.00703064

0

0

0

0.010349
8

0

0

0

0

0

0

0.0276876

0

0.00543897

0.334497

0.00894146
8

0

0

0

0

0

0

0.00543897

0

0

0

1.44242257027472e-15

0
4

0.683557
7

0.683557
7

0

0
4

0.0049252
7

0
7

0

0

0

0.0049252

0

0

0

0

0

0
4

0
6

0
6

0
6

0

0

0

0
4

0.033989
7

0.0197008
7

0.0142882
6

0

0

0
4

0.0180371
7

0.00503411

0.013003

0

0

0

0

0

0

0

0

0
4

0

0

0

0

0

0

0
4

0.0138667
1

0.0138667
1

0
4

0.00738779
6

0.00738779
6

0

0

0
4

0

0

0

0

0
4

0

0

0

0
4

1.15642
5

0.936482
7

0

0
6

0

0

0

0.0049252

0

0

0

0

0.0271145

0

0

0

0

0

0

0

0

0

0

0.0739865
6

0.0155325

0

0.0546195

0
6

0.00985039
5

0.0049252
6

0.019133
6

0.00985039
6

0
4

0
6

0
6

0
4

0.00520121

0.00520121

0

0
4

0

0

0

0
4

0

0

0

0
4

0

0

0

0
4

0

0

0

0
4

0

0

0

0
4

0.00351532

0.00351532

0
4

0

0

0
4

0

0

0
4

0.309574

0.0692535
1

0

0

0

0.0513095

0.0378621

0.0413059

0.0191396

0.0259803
7

0.0399407
2

0.0247823
8

0
6

4.16333634234434e-17

0
4

0

0

0
4

0

0

0
4

0

0

0
4

0

0

0
4

0

0

0
4

0

0

0
4

0

0

0
4

0.209162

0.209162

0
4

0

0

0
4

0

0

0
4

1.16874

0.00443786
5

0

1.15938

0
7

0
7

0

0

0.0049252

0

0

0
4

0

0

0
4

0

0

0
4

0

0

0
4

0

0

0
4

0

0

0
4

0

0

0
4

0

0

0
4

0

0

0
4

0

0

0
4

0

0

0
4

0.0496955

0.0311635
1

0.00447073
1

0

0.0140613

0

0

0

0

0
4

0

0

0
4

0
7

0
7

0
8

0

0

0

0

0
4

0.293116
6

0.257396
6

0.0167508
7

0.0189698

0

0

0

0

2.08166817117217e-17
6

0
4

0.405669
8

0.374833
8

0.0102214
8

0.0100682

0

0

0

0.010546

0
4

0.0587686

0.0321674

0

0.00443786

0

0

0.0221634

6.93889390390723e-18

0
4

0
4

17.3115

16.8654

16.4161

0

0

0

0

0

0

0

0

0

0

0

0.424719

0

0

0

0

0

0

0

0.0246072

0

0

0

0

0

0
4

0.446076
8

0.061234
8

0

0

0.00689936

0

0

0

0

0

0

0

0.241323
8

0

0

0

0

0

0

0.0196658

0

0

0

0.0721078
8

0

0

0.0448458

0

0

0

0

0

0
4

0

0

0
4

0

0

0
4

0

0

0
4

0

0

0
4

0

0

0
4

0

0

0
4

0

0

0
4

0

0

0
4

0

0

0
4

0

0

0
4

2.33146835171283e-15

0
4

6.80416

6.31491

2.97153

0
5

0

0

0

0

0

0

0

0

0

0

0.0127553

0

0

0

0

0

0

0

0

0

0

0
6

0

0

0

0

0

0

0

0

0

0

0
7

0

0

0.0255106

0

0

0.00738779

0

0

0

0

0.0295512

0

0

0

0

0

0

0

0

0

0

0

0

0

0.00985039
6

0
7

0.0049252

0
6

1.51722

0
7

0

0

0.019133
7

0

0

0

0

0

0

0.860572
7

0

0

0

0

0.0127553

0.036939

0

0.019133

0

0

0.635386

0.0221634

0

0

0

0

0.00738779

0

0

0

0

0
6

0.0049252

0

0

0

0.00520121

0

0

0

0

0

0.012313
7

0

0

0

0.0049252

0

0

0

0

0

0

0.0398439
1

0

0

0

0

0

0

0

0

0

0

0

0

0

0

0

0

0

0

0

0.0049252

0

0
7

0

0.0255106

0

0.0127553

0

0

0

0

0

0.012313

7.21644966006352e-16

0
4

0

0

0

0

0
4

0

0

0

0

0
4

0

0

0

0
4

0.0827923

0.0827923

0
4

0

0

0
4

0

0

0
4

0

0

0
4

0

0

0
4

0.139881

0.0911161

0

0

0

0

0

0.0049252

0.0049252

0

0.00443786

0

0.00738779
6

0

0

0

0

0

0

0

0

0

0

0.012313
6

0

0.0049252

0

0

0

0

0

0

0

0

0
6

0

0

0

0

0

0

0

0

0

0

0

0

0

0

0

0.00985039

0

0

5.20417042793042e-18

0
4

0.0258702
5

0
6

0.0049252

0.00985039

0

0

0

0.0110946

0

0

0

1.73472347597681e-18
5

0
4

0.0541771

0.0049252
6

0

0

0.0394016

0

0.0049252

0

0

0

0.0049252

0

0
4

0.159441
7

0
7

0.159441

0

0

0
4

0
6

0

0

0

0

0

0
4

0.0172382
7

0

0

0.0172382

0

0
4

0

0

0

0

0

0
4

0.00985039

0

0.0049252

0.0049252

0

0

0
4

0
4

1.17712

0

0

0
4

1.17712

1.17712

0
4

0
4

0

0

0

0
4

0

0

0
4

0
4

0.0127553

0.0127553

0

0.0127553

0
4

0
4

0

0

0

0

0

0
4

0
4

0

0

0

0

0
4

0
4

0

0

0

0

0
4

0
4

0

0

0

0

0
4

0
4

0

0

0

0

0
4

0

0

0
4

0
4

0

0

0

0
4

0
4

0.00665679

0.00665679

0.00665679

0
4

0
4

2.79537
7

2.71266
7

0.0521569
7

0

0

0

0

0

0

2.34955
7

0.286054
7

0.00520121
7

0

0.00738779

0.012313

0

0

0
4

0.0615199
6

0.0349187
6

0.0142882
7

0

0.012313

0

0

0

0

0
4

0.00887572

0.00443786

0.00443786

0
4

0

0

0

0
4

0
6

0

0

0

0
4

0.012313

0.012313

0

0
4

0

0

0
4

0

0

0
4

0

0

0
4

0
4

0.00689936

0.00689936

0.00689936

0
4

0

0

0
4

0
4

0

0

0

0
4

0

0

0
4

0
4

0

0

0

0

0
4

0
4

0

0

0

0
4

0
4

0

0

0

0
4

0
4

0

0

0

0
4

0
4

0.00447073

0.00447073

0.00447073

0
4

0
4

0

0

0

0
4

0
4

0

0

0

0
4

0
4

0

0

0

0
4

0
4

0.0382376
7

0.0333124
7

0.0333124
7

0

0

0

0

0
4

0
7

0
7

0

0

0

0

0

0
4

0.0049252
6

0

0.0049252

0

0
4

0

0

0
4

0
4

0

0

0

0
4

0
4

0

0

0

0
4

0
4

0

0

0

0
4

0
4

0

0

0

0
4

0
4

0.00447073

0.00447073

0.00447073

0
4

0
4

0

0

0

0
4

0
4

0.217939
6

0.0266097
6

0
6

0.0266097

0

0

0

0

0
4

0
7

0
7

0

0

0
4

0.19133
7

0
6

0.19133

0
4

0

0

0

0

0
4

0

0

0
4

0

0

0
4

0
4

0
6

0
6

0
6

0

0

0

0

0
4

0
4

0
2

0
2

0
2

0

0

0

0

0

0

0

0
4

0
4

0.0142882
6

0.0142882
6

0.0049252

0

0

0.0049252

0.00443786

0
4

0

0

0

0
4

0
4

0

0

0

0
4

0
4

0

0

0

0

0

0

0

0
4

0
4

0.993181

0.993181

0.073767

0.0321674
7

0
7

0.00665679

0

0.135355
7

0

0

0

0

0

0.635335
7

0

0

0

0

0

0

0.0049252

0

0

0

0.0938853

0

0

0

0

0

0

0

0

0

0

0

0

0

0

0

0

0

0

0

0

0

0
7

0

0

0

0

0

0

0

0

0

0

0

0

0

0

0

0

0

0

0

0

0.00665242

0
8

0

0

0

0

0

0

0

0

0

0

0

0

0

0

0

0

0.00443786

0

0

0

0

0

0

0

1.6826817716975e-16

0
4

0

0

0
4

0

0

0
4

0

0

0
4

0
4

4.49640324973188e-14

0
4

2.02649

0
7

0

0

0

0
4

0

0

0

0
4

0

0

0
4

0

0

0
4

0

0

0
4

0

0

0
4

0

0

0
4

0

0

0
4

0
4

0.509696
7

0.477808
7

0

0

0.00894146

0.0380012

0

0

0

0

0

0

0

0

0

0

0

0

0

0

0

0

0

0

0.102827

0

0

0

0.0255106

0

0

0

0.0637765

0

0

0.165417

0

0

0

0

0

0

0

0

0

0

0

0

0

0

0

0

0

0.047824

0

0

0

0

0

0

0

0

0

0

0

0

0

0

0

0.0255106

0

0

0

0

0

0

7.28583859910259e-17
7

0
4

0.0318883

0.0318883

0
4

6.93889390390723e-18
7

0
4

0
6

0
6

0
6

0

0
4

0
4

0

0

0

0
4

0
4

0

0

0

0
4

0
4

0.127553
7

0.127553
7

0.127553
7

0
4

0
4

0

0

0

0
4

0
4

0

0

0

0
4

0
4

0

0

0

0
4

0
4

0

0

0

0
4

0
4

0.0701542

0.0701542

0.0701542

0
4

0
4

0

0

0

0
4

0
4

0

0

0

0
4

0
4

1.31909

1.28082

0.59558

0.335989
7

0

0

0

0

0

0

0

0

0

0

0
6

0

0

0.349252

0

0

0

0

0

0

0

0

0

0

0

0
7

0

0
7

0

0

0

0
4

0.0382659

0.0382659

0

0

0
4

0

0

0
4

0

0

0
4

0

0

0
4

0

0

0

0
4

0

0

0
4

0

0

0
4

0

0

0
4

0
4

0
4

2.75012
4

2.60515
4

2.59995
4

0.183745
4

0.720675

0

0

0

0.0127553

0.0127553

0

0.0510212

0

0

0.0127553

1.07782

0

0

0

0

0.122078

0

0

0

0

0

0.0892871
7

0

0

0

0

0

0

0

0

0

0

0.0747043

0

0

0

0

0

0

0

0

0

0

0.229595

0

0

0

0

0

0.0127553

0

0

0

0
6

0

0

0

7.45931094670027e-17
4

0
4

0

0

0
4

0

0

0
4

0

0

0
4

0.00520121

0.00520121

0
4

0

0

0
4

0

0

0
4

0

0

0
4

0

0

0
4

0

0

0
4

0

0

0
4

0

0

0
4

0

0

0
4

0

0

0
4

0
4

0

0

0

0
4

0
4

0.00670609

0.00670609

0.00670609

0

0
4

0
4

0

0

0

0
4

0
4

0

0

0

0
4

0
4

0

0

0

0
4

0
4

0

0

0

0
4

0
4

0.0936218

0.0936218

0.0936218

0
4

0
4

0

0

0

0
4

0
4

0

0

0

0
4

0
4

0

0

0

0
4

0
4

0

0

0

0
4

0
4

0

0

0

0

0
4

0
4

0

0

0

0
4

0
4

0

0

0

0
4

0
4

0

0

0

0
4

0
4

0

0

0

0
4

0
4

0

0

0

0
4

0
4

0

0

0

0
4

0
4

0

0

0

0
4

0
4

0.0446436

0.0446436

0.0446436

0
4

0
4

0

0

0

0

0
4

0
4

0

0

0

0
4

0
4

0

0

0

0

0
4

0
4

0

0

0

0
4

0
4

0

0

0

0
4

0
4

0
4

396.487

0
1

0

0

0

0

0

0

0
4

0

0

0
4

0

0

0
4

0

0

0
4

0

0

0
4

0

0

0
4

0

0

0
4

0

0

0
4

0

0

0
4

0

0

0
4

0
4

0.707465

0.588113

0

0
2

0
3

0

0

0

0

0

0

0

0

0.165819

0

0

0

0

0

0

0

0

0

0

0
4

0

0

0

0

0

0

0

0.00503411

0

0

0.127553

0

0

0

0

0

0

0

0

0

0

0

0

0

0

0

0

0

0

0

0

0

0
4

0

0

0

0

0

0

0

0

0

0

0
3

0

0

0

0

0

0

0

0

0

0

0

0

0

0

0

0

0

0

0

0

0

0.289707
3

0

0

0

0

0

0

0

0

0

0
4

0
3

0
3

0

0

0

0

0
4

0.0127553

0.0127553

0
4

0.0127553

0.0127553

0
4

0

0

0
4

0

0

0
4

0

0

0
4

0.019133

0.019133

0
4

0

0

0
4

0

0

0
4

0

0

0
4

0

0

0
4

0
3

0
3

0
4

0.0109323

0.00728822

0.00364411

4.33680868994202e-19

0
4

0

0

0

0
4

0.0637765

0.0637765

0
4

0

0

0

0
4

0

0

0
4

0

0

0
4

0

0

0
4

5.55111512312578e-17

0
4

1.59634
1

0
1

0
1

0

0

0

0

0

0

0

0

0

0

0
1

0

0

0

0

0

0

0

0

0

0

0

0

0

0

0

0

0

0

0

0

0

0

0

0

0

0

0

0

0

0

0

0

0

0

0

0

0

0

0

0

0

0

0

0

0

0

0

0

0

0

0

0

0

0

0

0

0
4

0
2

0
2

0

0
4

0.769614

0.701627

0.0435118

0.0108779

0.0135974

0
4

0

0

0
4

0

0

0
4

0

0

0
4

0

0

0
4

0

0

0
4

0

0

0
4

0

0

0
4

0.00543897

0.00543897

0
4

0

0

0
4

0

0

0
4

0.821284

0.807687

0.00815845

0.00543897

6.33174068731535e-17

0
4

0

0

0
4

0

0

0
4

0

0

0
4

0

0

0

0
4

0

0

0
4

0

0

0
4

0

0

0
4

0

0

0
4

0
4

213.571

0.185756

0.0581195
2

0

0.073767

0

0

0

0

0

0.0104024

0

0

0
6

0

0

0

0

0

0

0

0

0

0

0
3

0.0382659

0

0

0

0

0

0

0

0

0

0
2

0

0.00520121

0

0

0

0

0

0

0

0

0
3

0

0

0

0

0

0

0

0

0

0

0
6

0

0

0

0

0

0

0

0
2

0

0
2

4.85722573273506e-17

0
4

208.749

203.064

1.1979
7

0.390146

0

0

0

0

0

0

0

0

0

0

0.0156036
7

0

0

0

0

0

0

0

0

0

0.0172382

0.185736

0

0

0

0

0

0

0

0

0

0.0135974

0.0127553
7

0

0.0765318

0

0

0.0236867

0

0

0

0

0

0

0

0

0

0

0

0

0

0

0

0

0.030791
7

0

0

0.00520121

0

0

0

0

0

0

0

0
6

0

0

0

0

0

0

0

0

0

0

0.0631504
7

0

0

0

0

0

0

0

0.0689527

0

0

0
7

0

0

0

0

0

0

0

0

0

0

0
7

0

0

0

0

0

0

0

0

0

0

0.125085
7

0.0314459
7

0

0

0

0

0.0147756

0

0

0

0.0127553

0.0127544

0.0398156
7

0

0.00364411

0.012313

0

0

0

0

0

0

0

0.0831629
7

0

0

0

0

0

0

0

0

0

0

0.15559
7

0

0

0

0

0

0

0

0

0

0

0
6

0

0

0

0

0

0

0.255106

0

0

0.0221634

0
2

0

0

0

0

0

0

0

0

0

0

0.0330081
7

0.0127553

0

0

0

0

0

0

0

0

0

0.00546617

0

0

0

0

0

0

0

0

0.00447073

0

0
7

0

0

0

0

0

0

0

0.00738779

0

0

0
6

0

0

0.0127553

0

0.00755116

0

0

0

0

0

0.11924
7

0
6

0.0049252

0

0

0.00503411

0

0

0

0

0

0.0049252

0.0182042
7

0

0

0

0

0

0

0

0

0

0

0.00447073

0

0

0

0

0

0

0

0

0

0

0.0127553
7

0

0

0

0

0

0

0.012313

0

0

0

0.267586
7

0

0

0

0

0

0

0

0

0

0.00543897

0.0049252
8

0

0

0

0

0.0049252

0

0

0

0

0

0.0110319
1

0

0

0

0

0

0

0

0

0

0

0.0127553
7

0

0

0

0

0

0

0

0

0

0

0

0

0

0

0

0

0

0

0

0

0

0
7

0

0

0

0

0

0

0

0

0.148153
7

0

0.0127553
7

0

0.0127553
7

0.00738779
7

0
6

0.0049252
8

0

0

0

0.265614
7

0.0241715
7

0.0255106
7

0

0
6

0.00520121

0

0

0.00780182

0.0127553

0

0.848862
7

0

0

0

0

0

0

0

0.0400467

0

0

0.481664
7

0

0

0

0

0

0.0423056

0

0

0

0.0446436

0.0630816

0

0

0.0364085

0

0.0172382

0

0.0104024

0

0

0

0.0961672

0.019133

0.0156036

0

0

0

0

0

0

0

0

3.00419411569663e-14

0
4

2.04549
7

2.04549
7

0

0

0
4

1.83231

0.556606

0.11075

0

0

0

0

0

0

0

0

0

0

0
6

0

0

0.0049252

0

0.0288695
4

0

0

0

0

0

0

0

0.00755116

0

0

0
2

0

0

0

0.00503411

0

0

0.00894146

0

0

0

0.0049252
3

0

0

0

0

0

0

0

0

0

0

0.0373565
6

0

0.012313

0

0

0

0

0

0.00447073

0

0

0.024589
1

0

0

0

0

0

0

0

0

0

0

0.0167837

0

0.945638

0

0

0

0

0

0

0

0

0
3

0

0

0.0469426

0

0

0

0

0

0

0

0
4

0

0.0111768

0

0.00543897

0

0

0

0

0

0

0
4

0.344234

0.262762

0.0814719
7

0

0
4

0

0

0
4

0

0

0

0
4

0.0109323

0.0109323

0
4

0

0

0
4

0

0

0
4

0

0

0
4

0

0

0
4

0

0

0
4

0

0

0
4

0.0418642

0.0418642

0
4

0.0676666

0.0485336
1

0

0

0

0

0

0

0

0

0.019133

0

0

0
4

0

0

0
4

0.00520121

0.00520121

0
4

0

0

0
4

0

0

0
4

0.0637765
7

0
7

0

0

0

0

0.0637765

0
4

0

0
1

0

0

0

0

0

0

0
4

0.0861909

0

0

0

0

0.0861909

0
4

0.133931

0

0

0.133931

0

0
4

0.0049252
1

0

0.0049252

0

0
4

0

0

0

0

0

0
4

0

0

0

0

0
4

4.08006961549745e-14

0
4

9.25162

9.19333

9.19333

0

0

0

0

0

0

0
4

0.0483514
1

0.0282331
2

0.00670609
1

0

0.0134122

3.46944695195361e-18
1

0
4

0

0

0
4

0

0

0
4

0

0

0
4

0

0

0

0
4

0

0

0

0
4

0

0

0
4

0

0

0

0
4

0

0

0
4

0

0

0
4

0

0

0
4

0

0

0

0
4

0

0

0

0
4

0

0

0

0
4

0

0

0
4

0

0

0
4

0

0

0
4

0

0

0
4

0

0

0
4

0

0

0
4

0

0

0
4

0

0

0
4

0.00447073
2

0.00447073
2

0

0
4

0

0

0
4

0

0

0
4

0

0

0
4

0.00546617

0.00546617

0
4

0

0

0
4

0

0

0
4

0

0

0
4

0

0

0
4

0

0

0
4

0

0

0
4

0
2

0
2

0
4

0

0

0
4

0

0

0
4

0

0

0
4

0

0

0
4

0

0

0
4

0

0

0
4

0

0

0
4

0

0

0
4

0

0

0
4

0

0

0

0
4

0

0

0
4

0

0

0

0
4

2.84060969191202e-15

0
4

0.10842
3

0.10842

0.10842

0

0

0

0
4

0

0

0

0

0
4

0

0

0
4

0

0

0

0
4

0

0

0

0

0
4

0

0

0
4

0

0

0

0
4

0

0

0
4

0

0

0
4

0

0

0
4

0

0

0
4

0
4

0.515851

0.284781

0.262176
8

0

0

0

0

0

0

0

0

0

0

0

0

0

0

0

0

0

0

0

0

0

0.0049252

0

0

0

0

0

0

0

0

0

0

0

0

0

0

0

0

0

0

0

0.0049252

0

0

0

0

0

0

0

0

0

0

0

0

0

0

0

0

0

0

0

0

0

0

0

0

0

0

0

0

0

0

0

0

0

0

0

0

0

0

0

0

0

0.0127553

0

0

0

0

0

0

0

0

0

0

0

0

0

0

0

0

0

0

0

0

0

0

0

0

0

0

0

0

0

0

0

0

0

0

0

1.90819582357449e-17

0
4

0

0

0

0

0

0

0

0

0
4

0

0

0
4

0

0

0

0

0

0
4

0

0

0
4

0

0

0

0
4

0

0

0
4

0

0

0

0

0
4

0

0

0

0

0
4

0

0

0

0

0
4

0.0127553

0

0.0127553

0
4

0

0

0
4

0

0

0

0

0

0
4

0

0

0

0

0
4

0

0

0
4

0

0

0
4

0

0

0

0

0
4

0

0

0

0

0
4

0

0

0
4

0

0

0
4

0

0

0

0
4

0

0

0
4

0

0

0

0
4

0

0

0

0
4

0

0

0
4

0

0

0
4

0

0

0
4

0

0

0
4

0

0

0
4

0

0

0
4

0

0

0
4

0

0

0
4

0

0

0
4

0

0

0
4

0

0

0

0

0

0

0

0
4

0

0

0
4

0

0

0
4

0

0

0
4

0

0

0
4

0

0

0
4

0

0

0
4

0

0

0
4

0

0

0
4

0

0

0
4

0

0

0
4

0.146899
7

0.146899
7

0

0
4

0.0714153

0.0714153

0
4

0

0

0
4

0

0

0
4

0

0

0
4

0

0

0
4

0

0

0
4

0

0

0
4

0

0

0
4

0

0

0
4

0

0

0

0

0

0

0
4

0

0

0
4

0

0

0

0

0
4

0

0

0

0
4

2.77555756156289e-17

0
4

0.641842

0

0

0

0

0

0

0

0

0

0

0

0

0

0

0

0

0

0

0

0

0

0

0

0

0

0

0

0

0

0

0

0

0
4

0.065594

0

0

0

0

0.065594

0
4

0

0

0
4

0.576248

0

0.576248

0
4

0

0

0
4

0

0

0
4

0

0

0
4

0

0

0
4

0
4

0.402221

0

0

0

0
4

0.125096

0.0135974

0.0979014

0

0

0.0135974

0

0

0

0

1.73472347597681e-18

0
4

0

0

0
4

0

0

0

0
4

0

0

0
4

0

0

0
4

0

0

0

0
4

0

0

0
4

0

0

0

0
4

0

0

0
4

0

0

0
4

0

0

0
4

0

0

0

0

0

0
4

0

0

0
4

0

0

0
4

0

0

0
4

0

0

0
4

0

0

0
4

0

0

0
4

0

0

0
4

0

0

0
4

0.149572

0

0

0

0.149572

0
4

0

0

0

0
4

0.127553

0.127553

0

0
4

0

0

0

0
4

0

0

0

0
4

0
2

0
2

0

0

0
4

0

0

0

0
4

0
4

4.65862

4.65862

4.65862

0

0

0

0

0

0

0

0

0
4

0

0

0

0
4

0

0

0

0
4

0

0

0
4

0

0

0
4

0

0

0
4

0
4

2.30694

0

0

0

0

0

0

0

0

0

0

0

0
4

2.30694

2.30694

0
4

0

0

0

0
4

0
4

12.9201

0
7

0

0

0

0

0

0

0

0

0

0

0

0

0

0

0

0

0

0

0

0

0

0

0

0

0

0

0

0

0

0

0

0

0
4

1.27703

0.697594
7

0

0

0

0

0

0

0

0.0127553

0

0.0127553

0.0573989
7

0.0382659

0

0

0.349842
7

0

0

0.10842

0

0

0

1.2490009027033e-16

0
4

0.752687

0.27267
7

0.135231
7

0

0

0

0

0

0

0

0

0

0

0.254859
7

0

0

0

0

0

0

0

0.00780182

0

0

0.0702163
6

0

0

0.00520121

0

0

0

0

0

0

0

0

0

0

0

0

0

0

0

0

0

0

0

0

0

0

0

0

0

0

0

0

0

0

0

0

0

0

0

0

0

0

0

0

0

0.00670609

0

0

0

1.58727198051878e-16

0
4

4.46073

1.55477

0.808951
7

0

0

0

0.0382659

0

0

0

0.0573989

0

0

0

0.165819
7

0

0

0

0

0

0

0

0

0

0.00689936

0
7

0

0

0

0

0

0

0

0.00689936

0

0

0.0508281
7

0

0.019133

0

0

0

0

0

0

0

0

0.0127553
6

0

0

0.0127553

0.0127553

0

0

0

0

0

0

0

0

0

0

0.0127553

0.019133

0.0382659

0

0

0

0

0

0

0

0

0

0.00543897

0

0

0

0
7

0

0

0.578013
7

0

0

0.0434798

0

0.00689936

0

0

0

0

0.0255106

0.178143

0

0.00447073

0

0

0

0

0

0

0

0

0
5

0

0

0.00520121

0

0

0.0469426

0

0.0108779

0

0

0.481692
7

0

0

0

0

0.0255106

0

0.0446436

0

0

0

0.0561176
7

0

0

0

0

0

0

0

0

0

0

0

0

0

0

0

0

0

0

0

0

0

0
7

0

0

0

0

0

0

0.019133

0

0

0.0127553

0.0907113

0

0

0

0

0

0

0

0

0.00780182

0

3.27515792264421e-15

0
4

0.573989
2

0.10842
2

0

0

0.0510212

0

0

0

0

0

0

0

0.267861
2

0.0127553

0

0

0

0

0

0

0

0

0

0

0.019133

0

0

0.0127553

0

0

0.019133

0.0255106

0

0

0.0127553
2

0

0

0

0

0

0

0

0

0

0

0

0

0

0.0446436

0

0

0

0

0

0

0

0

0

0

0

0

0

0

0

0

0

0

0

0

0
4

0.265633

0.265633

0
4

0.00447073
7

0.00447073
7

0

0

0
4

0.339936

0

0

0

0.00543897

0

0

0

0

0

0.149572

0

0.184925

0

0

0

0

0

2.77555756156289e-17

0
4

0.210737

0.00665242

0

0.0318883

0

0

0

0.0127553

0

0.0446436

0

0.0127553

0

0

0

0

0.0765318

0.0127553

0

0.0127553

0

0

0
4

0
3

0

0

0

0

0
4

0.0702163
6

0.0702163
6

0

0
4

0

0

0

0

0

0
4

0

0

0

0

0

0

0
4

0

0

0

0
4

0

0

0

0
4

0

0

0

0

0
4

0.00894146

0.00894146

0

0

0
4

0

0

0

0

0
4

0

0

0
4

0.306127

0.306127

0

0

0

0

0

0
7

0

0

0

0

0

0

0

0
4

0

0

0
4

0

0

0

0
4

0

0

0
4

0

0

0
4

0

0

0

0

0
4

0.153064

0

0

0.153064

0
4

0

0

0

0
4

0

0

0

0
4

0

0

0
4

0

0

0

0
4

0.153064
7

0.0701542
7

0.0829095
7

0

0

0

0

0

0
4

0

0

0

0
4

0

0

0

0
4

0

0

0

0
4

0

0

0
4

0

0

0

0
4

0

0

0

0
4

0

0

0
4

0

0

0
4

0

0

0
4

0

0

0
4

3.48214
7

3.40561
7

0.0765318

8.32667268468867e-17
7

0
4

0

0

0
4

0

0

0
4

0

0

0
4

0

0

0
4

0

0

0
4

0

0

0
4

0

0

0
4

0

0

0
4

0

0

0
4

0

0

0
4

0.660082
7

0.660082

0

0

0

0
4

0

0

0
4

0

0

0
4

0

0

0
4

0

0

0
4

0

0

0
4

0

0

0
4

0

0

0
4

0

0

0
4

0

0

0
4

0

0

0
4

0
7

0
7

0
4

0

0

0
4

0

0

0
4

0

0

0
4

0

0

0
4

0

0

0
4

0

0

0
4

0

0

0
4

0.0573989
7

0

0.0573989

0

0
4

0.0290597
5

0

0

0.0134122

0.0156475

0
4

0.114798
7

0.102042
7

0

0.0127553

5.20417042793042e-18
7

0
4

0
4

0.652588

0.652588

0.652588

0

0

0

0
4

0

0

0

0

0

0

0

0
4

0

0

0
4

0

0

0
4

0

0

0
4

0

0

0
4

0

0

0
4

0

0

0
4

0

0

0

0

0

0

0

0

0
4

0

0

0

0

0

0

0

0
4

0

0

0

0

0

0
4

0

0

0

0
4

0

0

0

0
4

0

0

0

0
4

0

0

0

0
4

0

0

0
4

0
4

144.19
7

48.623
7

9.30324
7

0.840724
7

0.0176347
7

0

0

0

0.00543897

0

0

0

0

0

0.00520121

0.0182042
7

0

0

0

0.013003

0

0.00520121

0

0

0

0

0.00520121
7

0

0

0

0

0

0

0

0

0

0

0
7

0

0

0

0

0

0

0

0.0458868

0

0

0
7

0

0

0.0318883

0

0

0

0

0

0

0

0.00520121

0

0

0

0

0

0

0

0

0

0

0
7

0

0

0

0

0

0

0

0

0

0

0.00520121
7

0

0

0

0

0

0

0

0

0

0

0
7

0

0

0

0

0

0

0

0

0

0.00443786

0

0

0

0

0

0

0

0

0

0

0

0.0173018
7

0.00780182
8

0.0156036

0

0

0

0

0

0

0

0

0

0.0701542
7

0

0

0

0

0

0

0

0

0

0

1.24992
8

0

0

0

0

0

0

0

0

0.0286067

0

0.0520121
7

0

0

0

0

0

0

0

0

0

0

0.0494115
7

0

0

0

0

0

0

0

0

0

0

0
7

0

0

0

0

0

0

0

0

0

0

0
7

0

0

0

0

0

0

0

0

0

0

0
7

0

0

0

0

0

0

0

0

0

0

0
7

0

0

0

0

0

0

0

0

0

0

0
7

0

0

0.0156036

0

0

0

0

0

0

0

1.62888
7

0.00665242
7

0

0

0

0

0

0

0

0

0

0

0
7

0

0

0

0

0

0.00689936

0

0

0

0

0.00520121
7

0

0

0

0

0

0

0.00520121

0

0

0

0
7

0

0

0

0

0

0

0

0

0

0

0
7

0

0

0

0

0

0

0

0

0.00780182

0.0312073

0
6

0

0

0

0

0.00543897

0

0

0

0

0

0
7

0

0

0

0

0

0

0

0

0

0

0.0127553
7

0

0

0

0

0

0

0

0

0

0

0.0179565
7

0

0

0

0

0

0

0

0

0

0

0.00780182
7

0

0

0

0

0

0

0

0

0

0

0.923441
7

0
7

0

0

0

0

0

0

0

0

0

0

0
7

0

0

0

0

0

0

0

0

0

0

0.00655526
7

0

0

0

0

0

0

0

0

0

0

0.0377218
7

0

0

0

0

0

0

0

0

0

0

0
6

0

0

0

0

0

0

0

0

0

0

0.00520121
7

0

0

0

0

0

0

0

0

0

0

0

0

0

0

0

0

0

0

0

0

0

0.0701542
7

0

0

0

0

0

0

0

0

0

0

0
7

0

0

0

0

0

0

0

0

0

0

0
7

0

0

0

0

0

0

0

0

0

0

0.241219
7

0.00655526
7

0

0

0

0

0

0.0338079

0

0

0

0.0956648

0
7

0

0

0

0

0

0

0

0

0

0

0
8

0

0

0

0

0

0

0

0

0

0

0
7

0

0

0

0

0

0

0

0

0

0.013003

0
6

0

0

0

0

0

0

0

0

0

0.0234054

0.0714153
6

0

0

0

0

0

0

0

0

0

0

0

0

0

0

0

0

0

0

0

0

0

0.0277786
7

0

0

0

0

0

0

0

0

0

0

0
8

0

0

0

0

0

0

0

0

0

0

0

0

0

0

0

0

0

0.019133

0

0

0

0.0104024
7

0
7

0

0.00665679

0

0

0

0

0.00543897

0

0

0

0
7

0

0

0

0

0

0

0

0

0

0

0
7

0

0.335058

0

0

0

0

0

0

0

0

0
7

0

0

0

0

0

0

0

0

0

0

0
7

0

0

0.0147756

0

0

0

0

0

0

0

0
7

0

0

0

0

0

0

0

0

0

0

0
7

0

0

0

0

0

0

0

0

0

0

0.0573989
7

0

0

0

0

0

0

0

0

0

0

0
6

0

0

0

0

0

0

0

0

0

0

0.0127553
7

0

0

0

0

0

0

0

0

0

0

0.397787
7

0
7

0.00520121

0

0

0

0

0

0

0

0

0.0104024

0
7

0

0

0

0

0

0

0

0

0

0

0
6

0

0

0

0

0

0

0

0

0

0

0.00815845
6

0

0

0

0.0127553

0

0

0

0

0

0

0.0119942

0

0

0

0

0

0

0

0

0

0

0
7

0

0

0

0

0

0

0

0

0

0

0

0

0

0

0

0

0

0

0

0

0

0
7

0

0

0

0

0

0

0

0

0

0

0
7

0

0

0

0

0

0

0

0

0

0

0.347307
7

0

0

0

0

0

0

0

0

0

0

0.14059
7

0
6

0

0

0

0

0

0

0

0

0

0

0.0156036
8

0

0

0

0

0

0

0

0

0

0

0.270463
7

0

0

0

0

0

0

0

0

0

0

0
7

0

0

0

0

0

0

0

0

0

0

0.0163881
6

0

0

0

0

0

0

0

0

0

0

0.00520121
7

0

0

0

0

0

0

0

0

0

0

0
7

0

0

0

0

0

0

0

0

0

0.0127553

0.0251716
7

0

0

0

0

0

0

0

0

0

0

0
7

0

0

0

0

0

0

0

0

0

0

0.0624145
8

0

0

0

0

0

0

0

0

0

0

0.174915
7

0.0104024
7

0

0

0

0

0

0

0

0

0

0

0
7

0

0

0

0

0

0

0

0

0

0

0
8

0

0

0

0

0

0

0

0

0

0

0
7

0

0

0

0

0

0

0

0.00780182

0

0

0
7

0

0

0

0

0

0

0

0

0

0

0
7

0

0

0.00520121

0

0

0

0

0

0

0

0
6

0

0

0

0

0

0

0

0

0

0

0
7

0

0

0

0

0

0

0

0

0

0

0.104024
7

0

0

0

0

0

0

0

0

0

0

0

0

0

0

0

0

0

0

0

0

0

0
7

0
6

0

0

0

0

0

0

0

0

0

0

0
7

0

0

0

0

0.0382659

0

0

0

0

0

0
6

0

0

0

0

0

0

0

0

0

0

0
7

0

0

0

0

0

0

0

0

0

0

0
7

0

0

0

0

0

0

0

0

0

0

0
7

0

0

0

0

0

0

0

0

0

0

0.696836

0

0

0

0

0

0

0

0

0

0

0
7

0

0

0

0

0

0

0

0

0

0

0
7

0

0

0

0

0

0

0

0

0

0

0.00520121
7

0

0

0

0

0

0

0

0

0

0

2.1453
7

0.013003
7

0
7

0

0.00780182

0

0

0

0

0

0

0

0

0
6

0

0

0

0

0

0

0

0

0

0

0

0

0

0

0

0

0

0

0

0

0

0.0104024
7

0

0

0

0

0

0

0

0

0

0

0.08582
7

0

0

0

0

0

0

0

0

0

0

0
7

0

0

0

0

0

0

0

0

0

0

0
7

0

0

0

0

0

0

0

0

0

0.00520121

0
8

0

0

0

0

0

0

0

0.019133

0

0

0.00520121
7

0

0

0

0

0

0

0

0

0

0.366685

0.00520121
7

0

0

0

0

0

0

0

0

0

0

0
7

0
7

0

0

0

0

0.00520121

0

0

0

0

0

0
7

0

0

0

0

0

0

0

0

0

0

0
7

0

0

0

0

0

0

0

0

0

0

0
7

0

0

0

0

0

0

0

0

0

0

0
7

0

0

0

0

0

0

0

0

0

0

0.00689936
6

0

0

0

0

0

0

0

0

0

0

0.0049252
6

0

0

0

0

0

0

0

0

0

0

0.00520121
6

0

0

0

0

0

0

0

0

0

0

0

0

0

0

0

0

0

0

0

0

0

0
7

0

0

0

0

0

0

0

0

0

0

0.0581206
7

0.00689936
7

0

0

0

0

0

0

0

0

0

0

0.0133136
7

0

0

0

0

0

0

0

0

0

0

0
6

0

0

0

0

0

0

0

0

0

0

0.00443786
7

0.00443786

0

0

0

0

0

0

0

0

0

0
7

0

0

0

0

0

0

0

0

0

0

0
7

0

0

0

0

0

0

0

0

0

0

0.0446436
7

0

0

0

0

0

0

0

0

0

0

0
7

0

0

0

0

0

0

0

0

0

0

0
7

0

0

0

0

0

0

0

0

0

0

0.0137987
8

0

0

0

0

0

0

0

0

0

0

0.0309595
7

0
6

0

0

0

0

0

0

0

0

0

0

0

0

0

0

0

0

0

0

0

0

0

0
8

0

0

0

0

0

0

0

0

0

0

0
7

0

0

0

0

0

0

0

0

0

0

0
8

0

0

0

0

0

0

0.00520121

0

0

0

0

0

0

0

0

0

0

0

0

0

0

0
6

0

0

0

0

0

0

0

0

0

0.00520121

0
7

0.00520121

0

0

0

0

0

0

0

0

0

0
7

0

0

0

0

0

0

0

0

0

0

0.00520121
6

0

0

0

0.0127553

0

0

0

0

0

0

0.11514
7

0

0

0

0

0

0

0

0

0

0

0.0379465

0.312073
7

0

0

0

0

0

0.0131105

0

0

0

0

0
6

0

0

0

0

0

0

0

0

0

0

0
7

0

0

0

0

0

0

0

0

0

0

0
7

0

0

0

0

0

0

0

0

0

0

0.0965911

0

0

0

0

0

0.00655526

0

0

0

0

0
7

0

0

0

0

0

0

0

0

0

0

0.00520121
7

0

0

0

0

0

0

0

0

0

0

0
8

0

0

0

0

0

0

0

0

0

0

0
7

0

0

0

0

0

0

0

0

0

0

0
7

0

0

0

0

0

0

0

0

0

0

0

0.013003
7

0

0

0

0

0

0

0

0

0

0

0
7

0

0

0

0

0

0

0

0

0

0

0

0

0

0

0

0

0

0

0

0

0

0
7

0

0.00689936

0

0

0

0

0

0

0

0

0

0

0

0

0

0

0

0

0

1.17634

0

0
6

0

0

0

0

0

0

0

0

0

0

0
7

0

0

0

0

0

0

0

0

0

0

0.013003

0

0

0

0

0

0.00985039

0

0

0

0

0.00520121
6

0

0

0

0

0

0

0

0

0

0

0.0156036
7

0

0

0

0

0

0

0.0133136

0

0

0

0

0
7

0

0

0

0

0

0

0

0

0

0

0.00520121
8

0

0

0

0

0

0

0

0

0

0

0
7

0

0

0

0

0

0

0

0

0

0

0
7

0

0

0

0

0

0

0

0

0

0

0.012313
6

0

0

0

0

0

0

0

0

0

0

0
7

0

0

0

0

0

0

0

0.00543897

0

0

0.207801
7

0

0

0

0

0

0

0

0

0

0

0

0

0

0

0

0

0

0

0

0

0

0

0.0104024

0

0

0

0

0

0

0

0

0

0

0
7

0.00447073

0.00689936

0.00780182

0

0

0

0

0

0

0

0.0127553
7

0

0

0

0

0

0

0

0

0

0

0
7

0

0

0

0

0

0

0

0

0

0

0.00520121
6

0

0

0

0

0

0

0

0

0

0

0

0.00543897

0

0

0

0

0

0

0

0

0

0
7

0

0

0

0

0

0

0

0

0

0

0

0

0

0

0

0

0

0

0

0

0

0

0

0

0

0

0

0

0

0

0

0

0

0

0

0

0

0

0

0

0

0

0

0.00780182

0

0

0

0

0

0

0

0

0

0

0.0227154
7

0.0377218

0.0865382
7

0

0
7

0.488914
7

0
7

0
7

0.121272
8

0

0
7

1.56015
7

0
7

0
6

0
7

0

0
7

0
7

0.010349
7

0
6

0
6

0
8

11.3723
7

0.0991914
6

0

0

0.0754176

0
6

0.00520121
7

0

0

0

0.00983289
8

0

0.104243
7

0.00655526

0.00520121
7

0

0.00520121

0
7

0.00983289

0

0
8

0
7

0
7

0.00520121
7

0
6

0
6

0

0.013003

0
6

0.181952
7

0
7

0.012313

0

0.0177804
8

0.013003
7

0
7

0
6

0
7

0
7

0
7

0

0

0

0

0

0.0135974
7

0.010349
8

0
7

0
6

0

0

0

0

0

0

0
7

0.0648462
7

0

0

0
6

0

0.013003

0

0

0

0

0

0.0392468
7

0
6

0

0
7

0
7

0

0

0

0

0.0269571

0

0.471946
5

0

0.133255

0

0

0

0.0140769

0

0.045006

0

0.0104024

0
7

0.152291

0

0.00689936

0.0133136

0

0

0.0359464

0

0

0

1.18647
7

0

0

0

0

0

0

0.00520121

0

0

0

2.47576
7

0.0720037
7

0

0

0

0

0

0

0

0

0

0

0
7

0

0

0

0

0

0

0

0

0

0.00520121

0.013003
7

0

0

0

0

0.0208048

0

0

0

0.00780182

0

0
7

0

0

0

0

0

0.0407923

0

0

0

0

3.06024
7

0

0

0

0.00443786

0

0

0

0

0

0.00780182

0.0646502
7

0

0.0182042

0

0

0

0

0

0.00543897

0

0

0.0312073
6

0

0

0

0

0.0182042

0

0

0

0.0144586

0.00520121

0.0207515
7

0

0

0

0

0

0

0

0

0.104024

0

0.0234054
7

0

0

0

0

0

0

0

0

0

0

0
7

0

0

0.0127553

0

0

0

0

0

0

0

0.185773
7

0
7

0

0

0

0.0104024

0

0

0

0

0

0

0.478511
7

0

0

0.00443786

0

0

0

0

0

0

0

0.0616196
7

0

0

0.0393315

0

0

0

0.0104024

0

0

0

0
7

0

0.0049252

0

0

0

0

0

0

0

0

0.0551833
7

0

0

0

0.0399145

0

0

0

0.00520121

0

0

0.0594534
7

0

0

0

0

0

0

0

0

0

0

0.00780182
7

0

0

0

0

0

0

0

0

0

0

0.00738779
7

0

0

0

0.00780182

0

0

0

0

0

0

0.156036
7

0.167457

0

0

0

0

0

0

0

0

0

0.013003
7

0

0

0

0

0

0

0

0

0

0

0.0925085
7

0.00447073
7

0

0

0

0

0

0

0

0

0

0

0.00543897
7

0

0.0260061

0.0104024

0

0

0

0

0

0.00655526

0.0156036

0.0193439
7

0

0

0

0

0

0

0

0.00665679

0

0

0.010349
7

0

0

0

0

0

0

0

0

0

0.0310471

0.0237621
7

0

0

0

0

0.0127553

0

0

0

0

0

0.0382075
7

0

0

0.00520121

0

0

0

0

0

0

0

0

0

0

0

0

0

0

0

0

0

0

0.0517146
7

0

0

0

0

0.0108779

0

0

0

0

0

0.0179565
8

0

0

0

0

0

0

0

0

0

0.00665242

0.00520121

0

0

0.00543897

0

0.0360539

0

0

0

0

0

0.250128
7

0.0104024

0

0

0

0

0.0110946

0

0

0

0

0

0.00655526

0

0

0

0

0

0

0

0

0

0

0
7

0

0

0

0

0

0

0

0

0

0

0.0633493
7

0

0

0

0

0.00543897

0

0

0

0.00780182

0

0.00738779
7

0

0

0

0

0

0

0

0

0

0

0
7

0

0

0

0

0

0

0

0

0

0

0.0208048
7

0

0.177514

0

0

0

0

0

0

0

0

0
7

0

0

0

0

0

0

0

0

0.00520121

0

0
7

0

0

0

0

0

0

0

0

0

0

0

0

0

0

0

0

0

0

0

0

0.00443786

0.013003
7

0.17369
7

0

0.00520121

0

0

0

0

0

0

0

0

0.033152
7

0

0

0

0

0

0.00665679

0

0

0

0

0
7

0

0

0

0

0

0

0

0

0

0

0.0221893
7

0

0

0.0127553

0

0

0

0

0

0.0127553

0

0.00738779
6

0

0

0

0

0.0399407

0

0.0344968

0

0

0

0.0117565
8

0

0

0

0

0

0

0

0

0

0

0
7

0

0

0

0

0.00520121

0

0

0

0

0

0
7

0

0

0

0

0

0

0

0

0

0

0
7

0

0

0

0

0

0

0

0

0

0

0.02573
7

0

0

0.00443786

0

0

0

0

0

0.012313

0

0.256451
7

0

0

0

0

0

0

0

0

0

0

0

0.0213955
7

0

0

0

0

0

0

0

0

0

0

0.013003
8

0

0

0

0

0

0

0

0

0

0

0.0133597
7

0

0

0

0

0

0

0

0

0

0

0
7

0

0

0

0

0

0

0

0

0

0

0
7

0

0

0

0

0

0

0

0

0

0

0
6

0

0

0

0

0

0

0

0

0.00520121

0

0
6

0

0

0

0.00815845

0

0

0

0

0

0

0
7

0

0

0

0

0

0

0

0

0

0

0.0463969
7

0

0

0

0

0

0

0

0

0

0

3.31401572850609e-14
7

0
4

0
6

0
6

0
4

0.0460677

0
7

0
5

0

0

0

0

0

0

0

0

0

0

0
5

0

0

0

0

0

0

0.0127553

0

0

0

0
6

0

0

0

0

0

0

0

0

0

0

0

0

0

0

0

0.0255106

0

0

0.00780182

0

0

0
5

0

0

0

0

0

0

0

0

0

0

0

0

0

0

0

0

0

0

0

0
6

0

0
6

3.46944695195361e-18

0
4

0

0

0

0

0

0

0
4

0.0127553
7

0

0

0

0

0

0

0

0

0

0

0

0.0127553

0

0

0

0

0

0

0

0

0

0

0

0

0

0

0
4

38.3191
7

1.92578
7

2.07052
7

0.0135974
7

0.00447073
6

34.3047
7

0

0

0

0

0

0

0

0

0

0

0

0
4

52.3147
7

49.2752
7

2.97474
7

0.0648255

0

0

0

7.49400541621981e-16
7

0
4

0.876589
7

0.476056

0.00983289
7

0

0

0

0

0

0

0

0

0

0

0
6

0

0.0208048

0

0

0.00689936

0

0

0

0

0

0.0413962

0

0

0.00689936

0

0

0

0.00543897

0

0

0

0
7

0

0

0

0

0

0

0

0

0

0
7

0

0

0
6

0

0
7

0.0189999
6

0

0

0

0

0

0

0

0

0

0

0
6

0

0

0

0

0

0

0

0.255276

0

0

0.00738779
6

0

0

0

0

0

0

0

0

0

0

0

0

0

0

0

0

0

0

0

0

0

0
7

0

0

0

0

0

0

0

0

0

0

0
7

0

0

0

0

0

0

0

0

0

0

0.0275974
7

0

0

0

0

0

0

0

0

0

0

0

0

0

0

0

0

0

0

0

0

0

3.46944695195361e-17
7

0
4

0.154049
7

0.154049
7

0
7

0
7

0
7

0

0

0

0

0

0
4

0.0208048
7

0.0208048
6

0

0
4

0.459654
7

0.459654

0

0
4

0
7

0

0

0

0

0
4

0
7

0

0

0

0
4

0

0

0

0
4

0

0

0

0

0

0

0
4

0

0

0

0

0
4

0

0

0

0

0
4

0

0

0
4

0.0510212

0.0510212

0
4

0.696188

0.639079

0.0462312

0

0

0

0.00543897

0

0.00543897

0

8.50014503228635e-17

0
4

0.00520121

0.00520121

0

0
4

0

0

0
4

0

0

0

0
4

0

0

0
4

0

0

0
4

0

0

0
4

0

0

0
4

0

0

0
4

0

0

0

0
4

0

0

0
4

0.329734
3

0.105716
3

0.208916

0

0.00503411

0

0

0.0100682

0

0

2.77555756156289e-17
3

0
4

0

0

0

0
4

0

0

0

0
4

0

0

0

0
4

0

0

0
4

0

0

0
4

0

0

0
4

0

0

0
4

0

0

0
4

0

0

0
4

0

0

0
4

0.00738779
7

0.00738779
7

0

0

0

0

0

0

0
4

0

0

0
4

0

0

0
4

0

0

0
4

0

0

0
4

0.0049252

0.0049252

0
4

0

0

0
4

0

0

0
4

0

0

0
4

0

0

0
4

0

0

0
4

0.355962
7

0.319374
7

0

0.0199573

0.0166311

0

2.08166817117217e-17
7

0
4

0

0

0
4

0

0

0
4

0

0

0
4

0

0

0
4

0

0

0
4

0

0

0
4

0

0

0
4

0

0

0
4

0.00543897

0.00543897

0
4

0

0

0
4

0.472819
7

0.472819
7

0

0

0

0
4

0

0

0
4

0.921556
7

0.0251705
7

0.896386
7

0

0
4

0
7

0
7

0

0
4

0.513479
3

0.513479
3

0

0
4

7.70494779089859e-14
7

0
4

0.0831553

0
2

0
2

0

0

0

0

0

0

0

0

0

0

0

0
4

0

0

0

0

0

0
4

0.0831553
2

0.0831553
2

0
4

0

0

0

0
4

0

0

0
4

0

0

0
4

0
4

0.208554

0.0218647

0
3

0

0

0

0

0

0

0

0

0

0

0
2

0

0

0

0

0

0

0

0

0

0

0
2

0

0

0

0

0

0

0

0

0

0

0
2

0

0.0218647

0

0

0

0

0
2

0
4

0

0

0

0
4

0

0

0

0
4

0

0

0

0
4

0

0

0

0
4

0

0

0
4

0

0

0
4

0.0309749
3

0
3

0.00364411

0

0

0.00546617

0

0.0182206

0

0

0

0

0

0

0

0.00364411

0

0

0

0

0

0

0

0

0

0

0

0
4

0.00364411

0

0

0

0

0
2

0
3

0

0

0.00364411

0

0

0

0
4

0

0
3

0

0

0

0

0

0

0

0

0

0

0
4

0.134832

0.0583058

0.0637719

0.0127544

0
4

0
2

0

0

0

0

0

0
4

0
2

0

0

0

0

0

0
4

0

0

0

0
4

0.0172382

0.0172382

0
4

0
4

0.0704775
2

0.0704775
2

0.0704775

0

0

0

0

0

0

0

0

0

0
4

0

0

0

0

0

0

0

0
4

0

0

0

0

0

0

0

0

0

0

0
4

0

0

0

0
4

0

0

0

0

0
4

0

0

0

0
4

0
4

0.00520121

0.00520121

0.00520121

0
4

0

0

0
4

0
4

0

0

0

0

0
4

0
4

0

0

0

0
4

0

0

0
4

0
4

0

0

0

0
4

0
4

0

0

0

0
4

0

0

0
4

0
4

0

0

0

0
4

0

0

0
4

0
4

0

0

0

0
4

0
4

0

0

0

0
4

0
4

0

0

0

0
4

0
4

0

0

0

0
4

0
4

0.00364411

0

0

0

0

0

0

0

0

0
4

0.00364411
3

0.00364411
2

0

0

0

0

0

0

0

0

0

0

0
4

0

0

0

0

0

0

0

0
4

0

0

0

0

0

0
4

0

0

0
4

0

0

0
4

0

0

0
4

0

0

0
4

0

0

0
4

0
4

0

0

0

0

0
4

0
4

0.0956648

0.0956648

0.0956648

0
4

0
4

0

0

0

0
4

0

0

0
4

0
4

0

0

0

0
4

0
4

0

0

0

0

0
4

0
4

0

0

0

0
4

0
4

0

0

0

0
4

0
4

0

0

0

0
4

0

0

0
4

0
4

0

0

0

0
4

0

0

0
4

0
4

0

0

0

0

0
4

0
4

0

0

0

0

0

0

0

0

0

0

0
4

0
7

0

0

0

0

0

0

0

0
4

0

0

0

0

0

0

0

0

0
4

0

0

0

0

0

0
4

0

0

0

0
4

0

0

0
4

0
4

0

0

0

0
4

0
4

0

0

0

0
4

0
4

0.00364411

0.00364411

0.00364411

0
4

0
4

0

0

0

0
4

0
4

0

0

0

0
4

0
4

0

0

0

0
4

0
4

0

0

0

0
4

0
4

0

0

0

0
4

0
4

0.0127553

0.0127553

0.0127553

0
4

0
4

0

0

0

0
4

0
4

0
2

0
1

0

0

0

0

0

0

0

0
4

0

0

0

0

0

0

0

0

0

0

0

0
4

0

0

0

0

0

0

0
4

0

0

0

0

0

0
4

0

0

0

0
4

0

0

0

0
4

0

0

0
4

0
4

0

0

0

0
4

0
4

0

0

0

0
4

0
4

0

0

0

0
4

0
4

0

0

0

0
4

0
4

0

0

0

0
4

0
4

0

0

0

0
4

0
4

0

0

0

0
4

0
4

0

0

0

0
4

0
4

0

0

0

0
4

0
4

0

0

0

0
4

0
4

0.090614

0

0
3

0

0

0

0
4

0

0

0

0

0

0

0

0
4

0.090614

0.090614

0

0
4

0

0

0

0
4

0

0

0
4

0

0

0
4

0
4

0

0

0

0
4

0
4

0

0

0

0
4

0
4

0

0

0

0
4

0
4

0

0

0

0
4

0
4

0

0

0

0
4

0
4

0

0

0

0
4

0
4

0

0

0

0
4

0
4

0

0

0

0
4

0
4

0

0

0

0
4

0
4

0

0

0

0
4

0
4

0.293998
7

0.281243
7

0.281243
7

0

0

0

0

0

0

0
4

0
7

0
7

0
4

0.0127553

0.0127553

0

0
4

0
4

0

0

0

0
4

0
4

0

0

0

0
4

0
4

0

0

0

0
4

0
4

0

0

0

0
4

0
4

0

0

0

0
4

0
4

0

0

0

0
4

0
4

0

0

0

0
4

0
4

0

0

0

0
4

0
4

0

0

0

0
4

0
4

0

0

0

0
4

0
4

0
3

0
3

0
3

0

0

0

0

0

0

0
4

0
4

0

0

0

0
4

0
4

0

0

0

0
4

0
4

0

0

0

0
4

0
4

0

0

0

0
4

0
4

0

0

0

0
4

0
4

0

0

0

0
4

0
4

0

0

0

0
4

0
4

0

0

0

0
4

0
4

0

0

0

0
4

0
4

0

0

0

0
4

0
4

0.299539

0
6

0

0

0

0

0
4

0.00447073

0.00447073

0
4

0

0

0
4

0

0

0
4

0.295068
7

0.295068

0

0
4

0

0

0

0

0

0

0
4

0

0

0

0

0
4

0

0

0

0
4

0

0

0

0
4

0

0

0
4

0

0

0

0
4

0

0

0
4

0
4

0.0100682

0.0100682

0.0100682

0
4

0
4

0

0

0

0
4

0
4

0

0

0

0
4

0
4

0

0

0

0
4

0
4

0

0

0

0
4

0
4

0

0

0

0
4

0
4

0

0

0

0
4

0
4

0

0

0

0
4

0
4

0

0

0

0
4

0
4

0

0

0

0
4

0
4

0.0881618
7

0.0755765
7

0.0250616
7

0.012589

0

0.0251705

0

0.0127553

0
4

0.0125853
7

0.00755116
6

0.00503411

0

0
4

0

0

0

0
4

0

0

0

0
4

0

0

0
4

0
4

0

0

0

0
4

0
4

0

0

0

0
4

0
4

0

0

0

0
4

0
4

0.0127553

0.0127553

0.0127553

0
4

0
4

0

0

0

0
4

0
4

0

0

0

0

0

0

0

0

0

0

0
4

0

0

0
4

0

0

0
4

0
4

0

0

0

0

0

0

0

0

0

0

0

0

0

0

0

0

0

0

0

0

0

0

0

0

0

0

0

0

0

0

0

0

0

0

0

0

0

0

0

0

0

0

0

0

0

0

0

0

0

0

0

0

0

0

0

0

0

0

0

0

0

0

0
4

0

0

0

0

0

0

0

0
4

0

0

0

0

0

0

0

0

0

0

0

0

0
4

0

0

0

0

0

0
4

0

0

0
4

0
4

0

0

0

0

0

0

0

0

0

0
4

0

0

0
4

0
4

0

0

0

0

0
4

0
4

0
4

0
4

0

0

0

0

0

0
4

0
4

0

0

0

0
4

0

0

0

0

0

0
4

0
4

0.253266

0.253266

0.222291

0.0127544

0

0.0145764

0.00364411

0

0
4

0
4

0

0

0

0

0

0

0
4

0

0

0

0

0
4

0

0

0

0

0
4

0

0

0
4

0
4

0

0

0

0

0

0

0
4

0

0

0
4

0
4

0
3

0
3

0
3

0

0

0

0

0
4

0

0

0

0
4

0

0

0
4

0
4

0.0413962
8

0.0413962
8

0.0413962

0

0

0

0

0
4

0

0

0
4

0
4

0
3

0
3

0
3

0
4

0
4

0
3

0
2

0
2

0

0
4

0
3

0

0

0

0
4

0

0

0
4

0
4

0
1

0
1

0

0

0

0

0

0

0

0

0

0

0

0
2

0

0

0

0

0

0

0

0

0
4

0

0

0
4

0

0

0
4

0
1

0
2

0

0

0

0

0

0

0

0

0

0

0
1

0

0

0

0

0

0

0

0

0

0

0
4

0
1

0

0

0

0

0

0

0

0

0

0

0
4

0

0

0

0

0
4

0

0

0

0
4

0

0

0

0
4

0

0

0

0
4

0

0

0

0
4

0

0

0

0
4

0
4

0
3

0
3

0
3

0

0

0

0

0
4

0

0

0

0

0

0
4

0
4

0.00985039

0

0

0

0

0

0
4

0

0

0

0

0

0

0
4

0

0

0
4

0

0

0

0
4

0

0

0

0
4

0.00985039

0.00985039

0
4

0
4

0

0

0

0

0

0

0
4

0

0

0

0

0

0

0
4

0
4

0

0

0

0

0

0

0
4

0
4

0
2

0

0

0

0

0

0

0
4

0

0

0
4

0

0

0
4

0
4

0

0

0

0

0
4

0

0

0

0
4

0

0

0

0
4

0
4

0

0

0
2

0

0

0
4

0

0

0
4

0

0

0
4

0
4

0
7

0

0

0

0
4

0

0

0

0

0
4

0

0

0

0
4

0

0

0
4

0
4

0.00364411
2

0.00364411

0

0

0.00364411

0
4

0
3

0

0

0

0

0
4

0

0

0
4

0
4

0.411254

0.0837283

0.036939

0.0197008

0.0221634

0.0049252

0
4

0.327525

0.317675

0.00985039

0
4

5.55111512312578e-17

0
4

0.249704

0.232478
7

0.192241
7

0.00447073

0

0.0312951

0

0

0.00447073

0

0

0

0

0

9.54097911787244e-18
7

0
4

0

0

0
4

0.0127553

0.0127553

0

0
4

0

0

0

0
4

0

0

0
4

0

0

0

0

0

0

0

0

0

0
4

0
1

0

0

0

0

0

0

0

0

0
4

0.00447073
6

0

0.00447073

0

0

0

0

0
4

0
6

0
6

0

0
4

0

0

0

0

0

0

0
4

0
7

0

0

0

0

0
4

0

0

0

0

0

0

0
4

0

0

0

0

0

0
4

0
4

0.351867
8

0.351867
8

0.351867
8

0
4

0

0

0
4

0

0

0
4

0
4

0.276952
3

0.276952

0.276952

0

0
4

0

0

0
4

0

0

0
4

0

0

0
4

0

0

0
4

0
4

0
7

0
7

0
7

0

0
4

0
4

0.0127553
7

0
7

0

0

0

0
4

0.0127553

0.0127553

0
4

0
4

0

0

0

0

0

0

0

0
4

0

0

0
4

0

0

0
4

0
4

0
2

0
2

0

0

0

0

0
4

0

0

0
4

0

0

0
4

0
4

0
4

0

0

0

0

0

0

0
4

0

0

0
4

0
4

0

0

0

0

0

0

0
4

0
4

0
7

0
7

0
7

0

0

0
4

0
4

0
4

0
4

0

0

0
4

0

0

0
4

0

0

0
4

0

0

0
4

0
4

0.213689
7

0.0347589
7

0.013003
7

0.0217559
6

0
8

0

0

0

0

0

0

0
4

0.0965069
7

0.039108
7

0.0573989

0

6.93889390390723e-18
7

0
4

0.0517146
7

0.0517146
7

0

0

0

0

0
4

0.0179536
7

0.0104024
8

0.00755116

0

0

0

0

0

8.67361737988404e-19
7

0
4

0.0127553

0

0.0127553

0

0
4

0

0

0

0

0
4

0

0

0

0
4

0

0

0
4

0
4

0

0

0

0

0

0

0
4

0

0

0

0

0
4

0

0

0
4

0
4

0

0

0

0

0
4

0
4

0

0

0

0
4

0
4

0

0

0

0

0

0

0
4

0
4

0
3

0
3

0

0

0

0
4

0

0

0
4

0
4

0

0

0

0

0
4

0

0

0
4

0

0

0
4

0
4

0

0

0

0

0

0

0
4

0
4

0

0

0

0

0
4

0

0

0

0
4

0
4

0

0

0

0

0
4

0

0

0

0
4

0
4

0

0

0

0

0

0
4

0

0

0
4

0
4

0
3

0
3

0
3

0

0

0

0

0
3

0

0

0

0

0

0

0

0
4

0

0

0

0
4

0

0

0
4

0
4

0

0

0

0

0

0

0

0
4

0

0

0
4

0
4

0

0

0

0

0

0
4

0
4

0

0

0

0

0
4

0

0

0

0
4

0

0

0
4

0
4

0

0

0

0

0
4

0

0

0
4

0

0

0

0
4

0
4

0.0427662

0.0427662

0.0127553

0.019133

0.00543897

0.00543897

5.20417042793042e-18

0
4

0
4

0.0127553
8

0.0127553

0

0.0127553

0
4

0

0

0
4

0

0

0
4

0
4

0

0

0

0
4

0

0

0
4

0

0

0
4

0
4

0

0

0

0

0

0
4

0

0

0
4

0
4

0

0

0

0

0
4

0
4

0

0

0

0

0
4

0

0

0

0
4

0

0

0
4

0
4

0.247969

0.196948

0.0344764

0

0

0

0

0.0221634

0

0.140308

0

0

0

0

0

0
4

0

0

0

0

0

0

0

0

0

0

0

0
4

0.0510212

0.0510212

0
4

0

0

0
4

0

0

0
4

0

0

0
4

0

0

0
4

0

0

0
4

0
4

0

0

0

0
4

0

0

0
4

0
4

0

0

0

0
4

0

0

0
4

0
4

0

0

0

0

0
4

0
4

0

0

0

0
4

0

0

0

0
4

0

0

0
4

0
4

0

0

0

0
4

0

0

0

0
4

0
4

0.00447073
7

0.00447073

0.00447073

0
4

0

0

0

0
4

0
4

0

0

0

0
4

0
4

0

0

0

0
4

0

0

0
4

0
4

0

0

0

0

0
4

0
4

0.0498448

0.0498448

0.0498448

0
4

0
4

0.830445

0.799603

0.698637

0

0

0.100967

0

0

0

0

0

0

0

0
4

0.00503411

0.00503411

0
4

0

0

0
4

0.00364411
7

0.00364411
7

0

0

0

0
4

0
7

0

0

0

0

0

0
4

0.0221634

0

0

0.0221634

0

0
4

0

0

0

0
4

0

0

0
4

0

0

0
4

0

0

0
4

0

0

0
4

3.46944695195361e-18

0
4

0

0

0

0
4

0
4

0

0

0

0

0

0
4

0
4

0

0

0

0
4

0
4

0

0

0

0

0
4

0
4

0

0

0

0

0
4

0

0

0
4

0
4

0

0

0

0

0
4

0

0

0
4

0
4

0

0

0

0

0
4

0

0

0
4

0
4

0.0127553

0.0127553

0.0127553

0

0
4

0
4

0

0

0

0

0

0
4

0
4

0

0

0

0
4

0
4

0

0

0

0

0

0

0

0

0

0

0

0

0

0

0

0

0

0

0

0

0

0

0

0

0

0

0

0

0

0
4

0

0

0

0
4

0
4

0

0

0

0
4

0
4

0

0

0

0

0

0
4

0
4

0

0

0

0

0
4

0

0

0
4

0
4

0.012313

0.012313

0

0.012313

0
4

0

0

0
4

0
4

0

0

0

0

0

0
4

0
4

0.0156036

0.0156036

0.0156036

0

0
4

0
4

0

0

0

0

0
4

0
4

0.00985039

0.0049252

0.0049252

0
4

0.0049252

0.0049252

0
4

0
4

0

0

0

0
4

0

0

0
4

0

0

0
4

0
4

0

0

0

0

0
4

0

0

0
4

0
4

0
3

0
3

0

0

0

0

0

0

0
4

0
4

0
3

0
3

0
3

0

0

0
4

0

0

0
4

0

0

0
4

0

0

0
4

0

0

0
4

0

0

0
4

0
4

0.622001

0.622001

0.299359
7

0
7

0

0

0

0

0

0

0

0

0

0

0
8

0

0

0

0

0

0

0

0

0

0

0
3

0

0

0

0

0

0

0

0

0

0

0

0

0

0

0

0

0

0

0

0

0

0

0

0

0

0

0

0

0

0

0

0

0
7

0

0

0

0

0

0

0

0

0

0

0

0

0

0

0

0

0

0

0

0

0

0
7

0

0

0

0

0

0

0

0

0

0

0

0

0

0

0

0

0

0

0

0

0

0

0

0

0

0

0

0

0
3

0
7

0

0

0
6

0

0
7

0
7

0

0

0

0.31599
7

0

0
7

0

0

0

0

0

0.00665242

0

0

0
7

0

0

0

0

0

0

0

0

0

0

0
6

0

0

0

0

0

0

0

0

0

0

0
3

0

0

0

0

0

0

0

0

0

0

0

0

0

0

0

0

0

0

0

0

0

0
3

0

0

0

0

0

0

0

0

0

0

0

0

0

0

0

0

0

0

0

0

0

0
4

0

0

0

0

0

0
4

0

0

0

0
4

0

0

0
4

0

0

0
4

0
4

0
3

0
4

0

0

0
4

0

0

0
4

0

0

0
4

0

0

0
4

0

0

0
4

0

0

0
4

0

0

0
4

0
4

0
4

0.562905
3

0.543772
3

0.140308

0.140308

0

0

0

0

0

0

0

0

0

0

0

0

0

0

0

0

0

0

0

0

0

0
4

0
5

0
5

0
6

0

0

0

0

0

0

0

0

0

0

0

0

0
4

0.403464
3

0

0.10842
3

0

0
4

0

0

0

0.0255106

0

0

0

0.10842

0

0

0
4

0

0

0

0

0

0

0

0

0

0

0
3

0

0

0

0

0

0

0

0

0

0

0
3

0

0

0

0

0

0

0

0

0

0

0
4

0

0

0

0

0

0

0

0

0

0

0

0

0

0

0

0

0

0

0

0

0.0765318

0
3

0

0

0

0

0

0

0

0

0

0

0

0

0

0

0

0

0

0

0

0

0

0
4

0

0

0

0

0

0

0

0

0

0

0
4

0

0

0

0

0

0

0

0

0

0

0

0
4

0

0

0

0

0

0

0

0

0

0

0
3

0

0

0

0

0

0

0

0

0

0

0
4

0

0

0

0

0

0

0

0

0

0

0

0

0

0

0

0

0

0

0

0

0

0.0446436

0

0

0

0

0

0

0

0

0

0

0
3

0

0

0

0

0

0

0

0

0

0

0

0

0

0

0

0

0
5

0.00520121
6

0

0

0
3

0

0

0

0
3

0

0

0
4

0

0

0

0.0156036
6

0
4

0

0

0
4

0.019133

0

0

0

0

0
4

0

0

0

0

0

0

0

0

0

0

0
3

0

0

0

0

0

0

0

0

0

0

0

0

0

0

0

0

0

0

0

0

0

0
3

0

0

0

0

0

0

0

0

0

0

0

0

0

0

0

0

0

0

0

0

0

3.46944695195361e-18
3

0
4

0

0

0

0
4

0

0

0
4

0

0

0
4

0

0

0
4

0

0

0
4

0

0

0

0
4

0

0

0

0
4

0

0

0
4

0

0

0
4

0

0

0
4

0

0

0
4

0

0

0
4

0

0

0
4

0
4

0
3

0
3

0
3

0

0

0

0

0

0

0

0

0

0

0

0

0

0

0

0

0

0

0

0
4

0
3

0

0

0

0

0

0
4

0
3

0

0
2

0

0

0

0

0

0

0

0
4

0
4

0

0

0

0

0

0

0

0

0

0

0

0

0

0

0

0

0
4

0

0

0
4

0
4

0
4

0
4

0
4

0
4

0

0

0
4

0
4

0

0

0

0

0

0

0

0
4

0

0

0
4

0
4

0

0

0

0
4

0
4

0

0

0

0

0

0
4

0
4

0

0

0

0

0
4

0

0

0
4

0

0

0
4

0
4

0

0

0

0

0

0

0
4

0

0

0

0
4

0
4

0

0

0

0

0
4

0
4

0

0

0

0

0

0
4

0

0

0
4

0

0

0
4

0
4

0

0

0

0
4

0

0

0
4

0
4

0

0

0

0

0
4

0
4

0
3

0
3

0
3

0

0

0

0

0

0

0

0
4

0

0

0
4

0

0

0
4

0

0

0

0

0

0

0

0
4

0
2

0

0

0

0

0
4

0
3

0

0

0

0
4

0

0

0
4

0

0

0
4

0

0

0
4

0

0

0
4

0

0

0
4

0
4

0

0

0

0
4

0
4

0

0

0

0
4

0
4

0

0

0

0
4

0

0

0
4

0
4

0

0

0

0
4

0
4

0

0

0

0
4

0

0

0
4

0
4

0

0

0

0

0
4

0

0

0
4

0
4

0

0

0

0
4

0

0

0
4

0
4

0

0

0

0
4

0
4

0

0

0

0
4

0

0

0
4

0

0

0
4

0
4

0

0

0

0

0
4

0
4

0
3

0
3

0
3

0

0

0

0

0

0
3

0
4

0

0

0

0

0

0

0
4

0

0

0
4

0

0

0
4

0

0

0

0
4

0
3

0

0

0

0

0

0

0
4

0

0

0
4

0

0

0

0
4

0

0

0

0
4

0

0

0

0
4

0

0

0
4

0

0

0
4

0
4

0

0

0

0
4

0

0

0
4

0

0

0
4

0
4

0

0

0

0
4

0
4

0.019133

0.019133

0.019133

0
4

0
4

0

0

0

0
4

0
4

0

0

0

0

0
4

0
4

0

0

0

0

0
4

0
4

0

0

0

0
4

0
4

0

0

0

0
4

0
4

0

0

0

0
4

0
4

0

0

0

0
4

0
4

0
4

0
4

0
4

0
4

0

0

0

0

0

0

0
4

0
4

0
4

0

0

0

0

0

0
4

0

0

0

0

0
4

0

0

0

0
4

0
4

0

0

0

0
4

0
4

0

0

0

0
4

0
4

0

0

0

0
4

0
4

0

0

0

0
4

0
4

0

0

0

0
4

0
4

0

0

0

0
4

0
4

0

0

0

0
4

0
4

0

0

0

0

0

0

0

0

0

0

0

0

0

0

0

0

0
4

0

0

0

0

0

0

0

0

0

0

0

0

0

0

0
4

0

0

0

0

0

0
4

0

0

0

0
4

0
4

0

0

0

0

0

0

0

0

0

0
4

0

0

0

0

0

0

0

0

0
4

0

0

0

0

0

0

0
4

0
4

0
4

0

0

0

0

0

0

0

0

0

0

0
4

0

0

0

0

0

0
4

0
4

0

0

0

0

0

0
4

0
4

0
4

0
4

0

0

0

0

0

0
4

0

0

0

0
4

0

0

0

0
4

0

0

0

0
4

0

0

0
4

0
4

4.5102810375397e-17
3

0
4

0

0

0

0

0

0

0

0

0

0

0

0

0

0

0
4

0

0

0

0

0

0
4

0

0

0

0

0

0
4

0

0

0

0
4

0
4

0
4

0
7

0
7

0
7

0
7

0

0

0
4

0
4

0
4

0

0

0

0

0
4

0
4

0
4

0

0

0

0

0

0
4

0
4

0
4

0

0

0

0

0

0
4

0
4

0
4

0

0

0

0

0
4

0
4

0
4

0

0

0

0

0
4

0
4

0
4

0

0

0

0

0
4

0
4

0
4

0.261484

0.261484

0.261484

0.261484

0
4

0
4

0
4

0

0

0

0

0
4

0
4

0
4

0

0

0

0

0
4

0
4

0
4

0.102071

0.102071

0.102071

0

0.102071

0
4

0
4

0
4

0
7

0
7

0
7

0
7

0

0

0
4

0
4

0
4

0

0

0

0

0

0
4

0
4

0
4

0

0

0

0

0
4

0
4

0
4

0

0

0

0

0
4

0
4

0
4

0

0

0

0

0
4

0
4

0
4

0

0

0

0

0
4

0
4

0
4

0

0

0

0

0
4

0
4

0
4

0

0

0

0

0
4

0
4

0
4

0

0

0

0

0
4

0

0

0
4

0
4

0
4

0

0

0

0

0
4

0
4

0
4

0

0

0

0

0
4

0
4

0
4

0
7

0
7

0
7

0
7

0
4

0

0

0
4

0
4

0
4

0

0

0

0

0
4

0

0

0
4

0
4

0
4

0

0

0

0

0
4

0
4

0
4

0

0

0

0

0
4

0

0

0
4

0
4

0
4

0

0

0

0

0
4

0

0

0
4

0
4

0
4

0

0

0

0

0
4

0
4

0
4

0

0

0

0

0

0
4

0
4

0
4

0

0

0

0

0
4

0
4

0
4

0

0

0

0

0
4

0
4

0
4

0

0

0

0

0
4

0
4

0
4

0

0

0

0

0
4

0
4

0
4

0

0

0

0

0

0

0
4

0

0

0

0
4

0
4

0

0

0

0
4

0

0

0
4

0
4

0
4

0

0

0

0

0

0
4

0
4

0
4

0

0

0

0

0

0
4

0
4

0
4

0.0127553

0.0127553

0.0127553

0.0127553

0
4

0
4

0
4

0

0

0

0

0
4

0

0

0
4

0
4

0
4

0

0

0

0

0
4

0

0

0
4

0
4

0
4

0

0

0

0

0
4

0
4

0
4

0

0

0

0

0
4

0
4

0
4

0.0318883

0.0318883

0.0318883

0.0318883

0
4

0
4

0
4

0

0

0

0

0
4

0
4

0
4

0.0108779

0.0108779

0.0108779

0.0108779

0
4

0
4

0
4

0

0

0

0

0

0

0

0

0

0

0
4

0

0

0

0

0

0
4

0

0

0
4

0
4

0
4

0

0

0

0

0
4

0
4

0
4

0

0

0

0

0
4

0
4

0
4

0

0

0

0

0
4

0
4

0
4

0

0

0

0

0
4

0
4

0
4

0

0

0

0

0
4

0
4

0
4

0

0

0

0

0
4

0
4

0
4

0

0

0

0

0
4

0
4

0
4

0

0

0

0

0
4

0
4

0
4

0

0

0

0

0
4

0
4

0
4

0

0

0

0

0
4

0
4

0
4

0
7

0
7

0

0

0

0
4

0

0

0

0

0
4

0

0

0
4

0

0

0
4

0
4

0
6

0

0

0

0

0
4

0

0

0
4

0

0

0
4

0
4

0
4

0

0

0

0

0
4

0
4

0
4

0

0

0

0

0
4

0
4

0
4

0

0

0

0

0
4

0
4

0
4

0

0

0

0

0
4

0
4

0
4

0

0

0

0

0
4

0
4

0
4

0

0

0

0

0
4

0
4

0
4

0

0

0

0

0
4

0
4

0
4

0

0

0

0

0
4

0
4

0
4

0

0

0

0

0
4

0
4

0
4

0

0

0

0

0
4

0
4

0
4

0
3

0
3

0
3

0
3

0

0
4

0

0

0
4

0
4

0
4

0

0

0

0

0
4

0
4

0
4

0

0

0

0

0
4

0
4

0
4

0

0

0

0

0
4

0
4

0
4

0

0

0

0

0
4

0
4

0
4

0

0

0

0

0
4

0
4

0
4

0

0

0

0

0
4

0
4

0
4

0

0

0

0

0
4

0
4

0
4

0

0

0

0

0
4

0
4

0
4

0

0

0

0

0
4

0
4

0
4

0

0

0

0

0
4

0
4

0
4

0.184952
5

0.184952
5

0.184952
5

0.184952
5

0

0

0
4

0

0

0
4

0

0

0
4

0
4

0
4

0

0

0

0

0
4

0
4

0
4

0

0

0

0

0
4

0
4

0
4

0

0

0

0

0
4

0
4

0
4

0

0

0

0

0
4

0
4

0
4

0

0

0

0

0
4

0
4

0
4

0

0

0

0

0
4

0
4

0
4

0

0

0

0

0
4

0
4

0
4

0

0

0

0

0
4

0
4

0
4

0

0

0

0

0
4

0
4

0
4

0

0

0

0

0
4

0
4

0
4

0

0

0

0

0

0

0

0
4

0

0

0

0
4

0
4

0
4

0

0

0

0

0
4

0
4

0
4

0

0

0

0

0
4

0
4

0
4

0

0

0

0

0
4

0
4

0
4

0

0

0

0

0
4

0
4

0
4

0

0

0

0

0
4

0
4

0
4

0

0

0

0

0
4

0
4

0
4

0

0

0

0

0
4

0
4

0
4

0

0

0

0

0
4

0
4

0
4

0

0

0

0

0
4

0
4

0
4

0

0

0

0

0
4

0
4

0
4

0

0

0

0

0

0

0

0
4

0

0

0
4

0
4

0
4

0

0

0

0

0
4

0
4

0
4

0

0

0

0

0
4

0
4

0
4

0

0

0

0

0
4

0
4

0
4

0

0

0

0

0
4

0
4

0
4

0

0

0

0

0
4

0
4

0
4

0

0

0

0

0
4

0
4

0
4

0

0

0

0

0
4

0
4

0
4

0

0

0

0

0
4

0
4

0
4

0

0

0

0

0
4

0
4

0
4

0

0

0

0

0
4

0
4

0
4

0

0

0

0

0

0

0

0

0

0

0
4

0

0

0

0

0

0
4

0

0

0

0

0
4

0

0

0
4

0
4

0
4

0
7

0
7

0
7

0
7

0
4

0
4

0
4

0

0

0

0

0
4

0
4

0
4

0

0

0

0

0
4

0
4

0
4

0

0

0

0

0
4

0
4

0
4

0

0

0

0

0
4

0
4

0
4

0

0

0

0

0
4

0
4

0
4

0

0

0

0

0
4

0
4

0
4

0

0

0

0

0
4

0
4

0
4

0

0

0

0

0
4

0
4

0
4

0

0

0

0

0
4

0
4

0
4

0

0

0

0

0
4

0
4

0
4

0

0

0

0

0

0

0
4

0
4

0
4

0

0

0

0

0
4

0
4

0
4

0

0

0

0

0
4

0
4

0
4

0

0

0

0

0
4

0
4

0
4

0

0

0

0

0
4

0
4

0
4

0

0

0

0

0
4

0
4

0
4

0

0

0

0

0
4

0
4

0
4

0

0

0

0

0
4

0
4

0
4

0.00447073

0.00447073

0.00447073

0.00447073

0
4

0
4

0
4

0

0

0

0

0
4

0
4

0
4

0

0

0

0

0
4

0
4

0
4

0

0

0

0

0

0
4

0
4

0
4

0

0

0

0

0
4

0
4

0
4

0

0

0

0

0
4

0
4

0
4

0

0

0

0

0
4

0
4

0
4

0

0

0

0

0
4

0
4

0
4

0

0

0

0

0
4

0
4

0
4

0

0

0

0

0
4

0
4

0
4

0

0

0

0

0
4

0
4

0
4

0

0

0

0

0
4

0
4

0
4

0

0

0

0

0
4

0
4

0
4

0

0

0

0

0
4

0
4

0
4

0
7

0

0

0

0

0

0
4

0
4

0

0

0

0
4

0

0

0
4

0
4

0
4

0

0

0

0

0
4

0
4

0
4

0

0

0

0

0
4

0
4

0
4

0

0

0

0

0
4

0
4

0
4

0

0

0

0

0
4

0
4

0
4

0

0

0

0

0
4

0
4

0
4

0.0234054

0.0234054

0.0234054

0.0234054

0
4

0
4

0
4

0

0

0

0

0
4

0
4

0
4

0

0

0

0

0
4

0
4

0
4

0

0

0

0

0
4

0
4

0
4

0

0

0

0

0
4

0
4

0
4

0
6

0
6

0
6

0
6

0
4

0
4

0
4

0

0

0

0

0
4

0
4

0
4

0

0

0

0

0
4

0
4

0
4

0

0

0

0

0
4

0
4

0
4

0

0

0

0

0
4

0
4

0
4

0

0

0

0

0
4

0
4

0
4

0

0

0

0

0
4

0
4

0
4

0

0

0

0

0

0

0
4

0
4

0

0

0

0

0
4

0
4

0
4

0.00887572
3

0.00887572
3

0.00887572
3

0.00887572

0

0

0

0
4

0
4

0
4

0.223652
6

0.223652
6

0.223652
6

0.223652

0

0

0
4

0
4

0
4

0
4

0
4

0
4

0
4

0
4

0

0

0
4

0
4

0
4

0
7

0
7

0
7

0
7

0
4

0
4

0
4

0
1

0
1

0
1

0
1

0

0

0

0

0
4

0

0

0
4

0
4

0
4

0
7

0
7

0
7

0
7

0
4

0
4

0
4

0
6

0
6

0
6

0
6

0

0
4

0
4

0
4

0

0

0

0

0

0

0

0
4

0
4

0
4

0

0

0
7

0
7

0

0
4

0

0

0
4

0
4

0
4

0

0

0

0

0

0

0
4

0

0

0

0

0
4

0
4

0

0

0

0
4

0

0

0
4

0
4

0
4

0
7

0
7

0
7

0
7

0

0
4

0
4

0
4

0

0

0

0

0

0
4

0
4

0
4

0

0

0

0

0

0
4

0

0

0

0
4

0
4

0
4

0

0

0

0

0

0

0
4

0
4

0
4

0

0

0

0

0

0
4

0

0

0

0
4

0

0

0
4

0

0

0
4

0
4

0
4

0
6

0
6

0
6

0
6

0

0
4

0
4

0
4

0.0591023
7

0.0591023
7

0.0591023
7

0

0

0

0.0591023

0
4

0
4

0
4

0
7

0
7

0
7

0
7

0

0
4

0
4

0
4

0

0

0

0

0

0
4

0

0

0
4

0
4

0
4

0.0701542

0.0701542

0.0701542

0.0701542

0

0
4

0
4

0
4

0

0

0

0

0

0

0
4

0

0

0
4

0

0

0
4

0
4

0
4

0.0177514

0.0177514

0.0177514

0.00665679

0.00665679

0

0.00443786

0
4

0
4

0
4

0.228007

0.228007

0.228007

0.228007

0
4

0
4

0
4

0

0

0

0

0

0
4

0
4

0
4

0
7

0
7

0
7

0
7

0
4

0
4

0
4

0

0

0

0

0

0

0

0
4

0
4

0
4

0.146686
5

0.0701542
5

0.0701542
5

0.0701542
5

0

0
4

0

0

0
4

0

0

0

0
4

0

0

0
4

0
4

0.0765318

0.0765318

0.0637765

0.0127553

0

5.20417042793042e-18

0
4

0
4

0
4

0

0

0

0

0

0

0

0
4

0
4

0
4

0
6

0
6

0
6

0
6

0
4

0
4

0
4

0

0

0

0

0

0
4

0

0

0
4

0
4

0
4

0

0

0

0

0

0

0
4

0
4

0
4

0

0

0

0

0
4

0

0

0

0
4

0
4

0
4

0

0

0

0

0

0
4

0

0

0

0
4

0
4

0
4

0

0

0

0

0

0

0

0
4

0
4

0
4

0

0

0

0

0

0
4

0

0

0
4

0
4

0
4

0.368835

0.368835

0.359894

0.359894

0
4

0

0

0
4

0.00447073

0.00447073

0
4

0.00447073

0.00447073

0
4

0
4

0
4

0

0

0

0

0

0

0
4

0
4

0
4

0
7

0
7

0
7

0
7

0

0

0
4

0
4

0
4

0

0

0

0

0

0

0
4

0
4

0
4

0.0892871

0.0892871

0.0892871

0.0892871

0

0
4

0
4

0
4

0
3

0
3

0

0

0
4

0

0

0
4

0
4

0
4

0

0

0

0

0
4

0
4

0
4

0

0

0

0

0
4

0
4

0
4

0

0

0

0

0
4

0
4

0
4

0

0

0

0

0

0
4

0
4

0
4

0

0

0

0

0
4

0
4

0
4

0

0

0

0

0

0
4

0
4

0
4

0

0

0

0

0

0
4

0
4

0
4

0

0

0

0

0

0

0

0

0

0

0

0

0
4

0

0

0

0

0

0

0
4

0

0

0

0
4

0

0

0

0
4

0
4

0
4

0

0

0

0

0

0

0
4

0
4

0
4

0

0

0

0

0

0
4

0
4

0
4

0

0

0

0

0
4

0
4

0
4

0

0

0

0

0

0

0
4

0
4

0
4

0

0

0

0

0
4

0
4

0
4

0

0

0

0

0
4

0
4

0
4

0

0

0

0

0

0
4

0

0

0

0
4

0
4

0
4

0

0

0

0

0
4

0

0

0
4

0
4

0

0

0

0
4

0
4

0
4

0

0

0

0

0

0
4

0
4

0
4

0

0

0

0

0
4

0
4

0
4

0
7

0
7

0
7

0
7

0

0

0

0

0
4

0
4

0
4

0

0

0

0

0

0
4

0

0

0
4

0
4

0
4

0

0

0

0

0
4

0
4

0
4

0

0

0

0

0
4

0
4

0
4

0

0

0

0

0
4

0
4

0
4

0

0

0

0

0

0
4

0
4

0
4

0

0

0

0

0
4

0
4

0
4

0

0

0

0

0

0
4

0
4

0
4

0

0

0

0

0

0

0
4

0
4

0
4

0

0

0

0

0

0
4

0

0

0
4

0
4

0
4

0

0

0

0

0
4

0

0

0
4

0
4

0
4

0.189844
6

0.189844
6

0.17164

0

0

0.17164

0

0

0
4

0.0182042

0.0182042

0

0

0

0
4

0

0

0
4

0
4

0
4

0

0

0

0

0
4

0
4

0
4

0

0

0

0

0
4

0
4

0
4

0

0

0

0

0
4

0

0

0
4

0
4

0
4

0

0

0

0

0

0

0
4

0
4

0
4

0

0

0

0

0
4

0
4

0

0

0

0
4

0
4

0
4

0

0

0

0

0
4

0
4

0
4

0

0

0

0

0

0
4

0

0

0
4

0
4

0
4

0

0

0

0

0
4

0
4

0
4

0

0

0

0

0

0
4

0
4

0
4

0

0

0

0

0
4

0
4

0
4

0
1

0

0

0

0
4

0
4

0

0

0

0
4

0
4

0

0

0

0
4

0
4

0

0

0

0

0
4

0
4

0

0

0

0
4

0
4

0

0

0

0
4

0
4

0

0

0

0
4

0
4

0

0

0

0
4

0

0

0
4

0
4

0

0

0

0
4

0
4

0

0

0

0

0
4

0
4

0

0

0

0
4

0
4

0
1

0
2

0

0

0

0

0

0

0

0

0

0
4

0

0

0
4

0
4

0
4

7.13631
7

0.279195

0.279195

0.273756

0.00543897

0
4

0
4

0

0

0

0
4

0
4

0

0

0

0
4

0
4

0

0

0

0
4

0
4

0

0

0

0
4

0
4

0

0

0

0
4

0
4

0.0127553

0.0127553

0.0127553

0
4

0
4

6.84436
7

6.52428
7

6.52428
7

0

0
4

0
7

0
7

0
4

0.320084

0.320084

0
4

0

0

0
4

0

0

0
4

0

0

0
4

0

0

0
4

0

0

0
4

0

0

0
4

0
4

0
4

0.331638
3

0.331638
3

0.331638
3

0.140308

0.019133

0

0

0

0

0

0

0

0

0

0.172197
3

0

0

0

0

0

0

0

0

0

0

0
2

0

0

0

0

0
3

0
2

0
3

0
2

0
3

0
2

0
4

0

0

0
4

0

0

0
4

0
4

0

0

0

0

0

0
4

0
4

0

0

0

0

0
4

0
4

0

0

0

0
4

0
4

0

0

0

0
4

0
4

0

0

0

0

0
4

0
4

0

0

0

0
4

0

0

0
4

0
4

0

0

0

0
4

0

0

0
4

0
4

0

0

0

0
4

0
4

0

0

0

0
4

0
4

0
4

0.704596
7

0

0

0

0
4

0
4

0

0

0

0
4

0
4

0

0

0

0
4

0
4

0

0

0

0
4

0
4

0

0

0

0
4

0
4

0

0

0

0
4

0
4

0

0

0

0
4

0
4

0

0

0

0
4

0
4

0

0

0

0
4

0
4

0

0

0

0
4

0
4

0

0

0

0
4

0
4

0.704596
7

0.704596
7

0.575691
7

0.0554732
5

0

0.0551949

0

0

0

0

0

0.00689936

0

0

0
7

0

0

0

0

0

0

0.00689936

0

0

0

0.00443786
6

0

0

0

0

0

0

0

0

0

0

0

0

0

0
7

0

0

0

0

0
4

0

0

0
4

0

0

0
4

0

0

0
4

0
4

0
4

0.317675

0.135443
5

0.135443
5

0.012313

0

0

0

0.12313
5

0
6

0

0

0

0

0

0

0
4

0

0

0
4

0
4

0.182232
5

0.182232
5

0.182232
5

0

0

0

0

0
4

0

0

0
4

0
4

0

0

0

0
4

0
4

0

0

0

0
4

0
4

0

0

0

0
4

0
4

0

0
7

0

0

0

0

0

0

0

0

0

0

0

0

0

0

0

0

0

0

0

0

0

0

0
4

0

0

0

0

0
4

0

0

0
4

0

0

0
4

0

0

0
4

0
4

0
4

0.570914

0

0

0

0

0

0

0

0

0

0

0

0
4

0

0

0

0
4

0

0

0
4

0
4

0.100682
5

0.100682
5

0.0679605
5

0

0.0100682

0

0.00503411
7

0

0.0125853

0

0

0

0.00503411

0

0
4

0
4

0

0

0

0

0
4

0

0

0
4

0
4

0

0

0

0
4

0
4

0

0

0

0
4

0
4

0

0

0

0
4

0
4

0

0

0

0
4

0
4

0

0

0

0
4

0
4

0

0

0

0

0
4

0
4

0

0

0

0

0
4

0
4

0.162984

0.162984

0.162984

0
4

0
4

0

0

0

0
4

0
4

0.302047
5

0.302047
5

0.276876
5

0.0251705

0

0

0

0

0

6.93889390390723e-18
5

0
4

0

0

0
4

0

0

0
4

0
4

0

0

0

0
4

0
4

0

0

0

0
4

0
4

0

0

0

0
4

0
4

0

0

0

0
4

0
4

0

0

0

0
4

0
4

0

0

0

0
4

0
4

0

0

0

0
4

0
4

0

0

0

0
4

0
4

0

0

0

0
4

0
4

0

0

0

0
4

0
4

0
7

0
7

0
7

0

0
7

0
7

0

0

0

0

0

0
4

0
4

0
5

0
5

0

0

0

0

0

0

0

0

0
4

0

0

0

0

0

0
4

0
4

0
5

0
5

0

0

0

0

0

0

0
4

0

0

0

0
4

0
4

0

0

0

0
4

0

0

0
4

0
4

0

0

0

0

0

0
4

0

0

0
4

0
4

0.00520121
7

0.00520121
7

0

0

0

0

0.00520121

0
4

0
4

0

0

0

0
4

0

0

0
4

0
4

6.24500451351651e-17

0
4

1.73819

0

0

0

0

0

0

0

0

0

0

0
4

0

0

0
4

0
4

0.0102353

0.0102353

0.00503411

0.00520121

0

0
4

0
4

0

0

0

0

0
4

0
4

0

0

0

0

0
4

0

0

0
4

0
4

0

0

0

0

0
4

0
4

0.0100682

0.0100682

0.0100682

0
4

0

0

0
4

0
4

0

0

0

0
4

0
4

0.178574

0.178574

0.178574

0
4

0
4

0.00520121

0.00520121

0.00520121

0
4

0
4

0

0

0

0
4

0

0

0
4

0
4

0

0

0

0
4

0
4

0

0

0

0

0

0
4

0
4

0

0

0

0
4

0
4

0

0

0

0
4

0
4

0

0

0

0
4

0
4

0

0

0

0
4

0
4

0

0

0

0
4

0
4

0

0

0

0
4

0
4

0

0

0

0
4

0
4

0

0

0

0
4

0
4

0

0

0

0
4

0
4

0

0

0

0
4

0
4

0.133931

0.0765318

0.0765318

0

0
4

0.0573989

0.0446436

0.0127553

0
4

6.93889390390723e-18

0
4

0

0

0

0
4

0
4

0

0

0

0
4

0
4

0

0

0

0
4

0
4

0

0

0

0
4

0
4

0

0

0

0
4

0
4

0

0

0

0
4

0
4

0

0

0

0

0
4

0
4

0

0

0

0

0

0
4

0

0

0
4

0
4

0

0

0

0

0
4

0

0

0
4

0

0

0
4

0
4

0.0104024

0.0104024

0.0104024

0
4

0

0

0

0
4

0
4

0

0

0

0

0
4

0
4

0

0

0

0

0
4

0

0

0

0
4

0
4

1.38978

1.04129

1.04129

0

0

0

0

0

0

0

0

0

0

0

0

0

0

0

0
3

0

0

0

0

0

0

0
4

0.34328

0.335478

0.00780182

0

0

0

0

0
4

0

0

0
4

0

0

0
4

0

0

0
4

0

0

0
4

0

0

0
4

0

0

0
4

0

0

0
4

0

0

0

0

0

0

0
4

0

0

0
4

0.00520121

0.00520121

0
4

0

0

0

0
4

0

0

0
4

0

0

0
4

0

0

0
4

0

0

0
4

0
4

0
4

0
5

0
6

0

0

0

0

0

0

0

0

0

0
4

0

0

0

0
4

0

0

0

0
4

0

0

0
4

0

0

0
4

0
4

0

0
5

0
6

0

0

0

0

0

0

0

0
4

0

0

0

0
4

0

0

0
4

0
4

0

0

0

0
4

0
4

0

0

0

0
4

0
4

0

0

0

0
4

0
4

0

0

0

0
4

0
4

0

0

0

0
4

0
4

0
5

0
5

0
6

0

0

0

0

0

0
4

0
4

0

0

0

0

0

0

0

0
4

0
4

0
5

0

0

0

0

0

0
4

0

0

0
4

0

0

0
4

0
4

0

0

0

0
4

0
4

0

0

0

0

0
4

0
4

0

0

0

0
4

0
4

0

0

0

0
4

0
4

0

0

0

0
4

0
4

0
4

0
3

0

0

0

0

0
4

0
4

0

0

0

0
4

0
4

0
3

0

0

0
4

0

0

0

0

0
4

0

0

0
4

0

0

0
4

0

0

0
4

0
4

0
4

4.14633

1.48131

1.48131

1.25726
7

0

0

0

0

0

0

0

0

0

0.102042
8

0.0127553
8

0.0327304
1

0.0765318

0

0

0

0

0
4

0

0

0

0

0
4

0

0

0

0
4

0
4

0.0318883

0.0318883

0.0127553

0.019133

3.46944695195361e-18

0
4

0
4

0

0

0

0

0

0
4

0

0

0
4

0
4

0

0

0

0

0
4

0

0

0

0
4

0
4

0

0

0

0
4

0
4

0

0

0

0
4

0
4

0

0

0

0

0
4

0

0

0
4

0
4

0

0

0

0
4

0
4

0

0

0

0

0
4

0
4

0

0

0

0
4

0
4

0

0

0

0
4

0

0

0
4

0
4

1.52295

0.0581444
7

0.0127553

0

0

0.00815845

0

0.0127553

0.00543897

0

0.00815845

0.00543897

0.00543897

1.73472347597681e-18
7

0
4

1.4648
1

1.4648
8

0

0

0
4

0

0

0
4

0

0

0
4

0
4

0

0

0

0
4

0
4

0

0

0

0

0
4

0
4

0

0

0

0
4

0
4

0

0

0

0
4

0
4

0

0

0

0
4

0
4

0

0

0

0
4

0
4

0

0

0

0
4

0
4

0

0

0

0
4

0
4

0

0

0

0
4

0
4

0

0

0

0
4

0
4

0

0

0

0

0

0

0
4

0

0

0

0
4

0

0

0
4

0

0

0
4

0
4

0

0

0

0
4

0
4

0

0

0

0
4

0
4

0

0

0

0
4

0
4

0

0

0

0
4

0
4

0

0

0

0
4

0
4

0

0

0

0
4

0
4

0

0

0

0
4

0
4

0

0

0

0
4

0
4

0.00364411

0.00364411

0.00364411

0
4

0
4

0

0

0

0
4

0
4

0.296469

0.241856

0.241856

0

0

0

0
4

0.0546127

0.013003

0.0416097

0
4

0
4

0.0226535

0

0

0

0
4

0

0

0

0

0

0
4

0.0226535

0.0226535

0

0
4

0
4

0
3

0
3

0

0

0

0

0

0

0

0

0
4

0

0

0
4

0

0

0
4

0
4

0.11075
3

0.11075
3

0

0

0

0

0.11075

0
4

0
4

0.0464244
6

0.0464244

0.0336691

0.0127553

0

5.20417042793042e-18

0
4

0

0

0
4

0

0

0
4

0
4

0

0

0

0

0

0
4

0
4

0.630233

0.588589

0
7

0

0

0

0

0

0

0.0573989

0.019133

0

0

0.0655439

0.312505

0

0.0127553

0

0

0

0

0

0

0

0

0

0.00364411

0

0

0

0

0.0255106

0

0

0

0

0

0

0

0

0

0

0

0

0

0

0
7

0

0

0

0

0

0

0

0

0

0

0.0793427

0

0

0

0

0

0

0

0

0.0127553

0

0

0

0

0

0
4

0

0

0

0

0
4

0

0

0
4

0

0

0
4

0

0

0

0
4

0.00520121

0.00520121

0

0
4

0

0

0
4

0

0

0
4

0

0

0
4

0

0

0
4

0

0

0
4

0

0

0
4

0
6

0
6

0

0

0
4

0

0

0
4

0

0

0
4

0

0

0
4

0.0255106

0.0255106

0
4

0

0

0
4

0

0

0

0

0

0
4

0

0

0

0

0
4

0
3

0

0

0

0
4

0

0

0

0
4

0.0109323

0.00728822

0.00364411

4.33680868994202e-19

0
4

0

0

0

0
4

0

0

0
4

6.93889390390723e-18

0
4

4.44089209850063e-16

0
4

0.992682
7

0.0399407
6

0.0399407
6

0.0399407
6

0

0
4

0
4

0.0687868
6

0.0687868
6

0.0687868
6

0
4

0
4

0

0

0

0
4

0
4

0

0

0

0
4

0
4

0

0

0

0
4

0
4

0

0

0

0
4

0
4

0.883954
7

0.883954
7

0.883954
7

0
4

0
4

0
4

0
4

42.105

0

0

0

0

0

0

0
4

0
4

0
4

25.7479
3

0.0765318

0.0765318

0

0

0

0

0

0

0

0

0

0

0.0765318
2

0

0

0

0

0

0

0

0
4

0

0

0
4

0

0

0
4

0
4

24.6071
3

0.787704

0

0

0.0208048

0

0

0

0

0

0

0

0

0

0

0

0

0

0

0

0

0

0

0

0

0

0

0

0

0

0

0

0

0

0

0.0753855

0

0

0

0

0

0

0

0

0

0

0

0

0

0

0

0

0

0

0

0

0

0

0

0
6

0

0

0

0

0

0

0

0

0

0

0
7

0

0

0

0

0

0.00655526

0

0

0

0.0104024

0

0

0

0

0

0

0

0

0

0

0

0

0

0

0

0

0

0

0

0

0

0

0

0

0

0

0

0

0

0

0

0

0

0

0
5

0

0

0

0

0

0

0

0

0

0

0

0

0

0

0

0

0

0

0

0

0

0
2

0

0

0

0

0

0

0

0

0

0

0

0

0

0

0

0

0

0

0

0

0

0.135231

0

0

0

0

0

0

0

0.00503411

0

0

0

0

0

0

0

0

0

0

0

0

0

0
6

0

0

0

0

0

0

0

0

0

0

0

0

0

0

0

0

0

0

0

0

0

0

0

0

0

0

0

0

0

0

0

0

0

0

0

0

0

0

0

0

0

0

0

0

0

0

0

0

0

0

0

0

0

0

0

0

0

0

0

0

0

0

0

0

0

0

0
2

0

0

0

0

0

0

0

0

0

0

0

0

0

0

0

0

0

0

0

0

0

0

0

0

0

0

0

0

0

0

0

0

0

0

0

0

0

0

0

0

0

0

0

0

0

0

0

0

0

0

0

0

0

0

0

0

0

0

0

0

0.244457

0

0

0
6

0

0

0
2

0

0

0
7

0

0

0

0

0

0

0

0

0

0

0

0

0

0

0

0

0

0

0

0.107501

0

0.00520121

0

0.0624145

0

0

0

0

0

0

0

0

0

0

0

0

0

0

0

0

0

0

0

0

0

0

0

0

0

0

0

0.114717

0

0

0

0

0
4

11.8183
4

0
3

0.699974
4

11.1147
3

0
6

0
4

0

0

0

0

0

0

0

0

0

0

0
4

0

0

0

0

0

0

0

0

0

0

0
4

0

0

0

0

0

0

0

0

0

0

0
4

0

0

0

0

0

0

0

0

0

0

0
4

0

0

0

0

0

0

0

0

0

0

0
2

0

0

0

0

0

0

0

0

0

0

0
3

0

0

0

0

0

0

0

0

0

0

0
4

0

0

0

0

0

0

0

0

0

0

0

0

0

0

0

0

0

0

0

0

0

0
2

0

0

0

0

0

0

0

0

0

0

0
3

0
4

0

0

0

0

0

0

0

0

0

0

0

0

0

0

0

0

0

0

0

0

0

0

0

0

0

0

0

0

0

0

0

0

0
4

0

0

0

0

0

0

0

0

0

0

0

0

0

0

0

0

0

0

0

0

0

0
3

0

0

0

0

0

0

0

0

0

0

0

0

0

0

0

0

0

0

0

0

0

0

0

0

0

0

0

0

0

0

0

0

0
4

0

0

0

0

0

0

0

0

0

0

0
4

0

0

0

0

0

0

0

0

0

0

0
4

0

0

0

0

0

0

0

0

0

0

0

0

0

0

0

0

0

0

0

0

0

0

0

0

0

0

0

0

0

0

0

0

0

0
4

0

0.00364411

0

0

0

0

0

0

0

0

0
3

0

0

0

0

0

0

0

0

0

0

0

0

0

0

0

0

0

0

0

0

0

0

0

0

0

0

0

0

0

0

0

0

0
4

0

0

0

0

0

0

0

0

0

0

0

0

0

0

0

0

0

0

0

0

0

0

0

0

0

0

0

0

0

0

0

0

0
4

0

0

0

0

0

0

0

0

0

0

0

0

0

0

0

0

0

0

0

0

0

0

0

0

0

0

0

0

0

0

0

0

0

0
2

0

0

0

0

0

0

0

0

0

0

0

0

0

0

0
4

0

0

0

0
3

0
4

0

0

0

0
4

0

0
4

0

0

0

0

0
4

0

0

0

0

0

0

0

0

0

0

0
4

0

0

0

0

0

0

0

0

0

0

0
3

0

0

0

0

0

0

0

0

0

0

0
3

0

0

0

0

0

0

0

0

0

0

2.02745806254789e-15
4

0
4

0.772608
3

0
3

0.518983
3

0.0176522
3

0
3

0
2

0
3

0
2

0.0573989
1

0

0

0

0

0

0

0

0

0

0

0.178574

0

0

0

0

0

0

0

0

0

0

0

0

0

0

0

0

0
8

0

0

0

0

0

0
4

9.43498

0.68341
7

0.176649
6

0

0.0573989

0.00703064

0.00665242

0

0

0

0

0

0

0.685512
7

0

0

0.0087883

0.0123036

0

0

0

0

0

0

0.0775818
7

0.00351532

0

0

0.0432407

0

0

0.0087883

0.0255106

0

0

0
5

0.0123036

0

0.0281226

0

0.0123036

0

0.019133

0.00703064

0

0

0.264554
6

0.0199573

0

0

0

0.00351532

0

0

0

0.0318883

0

0.0571485
7

0

0

0

0

0

0.00527298

0

0

0

0

0.0196658

0

0.0255106

0

0

0.00351532

0.309348

0

0

0

0

0

0

0

0.00351532

0

0

0.00527298

0

0

3.61783

0.0220931
8

0.0451318

0

0

0.0382659

0

0

0

0.0378142

0.0482689

1.29921

0.0203355

0.0344765

0

0.0119254

0

0.0123036

0.00655526

0

0

0

0
3

0

0.0199035

0.0333955

0

0

0.00351532

0.0205246

0

0.0171984

0

0.55067
7

0

0

0.00351532

0

0.348017

0

0

0.0205246

0.010546

0

0
3

0.0646552

0

0

0.00520121

0

0

0

0

0.0573989

0.00520121

0.0743225
7

0

0.0193343

0

0.0369109

0

0

0

0

0.0127553

0.0087883

0.105911
1

0

0

0

0

0

0

0

0

0

0.00351532

0

0.00997863

0

0.00351532

0

0.172251

0.00527298

0.00351532

0

0

0

0
4

0
2

0

0

0

0

0

0

0

0

0

0

0

0

0

0

0

0

0

0

0

0

0

0

0

0

0

0

0

0

0

0

0

0

0

0

0

0

0

0

0

0

0

0

0

0

0

0

0

0

0

0

0

0

0

0

0

0

0

0

0

0

0

0

0

0

0

0

0

0

0

0

0

0

0

0

0

0

0

0

0

0

0

0

0

0

0

0

0
4

0.374532
3

0.316877
3

0

0

0

0

0

0

0

0

0

0

0
4

0

0

0

0

0

0

0

0

0

0

0.057655
4

0

0

0

0

0

0

0
4

0
3

0
4

0
3

0
4

0

2.77555756156289e-17
3

0
4

0
3

0

0

0

0

0
4

0

0

0

0

0

0

0

0
4

0

0

0
4

0

0

0
4

0

0

0
4

0.121439

0.121439

0
4

0

0

0

0
4

0

0

0
4

0

0

0

0
4

0

0

0
4

0

0

0
4

0.00546617

0.00546617

0

0
4

0.0119558
3

0.0049252
4

0.00703064
3

0
3

0
3

0

0

0

0

0
4

0

0

0
4

0

0

0

0
4

0

0

0
4

0

0

0

0
4

0

0

0
4

0

0

0
4

0

0

0

0
4

0

0

0
4

0

0

0
4

0

0

0
4

0
3

0
3

0

0

0

0

0

0

0

0
4

0

0

0
4

0

0

0
4

0

0

0
4

0

0

0
4

0

0

0
4

0

0

0
4

0

0

0
4

0

0

0
4

0

0

0
4

0

0

0
4

0
2

0
2

0

0

0

0

0

0

0
4

0

0

0
4

0

0

0
4

0

0

0
4

0

0

0
4

0

0

0
4

0

0

0
4

0

0

0
4

0

0

0
4

0

0

0
4

0

0

0
4

0

0

0

0

0

0

0

0

0
4

0

0

0
4

0

0

0
4

0

0

0
4

0

0

0
4

0

0

0
4

0

0

0
4

0

0

0
4

0

0

0
4

0

0

0
4

0

0

0
4

0
4

0
4

0

0

0

0
4

0

0

0
4

0

0

0
4

0.0236867

0.0236867

0
4

0

0

0
4

0

0

0
4

0

0

0
4

0

0

0
4

0

0

0
4

0

0

0
4

0

0

0
4

0

0

0

0

0

0
4

0

0

0
4

0

0

0
4

0

0

0
4

0

0

0
4

0

0

0
4

0

0

0
4

0

0

0
4

0

0

0
4

0

0

0
4

0

0

0
4

0
3

0
3

0

0

0

0
4

0

0

0
4

0

0

0
4

0

0

0
4

0

0

0
4

0

0

0
4

0

0

0
4

0

0

0
4

0

0

0
4

0

0

0
4

0

0

0
4

0
4

0
4

0

0

0

0
4

0

0

0
4

0

0

0
4

0

0

0
4

0

0

0
4

0

0

0
4

0

0

0
4

0

0

0
4

0

0

0
4

0

0

0
4

0

0

0
4

0

0

0

0

0

0

0
4

0

0

0
4

0

0

0
4

0

0

0
4

0

0

0
4

0

0

0
4

0

0

0
4

0

0

0
4

0

0

0
4

0

0

0
4

0

0

0
4

0.0137135
3

0.00351532
3

0

0

0

0

0

0

0.0049252

0

0

0

0
4

0

0

0

0

0

0

0

0
4

0
4

0
3

0

0

0.00527298

0

8.67361737988404e-19
3

0
4

0

0

0

0

0

0
4

0

0

0
4

0

0

0
4

0

0

0
4

0

0

0
4

0

0

0
4

0.116612
3

0.116612
3

0

0

0
4

0
4

0

0

0

0

0

0
4

0
2

0

0

0

0

0

0

0

0
4

0

0

0

0

0

0

0

0

0

0
4

0

0

0

0
4

0
3

0

0

0

0

0

0

0
4

0
3

0

0

0

0

0

0

0

0
4

0

0

0

0
4

0
2

0

0

0

0

0

0
4

0.418135
3

0.413209
3

0

0

0

0

0

0

0

0

0

0.0049252

0
4

0

0

0
4

0

0

0

0

0

0
4

0
3

0

0

0

0

0

0
4

0
4

0

0

0

0

0

0
4

0

0

0
4

0
4

0
4

0

0

0

0
4

0

0

0

0
4

0

0

0

0

0
4

0
4

0
4

0

0
4

0
5

0
5

0

0
4

0
2

0

0

0

0

0

0
4

0
4

0
4

0
4

0
5

0
5

0

0

0

0

0

0

0

0

0

0

0

0

0

0

0

0

0

0

0

0

0

0

0

0

0
4

0
4

0

0

0
4

0

0

0

0

0
4

0

0

0
4

0

0

0

0

0
4

0
4

0

0

0
4

0

0

0

0

0

0
4

0

0

0

0
4

0

0

0

0

0

0
4

0

0

0
4

0

0

0
4

0
6

0

0

0

0

0

0

0

0

0

0

0

0

0

0

0

0

0

0

0

0

0

0

0

0

0

0

0

0

0

0

0

0

0
4

0
2

0

0

0
4

0

0

0

0

0
4

0

0

0

0
4

0.00364411

0

0

0.00364411

0
4

0

0

0

0

0
4

0

0

0

0

0
4

0

0

0

0
4

0

0

0
4

0

0

0

0
4

0

0

0

0

0
4

0.704303
2

0.517146
2

0.187157
2

0

0

0

0

0

0

0
4

0

0

0
4

0

0

0

0
4

0

0

0
4

0

0

0

0
4

0

0

0
4

0

0

0
4

0

0

0
4

0

0

0

0

0

0
4

0

0

0

0
4

0

0

0
4

0
3

0
4

0

0

0

0

0

0

0

0

0

0

0

0

0

0

0

0

0

0

0

0

0

0

0

0

0

0

0

0

0
4

0

0

0
4

0

0

0
4

0

0

0

0
4

0

0

0

0
4

0

0

0

0
4

0

0

0

0
4

0

0

0

0
4

0

0

0

0
4

0

0

0

0
4

0

0

0

0
4

0
6

0
6

0

0

0

0

0

0

0

0

0

0

0

0
4

0

0

0

0
4

0

0

0

0
4

0

0

0
4

0

0

0

0
4

0

0

0
4

0

0

0
4

0

0

0

0
4

0

0

0

0
4

0

0

0
4

0

0

0
4

0
4

0
4

0
4

0
4

0

0

0

0

0

0

0
4

0

0

0
4

0

0

0
4

0

0

0
4

0

0

0
4

0

0

0

0
4

0

0

0

0
4

0

0

0
4

0

0

0

0
4

0

0

0
4

0

0

0

0
4

1.32116539930394e-14
3

0
4

0
2

0
2

0
2

0

0

0

0

0

0

0

0

0

0

0

0

0

0

0

0

0

0

0

0

0

0
2

0

0

0

0

0

0

0

0

0

0

0

0

0

0

0

0

0

0

0

0

0

0

0

0

0

0

0

0

0

0

0

0

0

0

0

0

0

0

0

0

0

0

0

0

0

0

0

0

0

0

0

0

0
4

0
2

0

0

0

0

0

0

0

0

0

0

0

0
4

0

0

0

0

0

0

0

0

0
4

0

0

0

0

0

0
4

0

0

0
4

0
4

0

0

0
7

0

0

0

0

0

0
4

0

0

0
4

0

0

0
4

0
4

0

0

0

0
4

0

0

0
4

0
4

0

0

0

0
4

0

0

0
4

0
4

0

0

0

0
4

0

0

0
4

0
4

0

0

0

0

0
4

0
4

0

0

0

0
4

0

0

0
4

0
4

0

0

0

0
4

0
4

0

0

0

0
4

0
4

0

0

0

0
4

0
4

0

0

0

0
4

0

0

0
4

0
4

0

0

0

0
4

0
4

0

0

0

0

0

0

0

0

0
4

0
4

0

0

0

0

0
4

0
4

0

0

0

0
4

0

0

0
4

0
4

0.0637765

0.0637765

0

0.0637765

0
4

0
4

0

0

0

0
4

0
4

0

0

0

0

0
4

0
4

0

0

0

0
4

0
4

0

0

0

0
4

0
4

0

0

0

0
4

0
4

0

0

0

0
4

0
4

0

0

0

0

0
4

0
4

0.0382659
7

0.0382659
7

0

0

0.0382659

0

0

0

0
4

0
4

0.0182206

0.0182206

0.00364411

0.0145764

1.73472347597681e-18

0
4

0
4

0

0

0

0
4

0
4

0

0

0

0
4

0
4

0

0

0

0
4

0
4

0

0

0

0
4

0
4

0.0127553

0.0127553

0.0127553

0
4

0
4

0

0

0

0
4

0
4

0

0

0

0
4

0
4

0

0

0

0
4

0
4

0

0

0

0
4

0
4

0
2

0
2

0
2

0

0

0

0

0

0

0
4

0
4

0

0

0

0
4

0
4

0

0

0

0
4

0
4

0

0

0

0
4

0
4

0

0

0

0
4

0
4

0

0

0

0
4

0
4

0

0

0

0
4

0
4

0

0

0

0
4

0
4

0

0

0

0
4

0
4

0

0

0

0
4

0
4

0

0

0

0
4

0
4

0.0049252

0
5

0

0

0

0

0

0

0

0
4

0.0049252

0

0.0049252

0

0

0

0
4

0

0

0
4

0
4

0

0

0

0
4

0
4

0

0

0

0
4

0
4

0

0

0

0
4

0
4

0

0

0

0
4

0
4

0

0

0

0
4

0
4

0

0

0

0
4

0
4

0

0

0

0
4

0
4

0

0

0

0
4

0
4

0

0

0

0
4

0
4

0

0

0

0
4

0
4

0

0

0

0

0

0

0

0

0
4

0
4

0

0

0

0
4

0
4

0

0

0

0
4

0
4

0

0

0

0
4

0
4

0

0

0

0
4

0
4

0

0

0

0
4

0
4

0

0

0

0
4

0
4

0.00364411

0.00364411

0.00364411

0
4

0
4

0

0

0

0
4

0
4

0

0

0

0
4

0
4

0

0

0

0
4

0
4

0

0

0

0

0

0

0

0
4

0
4

0

0

0

0
4

0
4

0

0

0

0
4

0
4

0

0

0

0
4

0
4

0

0

0

0
4

0
4

0

0

0

0
4

0
4

0

0

0

0
4

0
4

0

0

0

0
4

0
4

0

0

0

0
4

0
4

0

0

0

0
4

0
4

0

0

0

0
4

0
4

0

0

0

0

0

0

0

0

0

0

0

0

0
4

0
4

0

0

0

0
4

0
4

0

0

0

0
4

0
4

0

0

0

0
4

0
4

0

0

0

0
4

0
4

0

0

0

0
4

0
4

0

0

0

0
4

0
4

0

0

0

0
4

0
4

0

0

0

0
4

0
4

0

0

0

0
4

0
4

0

0

0

0
4

0
4

0

0

0

0

0

0

0
4

0

0

0
4

0
4

0

0

0

0
4

0
4

0

0

0

0
4

0
4

0

0

0

0
4

0
4

0

0

0

0
4

0
4

0

0

0

0
4

0
4

0

0

0

0
4

0
4

0

0

0

0
4

0
4

0

0

0

0
4

0
4

0

0

0

0
4

0
4

0

0

0

0
4

0
4

0
6

0
6

0
6

0

0

0

0

0

0
4

0
4

0

0

0

0
4

0
4

0

0

0

0
4

0
4

0

0

0

0
4

0
4

0.0364085
2

0.0364085
2

0.0364085
2

0

0

0

0

0

0

0

0

0

0

0
2

0

0

0

0

0

0

0

0

0

0

0

0

0

0

0

0

0

0

0

0

0

0
2

0

0

0

0

0

0

0

0

0

0

0

0

0

0

0

0

0

0

0

0

0

0

0

0

0

0

0

0

0

0

0

0

0

0

0

0

0

0

0

0

0

0

0

0
2

0

0

0

0

0

0

0

0

0

0

0
4

0
2

0
2

0

0

0

0

0

0

0

0

0

0
4

0
2

0

0
2

0

0

0

0
4

0

0

0

0

0

0
4

0

0

0

0

0
4

0

0

0

0

0
4

0

0

0

0
4

0
4

0
2

0
2

0
2

0

0

0

0
4

0

0

0

0
4

0

0

0
4

0
4

0

0

0

0

0

0

0
4

0
4

0
5

0
5

0
5

0

0

0
4

0
4

0

0

0

0

0

0

0

0

0

0
4

0

0

0

0
4

0

0

0
4

0
4

0

0

0

0

0

0

0

0
4

0

0

0
4

0
4

0

0

0

0

0

0

0
4

0

0

0

0
4

0

0

0
4

0

0

0
4

0
4

0

0

0

0

0

0

0
4

0

0

0
4

0

0

0
4

0

0

0
4

0
4

0
5

0
5

0

0

0

0

0

0

0

0

0
4

0
4

0
4

0

0

0

0

0
4

0

0

0
4

0

0

0
4

0
4

0.11075
7

0.11075
7

0.11075
7

0

0

0
4

0
4

0
2

0
2

0
2

0

0

0

0

0

0

0

0

0
2

0

0

0

0

0

0

0

0
4

0
2

0
2

0

0

0

0
4

0

0

0

0

0

0

0

0

0

0

0
4

0

0

0

0
4

0
4

0

0

0

0

0

0

0
4

0
4

0
6

0
6

0
6

0
4

0
4

0
2

0

0

0

0

0

0
4

0

0

0

0
4

0

0

0
4

0
4

0.0573989
7

0.0573989
7

0.0573989
7

0

0
4

0
4

0.522967
7

0.522967
7

0.522967

0

0
4

0
4

0

0

0

0

0

0
4

0

0

0
4

0
4

0

0

0

0

0

0

0

0
4

0

0

0
4

0

0

0
4

0
4

0
6

0
6

0
6

0

0
4

0
4

0

0

0

0

0
4

0
4

0

0

0

0
4

0

0

0
4

0

0

0
4

0
4

0
2

0
2

0
2

0

0

0

0

0

0

0

0

0

0

0

0

0

0

0

0

0

0

0

0

0

0

0
4

0

0

0
4

0
2

0

0

0

0

0

0

0
4

0

0

0

0

0

0
4

0

0

0

0

0
4

0

0

0
4

0

0

0

0
4

0

0

0

0
4

0

0

0
4

0

0

0
4

0
4

0

0

0

0

0
4

0

0

0

0
4

0
4

0

0

0

0

0
4

0

0

0
4

0
4

0

0

0

0

0

0

0

0
4

0

0

0

0
4

0
4

0.0765318
6

0.0765318
6

0.0765318
6

0
4

0
4

0

0

0

0
4

0
4

0

0

0

0

0
4

0
4

0

0

0

0

0
4

0

0

0

0
4

0
4

0

0

0

0

0

0
4

0
4

0

0

0

0

0

0

0
4

0

0

0
4

0
4

0

0

0

0
4

0
4

0

0

0

0

0

0

0

0

0

0

0

0

0

0

0

0

0

0

0
4

0

0

0
4

0

0

0

0
4

0
4

0

0

0

0

0

0
4

0

0

0
4

0

0

0
4

0
4

0

0

0

0
4

0
4

0

0

0

0

0
4

0
4

0

0

0

0
4

0

0

0
4

0
4

0

0

0

0

0
4

0

0

0
4

0
4

0

0

0

0

0
4

0
4

0

0

0

0

0

0
4

0

0

0
4

0

0

0
4

0
4

0

0

0

0

0
4

0

0

0
4

0

0

0
4

0

0

0
4

0
4

0.0255106

0.0255106

0.0255106

0

0
4

0
4

0

0

0

0
4

0
4

0
2

0
2

0
2

0
2

0

0

0

0

0

0

0

0
4

0

0

0

0
4

0
4

0.00985039

0.0049252

0

0.0049252

0
4

0

0

0
4

0.0049252

0.0049252

0
4

0

0

0
4

0
4

0
7

0

0

0

0
4

0

0

0
4

0
4

0

0

0

0
4

0
4

0

0

0

0
4

0

0

0

0
4

0

0

0
4

0
4

0

0

0

0

0
4

0
4

0

0

0

0

0
4

0

0

0
4

0
4

0

0

0

0

0

0
4

0
4

0

0

0

0
4

0

0

0
4

0
4

0

0

0

0
4

0
4

0

0

0

0

0
4

0
4

0
2

0
2

0
2

0

0

0

0

0

0
4

0
2

0

0

0

0

0

0

0

0
4

0
2

0

0

0

0

0

0
4

0

0

0

0
4

0

0

0

0
4

0

0

0
4

0

0

0
4

0

0

0
4

0

0

0
4

0
4

0

0

0

0
4

0
4

0

0

0

0
4

0

0

0
4

0
4

0

0

0

0

0

0
4

0
4

0

0

0

0
4

0
4

0

0

0

0

0

0
4

0
4

0

0

0

0

0

0
4

0
4

0

0

0

0
4

0

0

0
4

0
4

0

0

0

0

0
4

0

0

0
4

0
4

0

0

0

0
4

0
4

0

0

0

0
4

0

0

0
4

0
4

0

0

0

0

0

0

0

0

0

0

0
4

0
4

0

0

0

0
4

0
4

0

0

0

0
4

0
4

0

0

0

0

0

0
4

0
4

0

0

0

0

0
4

0
4

0

0

0

0
4

0
4

0.0251705

0.0251705

0.0151023

0.0100682

0
4

0
4

0.045307

0.045307

0.045307

0
4

0

0

0
4

0
4

0.0127553

0.0127553

0

0.0127553

0
4

0
4

0

0

0

0
4

0
4

0

0

0

0
4

0

0

0
4

0
4

0

0

0

0

0

0

0

0

0

0

0
4

0

0

0
4

0

0

0

0
4

0
4

0

0

0

0
4

0

0

0
4

0
4

0

0

0

0
4

0
4

0

0

0

0

0
4

0
4

0

0

0

0

0
4

0
4

0

0

0

0
4

0
4

0

0

0

0
4

0

0

0
4

0
4

0

0

0

0
4

0

0

0
4

0
4

0

0

0

0

0
4

0
4

0

0

0

0

0
4

0
4

0

0

0

0
4

0

0

0
4

0
4

0

0

0

0

0

0

0

0

0
4

0
1

0
1

0
4

0

0

0
4

0

0

0

0
4

0

0

0

0
4

0

0

0

0
4

0

0

0
4

0
4

3.60649010655578e-15
3

0
4

0.345926
4

0
4

0

0

0

0
4

0

0

0
4

0

0

0
4

0

0

0
4

0
4

0.0532194

0.0532194

0.0532194

0
4

0
4

0.292707

0.292707

0.292707

0

0
4

0
4

0

0

0

0
4

0
4

0

0

0

0
4

0
4

0
4

8.60942
5

0
4

0
4

0
4

0

0

0

0

0

0

0

0

0

0

0

0

0

0

0

0

0

0

0

0

0

0

0

0

0

0

0

0

0

0

0

0

0

0

0

0

0

0

0

0

0

0

0

0

0

0

0
4

0

0

0

0
4

0

0

0

0

0

0

0

0

0

0

0

0
4

0

0

0

0

0

0

0

0

0

0

0
4

0

0

0

0

0

0

0

0

0

0

0
4

0

0

0

0

0

0

0

0

0

0

0

0

0

0

0

0

0

0

0

0

0

0
4

0

0

0

0

0

0

0

0

0

0

0
4

0

0

0

0

0

0

0

0

0

0

0
4

0

0

0

0

0

0

0

0

0

0

0
4

0
4

8.60942
5

3.20013

1.14256

0.0775485
2

0.389814

0
4

0

0
4

0

0

0

0

0

0

0

0.350824
7

0

0

0

0

0

0

0

0

0

0

0
3

0.086242

0

0

0

0

0

0

0

0

0

0
4

0

0

0

0

0

0

0

0

0

0

0

0

0.00689936

0

0

0

0.010349

0

0

0

0

0.925356

0

0

0.0586446

0

0

0

0

0

0

0

0

0

0

0

0

0

0

0

0

0

0

0.0760024

0

0

0

0

0.0655439

0

0

0

0.010349

0

0

0

0

0

6.31439345255558e-16

0
4

0.0892871

0.0892871

0

0

0

0
4

0

0

0
4

5.32
5

0.0955476
5

0
3

2.77677
3

0.127755
4

2.31992
5

0
4

0

0

0

0

0

0

0

0

0

0

0

0

0

0

0

0

0

0

0

0

0

0

0

0

0

0

0

0

0

0

0

0

0

0

0

0

0

0

0

0

0

0

0

0

0

0

0

0

0

0

0

0

0

0

0

0

0
4

0

0

0

0

0

0

0

0

0

0

0

0

0

0

0

0

0

0

0

0

0

0

0

0

0

0

0

0

0

0

0

0

0

0

0

0

0

0

0

0

0

0

0

0

0

0

0

0

0

0

0

0

0

0

0
4

0

0

0

0

0

0

0

0
3

0

0
4

0

0
4

0

0

0
4

0

0
4

0

0

0
4

0

0

0

0

0
4

0

0

0

0

0

0

0

0

0

0

0

0

0

0

0

0

0

0

0

0

0

0

0

0

0

0

0

0

0

0

0

0

0

0

0

0
4

0

0

0

0

0

0

0

0

0

0

0
3

0

0

0

0

0

0

0

0

0

0

0
4

0

0

0

0

0

0

0

0

0

0

0
4

0

0

0

0

0

0

0

0

0

0

0
4

0

0

0

0

0

0

0

0

0

0

0

0
4

0

0

0

0

0

0

0

0

0

0

0

0

0

0

0

0

0

0

0

0

0

0
4

0

0

0

0

0

0

0

0

0

0

0

0

0

0

0

0

0

0

0

0

0

0
4

0

0

0

0

0

0

0

0

0

0

0
4

0

0

0

0

0

0

0

0

0

0

0
4

0

0

0

0

0

0

0

0

0

0

0
4

0

0

0

0

0

0

0

0

0

0

0
4

0

0

0

0

0

0

0

0

0

0

0

0
4

0

0

0

0

0

0

0

0

0

0

0
4

0

0

0

0

0

0

0

0

0

0

0

0

0

0

0

0

0

0

0

0

0

0

0

0

0

0

0

0

0

0

0

0

0
4

0

0

0

0

0

0

0

0

0

0

0

0

0

0

0

0

0

0

0

0

0

0

0

0

0

0

0

0

0

0

0

0

0
4

0

0

0

0

0

0

0

0

0

0

0

0

0

0

0

0

0

0

0

0

0

0

0

0

0

0

0

0

0

0

0

0

0
6

0
3

0

0

0

0

0

0

0

0

0

0

0

0

0

0

0

0

0

0

0

0

0

0
3

0

0

0

0

0

0

0

0

0

0

0
4

0

0

0

0

0

0

0

0

0

0

0

0

0

0

0

0

0

0

0

0

0

0
4

0

0

0

0

0

0

0

0

0

0

0

0

0

0

0

0

0

0

0

0

0

0
4

0

0

0

0

0

0

0

0

0

0

0
4

0

0

0

0

0

0

0

0

0

0

0

0

0

0

0

0

0

0

0

0

0

0

0

0

0

0

0

0

0

0

0

0

0

0

0

0

0

0

0

0

0

0

0

0

0
4

0

0

0

0

0

0

0

0

0

0

0
4

0

0

0

0

0

0

0

0

0

0

0

0

0

0

0

0

0

0

0

0

0

0
4

0

0

0

0

0

0

0

0

0

0

0
3

0

0

0

0

0

0

0

0

0

0

0
3

0

0

0

0

0

0

0

0

0

0

0

0

0

0

0

0

0

0

0

0

0

0
4

0

0

0

0

0

0

0

0

0

0

0
4

0

0

0

0

0

0

0

0

0

0

0

0

0

0

0

0

0

0

0

0

0

0

0

0

0

0

0

0

0

0

0

0

0

0
4

0

0

0

0

0

0

0

0

0

0

0
4

0

0

0

0

0

0

0

0

0

0

0

0

0

0

0

0

0

0

0

0

0

0

0

0

0

0

0

0

0

0

0

0

0
4

0

0

0

0

0

0

0

0

0

0

0

0

0

0

0

0

0

0

0

0

0

0

0

0

0

0

0

0

0

0

0

0

0
4

0
4

0

0

0

0

0

0

0

0

0

0

0

0

0

0

0

0

0

0

0

0

0

0
4

0

0

0

0

0

0

0

0

0

0

0

0

0

0

0

0

0

0

0

0

0

0

0

0

0

0

0

0

0

0

0

0

0
3

0

0

0

0

0

0

0

0

0

0

0

0

0

0

0

0

0

0

0

0

0

0
4

0

0

0

0

0

0

0

0

0

0

0

0

0

0

0

0

0

0

0

0

0

0
4

0

0

0

0

0

0

0

0

0

0

0
3

0

0

0

0

0

0

0

0

0

0

0

0

0

0

0

0

0

0

0

0

0

0

0

0

0

0

0

0

0

0

0

0

0

0

0

0

0

0

0

0

0

0

0

0

0

0

0

0

0

0

0

0

0

0

0

0

0

0

0

0

0

0

0

0

0

0

0

0

0

0

0

0

0

0

0

0

0

0
4

0

0

0

0

0

0

0

0

0

0

0

0

0

0

0

0

0

0

0

0

0

0

0

0

0

0

0

0

0

0

0

0

0

0

0

0

0

0

0

0

0

0

0

0

0

0

0

0

0

0

0

0

0

0

0

0
4

0

0

0

0

0

0

0

0

0

0

0

0

0

0

0

0

0

0

0

0

0

0
4

0

0

0

0

0

0

0

0

0

0

0

0

0

0

0

0

0

0

0

0

0

0

0

0

0

0

0

0

0

0

0

0

0

0

0

0

0

0

0

0

0

0

0

0

0

0

0

0

0

0

0

0

0

0

0

0

0

0

0

0

0

0

0

0

0

4.44089209850063e-16
5

0
4

0

0

0

0
4

0
4

0

0
4

0

0

0

0
4

0

0
5

0

0

0

0

0

0

0

0

0

0

0

0

0

0

0
4

0

0

0
4

0

0

0
4

0

0

0
4

0

0

0
4

0

0

0
4

0

0

0
4

0

0

0
4

0

0

0
4

0
4

0

0

0

0
4

0
4

0

0

0

0
4

0
4

0

0

0

0
4

0
4

0

0

0

0
4

0
4

0
4

0
4

0

0

0
4

0
4

0

0

0

0
4

0

0

0
4

0

0

0
4

0
4

0
4

0
3

0

0

0

0

0

0

0
4

0
4

0

0

0

0
4

0
4

0

0

0

0
4

0
4

0

0

0

0

0
4

0
4

0

0

0

0

0
4

0
4

0

0

0

0
4

0
4

0

0

0

0
4

0
4

0

0

0

0
4

0
4

0

0

0

0
4

0
4

0

0

0

0
4

0
4

0

0

0

0
4

0
4

0
4

0.510996

0.510996

0.510996

0.15424

0

0

0

0

0

0

0

0

0

0

0
1

0

0

0

0.00447073

0

0

0

0.00447073

0

0

0

0

0.00447073

0

0.0236867

0

0

0

0.00670609

0

0

0.263773

0

0

0

0

0

0

0

0

0

0.0134122

0.0223536

0.0134122

7.97972798949331e-17

0
4

0
4

0

0

0

0
4

0
4

0
4

0.921019

0.90299

0.811888

0.154674
7

0

0

0

0

0

0.311571

0

0

0.191231

0

0.0140613
6

0

0

0

0

0

0

0

0

0

0.0728822

0

0

0

0

0

0

0

0

0

0.0100682

0

0

0

0

0

0

0

0

0

0

0

0

0.0382659
6

0

0

0

0

0

0

0

0

0.019133

0

0

0
4

0

0

0

0

0

0

0
4

0.0911028

0.0911028

0
4

0

0

0

0
4

0

0

0

0
4

0

0

0
4

0

0

0
4

0
4

0

0

0

0
4

0
4

0.00527298

0.00527298

0.00527298

0
4

0
4

0.0127553

0.0127553

0.0127553

0
4

0
4

6.76542155630955e-17

0
4

2.21304
7

2.21304
7

2.17478
7

1.47324
7

0.0637765

0.0127553

0

0.0127553

0.019133

0.0127553

0

0

0.0127553

0

0.140308
6

0.0637765
7

0.248728

0.019133

0.0255106

0.0318883

0

0.0382659

1.73472347597681e-16
7

0
4

0.0382659
6

0.0382659

0

0

0
4

0

0

0

0
4

2.08166817117217e-16
7

0
4

0
4

0.197008
7

0.197008
7

0.197008
7

0

0.00985039

0.187157

0

0

0
4

0
4

0
4

0

0

0

0

0
4

0
4

0
4

0

0

0

0

0
4

0
4

0
4

0

0

0

0

0
4

0
4

0
4

0

0

0

0

0
4

0
4

0
4

0

0

0

0

0
4

0
4

0
4

0

0

0

0

0
4

0
4

0
4

0

0

0

0

0
4

0
4

0
4

0

0

0

0

0
4

0
4

0
4

0

0

0

0

0
4

0
4

0
4

0

0

0

0

0
4

0
4

0
4

0.0589973
7

0.0589973
7

0.0589973
7

0.0163881
7

0.0426092
7

0

0
4

0

0

0
4

0
4

0
4

0

0

0

0

0
4

0
4

0
4

0

0

0

0

0
4

0
4

0
4

0

0

0

0

0
4

0
4

0
4

0

0

0

0

0
4

0
4

0
4

0

0

0

0

0
4

0
4

0
4

0

0

0

0

0
4

0
4

0
4

0

0

0

0

0
4

0
4

0
4

0

0

0

0

0
4

0
4

0
4

0

0

0

0

0
4

0
4

0
4

0.0127553

0.0127553

0.0127553

0.0127553

0
4

0
4

0
4

0.0382659

0.0382659

0.0382659

0.0382659

0

0

0
4

0

0

0
4

0
4

0
4

0

0

0

0

0
4

0
4

0
4

0

0

0

0

0
4

0
4

0
4

0

0

0

0

0
4

0
4

0
4

0

0

0

0

0
4

0
4

0
4

0

0

0

0

0
4

0
4

0
4

0

0

0

0

0
4

0
4

0
4

0

0

0

0

0
4

0
4

0
4

0

0

0

0

0
4

0
4

0
4

0

0

0

0

0
4

0
4

0
4

0

0

0

0

0
4

0
4

0
4

0.0213246
7

0.0213246
7

0.0176805
7

0

0.0127553

0.0049252

0
4

0.00364411

0.00364411

0

0

0
4

1.30104260698261e-18
7

0
4

0
4

0

0

0

0

0
4

0
4

0
4

0

0

0

0

0
4

0
4

0
4

0

0

0

0

0
4

0
4

0
4

0

0

0

0

0
4

0
4

0
4

0

0

0

0

0
4

0
4

0
4

0

0

0

0

0
4

0
4

0
4

0

0

0

0

0
4

0
4

0
4

0

0

0

0

0
4

0
4

0
4

0

0

0

0

0
4

0
4

0
4

0

0

0

0

0
4

0
4

0
4

0

0

0

0

0

0

0

0
4

0

0

0

0
4

0
4

0
4

0

0

0

0

0
4

0
4

0
4

0

0

0

0

0
4

0
4

0
4

0

0

0

0

0
4

0
4

0
4

0

0

0

0

0
4

0
4

0
4

0

0

0

0

0
4

0
4

0
4

0

0

0

0

0
4

0
4

0
4

0

0

0

0

0
4

0
4

0
4

0

0

0

0

0
4

0
4

0
4

0

0

0

0

0
4

0
4

0
4

0

0

0

0

0
4

0
4

0
4

0

0

0

0

0

0

0
4

0
4

0
4

0

0

0

0

0
4

0
4

0
4

0

0

0

0

0
4

0
4

0
4

0

0

0

0

0
4

0
4

0
4

0

0

0

0

0
4

0
4

0
4

0

0

0

0

0
4

0
4

0
4

0

0

0

0

0
4

0
4

0
4

0

0

0

0

0
4

0
4

0
4

0

0

0

0

0
4

0
4

0
4

0.0127553

0.0127553

0.0127553

0.0127553

0
4

0
4

0
4

0

0

0

0

0
4

0
4

0
4

0

0

0

0

0

0

0

0
4

0
4

0
4

0

0

0

0

0
4

0
4

0
4

0

0

0

0

0
4

0
4

0
4

0
7

0
7

0
7

0

0

0

0
4

0
4

0
4

0

0

0

0

0

0

0
4

0

0

0
4

0
4

0
4

0

0

0

0

0
4

0

0

0

0

0
4

0

0

0
4

0
4

0
4

2.21304
7

2.21304
7

2.21304
7

2.18753
7

0

0

0.0255106

2.42861286636753e-17
7

0
4

0
4

0

0

0

0
4

0
4

0
4

0

0

0

0

0

0

0
4

0
4

0
4

0

0

0

0

0

0
4

0
4

0
4

0.0446436

0.0446436

0.0446436

0.0446436

0
4

0
4

0
4

0

0

0

0

0
4

0
4

0
4

0

0

0

0

0
4

0

0

0
4

0
4

0

0

0

0
4

0

0

0
4

0
4

0
4

0

0

0

0

0

0
4

0
4

0
4

0

0

0

0

0

0
4

0
4

0
4

0

0

0

0

0

0
4

0
4

0
4

0

0

0

0

0
4

0
4

0
4

0

0

0

0

0
4

0
4

0
4

0
2

0
2

0
2

0
2

0

0

0

0

0
4

0
2

0

0

0

0

0

0

0
4

0

0

0
4

0

0

0
4

0
4

0
4

0

0

0

0

0

0

0
4

0
4

0
4

0

0

0

0

0
4

0
4

0
4

0

0

0

0

0
4

0

0

0
4

0

0

0
4

0
4

0
4

0

0

0

0

0

0
4

0
4

0
4

0

0

0

0

0

0

0

0
4

0
4

0
4

0

0

0

0

0
4

0
4

0
4

0.0127553

0.0127553

0.0127553

0

0.0127553

0
4

0

0

0
4

0
4

0

0

0

0
4

0
4

0
4

0

0

0

0

0

0
4

0

0

0
4

0
4

0
4

0

0

0

0

0
4

0
4

0
4

0

0

0

0

0

0

0
4

0
4

0
4

0

0

0

0

0

0

0

0

0

0
4

0
4

0
4

0.0446436

0.0446436

0.0446436

0.0446436

0

0
4

0

0

0
4

0
4

0
4

0

0

0

0

0
4

0
4

0
4

0

0

0

0

0
4

0
4

0
4

0

0

0

0

0
4

0

0

0
4

0
4

0
4

0

0

0

0

0

0
4

0

0

0
4

0
4

0
4

0

0

0

0

0

0
4

0
4

0
4

0

0

0

0

0

0
4

0
4

0
4

0

0

0

0

0
4

0
4

0
4

0

0

0

0

0

0
4

0
4

0
4

0

0

0

0

0

0
4

0

0

0
4

0
4

0
4

0
4

0
4

0
4

0

0

0

0

0

0

0
4

0

0

0
4

0
4

0
4

0

0

0

0

0
4

0
4

0

0

0

0
4

0
4

0
4

0.0127553

0.0127553

0

0

0
4

0

0

0
4

0.0127553

0.0127553

0
4

0
4

0
4

0

0

0

0

0

0
4

0
4

0
4

0

0

0

0

0

0
4

0
4

0
4

0

0

0

0

0

0
4

0
4

0
4

0

0

0

0

0
4

0

0

0
4

0
4

0
4

0

0

0

0

0
4

0

0

0
4

0
4

0
4

0

0

0

0

0
4

0
4

0
4

0

0

0

0

0
4

0

0

0
4

0
4

0
4

0

0

0

0

0
4

0

0

0
4

0
4

0
4

0.159177
7

0.159177
7

0.159177
7

0

0.0892871

0.0318883

0

0

0

0

0.0380012

0
4

0

0

0

0
4

0
4

0
4

0

0

0

0

0
4

0
4

0
4

0

0

0

0

0
4

0
4

0
4

0

0

0

0

0

0
4

0
4

0
4

0

0

0

0

0
4

0

0

0
4

0
4

0
4

0

0

0

0

0
4

0
4

0
4

0

0

0

0

0
4

0

0

0
4

0
4

0
4

0

0

0

0

0
4

0

0

0
4

0
4

0
4

0

0

0

0

0

0
4

0
4

0
4

0

0

0

0

0
4

0

0

0
4

0
4

0
4

0

0

0

0

0

0
4

0
4

0
4

0.0237934

0.0237934

0.0237934

0.012313

0

0

0.00655526

0.0049252

0

0
4

0

0

0
4

0
4

0

0

0

0

0
4

0
4

0
4

0

0

0

0

0
4

0
4

0
4

0

0

0

0

0
4

0

0

0
4

0
4

0
4

0

0

0

0

0
4

0
4

0
4

0

0

0

0

0

0
4

0
4

0
4

0

0

0

0

0
4

0
4

0
4

0

0

0

0

0
4

0

0

0
4

0
4

0
4

0

0

0

0

0
4

0
4

0
4

0

0

0

0

0
4

0

0

0
4

0
4

0
4

0

0

0

0

0
4

0

0

0
4

0
4

0
4

0.0197008

0.0197008

0.0197008

0.0197008

0

0
4

0
4

0
4

0
5

0
5

0
5

0
5

0

0

0
4

0

0

0

0

0

0
4

0
4

0
4

0

0

0

0

0

0
4

0
4

0
4

0

0

0

0

0

0
4

0
4

0
4

0

0

0

0

0

0
4

0
4

0
4

0.239777

0.239777

0.239777

0.239777

0
4

0
4

0
4

0

0

0

0

0
4

0

0

0
4

0
4

0
4

0

0

0

0

0
4

0
4

0
4

0

0

0

0

0
4

0
4

0
4

0

0

0

0

0
4

0
4

0
4

0

0

0

0

0
4

0

0

0
4

0
4

0
4

0

0

0

0

0
4

0
4

0
4

0.0318883
7

0.0318883
7

0.0318883
7

0.0318883
7

0

0
4

0

0

0

0
4

0

0

0
4

0
4

0

0

0

0
4

0
4

0
4

0

0

0

0

0

0
4

0
4

0
4

0

0

0

0

0
4

0
4

0
4

0.0127553

0.0127553

0.0127553

0.0127553

0
4

0
4

0
4

0

0

0

0

0
4

0
4

0
4

0

0

0

0

0
4

0
4

0
4

0.00503411

0.00503411

0.00503411

0.00503411

0
4

0
4

0
4

0

0

0

0

0
4

0
4

0
4

0

0

0

0

0
4

0
4

0
4

0

0

0

0

0
4

0
4

0
4

0

0

0

0

0
4

0
4

0
4

0.595632

0.595632

0.595632

0.0176194

0

0

0

0

0.0843677

0

0

0

0

0

0.0805457

0

0

0

0

0.093131

0

0

0

0

0

0
4

0

0

0

0

0

0

0

0

0

0

0

0

0

0

0

0

0.133931

0

0

0

0

0.0847249

0

0

0

0

0

0

0

0

0

0

0

0

0

0.00564832

0

0

0

0

0

0.0956648

0

0

0

0

0

0

0

0

0

0

0

0

0

0

0

0

0

0

0

0

0

0

2.77555756156289e-17

0
4

0

0

0

0

0

0
4

0

0

0
4

0

0

0
4

0

0

0
4

0

0

0
4

0

0

0
4

0

0

0

0

0
4

0

0

0

0
4

0

0

0

0

0
4

0

0

0

0

0
4

0

0

0

0
4

0

0

0
4

0

0

0
4

0

0

0
4

0
4

0

0

0

0

0

0

0
4

0
4

0

0

0

0

0

0
4

0

0

0
4

0
4

0

0

0

0

0
4

0
4

0

0

0

0
4

0
4

0

0

0

0
4

0
4

0
4

0

0

0

0

0
4

0
4

0
4

0
4

0.0823938
3

0.00780182
3

0.00780182
3

0

0

0

0

0

0

0

0

0

0

0

0

0
4

0
3

0
3

0

0

0
4

0

0

0
4

0

0

0

0

0

0

0

0

0

0
4

0

0

0

0

0

0
4

0

0

0
4

0

0

0
4

0.00780182

0.00780182

0
4

0

0

0
4

0

0

0

0
4

0

0

0
4

0

0

0

0
4

0

0

0

0
4

0

0

0
4

0

0

0

0
4

0

0

0
4

0

0

0
4

0
4

0

0

0

0

0

0

0

0

0
4

0
4

0

0

0

0

0
4

0
4

0

0

0

0
4

0
4

0

0

0

0
4

0
4

0
4

0

0

0

0

0

0

0
4

0
4

0
4

0.0701542

0.0701542

0.0701542

0.0701542

0
4

0
4

0
4

0

0

0

0

0
4

0
4

0
4

0

0

0

0

0
4

0
4

0
4

0

0

0

0

0
4

0
4

0
4

0

0

0

0

0
4

0
4

0
4

0

0

0

0

0
4

0
4

0
4

0

0

0

0

0
4

0
4

0
4

0

0

0

0

0
4

0
4

0
4

0

0

0

0

0
4

0
4

0
4

0

0

0

0

0

0
4

0
4

0
4

0

0

0

0

0

0
4

0
4

0
4

0

0

0

0

0
4

0
4

0
4

0

0

0

0

0
4

0
4

0
4

0.00443786

0.00443786

0.00443786

0.00443786

0
4

0
4

0
4

0

0

0

0

0
4

0
4

0
4

0

0

0

0

0
4

0
4

0
4

1.73472347597681e-18
3

0
4

195.971

0
4

0

0

0

0

0

0

0

0

0

0

0

0

0

0

0

0

0

0

0

0

0

0

0

0

0

0

0

0

0

0

0

0

0

0

0

0
4

0

0

0

0

0
4

0

0

0

0
4

0

0

0

0
4

0
4

0
4

0

0

0

0

0

0
4

0

0

0

0

0
4

0

0

0
4

0

0

0
4

0
4

0

0

0

0

0

0
4

0

0

0

0
4

0

0

0
4

0
4

0
6

0
6

0
6

0
4

0
4

0

0

0

0
4

0

0

0
4

0
4

0

0

0

0

0
4

0

0

0

0
4

0

0

0
4

0
4

0

0

0

0

0
4

0
4

0

0

0

0

0

0
4

0
4

0

0

0

0

0
4

0

0

0
4

0
4

0

0

0

0
4

0
4

0

0

0

0
4

0

0

0
4

0
4

0
4

0
4

0

0

0

0

0

0

0

0

0

0

0

0

0

0

0

0

0

0
4

0

0

0

0

0

0

0
4

0

0

0

0

0

0

0

0
4

0
4

0

0

0

0
4

0
4

0

0

0

0
4

0
4

0

0

0

0
4

0
4

0

0

0

0
4

0
4

0

0

0

0
4

0
4

0

0

0

0
4

0
4

0

0

0

0
4

0
4

0
4

0
4

0

0

0

0

0

0

0

0

0

0

0
4

0

0

0
4

0

0

0

0
4

0

0

0
4

0
4

0
4

0
4

0

0

0

0

0
4

0

0

0

0

0
4

0

0

0
4

0

0

0
4

0
4

0
4

0
4

0

0
4

0

0

0

0

0

0

0

0
4

0
4

0

0

0

0

0

0

0

0

0
4

0

0

0

0

0
4

0

0

0

0

0

0
4

0

0

0
4

0

0

0
4

0
4

0
6

0
6

0
6

0

0

0
4

0
4

0

0

0

0

0

0

0

0

0

0

0
4

0

0

0

0
4

0

0

0

0

0
4

0

0

0
4

0
4

0

0

0

0

0

0

0

0
4

0

0

0
4

0
4

0
4

0.312505
5

0.312505
5

0

0

0
4

0.312505
5

0.0829095
5

0

0

0

0.210462

0

0

0.019133

0

0

0

0

0
4

0
4

0
4

192.68
7

0
6

0
6

0

0

0
4

0

0

0
4

0
4

5.74914

0.178309

0.142486

0.0128359
1

0.0104024

0

0

0

0

0

0

0

0

0

0

0

0

0

0

0

0

0

0

0

0

0

0.0125853

0

0

0

0

0

0

0

0

0

0

0

0

0

0

0

0

0

0

0

0

0

0

0

0

0

0

0

0

0

0

0

0

0

0

0

0

0

0

0

0

0

0

0

3.64291929955129e-17

0
4

0.145299

0.0692963

0

0

0.0760024

0

0

0

0

0

0

0
4

1.56829

0.11389

0

0

0

0

0

0

0

0

0

0

0

0

0

0

0

0.0176194

0

0

0

0

0

0.0104024

0

0.0100682

0

0.00503411

0

0.0049252

0

0.0629264

0

0

0

0

0

0

0.0104024

0

0

0

0

0.013003

0

0

0

0

0

0.0147756

0

0

0

0

0

0

0

0

0

0.00520121

0

0.00755116

0

0

0.00780182

0

0

0

0.0780182

0

0.0049252

0

0

0

0.0255883
6

0.0911161

0

0

0

0

0

0.0182042

0.00780182

0

0

0.0221426
7

0

0

0

0

0

0

0

0

0

0

0.0179536
8

0

0

0.125853

0

0

0

0

0

0

0

0.0658084

0

0

0

0.00780182

0.0226535

0

0

0

0

0

0.0344764

0

0

0.0528581

0

0

0

0

0

0

0

0.00520121

0

0

0

0.0131105

0

0

0

0

0

0.0176194

0.0104024
7

0

0

0.0156036

0

0

0

0

0

0

0

0

0

0

0

0

0

0

0.647551

0

0

0

3.33066907387547e-16

0
4

0.0404262
6

0.00527298

0.010546

0

0.00527298

0.010546

0.0087883

0
4

0

0

0
4

0.00689936
7

0

0.00689936

0
4

0

0

0

0

0
4

0

0

0

0

0
4

0.0206981

0.0206981

0

0

0
4

0

0

0

0
4

0

0

0
4

0.0544875

0

0.0544875

0
4

0

0

0

0
4

1.87328

1.82729

0

0

0

0

0

0

0.014939

0

0.0310471

0

0
4

0

0

0
4

0

0

0

0
4

0.0158189

0.0158189

0
4

0

0

0

0
4

0

0

0
4

0

0

0
4

0

0

0
4

0

0

0
4

0

0

0
4

0.00527298

0.00527298

0
4

0.699549

0.0140613

0.493903

0.186312

0

0.00527298

0

0

0
4

0

0

0
4

0

0

0
4

0

0

0
4

0

0

0
4

0

0

0
4

0

0

0
4

0.00503411

0.00503411

0
4

0

0

0
4

0

0

0
4

0

0

0
4

0.151967

0.0275974
7

0

0.00689936

0.0551949

0.0622749
7

0

0

0

0

0

0

0
4

0

0

0
4

0

0

0
4

0

0

0
4

0

0

0
4

0

0

0
4

0

0

0
4

0

0

0
4

0

0

0
4

0

0

0
4

0

0

0
4

0.0286067

0

0

0

0

0

0

0.00520121
6

0.00520121

0

0

0.0104024

0

0

0.00780182

0
4

0

0

0
4

0

0

0
4

0

0

0
4

0.355317

0.231129

0.124189

0

0

0

0

0

0

0

0
4

0.124794

0.0703064
7

0.0087883

0

0.0246072

0.0210919

0

0
4

0.0128359

0.0128359

0

0

0

0

0
4

0.462257

0.455358

0

0

0.00689936

0

0

0

1.73472347597681e-17

0
4

0
4

183.763
7

0.0610849
6

0
6

0
7

0

0

0

0

0

0

0

0.0155325

0

0.0335305

0
6

0

0

0

0

0

0

0

0

0.00447073

0

0

0

0

0

0

0

0

0

0

0

0

0

0

0

0

0

0

0

0

0

0

0

0

0

0

0

0

0

0

0

0

0

0

0
5

0

0.00755116

0

0

0

0

0

0

0

0

0
6

0

0

0

0

0

0

0

0

0

0
6

0
6

3.46944695195361e-18
6

0
4

7.60457
7

7.60457
7

0

0

0

0
4

172.01
7

130.269
7

41.54
7

0.0558841

0

0

0

0

0

0.00527298

0

0

0

0

0.088496

0

0

0

0

0

0

0

0

0

0

0.0335305

0

0

0

0

0

0.00894146

0.00887572

0

0

0

0

0

0

0

0

0

0

0

0

1.8996956785422e-14
7

0
4

0
7

0

0

0

0

0

0
4

1.00046

0.990395

0

0.0100682

0

0
4

1.19287
7

0.290802
7

0

0.186262

0

0

0

0.010546

0

0

0.0131105

0

0.0151023

0.359939
7

0

0

0.0201364

0

0

0

0

0

0

0

0
6

0

0.0123036

0

0.00503411

0

0.00527298

0.269325

0.00503411

0

0

0

0

0
4

0
6

0
6

0

0

0

0
4

0
6

0
6

0

0

0

0

0

0
4

1.31835
6

0.251185
7

0.151565
6

0.0201364
7

0.0360539

0

0.00503411

0.00503411

0.00655526

0

0.0100682

0.0151023

0

0.19807

0

0

0.00655526

0

0

0

0

0

0

0

0.0851638
7

0

0

0

0

0.00503411

0

0

0.00503411

0.117603
6

0.00503411
7

0.115523
6

0.00655526
7

0.100212
7

0.172826
6

0
4

0.179097
7

0.174659
7

0

0

0

0

0

0

0.00443786

1.73472347597681e-18
7

0
4

0

0

0

0

0

0

0

0

0

0

0

0

0
4

0.0332839
6

0.0332839
6

0

0
4

0.0778942
6

0.0778942
6

0

0

0

0

0
4

0

0

0

0
4

0

0

0
4

0

0

0

0
4

0

0

0
4

0

0

0
4

0

0

0
4

0.00503411

0.00503411

0
4

0

0

0
4

0

0

0
4

0

0

0
4

0.198616
7

0.161705
7

0

0

0.0369109

0

1.38777878078145e-17
7

0
4

0

0

0
4

0

0

0
4

0

0

0
4

0

0

0
4

0

0

0
4

0

0

0
4

0

0

0
4

0

0

0
4

0

0

0
4

0

0

0
4

0
7

0
7

0

0
4

0

0

0
4

0

0

0
4

0

0

0
4

0

0

0
4

0

0

0
4

0

0

0
4

0

0

0
4

0

0

0
4

0

0

0
4

0

0

0
4

0.0637765

0.0637765

0

0

0

0
4

0

0

0
4

0

0

0
4

0

0

0
4

0

0

0
4

0

0

0
4

0.0175766
7

0.0175766
7

0
4

0

0

0
4

0

0

0
4

0

0

0

0
4

0

0

0
4

0
4

0
7

0
6

0

0
6

0

0

0

0
4

0

0

0

0
4

0
4

1.54244
7

0

0

0
4

1.52933
8

1.52933
8

0

0
4

0

0

0

0
4

0

0

0
4

0.0131105

0.0131105

0
4

0

0

0
4

0

0

0
4

0

0

0
4

0

0

0
4

0

0

0
4

7.11236625150491e-17
7

0
4

1.62516

1.27079

1.27079

0

0
4

0.284741

0.0123036

0.00527298

0

0.258376

0.0087883

0
4

0

0

0
4

0.0123036

0.0123036

0
4

0

0

0
4

0

0

0
4

0
5

0
5

0

0
4

0.010546
7

0.010546

0

0
4

0.00351532

0.00351532

0
4

0.0344764

0.00738779

0.0270886

3.46944695195361e-18

0
4

0

0

0

0
4

0.0087883

0.00527298

0.00351532

4.33680868994202e-19

0
4

0

0

0
4

0

0

0
4

0
4

0
7

0
7

0

0

0

0
6

0

0
4

0
4

0

0

0

0
4

0
4

0

0

0

0
4

0
4

0

0

0

0
4

0
4

0

0

0

0
4

0
4

0

0

0

0

0

0
4

0
4

0

0

0

0
4

0
4

0

0

0

0

0
4

0
4

0

0

0

0
4

0
4

0

0

0

0
4

0
4

0

0

0

0
4

0
4

0

0

0

0
4

0
4

0

0

0

0
4

0
4

2.79776202205539e-14
7

0
4

0.811436

0.811436

0.80597
7

0.285947

0.0528396

0.0049252

0

0

0

0

0

0

0

0.00738779

0.378387
6

0.0163985

0.0049252

0

0.0172382

0

0.0145764

0

0.00738779

0

0.00364411

0.012313

1.04083408558608e-17
7

0
4

0

0

0
4

0.00546617

0.00546617
7

0

0

0

0
4

0

0

0

0
4

0

0

0
4

0

0

0
4

0

0

0
4

0

0

0
4

4.85722573273506e-17

0
4

0

0

0

0

0

0

0

0

0
4

0
4

0

0

0

0
4

0
4

0

0

0

0
4

0
4

0

0

0

0
4

0
4

0

0

0

0

0
4

0
4

0

0

0

0
4

0

0

0
4

0
4

0

0

0

0
4

0
4

0

0

0

0
4

0

0

0
4

0
4

0

0

0

0
4

0
4

0

0

0

0
4

0
4

0

0

0

0
4

0
4

0

0

0

0
4

0
4

0
4

0.548478

0.548478

0.548478

0
6

0

0

0

0

0

0

0

0

0

0

0
6

0

0

0

0

0

0

0

0

0.357148

0

0

0.19133

0

0

0
4

0

0

0
4

0

0

0
4

0

0

0
4

0

0

0
4

0
4

0

0

0

0

0
4

0
4

0

0

0

0
4

0
4

0

0

0

0
4

0
4

0

0

0

0
4

0
4

0

0

0

0
4

0
4

0

0

0

0
4

0
4

0

0

0

0
4

0
4

0

0

0

0
4

0
4

0

0

0

0
4

0
4

0
4

0.695293

0

0

0

0

0
4

0
4

0.695293

0

0

0
4

0.695293
1

0.593251

0

0.102042

0

4.16333634234434e-17
1

0
4

0

0

0

0

0
4

0

0

0
4

0
4

0

0

0

0

0
4

0

0
1

0

0

0
4

0
4

0
4

0.412549
5

0.412549
5

0.368676

0.368676

0
7

0

0

0

0

0

0

0

0

0

0

0

0

0

0

0
4

0.0438723
6

0.00939592
6

0.0270886
7

0.00738779
6

0

0

0

0

0

2.60208521396521e-18
6

0
4

0

0

0
4

0

0

0
4

0

0

0
4

0

0

0
4

0

0

0
4

0

0

0
4

0
6

0
6

0

0
4

0

0

0

0

0

0

0
4

0

0

0

0

0
4

0

0

0

0

0
4

0

0

0

0
4

0

0

0
4

0

0

0
4

0

0

0
4

0
4

0

0

0

0

0
4

0
4

0

0

0

0

0
4

0
4

0

0

0

0
4

0
4

0

0

0

0
4

0
4

0

0

0

0
4

0
4

0

0

0

0
4

0
4

0

0

0

0
4

0
4

0
4

0

0

0

0

0

0

0

0

0

0

0

0
4

0

0

0

0

0

0
4

0

0

0

0

0

0
4

0

0

0
4

0

0

0
4

0
4

0
4

0
3

0
3

0
3

0
3

0

0
4

0
4

0
4

0
6

0
6

0
6

0
6

0

0
4

0

0

0
4

0
4

0
4

0.0892871

0.0892871

0.0255106

0

0.0127553

0.0127553

0

0
4

0.0637765

0.0446436

0.019133

3.46944695195361e-18

0
4

0
4

0
4

0

0

0

0

0

0

0
4

0
4

0
4

0
4

0
4

0
4

0
4

0

0

0
4

0

0

0
4

0
4

0
4

0.0382659

0.0382659

0.0382659

0.0255106

0.0127553

0

1.73472347597681e-18

0
4

0
4

0
4

0

0

0

0

0

0
4

0
4

0
4

0.317422

0.317422

0.317422

0.102827

0.143063

0.0715316

0

4.16333634234434e-17

0
4

0
4

0
4

0

0

0

0

0

0
4

0
4

0
4

0

0

0

0

0
4

0
4

0
4

0
6

0
6

0
6

0

0

0

0

0

0

0

0
4

0
4

0
4

0.0127553
6

0.0127553
6

0

0

0
4

0.0127553

0.0127553

0
4

0
4

0
4

0.00665242

0.00665242

0.00665242

0

0.00665242

0
4

0
4

0
4

0

0

0

0

0
4

0
4

0
4

0

0

0

0

0

0
4

0

0

0
4

0
4

0
4

0

0

0

0

0

0
4

0
4

0
4

0

0

0

0

0

0

0
4

0
4

0
4

0

0

0

0

0

0
4

0
4

0
4

0

0

0

0

0
4

0
4

0
4

0.0208048

0.0208048

0.0208048

0.0208048

0
4

0

0

0
4

0
4

0
4

0

0

0

0

0

0

0
4

0
4

0
4

0
4

0
4

0
4

0

0

0

0

0

0

0

0
4

0
4

0

0

0

0
4

0
4

0
4

0

0

0

0

0

0
4

0
4

0
4

0

0

0

0

0

0
4

0

0

0
4

0
4

0
4

0

0

0

0

0
4

0

0

0
4

0
4

0
4

0

0

0

0

0
4

0
4

0
4

0.0127553

0.0127553

0.0127553

0.0127553

0

0
4

0
4

0
4

0

0

0

0

0
4

0
4

0
4

0

0

0

0

0

0
4

0

0

0
4

0
4

0
4

0

0

0

0

0
4

0
4

0
4

0

0

0

0

0
4

0
4

0
4

0

0

0

0

0
4

0
4

0
4

0
4

0
4

0
4

0

0

0

0

0

0

0

0

0
4

0

0

0

0

0

0
4

0

0

0
4

0
4

0
4

0

0

0

0

0
4

0
4

0
4

0

0

0

0

0
4

0
4

0
4

0

0

0

0

0
4

0
4

0
4

0

0

0

0

0

0
4

0
4

0
4

0

0

0

0

0
4

0
4

0
4

0

0

0

0

0
4

0

0

0
4

0
4

0
4

0

0

0

0

0
4

0
4

0
4

0

0

0

0

0
4

0
4

0
4

0

0

0

0

0
4

0
4

0
4

0

0

0

0

0
4

0
4

0
4

0
4

0
4

0
4

0
4

0

0

0

0

0
4

0

0

0
4

0

0

0

0
4

0

0

0
4

0
4

0
4

0

0

0

0

0
4

0
4

0
4

0

0

0

0

0
4

0
4

0
4

0

0

0

0

0
4

0
4

0
4

0

0

0

0

0
4

0
4

0
4

0

0

0

0

0
4

0
4

0
4

0

0

0

0

0
4

0
4

0
4

0

0

0

0

0
4

0
4

0
4

0

0

0

0

0
4

0
4

0
4

0

0

0

0

0
4

0
4

0
4

0

0

0

0

0
4

0
4

0
4

0

0

0

0

0

0

0

0

0
4

0

0

0

0

0

0
4

0

0

0
4

0

0

0

0
4

0

0

0
4

0
4

0
4

0

0

0

0

0
4

0
4

0
4

0

0

0

0

0
4

0
4

0
4

0

0

0

0

0
4

0
4

0
4

0

0

0

0

0
4

0
4

0
4

0

0

0

0

0
4

0
4

0
4

0

0

0

0

0
4

0
4

0
4

0

0

0

0

0
4

0
4

0
4

0

0

0

0

0
4

0
4

0
4

0

0

0

0

0
4

0
4

0
4

0

0

0

0

0
4

0
4

0
4

0
7

0
7

0
7

0
7

0

0

0

0

0

0

0
4

0
4

0
4

0

0

0

0

0
4

0
4

0
4

0

0

0

0

0
4

0
4

0
4

0

0

0

0

0
4

0
4

0
4

0

0

0

0

0
4

0
4

0
4

0

0

0

0

0
4

0
4

0
4

0

0

0

0

0
4

0
4

0
4

0

0

0

0

0
4

0
4

0
4

0

0

0

0

0
4

0
4

0
4

0

0

0

0

0
4

0
4

0
4

0

0

0

0

0
4

0
4

0
4

0
7

0
7

0
7

0
7

0

0

0
4

0
4

0
4

0

0

0

0

0
4

0
4

0
4

0

0

0

0

0
4

0
4

0
4

0

0

0

0

0
4

0
4

0
4

0

0

0

0

0
4

0
4

0
4

0

0

0

0

0
4

0
4

0
4

0

0

0

0

0
4

0
4

0
4

0

0

0

0

0
4

0
4

0
4

0

0

0

0

0
4

0
4

0
4

0

0

0

0

0
4

0
4

0
4

0

0

0

0

0
4

0
4

0
4

0

0

0

0

0

0

0

0

0
4

0
4

0
4

0

0

0

0

0
4

0
4

0
4

0

0

0

0

0
4

0
4

0
4

0

0

0

0

0
4

0
4

0
4

0

0

0

0

0
4

0
4

0
4

0.0127553

0.0127553

0

0
8

0

0
4

0.0127553

0.0127553

0
4

0
4

0

0

0

0
4

0
4

0

0

0

0
4

0
4

0
4

3.55028506593413e-14

0
4

257.42

26.584
7

26.584
7

9.94741
7

9.94741
7

0

0
4

5.31256
7

1.44369
7

0.227577
7

0.629581
7

0

0

0

0

0

0

0

0

0

0.00443786

0.0304231
7

0.00447073

0

0

0

0

0

0

0.00738779

0

0

0
7

0

0

0

0

0

0

0

0.00447073

0

0

0.00443786
7

0

0

0

0

0

0

0

0

0

0

0.0133136
7

0

0

0

0

0

0

0

0

0

0

0
6

0

0

0

0

0

0

0

0

0

0

0
6

0

0

0

0

0

0

0

0

0

0

0.00665679
7

0

0

0

0

0

0

0.00543897

0

0

0

0.443533
7

0

0

0

0

0

0

0

0

0

0

0.910778
7

0

0

0

0

0

0

0

0

0

0

0.0558841
7

0
6

0

0

0

0

0

0

0

0

0

0

0.0135974
7

0

0

0

0

0

0

0

0

0

0

0.00665679
7

0

0

0

0

0

0

0

0

0

0

0
7

0

0

0

0

0

0

0

0

0

0

0
7

0

0

0

0

0

0

0

0

0

0

0.0143804
7

0

0

0

0

0

0

0

0

0

0

0
7

0

0

0.0134122

0

0

0

0

0

0

0

0
6

0

0

0

0

0

0

0

0

0

0

0
7

0

0.0099097
7

0.801088
7

0.00447073
6

0
6

0.167652
7

0
7

0

0
7

0.00447073
8

0
7

0
7

0
7

0.0201183
7

0

0

0
7

0

0
7

0.0571092

0.0311321

0

0

0
8

0.0754436
6

0

0.0435118

0

0

0

0

0.00543897

0

0

0

0

0

0

0

0

0

0

0

0

0.0127553

0

0.104468
6

0

0

0

0

0

0

0

0

0

0.0217559

0.0732247
6

0

0

0

0

0

0

0

0

0

0

0.0398784
7

0

0

0

0

0

0

0

0

0

0

2.85188539450587e-15
7

0
4

2.14305
7

0

0.448505
7

0
7

0
7

1.30792
7

0.386628
7

0

0

0

0

0

0

0

0
4

0
7

0
7

0

0

0
4

0.115384
7

0.115384
7

0

0

0

0

0

0

0

0

0

0

0

0

0

0
4

0

0

0

0

0
4

9.06118
7

1.04307
7

8.0181
7

0

0

0

0

0

0

0

0

0

0

0

0

0

0
4

0.00443786
7

0.00443786
7

0

0

0
4

0
8

0
8

0

0

0
4

0

0

0
4

0

0

0
4

0

0

0
4

2.55524768011384e-15
7

0
4

0

0

0

0
4

0
4

0

0

0

0

0
4

0
4

0

0

0

0
4

0
4

0

0

0

0
4

0
4

0

0

0

0
4

0
4

0

0

0

0
4

0
4

0

0

0

0
4

0
4

0

0

0

0
4

0
4

0
4

165.444

0.128538

0

0

0

0

0

0

0

0

0

0

0

0
4

0

0

0
4

0

0

0
4

0

0

0
4

0.0956648

0.0956648

0
4

0.0201183

0.0201183

0
4

0

0

0
4

0.0127553

0.0127553

0
4

0

0

0
4

0

0

0
4

0

0

0
4

0

0

0

0

0

0

0

0

0
4

0

0

0
4

0

0

0
4

0

0

0
4

0

0

0

0

0

0

0

0
4

0

0

0

0

0
4

0

0

0

0
4

0

0

0

0

0

0
4

0

0

0
4

0

0

0

0
4

0

0

0
4

5.20417042793042e-18

0
4

0

0

0

0
4

0
4

1.12913

0
3

0

0

0

0

0

0

0
4

0
3

0

0

0

0

0

0

0

0

0

0

0

0

0

0

0

0

0

0

0

0

0

0

0

0

0

0

0

0

0

0

0
4

0

0

0
4

0.133931
3

0.133931
3

0

0

0

0

0

0

0

0

0

0

0
4

0.434793

0
5

0

0

0

0

0

0

0

0

0

0

0.314804

0.0127553

0

0

0

0

0.019133

0
5

0

0.0753455

0

0

0.0127553

4.68375338513738e-17

0
4

0

0

0

0

0

0

0
4

0

0

0
4

0

0

0

0

0

0
4

0

0

0

0

0

0

0

0
4

0

0

0

0

0

0
4

0

0

0

0

0

0
4

0.0282416

0.0141208

0

0.00564832

0.00847249

0
4

0

0

0

0
4

0

0

0

0

0
4

0

0

0

0

0
4

0.350771
5

0.350771
5

0

0

0

0

0

0

0

0

0

0

0
4

0.0382659

0.0382659

0

0

0
4

0

0

0
4

0

0

0

0

0
4

0

0

0

0

0
4

0

0

0

0

0
4

0.0127553

0.0127553

0

0
4

0

0

0

0

0
4

0

0

0

0
4

0

0

0

0

0
4

0

0

0
4

0
5

0
5

0

0

0

0

0
4

0

0

0

0
4

0

0

0
4

0

0

0
4

0

0

0

0
4

0

0

0
4

0.0255106

0.0255106

0
4

0

0

0
4

0

0

0
4

0.00564832

0.00564832

0
4

0

0

0
4

0
3

0

0

0

0

0

0

0

0
4

0

0

0

0
4

0

0

0

0
4

0

0

0

0
4

0.0573989

0.0127553

0.0446436

0
4

0

0

0

0
4

0

0

0
4

0

0

0

0
4

0

0

0
4

0

0

0

0
4

0

0

0
4

0
5

0

0

0

0

0

0

0

0
4

0

0

0

0
4

0

0

0

0
4

0

0

0
4

0

0

0
4

0

0

0
4

0

0

0
4

0

0

0
4

0

0

0
4

0

0

0
4

0

0

0
4

0

0

0

0

0

0

0

0

0
4

0

0

0
4

0

0

0
4

0

0

0
4

0

0

0
4

0

0

0
4

0

0

0
4

0

0

0
4

0

0

0
4

0

0

0
4

0

0

0
4

0
5

0
5

0

0
4

0

0

0
4

0

0

0
4

0

0

0
4

0

0

0
4

0

0

0
4

0

0

0
4

0

0

0
4

0

0

0
4

0

0

0
4

0

0

0
4

0.0290597

0

0

0

0

0.0290597

0

0
4

0

0

0
4

0

0

0
4

0

0

0
4

0

0

0
4

0.0127553

0.0127553

0
4

0

0

0
4

0

0

0
4

0

0

0
4

0

0

0
4

0

0

0
4

0

0

0

0

0

0

0
4

0

0

0
4

0
4

9.94191
3

0

0

0
3

0

0

0

0

0

0

0

0

0

0

0

0

0

0

0

0
5

0

0

0

0

0

0

0

0

0

0

0

0

0

0

0

0

0

0

0

0

0

0
3

0

0

0

0

0

0

0

0

0

0

0

0

0

0

0

0

0

0

0

0

0

0
3

0

0

0

0

0

0

0

0

0

0

0
6

0

0

0

0

0

0

0

0

0

0

0

0

0

0

0

0

0

0

0

0

0

0

0

0

0

0

0

0

0

0

0

0

0
4

5.9284

0.771696
6

0.00670609

0.273348

0.0127553

0.364464

0.0318883

0

0.017226

0.0127553

0

0

0.0513003

0

0.0754176

0.0104024

0

0.0382659

0.0468109

0

0.0446436

0

0

0.0765318

0

0.172197

3.49495

0

0

0

0

0

0

0

0.0637765
7

0

0

0

0

0

0

0.0255106

0

0

0

0.146686

0

0

0

0

0

0

0

0

0

0

0
6

0

0

0.019133

0

0

0

0

0

0

0

0.00670609
7

0

0

0

0

0

0

0

0

0

0

0.0886939

0

0

0.0765318

0

0

0

0

0

0

0

0
3

0

7.63278329429795e-16

0
4

1.37351

0.187677

1.0753

0

0

0

0.0156475

0

0

0

0

0

0

0.0049252

0

0

0.00894146

0

0

0.0637765

0

0

0.0172382

0

0

0

0
4

0.205653
3

0
4

0
4

0

0

0

0

0

0

0

0

0

0

0

0
4

0

0

0

0

0

0

0

0

0

0

0
4

0

0

0

0

0

0

0

0

0

0

0

0

0

0

0

0

0

0

0

0

0

0
4

0

0

0

0

0

0

0

0

0

0

0

0

0

0

0

0

0

0

0

0

0

0

0

0

0

0

0

0

0

0

0

0

0

0

0

0

0

0

0

0

0

0

0

0

0

0

0

0

0

0

0

0

0

0

0

0

0

0

0

0

0

0

0

0.00447073

0

0

0

0

0

0

0

0

0

0

0

0

0

0

0

0

0

0

0

0

0

0

0

0

0

0

0

0

0

0

0

0

0

0

0

0

0

0

0

0

0

0

0

0

0

0

0

0

0

0

0

0

0

0

0

0

0

0

0

0

0

0

0

0

0

0

0

0

0

0

0

0

0

0

0

0

0

0

0

0

0

0

0

0

0

0

0

0

0

0

0

0

0

0

0

0

0

0

0

0

0

0
4

0

0

0

0

0

0

0

0

0

0

0

0

0

0

0

0

0

0

0

0

0

0

0

0

0

0

0

0

0

0

0

0

0

0
4

0

0

0

0

0

0

0

0

0

0

0

0

0

0

0

0

0

0

0

0

0

0

0

0

0

0

0

0

0

0

0

0

0

0

0

0

0

0

0

0

0

0

0

0

0

0

0

0

0

0

0

0

0

0

0

0

0

0

0

0

0

0

0

0

0

0

0

0

0

0

0

0

0

0

0

0

0

0

0

0

0

0

0

0

0

0

0

0

0
4

0

0

0

0

0

0

0

0

0

0

0
4

0

0

0

0

0

0

0

0

0

0

0

0

0

0

0

0

0

0

0

0

0

0

0

0

0

0

0

0

0

0

0

0

0

0

0

0

0

0

0

0

0

0

0

0

0

0

0

0

0

0

0

0

0

0

0

0

0

0

0

0

0

0

0

0

0

0

0

0

0

0

0

0

0

0

0

0

0

0

0

0

0

0

0

0

0

0

0

0

0

0

0

0

0

0

0

0

0

0

0

0

0

0

0

0

0

0

0

0

0

0

0
4

0

0

0

0

0

0

0

0

0

0

0
4

0

0

0

0

0

0

0

0

0

0

0

0

0

0

0

0

0

0

0

0

0

0

0

0

0

0

0

0

0

0

0

0

0

0

0

0

0

0

0

0

0

0

0

0

0

0

0

0

0

0

0

0

0

0

0

0

0

0

0

0

0

0

0

0

0

0

0

0

0

0

0

0

0

0

0

0

0

0

0

0

0

0

0

0

0

0

0

0

0

0

0

0

0

0

0

0

0

0

0

0

0

0

0

0

0

0

0

0

0

0

0.00670609
7

0

0

0

0

0

0

0

0

0

0

0

0

0

0

0

0

0

0

0

0

0

0

0

0

0

0

0

0

0

0

0

0

0

0

0

0

0

0

0

0

0

0

0

0

0

0

0

0

0

0

0

0

0

0

0

0

0

0

0

0

0

0

0

0

0

0

0

0

0

0

0

0

0

0

0

0
3

0

0

0

0

0

0

0

0

0

0

0

0

0

0

0

0

0

0

0

0

0

0

0

0

0

0

0

0

0

0

0.0178829

0

0

0

0.0223536

0

0

0

0

0

0

0

0

0

0

0

0

0

0

0

0

0

0

0

0

0
4

0

0

0

0

0

0

0

0

0

0

0
4

0

0

0

0

0

0

0

0

0

0

0
3

0

0

0

0

0

0

0

0

0

0

0

0

0

0

0

0

0

0

0

0

0

0

0

0

0

0

0

0

0

0

0

0

0
4

0

0

0

0

0

0

0

0

0

0

0
4

0

0

0

0

0

0

0

0

0

0

0
4

0
4

0

0

0

0

0

0

0

0

0

0

0
4

0

0

0

0

0

0

0

0

0

0

0

0

0

0

0

0

0

0

0

0

0

0

0

0

0

0

0

0

0

0

0

0

0

0

0

0

0

0

0

0

0

0

0.0156475

0
4

0

0

0

0

0

0

0

0

0

0

0
4

0

0

0

0

0

0

0

0

0

0

0
4

0

0

0.0178829

0

0

0

0

0

0

0

0
4

0

0

0

0

0

0

0

0

0

0

0
3

0

0

0

0

0

0

0

0

0

0

0
4

0

0

0

0

0

0

0

0

0

0

0

0
4

0

0

0

0

0

0

0

0

0

0

0
4

0

0

0

0

0

0

0

0

0

0

0.100591

0

0

0

0

0

0

0

0

0

0

0
4

0

0

0

0

0

0

0

0

0

0

0

0

0

0

0

0

0

0

0

0

0

0
3

0

0

0

0

0

0

0

0

0

0

0
4

0

0

0

0

0

0.0156475

0

0

0

0

0

0

0

0

0

0

0

0

0

0

0

0

0

0

0

0

0

0

0

0

0

0

0

0
4

0

0

0

0

0

0

0

0

0

0

0
4

0

0

0

0

0

0

0

0

0

0

0

0

0

0

0

0

0

0

0

0

0

0
4

0

0

0

0

0

0

0

0

0

0

0

0

0

0

0

0

0

0

0

0

0

0

0

0

0

0

0

0

0

0

0

0

0

0

0

0

0

0

0

0

0

0

0

0
4

0

0

0

0

0

0

0

0

0

0

0
7

0

0

0

0

0

0

0

0

0

0

0

0

0

0

0

0

0

0

0

0

0

0
4

0
4

0

0

0

0

0

0

0

0

0

0

0
4

0

0

0

0

0

0

0

0

0

0

0
4

0

0

0

0

0

0

0

0

0

0

0
4

0

0

0

0

0

0

0

0

0

0

0
4

0

0

0

0

0

0

0

0

0

0

0
4

0

0

0

0

0

0

0

0

0

0

0

0

0

0

0

0

0

0

0

0

0

0
4

0

0

0

0

0

0

0

0

0

0

0
4

0

0

0

0

0

0

0

0

0

0

0

0

0

0

0

0

0

0

0

0

0

0

0
4

0

0

0

0

0

0

0

0

0

0

0
4

0

0

0

0

0

0

0

0

0

0

0

0

0

0

0

0

0

0

0

0

0

0

0

0

0

0

0

0

0

0

0

0

0

0

0

0

0

0

0

0

0

0

0

0

0

0

0

0

0

0

0

0

0

0

0

0

0

0

0

0

0

0

0

0

0

0
4

0

0

0

0

0

0

0

0

0

0

0

0

0

0

0

0

0

0

0

0

0

0

0

0

0

0

0

0

0

0

0

0

0

0
4

0

0

0

0

0

0

0

0

0

0

0

0

0

0

0

0

0

0

0

0

0

0

0

0

0

0

0

0

0

0

0

0

0
4

0

0

0

0

0

0

0

0

0

0

0
4

0

0

0

0

0

0

0

0

0

0

0
4

0

0

0

0

0

0

0

0

0

0

0

0

0

0

0

0

0

0

0.00447073

0

0

0

0

0

0

0

0

0

0

0

0

0

0
3

0

0

0

0

0

0

0

0

0

0

0
4

0

0

0

0

0

0

0

0

0

0

0

0

0

0

0

0

0

0

0

0

0

0

0
4

0

0

0

0

0

0

0

0

0

0

0

0

0

0

0

0

0

0

0

0

0

0
4

0

0

0

0

0

0

0

0

0

0

0
4

0

0

0

0

0

0

0

0

0

0

0
4

0

0

0

0

0

0

0

0

0

0

0
4

0

0

0

0

0

0

0

0

0

0

0

0

0

0

0

0

0

0

0

0

0

0

0

0

0

0

0

0

0

0

0

0

0

0

0

0

0

0

0

0

0

0

0

0
4

2.23856

2.00896

0.0382659

0

0

0

0.178574

0

0

0

0

0

0.0127553

0

4.35415592470179e-16

0
4

0
4

0
4

0
4

0
4

0

0

0

0
4

0.0510212

0.0510212

0
4

0
4

0

0

0

0

0

0
4

0

0

0

0

0
4

0.0536487

0.024589

0.0201183

0.00447073

0.00447073

5.20417042793042e-18

0
4

0

0

0

0
4

0

0

0

0

0

0

0

0
4

0

0

0

0

0

0
4

0

0

0
4

0

0

0

0
4

0

0

0

0

0
4

0
3

0
3

0

0

0

0

0

0

0

0

0

0

0

0

0

0

0

0

0
4

0

0

0
4

0

0

0

0

0
4

0

0

0
4

0

0

0
4

0

0

0

0
4

0

0

0

0

0
4

0

0

0

0
4

0

0

0

0

0
4

0

0

0

0
4

0

0

0
4

0
3

0
4

0

0

0

0

0

0

0

0

0

0

0

0

0
4

0

0

0

0
4

0.00894146

0.00894146

0
4

0

0

0
4

0

0

0

0
4

0

0

0

0
4

0.0049252

0.0049252

0

0
4

0

0

0
4

0

0

0
4

0

0

0
4

0

0

0

0
4

0
3

0

0

0

0

0

0

0

0

0

0

0

0

0

0

0

0
4

0

0

0
4

0

0

0
4

0

0

0
4

0

0

0

0
4

0

0

0

0
4

0

0

0

0
4

0

0

0

0
4

0

0

0

0
4

0

0

0

0
4

0

0

0
4

0
3

0

0

0

0

0

0

0
4

0

0

0
4

0

0

0

0
4

0

0

0
4

0

0

0
4

0

0

0
4

0

0

0
4

0

0

0
4

0

0

0
4

0

0

0
4

0

0

0
4

0
4

0
4

0

0

0

0

0

0

0

0

0
4

0

0

0
4

0

0

0
4

0

0

0
4

0

0

0
4

0

0

0
4

0

0

0
4

0

0

0
4

0

0

0
4

0

0

0
4

0

0

0
4

0

0

0

0

0

0
4

0

0

0
4

0

0

0
4

0

0

0
4

0

0

0
4

0

0

0
4

0

0

0
4

0

0

0
4

0

0

0
4

0

0

0
4

0

0

0
4

0.0581195

0

0.0312951

0.0111768

0.0156475

0

0

0

0
4

0

0

0
4

0

0

0
4

0

0

0
4

0

0

0
4

0

0

0
4

0

0

0
4

0

0

0
4

0

0

0
4

0

0

0
4

0

0

0
4

0

0

0

0

0

0

0

0

0
4

0

0

0
4

0

0

0
4

0

0

0
4

0.019133

0.019133

0
4

0

0

0
4

0

0

0
4

0

0

0
4

0

0

0
4

0

0

0
4

0
4

0.017226
4

0
4

0
4

0

0

0

0

0

0

0

0

0

0

0

0

0

0

0

0

0

0

0

0

0

0

0

0

0

0

0

0

0

0

0

0

0

0

0

0

0

0

0

0

0

0

0

0

0
4

0.0127553
4

0.0127553
4

0

0
4

0

0

0
4

0

0

0
4

0.00447073

0.00447073

0
4

8.67361737988404e-19
4

0
4

12.6902

7.4587

1.03264
7

0.48748
7

0

0

0

0.0318883

0

0

0

0

0

0

0.0765318
8

0

0

0

0

0

0

0

0

0

0

0.0515429
7

0

0

0

0

0

0

0

0

0

0

0

0.0127553

0

0

0

0

0

0

0

0

0

0
7

0

0

0

0

0

0

0

0
7

0

0.225592
8

0
3

0.0203065
7

1.26007

0.0956648

0

0
7

0.0110946
8

0.0137987

0

0
7

0

0.0637765

0

0.452813
7

0.00443786

0

0

0

0
7

0

0.0133136

0.0299485

0

0.0318883

1.49167

0

0.0151023

0.049178

0.0127553

0

0.0573989

0.102042

0

0

0

0.337664
8

0.0637765

0

0

0

0

0

0.0670609

0.00689936

0

0

0
3

0

0

0

0

0

0.00670609

0

0

0

0

0
8

0

0

0

0

0.010349

0

0.0100682

0.0275974

0

0

0.878104
7

0

0

0

0

0

0

0

0

0.0127553

0

0.389558
7

0.00447073

0

0

0

0

0

0

0

0

0

6.96491475604688e-16

0
4

0

0

0

0
4

0
2

0

0

0

0

0

0

0

0

0

0

0
4

0.0481756
4

0

0
4

0

0

0

0

0

0

0

0.00447073

0

0

0.0127553

0

0

0

0

0

0
4

0

0

0

0

0

0

0

0

0

0

0
4

0

0

0

0

0

0

0

0

0

0

0
3

0

0

0.00543897

0

0.0127553

0

0

0

0

0

0
4

0

0

0

0

0

0

0

0

0

0

0

0

0

0

0

0

0

0

0

0

0

0

0

0

0

0

0

0

0

0

0

0

0.0127553
8

0

0

0

0

0

0

0

0

0

0

0

0

0

0

0

0

0

0

0

0

0

0
4

0
5

0

0

0

0

0

0

0

0

0

0

0

0

0

0

0

0

0

0

0
4

0.280283
6

0

0

0.0765318

0.00503411

0

0.00738779

0

0

0

0

0

0

0

0

0

0

0

0

0

0

0

0

0

0

0

0

0

0

0

0

0

0

0

0

0

0

0

0

0

0

0

0

0

0

0

0

0

0.0127553

0

0

0

0.178574

0

0

0

0

5.55111512312578e-17
6

0
4

1.98849
5

0
5

0.0134122

0

1.13109

0.00447073

0

0

0

0

0.0111768

0.019133

0

0.00447073

0

0

0

0

0

0

0

0

0

0.717552

0.00670609

0

0

0

0

0

0

0

0.0156475

0

0
5

0

0

0

0

0

0

0

0

0

0

0

0.0648255

0

0

0

0
4

0

0

0

0

0

0

0

0

0

0

0

0

0

0

0

0

0

0

0

0

0
4

0

0

0

0

0

0

0

0

0
4

0

0

0
4

0

0

0
4

0

0

0
4

0

0

0
4

0

0

0
4

0

0

0
4

0

0

0
4

0

0

0
4

0

0

0
4

0

0

0
4

0.0192248
7

0.00887572
7

0.010349

0

0

0

0
4

0

0

0
4

0

0

0
4

0

0

0
4

0

0

0
4

0

0

0
4

0

0

0
4

0

0

0
4

0

0

0
4

0

0

0
4

0

0

0
4

0
4

0

0

0

0
4

0.0382659

0.0382659

0
4

0

0

0
4

0.00447073

0.00447073

0
4

0.0127553

0.0127553

0
4

0

0

0
4

0.0260061

0.0260061

0
4

0

0

0
4

0

0

0
4

0

0

0
4

0

0

0
4

0

0

0

0

0

0

0

0

0
4

0

0

0
4

0

0

0
4

0

0

0
4

0

0

0
4

0

0

0
4

0

0

0
4

0

0

0
4

0
7

0

0

0

0

0
4

0
4

0

0

0

0

0

0
4

0
4

0

0

0

0

0
4

0

0

0

0
4

0.0134122
7

0.0134122
7

0
4

0.714297
7

0.714297
7

0
4

0
4

0
4

0
4

0
4

0

0

0

0
4

0

0

0

0
4

0
8

0

0

0
4

0
6

0

0

0

0
4

0.0382659

0.0255106

0

0

0.0127553

1.73472347597681e-18

0
4

0

0

0
4

0

0

0

0

0
4

0

0

0
4

0.0127553

0

0

0

0.0127553

0
4

0

0

0

0

0
4

0

0

0

0
4

1.70119
7

1.68843
7

0.0127553

0

0
4

0

0

0
4

0

0

0

0

0
4

0

0

0

0
4

0

0

0
4

0

0

0

0
4

0

0

0

0
4

0

0

0
4

0

0

0
4

0

0

0
4

0

0

0

0

0
4

0
7

0

0

0

0

0

0

0

0
4

0

0

0

0

0
4

0.0782377

0.0782377

0
4

0.00689936

0.00689936

0

0
4

0

0

0

0
4

0

0

0

0
4

0

0

0

0

0
4

0

0

0

0
4

0

0

0
4

0

0

0

0
4

0

0

0

0
4

0

0

0

0

0

0

0

0

0

0

0

0

0
4

0

0

0

0
4

0

0

0

0
4

0

0

0
4

0

0

0

0
4

0

0

0
4

0

0

0
4

0

0

0

0
4

0

0

0

0
4

0

0

0

0
4

0

0

0
4

0.184952
7

0.184952
7

0

0

0

0
4

0

0

0

0
4

0

0

0
4

0

0

0

0
4

0

0

0
4

0.0127553

0.0127553

0
4

0

0

0
4

0

0

0

0
4

0

0

0

0
4

0

0

0
4

0

0

0
4

0
3

0

0

0

0
4

0

0

0
4

0

0

0
4

0

0

0
4

0

0

0
4

0

0

0
4

0

0

0
4

0

0

0
4

0

0

0
4

0

0

0
4

0

0

0
4

0

0

0

0

0
4

0

0

0
4

0

0

0
4

0

0

0
4

0

0

0
4

0

0

0
4

0

0

0
4

0

0

0
4

0

0

0
4

0

0

0
4

0

0

0
4

0.0510212
7

0.0510212
7

0

0
4

0

0

0
4

0

0

0
4

0

0

0
4

0

0

0
4

0

0

0
4

0

0

0
4

0

0

0
4

0

0

0
4

0

0

0
4

0

0

0
4

0
4

25.6124

0
7

0
7

0

0

0

0

0

0

0

0

0
4

16.1665

1.66613
7

0.5421
7

0

0
3

0
6

0

0

0

0

0

0

0

0.0892871
3

0

0

0

0

0

0

0

0

0

0

0.0966682

0

0

0

0

0

0

0

0

0.00689936

0

0.278147
7

0

0

0

0

0

0

0

0

0

0.0127553

0.484988
7

0

0

0

0

0

0

0

0

0

0

0
6

0

0

0

0

0.0357658

0

0

0

0

0

1.62287

0.00503411

0

0

0

0

0

0

0

0

0

0.160511

0

0

0

0.0201183

0

0

0

0

0

0.00443786

0

0

0

0

0

0

0

0

0

0

0

0.0810055

0

0

0

0

0

0.00894146

0

0

0

0

0
3

0.0892871
7

0

0

0

0.0127553

0

0

0

0

0.0127553

0

0.491079
7

0

0

0

0

0

0

0

0

0

0

0
2

0

0

0.0956648

0

0

0

0

0

0

0

0
3

0

0

0

0.0255106

0

0

0

0

0

0.00670609

0
3

0

0

0

0

0

0

0

0

0

0

0.0510212
7

0

0

0

0

0

0

0

0

0.0178829

0

0.674124
7

0

0

0

0

0

0

0

0.019133

0

0

0
3

0

0

0

0.0229434

0

0.00670609

0

0

0

0

0.146686
7

0

0

0

0

0.0201183

0

0

0

0

0

0
2

0

0

0

0

0

0

0

0

0

0

3.16251
7

0.108573

0

0

0

0

0.0111768

0

0

0

0

0

0.121421
6

0

0.00447073

0

0

0

0

0

0

0

0

1.91124
2

0

0

0.00447073

0

0

0

0

0

0

0

0
3

0

0

0

0

0

0

0

0

0

0

0.340263
7

0

0

0

0.0127553

0

0

0

0

0

0.0111768

0
2

0

0

0

0

0

0

0.0127553

0

0

0

0

0

0

0

0

0

0

0

0

0

0

0.049178
5

0

0

0

0

0

0

0

0.0127553

0

0

0
5

0

0

0

0

0

0

0

0

0

0

0
3

0

0

0

0

0

0

0

0

0

0

0
4

0.0892871
6

0

0

0

0

0

0

0

0

0

0

0
3

0

0

0

0

0

0

0

0

0

0

0

0

0

0

0

0

0

0

0.0637765

0

0

0
3

0

0

0

0

0

0

0

0

0

0

0.0310471
7

0

0

0

0

0

0

0

0.00447073

0

0

0
3

0

0

0.0127553

0

0

0

0

0

0

0

0
3

0

0

0

0

0.0223536

0

0

0

0.049178

0

0
7

0

0

0

0

0

0

0

0

0

0

0
3

0.00670609

0

0

0

0

0

0.0134122

0.00689936

0

0

0.0760024
7

0

0

0

0

0

0

0

0

0

0

0.858334
7

0
2

0

0

0

0

0.00443786

0

0

0

0

0

0

0

0

0.00447073

0

0

0

0

0.0127553

0

0

0

0

0

0

0

0

0.0127553

0

0

0

0

0

0

0

0

0

0

0.00447073

0

0

0

0

0

0

0

0

0

0

0

0

0

0

0

0

0

0

0

0

0

0

0

0

0

0.0127553

0
3

0

0

0

0

0

0

0

0

0

0

0

0

0

0

0

0

0

0

0

0

0

0
5

0

0

0

0.0111768

0

0

0

0

0

0.019133

0
3

0.0127553

0

0

0

0

0

0

0

0

0

0.386607
7

0
3

0

0

0

0

0

0

0

0.0956648

0

0

0.00670609
3

0.0134122

0

0

0

0

0

0

0

0

0

0
3

0

0

0

0

0

0

0

0

0

0

0
6

0

0

0

0

0

0

0.0382659

0

0

0

0.0151023
7

0

0

0.0111768

0

0

0

0

0

0

0

0.0161288
7

0

0

0

0

0

0

0

0

0

0

0
7

0

0

0

0

0

0

0

0

0.00670609

0

0.178574
7

0.00665242

0

0

0

0

0

0

0

0

0

0.00894146
2

0

0

0

0

0

0

0.00447073

0

0

0

0
3

0

0

0

0

0

0

0

0

0.00670609

0

0.892871

0
2

0

0

0

0

0

0

0

0

0

0

0
3

0

0

0

0

0

0

0

0

0

0

0
3

0

0

0.019133

0

0

0

0

0

0

0

0

0

0

0

0

0.019133

0

0

0

0

0.0127553

0

0

0

0

0

0

0

0

0

0

0

0.0127553
6

0

0

0

0

0

0

0

0

0

0

0
3

0

0

0

0

0

0

0

0

0

0

0.00447073
2

0

0

0

0

0

0

0

0

0

0

0.0637765
7

0

0

0

0

0

0

0

0

0

0

0

0

0

0.0127553

0

0

0

0

0

0

0

0
3

0

0

0

0

0.00447073

0

0

0

0

0.0127553

0

0.134122
7

0

0

0

0

0

0.0956648

0

0

0

0

0

0

0

0

0

0

0

0

0

0

0

0

0

0

0

0

0

0

0

0

0

0.0829095
6

0

0.0510212
7

0

0.0151023
7

0

0.0335305

0

0
3

0

0

0

0

0
6

0.00443786
7

0

0

0
4

0

0

0

0

0

0

0

0

0

0

0

0

0

0

0

0

0
4

7.66869

5.11049

1.09305
7

0
4

0.0382659

0.113154

0

0

0

0

0

0.0255106

0

0
4

0

0.00997863

0

0

0

0

0

0

0

0

0.964696
7

0

0

0

0

0

0

0

0

0

0

0.0648154
7

0

0

0

0

0

0

0

0

0

0

0.229595
4

0

0

0

0

0

0.019133

0

0

0

0

0
7

0

0

0

0

0

0

0

0

0

0

0
4

0

0

0

0

0

0

0

0

0

0

0

0

0

0
7

2.67147415300428e-16

0
4

0.903151
7

0.279771
7

0.0462858

0.204085

0

0

0

0

0.00997863

0

0.00670609

0.019133

0.0127553
6

0.00447073

0.0127553

0

0.0127553

0

0

0.108303
7

0.0223961

0.024626
6

0.114798
7

0.00665242
7

0.0176805

0

0
4

0
4

0

0

0

0

0
4

0

0

0
4

0

0

0
4

0

0

0
4

0

0

0
4

0

0

0
4

0

0

0
4

0

0

0
4

0

0

0
4

0

0

0
4

0

0

0
4

0
4

0
4

0

0

0
4

0

0

0
4

0.0446436

0.0446436

0
4

0

0

0
4

0

0

0
4

0

0

0
4

0

0

0
4

0

0

0
4

0

0

0
4

0

0

0
4

0

0

0
4

0.0255106
7

0.0127553
7

0.0127553

0
4

0

0

0
4

0

0

0
4

0.0127553

0.0127553

0
4

0

0

0
4

0

0

0
4

0

0

0
4

0

0

0
4

0

0

0
4

0

0

0
4

0

0

0
4

0

0

0

0

0

0
4

0

0

0
4

0

0

0
4

0

0

0
4

0

0

0
4

0

0

0
4

0.00738779

0.00738779

0
4

0

0

0
4

0

0

0
4

0.0127553

0.0127553

0
4

0

0

0
4

0
3

0

0

0

0
4

0

0

0
4

0

0

0
4

0

0

0
4

0

0

0
4

0

0

0
4

0

0

0
4

0

0

0
4

0.0127553

0.0127553

0
4

0

0

0
4

0

0

0
4

0
2

0

0

0

0

0
4

0

0

0

0

0

0

0
4

0.0255106
8

0.0255106

0

0
4

0

0

0

0

0

0
4

0.0176805
7

0.0176805
7

0
4

0
4

0
4

0

0

0

0

0

0

0

0

0

0

0
4

0

0

0

0

0

0

0

0

0

0

0
4

0

0
4

0
4

0
4

0

0

0

0
4

0.0251705

0.0251705

0
4

0.019133

0.019133

0

0
4

0

0

0

0

0
4

0

0

0

0
4

0.153064

0.153064

0
4

0

0

0

0
4

0

0

0

0
4

0

0

0

0

0
4

0.197707

0.197707

0
4

0

0

0

0

0
4

0
4

0
4

0
4

0

0

0

0

0

0

0
4

0

0

0

0

0
4

0

0

0

0
4

0

0

0
4

0

0

0

0

0
4

0

0

0

0
4

0

0

0

0

0
4

0

0

0

0

0
4

0

0

0
4

0

0

0

0
4

0

0

0

0
4

0.0156036
5

0
7

0.0156036
5

0

0

0

0

0

0

0
4

0

0

0

0
4

0.0382659

0

0.0382659

0
4

0

0

0

0
4

0

0

0

0
4

0

0

0
4

0

0

0
4

0

0

0

0
4

0

0

0

0
4

0

0

0

0
4

0

0

0

0
4

0
3

0
3

0

0

0

0

0

0

0
4

0

0

0

0
4

0

0

0
4

0

0

0
4

0

0

0
4

0

0

0
4

0

0

0

0
4

0

0

0

0
4

0

0

0

0
4

0

0

0
4

0

0

0
4

0
3

0
3

0

0

0

0
4

0.0127553

0.0127553

0
4

0

0

0
4

0.00780182

0.00780182

0
4

0

0

0
4

0.0382659

0.0382659

0
4

0

0

0
4

0

0

0
4

0

0

0
4

0.0255106

0.0255106

0
4

0.0127553

0.0127553

0
4

0
4

0
4

0

0
4

0

0

0
4

0

0

0
4

0

0

0
4

0

0

0
4

0

0

0
4

0

0

0
4

0

0

0
4

0

0

0
4

0

0

0
4

0

0

0
4

0
3

0
4

0

0

0

0
4

0

0

0
4

0

0

0
4

0.00780182

0.00780182

0
4

0

0

0
4

0

0

0
4

0

0

0
4

0

0

0
4

0

0

0
4

0

0

0
4

0

0

0
4

0.161238
4

0.153436

0

0

0.00780182

0

0
4

0

0

0
4

0

0

0
4

0

0

0
4

0

0

0
4

0

0

0
4

0

0

0
4

0

0

0
4

0

0

0
4

0

0

0
4

0

0

0
4

9.6034291630076e-15

0
4

0.132217
3

0.119628
3

0.119628
3

0

0

0

0

0

0

0

0

0

0

0

0

0

0

0

0

0

0

0

0

0

0
4

0
4

0
4

0

0

0
4

0

0

0
4

0

0

0
4

0

0

0

0
4

0

0

0

0
4

0

0

0

0
4

0

0

0
4

0

0

0
4

0

0

0
4

0

0

0
4

0

0

0
4

0

0

0

0
4

0

0

0
4

0

0

0
4

0

0

0
4

0

0

0
4

0

0

0
4

0

0

0
4

0

0

0
4

0

0

0
4

0

0

0
4

0

0

0
4

0

0

0
4

0

0

0
4

0

0

0
4

0

0

0
4

0

0

0
4

0

0

0
4

0

0

0
4

0

0

0
4

0

0

0
4

0.00738779

0.00738779

0
4

0

0

0
4

0.00520121

0.00520121

0

0

0
4

0

0

0
4

0

0

0
4

0

0

0
4

0

0

0
4

0

0

0

0

0
4

0

0

0
4

0

0

0
4

0

0

0
4

0

0

0

0
4

6.07153216591882e-18
3

0
4

0.228668
2

0.228668
2

0
2

0
2

0

0.0134122

0

0

0

0

0

0

0

0

0

0

0

0

0

0.072817

0

0

0.0382659

0

0

0

0

0

0

0.0390091

0

0

0

0

0

0

0

0

0

0.0510212

0

0

0

0

0

0

0.00894146

0

0

0

0

0

0

0

0

0

0

0

0

0

0

0

0

0

0

0

0

0

0.00520121

0

0

0

0

0

0

0

0

0

0

0

0

0

0

0

0

0

0

0

0

0

0

0

0

0

0

0

0
2

0

0

0

0

0

0

0

0

0

0

0

0

0

0

0

0

0

0

0

0

0

1.90819582357449e-17
2

0
4

0

0

0
4

0

0

0
4

0
4

50.2862

19.9222
7

4.73109
7

0
7

0

0

0

0.0149038

0.00665242

0.0049252

0.0172382

0

0

0

0

0

0.0110946

0.0477623

0.0127553

0

0

0

0

0

0

0.0275916

0

0

0

0

0.00985039

0

0

0

0

0

0.00665679
6

0

0

0

0

0.00665679

0

0

0.0135974

0.0108779

0

0.0506212

0

0.852071

0.0135974

0

0

0

0

0

0.0400467

0

0.046767
7

0.0049252

0

0.019133

0.00665242

0

0.00689936

0

0

0.0295512

0

0.00543897
6

0

0

0

0

0.0137987

0

0

0

0

0

0.0559837
7

0.00543897

0

0

0

0

0

0

0

0

0

0.0500348
7

0

0

0

0

0

0

0

0

0

0

0.0241651
7

0

0

0

0

0

0

0

0.010349

0.0163985

0.0108779

9.39427
7

0.107579
6

0

0.00665242

0

0

0

0

0

0

0

0

0.00887572
7

0

0

0

0.0190364

0.010349

0

0

0

0.00364411

0.00997863

0.0426502
7

0

0

0

0

0.00543897

0.00364411

0

0

0

0

0.0103642
7

0.00670609

0

0

0.0118257

0.00665679

0

0.00364411

0

0.0271948

0.0147756

0.0324386
7

0

0

0

0

0.0190364

0

0

0

0

0

0.0149038
6

0

0

0

0

0.00738779

0

0

0

0

0

0.0133048
7

0

0

0

0

0

0.00689936

0

0

0

0

0.00665242
7

0

0

0

0

0

0

0

0

0.0365883

0

0.0332621
6

0

0

0

0

0.0489507

0

0.0255106

0

0

0

0
7

0

0

0

0.0309749

0

0

0

0

0

0

1.73446
7

0
5

0

0

0

0

0

0

0

0

0.00887572

0

0.0127553
7

0

0.0510212

0

0

0

0

0

0

0

0

0.0264101
7

0

0

0

0

0

0

0

0

0

0

0.0177773
7

0

0

0

0

0

0

0

0

0.0049252

0

0
7

0

0

0.00443786

0

0

0.00728822

0

0

0

0

0.00665679
7

0

0

0.00985039

0

0

0.00887572

0

0

0

0

0

0

0

0

0

0

0.0163169

0

0

0

0.00443786

0.0049252
7

0

0

0

0

0

0

0

0

0

0

0.0125963
6

0

0.00443786

0

0

0

0

0

0

0

0

0

0

0

0

0

0

0

0

0

0

0

0.281658
6

0
6

0

0

0

0

0

0

0

0

0

0

0

0

0

0

0

0

0

0

0

0.0221893

0

0
7

0

0

0.0127553

0

0

0

0

0

0

0

0.0177471
7

0

0

0

0

0

0

0.00364411

0

0

0

0

0

0

0

0

0

0

0

0

0

0

0
6

0

0

0

0.00443786

0

0

0

0

0.0163169

0.0190364

0.011582
6

0

0

0

0

0

0.00738779

0

0

0

0

0.0266271
7

0.00443786

0

0

0

0

0

0

0

0

0

0.00815845
7

0

0

0

0

0

0

0

0.0137987

0

0

0
7

0.00911028

0

0

0.00543897

0

0

0

0

0

0

0.0934098
7

0

0

0

0.00364411

0

0

0

0

0

0

0

0

0

0

0

0

0

0

0

0.00443786

0.00443786

0

0.0218647
7

0

0

0

0

0

0

0

0

0

0

0
6

0

0.0190364

0

0

0

0

0

0

0

0.0137987

0
7

0

0

0

0

0

0.00665679

0

0

0

0

0
7

0

0

0

0

0

0

0

0

0

0

0
6

0

0

0

0

0

0

0

0

0

0

0

0

0

0

0

0

0

0.00364411

0

0

0

0.012123
7

0

0

0

0

0.0049252

0

0

0

0

0

0
7

0

0

0

0

0

0

0

0

0

0

0.466145
7

0
7

0

0

0

0

0

0

0

0

0

0

0.011582
7

0.00887572

0

0

0

0.0177514

0

0

0

0

0

0.00985039
7

0.0236867

0

0

0

0

0

0

0

0

0

0
6

0.00985039

0

0

0

0

0

0

0

0

0

0

0

0

0
6

0

0.0133136
8

0.0508181
5

0
7

0

0.0209138
6

0

0.069313

0

0.0133136

0.0217559
7

0

0

0.167084
7

0.10694

0.00689936
7

0

0

0.00997863

0
7

0

0

0.00443786

0

0.0632067
7

0

0

0

0

0

0.00665242

0

0

0.0566397

0.021227

0
4

30.2493
7

30.2493
7

0

0
4

0
4

0
4

0

0

0

0

0

0

0

0

0

0

0

0

0

0

0

0
4

0

0

0
4

0

0

0
4

0

0

0
4

0

0

0
4

0

0

0
4

0

0

0
4

0

0

0
4

0

0

0
4

0

0

0
4

0

0

0
4

0.114717

0.114717

0
4

0

0

0

0
4

0

0

0
4

0

0

0
4

0

0

0
4

0

0

0
4

0

0

0
4

0

0

0

0
4

0

0

0
4

0

0

0
4

0

0

0
4

0

0

0
4

0
4

0.0151023
4

0.0151023
4

0
4

0
5

0

0

0

0

0

0

0

0

0

0

0
3

0

0

0

0

0

0

0

0

0

0

0
4

0

0

0

0

0

0

0

0

0

0

0
4

0

0

0

0

0

0

0

0

0

0

0

0

0

0

0

0

0

0

0

0

0

0

0

0

0

0

0

0

0

0

0

0

0
4

0

0

0

0

0

0

0

0

0

0

0
5

0

0

0

0

0

0

0

0

0

0

0

0

0

0

0

0

0

0

0

0

0

0

0.0151023

0

0

0

0

0

0

0

0

0

0

0
4

0

0

0

0

0

0

0

0

0

0

0

0

0

0

0

0

0

0

0

0

0

0

0

0

0

0

0

0

0

0

0

0

0

0

0

0

0

0

0

0

0

0

0

0

0

0

0

0

0

0

0

0

0

0

0

0

0

0

0

0

0

0

0

0

0

0

0

0

0

0

0

0

0

0

0

0

0

0

0

0

0

0

0

0

0

0

0

0

0

0

0

0

0

0

0

0

0

0

0
4

0

0

0

0

0

0

0

0

0

0

0

0

0

0

0

0

0

0

0

0

0

0

0

0

0

0

0

0

0

0

0

0

0

0

0

0

0

0

0

0

0

0

0

0

0

0

0

0

0

0

0

0

0

0

0

0

0

0

0

0

0

0

0

0

0

0

0
3

0

0

0

0

0

0

0

0

0

0

0

0

0

0

0

0

0

0

0

0

0

0

0

0

0

0

0

0

0

0

0

0

0

0

0

0

0

0

0

0

0

0

0

0

0

0

0

0

0

0

0

0

0

0

0

0

0

0

0

0

0

0

0

0

0

0

0

0

0

0

0

0

0

0

0

0

0

0

0

0

0

0

0

0

0

0

0

0

0

0

0

0

0

0

0

0

0

0

0

0

0

0

0

0

0

0

0

0

0

0

0

0

0

0

0

0

0

0

0

0

0

0
4

0

0

0

0

0

0

0

0

0

0

0

0

0

0

0

0

0

0

0

0

0

0

0

0

0

0
4

0
4

0

0

0

0

0

0

0

0

0

0

0
4

0

0

0

0

0

0

0

0

0

0

0
3

0

0

0

0

0

0

0

0

0

0

0

0

0

0

0

0

0

0

0

0

0

0
5

0

0

0

0

0

0

0

0

0

0

0
4

0
4

0
4

0

0

0

0

0

0

0

0

0

0

0

0

0

0

0

0

0

0

0
4

0

0

0

0

0
4

0

0

0

0
4

0

0

0

0
4

0

0

0

0

0

0
4

0

0

0

0
4

0

0

0
4

0

0

0

0
4

0

0

0

0

0
4

0

0

0

0
4

0

0

0

0

0
4

0
4

0
4

0

0

0
4

0
4

0

0

0

0

0

0

0
4

0

0

0

0
4

0

0

0
4

0

0

0

0
4

0

0

0
4

0

0

0
4

0

0

0

0
4

0

0

0
4

0

0

0

0
4

0

0

0

0
4

0

0

0
4

0
3

0
3

0

0

0

0

0

0
4

0

0

0
4

0

0

0
4

0

0

0
4

0

0

0
4

0

0

0
4

0

0

0
4

0

0

0
4

0

0

0
4

0

0

0
4

0

0

0
4

0
3

0
3

0
4

0

0

0
4

0

0

0
4

0

0

0
4

0

0

0
4

0

0

0
4

0

0

0
4

0

0

0
4

0

0

0
4

0

0

0
4

0

0

0
4

0

0

0

0

0
4

0

0

0

0

0
4

0

0

0

0

0
4

0

0

0

0

0

0
4

0

0

0

0

0

0
4

0
4

3.29826
4

2.37119
5

0

1.08543
5

0.0127553
4

0

0.196712

0

0

0.0127553

0

0

0

0.00447073

0

0.0446436
5

0

0

0

0

0.00894146

0

0

0

0

0

0
5

0

0

0

0

0.0127553

0

0

0

0

0

0

0

0

0

0

0.0382659

0

0

0

0

0.178574
5

0

0.019133

0

0

0

0

0

0.00894146

0.0127553

0

0.309603
5

0.0201183

0

0

0

0

0

0.00894146

0

0

0

0.10342
6

0.0573989

0.00447073

0

0

0

0

0

0

0

0

0.0424719
4

0

0

0

0

0

0

0

0

0

0

0.0318883
5

0

0

0

0

0

0

0

0

0

0

0.119724

0

0

0

0.019133

0

0.00670609

0

0

0

0

0.00447073
5

0

0

0

0

0

0

0

0

0

0

0

0

0

0

0

0

0

0

0

0.00670609

0

3.24393290007663e-16
5

0
4

0.805054
4

0
6

0
3

0.210649
4

0.0510212
5

0
5

0.159749
5

0
4

0
4

0
4

0
4

0

0

0

0

0

0

0

0

0

0

0
4

0

0

0

0

0

0

0

0

0

0

0

0

0

0

0

0

0

0

0

0

0

0
4

0

0

0

0

0

0

0

0

0

0

0

0

0

0

0

0

0

0

0

0

0

0

0

0

0

0

0

0

0

0

0

0

0
4

0

0

0

0

0

0

0

0

0

0

0

0

0

0

0

0

0

0

0

0

0

0

0

0

0

0

0

0

0

0

0

0

0

0

0

0

0

0

0

0

0

0

0

0.0286067
4

0
7

0

0

0

0

0

0

0

0

0

0

0

0

0

0

0

0

0

0

0

0

0

0

0

0

0

0

0

0

0

0

0

0

0

0

0

0

0

0

0

0

0

0

0

0

0

0

0

0

0

0

0

0

0

0

0
4

0

0

0

0

0

0

0

0

0

0

0

0

0

0

0

0

0

0

0

0

0

0

0

0

0

0

0

0

0

0

0

0

0

0

0
4

0

0

0

0

0

0

0.355029

0

0

0

0
3

0

0

0

0

0

0

0

0

0

0

0

0

0

0

0

0

0

0

0

0

0

0
4

0

0

0

0

0

0

0

0

0

0

0
4

0

0

0

0

0

0

0

0

0

0

0
4

0

0

0

0

0

0

0

0

0

0

0
3

0

0

0

0

0

0

0

0

0

0

0
4

0.10842

0.0382659
8

0.0127553

0

0

0

0

0

0

0

0

0

0.0573989
8

0

0

0

0

0

0

0

0

0

0

0

0

0

0

0

0

0

0

0

0

0

0

0

0

0

0

0

0

0
4

0

0

0

0

0

0

0

0

0
4

0

0

0
4

0

0

0
4

0

0

0
4

0.0135974

0.0135974

0
4

0

0

0
4

0

0

0
4

0

0

0
4

0

0

0
4

0

0

0
4

0

0

0
4

0
4

0
4

0

0

0

0
4

0

0

0
4

0

0

0
4

0

0

0
4

0

0

0
4

0
4

0
4

0

0

0
4

0
4

0

0

0

0

0
4

0

0

0

0

0
4

0

0

0
4

0

0

0
4

0

0

0
4

0

0

0
4

1.25073562617928e-15
4

0
4

0
3

0
4

0
4

0

0

0

0

0

0

0

0

0

0

0
4

0

0

0

0

0

0
4

0

0

0
4

0

0

0
4

0

0

0
4

0

0

0
4

0

0

0
4

0
3

0
4

0

0

0

0

0

0

0

0
4

0

0
4

0

0

0

0
4

0
3

0
3

0
4

0
4

0
4

0
4

0
4

0

0

0

0

0

0

0
4

0
3

0

0

0

0

0

0

0

0
4

0
4

0
4

0

0

0

0
4

0
4

0

0

0

0

0

0
4

0

0

0

0

0
4

0

0

0

0

0

0
4

0
4

1.08492

0.953973

0.518839

0

0.00447073

0.265622
7

0.0980293

0.024589

0.0424226

0

0

0

0

8.32667268468867e-17

0
4

0.0573311

0.0134122

0.0134122

0.0305067

0

0
4

0.0536487

0

0.0536487

0

0

0

0
4

0.00443786

0.00443786

0

0
4

0.0155325

0.0155325

0
4

0
4

0
4

0
4

0

0

0

0

0
4

0

0

0

0
4

0
4

0

0

0

0

0

0

0
4

0

0

0

0
4

0
4

0.344393

0.344393

0

0.344393

0
4

0

0

0

0
4

0

0

0
4

0
4

0

0

0

0

0

0
4

0
4

0
3

0
3

0
3

0

0
4

0

0

0
4

0
4

0.0754176
4

0.0702163

0.0702163

0

0
4

0.00520121

0.00520121

0
4

0

0

0
4

6.93889390390723e-18
4

0
4

0.0644699

0.0644699

0.0320138

0.0197008

0.0127553

0

1.73472347597681e-18

0
4

0

0

0
4

0
4

0

0

0

0

0
4

0

0

0

0

0
4

0

0

0
4

0
4

0
6

0
6

0
6

0
4

0
4

0

0

0

0
4

0
4

0
3

0
3

0
3

0

0

0
3

0

0

0

0

0

0

0

0
4

0

0

0

0

0

0
4

0

0

0

0

0
4

0
4

0

0

0

0

0

0
4

0

0

0
4

0
4

0
4

0

0

0

0

0
4

0

0

0
4

0

0

0
4

0
4

0.0787131

0.0787131

0.0721579

0.00655526

4.33680868994202e-18

0
4

0
4

0
3

0

0

0
4

0

0

0

0
4

0

0

0
4

0

0

0
4

0

0

0
4

0

0

0
4

0
4

0.0892871

0.0892871

0.0892871

0

0
4

0
4

0

0

0

0

0
4

0

0

0

0

0
4

0

0

0
4

0

0

0
4

0
4

0

0

0

0

0

0

0
4

0
4

0

0

0

0

0

0
4

0

0

0

0

0
4

0
4

0.246072

0.246072

0.240799

0.00527298

0
4

0
4

0

0

0

0

0
4

0
4

0
3

0
3

0
3

0

0
4

0

0

0

0

0

0

0

0
4

0

0

0

0

0
4

0

0

0

0
4

0

0

0

0
4

0

0

0

0
4

0
4

0

0

0

0

0
4

0

0

0

0
4

0

0

0
4

0
4

0

0

0

0

0

0

0

0

0
4

0
4

0
4

0
4

0

0

0

0
4

0

0

0
4

0

0

0
4

0
4

0

0

0

0

0

0
4

0

0

0

0
4

0
4

0.012313

0

0

0

0
4

0.012313

0.0049252

0.00738779

0
4

0

0

0
4

0
4

0

0

0

0

0

0

0
4

0

0

0
4

0
4

0

0

0

0

0

0
4

0

0

0
4

0

0

0
4

0

0

0
4

0
4

0
4

0
4

0
4

0
4

0

0

0
4

0

0

0
4

0
4

0
4

0
4

0

0

0

0
4

0
4

0

0

0

0

0
4

0

0

0

0
4

0
4

0.187244
3

0.187244
3

0.187244
3

0
3

0

0

0

0

0

0

0

0
4

0

0

0
4

0

0

0
4

0

0

0
4

0

0

0
4

0

0

0
4

0
4

0
4

0

0

0

0
4

0
4

0

0

0

0
4

0

0

0

0
4

0

0

0

0
4

0

0

0
4

0

0

0
4

0

0

0
4

0

0

0
4

0
4

0
6

0
6

0
6

0
4

0
4

0.00520121

0

0

0
4

0

0

0
4

0.00520121

0

0.00520121

0
4

0

0

0
4

0
4

0

0

0

0

0
4

0
4

0
3

0
3

0

0

0

0
4

0

0

0
4

0
4

0

0

0

0

0

0
4

0

0

0
4

0
4

0

0

0

0
4

0

0

0
4

0

0

0
4

0

0

0
4

0
4

0

0

0

0

0

0
4

0
4

0.00447073

0.00447073

0.00447073

0

0

0

0
4

0

0

0
4

0
4

0

0

0

0
4

0

0

0
4

0

0

0
4

0
4

0

0

0

0

0

0

0
4

0
4

0.0510212
5

0.0510212
5

0
5

0

0

0

0

0

0

0

0

0

0

0.0127553
5

0

0.019133
4

0

0

0

0

0

0.019133

0
4

0
4

0

0

0

0

0

0

0

0
4

0
4

0

0

0

0

0

0

0
4

0

0

0
4

0
4

0

0

0

0

0
4

0
4

0

0

0

0

0

0

0
4

0

0

0
4

0

0

0
4

0
4

0

0

0

0

0
4

0

0

0

0
4

0

0

0
4

0
4

0

0

0

0

0
4

0

0

0
4

0
4

0

0

0

0

0

0
4

0
4

0

0

0

0

0
4

0

0

0
4

0
4

0.0255106

0.0255106

0

0

0.0255106

0
4

0

0

0
4

0
4

0

0

0

0

0

0
4

0
4

0.157326
5

0.157326
5

0.147493
5

0.00983289

0

0

0

0

0

0

0

0
4

0
4

0

0

0

0

0
4

0

0

0
4

0

0

0
4

0

0

0
4

0
4

0

0

0

0

0

0
4

0

0

0

0
4

0
4

0

0

0

0

0

0
4

0

0

0

0
4

0
4

0

0

0

0

0
4

0

0

0
4

0

0

0
4

0
4

0

0

0

0

0
4

0

0

0
4

0

0

0
4

0

0

0
4

0
4

0.167159

0.167159

0

0.167159

0
4

0
4

0
4

0

0

0

0
4

0

0

0
4

0
4

0

0

0

0
4

0
4

0

0

0

0

0

0
4

0

0

0
4

0
4

0

0

0

0

0

0
4

0

0

0
4

0

0

0
4

0
4

0
4

0
4

0
4

0

0

0

0

0

0

0

0

0

0

0

0
4

0

0

0
4

0

0

0

0
4

0

0

0
4

0

0

0
4

0

0

0
4

0

0

0
4

0

0

0
4

0

0

0
4

0

0

0
4

0
4

0
4

0

0

0
4

0

0

0
4

0
4

0

0

0

0

0
4

0

0

0
4

0
4

0.0127553

0.0127553

0.0127553

0

0
4

0

0

0

0
4

0
4

0

0

0

0

0
4

0

0

0
4

0
4

0

0

0

0

0

0
4

0

0

0

0
4

0
4

0

0

0

0
4

0
4

0

0

0

0

0

0
4

0

0

0

0
4

0
4

0

0

0

0

0
4

0

0

0

0
4

0

0

0
4

0
4

0

0

0

0

0

0
4

0

0

0

0
4

0
4

0

0

0

0

0

0
4

0

0

0
4

0

0

0
4

0
4

0

0

0
6

0

0

0

0

0

0

0
4

0

0
6

0

0

0

0

0

0

0

0
4

0

0

0

0
4

0

0

0

0
4

0
4

0

0

0

0

0

0

0
4

0

0

0
4

0
4

0

0

0

0

0

0
4

0

0

0
4

0

0

0
4

0
4

0

0

0

0

0

0
4

0

0

0
4

0
4

0

0

0

0
4

0
4

0

0

0

0

0
4

0

0

0
4

0

0

0
4

0
4

0

0

0

0

0
4

0

0

0

0
4

0

0

0
4

0
4

0.019133

0.019133

0.019133

0
4

0
4

0

0

0

0

0

0
4

0
4

0

0

0

0

0
4

0
4

0

0

0

0

0

0

0
4

0
4

0.127553

0.127553
6

0.127553
6

0

0

0

0

0

0

0

0
4

0
2

0

0

0

0

0

0

0
4

0
2

0

0

0

0

0
4

0

0

0

0

0
4

0

0

0

0
4

0

0

0

0
4

0

0

0
4

0
4

0

0

0

0

0

0

0
4

0
4

0

0

0

0

0
4

0

0

0

0
4

0
4

0

0

0

0
4

0
4

0

0

0

0

0
4

0

0

0
4

0
4

0.0196658

0.0196658

0.0131105

0.00655526

0
4

0

0

0
4

0
4

0.019133

0.019133

0.019133

0

0
4

0
4

0

0

0

0

0

0
4

0
4

0

0

0

0

0
4

0

0

0
4

0
4

0

0

0

0
4

0

0

0

0
4

0
4

0

0

0

0
4

0

0

0
4

0
4

0.19133
5

0.19133
5

0.19133
5

0

0

0
4

0

0

0
4

0

0

0
4

0
4

0

0

0

0

0

0
4

0

0

0
4

0
4

0

0

0

0

0
4

0
4

0

0

0

0
4

0
4

0

0

0

0

0
4

0

0

0
4

0
4

0

0

0

0
4

0

0

0

0
4

0
4

0

0

0

0
4

0

0

0
4

0

0

0
4

0
4

0

0

0

0

0

0
4

0
4

0

0

0

0

0
4

0

0

0
4

0
4

0.0380012

0.0335305

0.0156475

0.00447073

0.0134122

3.46944695195361e-18

0
4

0.00447073

0.00447073

0
4

2.60208521396521e-18

0
4

0

0

0

0

0

0

0
4

0
4

0

0

0

0

0

0

0

0

0

0

0

0

0

0

0

0

0

0

0

0

0

0

0

0

0

0

0

0

0

0

0

0

0

0

0

0

0

0
4

0

0

0

0

0

0

0

0

0

0

0
4

0
4

0

0

0

0

0

0

0

0

0

0

0

0

0

0

0

0

0
4

0
4

0

0

0

0
4

0

0

0
4

0

0

0
4

0
4

0

0

0

0

0
4

0
4

0.0446436

0.0446436

0.0318883

0.0127553

0
4

0
4

0

0

0

0
4

0

0

0
4

0

0

0
4

0
4

0

0

0

0
4

0

0

0
4

0
4

0

0

0

0
4

0

0

0
4

0
4

0

0

0

0

0
4

0
4

0

0

0

0

0
4

0

0

0
4

0
4

0

0

0

0

0
4

0

0

0
4

0
4

0

0

0

0

0
4

0

0

0
4

0
4

0

0

0

0

0

0

0

0

0

0

0

0

0

0

0

0

0

0

0

0

0

0
4

0

0

0

0

0

0

0

0
4

0

0

0

0
4

0
4

0

0

0

0

0
4

0
4

0

0

0

0
4

0
4

0

0

0

0

0
4

0
4

0

0

0

0
4

0

0

0
4

0
4

0

0

0

0

0
4

0

0

0
4

0
4

0

0

0

0

0

0
4

0
4

0

0

0

0
4

0

0

0
4

0
4

0

0

0

0
4

0

0

0
4

0
4

0.0318883

0.0318883

0

0.0318883

0
4

0

0

0
4

0
4

0

0

0

0

0
4

0
4

0
3

0
3

0
3

0

0

0

0

0

0

0

0

0
4

0

0

0

0
4

0

0

0
4

0
4

0

0

0

0
4

0

0

0
4

0
4

0

0

0

0

0
4

0
4

0

0

0

0
4

0

0

0
4

0

0

0
4

0
4

0

0

0

0

0
4

0

0

0
4

0
4

0

0

0

0

0
4

0

0

0
4

0
4

0

0

0

0

0
4

0

0

0
4

0
4

0

0

0

0

0
4

0
4

0.172197

0.172197

0.172197

0
4

0
4

0

0

0

0

0
4

0
4

0

0

0

0

0
4

0

0

0
4

0
4

0
4

0
4

0

0

0

0

0

0

0

0

0
4

0
4

0
4

0

0

0

0

0
4

0

0

0

0

0

0
4

0

0

0

0
4

0

0

0
4

0
4

0

0

0

0
4

0

0

0
4

0
4

0

0

0

0
4

0

0

0
4

0

0

0
4

0
4

0

0

0

0

0
4

0
4

0

0

0

0

0
4

0
4

0

0

0

0
4

0
4

0

0

0

0
4

0

0

0
4

0
4

0

0

0

0
4

0
4

0

0

0

0

0
4

0
4

0

0

0

0

0

0
4

0
4

0

0

0

0

0
4

0
4

0

0
5

0

0

0

0

0

0

0

0

0
4

0

0

0

0

0
4

0

0

0

0
4

0

0

0
4

0
4

0

0

0

0

0
4

0
4

0

0

0

0
4

0

0

0
4

0
4

0

0

0

0
4

0
4

0

0

0

0
4

0
4

0.0357658

0.0357658

0.0357658

0
4

0
4

0

0

0

0

0
4

0
4

0

0

0

0
4

0

0

0
4

0
4

0

0

0

0

0
4

0
4

0

0

0

0

0
4

0
4

0

0

0

0
4

0

0

0
4

0
4

0
2

0
2

0

0

0

0

0
2

0

0

0

0

0

0

0

0
4

0

0

0

0
4

0
4

0

0

0

0
4

0
4

0

0

0

0

0
4

0
4

0

0

0

0
4

0
4

0

0

0

0

0
4

0
4

0

0

0

0

0
4

0
4

0

0

0

0

0
4

0
4

0

0

0

0

0
4

0
4

0

0

0

0
4

0

0

0
4

0
4

0

0

0

0

0
4

0
4

0

0

0

0
4

0

0

0
4

0
4

0
3

0
3

0
3

0

0

0

0

0

0

0

0
4

0
3

0

0

0

0

0

0

0

0
4

0
4

0

0

0

0
4

0
4

0

0

0

0
4

0

0

0
4

0
4

0

0

0

0
4

0

0

0
4

0
4

0

0

0

0
4

0

0

0
4

0
4

0

0

0

0

0
4

0
4

0

0

0

0
4

0

0

0
4

0
4

0

0

0

0
4

0
4

0

0

0

0

0
4

0
4

0

0

0

0

0
4

0
4

0

0

0

0
4

0

0

0
4

0
4

0
4

0
4

0
4

0

0

0

0

0

0

0

0
4

0

0

0

0

0

0

0
4

0

0

0

0

0
4

0

0

0

0
4

0

0

0

0
4

0

0

0

0
4

0

0

0
4

0
4

0

0

0

0
4

0

0

0
4

0
4

0

0

0

0

0
4

0
4

0

0

0

0

0
4

0
4

0

0

0

0
4

0

0

0
4

0
4

0

0

0

0
4

0

0

0
4

0
4

0

0

0

0
4

0

0

0
4

0
4

0

0

0

0

0
4

0
4

0

0

0

0

0
4

0
4

0

0

0

0
4

0

0

0
4

0
4

0

0

0

0
4

0
4

0
3

0
3

0
3

0
2

0

0

0

0

0
4

0
3

0
3

0

0

0

0
4

0

0

0

0
4

0

0

0

0

0

0
4

0

0

0
4

0
4

0

0

0

0

0
4

0
4

0

0

0

0
4

0
4

0

0

0

0

0
4

0
4

0

0

0

0
4

0

0

0
4

0
4

0.0573989

0.0573989

0.0127553

0.0446436

0
4

0
4

0

0

0

0

0
4

0
4

0

0

0

0
4

0
4

0

0

0

0

0
4

0
4

0

0

0

0

0
4

0
4

0

0

0

0

0
4

0
4

0
4

0
4

0
4

0

0

0

0

0

0

0

0
4

0

0

0
4

0

0

0
4

0
4

0

0

0

0
4

0
4

0

0

0

0
4

0

0

0
4

0
4

0

0

0

0

0
4

0
4

0

0

0

0
4

0
4

0

0

0

0
4

0

0

0
4

0
4

0

0

0

0

0
4

0
4

0

0

0

0
4

0

0

0
4

0
4

0

0

0

0

0
4

0
4

0

0

0

0

0
4

0
4

0

0

0

0
4

0

0

0
4

0
4

0
2

0
2

0
2

0

0

0

0

0

0
3

0

0

0

0

0

0

0
4

0
2

0
2

0

0

0

0

0

0

0

0

0

0

0

0
2

0
3

0

0

0

0

0

0
4

0

0

0

0
4

0

0

0

0

0

0
4

0

0

0
4

0

0

0
4

0
4

1.41939

1.41492

1.41492

0

0

0

0
4

0

0

0
4

0.00447073

0.00447073

0
4

1.4137996329211e-16

0
4

0

0

0

0
4

0

0

0
4

0
4

0

0

0

0
4

0
4

0

0

0

0
4

0

0

0
4

0
4

0

0

0

0
4

0
4

0

0

0

0
4

0

0

0
4

0
4

0

0

0

0
4

0
4

0

0

0

0
4

0

0

0
4

0
4

0

0

0

0
4

0
4

0

0

0

0
4

0
4

0

0

0

0
4

0
4

0.0765955
7

0.0676541
7

0.0351089
7

0
8

0.0236037

0.00894146

0

0

0

0

0

0
4

0

0

0

0

0

0
4

0.00894146

0.00447073

0.00447073

0
4

5.20417042793042e-18
7

0
4

0

0

0

0
4

0
4

0.00351532

0.00351532

0.00351532

0

0
4

0
4

0

0

0

0
4

0
4

0

0

0

0
4

0
4

0

0

0

0
4

0
4

0

0

0

0
4

0
4

0

0

0

0
4

0
4

0

0

0

0
4

0
4

0

0

0

0
4

0
4

0

0

0

0
4

0
4

0
2

0
2

0
2

0
2

0

0

0

0
4

0

0

0

0
4

0

0

0
4

0

0

0
4

0
4

0

0

0

0
4

0
4

0

0

0

0
4

0
4

0

0

0

0
4

0
4

0

0

0

0
4

0
4

0

0

0

0
4

0
4

0

0

0

0
4

0
4

0

0

0

0
4

0
4

0

0

0

0
4

0
4

0.00447073

0.00447073

0.00447073

0
4

0
4

0

0

0

0
4

0
4

0
4

0
4

0
4

0

0

0

0

0

0

0

0
4

0
4

0

0

0

0
4

0
4

0

0

0

0
4

0
4

0

0

0

0
4

0
4

0

0

0

0
4

0
4

0

0

0

0
4

0
4

0

0

0

0
4

0
4

0

0

0

0
4

0
4

0.0127553

0.0127553

0.0127553

0
4

0
4

0

0

0

0
4

0
4

0

0

0

0
4

0
4

0

0

0

0

0

0

0

0

0

0
4

0

0

0
4

0
4

0

0

0

0
4

0
4

0

0

0

0
4

0
4

0

0

0

0
4

0
4

0

0

0

0
4

0
4

0

0

0

0
4

0
4

0

0

0

0
4

0
4

0

0

0

0
4

0
4

0

0

0

0
4

0
4

0

0

0

0
4

0
4

0

0

0

0
4

0
4

0
3

0
3

0
3

0
3

0

0

0
4

0

0

0

0

0
4

0
4

0

0

0

0
4

0
4

0

0

0

0
4

0
4

0

0

0

0
4

0
4

0

0

0

0
4

0
4

0

0

0

0
4

0
4

0

0

0

0
4

0
4

0

0

0

0
4

0
4

0

0

0

0
4

0
4

0

0

0

0
4

0
4

0

0

0

0
4

0
4

0
4

0
4

0

0

0

0

0

0

0

0

0

0
4

0

0

0

0

0

0

0
4

0

0

0
4

0
4

0

0

0

0
4

0
4

0

0

0

0
4

0
4

0

0

0

0
4

0
4

0

0

0

0
4

0
4

0

0

0

0
4

0
4

0

0

0

0
4

0
4

0

0

0

0
4

0
4

0

0

0

0
4

0
4

0

0

0

0
4

0
4

0

0

0

0
4

0
4

0
3

0
3

0
3

0

0

0

0

0
4

0

0

0
4

0

0

0
4

0
4

0

0

0

0
4

0
4

0

0

0

0
4

0
4

0

0

0

0
4

0
4

0

0

0

0
4

0
4

0

0

0

0
4

0
4

0

0

0

0
4

0
4

0

0

0

0
4

0
4

0

0

0

0
4

0
4

0

0

0

0
4

0
4

0

0

0

0
4

0
4

0

0

0

0

0

0

0

0
4

0

0

0
4

0
4

0

0

0

0
4

0
4

0

0

0

0
4

0
4

0

0

0

0
4

0
4

0

0

0

0
4

0
4

0

0

0

0
4

0
4

0

0

0

0
4

0
4

0

0

0

0
4

0
4

0

0

0

0
4

0
4

0

0

0

0
4

0
4

0

0

0

0
4

0
4

0

0

0

0

0

0

0

0

0
4

0

0

0

0

0
4

0

0

0

0
4

0

0

0

0
4

0

0

0
4

0
4

0

0

0

0
4

0
4

0

0

0

0
4

0
4

0

0

0

0
4

0
4

0

0

0

0
4

0
4

0

0

0

0
4

0
4

0

0

0

0
4

0
4

0.0637765

0.0637765

0.0637765

0
4

0
4

0

0

0

0
4

0
4

0

0

0

0
4

0
4

0

0

0

0
4

0
4

0.0364085
3

0.0234054
3

0.0234054
5

0

0

0

0

0

0

0

0

0

0

0

0

0

0

0

0

0

0

0
4

0

0

0

0
4

0

0

0

0

0
4

0

0

0

0

0

0
4

0

0

0

0

0
4

0

0

0

0
4

0

0

0

0
4

0

0

0

0
4

0

0

0

0
4

0

0

0

0
4

0

0

0
4

0

0

0

0

0

0

0

0

0

0

0

0
4

0

0

0
4

0

0

0
4

0
3

0

0

0

0

0

0

0

0

0

0

0

0

0
4

0
3

0

0

0

0

0

0

0
4

0
4

0

0

0

0

0

0

0

0

0

0
4

0

0

0

0

0

0
4

0
5

0

0

0

0

0

0
4

0.013003
4

0

0

0

0.013003

0

0
4

0

0

0

0

0

0
4

0
4

0
3

0
3

0
3

0

0

0

0

0

0

0
4

0

0

0

0

0
4

0

0

0

0
4

0
4

0

0

0

0
4

0
4

0

0

0

0
4

0
4

0

0

0

0
4

0
4

0

0

0

0
4

0
4

0

0

0

0
4

0
4

0

0

0

0
4

0
4

0

0

0

0
4

0
4

0

0

0

0
4

0
4

0

0

0

0
4

0
4

0

0

0

0
4

0
4

0
4

0
4

0
4

0

0

0

0
4

0

0

0

0

0

0
4

0
4

0

0

0

0
4

0
4

0

0

0

0
4

0
4

0

0

0

0
4

0
4

0

0

0

0
4

0
4

0

0

0

0
4

0
4

0

0

0

0
4

0
4

0

0

0

0
4

0
4

0

0

0

0
4

0
4

0

0

0

0
4

0
4

0

0

0

0
4

0
4

0

0

0

0

0

0

0

0

0
4

0

0

0
4

0
4

0

0

0

0
4

0
4

0

0

0

0
4

0
4

0

0

0

0
4

0
4

0

0

0

0
4

0
4

0

0

0

0
4

0
4

0

0

0

0
4

0
4

0

0

0

0
4

0
4

0

0

0

0
4

0
4

0

0

0

0
4

0
4

0

0

0

0
4

0
4

0
4

0

0

0

0

0

0

0

0

0
4

0

0

0

0

0

0
4

0

0

0

0
4

0

0

0
4

0
4

0

0

0

0
4

0
4

0

0

0

0
4

0
4

0

0

0

0
4

0
4

0

0

0

0
4

0
4

0

0

0

0
4

0
4

0

0

0

0
4

0
4

0

0

0

0
4

0
4

0

0

0

0
4

0
4

0

0

0

0
4

0
4

0

0

0

0
4

0
4

0.00665679
3

0
3

0

0

0

0

0

0

0

0
4

0.00665679

0.00665679

0

0

0
4

0

0

0
4

0
4

0

0

0

0
4

0
4

0

0

0

0
4

0
4

0

0

0

0
4

0
4

0

0

0

0
4

0
4

0

0

0

0
4

0
4

0

0

0

0
4

0
4

0

0

0

0
4

0
4

0

0

0

0
4

0
4

0

0

0

0
4

0
4

0

0

0

0
4

0
4

0.461537
7

0.461537
7

0.461537
7

0
7

0
4

0
4

0

0

0

0
4

0
4

0

0

0

0
4

0
4

0

0

0

0
4

0
4

0

0

0

0
4

0
4

0

0

0

0
4

0
4

0

0

0

0
4

0
4

0

0

0

0
4

0
4

0

0

0

0
4

0
4

0

0

0

0
4

0
4

0

0

0

0
4

0
4

0
4

0
4

0
4

0

0

0

0

0

0

0
4

0

0

0

0

0
4

0

0

0

0
4

0
4

0

0

0

0
4

0
4

0

0

0

0
4

0
4

0

0

0

0
4

0
4

0

0

0

0
4

0
4

0

0

0

0
4

0
4

0

0

0

0
4

0
4

0

0

0

0
4

0
4

0

0

0

0
4

0
4

0

0

0

0
4

0
4

0

0

0

0
4

0
4

0
4

0
4

0
4

0

0

0

0

0

0
4

0
4

0

0

0

0
4

0

0

0
4

0

0

0
4

0

0

0
4

0
4

0

0

0

0
4

0
4

0

0

0

0
4

0
4

0

0

0

0
4

0
4

0

0

0

0
4

0
4

0

0

0

0
4

0
4

0.113839

0.113839

0.113839

0
4

0
4

0

0

0

0
4

0
4

0

0

0

0
4

0
4

0

0

0

0
4

0
4

0

0

0

0
4

0
4

0

0

0

0

0

0

0

0

0

0

0
4

0

0

0

0
4

0

0

0
4

0

0

0
4

0

0

0
4

0
4

0

0

0

0
4

0
4

0

0

0

0
4

0
4

0

0

0

0
4

0
4

0

0

0

0
4

0
4

0

0

0

0
4

0
4

0

0

0

0
4

0
4

0

0

0

0
4

0
4

0.00447073

0.00447073

0.00447073

0
4

0
4

0

0

0

0
4

0
4

0

0

0

0
4

0
4

0.0127553

0.0127553

0

0.0127553
7

0

0

0
4

0

0

0

0
4

0

0

0
4

0
4

0.00351532

0.00351532

0.00351532

0
4

0
4

0

0

0

0
4

0
4

0

0

0

0
4

0
4

0

0

0

0
4

0
4

0

0

0

0
4

0
4

0

0

0

0
4

0
4

0

0

0

0
4

0
4

0

0

0

0
4

0
4

0

0

0

0
4

0
4

0

0

0

0
4

0
4

0
4

0
4

0

0

0

0

0

0

0

0

0

0

0

0

0

0

0

0

0

0

0

0

0

0

0

0

0

0

0

0

0

0

0

0

0

0

0

0

0

0

0

0

0

0

0

0

0

0

0

0

0

0

0

0

0

0

0

0
4

0

0

0
4

0

0

0

0

0

0

0
4

0

0

0

0

0
4

0

0

0

0
4

0

0

0

0

0
4

0

0

0
4

0

0

0

0
4

0

0

0
4

0

0

0
4

0
4

0
4

0
4

0

0

0

0

0

0

0
4

0

0

0

0

0
4

0

0

0

0
4

0

0

0

0
4

0
4

0

0

0

0
4

0
4

0

0

0

0
4

0
4

0

0

0

0
4

0
4

0

0

0

0
4

0
4

0

0

0

0
4

0
4

0

0

0

0
4

0
4

0

0

0

0
4

0
4

0

0

0

0
4

0
4

0

0

0

0
4

0
4

0

0

0

0
4

0
4

0
4

0
4

0

0

0

0

0

0
4

0
3

0

0

0

0

0
4

0

0

0
4

0

0

0
4

0
4

0

0

0

0
4

0
4

0

0

0

0
4

0
4

0

0

0

0

0

0

0
4

0

0

0

0
4

0

0

0
4

0
4

0
4

0
4

0
4

0

0

0
4

0

0

0

0

0

0

0
4

0

0

0

0

0

0

0
4

0

0

0
4

0
4

0
4

0
4

0

0

0

0

0

0

0

0

0
4

0

0

0

0

0
4

0

0

0
4

0
4

0.0127553
3

0

0

0

0
4

0

0

0

0

0

0

0
4

0

0

0
4

0.0127553

0.0127553

0
4

0

0

0
4

0
4

0
2

0
2

0
2

0

0

0
4

0
4

0
3

0

0

0

0

0

0

0
4

0

0

0

0

0

0
4

0

0

0

0

0
4

0

0

0
4

0

0

0
4

0

0

0
4

0
4

0

0

0

0

0

0

0

0

0
4

0

0

0

0

0

0

0
4

0
4

0
3

0
4

0
4

0

0

0

0

0

0
4

0

0

0

0

0
4

0

0

0
4

0
4

0.135231
3

0.00520121
3

0.00520121
4

0

0

0

0

0

0

0

0

0

0

0

0
4

0

0

0
4

0

0

0
4

0

0

0
4

0.0962224
3

0.0910212
5

0

0

0

0

0

0

0

0

0.00520121

0

0

6.93889390390723e-18
3

0
4

0
4

0
4

0

0

0

0

0

0

0
4

0.0338079
3

0.0338079
7

0

0

0

0

0

0

0
4

0
5

0

0

0

0

0

0

0

0
4

0

0

0

0

0

0

0
4

0

0

0

0
4

0

0

0

0
4

0

0

0
4

0
4

0

0

0

0

0

0
4

0

0

0

0

0

0
4

0

0

0
4

0

0

0
4

0

0

0
4

0
4

0

0

0

0

0

0

0

0

0

0
4

0

0

0
4

0
4

0

0

0

0

0

0

0

0

0

0
4

0

0

0

0

0

0
4

0
4

0
4

0
4

0

0

0

0

0

0
4

0
4

0

0

0

0

0

0

0

0

0
4

0

0

0
4

0
4

0.0223536
7

0.0223536
7

0
7

0.0223536

0

0

0
4

0

0

0
4

0

0

0
4

0

0

0
4

0

0

0
4

0

0

0
4

0

0

0
4

0
4

0.114427
4

0.0494115
4

0.0442103

0

0.00520121

0

0

0

0

0
4

0.0650151

0

0.0650151

0
4

0
4

0
3

0
3

0
3

0
4

0

0

0
4

0
4

0
4

0

0

0

0

0

0
4

0

0

0

0

0
4

0

0

0

0

0
4

0

0

0
4

0
4

0

0

0

0

0

0

0

0

0
4

0

0

0

0
4

0
4

0.898568
7

0.861755
7

0.632147
7

0.0127553

0

0.0382659

0.0127553

0

0

0.0127553

0.0363261
7

0

0.0256233
7

0.0172484
7

0.0738779

0

0

0

0
4

0.0368134
7

0.0176805

0

0.019133

0

0
4

1.38777878078145e-17
7

0
4

0
4

0

0

0

0

0

0

0

0
4

0

0

0

0

0
4

0

0

0
4

0
4

0

0

0

0
4

0

0

0

0
4

0

0

0

0

0
4

0

0

0

0
4

0
4

0

0

0

0

0

0
4

0
4

0
4

0
4

0
4

0
4

0
4

0.00780182
4

0

0

0

0

0

0
4

0

0

0
4

0.00780182

0.00780182

0
4

0

0

0

0
4

0

0

0
4

0

0

0
4

0
4

0
2

0
2

0

0

0

0

0
4

0

0

0

0

0
4

0

0

0

0
4

0

0

0
4

0
4

0

0

0

0

0

0

0
4

0

0

0
4

0
4

0.026221
7

0.026221
7

0.026221
7

0

0
4

0

0

0

0
4

0
4

0

0

0

0

0
4

0

0

0

0
4

0

0

0
4

0
4

0
4

0

0

0

0

0

0

0
4

0

0

0

0
4

0
4

0
3

0
3

0
2

0
3

0
2

0

0

0

0

0

0
4

0

0

0

0

0

0

0
4

0

0

0

0

0

0
4

0

0

0

0

0
4

0

0

0
4

0

0

0
4

0

0

0
4

0
4

0
4

0

0

0

0

0
4

0

0

0

0

0
4

0

0

0
4

0

0

0
4

0

0

0
4

0
4

0
4

0

0

0

0

0

0

0
4

0

0

0

0

0

0
4

0

0

0
4

0

0

0
4

0

0

0
4

0
4

0.306245

0.073767

0.00894146

0.00670609

0.024589

0.00894146

0.0134122

0.00670609

0.00447073

0
4

0.232478

0.0156475

0.0290597

0.187771

0
4

5.55111512312578e-17

0
4

0
3

0
3

0
3

0

0

0

0
4

0

0

0

0
4

0

0

0
4

0

0

0
4

0
4

0

0

0

0

0

0

0

0
4

0

0

0

0
4

0
4

0
1

0

0

0

0

0

0

0
4

0

0

0
4

0

0

0
4

0

0

0
4

0

0

0
4

0

0

0
4

0
4

0
3

0

0

0

0

0

0

0
4

0
4

0

0

0

0

0

0
4

0
4

0

0

0

0

0
4

0

0

0

0
4

0

0

0

0

0
4

0
4

0

0

0

0

0

0

0

0

0
4

0
4

0

0

0

0

0

0

0

0
4

0

0

0
4

0

0

0

0
4

0

0

0
4

0

0

0
4

0

0

0
4

0
4

0
3

0
4

0
4

0

0

0

0

0

0

0

0

0

0

0

0

0

0

0
4

0
3

0
4

0

0

0

0

0

0
3

0

0

0

0

0

0

0

0
4

0
3

0

0

0
4

0

0

0
4

0
4

0
2

0
2

0
2

0

0

0

0
4

0
4

0.139204
7

0.0127553

0

0.0127553

0

0

0
4

0.00527298

0.00527298

0

0

0

0
4

0.121175

0.121175

0

0
4

0
4

0
4

0
4

0

0

0

0

0
4

0

0

0

0

0
4

0

0

0
4

0
4

0
7

0
7

0

0

0

0
4

0
4

0
4

0
4

0

0

0

0

0
4

0

0

0
4

0
4

0
4

0

0

0

0

0

0
4

0

0

0

0

0
4

0

0

0
4

0

0

0
4

0
4

0
3

0
3

0

0

0

0
4

0

0

0
4

0

0

0
4

0
4

0
2

0
2

0

0

0

0

0
4

0

0

0
4

0
4

0
3

0
3

0
3

0

0
4

0

0

0

0

0
4

0
4

0
4

0
4

0
4

0

0

0
4

0
4

0.312918

0.00443786

0.00443786

0

0
4

0.30848
6

0.30848

0

0
4

0

0

0

0

0
4

0

0

0
4

0

0

0
4

0

0

0
4

0
4

6.40646
3

6.20741
3

0
3

0.764955

1.42117
3

0.852698

0.397968
7

0

0

0

0

0.332681
7

0

0

0

0

0

0

0

0

0.13274
6

0

0

0

0

0

0

0

0

0

0

1.50308
7

0

0

0

0

0

0

0

0

0

0

0.0501801
7

0

0.124189

0

0

0

0

0

0

0

0

0
7

0

0

0

0

0

0

0

0

0.0701542

0

0.293372
7

0

0.00911028

0

0

0

0

0

0

0

0

0

0

0

0

0

0

0

0

0

0

0

0.0127553
7

0

0

0

0

0

0

0

0

0

0

0.0318883
2

0.210462

0

0

0

0

0

0

0

0

0

1.19348975147204e-15
3

0
4

0.160784

0.0630655
5

0

0.0834312
7

0.00689936
7

0

0.00738779

0

0

0

0

1.64798730217797e-17

0
4

0

0

0
4

0

0

0
4

0

0

0
4

0

0

0
4

0

0

0
4

0

0

0
4

0

0

0
4

0

0

0
4

0

0

0
4

0

0

0
4

0
4

0
4

0
4

0

0

0

0
4

0

0

0
4

0

0

0
4

0

0

0
4

0

0

0
4

0

0

0
4

0

0

0
4

0

0

0
4

0

0

0
4

0.019133
7

0
7

0.019133

0

0
4

0.019133
7

0

0

0

0.019133

0

0

0
4

0

0

0
4

0

0

0

0

0
4

0

0

0
4

0

0

0
4

0

0

0

0
4

0
4

0.613632

0.40087

0
3

0

0

0
4

0

0
6

0

0

0

0

0.140308

0

0

0

0

0

0

0

0

0

0

0

0

0

0

0

0

0

0

0

0

0

0

0

0

0

0

0

0

0

0.00447073

0

0

0.184952

0

0

0

0

0

0

0

0

0

0

0

0
4

0.0156475

0

0.019133

0

0

0

0

0.00447073

0

0

0

0

0

0

0

0

0

0

0

0

0

0

0

0.019133

0.0127553

0

0

0

0

0

0
4

0
3

0
3

0
2

0

0

0

0

0

0

0
4

0

0

0

0
4

0

0

0

0
4

0

0

0

0
4

0

0

0
4

0

0

0

0
4

0

0

0

0
4

0

0

0

0
4

0

0

0
4

0

0

0

0
4

0

0

0
4

0

0

0

0
4

0

0

0
4

0

0

0
4

0

0

0
4

0

0

0
4

0

0

0
4

0

0

0
4

0

0

0
4

0

0

0
4

0

0

0
4

0

0

0
4

0

0

0

0

0
4

0

0

0
4

0

0

0
4

0

0

0
4

0.0829095

0.0829095

0
4

0

0

0
4

0

0

0
4

0
4

0

0

0

0

0
4

0.0701542
7

0.0701542
7

0
4

0

0

0

0

0
4

0.0469426

0.0178829

0.0201183

0.00447073

0.00447073

8.67361737988404e-18

0
4

0.0127553

0.0127553

0

0

0
4

0

0

0
4

0
4

3.24684

3.2379

0.294252
6

0
4

0

0

0

0

0

0

0

0

0

0

0
3

0

0

0

0

0

0

0.0284028

0

0.00443786

0

0.00665679
7

0

0

0

0

0

0

0

0

0

0

0.00443786
6

0

0

0

0

0

0

0

0

0

0

0
2

0

0

0

0

0

0

0

0

0

0

0
4

0

0

0

0

0

0

0

0

0

0

0
3

0

0

0

0

0

0.0127553

0

0

0

0

0
4

0

0

0

0

0

0

0

0.0232835

0

0

0

0

0

0

0

0

0

0

0

0

0

0
3

0

0

0

0

0

0.0956648

0

0

0

0

0.905294
6

0
3

0

0

0

0

0

0

0

0

0

0

0.542346

0

0

0

0

0

0

0.019133

0

0

0

0
4

0

0

0

0

0.159441

0

0

0

0

0

0.0156036
6

0

0

0

0

0

0.0178829

0

0

0

0

0
4

0

0

0

0

0

0

0

0

0

0

0
5

0

0

0

0

0

0

0

0

0

0

0.100464
7

0

0

0

0

0

0

0

0

0

0

0

0

0

0

0

0

0

0

0

0

0

0
4

0

0

0

0

0

0

0

0

0

0

0.0510212
7

0

0

0

0

0

0

0

0

0

0

0
4

0
3

0

0

0

0

0

0

0

0.0127553

0

0

0

0

0

0

0

0

0

0.00447073

0

0

0

0
4

0

0

0

0

0

0

0

0

0

0

0
6

0

0

0

0

0

0

0

0

0

0

0
4

0

0.00447073

0

0

0

0

0

0

0

0

0

0

0

0

0

0.0127553

0

0

0

0

0

0
4

0

0

0

0

0

0

0

0.0111768

0

0

0

0

0

0

0

0

0

0

0

0

0

0

0

0

0

0

0

0

0

0

0

0

0

0

0

0

0

0

0

0

0

0

0

0.146686
7

0
3

0

0

0

0

0

0

0

0

0

0

0
3

0.0127553

0

0

0

0

0

0

0

0

0

0

0

0

0

0

0.00447073

0

0

0

0

0

0
3

0

0

0

0

0

0

0

0

0

0

0

0

0

0

0

0

0

0

0

0

0

0
4

0

0

0.0127553

0

0

0

0

0

0

0

0.0876265
7

0

0

0

0

0

0

0

0

0

0

0

0

0

0

0

0

0

0.0127553

0

0

0

0.0177514

0

0

0

0

0

0

0

0.00447073

0

0

0
4

0

0

0

0.0127553

0

0

0

0

0

0

0
4

0

0

0

0

0

0

0

0

0

0

0

0

0

0

0

0

0

0

0

0

0

0

0
2

0

0

0

0

0

0

0

0

0

0

0

0

0

0

0

0

0

0

0

0

0

0.00520121

0

0

0

0

0

0

0

0

0

0

0

0

0

0

0.0127553

0

0.00665242

0

0

0

0

0.019133

0

0

0

0

0

0

0

0

0

0

0
3

0

0

0.00443786

0

0

0

0

0

0

0

0

0

0

0

0

0

0

0.0127553

0

0

0

0
6

0

0

0

0

0

0

0

0

0

0

0

0

0

0

0

0

0

0

0

0

0

0

0

0

0

0

0

0

0

0

0

0

0

0

0

0

0

0

0

0

0

0

0

0

0
4

0

0

0.0134122

0

0

0

0

0

0

0

0
4

0

0

0

0

0

0

0

0

0

0

0

0

0

0

0

0

0

0

0

0

0

0
4

0

0

0

0

0

0

0

0

0

0

0

0

0

0

0

0

0

0

0

0

0

0

0

0

0

0

0

0

0

0

0

0

0

0

0

0

0

0

0

0

0

0

0

0.00443786
6

0

0

0

0

0

0

0

0

0

0

0

0.0134122

0

0.0127553

0

0

0

0

0

0

0

0

0

0

0

0

0.0255106

0

0

0

0.0127553

0.0255106

0

0

0.00447073

0

0

0

0

0

0

0

0

0

0.00443786

0.0127553

0

0

0

0

0

0.0446436

0.127553

0

0

0

0.16642
6

0

0

0

0

0

0

0

0

0

0

0
6

0

0

0

0

0

0

0

0

0

0.0701542

5.68989300120393e-16

0
4

0.00894146

0.00447073

0.00447073

0
4

0

0

0
4

0

0

0

0
4

0

0

0
4

0

0

0
4

0

0

0
4

0

0

0
4

0

0

0
4

0

0

0
4

7.26849136434282e-16

0
4

1.30833

0.0573989

0.0255106
7

0

0

0.0318883

0

0

0

0

0

0

0

0
7

0

0

0

0

0

0

0

0

0

0

0

0

0

0

6.93889390390723e-18

0
4

0.999746
7

0.921016
7

0

0

0

0.00894146
6

0.0402365
6

0.0295512

0

0

0

0

0

0
4

0

0

0
4

0.00738779
7

0

0.00738779

0
4

0.243797

0.243797

0

0
4

0

0

0

0

0
4

0

0

0
4

0

0

0
4

0

0

0
4

0

0

0
4

0

0

0
4

2.4980018054066e-16

0
4

3.10731

0.222296
3

0.0628549
3

0

0

0

0

0

0

0

0

0

0

0

0

0

0

0

0

0

0

0

0

0

0

0.0446436

0

0

0

0

0.114798

0

0

0

0

0

0

0

0

0

0

0

0

0
4

0.595697
7

0.551054
7

0

0

0.0446436

0

0

0

0
4

0
3

0

0

0

0

0
4

0

0

0
4

0

0

0
4

0

0

0
4

0

0

0
4

0

0

0
4

0

0

0
4

0

0

0
4

0.204085
7

0.0892871
6

0.114798

0
4

0
3

0

0

0

0

0

0
4

0.0581195

0.0268244

0.024589

0.00670609

0

0
4

0

0

0

0
4

0.0382659
7

0.0382659
7

0
4

0

0

0

0

0
4

0.073767

0.073767

0
4

0.019133
7

0

0.019133

0
4

0

0

0

0
4

0.0974027
6

0.0923686
6

0

0.00503411

0

0

0

0

0

0
4

0.0572133

0

0.0572133

0
4

0

0

0

0
4

0

0

0

0

0

0
4

0

0

0
4

0

0

0

0

0

0
4

0.0998518

0.0998518

0

0
4

0

0

0

0
4

0.102042

0.102042

0

0
4

0

0

0
4

0.00520121

0

0.00520121

0

0
4

0.465045
7

0.262083
7

0.0255106

0

0

0.0701542
7

0

0.107297

0

0

0

0

0

6.93889390390723e-17
7

0
4

0

0

0
4

0

0

0

0
4

0

0

0

0

0
4

0

0

0

0
4

0

0

0

0
4

0

0

0
4

0

0

0
4

0.0111768

0.0111768

0
4

0.0765318

0.0765318

0
4

0

0

0

0
4

0.0446436
8

0.0446436
8

0

0

0

0

0

0

0

0

0
4

0

0

0
4

0

0

0

0
4

0

0

0

0
4

0

0

0

0
4

0.0637765

0.0637765

0

0
4

0

0

0
4

0

0

0
4

0

0

0
4

0

0

0

0
4

0

0

0
4

0.599499
8

0.242351

0.280617

0

0.019133

0.0127553

0.019133

0.0127553

0.0127553

3.46944695195361e-17
8

0
4

0

0

0
4

0.126479

0.126479

0
4

0

0

0

0
4

0

0

0
4

0.0520121

0.0520121

0
4

0

0

0

0
4

0.0637765

0.0510212

0.0127553

0
4

0

0

0
4

0

0

0
4

0

0

0
4

0
3

0

0

0

0

0

0

0
4

0

0

0
4

0

0

0
4

0

0

0
4

0

0

0
4

0

0

0
4

0

0

0
4

0

0

0
4

0

0

0
4

0

0

0
4

0

0

0
4

0

0

0

0

0
4

0

0

0
4

0

0

0
4

0

0

0
4

0.0268244

0.0268244

0
4

0

0

0
4

0

0

0
4

0

0

0
4

0

0

0
4

0

0

0
4

0

0

0
4

0

0

0

0

0

0
4

0

0

0
4

0.00447073

0.00447073

0
4

0

0

0
4

0

0

0
4

0

0

0
4

0

0

0
4

0

0

0
4

0

0

0
4

0

0

0
4

0

0

0
4

0

0

0

0

0

0
4

0

0

0
4

0

0

0
4

0

0

0
4

0

0

0
4

0

0

0
4

0

0

0
4

0

0

0
4

0

0

0
4

0

0

0
4

0

0

0
4

8.97719398817998e-16

0
4

0.25566
3

0.251189
3

0
3

0
2

0
2

0.016945

0

0

0

0

0

0

0

0

0

0
3

0

0

0

0

0

0

0.00443786

0

0

0

0
3

0

0

0

0

0

0

0

0

0.0377218

0

0
2

0

0

0

0

0

0

0

0

0.0112966

0

0

0

0

0

0

0

0

0

0

0

0

0
2

0

0

0

0

0

0

0

0

0

0

0

0

0

0

0

0

0

0

0.0225933

0

0

0

0

0

0

0

0

0

0

0

0

0

0
2

0

0

0

0

0

0

0

0

0

0

0

0

0

0

0

0

0

0

0

0

0

0

0

0

0

0

0

0

0

0

0

0

0

0

0

0

0

0

0

0

0

0

0

0

0

0

0

0

0

0

0

0

0

0

0

0

0

0

0

0

0

0

0

0

0

0

0

0

0

0.00351532

0

0

0

0

0

0

0

0

0

0

0

0

0

0

0

0

0

0

0

0

0

0
2

0

0

0

0

0

0

0

0

0

0

0

0

0

0

0

0

0

0

0.0294987

0

0

0
3

0

0

0

0

0

0

0

0

0

0

0

0

0

0

0

0

0

0

0

0

0

0

0

0

0

0

0

0

0

0

0

0

0
3

0

0

0

0

0

0

0

0

0

0

0

0.12518

0

0

0

0

0

0

0

0

0

0
4

0

0

0

0

0

0
4

0

0

0

0

0
4

0

0

0
4

0

0

0

0

0
4

0

0

0

0

0
4

0

0

0

0
4

0

0

0
4

0

0

0

0
4

0

0

0

0
4

0

0

0

0
4

0

0

0
4

0
3

0

0

0

0
4

0

0

0

0
4

0

0

0
4

0

0

0

0
4

0

0

0
4

0

0

0

0
4

0

0

0
4

0

0

0
4

0

0

0
4

0

0

0
4

0

0

0
4

0
2

0

0

0

0
4

0

0

0
4

0

0

0
4

0

0

0
4

0

0

0
4

0

0

0
4

0

0

0
4

0

0

0
4

0

0

0
4

0

0

0
4

0

0

0
4

0

0

0
4

0

0

0
4

0

0

0
4

0

0

0
4

0

0

0
4

0

0

0
4

0

0

0
4

0

0

0
4

0.00447073

0.00447073

0
4

0

0

0
4

0

0

0
4

0

0

0

0
4

0

0

0
4

0

0

0
4

0

0

0
4

0

0

0
4

0

0

0
4

0

0

0
4

0

0

0
4

0

0

0
4

0

0

0
4

0

0

0
4

0

0

0
4

0

0

0
4

0

0

0
4

0

0

0

0

0
4

0

0

0

0

0
4

0

0

0

0

0
4

0
4

0.0299813
3

0
3

0
2

0

0

0

0

0

0

0

0

0

0

0
2

0

0

0

0

0

0

0

0

0

0

0
3

0

0

0

0

0

0

0

0

0

0

0

0

0

0

0

0

0

0

0

0

0

0

0

0
4

0

0

0

0

0

0

0
4

0

0

0

0
4

0

0

0

0

0
4

0.0255106

0.0255106

0

0
4

0

0

0
4

0

0

0
4

0

0

0
4

0

0

0
4

0.00447073

0.00447073

0
4

0
4

0.0403527
5

0.0403527
5

0.0231043
5

0.0172484
7

0

0

0

0

0

0

0

0

0

0

0

0

0

0

0

0

0

0

0

0

0

0

0

0

0

0

0

0

0
4

0

0

0
4

0

0

0
4

0

0

0
4

0

0

0
4

0

0

0
4

0

0

0
4

0

0

0
4

0
4

2.30914
4

1.69216
4

1.29286
4

0.31065
7

0

0

0

0

0

0

0

0

0

0

0

0

0

0

0

0

0

0

0

0

0

0

0

0

0

0

0.00655526

0

0.00665679

0.00887572

0

0

0
4

0

0

0

0

0

0

0

0

0

0

0.0177514
6

0

0

0

0

0

0.00443786

0

0

0

0

0.0110946
7

0

0

0

0

0

0

0

0

0

0

0.0244082
7

0

0

0

0

0

0

0

0

0

0

0
7

0

0

0

0

0

0

0

0

0

0

0
7

0

0

0

0

0

0

0

0

0

0

0
5

0

0

0

0

0

0

0

0.00887572

0

0

0
6

0

0

0

0

0

0

0

0

0

0

0
4

0.0510212
6

0.0382659
6

0

0

0

0

0

0

0

0

0

0

0.0127553
6

0

0

0

0

0

0

0

0

0

0

0
6

0

0

0

0

0

0

0

0

0

0

0

0

0

0

0

0

0

0

0

0

0

0

0

0

0

0

0

0

0

0

0

0

0

0

0

0

0

0

0

0

0

0

0

0

0

0

0

0

0
4

0
3

0

0

0

0

0

0

0

0

0

0

0
4

0

0

0
4

0

0

0

0
4

0

0

0
4

0

0

0

0
4

0

0

0

0
4

0

0

0
4

0

0

0

0
4

0

0

0

0
4

0

0

0

0
4

0

0

0
4

0
4

0
4

0
4

0

0

0

0

0

0
4

0

0

0

0
4

0

0

0

0
4

0

0

0
4

0

0

0
4

0

0

0

0
4

0

0

0

0
4

0

0

0

0
4

0

0

0

0
4

0

0

0
4

0

0

0

0
4

0
2

0

0
3

0

0

0

0

0

0

0
4

0

0

0
4

0

0

0

0
4

0

0

0
4

0

0

0
4

0

0

0

0
4

0

0

0

0
4

0

0

0

0
4

0

0

0

0
4

0

0

0
4

0

0

0
4

0

0

0

0

0

0

0

0

0

0

0
4

0

0

0

0
4

0

0

0

0
4

0

0

0

0
4

0

0

0
4

0

0

0
4

0

0

0
4

0

0

0
4

0

0

0
4

0

0

0
4

0

0

0
4

0.0956648
5

0.0956648
5

0

0

0

0

0

0

0

0

0

0
4

0

0

0
4

0

0

0
4

0

0

0
4

0

0

0
4

0

0

0
4

0

0

0
4

0

0

0
4

0

0

0
4

0

0

0
4

0

0

0
4

0.0382659

0.0382659

0

0

0
4

0

0

0
4

0

0

0
4

0

0

0
4

0

0

0
4

0

0

0
4

0

0

0
4

0

0

0
4

0

0

0
4

0

0

0
4

0

0

0
4

0

0

0

0

0

0

0
4

0

0

0
4

0

0

0
4

0

0

0
4

0

0

0
4

0

0

0
4

0

0

0
4

0

0

0
4

0

0

0
4

0

0

0
4

0

0

0
4

0

0

0

0

0

0
4

0

0

0
4

0

0

0
4

0

0

0
4

0

0

0
4

0

0

0
4

0

0

0
4

0

0

0
4

0

0

0
4

0

0

0
4

0

0

0
4

0
5

0
5

0

0
4

0

0

0
4

0

0

0
4

0

0

0
4

0

0

0
4

0

0

0
4

0

0

0
4

0

0

0
4

0

0

0
4

0

0

0
4

0

0

0
4

0

0
5

0

0

0

0

0

0
4

0

0

0
4

0

0

0
4

0

0

0
4

0.0127553

0.0127553

0
4

0

0

0
4

0

0

0
4

0

0

0
4

0

0

0
4

0

0

0
4

0

0

0
4

0
4

0
4

0

0

0

0

0

0

0

0

0

0

0
4

0

0

0

0

0

0

0

0

0

0

0

0

0

0

0

0

0
4

0

0

0

0
4

0

0

0
4

0

0

0
4

0

0

0
4

0

0

0
4

0

0

0
4

0

0

0
4

0

0

0
4

0

0

0
4

0

0

0
4

0

0

0
4

0
6

0

0

0

0

0

0

0

0

0
4

0

0

0
4

0

0

0
4

0

0

0
4

0

0

0
4

0

0

0
4

0

0

0
4

0

0

0
4

0

0

0
4

0

0

0
4

0

0

0
4

0
3

0

0

0

0

0

0

0
4

0

0

0
4

0

0

0
4

0

0

0
4

0

0

0
4

0

0

0
4

0

0

0
4

0

0

0
4

0

0

0
4

0

0

0
4

0

0

0
4

0
4

0

0

0

0

0

0

0

0

0
4

0

0

0
4

0

0

0
4

0

0

0
4

0

0

0
4

0

0

0
4

0

0

0
4

0

0

0
4

0

0

0
4

0

0

0
4

0

0

0
4

0
3

0

0

0

0

0

0
4

0

0

0
4

0

0

0
4

0

0

0
4

0

0

0
4

0

0

0
4

0

0

0
4

0

0

0
4

0

0

0
4

0

0

0
4

0
4

0
4

0

0

0

0
4

0
2

0

0

0

0

0

0

0
4

0

0

0

0

0

0

0
4

0
4

0

0

0

0

0

0

0
4

0

0

0

0

0
4

0
4

0
4

0

0

0

0

0

0

0

0

0

0

0
4

0

0

0

0

0

0
3

0
4

0

0

0

0

0

0
4

0
4

0
4

0

0

0

0
4

0
4

0

0

0

0

0

0

0
4

0

0

0

0

0

0

0

0
4

0

0

0

0

0

0

0

0

0
4

0.0110946
7

0.0110946
7

0

0

0
4

0
4

0
4

0

0
4

0

0

0

0

0
4

0.0446436
7

0.0446436
7

0
4

0
4

0

0

0

0
4

0

0

0

0

0

0
4

0
4

0
4

0

0

0

0

0
4

0
4

0

0

0

0

0

0

0
4

0.102042

0.102042

0

0
4

0

0

0

0

0
4

0
6

0
6

0
4

0
5

0
5

0
4

0

0

0

0
4

0

0

0

0
4

0

0

0
4

0

0

0

0

0

0
4

0

0

0

0

0

0
4

0

0

0

0

0

0
4

0.146686

0.114798

0

0.0318883

0

0

0

0

0

0

0

6.93889390390723e-18

0
4

0

0

0

0

0
4

0

0

0

0
4

0

0

0

0

0

0
4

0

0

0

0

0
4

0

0

0

0

0
4

0

0

0
4

0

0

0

0
4

0

0

0
4

0

0

0

0
4

0

0

0

0
4

0

0
3

0

0
2

0

0

0

0

0

0

0

0
4

0

0

0

0

0
4

0

0

0

0
4

0

0

0
4

0

0

0

0

0

0
4

0

0

0

0
4

0

0

0

0

0
4

0

0

0

0
4

0

0

0
4

0

0

0

0
4

0

0

0
4

0
4

0
4

0
4

0

0

0

0
4

0

0

0
4

0

0

0

0

0
4

0

0

0

0

0
4

0

0

0

0
4

0.114798

0.114798

0
4

0

0

0

0
4

0

0

0

0
4

0

0

0
4

0

0

0

0
4

0

0

0

0

0
4

0
4

0
4

0

0

0

0

0

0

0

0
4

0

0

0

0

0
4

0

0

0

0

0
4

0

0

0

0
4

0

0

0

0

0
4

0

0

0
4

0

0

0

0
4

0

0

0

0
4

0

0

0

0
4

0

0

0
4

0

0

0

0
4

0
6

0
6

0
4

0

0

0

0
4

0

0

0
4

0

0

0
4

0

0

0
4

0

0

0

0
4

0

0

0

0
4

0

0

0
4

0

0

0

0
4

0

0

0

0
4

0

0

0
4

0
4

0.704536

0.700065

0.527869

0.159441
6

0

0

0

0.0127553

0

0

0

0

0

0

0

0

0
4

0
6

0

0

0
4

0.00447073

0.00447073

0
4

0

0

0
4

0

0

0
4

0

0

0
4

3.03576608295941e-17

0
4

0.102042
3

0
3

0
3

0
4

0

0

0

0

0

0

0

0

0

0

0
4

0

0

0

0

0

0

0

0

0

0

0

0

0

0

0

0

0

0

0

0

0

0
4

0

0

0

0

0

0

0

0

0

0

0

0

0

0

0
4

0.102042
6

0.102042
6

0

0

0

0
4

0

0

0
4

0

0

0
4

0

0

0
4

0

0

0
4

0
4

0
4

0
4

0

0

0

0
4

0

0

0

0
4

0

0

0
4

0

0

0
4

0

0

0

0
4

0

0

0
4

0

0

0
4

0
4

15.4254

7.20155

4.04672

0.468462
7

0.0125853
6

0

0

0.0127553

0

0

0

0

0

0

0

0

0

0

0

0

0

0

0

0

0

0

0.165819
7

0.0127553

0.019133

0

0

0

0

0

0.140308

0

0

0.0689511

0

0.0127553

0

0

0

0

0

0

0

0

0

0

0

0

0

0

0

0

0

0.114798

0

0

0

0

0.0127553

0

0

0

0

0

0

0

0
5

0.0127553

0.0536487

0

0

0

0

0

0

0

0

0.233947
7

0
7

0.133048
7

0.0127553

0

0

0.0382659

0.0468109

0

0

0

0

0

0
6

0

0

0

0.0255106

0

0.0255106

0

0.019133

0

0

0.0637765
7

0.0255106

0

0

0

0

0

0

0.0318883

0

0

0

0

0.00447073

0

0

0

0

0.0382659

0.0266993

0

0.00738779

0.00780182
5

0

0

0

0

0

0

0

0

0

0

0
7

0

0.00689936

0

0

0

0

0

0

0

0.024589

0

0.0123036

0

0

0

0

0

0

0

0

0

1.23726

0

0.0127553

0

0

0.0127553

0

0

0

0

0

8.15320033709099e-16

0
4

0.732347
7

0.236421
7

0

0

0

0

0

0

0

0

0

0.179231
7

0.0329317
8

0.194477
7

0.0573989

0

0

0.0318883

0

2.08166817117217e-16
7

0
4

0

0

0
4

0

0

0
4

0.0127553

0.0127553

0
4

0

0

0
4

0

0

0
4

0

0

0
4

7.16187

7.16187

0

0

0
4

0.0318883

0

0.0318883

0

0

0

0

0

0

0
4

0.101424
4

0.101424
4

0

0

0

0
4

0.165819

0.165819

0

0

0
4

0.0177514

0.0177514

0

0
4

0

0

0
4

0

0

0
4

0

0

0
4

0
4

2.00029

1.9684

0.0382659

0

0

0

0

0

0

0

0

0

0

0

0.0510212
6

0

0

0.0127553

0

0

0

0

0

0

0

0
7

0

0

0

0

0

0

0

0

0

0

0

0

0

0

0

0

0

0

0.00447073

0

0

0

0

0

0

0

0

0

0

0

0

0

0
7

0

0

0

0

0

0

0

0

0

0

0.0318883

0

0

0

0

0

0

0

0

0

0

0

0

0

0

0

0

0

0

0

0

0

0

0

0

0

0

0

0

0

0

0

0

0
6

0

0

0

0.00447073

0.168839
7

0.574974

0
7

0

0

0
7

0.00963907
6

0

0
7

0

0.093131
6

0.197707

0

0

0

0.0127553

0

0

0.00447073

0

0

0

0.0956648

0

0.0529918

0

0

0

0

0.019133

0

0

0

0
7

0

0

0

0

0

0.00665679

0

0.0127553

0

0

0.120911
7

0

0

0

0

0

0

0

0

0

0

0.242351
7

0

0

0

0

0

0

0

0

0

0

0.153583
7

0

0.00894146

0

0

0

0

0

0

0

0

0.0510212
6

0

0

0

0

0

0

0

0

0

0

0
4

0.0318883
6

0.0318883
6

0
4

0

0

0
4

0

0

0
4

0

0

0
4

0

0

0
4

0

0

0
4

0

0

0
4

0

0

0
4

1.17961196366423e-16

0
4

0
3

0
3

0
3

0
3

0

0

0

0

0
4

0

0

0

0
4

0

0

0
4

0

0

0
4

0

0

0
4

0

0

0
4

0
4

9.97504

9.95969

5.12272

0.0111439
7

0

0

0

0

0

0.00894146

0.00894146

0

0

0

0.0590877
6

0

0

0

0.00447073

0

0.0127553

0

0

0

0

0
3

0

0

0

0

0

0

0

0.0127553

0

0

0
3

0

0

0

0

0

0

0

0

0

0

0.00365461
3

0

0

0

0

0

0

0

0

0

0

0
7

0

0

0

0.0127553

0

0

0

0

0

0

0.0127553
7

0

0

0

0

0

0

0

0

0.019133

0

0.0266341
7

0

0

0

0

0

0

0

0.00548192

0

0

0
3

0

0

0.0510212

0

0.0715316

0

0

0

0

0

0.0397377
6

0

0

0

0

0

0

0

0

0

0.0127553

2.91997
7

0.0140617

0.00443786

0.0510212

0

0

0

0

0.00364411

0

0

0

0.00689936
3

0.00983289

0

0.0172484

0.0127553

0

0

0

0

0.0127553

0

0

0

0

0

0

0

0

0

0

0

0

0
3

0

0

0

0

0

0

0.00365461

0.00670609

0

0

0

0

0

0

0

0

0

0

0

0.00447073

0

0.013003
7

0

0

0

0

0

0.019133

0

0

0

0.00447073

0.0113701
7

0

0

0

0

0

0

0.00520121

0

0.00670609

0

0.012188

0

0

0

0

0

0

0.0318883

0

0

0

0.0354642
4

0

0

0.00665679

0

0

0

0.019133

0

0

0.00447073

0.0255106

0

0

0

0

0.00730923

0

0

0.0127553

0

0

0.187821
7

0.139184
7

0.0127553

0.00447073

0

0

0

0

0.010349

0

0

0

0
3

0

0.0127553

0

0

0

0

0

0

0

0

0

0

0

0

0

0.00894146

0

0

0

0.00689936

0

0.0127553
7

0

0

0

0

0

0

0

0

0.00447073

0.019133

0
3

0

0

0

0

0

0

0

0

0

0

0

0

0

0

0

0

0

0

0

0

0

0.101102

0

0

0

0

0

0

0

0

0

0

0

0

0

0.00447073

0

0

0

0.00520121

0

0

0

0.00670609

0

0

0.00447073

0.00780182

0

0.0127553

0.0347188

0

0.0335305

0

0

0

0

0

0

0

0

0

0

0

0

0
4

0

0

0

0

0

0

0

0

0.0199704

0

0

0

0.00894146

0.0244082

0

0

0

0

0

0

0.0379465

0

0

0

0.00447073

0

0

0

0

0

0

0

0.00780182

0

0

0

0

0.0127553

0.0934615
7

0.00689936

0

0.036359

0

0.0201183

0

0.0135561

0.0127553

0

0

0.0196246
7

0.0127553

0

0.0382659

0

0

0

0

0

0.0156475

0

0.0111768
3

0.00365461

0

0

0.00670609

0

0

0.0134122

0

0

0

0.0427038
7

0

0

0

0

0

0.0155503

0

0

0

0.00670609

0
7

0

0

0

0.0446436

0.00447073

0

0.0156475

0

0

0

4.57966997657877e-16

0
4

0

0

0
4

0

0

0
4

0.0108779

0.0108779

0
4

0

0

0
4

0.00447073

0.00447073

0
4

0

0

0
4

0

0

0
4

0

0

0
4

1.08853898117545e-15

0
4

8.8807

0.786714
3

0.0446436
6

0.00738779
7

0

0

0

0

0

0

0.0127553

0

0

0

0.255106
6

0

0

0

0

0

0

0

0

0

0

0
3

0

0

0

0

0

0

0

0

0

0

0.00811484
6

0

0

0

0

0

0

0.00689936

0

0

0

0
2

0

0

0

0

0

0

0

0

0

0

0

0

0

0

0

0

0

0

0

0

0

0
4

0

0

0

0

0

0

0

0

0

0

0

0

0

0

0

0

0

0

0

0

0

0

0

0

0

0

0

0

0

0

0

0

0.0134122
7

0

0

0

0

0

0

0.0127553

0

0

0

0
2

0

0

0

0

0

0

0

0

0

0

0

0
6

0

0

0

0

0

0

0

0

0

0

0.0127553
6

0

0

0

0

0

0

0.0110946

0.019133

0

0

0.0255106
6

0

0

0

0

0

0

0

0

0

0

0

0

0.0724433

0

0

0

0

0

0

0

0

0
6

0

0

0

0

0

0

0

0

0

0

0

0

0

0

0

0

0

0

0

0

0

0
2

0

0

0

0

0

0

0

0

0

0

0
6

0

0

0

0

0

0

0

0

0

0

0.0172484
7

0

0

0

0

0

0

0

0

0

0

0.0495687
7

0

0

0

0

0

0

0

0

0

0

0

0
4

0

0

0

0

0

0

0

0

0

0

0.0363814
7

0

0

0

0

0

0

0

0

0

0

0
5

0

0

0

0

0

0

0

0

0

0

0

0

0

0

0

0

0

0

0

0

0

0
7

0

0

0

0

0

0

0

0

0

0

0

0

0

0

0

0

0

0

0

0

0

0.0382659

0

0

0

0

0

0

0

0

0

0

0

0

0

0

0

0

0

0

0

0

0

0

0.0127553

0

0

0

0

0

0

0

0

0

0

0
7

0

0

0

0

0

0

0

0

0.0127553

0

0

0

0

0

0

0

0

0

0

0

0

0

0

0

0

0.00780182

0

0

0

0

0

0

0

0

0

0

0

0

0

0

0

0

0

0

0

0

0

0

0

0

0

0

0

0

0

0

0

0

0

0

0

0

0

0

0

0

0

0

0

0

0

0

0

0

0

0

0

0

0

0

0

0

0

0

0

0

0

0

0

0

0

0

0

0

0

0

0

0

0

0

0

0

0

0

0

0

0

0

0

0
4

0
5

0

0

0

0

0

0

0

0

0

0

0

0

0

0

0

0

0

0

0

0

0

0

0

0

0

0

0

0

0

0

0

0

0

0

0

0

0

0

0

0

0

0

0

0

0

0

0

0

0

0

0

0

0

0

0

0

0

0

0

0

0

0

0.00364411

0

0

0

0

0

0

0

0

0

0

0

0

0

0

0

0

0

0

0

0

0

0

0

0

0

0

0

0

0

0

0

0

0

0

0

0

0

0

0

0

0

0

0

0

0

0

0
7

0

0

0

0

0

0

0

0

0

0

0

0
7

0

0

0

0

0

0

0

0

0

0
7

0

0

0

0
4

0

0

0
4

0.0187239
5

0

0

0

0.010349

0

0

0

0

0

0

0
2

0.0127553

0

0

0

0

0

0

0

0

0

0.0466638
6

0

0

0

0

0

0.0127553

0

0.00503411

0

0

0
4

1.13691

0.255763

0

0

0

0

0

0

0

0

0

0

0

0
6

0

0

0

0

0

0

0

0

0

0

0
7

0

0

0

0

0

0.0255106

0

0

0

0

0

0

0

0

0

0

0

0

0

0

0

0

0

0

0

0

0

0

0

0

0

0

0

0

0

0

0

0

0

0

0

0

0.0127553

0
7

0

0

0

0

0

0

0

0

0

0

0

0

0

0

0

0.0482955

0.0049252
7

0.158283
7

0

0

0

0.00985039

0

0.0127553
6

0

0

0

0

0.410778
7

0

0

0

0

0

0

0

0

0

0

0

0

0

0

0

0

0

0

0

0

0

0
5

0.165585

0

0

0

0

0.0255106

0

0

0.00689936

0

0
7

0

0

0

0

0

0

0

0

0

0

0

0

0

0

0

0

0

0

0

0

0

0

0

0

0

0

0

0

0

0

0

0

0

0

0

0

0

0

0

0

0

0

0

0
4

0
2

0
2

0

0

0

0
4

0.00665242

0

0.00665242

0
4

0

0

0

0
4

0

0

0
4

0

0

0

0
4

0

0

0
4

0

0

0
4

0

0

0

0
4

0

0

0

0
4

0

0

0

0
4

0

0

0
4

0
4

0
4

0

0

0

0

0

0

0

0

0

0
4

0

0

0
4

0

0

0

0
4

0

0

0

0
4

0

0

0
4

0

0

0

0
4

0

0

0
4

0

0

0
4

0

0

0
4

0

0

0
4

0

0

0
4

0.00520121
6

0.00520121
6

0

0

0

0

0

0

0

0

0
4

0

0

0
4

0

0

0
4

0

0

0
4

0

0

0
4

0

0

0
4

0

0

0
4

0

0

0
4

0

0

0
4

0

0

0
4

0

0

0
4

0
3

0
3

0

0

0

0

0

0

0

0

0

0
4

0

0

0
4

0

0

0
4

0

0

0
4

0

0

0
4

0

0

0
4

0

0

0
4

0

0

0
4

0

0

0
4

0

0

0
4

0

0

0
4

0.00689936
6

0
6

0

0

0.00689936

0

0

0
4

0

0

0
4

0

0

0
4

0

0

0
4

0

0

0
4

0

0

0
4

0

0

0
4

0

0

0
4

0

0

0
4

0

0

0
4

0

0

0
4

0
3

0
3

0

0

0

0

0

0
4

0

0

0
4

0

0

0
4

0

0

0
4

0

0

0
4

0

0

0
4

0

0

0
4

0

0

0
4

0

0

0
4

0

0

0
4

0

0

0
4

0

0

0

0

0
4

0

0

0
4

0

0

0
4

0

0

0
4

0

0

0
4

0

0

0
4

0

0

0
4

0

0

0
4

0

0

0
4

0

0

0
4

0

0

0
4

0
3

0
3

0

0

0

0

0

0

0

0

0

0
4

0

0

0
4

0

0

0
4

0

0

0
4

0

0

0
4

0

0

0
4

0

0

0
4

0

0

0
4

0

0

0
4

0

0

0
4

0

0

0
4

0

0

0

0

0

0

0

0

0
4

0

0

0
4

0

0

0
4

0

0

0
4

0

0

0
4

0

0

0
4

0

0

0
4

0

0

0
4

0

0

0
4

0

0

0
4

0

0

0
4

0.019133
5

0

0

0

0

0

0

0.019133

0

0

0
4

0

0

0
4

0

0

0
4

0

0

0
4

0

0

0
4

0

0

0
4

0

0

0
4

0

0

0
4

0

0

0
4

0

0

0
4

0

0

0
4

3.54425

2.28407

0

0

0

0

0

0

0

0

0

0

0.0155503
7

0

0

0

0

0

1.03318

0

0.189844

0.0216005

0

0

0

1.87350135405495e-16

0
4

0
4

0
4

0

0

0

0

0

0
4

0

0

0
4

0

0

0
4

0

0

0
4

0

0

0
4

0

0

0
4

0

0

0
4

0

0

0
4

0

0

0
4

0

0

0
4

0

0

0
4

0

0

0

0

0

0

0
4

0

0

0
4

0

0

0
4

0

0

0
4

0

0

0
4

0

0

0
4

0

0

0
4

0
6

0

0

0

0

0

0
4

0
4

0

0

0

0

0

0

0
4

0
4

0

0

0

0

0

0
4

0.133978

0.133978

0

0

0

0

0

0
4

0
4

0

0

0

0

0
4

0

0

0

0

0

0

0
4

0

0

0

0

0
4

0.00364411
6

0

0.00364411

0

0

0
4

0
4

0
4

0

0

0

0

0

0

0

0

0

0

0

0

0

0

0

0

0

0

0

0

0

0

0

0

0

0

0

0

0

0

0

0

0

0

0

0

0

0

0

0

0

0

0

0

0

0

0

0

0

0

0

0

0

0

0

0

0

0

0

0

0

0

0

0
4

0
6

0
6

0
4

0.306127
7

0.306127

0

0
4

0

0

0

0

0

0
4

0

0

0

0

0
4

0

0

0

0

0
4

0

0

0

0

0

0
4

0

0

0

0
4

0

0

0

0
4

0.0382659

0.0382659

0

0
4

0

0

0

0

0
4

0.294283

0.19133
7

0.0573989

0

0

0

0

0

0

0

0

0

0.00728822
6

0

0

0

0

0

0

0

0

0

0

0.0255106

0

0

0

0
2

0

0

0.0127553

2.25514051876985e-17

0
4

0

0

0

0

0
4

0

0

0

0

0
4

0.0137987

0.0137987
7

0

0
4

0

0

0

0

0

0
4

0.086242
7

0.086242

0

0

0

0
4

0

0

0

0

0
4

0

0

0

0

0
4

0

0

0

0

0

0
4

0.0109323

0.0109323

0

0

0
4

0

0

0

0
4

1.53627
6

1.49732
6

0

0

0

0.0286067

0.010349

0

0

0

0
4

0

0

0

0
4

0

0

0

0
4

0

0

0

0
4

0

0

0

0

0
4

0

0

0

0
4

0

0

0

0

0
4

0

0

0

0
4

0

0

0

0
4

0

0

0

0

0
4

0

0

0

0

0
4

0

0
6

0

0

0

0

0

0

0

0

0

0

0

0

0

0

0

0

0

0

0

0

0

0

0

0

0

0

0

0
4

0

0

0

0

0
4

0

0

0
4

0

0

0
4

0

0

0

0

0
4

0

0

0

0

0
4

0

0

0

0

0
4

0

0

0

0

0
4

0

0

0

0
4

0

0

0
4

0

0

0

0

0
4

0.421253
6

0.32526
6

0

0.00670609

0

0

0
7

0.0892871

0

0

0

0

0

0

0
4

0

0

0

0

0
4

0

0

0

0
4

0

0

0
4

0

0

0
4

0

0

0

0

0
4

0

0

0

0
4

0

0

0

0

0
4

0.0127553

0.0127553

0
4

0

0

0
4

0

0

0

0
4

0.420925

0.420925

0

0

0

0

0

0

0
4

0

0

0

0

0
4

0

0

0
4

0

0

0
4

0

0

0

0
4

0

0

0

0
4

0

0

0

0
4

0

0

0

0
4

0

0

0

0
4

0

0

0

0
4

0

0

0
4

0
3

0
3

0

0

0

0

0

0

0

0

0

0
4

0

0

0

0
4

0

0

0

0
4

0

0

0

0
4

0.0964601

0.0232835

0.0731766

0
4

0

0

0
4

0

0

0

0
4

0

0

0

0
4

0

0

0
4

0

0

0

0
4

0

0

0

0
4

0
4

0
4

1.27095
4

1.22729
4

1.22059
4

0.361236
4

0

0

0

0

0

0

0.00520121

0

0

0

0

0

0

0

0

0

0

0

0

0

0

0

0

0

0

0

0

0

0

0

0

0

0

0

0

0

0

0

0

0

0

0

0

0

0
4

0

0

0

0

0

0

0

0

0

0

0.00520121
4

0

0

0

0

0

0

0

0

0

0

0

0

0

0

0

0

0

0

0

0

0

0
4

0

0

0

0

0

0

0

0

0

0

0

0

0

0

0

0.00520121

0

0

0

0

0

0
4

0

0

0

0

0

0

0

0

0.641712
5

0.0260061

0

0

0

0.013003

0.0182042

0

0

0

0

0.0736747

0

0

0

0.00780182

0

0

0

0

0

0

0.019133

0

0

0

0

0

0

0.00520121

0

0

0

0
6

0

0

0

0

0

0

0

0

0

0

0

0

0

0

0.00520121

0

0

0

0

0

0

0

0.00520121

0

0

0

0

0

0

0

0

0

0
4

0

0

0

0

0

0

0

0

0

0

0
4

0.0234054

0

0

0

0

0.00520121

0

0

0

0

0
4

0
4

0

0

0

0

0

0

0

0

0
4

0

0

0
4

0

0

0

0

0

0
4

0

0

0

0

0

0
4

0

0

0

0
4

0

0

0

0
4

0

0

0

0
4

0.00670609

0.00670609

0
4

0

0

0
4

0

0

0
4

0
4

0

0

0

0

0

0

0

0
4

0
4

0

0

0

0

0
4

0
4

0

0

0

0

0
4

0

0

0
4

0
4

0

0

0

0

0
4

0
4

0

0

0

0

0
4

0
4

0

0

0

0
4

0
4

0

0

0

0
4

0
4

0

0

0

0
4

0
4

0.0141208

0.00564832

0.00564832

0
4

0

0

0
4

0.00847249

0.00847249

0
4

0
4

0

0

0

0
4

0

0

0
4

0
4

0

0

0

0
4

0

0

0
4

0
4

0
4

0
4

0

0

0

0

0

0

0
4

0
4

0

0

0

0
4

0
4

0

0

0

0
4

0

0

0
4

0
4

0

0

0

0
4

0
4

0

0

0

0
4

0
4

0

0

0

0

0
4

0
4

0

0

0

0

0
4

0
4

0

0

0

0
4

0
4

0

0

0

0
4

0
4

0

0

0

0
4

0
4

0

0

0

0
4

0
4

0

0

0

0

0

0

0
4

0

0

0
4

0

0

0
4

0

0

0
4

0
4

0

0

0

0
4

0
4

0

0

0

0
4

0
4

0

0

0

0
4

0
4

0

0

0

0
4

0
4

0

0

0

0
4

0
4

0

0

0

0
4

0
4

0

0

0

0
4

0
4

0.019133

0.019133

0.019133

0
4

0
4

0

0

0

0
4

0
4

0

0

0

0
4

0
4

0
6

0
6

0

0

0

0
4

0
4

0

0

0

0
4

0
4

0

0

0

0
4

0
4

0

0

0

0
4

0
4

0

0

0

0
4

0
4

0

0

0

0
4

0
4

0

0

0

0
4

0
4

0

0

0

0
4

0
4

0

0

0

0
4

0
4

0

0

0

0
4

0
4

0

0

0

0
4

0
4

0

0

0

0

0

0

0
4

0
4

0

0

0

0
4

0
4

0

0

0

0
4

0
4

0

0

0

0
4

0
4

0

0

0

0
4

0
4

0

0

0

0
4

0
4

0

0

0

0
4

0
4

0.0104024

0.0104024

0.0104024

0
4

0
4

0

0

0

0
4

0
4

0

0

0

0

0

0
4

0
4

0

0

0

0
4

0
4

0

0

0

0

0
4

0

0

0
4

0
4

1.2490009027033e-16
4

0
4

37.5437

0.184952
5

0
5

0
6

0
5

0
4

0.0637765

0.0637765

0

0

0

0

0
4

0

0

0
4

0

0

0
4

0

0

0
4

0

0

0
4

0

0

0
4

0

0

0
4

0

0

0
4

0

0

0
4

0

0

0
4

0.0127553

0.0127553

0
4

0
4

0

0

0

0

0

0

0
4

0

0

0
4

0

0

0
4

0

0

0
4

0

0

0

0
4

0.0956648

0.0956648

0

0
4

0

0

0

0
4

0.0127553

0.0127553

0
4

0

0

0

0
4

0

0

0
4

0

0

0
4

0
4

36.2449

4.26881

0.521891
7

0

0

0

0

0

0.133931

0

0.019133

0

0

0

0.159441

0.0127553

0

0

0.0127553

0.0127553

0

0

0

0

0

0.491079
7

0

0

0

0

0

0.0127553

0.00443786

0

0

0

0.0244082
7

0

0

0

0

0.0510212

0.0127553

0

0

0

0

0.829095
7

0

0

0

0

0

0

0

0.0382659

0

0

0

0

0

0

0

0

0

0

0

0

0.0127553

0.107611
7

0

0

0

0.0701542

0

0

0

0

0

0

0.0446436

0

0

0

0.0194121
6

0.0110946
6

0.0255106

0

0

0.0289833

0

0
7

0.0382659
7

0.0637765

0

0.197707
8

0

0

0.00443786

0

0

0

0

0.0127553

0

0

0.695164
7

0

0

0

0

0

0

0

0.0318883

0

0.0382659

0
7

0

0

0.0318883

0

0

0

0.019133

0

0.0382659

0

0

0.0127553

0

0

0

0

0.0255106

0

0

0

0

0

0.0127553

0.0127553

0

0

0.0133136

0

0.0127553

0

0.0127553

0

0

0

0.019133

0.0127553

0

0.0127553

0

0

0

0

0

0.293372
7

0

0

0

0

0

0

0

0

0

0

2.1094237467878e-15

0
4

0.695164

0.133931
7

0.0637765

0

0

0

0.0127553

0.0765318

0

0

0

0

0.0255106
7

0.0765318

0

0

0

0

0

0.0127553

0

0

0

0.153064
7

0

0

0

0

0.0892871

0.0255106

0

0

0

0

0.0255106
6

0

0

0

0

0

0

3.46944695195361e-17

0
4

19.898

1.00129
7

0

0.0446436

0.446436
7

0.019133
7

0.223218

0.0829095

10.1341

0.019133

0.164201

0.350771

0.389037
7

0

0.127553

0.0637765

0

0.019133

0.019133

0.197707

0

0.0147756

0.0127553

5.14676
8

0.0127553

0

0.019133

0

0.019133

0

0

0

0.0255106

0.0573989

0.676031
8

0

0

0

0.0127553

0

0.0049252

0.0382659

0

0

0.0127553

0.0892871
7

0.0318883

0.0127553

0.0382659

0.019133

0

0

0.120291
7

0.148389
7

0.0701542
7

0.0127553
7

7.36043170856959e-15

0
4

0.0829095

0

0

0

0

0

0

0.019133

0

0

0

0

0

0

0

0

0.0318883

0

0.0318883

0

0
4

8.95369

0
7

6.47629

2.0249

0

0.0520121

0.013003

0

0

0

0

0.382289

0

0

0

0.00520121

0

0

0

5.06539254985228e-16

0
4

1.14858

1.14858

0
4

1.06507
7

1.06507
7

0

0

0

0
4

0

0

0

0

0

0

0

0

0
4

0.132631

0.132631

0

0

0

0

0
4

0

0

0
4

0

0

0
4

0

0

0
4

0

0

0
4

0

0

0
4

0

0

0
4

0

0

0
4

0
4

0.42919

0.424719

0.362129

0.0111768

0.00447073

0.0312951

0

0.00447073

0.00447073

0.00670609

0
4

0.00447073

0

0.00447073

0

0
4

0
4

0

0

0

0

0
4

0

0

0
4

0
4

0

0

0

0
4

0
4

0

0

0

0

0
4

0

0

0
4

0
4

0

0

0

0

0

0
4

0
4

0

0

0

0
4

0

0

0
4

0
4

0

0

0

0
4

0

0

0
4

0
4

0

0

0

0
4

0
4

0

0

0

0

0
4

0
4

0

0

0

0
4

0
4

0

0

0

0
4

0

0

0
4

0
4

0
7

0
7

0

0
7

0

0

0

0

0
4

0

0

0
4

0
4

0

0

0

0
4

0
4

0

0

0

0
4

0
4

0

0

0

0
4

0
4

0

0

0

0
4

0
4

0

0

0

0
4

0
4

0.0127553

0.0127553

0.0127553

0
4

0
4

0

0

0

0
4

0
4

0

0

0

0
4

0
4

0.0127553

0.0127553

0.0127553

0
4

0
4

0

0

0

0
4

0
4

0

0

0

0

0
4

0

0

0
4

0
4

0.0692963

0.0692963

0.0692963

0
4

0
4

0

0

0

0
4

0
4

0

0

0

0

0

0
4

0

0

0
4

0
4

0

0

0

0
4

0
4

0.00443786
6

0.00443786

0

0.00443786

0

0
4

0

0

0
4

0

0

0
4

0
4

0.507428

0.496251

0.480603

0.00670609

0.00894146

0
4

0.0111768

0.00670609

0.00447073

0
4

1.56125112837913e-17

0
4

0.0780287

0.0780287

0.0704775

0.00755116

0
4

0
4

0

0

0

0

0
4

0
4

1.45300438347817e-14

0
4

0.69975

0.69975

0.677396

0.261513

0

0

0

0

0

0

0

0

0

0

0.0201364
1

0.0295512

0.356127

0.00503411

0

0

0.00503411

0

0
4

0.0223536

0.0178829

0.00447073

0
4

0

0

0

0
4

0

0

0
4

0

0

0
4

0

0

0
4

0

0

0
4

2.08166817117217e-17

0
4

0

0

0

0
4

0
4

0

0

0

0
4

0
4

0

0

0

0

0
4

0
4

0

0

0

0
4

0
4

0

0

0

0
4

0
4

0

0

0

0
4

0
4

0

0

0

0
4

0
4

0

0

0

0
4

0
4

0

0

0

0
4

0
4

0

0

0

0
4

0
4

0
4

0.0956648
5

0.0956648
5

0.0956648
5

0.0956648
5

0

0

0

0

0

0

0

0

0
4

0

0

0

0

0
4

0

0

0

0

0
4

0

0

0
4

0

0

0

0

0
4

0

0

0
4

0

0

0
4

0
4

0
4

0
4

0
4

0
4

0
4

0

0

0

0

0
4

0

0

0

0

0

0

0

0
4

0
4

0
4

0

0

0

0

0

0
4

0

0

0
4

0
4

0
4

0

0

0

0

0

0
4

0
4

0
4

0

0

0

0

0

0
4

0

0

0

0
4

0
4

0
4

0

0

0

0

0

0
4

0
4

0
4

0.0127553

0.0127553

0.0127553

0

0.0127553

0

0
4

0
4

0
4

0

0

0

0

0
4

0
4

0
4

0

0

0

0

0

0
4

0
4

0
4

0

0

0

0

0
4

0
4

0
4

0

0

0

0

0

0
4

0

0

0
4

0
4

0
4

0.163182

0.163182

0.163182

0.163182

0
4

0
4

0
4

0
2

0
2

0
2

0

0

0

0

0

0

0

0

0

0
4

0

0

0
4

0
4

0

0

0

0

0

0

0

0
4

0

0

0

0
4

0
4

0
4

0

0

0

0

0
4

0

0

0
4

0
4

0
4

0

0

0

0

0

0

0
4

0
4

0
4

0.0892871

0.0892871

0.0892871

0.0892871

0
4

0

0

0
4

0
4

0
4

0

0

0

0

0

0
4

0
4

0

0

0

0
4

0
4

0
4

0

0

0

0

0
4

0
4

0
4

0

0

0

0

0
4

0

0

0
4

0
4

0
4

0.0318883

0.0318883

0.0318883

0.0318883

0
4

0
4

0
4

0.0255106

0.0255106

0.0127553

0

0.0127553

0
4

0.0127553

0.0127553

0
4

0
4

0
4

0

0

0

0

0
4

0

0

0
4

0
4

0
4

0

0

0

0

0

0

0
4

0
4

0
4

0.133931
7

0.133931
7

0.0701542
7

0.0573989
7

0.0127553
7

0

0
4

0.0446436
7

0.019133
7

0.0255106

0

0
4

0

0

0

0

0
4

0.019133

0.019133

0

0
4

3.46944695195361e-18
7

0
4

0
4

0

0

0

0

0

0
4

0
4

0
4

0

0

0

0

0
4

0

0

0
4

0

0

0
4

0
4

0
4

0

0

0

0

0
4

0

0

0
4

0
4

0
4

0

0

0

0

0

0
4

0
4

0
4

0

0

0

0

0

0
4

0
4

0
4

0

0

0

0

0
4

0
4

0
4

0

0

0

0

0
4

0
4

0
4

0

0

0

0

0
4

0
4

0
4

0

0

0

0

0

0
4

0
4

0
4

0

0

0

0

0
4

0

0

0
4

0
4

0
4

0

0

0

0

0

0

0

0

0

0

0

0

0

0
4

0

0

0
4

0

0

0

0
4

0
4

0

0

0

0

0
4

0
4

0
4

0

0

0

0

0

0
4

0
4

0
4

0

0

0

0

0
4

0

0

0
4

0
4

0
4

0

0

0

0

0

0
4

0
4

0
4

0

0

0

0

0
4

0
4

0
4

0

0

0

0

0

0
4

0

0

0
4

0
4

0
4

0

0

0

0

0
4

0

0

0
4

0
4

0
4

0

0

0

0

0
4

0
4

0
4

0

0

0

0

0

0
4

0
4

0

0

0

0
4

0
4

0
4

0.111768

0.111768

0.105062

0.105062

0
4

0.00670609

0.00670609

0
4

0
4

0
4

0

0

0

0

0
4

0
4

0
4

0
2

0
2

0
2

0

0

0

0

0

0

0
4

0

0

0

0

0

0

0
4

0

0

0

0

0

0
4

0
4

0
4

0

0

0

0

0
4

0
4

0
4

0

0

0

0

0
4

0

0

0
4

0

0

0
4

0
4

0
4

0

0

0

0

0
4

0
4

0
4

0.0382659

0.0382659

0.0382659

0.0382659

0

0
4

0
4

0
4

0

0

0

0

0

0

0
4

0
4

0
4

0

0

0

0

0

0
4

0
4

0
4

0

0

0

0

0

0
4

0
4

0
4

0

0

0

0

0
4

0
4

0
4

0

0

0

0

0
4

0

0

0
4

0
4

0
4

0

0

0

0

0
4

0
4

0
4

0.0829095

0.0829095

0.0127553

0
6

0.0127553
7

0

0

0

0

0
4

0.0701542
6

0.0382659
6

0.019133

0.0127553

0

8.67361737988404e-18
6

0
4

0

0

0

0
4

0

0

0
4

0

0

0
4

0

0

0
4

0
4

0
4

0

0

0

0

0
4

0

0

0
4

0
4

0
4

0

0

0

0

0

0
4

0
4

0
4

0.00894146

0.00894146

0.00447073

0.00447073

0
4

0.00447073

0.00447073

0
4

0
4

0
4

0.73343

0.73343

0.73343

0

0.73343

0
4

0
4

0
4

0.0382659

0.0382659

0.0382659

0

0.0382659

0
4

0
4

0
4

0.00447073

0.00447073

0.00447073

0

0.00447073

0
4

0
4

0
4

0

0

0

0

0
4

0

0

0
4

0
4

0
4

0

0

0

0

0
4

0

0

0
4

0
4

0
4

0

0

0

0

0
4

0

0

0
4

0
4

0
4

0

0

0

0

0
4

0
4

0
4

0.0963216
7

0.0963216
7

0.0829095
7

0.0829095
7

0

0

0

0

0
4

0.0134122

0

0

0.0134122

0

0

0
4

3.46944695195361e-18
7

0
4

0
4

0

0

0

0

0
4

0

0

0
4

0
4

0
4

0

0

0

0

0
4

0

0

0
4

0
4

0
4

0

0

0

0

0

0
4

0
4

0
4

0

0

0

0

0

0
4

0
4

0
4

0

0

0

0

0

0
4

0
4

0
4

0

0

0

0

0
4

0
4

0
4

0.0131105

0.0131105

0.0131105

0.0131105

0
4

0
4

0
4

0

0

0

0

0
4

0

0

0
4

0
4

0
4

0

0

0

0

0
4

0

0

0
4

0
4

0
4

0

0

0

0

0
4

0
4

0
4

0.192139
7

0.192139
7

0.179383
7

0
7

0.179383

0

0

0

0

0
4

0.0127553

0.0127553

0

0

0
4

0

0

0
4

0
4

0
4

0

0

0

0

0
4

0

0

0
4

0
4

0
4

0

0

0

0

0
4

0
4

0
4

0

0

0

0

0
4

0

0

0
4

0
4

0
4

0

0

0

0

0

0
4

0
4

0
4

0

0

0

0

0
4

0
4

0
4

0

0

0

0

0

0
4

0
4

0
4

0

0

0

0

0

0
4

0
4

0
4

0

0

0

0

0

0
4

0
4

0
4

0

0

0

0

0

0
4

0
4

0
4

0

0

0

0

0
4

0

0

0
4

0
4

0
4

0.463122
7

0.463122
7

0.463122
7

0.463122
7

0

0

0
4

0

0

0
4

0

0

0
4

0
4

0
4

0

0

0

0

0

0
4

0
4

0
4

0

0

0

0

0
4

0
4

0
4

0

0

0

0

0
4

0
4

0
4

0

0

0

0

0
4

0

0

0
4

0
4

0
4

0

0

0

0

0
4

0
4

0
4

0

0

0

0

0

0
4

0
4

0
4

0

0

0

0

0
4

0

0

0
4

0
4

0
4

0

0

0

0

0
4

0
4

0
4

0

0

0

0

0
4

0
4

0
4

0

0

0

0

0
4

0
4

0
4

0.315059
7

0.315059
7

0.17528
7

0.166339
7

0
7

0

0

0.00894146

5.20417042793042e-18
7

0
4

0.139779
7

0.124131
7

0.0156475

0

0
4

2.77555756156289e-17
7

0
4

0
4

0

0

0

0

0
4

0

0

0
4

0
4

0
4

0

0

0

0

0

0
4

0
4

0
4

0

0

0

0

0
4

0
4

0
4

0

0

0

0

0
4

0

0

0
4

0
4

0
4

0.0573989

0.0573989

0.0573989

0.0573989

0
4

0
4

0
4

0

0

0

0

0

0
4

0
4

0
4

0

0

0

0

0
4

0

0

0
4

0
4

0
4

0

0

0

0

0
4

0
4

0
4

0

0

0

0

0
4

0

0

0
4

0
4

0
4

0

0

0

0

0
4

0

0

0
4

0
4

0
4

0.0701542
4

0.0701542
4

0

0

0

0

0

0

0

0

0

0

0

0

0

0

0

0

0

0

0
4

0.0701542
4

0.0701542

0

0

0

0

0

0

0

0

0

0

0

0

0

0

0
4

0

0

0

0

0

0

0

0
4

0

0

0

0
4

0
4

0

0

0

0
4

0
4

0
4

0

0

0

0

0

0

0

0

0

0

0
4

0

0

0

0

0
4

0
4

0
4

0

0

0

0

0
4

0
4

0
4

0

0

0

0

0

0
4

0
4

0
4

0

0

0

0

0
4

0
4

0
4

0

0

0

0

0

0
4

0
4

0
4

0

0

0

0

0

0
4

0
4

0
4

0

0

0

0

0
4

0
4

0
4

0

0

0

0

0
4

0

0

0
4

0
4

0
4

0

0

0

0

0
4

0
4

0
4

0

0

0

0

0

0
4

0
4

0
4

0.0447073

0.0447073

0.0447073

0.0178829

0.0268244

0
4

0
4

0
4

0.211314
7

0.211314
7

0.115649
7

0.0318883
7

0.083761
7

0

0
4

0.0956648

0.0956648

0

0
4

0
4

0

0

0

0
4

0
4

0
4

0

0

0

0

0
4

0
4

0
4

0

0

0

0

0
4

0
4

0
4

0

0

0

0

0
4

0
4

0
4

0

0

0

0

0
4

0
4

0
4

0

0

0

0

0
4

0
4

0
4

0

0

0

0

0
4

0
4

0
4

0

0

0

0

0
4

0
4

0
4

0

0

0

0

0
4

0
4

0
4

0

0

0

0

0
4

0
4

0
4

0

0

0

0

0
4

0
4

0
4

0

0

0

0
7

0

0

0
4

0

0

0
4

0

0

0
4

0

0

0
4

0
4

0
4

0

0

0

0

0
4

0
4

0
4

0

0

0

0

0
4

0
4

0
4

0

0

0

0

0
4

0
4

0
4

0

0

0

0

0
4

0
4

0
4

0

0

0

0

0
4

0
4

0
4

0

0

0

0

0
4

0
4

0
4

0

0

0

0

0
4

0
4

0
4

0

0

0

0

0
4

0
4

0
4

0

0

0

0

0
4

0
4

0
4

0

0

0

0

0
4

0
4

0
4

0
2

0
2

0
2

0
2

0

0

0

0

0

0
4

0
2

0

0

0

0
4

0

0

0
4

0
4

0
4

0

0

0

0

0
4

0
4

0
4

0

0

0

0

0
4

0
4

0
4

0

0

0

0

0
4

0
4

0
4

0

0

0

0

0
4

0
4

0
4

0

0

0

0

0
4

0
4

0
4

0

0

0

0

0
4

0
4

0
4

0

0

0

0

0
4

0
4

0
4

0

0

0

0

0
4

0
4

0
4

0

0

0

0

0
4

0
4

0
4

0

0

0

0

0
4

0
4

0
4

0
4

0
4

0
4

0

0

0

0

0

0

0

0

0

0
4

0
4

0
4

0
4

0
4

0

0

0

0
4

0
4

0
4

0

0

0

0

0
4

0
4

0
4

0

0

0

0

0
4

0
4

0
4

0

0

0

0

0
4

0
4

0
4

0

0

0

0

0
4

0
4

0
4

0

0

0

0

0
4

0
4

0
4

0

0

0

0

0
4

0
4

0
4

0

0

0

0

0
4

0
4

0
4

0

0

0

0

0
4

0
4

0
4

0

0

0

0

0
4

0
4

0
4

0

0

0

0

0
4

0
4

0
4

0.199935
5

0.199935
5

0.199935
5

0.199935
5

0

0

0
4

0
4

0
4

0

0

0

0

0
4

0
4

0
4

0

0

0

0

0
4

0
4

0
4

0.153064

0.153064

0.153064

0.153064

0
4

0
4

0
4

0

0

0

0

0
4

0
4

0
4

0

0

0

0

0
4

0
4

0
4

0

0

0

0

0
4

0
4

0
4

0

0

0

0

0
4

0
4

0
4

0

0

0

0

0
4

0
4

0
4

0.00447073

0.00447073

0.00447073

0.00447073

0
4

0
4

0
4

0

0

0

0

0
4

0
4

0
4

0.00894146
7

0.00894146
7

0.00894146
7

0.00447073
7

0.00447073

0
4

0

0

0
4

0
4

0

0

0

0

0

0
4

0
4

0
4

0

0

0

0

0
4

0
4

0
4

0

0

0

0

0
4

0
4

0
4

0

0

0

0

0
4

0
4

0
4

0

0

0

0

0
4

0
4

0
4

0

0

0

0

0
4

0
4

0
4

0

0

0

0

0
4

0
4

0
4

0

0

0

0

0
4

0
4

0
4

0

0

0

0

0
4

0
4

0
4

0

0

0

0

0
4

0
4

0
4

0

0

0

0

0
4

0
4

0
4

0

0

0

0

0

0

0

0

0

0

0
4

0
4

0
4

0

0

0

0

0
4

0
4

0
4

0

0

0

0

0
4

0
4

0
4

0

0

0

0

0
4

0
4

0
4

0

0

0

0

0
4

0
4

0
4

0

0

0

0

0
4

0
4

0
4

0

0

0

0

0
4

0
4

0
4

0

0

0

0

0
4

0
4

0
4

0

0

0

0

0
4

0
4

0
4

0

0

0

0

0
4

0
4

0
4

0

0

0

0

0
4

0
4

0
4

0

0

0

0

0

0

0

0

0
4

0
4

0
4

0

0

0

0

0
4

0
4

0
4

0

0

0

0

0
4

0
4

0
4

0

0

0

0

0
4

0
4

0
4

0

0

0

0

0
4

0
4

0
4

0

0

0

0

0
4

0
4

0
4

0

0

0

0

0
4

0
4

0
4

0

0

0

0

0
4

0
4

0
4

0

0

0

0

0
4

0
4

0
4

0

0

0

0

0
4

0
4

0
4

0

0

0

0

0
4

0
4

0
4

0
4

0
4

0
4

0
4

0

0

0

0
4

0
4

0
4

0

0

0

0

0
4

0
4

0
4

0

0

0

0

0
4

0
4

0
4

0

0

0

0

0
4

0
4

0
4

0

0

0

0

0
4

0
4

0
4

0

0

0

0

0
4

0
4

0
4

0

0

0

0

0
4

0
4

0
4

0

0

0

0

0
4

0
4

0
4

0

0

0

0

0
4

0
4

0
4

0

0

0

0

0
4

0
4

0
4

0

0

0

0

0
4

0
4

0
4

0
4

0
4

0
4

0
4

0

0

0

0

0

0

0

0

0

0

0
4

0

0

0
4

0

0

0

0

0

0

0
4

0
4

0

0

0

0

0

0

0
4

0

0

0

0
4

0

0

0
4

0
4

0
4

0.0637765
5

0.0637765
5

0.0637765
5

0
5

0

0.0637765

0

0

0

0

0
4

0

0

0
4

0
4

0
4

0

0

0

0

0
4

0
4

0
4

0

0

0

0

0
4

0
4

0
4

0

0

0

0

0
4

0
4

0
4

0

0

0

0

0
4

0
4

0
4

0

0

0

0

0
4

0
4

0
4

0

0

0

0

0
4

0
4

0
4

0

0

0

0

0
4

0
4

0
4

0

0

0

0

0
4

0
4

0
4

0

0

0

0

0
4

0
4

0
4

0

0

0

0

0
4

0
4

0
4

0
4

0
4

0
4

0

0

0

0

0

0

0

0
4

0
4

0
4

0.00894146

0.00894146

0.00894146

0.00894146

0
4

0
4

0
4

0

0

0

0

0
4

0
4

0
4

0

0

0

0

0
4

0
4

0
4

0

0

0

0

0
4

0
4

0
4

0

0

0

0

0
4

0
4

0
4

0

0

0

0

0
4

0
4

0
4

0

0

0

0

0
4

0
4

0
4

0

0

0

0

0
4

0
4

0
4

0

0

0

0

0
4

0
4

0
4

0

0

0

0

0
4

0
4

0
4

0

0

0
6

0

0

0

0
4

0

0

0

0

0
4

0

0

0
4

0

0

0

0
4

0

0

0
4

0

0

0
4

0
4

0
4

0

0

0

0

0
4

0
4

0
4

0

0

0

0

0
4

0
4

0
4

0

0

0

0

0
4

0
4

0
4

0.00503411

0.00503411

0.00503411

0.00503411

0
4

0
4

0
4

0

0

0

0

0
4

0
4

0
4

0

0

0

0

0
4

0
4

0
4

0

0

0

0

0
4

0
4

0
4

0

0

0

0

0
4

0
4

0
4

0

0

0

0

0
4

0
4

0
4

0

0

0

0

0
4

0
4

0
4

0.350771

0.350771

0.350771

0.331638
7

0

0.019133

0

0
4

0
4

0
4

0

0

0

0

0
4

0
4

0
4

0

0

0

0

0
4

0
4

0
4

0

0

0

0

0
4

0
4

0
4

0

0

0

0

0
4

0
4

0
4

0

0

0

0

0
4

0
4

0
4

0

0

0

0

0
4

0
4

0
4

0

0

0

0

0
4

0
4

0
4

0

0

0

0

0
4

0
4

0
4

0

0

0

0

0
4

0
4

0
4

0

0

0

0

0
4

0
4

0
4

0.286054
3

0.286054
3

0.286054
4

0.286054

0

0

0

0
4

0

0

0
4

0

0

0
4

0

0

0

0
4

0
4

0
4

0

0

0

0

0
4

0
4

0
4

0

0

0

0

0
4

0
4

0
4

0

0

0

0

0
4

0
4

0
4

0.019133

0.019133

0.019133

0.019133

0
4

0
4

0
4

0

0

0

0

0
4

0
4

0
4

0

0

0

0

0
4

0
4

0
4

0

0

0

0

0
4

0
4

0
4

0

0

0

0

0
4

0
4

0
4

0

0

0

0

0
4

0
4

0
4

0

0

0

0

0
4

0
4

0
4

0.101275
7

0.101275
7

0.0778562
7

0.057715

0.0201412

0

0
4

0.00703064

0

0.00703064

0

0

0
4

0.0163881

0.0163881

0
4

0
4

0
4

0

0

0

0

0
4

0
4

0
4

0

0

0

0

0
4

0
4

0
4

0

0

0

0

0
4

0
4

0
4

0

0

0

0

0
4

0
4

0
4

0

0

0

0

0
4

0
4

0
4

0

0

0

0

0
4

0
4

0
4

0

0

0

0

0
4

0
4

0
4

0

0

0

0

0
4

0
4

0
4

0

0

0

0

0
4

0
4

0
4

0

0

0

0

0
4

0
4

0
4

0

0

0

0

0

0

0
4

0

0

0

0
4

0
4

0

0

0

0

0
4

0

0

0
4

0
4

0
4

0

0

0

0

0
4

0
4

0
4

0

0

0

0

0
4

0
4

0
4

0

0

0

0

0
4

0
4

0
4

0

0

0

0

0
4

0
4

0
4

0

0

0

0

0
4

0
4

0
4

0

0

0

0

0
4

0
4

0
4

0.00520121

0.00520121

0.00520121

0.00520121

0
4

0
4

0
4

0

0

0

0

0
4

0
4

0
4

0

0

0

0

0
4

0
4

0
4

0

0

0

0

0
4

0
4

0
4

0
5

0
5

0
5

0
5

0

0
4

0
4

0
4

0

0

0

0

0
4

0
4

0
4

0

0

0

0

0
4

0
4

0
4

0

0

0

0

0
4

0
4

0
4

0

0

0

0

0
4

0
4

0
4

0

0

0

0

0
4

0
4

0
4

0

0

0

0

0
4

0
4

0
4

0

0

0

0

0
4

0
4

0
4

0

0

0

0

0
4

0
4

0
4

0

0

0

0

0
4

0
4

0
4

0

0

0

0

0
4

0
4

0
4

0
7

0
7

0

0

0

0
4

0

0

0

0
4

0
4

0
4

0

0

0

0

0
4

0
4

0
4

0

0

0

0

0
4

0
4

0
4

0

0

0

0

0
4

0
4

0
4

0

0

0

0

0
4

0
4

0
4

0

0

0

0

0
4

0
4

0
4

0

0

0

0

0
4

0
4

0
4

0

0

0

0

0
4

0
4

0
4

0

0

0

0

0
4

0
4

0
4

0

0

0

0

0
4

0
4

0
4

0

0

0

0

0
4

0
4

0
4

0
4

0
4

0
4

0

0

0

0
4

0
4

0
4

0
4

0

0

0
4

0

0

0
4

0
4

0
4

0

0

0

0

0
4

0
4

0
4

0

0

0

0

0
4

0
4

0
4

0

0

0

0

0
4

0
4

0
4

0

0

0

0

0
4

0
4

0
4

0

0

0

0

0
4

0
4

0
4

0

0

0

0

0
4

0
4

0
4

0

0

0

0

0
4

0
4

0
4

0

0

0

0

0
4

0
4

0
4

0

0

0

0

0
4

0
4

0
4

0

0

0

0

0
4

0
4

0
4

1.10016
7

1.10016
7

0.647389
7

0.577554
7

0.0379465

0
6

0

0

0.0318883

0
4

0.452768
7

0.021631
7

0.0992204
7

0.32526
7

0.00665679
7

0

0

0

6.33174068731535e-17
7

0
4

0
4

0
4

0

0

0

0

0

0

0

0
4

0

0

0

0
4

0
4

0
4

0

0

0

0

0
4

0
4

0
4

0

0

0

0

0
4

0
4

0
4

0

0

0

0

0
4

0
4

0
4

0

0

0

0

0
4

0
4

0
4

0

0

0

0

0
4

0
4

0
4

0

0

0

0

0
4

0
4

0
4

0

0

0

0

0
4

0
4

0
4

0

0

0

0

0
4

0
4

0
4

0

0

0

0

0
4

0
4

0
4

0

0

0

0

0
4

0
4

0
4

0.0510212
7

0.0510212
7

0.0510212
7

0.0318883
6

0.019133

0

0
4

0

0

0
4

0

0

0
4

0
4

0
4

0

0

0

0

0
4

0
4

0
4

0

0

0

0

0
4

0
4

0
4

0

0

0

0

0
4

0
4

0
4

0

0

0

0

0
4

0
4

0
4

0

0

0

0

0
4

0
4

0
4

0

0

0

0

0
4

0
4

0
4

0

0

0

0

0
4

0
4

0
4

0.0134122

0.0134122

0.0134122

0.0134122

0
4

0
4

0
4

0

0

0

0

0
4

0
4

0
4

0

0

0

0

0
4

0
4

0
4

0.143843
7

0.143843
7

0

0

0

0

0
4

0.143843

0.131088

0.0127553

0

0
4

0
4

0
4

0

0

0

0

0
4

0
4

0
4

0.0637765

0.0637765

0.0637765

0.0637765

0
4

0
4

0
4

0

0

0

0

0
4

0
4

0
4

0

0

0

0

0
4

0
4

0
4

0.146686
6

0.146686
6

0.146686
6

0.133931
6

0.0127553

0

0
4

0
4

0
4

0

0

0

0

0

0

0

0
4

0

0

0

0
4

0

0

0
4

0

0

0

0
4

0
4

0

0

0

0
4

0
4

0
4

0.146686
7

0.146686
7

0.146686
7

0.146686
7

0

0
4

0

0

0
4

0

0

0
4

0
4

0
4

0.0546127
7

0.0546127
7

0.0546127
7

0.0546127
7

0
4

0
4

0
4

0

0

0

0

0

0

0

0

0
4

0

0

0
4

0
4

0
4

0
4

0
4

0

0

0

0

0
4

0

0

0
4

0
4

0
4

0

0

0

0

0

0
4

0
4

0
4

0.0427366
5

0.0427366
5

0.00447073
6

0
6

0

0

0

0

0

0

0

0

0.00447073

0

0

0

0

0

0

0

0
4

0.0382659
5

0.0382659
7

0
6

0

0

0
4

0

0

0

0

0
4

0
4

0
4

0

0

0

0

0

0
4

0

0

0

0
4

0
4

0
4

0
4

0
4

0
4

0

0

0

0

0
4

0

0

0

0
4

0
4

0
4

0

0

0

0

0

0

0

0
4

0
4

0
4

0.0922431
7

0.0922431
7

0.0922431
7

0.0810663

0.0111768

0
4

0
4

0
4

0

0

0

0

0

0

0
4

0

0

0
4

0
4

0
4

0

0

0

0

0

0

0

0

0
4

0

0

0
4

0
4

0
4

0

0

0

0

0

0

0

0
4

0
4

0
4

0.0156036

0.0156036

0.0156036

0.0156036

0

0

0
4

0
4

0
4

0

0

0

0

0
4

0
4

0
4

0

0

0

0

0

0
4

0
4

0
4

0.0892871

0.0892871

0.0701542
5

0.0318883
6

0

0

0

0

0

0

0.0382659

0

0

0
4

0.019133
7

0
7

0

0.019133

0
4

0

0

0

0

0

0
4

0

0

0

0
4

3.46944695195361e-18

0
4

0

0

0

0
4

0
4

0
4

0

0

0

0

0

0

0
4

0

0

0

0
4

0
4

0

0

0

0
4

0
4

0
4

0
4

0
4

0
4

0
4

0

0
4

0
4

0
4

0
7

0
7

0
7

0
7

0

0
4

0
4

0
4

0

0

0

0

0

0
4

0

0

0
4

0
4

0
4

0.0123036

0.0123036

0.00703064

0.00351532

0.00351532

0
4

0

0

0
4

0.00527298

0

0.00527298

0
4

0

0

0
4

0
4

0
4

0

0

0

0

0

0
4

0
4

0
4

0
4

0
4

0

0

0

0
4

0

0

0

0
4

0

0

0
4

0
4

0
4

0

0

0

0

0

0

0
4

0
4

0
4

0
4

0
4

0

0

0
4

0

0

0
4

0

0

0
4

0
4

0
4

0
5

0
5

0
5

0
5

0
4

0
4

0
4

0.10842

0.10842

0

0

0

0

0
4

0

0

0
4

0

0

0
4

0.10842
4

0.10842
4

0

0

0

0

0
4

0

0

0

0

0
4

0

0

0
4

0

0

0
4

0

0

0
4

0

0

0
4

0

0

0
4

0

0

0
4

0
4

0
4

0

0

0

0

0

0
4

0
4

0
4

0

0

0

0

0

0

0
4

0

0

0

0
4

0
4

0
4

0.156332

0.156332

0.156332

0.156332

0
4

0
4

0
4

0

0

0

0

0

0
4

0

0

0

0
4

0
4

0
4

0.0255106

0.0255106

0.0255106

0

0.0255106

0
4

0
4

0
4

0

0

0

0

0

0

0

0

0
4

0

0

0
4

0
4

0
4

0

0

0

0

0

0

0
4

0
4

0
4

0

0

0

0

0
4

0

0

0

0
4

0
4

0
4

0.101841
7

0.101841
7

0.101841

0.101841

0
4

0

0

0
4

0
4

0
4

0

0

0

0

0

0
4

0

0

0

0
4

0
4

0
4

0

0

0

0

0

0

0

0

0

0

0

0

0

0

0
4

0

0

0

0

0

0

0

0

0
4

0

0

0

0

0

0

0

0

0

0
4

0
4

0
4

0

0

0

0

0

0

0
4

0
4

0
4

0
5

0
5

0
5

0

0

0
4

0
4

0
4

0.095414

0.095414

0.095414

0.095414

0
4

0
4

0
4

0

0

0

0

0
4

0
4

0
4

0

0

0

0

0

0
4

0
4

0
4

0

0

0

0

0
4

0
4

0
4

0

0

0

0

0

0
4

0

0

0
4

0
4

0
4

0.134122

0.134122

0.134122

0.12518

0.00447073

0.00447073

5.20417042793042e-18

0
4

0
4

0
4

0

0

0

0

0

0
4

0
4

0
4

0

0

0

0

0

0
4

0

0

0

0
4

0
4

0
4

0
7

0
7

0
7

0
7

0
7

0
7

0

0

0

0

0

0

0
4

0

0

0

0

0
4

0

0

0
4

0
4

0
4

0

0

0

0

0

0

0
4

0
4

0
4

0

0

0

0

0

0

0
4

0
4

0
4

0

0

0

0

0

0
4

0
4

0
4

0

0

0

0

0
4

0
4

0
4

0.00543897

0.00543897

0.00543897

0

0.00543897

0
4

0
4

0
4

0

0

0

0

0
4

0

0

0
4

0
4

0
4

0

0

0

0

0
4

0
4

0
4

0.0155325

0.0155325

0.0155325

0.0155325

0

0
4

0
4

0
4

0

0

0

0

0

0
4

0

0

0
4

0
4

0

0

0

0
4

0
4

0
4

0

0

0

0

0

0

0
4

0
4

0
4

0.0332723

0
4

0
4

0

0

0

0
4

0
4

0

0

0

0
4

0
4

0

0

0

0
4

0
4

0

0

0

0
4

0
4

0

0

0

0
4

0
4

0.00520121

0.00520121

0.00520121

0
4

0
4

0.00443786

0.00443786

0.00443786

0
4

0
4

0

0

0

0
4

0
4

0

0

0

0
4

0
4

0

0

0

0
4

0
4

0

0

0

0
4

0
4

0

0

0

0

0
4

0
4

0

0

0

0
4

0
4

0

0

0

0
4

0
4

0

0

0

0
4

0
4

0

0

0

0
4

0
4

0

0

0

0
4

0
4

0

0

0

0
4

0
4

0

0

0

0
4

0
4

0

0

0

0
4

0
4

0

0

0

0
4

0
4

0

0

0

0
4

0
4

0

0

0

0
4

0
4

0

0

0

0
4

0
4

0

0

0

0

0

0
4

0
4

0

0

0

0

0
4

0
4

0

0

0

0
4

0
4

0

0

0

0
4

0

0

0
4

0
4

0

0

0

0
4

0
4

0

0

0

0
4

0
4

0.0236332
3

0
2

0
3

0
2

0

0

0

0

0

0

0

0

0

0

0
2

0

0

0

0

0

0

0

0

0

0

0

0

0

0

0

0

0

0

0

0

0

0

0

0

0

0
4

0

0

0

0

0

0

0

0

0

0

0
4

0

0

0
4

0

0

0

0
4

0

0

0
4

0

0

0

0
4

0

0

0
4

0.00543897

0.00543897

0
4

0

0

0
4

0

0

0
4

0

0

0
4

0

0

0
4

0

0

0

0

0

0

0

0
4

0

0

0
4

0

0

0
4

0

0

0
4

0

0

0
4

0

0

0
4

0

0

0
4

0

0

0
4

0.0127553

0.0127553

0
4

0.00543897

0.00543897

0
4

0

0

0
4

0

0

0

0

0
4

0

0

0
4

0

0

0
4

0

0

0
4

0

0

0

0
4

0

0

0

0
4

0

0

0

0

0
4

0

0

0

0

0
4

0

0

0
4

0

0

0

0
4

0
4

0
4

0.591543
3

0

0

0

0

0

0

0

0

0
4

0

0

0

0

0
4

0

0

0
4

0
4

0.0446436
6

0.0446436
6

0.0446436
6

0
4

0
4

0

0

0

0
4

0
4

0

0

0

0
4

0
4

0

0

0

0
4

0
4

0

0

0

0
4

0
4

0

0

0

0
4

0
4

0

0

0

0
4

0
4

0

0

0

0
4

0
4

0

0

0

0
4

0
4

0

0

0

0
4

0
4

0

0

0

0
4

0
4

0

0

0

0

0

0

0

0
4

0

0

0

0
4

0

0

0
4

0
4

0

0

0

0
4

0
4

0

0

0

0
4

0
4

0

0

0

0
4

0
4

0

0

0

0
4

0
4

0

0

0

0
4

0
4

0

0

0

0
4

0
4

0

0

0

0
4

0
4

0.0127553

0.0127553

0.0127553

0
4

0
4

0

0

0

0
4

0
4

0

0

0

0
4

0
4

0

0

0

0

0
4

0

0

0

0
4

0
4

0

0

0

0
4

0
4

0

0

0

0
4

0
4

0

0

0

0
4

0
4

0

0

0

0
4

0
4

0

0

0

0
4

0
4

0

0

0

0
4

0
4

0

0

0

0
4

0
4

0

0

0

0
4

0
4

0

0

0

0
4

0
4

0

0

0

0
4

0
4

0

0

0

0

0

0

0

0
4

0

0

0
4

0
4

0

0

0

0
4

0
4

0

0

0

0
4

0
4

0

0

0

0
4

0
4

0

0

0

0
4

0
4

0

0

0

0
4

0
4

0

0

0

0
4

0
4

0

0

0

0

0

0
4

0

0

0

0
4

0

0

0
4

0
4

0
3

0

0

0

0

0

0
4

0

0

0

0
4

0

0

0
4

0

0

0
4

0

0

0
4

0
4

0
2

0

0

0

0

0
4

0

0

0
4

0

0

0

0
4

0

0

0
4

0

0

0
4

0
4

0

0

0

0

0

0

0
4

0
4

0

0

0

0

0
4

0
4

0
6

0
6

0
6

0
4

0
4

0
4

0
4

0
4

0
4

0

0

0
4

0

0

0
4

0

0

0
4

0
4

0
3

0

0

0

0

0

0
4

0

0

0
4

0

0

0
4

0
4

0
3

0

0

0

0

0
4

0

0

0
4

0

0

0
4

0

0

0
4

0

0

0
4

0
4

0

0

0

0

0
4

0

0

0
4

0
4

0

0

0

0

0
4

0
4

0

0

0

0

0
4

0
4

0

0

0

0
4

0

0

0
4

0
4

0

0

0

0
4

0

0

0

0
4

0
4

0

0

0

0

0

0
4

0

0

0
4

0
4

0
4

0
4

0

0

0

0

0

0

0
4

0
4

0
4

0

0

0

0

0

0
4

0

0

0

0
4

0
4

0
3

0
3

0

0

0

0

0
4

0

0

0

0

0
4

0

0

0
4

0
4

0

0

0

0

0
4

0

0

0
4

0
4

0

0

0

0

0

0

0
4

0
4

0.223218
6

0.223218
6

0.223218
6

0
4

0
4

0

0

0

0

0
4

0

0

0

0
4

0
4

0

0

0

0

0
4

0

0

0
4

0
4

0

0

0

0

0
4

0
4

0

0

0

0

0

0
4

0

0

0
4

0
4

0

0

0

0

0

0
4

0
4

0

0

0

0

0
4

0

0

0
4

0
4

0

0

0

0

0
4

0
4

0
2

0

0

0

0

0
4

0

0

0

0

0
4

0

0

0
4

0

0

0
4

0

0

0
4

0
4

0

0

0

0

0
4

0

0

0
4

0
4

0

0

0

0
4

0
4

0

0

0

0
4

0
4

0

0

0

0
4

0
4

0

0

0

0
4

0
4

0

0

0

0

0
4

0
4

0

0

0

0
4

0

0

0
4

0
4

0

0

0

0

0
4

0

0

0
4

0
4

0

0

0

0
4

0
4

0

0

0

0
4

0
4

0

0

0

0

0

0

0

0
4

0
4

0

0

0

0
4

0

0

0
4

0
4

0

0

0

0

0
4

0

0

0
4

0
4

0

0

0

0

0
4

0
4

0

0

0

0

0
4

0
4

0

0

0

0

0
4

0
4

0

0

0

0
4

0

0

0
4

0
4

0

0

0

0

0
4

0
4

0

0

0

0
4

0

0

0
4

0
4

0

0

0

0
4

0

0

0
4

0
4

0

0

0

0
4

0
4

0
4

0

0

0

0

0

0
4

0

0

0
4

0

0

0
4

0

0

0
4

0
4

0

0

0

0
4

0
4

0

0

0

0

0
4

0
4

0

0

0

0
4

0
4

0

0

0

0

0
4

0
4

0

0

0

0
4

0

0

0
4

0
4

0

0

0

0
4

0

0

0
4

0
4

0

0

0

0
4

0

0

0
4

0
4

0

0

0

0
4

0
4

0

0

0

0
4

0
4

0

0

0

0

0
4

0
4

0

0

0

0

0
4

0

0

0

0

0
4

0

0

0
4

0
4

0

0

0

0

0
4

0
4

0

0

0

0
4

0

0

0
4

0
4

0

0

0

0
4

0

0

0
4

0
4

0

0

0

0

0
4

0
4

0

0

0

0
4

0
4

0

0

0

0
4

0
4

0

0

0

0

0
4

0
4

0

0

0

0
4

0
4

0

0

0

0
4

0
4

0

0

0

0
4

0
4

0

0

0

0

0

0
4

0

0

0
4

0

0

0
4

0

0

0

0
4

0
4

0

0

0

0
4

0
4

0.019133

0.019133

0.019133

0
4

0
4

0

0

0

0
4

0
4

0

0

0

0
4

0
4

0

0

0

0
4

0
4

0

0

0

0
4

0
4

0

0

0

0
4

0
4

0

0

0

0
4

0
4

0

0

0

0
4

0
4

0

0

0

0
4

0
4

0
3

0

0

0

0
4

0

0

0

0

0
4

0

0

0
4

0

0

0
4

0
4

0

0

0

0
4

0
4

0

0

0

0
4

0
4

0

0

0

0
4

0
4

0

0

0

0
4

0
4

0

0

0

0
4

0
4

0

0

0

0
4

0
4

0

0

0

0
4

0
4

0

0

0

0
4

0
4

0

0

0

0
4

0
4

0

0

0

0
4

0
4

0.291793
3

0.27266
3

0
3

0
3

0

0

0

0

0

0

0.165819

0

0

0

0

0

0

0

0

0

0.00447073

0

0.0956648

0

0

0

0

0

0

0

0

0

0

0

0

0

0

0.00670609

0

0

0

0

0

0

0

0

0

0

0

0

0

1.30104260698261e-17
3

0
4

0
7

0
7

0
4

0

0

0
4

0

0

0
4

0.019133

0.019133

0
4

0

0

0
4

0

0

0
4

0

0

0
4

0

0

0
4

0

0

0
4

0

0

0
4

0

0

0
4

0

0

0
4

0

0

0
4

0

0

0
4

0

0

0
4

0

0

0

0
4

0

0

0
4

0

0

0
4

0

0

0
4

0

0

0
4

0
4

2.22044604925031e-16
3

0
4

14.5931
4

0.22078
7

0.22078
7

0.22078
7

0

0

0

0

0

0

0
4

0
4

0.262176
7

0.255276
7

0.255276
7

0
4

0.00689936

0.00689936

0
4

1.73472347597681e-17
7

0
4

0.00985039

0.00985039

0.0049252

0.0049252

0
4

0
4

0

0

0

0

0
4

0
4

0

0

0

0

0
4

0
4

0

0

0

0

0
4

0
4

0

0

0

0

0
4

0
4

0

0

0

0
4

0
4

0

0

0

0

0
4

0
4

0.0134122

0.0134122

0.00670609

0.00670609

0
4

0
4

0

0

0

0
4

0

0

0
4

0
4

0

0

0

0
4

0
4

0
4

0
4

0

0

0

0

0

0

0

0

0
4

0

0

0

0
4

0

0

0

0
4

0

0

0
4

0

0

0
4

0
4

0

0

0

0
4

0

0

0
4

0
4

0

0

0

0
4

0

0

0
4

0
4

0

0

0

0
4

0

0

0
4

0
4

0

0

0

0
4

0

0

0
4

0
4

0

0

0

0

0
4

0
4

0

0

0

0
4

0

0

0
4

0
4

0

0

0

0
4

0
4

0

0

0

0

0
4

0
4

0

0

0

0
4

0

0

0
4

0
4

0

0

0

0

0
4

0
4

1.29723
7

0.994914
7

0.293372
7

0.701542

0

1.11022302462516e-16
7

0
4

0

0

0

0
4

0.302313

0.302313

0
4

0
4

0

0

0

0
4

0

0

0
4

0
4

0

0

0

0
4

0
4

0

0

0

0
4

0
4

0

0

0

0
4

0

0

0
4

0
4

0

0

0

0
4

0
4

0

0

0

0
4

0

0

0
4

0
4

0

0

0

0
4

0

0

0
4

0
4

0

0

0

0
4

0
4

0

0

0

0

0
4

0
4

0

0

0

0
4

0

0

0
4

0
4

0
4

0
4

0

0

0

0

0

0
4

0

0

0

0
4

0

0

0

0
4

0
4

0.16045

0.16045

0.16045

0
4

0
4

0

0

0

0
4

0
4

0

0

0

0
4

0

0

0
4

0
4

0.0382659

0.0382659

0.0382659

0
4

0
4

0

0

0

0
4

0
4

0

0

0

0
4

0
4

0

0

0

0
4

0

0

0
4

0
4

0

0

0

0
4

0

0

0
4

0
4

0

0

0

0
4

0

0

0
4

0
4

0

0

0

0
4

0

0

0
4

0
4

0
7

0
7

0
7

0

0
4

0

0

0
4

0
4

0

0

0

0
4

0

0

0
4

0
4

0

0

0

0
4

0
4

0

0

0

0
4

0

0

0
4

0
4

0

0

0

0
4

0

0

0
4

0
4

0

0

0

0
4

0
4

0

0

0

0
4

0
4

0

0

0

0
4

0
4

0

0

0

0
4

0
4

0

0

0

0
4

0
4

0

0

0

0
4

0
4

0.0881946
7

0.0650903
6

0.0650903

0

0

0

0

0

0
4

0.010349

0.010349

0
4

0

0

0
4

0.0127553

0.0127553

0
4

1.21430643318376e-17
7

0
4

0

0

0

0
4

0
4

0

0

0

0
4

0
4

0

0

0

0
4

0
4

0

0

0

0
4

0
4

0

0

0

0
4

0
4

0

0

0

0
4

0
4

0

0

0

0
4

0
4

0

0

0

0
4

0
4

0

0

0

0
4

0
4

0

0

0

0
4

0
4

0

0

0

0

0

0

0

0
4

0

0

0

0

0

0
4

0

0

0

0

0
4

0
4

0

0

0

0
4

0
4

0

0

0

0
4

0
4

0

0

0

0
4

0
4

0.019133

0.019133

0.019133

0
4

0
4

0

0

0

0
4

0
4

0

0

0

0
4

0
4

0

0

0

0
4

0
4

0

0

0

0
4

0
4

0

0

0

0
4

0
4

0

0

0

0
4

0
4

0
5

0

0

0

0

0
4

0

0

0

0
4

0

0

0
4

0

0

0
4

0

0

0
4

0

0

0
4

0
4

0

0

0

0
4

0
4

0

0

0

0
4

0
4

0

0

0

0
4

0
4

0

0

0

0
4

0
4

0

0

0

0
4

0
4

0

0

0

0
4

0
4

0

0

0

0
4

0
4

0

0

0

0
4

0
4

0

0

0

0
4

0
4

0

0

0

0
4

0
4

0
4

0
4

0
6

0

0

0

0
4

0

0

0

0

0
4

0
4

0

0

0

0
4

0
4

0

0

0

0
4

0
4

0

0

0

0
4

0
4

0

0

0

0
4

0
4

0

0

0

0
4

0
4

0

0

0

0
4

0
4

0

0

0

0
4

0
4

0

0

0

0
4

0
4

0

0

0

0
4

0
4

0

0

0

0
4

0
4

0
4

0
4

0

0

0

0

0

0

0
4

0

0

0

0
4

0
4

0

0

0

0
4

0
4

0

0

0

0
4

0
4

0

0

0

0
4

0
4

0

0

0

0
4

0
4

0

0

0

0
4

0
4

0

0

0

0
4

0
4

0

0

0

0
4

0
4

0

0

0

0
4

0
4

0

0

0

0
4

0
4

0

0

0

0
4

0
4

0
4

0
4

0
4

0

0

0

0

0
4

0

0

0

0

0
4

0

0

0

0
4

0

0

0

0
4

0

0

0

0
4

0

0

0
4

0

0

0
4

0
4

0
4

0
4

0
4

0

0

0
4

0

0

0

0
4

0

0

0
4

0
4

0

0

0

0
4

0
4

0

0

0

0
4

0
4

0

0

0

0
4

0
4

0

0

0

0
4

0
4

0

0

0

0
4

0
4

0

0

0

0
4

0
4

0

0

0

0
4

0
4

0

0

0

0
4

0
4

0.00447073

0.00447073

0.00447073

0
4

0
4

0

0

0

0
4

0
4

0

0

0

0

0

0
4

0

0

0

0

0
4

0

0

0

0
4

0
4

0

0

0

0
4

0
4

0

0

0

0
4

0
4

0

0

0

0
4

0
4

0

0

0

0
4

0
4

0.552135

0.552135

0.552135

0
4

0
4

0

0

0

0
4

0
4

0

0

0

0
4

0
4

0

0

0

0
4

0
4

0

0

0

0
4

0
4

0

0

0

0
4

0
4

0

0

0

0

0

0

0

0

0
4

0

0

0

0

0
4

0
4

0

0

0

0
4

0
4

0

0

0

0
4

0
4

0

0

0

0
4

0
4

0

0

0

0
4

0
4

0.0127553

0.0127553

0.0127553

0
4

0
4

0

0

0

0
4

0
4

0

0

0

0
4

0
4

0

0

0

0
4

0
4

0

0

0

0
4

0
4

0

0

0

0
4

0
4

0
4

0

0

0

0

0

0
4

0

0

0

0

0
4

0

0

0
4

0

0

0
4

0
4

0

0

0

0
4

0
4

0

0

0

0
4

0
4

0

0

0

0
4

0
4

0

0

0

0
4

0
4

0

0

0

0
4

0
4

0

0

0

0
4

0
4

0

0

0

0
4

0
4

0

0

0

0
4

0
4

0

0

0

0
4

0
4

0

0

0

0
4

0
4

0
4

0

0

0

0

0
4

0

0

0

0
4

0

0

0
4

0

0

0
4

0
4

0

0

0

0
4

0
4

0.00520121

0.00520121

0.00520121

0
4

0
4

0

0

0

0
4

0
4

0

0

0

0
4

0
4

0

0

0

0
4

0
4

0

0

0

0
4

0
4

0

0

0

0
4

0
4

0

0

0

0
4

0
4

0

0

0

0
4

0
4

0.019133

0.019133

0.019133

0
4

0
4

0
4

0
4

0
4

0

0
4

0

0

0
4

0

0

0
4

0
4

0

0

0

0
4

0
4

0

0

0

0
4

0
4

0

0

0

0
4

0
4

0

0

0

0
4

0
4

0

0

0

0
4

0
4

0.00443786

0.00443786

0.00443786

0
4

0
4

0.0765318

0.0765318

0.0765318

0
4

0
4

0

0

0

0
4

0
4

0

0

0

0
4

0
4

0

0

0

0
4

0
4

0

0

0

0

0
4

0

0

0

0
4

0

0

0
4

0

0

0
4

0
4

0

0

0

0
4

0
4

0

0

0

0
4

0
4

0

0

0

0
4

0
4

0

0

0

0
4

0
4

0

0

0

0
4

0
4

0

0

0

0

0

0
4

0

0

0
4

0

0

0
4

0
4

0
4

0

0

0

0
4

0

0

0

0
4

0

0

0
4

0

0

0
4

0

0

0
4

0
4

0

0

0

0

0

0

0

0

0
4

0

0

0
4

0

0

0
4

0

0

0
4

0
4

0
4

0
4

0
4

0

0

0

0

0

0

0
4

0

0

0

0
4

0
4

0

0

0

0
4

0

0

0

0

0
4

0

0

0

0
4

0

0

0
4

0
4

0
3

0
3

0

0

0

0

0

0
4

0
4

0
3

0

0

0

0

0
4

0

0

0

0
4

0

0

0
4

0
4

0

0

0

0

0
4

0

0

0

0
4

0

0

0
4

0

0

0
4

0

0

0
4

0

0

0
4

0
4

0
4

0
4

0

0

0

0

0

0
4

0
4

0

0

0

0

0

0

0

0
4

0

0

0
4

0

0

0
4

0
4

0
3

0

0

0

0
4

0

0

0

0
4

0

0

0
4

0

0

0
4

0
4

0

0

0

0

0

0
4

0
4

0

0

0

0
4

0

0

0
4

0

0

0
4

0

0

0
4

0

0

0
4

0

0

0
4

0
4

0.0171932
6

0.0171932

0.0171932

0
4

0

0

0
4

0
4

0
7

0
7

0
7

0
6

0

0

0

0

0

0
4

0
4

0

0

0

0

0

0
4

0

0

0
4

0

0

0
4

0
4

0.0127553

0.0127553

0

0.0127553

0

0
4

0
4

0
3

0
3

0
3

0
4

0

0

0
4

0

0

0
4

0
4

0.00447073

0.00447073

0.00447073

0

0

0
4

0

0

0

0

0
4

0

0

0
4

0
4

0
5

0

0

0
4

0

0

0
4

0

0

0
4

0
4

0

0

0

0

0
4

0
4

0

0

0

0

0
4

0

0

0

0

0
4

0
4

0

0

0

0

0
4

0

0

0
4

0

0

0
4

0
4

0
4

0

0

0
4

0

0

0
4

0

0

0
4

0

0

0
4

0

0

0
4

0
4

0
4

0

0

0

0
4

0

0

0
4

0

0

0
4

0

0

0
4

0
4

0
4

0
4

0

0

0

0

0

0

0

0

0
4

0
4

0

0

0

0

0

0
4

0

0

0

0
4

0

0

0

0

0
4

0

0

0
4

0

0

0

0
4

0

0

0
4

0

0

0
4

0

0

0
4

0
4

0

0

0

0

0
4

0
4

0

0

0

0
4

0

0

0
4

0

0

0
4

0

0

0
4

0

0

0
4

0
4

0
4

0

0

0

0
4

0

0

0
4

0
4

0

0

0

0

0
4

0

0

0
4

0

0

0
4

0

0

0
4

0
4

0

0

0

0

0
4

0

0

0
4

0

0

0
4

0

0

0
4

0
4

0

0

0

0

0
4

0
4

0

0

0

0

0

0
4

0

0

0
4

0
4

0

0

0

0

0
4

0

0

0
4

0
4

0

0

0

0

0

0

0
4

0

0

0
4

0
4

0.115783

0.115783

0.0956648

0.0201183

0
4

0

0

0
4

0
4

0
4

0
4

0
4

0

0

0

0

0
4

0

0

0

0

0
4

0
4

0

0

0

0

0
4

0

0

0

0
4

0

0

0
4

0
4

0.0127553

0

0

0

0
4

0

0

0
4

0.0127553

0.0127553

0
4

0
4

0.0382659

0.0382659

0.0382659

0

0
4

0

0

0
4

0

0

0
4

0
4

0

0

0

0

0
4

0

0

0

0
4

0
4

0

0

0

0

0
4

0

0

0
4

0

0

0
4

0
4

0

0

0

0
4

0

0

0
4

0

0

0
4

0
4

0

0

0

0
4

0

0

0
4

0

0

0
4

0
4

0.0446436

0.0446436

0.0446436

0

0
4

0
4

0.0701542

0

0

0
4

0.0701542

0.0701542

0
4

0
4

0

0

0

0
4

0

0

0
4

0

0

0
4

0

0

0
4

0
4

0.210391
6

0.172197
7

0.172197
7

0

0

0

0
4

0.0381945
6

0.00689936
6

0.0312951

0

0

0
4

0
4

0

0

0

0
4

0

0

0
4

0

0

0
4

0
4

0

0

0

0
4

0
4

0

0

0

0
4

0

0

0
4

0

0

0
4

0
4

0

0

0

0
4

0

0

0
4

0
4

0

0

0

0

0
4

0

0

0
4

0
4

0

0

0

0

0
4

0

0

0
4

0
4

0

0

0

0
4

0
4

0

0

0

0
4

0

0

0
4

0

0

0
4

0
4

0

0

0

0

0
4

0

0

0
4

0
4

0

0

0

0

0
4

0
4

0.0573989
7

0.0573989
7

0.0573989
7

0

0

0

0
4

0

0

0
4

0
4

0

0

0

0
4

0
4

0

0

0

0

0
4

0
4

0

0

0

0
4

0

0

0
4

0
4

0

0

0

0
4

0

0

0
4

0
4

0

0

0

0
4

0

0

0
4

0

0

0
4

0
4

0

0

0

0

0
4

0

0

0
4

0
4

0

0

0

0

0
4

0
4

0

0

0

0
4

0

0

0
4

0
4

0

0

0

0

0
4

0

0

0
4

0
4

0

0

0

0

0
4

0

0

0
4

0
4

0
4

0

0

0

0

0
4

0

0

0

0

0
4

0

0

0

0
4

0

0

0

0
4

0

0

0
4

0

0

0
4

0

0

0
4

0
4

0

0

0

0

0
4

0

0

0
4

0
4

0

0

0

0
4

0

0

0
4

0
4

0

0

0

0
4

0
4

0.0127553

0

0

0

0
4

0.0127553

0.0127553

0
4

0
4

0.0049252

0.0049252

0.0049252

0
4

0
4

0

0

0

0

0

0
4

0
4

0

0

0

0
4

0

0

0
4

0

0

0
4

0
4

0

0

0

0
4

0

0

0
4

0

0

0
4

0
4

0

0

0

0

0
4

0

0

0
4

0
4

0

0

0

0

0
4

0
4

11.2074
4

11.0392
4

5.03
4

0
4

0
4

1.96072
7

0

0

0

0

0

0

0

0

0

0.0127553

0

0

0

0

0

0

0

0

0

0

0.0206981

0

0.00887572
7

0

0

0

0

0.00894146

0

0.00447073

0

0

0

0.0174453
6

0

0

0

0

0

0

0

0

0

0

0
7

0

0

0

0

0

0

0

0

0

0

0.0408297
7

0

0

0

0

0

0

0

0

0

0

0
7

0.0127553

0

0

0

0

0

0

0

0

0

0

0

0.0380728

0

0

0

0

0

0

0

0

0.0127553
6

0

0

0

0

0

0

0

0

0

0

0.0199704
7

0

0

0

0

0

0

0

0

0

0

3.02458
7

0
4

0

0

0

0

0

0

0

0

0

0

0
7

0

0

0

0.00443786

0

0

0

0

0

0

0.0313053
6

0

0

0

0

0

0

0

0.0229434

0

0.00670609

0
7

0

0

0

0

0

0

0

0

0

0

0.00655526
7

0

0

0

0.00447073

0.00447073

0

0

0

0.0127553

0

0
3

0

0

0

0

0

0

0

0

0

0

0
6

0

0

0

0.00689936

0

0

0

0

0

0

0

0.00655526

0

0

0

0

0

0

0

0

0

0
6

0

0.0765318

0

0

0

0

0

0

0

0

0
6

0

0

0

0

0

0

0

0

0

0

0.330644
7

0

0

0

0

0

0

0

0

0

0

0

0
6

0

0

0

0

0

0

0

0

0.00447073

0

0

0

0

0

0.0156475

0

0

0

0

0

0

0
3

0

0

0.00665679

0

0

0

0

0

0

0

0
4

0

0

0

0

0

0

0.00894146

0

0

0

0

0

0

0

0

0

0

0

0

0

0.0110946

0
6

0

0

0

0

0

0

0

0

0

0

0.00443786
7

0

0

0

0

0

0

0

0

0

0

0.00655526
7

0

0

0

0

0

0

0

0

0

0

0

0

0

0

0

0

0

0

0

0

0

0
4

0
7

0

0.00447073

0

0

0

0

0

0

0

0

0

0

0

0

0.0318883

0

0

0

0

0.0765318
7

0

0.0255106

0

0

0

0

0

0

0

0

0
3

0

0

0

0

0

0

0

0

0

0

0.0682144
7

0

0

0.0127553

0

0

0

0.0239321

0

0

0

0.0109931
7

0

0

0

0

0

0

0

0

0

0

0
4

0

0

0

0

0

0

0

0

0

0

7.91033905045424e-16
4

0
4

0
7

0
7

0

0

0

0
4

0

0

0

0

0
4

0

0

0
4

0

0

0
4

0

0

0
4

0

0

0
4

0

0

0
4

0.0127553

0.0127553

0

0

0

0
4

0

0

0

0
4

0

0

0

0
4

0

0

0

0
4

0

0

0

0

0
4

0

0

0

0
4

0.0392512

0.0392512

0
4

0

0

0

0
4

0

0

0
4

0

0

0

0

0

0
4

0

0

0

0
4

0.019133

0.019133

0
4

0

0

0
4

0

0

0

0
4

0

0

0

0
4

0

0

0
4

0

0

0

0
4

0

0

0

0
4

0

0

0

0
4

0

0

0

0
4

0.0470542
7

0.0470542

0

0

0
4

0

0

0

0
4

0

0

0
4

0

0

0

0
4

0

0

0
4

0

0

0
4

0

0

0
4

0

0

0
4

0

0

0
4

0

0

0
4

0

0

0
4

0.0255106
6

0.0255106
6

0
4

0

0

0
4

0

0

0
4

0

0

0
4

0

0

0
4

0

0

0
4

0

0

0
4

0

0

0
4

0

0

0
4

0

0

0
4

0

0

0
4

0
6

0
6

0
4

0

0

0
4

0

0

0
4

0

0

0
4

0

0

0
4

0

0

0
4

0

0

0
4

0

0

0
4

0

0

0
4

0

0

0
4

0

0

0
4

0
4

0
4

0
4

0

0

0
4

0

0

0
4

0

0

0
4

0

0

0
4

0

0

0
4

0

0

0
4

0

0

0
4

0

0

0
4

0

0

0
4

0

0

0
4

0

0

0

0

0
4

0

0

0
4

0

0

0
4

0

0

0
4

0

0

0
4

0

0

0
4

0

0

0
4

0

0

0
4

0

0

0
4

0.0244754

0.0244754

0
4

0

0

0
4

0

0

0

0

0
4

0

0

0
4

0

0

0
4

0

0

0
4

0

0

0
4

0

0

0
4

0

0

0
4

0

0

0
4

0

0

0
4

0

0

0
4

0

0

0
4

0

0

0

0
4

0

0

0
4

0

0

0
4

0

0

0
4

0

0

0
4

0

0

0
4

0

0

0
4

0

0

0
4

0

0

0
4

0

0

0
4

0

0

0
4

2.4980018054066e-16
4

0
4

0
4

3.24522
4

3.17954
4

0

0

0

0

0

0
4

2.78413
4

2.54816
4

0
4

0.235973
3

0
4

0
4

0
4

0
4

0
4

0
3

0

0

0

0

0

0

0

0

0

0

0

0

0
4

0.274239
3

0
3

0.274239
3

0
3

0
4

0
4

0

0
4

0

0

0

0

0

0

0

0
4

0

0

0

0

0

0

0

0

0

0

0
3

0

0

0

0

0

0

0

0

0

0

0
3

0

0

0

0

0

0

0

0

0

0

0

0

0

0

0

0

0

0

0

0

0

0
3

0

0

0

0

0

0

0

0

0

0

0
4

0

0

0

0

0

0

0

0

0

0

0
4

0
4

0
4

0
5

0
5

0
4

0

0

0
4

0

0

0
4

0

0

0
4

0

0

0
4

0

0

0
4

0

0

0
4

0

0

0
4

0

0

0
4

0

0

0
4

0

0

0
4

0

0

0

0
4

0.0127553

0.0127553

0
4

0

0

0
4

0

0

0
4

0

0

0
4

0

0

0
4

0

0

0
4

0

0

0
4

0

0

0
4

0

0

0
4

0.10842

0

0.10842

0
4

0

0

0
4

0

0

0
4

0

0

0
4

0

0

0
4

0

0

0
4

0

0

0
4

0
4

0
4

0
4

0

0

0

0

0

0

0

0

0
4

0

0

0

0
4

0

0

0
4

0
4

0
4

0

0

0

0

0
4

0

0

0
4

0
4

0
4

0

0

0
4

0

0

0
4

0

0

0
4

0

0

0
4

0
4

0

0

0

0
4

0

0

0

0
4

0

0

0
4

0
4

0

0

0

0
4

0

0

0
4

0
4

0

0

0

0

0

0

0
4

0
4

0.00443786

0.00443786

0.00443786

0
4

0

0

0
4

0

0

0
4

0
4

0

0

0

0
4

0

0

0
4

0

0

0
4

0
4

0

0

0

0

0
4

0
4

0

0

0

0

0

0
4

0
4

0

0

0

0
4

0
4

0
4

0
4

0
4

0

0
4

0

0

0

0
4

0

0

0
4

0
4

0

0

0

0
4

0

0

0
4

0

0

0
4

0
4

0

0

0

0
4

0

0

0
4

0

0

0
4

0
4

0

0

0

0

0
4

0
4

0

0

0

0

0
4

0

0

0
4

0
4

0

0

0

0

0
4

0
4

0

0

0

0

0
4

0

0

0
4

0
4

0

0

0

0
4

0
4

0

0

0

0
4

0
4

0

0

0

0

0
4

0
4

0

0

0

0
4

0
4

0
4

0
4

0
4

0

0

0
4

0

0

0

0
4

0

0

0
4

0
4

0

0

0

0
4

0
4

0

0

0

0
4

0
4

0

0

0

0
4

0
4

0.0395383

0.0395383

0.0197691

0.0197691

0
4

0
4

0

0

0

0
4

0
4

0

0

0

0
4

0

0

0
4

0
4

0

0

0

0

0
4

0
4

0

0

0

0

0
4

0
4

0

0

0

0
4

0

0

0
4

0
4

0

0

0

0
4

0
4

0
5

0

0

0

0
4

0

0

0

0

0

0
4

0
4

0

0

0

0
4

0
4

0

0

0

0
4

0
4

0

0

0

0
4

0
4

0

0

0

0
4

0
4

0

0

0

0
4

0
4

0

0

0

0
4

0
4

0

0

0

0
4

0
4

0

0

0

0
4

0
4

0

0

0

0
4

0
4

0

0

0

0
4

0
4

0
4

0
4

0
4

0

0

0
4

0

0

0
4

0

0

0
4

0
4

0

0

0

0
4

0
4

0

0

0

0
4

0
4

0

0

0

0
4

0
4

0

0

0

0
4

0
4

0

0

0

0
4

0
4

0

0

0

0
4

0
4

0

0

0

0
4

0
4

0

0

0

0
4

0
4

0

0

0

0
4

0
4

0

0

0

0
4

0
4

0
4

0
4

0

0

0

0

0
4

0
4

0

0

0

0
4

0
4

0

0

0

0
4

0
4

0

0

0

0
4

0
4

0

0

0

0
4

0
4

0

0

0

0
4

0
4

0

0

0

0
4

0
4

0

0

0

0
4

0
4

0

0

0

0
4

0
4

0

0

0

0
4

0
4

0

0

0

0
4

0
4

0

0

0

0

0
4

0

0

0
4

0

0

0
4

0

0

0
4

0
4

0

0

0

0
4

0
4

0

0

0

0
4

0
4

0

0

0

0
4

0
4

0

0

0

0
4

0
4

0

0

0

0
4

0
4

0

0

0

0
4

0
4

0

0

0

0
4

0
4

0

0

0

0
4

0
4

0.0127553

0.0127553

0.0127553

0
4

0
4

0

0

0

0
4

0
4

0

0

0

0

0

0
4

0

0

0
4

0
4

0

0

0

0
4

0
4

0

0

0

0
4

0
4

0

0

0

0
4

0
4

0

0

0

0
4

0
4

0

0

0

0
4

0
4

0

0

0

0
4

0
4

0

0

0

0
4

0
4

0.00894146

0.00894146

0.00894146

0
4

0
4

0

0

0

0
4

0
4

0

0

0

0
4

0
4

0
4

0
4

0
4

0

0
4

0
4

0

0

0

0
4

0
4

0

0

0

0
4

0
4

0

0

0

0
4

0
4

0

0

0

0
4

0
4

0

0

0

0
4

0
4

0

0

0

0
4

0
4

0

0

0

0
4

0
4

0

0

0

0
4

0
4

0

0

0

0
4

0
4

1.83880688453542e-16
4

0
4

0.559573
5

0

0

0

0

0

0
4

0

0

0
4

0
4

0.0127553

0.0127553

0.0127553

0
4

0
4

0

0

0

0
4

0
4

0

0

0

0
4

0
4

0

0

0

0
4

0
4

0

0

0

0
4

0
4

0.0110946

0.0110946

0.0110946

0
4

0
4

0

0

0

0
4

0
4

0

0

0

0
4

0
4

0

0

0

0
4

0
4

0

0

0

0
4

0
4

0
4

0
4

0

0

0

0

0
4

0

0

0

0
4

0

0

0
4

0
4

0

0

0

0
4

0
4

0

0

0

0
4

0
4

0

0

0

0
4

0
4

0.0637765
6

0.0637765
6

0

0.0446436

0.019133

3.46944695195361e-18
6

0
4

0
4

0.10842
7

0.10842
7

0.10842
7

0
4

0
4

0.184952
6

0.184952
6

0.184952
6

0

0
4

0
4

0

0

0

0
4

0

0

0
4

0
4

0

0

0

0
4

0
4

0

0

0

0

0
4

0
4

0

0

0

0
4

0
4

0.178574

0
5

0

0

0

0

0

0

0

0
4

0.0765318
7

0.0765318

0

0
4

0

0

0
4

0

0

0
4

0

0

0
4

0

0

0
4

0

0

0
4

0.102042

0.102042

0

0
4

0

0

0

0
4

0

0

0
4

0

0

0
4

0

0

0
4

0

0

0
4

0

0

0
4

0

0

0
4

1.38777878078145e-17

0
4

2.77555756156289e-17
5

0
4

2.8066438062524e-13

0
4

0.110062

0.110062

0.110062

0
3

0
3

0

0

0

0

0

0
4

0
3

0
3

0

0

0

0

0

0

0

0

0
2

0
3

0

0

0

0

0

0

0
4

0

0

0

0

0
4

0.110062
7

0

0

0

0

0

0

0

0

0

0

0.0382659
6

0.0382659

0

0

0.0335305

0

0

0

0
4

0

0

0
4

0

0

0
4

0

0

0
4

0
4

0
3

0

0

0

0
4

0

0

0

0

0

0
4

0

0

0

0

0
4

0

0

0
4

0
4

0

0

0

0
4

0
4

0

0

0

0
4

0
4

0

0

0

0
4

0
4

0

0

0

0
4

0

0

0
4

0
4

0
3

0
3

0

0

0

0
4

0

0

0

0
4

0

0

0
4

0
4

0

0

0

0

0
4

0
4

0
2

0
2

0
2

0

0
4

0
4

0

0

0

0

0
4

0
4

0

0

0

0
4

0
4

0

0

0

0
4

0
4

0

0

0

0
4

0
4

0
4

0
2

0
2

0
2

0

0

0

0

0

0
4

0
3

0

0

0

0

0

0
4

0

0

0
4

0
4

0
4

0

0

0

0

0
4

0
4

0
4

0

0

0

0

0

0
4

0
4

0
4

0

0

0

0

0

0

0
4

0
4

0
4

0

0

0

0

0
4

0
4

0
4

0

0

0

0

0
4

0
4

0
4

0
4

4.83057

4.83057

0.357248
3

0.357248
3

0

0

0

0.18639

0

0.0133136

0

0

0

0

0

0
3

0

0

0

0

0.00665679

0

0

0

0

0

0
3

0

0

0

0

0

0

0

0

0

0

0
3

0

0

0

0

0

0

0

0

0

0

0.150887

0

0

0

0

0

0

0

0

0

0

0
4

0

0

0

0
4

0

0
4

0
4

0.740744
3

0.740744
3

0.324485
1

0.00983289

0

0

0

0

0

0

0

0

0

0

0

0

0

0

0

0

0

0

0

0

0
4

0

0

0

0.00655526

0

0

0

0

0
4

0

0.399871

0

0

0

0
4

0
4

0.918382
6

0.918382
6

0.0573989
6

0

0.286994

0

0

0

0.0829095
7

0

0.357148

0

0

0
5

0

0

0

0

0

0

0

0

0

0

0
5

0

0

0

0

0

0

0

0

0

0

0
6

0

0

0

0

0

0.0701542
6

0
6

0.0637765
7

0
6

0
4

0

0

0
4

0
4

0.0637765
7

0.0637765
7

0.0255106
7

0.0382659
7

0
4

0
4

0.0127553
3

0
3

0

0

0

0

0

0

0

0

0

0

0

0

0

0

0

0

0

0

0

0

0

0

0

0

0

0

0

0

0

0

0

0

0

0

0

0

0

0

0

0

0

0

0

0

0

0

0

0

0

0

0

0

0

0

0

0

0

0

0

0

0

0

0

0

0

0

0

0

0

0

0

0

0

0

0

0

0

0

0

0

0

0

0

0

0

0

0

0

0

0
4

0.0127553

0.0127553

0

0

0

0
4

0

0

0
4

0

0

0
4

0

0

0

0

0
4

0

0

0
4

0

0

0

0
4

0

0

0

0
4

0

0

0
4

0

0

0
4

0

0

0
4

0

0

0
4

0
4

0
3

0
3

0

0

0
4

0
3

0
4

0

0

0

0

0

0

0

0

0

0

0
2

0

0

0

0

0

0

0

0

0

0

0

0

0

0

0

0

0

0

0

0

0

0
4

0

0

0

0

0

0

0

0

0

0

0

0

0

0

0

0

0

0

0

0

0

0
4

0

0

0

0

0

0

0

0

0

0
4

0

0

0

0
4

0

0

0
4

0

0

0
4

0

0

0

0

0
4

0

0

0

0

0

0

0

0

0

0

0

0

0
4

0

0

0

0

0
4

0

0

0

0

0

0

0

0

0

0

0

0

0

0

0

0

0
4

0

0

0
4

0

0

0
4

0
4

2.72135
5

0.391835
6

0.391835
6

0
4

2.32952
5

2.32952
5

0
4

0
7

0

0

0

0

0

0

0

0

0

0

0

0

0

0

0

0

0

0

0

0

0

0

0

0

0

0

0

0

0

0

0

0

0

0

0

0

0

0

0

0

0

0

0

0

0

0

0

0

0

0

0

0

0

0

0

0

0

0

0

0

0

0

0

0

0

0

0

0

0

0

0

0

0

0

0

0

0

0

0
4

0

0

0

0
4

0

0

0
4

0

0

0
4

0

0

0
4

0
4

0
3

0
3

0
4

0

0

0

0

0

0

0

0

0

0
4

0
4

0
3

0
3

0
3

0

0

0

0

0

0
3

0

0

0

0

0

0

0

0
4

0

0

0
4

0

0

0
4

0
4

0

0

0

0
4

0
4

0

0

0

0
4

0
4

0

0

0

0
4

0
4

0

0

0

0
4

0
4

0

0

0

0
4

0
4

0

0

0

0
4

0
4

0.0108779

0.0108779

0.0108779

0
4

0
4

0

0

0

0
4

0
4

0

0

0

0
4

0
4

0.00543897

0.00543897

0.00543897

0
4

0
4

0
3

0
3

0
3

0
4

0
4

0

0

0

0

0

0

0

0
4

0
4

0

0

0

0

0
4

0
4

0

0

0

0

0
4

0

0

0
4

0
4

0

0

0

0
4

0

0

0
4

0
4

0

0

0

0
4

0
4

0

0

0

0

0
4

0
4

0

0

0

0
4

0

0

0
4

0
4

1.55171014926125e-15

0
4

0
3

0
3

0
3

0
3

0
3

0

0

0

0

0
4

0

0

0
4

0

0

0

0
4

0
4

0
4

0

0

0

0

0

0

0
4

0

0

0
4

0
4

0
4

0

0

0

0

0

0
4

0
4

0
4

0

0

0

0

0

0
4

0
4

0
4

0

0

0

0

0

0
4

0
4

0
4

0

0

0

0

0
4

0

0

0
4

0
4

0
4

0

0

0

0

0
4

0
4

0
4

0

0

0

0

0

0
4

0
4

0
4

0

0

0

0

0
4

0
4

0
4

0

0

0

0

0
4

0
4

0
4

0

0

0

0

0
4

0
4

0
4

0
3

0
3

0
3

0
3

0

0

0

0
4

0
4

0
4

0

0

0

0

0
4

0
4

0
4

0

0

0

0

0
4

0
4

0
4

0

0

0

0

0
4

0
4

0
4

0

0

0

0

0
4

0
4

0
4

0

0

0

0

0
4

0
4

0
4

0

0

0

0

0
4

0
4

0
4

0

0

0

0

0
4

0
4

0
4

0

0

0

0

0
4

0
4

0
4

0

0

0

0

0
4

0
4

0
4

0

0

0

0

0
4

0
4

0
4

0

0

0

0

0

0

0

0

0
4

0

0

0
4

0
4

0
4

0

0

0

0

0
4

0
4

0
4

0

0

0

0

0
4

0
4

0
4

0

0

0

0

0
4

0
4

0
4

0

0

0

0

0

0
4

0
4

0
4

0

0

0

0

0

0

0
4

0

0

0
4

0

0

0
4

0
4

0
4

0

0

0

0

0

0
4

0
4

0
4

0

0

0

0

0

0

0

0
4

0

0

0
4

0
4

0
4

0

0

0

0

0

0

0
4

0
4

0
4

0

0

0

0

0

0

0
4

0

0

0
4

0
4

0
4

0
4

0.260351
3

0.260351
3

0
4

0
4

0
4

0
4

0
4

0

0

0

0

0

0

0

0

0

0

0

0

0

0

0

0

0

0

0

0
4

0

0

0

0

0

0

0

0

0

0

0

0

0

0

0
4

0

0

0

0
4

0
4

0

0

0

0
4

0
4

0

0

0

0
4

0

0

0

0
4

0
4

0.119822

0.119822
1

0.119822
1

0
4

0

0

0
4

0

0

0
4

0
4

0

0
4

0

0

0

0

0
4

0

0

0
4

0

0

0
4

0

0

0
4

0

0

0
4

0
4

0.127117
7

0.127117
7

0.127117
7

0
4

0
4

0
7

0
7

0
7

0

0

0

0
4

0
4

0
4

0
4

0
4

0

0

0

0

0

0

0

0
4

0

0

0
4

0

0

0
4

0
4

0
4

0
4

0

0

0

0
4

0
4

0

0

0

0
4

0
4

0

0

0

0
4

0
4

0.0134122
3

0.0134122
3

0

0
3

0

0
3

0

0

0.00670609

0

0

0

0.00670609

0

0

0

0

0

0

0

0

0

0

0

0

0

0

0

0

0

0

0

0

0

0

0

0

0

0

0

0

0

0

0
4

0

0

0
4

0
4

0
4

0
4

0
4

0
4

0
4

0
3

0

0

0

0

0

0
4

0
4

0

0

0

0

0
4

0
4

0
4

0

0

0

0

0
4

0
4

0
4

0

0

0

0

0
4

0
4

0
4

0
4

42.7085

0.90004

0

0

0

0
4

0
4

0

0
3

0
3

0

0

0

0

0

0

0

0

0

0

0
3

0

0

0

0

0

0

0

0

0
3

0

0

0

0

0

0

0
4

0

0

0
4

0

0

0

0

0

0

0
4

0
4

0.90004

0.102834

0.00520121

0.093195

0

0

0

0

0

0

0

0

0

0

0

0

0

0.00443786

0

0

0

0

0

1.73472347597681e-18

0
4

0.797206

0.797206

0

0

0

0

0
4

0
4

0

0

0

0
4

0
4

0

0

0

0
4

0
4

0
4

0.224723
1

0

0

0

0

0

0

0

0

0

0

0

0

0

0

0
4

0

0

0

0

0

0

0
4

0

0

0
4

0

0

0
4

0
4

0

0

0

0
4

0
4

0

0

0

0
4

0
4

0

0

0

0

0

0

0

0

0
4

0

0

0
4

0

0

0
4

0
4

0.0637765
8

0.0637765
8

0.0637765

0

0

0
4

0

0

0
4

0
4

0

0

0

0

0

0
4

0

0

0
4

0
4

0.0625902

0.0625902

0.0625902

0
4

0

0

0
4

0
4

0

0

0

0

0

0
4

0

0

0
4

0
4

0

0

0

0

0

0
4

0
4

0

0

0

0
4

0

0

0
4

0
4

0.098356

0.098356

0.098356

0
4

0
4

1.38777878078145e-17
1

0
4

0.470558
3

0.470558
3

0.419536
3

0

0

0

0

0

0

0

0

0

0

0

0

0.0131105

0

0

0

0

0

0

0

0

0

0.170437

0

0

0

0

0

0

0

0.00983289

0

0

0

0

0

0

0

0

0

0

0

0

0

0

0

0

0

0

0

0

0

0

0

0.226156

0

0

0
4

0
4

0
4

0
4

0

0

0

0

0

0

0

0

0

0

0
4

0

0

0

0

0

0

0

0

0

0

0
4

0

0

0

0

0

0

0

0

0

0

0
4

0

0

0

0

0

0

0

0

0

0

0

0

0

0

0

0

0

0

0

0

0

0
4

0

0

0

0

0

0

0

0

0

0

0
4

0

0

0

0

0

0

0

0

0

0

0
4

0

0

0

0

0

0

0

0

0

0

0
4

0

0

0

0

0

0

0

0

0

0

0
4

0

0

0

0

0

0

0

0

0

0

0
4

0
4

0

0

0

0

0

0

0

0

0

0

0
3

0
4

0
4

0
3

0

0
4

0

0

0
4

0

0
3

0

0

0

0

0

0

0

0

0
4

0

0

0

0

0

0

0

0

0

0

0
4

0

0

0

0

0

0

0

0

0

0

0
4

0

0

0

0

0

0

0

0

0

0

0
4

0

0

0

0

0

0

0

0

0

0

0
4

0

0

0

0

0

0

0

0

0

0

0
4

0

0

0

0

0

0

0

0

0

0

0
4

0
2

0
2

0

0

0
4

0.0510212
2

0

0

0.0382659

0

0

0

0

0

0

0

0

0

0

0

0

0

0.0127553

0

0

0

0

0

0

0

0

0

0

0

0

0

0

0

0

0

0

0

0

0

0

0

0

0

0

0

0

0
4

0

0

0

0

0
4

0

0

0

0

0

0

0

0

0

0

0

0
4

0

0

0
4

0

0

0
4

0

0

0
4

0

0

0
4

0

0

0

0

0

0

0

0
4

0

0

0

0
4

0

0

0

0
4

0

0

0

0

0
4

0

0

0

0
4

0

0

0
4

0

0

0
4

0

0

0
4

6.93889390390723e-18
3

0
4

0

0

0

0
4

0

0

0
4

0
4

0

0

0

0

0
4

0
4

0

0

0

0
4

0
4

0

0

0

0
4

0
4

0
4

2.56781
3

0.601247
3

0
3

0
2

0

0

0

0

0

0

0

0

0

0

0

0

0
3

0

0

0

0

0

0

0

0

0

0

0

0

0

0

0

0

0

0

0

0

0

0

0

0

0

0

0

0

0

0

0

0

0

0

0

0

0

0

0

0

0

0

0

0

0

0

0

0

0

0

0

0

0

0

0

0

0

0

0

0

0

0

0

0

0

0
4

0

0

0

0

0

0

0

0

0

0

0

0

0

0

0

0

0

0

0

0

0

0
4

0
4

0

0
4

0

0
4

0

0

0
4

0.00887572

0.00887572

0
4

0.375541

0.375541

0
4

0.21683

0.129651

0.0871792

0
4

0
4

0
4

0
4

0

0

0
4

0

0

0

0
4

0

0

0
4

0

0

0
4

0

0

0
4

0

0

0
4

5.55111512312578e-17
3

0
4

1.90887

1.90887

1.86933

0.0254175

0.00564832

0.00847249

4.85722573273506e-17

0
4

0

0

0
4

0
4

0

0

0

0
4

0

0

0
4

0
4

0

0

0

0

0

0
4

0
4

0

0

0

0
4

0
4

0

0

0

0
4

0
4

0

0

0

0
4

0
4

0

0

0

0
4

0
4

0.0576922

0.0576922

0.0576922

0
4

0
4

0

0

0

0
4

0
4

0

0

0

0
4

0
4

0

0

0

0
4

0
4

0

0

0

0

0
4

0

0

0
4

0
4

0

0

0

0
4

0
4

0

0

0

0
4

0
4

0

0

0

0
4

0
4

0

0

0

0
4

0
4

0

0

0

0
4

0
4

0

0

0

0
4

0
4

0

0

0

0
4

0
4

0

0

0

0
4

0
4

0

0

0

0
4

0
4

0

0

0

0
4

0
4

0

0

0

0

0

0

0
4

0

0

0
4

0
4

0

0

0

0
4

0

0

0

0

0
4

0
4

0

0

0

0

0
4

0
4

0

0

0

0

0

0
4

0
4

0

0

0

0

0
4

0
4

0

0

0

0
4

0
4

0

0

0

0
4

0
4

0
4

6.82994

0.29975
4

0.29975
5

0

0

0

0

0

0.121175

0

0.165819

0.0127553

0

0

4.68375338513738e-17
5

0
4

0

0

0

0
4

0

0

0
4

0
4

6.04695
7

0.283466
8

0.109225

0.174241
8

2.77555756156289e-17
8

0
4

0.010349
1

0.010349
1

0

0

0

0
4

0.184952
1

0.0127553

0.114798

0

0

0

0.0127553

0

0

0

0.0446436

0

0

0

6.93889390390723e-18
1

0
4

0

0

0
4

5.20739
7

0
7

3.87185
7

0.55942
7

0

0

0

0

0.00689936

0

0

0.0127553

0

0

0.0344968
7

0

0

0

0

0

0

0.00689936

0

0

0

0.684028
7

0

0

0

0

0.0241478
7

0
7

0

0

0

0.00689936

3.85108611666851e-16
7

0
4

0.335278

0.0327217

0.0392694
7

0

0

0

0.00755116

0.105716

0

0.130887

0.019133

0

0

0

0
4

0

0

0

0
4

0

0

0

0
4

0

0

0
4

0.0255106

0.0255106

0
4

6.34908792207511e-16
7

0
4

0
7

0
7

0
7

0

0

0
4

0
7

0
6

0

0
4

0
4

0.230805

0.121272
1

0.0327763

0

0.0721078

0

0

0

0

0

0

0

0

0

0

0

0

0

0

0

0

0

0

0

0.00983289

0

0

0

0

0

0

0

0

0

0

0

0

0

0

0

0

0

0

0

0

0

0.00655526

0

0

0

0

0

0

0

0

0

0

0

0

0

0
4

0

0

0

0

0

0

0

0

0

0

0

0

0
4

0

0

0

0

0

0

0

0

0

0

0
7

0

0
5

0

0

0

0

0
4

0.109533

0.109533

0
4

0

0

0
4

0
4

0

0

0

0

0

0

0
4

0

0

0
4

0
4

0

0

0

0
4

0
4

0.100591

0.100591

0.100591

0
4

0
4

0

0

0

0
4

0
4

0

0

0

0
4

0
4

0

0

0

0

0
4

0

0

0
4

0
4

0

0

0

0

0

0
4

0
4

0.0156475

0.00894146

0.00447073

0.00447073

0
4

0.00670609

0.00670609

0
4

8.67361737988404e-19

0
4

0

0

0

0
4

0
4

0

0

0

0
4

0
4

0.0223536

0.0223536

0.0178829

0.00447073

0
4

0
4

0

0

0

0
4

0
4

0

0

0

0
4

0
4

0.113839
7

0.113839
7

0.113839
7

0
4

0
4

1.29063426612674e-15

0
4

13.6148
3

0.0104024
3

0

0

0
4

0
3

0
3

0

0

0

0
4

0.0104024
2

0.0104024
2

0
2

0
2

0

0

0

0

0

0
4

0

0

0

0
4

0

0

0

0
4

0

0

0

0
4

0

0

0
4

0

0

0
4

0

0

0
4

0

0

0
4

0
4

0.959868
3

0.271082
7

0.258326
6

0.0127553

1.90819582357449e-17
7

0
4

0
3

0
3

0
4

0
3

0
3

0
2

0
4

0
3

0

0
3

0

0

0

0

0

0

0

0

0

0

0

0

0

0

0

0

0

0

0

0

0
4

0.669653
3

0

0

0

0

0

0

0

0

0

0

0

0

0

0.248728
7

0.420925
7

0

0

0

0

0

0
4

0.019133

0

0.019133

0
4

0

0

0
4

0

0

0
4

0

0

0
4

0

0

0
4

4.5102810375397e-17
3

0
4

1.10228
3

0

0

0

0

0

0

0

0
4

0
5

0

0

0

0

0

0

0

0

0

0
4

0.10842

0

0.0510212
6

0

0

0.0573989
6

0

0

0

0

0

0

0

0
4

0.993863
3

0
3

0.986062
4

0
4

0.00780182

0

0

0

0

0
4

0

0

0

0

0

0

0

5.55111512312578e-17
3

0
4

0

0

0

0
4

0

0

0

0
4

0

0

0
4

0

0

0
4

0

0

0
4

0

0

0
4

0

0

0

0

0
4

0

0

0

0
4

0

0

0
4

0

0

0
4

0

0

0
4

0

0

0
4

0

0

0
4

0

0

0
4

0
4

0.49252
4

0

0

0
4

0

0

0

0
4

0.49252
4

0.49252
4

0
4

0
4

0

0

0

0

0

0

0

0

0

0

0
4

0

0

0

0

0

0

0

0

0

0

0
4

0

0

0

0

0

0

0

0

0

0

0
4

0

0

0

0

0

0

0

0

0
4

0

0

0
4

0

0

0
4

0

0

0

0

0

0
4

0
4

0

0

0

0
4

0

0

0

0

0

0

0

0

0

0

0
4

0

0

0

0

0

0

0

0

0

0

0
4

0

0

0

0

0

0

0

0

0

0

0
4

0

0

0

0

0

0

0

0

0

0

0
3

0

0

0

0

0

0

0

0

0

0

0

0

0

0

0

0

0

0

0

0

0

0
4

0

0

0

0

0

0

0

0

0

0

0
4

0

0

0
4

0

0

0
4

0

0

0
4

0

0

0
4

0

0

0
4

0

0

0
4

0

0

0
4

0

0

0
4

0

0

0
4

0

0

0
4

0

0

0
4

0

0

0

0

0
4

0

0

0
4

0

0

0
4

0

0

0
4

0

0

0
4

0

0

0
4

0

0

0
4

0

0

0
4

0

0

0
4

0

0

0
4

0

0

0
4

0

0

0

0
4

0

0

0
4

0

0

0
4

0

0

0
4

0

0

0
4

0

0

0

0
4

0

0

0
4

0

0

0
4

0

0

0
4

0
4

8.5797

0.43611
6

0.206708
6

0.229402
6

0

0
6

0

0

0

0

0
4

0.00665242
3

0

0

0

0

0

0

0.00665242

0

0

0

0

0

0

0

0

0

0

0

0

0

0

0

0

0

0

0

0

0

0

0

0

0
4

0.0232835
3

0
3

0.0232835
2

0

0

0

0
2

0

0

0

0

0

0

0

0
4

0
2

0
2

0

0

0

0

0

0

0
2

0

0

0

0

0

0

0

0
4

6.95888

0.0049252
3

0.372325
7

0
3

0

6.51141

0.0702163
2

0

0

0

0

0

0

0

0

0

0

0

0

0

0

0

0

0

0

0

0

0

0

0

0

0

0

0
4

0.955029

0.947478

0

0

0

0

0

0

0.00755116

1.18828558104411e-16

0
4

0
6

0
6

0

0

0

0

0

0

0

0

0

0

0

0

0

0

0

0

0

0

0

0

0

0

0

0

0

0

0

0

0

0

0

0

0

0

0

0

0

0

0

0

0

0

0

0

0

0

0

0

0

0

0

0

0

0

0

0

0

0

0

0

0

0

0

0

0

0

0

0

0

0

0

0

0

0

0

0

0
6

0

0

0

0

0

0

0

0

0

0

0

0

0
4

0.129271
3

0
3

0.00997863
2

0

0

0

0

0

0

0

0

0

0

0
3

0

0

0

0

0

0

0

0

0

0

0.0698504
2

0.0427899

0

0

0

0

0

0

0

0

0

0.00665242

0

0

0

0

0

0

0

0

0

0

0

0

0

0

0

0

0

0

0

0

0

0

0

0

0

0

0

0

0

0

0

0

0

0

0

0

0

0
2

0

0
4

0
3

0

0

0
4

0

0

0

0

0

0

0

0

0

0

0
4

0

0

0

0

0

0

0
4

0

0

0

0

0

0

0
4

0

0

0

0
4

0

0

0
4

0

0

0
4

0

0

0
4

0

0

0
4

0

0

0
4

0

0

0
4

0

0

0
4

0

0

0
4

0

0

0
4

0.0704775

0.0704775

0
4

0

0

0
4

0

0

0
4

0

0

0
4

0

0

0
4

0

0

0
4

0

0

0
4

0

0

0

0
4

0

0

0
4

0

0

0
4

0

0

0
4

0

0

0
4

0
4

0
3

0

0

0
4

0
3

0
2

0

0

0

0

0
4

0
3

0
3

0

0

0

0

0

0

0

0

0

0
4

0

0

0
4

0

0

0
4

0

0

0
4

0

0

0
4

0

0

0
4

0

0

0

0
4

0

0

0
4

0

0

0
4

0

0

0
4

0

0

0
4

0

0

0
4

0
4

0

0

0

0

0
4

0
2

0

0

0

0
4

0

0

0
4

0

0

0
4

0

0

0
4

0
4

0
6

0
6

0
6

0

0

0

0
4

0
7

0
6

0
7

0

0

0

0

0

0

0

0
4

0
6

0

0

0

0

0

0

0

0

0
4

0
4

0

0

0

0
4

0
4

0

0

0

0
4

0

0

0
4

0
4

0

0

0

0

0

0
4

0

0

0
4

0
4

0

0

0

0

0

0
4

0

0

0
4

0

0

0
4

0
4

0

0

0

0

0
4

0

0

0
4

0

0

0
4

0
4

0

0

0

0

0
4

0

0

0
4

0

0

0
4

0
4

0

0

0

0

0

0
4

0
4

0.00665242

0.00665242

0

0.00665242

0
4

0
4

0

0

0

0

0
4

0
4

0

0

0

0

0
4

0
4

0
4

0
4

0
4

0

0

0
4

0

0

0
4

0
4

0.00520121

0.00520121

0.00520121

0

0
4

0
4

0

0

0

0
4

0
4

0

0

0

0

0
4

0
4

0

0

0

0
4

0
4

0

0

0

0

0
4

0
4

0

0

0

0
4

0
4

0

0

0

0
4

0
4

0

0

0

0

0
4

0
4

0

0

0

0

0
4

0
4

0

0

0

0
4

0
4

0
3

0
3

0
3

0
4

0

0

0

0

0
4

0

0

0

0
4

0

0

0
4

0
4

0

0

0

0
4

0
4

0

0

0

0
4

0
4

0

0

0

0
4

0
4

0

0

0

0
4

0
4

0

0

0

0
4

0
4

0

0

0

0
4

0
4

0

0

0

0
4

0
4

0

0

0

0
4

0
4

0

0

0

0
4

0
4

0

0

0

0
4

0
4

0
3

0
3

0
3

0

0

0

0
4

0
4

0

0

0

0
4

0
4

0

0

0

0
4

0
4

0

0

0

0
4

0
4

0

0

0

0
4

0
4

0

0

0

0
4

0
4

0

0

0

0
4

0
4

0

0

0

0
4

0
4

0

0

0

0
4

0
4

0

0

0

0
4

0
4

0

0

0

0
4

0
4

0

0

0

0

0
4

0
4

0

0

0

0
4

0
4

0

0

0

0
4

0
4

0

0

0

0
4

0
4

0

0

0

0
4

0
4

0

0

0

0
4

0
4

0

0

0

0
4

0
4

0

0

0

0
4

0
4

0

0

0

0
4

0
4

0

0

0

0
4

0
4

0

0

0

0
4

0
4

0

0

0

0

0

0

0
4

0
4

0

0

0

0
4

0
4

0
7

0
7

0

0

0
4

0
4

0

0

0

0
4

0
4

0
2

0
2

0

0

0
4

0
4

0.787264

0.76646

0
2

0
2

0

0

0.0765318
3

0.0110946
6

0.0829095

0

0

0
2

0

0
7

0

0.0970604
7

0

0

0

0.00443786

0

0

0

0

0

0

0.175825
7

0.10842

0

0

0

0

0

0

0

0

0

0.0829095

0

0

0

0

0

0

0

0

0

0

0.0177514
7

0

0

0

0

0

0

0

0

0

0

0

0.00665242

0

0.0199573

0

0

0.0127553

0

0

0

0

0.019133
2

0

0

0

0.0510212

0
5

2.84494650060196e-16

0
4

0.0208048
2

0.0208048
2

0

0

0

0

0

0
4

0

0

0

0
4

0

0

0
4

0

0

0
4

0
2

0
2

0

0

0
4

0

0

0

0

0
4

0
2

0

0

0

0
4

0

0

0
4

0

0

0
4

0

0

0

0
4

0

0

0
4

0

0

0

0
4

2.77555756156289e-17

0
4

0
4

0
4

0
4

0

0
4

0
7

0
7

0
7

0

0

0

0

0

0
4

0

0

0
4

0

0

0

0
4

0

0

0
4

0

0

0
4

0

0

0
4

0

0

0
4

0

0

0
4

0

0

0
4

0

0

0
4

0
4

0.10842

0.10842

0.0318883

0

0

0

0

0.0765318

0

0

0

0

0

0

0
4

0
3

0
3

0

0
4

0

0

0
4

0
4

0.184952

0.172197

0.172197

0
4

0

0

0
4

0

0

0
4

0

0

0
4

0

0

0
4

0

0

0
4

0.0127553

0.0127553

0
4

0
4

0

0

0

0

0

0

0

0

0

0

0

0

0

0

0

0

0

0
4

0

0

0
4

0

0

0
4

0

0

0
4

0

0

0
4

0
4

0
3

0
3

0
3

0
4

0
3

0

0
3

0
4

0

0

0

0

0

0
3

0

0

0

0

0

0

0

0

0

0

0
3

0

0

0

0

0

0

0

0

0

0

0
3

0

0

0

0

0

0

0

0
3

0
3

0
3

0
3

0

0
4

0
2

0
2

0

0

0

0

0
4

0

0

0
4

0

0

0
4

0

0

0
4

0

0

0
4

0
2

0

0

0

0

0

0

0
4

0

0

0

0
4

0

0

0
4

0

0

0
4

0

0

0

0
4

0

0

0

0
4

0

0

0

0
4

0

0

0
4

0
4

0.0377218
3

0.0377218
3

0
2

0

0

0.0377218

0

0

0

0

0

0

0

0
2

0

0

0

0

0

0

0

0

0

0

0
2

0

0
2

0

0

0

0
4

0

0

0

0
4

0

0

0
4

0

0

0
4

0

0

0
4

0

0

0
4

0

0

0
4

0

0

0
4

0

0

0
4

0

0

0
4

0
4

1.33983

1.33983

0
2

1.31986

0.0199704

0
4

0

0

0

0

0

0
4

0

0

0

0

0
4

0

0

0

0
4

0

0

0
4

0

0

0
4

0

0

0
4

0
4

0

0

0

0
4

0

0

0
4

0

0

0
4

0
4

0
4

0.00997863
2

0.00997863
2

0

0

0

0

0

0

0
4

0
2

0
2

0
4

0.00997863

0.00997863

0
4

0

0

0
4

0

0

0
4

0
4

0

0

0

0
4

0
4

0
4

3.92033
4

0

0

0

0

0
4

0
4

0
2

0
2

0
2

0

0
2

0

0

0

0

0

0
4

0

0

0
4

0

0

0

0

0
4

0

0

0

0
4

0
4

1.08867
4

1.08867
4

1.07592
7

0

0

0

0
7

0

0.0127553

0

0

0

0

0
4

0

0

0

0

0

0

0

0

0

0

0

0

0

0

0

0
4

0

0

0

0

0
4

0

0

0

0

0
4

0

0

0

0
4

0

0

0
4

0

0

0
4

0
4

0
7

0
7

0

0

0

0

0
4

0

0

0

0
4

0
4

0

0

0

0
4

0
4

0

0

0

0
4

0
4

0

0

0

0
4

0
4

0

0

0

0
4

0
4

0

0

0

0
4

0
4

0

0

0

0
4

0
4

0

0

0

0
4

0
4

0

0

0

0
4

0
4

0

0

0

0
4

0
4

0

0

0

0
4

0
4

0.293372
5

0.293372
7

0.293372
7

0
4

0

0

0
4

0

0

0
4

0
4

0

0

0

0
4

0
4

0

0

0

0
4

0
4

0

0

0

0
4

0
4

0

0

0

0
4

0
4

0

0

0

0
4

0
4

0

0

0

0
4

0
4

0

0

0

0
4

0
4

0

0

0

0
4

0
4

0

0

0

0
4

0
4

0

0

0

0
4

0
4

0.248728
7

0.248728
7

0.248728
7

0

0

0

0

0

0

0

0
4

0
4

0

0

0

0
4

0
4

0

0

0

0
4

0
4

0

0

0

0
4

0
4

0

0

0

0
4

0
4

0

0

0

0
4

0
4

0

0

0

0
4

0
4

0

0

0

0
4

0
4

0

0

0

0
4

0
4

0

0

0

0
4

0
4

0

0

0

0
4

0
4

0.0382659

0.0382659
7

0

0

0.0382659

0

0
4

0

0

0

0

0
4

0

0

0
4

0

0

0
4

0
4

0

0

0

0
4

0
4

0

0

0

0
4

0
4

0

0

0

0
4

0
4

0

0

0

0
4

0
4

0

0

0

0
4

0
4

0

0

0

0
4

0
4

0

0

0

0
4

0
4

0

0

0

0
4

0
4

0

0

0

0
4

0
4

0

0

0

0
4

0
4

0

0

0

0

0

0

0
4

0

0

0

0
4

0

0

0

0
4

0

0

0
4

0

0

0
4

0

0

0
4

0

0

0
4

0
4

0

0

0

0
4

0
4

0

0

0

0
4

0
4

0

0

0

0
4

0
4

0

0

0

0
4

0
4

0

0

0

0
4

0
4

0

0

0

0
4

0
4

0

0

0

0
4

0
4

0

0

0

0
4

0
4

0

0

0

0
4

0
4

0

0

0

0
4

0
4

0

0

0

0

0

0
4

0
4

0

0

0

0
4

0
4

0

0

0

0
4

0
4

0
7

0
7

0
7

0

0
4

0

0

0
4

0

0

0
4

0
4

0.0472521
5

0.0472521
7

0.0127553

0.0344968

0

0

0
4

0

0

0
4

0

0

0
4

0
4

0

0

0

0

0
4

0

0

0

0
4

0

0

0

0
4

0

0

0
4

0
4

0

0

0

0

0

0

0
4

0

0

0

0
4

0
4

0

0

0

0

0

0

0

0

0

0

0

0

0

0

0

0

0

0

0

0

0
4

0

0

0

0

0

0

0
4

0
4

0

0

0

0

0
4

0
4

0.0127553

0.0127553

0.0127553

0

0

0

0
4

0

0

0
4

0
4

0
4

0

0

0

0
4

0

0

0
4

0

0

0
4

0
4

0

0

0

0

0

0

0
4

0

0

0

0
4

0
4

0
3

0
3

0
3

0

0
4

0

0

0
4

0
4

0

0

0

0
4

0
4

0
2

0

0

0
4

0

0

0
4

0

0

0
4

0
4

0

0

0

0

0
4

0

0

0
4

0
4

0
6

0

0

0

0

0
4

0

0

0

0
4

0
4

0

0

0

0

0

0
4

0
4

0
4

0
4

0
4

0

0

0

0
4

0

0

0

0

0

0

0

0
4

0

0

0
4

0
4

0

0

0

0

0
4

0
4

0

0

0

0

0

0
4

0

0

0

0
4

0
4

0
5

0
5

0
5

0
4

0
4

0.102042

0.102042

0.102042

0
4

0

0

0
4

0

0

0
4

0

0

0
4

0

0

0
4

0
4

0
2

0

0

0

0

0
4

0

0

0
4

0

0

0
4

0
4

0

0

0

0
4

0
4

0

0

0

0

0
4

0

0

0
4

0

0

0
4

0

0

0
4

0
4

0

0

0

0

0
4

0
4

0

0

0

0

0

0
4

0
4

0

0

0

0
4

0
4

0.0389228
4

0

0

0

0

0

0

0

0

0

0
4

0.0389228

0.0389228

0

0

0

0
4

0

0

0

0

0
4

0

0

0
4

0

0

0
4

0
4

0

0

0

0
4

0
4

0

0

0

0

0
4

0

0

0
4

0
4

0

0

0

0

0
4

0
4

0

0

0

0

0
4

0
4

0

0

0

0

0
4

0

0

0

0
4

0
4

0.0290597

0.0290597

0.0134122

0.00447073

0.0111768

0
4

0
4

0

0

0

0

0
4

0

0

0
4

0
4

0

0

0

0
4

0
4

0

0

0

0
4

0

0

0

0
4

0
4

0

0

0

0

0
4

0
4

0.0573989
6

0.0573989
6

0.0573989
6

0

0

0
4

0
4

0

0

0

0

0
4

0
4

0.0255106

0.0255106

0.0255106

0
4

0
4

0

0

0

0
4

0

0

0
4

0
4

0

0

0

0
4

0
4

0

0

0

0
4

0
4

0

0

0

0

0

0
4

0
4

0

0

0

0
4

0

0

0
4

0
4

0

0

0

0

0
4

0

0

0
4

0
4

0

0

0

0

0
4

0
4

0

0

0

0
4

0

0

0
4

0

0

0
4

0
4

0.0221459
3

0.0221459

0.0147581

0.00738779
3

0

0

8.67361737988404e-19

0
4

0

0

0

0
4

0

0

0
4

0

0

0
4

0
4

0

0

0

0

0
4

0

0

0
4

0
4

0

0

0

0
4

0
4

0

0

0

0

0
4

0

0

0
4

0
4

0

0

0

0
4

0
4

0.114798

0.0446436

0.0446436

0
4

0.0701542

0.0701542

0
4

0
4

0

0

0

0
4

0
4

0

0

0

0
4

0

0

0
4

0
4

0

0

0

0
4

0

0

0
4

0
4

0

0

0

0
4

0

0

0
4

0
4

0

0

0

0
4

0
4

0.114798
6

0.114798
6

0.114798
6

0

0
4

0
4

0

0

0

0
4

0
4

0

0

0

0

0
4

0
4

0

0

0

0
4

0

0

0
4

0
4

0

0

0

0

0
4

0
4

0.0318883

0.0318883

0.0318883

0
4

0
4

0

0

0

0
4

0

0

0
4

0
4

0

0

0

0
4

0

0

0
4

0
4

0.0255106

0.0255106

0

0.0255106

0
4

0
4

0

0

0

0
4

0
4

0

0

0

0
4

0
4

0.0491644
2

0.0491644
2

0.0426092

0.00655526
2

0

0

0

0

0

0
4

0

0

0
4

0
4

0

0

0

0

0
4

0
4

0

0

0

0

0
4

0
4

0

0

0

0
4

0
4

0

0

0

0
4

0
4

0

0

0

0
4

0
4

0

0

0

0
4

0
4

0

0

0

0
4

0
4

0

0

0

0
4

0
4

0

0

0

0
4

0
4

0

0

0

0
4

0
4

0.0235708

0.00443786

0.00443786
6

0

0

0

0

0
4

0.019133

0.019133

0

0

0
4

0

0

0
4

0

0

0
4

0
4

0

0

0

0
4

0
4

0

0

0

0
4

0
4

0

0

0

0
4

0
4

0

0

0

0
4

0
4

0

0

0

0
4

0
4

0

0

0

0
4

0
4

0

0

0

0
4

0
4

0

0

0

0
4

0
4

0

0

0

0
4

0
4

0

0

0

0
4

0
4

1.55648

1.46048

0

1.23089

0.197707
2

0
3

0
3

0

0

0

0

0

0

0

0

0

0

0
3

0

0

0

0

0

0

0

0

0

0

0

0

0

0

0

0

0.0127553

0

0

0

0

0

0

0.019133

0

0

0

0

0

0

0

0

0

0

0

0

0

0

0

0
4

0

0

0

0
4

0

0

0
4

0

0

0

0
4

0

0

0
4

0

0

0
4

0

0

0

0
4

0

0

0
4

0

0

0
4

0

0

0
4

0

0

0

0
4

0

0

0
4

0

0

0
4

0

0

0
4

0

0

0
4

0

0

0
4

0

0

0
4

0

0

0
4

0

0

0
4

0.00670609

0.00670609

0
4

0

0

0
4

0

0

0
4

0

0

0
4

0

0

0

0

0

0

0
4

0

0

0
4

0

0

0
4

0

0

0
4

0

0

0
4

0

0

0
4

0

0

0
4

0

0

0
4

0

0

0
4

0

0

0
4

0

0

0
4

0
3

0
3

0
4

0

0

0
4

0

0

0
4

0

0

0
4

0

0

0
4

0

0

0
4

0

0

0
4

0

0

0
4

0

0

0

0
4

0

0

0
4

0.0892871

0.0892871

0

0
4

0

0

0

0
4

0

0

0

0

0
4

1.38777878078145e-16

0
4

0
4

0

0

0

0

0

0

0
4

0

0

0
4

0
4

0
4

2.36989
3

0.132681
3

0
3

0

0

0

0

0

0

0

0

0

0

0

0

0

0

0

0
4

0.102042
3

0
3

0
3

0

0

0
3

0

0

0
3

0

0

0

0
3

0

0
2

0

0

0

0

0

0.102042

0

0

0
3

0

0

0

0

0

0

0

0

0

0

0
3

0

0

0

0

0

0

0

0

0

0

0
3

0

0

0

0

0

0

0

0

0

0

0
3

0

0

0

0

0

0

0

0

0

0

0
3

0

0

0

0

0

0

0

0

0

0

0
3

0

0

0

0

0

0

0

0

0

0

0
2

0

0

0

0

0

0

0

0
4

0
3

0
3

0

0

0

0

0

0

0

0

0

0

0
3

0

0

0

0

0

0

0

0

0

0
3

0
3

0
2

0

0

0

0

0
4

0
2

0
3

0

0

0

0

0

0

0
4

0

0

0

0
4

0

0

0
4

0

0

0
4

0

0

0
4

0

0

0

0
4

0

0

0
4

0

0

0
4

0.0127553

0.0127553

0
4

0

0

0
4

0

0

0
4

0
2

0
3

0

0

0

0

0

0
4

0.0134122

0.0134122

0
4

0

0

0
4

0

0

0
4

0

0

0
4

0

0

0
4

0

0

0
4

0

0

0
4

0

0

0
4

0

0

0
4

0

0

0
4

0

0

0
4

0

0

0
4

0

0

0
4

0.00447073

0.00447073

0
4

0

0

0
4

0

0

0
4

0

0

0
4

0

0

0
4

0

0

0

0
4

0

0

0

0
4

0

0

0

0

0

0

0

0

0
4

0

0

0

0
4

0

0

0

0
4

0

0

0

0

0
4

0
4

1.41511
3

0.114004
3

0.114004
3

0

0

0

0

0

0

0

0

0
4

0
2

0

0

0

0

0

0

0

0

0

0

0
4

0

0

0

0

0
4

0.0447073

0

0.0447073

0
4

0

0

0

0
4

1.1416
2

0.338015
3

0
2

0
3

0

0

0

0

0

0

0

0

0

0

0

0

0

0

0

0

0

0

0

0

0

0
2

0

0

0

0

0

0

0

0

0

0

0
2

0

0

0

0

0

0

0

0

0

0

0
3

0

0

0

0

0

0

0

0

0

0.644143

0

0

0

0

0

0

0

0

0

0

0

0
2

0

0

0

0

0

0

0

0

0

0

0

0

0

0

0

0

0

0

0

0

0

0
2

0

0

0

0

0

0

0

0

0

0

0

0

0

0

0

0

0

0

0

0

0

0
3

0

0

0

0

0

0

0

0

0

0

0

0
2

0

0

0

0

0

0
2

0
2

0

0

0

0

0

0

0

0

0
2

0

0

0

0

0
2

0

0

0

0

0

0

0

0

0

0

0
3

0

0

0

0

0

0

0

0

0

0

0
2

0

0

0

0

0

0

0

0

0

0

0
3

0

0

0

0

0

0

0

0

0

0

0
2

0

0

0

0

0

0

0

0

0.121175

0

0

0.0382659

0

0

0

0

0

0

0

0

0

1.38777878078145e-16
2

0
4

0.0510212
3

0.0510212
3

0

0

0

0

0
4

0
3

0

0

0

0

0

0

0

0

0

0

0

0

0

0
4

0

0

0

0

0
4

0

0

0
4

0

0

0
4

0

0

0
4

0

0

0

0
4

0

0

0
4

0

0

0
4

0

0

0
4

0.0637765

0.0637765

0
4

0

0

0
4

0

0

0
4

0

0

0
4

6.93889390390723e-17
3

0
4

0.813163

0.451543

0.152005

0.0536487

0.00447073

0.241419

0

0

0

0

2.77555756156289e-17

0
4

0

0

0

0

0

0

0

0

0

0

0

0

0

0

0

0

0

0

0

0

0

0

0

0

0

0

0

0

0

0

0

0

0

0

0

0

0

0

0

0

0

0

0

0

0

0

0

0

0

0

0

0

0

0

0

0

0

0

0

0

0

0

0

0

0

0

0

0

0

0

0

0

0

0

0

0

0

0

0

0

0

0

0

0

0

0
4

0.121175

0.121175
7

0

0

0
4

0

0

0
4

0

0

0
4

0.00447073

0.00447073

0
4

0

0

0
4

0

0

0
4

0

0

0
4

0

0

0
4

0

0

0
4

0

0

0
4

0

0

0
4

0

0

0
4

0

0

0

0

0
4

0.140308
7

0.140308
7

0
4

0

0

0

0
4

0.0956648

0.0956648

0
4

0

0

0

0

0
4

0

0

0
4

0

0

0
4

2.77555756156289e-17

0
4

0
3

0
3

0
3

0

0
3

0
2

0
3

0

0

0

0

0

0
4

0
3

0
3

0

0
2

0

0

0

0

0

0

0

0
4

0

0

0

0

0
4

0
3

0

0

0

0

0

0

0

0
4

0

0

0

0

0

0
4

0

0

0

0

0
4

0
2

0

0

0

0

0
4

0

0

0

0
4

0

0

0

0
4

0
4

0

0

0

0
4

0

0

0

0
4

0
4

0

0

0

0

0
4

0

0

0
4

0
4

0

0

0

0
4

0
4

0

0

0

0
4

0
4

0

0

0

0

0
4

0
4

0

0

0

0
4

0
4

0

0

0

0
4

0
4

0

0

0

0
4

0
4

0

0

0

0

0
4

0
4

0

0

0

0
4

0
4

0

0

0

0

0

0

0

0

0

0

0

0

0

0

0

0

0

0

0

0

0

0

0

0

0
4

0

0

0

0

0
4

0

0

0
4

0
4

0

0

0

0
4

0
4

0

0

0

0
4

0
4

0.00894146

0.00894146

0.00894146

0
4

0
4

0

0

0

0
4

0
4

0

0

0

0
4

0
4

0

0

0

0
4

0
4

0

0

0

0
4

0
4

0

0

0

0
4

0
4

0

0

0

0
4

0
4

0

0

0

0
4

0
4

0

0

0

0

0

0

0

0

0

0

0

0
4

0

0

0

0

0

0
4

0
4

0

0

0

0
4

0
4

0

0

0

0
4

0
4

0

0

0

0
4

0
4

0

0

0

0
4

0
4

0
3

0
3

0
3

0

0

0

0

0

0

0

0

0
4

0
3

0
3

0

0
4

0

0

0
4

0

0

0
4

0

0

0
4

0
4

0
3

0
3

0

0

0

0

0

0

0

0
4

0
3

0

0

0

0

0
4

0

0

0
4

0

0

0

0
4

0
4

0
2

0
2

0

0

0

0

0

0

0
4

0

0

0

0

0
4

0

0

0

0
4

0

0

0
4

0

0

0
4

0

0

0
4

0
4

0

0

0

0

0

0

0

0
4

0

0

0

0

0

0
4

0

0

0
4

0

0

0
4

0
4

0

0

0

0

0
4

0

0

0

0
4

0

0

0
4

0
4

0

0

0

0
4

0

0

0
4

0
4

0

0

0

0
4

0
4

0
4

0.795295

0

0

0

0

0

0
4

0

0

0
4

0

0

0
4

0

0

0
4

0
4

0

0

0

0
4

0

0

0
4

0

0

0

0

0

0

0
4

0

0

0

0

0

0

0

0

0
4

0
4

0

0

0

0

0

0

0

0
4

0

0

0

0

0
4

0

0

0

0
4

0

0

0
4

0

0

0
4

0
4

0
5

0
5

0
5

0

0
4

0
4

0.612254

0.395414

0.280617

0.114798

0
4

0
7

0
7

0
4

0
7

0
7

0
4

0.10842
2

0.0765318
2

0

0

0

0

0

0

0.0318883

0

0

0

0

0

0

0

6.93889390390723e-18
2

0
4

0

0

0

0

0

0
4

0

0

0
4

0

0

0
4

0

0

0

0

0
4

0

0

0
4

0

0

0

0

0
4

0

0

0

0

0
4

0

0

0
4

0

0

0

0
4

0

0

0
4

0
2

0
2

0

0

0

0

0

0

0
4

0

0

0

0
4

0

0

0
4

0

0

0

0
4

0

0

0
4

0

0

0
4

0

0

0

0
4

0

0

0

0
4

0

0

0

0
4

0

0

0
4

0

0

0
4

0
2

0
2

0

0

0
4

0

0

0
4

0

0

0
4

0

0

0
4

0

0

0
4

0

0

0
4

0

0

0
4

0

0

0
4

0

0

0
4

0

0

0
4

0

0

0
4

0.10842
2

0.10842

0

0

0

0

0

0

0

0
4

0

0

0
4

0

0

0
4

0

0

0
4

0

0

0
4

0

0

0
4

0

0

0
4

0

0

0
4

0

0

0
4

0

0

0
4

0

0

0
4

0
2

0

0

0

0

0

0

0
4

0

0

0

0
4

0

0

0

0

0
4

0
2

0
2

0
4

0

0

0

0

0

0

0
4

0
4

0.106509
6

0.106509
6

0.106509
6

0
4

0

0

0
4

0
4

0

0

0

0
4

0
4

0

0

0

0
4

0
4

0

0

0

0
4

0
4

0

0

0

0
4

0
4

0.0765318
7

0.0765318
7

0.0765318
7

0
4

0
4

0

0

0

0
4

0
4

0

0

0

0

0

0
4

0
4

0

0

0

0

0
4

0

0

0
4

0
4

0

0

0

0
4

0
4

0

0

0

0

0
4

0
4

0

0

0

0
4

0
4

0

0

0

0
4

0
4

0
4

2.14584
6

2.14584
6

1.82208
7

0.848171
7

0
6

0

0

0

0

0

0
6

0.102042
6

0
7

0.00689936

0.414547
7

0

0.0448458
6

0

0.0226535

0.0547016
6

0

0

0

0.0127553

0

0

0

0

0

0.0110946

0.0302276
7

0

0

0

0.00447073

0

0

0

0

0

0

0.0640556
6

0.138975

0

0

0

0

0

0

0

0

0

0
6

0

0

0

0

0

0

0.00894146

0

0

0

0.0352388
6

0

0

0.00665679

0

0

0

0

0

0

0

0
6

0

0

0

0

0

0

0

0

0

0

0.00447073
7

0

0

0

0

0

0

0

0

0

0

0.00689936
6

0.00443786

0

0

0

0

0

0

0

0

0

0
4

0.272732
6

0.259976
6

0

0

0

0

0

0

0.0127553

1.90819582357449e-17
6

0
4

0.0255106
6

0

0

0.0255106

0

0

0

0

0

0
4

0.0255106
7

0.0127553
7

0

0

0

0

0

0.0127553

0
4

0
6

0

0

0

0

0

0

0
4

0

0

0

0

0
4

0

0

0

0

0

0
4

0

0

0

0

0
4

0

0

0
4

6.93889390390723e-18
6

0
4

0
4

0

0

0

0

0

0

0

0
5

0

0

0

0

0

0

0

0
4

0
2

0
2

0

0
4

0

0

0

0

0

0

0
4

0

0

0

0

0
4

0
4

0
7

0
7

0

0

0
4

0

0

0

0
4

0
4

0

0

0

0

0
4

0

0

0
4

0
4

0

0

0

0

0

0

0
4

0
4

0
4

0

0

0

0

0
4

0

0

0
4

0
4

0
4

0

0

0

0

0
4

0
4

0
4

0

0

0

0

0
4

0

0

0
4

0
4

0
4

0

0

0

0

0
4

0

0

0
4

0

0

0
4

0
4

0

0

0

0
4

0
4

0
4

0.669653

0.669653

0.669653

0.669653

0
4

0

0

0

0
4

0
4

0
4

0

0

0

0

0

0
4

0

0

0
4

0
4

0
4

0

0

0

0

0

0

0
4

0

0

0
4

0
4

0
4

0

0

0

0

0
4

0

0

0
4

0
4

0

0

0

0
4

0
4

0
4

0

0

0

0

0

0

0
4

0

0

0
4

0
4

0
4

0

0

0

0

0
4

0
4

0
4

0.0762747
6

0.0762747
6

0.055996
6

0.055996
6

0

0
4

0.0158079
6

0.00890859
7

0

0

0

0

0.00689936

0

0
4

0.00447073
6

0.00447073
7

0

0

0
4

0

0

0
4

0

0

0
4

9.54097911787244e-18
6

0
4

0
4

0

0

0

0

0

0
4

0
4

0
4

0

0

0

0

0
4

0
4

0
4

0

0

0

0

0
4

0
4

0
4

0

0

0

0

0
4

0

0

0
4

0
4

0
4

0

0

0

0

0

0
4

0

0

0
4

0
4

0
4

0

0

0

0

0
4

0
4

0
4

0

0

0

0

0
4

0

0

0
4

0
4

0

0

0

0
4

0
4

0
4

0

0

0

0

0

0

0
4

0
4

0
4

0

0

0

0

0
4

0

0

0
4

0
4

0
4

0.0399145

0.0399145

0.0399145

0.0399145

0

0
4

0
4

0
4

0
3

0
3

0
4

0
3

0

0

0

0

0

0

0

0

0

0

0
4

0
3

0

0

0

0

0
4

0

0

0
4

0
4

0
4

0

0

0

0

0
4

0
4

0
4

0

0

0

0

0
4

0
4

0
4

0

0

0

0

0
4

0

0

0
4

0

0

0
4

0
4

0
4

0

0

0

0

0
4

0

0

0
4

0
4

0
4

0

0

0

0

0

0
4

0
4

0
4

0

0

0

0

0
4

0

0

0
4

0
4

0
4

0

0

0

0

0
4

0
4

0
4

0

0

0

0

0
4

0
4

0
4

0

0

0

0

0
4

0

0

0
4

0
4

0
4

0

0

0

0

0
4

0
4

0
4

0.0435868

0.0308315

0.0239321

0.0127553
7

0

0.0111768

0

0

0

0

0

0

1.73472347597681e-18

0
4

0.00689936

0

0.00689936

0

0
4

0

0

0

0

0
4

0

0

0
4

0

0

0
4

0
4

0.0127553

0.0127553

0

0

0.0127553

0

0

0

0

0

0
4

0

0

0

0

0
4

0

0

0

0
4

0

0

0
4

0

0

0
4

0
4

0

0

0

0
4

0

0

0
4

0
4

0

0

0

0
4

0
4

0
4

0

0

0

0

0

0

0
4

0
4

0
4

0

0

0

0

0

0
4

0
4

0
4

0

0

0

0

0
4

0
4

0
4

0

0

0

0

0
4

0

0

0
4

0
4

0
4

0

0

0

0

0
4

0
4

0
4

0

0

0

0

0
4

0
4

0
4

0

0

0

0

0
4

0

0

0
4

0
4

0

0

0

0
4

0
4

0
4

0

0

0

0

0
4

0
4

0
4

0

0

0

0

0
4

0

0

0
4

0
4

0

0

0

0
4

0
4

0
4

0

0

0

0

0

0
4

0
4

0
4

0.102042

0.102042

0.102042

0.102042

0

0

0

0

0
4

0
5

0
5

0

0

0
4

0

0

0

0
4

0
4

0
4

0

0

0

0

0

0
4

0

0

0
4

0
4

0
4

0

0

0

0

0
4

0
4

0
4

0

0

0

0

0
4

0

0

0
4

0
4

0
4

0

0

0

0

0

0

0
4

0
4

0
4

0

0

0

0

0
4

0
4

0
4

0

0

0

0

0
4

0

0

0
4

0
4

0
4

0

0

0

0

0

0
4

0
4

0
4

0

0

0

0

0
4

0

0

0
4

0
4

0

0

0

0
4

0
4

0
4

0.102042

0.102042

0.102042

0.102042

0
4

0
4

0
4

0

0

0

0

0
4

0
4

0
4

0

0

0

0

0

0

0

0

0
4

0

0

0

0

0

0
4

0
4

0
4

0

0

0

0

0

0
4

0

0

0
4

0
4

0
4

0

0

0

0

0
4

0

0

0
4

0
4

0

0

0

0
4

0
4

0
4

0

0

0

0

0
4

0

0

0
4

0
4

0
4

0

0

0

0

0
4

0

0

0
4

0
4

0
4

0

0

0

0

0

0
4

0
4

0
4

0

0

0

0

0

0
4

0
4

0
4

0

0

0

0

0

0
4

0
4

0
4

0

0

0

0

0
4

0
4

0
4

0

0

0

0

0
4

0

0

0
4

0
4

0
4

0

0

0

0

0
4

0
4

0
4

0.0700058
6

0.0700058
6

0.0655679
6

0.0127553
6

0.00665242

0

0

0

0.00894146
7

0.0199704
6

0

0.0172484

0

0

0

0

6.93889390390723e-18
6

0
4

0.00443786

0.00443786

0
4

1.73472347597681e-18
6

0
4

0
4

0

0

0

0

0
4

0

0

0
4

0
4

0
4

0

0

0

0

0
4

0

0

0
4

0
4

0
4

0

0

0

0

0
4

0
4

0
4

0

0

0

0

0
4

0
4

0
4

0

0

0

0

0
4

0

0

0
4

0
4

0
4

0

0

0

0

0

0
4

0
4

0
4

0

0

0

0

0

0
4

0
4

0
4

0

0

0

0

0
4

0
4

0
4

0

0

0

0

0
4

0
4

0
4

0

0

0

0

0
4

0

0

0
4

0
4

0
4

0.0645197
7

0.0645197
7

0.0645197
7

0.0645197
7

0

0

0
4

0
4

0

0

0

0
4

0

0

0
4

0
4

0
4

0

0

0

0

0
4

0

0

0
4

0
4

0
4

0

0

0

0

0

0
4

0
4

0
4

0

0

0

0

0
4

0
4

0
4

0

0

0

0

0
4

0

0

0
4

0
4

0
4

0

0

0

0

0
4

0
4

0
4

0.0765318

0.0765318

0.0765318

0.0765318

0
4

0
4

0
4

0

0

0

0

0
4

0

0

0
4

0
4

0
4

0

0

0

0

0
4

0

0

0
4

0
4

0
4

0

0

0

0

0

0
4

0
4

0
4

0

0

0

0

0

0
4

0
4

0
4

0

0

0

0

0

0

0

0

0

0

0
4

0

0

0
4

0
4

0
4

0

0

0

0

0

0
4

0
4

0
4

0

0

0

0

0
4

0
4

0
4

0

0

0

0

0
4

0

0

0
4

0
4

0
4

0

0

0

0

0
4

0

0

0
4

0
4

0
4

0

0

0

0

0
4

0

0

0
4

0
4

0
4

0

0

0

0

0
4

0

0

0
4

0
4

0
4

0

0

0

0

0
4

0

0

0
4

0
4

0
4

0

0

0

0

0

0
4

0
4

0
4

0

0

0

0

0
4

0
4

0
4

0

0

0

0

0

0
4

0
4

0
4

0.0520313

0.0520313

0.00738779
5

0

0.00738779

0

0

0

0

0
4

0.0446436

0

0.0446436

0

0
4

0
3

0

0

0
4

0

0

0
4

0

0

0
4

0
4

0

0

0

0

0

0
4

0

0

0

0
4

0
4

0
4

0

0

0

0

0
4

0
4

0
4

0

0

0

0

0

0
4

0
4

0
4

0

0

0

0

0
4

0

0

0
4

0
4

0
4

0

0

0

0

0
4

0

0

0
4

0
4

0
4

0

0

0

0

0
4

0
4

0
4

0

0

0

0

0
4

0
4

0
4

0

0

0

0

0
4

0
4

0
4

0

0

0

0

0
4

0

0

0
4

0
4

0
4

0

0

0

0

0
4

0
4

0
4

0

0

0

0

0
4

0
4

0
4

0.759674

0.759674

0.754749

0.550252

0.0338079

0.00520121

0

0

0.0049252

0

0

0.00985039

0

0

0.0940726

0

0

0

0

0

0

0

0

0

0

0
3

0

0

0

0.0221634

0

0

0

0

0.0049252

0.0172382
3

0.012313
3

0
3

0

0

0

1.23165366794353e-16

0
4

0

0

0

0
4

0

0

0

0

0

0
4

0

0

0
4

0

0

0

0
4

0.0049252

0.0049252

0
4

0

0

0
4

0

0

0
4

0
4

0
4

0.0780287
7

0.0780287
7

0.0780287
7

0.0729946
7

0.00503411
7

0

0

0

0

0
4

0

0

0
4

0

0

0

0
4

0

0

0
4

0
4

0
4

0

0

0

0

0
4

0
4

0
4

0.0382659

0.0382659

0

0

0
4

0.0382659

0.0382659

0
4

0
4

0
4

0.0637765

0.0637765

0.0637765

0.0637765

0
4

0
4

0
4

0

0

0

0

0
4

0
4

0
4

0

0

0

0

0

0
4

0
4

0
4

0

0

0

0

0
4

0
4

0
4

0

0

0

0

0
4

0
4

0
4

0

0

0

0

0
4

0
4

0
4

0

0

0

0

0
4

0
4

0
4

0

0

0

0

0
4

0
4

0
4

0

0

0
2

0
2

0

0

0
4

0
5

0
5

0

0
4

0

0

0

0

0
4

0
4

0

0

0

0
4

0
4

0
4

0.0648255

0.0648255

0.0648255

0.0156475

0.049178

0
4

0
4

0
4

0.0255106

0.0255106

0.0255106

0.0255106

0
4

0
4

0
4

0

0

0

0

0
4

0

0

0
4

0
4

0
4

0

0

0

0

0

0
4

0
4

0
4

0

0

0

0

0
4

0
4

0
4

0

0

0

0

0
4

0
4

0
4

0

0

0

0

0
4

0
4

0
4

0

0

0

0

0
4

0
4

0
4

0

0

0

0

0
4

0
4

0
4

0.0127553

0.0127553

0.0127553

0.0127553

0
4

0
4

0
4

0

0

0

0

0

0

0

0

0

0

0
4

0
4

0
4

0

0

0

0

0
4

0
4

0
4

0

0

0

0

0
4

0
4

0
4

0

0

0

0

0
4

0
4

0
4

0

0

0

0

0
4

0
4

0
4

0

0

0

0

0
4

0
4

0
4

0

0

0

0

0
4

0
4

0
4

0

0

0

0

0
4

0
4

0
4

0

0

0

0

0
4

0
4

0
4

0

0

0

0

0
4

0
4

0
4

0.0255106

0.0255106

0.0255106

0.0255106

0
4

0
4

0
4

0.168639
6

0.168639
6

0.146449
6

0.146449
6

0

0
4

0.0177514
6

0

0.0177514

0

0
4

0.00443786

0

0.00443786

0
4

0

0

0
4

1.73472347597681e-18
6

0
4

0
4

0

0

0

0

0
4

0
4

0
4

0

0

0

0

0
4

0
4

0
4

0

0

0

0

0
4

0
4

0
4

0

0

0

0

0
4

0
4

0
4

0

0

0

0

0
4

0
4

0
4

0

0

0

0

0
4

0
4

0
4

0

0

0

0

0
4

0
4

0
4

0

0

0

0

0
4

0
4

0
4

0

0

0

0

0
4

0
4

0
4

0

0

0

0

0
4

0
4

0
4

0

0

0

0

0

0

0

0
4

0

0

0
4

0
4

0

0

0

0

0
4

0

0

0
4

0
4

0
4

0

0

0

0

0
4

0
4

0
4

0

0

0

0

0
4

0
4

0
4

0

0

0

0

0
4

0
4

0
4

0

0

0

0

0
4

0
4

0
4

0

0

0

0

0
4

0
4

0
4

0

0

0

0

0
4

0
4

0
4

0

0

0

0

0
4

0
4

0
4

0

0

0

0

0
4

0
4

0
4

0

0

0

0

0
4

0
4

0
4

0

0

0

0

0
4

0
4

0
4

0

0

0
2

0
2

0

0

0

0
4

0

0

0
4

0

0

0

0

0
4

0

0

0
4

0

0

0
4

0
4

0
4

0

0

0

0

0
4

0
4

0
4

0

0

0

0

0
4

0
4

0
4

0

0

0

0

0
4

0
4

0
4

0

0

0

0

0
4

0
4

0
4

0

0

0

0

0
4

0
4

0
4

0

0

0

0

0
4

0
4

0
4

0

0

0

0

0
4

0
4

0
4

0

0

0

0

0
4

0
4

0
4

0

0

0

0

0
4

0
4

0
4

0

0

0

0

0
4

0
4

0
4

0.0562451
7

0.0562451
7

0.0562451
7

0.0562451
7

0
4

0

0

0
4

0
4

0
4

0

0

0

0

0
4

0
4

0
4

0

0

0

0

0
4

0
4

0
4

0

0

0

0

0
4

0
4

0
4

0

0

0

0

0
4

0
4

0
4

0

0

0

0

0
4

0
4

0
4

0

0

0

0

0
4

0
4

0
4

0

0

0

0

0
4

0
4

0
4

0

0

0

0

0
4

0
4

0
4

0

0

0

0

0
4

0
4

0
4

0

0

0

0

0
4

0
4

0
4

0.80647
7

0.80647
7

0.648317
7

0.427846

0.0679662

0.152505

2.77555756156289e-17
7

0
4

0.0621316

0.0338899

0.0112966

0.016945

3.46944695195361e-18

0
4

0.0960215

0.0960215

0
4

0
4

0
4

0

0

0

0

0
4

0
4

0
4

0

0

0

0

0
4

0
4

0
4

0

0

0

0

0
4

0
4

0
4

0

0

0

0

0
4

0
4

0
4

0

0

0

0

0
4

0
4

0
4

0

0

0

0

0
4

0
4

0
4

0

0

0

0

0
4

0
4

0
4

0

0

0

0

0
4

0
4

0
4

0

0

0

0

0
4

0
4

0
4

0

0

0

0

0
4

0
4

0
4

0

0

0

0

0

0
4

0

0

0

0
4

0

0

0
4

0
4

0

0

0

0

0

0

0

0
4

0
4

0

0

0

0

0
4

0
4

0
4

0

0

0

0

0
4

0
4

0
4

0

0

0

0

0
4

0
4

0
4

0

0

0

0

0
4

0
4

0
4

0

0

0

0

0
4

0
4

0
4

0

0

0

0

0
4

0
4

0
4

0

0

0

0

0
4

0
4

0
4

0

0

0

0

0
4

0
4

0
4

0

0

0

0

0
4

0
4

0
4

0

0

0

0

0
4

0
4

0
4

0

0

0

0

0
4

0
4

0
4

0

0

0

0

0

0

0

0
4

0
4

0
4

0

0

0

0

0
4

0
4

0
4

0

0

0

0

0
4

0
4

0
4

0

0

0

0

0
4

0
4

0
4

0

0

0

0

0
4

0
4

0
4

0

0

0

0

0
4

0
4

0
4

0

0

0

0

0
4

0
4

0
4

0

0

0

0

0
4

0
4

0
4

0

0

0

0

0
4

0
4

0
4

0

0

0

0

0
4

0
4

0
4

0

0

0

0

0
4

0
4

0
4

0.280617
7

0.280617
7

0.280617
7

0.0956648
7

0

0

0

0

0

0

0

0

0

0

0.0956648
7

0

0

0

0

0

0

0

0

0

0

0.0255106
6

0

0

0

0

0

0

0

0

0

0

0.019133
7

0

0

0

0

0

0

0

0

0

0.019133

0

0

0

0.0255106

0

0

2.77555756156289e-17
7

0
4

0
6

0

0

0

0

0

0

0

0
4

0
4

0
6

0
6

0

0

0

0

0

0

0
4

0

0

0

0

0
4

0

0

0
4

0
4

0
4

0.363526
4

0.363526
4

0.363526

0.318883

0

0.0446436

0

0

0

0

1.38777878078145e-17

0
4

0

0

0

0

0
4

0

0

0

0
4

0

0

0
4

0

0

0
4

0
4

0
4

0

0

0

0

0
4

0
4

0
4

0

0

0

0

0
4

0
4

0
4

0

0

0

0

0
4

0
4

0
4

0

0

0

0

0
4

0
4

0
4

0

0

0

0

0
4

0
4

0
4

0

0

0

0

0
4

0
4

0
4

0

0

0

0

0
4

0
4

0
4

0

0

0

0

0
4

0
4

0
4

0

0

0

0

0
4

0
4

0
4

0

0

0

0

0
4

0
4

0
4

0
4

0
4

0
4

0

0

0

0

0

0

0
4

0
4

0

0

0

0

0

0
4

0

0

0
4

0
4

0
4

0

0

0

0

0
4

0
4

0
4

0

0

0

0

0
4

0
4

0
4

0.0701542

0.0701542

0.0701542

0.0701542

0
4

0
4

0
4

0

0

0

0

0
4

0
4

0
4

0

0

0

0

0
4

0
4

0
4

0

0

0

0

0
4

0
4

0
4

0

0

0

0

0
4

0
4

0
4

0

0

0

0

0
4

0
4

0
4

0

0

0

0

0
4

0
4

0
4

0.114798

0.114798

0.114798

0.114798

0
4

0
4

0
4

0.00665242
6

0.00665242
7

0

0

0

0

0
4

0.00665242

0.00665242

0

0

0
4

0

0

0

0
4

0
4

0

0

0

0

0
4

0

0

0
4

0
4

0
4

0

0

0

0

0
4

0
4

0
4

0.0765318

0.0765318

0.0765318

0.0765318

0
4

0
4

0
4

0

0

0

0

0
4

0
4

0
4

0

0

0

0

0
4

0
4

0
4

0

0

0

0

0
4

0
4

0
4

0

0

0

0

0
4

0
4

0
4

0

0

0

0

0
4

0
4

0
4

0

0

0

0

0
4

0
4

0
4

0

0

0

0

0
4

0
4

0
4

0

0

0

0

0
4

0
4

0
4

0

0

0
6

0
6

0

0
4

0

0

0

0
4

0

0

0
4

0
4

0
4

0

0

0

0

0
4

0
4

0
4

0

0

0

0

0
4

0
4

0
4

0

0

0

0

0
4

0
4

0
4

0

0

0

0

0
4

0
4

0
4

0

0

0

0

0
4

0
4

0
4

0

0

0

0

0
4

0
4

0
4

0

0

0

0

0
4

0
4

0
4

0

0

0

0

0
4

0
4

0
4

0

0

0

0

0
4

0
4

0
4

0

0

0

0

0
4

0
4

0
4

0.00364411

0.00364411

0.00364411
5

0.00364411

0

0

0

0
4

0

0

0

0

0
4

0

0

0

0

0
4

0

0

0
4

0

0

0
4

0
4

0
4

0

0

0

0

0
4

0
4

0
4

0

0

0

0

0
4

0
4

0
4

0

0

0

0

0
4

0
4

0
4

0

0

0

0

0
4

0
4

0
4

0

0

0

0

0
4

0
4

0
4

0

0

0

0

0
4

0
4

0
4

0

0

0

0

0
4

0
4

0
4

0

0

0

0

0
4

0
4

0
4

0

0

0

0

0
4

0
4

0
4

0

0

0

0

0
4

0
4

0
4

0
4

0
4

0
4

0

0

0

0

0
4

0

0

0

0

0

0
4

0
4

0
4

0

0

0

0

0
4

0
4

0
4

0

0

0

0

0
4

0
4

0
4

0

0

0

0

0
4

0
4

0
4

0

0

0

0

0
4

0
4

0
4

0

0

0

0

0
4

0
4

0
4

0

0

0

0

0
4

0
4

0
4

0

0

0

0

0
4

0
4

0
4

0

0

0

0

0
4

0
4

0
4

0

0

0

0

0
4

0
4

0
4

0

0

0

0

0
4

0
4

0
4

0.0127553

0

0

0

0

0

0

0

0

0
4

0

0

0

0

0
4

0

0

0

0
4

0

0

0
4

0

0

0
4

0
4

0

0

0

0
4

0
4

0.0127553

0.0127553

0.0127553

0
4

0
4

0
4

0.0127553

0.0127553

0.0127553

0.0127553

0
4

0
4

0
4

0

0

0

0

0
4

0
4

0
4

0

0

0

0

0
4

0
4

0
4

0

0

0

0

0
4

0
4

0
4

0

0

0

0

0
4

0
4

0
4

0

0

0

0

0
4

0
4

0
4

0

0

0

0

0
4

0
4

0
4

0

0

0

0

0
4

0
4

0
4

0

0

0

0

0
4

0
4

0
4

0

0

0

0

0
4

0
4

0
4

0

0

0

0

0

0
4

0

0

0

0

0

0

0
4

0
4

0
4

0

0

0

0

0
4

0
4

0
4

0

0

0

0

0
4

0
4

0
4

0

0

0

0

0
4

0
4

0
4

0

0

0

0

0
4

0
4

0
4

0

0

0

0

0
4

0
4

0
4

0

0

0

0

0
4

0
4

0
4

0

0

0

0

0
4

0
4

0
4

0

0

0

0

0
4

0
4

0
4

0

0

0

0

0
4

0
4

0
4

0

0

0

0

0
4

0
4

0
4

0

0

0

0

0

0
4

0

0

0
4

0
4

0
4

0

0

0

0

0
4

0
4

0
4

0

0

0

0

0
4

0
4

0
4

0

0

0

0

0
4

0
4

0
4

0

0

0

0

0
4

0
4

0
4

0

0

0

0

0
4

0
4

0
4

0

0

0

0

0
4

0
4

0
4

0

0

0

0

0
4

0
4

0
4

0

0

0

0

0
4

0
4

0
4

0

0

0

0

0
4

0
4

0
4

0

0

0

0

0
4

0
4

0
4

0

0

0

0

0

0

0

0

0

0
4

0

0

0

0
4

0
4

0
4

0

0

0

0

0
4

0
4

0
4

0.0127553

0.0127553

0.0127553

0.0127553

0
4

0
4

0
4

0

0

0

0

0
4

0
4

0
4

0

0

0

0

0
4

0
4

0
4

0

0

0

0

0
4

0
4

0
4

0

0

0

0

0
4

0
4

0
4

0

0

0

0

0
4

0
4

0
4

0

0

0

0

0
4

0
4

0
4

0

0

0

0

0
4

0
4

0
4

0

0

0

0

0
4

0
4

0
4

0.019133

0.019133

0

0

0

0

0

0

0

0

0

0

0

0

0

0

0

0

0

0

0

0

0

0

0

0

0

0

0

0

0
4

0

0

0

0

0

0

0
4

0

0

0

0

0

0

0
4

0

0

0

0

0

0

0

0
4

0

0

0

0
4

0.019133

0.019133

0
4

0
4

0

0

0

0

0

0

0

0

0

0

0

0

0

0

0

0

0

0

0

0

0
4

0

0

0

0

0

0

0

0

0

0
4

0

0

0

0

0

0

0
4

0

0

0

0

0
4

0

0

0
4

0

0

0
4

0
4

0

0

0

0

0

0
4

0
4

0

0

0

0
4

0

0

0
4

0
4

0
4

0
6

0
6

0
6

0

0

0

0
4

0

0

0
4

0
4

0
4

0

0

0

0

0
4

0
4

0
4

0

0

0

0

0
4

0
4

0
4

0

0

0

0

0
4

0
4

0
4

0

0

0

0

0
4

0
4

0
4

0.0127553

0

0

0

0

0

0
4

0

0

0
4

0
4

0.0127553

0.0127553

0.0127553

0
4

0
4

0
4

0
6

0
6

0
6

0
6

0
4

0
4

0
4

0

0

0

0

0

0

0
4

0

0

0

0
4

0

0

0

0

0
4

0
4

0
4

0.0163881
7

0.0163881
7

0.0163881
7

0

0.0163881

0

0

0
4

0
4

0
4

0
4

0
4

0

0

0

0

0
4

0

0

0

0

0
4

0

0

0
4

0

0

0
4

0
4

0

0

0

0

0
4

0

0

0
4

0

0

0
4

0
4

0
4

0

0

0

0

0

0
4

0

0

0
4

0
4

0

0

0

0
4

0
4

0
4

0
3

0
3

0
3

0

0

0

0
4

0

0

0

0

0

0
4

0
4

0
4

0

0

0

0

0

0

0

0

0
4

0

0

0
4

0
4

0
4

0
3

0
3

0
3

0

0

0

0

0

0
4

0
4

0
4

0.931137
7

0.931137
7

0.931137
7

0.931137
7

0

0

0

0

0

0

0

0

0

0

0

0
4

0

0

0
4

0

0

0
4

0

0

0
6

0

0

0

0

0
4

0
6

0
6

0

0
4

0

0

0
4

0

0

0
4

0

0

0
4

0

0

0
4

0

0

0
4

0

0

0
4

0
4

0
4

0

0

0

0

0

0
4

0
4

0
4

0
4

0
4

0

0

0

0

0

0
4

0

0

0

0

0

0
4

0
4

0
4

0

0

0

0

0

0

0

0

0
4

0
4

0
4

0
4

0
4

0
4

0

0

0
4

0

0

0

0
4

0

0

0
4

0

0

0
4

0
4

0
4

0

0

0

0

0

0

0
4

0

0

0
4

0
4

0
4

0
4

0
4

0
4

0

0

0

0
4

0
4

0

0

0

0
4

0
4

0
4

0

0

0

0

0

0

0

0

0
4

0
4

0
4

0

0

0

0

0

0

0

0

0
4

0

0

0
4

0
4

0
4

0

0

0

0

0

0
4

0

0

0
4

0
4

0
4

0

0

0

0

0

0

0

0
4

0
4

0
4

0.289265
5

0.289265
5

0.126589
6

0.122945
6

0

0

0

0

0

0

0

0

0

0.00364411

0

0

0

0

0

0

0
4

0.0137987

0

0

0.0137987

0

0

0

0

0

0

0

0
4

0.140308
4

0

0

0

0.140308

0

0

0

0

0
4

0

0

0

0

0

0

0
4

0.00364411

0

0

0.00364411

0
4

0.0049252

0.0049252

0
4

0

0

0
4

0

0

0
4

0
4

0
6

0

0

0

0

0

0
4

0

0

0

0

0

0
4

0

0

0

0

0

0
4

0

0

0
4

0
4

0

0

0

0
4

0

0

0
4

0
4

0
4

0

0

0

0

0

0

0
4

0
4

0
4

0.0510212

0.0510212

0.0510212

0.0510212

0

0
4

0

0

0
4

0

0

0
4

0
4

0
4

0
6

0
6

0

0

0

0
4

0

0

0

0
4

0
4

0
4

0.140308

0.140308

0.140308

0.140308

0

0
4

0
4

0

0

0

0
4

0

0

0
4

0
4

0
4

0

0

0

0

0

0
4

0
4

0

0

0

0
4

0
4

0

0

0

0
4

0
4

0
4

0

0

0

0

0

0

0

0
4

0
4

0
4

0.00443786

0.00443786

0

0

0

0
4

0.00443786

0.00443786

0
4

0
4

0
4

0

0

0

0

0

0
4

0
4

0
4

0
3

0
3

0
3

0

0

0
4

0

0

0
4

0
4

0
4

0

0

0

0

0

0

0
4

0

0

0
4

0
4

0
4

0.356407

0.356407

0.339169

0.339169

0

0

0
3

0

0
3

0
3

0

0

0

0

0
4

0

0

0
4

0.0172382
2

0.012313

0

0.0049252

0

0
4

0

0

0

0

0
4

0

0

0
4

0

0

0
4

0

0

0

0
4

0

0

0

0
4

0

0

0
4

0

0

0
4

6.24500451351651e-17

0
4

0
4

0

0

0

0

0

0
4

0

0

0
4

0

0

0

0
4

0
4

0
4

0

0

0

0

0

0

0
4

0

0

0
4

0

0

0
4

0
4

0
4

0

0

0

0

0

0
4

0

0

0
4

0
4

0

0

0

0
4

0
4

0
4

0.0127553
6

0.0127553

0

0

0

0
4

0.0127553

0.0127553

0
4

0

0

0
4

0
4

0

0

0

0
4

0
4

0
4

0
6

0
6

0
7

0
7

0
4

0

0

0
4

0
4

0
4

0

0

0

0

0

0

0
4

0
4

0
4

0

0

0

0

0

0

0
4

0

0

0
4

0
4

0

0

0

0

0
4

0
4

0
4

0.0255106
7

0.0255106
7

0.0255106

0

0.0255106

0
4

0

0

0
4

0
4

0
4

0

0

0

0

0

0
4

0
4

0

0

0

0
4

0

0

0
4

0
4

0
4

0

0

0

0

0
4

0

0

0
4

0
4

0
4

0.159441
7

0.0765318
6

0.0765318
7

0.0637765
6

0.0127553
7

0

0

0

0

0

5.20417042793042e-18
7

0
4

0
6

0
6

0

0

0

0

0
4

0

0

0

0
4

0
4

0.0829095
7

0.0829095
7

0.0829095

0

0

0

0

0

0

0

0

0

0

0
4

0

0

0

0

0
4

0

0

0

0
4

0

0

0
4

0
4

0
4

0

0

0

0

0
4

0
4

0
4

0

0

0

0

0

0
4

0

0

0

0
4

0

0

0
4

0
4

0
4

0.197485

0.197485

0.197485

0.197485

0

0
4

0
4

0
4

0

0

0

0

0

0
4

0

0

0
4

0
4

0
4

0

0

0

0

0
4

0

0

0
4

0
4

0
4

0.0127553

0.0127553

0

0

0

0
4

0.0127553

0.0127553

0
4

0
4

0

0

0

0
4

0
4

0

0

0

0
4

0
4

0
4

0

0

0

0

0
4

0

0

0
4

0
4

0
4

0

0

0

0

0

0

0
4

0

0

0
4

0
4

0
4

0

0

0

0

0
4

0

0

0
4

0

0

0
4

0
4

0
4

0

0

0

0

0

0

0
4

0
4

0
4

0
4

0
4

0
4

0
4

0

0

0

0

0

0

0
4

0

0

0
4

0
4

0
4

0

0

0

0

0

0

0

0

0

0
4

0

0

0

0

0
4

0

0

0

0

0

0
4

0

0

0
4

0
4

0

0

0

0

0

0
4

0

0

0
4

0

0

0
4

0
4

0

0

0

0
4

0

0

0
4

0
4

0
4

0
6

0
6

0
6

0
6

0
4

0
4

0
4

0

0

0

0

0
4

0
4

0
4

0.159441

0.159441

0.159441

0.159441

0
4

0
4

0
4

0

0

0

0

0
4

0

0

0
4

0
4

0
4

0

0

0

0

0

0
4

0
4

0
4

0

0

0

0

0

0
4

0

0

0

0
4

0
4

0
4

0

0

0

0

0
4

0
4

0
4

0

0

0

0

0

0
4

0
4

0
4

0

0

0

0

0

0

0
4

0
4

0
4

0

0

0

0

0
4

0
4

0
4

0.0648255
3

0.0268244
3

0.0178829
3

0

0

0.00670609

0.00670609

0

0
3

0
3

0

0

0

0.00447073

0

0

1.73472347597681e-18
3

0
4

0.00894146

0.00894146

0
4

0

0

0
4

0

0

0
4

0
4

0.0223536

0.0223536

0.0111768

0.00670609

0.00447073

0
4

0
4

0.0156475

0.0156475

0.0156475

0
4

0
4

3.46944695195361e-18
3

0
4

0
3

0

0

0

0
4

0
4

0

0

0

0

0
4

0
4

0

0

0

0
4

0
4

0

0

0

0
4

0
4

0

0

0

0
4

0
4

0
3

0
3

0

0

0

0

0

0

0

0

0

0

0

0

0

0

0

0

0

0

0

0

0

0

0

0

0

0

0

0

0

0

0

0
3

0

0

0

0

0
4

0

0

0

0

0
4

0

0

0
4

0

0

0
4

0

0

0
4

0
4

0
4

0
3

0

0

0

0
4

0
4

0
3

0
3

0

0

0

0

0

0

0
4

0

0

0
4

0

0

0
4

0
4

0
4

0
4

0
4

0
4

0

0

0

0

0

0

0

0
4

0

0

0

0
4

0
4

0

0

0
4

0
3

0

0

0

0

0

0

0

0

0

0

0

0

0

0

0

0

0
4

0

0

0
4

0

0

0
4

0

0

0
4

0
4

0

0

0

0

0
4

0
4

0

0

0

0
4

0
4

0

0

0

0
4

0
4

0

0

0

0
4

0
4

0

0

0

0
4

0
4

0

0

0

0
4

0
4

0

0

0

0
4

0
4

0
4

0.526497

0

0

0

0

0
4

0
4

0.46272

0.46272

0

0.46272

0
4

0
4

0

0

0

0
4

0
4

0

0

0

0

0
4

0
4

0

0

0

0
4

0
4

0.0637765

0.0637765

0.019133

0

0

0

0

0

0

0

0

0

0

0

0

0

0

0

0

0

0.0446436

0

0

0

0

0

0

0
4

0

0

0
4

0

0

0
4

0

0

0
4

0
4

0
4

0.1833
3

0.12071
3

0
2

0
2

0

0

0
4

0
3

0

0

0

0

0

0

0

0

0

0

0

0

0

0

0

0

0
4

0.12071

0.12071

0
4

0

0

0
4

0
4

0.0536487

0.0536487

0.049178

0.00447073

2.60208521396521e-18

0
4

0
4

0.00894146

0.00894146

0.00894146

0
4

0
4

0

0

0

0
4

0
4

0

0

0

0
4

0
4

0

0

0

0
4

0
4

1.90819582357449e-17
3

0
4

1.08676

0

0

0

0
4

0
4

1.08676

1.08676

1.08676

0
3

0

0

0

0

0

0

0

0

0
4

0

0

0
4

0
4

0
4

0
7

0
7

0
7

0
7

0
4

0
4

0
4

0.150771

0.150771

0

0

0

0

0
4

0

0

0
4

0
3

0
3

0
5

0
3

0
4

0.150771
3

0

0.150771
3

0
4

0
4

0

0

0

0

0

0

0
4

0

0

0
4

0

0

0

0
4

0

0

0
4

0

0

0
4

0

0

0
4

0
4

0

0

0

0

0
4

0
4

0

0

0

0
4

0
4

0

0

0

0
4

0
4

0

0

0

0
4

0
4

0

0

0

0
4

0
4

0
4

0.10842
2

0.10842
2

0.10842
2

0

0.0446436

0.0637765

0

0

0
4

0
4

0
4

0

0

0

0

0
4

0
4

0

0

0

0

0
4

0
4

0

0

0

0
4

0

0

0
4

0
4

0

0

0

0

0
4

0
4

0

0

0

0
4

0
4

0

0

0

0
4

0
4

0

0

0

0

0
4

0

0

0
4

0

0

0
4

0

0

0
4

0

0

0
4

0

0

0
4

0

0

0
4

0

0

0
4

0
4

0
4

2.47024622979097e-14

0
4

5.92485
7

5.92485
7

5.92485
7

0.00894146
6

0

0
7

0

0

0

0

0

0

0

0

0

0

0

0

0

0

0

0

0

0

0

0

0

0.00447073
7

0

0

0

0

0
6

0
6

0
6

0
6

0.00447073

0

0
4

0
7

0
7

0
7

0

0
4

5.91591
7

0.00447073

0.235973
7

2.7627
7

0.90524
7

0.19133
6

1.8162
7

0

0
6

0

0

0

0

0

2.22044604925031e-16
7

0
4

8.88178419700125e-16
7

0
4

0
4

0
4

0

0

0

0

0

0

0

0

0

0

0

0

0

0

0

0

0

0

0

0

0

0

0

0

0

0

0

0

0

0

0

0

0

0

0

0

0

0

0

0

0

0

0

0

0

0

0

0

0

0

0

0

0

0

0

0

0

0

0

0

0

0

0

0

0

0

0

0

0

0

0

0

0

0

0

0

0

0

0

0

0

0

0

0

0

0

0

0

0

0

0

0

0

0

0

0

0

0

0

0

0

0

0

0

0

0

0

0

0

0

0

0

0

0

0

0

0

0

0

0

0

0

0

0

0

0

0

0

0

0

0

0

0

0

0

0

0

0

0

0

0

0

0

0
4

0

0

0

0

0

0

0

0

0

0

0

0

0

0

0

0

0

0

0

0
4

0

0

0

0

0

0

0

0

0
4

0
4

0
4

0
4

0.26221
7

0.26221
7

0.26221
7

0.26221
7

0.26221
7

0

0

0

0
4

0

0

0

0

0

0
4

0
4

0
4

0
4

0

0

0

0

0

0
4

0

0

0
4

0

0

0
4

0
4

0
4

0
4

0

0

0

0

0

0

0
4

0

0

0
4

0

0

0
4

0
4

0
4

0
4

0

0

0

0

0

0

0
4

0
4

0
4

0
4

0

0

0

0

0

0

0
4

0

0

0
4

0
4

0
4

0
4

0
3

0
3

0
3

0
3

0

0

0
4

0
4

0
4

0
4

0

0

0

0

0

0

0
4

0
4

0
4

0
4

0

0

0

0

0

0

0
4

0
4

0
4

0
4

0

0

0

0

0

0

0
4

0
4

0
4

0
4

0

0

0

0

0

0
4

0
4

0
4

0
4

0

0

0

0

0

0
4

0

0

0
4

0

0

0
4

0
4

0
4

0
4

0
3

0
3

0
3

0
3

0
3

0

0

0

0

0

0
4

0

0

0

0
4

0

0

0

0
4

0

0

0

0
4

0

0

0
4

0
4

0
4

0
4

0

0

0

0

0

0

0
4

0
4

0
4

0
4

0

0

0

0

0

0

0
4

0
4

0
4

0
4

0

0

0

0

0

0
4

0
4

0
4

0
4

0

0

0

0

0

0

0
4

0
4

0
4

0
4

0

0

0

0

0

0

0
4

0
4

0
4

0
4

0

0

0

0

0

0
4

0
4

0
4

0
4

0

0

0

0

0

0
4

0
4

0
4

0
4

0

0

0

0

0

0
4

0

0

0
4

0

0

0
4

0

0

0
4

0
4

0
4

0
4

0

0

0

0

0

0
4

0
4

0
4

0
4

0.0133048

0.0133048

0.0133048

0.0133048

0

0.0133048

0
4

0
4

0
4

0
4

0
4

0
4

0
4

0
4

0
4

0

0

0

0
4

0
4

0
4

0
4

0
4

0
4

0
4

0

0

0
4

0

0

0

0

0

0

0
4

0
4

0
4

0
4

0

0

0

0

0

0
4

0
4

0
4

0
4

0

0

0

0

0

0

0
4

0

0

0
4

0
4

0
4

0
4

0

0

0

0

0

0

0
4

0
4

0
4

0
4

0

0

0

0

0

0

0
4

0
4

0
4

0
4

0

0

0

0

0

0
4

0

0

0
4

0
4

0
4

0
4

0

0

0

0

0

0
4

0
4

0
4

0
4

0

0

0

0

0

0

0
4

0
4

0
4

0
4

0

0

0

0

0

0
4

0
4

0
4

0
4

0

0

0

0

0

0
4

0
4

0
4

0
4

0

0

0

0

0

0

0
4

0
4

0
4

0
4

0
6

0
6

0
6

0
6

0
6

0

0

0

0

0

0

0

0
4

0

0

0

0
4

0
4

0
4

0
4

0

0

0

0

0

0

0
4

0

0

0
4

0
4

0
4

0
4

0

0

0

0

0

0

0

0
4

0

0

0
4

0
4

0
4

0
4

0

0

0

0

0

0

0
4

0
4

0
4

0
4

0

0

0

0

0

0

0

0
4

0

0

0
4

0
4

0
4

0
4

0

0

0

0

0

0
4

0
4

0
4

0
4

0

0

0

0

0

0
4

0
4

0
4

0
4

0

0

0

0

0

0

0
4

0
4

0
4

0
4

0

0

0

0

0

0

0

0
4

0
4

0
4

0
4

0.00665242

0.00665242

0.00665242

0

0

0

0
4

0.00665242

0.00665242

0
4

0
4

0
4

0
4

0

0

0

0

0

0
4

0
4

0
4

0
4

0

0

0

0

0

0

0

0

0

0

0

0

0
4

0

0

0

0

0

0

0

0
4

0

0

0

0

0
4

0
4

0
4

0

0

0

0

0

0

0
4

0

0

0

0
4

0
4

0
4

0

0

0

0

0
4

0
4

0
4

0
4

0

0

0

0

0

0
4

0

0

0
4

0

0

0
4

0
4

0
4

0
4

0

0

0

0

0

0

0
4

0
4

0
4

0

0

0

0

0
4

0
4

0
4

0
4

0

0

0

0

0

0
4

0

0

0
4

0
4

0
4

0
4

0

0

0

0

0

0

0
4

0
4

0
4

0
4

0

0

0

0

0

0

0
4

0
4

0
4

0
4

0

0

0

0

0

0
4

0

0

0
4

0
4

0
4

0
4

0.026221

0.026221

0.026221

0.026221

0.026221

0
4

0
4

0
4

0
4

0

0

0

0

0

0
4

0
4

0
4

0
4

0.103113

0.103113

0.103113

0.103113

0.103113

0
4

0
4

0
4

0
4

0

0

0

0

0

0
4

0
4

0
4

0
4

0
3

0
3

0
3

0
3

0
3

0
3

0

0

0

0

0

0

0
4

0

0

0

0
4

0
4

0
4

0

0

0

0

0
4

0
4

0
4

0
4

0

0

0

0

0

0

0
4

0

0

0
4

0
4

0
4

0
4

0

0

0

0

0

0
4

0
4

0
4

0
4

0

0

0

0

0

0

0
4

0

0

0
4

0
4

0
4

0
4

0.100591

0.100591

0.100591

0.100591

0.100591

0
4

0
4

0
4

0
4

0

0

0

0

0

0

0
4

0
4

0
4

0
4

0.0127553

0.0127553

0.0127553

0.0127553

0

0.0127553

0
4

0
4

0
4

0
4

0

0

0

0

0

0
4

0
4

0
4

0
4

0

0

0

0

0

0

0
4

0
4

0
4

0
4

0

0

0

0

0

0
4

0
4

0
4

0
4

0

0

0

0

0

0
4

0
4

0
4

0
4

0

0

0

0

0

0

0

0
4

0

0

0

0
4

0

0

0

0
4

0

0

0
4

0
4

0
4

0

0

0

0

0
4

0

0

0
4

0
4

0
4

0

0

0

0

0
4

0
4

0
4

0
4

0

0

0

0

0

0
4

0
4

0
4

0
4

0

0

0

0

0

0
4

0
4

0
4

0
4

0

0

0

0

0

0
4

0
4

0
4

0
4

0

0

0

0

0

0
4

0
4

0
4

0
4

0

0

0

0

0

0

0
4

0
4

0
4

0
4

0

0

0

0

0

0

0
4

0
4

0
4

0
4

0

0

0

0

0

0

0
4

0
4

0
4

0
4

0

0

0

0

0

0
4

0
4

0
4

0
4

0

0

0

0

0

0
4

0
4

0
4

0
4

0

0

0

0

0

0
4

0
4

0
4

0
4

0.0573989
7

0.0573989
7

0.0573989
7

0.0573989
7

0.0382659
7

0
7

0

0.019133

0

3.46944695195361e-18
7

0
4

0

0

0

0
4

0
4

0
4

0
4

0

0

0

0

0

0
4

0
4

0
4

0
4

0

0

0

0

0

0
4

0
4

0
4

0
4

0

0

0

0

0

0
4

0
4

0
4

0
4

0

0

0

0

0

0
4

0
4

0
4

0
4

0

0

0

0

0

0
4

0
4

0
4

0
4

0

0

0

0

0

0
4

0
4

0
4

0
4

0

0

0

0

0

0
4

0
4

0
4

0
4

0

0

0

0

0

0

0
4

0
4

0
4

0
4

0

0

0

0

0

0
4

0
4

0
4

0
4

0

0

0

0

0

0
4

0
4

0
4

0
4

0
4

0
4

0
4

0
4

0
4

0

0

0

0

0

0

0
4

0
4

0

0

0

0
4

0
4

0
4

0
4

0

0

0

0

0

0
4

0
4

0
4

0
4

0

0

0

0

0

0

0
4

0
4

0
4

0
4

0

0

0

0

0

0
4

0
4

0
4

0
4

0

0

0

0

0

0
4

0
4

0
4

0
4

0

0

0

0

0

0

0
4

0
4

0
4

0
4

0.010546

0.010546

0.010546

0.010546

0.00527298

0.00527298

0
4

0
4

0
4

0
4

0

0

0

0

0

0
4

0

0

0
4

0
4

0
4

0
4

0

0

0

0

0

0
4

0
4

0
4

0
4

0

0

0

0

0

0
4

0
4

0
4

0
4

0

0

0

0

0

0
4

0
4

0
4

0
4

0.0446436

0

0

0

0
1

0

0

0

0

0
4

0

0

0
4

0

0

0
4

0
4

0
4

0.0446436

0.0446436

0.0446436

0.0446436

0
4

0
4

0
4

0
4

0

0

0

0

0

0
4

0
4

0
4

0
4

0

0

0

0

0

0

0
4

0
4

0
4

0
4

0

0

0

0

0

0

0
4

0
4

0
4

0
4

0

0

0

0

0

0
4

0
4

0
4

0
4

0

0

0

0

0

0
4

0
4

0
4

0
4

0

0

0

0

0

0

0
4

0
4

0
4

0
4

0

0

0

0

0

0
4

0

0

0
4

0
4

0
4

0
4

0

0

0

0

0

0

0
4

0
4

0
4

0
4

0

0

0

0

0

0

0
4

0
4

0
4

0
4

0

0

0

0

0

0
4

0
4

0
4

0
4

4.06957
3

4.06957
3

4.06957
3

4.06957
3

2.72045
3

0.344393
4

0.0182042
5

0
4

0
2

0
4

0.0380012

0.00755116

0

0

0

0.189981
3

0

0

0

0

0

0

0

0

0

0

0.046567
3

0.478538
3

0.08124

0
3

0.0385078
3

0.106138

0
3

0
4

0
4

0
4

0
4

0
4

0
4

0
4

0
3

0
3

0
4

0
3

0
4

0

0
4

0
4

0

0

0

0
4

0

0

0

0
4

0

0

0
4

0

0

0
4

0

0

0

0
4

0
4

0
4

0
4

0

0

0

0

0

0
4

0
4

0
4

0
4

0

0

0

0

0

0
4

0
4

0
4

0
4

0

0

0

0

0

0

0
4

0
4

0
4

0
4

0

0

0

0

0

0
4

0
4

0
4

0
4

0

0

0

0

0

0
4

0
4

0
4

0
4

0

0

0

0

0

0
4

0
4

0
4

0
4

0

0

0

0

0

0
4

0
4

0
4

0
4

0

0

0

0

0

0
4

0
4

0
4

0
4

0

0

0

0

0

0
4

0
4

0
4

0
4

0

0

0

0

0

0
4

0

0

0
4

0
4

0
4

0
4

0

0

0

0

0

0

0

0

0

0

0

0
4

0

0

0
4

0
4

0
4

0
4

0

0

0

0

0

0

0
4

0
4

0
4

0
4

0

0

0

0

0

0
4

0
4

0
4

0
4

0

0

0

0

0

0
4

0
4

0
4

0
4

0

0

0

0

0

0

0
4

0
4

0
4

0
4

0

0

0

0

0

0
4

0
4

0
4

0
4

0

0

0

0

0

0
4

0
4

0
4

0
4

0

0

0

0

0

0
4

0
4

0
4

0
4

0

0

0

0

0

0

0
4

0
4

0
4

0
4

0

0

0

0

0

0
4

0

0

0
4

0
4

0
4

0
4

0

0

0

0

0

0
4

0

0

0
4

0
4

0
4

0
4

0
4

0
4

0
4

0
4

0
4

0

0

0

0

0

0

0

0
4

0
4

0
4

0
4

0

0

0

0

0

0
4

0
4

0
4

0
4

0

0

0

0

0

0

0
4

0
4

0
4

0
4

0

0

0

0

0

0
4

0
4

0
4

0
4

0

0

0

0

0

0
4

0
4

0
4

0
4

0

0

0

0

0

0
4

0
4

0
4

0
4

0

0

0

0

0

0
4

0
4

0
4

0
4

0

0

0

0

0

0
4

0
4

0
4

0
4

0

0

0

0

0

0
4

0
4

0
4

0
4

0

0

0

0

0

0
4

0
4

0
4

0
4

0

0

0

0

0

0
4

0
4

0
4

0
4

0

0

0

0

0

0

0

0

0

0

0

0
4

0

0

0

0

0
4

0
4

0
4

0
4

0

0

0

0

0

0
4

0
4

0
4

0
4

0

0

0

0

0

0
4

0
4

0
4

0
4

0

0

0

0

0

0
4

0
4

0
4

0
4

0

0

0

0

0

0
4

0
4

0
4

0
4

0

0

0

0

0

0
4

0
4

0
4

0
4

0

0

0

0

0

0
4

0
4

0
4

0
4

0

0

0

0

0

0
4

0
4

0
4

0
4

0

0

0

0

0

0
4

0
4

0
4

0
4

0

0

0

0

0

0
4

0
4

0
4

0
4

0

0

0

0

0

0
4

0
4

0
4

0
4

0.580366
7

0.580366
7

0.580366
7

0.580366
7

0.554856
7

0
7

0.0255106

2.42861286636753e-17
7

0
4

0
4

0
4

0
4

0

0

0

0

0

0
4

0
4

0
4

0
4

0

0

0

0

0

0
4

0
4

0
4

0
4

0

0

0

0

0

0
4

0
4

0
4

0
4

0

0

0

0

0

0
4

0
4

0
4

0
4

0

0

0

0

0

0
4

0
4

0
4

0
4

0

0

0

0

0

0
4

0
4

0
4

0
4

0

0

0

0

0

0
4

0
4

0
4

0
4

0

0

0

0

0

0
4

0
4

0
4

0
4

0

0

0

0

0

0
4

0
4

0
4

0
4

0

0

0

0

0

0
4

0
4

0
4

0
4

0

0

0

0

0

0

0

0

0

0

0
4

0

0

0

0

0

0

0
4

0
4

0
4

0

0

0

0

0

0

0

0
4

0

0

0
4

0

0

0
4

0
4

0
4

0
4

0

0

0

0

0

0
4

0
4

0
4

0
4

0

0

0

0

0

0
4

0
4

0
4

0
4

0

0

0

0

0

0
4

0
4

0
4

0
4

0

0

0

0

0

0
4

0
4

0
4

0
4

0

0

0

0

0

0
4

0
4

0
4

0
4

0

0

0

0

0

0
4

0
4

0
4

0
4

0

0

0

0

0

0
4

0
4

0
4

0
4

0

0

0

0

0

0
4

0
4

0
4

0
4

0

0

0

0

0

0
4

0
4

0
4

0
4

0

0

0

0

0

0
4

0
4

0
4

0
4

0
3

0
3

0
3

0
4

0
4

0
3

0

0
4

0

0

0

0
4

0
4

0
4

0
4

0.0288461

0.0288461

0.0288461

0.0288461

0.0288461

0
4

0
4

0
4

0
4

0

0

0

0

0

0
4

0
4

0
4

0
4

0

0

0

0

0

0
4

0
4

0
4

0
4

0

0

0

0

0

0
4

0
4

0
4

0
4

0

0

0

0

0

0
4

0
4

0
4

0
4

0

0

0

0

0

0
4

0
4

0
4

0
4

0

0

0

0

0

0
4

0
4

0
4

0
4

0

0

0

0

0

0
4

0
4

0
4

0
4

0

0

0

0

0

0
4

0
4

0
4

0
4

0

0

0

0

0

0
4

0
4

0
4

0
4

0

0

0

0

0

0

0

0

0

0

0

0
4

0
4

0
4

0
4

0

0

0

0

0

0
4

0
4

0
4

0
4

0

0

0

0

0

0
4

0
4

0
4

0
4

0

0

0

0

0

0
4

0
4

0
4

0
4

0

0

0

0

0

0
4

0
4

0
4

0
4

0

0

0

0

0

0
4

0
4

0
4

0
4

0

0

0

0

0

0
4

0
4

0
4

0
4

0

0

0

0

0

0
4

0
4

0
4

0
4

0

0

0

0

0

0
4

0
4

0
4

0
4

0

0

0

0

0

0
4

0
4

0
4

0
4

0

0

0

0

0

0
4

0
4

0
4

0
4

0
4

0
4

0
4

0
4

0
4

0

0
4

0

0

0

0
4

0

0

0

0
4

0

0

0
4

0

0

0
4

0

0

0
4

0
4

0
4

0

0

0

0

0
4

0
4

0
4

0

0

0

0

0
4

0
4

0
4

0
4

0

0

0

0

0

0
4

0
4

0
4

0
4

0

0

0

0

0

0
4

0
4

0
4

0
4

0

0

0

0

0

0
4

0
4

0
4

0
4

0

0

0

0

0

0
4

0
4

0
4

0
4

0.119628

0.119628

0.119628

0.119628

0.119628

0
4

0
4

0
4

0
4

0

0

0

0

0

0
4

0
4

0
4

0
4

0

0

0

0

0

0
4

0
4

0
4

0
4

0

0

0

0

0

0
4

0
4

0
4

0
4

0

0

0

0

0

0
4

0
4

0
4

0
4

0

0

0

0

0

0
4

0
4

0
4

0
4

0.0127553

0.0127553

0.0127553

0.0127553

0

0.0127553

0

0

0

0

0

0

0

0

0

0

0
4

0

0

0

0
4

0
4

0
4

0
4

0

0

0

0

0

0
4

0
4

0
4

0
4

0

0

0

0

0

0
4

0
4

0
4

0
4

0

0

0

0

0

0
4

0
4

0
4

0
4

0

0

0

0

0

0
4

0
4

0
4

0
4

0

0

0

0

0

0
4

0
4

0
4

0
4

0

0

0

0

0

0
4

0
4

0
4

0
4

0

0

0

0

0

0
4

0
4

0
4

0
4

0

0

0

0

0

0
4

0
4

0
4

0
4

0

0

0

0

0

0
4

0
4

0
4

0
4

0

0

0

0

0

0
4

0
4

0
4

0
4

0
4

0
4

0
4

0
4

0
4

0

0

0

0

0

0

0

0

0

0

0
4

0

0

0

0

0

0

0

0

0

0

0
4

0

0

0

0

0

0

0
4

0
3

0
4

0

0
3

0

0
4

0
4

0
4

0

0

0
4

0

0

0

0

0

0

0

0
4

0
4

0
4

0
4

0
4

0

0

0

0

0

0
4

0

0

0

0

0

0

0
4

0
4

0

0

0

0

0
4

0

0

0
4

0
4

0
4

0
4

0
4

0
4

0
4

0

0
4

0

0

0

0
4

0

0

0
4

0
4

0
4

0

0

0

0

0
4

0

0

0
4

0
4

0
4

0
4

0

0

0

0

0

0

0

0

0

0
4

0
4

0
4

0
4

0

0

0

0

0

0
4

0
4

0
4

0
4

0

0

0

0

0

0
4

0
4

0
4

0
4

0

0

0

0

0

0
4

0
4

0
4

0
4

0

0

0

0

0

0
4

0
4

0
4

0
4

0

0

0

0

0

0
4

0
4

0
4

0
4

0

0

0

0

0

0
4

0
4

0
4

0
4

0

0

0

0

0

0
4

0
4

0
4

0
4

0

0

0

0

0

0
4

0
4

0
4

0
4

0

0

0

0

0

0
4

0
4

0
4

0
4

0

0

0

0

0

0
4

0
4

0
4

0
4

0
5

0
5

0
5

0
5

0

0

0

0

0

0

0

0

0
4

0

0

0
4

0
4

0
4

0
4

0

0

0

0

0

0
4

0
4

0
4

0
4

0

0

0

0

0

0
4

0
4

0
4

0
4

0

0

0

0

0

0
4

0
4

0
4

0
4

0

0

0

0

0

0
4

0
4

0
4

0
4

0

0

0

0

0

0
4

0
4

0
4

0
4

0

0

0

0

0

0
4

0
4

0
4

0
4

0

0

0

0

0

0
4

0
4

0
4

0
4

0

0

0

0

0

0
4

0
4

0
4

0
4

0

0

0

0

0

0
4

0
4

0
4

0
4

0

0

0

0

0

0
4

0
4

0
4

0
4

0.072817
7

0.072817
7

0.072817
7

0.072817
7

0.0494115
7

0.0156036
7

0

0

0

0.00780182

6.93889390390723e-18
7

0
4

0
4

0
4

0
4

0

0

0

0

0

0
4

0
4

0
4

0
4

0

0

0

0

0

0
4

0
4

0
4

0
4

0

0

0

0

0

0
4

0
4

0
4

0
4

0

0

0

0

0

0
4

0
4

0
4

0
4

0

0

0

0

0

0
4

0
4

0
4

0
4

0

0

0

0

0

0
4

0
4

0
4

0
4

0

0

0

0

0

0
4

0
4

0
4

0
4

0

0

0

0

0

0
4

0
4

0
4

0
4

0

0

0

0

0

0
4

0
4

0
4

0
4

0

0

0

0

0

0
4

0
4

0
4

0
4

0

0

0

0

0

0

0

0

0

0

0

0

0

0

0

0

0

0
4

0

0

0

0

0

0
4

0

0

0

0
4

0

0

0
4

0
4

0
4

0
4

0

0

0

0

0

0
4

0
4

0
4

0
4

0

0

0

0

0

0
4

0
4

0
4

0
4

0

0

0

0

0

0
4

0
4

0
4

0
4

0

0

0

0

0

0
4

0
4

0
4

0
4

0

0

0

0

0

0
4

0
4

0
4

0
4

0

0

0

0

0

0
4

0
4

0
4

0
4

0

0

0

0

0

0
4

0
4

0
4

0
4

0

0

0

0

0

0
4

0
4

0
4

0
4

0

0

0

0

0

0
4

0
4

0
4

0
4

0.242351

0.242351

0.242351

0.242351

0.242351

0
4

0
4

0
4

0
4

0.0548988
7

0.0548988
7

0.0548988
7

0.0548988
7

0.0223536
6

0.00447073

0.019133

0.00447073

0.00447073

0

0

0

5.20417042793042e-18
7

0
4

0
4

0
4

0
4

0

0

0

0

0

0
4

0
4

0
4

0
4

0

0

0

0

0

0
4

0
4

0
4

0
4

0

0

0

0

0

0
4

0
4

0
4

0
4

0

0

0

0

0

0
4

0
4

0
4

0
4

0

0

0

0

0

0
4

0
4

0
4

0
4

0

0

0

0

0

0
4

0
4

0
4

0
4

0

0

0

0

0

0
4

0
4

0
4

0
4

0

0

0

0

0

0
4

0
4

0
4

0
4

0

0

0

0

0

0
4

0
4

0
4

0
4

0

0

0

0

0

0
4

0
4

0
4

0
4

0.750578

0.750578

0.750578

0.750578

0.731541

0.00543897

0

0

0

0.00815845

0.00543897

1.11889664200504e-16

0
4

0

0

0

0
4

0

0

0
4

0
4

0
4

0
4

0

0

0

0

0

0
4

0
4

0
4

0
4

0

0

0

0

0

0
4

0
4

0
4

0
4

0

0

0

0

0

0

0

0

0

0

0

0
4

0
4

0
4

0
4

0

0

0

0

0

0

0
4

0
4

0
4

0

0

0

0

0
4

0

0

0
4

0
4

0
4

0
4

0

0

0

0

0

0

0

0
4

0

0

0

0
4

0
4

0
4

0
4

0.0217559

0.0217559

0.0217559

0.0217559

0.0217559

0

0

0
4

0
4

0
4

0
4

0
4

0
4

0
4

0
4

0
4

0

0

0

0

0

0

0

0

0

0

0
4

0

0

0

0

0

0

0

0

0

0

0
4

0

0

0

0

0

0

0

0

0

0

0
4

0

0

0

0

0

0

0

0
4

0

0

0

0
4

0
4

0
4

0

0

0
4

0

0
4

0

0

0

0

0

0
4

0

0

0

0
4

0
4

0
4

0
4

0
3

0
3

0
3

0
3

0
3

0
4

0

0

0

0
4

0
4

0
4

0
4

0.990538

0.990538

0.990538

0.990538

0

0.987022

0

0.00351532

0

0
4

0
4

0
4

0
4

0

0

0

0

0

0

0
4

0
4

0
4

0

0

0

0

0
4

0
4

0
4

0
4

0

0

0

0

0

0

0
4

0
4

0
4

0
4

0
4

0
4

0
4

0
4

0
4

0

0
4

0

0

0
4

0
4

0
4

0
4

0

0

0

0

0

0
4

0
4

0
4

0
4

0
4

0
4

0

0

0

0

0

0

0
4

0
4

0

0

0

0
4

0
4

0
4

0

0

0

0

0

0
4

0

0

0
4

0
4

0
4

0
4

0
4

0
4

0
4

0
4

0
4

0

0
4

0

0

0

0
4

0
4

0
4

0
4

0

0

0

0

0

0

0

0

0
4

0

0

0
4

0
4

0
4

0
4

0

0

0

0

0

0

0

0

0

0
4

0

0

0

0
4

0

0

0
4

0
4

0
4

0
4

0
4

0
4

0
4

0
4

0
4

0

0

0

0

0

0

0

0

0

0

0
4

0

0

0

0

0

0

0

0

0

0

0

0
4

0
4

0

0

0

0

0
4

0
4

0
4

0
4

0

0

0

0

0

0

0

0
4

0
4

0
4

0
4

0
4

0

0

0

0
4

0

0

0

0
4

0

0

0
4

0

0

0
4

0
4

0
4

0
4

0
4

0
4

0
4

0

0

0

0
4

0

0

0

0
4

0

0

0

0
4

0
4

0
4

0

0

0

0

0
4

0
4

0
4

0
4

0
5

0
5

0
5

0
5

0

0

0

0

0
4

0
4

0
4

0
4

0.790564

0.790564

0.790564

0.790564

0.790564

0

0
4

0
4

0
4

0
4

0
5

0
5

0
5

0
5

0
4

0

0

0
4

0

0

0
4

0

0

0
4

0
4

0
4

0
4

0

0

0

0

0

0

0

0

0

0

0
4

0

0

0
4

0
4

0
4

0
4

0

0

0

0

0

0

0

0
4

0
4

0
4

0
4

0

0

0

0

0

0

0

0
4

0
4

0
4

0
4

0

0

0

0

0

0

0

0
4

0
4

0
4

0
4

0
3

0
3

0
3

0
3

0
3

0
4

0
4

0
4

0
4

0

0

0

0

0

0

0

0
4

0

0

0
4

0

0

0
4

0
4

0
4

0
4

0

0

0

0

0

0
4

0
4

0
4

0
4

0
4

0
4

0
4

0
4

0
4

0

0

0

0

0

0

0

0

0
4

0
4

0
4

0
4

0

0

0

0

0

0

0
4

0
4

0
4

0
4

0

0

0

0

0

0

0

0

0

0
4

0
4

0
4

0

0

0

0

0

0
4

0

0

0
4

0
4

0
4

0
4

0
7

0
7

0
7

0
7

0

0

0

0

0
4

0
4

0
4

0
4

0.0382659
7

0.0382659
7

0.0382659
7

0.0382659
7

0.0382659
7

0
4

0
4

0
4

0
4

0

0

0

0

0

0

0

0

0

0
4

0

0

0

0
4

0

0

0
4

0
4

0
4

0
4

0

0

0

0

0

0
4

0
4

0
4

0
4

0

0

0

0

0

0

0
4

0

0

0
4

0

0

0
4

0

0

0
4

0
4

0
4

0
4

0

0

0

0

0

0

0
4

0

0

0
4

0
4

0
4

0
4

0
4

0
4

0
4

0
4

0

0

0

0
4

0
4

0
4

0
4

0

0

0

0

0

0

0

0

0
4

0
4

0
4

0

0

0

0

0
4

0
4

0
4

0
4

0
4

0
4

0
4

0
4

0
4

0

0

0

0

0

0

0

0

0

0

0

0

0

0

0

0

0
4

0

0

0

0

0
4

0
3

0
3

0

0
4

0

0

0

0

0
4

0

0

0
4

0

0

0
4

0

0

0
4

0

0

0
4

0
4

0
4

0
4

0.00543897

0.00543897

0.00543897

0

0

0

0
4

0.00543897

0

0.00543897

0
4

0
4

0
4

0
4

0
4

0
4

0
4

0
4

0

0

0

0
4

0
4

0
4

0
4

0
4

0
4

0
4

0
4

0
4

0

0
4

0
4

0
4

0
4

0
4

0
4

0
4

0
4

0
4

0
4

0

0

0

0
4

0
4

0
4

0
4

0

0

0

0

0

0

0

0
4

0
4

0
4

0
4

0

0

0

0

0

0

0
4

0

0

0

0
4

0

0

0
4

0
4

0
4

0
4

0

0

0

0

0

0
4

0

0

0

0
4

0

0

0

0
4

0

0

0
4

0
4

0
4

0
4

0

0

0

0

0

0

0

0

0
4

0

0

0
4

0
4

0
4

0
4

0

0

0

0

0

0

0

0

0

0
4

0

0

0
4

0

0

0
4

0
4

0
4

0

0

0

0

0
4

0
4

0
4

0
4

0

0

0

0

0

0
4

0
4

0
4

0
4

0
4

0
4

0
4

0
4

0
4

0
3

0

0

0

0

0

0
4

0
4

0

0

0

0

0

0
4

0

0

0

0
4

0

0

0

0

0

0
4

0

0

0
4

0

0

0
4

0
4

0
4

0
4

0

0

0

0

0

0
4

0

0

0
4

0

0

0
4

0
4

0
4

0

0

0

0

0

0
4

0
4

0
4

0
4

0

0

0

0

0

0
4

0

0

0
4

0
4

0
4

0

0

0

0

0
4

0
4

0
4

0
4

0
7

0
7

0
7

0
7

0
7

0
4

0
4

0
4

0
4

0

0

0

0

0

0

0

0
4

0

0

0
4

0
4

0
4

0
4

0

0

0

0

0

0

0

0
4

0
4

0
4

0
4

0

0

0

0

0

0

0

0
4

0
4

0
4

0
4

0

0

0

0

0

0

0
4

0
4

0
4

0
4

0

0

0

0

0

0
4

0
4

0
4

0
4

0
3

0
3

0
3

0
3

0

0

0

0
4

0

0

0
4

0
4

0
4

0
4

0

0

0

0

0

0
4

0

0

0
4

0
4

0
4

0
4

0

0

0

0

0

0

0

0

0

0

0

0

0

0

0

0
4

0

0

0
4

0

0

0
4

0
4

0
4

0
4

0

0

0

0

0

0

0
4

0

0

0
4

0

0

0
4

0
4

0
4

0
4

0
2

0
2

0
2

0
2

0
2

0
4

0

0

0
4

0
4

0
4

0
4

0.0446436
7

0.0446436
7

0.0446436
7

0.0446436
7

0.0446436
7

0
4

0
4

0
4

0
4

0.111826
7

0.111826
7

0.111826
7

0.111826
7

0.111826
7

0
4

0
4

0
4

0
4

0

0

0

0

0

0

0

0
4

0

0

0
4

0

0

0
4

0
4

0
4

0
4

0

0

0

0

0

0

0
4

0

0

0
4

0
4

0
4

0
4

0

0

0

0

0

0

0
4

0
4

0
4

0
4

0

0

0

0

0

0

0
4

0

0

0
4

0

0

0
4

0
4

0
4

0
4

0

0

0

0

0

0
4

0
4

0
4

0
4

0

0

0

0

0

0

0
4

0
4

0
4

0
4

0.681243

0

0

0

0

0
4

0
4

0
4

0

0

0

0

0
4

0
4

0
4

0

0

0

0

0
4

0
4

0
4

0.681243

0
3

0
3

0
3

0

0
4

0

0

0
4

0

0

0

0
4

0
4

0

0

0

0
4

0
4

0

0

0

0
4

0
4

0

0

0

0
4

0
4

0

0

0

0

0
4

0

0

0
4

0

0

0
4

0
4

0

0

0

0

0
4

0
4

0

0

0

0

0
4

0
4

0

0

0

0
4

0
4

0

0

0

0
4

0

0

0
4

0

0

0
4

0
4

0

0

0

0
4

0
4

0

0

0

0

0
4

0
4

0

0

0

0
4

0
4

0.681243
6

0.681243
6

0.681243
6

0

0
4

0
4

0
4

0
4

0.605774

0.444704

0.444704

0.444704

0.00520121

0.439502

0
4

0
4

0
4

0.0884206

0.0884206

0.0884206

0.0494115

0

0.0390091

0
4

0
4

0
4

0

0

0

0

0
4

0

0

0
4

0
4

0
4

0.00520121

0.00520121

0.00520121

0.00520121

0
4

0
4

0
4

0

0

0

0

0
4

0
4

0
4

0

0

0

0

0
4

0
4

0
4

0.00503411

0.00503411

0.00503411

0.00503411

0
4

0
4

0
4

0

0

0

0

0
4

0
4

0
4

0.0624145

0.0520121

0.0520121

0.0156036

0.0364085

0
4

0
4

0.0104024

0.0104024

0

0.0104024

0
4

0
4

0

0

0

0
4

0
4

0

0

0

0
4

0
4

0

0

0

0
4

0
4

0

0

0

0

0
4

0

0

0
4

0

0

0
4

0

0

0
4

0
4

0
4

7.63278329429795e-17

0
4

0

0

0

0

0

0
4

0
4

0
4

0
4

0
3

0

0

0

0

0
4

0
4

0
4

0

0

0

0

0
4

0
4

0
4

0
3

0

0

0

0
4

0
4

0
3

0
3

0
2

0

0

0

0

0

0

0

0

0

0

0
2

0

0

0

0

0

0

0

0

0

0

0
2

0

0

0

0

0

0

0

0

0

0

0
2

0

0

0

0

0

0

0

0

0

0

0
2

0

0

0

0

0

0

0

0

0

0

0

0

0
2

0

0
4

0
4

0
4

0
4

0

0

0

0

0

0
4

0
4

0
4

0
4

0
2

0

0

0

0

0
4

0
4

0
4

0

0

0

0

0
4

0
4

0
4

0
2

0
2

0
2

0
2

0

0

0

0

0

0

0
4

0
4

0
2

0
2

0
2

0

0
4

0

0

0
4

0
4

0

0

0

0
4

0
4

0
3

0
3

0

0

0

0

0

0

0

0

0

0

0

0

0

0

0

0
4

0
4

0
4

0
4

2.69282

0.351428
7

0.351428
7

0.351428
7

0.00447073
7

0.338015

0

0

0

0

0.00894146

0

5.20417042793042e-18
7

0
4

0

0

0
4

0

0

0
4

0

0

0
4

0
4

0

0

0

0
4

0
4

0
4

0

0

0

0

0

0
4

0
4

0
4

0

0

0

0

0
4

0
4

0
4

0

0

0

0

0
4

0
4

0
4

0

0

0

0

0
4

0
4

0
4

0

0

0

0

0
4

0

0

0
4

0
4

0
4

0

0

0

0

0
4

0
4

0
4

0

0

0

0

0
4

0
4

0
4

0

0

0

0

0
4

0
4

0
4

0

0

0

0

0
4

0
4

0
4

0

0

0

0

0
4

0
4

0
4

0.0457549
7

0.0457549
7

0.019133
7

0.019133

0

0

0
4

0.0138667

0.00447073

0.00939592

0

0
4

0.0127553

0.0127553

0

0
4

0

0

0

0
4

0

0

0
4

0
4

0
4

0

0

0

0

0
4

0
4

0
4

0

0

0

0

0
4

0
4

0
4

0

0

0

0

0
4

0
4

0
4

0

0

0

0

0
4

0
4

0
4

0

0

0

0

0
4

0
4

0
4

0

0

0

0

0
4

0
4

0
4

0

0

0

0

0
4

0
4

0
4

0

0

0

0

0
4

0
4

0
4

0

0

0

0

0
4

0
4

0
4

0.0382659

0.0382659

0.0382659

0.0382659

0
4

0
4

0
4

0

0

0

0

0

0

0

0

0

0
4

0
4

0
4

0

0

0

0

0
4

0
4

0
4

0

0

0

0

0

0
4

0

0

0

0
4

0
4

0
4

0

0

0

0

0
4

0
4

0
4

0.127416

0.127416

0.127416

0.0134122

0.107297

0.00670609

0
4

0
4

0
4

0.0765318

0.0765318

0.0765318

0.0765318

0

0
4

0
4

0
4

0

0

0

0

0
4

0
4

0
4

0

0

0

0

0
4

0

0

0
4

0
4

0
4

2.05342

0

0

0

0
4

0
4

0

0

0

0
4

0

0

0
4

0
4

0

0

0

0
4

0
4

0

0

0

0
4

0
4

0

0

0

0
4

0
4

0

0

0

0
4

0
4

0

0

0

0
4

0
4

0

0

0

0
4

0
4

0

0

0

0
4

0
4

0

0

0

0
4

0
4

0

0

0

0
4

0
4

0.0637765
7

0.0637765
7

0.019133

0.0446436

0

0
4

0

0

0
4

0

0

0
4

0
4

0

0

0

0
4

0
4

0.0765318
7

0.0765318
7

0.0765318
7

0
4

0
4

0

0

0

0

0
4

0
4

0

0

0

0
4

0
4

0

0

0

0
4

0

0

0
4

0

0

0
4

0
4

0

0

0

0
4

0
4

0

0

0

0
4

0
4

0

0

0

0
4

0
4

1.91311

1.91311

0.255074
6

0.0637765

0.10842
7

0.0127553

0

0

0

0

0

0

0

0
5

0

0

0

0

0

0

0

0

0

0

0.274761
7

0

0

0

0

0

0

0

0

0

0

0.745514
6

0

0.0127553

0

0

0

0

0

0

0

0

0.331638
7

0

0

0

0

0

0

0

0

0

0

0
5

0

0

0

0

0

0

0

0

0

0

0.0956648
7

0

0

0

0

0

0

0

0.0127553

0

0

0
4

0

0

0

0
4

0

0

0
4

0

0

0
4

0

0

0
4

0

0

0
4

0

0

0
4

0

0

0
4

0

0

0
4

0
4

2.22044604925031e-16

0
4

0
4

0.270416

0
3

0
3

0
3

0

0
3

0

0

0

0

0

0
4

0

0

0

0

0
4

0

0

0

0
4

0
4

0

0

0

0

0

0
4

0

0

0
4

0
4

0

0

0

0

0
4

0

0

0
4

0
4

0

0

0

0
4

0

0

0
4

0
4

0
4

0

0

0

0

0
4

0
4

0
4

0

0

0

0

0

0

0
4

0
4

0
4

0

0

0

0

0
4

0
4

0
4

0

0

0

0

0

0
4

0
4

0
4

0

0

0

0

0

0
4

0
4

0
4

0

0

0

0

0
4

0
4

0
4

0

0

0

0

0

0
4

0
4

0
4

0

0

0

0

0
4

0
4

0
4

0.00520121

0.00520121

0.00520121

0

0.00520121

0
4

0
4

0
4

0

0

0

0

0

0
4

0
4

0
4

0
3

0
3

0
3

0
3

0

0

0

0

0

0

0
4

0

0

0
4

0

0

0
4

0

0

0
4

0
4

0
4

0

0

0

0

0
4

0
4

0
4

0

0

0

0

0
4

0
4

0
4

0

0

0

0

0
4

0

0

0
4

0
4

0
4

0

0

0

0

0

0
4

0
4

0
4

0

0

0

0

0
4

0
4

0
4

0

0

0

0

0
4

0

0

0
4

0
4

0
4

0

0

0

0

0
4

0
4

0
4

0

0

0

0

0
4

0
4

0
4

0

0

0

0

0
4

0
4

0
4

0

0

0

0

0
4

0
4

0
4

0.0829095
6

0.0829095
6

0.0829095
6

0.0829095
7

0

0

0

0

0

0
4

0
4

0
4

0

0

0

0

0
4

0
4

0
4

0

0

0

0

0
4

0
4

0
4

0

0

0

0

0
4

0
4

0
4

0

0

0

0

0
4

0
4

0
4

0

0

0

0

0
4

0
4

0
4

0

0

0

0

0
4

0
4

0
4

0

0

0

0

0
4

0
4

0
4

0

0

0

0

0
4

0
4

0
4

0

0

0

0

0
4

0
4

0
4

0

0

0

0

0
4

0
4

0
4

0.143033
7

0.143033
7

0.143033
7

0
6

0.143033

0

0

0

0
4

0

0

0
4

0
4

0
4

0

0

0

0

0
4

0
4

0
4

0

0

0

0

0
4

0
4

0
4

0

0

0

0

0
4

0
4

0
4

0

0

0

0

0
4

0
4

0
4

0

0

0

0

0
4

0
4

0
4

0

0

0

0

0
4

0
4

0
4

0

0

0

0

0
4

0
4

0
4

0

0

0

0

0
4

0
4

0
4

0

0

0

0

0
4

0
4

0
4

0

0

0

0

0

0

0
4

0
4

0
4

0

0

0

0

0

0
4

0
4

0
4

0.00665679

0

0

0

0

0

0
4

0
4

0.00665679

0.00665679

0.00665679

0
4

0
4

0
4

0
3

0
3

0
3

0

0

0

0
4

0
4

0

0

0

0
4

0
4

0
4

0

0

0

0

0
4

0

0

0
4

0

0

0
4

0
4

0
4

0.0326153
3

0

0

0

0

0

0

0

0
4

0
4

0

0

0

0
4

0
4

0

0

0

0
4

0
4

0

0

0

0
4

0

0

0
4

0
4

0

0

0

0
4

0
4

0

0

0

0
4

0
4

0

0

0

0
4

0
4

0

0

0

0
4

0
4

0

0

0

0
4

0
4

0

0

0

0
4

0
4

0

0

0

0
4

0
4

0

0

0

0

0

0
4

0
4

0

0

0

0
4

0
4

0

0

0

0
4

0
4

0

0

0

0
4

0
4

0

0

0

0
4

0
4

0

0

0

0
4

0
4

0

0

0

0
4

0
4

0

0

0

0
4

0
4

0

0

0

0
4

0
4

0

0

0

0
4

0
4

0

0

0

0
4

0
4

0

0

0

0

0
4

0

0

0
4

0
4

0

0

0

0
4

0
4

0

0

0

0
4

0
4

0

0

0

0
4

0
4

0

0

0

0
4

0
4

0

0

0

0

0
4

0

0

0
4

0
4

0

0

0

0

0
4

0

0

0
4

0
4

0

0

0

0
4

0

0

0
4

0

0

0
4

0
4

0

0

0

0

0
4

0
4

0

0

0

0
4

0
4

0

0

0

0

0
4

0
4

0.0326153
3

0.0326153
3

0.0229434
3

0
3

0.00520121
7

0

0

0

0

0

0.00447073

0

0
4

0

0

0
4

0

0

0
4

0

0

0
4

0

0

0
4

0
4

0
4

0
4

0.444646

0.281138
7

0.281138
7

0.274239
7

0.274239
7

0

0
4

0.00689936

0.00689936

0

0

0
4

1.73472347597681e-17
7

0
4

0
4

0

0

0

0

0
4

0
4

0
4

0

0

0

0

0
4

0
4

0
4

0

0

0

0

0
4

0
4

0
4

0

0

0

0

0
4

0
4

0
4

0

0

0

0

0
4

0
4

0
4

0.0598718

0.0598718

0.0598718

0.0598718

0
4

0
4

0
4

0.0137987

0.0137987
6

0.0137987
6

0.0137987
6

0

0

0

0

0

0
4

0
4

0

0

0

0
4

0
4

0
4

0
7

0
7

0
7

0
7

0

0

0
4

0
4

0
4

0

0

0

0

0
4

0

0

0
4

0
4

0
4

0

0

0

0

0
4

0

0

0
4

0
4

0
4

0

0

0

0

0
4

0

0

0
4

0
4

0
4

0

0

0

0

0

0
4

0
4

0
4

0

0

0

0

0

0
4

0
4

0
4

0

0

0

0

0
4

0
4

0
4

0.0898371
3

0.0194614
7

0.0127553

0.0127553

0

0
4

0.00670609

0.00670609

0
4

0

0

0
4

8.67361737988404e-19
7

0
4

0

0

0

0

0
4

0
4

0

0

0

0
4

0
4

0

0

0

0
4

0
4

0

0

0

0
4

0
4

0

0

0

0
4

0
4

0.0703757
3

0.0653416
3

0

0

0

0

0

0

0

0

0.0127553

0

0

0.0231043
7

0

0

0

0

0

0

0

0

0

0

0
5

0

0

0

0

0

0

0

0

0

0

0

0

0

0

0

0

0

0

0

0

0

0.010349
7

0

0

0

0

0

0

0

0

0

0

0
3

0

0

0

0

0

0

0

0

0

0

0

0

0

0

0

0

0

0.019133
7

0

3.46944695195361e-18
3

0
4

0.00503411

0.00503411

0

0
4

0

0

0
4

0

0

0
4

0
4

0
4

6.93889390390723e-17

0
4

0

0

0

0

0

0

0
4

0
4

0
4

0
4

1.2033
2

0

0

0

0

0

0

0
4

0
4

0
4

0

0

0

0

0
4

0
4

0
4

0

0

0

0

0

0

0

0
4

0

0

0
4

0
4

0
4

0

0

0

0

0
4

0
4

0
4

0

0

0

0

0
4

0
4

0
4

0

0

0

0

0
4

0
4

0
4

0

0

0

0

0
4

0
4

0
4

0

0

0

0

0
4

0
4

0
4

0

0

0

0

0
4

0
4

0
4

0

0

0

0

0
4

0
4

0
4

1.2033
2

0

0

0

0

0
4

0

0

0
4

0
4

0

0

0

0

0
4

0
4

0

0

0

0
4

0
4

0

0

0

0
4

0
4

0

0

0

0
4

0
4

0

0

0

0
4

0
4

0

0

0

0
4

0
4

0

0

0

0
4

0
4

0

0

0

0
4

0
4

1.2033
2

1.2033
2

0.526084

0.677214
2

0
2

1.11022302462516e-16
2

0
4

0

0

0

0
4

0

0

0
4

0

0

0
4

0

0

0
4

0

0

0
4

0

0

0
4

0

0

0
4

0

0

0
4

0

0

0
4

0

0

0
4

0

0

0
4

0
4

0
4

0
4

0
2

0
2

0
2

0
2

0

0

0

0

0

0

0

0

0

0

0

0
4

0
4

0
4

0
4

0

0

0

0

0

0
4

0
4

0
4

0
4

8.81866
2

0.0255106
2

0.0255106
2

0

0

0

0

0

0

0

0

0

0

0

0

0

0

0

0

0

0

0

0

0

0

0

0

0

0

0

0

0

0

0

0

0

0

0

0

0

0

0

0

0

0

0

0

0

0

0

0

0

0

0

0

0

0

0

0

0

0

0

0

0

0

0

0

0

0

0

0

0

0

0

0

0

0

0

0

0

0

0

0

0

0

0

0

0

0

0

0

0

0

0
4

0

0

0
4

0

0

0
4

0

0

0
4

0

0

0

0

0

0

0

0

0

0

0

0

0

0

0

0

0

0

0

0

0

0

0

0

0

0

0

0

0
4

0

0

0

0

0

0

0

0

0

0
4

0.0255106

0.0255106

0

0
4

0

0

0

0

0
4

0

0

0

0
4

0

0

0

0
4

0

0

0

0
4

0

0

0
4

0
4

0
4

0.0255106
4

0.0255106
4

0.0255106
4

0

0

0

0.0255106

0

0

0
4

0
4

0
4

0.0753455
7

0.0753455
7

0.0753455
7

0.0625902

0

0

0.0127553

5.20417042793042e-18
7

0
4

0
4

0
4

0
4

0
4

0

0

0

0

0
4

0

0

0
4

0

0

0
4

0

0

0
4

0
4

0
4

0

0

0

0

0

0
4

0
4

0
4

0

0

0

0

0

0

0
4

0

0

0

0
4

0
4

0
4

0.0648255

0.0648255

0.0648255

0.0648255

0
4

0
4

0
4

0

0

0

0

0

0
4

0

0

0
4

0
4

0
4

0

0

0

0

0
4

0

0

0
4

0
4

0
4

0

0

0

0

0
4

0

0

0
4

0
4

0

0

0

0
4

0
4

0
4

0

0

0

0

0
4

0
4

0
4

0.610348
3

0.586744
3

0

0

0

0

0

0

0

0

0

0

0

0

0

0

0

0

0

0

0

0
4

0.586744
3

0

0.586744

0

0

0
4

0

0

0

0
4

0

0

0
4

0

0

0
4

0

0

0
4

0
4

0.019133

0.019133
2

0
2

0

0

0

0.019133

0

0

0

0
4

0

0

0

0

0
4

0

0

0

0

0

0

0
4

0

0

0
4

0

0

0
4

0

0

0
4

0

0

0
4

0

0

0
4

0

0

0
4

0
4

0

0

0

0

0

0
4

0

0

0
4

0
4

0

0

0

0
4

0

0

0
4

0
4

0

0

0

0
4

0
4

0

0

0

0
4

0
4

0.00447073

0.00447073

0.00447073

0
4

0
4

7.54604712049911e-17
3

0
4

0

0

0

0

0

0
4

0

0

0
4

0
4

0
4

0.0573989

0.0573989

0.0573989

0.0573989

0
4

0
4

0
4

0

0

0

0

0
4

0

0

0
4

0

0

0
4

0
4

0
4

0.0446436

0.0446436

0.0446436

0.0446436

0
4

0
4

0
4

0

0

0

0

0
4

0

0

0
4

0
4

0
4

0.0892871

0.0892871

0.0892871

0.0892871

0
4

0
4

0
4

0

0

0

0

0
4

0
4

0
4

0

0

0

0

0
4

0

0

0
4

0
4

0
4

0.0127553

0.0127553

0

0

0
4

0.0127553

0.0127553

0
4

0
4

0
4

0

0

0

0

0
4

0
4

0
4

0

0

0

0

0

0

0

0

0

0

0

0

0

0

0

0

0
4

0

0

0

0
4

0

0

0
4

0

0

0
4

0
4

0

0

0

0

0

0

0
4

0

0

0
4

0
4

0
4

0

0

0

0

0

0
4

0
4

0
4

0

0

0

0

0

0
4

0
4

0
4

0

0

0

0

0
4

0

0

0
4

0
4

0
4

0

0

0

0

0
4

0

0

0
4

0
4

0
4

0.00738779

0.00738779

0.00738779

0.00738779

0
4

0
4

0
4

0

0

0

0

0
4

0
4

0
4

0

0

0

0

0
4

0
4

0
4

0

0

0

0

0
4

0
4

0
4

0

0

0

0

0
4

0
4

0
4

0

0

0

0

0
4

0
4

0
4

0

0

0

0

0

0

0

0

0

0

0

0

0

0
4

0

0

0

0

0

0
4

0

0

0

0
4

0

0

0
4

0
4

0

0

0

0
4

0
4

0

0

0

0
4

0
4

0
4

0

0

0

0

0
4

0
4

0
4

0.00815845

0.00815845

0.00815845

0.00815845

0
4

0
4

0
4

0

0

0

0

0
4

0
4

0
4

0

0

0

0

0
4

0
4

0
4

0

0

0

0

0
4

0
4

0
4

0

0

0

0

0
4

0
4

0
4

0

0

0

0

0
4

0
4

0
4

0

0

0

0

0
4

0
4

0
4

0

0

0

0

0
4

0
4

0
4

0

0

0

0

0
4

0
4

0
4

0.0510212

0.0510212

0.0510212

0

0

0

0.0510212

0
4

0

0

0
4

0
4

0
4

0.00447073

0.00447073

0.00447073

0.00447073

0
4

0
4

0
4

0

0

0

0

0
4

0
4

0
4

0

0

0

0

0
4

0
4

0
4

0

0

0

0

0
4

0
4

0
4

0

0

0

0

0
4

0
4

0
4

0

0

0

0

0
4

0
4

0
4

0

0

0

0

0
4

0
4

0
4

0

0

0

0

0
4

0
4

0
4

0

0

0

0

0
4

0
4

0
4

0

0

0

0

0
4

0
4

0
4

0

0

0

0

0

0

0

0
4

0

0

0

0
4

0

0

0
4

0
4

0
4

0

0

0

0

0
4

0
4

0
4

0

0

0

0

0
4

0
4

0
4

0

0

0

0

0
4

0
4

0
4

0

0

0

0

0
4

0
4

0
4

0.0127553

0.0127553

0.0127553

0.0127553

0
4

0
4

0
4

0.0127553

0.0127553

0.0127553

0.0127553

0
4

0
4

0
4

0

0

0

0

0
4

0
4

0
4

0

0

0

0

0
4

0
4

0
4

0

0

0

0

0
4

0
4

0
4

0

0

0

0

0
4

0
4

0
4

0
2

0
2

0
2

0

0

0
4

0

0

0

0
4

0

0

0
4

0

0

0
4

0
4

0
4

0

0

0

0

0
4

0
4

0
4

0

0

0

0

0
4

0
4

0
4

0

0

0

0

0
4

0
4

0
4

0

0

0

0

0

0

0
4

0
4

0
4

0

0

0

0

0

0

0
4

0
4

0
4

7.71649

0

0

0

0

0

0

0

0

0
4

0

0

0

0
4

0

0

0

0
4

0

0

0
4

0

0

0
4

0

0

0
4

0
4

0

0

0

0

0
4

0

0

0

0
4

0

0

0
4

0
4

0

0

0

0

0
4

0
4

0

0

0

0
4

0
4

0

0

0

0
4

0

0

0
4

0
4

0.0892871

0.0892871

0.0892871

0
4

0
4

0

0

0

0
4

0
4

0

0

0

0
4

0
4

0

0

0

0
4

0
4

0

0

0

0
4

0
4

0

0

0

0
4

0
4

0.440058
7

0.440058
7

0.40817
7

0.0318883

0

0

6.93889390390723e-18
7

0
4

0
4

0

0

0

0
4

0
4

0

0

0

0
4

0
4

0

0

0

0
4

0
4

0

0

0

0
4

0
4

0

0

0

0
4

0
4

0

0

0

0
4

0
4

0

0

0

0
4

0
4

0

0

0

0
4

0
4

0

0

0

0
4

0
4

0

0

0

0
4

0
4

0.0892871
6

0.0892871

0.0765318

0.0127553

0

5.20417042793042e-18

0
4

0

0

0

0
4

0
4

0.0127553

0.0127553

0.0127553

0
4

0
4

0

0

0

0
4

0
4

0

0

0

0
4

0
4

0

0

0

0
4

0
4

0

0

0

0

0

0

0
4

0

0

0
4

0
4

0.5421
7

0.5421
7

0.529345
7

0.0127553

0
4

0

0

0
4

0
4

0

0

0

0

0

0

0
4

0

0

0
4

0
4

5.61076
8

5.61076
8

5.60629
8

0.00447073

3.63424568217141e-16
8

0
4

0
4

0.127553

0.127553
6

0.127553

0

0
4

0

0

0
4

0

0

0
4

0
4

0.0127553
3

0

0

0

0

0
4

0

0

0
4

0.0127553

0.0127553

0
4

0
4

0.791932

0.785226

0.474628
7

0.306127

0

0

0

0

0

0

0

0

0

0

0

0

0

0

0

0

0

0

0

0

0

0

0

0

0

0

0

0

0

0

0.00447073

0

0

0

0

0

0

0

0
4

0.00670609
7

0.00670609
7

0
4

0
4

3.33066907387547e-16

0
4

0
4

0
7

0

0

0

0

0

0

0
4

0
4

0
4

0

0

0

0

0
4

0
4

0
4

0

0

0

0

0
4

0
4

0
4

0

0

0

0

0
4

0
4

0
4

0

0

0

0

0
4

0
4

0
4

0
7

0

0

0

0

0

0
4

0
4

0

0

0

0
4

0
4

0

0

0

0
4

0
4

0

0

0

0
4

0
4

0

0

0

0
4

0
4

0
7

0
7

0
7

0

0

0

0

0

0

0
4

0

0

0
4

0
4

0
4

0
4

0

0

0

0

0

0

0
4

0

0

0
4

0

0

0
4

0

0

0
4

0
4

0
4

0
4

0.0104024

0

0

0

0

0
4

0
4

0
4

0.0104024

0.0104024
7

0.0104024
7

0.0104024
7

0
4

0
4

0

0

0

0
4

0
4

0

0

0

0
4

0
4

0

0

0

0

0
4

0

0

0
4

0
4

0
4

0
4

0.27771
3

0

0

0

0

0
4

0
4

0
4

0

0

0

0

0
4

0
4

0
4

0.27771
3

0.27771
3

0.27771
3

0
3

0

0

0

0

0

0

0

0

0

0

0
3

0

0
3

0

0

0.27771

0

0

0
4

0
4

0
4

0
4

0
7

0
7

0
7

0
7

0

0

0

0

0

0

0

0

0

0

0

0

0

0

0

0

0

0
6

0

0

0

0

0
4

0
4

0
4

0
4

9.852

0
4

0
4

0
4

0
4

0

0

0

0

0

0

0

0

0

0

0

0

0

0

0

0

0
4

0
4

0
4

0

0

0

0

0
4

0
3

0
4

0

0

0

0

0
4

0

0

0
4

0
4

0
4

0
4

0

0

0

0

0

0

0

0

0

0

0

0
4

0

0

0

0

0

0
4

0

0

0

0

0

0

0
4

0

0

0

0
4

0

0

0
4

0

0

0
4

0

0

0
4

0
4

0

0

0

0
4

0

0

0
4

0
4

0

0

0

0
4

0
4

0
4

0
5

0
5

0
5

0
5

0

0

0

0

0
4

0
4

0

0

0

0

0
4

0

0

0
4

0
4

0
4

0

0

0

0

0
4

0

0

0
4

0
4

0

0

0

0
4

0
4

0

0

0

0
4

0
4

0
4

0

0

0

0

0

0
4

0

0

0
4

0
4

0

0

0

0
4

0
4

0
4

0

0

0

0

0
4

0

0

0
4

0
4

0
4

0

0

0

0

0

0
4

0

0

0
4

0
4

0
4

0

0

0

0

0

0
4

0

0

0
4

0
4

0
4

0

0

0

0

0

0
4

0
4

0
4

0

0

0

0

0

0
4

0
4

0
4

0

0

0

0

0

0
4

0

0

0
4

0

0

0
4

0
4

0
4

0

0

0

0

0
4

0
4

0
4

0

0

0

0

0

0
4

0
4

0
4

0

0

0

0

0

0

0
4

0

0

0

0

0

0

0
4

0
4

0
4

0

0

0

0

0

0
4

0

0

0

0
4

0
4

0
4

0

0

0

0

0

0
4

0

0

0
4

0
4

0
4

0

0

0

0

0

0

0
4

0
4

0
4

0

0

0

0

0

0
4

0
4

0
4

0

0

0

0

0
4

0

0

0
4

0
4

0
4

0.0318883

0.0318883

0.0318883

0.0318883

0
4

0

0

0
4

0
4

0
4

0

0

0

0

0
4

0
4

0
4

0

0

0

0

0

0
4

0
4

0
4

0

0

0

0

0
4

0

0

0
4

0
4

0

0

0

0
4

0
4

0
4

0

0

0

0

0
4

0
4

0
4

0

0

0

0

0

0

0

0

0
4

0

0

0

0

0
4

0

0

0
4

0

0

0
4

0
4

0
4

0

0

0

0

0
4

0

0

0
4

0
4

0

0

0

0
4

0
4

0
4

0

0

0

0

0
4

0
4

0
4

0

0

0

0

0
4

0
4

0
4

0

0

0

0

0

0

0
4

0
4

0
4

0

0

0

0

0

0
4

0
4

0
4

0

0

0

0

0
4

0
4

0
4

0

0

0

0

0

0
4

0

0

0
4

0
4

0
4

0

0

0

0

0

0
4

0
4

0
4

0

0

0

0

0

0
4

0
4

0

0

0

0
4

0
4

0
4

0

0

0

0

0

0
4

0
4

0
4

0
4

0
4

0
4

0
4

0

0

0
4

0

0

0
4

0

0

0
4

0

0

0
4

0

0

0
4

0

0

0
4

0
4

0
4

0

0

0

0

0
4

0
4

0
4

0

0

0

0

0
4

0
4

0
4

0

0

0

0

0
4

0

0

0
4

0

0

0
4

0
4

0
4

0

0

0

0

0
4

0
4

0
4

0

0

0

0

0
4

0

0

0
4

0
4

0
4

0

0

0

0

0

0
4

0
4

0
4

0

0

0

0

0
4

0

0

0
4

0
4

0
4

0

0

0

0

0
4

0
4

0
4

0

0

0

0

0
4

0
4

0
4

0

0

0

0

0

0
4

0

0

0
4

0
4

0
4

0

0

0

0

0

0

0
4

0

0

0

0

0
4

0
4

0
4

0

0

0

0

0

0
4

0
4

0

0

0

0
4

0
4

0
4

0

0

0

0

0

0
4

0
4

0
4

0

0

0

0

0
4

0
4

0
4

0

0

0

0

0
4

0
4

0
4

0

0

0

0

0

0
4

0
4

0
4

0

0

0

0

0
4

0

0

0
4

0
4

0
4

0

0

0

0

0
4

0
4

0
4

0

0

0

0

0

0
4

0

0

0
4

0
4

0
4

0

0

0

0

0
4

0
4

0
4

0.0494115

0.0494115

0.0494115

0.0494115

0
4

0

0

0
4

0
4

0
4

0
4

0
4

0
4

0
4

0

0
4

0
4

0
4

0

0

0

0

0
4

0

0

0
4

0
4
[truncated: 182,082 more chars]
