# Supplementary material for: Fungal communities decline with urbanization—more in air than in soil
Source: ISME J. 2020 Aug 5;14(11):2806–15. doi: 10.1038/s41396-020-0732-1 (PMC7784924; doi:10.1038/s41396-020-0732-1)
Supplement: Supplementary file 2 — Supplemental data [file 41396_2020_732_MOESM2_ESM.zip › Krona_PooledData.html]

Javascript must be enabled to view this page.

num
probth


1000000

375769

9314.45
3

9314.45
3

9310.96
3

9310.96
3

9271.19
3

8.26787

0.0732851

0.366426

0.119824

0.0565967

0.857939

0.107242

0.160864

0.116713

0.119824

0.325902

6.71016

0.414046

16.1363

0.0917376

0.0798828

0.077809

0.0917376

0.14657

0.0798828

0.137606

0.0798828

0.466029

0.0798828

0.159766

0.119824

0.0798828

0.0798828

0.199707

0.122142

0.643507

0.233721

0.27959

0.217883

1.19824

1.27812

2.08211226038202e-12
3

0
4

0
4

0.643454

0.428969

0.428969

0
4

0.214485

0.214485

0
4

2.77555756156289e-17

0
4

2.84192

2.84192

2.84192

0
4

0
4

2.42916797787984e-13
3

0
4

0
4

1.87455

1.87455

1.87455

1.87455

0.793077

1.08147

0
4

0
4

0
4

0
4

0.318211

0.281441

0.281441

0.281441

0.281441

0
4

0
4

0
4

0.0367698

0.0367698

0.0367698

0.0367698

0
4

0
4

0
4

6.93889390390723e-18

0
4

154386

381.375

341.675

242.873

12.7994
3

3.14345
2

0.265631

0.136044

0.764607

0.271308

0.192881

0.0347507

0.137103

0.322802

0.280203

0.451923

0.584993

4.56415

0.380801

0.0849438

0.327165

0.40817

0.258242

0.999396

0.0404642

0.0582163

0.054841

20.266
3

0.0599054

0.0404642

0.184115

0.0404642

0.0404642

0.0129576

0.065547

0.219364

0.144196

0.216294

1.56261

0.00364411

0.0599054

0.0397225

0.0887784

0.106147

0.658285

0.0168673

0.0163868

0.20715

0.0484412

1.00827

0.0409669

0.00897447

0.0129576

0.0599054

0.357411

0.0460286

0.0873245

0.193681

0.0163868

0.325521

2.70143

0.14617

0.00655526

0.129121

0.211081

0.0599054

0.192656

0.0125159

0.219428

0.129121

0.565392

7.43491
7

0.0582163

0.0129576

0.0173753

0.0877713

0.0434384

0.278058

0.113193

0.347468

0.0712667

0.465099

0.60847
7

0.343945

0.0163868

0.174649

0.045462

0.00458879

0.0245801

0.00408015

0.0163868

0.00780182

0.105778

1.70918
7

0.107242

0.0163868

0.0887784

0.110524

0.20597

0.054841

0.0404642

0.00997446

0.062322

0.00500636

3.02148

0.0582163

0.109682

0.0163868

0.0809283

0.310066

0.0595195

0.195564

0.0446436

0.0565967

0.0347507

17.4961

0.270396

7.86305

0.15413

0.0103722

0.0156497

0.0518304

0.129121

0.00458025

0.0127553

0.0950773

0.0163868

1.08383

0.00690984

0.0877713

0.00673085

0.013003

0.0340111

0.438857

0.0530735

0.0506019

0.0530735

0.0809283

0.403449
6

0.263314

0.0582163

0.0598143

0.0703603

0.0327735

0.0163868

0.119629

0.0316877

0.054841

0.131657

0.282642

0.0163868

0.00710613

1.83819

1.02618
6

3.87764
7

2.83648

0.431342

1.81838

6.3839
1

0.616401

13.8144
2

0.433219

0.920309

3.37563

0.236099

1.93339

4.4287

0.539429

0.105088

5.52662
3

2.64646

3.59424

0.168658

0.611272

0.61034

1.29272

0.213028

0.386981

0.500825

4.19643

10.8216
2

2.05654

6.50814

1.6145

0.0916979

0.245199

3.18298

1.59966

0.245801

1.10881

1.32577

7.88752
2

0.320775

0.258752

0.175516

0.123575

0.0207444

0.232382

0.341364

3.25404

5.0913

0.281543

2.65481
7

0.0388765

0.655481

0.700552

0.545039

0.566498

0.926608

0.0367103

2.21118

0.182089

1.14103

4.47895
6

0.438728

0.929243

0.114707

0.0572897

0.0557601

1.93097

0.0182206

0.0491603

0.458906

1.15152

4.13738
6

0.294223

0.183962

0.378025

0.203757

0.140539

0.884165

1.33483

0.815029

0.389385

0.172232

0
4

16.8131
3

14.7783
3

0.69043

1.26134

0.0829891

0
4

0.0351807

0.0351807

0
4

0.807802

0.807802

0
4

0.682776

0.682776

0
4

2.91674

2.91674

0
4

0.134278

0.0938138

0.0404642

0
4

0.0547087

0.00780182

0.0469069

0
4

0.423168

0.0387582

0.384409

0
4

0.276199

0.276199

0
4

0.18114

0.18114

0
4

0.204834

0.204834

0
4

3.17973
6

0.421385

0.0492752

2.62484

0.0842243

0
4

5.65117

5.65117

0
4

1.02773

0.00894146

1.01879

0
4

0.126934

0.0846225

0.0423112

0
4

1.26681

1.09253

0.174282

0
4

0.0768796

0.0768796

0
4

1.93816

1.86627

0.071891

0
4

0.030329

0.0168673

0.0134617

0
4

0.943374

0.943374

0
4

0.716162

0.716162

0
4

0.019133

0.019133

0
4

48.9409
2

44.6012

1.11831

0.451923

2.76947

0
4

0.0368421

0.0368421

0
4

0.145541

0.145541

0
4

0.027535

0.027535

0
4

0.00897447

0.00897447

0
4

0.0775164

0.0775164

0
4

0.536117

0.536117

0
4

0.00777913

0.00777913

0
4

0.123124

0.123124

0
4

0.184686

0.184686

0
4

0.0773585

0.0773585

0
4

0.17262
6

0.156233

0.0163868

0
4

0.0591856

0.0591856

0
4

0.0770648

0.0770648

0
4

0.100278

0.100278

0
4

0.0423112

0.0423112

0
4

0.290687

0.290687

0
4

0.00498723

0.00498723

0
4

0.177448

0.177448

0
4

0.0404642

0.0404642

0
4

0.20715

0.20715

0
4

2.03154

2.03154

0
4

0.262058

0.225887

0.0361715

0
4

0.069043

0.069043

0
4

0.0125159

0.0125159

0
4

0.0703603

0.0703603

0
4

0.169245

0.169245

0
4

0.0273308

0.0273308

0
4

0.0599054

0.0599054

0
4

0.119811

0.119811

0
4

0.00518609

0.00518609

0
4

0.269727

0.269727

0
4

0.00443786

0.00443786

0
4

0.453448

0.453448

0
4

0.536117

0.536117

0
4

0.732115

0.732115

0
4

0.00411208

0.00411208

0
4

3.42263
7

1.61671

1.80592

0
4

0.222498

0.222498

0
4

1.48489

0.451923

1.03297

0
4

0
4

4.65691
6

4.65691
6

4.65691
6

0
4

0
4

0.367379

0.206279

0.206279

0
4

0.1611

0.1611

0
4

0
4

1.90517

1.90517

1.90517

0
4

0
4

0.420657

0.420657

0.0367698

0.383887

0
4

0
4

0.0194364

0.0194364

0.0194364

0
4

0
4

1.70996

1.70996

1.70996

0
4

0
4

0.0259677

0.0259677

0.0259677

0
4

0
4

0.0112449

0.0112449

0.0112449

0
4

0
4

0.763337

0.763337

0.763337

0
4

0
4

0.123124

0.123124

0.123124

0
4

0
4

2.56494

2.56494

2.56494

0
4

0
4

2.20904
6

2.12312
6

2.12312
6

0
4

0.0809283

0.0809283

0
4

0.00498723

0.00498723

0
4

0
4

0.0633849

0.0633849

0.0633849

0
4

0
4

0.0259152

0.0259152

0.0259152

0
4

0
4

0.0255083

0.0255083

0.0255083

0
4

0
4

3.22

3.22

3.22

0
4

0
4

5.36117

5.36117

5.36117

0
4

0
4

0.00498723

0.00498723

0.00498723

0
4

0
4

0.0245801

0.0245801

0.0245801

0
4

0
4

0.184115

0.184115

0.184115

0
4

0
4

0.258242

0.258242

0.258242

0
4

0
4

1.0164

1.0164

0.995017

0.0213864

0
4

0
4

5.36219

5.08754

4.59504

0.369371

0.123124

4.16333634234434e-16

0
4

0.274652

0.274652

0
4

0
4

1.49575

1.49575

1.49575

0
4

0
4

6.12767

6.12767

6.12767

0
4

0
4

0.254349

0.254349

0.254349

0
4

0
4

1.07562

1.07562

1.07562

0
4

0
4

0.422742

0.422742

0.422742

0
4

0
4

4.50750547997814e-14

0
4

67041.4
7

4.95363

1.96168

1.18044

0.746065

0.0322355

0.0029305

3.07913416985883e-17

0
4

2.57335

1.58367

0.989678

3.33066907387547e-16

0
4

0.153701

0.0663766

0.0873245

0
4

0.206688

0.148471

0.0582163

6.93889390390723e-18

0
4

0.0582163

0.0582163

0
4

4.23272528138341e-16

0
4

64462.2
7

64459.9
7

6118.87
7

122.873
7

365.935
6

1.35074

0.0112377

0.0663141

0.0156497

0.0809283

0.0129576

0.00780182

0.0156497

0.0606962

0.007332

0.00736842

0.201283
7

0.0404642

0.0352388

0.0173753

0.00950508

0.0549439

0.00665679

0.00887572

0.00748085

0.216294

0.0199489

0.267188
7

0.0107775

0.0133136

0.010998

0.0591856

0.0469069

0.019133

0.121175

0.00615463

0.243255

0.0695014

0.436618

0.065547

0.0469069

0.00447073

0.0770648

0.0104881

0.266335

0.021996

0.0769775

0.0404642

0.0316877

0.935378
7

0.0091605

0.179716

0.288392

0.216294

0.464213

0.0633753

0.117267

0.0100616

0.0352157

0.0163169

1.44127

0.00527298

0.216294

0.00661392

0.0201232

0.0160762

0.0163868

0.0347507

0.00503411

0.236743

0.00458025

3.02824
7

0.01833

0.00520121

0.00665679

0.0327735

1.70996

0.00736842

0.0236854

0.0189229

0.0170056

0.118371

11.1913
8

0.0839044

0.416305

0.15413

0.0633753

0.007332

0.0404642

0.0049252

0.0324597

0.0189229

0.0255106

0.865608

0.0262201

0.00987078

0.00351532

0.0697535

0.136044

0.0142123

0.00748085

0.0107775

0.0163868

0.326431

7.98183

0.0255083

0.0163868

0.0173753

0.00475254

0.0255083

0.0606962

0.156378

0.0475315

0.00665242

0.0483291

4.48237
7

1.21437
7

0.0163868

0.0156497

0.114798

0.0599054

0.0469069

0.00332561

0.045462

0.017764

0.0172746

0.115072

1.34421

0.0163868

1.15357

0.00447073

0.0170056

0.0409669

0.00443786

0.00443786

0.10842

0.00888202

0.0852543

2.45588
6

0.00518609

0.0434384

0.0110526

0.144196

0.019133

0.127553

0.299527

0.121392

0.0382659

0.125263

1.21812
7

0.0404642

0.0170056

0.0148034

0.0232512

0.0809283

0.0340111

0.0129576

0.00443786

0.118371

0.36049

1.72937
7

0.0029305

0.0469069

0.552681

0.0761658

0.0881189

0.226535

0.0170056

0.0125853

0.0483291

0.0404642

1.40329
8

0.0591856

0.195045

0.00443786

0.0100616

0.0591856

0.0770648

0.0234908

0.00394831

0.0286603

0.0868184

5.58336
8

0.0877713

0.019133

0.00939633

0.0434384

0.00822417

0.0404642

0.032994

0.062599

0.0156497

0.026063

1.82796
7

0.0170056

0.0938138

0.169152

0.0425139

0.0104881

0.0255083

0.00923194

0.00703064

0.00612023

0.0129839

0.982328
7

0.0234746

0.221814

0.0118427

0.0901271

0.0378459

0.0244735

0.714822

0.0103648

0.0387519

0.0208048

7.26512
6

0.0156497

0.0391243

0.0245801

0.0310016

0.0591856

0.0189229

0.00835115

0.00670609

0.0173753

0.00997446

21.1092
6

0.483739
1

0.0155008

0.0080381

0.00498723

0.0938138

0.160604

0.0809283

0.031065

0.0173753

0.0850278

0.00443786

2.16067
7

0.36049

0.0318883

0.0425139

0.119811

0.295928

0.0283844

0.0599054

0.0155008

0.0302047

0.00592247

1.48595
7

0.0633849

0.029229

0.0898581

0.0163868

0.0624145

0.0483291

0.0382659

0.0377558

2.56494

0.147964

25.7005
1

0.0170056

0.0404642

0.331672

0.0391243

1.70996

0.0194758

0.0155008

0.0160762

0.634527

0.363526

1.51119
7

0.0194758

0.0122835

0.21317

0.170509

0.0255106

0.0173753

0.10116

0.0174553

0.0855799

0.0173753

2.10398
6

0.0255083

0.382258

0.605949

0.131094

0.00755116

0.00950508

0.0150924

0.115597

0.141625

0.0112377

7.76796
8

0.0387582

0.0156036

0.0598139

0.00525911

0.0469069

0.00887572

0.0173753

0.60979

0.0599054

0.00525911

2.54151
7

0.0156497

0.00498723

0.357411

0.0112449

0.0573536

0.0129839

0.0234746

0.119811

0.539453

0.00615463

0.346828

0.0379465

0.0201232

0.014664

0.0155008

0.0110946

0.0281121

0.0387582

0.00710613

0.00351532

0.0276876

2.35893
6

0.0118449

0.0483291

0.0251705

0.357411

0.0255083

1.51897

0.00703064

0.00525911

0.0599054

0.00780182

4.61899
7

0.537287

0.00503411

0.024626

0.175543

0.0161662

0.0189229

0.536117

0.0387582

0.0469069

0.00408015

1.80245

0.913348
6

0.0127553

0.0245801

1.25094

0.144196

0.0887784

0.0591856

0.0157321

0.0221893

0.0149617

0.0170056

1.59272
1

0.00939633

0.0201364

0.00458025

0.0510212

0.0300732

0.0114506

0.288392

0.00710613

0.0104881

2.0273

0.458057
7

0.0409669

0.0327735

1.25094

0.0724936

0.0316877

0.0129839

0.135654

0.0155008

0.00458025

0.0352743

0.538945
7

0.0637765

0.0549439

0.00777913

0.0173753

0.00408015

0.0877713

0.0851081

0.0483291

0.0469069

0.0792192

1.36366
7

0.00997863

0.0112377

0.0110232

0.119039

0.00738779

0.0114506

0.102042

0.0425139

0.0245801

0.0255106

11.1119
7

0.00443786

0.0170056

0.013003

0.0316877

0.00967569

0.118371

0.0387582

0.00518609

0.0129576

2.14447

0.521251
5

0.0310943

0.0129839

2.14447

0.00447073

0.0809283

0.00458025

0.0100064

0.026063

0.0599054

0.0189229

1.24311
7

0.007332

0.0347507

0.0483291

0.0491603

0.216294

0.256957

0.0892155

0.891439

0.0599054

0.536117

0.770218
6

0.0266271

0.00967569

0.681285

0.330419

0.0389321

0.0245801

0.00748085

0.0155008

0.0110946

1.10009

14.1616
7

0.335919
7

0.00498723

0.007332

0.00777913

0.0387582

0.065547

0.0712667

0.007332

0.0151023

0.024626

0.0968956

1.02437
7

0.0404642

0.0224897

0.0104024

0.617874
7

0.64061
7

1.64135
8

0.242938
7

12.1298
6

0.860521
6

0.567773
7

2.2729

45.0984
7

0.432672
7

1.04819
7

0.811579
7

0.821898
7

0.928652

0.759311

2.20141

0.605891
7

1.84673
7

0.211289

4.85523

0.575805
7

0.562944

0.377928
7

0.280417

0.32743
7

2.17147
7

0.226869
7

217.828
3

0.457044

0.385216
6

9.90533
7

0.832219
7

0.172543
7

0.542417

0.362194
7

0.276802

2.08549
7

0.689962
7

0.506482

582.562
6

0.957028
6

8.9073

1.31368
8

4.70009
6

0.213355
7

26.7218
6

1.67921

0.907523

1.03214

3.40277

0.202266
7

1.29694

8.34376
7

0.766185
7

0.216794
7

0.9417

0.336898
7

13.2651
6

0.338629
7

14.5898
2

0.498803

0.741807

1.30878
8

83.7326

6.61288
7

3.11507
6

52.3163
6

0.871472
8

2.21251
7

0.452849
7

0.616421
7

37.5206
8

143.917
6

1.51426

1.35629
6

6.50201

0.484462

1.04983

0.443723
6

5.22359
8

44.3591
6

0.804847
7

0.617028
7

1.35831
6

1.15858
8

1.84597
7

8.5127
7

1.03837
7

2.68062
8

0.536143

1.95177
6

0.567098
7

0.180537

0.273564
7

1.31802
7

4.28046
2

0.913741

5.72809
7

5.56612

1.06835

0.413186
6

3.597
7

4.05592
8

0.258092
7

0.637153

0.323663

1.33776
7

0.890154
7

8.08467

1.5636
1

3.48337
8

0.854069
7

6.15888

1.00514
7

4.2708
7

2.15017
6

1.14238
7

3.19594
7

1.89933
7

14.9836
7

0.463536
7

0.482079

0.206686
7

2.46266
6

0.585303
7

6.8031
6

0.35239

0.870909

0.228499
7

0.308719
7

6.38538
7

0.525866
6

0.463672
7

0.40798
7

0.244817
7

2.514

0.982827

2.07679
6

1.9996
8

16.3754
6

2.73016
7

14.2719
7

1.22629
7

0.370853
7

0.501482
7

0.498529
7

0.71573
6

0.513591
7

0.859079
7

1.16693
7

1.06704
7

0.293117
7

5.21801
7

0.603982

3.82112
6

2.3974
7

0.948099
6

0.190187
7

5.30847
6

2.12688
7

14.1595
6

0.505849
7

0.506904
7

10.2157
7

0.730582
7

1.14904
7

1.1512
7

1.78077

1.674
7

34.1115
6

1.18734
7

2.65248

0.576873
7

0.341103
7

46.782
7

8.29217
7

1.55044
6

0.49273

0.306229
7

5.5964
6

0.369515
7

0.901544

0.256617
6

0.341218
7

0.976204
5

1.64448
7

12.982
7

0.336715
7

0.125667
7

0.306431
7

1.39573
8

1.9325
8

2.19047
7

0.617405

0.596178
7

4.11139
1

2.0272

5.64751
7

0.915287
7

0.757932
7

1.10571
6

0.116551
7

2.30604
7

0.398009
5

0.313414

0.44134
7

2.84032
6

0.714754
6

16.07
7

1.48284
7

5.81436
8

1.92177
6

0.509134

0.493381
1

0.306968
7

2.12213
7

0.349711
7

0.313945

1.95811
7

6.66865
7

0.311566

3.68415
6

0.577837
7

2.5581
7

0.361918

0.579586
7

1.0455
7

0.608733

3.0186
6

1.07369
7

5.75189
7

0.678179
7

0.452148

1.02254
6

0.238839
7

1.337
7

0.579934
7

0.47098
7

0.429258
7

0.567368
7

36.4873
6

3.03814
7

0.446913

0.564318
7

0.139687
7

0.512632
7

0.512876
7

2.38571
6

3.23116
7

0.234854
7

1.87464
2

0.396727

19.1524
7

7.70038
1

0.576089
7

1.11385
7

0.664891

0.506688

3.34796
7

0.479949
7

0.995541
7

0.604968

0.40422
7

6.36487
7

0.891362

0.842227
7

2.92012
6

0.241513
7

0.322931
5

0.939
7

0.967343
7

1.68014
7

3.35481
2

1.34675

9.79143
7

0.436838
7

0.947204
7

0.515913
7

0.393714
5

0.402821
6

0.579682
7

0.918001
7

0.369738

2.28838
8

0.308393

78.1438
7

10.7421
7

0.941794
7

3.21316
7

0.320706
7

0.231834
6

0.584178
7

0.547934
7

0.146009
7

1.17649
7

1.24759
7

1.48252
7

5.48709

0.450083
7

0.262135
7

0.866278
7

0.341126
7

1.71473
8

1.34435

0.926921
7

0.630988
7

0.787529
7

0.641218

3.14548
7

1.91534
7

0.503719

0.354483
7

1.57663

1.4174
6

3.39662
2

1.14747
7

1.57705

0.957995
7

1.57635

3.10256

0.283211
7

0.287985
7

4.76145
6

0.322047
6

0.792333
6

98.0397
7

0.626458

2.9767

0.143091
7

0.725751
7

5.6115

0.295959

1.17603
7

0.586931
7

0.447205
7

1.08574
5

0.332962
7

1.21441
7

9.12254
2

1.68835
7

0.384883
7

15.1052
7

3.60481
6

0.532589

0.172354
7

5.74121
6

0.477588
7

0.165498
6

1.244

2.43125
7

0.488153
7

1.61793

6.03406
7

1.54195
7

0.277298

0.400403

7.23028
2

0.283109
7

0.822004
7

0.606709

0.325985

7.07966
6

0.797795

3.08081
7

0.216419
7

0.51343
7

0.306736
5

0.629713
7

1.40186
6

0.564932
7

0.613549

0.461226
7

1.15179
7

1.84881
7

2.35756
7

16.0838
4

1.0327
6

0.479789
7

2.26379
7

2.4537

2.46842
2

0.834839
7

0.0909186

0.200532
7

0.733545
7

55.8453
6

2.12451
6

5.8407

0.180575
7

3.20715
6

0.10702
7

0.132815
7

0.470811
7

0.918524
7

0.338215
7

0.470152
7

59.9174
7

4.79481

3.84684
6

0.328119
7

2.62189
7

0.311278

0.746252

0.403494
7

67.5872
7

0.325098

0.289119
7

0.435335
5

7.57995
7

0.937035
6

0.375081

0.634167

0.371279
6

0.736217
7

1.10155
6

1.24462
2

2.16432
7

2.69051
7

0.456984

6.96402
7

6.57414
4

4.31267
8

0.229279

1.95307

0.232986
7

6.94542
6

0.122898
7

0.65346
7

1.0674
8

1.36181
7

4.50412
7

0.517175
7

1.00939
7

0.742607
1

0.282002
7

0.462412

1.01311
6

1.44116
6

0.367594
7

0.662992
7

1.53187
8

7.89915
7

0.392676

1.58454
7

0.336889
7

0.620432

0.351461

0.379356
7

0.831945
7

2.07204
6

1.76528
6

0.296808

5.61187
7

0.129587
8

1.12974
8

2.4857
6

1.20955
7

2.18283
7

0.751947
7

2.77752
7

5.31572
6

0.560048
6

0.336318
7

2.11685

0.795428

0.672369

0.781452

1.37758
7

2.14407
7

0.273537
7

0.763619
7

0.145464
7

0.252931
7

0.287601
7

4.21279
7

0.315449

2.2726

0.410663
7

0.903063
7

1.43876
6

58.1888
8

0.603409

0.532166
7

0.641854
6

1.26522

6.83578

0.19695
7

0.629013
7

0.679696
7

0.683826

2.23196
7

8.22394
6

0.579177
7

6.41921
7

0.608467
7

1.47737

58.9673
7

0.145421
6

1.23981
7

0.627441
6

1.09494

0.260776
7

0.841736
1

0.2485
7

0.326179
6

0.909726
7

0.2447
6

31.0434
7

5.22366
7

0.848913
7

4.28099
8

1.1982
7

3.17074
6

1.31476
7

0.463538
7

0.494647
7

0.237578
7

0.179395

1.32753
6

12.1246
7

1.54882

0.241868
7

0.936309
7

0.243271
7

0.57073
7

2.23164
7

0.350753
7

0.761155

0.102281
7

0.241961

3.38519
7

0.247416
6

1.4266
6

3.2368

0.347729
7

0.236542
7

0.802493

2.01016
6

0.964182
7

1.18966

2.14051

6.78199
7

0.411656
7

0.396373
7

2.05814
8

0.0810701
7

0.451899

1.38216
6

0.744359
7

3.39302
5

0.20058
7

0.791032
6

6.5441
7

2.23529
7

1.52622
6

3.55785
7

0.281064
7

1.15862
7

0.207014
7

0.675755
6

2.01994
5

0.499073
8

0.128651
7

17.9262
7

0.24036
6

0.14275
8

0.305977
7

0.94853
7

1.2185
6

0.434534
7

2.71305
6

0.99749
7

0.817807

0.155133
7

5.7294
7

0.26616

0.569872
7

0.335414

0.344627
7

0.261816
7

0.0976487
7

0.453488
6

0.346044
7

0.165784
7

4.29281
7

20.6059
7

0.204621

0.410921
7

0.454438

0.396917
7

1.07272

0.478083
6

2.0199

0.289678

1.50197

0.551748
5

6.05973

0.213516

1.42658
6

0.558853
6

0.220669
1

0.436686
6

1.01905
7

3.46478
8

0.845752

0.291359

1.43593

4.7485
7

15.0568
2

0.588445

0.983508

18.0282

3.99394
7

1.08173
7

0.942501
7

0.226117
7

0.713065
7

0.178961
6

18.6402

12.5713
7

0.426195
7

0.369784

0.907648

2.58706
6

1.74088
7

0.220366
6

3.51759
6

0.333687
7

0.94149

0.226476
7

8.12278

0.493277
7

0.693029
7

2.64662

0.260645
7

0.235411

0.275246
7

0.639107
7

2.00209
8

0.610642

0.41699

6.87232
7

3.20679
6

0.337222
7

2.03797
7

0.67676

0.39027
5

0.157831
7

1.15904
7

1.05703
6

0.525558
6

45.6627
2

6.82859
7

0.707778
7

0.3527

0.628615
1

0.216784
6

0.142208
7

1.14941
6

0.906166
7

4.83779
2

0.856626

0.404166
7

3.12901
7

0.22397
7

0.726927
7

2.34519
8

0.0726304
7

0.322799
7

0.155288
7

1.86498
6

0.15308
7

0.862965
1

0.355284

3.83251
7

0.167543
7

0.36463
7

0.218299

0.126405

1.48176
6

0.590022
7

1.36015

0.433441
7

0.125979
7

0.29311
7

4.71066
7

0.171382
6

0.338238
6

55.9369
6

0.386079

0.236189
7

1.09429
7

0.570016

2.43905

0.770314
1

0.363392
7

1936.37
7

0.318399

25.4625
6

1.47397
8

2.31847

1.84201
8

0.852913
1

0.517455

0.385397
6

0.396982
7

0.329629
7

14.345
6

0.166238
7

0.839609

0.308135
7

0.434591

0.163853
7

0.0921685
7

0.462052

0.301711

0.909073

0.153721
7

7.30819
7

0.333236
7

0.403955
7

0.215546
7

0.766431
7

3.15877
6

7.57419
8

0.138962
8

0.318561

2.15417
7

89.7716
6

56.2022
7

2.21158

0.394881

1.09435
1

0.380466

0.2956
7

23.61
8

0.837356
5

5.89945
7

0.541811
6

0.738648
7

1.64138

5.83677

0.172599
7

0.406703
6

0.136283
7

0.172395
7

0.123094
7

1.17833
7

0.248662

3.26954
7

0.161064
7

0.602109

2.56356
7

0.384647
5

0.18798
7

0.57514
7

0.304906

0.451371
7

0.109189
7

0.41473
7

2.88564
7

5.87428
6

0.361384
6

7.35239
7

1.64541

0.221369
7

0.933779
7

0.458998
7

1.689
2

0.463076
6

0.456835
7

38.7636
6

0.094595
7

0.714125
8

2.72379
7

1.04154
7

0.205387
7

0.207439
7

0.190796
7

0.256413

8.34079
8

7.4335
4

0.277438

0.269571
7

1.48847
8

7.4105
7

0.770005
7

0.576261
6

0.12893
7

3.29537

0.153398
6

0.172422

4.51674
6

0.41036
7

0.326238

0.865393
7

11.7293

0.27877

0.635941
7

0.290286
7

1.22051

0.457791

0.12014
7

0.354318
7

0.149433
7

0.717392
7

0.913233

13.0562

0.490514
7

0.103891
7

0.449893
6

0.150843
7

0.0801508
7

0.167226
7

0.523481

0.160943
7

0.281998
7

0.285034
7

6.2903
6

0.245505
5

0.250837
6

0.0986404
6

0.377314
6

0.135674
5

3.43858
7

3.52806
6

0.122073

0.291606
7

0.394448

48.7236
6

14.8172
2

0.594842
6

0.567391
8

0.193439
7

0.36568
7

5.12807
6

0.229134
7

0.327469
7

0.204064

0.855033

31.038
7

33.3427
7

1.07307
6

0.214182

2.94349
6

0.759368

0.264509
6

0.168008

0.386493

3.02944
8

2.54526
8

0.690492
7

2.823
7

0.790062

0.471314

0.0819686
7

0.968008
6

0.474803
7

3.19405
6

1.15396
7

0.445962
6

0.226673
7

0.368232
6

4.06542
7

1.05162
8

2.25497
6

0.36728
7

0.268928
7

0.560392
7

0.34719
7

0.54541
6

0.117523

0.554335
6

2.56632
6

4.74385

0.53528
1

0.276652
5

0.731873
6

0.809784
7

0.761863
6

0.253537
6

0.257583

0.261806

0.798481
6

5.98039
2

38.6241

1.12042
6

1.75966
8

0.427433

0.2444
7

0.0943786
7

0.283023
7

0.428865
7

0.225704
7

0.343375
6

0.107551
7

72.2566
6

0.345009
7

2.74239
6

0.214097
7

0.659946
8

0.473628
7

0.58665
6

0.842383
5

0.641182
7

1.04005
5

0.152088
7

1.73226
7

0.284369
7

0.273711
7

0.575334

0.943402
7

0.409503
7

0.266313
7

0.255934
5

0.112763
7

0.303013

0.290323

38.7538
6

0.131416
7

0.371584

0.206015
6

8.19934
6

0.171548

11.9544
8

0.150552

1.0213

1.10259
8

0.136068
7

3.69724
7

1.29724
7

0.215608
6

0.364908
6

0.44056
6

42.0582
6

10.119
6

0.170215
7

0.335067
7

10.6364
8

0.732742

56.1307
4

0.18284
7

0.314091
5

1.49462
7

0.625622

0.264276
1

0.452093
6

0.856306
6

0.209344

1.08293
7

0.708554

50.142
7

8.58392

2.18877
7

6.63742
6

0.283086
7

2.12731
6

0.222728
6

0.284867
7

0.671649
7

0.217544

0.715501
6

0.220889

4.12005
7

0.556946

0.468159
6

0.428255

2.23715
7

0.345286
7

0.767982
7

0.0971119
7

1.13617
8

0.266541
7

6.00548
6

2.24545
7

0.822446
8

0.280988
7

0.465833
8

10.4771
6

0.34312
6

0.0991836

0.18058
6

1.06366
1

9.07914
7

0.738592
7

4.01493
7

1.93514
6

0.189575
7

0.288295
7

0.148635
7

0.606494
6

0.81965

0.451589
6

0.105432
7

0.191446
7

3.27138
8

2969.45
7

0.309552
8

0.454282
7

0.977662
7

0.240817
7

0.288953
6

3.18921
6

0.840349
8

13.9367
6

0.356613
6

1.55404
1

6.12265
7

0.888938

1.17803

0.152817
7

0.588526
7

5.81254
6

0.276302
7

0.205836
7

0.256281
7

7.45305

0.225816
7

5.19849
6

0.725866
7

0.632264
7

0.645791
7

0.0773472
7

0.165121

1.37932
7

0.248518

0.449885
7

1.05962
6

0.765872
1

15.6439
7

0.216422
7

0.245755
7

0.440517
7

0.129349
8

0.707385
7

0.339337

0.676567
6

8.62506
7

0.88071
7

0.916363
7

5.50053
6

1.6055
7

0.625584
7

0.433961

1.86762
7

0.422387
7

0.354228
8

1.05148
7

0.183716
7

0.504745
7

1.55432
7

8.20148
5

0.122938
7

1.42698
7

1.47419
2

0.271121
7

0.741879
6

0.402174
7

0.591238
7

2.01806
6

0.607132
7

1.0741

24851.9
7

76.8652
7

3.98271
6

2.24582
7

0.162135
7

0.191525
7

2.78134
7

31.0177
6

0.932786
7

0.162962
7

0.377938
7

0.125638
7

1.84494
6

261.809
8

2.21597
6

1.32464
6

1.82719
7

0.374645
7

0.651545

1.29974

0.252897
7

0.169102
5

1.22724

0.889546
7

1.76082

0.109938
7

0.462639
7

0.395727
6

1.85876
8

0.130668
7

0.733535
7

9.33343
4

1.39973
7

1.86452
6

0.986079
7

1.74209
7

1.12375
7

0.238129
7

2.64165
1

0.218594
7

0.70817
6

0.741516
6

0.104347
7

0.216122
6

0.274174
7

0.416307

5.9906
7

1.00425

0.447512
6

0.260505
8

0.0722462
7

4.19776
6

0.289628

0.0816168
7

0.354856

0.0991552
7

0.716516
8

9.62826
7

0.231256

0.219536
7

0.18877
7

0.574299

0.118427

0.165803
8

0.551408

0.112044
6

0.48536

0.57995

1.33156
7

1.28582
7

0.865195

0.197806
7

0.768888
7

0.186286
5

0.571326
6

0.278596
7

2.74356
6

0.333262

0.0770953
7

7.40315

0.474274
7

0.141195

0.725451
7

0.358879

0.154123

0.143656
7

0.248118
7

0.248937
6

0.114518
7

0.110083
7

3.27068

0.235195
6

0.159042

0.394666
6

2.84785
2

0.12601
7

0.170633
8

1.5122
6

17.3074
1

0.176145
6

0.239083
6

1.894
7

0.0956461
7

0.538827

0.641256
8

0.830485
7

0.571563
7

1.92546
6

0.185005
7

0.0470325

0.722894
7

0.103279

21.0165
7

5.52379

0.29437
7

2.28585
6

0.102579

0.38982
7

0.479728
8

0.123616
7

5.7927
7

1.01961
7

2.48697
6

0.269794
7

1.65401

0.0569747

0.450487

0.5106
6

0.059943
7

0.192328
7

0.127758
7

10.423
6

1.47547

0.368605
7

0.107596

3.38883
7

0.609205
8

0.0487669
7

0.69604
7

0.137843
6

0.663323
6

0.373301

0.361633

2.17914
6

0.14637
7

0.224599
7

4.07855
7

0.42429

0.942731

0.351964
5

0.199705

0.135213
7

0.447402

0.174194
7

0.480119
6

0.132774

0.0623792
7

2.46371
7

3.16291
2

1.08913
7

0.661886

0.497109

0.0752503

0.115601
7

0.110367
7

0.875386

0.5464
7

0.151549
7

7.25696
7

0.121488
7

0.133008
7

0.144029
7

0.982412
1

0.708234
7

9.07106

0.311144

0.394081

0.116218

0.235525
5

1.7573
7

0.283612
7

0.277139
7

0.359461
1

8.14443
6

0.59642
7

3.69064
4

0.272521

0.13351
8

1.21689
7

0.266451
7

13.9953
7

0.0798312

0.443512
7

0.125263
6

0.6331

0.476524
7

0.310631

0.13282
6

0.795373
2

0.161309
6

0.853437
8

2.27265
7

0.0907774

0.422457
7

2.72418
6

0.238196
1

0.537447

0.753113
7

0.763901
7

0.673006
7

0.446004

5.3283
8

4.27856

0.100574
7

0.186461
7

0.0667317
8

0.213898
7

0.684383

0.530691
8

0.186408
7

0.390655

0.196977

0.306164
8

30.9878
7

3.24928

0.0598131

0.104276
8

0.203356
7

0.231384
6

0.196057
8

1.21944
8

1.1757
6

0.238542
8

0.229626

1.82187
6

6.73075

0.841988

0.254088
7

1.57524

0.150201

0.150405
7

1.83961
6

0.74348
7

9.16708

0.108457
6

0.300439

5.01984
7

0.164989

0.133312
6

0.138543
7

0.301618
7

0.79519
6

0.321358

0.134948
7

0.454815
7

1.41154
8

0.166606
7

11.2208
7

0.166617
7

1.30408

0.317512

0.260936
7

1.43751
6

0.563422
6

10.945
6

0.111515

1.88628
6

1.15833
5

152.069
6

0.739751

0.142773

0.268402

0.455124

0.455812
7

0.106805

0.138276

0.644746
2

0.71775
1

0.0856296

2.61258
7

0.329968
7

0.223379
7

0.269841
6

0.207003

0.87258
7

0.279249

0.0981917

0.123072
7

3.48371
6

0.145715
7

12.3125
7

0.986055

0.877515

0.157857

1.31367

0.500935
7

0.148458
6

0.763255
7

0.328382
7

0.287673

0.574366

4.92534

0.395552

0.204457
7

1.92183

0.134734

0.238147
7

0.111271
7

0.208702
7

2.88889
6

0.417859
7

4.10136
6

9.36751
7

0.700575
5

0.14258
8

0.0368627

0.253393

0.439087

0.111995
7

0.360051

0.137608

0.545099

0.35791

14.4024
7

0.382966
6

0.106179
7

0.236535
5

0.236755
7

1.94088
6

0.305011
6

0.165305
7

0.107658
7

0.200088
7

1.88359
6

41.6685
7

4.39112
7

0.142837
7

0.410435
7

0.149479

1.29586

0.142794
7

0.209777
7

0.463927
5

0.112216
7

0.0530709
7

1.00177
7

2.96093

0.11732

0.864382
8

1.47433

0.126759
7

0.633162

0.176548
7

0.412254
7

2.73766
6

0.240036

0.067366
7

2.17288

1.91095
6

0.177326

0.12725
7

0.682418
8

0.49438
7

0.462046

0.186928
7

1.64266

0.21853
7

0.305125

2.37864

0.513367
6

0.375221
7

0.124129
6

0.264505

0.124341

2.64495

0.555282
1

0.693513

0.327982

0.355121
7

29.0411
7

0.122167

1.0834
6

0.646748
8

2.68077
6

0.88066
6

0.345094
7

1.39758

0.126028

0.44389
8

0.124653
6

4.81455
7

3.88272
1

0.341398
7

0.88913
1

0.15328

0.273195

0.853065
8

0.0623316
7

0.167236
7

3.4212
6

0.0786425
7

2.15576
7

0.235684
7

1.90888

0.224965
7

0.345371
6

0.147604
7

0.403928

0.507389
7

1.91113
6

0.239278

0.743718
6

10.3904
6

0.575398
6

4.62404
4

1.50556

0.596931
6

0.645527
7

2.75946
6

0.244801

0.130852
7

0.0824571
7

0.197058
8

5.31332
6

1.76502

0.276544
7

0.227081

0.730833

0.171426
6

0.196757
7

0.240159
1

0.331471
7

0.265022

49.1375
6

51.6477
6

0.214833
7

1.79039

0.362847
6

0.237912
7

0.398542
7

3.78207
7

0.0835867
7

0.261728
7

0.814389
8

0.0889978
7

36.1147
7

2.881

0.379213

0.27342

0.149828
6

3.57534
6

0.337153

0.381511
6

0.626031
1

0.46428

0.163724

0.124045
7

4.94029
7

0.161016

0.595385
6

0.11288

0.384243
7

0.0836796

0.124311
7

0.807587
8

0.25106

0.147641
6

0.458373

1.95586
7

0.218192
7

0.170983
6

2.39116

0.0657469
7

0.652357
7

0.389139
7

4.36076
6

0.285561
6

0.0781538
7

0.210341

8.73355
7

0.190141

9.46175

0.208077

0.0618298

0.0613446

0.472046

0.37946

0.65363

0.91462

0.222238

14.4432
7

0.279042

0.070633

0.0888096

0.0821458

0.214398

2.59875

0.13612

0.431067

0.488439

0.286812

1.43643
7

0.09342

0.604997

0.0666086

0.137858

0.717688

0.132613

0.125865

0.098055

2.42763

0.0955101

1.44822

0.281745

0.143155

0.0580219

0.0844482

2.10648

0.0242715

2.21865

0.0776091

0.225363

0.205314

4.65322
6

60.8624

0.125389

0.0907665

0.13798

0.808224

0.137654

0.0579593

2.05762

0.198478

0.0649982

3.9395

0.149068

0.0445114

0.412943

0.179281

0.165059

0.297966

0.150172

0.0452865

2.03049

0.089363

2.80637
7

0.163164

0.0684992

0.458856

0.0789228

0.148474

16.7143

0.125335

0.130567

0.136809

10.5376

21.2317
7

65.348
6

0.161643

3.57957

0.210323

0.0401315

0.300449

0.0377761

16.3177

0.669369

0.925155

0.119191

5.56353
7

0.0719466

0.304431

3.80758

0.0900121

0.0856994

0.105396

0.647567

0.529209

0.37714

0.138094

2.81619
7

0.107576

0.588763

0.0643774

0.138902

3.44891

0.155316

0.154788

0.438467

0.200754

0.12379

4.21292
7

0.106996

0.635028

0.0838883

6.00686

0.266961

88.8835

0.167076

0.0720805

0.169704

0.140937

2.6016
7

0.362258

0.513216

0.0562898

0.16339

0.141115

0.912499

0.299245

0.13853

0.257828

0.620166

2.28251

0.360127

0.177651

0.181108

0.166646

4.72614

1.80217

0.0964705

0.0660451

0.0775299

0.226191

5.45859
7

0.132663

1.15336

0.400728

6.93742

0.221291

0.269134

0.203499

0.497039

0.200137

0.20041

5.55897
7

0.230588

0.29676

1.20415

0.157015

0.116021

0.172375

0.0459743

52.0136

0.113114

0.19271

1.22931
7

0.0353973

0.0501761

0.981254

0.101555

0.390098

0.361859

1.85457

0.114041

0.26964

0.130096

7.04331
6

0.129447

5.34922

0.108973

1.79181

0.0966208

0.0884354

0.574025

0.203359

1.19322

0.947485

31.5593
7

29.9434
6

4.20061

0.0424419

0.424014

0.201864

0.0798775

0.247784

0.316448

2.10879

0.0510326

0.165353

3.99358
7

0.407435

0.181553

0.177119

0.302366

0.0977358

0.0744167

0.055735

0.0720354

4.95482

0.906077

2.31263
7

0.434855

0.0704694

0.932392

0.272729

0.142214

5.63841

0.384895

0.557941

0.214137

5.47896

3.91249
7

0.411441

0.23431

0.190051

0.276953

1.97991

0.264676

0.142423

0.0385975

0.578706

0.285357

1.44984

0.302281

0.0951779

0.248992

0.143701

0.490069

0.598219

0.207799

0.276692

5.25046

0.069285

2.72294
7

0.307327

0.269643

0.400965

0.0634286

0.0234103

0.728414

0.56854

0.472335

0.591351

1.87986

6.02561
7

0.0991791

0.143128

0.0530582

0.0964039

0.144347

0.126653

0.0449041

3.56386

0.0969451

0.181588

5.68307
7

0.123132

0.0895259

0.176232

0.228715

0.303553

0.170936

0.0725606

0.533218

0.629198

0.314757

2.5738

0.347768

0.239111

0.183251

0.392198

0.162522

0.432391

1.47009

0.588244

0.148355

0.166958

2.81733
7

0.40544

0.239134

0.211123

0.218917

0.156534

0.0976387

0.454282

0.124214

0.0949085

0.137085

45.3469
7

65.8955

0.133476

2.65399

0.0363182

0.0431824

0.0665059

0.0702199

0.21623

0.478271

0.17372

0.572365

5.05291
7

0.0863207

0.0865365

0.169251

0.503442

1.79101

9.76335

0.488004

0.308605

0.0587331

0.164829

5.55492
6

0.764935

0.733527

0.145365

1.61363

0.697368

0.0897121

0.0989192

0.0893967

0.123642

0.0966733

6.09575

0.934016

1.16237

0.0710332

0.10277

0.0650449

0.0709502

0.0763331

0.483384

0.197012

0.191301

2.42815

1.85412

0.110667

2.58958

0.567768

0.338786

0.206185

0.0526549

2.6889

0.201025

2.17206

12.5012

0.0969854

0.280754

0.0922387

0.0875305

0.173719

0.151807

3.19197

0.571142

2.0407

0.160674

2.73971
7

0.0965829

0.0886278

0.512659

0.161406

0.138836

0.077549

1.19709

0.0486271

0.0597055

2.67177

3.69112
7

0.277293

0.167761

0.079644

0.167315

0.142202

0.1102

0.150686

0.39831

0.300318

1.6891

6.76863
7

0.102173

0.0427823

0.368999

0.0641213

0.373522

0.17504

0.312463

0.158301

0.327604

0.36249

12.7189
6

0.242596

0.0431206

1.23351

0.198765

0.181699

0.672899

0.595607

0.418988

0.231416

0.118927

14.7613
7

5.67879
7

0.336845

0.334305

2.11618

0.115278

0.0573029

0.168254

0.190106

0.0673118

0.503367

1.78734

7.40716
7

0.230814

0.572025

0.0708126

0.429567

0.551871

0.413522

0.486173

0.412823

0.0990639

0.142155

9.95456
7

1.03885

0.131703

2.71095

0.284759

0.311287

0.18429

0.0360776

0.104847

0.488584

0.0539875

4.83287

0.0887361

0.196512

0.32836

0.110696

0.116066

0.641497

0.13685

0.533287

3.39371

0.279777

2.13005
7

0.192433

1.23505

0.865175

0.354164

1.79144

0.40146

0.232384

0.166789

0.0367694

0.239521

5.56271

4.30095

0.0686512

0.0603863

0.185681

0.268557

0.644609

0.164443

0.0479282

0.279901

0.58039

1.26329

0.1788

0.919745

0.0603518

3.73757

0.569654

1.13764

0.39364

0.04525

0.298524

0.276696

1.47098
7

0.102518

0.26574

1.74321

0.241665

0.0428906

0.0601621

3.89763

0.836818

0.518065

1.12159

5.24151
7

0.75726

0.278004

0.225939

0.153026

0.0958767

0.371693

0.495043

0.358266

0.23072

0.0745437

1.45099
7

0.760612

4.9238

0.0883408

0.350944

0.134524

0.350748

0.118005

0.0790725

0.511578

27.8919

50.0514
7

5.58196
7

0.431763

0.409182

0.273389

0.175411

5.19428

0.374712

0.64045

0.113536

6.77327

0.0658303

3.90526
6

0.416656

0.100117

0.269356

0.699919

0.141616

0.035154

0.380569

0.33944

0.295799

0.366028

17.246
7

0.144124

0.974186

0.190796

0.174035

0.128428

0.18375

0.0432017

0.1089

0.0986378

0.106847

1.53592

0.043595

0.48926

0.306558

0.259268

0.659769

0.167448

0.509804

0.0439763

0.194054

0.100063

10.1622
7

0.142744

0.187856

0.411057

7.78728

0.162819

1.97957

0.28656

0.0876381

0.579456

0.133546

12.181

0.42986

0.0714849

0.098758

0.167421

0.246916

0.35748

0.0458434

0.120955

0.0574505

0.11078

39.0863
7

1.78807

0.0340187

0.443811

0.245298

0.0302358

0.983754

0.691298

0.112228

0.0770525

0.356986

6.91361

0.0960007

0.256182

0.380136

0.248422

3.29567

0.0511359

0.0772376

0.045849

0.728739

0.0274374

26.8484
7

0.0328429

0.0904569

0.197618

0.170459

0.252977

0.152496

0.266736

0.38872

0.295271

0.0966442

1.71189
7

0.0402677

0.173187

0.818404

0.376475

0.623295

0.0535976

0.0287973

0.0259306

2.45339

0.331428

1462.45

28.6011
7

2.08583
7

0.076744

0.0851589

0.12401

1.40024

0.233571

8.06718

0.0362613

0.137879

0.0909259

0.292786

3.25627
7

2.15483

0.224246

0.74532

0.0535134

0.0393681

0.0757732

1.66648

0.161762

0.0638612

0.411295

2.52315
6

0.069151

0.238634

0.0217666

2.38149

0.0453285

0.382113

0.200096

1.95083

0.329809

0.0751353

2.3129
7

0.906512

0.405452

0.0933347

0.0306972

0.326519

0.101333

0.353915

0.0812424

0.444037

0.349125

2.18549
7

0.735112

0.316616

0.114071

0.0610446

70.6053

1.84584

0.322847

0.23979

0.147492

0.0469292

5.51889
7

1.93884

0.0883539

0.272408

0.260092

0.0378861

0.0296627

0.0785484

0.0871535

0.138776

0.078819

1.17301
7

0.132775

3.51824

16.2822

0.0709979

1.21285

0.106735

0.188443

1.8404

0.0963718

0.0482024

1.15873
7

0.101493

0.0544324

0.249766

10.9656

1.16911

0.138948

0.364286

0.324929

0.239952

0.0387962

1.36216

0.0738156

0.108962

0.337212

0.127998

9.79107

1.30776

0.278508

0.0632794

0.392351

0.0767418

5.60284
7

0.0434945

0.823432

2.66217

0.0271824

0.0475057

4.54664

0.033222

0.102013

0.243458

0.230665

23.7323
7

4.47269
7

0.070066

0.0429147

0.143603

0.0703223

0.0679575

0.0856183

0.0424185

0.0370688

0.846733

0.0699155

32.0575
6

0.165121

0.220592

0.0737892

0.604904

0.338457

0.21074

0.0841904

2.52392

0.0755266

1.74335

4.15277
7

0.113726

0.467117

0.0977067

0.0978672

0.966415

0.384975

0.0867253

0.123199

0.292623

0.162517

5.9923
7

0.106464

0.135231

0.166411

0.131952

0.0445428

0.0254941

0.0635801

0.0772043

0.31329

0.192953

6.01831
7

0.122641

0.0560216

0.126613

0.0334257

0.0705436

0.0341319

0.173949

0.119736

0.0363657

3.12415

3.35984
7

0.0979045

0.104096

0.647814

0.168726

0.0668862

4.21226

3.74278

0.194317

0.148615

0.0691597

1.88981
7

0.0434796

0.268314

0.155538

0.232235

0.0305983

0.174671

0.0270422

0.0793999

0.0337105

0.118032

3.35274
7

1.28849

0.101443

0.193368

0.20744

0.184316

0.183553

0.174162

14.4691

0.0380959

0.0464653

1.8831
7

0.0435955

0.232147

1.32138

0.187016

0.0887625

0.10389

0.112348

0.0785845

0.199301

0.116583

1.35467
7

2.98232

0.139538

0.102097

0.161977

0.116974

0.41167

0.298602

0.0342275

0.181551

0.112633

29.4779

3.24076

0.905214

0.037161

0.0781898

0.0152294

6.55935

3.24319

0.45401

0.0419522

0.419956

0.0307469

2.0441
7

0.141922

2.82617

0.0315662

0.0913508

0.0443783

0.125964

0.0338218

0.737859

0.190393

0.0683673

2.00646
7

0.344913

0.163185

0.0843918

0.06143

0.206932

0.0384967

0.257129

0.17228

6.87632

0.0545982

6.40091
7

0.548628

0.221107

0.0298518

0.252016

0.0373694

0.582585

0.61465

0.042447

0.0580744

1.33877

1.65126

0.270053

0.83416

0.218749

0.089831

0.0567129

0.0281468

0.175386

0.824111

0.0578957

0.179097

2.92986

0.0512321

0.119544

3.4424

0.048044

0.570541

0.0290065

0.657404

2.63906

0.26278

4.40766

2.93924
7

0.130545

0.0999229

0.198194

0.162115

0.156561

0.42489

0.111286

0.16711

0.57438

0.0931195

4.1194
7

0.0745885

0.0627318

0.0481212

0.153537

0.0309536

0.236077

0.65418

0.136011

0.374665

0.0988929

43.754
4

0.0885812

0.563908

0.267311

0.186137

0.451569

0.141642

1.11366

0.0284583

0.297899

0.0655124

1.71344
7

0.686467

0.639562

0.410969

0.33016

0.0567825

0.114666

0.125671

0.15131

0.173725

4.78624

287.947
6

1.89667
7

0.547443

0.302225

0.078827

0.813653

0.137119

0.117476

0.0937243

0.0455716

0.500878

0.165197

1.94503
5

0.199358

0.073399

0.197958

0.0903157

0.101265

0.0384818

5.07873

0.0282279

0.374759

0.231391

11.5004
7

0.163721

0.574051

0.411948

0.115906

0.16788

0.0234161

0.115593

1.69726

0.062821

0.131599

3.35514
7

0.0478972

0.976032

0.0863317

0.169306

1.63484

0.109846

0.254396

0.193583

1.08412

0.129322

4.45182
7

0.418799

0.0465023

0.294408

2.97437

0.149302

0.189015

0.0661474

2.65402

0.0510438

0.13204

0.813313

0.153864

0.089959

0.0926789

0.143998

0.129958

0.0394447

0.199449

0.143847

0.948208

83.9205

1.71215
7

0.067339

0.784962

0.113655

0.0673784

0.155206

7.12019

0.668917

0.0693281

0.0872906

0.0901819

3.02492
7

0.182527

0.132636

0.0830902

0.947376

0.054651

0.26305

0.143415

0.191966

0.0428678

0.386371

2.8761
7

0.0999511

0.0502119

0.0532441

0.0882071

0.114158

0.0807968

0.0206582

0.0839853

0.156052

1.65347

2.20166
7

0.222793

0.0317604

2.57364

0.0911059

0.100455

0.098824

0.204011

0.0814238

0.556247

0.122391

19.8902

4.12529
7

0.238616

0.778237

0.232808

0.0603079

1.03809

0.0343534

0.0762607

0.110445

0.428315

0.18627

7.78783
7

1.5429

0.0645342

0.174116

0.402647

0.101078

0.124838

0.0296637

0.518982

0.0887252

0.0480025

2.90834
7

0.0688016

0.180047

0.0162244

0.0543555

0.0756451

0.100159

0.0490178

0.0447563

2.00859

0.239841

67.4775
6

0.528632

0.379498

0.0728613

0.0477364

1.28447

0.123697

1.07966

0.0895661

0.0950936

0.149352

166.744
7

0.375469

0.559879

0.0709732

0.043374

0.359446

0.0512624

0.375527

1.3111

0.0660392

0.105712

15.7122
6

0.0490848

0.0909563

0.0381804

0.463217

0.258787

0.0502374

15.806

0.0962539

0.0185326

0.222553

7.32655
7

0.130709

0.205537

0.075977

0.0668853

0.0427619

1.34028

0.141814

0.411497

0.175004

4.10837

1.7785
7

0.473576

0.167848

0.181695

1.30023

0.22853

0.0728644

2.4505

0.0264726

0.0311445

0.159261

1.48287
7

0.0479785

0.620911

0.705277

0.112547

0.290328

0.234211

0.0522378

0.0258587

0.170125

0.185032

1.88821
7

0.190924

0.0463006

3.43279

0.134287

1.79013

0.272262

0.0635119

0.0726183

0.0897774

0.0520066

26.0593
7

3.78558

0.0774719

0.184868

0.0402685

0.0679604

0.616248

0.063691

0.209199

0.0863788

5.94835

0.271243

2.95094
7

0.190997

0.391116

0.101474

0.0946147

0.153951

0.0860682

0.084967

0.251337

0.0213853

0.135223

39.4317
5

0.120421

0.101483

0.150844

0.0698421

0.0519413

0.0703788

30.198

0.0377001

0.133238

0.683444

4.68734
6

0.889528

0.039506

2.05359

0.0883575

0.145599

0.676257

0.15616

0.133328

0.252411

1.37801

2.76936
7

0.0787874

0.14242

0.166009

0.244108

0.295833

0.139009

0.255704

0.585903

1.34028

0.260737

1.82729
7

0.0525771

0.115601

0.0372457

0.0493178

8.39722

5.16908

0.101204

1.12343

0.110604

0.0337883

10.1352
7

0.0393451

0.0341445

1.16924

0.292028

0.497715

0.191246

0.147748

0.206363

0.153282

2.31821

4.93742

0.192782

0.234817

0.259009

0.134469

0.166022

0.0728458

0.105377

0.0598224

0.482367

0.0655835

4.91649
7

0.103717

0.143416

0.10229

0.347954

0.107031

0.132879

0.0466414

0.822479

0.822693

0.649048

2.46927

0.052179

0.304202

0.313102

0.137104

0.0983553

0.199363

0.397212

0.0797659

0.148516

0.153342

20.0297
7

2.39286
7

0.504579

0.0441846

0.165082

0.174567

0.603573

0.169748

0.0853807

0.0332757

2.68234

0.0767734

1.68521
7

0.126125

0.157244

0.0364131

0.026342

0.155194

0.0869564

0.233681

0.117663

0.06455

0.0399351

12.3867
2

0.14586

0.0252416

0.441141

0.584494

0.0368275

0.0753243

0.721357

0.132572

0.0628952

0.191025

1.4456

0.174193

0.187146

0.536412

0.0825526

0.0925797

0.0496645

0.273908

0.159151

0.123292

0.0589113

5.13997
7

0.314768

0.106453

0.023097

2.12374

0.139543

1.44761

0.0511986

0.126056

0.246184

7.05934

2.38545
7

0.0390105

0.136199

0.0254876

0.172425

0.326472

0.109184

0.337256

1.93934

1.15848

0.340986

4.13576
7

0.0144266

0.569257

0.0490368

0.258442

0.0618059

0.0314523

0.917417

1.37012

0.0847791

0.133723

15.3546
7

24.2552

0.187741

0.124895

1.40738

0.132299

0.09241

0.0890685

0.0539134

0.0358901

0.0319763

2.41493

0.0391419

0.0285084

0.130065

0.0207121

12.0548

0.0362992

0.0539486

0.331824

2.67963

0.12824

6.34421

4.70176

0.0637743

0.394992

0.433404

0.0817566

0.238027

1.12559

0.606422

0.206707

0.122028

26.2911
7

5.81141
7

0.590282

0.0636646

0.0833998

0.0452018

0.142719

0.277372

0.0586063

0.0901767

0.0881793

0.170694

8.20837
7

0.220221

0.240912

0.0943703

0.0602933

0.0726016

1.11901

0.0955345

0.0440738

0.0399873

0.385998

2.53183

0.0321833

0.0748951

0.0921321

0.179712

0.0533031

0.0479171

4.76132

0.0595204

0.08468

0.234778

2.15311
7

0.0702742

7.17753

0.0474363

0.417947

7.86695

0.452704

0.359992

0.130302

0.104834

0.0498465

1.2707
7

0.0612565

0.091949

0.199467

0.215337

0.214319

0.218469

0.219039

0.495655

0.360818

0.111753

1.85077
7

0.0889467

0.266846

0.140264

0.185828

0.0899282

0.119401

1.71867

0.273556

0.0668973

0.244367

7.13348
5

0.608332

0.225167

0.0726944

1.27275

0.946688

0.120533

0.359581

0.0700225

1.79664

0.119227

3.29944
6

0.150736

0.0759162

0.575859

0.373803

1.89942

0.194453

0.228177

1.31714

0.323707

3.92265

10.4971
7

0.0298242

0.118686

1.72504

0.574949

0.0236015

0.0685838

0.299553

0.368925

0.0163144

0.01099

6.39272
7

0.0497854

0.0572147

0.0772808

0.298122

0.10193

0.0330595

0.0508206

0.0592969

0.231842

4.28176

38.716
7

6.85035
6

0.0333225

0.071487

0.017159

0.0612965

0.0373007

1.32465

0.0744197

0.0480744

0.1719

0.0623955

5027.74
6

0.0203717

0.0989125

0.123093

0.0648927

0.0556414

0.0297609

0.067008

0.603113

0.0785946

0.263243

3.56751
7

0.0354521

0.466697

0.0374541

0.143441

0.373452

0.177757

0.230196

0.404926

0.0139473

0.291907

6.97921
7

0.283051

0.0334185

0.0123996

0.0656428

0.117695

0.0985272

0.0328785

0.0208246

0.02023

0.0713306

1.1834

0.292046

0.0429659

0.0380465

0.330863

0.290697

0.396536

0.024438

0.0163066

0.147335

1.74523

2.94359

0.12934

0.104003

0.594544

0.156388

0.146256

0.0250669

0.581709

0.0961571

0.0478669

0.0549858

19.099
6

0.04085

0.0458203

0.023429

0.0596622

0.0689826

17.5948

0.228907

0.0617438

0.166497

0.0271972

2.64671
7

0.0342574

0.0388948

0.271961

1.19613

513.314

0.0856188

0.0463866

0.0340154

0.05823

0.139003

3.9902
7

0.0640799

0.0219928

0.374417

1.40848

0.548747

0.148359

0.115782

0.11203

0.303602

0.0232967

1.00933

0.0956621

0.0368064

0.365524

0.166422

0.177841

0.0142383

0.0401897

0.055817

2.60652

0.0965803

72.7245
7

1.41006
7

0.222799

0.0732203

0.201079

0.0863603

0.0230519

0.0949001

0.083672

0.469922

0.36049

0.0215387

3.30794
7

0.0316782

0.0500031

2.22232

0.742194

0.0276041

0.0213119

0.0257897

0.189288

3.73144

0.103552

3.78399

0.0928733

0.275853

0.041015

0.0367218

0.199399

38.0174

0.0527143

0.0281528

0.225991

0.143756

2.38111
7

0.0726607

0.209388

0.0483804

0.0210887

0.011209

0.77897

0.195888

0.273333

0.0203351

0.00978665

2.43578

1.08272

0.941707

0.119161

0.382047

0.46353

0.033325

0.204101

1.04509

0.066119

1.34126

1.86802
7

0.0589936

0.0150405

0.0737856

0.0113407

0.0858202

0.0803904

0.022942

0.15302

0.0459935

0.115298

12.8741

0.988829

0.117349

0.0228382

0.290034

0.166247

0.053171

0.359971

0.151583

0.159846

0.0794068

1.86989
8

0.278207

0.0213065

0.295092

0.0951612

0.775519

0.232681

0.0417493

0.100171

0.0269899

0.0433305

2.39066
7

0.0618802

0.240046

0.0461942

0.0642197

0.027638

0.0442507

2.23182

0.103925

0.0532195

0.147489

15.725
6

0.367814

0.0171543

0.0264988

0.0323573

0.0826602

0.144975

0.0293706

3.67948

0.0790087

0.0450639

2741.25

20.5901

1.31065
7

0.0236204

0.241835

0.0264137

0.32211

0.58886

0.208147

0.149647

0.0529322

0.0703935

0.0941022

4.00483
7

0.171615

0.0101733

0.148276

0.0372601

0.495327

0.144578

0.372373

1.73834

4.15005

0.243547

10.3006

0.294175

0.0979339

0.0853662

0.106245

0.0221516

0.0343705

0.612432

0.0651765

0.0761912

0.115373

2.64536

0.204903

0.362418

0.0201629

0.0790384

0.509872

0.0314698

0.075126

0.141719

0.0276909

0.0653346

1.46102

0.0572823

0.067883

0.0357672

1.17057

0.11397

0.291817

0.0762003

0.0569649

0.035851

0.0359356

192.437
6

0.0311866

0.03557

0.0281528

0.127622

0.0763814

1.10114

0.113359

0.391984

0.856462

0.0460677

1.44889
7

0.0617814

0.0637615

0.0829557

0.0273676

0.256471

0.0850398

0.0322575

0.0463709

0.308207

0.0482743

1.17308

0.0650915

0.0269348

0.0450629

0.0640834

0.110825

0.0574697

0.050177

0.763323

0.0416954

0.275479

2.08102
7

0.128581

0.053578

0.0156984

0.0622494

0.0819543

0.0768895

0.061634

0.0524934

0.209445

0.0353097

34.2795

0.0656182

0.0658126

0.0214928

0.377785

0.0232383

0.0633384

4.14212

0.305433

10.0934

0.0530046

11.5883
7

37.0695
6

1.12446

0.0776435

0.0892959

0.498132

0.128727

0.390185

0.0103873

0.0491903

0.0763416

0.320262

5.0712
7

0.0228903

0.0542797

0.330781

0.0201637

0.164831

0.0713018

0.0338698

0.259245

0.191306

0.00973656

5.31929

0.0128114

0.0660399

1.43987

0.11422

0.370888

0.102097

0.0128114

0.0257897

0.0285293

0.157153

1.81402
7

0.119402

0.0212006

0.0334621

0.0251926

0.0243934

0.172212

0.0223092

0.0178255

0.0982145

0.0177446

0.568948
7

0.00967194

0.128944

0.0513435

0.152951

0.0423592

0.260992

0.0138342

0.0844053

0.108234

0.154263

1.95438
7

0.0255526

0.0482779

0.0244576

0.0368808

0.0389363

0.0262511

0.0188064

0.177557

0.375499

2.0235

1.06475
7

0.127832

0.0295815

0.0979339

0.223872

0.072879

2.86773

0.0425162

0.0415646

0.0883222

0.0579265

6.65446
6

0.128124

0.046001

0.313543

0.271609

0.0930443

0.0368889

0.0330174

0.037875

0.156794

0.0204054

2.03303
7

0.327144

0.0295374

0.0758156

0.0808479

0.160513

0.0239316

0.0694027

0.515564

0.0515662

0.179483

15.6297
6

0.0354894

1.76986

0.0530029

0.114393

0.107176

0.0911157

0.0308066

0.00838617

0.368656

0.198449

18.9132

11.7702

1.71706

0.0432598

0.138524

0.520301

0.73874

0.198718

0.299876

0.0350083

0.0353096

0.674797

4.12028
7

0.0593027

0.131292

0.0174343

0.373476

0.066085

2.38367

0.292036

1.51527

0.0203065

3.83174

1.78375

0.370395

0.0261378

0.154889

0.0589006

0.0284874

0.0513198

0.365714

0.0302264

0.0501488

0.182357

0.694389
7

0.36049

0.155085

0.0296226

0.300728

0.0245305

0.0248389

0.0581446

0.106721

0.0254341

0.138282

0.742683

0.100752

0.0760313

0.0127406

0.0630731

0.0359987

0.112398

0.127888

0.035851

0.0191286

0.0640431

3.68841
7

0.135747

0.0793812

0.0264272

0.184395

0.167133

0.104533

0.0915931

0.123728

0.0475703

0.0953225

2.08299
7

0.0357902

0.0955644

0.0413126

0.138786

0.0729092

0.129304

0.555488

0.026805

0.152452

0.339646

5.63458

0.0823378

0.0632936

0.0961119

0.029018

0.0874434

0.0225766

0.0768595

0.020347

0.0721518

0.814245

6.02077
6

0.0301856

0.138768

0.20659

0.0367701

0.0102353

0.274239

0.0839158

0.0345713

0.429997

0.0214434

5.21902
7

0.301395

0.0901456

0.0948645

0.0418183

0.239785

0.0274436

0.0523975

0.056548

0.191103

0.0279173

33.4858
7

1.19601
7

0.070497

1.99533

0.0381077

1.7288

0.0361255

1.04349

0.0959554

0.0393391

0.106957

0.0504994

5.27639
7

0.181353

0.218324

0.0378096

0.0302631

0.121194

0.304316

0.110017

7.70863

0.103596

0.555593

2.4106

0.0353641

0.161201

1.32454

0.1927

0.109312

0.242775

0.230741

0.145709

0.373798

0.105961

11.1903
1

0.0291421

0.0782487

0.0458999

0.063912

0.0182042

0.394665

0.0181437

0.189378

0.02023

0.0730286

1.34458

0.076346

0.0127524

0.047287

0.10116

0.414932

0.024319

0.0392214

0.122651

0.0155885

0.0770917

3.91979
7

0.10814

0.0394895

0.147766

0.135839

0.191727

0.275479

1.02193

0.064253

0.169276

0.080036

3.29791
7

0.426037

0.0622165

0.0397917

0.0937657

0.0438631

0.0505445

0.254084

0.0101011

0.0179917

0.0680268

1.23204

0.0345588

0.0406378

0.953434

0.045887

1.23277

0.26427

0.108021

0.363566

0.0648983

0.0415857

2.622
7

0.221261

0.315914

0.114214

0.0254415

0.025536

3.7658

0.209396

0.0301505

0.00826786

0.0335464

2.69807
7

0.373245

0.0508112

0.508766

0.0813602

0.0596622

0.203246

0.100014

0.0643868

1.08348

0.0863173

44.5438
7

0.85721
7

0.0350043

0.0825589

0.0896118

0.795751

0.382922

0.0619191

0.0707066

3.32634

0.375991

0.0268417

1.43422
7

0.112616

0.233161

0.0314642

0.0392232

0.130452

1.95123

0.122192

0.10116

0.0103614

0.0253512

1.87533
7

0.39056

0.0577591

0.0940853

0.0548514

0.698923

0.0394579

0.0976599

0.0257937

0.163587

0.171725

2.44447
7

0.20291

0.497226

0.0817319

0.0662348

0.939075

0.0230435

1.60496

0.048754

2.9373

0.0189389

2.72089
7

0.190997

0.0974578

0.0241959

0.0300194

0.0293706

0.028972

0.0416782

0.0144442

0.176538

0.040326

13.5936
6

0.0245325

0.0693512

0.226946

0.0144332

0.475197

0.6021

0.065547

0.0378058

0.670316

0.0806052

5.42122
6

0.131225

0.0318883

0.0518321

0.0645197

0.0291421

0.0228235

0.0948454

0.212599

0.375031

0.0567688

1.25376

0.265765

0.0498448

0.492031

0.0117971

0.0536793

0.117919

0.159462

0.00956748

0.044443

0.148667

1.44502

0.326238

0.130322

0.0860971

0.067094

0.0267385

0.036687

0.0836479

0.152863

0.0203769

0.146609

4.02203
7

0.166941

0.201207

3.56389

0.36496

0.341611

0.124447

5.55329

0.425837

0.0736464

0.0724152

21.2607
7

1.10315
7

0.074729

0.0179086

0.0981093

0.0510189

0.049063

0.0902014

0.183124

0.011544

0.0533632

0.0650443

11.3028
7

0.429433

1.22567

0.0295593

0.0583532

0.0455471

0.0754122

0.726181

2.47162

0.104752

0.0993556

1.73218
7

0.104788

0.206768

0.0487555

0.208248

0.057561

0.130322

0.0854138

0.120823

4.6675

0.0651645

2.82327
7

0.159331

0.121616

0.136198

0.102573

0.557056

0.147788

0.022942

0.0155222

0.0218018

0.0362867

1.65648

0.0553752

0.0730695

0.111322

0.0458033

0.26904

0.113287

0.135979

0.0810424

0.0706395

94.0628

1.91306
7

0.0465023

0.0214434

0.0998206

0.102573

0.218718

0.0270543

0.0232884

0.535259

0.0850278

0.0407464

3.53615

0.0832523

0.204101

0.251116

0.0198614

0.177948

0.10761

0.0218132

0.0806047

0.0813885

0.120962

1.31417
7

0.0295885

0.0162946

0.153356

0.0377562

0.11002

0.152707

0.0708926

0.109453

0.730249

0.295498

1.04003
7

0.0424492

0.0542326

0.033395

0.105628

0.587293

0.0343037

0.0316782

0.0547052

0.0848439

0.017681

9.05809

0.048629

0.437681

0.161838

0.0202936

0.0216859

0.0218787

3.75259

0.238851

0.0466772

0.0551846

31.1522
7

0.914488
7

0.0328403

0.115791

0.301147

0.171747

0.0234929

0.25046

0.0933587

0.0919591

0.0942393

0.375014

1.48865

0.00993155

0.334665

0.295291

0.044473

0.109415

0.587308

1.97875

0.275261

0.0782354

0.0197611

4.24914
7

1.54595

0.213779

0.041971

0.0225405

0.0925234

0.0454705

0.387616

0.0257114

0.0800167

0.0832523

0.920586
7

0.254753

0.0998206

0.664651

0.307218

0.0104262

0.0236274

0.0223005

0.083672

0.0382588

0.0508666

1.33973
7

0.0708622

0.0358231

0.38292

0.0931662

0.314938

0.0438538

0.0519694

0.0152082

0.194424

0.0829816

1.91357
7

0.0335216

0.0392513

0.0256561

0.194008

0.0747696

0.397165

0.0570783

0.185108

0.0160198

1.73834

1.15006

0.135082

0.0829342

0.119761

0.0267456

0.106286

0.0340061

0.0125332

0.299629

0.117033

0.0442598

2.50301

0.148692

0.0382636

0.126577

0.023302

0.220732

0.0214209

10.7273

0.0827043

0.0728746

0.189612

90.3964
6

0.0271642

0.0738496

0.306842

0.0379596

0.0250437

0.227744

0.0788078

0.0134866

1.98944

0.832516

1.89085
7

0.138739

0.976032

0.0146514

0.0746158

0.0320527

0.171641

0.0582841

0.0267717

0.595551

1.24652

26.3861

1.38705

0.029411

0.0205909

0.119324

0.0274769

0.965593

0.159468

0.0533967

0.0648596

0.0274386

0.376334

0.534166

0.0532638

0.226127

0.593088

0.163119

0.0822983

0.0296226

0.0346655

0.0941543

0.0541344

0.0257897

1.54886
7

0.157434

0.0688569

0.0428859

0.87642

0.555536

0.0337548

0.159697

0.057838

0.314851

0.265588

2.18931
7

0.0891981

0.0217846

0.0389431

0.251647

0.182544

0.412927

0.0415638

0.39686

0.0652332

0.0361749

1.15646
7

0.0894933

0.0458346

0.10766

0.0286202

0.236957

0.241771

0.0489251

0.020702

0.149084

0.765123

0.726983

14.7885

29.2822

0.0793459

0.0637765

0.0347366

0.0355307

0.154258

0.203905

0.191071

0.0671712

4.24493
7

0.276425

0.0337367

0.132917

0.0231275

0.106909

2.45133

0.0473708

0.0261773

0.125702

0.0351872

1.54466
7

0.263314

0.011582

0.0514765

0.0379563

0.0324768

0.0311279

0.151998

0.10842

2.37415

0.0118151

1.52727

0.0547401

0.0874692

0.100803

0.0496408

0.264602

0.0858437

15.08

0.305627

0.146062

0.0625713

1.317

0.241001

0.067336

0.0157284

0.0925986

0.133578

0.0476926

0.0491603

0.171917

0.0647556

2.10113

23.8924
7

0.688316
7

0.0389281

2.59332

0.362612

0.0217385

0.0615622

0.321165

0.76395

0.0123661

0.462665

0.0205497

1.34474
7

0.0620416

5.43504

0.480119

0.09831

0.680635

0.0301306

0.156402

0.0542921

0.111368

0.0349446

5.45096
7

0.068949

0.0331691

0.05155

0.138288

1.72943

0.0392379

0.0634207

0.0288181

0.0724912

1.4173

6.06699

0.0859678

0.0639246

0.0284847

0.0246376

0.361991

0.0834255

0.253107

0.447538

0.0390197

1.76914

1.21758

0.022429

0.0267301

0.176009

0.0517069

0.0965149

0.0937036

0.0242891

0.065702

0.117163

0.0235389

1.50256
7

0.0783156

0.094522

0.382384

0.265202

0.103421

0.0699536

0.100005

0.445026

0.0458808

0.0255106

0.920228

0.0359888

0.0255106

0.379664

0.286073

0.0200896

0.107042

1.47216

0.41995

0.0340061

0.0366258

7.49104
5

0.137195

0.0768625

0.0604033

0.279726

0.117971

0.101401

0.0355781

0.0867128

0.406691

0.0849473

4.22671
7

0.220738

0.0374968

0.0270459

0.725143

0.0860245

0.0301606

0.206626

0.552984

0.0177894

0.0162718

6.34169
7

0.0511241

0.0398041

1.15446

0.0182569

0.0598804

0.0639491

0.0149259

0.296631

0.00926624

0.158623

98.2145
7

1.50174
7

0.0229911

0.352058

0.0371568

0.0649827

0.192353

0.0984558

0.0498823

0.748834

0.0376609

0.0310502

2.39863
7

0.0409026

0.123538

0.0761912

0.0867593

0.0875851

0.0973151

0.0758775

0.29283

0.019937

0.0149617

0.858978
7

0.0659725

0.175543

0.643363

0.0579472

0.233227

0.0181588

0.0911696

0.0257516

0.0870829

0.0285748

0.608556
7

0.364619

0.0196727

0.328856

0.120664

0.448974

0.178996

0.692334

0.0736801

0.0297609

0.162584

6.56497
6

0.0651856

0.0332967

0.0340092

0.13495

0.167671

0.268455

0.0616311

0.0214928

0.128692

0.0319844

1.09794
7

0.548426

3.48197

0.138873

0.50672

0.0470391

0.0162321

0.0937482

0.775519

0.044443

0.118034

0.721655
7

0.093926

0.1361

0.0365196

0.00962395

0.0150554

0.0100213

0.25901

1.81603

0.157153

0.0359019

4.28643
6

0.152716

0.242209

0.0914808

0.227183

0.0669679

1.17205

3.316

0.0561336

0.408824

0.920718

0.930488

0.307525

0.0393679

0.508198

0.0607184

0.0403122

0.0107349

0.0915492

0.0473176

0.190574

0.0343809

5.15415
7

0.086859

0.211378

0.0648813

0.127193

0.10559

0.0291946

0.0221916

0.0174494

0.0292807

0.0826718

565.616

20.6143
7

2.08221
7

0.226269

0.0263787

0.157697

0.0281764

0.0162807

0.0825747

0.139006

0.0733146

8.35795

0.0604055

6.08471
7

0.0580508

0.0978838

0.0201298

0.204863

0.0332875

0.948849

0.0319672

0.0760089

0.0573536

0.456307

22.3008
8

0.164832

0.0457739

0.0223626

0.304042

0.0565317

0.0253406

0.0360124

0.0742661

0.0983205

0.0915731

1.4931
6

0.105801

0.0792671

0.0479874

0.14074

0.0387435

0.897999

0.289738

0.0475673

0.0615709

0.180123

1.74186
6

0.0209138

0.0219556

0.119455

0.0926582

0.160583

0.164651

0.377865

0.0241886

0.0401657

0.114619

1.29146
7

2.57159

1.44697

0.0352235

1.46812

0.0542812

0.211548

7.2597

3.45121

0.102111

0.148684

3.41402

0.0425888

0.0150957

0.283173

0.101736

0.0288727

1.74185

0.139722

0.012515

0.0234456

0.0757176

9.22063

0.746331

0.0495119

0.00947197

0.0741987

0.731041

0.0380559

0.0688086

0.0491134

0.0756845

0.0150488

1.0611
7

0.117061

0.117185

0.0337856

0.235177

0.0299894

0.0877519

0.181926

0.0322602

0.147796

0.0175425

5.90177
7

0.104228

0.417889

0.0282432

0.451511

0.0169303

0.0312928

0.0634094

0.0362983

0.108933

0.365213

32.7801
7

3.61651
7

0.0268522

0.0283081

0.763427

0.14516

0.104376

0.0488264

0.08685

0.204014

0.0176805

0.157143

0.533966
7

0.185331

0.0600721

0.0665344

0.188023

0.0218007

0.0914164

6.55163

0.0102348

0.0796974

0.170914

0.307007
7

0.0670378

0.0318983

0.0356217

0.133646

0.0633753

0.0466548

0.162858

0.0537138

0.102048

0.490309

0.789857

0.0132533

0.0203065

0.164372

0.0414026

0.0923839

0.0776569

0.189011

0.128543

0.0238718

0.0446715

2.08521
7

0.123891

0.294399

0.0358366

0.19824

0.0291883

0.0663827

0.0286123

4.60157

0.0281765

0.0671426

1.10116
7

0.0419522

0.0241117

0.136835

0.0726758

0.181893

0.0567467

0.0210901

0.154195

0.0482995

0.727636

4.0342

0.041112

0.021374

0.0262618

0.18466

0.0540976

0.0198122

0.107444

0.310039

0.388156

0.0187185

1.5588

0.486998

0.0270628

0.0254826

0.231303

0.0610431

3.45121

0.0151328

0.0859624

3.41991

0.085141

20.9229

0.0286922

0.479775

0.0780341

0.183025

0.15312

0.15557

0.124861

0.0118593

5.98926

0.0505375

4.99103
6

0.634548

0.0841708

0.0439594

0.19007

0.722154

0.0149196

0.0681279

0.0906264

0.156008

0.124526

24.2585

2.07279
7

0.0252433

0.0500952

0.304784

0.0344237

0.0552692

0.041492

0.951665

2.98171

0.172789

0.0734436

9.27147
7

0.361849

0.0425105

0.0581518

0.110906

0.06606

0.0423019

0.0703815

0.0383032

0.0628438

0.0394771

1.72475
7

0.290355

0.08468

0.107603

0.0297071

0.0293962

0.0293962

0.0185793

0.0501274

0.285426

0.0271478

3.72988
7

0.0199767

0.0273886

0.0457574

0.233703

0.220071

0.00871653

0.0727693

0.0152076

0.0642527

0.53119

2.20706
7

0.167835

0.0395941

0.0587662

1.52034

0.0617713

0.0792161

0.460909

0.623691

0.0236195

0.0856809

1.03029

0.913004

0.165843

0.0668641

0.0157027

0.0218849

0.256758

1.45564

0.717523

0.0742646

0.0268265

1.28034
7

0.392177

0.0506012

0.220874

0.0564875

0.0257921

0.0466746

0.406216

0.191103

0.48789

0.0179565

2.33319

0.0237187

0.0324624

0.177629

0.107908

0.564501

2.0636

0.0844204

0.0987529

0.46621

0.0748354

2.53811
7

0.72098

0.583257

0.0727181

0.675622

0.0487519

0.015566

1.78407

0.361821

0.0343809

0.0673863

0.843807
7

3.45365

0.0161629

1.4765

0.11239

0.0959439

0.0409957

0.057196

0.11805

0.0384659

0.0410458

28.4935

2.88295

0.0491666

0.0444507

0.616173

5.95408

0.0245567

0.286732

0.0293552

0.0760887

0.0572703

0.0673729

0.671988

0.633323

0.0735741

0.263314

0.185073

0.0891692

0.0179016

0.0800419

0.0440549

0.131883

0.262192

0.34516
7

0.109792

0.0844204

0.0221228

0.0604891

0.119606

0.0199882

0.0614191

0.0750836

0.0734515

0.0288139

1.56329
7

0.0450032

1.16934

0.26594

0.0252928

0.0382636

0.246274

0.0214221

0.0113614

0.0293706

0.0480657

88.1875
7

0.0245567

0.0545227

0.0279251

0.0125544

0.150883

0.243488

0.0798433

0.00916182

0.495132

0.0630615

1.46443

0.0528347

0.124748

0.648893

0.326658

0.186616

0.252579

0.058309

0.152692

0.0737404

0.103799

1.07903

0.18475

0.0949176

0.330388

0.0610844

0.0137439

0.0189514

0.0443025

1.0163

0.0656988

0.223845

1.11906
7

0.386799

0.0850881

0.182899

0.0724905

0.0546527

0.104639

0.122419

0.0288869

0.117323

0.1194

3.51423

0.133433

2.57919

0.0575931

0.175295

0.0770241

0.0201232

0.0473073

0.0296123

0.00448724

0.00443786

5.639
7

0.0170056

0.026063

0.0245801

0.0170056

0.149764

0.172061

0.131657

0.0491603

0.0873245

0.0259677

26.0534
7

2.98387
7

0.357411

0.010998

0.0456992

0.00755116

0.0353533

0.155674

0.0106546

0.00351532

0.432588

0.0112377

0.588577
7

0.007332

0.0170056

0.00520121

0.0201232

0.00443786

0.865175

0.0404642

0.177557

0.504686

0.0129576

148.79
6

0.00710613

0.00458025

0.00458025

0.00738779

0.0608137

0.0610761

0.893528

0.00665679

0.00967569

0.021996

0.881089
7

0.00351532

0.0809283

0.0366292

0.00894146

0.0173753

0.0124681

0.026063

0.0674261

0.0327735

0.0049252

9.89881
7

0.00543897

0.0887784

0.0129576

0.00443786

0.00518609

0.0316877

0.0207444

0.0168673

0.00687037

0.119039

32.686
6

0.0968956

0.0110232

0.0404642

0.0606962

0.0332578

0.00443786

0.0170056

0.0245801

0.0819338

0.0132278

1.81546
6

0.0703603

0.007332

0.0775164

0.0245801

0.0126776

0.0170056

0.0829095

0.288392

0.0156036

0.0170056

3.01068
5

0.0245801

0.0366292

0.128202

0.639083

0.0404642

0.0704238

7.84226

1.54543

0.010998

0.019133

1.67775
7

0.648882

0.465338

0.007332

0.0276876

0.0255083

0.0127553

0.432588

6.45604

0.0127553

0.0877713

0.61103
7

0.007332

0.0156036

0.0224754

0.0562667

0.00351532

0.121392

0.0163868

0.793077

0.025662

0.00777913

19.4535
7

6.25843
7

0.00503411

0.0469069

0.0156497

0.117267

0.00498723

0.0284245

0.00690984

0.0157321

0.00503411

0.00665242

0.414566
7

0.0409669

0.0637765

0.0633753

0.095063

0.482591

0.0473073

0.019133

0.20715

0.0155008

0.00503411

0.848189
7

0.55585

0.0475315

0.0100616

0.0819338

0.0591856

0.0129839

0.00665679

0.85531

0.0163868

0.348824

7.27769
8

0.289974

0.00939633

0.00736842

0.0129576

0.0608137

0.0124681

0.00736842

0.0404642

0.00458025

0.078714

0.593852
7

0.0151023

0.0599054

0.00518609

0.039357

0.026063

0.00967569

0.00887572

0.251943

0.0775164

0.00351532

0.80995
7

0.212865

0.0899014

0.019133

0.00416335

0.0127553

0.00950508

0.0404642

0.0387582

0.139899

0.0600381

0.637176
7

0.0150095

0.0483291

0.00448724

0.0452773

0.0112449

0.0469492

0.107242

0.00661392

0.238078

0.0340111

22.5256
2

0.0704951

0.00518609

0.00665242

0.0154325

0.0680222

0.144196

0.00498723

0.0299143

0.144196

0.0173753

2.62892

1.70996

0.00736842

0.00440928

0.0104881

0.007332

0.0173753

0.209669

0.147964

0.00780182

0.121392

2.07048
7

0.159441

0.0469069

0.0562243

0.0245801

0.00815845

0.0127553

0.0340111

0.0200127

0.0703603

0.714822

15.2526
7

1.91039
7

0.536117

0.147964

0.0103648

0.0080381

0.0274298

0.00615279

0.0283844

0.025662

0.173753

0.905626

1.47546

0.0163868

0.0473073

0.0182042

0.561183

4.27489

0.0416097

1.58749

0.00661392

0.00498723

0.0475315

6.01267
7

0.0234746

0.052126

0.00661392

0.0245801

0.00710613

0.144196

0.0189229

0.00503411

0.0138197

0.00503411

0.888735
7

0.0255106

0.0201364

0.102033

0.0505696

0.0118427

0.280592

0.0581373

0.115597

0.0459313

0.0138191

1.19766
7

0.0726619

0.0468109

0.0100682

0.0049252

0.0599054

0.0591856

0.019133

0.14769

0.0255106

0.18614

4.19234
6

0.0737404

0.0118427

0.144196

0.865175

0.219428

0.0450286

0.147964

0.102033

0.026063

0.0129839

1.5547
7

0.0602858

0.0049252

0.0201232

0.0340111

0.130315

0.0737404

0.0366292

0.0107775

0.0127553

0.0190364

1.83315

0.00416335

0.029328

0.00755116

0.0147756

0.00498723

0.0156497

0.00408015

0.0150924

0.0973179

0.00748085

1.14831
7

0.00888202

0.0404642

0.0255106

0.0163169

0.00351532

0.00881856

0.026063

0.00755116

0.0123093

0.0232512

3.76093
6

0.0366292

0.0245801

0.144196

0.141922

0.0402465

0.093266

0.0316877

0.00923194

0.0484412

0.0109638

14.2119
7

1.87664
7

1.20637

0.00527298

0.0112377

0.359433

0.0170056

0.144196

0.0782487

0.00350607

0.144196

1.70996

0.757306
7

0.0201232

0.00498723

0.00443786

0.0724936

0.00750954

0.00332561

0.00689936

0.0409669

0.0469069

0.00815845

25.718
6

0.0106592

0.0127553

0.179716

0.0245801

0.0245801

0.00416335

0.0318883

0.0049252

0.187628

0.047824

6.08034

0.0604093

0.0150095

0.0732585

0.0316877

0.00532728

0.10116

0.0100616

0.0110232

0.0150924

0.0449507

13.3736
6

0.0469069

0.0133136

0.0606962

0.714822

0.0573989

0.0189229

0.0404642

5.98485

0.0112449

0.141625

130.478
6

0.0120572

0.116275

0.0368421

0.0156497

0.357411

0.00498723

0.140667

0.0129576

0.0697535

0.019133

4.13017
7

0.0547741

0.0127553

0.0465023

0.186532

0.0633753

0.026521

0.782487

0.357411

0.0173753

0.00518609

60.6838
8

0.00777913

0.0404642

0.0234746

0.0809283

0.0469069

0.263314

0.0249362

0.0189229

0.0087883

0.0761741

1.34651
7

1.43405

0.00612023

0.00612023

0.0562243

0.025662

0.00888202

0.045462

0.0276876

0.0312995

0.0125853

2.20746

0.16879

0.0156036

0.216294

2.56494

0.177557

0.00738779

0.0377558

0.237658

0.195445

0.0112449

7.84749

1.76509
7

0.032394

0.566498

0.0606962

0.0143923

0.216294

0.010998

0.0104024

0.0150924

0.0633753

0.00322316

5.03036

0.10116

0.504686

0.0129839

0.0387582

0.121392

0.121392

1.2329

0.00500636

0.0255106

0.0201232

1.20572

0.0510212

0.257988

0.00448724

0.00612023

0.00503411

0.0566397

0.0327735

0.0245801

0.0194364

0.0473787

11.0429
6

0.0174553

0.076525

0.182089

0.0606962

0.122901

0.0112449

0.0156497

0.0163868

0.209669

0.00712881

0.76163
6

0.0770648

0.0194758

0.0112377

0.0366292

0.0049252

0.144196

0.00701215

0.00518609

0.0156497

0.00443786

8.21155
6

0.0318883

0.0786604

0.0938138

0.248377

0.0259677

0.0850278

0.288392

0.0695014

0.295928

0.00525911

27.5442
6

0.0409669

0.0156497

0.018421

0.0155008

0.0190102

0.00408015

0.0573536

2.56494

0.00615279

0.0154325

2.16175

0.0163868

0.0129576

0.0286067

0.00748085

0.10842

0.0100064

0.0756917

5.12987

0.00443786

0.0409669

3.72717
8

0.0255106

0.00673085

0.00950508

1.51406

0.00458025

0.00710613

0.0125853

0.0232512

0.00665679

0.00408015

0.871229
7

0.0469069

0.0637765

0.00887572

0.031065

0.00592134

0.0104881

0.0338404

0.00503411

0.026139

0.0404642

17.9814

6.27578
6

0.0189229

0.00887572

0.0100616

0.0510167

0.0483291

0.0483291

0.0591856

0.648882

0.00592134

0.0281121

0.576301
7

0.025154

0.0765318

0.0156497

0.0898581

0.0213184

0.20715

0.0955644

0.00440928

0.950908

0.0599054

2.18932
7

0.0582163

0.0443268

0.0327735

0.0404642

0.0127553

0.0189229

0.0251705

0.144196

0.0170056

0.0404642

0.866147
6

0.0387582

0.29218

0.0245801

0.169152

0.0217157

0.0680222

0.00703064

0.0469069

0.0259677

0.386483

1.28395
7

0.0583091

0.141625

0.0483291

0.065547

0.0189229

0.0104881

0.0170056

0.00665679

0.00394831

0.149764

2.65198
7

0.0234746

0.0255106

0.0599054

0.00997446

0.576784

0.00443786

0.0340111

0.0104084

0.026063

0.357411

0.777707
7

1.22053

0.0163868

0.0898581

0.0150924

0.0324597

0.0102004

0.288392

0.0155008

0.00532728

0.0163868

2.54308
7

3.86982

0.0137407

0.237658

0.0327735

0.0337346

0.42634

0.0100682

2.56494

0.0127553

0.0868767

1.65741

0.216294

0.142595

0.186532

0.0150095

0.0770648

0.182089

0.0599054

0.119811

0.0947996

0.0255083

76.3874
6

0.0189229

0.0118427

0.00448724

0.262188

0.00416335

0.0792192

0.0404642

0.00592134

0.0446436

0.00592134

1504.76
7

9.73121

0.478646
7

0.0822527

0.0491603

0.144547

0.0132278

3.41991

0.00394831

0.00350607

0.0367082

1.32468

0.00748085

1.22245
7

0.00518609

0.00498723

0.030764

0.0163868

0.0391243

0.0173753

0.0606962

0.0352388

0.0955644

0.00543897

0.509758
6

0.110907

0.0402465

0.0298802

0.0127553

0.0404642

0.0347507

0.0301849

0.0469426

0.357411

0.234534

1.40345

0.0701542

0.00503411

0.0377558

16.2941

0.0163868

0.00498723

0.0127553

0.10116

0.0102004

0.0127553

1.57099
6

0.0281121

0.0915731

0.0427729

5.98485

0.0318883

0.357411

0.0110232

0.0189229

0.357411

0.161857

0.784308
7

0.0125853

0.0387582

0.0156497

0.0255106

0.00592134

0.0049252

0.0104881

0.0446436

0.0195035

0.0194364

1.33087
7

0.0104024

0.0156497

0.00498723

0.0547741

0.00939633

0.0469069

0.0127553

0.0735396

0.937273

0.0104024

103.402
8

0.00777913

0.0255083

0.0245801

2.58933

0.0662303

0.0110946

0.0168565

0.0391243

0.115597

4.27489

1.02781
7

0.0173753

0.504686

0.0189229

0.0118814

0.00518609

0.961275

0.00498723

0.0434384

2.19175

0.140863

1.06517
7

0.0591856

0.00939633

0.0634879

0.00394831

0.00498723

0.0118427

0.0194758

0.097998

0.0606962

0.893528

18.5323

1.29509
5

0.0703603

0.00498723

0.0163881

0.065547

0.00498723

0.0595195

0.0311165

0.117267

0.0112377

0.0781891

1.11684
7

0.00443786

0.00350607

0.163868

0.0323325

1.70996

0.00458025

0.0103722

0.0173753

0.0156497

0.0100616

0.662924
7

0.0519355

0.0157321

0.0366292

0.0966581

0.123614

0.0898581

0.0118449

0.0163868

0.0100682

0.00503411

1.35305
7

0.216294

0.122901

0.010998

0.0189229

0.0451866

0.652442

0.00665679

0.184115

0.110536

0.0127553

2.74697
7

0.00503411

0.0197008

0.121175

0.0633849

0.0347507

0.0475315

0.00624502

0.0080381

0.0151023

0.00665242

114.692
6

0.0321524

0.031065

0.00351532

0.0173753

0.389272

0.0966581

0.00724433

0.0792192

0.194636

0.0781891

2.90681

0.0581373

0.00498723

0.0938138

0.0606962

0.119811

0.00520121

0.532671

0.0283844

0.00498723

1.21246

5.50903
7

1.70996

0.0770648

0.00503411

0.00498842

0.0194758

0.00503411

0.0898581

0.155674

2.09084

0.318687

1.74124
7

0.0387582

0.0340111

0.00592134

0.00887572

0.00503411

0.0229012

0.0104881

0.0606962

0.00458025

0.0173753

1.0698

0.00755116

0.0860736

1.70996

0.00503411

0.0201232

0.0127553

0.0701542

0.0105182

0.0599054

0.0434322

9.19319
7

17.8746
6

0.00615463

0.00518609

0.924777

0.0151023

0.102042

0.0680222

0.234534

0.115597

0.0390091

0.0145135

2.22636
7

0.065547

0.007332

0.0316877

0.0591856

0.0091605

0.0127553

0.36642

0.0138197

0.561233

0.581373

4.81959
7

0.0483291

0.052126

0.0651483

0.0352388

0.007332

0.0599054

0.0170056

0.0129576

0.00350607

0.0276876

0.655932
7

0.0327735

0.095063

0.170056

0.0703603

0.00503411

0.0581373

0.288392

0.0409669

0.00527298

0.0283844

2.77928
7

0.149764

0.0312995

0.076525

0.00665679

0.118371

0.00458025

0.0129652

1.00937

0.127542

0.026063

0.546442
7

0.00780182

0.0606962

0.0422835

0.414258

0.0551547

0.131657

0.0465023

0.432588

0.0189229

0.0168673

1.47749
7

0.0151023

0.00665679

0.00365461

0.0792192

0.0591622

0.0245801

0.00780182

0.00612023

0.0781891

0.00411208

8.65551
7

0.0245801

0.01249

0.0581373

0.00520121

0.0347507

0.0299234

0.368203

0.0425139

0.0465975

0.0409669

0.885286

0.0909077

0.052126

0.0549439

0.0606962

0.0434384

0.0112377

0.0255106

0.0599054

0.00503411

0.0129576

4.02135

0.0104024

0.00665679

0.0129839

0.0173753

0.0316877

0.0133048

1.70996

0.0695014

0.0100616

0.0409669

19.6148
7

9.52112
6

0.0255106

0.0606962

0.0387582

0.0316877

0.0316877

0.147079

0.216294

0.0591856

0.0327217

0.0129839

1.90818
8

0.00612023

0.00520121

0.0104881

0.123614

0.0199489

0.374122

0.0168565

0.0469492

0.0155008

0.00520121

1.3619

0.139899

0.0318883

0.0163881

0.146353

0.0966581

0.0127553

0.052091

0.00923194

0.0156497

0.0314642

0.59342
6

0.0194758

2.85929

0.0877713

0.10116

0.0232512

0.0547741

0.142595

0.0387519

0.0213864

0.0340111

41.6047
6

0.536117

0.0968956

0.0503081

0.0112377

0.0829095

0.0404642

0.139287

0.0170056

0.288392

0.00364411

0.675253

0.0176194

0.0425139

0.0338649

0.0155008

0.0387582

0.00443786

0.10116

0.0327735

0.0107775

0.00503411

1.67642

0.0898581

0.00543897

0.0732585

0.0156497

0.357411

0.00443786

0.0599054

0.0168673

0.121392

0.0792192

0.945637
7

0.155033

0.0510167

0.026063

0.164174

0.0898581

0.0127553

0.00498723

0.120823

0.0697535

0.283249

2.78393
7

0.216294

0.144196

0.0221893

1.70996

0.0255106

0.00520121

0.00665679

0.0292553

0.00475254

0.0318883

20.1988
6

0.174412

0.0087883

0.00690984

0.0259677

0.361685

0.00525911

0.0434384

0.0388728

0.169152

0.0236854

18.0648

1.45374
7

0.0404642

0.032394

0.0262201

0.0340111

0.0606962

0.0255083

0.052126

0.121175

0.536117

0.424874

0.704237

0.0382659

0.0224754

0.121175

0.175532

0.01833

0.00815845

0.0189229

0.0157321

0.0189229

0.0251705

0.87929
7

0.0581373

0.0318883

0.0163868

0.0510167

0.0316877

0.00748085

0.0938138

0.0155008

0.0473073

0.0599054

1.84922
7

0.131101

0.0340111

0.0483291

0.0469069

0.0491603

0.00615463

0.00624502

0.00503411

1.42964

0.0281121

0.668877
7

0.0469069

0.0127553

0.0382659

0.0727532

0.010998

0.00440928

0.0049252

0.0599054

0.00950508

0.0156497

0.713297
7

0.0483291

0.010998

0.0770648

0.0173753

0.007332

0.00665679

0.0127553

0.00780182

0.0170056

0.0469069

0.754907
7

0.0168673

0.865175

0.0332578

0.0344764

0.00690984

0.0232512

0.0599054

0.285189

0.116275

0.00498723

1.7203
7

0.0591856

0.0173753

0.0606962

0.0255106

0.288392

0.0387519

0.00997446

0.0573536

0.00888202

0.0170056

1.5775
7

0.00777913

0.00887572

0.0983205

0.0404642

0.00443786

0.0314642

0.0199704

0.0209761

0.0110946

0.013003

1.15339
7

0.614399

0.0703603

0.0404642

0.0104084

0.0312073

0.00518609

0.00351532

0.11294

0.0353533

0.00503411

25.0125
7

2.52492
7

0.0150924

0.0127553

0.0316877

0.0316877

0.110309

0.0157053

0.0581373

0.0892871

0.0127553

0.0598139

2.84253
7

0.25421

0.0633753

0.0703603

0.0887784

0.007332

0.00815845

0.0091605

0.0103648

0.0809283

0.00443786

2.84654
6

0.0220464

0.288392

0.0469069

0.0163868

0.00592134

1.70996

0.288392

0.222553

0.0340111

0.0351532

1.94829

0.0782487

0.189229

0.0104881

0.0100682

0.0382659

0.0703603

0.00498723

0.0100682

0.100755

0.0157321

2.54427
7

0.0145764

0.0127553

0.131657

0.0245801

0.00411208

0.0695014

0.144196

0.130297

0.0591856

0.0189229

0.909308
7

0.142595

0.095063

0.0446436

0.0775164

0.492522

0.00503411

0.0173753

0.182089

0.0475315

0.389385

0.701547

0.0855237

0.00351532

0.0127553

0.0327735

0.00987078

0.357411

0.0382659

0.0655183

0.0868644

0.0703603

3.91665
7

0.0311165

0.0199704

0.01833

0.793077

0.0226535

0.0173753

0.007332

4.32588

0.0163868

0.389385

1.51536
7

0.0170056

0.00443786

0.0226535

0.154717

0.0606962

0.0296123

0.00967569

0.0591856

0.0189229

0.119811

2.15828

0.164174

0.609327

0.00498723

0.179768

0.00518609

0.0112181

0.144196

0.0172746

0.504686

0.0482286

35.1696
7

13.0174
7

0.0324597

0.0163868

0.216294

0.0327735

0.0114506

0.0124681

0.0390837

0.00458025

0.45415

0.0173753

1.72118
7

0.0194364

0.768084

0.0483291

0.00777913

0.00518609

0.144196

0.00755116

0.0898581

0.019133

0.014664

1.49547
7

0.00738779

0.00967569

0.0155325

0.0770648

0.00816031

0.714822

0.0460286

0.0599054

0.0571222

0.188448

3.84749
1

1.70996

0.0599054

0.402729

0.062599

67.5433

0.0234746

0.0591856

0.0347507

0.0129839

0.0935306

1.01176
6

0.0087883

0.00738779

0.0259152

0.007332

0.0182042

4.27489

0.177557

0.00815845

0.00364411

0.144196

0.551172
7

2.06263

0.00440928

0.0703603

0.029328

0.937273

0.36049

0.0269437

0.0404642

0.00498842

0.00615279

1.56957
6

0.746056

2.56494

0.428969

0.285189

0.0259677

0.0147756

0.0434384

0.147964

0.153064

0.007332

0.535453
7

0.0599054

0.0312073

0.0633849

0.0703603

0.118071

0.00498723

0.0100682

0.0112377

0.0898581

0.0124681

30.4045
6

0.00440928

0.0206981

0.00690984

0.290687

0.0173753

0.0469069

0.0129652

4.27489

0.0387582

0.0599054

1.45475
7

0.390837

0.0510167

0.0100616

0.110907

0.0127553

0.0157321

0.0110946

0.147964

0.0100616

0.0503411

21.1363
7

0.784602
7

0.0112377

0.026063

0.0163868

0.144196

0.0887784

0.0591856

0.0112449

0.0156497

0.019133

0.0316877

1.27157

2.59152

0.0938138

1.70996

0.0573536

0.0409669

0.115597

0.0201232

0.0194758

0.00503411

0.0327735

5.55852
7

1.70996

0.357411

0.0781891

0.052126

0.164174

0.118371

0.694637

0.00655526

0.00503411

0.00665242

1.20532
7

0.045307

0.0316877

0.00923194

0.0382659

0.00520121

0.257988

0.039007

0.207129

0.0127553

0.216294

1.80096
7

0.266335

0.288392

0.00661392

0.119811

0.0446436

1.70996

0.0155325

0.536117

0.301033

0.00592134

0.967776
7

0.00394831

0.0388728

3.41991

0.0104084

0.0738555

0.0404642

0.648882

0.0100616

0.00665679

0.0573536

1.84692
6

0.288392

0.0997735

0.288392

0.0133136

0.0950773

0.0887784

0.0125853

0.0387519

0.0938138

0.0129576

2.27454
7

0.0102004

0.014664

0.0163868

0.0599054

0.00655526

0.019133

0.0809283

0.0898581

1.70996

0.00777913

2.40798
7

0.0123036

0.0761741

0.0129839

0.325742

0.0283844

0.204517

1.70996

0.03666

0.00546617

1.70996

4.58484

0.0792192

0.0770648

0.00710613

0.0469069

0.0283844

0.019133

0.00498723

0.265168

0.337064

0.007332

10.9489
7

2.84154
6

0.0173753

0.0150924

0.0133136

0.0150095

0.0871792

0.00939633

0.00832669

0.0177653

2.36626

0.00543897

1.61553

0.893528

0.0156497

0.0404642

0.139287

0.328348

0.299527

0.00799092

0.0134617

0.0127553

0.0161662

2.71901

0.0245801

0.0127553

0.36049

0.239622

0.076525

0.0251705

0.0599054

0.00780182

0.0129576

0.0104881

0.487001
7

0.114707

0.00922919

0.00440928

0.028189

0.00967569

0.00887572

0.0106592

0.0170056

0.0125853

0.00394831

0.638638
7

0.193765

0.0399145

0.0112377

0.0189229

0.0449794

0.166017

0.00416335

0.00475254

0.0606962

0.0877713

0.502367

0.0446436

0.00440928

0.019133

0.00440928

0.0391243

0.093266

0.0469069

0.0173753

0.887784

0.0591856

0.59628

0.0680222

0.131657

0.0404642

0.00665679

0.0938138

0.0189229

0.0703603

0.0446436

0.0283844

0.152013

3.10671
7

0.0606962

0.00983289

0.0151023

0.0591856

0.010998

0.118071

0.10116

0.0637765

0.243255

0.0249362

0.591619
6

0.0809283

0.00997446

0.040326

0.0675082

0.0156036

0.3072

0.219428

0.0163868

0.0151023

0.0209761

4.17367
7

0.0251705

0.0469069

0.00416335

0.117267

0.216294

0.0864815

0.0125853

0.0156036

0.00520121

0.0259677

15.603

1.27252
7

0.135654

0.00458025

0.0255106

0.504686

0.00703064

0.0173753

0.372019

0.0129839

0.0039381

1.70996

0.480513

0.0161662

3.038

0.0877713

0.542615

0.0100127

0.0163868

0.414299

0.00364411

0.0149617

0.0163868

0.859055

0.536117

0.0110946

0.10116

0.770648

1.70996

0.0150924

0.0404642

0.00408015

0.0155008

0.029328

0.44375
7

0.0877713

0.00748085

0.00710613

0.00498723

0.119039

0.0127553

0.00712881

0.0316877

0.304696

0.0283844

0.82282
7

0.025662

0.209162

0.0173753

0.0819338

0.00615463

0.242206

0.0739218

0.0153866

0.062599

0.00518609

2.33098
7

0.0898581

0.0507772

0.00543897

0.144196

0.0150924

0.0168673

0.357411

0.00592247

0.0606962

0.0318883

2.83567
8

0.0419522

0.095063

0.106932

0.0452773

0.00503411

0.0968956

0.0104881

0.00615279

0.118371

0.432588

1.79271
7

0.0127553

0.0404642

0.0104881

0.0283844

0.0234746

0.0301849

0.147964

0.0701542

0.131657

0.0163868

5.23171
7

0.216294

0.0156497

0.0819338

0.00612023

0.0189229

0.0510212

0.777586

0.019133

0.0340111

0.00615463

1.67685

0.366292

0.0819338

0.0168565

0.0591856

0.130315

0.0868767

0.007332

0.00350607

0.00615279

0.00394831

275.435

12.1783
7

1.33837
7

0.0176194

0.0110946

1.96576

0.0170056

0.0599054

0.0819338

0.00939633

0.00888202

0.0469069

1.78706

2.55933
7

0.0174553

0.00881856

0.00712881

0.3072

0.0449507

0.095063

0.00440928

0.0123093

0.0398762

0.0150095

1.41654
7

0.0232512

0.00440928

0.0234746

0.0255083

0.0177514

0.0581373

0.267861

0.00832669

0.0163868

0.00592134

0.456931
7

0.00755116

0.0142576

0.0170056

4.15034

0.0578923

0.0387582

0.0127553

1.35681

0.019133

0.00527298

0.696544
7

0.00710613

0.00475254

0.026063

0.10116

0.00520121

0.0387582

0.0393319

0.0434384

0.216294

0.0674161

2.26357

0.0170056

0.0148034

0.119811

0.0591856

0.278575

0.0224754

0.0173753

1.15357

1.70996

0.0887784

0.489975
7

0.536117

0.0887784

0.00687037

0.0150924

0.0234054

0.0387582

0.144196

0.0173753

0.00503411

0.00443786

1.2707

0.0173753

0.539429

0.0547741

0.0327735

0.0255106

0.00744712

0.00748085

0.0469069

150.252

0.0938138

0.77624
7

0.0310016

0.0316877

0.179716

0.062599

0.0245801

0.03666

0.0471962

0.0131105

0.0606962

0.0163868

0.977247
1

0.0127553

0.0391243

0.0112377

0.0100682

0.483727

0.00518609

0.0599054

0.0868767

0.010998

0.00520121

69.0034
6

1.56244

21.6334

0.0966581

0.357411

0.0409669

0.72098

0.0106592

0.00394831

0.141625

0.0245801

0.0469492

2.81163

0.0201232

0.0340111

0.0110232

0.0195035

0.369904

0.0201004

0.0481639

0.00703064

0.10116

0.0491603

0.50669

0.0245801

0.00543897

0.0591856

0.026063

0.0430824

0.00665679

0.00527298

0.00520121

0.0112377

0.0133136

0.64299
7

0.0104881

0.0100616

0.216294

0.0819338

0.0633753

0.357411

0.0163868

0.0104881

0.0189229

0.00351532

0.824189

0.0127553

0.233165

0.115072

0.228522

0.0163868

0.0245801

0.216294

0.00475254

0.263314

0.00503411

1.19822
7

0.193791

0.0283844

0.629007

0.680635

0.00518609

0.144196

0.0150924

0.0110232

0.00518609

0.045307

0.911772

0.0375853

0.0100616

0.0761741

0.00518609

0.00687037

0.00520121

0.0189229

0.0163868

0.00967569

0.0382659

1.36953
7

0.00592134

0.007332

0.0946147

0.0127553

0.00440928

0.00503411

0.357411

0.0163868

0.00780182

0.00748085

3.36903
7

0.0145717

0.110309

0.0327735

0.121627

0.052126

0.110678

0.257988

0.00690984

0.259515

0.0157321

4.77987
5

0.00475254

0.0425139

0.0347507

0.0409669

1.1332

0.0578923

0.0324597

0.0189229

0.00305919

0.0549439

73.537
7

1.33971

0.0112449

0.0599054

0.893528

0.0104881

0.310066

0.179716

0.147481

0.0434384

0.00655526

0.00503411

4.31509
7

0.00665679

0.0104881

1.08147

0.0170056

0.0426368

0.0145135

0.0112377

0.179716

0.00443786

0.00710613

0.224304
7

0.885422

0.0633753

0.0124681

0.0173753

0.0316877

0.0163868

0.025662

0.0127553

0.0966581

0.00888202

5.4264
2

0.257988

0.452655

0.00500636

0.261974

0.0475315

0.127553

0.010998

0.714822

0.026063

3.57411

0.558059

0.0809283

0.00710613

0.0127553

0.0108779

0.0803844

0.0404642

0.216294

0.22588

0.0156036

0.0608137

2.1789
7

0.00394831

0.0851532

0.0168565

0.00615463

0.0283844

0.0389516

0.0129576

0.842869

0.264921

0.110536

3.20704
6

0.0100616

0.0255083

0.0316877

0.139899

0.0129576

0.0591856

0.217192

0.00923194

0.00498723

0.0703603

1.30436

0.00950508

0.0704951

0.0352157

0.0168565

0.161857

0.0319776

0.0340111

0.0137987

0.0714113

0.0781891

3.87073
6

0.0104881

0.0387582

0.147964

0.0483291

0.0608137

0.0127553

0.0831446

0.0100616

0.00416335

0.158438

1.07855
7

0.0127553

0.0112377

0.052126

0.0316877

0.0255083

0.0173753

0.00518609

0.0206752

0.0255106

0.0606962

13.4136
7

1.65018

0.00411208

0.007332

0.0156497

0.0510167

0.0898581

0.144196

0.0425139

0.0226535

0.00543897

0.0106592

0.673513

0.0140613

0.00543325

1.0967

0.784852

1.97923

0.144196

0.0337346

0.0189229

0.019133

0.00748085

6.39033
7

0.00500636

0.052126

0.576784

0.0111768

0.0680222

0.00615463

0.385089

0.0155008

0.109888

0.026063

1.47311
7

0.0724936

0.0176194

0.0819338

0.0150924

0.0129839

0.0189229

0.102033

0.0104881

0.0326412

0.007332

1.28958
7

0.010998

0.0404642

0.0170056

0.0469069

0.00755116

0.142823

0.0137987

0.00815845

0.0378459

0.0166339

0.601201
7

0.00888202

0.0173753

0.0104881

0.021996

0.141625

0.0378459

0.00520121

0.0775164

0.0403966

0.0140945

5.21165
6

0.0173753

0.007332

0.0168673

0.007332

0.00624502

0.0123363

0.0491603

0.0163868

0.00498723

0.0591856

0.725637
7

0.239622

0.0409669

0.0775164

0.0127553

0.0935306

0.216294

0.00443786

0.288392

0.236743

0.00503411

0.549516

0.0156497

0.00655526

0.019133

0.0409669

0.00443786

0.0269437

0.0255106

0.0271948

0.322802

0.0634669

2.78104

0.0173753

0.0599054

0.144196

0.0822434

0.00503411

0.0153866

0.28952

0.495197

0.0491603

0.254685

15.5714
7

0.716713

0.00923194

0.144196

0.029328

0.00443786

0.0125853

0.0134122

0.0173753

0.00616813

0.144196

0.019133

1.73268
7

0.0170056

0.38087

0.00780182

0.00447073

0.0606962

0.0404642

0.0364085

0.0327735

0.126751

0.0316877

2.09749
6

0.0140613

0.177557

0.010998

0.00690984

0.0770648

0.0599054

0.00689936

0.00881856

0.0301849

0.0340111

1.1987

0.0469069

0.0173753

0.118371

0.0404642

0.0125853

0.0209761

0.00351532

0.0276876

0.026063

0.007332

8.50306
6

0.0100616

0.0608137

0.00703064

0.0170056

0.0606962

0.0100064

0.0163868

0.0129839

0.0469069

0.00351532

4.58737
7

0.00518609

0.00518609

0.00736842

0.113655

0.104252

0.0127553

0.00665679

2.59087

0.0245801

0.0595195

0.526655
7

0.0382659

0.178558

0.0316877

3.038

0.10116

0.00503411

1.78706

0.0120572

0.188785

0.0175223

1.1673
7

0.0956648

0.118371

0.0469069

0.0166311

0.0234746

0.00687037

0.0245801

0.0591856

0.0170056

0.0170056

0.595736
7

0.0703603

0.019133

0.0312995

0.0174553

0.0446436

0.00416335

0.0599054

0.0770648

0.0118427

0.0157321

0.627741
7

0.0245801

0.0599054

0.119039

0.0377218

0.0245801

0.0316877

0.216294

0.0327217

0.0234746

0.00443786

12.0779
6

0.386053

0.0232512

0.00500636

0.0251705

0.0425139

0.222553

0.0168673

0.359433

0.012313

0.0327735

0.0324597

22.1044
6

0.288392

0.0523659

0.00832669

0.00755116

0.0955203

0.0701542

0.0316877

0.0129839

0.175543

0.0189229

2.62724
6

0.00592134

0.00624502

0.0160309

0.0168673

0.0173753

2.95602

0.0286067

0.0469492

0.0112377

0.0201232

9.1019
5

0.114707

0.00939633

0.0404642

0.00458025

0.0465975

0.0633753

0.149764

0.0177514

0.00498723

0.0140613

0.73278

0.00503411

0.202321

0.00897447

0.0704238

0.0608137

0.00518609

0.328348

0.0173753

0.00655526

0.00736842

0.935154
7

0.0145042

0.0142123

0.196641

0.0189229

0.0255083

0.0255106

0.0255106

0.0170056

0.0100616

0.0104881

0.791115

0.00503411

0.536117

0.357411

0.00362217

0.0176194

0.183146

0.0140613

0.00736842

0.0250534

0.013003

1.4809
6

0.264254

0.007332

0.0163868

0.147481

0.019133

0.0454436

0.53162

0.00351532

0.576784

0.00888202

0.317742

0.0125267

0.0809283

0.0387582

0.400005

0.0125853

0.0402465

0.0123036

0.00888202

0.00780182

0.0288461

0.651389
7

0.019133

0.0163868

0.0898581

0.36049

1.70996

0.687891

0.019133

0.00665679

0.00503411

0.0237627

38.82
6

2.76044
7

0.0259677

0.00475254

0.0163868

0.0582163

1.25094

0.00592134

0.0483291

0.00443786

0.00816031

0.0797524

0.926862
7

0.00503411

0.0100682

0.239622

0.0843952

0.0312995

0.0919245

0.00780182

0.0163868

0.0473073

0.0197008

2.43235
7

0.0475315

0.025664

0.0129576

0.0366292

0.0168673

0.0163868

0.153064

0.0127553

0.0938138

0.0606962

1.4416
6

0.0599054

0.0107775

0.0599054

0.0100682

0.0221634

0.0153866

0.0301849

0.0695014

0.0382659

0.0446436

4.33556
6

0.0232512

0.0125853

0.0244082

0.0100616

0.0176194

0.288392

0.0210919

0.385089

0.0404642

0.0550821

0.563854
7

0.0573536

0.27942

0.00503411

0.144196

0.0133136

0.174412

0.032994

0.021996

0.0140945

0.026063

0.551304

0.0187927

0.00748085

1.27268

0.0543897

0.0809283

0.0262201

0.0150924

0.00923194

0.0100616

0.0133136

1.73681
7

0.00503411

0.0156497

0.0637765

0.357411

0.0302047

0.0104024

0.00394831

0.0966581

0.093266

0.00777913

1.44551
7

0.144196

0.0312995

0.647427

0.0123093

0.0382659

0.0387582

0.0100064

0.0475315

2.56494

0.177557

1.14983
8

0.0606962

0.0475315

0.0829095

1.22567

0.00939633

0.110907

0.00780182

0.0327735

0.00447073

0.0255106

15.4014
7

1.11117
7

0.0382659

0.00475254

0.0703603

0.616825

0.00881856

0.109548

0.0138191

0.105647

0.404642

0.104252

2.13513
7

0.0173753

0.121392

0.0200127

0.0775164

0.0599054

0.0581373

0.10116

0.0770648

0.0156497

0.0104881

1.24286
7

0.0129839

0.264254

0.204834

0.432588

0.0312995

0.0549439

0.0156497

0.0389516

0.0127553

0.0681724

2.4533
6

0.00710613

0.01249

0.144196

0.00443786

0.0104881

0.0599054

0.0584274

0.0224897

0.0127553

0.00670609

1.35863
7

0.0449794

0.025154

0.0765318

0.0259152

0.714822

0.0174553

0.0316877

0.0152104

0.216294

0.357411

1.0368
7

0.0100064

0.052126

0.0775164

0.179716

0.0434384

0.0837283

0.00710613

0.0151023

0.00503411

0.0615768

0.670423
7

0.118371

0.0194758

0.0633753

0.121175

0.121392

0.0163868

0.288392

0.0129576

0.0446436

0.0168565

3.93617
7

0.0112377

0.0475315

0.0327735

0.0507067

0.0409669

0.0049252

0.0548351

0.095063

0.010998

0.0100616

3.55522
7

0.0510167

0.299527

0.00443786

0.134852

0.0377218

0.0158189

0.0189229

0.00503411

0.0483291

0.00443786

0.9342
7

0.0573536

0.0156497

0.00887572

0.0129839

0.140721

0.00411208

0.0255106

0.0100616

0.0163868

0.577986

4.78144
7

0.257603
7

0.0232512

0.00503411

0.149764

0.0129839

0.303481

0.0404642

0.0908871

0.0156497

0.0156497

0.0180541

3.37142
7

0.019133

0.00520121

0.014664

0.0327735

0.0251705

0.00527298

0.0201232

0.00394831

0.0163868

0.0107775

0.72967
7

0.0483291

0.0425139

0.0255106

0.0049252

0.0475315

0.00710613

0.0255106

0.0142123

0.0387582

0.921599

15.5364
7

0.0434384

0.0255083

0.00443786

0.0316877

0.00967569

0.010998

0.0446436

0.0615649

0.0100616

0.101606

1.46402
7

0.0770648

0.201461

0.0503081

0.195564

0.929061

0.0189229

0.00816031

0.00543897

0.0327735

0.0775164

2.40167
7

0.0110946

0.0129576

2.56494

0.0483291

0.10116

0.0127553

0.0851532

0.00518609

0.0935306

1.42964

16.5189
6

0.00394831

0.0138191

0.0128851

0.169152

0.432588

0.00665679

1.07223

0.00498723

0.0251705

0.00532728

1.63531
7

0.158614

0.0245801

0.0391243

0.0173753

0.104252

0.0389516

0.0080381

0.0156497

0.00351532

0.119811

0.8473
7

0.00939633

1.70996

0.00687037

0.144196

0.0338079

0.0163868

0.0786604

0.00967569

0.00615463

0.00590715

0.407558
7

0.0340111

0.0475315

0.0262201

0.0127553

0.0244754

0.159441

0.007332

0.0483785

0.0173753

0.0599054

18.5771
7

2.86842
7

0.0234746

0.0491603

0.0080381

0.0340111

0.0226535

0.0966581

0.00498723

0.00665679

0.00394831

0.0409669

5.03619
6

0.00362217

0.0318883

0.0510167

0.192662

0.0150924

0.0150924

0.0127553

30.5985

0.0327217

0.132461

27.6742
2

0.443892

0.0129839

0.0469069

0.00755116

0.065547

0.021555

0.0519355

0.144196

0.0127553

0.0155008

0.865805
6

0.00443786

0.00939633

0.00780182

0.144196

0.147964

0.0234746

0.0049252

0.0104881

0.447037

0.0269234

1.14145
7

0.110536

0.0156497

0.357411

0.0498932

0.239622

2.36715

0.110907

0.010546

0.0177514

0.216294

1.43697
7

0.0259152

0.00624502

0.00755116

0.0207247

0.0581373

0.0150924

0.0366292

0.00712881

0.0781891

0.0281121

34.7971
8

0.0452773

0.0129576

0.0150924

0.00543897

0.00748085

0.0147368

0.065547

0.0125853

0.0100682

0.0107775

0.789349
7

0.0150924

0.026063

0.0129839

0.00503411

0.0106592

0.0473073

0.0163868

0.019133

0.0112449

0.105823

3.68722

0.372326

0.0170056

0.00922919

0.0080381

0.0194758

0.00755116

0.0173753

0.00305919

0.0391243

0.357411

0.867106
7

0.0133048

0.00520121

0.00923194

0.0792192

0.0170056

0.0201232

0.116275

0.0327217

0.026063

0.00440928

105.924
7

14.0323
7

0.306278
7

0.0618072

0.0510212

0.0387582

0.0112449

0.0792192

0.222553

0.0283844

0.0573989

0.163868

0.00527298

0.799443
7

0.0049252

0.021555

0.0829095

0.0156497

0.0347507

0.0156497

0.00615463

0.333606

0.0483291

0.0148034

2.07702
7

0.0209761

0.0127553

0.00687037

0.0100616

0.119811

0.0127553

0.0898581

0.231194

0.142595

0.193316

0.822466

0.00543897

0.0049252

0.0887784

0.007332

0.0599054

0.0103722

0.144196

0.539149

0.00520121

0.0756917

0.918039

0.00416335

0.0232512

0.0404642

0.0129652

0.0103648

0.00527298

0.0156497

0.0434384

4.72284

0.0168673

0.515292

0.464199

0.00624502

0.172147

0.673199

0.00690984

0.0251705

0.0163868

0.117267

0.00498723

1.70996

0.703839
7

0.0898581

0.504686

0.0581373

0.00738779

0.234567

0.007332

0.00710613

0.0124681

0.158438

0.331638

1.06849

0.00503411

0.0170056

0.0127553

0.025154

0.0133182

0.0189229

0.007332

0.0423915

0.0199489

0.00520121

10.1119
6

0.0561884

0.280592

0.0049252

0.026063

0.0877713

0.076525

0.443071

0.00351532

0.0334046

0.283249

0.715546

0.0125853

0.00525911

0.147964

0.0591856

0.281725

0.0469069

0.0312995

0.0637765

0.122901

0.714822

11.3872
7

1.84504
7

0.230737

0.0491603

0.0737404

0.0316877

0.23439

0.504686

0.00411208

0.0547741

0.0100616

0.00458025

0.408776
7

0.288392

0.00394831

0.0234746

0.00967569

0.0255083

0.00690984

0.0469069

0.0142123

0.0155583

0.00710613

0.459366
7

0.0259152

0.0156497

0.0091605

0.00351532

0.842071

0.0316877

0.010998

0.076525

0.0100616

0.0599054

0.661747
5

0.357411

0.469069

0.10116

0.0100127

0.00475254

0.00748085

0.0739218

0.0173753

0.629718

0.566355

1.94238

0.147964

0.00443786

0.0156497

0.0170056

0.00939633

0.0173753

0.0855237

0.0868767

0.0150924

0.0877713

0.895237
7

0.126751

0.177557

0.00443786

0.0281121

0.019133

0.0473073

0.00750954

0.0775164

0.0124681

0.0157321

0.817876

0.0160762

0.0163868

0.0340111

0.0316877

0.0361715

0.0170056

0.0425139

0.0469069

0.0140613

0.029328

0.502449
7

0.755828

0.0147368

0.175543

0.0898581

0.0983205

0.0104881

0.00755116

0.0352388

9.40477

0.0100682

1.06222
7

0.00475254

0.00777913

0.0127553

0.00498723

0.0633753

0.0124681

0.0112377

0.0170056

0.019133

0.0877713

1.39128
7

0.01833

0.00701215

0.00939633

0.209669

0.00408015

0.00615463

0.0337346

0.0318883

0.0150924

0.0591856

24.6654
7

0.748838
7

1.52759

0.127542

0.0234746

0.15413

0.357411

0.0382659

0.0150924

0.0599054

0.937273

0.007332

2.28448
7

0.0608137

0.0404642

0.0606962

0.00440928

0.0234746

0.312505

0.20715

0.0155008

0.00443786

0.019133

1.93627
7

0.0327735

0.0129839

0.158438

0.010998

0.180254

0.0608137

0.054841

0.0232512

0.0324597

0.0425139

0.497814
7

0.0491603

0.116275

0.0147756

0.0049252

0.36049

0.0155008

0.0262201

2.10723

0.0510212

0.0283844

0.682894

0.0909077

0.0469069

0.00690984

0.0108779

0.0387582

0.019133

0.0221893

0.0551547

0.00592134

0.065547

8.79894
6

0.0633753

0.216294

0.556011

0.00777913

0.0189229

0.0170056

0.0127553

0.00967569

0.179716

0.142595

2.02112
7

0.170306

0.21317

0.010998

0.00520121

2.56494

0.0728586

0.363309

0.052126

0.0404642

0.0404642

2.08741

0.119811

0.0770648

0.0483689

0.443627

0.809283

0.0049252

0.065547

0.0316877

0.0599054

0.0118814

0.65988
7

0.00503411

0.0648786

0.0123093

0.0478785

0.0209761

0.0714113

0.0283844

0.0599054

0.0255083

0.0469069

3.39128
7

0.0378459

0.0327735

0.052126

0.0236899

0.00888202

0.254685

0.0100682

0.0465023

0.00498723

0.156125

16.905
7

4.57281
7

0.0606962

0.0387582

0.144196

0.714822

0.00789662

0.040326

0.0316877

0.00670609

0.0156497

0.00443786

0.88684

0.0523143

0.00394831

0.893528

0.0163868

0.0680955

1.07223

0.0163868

0.0226535

0.211081

0.144196

4.03051
6

0.0189229

0.0170056

2.42617

0.00503411

0.019133

0.236743

0.0901271

1.70996

0.357411

0.0182909

0.536411
7

0.0703603

0.591856

0.0168673

0.00408015

0.0473073

0.00520121

0.00543897

0.0224897

0.0724936

0.140863

13.7158
7

0.0288461

0.375255

0.0966581

0.0216596

0.0127553

0.10116

0.144547

0.0129839

0.0591856

0.0163868

1.13288
7

0.0127553

0.216294

0.0157321

0.0129839

0.11294

0.0091605

0.007332

0.00503411

0.0170056

0.21684

6.58169
4

0.0549439

0.00665242

0.117251

0.01833

0.269345

0.0312995

0.0129839

0.0156497

0.0599054

5.12987

0.587323

0.00673085

0.0484412

1.6388

0.0173753

0.144196

0.0737404

0.00440928

0.357411

0.00755116

0.264902

0.587821
7

0.290687

0.0145717

0.0599054

0.0506019

0.00760519

0.00527298

0.0510212

0.0234908

0.0150095

0.0364085

0.335901
7

0.00967569

0.15413

0.432588

0.0224897

0.118371

0.00503411

0.216294

0.00546617

0.0103722

0.0352388

16.0157
6

2.47534
7

0.0151023

1.70996

0.0163868

0.0163868

0.00543897

0.10116

0.0232512

0.00888202

0.0123093

0.0087883

6.55858
7

0.00520121

0.0266461

0.00543897

0.215005

0.112756

0.00458025

0.0170056

0.007332

0.0338649

0.288392

0.700825
7

0.00443786

0.460447

0.0887784

0.00498723

0.00905542

0.0473073

0.536117

5.98485

0.0475315

0.504686

0.667754
7

0.0190364

0.140721

0.0761741

0.0956648

0.0157321

0.093266

0.0573989

0.0732585

0.0125853

0.242785

1.07242
7

0.0434384

0.0100616

0.512963

0.0340111

0.0163868

0.0160309

0.0701542

0.0104881

0.0680222

0.0163868

2.38615
7

0.0189229

0.00443786

0.144196

0.0310016

0.00520121

0.0100616

0.432588

0.0312995

0.0163868

0.00712881

0.48658
7

0.00458025

0.00525911

0.0173753

0.026063

1.70996

0.0809283

0.216294

0.0049252

0.0177653

0.0127553

1.3865

0.0208048

0.0226535

0.0177514

0.0170056

0.12041

0.0112449

0.00985039

0.0722459

0.0127553

0.0173753

9.61223
7

0.0469069

0.0770648

0.0475315

0.007332

0.0703603

0.00520121

0.065547

0.0100064

0.0104881

0.032394

2.21517
7

0.00815845

0.0378459

0.174282

14.1665

0.0761741

0.0599054

0.00498723

0.026063

0.0175766

0.0469069

16.3305
7

2.32718
6

0.0255083

0.0129576

0.950908

0.182441

0.0147756

0.0469069

0.61791

0.242785

0.0163868

0.0510167

0.698168

0.00416335

0.00475254

0.0125853

0.0100616

0.132209

0.010998

7.491

0.00789662

0.0149617

0.00710613

0.481097
7

0.0404642

0.288392

0.0189229

0.0347507

0.0301849

207.363

0.0809283

0.0887784

0.0103648

0.00748085

1.68789
7

0.00923194

0.00592247

0.0409669

0.00394831

0.0591856

0.263586

0.0427729

0.0167023

0.0173753

0.026063

1.6022
7

0.0155008

0.00503411

0.438365

0.232549

0.385829

0.00475254

0.0425139

0.0434384

0.144196

0.0194758

2.49981
7

0.0155008

0.0153866

1.06409

0.0163169

0.155008

0.0955644

0.0217157

0.216294

0.0387582

0.180503

0.669064

0.357411

0.0283844

0.293372

0.00905542

0.00687037

0.0548351

0.00615279

0.00939633

0.119811

0.0792192

1.60274
7

0.019133

0.308259

0.0327735

0.00518609

0.00394831

0.0112377

0.0581373

0.0327735

0.0915731

0.0255083

0.454976
6

0.00498723

0.0232512

0.0210919

0.0581373

0.0387582

0.0110946

0.0491603

0.0599054

0.199381

0.144196

0.62321
7

0.0157321

0.338015

0.101849

0.121272

0.0770648

0.00748085

0.0581373

0.0765318

0.0280942

0.714822

14.4127
7

2.86868

0.0245801

0.0127553

0.0483191

0.0352475

0.0573536

0.0318883

0.0935306

0.0173753

0.0404642

0.15413

0.543094
7

0.0107775

0.0133136

0.0170056

0.0170056

0.0112449

0.019133

0.00503411

0.0127553

0.161857

0.131657

0.474203

0.0819338

0.102033

0.019133

0.0080381

0.208504

0.251928

0.007332

0.021996

0.216294

0.0469069

1.33747
6

0.0280942

0.0232512

0.0606962

0.00690984

0.0194364

0.0173753

0.0127553

0.0234746

0.0551547

1.82851

3.05518
7

0.0606962

0.0195035

0.00503411

0.0100616

0.047824

0.0434384

4.27489

0.00503411

0.00616813

0.122901

0.722323

0.00615463

0.118371

0.0209761

0.147964

0.0382659

0.0391243

0.0887784

0.0325674

0.139287

0.097379

1.20392
7

0.007332

0.203881

0.0765318

0.0127553

0.119811

0.0102802

0.00789662

0.00351532

0.0404642

0.007332

0.327351
7

0.019133

0.044445

0.0943925

0.0176194

0.00738779

0.0475315

0.095063

0.279134

0.0107775

0.0887784

1.0513

0.0770648

0.0104881

0.0107775

0.0245801

0.00498723

0.00780182

0.0637765

0.0475315

0.216294

0.00832669

0.860428

0.438342

0.00673085

0.0155008

0.00950508

0.0103722

0.950773

0.0226535

0.031065

0.131105

0.0434384

11.7129
7

0.457908

0.0729946

0.0155008

0.0245801

0.0599054

5.74902

0.0494115

0.0127553

0.0156497

1.69647

0.576784

4.42094
7

0.144196

0.0163868

0.106604

0.0132278

0.0510212

0.116275

0.0595195

0.0189229

0.0163868

0.0387582

1.17412
7

0.0440928

0.357411

0.00527298

0.0262201

0.0887784

0.0259152

0.025154

0.144987

0.149764

0.0446436

4.89797
6

0.026063

0.0189229

0.0127553

2.56494

0.0761741

0.19133

0.0112449

0.0444101

0.00835115

0.728355

2.39731
6

0.359433

0.0549439

0.06173

0.0168673

0.0703603

0.0232512

0.00687037

0.115597

0.031065

0.0301849

1.14966
5

0.00755116

0.0127553

0.161857

0.0761741

1.70996

0.0221893

0.0898581

0.36049

0.00443786

0.0173753

0.79112
6

0.495197

0.00351532

0.0475315

0.0225489

0.0168565

0.0347507

4.32588

0.0182042

0.00950508

0.0475315

1.10667
7

0.0281121

0.00665242

0.00755116

0.0173753

0.887784

0.0104881

0.0599054

0.144196

0.019133

0.0390648

0.504498

0.0125853

0.285189

0.0251705

0.355114

0.0595195

0.219775

0.140721

0.179716

0.0155325

0.0469069

1.24327

0.00939633

0.00738779

0.0808312

0.0104881

0.101606

0.0409669

0.00710613

0.144196

0.0898581

0.00670609

41.5977
7

0.800752
7

0.714822

0.0553916

0.0213091

1.70996

0.0153866

0.0916087

0.536117

0.00712881

0.0338404

0.0129839

2.73984
7

0.019133

0.032994

0.0220464

0.00394831

0.00362217

0.0562243

0.0251705

0.576784

0.0221893

0.0151023

2.1648
7

0.357411

0.00498723

0.0103722

0.0316877

0.0591856

0.373064

0.0387519

0.0189229

1.70996

0.045462

0.785666
7

0.179716

0.415723

0.36049

0.00592134

0.122999

0.144196

0.00710613

0.0469069

0.0388728

0.00351532

0.965714
7

0.0114506

0.174412

0.00997446

0.0737404

0.0898581

0.0491603

0.0966581

0.0491603

0.0189229

0.00520121

0.537253
7

0.0155008

0.0469069

0.0173753

0.109888

0.00592134

0.00661392

0.00448724

0.019133

0.0155008

0.745061

1.52861

0.104881

0.0551547

0.142595

0.0573989

0.0491603

0.0483291

0.0170056

0.0388728

0.0695014

0.144196

1.23735
7

0.0469492

0.144196

0.0573536

0.404468

0.0255106

0.00983289

0.217481

0.0129652

0.0480926

0.0255106

0.589832
7

0.0548595

0.0104881

0.00592134

0.0255106

0.0966581

0.0382659

0.0124681

0.052126

0.0156497

0.16549

1.03573

0.0404642

0.0382659

0.0877713

0.0217157

0.0578923

0.0898581

0.0510167

0.0391243

0.021996

0.0107775

4.29914
7

0.548123

0.0599054

0.216294

0.007332

0.0367082

0.216294

0.00755116

0.0194758

0.0166311

0.0163868

0.0163868

7.09548
2

1.70996

0.007332

0.0189229

0.213028

0.0103648

0.144196

0.0189229

0.0189229

0.00690984

0.00661392

0.869226
7

0.0104881

0.0662303

0.144196

0.0393319

0.0106592

0.007332

0.109548

0.0173753

0.0533022

0.014664

0.497525
7

0.00416335

0.0938138

0.199816

0.069654

0.00748085

0.0135974

0.0404642

0.00665679

0.288392

0.0156497

9.60485
6

0.0316877

0.0637765

0.0201232

0.00997446

0.0591856

0.0775164

0.00755116

1.62403

0.00736842

0.00520121

3.16038

0.0177674

0.0163868

0.0471962

0.00710613

0.0792192

0.0205604

0.0510212

0.0633753

0.0201364

0.175543

0.782953
7

0.280592

2.56494

0.00835115

0.03666

0.0898581

0.0127553

0.00592134

0.0404642

0.0170056

4.3499

0.306257
7

0.357411

0.0104881

0.0581373

0.536117

0.0127553

0.0112449

0.389385

0.0080381

0.0168673

0.0206111

2.94988

0.0491603

0.0170056

0.0606962

0.140721

0.0510212

0.0316877

0.00736842

0.00416335

0.00750954

0.0252757

0.286224
7

0.00789662

0.052126

0.00780182

0.0104084

0.147079

0.0366292

0.0310943

0.15413

0.0145135

0.0156497

340.484
7

8.2213
7

6.2627

0.485142

0.155674

0.0132278

0.114261

0.0448851

0.0633753

0.309466

0.0770648

0.0475315

0.00615279

0.753809

0.00520121

0.546266

0.0404642

0.0775164

0.0112449

0.0469069

3.41991

0.0224408

0.0938984

0.142595

0.737783
6

0.007332

0.0127553

0.010546

0.082918

0.0129839

0.0966581

0.00710613

0.0232512

0.00670609

0.0087883

0.578736
7

0.00520121

5.18246

0.0901271

0.00748085

0.0387582

0.0898581

0.0168565

0.0080381

0.0510212

0.0255106

4.87951
7

0.216294

0.194636

0.974531

0.00350607

0.133931

0.893528

46.1689

0.026063

0.714822

0.0680222

1.44738
7

0.0127553

0.0245801

0.00475254

0.72098

0.0387582

0.052126

0.0549439

0.0387582

0.00661392

0.266063

0.550752

0.0489507

0.00416335

0.937273

0.0194758

0.0469069

0.00661392

0.0637765

0.0347507

0.0404642

0.00520121

24.031
6

0.0483291

7.69481

0.0163868

0.026063

0.0127553

11.1147

0.00475254

0.00690984

0.0100579

0.0316877

11.2862
8

0.0104881

0.00755116

0.0255106

0.0127553

0.0387582

0.0104881

0.462389

0.0887784

0.00518609

0.019133

0.805666
7

0.0157321

0.0695014

0.052126

0.10842

0.00615279

0.571306

0.0340111

0.161857

0.0473073

0.00498723

8.02984

1.04553

0.0683439

0.526628

0.00592247

0.149764

0.116275

0.0127553

0.0129839

0.0283844

0.00447073

0.0367698

0.833385
7

0.131094

0.283249

0.357411

1.03855

0.0387519

0.0112449

0.0887784

0.00527298

0.0173753

0.0245801

2.23229
7

0.161857

0.0189229

0.144196

0.0170056

0.432588

0.025662

0.0469069

0.141625

0.0155008

0.0730916

1.56291
7

0.140721

0.019133

0.0173753

0.00950508

0.0933496

0.0377212

0.0127553

0.00592134

0.0163868

0.0510212

0.880144
7

0.0352157

0.0843179

0.065547

0.0732585

0.00520121

0.00351532

0.00815845

0.0374042

0.0127553

0.138086

10.1138
6

0.0100064

0.00351532

0.0112449

0.0129839

0.0168673

0.0340111

0.0148034

0.0173753

0.0283844

0.00832669

0.29378
7

0.662441

0.00440928

0.0125853

0.00520121

0.0606962

0.0259304

0.0049252

0.15413

0.36049

0.0189229

0.480669
7

0.0129839

0.026063

0.0425139

0.014664

0.116433

0.0599054

0.191041

0.0877713

0.00665679

0.0548351

3.61879
6

0.216294

0.0591856

0.404642

0.0245801

0.0378459

0.0194758

0.0100682

0.144196

0.116275

0.0201232

0.885863
7

0.0110232

0.0491603

0.216294

0.019133

0.287361

0.0245801

0.0573536

0.00887572

0.00498723

0.0327735

5.83377
7

0.257084
7

0.00661392

0.0704951

0.0316877

0.0245801

0.0599054

0.0809283

0.00503411

0.0316877

0.0697535

0.00780182

1.07787

0.0123036

0.010546

0.0283844

0.0387582

0.536117

61.0436

0.00665679

0.0170056

0.0170056

0.00447073

1.2626
7

0.00755116

0.00503411

0.0887784

0.0104024

0.0703603

0.0398762

0.0157321

0.00503411

0.119039

0.135654

1.74469
7

0.0703603

0.007332

0.0724936

0.121392

0.0255083

0.007332

0.0170056

0.0498868

0.0461597

0.062892

0.849171

0.0637765

0.229415

0.0189229

0.0378459

0.0100616

0.797417

0.00520121

0.0127553

0.0178829

0.0469069

1.34907
7

0.0173753

0.0166534

0.0968956

0.00503411

0.0255083

0.00665679

0.00939633

0.0100682

0.0260061

0.0166339

0.635981
7

0.093266

0.144196

0.00503411

0.0484412

0.0259152

0.0606962

0.0112449

0.0409669

0.0606962

0.00503411

0.671692
7

0.0155008

0.0127553

0.029328

0.00815845

0.0232512

0.00408015

0.0157321

0.0524403

0.303481

0.0107775

32.4034
6

0.013003

0.0259677

0.0112377

0.0599054

0.0591856

0.0887784

0.288392

0.0469069

1.40434

0.0170056

1.32045
7

0.0274298

0.10116

0.00500636

0.107242

0.00440928

0.00748085

0.357411

0.380252

0.0209761

0.281441

14.9853
7

9.09172
7

0.10116

0.0106592

0.288392

0.0340111

0.0549439

0.0347507

0.301033

0.0510167

0.177557

0.026063

0.391067
7

0.019133

0.36049

0.00475254

0.0391243

0.00615279

0.0877713

0.0387582

0.00394831

0.00351532

0.026063

2.72126
7

0.270381

0.014664

0.065547

0.00939633

0.792311

0.0547741

0.0194758

0.0150095

0.00411208

0.0182731

0.963474

0.0724936

0.0174553

0.0245801

0.0283844

0.0361715

0.00503411

0.0475315

0.0107775

0.141625

0.0327217

1.85428
7

0.0259677

23.0844

0.175543

0.0140613

0.0127553

0.0434384

0.0465023

0.141625

0.00503411

0.00518609

176.684
6

0.052126

0.0393319

0.019133

0.119811

0.0170056

0.0809283

0.0156497

0.0127553

0.0168565

0.00755116

0.875413
7

0.007332

0.00592134

0.141625

0.00394831

0.0387582

0.0312995

0.0091605

0.0649194

0.141625

0.093266

15.0585
2

0.177557

0.0259152

0.0314642

0.00483473

0.0170056

0.00394831

0.536117

0.0201232

0.0387582

1.08147

0.498889
7

0.00615279

0.0327735

0.0387582

0.00518609

0.0280942

0.170509

0.053042

0.0519355

0.0327735

0.0581373

0.19566

0.0606962

0.0112377

0.0471962

0.0515724

0.140721

0.0155008

2.56494

0.0434384

0.0318883

0.00673085

13.8573
7

2.23257
7

0.014664

0.0166311

0.00710613

0.0129839

0.0599054

0.0163868

0.00503411

0.013003

0.00710613

0.288392

1.43164
7

0.0434322

0.0194758

0.0387582

0.0483291

0.0367698

0.0338079

1.70996

0.0338079

0.0194364

0.0255106

0.392407
7

0.0127553

0.00500636

0.00673085

0.00475254

0.0398762

0.0127553

0.131094

0.0049252

0.0755116

0.0542527

2.8897
8

0.0633753

0.00520121

1.90462

0.0316877

0.0127553

0.0387582

0.0198418

0.0409669

0.00881856

0.327735

1.129

0.0148034

0.0163868

0.357411

0.0573536

0.00750954

16.977

0.00799092

0.0633849

0.0200426

0.0409669

0.394158

0.469492

0.288392

0.0149617

0.0129576

0.0155008

0.0283844

0.0129576

0.0165402

0.0615463

0.357411

0.461421

0.631387

0.0173753

0.010998

0.0434384

0.0606962

0.0316877

1.70996

0.216294

0.0465023

0.0312995

3.40344
7

0.357411

0.10116

0.00997446

0.0316877

0.00498723

0.0168565

0.00967569

0.0151023

0.00755116

0.0234746

3.50221
7

0.0336543

0.338303

0.0163868

0.0177653

0.0773585

0.00503411

0.0127553

0.0645319

0.00503411

0.0491603

0.362933
7

0.0898581

0.0107775

0.00351532

0.833827

0.3072

0.0154325

0.0201232

0.0318883

0.00416335

0.052126

11.4733
7

0.773273
7

0.0475315

0.216294

0.193316

0.502691

0.131657

0.00748085

0.0898581

0.0163868

0.0177653

0.0049252

1.56293
6

0.0127553

0.0366292

0.0276876

0.120737

0.36049

0.114261

0.0104881

0.596915

0.0127553

0.0104881

0.525981
7

0.0310016

0.140308

0.0581373

0.00498723

0.0156497

0.0327735

0.0703603

0.0232512

2.56494

0.0189229

1.47915
7

2.73059

0.0125853

0.00543897

0.0510167

0.140308

0.00520121

0.0104881

0.0176194

0.0391243

0.00887572

0.331521

0.0591856

0.0318883

0.00661392

0.0163868

0.500921

0.0155008

0.0498932

0.0983205

0.0127553

0.00592134

4.92866
7

0.0603697

0.0170056

0.0106592

0.0599054

0.769413

0.0100682

0.032394

0.00738779

0.00322316

0.449291

2.11611
7

0.0156497

0.0316877

0.0469069

0.0049252

0.214485

0.0100064

0.00670609

0.0224897

0.0157524

0.288392

0.404034
6

0.0366292

0.0129576

0.748818

0.0127553

0.00518609

0.00443786

0.085054

0.0581373

0.0701542

0.00498723

2.02472
7

0.0170056

0.0177674

0.0573989

0.0391243

0.314856

0.00416335

0.052126

0.0387582

0.0581373

0.120922

8.5632
7

0.0129839

0.0234746

0.0469069

0.0301849

0.00520121

0.00710613

0.0316877

0.0475315

0.00967569

0.019133

13.5275
7

1.58802
7

0.0522477

0.00443786

0.00416335

0.0809283

0.0773585

0.0877713

0.0209761

0.0633753

0.0156497

0.0173753

1.72873

0.00447073

0.00592247

0.0378459

0.0591856

0.290992

0.0387582

0.0151023

0.0255106

0.00592247

0.00498723

0.494034

0.147964

0.0255106

0.328348

0.0209761

0.0703603

0.0157321

0.0129839

0.144196

0.0868767

0.010546

1.32496
6

0.0100616

0.0125853

0.0491603

0.0340111

0.0232512

0.0194758

0.00527298

0.0104881

0.0318883

0.0194364

4.17472
7

0.0695014

0.0157321

0.0404642

0.0201232

0.216294

0.0404642

0.0173753

0.0156497

0.195564

0.00443786

0.548968

0.979293

0.030764

0.357411

0.0163868

0.0475315

0.0983205

0.158438

0.0404642

0.00881856

0.0591856

1.13417
7

0.0367698

0.0189229

0.0367082

0.0756917

0.0887784

0.0156497

0.0581373

0.432588

0.00997683

0.0127553

0.720463

3.2167

0.236743

0.00527298

0.0156036

0.00394831

0.0234054

0.0173753

0.026063

0.0170056

0.0127553

0.445873
7

0.0255106

0.0189229

0.010998

0.0107775

0.0898581

0.0316877

0.0953159

0.179716

0.0465023

0.00777913

1.79727
7

0.065547

0.357411

0.0898581

0.0938138

0.0318883

0.0163868

0.0127553

0.0280942

0.0209761

0.0606962

21.5987
7

1.20279
7

0.0422835

0.0163868

0.144196

0.0170056

0.0155008

0.0107775

0.0775164

0.0173753

0.357411

0.288392

0.63398
7

0.0255106

0.937273

0.00520121

0.2138

0.222553

0.091605

0.007332

0.0633753

0.0142123

0.00416335

3.47288
7

0.0100064

0.0124681

0.0434384

0.0761741

0.282642

0.00755116

0.0851532

0.0620031

0.00967569

0.0173753

1.33599
7

0.00832669

0.0199704

0.0255083

0.0892871

0.00738779

0.191129

0.216294

0.00661392

0.00416335

0.0581373

1.45043
7

0.123941

0.00440928

0.0503411

0.014664

0.00755116

0.00738779

0.0347507

0.216294

0.00351532

0.0378459

16.2286
8

0.0314642

0.0127553

0.119811

0.014664

0.0276876

0.136044

0.00520121

0.0049252

0.0703603

0.0157053

0.381771
7

0.0425139

0.0404642

0.0245801

0.00543897

0.0234746

0.0112449

0.00394831

0.0151023

0.047824

0.00447073

0.251176
7

0.0868767

0.00780182

0.0404642

0.0773684

0.222553

0.0923652

0.0163868

0.0234746

0.0409669

0.48557

2.96282
6

0.462389

0.007332

0.0154325

0.00475254

0.0311165

0.0173753

0.0387582

0.00789662

0.128694

0.0127553

1.23024
7

0.0765318

0.00525911

0.00498723

0.00755116

0.0100616

0.0402465

0.0112377

0.0662303

0.0226535

0.00440928

30.2725
7

0.493427
8

0.0224897

0.0469069

0.303993

0.129233

0.00498723

0.00440928

1.04794

0.0155008

0.010998

0.0966581

2.33331
7

0.013003

0.0850278

0.0129576

0.065988

0.0697535

0.0104881

0.0189229

0.00985039

0.00690984

0.0469492

1.37474
7

0.013003

0.0112377

0.177557

0.0674261

0.714822

0.00527298

0.0155008

0.0340111

0.0510167

0.0262201

0.775197

0.467653

0.0898581

0.0423915

0.0770648

0.0170056

0.0901271

0.144196

0.0125853

0.135654

0.00799092

3.79744
6

0.0591856

0.191163

0.0163868

0.00443786

0.00615463

0.0112449

0.0163868

0.0234054

0.0599054

0.0199489

1.02579
7

0.193681

0.0283844

0.136247

0.0104881

0.209669

0.0898581

0.432588

0.00518609

0.216294

0.00498723

0.84048
5

0.0868767

0.00687037

0.0387582

0.00503411

0.0189229

0.793077

0.0234746

0.0887784

0.065547

0.01833

1.07905
1

0.0387582

0.0633753

0.093266

0.00665679

0.00887572

0.576784

0.0469069

0.121627

0.0163169

0.143684

0.585432
7

0.0168673

0.0316877

0.007332

0.0633849

0.00690984

0.025154

0.0637765

0.00503411

0.0224897

0.144987

1.06492
6

0.00394831

0.0173753

0.00997446

0.216294

0.0573536

0.0475315

0.0132278

0.00518609

0.0809283

0.0703603

16.4659
7

1.45042
7

0.0177653

0.222553

0.288392

0.00997863

0.0271948

0.142595

0.0150924

0.00458025

0.0125853

0.0091605

1.04146
5

0.00322316

0.0127553

0.00518609

0.0127553

0.0809283

0.095063

0.00710613

0.0750954

0.0382659

1.70996

0.158562
7

0.0425139

0.0173753

0.019133

0.00503411

0.0127553

0.0255106

0.0155008

0.0915731

0.0127553

0.00789662

0.593485
7

0.00939633

0.00789662

0.0112449

0.648882

0.0810526

0.0104881

0.0387582

0.00616813

0.014664

0.121392

12.6673
8

0.033713

0.00518609

0.076525

0.0703603

0.504686

0.0100682

0.0168673

0.0189229

0.144196

0.118371

1.0259

0.0378459

0.0157321

0.140848

0.0255083

3.89329

0.0101402

0.0168673

0.0966581

0.0234746

0.114261

1.33203
7

5.12987

0.0168565

0.00615463

0.0112449

0.893528

0.00394831

0.0475315

0.0974271

0.0446436

0.00443786

3.20728
7

0.00447073

0.00888202

0.357411

0.00789662

0.134243

0.0155008

0.0938138

0.00443786

0.021555

0.0104024

3.77515
6

11.6159

0.144196

0.110125

0.0112377

0.144196

0.0104881

0.0107775

0.0387582

0.0765318

0.0318883

0.804141
7

0.0327735

0.00440928

0.0510354

0.0103648

0.382258

0.0232512

0.00724433

0.0387582

0.0209761

0.0224897

4.84268965553203e-09
7

0
4

1.18131
5

0.182646

0.993676

0.00498723

1.30104260698261e-17
5

0
4

0.063598
7

0.063598
7

0
4

0.296101
7

0.17169

0.124412

2.77555756156289e-17
7

0
4

0.13306

0.13306

0
4

0.136345

0.136345

0
4

0.288392

0.144196

0.144196

0
4

0.129121

0.129121

0
4

0.0606962

0.0606962

0
4

0.0163868

0.0163868

0
4

0
4

10.8505
7

10.8301
7

4.93721
7

3.45354
7

2.43415
7

0.00518609

1.40339129206524e-15
7

0
4

0.0204671

0.0204671

0
4

0
4

1121.32

115.71
7

56.4977
7

0.277978
7

0.233165

0.00712881

0.00408015

0.0080381

0.0114506

0.0156497

0.00411208

0.0103648

0.00458025

0.00690984

0.709644
7

2.56494

0.00458025

0.0306012

0.0404642

0.00665679

0.0155008

0.0173753

0.0809283

0.00592134

0.0100064

2.2258
6

0.00665679

0.0104881

0.357411

0.0163868

0.328348

0.00888202

0.0112377

0.00710613

0.0184584

0.0156497

0.110481

0.0542527

0.0316877

0.0404642

0.00458025

0.0112377

0.0310016

0.0704238

0.0435406

0.0469069

0.00712881

0.28778
7

3.23589
6

0.315232

1.53873
7

0.465846
6

0.0736538

7.9556
7

0.253186
7

0.263452

3.65966
7

1.31313

0.129964

0.460194

0.704394

0.201717

0.134701

0.0387823

5.20189
7

0.108211

0.353293

0.0713012

0.323419

0.0693192

0.0268084

0.210168

0.132422

0.0508922

0.381936

3.11304
6

0.0699261

0.0790991

0.0185232

0.055052

0.0888352

2.06643

0.049913

0.0861391

0.147704

0.0907938

3.42325
6

0.0570305

0.0318875

0.0221893

0.0784138

0.0384922

0.0141682

0.00869233

0.0299443

0.0659748

0.135355

0.693928
7

0.0620031

0.0288669

0.374417

0.314821

0.107861

0.0498526

0.0156755

0.0110946

0.0860736

0.0171405

1.167
7

0.0410165

0.0417956

0.00458025

0.0129839

0.0404642

0.0155008

0.0469069

0.00475254

0.0122405

0.00458025

3.90996
6

0.00411208

0.357411

0.0155008

0.019133

0.00665679

0.00736842

0.15413

0.0157321

0.00967569

0.0129839

5.81185
6

0.255083

0.0606962

0.00710613

0.0251914

0.0029305

0.0404642

0.0469069

0.0106592

0.0168673

0.00799092

1.77012918212149e-13
7

0
4

9.48398

1.28888

1.70779

6.43387

0.0129839

0.0404642

0
4

29.3352

29.3352

0
4

188.447

179.616

4.4238

3.8919
7

0.515976

1.15463194561016e-14

0
4

0.826138

0.271412

0.129121

0.283939

0.0799885

0.00835115

0.0533257

0
4

100.178
7

49.6408
7

0.307532

1.23761
6

4.35609
6

0.461625

0.173911

0.192113
5

0.0712667

0.401935

0.0785582

0.304722

11.0929

0.0557885

0.152261

1.52877

0.343945

0.0349246

0.125198

0.567363

0.20332

0.0965753

0.113353

8.64699
6

0.296866

0.0391923

0.209751

0.131057

0.106093

0.147308

0.117892

0.112348

0.07679

0.655955

1.20879
7

0.0632936

0.137651

0.0188392

0.00949426

0.189755

0.0835361

0.0534217

0.0876535

0.0475315

0.0245801

1.0292
7

0.0116951

0.00615279

0.0104881

0.0340111

0.0262201

1.10811

0.0163868

0.0168673

0.0388728

0.0112377

4.24335
7

0.36049

0.0194758

0.32513

0.0129576

0.00334147

0.0471962

0.0259152

0.0318883

0.0426287

0.0453516

4.13664
6

0.0224897

0.326431

0.00777913

0.0898581

0.0156497

0.00518609

0.0259152

0.0506019

0.0194364

0.0142123

2.83247
7

0.0129576

0.242785

0.0245801

0.0404642

0.00922919

0.135361

0.0469069

0.0194758

0.627563

1.02140518265514e-13
7

0
4

19.7617
6

5.45652
6

0.234567

0.00750954

0.144196

0.0163868

0.453268

0.0533257

1.70996

0.0163868

0.0469069

0.0799885

0.5625

0.0163868

8.44351
6

0.103039

0.19415

1.54109

0.191129

0.318296

0.17262

0
4

634.085
6

11.4062
6

4.82491
7

0.297251

0.0850404

0.0617825

0.0721518

0.0801998

0.00863407

0.107199

0.0764283

0.092258

0.0093712

1.83092
6

0.0100682

0.0561962

0.831759

2.14255

0.0597998

1.81199

0.0158179

0.0372934

0.0620031

0.0360294

35.4323

0.0561139

0.048555

0.0100334

0.00532728

0.0168673

0.0155008

0.0337346

0.0387582

0.0127553

0.00710613

18.8324
7

0.0366292

0.0404642

0.115597

0.0100064

0.0194364

0.0104881

0.0366292

0.0366292

0.0232512

0.00408015

6.86585
6

0.455417

0.00408015

0.0404642

0.0194758

0.0104881

0.0232512

0.0129839

0.00710613

0.00411208

0.0404642

16.2006

0.0404642

0.0155008

0.0195035

0.0585106

0.0112377

0.00665679

0.0316877

0.0404642

0.0155008

0.0129839

1.94654
7

0.0599054

0.00816031

0.0106592

0.0599054

0.147257

0.0155008

0.0366292

0.0599054

0.0404642

0.00503411

10.7912
6

0.00447073

0.0224754

0.0170056

0.00525911

0.45853

0.714822

0.00503411

0.0366292

0.0104881

0.00997863

4.21857
6

0.0168673

0.0112449

0.0164483

0.0156497

0.0168565

0.0232512

0.0155008

0.0310016

0.0449794

0.0159818

4.97877
7

0.144196

0.00799092

0.10116

0.0404642

0.103661

0.0633753

0.00710613

0.0104881

0.0606962

0.0449794

75.1027
6

0.974491
6

0.110907

0.423856

0.0404642

0.0155008

0.0449794

0.0142123

0.302265

0.0366292

0.357411

0.0080381

2.15468

0.0591856

0.0822417

0.0112377

0.0110526

0.0112449

0.0155008

0.0127553

0.00616813

0.0469069

0.0156497

0.619707
6

0.00532728

0.357411

0.0091605

0.0112449

0.0404642

0.0160762

0.00710613

0.0224754

0.0606962

0.0487588

0.887206
7

0.0133182

0.00687037

0.0404642

0.0378459

0.0404642

0.0112377

0.0168673

0.0404642

0.00687037

0.0633753

0.994861
6

0.0106592

0.0281121

0.0404642

0.0255083

0.161857

0.0155008

1.01413
7

1.0465
6

0.876033
6

0.916402
7

4.31428
6

27.0282
6

1.03822
6

0.298311
6

2.01555

1.90332
6

0.90484

0.390802
6

2.73699

0.196226
6

127.535
6

0.404463
7

50.9947
6

0.256736

5.3725
7

1.13448
6

0.181114
6

0.432401
7

2.01289
6

1.28649

0.145022

0.504706

0.0659238

7.43916

2.65677

2.55296

0.674866
7

0.133694

0.496534

0.154323
6

2.35028

2.11562

0.219016

0.128164

78.85
6

0.251374

1.00172

7.60038

0.23683

0.343116

0.0637071

0.311349

0.331588

0.0341485

0.499662

18.5921
6

0.0383239

0.0490132

8.47051

1.88575

0.12791

1.74647

0.219845

0.806934

1.83135

0.0360849

9.34038
7

0.074437

0.28197

0.0591046

0.266589

0.0477888

0.0731054

0.165809

0.43834

0.0408219

0.0345272

33.4051
7

0.200136

0.694555

0.0105182

0.0563032

0.11007

0.104279

0.0387414

0.0291792

0.025041

0.583313

0
4

3.05891

1.99125

0.217553

0.703536
5

0.100319

0.0319776

0.00690984

0.00736842

7.80625564189563e-17

0
4

0.0993305

0.0993305

0
4

0.0269234

0.00673085

0.0157053

0.00448724

2.60208521396521e-18

0
4

0.283323

0.283323

0
4

0.144089

0.144089

0
4

0.0809283

0.0404642

0.0404642

0
4

0.0577744

0.0577744

0
4

0.117267

0.117267

0
4

0.00448724

0.00448724

0
4

0.00532728

0.00532728

0
4

0.0194758

0.0194758

0
4

3.8874

3.88003

0.00736842

0
4

0.0647879

0.0647879

0
4

0.68663

0.68663

0
4

0.0469069

0.0469069

0
4

0.184686

0.184686

0
4

0.00447073

0.00447073

0
4

0.0469069

0.0469069

0
4

0.145676

0.145676

0
4

0.619239

0.585584

0.029167

0.00448724

0
4

2.74294

2.74294

0
4

0.756099

0.73815

0.00448724

0.00448724

0.00448724

0.00448724

0
4

0.0960882

0.0742453

0.0147368

0.00710613

9.54097911787244e-18

0
4

5.01702

5.01702

0
4

0.0393024

0.0348646

0.00443786

1.73472347597681e-18

0
4

5.2608

5.2608

0
4

4.34496882917301e-12

0
4

577.367

8.94828
7

8.94828
7

0
4

77.8746
5

74.0699
5

2.71463
5

0.271685

0.231869

0.0204671

0.357411

0.101849

0.0469069

0.0599054

2.61110577604029e-14
5

0
4

5.34496

5.12845

0.0914946

0.0177653

0.0150924

0.0770648

0.0150924

0
4

26.1477
7

25.8823
7

0.241975
7

0.0234746

3.78169717762944e-16
7

0
4

3.35413

3.08293

0.124962

0.128758

0.00416335

0.00416335

0.00498723

0.00416335

0
4

44.243
5

44.0882
5

0.0316877

0.123124

0
4

2.19507
7

2.11341
7

0.0749298

0.00673085

0
4

2.45236
5

1.02779
5

0.440807

0.305547

0.0890806

0.250605

0.325572

0.0129576

1.30104260698261e-16
5

0
4

3.22905
6

2.89912
6

0.295921

0.0340111

0
4

5.02842

4.96189

0.0260624

0.0404642

1.20042864537595e-15

0
4

2.50189
4

1.60988
4

0.426562

0.362523

0.0124681

0.0208779

0.0167023

0.0528713

2.15105711021124e-16
4

0
4

5.39875

3.47362

0.583917

0.131037

0.129121

0.0655183

1.01553

4.44089209850063e-16

0
4

55.9318

39.1112
5

0.0215348

0.0887784

0.325521

0.00923194

0.114261

12.9048
6

0.445725

0.442781

1.86813

0.173054

0.0930528

0.120466

0.213303

2.13717932240343e-15

0
4

2.44825
3

1.95322
4

0.495026

1.66533453693773e-16
3

0
4

21.2287

8.18275

13.046

0
4

1.39499

1.17833

0.140481

0.0761741

1.80411241501588e-16

0
4

1.08364
7

0.754213

0.329427

0
4

0.885422
5

0.426978

0.106855

0.0427571

0.101849

0.00500636

0.101849

0.00500636

0.0951208

0
4

6.07542

6.07542

0
4

4.04891

1.2804

2.62628

0.0657345

0.00416335

0.0170056

0.0553261

9.64506252643105e-16

0
4

2.67276
7

2.67276
7

0
4

1.85312
6

1.72354
6

0.129576

3.05311331771918e-16
6

0
4

0.718591

0.718591

0
4

22.166
5

10.2472
5

0.026063

0.00710613

0.00710613

0.0234746

0.0170056

0.185812

0.0966581

0.0599054

1.70996

0.0533257

7.80016
5

0.0553261

0.0404642

0.537317

0.575601

0.0262201

0.100346

0.451759

0.0670115

0.0781891

0
4

5.61832
4

0.165435

5.45289

0
4

1.06682

0.0792192

0.0170056

0.536117

0.0770648

0.357411

0
4

0.232671
7

0.154855

0.0216197

0.0157321

0.0404642

0
4

0.441047

0.161458

0.27959

0
4

0.533951

0.197805

0.336147

0
4

0.147921

0.147921

0
4

24.5103

24.2357

0.274652

0
4

0.894544

0.791728

0.0898581

0.0129576

8.84708972748172e-17

0
4

0.31136

0.31136

0
4

0.0847055

0.0785528

0.00615279

0
4

23.2588
7

8.32328
7

0.0156497

0.0367082

0.711314
7

12.7201
7

0.30372
7

0.509865
6

0.529094
7

0.0309555

0.0731694

0.00498723

0
4

6.04627

6.04627

0
4

0.122676

0.0916749

0.0310016

0
4

1.68209

1.68209

0
4

0.0502475

0.0502475

0
4

0.174276

0.00500636

0.0846347

0.0846347

0
4

1.09861

1.09861

0
4

0.597763

0.597763

0
4

0.0153956

0.00498723

0.00416335

0.00624502

8.67361737988404e-19

0
4

0.095112

0.0598645

0.0352475

0
4

0.0338404

0.0215348

0.00615279

0.00615279

0
4

33.9091
5

29.2281
5

1.54101
3

1.14882
5

0.460396

1.14501

0.123124

0.262628

0
4

0.278804

0.00922919

0.269574

0
4

0.52025

0.52025

0
4

0.0727014

0.0173753

0.0553261

0
4

0.236086

0.236086

0
4

0.30634

0.0316877

0.274652

0
4

1.78524

1.78524

0
4

0.40617

0.40617

0
4

0.204101

0.144196

0.0599054

0
4

0.0399035

0.0399035

0
4

0.586852

0.0375477

0.549304

0
4

30.2426

25.5713

2.35129
6

0.361339

1.25171

0.0660735

0.357411

0.257988

0.0255083

2.84147705365001e-15

0
4

0.168092

0.168092

0
4

0.0194364

0.0194364

0
4

0.0761741

0.0761741

0
4

0.0469069

0.0469069

0
4

0.00443786

0.00443786

0
4

0.063149

0.063149

0
4

0.0340111

0.0340111

0
4

2.19722

2.19722

0
4

0.56122

0.56122

0
4

0.0163868

0.0163868

0
4

97.3377

96.3542

0.00411208

0.0425139

0.0469069

0.763337

0.00615279

0.0316877

0.0887784

9.82547376793264e-15

0
4

0.262628

0.262628

0
4

0.00350607

0.00350607

0
4

0.0483291

0.0483291

0
4

0.0156497

0.0156497

0
4

0.0170056

0.0170056

0
4

0.00827008

0.00827008

0
4

0.0723307

0.0723307

0
4

0.0129576

0.0129576

0
4

0.0100616

0.0100616

0
4

0.0898581

0.0898581

0
4

3.49636

2.38394

0.0887784

0.00443786

0.456557

0.189717

0.182174

0.0377641

0.0561962

0.0234145

0.062892

0.0104881

1.56125112837913e-16

0
4

0.0469069

0.0469069

0
4

0.00967569

0.00967569

0
4

0.05102

0.05102

0
4

0.126298

0.126298

0
4

0.40816

0.40816

0
4

0.0387582

0.0387582

0
4

0.0655183

0.0655183

0
4

0.0157321

0.0157321

0
4

0.36049

0.36049

0
4

0.0887784

0.0887784

0
4

24.9428
5

18.5757

5.27037

0.374065

0.715589

0.00710613

2.95770352654046e-16
5

0
4

1.23593

1.23593

0
4

0.0195035

0.0195035

0
4

2.56494

2.56494

0
4

0
4

40.6735

40.6735

32.9159

7.21854

0.449141

0.0234746

0.0581004

0.00835115

3.89965837399586e-15

0
4

0
4

0.0458254

0.0458254

0.0458254

0
4

0
4

0.471288

0.471288

0.471288

0
4

0
4

0.188119

0.188119

0.152872

0.0352475

0
4

0
4

0.0225442

0.0225442

0.00458025

0.0129576

0.00500636

0
4

0
4

0.290687

0.290687

0.290687

0
4

0
4

0.425041

0.425041

0.0349106

0.390131

0
4

0
4

0.10139

0.10139

0.0827448

0.0186455

0
4

0
4

0.482338

0.482338

0.482338

0
4

0
4

0.564915

0.564915

0.564915

0
4

0
4

23.1863

23.1863

0.101849

23.0844

0
4

0
4

5.2546
3

5.1753
3

0.707429

0.123398

0.00733715

0.200527

0.0147258

0.0198122

0.22518

0.281984

0.12796

0.0599054

0.0316877

0.63571

0.00888202

0.0651483

0.00334147

0.0332578

0.00443786

0.00334147

0.079445

0.0651483

0.0533257

0.119824

0.195915
5

0.107261

0.00408015

0.244634

0.137606

0.214522

0.36769
3

0.283282
3

0.224588

0.0231548

0.192918

0.307842

2.16493489801906e-15
3

0
4

0.0793016

0.0793016

0
4

6.93889390390723e-16
3

0
4

0.164074

0.164074

0.0163868

0.147688

0
4

0
4

0.511089

0.352475

0.352475

0
4

0.158614

0.158614

0
4

2.77555756156289e-17

0
4

0.178269

0.0583091

0.0583091

0
4

0.11996

0.11996

0
4

0
4

0.397776

0.397776

0.397776

0
4

0
4

0.0292553

0.0292553

0.0292553

0
4

0
4

0.0170056

0.0170056

0.0170056

0
4

0
4

0.876329

0.876329

0.876329

0
4

0
4

0.421122

0.421122

0.421122

0
4

0
4

0.123536

0.123536

0.123536

0
4

0
4

0.437713

0.437713

0.437713

0
4

0
4

2.09609
3

2.05429
3

1.18864

0.83967

0.0185926

0.00738779

7.54604712049911e-17
3

0
4

0.00831403

0.00831403

0
4

0.0334842

0.0334842

0
4

2.56739074444567e-16
3

0
4

0.00701215

0.00701215

0.00701215

0
4

0
4

0.0292553

0.0292553

0.0292553

0
4

0
4

0.0714153

0.0714153

0.0714153

0
4

0
4

0.039007

0.039007

0.039007

0
4

0
4

1.60061

1.60061

1.60061

0
4

0
4

0.0312995

0.0312995

0.0312995

0
4

0
4

0.0310016

0.0310016

0.0310016

0
4

0
4

0.195266

0.195266

0.195266

0
4

0
4

0.0245801

0.0245801

0.0245801

0
4

0
4

0.0487588

0.0487588

0.0487588

0
4

0
4

0.327905
6

0.327905
6

0.210498
6

0.0129839

0.065547

0.0224897

0.0163868

6.93889390390723e-18
6

0
4

0
4

0.0606962

0.0606962

0.0606962

0
4

0
4

0.00498723

0.00498723

0.00498723

0
4

0
4

0.504686

0.504686

0.504686

0
4

0
4

0.136044

0.136044

0.136044

0
4

0
4

0.389385

0.389385

0.389385

0
4

0
4

2.25961

2.25961

2.25961

0
4

0
4

3.41991

3.41991

3.41991

0
4

0
4

4.13909

4.13909

4.13909

0
4

0
4

1.33763

1.33763

0.581044

0.304663

0.193681

0.258242

1.66533453693773e-16

0
4

0
4

0.200344
5

0.175126

0.175126

0
4

0.0157126

0.0157126

0
4

0.00950508

0.00950508

0
4

0
4

0.237212

0.237212

0.178902

0.0583091

6.93889390390723e-18

0
4

0
4

0.549726

0.549726

0.541946

0.00777913

0
4

0
4

52.5736

38.3276

37.8732

0.0898581

0.0310016

0.0356441

0.0898581

0.0195035

0.135654

0.0528713

0
4

6.95044
6

6.44316
6

0.0570135

0.0830627

0.125437

0.167805

0.00500636

0.0525668

0.0163868

0
4

0.0434384

0.0434384

0
4

0.119811

0.119811

0
4

0.290687

0.290687

0
4

2.15656
5

0.965833

0.12109

0.144014

0.663072

0.0150191

0.0966581

0.0440184

0.0487588

0.0581004

0
4

0.568191
7

0.403372
6

0.161313

0.00350607

3.46944695195361e-18
7

0
4

0.50332
7

0.471632
7

0.0316877

0
4

2.62396

0.0125159

2.61145

0
4

0.728014

0.728014

0
4

0.177726

0.177726

0
4

0.0404642

0.0404642

0
4

0.0434384

0.0434384

0
4

6.74460487459783e-15

0
4

99.8641

66.9449

4.34364

0.668313
5

1.09151
7

0.598838

6.55043

4.21974
5

0.176289

2.22313

0.521244
5

0.112169

0.105647

2.73396

0.157018

0.371886

0.113944

0.219749

0.0678866

6.19317

1.21439

1.46932

0.0868767

0.0234789

2.38253
6

3.24277

0.0194947

1.40068

0.437594

0.112405

0.0177344

0.0152076

0.209669

1.18268

0.0222131

7.94615
5

0.0693966

0.346099

1.47749

0.0100616

0.0292553

0.0124051

0.00500636

0.00475254

0.0100616

0.0599054

3.80914
6

0.00408015

0.763337

0.0100616

0.0163868

0.0125159

0.0599054

0.0606962

0.232549

1.92594

0.0599054

0.296913

0.007332

0.0150924

0.007332

0.144196

2.09695

0.00439576

0.00586972

0.0259152

0.0156497

0.211485

0.924256

0.0129839

0.00500636

0.763337

0.0155008

0.0703603

0.0100616

0.0150924

0.0281121

0.0404642

0.0232512

2.14242
7

0.0156497

0.0703603

0.0352475

0.0606962

0.00500636

0.193861

0.0599054

0.140721

0.250744

4.39648317751562e-14

0
4

6.94518

5.41544
7

1.52974

4.44089209850063e-16

0
4

0.420354

0.407396

0.0129576

1.90819582357449e-17

0
4

0.471883

0.471883

0
4

0.10116

0.10116

0
4

0.0404642

0.0404642

0
4

0.0996365

0.0996365

0
4

0.0425139

0.0425139

0
4

0.239622

0.239622

0
4

0.0129576

0.0129576

0
4

1.09299
5

0.924632
6

0.0681131

0.095236

0.00500636

0
4

1.2465
6

0.275505

0.828399

0.10384

0.0387582

0
4

20.4883
5

20.4883
5

0
4

0.269995
7

0.150185
7

0.0599054

0.0599054

5.55111512312578e-17
7

0
4

0.571024
7

0.571024
7

0
4

0.140765

0.053042

0.0877233

0
4

0.440839

0.300118

0.140721

2.77555756156289e-17

0
4

0.295011

0.295011

0
4

2.83106871279415e-14

0
4

31.5387

29.5709

5.29656
5

0.0347507

0.535102

0.789051

0.0809283

0.411978

0.00710613

0.0434384

0.0155008

0.72098

3.26431

7.99942
5

0.0195035

0.093266

0.00777913

0.0173753

0.0173753

0.093266

0.0695014

0.188448

0.0388728

0.0173753

4.24309

0.288392

0.0173753

0.093266

1.02919
6

2.79798

0.780141

0.104396

0.326431

0.128735

2.94209101525666e-15

0
4

1.29467

1.29467

0
4

0.597033

0.597033

0
4

0.0292553

0.0292553

0
4

0.0469069

0.0469069

0
4

4.5102810375397e-16

0
4

94.2656
5

93.8364
5

4.80701
5

0.38035
5

0.387257
5

0.428569
6

1.1647
5

0.219938

0.293491

0.0944971

0.13148

0.15288

0.140721

19.3933

0.0137664

0.0573536

0.0240923

0.0729092

0.109838

0.0974323

0.0465825

0.0292811

0.0777455

0.0278609

2.18202
6

0.584446

0.624031

0.129576

0.0183552

0.0907031

0.0512438

0.0470555

0.0323145

0.0324334

0.317391

3.2098
5

0.0173753

0.0112377

1.26997

0.0276876

0.0129576

0.00305919

0.253546

0.29211

0.0404642

0.0122368

3.73299
5

0.0245801

0.0501388

0.00615279

0.0606962

0.0129576

0.0140945

0.0501388

0.0409669

0.0173753

0.0404642

7.33373
5

0.0107072

0.0163868

0.00458879

0.0163868

0.0809283

0.0195035

0.0404642

0.0100127

0.0581004

0.00615279

2.73472
6

0.0245801

0.0129576

0.00764798

0.0155008

0.0129576

0.0292553

36.3787
4

5.5615
6

8.17124146124115e-14
5

0
4

0.312885

0.265353

0.0475315

1.38777878078145e-17

0
4

0.0873557

0.0292553

0.0581004

0
4

0.0120696

0.0120696

0
4

0.0168565

0.0168565

0
4

1.86656246015104e-14
5

0
4

183.588

163.77

90.0774

30.4905
5

0.445616

1.52009

1.01374

0.365985

0.0254735

0.0129576

0.0129576

0.239622

0.357411

0.823956

16.6545
5

0.118371

0.274652

0.076525

0.656697

0.146299

0.411978

0.116618

0.039007

0.373064

0.0129576

5.32438

0.0195035

0.0469069

0.664959

8.98926

0.803844

0.21306
7

1.55261

1.88865

0
4

4.72876
5

4.72876
5

0
4

2.02261

2.02261

0
4

0.14724

0.13973

0.00750954

0
4

0.0328967

0.00411208

0.00822417

0.0205604

0
4

0.112396

0.112396

0
4

5.26781

5.26781

0
4

0.0425139

0.0425139

0
4

0.648882

0.36049

0.288392

0
4

0.00411208

0.00411208

0
4

0.00615279

0.00615279

0
4

0.0112449

0.0112449

0
4

1.09844

0.492213

0.606229

0
4

0.0195035

0.0195035

0
4

0.0163868

0.0163868

0
4

0.792761

0.792761

0
4

0.0871506

0.0871506

0
4

0.0102004

0.0102004

0
4

0.0337346

0.0337346

0
4

0.00500636

0.00500636

0
4

0.0938138

0.0938138

0
4

0.357411

0.357411

0
4

0.0703603

0.0703603

0
4

0.577928

0.231073

0.256997

0.0898581

1.38777878078145e-17

0
4

0.0156497

0.0156497

0
4

0.0581004

0.0581004

0
4

0.0185044

0.0185044

0
4

0.00822417

0.00822417

0
4

0.119811

0.119811

0
4

0.318105

0.318105

0
4

0.0575692

0.0575692

0
4

0.0240859

0.00350607

0.00701215

0.0100616

0.00350607

0
4

0.0267285

0.0102802

0.00822417

0.00411208

0.00411208

3.46944695195361e-18

0
4

1.87111

1.87111

0
4

0.711842

0.711842

0
4

0.333537

0.333537

0
4

0.0678494

0.0370088

0.0308406

6.93889390390723e-18

0
4

0
4

174.571
5

40.1041
7

32.9692
7

6.77443
7

0.36049

4.38538094726937e-15
7

0
4

11.1868
4

10.8408
4

0.161286

0.184686

0
4

0.0129576

0.0129576

0
4

0.00408015

0.00408015

0
4

0.274652

0.274652

0
4

0.095063

0.095063

0
4

50.0946

43.967

6.12761

1.77635683940025e-15

0
4

64.8993
4

63.4029
4

0.94708

0.549304

0
4

1.87364

1.80123

0.0255106

0.0469069

0
4

0.689315

0.0926517

0.184686

0.411978

0
4

0.327921

0.327921

0
4

4.97476

4.97476

0
4

0.0207193

0.0207193

0
4

0.0129576

0.0129576

0
4

0
4

44.471

44.471

1.37376

5.58623
5

2.17356

2.21548

0.228492

0.288796

0.328348

0.310108

0.36049

0.154202

0.0513162

10.7305

0.120823

0.140721

0.0581373

0.0724936

0.0163868

0.648882

0.0163868

0.00440928

0.0966581

0.117267

1.26432

0.0938138

0.0469069

0.144196

0.0938138

0.0387582

0.00440928

0.0316877

0.0150095

0.211081

0.0316877

1.57287
7

0.0150095

5.9557
7

2.18105

3.56434

0.466299

3.64669

4.88498130835069e-15

0
4

0
4

35.8782
4

9.99708

2.71209

2.05989

5.21839

0.00670609

0
4

25.4906

24.5103

0.430933

0.549304

0
4

0.133739

0.133739

0
4

0.184686

0.184686

0
4

0.0591856

0.0591856

0
4

0.0129576

0.0129576

0
4

3.11382863937837e-15
4

0
4

55.3494
4

52.3426
4

1.82562
5

8.65072

0.329597

0.274652

0.149764

0.155674

0.246248

0.0898581

0.0469069

12.8152

17.6883

0.762392

0.980992

3.09305

2.74652

0.839153

1.64791

1.50990331349021e-14
4

0
4

2.85781

2.85781

0
4

0.123124

0.123124

0
4

0.0259152

0.0259152

0
4

0
4

2.51765186476405e-09
7

0
4

3798.05

1696.63

61.4505

51.5258
5

4.36843

0.707914

0.0511374

2.7553

0.605894

0.191753

0.126587

0.975974

0.125347

0.0163868

0
4

468.17

459.632

1.09078
7

0.10116

0.00710613

0.0501388

0.0104881

0.0156497

0.0367082

0.0163868

0.0469069

0.0319776

0.144196

4.93724
7

0.0501388

0.0703603

1.61297
7

0.216365

0.0255135

0.0419522

0.0104881

0.0163868

0.00500636

5.54469664626467e-14

0
4

783.626

587.31
7

0.893549
7

0.207234

0.0206884

0.0365659

0.132278

0.443892

0.0404642

0.00612023

0.0103722

0.288392

0.00612023

1.908
7

0.24673

0.0591856

0.00305919

0.0102004

0.0163868

0.0316877

0.0209761

0.0898581

0.0245801

0.0142123

1.52334
7

0.00408015

0.0591856

0.00520121

0.00408015

0.0122405

0.0224408

0.0316877

0.147964

0.0039381

0.0369278

0.68722
8

0.0156497

0.0404642

0.00736842

0.0104881

0.121392

0.090614

0.00447073

0.0693626

0.0156497

0.00416335

0.855022
7

0.0104881

0.118371

0.00447073

0.144196

0.0246798

0.0104881

0.00408015

1.70996

0.0156497

0.252596

0.613405
7

0.00816031

1.18371

0.0770648

0.00612023

0.0887784

0.00624502

0.0599054

0.0591856

0.029305

0.0244809

0.585429
1

0.288392

0.0366292

0.0234746

0.00416335

0.00447073

0.00624502

0.00615463

0.00416335

0.0403851

0.0591856

0.314571

0.00518609

0.222553

0.0122405

0.0591856

2.80103

0.00351532

0.0633753

0.00447073

0.00661392

0.0104881

0.715056
8

0.0591856

0.00448724

0.00416335

0.00612023

0.019133

0.00879151

0.00987078

0.0591856

0.00967569

0.131037

0.594486

0.0887784

0.00689936

0.0155008

0.00755116

0.00736842

0.0049252

0.144196

0.0655183

0.00816031

0.0460286

8.72234

0.26448

0.464531

0.00527298

0.00447073

0.00408015

0.118371

0.00408015

0.0102004

0.0591856

0.144196

0.0591856

0.269832
8

0.00624502

0.0591856

0.144196

0.0591856

0.0887784

0.0312995

0.015382

4.27489

0.0157321

0.266335

0.190183
7

0.00408015

0.0104881

0.0187351

0.00612023

0.0599054

0.0591856

0.00832669

0.0306012

0.00408015

0.00500636

0.213667
7

0.019133

0.0887784

0.229314

0.0732585

0.144196

0.00416335

0.00408015

0.00416335

0.00712881

0.0591856

0.43547
7

0.00416335

0.0151023

0.00394831

0.0606962

0.0873245

0.0156497

0.0310016

0.0952214

0.144892

0.874803
7

0.501444

1.8344

20.5572
7

0.108329
7

0.488346

0.307192

0.0952283

0.168091

0.211988

0.160517

0.663699

0.50548

0.0494744

70.8156

0.114042

0.298471

0.505923

0.0890553

0.173852

0.0680295

0.275482

0.0510294

0.237055

0.823211

6.94872
7

1.01603

0.610828

0.122936

0.97116

0.443892

0.164009

0.220975

1.4154

0.0356272

0.0204896

12.347
1

0.0979482

0.103225

0.0142805

0.364589

0.227517

0.348297

0.0362679

0.0565097

0.387806

0.0313818

14.6523
1

0.0662976

0.0739225

0.0087883

0.306233

0.178277

0.0696737

0.0662163

0.118371

0.862985

0.22609

12.7719
8

0.575046

0.0102004

0.246049

0.275479

0.122858

0.0490596

0.0399281

0.135983

0.015927

0.338906

1.23493
8

0.18059

0.0703935

0.0714261

0.275479

0.11637

0.153885

0.0942174

0.106717

0.154855

0.142583

0
4

25.7171
7

23.4808
7

2.23623

0
4

0.556205
7

0.201877
7

0.0156497

0.066453

0.0318875

0.0648306

0.0318875

0.0591856

0.0404642

0.0404642

0.00350607

0
4

16.811
6

10.1822
6

6.24736
6

0.0408175

0.0620031

0.278575

0
4

2.60005
7

0.549676
7

1.83185
6

0.0318875

0.186636

7.7715611723761e-16
7

0
4

1.09553
7

0.39262

0.013003

0.20787

0.144196

0.0475315

0.0591856

0.0591856

0.144196

0.0122405

0.0155008

3.64291929955129e-16
7

0
4

0.425029

0.0224362

0.285846

0.0695522

0.0404642

0.00673085

0
4

0.307468
1

0.307468
1

0
4

0.328789
6

0.228183
6

0.0456618

0.0549439

0
4

1.79423

1.30172

0.396177

0.0963278

1.2490009027033e-16

0
4

0.053042

0.0367214

0.0163206

0
4

82.62
5

54.0288
7

5.25966

0.2107
6

0.171773

0.244678

0.22108

0.422242

0.0647408

0.1065

0.0404642

0.0232512

12.0337

0.161286

0.0475315

0.0366292

0.0155008

0.0404642

0.119811

0.00475254

0.0310016

0.0327735

0.0366292

4.22188
6

0.0155325

0.141625

1.43842

1.03353
6

0.49698
5

1.04919
6

0.647534
6

0.221274
6

2.44526621173691e-14
5

0
4

0.257217
6

0.257217
6

0
4

0.12806

0.123572

0.00448724

0
4

0.325521

0.266335

0.0591856

0
4

0.387362

0.258242

0.129121

0
4

0.0262201

0.0157321

0.0104881

0
4

0.157123

0.118371

0.0387519

0
4

0.0598804

0.0232512

0.0366292

0
4

0.0634207

0.0634207

0
4

0.528903

0.528903

0
4

1.87455

1.87455

0
4

116.841

1.33277
1

2.6308

0.0180707

0.0591856

21.8116

1.32269

0.71433

0.350783

0.1465
7

8.25873
2

0.34568
8

79.8497
6

0
4

0.0484412

0.0484412

0
4

0.10116

0.10116

0
4

0.0104881

0.0104881

0
4

0.0591856

0.0591856

0
4

0.00888202

0.00888202

0
4

0.0887784

0.0887784

0
4

0.113193

0.113193

0
4

0.209162

0.209162

0
4

0.0104881

0.0104881

0
4

0.144196

0.144196

0
4

64.3818

19.3924
5

0.00710613

41.536

1.32608
7

1.64179
7

0.172516

0.0559649

0.0259013

0.143137

0.0809283

0
4

0.0975327

0.0975327

0
4

0.0347507

0.0347507

0
4

0.0129576

0.0129576

0
4

0.0460286

0.0460286

0
4

0.0404642

0.0404642

0
4

0.144196

0.144196

0
4

0.0387582

0.0387582

0
4

0.0404642

0.0404642

0
4

0.00816031

0.00816031

0
4

0.0591856

0.0591856

0
4

5.5048

2.13162
1

0.673457
1

0.244808

0.107905

0.263201

0.364177

1.70996

0.00967569

5.15212872365112e-16

0
4

0.0112181

0.0112181

0
4

4.05407
7

2.98984
7

0.393099
8

0.206267

0.16894

0.0591856

0.236743

3.33066907387547e-16
7

0
4

26.7978
6

26.6082
6

0.0616495
7

0.0293857

0.0210594

0.0549439

0.0155008

0.00710613

0
4

25.9057
8

25.5314
8

0.0700793
8

0.0345491

0.0195878

0.0232512

0.216294

0.010546

5.03069808033274e-16
8

0
4

2.4523

2.25471

0.0509522

0.0630379

0.0209761

0.0404642

0.0221634

0
4

1.64757096854373e-13

0
4

1214.18

1107.99

1060.88

1.93681

0.037564

0.322802

0.121392

2.30332

0.185812

0.00416335

0.221221

0.144196

0.144196

0.193681

2.70811

0.129121

0.0460286

0.0606962

0.0397225

0.21317

0.00498723

0.702607

0.148686

0.15722

1.67857

20.6299

3.93818

11.0398

0
4

97.8874
8

6.04308
8

0.321753

0.355114

0.223193

0.20715

0.0591856

0.0591856

0.0887784

0.00350607

0.0591856

0.216294

49.1196
8

5.23793

0.0591856

1.03575

0.118371

0.00518609

0.0173753

0.0196658

0.0591856

0.144196

0.0106592

11.0072
8

0.0887784

0.0887784

16.0792

1.61566

3.88732

1.33866

0.0852952

0.232974

3.77475828372553e-15
8

0
4

5.62081

5.62081

0
4

0.659306

0.659306

0
4

0.142612

0.142612

0
4

1.22665

1.22665

0
4

0.194962

0.194962

0
4

0.268848

0.268848

0
4

0.0168673

0.0168673

0
4

0.0581373

0.0581373

0
4

0.0752082

0.0752082

0
4

0.0409669

0.0409669

0
4

0
4

424.525

354.242

125.418

1.56902
5

0.0168673

0.00411208

0.0404642

0.00458025

0.0173753

0.00799092

0.0173753

0.00888202

0.121392

0.129121

1.06931

0.0434384

0.0129576

0.0404642

0.00532728

0.0194758

0.0599054

0.0973179

0.0404642

0.0163868

0.0404642

3.30913
6

0.221022

0.0173753

0.0404642

0.0469069

0.0112377

0.0123056

0.0327735

0.0819338

0.129121

0.0347507

1.46693
7

0.0163868

0.0404642

0.0255106

0.0259152

0.0163868

0.00738779

0.0143923

0.0469069

0.00687037

0.0129576

0.521929

0.229314

0.00611838

0.0133182

0.0112377

0.0404642

0.269727

0.269727

0.357411

0.0633753

0.0606962

1.00245

0.0100064

0.018321

0.236132
6

0.303905
7

0.0363682

0.21697
6

56.6768

0.63296
7

0.233194

1.01029

0.136493
7

0.098302

0.329084

1.07779

0.772521

0.0923066

2.40974

91.231
7

0.209127

1.93114

0.177928

0.289468

0.311519

0.0774031

0.0696331

0.0947053

0.0188843

0.113151

35.5004

0.035121

0.23417

2.81501

2.23469

0.217077

0.0119766

0.263017

0.0273817

0.068598

0.137264

3.60484
6

0.0453894

0.205455

0.121603

0.0454514

0.0456654

0.0543165

0.0578395

0.10037

0.0578395

0.0570164

2.88818
7

0.0216737

0.0276393

0.0126269

0.0648306

0.0219522

0.11821

0.0170423

0.170432

0.220041

0.0785946

2.19846
1

0.125732

0.0102004

0.0112449

0.0156497

0.288392

0.00518609

0.0404642

0.00498723

0.00416335

0.00518609

2.72484

0.0809283

0.0887784

0.00458025

0.0155008

0.0581373

0.0809283

0.00458025

0.0887784

0.0049252

1.70996

0.703361
7

0.0606962

0.0255106

0.0266461

0.0127553

0.0137407

0.0898581

0.00939633

0.0155008

0.0404642

0.012313

4.83854276089879e-13

0
4

0.236442

0.106119

0.0404642

0.0898581

0
4

0.0347139

0.0118427

0.0189229

0.00394831

2.60208521396521e-18

0
4

0.301769

0.263017

0.0387519

0
4

0.0827923

0.0827923

0
4

0.0404642

0.0404642

0
4

0.144196

0.144196

0
4

0.0809283

0.0809283

0
4

0.00408015

0.00408015

0
4

59.4433

24.5166

2.02814

1.31237

0.499036

0.112499

0.875883

0.0484485

0.0453894

0.202833

0.0174217

0.0606962

4.37582
6

0.121392

0.0938138

0.58673

0.00305919

0.0173753

0.0107775

0.0316877

0.0469069

0.113193

0.00967569

6.01421
6

0.0316877

0.0049252

0.0606962

0.202321

0.0469069

0.0404642

0.0404642

0.0404642

0.0848951

0.242785

4.5199
6

0.0404642

0.0606962

0.141625

0.0973179

0.536117

0.0404642

0.00799092

0.0461898

0.0606962

0.0103722

1.55411

0.0404642

0.0404642

1.25094

7.16279

0.120376

0.177819

1.59488

4.01900734914307e-14

0
4

3.08855
5

2.40085
6

0.0049252

0.0675352

0.0341601

0.36081

0.0950442

0.0903138

0.0104881

0.00498723

0.0194364

3.2612801348364e-16
5

0
4

0.795555

0.331675
6

0.0409669

0.00305919

0.156722

0.0534217

0.0127043

0.0404642

0.00305919

0.0194364

0.0049252

0.129121

0
4

1.76349
7

1.55979
7

0.159441

0.0312995

0.0129576

0
4

3.13689
6

0.0935271

0.0804009

0.368656

2.58132

0.0129839

0
4

0.406622
7

0.058002

0.121392

0.0172382

0.20999

0
4

0.531575

0.303483

0.0873711

0.0469069

0.0938138

0
4

0.191939

0.10116

0.0049252

0.0049252

0.0404642

0.0404642

1.38777878078145e-17

0
4

0
4

1.76656

0.589437

0.589437

0
4

1.17712

1.17712

0
4

0
4

0.0955183

0.0781429

0.0781429

0
4

0.0173753

0.0173753

0
4

0
4

0.0531889

0.0531889

0.0404336

0.0127553

0
4

0
4

4.21439

4.21439

0.0484412

4.11751

0.0484412

0
4

0
4

0.075211

0.075211

0.0721518

0.00305919

4.33680868994202e-18

0
4

0
4

0.968406

0.968406

0.774725

0.193681

0
4

0
4

0.438897

0.438897

0.341364

0.0975327

0
4

0
4

2.8955

2.76638

0.054841

2.71154

0
4

0.129121

0.129121

0
4

0
4

0.0276244

0.0276244

0.0276244

0
4

0
4

0.0236623

0.0236623

0.0236623

0
4

0
4

136.675
7

40.6595
7

0.429157
7

0.00592134

0.0703603

0.0259152

0.00592134

0.00822417

0.129121

35.1317
7

4.59429
7

0.0826646
7

0.0537386

0.0204063

0.0314798

0.044251

0.0262735

0
4

87.3102
6

84.6483
6

0.0280114
7

0.0247222

0.0300277

2.42495

0.0668146

0.0404642

0.0469069

5.13547537828174e-14
6

0
4

0.180104

0.105195

0.074909

0
4

3.80869

2.54691

1.26178

2.22044604925031e-16

0
4

4.64805
6

0.30402

0.0691316

4.27489

8.88178419700125e-16
6

0
4

0.0379091

0.0266642

0.0112449

5.20417042793042e-18

0
4

0.00687037

0.00687037

0
4

0.00687037

0.00687037

0
4

0.0163868

0.0163868

0
4

0
4

0.0956778

0.00689936

0.00689936

0
4

0.0887784

0.0887784

0
4

0
4

0.159117

0.00498723

0.00498723

0
4

0.15413

0.15413

0
4

0
4

0.077083

0.077083

0.0606962

0.0163868

3.46944695195361e-18

0
4

0
4

3.29911

3.29911

3.29911

0
4

0
4

1.7229

1.7229

1.7229

0
4

0
4

0.00736842

0.00736842

0.00736842

0
4

0
4

0.00447073

0.00447073

0.00447073

0
4

0
4

0.0170056

0.0170056

0.0170056

0
4

0
4

0.164174

0.164174

0.164174

0
4

0
4

0.164436

0.164436

0.164436

0
4

0
4

5.87265
7

2.88966
7

0.834337
7

1.8277

0.115002

0.0809283

0.0316877

0
4

2.09884
7

1.29121
7

0.27633

0.119882

0.0173753

0.357411

0.0366292

0
4

0.843683
6

0.58077

0.0049252

0.257988

0
4

0.0404642

0.0404642

0
4

8.32667268468867e-17
7

0
4

0.129121

0.129121

0.129121

0
4

0
4

0.0501388

0.0501388

0.0501388

0
4

0
4

0.0388728

0.0388728

0.0388728

0
4

0
4

0.161286

0.161286

0.161286

0
4

0
4

0.00447073

0.00447073

0.00447073

0
4

0
4

0.093266

0.093266

0.093266

0
4

0
4

14.1847
6

11.4819
6

3.59856
6

6.13413

1.17077

0.0404642

0.234534

0.303481

0
4

1.12572
7

1.01849
7

0.0898581

0.0173753

7.97972798949331e-17
7

0
4

1.18296
7

0.991634
6

0.19133

0
4

0.111661

0.0156497

0.0898581

0.00615279

9.54097911787244e-18

0
4

0.222553

0.222553

0
4

0.0599054

0.0599054

0
4

0
4

9.43595
6

9.43595
6

8.28821
6

0.73286

0.357411

0.0170056

0.0404642

0
4

0
4

27.8551
2

27.8551
2

22.811
2

0.645604

0.069043

4.13187

0.0726619

0.0397225

0.0367698

0.0484412

1.86656246015104e-15
2

0
4

0
4

1.31437
6

0.653895
6

0.541042

0.0938138

0.00967569

0.0049252

0.00443786

0
4

0.660471

0.629007

0.0314642

4.16333634234434e-17

0
4

0
4

1.11861

1.11861

1.11861

0
4

0
4

7.46687

7.46687

6.7186

0.110907

0.110907

0.300833

0.225625

3.33066907387547e-16

0
4

0
4

242.051

241.658

93.0601

0.159648
7

0.334789
7

0.103125

0.183642

0.544762
7

0.210118

0.190066

0.256117

0.149967

0.575491

20.5193
7

0.152977

0.671138

0.120365

0.245949

0.0163206

0.224954

0.14655

0.645951

0.117944

0.0793666

109.131

0.111076

0.0809283

0.0109505

0.0122405

0.0595195

0.0183607

0.037564

0.09636

1.10839

0.0086604

1.54748

0.0853976

0.077083

0.100119

0.718733

0.121392

0.137779

0.0086604

0.0114506

0.00816031

0.0724936

3.86421
7

0.0163206

0.0501388

0.00408015

0.0404642

0.0163206

0.10116

0.0404642

0.00408015

0.0163868

0.0163868

0.220929

0.00408015

0.100278

0.0501388

0.0340111

0.0122405

0.0483291

0.0245801

0.300833

0.0387582

0.00665242

2.42359
8

0.00408015

0.0129839

0.0163868

0.0404642

0.00408015

0.0129839

0.00408015

0.00408015

0.0163868

0.606962

0.0693626

0.0966581

0.00408015

0.103661

0.168449

0.0163868

0.00443786

0.0489618

0.0163868

0.00612023

0.11996

0.473106

0.0606962

0.0404642

0
4

0.310982

0.310982

0
4

0.0409669

0.0409669

0
4

0.0404642

0.0404642

0
4

0
4

2.8421709430404e-14

0
4

127.207

0.845475
7

0.0860541

0.0798091

0.00624502

6.93889390390723e-18

0
4

0.200127

0.120076

0.0800508

0
4

0.0779032

0.0779032

0
4

0.0779032

0.0779032

0
4

0.110458

0.110458

0
4

0.245156

0.245156

0
4

0.0366292

0.0366292

0
4

0.0112449

0.0112449

0
4

8.50014503228635e-17
7

0
4

14.5465
7

14.5146
7

1.51396

0.479243

0.0240339

0.0380012

0.352315

0.167341

1.83853

0.451225

0.230692

0.285189

0.062599

0.676214

0.0764283

0.0553389

0.351294

0.0907031

0.192662

0.0224897

0.160485

0.222553

0.0606962

0.123097

0.50246

0.304895

0.0327735

0.0606962

0.0255106

0.0938138

0.269727

0.0189229

0.0637765

0.0469069

0.222553

0.84436

0.124377

0.0792192

0.0155008

0.0606962

0.095063

0.0434384

0.0703603

0.0168673

0.0163868

0.0770648

0.156952

0.0581373

0.0483785

0.039357

0.0387582

0.629007

0.0112449

0.047824

0.0704238

0.0283844

0.0168673

0.0967377

0.216294

0.0283844

0.164174

0.052126

0.136331

0.0168673

0.0112449

0.0606962

0.232217

0.0316877

1.2736

0.0255106

0.0245801

0.0112449

0.0283844

0.0606962

0.229528

0.10846

0
4

0.0318883

0.0318883

0
4

0
4

3.85388
6

3.85388
6

3.81342
6

0.0404642

0
4

0
4

0.0843952

0.0843952

0.0843952

0
4

0
4

0.693231

0.693231

0.693231

0
4

0
4

11.2482
7

11.2482
7

11.2482
7

0
4

0
4

0.345292

0.345292

0.345292

0
4

0
4

0.0606962

0.0606962

0.0606962

0
4

0
4

0.129121

0.129121

0.129121

0
4

0
4

0.095063

0.095063

0.095063

0
4

0
4

0.0701542

0.0701542

0.0701542

0
4

0
4

0.182089

0.182089

0.182089

0
4

0
4

0.115597

0.115597

0.115597

0
4

0
4

94.9369

88.3537

33.1546

18.5047
7

4.30005

0.222445

0.0373079

1.02218

0.0105182

0.0584274

0.0355281

0.0887784

0.0337346

0.0737404

8.63068
6

0.979293

0.149764

0.349252

0.0583091

0.117267

1.09753

1.70996

0.0770648

0.0350607

0.0606962

6.00801

0.0327735

0.0809283

0.0214143

1.08193
7

0.348366

7.69325
7

0.518098

1.61701

0.145007

0
4

3.05967

3.0474

0.00876519

0.00350607

1.83880688453542e-16

0
4

0.890343

0.890343

0
4

1.20616

1.20616

0
4

0.769694

0.769694

0
4

0.0157773

0.00525911

0.0105182

0
4

0.557176

0.557176

0
4

0.0809283

0.0809283

0
4

0.00350607

0.00350607

0
4

0
4

7.105427357601e-14

0
4

47371.7
4

47336.7
4

47330.5
4

47263
4

12.1311

0.401685

4.53756

0.50937

0.0267796

0.0552692

0.501097

0.0765295

0.140721

0.278328

0.128464

25.8048

4.12805

0.237608

0.676838

0.032394

0.223238

0.123124

0.0155583

0.0387582

0.0737404

0.0565967

0.980522
7

0.763337

0.185812

0.185812

0.0565967

0.221304

0.0170056

0.274652

0.0100616

0.00525911

0.115072

0.155633

0.0565967

0.0259152

3.03434

0.113193

0.123124

0.00748085

0.0301849

0.139287

0.0100616

0.216294

0.402323

0.0112449

0.115072

0.0556337

0.193681

0.00305919

0.0127553

0.0434384

0.0848951

0.0761741

0.868043
6

5.48396

2.00137

1.19028

2.56177301594107e-11
4

0
4

0.0647879

0.0647879

0
4

0.0968956

0.0968956

0
4

1.70996

1.70996

0
4

0.00520121

0.00520121

0
4

0.10116

0.10116

0
4

3.91083

3.91083

0
4

0.0112449

0.0112449

0
4

0.0501388

0.0501388

0
4

0.193681

0.193681

0
4

0.0168565

0.0168565

0
4

0.00518609

0.00518609

0
4

0.0201232

0.0201232

0
4

0.0224754

0.0224754

0
4

0
4

4.93722

4.93722

4.93722

0
4

0
4

0.0240814

0.0240814

0.00670609

0.0173753

0
4

0
4

0.58957

0.58957

0.58957

0
4

0
4

0.411978

0.411978

0.411978

0
4

0
4

0.0327735

0.0327735

0.0327735

0
4

0
4

0.0491603

0.0491603

0.0491603

0
4

0
4

0.0936218

0.0936218

0.0936218

0
4

0
4

0.192662

0.192662

0.192662

0
4

0
4

0.246248

0.246248

0.246248

0
4

0
4

0.134838

0.134838

0.134838

0
4

0
4

0.00777913

0.00777913

0.00777913

0
4

0
4

0.716264

0.716264

0.464073

0.252192

0
4

0
4

0.0170056

0.0170056

0.0170056

0
4

0
4

0.0599054

0.0599054

0.0599054

0
4

0
4

0.0168565

0.0168565

0.0168565

0
4

0
4

0.903846

0.903846

0.903846

0
4

0
4

0.0168565

0.0168565

0.0168565

0
4

0
4

0.231194

0.231194

0.231194

0
4

0
4

21.4024

21.4024

21.4024

0
4

0
4

0.248359

0.248359

0.248359

0
4

0
4

0.0907031

0.0907031

0.0647879

0.0259152

0
4

0
4

0.025154

0.025154

0.025154

0
4

0
4

3.4363

3.4363

3.41991

0.0163868

0
4

0
4

1.10267

1.10267

1.10267

0
4

0
4

0.0459884

0.0459884

0.0459884

0
4

0
4

3.27973759262079e-13
4

0
4

30104.5

16.577
1

4.31668

3.56337

0.0633849

0.25354

0.348617

0.0877713

1.38777878078145e-16

0
4

0.0633849

0.0633849

0
4

0.710228

0.710228

0
4

6.65432

6.65432

0
4

1.51403

1.51403

0
4

0.94697

0.94697

0
4

1.71139

1.71139

0
4

0.0877713

0.0877713

0
4

0.484478

0.484478

0
4

0.0877713

0.0877713

0
4

5.63438184997267e-15
1

0
4

309.574

258.335

27.2356

3.35479
2

0.458081
3

0.478018

1.22371

0.26502

0.550126

0.276623

0.200248

3.08214

1.06499

46.2584

0.216407

0.270321

0.261726

0.173268

7.78978

0.177091

0.142735

0.316525

0.25883

0.206941

36.104
4

0.243255

0.530228

0.0909637

0.224485

0.0982732

0.350972

0.134773

0.00503411

0.200555

0.00592247

8.2964

0.0651483

0.0460286

0.126952

0.077809

0.0501388

0.120823

0.0155008

8.54979

0.253158

0.0898581

20.6082

0.0633849

0.126952

0.285232

0.0735396

0.0155008

0.0104881

0.0501388

0.114261

0.108581

1.98149

58.735
4

0.0877713

0.115072

4.3573

0.364177

0.380856

0.357411

0.0633849

0.0423112

0.0143923

0.0404642

0.610511
3

0.0423112

1.70996

0.00411208

0.0633849

0.0651483

0.00362217

0.0633849

0.0846225

0.0434322

0.00448724

5.02282

0.0651483

0.141625

0.0423112

0.0822615

0.0423112

0.00897447

0.14769

0.0551547

0.0434322

0.126952

10.0697
3

0.667604

0.0367698

0.506914

0.228522

0.0950773

0.0633849

0.0423112

0.126952

0.0868644

0
4

10.9787
3

5.44524
3

4.47386

0.337786

0.188583

0.533219

0
4

0.0127553

0.0127553

0
4

0.0127553

0.0127553

0
4

0.0367698

0.0367698

0
4

0.0475315

0.0475315

0
4

0.0404642

0.0404642

0
4

0.019133

0.019133

0
4

0.114261

0.114261

0
4

1.70996

1.70996

0
4

1.70996

1.70996

0
4

3.33669

3.33669

0
4

27.2366
3

27.2366
3

0
4

1.64206

1.63841

0.00364411

2.90566182226115e-17

0
4

0.142147

0.0102802

0.131867

0
4

2.08698

2.08698

0
4

1.84221

1.82165

0.0205604

1.00613961606655e-16

0
4

0.131657

0.131657

0
4

0.131657

0.131657

0
4

0.00616813

0.00616813

0
4

0
4

292.325
1

112.367
1

41.2501
1

0.252673

0.216243

0.469646

0.205388

0.356518

0.187885

0.290834

0.621449

0.0518304

0.0453516

43.3244
1

0.0647879

0.149107

0.317766

0.032394

0.0583091

0.0864972

0.0777455

0.0388728

0.116618

0.187885

0.886994

0.209764

0.0787257

0.288731

0.0453516

0.405349

0.0129576

0.0129576

0.651042

0.0129576

0.12041

0.0583091

0.0259152

0.0129576

0.0129576

0.00777913

1.16713

0.0367698

0.0103722

0.0259152

0.0129576

0.0194364

0.100506

0.147079

0.0367698

0.0919245

0.110309

0.0259152

0.0129576

0.0982775

0.0194364

0.0129576

0.114261

1.24485

0.0129576

0.0194364

0.0129576

0.0194364

0.0388728

0.0259152

0.0259152

0.0129576

0.032394

0.41837

17.1392

0.0756426

0
4

159.414
2

158.671
2

0.742735

3.33066907387547e-15
2

0
4

4.57891

4.51093

0.0435118

0.0108779

0.0135974

0
4

0.667415

0.667415

0
4

0.159117

0.159117

0
4

0.101849

0.101849

0
4

0.0155008

0.0155008

0
4

0.093266

0.093266

0
4

0.120694

0.120694

0
4

0.152348

0.152348

0
4

0.00543897

0.00543897

0
4

0.105778

0.105778

0
4

0.149012

0.149012

0
4

4.85224

4.83865

0.00815845

0.00543897

5.07406616723216e-16

0
4

0.0666674

0.0666674

0
4

3.53686

3.53686

0
4

2.62801

2.62801

0
4

3.16122

3.08505

0.0761741

1.80411241501588e-16

0
4

0.09597

0.09597

0
4

0.00518609

0.00518609

0
4

0.0155008

0.0155008

0
4

0.032394

0.032394

0
4

0
4

6920.35

102.106

30.9029
2

1.04569

0.292306

1.99257

1.92214

0.302844

0.110365

0.133916

0.0683571

0.141742

0.0809184

13.175
6

0.173776

0.526628

0.207861

0.110692

0.0609897

0.160172

0.0337621

0.142606

0.00362217

0.269318

7.65058
3

0.0382659

0.00498723

0.0551547

0.026063

0.052126

0.0404642

0.0367698

0.274176

0.0887784

0.0507772

5.06295
2

0.0761658

0.00520121

0.0404642

0.0634669

0.0761658

0.101554

0.0481639

0.165464

0.126943

0.288392

15.1361
3

0.0761658

0.0469069

0.768819

0.0163868

0.0551547

0.0481639

0.0608137

0.0548351

0.0245801

0.0548351

12.9113
6

0.0367698

0.0919245

0.710881

0.00615463

0.0943925

0.122901

0.0173753

1.37658
2

3.51776

0.64569
2

1.48436818392383e-13

0
4

6533.35

6289.53

11.8326
7

8.53708

0.477784

0.163672

0.053448

0.0274936

1.93045

0.0707579

0.0898362

0.116256

0.314506

0.152857

2.63785
7

0.0303573

0.0478845

0.0748354

0.0604021

0.025109

0.0396852

3.26337

0.042936

0.0779085

0.197156

3.19369

0.57582

0.0311505

0.20816

0.0646343

0.0727806

0.029862

0.0471885

0.0852764

0.0568314

0.0483481

1.90855
7

0.0225414

0.102752

0.0534217

0.2374

0.0236867

0.0114506

0.0112377

0.10116

0.0232512

0.0173753

2.10183

0.240375

0.190435

0.114261

0.00736842

0.0312995

0.06398

0.029328

0.0163868

0.0761741

0.0173753

1.08655
7

0.0307731

0.0155008

0.00520121

0.0483291

0.0148034

0.0966581

0.137357

0.0155008

0.0129839

0.0887784

0.596071
6

0.0404642

0.0387519

0.0209761

0.00592134

0.0310016

0.0484412

0.0387519

0.0163868

0.0580542

1.15357

0.551988
7

0.0761741

0.0120572

0.00518609

0.152348

0.0919191

0.0100616

0.0194364

0.0689527

5.90349

0.104252

1.62296
7

0.0145135

0.0100616

0.0761741

0.0327735

0.0518304

0.00498723

0.0262201

0.131657

0.00518609

0.0449794

2.03044
7

0.621449

0.0761741

0.0245801

0.0724936

0.0310016

0.00518609

0.146299

0.500948

0.0193514

0.0340111

33.0706
7

2.22703
7

0.190435

0.00736842

0.00690984

0.0484987

0.0147756

0.00475254

0.0761741

0.0163868

0.0127553

0.0127544

0.439338
7

0.0163868

0.00364411

0.012313

0.327003

0.0156497

0.0259677

0.152348

0.0483291

0.0404642

0.144987

1.20861
7

0.0234746

0.121392

0.0104881

0.0591856

0.0404642

0.0155008

0.0761741

0.0469069

0.0129839

0.0163868

1.40047
7

0.0157321

0.142595

0.0103722

0.0170056

0.257988

0.0484412

0.09597

0.0761741

0.00888202

0.105743

10.4968
6

0.152348

0.0163868

0.290647

0.0327735

0.373064

0.0506019

0.255106

0.0155008

0.0163868

0.0221634

2.93141
2

0.0232512

0.0761741

0.0469069

0.38087

0.0155008

0.0887784

0.00615463

0.480751

0.0110526

4.97789

0.35781
7

0.0127553

0.00710613

0.152348

0.0145135

0.0469069

0.00710613

0.0163868

0.0112377

0.0404642

0.589923

0.272821

0.018421

0.0404642

0.216294

0.00967569

0.0703603

0.0860736

0.00319998

0.0775039

0.00447073

0.0761741

0.683753
7

0.0232512

0.0366292

0.0104881

0.0129839

0.0129576

0.0761741

0.0163868

0.00738779

0.0591856

0.0595432

0.224058
6

0.0469069

0.0966581

0.0127553

0.0591856

0.00755116

0.00939633

0.0495996

0.0809283

0.0404642

0.0404642

7.14996
7

0.236716
6

0.0049252

0.128202

0.093266

0.00503411

0.0377212

0.0155008

0.114261

0.0404642

0.0316877

0.0049252

0.975534
7

0.0100616

0.0434322

0.0225286

0.0155008

0.0549439

0.0491603

0.00748085

0.0155008

0.0138197

0.00690984

1.50949

0.0452773

0.0968825

1.84077

0.0100616

0.0112377

0.0209761

0.0156497

0.0484412

0.114261

0.219428

0.433172
7

0.0163868

0.0173753

0.00498723

0.3199

0.0106592

0.0224897

0.012313

0.0112377

0.00615463

0.0080381

0.63992
7

0.131037

0.0155008

0.0775039

0.0173753

0.0606962

0.0737404

0.0391243

0.0232512

0.0606962

0.00543897

1.92803
8

0.09597

0.0112377

0.222553

0.0404642

0.0049252

0.0155008

0.0241892

0.0542527

0.0435406

0.0469069

0.405463
1

0.0777455

0.0156497

0.0173753

0.00777913

0.09597

0.00710613

0.00690984

0.0404642

0.0163868

0.0232512

0.221376
7

0.0314642

0.0255083

0.0779032

0.00518609

0.0404642

0.0352157

0.00690984

0.161857

0.00710613

0.0404642

0.20258

0.0129839

0.0155008

0.0112377

0.00777913

0.00532728

0.266609

0.0106592

0.00967569

0.121392

0.0103648

0.487824
7

0.0761741

0.0310016

0.0460286

0.0968825

0.00967569

0.00777913

0.0156497

0.0100616

8.16651
7

0.410707

0.0811675
7

1.47694

0.166785
7

0.514724
7

0.470182
6

0.429798
8

0.214104

0.45921

0.191651

8.46272
7

0.100501
7

0.246741
7

0.0851005

10.5036
6

0.167262

2.03385

1.98117

0.119753

0.130965

0.134593

22.3047
7

0.100094

0.196405

0.114704

0.144697

0.796743

0.249321

0.0552115

0.141098

0.0468337

2.51629

11.2783
7

0.253129

0.0700412

0.0620031

0.171188

0.0814864

0.0423056

0.235131

0.0781756

0.497256

0.0686538

7.83973

0.0287819

0.0538319

0.0754367

2.48698

1.02661

0.0470454

0.0140246

1.80435

0.196683

0.154977

11.1314

0.0984

0.0863615

0.118371

0.184442

0.295325

0.0350863

0.114941

0.0285235

0.032394

0.0389833

0
4

73.2762
7

73.1698
7

0.0515724

0.0548351

3.05311331771918e-15
7

0
4

173.576

32.6217

28.0354

0.00777913

0.175543

0.0163868

0.00673085

0.00736842

0.0163868

14.5936

0.188448

2.57817

6.22605

0.909587
6

2.11699

0.315033

0.190663

0.738719

13.8719
4

0.216328

16.176

0.718545

0.0757369

0.122398

0.0809283

0.0529645

0.0693584

0.609393

0.0670833

4.48932
2

0.204735

0.152348

0.74313

0.0155222

0.152348

0.133872

0.0134287

0.0918238

0.0318875

0.0483291

5.68022
3

0.0181513

0.0409669

0.0181513

0.0255083

0.0483291

0.06398

0.0483291

0.0761741

0.0245801

0.0751983

12.2717
6

0.0163868

0.012313

0.0409669

0.0761741

0.524348

0.12796

0.0163868

0.00447073

0.141625

0.0232512

0.634035
1

0.039357

0.0483291

0.0340111

0.141492

0.278718

0.114261

0.64748

0.06398

0.16979

0.0809283

0.518195

0.00897447

0.945638

0.00967569

0.00479996

0.53162

0.262073

0.714822

0.0091605

0.0155008

0.0434322

17.6512
3

0.0255083

0.0724936

0.0469426

0.0975327

0.0112377

0.0207444

0.0104881

0.00319998

0.00498723

0.053673

2.75425
4

1.02197

0.0111768

0.0259152

0.00543897

0.0245801

0.065547

0.0599054

0.0170056

0.00777913

0.0112449

9.44244682443696e-14

0
4

26.0428

24.0418

1.9777
7

0.0232512

0
4

0.0454803

0.0454803

0
4

0.160583

0.0163868

0.144196

0
4

0.0691487

0.0691487

0
4

0.228522

0.228522

0
4

0.121392

0.121392

0
4

0.00543325

0.00543325

0
4

0.144196

0.144196

0
4

0.0173753

0.0173753

0
4

0.0591856

0.0591856

0
4

0.0418642

0.0418642

0
4

1.37712

0.402303
1

0.0194364

0.0404642

0.0761741

0.107235

0.326431

0.172144

0.0449507

0.12069

0.0361385

0.0155008

0.0156497

0
4

0.0163868

0.0163868

0
4

0.00520121

0.00520121

0
4

0.0530735

0.0530735

0
4

0.0352475

0.0352475

0
4

3.35214
7

2.14577
7

0.474086

0.459849

0.149475

0.0591856

0.0637765

0
4

1.43258

0.623122
1

0.245614

0.114261

0.238877

0.139899

0.0224754

0.0483291

2.63677968348475e-16

0
4

1.45447

0.594756

0.177898

0.480917

0.114707

0.0861909

2.77555756156289e-17

0
4

2.3772

0.404126

1.31458

0.286981

0.371514

0
4

0.292383
1

0.276272

0.00900535

0.00710613

0
4

0.528804

0.00777913

0.152348

0.06398

0.304696

5.55111512312578e-17

0
4

0.179062

0.0938138

0.0591856

0.026063

0
4

4.87296314410912e-12

0
4

1088.64

1030.09

1020.05

0.0510167

0.789999

0.792761

0.0950773

0.539429

7.77507

6.03961325396085e-14

0
4

2.36669
1

1.83305
2

0.467567
1

0.0526513

0.0134122

0
4

2.85232

2.85232

0
4

3.60578

3.60578

0
4

0.306012

0.306012

0
4

0.121392

0.0809283

0.0404642

0
4

0.316924

0.221847

0.0950773

0
4

0.727704

0.727704

0
4

0.294921

0.20715

0.0877713

0
4

2.14151

2.14151

0
4

0.148673

0.148673

0
4

0.478586

0.478586

0
4

2.4564

2.32728

0.129121

0
4

0.0206869

0.00518609

0.0155008

0
4

0.017764

0.0118427

0.00592134

0
4

0.435219

0.435219

0
4

0.142595

0.142595

0
4

0.0104881

0.0104881

0
4

0.0170056

0.0170056

0
4

0.093266

0.093266

0
4

0.0404642

0.0404642

0
4

0.069043

0.069043

0
4

2.58242

2.58242

0
4

2.66751
2

1.61426
2

1.05326

0
4

0.097998

0.097998

0
4

0.0734164

0.0734164

0
4

0.0761741

0.0761741

0
4

0.00546617

0.00546617

0
4

2.59695

2.59695

0
4

1.70996

1.70996

0
4

0.346792

0.346792

0
4

0.114261

0.114261

0
4

0.216294

0.216294

0
4

0.093266

0.093266

0
4

22.3939
2

22.3939
2

0
4

0.187797

0.187797

0
4

0.144196

0.144196

0
4

0.15413

0.15413

0
4

0.160864

0.160864

0
4

0.0155008

0.0155008

0
4

0.0877713

0.0877713

0
4

0.0877713

0.0877713

0
4

0.0346813

0.0346813

0
4

3.61426

3.61426

0
4

0.276851

0.16259

0.114261

1.38777878078145e-17

0
4

3.99332

3.99332

0
4

0.158935

0.151156

0.00777913

0
4

3.15608650325316e-13

0
4

175.095
3

160.908

157.454

3.40096

0.0142805

0.0387582

0
4

2.28186

1.07754

0.25354

0.950773

0
4

0.00612023

0.00612023

0
4

10.0506

8.96585

1.08471

8.88178419700125e-16

0
4

0.0652825

0.00612023

0.026521

0.0326412

6.93889390390723e-18

0
4

0.0889752

0.0889752

0
4

0.158462

0.0950773

0.0633849

0
4

0.0722459

0.0722459

0
4

1.39627

1.39627

0
4

0.00408015

0.00408015

0
4

0.0633849

0.0633849

0
4

3.26960680752109e-14
3

0
4

199.595

152.983

2.68128
8

5.02622

0.0104881

0.0153866

0.00411208

0.0665157

0.0332578

0.00736842

0.0483291

0.120823

0.0761741

0.152348

4.01656

0.0460286

0.00616813

0.00736842

0.0761741

0.0157321

0.0387582

0.120823

0.0316877

0.0761741

0.0049252

0.511552

0.0156497

7.57932

0.24942

0.21032

2.87578

0.399094

1.23239

2.36746

10.9774

0.290687

5.41116

0.382828

0.38087

1.44987

0.199547

7.70556

0.135163

4.01056

3.8208

4.63947

0.40657

0.233427

0.0149524

0.0147368

0.169152

0.0155583

15.08

0.0580196

0.144987

5.9928

1.58957

0.528519

0.486303

0.415723

0.443524

0.258726

0.0297196

0.0380138

0.761741

2.66118

0.338303

0.410935

0.914089

0.859018

0.015477

0.152348

0.0332578

0.628335

0.0245801

0.0633753

0.0164483

0.00416335

12.2225

0.0404642

0.110883

0.184804

0.0123363

0.0761741

0.0460286

0.0761741

0.0127553

0.0316877

0.0316877

4.61622

0.0997735

0.069043

0.0332578

0.095063

0.536117

0.077809

0.0483291

0.147844

0.0761741

0.0104881

3.76479

0.0460286

0.258726

0.0595838

0.114261

0.114261

0.0316877

0.00416335

0.0483291

0.517453

0.069043

23.5525

0.184804

0.147844

0.0761741

4.72279

0.0724936

0.00518609

0.114261

0.0483291

0.00518609

0.0387582

1.63202784619898e-14

0
4

6.8491

4.16626

1.72173

0.406584

0.0792192

0.126751

0.0316877

0.316877

1.16573417585641e-15

0
4

0.246469

0.246469

0
4

0.887255

0.649597

0.0475315

0.0316877

0.158438

0
4

0.153083

0.153083

0
4

0.269345

0.237658

0.0316877

0
4

0.877094

0.877094

0
4

0.147615

0.0792192

0.0367082

0.0316877

0
4

0.0583091

0.032394

0.0129576

0.0129576

0
4

0.163571

0.105551

0.0475315

0.0104881

3.46944695195361e-18

0
4

1.63132

1.61857

0.0127553

0
4

0.166301

0.166301

0
4

0.0863538

0.0616813

0.0143923

0.00411208

0.00616813

0
4

0.299321

0.0332578

0.232805

0.0332578

0
4

0.217665

0.217665

0
4

0.269122

0.269122

0
4

0.0631518

0.0316877

0.0209761

0.0104881

3.46944695195361e-18

0
4

5.02194

0.0332578

4.90553

0.0831446

5.55111512312578e-17

0
4

4.79391

4.79391

0
4

0.120823

0.120823

0
4

0.0123996

0.00615463

0.00624502

0
4

0.174282

0.174282

0
4

0.160722

0.0642886

0.0964329

0
4

11.9066

11.2592

0.64748

1.11022302462516e-16

0
4

0.478629

0.478629

0
4

0.158438

0.158438

0
4

0.0901271

0.0901271

0
4

0.0352475

0.0352475

0
4

0.0599054

0.0599054

0
4

0.166289

0.166289

0
4

0.00498723

0.00498723

0
4

0.0316877

0.0316877

0
4

0.228522

0.228522

0
4

0.0316877

0.0316877

0
4

1.70014

1.45071

0.0831446

0.0498868

0.0332578

0.0332578

0.0498868

1.59594559789866e-16

0
4

0.0143923

0.0143923

0
4

0.144196

0.144196

0
4

0.129121

0.129121

0
4

0.0194364

0.0194364

0
4

0.0173753

0.0173753

0
4

0.0595838

0.0595838

0
4

0.0663141

0.0663141

0
4

1.3926

1.3926

0
4

0.00411208

0.00411208

0
4

0.0316877

0.0316877

0
4

1.30654
7

1.01815
7

0.288392

1.66533453693773e-16
7

0
4

0.0714153

0.0714153

0
4

0.118371

0.118371

0
4

3.15977

3.15977

0
4

0.214485

0.214485

0
4

0.026063

0.026063

0
4

0.0831446

0.0831446

0
4

0.0316877

0.0316877

0
4

0.0104881

0.0104881

0
4

0.00416335

0.00416335

0
4

0.0884098

0.0328967

0.0123363

0.0164483

0.0123363

0.0143923

3.46944695195361e-18

0
4

0.803959

0.803959

0
4

0.71116

0.672036

0.0234746

0.0156497

6.59194920871187e-17

0
4

0.542005

0.523854

0.0181513

4.16333634234434e-17

0
4

0
4

39.0227

32.197

18.486

5.88855

0.0724936

0.966581

0.0483291

0.0483291

0.837915

0.0966581

0.14617

0.115597

0.266335

0.231194

0.238834

0.0483291

0.0483291

0.712581

0.115597

0.0730852

0.0591856

0.310612

0.00690984

0.169152

0.555784

0.605017

0.144196

1.11157

0.0676807

0.147964

0.0241844

0.352852

0.200984

8.10462807976364e-15

0
4

1.48837

1.14249

0.0898581

0.114261

0.0761741

0.065594

0
4

0.0322355

0.0322355

0
4

0.652422

0.0761741

0.576248

0
4

0.38087

0.38087

0
4

0.15413

0.15413

0
4

4.0578

4.0578

0
4

0.0599054

0.0599054

0
4

4.49640324973188e-15

0
4

107.081

26.7987

25.8677

0.930981

2.22044604925031e-15

0
4

1.92384

0.667603

0.107963

0.865175

0.025154

0.0135974

0.0100616

0.144196

0.0599054

0.0301849

9.0205620750794e-17

0
4

11.0924

11.0924

0
4

1.54783

1.53734

0.0104881

0
4

2.258

2.258

0
4

0.294158

0.294158

0
4

2.66122

0.161286

2.49993

0
4

0.136044

0.136044

0
4

1.29776

1.15357

0.144196

0
4

0.0633849

0.0633849

0
4

0.0599054

0.0599054

0
4

0.553055

0.553055

0
4

11.5994

9.34927

2.0917

0.0950773

0.0633849

0
4

0.0163868

0.0163868

0
4

0.536117

0.536117

0
4

0.216294

0.216294

0
4

0.170056

0.170056

0
4

0.0100616

0.0100616

0
4

1.25094

1.25094

0
4

0.0599054

0.0599054

0
4

0.357411

0.357411

0
4

1.02927

0.467865

0.381645

0.0201232

0.159633

0
4

4.09858

0.99837

3.10021

0
4

15.4256

15.1028

0.322802

0
4

0.241956

0.212628

0.029328

0
4

0.274303

0.227396

0.0469069

0
4

22.8075
2

22.1751
2

0.139899

0.492495

0
4

0.301175

0.262417

0.0387582

2.08166817117217e-17

0
4

1.11577413974828e-14

0
4

1309.31

1306.68

1304.04

0.430902

0.217963

0.0935306

0.0761741

0.0156497

0.0340111

1.70996

0.06398

4.11434775138275e-13

0
4

0.38087

0.304696

0.0761741

1.38777878078145e-17

0
4

0.685567

0.571306

0.114261

0
4

1.42097

1.42097

0
4

0.0163868

0.0163868

0
4

0.12796

0.12796

0
4

0
4

47.9639

3.80198

0.106963

0.0104881

1.23705

0.257186

1.80377

0.204517

0.123081

0.0029305

0.0530735

0.0029305

0
4

43.7858

43.7858

0
4

0.376163

0.288392

0.0877713

0
4

5.44009282066327e-15

0
4

872.545

9.89126
7

0.633758

0.0483785

1.70996

0.0606962

0.0798828

0.0809283

0.80918

0.062892

0.0404642

0.686974

0.00967569

0.464107

0.0290271

0.0404642

0.00967569

0.121392

0.0606962

0.144196

0.142595

0.0475315

0.364177

0.0606962

0.627194

0.0404642

0.0404642

0.00967569

0.616518

0.364177

0.0792192

0.0290271

1.66235

0.714822

0
4

20.9989

2.3405
7

0.0367698

0.0739218

0.0404642

0.00736842

0.147844

0.00518609

0.09597

0.0127553

0.0739218

0.0127553

4.00729
7

0.0382659

0.754728

0.0233374

0.795086
7

2.11828

2.84711

4.55524

2.21765

0.15889

0.63556

2.66453525910038e-15

0
4

265.844

161.392
7

35.6221
7

0.144225

0.12831

0.151201

0.187628

2.95602

0.0972266

0.161961

0.0144381

0.329332

0.0414474

45.6662
7

0.032994

0.150856

0.0323681

0.140721

0.571306

0.0294737

0.00736842

0.00780182

0.144196

0.144196

0.815454
6

0.0469069

0.0938138

0.00520121

0.211081

0.00394831

0.010998

0.0283844

0.211081

0.191944

0.007332

1.20463

0.0501388

0.0213184

0.117267

0.0378459

0.0404642

0.010998

0.007332

0.007332

0.117267

0.109682

5.50877

0.0796102

0.36049

0.0155008

0.00710613

0.36049

0.0404642

0.0404642

0.0633849

0.0110526

0.0938138

0.422059

0.0155008

0.010998

0.0907031

0.182089

0.00498723

0.504686

0.0703603

0.0606962

1.28691

0.007332

3.55826

0.00670609

0.0404642

0.732252

0.764906

0
4

234.656

112.297

22.1515
7

0.310288

0.154593

0.0100616

0.0382659

0.35556

0.00967569

0.0294737

0.0573989

0.0194364

0.0724936

0.0170056

2.09116
7

0.0194364

0.00777913

0.00350607

0.0404642

0.0262956

0.0549439

0.0156497

0.311349

0.0316877

0.00689936

1.5837
7

0.014664

0.0232512

0.0542527

0.121392

0.00498723

0.724936

0.007332

0.00689936

0.0156497

0.0483291

0.563786
7

0.0155008

0.019133

0.0104881

0.0316877

0.0366292

0.00736842

0.0173753

0.01833

0.0633849

0.0155008

4.35541
6

0.0155008

0.00350607

0.0127553

0.0127553

0.269727

0.00525911

0.155033

0.00518609

0.144196

0.0724936

1.46832

0.0549439

0.0157321

0.584154

0.0127553

0.019133

0.0382659

0.014664

0.0434322

0.0366292

0.00710613

0.547227

0.333337

0.115597

0.00736842

0.0129576

0.00543897

0.00923194

0.144196

0.0104881

0.920892
7

0.590849

0.113949

14.5247
7

0.665339

7.4448

0.171254

8.57202

0.31646

1.65225

0.149105

6.2801

0.555784

0.107609

3.17377

0.0876101

0.373127

4.34278

0.32887

2.37923

0.324192

0.0351877

1.11276

1.85215

0.159846

9.28219
5

0.014664

0.0676276

0.0164461

0.086159

0.36049

0.431352

0.194519

0.0205536

0.115926

2.77433

2.6628
7

0.831594

0.153428

0.865175

0.0989819

0.0375678

0.0294619

0.0446436

0.133287

0.0770934

0.0208779

0.490605
7

0.109888

0.109888

0.36049

0.181616

0.137642

0.270911

0.252883

0.246423

0.022755

0.0843364

0.43646

0.0950773

0.00615463

0.0120572

0.108581

0.00615463

0.01833

0.0483291

0.0100616

0.115597

0.346792

3.33809
7

0.0868644

2.14447

0.010998

0.00416335

0.007332

0.010998

0.019133

0.00923194

0.0209761

0.0127553

0.567728

0.0591856

0.0855237

0.0104881

0.007332

0.0156497

0.0761741

0.0175304

0.00615463

0.00780182

0.0434322

1.26967880653694e-13

0
4

88.3676
2

17.006
2

4.11366

3.35166

0.631305

0.441753

0.215762

3.08149

0.0938138

0.308179

0.119568

0.422853

35.5112
2

0.0643277

0.143497

0.0827985

0.340929

3.02392

0.294158

0.0367698

0.183849

0.117267

0.0551547

0.818661

0.019133

0.0920573

0.0367698

0.0127553

0.40657

0.128931

0.019133

0.0255106

0.0633849

0.0919245

1.84881
2

0.175543

0.0877713

0.0735396

0.0337096

0.00777913

0.0735396

0.438857

0.128694

0.161857

0.110883

1.39812

0.0919245

0.116713

0.0446436

0.0877713

0.110309

0.0367698

0.0398762

0.0739218

0.351085

0.131657

5.50871

0.0633849

0.00411208

0.115072

0.0761741

0.0919245

0.0367698

0.0367698

0.0367698

0.414258

2.40387

1.36545

1.23579

0
4

13.1656

13.1656

0
4

20.6234
7

20.5392
7

0.0524403

0.0316877

3.7470027081099e-16
7

0
4

14.1886

3.52389

0.432588

0.00518609

0.00543897

0.0549439

0.0366292

0.128202

0.0366292

0.0366292

0.456382

4.26731

0.592245

0.798022

0.871287

2.77835

0.0915731

0.0732585

0
4

32.7215

16.7715

0.400827

0.0318883

0.0129839

0.0419522

0.0950773

0.0127553

0.139507

0.0446436

0.00827008

0.0127553

0.358992

0.0232512

0.0542527

1.84605

0.294004

0.253186

0.710406

0.15114

11.2382

0.219832

0
4

2.01361
3

0.199771

1.21496

0.291674

0.3072

1.66533453693773e-16
3

0
4

1.99332
6

1.98208
6

0.0112449

0
4

0.488158

0.340289

0.0165402

0.0157321

0.115597

5.55111512312578e-17

0
4

0.627817

0.575699

0.0183607

0.00408015

0.0102004

0.0194758

8.32667268468867e-17

0
4

4.04929

3.51318

0.536117

0
4

0.0801856

0.0637373

0.0164483

3.46944695195361e-18

0
4

1.17423

0.634781

0.423856

0.115597

1.11022302462516e-16

0
4

0.154735

0.0139478

0.0209761

0.119811

0
4

0.266779

0.0839044

0.175543

0.007332

3.98986399474666e-17

0
4

1.40922

1.40922

0
4

63.8783

26.7413

0.00827008

0.007332

0.109888

0.00350607

0.10116

22.099
7

10.7347

0.238927

0.117529

3.3544

0.306012

0.0157321

0.0404642

4.27435864480685e-15

0
4

0.177574

0.177574

0
4

0.172791

0.132327

0.0404642

0
4

0.510522

0.510522

0
4

0.475315

0.475315

0
4

0.729103

0.714822

0.00408015

0.0102004

0
4

0.520885

0.007332

0.36049

0.153064

5.55111512312578e-17

0
4

2.21464

1.70996

0.504686

0
4

0.0595739

0.0112449

0.0483291

0
4

0.326699

0.326699

0
4

0.222553

0.121392

0.10116

0
4

2.77291
7

1.67425
7

0.916353
7

0.145211

0.0215442

0.00518609

0.00518609

0.00518609

0
4

1.43698

0.007332

1.42964

0
4

0.025662

0.01833

0.007332

1.73472347597681e-18

0
4

0.025154

0.0100616

0.0150924

0
4

0.544786

0.544786

0
4

0.788103

0.770648

0.0174553

0
4

0.0654693

0.007332

0.0581373

0
4

0.0633753

0.0633753

0
4

0.00416335

0.00416335

0
4

0.097998

0.097998

0
4

0.00701215

0.00701215

0
4

45.0283
7

44.7753
7

0.25304

0
4

0.00777913

0.00777913

0
4

0.010998

0.010998

0
4

0.142974

0.142974

0
4

0.222553

0.222553

0
4

0.146517

0.146517

0
4

3.24441

3.24441

0
4

0.505802

0.505802

0
4

0.0276393

0.0276393

0
4

0.044445

0.044445

0
4

0.216294

0.216294

0
4

21.0977
7

12.7479

2.87963

5.09509

0.37515

4.38538094726937e-15
7

0
4

1.59966

1.59966

0
4

0.144196

0.144196

0
4

0.0387582

0.0387582

0
4

0.00624502

0.00624502

0
4

1.29776

1.29776

0
4

0.045462

0.045462

0
4

0.0850278

0.0850278

0
4

0.09869

0.09869

0
4

0.0809283

0.0809283

0
4

0.0124681

0.0124681

0
4

1.25733
7

1.25733
7

0
4

0.010998

0.010998

0
4

0.0170056

0.0170056

0
4

0.0633753

0.0633753

0
4

0.007332

0.007332

0
4

0.00518609

0.00518609

0
4

0.0468881

0.0468881

0
4

0.144196

0.144196

0
4

3.26583
7

2.9601

0.254708

0.0510167

3.12250225675825e-16
7

0
4

2.6651
5

0.726698

1.90934

0.0134122

0.0156475

9.71445146547012e-17
5

0
4

3.12345
7

3.10551
7

0.00518609

0.0127553

0
4

0
4

81.0419

51.2487

47.5597

0.190155

3.17598

0.322802

2.77555756156289e-15

0
4

2.86574

2.12885

0.0842977

0.0226165

0.387362

0.236445

0.00616813

6.41847686111419e-17

0
4

0.193681

0.193681

0
4

0.0123363

0.0123363

0
4

0.093266

0.093266

0
4

0.00616813

0.00616813

0
4

3.0989

3.0989

0
4

0.093266

0.093266

0
4

20.1254

16.3481

0.279798

0.233165

0.139899

0.093266

0.326431

2.70471

0
4

1.95859

0.373064

0.186532

0.279798

0.419697

0.326431

0.373064

0
4

0.0164483

0.00411208

0.00411208

0.00411208

0.00411208

0
4

0.46633

0.186532

0.279798

0
4

0.756624

0.748399

0.00822417

0
4

0.00822417

0.00411208

0.00411208

0
4

0.0920573

0.0460286

0.0460286

0
4

0.00616813

0.00616813

0
4

1.13277442981286e-15

0
4

14479.7
7

10023.2
7

1550.92
7

174.448
7

1.40876
7

0.594841

0.285337

0.0223626

0.0653444

0.13277

0.0327735

0.0241062

0.0536363

0.0581446

0.0225766

1.18917
7

0.161857

0.10247

0.0642822

0.0234911

0.499742

0.18729

0.0608137

0.0773625

0.155441

0.00908513

1.67207
7

0.460699

0.0755724

0.107313

2.74891

0.247614

0.132444

0.149202

0.124334

0.106093

0.247582

1.11171
7

0.0643184

0.212623

0.0809283

0.0755724

0.154684

0.275479

0.455067

0.105072

0.0162577

0.0482743

2.77444
7

0.203382

0.0479811

0.0318883

0.850758

0.0240102

0.147964

0.595302

0.049903

0.769413

0.118371

0.26238

0.0695014

0.0722164

1.07896

0.134758

0.0844855

0.114694

0.052126

0.10037

0.147964

0.0702749

0.799258
7

0.104881

0.430686

0.0928501

0.0500031

0.104279

0.0306294

0.0124681

0.0298651

0.14044

0.0635949

2.78096
7

0.106093

0.0823951

0.0103161

0.119091

0.0491603

0.0155008

0.0163868

0.00498723

17.6669

0.0591856

3.04608
7

0.0280942

0.0245801

0.0591856

0.0591856

0.0173753

0.0599054

0.147964

0.0155008

0.714822

0.00443786

0.322126

0.00532728

0.0591856

0.0488992

0.178259

0.0157321

0.0337346

0.0404642

0.0366292

0.0469069

0.00498723

10.3958
7

2.43319
8

0.0156036

0.0387582

0.0367698

0.00939633

0.207129

0.0633753

0.0163868

0.0149617

0.0591856

0.00500636

0.837677
7

0.0599054

0.0129576

0.144196

0.0129576

0.0189229

0.0547741

0.0404642

0.0163868

0.132461

0.0163868

65.5299
8

0.0283844

0.144196

0.144196

0.0887784

0.0118427

0.144196

0.0591856

0.0633753

0.0286067

0.0163868

2.40363
7

0.0599054

0.00748085

0.0591856

0.0581373

0.0245801

0.0404642

0.32948

0.0591856

0.0155008

0.0469069

1.41413
7

0.0661607

0.026063

0.0150191

0.865175

0.0606962

0.255763

0.00615279

0.0851532

0.0378459

0.0299234

3.2559
7

0.144196

0.0887784

0.0469069

0.144196

0.0591856

0.0469069

0.164174

0.0327735

0.0173753

0.432588

4.80862
7

0.0155008

0.0404642

0.144196

0.00498723

0.0469069

0.110907

0.0155008

0.0173753

0.0327735

0.00498723

3.82258
7

0.144196

0.144196

0.0145717

0.0112449

0.0209761

0.477206

0.288392

0.432588

0.0404642

0.0591856

3.10865
7

0.0938138

0.0599054

0.0469069

0.0124681

0.0809283

0.00416335

0.0155008

0.026063

0.0173753

0.140721

1.30543
7

0.0599054

0.36049

0.0156036

0.0173753

0.0163868

0.0591856

0.0591856

0.00499292

0.052126

0.651042

50.7682
7

0.596503
7

0.00615463

0.0234746

0.0591856

0.065547

0.0112449

0.357411

0.0809283

0.0606962

0.0434384

0.0469069

8.96321
7

0.0155008

0.0404642

0.0591856

0.216294

1.70996

0.00689936

0.0209761

0.0703603

0.00748085

0.0156497

0.704308
7

0.0469069

0.026063

0.357411

0.137664

0.187061

0.026063

0.00520121

0.0245801

0.0104881

0.00690984

4.40124
7

0.0189229

0.118371

0.0938138

0.0955644

0.288392

0.00448724

0.0163868

0.0591856

0.36049

0.0155008

1.19327
7

0.0591856

0.0168673

0.0887784

0.120823

0.0163868

0.0703603

0.118371

0.0434384

0.00780182

0.0312073

0.479636
6

0.0409669

0.104252

0.357411

0.144196

0.00543897

0.0606962

0.0245801

0.00350607

1.70996

0.0283844

1.52606
7

0.0599054

0.144196

0.0606962

0.0591856

0.0245801

0.0155008

0.0591856

0.007332

0.0404642

0.0173753

1.26658
7

0.0404642

0.00498723

0.0469069

0.0887784

0.0100127

0.0245801

0.0155008

0.0404642

0.00498723

0.144196

1.6923
7

0.0124681

0.104252

0.599054

0.0887784

0.147964

0.110907

0.00615279

0.216294

0.0349106

0.187628

0.633264
7

0.144196

0.144196

0.10116

0.0104881

0.144196

0.0887784

0.161857

0.0112449

0.0983205

0.0761741

553.916
7

0.795454
7

0.0316877

0.0599054

0.00448724

0.0129576

0.0112449

0.0189229

0.144196

0.0409669

0.0591856

0.0232512

0.911587
7

0.0104084

0.117267

0.0163868

0.0156497

0.140721

0.0491603

0.00498723

0.0104881

0.0283844

0.0887784

0.778179
7

0.0591856

0.118371

0.0387519

0.0809283

0.144196

0.0189229

0.00440928

0.0112449

0.0938138

0.0163868

0.922808
7

0.0224897

0.0703603

0.0245801

0.0100616

0.0314642

0.0938138

0.0112449

0.026063

0.0173753

0.0404642

37.6966
6

0.00498723

0.144196

0.0194364

0.026063

0.0259677

0.0469069

0.0404642

0.0112449

0.0887784

0.0599054

0.760809
7

0.0173753

0.0599054

0.0591856

0.0327735

0.144196

0.144196

0.0104881

0.00624502

0.0104084

0.0316877

25.0182

0.352315

0.0173753

0.0163868

0.144196

1.70996

0.0529114

0.0104881

0.177557

1.44196

0.0404642

0.638202
7

0.0434384

0.026063

0.0599054

0.0163868

0.216294

0.0938138

0.0737404

0.0573536

0.0104881

0.0591856

5.28056
7

0.0387582

0.0387582

0.0599054

0.355114

0.0469069

0.216294

0.0404642

0.216294

0.144196

0.144196

0.864685
7

0.0173753

0.0591856

0.0155008

0.118371

0.0173753

0.0104881

0.251713

0.0591856

0.0163868

0.0591856

52.7172
7

1.68171
7

0.0591856

0.0469069

0.0591856

0.00416335

0.0591856

0.0338079

0.144196

0.0703603

0.144196

0.0956648

1.36672
7

0.0469069

0.00498723

0.0163868

0.0591856

0.00710613

0.119811

0.0599054

0.00615463

0.0591856

0.0173753

5.18558
8

0.00736842

0.0606962

0.0591856

0.0245801

0.0168673

0.164174

0.0703603

0.36049

0.0968956

0.295928

1.5416
7

0.536117

0.0163868

0.953298

0.0400509

0.0475315

0.144196

0.0112449

0.00748085

0.714822

0.013003

2.199
6

0.0189229

0.0112449

0.0388728

0.00897447

0.0591856

0.0173753

0.0966581

0.0591856

0.0591856

0.0234054

2.26374
6

0.0163868

0.0591856

0.0404642

0.0104881

0.0155008

0.0898581

0.0316877

0.36049

0.0157321

0.0732585

1.17516

0.443892

0.165464

0.0898581

0.0887784

0.0591856

0.00362217

0.119811

0.0129576

0.0409669

0.0809283

0.695995
7

0.116275

0.0591856

0.0898581

0.20715

0.0465023

0.849747

0.00498723

0.0189229

0.0591856

0.0163868

9.40285
8

0.0469069

0.00615463

0.0245801

0.0573536

0.0599054

0.0469069

0.0591856

0.0245801

0.102033

0.144196

1.20551

0.0591856

0.144196

0.00416335

0.0770648

0.0163868

0.0591856

0.019133

0.0107775

0.0404642

0.0155008

23.0391
7

1.11933
7

0.0155008

0.00665679

0.140721

0.0599054

0.288392

0.0163868

0.00543897

0.868044

0.288392

0.00440928

1.64398
7

0.0606962

0.0367082

0.0173753

0.0404642

0.0591856

0.0404642

0.0163868

0.00458025

0.0591856

0.0104881

2.82971
7

0.0388728

0.335058

0.144196

0.0591856

0.0173753

0.00827008

0.0591856

0.0142123

0.0469069

0.0103648

0.583038
7

0.876773

0.140721

0.0591856

0.00881856

0.0591856

0.118371

0.0155008

1.25094

0.0163868

0.007332

0.905996
7

0.0241143

0.714822

0.0147756

0.0173753

0.0591856

0.0591856

0.026063

0.0756917

0.117996

0.0163868

1.14465
7

0.288392

0.0809283

0.0106592

15.2995

0.0469069

0.0591856

0.0975176

0.0156497

0.0469069

0.144196

2.50674
7

0.0029305

0.00416335

0.0887784

0.0606962

0.0599054

0.239622

0.0189229

0.0189229

0.0955644

0.0189229

0.599638
7

0.118371

0.0469069

0.00416335

0.0157321

0.0155008

0.0173753

0.0163868

0.0591856

0.0155008

0.0899589

3.00362
6

0.00897447

0.0599054

0.0104881

0.0599054

0.0156497

0.0366292

0.216294

0.00922919

0.0591856

0.216294

0.925042
7

0.216294

0.216294

0.357411

0.0283844

0.0387582

0.310016

0.0163868

4.49811

0.0189229

0.0506019

20.1717
7

0.807398
7

0.00520121

0.032394

0.0327735

0.0792192

0.0591856

0.0703603

0.117267

0.00498723

0.0703603

0.0104024

1.08568
7

0.144196

0.0173753

0.0469069

0.976563

0.00748085

0.0599054

0.0327735

0.0209761

0.0938138

0.144196

0.69428
6

0.0281121

0.0404642

0.216294

0.357411

0.00690984

0.539429

0.0104881

0.0409669

0.0591856

0.0599054

1.4697
6

0.0591856

0.0591856

0.00897447

0.0127553

0.116275

0.00690984

0.599054

0.0209761

0.0327735

0.36049

0.857058

0.0157321

0.0404642

0.0819338

0.0898581

0.0703603

0.144196

0.0404642

0.0310016

0.0606962

0.0156497

0.655225
7

0.00500636

0.144196

0.0104881

0.0469069

0.0163868

0.0562243

0.0245801

0.0938138

0.0189229

0.069043

0.411213

0.0245801

0.0283844

0.144196

0.177557

0.0404642

0.0606962

0.00498723

0.0938138

0.182089

0.0249362

1.28945
7

0.0259152

0.0591856

0.00592134

0.0599054

0.01249

0.0404642

0.0163868

0.106514

0.0163868

0.216294

1.85555
7

0.0189229

0.0946147

0.0938138

0.0578906

0.236743

0.0100064

0.00997446

0.0591856

0.0129576

0.0163868

1.7678
7

0.0245801

0.0104881

0.0245801

0.0591856

0.0599054

0.0176371

0.0404642

0.0163868

0.00750954

0.0163868

11.7987
7

1.16437
6

0.0404642

0.106514

0.118371

0.0347507

0.0122405

0.00416335

0.0124681

0.144196

0.00615279

0.144196

1.1517
8

0.0378459

0.0887784

0.0168673

0.0155008

0.0163868

0.144196

0.00448724

0.288392

0.0868767

0.0591856

1.18048
7

0.144196

0.0104881

0.0173753

0.0887784

0.0887784

0.0104881

0.00498723

0.357411

0.0245801

0.216294

0.519579
7

0.0232512

0.0599054

0.0104881

0.0473073

0.0112449

0.0327735

0.0347507

0.393282

0.147964

0.0591856

3.85754
6

0.0173753

0.0204008

0.288392

0.0898581

0.0591856

0.0404642

0.0189229

0.216294

0.0469069

0.562264

0.782568
7

0.163868

0.144196

0.0887784

0.0163868

0.0469069

0.0591856

0.0129576

0.793077

0.0163868

0.0491603

1.22375
7

0.0475315

0.0129576

0.36049

0.0591856

0.504686

0.00440928

0.0163868

1.00937

0.0189229

0.0127553

0.40651
7

0.0155008

0.00748085

0.0887784

3.82119

0.095063

0.216294

0.144196

0.0173753

0.0104881

0.00362217

0.335073
7

0.0194364

0.00498723

0.0173753

0.0104881

0.0104881

0.0434384

0.00592247

0.0887784

0.135654

0.00448724

1.01681
8

0.473485

0.0469069

0.0591856

0.0599054

0.147964

0.0591856

0.0245801

1.70996

0.0469069

0.00748085

25.2991
7

1.1166
7

0.0112449

0.026063

0.288392

0.0170056

0.0224897

0.0327735

0.00500636

0.0173753

0.0367082

0.199816

0.844704
7

0.0104881

0.0591856

0.0542527

0.0404642

0.0469069

0.0125159

0.144196

0.0112449

0.266335

0.288392

6.45486
8

0.0129576

0.0491603

0.0155008

0.0591856

0.144196

0.0173753

0.144196

0.007332

0.00832669

0.0582868

0.881149
7

0.0104881

0.0245801

0.0163868

0.00416335

0.144196

0.144196

0.0245801

0.00780182

0.0245801

0.0898581

0.304482
7

0.0163868

1.70996

0.0469069

0.0591856

0.0573536

0.0542527

0.00416335

0.428969

0.0469069

0.0173753

1.49744
7

0.00897447

0.0404642

0.00520121

0.0898581

0.01249

0.0591856

0.0173753

1.70996

0.0245801

0.0080381

7.33319
6

0.288392

0.0469069

0.0310016

0.0283844

0.00592134

0.144196

0.0163868

0.00997446

0.0469069

0.0703603

0.623774
7

0.0104881

0.288392

0.065547

0.0591856

0.155674

0.0173753

0.36049

0.121392

0.0163868

0.0712667

2.50584
7

0.147964

0.216294

0.0249362

0.147964

0.0283844

0.0163868

0.216294

0.357411

0.0134617

0.0259152

0.486068

0.0163868

0.0173753

0.10116

0.052126

0.00498723

0.288392

0.0112449

0.0591856

0.00475254

0.0599054

12.3807
7

0.702371
6

0.0163868

0.0809283

0.0112449

0.00832669

0.00950508

0.0703603

0.216294

0.0475315

0.0887784

0.15413

0.611142
7

0.162565

0.0887784

0.161857

0.0276958

0.0382659

0.00440928

0.144196

0.0591856

0.00923194

0.0591856

0.292814
6

0.0503081

0.021996

0.177557

0.0245801

0.144196

0.118371

0.144196

0.0503081

0.360509

0.0703603

2.47419
7

0.0591856

0.052126

0.216294

0.0591856

0.0703603

0.0173753

0.00498723

0.00750954

0.0547741

0.0366292

0.540118
7

0.0163868

0.0245801

0.0469069

0.0259152

0.0347507

0.0591856

0.0591856

0.891231

0.0173753

0.216294

0.985659
7

0.0591856

0.00498723

0.0887784

0.162758

0.144196

0.0347507

0.0104881

0.0163868

0.00987078

0.0409669

0.696836

0.288392

0.00748085

0.0262201

0.0378459

0.115597

0.0112449

0.0224897

0.0703603

0.026063

0.0549439

2.05041
7

0.0591856

0.0898581

0.0473073

0.026063

0.06398

0.00543325

0.0189229

0.0938138

0.10116

0.144196

1.00803
7

0.164174

0.0163868

0.0599054

0.00498723

0.216294

0.00498723

0.0839044

0.00498723

0.0245801

0.0887784

0.87703
7

0.0163868

0.0770648

0.0409669

0.0404642

0.0404642

0.0224897

0.0591856

0.144196

0.00416335

0.10116

1161.73
7

41.1634
7

2.80748
7

0.0262201

0.00780182

0.007332

1.70996

0.0163868

0.0703603

0.216294

0.00687037

0.0155008

0.0591856

30.1425
6

0.0173753

0.00498723

0.0245801

0.144196

0.00832669

0.0404642

0.0150191

0.192662

0.0591856

0.0163868

0.289126

0.0173753

0.0591856

0.0170056

0.144196

0.0591856

0.149764

0.0449794

0.0232512

0.00498723

0.117267

1.40245
7

0.473485

0.0168673

0.0163868

0.0310016

0.0163868

0.144196

0.00416335

0.0403851

0.125198

1.24303

1.66341
7

0.0173753

0.147964

0.288392

3.41991

0.224897

0.0283844

0.0591856

0.0163868

0.0737404

0.0209761

0.331961
7

0.0469069

0.00615279

0.0599054

0.118371

0.144196

0.00923194

0.0209761

0.0310016

0.144196

0.0100064

4.66125
7

0.00592134

0.0155008

0.0161662

0.0606962

0.0887784

0.0194364

0.20715

0.118371

0.0173753

0.00520121

49.1303
8

0.0591856

0.0695014

0.0673276

0.0155008

0.0189229

0.0523659

0.0599054

0.019133

0.052126

0.0887784

0.243593
7

0.030865

0.0573536

0.144196

0.0475315

0.126773

0.0469069

0.0316877

0.0173753

0.0189229

0.366685

0.514367
7

0.0316877

0.0591856

0.0245801

0.0173753

0.0163868

0.0156497

0.0852543

0.393282

0.0149617

0.144196

6.60403
7

0.510688
7

0.00408015

0.0163868

0.00498723

0.0471962

0.00520121

0.0591856

0.00624502

0.0409669

0.0591856

0.0887784

1.69349
7

0.0163868

0.0887784

0.0404642

0.0299234

0.0194364

0.0591856

0.144196

0.0404642

0.0404642

0.0591856

0.206191
7

0.0173753

0.0232512

0.0606962

0.20715

0.0469069

0.00498723

0.0378459

0.0104881

0.0104881

0.0434384

0.761951
7

0.144196

0.432588

0.0173753

0.026063

0.0104881

0.0591856

0.0404642

0.0281121

0.0104084

0.0155008

1.58737
7

0.0316877

0.144196

0.36049

0.0404642

0.0703603

0.0378459

0.144196

0.0591856

0.0599054

0.0173753

0.299589
6

0.0316877

1.96576

0.0155008

0.0378459

0.0163868

0.00498723

0.504686

0.0245801

0.283177

0.0547741

2.72863
6

0.0606962

0.0173753

0.0606962

0.0591856

0.0469069

0.0887784

1.42964

0.0786604

0.0703603

0.0591856

5.6579
6

0.0591856

0.0606962

0.026063

0.0599054

0.0316877

0.0232512

0.140721

0.026063

0.0404642

0.0938138

0.831849

0.144196

0.026063

0.144196

0.0283844

0.504686

0.00362217

0.144196

0.357411

0.00408015

0.144196

0.832814
7

0.0599054

0.095063

0.0129576

0.0591856

0.0606962

0.0591856

0.0245801

0.0104881

0.00416335

0.0232512

5.53548
7

2.30587
7

0.0327735

0.0599054

0.0104881

0.0484987

0.48557

0.0366292

0.0955644

0.0173753

0.119811

0.0591856

0.648835
7

0.00498723

0.236743

0.0404642

0.144196

0.0155008

0.0245801

0.0173753

0.00498723

0.216294

0.36049

2.24555
6

0.144196

0.0591856

0.0157321

0.026063

0.0163868

0.0104881

0.144196

0.106514

0.0599054

0.00750954

0.413022
7

0.00443786

0.0465023

0.0245801

0.0316877

0.0591856

0.00615463

0.0107775

0.0599054

0.0591856

0.026063

0.622089
7

0.0591856

0.0175223

0.0245801

0.0112449

0.216294

0.0573536

0.0606962

0.0599054

0.00624502

0.0469069

0.308829
7

0.266335

0.0465023

0.0898581

0.144196

0.00967569

0.00710613

0.118371

0.00615279

0.0163868

0.0245801

0.559162
7

0.0029305

0.0599054

0.169152

0.121392

0.0938138

0.0606962

0.288392

0.0281121

0.144196

0.0469069

0.588996
7

0.0173753

0.0155008

0.0245801

0.0887784

0.0606962

0.0599054

0.0314642

0.0183607

0.0248714

0.0519355

0.573613
7

0.937273

0.00710613

0.0404642

0.00500636

0.00615279

0.0245801

0.00967569

0.473485

0.503078

0.0173753

0.647327
8

0.0232512

0.00500636

0.0404642

0.0104881

0.00416335

0.144196

0.536117

0.0262201

0.144196

0.144547

52.1091
7

1.82656
6

0.00673085

0.0620031

0.893528

0.0404642

0.0173753

0.0104881

0.118371

0.0898581

0.357411

0.00736842

0.361154

0.0245801

2.56494

0.0434384

0.20715

0.149764

0.0163868

0.0173753

0.0898581

0.0366292

0.00498723

36.1648
8

0.0327735

0.0129576

0.0409669

0.0327735

0.144196

0.0756917

0.0316877

0.0173753

0.257988

0.0819338

1.49262
7

0.0887784

0.0591856

0.021555

0.182089

0.0163868

0.0163868

0.0599054

0.144196

0.144196

0.0314642

0.525873
8

0.0599054

0.0898581

0.00416335

0.0404642

0.0155008

0.0599054

0.00520121

0.0245801

0.065547

0.052126

0.438523

0.0163868

0.0163868

0.00498723

0.0184639

0.0599054

0.147964

0.032394

0.0404642

0.0163868

0.0112449

4.16841
6

0.0112449

0.10116

0.0402465

0.266335

0.0112449

0.179716

0.0809283

0.0123056

0.0378459

0.00520121

1.1522
7

0.00520121

0.0599054

0.0591856

0.144196

0.0938138

0.0469069

0.216294

0.00923194

0.0314642

0.0173753

0.521848
7

0.0366292

0.0245801

0.0245801

0.0173753

0.0104881

0.216294

0.0465023

0.00416335

0.144196

0.0107775

1.84787
6

0.144196

0.0591856

0.00475254

0.0127553

0.0938984

0.0173753

0.0703603

0.118371

0.0155008

0.0469069

47.934
7

0.318398

0.00416335

0.0112449

0.0163868

0.0170056

0.0140945

0.118371

1.70996

0.576784

0.00690984

0.0379465

0.648949
7

0.032417

0.0189229

0.144196

0.216294

0.0327735

0.0131105

0.161857

0.216294

0.118371

0.0173753

0.751088
6

0.0163868

0.0232512

0.0249362

0.0469069

0.0209761

0.0404642

0.0591856

0.0606962

0.0155008

0.00690984

0.621323
7

0.00525911

0.0163868

0.384707

0.0703603

0.0168673

0.0104881

0.00690984

0.0695014

0.0163868

0.0232512

1.15278
7

0.0573536

0.00500636

0.0599054

0.0163868

0.0163868

0.216294

0.0163868

0.0245801

0.144196

0.00416335

0.0965911

0.0163868

1.42964

0.00732626

0.0232512

0.0966581

0.00655526

0.0647879

0.0404642

0.0157321

0.144196

3.57049
7

0.0887784

0.0703603

0.0245801

0.0173753

0.0887784

0.144196

0.00498723

0.0163868

0.0469069

0.0404642

0.250702
7

0.00615279

0.118371

0.0703603

0.144196

0.288392

0.0409669

0.0618467

0.0163868

0.0591856

0.0599054

8.4961
8

0.0163868

0.0775039

0.0163868

0.0163868

0.0703603

0.0378459

0.0189229

0.0387582

0.216294

0.0366292

0.702633
7

0.00997446

0.0232512

0.0434384

4.32588

0.0591856

0.0245801

0.0245801

0.0591856

0.0173753

0.0599054

35.0516
7

0.259109

0.0378459

0.0469069

0.164174

0.0469069

0.025154

0.0591856

0.0327735

0.0314642

0.144196

0.0591856

0.213092
7

0.00416335

0.0491603

0.0454854

0.0591856

0.0404642

0.216294

0.0469069

0.144196

3.41991

0.0887784

0.172354
7

0.0775164

0.00615463

0.0316877

0.0469069

0.928534

0.026063

0.0100616

0.0599054

0.0591856

0.0245801

0.512519

0.0809283

0.0703603

0.00448724

0.0887784

0.0887784

1.70996

0.0983205

0.0887784

0.106514

0.144196

3.80219
7

0.0259152

0.00689936

0.0100064

0.0173753

0.0404642

0.144196

0.118371

0.0469069

0.20715

0.00624502

0.294459

0.0155008

0.0224897

0.0314642

0.0938138

0.0887784

0.0155008

0.0173753

0.0599054

1.17634

0.116275

1.95031
6

0.144196

0.216294

1.22567

0.00897447

0.0173753

0.0591856

0.0104881

0.0852543

0.144196

0.09597

0.413735
7

0.0173753

0.121392

0.0194364

0.0123056

0.0366292

0.0591856

0.0387519

0.0163868

0.00416335

0.0819338

1.96053

0.0404642

0.0163868

0.0591856

0.01249

0.504686

0.00985039

0.36049

0.0155008

0.14769

0.144196

0.745389
6

0.144196

0.0606962

0.141625

0.144196

0.0122368

0.0591856

0.144196

0.144196

0.0157321

0.00997446

15.3222
7

7.87273

2.14447

0.0887784

0.357411

0.0387519

0.0475315

0.0133136

0.0964756

0.0245801

0.216294

0.0591856

0.392237
7

0.0129576

0.0469069

0.021555

0.0225286

0.216294

0.0163868

0.340613

0.0469069

0.00592134

0.144196

265.431
8

0.0599054

0.117267

0.164174

0.0080381

0.257988

0.0573536

1.70996

0.00615463

0.026063

0.0475315

1.86106
7

0.0398438

0.0387519

0.0245801

0.118371

0.0775164

0.0469069

0.0155008

0.0955644

0.00710613

0.144196

2.96681
7

0.0155008

0.0157321

0.0157321

0.0112449

0.0366292

0.0163868

0.0163868

0.032394

0.182089

0.0245801

6.00812
6

0.0234746

0.015296

0.0367082

0.0809283

0.0623404

0.0968956

0.0633753

0.00661392

0.0173753

0.0404642

0.481116
7

0.0110232

0.0163868

0.128202

0.0232512

1.78706

0.0224425

0.0737404

0.00543897

0.0567688

0.00498723

9.51104
7

0.144196

0.0599054

0.00500636

0.216294

0.0209761

0.00748085

0.0327735

0.0249801

0.0591856

0.0938138

0.272689

0.00687037

0.00500636

0.0312995

0.0591856

0.0887784

0.216294

0.0591856

0.0581373

0.865175

0.118371

0.280884

0.0104024

0.0163868

0.0591856

0.0137407

0.0189229

0.0168673

0.357411

0.0163868

0.0104881

0.144196

12.171

9.77867
7

0.00447073

0.00689936

0.00780182

0.216294

0.20715

0.00500636

0.144196

0.0173753

0.288392

0.00673085

0.136206
7

0.23809

0.0173753

0.118371

0.0163868

0.0163868

0.357411

0.0163868

0.0168673

0.062599

0.0327735

0.37308
7

0.00543325

0.144196

0.144196

0.118371

0.0591856

0.144196

0.00673085

0.0189229

0.0347507

0.0327735

0.378902
6

1.52759

0.0129576

0.0163868

0.0232512

0.0163868

0.0163868

0.026063

0.144196

0.0104881

0.144196

0.8123

0.00543897

0.0591856

0.357411

0.144196

0.0591856

0.0129576

0.0155008

0.0792192

0.591856

0.00923194

0.536155
7

0.357411

0.00362217

0.147964

0.0737404

0.0112449

0.0189229

0.144196

0.0280942

0.0163868

0.144196

0.232906

0.00408015

0.0245801

0.06398

0.893528

0.0245801

0.0591856

0.648882

0.0316877

0.0189229

0.0615794

0.44154

0.0327735

0.0599054

0.0898581

0.0163868

0.0868767

0.0404642

0.117267

1.70996

0.0189229

0.0473073

1.35723

0.142595

0.0163868

0.0232512

0.00416335

0.0173753

0.187628

0.0259152

0.00475254

0.0173753

0.0163868

0.08943

0.144196

0.052126

0.0103648

0.00500636

0.0404642

0.0591856

0.0469069

0.0475315

0.0232512

1.44196

10.7452
7

0.0889772

0.196156
7

0.572821

0.577432
7

0.882886
7

0.23221
7

0.631434
7

0.511037
8

3.26223

0.964071
7

47.7114
7

0.129034
7

0.194077
6

0.181803
7

0.335485

0.482448
7

1.35987
7

0.131941
7

1.21967
6

0.320382
6

4.51131
8

1330.14
7

798.316
6

0.716683

0.187415

0.23045

2.71299
6

0.212561
7

0.198052

0.586336

0.403636

0.245737
8

0.992742

15.8059
7

0.642977

0.208217
7

0.278349

0.307846

0.961508
7

0.1577

0.388018

2.79283
8

3.51741
7

0.633656
7

2.27239
7

0.615253
6

0.207221
6

0.136345

0.311135

1.01403
6

0.373939
7

0.509544
7

0.295962

0.600109

0.131807
8

2.81647
7

0.22842
7

4.40236
6

0.525611
7

0.421233
7

0.213208
7

17.3713

0.514523

0.505761

0.103327

0.135365

4.0611
7

145.834
8

0.370323
7

9.68779
6

0.435116

0.401114

0.838154

0.234508

0.43804

49.769

0.192774
7

2.5542
7

0.0894146

0.313942

0.605137
6

0.23105

0.917659

0.300597

0.23002

0.270381

0.511748

0.215516

10.2775
7

0.215502
6

6.24991

0.60765
7

0.126964
7

0.264481

0.218711

0.102302

0.210417

0.339452

0.172938

171.571
5

0.267055

0.133255

0.517477

7.90032

1.43913

0.0732626

0.104845

0.108915

0.239924

0.0828593

18.3255
7

0.666327

0.663843

0.0556118

0.166552

0.514534

0.139011

0.0570196

0.154336

0.37023

0.449595

67.7563
7

0.0363148

0.0970557

0.213709

0.105144

16.9632

0.357918

0.108286

6.91845

0.248255

0.514422

1093.16
7

14.0734
7

0.549614

0.119279

0.883371

0.0964908

0.127519

0.324585

0.70853

0.255557

1.47069

0.432548

33.2795
7

3.37486

0.26546

0.12064

0.341356

0.29987

0.230401

0.223179

0.425398

0.149472

0.176503

4.0146
7

0.245777

0.159008

0.321651

0.192718

0.384932

0.63134

0.596556

0.193476

0.0887354

0.401007

6.28739
7

0.56646

0.0692875

0.672789

0.345318

0.304259

0.084831

0.14302

0.329407

0.290487

0.200568

56.3785
7

0.271184

0.183479

0.114733

0.319436

0.0457641

11.5504

0.44968

0.182437

0.225684

0.330473

1.00073
7

0.349536

0.645982

0.666942

0.257831

0.220818

0.27338

11.2712

0.547433

0.798985

0.121317

10.531
6

0.925366

0.713133

0.223536

0.275132

0.441019

0.34082

0.182238

7.47405

0.0335628

0.196817

3.03434
7

0.076283

0.341814

8.03136

0.313956

0.0474183

0.343445

0.307884

0.17546

0.353092

0.146339

6.08441
7

0.0359962

0.238652

0.389179

0.104656

0.322386

0.437575

0.398378

0.156503

0.39759

0.159322

3.84132
7

0.356503

0.193436

0.204259

0.0449363

0.440784

0.850851

10.9381

0.0759901

0.15369

0.348349

25.8011
7

2.47025
7

0.104746

0.153421

0.0892633

0.0869634

0.0251615

0.222256

0.642295

0.145047

0.0425467

0.345787

93.6527
7

0.15341

0.127788

0.566701

0.133743

0.292967

0.214627

0.0705984

0.0738186

0.176533

1.84022

3.47619
7

0.53627

0.0866154

0.0539829

2.67677

0.313396

0.111728

0.163815

0.348297

0.177004

0.31535

1.98575
7

0.180483

0.138797

0.45869

0.242133

0.273742

1.48883

0.138425

0.209089

0.259555

0.443892

8.62779
7

0.306541

0.214231

0.11641

0.225718

0.468737

0.0582479

0.150059

2.14042

0.621281

0.0963064

5.19412
7

3.96886

0.0922289

3.16594

8.00667

0.579768

0.0765736

0.284226

0.423462

0.0454299

0.177557

7.93006
7

0.226717

0.791766

0.195333

0.0781891

0.190325

0.175579

0.27758

0.340035

0.0625613

0.10726

10.5812
7

1.05859

0.39371

0.40912

0.0346841

0.0691601

3.22778

0.430446

0.366411

0.110511

0.0345943

3.89612
7

0.201219

15.3949

0.219213

0.294023

0.302572

0.381032

0.378155

0.164174

0.541051

0.0555203

2.87952
7

0.0471197

0.0276393

0.0353442

0.0304129

0.677663

0.323507

0.0700795

0.212573

0.404642

0.0392743

25.7498
7

6.48689
7

0.278575

0.128637

0.120262

0.320508

0.0573536

0.0391256

0.313875

0.117478

0.212356

0.0570863

11.1527
7

0.192141

0.0260061

0.674785

0.309576

0.584005

0.50643

1.78625

1.15359

0.145574

0.0205909

9.13144
7

0.0901577

0.0548327

0.0713157

0.15001

0.109652

0.516353

0.153131

0.0549311

0.0979192

0.160275

8.54947
7

0.303171

0.235639

0.0835193

0.104541

0.065547

0.389747

1.12404

0.227637

0.0678362

0.0310471

1.37361
7

0.155833

0.116539

0.0909595

0.404545

0.0695241

0.224313

0.249361

0.443892

2.7183

0.0365281

0.445746
7

0.104495

0.209894

0.509887

0.0839025

0.0811967

0.0347507

0.0333923

0.151106

0.106154

0.0238676

0.88951

0.0934697

0.0321188

0.0208528

0.057395

0.171626

0.0837658

0.191392

0.127844

0.0391243

0.242599

8.47717
7

0.0569196

0.376877

1.15357

0.147481

0.0703974

0.828599

0.116216

0.0578395

0.0456502

0.232681

6.40028
8

0.20715

0.420796

0.380938

0.0293573

0.0990038

0.0452387

0.204101

0.510941

0.00997446

0.0954309

0.886044

0.368188

0.416597

0.0286901

0.221281

0.0952395

0.0208814

0.203382

0.0858611

0.191103

13.507

25.69
7

0.748732

0.166887

0.0110232

0.136678

0.744231

0.299486

0.02781

0.0819338

0.0865217

0.149183

0.628959

0.761308

0.156081

0.122901

0.110201

0.656459

0.106514

0.0873711

0.101663

0.10482

0.0994376

0.0491603

5.93478
7

0.10116

0.195012

0.18466

0.38507

0.232974

0.0289026

0.251045

0.37873

0.0824368

0.234054

0.552982
7

0.213028

0.0772808

0.105646

0.223536

0.0596917

0.0179689

0.0946998

0.0872798

0.0241886

0.304226

1.42276
7

0.0276316

0.149183

26.8955

0.540197

0.309982

0.221174

0.161272

0.165278

0.0986636

5.56793

3.4934
7

0.368572

0.0531519

0.160922

0.288392

0.46076

0.062249

0.143062

0.16447

0.0629283

0.0262639

4.24889
7

0.205248

0.90963

0.404318

0.0327735

0.491773

0.30865

0.0602649

0.0299234

0.403921

0.0492936

3.71524
7

0.0951953

0.247593

0.0620031

0.0305503

0.132999

0.499333

0.0391685

0.797487

0.0126821

0.124817

1.19536
7

0.0714657

0.0253577

0.106812

0.162982

0.0327735

0.0431383

0.0123394

0.183841

0.0949312

0.105059

0.455133

0.0337621

0.240119

0.157153

0.351948

0.0704305

0.365496

0.160275

0.0190778

0.0491603

0.0643433

35.4131
7

13.0987
7

0.0727989

0.149397

0.141625

0.0696737

0.169704

0.160583

0.155033

0.288392

0.114205

0.0169321

0.502486
7

0.20715

0.0908733

0.0491603

0.0310016

0.136765

0.150853

0.0755724

0.0946561

0.0516356

0.0430208

1.15029
7

0.110201

0.225124

0.72098

0.296648

0.123529

0.188931

0.0258056

0.0314107

0.437575

1.08147

1.26352
7

0.0935306

0.118371

0.0719409

0.174621

0.163868

0.0506106

0.0357867

0.140721

0.101534

0.0138197

18.1324
6

0.196641

0.45811

0.0559623

0.018351

0.120869

0.83209

0.0344968

0.0939363

0.0583422

0.0832194

9.53502
8

0.385108

0.0568509

0.262745

0.0309618

0.0765131

0.0992665

0.0474857

0.311643

0.052126

0.0668153

3.34344
7

0.714355

0.105165

0.0612094

0.0604033

0.0213675

0.137294

0.0387519

0.133872

0.0518304

0.0811378

2.04298
7

0.13625

0.241994

0.130322

0.0426069

4.52678

0.013837

0.0937437

0.717901

0.132926

0.0252053

1.46078
7

6.5776

0.0274936

0.682074

0.0169312

0.093835

0.020967

0.0207193

0.351599

0.0277631

0.708067

0.361195
7

0.158835

0.189412

0.0214434

0.0213931

0.558559

0.00947447

0.135747

0.18466

0.0714986

0.417317

83.6183
7

1.00265

0.362398

0.118371

0.266335

0.295928

0.269125

0.117163

0.182561

0.0400809

0.139003

0.397875

0.436869
7

0.106154

0.410531

1.02897

0.0568509

0.0920785

0.0848445

0.0143837

0.202258

0.0174553

0.0711503

4.18742
8

0.168587

0.0335726

0.583024

77.1763

0.149044

0.171532

0.223536

0.0295674

0.106154

0.0696737

1.28397
7

0.0454514

0.408094

0.032394

0.154903

0.154684

0.0388765

0.0453516

0.360509

0.19667

0.604479

1.72045
7

0.064661

0.131094

0.0628081

0.0162321

0.118371

0.23801

0.288392

0.0471885

0.893528

0.583024

2.04086
7

0.109313

0.501607

0.160275

146.043

0.121552

0.0632936

0.2217

0.115954

0.0501488

0.140834

9.54457
6

1.69495

0.72846

0.0452819

0.199906

0.0721116

0.021374

0.150316

0.455594

0.236396

1.13098

3.58955
6

0.032553

0.146756

0.0755724

0.0327386

29.9406

0.0755724

0.0440184

0.0215387

0.157726

0.0944384

7.61264
7

0.349088

0.064811

0.135747

0.295928

1.90282

0.0601789

0.0962593

0.415411

0.0303329

45.8406

3.54373
7

0.0992665

0.436751

0.161857

3.7804

0.117288

0.0409053

0.0633753

0.140834

0.106154

0.00997446

2.33807551444642e-10
7

0
4

235.719
6

235.719
6

0
4

241.44

55.6593
7

69.498
5

1.23478

0.228518

0.0446771

1.75707

1.04938

0.116799

0.129864

0.477758

0.04839

0.0456502

49.1117
5

0.0918626

0.0400388

0.0388957

0.732198

0.0579195

1.75749

0.0127553

0.0475315

0.00518609

0.00835115

3.15165
6

1.25094

0.322802

0.026063

0.0633753

0.129121

0.0250534

0.00835115

0.0887784

0.00881856

0.00518609

6.4818

0.129121

0.00498723

0.00777913

0.0233374

0.0255106

0.00518609

0.0599054

0.00780182

0.190126

1.70996

23.8626
5

0.0316877

0.0208779

0.00835115

0.00748085

0.0387582

0.00498723

0.82388

0.0316877

0.0125267

0.00835115

4.49453

0.193681

0.00518609

0.0316877

0.0544539

0.0250534

0.0129652

0.00777913

0.0103722

2.10277
6

12.5967

1.13362
6

0
4

4.71758

4.61315

0.0104881

0.0591856

0.0173753

0.0173753

0
4

8.72353
7

1.6059

0.0163868

0.0245801

0.0163868

0.0591856

0.0245801

0.0173753

0.243255

0.0163868

2.56494

0.0163868

2.07749

0.0163868

0.0599054

0.144196

0.0409669

0.609393

0.0157321

0.0491603

0.360509

0.305692

0.196223

0.0491603

0.0245801

0.0245801

0.144196

1.58206781009085e-15
7

0
4

2634.13
7

152.889
7

48.7438
7

17.5281
7

12.3901
6

2401.15
7

0.775996

0.0138197

0.0404642

0.191343

0.0578395

0.161857

0.0316877

0.110907

0.00967569

0.0163868

0.0173753

0
4

1036.91
7

1026.86
7

8.70128
7

0.270348

0.0404642

1.04056

0.00518609

1.38626957135735e-13
7

0
4

120.168
7

12.9032

1.34755
7

0.00816031

0.0157321

0.0170056

0.0173753

0.0155008

0.0328872

0.0155008

0.0173753

0.0173753

0.00394831

1.11942
6

0.357411

0.0208048

0.0163868

0.00408015

0.00689936

1.70996

0.0232512

0.00615463

0.0469069

0.0244809

0.984777

0.0465023

0.144196

0.00689936

0.0100064

0.026063

0.0404642

0.00543897

0.0404642

0.00615279

0.110907

0.429884
7

0.00448724

0.0232512

0.0173753

0.0703603

0.0155008

0.0163868

0.0693626

0.0591856

0.00448724

1.95477
7

0.261167

0.735329

11.4499
6

0.121627

3.06624
7

6.41205
6

0.899531

0.139029

0.835256

0.810989

0.459826

1.5016

1.10921

1.6163

0.167495

1.92145

4.98955
6

0.0592762

0.680635

0.0122405

0.103393

0.739875

1.34904

0.052223

2.85222

0.142457

0.0400809

31.4244
6

0.318315

0.266335

0.186789

0.0545587

0.417317

0.0701836

0.184288

0.0149617

0.08622

0.166884

1.57195

0.08757

0.155894

0.0421741

0.0228328

1.71996

0.374434

0.0806048

0.277108

0.0139617

0.373798

10.818
7

0.0599054

0.0469069

0.0163868

0.94842

0.0887784

0.0404642

0.0599054

0.00408015

0.314139

0.00408015

0.506071
7

0.00448724

0.0591856

0.052126

0.0189229

0.0100616

0.0104881

0.0323325

0.357411

0.0316877

0.0173753

2.25313
7

0.0347507

0.216294

0.06398

0.0434384

0.0107775

0.0129576

0.211081

0.417862

0.0469069

0.0155008

0.411249

0.0189229

0.0761741

0.0983205

0.0955854

0.146997

0.0209761

0.00624502

0.0887784

0.0633753

0.0409669

6.99856839148083e-14
7

0
4

31.5568
7

22.9541
7

1.50597
7

1.12401
7

0.526756
7

0.126181

0.157196

0.0277096

5.12987

0.00498723

0
4

0.887437
7

0.87666
6

0.0107775

6.93889390390723e-18
7

0
4

2.91445
7

1.19829

1.71615

0
4

0.575133
7

0.171591

0.218882

0.144196

0.0404642

0
4

0.0955782
7

0.0551436

0.0179448

0.0224897

6.93889390390723e-18
7

0
4

0.0665356

0.061783

0.00475254

4.33680868994202e-18

0
4

6.89489

0.0358731

6.83983

0.0103648

0.00440928

0.00440928

1.12410081243297e-15

0
4

1.39491

1.28196

0.0761741

0.0367698

0
4

0.0285235

0.00777913

0.00777913

0.0129652

0
4

2.89645

2.89645

0
4

0.333172

0.333172

0
4

21.6396

19.8404

0.340054

0.0809283

1.19824

0.147706

0.00543897

0.00525911

0.00543897

0.0161662

0
4

0.0252101

0.021588

0.00362217

1.30104260698261e-18

0
4

0.113434

0.113434

0
4

0.112616

0.0721518

0.0404642

0
4

0.482145

0.482145

0
4

0.319618

0.319618

0
4

0.0586155

0.0586155

0
4

0.0580343

0.0580343

0
4

0.0355604

0.0355604

0
4

0.115894

0.00498723

0.110907

0
4

0.134278

0.134278

0
4

37.6001
3

28.6516
3

1.83176

2.60545

0.556084

3.62392

0.0877713

0.0100682

0.077809

0.155618

0
4

0.953434

0.893528

0.0599054

0
4

0.0642822

0.0469069

0.0173753

3.46944695195361e-18

0
4

0.0351533

0.0156497

0.0195035

0
4

0.0599054

0.0599054

0
4

0.00586101

0.00586101

0
4

0.0177674

0.0177674

0
4

0.0877713

0.0877713

0
4

0.0170056

0.0170056

0
4

0.0245801

0.0245801

0
4

0.0234746

0.0234746

0
4

6.32305
7

5.78159
7

0.117142

0.266335

0.00500636

0.00500636

0.0591856

0.0887784

5.13478148889135e-16
7

0
4

0.0029305

0.0029305

0
4

0.0170056

0.0170056

0
4

0.20715

0.20715

0
4

0.0155008

0.0155008

0
4

0.0049252

0.0049252

0
4

0.319531

0.319531

0
4

0.00905542

0.00905542

0
4

0.147964

0.147964

0
4

0.114261

0.114261

0
4

0.0387582

0.0387582

0
4

8.54746
7

8.26295
7

0.0821109

0.0199573

0.158256

0.0241844

0
4

0.0591856

0.0591856

0
4

0.0316877

0.0316877

0
4

0.15921

0.15921

0
4

0.0877713

0.0877713

0
4

0.0209761

0.0209761

0
4

0.007332

0.007332

0
4

0.0103722

0.0103722

0
4

3.02117

3.02117

0
4

0.00543897

0.00543897

0
4

0.0404642

0.0404642

0
4

6.62523
7

6.39783
7

0.161857

0.0245801

0.0409669

0
4

0.0404642

0.0404642

0
4

2.31076
7

0.88551
7

1.40975
7

0.0155008

6.22765727875674e-16
7

0
4

2.96615
7

2.84778
7

0.118371

1.38777878078145e-16
7

0
4

33.9878
3

33.9633
3

0.0245801

0
4

0
4

525.011

357.96
2

340.711
2

8.95624

0.137103

2.71154

2.4533

2.16787

0.118299

0.147844

0.0739218

0.129121

0.279798

0.0739218

0
4

150.645

147.717

1.29972

1.55246

0.0761741

1.32810429320784e-14

0
4

14.856
2

14.856
2

0
4

1.37511

1.11919

0.25592

0
4

0.129121

0.129121

0
4

0.0460286

0.0460286

0
4

2.36026476141404e-13

0
4

187.804

68.9348

14.707
3

0.98762

0.38087

0.295628

0.923532

0.0648261

0.190435

0.0761741

0.0663141

0.0642886

0.0761741

4.80445
2

2.9327

0.219319

1.25997

4.26575

0.0484412

0.0530735

0.492522

0.0483291

0.0582163

0.0968825

8.78106
2

0.190435

0.64748

0.06398

0.00518609

0.266609

0.00518609

0.193681

0.0761741

0.228522

0.113655

10.1808
2

0.152348

0.0218647

5.84959

1.65158

4.67704

3.4041

0.312949
2

1.26010313294955e-14

0
4

0.316754

0.123073

0.193681

2.77555756156289e-17

0
4

0.36115

0.132628

0.228522

0
4

0.145541

0.0582163

0.0873245

0
4

0.92656

0.829028

0.0975327

5.55111512312578e-17

0
4

0.0761741

0.0761741

0
4

0.0761741

0.0761741

0
4

39.3693
3

22.8128
3

0.742963

0.0975327

0.291082

0.00546617

0.0761741

0.0182206

0.0703603

0.0982775

0.76776

0.243832

3.34986

1.21916

0.0773585

0.00364411

0.0761741

0.0761658

0.0799732

0.152348

4.92358

0.767699

0.494694

1.8214

0.3199

0.487663

0.295236

0
4

51.9905

20.5369

0.09597

0.0761741

0.0873245

13.9986
2

13.1148
3

2.56618

0.304696

0.916525

0.114261

0.114261

0.0648786

0
4

6.64563

0.789845
3

0.0761741

0.131037

0.541107

3.09135

0.155784

0.0530735

1.29121

0.229314

0.0582163

0.228522

0
4

1.39604

0.55469

0.192349

0.649004

1.11022302462516e-16

0
4

12.6413
2

5.03494

0.5555

0.110524

0.198942

6.74141

8.88178419700125e-16
2

0
4

1.42142
2

0.692059

0.276851

0.120383

0.266609

0.0655183

4.16333634234434e-17
2

0
4

3.40006

3.19463

0.205422

2.4980018054066e-16

0
4

0.102492

0.102492

0
4

1.3150591726685e-13

0
4

43.5269
2

33.6674
2

3.40269

1.81869

20.8717

5.99604

0.914089

0.266609

0.054841

0.0761741

0.190435

0.0761741

0
4

3.8087

2.70418

0.495132

0.342783

0.114261

0.0761741

0.0761741

4.71844785465692e-16

0
4

4.26575

0.152348

0.0761741

2.81844

0.0761741

0.418957

0.0761741

0.228522

0.152348

0.266609

6.10622663543836e-16

0
4

0.457045

0.342783

0.114261

1.38777878078145e-17

0
4

0.457045

0.0761741

0.304696

0.0761741

1.38777878078145e-17

0
4

0.870911

0.495132

0.37578

0
4

1.57651669496772e-14
2

0
4

0.116461

0.0451944

0.0451944

0
4

0.0712667

0.0712667

0
4

1.38777878078145e-17

0
4

1.752

1.752

0.0761741

1.67583

0
4

0
4

0.0732585

0.0366292

0.0366292

0
4

0.0366292

0.0366292

0
4

0
4

0.424874

0.424874

0.424874

0
4

0
4

0.0334151

0.0156497

0.0156497

0
4

0.0177653

0.0177653

0
4

3.46944695195361e-18

0
4

0.0143923

0.00616813

0.00616813

0
4

0.00822417

0.00822417

0
4

0
4

0.202321

0.202321

0.202321

0
4

0
4

1.00937

1.00937

1.00937

0
4

0
4

0.204134

0.204134

0.204134

0
4

0
4

1.16436

1.16436

1.16436

0
4

0
4

82.3792

74.3296

70.3044

0.306486

2.0146

0.0663141

0.114261

1.37113

0.152348

1.99562588676372e-14

0
4

1.75303
3

0.631422
2

0.0397225

0.0761741

0.364511

0.0449086

0.218662

0.0967703

0.124503

0.0483291

0.0484412

0.0595838

1.52655665885959e-16
3

0
4

4.90497

0.360405

4.04704

0.142488

0.196558

0.114261

0.0442094

0
4

1.10452

0.152348

0.152348

0.685567

0.114261

1.80411241501588e-16

0
4

0.0397225

0.0397225

0
4

0.0761741

0.0761741

0
4

0.0442094

0.0442094

0
4

0.0507772

0.0507772

0
4

0.0761741

0.0761741

0
4

6.07985883860351e-14

0
4

0.00701215

0.00701215

0.00350607

0.00350607

0
4

0
4

0.0956648

0.0956648

0.0956648

0
4

0
4

0.933992

0.893528

0.893528

0
4

0.0404642

0.0404642

0
4

0
4

0.773265

0.773265

0.773265

0
4

0
4

2.54679

2.54679

0.0887784

2.45801

0
4

0
4

0.10116

0.10116

0.10116

0
4

0
4

2.45133

2.45133

2.45133

0
4

0
4

0.530735

0.477661

0.477661

0
4

0.0530735

0.0530735

0
4

0
4

0.110309

0.0735396

0.0735396

0
4

0.0367698

0.0367698

0
4

6.93889390390723e-18

0
4

0.0209761

0.0209761

0.0104881

0.0104881

0
4

0
4

370.254

161.161

158.751

0.237078

0.377138

0.267031

0.119811

0.190435

1.14261

0.0761741

0
4

34.7807
7

1.29006

17.6896

15.3872

0.388202

0.010998

0.007332

0.007332

0
4

104.208

48.9038

0.388202

53.7783

0.190435

0.609393

0.304696

0.032994

0
4

69.9091

69.4703

0.175543

0.131657

0.131657

0
4

0.13608

0.0761741

0.0599054

1.38777878078145e-17

0
4

0.0591856

0.0591856

0
4

3.25156568337093e-14

0
4

0.0735396

0.0735396

0.0735396

0
4

0
4

0.00525911

0.00525911

0.00525911

0
4

0
4

0.00364411

0.00364411

0.00364411

0
4

0
4

0.0761741

0.0761741

0.0761741

0
4

0
4

0.0091605

0.0091605

0.0091605

0
4

0
4

0.0877713

0.0877713

0.0877713

0
4

0
4

0.0524403

0.0524403

0.0524403

0
4

0
4

0.0316877

0.0316877

0.0316877

0
4

0
4

0.0127553

0.0127553

0.0127553

0
4

0
4

0.0909239

0.0909239

0.0909239

0
4

0
4

88.8296
2

17.3967
1

10.7193

1.11412

2.27865

0.131657

1.66765

0.304696

1.1807

0
4

4.02783

1.98892

0.64748

0.11605

0.0877713

0.357411

0.190435

0.357411

0.0761741

0.0582163

0.147964

1.94289029309402e-16

0
4

0.990417

0.145988

0.239673

0.279931

0.266609

0.0582163

0
4

64.796

64.2255

0.131657

0.351085

0.0877713

0
4

1.36782

1.23616

0.131657

2.77555756156289e-17

0
4

0.192607

0.0761741

0.116433

0
4

0.0582163

0.0582163

0
4

1.27467481014776e-14
2

0
4

0.139899

0.139899

0.139899

0
4

0
4

0.0316877

0.0316877

0.0316877

0
4

0
4

0.0950773

0.0950773

0.0950773

0
4

0
4

0.0434384

0.0434384

0.0434384

0
4

0
4

0.0170056

0.0170056

0.0170056

0
4

0
4

0.418957

0.418957

0.418957

0
4

0
4

0.0355306

0.0355306

0.0355306

0
4

0
4

0.0582163

0.0582163

0.0582163

0
4

0
4

0.0155008

0.0155008

0.0155008

0
4

0
4

0.10116

0.10116

0.10116

0
4

0
4

78.8363

37.9407

16.2712
3

8.01439

1.68546

11.9697

7.105427357601e-15

0
4

30.2991

29.0274

0.281469

0.266609

0.418957

0.152348

0.152348

5.55111512312578e-17

0
4

2.07134

0.547857

1.52348

0
4

0.319556

0.243382

0.0761741

1.38777878078145e-17

0
4

8.12932

8.12932

0
4

0.0761741

0.0761741

0
4

4.94743135348585e-14

0
4

0.0606962

0.0606962

0.0606962

0
4

0
4

0.110309

0.110309

0.110309

0
4

0
4

0.0633849

0.0633849

0.0633849

0
4

0
4

0.155491

0.155491

0.155491

0
4

0
4

0.054841

0.054841

0.054841

0
4

0
4

0.0155008

0.0155008

0.0155008

0
4

0
4

0.289974

0.289974

0.289974

0
4

0
4

0.0877713

0.0877713

0.0877713

0
4

0
4

0.0518304

0.0518304

0.0518304

0
4

0
4

1.79009

1.79009

1.79009

0
4

0
4

7.25925
7

6.43773
7

4.13891
7

0.311758

0.109112

1.71993

0.0163868

0.0809283

0.0606962

4.02455846426619e-16
7

0
4

0.566772
7

0.566772
7

0
4

0.254749

0.0726607

0.182089

0
4

0
4

0.0108665

0.0108665

0.0108665

0
4

0
4

0.139899

0.139899

0.139899

0
4

0
4

0.0909077

0.0909077

0.0909077

0
4

0
4

0.332648

0.332648

0.332648

0
4

0
4

0.09597

0.09597

0.09597

0
4

0
4

0.0975327

0.0975327

0.0975327

0
4

0
4

0.0232512

0.0232512

0.0232512

0
4

0
4

0.0201232

0.0201232

0.0201232

0
4

0
4

0.0873245

0.0873245

0.0873245

0
4

0
4

0.0175304

0.0175304

0.0175304

0
4

0
4

113.522
3

113.522
3

112.325
3

0.408318

0.233576

0.379434

0.0501388

0.0501388

0.0752082

1.26010313294955e-14
3

0
4

0
4

0.0516798

0.0516798

0.0516798

0
4

0
4

0.0606962

0.0606962

0.0606962

0
4

0
4

0.349565

0.349565

0.349565

0
4

0
4

0.0129839

0.0129839

0.0129839

0
4

0
4

1.70996

1.70996

1.70996

0
4

0
4

0.0168565

0.0168565

0.0168565

0
4

0
4

0.0591856

0.0591856

0.0591856

0
4

0
4

0.484779

0.484779

0.484779

0
4

0
4

0.119811

0.119811

0.119811

0
4

0
4

0.0194364

0.0194364

0.0194364

0
4

0
4

28.908

2.83889
6

1.04848

0.684476

0.950908

0.155033

5.55111512312578e-17
6

0
4

0.00447073

0.00447073

0
4

0.010998

0.010998

0
4

0.288392

0.288392

0
4

2.12071
7

2.09474

0.0259677

1.04083408558608e-17
7

0
4

21.0146

17.2471

0.219428

0.0404642

0.0877713

3.41991

0
4

1.02171

1.00937

0.007332

0.00500636

0
4

0.156012

0.13035

0.025662

1.38777878078145e-17

0
4

0.014664

0.007332

0.007332

0
4

1.1296

1.1296

0
4

0.029328

0.007332

0.021996

0
4

0.278575

0.278575

0
4

0
4

0.0100682

0.0100682

0.0100682

0
4

0
4

0.0877713

0.0877713

0.0877713

0
4

0
4

0.0389321

0.0389321

0.0389321

0
4

0
4

0.0367698

0.0367698

0.0367698

0
4

0
4

0.152348

0.152348

0.152348

0
4

0
4

0.141625

0.141625

0.141625

0
4

0
4

0.0245801

0.0245801

0.0245801

0
4

0
4

2.23504

2.23504

2.23504

0
4

0
4

0.221765

0.221765

0.221765

0
4

0
4

0.0129576

0.0129576

0.0129576

0
4

0
4

1.98338
7

0.807692
7

0.061239
7

0.113344

0.538916

0.0497507

0.0316877

0.0127553

8.15320033709099e-17
7

0
4

0.237386
7

0.19889
6

0.0296142

0.00888202

1.73472347597681e-18
7

0
4

0.320571

0.233545

0.0870262

0
4

0.601344

0.0966581

0.504686

0
4

0.0163868

0.0163868

0
4

0
4

0.0418477

0.0418477

0.0418477

0
4

0
4

0.0704238

0.0704238

0.0704238

0
4

0
4

0.0366292

0.0366292

0.0366292

0
4

0
4

0.0127553

0.0127553

0.0127553

0
4

0
4

0.763337

0.763337

0.763337

0
4

0
4

8.47041

8.20381

4.36652

0.455176

2.07417

0.577798

0.387362

0.0761741

0.152348

0.114261

0
4

0.190435

0.190435

0
4

0.0761741

0.0761741

0
4

6.24500451351651e-16

0
4

269.997

266.646

173.185

1.60749

0.754439

0.122852

0.153972

0.266609

0.952176

0.342783

0.206085

1.29496

0.029328

5.55777

0.114261

0.114261

0.266609

0.799828

0.114261

0.0761741

0.495132

0.562264

0.007332

0.114261

11.5078

0.0898581

0.418957

0.114261

0.228522

0.03666

0.357411

0.38087

0.0761741

0.119811

0.418957

2.45041

0.0599054

39.4582

0.007332

0.190435

2.13287

0.152348

0.0599054

0.149764

0.114261

0.28952

0.167469

0.533219

0.0761741

0.0761741

0.152348

0.0761741

0.0761741

0.209669

0.007332

0.0761741

0.114261

1.14162

1.71392

0.50613

0.837915

14.929

0
4

1.44961

0.249995

0.15218

0.204119

0.485868

0.342783

0.014664

7.97972798949331e-17

0
4

1.22708

0.117267

0.01833

0.0761741

0.0599054

0.025662

0.0761741

0.0761741

0.114261

0.0156497

0.0761741

0.571306

1.11022302462516e-16

0
4

0.499254

0.141807

0.007332

0.342783

0.007332

1.21430643318376e-17

0
4

0.175543

0.175543

0
4

0
4

13.0809

12.4788

5.89299

1.54669

0.190155

0.0633849

4.37356

0.190155

0.221847

0
4

0.602156

0.602156

0
4

0
4

7.86697

7.86697

7.33689

0.530079

0
4

0
4

19.0716
4

9.40624
4

3.15767

1.6066

2.67168

0.801847

1.16844

8.88178419700125e-16
4

0
4

7.48663
4

2.3359

1.63578

3.51495

0
4

2.17869

1.19386

0.108496

0.29211

0.58422

1.11022302462516e-16

0
4

1.33226762955019e-15
4

0
4

5.12715

5.12715

2.75099

0.207245

1.49799

0.353838

0.120358

0.196734

6.10622663543836e-16

0
4

0
4

138.289

3.09023

1.05547

1.05547

0.886027

0.093266

1.80411241501588e-16

0
4

97.9831

89.9794

7.91039

0.093266

6.50868248186498e-15

0
4

36.5162

35.0214

1.40154

0.093266

4.5102810375397e-15

0
4

0.699495

0.699495

0
4

0
4

12.9935

9.39993

2.26374

5.76769

1.14597

0.222535

0
4

3.59355

3.59355

0
4

1.77635683940025e-15

0
4

48.2171
3

47.6077
3

26.5323
3

19.9708

0.0761741

0.952176

0.0761741

1.20597976049908e-14
3

0
4

0.418957

0.266609

0.152348

2.77555756156289e-17

0
4

0.190435

0.190435

0
4

0
4

0.407427
8

0.399101
8

0.230403

0.100109

0.0479529

0.0144813

0.00615463

0
4

0.00832669

0.00832669

0
4

8.67361737988404e-18
8

0
4

7.37895
3

7.37895
3

7.37895
3

0
4

0
4

32.9566
3

25.786
2

25.7492
2

0.0367698

1.3392065234541e-15
2

0
4

7.08035
3

0.334471

3.07817

3.66772

0
4

0.0902983

0.0902983

0
4

3.76088049591772e-15
3

0
4

163.749
1

51.2599
1

14.9708

0.0873245

0.144196

0.571306

0.0877713

0.0582163

0.349298

0.342783

0.228522

0.131657

0.0582163

29.9281
2

0.114261

0.738629

1.25673

0.853408

0.286739

0.292468

0.526628

0.232865

3.94129173741931e-15
1

0
4

0.216294

0.216294

0
4

0.0761741

0.0761741

0
4

98.4192
1

68.0709
2

0.799828

0.0591856

0.118371

0.0582163

3.59862

0.266609

1.00937

0.116433

6.80228

0.116433

6.03748
1

0.70217

0.291082

0.0873245

1.5182

1.84635

2.00794

3.15753

1.36035

0.3072

0.0873245

2.07889261361061e-14
1

0
4

9.80823
1

4.6872

0.228522

2.25895

0.837915

0.205296

0.0591856

0.64748

0.288392

0.0591856

0.536117

0
4

1.17283

0.468642

0.526628

0.177557

0
4

1.46619

0.597558

0.868629

0
4

0.615406

0.526628

0.0887784

2.77555756156289e-17

0
4

0.231967

0.144196

0.0877713

0
4

0.175543

0.0877713

0.0877713

0
4

0.3072

0.175543

0.131657

2.77555756156289e-17

0
4

0
4

9.87313
3

9.01587
3

5.32687
3

2.42991

0.139899

1.02593

0.093266

0
4

0.857257

0.274652

0.309187

0.177448

0.09597

0
4

0
4

15.7064

13.5891

6.27635

0.0761741

0.114261

7.12228

0
4

1.56157

0.0761741

1.02835

0.190435

0.114261

0.152348

2.77555756156289e-17

0
4

0.228522

0.228522

0
4

0.190435

0.0761741

0.114261

0
4

0.126951

0.0761741

0.0507772

6.93889390390723e-18

0
4

0.00985039

0.00985039

0
4

0
4

12.548

2.95002

1.39361

0.246785

1.11919

0.190435

2.22044604925031e-16

0
4

9.59793

0.685567

0.266609

0.0761741

0.0761741

8.49341

1.77635683940025e-15

0
4

1.77635683940025e-15

0
4

1.32778

1.32778

1.22213

0.0804929

0.0100616

0.0150924

9.36750677027476e-17

0
4

0
4

45.8859
2

42.9221

5.0588

29.4649

5.44645

1.54273

1.40922

1.11022302462516e-15

0
4

2.43303

2.43303

0
4

0.530735

0.530735

0
4

4.21884749357559e-15
2

0
4

17.7104

15.8822

12.4925

3.38975

4.44089209850063e-16

0
4

1.06644

0.342783

0.723654

0
4

0.761741

0.609393

0.152348

0
4

0
4

16.1123

15.701

2.39912
2

12.9897

0.312208

0
4

0.329046

0.329046

0
4

0.0822615

0.0822615

0
4

0
4

4.81422
7

1.78546

1.67464

0.110825

0
4

0.22057

0.153885

0.0404642

0.0262201

0
4

2.72041

0.155476

2.56494

0
4

0.0877713

0.0877713

0
4

0
4

6.22556
2

4.53966

4.28613

0.249881

0.00364411

1.67834496300756e-16

0
4

1.61324
3

1.32842

0.174649

0.054841

0.0553261

1.45716771982052e-16
3

0
4

0.0726619

0.0726619

0
4

0
4

0.815167

0.0837283

0.036939

0.0197008

0.0221634

0.0049252

0
4

0.731439

0.721589

0.00985039

0
4

1.11022302462516e-16

0
4

43.2036

23.3118
7

21.0281
7

0.00447073

0.00997446

0.0312951

0.37271

0.0332337

0.00947709

0.161857

0.114707

0.647427

0.893528

0.00498723

0
4

0.221221

0.221221

0
4

0.194844

0.0127553

0.182089

0
4

0.222553

0.182089

0.0404642

0
4

0.0194758

0.0194758

0
4

4.09447

0.431899

0.424874

0.256494

0.58673

1.99662

0.316924

0.0404642

0.0404642

6.10622663543836e-16

0
4

5.27369
1

2.06306

0.302734

0.336174

0.195042

2.01874

0.175543

0.0950773

0.0873245

2.91433543964104e-16
1

0
4

1.09239
6

0.386195

0.0118027

0.0122405

0.667873

0.00816031

0.00612023

8.50014503228635e-17
6

0
4

1.0278
6

0.998475
6

0.029328

4.85722573273506e-17
6

0
4

2.50465

0.263017

1.95194

0.202321

0.0469069

0.0404642

0
4

3.86903
7

0.432588

2.57792

0.203382

0.655141

0
4

0.224078

0.0259677

0.0104881

0.131657

0.0404642

0.0155008

1.21430643318376e-17

0
4

1.14767

0.0327735

0.954315

0.0163868

0.144196

0
4

0
4

2.69765
8

2.6781
8

2.6781
8

0
4

0.0133953

0.0133953

0
4

0.00615463

0.00615463

0
4

0
4

3.76168
3

0.364277

0.276952

0.0873245

2.77555756156289e-17

0
4

0.179987

0.179987

0
4

3.1118

3.1118

0
4

0.054841

0.054841

0
4

0.0507772

0.0507772

0
4

3.60822483003176e-16
3

0
4

1.46245
7

1.46245
7

1.45196
7

0.0104881

0
4

0
4

0.439133
7

0.426378
7

0.327783

0.0885339

0.0100616

0
4

0.0127553

0.0127553

0
4

1.90819582357449e-17
7

0
4

1.03059

0.383109

0.032994

0.0761741

0.114261

0.007332

0.152348

0
4

0.190435

0.190435

0
4

0.457045

0.457045

0
4

0
4

2.26396
2

0.765541
2

0.293262

0.137416

0.132628

0.202234

2.77555756156289e-17
2

0
4

1.37759

1.37759

0
4

0.120823

0.120823

0
4

0
4

3.59043
4

2.56378

0.213303

0.784976

0.154593

0.479931

0.930982

0
4

1.02665

1.02665

0
4

2.22044604925031e-16
4

0
4

3.34259

3.34259

1.43858

0.232712

1.26934

0.401957

0
4

0
4

0.468573
7

0.468573
7

0.31939
7

0.144196

0.00498723

0
4

0
4

24.2032
4

22.6456
4

22.5071

0.138508

1.11022302462516e-16
4

0
4

0.399414

0.399414

0
4

0.0501388

0.0501388

0
4

1.10807

1.10807

0
4

1.33226762955019e-15
4

0
4

57.5246
7

20.2669
7

4.84254
7

2.52594
6

10.0894
8

2.05106

0.362839

0.224061

0.0446715

0.0363026

0.0901271

0
4

33.2772
7

31.8681
7

1.38916

0.0199489

0
4

1.02011
7

0.406426
7

0.242983

0.0634529

0.302265

0.00498723

0
4

2.48549
7

1.0971
8

0.529898

0.38258

0.300092

0.0761741

0.0591856

0.0404642

0
4

0.229165

0.205165

0.0127553

0.0112449

0
4

0.130651

0.0310016

0.0404642

0.0591856

0
4

0.0350682

0.0104881

0.0245801

0
4

0.07993

0.07993

0
4

2.16493489801906e-15
7

0
4

14.0649

0.507079

0.0633849

0.25354

0.0950773

0.0950773

0
4

13.4944

11.7579

0.0950773

1.64144

0
4

0.0633849

0.0633849

0
4

0
4

1.62312

1.62312

1.087

0.536117

0
4

0
4

0.762435

0.762435

0.762435

0
4

0
4

1.8579

1.8579

1.25869

0.0352475

0.0704951

0.493466

0
4

0
4

1.91723
3

1.91282
3

1.48319

0.332094

0.0975327

5.55111512312578e-17
3

0
4

0.00440928

0.00440928

0
4

0
4

1.07226

0.749759

0.602802

0.146957

0
4

0.0591856

0.0591856

0
4

0.263314

0.263314

0
4

2.77555756156289e-16

0
4

1.0017

1.0017

0.761325

0.164174

0.0155008

0.0606962

0
4

0
4

0.763427

0.430386

0.301265

0.129121

2.77555756156289e-17

0
4

0.333041

0.281469

0.0515724

0
4

0
4

8.74945

6.21704

6.05484

0.162197

0
4

2.53241

2.47419

0.0582163

0
4

2.22044604925031e-15

0
4

1.40434

1.31657

0.921599

0.263314

0.131657

8.32667268468867e-17

0
4

0.0877713

0.0877713

0
4

0
4

105.934
3

104.75
3

82.0796
3

0.0798828

0.150416

0.0501388

0.236598

8.96288
3

5.52192

3.15101

4.05778

0.145251

0.125347

0.138508

0.0501388

2.57224797017841e-14
3

0
4

0.381025

0.330886

0.0501388

0
4

0.803474

0.803474

0
4

0
4

0.66622

0.608083

0.144196

0.144196

0.0312995

0.144196

0.144196

5.55111512312578e-17

0
4

0.0581373

0.0581373

0
4

0
4

1.06513

1.06513

0.269994

0.721856

0.0732851

9.71445146547012e-17

0
4

0
4

0.551806

0.482903

0.406729

0.0761741

1.38777878078145e-17

0
4

0.0542389

0.0469069

0.007332

0
4

0.014664

0.014664

0
4

0
4

1.75349

0.63577

0.0761741

0.559596

0
4

0.792761

0.792761

0
4

0.324955

0.228522

0.0964329

0
4

1.11022302462516e-16

0
4

7.76775

7.76775

7.73774

0.019133

0.00543897

0.00543897

6.99093560818653e-16

0
4

0
4

1.36654
8

0.316648

0.303893

0.0127553

0
4

0.968961

0.968961

0
4

0.0809283

0.0809283

0
4

0
4

0.751954

0.243329

0.243329

0
4

0.43534

0.43534

0
4

0.0732851

0.0732851

0
4

0
4

0.19627

0.0987373

0.0663433

0.0129576

0.0194364

1.04083408558608e-17

0
4

0.0975327

0.0975327

0
4

0
4

1.3003

1.3003

1.24723

0.0530735

6.24500451351651e-17

0
4

0
4

3.85238

0.105778

0.0423112

0.0634669

0
4

3.49068

3.42721

0.0634669

9.71445146547012e-17

0
4

0.25592

0.25592

0
4

1.66533453693773e-16

0
4

42.1223

27.2005

19.6046

0.00458025

0.0724936

0.314139

0.0966581

1.88161

0.298886

0.952199

0.173723

3.58976

0.0873659

0.0761741

0.0483291

0
4

12.4775

9.85238

0.0761741

1.1034

0.512292

0.420697

0.266609

0.0163868

0.0156497

0.190435

0.0234746

0
4

1.92256

1.92256

0
4

0.349565

0.349565

0
4

0.0606962

0.0606962

0
4

0.0155008

0.0155008

0
4

0.0163868

0.0163868

0
4

0.0796102

0.0796102

0
4

0
4

3.88248

3.85969

3.85969

0
4

0.0227895

0.0227895

0
4

1.14491749414469e-16

0
4

0.674197

0.229998

0.229998

0
4

0.444198

0.444198

0
4

0
4

2.37923

2.37923

2.23504

0.144196

0
4

0
4

15.6988

13.8239

13.8239

0
4

1.79874

1.70996

0.0887784

2.77555756156289e-17

0
4

0.0761741

0.0761741

0
4

0
4

1.2478

0.799535

0.799535

0
4

0.448261

0.0877713

0.36049

0
4

1.11022302462516e-16

0
4

0.17838
7

0.153673

0.153673

0
4

0.0247073

0.007332

0.0173753

3.46944695195361e-18

0
4

0
4

2.20347

2.20347

2.20347

0
4

0
4

0.254609

0.239108

0.239108

0
4

0.0155008

0.0155008

0
4

1.21430643318376e-17

0
4

0.188032

0.188032

0.17802

0.0100127

5.20417042793042e-18

0
4

0
4

0.0498448

0.0498448

0.0498448

0
4

0
4

97.9085

82.3269

36.371

0.0483291

0.0155008

43.114

0.510804

0.714754

0.20353

0.0606962

0.01249

1.25094

0.0248714

3.50414142147315e-15

0
4

0.00503411

0.00503411

0
4

0.152348

0.152348

0
4

9.69745
7

2.24032
7

7.39985

0.052093

0.00518609

7.58074159001865e-16
7

0
4

4.03293
7

1.68121

2.10094

0.0484551

0.161857

0.0404642

7.21644966006352e-16
7

0
4

1.15402

0.931181

0.152348

0.0221634

0.0483291

6.93889390390723e-17

0
4

0.327877

0.31314

0.0147368

0
4

0.0686027

0.0686027

0
4

0.100755

0.100755

0
4

0.0316877

0.0316877

0
4

0.0108665

0.0108665

0
4

0
4

0.434779

0.434779

0.434779

0
4

0
4

0.426559

0.426559

0.266609

0.06398

0.09597

1.38777878078145e-17

0
4

0
4

0.973194

0.973194

0.973194

0
4

0
4

0.399614

0.399614

0.36049

0.0391243

1.38777878078145e-17

0
4

0
4

7.3261

3.99914

0.0761741

3.92297

0
4

3.32696

3.32696

0
4

0
4

0.207189

0.131015

0.054841

0.0761741

0
4

0.0761741

0.0761741

0
4

1.38777878078145e-17

0
4

0.158614

0.105743

0.0528713

0.0528713

0
4

0.0528713

0.0528713

0
4

1.38777878078145e-17

0
4

0.116604

0.116604

0.0532195

0.0633849

0
4

0
4

0.322287

0.322287

0.0722459

0.113655

0.136386

0
4

0
4

0.287019

0.287019

0.287019

0
4

0
4

105.375

104.447

18.5432

0.474136

1.72542

0.718496

0.419697

0.326431

0.886027

0.139899

0.233165

0.139899

0.093266

2.68762

0.093266

2.09848

0.186532

0.233165

0.139899

0.152348

0.0761741

0.139899

38.0908

1.92497

0.886027

29.8195

0.534349

1.07256

2.61145

4.35207425653061e-14

0
4

0.928017

0.788118

0.139899

5.55111512312578e-17

0
4

0
4

1.77306

1.77306

1.77306

0
4

0
4

0.343945

0.343945

0.161857

0.0404642

0.141625

0
4

0
4

0.155564

0.1151

0.0681929

0.0469069

0
4

0.0404642

0.0404642

0
4

0
4

0.0226436

0.0167223

0.00440928

0.012313

1.73472347597681e-18

0
4

0.00592134

0.00592134

0
4

0
4

0.236026

0.236026

0.0236899

0.20715

0.00518609

1.56125112837913e-17

0
4

0
4

0.0565705

0.0565705

0.0401838

0.0163868

0
4

0
4

0.0518365

0.0518365

0.0318875

0.0199489

0
4

0
4

0.172609

0.167683

0.167683

0
4

0.0049252

0.0049252

0
4

0
4

0.511802

0.182089

0.182089

0
4

0.25354

0.25354

0
4

0.0761741

0.0761741

0
4

1.38777878078145e-17

0
4

0.0707971

0.0534217

0.0129576

0.0404642

0
4

0.0173753

0.0173753

0
4

3.46944695195361e-18

0
4

16.5674
3

16.5674
3

10.9062

4.78361

0.4352

0.0739218

0.266609

0.101849

5.17641485231479e-15
3

0
4

0
4

389.197
3

388.441
3

284.416
3

103.948

0.0761741

3.1710745140856e-14
3

0
4

0.0761741

0.0761741

0
4

0.495132

0.495132

0
4

0.0168565

0.0168565

0
4

0.0761741

0.0761741

0
4

0.0920573

0.0920573

0
4

0
4

753.811

748.468

38.8051
7

6.66418
7

0.0770648

0.0633849

0.0173753

0.0633849

0.182089

0.00612023

0.09597

0.00592134

0.0469069

0.0163868

1.23508
8

0.0542527

0.0469069

0.0595195

0.0469492

0.533162

0.0703603

0.00592134

0.0404642

0.00416335

1.43648

15.2794
3

0.0404642

0.288392

0.026063

0.0155008

0.0703603

0.0156497

0.0236854

0.0163868

0.0201232

0.0950773

0.341745

0.00592134

0.0234746

0.0938138

0.0156497

0.0166534

0.0469069

0.0562528

0.00592134

0.00592134

0.00592134

0.246561

0.0201232

0.57582

0.144196

0.0173753

0.0469069

0.0877713

0.0469069

0.0118427

0.0155008

1.37326

0.551807
7

0.0201232

0.0249801

0.0469069

0.0155008

0.0469069

0.0207247

0.937273

0.0118427

0.0163868

3.77417

18.3574

0.144196

0.0469069

0.0633849

0.0156497

0.0469069

0.140721

1.22567

0.0770648

0.09597

0.00592134

0.70134
7

0.00592134

0.0100616

0.0255083

0.186532

0.22393

0.017764

0.0148034

0.0156497

0.00888202

0.28791

0.708429

0.0310016

0.0245801

0.0469069

0.709793

0.0163868

0.093266

0.076525

0.274652

0.0232512

0.0327735

1.8383

0.0296067

0.0469069

0.0770648

0.0112377

0.00592134

0.115597

249.648
3

0.128599
7

0.244836

1.53538

8.56937
6

0.181567

0.152452
7

0.101818
7

1.18709

4.75208

0.822191

65.4506
7

5.57294

0.231142
7

0.408564

0.937273

0.0584393

4.89128

2.76223

0.0327431

0.0299304

0.516513

14.5709
7

2.11081

0.141847

0.0518407

0.0834252

0.0307863

2.88785

7.17743

0.225005

0.575097

0.11059

8.03262
6

0.247308

0.0361998

0.03111

0.273489

0.0705923

0.0860101

0.36049

0.582619

0.113336

2.14512

15.5407
3

5.69457

0.251545

1.17346

0.181185

0.0361754

0.0829861

0.204772

0.0248881

0.0214221

0.117267

13.0933

0.187628

0.235942

0.0810131

0.0148034

4.18168

0.0417128

0.169779

0.01249

2.32529

0.253513

207.679
3

1.37801

0.017764

0.0607781

0.36049

0.109315

0.504686

2.65808

0.0148034

0.00592134

0.0703603

1.46053

0.0469069

0.937273

0.0155008

0.0877713

0.0938138

0.68015

0.00592134

0.0592134

0.103624

0.0120572

2.66347707778003e-13

0
4

4.85146

0.157246

3.5189

0.763337

0.411978

5.55111512312578e-17

0
4

0.347054

0.123124

0.22393

0
4

0.139899

0.139899

0
4

0.00518609

0.00518609

0
4

2.01106492569991e-14

0
4

6.1385
3

3.22505
4

0.310752

2.91429

0
4

2.66141

2.66141

0
4

0.0316877

0.0316877

0
4

0.00475254

0.00475254

0
4

0.144196

0.144196

0
4

0.0397225

0.0397225

0
4

0.0316877

0.0316877

0
4

6.86950496486816e-16
3

0
4

7.41263050940688e-10

0
4

2778.39
3

2337.67
3

59.6151

56.0738

0.540239

0.0914821

0.119824

0.0683282

0.0125159

0.438165

0.129121

0.0404642

0.0460286

0.155618

0.153738

0.454241

0.0617678

0.0798828

0.214531

0.360734

0.151349

0.0733345

0.110338

0.0454705

0.194088

8.74300631892311e-15

0
4

78.5092
5

73.4415
5

0.99759
6

0.0533257

0.00500636

0.0404642

0.0404642

0.452308

0.885621

0.100634

0.192063

1.81307

0.0991568

0.32948

0.0584274

4.9890647169093e-15
5

0
4

2197.49
3

25.7017

139.699
3

217.946

16.1102
4

0.106093

1.98494

0.967591

0.0438406

1.44603

0.147481

0.581044

0.178074

1.2015

2.72054

126.282
4

0.823956

0.188483

0.470887

0.38087

2.07696

0.122142

0.129121

2.45441

0.0488567

0.0761741

115.78
3

0.077809

0.026063

1.03491

0.0920573

0.0808509

0.129121

0.0846347

0.401491

0.399414

0.126952

5.6622
3

0.0873245

0.165978

0.021996

0.101849

0.161286

0.58422

0.0501388

0.0553261

0.411978

0.0599054

197.828
4

0.146299

0.123124

0.043992

0.430933

6.48837

0.129121

0.114261

0.516483

0.212569

0.0397225

1.21559

0.977135

0.0129839

0.274652

0.007332

2.05989

0.07653

0.278718

0.154593

0.0846347

0.0765318

3.0551
3

0.121276

1.51059

0.299527

0.153972

0.007332

0.129121

0.0808509

0.119811

0.229344

0.014664

2.97842

2.06593

0.0156497

0.309187

0.484779

0.58422

0.341997

0.414299

0.195065

0.229344

0.868263

11.1049
4

0.0189229

0.0245801

0.126952

1.23593

0.101849

0.430933

0.387362

1.70996

0.0553261

0.274652

284.37
4

0.274652

0.0200127

0.29211

0.15995

0.043992

0.139084

0.763337

0.021996

0.119824

0.007332

45.2615

27.938
4

0.274652

0.014664

0.183475

0.0300191

0.0469069

0.387362

0.0799732

1.22665

0.007332

0.438165

2.17937
3

0.245801

19.2256

0.876329

0.733902

0.249849

0.211587

0.06398

0.0975327

0.0378459

0.357411

17.9012
4

0.202127

1.14501

0.239648

0.390854

0.274652

0.229344

0.021996

0.341364

0.129121

0.193681

6.75807

0.266609

15.1897

0.07653

0.214522

0.0403499

0.0163868

0.0887784

0.0703603

0.079445

0.387362

24.6301

0.119824

0.387362

0.00500636

0.27959

4.19836

0.68663

0.007332

1.70996

0.274652

0.0312995

6.87999
3

1.52667

0.184686

0.975327

0.763337

0.0391243

0.0798828

0.174412

0.175085

0.241929

0.549304

1.25943

0.0846347

0.0662303

0.0846347

0.357411

0.007332

4.45026
5

1.57869
6

6.74145

22.3707

9.15734
3

37.5621

77.7196

1.59841

4.96896
3

39.6182

6.18044

8.01987
4

1.74779

2.48652

7.69711

4.52208
6

29.9587
4

12.4147

241.769

2.23936
4

0.460597

1.61468

3.93067

1.15236

6.66799

11.3745
4

0.378083

15.3287

1.35577

1.52849

1.82348

0.603454

6.19067

2.06593

1.67434

8.73482

45.2549
3

4.61569

1.67359

6.40692

1.25319

0.414915

0.0521608

6.1067

4.19836

0.451923

0.551642

20.9896

1.58658

41.3897

1.03221

0.464502

0.0842568

0.198613

0.442689

25.4822

2.02147

0.234567

28.8726
3

0.959679

0.14657

0.181822

0.106147

0.106346

0.297919

0.062599

0.674938

1.23593

0.265526

16.8946

1.52667

0.68663

1.43949

1.56867

0.045222

0.341997

0.200835

4.19836

0.01833

0.19229

1.79412040779425e-13
3

0
4

0.122565

0.0756579

0.0469069

0
4

0.27959

0.27959

0
4

0.247815

0.247815

0
4

0.10116

0.10116

0
4

0.0404642

0.0404642

0
4

0.244927

0.149864

0.095063

0
4

0.239622

0.149764

0.0898581

1.38777878078145e-17

0
4

0.200626

0.200626

0
4

0.0809283

0.0809283

0
4

0.0917376

0.0917376

0
4

0.0469069

0.0469069

0
4

0.357411

0.357411

0
4

0.00500636

0.00500636

0
4

2.67291570821282e-12
3

0
4

95.0135
3

62.9743
3

32.0792
3

0.304026

0.282502

0.15995

0.968935

0.0882824

0.469034

1.91102

0.184686

0.262628

0.438897

5.82284

0.0434322

0.160722

0.0482912

0.716632

0.00322127

0.292598

0.108581

0.0434322

12.7352
4

2.54231
3

0.988072

0.681754

0.088585

0.810175

0.739342

0
4

23.3682
3

12.2878

8.00425
2

1.28125

0.325742

0.977225

0.137103

0.0434322

0.0724369

0.238877

0
4

8.49246
4

0.533241

0.0868644

2.67168

0.045462

0.152013

0.108581

0.39089

1.52667

0.952653

1.28605

0.101159

0.06398

0.33163

0.0528677

0.0868644

0.101849

3.05311331771918e-15
4

0
4

0.17857

0.17857

0
4

2.50632847809129e-14
3

0
4

3.35975
4

3.11511
4

3.11511
4

0
4

0.244634

0.244634

0
4

0
4

2.5041

2.46756

1.31169

0.535306

0.28791

0.184804

0.147844

1.94289029309402e-16

0
4

0.0365426

0.0365426

0
4

2.56739074444567e-16

0
4

0.4814

0.4814

0.4814

0
4

0
4

2.46084

2.46084

2.29631

0.0822615

0.0822615

2.77555756156289e-17

0
4

0
4

5.50345

4.61699

4.49387

0.123124

8.32667268468867e-17

0
4

0.763337

0.763337

0
4

0.123124

0.123124

0
4

0
4

42.4085

1.21143

0.534251

0.123124

0.184686

0.369371

1.66533453693773e-16

0
4

41.197

0.123124

41.0739

0
4

0
4

0.871583

0.871583

0.779845

0.0917376

2.77555756156289e-17

0
4

0
4

0.49947

0.39089

0.195445

0.152013

0.0434322

0
4

0.0651483

0.0651483

0
4

0.0434322

0.0434322

0
4

0
4

0.868644

0.825212

0.825212

0
4

0.0434322

0.0434322

0
4

3.46944695195361e-17

0
4

0.170685

0.170685

0.130962

0.0397225

0
4

0
4

126.777
3

124.953
3

122.628
3

0.578945

0.933793

0.12796

0.09597

0.09597

0.369174

0.123124

7.91033905045424e-15
3

0
4

0.07653

0.07653

0
4

0.0651483

0.0651483

0
4

0.315392

0.112849

0.125734

0.0365426

0.0144957

0.00644255

0.0193276

1.38777878078145e-17

0
4

0.362358
2

0.173608

0.127467

0.054841

0.00644255

2.68882138776405e-17
2

0
4

0.297827
3

0.135304

0.109655

0.0528677

2.08166817117217e-17
3

0
4

0.49947

0.49947

0
4

0.0144957

0.0144957

0
4

0.0651483

0.0651483

0
4

0.0642886

0.0642886

0
4

0.06398

0.06398

0
4

2.79359868571305e-14
3

0
4

0.125903

0.125903

0.125903

0
4

0
4

0.155111

0.155111

0.155111

0
4

0
4

0.582163

0.291082

0.291082

0
4

0.291082

0.291082

0
4

0
4

0.743697

0.743697

0.743697

0
4

0
4

0.294584

0.291363

0.291363

0
4

0.00322127

0.00322127

0
4

5.20417042793042e-18

0
4

0.146708

0.0965695

0.00483191

0.0917376

0
4

0.0501388

0.0501388

0
4

6.93889390390723e-18

0
4

0.303602

0.185303

0.185303

0
4

0.118299

0.118299

0
4

0
4

0.7653

0.7653

0.7653

0
4

0
4

0.891915

0.0642886

0.0642886

0
4

0.0642886

0.0642886

0
4

0.763337

0.763337

0
4

0
4

0.594246

0.594246

0.580426

0.0138197

0
4

0
4

11.9478
3

8.48315
3

0.734989
3

0.0965825

0.175085

0.192866

0.0434322

0.054841

2.85729
3

2.55168
4

0.789811

0.0965825

0.0434322

0.763337

0.0283844

0.054841

0
4

0.00805318

0.00805318

0
4

0.0112745

0.0112745

0
4

1.50109

1.49626

0.00483191

0
4

1.23683
3

0.14945

0.164442

0.05102

0.0651483

0.763337

0.0434322

1.45716771982052e-16
3

0
4

0.128955

0.128955

0
4

0.304995

0.0128851

0.29211

0
4

0.0868644

0.0434322

0.0434322

0
4

0.0715909

0.0651483

0.00644255

6.07153216591882e-18

0
4

0.108581

0.108581

0
4

0.00644255

0.00644255

0
4

5.22411974790415e-15
3

0
4

0.338539

0.0846347

0.0846347

0
4

0.169269

0.169269

0
4

0.0846347

0.0846347

0
4

0
4

0.0949499

0.0949499

0.0949499

0
4

0
4

0.376544

0.376544

0.376544

0
4

0
4

0.108581

0.108581

0.108581

0
4

0
4

0.207597

0.207597

0.182089

0.0255083

1.04083408558608e-17

0
4

0
4

0.247071

0.247071

0.0926517

0.15442

0
4

0
4

0.0548351

0.0548351

0.0548351

0
4

0
4

0.00750954

0.00750954

0.00750954

0
4

0
4

0.236598

0.236598

0.236598

0
4

0
4

0.0163868

0.0163868

0.0163868

0
4

0
4

118.165
4

106
4

10.4505
4

21.595
4

54.3294

6.66076

9.66484

0.369371

1.14501

1.7853

1.4432899320127e-14
4

0
4

7.55087
4

4.83623
4

2.22215

0.123124

0.123124

0.123124

0.123124

0
4

1.50208

0.492495

0.246248

0.763337

1.11022302462516e-16

0
4

3.11209

1.57304

1.53905

0
4

0
4

0.185303

0.185303

0.185303

0
4

0
4

0.0599054

0.0599054

0.0599054

0
4

0
4

0.06398

0.06398

0.06398

0
4

0
4

0.0434322

0.0434322

0.0434322

0
4

0
4

0.0156497

0.0156497

0.0156497

0
4

0
4

0.0770648

0.0770648

0.0770648

0
4

0
4

0.00966382

0.00966382

0.00966382

0
4

0
4

14.0057

4.05582

0.896399

0.0397225

0.0397225

0.0434322

0.079445

0.0651483

0.447848

0.15889

0.369174

0.0993063

0.079445

1.60699

0.0651483

0.0651483

0
4

9.39265

2.46503

0.0595838

0.0868644

1.19168

0.0434322

0.325742

1.53786

3.08369

0.173729

0.195445

0.0868644

0.0993063

0.0434322

0
4

0.318322

0.130297

0.079445

0.0651483

0.0434322

2.08166817117217e-17

0
4

0.238877

0.108581

0.130297

0
4

0
4

1.87596

0.620131

0.186171

0.136042

0.0397225

0.0595838

0.0397225

0.079445

0.079445

1.66533453693773e-16

0
4

0.868644

0.0651483

0.304026

0.0434322

0.0651483

0.130297

0.0868644

0.173729

0
4

0.38718

0.0868644

0.0434322

0.0397225

0.0868644

0.130297

0
4

3.33066907387547e-16

0
4

3.00041
4

1.17794

0.325742

0.152013

0.184686

0.0868644

0.15306

0.0434322

0.0868644

0.0434322

0.101849

0
4

1.82246

1.65907

0.07653

0.0434322

0.0434322

0
4

6.66133814775094e-16
4

0
4

2.51081

2.51081

1.60504

0.614304

0.29147

0
4

0
4

1.54444
4

0.85666
4

0.226727

0.253172

0.05102

0.238877

0.0868644

0
4

0.173729

0.108581

0.0651483

0
4

0.195445

0.130297

0.0651483

0
4

0.16659

0.0482912

0.118299

0
4

0.152013

0.152013

0
4

0
4

0
4

601.07

601.07

594.999

583.249

0.258242

0.387362

2.51786

1.16209

0.581044

1.35577

0.581044

2.84066

0.516483

1.54945

2.25153229393982e-13

0
4

2.64929

0.970721

1.22665

0.258242

0.193681

0
4

2.84066

1.35577

0.129121

0.322802

1.03297

0
4

0.581044

0.129121

0.451923

0
4

0
4

0
4

0.935847
7

0.935847
7

0.935847
7

0.84267
7

0.0737404

0.0194364

1.73472347597681e-17
7

0
4

0
4

0
4

0.0281014

0.0281014

0.0281014

0.0281014

0
4

0
4

0
4

1.18013

1.18013

1.18013

1.10025

0.0798828

0
4

0
4

0
4

0.157597

0.157597

0.157597

0.0926517

0.0649455

0
4

0
4

0
4

0.625281

0.625281

0.625281

0.625281

0
4

0
4

0
4

0.47985

0.47985

0.47985

0.47985

0
4

0
4

0
4

0.0629283

0.0629283

0.0629283

0.0629283

0
4

0
4

0
4

0.261484

0.261484

0.261484

0.261484

0
4

0
4

0
4

0.117267

0.117267

0.117267

0.117267

0
4

0
4

0
4

0.891439

0.891439

0.891439

0.891439

0
4

0
4

0
4

0.38532

0.38532

0.38532

0.283249

0.102071

0
4

0
4

0
4

4.76078
7

4.76078
7

4.76078
7

4.35686
7

0.263201

0.140721

6.38378239159465e-16
7

0
4

0
4

0
4

0.115072

0.115072

0.115072

0.069043

0.0460286

6.93889390390723e-18

0
4

0
4

0
4

0.943078

0.943078

0.943078

0.943078

0
4

0
4

0
4

0.891915

0.891915

0.891915

0.891915

0
4

0
4

0
4

0.0666256

0.0666256

0.0666256

0.0666256

0
4

0
4

0
4

0.0084933

0.0084933

0.0084933

0.0084933

0
4

0
4

0
4

1.7228

1.7228

1.7228

1.7228

0
4

0
4

0
4

0.369287

0.369287

0.369287

0.369287

0
4

0
4

0
4

0.234534

0.234534

0.0938138

0.0938138

0
4

0.140721

0.140721

0
4

0
4

0
4

0.623115

0.623115

0.623115

0.623115

0
4

0
4

0
4

0.0680282

0.0680282

0.0680282

0.0680282

0
4

0
4

0
4

7.99751
7

7.99751
7

7.75292
7

7.75292
7

0
4

0.244592

0.244592

0
4

3.33066907387547e-16
7

0
4

0
4

0.0215407

0.0215407

0.0104881

0.0104881

0
4

0.0110526

0.0110526

0
4

0
4

0
4

0.68663

0.68663

0.68663

0.68663

0
4

0
4

0
4

1.84043

1.84043

1.83451

1.83451

0
4

0.00592134

0.00592134

0
4

0
4

0
4

0.188315

0.188315

0.125543

0.125543

0
4

0.0627716

0.0627716

0
4

0
4

0
4

1.38936

1.38936

1.38936

1.38936

0
4

0
4

0
4

0.0394338

0.0394338

0.0394338

0.0112449

0.028189

0
4

0
4

0
4

1.40778

1.40778

1.40778

1.40778

0
4

0
4

0
4

3.93897

3.93897

3.93897

3.93897

0
4

0
4

0
4

0.791069

0.791069

0.791069

0.791069

0
4

0
4

0
4

0.0313917

0.0313917

0.0313917

0.0313917

0
4

0
4

0
4

9.118

8.00529

7.78592

7.6315

0.0617678

0.0926517

0
4

0.219365

0.15442

0.0649455

1.38777878078145e-17

0
4

0
4

1.11271

0.989177

0.989177

0
4

0.123536

0.123536

0
4

5.55111512312578e-17

0
4

0
4

0.191923

0.191923

0.191923

0.0548351

0.137088

0
4

0
4

0
4

0.72098

0.72098

0.72098

0.144196

0.576784

0
4

0
4

0
4

0.548872

0.548872

0.548872

0.548872

0
4

0
4

0
4

0.123536

0.123536

0.0617678

0.0617678

0
4

0.0617678

0.0617678

0
4

0
4

0
4

0.387362

0.387362

0.129121

0.129121

0
4

0.258242

0.258242

0
4

0
4

0
4

0.0761741

0.0761741

0.0761741

0.0761741

0
4

0
4

0
4

0.097998

0.097998

0.097998

0.097998

0
4

0
4

0
4

0.0318883

0.0318883

0.0318883

0.0318883

0
4

0
4

0
4

0.0724936

0.0724936

0.0724936

0.0724936

0
4

0
4

0
4

0.0108779

0.0108779

0.0108779

0.0108779

0
4

0
4

0
4

11.7647

11.7647

7.25093

1.80989

0.30781

3.65575

0.861867

0.30781

0.184686

0.123124

0
4

4.32912

1.23124

1.37326

0.763337

0.961282

0
4

0.184686

0.184686

0
4

1.49880108324396e-15

0
4

0
4

0.526034

0.526034

0.526034

0.526034

0
4

0
4

0
4

0.0157321

0.0157321

0.0157321

0.0157321

0
4

0
4

0
4

0.00777913

0.00777913

0.00777913

0.00777913

0
4

0
4

0
4

0.0170056

0.0170056

0.0170056

0.0170056

0
4

0
4

0
4

0.0770648

0.0770648

0.0770648

0.0770648

0
4

0
4

0
4

0.357411

0.357411

0.357411

0.357411

0
4

0
4

0
4

2.58723

2.58723

2.58723

2.58723

0
4

0
4

0
4

0.129121

0.129121

0.129121

0.129121

0
4

0
4

0
4

0.0963278

0.0963278

0.0963278

0.0963278

0
4

0
4

0
4

0.269574

0.269574

0.269574

0.269574

0
4

0
4

0
4

3.94003
7

2.08766
7

1.41528

1.36199

0.0532921

1.38777878078145e-16

0
4

0.166877

0.0633753

0.0565951

0.0469069

0
4

0.00458025

0.00458025

0
4

0.500921

0.500921

0
4

2.22044604925031e-16
7

0
4

1.85237
6

1.42231

1.08984

0.0173753

0.315097

0
4

0.389596

0.389596

0
4

0.0404642

0.0404642

0
4

8.32667268468867e-17
6

0
4

0
4

0.0475315

0.0475315

0.0475315

0.0475315

0
4

0
4

0
4

0.0510167

0.0510167

0.0510167

0.0510167

0
4

0
4

0
4

0.102033

0.102033

0.102033

0.102033

0
4

0
4

0
4

2.63338

2.63338

2.63338

2.63338

0
4

0
4

0
4

0.0163868

0.0163868

0.0163868

0.0163868

0
4

0
4

0
4

0.400509

0.400509

0.400509

0.400509

0
4

0
4

0
4

0.411978

0.411978

0.411978

0.411978

0
4

0
4

0
4

0.0761741

0.0761741

0.0761741

0.0761741

0
4

0
4

0
4

0.0232512

0.0232512

0.0232512

0.0232512

0
4

0
4

0
4

3.84522

3.84522

3.84522

3.84522

0
4

0
4

0
4

55.4131
3

55.4131
3

55.0364
3

54.9586
3

0.077809

0
4

0.376689

0.376689

0
4

6.32827124036339e-15
3

0
4

0
4

0.0877713

0.0877713

0.0877713

0.0877713

0
4

0
4

0
4

0.138508

0.138508

0.138508

0.138508

0
4

0
4

0
4

0.0633849

0.0633849

0.0633849

0.0633849

0
4

0
4

0
4

0.0752082

0.0752082

0.0752082

0.0752082

0
4

0
4

0
4

1.11965

1.11965

1.11965

1.11965

0
4

0
4

0
4

0.125159

0.125159

0.125159

0.125159

0
4

0
4

0
4

0.965349

0.965349

0.965349

0.965349

0
4

0
4

0
4

0.202321

0.202321

0.202321

0.202321

0
4

0
4

0
4

0.00500636

0.00500636

0.00500636

0.00500636

0
4

0
4

0
4

0.158462

0.158462

0.158462

0.158462

0
4

0
4

0
4

26.8374
5

26.8374
5

25.1104
5

21.4607
5

3.54247

0.107242

1.70696790036118e-15
5

0
4

1.70996

1.70996

0
4

0.0170056

0.0170056

0
4

0
4

0
4

0.0975327

0.0975327

0.0975327

0.0975327

0
4

0
4

0
4

0.163796

0.163796

0.163796

0.163796

0
4

0
4

0
4

0.0404642

0.0404642

0.0404642

0.0404642

0
4

0
4

0
4

0.093266

0.093266

0.093266

0.093266

0
4

0
4

0
4

4.66908

4.66908

4.66908

4.66908

0
4

0
4

0
4

0.54383

0.54383

0.54383

0.54383

0
4

0
4

0
4

0.12796

0.12796

0.12796

0.12796

0
4

0
4

0
4

0.585196

0.585196

0.585196

0.585196

0
4

0
4

0
4

0.076525

0.076525

0.076525

0.076525

0
4

0
4

0
4

0.0409669

0.0409669

0.0409669

0.0409669

0
4

0
4

0
4

122.844

122.844

122.446

118.383

0.582462

1.87996

1.60061

0
4

0.397776

0.123124

0.274652

0
4

9.99200722162641e-16

0
4

0
4

0.0868644

0.0868644

0.0868644

0.0868644

0
4

0
4

0
4

0.0809283

0.0809283

0.0809283

0.0809283

0
4

0
4

0
4

7.2517

7.2517

7.2517

7.2517

0
4

0
4

0
4

0.36049

0.36049

0.36049

0.36049

0
4

0
4

0
4

0.0599054

0.0599054

0.0599054

0.0599054

0
4

0
4

0
4

0.0488567

0.0488567

0.0488567

0.0488567

0
4

0
4

0
4

0.0152104

0.0152104

0.0152104

0.0152104

0
4

0
4

0
4

0.121392

0.121392

0.121392

0.121392

0
4

0
4

0
4

0.0737404

0.0737404

0.0737404

0.0737404

0
4

0
4

0
4

0.117267

0.117267

0.117267

0.117267

0
4

0
4

0
4

30.0893

30.0893

28.9443

26.811

0.886461

0.101849

1.14501

0
4

1.14501

1.14501

0
4

0
4

0
4

2.90522

2.90522

2.90522

2.90522

0
4

0
4

0
4

0.100278

0.100278

0.100278

0.100278

0
4

0
4

0
4

0.357411

0.357411

0.357411

0.357411

0
4

0
4

0
4

0.0170056

0.0170056

0.0170056

0.0170056

0
4

0
4

0
4

0.0703603

0.0703603

0.0703603

0.0703603

0
4

0
4

0
4

0.0834505

0.0834505

0.0834505

0.0834505

0
4

0
4

0
4

0.0712667

0.0712667

0.0712667

0.0712667

0
4

0
4

0
4

0.0460286

0.0460286

0.0460286

0.0460286

0
4

0
4

0
4

0.123124

0.123124

0.123124

0.123124

0
4

0
4

0
4

0.0112449

0.0112449

0.0112449

0.0112449

0
4

0
4

0
4

102.66

102.66

95.6941

38.6711

6.3407

41.4281

1.16111

7.72364

0.123124

0.246248

0
4

3.31953

2.45767

0.615619

0.123124

0.123124

0
4

3.04963

0.961282

0.549304

1.53905

0
4

0.596664

0.596664

0
4

3.36397576461422e-14

0
4

0
4

5.96872
7

5.96872
7

5.96872
7

5.96872
7

0
4

0
4

0
4

0.119811

0.119811

0.119811

0.119811

0
4

0
4

0
4

0.00922919

0.00922919

0.00922919

0.00922919

0
4

0
4

0
4

0.106514

0.106514

0.106514

0.106514

0
4

0
4

0
4

0.0404642

0.0404642

0.0404642

0.0404642

0
4

0
4

0
4

0.144196

0.144196

0.144196

0.144196

0
4

0
4

0
4

0.152774

0.152774

0.152774

0.152774

0
4

0
4

0
4

0.0599054

0.0599054

0.0599054

0.0599054

0
4

0
4

0
4

0.53643

0.53643

0.53643

0.53643

0
4

0
4

0
4

0.00448724

0.00448724

0.00448724

0.00448724

0
4

0
4

0
4

0.763337

0.763337

0.763337

0.763337

0
4

0
4

0
4

12.5188

12.5188

12.5188

2.29523

3.72583

6.49771

1.77635683940025e-15

0
4

0
4

0
4

0.193791

0.193791

0.193791

0.193791

0
4

0
4

0
4

0.214522

0.214522

0.214522

0.214522

0
4

0
4

0
4

0.0842243

0.0842243

0.0842243

0.0842243

0
4

0
4

0
4

6.1067

6.1067

6.1067

6.1067

0
4

0
4

0
4

0.0404642

0.0404642

0.0404642

0.0404642

0
4

0
4

0
4

0.0505696

0.0505696

0.0505696

0.0505696

0
4

0
4

0
4

0.216294

0.216294

0.216294

0.216294

0
4

0
4

0
4

0.00447073

0.00447073

0.00447073

0.00447073

0
4

0
4

0
4

0.141625

0.141625

0.141625

0.141625

0
4

0
4

0
4

0.0798828

0.0798828

0.0798828

0.0798828

0
4

0
4

0
4

13.3685

13.3685

13.3685

6.26092

7.10757

0
4

0
4

0
4

0.0898581

0.0898581

0.0898581

0.0898581

0
4

0
4

0
4

1.45541

1.45541

1.45541

1.45541

0
4

0
4

0
4

0.15413

0.15413

0.15413

0.15413

0
4

0
4

0
4

0.507079

0.507079

0.507079

0.507079

0
4

0
4

0
4

0.0898581

0.0898581

0.0898581

0.0898581

0
4

0
4

0
4

0.0984447

0.0984447

0.0984447

0.0984447

0
4

0
4

0
4

0.0316877

0.0316877

0.0316877

0.0316877

0
4

0
4

0
4

0.0773585

0.0773585

0.0773585

0.0773585

0
4

0
4

0
4

0.06398

0.06398

0.06398

0.06398

0
4

0
4

0
4

0.163089

0.163089

0.163089

0.163089

0
4

0
4

0
4

5.69367
7

2.6634

2.6634

2.20483

0.397875

0.0606962

0
4

0
4

3.03027

0.465338

0.465338

0
4

2.56494

2.56494

0
4

0
4

0
4

0.144196

0.144196

0.144196

0.144196

0
4

0
4

0
4

0.27201

0.27201

0.27201

0.27201

0
4

0
4

0
4

0.0110526

0.0110526

0.0110526

0.0110526

0
4

0
4

0
4

1.25094

1.25094

1.25094

1.25094

0
4

0
4

0
4

0.232865

0.232865

0.232865

0.232865

0
4

0
4

0
4

0.0234054

0.0234054

0.0234054

0.0234054

0
4

0
4

0
4

2.29314

2.29314

2.29314

2.29314

0
4

0
4

0
4

0.473485

0.473485

0.473485

0.473485

0
4

0
4

0
4

0.143684

0.143684

0.143684

0.143684

0
4

0
4

0
4

0.0898581

0.0898581

0.0898581

0.0898581

0
4

0
4

0
4

13.3898
6

13.3898
6

13.3898
6

13.3898
6

0
4

0
4

0
4

0.0655183

0.0655183

0.0655183

0.0655183

0
4

0
4

0
4

0.0582163

0.0582163

0.0582163

0.0582163

0
4

0
4

0
4

0.0100064

0.0100064

0.0100064

0.0100064

0
4

0
4

0
4

0.0752082

0.0752082

0.0752082

0.0752082

0
4

0
4

0
4

0.106826

0.106826

0.106826

0.106826

0
4

0
4

0
4

0.0971819

0.0971819

0.0971819

0.0971819

0
4

0
4

0
4

4.44506

3.56043

3.56043

2.54317

0.38753

0.629736

1.11022302462516e-16

0
4

0
4

0.884627

0.884627

0.815584

0.069043

0
4

0
4

0
4

2.68191
3

2.68191
3

2.68191
3

1.44593

0.437272

0.0491603

0.749547

0
4

0
4

0
4

19.4855
6

19.4855
6

19.4855
6

1.80073

4.37544

13.3093

0
4

0
4

0
4

33.3806
4

33.3806
4

32.7943
4

32.7943
4

0
4

0.586281

0.586281

0
4

0
4

0
4

0.909496
7

0.909496
7

0.909496
7

0.909496
7

0
4

0
4

0
4

5.90038
1

5.90038
1

5.88989
1

5.23854
1

0.295687

0.0198674

0.00690984

0.328884

4.44089209850063e-16
1

0
4

0.0104881

0.0104881

0
4

8.67361737988404e-17
1

0
4

0
4

4.0992
7

4.0992
7

4.0992
7

4.0992
7

0
4

0
4

0
4

6.6911
6

6.6911
6

6.6911
6

6.3985
6

0.292598

0
4

0
4

0
4

1.18056

1.18056

1.18056

0.970591

0.100297

0.0548351

0.0548351

0
4

0
4

0
4

3.95751

3.95751

2.03494
7

1.9248
7

0.11014

0
4

1.92256

1.92256

0
4

0
4

0
4

7.31948

6.39297

5.49733

5.12673

0.123536

0.247071

0
4

0.895633

0.401491

0.185303

0.308839

5.55111512312578e-17

0
4

0
4

0.926517

0.0617678

0.0617678

0
4

0.864749

0.864749

0
4

0
4

0
4

1.36156
7

1.36156
7

1.36156
7

1.35655
7

0.00500636

0
4

0
4

0
4

2.22176

2.22176

2.22176

0.155603

2.06615

0
4

0
4

0
4

3.15362

3.15362

1.35273

1.27276

0.0799732

1.11022302462516e-16

0
4

1.80089

1.67231

0.128577

2.77555756156289e-17

0
4

2.22044604925031e-16

0
4

0
4

0.794544

0.794544

0.794544

0.620132

0.0775164

0.0968956

8.32667268468867e-17

0
4

0
4

0
4

7.07457

7.07457

6.21753

6.03284

0.184686

6.10622663543836e-16

0
4

0.492495

0.369371

0.123124

0
4

0.246248

0.246248

0
4

0.118299

0.118299

0
4

0
4

0
4

4.22913
6

4.22913
6

4.22913
6

4.10159
6

0.127542

0
4

0
4

0
4

1.34144
7

1.34144
7

1.34144
7

1.06511

0.201251

0.0159818

0.0591023

0
4

0
4

0
4

2.79865
7

2.79865
7

2.79865
7

2.71671
7

0.0819338

1.11022302462516e-16
7

0
4

0
4

0
4

0.91276

0.91276

0.637318

0.239442

0.397875

0
4

0.275443

0.275443

0
4

1.11022302462516e-16

0
4

0
4

0.227804

0.227804

0.227804

0.18734

0.0404642

0
4

0
4

0
4

0.103095

0.103095

0.0275951

0.0127917

0.00592134

0.00888202

0
4

0.0606962

0.0606962

0
4

0.0148034

0.0148034

0
4

0
4

0
4

0.0516157

0.0516157

0.0516157

0.0127752

0.0261326

0.00827008

0.00443786

3.46944695195361e-18

0
4

0
4

0
4

0.228007

0.228007

0.228007

0.228007

0
4

0
4

0
4

3.57193

3.57193

3.57193

3.51711

0.0548139

2.15105711021124e-16

0
4

0
4

0
4

2.15675
7

2.15675
7

2.15675
7

2.15675
7

0
4

0
4

0
4

8.83271

8.83271

8.83271

0.407376

8.03885

0.23189

0.154593

3.21964677141295e-15

0
4

0
4

0
4

18.6926
5

13.6829
5

8.56765
5

8.51189
5

0.0557638

4.02455846426619e-16
5

0
4

3.06654

3.06654

0
4

2.03168

0.321727

1.70996

0
4

0.0170056

0.0170056

0
4

1.59594559789866e-16
5

0
4

5.00968

5.00968

4.88968

0.0127553

0.107242

5.55111512312578e-16

0
4

0
4

0
4

1.93625

1.93625

1.93625

0.189377

1.46489

0.246733

0.0352475

2.77555756156289e-17

0
4

0
4

0
4

4.47856
6

4.47856
6

4.47856
6

4.47856
6

0
4

0
4

0
4

12.7135

12.7135

12.4214

10.4371

1.98433

0
4

0.29211

0.29211

0
4

0
4

0
4

0.730533

0.730533

0.730533

0.293745

0.23309

0.203698

0
4

0
4

0
4

0.22721

0.22721

0.157799

0.157799

0
4

0.0694108

0.0120572

0.0573536

0
4

1.38777878078145e-17

0
4

0
4

0.332325

0.332325

0.32235

0.304895

0.0174553

1.38777878078145e-17

0
4

0.00997446

0.00498723

0.00498723

0
4

2.60208521396521e-17

0
4

0
4

2.4165

2.4165

2.4165

0.644401

0.253158

0.0460286

1.47292

0
4

0
4

0
4

0.621865

0.621865

0.182968

0.0453616

0.137606

0
4

0.438897

0.438897

0
4

0
4

0
4

2.15817

2.15817

2.10638

2.10638

0
4

0.0428416

0.0428416

0
4

0.00447073

0.00447073

0
4

0.00447073

0.00447073

0
4

1.50920942409982e-16

0
4

0
4

0.202038

0.202038

0.202038

0.00997446

0.187076

0.00498723

1.30104260698261e-17

0
4

0
4

0
4

2.36092
7

2.36092
7

2.36092
7

2.32175
7

0.0316877

0.00748085

0
4

0
4

0
4

0.641495

0.641495

0.641495

0.309656

0.19194

0.139899

5.55111512312578e-17

0
4

0
4

0
4

0.309566

0.309566

0.309566

0.296609

0.0129576

1.90819582357449e-17

0
4

0
4

0
4

0.6621
3

0.6621
3

0.61108

0.61108

0
4

0.05102

0.05102

0
4

0
4

0
4

29.7806

29.7806

29.7806

29.7806

0
4

0
4

0
4

1.49728

1.49728

1.49728

1.49728

0
4

0
4

0
4

14.8221

14.8221

14.8221

14.8221

0
4

0
4

0
4

0.094732

0.094732

0.094732

0.0733389

0.0213931

0
4

0
4

0
4

0.414614

0.414614

0.414614

0.414614

0
4

0
4

0
4

0.234133

0.234133

0.234133

0.226623

0.00750954

0
4

0
4

0
4

1.65458

1.65458

1.65458

1.40389

0.250694

5.55111512312578e-17

0
4

0
4

0
4

26.5959

26.5959

1.34706

0.193101

0.225091

0.193101

0.28791

0.12796

0.06398

0.06398

0.09597

0.09597

0
4

24.737

21.5624

1.31914

0.06398

1.2796

0.51184

0
4

0.38388

0.28791

0.09597

0
4

0.12796

0.06398

0.06398

0
4

0
4

0
4

2.09589

2.09589

2.09589

0.929845

0.0938138

1.07223

0
4

0
4

0
4

0.359652

0.359652

0.359652

0.125263

0.234389

0
4

0
4

0
4

3.82119

3.82119

3.82119

3.82119

0
4

0
4

0
4

0.0836407

0.0836407

0.0836407

0.0274815

0.00458025

0.0515789

6.93889390390723e-18

0
4

0
4

0
4

0.633561

0.633561

0.633561

0.633561

0
4

0
4

0
4

0.0370088

0.0370088

0.0370088

0.0370088

0
4

0
4

0
4

0.60781

0.60781

0.44786

0.25592

0.19194

0
4

0.15995

0.09597

0.06398

1.38777878078145e-17

0
4

0
4

0
4

1.58469

0.316761

0.2025

0.2025

0
4

0.114261

0.114261

0
4

1.38777878078145e-17

0
4

1.26792

1.26792

1.26792

0
4

0
4

0
4

1.22716

1.22716

1.22716

1.09607

0.131094

1.66533453693773e-16

0
4

0
4

0
4

6.03062

6.03062

6.03062

6.03062

0
4

0
4

0
4

3.21529
7

3.21529
7

3.21529
7

2.66113
7

0.0683992

0.390185

0.0843364

0.0112377

1.05818132034585e-16
7

0
4

0
4

0
4

17.6238

17.6238

17.4076

9.56308

7.84451

0
4

0.216187

0.216187

0
4

0
4

0
4

1.10169

1.10169

1.10169

1.10169

0
4

0
4

0
4

2.20248

2.20248

2.20248

2.20248

0
4

0
4

0
4

3.49789

3.49789

3.49789

3.49789

0
4

0
4

0
4

1.06418

1.06418

1.06418

0.0656434

0.998535

1.11022302462516e-16

0
4

0
4

0
4

1.60835

1.60835

1.60835

1.60835

0
4

0
4

0
4

4.76203

4.76203

4.76203

4.75202

0.0100127

0
4

0
4

0
4

0.0199489

0.0199489

0.0199489

0.00498723

0.00997446

0.00498723

8.67361737988404e-19

0
4

0
4

0
4

0.516667

0.516667

0.491159

0.0792192

0.41194

0
4

0.0255083

0.0255083

0
4

1.04083408558608e-17

0
4

0
4

2.65492

2.65492

2.51502

2.51502

0
4

0.139899

0.139899

0
4

5.55111512312578e-17

0
4

0
4

8.7918
6

8.7918
6

4.9641

1.58042

2.46869

0.600684

0.0259152

0.288392

0
4

0.40778

0.125465

0.0469069

0.115597

0.119811

1.38777878078145e-17

0
4

3.41991

3.41991

0
4

8.88178419700125e-16
6

0
4

0
4

0.654419

0.654419

0.654419

0.654419

0
4

0
4

0
4

0.175586

0.175586

0.175586

0.175586

0
4

0
4

0
4

1.19001

1.19001

0.900041

0.900041

0
4

0.289974

0.289974

0
4

0
4

0
4

2.95746

2.95746

2.95746

0.0846347

0.0798828

2.79294

0
4

0
4

0
4

0.737937

0.661772

0.661772

0.661772

0
4

0
4

0.0761658

0.0761658

0.0761658

0
4

0
4

1.38777878078145e-17

0
4

1.09475

1.09475

1.09475

1.09475

0
4

0
4

0
4

0.759593

0.759593

0.310612

0.0548139

0.255798

0
4

0.448981

0.448981

0
4

5.55111512312578e-17

0
4

0
4

0.177224

0.177224

0.177224

0.177224

0
4

0
4

0
4

0.749287

0.749287

0.749287

0.21317

0.536117

0
4

0
4

0
4

0.241896

0.241896

0.241896

0.241896

0
4

0
4

0
4

9.83028
1

1.36876

1.36876

1.36876

0
4

0
4

1.29776

1.29776

1.29776

0
4

0
4

0.385324

0.385324

0.385324

0
4

0
4

0.032994

0.032994

0.025662

0.007332

0
4

0
4

0.30983

0.30983

0.30983

0
4

0
4

1.83145

1.83145

1.83145

0
4

0
4

0.344978

0.344978

0.344978

0
4

0
4

0.407432

0.0481639

0.0481639

0
4

0.359268

0.359268

0
4

0
4

0.72098

0.72098

0.72098

0
4

0
4

0.084709

0.084709

0.0327735

0.0519355

0
4

0
4

0.258242

0.258242

0.258242

0
4

0
4

2.78782
1

2.67768
2

0.722459

1.22818

0.142612

0.0481639

0.0481639

0.216294

0.168574

0.0404642

0.0627716

0
4

0.11014

0.11014

0
4

0
4

4.44089209850063e-16
1

0
4

240.516
7

0.279195

0.279195

0.273756

0.00543897

0
4

0
4

0.129531

0.129531

0.129531

0
4

0
4

0.0582163

0.0582163

0.0582163

0
4

0
4

0.00822417

0.00822417

0.00822417

0
4

0
4

0.00475254

0.00475254

0.00475254

0
4

0
4

0.0770648

0.0770648

0.0770648

0
4

0
4

0.0127553

0.0127553

0.0127553

0
4

0
4

239.946
7

234.465
7

234.215
7

0.250694

4.27435864480685e-15
7

0
4

4.01203
7

4.01203
7

0
4

0.320084

0.320084

0
4

0.798483

0.798483

0
4

0.0549439

0.0549439

0
4

0.0366292

0.0366292

0
4

0.219775

0.219775

0
4

0.0155008

0.0155008

0
4

0.0232512

0.0232512

0
4

0
4

0
4

143.747
3

134.396
3

134.115
3

61.097

0.23368

0.355811

0.386612

0.179382

0.24499

0.097466

0.206854

0.151349

0.449848

0.239257

59.3896
3

0.054841

1.32275

0.077809

0.0515724

0.155618

0.190155

0.077809

0.0950773

0.0598143

0.0724936

2.64966
2

0.0681929

0.0877713

0.155618

0.0423112

1.86538
3

1.52483
2

0.466028
3

0.564291
2

0.572065
3

0.928842
2

4.88498130835069e-14
3

0
4

0.164174

0.164174

0
4

0.116618

0.116618

0
4

4.8988590961585e-15
3

0
4

2.18254

2.18254

1.78122

0.388357

0.0129576

0
4

0
4

5.35931

5.35931

5.26288

0.0964329

0
4

0
4

0.0907031

0.0907031

0.0907031

0
4

0
4

0.231323

0.231323

0.231323

0
4

0
4

0.815672

0.815672

0.0964329

0.719239

0
4

0
4

0.451397

0.356334

0.356334

0
4

0.095063

0.095063

0
4

0
4

0.0973151

0.0809283

0.0809283

0
4

0.0163868

0.0163868

0
4

3.46944695195361e-18

0
4

0.0460286

0.0460286

0.0460286

0
4

0
4

0.0770648

0.0770648

0.0770648

0
4

0
4

0
4

111.665
7

0.0767062

0.0767062

0.0767062

0
4

0
4

0.0409669

0.0409669

0.0409669

0
4

0
4

0.181406

0.181406

0.181406

0
4

0
4

0.305067

0.305067

0.305067

0
4

0
4

0.0259152

0.0259152

0.0259152

0
4

0
4

0.00448724

0.00448724

0.00448724

0
4

0
4

0.0404642

0.0404642

0.0404642

0
4

0
4

0.0809283

0.0809283

0.0809283

0
4

0
4

0.0606962

0.0606962

0.0606962

0
4

0
4

0.0404642

0.0404642

0.0404642

0
4

0
4

0.0404642

0.0404642

0.0404642

0
4

0
4

110.767
7

109.235
7

87.3868
7

8.71844
5

0.083451

0.113332

0.102705

0.0784464

0.65547

0.211081

0.299604

0.00689936

0.0259152

0.0419522

0.561563
7

0.359433

0.0327735

0.0404642

0.0674692

0.10116

0.0599054

0.00689936

0.119694

0.0112449

0.269727

3.69002
6

0.0852543

0.0714027

0.0571222

0.82388

0.357411

0.157873

0.139003

0.0581373

0.179716

0.0599054

1.08021

0.0732585

0.105743

0.871877
7

1.28192

0.325047

0.138793

0.324536

0
4

1.43278

1.43278

0
4

0.0583091

0.0583091

0
4

0.0404642

0.0404642

0
4

1.74929515317501e-14
7

0
4

0
4

67.6903

21.6016
5

21.4929
5

10.2487

0.0194758

0.0404642

0.00458879

5.24674
5

5.07675
6

0.399837

0.0988051

0.335437

0.00611838

0.00305919

0.0129576

0
4

0.108726

0.108726

0
4

0
4

35.2024
5

35.1993
5

35.1134
5

0.00615279

0.0404642

0.00764798

0.0316877

0
4

0.00305919

0.00305919

0
4

0
4

0.329317

0.329317

0.329317

0
4

0
4

0.763337

0.763337

0.763337

0
4

0
4

0.449291

0.449291

0.449291

0
4

0
4

9.34431

8.22147
7

0.2109

0.00748085

0.0233374

0.00448724

0.00518609

0.00518609

0.0426287

0.346792

0.00673085

0.106826

0.00777913

1.34218

0.00673085

0.00448724

0.0134617

0.271478

2.77467

0.385324

2.40324

0.159591

0.0674692

0.0255083

4.60395610524245e-15
7

0
4

0.0605777

0.047116

0.00448724

0.00897447

0
4

0.00448724

0.00448724

0
4

1.04655

1.04655

0
4

0.0112181

0.0112181

0
4

0
4

1.59872115546023e-14

0
4

81.3226

2.8648

2.75813

0.552358

0.488287

0.0679262

0.699268

0.822613

0.0177653

0.0380203

0.0473073

0.0245801

8.32667268468867e-17

0
4

0.0583418

0.0533354

0.00500636

0
4

0.0483291

0.0483291

0
4

6.93889390390723e-17

0
4

12.9189
5

12.9189
5

10.8076
5

0.0898581

0.0100682

0.0898581

1.13712
7

0.162946

0.0125853

0.253157

0.0100064

0.0112377

0.00503411

0.32948

0
4

0
4

0.136677

0.13169

0.101766

0.0299234

0
4

0.00498723

0.00498723

0
4

1.30104260698261e-17

0
4

0.700576

0.700576

0.700576

0
4

0
4

0.174412

0.174412

0.174412

0
4

0
4

0.149012

0.149012

0.149012

0
4

0
4

0.522171

0.522171

0.522171

0
4

0
4

6.13898

6.13898

6.13898

0
4

0
4

0.220279

0.220279

0.207321

0.0129576

0
4

0
4

0.218562

0.218562

0.00748085

0.211081

0
4

0
4

0.162984

0.162984

0.162984

0
4

0
4

0.0581373

0.0581373

0.0581373

0
4

0
4

22.6258
5

20.8552
5

18.6156
5

1.89476

0.0703603

0.0581004

0.119811

0.0809283

0.0156497

0
4

0.0605874

0.0605874

0
4

1.70996

1.70996

0
4

5.99520433297585e-15
5

0
4

0.0107775

0.0107775

0.0107775

0
4

0
4

0.0622331

0.0622331

0.0622331

0
4

0
4

0.0591856

0.0591856

0.0591856

0
4

0
4

0.0100616

0.0100616

0.0100616

0
4

0
4

1.70996

1.70996

1.70996

0
4

0
4

0.308259

0.308259

0.308259

0
4

0
4

0.0213184

0.0213184

0.0213184

0
4

0
4

0.010272

0.010272

0.010272

0
4

0
4

0.0599054

0.0599054

0.0599054

0
4

0
4

0.290687

0.290687

0.290687

0
4

0
4

1.92708
7

1.92708
7

0.761436
7

0.167721

0.289806
7

0.27515
7

0.0220942

0.0791236

0.307825

0.0156497

0.00827008

2.4459601011273e-16
7

0
4

0
4

7.62144
5

4.26691
5

0.670328

0.108405

1.17441

0.0590325

1.97515

0.193316

0.0469069

0.039357

0
4

3.35453

1.05638

0.039007

0.0843364

2.17481

0
4

1.77635683940025e-15
5

0
4

17.8031
5

17.7931
5

17.2776

0.125913

0.0678326

0.203351

0.0483291

0.070089

0
4

0.0100127

0.00500636

0.00500636

0
4

0
4

0.609836

0.604849

0.604849

0
4

0.00498723

0.00498723

0
4

1.30104260698261e-17

0
4

2.78538

2.78099

2.7634

0.0102568

0.00732626

0
4

0.00439576

0.00439576

0
4

9.54097911787244e-18

0
4

0.11589
7

0.11589
7

0.0506431

0.0388957

0.0107775

0.0103722

0.00520121

3.29597460435593e-17
7

0
4

0
4

1.02593

0.886027

0.886027

0
4

0.139899

0.139899

0
4

0
4

2.1316282072803e-14

0
4

418.941

13.1557

13.1075

8.52319

1.07106

0.238118

2.02288

0.0963278

1.01144

0.0963278

0.0481639

0
4

0.0481639

0.0481639

0
4

0
4

0.0421348

0.0421348

0.0153989

0.00520121

0.0215348

0
4

0
4

0.909077

0.909077

0.363631

0.545446

0
4

0
4

2.01113

1.89072

1.81848

0.0722459

6.93889390390723e-17

0
4

0.12041

0.12041

0
4

0
4

0.312481

0.312481

0.248192

0.0642886

0
4

0
4

0.020433

0.0100682

0.0100682

0
4

0.0103648

0.0103648

0
4

0
4

0.119615

0.119615

0.119615

0
4

0
4

0.244888

0.244888

0.244888

0
4

0
4

0.00882338

0.00882338

0.00882338

0
4

0
4

1.22027

0.0518304

0.0518304

0
4

1.16844

1.16844

0
4

0
4

0.32394

0.32394

0.32394

0
4

0
4

2.09118

2.09118

1.08488

0.965837

0.0404642

0
4

0
4

0.0316877

0.0316877

0.0316877

0
4

0
4

1.32905

1.32905

1.32905

0
4

0
4

0.0739218

0.0739218

0.0739218

0
4

0
4

0.0704951

0.0704951

0.0704951

0
4

0
4

0.174282

0.174282

0.174282

0
4

0
4

0.0103648

0.0103648

0.0103648

0
4

0
4

0.00690984

0.00690984

0.00690984

0
4

0
4

1.03491

1.03491

1.03491

0
4

0
4

0.079445

0.079445

0.079445

0
4

0
4

0.0104881

0.0104881

0.0104881

0
4

0
4

1.10654

0.91651

0.319684

0.596827

0
4

0.190027

0.177272

0.0127553

0
4

0
4

0.115597

0.115597

0.115597

0
4

0
4

0.0367698

0.0367698

0.0367698

0
4

0
4

4.7272

4.7272

4.7272

0
4

0
4

0.125857

0.125857

0.125857

0
4

0
4

2.56494

2.56494

2.56494

0
4

0
4

1.70996

1.70996

1.70996

0
4

0
4

0.272109

0.272109

0.226758

0.0453516

0
4

0
4

10.7619

10.7165

0.0963278

10.572

0.0481639

0
4

0.045462

0.045462

0
4

8.32667268468867e-16

0
4

0.337019

0.277118

0.134585

0.142533

0
4

0.0194364

0.0194364

0
4

0.0404642

0.0404642

0
4

0
4

0.0618186

0.0393798

0.0393798

0
4

0.0224388

0.0170056

0.00543325

0
4

0
4

1.1599

1.1599

1.11157

0.0483291

9.0205620750794e-17

0
4

0
4

0.259498

0.117708

0.0722459

0.045462

0
4

0.14179

0.045462

0.0963278

1.38777878078145e-17

0
4

0
4

372.42

358.428

327.421

7.86919

0.0649194

0.00500636

0.0397225

0.00687037

0.0397225

0.0397225

0.0352475

0.193641

1.61857

0.261974

8.69923

0.0397225

0.318177

0.0595838

6.77539
3

1.22703

0.161184

3.19075

0.223054

0.0993063

0.0397225

0
4

0.484473

0.408797

0.0377605

0.0114506

0.00922919

0.00687037

0.0103648

0
4

0.00362217

0.00362217

0
4

0.432588

0.432588

0
4

0.432588

0.432588

0
4

0.0208779

0.0208779

0
4

0.032394

0.032394

0
4

5.99826

5.99826

0
4

0.0340111

0.0340111

0
4

2.21829

2.08231

0.0129576

0.0975327

0.0129576

0.0125267

0
4

3.04541

3.04541

0
4

0.0217188

0.0217188

0
4

0.0453516

0.032394

0.0129576

0
4

0.370099

0.370099

0
4

0.722169

0.722169

0
4

0.122949

0.122949

0
4

0.00690984

0.00690984

0
4

0
4

0
4

18.1173
5

1.06911
6

1.03105

0.371444

0.114706

0.0174553

0.427968

0.00710613

0.00448724

0.0497429

0.0381415

2.15105711021124e-16

0
4

0.0111056

0.00611838

0.00498723

8.67361737988404e-19

0
4

0.0112181

0.00673085

0.00448724

8.67361737988404e-19

0
4

0.0112449

0.0112449

0
4

0.00448724

0.00448724

0
4

1.01481323344643e-16
6

0
4

10.251

6.20159
5

5.39978
6

0.170996

0.422892

0.0793941

0.01054

0.0829123

0.0104881

0.0245801

1.0096090630185e-15
5

0
4

4.04485

3.97659

0.0682623

3.05311331771918e-16

0
4

0.00458879

0.00458879

0
4

0
4

0.0907031

0.0907031

0.0907031

0
4

0
4

0.0724936

0.0724936

0.0724936

0
4

0
4

0.0177653

0.0177653

0.0177653

0
4

0
4

0.00917758

0.00917758

0.00917758

0
4

0
4

0.00305919

0.00305919

0.00305919

0
4

0
4

3.55135
5

3.55135
5

2.00049
6

0.382964

0.176375

0.880691

0.0501388

0.0606962

0
4

0
4

2.2234

2.2234

0.153722

0.632207

0.198908

1.1599

0.0786638

5.55111512312578e-17

0
4

0
4

0.132718
5

0.0527734

0.0240923

0.0195035

0.00611838

0.00305919

2.60208521396521e-18

0
4

0.0749569

0.0749569

0
4

0.00498723

0.00498723

0
4

1.30104260698261e-17
5

0
4

0.214163

0.214163

0.214163

0
4

0
4

0.10116

0.10116

0.0404642

0.0606962

0
4

0
4

0.323713

0.323713

0.323713

0
4

0
4

0.0404642

0.0404642

0.0404642

0
4

0
4

0.0170056

0.0170056

0.0170056

0
4

0
4

0
4

8.09547
3

0.248035

0.248035

0.178992

0.069043

0
4

0
4

0.049085

0.049085

0.049085

0
4

0
4

7.79835
3

4.08329

4.08329

0
4

3.41745

1.33483

2.02303

0.0595838

0
4

0.116921

0.116921

0
4

0.140221

0.140221

0
4

0.0404642

0.0404642

0
4

0
4

8.88178419700125e-16
3

0
4

218.405

28.5329

28.4368

23.8224
7

0.0201232

0.0452773

0.0100616

0.00394831

0.0402465

0.0173753

0.0549439

0.0201232

0.0100616

0.863408
8

1.66839
8

1.56915
1

0.186325

0.0452773

0.0138191

0.0317773

0.0140099

1.19036724921529e-14

0
4

0.064318

0.0503081

0.0100616

0.00394831

0
4

0.0317773

0.0217157

0.0100616

3.46944695195361e-18

0
4

0
4

0.516324

0.516324

0.497192

0.019133

0
4

0
4

5.97714

5.86042

5.627

0.155618

0.077809

2.91433543964104e-16

0
4

0.116713

0.116713

0
4

0
4

0.294833

0.131037

0.0655183

0.0655183

0
4

0.163796

0.0655183

0.0982775

1.38777878078145e-17

0
4

2.77555756156289e-17

0
4

4.80864

4.80864

4.80864

0
4

0
4

0.397225

0.397225

0.397225

0
4

0
4

0.15889

0.0993063

0.0595838

0.0397225

0
4

0.0595838

0.0595838

0
4

0
4

0.0807932

0.0807932

0.0807932

0
4

0
4

0.266609

0.266609

0.114261

0.152348

0
4

0
4

1.14754

1.14754

1.14754

0
4

0
4

0.702562

0.00543325

0.00543325

0
4

0.697129

0.697129

0
4

0
4

32.8459

16.0808
7

0.0635734

0.140863

0.00592247

1.38253

0.240509

0.0267652

0.0295105

14.172

0.00815845

0.00543897

0.00543897

0
4

16.5754
1

13.9555
8

2.59468

0.025154

2.05044314860459e-15
1

0
4

0.185812

0.185812

0
4

0.00394831

0.00394831

0
4

0
4

0.0801856

0.0801856

0.0801856

0
4

0
4

2.2759

2.2759

2.22106

0.054841

0
4

0
4

0.0388728

0.0388728

0.0388728

0
4

0
4

0.0185044

0.0185044

0.0185044

0
4

0
4

0.0483291

0.0483291

0.0483291

0
4

0
4

0.0843952

0.0843952

0.0843952

0
4

0
4

0.0129576

0.0129576

0.0129576

0
4

0
4

0.0215348

0.0215348

0.0215348

0
4

0
4

0.0327735

0.0327735

0.0327735

0
4

0
4

0.0809283

0.0809283

0.0809283

0
4

0
4

1.10109

0.972508

0.347471

0.608589

0.0123363

0.00411208

0
4

0.0328967

0.0287846

0.00411208

8.67361737988404e-19

0
4

0.0915731

0.0915731

0
4

0.00411208

0.00411208

0
4

0
4

0.0887784

0.0887784

0.0887784

0
4

0
4

0.402922

0.402922

0.402922

0
4

0
4

0.0129576

0.0129576

0.0129576

0
4

0
4

0.0877713

0.0877713

0.0877713

0
4

0
4

0.152348

0.152348

0.152348

0
4

0
4

0.0809283

0.0809283

0.0809283

0
4

0
4

0.0964329

0.0964329

0.0964329

0
4

0
4

0.0129576

0.0129576

0.0129576

0
4

0
4

0.00364411

0.00364411

0.00364411

0
4

0
4

1.70996

1.70996

1.70996

0
4

0
4

0.895431

0.660565

0.636933

0.00362217

0.00362217

0.0163868

0
4

0.234867

0.0457765

0.18909

0
4

0
4

7.30619

2.19364

2.11138

0.0822615

0
4

4.75811

4.31536

0.144196

0.0822615

0.216294

9.99200722162641e-16

0
4

0.354436

0.342195

0.0122405

0
4

0
4

1.24571
3

1.00789
3

0.348665

0.234065

0.0367698

0.0434322

0.0582163

0.0582163

0.114261

0.114261

0
4

0.161649

0.161649

0
4

0.0761741

0.0761741

0
4

1.80411241501588e-16
3

0
4

4.19572
3

4.19572
3

1.73524

2.29238

0.0409669

0.0163868

0.11075

7.21644966006352e-16
3

0
4

0
4

2.23726
6

0.289209

0.23599

0.0127553

0.0404642

0
4

1.70996

1.70996

0
4

0.23809

0.23809

0
4

0
4

4.75941

4.75941

4.53089

0.152348

0.0761741

0
4

0
4

115.592

87.587

4.14
7

1.76208

0.0894233

0.0919557

0.284666

0.0774299

2.76619

0.0573989

0.0291946

0.433784

1.752

23.8172

0.519655

0.288392

0.0127553

0.0950773

0.219428

0.00750954

0.239715

0.014664

0.131657

0.144196

0.24735

0.0633849

0.00364411

0.315276

0.0102802

0.129576

0.0156497

0.0255106

0.0877713

0.0366292

0.0877713

0.506164

0.0259677

0.00701215

0.0123363

0.0404642

0.0245801

0.0469492

0.175543

0.0591856

0.0877713

0.129121

1.98159
7

0.0404642

0.196734

0.263314

0.0259152

0.0404642

0.0232512

0.0877713

0.685567

0.0633849

0.010998

30.6886

1.28072

1.70996

0.129121

0.0877713

3.80309

0.0722459

0.0173753

0.36049

0.0127553

0.0606962

3.65324

0.0173753

0.444991

2.71675

3.99680288865056e-14

0
4

8.82992

7.74285

0.71171

0.375354

3.88578058618805e-16

0
4

0.131094

0.131094

0
4

0.151031

0.151031

0
4

0.12677

0.0633849

0.0633849

0
4

0.087135

0.00520121

0.0819338

0
4

0.0582163

0.0582163

0
4

0.00750954

0.00750954

0
4

3.11212

3.11212

0
4

0.38622

0.38622

0
4

2.04819

2.04819

0
4

0.114261

0.114261

0
4

2.53702
6

2.5084
6

0.0112449

0.0173753

0
4

0.0173753

0.0173753

0
4

2.76441

2.76441

0
4

0.0259152

0.0259152

0
4

0.0255106

0.0255106

0
4

0.0724936

0.0724936

0
4

0.747557

0.0659281

0.103344

0.522061

0.0562243

3.46944695195361e-17

0
4

3.36131

3.11884

0.0982775

0.144196

0
4

1.8546
3

0.714743

1.00547

0.13439

1.66533453693773e-16
3

0
4

0.0582313

0.0253552

0.0328761

6.93889390390723e-18

0
4

0.502768

0.499124

0.00364411

0
4

0.337641

0.238335

0.0993063

2.77555756156289e-17

0
4

0.647653

0.647653

0
4

8.04911692853238e-14

0
4

0
4

39.9102
7

0.958539
6

0.958539
6

0.601128
6

0.357411

0
4

0
4

1.26386
6

1.26386
6

1.26386
6

0
4

0
4

0.157086

0.157086

0.157086

0
4

0
4

0.0173753

0.0173753

0.0173753

0
4

0
4

0.0110526

0.0110526

0.0110526

0
4

0
4

0.0284245

0.0284245

0.0284245

0
4

0
4

37.4738
7

37.4738
7

37.4738
7

0
4

0
4

0
4

0
4

57460.5

4.05663

4.05663

4.05663

1.42616

0.443694

2.18678

4.44089209850063e-16

0
4

0
4

0
4

29728.5
3

118.269

117.88

91.2508

6.625

0.191944

0.054841

0.0993063

0.0877713

0.0129576

0.0877713

0.373064

0.10116

3.56709
2

7.78367

5.54129

0.82434

0.44786

0.534476

0.175543

0.121627

0
4

0.274652

0.274652

0
4

0.114261

0.114261

0
4

0
4

28158
3

747.331

1.4348

66.8538

3.1294

0.359433

0.109888

0.38087

0.820726

0.433564

0.883861

0.356382

0.266609

0.135685

0.28239

8.19205

0.220675

5.52554

0.0259152

0.397776

2.14707

0.780261

0.0335696

7.70508

2.71395

0.0599005

20.3077

0.129573

0.823956

0.0349372

0.102492

0.303481

24.0165

0.457045

1.03013

0.0753855

0.421391

27.2055

0.0252756

2.33165

0.178464

0.0406641

0.210565

0.0583091

1.20153

2.53932

0.481413

0.291904

7.83115

1.80175

0.131963

0.136765

4.64662

0.349298

0.0259152

0.119811

4.79897

0.0518304

0.0663141

9.21191
6

0.274652

0.032394

0.0475315

0.209669

0.0512461

0.479243

0.0129576

0.0582163

0.0599054

0.397885

2.32341
7

0.0606962

0.063149

0.0512461

0.0156497

0.266609

0.00655526

0.723654

2.91109

0.114261

0.0104024

1.90872

0.0194364

0.0195035

0.0453516

0.152348

0.00615279

0.114261

0.0761741

0.0366292

0.20715

0.0599054

0.953364

0.032394

0.114261

0.0599054

0.0599054

0.0245801

0.0761741

0.0129576

0.114261

0.0453516

0.378406

5.03867

0.0633753

0.763337

0.0129576

0.0129576

0.0173753

0.0163868

0.0599054

0.0173753

0.0460286

0.0129576

14.7509

2.64157
5

0.0129576

0.0434384

0.146299

0.0259152

0.0591622

0.06398

0.0112449

0.0761741

0.0761741

0.0129576

30.9167

0.0761741

0.00615279

0.207321

0.0391243

1.60835

2.56494

0.0453516

0.06398

2.56494

0.0184584

113.467
2

0.0129576

0.0369167

0.0975327

1.70996

0.09597

0.093266

0.0194364

0.063149

0.0469069

0.0316877

0.328357

1.25687

0.0259152

2.7761

0.0915731

0.0599054

0.0777455

0.0129576

0.093266

0.0674192

0.0599054

7.49272

0.0475315

0.358886

0.00799092

0.0404642

0.0585106

0.00827008

0.0129576

0.00503411

0.0366292

0.0129576

0.882828

0.00777913

0.0129576

0.0591856

0.193681

0.0129576

0.610345

0.0404642

0.0404642

0.0404642

0.0442094

3.806
6

0.0156497

0.06398

0.00835115

0.0761741

0.114261

0.093266

0.0898581

0.152348

0.0898581

0.278588

0.794022

0.0761741

0.0259152

0.0761741

0.0761741

0.0873245

0.0129576

0.161857

0.0129576

0.0129576

0.0873245

1.22645

0.0155008

0.266609

0.147964

0.0761741

0.0599054

0.274652

0.25592

0.0703603

0.0761741

0.0195035

2.51398

0.0194364

0.0129576

0.0591856

0.0775164

0.152348

0.0194364

0.147964

0.0341641

0.0259152

0.237064

18.0761

0.977785

0.114261

0.0194364

0.0761741

0.209669

0.451923

0.108505

0.228522

0.121392

0.039007

0.119811

3.52808

0.015382

0.0129576

0.0163868

0.0129576

0.0129576

0.0404642

0.0129576

0.0245801

0.144196

0.0194364

14.3143
2

0.114261

0.0129576

0.0898581

0.238831

0.190435

0.114261

0.0469069

0.0129576

0.0129576

0.0184584

7.43028

0.152348

0.0259152

0.152348

0.0777455

0.00748085

0.0907031

0.114261

0.032394

0.0599054

0.0195035

0.497035

0.0469069

0.190435

0.0599054

0.279798

0.06398

0.0530735

0.0761741

0.0549439

0.0761741

0.0091605

2.14707

0.207321

0.0761741

0.0582163

0.0703603

0.114261

0.032394

1.39719

0.0194364

0.0530735

0.239622

11.6165

0.68663

0.799828

0.0129576

0.0598143

0.533219

0.3199

0.0599054

0.149764

0.0129576

0.0907031

0.939872

0.68663

0.0129576

7.6555

4.26256

2.62318

39.6568

2.06233

0.218264

2.60408
6

3.00888

0.456308

0.551501
2

1.47747

0.665763

0.329186
7

3.86373

17.0948

0.528128

1.14611

0.597265

3.35464

0.832038

5.2941

1.55583

0.2138

5.52578

0.192674

19.537

0.103661

29.0754

5.54998

0.668409

6.30042

0.142533

0.370439

0.142884

1.02237

0.509196

19.7518

1.19983

0.38087

1.78524

0.0372909

2.07482

0.349298

0.266609

0.38087

0.0480437

10.512

22.4105

1.09101

0.38087

0.0273805

2.92713

0.357812

0.0908374

0.204283

0.886027

0.959871

1.25687

5.06301

1.10753

0.884087

0.170655

0.0578395

0.288331

0.114717

0.621637

0.553055

0.238831

0.119091

0
4

22265.9
4

3605.46
3

9276.33
4

5138.94
3

27.2294
6

56.6826
4

0.352041

0.433984

0.130315

0.431993

0.685567

0.459822

0.332541

1.68133

0.5209

0.536111

48.461
4

0.774946

0.750827

3.40184

2.77755

0.804457

0.350972

0.493162

13.6318

0.357694

4.51705

317.063
4

0.14769

0.286693

1.75085

0.415224

0.0343809

2.1468

0.195455

1.40922

1.13293

0.398018

153.196
4

0.435938

0.8838

0.334851

27.6552

0.35329

0.940758

0.131534

0.800305

0.163838

14.7335

32.1329
4

0.116433

0.386842

1.66443

0.591631

0.10619

1.30069

0.342783

2.44309

0.858088

2.89429

271.39
2

17.3291

6.12773

0.644133

0.0608137

0.850263

0.765687

0.354366

0.984397

0.0649673

0.121627

16.1242
3

5.23355

16.3367

0.158414

2.17203

0.221047

0.0674692

0.571306

0.142835

0.865186

0.229314

11.1419
4

2.52448

0.304696

0.654762

8.73318

0.375413

1.62768

0.052126

0.0345492

103.814

0.122993

0.601417

0.918784

1.06888

2.19722

0.271239

0.321783

0.133314

1.92594

0.110524

0.0761741

0.160891

25.8591
2

0.152348

0.0752082

1.48823

0.0873245

0.225625

0.207762

0.0582163

0.0850278

0.29211

8.61724

60.4882
3

34.2561
4

0.160891

0.0642886

1.09861

0.137606

0.108496

0.965349

2.24714

0.0695014

0.0582163

0.0723307

4.06845

0.152774

0.026063

0.0442094

0.107261

0.00690984

0.549304

0.177448

0.0582163

0.107261

0.160891

6.50361

0.574722

0.429044

0.858088

0.0442094

0.154593

0.119811

0.0173753

0.305547

0.214522

0.228522

17.9224
4

0.482674

0.185812

0.549304

0.0501388

0.0723307

0.29211

0.0598143

0.0663141

2.67168

0.0129576

5.41771

0.0665157

0.0173753

1.01297

0.198942

41.1721

1.15945

1.26943

0.0442094

0.160891

0.160891

3.53254
3

0.152348

0.0884188

0.68663

3.81669

1.52667

0.875427

0.185812

0.0884188

0.154593

0.0582163

1.71392

0.101849

0.0533257

1.75085

0.0655183

0.144661

0.275213

0.00518609

0.0488567

0.0104881

0.371625

2.5005

0.152348

0.0732851

0.137606

0.107261

0.64748

0.418957

0.101849

0.131037

0.00394831

0.858088

2.84993
4

0.0442094

0.110125

0.199707

3.57921

0.026063

0.509245

0.350171

0.319954

4.46672

0.327592

201.171
4

0.262628

0.145251

0.761796

0.100278

0.495197

0.429044

0.107261

0.268152

0.175085

0.119824

358.029
4

4.62218

0.0173753

0.885304

0.434131

0.163796

1.17732

0.160891

0.138315

0.479931

0.0582163

0.241929

1.07474

0.216187

0.0173753

0.253157

0.0752082

0.199816

0.927568

0.0663141

0.0112449

0.0442094

0.962969

35.8958

0.0104881

0.00592247

0.107261

0.185812

0.123124

0.0530735

0.0723307

0.107261

0.0582163

0.0442094

19.6534
4

0.29211

0.00364411

0.0634669

0.321783

0.0974182

3.04448

0.107261

0.107261

0.159766

0.192866

1.55528
3

0.0752082

0.175085

0.0404642

0.0488567

0.05102

0.0917376

0.116433

0.0442094

0.0173753

1.39439

1.11849

0.221047

1.35577

0.0442094

0.100278

0.0347507

0.150416

0.0104881

0.0663141

0.268152

0.116201

0.6342

0.152332

0.763337

0.108496

0.0975327

0.557437

0.229344

0.185812

0.185812

0.0442094

0.0582163

3.86314
4

0.482674

0.110652

0.0947236

0.114261

0.0761741

0.0633753

0.289299

0.0977135

0.262628

0.0829891

0.662612

0.190435

0.0846347

0.268152

0.107261

0.125347

0.0582163

0.110524

6.36726

1.39795

0.125347

3.03826

0.026063

0.0767289

0.0501388

0.29211

0.123124

0.243832

1.28433

0.0398762

0.160891

0.188785

55.6634
4

0.887909

0.180827

0.0442094

0.0442094

0.0695014

1.90834

0.121392

7.35989

0.0752082

0.0663141

0.292598

5.38047

0.509245

0.138508

0.0103648

0.429044

6.87004

2.20496

0.0173753

0.371625

1.53222

0.536305

1.06246

0.375413

0.0488567

1.02835

0.00319998

0.0442094

0.146997

0.81945

0.0617678

0.0105182

0.0442094

5.25743
2

0.0434384

0.107261

0.00987078

1.04446

0.241929

0.152348

0.184686

0.228522

0.107261

0.0761741

2.6541

0.289323

0.578842

0.0488567

2.23767
4

20.6185

0.269318

2.09995

1.20561
3

205.673
4

1.28555

13.9633

0.739965

39.1457
4

1.18679

0.771976
4

0.208504

0.11294

2.94585

2.40108

271.405
4

8.47834

8.91239

3.57156

938.619

0.352714

2.42095

1.14024

196.77

3.35037

28.0426

43.1079
4

6.79667

2.78741

1.29702

0.940216

1.85224

17.6138

0.448003

2.27543

0.809473

2.1865

7.98927
3

1.12748

0.187154

1.08916

0.139003

0.450523

0.121627

6.34521

8.92172

61.7612

1.18428

38.241
3

1.32107

0.319326

1.1127

22.5975

9.40084

0.662042

3.50557

2.51348

0.551523

2.80958

1.36912703396774e-12
4

0
4

1505.75
3

5.05816
3

225.765
3

676.417
3

85.2415
3

443.382
2

45.8692
3

4.89565
2

4.39254
1

0.0582163

0.14099

0.115597

0.0442094

0.0663141

0.0975327

1.37465

0.0582163

0.288392

0.0442094

6.54068

0.00408015

0.00835115

0.0397225

0.0403499

0.246248

0.0975327

0.984991

0.322572

0.0996905

0.0442094

0.569998

0.144196

0.0873245

0.0155008

0.0917376

0.0655183

0.950379
8

0.243797

0.397225

1.07505

0.30781

0.062599

0
4

215.692

20.554
7

2.73439
6

0.0100064

0.0573989

0.00703064

0.00665242

0.0642886

0.0229265

0.192662

0.0724163

0.0532163

0.15413

2.90585
7

0.0170056

0.06398

0.0087883

0.0123036

0.06398

0.803608

0.147079

0.0129576

0.00799092

0.0964329

0.15289
7

0.00351532

0.00475254

0.0591856

0.0432407

0.110309

0.0913565

0.0087883

0.0255106

0.00750954

0.0761741

5.53451
5

0.0123036

0.893528

0.0281226

0.0163868

0.0123036

0.0761741

0.019133

0.00703064

0.0730916

0.0387582

3.69869
6

0.0199573

0.128577

0.121392

0.152348

0.00351532

0.0080381

0.0367698

0.0237627

0.0318883

0.523947

0.271239
7

0.0965551

0.0501388

0.0703603

0.357411

0.0112377

0.00527298

0.0482912

0.0365426

2.56494

0.00690984

0.117431

0.0642886

0.0255106

0.165464

0.0642886

0.00351532

0.309348

0.00750954

0.00615279

0.289299

0.228522

4.15149

0.175085

0.0133182

0.00351532

0.0080381

0.107242

0.00527298

0.492366

0.827936

57.2848

0.0854054
8

0.17957

0.543417

1.37152

0.104645

0.162035

0.336897

0.188776

0.194123

0.131484

14.24

0.0571053

0.0398038

2.36073

0.0670801

0.597033

0.133268

0.599625

4.4117

0.79348

3.32626

15.5059
3

0.245692

1.27084

0.0680229

0.137982

0.0200254

0.0194972

0.0276307

0.955323

0.0422143

0.504547

8.47581
7

0.232737

1.39409

0.0274881

0.045332

0.348017

0.758598

0.0573163

0.0205246

0.0283113

2.10102

34.5379
3

0.0646552

0.0921133

0.0843364

0.258741

0.429715

0.183391

0.095063

0.0511187

0.19812

0.134322

1.29137
7

0.0552897

0.0193343

0.00687037

0.0369109

0.0155008

0.0919245

0.110309

0.0310943

0.0127553

0.0087883

0.443006
1

1.70996

0.0482775

0.0735396

0.0100064

0.0482775

0.0730852

0.0642886

0.00411208

0.200984

0.00351532

4.55049

0.00997863

0.00500636

0.00351532

0.0404642

0.172251

0.00527298

0.00351532

0.0367698

0.0365426

0.147079

0
4

244.801
2

26.8667

6.29325

3.61653

22.6691

0.490302

18.1735

2.33038

13.113

0.467909

0.568218

0.443444

11.4956

1.64112

0.76776

0.494839

2.38955

1.7902

0.35189

10.7939

0.556115

0.243832

0.460089

17.0189

0.190799

0.195065

0.243832

0.28791

1.82459

0.522725

0.0842243

0.157246

0.3199

0.0975327

2.68746

0.233165

0.09597

0.093266

0.0582163

0.06398

0.0582163

0.146299

0.195065

0.06398

0.0582163

44.0049

0.15995

0.263017

0.06398

0.292598

0.195065

0.146299

0.195065

4.4865

0.438897

0.146299

2.74399

0.093266

0.233165

0.174649

0.89572

0.146299

0.0907031

0.093266

0.139899

0.093266

0.06398

11.3442

0.144196

0.715005

0.0582163

0.119168

0.292598

0.6398

0.0761741

0.139899

0.0873245

0.09597

1.12775

0.146299

0.146299

0.0975327

0.093266

0.0582163

0.0975327

0.698596

22.7489

0
4

624.791
3

523.238
3

9.79174

0.168572

0.834474

0.450138

0.0479112

0.0536449

0.0703935

0.205485

0.0111805

0.0501388

24.7136
4

0.411978

0.274652

0.0971819

0.107261

0.868263

0.243832

0.0129576

0.0404642

0.0975327

0.0792192

6.62995
4

0.032394

0.05102

0.100278

0.0501388

0.15306

0.0122713

31.3194
4

2.70298
3

9.75365
4

3.13189
3

8.96104
4

0.0247098

1.87537485540901e-13
3

0
4

16.5447
3

3.01607

0.0210364

0.40111

0.274652

4.05778
4

0.458833

2.07687

3.49883

2.62204

0.0171524

0.0501388

0.0501388

0
4

0.449664

0.449664

0
4

4.32752

4.32752

0
4

4.0517

4.0517

0
4

0.356006

0.356006

0
4

0.133335

0.044445

0.0888899

0
4

0.269654

0.269654

0
4

0.0551451

0.00500636

0.0501388

0
4

0.0744307

0.0744307

0
4

0.203097

0.203097

0
4

4.0827

0.00546617

4.07723

0
4

5.31612
3

1.59358
4

0.637604
3

1.00621
3

0.497223
3

0.436539

0.445043

0.395137

0.304785

1.66533453693773e-16
3

0
4

4.74121

4.74121

0
4

0.453268

0.106651

0.346617

0
4

0.266609

0.266609

0
4

0.139788

0.0599054

0.0798828

0
4

0.261169

0.261169

0
4

3.59473

3.59473

0
4

0.37259

0.284171

0.0884188

0
4

0.0029305

0.0029305

0
4

0.998535

0.998535

0
4

1.70996

1.70996

0
4

156.408
3

146.933
3

2.82566

1.99599

2.48909

0.207762

0.138508

0.330374

1.48823

0
4

0.447037

0.447037

0
4

0.326431

0.326431

0
4

0.0352475

0.0352475

0
4

0.144661

0.144661

0
4

1.55528

1.55528

0
4

1.02197

1.02197

0
4

0.477661

0.477661

0
4

0.596827

0.596827

0
4

0.0798828

0.0798828

0
4

0.114261

0.114261

0
4

72.7839
2

4.26304
2

7.82933

4.74944

43.3567

5.21773

7.10239

0.265256

0
4

0.00876519

0.00876519

0
4

1.04655

1.04655

0
4

1.1583

1.1583

0
4

0.0599054

0.0599054

0
4

0.119811

0.119811

0
4

0.0129576

0.0129576

0
4

1.39795

1.39795

0
4

0.0798828

0.0798828

0
4

0.00732626

0.00732626

0
4

0.0798828

0.0798828

0
4

12.7025

11.0475

1.02097

0.177845

0.309466

0.0582163

0.0442094

0.0442094

0
4

0.133314

0.133314

0
4

0.0761741

0.0761741

0
4

1.33305

1.33305

0
4

0.0975327

0.0975327

0
4

0.360351

0.360351

0
4

2.69164

2.69164

0
4

0.262628

0.262628

0
4

0.0173753

0.0173753

0
4

0.00319998

0.00319998

0
4

2.13287

2.13287

0
4

80.7189
4

63.212
4

16.5827

0.763337

0.160891

1.69864122767649e-14
4

0
4

2.43832

2.43832

0
4

0.228522

0.228522

0
4

0.0236867

0.0236867

0
4

0.342783

0.342783

0
4

1.40922

1.40922

0
4

0.0761741

0.0761741

0
4

0.0761741

0.0761741

0
4

0.0761741

0.0761741

0
4

0.294833

0.294833

0
4

0.763337

0.763337

0
4

5.35015

4.36189

0.70821

0.0488567

0.231194

9.43689570931383e-16

0
4

0.00690984

0.00690984

0
4

0.196555

0.196555

0
4

0.326179

0.326179

0
4

1.16582

1.16582

0
4

0.0655183

0.0655183

0
4

0.239648

0.239648

0
4

0.589665

0.589665

0
4

1.12812

1.12812

0
4

0.0799885

0.0799885

0
4

0.180827

0.180827

0
4

8.02055
3

7.63706
3

0.277017

0.0511526

0.0553261

7.21644966006352e-16
3

0
4

0.19194

0.19194

0
4

0.0761741

0.0761741

0
4

0.720702

0.720702

0
4

2.04579

2.04579

0
4

1.70996

1.70996

0
4

0.173753

0.173753

0
4

0.139029

0.139029

0
4

0.0599054

0.0599054

0
4

2.62628

2.62628

0
4

0.0797524

0.0797524

0
4

24.5086
4

9.86297
4

1.97801

12.6026

0.0649455

3.6498581934552e-15
4

0
4

0.268152

0.268152

0
4

0.0649455

0.0649455

0
4

0.254623

0.254623

0
4

0.0975327

0.0975327

0
4

0.0761741

0.0761741

0
4

0.169269

0.169269

0
4

0.0666674

0.0666674

0
4

0.095063

0.095063

0
4

0.123124

0.123124

0
4

0.0390837

0.0390837

0
4

12.9916

7.034

1.55171

2.3189

1.00486

1.08215

0
4

2.12935

2.12935

0
4

0.0157773

0.0157773

0
4

0.0475315

0.0475315

0
4

0.0530735

0.0530735

0
4

0.994712

0.994712

0
4

0.0157321

0.0157321

0
4

0.0770648

0.0770648

0
4

2.67168

2.67168

0
4

0.0404642

0.0404642

0
4

0.0975327

0.0975327

0
4

61.5294
3

25.9231
3

0.843214

0.0709862

0.366956

0.753342

0.0975327

0.0501388

0.0049252

0.00350607

0.0723307

0.00525911

14.5299
4

0.00525911

0.0501388

0.0975327

0.00507012

0.00701215

0.107261

0.0039381

2.79481
4

8.68203
4

1.79552
3

1.77764

3.06709

0.336829

0.0780946

0
4

54.003

29.7776

23.8657

0.279801

0.0798828

3.31679128606766e-15

0
4

0.0229265

0.0229265

0
4

0.342783

0.342783

0
4

0.0173753

0.0173753

0
4

0.116275

0.116275

0
4

0.262073

0.262073

0
4

14.3488
3

12.3042
3

0.396664

1.64791

4.44089209850063e-16
3

0
4

9.7122
4

8.34783

1.06028

0.289421

0.007332

0.007332

0
4

65.2392
2

51.6309

6.16997

1.027

2.12205

0.262073

3.78406

0.243152

2.52575738102223e-15
2

0
4

3.31113

0.641088

0.664186

0.426566

0.399414

0.268152

0.429044

0.107261

0.375413

2.77555756156289e-16

0
4

3.73386

3.67304

0.0608137

0
4

1.75784
3

0.566212

0.488688

0.259125

0.277017

0.101849

0.0649455

0
4

2.07818
3

0.736354

0.475907

0.415525

0.138508

0.101849

0.0917376

0.118299

0
4

6.73086

5.05593

1.67493

0
4

0.941859
2

0.672987

0.0761741

0.0761741

0.0403499

0.0761741

0
4

762.874
3

720.191
3

11.6993

0.260461

1.35906

0.0536449

0.519595

0.101849

0.274652

0.150416

0.100278

0.0049252

5.67731
4

0.438897

5.09586

7.32795
4

0.317638

0.524147

3.9616

3.30845

1.50715

0
4

2.84168
3

0.722728

1.63702

0.263448

0.0799732

0.138508

5.55111512312578e-16
3

0
4

5.35797
4

2.47526

1.52667

0.0501388

0.00350607

1.30239

2.22044604925031e-16
4

0
4

3.79308

3.79308

0
4

2.34142
4

1.3754
4

0.214522

0.0511526

0.700341

2.22044604925031e-16
4

0
4

11.7191

10.942

0.777151

8.88178419700125e-16

0
4

25.8334

25.5704

0.154593

0.108496

0
4

16.6541
4

10.2454
4

6.40873

0
4

2.92102
5

2.91595
5

0.00507012

3.11382863937837e-16
5

0
4

12.4291
2

0.442474

0.0442094

0.378406

11.4985

0.0655183

5.41233724504764e-16
2

0
4

5.8682
4

5.8682
4

0
4

12.943
5

7.20463
5

0.145874

0.0430686

0.0434384

0.187634

0.0173753

0.139003

0.0173753

0.0173753

0.495197

0.0347507

1.45561

0.0177674

0.173753

0.026063

0.00394831

0.0173753

0.0173753

1.51215

0.428123

0.467318

0.130315

0.121627

0.11294

0.11294

2.44249065417534e-15
5

0
4

8.34941
4

7.39845

0.950965

0
4

0.330606

0.110796

0.119532

0.100278

1.38777878078145e-17

0
4

0.665441

0.665441

0
4

0.759098

0.213091

0.439355

0.106651

0
4

0.668434
4

0.156179

0.512256

1.11022302462516e-16
4

0
4

1.26788

0.68663

0.183475

0.123124

0.274652

0
4

0.588576

0.474315

0.114261

1.38777878078145e-17

0
4

0.631309

0.186696

0.299072

0.0582163

0.0873245

0
4

18.4957

18.4957

0
4

2.40147

2.40147

0
4

46.9979
6

43.4968

0.0772808

0.0347507

0.0434384

0.0608137

0.0434384

0.0173753

0.0173753

0.0173753

0.0173753

0.0173753

0.381097

0.0434384

0.0173753

0.026063

0.026063

0.0173753

0.0173753

0.0608137

0.026063

0.0347507

0.0173753

0.104252

0.0347507

0.0173753

0.243255

0.353357

0.495197

0.11964

0.163249

0.920893

0.0347507

0
4

1.25216
2

0.473464

0.778693

1.11022302462516e-16
2

0
4

5.40836

3.65636

1.67583

0.0761741

1.80411241501588e-16

0
4

0.8229

0.807168

0.0157321

7.97972798949331e-17

0
4

4.2237

4.11978

0.100278

0.00364411

0
4

1.20893

0.174282

0.763337

0.271308

0
4

0.401775

0.212539

0.093266

0.09597

4.16333634234434e-17

0
4

1.08821

0.078622

1.00958

2.22044604925031e-16

0
4

0.301316

0.301316

0
4

0.338548

0.143483

0.195065

0
4

3.22611

2.87131

0.175085

0.179716

0
4

513.217
2

472.388
2

38.6955
2

0.783099

0.712254

0.304696

0.12796

0.0761741

0.129121

0
4

1.40922

1.40922

0
4

0.910208

0.553737

0.356472

0
4

0.261405

0.261405

0
4

7.5106

0.2589

7.2517

0
4

0.947122

0.947122

0
4

0.620971

0.620971

0
4

1.0719

1.0719

0
4

1.83789

1.50813

0.114261

0.152348

0.063149

4.16333634234434e-17

0
4

0.309488

0.180367

0.129121

0
4

0.0489653

0.0489653

0
4

146.726
3

11.45
4

0.160891

0.0917376

0.0553261

0.0511526

0.29211

0.163089

0.275213

0.0723307

8.7954

0.145251

73.0207

1.73579

0.125347

0.709793

3.25495

0.107261

0.0553261

0.101849

5.54057

0.0798828

0.68663

4.51754

28.6661

0.754102

0.768579

0.503943

2.89714

1.64791

0
4

0.78433

0.78433

0
4

3.59486

3.59486

0
4

0.216294

0.021996

0.194298

0
4

5.02167

4.66132

0.360351

0
4

0.117653

0.0675141

0.0501388

0
4

0.431687

0.157035

0.274652

0
4

1.24442

1.13016

0.114261

6.93889390390723e-17

0
4

11.4845

10.9997

0.484779

6.10622663543836e-16

0
4

1.37578

1.33532

0.0404642

0
4

0.255954

0.190435

0.0655183

0
4

105.843
6

102.919
6

0.0347507

0.200555

0.0173753

1.42588

0.617819

0.173753

0.349666

0.0173753

0.052126

0.0173753

0.0173753

1.1220191442618e-14
6

0
4

4.59591

4.5517

0.0442094

1.38777878078145e-17

0
4

0.21317

0.174412

0.0387582

0
4

0.210322

0.210322

0
4

0.325502

0.150416

0.175085

2.77555756156289e-17

0
4

1.91355

1.91355

0
4

2.53668

2.53668

0
4

0.107358

0.063149

0.0442094

0
4

0.0347507

0.0173753

0.0173753

0
4

0.132628

0.132628

0
4

0.552344

0.552344

0
4

54.321
4

47.1405
4

2.83001
4

1.59474
4

0.604483

0.870598

0.00350607

0.101849

0.411978

0.763337

0
4

0.131832

0.131832

0
4

0.408733

0.408733

0
4

3.05335

3.05335

0
4

0.16979

0.16979

0
4

9.16005

0.763337

8.39671

0
4

0.74466

0.712973

0.0316877

4.85722573273506e-17

0
4

0.196555

0.196555

0
4

0.374585

0.294833

0.0797524

0
4

0.956978

0.956978

0
4

16.4921

0.0129576

16.4791

0
4

1.34320998768089e-10
3

0
4

260.865
2

253.977
2

94.9733
2

1.53653

2.78864

8.05414

2.45083

2.34977

4.63104

3.34508

2.45518

0.321668

0.721184

30.4574

0.220251

0.327592

0.327592

0.197848

0.163796

0.12453

0.156494

0.982775

0.590261

0.109728

36.9884
2

0.556906

0.0655183

0.294833

0.0982775

0.0982775

0.0582163

0.0655183

0.0982775

0.174649

0.0582163

7.92632

0.0442094

0.0663141

0.0655183

3.63107

0.0655183

0.0582163

0.163796

0.0797524

0.0982775

0.063149

7.64718

0.0655183

0.556906

0.145541

0.0582163

0.989678

6.48632

4.88112

0.0982775

0.0655183

0.0884188

2.05653

0.0982775

0.0398762

0.294833

0.327592

0.0655183

0.198942

0.0655183

0.0663141

0.950016

0.146299

3.09156

0.163796

7.69841

0.0442094

0.116433

0.0442094

0.0947236

7.24322

1.98298

5.75095526755831e-14
2

0
4

5.59582
2

2.71901

0.163796

0.0655183

0.356797

1.41072

0.0840856

0.191816

0.414816

0.0655183

0.0582163

0.0655183

0
4

1.01316

0.418567

0.123735

0.0582163

0.0982775

0.0982775

0.157873

0.0582163

3.46944695195361e-17

0
4

0.212949

0.0442094

0.0442094

0.0582163

0.0663141

1.38777878078145e-17

0
4

0.0663141

0.0663141

0
4

1.51129109227099e-14
2

0
4

1.93886

1.91294

0.593007
7

0.883188

0.237733

0.175658

0.0103722

0.0129839

9.54097911787244e-17

0
4

0.0209134

0.0209134

0
4

0.00500636

0.00500636

0
4

0
4

0.797429

0.217481

0.217481

0
4

0.579949

0.579949

0
4

0
4

0.240764

0.196555

0.196555

0
4

0.0442094

0.0442094

0
4

1.38777878078145e-17

0
4

0.0884188

0.0442094

0.0442094

0
4

0.0442094

0.0442094

0
4

0
4

0.0518304

0.0518304

0.0388728

0.0129576

0
4

0
4

0.161821

0.0975327

0.0975327

0
4

0.0642886

0.0642886

0
4

0
4

0.0881189

0.0881189

0.0881189

0
4

0
4

1.71392

1.71392

1.71392

0
4

0
4

0.685567

0.685567

0.685567

0
4

0
4

4.89351

4.76088

4.76088

0
4

0.132628

0.132628

0
4

0
4

0.878711

0.878711

0.878711

0
4

0
4

6.67115

6.67115

6.27177

0.308646

0.0388728

0.0129576

0.0259152

0.0129839

0
4

0
4

0.628335

0.628335

0.554413

0.0739218

1.38777878078145e-17

0
4

0
4

1.42708

0.55918

0.55918

0
4

0.867896

0.867896

0
4

0
4

0.0794262

0.0794262

0.0156497

0.0637765

1.38777878078145e-17

0
4

0
4

0.142595

0.142595

0.142595

0
4

0
4

0.100278

0.100278

0.0501388

0.0501388

0
4

0
4

3.98431

3.98431

3.98431

0
4

0
4

0.180827

0.180827

0.180827

0
4

0
4

0.176042

0.176042

0.176042

0
4

0
4

0.968406

0.968406

0.968406

0
4

0
4

0.0966581

0.0966581

0.0483291

0.0483291

0
4

0
4

9.12963
7

9.12963
7

8.90079

0.0674801

0.0965751

0.0129576

0.0129576

0.0388728

0
4

0
4

0.0182206

0.0182206

0.00364411

0.0145764

1.73472347597681e-18

0
4

0
4

0.00690984

0.00690984

0.00690984

0
4

0
4

0.0938138

0.0938138

0.0938138

0
4

0
4

0.0975327

0.0975327

0.0975327

0
4

0
4

0.0993063

0.0993063

0.0993063

0
4

0
4

0.0127553

0.0127553

0.0127553

0
4

0
4

0.0404642

0.0404642

0.0404642

0
4

0
4

0.0582163

0.0582163

0.0582163

0
4

0
4

0.233165

0.233165

0.233165

0
4

0
4

0.0398762

0.0398762

0.0398762

0
4

0
4

25.4007
2

25.4007
2

20.8515
2

1.71287

0.755505

1.02835

0.129121

0.390131

0.533219

0
4

0
4

0.114261

0.114261

0.114261

0
4

0
4

0.15413

0.15413

0.15413

0
4

0
4

0.192662

0.192662

0.192662

0
4

0
4

0.0798828

0.0798828

0.0798828

0
4

0
4

0.140721

0.140721

0.140721

0
4

0
4

0.798828

0.798828

0.798828

0
4

0
4

0.117267

0.117267

0.117267

0
4

0
4

0.0761741

0.0761741

0.0761741

0
4

0
4

1.90834

1.90834

1.90834

0
4

0
4

0.0156497

0.0156497

0.0156497

0
4

0
4

3.39854

1.06237
5

0.786381

0.0103648

0.241388

0.00690984

0.00690984

0.00690984

0.00350607

3.46944695195361e-16
5

0
4

2.18917

0.311715

1.75535

0.0770648

0.00458025

0.0404642

0
4

0.146997

0.146997

0
4

0
4

0.00525911

0.00525911

0.00525911

0
4

0
4

0.0316877

0.0316877

0.0316877

0
4

0
4

0.0316877

0.0316877

0.0316877

0
4

0
4

0.00687037

0.00687037

0.00687037

0
4

0
4

0.117267

0.117267

0.117267

0
4

0
4

0.0442094

0.0442094

0.0442094

0
4

0
4

0.0798828

0.0798828

0.0798828

0
4

0
4

0.107261

0.107261

0.107261

0
4

0
4

0.438165

0.438165

0.438165

0
4

0
4

0.0796102

0.0796102

0.0796102

0
4

0
4

16.0816

16.0816

12.9204

2.28522

0.342783

0.342783

0.0761741

0.114261

0
4

0
4

0.0770648

0.0770648

0.0770648

0
4

0
4

0.0501388

0.0501388

0.0501388

0
4

0
4

0.357411

0.357411

0.357411

0
4

0
4

0.00876519

0.00876519

0.00876519

0
4

0
4

0.29211

0.29211

0.29211

0
4

0
4

0.0475315

0.0475315

0.0475315

0
4

0
4

0.00364411

0.00364411

0.00364411

0
4

0
4

0.0442094

0.0442094

0.0442094

0
4

0
4

0.0663141

0.0663141

0.0663141

0
4

0
4

0.0655183

0.0655183

0.0655183

0
4

0
4

3.50534

3.50534

2.23826

0.778604

0.44138

0.0289471

0.0181513

2.98372437868011e-16

0
4

0
4

0.00458025

0.00458025

0.00458025

0
4

0
4

0.15922

0.15922

0.15922

0
4

0
4

0.0739218

0.0739218

0.0739218

0
4

0
4

0.0442094

0.0442094

0.0442094

0
4

0
4

0.00701215

0.00701215

0.00701215

0
4

0
4

0.0642886

0.0642886

0.0642886

0
4

0
4

0.438165

0.438165

0.438165

0
4

0
4

0.0112377

0.0112377

0.0112377

0
4

0
4

0.0761741

0.0761741

0.0761741

0
4

0
4

0.0501388

0.0501388

0.0501388

0
4

0
4

4.03991

4.03991

0.761741

0.152348

0.272697

1.56157

0.457045

0.190435

0.249881

0.203757

0.114261

0.0761741

0
4

0
4

0.0761741

0.0761741

0.0761741

0
4

0
4

0.0168673

0.0168673

0.0168673

0
4

0
4

0.0316877

0.0316877

0.0316877

0
4

0
4

0.190435

0.190435

0.190435

0
4

0
4

0.46633

0.46633

0.46633

0
4

0
4

0.0761741

0.0761741

0.0761741

0
4

0
4

0.0799732

0.0799732

0.0799732

0
4

0
4

0.0851532

0.0851532

0.0851532

0
4

0
4

0.0469069

0.0469069

0.0469069

0
4

0
4

0.29211

0.29211

0.29211

0
4

0
4

4.31953

4.25401

4.01384

0.116433

0.0655183

0.0582163

8.81239525796218e-16

0
4

0.0655183

0.0655183

0
4

0
4

0.0982775

0.0982775

0.0982775

0
4

0
4

0.0761741

0.0761741

0.0761741

0
4

0
4

0.299527

0.299527

0.299527

0
4

0
4

0.171759

0.171759

0.171759

0
4

0
4

0.00690984

0.00690984

0.00690984

0
4

0
4

0.118371

0.118371

0.118371

0
4

0
4

1.29121

1.29121

1.29121

0
4

0
4

0.199707

0.199707

0.199707

0
4

0
4

0.093266

0.093266

0.093266

0
4

0
4

0.0398762

0.0398762

0.0398762

0
4

0
4

3.84191
6

3.84191
6

3.26808
6

0.459802

0.0306347

0.0140945

0.0599054

0.00939633

2.8275992658422e-16
6

0
4

0
4

0.114261

0.114261

0.114261

0
4

0
4

0.14657

0.14657

0.14657

0
4

0
4

0.0761741

0.0761741

0.0761741

0
4

0
4

190.954
2

165.84
2

72.4453
2

1.27168

4.2992

1.43756

0.884498

0.198942

0.163796

0.31048

0.131037

3.24316

0.218361

19.9036
2

0.174503

0.198942

0.196555

0.0398762

0.552618

0.243152

0.131037

0.131037

0.229314

0.0398762

4.34372

0.0655183

0.0884188

7.3053

0.131037

0.0884188

0.137103

0.0655183

0.37578

1.06644

0.0442094

6.98971
2

0.294833

0.0582163

0.163796

3.98782

0.198942

0.131037

0.442094

0.884498

0.0982775

0.0442094

2.45193

0.0982775

0.0655183

0.063149

0.131037

0.0655183

0.110524

0.262073

0.163796

0.0655183

0.174649

7.51503

0.132628

0.0398762

0.0982775

0.906293

0.262073

0.327592

0.0655183

0.196555

0.0982775

0.265256

1.60093

0.196555

0.131037

0.203757

0.0655183

0.219364

0.75156

0.126298

5.60182

0.116433

0.508408

7.71885
2

0.0442094

0.0982775

0.0655183

0.132628

0.233165

0.0189229

0.0797524

0.0655183

0.0442094

0.808026

1.52655665885959e-13
2

0
4

17.4194
2

12.0031
2

0.163796

1.92055

0.929735

1.02588

0.622424

0.131534

0.252215

0.0655183

0.304696

0
4

2.11595
2

0.881943

0.530083
2

0.458628

0.131037

0.114261

0
4

3.90443

1.60379

0.844137

1.34007

0.116433

0
4

0.349298

0.145541

0.116433

0.0873245

0
4

0.418761

0.189447

0.131037

0.0982775

0
4

0.906293

0.0663141

0.839979

0
4

1.29896093881143e-14
2

0
4

5.89027
2

5.46604
2

2.53569
2

2.31783

0.319919

0.292598

0
4

0.13632

0.09597

0.0403499

0
4

0.28791

0.28791

0
4

6.66133814775094e-16
2

0
4

0.397083

0.397083

0.35166

0.0263745

0.0161178

0.0029305

0
4

0
4

2.66641
5

2.66641
5

2.51957
5

0.143327

0.00350607

0
4

0
4

0.641299

0.588781

0.0793722

0.0473867

0.144925

0.136238

0.0580485

0.00987078

0.11294

0
4

0.0485698

0.0406732

0.00789662

1.73472347597681e-18

0
4

0.00394831

0.00394831

0
4

1.64798730217797e-17

0
4

2.76023

2.45105

1.48597

0.38106

0.272038

0.239648

0.0723307

0
4

0.309187

0.309187

0
4

0
4

1.73111

0.846421

0.47541

0.180575

0.114261

0.0761741

0
4

0.589855

0.247072

0.342783

0
4

0.228522

0.228522

0
4

0.0663141

0.0663141

0
4

1.52655665885959e-16

0
4

2.66609

2.09479

1.40922

0.304696

0.304696

0.0761741

2.91433543964104e-16

0
4

0.266609

0.266609

0
4

0.0761741

0.0761741

0
4

0.228522

0.228522

0
4

2.4980018054066e-16

0
4

2.37059
5

2.37059
5

1.88584

0.214485

0.0251765

0.160864

0.0423112

0.0168565

0.0138197

0.0112377

1.28369537222284e-16
5

0
4

0
4

5.84541
4

5.36307

3.89565

0.704088

0.763337

0
4

0.411978

0.411978

0
4

0.0703603

0.0703603

0
4

7.49400541621981e-16
4

0
4

1.54956
7

1.54956
7

1.37626
7

0.168299

0.00500636

0
4

0
4

169.117
2

157.336
2

97.2288
2

0.0761741

0.0761741

0.533219

0.152348

0.457045

0.457045

0.0598143

0.152348

52.7269
2

1.14261

0.495132

0.228522

0.469219

0.190435

2.6996

0.190435

3.68594044175552e-14
2

0
4

5.31408
2

3.74908
2

1.20011

0.288713

0.0761741

6.93889390390723e-17
2

0
4

6.27473

2.47566

2.47589

0.142488

0.190435

0.457045

0.0761741

0.0761741

0.190435

0.190435

1.11022302462516e-16

0
4

0.192607

0.116433

0.0761741

0
4

6.32827124036339e-15
2

0
4

2.69183

2.69183

1.46173

0.364775

0.451249

0.414068

0
4

0
4

12.6821
6

12.6821
6

12.6821
6

0
4

0
4

12.0333
2

3.84857

3.55759

0.138627

0.0761741

0.0761741

1.38777878078145e-16

0
4

8.11919

8.11439

0.00479996

1.56992474575901e-16

0
4

0.0655183

0.0655183

0
4

2.31759056390501e-15
2

0
4

11.1576
7

11.1576
7

11.1058
7

0.0518304

0
4

0
4

7.0645
7

7.0645
7

5.50818

1.55632

0
4

0
4

7.09512

7.0692

7.04329

0.0129576

0.0129576

0
4

0.0259152

0.0259152

0
4

0
4

0.443971

0.0205135

0.00732626

0.00439576

0.0029305

0.0029305

0.0029305

2.60208521396521e-18

0
4

0.417597

0.417597

0
4

0.00586101

0.00586101

0
4

6.07153216591882e-18

0
4

14.9928
6

14.9928
6

14.8107
6

0.182089

0
4

0
4

3.22023

3.22023

1.06723

2.153

0
4

0
4

1.59796

0.293798

0.293798

0
4

1.19842

1.19842

0
4

0.105743

0.105743

0
4

0
4

210.111
2

168.388
2

107.646
2

8.28807

0.272927

1.60115

0.556906

0.28388

0.327592

0.262073

0.0655183

0.291082

0.0655183

12.7829

0.524147

0.0947236

0.0655183

0.0982775

1.37531

13.0066

15.4082

1.20114

0.436623

3.60351

0.131037

7.74380559676047e-15
2

0
4

0.0655183

0.0655183

0
4

14.8565
2

13.0292

0.673751

0.284675

0.549419

0.122963

0.196555

0
4

1.20341

0.503335

0.20869

0.131037

0.360351

0
4

23.4381

0.363423

0.110524

22.9642

0
4

0.550101

0.550101

0
4

1.2339

0.64423

0.589665

1.11022302462516e-16

0
4

0.193443

0.129463

0.06398

1.38777878078145e-17

0
4

0.0655183

0.0655183

0
4

0.116433

0.116433

0
4

6.77374822899424e-14
2

0
4

2.89221

0.257421

0.206405

0.0510167

3.46944695195361e-17

0
4

2.63479

1.10811

1.52667

0
4

0
4

1.87395

1.82974

1.63356

0.196185

5.55111512312578e-17

0
4

0.0442094

0.0442094

0
4

1.38777878078145e-17

0
4

0.773222

0.533257

0.213303

0.0799885

0.0799885

0.0799885

0.0799885

0
4

0.239966

0.106651

0.133314

0
4

0
4

4.46387
6

4.46387
6

4.46387
6

0
4

0
4

2.56801

2.56801

2.56801

0
4

0
4

0.344647

0.344647

0.333988

0.0106592

0
4

0
4

5.80021

5.77429

5.57345

0.200843

3.33066907387547e-16

0
4

0.0259152

0.0129576

0.0129576

0
4

0
4

0.120836

0.120836

0.051335

0.052126

0.0173753

0
4

0
4

0.344719

0.286581

0.194875

0.0229265

0.0229265

0.045853

2.08166817117217e-17

0
4

0.0581373

0.0581373

0
4

0
4

4.88228

4.88228

4.88228

0
4

0
4

59.2875

58.9142

52.7206

0.0799885

0.0533257

0.0533257

0.0798828

0.0799885

0.119824

0.0798828

3.78612

0.186534

0.359578

0.342511

0.239648

0.453268

0.0799885

0.199707

0
4

0.266628

0.266628

0
4

0.106651

0.0533257

0.0533257

0
4

1.1518563880486e-15

0
4

0.575908

0.392433

0.0917376

0.163089

0.137606

2.77555756156289e-17

0
4

0.0917376

0.0917376

0
4

0.0917376

0.0917376

0
4

5.55111512312578e-17

0
4

3.74577

3.74577

3.74577

0
4

0
4

4.87311

4.87311

4.8289

0.0442094

1.38777878078145e-17

0
4

0
4

0.282469

0.120613

0.120613

0
4

0.161857

0.161857

0
4

0
4

1.83587

1.23675

0.92756

0.309187

1.11022302462516e-16

0
4

0.599121

0.599121

0
4

0
4

1.46668

1.46668

0.466672

1.00001

0
4

0
4

1.23102

0.783844

0.114261

0.0982775

0.571306

1.11022302462516e-16

0
4

0.152348

0.152348

0
4

0.294833

0.294833

0
4

0
4

0.387785

0.131037

0.0655183

0.0655183

0
4

0.114261

0.114261

0
4

0.0442094

0.0442094

0
4

0.0982775

0.0982775

0
4

0
4

1.92664

1.92664

0.101085

1.82555

0
4

0
4

6.26056

6.26056

6.26056

0
4

0
4

23.688
2

20.5486
2

8.78551
2

6.46359
2

2.17093

0.38087

1.34313

0.542202

0.0655183

0.720702

0.0761741

0
4

3.13938

1.25448

1.8849

0
4

0
4

0.190263

0.0689052

0.06398

0.0049252

0
4

0.0582163

0.0582163

0
4

0.0049252

0.0049252

0
4

0.0582163

0.0582163

0
4

6.93889390390723e-18

0
4

0.123945
7

0.120439

0.115859

0.00458025

0
4

0.00350607

0.00350607

0
4

3.46944695195361e-18
7

0
4

2.14572

2.14572

2.14572

0
4

0
4

1.1807

0.266609

0.266609

0
4

0.723654

0.304696

0.418957

0
4

0.190435

0.190435

0
4

0
4

0.151148

0.151148

0.141988

0.0091605

6.93889390390723e-18

0
4

0
4

0.457045

0.342783

0.266609

0.0761741

1.38777878078145e-17

0
4

0.114261

0.114261

0
4

1.38777878078145e-17

0
4

0.436577

0.436577

0.211143

0.152348

0.0730852

1.38777878078145e-17

0
4

0
4

1.67392

1.63405

1.63405

0
4

0.0398762

0.0398762

0
4

0
4

1.54696

1.54696

1.54696

0
4

0
4

0.323713

0.323713

0.283249

0.0404642

0
4

0
4

102
2

47.2396
2

8.35864
2

1.05235

25.4169

0.84367

0.528126

11.0398

1.77635683940025e-15
2

0
4

51.3326
2

0.389592

0.990334

1.3433

0.8406

47.468

0.221022

0.0797524

0
4

1.97706
2

1.20281

0.220056

0.0398762

0.116433

0.397885

2.77555756156289e-16
2

0
4

0.39311

0.327592

0.0655183

0
4

0.182747

0.0663141

0.116433

0
4

0.536393

0.536393

0
4

0.203757

0.203757

0
4

0.0761741

0.0761741

0
4

0.0582163

0.0582163

0
4

3.1412372702988e-14
2

0
4

0.294697

0.294697

0.294697

0
4

0
4

1.7037

0.293798

0.293798

0
4

1.4099

1.4099

0
4

0
4

1.6413

1.6413

1.49981

0.0565967

0.0848951

0
4

0
4

6.06747

6.06747

6.06747

0
4

0
4

1.1257

1.1257

0.95669

0.00448724

0.164523

0
4

0
4

0.0486477

0.0486477

0.0401353

0.00500636

0.00350607

1.73472347597681e-18

0
4

0
4

5.33328

5.235

5.235

0
4

0.0982775

0.0982775

0
4

0
4

2.06529

1.79089

1.70996

0.0809283

0
4

0.274404

0.274404

0
4

5.55111512312578e-17

0
4

0.0987733

0.0987733

0.0987733

0
4

0
4

2.45614

2.23359

2.23359

0
4

0.222553

0.222553

0
4

1.66533453693773e-16

0
4

18.8435

18.8435

16.7986

0.449567

0.445832

0.265256

0.508408

0.110524

0.221047

0.0442094

0
4

0
4

0.441179

0.441179

0.441179

0
4

0
4

0.185417

0.185417

0.185417

0
4

0
4

0.105018

0.105018

0.0598143

0.00532728

0.0398762

1.38777878078145e-17

0
4

0
4

0.161128

0.161128

0.141625

0.0195035

0
4

0
4

0.54323

0.54323

0.54323

0
4

0
4

0.0251705

0.0251705

0.0151023

0.0100682

0
4

0
4

0.322103

0.0793181

0.0793181

0
4

0.242785

0.242785

0
4

2.77555756156289e-17

0
4

0.0285732

0.0285732

0.0158179

0.0127553

0
4

0
4

0.397885

0.397885

0.397885

0
4

0
4

1.09299

0.120383

0.120383

0
4

0.972607

0.972607

0
4

0
4

6.95234

1.47717

1.24393

0.0647879

0.0583091

0.0388728

0.0129576

0.0259152

0.0194364

0.0129576

2.51534904016637e-16

0
4

5.44926

5.44926

0
4

0.0259152

0.0129576

0.0129576

0
4

0
4

15.1529

15.0767

15.0767

0
4

0.0761741

0.0761741

0
4

0
4

5.69443

5.69443

5.69443

0
4

0
4

2.37828

2.37828

0.139899

2.23838

0
4

0
4

0.149259

0.149259

0.0761741

0.0730852

0
4

0
4

0.331956

0.331956

0.331956

0
4

0
4

1.77065

1.70996

1.70996

0
4

0.0606962

0.0606962

0
4

0
4

0.171871

0.0582163

0.0582163

0
4

0.113655

0.113655

0
4

0
4

0.17088

0.17088

0.141625

0.0292553

0
4

0
4

0.106147

0.106147

0.0530735

0.0530735

0
4

0
4

0.152348

0.0761741

0.0761741

0
4

0.0761741

0.0761741

0
4

0
4

98.9165

78.7232

78.4081

0.0646827

0.0761741

0.054841

0.00518609

0.114261

8.93729534823251e-15

0
4

1.2337
1

1.2337
1

0
4

2.47165

2.47165

0
4

13.0568

12.9425

0.114261

6.93889390390723e-17

0
4

0.64748

0.533219

0.114261

0
4

0.21872

0.190625

0.0280942

6.93889390390723e-18

0
4

2.56494

2.56494

0
4

0
4

6.82121026329696e-11
3

0
4

30.3511
4

27.8937
4

0.59172

0.484478

0.107242

0
4

26.6308

26.6308

0
4

0.575226

0.575226

0
4

0.09597

0.09597

0
4

0
4

2.07183

2.07183

2.07183

0
4

0
4

0.334554

0.334554

0.292707

0.0418477

0
4

0
4

0.0340111

0.0340111

0.0340111

0
4

0
4

0.0170056

0.0170056

0.0170056

0
4

0
4

0
4

27063.9
5

2279.96
4

2279.96
4

474.129
4

14.1732

0.123124

0.123124

0.123124

0.0723307

0.123124

0.184686

0.123124

0.118299

0.29211

0.177448

4.98717

0.438165

0.29211

0.0999396

0.123124

0.0723307

0.118299

0.118299

0.196734

0.29211

0.154593

4.55636

0.246248

0.123124

0.108496

0.154593

0.123124

0.29211

0.118299

0.0129839

7.88696

0.108496

188.455

0.473196

0.123124

0.118299

0.0723307

0.123124

0.438165

0.29211

0.295747

0.295747

0.123124

1.65392

0.438165

23.9401
4

0.772456

21.4392

41.2369

20.859
4

22.059

1.31094

1.189

5.11239

12.882

0.901716

9.38323

3.95183

4.90041

423.776

2.31889

39.8413
4

0.745878

168.722

1.07906

1.57075

0.925835

0.660294

1.47934

0.902904

1.08215

3.42701

332.485
4

1.33019

6.33334

1.23675

0.684412

0.426108

0.925835

1.1382

0.606022

67.3144

0.922876

99.5531
4

0.850263

1.35907

0.742695

0.784605

0.533532

0.522884

1.0369

4.08614

0.469558

0.359722

15.4906

1.02238

0.241423

0.618373

0.415234

6.45676

0.241423

0.449695

0.702518

0.309187

0.421284

9.29889
4

0.254533

0.77618

0.538357

0.730274

0.840155

0.154593

0.0723307

0.196734

0.184686

0.23189

7.67459
4

0.123124

0.618373

0.29211

0.0723307

0.118299

0.154593

0.199879

0.123124

0.123124

0.118299

153.739
4

0.473196

31.5479

0.184686

0.23189

0.123124

0.29211

0.29211

0.0723307

0.154593

0.123124

5.09700615047848e-12
4

0
4

0
4

24710.5
5

239.851

31.8888

117.265
2

6.89132

11.7249
4

0.934835

2.09951
4

0.113622

1.02705

1.15347

5.37338

1.15027

7.84157

1.14124

2.14161
7

0.487729

0.227145

0.536173

0.21317

0.116458

0.174282

0.110907

0.68857

1.71776

2.44458

6.48347
3

0.086242

0.0229265

0.0475315

1.01445

0.763337

0.279906

0.29211

0.107261

0.196734

0.0229265

7.55092
4

0.00448724

0.0475315

0.0343898

0.0556337

0.107261

0.144196

0.0633753

0.196734

0.0469069

0.310066

1.76534

0.107261

0.00689936

0.0475315

0.118299

0.137559

0.010349

0.158438

0.0530735

0.0343898

0.0283844

10.0516

0.0207444

0.0316877

0.0586446

0.045853

0.093266

0.177557

0.513944

1.47551

0.266335

0.0511526

1.19218

0.0316877

0.0316877

0.169269

0.763337

0.0846347

0.0343898

0.0799732

0.126952

0.0591856

0.0917376

0.118452

0.0792192

0.11996

0.0343898

0.0316877

0.0655439

0.295101

0.239622

0.0767289

0.010349

0.0530735

4.03971

1.38626

0.0229265

0.0173753

3.25919846666523e-14

0
4

7.18541

0.0892871

2.80868

4.16914

0.118299

1.66533453693773e-16

0
4

0.0926517

0.0926517

0
4

24329.4
5

10252.4
5

110.71
3

3495.93
3

5476.05
4

937.155
5

154.547
4

30.8247

0.469633

0.0773585

0.0229265

0.160891

0.652357

0.366824

0.126096

0.0846347

0.0581004

0.126952

0.0846347

0.928524

0.045853

0.05102

0.268152

0.119824

1.07261

0.0511526

0.0343898

0.0343898

0.49292

0.0798828

1.40998

4.19836

0.126096

0.244634

0.0501388

0.27959

0.163089

0.0343898

0.0229265

0.0799732

0.447067

0.413461

0.0283844

0.0229265

0.244634

0.252192

0.309508

0.0343898

0.045853

0.550126

3.85188

0.332492

19.8798

0.045853

0.0917376

0.114633

0.244634

0.0343898

0.588255

0.0229265

0.0802428

0.0229265

0.0798828

1.00742
4

0.119824

0.268152

0.589935

0.163089

0.389751

0.0573163

0.126952

0.0343898

0.0229265

0.0802428

0.736561

0.160891

0.174301

0.045853

0.0798828

0.126952

0.252192

0.0581004

0.091706

0.0343898

0.0511526

1.19218

0.0229265

0.0229265

0.0229265

0.045853

1.12624

0.0581004

0.0229265

0.163089

0.045853

0.0511526

1.91279

0.114633

0.0229265

0.126096

0.137606

0.137559

0.196734

0.214522

0.160486

0.107261

0.262628

1.44578

0.045853

0.126096

0.163089

0.0573163

0.0802428

0.196734

0.091706

0.0229265

0.107261

0.163089

15.9284
4

0.974377

0.0229265

0.149022

0.107261

0.0229265

0.0767289

0.0581004

0.465493
3

0.413552

1.34009
4

0.722185

0.738555
4

0.909713

0.859744

0.780577
4

0.56027

9.72958
4

0.945194

1.30681

1.71189
4

0.59817

1.39214

0.55102

0.670416

1.97698
4

3.04358

0.95145

26.996

3.22358

0.714721

3.78888

0.217802

2.07211

2.20095

0.408935

0.480141

4.24618

0.580769

14.7082

0.194875

1.59329

0.515847

0.997303

0.447067

0.183412

0.149022

0.504383

0.584626

2.4981

61.936

0.902483

0.427316

0.667446

2.27439

0.320971

0.231869

0.352611

0.922284

0.607149

0.68409

34.7057
4

0.641942

2.46341

0.309508

0.654636

3.90699

0.692542

0.641942

0.378287

0.733648

0.457132

10.5803
3

0.250894

0.477586

0.230187

1.54754

0.298712

0.277362

0.298045

0.574358

0.741713

0.905597

24.7829
4

0.406061

0.695968

0.217802

0.714197

0.306968

1.23803

0.126096

0.269251

0.206339

1.15238

10.009
4

0.547034

1.08901

0.194875

0.597196

0.674677

0.367852

0.699259

0.596089

0.859744

0.333098

67.3546
4

14.9595

0.435161

0.923638

0.294405

0.149022

0.451965

0.816609

0.628715

0.298045

1.19769

1.80052

10.0195
4

0.719396

0.355361

0.481457

0.413662

0.314102

0.42414

0.8199

0.471943

1.02104

0.447067

8.91841

0.590203

0.370833

0.298045

0.504383

0.603097

0.722185

0.091706

0.905597

7.10178

0.802428

9.24875
4

0.448859

5.2982

1.58193

0.0802428

0.229265

0.814675

0.332434

0.309508

0.286581

0.45202

9.12439

0.091706

1.73095

1.04835

0.199707

0.286581

0.373475

0.169963

0.29183

0.0573163

0.0687795

5.45502
4

0.653406

0.194875

0.240728

0.813891

0.268152

0.378287

0.398408

0.298045

0.140721

0.311847

79.1955
4

0.045853

0.321783

0.316495

0.194875

0.538773

0.98584

9.10424

0.103169

0.217802

0.254827

5.33103
4

0.391238

0.133208

2.33819

0.045853

0.0573163

0.27959

0.0802428

0.378287

0.197479

0.114633

5.41871
4

0.214103

0.252192

0.091706

1.27242

0.508927

0.234534

0.0519355

0.883214

0.928524

0.576673

18.6321
4

0.756575

0.167353

0.126096

0.194875

0.522904

0.364995

0.5617

0.103169

0.275118

0.137559

40.0161

19.5353
4

0.0802428

0.282914

1.03417

0.229265

0.240728

0.091706

0.723043

0.0687795

0.045853

0.448735

21.0893
4

0.965349

0.326179

0.0573163

0.0802428

0.183412

0.52731

0.5617

0.255763

0.240774

0.289787

7.56575

0.137559

0.33769

0.187249

0.45853

0.65776

0.275118

0.114633

0.126096

0.326179

0.14289

5.59928

0.379883

1.17317

0.558047

0.194875

0.0802428

0.905597

0.282914

0.217802

0.204459

0.361165

5.56749
4

0.194875

0.149022

0.745112

0.0928667

1.32309

0.153458

0.091706

0.495383

0.160486

0.412819

6.18183

0.103169

0.399058

1.74301

0.0687795

0.0687795

1.00205

0.103169

0.0573163

1.38144

0.355096

2.44167

0.707092

0.204154

0.675876

0.366824

0.14289

0.245769

0.091706

0.480054

0.125736

0.0687795

17.084
4

0.967856

0.526991

0.355361

0.0573163

0.102809

0.346271

0.103169

0.495238

0.407723

4.63884

2.87728

0.134773

0.285113

0.0802428

0.653406

0.676332

0.126096

0.315023

0.290736

0.504383

0.045853

4.63116

1.39852

0.91706

0.489268

0.393468

0.196734

0.107261

0.263655

0.0798828

0.0767289

0.281339

1748.75
6

8.62091
3

0.0581004

0.0917376

0.116201

0.0343898

0.107261

0.196734

0.0802428

0.0573163

0.0501388

0.0581004

3.10654

0.0229265

0.045853

0.0802428

0.171949

0.160891

1.07421

0.0846347

0.103169

0.091706

0.0343898

9.49273
3

0.163089

0.175486

0.114633

0.0511526

0.0229265

0.0573163

0.196734

0.0722459

0.0229265

0.0229265

6.34064
4

0.0343898

0.163089

0.160891

0.138508

0.0343898

0.114633

0.0229265

0.911718

0.0871506

0.366824

4.60921

0.0343898

0.0752082

0.0229265

0.0573163

0.045853

0.0798828

0.0573163

0.0229265

0.091706

0.217802

36.084
4

0.261452

0.045853

0.126952

0.0802428

0.858088

0.295101

0.0229265

0.160891

0.0687795

0.0798828

2.07485

0.0917376

0.0343898

0.199707

0.0846347

0.05102

0.196734

0.407723

1.72767

0.107261

0.0343898

5.70661
4

0.149022

0.163089

0.0573163

0.0581004

0.0687795

0.103169

0.103169

0.0767289

1.8384

0.045853

4.55667
4

0.100278

0.149022

0.0687795

0.0229265

10.6236

0.309508

0.137559

0.0343898

0.244634

0.326179

2.71679

0.343898

0.0846347

0.137606

0.107261

0.0649455

0.0229265

0.138508

0.107261

0.107261

0.119824

82.4441

5.0553

0.0229265

0.196734

0.045853

0.0343898

0.0687795

0.0343898

0.0974182

0.875427

0.0798828

0.0687795

17.7926

0.179034

0.214522

0.100278

0.733648

0.244634

0.103169

0.196734

0.103169

0.0229265

0.275213

7.98787
4

0.0347274

0.0798828

0.0802428

0.160486

0.091706

0.045853

0.159766

0.196734

0.407723

0.0533257

2.75872
4

0.0229265

0.263655

0.0649455

0.401214

1.22011

0.114633

0.0798828

0.321783

0.149022

0.045853

1.29535

0.091706

0.0343898

0.0648786

0.401214

0.0573163

0.252192

0.209669

0.145251

0.0511526

0.0229265

2.91124
4

0.871207

0.196734

0.0798828

0.0343898

0.0229265

0.29211

0.607553

0.206339

0.0573163

0.0511526

3.95722
3

0.0343898

0.761796

0.163089

0.107261

0.491835

0.0343898

0.045853

0.0343898

0.116201

0.18664

1.35503
3

0.045853

0.0511526

0.885304

0.108496

0.0229265

0.045853

0.137606

0.126952

0.107261

0.174301

6.90088

0.196734

0.196734

0.045853

0.126952

0.0687795

0.0802428

0.0343898

1.59766

0.332434

0.0687795

3.34235
4

0.0581004

0.0229265

0.137559

0.36695

0.107261

0.0343898

0.239648

0.0846347

0.0343898

0.917956

279.911
4

1.7768

0.263655

0.0229265

0.137559

0.0501388

0.0687795

0.0798828

0.091706

0.375413

0.0581004

0.126096

2.02053

0.0343898

0.152774

0.0723307

0.677078

0.196734

0.277017

0.045853

0.268152

0.0343898

0.138508

0.756575

0.163089

0.0767289

1.02023

0.091706

0.0343898

0.0229265

1.34076

0.0687795

0.0798828

0.102305

1.55549
4

0.0723307

0.0343898

0.107261

0.0229265

0.107261

0.162364

0.206339

0.160891

0.160486

0.0573163

3.84886
4

0.0343898

0.163089

0.091706

0.42414

0.171949

0.229265

0.0798828

0.119824

0.0229265

0.207762

1.21439

0.0343898

0.326179

0.0846347

0.309508

0.107261

0.0573163

0.119824

0.0229265

0.0846347

0.0798828

1.15779

0.138508

0.412677

0.045853

0.332492

0.375413

0.0687795

0.438165

0.127882

0.0846347

0.126952

13.1719
4

0.103169

0.0343898

0.214522

0.0229265

0.0229265

0.207762

0.0229265

0.0343898

0.137606

0.196734

0.515847

0.0581004

0.0723307

0.0767289

0.298045

0.0343898

0.393468

0.0798828

0.0343898

0.0798828

0.244634

0.481457

0.103169

0.261452

0.160486

0.184686

0.0687795

0.0846347

0.138508

0.0229265

0.389751

0.469993

18.169
4

4.71319
4

0.163089

0.175085

0.0846347

0.0649455

0.0229265

0.160486

1.42144

0.194875

0.045853

0.0229265

0.768038

0.0917376

0.196734

0.0229265

0.0229265

0.0343898

0.0573163

0.0343898

0.0798828

0.375413

0.177448

9.44622
4

0.0511526

0.045853

0.263655

0.0871506

0.0798828

0.107261

0.126096

0.0798828

0.0917376

0.149022

4.68847

0.0798828

0.118299

0.0573163

0.0846347

0.103169

1.17987

0.107261

0.126096

0.23189

0.0343898

1.74725

0.0917376

0.119824

0.407723

0.0229265

0.160891

0.149022

0.813891

0.240728

0.206339

0.0229265

40.6505
3

0.393468

0.0846347

0.045853

0.0229265

0.163089

0.09597

0.779501

0.0229265

0.107261

0.0229265

1.23803

0.0573163

0.573163

0.107261

0.0343898

0.114633

0.0229265

0.091706

0.161286

0.0533257

0.309508

1.5668
4

0.0581004

0.163089

0.163089

0.0802428

0.160891

0.119824

0.0687795

0.0343898

0.0533257

0.137559

0.939987

0.0343898

0.0798828

0.126096

0.244634

0.206339

0.0802428

0.375413

0.0687795

0.00305919

0.196734

3.55538
4

0.0802428

0.163089

0.144661

0.0802428

0.229265

0.263655

0.0343898

0.232402

0.045853

0.0229265

398.459
3

17.8406

0.0548351

0.244634

0.091706

0.0229265

0.203351

0.0229265

0.045853

0.263655

0.0423112

0.126096

8.44018

0.0687795

0.0229265

0.0343898

0.0573163

0.0229265

0.123124

0.119824

0.0229265

0.217802

0.137559

0.332434

2.03796

0.169269

0.0229265

0.0229265

0.52731

0.107261

0.207762

0.319531

0.126096

0.0767289

1.40998

0.045853

0.196734

0.114633

0.0802428

0.0229265

0.289421

0.0917376

0.607553

0.045853

1.02023

6.90527

0.0511526

0.19194

0.0798828

0.163089

0.0687795

0.438165

0.160486

0.45853

0.045853

0.119824

4.89337

0.0229265

0.423173

0.0752082

0.045853

0.179034

0.0581004

0.119824

0.0189229

0.126096

0.0834505

1.87997

0.149022

0.160486

0.0343898

0.103169

0.309508

0.0229265

0.107261

0.0573163

0.0229265

0.045853

10.1262
4

0.091706

0.0556337

0.0798828

0.0501388

0.163089

0.045853

0.0343898

0.0581004

0.114633

0.0846347

1.20364

0.0846347

0.169269

0.206339

0.05102

0.0917376

0.346271

0.0581004

0.137606

0.244634

0.0229265

0.229265

0.103169

0.206339

0.0229265

0.137559

0.0687795

0.0802428

0.288984

0.0846347

0.0229265

0.045853

93.8368

3.55361

10.5118

0.481457

0.0229265

1.63925

0.504383

0.0573163

0.0229265

0.0533257

0.045853

0.538773

1.1021

0.179034

0.0229265

0.0511526

0.126096

0.0229265

0.277017

0.175085

0.515847

0.00408015

0.159766

2.37492
4

0.117267

0.652357

0.153458

0.217802

0.0798828

0.0573163

0.815446

0.0229265

0.119824

0.0802428

0.720231

0.163089

0.0798828

0.0752082

0.107261

0.0501388

0.429044

0.0573163

0.326179

0.0229265

0.0846347

5.29079
4

0.733648

0.214522

0.0573163

0.0229265

0.0511526

0.275118

0.0229265

0.107261

0.163089

0.262628

3.40467

0.298045

0.0343898

0.0343898

0.103169

0.107261

0.159766

0.0343898

0.0798828

0.320971

0.0799885

2.49899

0.0229265

0.107261

0.298045

0.0343898

0.0343898

0.0343898

0.473196

0.194875

0.0511526

0.199707

1.02542

0.045853

0.138508

0.0229265

0.119824

0.0581004

0.0798828

0.0752082

0.0846347

0.160891

0.179034

1.36413

0.103169

0.0802428

0.309508

2.44167

0.0343898

0.163089

0.0229265

0.319552

0.00458879

0.0229265

0.297685

0.0846347

0.107261

0.171949

0.0687795

0.0917376

0.045853

0.160891

0.275118

0.00305919

0.0752082

0
4

4.43127

4.26998

0.161286

0
4

97.7566
4

1.45304

95.6148
4

0.154717

0.241929

0.29211

6.49480469405717e-15
4

0
4

22.4729

16.4255
5

1.93612

0.103871

0.0553261

0.180503

0.0553261

1.10881

1.41743

0.228035

0.138315

0.0259677

0.0584274

0.495675

0.196734

0.0469069

5.11396480717963e-15

0
4

2.03796

2.03796

0
4

6.24083

6.24083

0
4

0.331189

0.331189

0
4

0.0798828

0.0798828

0
4

0.193681

0.193681

0
4

0.0917376

0.0917376

0
4

0.283249

0.283249

0
4

0.00590715

0.00590715

0
4

0
4

2.77029

2.77029

2.77029

0
4

0
4

0.025154

0.025154

0.025154

0
4

0
4

0.29211

0.29211

0.29211

0
4

0
4

2.28325

2.28325

2.28325

0
4

0
4

68.1387
4

66.9239
4

0.192662

0.393493

66.3377
4

1.4210854715202e-14
4

0
4

0.965484

0.746056

0.219428

0
4

0.131037

0.131037

0
4

0.118299

0.118299

0
4

0
4

1.01039177025086e-11
5

0
4

8.98332
3

3.16066

3.16066

1.14445

1.64794

0.325959

0.0423112

0
4

0
4

0.160864

0.160864

0.160864

0
4

0
4

0.0808509

0.0808509

0.0808509

0
4

0
4

3.82036

3.82036

3.73574

0.0846225

5.55111512312578e-17

0
4

0
4

0.281651

0.281651

0.230078

0.0515724

0
4

0
4

0.806238

0.806238

0.806238

0
4

0
4

0.123124

0.123124

0.123124

0
4

0
4

0.177448

0.177448

0.177448

0
4

0
4

0.0488567

0.0488567

0.0488567

0
4

0
4

0.079445

0.079445

0.079445

0
4

0
4

0.243832

0.243832

0.243832

0
4

0
4

0
4

34.6922

34.2905

34.2905

2.30262

2.4004

0.309306

2.71366

1.18939

0.165162

0.0485602

0.418866

2.97924

0.266335

0.206186

3.20348
1

0.0259152

0.462389

0.536117

0.00447073

0.0194364

0.0129576

0.207321

0.00447073

0.0129576

0.0129576

1.45961

0.355114

0.00447073

0.714822

0.0236867

0.0129576

0.0129576

0.239622

0.00670609

0.179716

0.0129576

4.22255

0.0129576

0.0194364

0.032394

0.0388728

0.616518

0.0129576

0.0129576

4.38493

3.38147

0.392296

0.164914

0.473407

7.27196081129478e-15

0
4

0
4

0.401685

0.401685

0.401685

0
4

0
4

0
4

147.863

141.587

109.232

1.16268
7

5.05442

4.24267

0.806001

2.68822

16.5841

2.26048

0.79975

1.24761

0.191231

1.31669

1.81779
6

0.121392

22.53

0.44786

0.243832

2.53248

0.243832

0.15995

5.95014

0.390131

0.214507

14.6787

0.51184

0.15995

0.0926517

0.09597

0.09597

0.06398

0.0975327

0.06398

0.0100682

0.06398

7.32028

0.09597

0.612799

0.0975327

0.06398

0.146299

0.787884

0.06398

0.0809283

0.0404642

0.0617678

5.95256
6

0.0975327

0.06398

0.06398

0.19194

0.12796

0.12796

0.0975327

1.59054

1.88937

2.13099

0.585196

7.27196081129478e-14

0
4

12.0922

9.9169

0.25592

0.92771

0.09597

0.89572

4.44089209850063e-16

0
4

1.25319

1.25319

0
4

18.2343

18.0104

0.22393

0
4

0.28791

0.06398

0.22393

2.77555756156289e-17

0
4

0.12796

0.12796

0
4

0.359433

0.359433

0
4

0
4

6.25765

6.25765

6.25765

0
4

0
4

0.00527298

0.00527298

0.00527298

0
4

0
4

0.0127553

0.0127553

0.0127553

0
4

0
4

0
4

31.9838
7

31.9838
7

29.0461
7

9.83864
7

0.0637765

0.166885

0.0114506

0.0127553

0.019133

0.0127553

0.00362217

0.00518609

0.0127553

0.192662

11.5204
6

3.16451
7

0.614185

0.42317

2.75479

0.142964

0.0441023

0.042378

0
4

2.84657
6

2.18273

0.553882

0.109952

8.32667268468867e-17
6

0
4

0.0911168

0.0840107

0.00710613

4.33680868994202e-18

0
4

0
4

0
4

1.70004
7

1.70004
7

1.70004
7

1.40731

0.0705466

0.187157

0.0250159

0.0100064

1.04083408558608e-16
7

0
4

0
4

0
4

0.0582163

0.0582163

0.0582163

0.0582163

0
4

0
4

0
4

0.00408015

0.00408015

0.00408015

0.00408015

0
4

0
4

0
4

0.599121

0.599121

0.599121

0.599121

0
4

0
4

0
4

1.01412

1.01412

1.01412

1.01412

0
4

0
4

0
4

1.03663

1.03663

1.03663

1.03663

0
4

0
4

0
4

0.0163868

0.0163868

0.0163868

0.0163868

0
4

0
4

0
4

0.161857

0.161857

0.161857

0.161857

0
4

0
4

0
4

0.0947236

0.0947236

0.0947236

0.0947236

0
4

0
4

0
4

0.0770648

0.0770648

0.0770648

0.0770648

0
4

0
4

0
4

0.129121

0.129121

0.129121

0.129121

0
4

0
4

0
4

1.03608
7

1.03608
7

1.03109
7

0.186859
7

0.739721
7

0.104511

0
4

0.00498723

0.00498723

0
4

1.30104260698261e-17
7

0
4

0
4

1.70996

1.70996

1.70996

1.70996

0
4

0
4

0
4

2.56494

2.56494

2.56494

2.56494

0
4

0
4

0
4

0.0761741

0.0761741

0.0761741

0.0761741

0
4

0
4

0
4

0.007332

0.007332

0.007332

0.007332

0
4

0
4

0
4

0.123124

0.123124

0.123124

0.123124

0
4

0
4

0
4

0.093266

0.093266

0.093266

0.093266

0
4

0
4

0
4

0.0481639

0.0481639

0.0481639

0.0481639

0
4

0
4

0
4

1.70996

1.70996

1.70996

1.70996

0
4

0
4

0
4

0.501388

0.501388

0.501388

0.501388

0
4

0
4

0
4

0.0127553

0.0127553

0.0127553

0.0127553

0
4

0
4

0
4

0.453972

0.453972

0.437941

0.41275

0.00687037

0.018321

2.77555756156289e-17

0
4

0.0160309

0.0160309

0
4

0
4

0
4

0.107261

0.107261

0.107261

0.107261

0
4

0
4

0
4

0.0669684

0.0669684

0.0669684

0.0669684

0
4

0
4

0
4

0.16497

0.16497

0.16497

0.16497

0
4

0
4

0
4

0.0259304

0.0259304

0.0259304

0.0259304

0
4

0
4

0
4

1.70996

1.70996

1.70996

1.70996

0
4

0
4

0
4

0.0168565

0.0168565

0.0168565

0.0168565

0
4

0
4

0
4

0.054963

0.054963

0.054963

0.054963

0
4

0
4

0
4

0.763337

0.763337

0.763337

0.763337

0
4

0
4

0
4

0.09597

0.09597

0.09597

0.09597

0
4

0
4

0
4

0.525256

0.525256

0.525256

0.525256

0
4

0
4

0
4

0.333603
7

0.333603
7

0.305775
7

0.211802

0.0890475

0.0049252

0
4

0.0278285

0.0140089

0.00690984

0.00690984

0
4

0
4

0
4

0.0798828

0.0798828

0.0798828

0.0798828

0
4

0
4

0
4

1.3896

1.3896

1.3896

1.3896

0
4

0
4

0
4

0.00500636

0.00500636

0.00500636

0.00500636

0
4

0
4

0
4

3.41991

3.41991

3.41991

3.41991

0
4

0
4

0
4

0.0686787

0.0686787

0.0686787

0.0686787

0
4

0
4

0
4

0.177448

0.177448

0.177448

0.177448

0
4

0
4

0
4

0.0770648

0.0770648

0.0770648

0.0770648

0
4

0
4

0
4

0.107261

0.107261

0.107261

0.107261

0
4

0
4

0
4

0.0442094

0.0442094

0.0442094

0.0442094

0
4

0
4

0
4

0.0442094

0.0442094

0.0442094

0.0442094

0
4

0
4

0
4

0.368798

0.368798

0.296395

0.185522

0.0423112

0.00518609

0.0633753

0
4

0.072403

0.0248714

0.0475315

0
4

2.77555756156289e-17

0
4

0
4

0.516483

0.516483

0.516483

0.516483

0
4

0
4

0
4

0.0877713

0.0877713

0.0877713

0.0877713

0
4

0
4

0
4

0.0752082

0.0752082

0.0752082

0.0752082

0
4

0
4

0
4

0.0549439

0.0549439

0.0549439

0.0549439

0
4

0
4

0
4

0.00690984

0.00690984

0.00690984

0.00690984

0
4

0
4

0
4

0.0163868

0.0163868

0.0163868

0.0163868

0
4

0
4

0
4

0.0425139

0.0425139

0.0425139

0.0425139

0
4

0
4

0
4

0.246248

0.246248

0.246248

0.246248

0
4

0
4

0
4

0.914685

0.914685

0.914685

0.914685

0
4

0
4

0
4

0.0229265

0.0229265

0.0229265

0.0229265

0
4

0
4

0
4

31.3148

31.3148

31.3148

31.0467

0.107242

0.160864

4.44089209850063e-16

0
4

0
4

0
4

0.72098

0.72098

0.72098

0.72098

0
4

0
4

0
4

0.536117

0.536117

0.536117

0.536117

0
4

0
4

0
4

0.0799732

0.0799732

0.0799732

0.0799732

0
4

0
4

0
4

0.0761741

0.0761741

0.0761741

0.0761741

0
4

0
4

0
4

0.0163868

0.0163868

0.0163868

0.0163868

0
4

0
4

0
4

0.0761741

0.0761741

0.0761741

0.0761741

0
4

0
4

0
4

0.0332578

0.0332578

0.0332578

0.0332578

0
4

0
4

0
4

0.0163868

0.0163868

0.0163868

0.0163868

0
4

0
4

0
4

0.0127553

0.0127553

0.0127553

0.0127553

0
4

0
4

0
4

0.357411

0.357411

0.357411

0.357411

0
4

0
4

0
4

1.36671

1.36671

1.36671

0.967696

0.177406

0.0599054

0.161702

1.66533453693773e-16

0
4

0
4

0
4

0.00690984

0.00690984

0.00690984

0.00690984

0
4

0
4

0
4

0.0442094

0.0442094

0.0442094

0.0442094

0
4

0
4

0
4

1.14197
7

1.14197
7

1.14197
7

0.983203

0.139287

0.0194758

0
4

0
4

0
4

2.32624

2.32624

2.24325

1.85312

0.146299

0.243832

2.77555756156289e-17

0
4

0.0829891

0.0829891

0
4

2.63677968348475e-16

0
4

0
4

0.487133

0.487133

0.225625

0.225625

0
4

0.238582

0.0129576

0.125347

0.100278

0
4

0.0229265

0.0229265

0
4

7.97972798949331e-17

0
4

0
4

44.4087
7

44.1756
7

44.1756
7

43.4352
7

0.357411

0.357411

0.0255106

5.24233434440191e-15
7

0
4

0
4

0.233165

0.233165

0.233165

0
4

0
4

0
4

1.23124

1.23124

1.23124

0.479183

0.215757

0.536305

0
4

0
4

0
4

25.4567

25.4567

25.4567

25.3634

0.093266

0
4

0
4

0
4

1.11406

1.11406

1.11406

1.11406

0
4

0
4

0
4

2.72789

2.72789

2.72789

2.72789

0
4

0
4

0
4

2.02297

0.117449

0.094522

0.094522

0
4

0.0229265

0.0229265

0
4

0
4

1.90552

0.195564

0.195564

0
4

1.70996

1.70996

0
4

0
4

0
4

1.44777

1.44777

1.44777

1.23329

0.214485

8.32667268468867e-17

0
4

0
4

0
4

0.084031

0.084031

0.084031

0.0645552

0.0194758

6.93889390390723e-18

0
4

0
4

0
4

0.0712667

0.0712667

0.0712667

0.0583091

0.0129576

0
4

0
4

0
4

2.06371

2.06371

2.06371

2.06371

0
4

0
4

0
4

10.6835

10.6835

10.6835

10.6835

0
4

0
4

0
4

53.1034
2

53.1034
2

37.198
2

27.2081
2

5.00053

0.0761741

4.83705

0.0761741

0
4

15.3341
2

5.66017

1.48057

1.25687

6.64972

0.0582163

0.228522

0
4

0.38087

0.38087

0
4

0.190435

0.190435

0
4

1.60982338570648e-14
2

0
4

0
4

0.285067

0.285067

0.285067

0.239715

0.032394

0.0129576

0
4

0
4

0
4

0.395384

0.395384

0.395384

0.395384

0
4

0
4

0
4

0.26291

0.26291

0.145857

0.145857

0
4

0.0530735

0.0530735

0
4

0.06398

0.06398

0
4

0
4

0
4

0.221356

0.221356

0.221356

0.206263

0.0150924

0
4

0
4

0
4

0.226299

0.226299

0.226299

0.0724936

0.00881856

0.0724936

0.0724936

0
4

0
4

0
4

0.0946041

0.0946041

0.0946041

0.0946041

0
4

0
4

0
4

0.496251

0.370904

0.0700716

0.0573163

0.0127553

0
4

0.300833

0.300833

0
4

0
4

0.125347

0.125347

0.125347

0
4

0
4

0
4

1.36095

1.36095

1.35577

0.193681

1.16209

0
4

0.00518609

0.00518609

0
4

7.11236625150491e-17

0
4

0
4

3.91276

3.91276

3.91276

3.91276

0
4

0
4

0
4

0.192071

0.192071

0.192071

0.0761741

0.0761741

0.0397225

0
4

0
4

0
4

21.973

21.973

21.973

9.749

7.20311

2.26955

0.652862

0.093266

2.00522

0
4

0
4

0
4

0.236692

0.236692

0.0681182

0.0446436

0.0234746

3.46944695195361e-18

0
4

0.168574

0.168574

0
4

0
4

0
4

0.241994

0.241994

0.241994

0.241994

0
4

0
4

0
4

0.0435124

0.0435124

0.0435124

0.0435124

0
4

0
4

0
4

0.396074

0.396074

0.3199

0.3199

0
4

0.0761741

0.0761741

0
4

1.38777878078145e-17

0
4

0
4

3.1761

3.1761

3.06884

2.9713

0.0975327

0
4

0.107261

0.107261

0
4

0
4

0
4

1.42126

1.42126

1.42126

0.633373

0.787884

0
4

0
4

0
4

1.65366

1.65366

1.65366

0.319618

1.33404

2.22044604925031e-16

0
4

0
4

0
4

0.174591

0.174591

0.174591

0.174591

0
4

0
4

0
4

0.487096

0.487096

0.487096

0.111841

0.375255

0
4

0
4

0
4

0.331491

0.331491

0.224248

0.0633849

0.160864

0
4

0.107242

0.107242

0
4

0
4

0
4

20.0583
4

20.0583
4

19.7505
4

17.7028

1.51849

0.154812

0.00507012

0.184686

0.184686

0
4

0.30781

0.30781

0
4

0
4

0
4

0.661082

0.581093

0.581093

0.581093

0
4

0
4

0.0799885

0.0799885

0.0799885

0
4

0
4

2.77555756156289e-17

0
4

1.0406

1.0406

0.927568

0.927568

0
4

0.100278

0.100278

0
4

0.0127553

0.0127553

0
4

0
4

0
4

2.30783

2.30783

2.30783

2.23166

0.0761741

1.80411241501588e-16

0
4

0
4

0
4

0.00851243

0.00851243

0.00851243

0.00500636

0.00350607

0
4

0
4

0
4

0.00701215

0.00701215

0.00701215

0.00350607

0.00350607

0
4

0
4

0
4

0.248036

0.248036

0.0724936

0.0724936

0
4

0.175543

0.175543

0
4

0
4

0
4

0.38087

0.38087

0.114261

0.114261

0
4

0.266609

0.266609

0
4

0
4

0
4

0.326431

0.326431

0.326431

0.326431

0
4

0
4

0
4

0.0160309

0.0160309

0.00687037

0.00687037

0
4

0.0091605

0.0091605

0
4

0
4

0
4

1.97872

1.97872

1.96576

1.96576

0
4

0.0129576

0.0129576

0
4

7.45931094670027e-17

0
4

0
4

1.08931
7

1.08931
7

0.992927
7

0.038896

0.210445

0.120667

0.131935

0.400434

0.02009

0.0324597

0.0380012

8.32667268468867e-17
7

0
4

0.0963868

0.0793812

0.0170056

0
4

1.38777878078145e-16
7

0
4

0
4

0.55918

0.55918

0.55918

0.55918

0
4

0
4

0
4

0.0984527

0.0984527

0.0984527

0.0984527

0
4

0
4

0
4

0.195065

0.195065

0.195065

0.0975327

0.0975327

0
4

0
4

0
4

0.472952

0.472952

0.459994

0.459994

0
4

0.0129576

0.0129576

0
4

1.90819582357449e-17

0
4

0
4

0.550073

0.550073

0.550073

0.550073

0
4

0
4

0
4

0.200555

0.200555

0.125347

0.125347

0
4

0.0752082

0.0752082

0
4

0
4

0
4

0.460464

0.460464

0.185812

0.185812

0
4

0.274652

0.274652

0
4

0
4

0
4

0.120823

0.120823

0.120823

0.0483291

0.0724936

0
4

0
4

0
4

0.282803

0.282803

0.097998

0.097998

0
4

0.184804

0.184804

0
4

0
4

0
4

1.29468

1.29468

1.29468

0.357411

0.937273

0
4

0
4

0
4

1.02267

0.83112

0.81547

0.263831

0.328387

0.0180906

0.00655526

0.0049252

0.193681

1.11022302462516e-16

0
4

0.0156497

0.0156497

0
4

4.85722573273506e-17

0
4

0.191555

0.191555

0.17418

0.0173753

1.04083408558608e-17

0
4

0
4

0
4

0.412819

0.412819

0.412819

0.412819

0
4

0
4

0
4

0.0520819

0.0520819

0.0391243

0.0391243

0
4

0.0129576

0.0129576

0
4

0
4

0
4

0.318234

0.318234

0.318234

0.318234

0
4

0
4

0
4

0.175486

0.175486

0.175486

0.125347

0.0501388

0
4

0
4

0
4

0.233165

0.233165

0.233165

0.233165

0
4

0
4

0
4

1.81245

1.81245

0.102492

0.102492

0
4

1.70996

1.70996

0
4

0
4

0
4

0.100278

0.100278

0.100278

0.100278

0
4

0
4

0
4

20.9385

20.9385

0.0262201

0.0262201

0
4

20.9123

20.9123

0
4

0
4

0
4

0.341396

0.341396

0.144661

0.144661

0
4

0.196734

0.196734

0
4

0
4

0
4

0.204818

0.204818

0.204818

0.0197008

0.185118

0
4

0
4

0
4

1.78515
5

1.78515
5

0.791873
5

0.389892
5

0.398475

0.00350607

5.89805981832114e-17
5

0
4

0.993279

0.793949

0.0665208

0.00525911

0.12755

0
4

0
4

0
4

0.786339

0.786339

0.786339

0.0761741

0.710164

0
4

0
4

0
4

0.373064

0.373064

0.373064

0.279798

0.093266

1.38777878078145e-17

0
4

0
4

0
4

0.331839

0.331839

0.331839

0.139899

0.19194

0
4

0
4

0
4

0.239777

0.239777

0.239777

0.239777

0
4

0
4

0
4

0.19194

0.19194

0.12796

0.12796

0
4

0.06398

0.06398

0
4

1.38777878078145e-17

0
4

0
4

0.63118

0.63118

0.63118

0.63118

0
4

0
4

0
4

0.455867

0.455867

0.455867

0.455867

0
4

0
4

0
4

0.106314

0.106314

0.106314

0.106314

0
4

0
4

0
4

0.792761

0.792761

0.279798

0.279798

0
4

0.512963

0.512963

0
4

0
4

0
4

0.0282575

0.0282575

0.0282575

0.0282575

0
4

0
4

0
4

2.81596
7

2.81245
7

2.20869
7

1.65557
7

0.553122

0
4

0.0538193

0.0368138

0.0170056

0
4

0.549937

0.549937

0
4

4.44089209850063e-16
7

0
4

0.00350607

0.00350607

0.00350607

0
4

0
4

1.14491749414469e-16
7

0
4

0.154107

0.154107

0.154107

0.105778

0.0483291

0
4

0
4

0
4

0.0316877

0.0316877

0.0316877

0.0316877

0
4

0
4

0
4

0.0127553

0.0127553

0.0127553

0.0127553

0
4

0
4

0
4

0.459471

0.459471

0.459471

0.459471

0
4

0
4

0
4

0.536117

0.536117

0.536117

0.536117

0
4

0
4

0
4

0.00503411

0.00503411

0.00503411

0.00503411

0
4

0
4

0
4

0.129121

0.129121

0.129121

0.129121

0
4

0
4

0
4

0.0583091

0.0583091

0.0583091

0.0583091

0
4

0
4

0
4

0.0798828

0.0798828

0.0798828

0.0798828

0
4

0
4

0
4

0.0642886

0.0642886

0.0642886

0.0642886

0
4

0
4

0
4

96.4956

95.1674

76.9674

2.68295

0.109669

0.0523659

0.285067

1.14555

1.19141

0.464867

0.325902

0.202379

1.02785

0.199747

7.426

4.23637

4.84968

2.09479

0.576596

0.127142

0.20597

0.596022

0.418107

2.27611

0.0704951

6.47876
4

0.251341

0.0599005

4.19836

0.0293706

0.250694

0.165843

1.94856

0.42663

0.228522

0.0501388

2.1562

1.25094

0.014664

0.0173753

0.119824

0.536117

0.133931

0.0469069

0.0761741

0.0352475

0.53643

0.267166

0.303481

0.114261

0.007332

0.159505

0.0316877

0.01833

1.0487

0.357411

0.025662

0.0398762

3.04696

0.0761741

0.0129576

0.00564832

0.763337

0.00319998

0.0649194

0.146997

0.0327735

0.0956648

0.0528713

9.9792

0.27495

0.107261

0.0501388

0.0194364

1.19842

0.0324597

1.05291

0.0112449

0.0506019

0.119824

1.57831

0.0761741

0.504686

2.29001

0.424874

0.437713

1.60444

0.221814

0.274652

0.406369

2.88657986402541e-15

0
4

0.802979

0.721056

0.0345492

0.0404642

0.00690984

0
4

0.0475315

0.0475315

0
4

0.19194

0.19194

0
4

0.119811

0.119811

0
4

0.0380041

0.0380041

0
4

0.00690984

0.00690984

0
4

13.0214

11.5361

1.37113

0.114261

6.93889390390723e-17

0
4

1.35801

0.998535

0.359473

5.55111512312578e-17

0
4

0.821931

0.669583

0.0761741

0.0761741

0
4

0.466414

0.445684

0.0138197

0.00690984

0
4

0.248956

0.214205

0.0347507

0
4

0.984991

0.984991

0
4

0.0761741

0.0761741

0
4

0.0149617

0.0149617

0
4

2.602605631008e-14

0
4

0.568387

0.568387

0.43532

0.0276393

0.095063

0.0103648

0
4

0
4

0.0518238

0.0380041

0.0207295

0.00690984

0.0103648

3.46944695195361e-18

0
4

0.0138197

0.0138197

0
4

1.73472347597681e-18

0
4

0.685567

0.685567

0.457045

0.228522

0
4

0
4

0.0173753

0.0173753

0.0173753

0
4

0
4

0.00498723

0.00498723

0.00498723

0
4

0
4

4.07573280680751e-15

0
4

0.0761741

0.0761741

0.0761741

0.0761741

0
4

0
4

0
4

4.36404426484849e-10

0
4

331.588
3

316.106
3

257.78
3

29.2374

8.55714

1.4795

1.71942

9.31688

0.319893

6.10797

1.23329

0.23992

0.107242

0.0799732

0.0761658

8.46545056276682e-16

0
4

188.322
3

188.163
3

0.11996

0.0388728

0
4

14.5231

14.5231

0
4

7.03465

3.4241

3.25431

0.0565967

0.0507772

0.0848951

0.0565967

0.0565967

0.0507772

9.36750677027476e-16

0
4

2.40793

0.775882

1.17671

0.375348

0.0799885

1.38777878078145e-16

0
4

0.00416335

0.00416335

0
4

6.13324

6.13324

0
4

0.00780182

0.00780182

0
4

0.185812

0.185812

0
4

0.195677

0.191514

0.00416335

4.33680868994202e-18

0
4

1.30172

1.30172

0
4

0.650343

0.464531

0.185812

0
4

1.3182

1.08804

0.230163

5.55111512312578e-17

0
4

2.43311

2.43311

0
4

0.253964

0.00416335

0.249801

0
4

1.30172

1.30172

0
4

2.46902

2.46902

0
4

0
4

25.6145

25.6145

14.0907

5.47182

5.01244

0.601646

0.371625

0.0663141

0
4

0
4

27.8619

27.8619

26.6541

1.20778

1.55431223447522e-15

0
4

0
4

4.58012

4.58012

4.58012

0
4

0
4

0.269574

0.269574

0.269574

0
4

0
4

1.41553435639707e-13
3

0
4

0.74178

0.74178

0.74178

0.666555

0.0112449

0.06398

1.38777878078145e-17

0
4

0
4

0
4

0.0701542

0.0701542

0.0701542

0.0701542

0
4

0
4

0
4

2.00002

2.00002

2.00002

2.00002

0
4

0
4

0
4

2.0793

2.0793

2.0793

2.0793

0
4

0
4

0
4

0.35988

0.35988

0.35988

0.35988

0
4

0
4

0
4

0.369371

0.369371

0.369371

0.369371

0
4

0
4

0
4

1.71942

1.71942

1.71942

1.71942

0
4

0
4

0
4

5.73288

5.73288

5.73288

5.73288

0
4

0
4

0
4

0.05102

0.05102

0.05102

0.05102

0
4

0
4

0
4

0.0910405

0.0910405

0.0910405

0.0910405

0
4

0
4

0
4

0.375413

0.375413

0.375413

0.107261

0.268152

0
4

0
4

0
4

0.0220119

0.0220119

0.0220119

0.0170056

0.00500636

0
4

0
4

0
4

1.32763

1.32763

1.32763

1.32763

0
4

0
4

0
4

0.357411

0.357411

0.357411

0.357411

0
4

0
4

0
4

0.00443786

0.00443786

0.00443786

0.00443786

0
4

0
4

0
4

0.0798828

0.0798828

0.0798828

0.0798828

0
4

0
4

0
4

0.100278

0.100278

0.100278

0.100278

0
4

0
4

0
4

8.11711808879068e-14
3

0
4

5614.29

929.101
4

209.199

200.264

27.639

0.482674

2.30611

0.545426

1.60891

0.268152

1.23691

0.429044

0.107261

5.14853

0.107261

136.484

0.107261

0.160891

0.29211

0.107261

0.482674

0.107261

0.160891

0.858088

0.107261

1.82344

3.74842

0.107261

0.804457

0.268152

0.321783

5.22611

4.12724

1.12052

0.429044

0.375413

3.1642

0
4

1.9307

1.34076

0.482674

0.107261

6.93889390390723e-17

0
4

0.429044

0.268152

0.160891

0
4

6.576

5.84572

0.730274

4.44089209850063e-16

0
4

0
4

33.7177
4

9.4182

1.36428

2.71382

0.262628

5.07747

8.88178419700125e-16

0
4

23.424

1.66331

16.3331

5.42764

0
4

0.175085

0.175085

0
4

0.700341

0.700341

0
4

0
4

90.6058

89.992

88.8121

0.160891

1.01898

6.21724893790088e-15

0
4

0.506632

0.399371

0.107261

0
4

0.107261

0.107261

0
4

1.50573997714787e-14

0
4

0.85488
6

0.85488
6

0.85488
6

0
4

0
4

0.618857

0.263298

0.263298

0
4

0.35556

0.35556

0
4

0
4

3.32455

2.3919

2.31537

0.07653

4.16333634234434e-17

0
4

0.881636

0.763337

0.118299

5.55111512312578e-17

0
4

0.05102

0.05102

0
4

0
4

1.60002

1.60002

1.55557

0.044445

8.32667268468867e-17

0
4

0
4

0.400309

0.400309

0.175085

0.0501388

0.175085

0
4

0
4

0.370607

0.185303

0.0617678

0.123536

0
4

0.185303

0.185303

0
4

0
4

0.0336511

0.0336511

0.0336511

0
4

0
4

0.0888899

0.044445

0.044445

0
4

0.044445

0.044445

0
4

0
4

233.466
4

223.512
4

151.413

5.9529

0.437713

0.175085

2.01348

0.262628

0.262628

0.262628

0.437713

13.5074

29.3191

3.23908

9.04236

1.84657

4.20205

0.787884

0.350171

0
4

6.81087

5.55299

0.612799

0.29491

0.175085

0.175085

1.16573417585641e-15

0
4

3.14266

2.40074

0.199707

0.0798828

0.0798828

0.262628

0.119824

0
4

0
4

1.10537

1.10537

1.10537

0
4

0
4

0.235063

0.235063

0.235063

0
4

0
4

0.0573536

0.0573536

0.0573536

0
4

0
4

0.0104881

0.0104881

0.0104881

0
4

0
4

0.160864

0.160864

0.160864

0
4

0
4

0.175085

0.175085

0.175085

0
4

0
4

0.0491603

0.0491603

0.0491603

0
4

0
4

22.5158
4

19.5806
4

7.25189

0.241929

1.44915

1.46904

0.639745

0.514309

0.161286

6.9353

0.763337

0.154593

3.33066907387547e-15
4

0
4

2.02959

2.02959

0
4

0.596446

0.158281

0.438165

0
4

0.309187

0.309187

0
4

2.44249065417534e-15
4

0
4

10.8745
4

3.27672
4

1.58616

0.89642

0.738813

0.0553261

3.33066907387547e-16
4

0
4

5.70521

5.0739

0.399414

0.23189

0
4

1.8481

1.8481

0
4

0.044445

0.044445

0
4

0
4

31.1218
4

31.1218
4

16.1487

8.11194
4

3.54759

2.29253

0.340441

0.161286

0.161286

0.161286

0.196734

5.27355936696949e-15
4

0
4

0
4

71.5374

68.7246

66.8772

0.107261

0.429044

0.482674

0.536305

0.29211

5.38458166943201e-15

0
4

0.989306

0.667523

0.160891

0.160891

0
4

1.39439

0.804457

0.107261

0.321783

0.160891

5.55111512312578e-17

0
4

0.107261

0.107261

0
4

0.321783

0.321783

0
4

1.72639680329212e-14

0
4

1.39685
6

1.39685
6

1.35278
6

0.0367082

0.00736842

1.52655665885959e-16
6

0
4

0
4

210.647

185.853

183.402

0.612799

0.262628

0.175085

0.350171

0.525256

0.262628

0.262628

0
4

22.3018

20.5509

1.75085

1.33226762955019e-15

0
4

2.05409

1.35375

0.525256

0.175085

0
4

0.437713

0.437713

0
4

3.94129173741931e-15

0
4

4.93463

4.87286

2.77021

0.958419

0.835392

0.0926517

0.216187

0
4

0.0617678

0.0617678

0
4

3.46944695195361e-17

0
4

0
4

70.6104
5

70.6104
5

0.135172

0.135172

0
4

70.4753
5

23.0593
5

37.8989

0.697196

0.743249

0.661404

1.3101

4.16562

0.13473

0.695993

0.0617678

0.308259

0.738743

0
4

1.4210854715202e-14
5

0
4

0
4

4015.78
7

5.85533
6

5.85032
6

4.37974

1.47058

0
4

0.00500636

0.00500636

0
4

0
4

201.535

22.2432

10.4101

0.17951
1

0.113686

0.24379

0.0262956

0.049085

0.0217228

0.0771336

0.130735

0.240166

0.0105182

1.53566

0.152514

0.0280486

0.00701215

0.045579

0.0403199

0.0140243

0.108688

0.00701215

0.00525911

0.00525911

1.20418

0.0122713

0.0125853

0.0239728

0.00525911

0.0245425

0.0280486

0.00525911

0.0298016

0.00876519

0.00350607

5.45855

0.00350607

0.0403199

0.00350607

0.0105182

0.00710613

0.00350607

0.00350607

0.00592247

0.0631093

0.0404642

0.361126

0.0140243

0.00350607

0.211081

0.00350607

0.0262956

0.0262956

0.0210364

0.0105182

0.00876519

0.00350607

0.0403199

0.0157773

0.0245801

0.164174

0.105743

0.00350607

0.0438259

0.00350607

0.0110526

0.00701215

0.440012

0.124466

0.119207

0
4

5.34794

0.615562

0.0484412

0.847722

0.784125

1.01727

0.0809283

1.81655

0.0484412

0.0484412

0.0404642

1.94289029309402e-16

0
4

60.1432

14.1603

0.213523

0.00736842

0.0770648

0.125643

0.128202

0.146088

0.0404642

0.0881189

0.266335

0.0112449

0.0112449

0.211848

0.00498723

0.101521

0.0246725

0.0176194

0.0104881

1.29776

2.1446

0.00592247

0.0404642

0.0104024

0.0868508

0.0100682

0.0589474

0.00503411

0.0224897

0.0049252

0.0515789

0.0629264

0.0404642

0.0194364

0.00458025

0.871544

0.00710613

0.00448724

0.0104024

0.0215412

0.0137407

0.0327735

0.141625

0.013003

0.0150095

0.202321

0.0197636

0.0887784

0.0110526

0.0147756

0.00748085

0.0405263

0.577986

0.00799092

0.00748085

0.0184584

0.0138191

0.180072

0.0262201

0.00520121

0.182089

0.00755116

0.00592247

0.0458025

0.00780182

0.385324

0.0147368

0.115597

0.166137

0.519474

0.0049252

0.0444196

0.0110544

0.167087

6.58524
6

0.232106

2.84411

0.0516024

0.0767946

0.159264

0.623834

0.0223163

0.0122891

0.0368117

0.00448724

0.990479
7

0.00748085

0.53643

0.251713

0.0404642

0.317228

0.0404642

0.0246185

0.144196

0.327003

0.00394831

1.50874
8

0.025154

0.00736842

0.125853

0.032394

0.0549439

2.56494

0.0294737

0.0100616

0.357411

0.0887784

2.40314

1.73996

1.61836

0.917377

0.00780182

0.0226535

0.00532728

0.0104881

0.528713

0.0381415

0.0104881

3.6694

0.0112449

0.479431

0.0528581

0.141625

0.00710613

0.0259152

0.0491603

0.0598468

0.0134617

0.0129576

0.58587

0.0352475

0.0434384

0.0281121

0.0131105

0.0809283

0.0404642

0.00736842

0.00394831

0.0336543

0.0176194

0.322707
7

0.0352475

0.0194364

0.0156036

0.236743

0.00448724

0.0409669

0.0770648

0.303611

0.0173753

0.0639551

0.281495

0.0938138

2.22553

0.0352475

0.718432

0.00498723

0.299604

0.647551

0.122527

0.16154

0.0177653

0
4

2.03414
6

0.023516

0.0235036

1.96251

0.00527298

0.010546

0.0087883

0
4

2.89138

2.89138

0
4

0.672555
7

0.658146

0.0144089

0
4

0.169212

0.104029

0.0606962

0.00448724

6.07153216591882e-18

0
4

0.304547

0.292598

0.00736842

0.00458025

0
4

0.192429

0.0206981

0.144196

0.027535

1.04083408558608e-17

0
4

0.993489

0.977839

0.0156497

4.85722573273506e-17

0
4

0.062599

0.062599

0
4

0.101394

0.0469069

0.0544875

0
4

0.0150191

0.0100127

0.00500636

0
4

82.0957

79.2954

0.0104881

0.0809283

0.471292

0.108046

0.233041

1.819

0.014939

0.0104881

0.0310471

0.0209761

2.59965660109884e-14

0
4

0.0454705

0.0454705

0
4

0.0934515

0.0163868

0.0770648

0
4

0.0967473

0.0967473

0
4

1.75042

1.70996

0.0404642

0
4

0.0387615

0.0387615

0
4

0.0129839

0.0129839

0
4

0.0112449

0.0112449

0
4

0.00500636

0.00500636

0
4

0.0129839

0.0129839

0
4

0.00527298

0.00527298

0
4

3.783

1.24209

1.82837

0.657035

0.0337346

0.00527298

0.00350607

0.0129839

9.19403442267708e-16

0
4

0.0129576

0.0129576

0
4

0.00394831

0.00394831

0
4

0.0112449

0.0112449

0
4

0.053042

0.053042

0
4

0.0842243

0.0842243

0
4

0.00411208

0.00411208

0
4

0.00503411

0.00503411

0
4

0.0404642

0.0404642

0
4

0.10116

0.10116

0
4

0.0404642

0.0404642

0
4

2.29644

0.227174
7

0.00750954

0.00689936

0.866867

0.405351
7

0.635323

0.0125159

0.0878143

0.00673085

0.00500636

0.0352475

0
4

0.00750954

0.00750954

0
4

0.182089

0.182089

0
4

0.123366

0.123366

0
4

0.00448724

0.00448724

0
4

0.546266

0.546266

0
4

0.357411

0.357411

0
4

0.0245801

0.0245801

0
4

0.00394831

0.00394831

0
4

0.357411

0.357411

0
4

0.00500636

0.00500636

0
4

4.1801

1.73834

0.00615463

0.0104881

0.0129576

0.10116

0.195065

0.670184
6

0.421993

0.506881

0.148903

0.0313785

0.0209761

0.30781

0.00780182

1.66533453693773e-16

0
4

0.346792

0.346792

0
4

0.174927

0.174927

0
4

0.109548

0.109548

0
4

3.26687

2.08207

0.190856

0.17113

0.487952

0.182732

0.0390837

0.0704951

0.0300382

0.0125159

0
4

2.96087

1.96919
7

0.837816

0.10116

0.0246072

0.0210919

0.00701215

6.24500451351651e-17

0
4

2.17302

0.295713

0.0179448

1.76019

0.0881189

0.0110526

0
4

0.935685

0.768377

0.0498632

0.0477634

0.0219184

0.0352475

0.00500636

0.00750954

1.55257751099924e-16

0
4

4.41646719195887e-13

0
4

3742.18
7

129.35
6

28.9153
6

9.56879
7

4.18527

2.08002

0.739831

0.223403

0.416772

0.466245

0.599159

0.657818

3.88089

0.378767

9.94615
6

1.77455

0.136055

1.77772

0.239622

0.353778

0.0122713

0.19667

2.60589

0.0194898

0.149764

0.17881

0.0915931

0.0982093

0.0894049

0.391642

0.149764

3.45507

0.119811

0.0599054

0.0898581

0.209669

2.53037

0.00967569

0.0898581

0.119811

0.411964

0.0163868

0.00997446

0.119811

0.239622

0.0469069

0.0599054

1.09516

0.0404642

0.0599054

0.0599054

0.0968956

0.0599054

0.0250534

0.599054

0.0898581

0.0129839

0.119811

3.46765
5

0.0599054

0.00755116

0.0599054

0.0606962

0.0469069

0.0469069

0.00701215

0.0599054

0.0316877

0.0159983

6.74324
6

0.00525911

0.0599054

0.00350607

0.0703603

0.00350607

0.0599054

0.0475315

0.0475315

0.269574

37.4604
6

0.544331
6

5.6621374255883e-14
6

0
4

13.2925
7

13.0509
7

0.00750954

0.144196

0.0898581

0
4

3072.99
7

2478.34
7

586.558
7

0.210892

0.01833

0.272681

0.082434

0.115773

0.198185

0.00527298

0.0259152

0.536117

0.0173753

0.0839044

0.203266

0.0194758

0.257988

0.097998

1.52667

0.107242

0.0547741

0.00498723

0.31191

0.00500636

0.291731

0.0335305

0.0245801

0.01833

0.0163868

0.0703603

0.0483689

0.00894146

0.00887572

0.0156497

0.032394

0.0469069

0.248269

0.0404642

0.0224362

0.357411

0.453446

1.82566

0.186009

0.13019

0.0562243

1.51998552633259e-12
7

0
4

2.39855
7

1.88223

0.440657

0.0387519

0.00835115

0.0285611

0
4

21.8359

21.3408

0.438165

0.0100682

0.0469069

0
4

93.6335
7

87.5172
7

0.197266

0.273633

0.179752

0.0298778

0.15489

0.0876796

0.113796

0.0183438

0.0167327

0.0106592

0.0151023

0.777633
7

0.00710613

0.0404642

0.0201364

0.00827008

0.0316877

0.0155008

0.00500636

0.056849

0.0104881

0.00500636

2.35706
6

0.0454854

0.0123036

0.0316877

0.00503411

0.0156497

0.00527298

0.731779

0.0305603

0.12346

0.0546764

0.212817

0.414661

3.23630011678233e-14
7

0
4

198.991
6

193.068
6

5.56557

0.195527

0.161857

0
4

91.9969
6

91.6177
6

0.140721

0.0209761

0.0469069

0.0165402

0.15413

0
4

57.6021
6

3.05972
7

3.35054
6

0.135283
7

0.619368

0.0816195

0.622333

0.315227

0.0260588

0.0387938

1.24589

0.0276182

0.0124051

5.41882

0.00615279

0.0112449

0.00655526

0.30781

0.0112449

0.0404642

0.0319776

0.0103648

0.0156497

0.0173753

0.710212
7

0.00750954

0.00362217

0.0316877

0.0173753

0.00503411

0.0245801

0.144196

0.00503411

26.7708
6

1.12049
7

7.16526
6

1.7125
7

0.262184
7

4.20916
6

1.50990331349021e-14
6

0
4

2.02374
7

1.52136
7

0.240729

0.134645

0.0129576

0.0888266

0.00612023

0.014664

0.00443786

0
4

2.84834

0.61791

0.0898581

0.0475315

0.966474

0.0152104

0.110907

0.539149

0.00507012

0.0633753

0.32948

0.0633753

0
4

11.1828
6

11.1551
6

0.0276876

1.56125112837913e-16
6

0
4

15.4514
6

14.6512
6

0.0459732

0.00500636

0.00827008

0.740941

5.55111512312578e-16
6

0
4

2.68215

2.53585

0.146299

0
4

0.629007

0.629007

0
4

0.251545

0.0404642

0.211081

0
4

0.780261

0.780261

0
4

1.64004

1.64004

0
4

0.0126753

0.0126753

0
4

0.00503411

0.00503411

0
4

0.484478

0.484478

0
4

0.10116

0.10116

0
4

0.00458879

0.00458879

0
4

2.03398
7

1.52945
7

0.367252

0.0599054

0.0369109

0.0404642

0
4

0.00350607

0.00350607

0
4

0.656697

0.656697

0
4

0.206119

0.206119

0
4

0.114261

0.114261

0
4

0.0250318

0.0250318

0
4

0.0404642

0.0404642

0
4

0.349789

0.349789

0
4

1.02409

1.02409

0
4

0.0129839

0.0129839

0
4

0.0102802

0.0102802

0
4

4.25584
7

4.16459
7

0.0912536

0
4

0.00710613

0.00710613

0
4

0.0599054

0.0599054

0
4

0.00408015

0.00408015

0
4

0.0129576

0.0129576

0
4

0.0245801

0.0245801

0
4

0.504686

0.504686

0
4

0.808723

0.808723

0
4

0.049085

0.049085

0
4

0.0316877

0.0316877

0
4

0.107261

0.107261

0
4

0.549926

0.123296

0.0591856

0.308259

0.0591856

4.16333634234434e-17

0
4

0.00408015

0.00408015

0
4

0.0770648

0.0770648

0
4

0.00500636

0.00500636

0
4

0.0681724

0.0681724

0
4

0.0316877

0.0316877

0
4

2.43631
7

2.43631
7

0
4

3.82375

3.82375

0
4

3.98729

3.98729

0
4

0.448032

0.431645

0.0163868

0
4

0.21317

0.21317

0
4

1.77075021312589e-12
7

0
4

3.95991
7

3.72019
6

1.49533

2.09377
6

0.119811

0.00805789

0.00322316

0
4

0.239715

0.116618

0.123097

1.38777878078145e-17

0
4

2.77555756156289e-16
7

0
4

5.13613
7

1.72474

1.72474

0
4

2.99242
8

2.92813
8

0.0642886

0
4

0.0633753

0.0316877

0.0316877

0
4

0.007332

0.007332

0
4

0.0131105

0.0131105

0
4

0.0404642

0.0404642

0
4

0.0227895

0.0227895

0
4

0.0129576

0.0129576

0
4

0.195564

0.195564

0
4

0.0633753

0.0633753

0
4

6.24500451351651e-16
7

0
4

42.3635

31.1844

24.6575

6.52686

5.32907051820075e-15

0
4

1.31325

0.693659

0.19171

0.147733

0.258376

0.0217722

1.52655665885959e-16

0
4

0.00408015

0.00408015

0
4

0.0123036

0.0123036

0
4

0.200843

0.200843

0
4

0.0112449

0.0112449

0
4

6.5298
5

6.46642
5

0.0633753

0
4

0.801523
7

0.393563

0.40796

0
4

0.044022

0.044022

0
4

0.157475

0.130387

0.0270886

0
4

0.233226

0.20027

0.0329558

0
4

0.0825287

0.0790134

0.00351532

0
4

1.77876

1.77876

0
4

0.0101402

0.0101402

0
4

5.82867087928207e-16

0
4

13.3872
7

13.3872
7

6.62816

1.28328

0.317461

5.12666
6

0.0316877

1.71390679426509e-15
7

0
4

0
4

0.510674

0.510674

0.510674

0
4

0
4

0.0157321

0.0157321

0.0157321

0
4

0
4

0.0106592

0.0106592

0.0106592

0
4

0
4

0.0562243

0.0562243

0.0562243

0
4

0
4

0.117195

0.117195

0.0334842

0.0502263

0.0334842

0
4

0
4

0.292598

0.292598

0.292598

0
4

0
4

0.0534217

0.0534217

0.0404642

0.0129576

0
4

0
4

0.195065

0.195065

0.195065

0
4

0
4

0.0387582

0.0387582

0.0387582

0
4

0
4

0.0245801

0.0245801

0.0245801

0
4

0
4

0.0347507

0.0347507

0.0347507

0
4

0
4

0.0129576

0.0129576

0.0129576

0
4

0
4

3.78415875024185e-12
7

0
4

24.4499

21.1235

8.64135
7

1.7395

0.0528396

0.0049252

0.0877659

0.321727

0.0469069

0.00888202

0.0898581

0.0599054

0.281441

0.00738779

5.04707
6

0.0163985

0.0049252

0.0599054

0.0172382

0.0585106

0.0614833

0.346434

0.00738779

0.304895

0.00364411

0.012313

0
4

1.61143

1.61143

0
4

9.44267

0.223326
7

6.43716

1.07223

1.70996

0
4

0.0797957

0.0748085

0.00498723

0
4

0.0163868

0.0163868

0
4

0.375348

0.375348

0
4

0.70463

0.70463

0
4

0.251928

0.251928

0
4

0
4

0.294021

0.294021

0.0333077

0.188839

0.0420729

0.0105182

0.0157773

0.00350607

0
4

0
4

0.0475315

0.0475315

0.0475315

0
4

0
4

0.0475315

0.0475315

0.0475315

0
4

0
4

0.0387582

0.0387582

0.0387582

0
4

0
4

0.92656

0.92656

0.829028

0.0975327

5.55111512312578e-17

0
4

0
4

0.397309

0.0398978

0.0398978

0
4

0.357411

0.357411

0
4

0
4

1.07242

1.07242

1.07242

0
4

0
4

0.108988

0.00532728

0.00532728

0
4

0.103661

0.103661

0
4

0
4

0.184686

0.184686

0.184686

0
4

0
4

0.0475315

0.0475315

0.0475315

0
4

0
4

0.14463

0.14463

0.14463

0
4

0
4

0.0163868

0.0163868

0.0163868

0
4

0
4

0
4

36.7768

32.0505

29.6982

4.57574
6

0.389385

0.0633753

0.0404642

4.61439

0.0599054

0.0583091

1.31657

0.159946

0.0163868

0.0173753

5.47853
6

0.0327735

0.00305919

0.115597

0.0404642

2.06263

0.0404642

0.0173753

5.11447

1.34185

0.408115

0.898879

0.231794

2.56143

0.0388728

2.32452945780892e-15

0
4

2.15467

2.15467

0
4

0.0107072

0.0107072

0
4

0.0552787

0.0552787

0
4

0.131657

0.131657

0
4

0
4

0.0773684

0.0773684

0.0663158

0.0110526

0
4

0
4

1.41422

1.41422

1.41422

0
4

0
4

0.554057

0.554057

0.554057

0
4

0
4

0.394971

0.394971

0.394971

0
4

0
4

0.176201

0.176201

0.176201

0
4

0
4

0.246248

0.246248

0.246248

0
4

0
4

1.75266

1.75266

1.75266

0
4

0
4

0.0207295

0.0207295

0.0207295

0
4

0
4

0.0898581

0.0898581

0.0898581

0
4

0
4

0
4

23.7771

0.142595

0.142595

0.110907

0.0316877

0
4

0
4

9.51511

1.07717

1.07717

0
4

6.87327
1

0.593251

0.301033

5.77728

0.201698

9.15933995315754e-16
1

0
4

1.37937

1.26486

0.0526729

0.0618334

0
4

0.185303

0.185303

0
4

0
4

14.1194

0.288392

0.144196

0.144196

0
4

13.831

8.26021
1

4.98972

0.581044

8.88178419700125e-16

0
4

0
4

0
4

122.438
5

119.483
5

53.7279

22.2794

25.1835
7

0.00498723

0.0915731

0.0157321

1.16968

0.00305919

0.137675

0.462038

3.96758

0.318032

0.0224425

0.0125217

0.0310694

0.0209761

0.00764798

7.48880124579188e-15

0
4

5.06613
6

2.22415
6

0.369816
7

1.62273
6

0.0519133

0.718865

0.00750954

0.0599054

0.0112449

0
4

0.184686

0.184686

0
4

0.175486

0.175486

0
4

0.185812

0.185812

0
4

0.185812

0.185812

0
4

1.31807

1.31807

0
4

0.301033

0.301033

0
4

15.6346
6

15.6182
6

0.0163868

7.11236625150491e-16
6

0
4

11.033

2.79571

0.929061

0.650343

0.15442

6.50343

0
4

0.122187

0.0922638

0.0249362

0.00498723

2.60208521396521e-18

0
4

9.17144

1.56647

0.185812

7.41916

0
4

0.216187

0.123536

0.0926517

0
4

1.61696

1.61696

0
4

0.555114

0.555114

0
4

19.9885

19.9885

0
4

7.46069872548105e-14
5

0
4

0.608442

0.608442

0.59348

0.0149617

0
4

0
4

2.17198

2.17198

0.460513

1.71147

0
4

0
4

0.0633753

0.0633753

0.0633753

0
4

0
4

0.0475315

0.0475315

0.0475315

0
4

0
4

0.0469069

0.0469069

0.0469069

0
4

0
4

0.00997446

0.00997446

0.00997446

0
4

0
4

0.00690984

0.00690984

0.00690984

0
4

0
4

0
4

2.38642

2.38642

1.73971

1.35666

0.3217

0.0122713

0.0122713

0.0210364

0.00701215

0.00350607

0.00525911

0
4

0.147255

0.0508381

0.0227895

0.0683684

0.00525911

2.42861286636753e-17

0
4

0.0403199

0.0227895

0.00525911

0.00525911

0.00701215

0
4

0.0140243

0.0140243

0
4

0.445106

0.445106

0
4

0
4

0
4

74.8308
3

74.8308
3

74.8308
3

74.5977
3

0.233165

3.85802501057242e-15
3

0
4

0
4

0
4

1.53984
6

1.53984
6

1.46948
6

1.4531
6

0.0163868

0
4

0.0703603

0.0703603

0
4

1.38777878078145e-16
6

0
4

0
4

4.01768

4.01768

3.9539

3.67572

0.0451493

0.220077

0.0129576

6.29704621779581e-16

0
4

0.0637765

0.0446436

0.019133

3.46944695195361e-18

0
4

3.33066907387547e-16

0
4

0
4

3.38171

3.38171

3.38171

3.2708

0.0792192

0.0316877

2.84494650060196e-16

0
4

0
4

0
4

9.1819
4

9.1819
4

9.00445
4

8.27536
4

0.610794

0.118299

0
4

0.177448

0.177448

0
4

1.94289029309402e-16
4

0
4

0
4

0.627971

0.627971

0.627971

0.544856

0.0127553

0.0703603

0
4

0
4

0
4

0.595801

0.595801

0.595801

0.588891

0.00690984

0
4

0
4

0
4

0.383949

0.383949

0.383949

0.143291

0.143063

0.0715316

0.026063

0
4

0
4

0
4

0.186239

0.186239

0.186239

0.147481

0.0387582

0
4

0
4

0
4

1.30116

1.30116

1.30116

1.30116

0
4

0
4

0
4

3.16045
6

3.16045
6

3.16045
6

0.361574

0.827008

1.86957

0.0724564

0.00498723

0.0173753

0.00748085

1.87350135405495e-16
6

0
4

0
4

0
4

7.40487
6

7.40487
6

7.38713

7.38713

0
4

0.0177425

0.0177425

0
4

1.97758476261356e-16
6

0
4

0
4

0.789413

0.789413

0.789413

0.782761

0.00665242

0
4

0
4

0
4

0.283249

0.283249

0.283249

0.283249

0
4

0
4

0
4

1.14268

1.14268

0.277506

0.269727

0.00777913

0
4

0.865175

0.865175

0
4

0
4

0
4

1.9307

1.9307

1.9307

1.50165

0.429044

0
4

0
4

0
4

0.0449139

0.0449139

0.0449139

0.0172746

0.00690984

0.0207295

0
4

0
4

0
4

6.03108

6.03108

6.03108

5.26774

0.763337

0
4

0
4

0
4

0.507008

0.507008

0.507008

0.507008

0
4

0
4

0
4

0.344518

0.344518

0.223126

0.223126

0
4

0.121392

0.121392

0
4

0
4

0
4

0.439355

0.439355

0.439355

0.239648

0.119824

0.0798828

0
4

0
4

0
4

24.3039
4

24.3039
4

18.2455
4

6.64034

9.53608

0.811629

0.643929

0.185812

0.185812

0.241929

0
4

6.0584
4

5.78853

0.11996

0.149909

0
4

8.88178419700125e-16
4

0
4

0
4

0.758887

0.758887

0.758887

0.519238

0.239648

0
4

0
4

0
4

0.121392

0.121392

0.0809283

0.0404642

0.0404642

0
4

0.0404642

0.0404642

0
4

0
4

0
4

2.58421

2.58421

2.00214

2.00214

0
4

0.582074

0.582074

0
4

0
4

0
4

0.123385

0.123385

0.123385

0.123385

0
4

0
4

0
4

1.75511

1.75511

1.75511

0.0451493

1.70996

0
4

0
4

0
4

0.539321

0.539321

0.539321

0.539321

0
4

0
4

0
4

0.948269

0.948269

0.598098

0.277017

0.321081

0
4

0.350171

0.350171

0
4

1.11022302462516e-16

0
4

0
4

0.569959

0.569959

0.569959

0.569959

0
4

0
4

0
4

0.97241

0.97241

0.97241

0.97241

0
4

0
4

0
4

0.267445

0.267445

0.267445

0.267445

0
4

0
4

0
4

30.9352
4

30.9352
4

19.0944
4

4.02223

4.36148

8.17907

0.403215

0.289863

0.23189

1.31449

0.29211

5.55111512312578e-17
4

0
4

11.6862

8.6638

2.18489

0.107261

0.730274

0
4

0.154593

0.154593

0
4

6.10622663543836e-15
4

0
4

0
4

0.849201

0.849201

0.849201

0.849201

0
4

0
4

0
4

2.38874

2.38874

2.38874

2.38874

0
4

0
4

0
4

13.8269

13.8269

13.8269

13.8269

0
4

0
4

0
4

0.150416

0.150416

0.150416

0.0501388

0.100278

0
4

0
4

0
4

0.0714657

0.0714657

0.0714657

0.0714657

0
4

0
4

0
4

0.338452

0.338452

0.322802

0.322802

0
4

0.0156497

0.0156497

0
4

0
4

0
4

0.0694547

0.0694547

0.0694547

0.0694547

0
4

0
4

0
4

0.0469069

0.0469069

0.0469069

0.0469069

0
4

0
4

0
4

0.0194364

0.0194364

0.0194364

0.0194364

0
4

0
4

0
4

0.0347274

0.0347274

0.0347274

0.0347274

0
4

0
4

0
4

105.528
4

105.528
4

97.5349
4

95.634
4

0.106346

1.23675

0.154593

0.403215

0
4

3.99215

3.99215

0
4

2.08994

0.161286

1.92866

0
4

1.91113

1.91113

0
4

2.22044604925031e-16
4

0
4

0
4

0.0194364

0.0194364

0.0194364

0.0194364

0
4

0
4

0
4

0.0137664

0.0137664

0.0137664

0.0137664

0
4

0
4

0
4

0.0229265

0.0229265

0.0229265

0.0229265

0
4

0
4

0
4

0.0943925

0.0943925

0.0943925

0.0943925

0
4

0
4

0
4

0.125347

0.125347

0.125347

0.125347

0
4

0
4

0
4

0.00498723

0.00498723

0.00498723

0.00498723

0
4

0
4

0
4

0.308259

0.308259

0.308259

0.308259

0
4

0
4

0
4

0.0327735

0.0327735

0.0327735

0.0327735

0
4

0
4

0
4

0.00736842

0.00736842

0.00736842

0.00736842

0
4

0
4

0
4

0.00458879

0.00458879

0.00458879

0.00458879

0
4

0
4

0
4

2.94029

2.94029

2.0687

2.04941

0.00350607

0.00350607

0.00525911

0.00701215

0
4

0.0262956

0.00525911

0.00701215

0.00876519

0.00525911

0
4

0.829515

0.829515

0
4

0.0122713

0.00525911

0.00701215

0
4

0.00350607

0.00350607

0
4

0
4

0
4

0.0388728

0.0388728

0.0388728

0.0388728

0
4

0
4

0
4

0.0606962

0.0606962

0.0606962

0.0606962

0
4

0
4

0
4

0.0229265

0.0229265

0.0229265

0.0229265

0
4

0
4

0
4

0.0481639

0.0481639

0.0481639

0.0481639

0
4

0
4

0
4

1.54653

1.54653

1.54653

1.54653

0
4

0
4

0
4

0.107242

0.107242

0.107242

0.107242

0
4

0
4

0
4

0.00448724

0.00448724

0.00448724

0.00448724

0
4

0
4

0
4

0.00750954

0.00750954

0.00750954

0.00750954

0
4

0
4

0
4

0.0606962

0.0606962

0.0606962

0.0606962

0
4

0
4

0
4

1.70996

1.70996

1.70996

1.70996

0
4

0
4

0
4

1.37026
7

1.37026
7

1.37026
7

1.1255
7

0.077958

0.131094

0.00416335

0.0110526

0.00411208

0.0163868

0
4

0
4

0
4

0.25592

0.25592

0.25592

0.25592

0
4

0
4

0
4

3.05335

3.05335

3.05335

3.05335

0
4

0
4

0
4

0.536117

0.536117

0.536117

0.536117

0
4

0
4

0
4

0.0327735

0.0327735

0.0327735

0.0327735

0
4

0
4

0
4

0.0172746

0.0172746

0.0172746

0.0172746

0
4

0
4

0
4

0.7653

0.7653

0.7653

0.7653

0
4

0
4

0
4

0.274652

0.274652

0.274652

0.274652

0
4

0
4

0
4

0.0112377

0.0112377

0.0112377

0.0112377

0
4

0
4

0
4

0.0105182

0.0105182

0.0105182

0.0105182

0
4

0
4

0
4

0.163796

0.163796

0.163796

0.163796

0
4

0
4

0
4

0.986313
7

0.986313
7

0.986313
7

0.850675
7

0.127858

0.00777913

0
4

0
4

0
4

0.0582163

0.0582163

0.0582163

0.0582163

0
4

0
4

0
4

0.0484412

0.0484412

0.0484412

0.0484412

0
4

0
4

0
4

0.0118449

0.0118449

0.0118449

0.0118449

0
4

0
4

0
4

0.141589

0.141589

0.141589

0.141589

0
4

0
4

0
4

0.40379

0.40379

0.40379

0.40379

0
4

0
4

0
4

0.0404642

0.0404642

0.0404642

0.0404642

0
4

0
4

0
4

0.321808

0.321808

0.321808

0.321808

0
4

0
4

0
4

0.0129652

0.0129652

0.0129652

0.0129652

0
4

0
4

0
4

1.44196

1.44196

1.44196

1.44196

0
4

0
4

0
4

0.536117

0.536117

0.536117

0.536117

0
4

0
4

0
4

61.4848

61.4848

61.4848

57.8916

1.50165

1.2335

0.107261

0.750827

0
4

0
4

0
4

0.0561884

0.0561884

0.0561884

0.0561884

0
4

0
4

0
4

0.0518304

0.0518304

0.0518304

0.0518304

0
4

0
4

0
4

0.0860736

0.0860736

0.0860736

0.0860736

0
4

0
4

0
4

0.0453516

0.0453516

0.0453516

0.0453516

0
4

0
4

0
4

4.24805

4.01229

3.99954

3.63017
8

0.369371

0
4

0.0127553

0.0127553

0
4

1.85615411929518e-16

0
4

0.177448

0.177448

0.177448

0
4

0
4

0.0583091

0.0583091

0.0583091

0
4

0
4

1.73472347597681e-16

0
4

1.13242748511766e-12

0
4

90345.4

2385.81
7

2385.61
7

1143.83
7

1143.79
7

0.0307731

5.7145260745628e-14
7

0
4

537.951
7

64.038
7

57.283
7

2.0069
7

0.0140929

0.0766075

0.0354888

0.0734467

0.0179689

0.0361246

0.0939211

0.014451

0.0912097

0.011544

0.872994
7

0.0113411

0.0248714

0.0506019

0.116938

0.0629284

0.276842

0.0317536

0.0230375

0.0598804

0.0181687

1.36352
7

0.165413

0.269727

0.0118427

0.0181606

0.0261378

0.0686254

0.369927

0.020637

0.0408038

0.0505828

5.78027
7

0.178069

0.0401185

0.0430824

0.0312178

0.0319776

1.96515

0.0387519

0.0542527

0.15413

0.00736842

0.900042
7

0.0926176

0.00500636

0.0157321

0.0160309

0.0310016

0.0581373

0.393896

0.0163868

0.00615463

0.0430824

6.71007
6

0.0155008

0.0770648

0.0104881

0.00710613

0.0599054

0.0100616

0.0770648

0.0224425

0.00615463

0.0155008

0.538139
6

0.0103648

0.00615463

0.0104881

0.0104881

0.0573536

0.0091605

0.0262201

0.00592134

0.0104881

0.0387519

0.33386
7

0.00710613

0.0107775

0.0112181

0.0155008

0.00888202

0.0138197

0.00543897

0.0156497

0.00500636

0.0107775

3.23759
7

0.00458025

0.0232512

0.0173753

0.0157321

0.00500636

0.0107775

0.0232512

0.0100616

0.10116

0.357411

10.8863
7

0.0310016

0.0366292

0.0201232

0.847713

0.10116

0.00888202

0.0106592

1.70996

0.00736842

0.0935306

18.5921
7

4.41915
6

0.0461597

0.017764

0.0770648

0.0155008

1.07223

0.0155008

0.00394831

0.0366292

0.269727

0.00710613

0.375154
7

0.0434384

0.00710613

0.0469069

8.28446

0.00750954

0.0307731

0.0155008

0.00710613

0.0118427

0.0168673

0.206884
7

0.0184639

0.0599054

0.00458025

0.0419522

0.0770648

0.0104881

0.00500636

0.0213184

0.0173753

0.021555

0.470837
7

0.00458025

0.536117

0.00710613

0.0156497

0.161857

0.00615463

0.0491603

0.0118427

0.0599054

0.0100127

23.9211
7

0.0366292

0.0209761

0.0104881

0.0100616

0.00615463

0.0173753

0.00448724

0.0155008

0.026063

0.0104881

0.244749
7

0.00710613

0.0155008

0.0523143

0.0106592

0.0887784

0.00458025

0.00615463

0.00500636

0.0091605

0.0269437

0.504408
7

0.00592134

0.0184639

0.0134122

0.00710613

0.0155008

0.0112449

0.0155008

0.146517

0.115597

0.0157321

12.5936
6

0.00500636

0.00710613

0.00687037

0.0155008

0.0156497

0.0123093

0.330374

0.00710613

0.0157321

0.15413

0.639627
7

3.00553

0.0907926
7

98.2996
7

2.78321
6

3.73098
6

3.02662
7

0.15303
7

2.14958

0.149216
7

0.046378
8

0.0725976
7

0.0823391
7

0.0758081
7

27.3886
7

0.217032

1.28811

0.139647
7

0.13027

0.0608372
7

3.48944

0.107394

0.138369

0.120187

0.802001
8

12.001
6

0.340127

0.488617

0.0675005

49.2482

0.386253

0.127252

0.0587349

0.0485695

0.212256

1.11744

49.5713

0.0769328

0.471304

0.071171

0.085374

0.110657

0.192573

0.167106

0.339491

0.127189

0.0385705

6.96905
6

3.93435

0.879846

0.17262

0.0597998

0.0620486

0.0389444

0.118071

0.455875

0.037465

0.556533

5.36741
6

0.346792

0.0316618

0.0395253

6.64208

0.121884

0.587015

0.031524

4.00737

0.0524543

0.256225

4.62715
7

0.0930047

0.0390837

0.166017

0.0582009

0.342957

0.062656

0.0230103

0.192634

0.770648

0.0691349

0
4

210.242
7

0.648261

27.0579
7

2.19725
7

50.1687
7

69.9427
7

57.5859
7

0.797524

0.163796

1.2561

0.0398762

0.0248714

0.31901

0.0398762

0
4

38.1598
7

33.5333
7

4.27489

0.3516

0
4

13.0184
7

10.97
7

0.00592134

0.122901

0.00458025

0.00458025

0.384607

0.271634

0.0542527

0.0387519

0.0310016

0.927924

0.0704446

0.108505

0.0232512

2.77555756156289e-17
7

0
4

6.16988

2.83893

3.27025

0.0606962

1.80411241501588e-16

0
4

409.095
7

91.237
7

314.892
7

2.21583

0.0155008

0.0542527

0.0155008

0.237658

0.0387519

0.0387519

0.0775039

0.0465023

0.108505

0.0156497

0.0549439

0.0465023

2.73461808752984e-14
7

0
4

1.13794
7

1.10007
7

0.00687037

0.0310016

1.49186218934005e-16
7

0
4

25.9216
8

25.7719
8

0.110907

0.0387519

0
4

0.0633753

0.0633753

0
4

0.0129839

0.0129839

0
4

0.0091605

0.0091605

0
4

0
4

0.0172426

0.0172426

0.0172426

0
4

0
4

0.0232641

0.0232641

0.0129839

0.0102802

1.73472347597681e-18

0
4

0
4

0.00518609

0.00518609

0.00518609

0
4

0
4

0.0591856

0.0591856

0.0591856

0
4

0
4

0.0599054

0.0599054

0.0599054

0
4

0
4

0.0155008

0.0155008

0.0155008

0
4

0
4

0.00458025

0.00458025

0.00458025

0
4

0
4

0.0234746

0.0234746

0.0234746

0
4

0
4

0
4

52303.5

58.5448

6.08365

3.90203

0.0469069

1.54976

0.295479

0.0955813

0.0649194

0.0209761

0.0506019

0.0469069

0.0104881

1.0096090630185e-15

0
4

1.39683

1.39683

0
4

2.95513

2.95513

0
4

0.0194758

0.0194758

0
4

0.0956648

0.0956648

0
4

0.0201183

0.0201183

0
4

2.35464

2.35464

0
4

0.0127553

0.0127553

0
4

1.70996

1.70996

0
4

0.0129839

0.0129839

0
4

0.0519355

0.0519355

0
4

10.8195

5.55138

0.292137

0.584274

0.0454436

4.30735

0.0129839

0.0259677

1.17614251671228e-15

0
4

0.0556337

0.0556337

0
4

1.70996

1.70996

0
4

0.0314642

0.0314642

0
4

2.48045

2.05262

0.193786

0.0195035

0.0195035

0.16578

0.0292553

2.22044604925031e-16

0
4

21.5817

5.8301

12.9566

2.79492

1.33226762955019e-15

0
4

2.78583

2.75771

0.0281121

1.90819582357449e-16

0
4

2.43916

1.99354

0.281441

0.0938138

0.0703603

8.32667268468867e-17

0
4

1.86986

1.86986

0
4

0.0299639

0.0104881

0.0194758

0
4

0.0281121

0.0281121

0
4

0
4

0.233802

0.233802

0.233802

0
4

0
4

815.984

6.31258
3

1.46415

4.49643

0.0973179

0.0507772

0.0966581

0.107242

2.77555756156289e-16
3

0
4

70.1048
3

9.83956

4.39937

0.0156497

0.139899

0.0232512

0.0533257

0.0767289

0.279798

0.093266

0.093266

0.093266

31.8937

0.127882

0.186532

0.093266

0.19194

0.106651

0.279798

12.4976

0.0189229

0.20461

0.139899

0.357142

0.0511526

0.434797

5.0764

0.725787

1.21618

1.07256

0.322572

2.56461518688411e-14
3

0
4

0.0327735

0.0327735

0
4

459.128
3

456.16
3

1.17651

0.0926517

0.100278

0.175085

0.281339

0.0310016

0.0617678

0.818442

0.129121

0.101849

0
4

33.5057

16.9478
5

2.91174

0.177755

0.15705

0.131673

0.219031

0.357411

0.451249

0.0581373

0.00703794

0.100278

5.45602

0.0127553

0.045853

0.0194364

0.0129839

0.0752082

2.85039

1.24177
5

0.854544

0.184817

0.776749

0.371947

0.084022

0
4

0.737308

0.193478

0.22393

0.09597

0.12796

0.09597

0
4

1.20103

1.20103

0
4

2.89012

1.509

0.127882

0.9719

0.281339

0
4

1.20209

0.179034

0.332492

0.127882

0.127882

0.230187

0.20461

0
4

2.71772

0.162257

1.94848

0.063149

0.54383

1.11022302462516e-16

0
4

0.580168

0.275472

0.114261

0.0761741

0.114261

2.77555756156289e-17

0
4

0.0420613

0.0210306

0.00690984

0.00564832

0.00847249

5.20417042793042e-18

0
4

0.848293

0.750295

0.097998

0
4

2.10317

0.27762

1.70996

0.115597

0
4

0.325902

0.225625

0.0501388

0.0501388

0
4

144.681
5

140.823
5

0.0501388

0.125347

1.18647

1.84022

0.127655

0.0469069

0.0501388

0.357411

0.0229265

0.0501388

0
4

3.52658

3.45818

0.0340111

0.0343898

2.77555756156289e-17

0
4

0.854342

0.854342

0
4

0.868637

0.356797

0.3199

0.19194

0
4

0.862877

0.0947236

0.757789

0.0103648

7.11236625150491e-17

0
4

2.40315

0.0767289

0.30781

2.01861

4.44089209850063e-16

0
4

0.263449

0.188241

0.0752082

2.77555756156289e-17

0
4

2.45657

0.594265

0.152348

1.70996

0
4

0.325902

0.175486

0.150416

0
4

1.79129

0.876329

0.730274

0.184686

5.55111512312578e-17

0
4

0.475704

0.475704

0
4

26.2284
5

25.2564
5

0.464891

0.404318

0.0129839

0.0898581

5.13478148889135e-16
5

0
4

0.226387

0.16979

0.0565967

0
4

2.56189

2.56189

0
4

0.0297716

0.0297716

0
4

0.126298

0.063149

0.063149

0
4

4.31276

4.31276

0
4

0.0255106

0.0255106

0
4

0.376041

0.376041

0
4

0.305547

0.305547

0
4

0.019468

0.019468

0
4

0.0730653

0.0730653

0
4

6.07359
3

1.08063

0.196555

0.644308

2.75808

0.0655183

0.0655183

1.26298

0
4

0.126298

0.063149

0.063149

0
4

0.701943

0.250694

0.451249

5.55111512312578e-17

0
4

0.253904

0.169269

0.0846347

0
4

0.0573989

0.0127553

0.0446436

0
4

0.613831

0.434797

0.179034

0
4

0.131287

0.131287

0
4

0.120728

0.0482912

0.0724369

0
4

0.478905

0.478905

0
4

1.22649

1.03985

0.18664

8.32667268468867e-17

0
4

0.127882

0.127882

0
4

0.564797
5

0.184991

0.162378

0.0501388

0.0501388

0.00710613

0.0599054

0.0501388

9.0205620750794e-17
5

0
4

0.0371008

0.0170056

0.0200953

0
4

0.159977

0.0533257

0.106651

1.38777878078145e-17

0
4

0.0281121

0.0281121

0
4

0.129121

0.129121

0
4

0.0501388

0.0501388

0
4

0.0255083

0.0255083

0
4

0.175085

0.175085

0
4

0.129121

0.129121

0
4

1.05051

1.05051

0
4

0.0501388

0.0501388

0
4

3.03746

1.90435

0.38087

0.389436

0.152348

0.0581004

0.0761741

0.0761741

1.94289029309402e-16

0
4

0.0129576

0.0129576

0
4

0.357411

0.357411

0
4

0.0170056

0.0170056

0
4

0.612799

0.612799

0
4

0.0129839

0.0129839

0
4

0.371625

0.371625

0
4

0.0103648

0.0103648

0
4

0.144196

0.144196

0
4

0.0965825

0.0965825

0
4

0.0501388

0.0501388

0
4

15.8837
5

15.7958
5

0.0879209

1.29063426612674e-15
5

0
4

0.0387582

0.0387582

0
4

0.063149

0.063149

0
4

0.0129839

0.0129839

0
4

0.0507772

0.0507772

0
4

1.74783

1.74783

0
4

0.169269

0.169269

0
4

0.125347

0.125347

0
4

0.0501388

0.0501388

0
4

0.0129576

0.0129576

0
4

0.525256

0.525256

0
4

1.14988

0.709694

0.165736

0.0752082

0.0968956

0.0290597

0.0732851

2.91433543964104e-16

0
4

0.0556337

0.0556337

0
4

0.262628

0.262628

0
4

0.329046

0.329046

0
4

0.0434322

0.0434322

0
4

0.0127553

0.0127553

0
4

1.20965

1.20965

0
4

0.063149

0.063149

0
4

0.00475254

0.00475254

0
4

0.0793016

0.0793016

0
4

0.149012

0.149012

0
4

2.02601

0.884463

0.723654

0.152348

0.152348

0.113193

0
4

0.357411

0.357411

0
4

0
4

9461.45
3

173.23

2.06599

2.4086
3

0.184804

0.120823

0.115597

0.0648786

0.0484412

0.00799092

2.09503

0.26614

0.66501

0.545364

17.4526

0.144068

0.238596

1.56157

1.94428

11.7112
5

0.305587

0.178159

0.350333

0.116863

0.150314

0.53162

0.0331074

0.504686

0.195025

0.0657044

11.9978

2.46478

0.0465023

2.32836

0.1361

1.18491

0.346792

0.0954469

0.210186

0.0966581

0.0724936

8.89591
3

0.0483291

0.0877713

0.394971

0.0221053

0.219428

0.397819

0.0209761

0.0966581

2.56494

0.0110526

2.58332

0.0724936

0.00532728

0.0739218

0.0724936

0.0606962

0.0724936

0.0173753

0.0483291

0.228522

0.0966581

20.1493
3

0.322802

0.0726619

0.314139

0.026063

0.0404642

0.0173753

0.266609

0.0387582

0.321444

0.0173753

29.2718
6

0.15413

0.144987

0.182089

0.0966581

0.0434384

0.0966581

0.114261

0.0483291

0.0347507

0.00532728

18.09

0.0173753

0.314139

0.114261

0.357411

0.434961

0.0419522

1.66765

0.0104881

0.38753

0.0608137

17.6271

0.121392

0.120823

0.0724936

0.0483291

0.272331

0.10116

0.576784

1.70996

0.110883

0.0483291

0
4

145.648

41.5234
6

0.27799

0.324365

0.378903

2.21517

2.95424

1.66788

0.426141

0.235766

0.790312

0.124006

2.17365

3.65835

0.626942

0.0614191

0.0358797

0.116515

0.0468109

0.140145

2.96699

4.12372

0.813394

12.8071

0.0469492

0.518673

3.58848

0.090521

0.414046

0.432618

0.242422

1.10449

3.76924

0.270947

1.13577
7

0.0470882

0.591494

0.0391243

0.0155008

0.077809

0.116713

0.0255106

0.291546

0.126943

0.236598

2.06766

0.0129576

0.0156497

0.0156497

0.0155008

0.129121

11.9697

0.0469492

0.0547741

0.0465023

0.0868644

0.583666
6

0.174282

0.185812

0.019133

0.0103648

0.15995

0.0340111

0.0483291

0.0156497

0.095063

0.152013

0.830048
7

0.152013

0.250396

0.0761658

0.0234746

0.0770648

0.0156497

0.0155008

0.0423112

0.0156497

0.0340111

0.248716

0.0234746

0.0633753

0.0765318

0.0156497

0.129121

0.484779

0.0868644

0.185812

0.0170056

0.0156497

35.0177
3

0.139899

5.73430192218893e-14

0
4

313.57

1.40976

303.556

0.173729

0.121276

0.0316877

0.0156475

0.161286

0.0129576

0.500921

0.236598

0.0172746

0.130297

0.0049252

2.05398

0.30781

0.00894146

0.236598

0.277017

0.0637765

2.57535

0.179098

0.0172382

0.0483291

0.893528

0.536117

2.54241072639161e-14

0
4

8329.89
3

773.775
4

41.3693
4

3.20122

4.24777

0.0488567

0.118299

0.130297

0.152774

0.623287

0.0434322

1.4471

0.11996

0.0434322

6.94
4

0.260593

0.289421

0.161286

1.09861

0.123124

0.0634669

0.0651483

0.108581

11.8663

0.123124

5.12043
4

0.123124

0.439711

0.0434322

0.873913

0.946391

0.179443

0.118299

0.92656

0.0488567

0.125347

11.4308

0.173729

0.289421

0.195445

0.155618

1.39915

0.542903

0.123124

0.123124

0.118299

0.0798828

87.8639
4

0.0434322

0.155618

0.123124

0.0488567

0.591494

0.173729

0.0647879

0.108581

0.0651483

0.125347

0.825212

0.439551

0.184686

0.289421

0.246248

0.0868644

0.107261

0.123124

0.0848951

0.946391

0.0655183

3.9346

0.100278

0.551527

0.506315

0.169269

0.0752082

0.108581

0.0434322

0.434131

0.0868644

0.274652

14.6812

0.0434322

0.123124

0.0868644

0.716632

0.195445

0.0917376

0.0752082

0.118299

0.116201

0.130297

0.984991

0.118299

0.0868644

0.0871506
[truncated: 877,022 more chars]
