# Supplementary material for: Fungal communities decline with urbanization—more in air than in soil
Source: ISME J. 2020 Aug 5;14(11):2806–15. doi: 10.1038/s41396-020-0732-1 (PMC7784924; doi:10.1038/s41396-020-0732-1)
Supplement: Supplementary file 2 — Supplemental data [file 41396_2020_732_MOESM2_ESM.zip › Krona_SoilNaturalCore.html]

Javascript must be enabled to view this page.

num
probth


349156

165125

5076.54
3

5076.54
3

5076.54
3

5076.54
3

5074.18
3

0

0

0

0

0.0565967

0

0

0

0

0

0.325902

0

0.414046

0

0

0

0

0

0

0

0

0

0.466029

0

0

0

0

0

0

0

0.643507

0.233721

0

0.217883

0

0

0
4

0
4

0

0

0

0
4

0

0

0
4

0
4

0

0

0

0
4

0
4

0
4

0
4

0

0

0

0

0

0

0
4

0
4

0
4

0
4

0

0

0

0

0

0
4

0
4

0
4

0

0

0

0

0
4

0
4

0
4

0
4

52473.3

23.3188

10.9425

4.6375

0
3

0.257154
2

0

0

0

0

0

0

0

0

0

0

0

0

0

0

0

0

0

0.999396

0

0

0

0
3

0

0

0

0

0

0

0

0

0

0

0

0

0

0

0

0

0

0

0

0

0

0

0

0

0

0

0

0

0

0

0

0

0

0

0

0

0

0

0

0

0

0

0

0
7

0

0

0

0

0

0

0.113193

0

0

0

0
7

0

0

0

0

0

0

0

0

0

0

0
7

0

0

0

0

0

0

0

0

0

0

0

0

0

0

0

0

0

0

0

0.0565967

0

0.311282

0

0

0

0

0

0

0

0

0

0

0

0

0

0

0

0

0

0

0

0

0

0

0
6

0

0

0

0

0

0

0

0

0

0

0

0

0

0

0
6

0
7

0

0

0

0
1

0.367879

0
2

0

0

0

0

0

0

0

0

0.713207
3

0

0

0

0

0

0

0

0

0

0

0
2

0

0

0

0

0

0

0

0

0

0.0848951

0
2

0.0642886

0

0

0

0

0

0

0

1.6696

0

0
7

0

0

0

0

0

0

0

0

0

0

0
6

0

0

0

0

0

0

0

0

0

0

0
6

0

0

0

0

0

0

0

0

0

0

0
4

2.03483
3

1.95184
3

0

0

0.0829891

2.63677968348475e-16
3

0
4

0

0

0
4

0

0

0
4

0

0

0
4

0

0

0
4

0

0

0

0
4

0

0

0

0
4

0

0

0

0
4

0

0

0
4

0

0

0
4

0

0

0
4

0
6

0

0

0

0

0
4

0

0

0
4

0

0

0

0
4

0

0

0

0
4

0

0

0

0
4

0

0

0
4

0

0

0

0
4

0

0

0

0
4

0

0

0
4

0

0

0
4

0

0

0
4

1.65308
2

0.933846

0.719239

0

0

1.11022302462516e-16
2

0
4

0

0

0
4

0

0

0
4

0

0

0
4

0

0

0
4

0

0

0
4

0

0

0
4

0

0

0
4

0.123124

0.123124

0
4

0.184686

0.184686

0
4

0

0

0
4

0
6

0

0

0
4

0

0

0
4

0

0

0
4

0.100278

0.100278

0
4

0

0

0
4

0

0

0
4

0

0

0
4

0.177448

0.177448

0
4

0

0

0
4

0

0

0
4

2.03154

2.03154

0
4

0

0

0

0
4

0

0

0
4

0

0

0
4

0

0

0
4

0

0

0
4

0

0

0
4

0

0

0
4

0

0

0
4

0

0

0
4

0

0

0
4

0

0

0
4

0

0

0
4

0

0

0
4

0

0

0
4

0

0

0
4

0
7

0

0

0
4

0

0

0
4

0

0

0

0
4

0
4

0
6

0
6

0
6

0
4

0
4

0

0

0

0
4

0

0

0
4

0
4

0

0

0

0
4

0
4

0

0

0

0

0
4

0
4

0

0

0

0
4

0
4

0

0

0

0
4

0
4

0

0

0

0
4

0
4

0

0

0

0
4

0
4

0.763337

0.763337

0.763337

0
4

0
4

0.123124

0.123124

0.123124

0
4

0
4

0

0

0

0
4

0
4

0
6

0
6

0
6

0
4

0

0

0
4

0

0

0
4

0
4

0

0

0

0
4

0
4

0

0

0

0
4

0
4

0

0

0

0
4

0
4

0

0

0

0
4

0
4

0

0

0

0
4

0
4

0

0

0

0
4

0
4

0

0

0

0
4

0
4

0

0

0

0
4

0
4

0

0

0

0
4

0
4

0

0

0

0

0
4

0
4

5.36219

5.08754

4.59504

0.369371

0.123124

4.16333634234434e-16

0
4

0.274652

0.274652

0
4

0
4

0

0

0

0
4

0
4

6.12767

6.12767

6.12767

0
4

0
4

0

0

0

0
4

0
4

0

0

0

0
4

0
4

0

0

0

0
4

0
4

2.66453525910038e-15

0
4

1678.8
7

0

0

0

0

0

0

0
4

0

0

0

0
4

0

0

0

0
4

0

0

0

0
4

0

0

0
4

0
4

977.269
7

976.307
7

54.6117
7

0
7

0
6

0

0

0

0

0

0

0

0

0

0

0

0
7

0

0

0

0

0

0

0

0

0

0

0
7

0

0

0

0

0

0

0

0

0

0

0

0

0

0

0

0

0

0

0

0

0

0
7

0

0

0

0

0

0

0

0

0

0

0.14009

0

0

0

0

0

0

0

0

0

0

0
7

0

0

0

0

0

0

0

0

0

0

0.173729
8

0

0

0

0

0

0

0

0

0

0

0

0

0

0

0

0

0

0

0

0

0

0

0

0

0

0

0

0

0

0

0

0.0483291

0
7

0
7

0

0

0

0

0

0

0

0

0

0

0.0724936

0

0

0

0

0

0

0

0

0

0

0
6

0

0

0

0

0

0

0

0

0

0

0
7

0

0

0

0

0

0

0

0

0

0

0
7

0

0

0

0

0

0

0

0

0.0483291

0

0
8

0

0

0

0

0

0

0

0

0

0

0
8

0

0

0

0

0

0

0

0

0

0

0
7

0

0

0.169152

0

0

0

0

0

0

0

0
7

0

0

0

0

0

0

0

0

0

0

0
6

0

0

0

0

0

0

0

0

0

0

0
6

0
1

0

0

0

0

0

0

0

0

0

0

0.0966581
7

0

0

0

0

0

0

0

0

0

0

0
7

0

0

0

0

0

0.0483291

0

0

0

0

0
1

0

0

0

0

0

0

0

0

0

0

0
7

0

0

0

0

0

0

0

0

0

0

0
6

0

0

0

0

0

0

0

0

0

0

0.241645
8

0

0

0

0

0

0

0

0

0

0

0
7

0

0

0

0

0

0

0

0

0

0

0.0483291

0

0

0

0

0

0

0

0

0

0

0
6

0

0.0483291

0

0

0

0

0

0

0

0

0
7

0.0483291

0

0

0

0

0

0

0

0

0

0

0
6

0

0

0

0

0

0

0

0

0

0

0
1

0

0

0

0

0

0

0

0

0

0

0
7

0

0

0

0.0724936

0

0

0

0

0

0

0
7

0

0

0

0

0

0

0

0.0483291

0

0

0
7

0

0

0

0

0

0

0

0

0

0

0
7

0

0

0

0

0

0

0

0

0

0

0
5

0

0

0

0

0

0

0

0

0

0

0
7

0

0

0.0483291

0

0

0

0

0

0

0

0
6

0

0

0

0

0

0

0

0

0

0

0
7

0
7

0

0

0

0

0

0

0

0

0

0

0
7

0

0

0

0
7

0
7

0
8

0
7

0
6

0
6

0.0483291
7

0

0.39089
7

0
7

0
7

0
7

0
7

0

0

0.821594

0
7

0
7

0.0483291

0

0
7

0

0
7

0.0483291

0
7

0
7

0
7

84.8514
3

0.0483291

0
6

0
7

0
7

0
7

0

0
7

0

0
7

0
7

0

0.362468
6

0
6

0.354964

0
8

0
6

0
7

0
6

0

0

0

0

0
7

0

0
7

0
7

0
7

0.14009

0
7

0
6

0
7

0.464531
2

0

0.342881

0
8

7.40957

0
7

0
6

0
6

0
8

0.0724936
7

0
7

0
7

0
8

0
6

0

0
6

0

0

0

0
6

0
8

0
6

0
7

0
7

0
6

0
8

0
7

0.0483291
7

0
7

0
8

0.0724936

0
6

0
7

0

0
7

0
7

0.193316
2

0

0
7

2.12818

0

0
6

0
7

0
8

0
7

0.144987

0

0
7

0
7

0.845758

0
1

0
8

0
7

0

0
7

0
7

0
6

0
7

0
7

0
7

0
7

0
7

0

0
7

0.0434322
6

0
7

0
6

0

0

0
7

0
7

0.321164
7

0
6

0
7

0
7

0
7

0

0

0
6

0
8

0
6

0
7

0
7

0
7

0
7

0
7

0
7

0
6

0
7

0
7

0.0724936
7

0
7

0
7

0
7

0

0
6

0.0483291
7

0.0483291
6

0
7

0
6

0
7

0
6

0
7

0
7

0
7

0
7

0
7

0
7

0

0
7

0
6

0
7

0.169152

0
7

0
7

1.14282
7

0
7

0
6

0

0
7

0.0483291
6

0
7

0

0
6

0
7

0.763337
5

0
7

0
7

0
7

0
7

0
7

0
8

0
8

0
7

0

0
7

0
1

0

0
7

0
7

0
7

0
6

0
7

0
7

0
5

0.120823

0
7

0
6

0
6

0
7

0
7

0
8

0
6

0

0
1

0
7

0
7

0
7

0.161806

0
7

0
7

0

0
6

0
7

0
7

0

0
7

0
7

0

0
6

0
7

0.193316
7

0
7

0.0724936

0
6

0
7

0
7

0
7

0
7

0
7

0
7

0
6

0.0966581
7

0

0
7

0
7

0
7

0
7

0
6

0
7

0
7

0
2

0.144987

0
7

0
1

0
7

0
7

0.338303

0

0
7

0
7

0
7

0.0966581

0
7

0.0651483
7

0

0
7

0
6

0
7

0.0966581
5

0
7

0
7

0
7

0.0724936
2

0

0
7

0
7

0
7

0
7

0.0483291
5

0
6

0
7

0
7

0

0
8

0

0
7

0.193316
7

0
7

0
7

0
7

0
6

0
7

0
7

0
7

0
7

0
7

0
7

0.0724936

0
7

0
7

0
7

0
7

0
8

0.771624

0
7

0
7

0
7

0

0
7

0
7

0.0483291

0
7

0.797429

0
6

0.0966581
2

0
7

0

0
7

0

0.183523

0
7

0
7

0
6

0
6

0
6

0
7

0.120823

0

0
7

0
7

0.797429

0.0966581

0
7

0
7

0
7

0.773265
5

0
7

0
7

0.0651483
2

0
7

0
7

0
7

0
6

0

0
7

0
6

0
7

0
6

0.260593

0
7

0
7

0

0
7

0
7

0

0

0
2

0
7

0
7

0

0

0
6

0

0.159358
7

0
7

0
7

0.0724936
5

0
7

0
6

0
7

0.410797

0
7

0
7

0
7

0
7

15.7442
4

0
6

0
7

0
7

0.0724936

0
2

0
7

0

0
7

0
7

0.434961
6

0
6

0

0
7

0
6

0
7

0
7

0
7

0.0483291
7

0
7

0
7

0.289974
7

0.169152

0
6

0
7

0
7

0

0

0
7

0
7

0

0
7

0
5

0
7

0
6

0

0.349565

0
6

0
7

0
6

0
2

0
7

0
7

0.241645

0.144987
7

6.25861
4

0
8

0

0

0
7

0
6

0
7

0
7

0
8

0
7

0.35875
7

0
7

0
7

0
1

0
7

0

0
6

0.0483291
6

0
7

0
7

0
8

0
7

0.0868644

0
7

0
7

0

0

0
7

0
7

0
6

0
6

0

0
7

0
8

0
8

0
6

0
7

0
7

0
7

0
7

0
6

0
6

0
7

0.434961

0

0.0724936

0

0
7

0
7

0
7

0
7

0
7

0
7

0
7

0.0966581
7

0

0

0
7

0
7

0
6

0
8

0

0
7

0
6

0.354964

0.966581

0
7

0
7

0
7

0

0
7

0
6

0
7

0
7

0
7

0

0.120823
7

0
6

0
7

0
6

0

0
7

0
1

0
7

0
6

0.0483291
7

0
6

0
7

0.0483291
7

0
7

0
8

0
7

0
6

0
7

0
7

0
7

0
7

0

0
6

0
7

1.06324

0
7

0
7

0
7

0
7

0
7

0
7

0

0
7

0.0724936

0
7

0
6

0
6

0

0
7

0
7

0

0
6

0
7

0

0

0
7

0
7

0
7

0
8

0
7

0

0
6

0
7

0
5

0
7

0
6

0
7

0
7

0
6

0
7

0
7

0
7

0
7

0
6

1.71557
5

0
8

0
7

0
7

0
6

0
8

0
7

0
7

0
6

0
7

0
6

0
7

0

0
7

0
7

0.0966581

0
7

0.0966581

0
7

0
7

0
7

0
6

0
7

0
7

0
7

0.185971
7

0

0
7

0.217481

0
7

0

0
6

0

0

0

0.352674
5

0

0.0966581

0
6

0
6

0
1

0
6

0
7

0
8

0

0

0

0
7

0
2

0

0

0.0483291

0
7

0
7

0
7

0
7

0
7

0
6

0.304758

0.0966581
7

0
7

0

0

0
6

0
7

0
6

0.0483291
6

0
7

0

0
7

0.0724936

0
7

0
7

0

0
7

0

0
7

0
7

0
8

0

0.144987

0.0651483
7

0
6

0
7

0
7

0.0966581

0.169152
5

0
7

0
7

0
6

0
6

0
2

0
7

0
7

0

0
1

0
6

0
7

0
6

0
7

0
2

0

0
7

0
7

0
7

0
7

0
8

0
7

0
7

0
7

0
6

0
7

0
1

0

0
7

0
7

0
7

0

0.0483291

0
6

0
7

0.144987

0
7

0
7

0
7

0.0483291
7

0
6

0
6

0
6

0

0
7

0
7

0.0724936

0

0
1

0
7

0
7

0.0483291

0
6

0
8

0

0
8

0
1

0

0
6

0
7

0
7

0
6

0
7

0.0724936

0
7

0

0
7

0
7

0

0.101849

0

0
7

0
7

0
7

0
7

0
7

0
7

0
6

0
8

0
8

0.193316

0
7

0
6

0.894087
7

0.258465

0.0966581

0
1

0

0
7

0
8

0.185812
5

0
7

0
6

0
7

0

0.144987

0
7

0
6

0
7

0
7

0
7

0
7

0

0
7

0
7

0

0
7

0
5

0
7

0
7

0

0
7

0
7

0
7

0
7

0
6

0
6

0
7

0.144987

0
7

0
7

0
7

0
2

0
6

0
7

0
6

0
7

0
8

0.212584
7

0
7

0
7

0
7

0
7

0

0.0724936
8

7.29769
4

0

0
7

0
8

0
7

0.0483291
7

0
6

0
7

0

0
6

0

0
6

0
7

0

0
7

0.724936

0

0
7

0
7

0

0.0724936

0
7

0
7

0
7

0
7

0

0.169152

0
7

0
7

0
6

0
7

0
7

0
7

0.0483291

0
7

0
7

0
7

0
6

0.0483291
5

0
6

0
6

0
6

0.0483291
5

0
7

0
6

0

0
7

0

0.241645
6

0
2

0
6

0
8

0
7

0
7

0
6

0
7

0
7

0

0.144987

0
7

0.53162
7

0
6

0

0
6

0

0
6

0

0

0
8

0
8

0
7

0.0724936
7

0

0.0868644

0
7

0
6

0
7

0
6

0
7

0
6

0
7

0
6

0
7

0
8

0
6

0
7

0
7

0
7

0
7

0
6

0.0483291

0
6

0
6

0.0966581

0
1

0.144987
5

0
6

0
7

0
6

0
6

0

0

0
6

0
2

0.0434322

0
6

0
8

0

0
7

0
7

0
7

0
7

0
7

0
6

0
7

0
6

0
7

0
6

0
7

0
8

0
7

0
6

0.0966581
5

0
7

0.144987
5

0
7

0
7

0
7

0
7

0

0
7

0
7

0
7

0.0434322
5

0
7

0.185971

0

0.0966581
6

0
7

0.193316

0
6

0
6

0

0
8

0

0.0651483

0
8

0
7

0
7

0
7

0
6

0
6

0
6

0
6

0
6

0
7

0
7

0
8

0.0724936

0
4

0
7

0
5

0.0434322
7

0

0
1

0
6

0
6

0

0
7

0

0
7

0.0483291

0
7

0
6

0
7

0
6

0
6

0
7

0
7

0

0
6

0

0
7

0

0
6

0

0
7

0
7

0
7

0
7

0
8

0
7

0
6

0.0483291
7

0
8

0
7

0
8

0
6

0
6

0

0
6

0
1

0
7

0
7

0.0868644
7

0
6

0
7

0
7

0
7

0
6

0.483291

0
6

0
7

0
7

0
8

0.222058
7

0
8

0
7

0
7

0
7

0
6

0
6

0
8

0
6

0
6

0
1

0
7

0

0

0
7

0
7

0
6

0
7

0
7

0
7

0

0
7

0.0483291
6

0
7

0
7

0
7

0
7

0

0
7

0

0
7

0
6

0
1

0
7

0
7

0
7

0
7

0
8

0
7

0

0
6

0
7

0
7

0
7

0
6

0
7

0
7

0.0434322

0
7

0
7

0
8

0
7

0
7

0
7

0
7

2.65075
5

0
7

0
7

0
2

0
7

0
6

0
7

0
7

0
6

0
7

0

366.421
7

1.9166
7

0.0651483
6

0
7

0
7

0
7

0
7

0
6

0
7

0
7

0
7

0
7

0
6

0.144987
8

0
6

0
6

0
7

0
7

0.0724936

0

0
7

0.0483291
5

0

0
7

0.236748

0
7

0
7

0
6

0
8

0
7

0
7

8.28843
4

0
7

0
6

0
7

0
7

0
7

0
7

0
1

0
7

0
6

0
6

0
7

0
6

0
7

0

0
7

0

0
6

0
8

0
7

0
6

0

0
7

0

0
7

0
8

0
7

0

0
7

0
7

0

0

0
8

0

0
6

0

0

0
7

0
7

0.144987

0
7

0
7

0.0434322
5

0
6

0
7

0
6

0

0
7

0

0
7

0

0
7

0

0.0724936

0
7

0
7

0
6

0
7

0
7

0

0
6

0.0483291

0
6

0
2

0
7

0
8

0
6

0
1

0
6

0
6

0
7

0
7

0

0
8

0
7

0
7

0
6

0
7

0

0
7

0

0.0483291
7

0

0
7

0
6

0

0
7

0
8

0
7

0
7

0
7

0
6

0
7

0.0483291

0

0

0
6

0
7

0
7

0
7

0
6

0

0
7

0

0.0483291
7

0
8

0
7

0
7

0
6

0
6

0

0

0
6

0
7

0
7

0
7

0

0

0.0483291
5

0

0
7

0

0
7

0
6

0.0434322

0
7

0.144987
7

0
2

0
7

0

0

0.0483291

0
7

0
7

0

0
7

0
7

0
7

0
7

0
7

0
7

0
1

0
7

0

0

0

0

0.120823
5

0
7

0
7

0
7

0
1

0
6

0
7

3.55219
4

0

0
8

0
7

0
7

0
7

0

0
7

0
6

0

0
7

0

0
6

0
2

0
6

0
8

0
7

0

0
7

0
6

0
1

0

0
7

0
7

0
7

0

0
8

0

0
7

0
7

0
8

0
7

0

0
8

0
7

0

0

0
8

0.120823
7

0.120823

0

0
8

0
7

0
6

0
8

0
8

0
6

0
8

0

0
6

0

0

0
7

0

0

0
7

0
6

0
7

0

0
6

0

0.0724936
7

0

0
6

0
7

0
7

0
6

0.0483291

0
7

0
7

0
8

0
7

0.0483291
7

0
7

0

0

0
7

0
6

0
6

0.289974
6

0.0483291

0
6

0
5

0
6

0

0

0.0483291

0

0
7

0

0

0
2

0
1

0

0
7

0
7

0
7

0
6

0.0483291

0
7

0

0

0
7

0
6

0
7

0
7

0

0

0

0

0
7

0
6

0
7

0
7

0

0

0

0

0
7

0.0483291

0.0724936

0
7

0
7

0
7

0
6

0
7

0
6

0.205239
7

0.412606
5

0
8

0

0

0

0
7

0

0

0

0

0
7

0
6

0
7

0.0434322
5

0
7

0
6

0
6

0
7

0
7

0
7

0
6

0
7

0
7

0
7

0
7

0

0

0
7

0
7

0.0724936
5

0
7

0
7

0
7

0

0

0
8

0

0
7

0

0
7

0
7

0
6

0

0
7

0

0
6

0

0
7

0
8

0
7

0

0
7

0

0
7

0

0.169152

0
6

0
7

0
6

0

0

0

0
1

0

0

0
7

0
7

0

0
6

0
8

0
6

0
6

0
7

0

0.0651483

0
8

0
6

0.0966581
7

0
1

0
7

0
1

0

0

0
8

0
7

0
7

0
6

0
7

0
7

0
7

0

0
7

0
6

0
7

0

0
7

0
6

0

0
6

0
6

0
6

4.19836
4

0

0
6

0
7

0
6

0

0
7

0
7

0
8

0
6

0

0
7

0

0

0
6

0
7

0
1

0
7

0

0
6

0
6

0
7

0

0
6

0
7

0
7

0
7

0
7

0
7

0
8

0
7

0
7

0

0

0.0434322

0
6

0.0966581
6

0

0
6

0
1

0.0724936

0

0
7

0.26581
7

0

0
6

0

0
7

0

0
7

0
8

0.0434322

0
6

0

0
7

0
7

0
6

0

0
7

0
7

0
7

0
6

0
6

0
7

0

0
7

0

9.30334

0

0

0

0

0

0

0

0

0
7

0

0

0

0

0

0

0

0

0.0966581

0.169152

0
7

0

0

0

0

0

0

0

0

0

0

0

0

0

0

0

0

0

0

0

0

0

0
6

0

0

0

0

0

0

0

0

0

0

0

0

0

0

0

0

0

0

0

0

0

0
7

0

0

0

0

0

0

0

0

0

0

0
7

0
6

0

0

0

0

0

0

0

0

0

0

0
7

0

0

0

0

0

0

0

0

0

0.0724936

0.0966581
7

0

0

0

0

0.877249

0

0.0483291

0

0

0.0966581

0
7

0

0

0

0

0

0

0

0

0

0

0
7

0

0

0

0

0

0

0

0

0

0

0

0

0

0

0

0

0

0

0

0

0

0
7

0

0.144987

0

0

0

0

0

0

0

0

0.0724936
7

0

0

0

0.0651483

0

0

0

0

0

0

0
7

0

0

0

0

0

0

0

0

0

0

0
6

0

0

0

0

0

0

0

0

0.193316

0

0.0966581
7

0
6

0

0

0

0

0

0

0

0

0

0

0
7

0

0

0

0

0

0

0

0

0

0

0.0483291
7

0

0

0

0

0

0

0

0

0

0

0
7

0

0

0

0

0

0

0

0

0

0

0

0

0

0

0.0483291

0

0

0

0

0

0

0
7

0

0

0

0

0

0

0

0

0

0

0.193316
7

0

0

0

0

0

0

0

0

0

0

0.0483291
7

0

0

0

0

0

0

0

0

0

0.144987

0

0

0

0

0

0

0

0

0

0

0

0.0966581
7

0

0

0

0.0483291

0.0483291

0

0

0

0

0

0
7

32.6221

0.0434322

0

0

0

0

0

0

0

0.0434322

0

0
7

0

0

0

0

0

0

0

0

0

0

0
6

0

0

0

0

0

0

0

0

0

0

0

0

0

0

0

0

0

0

0

0

0

0

0

0

0

0

0

0.0966581

0

0

0

0

0.524348

0

0

0

0

0

0

0

0

0.144987

0

0
7

0

0

0

0

0.0724936

0

0

0

0

0

0
7

0

0

0

0

0

0

0

0

0

0

0
7

0

0

0

0

0

0

0

0

0

0

0
6

0

0

0

0

0

0

0

0

0

0

0
7

0
7

0

0

0

0

0

0

0.0483291

0

0.0724936

0

0
7

0

0

0

0

0

0.26581

0

0

0

0

0.193316
7

0

0

0

0

0

0

0

0

0

0

0.0724936

0

0

0

0

0

0

0

0

0

0

0
7

0

0

0

0

0

0

0

0

0

0

0.314139

0

0

0

0

0

0

0

0

0

0

0

0

0

0

0

0

1.06324

0

0

0

0

0
7

0

0.0483291

0

0

0

0

0

0

0

0

0.0434322
7

0

0

0

0

0

0.314139

0

0

0

0

0
7

0

3.818

0.0483291

0

0

0

0

0

0

27.2334

0
7

0
7

0

0

0

0

0

0

0

0

0

0

0
6

0

0

0

0

0

0

0

0.0565967

0

0

0
7

0

0

0

0

0

0

0

0

0

0

0

0

0

0

0

0

0

0

0

0

0

0
7

0

0

0

0

0

0

0

0

0

0

8.05668

0

0

0

0

0

0

0

0

0

0

0.120823
7

0

0

0

0

0

0

0

0

0

0

0.169152

0

0

0

0.0868644

0

0

0

0

0

0

0
7

0

0

0

0.144987

0

0

0

0

0

0

0
7

0

0

0

0

0

0

0

0

0

0

75.5219

0
7

0
7

0

0

0

0

0

0

0

0

0

0.185812

0
7

0

0.0483291

0

0

0

0

1.62958

0

0

0

0
6

0

0

0

0

0

0

0

0

0

0

0
7

0

0

0

0

0

0

0

0

0

0

0
7

0

0

0

0

0

0

0

0

0

0

0.459126
7

0

0

0

0

0

0

0

0

0

0

0
7

0

0

0

0

0

0

0.0483291

0

0

0

0
7

0

0

0

0

0

0.0724936

0

0

0

0

0

0

0.0724936

0

0

0

0

0

0

0

0

0
7

0

0

0

0

0

0

0

0

0

0

0
7

0
7

0

0

0

0

0

0

0

0

0

0

0
6

0

0

0

0

0

0

0

0

0

0

0
7

0

0

0

0

0

0

0

0

0

0.0483291

0
7

0

0

0

0

0

0

0

0

0

0.0483291

0.26581
7

0

0

0

0

0

0

0

0

0

0

0
7

0

0

0

0

0

0

0

0

0

0

0
7

0

0

0

0

0

0

0

0

0

0

0
7

0

0

0

0

0

0

0

0

0

0

0
7

0

0.144987

0

0

0

0

0

0

0

0

0
7

0

0.0966581

0

0

0

0

0

0

0

0

1.93406

0.144987

0

0

0

0

0

0

0

0

0

0

0
7

0

0

0

0

0

0

0

0

0

0

0
7

0.289974

0.0966581

0

0

0

0

0

0.0868644

0

0

0
7

0

0

0

0

0

0

0

0

0

0

0.0966581

0.120823

0

0

0

0

0

0

0

0

0

0.0724936

0

0

0

0

0

0

0.459126

0

0

0

0
7

0

0

0

0

0

0

0

0

0

0

0
7

0

0

0

0

0

0

0

0

0

0

42.3846
4

0

0

0

0

0

0

0

0

0

0

0
7

0

0

0

0

0

0

0

0

0

0

0
6

0
7

0

0

0

0

0.0483291

0

0

0

0

0

0.933793
5

0

0

0

0

0

0

0

0

0

0

0
7

0

0

0

0

0

0

0

0

0

0

0
7

0

0

0

0

0

0

0

0

0

0

0
7

0

0

0

0

0

0

0

0

0

0

0

0

0

0

0

0

0

0

0.0966581

0

0

0
7

0

0

0

0

0

0

0

0

0

0

0
7

0

0

0

0

0

0

0

0.120823

0

0

0
7

0

0

0

0

0

0.0483291

0

0

0

0

0
7

0

0

0

0.0724936

0.0483291

0

0

0

0

0

0.231852

0.0724936
7

0

0

0

0

0

0

0

0

0

0

0
7

0

0

0

0

0

0

0

0

0

0

0
7

0

0

0

0

0

0

0

0

0

0

0
6

0

0

0

0

0

0

0

0.0724936

0

0

0
7

0

0

0

0

0

0

0

0

0.0483291

0

0
6

0

0

0

0

0

0

0

0

0

0

0
7

0.0483291

0

0

0

0

0

0

0

0

0

0.0483291
7

0

0

0

0

0

0

0

0

0

0

0
7

0

0

0

0

0

0

0

0

0

0

0
7

0

0

0

0

0

0

0

0

0

0

0
7

0

0

0

0

0

0

0

0

0

0

0

0
7

0

0

0

0

0

0

0

0

0

0

31.4139
5

0

0

0

0

0

0

0

0

0

0

0
6

0

0

0

0

0.0966581

0

0

0

0

0

0
7

0

0

0

0

0

0

0

0

0

0

0
7

0

0

0

0

0

0

0

0

0

0

0.0724936
7

0

0

0

0

0

0

0

0

0

1.43326

0

0

0.120823

0

0

0

0

0

0

0

0

0
7

0

0

0

0

0

0

0

0.0966581

0

0

0.241645

0

0

0

0

0

0

0

0

0

0.0724936

0.0483291
7

0
7

0

0

0

0

0

0

0

0

0

0

0
7

0.0724936

0

0

0

0.120823

0

0

0

0

0

0
2

0

0

0

0

0

0

0

0

0

0

0.0483291

0.0724936

0.0434322

0

0

0.0483291

0

0

0

0

0

0
7

0

0

0

0

0

0

0

0

0

0

0
7

0

0

0

0

0

0

0

0

0

0

0
7

0

0

0

0

0

0

0

0

0

0

0
7

0

0

0

1.32905

0

0

0

0

0

0

0

0

0

0

0

0

0

0

0

0

0

0

0

0

0

0

0

0

0

0

0

0.0483291

0.26581
7

0
7

0

0

0

0

0

0

0

0

0

0

0
7

0

0

0

0

0

0.0966581

0

0

0

0

0.604113

0

0

0

0

0

0

0

0

0

0

0
7

0

0

0

0

0

0

0

0

0

0

0
7

0

0

0

0

0

0

0

0.0642886

0

0

0
7

0

0

0

0

0

0

0

0

0

0.0724936

0.773265
5

0

0

0

0

0

0

0

0

0

0

0
6

0

0

0

0

0

0

0

0

0

0

0
7

0

0

0

0

0

0

0

0

0

0

0
7

0

0

0

0

0

0

0

0

0

0

0.351212
7

0
6

0

0

0

0

0

0

0

0

0

0

0
6

0

0

0

0

0

0

0

0

0

0

0
7

0

0

0

0

0

0

0

0

0

0

0.483291
7

0

0

0

0

0

0

0

0

0

0

0

0

0

0

0

0

0

0

0

0

0

0

0

0

0

0

0

0

0

0

0

0.0483291

0
6

0

0

0

0

0

0

0

0

0

0

0
7

0

0

0

0

0

0

0

0

0

0

0
7

0

0

0

0

0

0

0

0

0

0

0.0966581

0

0

0

0

0

0

0

0

0

0

0
7

0.0483291
7

0

0

0

0

0

0

0

0

0

0

0
7

0

0

0

0

0

0

0

0.169152

0

0

1.1599

0

0

0

0

0

0

0

0

0

0

0
7

0

0

0

0

0

0

0

0.241645

0

0

0.169152

0

0

0

0

0

0

0

0

0

0

0
7

0

0

0

0

0

0

0

0

0

0

0.362468

0

0

0

0

0

0

0

0

0

0

0
8

0

0

0

0

0

0

0

0

0

0

0
7

0

0

0

0

0

0

0

0

0

0

0
6

0

0

0

0

0

0

0

0

0

0

28.7607

1.60125

0
7

0

0

0

0

0

0.0724936

0

0

0

0

0
7

0

0

0

0

0

0

0

0

0

0

0

0

0

0

0

0

0

0

0

0

0

0.0483291

0

0

0

0

0

0

0

0

0

0.0483291

0

0

0

0

0

0

0

0

0

0

0

0.0724936
6

0

0

0

0

0

0

0

0

0

0

0
7

0

0.0483291

0

0

0

0

0

0

0

0

0

0

0

0

0

0

0

0

0

0

0

0
7

0

0

0

0

0

0

0

0

0

0

0

0

0

0

0

0

0

0

0

0

0

0.0483291
7

0
6

0

0

0.0483291

0

0

0

0

0

0

0

0.0724936
7

0

0

0

0

0

0

0

0

0

0

0.0724936

0

0

0

0

0

0

0

0

0

0

0.0434322
7

0

0

0

0

0

0

0

0

0

0

0
7

0

0

0

0

0

0

0

0

0.0483291

0

0.0483291
7

0

0

0

0

0

0

0

0

0

0

0
7

0

0

0

0.0483291

0

0

0

0

0

0

0
6

0

0

0

0

0

0

0

0

0

0

0
7

0

0

0

0

0

0

0

0

0

0

0
6

0

0

0

0

0

0

0

0

0

0

0

0

0

0

0

0

0

0

0

0

0

0

0.0724936
7

0

0

0

0

0

0

0

0

0

0

0

0

0

0

0

0

0

0

0

0

0

0
7

0

0

0

0

0

0

0

0

0

0

0.0483291

0

0

0

0

0

0

0

0

0

0

0
7

0

0

0

0

0

0

0

0

0

0

0
7

0

0

0

0

0.0483291

0

0

0

0

0

0

0

0

0

0

0

0

0

0

0

0

0
6

0

0

0

0

0

0

0

0

0

0

0
7

0

0

0

0

0

0

0

0

0

0

0.676607
7

0
7

0

0

0

0

0

0

0

0

0

0

0
7

0

0

0

0

0

0

0

0

0

0

0.0434322

0

0

0

0

0

0

0

0

0

0

0
1

0

0

0

0

0

0

0

0

0

0

0

0

0

0

0

0

0

0

0.0434322

0

0

0
7

0

0

0.120823

0

0

0

0

0

0

0

0
7

0

0

0

0

0

0

0

0

0

0

0.120823

0

0

0

0

0

0

0

0

0

0

0
7

0

0

0

0

0

0

0

0

0

0

0
7

0

0

0

0

0

0

0

0

0

0

0
7

0
7

0

0

0

0

0

0

0

0

0

0

0
7

0

0

0

0

0

0

0

0

0

0

0
7

0

0

0

0

0

0

0

0

0

0

0
7

0

0

0

0

0

0

0

0

0

0

0
7

0

0

0

0

0

0

0

0

0

0

0
6

0

0

0

0

0

0

0

0

0

0

0
6

0

0

0

0

0

0

0

0

0

0

0

0

0

0

0

0

0

0

0

0

0

0

0

0

0

0

0

0

0

0

0

0

0
7

0

0

0

0

0

0

0

0

0

0

0
7

0
7

0

0

0.0868644

0

0

0

0

0

0.0483291

0

0
7

0

0

0

0

0

0

0

0

0

0

0.0434322
7

0

0

0

0

0.0483291

0

0

0.120823

0

0

0
7

0

0

0

0

0

0

0

0

0

0

0.169152

0

0

0.0966581

0

0

0

0

0

0

0

0
7

0

0

0

0

0

0

0

0

0

0

0

0

0

0

0

0

0

0

0

0

0

0
7

0

0

0

0

0

0

0

0

0

0

0
7

0

0

0

0

0

0

0

0

0

0

0.115926

0

0

0

0

0

0

0

0

0

0

0.362468
7

0
7

0

0.0966581

0

0

0

0

0

0

0

0

0

0

0

0

0

0

0

0

0

0

0

0
7

0

0

0

0

0

0

0

0

0.0483291

0

0
7

0.120823

0

0

0

0

0

0

0

0

0

0
7

0

0

0

0

0.0483291

0

0

0

0

0.0724936

0
7

0

0

0

0

0

0

0

0

0

0

0.120823

0

0

0

0

0

0

0

0

0

0

0.0434322

0

0

0

0

0

0

0

0

0

0

0
6

0

0

0

0

0

0

0

0

0

0

0
7

0

0

0

0

0

0

0

0

0

0

1.75236

0

0

0

0

0

0

0

0

0

0

0

0.0483291

0

0

0

0

0

0

0

0

0

0

0
7

0

0

0

0

0

0

0

0

0

0

0
7

0

0

0

0

0

0

0

0

0

0

0
7

0

0

0

0

0

0

0

0

0

0

0.0483291

0.241645

0

0

0

0

0

0

0

0.185812

0

0
7

0

0

0

0

0

0

0

0

0

0

0
7

0

0

0

0

0

0

0

0

0

0

0

0

0

0

0

0

0

0

0

0

0

0

0

0

0

0

0.120823

0

0

0

0

2.08475

0
7

0
7

0

0

0

0

0

0

0

0

0

0

0.0966581
7

0

0

0

0

0

0

0

0

0

0

0
7

0

0

0

0.0483291

0

0

0

0

0

0

0.108276

0

0

0

0

0

0

0

0

0

0

0.0724936

0

0

0

0

0

0

0

0

0

0

0
7

0

0

0

0

0

0

0

0

0

0

0.169152

0

0

0

0

0

0

0

0

0

0

0.0483291
5

0

0

0

0

0

0

0

0

0

0

0
7

0

0

0

0

0

0

0

0

0

0

0
7

0

0

0

0

0

0

0

0.289974

0

0

0.386632
7

0
7

0

0

0

0

0

0

0

0

0

0

0
7

0

0

0

0

0

0

0

0

0

0

0
7

0

0

0

0

0

0

0

0

0

0

0
7

0

0

0

0

0

0

0

0

0

0

0
6

0.0483291

0

0

0

0

0

0

0

0

0

0.0651483
7

0

0

0

0

0

0

0

0

0

0

0
7

0

0.0483291

0

0

0

0

0.169152

0

0

0

0.120823
6

0

0

0

0

0

0

0

0

0

0

0.212584

0

0

0

0

0

0

0

0

0

0

0
7

0

0

0

0

0

0

0

0

0

0

7.33044

0.616036
7

0.0724936
7

0.217481

0

0

0

0

0

0

0

0

0

0
7

0

0

0

0

0

0

0

0.0724936

0

0

0
8

0

0

0

0

0

0

0

0

0

0

0
6

0

0

0

0

0

0

0

0

0

0

0
6

0

0

0

0

0

0

0

0

0

0

0
7

0

0

0

0

0

0

0

0

0

0

0

0

0

0

0

0

0

0

0

0

0

0.0483291

0.241645

0

0

0

0

0

0

0

0

0

0.0553261
7

0

0

0

0

0

0

0

0

0

0

0
7

0

0

0

0

0

0

0

0

0

0

0
7

0
7

0

0

0

0

0

0

0

0

0

0

0
7

0

0

0

0

0

0

0

0

0

0

0
7

0

0

0

0

0

0

0

0

0

0

0

0

0

0

0

0

0

0

0

0

0

0
7

0

0

0

0

0

0

0

0

0

0

0
7

0

0

0

0

0

0

0

0

0

0

0

0

0

0

0

0

0

0

0

0

0

0.521826

0

0

0

0

0

0

0

0

0

0

0

0

0

0

0

0

0

0.0483291

0

0

0

0
6

0

0

0

0

0

0

0

0

0

0

2.09131

0
7

0

0

0

0

0

0

0

0

0

0

0
7

0

0

0

0

0

0

0

0

0

0

0
7

0

0

0

0

0

0

0

0

0.169152

0

0
7

0

0

0

0

0

0

0

0

0

0

0
7

0

0

0

0

0

0

0

0

0

0

0.0966581

0

0

0

0

0

0

0

0

0

0

0
7

0

0

0

0.0483291

0

0

0

0

0

0

0.0966581

0

0

0

0

0

0

0

0

0.434322

0

0
7

0

0

0

0

0

0

0

0

0

0

0.0483291
7

0

0

0

0

0

0

0

0

0

0

2.61095

0

0

0

0

0

0

0

0

0

0

0

0.0483291

0

0

0

0

0

0

0

0

0

0

0
7

0.0651483

0

0

0

0.0434322

0

0

0

0

0

0
7

0

0

0

0

0

0

0

0

0

0

0
7

0

0

0

0

0

0

0

0

0

0

0

0

0

0

0

0

0

0

0

0

0

0.0966581

0

0

0

0.0483291

0

0

0

0

0

0

0
7

0

0

0

0

0

0

0

0

0

0.0724936

0

0

0

0

0

0

0

0

0

0

0

0
7

0

0

0

0

0

0

0

0

0

0

0.362468
7

0
7

0

0

0

0

0

0

0

0

0

0

0
7

0

0

0

0

0

0

0

0

0

0

0
6

0

0

0

0

0

0

0

0

0

0

0
7

0

0

0

0

0

0

0

0

0

0

0
7

0

0

0

0

0

0

0

0

0

0

0
6

0

0

0

0

0

0

0

0

0

0

0
6

0

0

0

0

0

0

0

0

0

0

0
5

0

0

0

0

0

0

0

0

0

0

0.120823
7

0

0

0

0

0

0

0

0

0

0

0
7

0

0

0

0

0

0

0

0

0

0

0
7

0
7

0

0

0

0

0

0

0

0

0

0

0
7

0

0

0

0

0

0

0

0

0

0

0
7

0

0

0

0

0

0

0

0

0

0

0
8

0.289974

0

0

0

0

0

0

0

0

0

0
7

0

0

0

0

0

0

0

0

0

0

0
7

0

0

0

0

0

0

0

0

0

0

0
7

0

0.0483291

0

0

0

0

0

0

0

0

0
2

0

0

0

0

0

0

0

0

0

0

0

0

0

0

0

0

0

0

0

0

0

0
7

0

0

0

0

0

0

0

0

0

0

0.0966581
7

0
7

0

0

0

0

0

0

0

0

0

0

0.656828

0

0

0

0

0

0

0

0

0

0

0
7

0

0

0

0

0

0

0

0

0

0

0
7

0

0

0

0

0

0

0

0

0

0

0
7

0

0

0

0

0

0

0

0

0

0

0
6

0

0

0

0

0

0

0

0

0

0

0
7

0

0

0

0

0

0

0

0

0

0

0

0

0

0

0

0

0

0

0

0

0

0
7

0

0

0

0

0

0

0

0

0

0

0
6

0

0

0

0

0

0

0

0

0

0

0
7

0
7

0

0

0

0

0

0

0

0

0

0

0.0483291
7

0

0

0

0.0724936

0

0

0

0

0

0

0
6

0

0

0

0

0

0

0

0

0

0

0

0

0

0

0

0

0

0

0

0

0

0
6

0

0

0

0

0

0

0

0

0

0

0
6

0

0

0

0

0

0

0

0

0

0

0
7

0

0

0

0

0

0

0

0

0

0

0
8

0

0

0

0

0

0

0

0

0

0

0
7

0

0

0

0

0

0

0

0

0

0

0

0

0

0

0

0

0

0

0

0.195445

0

0.0483291

0
7

0

0

0

0

0

0

0

0

0

0

0

0

0

0

0

0

0

0

0

0

0

0

0

0

0

0

0

0

0

0

0

0

0
6

0

0

0

0

0

0

0

0

0

0

0
6

0

0

0

0

0

0

0

0

0

0

0
6

0

0

0

0

0

0

0

0

0

0

0.152013
6

0

0

0

0

0

0

0

0

0

0

0

0

0

0

0

0

0

0

0

0

0

0
8

0

0

0

0

0

0

0

0

0

0

0
7

0

0

0

0

0

0

0

0

0

0

1.71472

0
6

0

0

0

0

0.0483291

0.0483291

0

0

0

0

0
7

0

0

0

0

0

0

0

0

0

0

0
7

0

0

0

0

0

0

0

0

0

0

0
6

0

0

0

0.169152

0

0

0

0

0

0

0
7

0

0

0.0483291

0

0

0

0

0

0

0

0
7

0

0

0

0

0

0

0

0

0

0

0
7

0

0

0

0

0

0

0

0

0

0

0
7

0

0

0

0

0

0

0

0

0

0

0.217481

0

0

0

0

0

0

0

0

0

0

0
6

0

0

0

0

0

0

0

0

0

0

1.60647
7

0.773265

0
7

0

0

0

0

0

0

0

0

1.32468

0

0
7

0

0

0

0

0

0

0

0

0

0

0
6

0

0

0

0

0

0

0

0

0

0

0

0

0

0

0

0

0

0

0

0

0

0
6

0

0

0

0

0

0

0

0

0

0

0
7

0

0

0

0

0

0

0

0

0

0

0
7

0

0

0

0

0

0

0

0

0

0

0
8

0

0

0

0

0

0

0

0

0

0

0
7

0

0

0

0

0

0

0

0

0

0

0
7

0

0

0

0

0

0

0

0

0

0

0.7491

0.173729
5

0

0

0

0

0

0

0

0

0

0

0
7

0

0

0

0

0

0

0

0

0

0

0
7

0

0

0

0.0966581

0

0

0

0

0

0

0
7

0

0

0

0

0

0.652442

0

0

0

0

0
7

0

0

0

0

0

0

0

0

0

0

0
6

0

0

0

0

0

0.0966581

0

0

0

0

0.0966581

0

0

0

0

0

0

0

0

0

0

0
7

0

0

0

0

0

0

0

0

0

0

0
7

0

0

0

0

0

0

0

0

0

0

0

0

0

0

0

0

0

0

0

0

0.0434322

0
7

0
6

0

0

0

0

0

0

0

0

0

0

0
7

0

0

0

0

0

0

0

0

0

0

0
7

0.0483291

0

0.0651483

0

0

0

0

0

0

0

0
7

0

0

0

0

0

0

0

0

0

0

0
7

0

0

0

0

0

0

0

0

0

0

0
7

0

0

0

0

0

0

0

0

0

0

0
7

0

0

0

0

0

0

0

0

0

0

0
7

0

0

0

0

0

0

0

0

0

0

0.0966581

0

0

0

0

0

0

0

0

0

0

0

0

0

0

0

0

0

0

0

0

0

0.169152
7

0
6

0

0

0

0

0

0

0

0

0

0

0
8

0

0

0

0

0

0

0

0

0

0

0

0

0

0

0

0.0966581

0

0

0

0

0

0
6

0

0

0

0

0

0

0

0

0

0

0
6

0

0

0

0

0

0

0

0

0

0

0

0

0

0

0

0

0

0

0

0

0

0.0724936

0

0

0

0

0

0

0

0

0

0

0
7

0

0

0

0

0

0

0

0.120823

0

0

0.0724936
7

0

0

0

0

0

0

0

0

0

0

0
6

0

0

0

0

0

0

0

0

0.169152

0

0.419951

0
7

0

0

0

0

0

0

0

0

0

0

0

0

0

0

0

0

0

0

0

0

0

0
7

0

0

0

0

0

0

0

0

0

0

0.144987
7

0

0

0.0483291

0

0

0

0

0

0

0

0
7

0

0

0

0

0

0

0

0

0

0

0
7

0.0483291

0

0

0

0

0

0

0

0

0

0
7

0

0

0

0

0

0

0

0

0

0

0
7

0

0

0

0

0

0

0

0

0

0

0
7

0

0

0

0

0

0

0

0

0

0

0.0966581
7

0

0

0

0

0

0

0

0

0

0

0
7

0
7

0

0

0

0

0

0

0

0

0

0

0
7

0

0

0

0

0

0

0

0

0

0

0
6

0

0

0

0

0

0

0

0

0

0

0.0483291

0

0

0

0

0

0

0

0

0

0

0.0724936
7

0

0

0

0

0

0

0

0.130297

0

0

0
7

0

0

0

0

0

0

0

0

0

0

0

0

0

0

0

0

0

0

0

0.0868644

0

0
7

0

0

0

0

0

0

0

0

0

0

0
7

0

0

0

0

0

0

0

0

0

0

1.69152

0

0

0

0

0

0

0

0

0

0

0.238877
7

0
7

0

0

0

0

0

0

0

0

0

0

0
7

0

0

0.0483291

0

0

0

0

0

0

0

0.0483291
7

0

0

0

0

0

0

0

0

0

0

0
1

0

0

0

0

0

0

0

0

0

0

0
6

0

0

0

0

0

0

0

0

0

0

0
7

0

0

0

0

0

0

0

0

0

0

0
6

0

0

0

0

0

0

0

0

0

0

0
7

0

0

0

0

0

0

0

0

0

0

0.836463
6

0

0

0

0

0

0

0

0

0

0

0
7

0

0

0

0

0

0

0

0

0

0

0
7

0
7

0

0

0

0

0

0

0

0

0

0

0.113193

0

0

0

0

0

0

0

0

0

0

0
7

0

0

0

0

0

0

0

0

0

0

0
7

0

0

0

0

0

0

0

0

0

0

0
7

0

0

0

0

0

0

0

0

0

0

0
7

0

0

0

0

0

0

0

0

0

0

0
6

0

0

0

0

0

0

0

0

0

0

0
7

0

0

0

0

0

0

0

0

0

0

0
7

0

0

0

0.325742

0

0

0

0

0

0

0.0724936

0

0

0

0

0

0

0

0

0

0

0.289974
7

0
6

0

0

0

0

0

0

0

0

0

0

0

0

0

0

0

0

0

0

0

0

0

0.289974

0

0

0

0

0

0

0

0

0

0

0
7

0

0

0

0

0

0

0

0

0

0

0
7

0

0

0

0

0

0

0

0

0

0

0

0

0

0

0

0

0

0

0

0

0

0

0

0

0

0

0

0

0

0

0

0.152013

0.0483291
7

0

0

0

0

0

0

0

0

0

0

0.0483291
6

0

0

0

0

0

0

0

0

0

0

0.0483291
7

0

0

0

0

0

0

0

0

0

0

1.28622

0
7

0

0

0

0

0

0

0

0

0

0

0

0

0

0

0

0

0

0

0

0

0

0

0

0

0

0

0

0

0

0

0

0

0
7

0

0

0

0

0

0

0

0

0

0

0
7

0

0

0

0

0

0

0

0

0

0

0
7

0

0

0

0

0

0

0

0

0

0

0
8

0

0

0

0

0

0

0

0

0

0

0
7

0

0

0

0

0

0

0

0

0

0

0.217481
7

0

0

0

0

0

0

0

0

0

0

0

0

0

0

0

0

0

0

0

0

0

7.26969

0.0434322
7

0
7

0

0

0

0

0

0

0

0

0

0

0
7

0

0

0

0

0

0

0

0

0

0

0
7

0

0

0

0

0

0

0

0

0

0

0
7

0

0

0

0

0

0

0

0

0

0

0
7

0

0

0

0

0

0

0

0

0

0

0

0

0

0

0

0

0

0

0

0

0

0
7

0

0

0

0

0

0

0

0

0

0

0.967566

0

0

0

0

0

0

0

0

0

0

0
7

0

0

0

0

0

0

0

0

0

0

0
1

0

0

0

0

0

0

0

0

0

0

0.333407
6

0.0724936

0

0.0966581

0

0

0

0

0

0

0

0

0.0966581

0

0

0

0

0

0

0

0

0

0

0.0724936

0

0

0

0

0

0

0

0

0

0

0
7

0

0

0

0

0

0

0

0

0

0

0.0483291

0

0

0

0

0

0

0

0

0

0

0
7

0

0

0

0

0

0

0

0

0

0

0

0

0

0

0

0

0

0

0

0

0

0
7

0

0

0

0

0

0

0

0

0

0

0
7

0

0

0

0

0

0

0

0

0

0

0
5

0

0

0

0

0

0

0

0

0

0

0
7

0.0724936

0

0

0

0

0

0

0

0

0

0

0
7

0

0

0

0

0

0

0

0

0

0

0
7

0

0

0

0

0

0

0

0

0.0966581

0

0
2

0

0

0

0

0

0

0

0

0

0

0.144987

0

0

0

0

0

0

0

0

0

0

0
7

0

0

0

0

0

0

0

0

0

0

0
6

0

0

0

0

0

0

0

0

0

0

0

0

0

0

0

0

0

0

0

0

0

0
6

0

0

0

0.0483291

0

0

0

0

0

0

0
7

0

0

0

0

0

0

0

0

0

0

0.14009
7

0

0

0

0

0

0

0

0

0

0

0

0

0

0

0

0

0

0

0

0

0

0

0
7

0

0

0

0

0

0

0

0

0

0

0
7

0.0724936

0

0

0

0

0

0

0

0

0

0
7

0

0

0

0

0

0

0

0

0

0

0
7

0

0

0

0

0

0

0

0

0

0

0
6

0

0

0

0

0

0

0

0

0

0

0
7

0

0

0

0

0

0

0

0

0

0

0

0

0

0

0

0

0

0

0

0

0

0

0

0

0

0

0

0

0

0

0

0.254685

0.700771
7

0

0

0

0

0

0

0

0

0

0

0

0
7

0

0

0

0

0

0

0

0

0

0

0
6

0

0

0

0

0

0

0

0

0

0

0

0

0

0

0

0

0

0

0

0

0

0.201103
6

0

0

0

0

0

0

0

0

0

0

0
7

0

0

0

0

0

0

0

0

0

0

0
7

0

0

0

0

0

0

0

0

0

0

0
7

0

0

0

0

0

0

0

0

0

0

0
7

0

0

0

0

0

0

0

0

0

0

0
7

0

0

0

0

0

0

0

0

0

0

0
6

0.0483291

0

0

0

0

0

0

0

0

0

0

0
6

0

0

0

0

0

0

0

0

0

0

0
6

0

0

0

0

0

0

0

0

0

0

7.82931
5

0

0

0

0

0

0

0

0

0

0

0

0

0

0

0

0

0

0

0

0

0

0
7

0

0

0

0

0

0

0

0

0

0

0.144987

0

0

0

0

0

0

0

0

0

0

0.0483291
6

0

0

0

0

0

0

0.53162

0

0

0

0

0

0

0

0

0

0

0

0

0

0

0
7

0

0

0

0

0

0

0

0

0

0

0
6

0
7

0

0

0

0

0

0

0.0483291

0

0

0

0
7

0

0

0

0

0

0

0

0

0

0

0
7

0

0

0

0

0

0

0

0

0

0

0
6

0

0

0

0

0

0

0

0

0

0

0
6

0

0

0

0

0

0

0

0

0

0

0
7

0

0

0

0

0

0

0

0

0

0

0

0

0

0

0

0

0

0

0

0

0

0
7

0

0

0

0

0

0

0

0.0966581

0

0

0
7

0

0

0

0

0

0

0

0

0

0

0.0483291
8

0

0

0

0

0

0

0

0

0

0

0.217481
7

0.0483291
7

0

0

0

0

0

0

0

0

0

0

0.0434322
7

0

0

0

0

0

0

0

0

0

0

0
7

0

0

0

0

0

0

0

0

0

0

0
6

0

0

0

0

0

0

0

0

0

0

0
7

0

0

0

0

0

0

0

0

0

0

0
7

0

0

0

0

0

0

0

0

0

0

0
7

0

0

0

0

0

0

0

0

0

0

0
7

0

0

0

0

0

0

0

0

0

0

0.195445
7

0

0

0

0

0

0

0

0

0.0483291

0

0
7

0

0

0

0

0

0

0

0

0

0

0
7

0
7

0

0

0

0

0

0

0

0

0

0

0.169152
7

0

0

0

0

0

0

0

0

0

0

0
7

0.0483291

0

0

0

0

0

0

0

0

0

0
7

0

0

0

0

0

0

0

0

0

0

0
7

0

0

0

0

0.929061

0

0

0

0

0

0
7

0

0

0

0.0483291

0

0

0

0

0

0

0
6

0

0

0

0.169152

0

0

0

0

0

0

0.0434322
7

0

0

0

0

0

0

0

0

0

0

0
7

0

0

0

0

0

0

0

0

0

0

0
7

0

0

0

0

0

0

0

0

0

0

0.120823
7

0
7

0

0

0

0

0

0.0966581

0

0

0

0

0.193316
6

0

0

0

0

0

0

0

0

0

0

0
2

0

0

0

0

0

0

0

0

0

0

0
6

0

0

0

0

0

0

0

0

0

0

0
7

0

0

0

0

0

0

0

0

0

0

0
7

0

0

0

0

0

0

0

0

0

0

0
8

0

0

0

0

0

0

0

0

0

0

0
7

0

0

0

0

0

0

0

0

0

0

0

0

0

0

0

0

0

0

0

0

0

0.0483291
7

0

0

0

0

0

0

0

0

0

0

0.231852
7

0
7

0
7

0

0

0

0

0

0

0

0

0

0

0
7

0

0

0

0

0

0

0

0

0.0483291

0

0
7

0

0

0

0

0

0

0

0

0

0.193316

0

0

0

0

0

0

0

0

0

0

0

0

0

0

0

0

0

0

0

0

0

0

0

0

0

0

0.673199

0

0

0

0

0

0

0
7

0

0

0

0

0

0

0

0

0

0

0

0

0

0

0

0

0

0

0

0

0

0
6

0

0

0

0

0

0

0

0

0

0

0

0

0

0

0

0

0

0

0

0

0

0.442716
7

0
7

0

0

0

0

0

0

0

0

0

0

0
7

0

0

0

0

0

0

0

0

0

0

0
7

0

0

0

0

0

0

0

0

0

0

0.0966581
5

0

0

0

0

0

0

0

0

0

0

0

0

0

0

0

0

0

0

0

0

0

0
7

0

0

0

0

0

0

0

0

0

0

0.0434322

0

0

0

0

0

0

0

0

0

0

0
7

0

0

0

0

0

0

0

0

0

0

0
7

0

0

0

0

0

0

0

0

0

0

0
7

0

0

0

0

0

0

0

0

0

0

0.217481
7

0
7

0

0

0

0

0

0

0

0

0

0

0
7

0

0

0

0

0

0

0

0

0

0

0
7

0

0

0

0

0

0

0

0

0

0

0
7

0

0

0

0

0

0

0

0

0

0

0.193316

0

0

0

0

0

0

0

0

0

0

0
6

0

0

0

0

0

0

0

0

0

0

0
7

0

0

0

0

0

0

0

0

0

0

0.169152

0

0

0

0

0

0

0

0

0

0

0
7

0

0

0

0

0

0

0

0

0

0

0
7

0

0

0

0

0

0.254685

0

0

0

0

0
7

0
7

0

0

0

0

0

0

0

0

0

0

0

0

0

0

0

0

0

0

0

0

0

0.0724936
6

0

0

0

0

0

0

0

0

0

0

0
7

0

0

0

0

0

0

0

0

0.0724936

0

0
7

0

0

0.0966581

0

0

0

0

0

0

0

0
7

0

0

0

0

0

0

0

0

0

0

0
4

0

0

0

0

0

0

0

0

0

0

0

0

0

0

0

0

0

0

0

0

0

0
7

0

0

0

0

0

0

0

0

0

0

0
7

0

0

0

0

0

0

0

0

0

0

0.0966581
6

0
7

0

0

0

0

0

0

0

0

0

0

0
7

0

0

0

0

0

0

0

0

0

0

0
7

0

0

0

0

0

0

0

0

0

0

0
7

0

0

0

0

0

0

0

0

0

0

0
7

0

0

0

0

0

0

0

0

0

0

0
7

0

0

0

0

0

0

0

0

0

0

0
7

0

0

0

0

0

0

0

0

0

0

0.49947

0

0

0

0

0

0

0

0

0

0

0
7

0

0

0

0

0

0

0

0

0

0

0
7

0

0

0

0

0

0

0

0

0

0

0.169152
7

0.0483291
6

0

0

0

0

0

0

0

0

0

0

0

0

0

0

0

0

0

7.491

0

0

0

0
7

0

0

0

0

0

0

0

0

0

0

0
7

0

0

0

0

0

0

0

0

0

0

0
7

0

0

0

0

0

0

0

0

0

0

0
7

0

0

1.06409

0

0

0

0

0

0

0

0

0

0

0

0

0

0

0

0

0

0

0
7

0

0

0

0

0

0

0

0

0

0

0
6

0

0

0

0

0

0

0

0

0

0

0
7

0

0

0.101849

0

0

0

0

0

0

0

0
7

0.120823

0

0

0

0

0

0

0

0

0

0

0
7

0

0

0

0

0

0

0

0

0

0

0

0

0

0

0

0

0

0

0

0

0

0
6

0

0

0

0

0

0

0

0

0

0

0
7

0

0

0

0

0

0

0

0

0

0

0

0

0

0

0

0

0

0

0

0

0

0
7

0

0

0

0

0

0

0

0

0

0

0
7

0

0

0

0

0

0

0

0

0

0

0

0

0

0

0

0

0

0

0

0

0

0.0483291

0

0

0

0

0

0

0

0

0

0

0
7

0

0

0

0

0

0

0

0

0

0

0

0
7

0

0

0

0

0

0

0

0

0

0

0
7

0

0

0

0

0

0

0

0.144987

0

0

0
6

0

0

0

0

0

0

0

0

0

0

0
6

0

0

0

0

0

0

0

0

0

0

0.289974
5

0

0

0

0

0

0

0

0

0

0

0
6

0

0

0

0

0

0

0

0

0

0

0
7

0

0

0

0

0

0

0

0

0

0

0

0

0

0

0

0

0

0

0

0

0

0.120823

0

0

0

0

0

0

0

0

0

0

0.347146
7

0
7

0

0

0

0

0

0

0

0

0

0

0
7

0

0

0

0

0

0

0

0

0

0

0
7

0

0

0

0

0

0

0

0

0

0

0
7

0

0

0

0

0

0

0

0

0

0

0
7

0

0

0

0

0

0

0.0966581

0

0

0

0
7

0

0

0

0

0

0

0

0

0

0

0

0

0

0

0

0

0.0483291

0

0

0

0

0
7

0

0

0

0

0

0

0.217481

0

0

0

0
7

0

0

0

0

0.0966581

0

0

0

0

0

0.120823

0

0

0

0

0

0

0

0

0

0

0
7

0.26581

0

0

0

0

0

0

0

0

0

0

0.0724936
2

0

0

0

0

0

0

0

0

0

0

0
7

0

0

0

0

0

0

0

0

0

0

0
7

0

0

0

0

0

0

0

0

0

0

0
6

0

0

0

0

0

0

0

0

0

0

0

0

0

0

0

0

0

0

0

0

0

0
7

0

0

0

0

0

0

0

0

0

0

0
7

0

0

0

0

0

0

0

0

0

0

1.0874

0

0

0

0

0

0

0

0

0

0

0
7

0

0

0

0

0

0

0

0

0

0

0
7

0
7

4.5524

0

0

0

0

0

0

0

0

0

0

0

0

0

0

0

0

0

0

0

0

0

0
6

0

0

0

0

0

0.0966581

0

0

0

0

0
7

0

0

0

0

0

0

0

0

0

0

0
7

0

0

0

0

0

0

0

0

0

0

0
7

0

0

0

0

0

0

0

0

0

0

0

0

0

0

0

0

0

0

0

0

0

0
6

0.0483291

0

0

0

0

0

0

0

0

0

0
8

0

0

0

0

0

0

0

0

0

0

0
7

0

0

0

0

0

0

0

0

0

0

0.0868644

0

0

0

0

0

0

0

0

0

0

0

0.0483291
7

0

0

0

0

0

0

0

0

0

0

0
7

0

0

0

0

0

0

0

0

0

0

0
7

0

0

0

0

0

0

0

0

0

0

0
7

0

0

0

0

0

0

0

0

0

0

0
6

0

0

0

0

0

0

0

0

0

0

0
7

0

0

0

0

0

0

0

0

0

0

0
7

0

0

0

0

0

0

0

0

0

0

0
6

0

0

0

0

0

0

0

0

0

0

0
7

0

0

0

0

0

0

0

0

0

0

0.270387
7

0
7

0

0

0

0

0

0

0

0

0

0

0.120823

0

0

0

0

0

0

0

0

0

0

0
7

0

0

0

0

0

0

0

0

0

0

0.0483291
7

0

0

0.0724936

0

0

0

0

0

0

0

0

0

0

0

0

0

0

0

0

0

0

0
7

0

0

0

0

0

0

0

0

0

0

0
7

0

0

0

0

0

0

0

0

0

0

0
7

0

0

0

0

0

0

0

0

0

0

0
6

0

0

0

0

0

0

0

0

0

0

0
7

0

0

0

0

0

0

0

0

0

0

0.0724936
7

0
7

0

0

0

0

0

0

0

0

0

0

0
7

0

0

0

0

0

0

0

0

0

0

0
7

0

0

0

0

0

0

0

0

0

0

0

0.0724936

0

0

0

0

0

0

0

0

0

0
7

0

0

0

0

0

0

0

0

0

0

0
6

0

0

0

0

0

0

0

0

0

0

0
7

0

0

0

0

0

0

0

0

0

0

0
2

0

0

0

0

0

0

0

0

0

0

0
7

0

0

0

0

0

0

0

0

0

0

0.0434322

0

0

0

0

0

0

0

0

0

0

0
7

0
7

0

0

0

0

0

0

0

0

0

0

0
7

0.0434322

0

0

0.0483291

0

0

0

0

0

0

0
7

0

0

0

0

0

0

0

0

0

0

0
8

0

0

0

0

0

0

0

0

0

0

0

0

0

0

0

0

0

0

0

0

0

0

0

0

0

0

0

0

0

0

0

0

0

0

0

0

0

0

0

0

0

0

0

0
7

0

0

0

0

0

0

0

0

0

0

0
7

0

0.338303

0

0

0

0

0

0

0

0

0
7

0

0

0

0

0

0

0

0

0

0

0.407723
7

0
7

0

0

0.193316

0

0

0

0

0

0

0

0
6

0

0

0

0

0

0

0

0

0

0

0
7

0

0

0

0

0

0

0

0

0

0

0
7

2.73059

0

0

0

0

0

0

0

0

0

0.0483291

0

0

0

0

0

0

0

0

0

0

0
7

0

0

0

0

0

0

0

0

0

0

0.0724936
7

0

0

0

0

0

0

0

0

0

0

0
6

0

0

0

0

0

0

0

0

0

0

0
7

0

0

0

0

0

0

0

0

0

0

0
7

0

0

0

0

0

0

0

0

0

0

0
7

0
7

0

0

0

0

0

0

0

0

0

0

0.193316

0

0

0

0

0

0

0

0

0

0

0

0

0

0

0

0

0

0

0

0

0

0
6

0

0

0

0

0

0

0

0

0

0

0
7

0

0

0

0

0

0

0

0

0

0

0.0966581

0

0

0

0

0

0

0

0

0

0

0
7

0

0

0

0

0

0

0

0

0

0

0.193316

0

0

0

0

0

0

0

0

0

0

0
7

0

0

0

0

0

0

0

0

0

0

0
7

0

0

0

0

0

0

0

0

0

0

0
7

0
7

0

0

0

0

0

0

0

0

0

0

0
7

0

0

0

0

0

0

0

0

0

0

0
7

0

0

0

0

0

0

0

0

0

0

0
7

0

0

0

0

0

0

0

0

0

0

0
7

0

0

0

0

0

0

0

0

0

0

0
8

0

0

0

0

0

0

0

0

0

0

0
7

0

0

0

0

0

0

0

0

0

0

0
7

0

0

0

0

0

0

0

0

0

0

0.0483291
6

0

0

0

0

0

0

0

0

0

0

0
7

0

0

0

0

0

0

0

0

0

0

0
7

0
8

0

0

0

0

0

0

0

0

0

0.0966581

0
7

0

0

0

0

0

0

0

0

0

0

0
7

0

0

0

0

0

0

0

0

0

0

0.137642

0

0

0

0

0

0

0

0

0

0

0
6

0

0

0

0

0

0

0

0

0

0

0.0483291
7

0

0

0

0

0

0

0

0

0

0

0.0724936
5

0

0

0

0

0

0

0

0

0

0

0
1

0

0

0

0

0

0

0

0

0

0

0
7

0

0

0

0

0

0

0

0

0

0.144987

0
6

0

0

0

0

0

0

0

0

0

0

0.120823
7

0
7

0

0

0

0

0

0

0

0

0

0

0
5

0

0

0

0

0

0

0

0

0

0

0
7

0

0

0

0

0

0

0

0

0

0

0
7

0

0

0

0

0

0

0

0

0

0

0
8

0

0

0

0

0

0

0

0

0

0

0.0724936

0

0

0

0

0

0

0

0.0966581

0

0

0
7

0

0

0

0

0

0

0

0

0

0

0
7

0

0

0

0

0

0

0

0

0

0

0
6

0

0

0

0

0

0

0

0

0

0

0
7

0

0

0

0

0

0

0

0

0

0

1.09959263916437e-12
7

0
4

0.961282
5

0

0.961282

0

0
4

0
7

0
7

0
4

0
7

0

0

0
4

0

0

0
4

0

0

0
4

0

0

0

0
4

0

0

0
4

0

0

0
4

0

0

0
4

1.18793863634892e-13
7

0
4

0
7

0
7

0
7

0
7

0
7

0

0
4

0

0

0
4

0
4

73.8509

1.2799
7

0
7

0
7

0

0

0

0

0

0

0

0

0

0

0
7

0

0

0

0

0

0

0

0

0

0

0
6

0

0

0

0

0

0

0

0

0

0

0

0

0

0

0

0

0

0

0

0

0

0
7

0
6

0

0
7

0
6

0

0
7

0
7

0

0
7

0.923429

0

0

0

0

0

0

0
7

0

0

0

0

0

0

0

0

0

0

0
6

0

0

0

0

0

0.356472

0

0

0

0

0
6

0

0

0

0

0

0

0

0

0

0

0
7

0

0

0

0

0

0

0

0

0

0

0
7

0

0

0

0

0

0

0

0

0

0

0
6

0

0

0

0

0

0

0

0

0

0

0
6

0

0

0

0

0

0

0

0

0

0

5.55111512312578e-17
7

0
4

0

0

0

0

0

0

0
4

0

0

0
4

66.748

65.6494

0.823956

0.274652
7

0

0
4

0

0

0

0

0

0

0

0
4

1.10811
7

0
7

0

0
6

0
6

0

0

0
5

0

0

0

0

0

0

0

0

0

0

0

0

0

0

0

0
6

0

0

0

0

0

0

0

0

0

0

0
7

0

0

0

0

0

0

0

0

0

0

0
7

0

0

0

0

0

1.10811

0

0

0

0

0
7

0

0

0

0

0

0

0

0

0

0

0
6

0

0

0

0

0

0

0

0

0

0

0
7

0

0

0

0

0

0

0

0

0

0
4

0
6

0
6

0

0

0

0

0

0

0

0

0

0

0

0

0
6

0

0

0

0

0

0

0
4

2.33774
6

0
6

0
7

0

0

0

0

0

0

0

0

0

0

0
6

0

0

0

0

0

0

0

0

0

0

1.57387

0

0

0

0

0

0

0

0

0

0

0
7

0

0

0

0

0

0

0

0

0

0

0
6

0

0

0

0

0

0

0

0

0

0

0

0

0

0

0

0

0

0

0

0

0

0
7

0

0

0

0

0

0

0

0

0

0

0
6

0

0

0

0

0

0

0

0

0

0

0
6

0

0

0

0

0

0

0

0

0

0

0
7

0

0

0

0

0

0

0

0

0

0

0
6

0
6

0

0

0

0

0

0

0

0

0

0

0

0

0

0

0

0

0

0

0

0

0

0
6

0

0

0

0

0

0

0

0

0

0

0
7

0

0

0

0

0

0

0

0

0

0

0
6

0

0

0

0

0

0

0
7

0
6

0
6

0
7

0
6

0
6

0
6

0
6

0

0
6

0

0
6

0

0
6

0
6

0
7

0
6

0

0
7

0
6

0
6

0
7

0
6

0

0

0

0

0

0.152774

0

0
7

0

0

0
6

0

0

0

0

0
6

0

0

0.611094

0

0

0

0

0

0

0

0
6

0

0

0

0

0

0

0

0

0

0

0
7

0

0

0

0

0

0

0

0

0

0

0
7

0

0

0

0

0

0

0

0

0

0

2.22044604925031e-16
6

0
4

0

0

0

0
5

0

0

0

0

0
4

0

0

0
4

0

0

0

0

0
4

0

0

0
4

0

0

0
4

0

0

0

0
4

0

0

0
4

0

0

0
4

0

0

0
4

0

0

0
4

0

0

0
4

0

0

0

0
4

0

0

0
4

0.68663

0.68663

0
4

0

0

0
4

0.184686

0.184686

0
4

0

0

0
4

0

0

0
4

0

0

0
4

0

0

0

0

0
4

1.50589

1.50589

0
4

0

0

0

0

0

0

0
4

0

0

0

0

0
4

0

0

0
4

0

0

0

0
4

0

0

0
4

0
4

203.012

0
7

0
7

0
4

39.0865
5

38.1607
5

0.823956
5

0

0

0

0

0.101849

0

0

0
4

2.44392

2.39559

0.0483291

0

0

0

0

9.0205620750794e-17

0
4

0
7

0
7

0
7

0

0
4

0

0

0

0

0

0

0

0

0
4

28.4289
5

28.3058
5

0

0.123124

1.85962356624714e-15
5

0
4

0
7

0
7

0

0

0
4

2.02158
5

0.911041
5

0.295747

0.305547

0

0.203698

0.305547

0

1.11022302462516e-16
5

0
4

0.184686
6

0
6

0.184686

0

0
4

0

0

0

0

0
4

2.23119
4

1.46758
4

0.414046

0.349565

0

0

0

0

1.66533453693773e-16
4

0
4

0

0

0

0

0

0

0

0
4

7.93566

7.0854
5

0

0

0

0

0

0.0556337
6

0.166901

0.349565

0.111267

0.166901

0

0

0

0
4

1.96845
3

1.66788
4

0.300572

2.77555756156289e-16
3

0
4

21.2287

8.18275

13.046

0
4

0

0

0

0

0
4

0
7

0

0

0
4

0.717525
5

0.411978

0.101849

0

0.101849

0

0.101849

0

0

0
4

2.88804

2.88804

0
4

1.16185

1.0512

0

0.0553261

0

0

0.0553261

0
4

0
7

0
7

0
4

0
6

0
6

0

0
4

0.393468

0.393468

0
4

5.51266
5

1.78817
5

0

0

0

0

0

0.185812

0.0966581

0

0

0

3.03406
5

0.0553261

0

0.0483291

0.304294

0

0

0

0

0

2.77555756156289e-16
5

0
4

2.21304
4

0.0553261

2.15772

0
4

0

0

0

0

0

0

0
4

0
7

0

0

0

0

0
4

0

0

0

0
4

0.27663

0.193641

0.0829891

0
4

0

0

0
4

24.5103

24.2357

0.274652

0
4

0

0

0

0

0
4

0.123124

0.123124

0
4

0

0

0

0
4

0
7

0
7

0

0

0
7

0
7

0
7

0
6

0
7

0

0

0

0
4

0

0

0
4

0

0

0

0
4

0

0

0
4

0

0

0
4

0.169269

0

0.0846347

0.0846347

0
4

1.09861

1.09861

0
4

0

0

0
4

0

0

0

0

0
4

0

0

0

0
4

0

0

0

0

0
4

22.2869
5

18.8825
5

0.892029
3

0.948023
5

0.296221

1.14501

0.123124

0

1.85962356624714e-15
5

0
4

0

0

0

0
4

0

0

0
4

0.0553261

0

0.0553261

0
4

0

0

0
4

0.274652

0

0.274652

0
4

1.78524

1.78524

0
4

0

0

0
4

0

0

0

0
4

0

0

0
4

0.549304

0

0.549304

0
4

7.24969

6.04905

0.0556337
6

0

1.14501

0

0

0

0

4.44089209850063e-16

0
4

0

0

0
4

0

0

0
4

0

0

0
4

0

0

0
4

0

0

0
4

0

0

0
4

0

0

0
4

2.19722

2.19722

0
4

0.56122

0.56122

0
4

0

0

0
4

16.6842

15.9209

0

0

0

0.763337

0

0

0

2.55351295663786e-15

0
4

0

0

0
4

0

0

0
4

0.0483291

0.0483291

0
4

0

0

0
4

0

0

0
4

0

0

0
4

0

0

0
4

0

0

0
4

0

0

0
4

0

0

0
4

0

0

0

0

0

0

0

0

0

0

0

0

0
4

0

0

0
4

0

0

0
4

0.05102

0.05102

0
4

0

0

0
4

0.40816

0.40816

0
4

0

0

0
4

0

0

0
4

0

0

0
4

0

0

0
4

0

0

0
4

5.03023
5

4.9749

0

0

0.0553261

0

1.11022302462516e-16
5

0
4

1.23593

1.23593

0
4

0

0

0
4

0

0

0
4

0
4

9.72806

9.72806

9.66996

0

0

0

0.0581004

0

6.93889390390723e-16

0
4

0
4

0

0

0

0
4

0
4

0.128577

0.128577

0.128577

0
4

0
4

0

0

0

0

0
4

0
4

0

0

0

0

0

0
4

0
4

0

0

0

0
4

0
4

0

0

0

0

0
4

0
4

0

0

0

0

0
4

0
4

0.411978

0.411978

0.411978

0
4

0
4

0

0

0

0
4

0
4

0.101849

0.101849

0.101849

0

0
4

0
4

3.06359
3

2.98429
3

0.37371

0.0511526

0

0.107261

0

0

0.22518

0.274652

0

0

0

0.28703

0

0.0651483

0

0

0

0

0

0.0651483

0

0

0.108194
5

0.107261

0

0.244634

0

0.214522

0.183447
3

0.0917613
3

0.107261

0

0.185812

0.29211

0
4

0.0793016

0.0793016

0
4

2.4980018054066e-16
3

0
4

0

0

0

0

0
4

0
4

0

0

0

0
4

0

0

0
4

0
4

0

0

0

0
4

0

0

0
4

0
4

0.397776

0.397776

0.397776

0
4

0
4

0

0

0

0
4

0
4

0

0

0

0
4

0
4

0.876329

0.876329

0.876329

0
4

0
4

0

0

0

0
4

0
4

0.123536

0.123536

0.123536

0
4

0
4

0

0

0

0
4

0
4

1.15183
3

1.15183
3

1.15183

0

0

0

0
4

0

0

0
4

0

0

0
4

0
4

0

0

0

0
4

0
4

0

0

0

0
4

0
4

0

0

0

0
4

0
4

0

0

0

0
4

0
4

1.60061

1.60061

1.60061

0
4

0
4

0

0

0

0
4

0
4

0

0

0

0
4

0
4

0

0

0

0
4

0
4

0

0

0

0
4

0
4

0

0

0

0
4

0
4

0
6

0
6

0
6

0

0

0

0

0
4

0
4

0

0

0

0
4

0
4

0

0

0

0
4

0
4

0

0

0

0
4

0
4

0

0

0

0
4

0
4

0

0

0

0
4

0
4

0

0

0

0
4

0
4

0

0

0

0
4

0
4

0

0

0

0
4

0
4

0

0

0

0

0

0

0
4

0
4

0
5

0

0

0
4

0

0

0
4

0

0

0
4

0
4

0

0

0

0

0
4

0
4

0

0

0

0

0
4

0
4

4.74798

3.43427

3.43427

0

0

0

0

0

0

0

0
4

0.196734
6

0.196734
6

0

0

0

0

0

0

0

0
4

0

0

0
4

0

0

0
4

0

0

0
4

1.11698
5

0.846022

0.0581004

0.0581004

0

0

0.0966581

0

0

0.0581004

0
4

0
7

0
6

0

0

0
4

0
7

0
7

0

0
4

0

0

0

0
4

0

0

0
4

0

0

0
4

0

0

0
4

0

0

0
4

0
4

35.7364

25.1029

0.68857

0
5

0
7

0.295124

4.28857

1.14501
5

0

2.19722

0.470388
5

0

0

0

0

0

0

0.193316

0

6.16077

0

0

0

0

0
6

0

0

0

0

0

0

0

0

0.763337

0

5.89641
5

0

0

1.47749

0

0

0

0

0

0

0

0
6

0

0.763337

0

0

0

0

0

0

0

0

0

0

0

0

0

0

0

0

0

0

0

0

0

0

0.763337

0

0

0

0

0

0

0

0
7

0

0

0

0

0

0

0

0

0

0
4

0

0
7

0

0
4

0.407396

0.407396

0

0
4

0.411978

0.411978

0
4

0

0

0
4

0

0

0
4

0

0

0
4

0

0

0
4

0

0

0
4

0

0

0
4

0.106429
5

0
6

0.0581004

0.0483291

0

6.93889390390723e-18
5

0
4

0
6

0

0

0

0

0
4

9.70769
5

9.70769
5

0
4

0
7

0
7

0

0

0
4

0
7

0
7

0
4

0

0

0

0
4

0

0

0

0
4

0

0

0
4

0
4

6.6017

6.6017

1.02197
5

0

0.535102

0

0

0.411978

0

0

0

0

0

1.20778
5

0

0

0

0

0

0

0

0

0

0

3.42488

0

0

0

0
6

0

0

0

0

0

4.44089209850063e-16

0
4

0

0

0
4

0

0

0
4

0

0

0
4

0

0

0
4

0
4

52.3655
5

52.3074
5

1.47337
5

0.204649
5

0.325902
5

0
6

0.145251
5

0

0

0.0501388

0

0.125347

0

4.97588

0

0

0

0.0483291

0

0.0581004

0

0

0

0

0
6

0.0483291

0

0

0

0

0

0

0

0

0

2.35307
5

0

0

0

0

0

0

0

0.29211

0

0

2.05398
5

0

0.0501388

0

0

0

0

0.0501388

0

0

0

4.22879
5

0

0

0

0

0

0

0

0

0.0581004

0

0
6

0

0

0

0

0

0

35.7658
4

0
6

0
4

0

0

0

0
4

0.0581004

0

0.0581004

0
4

0

0

0
4

0

0

0
4

0
4

89.0094

81.2543

36.2743

18.934
5

0

1.52009

0.549304

0

0

0

0

0

0

0.823956

14.9652
5

0

0.274652

0

0

0

0.411978

0

0

0

0

0

0

0

0.369371

3.36695

0.411978

0
7

1.46389

1.88865

1.19904086659517e-14

0
4

0.763337
5

0.763337
5

0
4

0

0

0
4

0

0

0

0
4

0

0

0

0

0
4

0

0

0
4

5.26781

5.26781

0
4

0

0

0
4

0

0

0

0
4

0

0

0
4

0

0

0
4

0

0

0
4

0

0

0

0
4

0

0

0
4

0

0

0
4

0

0

0
4

0.0871506

0.0871506

0
4

0

0

0
4

0

0

0
4

0

0

0
4

0

0

0
4

0

0

0
4

0

0

0
4

0

0

0

0

0
4

0

0

0
4

0.0581004

0.0581004

0
4

0

0

0
4

0

0

0
4

0

0

0
4

0

0

0
4

0

0

0
4

0

0

0

0

0

0
4

0

0

0

0

0

0
4

1.37496

1.37496

0
4

0

0

0
4

0.203698

0.203698

0
4

0

0

0

0
4

7.35522753814166e-15

0
4

127.025
5

0
7

0
7

0
7

0

0
4

11.1269
4

10.7809
4

0.161286

0.184686

0
4

0

0

0
4

0

0

0
4

0.274652

0.274652

0
4

0

0

0
4

50.0946

43.967

6.12761

1.77635683940025e-15

0
4

64.8394
4

63.343
4

0.94708

0.549304

0
4

0

0

0

0

0
4

0.689315

0.0926517

0.184686

0.411978

0
4

0

0

0
4

0

0

0
4

0

0

0
4

0

0

0
4

0
4

8.13695

8.13695

1.06324

1.83065
5

1.47404

0

0

0

0

0

0

0

0

1.1599

0.120823

0

0

0.0724936

0

0

0

0

0.0966581

0

0.845758

0

0

0

0

0

0

0

0

0

0

0.0483291
7

0

0.0483291
7

0

0

0.386632

0.990106

1.55431223447522e-15

0
4

0
4

34.6015
4

8.92619

1.64791

2.05989

5.21839

0

0
4

25.4906

24.5103

0.430933

0.549304

0
4

0

0

0
4

0.184686

0.184686

0
4

0

0

0
4

0

0

0
4

3.27515792264421e-15
4

0
4

48.86
4

48.7369
4

0.735116
5

8.65072

0

0.274652

0

0

0.246248

0

0

12.8152

17.6883

0

0

3.09305

2.74652

0.839153

1.64791

9.76996261670138e-15
4

0
4

0

0

0
4

0.123124

0.123124

0
4

0

0

0
4

0
4

4.37694325228222e-12
7

0
4

198.201

121.856

33.4997

32.898
5

0

0

0

0

0.476319

0

0

0

0.125347

0

1.13797860024079e-15

0
4

57.8052

57.7049

0
7

0

0

0.0501388

0

0

0

0

0

0

0

0
7

0.0501388

0

0
7

0

0

0

0

0

0

0
4

5.29565

4.4595
7

0
7

0

0

0

0

0

0

0

0

0

0

0
7

0

0

0

0

0

0

0

0

0

0

0
7

0

0

0

0

0

0

0

0

0

0

0
8

0

0

0

0

0

0

0

0

0

0

0
7

0

0

0

0

0

0

0

0

0

0

0
7

0

0

0

0

0

0

0

0

0

0

0
1

0

0

0

0

0

0

0

0

0

0

0

0

0

0

0

0

0

0

0

0

0

0
8

0

0

0

0

0

0

0

0

0

0

0

0

0

0

0

0

0

0

0

0

0

0

0

0.464531

0

0

0

0

0

0

0

0

0

0
8

0

0

0

0

0

0

0

0

0

0

0
7

0

0

0

0

0

0

0

0

0

0

0
7

0

0

0

0

0

0

0

0

0

0

0
7

0

0

0

0

0

0

0

0

0

0
7

0

0

0.371625
7

0
7

0

0

0

0

0

0

0

0

0

0

0

0

0

0

0

0

0

0

0

0

0
7

0

0

0

0

0

0

0

0

0

0

0
1

0

0

0

0

0

0

0

0

0

0

0
1

0

0

0

0

0

0

0

0

0

0

0
8

0

0

0

0

0

0

0

0

0

0

0
8

0

0

0

0

0

0

0

0

0

0

3.88578058618805e-16

0
4

0
7

0
7

0

0
4

0
7

0
7

0

0

0

0

0

0

0

0

0

0
4

0
6

0
6

0
6

0

0

0

0
4

0
7

0
7

0
6

0

0

0
4

0
7

0

0

0

0

0

0

0

0

0

0

0
4

0

0

0

0

0

0

0
4

0
1

0
1

0
4

0
6

0
6

0

0

0
4

1.6979

1.30172

0.396177

0

1.11022302462516e-16

0
4

0

0

0

0
4

7.46261
5

3.80915
7

0

0
6

0

0

0

0

0

0

0

0

3.14508

0.161286

0

0

0

0

0

0

0

0

0

0
6

0

0

0.161286

0
6

0.185812
5

0
6

0
6

0
6

1.94289029309402e-16
5

0
4

0
6

0
6

0
4

0

0

0

0
4

0

0

0

0
4

0

0

0

0
4

0

0

0

0
4

0

0

0

0
4

0

0

0

0
4

0

0

0
4

0

0

0
4

0

0

0
4

5.57437

0
1

0

0

0

5.57437

0

0

0

0
7

0
2

0
8

0
6

0
4

0

0

0
4

0

0

0
4

0

0

0
4

0

0

0
4

0

0

0
4

0

0

0
4

0.113193

0.113193

0
4

0

0

0
4

0

0

0
4

0

0

0
4

9.68196

4.92403
5

0

4.75794

0
7

0
7

0

0

0

0

0

0
4

0

0

0
4

0

0

0
4

0

0

0
4

0

0

0
4

0

0

0
4

0

0

0
4

0

0

0
4

0

0

0
4

0

0

0
4

0

0

0
4

0.161286

0
1

0
1

0.161286

0

0

0

0

0

0
4

0

0

0
4

0
7

0
7

0
8

0

0

0

0

0
4

0
6

0
6

0
7

0

0

0

0

0

0
4

0
8

0
8

0
8

0

0

0

0

0

0
4

0.564501

0.564501

0

0

0

0

0

0
4

2.20934381900406e-14

0
4

3.91151

3.8363

0

0

0

0

0

0

0.185812

0

0

0

0

0

0

0

0

0

0

0

0

0

0

0

0

3.65049

0

0

0
4

0
8

0
8

0

0

0

0

0

0

0

0

0

0

0
8

0

0

0

0

0

0

0

0

0

0

0
8

0

0

0

0

0

0

0

0

0
4

0

0

0
4

0

0

0
4

0

0

0
4

0

0

0
4

0

0

0
4

0

0

0
4

0

0

0
4

0

0

0
4

0.0752082

0.0752082

0
4

0

0

0
4

1.11022302462516e-16

0
4

37.8909

17.8197

11.3825

0
5

0

0

0

0

0

0

0

0

0

0

0

0

0

0

0

0

0

0

0

0

0

0
6

0

0

0

0

0

0

0

0

0

0

0
7

0

0

0

0

0

0

0

0

0

0

0

0

0

0

0

0

0

0

0

0

0

0

0

0

0
6

0
7

0

0
6

5.04261

0
7

0

0

0
7

0

0

0

0

0

0

1.20778
7

0

0

0

0

0

0

0

0

0

0

0

0

0

0

0

0

0

0

0

0

0

0
6

0

0

0.0848951

0

0

0

0

0

0

0

0.101849
7

0

0

0

0

0

0

0

0

0

0

0
1

0

0

0

0

0

0

0

0

0

0

0

0

0

0

0

0

0

0

0

0

0

0
7

0

0

0

0

0

0

0

0

0

0

0
4

0

0

0

0

0
4

0

0

0

0

0
4

0

0

0

0
4

0

0

0
4

0

0

0
4

0

0

0
4

0

0

0
4

0

0

0
4

19.7216

15.9013

0

0

0

0

0

0

0

0

0

0

0.16979
6

0

0

0

0

0

0

0

0

0.113193

0

0
6

0

0

0

0

0

0

0

0

0.0848951

0

0
6

0

0

0

0

0

0

0

0

0

0

0

0

0

0

3.28261

0.0565967

0.113193

0

5.55111512312578e-16

0
4

0.349565
5

0
6

0

0

0

0.349565

0

0

0

0

0

0
4

0

0
6

0

0

0

0

0

0

0

0

0

0

0
4

0
7

0
7

0

0

0

0
4

0
6

0

0

0

0

0

0
4

0
7

0

0

0

0

0
4

0

0

0

0

0

0
4

0

0

0

0

0

0

0
4

0
4

0

0

0

0
4

0

0

0
4

0
4

0

0

0

0
4

0

0

0
4

0
4

0

0

0

0

0
4

0
4

0

0

0

0

0

0
4

0
4

0

0

0

0

0
4

0
4

0

0

0

0

0
4

0
4

0

0

0

0

0
4

0
4

0

0

0

0

0
4

0

0

0
4

0
4

0

0

0

0
4

0
4

0

0

0

0
4

0
4

0
7

0
7

0
7

0

0

0

0

0

0

0
7

0
7

0
7

0

0

0

0

0

0
4

0
6

0
6

0
7

0

0

0

0

0

0

0
4

0

0

0

0
4

0

0

0

0
4

0
6

0

0

0

0
4

0

0

0

0
4

0

0

0
4

0

0

0
4

0

0

0
4

0
4

0

0

0

0
4

0

0

0
4

0
4

0

0

0

0
4

0

0

0
4

0
4

0

0

0

0

0
4

0
4

0

0

0

0
4

0
4

0

0

0

0
4

0
4

0

0

0

0
4

0
4

0

0

0

0
4

0
4

0

0

0

0
4

0
4

0

0

0

0
4

0
4

0

0

0

0
4

0
4

0
7

0
7

0
7

0

0

0

0

0
4

0
7

0
7

0

0

0

0

0

0
4

0
6

0

0

0

0
4

0

0

0
4

0
4

0

0

0

0
4

0
4

0.0501388

0.0501388

0.0501388

0
4

0
4

0

0

0

0
4

0
4

0.161286

0.161286

0.161286

0
4

0
4

0

0

0

0
4

0
4

0

0

0

0
4

0
4

0
6

0
6

0
6

0

0

0

0

0

0
4

0
7

0
7

0

0

0
4

0
7

0
6

0

0
4

0

0

0

0

0
4

0

0

0
4

0

0

0
4

0
4

0
6

0
6

0
6

0

0

0

0

0
4

0
4

0.992768
2

0.992768
2

0.992768
2

0

0

0

0

0

0

0

0
4

0
4

0
6

0
6

0

0

0

0

0

0
4

0

0

0

0
4

0
4

0

0

0

0
4

0
4

7.24506

7.24506

6.7186

0

0

0.300833

0.225625

1.11022302462516e-16

0
4

0
4

26.0932

26.0932

11.1078

0
7

0
7

0

0

0
7

0

0

0.0966581

0

0.175486

0
7

0

0

0

0.125347

0

0

0

0.250694

0.0984678

0

13.1575

0

0

0

0

0

0

0

0

0

0

0

0

0

0

0

0

0

0

0

0

0.0724936

0.314139
7

0

0.0501388

0

0

0

0

0

0

0

0

0

0

0.100278

0.0501388

0

0

0.0483291

0

0.300833

0

0

0
8

0

0

0

0

0

0

0

0

0

0

0

0.0966581

0

0

0

0

0

0

0

0

0

0.0483291

0

0

1.15879528195251e-15

0
4

0

0

0
4

0

0

0
4

0

0

0
4

0
4

1.77635683940025e-14

0
4

3.84826

0
7

0

0

0

0
4

0

0

0

0
4

0

0

0
4

0

0

0
4

0

0

0
4

0

0

0
4

0

0

0
4

0

0

0
4

0
4

0
7

0
7

0

0

0

0

0

0

0

0

0

0

0

0

0

0

0

0

0

0

0

0

0

0

0

0

0

0

0

0

0

0

0

0

0

0

0

0

0

0

0

0

0

0

0

0

0

0

0

0

0

0

0

0

0

0

0

0

0

0

0

0

0

0

0

0

0

0

0

0

0

0

0

0

0

0

0
4

0

0

0
4

0
4

0
6

0
6

0
6

0

0
4

0
4

0

0

0

0
4

0
4

0

0

0

0
4

0
4

0.414046
7

0.414046
7

0.414046
7

0
4

0
4

0

0

0

0
4

0
4

0

0

0

0
4

0
4

0

0

0

0
4

0
4

0

0

0

0
4

0
4

0

0

0

0
4

0
4

0

0

0

0
4

0
4

0

0

0

0
4

0
4

3.43422

3.07932

1.56871

0
7

0

0

0

0

0

0

0

0

0

0

0
6

0

0

0

0

0

0

0

0

0

0

1.51061

0

0

0

0
7

0

0
7

0

0

0

0
4

0.354897

0.354897

0

0

0
4

0

0

0
4

0

0

0
4

0

0

0
4

0

0

0

0
4

0

0

0
4

0

0

0
4

0

0

0
4

0
4

0
4

46961.8
4

46933.7
4

46929.8
4

46896
4

2.29001

0

4.53756

0.50937

0

0

0

0

0

0

0

13.3584

4.12805

0

0

0

0

0.123124

0

0

0

0.0565967

0
7

0.763337

0.185812

0.185812

0.0565967

0.221304

0

0.274652

0

0

0

0

0.0565967

0

0

0.113193

0.123124

0

0

0

0

0

0

0

0

0.0556337

0

0

0

0

0.0848951

0

0
6

5.48396

0

1.14501

3.13415959851682e-12
4

0
4

0

0

0
4

0

0

0
4

0

0

0
4

0

0

0
4

0

0

0
4

3.91083

3.91083

0
4

0

0

0
4

0.0501388

0.0501388

0
4

0

0

0
4

0

0

0
4

0

0

0
4

0

0

0
4

0

0

0
4

1.09339620690818e-12
4

0
4

4.93722

4.93722

4.93722

0
4

0
4

0

0

0

0

0
4

0
4

0

0

0

0
4

0
4

0.411978

0.411978

0.411978

0
4

0
4

0

0

0

0
4

0
4

0

0

0

0
4

0
4

0

0

0

0
4

0
4

0

0

0

0
4

0
4

0.246248

0.246248

0.246248

0
4

0
4

0

0

0

0
4

0
4

0

0

0

0
4

0
4

0

0

0

0

0
4

0
4

0

0

0

0
4

0
4

0

0

0

0
4

0
4

0

0

0

0
4

0
4

0

0

0

0
4

0
4

0

0

0

0
4

0
4

0

0

0

0
4

0
4

21.4024

21.4024

21.4024

0
4

0
4

0

0

0

0
4

0
4

0

0

0

0

0
4

0
4

0

0

0

0
4

0
4

0

0

0

0

0
4

0
4

1.10267

1.10267

1.10267

0
4

0
4

0

0

0

0
4

0
4

0
4

1105.24

0
1

0

0

0

0

0

0

0
4

0

0

0
4

0

0

0
4

0

0

0
4

0

0

0
4

0

0

0
4

0

0

0
4

0

0

0
4

0

0

0
4

0

0

0
4

0
4

88.777

82.7655

2.08475

0.0868644
2

0.0651483
3

0.152013

0.608051

0

0.550126

0.0651483

0.0434322

0

0

8.59958

0

0.152013

0.261726

0

0

0.177091

0.142735

0.0846347

0.195445

0.126952

19.0021
4

0

0

0.0434322

0

0.0434322

0.350972

0.134773

0

0.200555

0

3.58316

0.0651483

0

0.126952

0

0.0501388

0.120823

0

0

0

0

7.77694

0

0.126952

0

0

0

0

0.0501388

0

0.108581

1.98149

29.1524
4

0

0

0

0

0.380856

0

0

0

0

0

0.0651483
3

0

0

0

0

0.0651483

0

0

0

0.0434322

0

1.04237

0.0651483

0

0

0

0

0

0

0

0.0434322

0.126952

3.73795
3

0.667604

0

0

0

0

0

0

0.126952

0.0868644

3.99263955230822e-14

0
4

1.59049
3

1.37301
3

0.0483291

0.0724936

0.0966581

0

0
4

0

0

0
4

0

0

0
4

0

0

0
4

0

0

0
4

0

0

0
4

0

0

0
4

0

0

0
4

0

0

0
4

0

0

0
4

0

0

0
4

2.94551
3

2.94551
3

0
4

1.47551

1.47551

0

0
4

0

0

0

0
4

0

0

0
4

0

0

0

0
4

0

0

0
4

0

0

0
4

0

0

0
4

1.53210777398272e-14

0
4

0.203698
1

0
1

0
1

0

0

0

0

0

0

0

0

0

0

0
1

0

0

0

0

0

0

0

0

0

0

0

0

0

0

0

0

0

0

0

0

0

0

0

0

0

0

0

0

0

0

0

0

0

0

0

0

0

0

0

0

0

0

0

0

0

0

0

0

0

0

0

0

0

0

0

0

0
4

0
2

0
2

0

0
4

0

0

0

0

0

0
4

0

0

0
4

0

0

0
4

0.101849

0.101849

0
4

0

0

0
4

0

0

0
4

0

0

0
4

0

0

0
4

0

0

0
4

0

0

0
4

0

0

0
4

0.101849

0.101849

0

0

0
4

0

0

0
4

0

0

0
4

0

0

0
4

0

0

0

0
4

0

0

0
4

0

0

0
4

0

0

0
4

0

0

0
4

0
4

132.505

0

0
2

0

0

0

0

0

0

0

0

0

0

0
6

0

0

0

0

0

0

0

0

0

0

0
3

0

0

0

0

0

0

0

0

0

0

0
2

0

0

0

0

0

0

0

0

0

0

0
3

0

0

0

0

0

0

0

0

0

0

0
6

0

0

0

0

0

0

0

0
2

0

0
2

0
4

82.2847

71.6828

0.113477
7

0.575052

0

0

0

0

1.57304

0

0

0

0

0

0
7

0

0

0

0

0

0

0

0

0

0

1.01491

0

0

0

0

0

0

0

0

0

0

0
7

0

0

0

0

0

0

0

0

0

0

0

0

0

0

0

0

0

0

0

0

0

0
7

0

0

0

0.0483291

0

0.0966581

0

0

0

0

0
6

0

0

0

0

0

0

0

0

0

0

0
7

0

0

0

0

0

0

0

0

0

0

0
7

0

0

0

0

0

0

0

0

0

0

0
7

0

0

0

0.0724936

0

0

0

0.500948

0

0

0.0724936
7

0
7

0

0

0

0

0

0

0

0

0

0

0
7

0

0

0

0

0

0

0

0.0483291

0

0.144987

0
7

0

0

0

0

0

0

0

0

0

0

0
7

0

0

0

0

0

0

0

0

0

0

0
6

0

0

0

0

0

0

0

0

0

0

0
2

0

0

0

0

0

0

0

0

0

4.97789

0
7

0

0

0

0

0

0

0

0

0

0

0

0

0

0

0

0

0

0

0

0

0

0
7

0

0

0

0

0

0

0

0

0

0

0
6

0

0.0966581

0

0

0

0

0

0

0

0

0
7

0
6

0

0

0

0

0

0

0

0

0

0

0
7

0

0.0434322

0

0

0

0

0

0

0

0

0

0

0

0

0

0

0

0

0

0

0

0
7

0

0

0

0

0

0

0

0

0

0

0
7

0

0

0

0

0

0

0

0

0

0

0
8

0

0

0

0

0

0

0

0

0

0

0
1

0

0

0

0

0

0

0

0

0

0

0
7

0

0

0

0

0

0

0

0

0

0

0

0

0

0

0

0

0

0

0

0

0

0
7

0

0

0

0

0

0

0

0

0
7

0

0
7

0

0
7

0
7

0
6

0
8

0

0

0

0
7

0
7

0
7

0

0
6

0

0

0

0

0

0

0
7

0

0

0

0

0

0

0

0

0

0

0
7

0

0

0

0

0

0

0

0

0

0

1.22317

0

0

0

0

0

0

0

0

0

0

0

0

0

0

0

0

0

0

0

0

0

9.76996261670138e-15

0
4

0
7

0
7

0

0

0
4

44.0769

19.4734

0.185812

0

0

0

0

0

0

0

0

1.30488

0.13244

0
6

0

0

0

0

13.5333
4

0

0

0

0

0

0

0

0

0

0

0.27167
2

0.185812

0

0

0

0

0

0

0

0

0.0483291

0.999737
3

0

0

0

0

0.0483291

0

0.0483291

0

0

0

0.459126
6

0

0

0

0

0.524348

0

0

0

0

0

0
1

0

0.0483291

0

0.141492

0.278718

0

0

0

0.16979

0

0

0

0

0

0

0.53162

0

0

0

0

0.0434322

1.91474
3

0

0.0724936

0

0

0

0

0

0

0

0.053673

2.58511
4

1.02197

0

0

0

0

0

0

0

0

0

0
4

5.34336

5.34336

0
7

0

0
4

0

0

0
4

0

0

0

0
4

0

0

0
4

0

0

0
4

0

0

0
4

0

0

0
4

0

0

0
4

0

0

0
4

0

0

0
4

0

0

0
4

0

0
1

0

0

0

0

0

0

0

0

0

0

0

0
4

0

0

0
4

0

0

0
4

0

0

0
4

0

0

0
4

0
7

0
7

0

0

0

0

0

0
4

0.287206

0
1

0

0

0.238877

0

0

0.0483291

6.93889390390723e-18

0
4

0.464531

0

0

0.464531

0

0

0
4

0.0483291

0.0483291

0

0

0

0
4

0
1

0

0

0

0
4

0

0

0

0

0

0
4

0

0

0

0

0
4

0
4

3.05528

1.09861

1.09861

0

0

0

0

0

0

0
4

0
1

0
2

0
1

0

0

0
4

0

0

0
4

0

0

0
4

0

0

0
4

0

0

0

0
4

0

0

0

0
4

0

0

0
4

0

0

0

0
4

1.90834

1.90834

0
4

0

0

0
4

0

0

0
4

0

0

0

0
4

0

0

0

0
4

0

0

0

0
4

0

0

0
4

0

0

0
4

0

0

0
4

0

0

0
4

0

0

0
4

0

0

0
4

0

0

0
4

0

0

0
4

0
2

0
2

0

0
4

0

0

0
4

0

0

0
4

0

0

0
4

0

0

0
4

0

0

0
4

0

0

0
4

0

0

0
4

0

0

0
4

0

0

0
4

0

0

0
4

0
2

0
2

0
4

0

0

0
4

0

0

0
4

0

0

0
4

0

0

0
4

0

0

0
4

0

0

0
4

0

0

0
4

0

0

0
4

0

0

0
4

0.0483291

0.0483291

0

0
4

0

0

0
4

0

0

0

0
4

9.0205620750794e-17

0
4

8.02441
3

8.02441

8.02441

0

0

0

0
4

0

0

0

0

0
4

0

0

0
4

0

0

0

0
4

0

0

0

0

0
4

0

0

0
4

0

0

0

0
4

0

0

0
4

0

0

0
4

0

0

0
4

0

0

0
4

0
4

25.682

24.9623

0
8

5.02622

0

0

0

0

0

0

0.0483291

0.120823

0

0

0

0

0

0

0

0

0

0.120823

0

0

0

0.410797

0

0

0

0

0

0

1.23239

1.4982

0

0

3.70958

0

0

1.44987

0

0

0

0

0

0

0

0

0

0

0.169152

0

0

0

0.144987

5.9928

1.01491

0

0

0

0

0

0

0

0

0

0.338303

0

0

0

0

0

0

0

0

0

0

0

0

0

0

0

0

0

0

0

0

0

0

3.46765

0

0

0

0

0

0

0.0483291

0

0

0

0

0

0

0

0

0

0

0

0.0483291

0

0

0

0

0

0

0

0.0724936

0

0

0.0483291

0

0

0
4

0

0

0

0

0

0

0

0

0
4

0

0

0
4

0

0

0

0

0

0
4

0

0

0
4

0

0

0

0
4

0

0

0
4

0

0

0

0

0
4

0

0

0

0

0
4

0

0

0

0

0
4

0

0

0

0
4

0

0

0
4

0

0

0

0

0

0
4

0

0

0

0

0
4

0

0

0
4

0

0

0
4

0

0

0

0

0
4

0

0

0

0

0
4

0

0

0
4

0.120823

0.120823

0
4

0

0

0

0
4

0

0

0
4

0.160722

0.0642886

0.0964329

0
4

0

0

0

0
4

0.438165

0.438165

0
4

0

0

0
4

0

0

0
4

0

0

0
4

0

0

0
4

0

0

0
4

0

0

0
4

0

0

0
4

0

0

0
4

0

0

0
4

0

0

0

0

0

0

0

0
4

0

0

0
4

0

0

0
4

0

0

0
4

0

0

0
4

0

0

0
4

0

0

0
4

0

0

0
4

0

0

0
4

0

0

0
4

0

0

0
4

0
7

0
7

0

0
4

0

0

0
4

0

0

0
4

0

0

0
4

0

0

0
4

0

0

0
4

0

0

0
4

0

0

0
4

0

0

0
4

0

0

0
4

0

0

0

0

0

0

0
4

0

0

0
4

0

0

0

0

0
4

0

0

0

0
4

5.21804821573824e-15

0
4

12.5656

12.5656

3.23805

5.72699

0.0724936

0.966581

0.0483291

0.0483291

0

0.0966581

0

0

0

0

0

0.0483291

0.0483291

0

0

0

0

0

0

0.169152

0.555784

0.434961

0

1.11157

0

0

0

0

0

3.77475828372553e-15

0
4

0

0

0

0

0

0

0
4

0

0

0
4

0

0

0

0
4

0

0

0
4

0

0

0
4

0

0

0
4

0

0

0
4

0
4

7.71182

0

0

0

0
4

0

0

0

0

0

0

0

0

0

0

0
4

0

0

0
4

0

0

0

0
4

2.258

2.258

0
4

0

0

0
4

2.66122

0.161286

2.49993

0
4

0

0

0
4

0

0

0

0
4

0

0

0
4

0

0

0
4

0

0

0
4

0

0

0

0

0

0
4

0

0

0
4

0

0

0
4

0

0

0
4

0

0

0
4

0

0

0
4

0

0

0
4

0

0

0
4

0

0

0
4

0

0

0

0

0

0
4

0

0

0

0
4

1.06324

1.06324

0

0
4

0

0

0

0
4

0

0

0

0
4

1.72935
2

1.23686
2

0

0.492495

0
4

0

0

0

0
4

2.22044604925031e-16

0
4

221.567

221.567

221.567

0

0

0

0

0

0

0

0

0
4

0

0

0

0
4

0

0

0

0
4

0

0

0
4

0

0

0
4

0

0

0
4

0
4

0

0

0

0

0

0

0

0

0

0

0

0

0
4

0

0

0
4

0

0

0

0
4

0
4

29.0582

0
7

0

0

0

0

0

0

0

0

0

0

0

0

0

0

0

0

0

0

0

0

0

0

0

0

0

0

0

0

0

0

0

0

0
4

0

0
7

0

0

0

0

0

0

0

0

0

0

0
7

0

0

0

0
7

0

0

0

0

0

0

0
4

0.0501388

0
7

0
7

0

0

0

0

0

0

0

0

0

0

0
7

0

0

0

0

0

0

0

0

0

0

0
6

0

0

0

0

0

0

0

0

0

0

0

0.0501388

0

0

0

0

0

0

0

0

0

0

0

0

0

0

0

0

0

0

0

0

0

0

0

0

0

0

0

0

0

0

0

0

0

0

0

0

0
4

25.4558

3.09819

0.386632
7

0

0

0

0

0

0

0

0

0

0.0724936

0

0
7

0

0

0

0

0

0

0

0

0

0

0
7

0

0

0

0

0

0.724936

0

0

0

0.0483291

0
7

0

0

0

0

0

0

0

0

0

0

0
6

0

0

0

0

0

0

0

0

0

0.0724936

0

0

0

0.584154

0

0

0

0

0.0434322

0

0

0

0

0

0

0

0

0

0

0

0
7

0

0

0
7

0

7.20103

0

0

0

1.61562

0

1.97617

0.555784

0

0.434961

0

0

0

0

0

0

0

0

1.8365

0

5.66025
5

0

0

0

0

0

0

0

0

0.115926

0

0.0483291
7

0

0

0

0

0

0

0

0.0966581

0

0

0
7

0

0

0

0.144987

0.137642

0

0.241645

0

0

0

0

0

0

0

0.108581

0

0

0.0483291

0

0

0

0
7

0.0868644

0

0

0

0

0

0

0

0

0

0.0724936

0

0

0

0

0

0

0

0

0

0.0434322

2.19962936753859e-15

0
4

0
2

0
2

0

0

0

0

0

0

0

0

0

0

0
2

0

0

0

0

0

0

0

0

0

0

0

0

0

0

0

0

0

0

0

0

0

0
2

0

0

0

0

0

0

0

0

0

0

0

0

0

0

0

0

0

0

0

0

0

0

0

0

0

0

0

0

0

0

0

0

0

0

0
4

0

0

0
4

0
7

0
7

0

0

0
4

1.44987

0

0

0

0

0

0

0

0

0

0

0

0

0.700771

0.7491

0

0

0

0
4

0.53162

0.53162

0

0

0

0

0

0

0

0

0

0

0

0

0

0

0

0

0

0

0

0

0
4

0
3

0

0

0

0

0
4

0
6

0
6

0

0
4

0

0

0

0

0

0
4

0

0

0

0

0

0

0
4

0

0

0

0
4

0

0

0

0
4

0

0

0

0

0
4

0

0

0

0

0
4

0

0

0

0

0
4

0

0

0
4

0

0

0

0

0

0

0

0
7

0

0

0

0

0

0

0

0
4

0

0

0
4

0

0

0

0
4

0

0

0
4

0

0

0
4

0

0

0

0

0
4

0

0

0

0

0
4

0

0

0

0
4

0.0483291

0

0.0483291

0
4

0

0

0
4

0

0

0

0
4

0
7

0
7

0
7

0

0

0

0

0

0
4

0

0

0

0
4

0

0

0

0
4

0

0

0

0
4

0

0

0
4

0

0

0

0
4

0

0

0

0
4

0

0

0
4

0

0

0
4

0

0

0
4

0

0

0
4

1.42571
7

1.42571
7

0

0
4

0

0

0
4

0

0

0
4

0

0

0
4

0

0

0
4

0

0

0
4

0

0

0
4

0

0

0
4

0

0

0
4

0

0

0
4

0

0

0
4

0
7

0

0

0

0

0
4

0

0

0
4

0

0

0
4

0

0

0
4

0

0

0
4

0

0

0
4

0

0

0
4

0

0

0
4

0

0

0
4

0

0

0
4

0

0

0
4

0
7

0
7

0
4

0

0

0
4

0

0

0
4

0

0

0
4

0

0

0
4

0

0

0
4

0

0

0
4

0

0

0
4

0
7

0

0

0

0
4

0.0966581
5

0

0.0966581

0

0

0
4

0
7

0
7

0

0

0
4

0
4

0

0

0

0

0

0

0
4

0

0

0

0

0

0

0

0
4

0

0

0
4

0

0

0
4

0

0

0
4

0

0

0
4

0

0

0
4

0

0

0
4

0

0

0

0

0

0

0

0

0
4

0

0

0

0

0

0

0

0
4

0

0

0

0

0

0
4

0

0

0

0
4

0

0

0

0
4

0

0

0

0
4

0

0

0

0
4

0

0

0
4

0
4

116.83
7

61.8885
7

0
7

0
7

0
7

0

0

0

0

0

0

0

0

0

0

0
7

0

0

0

0

0

0

0

0

0

0

0
7

0

0

0

0

0

0

0

0.0651483

0

0

0
7

0

0

0

0

0

0

0

0

0

0

0
7

0

0

0

0

0

0

0

0

0

0

0.0483291

0

0

0

0

0

0

0

0

0

0

0
7

0

0

0

0

0

0

0

0

0

0

0
7

0

0

0

0

0

0

0

0

0

0

0
7

0

0

0

0

0

0

0

0

0

0

0.0483291

0

0

0

0

0

0

0

0

0

0

0
7

0
8

0

0

0

0

0

0

0

0

0

0

0
7

0

0

0

0

0

0

0

0

0

0

0
8

0

0

0

0

0

0

0

0

0

0

0
7

0

0

0

0

0

0

0

0

0

0

0
7

0

0

0

0

0

0

0

0

0

0

0
7

0

0

0

0

0

0

0

0

0

0

0
7

0

0

0

0

0

0

0

0

0

0

0
7

0

0

0

0

0

0

0

0

0

0

0
7

0

0

0

0

0

0

0

0

0

0

0
7

0

0

0

0

0

0

0

0

0

0

0
7

0
7

0

0

0

0

0

0

0

0

0

0

0
7

0

0

0

0

0

0

0

0

0

0

0
7

0

0

0

0

0

0

0

0

0

0

0
7

0

0

0

0

0

0

0

0

0

0

0
7

0

0

0

0.120823

0

0

0

0

0

0

0
6

0

0

0

0

0

0

0

0

0

0

0
7

0

0

0

0

0

0

0

0

0

0

0
7

0

0

0

0

0

0

0

0

0

0

0
7

0

0

0

0

0

0

0

0

0

0

0
7

0

0

0

0

0

0

0

0

0

0

0
7

0
7

0

0

0

0

0

0

0

0

0

0

0
7

0

0

0

0

0

0

0

0

0

0

0
7

0

0

0

0

0

0

0

0

0

0

0
7

0

0

0

0

0

0

0

0

0

0

0
6

0

0

0

0

0

0

0

0

0

0

0
7

0

0

0

0

0

0

0

0

0

0

0

0

0

0

0

0

0

0

0

0

0

0
7

0

0

0

0

0

0

0

0

0

0

0
7

0

0

0

0

0

0

0

0

0

0

0
7

0

0

0

0

0

0

0

0

0

0

0
7

0
7

0

0

0

0

0

0

0

0

0

0

0
7

0

0

0

0

0

0

0

0

0

0

0
8

0

0

0

0

0

0

0

0

0

0

0
7

0

0

0

0

0

0

0

0

0

0

0
6

0

0

0

0

0

0

0.0966581

0

0

0

0
6

0

0

0

0

0

0

0

0

0

0

0

0

0

0

0

0

0

0

0

0

0

0
7

0

0

0

0

0

0

0

0

0

0

0
8

0

0

0

0

0

0

0

0

0

0

0

0

0

0

0

0

0

0

0

0

0

0.724936
7

0
7

0

0

0

0

0

0

0

0

0

0

0
7

0

0

0

0

0

0

0

0

0

0

0
7

0

0

0

0

0

0

0

0

0

0

0
7

0

0

0

0

0

0

0

0

0

0

0
7

0

0

0

0

0

0

0

0

0

0

0
7

0

0

0

0

0

0

0

0

0

0

0
7

0

0

0

0

0

0

0

0

0

0

0
7

0

0

0

0

0

0

0

0

0

0

0
6

0

0

0

0

0

0

0

0

0

0

0
7

0

0

0

0

0

0

0

0

0

0

0
7

0
7

0

0

0

0

0

0

0

0

0

0

0
7

0

0

0

0

0

0

0

0

0

0

0
6

0

0

0

0

0

0

0

0

0

0

0.0553261
6

0

0

0

0

0

0

0

0

0

0

0

0

0

0

0

0

0

0

0

0

0

0
7

0

0

0

0

0

0

0

0

0

0

0.0651483

0

0

0

0

0

0

0

0

0

0

0
7

0

0

0

0

0

0

0

0

0

0

0
7

0

0

0

0

0

0

0

0

0

0

0
7

0

0

0

0

0

0

0

0

0

0

0
7

0
6

0

0

0

0

0

0

0

0

0

0

0
8

0

0

0

0

0

0

0

0

0

0

0
7

0

0

0

0

0

0

0

0

0

0

0
7

0

0

0

0

0

0

0

0

0

0

0
6

0

0

0

0

0

0

0

0

0

0

0
7

0

0

0

0

0

0

0

0

0

0

0
7

0

0

0

0

0

0

0

0

0

0

0
7

0

0

0

0

0

0

0

0

0

0

0
7

0

0

0

0

0

0

0

0

0

0

0
8

0

0

0

0

0

0

0

0

0

0

0
7

0
7

0

0

0

0

0

0

0

0

0

0

0
7

0

0

0

0

0

0

0

0

0

0

0
8

0

0

0

0

0

0

0

0

0

0

0
7

0

0

0

0

0

0

0

0

0

0

0
7

0

0

0

0

0

0

0

0

0

0

0
7

0

0

0

0

0

0

0

0

0

0

0
6

0

0

0

0

0

0

0

0

0

0

0
7

0

0

0

0

0

0

0

0

0

0

0
7

0

0

0

0

0

0

0

0

0

0

0

0

0

0

0

0

0

0

0

0

0

0
7

0
6

0

0

0

0

0

0

0

0

0

0

0
7

0

0

0

0

0

0

0

0

0

0

0
6

0

0

0

0

0

0

0

0

0

0

0
7

0

0

0

0

0

0

0

0

0

0

0
7

0

0

0

0

0

0

0

0

0

0

0
7

0

0

0

0

0

0

0

0

0

0

0

0

0

0

0

0

0

0

0

0

0

0
7

0

0

0

0

0

0

0

0

0

0

0
7

0

0

0

0

0

0

0

0

0

0

0
7

0

0

0

0

0

0

0

0

0

0

23.0522
7

0
7

0
7

0

0

0

0

0

0

0

0

0

0

0
6

0

0

0

0

0

0

0

0

0

0

0

0

0

0

0

0

0

0

0

0

0

0
7

0

0

0

0

0

0

0

0

0

0

0
7

0

0

0

0

0

0

0

0

0

0

0
7

0

0

0

0

0

0

0

0

0

0

0
7

0

0

0

0

0

0

0

0

0

0

0
8

0

0

0

0

0

0

0

0

0

0

0
7

0

0

0

0

0

0

0

0

0

0

0
7

0

0

0

0

0

0

0

0

0

0

0
7

0
7

0

0

0

0

0

0

0

0

0

0

0
7

0

0

0

0

0

0

0

0

0

0

0
7

0

0

0

0

0

0

0

0

0

0

0
7

0

0

0

0

0

0

0

0

0

0

0
7

0

0

0

0

0

0

0

0

0

0

0
6

0

0

0

0

0

0

0

0

0

0

0
6

0

0

0

0

0

0

0

0

0

0

0
6

0

0

0

0

0

0

0

0

0

0

0

0

0

0

0

0

0

0

0

0

0

0
7

0

0

0

0

0

0

0

0

0

0

0
7

0
7

0

0

0

0

0

0

0

0

0

0

0
7

0

0

0

0

0

0

0

0

0

0

0
6

0

0

0

0

0

0

0

0

0

0

0
7

0

0

0

0

0

0

0

0

0

0

0
7

0

0

0

0

0

0

0

0

0

0

0
7

0

0

0

0

0

0

0

0

0

0

0
7

0

0

0.169152

0

0

0

0

0

0

0

0
7

0

0

0

0

0

0

0

0

0

0

0
7

0

0

0

0

0

0

0

0

0

0

0
8

0

0

0

0

0

0

0

0

0

0

0
7

0
6

0

0

0

0

0

0

0

0

0

0

0

0

0

0

0

0

0

0

0

0

0

0
8

0

0

0

0

0

0

0

0

0

0

0
7

0

0

0

0

0

0

0

0

0

0

0
8

0

0

0

0

0

0

0

0

0

0

0.193316

0

0

0

0

0

0

0

0

0

0

0
6

0

0

0

0

0

0

0

0

0

0

0
7

0

0

0

0

0

0

0

0

0

0

0
7

0

0

0

0

0

0

0

0

0

0

0
6

0

0

0

0

0

0

0

0

0

0

0
7

0

0

0

0

0

0

0

0

0

0

0

0
7

0

0

0

0

0

0

0

0

0

0

0
6

0

0

0

0

0

0

0

0

0

0

0
7

0

0

0

0

0

0

0

0

0

0

0
7

0

0

0

0

0

0

0

0

0

0

0

0

0

0

0

0.0966581

0

0

0

0

0

0
7

0

0

0

0

0

0

0

0

0

0

0
7

0

0

0

0

0

0

0

0

0

0

0
8

0

0

0

0

0

0

0

0

0

0

0
7

0

0

0

0

0

0

0

0

0

0

0
7

0

0

0

0

0

0

0

0

0

0

0

0
7

0

0

0

0

0

0

0

0

0

0

0
7

0

0

0

0

0

0

0

0

0

0

0

0

0

0

0

0

0

0

0

0

0

0
7

0

0

0

0

0

0

0

0

0

0

0

0

0

0

0

0

0

0

0

0

0

0
6

0

0

0

0

0

0

0

0

0

0

0
7

0

0

0

0

0

0

0

0

0

0

0

0

0

0

0

0

0

0

0

0

0

0
6

0

0

0

0

0

0

0

0

0

0

0
7

0

0

0

0

0

0

0

0

0

0

0

0
7

0

0

0

0

0

0

0

0

0

0

0
8

0

0

0

0

0

0

0

0

0

0

0
7

0

0

0

0

0

0

0

0

0

0

0
7

0

0

0

0

0

0

0

0

0

0

0
6

0

0

0

0

0

0

0

0

0

0

0
7

0

0

0

0

0

0

0

0

0

0

0
7

0

0

0

0

0

0

0

0

0

0

0

0

0

0

0

0

0

0

0

0

0

0

0

0

0

0

0

0

0

0

0

0

0.990746

0
7

0

0

0

0

0

0

0

0

0

0

0
7

0

0

0

0

0

0

0

0

0

0

0
7

0

0

0

0

0

0

0

0

0

0

0
6

0

0

0

0

0

0

0

0

0

0

0

0

0

0

0

0

0

0

0

0

0

0
7

0

0

0

0

0

0

0

0

0

0

0

0

0

0

0

0

0

0

0

0

0

0

0

0

0

0

0

0

0

0

0

0

0

0

0

0

0

0

0

0

0

0

0

0

0

0

0

0

0

0

0

0

0

0

0
7

0

0
7

0

0
7

0
7

0
7

0
7

0
8

0

0
7

0
7

0
7

0
6

0
7

0

0
7

0
7

0
7

0
6

0
6

0
8

0
7

0
6

0

0

0

0
6

0
7

0

0

0

0
8

0

0
7

0

0
7

0

0

0
7

0

0

0
8

0
7

0
7

0
7

0
6

0
6

0

0

0
6

0
7

0
7

0

0

0
8

0
7

0
7

0
6

0
7

0
7

0
7

0

0

0

0

0

0
7

0
8

0
7

0
6

0

0

0

0

0

0

0
7

0
7

0

0

0
6

0

0

0

0

0

0

0.0724936

0
7

0
6

0

0
7

0
7

0

0

0

0

0

0

26.2401
5

0

0

0

0

0

0

0

0

0

0

0
7

0

0

0

0

0

0

0

0

0

0

0
7

0

0

0

0

0

0

0

0

0

0

0.115926
7

0
7

0

0

0

0

0

0

0

0

0

0

0
7

0

0

0

0

0

0

0

0

0

0

0
7

0

0

0

0

0

0

0

0

0

0

0
7

0

0

0

0

0

0

0

0.0483291

0

0

0
7

0

0

0

0

0

0

0

0

0

0

0
7

0

0

0

0

0

0

0

0

0

0

0
6

0

0

0

0

0

0

0

0

0

0

0
7

0

0

0

0

0

0

0

0

0

0

0
7

0

0

0

0

0

0

0

0

0

0

0
7

0

0

0

0

0

0

0

0

0

0

0
7

0
7

0

0

0.0483291

0

0

0

0

0

0

0

4.27368
7

0

0

0

0

0

0

0

0

0

0

0
7

0

0

0

0

0

0

0

0

0

0

0
7

0

0

0

0

0

0

0

0

0

0

0
7

0.169152

0

0

0

0

0

0

0

0

0

0
7

0

0

0

0

0

0

0

0

0

0

0
7

0

0

0

0

0

0

0

0

0

0

0
7

0

0

0

0

0

0

0

0

0

0

0
7

0

0

0

0

0

0

0

0

0

0

0
7

0

0

0

0

0

0

0

0

0

0

0
7

0
7

0

0

0

0

0

0

0

0

0

0

0
7

0

0

0

0

0

0

0

0

0

0

0
7

0

0

0

0

0

0

0

0

0.0724936

0

0
7

0

0

0

0

0

0

0

0

0

0

0
7

0

0

0

0

0

0

0

0

0

0

0
7

0

0

0

0

0

0

0

0

0

0

0.0651483

0

0

0

0

0

0

0

0

0

0

0
7

0

0

0

0

0

0

0

0

0

0

0
8

0

0

0

0

0

0

0

0

0

0

0.185812

0

0

0

0

0

0

0

0

0

0

0
7

0.0966581

0

0

0

0

0

0

0

0

0

0

0.0434322

0

0

0

0

0

0

0

0

0

0

0
7

0

0

0

0

0

0

0

0

0

0

0
7

0

0

0

0

0

0

0

0

0

0

0
7

0

0

0

0

0

0

0.0724936

0

0

0

0
7

0

0

0

0

0

0

0

0

0

0

0
7

0

0

0

0

0

0

0

0

0

0

0
7

0

0

0

0

0

0

0

0

0

0

0
7

0

0

0

0

0

0

0

0

0

0

0.144987

0

0

0

0

0

0

0

0

0

0

0
7

0
7

0

0

0

0

0

0

0

0

0

0

0
7

0

0

0

0

0

0

0

0

0

0

0
7

0

0

0

0

0

0

0

0

0

0

0
7

0

0

0

0

0

0

0

0

0

0

0
6

0

0.442609

0

0

0

0

0

0

0

0

0
8

0

0

0.120823

0

0

0

0

0

0

0

0
7

0

0

0

0

0

0

0

0

0

0

0
7

0

0

0

0

0

0

0

0

0

0

0
7

0

0

0

0

0

0

0

0

0

0

0
7

0

0

0

0

0

0

0

0

0

0

3.80915
7

0.14009

0

0

0

0

0

0

0

0

0

0

0
7

0

0

0

0

0

0

0

0

0

0

0
8

0

0

0

0

0

0

0

0

0

0

0
7

0

0

0

0

0

0

0

0

0

0

0
7

0

0

0

0

0

0

0

0

0

0

0
7

0

0

0

0

0

0

0

0

0

0

0
6

0

0

0

0

0

0

0

0

0

0

0
6

0

0

0

0

0

0

0

0

0

0

0
7

0

0

0

0

0

0

0

0

0

0

0
7

0

0

0

0

0

0

0

0

0

0

8.65973959207622e-15
7

0
4

0
6

0
6

0
4

0

0
7

0
5

0

0

0

0

0

0

0

0

0

0

0
5

0

0

0

0

0

0

0

0

0

0

0
6

0

0

0

0

0

0

0

0

0

0

0

0

0

0

0

0

0

0

0

0

0

0
5

0

0

0

0

0

0

0

0

0

0

0

0

0

0

0

0

0

0

0

0
6

0

0
6

0
4

0

0

0

0

0

0

0
4

0
7

0

0

0

0

0

0

0

0

0

0

0

0

0

0

0

0

0

0

0

0

0

0

0

0

0

0

0
4

0
7

0
7

0
7

0
7

0
6

0
7

0

0

0

0

0

0

0

0

0

0

0

0
4

0
7

0
7

0
7

0

0

0

0

0
4

0.652442
7

0

0
7

0

0

0

0

0

0

0

0

0

0

0
6

0

0

0

0

0

0

0

0

0

0

0

0

0

0

0

0

0

0

0

0

0

0
7

0

0

0

0

0

0

0

0

0

0
7

0

0

0
6

0

0
7

0
6

0

0

0

0

0

0

0

0

0

0

0
6

0

0

0

0

0

0

0

0

0.0724936

0

0.26581
6

0

0

0

0

0

0

0

0

0

0

0

0

0

0

0

0

0

0

0

0

0

0
7

0

0

0

0

0

0

0

0

0.314139

0

0
7

0

0

0

0

0

0

0

0

0

0

0
7

0

0

0

0

0

0

0

0

0

0

0

0

0

0

0

0

0

0

0

0

0

0
4

0
7

0
7

0
7

0
7

0
7

0

0

0

0

0

0
4

0
7

0
6

0

0
4

0
7

0

0

0
4

0
7

0

0

0

0

0
4

0
7

0

0

0

0
4

0

0

0

0
4

0

0

0

0

0

0

0
4

0

0

0

0

0
4

0

0

0

0

0
4

0

0

0
4

0

0

0
4

3.91343

3.91343

0

0

0

0

0

0

0

0

0
4

0

0

0

0
4

0

0

0
4

0

0

0

0
4

0.177448

0.177448

0
4

0

0

0
4

0

0

0
4

0

0

0
4

0

0

0
4

0

0

0

0
4

0

0

0
4

22.5157
3

16.1051
3

1.5794

2.60137

0.278718

1.95103

0

0

0

0

0
4

0

0

0

0
4

0

0

0

0
4

0

0

0

0
4

0

0

0
4

0

0

0
4

0

0

0
4

0

0

0
4

0

0

0
4

0

0

0
4

0

0

0
4

0.0434322
7

0.0434322
7

0

0

0

0

0

0

0
4

0

0

0
4

0

0

0
4

0

0

0
4

0

0

0
4

0

0

0
4

0

0

0
4

0

0

0
4

0

0

0
4

0

0

0
4

0

0

0
4

0
7

0
7

0

0

0

0

0
4

0

0

0
4

0

0

0
4

0

0

0
4

0

0

0
4

0

0

0
4

0

0

0
4

0

0

0
4

3.02117

3.02117

0
4

0

0

0
4

0

0

0
4

0
7

0
7

0

0

0

0
4

0

0

0
4

0
7

0
7

0
7

0

0
4

0
7

0
7

0

0
4

24.6176
3

24.6176
3

0

0
4

0
4

0.118299

0.118299
2

0
2

0

0

0

0

0

0.118299

0

0

0

0

0

0
4

0

0

0

0

0

0
4

0
2

0
2

0
4

0

0

0

0
4

0

0

0
4

0

0

0
4

0
4

12.9899

5.85673

2.43011
3

0

0

0

0.314139

0

0

0

0

0.0642886

0

0
2

0

0

0

0

0

0

0

0.0483291

0

0

0
2

0

0

0

0

0

0

0

0

0

0

0
2

0

0

0.707175

1.27071

0

1.02197

0
2

6.66133814775094e-16

0
4

0

0

0

0
4

0

0

0

0
4

0

0

0

0
4

0

0

0

0
4

0

0

0
4

0

0

0
4

4.08394
3

2.60137
3

0.739319

0

0

0

0

0

0

0

0

0

0.371625

0

0

0

0

0

0

0

0

0.371625

0

0

0

0

0

1.11022302462516e-16
3

0
4

1.73969

0

0

0

0

0.304294
2

0.522514
3

0

0

0.912881

0

0

0

1.11022302462516e-16

0
4

0

0
3

0

0

0

0

0

0

0

0

0

0

0
4

1.26121

0.496384

0.128577

0.63625

0
4

0
2

0

0

0

0

0

0
4

0.0483291
2

0

0.0483291

0

0

0

0
4

0

0

0

0
4

0

0

0
4

0
4

0
2

0
2

0

0

0

0

0

0

0

0

0

0

0
4

0

0

0

0

0

0

0

0
4

0

0

0

0

0

0

0

0

0

0

0
4

0

0

0

0
4

0

0

0

0

0
4

0

0

0

0
4

0
4

0

0

0

0
4

0

0

0
4

0
4

0

0

0

0

0
4

0
4

0

0

0

0
4

0

0

0
4

0
4

0

0

0

0
4

0
4

0

0

0

0
4

0

0

0
4

0
4

0

0

0

0
4

0

0

0
4

0
4

0

0

0

0
4

0
4

0

0

0

0
4

0
4

0

0

0

0
4

0
4

0

0

0

0
4

0
4

5.36234

5.16203

5.16203

0

0

0

0

0

0

0
4

0.200313
3

0.0553261
2

0

0

0

0

0

0.0483291

0.0483291

0.0483291

0

0

0
4

0

0

0

0

0

0

0

0
4

0

0

0

0

0

0
4

0

0

0
4

0

0

0
4

0

0

0
4

0

0

0
4

0

0

0
4

3.88578058618805e-16

0
4

0

0

0

0

0
4

0
4

0

0

0

0
4

0
4

0

0

0

0
4

0

0

0
4

0
4

0.773265

0.773265

0.773265

0
4

0
4

0

0

0

0

0
4

0
4

0

0

0

0
4

0
4

0

0

0

0
4

0
4

0

0

0

0
4

0

0

0
4

0
4

0

0

0

0
4

0

0

0
4

0
4

0

0

0

0

0
4

0
4

0

0

0

0

0

0

0

0

0

0

0
4

0
7

0

0

0

0

0

0

0

0
4

0

0

0

0

0

0

0

0

0
4

0

0

0

0

0

0
4

0

0

0

0
4

0

0

0
4

0
4

0

0

0

0
4

0
4

0

0

0

0
4

0
4

0

0

0

0
4

0
4

0

0

0

0
4

0
4

0

0

0

0
4

0
4

0

0

0

0
4

0
4

0

0

0

0
4

0
4

0

0

0

0
4

0
4

0

0

0

0
4

0
4

0

0

0

0
4

0
4

0
2

0
1

0

0

0

0

0

0

0

0
4

0

0

0

0

0

0

0

0

0

0

0

0
4

0

0

0

0

0

0

0
4

0

0

0

0

0

0
4

0

0

0

0
4

0

0

0

0
4

0

0

0
4

0
4

0

0

0

0
4

0
4

0

0

0

0
4

0
4

0

0

0

0
4

0
4

0

0

0

0
4

0
4

0

0

0

0
4

0
4

0

0

0

0
4

0
4

0

0

0

0
4

0
4

0

0

0

0
4

0
4

0

0

0

0
4

0
4

0

0

0

0
4

0
4

2.13005

2.13005

0
3

2.13005

0

0

0
4

0

0

0

0

0

0

0

0
4

0

0

0

0
4

0

0

0

0
4

0

0

0
4

0

0

0
4

0
4

0

0

0

0
4

0
4

0

0

0

0
4

0
4

0

0

0

0
4

0
4

0

0

0

0
4

0
4

0

0

0

0
4

0
4

0

0

0

0
4

0
4

0.289974

0.289974

0.289974

0
4

0
4

0

0

0

0
4

0
4

0

0

0

0
4

0
4

0

0

0

0
4

0
4

0
7

0
7

0
7

0

0

0

0

0

0

0
4

0
7

0
7

0
4

0

0

0

0
4

0
4

0

0

0

0
4

0
4

0

0

0

0
4

0
4

0

0

0

0
4

0
4

0

0

0

0
4

0
4

0

0

0

0
4

0
4

0

0

0

0
4

0
4

0

0

0

0
4

0
4

0

0

0

0
4

0
4

0

0

0

0
4

0
4

0

0

0

0
4

0
4

46.4945
3

46.4945
3

45.7675
3

0.200555

0.100278

0.250694

0.0501388

0.0501388

0.0752082

0
4

0
4

0

0

0

0
4

0
4

0

0

0

0
4

0
4

0.349565

0.349565

0.349565

0
4

0
4

0

0

0

0
4

0
4

0

0

0

0
4

0
4

0

0

0

0
4

0
4

0

0

0

0
4

0
4

0

0

0

0
4

0
4

0

0

0

0
4

0
4

0

0

0

0
4

0
4

0

0
6

0

0

0

0

0
4

0

0

0
4

0

0

0
4

0

0

0
4

0
7

0

0

0
4

0

0

0

0

0

0

0
4

0

0

0

0

0
4

0

0

0

0
4

0

0

0

0
4

0

0

0
4

0

0

0

0
4

0

0

0
4

0
4

0

0

0

0
4

0
4

0

0

0

0
4

0
4

0

0

0

0
4

0
4

0

0

0

0
4

0
4

0

0

0

0
4

0
4

0

0

0

0
4

0
4

0

0

0

0
4

0
4

0

0

0

0
4

0
4

0

0

0

0
4

0
4

0

0

0

0
4

0
4

0.0966581
7

0
7

0
7

0

0

0

0

0

0
4

0
7

0
6

0

0

0
4

0

0

0

0
4

0.0966581

0.0966581

0

0
4

0

0

0
4

0
4

0

0

0

0
4

0
4

0

0

0

0
4

0
4

0

0

0

0
4

0
4

0

0

0

0
4

0
4

0.763337

0.763337

0.763337

0
4

0
4

0

0

0

0

0

0

0

0

0

0

0
4

0

0

0
4

0

0

0
4

0
4

0

0

0

0

0

0

0

0

0

0

0

0

0

0

0

0

0

0

0

0

0

0

0

0

0

0

0

0

0

0

0

0

0

0

0

0

0

0

0

0

0

0

0

0

0

0

0

0

0

0

0

0

0

0

0

0

0

0

0

0

0

0

0
4

0

0

0

0

0

0

0

0
4

0

0

0

0

0

0

0

0

0

0

0

0

0
4

0

0

0

0

0

0
4

0

0

0
4

0
4

0

0

0

0

0

0

0

0

0

0
4

0

0

0
4

0
4

0

0

0

0

0
4

0
4

17.2819
4

9.40624
4

3.15767

1.6066

2.67168

0.801847

1.16844

8.88178419700125e-16
4

0
4

5.8543
4

0.923429

1.41592

3.51495

0
4

2.02134

1.14501

0

0.29211

0.58422

0
4

0
4

0.590203

0.590203

0

0

0.393468

0

0

0.196734

0
4

0
4

4.03738

0

0

0

0

0

0
4

2.63583

2.63583

0

0

0
4

1.40154

0

1.40154

0

0
4

0

0

0
4

0
4

12.9935

9.39993

2.26374

5.76769

1.14597

0.222535

0
4

3.59355

3.59355

0
4

1.77635683940025e-15

0
4

4.92403
3

4.92403
3

4.92403
3

0

0

0

0

0
4

0

0

0

0
4

0

0

0
4

0
4

0
8

0
8

0

0

0

0

0

0
4

0

0

0
4

0
4

0.226955
3

0.226955
3

0.226955
3

0
4

0
4

0
3

0
2

0
2

0

0
4

0
3

0

0

0

0
4

0

0

0
4

0
4

0
1

0
1

0

0

0

0

0

0

0

0

0

0

0

0
2

0

0

0

0

0

0

0

0

0
4

0

0

0
4

0

0

0
4

0
1

0
2

0

0

0

0

0

0

0

0

0

0

0
1

0

0

0

0

0

0

0

0

0

0

0
4

0
1

0

0

0

0

0

0

0

0

0

0

0
4

0

0

0

0

0
4

0

0

0

0
4

0

0

0

0
4

0

0

0

0
4

0

0

0

0
4

0

0

0

0
4

0
4

4.39692
3

3.94482
3

1.6548
3

2.29001

0

0

0

0
4

0.4521

0.274652

0

0.177448

0

0
4

0
4

0

0

0

0

0

0

0
4

0

0

0

0

0

0

0
4

0

0

0
4

0

0

0

0
4

0

0

0

0
4

0

0

0
4

0
4

0

0

0

0

0

0

0
4

0

0

0

0

0

0

0
4

0
4

0

0

0

0

0

0

0
4

0
4

0
2

0

0

0

0

0

0

0
4

0

0

0
4

0

0

0
4

0
4

0

0

0

0

0
4

0

0

0

0
4

0

0

0

0
4

0
4

0.0724936

0.0724936

0.0724936
2

0

0

0
4

0

0

0
4

0

0

0
4

0
4

0
7

0

0

0

0
4

0

0

0

0

0
4

0

0

0

0
4

0

0

0
4

0
4

0.241138
2

0

0

0

0

0
4

0.241138
3

0.185812

0

0

0.0553261

0
4

0

0

0
4

0
4

0

0

0

0

0

0

0
4

0

0

0

0
4

0
4

0

0
7

0
7

0

0

0

0

0

0

0

0

0

0

0

0
4

0

0

0
4

0

0

0

0
4

0

0

0

0
4

0

0

0
4

0

0

0

0

0

0

0

0

0

0
4

0
1

0

0

0

0

0

0

0

0

0
4

0
6

0

0

0

0

0

0

0
4

0
6

0
6

0

0
4

0

0

0

0

0

0

0
4

0
7

0

0

0

0

0
4

0

0

0

0

0

0

0
4

0

0

0

0

0

0
4

0
4

0
8

0
8

0
8

0
4

0

0

0
4

0

0

0
4

0
4

1.83223
3

0

0

0

0
4

0

0

0
4

1.83223

1.83223

0
4

0

0

0
4

0

0

0
4

0
4

0
7

0
7

0
7

0

0
4

0
4

0
7

0
7

0

0

0

0
4

0

0

0
4

0
4

0

0

0

0

0

0

0

0
4

0

0

0
4

0

0

0
4

0
4

0.120823
2

0
2

0

0

0

0

0
4

0

0

0
4

0.120823

0.120823

0
4

0
4

1.73308
4

1.01562

0

0.0846347

0

0

0.930982

1.11022302462516e-16

0
4

0.71746

0.71746

0
4

0
4

0

0

0

0

0

0

0
4

0
4

0
7

0
7

0
7

0

0

0
4

0
4

8.605
4

8.55486
4

8.55486

0

0
4

0

0

0
4

0.0501388

0.0501388

0
4

0

0

0
4

4.85722573273506e-17
4

0
4

0.0483291
7

0.0483291
7

0.0483291
7

0
6

0
8

0

0

0

0

0

0

0
4

0
7

0
7

0

0

0
4

0
7

0
7

0

0

0

0

0
4

0
7

0
8

0

0

0

0

0

0

0
4

0

0

0

0

0
4

0

0

0

0

0
4

0

0

0

0
4

0

0

0
4

0
4

0

0

0

0

0

0

0
4

0

0

0

0

0
4

0

0

0
4

0
4

0

0

0

0

0
4

0
4

0

0

0

0
4

0
4

0

0

0

0

0

0

0
4

0
4

0.483291
3

0.483291
3

0.483291

0

0

0
4

0

0

0
4

0
4

0

0

0

0

0
4

0

0

0
4

0

0

0
4

0
4

0

0

0

0

0

0

0
4

0
4

0

0

0

0

0
4

0

0

0

0
4

0
4

0

0

0

0

0
4

0

0

0

0
4

0
4

0

0

0

0

0

0
4

0

0

0
4

0
4

41.6296
3

40.9299
3

30.7606
3

0

0.150416

0.0501388

0.236598

4.49571
3

1.58919

3.15101

0.175486

0.145251

0.125347

0

0.0501388

0
4

0.173263

0.123124

0.0501388

0
4

0.526457

0.526457

0
4

1.22124532708767e-15
3

0
4

0

0

0

0

0

0

0

0
4

0

0

0
4

0
4

0.771994

0.771994

0.0501388

0.721856

0

0
4

0
4

0

0

0

0

0
4

0

0

0

0
4

0

0

0
4

0
4

0.0964329

0

0

0

0
4

0

0

0
4

0.0964329

0

0.0964329

0
4

0
4

7.63337

7.63337

7.63337

0

0

0

0
4

0
4

0
8

0

0

0

0
4

0

0

0
4

0

0

0
4

0
4

0

0

0

0
4

0

0

0
4

0

0

0
4

0
4

0

0

0

0

0

0
4

0

0

0
4

0
4

0

0

0

0

0
4

0
4

0

0

0

0

0
4

0

0

0

0
4

0

0

0
4

0
4

15.8879

9.27399

8.4524

0

0.0724936

0.314139

0.0966581

0.0483291

0.144987

0

0.0966581

0

0

0

0.0483291

1.88044024795886e-15

0
4

6.26439

6.26439

0

0

0

0

0

0

0

0

0

0
4

0

0

0
4

0.349565

0.349565

0
4

0

0

0
4

0

0

0
4

0

0

0
4

0

0

0
4

2.22044604925031e-16

0
4

0

0

0

0
4

0

0

0
4

0
4

0

0

0

0
4

0

0

0
4

0
4

0

0

0

0

0
4

0
4

0

0

0

0
4

0

0

0

0
4

0

0

0
4

0
4

0

0

0

0
4

0

0

0

0
4

0
4

0
7

0

0

0
4

0

0

0

0
4

0
4

0.691772

0.691772

0.691772

0
4

0
4

0

0

0

0
4

0

0

0
4

0
4

0

0

0

0

0
4

0
4

0

0

0

0
4

0
4

23.073

23.0247

2.77543

0.0483291

0

20.1284

0

0

0.0724936

0

0

0

0

0
4

0

0

0
4

0

0

0
4

0
7

0
7

0

0

0

0
4

0
7

0

0

0

0

0

0
4

0.0483291

0

0

0

0.0483291

0
4

0

0

0

0
4

0

0

0
4

0

0

0
4

0

0

0
4

0

0

0
4

9.0205620750794e-17

0
4

0

0

0

0
4

0
4

0

0

0

0

0

0
4

0
4

0

0

0

0
4

0
4

0

0

0

0

0
4

0
4

0

0

0

0

0
4

0

0

0
4

0
4

0

0

0

0

0
4

0

0

0
4

0
4

0

0

0

0

0
4

0

0

0
4

0
4

0

0

0

0

0
4

0
4

0

0

0

0

0

0
4

0
4

0

0

0

0
4

0
4

2.50726

2.50726

1.63936

0

0

0.578598

0

0

0

0

0

0

0

0

0

0

0

0

0

0

0

0

0

0.0642886

0

0

0.22501

0

0

0
4

0

0

0

0
4

0
4

1.23593

1.23593

1.23593

0
4

0
4

0

0

0

0

0

0
4

0
4

0

0

0

0

0
4

0

0

0
4

0
4

0

0

0

0

0
4

0

0

0
4

0
4

0

0

0

0

0

0
4

0
4

0

0

0

0

0
4

0
4

0

0

0

0

0
4

0
4

0

0

0

0
4

0

0

0
4

0
4

0

0

0

0
4

0

0

0
4

0

0

0
4

0
4

0

0

0

0

0
4

0

0

0
4

0
4

4.95892
3

4.95892
3

4.85707

0

0

0

0

0.101849

1.80411241501588e-16
3

0
4

0
4

6.15038
3

6.15038
3

6.15038
3

0

0

0
4

0

0

0
4

0

0

0
4

0

0

0
4

0

0

0
4

0

0

0
4

0
4

227.416

226.118

0
7

0
7

0

0

0

0

0

0

0

0

0

0

0
8

0

0

0

0

0

0

0

0

0

0

13.7959
3

0

0

0

0

0

0

0

0

0

0

0

0

0

0

0

0

0

0

0

0

0

0

0

0

0

0

0

0

0

0

0

1.37326

0
7

0

0

0

0

0

0

0

0

0

0

0

0

0

0

0

0

0

0

0

0

0

0
7

0

0

0

0

0

0

0

0

0

0

0

0

0

0

0.709793

0

0

0

0.274652

0

0

0

0

0

0

0

0

0

124.552
3

0
7

0

0

0
6

0

0
7

0
7

0

4.6881

0

0
7

0

0
7

0

0

0

0

0

0

0

0

0
7

0

0

0

0

0

0

0

0

0.184686

0

0
6

0

0

0

0

0

0

0

0

0

0

4.80584
3

0

0

0

0

0

0

0

0

0

0

0

0

0

0

0

0

0

0

0

0

0

74.3551
3

1.37801

0

0

0

0

0

0

0

0

0

0

0

0

0

0

0

0

0

0

0

0

1.33226762955019e-15

0
4

1.17532

0

0

0.763337

0.411978

5.55111512312578e-17

0
4

0.123124

0.123124

0

0
4

0

0

0
4

0

0

0
4

2.00672811700997e-14

0
4

0.253904
3

0.253904
4

0

0.253904

0
4

0

0

0
4

0

0

0
4

0

0

0
4

0

0

0
4

0

0

0
4

0

0

0
4

0
4

1.91391347215131e-12

0
4

1952.59
3

1619.33
3

10.8431

10.156

0.187182

0

0

0

0

0.438165

0

0

0

0

0

0

0.0617678

0

0

0

0

0

0

0

0

7.56339435525888e-16

0
4

23.9331
5

23.9331
5

0
6

0

0

0

0

0

0

0

0

0

0

0

0

0
4

1584.55
3

15.4066

114.494
3

54.1714

14.872
4

0

1.78524

0.192866

0

1.44603

0

0

0

1.2015

2.67168

125.216
4

0.823956

0.128577

0.470887

0

2.07696

0

0

2.45441

0

0

58.7909
3

0

0

0

0

0

0

0.0846347

0.401491

0

0.126952

2.89153
3

0

0.165978

0

0.101849

0.161286

0.58422

0.0501388

0.0553261

0.411978

0

195.573
4

0

0.123124

0

0.430933

6.48837

0

0

0

0

0

0

0

0

0.274652

0

2.05989

0.07653

0.278718

0

0.0846347

0

1.46943
3

0

1.51059

0

0

0

0

0

0

0

0

0

0

0

0

0

0.58422

0

0

0

0

0

2.73253
4

0

0

0.126952

1.23593

0.101849

0.430933

0

0

0.0553261

0.274652

282.158
4

0.274652

0

0.29211

0

0

0.139084

0.763337

0

0

0

45.2615

8.03419
4

0.274652

0

0

0

0

0

0

0

0

0.438165

1.39713
3

0

19.2256

0.876329

0.733902

0.249849

0.211587

0

0

0

0

17.6931
4

0

1.14501

0

0

0.274652

0

0

0

0

0

0.327507

0

15.1897

0.07653

0.214522

0

0

0

0

0

0

0

0

0

0

0

4.19836

0.68663

0

0

0.274652

0

3.86277
3

1.52667

0.184686

0

0.763337

0

0

0

0

0.241929

0.549304

0.978536

0.0846347

0

0.0846347

0

0

0.821594
5

0
6

0

11.8156

6.89084
3

0

77.7196

1.37993

2.60266
3

39.1892

0.358068

0.405199
4

0

0

0.815446

0
6

21.6509
4

12.4147

241.769

1.86263
4

0

1.61468

0

0

6.66799

3.51115
4

0.163089

3.71436

0

1.5155

1.59159

0.211587

6.17967

0

1.67434

0.185029

12.6507
3

0

0

0

0

0.295747

0

6.1067

4.19836

0

0

0.555784

1.52667

41.3897

1.03221

0.184912

0

0

0.0553261

25.4822

1.37801

0

7.51794
3

0

0

0.101849

0

0.106346

0

0

0

1.23593

0

3.50179

1.52667

0.68663

0

0

0

0

0.200835

4.19836

0

0.0556337

1.8917020727649e-12
3

0
4

0

0

0

0
4

0

0

0
4

0

0

0
4

0

0

0
4

0

0

0
4

0

0

0

0
4

0

0

0

0
4

0

0

0
4

0

0

0
4

0

0

0
4

0

0

0
4

0

0

0
4

0

0

0
4

0
4

55.8686
3

31.9958
3

10.1052
3

0.304026

0

0

0

0.0834505

0

1.91102

0.184686

0

0

5.82284

0.0434322

0.160722

0.0482912

0.716632

0

0

0.108581

0.0434322

10.3715
4

1.31704
3

0

0.236598

0

0

0.538357

0
4

15.628
3

11.9588

0.730274
2

1.28125

0.325742

0.977225

0

0.0434322

0.0724369

0.238877

0
4

8.06623
4

0.30781

0.0868644

2.67168

0

0.152013

0.108581

0.39089

1.52667

0.916111

1.23124

0.101159

0

0.33163

0.0528677

0.0868644

0.101849

0
4

0.17857

0.17857

0
4

5.52335954751015e-15
3

0
4

1.8061
4

1.56147
4

1.56147
4

0
4

0.244634

0.244634

0
4

0
4

0

0

0

0

0

0

0

0
4

0

0

0
4

0
4

0

0

0

0
4

0
4

0

0

0

0

0

0
4

0
4

5.50345

4.61699

4.49387

0.123124

8.32667268468867e-17

0
4

0.763337

0.763337

0
4

0.123124

0.123124

0
4

0
4

42.4085

1.21143

0.534251

0.123124

0.184686

0.369371

1.66533453693773e-16

0
4

41.197

0.123124

41.0739

0
4

0
4

0.489268

0.489268

0.489268

0

0
4

0
4

0.49947

0.39089

0.195445

0.152013

0.0434322

0
4

0.0651483

0.0651483

0
4

0.0434322

0.0434322

0
4

0
4

0.868644

0.825212

0.825212

0
4

0.0434322

0.0434322

0
4

3.46944695195361e-17

0
4

0

0

0

0

0
4

0
4

79.2562
3

78.3603
3

76.9342
3

0

0.933793

0

0

0

0.369174

0.123124

0
4

0.07653

0.07653

0
4

0.0651483

0.0651483

0
4

0

0

0

0

0

0

0

0
4

0
2

0

0

0

0

0
4

0.125305
3

0.0724369

0

0.0528677

0
4

0.49947

0.49947

0
4

0

0

0
4

0.0651483

0.0651483

0
4

0.0642886

0.0642886

0
4

0

0

0
4

9.10382880192628e-15
3

0
4

0

0

0

0
4

0
4

0

0

0

0
4

0
4

0

0

0

0
4

0

0

0
4

0
4

0

0

0

0
4

0
4

0

0

0

0
4

0

0

0
4

0
4

0.0501388

0

0

0

0
4

0.0501388

0.0501388

0
4

0
4

0.303602

0.185303

0.185303

0
4

0.118299

0.118299

0
4

0
4

0.7653

0.7653

0.7653

0
4

0
4

0.891915

0.0642886

0.0642886

0
4

0.0642886

0.0642886

0
4

0.763337

0.763337

0
4

0
4

0

0

0

0

0
4

0
4

9.11123
3

6.87052
3

0.345659
3

0.0965825

0

0.192866

0.0434322

0

2.17566
3

2.55168
4

0.561288

0.0965825

0.0434322

0.763337

0

0

0
4

0

0

0
4

0

0

0
4

0.615619

0.615619

0

0
4

1.07239
3

0.14945

0

0.05102

0.0651483

0.763337

0.0434322

3.46944695195361e-17
3

0
4

0

0

0
4

0.29211

0

0.29211

0
4

0.0868644

0.0434322

0.0434322

0
4

0.0651483

0.0651483

0

0
4

0.108581

0.108581

0
4

0

0

0
4

2.4980018054066e-16
3

0
4

0.338539

0.0846347

0.0846347

0
4

0.169269

0.169269

0
4

0.0846347

0.0846347

0
4

0
4

0

0

0

0
4

0
4

0

0

0

0
4

0
4

0.108581

0.108581

0.108581

0
4

0
4

0

0

0

0

0
4

0
4

0.247071

0.247071

0.0926517

0.15442

0
4

0
4

0

0

0

0
4

0
4

0

0

0

0
4

0
4

0.236598

0.236598

0.236598

0
4

0
4

0

0

0

0
4

0
4

118.118
4

105.953
4

10.4505
4

21.5475
4

54.3294

6.66076

9.66484

0.369371

1.14501

1.7853

1.4432899320127e-14
4

0
4

7.55087
4

4.83623
4

2.22215

0.123124

0.123124

0.123124

0.123124

0
4

1.50208

0.492495

0.246248

0.763337

1.11022302462516e-16

0
4

3.11209

1.57304

1.53905

0
4

0
4

0.185303

0.185303

0.185303

0
4

0
4

0

0

0

0
4

0
4

0

0

0

0
4

0
4

0.0434322

0.0434322

0.0434322

0
4

0
4

0

0

0

0
4

0
4

0

0

0

0
4

0
4

0

0

0

0
4

0
4

9.21911

3.08262

0.697787

0

0

0.0434322

0

0.0651483

0.16979

0

0.369174

0

0

1.60699

0.0651483

0.0651483

0
4

5.65874

0.836412

0

0.0868644

0

0.0434322

0.325742

0.783128

3.08369

0.173729

0.195445

0.0868644

0

0.0434322

0
4

0.238877

0.130297

0

0.0651483

0.0434322

6.93889390390723e-18

0
4

0.238877

0.108581

0.130297

0
4

0
4

1.35956

0.143461

0.0868644

0.0565967

0

0

0

0

0

1.38777878078145e-17

0
4

0.868644

0.0651483

0.304026

0.0434322

0.0651483

0.130297

0.0868644

0.173729

0
4

0.347458

0.0868644

0.0434322

0

0.0868644

0.130297

0
4

0
4

2.82532
4

1.17794

0.325742

0.152013

0.184686

0.0868644

0.15306

0.0434322

0.0868644

0.0434322

0.101849

0
4

1.64738

1.48398

0.07653

0.0434322

0.0434322

0
4

6.66133814775094e-16
4

0
4

1.2272

1.2272

0.888664

0.126952

0.211587

0
4

0
4

1.52833
4

0.840554
4

0.210621

0.253172

0.05102

0.238877

0.0868644

0
4

0.173729

0.108581

0.0651483

0
4

0.195445

0.130297

0.0651483

0
4

0.16659

0.0482912

0.118299

0
4

0.152013

0.152013

0
4

0
4

1.32316380074826e-12
3

0
4

0

0

0

0

0

0

0

0

0

0

0

0

0

0

0
4

0

0

0

0

0

0
4

0

0

0

0

0

0
4

0

0

0

0
4

0
4

0
4

0
7

0
7

0
7

0
7

0

0

0
4

0
4

0
4

0

0

0

0

0
4

0
4

0
4

1.10025

1.10025

1.10025

1.10025

0

0
4

0
4

0
4

0.0926517

0.0926517

0.0926517

0.0926517

0

0
4

0
4

0
4

0

0

0

0

0
4

0
4

0
4

0

0

0

0

0
4

0
4

0
4

0

0

0

0

0
4

0
4

0
4

0

0

0

0

0
4

0
4

0
4

0

0

0

0

0
4

0
4

0
4

0

0

0

0

0
4

0
4

0
4

0

0

0

0

0

0
4

0
4

0
4

0
7

0
7

0
7

0
7

0

0

0
4

0
4

0
4

0

0

0

0

0

0
4

0
4

0
4

0

0

0

0

0
4

0
4

0
4

0.891915

0.891915

0.891915

0.891915

0
4

0
4

0
4

0

0

0

0

0
4

0
4

0
4

0

0

0

0

0
4

0
4

0
4

0.275763

0.275763

0.275763

0.275763

0
4

0
4

0
4

0

0

0

0

0
4

0
4

0
4

0

0

0

0

0
4

0

0

0
4

0
4

0
4

0

0

0

0

0
4

0
4

0
4

0

0

0

0

0
4

0
4

0
4

0
7

0
7

0
7

0
7

0
4

0

0

0
4

0
4

0
4

0

0

0

0

0
4

0

0

0
4

0
4

0
4

0.68663

0.68663

0.68663

0.68663

0
4

0
4

0
4

0

0

0

0

0
4

0

0

0
4

0
4

0
4

0

0

0

0

0
4

0

0

0
4

0
4

0
4

0

0

0

0

0
4

0
4

0
4

0

0

0

0

0

0
4

0
4

0
4

0

0

0

0

0
4

0
4

0
4

3.93897

3.93897

3.93897

3.93897

0
4

0
4

0
4

0

0

0

0

0
4

0
4

0
4

0

0

0

0

0
4

0
4

0
4

8.33865

7.87539

7.72098

7.56656

0.0617678

0.0926517

0
4

0.15442

0.15442

0

0
4

2.77555756156289e-17

0
4

0.463259

0.339723

0.339723

0
4

0.123536

0.123536

0
4

0
4

0
4

0

0

0

0

0

0
4

0
4

0
4

0

0

0

0

0

0
4

0
4

0
4

0

0

0

0

0
4

0
4

0
4

0.123536

0.123536

0.0617678

0.0617678

0
4

0.0617678

0.0617678

0
4

0
4

0
4

0

0

0

0

0
4

0

0

0
4

0
4

0
4

0

0

0

0

0
4

0
4

0
4

0

0

0

0

0
4

0
4

0
4

0

0

0

0

0
4

0
4

0
4

0.0724936

0.0724936

0.0724936

0.0724936

0
4

0
4

0
4

0

0

0

0

0
4

0
4

0
4

11.7647

11.7647

7.25093

1.80989

0.30781

3.65575

0.861867

0.30781

0.184686

0.123124

0
4

4.32912

1.23124

1.37326

0.763337

0.961282

0
4

0.184686

0.184686

0
4

1.49880108324396e-15

0
4

0
4

0

0

0

0

0
4

0
4

0
4

0

0

0

0

0
4

0
4

0
4

0

0

0

0

0
4

0
4

0
4

0

0

0

0

0
4

0
4

0
4

0

0

0

0

0
4

0
4

0
4

0

0

0

0

0
4

0
4

0
4

0

0

0

0

0
4

0
4

0
4

0

0

0

0

0
4

0
4

0
4

0

0

0

0

0
4

0
4

0
4

0

0

0

0

0
4

0
4

0
4

0
7

0
7

0

0

0

0
4

0

0

0

0

0
4

0

0

0
4

0

0

0
4

0
4

0
6

0

0

0

0

0
4

0

0

0
4

0

0

0
4

0
4

0
4

0

0

0

0

0
4

0
4

0
4

0

0

0

0

0
4

0
4

0
4

0

0

0

0

0
4

0
4

0
4

0

0

0

0

0
4

0
4

0
4

0

0

0

0

0
4

0
4

0
4

0

0

0

0

0
4

0
4

0
4

0.411978

0.411978

0.411978

0.411978

0
4

0
4

0
4

0

0

0

0

0
4

0
4

0
4

0

0

0

0

0
4

0
4

0
4

3.84522

3.84522

3.84522

3.84522

0
4

0
4

0
4

3.85732
3

3.85732
3

3.85732
3

3.85732
3

0

0
4

0

0

0
4

0
4

0
4

0

0

0

0

0
4

0
4

0
4

0

0

0

0

0
4

0
4

0
4

0

0

0

0

0
4

0
4

0
4

0.0752082

0.0752082

0.0752082

0.0752082

0
4

0
4

0
4

0

0

0

0

0
4

0
4

0
4

0

0

0

0

0
4

0
4

0
4

0.965349

0.965349

0.965349

0.965349

0
4

0
4

0
4

0

0

0

0

0
4

0
4

0
4

0

0

0

0

0
4

0
4

0
4

0

0

0

0

0
4

0
4

0
4

0
5

0
5

0
5

0
5

0

0

0
4

0

0

0
4

0

0

0
4

0
4

0
4

0

0

0

0

0
4

0
4

0
4

0

0

0

0

0
4

0
4

0
4

0

0

0

0

0
4

0
4

0
4

0

0

0

0

0
4

0
4

0
4

4.66908

4.66908

4.66908

4.66908

0
4

0
4

0
4

0

0

0

0

0
4

0
4

0
4

0

0

0

0

0
4

0
4

0
4

0

0

0

0

0
4

0
4

0
4

0

0

0

0

0
4

0
4

0
4

0

0

0

0

0
4

0
4

0
4

122.844

122.844

122.446

118.383

0.582462

1.87996

1.60061

0
4

0.397776

0.123124

0.274652

0
4

9.99200722162641e-16

0
4

0
4

0.0868644

0.0868644

0.0868644

0.0868644

0
4

0
4

0
4

0

0

0

0

0
4

0
4

0
4

7.2517

7.2517

7.2517

7.2517

0
4

0
4

0
4

0

0

0

0

0
4

0
4

0
4

0

0

0

0

0
4

0
4

0
4

0

0

0

0

0
4

0
4

0
4

0

0

0

0

0
4

0
4

0
4

0

0

0

0

0
4

0
4

0
4

0

0

0

0

0
4

0
4

0
4

0

0

0

0

0
4

0
4

0
4

30.0893

30.0893

28.9443

26.811

0.886461

0.101849

1.14501

0
4

1.14501

1.14501

0
4

0
4

0
4

0

0

0

0

0
4

0
4

0
4

0.100278

0.100278

0.100278

0.100278

0
4

0
4

0
4

0

0

0

0

0
4

0
4

0
4

0

0

0

0

0
4

0
4

0
4

0

0

0

0

0
4

0
4

0
4

0.0834505

0.0834505

0.0834505

0.0834505

0
4

0
4

0
4

0

0

0

0

0
4

0
4

0
4

0

0

0

0

0
4

0
4

0
4

0.123124

0.123124

0.123124

0.123124

0
4

0
4

0
4

0

0

0

0

0
4

0
4

0
4

102.66

102.66

95.6941

38.6711

6.3407

41.4281

1.16111

7.72364

0.123124

0.246248

0
4

3.31953

2.45767

0.615619

0.123124

0.123124

0
4

3.04963

0.961282

0.549304

1.53905

0
4

0.596664

0.596664

0
4

3.36397576461422e-14

0
4

0
4

0
7

0
7

0
7

0
7

0
4

0
4

0
4

0

0

0

0

0
4

0
4

0
4

0

0

0

0

0
4

0
4

0
4

0

0

0

0

0
4

0
4

0
4

0

0

0

0

0
4

0
4

0
4

0

0

0

0

0
4

0
4

0
4

0.152774

0.152774

0.152774

0.152774

0
4

0
4

0
4

0

0

0

0

0
4

0
4

0
4

0

0

0

0

0
4

0
4

0
4

0

0

0

0

0
4

0
4

0
4

0.763337

0.763337

0.763337

0.763337

0
4

0
4

0
4

0

0

0

0

0

0

0
4

0
4

0
4

0

0

0

0

0
4

0
4

0
4

0.214522

0.214522

0.214522

0.214522

0
4

0
4

0
4

0

0

0

0

0
4

0
4

0
4

6.1067

6.1067

6.1067

6.1067

0
4

0
4

0
4

0

0

0

0

0
4

0
4

0
4

0

0

0

0

0
4

0
4

0
4

0

0

0

0

0
4

0
4

0
4

0

0

0

0

0
4

0
4

0
4

0

0

0

0

0
4

0
4

0
4

0

0

0

0

0
4

0
4

0
4

2.65991

2.65991

2.65991

1.34076

1.31915

0
4

0
4

0
4

0

0

0

0

0
4

0
4

0
4

0

0

0

0

0
4

0
4

0
4

0

0

0

0

0
4

0
4

0
4

0

0

0

0

0
4

0
4

0
4

0

0

0

0

0
4

0
4

0
4

0

0

0

0

0
4

0
4

0
4

0

0

0

0

0
4

0
4

0
4

0

0

0

0

0
4

0
4

0
4

0

0

0

0

0
4

0
4

0
4

0.163089

0.163089

0.163089

0.163089

0
4

0
4

0
4

0
7

0

0

0

0

0

0
4

0
4

0

0

0

0
4

0

0

0
4

0
4

0
4

0

0

0

0

0
4

0
4

0
4

0

0

0

0

0
4

0
4

0
4

0

0

0

0

0
4

0
4

0
4

0

0

0

0

0
4

0
4

0
4

0

0

0

0

0
4

0
4

0
4

0

0

0

0

0
4

0
4

0
4

0

0

0

0

0
4

0
4

0
4

0

0

0

0

0
4

0
4

0
4

0

0

0

0

0
4

0
4

0
4

0

0

0

0

0
4

0
4

0
4

0
6

0
6

0
6

0
6

0
4

0
4

0
4

0

0

0

0

0
4

0
4

0
4

0

0

0

0

0
4

0
4

0
4

0

0

0

0

0
4

0
4

0
4

0.0752082

0.0752082

0.0752082

0.0752082

0
4

0
4

0
4

0

0

0

0

0
4

0
4

0
4

0

0

0

0

0
4

0
4

0
4

0

0

0

0

0

0

0
4

0
4

0

0

0

0

0
4

0
4

0
4

0.749547
3

0.749547
3

0.749547
3

0

0

0

0.749547

0
4

0
4

0
4

0.858088
6

0.858088
6

0.858088
6

0.858088

0

0

0
4

0
4

0
4

18.7294
4

18.7294
4

18.7294
4

18.7294
4

0
4

0

0

0
4

0
4

0
4

0
7

0
7

0
7

0
7

0
4

0
4

0
4

0
1

0
1

0
1

0
1

0

0

0

0

0
4

0

0

0
4

0
4

0
4

0
7

0
7

0
7

0
7

0
4

0
4

0
4

0
6

0
6

0
6

0
6

0

0
4

0
4

0
4

0

0

0

0

0

0

0

0
4

0
4

0
4

1.92256

1.92256

0
7

0
7

0

0
4

1.92256

1.92256

0
4

0
4

0
4

7.31948

6.39297

5.49733

5.12673

0.123536

0.247071

0
4

0.895633

0.401491

0.185303

0.308839

5.55111512312578e-17

0
4

0
4

0.926517

0.0617678

0.0617678

0
4

0.864749

0.864749

0
4

0
4

0
4

0
7

0
7

0
7

0
7

0

0
4

0
4

0
4

0

0

0

0

0

0
4

0
4

0
4

2.51383

2.51383

0.712943

0.712943

0

0
4

1.80089

1.67231

0.128577

2.77555756156289e-17

0
4

2.22044604925031e-16

0
4

0
4

0

0

0

0

0

0

0
4

0
4

0
4

7.07457

7.07457

6.21753

6.03284

0.184686

6.10622663543836e-16

0
4

0.492495

0.369371

0.123124

0
4

0.246248

0.246248

0
4

0.118299

0.118299

0
4

0
4

0
4

0
6

0
6

0
6

0
6

0

0
4

0
4

0
4

0
7

0
7

0
7

0

0

0

0

0
4

0
4

0
4

0
7

0
7

0
7

0
7

0

0
4

0
4

0
4

0

0

0

0

0

0
4

0

0

0
4

0
4

0
4

0

0

0

0

0

0
4

0
4

0
4

0

0

0

0

0

0

0
4

0

0

0
4

0

0

0
4

0
4

0
4

0

0

0

0

0

0

0

0
4

0
4

0
4

0

0

0

0

0
4

0
4

0
4

0

0

0

0

0

0
4

0
4

0
4

0
7

0
7

0
7

0
7

0
4

0
4

0
4

0.175486

0.175486

0.175486

0.175486

0

0

0

0
4

0
4

0
4

0
5

0
5

0
5

0
5

0

0
4

0

0

0
4

0

0

0

0
4

0

0

0
4

0
4

0

0

0

0

0

0
4

0
4

0
4

0

0

0

0

0

0

0

0
4

0
4

0
4

0
6

0
6

0
6

0
6

0
4

0
4

0
4

12.7135

12.7135

12.4214

10.4371

1.98433

0
4

0.29211

0.29211

0
4

0
4

0
4

0.288593

0.288593

0.288593

0.0848951

0

0.203698

0
4

0
4

0
4

0

0

0

0

0
4

0

0

0

0
4

0
4

0
4

0

0

0

0

0

0
4

0

0

0

0
4

0
4

0
4

0

0

0

0

0

0

0

0
4

0
4

0
4

0

0

0

0

0

0
4

0

0

0
4

0
4

0
4

0

0

0

0

0
4

0

0

0
4

0

0

0
4

0

0

0
4

0
4

0
4

0

0

0

0

0

0

0
4

0
4

0
4

0
7

0
7

0
7

0
7

0

0

0
4

0
4

0
4

0.123124

0.123124

0.123124

0.123124

0

0

0
4

0
4

0
4

0

0

0

0

0

0
4

0
4

0
4

0.169319
3

0.169319
3

0.118299

0.118299

0
4

0.05102

0.05102

0
4

1.38777878078145e-17
3

0
4

0
4

29.7806

29.7806

29.7806

29.7806

0
4

0
4

0
4

0

0

0

0

0
4

0
4

0
4

14.8221

14.8221

14.8221

14.8221

0
4

0
4

0
4

0

0

0

0

0

0
4

0
4

0
4

0

0

0

0

0
4

0
4

0
4

0

0

0

0

0

0
4

0
4

0
4

1.65458

1.65458

1.65458

1.40389

0.250694

5.55111512312578e-17

0
4

0
4

0
4

0

0

0

0

0

0

0

0

0

0

0

0

0
4

0

0

0

0

0

0

0
4

0

0

0

0
4

0

0

0

0
4

0
4

0
4

0

0

0

0

0

0

0
4

0
4

0
4

0

0

0

0

0

0
4

0
4

0
4

0

0

0

0

0
4

0
4

0
4

0

0

0

0

0

0

0
4

0
4

0
4

0

0

0

0

0
4

0
4

0
4

0

0

0

0

0
4

0
4

0
4

0

0

0

0

0

0
4

0

0

0

0
4

0
4

0
4

0

0

0

0

0
4

0

0

0
4

0
4

0

0

0

0
4

0
4

0
4

0

0

0

0

0

0
4

0
4

0
4

0

0

0

0

0
4

0
4

0
4

0
7

0
7

0
7

0
7

0

0

0

0

0
4

0
4

0
4

16.5847

16.5847

16.3685

8.52396

7.84451

1.77635683940025e-15

0
4

0.216187

0.216187

0
4

0
4

0
4

0

0

0

0

0
4

0
4

0
4

0

0

0

0

0
4

0
4

0
4

3.49789

3.49789

3.49789

3.49789

0
4

0
4

0
4

0

0

0

0

0

0
4

0
4

0
4

0

0

0

0

0
4

0
4

0
4

3.86368

3.86368

3.86368

3.86368

0

0
4

0
4

0
4

0

0

0

0

0

0

0
4

0
4

0
4

0

0

0

0

0

0
4

0

0

0
4

0
4

0
4

2.51502

2.51502

2.51502

2.51502

0
4

0

0

0
4

0
4

0
4

0.536305
6

0.536305
6

0.429044

0

0

0.429044

0

0

0
4

0.107261

0.107261

0

0

0

0
4

0

0

0
4

1.38777878078145e-17
6

0
4

0
4

0

0

0

0

0
4

0
4

0
4

0

0

0

0

0
4

0
4

0
4

1.19001

1.19001

0.900041

0.900041

0
4

0.289974

0.289974

0
4

0
4

0
4

2.87758

2.87758

2.87758

0.0846347

0

2.79294

0
4

0
4

0
4

0

0

0

0

0
4

0
4

0

0

0

0
4

0
4

0
4

0

0

0

0

0
4

0
4

0
4

0

0

0

0

0

0
4

0

0

0
4

0
4

0
4

0

0

0

0

0
4

0
4

0
4

0

0

0

0

0

0
4

0
4

0
4

0

0

0

0

0
4

0
4

0
4

0
1

0

0

0

0
4

0
4

0

0

0

0
4

0
4

0

0

0

0
4

0
4

0

0

0

0

0
4

0
4

0

0

0

0
4

0
4

0

0

0

0
4

0
4

0

0

0

0
4

0
4

0

0

0

0
4

0

0

0
4

0
4

0

0

0

0
4

0
4

0

0

0

0

0
4

0
4

0

0

0

0
4

0
4

0
1

0
2

0

0

0

0

0

0

0

0

0

0
4

0

0

0
4

0
4

0
4

2.4255
7

0

0

0

0

0
4

0
4

0

0

0

0
4

0
4

0

0

0

0
4

0
4

0

0

0

0
4

0
4

0

0

0

0
4

0
4

0

0

0

0
4

0
4

0

0

0

0
4

0
4

2.4255
7

2.4255
7

2.17481
7

0.250694

0
4

0
7

0
7

0
4

0

0

0
4

0

0

0
4

0

0

0
4

0

0

0
4

0

0

0
4

0

0

0
4

0

0

0
4

0
4

0
4

18.7281
3

12.5531
3

12.5531
3

5.43478

0

0

0.0483291

0

0

0

0.152013

0

0

0

6.20773
3

0

0

0

0

0

0

0

0

0

0.0724936

0.193316
2

0

0

0

0

0.236748
3

0
2

0.120823
3

0
2

0.0868644
3

0
2

9.15933995315754e-16
3

0
4

0

0

0
4

0

0

0
4

0
4

0

0

0

0

0

0
4

0
4

5.35931

5.35931

5.26288

0.0964329

0
4

0
4

0

0

0

0
4

0
4

0

0

0

0
4

0
4

0.815672

0.815672

0.0964329

0.719239

0
4

0
4

0

0

0

0
4

0

0

0
4

0
4

0

0

0

0
4

0

0

0
4

0
4

0

0

0

0
4

0
4

0

0

0

0
4

0
4

0
4

3.65137
7

0

0

0

0
4

0
4

0

0

0

0
4

0
4

0

0

0

0
4

0
4

0.29211

0.29211

0.29211

0
4

0
4

0

0

0

0
4

0
4

0

0

0

0
4

0
4

0

0

0

0
4

0
4

0

0

0

0
4

0
4

0

0

0

0
4

0
4

0

0

0

0
4

0
4

0

0

0

0
4

0
4

3.35926
7

3.35926
7

1.46055
7

1.89871
5

0

0

0

0

0

0

0

0

0

0

0
7

0

0

0

0

0

0

0

0

0

0

0
6

0

0

0

0

0

0

0

0

0

0

0

0

0

0
7

0

0

0

0

0
4

0

0

0
4

0

0

0
4

0

0

0
4

0
4

4.44089209850063e-16
7

0
4

20.2766

7.05073
5

7.05073
5

4.2031

0

0

0

2.2296
5

0
6

0.300833

0.0793016

0.237905

0

0

0

0
4

0

0

0
4

0
4

11.416
5

11.416
5

11.416
5

0

0

0

0

0
4

0

0

0
4

0
4

0

0

0

0
4

0
4

0.763337

0.763337

0.763337

0
4

0
4

0

0

0

0
4

0
4

1.04655

0
7

0

0

0

0

0

0

0

0

0

0

0

0

0

0

0

0

0

0

0

0

0

0

0
4

0

0

0

0

0
4

0

0

0
4

1.04655

1.04655

0
4

0

0

0
4

0
4

0
4

20.4977

0.0966581

0

0

0

0

0

0

0

0

0

0

0
4

0.0483291

0.0483291

0

0
4

0.0483291

0.0483291

0
4

0
4

3.28886
5

3.28886
5

3.28886
5

0

0

0

0
7

0

0

0

0

0

0

0

0
4

0
4

0

0

0

0

0
4

0

0

0
4

0
4

0

0

0

0
4

0
4

0

0

0

0
4

0
4

0

0

0

0
4

0
4

0

0

0

0
4

0
4

0

0

0

0
4

0
4

0

0

0

0

0
4

0
4

0

0

0

0

0
4

0
4

0

0

0

0
4

0
4

0

0

0

0
4

0
4

3.91476
5

3.91476
5

3.85666
5

0

0

0.0581004

0

0

0

0
4

0

0

0
4

0

0

0
4

0
4

0

0

0

0
4

0
4

0

0

0

0
4

0
4

0

0

0

0
4

0
4

0

0

0

0
4

0
4

0

0

0

0
4

0
4

0

0

0

0
4

0
4

0

0

0

0
4

0
4

0

0

0

0
4

0
4

0

0

0

0
4

0
4

0

0

0

0
4

0
4

0
7

0
7

0
7

0

0
7

0
7

0

0

0

0

0

0
4

0
4

5.52456
5

2.42015
5

0.203351

0.0483291

0

0

1.97515

0.193316

0

0

0
4

3.10441

0.929606

0

0

2.17481

0
4

0
4

7.67283
5

7.67283
5

7.27616

0.0966581

0.0483291

0.203351

0.0483291

0

1.45716771982052e-16
5

0
4

0

0

0

0
4

0
4

0

0

0

0
4

0

0

0
4

0
4

0

0

0

0

0

0
4

0

0

0
4

0
4

0
7

0
7

0

0

0

0

0

0
4

0
4

0

0

0

0
4

0

0

0
4

0
4

0
4

28.1568

0

0

0

0

0

0

0

0

0

0

0
4

0

0

0
4

0
4

0

0

0

0

0

0
4

0
4

0

0

0

0

0
4

0
4

0

0

0

0

0
4

0

0

0
4

0
4

0.312481

0.312481

0.248192

0.0642886

0
4

0
4

0

0

0

0
4

0

0

0
4

0
4

0.119615

0.119615

0.119615

0
4

0
4

0

0

0

0
4

0
4

0

0

0

0
4

0
4

1.16844

0

0

0
4

1.16844

1.16844

0
4

0
4

0

0

0

0
4

0
4

0.730274

0.730274

0.730274

0

0

0
4

0
4

0

0

0

0
4

0
4

1.32905

1.32905

1.32905

0
4

0
4

0

0

0

0
4

0
4

0

0

0

0
4

0
4

0

0

0

0
4

0
4

0

0

0

0
4

0
4

0

0

0

0
4

0
4

0

0

0

0
4

0
4

0

0

0

0
4

0
4

0

0

0

0
4

0
4

0

0

0

0

0
4

0

0

0

0
4

0
4

0

0

0

0
4

0
4

0

0

0

0
4

0
4

0

0

0

0
4

0
4

0

0

0

0
4

0
4

0

0

0

0
4

0
4

0

0

0

0
4

0
4

0

0

0

0

0
4

0
4

0

0

0

0

0

0
4

0

0

0
4

0
4

0

0

0

0

0
4

0

0

0
4

0

0

0
4

0
4

0

0

0

0
4

0

0

0

0
4

0
4

1.1599

1.1599

1.11157

0.0483291

9.0205620750794e-17

0
4

0
4

0

0

0

0

0
4

0

0

0

0
4

0
4

23.337

22.6149

7.96787

5.58599

0

0

0

0

0

0

0

0.193641

0

0

0

0

0

0

5.38928
3

0

0.0642886

3.19075

0.223054

0

0

0
4

0

0

0

0

0

0

0

0
4

0

0

0
4

0

0

0
4

0

0

0
4

0

0

0
4

0

0

0
4

0

0

0
4

0

0

0
4

0

0

0

0

0

0

0
4

0

0

0
4

0

0

0
4

0

0

0

0
4

0

0

0
4

0.722169

0.722169

0
4

0

0

0
4

0

0

0
4

5.77315972805081e-15

0
4

0
4

4.89456
5

0
6

0

0

0

0

0

0

0

0

0

0
4

0

0

0

0
4

0

0

0

0
4

0

0

0
4

0

0

0
4

0
4

1.82296

0.840844
5

0.391964
6

0.116201

0.232402

0.0501388

0

0.0501388

0

0

1.52655665885959e-16
5

0
4

0.982114

0.982114

0

0
4

0

0

0
4

0
4

0

0

0

0
4

0
4

0.0724936

0.0724936

0.0724936

0
4

0
4

0

0

0

0
4

0
4

0

0

0

0
4

0
4

0

0

0

0
4

0
4

1.05618
5

1.05618
5

0.125347
6

0

0

0.880691

0.0501388

0

0
4

0
4

1.88483

1.88483

0.0483291

0.604113

0.0724936

1.1599

0

0
4

0
4

0.0581004
5

0

0

0

0

0

0
4

0.0581004

0.0581004

0
4

0

0

0
4

0
4

0

0

0

0
4

0
4

0

0

0

0

0
4

0
4

0

0

0

0
4

0
4

0

0

0

0
4

0
4

0

0

0

0
4

0
4

6.93889390390723e-16
5

0
4

0
3

0

0

0

0

0
4

0
4

0

0

0

0
4

0
4

0
3

0

0

0
4

0

0

0

0

0
4

0

0

0
4

0

0

0
4

0

0

0
4

0
4

0
4

6.42998

0

0

0
7

0

0

0

0

0

0

0

0

0

0
8

0
8

0
1

0

0

0

0

0

0
4

0

0

0

0

0
4

0

0

0

0
4

0
4

0

0

0

0

0
4

0
4

0

0

0

0

0

0
4

0

0

0
4

0
4

0

0

0

0

0
4

0

0

0

0
4

0
4

0

0

0

0
4

0
4

0

0

0

0
4

0
4

0

0

0

0

0
4

0

0

0
4

0
4

0

0

0

0
4

0
4

0

0

0

0

0
4

0
4

0

0

0

0
4

0
4

0

0

0

0
4

0

0

0
4

0
4

0.185812

0
7

0

0

0

0

0

0

0

0

0

0

0

0
4

0
1

0
8

0

0

0
4

0.185812

0.185812

0
4

0

0

0
4

0
4

0

0

0

0
4

0
4

0

0

0

0

0
4

0
4

0

0

0

0
4

0
4

0

0

0

0
4

0
4

0.0483291

0.0483291

0.0483291

0
4

0
4

0

0

0

0
4

0
4

0

0

0

0
4

0
4

0

0

0

0
4

0
4

0

0

0

0
4

0
4

0

0

0

0
4

0
4

0

0

0

0

0

0

0
4

0

0

0

0
4

0

0

0
4

0

0

0
4

0
4

0

0

0

0
4

0
4

0

0

0

0
4

0
4

0

0

0

0
4

0
4

0

0

0

0
4

0
4

0

0

0

0
4

0
4

0

0

0

0
4

0
4

0.0964329

0.0964329

0.0964329

0
4

0
4

0

0

0

0
4

0
4

0

0

0

0
4

0
4

0

0

0

0
4

0
4

0

0

0

0

0

0

0
4

0

0

0

0
4

0
4

0

0

0

0

0
4

0

0

0

0

0

0
4

0

0

0

0
4

0
4

0.0434322
3

0.0434322
3

0

0

0

0.0434322

0

0

0

0

0
4

0

0

0
4

0

0

0
4

0
4

0.483858
3

0.483858
3

0.161286

0.322572

0

0

0

0
4

0
4

0
6

0

0

0

0

0
4

0

0

0
4

0

0

0
4

0
4

0

0

0

0

0

0
4

0
4

5.57211

1.47745

0
7

0

0

0

0

0

0

0

0

0

0

0

0

0

0

0

0

0

0

0

0

0

0

0

0

0

0

0

0

0

0

0

0

0

0

0

0

0

0

0

0

0

0

0

0
7

0

0.196734

0

0

0

0

0

0

0

0

0

1.28072

0

0

0

0

0

0

0

0

0

0

0

0

0

0
4

0

0

0

0

0
4

0

0

0
4

0

0

0
4

0

0

0

0
4

0

0

0

0
4

0

0

0
4

0

0

0
4

0

0

0
4

0

0

0
4

0

0

0
4

0

0

0
4

0
6

0
6

0

0

0
4

0

0

0
4

2.76441

2.76441

0
4

0

0

0
4

0

0

0
4

0.0724936

0.0724936

0
4

0

0

0

0

0

0
4

0

0

0

0

0
4

0.76592
3

0.0651483

0.700771

0

0
4

0

0

0

0
4

0.491835

0.491835

0

0
4

0

0

0

0
4

0

0

0
4

2.22044604925031e-16

0
4

0
4

0
7

0
6

0
6

0
6

0

0
4

0
4

0
6

0
6

0
6

0
4

0
4

0

0

0

0
4

0
4

0

0

0

0
4

0
4

0

0

0

0
4

0
4

0

0

0

0
4

0
4

0
7

0
7

0
7

0
4

0
4

0
4

8.29514235078932e-11

0
4

30444.2

0

0

0

0

0

0

0
4

0
4

0
4

14554
3

4.53686

4.26221

2.18406

0.942417

0

0

0

0

0

0

0

0

0
2

1.13573

0

0

0

0

0

0

0
4

0.274652

0.274652

0
4

0

0

0
4

0
4

14509.3
3

42.5687

0

2.25248

0

0

0

0

0

0

0.823956

0

0

0

0

0

0.214522

0

0

0.397776

0

0

0

0

0

0

0

0

0.823956

0

0

0

0

0

0

0

0

16.4679

0

0

0

0

0

0

0

0

0

0

0

0

0

0

0

0

0

0

0

0

0

0
6

0.274652

0

0

0

0

0

0

0

0

0

0
7

0

0

0

0

0

0

0

0

0

0

0

0

0

0

0

0

0

0

0

0

0

0

0

0

0

0

0

0

0

0

0

0

0

0

0.763337

0

0

0

0

0

0

0

0

0.804457

2.34572
5

0

0

0

0

0

0

0

0

0

0

0

0

0

0

0

0

0

0

0

0

0

0
2

0

0

0

0

0

0

0

0

0

0

0

0

0

0

0

0

0

0

0

0

0

0

0

0

0

0

0

0

0

0

0

0

0

0

0

0

0

0

0

0

0

0

0

0
6

0

0

0

0

0

0

0

0

0

0

0

0

0

0

0

0

0

0

0

0

0

0

0

0

0

0

0

0.274652

0

0

0

0

0

0

0

0

0

0

0

0

0

0

0

0

0

0

0

0

0

0

0

0

0

0

0

0

0

0

0

0

0

0

0

0

0

0

0
2

0

0

0

0

0

0

0

0

0

0

0

0

0

0

0

0

0

0

0

0

0

0

0

0

0

0

0

0

0

0

0

0

0

0

0

0

0

0

0

0

0

0

0

0

0.68663

0

0

0

0

0

0

0

0

0

0

0.68663

0

0

0

0

0.817456

1.32404

0

0
6

0

0

0
2

0

0

0
7

0

0

0

0

0

0

0

0

0

0

0

0

11.1529

0

0

0

0

0

0

0

0

0

0

0.549304

0

0

1.78524

0

0

0

0

0

0

0

0

0

0

0

0

0

0

0

0

0

0

0

0

0

0.123124

0

0

0

0

0

0

0

0
4

12717.3
4

2767.89
3

4322.89
4

3653.4
3

1.27854
6

39.432
4

0.254623

0

0

0.431993

0

0

0

1.32413

0.5209

0

6.26297
4

0

0.750827

3.40184

2.77755

0.804457

0.350972

0.482674

7.59744

0

1.44802

104.047
4

0

0

0

0.415224

0

0

0.123124

0

0

0

150.726
4

0.435938

0.608587

0

27.6552

0

0

0

0.800305

0

0

29.6873
4

0

0

1.66443

0.591631

0

1.30069

0

0

0.858088

0

0
2

17.3291

6.12773

0.644133

0

0

0.611094

0

0

0

0

6.7738
3

5.23355

0

0.158414

0

0

0

0

0

0.865186

0

10.6916
4

0

0

0

8.73318

0.375413

1.62768

0

0

103.814

0.107261

0

0.858088

1.06888

2.19722

0

0.321783

0

0

0

0

0.160891

0
2

0

0.0752082

0

0

0.225625

0

0

0

0.29211

8.61724

30.8476
3

32.4933
4

0.160891

0.0642886

1.09861

0

0

0.965349

0

0

0

0

0

0.152774

0

0

0.107261

0

0.549304

0.177448

0

0.107261

0.160891

3.17527

0

0.429044

0.858088

0

0

0

0

0.305547

0.214522

0

14.7985
4

0.482674

0.185812

0.549304

0.0501388

0

0.29211

0

0

2.67168

0

5.01073

0

0

0

0

0

0

0

0

0.160891

0.160891

0.99587
3

0

0

0.68663

3.81669

1.52667

0

0.185812

0

0

0

0

0.101849

0

0

0

0

0

0

0

0

0.371625

0

0

0

0

0.107261

0

0

0.101849

0

0

0.858088

2.64862
4

0

0

0

0

0

0.509245

0

0

0

0

199.763
4

0

0.145251

0

0.100278

0

0.429044

0.107261

0.268152

0

0

262.122
4

3.85892

0

0.885304

0

0

0

0.160891

0.138315

0

0

0.241929

0

0.216187

0

0

0.0752082

0

0.927568

0

0

0

0

23.4074

0

0

0.107261

0.185812

0.123124

0

0

0.107261

0

0

19.194
4

0.29211

0

0

0.321783

0

0

0.107261

0.107261

0

0.192866

1.41677
3

0.0752082

0

0

0

0.05102

0

0

0

0

1.39439

0.556925

0

0

0

0.100278

0

0.150416

0

0

0.268152

0.116201

0

0

0.763337

0

0

0.557437

0

0.185812

0.185812

0

0

3.33727
4

0.482674

0.110652

0

0

0

0

0.289299

0

0

0.0829891

0

0

0.0846347

0.268152

0.107261

0.125347

0

0

0

0

0.125347

0

0

0.0767289

0.0501388

0.29211

0.123124

0

0

0

0.160891

0

42.5429
4

0

0

0

0

0

1.90834

0

0

0.0752082

0

0

2.98031

0.509245

0

0

0.429044

6.87004

0

0

0.371625

1.53222

0.536305

0.82415

0.375413

0

0

0

0

0

0.81945

0.0617678

0

0

0
2

0

0.107261

0

0

0.241929

0

0.184686

0

0.107261

0

0

0

0

0

1.66281
4

19.8588

0

0

0.513073
3

181.097
4

0

0

0.464531

10.7677
4

0

0.441212
4

0

0

2.7248

2.05091

39.9862
4

3.97682

0

3.0044

100.955

0

0

1.14024

162.416

3.19577

27.8618

41.3062
4

0

0

0

0

0

0.634413

0

0

0.809473

1.33624

4.67127
3

1.01898

0.100278

1.08916

0

0.450523

0

0

0.161286

61.7612

0.976522

19.9985
3

1.32107

0

1.04639

4.81981

9.34751

0

3.50557

0

0

0

0
4

103.498
3

0
3

11.9195
3

44.7627
3

15.2591
3

10.6756
2

19.0192
3

0
2

0
1

0

0

0

0

0

0

0

0

0

0

0

0

0

0

0

0.246248

0

0.984991

0.322572

0

0

0

0

0

0

0

0

0
8

0

0

0

0.30781

0

0
4

37.9887

0
7

0
6

0

0

0

0

0.0642886

0

0

0

0

0

0
7

0

0

0

0

0

0.803608

0

0

0

0.0964329

0
7

0

0

0

0

0

0

0

0

0

0

4.6372
5

0

0

0

0

0

0

0

0

0

0

0
6

0

0.128577

0

0

0

0

0

0

0

0

0
7

0

0.0501388

0

0

0

0

0.0482912

0

0

0

0

0.0642886

0

0

0.0642886

0

0

0

0

0.289299

0

0

0

0

0

0

0

0

0

0.482165

0

0
8

0

0.257154

0

0

0

0

0

0

0

0

0

0

0

0

0

0

0

0

0.763337

0

9.61115
3

0.0964329

0

0

0.0964329

0

0

0

0

0

0.0642886

0
7

0

1.31792

0

0

0

0.0642886

0

0

0

0

16.8355
3

0

0

0

0

0.0642886

0.128577

0

0

0

0

0
7

0

0

0

0

0

0

0

0

0

0

0
1

0

0

0

0

0

0

0.0642886

0

0

0

1.89651

0

0

0

0

0

0

0

0

0

0

1.99840144432528e-15

0
4

0
2

0

0

0

0

0

0

0

0

0

0

0

0

0

0

0

0

0

0

0

0

0

0

0

0

0

0

0

0

0

0

0

0

0

0

0

0

0

0

0

0

0

0

0

0

0

0

0

0

0

0

0

0

0

0

0

0

0

0

0

0

0

0

0

0

0

0

0

0

0

0

0

0

0

0

0

0

0

0

0

0

0

0

0

0

0

0

0
4

492.104
3

400.616
3

9.33817

0.0752082

0.823956

0.450138

0

0.0501388

0

0

0

0.0501388

23.1835
4

0.411978

0.274652

0

0.107261

0

0

0

0

0

0

5.86679
4

0

0.05102

0.100278

0.0501388

0.15306

0

28.765
4

1.40541
3

9.6697
4

1.85155
3

8.80949
4

0

3.5527136788005e-15
3

0
4

11.0717
3

1.73621

0

0.40111

0.274652

3.61141
4

0.286238

0.68663

3.49883

0.476319

0

0.0501388

0.0501388

8.74300631892311e-16
3

0
4

0.449664

0.449664

0
4

4.32752

4.32752

0
4

0

0

0
4

0

0

0
4

0

0

0

0
4

0

0

0
4

0.0501388

0

0.0501388

0
4

0

0

0
4

0.123124

0.123124

0
4

4.07723

0

4.07723

0
4

3.56671
3

1.57053
4

0.377612
3

0.650693
3

0.175486
3

0.274652

0.225625

0

0.29211

1.11022302462516e-16
3

0
4

2.29001

2.29001

0
4

0

0

0

0
4

0

0

0
4

0

0

0

0
4

0.261169

0.261169

0
4

0

0

0
4

0

0

0

0
4

0

0

0
4

0

0

0
4

0

0

0
4

53.2303
3

47.5698
3

2.82566

0.497935

2.33688

0

0

0

0

4.44089209850063e-16
3

0
4

0

0

0
4

0

0

0
4

0

0

0
4

0

0

0
4

1.55528

1.55528

0
4

1.02197

1.02197

0
4

0

0

0
4

0

0

0
4

0

0

0
4

0

0

0
4

0
2

0
2

0

0

0

0

0

0

0
4

0

0

0
4

1.04655

1.04655

0
4

0

0

0
4

0

0

0
4

0

0

0
4

0

0

0
4

0

0

0
4

0

0

0
4

0

0

0
4

0

0

0
4

0

0

0

0

0

0

0

0

0
4

0

0

0
4

0

0

0
4

0

0

0
4

0

0

0
4

0

0

0
4

0

0

0
4

0

0

0
4

0

0

0
4

0

0

0
4

0

0

0
4

74.4232
4

56.9163
4

16.5827

0.763337

0.160891

9.88098491916389e-15
4

0
4

0

0

0
4

0

0

0
4

0

0

0
4

0

0

0
4

0

0

0
4

0

0

0
4

0

0

0
4

0

0

0
4

0

0

0
4

0.763337

0.763337

0
4

2.33176

2.12018

0.211587

0

0

0
4

0

0

0
4

0

0

0
4

0.326179

0.326179

0
4

0

0

0
4

0

0

0
4

0

0

0
4

0

0

0
4

1.12812

1.12812

0
4

0

0

0
4

0

0

0
4

1.78559
3

1.67911
3

0

0.0511526

0.0553261

0
4

0

0

0
4

0

0

0
4

0

0

0
4

0

0

0
4

0

0

0
4

0

0

0
4

0

0

0
4

0

0

0
4

0

0

0
4

0

0

0
4

14.8823
4

2.17777
4

0.101849

12.6026

0

0
4

0.268152

0.268152

0
4

0

0

0
4

0.254623

0.254623

0
4

0

0

0
4

0

0

0
4

0.169269

0.169269

0
4

0

0

0
4

0

0

0
4

0.123124

0.123124

0
4

0

0

0
4

0

0

0

0

0

0

0
4

0

0

0
4

0

0

0
4

0

0

0
4

0

0

0
4

0

0

0
4

0

0

0
4

0

0

0
4

2.67168

2.67168

0
4

0

0

0
4

0

0

0
4

42.8398
3

11.3548
3

0.823956

0.0501388

0.123124

0.411978

0

0.0501388

0

0

0

0

12.8505
4

0

0.0501388

0

0

0

0.107261

0

2.0998
4

8.64652
4

1.36239
3

1.72723

3.05657

0.0752082

0.0501388

6.03683769639929e-16
3

0
4

0

0

0

0

0

0
4

0

0

0
4

0

0

0
4

0

0

0
4

0

0

0
4

0

0

0
4

10.107
3

8.06238
3

0.396664

1.64791

4.44089209850063e-16
3

0
4

9.11117
4

8.34783

0.763337

0

0

0

0
4

0
2

0

0

0

0

0

0

0

0
4

2.91171

0.641088

0.664186

0.426566

0

0.268152

0.429044

0.107261

0.375413

5.55111512312578e-17

0
4

3.05335

3.05335

0

0
4

0.598272
3

0.330286

0.101849

0.0642886

0

0.101849

0

1.38777878078145e-17
3

0
4

0.730638
3

0.459338

0.0511526

0

0

0.101849

0

0.118299

0
4

0

0

0

0
4

0
2

0

0

0

0

0

0
4

576.019
3

538.179
3

11.6993

0.200555

1.35906

0.0501388

0.275763

0.101849

0.274652

0.150416

0.100278

0

5.39548
4

0

5.09586

7.21751
4

0

0.501388

3.9616

0

1.45631

0
4

0.991616
3

0.58422

0.407396

0

0

0

0
4

3.70929
4

2.13247

1.52667

0.0501388

0

0

4.85722573273506e-17
4

0
4

3.79308

3.79308

0
4

1.07915
4

0.813476
4

0.214522

0.0511526

0

0
4

9.88074

9.10359

0.777151

8.88178419700125e-16

0
4

0

0

0

0

0
4

11.0606
4

4.74358
4

6.317

8.88178419700125e-16
4

0
4

2.29001
5

2.29001
5

0

0
4

0
2

0

0

0

0

0

0
4

3.0612
4

3.0612
4

0
4

2.03062
5

2.03062
5

0

0

0

0

0

0

0

0

0

0

0

0

0

0

0

0

0

0

0

0

0

0

0

0

0
4

8.07887
4

7.31553

0.763337

0
4

0.307816

0.100278

0.107261

0.100278

1.38777878078145e-17

0
4

0

0

0
4

0

0

0

0

0
4

0.635379
4

0.123124

0.512256

1.11022302462516e-16
4

0
4

1.08441

0.68663

0

0.123124

0.274652

0
4

0

0

0

0
4

0

0

0

0

0

0
4

0

0

0
4

1.96734

1.96734

0
4

0.100278
6

0.0501388

0

0

0

0

0

0

0

0

0

0

0

0

0

0

0

0

0

0

0

0

0

0

0

0

0

0

0

0.0501388

0

0

0

0
4

0
2

0

0

0
4

0

0

0

0

0
4

0.807168

0.807168

0

0
4

4.22006

4.11978

0.100278

0

0
4

0.763337

0

0.763337

0

0
4

0

0

0

0

0
4

1.00958

0

1.00958

0
4

0

0

0
4

0

0

0

0
4

1.90834

1.90834

0

0

0
4

3.2376
2

2.99595
2

0.241645
2

0

0

0

0

0

0

0
4

0

0

0
4

0.780317

0.423846

0.356472

0
4

0

0

0
4

7.47821

0.226506

7.2517

0
4

0

0

0
4

0

0

0
4

0

0

0
4

0

0

0

0

0

0
4

0

0

0

0
4

0

0

0
4

114.996
3

9.48601
4

0.160891

0

0.0553261

0.0511526

0.29211

0.163089

0

0

8.7954

0.145251

65.5627

1.73579

0.125347

0.709793

0

0.107261

0.0553261

0.101849

5.54057

0

0.68663

0.297972

14.6411

0.754102

0.768579

0.214522

2.89714

1.64791

8.43769498715119e-15
3

0
4

0

0

0
4

3.43502

3.43502

0
4

0

0

0

0
4

0

0

0

0
4

0.100278

0.0501388

0.0501388

0
4

0.399999

0.125347

0.274652

0
4

0

0

0

0
4

10.9997

10.9997

0

0
4

0

0

0

0
4

0

0

0

0
4

6.69353
6

6.3927
6

0

0.200555

0

0

0.0501388

0

0.0501388

0

0

0

0

4.85722573273506e-16
6

0
4

0

0

0

0
4

0

0

0

0
4

0.150416

0.150416

0
4

0.150416

0.150416

0

0
4

1.91355

1.91355

0
4

0

0

0
4

0

0

0

0
4

0

0

0

0
4

0

0

0
4

0

0

0
4

49.3609
4

42.6864
4

2.66443
4

1.25779
4

0.604483

0.870598

0

0.101849

0.411978

0.763337

0
4

0

0

0
4

0.248967

0.248967

0
4

3.05335

3.05335

0
4

0.16979

0.16979

0
4

9.16005

0.763337

8.39671

0
4

0

0

0

0
4

0

0

0
4

0

0

0

0
4

0.697196

0.697196

0
4

16.4791

0

16.4791

0
4

2.19664286760235e-11
3

0
4

0
2

0
2

0
2

0

0

0

0

0

0

0

0

0

0

0

0

0

0

0

0

0

0

0

0

0

0
2

0

0

0

0

0

0

0

0

0

0

0

0

0

0

0

0

0

0

0

0

0

0

0

0

0

0

0

0

0

0

0

0

0

0

0

0

0

0

0

0

0

0

0

0

0

0

0

0

0

0

0

0

0
4

0
2

0

0

0

0

0

0

0

0

0

0

0

0
4

0

0

0

0

0

0

0

0

0
4

0

0

0

0

0

0
4

0

0

0
4

0
4

0

0

0
7

0

0

0

0

0

0
4

0

0

0
4

0

0

0
4

0
4

0.797429

0.217481

0.217481

0
4

0.579949

0.579949

0
4

0
4

0

0

0

0
4

0

0

0
4

0
4

0

0

0

0
4

0

0

0
4

0
4

0

0

0

0

0
4

0
4

0.0642886

0

0

0
4

0.0642886

0.0642886

0
4

0
4

0

0

0

0
4

0
4

0

0

0

0
4

0
4

0

0

0

0
4

0
4

0

0

0

0
4

0

0

0
4

0
4

0

0

0

0
4

0
4

1.47749

1.47749

1.47749

0

0

0

0

0

0
4

0
4

0

0

0

0

0
4

0
4

0.867896

0

0

0
4

0.867896

0.867896

0
4

0
4

0

0

0

0

0
4

0
4

0

0

0

0
4

0
4

0.100278

0.100278

0.0501388

0.0501388

0
4

0
4

0

0

0

0
4

0
4

0

0

0

0
4

0
4

0

0

0

0
4

0
4

0

0

0

0
4

0
4

0.0966581

0.0966581

0.0483291

0.0483291

0
4

0
4

0
7

0
7

0

0

0

0

0

0

0
4

0
4

0

0

0

0

0
4

0
4

0

0

0

0
4

0
4

0

0

0

0
4

0
4

0

0

0

0
4

0
4

0

0

0

0
4

0
4

0

0

0

0
4

0
4

0

0

0

0
4

0
4

0

0

0

0
4

0
4

0

0

0

0
4

0
4

0

0

0

0
4

0
4

0
2

0
2

0
2

0

0

0

0

0

0

0
4

0
4

0

0

0

0
4

0
4

0

0

0

0
4

0
4

0

0

0

0
4

0
4

0

0

0

0
4

0
4

0

0

0

0
4

0
4

0

0

0

0
4

0
4

0

0

0

0
4

0
4

0

0

0

0
4

0
4

1.90834

1.90834

1.90834

0
4

0
4

0

0

0

0
4

0
4

0

0
5

0

0

0

0

0

0

0

0
4

0

0

0

0

0

0

0
4

0

0

0
4

0
4

0

0

0

0
4

0
4

0

0

0

0
4

0
4

0

0

0

0
4

0
4

0

0

0

0
4

0
4

0

0

0

0
4

0
4

0

0

0

0
4

0
4

0

0

0

0
4

0
4

0.107261

0.107261

0.107261

0
4

0
4

0.438165

0.438165

0.438165

0
4

0
4

0

0

0

0
4

0
4

0.123124

0.123124

0.123124

0

0

0

0

0

0
4

0
4

0

0

0

0
4

0
4

0.0501388

0.0501388

0.0501388

0
4

0
4

0

0

0

0
4

0
4

0

0

0

0
4

0
4

0.29211

0.29211

0.29211

0
4

0
4

0

0

0

0
4

0
4

0

0

0

0
4

0
4

0

0

0

0
4

0
4

0

0

0

0
4

0
4

0

0

0

0
4

0
4

0

0

0

0

0

0

0

0
4

0
4

0

0

0

0
4

0
4

0

0

0

0
4

0
4

0

0

0

0
4

0
4

0

0

0

0
4

0
4

0

0

0

0
4

0
4

0.0642886

0.0642886

0.0642886

0
4

0
4

0.438165

0.438165

0.438165

0
4

0
4

0

0

0

0
4

0
4

0

0

0

0
4

0
4

0.0501388

0.0501388

0.0501388

0
4

0
4

0

0

0

0

0

0

0

0

0

0

0

0

0
4

0
4

0

0

0

0
4

0
4

0

0

0

0
4

0
4

0

0

0

0
4

0
4

0

0

0

0
4

0
4

0

0

0

0
4

0
4

0

0

0

0
4

0
4

0

0

0

0
4

0
4

0

0

0

0
4

0
4

0

0

0

0
4

0
4

0.29211

0.29211

0.29211

0
4

0
4

0

0

0

0

0

0

0
4

0

0

0
4

0
4

0

0

0

0
4

0
4

0

0

0

0
4

0
4

0

0

0

0
4

0
4

0

0

0

0
4

0
4

0

0

0

0
4

0
4

0

0

0

0
4

0
4

0

0

0

0
4

0
4

0

0

0

0
4

0
4

0

0

0

0
4

0
4

0

0

0

0
4

0
4

0
6

0
6

0
6

0

0

0

0

0

0
4

0
4

0

0

0

0
4

0
4

0

0

0

0
4

0
4

0

0

0

0
4

0
4

0
2

0
2

0
2

0

0

0

0

0

0

0

0

0

0

0
2

0

0

0

0

0

0

0

0

0

0

0

0

0

0

0

0

0

0

0

0

0

0
2

0

0

0

0

0

0

0

0

0

0

0

0

0

0

0

0

0

0

0

0

0

0

0

0

0

0

0

0

0

0

0

0

0

0

0

0

0

0

0

0

0

0

0

0
2

0

0

0

0

0

0

0

0

0

0

0
4

0
2

0
2

0

0

0

0

0

0

0

0

0

0
4

0
2

0

0
2

0

0

0

0
4

0

0

0

0

0

0
4

0

0

0

0

0
4

0

0

0

0

0
4

0

0

0

0
4

0
4

0
2

0
2

0
2

0

0

0

0
4

0

0

0

0
4

0

0

0
4

0
4

0

0

0

0

0

0

0
4

0
4

2.13782
5

2.13782
5

2.01027
5

0.12755

0

0
4

0
4

0

0

0

0

0

0

0

0

0

0
4

0

0

0

0
4

0

0

0
4

0
4

0

0

0

0

0

0

0

0
4

0

0

0
4

0
4

0

0

0

0

0

0

0
4

0

0

0

0
4

0

0

0
4

0

0

0
4

0
4

0

0

0

0

0

0

0
4

0

0

0
4

0

0

0
4

0

0

0
4

0
4

0
5

0
5

0

0

0

0

0

0

0

0

0
4

0
4

5.70941
4

5.29743

3.83001

0.704088

0.763337

0
4

0.411978

0.411978

0
4

0

0

0
4

6.10622663543836e-16
4

0
4

0
7

0
7

0
7

0

0

0
4

0
4

0
2

0
2

0
2

0

0

0

0

0

0

0

0

0
2

0

0

0

0

0

0

0

0
4

0
2

0
2

0

0

0

0
4

0

0

0

0

0

0

0

0

0

0

0
4

0

0

0

0
4

0
4

1.50194

1.50194

0.323679

0.325902

0.451249

0.40111

0
4

0
4

0
6

0
6

0
6

0
4

0
4

0
2

0

0

0

0

0

0
4

0

0

0

0
4

0

0

0
4

0
4

0
7

0
7

0
7

0

0
4

0
4

0
7

0
7

0

0

0
4

0
4

1.47749

1.47749

1.47749

0

0

0
4

0

0

0
4

0
4

0

0

0

0

0

0

0

0
4

0

0

0
4

0

0

0
4

0
4

0
6

0
6

0
6

0

0
4

0
4

0

0

0

0

0
4

0
4

0

0

0

0
4

0

0

0
4

0

0

0
4

0
4

0
2

0
2

0
2

0

0

0

0

0

0

0

0

0

0

0

0

0

0

0

0

0

0

0

0

0

0

0
4

0

0

0
4

0
2

0

0

0

0

0

0

0
4

0

0

0

0

0

0
4

0

0

0

0

0
4

0

0

0
4

0

0

0

0
4

0

0

0

0
4

0

0

0
4

0

0

0
4

0
4

2.63479

0

0

0

0
4

2.63479

1.10811

1.52667

0
4

0
4

0

0

0

0

0
4

0

0

0
4

0
4

0

0

0

0

0

0

0

0
4

0

0

0

0
4

0
4

0
6

0
6

0
6

0
4

0
4

0

0

0

0
4

0
4

0

0

0

0

0
4

0
4

5.08106

5.08106

5.08106

0

0
4

0

0

0

0
4

0
4

0

0

0

0

0

0
4

0
4

0

0

0

0

0

0

0
4

0

0

0
4

0
4

0

0

0

0
4

0
4

0

0

0

0

0

0

0

0

0

0

0

0

0

0

0

0

0

0

0
4

0

0

0
4

0

0

0

0
4

0
4

0.163089

0.163089

0

0.163089

0

0
4

0

0

0
4

0

0

0
4

0
4

3.74577

3.74577

3.74577

0
4

0
4

0

0

0

0

0
4

0
4

0

0

0

0
4

0

0

0
4

0
4

0

0

0

0

0
4

0

0

0
4

0
4

0

0

0

0

0
4

0
4

0

0

0

0

0

0
4

0

0

0
4

0

0

0
4

0
4

0

0

0

0

0
4

0

0

0
4

0

0

0
4

0

0

0
4

0
4

0

0

0

0

0
4

0
4

0

0

0

0
4

0
4

0
2

0
2

0
2

0
2

0

0

0

0

0

0

0

0
4

0

0

0

0
4

0
4

0

0

0

0

0
4

0

0

0
4

0

0

0
4

0

0

0
4

0
4

0
7

0

0

0

0
4

0

0

0
4

0
4

0

0

0

0
4

0
4

0

0

0

0
4

0

0

0

0
4

0

0

0
4

0
4

0

0

0

0

0
4

0
4

0

0

0

0

0
4

0

0

0
4

0
4

0

0

0

0

0

0
4

0
4

0

0

0

0
4

0

0

0
4

0
4

1.52667

1.52667

1.52667

0
4

0
4

0

0

0

0

0
4

0
4

0
2

0
2

0
2

0

0

0

0

0

0
4

0
2

0

0

0

0

0

0

0

0
4

0
2

0

0

0

0

0

0
4

0

0

0

0
4

0

0

0

0
4

0

0

0
4

0

0

0
4

0

0

0
4

0

0

0
4

0
4

0

0

0

0
4

0
4

0

0

0

0
4

0

0

0
4

0
4

1.6413

1.6413

1.49981

0.0565967

0.0848951

0
4

0
4

0

0

0

0
4

0
4

0

0

0

0

0

0
4

0
4

0

0

0

0

0

0
4

0
4

0

0

0

0
4

0

0

0
4

0
4

0

0

0

0

0
4

0

0

0
4

0
4

0

0

0

0
4

0
4

2.06578

2.06578

2.06578

0
4

0

0

0
4

0
4

0

0

0

0

0

0

0

0

0

0

0
4

0
4

0

0

0

0
4

0
4

0

0

0

0
4

0
4

0

0

0

0

0

0
4

0
4

0

0

0

0

0
4

0
4

0

0

0

0
4

0
4

0

0

0

0

0
4

0
4

0

0

0

0
4

0

0

0
4

0
4

0

0

0

0

0
4

0
4

0

0

0

0
4

0
4

0

0

0

0
4

0

0

0
4

0
4

5.12532

0

0

0

0

0

0

0

0

0

0
4

5.12532

5.12532

0
4

0

0

0

0
4

0
4

0.184686

0.184686

0.184686

0
4

0

0

0
4

0
4

0

0

0

0
4

0
4

0

0

0

0

0
4

0
4

0

0

0

0

0
4

0
4

0

0

0

0
4

0
4

0

0

0

0
4

0

0

0
4

0
4

0

0

0

0
4

0

0

0
4

0
4

0

0

0

0

0
4

0
4

0

0

0

0

0
4

0
4

0

0

0

0
4

0

0

0
4

0
4

0

0

0

0

0

0

0

0

0
4

0
1

0
1

0
4

0

0

0
4

0

0

0

0
4

0

0

0

0
4

0

0

0

0
4

0

0

0
4

0
4

3.64391850027346e-12
3

0
4

0
4

0
4

0

0

0

0
4

0

0

0
4

0

0

0
4

0

0

0
4

0
4

0

0

0

0
4

0
4

0

0

0

0

0
4

0
4

0

0

0

0
4

0
4

0

0

0

0
4

0
4

0
4

15804.8
5

1958.85
4

1958.85
4

464.154
4

0.236598

0.123124

0.123124

0.123124

0

0.123124

0.184686

0.123124

0.118299

0.29211

0.177448

4.85385

0.438165

0.29211

0.0999396

0.123124

0

0.118299

0.118299

0.196734

0.29211

0

4.51091

0.246248

0.123124

0

0

0.123124

0.29211

0.118299

0

7.88696

0

188.455

0.473196

0.123124

0.118299

0

0.123124

0.438165

0.29211

0.295747

0.295747

0.123124

1.6006

0.438165

23.2159
4

0

21.2792

41.0769

2.04722
4

3.34448

1.31094

1.189

4.63643

12.882

0.901716

9.24991

0.118299

4.50259

422.033

2.31889

37.4375
4

0.706156

168.722

1.00673

1.49076

0.925835

0.660294

1.31936

0.902904

0

3.42701

320.841
4

1.33019

6.22485

0

0.684412

0.426108

0.925835

0.849804

0

67.0392

0.149909

2.38283
4

0

1.28674

0.662706

0.784605

0.533532

0.469558

1.0369

4.08614

0.469558

0.359722

15.4906

1.02238

0.241423

0

0.415234

6.45676

0.241423

0.295101

0.702518

0

0.421284

9.04533
4

0.0999396

0.77618

0.538357

0.730274

0.840155

0

0

0.196734

0.184686

0

7.60226
4

0.123124

0

0.29211

0

0.118299

0

0.199879

0.123124

0.123124

0.118299

23.7818
4

0.473196

31.5479

0.184686

0

0.123124

0.29211

0.29211

0

0

0.123124

2.67794120212272e-12
4

0
4

0
4

13799.8
5

65.1171

19.3272

0.464531
2

0.85067

11.428
4

0.695187

1.84519
4

0

0.94708

1.0204

2.21443

0

7.44899

0

0.149909
7

0.212516

0.227145

0.536173

0

0

0

0

0.68857

0.118299

0.295101

1.41392
3

0

0

0

0

0.763337

0

0.29211

0.107261

0.196734

0

5.51239
4

0

0

0

0.0556337

0.107261

0

0

0.196734

0

0

0

0.107261

0

0

0.118299

0

0

0

0

0

0

1.32597

0

0

0

0

0

0

0

1.47551

0

0.0511526

0

0

0

0.169269

0.763337

0.0846347

0

0

0.126952

0

0

0

0

0

0

0

0

0.295101

0

0.0767289

0

0

2.02184

1.38626

0

0

0
4

0.118299

0

0

0

0.118299

0
4

0.0926517

0.0926517

0
4

13630.5
5

6395.87
5

41.1383
3

1920.45
3

3844.37
4

216.603
5

119.329
4

0

0

0

0

0.160891

0.652357

0

0

0.0846347

0.0581004

0.126952

0.0846347

0

0

0.05102

0.268152

0

1.07261

0.0511526

0

0

0

0

0

4.19836

0

0.244634

0.0501388

0

0.163089

0

0

0

0

0.0581004

0

0

0.244634

0

0

0

0

0.550126

0

0.332492

19.8798

0

0

0

0.244634

0

0.588255

0

0

0

0

0.290502
4

0

0.268152

0.589935

0.163089

0

0

0.126952

0

0

0

0.644824

0.160891

0.174301

0

0

0.126952

0

0.0581004

0

0

0.0511526

0

0

0

0

0

1.12624

0.0581004

0

0.163089

0

0.0511526

0.750827

0

0

0

0

0

0.196734

0.214522

0

0.107261

0

1.12625

0

0

0.163089

0

0

0.196734

0

0

0.107261

0.163089

13.9739
4

0

0

0

0.107261

0

0.0767289

0.0581004

0.134829
3

0.253786

1.07121
4

0

0.411256
4

0.909713

0

0.642971
4

0.359823

7.12231
4

0.326179

1.21508

1.09409
4

0.334707

1.3004

0.0581004

0

1.91203
4

3.04358

0

0

1.98513

0.296221

3.69714

0

1.37177

0

0.271328

0.377331

4.12636

0.44226

0.126952

0

1.59329

0

0

0

0

0

0

0

2.43315

0

0.8226

0.354985

0.587457

2.07226

0

0.163089

0.272622

0.922284

0.607149

0.341474

28.0209
4

0

2.38353

0

0.268152

3.69923

0

0

0

0

0.457132

8.67273
3

0.163089

0.477586

0.230187

0

0.0871506

0.163089

0

0.163089

0.741713

0

22.3275
4

0.326179

0.524348

0

0.451569

0.107261

0

0

0.177513

0

0.53913

8.28772
4

0.375413

0

0

0

0.423173

0

0

0

0

0.268152

44.6767
4

0

0

0.244634

0.214522

0

0.451965

0.816609

0.399371

0

1.06008

0.0581004

8.37176
4

0.442379

0

0

0.230187

0.0556337

0

0.711404

0.107261

0

0

0

0.590203

0.370833

0

0

0.465491

0

0

0

6.0343

0

6.61111
4

0.169269

5.2982

0

0

0

0.0581004

0

0

0

0

0

0

0

0

0

0

0.373475

0.0501388

0.211587

0

0

4.79645
4

0

0

0

0

0.268152

0

0.145251

0

0

0.203351

74.9013
4

0

0.321783

0.0871506

0

0

0

9.10424

0

0

0.163089

4.51874
4

0

0

2.33819

0

0

0

0

0

0.163089

0

4.39578
4

0.214103

0

0

0

0.429044

0

0

0.643566

0

0.438165

18.1077
4

0

0.167353

0

0

0.522904

0.125347

0

0

0

0

1.72277

17.6336
4

0

0.163089

1.03417

0

0

0

0.723043

0

0

0.448735

15.9811
4

0.965349

0.326179

0

0

0

0

0

0.255763

0.160891

0.0501388

0

0

0.0581004

0.107261

0

0.482674

0

0

0

0.326179

0.0511526

0.326179

0.379883

1.17317

0.489268

0

0

0

0.163089

0

0.0846347

0.361165

4.70634
4

0

0

0

0

0.0846347

0.153458

0

0.174301

0

0

0

0

0.261452

0

0

0

1.00205

0

0

1.38144

0

0

0.591494

0.204154

0.675876

0

0.0511526

0.107261

0

0.407723

0

0

5.37159
4

0.967856

0.295101

0

0

0

0

0

0.375413

0.407723

4.63884

0

0.134773

0.125347

0

0

0

0

0.107261

0.107261

0

0

0

0

0

0.489268

0.393468

0.196734

0.107261

0

0

0.0767289

0.281339

14.5358
6

6.70826
3

0.0581004

0

0.116201

0

0.107261

0.196734

0

0

0.0501388

0.0581004

0

0

0

0

0

0.160891

1.07421

0.0846347

0

0

0

4.9399
3

0.163089

0.175486

0

0.0511526

0

0

0.196734

0

0

0

6.07795
4

0

0.163089

0.160891

0

0

0

0

0.911718

0.0871506

0

0

0

0.0752082

0

0

0

0

0

0

0

0

28.1305
4

0.261452

0

0.126952

0

0.858088

0.295101

0

0.160891

0

0

0

0

0

0

0.0846347

0.05102

0.196734

0.407723

0

0.107261

0

2.35222
4

0

0.163089

0

0.0581004

0

0

0

0.0767289

0

0

3.19119
4

0.100278

0

0

0

10.6236

0

0

0

0.244634

0.326179

0

0

0.0846347

0

0.107261

0

0

0

0.107261

0.107261

0

0.160891

0

0

0.196734

0

0

0

0

0

0

0

0

0.643566

0.179034

0.214522

0.100278

0

0.244634

0

0.196734

0

0

0

7.47199
4

0

0

0

0

0

0

0

0.196734

0.407723

0

2.55901
4

0

0

0

0

1.22011

0

0

0.321783

0

0

0

0

0

0

0

0

0

0

0.145251

0.0511526

0

1.32677
4

0

0.196734

0

0

0

0.29211

0

0

0

0.0511526

2.65375
3

0

0

0.163089

0.107261

0.491835

0

0

0

0.116201

0

0.678445
3

0

0.0511526

0.885304

0

0

0

0

0.126952

0.107261

0.174301

0

0.196734

0.196734

0

0.126952

0

0

0

0

0

0

2.16627
4

0.0581004

0

0

0

0.107261

0

0

0.0846347

0

0.917956

260.558
4

0

0

0

0

0.0501388

0

0

0

0.375413

0.0581004

0

2.02053

0

0.152774

0

0.677078

0.196734

0

0

0.268152

0

0

0

0.163089

0.0767289

0

0

0

0

1.34076

0

0

0.102305

1.0004
4

0

0

0.107261

0

0.107261

0

0

0.160891

0

0

3.61862
4

0

0.163089

0

0

0

0

0

0

0

0

0

0

0.326179

0.0846347

0

0.107261

0

0

0

0.0846347

0

0

0

0

0

0.332492

0.375413

0

0.438165

0.127882

0.0846347

0.126952

12.94
4

0

0

0.214522

0

0

0

0

0

0

0.196734

0

0.0581004

0

0.0767289

0

0

0.393468

0

0

0

0.244634

0

0

0.261452

0

0.184686

0

0.0846347

0

0

0

0

15.4669
4

3.8318
4

0.163089

0

0.0846347

0

0

0

0

0

0

0

0

0

0.196734

0

0

0

0

0

0

0.375413

0.177448

9.44622
4

0.0511526

0

0

0.0871506

0

0.107261

0

0

0

0

0

0

0.118299

0

0.0846347

0

1.17987

0.107261

0

0

0

0

0

0

0.407723

0

0.160891

0

0

0

0

0

18.8814
3

0.393468

0.0846347

0

0

0.163089

0

0

0

0.107261

0

0

0

0

0.107261

0

0

0

0

0.161286

0

0

0.915879
4

0.0581004

0.163089

0.163089

0

0.160891

0

0

0

0

0

0

0

0

0

0.244634

0

0

0.375413

0

0

0.196734

2.98962
4

0

0.163089

0

0

0

0

0

0.232402

0

0

153.161
3

0

0

0.244634

0

0

0.203351

0

0

0

0

0

8.44018

0

0

0

0

0

0.123124

0

0

0

0

0

2.03796

0.169269

0

0

0

0.107261

0

0

0

0.0767289

0

0

0.196734

0

0

0

0

0

0

0

0

0.0846347

0.0511526

0

0

0.163089

0

0.438165

0

0

0

0

3.19622

0

0.423173

0.0752082

0

0.179034

0.0581004

0

0

0

0.0834505

0

0

0

0

0

0

0

0.107261

0

0

0

9.51619
4

0

0.0556337

0

0.0501388

0.163089

0

0

0.0581004

0

0.0846347

0

0.0846347

0.169269

0

0.05102

0

0

0.0581004

0

0.244634

0

0

0

0

0

0

0

0

0

0.0846347

0

0

3.96492

0

0

0

0

0

0

0

0

0

0

0

1.02222

0.179034

0

0.0511526

0

0

0

0

0

0

0

0.375413
4

0

0.652357

0.153458

0

0

0

0.815446

0

0

0

0.560466

0.163089

0

0.0752082

0.107261

0.0501388

0.429044

0

0.326179

0

0.0846347

4.94755
4

0

0.214522

0

0

0.0511526

0

0

0.107261

0.163089

0

0

0

0

0

0

0.107261

0

0

0

0

0

0

0

0.107261

0

0

0

0

0.473196

0

0.0511526

0

0

0

0

0

0

0.0581004

0

0.0752082

0.0846347

0.160891

0.179034

0

0

0

0

0

0

0.163089

0

0.319552

0

0

0

0.0846347

0.107261

0

0

0

0

0.160891

0

0

0.0752082

0
4

0.161286

0

0.161286

0
4

91.1768
4

0

90.6428
4

0

0.241929

0.29211

3.1641356201817e-15
4

0
4

4.3433

1.90875
5

1.82576

0

0.0553261

0

0.0553261

0

0

0.163089

0.138315

0

0

0

0.196734

0

1.66533453693773e-16

0
4

2.03796

2.03796

0
4

6.24083

6.24083

0
4

0

0

0
4

0

0

0
4

0

0

0
4

0

0

0
4

0

0

0
4

0

0

0
4

4.34390301506937e-11
5

0
4

2.77029

2.77029

2.77029

0
4

0
4

0

0

0

0
4

0
4

0.29211

0.29211

0.29211

0
4

0
4

2.28325

2.28325

2.28325

0
4

0
4

40.7841
4

40.6658
4

0

0

40.6658
4

0
4

0

0

0

0
4

0

0

0
4

0.118299

0.118299

0
4

2.83106871279415e-15
4

0
4

1.4317436125566e-11
5

0
4

1.10681
3

0

0

0

0

0

0

0
4

0
4

0

0

0

0
4

0
4

0

0

0

0
4

0
4

0

0

0

0

0
4

0
4

0

0

0

0

0
4

0
4

0.806238

0.806238

0.806238

0
4

0
4

0.123124

0.123124

0.123124

0
4

0
4

0.177448

0.177448

0.177448

0
4

0
4

0

0

0

0
4

0
4

0

0

0

0
4

0
4

0

0

0

0
4

0
4

0
4

5.01858

5.01858

5.01858

1.2928

0

0

1.30129

0

0

0

0.392951

0

0

0

0
1

0

0

0

0

0

0

0

0

0

0

0

0

0

0

0

0

0

0

0

0

0

1.90842

0

0

0

0

0

0

0

0

0

0

0.123124

0

0
4

0
4

0

0

0

0
4

0
4

0
4

4.81789

4.81789

4.81789

0
7

0

0

0

0

0

0

0

0

0

0

0
6

0

0

0

0

2.53248

0

0

0

0

0

0

0

0

0.0926517

0

0

0

0

0

0

0

0

0

0

0

0

0

0

0

0

0

0.0617678

0
6

0

0

0

0

0

0

0

0

0

2.13099

0

0
4

0

0

0

0

0

0

0
4

0

0

0
4

0

0

0

0
4

0

0

0

0
4

0

0

0
4

0

0

0
4

0
4

0

0

0

0
4

0
4

0

0

0

0
4

0
4

0

0

0

0
4

0
4

0
4

0
7

0
7

0
7

0
7

0

0

0

0

0

0

0

0

0

0

0
6

0
7

0

0

0

0

0

0

0
4

0
6

0

0

0

0
4

0

0

0

0
4

0
4

0
4

0
7

0
7

0
7

0

0

0

0

0

0
4

0
4

0
4

0

0

0

0

0
4

0
4

0
4

0

0

0

0

0
4

0
4

0
4

0

0

0

0

0
4

0
4

0
4

1.01412

1.01412

1.01412

1.01412

0
4

0
4

0
4

0

0

0

0

0
4

0
4

0
4

0

0

0

0

0
4

0
4

0
4

0

0

0

0

0
4

0
4

0
4

0

0

0

0

0
4

0
4

0
4

0

0

0

0

0
4

0
4

0
4

0

0

0

0

0
4

0
4

0
4

0
7

0
7

0
7

0
7

0
7

0

0
4

0

0

0
4

0
4

0
4

0

0

0

0

0
4

0
4

0
4

0

0

0

0

0
4

0
4

0
4

0

0

0

0

0
4

0
4

0
4

0

0

0

0

0
4

0
4

0
4

0.123124

0.123124

0.123124

0.123124

0
4

0
4

0
4

0

0

0

0

0
4

0
4

0
4

0

0

0

0

0
4

0
4

0
4

0

0

0

0

0
4

0
4

0
4

0.501388

0.501388

0.501388

0.501388

0
4

0
4

0
4

0

0

0

0

0
4

0
4

0
4

0

0

0

0

0

0

0
4

0

0

0
4

0
4

0
4

0.107261

0.107261

0.107261

0.107261

0
4

0
4

0
4

0

0

0

0

0
4

0
4

0
4

0

0

0

0

0
4

0
4

0
4

0

0

0

0

0
4

0
4

0
4

0

0

0

0

0
4

0
4

0
4

0

0

0

0

0
4

0
4

0
4

0

0

0

0

0
4

0
4

0
4

0.763337

0.763337

0.763337

0.763337

0
4

0
4

0
4

0

0

0

0

0
4

0
4

0
4

0

0

0

0

0
4

0
4

0
4

0
7

0
7

0
7

0

0

0

0
4

0

0

0

0

0
4

0
4

0
4

0

0

0

0

0
4

0
4

0
4

0

0

0

0

0
4

0
4

0
4

0

0

0

0

0
4

0
4

0
4

0

0

0

0

0
4

0
4

0
4

0

0

0

0

0
4

0
4

0
4

0.177448

0.177448

0.177448

0.177448

0
4

0
4

0
4

0

0

0

0

0
4

0
4

0
4

0.107261

0.107261

0.107261

0.107261

0
4

0
4

0
4

0

0

0

0

0
4

0
4

0
4

0

0

0

0

0
4

0
4

0
4

0

0

0

0

0

0

0

0
4

0

0

0

0
4

0
4

0
4

0

0

0

0

0
4

0
4

0
4

0

0

0

0

0
4

0
4

0
4

0.0752082

0.0752082

0.0752082

0.0752082

0
4

0
4

0
4

0

0

0

0

0
4

0
4

0
4

0

0

0

0

0
4

0
4

0
4

0

0

0

0

0
4

0
4

0
4

0

0

0

0

0
4

0
4

0
4

0.246248

0.246248

0.246248

0.246248

0
4

0
4

0
4

0

0

0

0

0
4

0
4

0
4

0

0

0

0

0
4

0
4

0
4

0

0

0

0

0

0

0
4

0
4

0
4

0

0

0

0

0
4

0
4

0
4

0

0

0

0

0
4

0
4

0
4

0

0

0

0

0
4

0
4

0
4

0

0

0

0

0
4

0
4

0
4

0

0

0

0

0
4

0
4

0
4

0

0

0

0

0
4

0
4

0
4

0

0

0

0

0
4

0
4

0
4

0

0

0

0

0
4

0
4

0
4

0

0

0

0

0
4

0
4

0
4

0

0

0

0

0
4

0
4

0
4

0

0

0

0

0

0

0

0
4

0
4

0
4

0

0

0

0

0
4

0
4

0
4

0

0

0

0

0
4

0
4

0
4

0
7

0
7

0
7

0

0

0

0
4

0
4

0
4

0.0829891

0.0829891

0

0

0

0

0
4

0.0829891

0.0829891

0
4

0
4

0
4

0.451249

0.451249

0.225625

0.225625

0
4

0.225625

0

0.125347

0.100278

0
4

0

0

0
4

0
4

0
4

0
7

0
7

0
7

0
7

0

0

0

0
4

0
4

0

0

0

0
4

0
4

0
4

1.12275

1.12275

1.12275

0.479183

0.107261

0.536305

1.11022302462516e-16

0
4

0
4

0
4

0

0

0

0

0

0
4

0
4

0
4

0

0

0

0

0
4

0
4

0
4

0

0

0

0

0
4

0
4

0
4

0

0

0

0

0
4

0

0

0
4

0
4

0

0

0

0
4

0

0

0
4

0
4

0
4

0

0

0

0

0

0
4

0
4

0
4

0

0

0

0

0

0
4

0
4

0
4

0

0

0

0

0

0
4

0
4

0
4

1.87707

1.87707

1.87707

1.87707

0
4

0
4

0
4

10.6835

10.6835

10.6835

10.6835

0
4

0
4

0
4

1.13573
2

1.13573
2

0.821594
2

0.821594
2

0

0

0

0

0
4

0.314139
2

0.314139

0

0

0

0

0

0
4

0

0

0
4

0

0

0
4

5.55111512312578e-17
2

0
4

0
4

0

0

0

0

0

0

0
4

0
4

0
4

0

0

0

0

0
4

0
4

0
4

0

0

0

0

0
4

0

0

0
4

0

0

0
4

0
4

0
4

0

0

0

0

0

0
4

0
4

0
4

0.217481

0.217481

0.217481

0.0724936

0

0.0724936

0.0724936

0
4

0
4

0
4

0

0

0

0

0
4

0
4

0
4

0.42618

0.300833

0

0

0

0
4

0.300833

0.300833

0
4

0
4

0.125347

0.125347

0.125347

0
4

0
4

0
4

0

0

0

0

0

0
4

0

0

0
4

0
4

0
4

0

0

0

0

0
4

0
4

0
4

0

0

0

0

0

0

0
4

0
4

0
4

0

0

0

0

0

0

0

0

0

0
4

0
4

0
4

0

0

0

0

0

0
4

0

0

0
4

0
4

0
4

0

0

0

0

0
4

0
4

0
4

0

0

0

0

0
4

0
4

0
4

0

0

0

0

0
4

0

0

0
4

0
4

0
4

3.07857

3.07857

2.9713

2.9713

0

0
4

0.107261

0.107261

0
4

0
4

0
4

0

0

0

0

0

0
4

0
4

0
4

0

0

0

0

0

0
4

0
4

0
4

0

0

0

0

0
4

0
4

0
4

0

0

0

0

0

0
4

0
4

0
4

0

0

0

0

0

0
4

0

0

0
4

0
4

0
4

19.2782
4

19.2782
4

18.9703
4

17.2419

1.23593

0.123124

0

0.184686

0.184686

0
4

0.30781

0.30781

0
4

0
4

0
4

0.507808

0.507808

0.507808

0.507808

0
4

0
4

0

0

0

0
4

0
4

0
4

1.02785

1.02785

0.927568

0.927568

0
4

0.100278

0.100278

0
4

0

0

0
4

0
4

0
4

0

0

0

0

0

0
4

0
4

0
4

0

0

0

0

0

0
4

0
4

0
4

0

0

0

0

0

0
4

0
4

0
4

0.0724936

0.0724936

0.0724936

0.0724936

0
4

0

0

0
4

0
4

0
4

0

0

0

0

0
4

0

0

0
4

0
4

0
4

0

0

0

0

0
4

0
4

0
4

0

0

0

0

0
4

0

0

0
4

0
4

0
4

0

0

0

0

0
4

0

0

0
4

0
4

0
4

0
7

0
7

0
7

0

0

0

0

0

0

0

0

0
4

0

0

0

0
4

0
4

0
4

0

0

0

0

0
4

0
4

0
4

0.0642886

0.0642886

0.0642886

0.0642886

0
4

0
4

0
4

0

0

0

0

0

0
4

0
4

0
4

0

0

0

0

0
4

0

0

0
4

0
4

0
4

0

0

0

0

0
4

0
4

0
4

0.200555

0.200555

0.125347

0.125347

0
4

0.0752082

0.0752082

0
4

0
4

0
4

0.460464

0.460464

0.185812

0.185812

0
4

0.274652

0.274652

0
4

0
4

0
4

0.120823

0.120823

0.120823

0.0483291

0.0724936

0
4

0
4

0
4

0

0

0

0

0
4

0

0

0
4

0
4

0
4

0

0

0

0

0

0
4

0
4

0
4

0.230426

0.128577

0.128577

0

0.128577

0

0

0

0

0
4

0

0

0
4

0
4

0.101849

0.101849

0.101849

0

0
4

0
4

0
4

0

0

0

0

0
4

0
4

0
4

0

0

0

0

0
4

0

0

0
4

0
4

0
4

0

0

0

0

0
4

0
4

0
4

0.175486

0.175486

0.175486

0.125347

0.0501388

0
4

0
4

0
4

0

0

0

0

0
4

0
4

0
4

0

0

0

0

0
4

0

0

0
4

0
4

0
4

0.100278

0.100278

0.100278

0.100278

0
4

0
4

0
4

0

0

0

0

0
4

0

0

0
4

0
4

0
4

0.196734

0.196734

0

0

0
4

0.196734

0.196734

0
4

0
4

0
4

0

0

0

0

0

0
4

0
4

0
4

1.48653
5

1.48653
5

0.521018
5

0.12755
5

0.393468

0

0
4

0.965507

0.786937

0.05102

0

0.12755

5.55111512312578e-17

0
4

0
4

0
4

0

0

0

0

0

0
4

0
4

0
4

0

0

0

0

0

0
4

0
4

0
4

0

0

0

0

0

0
4

0
4

0
4

0

0

0

0

0
4

0
4

0
4

0

0

0

0

0
4

0

0

0
4

0
4

0
4

0

0

0

0

0
4

0
4

0
4

0

0

0

0

0
4

0
4

0
4

0

0

0

0

0
4

0
4

0
4

0

0

0

0

0
4

0

0

0
4

0
4

0
4

0

0

0

0

0
4

0
4

0
4

0
7

0
7

0
7

0
7

0

0
4

0

0

0

0
4

0

0

0
4

0
4

0

0

0

0
4

0
4

0
4

0.0483291

0.0483291

0.0483291

0

0.0483291

0
4

0
4

0
4

0

0

0

0

0
4

0
4

0
4

0

0

0

0

0
4

0
4

0
4

0

0

0

0

0
4

0
4

0
4

0

0

0

0

0
4

0
4

0
4

0

0

0

0

0
4

0
4

0
4

0

0

0

0

0
4

0
4

0
4

0

0

0

0

0
4

0
4

0
4

0

0

0

0

0
4

0
4

0
4

0.0642886

0.0642886

0.0642886

0.0642886

0
4

0
4

0
4

28.2105

28.2105

27.2255

0

0

0

0

0

0

0

0.325902

0

1.02785

0

0

4.23637

0

0

0.576596

0

0

0

0

0

0

6.16832
4

0

0

4.19836

0

0.250694

0

0

0

0

0.0501388

1.82562

0

0

0

0

0

0

0

0

0

0

0

0

0

0

0

0

0

1.0487

0

0

0

0

0

0

0

0.763337

0

0

0

0

0

0

0

0

0.107261

0.0501388

0

0

0

1.05291

0

0

0

1.0391

0

0

2.29001

0

0

1.60444

0

0.274652

0.335102

0
4

0

0

0

0

0

0
4

0

0

0
4

0

0

0
4

0

0

0
4

0

0

0
4

0

0

0
4

0

0

0

0

0
4

0

0

0

0
4

0

0

0

0

0
4

0

0

0

0

0
4

0

0

0

0
4

0.984991

0.984991

0
4

0

0

0
4

0

0

0
4

0
4

0

0

0

0

0

0

0
4

0
4

0

0

0

0

0

0
4

0

0

0
4

0
4

0

0

0

0

0
4

0
4

0

0

0

0
4

0
4

0

0

0

0
4

0
4

0
4

0

0

0

0

0
4

0
4

0
4

8.86934969912545e-11

0
4

175.531
3

174.635
3

124.187
3

0.123124

0

0

0

0

0

0.123124

0

0

0

0

0

0
4

99.1757
3

99.1757
3

0

0

0
4

14.5157

14.5157

0
4

6.9331

3.4241

3.25431

0.0565967

0

0.0848951

0.0565967

0.0565967

0

1.66533453693773e-16

0
4

0

0

0

0

0

0
4

0

0

0
4

0

0

0
4

0

0

0
4

0.185812

0.185812

0
4

0

0

0

0
4

1.30172

1.30172

0
4

0.650343

0.464531

0.185812

0
4

0

0

0

0
4

0

0

0
4

0

0

0

0
4

1.30172

1.30172

0
4

0

0

0
4

0
4

24.1556

24.1556

13.9359

4.36659

4.92403

0.557437

0.371625

0

0
4

0
4

26.2924

26.2924

25.0847

1.20778

1.55431223447522e-15

0
4

0
4

0

0

0

0
4

0
4

0

0

0

0
4

0
4

7.105427357601e-15
3

0
4

0

0

0

0

0

0

0
4

0
4

0
4

0

0

0

0

0
4

0
4

0
4

0

0

0

0

0
4

0
4

0
4

0

0

0

0

0
4

0
4

0
4

0

0

0

0

0
4

0
4

0
4

0.369371

0.369371

0.369371

0.369371

0
4

0
4

0
4

0

0

0

0

0
4

0
4

0
4

0

0

0

0

0
4

0
4

0
4

0.05102

0.05102

0.05102

0.05102

0
4

0
4

0
4

0

0

0

0

0
4

0
4

0
4

0.375413

0.375413

0.375413

0.107261

0.268152

0
4

0
4

0
4

0

0

0

0

0

0
4

0
4

0
4

0

0

0

0

0
4

0
4

0
4

0

0

0

0

0
4

0
4

0
4

0

0

0

0

0
4

0
4

0
4

0

0

0

0

0
4

0
4

0
4

0.100278

0.100278

0.100278

0.100278

0
4

0
4

0
4

6.07847105982273e-15
3

0
4

867.974

433.527
4

209.199

200.264

27.639

0.482674

2.30611

0.545426

1.60891

0.268152

1.23691

0.429044

0.107261

5.14853

0.107261

136.484

0.107261

0.160891

0.29211

0.107261

0.482674

0.107261

0.160891

0.858088

0.107261

1.82344

3.74842

0.107261

0.804457

0.268152

0.321783

5.22611

4.12724

1.12052

0.429044

0.375413

3.1642

0
4

1.9307

1.34076

0.482674

0.107261

6.93889390390723e-17

0
4

0.429044

0.268152

0.160891

0
4

6.576

5.84572

0.730274

4.44089209850063e-16

0
4

0
4

0.0501388
4

0

0

0

0

0

0
4

0.0501388

0

0.0501388

0

0
4

0

0

0
4

0

0

0
4

0
4

90.6058

89.992

88.8121

0.160891

1.01898

6.21724893790088e-15

0
4

0.506632

0.399371

0.107261

0
4

0.107261

0.107261

0
4

1.50573997714787e-14

0
4

0
6

0
6

0
6

0
4

0
4

0

0

0

0
4

0

0

0
4

0
4

1.00919

0.07653

0

0.07653

0
4

0.881636

0.763337

0.118299

5.55111512312578e-17

0
4

0.05102

0.05102

0
4

0
4

0

0

0

0

0
4

0
4

0.0501388

0.0501388

0

0.0501388

0

0
4

0
4

0.370607

0.185303

0.0617678

0.123536

0
4

0.185303

0.185303

0
4

0
4

0

0

0

0
4

0
4

0

0

0

0
4

0

0

0
4

0
4

1.07798
4

0.952637
4

0.752082

0

0

0

0

0

0

0

0

0

0

0

0.200555

0

0

0

0

5.55111512312578e-17
4

0
4

0.125347

0.125347

0

0

0

0

0
4

0

0

0

0

0

0

0

0
4

0
4

1.10537

1.10537

1.10537

0
4

0
4

0

0

0

0
4

0
4

0

0

0

0
4

0
4

0

0

0

0
4

0
4

0

0

0

0
4

0
4

0

0

0

0
4

0
4

0

0

0

0
4

0
4

18.4883
4

15.8622
4

4.27685

0.241929

1.44915

1.46904

0.05102

0.514309

0.161286

6.9353

0.763337

0

0
4

2.02959

2.02959

0
4

0.596446

0.158281

0.438165

0
4

0

0

0
4

3.66373598126302e-15
4

0
4

3.14625
4

1.45274
4

0.813197

0

0.58422

0.0553261

0
4

0

0

0

0

0
4

1.6935

1.6935

0
4

0

0

0
4

0
4

30.6778
4

30.6778
4

16.1487

8.11194
4

3.54759

2.13793

0.05102

0.161286

0.161286

0.161286

0.196734

3.05311331771918e-15
4

0
4

0
4

71.5374

68.7246

66.8772

0.107261

0.429044

0.482674

0.536305

0.29211

5.38458166943201e-15

0
4

0.989306

0.667523

0.160891

0.160891

0
4

1.39439

0.804457

0.107261

0.321783

0.160891

5.55111512312578e-17

0
4

0.107261

0.107261

0
4

0.321783

0.321783

0
4

1.72639680329212e-14

0
4

0
6

0
6

0
6

0

0

0
4

0
4

1.35375

0

0

0

0

0

0

0

0

0

0
4

0

0

0

0
4

1.35375

1.35375

0

0

0
4

0

0

0
4

0
4

4.85465

4.79288

2.69023

0.958419

0.835392

0.0926517

0.216187

0
4

0.0617678

0.0617678

0
4

3.46944695195361e-17

0
4

0
4

54.1804
5

54.1804
5

0

0

0
4

54.1804
5

14.8962
5

36.3794

0.697196

0.743249

0

0

0.663913

0

0

0.0617678

0

0.738743

0
4

0
4

0
4

67.0789
7

0
6

0
6

0

0

0
4

0

0

0
4

0
4

4.43246

0

0

0
1

0

0

0

0

0

0

0

0

0

0

0

0

0

0

0

0

0

0

0

0

0

0

0

0

0

0

0

0

0

0

0

0

0

0

0

0

0

0

0

0

0

0

0

0

0

0

0

0

0

0

0

0

0

0

0

0

0

0

0

0

0

0

0

0

0

0

0
4

0

0

0

0

0

0

0

0

0

0

0

0
4

1.84686

1.60061

0

0

0

0

0

0

0

0

0

0

0

0

0

0

0

0

0

0

0

0

0

0

0

0

0

0

0

0

0

0

0

0

0

0

0

0

0

0

0

0

0

0

0

0

0

0

0

0

0

0

0

0

0

0

0

0

0

0

0

0

0

0

0

0

0

0

0

0

0

0

0

0

0.246248
6

0

0

0

0

0

0

0

0

0

0

0
7

0

0

0

0

0

0

0

0

0

0

0
8

0

0

0

0

0

0

0

0

0

0

0

0

0

0

0

0

0

0

0

0

0

0

0

0

0

0

0

0

0

0

0

0

0

0

0

0

0

0

0

0

0

0

0

0
7

0

0

0

0

0

0

0

0

0

0

0

0

0

0

0

0

0

0

0

0

0

5.55111512312578e-17

0
4

0
6

0

0

0

0

0

0

0
4

0

0

0
4

0
7

0

0

0
4

0

0

0

0

0
4

0

0

0

0

0
4

0

0

0

0

0
4

0

0

0

0
4

0

0

0
4

0

0

0

0
4

0

0

0

0
4

2.27779

2.27779

0

0

0

0

0

0

0

0

0

0

0
4

0

0

0
4

0

0

0

0
4

0

0

0
4

0

0

0

0
4

0

0

0
4

0

0

0
4

0

0

0
4

0

0

0
4

0

0

0
4

0

0

0
4

0

0

0

0

0

0

0

0

0
4

0

0

0
4

0

0

0
4

0

0

0
4

0

0

0
4

0

0

0
4

0

0

0
4

0

0

0
4

0

0

0
4

0

0

0
4

0

0

0
4

0

0
7

0

0

0

0
7

0

0

0

0

0

0

0
4

0

0

0
4

0

0

0
4

0

0

0
4

0

0

0
4

0

0

0
4

0

0

0
4

0

0

0
4

0

0

0
4

0

0

0
4

0

0

0
4

0.30781

0

0

0

0

0

0

0
6

0

0

0

0

0

0.30781

0

0
4

0

0

0
4

0

0

0
4

0

0

0
4

0

0

0

0

0

0

0

0

0

0

0
4

0

0
7

0

0

0

0

0

0
4

0

0

0

0

0

0

0
4

0

0

0

0

0

0

0

0

0
4

0
4

59.1471
7

1.14501
6

0
6

0
7

0

0

0

0

0

0

0

0

0

0

0
6

0

0

0

0

0

0

0

0

0

0

0

0

0

0

0

0

0

0

0

0

0

0

0

0

0

0

0

0

0

0

0

0

0

0

0

0

0

0

0

0

0

0

0

1.14501
5

0

0

0

0

0

0

0

0

0

0

0
6

0

0

0

0

0

0

0

0

0

0
6

0
6

0
4

0
7

0
7

0

0

0

0
4

37.7904
7

2.63479
7

31.8033
7

0

0

0

0

0

0

0

0

0

0

0

0

0

0

0

1.52667

0

0

0

0

0

0

0

0

0

0

0

0

0

0

0

0

0

0

0

0

0

0

1.82566

0

0

0

1.22124532708767e-14
7

0
4

0
7

0

0

0

0

0

0
4

16.3072

15.869

0.438165

0

0

8.32667268468867e-16

0
4

0
7

0
7

0

0

0

0

0

0

0

0

0

0

0

0
7

0

0

0

0

0

0

0

0

0

0

0
6

0

0

0

0

0

0

0

0

0

0

0

0

0
4

0
6

0
6

0

0

0

0
4

0
6

0
6

0

0

0

0

0

0
4

3.44747
6

0
7

0
6

0
7

0

0

0

0

0

0

0

0

0

3.13966

0

0

0

0.30781

0

0

0

0

0

0

0
7

0

0

0

0

0

0

0

0

0
6

0
7

0
6

0
7

0
7

0
6

2.22044604925031e-16
6

0
4

0
7

0
7

0

0

0

0

0

0

0

0
4

0

0

0

0

0

0

0

0

0

0

0

0

0
4

0
6

0
6

0

0
4

0
6

0
6

0

0

0

0

0
4

0

0

0

0
4

0

0

0
4

0

0

0

0
4

0

0

0
4

0

0

0
4

0

0

0
4

0

0

0
4

0

0

0
4

0

0

0
4

0

0

0
4

0
7

0
7

0

0

0

0

0
4

0

0

0
4

0

0

0
4

0

0

0
4

0

0

0
4

0

0

0
4

0

0

0
4

0.349789

0.349789

0
4

0

0

0
4

0

0

0
4

0

0

0
4

0
7

0
7

0

0
4

0

0

0
4

0

0

0
4

0

0

0
4

0

0

0
4

0

0

0
4

0

0

0
4

0

0

0
4

0

0

0
4

0

0

0
4

0.107261

0.107261

0
4

0

0

0

0

0

0
4

0

0

0
4

0

0

0
4

0

0

0
4

0

0

0
4

0

0

0
4

0
7

0
7

0
4

0

0

0
4

0

0

0
4

0

0

0

0
4

0

0

0
4

0
4

0
7

0
6

0

0
6

0

0

0

0
4

0

0

0

0
4

0
4

0.0642886
7

0

0

0
4

0.0642886
8

0
8

0.0642886

0
4

0

0

0

0
4

0

0

0
4

0

0

0
4

0

0

0
4

0

0

0
4

0

0

0
4

0

0

0
4

0

0

0
4

0
4

3.43502

0

0

0

0
4

0

0

0

0

0

0

0
4

0

0

0
4

0

0

0
4

0

0

0
4

0

0

0
4

2.29001
5

2.29001
5

0

0
4

0
7

0

0

0
4

0

0

0
4

0

0

0

0
4

0

0

0

0
4

0

0

0

0
4

1.14501

1.14501

0
4

0

0

0
4

2.22044604925031e-16

0
4

0
7

0
7

0

0

0

0
6

0

0
4

0
4

0

0

0

0
4

0
4

0

0

0

0
4

0
4

0

0

0

0
4

0
4

0

0

0

0
4

0
4

0

0

0

0

0

0
4

0
4

0

0

0

0
4

0
4

0

0

0

0

0
4

0
4

0

0

0

0
4

0
4

0

0

0

0
4

0
4

0

0

0

0
4

0
4

0

0

0

0
4

0
4

0

0

0

0
4

0
4

5.32907051820075e-15
7

0
4

0.184686

0

0
7

0

0

0

0

0

0

0

0

0

0

0

0
6

0

0

0

0

0

0

0

0

0

0

0

0
4

0

0

0
4

0

0
7

0

0

0

0
4

0

0

0

0
4

0

0

0
4

0

0

0
4

0

0

0
4

0

0

0
4

0
4

0

0

0

0

0

0

0

0

0
4

0
4

0

0

0

0
4

0
4

0

0

0

0
4

0
4

0

0

0

0
4

0
4

0

0

0

0

0
4

0
4

0

0

0

0
4

0

0

0
4

0
4

0

0

0

0
4

0
4

0

0

0

0
4

0

0

0
4

0
4

0.184686

0.184686

0.184686

0
4

0
4

0

0

0

0
4

0
4

0

0

0

0
4

0
4

0

0

0

0
4

0
4

0
4

7.95763

5.40467

3.25

0
6

0

0

0

0

0

0

0

0

0

0

0
6

0

0

0

0

0

0

0

0

0.68857

0

0

0

2.56143

0

0
4

2.15467

2.15467

0
4

0

0

0
4

0

0

0
4

0

0

0
4

0
4

0

0

0

0

0
4

0
4

0

0

0

0
4

0
4

0.554057

0.554057

0.554057

0
4

0
4

0

0

0

0
4

0
4

0

0

0

0
4

0
4

0.246248

0.246248

0.246248

0
4

0
4

1.75266

1.75266

1.75266

0
4

0
4

0

0

0

0
4

0
4

0

0

0

0
4

0
4

2.22044604925031e-16

0
4

0.185303

0

0

0

0

0
4

0
4

0.185303

0

0

0
4

0
1

0

0

0

0

0
4

0

0

0

0

0
4

0.185303

0.185303

0
4

0
4

0

0

0

0

0
4

0

0
1

0

0

0
4

0
4

0
4

22.6175
5

22.6175
5

1.54505

0.278718

0
7

0

0

0

1.16968

0

0

0

0.0966581

0

0

0

0

0

0

6.93889390390723e-17

0
4

0
6

0
6

0
7

0
6

0

0

0

0

0

0
4

0.184686

0.184686

0
4

0.175486

0.175486

0
4

0.185812

0.185812

0
4

0.185812

0.185812

0
4

0

0

0
4

0

0

0
4

0
6

0
6

0

0
4

11.033

2.79571

0.929061

0.650343

0.15442

6.50343

0
4

0

0

0

0

0
4

9.09147

1.4865

0.185812

7.41916

0
4

0.216187

0.123536

0.0926517

0
4

0

0

0
4

0

0

0
4

0

0

0
4

1.66533453693773e-15
5

0
4

0

0

0

0

0
4

0
4

0

0

0

0

0
4

0
4

0

0

0

0
4

0
4

0

0

0

0
4

0
4

0

0

0

0
4

0
4

0

0

0

0
4

0
4

0

0

0

0
4

0
4

0
4

0

0

0

0

0

0

0

0

0

0

0

0
4

0

0

0

0

0

0
4

0

0

0

0

0

0
4

0

0

0
4

0

0

0
4

0
4

0
4

39.4888
3

39.4888
3

39.4888
3

39.4888
3

0

0
4

0
4

0
4

0
6

0
6

0
6

0
6

0

0
4

0

0

0
4

0
4

0
4

0

0

0

0

0

0

0

0
4

0

0

0

0
4

0
4

0
4

0

0

0

0

0

0

0
4

0
4

0
4

9.04339
4

9.04339
4

8.86594
4

8.13685
4

0.610794

0.118299

0
4

0.177448

0.177448

0
4

1.94289029309402e-16
4

0
4

0
4

0

0

0

0

0

0

0
4

0
4

0
4

0

0

0

0

0

0
4

0
4

0
4

0

0

0

0

0

0

0

0
4

0
4

0
4

0

0

0

0

0

0
4

0
4

0
4

0

0

0

0

0
4

0
4

0
4

0
6

0
6

0
6

0

0

0

0

0

0

0

0
4

0
4

0
4

0
6

0
6

0

0

0
4

0

0

0
4

0
4

0
4

0

0

0

0

0

0
4

0
4

0
4

0

0

0

0

0
4

0
4

0
4

0

0

0

0

0

0
4

0

0

0
4

0
4

0
4

1.9307

1.9307

1.9307

1.50165

0.429044

0
4

0
4

0
4

0

0

0

0

0

0

0
4

0
4

0
4

6.03108

6.03108

6.03108

5.26774

0.763337

0
4

0
4

0
4

0

0

0

0

0
4

0
4

0
4

0

0

0

0

0
4

0

0

0
4

0
4

0
4

0

0

0

0

0

0

0
4

0
4

0
4

23.0643
4

23.0643
4

17.1259
4

6.64034

8.41645

0.811629

0.643929

0.185812

0.185812

0.241929

0
4

5.93844
4

5.78853

0

0.149909

0
4

1.77635683940025e-15
4

0
4

0
4

0

0

0

0

0

0
4

0
4

0
4

0

0

0

0

0

0
4

0

0

0
4

0
4

0
4

0

0

0

0

0
4

0

0

0
4

0
4

0
4

0

0

0

0

0
4

0
4

0
4

0

0

0

0

0

0
4

0
4

0
4

0

0

0

0

0
4

0
4

0
4

0

0

0

0

0

0
4

0

0

0
4

0
4

0
4

0

0

0

0

0
4

0
4

0
4

0.649608

0.649608

0.649608

0.649608

0
4

0
4

0
4

0

0

0

0

0
4

0
4

0
4

27.7992
4

27.7992
4

16.113
4

3.44339

2.19082

8.17907

0.403215

0.289863

0

1.31449

0.29211

0
4

11.6862

8.6638

2.18489

0.107261

0.730274

0
4

0

0

0
4

5.32907051820075e-15
4

0
4

0
4

0

0

0

0

0
4

0
4

0
4

0

0

0

0

0
4

0
4

0
4

13.8269

13.8269

13.8269

13.8269

0
4

0
4

0
4

0.150416

0.150416

0.150416

0.0501388

0.100278

0
4

0
4

0
4

0

0

0

0

0
4

0
4

0
4

0

0

0

0

0
4

0

0

0
4

0
4

0
4

0

0

0

0

0
4

0
4

0
4

0

0

0

0

0
4

0
4

0
4

0

0

0

0

0
4

0
4

0
4

0

0

0

0

0
4

0
4

0
4

92.4619
4

92.4619
4

90.1674
4

89.6578
4

0.106346

0

0

0.403215

0
4

0.20461

0.20461

0
4

2.08994

0.161286

1.92866

0
4

0

0

0
4

0
4

0
4

0

0

0

0

0
4

0
4

0
4

0

0

0

0

0
4

0
4

0
4

0

0

0

0

0
4

0
4

0
4

0

0

0

0

0
4

0
4

0
4

0.125347

0.125347

0.125347

0.125347

0
4

0
4

0
4

0

0

0

0

0
4

0
4

0
4

0

0

0

0

0
4

0
4

0
4

0

0

0

0

0
4

0
4

0
4

0

0

0

0

0
4

0
4

0
4

0

0

0

0

0
4

0
4

0
4

0

0

0

0

0

0

0

0

0
4

0

0

0

0

0

0
4

0

0

0
4

0

0

0

0
4

0

0

0
4

0
4

0
4

0

0

0

0

0
4

0
4

0
4

0

0

0

0

0
4

0
4

0
4

0

0

0

0

0
4

0
4

0
4

0

0

0

0

0
4

0
4

0
4

1.54653

1.54653

1.54653

1.54653

0
4

0
4

0
4

0

0

0

0

0
4

0
4

0
4

0

0

0

0

0
4

0
4

0
4

0

0

0

0

0
4

0
4

0
4

0

0

0

0

0
4

0
4

0
4

0

0

0

0

0
4

0
4

0
4

0
7

0
7

0
7

0
7

0

0

0

0

0

0

0
4

0
4

0
4

0

0

0

0

0
4

0
4

0
4

3.05335

3.05335

3.05335

3.05335

0
4

0
4

0
4

0

0

0

0

0
4

0
4

0
4

0

0

0

0

0
4

0
4

0
4

0

0

0

0

0
4

0
4

0
4

0.7653

0.7653

0.7653

0.7653

0
4

0
4

0
4

0.274652

0.274652

0.274652

0.274652

0
4

0
4

0
4

0

0

0

0

0
4

0
4

0
4

0

0

0

0

0
4

0
4

0
4

0

0

0

0

0
4

0
4

0
4

0
7

0
7

0
7

0
7

0

0

0
4

0
4

0
4

0

0

0

0

0
4

0
4

0
4

0

0

0

0

0
4

0
4

0
4

0

0

0

0

0
4

0
4

0
4

0

0

0

0

0
4

0
4

0
4

0

0

0

0

0
4

0
4

0
4

0

0

0

0

0
4

0
4

0
4

0

0

0

0

0
4

0
4

0
4

0

0

0

0

0
4

0
4

0
4

0

0

0

0

0
4

0
4

0
4

0

0

0

0

0
4

0
4

0
4

61.4848

61.4848

61.4848

57.8916

1.50165

1.2335

0.107261

0.750827

0
4

0
4

0
4

0

0

0

0

0
4

0
4

0
4

0

0

0

0

0
4

0
4

0
4

0

0

0

0

0
4

0
4

0
4

0

0

0

0

0
4

0
4

0
4

0.54682

0.369371

0.369371

0
8

0.369371

0
4

0

0

0
4

0
4

0.177448

0.177448

0.177448

0
4

0
4

0

0

0

0
4

0
4

0
4

9.55346912689947e-13

0
4

51391.5

0
7

0
7

0
7

0
7

0

0
4

0
7

0
7

0
7

0
7

0

0

0

0

0

0

0

0

0

0

0
7

0

0

0

0

0

0

0

0

0

0

0
7

0

0

0

0

0

0

0

0

0

0

0
7

0

0

0

0

0

0

0

0

0

0

0
7

0

0

0

0

0

0

0

0

0

0

0
6

0

0

0

0

0

0

0

0

0

0

0
6

0

0

0

0

0

0

0

0

0

0

0
7

0

0

0

0

0

0

0

0

0

0

0
7

0

0

0

0

0

0

0

0

0

0

0
7

0

0

0

0

0

0

0

0

0

0

0
7

0
6

0

0

0

0

0

0

0

0

0

0

0
7

0

0

0

0

0

0

0

0

0

0

0
7

0

0

0

0

0

0

0

0

0

0

0
7

0

0

0

0

0

0

0

0

0

0

0
7

0

0

0

0

0

0

0

0

0

0

0
7

0

0

0

0

0

0

0

0

0

0

0
7

0

0

0

0

0

0

0

0

0

0

0
6

0

0

0

0

0

0

0

0

0

0

0
7

0

0
7

0
7

0
6

0
6

0
7

0
7

0

0
7

0
8

0
7

0
7

0
7

0
7

0

0

0
7

0

0
7

0

0

0

0

0
8

0
6

0

0

0

0

0

0

0

0

0

0

0

0

0

0

0

0

0

0

0

0

0

0
6

0

0

0

0

0

0

0

0

0

0

0
6

0

0

0

0

0

0

0

0

0

0

0
7

0

0

0

0

0

0

0

0

0

0

0
4

0
7

0

0
7

0
7

0
7

0
7

0
7

0

0

0

0

0

0

0

0
4

0
7

0
7

0

0

0
4

0
7

0
7

0

0

0

0

0

0

0

0

0

0

0

0

0

0
4

0

0

0

0

0
4

0
7

0
7

0
7

0

0

0

0

0

0

0

0

0

0

0

0

0

0
4

0
7

0
7

0

0

0
4

0
8

0
8

0

0

0
4

0

0

0
4

0

0

0
4

0

0

0
4

0
4

0

0

0

0
4

0
4

0

0

0

0

0
4

0
4

0

0

0

0
4

0
4

0

0

0

0
4

0
4

0

0

0

0
4

0
4

0

0

0

0
4

0
4

0

0

0

0
4

0
4

0

0

0

0
4

0
4

0
4

25608.4

3.45728

1.60632

1.09742

0

0.450798

0.0581004

0

0

0

0

0

0

0
4

0

0

0
4

0

0

0
4

0

0

0
4

0

0

0
4

0

0

0
4

0

0

0
4

0

0

0
4

0

0

0
4

0

0

0
4

0

0

0
4

0

0

0

0

0

0

0

0

0
4

0.0556337

0.0556337

0
4

0

0

0
4

0

0

0
4

0

0

0

0

0

0

0

0
4

0.871506

0.871506

0

0

0
4

0.923817

0.923817

0

0
4

0

0

0

0

0

0
4

0

0

0
4

0

0

0

0
4

0

0

0
4

0
4

0

0

0

0
4

0
4

479.591

0.942417
3

0

0.845758

0

0

0.0966581

0

0
4

39.7005
3

0

0

0

0

0

0

0.0767289

0

0

0

0

31.8937

0.127882

0

0

0

0

0

0

0

0.20461

0

0

0.0511526

0.434797

5.0764

0.725787

0.786937

0

0.322572

0
4

0

0

0
4

309.908
3

307.275
3

1.17651

0.0926517

0.100278

0

0.281339

0

0.0617678

0.818442

0

0.101849

0
4

5.68749

2.31125
5

0

0

0.0846347

0

0

0

0.451249

0

0

0.100278

0.802221

0

0

0

0

0.0752082

1.16071

0.225625
5

0.476319

0

0

0

0

0
4

0

0

0

0

0

0

0
4

1.20103

1.20103

0
4

2.89012

1.509

0.127882

0.9719

0.281339

0
4

1.20209

0.179034

0.332492

0.127882

0.127882

0.230187

0.20461

0
4

0

0

0

0

0

0
4

0.123124

0.123124

0

0

0

0
4

0

0

0

0

0

0
4

0

0

0

0
4

0.200555

0.200555

0

0

0
4

0.325902

0.225625

0.0501388

0.0501388

0
4

95.7612
5

95.3099
5

0.0501388

0.125347

0.125347

0

0.0501388

0

0.0501388

0

0

0.0501388

1.87211357527417e-14
5

0
4

0

0

0

0

0
4

0.854342

0.854342

0
4

0

0

0

0

0
4

0

0

0

0

0
4

0.384539

0.0767289

0.30781

0

0
4

0.250694

0.175486

0.0752082

0
4

0.594265

0.594265

0

0

0
4

0.325902

0.175486

0.150416

0
4

1.79129

0.876329

0.730274

0.184686

5.55111512312578e-17

0
4

0

0

0
4

3.07151
5

3.07151
5

0

0

0

0

0
4

0.226387

0.16979

0.0565967

0
4

2.56189

2.56189

0
4

0

0

0
4

0

0

0

0
4

1.74783

1.74783

0
4

0

0

0
4

0.376041

0.376041

0
4

0.305547

0.305547

0
4

0

0

0
4

0.0501388

0.0501388

0
4

1.70279
3

0.0581004

0

0.524348

1.12034

0

0

0

2.22044604925031e-16
3

0
4

0

0

0

0
4

0.701943

0.250694

0.451249

5.55111512312578e-17

0
4

0.253904

0.169269

0.0846347

0
4

0

0

0

0
4

0.613831

0.434797

0.179034

0
4

0

0

0
4

0.120728

0.0482912

0.0724369

0
4

0.265602

0.265602

0
4

0

0

0

0
4

0.127882

0.127882

0
4

0.451249
5

0.175486

0.125347

0.0501388

0.0501388

0

0

0.0501388

0
4

0

0

0

0
4

0

0

0

0
4

0

0

0
4

0

0

0
4

0.0501388

0.0501388

0
4

0

0

0
4

0

0

0
4

0

0

0
4

0

0

0
4

0.0501388

0.0501388

0
4

0.447536

0

0

0.389436

0

0.0581004

0

0

0
4

0

0

0
4

0

0

0
4

0

0

0
4

0

0

0
4

0

0

0
4

0.371625

0.371625

0
4

0

0

0
4

0

0

0
4

0.0965825

0.0965825

0
4

0.0501388

0.0501388

0
4

0
5

0
5

0

0
4

0

0

0
4

0

0

0
4

0

0

0
4

0

0

0
4

1.74783

1.74783

0
4

0.169269

0.169269

0
4

0.125347

0.125347

0
4

0.0501388

0.0501388

0
4

0

0

0
4

0

0

0
4

0.125347

0

0.0501388

0.0752082

0

0

0

0
4

0.0556337

0.0556337

0
4

0

0

0
4

0

0

0
4

0.0434322

0.0434322

0
4

0

0

0
4

1.20965

1.20965

0
4

0

0

0
4

0

0

0
4

0.0793016

0.0793016

0
4

0

0

0
4

0.197828

0.0846347

0

0

0

0.113193

1.38777878078145e-17

0
4

0

0

0
4

0
4

6600.3
3

51.4783

0.894087

0.869923
3

0

0.120823

0

0

0

0

0

0.0966581

0.185812

0

16.3835

0.0483291

0

0

0

3.02057
5

0

0.0966581

0

0

0.144987

0.53162

0

0

0

0.0483291

11.5023

2.46478

0

1.88483

0.0483291

0

0

0

0.0483291

0.0966581

0.0724936

1.67231
3

0.0483291

0

0

0

0

0

0

0.0966581

0

0

0.144987

0.0724936

0

0

0.0724936

0

0.0724936

0

0.0483291

0

0.0966581

2.13684
3

0

0

0.314139

0

0

0

0

0

0

0

0
6

0

0.144987

0

0.0966581

0

0.0966581

0

0.0483291

0

0

6.64524

0

0.314139

0

0

0.434961

0

0

0

0

0

0.0724936

0

0.120823

0.0724936

0.0483291

0

0

0

0

0

0.0483291

0
4

13.385

0
6

0

0

0

0

0

1.66788

0

0

0

0

0

0

0

0

0

0

0

0.0483291

0

0.611971

0

0

0

0

0

0

0.414046

0

0

0

3.76924

0

0
7

0

0.591494

0

0

0

0

0

0

0

0.236598

0

0

0

0

0

0

0

0

0

0

0.0868644

0
6

0

0.185812

0

0

0

0

0.0483291

0

0

0.152013

0
7

0.152013

0

0

0

0

0

0

0

0

0

0

0

0

0

0

0

0

0.0868644

0.185812

0

0

5.14778
3

0

0
4

113.094

1.22209

108.524

0.173729

0

0

0

0.161286

0

0

0.236598

0

0.130297

0

2.05398

0.30781

0

0.236598

0

0

0

0

0

0.0483291

0

0

5.34294830600857e-16

0
4

6080.1
3

591.782
4

34.7614
4

3.20122

4.24777

0

0.118299

0.130297

0.152774

0

0.0434322

0

0

0.0434322

6.21498
4

0.260593

0

0.161286

1.09861

0.123124

0

0.0651483

0.108581

0

0.123124

1.11571
4

0.123124

0

0.0434322

0.873913

0.946391

0

0.118299

0

0

0.125347

11.4308

0.173729

0

0.195445

0

1.39915

0.542903

0.123124

0.123124

0.118299

0

84.2911
4

0.0434322

0

0.123124

0

0.591494

0.173729

0

0.108581

0.0651483

0.125347

0.825212

0

0.184686

0

0.246248

0.0868644

0.107261

0.123124

0.0848951

0.946391

0

0.250694

0.100278

0.551527

0

0.169269

0.0752082

0.108581

0.0434322

0

0.0868644

0.274652

0

0.0434322

0.123124

0.0868644

0.716632

0.195445

0

0.0752082

0.118299

0.116201

0.130297

0.984991

0.118299

0.0868644

0.0871506

0

0.49947

0.123124

0

0.184686

0.177448

0

0

0

0.152013

1.52667

0

0.123124

0.0651483

0.0434322

0

0

0.0565967

3.67002

2.44661

0

0.0434322

0

0.177448

0

0

0

0.524348

0.29211

0.123124

1.32176

0.0434322

0.123124

0.123124

0

0

0.241929

0

0.0556337

0

0

0

0.0434322

0.160891

0.0501388

0.823956

0.0434322

0

0.145251

0.107261

0.0501388

0.195445

0.704561

0.0434322

0

0

0.0868644

0

0.214522

0

0.0434322

0.338539

0

2.28019

0

0

0.132169

0.108581

0

0.195445

0.108581

0.0651483

0.30781

0

0.163089

0.118299

0.0846347

0.887073

0.0793016

0.123124

0.354897

0.304026

0

0.0434322

0.0434322

6.71852

0.173729

0.246248

0

1.69386

0.0752082

0.0651483

0

0

0.130297

0.246248

0

0.0868644

0.217161

0.369371

0.694915

0.0651483

0.0651483

0

0.152013

0

0.0434322

0

0.0434322

0

0.160891

0

2.30191

0.0793016

0.304026

0.108581

0.0651483

0.107261

42.6734
4

0.260593

0.107261

0

0.118299

0.282309

0.524348

0.0501388

0.0553261

0

0.130297

5.05985

303.331

0

0

0.0868644

0

0.195445

0.0434322

0

0

0.80403

0

3.82416

0

0

0

0

0.0482912

0.0651483

0

0

0

0.211587

7.16305
4

0.184686

0

0.145251

0.0651483

0

0.0434322

0.0752082

0.123124

0.123124

0.177448

3.64251

0.246248

0.118299

0

0.214522

0

0.0553261

0

0.0868644

0

0.145251

0.794168

0

0.0434322

0

0.369371

0

0.152013

0.0868644

0.152013

0.0565967

0.275763

0

0

2.85406

0.107261

0

0.107261

0.268152

0.0868644

0.30781

0

0.0752082

0.887242

0.0434322

0.29211

0

0.733902

0.123124

0.0434322

0.0651483

0

0.274652

0.107261

0

0.58422

0.184686

0.0553261

0.264339

0.118299

0.0434322

0

0

0.118299

0.0868644

5.21932

0.214522

0

0.108581

0.238877

0.118299

0.108581

0

0.430933

0.0651483

0.998941

4.92956

3.47002

0.438165

0

0

0.0434322

0.0793016

0

0

0

0.0868644

69.2572

0.0846347
4

0

0

0

0

0

0

0.268152

1.16201

2.93561

0.0651483

22.576
4

0.0434322

0

0.138315

0.0434322

0

11.6181

0.652357

0.152013

0.0868644

0.123124

35.8055

1.52667

0.0651483

0.0868644

0.130297

0

0.123124

0

0

0

0.130297

0.583893

0

0.108581

0.107261

0.22959

0.123124

0

0.0868644

0.369371

0.0651483

0

0.274652

0.130297

0.0556337

0

0.116201

0.126952

0.29211

0.195445

0.173729

0.130297

0.0829891

0.738743

0.0434322

0.173729

0.414046

0

1.72373

0.107261

0.123124

0.0581004

0

1.1572

0.880776

0.30781

0

0.0501388

0

0.128577

0

0

0.123124

0

0

0.716632

0.123124

31.4084

0.163089

0.0868644

0

0.0846347

0

0.217161

0

0

0

0.238877

0.29211

0.116201

0.123124

0

0.0511526

0

0.123124

0

0.554057

0.434322

0

0.118299

0.451249

0.130297

0

14.5566

0.184686

0.246248

0

0.238877

6.57998

3.99576

0.150416

4.25585

0

0.184686

0.0651483

0.152013

0.108581

22.1112

0.0868644

0

0.701758
4

0.198088

0.184686

0.160891

0.29211

0.217161

0

0.214522

0.123124

0

0.130297

1.28767
4

0.0434322

0

0.0501388

0

0.0846347

0.0434322

0.150416

0.118299

6.36048

0.0793016

0.347458

0.184686

0

0

0

0.0501388

0.0581004

0

0.0434322

0.0651483

0

0.260593

0

0

0.49947

0

0

0

0.30781

0.108581

0.0434322

0.184686

1.92261

0.0868644

0.0434322

0

0.130297

0.126952

0

0.304026

13.2903

0.699131

0.169269

89.8804

0.0553261

0

0.108581

17.4163

0

0

0

0.0846347

0

0.0434322

0

0.107261

0

0.107261

4.06309

0.0434322

0

0

0

0.123124

0.347458

6.15619

0

0.282309

0.238877

0.0434322

0.0752082

0.123124

0.196734

0.130297

0

0

0.542903

1.47874

0

0.0501388

0

1.08486

0.0434322

0.0553261

0

0.0434322

0

0

33.1074

0.100278

0.0651483

0

0.331957

0.123124

0

0

0.0651483

0

0

0

0.0434322

1.84686

0.0434322

0

0

0.123124

0.629767

0

0.123124

0.0528677

0
7

0.185037

0.123124

0.0846347

0.195445

0.0752082

0.861867

0.177448

0.0434322

0

0

0.477754

0.0724369

0.236598

0.260593

3.19227

0.0651483

0.763337

0.438165

0.173729

0

0.184686

0.325742

0

0

0.0434322

0.184686

0.354897

0.260593

0.0434322

0.160891

0.0434322

0.195445

0

0.107261

0.217161

0.217161

0

0

0

0

0

0.282309

0.107261

0.391017

0

0.0434322

0.0565967

0.0434322

0

3.29582

1.24737

0

0.476632

305.546

1.54184

0

0.238877

8.16526

0

0.746173

5.64783

7.40108

0

1.99454

2.32362

0.651483

0

0

0

0

0.845952

0.304026

0

0.277422

0.89036

15.3591
3

0.451249

0.206894

0

0

0.521187

4.53867

0

2.46482

0.275763

0.923429

2.30191

0.48595

0.221612

0

33.4807

0.447985

10.968

1.48136

0.803496

0

0.49947

99.3055

0

0.542903

4.78829

1.65042

0

2.4322

0.217161

13.2757

0

0.666479

0.304026

0

0

0.738743

0

0.350972

0

0.298181

0

0.521187

1.02066

7.87993

0

0.778593

0

1.53905

0

0.672428

0

3.05335

66.6614

1.83679

1.35436

230.099
4

0.629767

1.71557

0.238877

0.825212

0.173729

0.1574

0.451249

5.42903

0.542804

1.15797

37.5025
4

0

0

0.369174

0

0.477754

3.65137

1.16968

6.86229

0.760064

0.436655

5.98465
3

0

0.169269

0.250352

0

0.586335

0.521187

6.96896

0.469649

0.282309

0

1.08581

0

0.0482912

0.716632

0.456221

0.977859

1.53905

0.564619

0.650644

0.367601

0.49947

0

0.562777

0

0.152013

0

0.291947

0.238877

0.586335

0

0.929634

0.120728

4.57962
4

0.227587

0.173729

0.846928

1.24013

0.260593

0.504238

0

0.325742

0

0.161805

7.4308
4

0

1.23782

0.504534

0

2.48593

0

0.861867

0.944796

0

0.369174

1451.93
4

3.56166
4

0.832917

1.06854

0

0

0.492495

0.173729

0.184686

0.152013

0.118299

0

2.1471
4

0.217161

0

0.195445

0.0511526

0.430933

0.620951

0

0.403073

0.29211

0.342775

0

0.300572

0.195445

0

0

0.375413

0.921815

0

0.304026

0.191896

0

0

0

0.510457

0.430933

0

0.434322

0.126952

8.37689

2.516

0.130297

0.27919

1.41155

0.160891

0.69882

0

0.22556

0.14945

0.321783

3.22572

0.430933

0.391276

0

4.90611
4

0.542903

0.156536

0.438165

0

0.184686

0.214154

0.47802

0

0

0.108581

7.12541
4

0.130297

0.29211

1.53088

0.30781

0

0.412606

0.697196

0.489268

0

0.933793

3.95804
4

0.0868644

0.214154

0

0.27919

0

0.477754

0.125347

0

0

0.451249

5.27714
4

0

0

4.25876

0.998941

0.241929

0.282309

0.260593

0.214522

0.5209

0.825212

3.33875
3

0.196734

0.30781

0.108581

0.241423

0.364547

0.49947

0

0.515568

0

0

593.903
4

0

0.130297

0.217161

0.399758

0

0.884412

0.304026

0

0.861867

0

0.141069

3.0681
4

0.349565

0.78178

0.0501388

0.152013

0.284709

0

0

0.421284

0.125347

0.0846347

4.37717
4

1.94002

0.407396

0.304026

0

0.542903

0.369371

0.313665

0.763337

0.152013

0

0

0.107261

0.152013

0

2.90849

1.28831

0.434322

1.39826

0

0.274652

0.173729

1.48702
4

0.195445

0.195445

8.99457

0.30781

0

0.123124

0.416993

3.84858

0.304026

0.456038

26.5827

7.89788

17.8053

0

0.107261

0

0

0.173729

149.041

0.268152

0.369174

15.7928
3

1.2777

0.173729

0.238877

0

0.434322

0.173729

0

3.07349

0.30781

0.475188

9.26117
4

0

0.227587

0.238877

0

0.426108

0

0

0.241423

0.639104

0.123124

0

0.238877

0

0.130297

0.173263

0.549304

0.238877

0

0.225625

0

0.0556337

75.9629

0.198088

0.156536

0.438165

0

0.250076

0.214522

0.802221

0.130297

3.68229

0

282.505

25.5758
4

0.556464

0

0.150416

0.236598

0.369371

3.83093

0

0.246248

0.58422

0.130297

1.45336
4

0.123124

0.118299

0

0.0501388

0.123124

0

0

0

0.173729

0.260593

1.06951

0

0.123124

0

3.84471

0

0.274652

0.0752082

0

0.184686

0.108581

2.10771
4

0

3.99576

0.0501388

0.0868644

0.177448

0

0.161286

0.123124

0.107261

0

0.673199

0

0

0

0

0.214522

1.35436

0

0

0.173729

4.14778

0

0.0834505

0

0.29211

0

0.0434322

0.0642886

0.321783

0.0868644

0

0.118299

2.90996

0.58422

0.163089

0

7.94149

0.152013

0

10.3424

0.175486

0.29211

0.260593

24.8561
4

0.430933

0

0

0

0

0.161286

3.36982

0.0434322

0

0

0
7

0

0.492495

0

0

0.411978

0

0.225625

0

0

0.677181

0

0

0.0434322

0

0.108581

0.30781

0

0.0528677

0

0.0434322

0.177448

34.012
4

24.9564
4

0

0

1.47749

0.108581

0.113193

0

0

0.349565

0.369174

0

2.3026
4

0

0

0.0434322

0.27663

0.152013

0

0

0

0.217161

2.15597

4.24087
4

0

0

0.282309

0

0

0.246248

0

0.150416

0.0434322

0.326179

5.50497
4

0.118299

0.0752082

0.130297

0.123124

0.0501388

0.0565967

0.492495

0

0

0.161286

2.75749
4

0.0868644

0.195445

0

0.184686

0

0.0565967

0.0999396

0

0.0434322

0.118299

6.29447
4

0.118299

0

0

0

0.524348

0.0434322

0

0.195445

0.123124

0.130297

0.591494

0.236598

0

0.0434322

0.0752082

0

0.0793016

0

1.36044

0.107261

0

1.2698
4

0.0868644

0

0.0868644

0

0

0.274652

0.0651483

0.0434322

0

0.0528677

8.44986
4

0.0651483

0.0752082

0.110652

0.184686

0

0.0868644

0

0.876329

2.9211

0

2.08933

0.108581

0

0.0651483

0.177448

0.246248

0.0434322

0.0434322

0.107261

1.65042

0.123124

9.33793

4.58641
4

0

0

0.102305

0.184686

0.184686

0

0.244634

0.100278

0

0

4.25724
4

0.0868644

0

0

0.430933

0.763337

0.0868644

0.0871506

0.108581

0

8.51421

0.977225

0.0434322

0.196734

0.0793016

0

0.0642886

0.0434322

0.0434322

0

0.184686

0

0.456038

0.412606

0

0.0868644

0.967566

0.326179

0.0846347

0.107261

0.0434322

0

0.260593

0

0.107261

0

0

0

0.211471

0.369371

0

0

0.123124

0

0

0.244634

0.107261

0.0528677

0.0868644

0

0.0868644

1.19612

0

0.275763

0.130297

0.564619

2.40091

2.77504

0.108581

0.160891

0

0.0829891

0

1.41959

0.0434322

0

2.01083
4

0

0.123124

0

0

0.108581

0.196734

0

0.123124

0.0868644

0.130297

0.884318

0

0.214522

0.0868644

0

0

0.295747

0

0.260593

0

0

0

0.123124

0.0581004

0.0651483

0.238877

0

0.246248

0

0.125347

0

0

41.3475

51.7717
4

0.169269

0.107261

0.369371

0.125347

0.160891

0

0

0.0434322

0.0501388

0.107261

1.23782

0.0434322

0.0434322

0

0

0.482674

0.196734

0.0434322

0

0

0.0434322

0

0.0434322

1.0487

2.77504

0.125347

1.45498

0

0.0434322

0.107261

3.63929

0.0651483

7.33193
4

0.141492

0.763337

0.0617678

0.0434322

0.195445

0.130297

0.108581

0.369371

0.0868644

0.477754

2.4777
4

0.130297

0.160891

0.325742

0

0

0.0501388

0.217161

0.0651483

0.177448

0.107261

7.2192
4

0.123124

0.29211

0.123124

0.456038

2.41049

0

0.0846347

0

0.407723

0.125347

0

0

0

0.108581

0.108581

0.0651483

0.464803

0

0

1.50165

0

1.99711

0.0501388

0.0434322

0.195445

1.52667

0.238877

0.0846347

0.130297

0.0868644

0.184686

0

2.48073
3

0

0.0642886

0

14.5064

1.90834

0.0434322

0

0.107261

0.123124

0.0651483

10.6729
4

0.163089

0.268152

0.152013

0.123124

0

0.118299

0

0

0.246248

0

86.9079

1.21982

0.393468

0.184686

0.0434322

0.0434322

0.108581

0

2.41337

0.161286

0

0.244634

1.28169
4

0

0

0.123124

0.0651483

0

0.161286

0.0434322

0

0.246248

0.184686

0

0.152013

0.238877

0

0

0.0846347

0.673199

0.0868644

0.173729

0.125347

0.0752082

1.86733
4

0.173729

0

0

0

0.118299

0.152013

0.173729

0.0501388

0.107261

0

3.49546
4

0.0434322

0

0.123124

0.123124

0

0.0651483

0.0434322

0.246248

0

0.649608

23.9776
4

0.49947

0.586335

0.300833

0

0

0.68663

0

0

0.0434322

0.107261

1.63334
4

0.123124

0

0.0434322

0.0651483

0.295747

0.166901

0.0651483

0

0.0651483

0.123124

27.7539

0.0868644

0.0434322

0.0553261

0.0651483

0.0642886

0

0.0434322

0.0434322

0

0.123124

0

0

13.1127

0

0.0434322

0.0434322

0.195445

0

0

0

0

0

3.9089

99.6072

1.2928

0.107261

0.123124

0.152013

0.0868644

4.64804

0.123124

0.175486

0
4

5.82888

5.09706

0

0.246248

0.116201

0.369371

0

0

0

0

0

0

0

0

0
4

54.3956
4

26.6898
4

20.8311
4

2.28202
4

4.59271

0

0

0
4

0

0

0
4

70.9553
4

70.8322

0

0

0

0.123124

0
4

0

0

0

0

0
4

0

0

0

0

0

0
4

2.19783

2.04477

0.15306

5.55111512312578e-17

0
4

2.80339

2.00308

0

0

0

0.430933

0.369371

2.22044604925031e-16

0
4

0

0

0

0

0

0
4

0

0

0
4

1.23143

1.23143

0

0
4

0.397776

0

0.123124

0.274652

0
4

5.89846
3

1.61951
3

0.274652

0.411978

0

0

0.492495

0.184686

0.274652

0.102305

1.23593

0.411978

0.184686

0

0.430933

0

0.274652

0

0
4

2.13096

2.13096

0
4

0

0

0

0

0
4

0.610742

0.610742

0
4

0

0

0
4

0

0

0

0
4

0.397776

0.274652

0

0.123124

0
4

1.2928

1.10811

0.184686

0
4

0.123124

0

0.123124

0

0
4

0.212028

0.128577

0.0834505

1.38777878078145e-17

0
4

0.349362

0.349362

0
4

130.255
3

127.684
4

0.123124

0.184686

0.123124

0

0

1.89416

0

0

0

0

0.246248

0

2.40363284831346e-14
3

0
4

3.88304

3.63679

0.246248

0
4

0

0

0
4

1.26909

1.26909

0
4

2.40091

1.53905

0.861867

0
4

0.1574

0.0501388

0.107261

1.38777878078145e-17

0
4

0

0

0

0
4

0.395406

0.395406

0
4

0.0868644

0.0868644

0
4

0

0

0
4

0.397776

0.123124

0.274652

0
4

6.21288
3

1.78524

0.30781

0

0

0

0.554057

0

1.37326

0.30781

1.33065

0

0.369371

0.184686

0

0

0
4

0.857114

0.857114

0
4

0

0

0
4

0.535102

0.535102

0
4

0.187412

0.123124

0.0642886

0
4

0

0

0

0
4

0

0

0

0
4

0.0434322

0

0.0434322

0
4

0.423683

0.260593

0.163089

0
4

0.545245

0.429044

0.116201

2.77555756156289e-17

0
4

0

0

0
4

1.77779
3

1.32718

0.0846347

0.238093

0.127882

0

0

0
4

0

0

0
4

0.554057

0.246248

0.30781

5.55111512312578e-17

0
4

0.0581004

0.0581004

0
4

0

0

0
4

0

0

0
4

0.274652

0.274652

0
4

0

0

0
4

0

0

0
4

0.0651483

0.0651483

0
4

0.0846347

0.0846347

0
4

29.9426
4

25.5244
4

0.677181

0

1.64791

0.984991

0.123124

0

0.984991

0

0
4

0.0434322

0.0434322

0
4

0.29211

0.29211

0
4

0.246248

0.246248

0
4

0.823956

0.823956

0
4

0

0

0
4

0.0651483

0.0651483

0
4

0

0

0
4

0

0

0
4

0.211471

0.211471

0
4

0

0

0
4

0

0

0

0

0

0
4

0

0

0
4

0

0

0
4

0.274652

0.274652

0
4

0.0511526

0.0511526

0
4

0

0

0
4

0

0

0
4

0

0

0
4

0

0

0
4

0.0868644

0.0868644

0
4

0

0

0
4

0

0

0

0

0

0

0

0

0
4

0.369371

0.369371

0
4

0

0

0
4

0

0

0
4

0.29211

0.29211

0
4

0.589935

0.589935

0
4

0.730274

0.730274

0
4

0

0

0
4

0

0

0
4

0

0

0
4

0

0

0
4

6.69112

2.60919

0.923429

1.92256

0

0

0.961282

0.274652

0
4

0

0

0
4

1.52667

1.52667

0
4

0

0

0
4

0

0

0
4

0

0

0
4

0

0

0
4

0.58422

0.58422

0
4

0.0501388

0.0501388

0
4

0.0846347

0.0846347

0
4

0
4

136.653
4

87.9042
4

23.6174
4

1.8925

0

0

0.806997

0.381913

0.549304

0

0.438165

0

1.14501

3.25663

0.0581004

0.05102

0.160891

0.123124

0

0

0.0581004

0.0871506

0.174301

0.174301

1.37074

0.0871506

0.68663

0.274652

0

0.0581004

0

0.29211

0.411978

0.177448

0.68663

1.67906

1.16844

0

0

0

26.9159

0.290502

1.41187

1.25343

16.7254

0.969207

0.470078

7.20534742981727e-14
4

0
4

48.7491
4

47.9251
4

0.823956

0
4

0

0

0
4

0

0

0
4

0

0

0
4

0
4

289.939

15.2699

0
7

0
7

0

0

0

0

0

0

0

0

0

0

0
8

0

0

0

0

0

0

0.0724936

0

0

0.241929

0
7

0

0

0

0

0

0

0.338303

0

0

0

0

0

0

0

0

0

0

0

0

0

0

0
7

0

0

0

0

0

0

0

0
7

0

0
8

0
3

0
7

0

0

0

0
7

0
8

0

0

0
7

0

0

0

0
7

0

0

0

0

0
7

0

0

0

0

0

0

0

0

0

0

0

0

0

0

0

0

0
8

0

0.0834505

0

0

0

0

0

0

0.152013

0

14.2982
3

0

0

0

0

0

0

0

0

0

0

0
8

0

0

0

0

0

0

0

0

0

0

0
7

0

0

0.0834505

0

0

0

0

0

0

0

0
7

0

0

0

0

0

0

0

0

0

0

3.31679128606766e-15

0
4

0

0

0

0
4

0
2

0

0

0

0

0

0

0

0

0

0

0
4

120.421
4

0

18.9238
4

0.35714

0

0.12755

0.58422

0

0

0.12755

0

0

3.73545

0

0

0

0

0.2551

2.33688

2.53208
4

0.952859

0.113193

0

0.132164

0

0.106654

0

1.46055

0

0

67.8039
4

0

3.79743

0

0

0

0.13463

8.61724

0.141492

2.04477

0

0.407723
3

0

0

0

0.58422

0

0

0.05102

0

0

0.29211

0.34313
4

0

0

0

0.07653

0.141492

0

0

0

0

0

0.58422

0

0

0

0.05102

0.438165

0

0

0.05102

0

0

0.05102

0

0.184686

0

0

0

0.12755

0

0

0

0

0
8

0.29211

0.111267

0

0

0

0

0

0.33958

0.07653

0

0.07653

0.05102

0

0

0.876329

0

0

0.29211

0

0.58422

0.0528677

0
4

9.52347
5

0.257154

0.738743

0.0642886

0

0

0

0

0.524348

0

0

0.916111

0.152774

5.8422

0.435543

0.107261

0.128577

0.356472

0

2.16493489801906e-15
5

0
4

0.619247
6

0

0

0

0

0

0

0

0

0

0

0

0

0

0

0

0.108581

0

0.0834505

0

0

0.0501388

0

0

0

0

0

0

0.0556337

0

0

0

0

0

0

0

0

0

0

0

0.0483291

0

0

0

0

0

0.0556337

0

0

0

0.217481

0

0

0

0

0

0

2.77555756156289e-17
6

0
4

16.8019
5

6.25737
5

0

0

0

0

0

0.0565967

0

0

0

0

6.16319

0

0

0

0.118299

0.118299

0

0.177448

0

0

0

0

0

0

0

0

0

0

0.349565

0

0

0

1.95193
5

0

0

0

0

1.14501

0

0.0501388

0

0

0

0.295747

0

0.118299

0

0

0
4

43.1043

35.1181

0.160722

0.0964329

0.0642886

0.0642886

0.0642886

0.0964329

0.0964329

0.0642886

0.0642886

0.0642886

1.92561

1.02862

0.385732

0.417876

0.160722

0.546453

2.08938

0.596095

1.14352971536391e-14

0
4

1.85696

0.548004

0.45002

0.546453

0.0964329

0.0553261

0.0964329

0.0642886

2.22044604925031e-16

0
4

0

0

0
4

0

0

0
4

0

0

0
4

0

0

0
4

0

0

0
4

0

0

0
4

0

0

0
4

0

0

0
4

0

0

0
4

0

0

0
4

0
7

0
7

0

0

0

0

0
4

0

0

0
4

0

0

0
4

0

0

0
4

0

0

0
4

0

0

0
4

0

0

0
4

0

0

0
4

0.05102

0.05102

0
4

0

0

0
4

0

0

0
4

4.99155
4

4.69944

0.29211

0

4.9960036108132e-16
4

0
4

0

0

0
4

0.0724936

0.0724936

0
4

0

0

0
4

0

0

0
4

0

0

0
4

0

0

0
4

1.52667

1.52667

0
4

0

0

0
4

0

0

0
4

0.198088

0.198088

0
4

1.09291

0.160722

0.128577

0.160722

0.22501

0.0642886

0.0642886

0.289299

5.55111512312578e-17

0
4

0

0

0
4

0.185812

0.185812

0
4

0.0483291

0.0483291

0
4

0.120823

0.120823

0
4

0

0

0
4

0

0

0
4

0.0724936

0.0724936

0
4

0
7

0

0

0

0

0
4

24.3912
4

23.8069

0.58422

0

0

0

0
4

0.752367
4

0.327623

0.102173

0.322572

0

0
4

0

0

0

0
4

0
7

0
7

0
4

0
7

0
7

0
4

17.877
4

14.0475
4

1.34417
4

2.04712
4

0.438165

0

0

1.27675647831893e-15
4

0
4

0.474018

0.277284

0.196734

0
4

0
8

0

0

0
4

0
6

0

0

0

0
4

0

0

0

0

0

0
4

5.95705

5.95705

0
4

2.69428

0.650343

1.67231

0.371625

0
4

0.332441

0.332441

0
4

0

0

0

0

0

0
4

0

0

0

0

0
4

0.483291

0.410797

0.0724936

1.38777878078145e-17

0
4

0
7

0
7

0

0

0
4

0.150331

0.150331

0
4

0

0

0

0

0
4

2.53133

2.53133

0

0
4

0.911718

0.911718

0
4

0

0

0

0
4

0.305985

0.139084

0.166901

2.77555756156289e-17

0
4

0

0

0
4

0

0

0
4

0.829803

0.829803

0
4

0

0

0

0

0
4

0
7

0

0

0

0

0

0

0

0
4

0

0

0

0

0
4

0

0

0
4

0

0

0

0
4

3.0659

2.88009

0.185812

1.94289029309402e-16

0
4

0

0

0

0
4

0

0

0

0

0
4

0

0

0

0
4

0

0

0
4

0.173263

0.0501388

0.123124

0
4

0

0

0

0
4

1.12505

0.353587

0.128577

0.0642886

0.128577

0.0642886

0.0642886

0.0642886

0.0642886

0.0642886

0.0642886

0.0642886

0
4

0

0

0

0
4

0.0966581

0.0966581

0

0
4

0

0

0
4

0

0

0

0
4

0

0

0
4

0

0

0
4

0

0

0

0
4

0

0

0

0
4

0

0

0

0
4

0.140221

0.140221

0
4

0
7

0
7

0

0

0

0
4

0

0

0

0
4

0

0

0
4

0

0

0

0
4

0.257154

0.257154

0
4

0

0

0
4

0.154027

0.154027

0
4

1.05908

0.763337

0.295747

0
4

0.434961

0.434961

0

0
4

0.724936

0.724936

0
4

0

0

0
4

0.273463
3

0.273463

0

0

0
4

1.09861

1.09861

0
4

0

0

0
4

0

0

0
4

0

0

0
4

0.68663

0.68663

0
4

0.0999396

0.0999396

0
4

0

0

0
4

0

0

0
4

1.51059

1.51059

0
4

0

0

0
4

3.96383

0.183523

1.29136

2.48895

0
4

0

0

0
4

0

0

0
4

0

0

0
4

0.438165

0.438165

0
4

0.438165

0.438165

0
4

0.185812

0.185812

0
4

0

0

0
4

0

0

0
4

0

0

0
4

0

0

0
4

0
7

0
7

0

0
4

0

0

0
4

0.0724936

0.0724936

0
4

0

0

0
4

0

0

0
4

0

0

0
4

0.29211

0.29211

0
4

0

0

0
4

0

0

0
4

0

0

0
4

0

0

0
4

0
4

1170.07

0
7

0
7

0

0

0

0

0

0

0

0

0
4

926.228

0
7

0
7

0

1.18406
3

0
6

5.76783

0

0.433392

0

0

0.26581

0.0724936

295.631
3

0

0

1.47749

0

0

0

0

0

0

0

4.75794

0.0556337

0.929061

0

0

0

0

0

21.4339

0

1.40066

0
7

0

0

0

0

0.700771

0.309348

7.86588

0

0

0

0
7

0

0

0.489268

0

0

0

0

0

0.423706

0

0
6

0

0

0

0

0

0.806688

0

0

17.6522

0

0

0

0

0

0.4059

0

0

0

0

0

0.650343

8.6225

0

0.101849

0

0

0

0

0

0.15442

4.77357

0

0

0

0

0

0

0

0

0

0

0

0

0

0

0

0

0

0

0

0

0

0

0

39.6423
3

0
7

0

1.08374

0

0

0.0483291

0

0

0

0

0.278718

0
7

0.193316

0

3.16555

0.214541

0

0.0966581

0.120823

0

0.110097

0

1.52237
2

0

0

0

0.650343

0

0

0

0.0966581

0.101849

0

6.30837
3

0.0724936

3.43753

0

0

0

0

0

0

0.322572

0

1.74783
3

0

0.557437

0

0

0.0966581

0.0556337

0

0

0

0.24758

0
7

0

0

0

0.650343

0

0

0

0

0

0

0
7

0.0483291

0

0

0.68663

1.39359

0

0

0

0.371625

0

1.03621
3

0

0.0966581

0

0

0.110097

0

0.120823

0

0

5.6545

0
7

14.4005

0

0.0617678

0

0

0

0.185812

0

0

0

0
2

0

0

0.314139

0

0

0

0.161286

0.185812

0

0

0
7

5.40308

0

0

0

0.0966581

0

0

0

0.0617678

0

0

0
6

0.120823

0

11.2365

0

0

0

0

0

0.652442

0.0483291

1.76401
2

0

3.34462

0

0

0.120823

0

0

0

0

0

0.960794
3

0.278718

0

0.0483291

0

0

0.0483291

0

0

0.185812

0

0.193316
7

0

0

0

0

0

0.185303

0.836155

0

0

0

0.314139
2

0

0

0

0

0

0

0

0

0

0

0

0.862322

0

0

0

0.763337

0

0

0

0

0.411978

16.9937
5

0

0

0

0

0

0

0

0

0.0483291

0

0
5

0

0

0

0

0

0

0

0

0

0

4.46039
3

0.0724936

0

0

0

0.339723

0

0

0

0

0

280.831
4

0
6

0

0

0

0

0

0

0

0

0

0.773265

1.07057
3

0

0

0

0

0

0

0

0

2.09739

0

0

0

0

0

0

0

0

0.524348

0

0

0.0617678

5.03574
3

0

0.386632

0

0

0.241645

0.0617678

0

0

0

0

0
7

0

0

0

0

0.26581

0

0

0

0

0

6.88504
3

0

0

0

0

0

0

0

0

0

0.185812

27.5286
3

0.0724936

0.551954

0.169152

0.0966581

0

0.185812

0

0

0

0

0
7

0

0

0

0

0.0434322

0

0.0483291

0

0

0

1.09126
3

0

0

0

0.111267

0

0

0

0

0.0617678

0

0
7

0

0

0

0.185812

0

0

0.185812

0

0.185812

0

0
7

0
2

0

0

0

0

0

0

0

0

0

0

1.94757

0

0

0

0

0.0483291

0.0724936

0.277955

0

0

0

0

0

0

0

0

0

0

0

0

0.116201

0

0.0483291

0

0

0.278718

0

0

0

0

0

0

0.185812

0

0

0.185812

0

0

0

0

0

0

0

0.277955

2.36443

0.217481

0

0

0

0

0

0

0

0

0

1.49
3

0.152774

0

0.401491

0.203698

0

0.111267

0

0

0

0

0

0

0

0

0

0

0.0617678

3.62334

0

0

0

1.01307
5

0

0

0

0

0

0

0.185812

0

0.0483291

0

0.88476
3

0

0.0966581

0

0

0

0

0.0724936

0.185812

0

0

0
7

0.303413
3

0

0.0724936

0

0.0483291

0

0

0

0

0

0

0.459126
3

0

0.371625

0

0

0.0483291

0.169152

0

0

0

0

0.920324
3

0.0483291

0.120823

0

0

0

0.296221

0

1.20778

0

0

0
6

0

0.185812

0

0.0793016

0.278718

0

0

0.185303

0

0.0834505

0
7

0

0

0

0

0

0

0

0

0

0

0
7

0

0

0.0834505

0

0

0

0

0.0834505

0

0

0
7

0

0

0

0

0.217481

0

0

0

0

0

0
7

0

0

0

0

0.0581004

0

0

0.362468

0

0

0
2

0.0926517

0

0

0

0

0

0

0

0

0

1.36101
3

0

0

0

0

0.464531

0

1.17359

0

0

0

0

0
2

0

0

0

0

0.0483291

0

0

0

0

0

0.225385
3

0

0.26581

0.0483291

0.0483291

0.0483291

0

0

0

0

0

0.362468
3

0

0

0

0

0

0

0

0

0

0

0

0

0.0617678

0.0724936

0

0

0

0

0

0

0

0.942417

0.278718

0

0

0

0

0

0

0

0

0

0
6

0

0.0724936

0

0

0.0556337

0

0.185812

0

0

0

0.464022
3

0.0556337

0

0.0556337

0

0

0

0.0483291

0

0

0

0
2

0

0

0

0

0

0

1.02197

0

0

0.53162

0
7

0

0

0

0.166901

0

0

0

0.0724936

0

0

0

0.339723

0

0

0.0483291

0

0.0966581

0

0.0617678

0

0

4.45954
3

0

0

0

0

0

0

0

0

0.185812

0

0.120823

0
7

0

0

0

0.0483291

0

0

0

0.483858

0

0

0.118299

0

0

0.0834505

0.15442

0

0

0

0

0.123536

0.185812

0

0.0556337

0

0

0

0

0

0

0.305985

0.166901

0
6

0.0724936

0
7

0

0
7

0

22.1951

1.53444

0.375907
3

0

0

0

0.940518

0
6

0
7

0

24.8039

7.56728013584507e-13

0
4

21.1694

9.70424

0

0.0966581

0.0868644

0

0.483291

0.0483291

5.70698

0

0

4.53559

0.0483291

0.410797

0.0483291

0

9.78384040450919e-16

0
4

140.688

94.828

0
7

1.09999
4

0

0

0

0

0.356731

2.18223

0.20408

0

1.6066

29.5009
4

0

0

0.374098

0.492495

1.31449

0.22501

0.221304

0.744941

0

0

0
7

0.557635

0.521154

0

0.0617678

0

0

0.385732

0

0

0.184686

0
7

0.160722

0.349565

0

0.29211

0

0

0

0

0.107261

0

0
4

0

0

0.184686

0.244634

0

0

0.274652

0

0

0.268152

0
7

0

0

0.123124

0

1.08204

0.0528677

0

0

0

0

1.84074
4

0

0.268152

0

0

0

0.470272

0

0

0.107261

0

0

0

0

0
7

0
4

0
7

0
7

0

0

0

0

0

0

0

0

0

0

0
6

0

0

0

0

0

0

0
7

0

0
6

0
7

0
7

0

0

0
4

2.68524
4

1.6016

0.965349

0

0.118299

0
4

0.05102

0.05102

0
4

0.248967

0.248967

0
4

1.23124

1.23124

0
4

0

0

0
4

0

0

0
4

0.0483291

0.0483291

0
4

0

0

0
4

0.0752082

0.0752082

0
4

0

0

0
4

0

0

0
4

2.67741
4

2.67741
4

0

0

0
4

0

0

0
4

0

0

0
4

0

0

0
4

0

0

0
4

0

0

0
4

0

0

0
4

0

0

0
4

0.05102

0.05102

0
4

0

0

0
4

0

0

0
4

0
7

0
7

0

0
4

0

0

0
4

0

0

0
4

0

0

0
4

0

0

0
4

0

0

0
4

0

0

0
4

0.123124

0.123124

0
4

0

0

0
4

0

0

0
4

0

0

0
4

0

0

0

0

0

0
4

0

0

0
4

0.110652

0.110652

0
4

0.0848951

0.0848951

0
4

0.0553261

0.0553261

0
4

0

0

0
4

0

0

0
4

0.763337

0.763337

0
4

0

0

0
4

0

0

0
4

0

0

0
4

3.41343
3

3.05361

0.359823

0

5.55111512312578e-17
3

0
4

0

0

0
4

0

0

0
4

0.0848951

0.0848951

0
4

0

0

0
4

0

0

0
4

0

0

0
4

0

0

0
4

0

0

0
4

0

0

0
4

0

0

0
4

0.0724936
2

0.0724936

0

0

0

0
4

0.380856

0.380856

0

0

0

0

0
4

0
8

0

0

0
4

1.31449

1.02238

0.29211

0

0

5.55111512312578e-17

0
4

0
7

0
7

0
4

36.5622
4

17.7141
4

2.12287

0

0.405525

0.192866

0.268152

0

0

0.107261

0

0.107261

3.11324
4

0.107261

0.163089

0

0.532345

0.107261

0.05102

0.160891

0.107261

0.107261

1.55528

2.99666
4

0.161286

1.38296
4

0.357918
4

0.87613
4

2.85356

0.503821

0.506965

3.21964677141295e-15
4

0
4

0

0

0
4

0

0

0

0
4

0.238171

0.123536

0.0617678

0.0528677

0
4

0.820652

0.0565967

0.764056

0
4

0

0

0
4

0

0

0

0
4

0

0

0

0
4

0

0

0

0

0
4

0

0

0
4

0.1574

0.0501388

0.107261

0

1.38777878078145e-17

0
4

12.0166
4

1.51968
4

5.99778
4

2.04545

0.241929

0.483069

1.62144

0.107261

0

1.51267887105178e-15
4

0
4

0.0829891

0

0

0.0829891

0
4

0.650343

0

0.650343

0
4

0.0556337

0.0556337

0
4

0.555111

0.0528677

0.502244

0

0
4

0

0

0

0
4

0.745111

0.160891

0.58422

0

0
4

0

0

0

0

0
4

0

0

0
4

0

0

0

0
4

0.160891

0

0.160891

0
4

0
5

0
7

0
5

0

0

0

0

0

0

0
4

0

0

0

0
4

0

0

0

0
4

0

0

0

0
4

0

0

0

0
4

0

0

0
4

0

0

0
4

0.163089

0

0.163089

0
4

0

0

0

0
4

0

0

0

0
4

0

0

0

0
4

6.39495
3

6.17242
3

0.166901

0.0556337

0

0

0

0

5.20417042793042e-16
3

0
4

0

0

0

0
4

0.0483291

0.0483291

0
4

0.107261

0.107261

0
4

0

0

0
4

0.171953

0.171953

0
4

0.179169

0.123536

0.0556337

0
4

0

0

0

0
4

1.0507

0.246248

0.804457

0
4

0.0724936

0.0724936

0
4

0.30612

0.30612

0
4

0
3

0
3

0

0

0

0
4

0

0

0
4

0

0

0
4

0

0

0
4

0

0

0
4

0

0

0
4

0

0

0
4

0.185812

0.185812

0
4

0.126952

0.126952

0
4

0

0

0
4

0

0

0
4

1.82965
4

1.82965
4

0

0
4

0.29211

0.29211

0
4

0.193641

0.193641

0
4

0.116201

0.116201

0
4

2.40894

2.40894

0
4

0

0

0
4

0.185812

0.185812

0
4

0.0553261

0.0553261

0
4

0

0

0
4

0

0

0
4

0

0

0
4

0.943294
3

0.830101
4

0.113193

0

0

0
4

0.0617678

0.0617678

0
4

0.278718

0.278718

0
4

0

0

0
4

0.128577

0.128577

0
4

0.58422

0.58422

0
4

0

0

0
4

0.195445

0.195445

0
4

0

0

0
4

0

0

0
4

0

0

0
4

0
4

0

0

0

0

0

0
4

0.05102

0.05102

0
4

0

0

0
4

0

0

0
4

0

0

0
4

0

0

0
4

0.0553261

0.0553261

0
4

0

0

0
4

0.214522

0.214522

0
4

0

0

0
4

0.0642886

0.0642886

0
4

0
4

188.014
3

181.291
3

116.418
3

61.9889

0

0

0

0

0

0

0

0.0556337

0

0

0.923429

0.691576

0.677181

0

0

0

0

0.123124

0.414046

0

0
4

1.7398
4

1.19209
4

0.497567

0.0501388

4.85722573273506e-17
4

0
4

0

0

0
4

0

0

0
4

0.158603

0.0528677

0.105735

0
4

0

0

0

0
4

0.213228

0.0501388

0.163089

0
4

0

0

0
4

0

0

0
4

0

0

0
4

0

0

0
4

0

0

0
4

0

0

0

0
4

0

0

0
4

0

0

0
4

0

0

0
4

0.0793016

0.0793016

0
4

0.177448

0.177448

0
4

0.0528677

0.0528677

0
4

0

0

0
4

0

0

0
4

0.322572

0.322572

0
4

0

0

0
4

0

0

0
4

0

0

0
4

0.763337

0.763337

0
4

0

0

0
4

0.0501388

0.0501388

0
4

0

0

0
4

0

0

0
4

0

0

0
4

0

0

0
4

0

0

0
4

0.590203

0.590203

0
4

0

0

0

0

0
4

0

0

0
4

0

0

0
4

0

0

0
4

0

0

0
4

2.14971

0.174301

0.0581004

1.91731

0
4

0.375413

0.375413

0
4

0.0501388

0.0501388

0
4

0

0

0
4

0

0

0

0
4

0
4

11.8276
2

11.8276
2

0
2

0
2

0

0

0

0

0

0

0

0

0

0

1.54293

0

0

0.05102

0

0

0

0

0

0

0

0

0.84183

0

0

0

0

1.94405

0

0

0

0

0

0

0

0

0

0

0

0

0

0

0

0

0

0

0

0

0.829891

0

0

0

0

0

0

0

0.0553261

0

0

0

0

0

0.0964329

0

0

3.81669

0

0

0.185812

0

0

0.0553261

0

0

0

0

1.99295

0

0.185812

0

0

0

0

0

0.05102

0.05102

0

0

0

0

0

0

0
2

0

0

0

0

0

0

0.07653

0

0

0

0

0

0

0

0

0

0

0

0

0.05102

0

0
4

0

0

0
4

0

0

0
4

0
4

829.055

63.906
7

5.61925
7

0
7

0

2.90315

0

0

0

0

0

0

0

0

2.33865

0

0

0

0

0

0

0

0

0

0

0.0987583

0

0

0

0

0

0

0

0

0

0

0
6

0

0

0

0

0

0

0

0

0

0

0.0917613

0

0

0

0

0

0

0

0

0

0

0
7

0

0

0

0

0

0

0

0

0

0

0
6

0

0

0

0

0

0

0

0

0

0

0
7

0

0

0

0

0

0

0.0868644

0

0

0

0
7

0

0

0

0

0

0

0

0

0

0

0.0651483
7

0

0.130297

0

0

0

0

0

0

0

0

27.6545
7

0
6

0

0

0

0

0

0

0

0.185812

0

0

0.0434322
7

0

0

0

0

0

0

0

0

0

0

0
7

0

0

0

0

0

0

0

0

0

0

0
7

0

0.349565

0

0

0

0

0

0

0

0

0
7

0.0724936

0

0

0

0

0

0

0.304294

0

0

0
6

0

0

0

0

0

0

0

0

0

0

0.0651483
7

0

0

0

0

0

0

0

0

0

0

0
7

0

0

0

0

0

0.349565

0

0

0

0

0
6

0

0

0

0

0

0

0

0

0

0

0
7

0

0

0

0

0

0

0

0

0

0

0.464531
7

0.349565
5

0

0

0

0

0

0

0

0

0

0

0
7

0

0

0

0

0

0

0

0

0

0

0
7

0

0

0

0

0

0

0

0

0

0

0
7

0

0

0

0

0

0

0

0

0

0

0
7

0

0

0

0

0.0553261

0

0

0

0

0

0
7

0

0

0

0

0

0

0

0

0

0

10.9566

0

0

0

0

0

0

0

0

0

0

0
7

0

0

0

0

0

0

0

0

0

0

0
6

0

0

0

0

0

0

0

0.0868644

0

0

0

0

0

0.464531

0

0

0

0

0

0

0

0.294393
6

0
6

0

0

0

0

0

0

0

0

0

0

1.87382

0

0

0

0

0

0

0

0

0

0

0
7

0

0

0

0

0

0

0

0

0

0

0
7

0

0

0

0

0.0724936

0

0

0

0

0

0

0.185812

0

0

0

0

0

0

0

0

0.0651483

0
6

0

0

0

0

0

0

0

0

0

0

0
6

0

0

0.699131

0

0

0

0

0

0

0

0
7

0

0

0

0

0

0

0

0

0

0

0
7

0

0

0.138315

0

0

0

0

0

1.20778

0

0
7

0

0

0

0

0

0

0

0

0

0

0.0483291
7

0

0

0

0

0

0

1.69897

0

0

0

0

0

0

0

0

0

0

0

0

0

0

0

0
7

0

0

0

0

0

0

0

0

0

0

0
6

0

0

0

0

0

0

0

0

0

0

0
7

0

0

0

0

0

0

0

0.0434322

0

0

0
7

0

0

0

0

0

0

0

0

0

0

0
6

0

0

0

0

0.173729

0

0

0

0

0

0

0

0

0

0

0

0

0

0

0

0

0
7

0

0

0

0

0

0

0

0

0

0

0
7

0

0

0

0

0

0

0

0

0

0

0
7

0
7

0.542903

0.0434322

0

0

0

0

0

0

0

0

0
7

0

0

0

0

0

0

0

0

0

0

0
7

0

0

0

0

0

0

0

0

0

0

0.0434322
6

0

0

0

0

0

0

0

0

0

0

0

0

0

0
6

0

0
8

0.464531
5

0.0434322
7

3.18125

0
6

0

0

0

0

0
7

0

0

0
7

0

0
7

0

0

0

0
7

0

0

0

0

0
7

0

0

0.349565

0

0

0

0

0

0

0

9.99200722162641e-15
7

0
4

0.115926
7

0.115926
7

0

0
4

758.004
4

756.14
4

0

0

0

0

0

0.464531

1.39915

0

0

0

0

0

0

0

0

0
4

0

0

0
4

0

0

0
4

0

0

0
4

0.0868644

0.0868644

0
4

0.0434322

0.0434322

0
4

1.90834

1.90834

0
4

0

0

0
4

0

0

0
4

0

0

0
4

3.43115

3.43115

0
4

0

0

0
4

0

0

0

0
4

0

0

0
4

0

0

0
4

1.14501

1.14501

0
4

0

0

0
4

0

0

0
4

0.0434322

0

0.0434322

0
4

0.371625

0.371625

0
4

0

0

0
4

0

0

0
4

0

0

0
4

2.45692355349547e-13

0
4

485.675
4

453.528
4

19.9685
4

15.8866
5

0

0.295101

0.0752082

0.438165

0.163089

0

0

0.247071

0

0.199879

43.0701
3

0

0.249849

0

0

0

0.196734

0

4.17655

0

0

25.7456
4

0

0

0.295101

0

2.33688

0

0.216187

0.0793016

0

0.0501388

0.491835
4

0.34364

0.241929

1.90834

0.401491

0.330555

0

0

0.295101

0

0

8.66827

0.196734

0.196734

0

2.82842

0

0

0.120728

0.150416

0.105735

0

4.13844

0.196734

0

0

0.246248

0

0

0

0

1.4876

0

7.05711
4

0

0.295101

0.409221

0

1.61286

1.47551

0

0.491835

0

0

2.07553
5

0

0

0

0

0.185303

0

0

0

0

0

1.30157

0

0

0

0.105735

0

0

0

0.132169

0

0

0.68857

0

0

0

0.160722

0

0

0

0

0

0.241929

0

1.9243
4

0

0

0

0.100278

0

0.277955

0

0

0.885304

0

0

0

0

0

0.158603

0

0.0793016

0.0752082

0.196734

0

0

1.56947

0

0

0.0501388

0

0

0

0.0528677

0.0868644

0.0528677

0

0.885304

8.38957

0.161286

0.0482912

0.0528677

0.0752082

0

0

0

0

0

1.8353

0

0

0.123124

0

0

0

0.733902

0.403215

0

0

0

0

0

4.69435

0.102305

3.73534

0

0.525026

0

0

0

0.590203

0

0

0

0

0.0752082

0

0

0.0553261

0

0.0565967

0.200555

0

0.295101

0

0.116201

0

0

0

0

0.161286

0.0581004

1.74464

0.100278

0

0

0

0

0

0

0

0

0

5.10625
4

0

0.0501388

0

0

0

0.161286

0

0

0

0

0

0.196734

0

0

0

0

0

0

0

0

0.185812

0

12.4017

0

0

0

0.0528677

0.123124

0

0

0.125347

0

0.150416

0

0

0

0.184686

0

0

0

0

0

0

0

0.116201

0

0

0.163089

0

0

0

0.295101

0

0

0

0

0

0

0.29211

0

0

0

0

0

0

0

2.22046
3

0.123536

0

0

0

0

0

0

0.0724369

2.5856

0

1.88336

0

0

0

0

0

0

0.0528677

0

0

0.0528677

31.6742

0

0

0

0

0

0

0

9.63997

0

0.763337

9.58617

0

0

0

0

0

0

0.295101

0

0

0

9.32694

0.0793016

0

0.0501388

0.0501388

0

0

0.295101

0.185303

0

0

0

0

0.0793016

0.652357

0

0.0999396

0

0.0724369

0

0

0

0

0.319842

0

0

0.132169

0.196734

0

0

0

0

0

0

3.51728

0

0

0.105735

0

0

0

0

0

0

0.247071

17.7827

0.0528677

0.107261

0

0

1.79891

0.0482912

0.184686

0

0

0.0926517

0

0

0

0

0

0

0.15442

0.149909

0

0

0

16.8665

0

0

0

0.0767289

0

0

0.0501388

0.241929

0

0.0501388

0.0871506
4

0

0

0

0

0

0

0

0

0

0

0

0.0501388

0

0

0

0

0

0

0

0

0.166901

1.10879

0

0

0

1.29029
4

1.86897
4

0.277955

0

2.66122

14.9298

0

0

1.15319

3.79169

0

1.27877

65.4141
4

0

0

0

0.55884

0

1.04653

0.0501388

0

0

0

20.2186
3

0

0.295101

0

0.797189

2.77955

0

0

0

0.179034

1.37714

0

0.35962

1.52667

0

1.49981

0

0

0

2.37905

0

0.295101

6.24504
5

0.58422

0.0617678

0.749805

0.207287

0.551527

0

0

0

3.06443

0

0
4

6.00746
4

5.33222
4

0

0

0

0

0

0

0

0

0

0

0.163089

0.261452

0.175486

0

0

0

0

0.0752082

5.55111512312578e-17
4

0
4

0

0

0

0

0
4

0

0

0

0
4

0.852588

0.699131

0.153458

0
4

0

0

0

0

0

0
4

0

0

0

0
4

0

0

0
4

0

0

0

0
4

0.738743

0.123124

0.184686

0.430933

5.55111512312578e-17

0
4

1.16023

0

1.16023

0
4

0

0

0

0

0
4

12.7188
4

9.48214
4

0

0

1.91611
4

0.879529
4

0.244275

0.196734

0

0

0

0

0
4

0.0511526

0

0.0511526

0
4

0

0

0
4

0

0

0

0
4

0.835752

0.835752

0
4

0

0

0
4

0

0

0

0
4

0

0

0
4

0

0

0

0
4

0.707175

0.289299

0.417876

0
4

0

0

0
4

0
3

0
3

0

0

0

0

0

0
4

0

0

0
4

0

0

0
4

2.09739

2.09739

0
4

0

0

0
4

0

0

0
4

0

0

0
4

1.52667

1.52667

0
4

1.14501

1.14501

0
4

1.44649

1.44649

0
4

0.0501388

0.0501388

0
4

0.652442
3

0.652442
3

0
4

0

0

0
4

0

0

0
4

0

0

0
4

0

0

0
4

0.354897

0.354897

0
4

0.0926517

0.0926517

0
4

0

0

0
4

0

0

0
4

0.199879

0.199879

0
4

0

0

0
4

0

0

0

0

0
4

0.393468

0.393468

0

0

0
4

0.163089

0

0

0.163089

0
4

0.271942

0

0.0752082

0.196734

0

0
4

0.68117

0.216187

0.0617678

0.241929

0.161286

0
4

1.23234755733392e-14
4

0
4

6031.74
4

322.468
5

85.8636

26.0581
5

0.526457
4

1.93034

0

0.0752082

0

0

0.185812

0.0501388

0.214522

0

9.90241

1.2284
5

0.152774

0.0501388

0.0752082

0.0752082

0

0.118299

0

0.100278

0.0501388

0

0.551527
5

0.464531

0.0501388

0.0752082

0.0501388

0

0.0752082

0.0501388

0.101849

0.101849

0.0617678

0.250694

8.75412

0.185812

0.0501388

1.60444

0.576596

0.300833

6.01665

19.1387

3.62334

15.985
5

0.350972

0

0.0501388

0.626735

0

1.67187

0.225625

0.77438

0.676874

0.929061

77.5647
5

0

0

0.557437

1.95541

0.200555

0.177448

1.02785

0.200555

0.225625

0.125347

0.19686
6

0

0.325902

0.557437

0.371625

0.371625

4.27368

1.30069

0.471233

0.175486

0.100278

4.88853
4

0

0.150416

0.184686

27.2504

0.0581004

0.0752082

0.200555

0.0501388

0.0617678

0

1.2284
5

0.118299

0.0752082

1.57937

0

0

0

0.0501388

0.42618

0.0501388

0.0752082

1.25347

0

0

0

0

0.0501388

0

0.557437

0.0501388

0

0

0.349124
5

0.0501388

0.0752082

0

0.225625

0.350972

0

0.175486

0.123536

0

0.0617678

1.16087

0.236598

0.100278

0.40111

0.125347

0.100278

0

0.0501388

0.185812

0

0.0501388

0
4

5674.28
4

0
6

519.345
3

1733
4

0
5

20.799
5

13.5526
5

175.77
4

70.8531
4

605.302
4

5.76759
4

0.68663

0.278718

0.376041

0.247071

0

0

0

0

0

0.0617678

2.32153
4

0.438165

0.274652

0.274652

0

0.557437

0

0.0642886

0.185812

0.411978

0

40.6936

0.549304

0.274652

0.823956

0.411978

0.278718

0.105735

0

0.128577

0.305547

0

1.76408
4

0

0.68663

0

0.0482912

0

0.68663

0

0.274652

0

1.37326

2.79279

0.0501388

0.123536

0.0926517

0.118299

0.185812

0.0553261

63.17

0.278718

0.464531

0.611094

2.13684

0

0

0.274652

0

1.23593

0.557437

0.274652

1.64791

0

0.185812

1.38696
4

0.0617678

0.274652

0.411978

0.557437

0

0.107261

0.0829891

0

0.274652

0.68663

3.91703

0.0553261

0

0

0.0926517

0

0.274652

0.549304

0.141492

0.549304

0

6.18374

0

0.396508

0

0.216187

0.961282

0.278718

12.7713

0.161286

0.105735

0.185812

0

1.30069

0

1.06941

0.185812

0.177448

0.0617678

0.222535

0.274652

0.278718

0.225625

728.609
4

0
7

1.23593

0

0.0617678

0.123536

1.32404

0.185812

0.107261

0

0.274652

0.0556337

0

0.185812

0

2.22975

0.123536

0.29211

0.278718

0

0.549304

0

0.278718

3.25617

1.85812

0.411978

0.203698

21.1482

0.549304

0.464531

0.349565

0.185812

0.118299

0.278718

12.7003

0.185812

0.0793016

0.411978

0.0829891

0.27663

0.411978

0.295101

0

0.411978

1.0487

3.81561

0

0.743249

0.274652

0.823956

0.549304

0

0

0.111267

0

0.0511526

9.2231
4

0.549304

0.194718

0

0.110652

0

0.464531

0

0

0.278718

0.0617678

10.787

0.274652

0.0617678

0.0829891

0.438165

0.0926517

0.0617678

0.411978

0.411978

0

0

0.117094

0.0617678

0

0.473196

0

0.110652

0.464531

0.411978

0.411978

0.823956

0

0.474592

1.01956

953.835
4

150.164

3.5915

1.12384

26.8744

5.17372

2.29101

0

0.185037

1.8845

1.01917

96.6231
3

1.27175

0.44063

0.466456

1.19519

1.74672

1.39359

0.739183

0

0.193641

0.433392

8.06417

0.208694

1.13836

0.101849

0

0

0.117401

0.823956

0.836155

191.316

45.7673

12.9759
4

0.177448

0.549304

1.78524

0.573264

0.0752082

0.185812

0.185812

0.193641

0.823956

0.600831

6.17175
4

1.5794

0.274652

0.719893

0.570116

2.19722

0.0617678

0.152774

0.24758

0.923863

0.530277

8.1766
4

1.43631

0

0.34364

0.68663

2.7945

0

0.185812

0

0.274652

0

2.18817
3

0.110652

0.823956

1.02197

0.177448

0.549304

0

0

1.92256

0

0.411978

1.82004411541925e-12
4

0
4

4.52209

0
8

0

0

0

0

0

0

0

0.0834505

0

0

0
8

0.107261

0

0.236598

0.185812

0.0553261

0

0

0.274652

0

0

0

0.274652

0.118299

0

0.185812

0.185812

0

0

0.278718

1.56947

0

0

0

0

0.218386

0

0

0.747848

0
4

0

0

0

0

0

0

0

0

0
4

0.0483291

0.0483291

0
4

0.118299

0.118299

0
4

0

0

0
4

0

0

0
4

0

0

0
4

0

0

0
4

0.58422

0.58422

0
4

0

0

0
4

0

0

0
4

0.0724936

0.0724936

0
4

3.21088
4

2.28135
4

0.525598

0.348602

0.0553261

7.7715611723761e-16
4

0
4

0

0

0
4

0

0

0
4

0.0483291

0.0483291

0
4

0

0

0
4

21.6562
4

21.5456
4

0.110652

0

1.59594559789866e-15
4

0
4

2.70707
4

0.790045

0.0553261

0.2551

1.6066

2.22044604925031e-16
4

0
4

0.442609

0.0829891

0.27663

0.0829891

0
4

1.13573

1.13573

0
4

0

0

0
4

0.442609

0.442609

0
4

0

0

0
4

0
4

182.339
3

46.6549
4

32.8389
4

0.464531

0.278718

8.64731

0.569713

0.163858

0

0.0926517

0.185812

0.254623

3.15881

0
4

1.20778

0.557437

0.464531

0.185812

0

8.32667268468867e-17

0
4

0.0617678

0.0617678

0
4

0

0

0
4

0

0

0
4

0.0528677

0.0528677

0
4

0

0

0
4

68.5321
3

11.5796
4

0

0

0.0528677

0.0617678

0.0528677

0.401491

15.3302

2.73326
4

4.96957

31.8854
4

0.114636

0.130322

1.22011

0
4

26.3997
3

23.5882
3

0.758571
4

2.05295
4

3.99680288865056e-15
3

0
4

13.0951
4

11.1438
4

0.606023

0.344416

0.689645

0.141492

0.16979

0

0
4

1.41712
3

0.152774

0.967566

0.217481

0.0793016

0

0

0

0
4

1.32616
4

0.876435
4

0.249849

0

0.199879

1.94289029309402e-16
4

0
4

7.43405
4

6.62675

0.560491

0

0.185037

0.0617678

0
4

14.5863

14.4005

0

0.185812

0
4

1.57072

0.401491

0.34364

0.268152

0.557437

1.11022302462516e-16

0
4

0
4

0

0

0

0

0

0
7

0

0

0

0

0

0

0

0
4

0

0

0

0

0

0
4

0

0

0

0

0

0

0
4

0

0

0

0
4

0

0

0
4

0
4

1.78226
4

1.51351
4

1.05255

0.193937

0.161286

0.105735

0
4

0.268754

0.0834505

0.185303

2.77555756156289e-17

0
4

1.11022302462516e-16
4

0
4

0

0

0

0

0

0

0
4

0

0

0

0
4

0
4

0

0

0

0

0
4

0

0

0

0
4

0

0

0
4

0
4

0

0

0

0

0

0
4

0
4

0
3

0
3

0
3

0

0
4

0

0

0
4

0
4

0.289299
4

0

0

0

0
4

0.289299

0.289299

0
4

0

0

0
4

0
4

0

0

0

0

0

0

0
4

0

0

0
4

0
4

1.83537

1.47089

0.768943

0.701943

0
4

0.364479

0.127882

0.118299

0.118299

0
4

0

0

0
4

0
4

0
6

0
6

0
6

0
4

0
4

9.94682

9.94682

9.94682

0
4

0
4

25.1358
3

24.5484
3

22.9669
3

0.0966581

0.185303

0.300587
3

0.179516

0.166968

0

0

0.652442

0

0

0
4

0

0

0

0

0

0
4

0.587366

0.504377

0

0.0829891

4.16333634234434e-17

0
4

2.22044604925031e-16
3

0
4

0

0

0

0

0

0
4

0

0

0
4

0
4

2.60226
4

0.580924

0.110652

0

0.470272

5.55111512312578e-17

0
4

0.876329

0.876329

0
4

1.14501

1.14501

0
4

0
4

0

0

0

0

0
4

0
4

0.775669
3

0

0

0
4

0.554057

0

0.554057

0
4

0.110652

0.110652

0
4

0.0556337

0.0556337

0
4

0.0553261

0.0553261

0
4

0

0

0
4

3.46944695195361e-17
3

0
4

0

0

0

0

0
4

0
4

0

0

0

0

0
4

0

0

0

0

0
4

0

0

0
4

0

0

0
4

0
4

14.4511

14.4511

12.4143

1.23653

0.492495

0.30781

0
4

0
4

0

0

0

0

0

0
4

0

0

0

0

0
4

0
4

0

0

0

0

0
4

0
4

0

0

0

0

0
4

0
4

28.4716
3

26.9449
3

8.63421
3

0

1.99623
4

14.1217

0

1.07984

0.349565

0

0

0.763337

0
4

0

0

0

0

0
4

0.763337

0.763337

0

0
4

0

0

0

0
4

0.763337

0.763337

0

0
4

1.55431223447522e-15
3

0
4

0.0528677

0

0

0

0
4

0

0

0

0
4

0.0528677

0.0528677

0
4

0
4

0

0

0

0

0

0

0

0

0
4

0
4

5.44115
4

5.39102
4

4.7899

0.107261

0.493853

0
4

0

0

0
4

0.0501388

0.0501388

0
4

4.85722573273506e-17
4

0
4

0.869923

0.869923

0.869923

0

0

0
4

0

0

0

0
4

0
4

0

0

0

0

0
4

0

0

0

0
4

0

0

0
4

0
4

2.32362

2.32362

2.32362

0

0

0

0
4

0

0

0
4

0
4

0.271639

0

0

0

0

0
4

0.16979

0.16979

0
4

0.101849

0.101849

0
4

0

0

0
4

1.38777878078145e-17

0
4

27.8213
4

27.8213
4

27.8213
4

0
4

0

0

0
4

0

0

0
4

0
4

0.613056
4

0.613056
4

0.342349

0.270707

0

0
4

0
4

0.0926517

0.0926517

0.0926517

0

0
4

0

0

0

0
4

0
4

105.848
3

67.0129
3

54.0494
3

3.85072
3

2.32203

5.07479

0

0

0.401491

1.02238

0.29211

6.27276008913213e-15
3

0
4

0

0

0
4

0.211471

0.211471

0
4

0.29211

0.29211

0
4

0

0

0
4

0.132169

0.132169

0
4

30.4784
4

30.3462
4

0

0.132169

0

0
4

7.33263
4

7.17821

0

0.15442

2.77555756156289e-17
4

0
4

0

0

0

0
4

0

0

0

0
4

0

0

0
4

0.107261

0.107261

0
4

0.126952

0.126952

0
4

0.15442

0.15442

0
4

3.22797344409764e-14
3

0
4

0
6

0
6

0
6

0
4

0
4

0.295101

0

0

0
4

0

0

0
4

0

0

0

0
4

0.295101

0.295101

0
4

0
4

1.54385

1.54385

1.25174

0.29211

0
4

0
4

0
3

0
3

0

0

0

0
4

0

0

0
4

0
4

0.0926517

0.0926517

0.0926517

0

0

0
4

0

0

0
4

0
4

2.3922

0

0

0
4

0.0553261

0.0553261

0
4

2.33688

2.33688

0
4

0

0

0
4

0
4

0

0

0

0

0

0
4

0
4

0

0

0

0

0

0

0
4

0

0

0
4

0
4

0.26581

0.144987

0.144987

0
4

0.120823

0.120823

0
4

0

0

0
4

1.38777878078145e-17

0
4

0

0

0

0

0

0

0
4

0
4

5.81454
5

5.81454
5

2.81743
5

0.763337

0

0

0

0

0

0

0

0

0

0.763337
5

0

0
4

1.23593

0.118299

0.116201

0

0

0

9.15933995315754e-16
5

0
4

0
4

0

0

0

0

0

0

0

0
4

0
4

0.240012

0.0553261

0.0553261

0

0

0

0
4

0.184686

0.184686

0
4

0
4

0

0

0

0

0
4

0
4

23.3689

0.22501

0.0642886

0

0.0642886

0.0964329

0
4

0.128577

0.128577

0
4

23.0153

23.0153

0
4

3.5527136788005e-15

0
4

2.53596

0.15442

0.0617678

0.0926517

0
4

0.123536

0.0617678

0.0617678

0
4

2.258

2.258

0
4

0
4

2.00566

0

0

0

0
4

2.00566

2.00566

0
4

0
4

1.19176

1.19176

0.886212

0.305547

0

1.11022302462516e-16

0
4

0
4

0

0

0

0

0
4

0

0

0
4

0
4

0

0

0

0

0

0
4

0

0

0
4

0
4

0

0

0

0

0

0
4

0
4

4.92598
5

4.92598
5

4.20045
5

0.53363

0

0.0846347

0

0

0

0

0.107261

2.77555756156289e-17
5

0
4

0
4

0.374791

0.207287

0.0528677

0.15442

0
4

0.0528677

0.0528677

0
4

0.0528677

0.0528677

0
4

0.0617678

0.0617678

0
4

0
4

0

0

0

0

0

0
4

0

0

0

0
4

0
4

0.786937

0

0

0

0

0
4

0.786937

0

0.786937

0
4

0
4

3.29681

1.98507

1.28594

0.699131

0
4

1.20447

1.20447

0
4

0.107261

0.107261

0
4

1.80411241501588e-16

0
4

0

0

0

0

0
4

0

0

0
4

0

0

0
4

0

0

0
4

0
4

0

0

0

0

0
4

0
4

1.15838
4

0.90376

0.346323

0.557437

1.11022302462516e-16

0
4

0.254623

0.254623

0
4

0
4

0

0

0

0
4

0
4

0.321783

0.107261

0

0

0.107261

0
4

0.214522

0.214522

0
4

0
4

2.70039

2.3756

0.123124

0.375413

1.87707

0
4

0.0501388

0.0501388

0
4

0.274652

0.274652

0
4

5.55111512312578e-17

0
4

30.6499
4

30.4964
4

23.1708
4

0

0

0

0

0

1.05826

0

0

6.26735

0

0

1.77635683940025e-15
4

0
4

0

0

0
4

0.0767289

0.0767289

0

0
4

0

0

0
4

0

0

0
4

0

0

0
4

0

0

0
4

0

0

0
4

0

0

0
4

0.0767289

0.0767289

0
4

3.49720252756924e-15
4

0
4

2.29303
4

2.17473

2.17473

0
4

0.118299

0.118299

0
4

0
4

0

0

0

0

0
4

0

0

0
4

0
4

0

0

0

0

0
4

0

0

0

0
4

0
4

0.113193

0.113193

0.113193

0

0
4

0

0

0
4

0
4

0

0

0

0

0

0
4

0

0

0

0
4

0
4

0

0

0

0
4

0
4

0

0

0

0

0

0
4

0

0

0

0
4

0
4

0

0

0

0

0
4

0

0

0

0
4

0

0

0
4

0
4

0.502244

0.449376

0

0

0.449376

0
4

0.0528677

0

0.0528677

0
4

2.08166817117217e-17

0
4

2.46248

1.96998

0.30781

0.184686

1.47749

0
4

0

0

0
4

0.492495

0.492495

0
4

0
4

0

0

0
6

0

0

0

0

0

0

0
4

0

0
6

0

0

0

0

0

0

0

0
4

0

0

0

0
4

0

0

0

0
4

0
4

0.92592

0.825642

0.123124

0.118299

0.29211

0.29211

0
4

0.100278

0.100278

0
4

2.77555756156289e-17

0
4

0

0

0

0

0

0
4

0

0

0
4

0

0

0
4

0
4

0

0

0

0

0

0
4

0

0

0
4

0
4

0

0

0

0
4

0
4

0

0

0

0

0
4

0

0

0
4

0

0

0
4

0
4

0.572148

0.214522

0.107261

0.107261

0
4

0.357626

0.160891

0.196734

0
4

0

0

0
4

5.55111512312578e-17

0
4

0

0

0

0
4

0
4

0.555784

0.555784

0.338303

0.0966581

0.120823

0
4

0
4

0

0

0

0

0
4

0
4

0

0

0

0

0

0

0
4

0
4

0

0
6

0
6

0

0

0

0

0

0

0

0
4

0
2

0

0

0

0

0

0

0
4

0
2

0

0

0

0

0
4

0

0

0

0

0
4

0

0

0

0
4

0

0

0

0
4

0

0

0
4

0
4

0.66865

0.66865

0.0501388

0.107261

0.236598

0.274652

0
4

0
4

0.185303

0.185303

0.185303

0

0
4

0

0

0

0
4

0
4

1.11703

1.11703

1.11703

0
4

0
4

0.587448

0.402762

0.347436

0.0553261

0
4

0.184686

0.184686

0
4

0
4

0

0

0

0

0
4

0

0

0
4

0
4

0

0

0

0

0
4

0
4

0

0

0

0

0

0
4

0
4

1.73401

1.73401

1.67224

0.0617678

3.46944695195361e-17

0
4

0

0

0
4

0
4

0.200555

0

0

0
4

0.200555

0.100278

0.100278

0
4

0
4

1.30654

1.23001

1.23001

0
4

0.07653

0.07653

0
4

4.16333634234434e-17

0
4

42.8125
5

42.8125
5

42.8125
5

0

0

0
4

0

0

0
4

0

0

0
4

0
4

0

0

0

0

0

0
4

0

0

0
4

0
4

0.101291

0.101291

0.101291

0

0
4

0
4

0

0

0

0
4

0
4

0.163089

0.163089

0.163089

0

0
4

0

0

0
4

0
4

0

0

0

0
4

0

0

0

0
4

0
4

0.138315

0

0

0
4

0.138315

0.138315

0
4

0

0

0
4

0
4

20.5617

20.5617

20.1923

0.246248

0.123124

0
4

0
4

0

0

0

0

0
4

0

0

0
4

0
4

0

0

0

0

0

0
4

0

0

0
4

0
4

0.222535

0.222535

0.0834505

0.0834505

0

0.0556337

0
4

0
4

0.287957

0.287957

0

0

0

0

0

0

0

0

0

0

0

0.287957

0

0

0

0

0

0

0

0

0

0

0

0

0

0

0

0

0

0

0

0

0

0

0

0
4

0

0

0

0

0

0

0

0

0

0

0
4

0
4

0

0

0

0

0

0

0

0

0

0

0

0

0

0

0

0

0
4

0
4

0

0

0

0
4

0

0

0
4

0

0

0
4

0
4

0.173263

0.173263

0.173263

0

0
4

0
4

0

0

0

0

0
4

0
4

0.567471

0.0581004

0.0581004

0
4

0.452774

0.452774

0
4

0.0565967

0.0565967

0
4

0
4

0.631806

0.193641

0.193641

0
4

0.438165

0.438165

0
4

0
4

2.07432

1.90834

1.90834

0
4

0.165978

0.165978

0
4

0
4

0.300833

0.300833

0.125347

0.175486

0
4

0
4

0

0

0

0

0
4

0

0

0
4

0
4

0.433332

0.236598

0.118299

0.118299

0
4

0.196734

0.196734

0
4

0
4

0

0

0

0

0
4

0

0

0
4

0
4

0

0

0

0

0

0

0

0

0

0

0

0

0

0

0

0

0

0

0

0

0

0
4

0

0

0

0

0

0

0

0
4

0

0

0

0
4

0
4

0

0

0

0

0
4

0
4

0

0

0

0
4

0
4

0

0

0

0

0
4

0
4

0.396508

0.237905

0.237905

0
4

0.158603

0.158603

0
4

0
4

0.335329

0.335329

0.250694

0.0846347

1.38777878078145e-17

0
4

0

0

0
4

0
4

0

0

0

0

0

0
4

0
4

0

0

0

0
4

0

0

0
4

0
4

0.281372

0.18872

0.18872

0
4

0.0926517

0.0926517

0
4

0
4

0

0

0

0

0
4

0

0

0
4

0
4

0.33958

0.33958

0.226387

0.113193

0
4

0
4

5.37026
3

5.37026
3

5.37026
3

0

0

0

0

0

0

0

0

0
4

0

0

0

0
4

0

0

0
4

0
4

0

0

0

0
4

0

0

0
4

0
4

0.185812

0.185812

0.185812

0

0
4

0
4

0

0

0

0
4

0

0

0
4

0

0

0
4

0
4

0

0

0

0

0
4

0

0

0
4

0
4

0

0

0

0

0
4

0

0

0
4

0
4

0.337233

0.286213

0.123124

0.163089

0
4

0.05102

0.05102

0
4

1.38777878078145e-17

0
4

0.173263

0.173263

0.123124

0.0501388

0
4

0
4

0

0

0

0
4

0
4

0

0

0

0

0
4

0
4

0

0

0

0

0
4

0

0

0
4

0
4

129.76
4

2.10043
4

0.614799

0.382783

0.0848951

0.306253

0.314732

0.20408

0.105735

0.0871506

1.66533453693773e-16
4

0
4

126.416
4

125.944
4

0.318459

0.07653

0.07653

0

1.16850973341798e-14
4

0
4

0.587978

0.238498

0.144874

0.0724369

0.132169

8.32667268468867e-17

0
4

0.392026

0.118365

0.273661

0
4

0.264339

0.264339

0
4

1.56541446472147e-14
4

0
4

0

0

0

0
4

0

0

0
4

0
4

0

0

0

0
4

0

0

0
4

0

0

0
4

0
4

0

0

0

0

0
4

0
4

0.351282

0.351282

0.185303

0.165978

0
4

0
4

2.17948

2.17948

2.17948

0
4

0
4

0.87584

0.629592

0.629592

0
4

0.246248

0.246248

0
4

5.55111512312578e-17

0
4

0

0

0

0
4

0
4

0

0

0

0

0
4

0
4

0.999396

0.999396

0.899457

0

0.0999396

2.77555756156289e-17

0
4

0
4

0

0

0

0

0
4

0
4

10.9799

8.78269
5

8.78269

0

0

0

0

0

0

0

0
4

2.19722

2.19722

0

0

0
4

0

0

0

0
4

0

0

0
4

0
4

0

0

0

0

0
4

0
4

0.214522

0.107261

0.107261

0
4

0.107261

0.107261

0
4

0
4

0

0

0

0
4

0
4

0.24758

0.24758

0.24758

0
4

0
4

0

0

0

0
4

0
4

0.375413

0.375413

0.268152

0.107261

1.38777878078145e-17

0
4

0
4

0

0

0

0
4

0

0

0
4

0
4

0

0

0

0

0
4

0
4

0

0

0

0

0
4

0
4

0.232402

0.116201

0.116201

0
4

0.116201

0.116201

0
4

0
4

0
2

0
2

0

0

0

0

0
2

0

0

0

0

0

0

0

0
4

0

0

0

0
4

0
4

0

0

0

0
4

0
4

0

0

0

0

0
4

0
4

0.0848951

0.0848951

0.0848951

0
4

0
4

0.58422

0.58422

0.29211

0.29211

0
4

0
4

0.16352

0.16352

0.110652

0.0528677

6.93889390390723e-18

0
4

0
4

0

0

0

0

0
4

0
4

0

0

0

0

0
4

0
4

0.281344

0.184686

0.184686

0
4

0.0966581

0.0966581

0
4

1.38777878078145e-17

0
4

0

0

0

0

0
4

0
4

0

0

0

0
4

0

0

0
4

0
4

0.282984
3

0.282984
3

0.226387
3

0

0

0

0.0565967

0

0

0

0
4

0
3

0

0

0

0

0

0

0

0
4

0
4

0.60728

0.60728

0.60728

0
4

0
4

0.370607

0.0617678

0.0617678

0
4

0.308839

0.308839

0
4

0
4

0

0

0

0
4

0

0

0
4

0
4

10.3459

10.0778

10.0778

0
4

0.268152

0.268152

0
4

6.10622663543836e-16

0
4

1.52667

1.52667

0.763337

0.763337

0
4

0
4

0

0

0

0
4

0

0

0
4

0
4

0.0483291

0.0483291

0.0483291

0
4

0
4

0

0

0

0

0
4

0
4

0.453001

0.453001

0.160891

0.29211

0
4

0
4

0

0

0

0
4

0

0

0
4

0
4

35.5482
4

24.726
4

12.6356
4

10.4568

0.694895

0.0553261

0.0553261

0.295747

0.354897

0.177448

0
4

8.42668

7.69033

0.414946

0.127763

0.0553261

0.138315

0
4

0.470272

0.193641

0.0829891

0.193641

0
4

1.39959

0.63625

0.763337

0
4

0.331957

0.193641

0.138315

0
4

0.193641

0.110652

0.0829891

0
4

0

0

0
4

0
4

0.371625

0

0

0
4

0.371625

0.371625

0
4

0
4

0.912881

0.912881

0.0553261

0.857554

0
4

0
4

0

0

0

0

0
4

0
4

0.203694

0.0964329

0.0964329

0
4

0.107261

0.107261

0
4

1.38777878078145e-17

0
4

1.21364

0.0846347

0.0846347

0
4

1.129

1.129

0
4

0
4

0

0

0

0
4

0

0

0
4

0
4

0

0

0

0

0
4

0
4

0.278811

0.278811

0.0642886

0.214522

2.77555756156289e-17

0
4

0
4

0

0

0

0
4

0

0

0
4

0
4

0.22501

0.22501

0.22501

0
4

0
4

2.00566
3

0
3

0
3

0
2

0

0

0

0

0
4

1.25656
3

1.25656
3

0

0

0

0
4

0.7491

0.7491

0

0
4

0

0

0

0

0

0
4

0

0

0
4

0
4

0.295747

0.295747

0.118299

0.177448

2.77555756156289e-17

0
4

0
4

0

0

0

0
4

0
4

0

0

0

0

0
4

0
4

0

0

0

0
4

0

0

0
4

0
4

0

0

0

0

0
4

0
4

0

0

0

0

0
4

0
4

0

0

0

0
4

0
4

0

0

0

0

0
4

0
4

0

0

0

0

0
4

0
4

0.367879

0.367879

0.0565967

0.311282

0
4

0
4

197.256
4

195.817
4

193.672
4

0

0.743249

0.609661

0.0483291

0.557437

0.185812

0

0
4

0

0

0
4

1.43879

1.43879

0
4

6.43929354282591e-15
4

0
4

0

0

0

0
4

0
4

0.29211

0

0

0
4

0.29211

0.29211

0
4

0
4

0

0

0

0

0
4

0
4

0

0

0

0
4

0
4

0

0

0

0
4

0

0

0
4

0
4

0.108581

0.108581

0.108581

0

0
4

0
4

0

0

0

0
4

0

0

0
4

0
4

0.241423

0.241423

0.118299

0.123124

0
4

0
4

0

0

0

0

0
4

0
4

1.27311

1.27311

1.27311

0
4

0

0

0
4

0
4

3.90785
2

3.60237
2

3.34145
2

0

0

0

0

0

0.260913
3

0

0

0

0

0

0

0
4

0.305484
2

0.112618
2

0

0

0

0

0

0

0

0

0

0

0

0
2

0.192866
3

0

0

0

0

0

2.77555756156289e-17
2

0
4

0

0

0

0
4

0

0

0

0

0

0
4

0

0

0
4

0

0

0
4

0
4

0

0

0

0

0

0

0
4

0

0

0
4

0

0

0
4

0
4

0

0

0

0
4

0

0

0
4

0
4

0.118299

0.118299

0.118299

0
4

0
4

0

0

0

0
4

0

0

0
4

0
4

0

0

0

0
4

0
4

0

0

0

0
4

0

0

0
4

0
4

0

0

0

0
4

0
4

0

0

0

0
4

0

0

0
4

0
4

2.34534

2.34534

2.34534

0
4

0
4

13.7401

13.7401

13.7401

0
4

0
4

0

0

0

0
4

0
4

0
7

0
7

0
7

0
8

0

0

0

0

0

0

0

0
4

0

0

0

0

0

0
4

0

0

0

0
4

0
4

0

0

0

0
4

0
4

0

0

0

0

0
4

0
4

0

0

0

0
4

0
4

0

0

0

0
4

0
4

0

0

0

0
4

0
4

1.32783

1.32783

1.32783

0
4

0
4

0.108581

0.108581

0.108581

0
4

0
4

0.0651483

0.0651483

0.0651483

0
4

0
4

0.184686

0.184686

0.184686

0
4

0
4

0

0

0

0
4

0
4

0
2

0
2

0
2

0
2

0

0

0

0
4

0

0

0

0
4

0

0

0
4

0

0

0
4

0
4

0

0

0

0
4

0
4

0

0

0

0
4

0
4

0

0

0

0
4

0
4

0.185812

0.185812

0.185812

0
4

0
4

0.214522

0.214522

0.214522

0
4

0
4

0

0

0

0
4

0
4

0

0

0

0
4

0
4

0.177448

0.177448

0.177448

0
4

0
4

0

0

0

0
4

0
4

0.196734

0.196734

0.196734

0
4

0
4

54.768
4

54.768
4

38.6568
4

8.60486

1.70973

0.887242

0.118299

0.650644

0.768943

3.37152

0
4

0
4

0

0

0

0
4

0
4

0

0

0

0
4

0
4

0.0434322

0.0434322

0.0434322

0
4

0
4

0.0829891

0.0829891

0.0829891

0
4

0
4

0

0

0

0
4

0
4

0.0999396

0.0999396

0.0999396

0
4

0
4

0

0

0

0
4

0
4

0

0

0

0
4

0
4

0

0

0

0
4

0
4

0

0

0

0
4

0
4

89.6544

89.3757

86.4027

1.85812

0.185812

0.185812

0.185812

0.371625

0.185812

1.72362124573056e-14

0
4

0.278718

0.278718

0
4

0
4

0

0

0

0
4

0
4

0

0

0

0
4

0
4

0

0

0

0
4

0
4

0

0

0

0
4

0
4

0

0

0

0
4

0
4

0

0

0

0
4

0
4

0

0

0

0
4

0
4

0

0

0

0
4

0
4

0

0

0

0
4

0
4

0

0

0

0
4

0
4

6.51402
3

6.09879
3

4.01894
3

0.123124
3

1.89244

0.0642886

0
4

0.415234

0.123124

0.29211

0

5.55111512312578e-17

0
4

0
4

0

0

0

0
4

0
4

0.123124

0.123124

0.123124

0
4

0
4

0

0

0

0
4

0
4

0

0

0

0
4

0
4

0

0

0

0
4

0
4

0

0

0

0
4

0
4

0

0

0

0
4

0
4

0

0

0

0
4

0
4

0

0

0

0
4

0
4

0

0

0

0
4

0
4

0.965798
4

0.178862
4

0.0553261

0.123536

0

0

0

0

0

0

0

0
4

0.786937

0

0.786937

0

0

0

0
4

0

0

0
4

1.11022302462516e-16
4

0
4

0

0

0

0
4

0
4

0.110652

0.110652

0.110652

0
4

0
4

0

0

0

0
4

0
4

0

0

0

0
4

0
4

0

0

0

0
4

0
4

0

0

0

0
4

0
4

0

0

0

0
4

0
4

0

0

0

0
4

0
4

0

0

0

0
4

0
4

0

0

0

0
4

0
4

10.796
3

10.796
3

10.7476
3

0

0

0

0.0483291

9.0205620750794e-17
3

0
4

0

0

0
4

0

0

0
4

0
4

0

0

0

0
4

0
4

0

0

0

0
4

0
4

0

0

0

0
4

0
4

0

0

0

0
4

0
4

0

0

0

0
4

0
4

0

0

0

0
4

0
4

0

0

0

0
4

0
4

0

0

0

0
4

0
4

0

0

0

0
4

0
4

0

0

0

0
4

0
4

20.377

20.377

16.9295

2.46248

0.615619

0.123124

0.246248

0
4

0

0

0
4

0
4

0

0

0

0
4

0
4

0.0642886

0.0642886

0.0642886

0
4

0
4

0

0

0

0
4

0
4

0

0

0

0
4

0
4

0.0642886

0.0642886

0.0642886

0
4

0
4

0.0556337

0.0556337

0.0556337

0
4

0
4

0

0

0

0
4

0
4

0

0

0

0
4

0
4

0

0

0

0
4

0
4

0

0

0

0
4

0
4

0

0

0

0

0

0

0

0

0
4

0

0

0

0

0
4

0

0

0

0
4

0

0

0

0
4

0

0

0
4

0
4

0.774565

0.774565

0.774565

0
4

0
4

0

0

0

0
4

0
4

0

0

0

0
4

0
4

0

0

0

0
4

0
4

0.0752082

0.0752082

0.0752082

0
4

0
4

0.175486

0.175486

0.175486

0
4

0
4

0

0

0

0
4

0
4

0

0

0

0
4

0
4

0

0

0

0
4

0
4

0

0

0

0
4

0
4

18.7753
3

1.98453
3

0.869073
5

0

0

0.0528677

0.0926517

0.0617678

0

0

0.149909

0.105735

0

0.0565967

0

0

0

0.278718

0.317206

0

0

1.11022302462516e-16
3

0
4

0.648562

0.586794

0.0617678

3.46944695195361e-17

0
4

0.101159

0.0528677

0

0.0482912

0
4

0.0565967

0

0

0

0.0565967

0
4

0.0528677

0

0

0.0528677

0
4

0.479944

0.318658

0.161286

0
4

0.211471

0.0528677

0.158603

0
4

0.321231

0.0793016

0.241929

0
4

0.105735

0.0528677

0.0528677

0
4

0

0

0

0
4

0.29211

0.29211

0
4

0.953945

0.557437

0

0

0

0

0

0

0

0.396508

0

0
4

0.105735

0.105735

0
4

0

0

0
4

2.86101
3

0.494142

0

0.0528677

1.16844

0

0.0617678

0

0

0

0

0

1.08379

0
4

0.878802
3

0.571249

0

0.109464

0.198088

0

0

8.32667268468867e-17
3

0
4

4.41334
4

3.29902

0.364195

0.288155

0.307553

0.0617678

0

0

0.0926517

0

0
4

3.95314

3.24281

0.494142

0

0.216187

0
4

0.424279
5

0

0.0528677

0.29211

0.0793016

0

0
4

0.816205
4

0.0528677

0

0

0

0.763337

0
4

0.114636

0.0528677

0

0

0.0617678

0
4

0
4

1.20109
3

1.11423
3

0.644158
3

0.0434322

0.259734

0.166901

0

0

0

0
4

0

0

0

0

0
4

0.0868644

0.0434322

0.0434322

0
4

6.93889390390723e-17
3

0
4

1.04937

1.04937

1.04937

0
4

0
4

0

0

0

0
4

0
4

0

0

0

0
4

0
4

0

0

0

0
4

0
4

0

0

0

0
4

0
4

0

0

0

0
4

0
4

0

0

0

0
4

0
4

0

0

0

0
4

0
4

0.160722

0.160722

0.160722

0
4

0
4

0

0

0

0
4

0
4

20.2761
4

9.19492
4

8.64568
4

0.118299

0.30781

0.123124

9.15933995315754e-16
4

0
4

11.0812

4.86243

1.52667

4.49536

0.196734

1.0547118733939e-15

0
4

0
4

0.196734

0.196734

0.196734

0
4

0
4

0.0617678

0.0617678

0.0617678

0
4

0
4

0

0

0

0
4

0
4

0

0

0

0
4

0
4

0.0617678

0.0617678

0.0617678

0
4

0
4

0

0

0

0
4

0
4

1.0487

1.0487

1.0487

0
4

0
4

0.07653

0.07653

0.07653

0
4

0
4

0

0

0

0
4

0
4

0

0

0

0
4

0
4

21.4081

21.4081

19.7687

0.546453

0.289299

0.321443

0.160722

0.321443

2.83106871279415e-15

0
4

0

0

0
4

0
4

0

0

0

0
4

0
4

0

0

0

0
4

0
4

0.163089

0.163089

0.163089

0
4

0
4

0.0528677

0.0528677

0.0528677

0
4

0
4

0

0

0

0
4

0
4

0.107261

0.107261

0.107261

0
4

0
4

0

0

0

0
4

0
4

0.0642886

0.0642886

0.0642886

0
4

0
4

0

0

0

0
4

0
4

0.160891

0.160891

0.160891

0
4

0
4

14.9314
4

5.39355

4.66777

0

0

0.161286

0.403215

0.161286

0

0
4

6.47342

5.49105

0.241929

0.322572

0.417876

2.22044604925031e-16

0
4

3.06443

0

3.06443

0
4

0

0

0
4

0
4

0

0

0

0
4

0
4

0

0

0

0
4

0
4

0

0

0

0
4

0
4

0

0

0

0
4

0
4

0

0

0

0
4

0
4

0

0

0

0
4

0
4

0

0

0

0
4

0
4

0

0

0

0
4

0
4

0

0

0

0
4

0
4

0

0

0

0
4

0
4

0.554057
3

0.554057
3

0.554057

0

0

0

0

0

0

0
4

0

0

0

0

0
4

0

0

0
4

0
4

0

0

0

0
4

0
4

0

0

0

0
4

0
4

0.0724936

0.0724936

0.0724936

0
4

0
4

0.0752082

0.0752082

0.0752082

0
4

0
4

0

0

0

0
4

0
4

0.107261

0.107261

0.107261

0
4

0
4

0.101849

0.101849

0.101849

0
4

0
4

0

0

0

0
4

0
4

0

0

0

0
4

0
4

0

0

0

0
4

0
4

0
7

0
7

0
7

0
7

0
4

0
4

0.236598

0.236598

0.236598

0
4

0
4

0

0

0

0
4

0
4

0.185812

0.185812

0.185812

0
4

0
4

0

0

0

0
4

0
4

0

0

0

0
4

0
4

0

0

0

0
4

0
4

0.15442

0.15442

0.15442

0
4

0
4

0

0

0

0
4

0
4

0.0501388

0.0501388

0.0501388

0
4

0
4

0.123124

0.123124

0.123124

0
4

0
4

1.85361
4

1.29403
4

0.435293
4

0.22959

0.415839

0.0556337

0.0556337

0.05102

0.05102

2.77555756156289e-17
4

0
4

0.30665

0.05102

0.20461

0.05102

0
4

0.252933

0.176403

0.07653

1.38777878078145e-17

0
4

1.11022302462516e-16
4

0
4

0.290502

0.290502

0.290502

0
4

0
4

0.0553261

0.0553261

0.0553261

0
4

0
4

0.29211

0.29211

0.29211

0
4

0
4

0

0

0

0
4

0
4

0.0501388

0.0501388

0.0501388

0
4

0
4

0.0642886

0.0642886

0.0642886

0
4

0
4

0

0

0

0
4

0
4

0.0434322

0.0434322

0.0434322

0
4

0
4

0

0

0

0
4

0
4

0

0

0

0
4

0
4

4.4709
4

2.40193
4

1.98526
4

0

0

0

0.354897

0.0617678

0
4

1.90512
4

1.10957

0.246248

0.549304

1.11022302462516e-16
4

0
4

0

0

0
4

0.0565967

0.0565967

0
4

0.107261

0.107261

0
4

6.93889390390723e-17
4

0
4

0

0

0

0
4

0
4

0.123124

0.123124

0.123124

0
4

0
4

0

0

0

0
4

0
4

0

0

0

0
4

0
4

0

0

0

0
4

0
4

0

0

0

0
4

0
4

0

0

0

0
4

0
4

0

0

0

0
4

0
4

0

0

0

0
4

0
4

0.0511526

0.0511526

0.0511526

0
4

0
4

0

0

0

0

0

0

0

0

0

0

0
4

0

0

0

0
4

0

0

0
4

0

0

0
4

0

0

0
4

0
4

0

0

0

0
4

0
4

0

0

0

0
4

0
4

0

0

0

0
4

0
4

0.449376

0.449376

0.449376

0
4

0
4

0

0

0

0
4

0
4

0

0

0

0
4

0
4

0.438165

0.438165

0.438165

0
4

0
4

0

0

0

0
4

0
4

0

0

0

0
4

0
4

0

0

0

0
4

0
4

0

0

0

0
7

0

0

0
4

0

0

0

0
4

0

0

0
4

0
4

0

0

0

0
4

0
4

0.0848951

0.0848951

0.0848951

0
4

0
4

0

0

0

0
4

0
4

0

0

0

0
4

0
4

0

0

0

0
4

0
4

0.107261

0.107261

0.107261

0
4

0
4

0.763337

0.763337

0.763337

0
4

0
4

0

0

0

0
4

0
4

0.185812

0.185812

0.185812

0
4

0
4

0

0

0

0
4

0
4

19.322
4

19.0713
4

0.326179

0.20461

1.71244

0

0

0

0

0

0

0

0

1.81592

0

0

0

0

0

0

0

0

0

0

3.00833

0

0

0

0

0

0

0

0

0

0

0

0

0

0

0

0

0

0

0

0

0

7.25747

0

0

0.102305

0

0

0.244634

1.86741

2.53201

0

0

0
4

0

0

0
4

0

0

0

0

0

0

0
4

0

0

0

0

0
4

0

0

0

0
4

0

0

0

0

0
4

0

0

0
4

0.100278

0.100278

0

0
4

0

0

0
4

0.150416

0.150416

0
4

0
4

0.390011
4

0.264664
4

0.102305

0.0871506

0

0

0

0.0752082

0
4

0.0501388

0

0.0501388

0

0
4

0

0

0

0
4

0.0752082

0.0752082

0

0
4

0
4

1.14501

1.14501

1.14501

0
4

0
4

0

0

0

0
4

0
4

0

0

0

0
4

0
4

0

0

0

0
4

0
4

0

0

0

0
4

0
4

0

0

0

0
4

0
4

0

0

0

0
4

0
4

0

0

0

0
4

0
4

0.238877

0.238877

0.238877

0
4

0
4

0.0434322

0.0434322

0.0434322

0
4

0
4

7.92933
4

5.69985
4

1.41819

0.0501388

0.296386

0.118299

3.81684

4.44089209850063e-16
4

0
4

1.34302
3

0.997074

0.185052

0.160891

0

0
4

0.123124

0.123124

0
4

0.763337

0.763337

0
4

6.66133814775094e-16
4

0
4

0

0

0

0
4

0
4

0

0

0

0
4

0
4

0

0

0

0

0

0

0
4

0

0

0

0
4

0

0

0
4

0
4

66.0488
4

16.9937
4

15.2786
4

1.46614

0.248967

1.47104550762833e-15
4

0
4

48.2652

7.81082

0.118299

0.0553261

40.1625

0.118299

2.83106871279415e-15

0
4

0.553261

0.138315

0.110652

0.110652

0.110652

0.0829891

0
4

0.236598

0.236598

0
4

0
4

2.25916
4

2.02048
4

0.639238

0.153179

0.113193

0

0.371625

0.743249

0

0

0
4

0.185812

0

0.185812

0

0
4

0.0528677

0.0528677

0
4

3.2612801348364e-16
4

0
4

0
3

0

0

0

0
4

0

0

0

0

0

0

0
4

0

0

0
4

0

0

0
4

0

0

0
4

0
4

1.32905
2

1.32905
2

1.32905
2

0

0

0
4

0
4

0.771184
3

0.185812

0

0

0.185812

0

0

0
4

0.107261

0

0

0.107261

0

0
4

0.107261

0

0.107261

0

0
4

0.185812

0.185812

0
4

0

0

0
4

0.185037

0.185037

0
4

2.77555756156289e-17
3

0
4

0.612818

0.152444

0.0511526

0.0501388

0

0

0.0511526

0

0
4

0.460374

0

0

0.460374

0

0

0
4

5.55111512312578e-17

0
4

3.54591
3

2.49889
4

0.561766
4

1.33475

0.0482912

0.132169

0.132169

0.289747

0
4

0.0793016

0

0.0793016

0

0
4

0.967716

0.967716

0
4

1.11022302462516e-16
3

0
4

27.8746
3

5.82832
3

3.05335
4

0

0.216187

0

0

0

0.804457

0.185037

0.0528677

0.0793016

1.14501

0.29211

0
4

0.107261

0.107261

0
4

0.236598

0.236598

0
4

0.29211

0.29211

0
4

8.94716
3

4.58002
5

0

0.0793016

0

0

2.62899

1.52667

0.0793016

0

0

0

0.0528677

0
4

2.14172
4

1.5063
4

0.273661

0.12755

0.05102

0.05102

0.132169

0

0
4

4.07679
3

0
7

0

0.453001

2.77504

0.449376

0.107261

0.29211

5.55111512312578e-16
3

0
4

3.80998
5

0.158603

0.58422

0

0

1.75266

1.31449

0

0
4

0.185303

0.123536

0

0

0.0617678

0

6.93889390390723e-18

0
4

1.90443

1.79717

0.107261

0
4

0.29211

0

0.29211

0
4

0.0528677

0.0528677

0
4

0
4

158.219

128.675

36.605

2.32265

89.7473

1.4210854715202e-14

0
4

28.3364

2.69428

24.4343

0.836155

0.371625

0
4

0.929061

0.929061

0
4

0.278718

0.278718

0
4

0

0

0
4

2.4980018054066e-15

0
4

2.57779

2.57779

2.57779

0

0

0

0

0

0

0
4

0

0

0
4

0
4

36.4516

33.9107

18.1167

0.278718

0.185812

0.185812

14.6792

0.185812

0.278718

1.83186799063151e-15

0
4

2.54085

1.21906

0.299819

0.836155

0.185812

8.32667268468867e-17

0
4

0
4

11.1663
4

11.1663
4

3.53275

5.12165

2.51194

0

0

0
4

0
4

0

0

0

0

0

0

0

0

0
4

0

0

0
4

0
4

0
7

0
7

0
7

0

0

0

0
4

0

0

0
4

0

0

0
4

0

0

0
4

0

0

0
4

0

0

0
4

0

0

0
4

0
4

1.9466
4

1.9466
4

1.10025

0.677078

0.0846347

0

0

0

0.0846347

2.77555756156289e-17
4

0
4

0

0

0

0
4

0
4

18.7346
3

18.7346
3

18.7346
3

0
4

0

0

0
4

0
4

0.345925
4

0.244634

0

0

0

0.244634

0
4

0.0511526

0.0511526

0

0

0
4

0.0501388

0

0.0501388

0

0
4

0

0

0
4

0
4

0

0

0

0

0

0

0

0

0
4

0

0

0

0
4

0
4

0
7

0
7

0
7

0

0

0

0

0

0

0

0
7

0

0
7

0
7

0

0

0

0

0
4

0
7

0

0

0

0

0
4

0
4

0.216478
4

0.216478

0.116201

0.0501388

0

0

0

0.0501388

0
4

0

0

0

0

0
4

0

0

0
4

0
4

0.287957

0.287957

0.287957

0
4

0

0

0

0
4

0

0

0

0

0
4

0

0

0

0
4

0
4

0

0

0

0

0

0
4

0
4

0
4

0
4

0
4

0
4

0
4

0.640834
4

0.0846347

0.0846347

0

0

0

0
4

0.471564

0.471564

0
4

0

0

0
4

0.0846347

0.0846347

0

0
4

0

0

0
4

0

0

0
4

0
4

0.0724936
2

0
2

0

0

0

0

0
4

0

0

0

0

0
4

0.0724936

0

0.0724936

0
4

0

0

0
4

0
4

13.531

13.4253

13.0904

0.15442

0.0482912

0.132169

0
4

0.105735

0.105735

0
4

4.71844785465692e-16

0
4

0
7

0
7

0
7

0

0
4

0

0

0

0
4

0
4

0

0

0

0

0
4

0

0

0

0
4

0

0

0
4

0
4

2.25771
4

1.81576

0.945806

0.482674

0.0829891

0.165978

0.138315

1.11022302462516e-16

0
4

0.441951

0.313374

0.128577

2.77555756156289e-17

0
4

0
4

3.16555
3

0.918252
3

0
2

0
3

0
2

0.918252

0

0

0

0

0
4

0

0

0

0

0

0

0
4

0.652442

0.652442

0

0

0

0
4

0.169152

0

0

0.169152

0
4

1.42571

1.42571

0
4

0

0

0
4

0

0

0
4

0
4

16.6461
4

16.6461

16.4044

0.145251

0.0964329

0
4

0

0

0

0

0
4

0

0

0
4

0

0

0
4

0

0

0
4

0
4

6.30887
4

1.02737

0.468701

0.274652

0.123124

0

0.160891

0
4

3.90824

0.123124

1.35436

0.214522

2.21623

0
4

0.274652

0.274652

0
4

1.09861

1.09861

0
4

0

0

0
4

4.44089209850063e-16
4

0
4

0

0

0

0

0

0

0

0

0

0
4

0

0

0

0

0
4

0
4

0
3

0
3

0
3

0

0

0

0
4

0

0

0

0
4

0

0

0
4

0

0

0
4

0
4

2.06303

1.82415

1.759

0

0

0.0651483

0

0
4

0.238877

0.173729

0.0651483

0
4

0
4

0
1

0

0

0

0

0

0

0
4

0

0

0
4

0

0

0
4

0

0

0
4

0

0

0
4

0

0

0
4

0
4

0.685238
3

0

0

0

0

0

0

0
4

0.685238
4

0.208431

0

0.198088

0.278718

0

0
4

0
4

0.274652

0.274652

0

0.274652

0
4

0

0

0

0
4

0

0

0

0

0
4

0
4

0

0

0

0

0

0

0

0

0
4

0
4

0.101849

0

0

0

0

0

0

0
4

0

0

0
4

0

0

0

0
4

0.101849

0.101849

0
4

0

0

0
4

0

0

0
4

0
4

5.35332
3

3.4876
4

1.53387
4

0

0

0

0

0

0

0.464531

0.46558

0

0.885304

0.0553261

0

0

0.0829891

0
4

1.08266
3

0
4

0

0

0.403215

0

0

0.494142
3

0

0

0.185303

0

0

0

0

0
4

0.590203
3

0

0.590203

0
4

0.192866

0.192866

0
4

0
4

0
2

0
2

0
2

0

0

0

0
4

0
4

0
7

0

0

0

0

0

0
4

0

0

0

0

0

0
4

0

0

0

0
4

0
4

24.7679
4

24.2823
4

23.3454

0.118299

0.763337

0.0553261

1.11022302462516e-15
4

0
4

0.319587

0.0829891

0.118299

0.118299

0
4

0.165978

0.165978

0
4

3.5527136788005e-15
4

0
4

0
7

0
7

0

0

0

0
4

0
4

6.74862
4

6.63032
4

2.73597

0.184686

3.43502

0.274652

0
4

0.118299

0.118299

0
4

1.66533453693773e-16
4

0
4

0.0581004
4

0

0

0

0

0

0
4

0

0

0

0

0
4

0.0581004

0.0581004

0
4

0

0

0
4

0
4

0
3

0
3

0

0

0

0
4

0

0

0
4

0

0

0
4

0
4

0
2

0
2

0

0

0

0

0
4

0

0

0
4

0
4

0.33958
3

0.33958
3

0.33958
3

0

0
4

0

0

0

0

0
4

0
4

1.68677
4

1.68677
4

1.17631
4

0.160891

0.349565

0
4

0
4

0.0482912

0.0482912

0.0482912

0

0
4

0
6

0

0

0
4

0

0

0

0

0
4

0

0

0
4

0

0

0
4

0

0

0
4

0
4

869.822
3

849.618
3

555.036
3

4.46622

273.606
3

5.82593

0
7

0.185812

0

0

0

0
7

1.81686

0.169152

2.35743

0

0.185812

0.0483291

0

0

0
6

0

0

0

0

0

0

0

0

0

0

0
7

0

0

0.120823

0

0.241138

0.0617678

0

0

0.0617678

0.0966581

0
7

0

0

0

0

0

0

0.0553261

0

0.0651483

0

0
7

0

0

0.0829891

0.120823

0

0

0.185037

0

0

0

0
7

0

0

0

0.0483291

0

0.144987

0

0.29211

0

0

2.59843

0.0434322

0

0.118299

0.763337

0

0

0.120823

0

0

0.0926517

0
7

0

0

0.120823

0

0

0

0.149909

0

0

0.0434322

0
2

0

0.29211

0

0

0

0

0

0

0

0

3.44946293751036e-13
3

0
4

0

0
5

0

0
7

0
7

0

0

0

0

0

0

0
4

0

0

0
4

0

0

0
4

0

0

0
4

1.08094

1.08094

0
4

0

0

0
4

0

0

0
4

0.149909

0.149909

0
4

0

0

0
4

0.730274

0.730274

0
4

0

0

0
4

14.0511
4

7.95448
4

5.92509
4

0.116201

0

0.0553261

0
4

0

0

0
4

0

0

0
4

0

0

0
4

0

0

0
4

0

0

0
4

0

0

0
4

0

0

0
4

0

0

0
4

0
7

0
7

0

0

0
4

0
7

0

0

0

0

0

0

0
4

3.02122

3.02122

0
4

0

0

0

0

0
4

0.533997

0.533997

0
4

0.0501388

0.0501388

0
4

0.586794

0.586794

0

0
4

0
4

67.3441

52.9548

0.678122
3

0

0

1.4616
4

0

0
6

0

0

0

0

0

0

0

0

0.354897

0.279312

0

0

0

0

0

0.0834505

0

0

4.07399

0

0

0

0

0

0

0

0

0

0

0

0

0.118299

0

0

0

0

0

0

16.8851

0

0

0

0.0767289

0

0

0

0

0

0

28.4862
4

0

0

0

0

0

0

0

0

0

0

0

0

0

0

0.0752082

0

0

0

0

0

0.0965825

0

0

0

0

0

0

0.118299

0.166901

0

0
4

6.12802
3

5.77065
3

0
2

0

0.229245

0.0724936

0

0

0.0556337

2.28983498828939e-16
3

0
4

0

0

0

0
4

0

0

0

0
4

1.11157

0.483291

0.628278

0
4

0

0

0
4

0

0

0

0
4

0

0

0

0
4

0.885655

0.639408

0.246248

0
4

0

0

0
4

0.161806

0.0651483

0.0966581

1.38777878078145e-17

0
4

0

0

0
4

0

0

0

0
4

0.207759

0.207759

0
4

0

0

0
4

0

0

0
4

0.123124

0.123124

0
4

1.22394

1.22394

0
4

0

0

0
4

1.16831

1.16831

0
4

0

0

0
4

0

0

0
4

0.123124

0.123124

0
4

0

0

0

0

0
4

1.75266

1.75266

0
4

0

0

0
4

0

0

0
4

0

0

0
4

0

0

0
4

0

0

0
4

1.50338
4

1.40672

0.0483291

0.0483291

0

1.80411241501588e-16
4

0
4

0
7

0
7

0
4

0

0

0

0

0
4

0

0

0

0

0

0
4

0

0

0

0

0
4

0

0

0
4

4.66293670342566e-15

0
4

605.661

604.512

0
6

132.892
4

0.159949

0.241645

0

0.253904

0

0.423173

0

0

0

0.111267

34.3414
3

0

0.356472

0.108501

0

0

0

0

0.185812

0

0

0
7

0.241645

0

0

0.0528677

5.94261

71.817

0

0

12.5843

0.737694

0
6

0

0

0.782399

0

0

0

0.253904

0

0

0

0.0846347
2

0

0

0

0.071564

0.773265

0

0

0

0.0581004

0

5.17042
4

0.332359

0

0.592443

0

0

0

0

0

0

0

10.3637
3

0

0

1.49369

0.0724936

0

0

0

1.04655

0.0846347

0

2.96127
4

0

0

0

0

0

13.7847

0.136084

0

0

0

0

0

0

0

0

0

0

0

0

0

0

6.87004
3

0

0

0

0

0

0

0

0.10204

0

0

0.407723
6

2.22158
3

0

0.550126

0

0

0

0

0

0

0

0

0

0.169269

0

0

0

0

0.142735

0

0.700771

0

0

6.20522
4

0

0

0

3.80856

0

0

0

0

0

0

0
6

0

0

0.126952

0

1.43879

0

0

0.101849

0

0

9.05119
4

0.17857

0

0

0

0

0.101849

0.253904

0

0

0

0
5

0

0

0

0

0

1.93513

0

0

0.0846347

0

0.326179
7

0

0

0

0

0.150416

0.0871506

0

0

0

0

0

0.916641

0.116201

0

0

0

0

0

0

0

0

4.57167
4

0

0

0

0

0

0.0846347

0

0

0.0617678

0

0
7

0.333802

0

0

0.101849

0

0

0

0

0

0

82.3066
4

20.5172
3

0.101849

0.152774

0.0834505

0.0724936

0

0.0846347

0

0

0

0

0

0

0

0.0617678

0

0

0

0

0

0

0

1.05593
4

0.465491

0

0.0846347

1.03907

0.163089

0

1.94636

0

0

0.0483291

0
6

0.116201

0

0

0

0.101849

0

0

0

0

0.0511526

0.973299
4

0.846347

0

0

0.0501388

0.0846347

0.0871506

0

0

0

0

0

0

0

0

0

0

0.0483291

0

0.0556337

0.0846347

0

1.76469
4

0

0

1.86196

0

0

0

0

0

0

0

1.5596

0

0

0

0

0

0

0

0.053673

0.0846347

0

1.35321

0.0871506

0

0

0

0

0.489268

0

0

0

0.0483291

0

0

0

1.18041

0.0483291

0

0

0.0483291

0

0

0

0
7

0.735178
3

0

0

0

0

0.296221

0

0

0

0

1.18489

0.504878
3

0

0

0

0

0.217481

0

0

0

0

0

0

0

0

0

0

0

0

0

0

0

0.0724936

0.265707
3

0

0

0

0

0

0

0

0

0

0

0

0

0

0

0.169269

0.429044

0.0846347

0

0

0

0

1.39404
4

0

0

0

0.319552

0

0

0

0

0

0

0
7

0

0.0846347

0

0

0

0.338539

0

0

0

0

0

0

0.0846347

0

0

0

0.101849

0

0.126952

0

0

0

0

0

0

0

0

0

0

0

0

0

0.471847
4

0.0483291

0

0

0

0

0

0

0

0

0

23.9011
4

0

0

0

0.0528677

0

0

0

0

0

0

0.0966581

0

0

0

0

0

0

0

0.26581

0

0.126952

0

0.0848951
2

0

0

0

0

0

0

2.27331

0

0.126952

0

5.94261

0.0846347

0

0

0

0

0

0

0.0846347

0

0

0

0

0

0

0

0

0

0

0

0

0

0

0

0.211587

0

0

0

0

0

0

0

0

0.053673

0

0

0

0

0

0

0

0

0

0

0.211587
3

0.0724936

0

0

0

0

0

0

0

0

0.0556337

0

0

0

0

0

0

0

0

0

0

0

0
6

0

0

0

0.0846347

0

0

0

0

0

0

66.2833

7.47982

0

0

0

0

0

0

0

0.0581004

0.126952

0

0

0.175486

0

0

0.253904

0

0

0.56017

0.719395

0

0

0.338539

0

0

0

0

0

0.0556337

0.105735

0

0.0793016

0

12.2042
4

0.300833

0.295101

0

0.0617678

0

0

0.169269

0

0

0

0.400586
4

0.489268

0

0

0

0

0

0.203351

0

0

0

0

0

0

0

0

0

0

0

0

0

0

0.586263
4

0.406703

0

0

0.0724936

0.0581004

0

0

0

0.126952

0

0

0

0

0

0

0

0

0

0

0.0846347

0

0.338303

0

0

0

0.0846347

0

1.10025

0

0

0

0

1.64597

0

0.877249

0

0.0966581

0

0

0

0

0

0

0
6

0

0

0

0

0

0

0

0

0

0.712943

0.0846347

0

0

0

0

0

0

0.144987

0.407723

0

0

0

0

0.0848951

0

0.250694

0

0.152774

0

0

0

0

0

0.492495

0

0

0.0846347

0

0

0

0

0

0

0

0

0

0

0

0

0

0

0

0

2.42444

0.0846347

0

0
6

0.26581

0

0.142735

0

0

1.88421

0

0

0

1.05793

0
6

0

0.29211

0.0565967

0

0.169269

0

0.243153

0

0

0

0
4

0

0

0

0
4

0

0

0
4

0.241645

0.193316

0.0483291

6.93889390390723e-18

0
4

0.123124

0.123124

0
4

0

0

0
4

0

0

0
4

0

0

0
4

0.414946

0.414946

0
4

0.369371

0.369371

0
4

0
4

0.251952

0.251952

0
7

0

0

0

0

0

0

0

0

0

0

0
7

0

0

0

0

0.108581

0.0999396

0.0434322

0

0

0

0

0

0

0

0
4

0
7

0
7

0

0

0

0
6

0
6

0

0

0

0

0

0

0
4

0

0

0
4

0
7

0

0

0
4

0

0

0

0
4

0

0

0

0

0
4

0

0

0
4

0

0

0
4

0

0

0
4

0

0

0
4

0

0

0
4

0
4

42.2101

13.0649
3

10.9963
3

0.362279

0

0

0

0.0556337

0.118299

0

0.918252

0.177448

0

0

0.169095

0.0483291

0

0

0

0

0.0483291

0

0

0

0.0501388

0

0

0

0

0

0

0

0

0

0

0

0.120823

0

0

0

0

0

0

0
4

0
7

0
7

0

0

0

0

0

0

0
4

0.500703
3

0.417253

0

0.0834505

0

4.16333634234434e-17
3

0
4

0

0

0
4

0

0

0
4

0

0

0
4

0

0

0
4

0

0

0
4

0.161286

0.161286

0
4

0.0483291

0.0483291

0
4

0
7

0
6

0

0
4

4.05002
3

0.0434322

3.30636

0

0

0.700223

0
4

0

0

0

0

0

0
4

0

0

0

0
4

0
7

0
7

0
4

0.763337

0

0

0.763337

0
4

0

0

0
4

0
7

0

0

0
4

0

0

0

0
4

0
6

0
6

0

0

0

0

0

0

0

0
4

0.918252

0.918252

0

0
4

0

0

0

0
4

7.39434

0.0483291

0.0483291

7.24936

0.0483291

9.0205620750794e-17

0
4

2.2656

2.2656

0
4

0

0

0

0

0

0
4

0

0

0

0
4

0

0

0

0
4

0

0

0

0
4

0

0

0
4

0

0

0

0

0
4

0.966581
7

0.966581
7

0

0

0

0
7

0

0

0

0

0

0

0

0
4

0

0

0
4

0.120823

0.120823

0

0
4

0

0

0

0

0
4

0

0

0

0
4

0

0

0

0
4

0

0

0
4

0

0

0
4

0

0

0
4

0

0

0
4

0

0

0

0
4

0
8

0
8

0

0

0

0

0

0

0

0

0
4

0

0

0
4

0.125347

0

0.125347

0
4

0

0

0

0
4

0

0

0

0
4

0

0

0

0
4

0.403215

0.403215

0
4

0.532345

0.532345

0
4

0.56017

0.56017

0
4

1.01491

0.555784

0.459126

0
4

0

0

0
4

0
8

0

0

0

0

0

0

0

0

0
4

0.483858

0.483858

0
4

0

0

0
4

0

0

0

0
4

0.241645

0.241645

0
4

0

0

0
4

0

0

0

0
4

0

0

0

0
4

0

0

0
4

0

0

0
4

0

0

0
4

0.669772
3

0.113477

0.241929

0.0724369

0

0.241929

0

0
4

0

0

0
4

0

0

0
4

0

0

0
4

0

0

0
4

0

0

0
4

0

0

0
4

0.0724936

0.0724936

0
4

0

0

0
4

0

0

0
4

0

0

0
4

1.69023

1.69023

0

0

0
4

0

0

0
4

0.0724936

0.0724936

0
4

0

0

0
4

0

0

0
4

0.0724936

0.0724936

0
4

0.0483291

0.0483291

0
4

0

0

0
4

0

0

0
4

0.0483291

0.0483291

0
4

0

0

0
4

0

0

0

0

0

0
4

0

0

0
4

0

0

0
4

0.0724936

0.0724936

0
4

0

0

0
4

0

0

0
4

0

0

0
4

0

0

0
4

0

0

0
4

0

0

0
4

0

0

0
4

5.43702

1.25656

3.4072

0.724936

0.0483291

0
4

0

0

0
4

0

0

0
4

0.0724936

0.0724936

0
4

0

0

0
4

0.120823

0.120823

0
4

0.0966581

0.0966581

0
4

0

0

0
4

0

0

0
4

0.0483291

0.0483291

0
4

0.0724936

0.0724936

0
4

8.17401701880272e-15

0
4

308.867
3

302.891
3

261.009
3

0
2

0.0553261
2

0

0

0

0.0483291

0.0793016

0

0

0

0

0.123124

0.206525
3

0

0

0.185812

0.278718

0

0

0

0

0

0

0.76231
3

0

0

0

0

0

0.0846347

0

0

0

0

0
2

0

0.0483291

0.108581

0

0.0511526

0

0.0483291

0

0

0.0617678

0

0

0

0.160722

0

0

0

0.274652

1.63685

0.0483291

0

0
2

0.185812

0

0

0.0868644

0.0511526

0.185812

0

1.50165

0.185812

0

0

0.438165

0

0

0

0.0617678

0

0

0

0

0

0.247071

0

0

0

0

0

0.0793016

0

0

0

0.185812

0
2

0

0

0

0.464531

0

0

0

0

0.185812

0

0

0

0.0767289

0.278718

0

0

0

0

1.58573

0.78178

0

5.80923

0

0

0

0

0.0483291

0

0

0

0.185812

0

0

1.46543

0

0

0

0.29211

0.0501388

0

0.127882

0

0

0

0.144987

0

0

0

0

0

0

0

0

0

0.278718

0

0

0

0

0.0556337

0

0

0

0

0.130297

0.846928

0

0.185812

0

0

0

0.120823

0.29211

0

0.69056

0.730274

0.0511526

0

0

0

0.184686

0.557437

0

0

0

0

0

0

0.304026

0

0

0
2

0

0

0.0617678

0.144987

0

0

0

0

0

0.0966581

0

1.84548

0

0

1.81396

0

0

0

0

0.650343

0.0617678

0
3

4.84317

0

0

0

0

0.456038

0

0

0

0.195445

0.370607

0.0999396

1.53043

0

0

0

0

0

0

0

0

0

0

0.650343

0

0

0

0.589935

0

3.34462

0

0

0.123536
3

0

0.464531

0

0.304026

0

0

0

0.163089

0.111923

0

0

0

0

0

0

0

0

0.0966581

0

0.464531

0

0
4

0

0

0

0

0

0
4

0

0

0

0

0
4

0.319172

0.319172

0
4

0.483634

0.105735

0.322572

0.0553261

0
4

0

0

0

0

0
4

0.120823

0

0.120823

0
4

0.154908

0.154908

0
4

0

0

0

0
4

0

0

0

0
4

0

0

0

0
4

0

0

0
4

0
3

0

0

0

0
4

0

0

0

0
4

0

0

0
4

0.0966581

0.0483291

0.0483291

0
4

0

0

0
4

0

0

0

0
4

0

0

0
4

0

0

0
4

0

0

0
4

0

0

0
4

0

0

0
4

0
2

0

0

0

0
4

0.0846347

0.0846347

0
4

0.0483291

0.0483291

0
4

0

0

0
4

0

0

0
4

0

0

0
4

0

0

0
4

0

0

0
4

0.236598

0.236598

0
4

0

0

0
4

0

0

0
4

0.408934

0.408934

0
4

0

0

0
4

0.184686

0.184686

0
4

0.430933

0.430933

0
4

0

0

0
4

0

0

0
4

0

0

0
4

0

0

0
4

0

0

0
4

0

0

0
4

0

0

0
4

0

0

0

0
4

0

0

0
4

0

0

0
4

0

0

0
4

0.0556337

0.0556337

0
4

0

0

0
4

0

0

0
4

0

0

0
4

0

0

0
4

0

0

0
4

0

0

0
4

0

0

0
4

0.110652

0.110652

0
4

0

0

0
4

0.740466

0.375413

0.241929

0.123124

5.55111512312578e-17

0
4

2.50038

2.07151

0.0793016

0.349565

0
4

0

0

0

0

0
4

0
4

4.89696
3

4.7897
3

0.873913
2

1.92261

0

0

0

0

0

0

0

0

0

0
2

0

0

0

0

0

0

0

0

0

0

0.699131
3

0.102305

0

0

0

0.58422

0

0

0

0

0

0.349565

0

0

0

0.214522

0

0.0434322

0

0

0

0

0

0

1.73472347597681e-16
3

0
4

0

0

0

0

0

0

0
4

0

0

0

0
4

0

0

0

0

0
4

0

0

0

0
4

0

0

0
4

0

0

0
4

0

0

0
4

0.107261

0.107261

0
4

0

0

0
4

4.02455846426619e-16
3

0
4

90.5507
5

90.4755
5

89.6216
5

0
7

0.0501388

0

0

0

0

0.701943

0

0

0

0

0

0

0.101849

0

0

0

0

0

0

0

0

0

0

0

0

0

0

0

0
4

0

0

0
4

0

0

0
4

0

0

0
4

0

0

0
4

0.0752082

0.0752082

0
4

0

0

0
4

0

0

0
4

0
4

2528.61
4

1727.72
4

1448.56
4

0
7

0

0

0

0.0501388

0

0

0

0.100278

0.0565967

0.123536

0.0964329

4.28222

0

0

0

0

0.268152

0.464531

0.268152

0.650343

0

0

50.3597

0

0.815446

5.118

0

1.72802

0

0

0.942255

0

183.75
4

0

0

0.300833

0.549304

0.125347

0

0

0

0

0.123536

0
6

0.530277

0.399371

0

0.651804

0.31439

0

0.340486

0.852359

5.88666

0

0
7

0

0.192866

0

0

0

0

0

3.88946

0

0.0501388

0
7

0.411978

0

0.10204

0

0

0

0.274652

0

3.05622

0.0501388

0.628278
7

0.192866

0

0.225625

0

2.18581

0.107261

0.200555

0

0.0642886

0

0
7

0

0

0

1.14501

0.699131

0.177448

0

0.0501388

0.184686

0

0.225625
5

0

0

0

3.84858

0

0

0

0

0

0

0
6

1.23593

0.0642886

0

0.184686

0.12755

0

0.29211

0.0642886

0

0.0848951

5.26023669067399e-13
4

0
4

1.11487
6

0.650343
6

0

0

0

0

0

0

0

0

0

0

0
6

0

0

0.464531

0

0

0

0

0

0

0

0
6

0

0

0

0

0

0

0

0

0

0

0

0

0

0

0

0

0

0

0

0

0

0

0

0

0

0

0

0

0

0

0

0

0

0

0

0

0

0

0

0

0

0

0

0

0

0

0

0

0
4

0
3

0

0

0

0

0

0

0

0

0

0

0
4

0

0

0
4

0.300833

0.150416

0.150416

0
4

0

0

0
4

0

0

0

0
4

0

0

0

0
4

1.09861

1.09861

0
4

0.848573

0.730274

0.118299

0
4

0.701943

0.150416

0.551527

0
4

0.128577

0.0642886

0.0642886

0
4

0

0

0
4

42.0185
4

40.5099
4

0.449433
4

0.536398

0.287957

0.184686

0.0501388

0

0
4

0

0

0

0
4

0.376041

0.300833

0.0752082

0
4

0

0

0
4

0.566762

0.566762

0
4

6.79381

6.29242

0.501388

0
4

0.123124

0.123124

0

0
4

0.214522

0.107261

0.107261

0
4

0.846325

0.438165

0.40816

0
4

0

0

0
4

0

0

0

0
4

0
2

0

0
3

0

0

0

0

0

0

0
4

12.0541

12.0541

0
4

0

0

0

0
4

0

0

0
4

0

0

0
4

0

0

0

0
4

0

0

0

0
4

0.17845

0.0553261

0.123124

0
4

0.576596

0.451249

0.125347

2.77555756156289e-17

0
4

1.10305

1.10305

0
4

0

0

0
4

0

0

0

0

0

0

0

0

0

0

0
4

0

0

0

0
4

0

0

0

0
4

0.104888

0.0565967

0.0482912

6.93889390390723e-18

0
4

0

0

0
4

0.0553261

0.0553261

0
4

0

0

0
4

0

0

0
4

0

0

0
4

0

0

0
4

0

0

0
4

5.85309
5

5.85309
5

0

0

0

0

0

0

0

0

0

0
4

0

0

0
4

0.107261

0.107261

0
4

0

0

0
4

0.274652

0.274652

0
4

0.0617678

0.0617678

0
4

0.107261

0.107261

0
4

0

0

0
4

0

0

0
4

0.0501388

0.0501388

0
4

0

0

0
4

0

0

0

0

0
4

0

0

0
4

0.107261

0.107261

0
4

0.107261

0.107261

0
4

0.10204

0.10204

0
4

0

0

0
4

0.160722

0.160722

0
4

0

0

0
4

0.246248

0.246248

0
4

0

0

0
4

0

0

0
4

0

0

0

0

0

0

0
4

0

0

0
4

0

0

0
4

0

0

0
4

0

0

0
4

0.150416

0.150416

0
4

0

0

0
4

0

0

0
4

0

0

0
4

0

0

0
4

0

0

0
4

9.17385

3.87318

3.51495

1.02238

0.763337

0
4

0

0

0
4

0

0

0
4

0.123124

0.123124

0
4

0

0

0
4

0

0

0
4

0.55591

0.55591

0
4

0

0

0
4

2.01608

2.01608

0
4

0

0

0
4

0.100278

0.100278

0
4

2.67341
5

2.67341
5

0

0
4

0

0

0
4

0.123124

0.123124

0
4

0

0

0
4

0

0

0
4

1.46055

1.46055

0
4

0

0

0
4

0.68663

0.68663

0
4

0

0

0
4

0.43367

0.43367

0
4

0

0

0
4

1.28713

0.965349
5

0.321783

0

0

0

0

0
4

0

0

0
4

0

0

0
4

0.464531

0.464531

0
4

0

0

0
4

0

0

0
4

0

0

0
4

0

0

0
4

0

0

0
4

0.0642886

0.0642886

0
4

0

0

0
4

177.268
4

156.142
4

3.11057

1.39439

0.631609

1.31449

0.510476

0.319172

0.107261

0

0.375413

0.160891

7.90316
4

0

0

1.07261

0.29211

0.160722

0.268152

0.268152

0

0.107261

0.929397

0.482674

0.343361

0.564107

0.372803

0.32992

0.107261

6.28525009815917e-14
4

0
4

3.25917

2.699

0.56017

0
4

0

0

0
4

0

0

0
4

0

0

0
4

0

0

0
4

0

0

0
4

0.278718

0.278718

0
4

0

0

0
4

0

0

0
4

0.438165

0.438165

0
4

0

0

0
4

0
6

0

0

0

0

0

0

0

0

0
4

0.275763

0.275763

0
4

0.274652

0.274652

0
4

0

0

0
4

0.33958

0.33958

0
4

0

0

0
4

0

0

0
4

0.482674

0.482674

0
4

0.0752082

0.0752082

0
4

0

0

0
4

0.0501388

0.0501388

0
4

0
3

0

0

0

0

0

0

0
4

0

0

0
4

0.107261

0.107261

0
4

0.05102

0.05102

0
4

0

0

0
4

0

0

0
4

0

0

0
4

0

0

0
4

0

0

0
4

0.29211

0.29211

0
4

0.29211

0.29211

0
4

6.2513
4

1.37801

2.38246

1.78524

0

0.430933

0

0

0.274652

5.55111512312578e-16
4

0
4

0

0

0
4

0.268152

0.268152

0
4

0.10204

0.10204

0
4

0

0

0
4

0

0

0
4

0

0

0
4

0

0

0
4

0.29211

0.29211

0
4

0.763337

0.763337

0
4

0

0

0
4

2.92242
3

0.407396

1.86196

0.169269

0.145251

0.338539

0
4

0

0

0
4

0

0

0
4

0.128577

0.128577

0
4

0.236598

0.236598

0
4

0.161286

0.161286

0
4

0

0

0
4

0

0

0
4

0

0

0
4

0.823956

0.823956

0
4

218.024
4

217.142
4

0.429044

0.29211

0.160891

0
4

0.0565967
2

0

0

0.0565967

0

0

0

0
4

0

0

0

0

0

0

0
4

1.84148
4

0.826024

0.30781

0.224973

0

0

0.482674

1.66533453693773e-16
4

0
4

0

0

0

0

0
4

33.1797
4

4.49193
4

0.338575

0.174301

0

0.430933

0

0.0501388

0

0.236598

0.126952

0.295747

20.4047
4

0.0553261

0.100278

0.123124

0.175486

0

0.523332
3

2.07073
4

0.96311

0.864302

0.913143

0.677888

0.163089

3.88578058618805e-15
4

0
4

7.80299
4

6.93339
4

0.0767289

0

0.792866

2.22044604925031e-16
4

0
4

0
4

0

0

0

0

0

0

0
4

2.23545

0.371085

0.321443

0.353587

0.45002

0.0642886

0.67503

0
4

0

0

0

0

0

0

0

0

0
4

0
7

0
7

0

0

0
4

10.7916
4

10.7916
4

0

0
4

1.17987

1.17987

0

0

0
4

0
7

0
7

0
4

2.59101
4

0.268152

2.02775

0.295101

2.77555756156289e-16
4

0
4

1.63967

0.763337

0.438165

0

0.438165

0
4

112.247
4

77.1515
4

0.858088

0.268152

0.107261

0.160891

20.9327
4

3.9042
4

1.8299

0.489342

0.766807

0.763337

4.90777

0.107261

0
4

0

0

0

0
4

0

0

0

0

0
4

0
6

0
6

0
4

3.37503
5

3.37503
5

0
4

0

0

0

0
4

0.594265

0.396177

0.198088

0
4

12.5992

12.5992

0
4

1.23593

0.68663

0.549304

0

0

0
4

3.71625

3.71625

0

0

0

0
4

0

0

0

0

0

0
4

0

0

0

0

0

0

0

0

0

0

0

0
4

4.62071

4.24254

0.285518

0.0926517

1.66533453693773e-16

0
4

0.127882

0

0.127882

0
4

0.665505

0.116201

0.549304

0

0

0
4

0

0

0

0

0
4

0.24758

0

0.0617678

0.185812

0
4

0

0

0
4

0

0

0

0
4

3.51379

3.51379

0
4

0

0

0

0
4

0

0

0

0
4

0

0
3

0

0
2

0

0

0

0

0

0

0

0
4

0.274652

0.274652

0

0

0
4

0.150416

0

0.150416

0
4

0

0

0
4

0.451249

0.125347

0.100278

0.100278

0.125347

0
4

0

0

0

0
4

0

0

0

0

0
4

0.254685

0.16979

0.0848951

0
4

0

0

0
4

0

0

0

0
4

0.293785

0.293785

0
4

31.6924
4

15.5169
4

15.8001
4

0.160891

0

0.214522

2.80331313717852e-15
4

0
4

0.116201

0.116201

0
4

0

0

0

0

0
4

0

0

0

0

0
4

0.719395

0.63476

0.0846347

0
4

0

0

0
4

0.50937

0.226387

0.282984

0
4

0.0501388

0

0.0501388

0
4

0

0

0
4

0

0

0

0
4

0.148923

0.0642886

0

0.0846347

1.38777878078145e-17

0
4

42.9041
4

40.3832
4

0

0.224973

1.81965

0.0642886

0.411978

0

0

4.71844785465692e-15
4

0
4

0.814173

0.107261

0.0617678

0.645144

0
4

0

0

0

0

0
4

6.72897

1.64791

5.08106

0
4

0

0

0

0

0
4

0.524039

0.524039

0
4

0.407094

0.265602

0.141492

0
4

0.325902

0.175486

0.150416

0
4

0

0

0

0
4

0

0

0
4

0.165978

0.165978

0

0
4

0
6

0
6

0
4

0

0

0

0
4

0.321783

0.321783

0
4

0.321783

0.321783

0
4

0

0

0
4

0

0

0

0
4

0

0

0

0
4

0.29211

0.29211

0
4

0

0

0

0
4

0

0

0

0
4

0

0

0
4

0
4

41.5792

41.5792

41.5792

0
6

0

0

0

0

0

0

0

0

0

0

0

0

0
4

0
6

0

0

0
4

0

0

0
4

0

0

0
4

0

0

0
4

0

0

0
4

0
4

349.264
3

341.392
3

125.156
3

173.24
4

0

4.9947

0

0.833409

0

0

0

0.876329

0

0

16.2707
4

0

0

0.141492

0

0.0651483

0.876329

0

0

0

0

8.62129

0

0

0.730274

0

0

0.138315

0

0

0

0.29211

7.23797
4

0

0

0

0.0434322

0.141492

0

0

0.0829891

0

1.43326

0.0642886

0

0

0.152013

0
4

0
6

0
6

0

0

0

0
4

0

0

0
4

0

0

0
4

0

0

0
4

0

0

0
4

7.30668
4

7.30668
4

0
4

0

0

0

0
4

0

0

0

0
4

0

0

0
4

0

0

0
4

0.565967

0.0565967

0.50937

0
4

0

0

0
4

0

0

0
4

0
4

45.7334

43.6862

21.1706

0
7

0
6

0

0

0

0

0

0

0.0483291

0

0

0

0

0

0

0

0.116201

0

0

0.0483291

0

0.0966581

0

0
7

0

0

0

0

0

0

0

0

0

0

0

0

0

0

0.724936

0

0

0

0

0

0

0

0

0

0

0

0

0

0

0

0

0.0483291

0.549668

0

0

0

0

0

0

0

0

0.29211

0

0
5

0

0

0

0

0

0

0.0483291

0

0

0

0
7

0
7

0
7

0

0

0

0

0

0

0.0483291

0

0

0

0
6

0

0.241645

0

0

0

0

1.40154

0

0

0

0
7

0

0.289974

0.371625

0

0

0

0

0

0

0

3.6478

0.7491

0

0

0

0

0

0

0

0

0

0
5

10.8934

0

0

0

0

1.20778

0

0

0

0

0
7

0

0

0

0

0

0

0

0

0

0

0

0

0.0483291

0

0

0

0.0483291

0

1.59486

0

0

0

0

0

0

0

0

0

0

0

0

0

0
4

0
7

0
7

0

0

0

0

0

0

0

0

0

0
7

0
8

0
7

0

0

0

0

0

0
4

0

0

0
4

0

0

0
4

0

0

0
4

0

0

0
4

0

0

0
4

0.0483291

0.0483291

0
4

0

0

0

0

0
4

0

0

0

0

0

0

0

0

0

0
4

0
4

0
4

0

0

0

0
4

0

0

0

0

0
4

0

0

0

0
4

1.99879

1.99879

0
4

0

0

0
4

0

0

0
4

2.66453525910038e-15

0
4

64.5843

62.68

0

0

0.417253

0.144987

0

0

0.217481

0.120823

0.0556337

0

0

0.144987

0
6

0

0

0

0.459126

0

0

0

0

0

0

0
7

0.0871506

0.0724936

0

0.676607

0

0.169152

0

0.0724936

0.0724936

0

0

0.0483291

0

0

0

3.03569

0

0

0

0.0483291

0.306916

0

0

0

0

0.0724936

0.26581

0

0

0

0

0.289974

0
7

1.06324

0

0

0

0.144987

0.0966581

0

0

0.0724936

0.217481

0

0

0

0

0

0

0

0

0

0

0.966581

4.9054

0.0724936

0

0

0

0.0483291

0.169152

0.314139

0

0

0.26581

0

0

0

0

0.217481

0.0483291

0

0

0

0.0483291

0

0
6

0

0

0

0

0
7

0

0
7

2.60977

0

0
7

0
6

0

0
7

0

0
6

0

0

0

0

0

0

0.386632

0

0

0.966581

0.555784

0

0

0

0

0

23.1012

0

0

0.26581

0

0.373288

0
7

0

0

0

0.264327

15.5136

0

0

0

0

0

0
7

0.120823

0.314139

0

0

0

0

0.0966581

0.169152

0.338303

0

0
7

0.217481

0.193316

0

0

0

0.169152

0.120823

0

0

0

0
7

0

0

0

0.362468

0

0

0

0

0

0

0
6

0

0.362468

0.0483291

0

0

0.459126

0

0

0.241645

0

2.83384427035571e-14

0
4

0
6

0
6

0
4

1.53905

1.53905

0
4

0.193316

0.193316

0
4

0.120823

0.120823

0
4

0

0

0
4

0

0

0
4

0

0

0
4

0.0511526

0.0511526

0
4

0
4

45.9553
3

45.8828
3

43.7673
3

1.83887
3

0.0553261

0

0

0.221304

0
4

0.0724936

0.0724936

0

0
4

0

0

0
4

0

0

0
4

0

0

0
4

0

0

0
4

3.78863607153335e-15
3

0
4

39.7044

39.4215

15.998

0
7

0

0

0

0

0.247071

0

0

0

0

0.107346

0
6

0

0

0

0

0

0

0.639408

0

0

0

0.632851
3

0

0

0

0

0

0

0.0868644

0

0

0

0.170348
3

0

0

0

0

0

0

0

0

0

0

0.0434322
3

0

0

0

0

0

0

0

0

0

0

0
7

0

0

0

0

0

0

0

0

0.652442

0

0
7

0

0

0

0

0

0

0

0

0

0

0
7

0

0

0

0.617678

0

0

0

0

0

0

0.035782
3

0

0

0

0

0

0

0

0

0

0

0
6

0

0

0

0

0

0

0

0

0

0

0
7

0.0767289

0

0

0

0

0

0

0

0

0

0

0
3

0

0

0

0

0

0

0

0

0

0

0

0

0

0

0

0

0

0.0434322

0

0

1.44649

0.160722
3

0

0.152013

0

0

0

0

0

0

0

0

0

0

0.0511526

0

0

0

0

0

0.7491

0

0

0
7

0

0

0

0

0

0

0

0

0.0767289

0

0
7

0

0

0

1.64319

0

0

0

0

0

0

0.0767289

0.0434322

0

0

0

0

0

0

0

0

0

1.17651
4

0

0

0

0

0

0

0

0

0.102305

0

0

0

0

0

0

0

0

0

0

0

0

0
7

0
7

0

0

0

0

0

0

0

0

0

0

0.0511526
3

0

0

0

2.17736

0

0

0

0

0

0

0

0.0767289

0

0

0

0

0

0

0

0

0

0
7

0

0

0

0

0

0

0

0

0

0

0
3

0

0

0

0

0

0

0

0

0.216187

0

0

0.0617678

0

0.278718

0

0.0767289

0

0

0

0

0

0

0

0

0

1.57508

0

0

0.0767289

0

0

0

0

0

0

0

0

0

0

0

0

0

0.0767289

0

0

0

0

0

0

0

0

0

0

0

0

0

0

0

0

0

0

0

0

0

0

5.74897
4

0

0

0

0

0

0

0

0

0

0.0617678

0

0

0

0

0.089455

0

0

0

0

0

0

0.0434322

0

0

0

0

0

0

0

0

0.0511526

0

0

0

0

0

0.101849

0

0
7

0.0511526

0

0

0

0.338303

0

0

0

0

0

0
7

0

0

0

0

0

0

0.803496

0

0

0.101849

0.101849
3

0

0

0

0

0

0

0

0

0

0

0
7

0.127882

0

0

0

0.254623

0

0

0

0

0

0
7

0

0

0

0

0.339723

1.509

0

0

0

0

1.35447209004269e-14

0
4

0

0

0
4

0.199879

0.199879

0
4

0

0

0
4

0

0

0
4

0

0

0
4

0

0

0
4

0.0829891

0.0829891

0
4

0

0

0
4

0
4

2547.1

160.629
3

0
6

0
7

0.130297

0

0.926468

0

0

3.21321

0

0

0

0

0
6

0

0.361015

0

0

0

0

0.152013

0

0

0

2.91593
3

0

0.217161

0

0

0

2.09739

0

0

0.410822

0

0
6

1.16844

0

0

0

0.305547

0.128577

0.203698

0

1.58105

0

8.01773
2

0

0.628284

0

0

0

0

0

0

0

0.101849

0

0

0

0.101849

0.29211

0

0.101849

0

0

0

0

6.22037
4

0

0

0

0

0

0

0.876329

0

0

0

0

0

0.58422

0

3.05335

0

0

0

0

0

0.263367

0

0

0

0

0

0

0.101849

0

0

0.58422

0

0
7

2.19924

0

0

0

0

0

0

0

0

0

5.85028
2

7.29296

0

2.2501

0

0.152774

0

0.0642886

0

0.173729

0.549304

0

0
6

0.549304

0

0

0

0.0642886

0

0

0

0

0.402

0
6

0

0

0

0

0.481072

0.236598

0

0.254623

0.730274

0

0
6

0

0

0

0.274652

0

0

0

0

0

0

0

0

0

0.29211

0

0

0

0

5.59304

0.49947

0

0
6

0

0

0

0

0

0.0565967

0

0.29211

0.101849

0

0

0.152774

0

0

0.763337

0

0

0

0

0

0.458321

0
2

0

0

0

0

0

0.0434322

0.29211

0

0

0.611094

0
6

0.101849

0

0.58422

0

0

0

0

0

0

0

0
7

0

0

0

0.58422

0

0

0

0

0

0

0.152774
7

3.84086

0

0

0

0

0

0

0.101849

0

0

0

2.55095
4

0

0

0

0.29211

0

0

0.0565967

0

0

0

0
7

0

0

0.29211

0

0

0

0.29211

0

0

0.0434322

0.56017
5

0

0

0.163089

0

0

0

0

0.101849

0.961282

0

0

0

0

0

0.411978

0

0

0

0.113193

0

0

0
7

0

0

0

0

0

0.16979

0.101849

3.7078

0

0

1.84375

0

0

0

0

0

0.0999396

0

0

0

0

0

0

0

0.249849

0

0

0.250694

0

0.0793016

0

0

0

0

0

0

0

0

0

0.107261

0

0

0

0.128577

0

0

0

0.29211

0.0848951

0

0.763337

0

0

0

16.0032

0
7

0.101849

0

0

0

0

0

0.295747

0

0

0.0434322

0.101849

0.0848951

0

0

0

0

0.274652

0

0

0

0

0.916641

0

0.101849

0

0

0

0.438165

0.438165

0.0434322

0

0

0

0

0.101849

0

0

0

0.282309

0

0

0

0

0

0

0

0

0.438165

0

0

0

0

0

0.712943

0.305547

0

0

0

0

0

0

0.356472

0.489268

0.274652

0

2.95973

0

0

0

0.150416

0

0

0

0

0

0

0

0.438165

0.29211

0.0501388

0

0

0

0

0.203698

0

0

0

0

0

0

0

0

0.29211

0.29211

0

0.0999396

0

0

0

0

0.763337

0

0.0528677

0

0.152774

0

0

0

12.5205
4

1.01849
5

0

0

0

0

0

0

0

0

0

0

0

0.29211

0

0.128577

0.118299

0

0

0

0.101849

0

0.0434322

0

0

0

0

0

0.0553261

0

0

0

0

0.763337

0

0

0

0

0

0

0

0

0

0

0

1.94081

0.0501388

0

0

0

0

0

0

0

0

0

7.92685

0.699131

0

0.254623

0

0

0

0

0

0.411978

0

0

0.0846347

0.29211

0.128577

0.58422

0

0

0.195445

0

0

0

0

0

0

0

0

0

0

0

0

0

0.101849

0.356472

0

0

0

0

0

0.05102

0

0.29211

0

0.0752082

0

0

6.31464

0

0

0

0

0

0

0

0

0
7

0

0

0.20408

0

0

0.0617678

0

0

0

0

0

0
7

0

0

0.268152

0

0

0

0

0

0

0
7

2.09416

0

0

1.24648
4

0

0

0.603065
4

2.44438
5

0

0

0

0.509245

0

0

0

0

0

1.23593

0.118299
2

0

0

0

0

0

0

0

0

0

0

0
6

0

0.356472

1.46055

0

2.22489

0

0

0

0.942884

0

5.62883073484954e-14
3

0
4

165.639

138.118

0

0

0

0

0

0

0

0

0

0

0

0
6

0

0

0

0

0

0.101849

0

0

0

0

0
7

0

0

0

0

0

0

0

0

0.203698

0

0

0

0.152774

0.916641

0

0

0

0

0.254623

0

0

0

0

0

0

0

0

0

0

0

0

0

0

0

0

0

0

0

0

0

0

0

0

0
7

0

0

0

0

0

0

0

0

0

0

0

0

0

0

0

0

0
7

0.305547
7

0

0

0

0

0

0
6

0

2.33

0.101849

0

0
7

0

0

0

0

0.254623

0

13.2913

0

0

0.152774

0

0.803608

0

0

0

0

0

0

0

0.712943

0

5.14725
5

0

0

0

0

0

0

0

0

0

0

0.152774
7

0.152774

0

0

0

0

0

0

0

0

0

0

0

0

0

0

0

0

0

0

0

0

0

0

0

0

0

0

0

0

0

0

0

2.28225

0

0

0.203698

0

0

0

0

0

0

0

3.111400026512e-14

0
4

0.612051
2

0.612051
2

0

0

0

0
4

0

0

0

0
4

0

0

0

0
4

0

0

0
4

0

0

0

0
4

0

0

0
4

0

0

0
4

0

0

0

0
4

1.74894

0

1.74894

0
4

0

0

0

0
4

0

0

0
4

383.064
4

327.498
4

1.89871

46.1533

1.16844

3.79743

1.31449

0.29211

0.438165

0.0651483

0.438165

2.47024622979097e-14
4

0
4

0

0

0
4

1.72973

0.123124

1.6066

0
4

0.549304

0.274652

0.274652

0
4

0

0

0
4

0.938823

0.175486

0.763337

0
4

0

0

0
4

0

0

0
4

0

0

0
4

0

0

0
4

0

0

0
4

0
6

0
6

0

0

0

0

0

0

0

0

0
4

0.101849

0.101849

0
4

0

0

0
4

0.0501388

0.0501388

0
4

0

0

0
4

0

0

0
4

1.00785

1.00785

0
4

0

0

0
4

0.438165

0.438165

0
4

0.0964329

0.0964329

0
4

0.107261

0.107261

0
4

0
3

0
3

0

0

0

0

0

0

0

0

0

0
4

0

0

0
4

0.107261

0.107261

0
4

0

0

0
4

0.113193

0.113193

0
4

0

0

0
4

0.0848951

0.0848951

0
4

0.29211

0.29211

0
4

0.185812

0.185812

0
4

0.0752082

0.0752082

0
4

0

0

0
4

0
6

0
6

0

0

0

0

0

0
4

0.0752082

0.0752082

0
4

0.763337

0.763337

0
4

0

0

0
4

0.29211

0.29211

0
4

0.438165

0.438165

0
4

0

0

0
4

0

0

0
4

0

0

0
4

0.0829891

0.0829891

0
4

0

0

0
4

37.5061
3

36.7374
3

0.378125

0.333987

0.0565967

0

0

7.43849426498855e-15
3

0
4

1.14501

1.14501

0
4

0

0

0
4

0

0

0
4

0

0

0
4

0

0

0
4

0.0964329

0.0964329

0
4

1.52667

1.52667

0
4

0

0

0
4

0.113193

0.113193

0
4

0

0

0
4

0

0

0

0

0
4

0

0

0
4

3.65137

3.65137

0
4

0

0

0
4

0

0

0
4

0

0

0
4

51.6884

51.6884

0
4

0

0

0
4

0

0

0
4

0.289299

0.289299

0
4

0

0

0
4

0.546453
3

0.192866
3

0

0

0

0

0

0.353587

0

0

0

5.55111512312578e-17
3

0
4

0

0

0
4

0.257154

0.257154

0
4

0

0

0
4

0

0

0
4

0.0724369

0.0724369

0
4

0

0

0
4

0.0752082

0.0752082

0
4

0

0

0
4

0

0

0
4

0

0

0
4

5.72169

0

0

0

5.72169

0

0

0

0
4

0.356472

0.356472

0
4

0

0

0
4

0.0501388

0.0501388

0
4

0

0

0
4

0

0

0
4

5.0687

5.0687

0
4

0.0434322

0.0434322

0
4

0.876329

0.876329

0
4

0

0

0
4

0

0

0
4

0.101849
5

0

0

0

0

0

0

0

0.101849

0

0
4

0

0

0
4

0

0

0
4

0

0

0
4

0

0

0
4

0

0

0
4

0

0

0
4

0

0

0
4

0

0

0
4

0

0

0
4

0

0

0
4

168.503

151.134

0

1.01158

3.31086

2.74992

0

0

9.78783

0

0

0

0
7

0.101849

0

0

0

0

0

0

0.101849

0

0

0.203698

0.101849

0
4

125.596
4

18.1885
4

106.036

0.163089

0

0.185812

1.02238

1.99840144432528e-14
4

0
4

0

0

0
4

0

0

0
4

0

0

0
4

0

0

0
4

0

0

0
4

0

0

0
4

0.150416

0.150416

0
4

0

0

0
4

0

0

0
4

0

0

0
4

59.312

56.1303

1.76621

0.904201

0.274652

0.236598

0
4

0

0

0
4

0

0

0
4

0

0

0
4

0

0

0
4

0

0

0
4

0.123124

0.123124

0
4

0
6

0

0

0

0

0

0
4

5.37444
4

1.15629

0.591494

0.0752082

0.118299

2.88385

0.549304

1.11022302462516e-16
4

0
4

5.951
4

5.24497

0.101849

0.254623

0

0.349565

6.10622663543836e-16
4

0
4

1.74783

0

0

1.74783

0

0

0

0
4

328.188
4

287.096

40.6033

0.438165

0.0501388

0
4

11.8598

6.20951

1.31449

1.41469

1.46055

1.46055

8.88178419700125e-16

0
4

0

0

0

0

0
4

0
6

0

0

0

0

0
4

820.843
4

512.077
4

12.6654

0.438165

0.730274

1.50633

2.48729

128.82

1.31449

0.730274

1.02238

0.0651483

13.2034

0.0868644

0.29211

0.29211

0.58422

0.730274

0.29211

0.0434322

0.29211

1.46055

0.438165

12.1292

0.58422

0.438165

0.29211

0.876329

0.29211

0.58422

0.438165

1.6066

0.0434322

0.29211

8.94314

0.29211

0.438165

1.89871

0.438165

0.730274

0.438165

3.35926

0

0.438165

0.876329

62.9192

0.438165

0.730274

0

1.14501

0.0651483

0

0.163089

17.2345

0.0651483

0.730274

3.21321

0.0434322

0.730274

0.438165

0.0434322

0.438165

0.803496

16.3491

0.29211

0
4

0
6

0
6

0
4

0
7

0

0

0
4

0.78178

0.738348

0

0.0434322

0

3.46944695195361e-17

0
4

9.0554

6.1343

1.16844

1.75266

0
4

2.17161

1.02066

1.04237

0.108581

0
4

9.70011

6.8663

1.46055

1.09861

0.274652

0
4

4.45424

2.11736

2.33688

0
4

1.29922

0

1.29922

0
4

0

0

0

0
4

0

0

0

0

0
4

0.108581

0
7

0

0

0.108581

0

0

0

0

0

0

0

0
6

0

0

0

0

0

0

0

0

0

0

0

0

0

0

0
2

0

0

0

0
4

0

0

0

0

0
4

0

0

0

0

0
4

0.128577

0
7

0.128577

0
4

0

0

0

0

0

0
4

0
7

0

0

0

0

0
4

0.720304

0.346121

0.196734

0.177448

0
4

0.332492

0

0.332492

0

0
4

1.70688

0.342249

0.0501388

1.31449

0

0
4

0

0

0

0

0
4

0.325742

0.0868644

0.238877

0
4

0
6

0
6

0

0

0

0

0

0

0

0

0
4

0

0

0

0
4

0

0

0

0
4

0

0

0

0
4

0.369174

0.217161

0.108581

0.0434322

0
4

0

0

0

0
4

0

0

0

0

0
4

4.38492

4.27766

0.107261

4.02455846426619e-16

0
4

0.192866

0.192866

0

0
4

0

0

0

0

0
4

0.0752082

0

0.0752082

0

0
4

0.835752

0
6

0

0

0

0

0.160722

0

0.0642886

0.610742

0

0

0

0

0

0

0

0

0

0

0

0

0

0

0

0

0

0

0

0
4

0

0

0

0

0
4

0

0

0
4

0

0

0
4

0

0

0

0

0
4

0

0

0

0

0
4

0

0

0

0

0
4

0

0

0

0

0
4

0

0

0

0
4

10.5741

10.5741

0
4

0

0

0

0

0
4

0
6

0
6

0

0

0

0

0
7

0

0

0

0

0

0

0

0
4

0

0

0

0

0
4

0

0

0

0
4

0.697196

0.697196

0
4

0

0

0
4

2.70663

2.62174

0.0848951

0

5.55111512312578e-17

0
4

0.353587

0.353587

0

0
4

0

0

0

0

0
4

0

0

0
4

0.27658

0.27658

0
4

0

0

0

0
4

1.04237

1.04237

0

0

0

0

0

0

0
4

0

0

0

0

0
4

0

0

0
4

0

0

0
4

2.85813

1.6696

1.18853

0
4

0

0

0

0
4

0.517734

0.29211

0.225625

0
4

0

0

0

0
4

4.11978

0.823956

3.29582

0
4

0

0

0

0
4

0.324791

0.324791

0
4

11.6142
3

8.01931
3

0.282984

0.44718

1.12132

0

0.249092

0.16979

0

0.594265

0.730274

1.22124532708767e-15
3

0
4

0.412606

0.152013

0.260593

0
4

0

0

0

0
4

0.0642886

0.0642886

0

0
4

0

0

0

0
4

0

0

0
4

0

0

0

0
4

0.876329

0.438165

0.438165

0
4

117.963

117.963

0
4

0

0

0

0
4

0

0

0

0
4

1.406874616805e-12

0
4

7.29414750821888e-10

0
4

2280.22
4

2249.25
4

2175.73
4

757.754
4

7.03093

0

0.763337

0.0846347

0

0

0

0.763337

0.763337

0

0

0

0.524348

0

0.763337

0

0

0.763337

0.123124

1.52667

0

0

0

1.52667

0.763337

0.763337

0.160891

0

0

0.763337

0

0

0

7.41479

0.349565

0

1.14501

0.763337

6.1067

0

0

0

0

0.214522

1.67166
4

0.763337

0

0.763337

0.763337

0.763337

0

0

0.763337

0

0

1.99068
4

0

0

0

0

0

0

0

0.763337

0.763337

0

1.39826

1.41155

0.763337

0.0846347

2.29001

1.90834

1.90834

0.763337

0.68663

0

1.0487

2.92288
4

0.17857

0

3.05335

0.0617678

0

0

0

0

0

0

16.7934

0.0581004

0.38265

0

0.763337

0

0.163089

0.0926517

0

0.138315

1.14501

1.17724
4

0

0.524348

18.7018

0

0.763337

0.349565

0.763337

0

395.912
5

0.203351

0

0

0

0.981174

0

11.4501

0

0

42.7469

1.52667

0

0

17.9384

0

462.201

0

44.6552

2.67168

0

0

0

2.67168

4.96169

0.352787

1.90834

0

0

0

0.364547

3.81669

3.43502

0
6

0.763337

1.52667

1.52667

0

0

4.96169

4.96169

5.02346

1.90834

0

31.6785

0

0

2.29001

0

0

3.05335

1.1068

6.40298

4.19836

1.52667

29.0686

0

0

1.52667

0

0

9.92339

0

1.52667

0

0

182.988
4

0.763337

0

1.52667

3.43502

2.67168

0

0

0.763337

1.14501

0.0553261

9.28868
4

0

0

0.123124

0

1.14501

0

0

0

0.763337

0.160891

0
4

63.854
4

36.5287

0

23.7797

0

0

0.524348

0.823956

2.19722

0
4

0

0

0
4

0

0

0

0

0

0
4

4.80641

0.549304

0

4.25711

0

0
4

2.33454

1.64791

0.68663

0
4

0.873913

0.873913

0

0
4

1.64791

1.64791

0

0
4

0

0

0
4

0

0

0
4

0

0

0
4

0
4

0

0

0

0

0

0

0

0
4

0
4

0

0

0

0

0
4

0
4

0.747848

0.274652

0.274652

0

0
4

0.473196

0.473196

0
4

0
4

0.697205

0.697205

0.697205

0

0
4

0
4

0

0

0

0

0
4

0
4

0

0

0

0
4

0
4

0

0

0

0
4

0
4

0

0

0

0
4

0
4

0

0

0

0
4

0

0

0
4

0

0

0
4

0
4

0.557182

0.0926517

0.0926517

0
4

0.464531

0.464531

0
4

5.55111512312578e-17

0
4

0

0

0

0
4

0

0

0
4

0
4

14.9635
4

14.9635
4

12.6858

0.535102

0.184686

1.37326

0

0.184686

1.66533453693773e-16
4

0
4

0
4

0

0

0

0
4

0
4

0

0

0

0
4

0

0

0
4

0
4

0

0

0

0
4

0
4

0

0

0

0
4

0
4

0

0

0

0

0
4

0
4

0

0

0

0

0
4

0
4

0.05102

0.05102

0.05102

0
4

0
4

0

0

0

0
4

0
4

0

0

0

0
4

0
4

0.123124

0.123124

0.123124

0
4

0
4

0

0

0

0

0

0

0
4

0

0

0
4

0

0

0
4

0

0

0
4

0
4

0.371625

0.371625

0.371625

0
4

0
4

0

0

0

0
4

0
4

0.161286

0.161286

0.161286

0
4

0
4

0

0

0

0
4

0
4

0

0

0

0
4

0
4

0

0

0

0
4

0
4

0

0

0

0
4

0
4

0

0

0

0
4

0
4

0

0

0

0
4

0
4

0.0724936

0.0724936

0.0724936

0
4

0
4

0
6

0
6

0

0

0

0
4

0
4

0

0

0

0
4

0
4

0

0

0

0
4

0
4

0

0

0

0
4

0
4

0

0

0

0
4

0
4

0.0846347

0.0846347

0.0846347

0
4

0
4

0

0

0

0
4

0
4

0

0

0

0
4

0
4

0

0

0

0
4

0
4

0

0

0

0
4

0
4

0

0

0

0
4

0
4

0

0

0

0

0

0

0
4

0
4

0

0

0

0
4

0
4

0

0

0

0
4

0
4

0

0

0

0
4

0
4

0

0

0

0
4

0
4

0

0

0

0
4

0
4

0

0

0

0
4

0
4

0

0

0

0
4

0
4

11.5538

11.5538

11.5538

0
4

0
4

0

0

0

0

0

0
4

0
4

0.719239

0.719239

0.719239

0
4

0
4

0.866373

0.743249

0.557437

0.185812

0
4

0.123124

0.123124

0
4

0
4

7.60280727263307e-13
4

0
4

290.356

191.929
5

131.767
5

0.814792
6

130.952
5

0
4

0

0

0

0

0

0

0
4

0

0

0
4

0

0

0
4

0

0

0
4

0

0

0
4

0

0

0
4

0

0

0
4

0

0

0
4

0

0

0
4

0

0

0
4

0

0

0
4

32.0958
4

32.0958

0

0

0

0

0

0
4

0

0

0
4

0

0

0
4

0

0

0
4

26.817

26.817

0

0
4

0

0

0

0
4

1.24916

0.987707

0.261452

1.11022302462516e-16

0
4

0

0

0
4

0

0

0

0
4

0

0

0
4

0

0

0
4

3.10862446895044e-15
5

0
4

86.8465

28.821

0
7

3.86049

0

0.161286

0

0

0

0

0

0

0.0926517

0.0617678

0

0

0

0

0

0

0

0

0

0

0

0
7

0

0

0

0

0

0

0

0

0

0.161286

0
7

0

0

0

0

0

0

0

0

0

0

0
7

0

0

0

0

0

0

0

0

0

0

0

0

0

0

0

0

0

0

0

0

0

0
7

0

0

0

0

0

0

0

0

0

0

0

0

4.87966

0

0
6

0
6

0

0

0.371625

0

3.87606

0
7

0
7

0

0.589027

0
8

0

0.161286

0

0

0

0

0

0

0

0

0
7

0

0

0

0

0

0.15442

0

0

0

0

0
7

0

0.15442

0

0

0

0

0

0.836155

0

0

11.1071

0

0

0.15442

0

0

0

0.403215

0

0.277955

0

0

0

0

0

0

0

0

0

0

0

0

1.51809

0

0

0

0

0

0

0

0

0

0

0
7

0

0

0

0

0

0

0

0

0

0

0
4

4.52257

0
7

0

0

0.958548

0

0

0

0

0

0

0

0
7

0

0

0

0

0

0

0

0

0

0

0
7

0

0

0

0

0

0

0

0

0

0

0
6

0

0

3.56402

0

0

0

0
4

0

0
7

0

0

0
7

0
7

0

0

0

0

0

0

0
7

0

0

0

0

0

0

0

0

0

0

0
8

0

0

0

0

0

0

0

0

0

0

0
8

0

0

0

0

0

0

0

0

0

0

0
7

0

0

0

0

0

0

0
7

0
7

0
7

0
7

0
4

0

0

0

0

0

0

0

0

0

0

0

0

0

0

0

0

0

0

0

0

0
4

52.7961

0
7

45.5209

6.10174

0

0

0

0

0

0

0

0

0

0

0

0

1.17346

0

0

1.11022302462516e-15

0
4

0

0

0
4

0
7

0
7

0

0

0

0
4

0

0

0

0

0

0

0

0

0
4

0

0

0

0

0

0

0
4

0.15694

0.15694

0
4

0.35714

0.35714

0
4

0.192866

0.192866

0
4

0

0

0
4

0

0

0
4

0

0

0
4

0

0

0
4

0
4

0

0

0

0

0

0

0

0

0

0

0
4

0

0

0

0

0
4

0
4

0

0

0

0

0
4

0

0

0
4

0
4

0

0

0

0
4

0
4

0

0

0

0

0
4

0

0

0
4

0
4

0

0

0

0

0

0
4

0
4

0.241929

0.241929

0.241929

0
4

0

0

0
4

0
4

0

0

0

0
4

0

0

0
4

0
4

0

0

0

0
4

0
4

0

0

0

0

0
4

0
4

0

0

0

0
4

0
4

0

0

0

0
4

0

0

0
4

0
4

0
7

0
7

0

0
7

0

0

0

0

0
4

0

0

0
4

0
4

0

0

0

0
4

0
4

0

0

0

0
4

0
4

0

0

0

0
4

0
4

0

0

0

0
4

0
4

0

0

0

0
4

0
4

0

0

0

0
4

0
4

0

0

0

0
4

0
4

1.0487

1.0487

1.0487

0
4

0
4

0

0

0

0
4

0
4

0

0

0

0
4

0
4

10.2899

8.91899

8.91899

0

0
4

1.37093

1.37093

0
4

0
4

0

0

0

0
4

0
4

0

0

0

0
4

0
4

0

0

0

0

0

0
4

0

0

0
4

0
4

0

0

0

0
4

0
4

0
6

0

0

0

0

0
4

0

0

0
4

0

0

0
4

0
4

0

0

0

0

0

0
4

0

0

0

0
4

0
4

0

0

0

0

0
4

0
4

0

0

0

0

0
4

0
4

1.26121335597418e-13

0
4

0.241836

0.166628

0.0483291

0

0

0

0

0

0.0483291

0

0

0

0

0

0
1

0

0

0

0

0

0

0

0
4

0

0

0

0
4

0

0

0

0
4

0

0

0
4

0.118299

0.118299

0
4

0

0

0
4

0

0

0
4

0
4

0

0

0

0
4

0
4

0

0

0

0
4

0
4

0

0

0

0

0
4

0
4

0

0

0

0
4

0
4

0

0

0

0
4

0
4

0.0752082

0.0752082

0.0752082

0
4

0
4

0

0

0

0
4

0
4

0

0

0

0
4

0
4

0

0

0

0
4

0
4

0

0

0

0
4

0
4

0
4

35.6827
5

35.6827
5

29.8564
5

27.1759
5

1.22011

0

1.37326

0.0871506

0

0

0

0

0
4

1.14501

0

0

1.14501

0
4

2.29001

2.29001

0

0

0
4

2.15467

2.15467

0
4

0

0

0

0

0
4

0.236598

0.236598

0
4

0

0

0
4

5.99520433297585e-15
5

0
4

0
4

6.45978
4

6.45978
4

6.45978
4

2.52409
4

0

0

0.0556337

0

3.51919
4

0

0.196734

0.0556337

0.108501

0

0

0

5.13478148889135e-16
4

0
4

0
4

0
4

0

0

0

0

0

0
4

0

0

0
4

0
4

0
4

0

0

0

0

0

0
4

0
4

0
4

0

0

0

0

0

0
4

0

0

0

0
4

0
4

0
4

0

0

0

0

0

0
4

0
4

0
4

0

0

0

0

0

0

0
4

0
4

0
4

0

0

0

0

0
4

0
4

0
4

0

0

0

0

0

0
4

0
4

0
4

11.9311

11.9311

11.9311

11.9311

0
4

0
4

0
4

0

0

0

0

0

0
4

0

0

0
4

0
4

0
4

0

0

0

0

0
4

0
4

0
4

0
2

0
2

0
2

0

0

0

0

0

0

0

0

0

0
4

0

0

0
4

0
4

0

0

0

0

0

0

0

0
4

0

0

0

0
4

0
4

0
4

0

0

0

0

0
4

0

0

0
4

0
4

0
4

4.40091

4.40091

4.40091

2.67168

1.14501

0.58422

3.33066907387547e-16

0
4

0
4

0
4

0

0

0

0

0
4

0

0

0
4

0
4

0
4

1.22173

1.22173

1.22173

1.09861

0.123124

8.32667268468867e-17

0
4

0
4

0

0

0

0
4

0
4

0
4

0

0

0

0

0
4

0
4

0
4

0

0

0

0

0
4

0

0

0
4

0
4

0
4

0

0

0

0

0
4

0
4

0
4

0

0

0

0

0

0
4

0

0

0
4

0
4

0
4

0

0

0

0

0
4

0

0

0
4

0
4

0
4

0

0

0

0

0

0

0
4

0
4

0
4

0
7

0
7

0
7

0
7

0
7

0

0
4

0
7

0
7

0

0

0
4

0

0

0

0

0
4

0

0

0

0
4

0
4

0
4

0.28061

0.28061

0.28061

0.17857

0.10204

0
4

0
4

0
4

0

0

0

0

0
4

0

0

0
4

0

0

0
4

0
4

0
4

0

0

0

0

0
4

0

0

0
4

0
4

0
4

0

0

0

0

0

0
4

0
4

0
4

0.275763

0.275763

0.275763

0.200555

0.0752082

0
4

0
4

0
4

0

0

0

0

0
4

0
4

0
4

4.39443

4.39443

4.39443

4.39443

0
4

0
4

0
4

0

0

0

0

0
4

0
4

0
4

1.36194

1.36194

1.36194

1.06983

0.29211

5.55111512312578e-17

0
4

0
4

0
4

0

0

0

0

0
4

0

0

0
4

0
4

0
4

14.7742

14.7742

3.02441

3.02441

0

0

0

0

0

0

0

0

0

0
4

0.204154

0.204154

0
4

11.5456

0.591494

10.9541

0
4

0
4

0

0

0

0

0
4

0
4

0
4

0.0724369

0.0724369

0.0724369

0

0.0724369

0
4

0
4

0
4

0.556337

0.556337

0.556337

0.556337

0
4

0

0

0
4

0
4

0
4

1.72464

1.72464

1.72464

0

1.72464

0
4

0
4

0
4

1.01898

1.01898

1.01898

1.01898

0
4

0
4

0
4

0

0

0

0

0

0
4

0

0

0
4

0
4

0
4

0

0

0

0

0
4

0

0

0
4

0
4

0
4

0

0

0

0

0
4

0
4

0
4

4.58002

3.81669

3.81669

3.05335

0.763337

0
4

0
4

0.763337

0.763337

0.763337

0
4

0
4

3.33066907387547e-16

0
4

0

0

0

0

0
4

0

0

0
4

0
4

0
4

0

0

0

0

0
4

0
4

0
4

0
2

0
2

0
2

0

0

0

0

0

0

0
4

0

0

0

0

0

0

0
4

0

0

0

0

0

0
4

0
4

0
4

2.96872

2.96872

2.96872

2.96872

0
4

0
4

0
4

6.68892

6.68892

0

0

0
4

6.48837

6.48837

0
4

0.200555

0.200555

0
4

3.88578058618805e-16

0
4

0
4

0

0

0

0

0
4

0
4

0
4

0

0

0

0

0

0
4

0
4

0
4

0.727773

0.727773

0.727773

0.221304

0.321783

0.184686

0
4

0
4

0
4

0

0

0

0

0

0
4

0
4

0
4

0.0724936

0.0724936

0.0724936

0

0.0724936

0
4

0
4

0
4

0

0

0

0

0
4

0
4

0
4

0

0

0

0

0
4

0

0

0
4

0
4

0
4

0.238817

0.238817

0.238817

0.238817

0
4

0
4

0
4

0

0

0

0
6

0
7

0

0

0

0

0
4

0
6

0
6

0

0

0

0
4

0

0

0

0
4

0

0

0
4

0

0

0
4

0

0

0
4

0
4

0
4

0

0

0

0

0
4

0

0

0
4

0
4

0
4

0.464531

0.464531

0.464531

0.278718

0.185812

2.77555756156289e-17

0
4

0
4

0
4

0

0

0

0

0
4

0

0

0
4

0
4

0
4

0

0

0

0

0

0
4

0
4

0
4

0

0

0

0

0

0
4

0
4

0
4

0

0

0

0

0

0
4

0
4

0
4

0.238877

0.238877

0.0434322

0.0434322

0
4

0.195445

0.195445

0
4

0
4

0
4

0.866146

0.866146

0.274652

0.274652

0
4

0.591494

0.591494

0
4

0
4

0
4

0

0

0

0

0
4

0

0

0
4

0
4

0
4

0.152807

0.152807

0.152807

0.152807

0
4

0
4

0
4

0.0966581
7

0.0966581
7

0
7

0
7

0

0

0

0

0
4

0.0966581

0

0

0

0.0483291

0.0483291

0
4

0
4

0
4

0.161286

0.161286

0.161286

0.161286

0
4

0

0

0
4

0
4

0
4

0.274652

0.274652

0.274652

0.274652

0
4

0

0

0
4

0
4

0
4

0

0

0

0

0

0
4

0
4

0
4

0

0

0

0

0

0
4

0
4

0
4

0.108581

0.108581

0.108581

0.0434322

0.0651483

0
4

0
4

0
4

0

0

0

0

0
4

0
4

0
4

0

0

0

0

0
4

0
4

0
4

0.274652

0.274652

0

0

0
4

0.274652

0.274652

0
4

0
4

0
4

0

0

0

0

0
4

0

0

0
4

0
4

0
4

0

0

0

0

0
4

0
4

0
4

0
7

0
7

0
7

0
7

0

0

0

0

0

0
4

0

0

0

0

0
4

0

0

0
4

0
4

0
4

0

0

0

0

0
4

0

0

0
4

0
4

0
4

0

0

0

0

0
4

0
4

0
4

0.813476

0.813476

0.763337

0.763337

0
4

0.0501388

0.0501388

0
4

4.85722573273506e-17

0
4

0
4

0.549304

0.549304

0.549304

0.274652

0.274652

0
4

0
4

0
4

0.68663

0.68663

0.68663

0.68663

0
4

0
4

0
4

0

0

0

0

0

0
4

0
4

0
4

0

0

0

0

0

0
4

0
4

0
4

0

0

0

0

0

0
4

0
4

0
4

0

0

0

0

0

0
4

0
4

0
4

0

0

0

0

0
4

0

0

0
4

0
4

0
4

0
7

0
7

0
7

0
7

0

0

0
4

0

0

0
4

0

0

0
4

0
4

0
4

0

0

0

0

0

0
4

0
4

0
4

0

0

0

0

0
4

0
4

0
4

0

0

0

0

0
4

0
4

0
4

0.161286

0.161286

0

0

0
4

0.161286

0.161286

0
4

0
4

0
4

0

0

0

0

0
4

0
4

0
4

0.557437

0.557437

0.557437

0.278718

0.278718

0
4

0
4

0
4

0

0

0

0

0
4

0

0

0
4

0
4

0
4

0

0

0

0

0
4

0
4

0
4

0

0

0

0

0
4

0
4

0
4

0

0

0

0

0
4

0
4

0
4

0
7

0
7

0
7

0
7

0
7

0

0

0

0
4

0
7

0
7

0

0

0
4

0
4

0
4

0

0

0

0

0
4

0

0

0
4

0
4

0
4

0

0

0

0

0

0
4

0
4

0
4

0

0

0

0

0
4

0
4

0
4

0

0

0

0

0
4

0

0

0
4

0
4

0
4

0

0

0

0

0
4

0
4

0
4

0

0

0

0

0

0
4

0
4

0
4

0

0

0

0

0
4

0

0

0
4

0
4

0
4

4.18318

4.18318

4.18318

4.18318

0
4

0
4

0
4

0

0

0

0

0
4

0

0

0
4

0
4

0
4

0

0

0

0

0
4

0

0

0
4

0
4

0
4

0
4

0
4

0

0

0

0

0

0

0

0

0

0

0

0

0

0

0

0

0

0

0
4

0
4

0

0

0

0

0

0

0

0

0

0

0

0

0

0

0

0
4

0

0

0

0

0

0

0

0
4

0

0

0

0
4

0
4

0

0

0

0
4

0
4

0
4

0

0

0

0

0

0

0

0

0

0

0
4

0

0

0

0

0
4

0
4

0
4

0

0

0

0

0
4

0
4

0
4

0

0

0

0

0

0
4

0
4

0
4

0

0

0

0

0
4

0
4

0
4

0

0

0

0

0

0
4

0
4

0
4

0.371625

0.371625

0.371625

0.185812

0.185812

0
4

0
4

0
4

0

0

0

0

0
4

0
4

0
4

0

0

0

0

0
4

0

0

0
4

0
4

0
4

0

0

0

0

0
4

0
4

0
4

0

0

0

0

0

0
4

0
4

0
4

0

0

0

0

0

0
4

0
4

0
4

0
7

0
7

0
7

0
7

0
7

0

0
4

0

0

0

0
4

0
4

0

0

0

0
4

0
4

0
4

0

0

0

0

0
4

0
4

0
4

0

0

0

0

0
4

0
4

0
4

0.601665

0.601665

0.601665

0.601665

0
4

0
4

0
4

0

0

0

0

0
4

0
4

0
4

0

0

0

0

0
4

0
4

0
4

0

0

0

0

0
4

0
4

0
4

0

0

0

0

0
4

0
4

0
4

0

0

0

0

0
4

0
4

0
4

0

0

0

0

0
4

0
4

0
4

0

0

0

0

0
4

0
4

0
4

13.8162

13.8162

13.8162

0
7

6.74919

7.067

0
4

0

0

0
4

0

0

0
4

0

0

0
4

0
4

0
4

0

0

0

0

0
4

0
4

0
4

0.100278

0.100278

0.100278

0.100278

0
4

0
4

0
4

0

0

0

0

0
4

0
4

0
4

4.38165

4.38165

4.38165

4.38165

0
4

0
4

0
4

0

0

0

0

0
4

0
4

0
4

0

0

0

0

0
4

0
4

0
4

0.763337

0.763337

0.763337

0.763337

0
4

0
4

0
4

0

0

0

0

0
4

0
4

0
4

0.699131

0.699131

0.699131

0.699131

0
4

0
4

0
4

0

0

0

0

0
4

0
4

0
4

0.0483291
2

0.0483291
2

0
2

0
2

0

0

0

0

0

0
4

0.0483291
2

0

0

0.0483291

0
4

0

0

0
4

0
4

0
4

0

0

0

0

0
4

0
4

0
4

0

0

0

0

0
4

0
4

0
4

0

0

0

0

0
4

0
4

0
4

0

0

0

0

0
4

0
4

0
4

0

0

0

0

0
4

0
4

0
4

0

0

0

0

0
4

0
4

0
4

1.52667

1.52667

1.52667

1.52667

0
4

0
4

0
4

0

0

0

0

0
4

0
4

0
4

0

0

0

0

0
4

0
4

0
4

0

0

0

0

0
4

0
4

0
4

331.776
4

331.776
4

4.48859
4

1.07647

0.614315

0.857264

0.35021

0.977132

0.232402

0.0581004

0.145251

0.177448

0
4

327.287
4

327.287
4

0
4

5.6843418860808e-14
4

0
4

0

0

0

0
4

0
4

0
4

0

0

0

0

0
4

0
4

0
4

0.0651483

0.0651483

0.0651483

0.0651483

0
4

0
4

0
4

0.175486

0.175486

0.175486

0.175486

0
4

0
4

0
4

0.0966581

0.0966581

0.0966581

0.0966581

0
4

0
4

0
4

0.0501388

0.0501388

0.0501388

0.0501388

0
4

0
4

0
4

0.10204

0.10204

0.10204

0.10204

0
4

0
4

0
4

0

0

0

0

0
4

0
4

0
4

0

0

0

0

0
4

0
4

0
4

0

0

0

0

0
4

0
4

0
4

0

0

0

0

0
4

0
4

0
4

10.4432
5

10.4432
5

10.4432
5

10.3413
5

0.101849

0

1.80411241501588e-16
5

0
4

0
4

0
4

0.0829891

0.0829891

0.0829891

0.0829891

0
4

0
4

0
4

0.0871506

0.0871506

0.0871506

0.0871506

0
4

0
4

0
4

0

0

0

0

0
4

0
4

0
4

0

0

0

0

0
4

0
4

0
4

1.95193

1.95193

1.95193

1.95193

0
4

0
4

0
4

0.160891

0.160891

0.160891

0.160891

0
4

0
4

0
4

0

0

0

0

0
4

0
4

0
4

0.730274

0.730274

0.730274

0.730274

0
4

0
4

0
4

0

0

0

0

0
4

0
4

0
4

0

0

0

0

0
4

0
4

0
4

0
7

0
7

0
7

0
7

0

0
4

0

0

0
4

0
4

0

0

0

0

0

0
4

0
4

0
4

0

0

0

0

0
4

0
4

0
4

1.31449

1.31449

1.31449

1.31449

0
4

0
4

0
4

0.549304

0.549304

0.549304

0.549304

0
4

0
4

0
4

0

0

0

0

0
4

0
4

0
4

0

0

0

0

0
4

0
4

0
4

0

0

0

0

0
4

0
4

0
4

0

0

0

0

0
4

0
4

0
4

0

0

0

0

0
4

0
4

0
4

0.29211

0.29211

0.29211

0.29211

0
4

0
4

0
4

0

0

0

0

0
4

0
4

0
4

0

0

0

0

0

0

0

0

0

0

0
4

0
4

0
4

0

0

0

0

0
4

0
4

0
4

0

0

0

0

0
4

0
4

0
4

0

0

0

0

0
4

0
4

0
4

0.677078

0.677078

0.677078

0.677078

0
4

0
4

0
4

0

0

0

0

0
4

0
4

0
4

0

0

0

0

0
4

0
4

0
4

0

0

0

0

0
4

0
4

0
4

0

0

0

0

0
4

0
4

0
4

0

0

0

0

0
4

0
4

0
4

0

0

0

0

0
4

0
4

0
4

0

0

0

0

0

0

0

0

0
4

0
4

0
4

0

0

0

0

0
4

0
4

0
4

0

0

0

0

0
4

0
4

0
4

0

0

0

0

0
4

0
4

0
4

0

0

0

0

0
4

0
4

0
4

0.0651483

0.0651483

0.0651483

0.0651483

0
4

0
4

0
4

0.05102

0.05102

0.05102

0.05102

0
4

0
4

0
4

0

0

0

0

0
4

0
4

0
4

0

0

0

0

0
4

0
4

0
4

0

0

0

0

0
4

0
4

0
4

0

0

0

0

0
4

0
4

0
4

174.758
4

174.758
4

174.758
4

143.494
4

29.7963

1.05545

0.411978

1.26010313294955e-14
4

0
4

0
4

0
4

0.161286

0.161286

0.161286

0.161286

0
4

0
4

0
4

0

0

0

0

0
4

0
4

0
4

0

0

0

0

0
4

0
4

0
4

0

0

0

0

0
4

0
4

0
4

0

0

0

0

0
4

0
4

0
4

0

0

0

0

0
4

0
4

0
4

0

0

0

0

0
4

0
4

0
4

0

0

0

0

0
4

0
4

0
4

0.0483291

0.0483291

0.0483291

0.0483291

0
4

0
4

0
4

0

0

0

0

0
4

0
4

0
4

352.207
4

352.207
4

335.193
4

227.107
4

0.566762

0.0581004

2.47187

0.274652

1.14501

0.107261

0.29211

0.116201

0.274652

0.274652

32.4634
4

0.123124

0.961282

7.22145
4

16.5294

2.69634

2.20678

39.6872

0.499129

0.116201

1.69725344889571e-13
4

0
4

16.6025
4

2.37215

0.900729

0.730274

0.876329

0.184686

11.5383

0
4

0.116201

0.0581004

0.0581004

0
4

0.295747

0.295747

0
4

0
4

0
4

1.96994
5

1.96994
5

1.96994
5

1.71532
5

0.254623

0

0

0

0

0

1.66533453693773e-16
5

0
4

0

0

0
4

0
4

0
4

0

0

0

0

0
4

0
4

0
4

0

0

0

0

0
4

0
4

0
4

0

0

0

0

0
4

0
4

0
4

0

0

0

0

0
4

0
4

0
4

0.0553261

0.0553261

0.0553261

0.0553261

0
4

0
4

0
4

8.01504

8.01504

8.01504

8.01504

0
4

0
4

0
4

0.101849

0.101849

0.101849

0.101849

0
4

0
4

0
4

0

0

0

0

0
4

0
4

0
4

0

0

0

0

0
4

0
4

0
4

3.05335

3.05335

3.05335

3.05335

0
4

0
4

0
4

23.8767
4

23.8767
4

23.8767
4

18.4405

4.30447

0

0.411978

0.184686

0.123124

0.411978

2.05391259555654e-15
4

0
4

0
4

0
4

0

0

0

0

0
4

0
4

0
4

0.961282

0.961282

0.961282

0.961282

0
4

0
4

0
4

0

0

0

0

0
4

0
4

0
4

0

0

0

0

0
4

0
4

0
4

0

0

0

0

0
4

0
4

0
4

0

0

0

0

0
4

0
4

0
4

0

0

0

0

0
4

0
4

0
4

0

0

0

0

0
4

0
4

0
4

0.0434322

0.0434322

0.0434322

0.0434322

0
4

0
4

0
4

0

0

0

0

0
4

0
4

0
4

1.86759

1.86759

0
6

0

0

0

0
4

1.86759

0

1.86759

0

0
4

0

0

0
4

0

0

0

0
4

0

0

0
4

0

0

0
4

0
4

0
4

0

0

0

0

0
4

0
4

0
4

0

0

0

0

0
4

0
4

0
4

0.125347

0.125347

0.125347

0.125347

0
4

0
4

0
4

0

0

0

0

0
4

0
4

0
4

0

0

0

0

0
4

0
4

0
4

0

0

0

0

0
4

0
4

0
4

0

0

0

0

0
4

0
4

0
4

0.438165

0.438165

0.438165

0.438165

0
4

0
4

0
4

0

0

0

0

0
4

0
4

0
4

0

0

0

0

0
4

0
4

0
4

0

0

0

0
7

0

0

0

0
4

0
4

0
4

0

0

0

0

0
4

0
4

0
4

0

0

0

0

0
4

0
4

0
4

0

0

0

0

0
4

0
4

0
4

0.254623

0.254623

0.254623

0.254623

0
4

0
4

0
4

0.438165

0.438165

0.438165

0.438165

0
4

0
4

0
4

0

0

0

0

0
4

0
4

0
4

0

0

0

0

0
4

0
4

0
4

0

0

0

0

0
4

0
4

0
4

0

0

0

0

0
4

0
4

0
4

0.763337

0.763337

0.763337

0.763337

0
4

0
4

0
4

45.8339
3

45.8339
3

42.6204
4

0

36.2216

6.1067

0.29211

0
4

0

0

0
4

3.21349

3.21349

0
4

0

0

0

0
4

1.33226762955019e-15
3

0
4

0
4

0

0

0

0

0
4

0
4

0
4

0

0

0

0

0
4

0
4

0
4

0.100278

0.100278

0.100278

0.100278

0
4

0
4

0
4

0

0

0

0

0
4

0
4

0
4

0

0

0

0

0
4

0
4

0
4

0

0

0

0

0
4

0
4

0
4

0

0

0

0

0
4

0
4

0
4

0.0556337

0.0556337

0.0556337

0.0556337

0
4

0
4

0
4

0

0

0

0

0
4

0
4

0
4

0

0

0

0

0
4

0
4

0
4

0
7

0
7

0
7

0

0

0

0
4

0

0

0

0

0

0
4

0

0

0
4

0
4

0
4

0

0

0

0

0
4

0
4

0
4

0

0

0

0

0
4

0
4

0
4

0

0

0

0

0
4

0
4

0
4

0

0

0

0

0
4

0
4

0
4

0.07653

0.07653

0.07653

0.07653

0
4

0
4

0
4

0

0

0

0

0
4

0
4

0
4

2.67168

2.67168

2.67168

2.67168

0
4

0
4

0
4

0

0

0

0

0
4

0
4

0
4

0

0

0

0

0
4

0
4

0
4

0.411978

0.411978

0.411978

0.411978

0
4

0
4

0
4

1.14257

1.14257

1.14257

1.14257

0

0

0
4

0

0

0

0
4

0
4

0

0

0

0

0
4

0

0

0
4

0
4

0
4

0.200555

0.200555

0.200555

0.200555

0
4

0
4

0
4

0.118299

0.118299

0.118299

0.118299

0
4

0
4

0
4

0

0

0

0

0
4

0
4

0
4

0

0

0

0

0
4

0
4

0
4

0

0

0

0

0
4

0
4

0
4

0

0

0

0

0
4

0
4

0
4

0

0

0

0

0
4

0
4

0
4

0

0

0

0

0
4

0
4

0
4

0

0

0

0

0
4

0
4

0
4

0

0

0

0

0
4

0
4

0
4

3.86867
5

3.86867
5

3.86867
5

3.56312
5

0.305547

2.22044604925031e-16
5

0
4

0
4

0
4

0

0

0

0

0
4

0
4

0
4

0

0

0

0

0
4

0
4

0
4

0.169269

0.169269

0.169269

0.169269

0
4

0
4

0
4

0

0

0

0

0
4

0
4

0
4

0.66326

0.66326

0.66326

0.66326

0
4

0
4

0
4

0.248967

0.248967

0.248967

0.248967

0
4

0
4

0
4

0

0

0

0

0
4

0
4

0
4

0

0

0

0

0
4

0
4

0
4

0.20408

0.20408

0.20408

0.20408

0
4

0
4

0
4

0

0

0

0

0
4

0
4

0
4

0
7

0
7

0

0

0

0
4

0

0

0

0
4

0
4

0
4

0.0556337

0.0556337

0.0556337

0.0556337

0
4

0
4

0
4

0

0

0

0

0
4

0
4

0
4

0

0

0

0

0
4

0
4

0
4

0

0

0

0

0
4

0
4

0
4

1.52667

1.52667

1.52667

1.52667

0
4

0
4

0
4

0

0

0

0

0
4

0
4

0
4

0

0

0

0

0
4

0
4

0
4

0

0

0

0

0
4

0
4

0
4

0

0

0

0

0
4

0
4

0
4

0.05102

0.05102

0.05102

0.05102

0
4

0
4

0
4

1.49371
4

1.49371
4

1.35739
4

0.167864

1.18952

0

0
4

0.136318
4

0.136318
4

0
4

0

0

0
4

0

0

0
4

0
4

0
4

0

0

0

0

0
4

0
4

0
4

0.0829891

0.0829891

0.0829891

0.0829891

0
4

0
4

0
4

0

0

0

0

0
4

0
4

0
4

0

0

0

0

0
4

0
4

0
4

0

0

0

0

0
4

0
4

0
4

0

0

0

0

0
4

0
4

0
4

0.730274

0.730274

0.730274

0.730274

0
4

0
4

0
4

0

0

0

0

0
4

0
4

0
4

0

0

0

0

0
4

0
4

0
4

0

0

0

0

0
4

0
4

0
4

0
7

0
7

0
7

0
7

0

0
6

0

0

0

0
4

0
7

0
7

0
7

0
7

0
7

0

0

0

0
4

0
4

0
4

0

0

0

0

0

0

0

0
4

0

0

0

0
4

0
4

0
4

0

0

0

0

0
4

0
4

0
4

0

0

0

0

0
4

0
4

0
4

0

0

0

0

0
4

0
4

0
4

0.246248

0.246248

0.246248

0.246248

0
4

0
4

0
4

0

0

0

0

0
4

0
4

0
4

0.27663

0.27663

0.27663

0.27663

0
4

0
4

0
4

0

0

0

0

0
4

0
4

0
4

0

0

0

0

0
4

0
4

0
4

0

0

0

0

0
4

0
4

0
4

0.0581004

0.0581004

0.0581004

0.0581004

0
4

0
4

0
4

0
7

0
7

0
7

0
6

0

0

0
4

0

0

0
4

0

0

0
4

0
4

0
4

0

0

0

0

0
4

0
4

0
4

0

0

0

0

0
4

0
4

0
4

0

0

0

0

0
4

0
4

0
4

0

0

0

0

0
4

0
4

0
4

0.161286

0.161286

0.161286

0.161286

0
4

0
4

0
4

0

0

0

0

0
4

0
4

0
4

0

0

0

0

0
4

0
4

0
4

0

0

0

0

0
4

0
4

0
4

0.94387

0.94387

0.94387

0.94387

0
4

0
4

0
4

0

0

0

0

0
4

0
4

0
4

0
7

0
7

0

0

0

0

0
4

0

0

0

0

0
4

0
4

0
4

1.02238

1.02238

1.02238

1.02238

0
4

0
4

0
4

0

0

0

0

0
4

0
4

0
4

0.0483291

0.0483291

0.0483291

0.0483291

0
4

0
4

0
4

0.161286

0.161286

0.161286

0.161286

0
4

0
4

0
4

0
6

0
6

0
6

0
6

0

0

0
4

0
4

0
4

0

0

0

0

0

0

0

0
4

0

0

0

0
4

0

0

0
4

0

0

0

0
4

0
4

0

0

0

0
4

0
4

0
4

0.0966581
7

0.0966581
7

0.0483291
7

0.0483291
7

0

0
4

0

0

0
4

0.0483291

0.0483291

0
4

0
4

0
4

0
7

0
7

0
7

0
7

0
4

0
4

0
4

0

0

0

0

0

0

0

0

0
4

0

0

0
4

0
4

0
4

20.9098
4

20.9098
4

20.2466

18.577

1.25765

0.411978

1.66533453693773e-16

0
4

0.66326

0.66326

0
4

1.33226762955019e-15
4

0
4

0
4

0

0

0

0

0

0
4

0
4

0
4

1.52013
5

1.52013
5

0
6

0
6

0

0

0

0

0

0

0

0

0

0

0

0

0

0

0

0

0
4

1.52013
5

0
7

0
6

1.52013

0

0
4

0

0

0

0

0
4

0
4

0
4

0

0

0

0

0

0
4

0

0

0

0
4

0
4

0
4

5.34762
4

5.34762
4

5.13258
4

5.13258

0

0

0

0
4

0.215042

0.101849

0.113193

1.38777878078145e-17

0
4

8.32667268468867e-16
4

0
4

0
4

0

0

0

0

0

0

0

0
4

0
4

0
4

0
7

0
7

0
7

0

0

0
4

0
4

0
4

0

0

0

0

0

0

0
4

0

0

0
4

0
4

0
4

0

0

0

0

0

0

0

0

0
4

0

0

0
4

0
4

0
4

1.00278

1.00278

1.00278

0.952637

0

0.0501388

0

4.85722573273506e-17

0
4

0
4

0
4

0

0

0

0

0

0

0
4

0
4

0
4

0

0

0

0

0
4

0
4

0
4

0

0

0

0

0

0
4

0
4

0
4

12.0671

11.9598

10.0128
5

0
6

0

9.09333

0

0.507808

0.234213

0

0

0

0.177448

1.13797860024079e-15
5

0
4

0
7

0
7

0

0

0
4

1.56617

1.56617

0

0

0

0
4

0.380856

0.126952

0.253904

0
4

4.44089209850063e-16

0
4

0.107261

0.107261

0.107261

0
4

0
4

2.17881268582687e-15

0
4

0.476319

0.376041

0.175486

0.0501388

0.0501388

0.0752082

0
4

0.200555

0.125347

0.0752082

0
4

2.77555756156289e-17

0
4

0.100278

0.100278

0.100278

0
4

0
4

2.77555756156289e-17

0
4

0.36412
4

0.36412
4

0.36412
4

0.308794
4

0.0553261

0
4

0
4

0
4

0
7

0
7

0
7

0
7

0

0
4

0
4

0
4

1.26952

1.26952

1.10025

1.01562

0.0846347

0
4

0.169269

0.169269

0
4

0
4

0
4

0

0

0

0

0

0
4

0

0

0
4

0

0

0

0
4

0

0

0
4

0
4

0
4

3.29582

3.29582

3.29582

3.29582

0

0
4

0
4

0
4

1.79748
4

1.79748
4

1.4449

0.47718

0.967716

0
4

0.352581

0.110652

0.241929

2.77555756156289e-17

0
4

0

0

0
4

5.55111512312578e-17
4

0
4

0
4

0

0

0

0

0

0

0
4

0
4

0
4

7.75271
4

7.75271
4

7.75271

7.75271

0
4

0

0

0
4

0

0

0
4

0
4

0
4

10.2123
5

10.2123
5

10.2123
5

10.2123
5

0
4

0
4

0
4

33.0861

33.0861

25.4527

4.71294

20.7398

0

0
4

0

0

0
4

1.14501

1.14501

0
4

0
4

0
4

0

0

0

0

0
4

0

0

0

0

0
4

0

0

0
4

0

0

0
4

0

0

0
4

0

0

0
4

0

0

0
4

6.48837

6.48837

0
4

8.88178419700125e-16

0
4

0
4

1.1599

1.1599

1.1599

1.1599

0

0
4

0
4

0
4

0.804457

0.804457

0

0

0

0

0
4

0.804457

0.804457

0

0
4

0
4

0
4

0

0

0

0

0
4

0
4

0
4

39.8794

39.8794

35.9359

34.7675

1.16844

4.44089209850063e-16

0
4

3.94348

3.65137

0.29211

5.55111512312578e-17

0
4

0
4

0
4

0

0

0

0

0

0
4

0
4

0
4

0

0

0

0

0

0

0

0

0
4

0

0

0
4

0
4

0
4

0

0

0

0

0

0

0
4

0
4

0
4

0

0

0

0

0
4

0

0

0

0
4

0
4

0
4

0
7

0
7

0

0

0
4

0

0

0
4

0
4

0
4

1.1233

1.1233

0.316873

0.265721

0.0511526

0
4

0.80643

0.564501

0.241929

2.77555756156289e-17

0
4

0
4

0
4

0.0846347

0.0846347

0.0846347

0

0

0

0

0

0

0

0

0.0846347

0

0

0
4

0

0

0

0

0

0

0

0

0
4

0

0

0

0

0

0

0

0

0

0
4

0
4

0
4

0

0

0

0

0

0

0
4

0
4

0
4

10.2629
5

10.2629
5

10.2629
5

10.2629

0

0
4

0
4

0
4

0

0

0

0

0
4

0
4

0
4

0

0

0

0

0
4

0
4

0
4

2.52404

2.52404

2.52404

2.52404

0

0
4

0
4

0
4

0

0

0

0

0
4

0
4

0
4

0

0

0

0

0

0
4

0

0

0
4

0
4

0
4

0

0

0

0

0

0

0
4

0
4

0
4

0

0

0

0

0

0
4

0
4

0
4

0.246248

0.246248

0

0

0

0
4

0.246248

0.123124

0.123124

0
4

0
4

0
4

0
7

0
7

0
7

0
7

0
7

0
7

0

0

0

0

0

0

0
4

0

0

0

0

0
4

0

0

0
4

0
4

0
4

10.3882

10.3882

10.3882

8.05363

1.23593

1.09861

0
4

0
4

0
4

0

0

0

0

0

0

0
4

0
4

0
4

1.12687

1.12687

1.12687

0.695935

0.430933

1.11022302462516e-16

0
4

0
4

0
4

0

0

0

0

0
4

0
4

0
4

0.229886

0.229886

0.229886

0.229886

0

0
4

0
4

0
4

2.33688

2.33688

0

0

0
4

2.33688

2.33688

0
4

0
4

0
4

3.30797

3.30797

3.30797

3.30797

0
4

0
4

0
4

0

0

0

0

0

0
4

0
4

0
4

0

0

0

0

0

0
4

0

0

0
4

0
4

0

0

0

0
4

0
4

0
4

0

0

0

0

0

0

0
4

0
4

0
4

16.226

1.13261
4

1.13261
4

0.784605

0.247724

0.100278

0
4

0
4

0

0

0

0
4

0
4

0.203351

0.203351

0.203351

0
4

0
4

0

0

0

0
4

0
4

0

0

0

0
4

0
4

0

0

0

0
4

0
4

0

0

0

0
4

0
4

0

0

0

0
4

0
4

0.0617678

0.0617678

0.0617678

0
4

0
4

0

0

0

0
4

0
4

0

0

0

0
4

0
4

1.03438

1.03438

0.283554

0.750827

0
4

0
4

0

0

0

0
4

0
4

0.349565

0.349565

0.349565

0
4

0
4

0

0

0

0
4

0
4

0.0793016

0.0793016

0.0793016

0
4

0
4

0.407723

0.407723

0.407723

0
4

0
4

0.0501388

0.0501388

0.0501388

0
4

0
4

0.15306

0.15306

0.15306

0
4

0
4

0

0

0

0
4

0
4

0

0

0

0
4

0
4

0.349565

0.349565

0.349565

0
4

0
4

0

0

0

0
4

0
4

0.152774

0.152774

0.152774

0
4

0
4

0

0

0

0

0

0
4

0
4

0.58422

0.58422

0.29211

0.29211

0
4

0
4

0.211558

0.211558

0.211558

0
4

0
4

0

0

0

0
4

0

0

0
4

0
4

0.126952

0.126952

0.126952

0
4

0
4

0

0

0

0
4

0
4

11.3291
3

5.71311
2

0.843751
3

1.69152
2

0

0

0

0.198088

0

0

0

0

0

0.0848951

1.86067
2

0

0

0

0

0

0

0.141492

0.120823

0

0

0

0

0

0

0

0

0.0834505

0

0

0.688421

0

0

0

0

0

0
4

0

0

0

0

0

0

0

0

0

0

0
4

0.464531

0.464531

0
4

0

0

0

0
4

0.68663

0.68663

0
4

0

0

0

0
4

0

0

0
4

0

0

0
4

0

0

0
4

0

0

0
4

0.29211

0.29211

0
4

0.185812

0.185812

0
4

0

0

0

0

0

0

0

0
4

0

0

0
4

0

0

0
4

0.322572

0.322572

0
4

0.0483291

0.0483291

0
4

0

0

0
4

0.371625

0.371625

0
4

0

0

0
4

0

0

0
4

0

0

0
4

0

0

0
4

0

0

0

0

0
4

0

0

0
4

0.0565967

0.0565967

0
4

0

0

0
4

0

0

0

0
4

0

0

0

0
4

2.98405

2.88009

0.0483291

0.0556337

0
4

0.203698

0.101849

0

0.101849

0
4

0

0

0
4

0

0

0

0
4

1.2490009027033e-15
3

0
4

3.5527136788005e-15

0
4

536.723
3

0

0

0

0

0

0

0

0

0
4

0

0

0

0

0
4

0

0

0
4

0
4

0
6

0
6

0
6

0
4

0
4

0.371625

0.371625

0.371625

0
4

0
4

0.274652

0.274652

0.274652

0
4

0
4

0.05102

0.05102

0.05102

0
4

0
4

0.274652

0.274652

0.274652

0
4

0
4

0

0

0

0
4

0
4

0

0

0

0
4

0
4

0

0

0

0
4

0
4

0

0

0

0
4

0
4

0

0

0

0
4

0
4

0.438165

0.438165

0.438165

0
4

0
4

38.1669

34.3502

20.9918

9.16005

1.14501

0.763337

2.29001

0
4

2.29001

0.763337

1.52667

0
4

1.52667

1.52667

0
4

0
4

0.0565967

0.0565967

0.0565967

0
4

0
4

0.184686

0.184686

0.184686

0
4

0
4

0

0

0

0
4

0
4

0

0

0

0
4

0
4

0.0829891

0.0829891

0.0829891

0
4

0
4

0

0

0

0
4

0
4

0

0

0

0
4

0
4

0

0

0

0
4

0
4

0.375413

0.375413

0.375413

0
4

0
4

0

0

0

0
4

0
4

0.731933

0.731933

0.676607

0.0553261

0
4

0

0

0

0
4

0
4

0

0

0

0
4

0
4

0.07653

0.07653

0.07653

0
4

0
4

0

0

0

0
4

0
4

0

0

0

0
4

0
4

0

0

0

0
4

0
4

0

0

0

0
4

0
4

0

0

0

0
4

0
4

0

0

0

0
4

0
4

0

0

0

0
4

0
4

0

0

0

0
4

0
4

0

0

0

0

0

0

0

0
4

0

0

0
4

0
4

0

0

0

0
4

0
4

0

0

0

0
4

0
4

0.0434322

0.0434322

0.0434322

0
4

0
4

0

0

0

0
4

0
4

2.33688

2.33688

2.33688

0
4

0
4

0

0

0

0
4

0
4

0

0

0

0

0

0
4

0

0

0

0
4

0

0

0
4

0
4

0.442609
3

0

0

0

0

0

0
4

0

0

0

0
4

0

0

0
4

0.442609

0.442609

0
4

0

0

0
4

0
4

0
2

0

0

0

0

0
4

0

0

0
4

0

0

0

0
4

0

0

0
4

0

0

0
4

0
4

3.25255

3.25255

2.69576

0.184686

0.30781

0.0642886

0
4

0
4

0

0

0

0

0
4

0
4

0
6

0
6

0
6

0
4

0
4

313.177
4

312.429
4

312.429
4

0
4

0.163089

0.163089

0
4

0.29211

0.29211

0
4

0.29211

0.29211

0
4

3.99680288865056e-15
4

0
4

0.327578
3

0.279249

0.279249

0

0

0

0
4

0

0

0
4

0.0483291

0.0483291

0
4

0
4

0.802228
3

0

0

0

0

0
4

0.802228

0.802228

0
4

0

0

0
4

0

0

0
4

0

0

0
4

0
4

44.3301

38.605

15.7049

22.9001

0
4

5.72503

5.72503

0
4

0
4

0

0

0

0

0
4

0
4

0

0

0

0

0
4

0
4

0

0

0

0
4

0

0

0
4

0
4

0

0

0

0
4

0

0

0

0
4

0
4

12.3797

11.5034

4.63884

5.55009

1.31449

4.44089209850063e-16

0
4

0.876329

0.876329

0
4

0
4

0.891481
4

0.891481
4

0.211587

0.123124

0.214522

0.29211

0

0.0501388

1.04083408558608e-16
4

0
4

0
4

1.93004
4

1.4108

0.639104

0.411978

0.236598

0.123124

0
4

0.519239

0.107261

0.411978

0
4

1.11022302462516e-16
4

0
4

0.278718
3

0
3

0

0

0

0

0
4

0

0

0

0

0
4

0.278718

0.278718

0
4

0
4

0

0

0

0

0
4

0

0

0
4

0
4

0

0

0

0

0

0

0
4

0
4

0
6

0
6

0
6

0
4

0
4

11.1487

1.39359

1.20778

0.185812

0
4

9.75515

9.75515

0

0
4

0
4

0.257154

0

0

0

0
4

0.257154

0.257154

0
4

0
4

0

0

0

0

0
4

0
4

0.0829891

0

0

0

0

0
4

0.0829891

0.0829891

0
4

0
4

0

0

0

0

0

0
4

0
4

3.81361

3.81361

2.06578

1.74783

0
4

0

0

0
4

0
4

0

0

0

0

0
4

0
4

0
2

0

0

0

0

0
4

0

0

0

0

0
4

0

0

0
4

0

0

0
4

0

0

0
4

0
4

2.51502

2.45073

0.160722

2.29001

0
4

0.0642886

0.0642886

0
4

0
4

0

0

0

0
4

0
4

0

0

0

0
4

0
4

0

0

0

0
4

0
4

0.534251

0.534251

0.534251

0
4

0
4

0

0

0

0

0
4

0
4

0.217481

0.169152

0.169152

0
4

0.0483291

0.0483291

0
4

6.93889390390723e-18

0
4

1.06102

0.876329

0.438165

0.438165

0
4

0.184686

0.184686

0
4

5.55111512312578e-17

0
4

0

0

0

0
4

0
4

0

0

0

0
4

0
4

0.763337

0.763337

0.763337

0

0

0

0

0
4

0
4

0.0926517

0

0

0
4

0.0926517

0.0926517

0
4

0
4

0

0

0

0

0
4

0

0

0
4

0
4

1.92256

1.92256

0.823956

1.09861

0
4

0
4

2.29001

2.29001

1.52667

0.763337

0
4

0
4

0.743249

0.743249

0.278718

0.464531

5.55111512312578e-17

0
4

0
4

0

0

0

0
4

0

0

0
4

0
4

1.90834

1.90834

1.14501

0.763337

1.11022302462516e-16

0
4

0
4

0

0

0

0
4

0

0

0
4

0
4

0

0

0

0
4

0

0

0
4

0
4

0

0

0

0
4

0
4

1.36731
4

0.123124

0

0

0.123124

0

0
4

0.150416

0.150416

0
4

0.932878

0.932878

0
4

0.160891

0.160891

0
4

0
4

0

0

0

0
4

0
4

0

0

0

0

0
4

0
4

0.738348

0.738348

0.738348

0
4

0
4

0

0

0

0

0
4

0
4

0.522332

0.214522

0.214522

0
4

0.30781

0.30781

0
4

0
4

0

0

0

0
4

0

0

0
4

0
4

0.123124

0.123124

0.123124

0
4

0

0

0
4

0
4

0

0

0

0
4

0
4

0

0

0

0
4

0
4

0

0

0

0

0
4

0
4

12.1707

12.1707

10.3126

1.85812

0
4

0

0

0

0

0
4

0

0

0
4

0
4

0

0

0

0

0
4

0
4

0

0

0

0
4

0

0

0
4

0
4

0.464531

0.278718

0.278718

0
4

0.185812

0.185812

0
4

2.77555756156289e-17

0
4

0

0

0

0

0
4

0
4

0

0

0

0
4

0
4

0

0

0

0
4

0
4

1.89871

1.89871

1.6066

0.29211

5.55111512312578e-17

0
4

0
4

3.05335

3.05335

3.05335

0
4

0
4

2.03796

2.03796

2.03796

0
4

0
4

0.0642886

0.0642886

0.0642886

0
4

0
4

0

0

0

0

0

0
4

0

0

0
4

0

0

0
4

0

0

0

0
4

0
4

0

0

0

0
4

0
4

0

0

0

0
4

0
4

0.868644

0.868644

0.868644

0
4

0
4

0.0724936

0.0724936

0.0724936

0
4

0
4

0

0

0

0
4

0
4

0

0

0

0
4

0
4

0

0

0

0
4

0
4

0

0

0

0
4

0
4

0.0565967

0.0565967

0.0565967

0
4

0
4

0

0

0

0
4

0
4

2.13335
3

0

0

0

0
4

0.0964329

0

0

0.0964329

0
4

0.128577

0.128577

0
4

1.90834

1.90834

0
4

0
4

0

0

0

0
4

0
4

0

0

0

0
4

0
4

0.464531

0.464531

0.464531

0
4

0
4

0

0

0

0
4

0
4

0

0

0

0
4

0
4

0.549304

0.549304

0.549304

0
4

0
4

0

0

0

0
4

0
4

0

0

0

0
4

0
4

0

0

0

0
4

0
4

0

0

0

0
4

0
4

61.5094
3

58.4804
3

29.8135
3

0.371565
3

0

0.1052

0

0.100278

0.148632

0

0

0

0

0

0

0

0

0.0829891

0

0

0

0.278718

0

0.278718

0

0

0

0

0

0

0.339723

0

0.0483291

0

0

0.278718

0

0

0.438165

0

0.557437

0

0

0.278718

0

0

0

0

0

25.3597

0

1.06581410364015e-14
3

0
4

0
7

0
7

0
4

0.247071

0.247071

0
4

0

0

0
4

0

0

0
4

0

0

0
4

0

0

0
4

0

0

0
4

0

0

0
4

0

0

0
4

0.116201

0.116201

0
4

0.0553261

0.0553261

0
4

1.02345

1.02345

0
4

0

0

0
4

0.78547

0.78547

0
4

0

0

0
4

0

0

0

0
4

0.107261

0.107261

0
4

0

0

0
4

0.144987

0.144987

0
4

0.549304

0.549304

0
4

3.33066907387547e-16
3

0
4

0
4

14541.5
4

0
7

0
7

0
7

0

0

0

0

0

0

0
4

0
4

0
7

0
7

0
7

0
4

0

0

0
4

0
4

0

0

0

0

0
4

0
4

0

0

0

0

0
4

0
4

0

0

0

0

0
4

0
4

3.55698

3.55698

2.67168

0.885304

0
4

0
4

0.100278

0.100278

0.0501388

0.0501388

0
4

0
4

0

0

0

0
4

0
4

4.58002

4.58002

3.43502

1.14501

0
4

0
4

0

0

0

0

0
4

0
4

0

0

0

0
4

0

0

0
4

0
4

0

0

0

0
4

0
4

6.06509
4

4.88895
4

0.706245

0.430933

3.20122

0.184686

0

0.246248

0.0553261

0.0642886

4.44089209850063e-16
4

0
4

0.0556337

0

0.0556337

0
4

0.468701

0.160891

0.30781

0
4

0.175486

0.175486

0
4

0.476319

0.476319

0
4

0
4

0

0

0

0
4

0

0

0
4

0
4

0.243711

0.160722

0.160722

0
4

0.0829891

0.0829891

0
4

0
4

0.278464

0.0926517

0.0926517

0
4

0.185812

0.185812

0
4

2.77555756156289e-17

0
4

0.523619

0.248967

0.248967

0
4

0.274652

0.274652

0
4

0
4

3.87263

3.87263

0.0752082

3.79743

0
4

0
4

0.123124

0.123124

0.123124

0
4

0

0

0
4

0
4

0

0

0

0
4

0
4

0.112788

0.112788

0.05102

0.0617678

0
4

0
4

0.128577

0

0

0
4

0.128577

0.128577

0
4

0
4

0

0

0

0

0
4

0
4

0
7

0
7

0
7

0

0

0
4

0

0

0

0
4

0

0

0
4

0
4

0.371625

0.185812

0.185812

0
4

0.185812

0.185812

0
4

0
4

1.78524

1.78524

1.78524

0
4

0
4

0.890289

0.890289

0.890289

0
4

0
4

0

0

0

0
4

0

0

0
4

0
4

0.650343

0.650343

0.650343

0
4

0
4

0.243341

0.101849

0.101849

0
4

0.141492

0.141492

0
4

0
4

0.706831

0.152774

0.152774

0
4

0.554057

0.554057

0
4

0
4

0

0

0

0
4

0
4

0.183791

0.183791

0.07653

0.107261

0
4

0
4

2.35391

0.163089

0.163089

0
4

2.19082

2.19082

0
4

0
4

41.8781
4

41.0525
4

39.9736

0.304294

0.719239

0

0.0553261

1.88737914186277e-15
4

0
4

0.387283

0.138315

0.248967

2.77555756156289e-17

0
4

0.438303

0.387283

0.05102

1.38777878078145e-17

0
4

1.49880108324396e-15
4

0
4

0

0

0

0
4

0
4

0.415514

0.415514

0.415514

0
4

0
4

0

0

0

0
4

0

0

0
4

0
4

0

0

0

0
4

0
4

0

0

0

0
4

0
4

0

0

0

0
4

0
4

0

0

0

0
4

0

0

0
4

0
4

0

0

0

0
4

0

0

0
4

0
4

0.05102

0

0

0
4

0.05102

0.05102

0
4

0
4

0.225625

0.175486

0.175486

0
4

0.0501388

0.0501388

0
4

0
4

0
7

0
7

0
7

0

0
4

0

0

0
4

0
4

0

0

0

0
4

0

0

0
4

0
4

0

0

0

0
4

0
4

0

0

0

0
4

0

0

0
4

0
4

0.0793016

0

0

0
4

0.0793016

0.0793016

0
4

0
4

0.185812

0.185812

0.185812

0
4

0
4

0.169152

0.169152

0.169152

0
4

0
4

0

0

0

0
4

0
4

0

0

0

0
4

0
4

0.110652

0.110652

0.110652

0
4

0
4

0

0

0

0
4

0
4

0
7

0
6

0

0

0

0

0

0

0
4

0

0

0
4

0

0

0
4

0

0

0
4

0
4

0.193316

0.193316

0.193316

0
4

0
4

0

0

0

0
4

0
4

0.549304

0.549304

0.549304

0
4

0
4

0.0926517

0.0926517

0.0926517

0
4

0
4

0

0

0

0
4

0
4

0

0

0

0
4

0
4

0

0

0

0
4

0
4

0

0

0

0
4

0
4

0.278718

0.278718

0.278718

0
4

0
4

0

0

0

0
4

0
4

0

0

0

0

0

0

0

0
4

0

0

0

0

0

0
4

0

0

0

0

0
4

0
4

0

0

0

0
4

0
4

0.0553261

0.0553261

0.0553261

0
4

0
4

0

0

0

0
4

0
4

0

0

0

0
4

0
4

0

0

0

0
4

0
4

0

0

0

0
4

0
4

0.411978

0.411978

0.411978

0
4

0
4

0

0

0

0
4

0
4

0

0

0

0
4

0
4

0

0

0

0
4

0
4

0.429044
5

0

0

0

0

0
4

0

0

0

0
4

0

0

0
4

0

0

0
4

0.429044

0.429044

0
4

0

0

0
4

0
4

0.0724936

0.0724936

0.0724936

0
4

0
4

0

0

0

0
4

0
4

0

0

0

0
4

0
4

0.110652

0.110652

0.110652

0
4

0
4

0

0

0

0
4

0
4

0.161286

0.161286

0.161286

0
4

0
4

0.0752082

0.0752082

0.0752082

0
4

0
4

0.0848951

0.0848951

0.0848951

0
4

0
4

0.463259

0.463259

0.463259

0
4

0
4

0.07653

0.07653

0.07653

0
4

0
4

49.3855
4

43.2788
4

0
6

42.8406

0

0.438165

0
4

6.1067

2.29001

2.29001

1.52667

0
4

0
4

0

0

0

0
4

0
4

0

0

0

0
4

0
4

0

0

0

0
4

0
4

0

0

0

0
4

0
4

0

0

0

0
4

0
4

0.0565967

0.0565967

0.0565967

0
4

0
4

0.0553261

0.0553261

0.0553261

0
4

0
4

0

0

0

0
4

0
4

0

0

0

0
4

0
4

1.14501

1.14501

1.14501

0
4

0
4

0.317654
4

0.317654
4

0.05102

0.266634

0

0

0

0

0
4

0

0

0

0
4

0
4

0

0

0

0
4

0
4

0

0

0

0
4

0
4

0

0

0

0
4

0
4

0

0

0

0
4

0
4

0.161286

0.161286

0.161286

0
4

0
4

0

0

0

0
4

0
4

0

0

0

0
4

0
4

0.107261

0.107261

0.107261

0
4

0
4

0

0

0

0
4

0
4

0.101849

0.101849

0.101849

0
4

0
4

20.8836
4

16.6939
4

11.8815
4

2.79758

0.763337

1.16844

0.0829891

0
4

0.999453

0.999453

0

0

0
4

1.90834

0.763337

1.14501

2.22044604925031e-16

0
4

0

0

0

0
4

0.27663

0

0.27663

0
4

0.877429

0.877429

0
4

0.127882

0.127882

0
4

8.52096171399808e-15
4

0
4

42.6216
4

41.8716
4

41.6779
4

0.0553261

0.138315

4.44089209850063e-16
4

0
4

0.580924

0.497935

0.0829891

0
4

0.169152

0.169152

0
4

0
4

0

0

0

0
4

0
4

0

0

0

0
4

0
4

0

0

0

0
4

0
4

0

0

0

0
4

0
4

0

0

0

0
4

0
4

0

0

0

0
4

0
4

0.371625

0.371625

0.371625

0
4

0
4

0

0

0

0
4

0
4

0

0

0

0
4

0
4

0.22959

0.22959

0.22959

0
4

0
4

0

0

0

0

0

0
4

0

0

0

0

0
4

0

0

0

0
4

0
4

1.02197

1.02197

1.02197

0
4

0
4

0

0

0

0
4

0
4

0

0

0

0
4

0
4

0

0

0

0
4

0
4

0

0

0

0
4

0
4

0

0

0

0
4

0
4

0

0

0

0
4

0
4

0.763337

0.763337

0.763337

0
4

0
4

0

0

0

0
4

0
4

0

0

0

0
4

0
4

18.9871

2.04477

0

0

0

0

1.75266

0.29211

5.55111512312578e-17

0
4

16.9424

15.92

0.29211

0.730274

0
4

0
4

0

0

0

0
4

0
4

0.0483291

0.0483291

0.0483291

0
4

0
4

0

0

0

0
4

0
4

0

0

0

0
4

0
4

0

0

0

0
4

0
4

0

0

0

0
4

0
4

0.0511526

0.0511526

0.0511526

0
4

0
4

0

0

0

0
4

0
4

0.30781

0.30781

0.30781

0
4

0
4

0.464531

0.464531

0.464531

0
4

0
4

0.783287
4

0.725187

0.45474

0.185812

0

0.0846347

0
4

0.0581004

0

0.0581004

0

0
4

0

0

0
4

0

0

0
4

2.77555756156289e-17
4

0
4

0

0

0

0
4

0
4

0

0

0

0
4

0
4

0

0

0

0
4

0
4

0

0

0

0
4

0
4

0

0

0

0
4

0
4

0

0

0

0
4

0
4

0.0724936

0.0724936

0.0724936

0
4

0
4

0.0528677

0.0528677

0.0528677

0
4

0
4

0.29211

0.29211

0.29211

0
4

0
4

0.123536

0.123536

0.123536

0
4

0
4

1.27757
4

0.761712

0

0.761712

0

0
4

0.28547

0.116201

0.169269

0
4

0.107261

0.107261

0
4

0.123124

0.123124

0
4

0
4

0

0

0

0
4

0
4

0

0

0

0
4

0
4

0

0

0

0
4

0
4

0

0

0

0
4

0
4

0

0

0

0
4

0
4

0.0724936

0.0724936

0.0724936

0
4

0
4

0

0

0

0
4

0
4

0

0

0

0
4

0
4

0.184686

0.184686

0.184686

0
4

0
4

0

0

0

0
4

0
4

10.1582
4

9.84696
4

9.73376
4

0.113193

6.66133814775094e-16
4

0
4

0.311282

0.311282

0
4

0

0

0
4

5.55111512312578e-17
4

0
4

0

0

0

0
4

0
4

0

0

0

0
4

0
4

0

0

0

0
4

0
4

0

0

0

0
4

0
4

0

0

0

0
4

0
4

0

0

0

0
4

0
4

0

0

0

0
4

0
4

0.763337

0.763337

0.763337

0
4

0
4

0

0

0

0
4

0
4

0

0

0

0
4

0
4

7.18577

2.0635

1.30016

0.763337

0
4

0.956979

0.193641

0.763337

0
4

2.63862

2.63862

0
4

1.52667

1.52667

0
4

0
4

0.763337

0.763337

0.763337

0
4

0
4

0

0

0

0
4

0
4

0

0

0

0
4

0
4

0

0

0

0
4

0
4

0

0

0

0
4

0
4

1.31729

1.06668

0.983686

0.0829891

0

4.16333634234434e-17

0
4

0.139961

0.139961

0
4

0.110652

0.110652

0
4

9.71445146547012e-17

0
4

3.60397
4

3.43753

3.15881

0.278718

0
4

0.0829891

0

0.0829891

0
4

0.0834505

0.0834505

0
4

0

0

0
4

0

0

0
4

2.77555756156289e-17
4

0
4

2.99473

2.49997

0.29211

1.02238

0.29211

0.29211

0.438165

0.163089

5.55111512312578e-17

0
4

0.438165

0.438165

0
4

0

0

0
4

0.0565967

0.0565967

0
4

0
4

3.89308
4

3.89308
4

3.30886
4

0.29211

0

0

0

0.29211

0

0
4

0

0

0

0
4

0
4

7.13435

3.05335

3.05335

0
4

2.67168

0.763337

0.763337

1.14501

2.22044604925031e-16

0
4

0.645987

0.0617678

0.58422

0
4

0.763337

0.763337

0
4

4.44089209850063e-16

0
4

1.7132
3

1.7132
3

1.30231

0.0581004

0

0.116201

0.236598

8.32667268468867e-17
3

0
4

0
4

0.269633
3

0.221304

0

0

0.221304

0
4

0.0483291

0.0483291

0

0
4

0

0

0
4

6.93889390390723e-18
3

0
4

0.186644

0

0

0

0
4

0.138315

0.138315

0

0
4

0

0

0
4

0.0483291

0.0483291

0
4

0

0

0
4

0

0

0
4

6.93889390390723e-18

0
4

5.30668
4

5.30668
4

4.46049

0.338539

0.0501388

0.296221

0.161286

0
4

0
4

0.0846347

0.0846347

0

0

0.0846347

0

0

0
4

0

0

0
4

0

0

0
4

0
4

0.19037
3

0

0

0

0
4

0.19037

0.105735

0.0846347

0
4

0

0

0
4

0

0

0
4

0
4

4.98579

4.98579

0.433392

2.69428

1.85812

8.88178419700125e-16

0
4

0
4

1.9495

0

0

0
4

0.0617678

0.0617678

0
4

0

0

0
4

1.39359

1.39359

0
4

0

0

0
4

0.494142

0.494142

0
4

0
4

0
6

0

0

0
4

0

0

0
4

0
4

0
7

0
7

0
7

0
6

0

0

0

0

0

0
4

0
4

1.85753

1.42439

1.20778

0.0553261

0.161286

0
4

0.15442

0.15442

0
4

0.278718

0.278718

0
4

5.55111512312578e-17

0
4

0

0

0

0

0

0
4

0
4

1.42985
3

0.779503
3

0.779503
3

0
4

0

0

0
4

0.650343

0.650343

0
4

0
4

0

0

0

0

0

0
4

0

0

0

0

0
4

0

0

0
4

0
4

0
5

0

0

0
4

0

0

0
4

0

0

0
4

0
4

1.61301

1.61301

1.61301

0

0
4

0
4

1.49469

0.557437

0.371625

0.185812

2.77555756156289e-17

0
4

0.937254

0.29211

0.403215

0.241929

0
4

0
4

4.42565

2.8174

2.69428

0.123124

8.32667268468867e-17

0
4

0.772098

0.772098

0
4

0.836155

0.836155

0
4

0
4

1.98582
4

0.482086

0.482086

0
4

0.570813

0.570813

0
4

0.876329

0.876329

0
4

0

0

0
4

0.0565967

0.0565967

0
4

2.22044604925031e-16
4

0
4

1.44444
4

0.200083

0.144757

0.0553261

0
4

0.05102

0.05102

0
4

0.0483291

0.0483291

0
4

1.14501

1.14501

0
4

0
4

37.3749
4

24.8727
4

1.44056

0.371625

20.7838

1.11487

0.0553261

1.10652

0

0

0
4

3.99012
4

3.80763

0

0.0617678

0.120728

0

1.38777878078145e-16
4

0
4

0.183791

0.183791

0

0
4

0.20408

0.20408

0

0

0
4

0

0

0
4

6.47928

0.0528677

6.42641

0
4

0.836155

0.836155

0
4

0.370607

0.370607

0
4

0.438165

0.438165

0
4

0
4

0.486861

0.486861

0.425093

0.0617678

0
4

0
4

0

0

0

0
4

0

0

0
4

0

0

0
4

0

0

0
4

0

0

0
4

0
4

1.10857
4

0.829854

0.411978

0.417876

5.55111512312578e-17

0
4

0.278718

0.278718

0
4

5.55111512312578e-17
4

0
4

0.0617678

0

0

0

0
4

0

0

0
4

0

0

0
4

0.0617678

0.0617678

0
4

0
4

1.09861

0

0

0

0
4

0

0

0
4

0

0

0
4

1.09861

1.09861

0
4

0
4

0

0

0

0

0
4

0
4

0.337062

0.185303

0.0926517

0.0926517

0

0
4

0.151759

0.151759

0
4

0
4

1.85787

1.76522

1.39359

0.371625

0
4

0.0926517

0.0926517

0
4

5.55111512312578e-17

0
4

0.0829891

0

0

0

0

0

0
4

0.0829891

0.0829891

0
4

0
4

0

0

0

0

0
4

0

0

0
4

0
4

10.6825
4

10.3712
4

10.0373
4

0.135898

0.141492

0

0.0565967

1.30451205393456e-15
4

0
4

0.311282

0.0565967

0.113193

0.141492

0
4

1.83186799063151e-15
4

0
4

0

0

0

0

0
4

0

0

0

0
4

0

0

0
4

0
4

0

0

0

0

0
4

0

0

0
4

0

0

0
4

0
4

0

0

0

0

0
4

0

0

0
4

0

0

0
4

0
4

0.438165

0.438165

0

0.438165

0
4

0

0

0

0
4

0
4

0.165978

0.165978

0.0553261

0.110652

0
4

0

0

0
4

0

0

0
4

0
4

0.39415

0.39415

0.39415

0
4

0

0

0
4

0

0

0
4

0
4

0.111267

0

0

0
4

0.111267

0.111267

0
4

0

0

0
4

0
4

0

0

0

0

0
4

0
4

0

0

0

0
4

0

0

0
4

0
4

0.351067

0

0

0
4

0

0

0
4

0.289299

0.289299

0
4

0.0617678

0.0617678

0
4

0
4

0
6

0
7

0
7

0

0

0

0
4

0
6

0
6

0

0

0

0
4

0
4

0.429019

0.318922

0.318922

0
4

0.0483291

0.0483291

0
4

0.0617678

0.0617678

0
4

0
4

0.20461

0.20461

0.20461

0
4

0
4

28.0052

0.158414

0.158414

0
4

0.160722

0.160722

0
4

27.686

27.686

0
4

0
4

0

0

0

0
4

0

0

0
4

0
4

0

0

0

0

0
4

0

0

0
4

0
4

0

0

0

0

0
4

0

0

0
4

0
4

1.02197

1.02197

1.02197

0
4

0
4

0.24758

0

0

0
4

0.0617678

0.0617678

0
4

0.185812

0.185812

0
4

0
4

0.327783

0.27663

0.221304

0.0553261

0
4

0.0511526

0.0511526

0
4

5.55111512312578e-17

0
4

1.6223

1.6223

1.49918

0.123124

8.32667268468867e-17

0
4

0
4

0
7

0
7

0
7

0

0

0

0
4

0

0

0
4

0
4

0.96541

0.96541

0.96541

0
4

0
4

0

0

0

0

0
4

0
4

1.65267

1.09861

1.09861

0
4

0.554057

0.554057

0
4

1.11022302462516e-16

0
4

0.314139

0.314139

0.314139

0
4

0

0

0
4

0
4

0.07653

0

0

0
4

0

0

0
4

0.07653

0.07653

0
4

0
4

0.823956

0.823956

0

0.823956

0
4

0

0

0
4

0
4

0

0

0

0

0
4

0
4

0.742123

0.557437

0.557437

0
4

0.184686

0.184686

0
4

5.55111512312578e-17

0
4

0

0

0

0

0
4

0

0

0
4

0
4

0

0

0

0

0
4

0

0

0
4

0
4

4.90221
4

0.132164

0

0

0.132164

0
4

4.32655

3.53043

0.743249

0.0528677

4.64905891561784e-16

0
4

0.111907

0.0501388

0.0617678

0
4

0

0

0

0
4

0.278718

0.278718

0
4

0.0528677

0.0528677

0
4

0

0

0
4

0
4

0.774784

0.506632

0.214522

0.29211

0
4

0.268152

0.268152

0
4

0
4

0

0

0

0
4

0

0

0
4

0
4

0.183184

0.183184

0.183184

0
4

0
4

0.26581

0.26581

0.144987

0.120823

1.38777878078145e-17

0
4

0

0

0
4

0
4

0

0

0

0
4

0
4

0.0617678

0.0617678

0

0.0617678

0

0
4

0
4

0.0966581

0

0

0
4

0.0483291

0.0483291

0
4

0.0483291

0.0483291

0
4

0
4

0.139084

0

0

0
4

0

0

0
4

0.139084

0.139084

0
4

0
4

0.399541

0.120823

0.120823

0

0
4

0.278718

0.278718

0
4

0
4

1.66255

1.66255

1.55528

0.107261

0
4

0
4

14162.4
4

14094.7
4

6981.25
4

2922.58
4

3695.94
4

0
7

97.2666

0.185303

0

0

0

0

1.08173

0.583893

0

0

6.45432

0.192866

0.56017

3.43502

0.119615

0

0

0

0

0.279585

0

0.107261

0
7

0

0.685634

0.257154

0

0

0

0

0.415234

0

0.244634

0
6

0

1.07306

0.423632

0.203698

0

0.677121

0

0.29211

0.0482912

0

0
7

0

0

0

0.110652

0

0.0793016

0.349565

0

0

0.645144

0
7

0

0.118299

0

0

0

0

0.26581

0.165978

0.0846347

0

0
7

0

0

0

0

0

0

0.617678

0

0.730274

0

0

0

0

0.0553261

0.349565

0

0.138315

0

0

0

0.107261

0
6

0.295101

0.0617678

0

1.52667

0.0767289

0.05102

0.07653

0

0

0

0
7

0.0553261

0

0.107261

0

0

0.763337

0

0.05102

0

0.101849

0
7

135.28
4

0

0

0.0501388

0.123536

0

0

0.699131

0

0.193641

0

0
7

0

0

0

0

0

0

0

0

0.29211

0

0
6

0

0

0.951619

1.52667

0

0

0.0846347

0

0.107261

0

0
7

0

0.247071

0

0.305547

0

0.123536

0.0617678

0

0

0

0
7

0

0

0.107261

0

0

0.247071

0

0.0482912

0

0.0553261

1.38441
3

0

0

0.241929

0

0

0.876329

0

0.141492

0.101849

0.0829891

0
6

0

0.0834505

0.268152

0

0.107261

0

0.185303

0

0.0482912

0.308839

0

0

0.246248

0.0793016

0

0

0.184686

0

0.203698

0

0.362184

0
6

0

0

0.887242

0.0553261

0

0

0

0

0

0.0553261

0
6

0

0

0

0.0829891

0.33163

0

0

0

0

0

0
7

0

0

0

0

0

0.29211

0.214522

0.161286

0

0

0.101849

0
6

0

0

0.123536

0

0

0

0

0

0

0

0.385732

0

0

0.107261

0

0.247071

0.123124

0

0

0

0

0.389467
3

0.15306

0

0

0.0617678

0

0

0

0

0

0.0829891

0
4

0

0

0.650644

0.110652

0.161286

0

0

0

0

0.354897

0.255299

0

0

0.0926517

0

0

0

0

0

0.0829891

0

0
6

0

0

0

0

0.0553261

0.118299

0

0.0553261

0

0

0
7

0.225625

0

0

0

0.58422

0

0

0.0617678

0.111267

0

0
7

0

0.0617678

0

0

0

0

0

0

0

0

0.39197

0

0

0

0.278718

0.0617678

0

0.0834505

0.763337

2.67168

0

126.892
4

0
7

0.184686

0

0

0

0.123124

0

0

0.464531

0

0

0

0

4.87936

0

0

0

2.17388

0.689485

0

0
7

0

0

0.162587

0

0.160891

0

0

0

0.0642886

0

2.6586
3

1.94024

0.549304

0.257154

0

0

0

0

0.641612

0

0

0
7

1.39826

0.0642886

0

0

0.0642886

2.80811

0

0

0

0

0
7

0.278464

0

0.349565

0

0.274652

0.0846347

0

0.254623

0

0

68.5028
4

0.171953

0.169319

0

0

0

0.587211

0

0

0

0

0
4

0
7

0
7

0

0

0

0
4

1.82054

0.0553261

1.30069

0.464531

0
4

0

0

0
4

0

0

0
4

1.57304

1.57304

0
4

0

0

0
4

0

0

0
4

0

0

0

0

0

0
4

2.32265

2.32265

0

0
4

2.37798

2.32265

0.0553261

1.11022302462516e-16

0
4

1.34756

1.05545

0.29211

5.55111512312578e-17

0
4

2.37402

1.29029

1.02197

0.0617678

2.56739074444567e-16

0
4

2.60112

2.50847

0.0926517

5.55111512312578e-17

0
4

0

0

0
4

0.429044

0.107261

0.321783

0
4

6.37392

6.37392

0
4

3.07664

2.41531

0.29022

0.185303

0.185812

2.77555756156289e-16

0
4

1.67231

1.4865

0.185812

0
4

0

0

0
4

0.206113

0.206113

0
4

0.230385

0.107261

0.123124

0
4

2.88009

2.88009

0

0
4

0

0

0
4

0.650343

0.650343

0

0
4

0.248967

0

0.248967

0
4

0

0

0

0
4

0.415234

0.123124

0.29211

5.55111512312578e-17

0
4

0
7

0

0

0

0
4

0.424475

0

0.424475

0
4

0.268801

0.268801

0
4

0

0

0

0
4

0.203698

0.203698

0
4

0

0

0
4

0.338539

0.338539

0
4

0.123124

0.123124

0
4

0

0

0
4

0

0

0
4

0.163089

0.163089

0
4

0
6

0
6

0
4

0

0

0
4

0

0

0
4

0

0

0
4

0

0

0
4

0

0

0
4

0

0

0
4

0

0

0
4

0

0

0
4

0

0

0
4

0.05102

0.05102

0
4

0
6

0
6

0
4

0

0

0
4

2.04707

2.04707

0
4

0.138315

0.138315

0
4

0.28061

0.28061

0
4

0.58422

0.58422

0
4

0.0642886

0.0642886

0
4

0

0

0
4

0

0

0
4

0

0

0
4

0

0

0
4

15.7239
4

15.7239
4

0
4

0

0

0
4

0

0

0
4

0

0

0
4

0.29211

0.29211

0
4

0.185812

0.185812

0
4

0

0

0
4

0.29211

0.29211

0
4

0.0846347

0.0846347

0
4

3.81669

3.81669

0
4

0

0

0
4

4.13085

2.81636

0.438165

0.876329

0
4

0.161286

0.161286

0
4

0.185812

0.185812

0
4

0

0

0
4

0.185812

0.185812

0
4

0.123124

0.123124

0
4

0

0

0
4

0.278718

0.278718

0
4

0.0642886

0.0642886

0
4

0

0

0
4

0.100278

0.100278

0
4

3.91489

0.570266

3.34462

0

0
4

1.85812

1.85812

0
4

0

0

0
4

0

0

0
4

0

0

0
4

0

0

0
4

0

0

0
4

0.123124

0.123124

0
4

0

0

0
4

0

0

0
4

0.05102

0.05102

0
4

0

0

0

0
4

0

0

0
4

0.304294

0.304294

0
4

0

0

0
4

0

0

0
4

0.29211

0.29211

0
4

0.110652

0.110652

0
4

0.123124

0.123124

0
4

0

0

0
4

0

0

0
4

0

0

0
4

0
4

0
4

6778.28
4

6707.18
4

0

0

0

0

0

0
4

4435.52
4

568.229
4

1503.44
4

119.63
3

624.054
4

179.547
4

1067.35
4

98.6384
4

245.988
4

28.2011
3

0.165978

0

0

0

0

0.0501388

0

0

0.0553261

0.128577

0.0501388

0

0
4

2266.43
3

1777.89
3

166.207
3

207.192
3

15.8436
4

2.63608
4

1.06581

0.828496
4

0

0.86206

0.22556

0.904674

0.755426

0.699131

0.166901

17.6324
4

0.69148

0.20911

0.0834505

1.50148

0.433016

0

0

0.291858

0.200929

0.763337

4.68657
3

0.0565967

0

0.0556337

0

0.0848951

0.349565

0.0617678

0.161286

0.0556337

0.110652

1.94218
3

0

0.763337

0.05102

0.0565967

0.101849

0.214522

0.369371

0

0.0553261

0

0

0.0617678

0.05102

0.0565967

0.349565

0.0565967

0.0846347

0.101849

0.0556337

0

0.0565967

3.94551
3

0.0848951

0

0

0.05102

0.0565967

0.0617678

0.349565

0.0848951

0.278718

0.254623

50.2149
4

0

0.0999396

0.0565967

0

0

0

0.349565

0.185812

0.126952

0.07653

1.50025
4

1.50911
4

1.58895119284352e-12
3

0
4

0
5

0
5

0
4

0.0501388

0.0501388

0
4

0.274652

0.274652

0
4

0.0834505

0.0834505

0
4

0.275763

0.275763

0
4

0.836155

0.836155

0
4

0

0

0
4

0

0

0
4

0

0

0
4

0.524348

0.524348

0
4

0

0

0
4

1.77332

1.77332

0

0
4

0

0

0
4

0

0

0
4

0

0

0
4

0

0

0
4

0

0

0
4

0

0

0
4

0.200555

0.200555

0
4

0.0834505

0.0834505

0
4

0.551954

0.551954

0
4

0

0

0

0
4

0.576596

0.576596

0
4

0

0

0
4

0

0

0
4

0

0

0
4

0

0

0
4

0

0

0
4

0
4

0.752879
4

0.70274
4

0.225625

0

0

0.12755

0

0

0.349565

0

5.55111512312578e-17
4

0
4

0.0501388

0

0.0501388

0
4

0

0

0
4

4.85722573273506e-17
4

0
4

0.478723
4

0.414435

0.321783

0

0.0926517

0
4

0.0642886

0.0642886

0
4

0
4

2.45728
4

1.49561

1.49561

0
4

0.0752082

0.0752082

0
4

0.763337

0.763337

0
4

0.123124

0.123124

0
4

8.32667268468867e-17
4

0
4

15.7971

0.857264

0.857264

0
4

0.821438

0.763337

0.0581004

2.77555756156289e-17

0
4

14.1184

14.1184

0
4

0
4

0.370105

0.28547

0.28547

0
4

0.0846347

0.0846347

0
4

0
4

0

0

0

0

0

0

0
4

0
4

0.0642886

0

0

0
4

0

0

0
4

0.0642886

0.0642886

0
4

0
4

2.21803

0.173263

0.173263

0
4

0.29211

0.29211

0
4

1.75266

1.75266

0
4

0
4

0.893352

0.893352

0.818144

0.0752082

0
4

0
4

1.98645

1.98645

1.53222

0.05102

0.403215

5.55111512312578e-17

0
4

0
4

0

0

0

0
4

0
4

1.49332
4

1.15779
4

0.93649
4

0.221304

0
4

0.260321

0.209301

0.05102

0
4

0.0752082

0.0752082

0
4

2.22044604925031e-16
4

0
4

0.403377

0.29211

0.29211

0
4

0

0

0
4

0.111267

0.111267

0
4

0
4

0.835136

0.0553261

0.0553261

0
4

0.613831

0.613831

0
4

0.165978

0.165978

0
4

5.55111512312578e-17

0
4

0

0

0

0

0
4

0
4

0.663531

0.395379

0.100278

0.295101

0
4

0.268152

0.268152

0
4

5.55111512312578e-17

0
4

0.0553261

0.0553261

0

0.0553261

0
4

0
4

0.268068

0.211471

0.211471

0

0
4

0.0565967

0.0565967

0
4

0
4

0.101159

0.101159

0.101159

0
4

0
4

3.8963

3.8963

3.8963

0
4

0
4

0.0501388

0.0501388

0.0501388

0

0
4

0
4

0.262541

0.262541

0.262541

0
4

0
4

0.871269
4

0.625372
4

0.497689
4

0.0511526

0.07653

4.16333634234434e-17
4

0
4

0.245898

0.13463

0.111267

0
4

0

0

0
4

2.77555756156289e-17
4

0
4

0.557437

0.557437

0.557437

0
4

0
4

0

0

0

0
4

0
4

4.72563

4.72563

4.72563

0
4

0
4

0

0

0

0

0
4

0
4

0.246248

0.246248

0.246248

0
4

0
4

0.193641

0.0829891

0.0829891

0
4

0.110652

0.110652

0
4

0
4

0

0

0

0

0
4

0
4

0

0

0

0

0
4

0
4

0.566762

0.29211

0.29211

0
4

0.274652

0.274652

0
4

5.55111512312578e-17

0
4

0

0

0

0
4

0
4

19.4584
5

18.4029

0

18.4029

0
4

1.05545

0

0.763337

0

0.29211

0
4

2.22044604925031e-15
5

0
4

0.107261

0.107261

0.107261

0
4

0
4

0.0501388

0.0501388

0.0501388

0
4

0
4

0

0

0

0
4

0
4

0

0

0

0
4

0
4

0

0

0

0
4

0
4

0.248967

0.248967

0.248967

0
4

0
4

0

0

0

0
4

0
4

0

0

0

0
4

0
4

0.0848951

0.0848951

0.0848951

0
4

0
4

0

0

0

0
4

0
4

2.09963
4

1.86586
4

1.81484
4

0

0.05102

0
4

0.110652

0.110652

0
4

0.123124

0.123124

0
4

1.2490009027033e-16
4

0
4

0

0

0

0
4

0
4

0

0

0

0
4

0
4

0

0

0

0
4

0
4

0

0

0

0
4

0
4

0

0

0

0
4

0
4

0

0

0

0
4

0
4

0.524348

0.524348

0.524348

0
4

0
4

0

0

0

0
4

0
4

0

0

0

0
4

0
4

0

0

0

0
4

0
4

2.08988
4

2.08988
4

0.580794

1.11823

0.116201

0.274652

0
4

0
4

0.126952

0.126952

0.126952

0
4

0
4

0

0

0

0
4

0
4

0.0846347

0.0846347

0.0846347

0
4

0
4

0

0

0

0
4

0
4

0.118299

0.118299

0.118299

0
4

0
4

0

0

0

0
4

0
4

0

0

0

0
4

0
4

0

0

0

0
4

0
4

0

0

0

0
4

0
4

0.29211

0.29211

0.29211

0
4

0
4

0.626007

0.3148

0.100278

0.214522

0
4

0.110652

0.110652

0
4

0.125347

0.125347

0
4

0.0752082

0.0752082

0
4

2.77555756156289e-17

0
4

0.107261

0.107261

0.107261

0
4

0
4

0

0

0

0
4

0
4

0

0

0

0
4

0
4

0

0

0

0
4

0
4

0

0

0

0
4

0
4

0

0

0

0
4

0
4

0.549304

0.549304

0.549304

0
4

0
4

0

0

0

0
4

0
4

0

0

0

0
4

0
4

0.532345

0.532345

0.532345

0
4

0
4

0.681888

0.626254

0.542804

0.0834505

0

0
4

0.0556337

0.0556337

0
4

0
4

0

0

0

0
4

0
4

0.185812

0.185812

0.185812

0
4

0
4

0.05102

0.05102

0.05102

0
4

0
4

0

0

0

0
4

0
4

0.107261

0.107261

0.107261

0
4

0
4

0

0

0

0
4

0
4

0

0

0

0
4

0
4

0

0

0

0
4

0
4

0

0

0

0
4

0
4

0

0

0

0
4

0
4

2.04642
4

2.04642
4

1.98832
4

0.0581004

4.71844785465692e-16
4

0
4

0
4

0

0

0

0
4

0
4

0

0

0

0
4

0
4

0

0

0

0
4

0
4

0.0846347

0.0846347

0.0846347

0
4

0
4

0.123124

0.123124

0.123124

0
4

0
4

0.349565

0.349565

0.349565

0
4

0
4

0

0

0

0
4

0
4

0.160891

0.160891

0.160891

0
4

0
4

0

0

0

0
4

0
4

0
4

55.2246
5

17.1616

17.0088

14.5135

2.24068

0.254623

0
4

0.152774

0.152774

0
4

7.7715611723761e-16

0
4

0

0

0

0
4

0
4

0

0

0

0
4

0
4

0

0

0

0
4

0
4

0

0

0

0
4

0
4

0

0

0

0
4

0
4

0

0

0

0
4

0
4

0.107261

0.107261

0.107261

0
4

0
4

0

0

0

0
4

0
4

0.241929

0.241929

0.241929

0
4

0
4

0

0

0

0
4

0
4

15.301
4

15.301
4

15.301

0

0

0

0
4

0

0

0

0
4

0

0

0
4

0
4

0

0

0

0
4

0
4

0

0

0

0
4

0
4

0

0

0

0
4

0
4

0
6

0
6

0

0

0

0
4

0
4

0
7

0
7

0
7

0
4

0
4

0
6

0
6

0
6

0

0
4

0
4

0

0

0

0
4

0

0

0
4

0
4

19.4532

19.4532

19.4532

0
4

0
4

0

0

0

0

0
4

0
4

0

0

0

0
4

0
4

2.95969

1.11271
5

0

0.750827

0

0.254623

0

0

0.107261

1.38777878078145e-17
5

0
4

0
7

0

0

0
4

0

0

0
4

0

0

0
4

0

0

0
4

0

0

0
4

0

0

0
4

0

0

0

0
4

1.56826

1.56826

0

0
4

0

0

0
4

0

0

0
4

0

0

0
4

0.278718

0.278718

0
4

0

0

0
4

0

0

0
4

2.77555756156289e-16

0
4

2.66453525910038e-15
5

0
4

9.97907534383558e-10

0
4

42.0525

41.8596

17.8663

0
3

0
3

0

0

0

0

0

0
4

7.20157
3

5.10899
3

0

0

0

0

0

0

0

0.248967

0
2

0.257154
3

1.39359

0

0.0642886

0

0

0.128577

0
4

10.6647

10.6647

0

0

0
4

0
7

0

0

0

0

0

0

0

0

0

0

0
6

0

0

0

0

0

0

0

0
4

0

0

0
4

0

0

0
4

0

0

0
4

1.77635683940025e-15

0
4

10.4614
3

0

0

0

0
4

2.3289

0.663913

0

0.138315

1.52667

0
4

8.13248

4.75583

0.499698

2.87696

4.44089209850063e-16

0
4

0

0

0
4

0
4

0.0553261

0.0553261

0.0553261

0
4

0
4

0

0

0

0
4

0
4

0

0

0

0
4

0
4

0

0

0

0
4

0

0

0
4

0
4

13.4766
3

9.24612
3

0

5.70525

3.54087

0
4

4.0645

1.77448

2.29001

4.44089209850063e-16

0
4

0.165978

0.165978

0
4

0
4

0

0

0

0

0
4

0
4

0
2

0
2

0
2

0

0
4

0
4

0

0

0

0

0
4

0
4

0

0

0

0
4

0
4

0

0

0

0
4

0
4

0

0

0

0
4

0
4

1.4210854715202e-14

0
4

0.192866
2

0.192866
2

0.128577
2

0.128577

0

0

0

0

0
4

0.0642886
3

0.0642886

0

0

0

0

0
4

0

0

0
4

0
4

0
4

0

0

0

0

0
4

0
4

0
4

0

0

0

0

0

0
4

0
4

0
4

0

0

0

0

0

0

0
4

0
4

0
4

0

0

0

0

0
4

0
4

0
4

0

0

0

0

0
4

0
4

0
4

4.57966997657877e-15

0
4

950.108

943.528

10.0102
3

10.0102
3

0

0

0

0

0

0

0.152013

0.410797

0

0

0.05102

0.483291
3

0

0.17857

0

0

0

0

0.0724936

0

0

0

4.52044
3

0

0

0

0

0

0

0

0.05102

0

0

2.31737
3

0

0

0

0

0

0

0

0

0

0

0

0.07653

0

0.12755

0

0

0

0

0

0

0.483291

0.521187
4

0

0

0

0.564619
4

0

0
4

0
4

156.881
3

156.881
3

0
1

0

2.83732

0.723238

0.278168

1.27957

0.278168

0.445069

0.584154

0

0.222535

22.7316

0.139084

0

0.0556337

0.111267

0

0.222535

0.361619

0

0.250352

2.11408

16.9415
4

0

0

0.222535

0

0

0.0834505

0.349565

0.139084

78.3002
4

22.5595

0

5.08106

0

0.570018

2.19824158875781e-14
3

0
4

0
4

8.35302
6

8.35302
6

0
6

0.0999396

0

0

0

0

0
7

0

0

0

0

1.19928
5

0.594265

3.31144

0.849487

0

0

0

0

0

0

0

1.84888
5

0

0

0

0

0

0

0

0

0.0999396

0

0
6

0

0

0

0

0.349789

0
6

0
6

0
7

0
6

6.10622663543836e-16
6

0
4

0

0

0
4

0
4

0
7

0
7

0
7

0
7

0
4

0
4

0.887073
3

0
3

0

0

0

0

0

0

0

0

0

0

0

0

0

0

0

0

0

0

0

0

0

0

0

0

0

0

0

0

0

0

0

0

0

0

0

0

0

0

0

0

0

0

0

0

0

0

0

0

0

0

0

0

0

0

0

0

0

0

0

0

0

0

0

0

0

0

0

0

0

0

0

0

0

0

0

0

0

0

0

0

0

0

0

0

0

0

0

0

0

0
4

0

0

0

0

0

0
4

0

0

0
4

0

0

0
4

0.403215

0.403215

0

0

0
4

0.483858

0.483858

0
4

0

0

0

0
4

0

0

0

0
4

0

0

0
4

0

0

0
4

0

0

0
4

0

0

0
4

5.55111512312578e-17
3

0
4

174.862
3

1.83507
3

1.83507

0

0
4

78.2127
3

29.0281
4

0.584154

0.869801

1.67497

1.20064

8.34012

0

3.67044

0

0

0

0
2

0.178758

2.37273

0

0

0

0.234391

0.0511526

0

0.0556337

0

0.59601

1.0487

0

0

0

0

0.246248

0

0

0

0

25.6402
4

0.0834505

0

0

0

0.123124

0.349565

0

0

0

0

0

0.0511526

0

0

0

0

0.166901

0

0

0

0

1.59528
4

0

0

0

0

0.0511526

0

0

0

0

1.75970349403087e-14
3

0
4

0

0

0

0
4

0

0

0
4

57.7094

57.7094

0
4

0

0

0

0

0
4

0

0

0

0

0

0

0

0

0

0

0

0

0
4

0

0

0

0

0
4

37.1048

22.4772

0

0

0.161286

0

0.0528677

0.161286

0

0

0

13.0047

0

0

1.10596

0.141492

3.88578058618805e-16

0
4

0

0

0
4

0

0

0
4

7.105427357601e-15
3

0
4

142.462
5

0
6

0
6

0
4

142.462
5

142.462
5

0
4

0
7

0

0

0

0

0

0

0

0

0

0

0

0

0

0

0

0

0

0

0

0

0

0

0

0

0

0

0

0

0

0

0

0

0

0

0

0

0

0

0

0

0

0

0

0

0

0

0

0

0

0

0

0

0

0

0

0

0

0

0

0

0

0

0

0

0

0

0

0

0

0

0

0

0

0

0

0

0

0

0
4

0

0

0

0
4

0

0

0
4

0

0

0
4

0

0

0
4

0
4

410.733
3

410.733
3

410.733
4

0

0

0

0

0

0

0

0

0

0
4

0
4

19.5507
3

19.5507
3

16.1125
3

0

2.09672

0

0.226387

0

0.331076
3

0

0.679161

0.0482912

0

0

0.0565967

0

5.89805981832114e-16
3

0
4

0

0

0
4

0

0

0
4

0
4

0.0556337

0.0556337

0.0556337

0
4

0
4

0

0

0

0
4

0
4

0

0

0

0
4

0
4

0

0

0

0
4

0
4

0

0

0

0
4

0
4

0

0

0

0
4

0
4

0

0

0

0
4

0
4

0

0

0

0
4

0
4

0

0

0

0
4

0
4

0

0

0

0
4

0
4

19.5291
3

19.5291
3

19.5291
3

0
4

0
4

0

0

0

0

0

0

0

0
4

0
4

0

0

0

0

0
4

0
4

0

0

0

0

0
4

0

0

0
4

0
4

0

0

0

0
4

0

0

0
4

0
4

0

0

0

0
4

0
4

0

0

0

0

0
4

0
4

0.203698

0.203698

0.203698

0
4

0

0

0
4

0
4

3.1399882693961e-13

0
4

2.35911
3

2.35911
3

2.35911
3

1.97994
3

0.0565967
3

0.322572

0

0

0

0
4

0

0

0
4

0

0

0

0
4

0
4

0
4

0

0

0

0

0

0

0
4

0

0

0
4

0
4

0
4

0

0

0

0

0

0
4

0
4

0
4

0

0

0

0

0

0
4

0
4

0
4

0

0

0

0

0

0
4

0
4

0
4

0

0

0

0

0
4

0

0

0
4

0
4

0
4

0

0

0

0

0
4

0
4

0
4

0

0

0

0

0

0
4

0
4

0
4

0.890139

0.890139

0.890139

0.890139

0
4

0
4

0
4

0

0

0

0

0
4

0
4

0
4

0

0

0

0

0
4

0
4

0
4

0.792651
3

0.792651
3

0.792651
3

0.7092
3

0

0

0.0834505

0
4

0
4

0
4

0

0

0

0

0
4

0
4

0
4

0

0

0

0

0
4

0
4

0
4

0

0

0

0

0
4

0
4

0
4

1.01849

1.01849

1.01849

1.01849

0
4

0
4

0
4

0

0

0

0

0
4

0
4

0
4

0

0

0

0

0
4

0
4

0
4

0

0

0

0

0
4

0
4

0
4

0

0

0

0

0
4

0
4

0
4

0

0

0

0

0
4

0
4

0
4

0

0

0

0

0
4

0
4

0
4

0

0

0

0

0

0

0

0

0
4

0

0

0
4

0
4

0
4

0

0

0

0

0
4

0
4

0
4

0

0

0

0

0
4

0
4

0
4

0

0

0

0

0
4

0
4

0
4

0

0

0

0

0

0
4

0
4

0
4

0.712943

0.712943

0.407396

0.101849

0.203698

0.101849

0
4

0.305547

0.305547

0
4

0

0

0
4

0
4

0
4

0

0

0

0

0

0
4

0
4

0
4

0

0

0

0

0

0

0

0
4

0

0

0
4

0
4

0
4

0.806688

0.806688

0.806688

0.695421

0.0556337

0.0556337

0
4

0
4

0
4

0

0

0

0

0

0

0
4

0

0

0
4

0
4

0
4

0
4

324.136
3

323.69
3

25.4948
4

25.4948
4

25.4948
4

0
4

0
4

0

0

0

0

0

0

0

0

0

0

0

0

0

0

0

0

0

0

0

0
4

0

0

0

0

0

0

0

0

0

0

0

0

0

0

0
4

0

0

0

0
4

0
4

0

0

0

0
4

0
4

0

0

0

0
4

0

0

0

0
4

0
4

8.84889

0
1

0
1

0
4

0

0

0
4

0

0

0
4

8.84889
4

3.60626

1.37243
4

0

3.78556

0

0.0846347

8.46545056276682e-16
4

0
4

0

0

0
4

0

0

0
4

0

0

0
4

0

0

0
4

0
4

0
7

0
7

0
7

0
4

0
4

0
7

0
7

0
7

0

0

0

0
4

0
4

85.9631
4

85.9078
4

83.2185
4

0.578594

0.125347

0.322572

1.04836

0.317137

0.241929

0.0553261

0
4

0.0553261

0.0553261

0
4

0

0

0
4

0
4

14.5347
4

14.5347
4

7.97054

5.95196

0.61224

0
4

0
4

0

0

0

0
4

0
4

0.800305

0.800305

0.800305

0
4

0
4

79.418
3

79.418
3

0

12.0834
3

0

35.2352
3

0

0

0.804457

0

0

0

0.214522

0

0

0

0

0.05102

0.160891

0

0

0

0

0.107261

0

0.107261

0

0

0

0.321783

0

0.0752082

0.107261

0

0.05102

0

0.107261

1.30069

1.11487

0.836155

26.7397

0

0

1.4210854715202e-14
3

0
4

0

0

0
4

0
4

108.63
4

108.63
4

60.0259
4

32.1866
4

0.300833
4

0
3

15.5652

0.100278

0.376041

0

0.0752082

8.77076189453874e-15
4

0
4

0
4

0

0

0

0

0
4

0
4

0
4

0.445518

0.445518

0.445518

0.445518

0
4

0
4

0
4

0

0

0

0

0
4

0
4

0
4

3.09752223870419e-14
3

0
4

18938.2

51.032

0.942417

0.942417

0.942417

0
4

0
4

48.1564

45.9888
3

18.0177
3

0

0.244634

0

0

0

0

0

0.163089

0

0

24.1311
3

0.0553261

0

0

0

0

0

0

0

2.07226
3

0.652357

0

0

0.407723

0

0.244634

0
4

0

0

0
4

2.16758

2.16758

0

0

0

0

0
4

0
4

0

0

0

0

0

0

0

0

0

0

0

0

0

0

0

0

0

0

0

0

0

0

0

0
4

0

0

0

0

0

0

0
4

0
4

0

0

0

0
4

0
4

1.93316

1.93316

1.93316

0
4

0
4

3.5527136788005e-15

0
4

0.459126
1

0

0

0

0

0

0

0

0

0

0

0

0

0

0

0
4

0

0

0

0

0

0

0
4

0

0

0
4

0

0

0
4

0
4

0

0

0

0
4

0
4

0

0

0

0
4

0
4

0

0

0

0

0

0

0

0

0
4

0

0

0
4

0

0

0
4

0
4

0
8

0
8

0

0

0

0
4

0

0

0
4

0
4

0

0

0

0

0

0
4

0

0

0
4

0
4

0.459126

0

0

0
4

0.459126

0.459126

0
4

0
4

0

0

0

0

0

0
4

0

0

0
4

0
4

0

0

0

0

0

0
4

0
4

0

0

0

0
4

0

0

0
4

0
4

0

0

0

0
4

0
4

0
4

2216.13
3

2216.13
3

149.258
3

36.2158

3.93577

0.545762

0.772648

1.90834

0.287957

0

0

4.64381

0.228801

0.966581

7.12459

0

3.85474

0

0.169152

3.35887

0.507808

0.58422

0

0.130297

0

0

0.434797

0

0

0

0

0.179034

0

0

8.61724

0

0.604113

0

0.10204

0

0.0434322

0

0.0966581

0.0483291

0.58422

0

0

0

0.0434322

0

0

0.152774

0

21.7391

0

0

4.97789

0

2.50413

43.8961

2.1316282072803e-14
3

0
4

2056.72
4

528.5
4

2.05621
4

0

0.0501388

0.139084

0.0556337

0

0

0

0.126952

0.296221

0.152774

3.03818
4

0

0.111267

0

0.0871506

0.101849

3.00453

0.338539

0

0.0846347

0

3.52229
4

0.369371

0

0.0871506

0

0.196734

0.166901

0.169269

0

0

0.126952

2.97245
4

0

0.0581004

0.139084

0.58422

0

0

0.126952

0

0

0

0.909376

0

0.338539

0.349565

0.0556337

0

0.0556337

0

1.14501

0

0.0556337

2.46095
4

0

0.0556337

0.211587

0.196734

0.458321

0.0556337

0

0

0.0556337

0

2.30445
4

0

0

0.445069

0.139084

0.05102

5.72503

0.126952

0.0556337

0

0.126952

6.30354
4

0.196734

0.0846347

0

0

0

0.169269

0

0.0556337

4.89576

0.196734

0.296221
4

0.611094

0.0846347

0

0

1.14501

0.0724936

0

0

0.107261

0.127882

1.22334
4

0.0846347

0.377653

0

0

0

0.389436

0

0

1.57866

0

1021.21
4

5.07554
4

0.111267

0

0.0846347

0.116201

0

0.107261

0.319552

0.193641

0

0.417823

0.883967
3

23.0626
4

0.897988
4

1.0085
3

8.03907

1.26523
4

0.568128

2.59019

221.025
4

5.98869

0.160891
3

4.08069

1.38818

0.817346

0.653059

0.166901

0.0483291

0.77266

0.57439

15.7862
4

5.10717

1.30739

0.583016

2.05079

6.37201

0.500703

2.29001

0.525022

1.76164

0

16.8945
4

0.412639

5.61989

0.161364

0.423892

0.695377

0.222535

0.376744

0.389436

0

0.400432

34.9575
4

1.61235

0.153458

2.02454

3.92659

0.102305

3.43502

0.338539

0.423173

2.21078

0.141551

5.50365
4

0

0

0.548129

0.171785

0.695729

0.58422

0

1.80891

0.0556337

0

4.02309
4

0.139084

0.0483291

0.799918

0.152774

0.207759

0.389436

0.244342

0.26722

3.88213

0.283885

40.2036
4

1.62681

0.140268

0

0.346086

1.27196

0.211587

0

0.238219

0

0

2.12857509396258e-13
4

0
4

8.22369
2

8.22369
2

0

0

0
4

0
2

0

0

0

0

0

0

0

0

0

0

0

0

0

0

0

0

0

0

0

0

0

0

0

0

0

0

0

0

0

0

0

0

0

0

0

0

0

0

0

0

0

0

0

0

0

0
4

0.66326

0

0.66326

0

0
4

0

0

0

0

0

0

0

0

0

0

0

0
4

0

0

0
4

0.125347

0.125347

0
4

0

0

0
4

0.169152

0.169152

0
4

0

0

0

0

0

0

0

0
4

0

0

0

0
4

0

0

0

0
4

0

0

0

0

0
4

0.0483291

0

0.0483291

0
4

0

0

0
4

0

0

0
4

0.923429

0.923429

0
4

1.29563026973756e-13
3

0
4

0

0

0

0
4

0

0

0
4

0
4

0

0

0

0

0
4

0
4

0

0

0

0
4

0
4

0

0

0

0
4

0
4

0
4

50.6971
3

37.7082
3

19.837
3

0
2

0

0

0

0.924052

0

0

0

0

1.9014

0

0

0

0
3

1.50165

0.709389

0

0

0

0

0

0

0

0

0

0

0

0

0

0

0

0

0

0

0

0

0

0.196734

0

0

0

0

0

0

0

0

0

0

0

0

0

0

0

0

0

0

0

0

0

0

0

0

0

0.0483291

0

0

0

0

0

0

0

0

0

0

0

0

0

0

0

10.1603
4

4.19836

0

0

0

0

0

0

0

0

0.196734

0

0

0

0

0

0

0

0

0

0

0

0
4

7.59936
4

4.84558

1.80536
4

0.948424

3.33066907387547e-16
4

0
4

0

0

0
4

0

0

0
4

0

0

0
4

0

0

0

0
4

9.51312
4

9.51312
4

0
4

0

0

0
4

0

0

0

0
4

0.651483

0.651483

0
4

0

0

0
4

0

0

0
4

0.107261

0.107261

0
4

0
4

0.367879

0

0

0

0

0

0
4

0.367879

0.367879

0
4

0
4

0

0

0

0
4

0

0

0
4

0
4

0

0

0

0

0

0
4

0
4

0

0

0

0
4

0
4

2.47187

2.47187

2.47187

0
4

0
4

0

0

0

0
4

0
4

0.800305

0.800305

0.800305

0
4

0
4

0

0

0

0
4

0
4

0

0

0

0
4

0
4

0

0

0

0
4

0
4

2.15064

2.15064

2.15064

0
4

0
4

0

0

0

0

0
4

0

0

0
4

0
4

0

0

0

0
4

0
4

0

0

0

0
4

0
4

0

0

0

0
4

0
4

1.129

1.129

1.129

0
4

0
4

0

0

0

0
4

0
4

0

0

0

0
4

0
4

0

0

0

0
4

0
4

0

0

0

0
4

0
4

0

0

0

0
4

0
4

0

0

0

0
4

0
4

2.15064

2.15064

1.76401

0.289974

0.0483291

0.0483291

2.91433543964104e-16

0
4

0

0

0
4

0
4

0

0

0

0
4

0

0

0

0

0
4

0
4

0

0

0

0

0
4

0
4

0.988814

0.988814

0.478335

0.333031

0.177448

0
4

0
4

0

0

0

0

0
4

0
4

0.869923

0.869923

0.869923

0
4

0
4

2.05989

2.05989

2.05989

0
4

0
4

3.99680288865056e-15
3

0
4

15.9762

9.30949
4

4.54306
5

1.88483

0

0

0

0

0

0.55591

0

0

2.10231

0

0
4

4.33406

3.36635

0.967716

3.33066907387547e-16

0
4

0.432375

0.432375

0
4

0
4

1.59957
7

0
8

0

0
8

0
4

0
1

0
1

0

0

0

0
4

0
1

0

0

0

0

0

0

0

0

0

0

0

0

0

0
4

0

0

0
4

0
7

0
7

0
7

0
7

0

0

0

0

0

0

0

0

0

0

0
7

0

0

0

0

0

0

0

0

0

0

0
7

0

0

0

0

0
7

0
7

0

0

0

0

0
4

1.37456

0.898752

0
7

0

0

0

0.47581

0

0

0

0

0

0

0

1.11022302462516e-16

0
4

0

0

0

0
4

0

0

0

0
4

0.22501

0.22501

0
4

0

0

0
4

5.55111512312578e-17
7

0
4

0
7

0
7

0
7

0

0

0
4

0
7

0
6

0

0
4

0
4

3.6463

0
1

0

0

0

0

0

0

0

0

0

0

0

0

0

0

0

0

0

0

0

0

0

0

0

0

0

0

0

0

0

0

0

0

0

0

0

0

0

0

0

0

0

0

0

0

0

0

0

0

0

0

0

0

0

0

0

0

0

0

0
4

0

0

0

0

0

0

0

0

0

0

0

0

0
4

3.6463

0

0

0

0

0

0.123124

0

0

0

0
7

0

2.96912
5

0

0.554057

0

0

1.11022302462516e-16

0
4

0

0

0
4

0

0

0
4

0
4

0

0

0

0

0

0

0
4

0

0

0
4

0
4

0

0

0

0
4

0
4

0

0

0

0
4

0
4

0.0617678

0.0617678

0.0617678

0
4

0
4

0

0

0

0
4

0
4

0

0

0

0

0
4

0

0

0
4

0
4

0

0

0

0

0

0
4

0
4

0

0

0

0

0
4

0

0

0
4

0
4

0

0

0

0
4

0
4

1.35906

1.35906

1.35906

0
4

0
4

0

0

0

0

0
4

0
4

0

0

0

0
4

0
4

0

0

0

0
4

0
4

0
7

0
7

0
7

0
4

0
4

2.66453525910038e-15

0
4

6520.15
3

10.9337
3

0.564501

0.564501

0
4

9.11266
3

7.98366
3

1.129

0

0

6.66133814775094e-16
3

0
4

1.25656
2

1.03907
2

0.0966581
2

0.120823
2

0

0

0

0

0

8.32667268468867e-17
2

0
4

0

0

0

0
4

0

0

0

0
4

0

0

0

0
4

0

0

0
4

0

0

0
4

0

0

0
4

0

0

0
4

0
4

141.246
3

0
7

0
6

0

0
4

12.9241
3

12.9241
3

0
4

91.709
3

91.709
3

0
2

0
4

8.86475
3

0.264339

8.60041
3

0

0

0

0

0

0

0

0

0

0

0

0

0

0

0

0

0

0

0

0

1.77635683940025e-15
3

0
4

26.7336
3

0.725787

0.274652

2.88385

0

0

0

0

0

0

0.549304

0

8.30898

0

0
7

0
7

10.0801

0.725787

2.59499

0

0.590203

5.88418203051333e-15
3

0
4

0

0

0

0
4

0

0

0
4

0

0

0
4

1.01412

1.01412

0
4

0

0

0
4

2.79776202205539e-14
3

0
4

316.604
3

3.04342

2.67342

0.26274

0.107261

0

0

0

0
4

11.5573
5

0

0

0

0.870598

0

10.6867

0

0

0

1.77635683940025e-15
5

0
4

0.94737

0

0
6

0.107261

0.107261

0
6

0

0.282458

0

0.34313

0

0.107261

0

0
4

300.959
3

31.2199
3

128.563
4

86.5196
4

0

5.34336

12.5951

0.185812

0

25.8634
4

6.31762

1.7137

1.6066

0

0.107261

0.160891

0.763337

3.1863400806742e-14
3

0
4

0

0

0

0
4

0

0

0

0
4

0

0

0
4

0

0

0
4

0

0

0
4

0

0

0
4

0

0

0

0

0
4

0

0

0

0
4

0.0965825

0.0965825

0
4

0

0

0
4

0

0

0
4

0

0

0
4

0

0

0
4

0

0

0
4

0
4

5266.21
4

0

0

0
4

4.97923

1.98648

2.99275

0
4

5237.76
4

4794.74
4

100.142
4

0.101849
4

0

0

0

0.524348

0.0834505

0

0

0

0.101849

0

0.585416
4

1.92261

0

0

0

0

0

0.118299

0

0

0

39.7893
4

0

0.699131

0

0

0

0

0

0

0

0.12755

0.679409
4

0.101849

0

0

0

0

0

0.29211

0

0.458321
4

0

0.0511526

0.253837
4

0.152774

0.356472

5.04705
4

0.29211

0.370001

0

0

0.828092

0.407852
4

0.202912
4

0

39.1039

0.40661

163.358
4

0

1.52667

0

0

0.356472

3.32087

0.152774

0

23.3521

0.152774

3.4878
4

0

0

0.152774

0

0

0

0

0

18.3363

0

6.97731
4

0

0

0

0.177448

0

0

0

6.65018

0

0

0.582096
4

0

0.152774

0

0.211471

0.865186

0

0

0

0

0.177448

16.6053
3

0

0

0

0

0

3.00331

0

0

0

0

0

0

0

0

0

0

0

0

0

0.107261

0

0
4

0

0

0

0

0

0

0

0

0.107261

0

0
4

0

0

0
4

0

0

0
4

0.0846347

0.0846347

0
4

0.0556337

0.0556337

0
4

1.14501

1.14501

0
4

0

0

0
4

0

0

0
4

1.5794

1.5794

0
4

0

0

0
4

0.160891

0.160891

0
4

0.111267

0.111267

0
4

1.98639

1.80895

0.177448

0

0
4

0

0

0
4

12.9339

12.9339

0
4

0

0

0
4

0.0834505

0.0834505

0
4

0.0556337

0.0556337

0
4

0

0

0
4

1.14501

1.14501

0
4

0

0

0
4

0.160891

0.160891

0
4

0

0

0
4

0.716137

0.332492

0.383645

0
4

0

0

0
4

0.196734

0.196734

0
4

1.27722

1.27722

0
4

0

0

0
4

0.268152

0.268152

0

0
4

1.51059

1.51059

0
4

0

0

0
4

0

0

0
4

3.03090885722668e-13
4

0
4

318.517

0
6

0
6

0
6

0

0
6

0

0

0

0

0
4

2.36112
3

0

0

0

0.452774

0

0

0

0

0

0

0

0

0

0

0

0

0

0

0

0

0

0

1.90834

0

0

0

0

0

0

0

0

0
4

36.8785
3

36.661
3

0
2

0

0

0

0.0483291
2

0

0

0

0

0.169152

0

0

3.96904731303493e-15
3

0
4

0
2

0
2

0

0

0

0

0

0

0
2

0

0

0

0

0

0

0

0
4

131.998

92.5738
3

1.22132
7

0.579949
3

0

36.1523

0
2

0

0

0

0

0

0

0

0

0.0651483

0

0

0

0

0

0.873913

0

0

0

0.0724936

0

0.0483291

0.410797

0

0

0

0

1.23234755733392e-14

0
4

54.637

54.637

0

0

0

0

0

0

0

0
4

1.59586
6

0
6

0

0

0

0

0

0

0

0

0

0

0

0

0

0

0

0

0

1.04655

0

0

0

0

0

0

0

0

0

0

0

0

0

0

0

0

0

0

0

0.274652

0

0

0

0

0

0

0

0

0

0

0

0

0

0

0

0

0

0

0

0

0

0.274652

0

0

0

0

0

0

0

0

0

0

0

0

0

0

0

0

0
6

0

0

0

0

0

0

0

0

0

0

0

0

0
4

21.9142
3

4.40062
3

0.773265
2

0

0

0

0

0.966581

0

0

1.90834

0

0

0.193316
3

0

0

0

0

0

0

0

0

0

0

0.0483291
2

0

1.09861

0

0

0

0

0

0

0

0

0.0724936

0

0

0

0

0

0

0

0

0

0

0

0

0

0

0

0

0

0

0.0511526

0.241645

11.8852

0

0

0

0

0

0

0

0

0.274652

0

0

0

0

0

0

0

0
2

0

8.88178419700125e-16
3

0
4

67.805
3

0

0

6.59758
4

6.53085

14.0939

0.0501388

0

0

0.05102

0

0.246248

0

0

19.0814
4

0.411978

0.615619

0.274652

0.0752082

1.66217

0.411978

9.17946
4

2.22213

2.74805

0.376782

2.19082

0.984991

0

0
4

0

0

0

0
4

0

0

0
4

0

0

0
4

0

0

0
4

0

0

0
4

0

0

0
4

0

0

0
4

0

0

0
4

0

0

0
4

1.14163

1.14163

0
4

0

0

0
4

0

0

0
4

0

0

0
4

0

0

0
4

0

0

0
4

0

0

0
4

0

0

0
4

0.185812

0.185812

0

0
4

0

0

0
4

0

0

0
4

0

0

0
4

0

0

0
4

7.90201237776955e-14

0
4

26.1564
3

0

0

0
4

0.763337
3

0.763337
2

0

0

0

0

0
4

23.5514
3

22.9612
3

0

0.590203

0

0

0

0

0

0

0

1.4432899320127e-15
3

0
4

0

0

0
4

0

0

0
4

0

0

0
4

0.410797

0.410797

0
4

0

0

0
4

0

0

0

0
4

0

0

0
4

0

0

0
4

0.438165

0.438165

0
4

0.869595

0.869595

0
4

0.123124

0.123124

0
4

0
4

0

0

0

0

0
4

0
2

0

0

0

0
4

0

0

0
4

0

0

0
4

0

0

0
4

0
4

0
6

0
6

0
6

0

0

0

0
4

0
7

0
6

0
7

0

0

0

0

0

0

0

0
4

0
6

0

0

0

0

0

0

0

0

0
4

0
4

0

0

0

0
4

0
4

1.88681

0.249429

0.249429

0
4

1.63738

1.63738

0
4

0
4

0

0

0

0

0

0
4

0

0

0
4

0
4

0.414946

0

0

0

0

0
4

0

0

0
4

0.414946

0.414946

0
4

0
4

0.546299

0.349565

0.349565

0

0
4

0

0

0
4

0.196734

0.196734

0
4

0
4

0

0

0

0

0
4

0

0

0
4

0

0

0
4

0
4

0

0

0

0

0

0
4

0
4

0

0

0

0

0
4

0
4

0

0

0

0

0
4

0
4

0

0

0

0

0
4

0
4

29.7282
4

29.7282
4

29.029
4

0.699131

0

4.44089209850063e-16
4

0
4

0

0

0
4

0
4

0

0

0

0

0
4

0
4

0

0

0

0
4

0
4

0

0

0

0

0
4

0
4

0

0

0

0
4

0
4

0.217883

0.217883

0.0565967

0.161286

0
4

0
4

1.97943

1.97943

1.97943

0
4

0
4

0

0

0

0
4

0
4

0

0

0

0

0
4

0
4

0

0

0

0

0
4

0
4

0

0

0

0
4

0
4

8.00582
3

5.40968
3

5.40968
3

0
4

0.785902

0.392951

0.274652

0.118299

0
4

1.39826

0

1.39826

0
4

0.411978

0.411978

0
4

1.0547118733939e-15
3

0
4

0

0

0

0
4

0
4

0

0

0

0
4

0
4

0.750827

0.750827

0.750827

0
4

0
4

0

0

0

0
4

0
4

0

0

0

0
4

0
4

0

0

0

0
4

0
4

0

0

0

0
4

0
4

0

0

0

0
4

0
4

0

0

0

0
4

0
4

0

0

0

0
4

0
4

41.2311
3

41.2311
3

38.9036
3

0

2.27217

0.0553261

0
4

0
4

0

0

0

0
4

0
4

0.0724369

0.0724369

0.0724369

0
4

0
4

0

0

0

0
4

0
4

0

0

0

0
4

0
4

15.1059

15.1059

15.1059

0
4

0
4

0

0

0

0
4

0
4

0.929061

0.929061

0.929061

0
4

0
4

0

0

0

0
4

0
4

0.123536

0.123536

0.123536

0
4

0
4

0

0

0

0
4

0
4

0

0

0

0

0
4

0
4

0

0

0

0
4

0
4

1.14501

1.14501

1.14501

0
4

0
4

0

0

0

0
4

0
4

0

0

0

0
4

0
4

0

0

0

0
4

0
4

0

0

0

0
4

0
4

0

0

0

0
4

0
4

0

0

0

0
4

0
4

0

0

0

0
4

0
4

0

0

0

0
4

0
4

0

0

0

0

0

0

0
4

0
4

0.123124

0.123124

0.123124

0
4

0
4

0
7

0
7

0

0

0
4

0
4

0

0

0

0
4

0
4

0
2

0
2

0

0

0
4

0
4

23.6505

21.9832

0.325742
2

2.73059
2

1.69152

0

3.31054
3

0
6

0.990746

0

0

0
2

0

0
7

0

0.0483291
7

0

0

0

0

0.459126

0

0.115926

0

0

0

0.0966581
7

1.11157

0

0

0

0

0

0.918252

0

0

0

5.26787

0

0

0

0.766582

0

0

0

0

0

0

0.0483291
7

0

0

0

0

0

0

0

0.456038

0

0

0.687281

0

0

0

0.338303

0

0

0

0

0

0

0.20279
2

0

0

0

1.74559

0.67139
5

0
4

1.40154
2

1.40154
2

0

0

0

0

0

0
4

0

0

0

0
4

0

0

0
4

0

0

0
4

0
2

0
2

0

0

0
4

0

0

0

0

0
4

0
2

0

0

0

0
4

0.26581

0.26581

0
4

0

0

0
4

0

0

0

0
4

0

0

0
4

0

0

0

0
4

2.05391259555654e-15

0
4

133.647
4

130.448
4

130.448
4

0

0
4

0
7

0
7

0
7

0

0

0

0

0

0
4

0

0

0
4

0

0

0

0
4

2.21406

2.21406

0
4

0

0

0
4

0

0

0
4

0.984991

0.984991

0
4

0

0

0
4

0

0

0
4

0

0

0
4

3.64153152077051e-14
4

0
4

9.01337

8.16761

0.362468

0.0724936

0

0

2.94807

4.27712

0.362468

0

0.0483291

0.0483291

0

0.0483291

9.85322934354826e-16

0
4

0.845758
3

0.845758
3

0

0
4

0

0

0
4

8.88178419700125e-16

0
4

2.96893

0

0

0
4

0.274652

0.274652

0
4

0

0

0
4

0

0

0
4

0

0

0
4

2.69428

2.69428

0
4

0

0

0
4

0
4

0

0

0

0

0

0

0

0

0

0

0

0

0

0

0

0

0

0
4

0

0

0
4

0

0

0
4

0

0

0
4

0

0

0
4

0
4

152.256
3

151.04
3

41.2172
3

2.33134
4

0.140981
3

0

0.220779
3

34.6476
4

0.185303

0.110097

0.194718

0.185812

0.101849

41.0394
3

0.918252

0

0.0617678

0.185303

1.11487

0.101849

0

0

0

0

8.431
3

0

0

0

0

2.04394

0.278718

0

0.0617678

0.0868644

0.0617678

0.886786
3

0.0556337

0

0

0

0

0.185812

1.5794

1.09559
3

2.1479
3

2.249
3

9.11819
3

0

6.21724893790088e-14
3

0
4

0
2

0
2

0

0

0

0

0
4

0

0

0
4

0

0

0
4

0

0

0
4

0

0

0
4

0
2

0

0

0

0

0

0

0
4

0

0

0

0
4

1.2161

1.2161

0
4

0

0

0
4

0

0

0

0
4

0

0

0

0
4

0

0

0

0
4

0

0

0
4

0
4

3.86249
3

3.46632
3

0
2

0

0

0

0

1.39232

0

0

0

0

0

0
2

0

0

0

0

0

0

0

0

0

0

0
2

0

0
2

2.074

0

0

0
4

0.396177

0.33958

0.0565967

0
4

0

0

0
4

0

0

0
4

0

0

0
4

0

0

0
4

0

0

0
4

0

0

0
4

0

0

0
4

0

0

0
4

0
4

12.8298

12.2243

0
2

12.1759

0.0483291

9.0205620750794e-17

0
4

0

0

0

0

0

0
4

0

0

0

0

0
4

0.552696

0.552696

0

0
4

0

0

0
4

0

0

0
4

0.0528677

0.0528677

0
4

0
4

0

0

0

0
4

0

0

0
4

0

0

0
4

0
4

8.5265128291212e-14
3

0
4

2.50525
2

2.50525
2

2.34396

0.7491

0.314139

1.28072

0

0

0
4

0
2

0
2

0
4

0

0

0
4

0

0

0
4

0.161286

0.161286

0
4

0
4

0

0

0

0
4

0
4

0
4

6297.01
4

0

0

0

0

0
4

0
4

0
2

0
2

0
2

0

0
2

0

0

0

0

0

0
4

0

0

0
4

0

0

0

0

0
4

0

0

0

0
4

0
4

1547.65
4

955.99
4

0
7

0

0

582.337

0
7

371.364

0

0.763337

0

0

1.52667

0
4

574.104

339.185

1.14501

0.763337

33.9685

1.14501

7.2517

3.05335

5.34336

5.15461

80.5321

89.6921

3.05335

1.14501

2.67168

1.67421632113474e-13

0
4

12.9767

11.4501

0.763337

0.763337

1.55431223447522e-15

0
4

0

0

0

0

0
4

3.81669

1.52667

2.29001

0
4

0.763337

0.763337

0
4

0

0

0
4

8.84070594509012e-13
4

0
4

0
7

0
7

0

0

0

0

0
4

0

0

0

0
4

0
4

0

0

0

0
4

0
4

0

0

0

0
4

0
4

0

0

0

0
4

0
4

0.185303

0.185303

0.185303

0
4

0
4

0.29211

0.29211

0.29211

0
4

0
4

0

0

0

0
4

0
4

0.876329

0.876329

0.876329

0
4

0
4

0

0

0

0
4

0
4

0

0

0

0
4

0
4

0

0

0

0
4

0
4

0
5

0
7

0
7

0
4

0

0

0
4

0

0

0
4

0
4

0

0

0

0
4

0
4

0

0

0

0
4

0
4

0

0

0

0
4

0
4

0.615619

0.615619

0.615619

0
4

0
4

0

0

0

0
4

0
4

0

0

0

0
4

0
4

0

0

0

0
4

0
4

0

0

0

0
4

0
4

0

0

0

0
4

0
4

0

0

0

0
4

0
4

0
7

0
7

0
7

0

0

0

0

0

0

0

0
4

0
4

0

0

0

0
4

0
4

0

0

0

0
4

0
4

0

0

0

0
4

0
4

0

0

0

0
4

0
4

0

0

0

0
4

0
4

0

0

0

0
4

0
4

0.274652

0.274652

0.274652

0
4

0
4

0

0

0

0
4

0
4

0

0

0

0
4

0
4

0

0

0

0
4

0
4

0

0
7

0

0

0

0

0
4

0

0

0

0

0
4

0

0

0
4

0

0

0
4

0
4

0.763337

0.763337

0.763337

0
4

0
4

0

0

0

0
4

0
4

0

0

0

0
4

0
4

0

0

0

0
4

0
4

0

0

0

0
4

0
4

0

0

0

0
4

0
4

0

0

0

0
4

0
4

0

0

0

0
4

0
4

0

0

0

0
4

0
4

0

0

0

0
4

0
4

0

0

0

0

0

0

0
4

0

0

0

0
4

0

0

0

0
4

0

0

0
4

0

0

0
4

0

0

0
4

0

0

0
4

0
4

0

0

0

0
4

0
4

0

0

0

0
4

0
4

0

0

0

0
4

0
4

0

0

0

0
4

0
4

0

0

0

0
4

0
4

0

0

0

0
4

0
4

0

0

0

0
4

0
4

0

0

0

0
4

0
4

0

0

0

0
4

0
4

0.0482912

0.0482912

0.0482912

0
4

0
4

0

0

0

0

0

0
4

0
4

0

0

0

0
4

0
4

0.0501388

0.0501388

0.0501388

0
4

0
4

0
7

0
7

0
7

0

0
4

0

0

0
4

0

0

0
4

0
4

0
5

0
7

0

0

0

0

0
4

0

0

0
4

0

0

0
4

0
4

0

0

0

0

0
4

0

0

0

0
4

0

0

0

0
4

0

0

0
4

0
4

0.125347

0.125347

0

0

0.125347

0

0
4

0

0

0

0
4

0
4

213.951

201.356

19.8468

6.1067

3.05335

2.29001

1.90834

1.14501

0.763337

1.14501

0.763337

0.763337

21.3404

24.0451

10.5972

25.1901

66.792

5.68234

3.05335

6.87004

6.30606677987089e-14

0
4

12.5951

3.43502

2.67168

2.67168

3.05335

0.763337

1.66533453693773e-15

0
4

0
4

1.72979

1.72979

0.727012

1.00278

0
4

0
4

0

0

0

0

0

0

0
4

0

0

0
4

0
4

5.58158
4

1.28295

1.0083

0.274652

0
4

4.29863

4.29863

0
4

0

0

0
4

0
4

14.1217

6.87004

1.90834

1.90834

1.14501

1.90834

0
4

7.2517

4.96169

2.29001

0
4

1.77635683940025e-15

0
4

0.0964329
3

0.0964329
3

0.0964329
3

0

0
4

0

0

0
4

0
4

1.59096

1.59096

1.59096

0
4

0
4

0
2

0

0

0
4

0

0

0
4

0

0

0
4

0
4

0

0

0

0

0
4

0

0

0
4

0
4

0
6

0

0

0

0

0
4

0

0

0

0
4

0
4

0

0

0

0

0

0
4

0
4

114.593
4

114.593
4

103.953
4

0.123124

0.861867

0.184686

7.98495
4

0.667741

0.397776

0

0.0511526

0.29211

0.0767289

0

2.85882428840978e-15
4

0
4

0

0

0
4

0
4

0

0

0

0

0
4

0
4

2333.81

2333.52

2327.8

2.67168

3.05335

0
4

0.29211

0.29211

0

0
4

6.8889338677991e-14

0
4

2.33454
5

2.33454
5

2.33454
5

0
4

0
4

0

0

0

0
4

0

0

0
4

0

0

0
4

0

0

0
4

0

0

0
4

0
4

0
2

0

0

0

0

0
4

0

0

0
4

0

0

0
4

0
4

0

0

0

0
4

0
4

0

0

0

0

0
4

0

0

0
4

0

0

0
4

0

0

0
4

0
4

0.369174

0.369174

0.304026

0.0651483

0
4

0
4

0

0

0

0

0

0
4

0
4

1.83672

1.83672

1.83672

0
4

0
4

1514.47
4

1398.44

1267.9

99.2412

2.67168

1.90834

1.52667

0.763337

23.6635

0.763337

1.64201985342061e-13

0
4

60.6853

3.05335

51.5253

4.58002

1.52667

4.44089209850063e-15

0
4

9.92339

2.67168

4.19836

3.05335

0
4

42.7469

42.7469

0
4

2.67168

2.67168

0
4

1.21236354289067e-13
4

0
4

12.4977

12.4977

12.4977

0
4

0
4

4.58002

3.81669

3.05335

0.763337

0
4

0.763337

0.763337

0
4

3.33066907387547e-16

0
4

0

0

0

0

0
4

0
4

0

0

0

0

0
4

0
4

16.169

15.8944

2.29001

13.6044

0
4

0.274652

0

0.274652

0
4

0
4

0

0

0

0

0

0
4

0
4

0

0

0

0

0
4

0

0

0
4

0
4

0

0

0

0
4

0
4

0

0

0

0
4

0

0

0

0
4

0
4

0

0

0

0

0
4

0
4

0
6

0
6

0
6

0

0

0
4

0
4

0.718755

0.718755

0.667603

0.0511526

5.55111512312578e-17

0
4

0
4

0

0

0

0
4

0
4

1.75583

1.44802

1.44802

0
4

0.30781

0.30781

0
4

0
4

0

0

0

0
4

0
4

0

0

0

0
4

0
4

0

0

0

0

0

0
4

0
4

0

0

0

0
4

0

0

0
4

0
4

3.81669

3.81669

0.763337

3.05335

0
4

0

0

0
4

0
4

0

0

0

0

0
4

0
4

0.15306

0

0

0
4

0

0

0
4

0.15306

0.15306

0
4

0
4

0.0964329
3

0.0964329

0.0964329

0
3

0

0

0
4

0

0

0

0
4

0

0

0
4

0

0

0
4

0
4

0

0

0

0

0
4

0

0

0
4

0
4

0

0

0

0
4

0
4

7.63337

6.87004

5.72503

1.14501

0
4

0.763337

0.763337

0
4

0
4

0

0

0

0
4

0
4

0

0

0

0
4

0

0

0
4

0
4

0

0

0

0
4

0
4

0

0

0

0
4

0

0

0
4

0
4

0

0

0

0
4

0

0

0
4

0
4

0

0

0

0
4

0

0

0
4

0
4

0.349565

0.349565

0.349565

0
4

0
4

0
6

0
6

0
6

0

0
4

0
4

0

0

0

0
4

0
4

3.52038

3.52038

2.47447

1.04591

0
4

0
4

0.0964329

0

0

0
4

0.0964329

0.0964329

0
4

0
4

2.09739

2.09739

0

2.09739

0
4

0
4

0

0

0

0
4

0
4

0

0

0

0
4

0

0

0
4

0
4

0

0

0

0
4

0

0

0
4

0
4

0

0

0

0

0
4

0
4

0

0

0

0
4

0
4

0

0

0

0
4

0
4

0.0642886
2

0.0642886
2

0.0642886

0
2

0

0

0

0

0

0
4

0

0

0
4

0
4

0

0

0

0

0
4

0
4

0.586794

0.586794

0.308839

0.277955

5.55111512312578e-17

0
4

0
4

0.634358

0.634358

0.634358

0
4

0
4

0.773265

0.773265

0.773265

0
4

0
4

0

0

0

0
4

0
4

0

0

0

0
4

0
4

0

0

0

0
4

0
4

0

0

0

0
4

0
4

0

0

0

0
4

0
4

0

0

0

0
4

0
4

0

0

0
6

0

0

0

0

0
4

0

0

0

0

0
4

0

0

0
4

0

0

0
4

0
4

0

0

0

0
4

0
4

0

0

0

0
4

0
4

0

0

0

0
4

0
4

0

0

0

0
4

0
4

0

0

0

0
4

0
4

0

0

0

0
4

0
4

0

0

0

0
4

0
4

0

0

0

0
4

0
4

0

0

0

0
4

0
4

0

0

0

0
4

0
4

486.092

461.169

60.1452

163.258

0.546478
2

0.0483291
3

23.9593
3

0

0

0

0

7.63337

0

0

0

1.2928

0

50.4028
3

0

0

0

0

0

0

0

0

0

0

128.619

0

11.8317

0.763337

0

8.39671

0

0

0

0

0

0

0.260593

0

0

0.274652

0

0

0.123124

0

0

0

0

1.85921

0

0

0.750791

1.00449

0

0
4

4.99115

4.4371

0.554057

0
4

0

0

0
4

0

0

0

0
4

0

0

0
4

0

0

0
4

1.2928

1.04655

0.246248

5.55111512312578e-17

0
4

0

0

0
4

1.51078

1.51078

0
4

0

0

0
4

0

0

0

0
4

0

0

0
4

0

0

0
4

0

0

0
4

0

0

0
4

0

0

0
4

0

0

0
4

0

0

0
4

0

0

0
4

0

0

0
4

0

0

0
4

0

0

0
4

0

0

0
4

0

0

0

0

0

0

0
4

0

0

0
4

0

0

0
4

0

0

0
4

0

0

0
4

0.411978

0.411978

0
4

0

0

0
4

0

0

0
4

0

0

0
4

0

0

0
4

0

0

0
4

7.70088
3

7.70088
3

0
4

0.163089

0.163089

0
4

0

0

0
4

0

0

0
4

0.411978

0.411978

0
4

0

0

0
4

0

0

0
4

0

0

0
4

0

0

0

0
4

0

0

0
4

0

0

0

0
4

4.06916

3.88448

0.184686

0
4

4.3709

0.492495

1.23124

2.64716

0
4

1.50990331349021e-13

0
4

3.18323145620525e-12
4

0
4

0.393468

0.393468

0.196734

0

0

0.196734

0
4

0.196734

0.196734

0
4

0
4

0
4

242.878
3

83.3833
3

4.33728
3

0

0

0

0.0434322

0

0.0868644

0

0.173729

0

3.99747

0

0

0

0

0.035782

0
4

42.3324
3

0.644076
3

1.12756
3

0

0

0.130297
3

1.08954

0

0.127543
3

1.02238

0

0

8.89921
3

0.26581

0
2

0

0

0

0

0.82729

0

0

0.0966581

4.60655
3

0

0

0.0724936

0

0

0.035782

0

0

0

1.89871

7.44984
3

0

0

0.0724936

0.304026

0

0

0

0

0.0483291

0

0.805095
3

0.0724936

0

0

0

0

0

0

0.0483291

0

0

3.16866
3

0

0.894087

0.0483291

0

0.241456

0

0.0483291

0

0

0

1.45407
3

0

0

0

0

0

0

0

0

0.0434322

1.37882

5.22958
3

0

0

0

0.0724936

0

0

0

0

0

0

0.108581
2

0

0

0

0

0

0

0

2.13579154362264e-14
3

0
4

33.3703
3

26.8009
3

0.364603

0

0.0434322

0

0

0

0

0.169152

0

0

1.68078
3

0

0

0

0

0

0.0483291

0

0

0

0.78178
3

2.83899
3

0.130297
2

0.181074

0.185971

0.0966581

0.0483291

7.8756445809347e-15
3

0
4

0.845758
2

0.845758
3

0

0

0

0

0

0

0
4

0.349789

0

0.349789

0
4

0

0

0
4

0

0

0
4

0.217311

0.217311

0
4

0

0

0

0
4

0

0

0
4

0.225625

0.225625

0
4

0

0

0
4

0

0

0
4

0

0

0
4

1.0874
2

1.0874
3

0

0

0

0

0

0
4

0

0

0
4

0

0

0
4

0

0

0
4

0

0

0
4

0.0617678

0.0617678

0
4

0

0

0
4

0

0

0
4

0

0

0
4

0

0

0
4

0

0

0
4

0

0

0
4

0

0

0
4

0

0

0
4

0

0

0
4

0

0

0
4

0

0

0
4

0

0

0
4

0

0

0
4

0.555784

0.555784

0

0
4

0

0

0

0
4

0

0

0

0

0

0

0

0

0
4

0

0

0

0
4

0

0

0

0
4

0

0

0

0

0
4

0
4

103.087
3

59.0286
3

59.0286
3

0

0

0

0

0

0

0

0

0
4

0
2

0

0

0

0

0

0

0

0

0

0

0
4

0

0

0

0

0
4

0

0

0

0
4

0

0

0

0
4

19.5673
2

6.25665
3

1.80148
2

0.7491
3

0

0

0

0

0

0

0

0

0

0

0

0

0

0

0

0

0

0

0

0

0

0
2

0

0

0

0

0

0

0

0

0

0

0
2

0

0

0

0

0

0.410797

0

1.27026

0

0

0.217481
3

0

0

0

0

0

0

0

0

0

0

0

0

0.161019

0

0

0

0

0.035782

0

0

0

0
2

0

0

0

0

0

0

0

0

0

0

0

0

0

0

0

0

0

0

0

0

0.0483291

0
2

0.0651483

0

0

0

0

0

0

0

0

0

0.977225

0

0

0

0

0

0

0

0

0

0

4.26714
3

0

0.053673

1.25953

0

0

0

0

0

0

0

0

0
2

0

0

0

0

0

0
2

0
2

0

0

0

0.679858

0

0

0

0

0
2

0

0

0

0.0868644

0
2

0

0

0

0

0

0

0

0

0

0

0
3

0

0

0

0

0

0

0.0434322

0

0

0

0
2

0

0.0483291

0

0

0

0

0

0

0

0

0.178626
3

0

0.159358

0.0966581

0

0

0

0.483057

0

0

0

0
2

0

0.0724936

0

0.0966581

0

0

0

0

0

0

0

0

0

0

0

0

0.0483291

0

0

0

0

0
4

23.3248
3

23.3248
3

0

0

0

0

0
4

0.15096
3

0

0.0999396

0

0.05102

0

0

0

0

0

0

0

0

0

0
4

0

0

0

0

0
4

0

0

0
4

0

0

0
4

0

0

0
4

1.01491

1.01491

0

0
4

0

0

0
4

0

0

0
4

0

0

0
4

0

0

0
4

0

0

0
4

0

0

0
4

0

0

0
4

1.02140518265514e-14
3

0
4

0.0434322

0

0

0

0

0

0

0

0

0

0
4

0

0

0

0

0

0

0

0

0

0

0

0

0

0

0

0

0

0

0

0

0

0

0

0

0

0

0

0

0

0

0

0

0

0

0

0

0

0

0

0

0

0

0

0

0

0

0

0

0

0

0

0

0

0

0

0

0

0

0

0

0

0

0

0

0

0

0

0

0

0

0

0

0

0

0

0

0

0

0

0

0

0

0

0

0

0
4

0

0
7

0

0

0
4

0

0

0
4

0

0

0
4

0

0

0
4

0

0

0
4

0

0

0
4

0

0

0
4

0

0

0
4

0

0

0
4

0

0

0
4

0

0

0
4

0

0

0
4

0

0

0

0

0
4

0
7

0
7

0
4

0.0434322

0.0434322

0

0
4

0

0

0
4

0

0

0

0

0
4

0

0

0
4

0

0

0
4

0
4

5.67183
3

2.26167
3

0.0724936
3

0

2.10231
3

0.0868644
2

0
3

0

0

0

0

0

0
4

0.53162
3

0.483291
3

0

0
2

0

0

0

0

0.0483291

0

0

3.46944695195361e-17
3

0
4

0

0

0

0

0
4

0.0724936
3

0

0

0

0.0724936

0

0

0

0
4

0.108581

0

0

0

0.108581

0
4

2.57664

2.49365

0

0.0829891

0
4

0
2

0

0

0

0

0
4

0

0

0

0
4

0.120823

0

0.120823

0
4

6.80011602582908e-16
3

0
4

0.496039

0.117094

0.117094

0
4

0.378945

0.193641

0.185303

2.77555756156289e-17

0
4

0
4

2.68226

0.144987

0.0724936

0.0724936

0
4

2.53728

2.53728

0
4

0
4

2.45578

2.45578

2.45578

0
4

0
4

0.289974

0.289974

0.289974

0
4

0
4

0.0483291

0.0483291

0.0483291

0

0
4

0
4

0.117094

0.117094

0.117094

0
4

0
4

0.0752082

0.0752082

0.0752082

0
4

0
4

0.525026

0.525026

0.525026

0
4

0
4

9.88747

9.88747

0.549304

9.33817

0
4

0
4

0

0

0

0
4

0
4

13.3343

13.2909

7.59638

1.08581

0.0434322

0

0.108581

0

0

0

0

0

0

0.0434322

0

0

0

0.0868644

0.0868644

0.0483291

0.542903

3.64831

0

0

1.33226762955019e-15

0
4

0.0434322

0

0

0.0434322

0
4

0

0

0
4

0
4

0

0

0

0
4

0
4

0

0

0

0
4

0
4

0

0

0

0
4

0
4

0

0

0

0
4

0
4

0

0

0

0
4

0
4

0

0

0

0
4

0
4

0

0

0

0
4

0
4

0

0

0

0
4

0
4

0

0

0

0
4

0
4

5.86794

5.86794

5.86794

0
4

0
4

0

0

0

0

0

0

0

0

0

0

0

0
4

0

0

0

0

0

0
4

0
4

0

0

0

0
4

0
4

0.0483291

0.0483291

0.0483291

0
4

0
4

0

0

0

0
4

0
4

0

0

0

0
4

0
4

9.39106
3

2.95648
3

2.88398
3

0

0

0

0

0

0.0724936

0

0

2.35922392732846e-16
3

0
4

6.43458
3

3.04473
3

3.38985

0
4

0

0

0
4

0

0

0
4

0

0

0
4

1.77635683940025e-15
3

0
4

4.99152
3

4.62692
3

2.36812

0.449013

0

1.73729

0

0.0724936

0

0
4

0.113477
3

0.0483291

0

0.0651483

0

0
4

0.120823

0.120823

0
4

0.130297

0.0651483

0.0651483

0
4

0
4

0.222058
2

0.0434322
2

0

0

0

0

0

0.0434322

0
4

0.0434322

0.0434322

0

0

0
4

0

0

0

0
4

0.0483291

0.0483291

0
4

0

0

0
4

0.0868644

0.0868644

0
4

0
4

0.260593

0.130297

0.0434322

0

0.0434322

0.0434322

0

0
4

0.130297

0.130297

0

0

0

0
4

0

0

0
4

0

0

0
4

0
4

0

0

0

0

0
4

0

0

0

0
4

0

0

0
4

0
4

0

0

0

0
4

0

0

0
4

0
4

0

0

0

0
4

0
4

0
4

59.2882

28.6251

0

0

0

0

0
4

13.3584

13.3584

0
4

15.2667

15.2667

0
4

0

0

0
4

0
4

0

0

0

0
4

0

0

0
4

0

0

0

0

0

0

0
4

0

0

0

0

0

0

0

0

0
4

0
4

0

0

0

0

0

0

0

0
4

0

0

0

0

0
4

0

0

0

0
4

0

0

0
4

0

0

0
4

0
4

15.6987
5

15.6987
5

15.6987
5

0

0
4

0
4

14.9643

0

0

0

0
4

0
7

0
7

0
4

0
7

0
7

0
4

1.54913
2

1.43848
2

0

0

0

0

0

0

0

0.110652

0

0

0

0

0

0

4.16333634234434e-17
2

0
4

0

0

0

0

0

0
4

0

0

0
4

0

0

0
4

0

0

0

0

0
4

1.59903

1.59903

0
4

0

0

0

0

0
4

0

0

0

0

0
4

0

0

0
4

0

0

0

0
4

2.60324

2.60324

0
4

0
2

0
2

0

0

0

0

0

0

0
4

0

0

0

0
4

0

0

0
4

0

0

0

0
4

0

0

0
4

0

0

0
4

0

0

0

0
4

0

0

0

0
4

0

0

0

0
4

3.55163

3.55163

0
4

0

0

0
4

0
2

0
2

0

0

0
4

0

0

0
4

0

0

0
4

0

0

0
4

0

0

0
4

2.19722

2.19722

0
4

0

0

0
4

0

0

0
4

0

0

0
4

0

0

0
4

1.74894

1.74894

0
4

1.71511
2

0

1.71511

0

0

0

0

0

0

0
4

0

0

0
4

0

0

0
4

0

0

0
4

0

0

0
4

0

0

0
4

0

0

0
4

0

0

0
4

0

0

0
4

0

0

0
4

0

0

0
4

0
2

0

0

0

0

0

0

0
4

0

0

0

0
4

0

0

0

0

0
4

0
2

0
2

0
4

0

0

0

0

0

0

0
4

0
4

0
6

0
6

0
6

0
4

0

0

0
4

0
4

0

0

0

0
4

0
4

0

0

0

0
4

0
4

0

0

0

0
4

0
4

0

0

0

0
4

0
4

0
7

0
7

0
7

0
4

0
4

0

0

0

0
4

0
4

0

0

0

0

0

0
4

0
4

0

0

0

0

0
4

0

0

0
4

0
4

0

0

0

0
4

0
4

0

0

0

0

0
4

0
4

0

0

0

0
4

0
4

0

0

0

0
4

0
4

1.4210854715202e-14

0
4

0
6

0
6

0
7

0
7

0
6

0

0

0

0

0

0
6

0
6

0
7

0

0
7

0

0
6

0

0

0
6

0

0

0

0

0

0

0

0

0

0

0
7

0

0

0

0

0

0

0

0

0

0

0
6

0

0

0

0

0

0

0

0

0

0

0
6

0

0

0

0

0

0

0

0

0

0

0
6

0

0

0

0

0

0

0

0

0

0

0
6

0

0

0

0

0

0

0

0

0

0

0
7

0

0

0

0

0

0

0

0

0

0

0
6

0

0

0

0

0

0

0

0

0

0

0
4

0
6

0
6

0

0

0

0

0

0

0

0
4

0
6

0

0

0

0

0

0

0

0

0
4

0
7

0
7

0

0

0

0

0

0

0
4

0
6

0

0

0

0

0

0

0
4

0

0

0

0

0
4

0

0

0

0

0

0
4

0

0

0

0

0
4

0

0

0
4

0
4

0
4

0

0

0

0

0

0

0

0
5

0

0

0

0

0

0

0

0
4

0
2

0
2

0

0
4

0

0

0

0

0

0

0
4

0

0

0

0

0
4

0
4

0
7

0
7

0

0

0
4

0

0

0

0
4

0
4

0

0

0

0

0
4

0

0

0
4

0
4

0

0

0

0

0

0

0
4

0
4

0
4

0.107261

0.107261

0

0

0
4

0.107261

0.107261

0
4

0
4

0
4

0

0

0

0

0
4

0
4

0
4

0

0

0

0

0
4

0

0

0
4

0
4

0
4

0

0

0

0

0
4

0

0

0
4

0

0

0
4

0
4

0

0

0

0
4

0
4

0
4

0

0

0

0

0
4

0

0

0

0
4

0
4

0
4

0

0

0

0

0

0
4

0

0

0
4

0
4

0
4

0

0

0

0

0

0

0
4

0

0

0
4

0
4

0
4

0.449026

0.325902

0.275763

0.275763

0
4

0.0501388

0.0501388

0
4

0
4

0.123124

0.123124

0.123124

0
4

0
4

2.77555756156289e-17

0
4

0

0

0

0

0

0

0
4

0

0

0
4

0
4

0
4

0

0

0

0

0
4

0
4

0
4

0
6

0
6

0
6

0
6

0

0
4

0
6

0
7

0

0

0

0

0

0

0
4

0
6

0
7

0

0

0
4

0

0

0
4

0

0

0
4

0
4

0
4

0.438165

0.438165

0.438165

0.438165

0

0
4

0
4

0
4

0

0

0

0

0
4

0
4

0
4

0.48469

0.48469

0.48469

0.48469

0
4

0
4

0
4

0

0

0

0

0
4

0

0

0
4

0
4

0
4

0

0

0

0

0

0
4

0

0

0
4

0
4

0
4

0

0

0

0

0
4

0
4

0
4

0

0

0

0

0
4

0

0

0
4

0
4

0

0

0

0
4

0
4

0
4

0

0

0

0

0

0

0
4

0
4

0
4

0

0

0

0

0
4

0

0

0
4

0
4

0
4

0

0

0

0

0

0
4

0
4

0
4

81.4743
3

81.4743
3

74.7329
4

43.6095
3

1.16968

0.800305

0.610794

15.3198

9.61881

0.450858

1.22173

0.669944

0.768943

0.492495

1.04360964314765e-14
4

0
4

6.69032
3

4.18764

1.72409

0.53717

0.241423

0
4

0.0511526

0.0511526

0
4

0
4

0
4

0

0

0

0

0
4

0
4

0
4

0

0

0

0

0
4

0
4

0
4

0.100278

0.100278

0.100278

0.100278

0
4

0

0

0
4

0

0

0
4

0
4

0
4

0

0

0

0

0
4

0

0

0
4

0
4

0
4

0

0

0

0

0

0
4

0
4

0
4

0

0

0

0

0
4

0

0

0
4

0
4

0
4

0

0

0

0

0
4

0
4

0
4

10.3051

10.3051

10.3051

10.3051

0
4

0
4

0
4

0.211587

0.211587

0

0

0
4

0.211587

0.211587

0
4

0
4

0
4

1.60444

1.60444

1.60444

1.60444

0
4

0
4

0
4

0

0

0

0
7

0

0

0

0

0

0

0

0

0
4

0

0

0

0

0
4

0

0

0

0

0
4

0

0

0
4

0

0

0
4

0
4

0

0

0

0

0

0

0

0

0

0

0
4

0

0

0

0

0
4

0

0

0

0
4

0

0

0
4

0

0

0
4

0
4

0

0

0

0
4

0

0

0
4

0
4

0

0

0

0
4

0
4

0
4

0.397776

0.397776

0.397776

0.274652

0

0.123124

0
4

0
4

0
4

0.869923

0.869923

0.869923

0.652442

0.217481

0
4

0
4

0
4

0

0

0

0

0
4

0
4

0
4

0.357948

0.357948

0.357948

0.357948

0
4

0

0

0
4

0
4

0
4

0

0

0

0

0
4

0
4

0
4

0

0

0

0

0
4

0
4

0
4

0.768679

0.692149

0.07653

0.07653

0
4

0.615619

0.615619

0
4

0
4

0.07653

0.07653

0.07653

0
4

0
4

4.16333634234434e-17

0
4

2.09311

2.09311

2.09311

2.09311

0
4

0
4

0
4

0.189636

0.139497

0.0642886

0.0642886

0
4

0.0752082

0.0752082

0
4

0
4

0.0501388

0.0501388

0.0501388

0
4

0
4

0
4

0

0

0

0

0

0
4

0
4

0
4

0

0

0

0

0

0

0

0

0
4

0
5

0
5

0

0

0
4

0

0

0

0
4

0
4

0
4

0

0

0

0

0

0
4

0

0

0
4

0
4

0
4

0

0

0

0

0
4

0
4

0
4

0

0

0

0

0
4

0

0

0
4

0
4

0
4

1.53392

1.53392

1.53392

1.10025

0.43367

0

0
4

0
4

0
4

1.13349

1.13349

1.13349

1.13349

0
4

0
4

0
4

0

0

0

0

0
4

0

0

0
4

0
4

0
4

0

0

0

0

0

0
4

0
4

0
4

0.549304

0.549304

0

0

0
4

0.549304

0.549304

0
4

0
4

0

0

0

0
4

0
4

0
4

0

0

0

0

0
4

0
4

0
4

0

0

0

0

0
4

0
4

0
4

0

0

0

0

0

0

0

0

0
4

0

0

0

0

0

0
4

0
4

0
4

2.67731

2.67731

0.840586

0.764056

0.07653

4.16333634234434e-17

0
4

1.83672

1.83672

0
4

0
4

0
4

0.308002

0.111267

0

0

0
4

0.111267

0.111267

0
4

0
4

0.196734

0.196734

0.196734

0
4

0
4

0
4

0.869923

0.869923

0.797429

0.797429

0
4

0.0724936

0.0724936

0
4

1.38777878078145e-17

0
4

0
4

0.293785

0.293785

0.175486

0.175486

0
4

0.118299

0.118299

0
4

0
4

0
4

0

0

0

0

0

0
4

0
4

0
4

0

0

0

0

0

0
4

0
4

0
4

0.110652

0.110652

0.110652

0.0553261

0.0553261

0
4

0
4

0
4

0

0

0

0

0
4

0
4

0
4

0

0

0

0

0
4

0

0

0
4

0
4

0
4

0

0

0

0

0
4

0
4

0
4

0
6

0
6

0
6

0
6

0

0

0

0

0
7

0
6

0

0

0

0

0

0

0
4

0

0

0
4

0
4

0
4

0.0528677

0.0528677

0.0528677

0.0528677

0
4

0

0

0
4

0
4

0
4

0

0

0

0

0
4

0

0

0
4

0
4

0
4

0

0

0

0

0
4

0
4

0
4

0

0

0

0

0
4

0
4

0
4

0

0

0

0

0
4

0

0

0
4

0
4

0
4

0

0

0

0

0

0
4

0
4

0
4

0.0617678

0.0617678

0.0617678

0.0617678

0

0
4

0
4

0
4

0

0

0

0

0
4

0
4

0
4

0.120823

0.120823

0.120823

0.120823

0
4

0
4

0
4

0.125347

0.125347

0.0752082

0.0752082

0
4

0.0501388

0.0501388

0
4

0
4

0
4

0
7

0
7

0
7

0
7

0

0

0
4

0
4

0

0

0

0
4

0

0

0
4

0
4

0
4

0.300833

0.300833

0.0501388

0.0501388

0
4

0.250694

0.250694

0
4

0
4

0
4

0

0

0

0

0

0
4

0
4

0
4

0

0

0

0

0
4

0
4

0
4

0

0

0

0

0
4

0

0

0
4

0
4

0
4

0

0

0

0

0
4

0
4

0
4

0

0

0

0

0
4

0
4

0
4

12.2134

12.2134

2.67168

2.67168

0
4

9.54172

9.54172

0
4

0
4

0
4

0.730274

0.730274

0.730274

0.730274

0
4

0

0

0
4

0
4

0
4

0

0

0

0

0

0
4

0
4

0
4

0

0

0

0

0

0
4

0
4

0
4

0

0

0

0

0

0

0

0

0

0

0
4

0

0

0
4

0
4

0
4

0.05102

0.05102

0.05102

0

0.05102

0
4

0
4

0
4

0.0752082

0.0752082

0.0752082

0.0752082

0
4

0
4

0
4

0

0

0

0

0
4

0

0

0
4

0
4

0
4

0.0511526

0.0511526

0.0511526

0.0511526

0
4

0

0

0
4

0
4

0
4

0.244634

0.244634

0.244634

0.244634

0
4

0

0

0
4

0
4

0
4

0

0

0

0

0
4

0

0

0
4

0
4

0
4

0.58422

0.58422

0.29211

0.29211

0
4

0.29211

0.29211

0
4

0
4

0
4

0

0

0

0

0

0
4

0
4

0
4

0

0

0

0

0
4

0
4

0
4

0.615619

0.615619

0.615619

0.615619

0

0
4

0
4

0
4

0

0

0
5

0

0

0

0

0

0

0
4

0

0

0

0

0
4

0
3

0

0

0
4

0

0

0
4

0

0

0
4

0
4

0

0

0

0

0

0
4

0

0

0

0
4

0
4

0
4

0

0

0

0

0
4

0
4

0
4

0.339723

0.339723

0.339723

0.277955

0.0617678

0
4

0
4

0
4

0

0

0

0

0
4

0

0

0
4

0
4

0
4

0

0

0

0

0
4

0

0

0
4

0
4

0
4

0

0

0

0

0
4

0
4

0
4

0.55591

0.55591

0.55591

0.55591

0
4

0
4

0
4

0

0

0

0

0
4

0
4

0
4

0.0966581

0.0966581

0

0

0
4

0.0966581

0.0966581

0
4

0
4

0
4

0

0

0

0

0
4

0
4

0
4

0

0

0

0

0
4

0
4

0
4

19.2544

19.2544

18.933

11.2184

0

0

0

0

0

0

0.0642886

0

1.02862

0

5.20738

0

0

0

0

0

0

0.0964329

0

0

0

0.67503
3

0

0.0642886

0

0

0

0

0

0

0

0.0964329
3

0
3

0
3

0

0

0.482165

0
4

0

0

0

0
4

0.257154

0.0642886

0.192866

0

0

0
4

0

0

0
4

0.0642886

0

0.0642886

0
4

0

0

0
4

0

0

0
4

0

0

0
4

1.60982338570648e-15

0
4

0
4

0
7

0
7

0
7

0
7

0
7

0

0

0

0

0
4

0

0

0
4

0

0

0

0
4

0

0

0
4

0
4

0
4

0

0

0

0

0
4

0
4

0
4

0

0

0

0

0
4

0

0

0
4

0
4

0
4

0

0

0

0

0
4

0
4

0
4

0

0

0

0

0
4

0
4

0
4

0

0

0

0

0

0
4

0
4

0
4

0

0

0

0

0
4

0
4

0
4

0

0

0

0

0
4

0
4

0
4

0

0

0

0

0
4

0
4

0
4

0

0

0

0

0
4

0
4

0
4

36.1977

36.1977

36.1977

36.1977

0
4

0
4

0
4

0

0

0
2

0
2

0

0

0
4

0
5

0
5

0

0
4

0

0

0

0

0
4

0
4

0

0

0

0
4

0
4

0
4

0

0

0

0

0

0
4

0
4

0
4

0

0

0

0

0
4

0
4

0
4

0

0

0

0

0
4

0

0

0
4

0
4

0
4

0.349565

0.349565

0.349565

0

0.349565

0
4

0
4

0
4

0

0

0

0

0
4

0
4

0
4

0

0

0

0

0
4

0
4

0
4

0

0

0

0

0
4

0
4

0
4

0.58422

0.58422

0.58422

0.58422

0
4

0
4

0
4

0.497935

0.497935

0.497935

0.497935

0
4

0
4

0
4

0

0

0

0

0
4

0
4

0
4

46.0593

46.0593

46.0593

8.77838

23.9225

5.72503

1.90834

2.67168

1.90834

1.14501

2.22044604925031e-16

0
4

0
4

0
4

0

0

0

0

0
4

0
4

0
4

0

0

0

0

0
4

0
4

0
4

0

0

0

0

0
4

0
4

0
4

1.11157

1.11157

1.11157

1.11157

0
4

0
4

0
4

0

0

0

0

0
4

0
4

0
4

0

0

0

0

0
4

0
4

0
4

0

0

0

0

0
4

0
4

0
4

0

0

0

0

0
4

0
4

0
4

0

0

0

0

0
4

0
4

0
4

0

0

0

0

0
4

0
4

0
4

0
6

0
6

0
6

0
6

0

0
4

0
6

0

0

0

0
4

0

0

0

0
4

0

0

0
4

0
4

0
4

0

0

0

0

0
4

0
4

0
4

0

0

0

0

0
4

0
4

0
4

0

0

0

0

0
4

0
4

0
4

0.349565

0.349565

0.349565

0.349565

0
4

0
4

0
4

0

0

0

0

0
4

0
4

0
4

0

0

0

0

0
4

0
4

0
4

0

0

0

0

0
4

0
4

0
4

0.123124

0.123124

0.123124

0.123124

0
4

0
4

0
4

0.237905

0.237905

0.237905

0.237905

0
4

0
4

0
4

0

0

0

0

0
4

0
4

0
4

0

0

0

0

0

0

0

0
4

0

0

0
4

0
4

0

0

0

0

0
4

0

0

0
4

0
4

0
4

0.118299

0.118299

0.118299

0.118299

0
4

0
4

0
4

0

0

0

0

0
4

0
4

0
4

0

0

0

0

0
4

0
4

0
4

0

0

0

0

0
4

0
4

0
4

0

0

0

0

0
4

0
4

0
4

0

0

0

0

0
4

0
4

0
4

0.0871506

0.0871506

0.0871506

0.0871506

0
4

0
4

0
4

0

0

0

0

0
4

0
4

0
4

0

0

0

0

0
4

0
4

0
4

0

0

0

0

0
4

0
4

0
4

0

0

0
2

0
2

0

0

0

0
4

0

0

0
4

0

0

0

0

0
4

0

0

0
4

0

0

0
4

0
4

0
4

0.107261

0.107261

0.107261

0.107261

0
4

0
4

0
4

0.30781

0.30781

0.30781

0.30781

0
4

0
4

0
4

0

0

0

0

0
4

0
4

0
4

0

0

0

0

0
4

0
4

0
4

0

0

0

0

0
4

0
4

0
4

0.58422

0.58422

0.58422

0.58422

0
4

0
4

0
4

0

0

0

0

0
4

0
4

0
4

0

0

0

0

0
4

0
4

0
4

0

0

0

0

0
4

0
4

0
4

1.14501

1.14501

1.14501

1.14501

0
4

0
4

0
4

0
7

0
7

0
7

0
7

0
4

0

0

0
4

0
4

0
4

0

0

0

0

0
4

0
4

0
4

0

0

0

0

0
4

0
4

0
4

0.120823

0.120823

0.120823

0.120823

0
4

0
4

0
4

0

0

0

0

0
4

0
4

0
4

0

0

0

0

0
4

0
4

0
4

0

0

0

0

0
4

0
4

0
4

0

0

0

0

0
4

0
4

0
4

0

0

0

0

0
4

0
4

0
4

0.15306

0.15306

0.15306

0.15306

0
4

0
4

0
4

0

0

0

0

0
4

0
4

0
4

0
7

0
7

0
7

0

0

0

0
4

0

0

0

0

0
4

0

0

0
4

0
4

0
4

0

0

0

0

0
4

0
4

0
4

0

0

0

0

0
4

0
4

0
4

0

0

0

0

0
4

0
4

0
4

0

0

0

0

0
4

0
4

0
4

0

0

0

0

0
4

0
4

0
4

0

0

0

0

0
4

0
4

0
4

0

0

0

0

0
4

0
4

0
4

0

0

0

0

0
4

0
4

0
4

0.0724936

0.0724936

0.0724936

0.0724936

0
4

0
4

0
4

0.123536

0.123536

0.123536

0.123536

0
4

0
4

0
4

0

0

0

0

0

0
4

0

0

0

0
4

0

0

0
4

0
4

0

0

0

0

0

0

0

0
4

0
4

0

0

0

0

0
4

0
4

0
4

0

0

0

0

0
4

0
4

0
4

0

0

0

0

0
4

0
4

0
4

0

0

0

0

0
4

0
4

0
4

0

0

0

0

0
4

0
4

0
4

0

0

0

0

0
4

0
4

0
4

0

0

0

0

0
4

0
4

0
4

0

0

0

0

0
4

0
4

0
4

0

0

0

0

0
4

0
4

0
4

1.6066

1.6066

1.6066

1.6066

0
4

0
4

0
4

0

0

0

0

0
4

0
4

0
4

0

0

0

0

0

0

0

0
4

0
4

0
4

0

0

0

0

0
4

0
4

0
4

0

0

0

0

0
4

0
4

0
4

0

0

0

0

0
4

0
4

0
4

0

0

0

0

0
4

0
4

0
4

0.123124

0.123124

0.123124

0.123124

0
4

0
4

0
4

0.873913

0.873913

0.873913

0.873913

0
4

0
4

0
4

0

0

0

0

0
4

0
4

0
4

0

0

0

0

0
4

0
4

0
4

0

0

0

0

0
4

0
4

0
4

0.438165

0.438165

0.438165

0.438165

0
4

0
4

0
4

0
7

0
7

0
7

0
7

0

0

0

0

0

0

0

0

0

0

0
7

0

0

0

0

0

0

0

0

0

0

0
6

0

0

0

0

0

0

0

0

0

0

0
7

0

0

0

0

0

0

0

0

0

0

0

0

0

0

0

0

0
4

0
6

0

0

0

0

0

0

0

0
4

0
4

0
6

0
6

0

0

0

0

0

0

0
4

0

0

0

0

0
4

0

0

0
4

0
4

0
4

7.92548
4

7.92548
4

0.763337

0

0

0

0

0.763337

0

0

0
4

6.39881

4.96169

0.29211

1.14501

0
4

0

0

0

0
4

0

0

0
4

0.763337

0.763337

0
4

0
4

0
4

0

0

0

0

0
4

0
4

0
4

0.116201

0.116201

0.116201

0.116201

0
4

0
4

0
4

0

0

0

0

0
4

0
4

0
4

0

0

0

0

0
4

0
4

0
4

0.107261

0.107261

0.107261

0.107261

0
4

0
4

0
4

0.0724936

0.0724936

0.0724936

0.0724936

0
4

0
4

0
4

0.192866

0.192866

0.192866

0.192866

0
4

0
4

0
4

0

0

0

0

0
4

0
4

0
4

0

0

0

0

0
4

0
4

0
4

0

0

0

0

0
4

0
4

0
4

90.6313
4

54.561
4

54.561
4

53.5857

0

0.430933

0.236598

0.123124

0.184686

2.58126853225349e-15
4

0
4

0
4

36.0703

0.640206

0.101849

0.29211

0.246248

0
4

35.4301

35.4301

0
4

0
4

0
4

0

0

0

0

0
4

0
4

0
4

0.0964329

0.0964329

0.0964329

0.0964329

0
4

0
4

0
4

0

0

0

0

0
4

0
4

0
4

0

0

0

0

0
4

0
4

0
4

0

0

0

0

0
4

0
4

0
4

0

0

0

0

0
4

0
4

0
4

0

0

0

0

0
4

0
4

0
4

0.175486

0.175486

0.175486

0.175486

0
4

0
4

0
4

0

0

0

0

0
4

0
4

0
4

0

0

0

0

0
4

0
4

0
4

0
6

0
7

0

0

0

0

0
4

0

0

0

0

0
4

0

0

0

0
4

0
4

0

0

0

0

0
4

0

0

0
4

0
4

0
4

0

0

0

0

0
4

0
4

0
4

0

0

0

0

0
4

0
4

0
4

0

0

0

0

0
4

0
4

0
4

0

0

0

0

0
4

0
4

0
4

0

0

0

0

0
4

0
4

0
4

0

0

0

0

0
4

0
4

0
4

0

0

0

0

0
4

0
4

0
4

0.123124

0.123124

0.123124

0.123124

0
4

0
4

0
4

0

0

0

0

0
4

0
4

0
4

0

0

0

0

0
4

0
4

0
4

0

0

0
6

0
6

0

0
4

0

0

0

0
4

0

0

0
4

0
4

0
4

0

0

0

0

0
4

0
4

0
4

0.118299

0.118299

0.118299

0.118299

0
4

0
4

0
4

0

0

0

0

0
4

0
4

0
4

0

0

0

0

0
4

0
4

0
4

0.29211

0.29211

0.29211

0.29211

0
4

0
4

0
4

0.385732

0.385732

0.385732

0.385732

0
4

0
4

0
4

0.05102

0.05102

0.05102

0.05102

0
4

0
4

0
4

0

0

0

0

0
4

0
4

0
4

0

0

0

0

0
4

0
4

0
4

0

0

0

0

0
4

0
4

0
4

0

0

0
5

0

0

0

0

0
4

0

0

0

0

0
4

0

0

0

0

0
4

0

0

0
4

0

0

0
4

0
4

0
4

0

0

0

0

0
4

0
4

0
4

0

0

0

0

0
4

0
4

0
4

0

0

0

0

0
4

0
4

0
4

0

0

0

0

0
4

0
4

0
4

0

0

0

0

0
4

0
4

0
4

0

0

0

0

0
4

0
4

0
4

0

0

0

0

0
4

0
4

0
4

0

0

0

0

0
4

0
4

0
4

0.0434322

0.0434322

0.0434322

0.0434322

0
4

0
4

0
4

0

0

0

0

0
4

0
4

0
4

242.509
4

242.509
4

178.434
4

176.082

1.20659

0

1.14501

1.53210777398272e-14
4

0
4

64.0753

37.8831

24.856

1.06156

0.274652

0
4

0
4

0
4

0

0

0

0

0
4

0
4

0
4

0

0

0

0

0
4

0
4

0
4

0

0

0

0

0
4

0
4

0
4

0

0

0

0

0
4

0
4

0
4

0

0

0

0

0
4

0
4

0
4

0

0

0

0

0
4

0
4

0
4

0

0

0

0

0
4

0
4

0
4

0

0

0

0

0
4

0
4

0
4

0

0

0

0

0
4

0
4

0
4

0

0

0

0

0
4

0
4

0
4

36.6402

35.8769

11.8317

1.90834

1.14501

4.58002

1.52667

0.763337

1.90834

1.77635683940025e-15

0
4

17.5568

1.52667

15.2667

0.763337

7.7715611723761e-16

0
4

3.05335

1.52667

1.52667

0
4

1.90834

1.90834

0
4

1.52667

1.52667

0
4

0
4

0.763337

0.763337

0.763337

0
4

0
4

0

0

0

0
4

0
4

0
4

0

0

0

0

0
4

0
4

0
4

0

0

0

0

0
4

0
4

0
4

0

0

0

0

0
4

0
4

0
4

0

0

0

0

0
4

0
4

0
4

0

0

0

0

0
4

0
4

0
4

0

0

0

0

0
4

0
4

0
4

0

0

0

0

0
4

0
4

0
4

0

0

0

0

0
4

0
4

0
4

0

0

0

0

0
4

0
4

0
4

0

0

0

0

0
4

0
4

0
4

0

0

0

0

0

0
4

0

0

0

0

0

0

0
4

0
4

0
4

0.0642886

0.0642886

0.0642886

0.0642886

0
4

0
4

0
4

0.615619

0.615619

0.615619

0.615619

0
4

0
4

0
4

1.66217

1.66217

1.66217

1.66217

0
4

0
4

0
4

1.14501

1.14501

1.14501

1.14501

0
4

0
4

0
4

1.90834

1.90834

1.90834

1.90834

0
4

0
4

0
4

0

0

0

0

0
4

0
4

0
4

0

0

0

0

0
4

0
4

0
4

0.371625

0.371625

0.371625

0.371625

0
4

0
4

0
4

0.0565967

0.0565967

0.0565967

0.0565967

0
4

0
4

0
4

0

0

0

0

0
4

0
4

0
4

0

0

0

0

0

0
4

0

0

0
4

0
4

0
4

0.29211

0.29211

0.29211

0.29211

0
4

0
4

0
4

0.763337

0.763337

0.763337

0.763337

0
4

0
4

0
4

0.128577

0.128577

0.128577

0.128577

0
4

0
4

0
4

0.29211

0.29211

0.29211

0.29211

0
4

0
4

0
4

0

0

0

0

0
4

0
4

0
4

0

0

0

0

0
4

0
4

0
4

0

0

0

0

0
4

0
4

0
4

0

0

0

0

0
4

0
4

0
4

0

0

0

0

0
4

0
4

0
4

0

0

0

0

0
4

0
4

0
4

0

0

0

0

0

0

0

0

0

0
4

0

0

0

0
4

0
4

0
4

0

0

0

0

0
4

0
4

0
4

0

0

0

0

0
4

0
4

0
4

0

0

0

0

0
4

0
4

0
4

0.0724936

0.0724936

0.0724936

0.0724936

0
4

0
4

0
4

0.0565967

0.0565967

0.0565967

0.0565967

0
4

0
4

0
4

0

0

0

0

0
4

0
4

0
4

0

0

0

0

0
4

0
4

0
4

0

0

0

0

0
4

0
4

0
4

0

0

0

0

0
4

0
4

0
4

0.371625

0.371625

0.371625

0.371625

0
4

0
4

0
4

1883.19

1563.68

236.621

54.4891

3.81669

1.05545

3.81669

0.29211

0.763337

1.14501

0.763337

1.14501

2.67168

0.763337

75.4439

1.14501

13.3584

3.05335

1.14501

1.90834

0.763337

1.90834

1.90834

17.5568

10.6867

11.8317

5.34336

4.96169

3.81669

11.0684

1.4210854715202e-14

0
4

101.524

25.1901

25.1901

46.9452

0.763337

3.43502

8.88178419700125e-16

0
4

211.444

9.92339

199.231

0.763337

0.763337

0.763337

0
4

1012.19

3.43502

985.85

19.8468

1.14501

0.763337

1.14501

1.28119737041743e-13

0
4

1.90834

0.763337

1.14501

2.22044604925031e-16

0
4

0

0

0
4

3.94795307556706e-13

0
4

308.704

69.1055

7.2517

2.67168

1.52667

1.14501

0.763337

0.763337

0.763337

0.763337

0.763337

31.6785

3.34546

3.49151

2.29001

3.05335

3.81669

1.90834

1.52667

1.58317

2.44249065417534e-15

0
4

9.16005

1.90834

1.90834

1.14501

0.763337

0.763337

0.763337

0.763337

1.14501

0
4

222.513

7.63337

23.2818

188.544

0.763337

2.29001

0
4

6.87004

4.19836

1.90834

0.763337

0
4

0.29211

0.29211

0
4

0.763337

0.763337

0
4

0
4

9.92339

9.92339

8.01504

1.14501

0.763337

5.55111512312578e-16

0
4

0
4

0.876329

0

0

0
4

0.876329

0.876329

0
4

0
4

2.16171525124764e-12

0
4

0
6

0
6

0
6

0

0

0

0
4

0

0

0
4

0
4

0
4

0

0

0

0

0
4

0
4

0
4

0

0

0

0

0
4

0
4

0
4

0.0483291

0.0483291

0.0483291

0.0483291

0
4

0
4

0
4

0

0

0

0

0
4

0
4

0
4

61.6181

61.6181

55.4549

18.0514

30.1518

7.2517

8.88178419700125e-16

0
4

6.16319

6.16319

0
4

0
4

0

0

0

0
4

0
4

0
4

0
6

0
6

0
6

0
6

0
4

0
4

0
4

0

0

0

0

0

0

0
4

0

0

0

0
4

0

0

0

0

0
4

0
4

0
4

0
7

0
7

0
7

0

0

0

0

0
4

0
4

0
4

2.52911
4

0.660138
4

0

0

0

0

0
4

0.660138

0.196734

0.278718

0.184686

0
4

0

0

0
4

0

0

0
4

0
4

1.86897

0

0

0

0
4

1.86897

1.86897

0
4

0

0

0
4

0
4

0
4

0

0

0

0

0

0
4

0

0

0
4

0
4

0

0

0

0
4

0
4

0
4

0
3

0
3

0
3

0

0

0

0
4

0

0

0

0

0

0
4

0
4

0
4

29.8608

29.8608

29.5862

18.0602

0.246248

10.4368

0.411978

0.430933

1.38777878078145e-15

0
4

0.274652

0.274652

0
4

0
4

0
4

0
3

0
3

0
3

0

0

0

0

0

0
4

0
4

0
4

0.203698
7

0.203698
7

0
7

0
7

0

0

0

0

0

0

0

0

0

0

0

0
4

0

0

0
4

0

0

0
4

0

0

0
6

0

0

0

0

0
4

0
6

0
6

0

0
4

0

0

0
4

0.203698

0.203698

0
4

0

0

0
4

0

0

0
4

0

0

0
4

0

0

0
4

0
4

0
4

6.8286

6.8286

6.8286

6.39044

0.438165

8.32667268468867e-16

0
4

0
4

0
4

1.04976
4

1.04976
4

0.80351

0.141492

0

0.662019

0

0
4

0.246248

0

0

0

0.246248

0
4

5.55111512312578e-17
4

0
4

0
4

0

0

0

0

0

0

0

0

0
4

0
4

0
4

1.26287
4

1.26287
4

0.696334
4

0.578035

0.118299

0
4

0.223401

0.123124

0.100278

0
4

0.05102

0.05102

0
4

0.29211

0.29211

0
4

0
4

0
4

49.9697

49.9697

49.5577

47.3775

1.63095

0.549304

0
4

0.411978

0.411978

0
4

0
4

0
4

20.32
4

20.32
4

20.32
4

6.77633

13.4205

0.123124

0
4

0
4

0

0

0

0
4

0
4

0
4

0

0

0

0

0

0

0

0

0
4

0
4

0
4

42.9398

42.9398

28.0547

2.67168

18.3201

3.05335

1.52667

2.48293

0
4

14.8851

14.8851

0
4

0
4

0
4

0

0

0

0

0

0
4

0

0

0
4

0
4

0
4

0

0

0

0

0

0

0

0
4

0
4

0
4

0
5

0
5

0
6

0
6

0

0

0

0

0

0

0

0

0

0

0

0

0

0

0

0

0
4

0

0

0

0

0

0

0

0

0

0

0

0
4

0
4

0

0

0

0

0

0

0

0

0
4

0

0

0

0

0

0

0
4

0

0

0

0

0
4

0

0

0
4

0

0

0
4

0

0

0
4

0
4

0
6

0

0

0

0

0

0
4

0

0

0

0

0

0
4

0

0

0

0

0

0
4

0

0

0
4

0
4

0

0

0

0
4

0

0

0
4

0
4

0
4

0

0

0

0

0

0

0
4

0
4

0
4

5.03895

5.03895

4.68798

4.51249

0.175486

0
4

0.300833

0.300833

0
4

0.0501388

0.0501388

0
4

1.04083408558608e-16

0
4

0
4

0
6

0
6

0

0

0

0
4

0

0

0

0
4

0
4

0
4

39.2924

38.2395

38.2395

37.9888

0.250694

0
4

0
4

1.05291

0.125347

0.125347

0
4

0.927568

0.927568

0
4

0
4

4.88498130835069e-15

0
4

0

0

0

0

0

0
4

0
4

0

0

0

0
4

0
4

0

0

0

0
4

0
4

0
4

0

0

0

0

0

0

0

0
4

0
4

0
4

0

0

0

0

0

0
4

0

0

0
4

0
4

0
4

1.96627

1.96627

1.96627

1.85901

0.107261

0
4

0
4

0
4

2.20982
3

2.20982
3

1.94167
3

1.4352

0.506469

0
4

0.268152

0.268152

0
4

1.66533453693773e-16
3

0
4

0
4

0

0

0

0

0

0

0
4

0

0

0
4

0
4

0
4

25.2831

25.2831

21.1831

21.0224

0

0

0.0964329
3

0

0
3

0
3

0

0.0642886

0

0

6.10622663543836e-16

0
4

0

0

0
4

0
2

0

0

0

0

0
4

0

0

0

0

0
4

2.95497

2.95497

0
4

0

0

0
4

0

0

0

0
4

1.14501

1.14501

0

0
4

0

0

0
4

0

0

0
4

1.11022302462516e-15

0
4

0
4

0

0

0

0

0

0
4

0

0

0
4

0

0

0

0
4

0
4

0
4

9.16005

9.16005

7.2517

4.96169

1.52667

0.763337

0
4

0.763337

0.763337

0
4

1.14501

1.14501

0
4

6.66133814775094e-16

0
4

0
4

0.0581004

0

0

0

0

0
4

0

0

0
4

0
4

0.0581004

0.0581004

0.0581004

0
4

0
4

0
4

0
6

0

0

0

0

0
4

0

0

0
4

0

0

0
4

0
4

0

0

0

0
4

0
4

0
4

0
6

0
6

0
7

0
7

0
4

0

0

0
4

0
4

0
4

27.8618

27.8618

27.8618

15.6484

7.2517

4.96169

1.77635683940025e-15

0
4

0
4

0
4

0

0

0

0

0

0

0
4

0

0

0
4

0
4

0

0

0

0

0
4

0
4

0
4

0
7

0
7

0

0

0

0
4

0

0

0
4

0
4

0
4

0.978053

0.211471

0.211471

0.0793016

0.132169

0
4

0
4

0.766582

0

0

0
4

0.766582

0.766582

0
4

0
4

0
4

8.22473

8.22473

8.22473

8.22473

0
4

0

0

0
4

0
4

0
4

0
7

0
6

0
7

0
6

0
7

0

0

0

0

0

0
4

0
6

0
6

0

0

0

0

0
4

0

0

0

0
4

0
4

0
7

0
7

0

0

0

0

0

0

0

0

0

0

0

0
4

0

0

0

0

0
4

0

0

0

0
4

0

0

0
4

0
4

0
4

0

0

0

0

0
4

0
4

0
4

0.456977

0.456977

0.0752082

0

0.0752082

0
4

0.33163

0.33163

0

0
4

0.0501388

0.0501388

0
4

0
4

0
4

0

0

0

0

0

0
4

0
4

0
4

0

0

0

0

0

0
4

0

0

0
4

0
4

0
4

0

0

0

0

0
4

0

0

0
4

0
4

0
4

0

0

0

0

0

0
4

0

0

0
4

0
4

0

0

0

0
4

0
4

0

0

0

0
4

0
4

0
4

0.526457

0.526457

0.250694

0.250694

0
4

0.275763

0.275763

0
4

0
4

0
4

0

0

0

0

0

0

0
4

0

0

0
4

0
4

0
4

0

0

0

0

0
4

0

0

0
4

0

0

0
4

0
4

0
4

1.07312

1.07312

1.07312

0.331957

0.676871

0.0642886

1.11022302462516e-16

0
4

0
4

0
4

199.531
4

133.768
4

133.494
4

112.012
4

12.2134

6.59671

0

0

1.90834

0.763337

0
4

0.274652

0.274652

0
4

1.33226762955019e-14
4

0
4

54.9716
4

50.4116

39.8659

2.47187

0.961282

0.68663

0.549304

3.84513

0.123124

1.90834

0
4

2.38154

0

0.473196

1.90834

0
4

1.41514

0.451249

0.763337

0.200555

0

5.55111512312578e-17

0
4

0.763337

0.763337

0
4

0
4

8.1198

7.86911

5.29696

2.47187

0.100278

0
4

0.100278

0.100278

0
4

0.150416

0.150416

0
4

0
4

2.67168

0

0

0
4

2.67168

2.67168

0
4

0
4

0
4

0
6

0
6

0
6

0
6

0
4

0
4

0
4

0

0

0

0

0
4

0
4

0
4

0

0

0

0

0
4

0
4

0
4

3.31138

3.31138

3.31138

3.31138

0
4

0

0

0
4

0
4

0
4

0

0

0

0

0

0
4

0
4

0
4

0

0

0

0

0

0
4

0

0

0

0
4

0
4

0
4

0

0

0

0

0
4

0
4

0
4

0

0

0

0

0

0
4

0
4

0
4

0

0

0

0

0

0

0
4

0
4

0
4

0

0

0

0

0
4

0
4

0
4

2.16266
3

2.16266
3

1.87055
3

0

0

0

0

0

0.786937
3

0.983671
3

0

0

0

0

0

0.0999396

2.77555756156289e-17
3

0
4

0

0

0
4

0.29211

0.29211

0
4

0

0

0
4

5.55111512312578e-17
3

0
4

0

0

0

0

0

0
4

0
4

0

0

0

0
4

0
4

0
4

32.3121
3

0

0

0

0
4

0
4

0

0

0

0

0
4

0
4

0.0724936

0.0724936

0.0724936

0
4

0
4

0.0926517

0.0926517

0.0926517

0
4

0
4

0

0

0

0
4

0
4

32.147
3

32.147
3

0

0.120823

0.773265

0

0

0

12.3119

0

0

0.0483291

0

6.41142

0

0

0

0

0

0

0

0

0

0

1.8248

0

0

0

0

0

0.0966581

0

5.55771

0.652442
3

0.314139

3.93882

0.0966581

0

3.8441472227646e-15
3

0
4

0

0

0

0

0
4

0

0

0
4

0

0

0
4

0

0

0
4

0
4

7.105427357601e-15
3

0
4

6.12976
3

0.161286

0.161286

0.161286

0
4

0
4
[truncated: 251,781 more chars]
