# Supplementary material for: Fungal communities decline with urbanization—more in air than in soil
Source: ISME J. 2020 Aug 5;14(11):2806–15. doi: 10.1038/s41396-020-0732-1 (PMC7784924; doi:10.1038/s41396-020-0732-1)
Supplement: Supplementary file 2 — Supplemental data [file 41396_2020_732_MOESM2_ESM.zip › Krona_SoilNaturalEdge.html]

Javascript must be enabled to view this page.

num
probth


82022

41564.6

2974.04
3

2974.04
3

2970.56
3

2970.56
3

2933.33
3

8.26787

0.0732851

0.366426

0.119824

0

0.857939

0.107242

0.160864

0

0.119824

0

6.71016

0

16.1363

0.0917376

0.0798828

0

0.0917376

0.14657

0.0798828

0.137606

0.0798828

0

0.0798828

0.159766

0.119824

0.0798828

0.0798828

0.199707

0.122142

0

0

0.27959

0

1.19824

1.27812

1.09445785767548e-12
3

0
4

0
4

0.643454

0.428969

0.428969

0
4

0.214485

0.214485

0
4

2.77555756156289e-17

0
4

2.84192

2.84192

2.84192

0
4

0
4

0
4

0
4

0

0

0

0

0

0

0
4

0
4

0
4

0
4

0

0

0

0

0

0
4

0
4

0
4

0

0

0

0

0
4

0
4

0
4

0
4

1360.46

8.82132

8.82132

8.82132

3.90702
3

0
2

0

0

0

0

0

0

0

0

0

0

0

0

0

0

0

0

0

0

0

0

0

4.80706
3

0

0

0

0

0

0

0

0

0

0

0

0

0

0

0

0

0

0

0

0

0

0

0

0

0

0

0

0

0

0

0

0

0

0

0

0

0

0

0

0

0

0

0

0
7

0

0

0

0

0

0

0

0

0

0

0
7

0

0

0

0

0

0

0

0

0

0

0
7

0.107242

0

0

0

0

0

0

0

0

0

0

0

0

0

0

0

0

0

0

0

0

0

0

0

0

0

0

0

0

0

0

0

0

0

0

0

0

0

0

0

0

0

0

0

0
6

0

0

0

0

0

0

0

0

0

0

0

0

0

0

0
6

0
7

0

0

0

0
1

0

0
2

0

0

0

0

0

0

0

0

0
3

0

0

0

0

0

0

0

0

0

0

0
2

0

0

0

0

0

0

0

0

0

0

0
2

0

0

0

0

0

0

0

0

0

0

0
7

0

0

0

0

0

0

0

0

0

0

0
6

0

0

0

0

0

0

0

0

0

0

0
6

0

0

0

0

0

0

0

0

0

0

0
4

0
3

0
3

0

0

0

0
4

0

0

0
4

0

0

0
4

0

0

0
4

0

0

0
4

0

0

0

0
4

0

0

0

0
4

0

0

0

0
4

0

0

0
4

0

0

0
4

0

0

0
4

0
6

0

0

0

0

0
4

0

0

0
4

0

0

0

0
4

0

0

0

0
4

0

0

0

0
4

0

0

0
4

0

0

0

0
4

0

0

0

0
4

0

0

0
4

0

0

0
4

0

0

0
4

0
2

0

0

0

0

0
4

0

0

0
4

0

0

0
4

0

0

0
4

0

0

0
4

0

0

0
4

0

0

0
4

0

0

0
4

0

0

0
4

0

0

0
4

0

0

0
4

0
6

0

0

0
4

0

0

0
4

0

0

0
4

0

0

0
4

0

0

0
4

0

0

0
4

0

0

0
4

0

0

0
4

0

0

0
4

0

0

0
4

0

0

0
4

0

0

0

0
4

0

0

0
4

0

0

0
4

0

0

0
4

0

0

0
4

0

0

0
4

0

0

0
4

0

0

0
4

0

0

0
4

0

0

0
4

0

0

0
4

0

0

0
4

0

0

0
4

0

0

0
4

0

0

0
4

0
7

0

0

0
4

0

0

0
4

0

0

0

0
4

0
4

0
6

0
6

0
6

0
4

0
4

0

0

0

0
4

0

0

0
4

0
4

0

0

0

0
4

0
4

0

0

0

0

0
4

0
4

0

0

0

0
4

0
4

0

0

0

0
4

0
4

0

0

0

0
4

0
4

0

0

0

0
4

0
4

0

0

0

0
4

0
4

0

0

0

0
4

0
4

0

0

0

0
4

0
4

0
6

0
6

0
6

0
4

0

0

0
4

0

0

0
4

0
4

0

0

0

0
4

0
4

0

0

0

0
4

0
4

0

0

0

0
4

0
4

0

0

0

0
4

0
4

0

0

0

0
4

0
4

0

0

0

0
4

0
4

0

0

0

0
4

0
4

0

0

0

0
4

0
4

0

0

0

0
4

0
4

0

0

0

0

0
4

0
4

0

0

0

0

0

0
4

0

0

0
4

0
4

0

0

0

0
4

0
4

0

0

0

0
4

0
4

0

0

0

0
4

0
4

0

0

0

0
4

0
4

0

0

0

0
4

0
4

0
4

342.012
7

0

0

0

0

0

0

0
4

0

0

0

0
4

0

0

0

0
4

0

0

0

0
4

0

0

0
4

0
4

283.771
7

283.771
7

17.5889
7

0
7

0
6

0

0

0

0

0

0

0

0

0

0

0

0
7

0

0

0

0

0

0

0

0

0

0

0
7

0

0

0

0

0

0

0

0

0

0

0

0

0

0

0

0

0

0

0

0

0

0
7

0

0

0

0

0

0

0

0

0

0

0

0

0

0

0

0

0

0

0

0

0

0
7

0

0

0

0

0

0

0

0

0

0

0
8

0

0

0

0

0

0

0

0

0

0

0.175085

0

0

0

0

0

0

0

0

0

0

0

0

0

0

0

0

0

0

0

0

0

0
7

0
7

0

0

0

0

0

0

0

0

0

0

0

0

0

0

0

0

0

0

0

0

0

0
6

0

0

0

0

0

0

0

0

0

0

0
7

0

0

0

0

0

0

0

0

0

0

0
7

0

0

0

0.0761658

0

0

0

0

0

0

0
8

0

0

0

0

0

0

0

0

0

0

0
8

0

0

0

0

0

0

0

0

0

0

0
7

0

0

0

0

0

0

0

0

0

0

0
7

0

0

0

0

0

0

0

0

0

0

0
6

0

0

0

0

0

0

0

0

0

0

0
6

0
1

0

0

0

0

0

0

0

0

0

0

0
7

0

0

0

0

0

0

0

0

0

0

0
7

0

0

0

0

0

0

0

0

0

0

0
1

0

0

0

0

0

0

0

0

0

0

0
7

0

0

0

0

0

0

0

0

0

0

0
6

0

0

0

0

0

0

0

0

0

0

0
8

0

0

0

0

0

0

0

0

0

0

0
7

0

0

0

0

0

0

0

0

0

0

0

0

0

0

0

0

0

0

0

0

0

0
6

0

0

0

0

0

1.51897

0

0

0

0

0
7

0

0

0

0

0

0

0

0

0

0

0

0
6

0

0

0

0

0

0

0

0

0

0

0
1

0

0

0

0

0

0

0

0

0

0

0
7

0

0

0

0

0

0

0

0

0

0

0
7

0

0

0

0

0

0

0

0

0

0

0
7

0

0

0

0

0

0

0

0

0

0

0
7

0

0

0

0

0

0

0

0

0

0

0.0507772
5

0

0

0

0

0

0

0

0

0

0

0
7

0

0

0

0

0

0

0

0

0

0

0
6

0

0

0

0

0

0

0

0

0

0

0
7

0
7

0

0

0

0

0

0

0

0

0

0

0
7

0

0

0

0
7

0
7

0
8

0
7

0
6

0
6

0
7

0

0.871507
7

0
7

0
7

0
7

0
7

0

0

0

0
7

0
7

0

0

0
7

0

0
7

0

0
7

0
7

0
7

0
3

0

0
6

0
7

0
7

0
7

0

0
7

0

0
7

0
7

0

0
6

0
6

0

0
8

0
6

0
7

0
6

0.391992

0

0

0.152332

0
7

0

0
7

0
7

0
7

0

0
7

0
6

0
7

0
2

0

0

0
8

0.214485

0
7

0
6

0
6

0
8

0
7

0
7

0
7

0
8

0
6

0

0
6

0

0

0

0
6

0
8

0
6

0
7

0
7

0
6

0
8

0
7

0
7

0
7

0
8

0

0
6

0
7

0

0
7

0
7

0
2

0

0
7

0

0

0
6

0
7

0
8

0
7

0

0

0
7

0
7

0

0
1

0
8

0
7

0

0
7

0
7

0
6

0
7

0
7

0
7

0
7

0
7

0

0
7

0
6

0
7

0
6

0

0

0
7

0
7

0
7

0
6

0
7

0
7

0
7

0

0

0
6

0
8

0
6

0
7

0
7

0
7

0
7

0
7

0
7

0
6

0
7

0
7

0
7

0
7

0
7

0
7

0

0
6

0
7

0
6

0
7

0
6

0
7

0
6

0
7

0
7

0
7

0
7

0
7

0
7

1.1828

0
7

0
6

0
7

0.431606

0
7

0
7

0.0761658
7

0
7

0
6

0

0
7

0
6

0
7

0

0
6

0
7

0
5

0
7

0
7

0
7

0
7

0
7

0
8

0
8

0
7

0.406218

0
7

0
1

0

0
7

0
7

0
7

0
6

0
7

0
7

0.0649455
5

0

0
7

0
6

0
6

0
7

0
7

0
8

0
6

0

0
1

0
7

0
7

0
7

0

0
7

0
7

0

0
6

0
7

0
7

0

0
7

0
7

0

0
6

0
7

0
7

0
7

0

0
6

0
7

0
7

0
7

0
7

0
7

0
7

0
6

0.0507772
7

0

0
7

0
7

0
7

0
7

0
6

0
7

0
7

0
2

0

0
7

0
1

0
7

0
7

0

0

0
7

0
7

0
7

0

0
7

0
7

0

0
7

0
6

0
7

0
5

0
7

0
7

0
7

0
2

0

0
7

0
7

0
7

0
7

0
5

0
6

0
7

0
7

0

0
8

0

0
7

0
7

0
7

0
7

0
7

0
6

0
7

0
7

0
7

0
7

0
7

0
7

0

0
7

0
7

0
7

0
7

0
8

0

0
7

0
7

0
7

0.440991

0
7

0
7

0

0
7

0

0
6

0
2

0
7

0

0
7

0

0

0
7

0
7

0
6

0
6

0
6

0
7

0

0

0
7

0
7

0

0

0
7

0
7

0
7

0
5

0
7

0
7

0
2

0
7

0
7

0
7

0
6

0

0
7

0
6

0
7

0
6

0

0
7

0
7

0

0
7

0
7

0

0

0
2

0
7

0
7

0

0

0
6

0

0
7

0
7

0
7

0
5

0
7

0
6

0
7

0

0
7

0
7

0
7

0
7

0
4

0
6

0
7

0
7

0

0
2

0
7

0

0
7

0
7

0
6

0
6

0

0
7

0
6

0
7

0
7

0
7

0
7

0
7

0
7

0
7

0

0
6

0
7

0
7

0

0

0
7

0
7

0

0
7

0.0724163
5

0
7

0
6

0

0

0
6

0
7

0
6

0
2

0
7

0
7

0

0
7

0
4

0
8

0.0507772

0

0
7

0
6

0
7

0
7

0
8

0
7

0
7

0
7

0
7

0
1

0
7

0

0
6

0
6

0
7

0
7

0
8

0
7

0

0
7

0
7

0

0

0
7

0
7

0
6

0
6

0.0649455

0
7

0
8

0
8

0
6

0
7

0
7

0
7

0
7

0
6

0
6

0
7

0

0

0

0

0
7

0
7

0
7

0
7

0
7

0
7

0
7

0
7

0

0

0
7

0
7

0
6

0
8

0

0
7

0
6

0

0

0
7

0
7

0
7

0

0
7

0
6

0
7

0
7

0
7

0

0
7

0
6

0
7

0
6

0

0
7

0
1

0
7

0
6

0
7

0
6

0
7

0
7

0
7

0
8

0
7

0
6

0
7

0
7

0
7

0
7

0

0
6

0
7

0

0
7

0
7

0
7

0
7

0
7

0
7

0

0
7

0

0
7

0
6

0
6

2.30571

0
7

0
7

0

0.0761658
6

0
7

0

0

0
7

0
7

0
7

0
8

0
7

0

0
6

0
7

1.65026
5

0
7

0
6

0
7

0
7

0
6

0
7

0
7

0
7

0
7

0
6

0
5

0
8

0
7

0
7

0
6

0
8

0
7

0
7

0
6

0
7

0
6

0
7

0

0
7

0
7

0

0
7

0

0
7

0
7

0
7

0
6

0
7

0
7

0
7

0
7

0

0
7

0

0
7

0

0
6

0

0

0

0
5

0

0

0
6

0
6

0
1

0
6

0
7

0
8

0

0

0

0
7

1.08215
2

0

0

0

0
7

0
7

0.0761658
7

0
7

0
7

0
6

0

0
7

0
7

0

0

0
6

0
7

0
6

0
6

0
7

0

0
7

0

0
7

0
7

0

0
7

0

0
7

0
7

0
8

0

0

0.0507772
7

0
6

0
7

0
7

0

0
5

0
7

0
7

0
6

0
6

0
2

0
7

0
7

0

0
1

0
6

0
7

0
6

0
7

0
2

0

0
7

0
7

0
7

0
7

0
8

0
7

0
7

0
7

0
6

0
7

0
1

0

0.175085
7

0
7

0
7

0

0

0
6

0
7

0

0
7

0
7

0
7

0
7

0
6

0
6

0
6

0

0
7

0
7

0

0

0
1

0
7

0.160864
7

0

0
6

0
8

0

0
8

0
1

0

0
6

0
7

0
7

0
6

0
7

0

0
7

0

0
7

0
7

0

0

0

0
7

0
7

0
7

0
7

0
7

0
7

0
6

0
8

0
8

0

0
7

0
6

0.237029
7

0

0

0
1

0

0
7

0
8

0
5

0
7

0
6

0
7

0

0

0
7

0
6

0
7

0
7

0
7

0
7

0

0
7

0
7

0

0
7

0.309187
5

0
7

0
7

0

0
7

0
7

0
7

0
7

0
6

0
6

0
7

0

0
7

0
7

0
7

0
2

0
6

0
7

0
6

0
7

0
8

0
7

0
7

0
7

0
7

0
7

0

0
8

0
4

0

0
7

0
8

0
7

0
7

0
6

0
7

0

0
6

0

0
6

0
7

0

0
7

0

0

0
7

0
7

0

0

0
7

0
7

0
7

0
7

0

0

0
7

0
7

0
6

0
7

0
7

0
7

0

0
7

0
7

0
7

0
6

0
5

0
6

0
6

0
6

0
5

0
7

0
6

0

0
7

0

0
6

0
2

0
6

0
8

0
7

0
7

0
6

0
7

0
7

0

0

0.0507772
7

0
7

0
6

0

0
6

0

0
6

0

0

0
8

0
8

0
7

0
7

0

0

0
7

0
6

0
7

0
6

0
7

0
6

0
7

0
6

0
7

0
8

0
6

0
7

0
7

0
7

0
7

0
6

0

0
6

0
6

0

0
1

0
5

0
6

0
7

0
6

0
6

0.0917376

0

0
6

0
2

0

0
6

0
8

0

0
7

0
7

0
7

0
7

0
7

0
6

0
7

0
6

0
7

0
6

0
7

0
8

0
7

0
6

0
5

0
7

0
5

0
7

0
7

0
7

0
7

0

0
7

0
7

0
7

0
5

0
7

0

0

0
6

0
7

0

0
6

0
6

0

0
8

0

0

0
8

0
7

0
7

0
7

0
6

0
6

0
6

0
6

0
6

0
7

0
7

0
8

0

54.3719
4

0
7

0.265526
5

0
7

0

0
1

0
6

0
6

0

0
7

0

0
7

0

0
7

0
6

0
7

0
6

0
6

0
7

0
7

0

0
6

0

0
7

0

0
6

0

0
7

0
7

0.0507772
7

0
7

0
8

0
7

0
6

0
7

0
8

0
7

0
8

0
6

0
6

0

0
6

0
1

0
7

0
7

0
7

0
6

0
7

0
7

0
7

0
6

0

0
6

0
7

0
7

0
8

0
7

0
8

0
7

0
7

0
7

0
6

0
6

0
8

0
6

0
6

0
1

0
7

0

0

0
7

0
7

0
6

0
7

0
7

0
7

0

0
7

0
6

0
7

0
7

0
7

0
7

0

0
7

0

0
7

0
6

0
1

0
7

0
7

0
7

0
7

0
8

0
7

0

0
6

0
7

0
7

0
7

0
6

0
7

0
7

0

0
7

0
7

0
8

0
7

0
7

0
7

0
7

0
5

0
7

0
7

0
2

0
7

0
6

0
7

0
7

0
6

0
7

0

137.499
7

0.577134
7

0
6

0
7

0
7

0
7

0
7

0
6

0
7

0
7

0
7

0
7

0
6

0.0507772
8

0
6

0
6

0
7

0
7

0

0

0
7

0
5

0

0
7

0.203109

0
7

0
7

0
6

0
8

0
7

0
7

0.734985
4

0
7

0
6

0
7

0
7

0
7

0
7

0
1

0
7

0
6

0
6

0
7

0
6

0
7

0

0
7

0

0
6

0
8

0
7

0
6

0

0
7

0

0
7

0
8

0
7

0

0
7

0
7

0

0

0
8

0.0507772

0
6

0

0

0
7

0
7

0

0
7

0
7

0
5

0
6

0
7

0
6

0

0
7

0

0
7

0

0
7

0

0

0
7

0
7

0
6

0
7

0
7

0

0
6

0

0
6

0
2

0
7

0
8

0
6

0
1

0
6

0
6

0
7

0
7

0

0
8

0
7

0
7

0
6

0
7

0

0
7

0

0
7

0

0
7

0
6

0

0
7

0
8

0
7

0
7

0
7

0
6

0
7

0

0

0

0
6

0
7

0
7

0
7

0
6

0

0
7

0

0
7

0
8

0
7

0
7

0
6

0
6

0

0

0
6

0
7

0
7

0
7

0

0

0
5

0

0
7

0

0
7

0
6

0

0
7

0
7

0
2

0
7

0

0

0

0
7

0
7

0

0
7

0
7

0.101554
7

0
7

0
7

0
7

0
1

0
7

0

0

0

0

0
5

0
7

0
7

0
7

0
1

0
6

0
7

0
4

0

0
8

0
7

0
7

0
7

0

0
7

0
6

0

0
7

0

0
6

0
2

0
6

0
8

0
7

0

0
7

0
6

0
1

0

0
7

0
7

0
7

0

0
8

0

0
7

0
7

0
8

0
7

0

0
8

0
7

0

0

0
8

0
7

0

0

0
8

0
7

0
6

0
8

0
8

0
6

0
8

0

0
6

0

0

0
7

0

0

0
7

0
6

0
7

0

0
6

0

0
7

0

0
6

0
7

0
7

0
6

0

0
7

0
7

0
8

0
7

0
7

0
7

0

0

0
7

0
6

0
6

0
6

0

0
6

1.01554
5

0
6

0

0

0

0

0
7

0

0

0
2

0
1

0

0
7

0
7

0
7

0
6

0

0
7

0

0

0
7

0
6

0
7

0
7

0

0

0

0

0
7

0
6

0
7

0
7

0

0

0

0

0
7

0

0

0
7

0
7

0
7

0
6

0
7

0
6

0.0917376
7

0
5

0
8

0

0

0

0
7

0

0

0

0

0
7

0
6

0
7

0
5

0
7

0
6

0
6

0
7

0
7

0
7

0
6

0
7

0
7

0
7

0
7

0

0.965181

0
7

0
7

0
5

0
7

0
7

0
7

0.292834

0

0
8

0

0
7

0

0
7

0
7

0
6

0

0
7

0

0
6

0

0
7

0
8

0
7

0

0
7

0

0
7

0.152332

0.0761658

0
6

0
7

0
6

0

0

0

0
1

0

0

0
7

0
7

0

0
6

0
8

0
6

0
6

0
7

0

0

0
8

0
6

0
7

0
1

0
7

0
1

0

0

0
8

0
7

0
7

0
6

0
7

0
7

0
7

0

0
7

0
6

0
7

0

0
7

0
6

0

0
6

0
6

0
6

0.406218
4

0

0
6

0
7

0
6

0

0
7

0
7

0
8

0
6

0

0
7

0

0

0
6

0
7

0
1

0
7

0

0
6

0.268106
6

0
7

0

0
6

0
7

0
7

0
7

0
7

0
7

0
8

0
7

0.0761658
7

0

0

0

0
6

0
6

0

0
6

0
1

0

0.119824

0
7

0
7

0

0
6

0

0
7

0

0
7

0
8

0

0
6

0

0
7

0
7

0
6

0

0
7

0
7

0
7

0
6

0
6

0
7

0

0.194836
7

0

0

0

0

0

0

0

0

0

0

0
7

0

0

0

0

0

0

0

0

0

0

0
7

0

0

0

0

0

0

0

0

0

0

0

0

0

0

0

0

0

0

0

0

0

0
6

0

0

0

0

0

0

0

1.80259

0

0

0

0

0

0

0

0

0

0

0

0

0

0
7

0

0

0

0

0

0

0

0

0

0

0
7

0
6

0

0

0

0

0

0

0

0

0

0

0
7

0

0

0

0

0

0

0

0

0

0

0
7

0

0

0

0

0

0

0

0

0

0

0
7

0

0

0

0

0

0

0

0

0

0

0
7

0

0

0

0

0

0

0

0

0

0

0

0

0

0

0

0

0

0

0

0

0

0.0507772
7

0

0

0

0

0

0

0

0

0

0

0
7

0

0

0

0

0

0

0

0

0

0

0
7

0

0

0

0

0

0

0

0

0

0

0
6

0

0

0

0

0

0

0

0

0

0

0.0507772
7

0
6

0

0

0

0

0

0

0

0

0

0

0
7

0

0

0

0

0

0

0

0

0

0

0
7

0

0

0

0

0

0

0

0

0

0

0
7

0

0

0

0

0

0

0

0

0

0

0

0

0

0

0

0

0

0

0

0

0

0
7

0

0

0

0

0

0

0

0

0

0

0
7

0

0

0

0

0

0

0

0

0

0

0
7

0

0

0

0

0.0507772

0

0

0

0

0

0

0

0

0

0

0

0

0

0

0

0

0
7

0.355441

0

0

0

0

0

0

0

0

0

0
7

0

0

0

0

0

0

0

0

0

0

0

0
7

0

0

0

0

0

0

0

0

0

0

0
6

0

0

0

0

0

0

0

0

0

0

0.0507772

0

0

0

0

0

0

0

0

0

0

0

0.107242

0

0

0

0

0

0

0

0

0

0

0

0

0

0

0

0

0

0

0

0

0
7

0

0

0

0

0

0

0

0

0

0

0
7

0

0

0

0

0

0

0

0

0

0

0.0482775
7

0

0

0

0

0

0

0

0

0

0

0
6

0

0

0

0

0

0

0

0

0

0

0
7

0
7

0

0

0

0

0

0

0

0

0

0

0
7

0

0

0

0

0

0

0

0

0

0

0
7

0

0

0

0

0

0

0

0

0

0

0

0

0

0

0

0

0

0

0

0

0

0
7

0

0

0

0

0

0

0

0

0

0

0

0

0

0

0

0

0

0

0

0

0

0

0

0

0

0

0

0

0

0

0

0

0
7

0

0

0

0

0

0

0

0

0

0

0
7

0

0

0

0

0

0

0

0

0

0

0
7

0

0

0

0

0

0

0

0

0

0

0
7

0
7

0

0

0

0

0

0

0

0

0

0

0
6

0

0

0

0

0

0

0

0

0

0

0
7

0

0

0

0

0

0

0

0

0

0

0

0

0

0

0

0

0

0

0

0

0

0
7

0

0

0

0

0

0

0

0

0

0

0

0

0

0

0

0

0

0

0

0

0

0
7

0

0

0

0

0

0

0

0

0

0

0

0

0

0

0

0

0

0

0

0

0

0
7

0

0

0

0

0

0

0

0

0

0

0
7

0

0

0

0

0

0

0

0

2.00896

0

11.5589

0
7

0
7

0

0

0

0

0

0

0

0

0

0

0
7

0

0

0

0

0

0

0

0

0

0

0
6

0

0

0

0

0

0

0

0

0

0

0
7

0

0

0

0

0

0

0

0

0

0

0
7

0

0

0

0

0

0

0

0

0

0

0
7

0

0

0

0

0

0

0

0

0

0

0
7

0

0

0

0

0

0

0

0

0

0

0
7

0

0

0

0

0

0

0

0

0

0

0

0

0

0

0

0

0

0

0

0

0

0
7

0

0

0

0

0

0

0

0

0

0

0
7

0
7

0

0

0

0

0

0

0

0

0

0

0
6

0

0

0

0

0

0

0

0

0

0

0
7

0

0

0

0

0

0

0

0

0

0

0
7

0

0

0

0

0

0

0

0

0

0

0
7

0

0

0

0

0

0

0

0

0

0

0
7

0

0

0

0

0

0

0

0

0

0

0
7

0

0

0

0

0

0

0

0

0

0

0
7

0

0

0

0

0

0

0

0

0

0

0
7

0

0

0

0

0

0

0

0

0

0

0
7

0

0

0

0

0

0

0

0

0

0

0.465527

0

0

0

0

0

0

0

0

0

0

0

0
7

0

0

0

0

0

0

0

0

0

0

0
7

0

0

0

0

0

0

0

0

0

0

0
7

0

0

0

0

0

0

0

0

0

0

0.0761658

0

0

0

0

0

0

0

0

0

0

0

0

0

0

0

0

0

0

0

0

0

0
7

0

0

0

0

0

0

0

0

0

0

0
7

0

0

0

0

0

0

0

0

0

0

0
4

0

0

0

0

0

0

0

0

0

0

0
7

0

0

0

0

0

0

0

0

0

0

0
6

0
7

0

0

0

0

0

0

0

0

0

0

0
5

0

0

0

0

0

0

0

0

0

0

0
7

0

0

0

0

0

0

0

0

0

0

0
7

0

0

0

0

0

0

0

0

0

0

0
7

0

0

0

0

0

0

0

0

0

0

0

0

0

0

0

0

0

0

0

0

0

0
7

0

0

0

0

0

0

0

0

0

0

0
7

0

0

0

0

0

0

0

0

0

0

0
7

0

0

0

0

0

0

0

0

0

0

0
7

0

0

0

0

0

0

0

0

0

0

1.17598

0
7

0

0

0

0

0

0

0

0

0

0

0
7

0

0

0

0

0

0

0

0

0

0

0
7

0

0

0

0

0

0

0

0

0

0

0
6

0

0

0

0

0

0

0

0

0

0

0
7

0

0

0

0

0

0

0

0

0

0

0
6

0

0

0

0

0

0

0

0

0

0

0
7

0

0

0

0

0

0

0

0

0

0

0
7

0

0

0

0

0

0

0

0

0

0

0
7

0

0

0

0

0

0

0

0

0

0

0
7

0

0

0

0.0761658

0

0

0

0

0

0

0
7

0

0

0

0

0

0

0

0

0

0

0

0
7

0

0

0

0

0

0

0

0

0

0

0
5

0

0

0

0

0

0

0.0507772

0

0

0

0
6

0

0

0

0

0

0

0

0

0

0

0
7

0

0

0

0

0

0

0

0

0

0

0
7

0

0

0

0

0

0

0

0

0

0

0
7

0

0

0

0

0

0

0

0

0

0

0

0

0

0

0

0

0

0

0

0

0

0
7

0

0

0

0

0

0

0

0

0

0

0

0

0

0

0

0

0

0

0

0

0

0
7

0
7

0

0

0

0

0

0

0

0

0

0

0
7

0

0

0

0

0

0

0

0

0

0

0
2

0

0

0

0.558549

0

0

0

0

0

0

0

0

0

0

0

0

0

0

0

0

0

0
7

0

0

0

0

0

0

0

0

0

0

0
7

0

0

0

0

0

0

0

0

0

0

0
7

0

0.121276

0

0

0

0

0

0

0

0

0
7

0

0

0

0

0

0

0

0

0

0

0

0

0

0

0

0

0

0

0

0

0

0

0

0

0

0

0

0

0

0

0

0

0
7

0
7

0

0

0

0

0

0

0

0

0

0

0
7

0

0

0

0

0

0

0

0

0

0

0

0

0

0

0

0

0

0

0

0

0

0
7

0

0

0

0

0

0

0

0

0

0

0
7

0

0

0

0

0

0

0

0

0

0

0
7

0

0

0

0

0

0

0

0

0

0

0
5

0

0

0

0

0

0

0

0

0

0

0
6

0

0

0

0

0

0

0

0

0

0

0
7

0

0

1.46997

0.525256

0

0

0

0

0

0

0
7

0

0

0

0

0

0

0

0

0

0

0.0507772
7

0
6

0

0

0

0

0

0.097998

0

0

0

0

0.482591
6

0

0

0

0

0

0

0

0

0

0

0
7

0

0

0

0

0

0

0

0

0

0

0
7

0

0

0

0

0

0

0

0

0

0

0

0

0

0

0

0

0

0

0

0

0

0

0

0

0

0

0

0

0

0

0

0

0
6

0

0

0

0

0

0

0

0

0

0

0
7

0

0

0

0

0

0

0

0

0

0

0
7

0

0

0

0

0

0

0

0

0

0

0

0

0

0

0

0

0

0

0

0

0

0
7

0
7

0

0

0

0

0

0

0

0

0

0

0
7

0

0

0

0

0

0

0

0

0

0

0

0

0

0

0

0

0

0

0

0

0

0
7

0

0

0

0

0

0

0

0

0

0

0

0

0

0

0

0

0

0

0

0

0

0
7

0

0

0

0

0

0

0

0

0

0

0

0

0

0

0

0

0

0

0

0

0

0
8

0

0

0

0

0

0

0

0

0

0

0
7

0

0

0

0

0

0

0

0

0

0

0
6

0

0

0

0

0

0

0

0

0

0

1.4849

0.617859

0
7

0

0

0

0

0

0

0

0

0

0

0
7

0

0

0

0

0

0

0

0

0

0

0

0

0

0

0

0

0

0

0

0

0

0

0

0

0

0

0

0

0

0

0

0

0

0

0

0

0

0

0

0

0

0

0

0
6

0

0

0

0

0

0

0

0

0

0

0
7

0

0

0

0

0

0

0

0

0

0

0

0

0

0

0

0

0

0

0

0

0

0
7

0

0

0

0

0

0

0

0

0

0

0

0

0

0

0

0

0

0

0

0

0

0
7

0
6

0

0

0

0

0

0

0

0

0

0

0
7

0

0

0

0

0

0

0

0

0

0

0

0

0

0

0

0

0

0

0

0

0

0
7

0

0

0

0

0

0

0

0

0

0

0
7

0

0

0

0

0

0

0

0

0

0

0
7

0

0

0

0

0

0

0

0

0

0

0
7

0

0

0

0

0

0

0

0

0

0

0
6

0

0

0

0

0

0

0

0

0

0

0
7

0

0

0

0

0

0

0

0

0

0

0
6

0

0

0

0

0

0

0

0

0

0

0

0

0

0

0

0

0

0

0

0

0

0

0
7

0

0

0

0

0

0

0

0

0

0

0

0

0

0

0

0

0

0

0

0

0

0
7

0

0

0

0

0

0

0

0

0

0

0.097998

0

0

0

0

0

0

0

0

0

0

0
7

0

0

0

0

0

0

0

0

0

0

0
7

0

0

0

0

0

0

0

0

0

0

0

0

0

0

0

0

0

0

0

0

0

0
6

0

0

0

0

0

0

0

0

0

0

0
7

0

0

0

0

0

0

0

0

0

0

0
7

0
7

0

0

0

0

0

0

0

0

0

0

0
7

0

0

0

0

0

0

0

0

0

0

0

0

0

0

0

0

0

0

0

0

0

0
1

0

0

0

0

0

0

0

0

0

0

0

0

0

0

0

0

0

0

0

0

0

0
7

0

0

0

0

0

0

0

0

0

0

0
7

0

0

0

0

0

0

0

0

0

0

0

0

0

0

0

0

0

0

0

0

0

0
7

0

0

0

0

0

0

0

0

0

0

0
7

0

0

0

0

0

0

0

0

0

0

0
7

0
7

0

0

0

0

0

0

0

0

0

0

0
7

0

0

0

0

0

0

0

0

0

0

0
7

0

0

0

0

0

0

0

0

0

0

0
7

0

0

0

0

0

0

0

0

0

0

0
7

0

0

0

0

0

0

0

0

0

0

0
6

0

0

0.175085

0

0

0

0

0

0

0

0
6

0

0

0

0

0

0

0

0

0

0

0

0

0

0

0

0

0

0

0

0

0

0

0

0

0

0

0

0

0

0

0

0

0
7

0

0

0

0

0

0

0

0

0

0

0
7

0
7

0

0

0

0

0

0

0

0

0

0

0.832983
7

0

0

0

0

0

0

0

0.761658

0

0

0
7

0

0

0

0

0

0

0

0

0

0

0
7

0

0

0

0

0

0

0

0

0

0

0

0

0

0

0

0

0

0

0

0

0

0
7

0

0

0

0

0

0

0

0

0

0

0

0

0

0

0

0

0

0

0

0

0

0
7

0

0

0

0

0

0

0

0

0

0

0
7

0

0

0

0

0

0

0

0

0

0

0

0

0

0

0

0

0

0

0

0

0

0
7

0
7

0

0

0

0

0

0

0

0

0

0

0

0

0

0

0

0

0

0

0

0

0

0
7

0

0

0

0

0

0

0

0

0

0

0
7

0

0

0

0

0

0

0

0

0

0

0
7

0

0

0

0

0

0

0

0

0

0

0
7

0

0

0

0.175085

0

0

0

0

0

0

0.175085

0

0

0

0

0

0

0

0

0

0

0

0

0

0

0

0

0

0

0

0

0

0
6

0

0

0

0

0

0

0

0

0

0

0
7

0

0

0

0

0

0

0

0

0

0

0.648935

0

0

0

0

0

0

0

0

0

0

0

0

0

0

0

0

0

0

0

0

0

0

0
7

0

0

0

0

0

0

0

0

0

0

0
7

0

0

0

0

0

0

0

0

0

0

0
7

0

0

0

0

0

0

0

0

0

0

0

0

0

0

0

0

0

0

0

0

0

0
7

0

0

0

0

0

0

0

0

0

0

0
7

0

0

0

0

0

0

0

0

0

0

0

0

0

0

0

0

0

0

0

0

0

0.175085

0

0

0

0

0

0

0

0

0

0

0
7

0
7

0

0

0

0

0

0

0

0

0

0

0
7

0

0

0

0

0

0

0

0

0

0

0
7

0

0

0

0

0

0

0

0

0

0

0

0

0

0

0

0

0

0

0

0

0

0

0

0

0

0

0

0

0

0

0

0

0
7

0

0

0

0

0

0

0

0

0

0

0

0

0

0

0

0

0

0

0

0

0

2.61503
5

0

0

0

0

0

0

0

0

0

0

0
7

0

0

0

0

0

0

0

0

0

0

0.195996
7

0

0

0

0

0

0

0

0

0

0

0
7

0
7

0

0

0

0

0

0

0

0

0

0

0
7

0

0

0

0

0

0

0

0

0

0

0
7

0

0

0

0

0

0

0

0

0

0

0
7

0

0

0

0

0

0

0

0

0

0

0
6

0

0

0

0

0

0

0

0

0

0

0
7

0

0

0

0

0

0

0

0

0

0

0
7

0

0

0

0

0

0

0

0

0

0

0
6

0

0

0

0

0

0

0

0

0

0

0

0

0

0

0

0

0

0.0724163

0

0

0

0
7

0

0

0

0

0

0

0

0

0

0

3.73544

0
7

0
7

0

0

0

0

0

0

0

0

0

0

0
7

0

0

0

0

0

0

0

0

0

0

0
8

0

0

0

0

0

0

0

0

0

0

0
6

0

0

0

0

0

0

0

0

0

0

0
6

0

0

0

0

0

0

0

0

0

0

0
7

0

0

0

0

0

0

0

0

0

0

0

0

0

0

0

0

0

0

0

0

0

0.0798828

0

0

0

0

0

0

0

0

0

0

0
7

0

0

0

0

0

0

0

0

0

0

0
7

0

0

0

0

0

0

0

0

0

0

0
7

0
7

0

0

0

0

0

0

0

0

0

0

0
7

0

0

0

0

0

0

0

0

0

0

0
7

0

0

0

0

0

0

0

0

0

0

0

0

0

0

0

0

0

0

0

0

0

0
7

0

0

0

0

0

0

0

0

0

0

0
7

0

0

0

0

0

0

0

0

0

0

0.434131

0

0

0

0

0

0

0

0

0

0

0

0

0

0

0

0

0

0

0

0

0

0

0

0

0

0

0

0

0

0

0

0

0
6

0

0

0

0

0

0

0

0

0

0

0.693312

0
7

0

0

0

0

0

0

0

0

0

0

0.0507772
7

0

0

0

0

0

0

0

0

0

0

0
7

0

0

0

0

0

0

0

0

0

0

0
7

0

0

0

0

0

0

0

0

0

0

0
7

0

0

0

0

0

0

0

0

0

0

0

0

0

0

0

0

0

0

0

0

0

0
7

0

0

0

0

0

0

0

0

0

0

0

0

0

0

0

0

0

0

0

0

0

0
7

0

0

0

0

0

0

0

0

0

0

0
7

0

0

0

0

0

0

0

0

0

0

1.12015

0.330052

0

0

0

0

0

0

0

0

0

0

0

0

0

0

0

0

0

0

0

0

0

0
7

0

0

0

0

0

0

0

0

0

0

0
7

0

0

0

0

0

0

0

0

0

0

0
7

0

0

0

0

0

0

0

0

0

0

0

0

0

0

0

0

0

0

0

0

0

0

0

0

0

0

0

0

0

0

0

0

0
7

0

0

0

0

0

0

0

0

0

0

0

0

0

0

0

0

0

0

0

0

0

0
7

0

0

0

0

0

0

0

0

0

0

0
7

0
7

0

0

0

0

0

0

0

0

0

0

0
7

0

0

0

0

0

0

0

0

0

0

0
6

0

0

0

0

0

0

0

0

0

0

0
7

0

0

0

0

0

0

0

0

0

0

0
7

0

0

0

0

0

0

0

0

0

0

0
6

0

0

0

0

0

0

0

0

0

0

0
6

0

0

0

0

0

0

0

0

0

0

2.20881
5

0

0

0

0

0

0

0

0

0

0

0
7

0

0

0

0

0

0

0

0

0

0

0
7

0

0

0

0

0

0

0

0

0

0

0
7

0
7

0

0

0

0

0

0

0

0

0

0

0
7

0

0

0

0

0.482591

0

0

0

0

0

0
7

0

0

0

0

0

0

0

0

0

0

0
8

0

0

0

0

0

0

0

0

0

0

0
7

0

0

0

0

0

0

0

0

0

0

0
7

0

0

0

0

0

0

0

0

0

0

0
7

0

0

0

0

0

0

0.107242

0

0

0

0
2

0

0

0

0

0

0

0

0

0

0

0

0

0

0

0

0

0

0

0

0

0

0
7

0

0

0

0

0

0

0

0

0

0

0
7

0
7

0

0

0

0

0

0

0

0

0

0

0

0

0

0

0

0

0

0

0

0

0

0
7

0

0

0

0

0

0

0

0

0

0

0
7

0

0

0

0

0

0

0

0

0

0

0
7

0

0

0

0

0

0

0

0

0

0

0
6

0

0

0

0

0

0

0

0

0

0

0
7

0

0

0

0

0

0

0

0

0

0

0

0

0

0

0

0

0

0

0

0

0

0
7

0

0

0

0

0

0

0

0

0

0

0
6

0

0

0

0

0

0

0

0

0

0

0
7

0
7

0

0

0

0

0

0

0

0

0

0

0
7

0

0

0

0

0

0

0

0

0

0

0
6

0

0

0

0

0

0

0

0

0

0

0

0

0

0

0

0

0

0

0

0

0

0
6

0

0

0

0

0

0

0

0

0

0

0.46378
6

0

0

0

0

0

0

0

0

0

0

0
7

0

0

0

0

0

0

0

0

0

0

0
8

0

0

0

0

0

0

0

0

0

0

0
7

0

0

0

0

0

0

0

0

0

0

0

0

0

0

0

0

0

0

0

0

0

0

0
7

0

0

0

0

0

0

0

0

0

0

0

0

0

0

0

0

0

0

0

0

0

0

0

0

0

0

0

0

0

0

0

0

0
6

0

0

0

0

0

0

0

0

0

0

0
6

0

0

0

0

0

0

0

0

0

0

0
6

0

0

0

0

0

0

0

0

0

0

0.0507772
6

0

0

0

0

0

0

0

0

0

0

0

0

0

0

0

0

0

0

0

0

0

0
8

0

0

0

0

0

0

0

0

0

0

0
7

0

0

0

0

0

0

0

0

0

0

0.199552

0
6

0

0

0

0

0

0

0

0

0

0

0
7

0

0

0

0

0

0

0

0

0

0

0
7

0

0

0

0

0

0

0

0

0

0

0
6

0

0

0

0

0

0

0

0

0

0.386483

0
7

0

0

0

0

0

0

0

0

0

0

0
7

0

0

0

0

0

0

0

0

0

0

0
7

0

0

0

0

0

0

0

0

0

0

0
7

0

0

0

0

0

0

0

0

0

0

0

0

0

0

0

0

0

0

0

0

0

0
6

0

0

0

0

0

0

0

0

0

0

1.4699
7

0

0
7

0

0

0

0

0

0

0

0

0

0

0
7

0

0

0

0

0

0

0

0

0

0

0
6

0

0

0

0

0

0

0

0

0

0

0

0

0

0

0

0

0

0

0

0

0

0
6

0

0

0

0

0

0

0

0

0

0

0
7

0

0

0

0

0

0

0

0

0

0

0
7

0

0

0

0

0

0

0

0

0

0

0
8

0

0

0

0

0

0

0

0

0

0

0
7

0

0

0

0

0

0

0

0

0

0

0
7

0

0

0

0

0

0

0

0.097998

0

0

0.126943

0
5

0

0

0

0

0

0

0

0

0

0

0
7

0

0

0

0

0

0

0

0

0

0

0
7

0

0

0

0

0

0

0

0

0

0

0
7

0

0

0

0

0

0

0

0

0

0

0
7

0

0

0

0

0

0

0

0

0

0

0
6

0

0

0

0

0

0

0

0

0

0

0

0

0

0

0

0

0

0

0

0

0

0
7

0

0

0

0

0

0

0

0

0

0

0
7

0

0

0

0

0

0

0

0

0

0

0

0

0

0

0

0

0

0

0

0

0

0
7

0
6

0

0

0

0

0

0

0

0

0

0

0
7

0

0

0

0

0

0

0

0

0

0

0
7

0

0

0

0

0

0

0

0

0

0

0
7

0

0

0

0

0

0

0

0

0

0

0
7

0

0

0

0

0

0

0

0

0

0

0
7

0

0

0

0

0

0

0

0

0

0

0
7

0

0

0

0

0

0

0

0

0

0

0
7

0

0

0

0

0

0

0

0

0

0

0

0

0

0

0

0

0

0

0

0

0

0

0

0

0

0

0

0

0

0

0

0

0.0761658
7

0
6

0

0

0

0

0

0

0

0

0

0

0
8

0

0

0

0

0

0

0

0

0

0

0

0

0

0

0

0

0

0

0

0

0

0
6

0

0

0

0

0

0

0

0

0

0

0
6

0

0

0

0

0

0

0

0

0

0

0

0

0

0

0

0

0

0

0

0

0

0

0

0

0

0

0

0

0

0

0

0

0
7

0

0

0

0

0

0

0

0

0

0

0
7

0

0

0

0

0

0

0

0

0

0

0
6

0

0

0

0

0

0

0

0

0

0

0.569925

0
7

0

0

0

0

0

0

0

0

0

0

0

0

0

0

0

0

0

0

0

0

0

0.0507772
7

0

0

0

0

0

0

0

0

0

0

0
7

0

0

0

0

0

0

0

0

0

0

0
7

0

0

0

0

0

0

0

0

0

0

0
7

0

0

0

0

0

0

0

0

0

0

0
7

0

0

0

0

0

0

0

0

0

0

0
7

0

0

0

0

0

0

0

0

0

0

0
7

0

0

0

0

0

0

0

0

0

0

0
7

0

0

0

0

0

0

0

0

0

0

0
7

0
7

0

0

0

0

0

0

0

0

0

0

0
7

0

0

0

0

0

0

0

0

0

0

0
6

0

0

0

0

0

0

0

0

0

0

0

0

0

0

0

0

0

0

0

0

0

0
7

0

0

0

0

0

0

0

0

0

0

0
7

0

0

0

0

0

0

0

0

0

0

0

0

0

0

0

0

0

0

0

0

0

0
7

0

0

0

0

0

0

0

0

0

0

0
7

0

0

0

0

0

0

0

0

0

0

0

0

0.609327

0

0

0

0

0

0

0

0

0
7

0
7

0

0

0

0

0

0

0

0

0

0

0
7

0

0.768084

0

0

0

0

0

0

0

0

0
7

0

0

0

0

0

0

0

0

0

0

0
1

0

0

0

0

0

0

0

0

0

0

0
6

0

0

0

0

0

0

0

0

0

0

0
7

0

0

0

0

0

0

0

0

0

0

0
6

0

0

0.428969

0

0

0

0

0

0

0

0
7

0

0

0

0

0

0

0

0

0

0

0
6

0

0

0

0

0

0

0

0

0

0

0
7

0

0

0

0

0

0

0

0

0

0

0
7

0
7

0

0

0

0

0

0

0

0

0

0

0

0

0

0

0

0

0

0

0

0

0

0
7

0

0

0

0

0

0

0

0

0

0

0
7

0

0

0

0

0

0

0

0

0

0

0
7

0

0

0

0

0

0

0

0

0

0

0
7

0

0

0

0

0

0

0

0

0

0

0
6

0

0

0

0

0

0

0

0

0

0

0
7

0

0

0

0

0

0

0

0

0

0

0
7

0

0

0

0

0

0

0

0

0

0

0.0507772

0

0

0

0

0

0

0

0

0

0

0
7

0
6

0

0

0

0

0

0

0

0

0

0

0

0

0

0

0

0

0

0

0

0

0

0

0

0

0

0

0

0

0

0

0

0

0
7

0

0

0

0

0

0

0

0

0

0

0
7

0

0

0

0

0

0

0

0

0

0

0

0

0

0

0

0

0

0

0

0

0

0

0

0

0

0

0

0

0

0

0

0

0
7

0

0

0

0

0

0

0

0

0

0

0
6

0

0

0

0

0

0

0

0

0

0

0
7

0

0

0

0

0

0

0

0

0

0

0.203109

0
7

0

0

0

0

0

0

0

0

0

0

0

0

0

0

0

0

0

0

0

0

0

0

0

0

0

0

0

0

0

0

0

0

0
7

0

0

0

0

0

0

0

0

0

0

0
7

0

0

0

0

0

0

0

0

0

0

0
7

0

0.0507772

0

0

0

0

0

0

0

0

0
8

0

0

0

0

0

0

0

0

0

0

0
7

0

0

0

0

0

0

0

0

0

0

0
7

0

0

0

0

0

0

0

0

0

0

0

0

0

0

0

0

0

0

0

0

0

0.279275

0
7

0
7

0

0

0

0

0

0

0

0

0

0

0
7

0

0

0

0

0

0

0

0

0

0

0
7

0

0

0

0

0

0

0

0

0

0

0
7

0

0

0

0

0

0

0

0

0

0

0
7

0

0

0

0

0

0

0

0

0

0

0

0

0

0

0

0

0

0

0

0

0

0
7

0

0

0

0

0

0

0

0

0

0

0

0

0

0

0

0

0

0

0

0

0

0
7

0

0

0

0

0

0

0

0

0

0

0
1

0

0

0

0

0

0

0

0

0

0

0
6

0

0

0

0

0

0

0

0

0

0

0

0

0

0

0

0

0

0

0

0

0

0

0

0

0

0

0

0

0

0

0

0

0

0
7

0

0

0

0

0

0

0

0

0

0

0

0

0

0

0

0

0

0

0

0

0

0
7

0

0

0

0

0

0

0

0

0

0

0

0

0

0

0

0

0

0

0

0

0

0
7

0

0

0

0

0

0

0

0

0

0

0.0761658
7

0

0

0

0

0

0

0

0

0

0

0.587988
5

0

0

0

0

0

0

0

0

0

0

0
7

0

0

0

0

0

0

0

0

0

0

0

0.0507772
7

0

0

0

0

0

0

0

0

0

0

0
7

0

0

0

0

0

0

0

0

0

0

0
2

0

0

0

0

0

0

0

0

0

0

0

0

0

0

0

0

0

0

0

0

0

0
7

0

0

0

0

0

0

0

0

0

0

0
6

0

0

0

0

0

0

0

0

0

0

0

0

0

0

0

0

0

0

0

0

0

0
6

0

0

0

0

0

0

0

0

0

0

0
7

0

0

0

0

0

0

0

0

0

0

0
7

0

0

0

0

0

0

0

0

0

0

0

0

0

0

0

0

0

0

0

0

0

0

0
7

0

0

0

0

0

0

0

0

0

0

0
7

0

0

0

0

0

0

0

0

0

0

0
7

0

0

0

0

0

0

0

0

0

0

0
7

0

0

0

0

0

0

0

0

0

0

0
6

0

0

0

0

0

0

0

0

0

0

0
7

0

0

0

0

0

0

0

0

0

0

0

0

0

0

0

0

0

0

0

0

0

0

0

0

0

0

0

0

0

0

0

0

0
7

0

0

0

0

0

0

0

0

0

0

0

0
7

0

0

0

0

0

0

0

0

0

0

0
6

0

0

0

0

0

0

0

0

0

0

0.504557

0

0

0

0

0

0

0

0

0

0

0
6

0

0

0

0

0

0

0

0

0

0

0
7

0

0

0

0

0

0

0

0

0

0

0
7

0

0

0

0

0

0

0

0

0

0

0
7

0

0

0

0

0

0

0

0

0

0

0
7

0

0

0

0

0

0

0

0

0

0

0
7

0

0

0

0

0

0

0

0

0

0

0
6

0

0

0

0

0

0

0

0

0

0

0

0
6

0

0

0

0

0

0

0

0

0

0

0
6

0

0

0

0

0

0

0

0

0

0

0
5

0

0

0

0

0

0

0

0

0

0

0

0

0

0

0

0

0

0

0

0

0

0
7

0

0

0

0

0

0

0

0

0

0

0

0

0

0

0

0

0

0

0

0

0

0
6

0

0

0

0

0

0

0

0

0

0

0

0

0

0

0

0

0

0

0

0

0

0
7

0

0

0

0

0

0

0

0

0

0

0
6

0
7

0

0

0

0

0

0

0

0

0

0

0
7

0

0

0

0

0

0

0

0

0

0

0
7

0

0

0

0

0

0

0

0

0

0

0
6

0

0

0

0

0

0

0

0

0

0

0
6

0

0

0

0

0

0

0

0

0

0

0
7

0

0

0

0

0

0

0

0

0

0

0

0

0

0

0

0

0

0

0

0

0

0
7

0

0

0

0

0

0

0

0

0

0

0
7

0

0

0

0

0

0

0

0

0

0

0
8

0

0

0

0

0

0

0

0

0

0

0
7

0
7

0

0

0

0

0

0

0

0

0

0

0
7

0

0

0

0

0

0

0

0

0

0

0
7

0

0

0

0

0

0

0

0

0

0

0
6

0

0

0

0

0

0

0

0

0

0

0
7

0

0

0

0

0

0

0

0

0

0

0
7

0

0

0

0

0

0

0

0

0

0

0
7

0

0

0

0

0

0

0

0

0

0

0
7

0

0

0

0

0

0

0

0

0

0

0
7

0

0

0

0

0

0

0

0

0

0

0
7

0

0

0

0

0

0

0

0

0

0

0
7

0
7

0

0

0

0

0

0

0

0

0

0

0
7

0

0

0

0

0

0

0

0

0

0

0
7

0

0

0

0

0

0

0

0

0

0

0
7

0

0

0

0

0

0

0

0

0

0

0
7

0

0

0

0

0

0

0

0

0

0

0
7

0

0

0

0

0

0

0

0

0

0

0
6

0

0

0

0

0

0

0

0

0

0

0
7

0

0

0

0

0

0

0

0

0

0

0
7

0

0

0

0

0

0

0

0

0

0

0
7

0

0

0

0

0

0

0

0

0

0

0
7

0
7

0

0

0

0

0

0

0

0

0

0

0
6

0

0

0

0

0

0

0

0

0

0

0
2

0

0

0

0

0

0

0

0

0

0

0
6

0

0

0

0

0

0

0

0

0

0

0
7

0

0

0

0

0

0

0

0

0

0

0
7

0

0

0

0

0

0

0

0

0

0

0
8

0

0

0

0

0

0

0

0

0

0

0
7

0

0

0

0

0

0

0

0

0

0

0

0

0

0

0

0

0

0

0

0

0

0
7

0

0

0

0

0

0

0

0

0

0

0
7

0.253886
7

0
7

0

0

0

0

0

0

0

0

0

0

0
7

0

0

0

0

0

0

0

0

0

0

0
7

0

0

0

0

0

0

0

0

0

0

0

0

0

0

0

0

0

0

0

0

0

0

0

0

0

0

0

0

0

0

0

0

0

0

0

0

0

0

0

0

0

0

0

0
7

0

0

0

0

0

0

0

0

0

0

0

0

0

0

0

0

0

0

0

0

0

0
6

0

0

0

0

0

0

0

0

0

0

0

0

0

0

0

0

0

0

0

0

0

0
7

0
7

0

0

0

0

0

0

0

0

0

0

0
7

0

0

0

0

0

0

0

0

0

0

0
7

0

0

0

0

0

0

0

0

0

0

0
5

0

0

0

0

0

0

0

0

0

0

0

0

0

0

0

0

0

0

0

0

0

0
7

0

0

0

0

0

0

0

0

0

0

0

0

0

0

0

0

0

0

0

0

0

0
7

0

0

0

0

0

0

0

0

0

0

0
7

0

0

0

0

0

0

0

0

0

0

0
7

0

0

0

0

0

0

0

0

0

0

0
7

0
7

0

0

0

0

0

0

0

0

0

0

0
7

0

0

0

0

0

0

0

0

0

0

0
7

0

0

0

0

0

0

0

0

0

0

0
7

0

0

0

0

0

0

0

0

0

0

0

0

0

0

0

0

0

0

0

0

0

0
6

0

0

0

0

0

0

0

0

0

0

0
7

0

0

0

0

0

0

0

0

0

0

0

0

0

0

0

0

0

0

0

0

0

0
7

0

0

0

0

0

0

0

0

0

0

0
7

0

0

0

0

0

0

0

0

0

0

0
7

0
7

0

0

0

0

0

0

0

0

0

0

0

0

0

0

0

0

0

0

0

0

0

0
6

0

0

0

0

0

0

0

0

0

0

0
7

0

0

0

0

0

0

0

0

0

0

0
7

0

0

0

0

0

0

0

0

0

0

0
7

0

0

0

0

0

0

0

0

0

0

6.2456
4

0

0

0

0

0

0

0

0

0

0

0

0

0

0

0

0

0

0

0

0

0

0
7

0

0

0

0

0

0

0

0

0

0

0
7

0

0

0

0

0

0

0

0

0

0

0
6

0
7

0

0

0

0

0

0

0

0

0

0

0
7

0

0

0

0

0

0

0

0

0

0

0
7

0

0

0

0

0

0

0

0

0

0

0
7

0

0

0

0

0

0

0

0

0

0

0
7

0

0

0

0

0

0

0

0

0

0

0
7

0

0

0

0

0

0

0

0

0

0

0
7

0

0

0

0

0

0

0

0

0

0

0

0

0

0

0

0

0

0

0

0

0

0
7

0

0

0

0

0

0

0

0

0

0

0
7

0

0

0

0

0

0

0

0

0

0

0
7

0
6

0

0

0

0

0

0

0

0

0

0

0

0

0

0

0

0

0

0

0

0

0

0
7

0

0

0

0

0

0

0

0

0

0

0
7

0

0

0

0

0

0

0

0

0

0

0
7

0

0

0

0

0

0

0

0

0

0

0
7

0

0

0

0

0

0

0

0

0

0

0

0

0

0

0

0

0

0

0

0

0

0
7

0

0

0

0

0

0

0

0

0

0

0
6

0

0

0

0

0

0

0

0

0

0

0
7

0

0

0

0

0

0

0

0

0

0

0
7

0

0

0

0

0

0

0

0

0

0

0

0
7

0

0

0

0

0

0

0

0

0

0

0

0

0

0

0

0

0

0

0

0

0

0
6

0

0

0

0

0

0

0

0

0

0

0
7

0

0

0

0

0

0

0

0

0

0

0

0

0

0

0

0

0

0

0

0

0

0
7

0

0

0

0

0

0

0

0

0

0

0
7

0

0

0

0

0

0

0

0

0

0

0

0

0

0

0

0

0

0

0

0

0

0

0

0

0

0

0

0

0

0

0

0

0
7

0

0

0

0

0

0

0

0

0

0

0

0
7

0

0

0

0

0

0

0

0

0

0

0
7

0

0

0

0

0

0

0

0

0

0

0
6

0

0

0

0

0

0

0

0

0

0

0
6

0

0

0

0

0

0

0

0

0

0

0
5

0

0

0

0

0

0

0

0

0

0

0
6

0

0

0

0

0

0

0

0

0

0

0
7

0

0

0

0

0

0

0

0

0

0

0

0

0

0

0

0

0

0

0

0

0

0

0

0

0

0

0

0

0

0

0

0

2.89421
7

0
7

0

0

0

0

0

0

0

0

0

0

0
7

0

0

0

0

0

0

0

0

0

0

0
7

0

0

0

0

0

0

0

0

0

0

0
7

0

0

0

0

0

0

0

0

0

0

0
7

0

0

0

0

0

0

0

0

0

0

0
7

0

0

0

0

0

0

0

0

0

0

0

0

0

0

0

0

0

0

0

0

0

0
7

0

0

0

0

0

0

0

0

0

0

0
7

0

0

0

0

0

0

0

0

0

0

0

0

0

0

0

0

0

0

0

0

0

0
7

0

0

0

0

0

0

0

0

0

0

0

0
2

0

0

0

0

0

0

0

0

0

0

0
7

0

0

0

0

0

0

0

0

0

0

0
7

0

0

0

0

0

0

0

0

0

0

0
6

0

0

0

0

0

0

0

0

0

0

0

0

0

0

0

0

0

0

0

0

0

0
7

0

0

0

0

0

0

0

0

0

0

0
7

0

0

0

0

0

0

0

0

0

0

0

0

0

0

0

0

0

0

0

0

0

0
7

0

0

0

0

0

0

0

0

0

0

0
7

0
7

0

0

0

0

0

0

0

0

0

0

0

0

0

0

0

0

0

0

0

0

0

0

0
6

0

0

0

0

0

0

0

0

0

0

0
7

0

0

0

0

0

0

0

0

0

0

0
7

0

0

0

0

0

0

0

0

0

0

0
7

0

0

0

0

0

0

0

0

0

0

0

0

0

0

0

0

0

0

0

0

0

0
6

0

0

0

0

0

0

0

0

0

0

0
8

0

0

0

0

0

0

0

0

0

0

0
7

0

0

0

0

0

0

0

0

0

0

0

0

0

0

0

0

0

0

0

0

0

0

0
7

0

0

0

0

0

0

0

0

0

0

0
7

0

0

0

0

0

0

0

0

0

0

0
7

0

0

0

0

0

0

0

0

0

0

0
7

0

0

0

0

0

0

0

0

0

0

0
6

0

0

0

0

0

0

0

0

0

0

0
7

0

0

0

0

0

0

0

0

0

0

0
7

0

0

0

0

0

0

0

0

0

0

0
6

0

0

0

0

0

0

0

0

0

0

0
7

0

0

0

0

0

0

0

0

0

0

0
7

0
7

0

0

0

0

0

0

0

0

0

0

0

0

0

0

0

0

0

0

0

0

0

0
7

0

0

0

0

0

0

0

0

0

0

0
7

0

0

0

0

0

0

0

0

0

0

0

0

0

0

0

0

0

0

0

0

0

0
7

0

0

0

0

0

0

0

0

0

0

0
7

0

0

0

0

0

0

0

0

0

0

0
7

0

0

0

0

0

0

0

0

0

0

0
6

0

0

0

0

0

0

0

0

0

0

0
7

0

0

0

0.107242

0

0

0

0

0

0

0
7

0
7

0

0

0

0

0

0

0

0

0

0

0
7

0

0

0

0

0

0

0

0

0

0

0
7

0

0

0

0

0

0

0

0

0

0

0

0

0

0

0

0

0

0

0

0

0

0
7

0

0

0

0

0

0

0

0

0

0

0
6

0

0

0

0

0

0

0

0

0

0

0
7

0

0

0

0

0

0

0

0

0

0

0
2

0

0

0

0

0

0

0

0

0

0

0
7

0

0

0

0

0

0

0

0

0

0

0

0

0

0

0

0

0

0

0

0

0

0
7

0
7

0

0

0

0

0

0

0

0

0

0

0
7

0

0

0

0

0

0

0

0

0

0

0
7

0

0

0

0

0

0

0

0

0

0

0
8

0

0

0

0

0

0

0

0

0

0

0

0

0

0

0

0

0

0

0

0

0

0

0

0

0

0

0

0

0

0

0

0

0

0

0

0

0

0

0

0

0

0

0

0
7

0

0

0

0

0

0

0

0

0

0

0
7

0

0

0

0

0

0

0

0

0

0

0
7

0

0

0

0

0

0

0

0

0

0

0.342993
7

0
7

0

0

0

0

0

0

0

0

0

0

0
6

0

0

0

0

0

0

0

0

0

0

0
7

0

0

0

0

0

0

0

0

0

0

0
7

0

0

0

0

0

0

0

0

0

0

0

0

0

0

0

0

0

0

0

0

0

0
7

0

0

0

0

0

0

0

0

0

0

0
7

0

0

0

0

0.214485

0

0

0

0

0

0
6

0

0

0

0

0

0

0

0

0

0

0
7

0

0

0

0

0

0

0

0

0

0

0
7

0

0

0

0

0

0

0

0

0

0

0
7

0
7

0

0

0

0

0

0

0

0

0

0

0

0

0

0

0

0

0

0

0

0

0

0

0

0

0

0

0

0

0

0

0

0

0
6

0

0

0

0

0

0

0

0

0

0

0
7

0

0

0

0

0

0

0

0

0

0

0

0

0

0

0

0

0

0

0

0

0

0
7

0

0

0

0

0

0

0

0

0

0

0

0

0

0

0

0

0

0

0

0

0

0
7

0

0

0

0

0

0

0

0

0

0

0
7

0

0

0

0

0

0

0

0

0

0

0
7

0
7

0

0

0

0

0

0

0

0

0

0

0
7

0

0

0

0

0

0

0

0

0

0

0
7

0

0

0

0

0

0

0

0

0

0

0
7

0

0

0

0

0

0

0

0

0

0

0
7

0

0

0

0

0

0

0

0

0

0

0
8

0

0

0

0

0

0

0

0

0

0

0
7

0

0

0

0

0

0

0

0

0

0

0
7

0

0

0

0

0

0

0

0

0

0

0
6

0

0

0

0

0

0

0

0

0

0

0.097998
7

0

0

0

0

0

0

0

0

0

0

0
7

0
8

0

0

0

0

0

0

0

0

0

0

0
7

0

0

0

0

0

0

0

0

0

0

0
7

0

0

0

0

0

0

0

0

0

0

0

0

0

0

0

0

0

0

0

0

0

0
6

0

0

0

0

0

0

0

0

0

0

0
7

0

0

0

0

0

0

0

0

0

0

0
5

0

0

0

0

0

0

0

0

0

0

0
1

0

0

0

0

0

0

0

0

0

0

0
7

0

0

0

0

0

0

0

0

0

0

0
6

0

0

0

0

0

0

0

0

0

0

0
7

0
7

0

0

0

0

0

0

0

0

0

0

0.293994
5

0

0

0

0

0

0

0

0

0

0

0
7

0

0

0

0

0

0

0

0

0

0

0
7

0

0

0

0

0

0

0

0

0

0

0
8

0

0

0

0

0

0

0

0

0

0

0

0

0

0

0

0

0

0

0

0

0

0
7

0

0

0

0

0

0

0

0

0

0

0
7

0

0

0

0

0

0

0

0

0

0

0
6

0

0

0

0

0

0

0

0

0

0

0
7

0

0

0

0

0

0

0

0

0

0

0
4

0
5

0

0

0

0
4

0
7

0
7

0
4

0
7

0

0

0
4

0

0

0
4

0

0

0
4

0

0

0

0
4

0

0

0
4

0

0

0
4

0

0

0
4

0
4

0
7

0
7

0
7

0
7

0
7

0

0
4

0

0

0
4

0
4

13.8347

0
7

0
7

0
7

0

0

0

0

0

0

0

0

0

0

0
7

0

0

0

0

0

0

0

0

0

0

0
6

0

0

0

0

0

0

0

0

0

0

0

0

0

0

0

0

0

0

0

0

0

0
7

0
6

0

0
7

0
6

0

0
7

0
7

0

0
7

0

0

0

0

0

0

0

0
7

0

0

0

0

0

0

0

0

0

0

0
6

0

0

0

0

0

0

0

0

0

0

0
6

0

0

0

0

0

0

0

0

0

0

0
7

0

0

0

0

0

0

0

0

0

0

0
7

0

0

0

0

0

0

0

0

0

0

0
6

0

0

0

0

0

0

0

0

0

0

0
6

0

0

0

0

0

0

0

0

0

0

0
4

0

0

0

0

0

0

0
4

5.82245

5.82245

0
4

0

0

0

0
7

0

0
4

0.133314

0

0

0

0.0799885

0

0.0533257

0
4

3.91426
7

1.99707
7

0

0
6

0
6

0

0

0.119824
5

0

0

0

0

1.55771

0

0

0

0

0

0

0

0

0

0

0.0798828
6

0.0798828

0

0

0

0

0

0

0

0

0

0.0798828
7

0

0

0

0

0

0

0

0

0

0

0
7

0

0

0

0

0

0

0

0

0

0

0
7

0

0

0

0

0

0

0

0

0

0

0
6

0

0

0

0

0

0

0

0

0

0

0
7

0

0

0

0

0

0

0

0

0

0
4

0.586582
6

0
6

0

0

0

0

0.453268

0.0533257

0

0

0

0.0799885

0

0

0
6

0

0

0

0

0

0

2.77555756156289e-17
6

0
4

1.15729
6

0
6

0
7

0

0

0

0

0

0

0

0

0

0

0
6

0

0

0

0

0

0

0

0

0

0

1.15729

0

0

0

0

0

0

0

0

0

0

0
7

0

0

0

0

0

0

0

0

0

0

0
6

0

0

0

0

0

0

0

0

0

0

0

0

0

0

0

0

0

0

0

0

0

0
7

0

0

0

0

0

0

0

0

0

0

0
6

0

0

0

0

0

0

0

0

0

0

0
6

0

0

0

0

0

0

0

0

0

0

0
7

0

0

0

0

0

0

0

0

0

0

0
6

0
6

0

0

0

0

0

0

0

0

0

0

0

0

0

0

0

0

0

0

0

0

0

0
6

0

0

0

0

0

0

0

0

0

0

0
7

0

0

0

0

0

0

0

0

0

0

0
6

0

0

0

0

0

0

0
7

0
6

0
6

0
7

0
6

0
6

0
6

0
6

0

0
6

0

0
6

0

0
6

0
6

0
7

0
6

0

0
7

0
6

0
6

0
7

0
6

0

0

0

0

0

0

0

0
7

0

0

0
6

0

0

0

0

0
6

0

0

0

0

0

0

0

0

0

0

0
6

0

0

0

0

0

0

0

0

0

0

0
7

0

0

0

0

0

0

0

0

0

0

0
7

0

0

0

0

0

0

0

0

0

0

0
4

1.03797

0.265526

0.0724163

0.627608
5

0.0724163

0

0

0

0
4

0

0

0
4

0

0

0

0

0
4

0

0

0
4

0

0

0
4

0

0

0

0
4

0

0

0
4

0

0

0
4

0

0

0
4

0

0

0
4

0

0

0
4

1.13452

1.13452

0

0
4

0

0

0
4

0

0

0
4

0

0

0
4

0

0

0
4

0

0

0
4

0

0

0
4

0

0

0
4

0

0

0

0

0
4

0

0

0
4

0

0

0

0

0

0

0
4

0.0482775

0.0482775

0

0

0
4

0

0

0
4

0

0

0

0
4

0

0

0
4

5.21804821573824e-15

0
4

27.8818

0
7

0
7

0
4

0
5

0
5

0
5

0

0

0

0

0

0

0

0
4

0

0

0

0

0

0

0

0
4

0
7

0
7

0
7

0

0
4

0

0

0

0

0

0

0

0

0
4

6.19487
5

6.19487
5

0

0

0
4

0
7

0
7

0

0

0
4

0
5

0
5

0

0

0

0

0

0

0
4

0
6

0
6

0

0

0
4

0

0

0

0

0
4

0
4

0
4

0

0

0

0

0

0

0
4

0

0

0

0

0

0

0

0
4

0

0
5

0

0

0

0

0

0
6

0

0

0

0

0

0

0

0
4

0.175085
3

0.175085
4

0

0
4

0

0

0

0
4

0

0

0

0

0
4

0
7

0

0

0
4

0
5

0

0

0

0

0

0

0

0

0
4

0

0

0
4

2.80136

0.175085

2.62628

0

0

0

0

0
4

0
7

0
7

0
4

0
6

0
6

0

0
4

0

0

0
4

3.84122
5

0
5

0

0

0

0

0

0

0

0

0

0.0533257

3.78789
5

0

0

0

0

0

0

0

0

0

0
4

3.23142
4

0.0798828

3.15154

0
4

0

0

0

0

0

0

0
4

0
7

0

0

0

0

0
4

0.399414

0.119824

0.27959

0
4

0.253157

0

0.253157

0
4

0

0

0
4

0

0

0

0
4

0

0

0

0

0
4

0

0

0
4

0

0

0

0
4

0
7

0
7

0

0

0
7

0
7

0
7

0
6

0
7

0

0

0

0
4

0

0

0
4

0

0

0

0
4

0

0

0
4

0

0

0
4

0

0

0

0

0
4

0

0

0
4

0

0

0
4

0

0

0

0

0
4

0

0

0

0
4

0

0

0

0

0
4

5.51921
5

4.88546
5

0.371124
3

0
5

0

0

0

0.262628

0
4

0

0

0

0
4

0

0

0
4

0

0

0

0
4

0

0

0
4

0

0

0

0
4

0

0

0
4

0

0

0
4

0

0

0

0
4

0

0

0
4

0

0

0

0
4

0

0

0
6

0

0

0

0

0

0

0
4

0.159766

0.159766

0
4

0

0

0
4

0

0

0
4

0

0

0
4

0

0

0
4

0

0

0
4

0

0

0
4

0

0

0
4

0

0

0
4

0

0

0
4

0

0

0

0

0

0

0

0

0

0
4

0.262628

0.262628

0
4

0

0

0
4

0

0

0
4

0

0

0
4

0

0

0
4

0

0

0
4

0.0723307

0.0723307

0
4

0

0

0
4

0

0

0
4

0

0

0
4

0

0

0

0

0

0

0

0

0

0

0

0

0
4

0

0

0
4

0

0

0
4

0

0

0
4

0

0

0
4

0

0

0
4

0

0

0
4

0

0

0
4

0

0

0
4

0

0

0
4

0

0

0
4

4.97136
5

0.426605

4.54476

0

0

0

0
4

0

0

0
4

0

0

0
4

0

0

0
4

7.105427357601e-15

0
4

9.61044

9.61044

9.42696

0.183475

0

0

0

0

0
4

0
4

0

0

0

0
4

0
4

0

0

0

0
4

0
4

0

0

0

0

0
4

0
4

0

0

0

0

0

0
4

0
4

0

0

0

0
4

0
4

0

0

0

0

0
4

0
4

0

0

0

0

0
4

0
4

0

0

0

0
4

0
4

0

0

0

0
4

0
4

0

0

0

0

0
4

0
4

0.489667
3

0.489667
3

0

0

0

0

0

0

0

0

0

0

0

0

0

0

0

0

0

0

0

0

0.0533257

0.119824

0
5

0

0

0

0.137606

0

0.0724163
3

0
3

0.106495

0

0

0

2.77555756156289e-17
3

0
4

0

0

0
4

0
4

0

0

0

0

0
4

0
4

0

0

0

0
4

0

0

0
4

0
4

0

0

0

0
4

0

0

0
4

0
4

0

0

0

0
4

0
4

0

0

0

0
4

0
4

0

0

0

0
4

0
4

0

0

0

0
4

0
4

0

0

0

0
4

0
4

0

0

0

0
4

0
4

0.437713

0.437713

0.437713

0
4

0
4

0.0649455
3

0.0649455
3

0

0.0649455

0

0

0
4

0

0

0
4

0

0

0
4

0
4

0

0

0

0
4

0
4

0

0

0

0
4

0
4

0

0

0

0
4

0
4

0

0

0

0
4

0
4

0

0

0

0
4

0
4

0

0

0

0
4

0
4

0

0

0

0
4

0
4

0

0

0

0
4

0
4

0

0

0

0
4

0
4

0

0

0

0
4

0
4

0
6

0
6

0
6

0

0

0

0

0
4

0
4

0

0

0

0
4

0
4

0

0

0

0
4

0
4

0

0

0

0
4

0
4

0

0

0

0
4

0
4

0

0

0

0
4

0
4

0

0

0

0
4

0
4

0

0

0

0
4

0
4

0

0

0

0
4

0
4

0

0

0

0

0

0

0
4

0
4

0.17162
5

0.17162

0.17162

0
4

0

0

0
4

0

0

0
4

0
4

0

0

0

0

0
4

0
4

0

0

0

0

0
4

0
4

0.253157

0.253157

0.253157

0

0

0

0

0

0

0

0
4

0
6

0
6

0

0

0

0

0

0

0

0
4

0

0

0
4

0

0

0
4

0

0

0
4

0
5

0

0

0

0

0

0

0

0

0

0
4

0
7

0
6

0

0

0
4

0
7

0
7

0

0
4

0

0

0

0
4

0

0

0
4

0

0

0
4

0

0

0
4

0

0

0
4

0
4

5.49654

5.20722

0.54248

0.470149
5

0
7

0

0

0
5

0

0

0
5

0

0

0.867968

0

0

0

0

0

0

0

0

0

0

0
6

0

0

1.40068

0

0

0

0

0

0

0

0
5

0

0

0

0

0

0

0

0

0

0

0
6

0

0

0

0

0

0

0

0

1.92594

0

0

0

0

0

0

0

0

0

0

0

0

0

0

0

0

0

0

0

0

0

0

0

0
7

0

0

0

0

0

0

0

0

0

0
4

0.289323

0.144661
7

0.144661

0
4

0

0

0

0
4

0

0

0
4

0

0

0
4

0

0

0
4

0

0

0
4

0

0

0
4

0

0

0
4

0

0

0
4

0
5

0
6

0

0

0

0
4

0
6

0

0

0

0

0
4

0
5

0
5

0
4

0
7

0
7

0

0

0
4

0
7

0
7

0
4

0

0

0

0
4

0

0

0

0
4

0

0

0
4

5.55111512312578e-16

0
4

0

0

0
5

0

0

0

0

0

0

0

0

0

0

0
5

0

0

0

0

0

0

0

0

0

0

0

0

0

0

0
6

0

0

0

0

0

0
4

0

0

0
4

0

0

0
4

0

0

0
4

0

0

0
4

0
4

0
5

0
5

0
5

0
5

0
5

0
6

0
5

0

0

0

0

0

0

0

0

0

0

0

0

0

0

0

0

0

0
6

0

0

0

0

0

0

0

0

0

0

0
5

0

0

0

0

0

0

0

0

0

0

0
5

0

0

0

0

0

0

0

0

0

0

0
5

0

0

0

0

0

0

0

0

0

0

0
6

0

0

0

0

0

0

0
4

0
6

0
4

0

0

0

0
4

0

0

0

0
4

0

0

0
4

0

0

0
4

0
4

0

0

0

0
5

0

0

0

0

0

0

0

0

0

0

0
5

0

0

0

0

0

0

0

0

0

0

0

0

0

0

0

0

0
7

0

0

0
4

0
5

0
5

0
4

0

0

0
4

0

0

0

0
4

0

0

0

0

0
4

0

0

0
4

0

0

0
4

0

0

0
4

0

0

0

0
4

0

0

0
4

0

0

0
4

0

0

0
4

0

0

0

0
4

0

0

0
4

0

0

0
4

0

0

0
4

0

0

0
4

0

0

0
4

0

0

0
4

0

0

0
4

0

0

0
4

0

0

0
4

0

0

0
4

0

0

0

0

0
4

0

0

0
4

0

0

0
4

0

0

0
4

0

0

0
4

0

0

0
4

0

0

0
4

0

0

0
4

0

0

0

0

0

0
4

0

0

0

0

0

0
4

0

0

0
4

0

0

0
4

0

0

0
4

0

0

0

0
4

0
4

0
5

0
7

0
7

0
7

0

0
4

0
4

0
4

0

0

0
4

0

0

0
4

0

0

0
4

0

0

0
4

0

0

0
4

0

0

0

0
4

0
4

0
4

0

0

0
4

0

0

0

0

0
4

0

0

0

0

0
4

0

0

0
4

0

0

0
4

0

0

0
4

0

0

0
4

0
4

0

0

0

0
5

0

0

0

0

0

0

0

0

0

0

0

0

0

0

0

0

0

0

0

0

0

0

0

0

0

0

0

0

0

0

0

0
7

0

0
7

0

0

0

0

0
4

0
4

0
4

0

0

0

0

0

0
4

0

0

0

0

0
4

0

0

0
4

0

0

0
4

0

0

0
4

0

0

0
4

0
4

0
4

0
4

0
5

0

0

0

0

0

0

0

0

0

0

0

0

0

0

0

0

0
4

0

0

0
4

0

0

0
4

0

0

0
4

0
4

0
4

1.74499

0.359473

0

0
5

0

0

0

0

0

0

0

0

0

0

0
4

0

0

0
7

0

0

0

0

0

0

0

0

0

0

0
7

0

0

0
7

0

0

0

0

0

0

0
4

0.359473

0.359473
7

0
7

0

0

0

0

0

0

0

0

0

0

0
7

0

0

0

0

0

0

0

0

0

0

0
7

0

0

0

0

0

0

0

0

0

0

0
8

0

0

0

0

0

0

0

0

0

0

0
7

0

0

0

0

0

0

0

0

0

0

0
7

0

0

0

0

0

0

0

0

0

0

0
1

0

0

0

0

0

0

0

0

0

0

0

0

0

0

0

0

0

0

0

0

0

0
8

0

0

0

0

0

0

0

0

0

0

0

0

0

0

0

0

0

0

0

0

0

0

0

0

0

0

0

0

0

0

0

0

0

0
8

0

0

0

0

0

0

0

0

0

0

0
7

0

0

0

0

0

0

0

0

0

0

0
7

0

0

0

0

0

0

0

0

0

0

0
7

0

0

0

0

0

0

0

0

0

0
7

0

0

0
7

0
7

0

0

0

0

0

0

0

0

0

0

0

0

0

0

0

0

0

0

0

0

0
7

0

0

0

0

0

0

0

0

0

0

0
1

0

0

0

0

0

0

0

0

0

0

0
1

0

0

0

0

0

0

0

0

0

0

0
8

0

0

0

0

0

0

0

0

0

0

0
8

0

0

0

0

0

0

0

0

0

0

0
4

0
7

0
7

0

0
4

0
7

0
7

0

0

0

0

0

0

0

0

0

0
4

0
6

0
6

0
6

0

0

0

0
4

0
7

0
7

0
6

0

0

0
4

0
7

0

0

0

0

0

0

0

0

0

0

0
4

0

0

0

0

0

0

0
4

0
1

0
1

0
4

0
6

0
6

0

0

0
4

0

0

0

0

0
4

0

0

0

0
4

0
5

0
7

0

0
6

0

0

0

0

0

0

0

0

0

0

0

0

0

0

0

0

0

0

0

0
6

0

0

0

0
6

0
5

0
6

0
6

0
6

0
4

0
6

0
6

0
4

0

0

0

0
4

0

0

0

0
4

0

0

0

0
4

0

0

0

0
4

0

0

0

0
4

0

0

0

0
4

0

0

0
4

0

0

0
4

0

0

0
4

0

0
1

0

0

0

0

0

0

0

0
7

0
2

0
8

0
6

0
4

0

0

0
4

0

0

0
4

0

0

0
4

0

0

0
4

0

0

0
4

0

0

0
4

0

0

0
4

0

0

0
4

0

0

0
4

0

0

0
4

0

0
5

0

0

0
7

0
7

0

0

0

0

0

0
4

0

0

0
4

0

0

0
4

0

0

0
4

0

0

0
4

0

0

0
4

0

0

0
4

0

0

0
4

0

0

0
4

0

0

0
4

0

0

0
4

0

0
1

0
1

0

0

0

0

0

0

0
4

0

0

0
4

0
7

0
7

0
8

0

0

0

0

0
4

0
6

0
6

0
7

0

0

0

0

0

0
4

0
8

0
8

0
8

0

0

0

0

0

0
4

0

0

0

0

0

0

0

0
4

0
4

0.718945

0.718945

0.718945

0

0

0

0

0

0

0

0

0

0

0

0

0

0

0

0

0

0

0

0

0

0

0

0

0

0
4

0
8

0
8

0

0

0

0

0

0

0

0

0

0

0
8

0

0

0

0

0

0

0

0

0

0

0
8

0

0

0

0

0

0

0

0

0
4

0

0

0
4

0

0

0
4

0

0

0
4

0

0

0
4

0

0

0
4

0

0

0
4

0

0

0
4

0

0

0
4

0

0

0
4

0

0

0
4

0
4

0.666571

0.666571

0

0.346617
5

0

0

0

0

0

0

0

0

0

0

0

0

0

0

0

0

0

0

0

0

0

0
6

0

0

0

0

0

0

0

0

0

0

0
7

0

0

0

0

0

0

0

0

0

0

0

0

0

0

0

0

0

0

0

0

0

0

0

0

0
6

0
7

0

0
6

0.213303

0
7

0

0

0
7

0

0

0

0

0

0

0
7

0

0

0

0

0

0

0

0

0

0.0533257

0

0

0

0

0

0

0

0

0

0

0

0.0533257
6

0

0

0

0

0

0

0

0

0

0

0
7

0

0

0

0

0

0

0

0

0

0

0
1

0

0

0

0

0

0

0

0

0

0

0

0

0

0

0

0

0

0

0

0

0

0
7

0

0

0

0

0

0

0

0

0

0

6.93889390390723e-17

0
4

0

0

0

0

0
4

0

0

0

0

0
4

0

0

0

0
4

0

0

0
4

0

0

0
4

0

0

0
4

0

0

0
4

0

0

0
4

0

0

0

0

0

0

0

0

0

0

0

0

0
6

0

0

0

0

0

0

0

0

0

0

0
6

0

0

0

0

0

0

0

0

0

0

0
6

0

0

0

0

0

0

0

0

0

0

0

0

0

0

0

0

0

0

0
4

0
5

0
6

0

0

0

0

0

0

0

0

0

0
4

0

0
6

0

0

0

0

0

0

0

0

0

0

0
4

0
7

0
7

0

0

0

0
4

0
6

0

0

0

0

0

0
4

0
7

0

0

0

0

0
4

0

0

0

0

0

0
4

0

0

0

0

0

0

0
4

0
4

0

0

0

0
4

0

0

0
4

0
4

0

0

0

0
4

0

0

0
4

0
4

0

0

0

0

0
4

0
4

0

0

0

0

0

0
4

0
4

0

0

0

0

0
4

0
4

0

0

0

0

0
4

0
4

0

0

0

0

0
4

0
4

0

0

0

0

0
4

0

0

0
4

0
4

0

0

0

0
4

0
4

0

0

0

0
4

0
4

0
7

0
7

0
7

0

0

0

0

0

0

0
7

0
7

0
7

0

0

0

0

0

0
4

0
6

0
6

0
7

0

0

0

0

0

0

0
4

0

0

0

0
4

0

0

0

0
4

0
6

0

0

0

0
4

0

0

0

0
4

0

0

0
4

0

0

0
4

0

0

0
4

0
4

0

0

0

0
4

0

0

0
4

0
4

0

0

0

0
4

0

0

0
4

0
4

0

0

0

0

0
4

0
4

0

0

0

0
4

0
4

0

0

0

0
4

0
4

0

0

0

0
4

0
4

0

0

0

0
4

0
4

0

0

0

0
4

0
4

0

0

0

0
4

0
4

0

0

0

0
4

0
4

0
7

0
7

0
7

0

0

0

0

0
4

0
7

0
7

0

0

0

0

0

0
4

0
6

0

0

0

0
4

0

0

0
4

0
4

0

0

0

0
4

0
4

0

0

0

0
4

0
4

0

0

0

0
4

0
4

0

0

0

0
4

0
4

0

0

0

0
4

0
4

0

0

0

0
4

0
4

0
6

0
6

0
6

0

0

0

0

0

0
4

0
7

0
7

0

0

0
4

0
7

0
6

0

0
4

0

0

0

0

0
4

0

0

0
4

0

0

0
4

0
4

0
6

0
6

0
6

0

0

0

0

0
4

0
4

0
2

0
2

0
2

0

0

0

0

0

0

0

0
4

0
4

0
6

0
6

0

0

0

0

0

0
4

0

0

0

0
4

0
4

0

0

0

0
4

0
4

0

0

0

0

0

0

0

0
4

0
4

0

0

0

0
7

0
7

0

0

0
7

0

0

0

0

0

0
7

0

0

0

0

0

0

0

0

0

0

0

0

0

0

0

0

0

0

0

0

0

0

0

0

0

0

0

0

0

0

0

0

0
7

0

0

0

0

0

0

0

0

0

0

0

0

0

0

0

0

0

0

0

0

0

0
8

0

0

0

0

0

0

0

0

0

0

0

0

0

0

0

0

0

0

0

0

0

0

0

0

0
4

0

0

0
4

0

0

0
4

0

0

0
4

0
4

0
4

3.71455

0
7

0

0

0

0
4

0

0

0

0
4

0

0

0
4

0

0

0
4

0

0

0
4

0

0

0
4

0

0

0
4

0

0

0
4

0
4

0
7

0
7

0

0

0

0

0

0

0

0

0

0

0

0

0

0

0

0

0

0

0

0

0

0

0

0

0

0

0

0

0

0

0

0

0

0

0

0

0

0

0

0

0

0

0

0

0

0

0

0

0

0

0

0

0

0

0

0

0

0

0

0

0

0

0

0

0

0

0

0

0

0

0

0

0

0

0
4

0

0

0
4

0
4

0
6

0
6

0
6

0

0
4

0
4

0

0

0

0
4

0
4

0

0

0

0
4

0
4

0
7

0
7

0
7

0
4

0
4

0

0

0

0
4

0
4

0

0

0

0
4

0
4

0

0

0

0
4

0
4

0

0

0

0
4

0
4

0

0

0

0
4

0
4

0

0

0

0
4

0
4

0

0

0

0
4

0
4

3.71455

3.71455

1.79736

0
7

0

0

0

0

0

0

0

0

0

0

0
6

0

0

0

0

0

0

0

0

0

0

1.91719

0

0

0

0
7

0

0
7

0

0

0

0
4

0

0

0

0

0
4

0

0

0
4

0

0

0
4

0

0

0
4

0

0

0

0
4

0

0

0
4

0

0

0
4

0

0

0
4

0
4

0
4

220.961
4

220.961
4

220.961
4

220.602
4

0

0

0

0

0

0

0.359473

0

0

0

0

0

0

0

0

0

0

0

0

0

0

0

0
7

0

0

0

0

0

0

0

0

0

0

0

0

0

0

0

0

0

0

0

0

0

0

0

0

0

0

0

0

0

0

0

0
6

0

0

0

4.94049245958195e-15
4

0
4

0

0

0
4

0

0

0
4

0

0

0
4

0

0

0
4

0

0

0
4

0

0

0
4

0

0

0
4

0

0

0
4

0

0

0
4

0

0

0
4

0

0

0
4

0

0

0
4

0

0

0
4

0
4

0

0

0

0
4

0
4

0

0

0

0

0
4

0
4

0

0

0

0
4

0
4

0

0

0

0
4

0
4

0

0

0

0
4

0
4

0

0

0

0
4

0
4

0

0

0

0
4

0
4

0

0

0

0
4

0
4

0

0

0

0
4

0
4

0

0

0

0
4

0
4

0

0

0

0
4

0
4

0

0

0

0

0
4

0
4

0

0

0

0
4

0
4

0

0

0

0
4

0
4

0

0

0

0
4

0
4

0

0

0

0
4

0
4

0

0

0

0
4

0
4

0

0

0

0
4

0
4

0

0

0

0
4

0
4

0

0

0

0
4

0
4

0

0

0

0

0
4

0
4

0

0

0

0
4

0
4

0

0

0

0

0
4

0
4

0

0

0

0
4

0
4

0

0

0

0
4

0
4

0
4

477.675

0
1

0

0

0

0

0

0

0
4

0

0

0
4

0

0

0
4

0

0

0
4

0

0

0
4

0

0

0
4

0

0

0
4

0

0

0
4

0

0

0
4

0

0

0
4

0
4

72.2911

63.7701

0

0
2

0
3

0

0

0

0

0

0

1.28691

1.0188

0.734985

0

0

0

0

0

0

0

0.23189

0

0.0799885

16.9577
4

0

0

0

0

0

0

0

0

0

0

0.538989

0

0

0

0

0

0

0

0

0

0

12.8312

0

0

0

0

0

0

0

0

0

0

29.5827
4

0

0

0

0

0

0

0

0

0

0

0
3

0

0

0

0

0

0

0

0

0

0

0

0

0

0

0

0

0

0

0

0

0

0
3

0

0

0.506914

0

0

0

0

0

0

0
4

3.45184
3

3.33115
3

0.120694

0

0

0

2.77555756156289e-17
3

0
4

0

0

0
4

0

0

0
4

0

0

0
4

0

0

0
4

0

0

0
4

0

0

0
4

0

0

0
4

0

0

0
4

0

0

0
4

0

0

0
4

5.06914
3

5.06914
3

0
4

0

0

0

0
4

0

0

0

0
4

0

0

0
4

0

0

0

0
4

0

0

0
4

0

0

0
4

0

0

0
4

0
4

0.120694
1

0
1

0
1

0

0

0

0

0

0

0

0

0

0

0
1

0

0

0

0

0

0

0

0

0

0

0

0

0

0

0

0

0

0

0

0

0

0

0

0

0

0

0

0

0

0

0

0

0

0

0

0

0

0

0

0

0

0

0

0

0

0

0

0

0

0

0

0

0

0

0

0

0
4

0
2

0
2

0

0
4

0

0

0

0

0

0
4

0

0

0
4

0

0

0
4

0

0

0
4

0

0

0
4

0

0

0
4

0.120694

0.120694

0
4

0

0

0
4

0

0

0
4

0

0

0
4

0

0

0
4

0

0

0

0

0
4

0

0

0
4

0

0

0
4

0

0

0
4

0

0

0

0
4

0

0

0
4

0

0

0
4

0

0

0
4

0

0

0
4

0
4

41.2719

10.7902

0
2

1.04093

0

0

0

0.0507772

0.0507772

0

0

0

0.0761658

0
6

0

0

0.203109

0

0

0

0

0

0

0

1.72643
3

0

0

0

0

0

0

0

0

0

0.0507772

0
2

0.0761658

0

0

0

0.0761658

0.101554

0

0

0.126943

0

2.99586
3

0.0761658

0

0

0

0

0

0

0

0

0

0
6

0

0

0.710881

0

0

0

0

0
2

3.42746

0
2

0
4

30.4817

30.4817

0
7

0

0

0

0

0

0

0

0

0

0

0

0
7

0

0

0

0

0

0

0

0

0

0

0

0

0

0

0

0

0

0

0

0

0

0
7

0

0

0

0

0

0

0

0

0

0

0

0

0

0

0

0

0

0

0

0

0

0
7

0

0

0

0

0

0

0

0

0

0

0
6

0

0

0

0

0

0

0

0

0

0

0
7

0

0

0

0

0

0

0

0

0

0

0
7

0

0

0

0

0

0

0

0

0

0

0
7

0

0

0

0

0

0

0

0

0

0

0
7

0
7

0

0

0

0

0

0

0

0

0

0

0
7

0

0

0

0

0

0

0

0

0

0

0
7

0

0

0

0

0

0

0

0

0

0

0
7

0

0

0

0

0

0

0

0

0

0

0
6

0

0

0

0

0

0

0

0

0

0

0
2

0

0

0

0

0

0

0

0

0

0

0
7

0

0

0

0

0

0

0

0

0

0

0

0

0

0

0

0

0

0

0

0

0

0
7

0

0

0

0

0

0

0

0

0

0

0
6

0

0

0

0

0

0

0

0

0

0

0
7

0
6

0

0

0

0

0

0

0

0

0

0

0
7

0

0

0

0

0

0

0

0

0

0

0

0

0

0

0

0

0

0

0

0

0

0
7

0

0

0

0

0

0

0

0

0

0

0
7

0

0

0

0

0

0

0

0

0

0

0
8

0

0

0

0

0

0

0

0

0

0

0
1

0

0

0

0

0

0

0

0

0

0

0
7

0

0

0

0

0

0

0

0

0

0

0

0

0

0

0

0

0

0

0

0

0

0
7

0

0

0

0

0

0

0

0

0
7

0

0
7

0

0
7

0
7

0
6

0
8

0

0

0

0
7

0
7

0
7

0

0
6

0

0

0

0

0

0

0
7

0

0

0

0

0

0

0

0

0

0

0
7

0

0

0

0

0

0

0

0

0

0

0

0

0

0

0

0

0

0

0

0

0

0

0

0

0

0

0

0

0

0

0

0

0
4

0
7

0
7

0

0

0
4

0

0

0

0

0

0

0

0

0

0

0

0

0

0
6

0

0

0

0

0
4

0

0

0

0

0

0

0

0

0

0

0
2

0

0

0

0

0

0

0

0

0

0

0
3

0

0

0

0

0

0

0

0

0

0

0
6

0

0

0

0

0

0

0

0

0

0

0
1

0

0

0

0

0

0

0

0

0

0

0

0

0

0

0

0

0

0

0

0

0

0
3

0

0

0

0

0

0

0

0

0

0

0
4

0

0

0

0

0

0

0

0

0

0

0
4

0

0

0
7

0

0
4

0

0

0
4

0

0

0

0
4

0

0

0
4

0

0

0
4

0

0

0
4

0

0

0
4

0

0

0
4

0

0

0
4

0

0

0
4

0

0

0
4

0

0
1

0

0

0

0

0

0

0

0

0

0

0

0
4

0

0

0
4

0

0

0
4

0

0

0
4

0

0

0
4

0
7

0
7

0

0

0

0

0

0
4

0

0
1

0

0

0

0

0

0

0
4

0

0

0

0

0

0

0
4

0

0

0

0

0

0
4

0
1

0

0

0

0
4

0

0

0

0

0

0
4

0

0

0

0

0
4

3.5527136788005e-15

0
4

10.6309

7.77507

0

0

0

0

0

0

7.77507

0
4

0
1

0
2

0
1

0

0

0
4

0

0

0
4

0

0

0
4

0

0

0
4

0

0

0

0
4

0

0

0

0
4

0

0

0
4

0

0

0

0
4

0

0

0
4

0

0

0
4

0

0

0
4

0

0

0

0
4

0

0

0

0
4

0

0

0

0
4

0

0

0
4

0

0

0
4

0

0

0
4

0

0

0
4

0

0

0
4

0

0

0
4

0

0

0
4

0

0

0
4

0
2

0
2

0

0
4

0.097998

0.097998

0
4

0

0

0
4

0

0

0
4

0

0

0
4

2.59695

2.59695

0
4

0

0

0
4

0

0

0
4

0

0

0
4

0

0

0
4

0

0

0
4

0
2

0
2

0
4

0

0

0
4

0

0

0
4

0

0

0
4

0.160864

0.160864

0
4

0

0

0
4

0

0

0
4

0

0

0
4

0

0

0
4

0

0

0
4

0

0

0

0
4

0

0

0
4

0

0

0

0
4

0
4

9.06271
3

9.06271

9.06271

0

0

0

0
4

0

0

0

0

0
4

0

0

0
4

0

0

0

0
4

0

0

0

0

0
4

0

0

0
4

0

0

0

0
4

0

0

0
4

0

0

0
4

0

0

0
4

0

0

0
4

0
4

0.214485

0

0
8

0

0

0

0

0

0

0

0

0

0

0

0

0

0

0

0

0

0

0

0

0

0

0

0

0

0

0

0

0

0

0

0

0

0

0

0

0

0

0

0

0

0

0

0

0

0

0

0

0

0

0

0

0

0

0

0

0

0

0

0

0

0

0

0

0

0

0

0

0

0

0

0

0

0

0

0

0

0

0

0

0

0

0

0

0

0

0

0

0

0

0

0

0

0

0

0

0

0

0

0

0

0

0

0

0

0

0

0

0

0

0

0

0

0

0

0

0

0

0

0
4

0

0

0

0

0

0

0

0

0
4

0

0

0
4

0

0

0

0

0

0
4

0

0

0
4

0

0

0

0
4

0

0

0
4

0

0

0

0

0
4

0

0

0

0

0
4

0

0

0

0

0
4

0

0

0

0
4

0

0

0
4

0

0

0

0

0

0
4

0

0

0

0

0
4

0

0

0
4

0

0

0
4

0

0

0

0

0
4

0

0

0

0

0
4

0

0

0
4

0

0

0
4

0

0

0

0
4

0

0

0
4

0

0

0

0
4

0

0

0

0
4

0

0

0
4

0

0

0
4

0

0

0
4

0

0

0
4

0

0

0
4

0

0

0
4

0

0

0
4

0

0

0
4

0

0

0
4

0

0

0
4

0

0

0

0

0

0

0

0
4

0

0

0
4

0

0

0
4

0

0

0
4

0

0

0
4

0

0

0
4

0

0

0
4

0

0

0
4

0

0

0
4

0

0

0
4

0

0

0
4

0
7

0
7

0

0
4

0

0

0
4

0

0

0
4

0

0

0
4

0.214485

0.214485

0
4

0

0

0
4

0

0

0
4

0

0

0
4

0

0

0
4

0

0

0
4

0

0

0

0

0

0

0
4

0

0

0
4

0

0

0

0

0
4

0

0

0

0
4

0
4

1.14249

0

0

0

0

0

0

0

0

0

0

0

0

0

0

0

0

0

0

0

0

0

0

0

0

0

0

0

0

0

0

0

0

0
4

1.14249

1.14249

0

0

0

0

0
4

0

0

0
4

0

0

0

0
4

0

0

0
4

0

0

0
4

0

0

0
4

0

0

0
4

0
4

0.930981

0.930981

0

0.930981

0
4

0

0

0

0

0

0

0

0

0

0

0
4

0

0

0
4

0

0

0

0
4

0

0

0
4

0

0

0
4

0

0

0

0
4

0

0

0
4

0

0

0

0
4

0

0

0
4

0

0

0
4

0

0

0
4

0

0

0

0

0

0
4

0

0

0
4

0

0

0
4

0

0

0
4

0

0

0
4

0

0

0
4

0

0

0
4

0

0

0
4

0

0

0
4

0

0

0

0

0

0
4

0

0

0

0
4

0

0

0

0
4

0

0

0

0
4

0

0

0

0
4

0
2

0
2

0

0

0
4

0

0

0

0
4

0
4

90.5016

89.0806

89.0806

0

0

0

0

0

0

0

0

0
4

0

0

0

0
4

0

0

0

0
4

1.42097

1.42097

0
4

0

0

0
4

0

0

0
4

0
4

1.71588

0

0

0

0

0

0

0

0

0

0

0

0
4

1.71588

1.71588

0
4

0

0

0

0
4

0
4

31.969

0.0798828
7

0

0

0

0

0.0798828

0

0

0

0

0

0

0

0

0

0

0

0

0

0

0

0

0

0

0

0

0

0

0

0

0

0

0

0
4

0

0
7

0

0

0

0

0

0

0

0

0

0

0
7

0

0

0

0
7

0

0

0

0

0

0

0
4

9.43733

8.15042
7

0
7

0

0

0

0

0

0

0

0

0

0

0
7

0

0

0

0

0

0

0

0

0

0

0
6

0

0

0

0

0

0

0

0

0

0

0

0

0

0

0

0

0

0

0

0

0

0

0

0

0

0

0

0

0

0

0

0

0

0

0

0

0

0

0

0

0

1.28691

0

0

0

0

0

0

0
4

5.40947

5.25487

0
7

0

0.154593

0

0

0

0

0

0

0

0

0

0
7

0

0

0

0

0

0

0

0

0

0

0
7

0

0

0

0

0

0

0

0

0

0

0
7

0

0

0

0

0

0

0

0

0

0

0
6

0

0

0

0

0

0

0

0

0

0

0

0

0

0

0

0

0

0

0

0

0

0

0

0

0

0

0

0

0

0

0
7

0

0

0
7

0

0

0

0

0

0

0

0

0

0

0

0

0

0

0

0

0

0

0

0

0

0
5

0

0

0

0

0

0

0

0

0

0

0
7

0

0

0

0

0

0

0

0

0

0

0
7

0

0

0

0

0

0

0

0

0

0

0

0

0

0

0

0

0

0

0

0

0

0
7

0

0

0

0

0

0

0

0

0

0

0

0

0

0

0

0

0

0

0

0

0

0
4

0
2

0
2

0

0

0

0

0

0

0

0

0

0

0
2

0

0

0

0

0

0

0

0

0

0

0

0

0

0

0

0

0

0

0

0

0

0
2

0

0

0

0

0

0

0

0

0

0

0

0

0

0

0

0

0

0

0

0

0

0

0

0

0

0

0

0

0

0

0

0

0

0

0
4

0

0

0
4

0
7

0
7

0

0

0
4

0

0

0

0

0

0

0

0

0

0

0

0

0

0

0

0

0

0

0
4

4.71866

4.07521

0

0

0

0

0

0

0

0

0

0

0

0

0

0

0

0.107242

0.536212

0

0

0

4.44089209850063e-16

0
4

1.0188
3

0

1.0188

0

0

0
4

0
6

0
6

0

0
4

0.107242

0.107242

0

0

0

0
4

0

0

0

0

0

0

0
4

0

0

0

0
4

0

0

0

0
4

0

0

0

0

0
4

0

0

0

0

0
4

0

0

0

0

0
4

0

0

0
4

10.7242

0

0

0

0

0

0

0
7

10.7242

0

0

0

0

0

0

0
4

0

0

0
4

0

0

0

0
4

0

0

0
4

0

0

0
4

0

0

0

0

0
4

0

0

0

0

0
4

0

0

0

0
4

0

0

0

0
4

0

0

0
4

0

0

0

0
4

0
7

0
7

0
7

0

0

0

0

0

0
4

0

0

0

0
4

0

0

0

0
4

0

0

0

0
4

0

0

0
4

0

0

0

0
4

0

0

0

0
4

0

0

0
4

0

0

0
4

0.097998

0.097998

0
4

0

0

0
4

0
7

0
7

0

0
4

0

0

0
4

0

0

0
4

0

0

0
4

0

0

0
4

0

0

0
4

0

0

0
4

0

0

0
4

0

0

0
4

0

0

0
4

0

0

0
4

0
7

0

0

0

0

0
4

0

0

0
4

0

0

0
4

0

0

0
4

0

0

0
4

0

0

0
4

0

0

0
4

0

0

0
4

0

0

0
4

0

0

0
4

0

0

0
4

0
7

0
7

0
4

0

0

0
4

0

0

0
4

0

0

0
4

0

0

0
4

0

0

0
4

0

0

0
4

0

0

0
4

0
7

0

0

0

0
4

0.375348
5

0.375348

0

0

0

0
4

0
7

0
7

0

0

0
4

0
4

6.54593

6.54593

6.54593

0

0

0

0
4

0

0

0

0

0

0

0

0
4

0

0

0
4

0

0

0
4

0

0

0
4

0

0

0
4

0

0

0
4

0

0

0
4

0

0

0

0

0

0

0

0

0
4

0

0

0

0

0

0

0

0
4

0

0

0

0

0

0
4

0

0

0

0
4

0

0

0

0
4

0

0

0

0
4

0

0

0

0
4

0

0

0
4

0
4

41.1215
7

5.73746
7

2.14485
7

0
7

0
7

0

0

0

0

0

0

0

0

0

0

0
7

0

0

0

0

0

0

0

0

0

0

0
7

0

0

0

0

0

0

0

0

0

0

0
7

0

0

0

0

0

0

0

0

0

0

0
7

0

0

0

0

0

0

0

0

0

0

0

0

0

0

0

0

0

0

0

0

0

0
7

0

0

0

0

0

0

0

0

0

0

0
7

0

0

0

0

0

0

0

0

0

0

0
7

0

0

0

0

0

0

0

0

0

0

0

0

0

0

0

0

0

0

0

0

0

0
7

0
8

0

0

0

0

0

0

0

0

0

0

0
7

0

0

0

0

0

0

0

0

0

0

0
8

0

0

0

0

0

0

0

0

0

0

0
7

0

0

0

0

0

0

0

0

0

0

0
7

0

0

0

0

0

0

0

0

0

0

0
7

0

0

0

0

0

0

0

0

0

0

0
7

0

0

0

0

0

0

0

0

0

0

0
7

0

0

0

0

0

0

0

0

0

0

0
7

0

0

0

0

0

0

0

0

0

0

0
7

0

0

0

0

0

0

0

0

0

0

0
7

0
7

0

0

0

0

0

0

0

0

0

0

0
7

0

0

0

0

0

0

0

0

0

0

0
7

0

0

0

0

0

0

0

0

0

0

0
7

0

0

0

0

0

0

0

0

0

0

0
7

0

0

0

0

0

0

0

0

0

0

0
6

0

0

0

0

0

0

0

0

0

0

0
7

0

0

0

0

0

0

0

0

0

0

0
7

0

0

0

0

0

0

0

0

0

0

0
7

0

0

0

0

0

0

0

0

0

0

0
7

0

0

0

0

0

0

0

0

0

0

0
7

0
7

0

0

0

0

0

0

0

0

0

0

0
7

0

0

0

0

0

0

0

0

0

0

0
7

0

0

0

0

0

0

0

0

0

0

0
7

0

0

0

0

0

0

0

0

0

0

0
6

0

0

0

0

0

0

0

0

0

0

0
7

0

0

0

0

0

0

0

0

0

0

0

0

0

0

0

0

0

0

0

0

0

0
7

0

0

0

0

0

0

0

0

0

0

0
7

0

0

0

0

0

0

0

0

0

0

0
7

0

0

0

0

0

0

0

0

0

0

0
7

0
7

0

0

0

0

0

0

0

0

0

0

0
7

0

0

0

0

0

0

0

0

0

0

0
8

0

0

0

0

0

0

0

0

0

0

0
7

0

0

0

0

0

0

0

0

0

0

0
6

0

0

0

0

0

0

0

0

0

0

0
6

0

0

0

0

0

0

0

0

0

0

0

0

0

0

0

0

0

0

0

0

0

0
7

0

0

0

0

0

0

0

0

0

0

0
8

0

0

0

0

0

0

0

0

0

0

0

0

0

0

0

0

0

0

0

0

0

0
7

0
7

0

0

0

0

0

0

0

0

0

0

0
7

0

0

0

0

0

0

0

0

0

0

0
7

0

0

0

0

0

0

0

0

0

0

0
7

0

0

0

0

0

0

0

0

0

0

0
7

0

0

0

0

0

0

0

0

0

0

0
7

0

0

0

0

0

0

0

0

0

0

0
7

0

0

0

0

0

0

0

0

0

0

0
7

0

0

0

0

0

0

0

0

0

0

0
6

0

0

0

0

0

0

0

0

0

0

0
7

0

0

0

0

0

0

0

0

0

0

0
7

0
7

0

0

0

0

0

0

0

0

0

0

0
7

0

0

0

0

0

0

0

0

0

0

0
6

0

0

0

0

0

0

0

0

0

0

0
6

0

0

0

0

0

0

0

0

0

0

0

0

0

0

0

0

0

0

0

0

0

0
7

0

0

0

0

0

0

0

0

0

0

0

0

0

0

0

0

0

0

0

0

0

0
7

0

0

0

0

0

0

0

0

0

0

0
7

0

0

0

0

0

0

0

0

0

0

0
7

0

0

0

0

0

0

0

0

0

0

0
7

0
6

0

0

0

0

0

0

0

0

0

0

0
8

0

0

0

0

0

0

0

0

0

0

0
7

0

0

0

0

0

0

0

0

0

0

0
7

0

0

0

0

0

0

0

0

0

0

0
6

0

0

0

0

0

0

0

0

0

0

0
7

0

0

0

0

0

0

0

0

0

0

0
7

0

0

0

0

0

0

0

0

0

0

0
7

0

0

0

0

0

0

0

0

0

0

0
7

0

0

0

0

0

0

0

0

0

0

0
8

0

0

0

0

0

0

0

0

0

0

0
7

0
7

0

0

0

0

0

0

0

0

0

0

0
7

0

0

0

0

0

0

0

0

0

0

0
8

0

0

0

0

0

0

0

0

0

0

0
7

0

0

0

0

0

0

0

0

0

0

0
7

0

0

0

0

0

0

0

0.428969

0

0

0
7

0

0

0

0

0

0

0

0

0

0

0
6

0

0

0

0

0

0

0

0

0

0

0
7

0

0

0

0

0

0

0

0

0

0

0
7

0

0

0

0

0

0

0

0

0

0

0

0

0

0

0

0

0

0

0

0

0

0
7

0
6

0

0

0

0

0

0

0

0

0

0

0
7

0

0

0

0

0

0

0

0

0

0

0
6

0

0

0

0

0

0

0

0

0

0

0
7

0

0

0

0

0

0

0

0

0

0

0
7

0

0

0

0

0

0

0

0

0

0

0
7

0

0

0

0

0

0

0

0

0

0

0

0

0

0

0

0

0

0

0

0

0

0
7

0

0

0

0

0

0

0

0

0

0

0
7

0

0

0

0

0

0

0

0

0

0

0
7

0

0

0

0

0

0

0

0

0

0

0
7

0
7

0
7

0

0

0

0

0

0

0

0

0

0

0
6

0

0

0

0

0

0

0

0

0

0

0

0

0

0

0

0

0

0

0

0

0

0
7

0

0

0

0

0

0

0

0

0

0

0
7

0

0

0

0

0

0

0

0

0

0

0
7

0

0

0

0

0

0

0

0

0

0

0
7

0

0

0

0

0

0

0

0

0

0

0
8

0

0

0

0

0

0

0

0

0

0

0
7

0

0

0

0

0

0

0

0

0

0

0
7

0

0

0

0

0

0

0

0

0

0

0
7

0
7

0

0

0

0

0

0

0

0

0

0

0
7

0

0

0

0

0

0

0

0

0

0

0
7

0

0

0

0

0

0

0

0

0

0

0
7

0

0

0

0

0

0

0

0

0

0

0
7

0

0

0

0

0

0

0

0

0

0

0
6

0

0

0

0

0

0

0

0

0

0

0
6

0

0

0

0

0

0

0

0

0

0

0
6

0

0

0

0

0

0

0

0

0

0

0

0

0

0

0

0

0

0

0

0

0

0
7

0

0

0

0

0

0

0

0

0

0

0.214485
7

0
7

0

0

0

0

0

0

0

0

0

0

0
7

0

0

0

0

0

0

0

0

0

0

0
6

0

0

0

0

0

0

0

0

0

0

0
7

0

0

0

0

0

0

0

0

0

0

0
7

0

0

0

0

0

0

0

0

0

0

0
7

0

0

0

0

0

0

0

0

0

0

0
7

0

0

0

0

0

0

0

0

0

0

0
7

0

0

0

0

0

0

0

0

0

0

0
7

0

0

0

0

0

0

0

0

0

0

0
8

0

0

0

0

0

0

0

0

0

0

0
7

0
6

0

0

0

0

0

0

0

0

0

0

0

0

0

0

0

0

0

0

0

0

0

0
8

0

0

0

0

0

0

0

0

0

0

0
7

0

0

0

0

0

0

0

0

0

0

0
8

0

0

0

0

0

0

0

0

0

0

0

0

0

0

0

0

0

0

0

0

0

0
6

0

0

0

0

0

0

0

0

0

0

0
7

0

0

0

0

0

0

0

0

0

0

0
7

0

0

0

0

0

0

0

0

0

0

0
6

0

0

0

0

0

0

0

0

0

0

0
7

0

0

0

0

0

0

0

0

0

0

0

0
7

0

0

0

0

0

0

0

0

0

0

0
6

0

0

0

0

0

0

0

0

0

0

0
7

0

0

0

0

0

0

0

0

0

0

0
7

0

0

0

0

0

0

0

0

0

0

0

0

0

0

0

0

0

0

0

0

0

0
7

0

0

0

0

0

0

0

0

0

0

0
7

0

0

0

0

0

0

0

0

0

0

0
8

0

0

0

0

0

0

0

0

0

0

0
7

0

0

0

0

0

0

0

0

0

0

0
7

0

0

0

0

0

0

0

0

0

0

0

0
7

0

0

0

0

0

0

0

0

0

0

0
7

0

0

0

0

0

0

0

0

0

0

0

0

0

0

0

0

0

0

0

0

0

0
7

0

0

0

0

0

0

0

0

0

0

0

0

0

0

0

0

0

0

0

0

0

0
6

0

0

0

0

0

0

0

0

0

0

0
7

0

0

0

0

0

0

0

0

0

0

0

0

0

0

0

0

0

0

0

0

0

0
6

0

0

0

0

0

0

0

0

0

0

0
7

0

0

0

0

0

0

0

0

0

0

0

0
7

0

0

0

0

0

0

0

0

0

0

0
8

0

0

0

0

0

0

0

0

0

0

0
7

0

0

0

0

0

0

0

0

0

0

0
7

0

0

0

0

0

0

0

0

0

0

0
6

0

0

0

0

0

0

0

0

0

0

0
7

0

0

0

0

0

0

0

0

0

0

0
7

0

0

0

0

0

0

0

0

0

0

0

0

0

0

0

0

0

0

0

0

0

0

0

0

0

0

0

0

0

0

0

0

0

0
7

0

0

0

0

0

0

0

0

0

0

0
7

0

0

0

0

0

0

0

0

0

0

0
7

0

0

0

0

0

0

0

0

0

0

0
6

0

0

0

0

0

0

0

0

0

0

0

0

0

0

0

0

0

0

0

0

0

0
7

0

0

0

0

0

0

0

0

0

0

0

0

0

0

0

0

0

0

0

0

0

0

0

0

0

0

0

0

0

0

0

0

0

0

0

0

0

0

0

0

0

0

0

0

0

0

0

0

0

0

0

0

0

0

0
7

0

0
7

0

0
7

0
7

0
7

0
7

0
8

0

0
7

0
7

0
7

0
6

0
7

0

0
7

0
7

0
7

0
6

0
6

0
8

0
7

0
6

0

0

0

0
6

0
7

0

0

0

0
8

0

0
7

0

0
7

0

0

0
7

0

0

0
8

0
7

0
7

0
7

0
6

0
6

0

0

0
6

0
7

0
7

0

0

0
8

0
7

0
7

0
6

0
7

0
7

0
7

0

0

0

0

0

0
7

0
8

0
7

0
6

0

0

0

0

0

0

0
7

0
7

0

0

0
6

0

0

0

0

0

0

0

0
7

0
6

0

0
7

0
7

0

0

0

0

0

0

0
5

0

0

0

0

0

0

0

0

0

0

0
7

0

0

0

0

0

0

0

0

0

0

2.94916
7

0

0

0

0

0

0

0

0

0

0

0
7

0
7

0

0

0

0

0

0

0

0

0

0

0
7

0

0

0

0

0

0

0

0

0

0

0
7

0

0

0

0

0

0

0

0

0

0

0
7

0

0

0

0

0

0

0

0

0

0

0
7

0

0

0

0

0

0

0

0

0

0

0
7

0

0

0

0

0

0

0

0

0

0

0
6

0

0

0

0

0

0

0

0

0

0

0
7

0

0

0

0

0

0

0

0

0

0

0
7

0

0

0

0

0

0

0

0

0

0

0
7

0

0

0

0

0

0

0

0

0

0

0
7

0
7

0

0

0

0

0

0

0

0

0

0

0
7

0

0

0

0

0

0

0

0

0

0

0
7

0

0

0

0

0

0

0

0

0

0

0
7

0

0

0

0

0

0

0

0

0

0

0
7

0

0

0

0

0

0

0

0

0

0

0
7

0

0

0

0

0

0

0

0

0

0

0
7

0

0

0

0

0

0

0

0

0

0

0
7

0

0

0

0

0

0

0

0

0

0

0
7

0

0

0

0

0

0

0

0

0

0

0
7

0

0

0

0

0

0

0

0

0

0

0
7

0
7

0

0

0

0

0

0

0

0

0

0

0
7

0

0

0

0

0

0

0

0

0

0

0
7

0

0

0

0

0

0

0

0

0

0

0
7

0

0

0

0

0

0

0

0

0

0

0
7

0

0

0

0

0

0

0

0

0

0

0
7

0

0

0

0

0

0

0

0

0

0

0

0

0

0

0

0

0

0

0

0

0

0
7

0

0

0

0

0

0

0

0

0

0

0
8

0

0

0

0

0

0

0

0

0

0

0

0

0

0

0

0

0

0

0

0

0

0
7

0

0

0

0

0

0

0

0

0

0

0

0

0

0

0

0

0

0

0

0

0

0

0
7

0

0

0

0

0

0

0

0

0

0

0
7

0

0

0

0

0

0

0

0

0

0

0
7

0

0

0

0

0

0

0

0

0

0

0
7

0

0

0

0

0

0

0

0

0

0

0
7

0

0

0

0

0

0

0

0

0

0

0
7

0

0

0

0

0

0

0

0

0

0

0
7

0

0

0

0

0

0

0

0

0

0

0

0

0

0

0

0

0

0

0

0

0

0
7

0
7

0

0

0

0

0

0

0

0

0

0

0
7

0

0

0

0

0

0

0

0

0

0

0
7

0

0

0

0

0

0

0

0

0

0

0
7

0

0

0

0

0

0

0

0

0

0

0
6

0

0

0

0

0

0

0

0

0

0

0
8

0

0

0

0

0

0

0

0

0

0

0
7

0

0

0

0

0

0

0

0

0

0

0
7

0

0

0

0

0

0

0

0

0

0

0
7

0

0

0

0

0

0

0

0

0

0

0
7

0

0

0

0

0

0

0

0

0

0

0
7

0

0

0

0

0

0

0

0

0

0

0

0
7

0

0

0

0

0

0

0

0

0

0

0
8

0

0

0

0

0

0

0

0

0

0

0
7

0

0

0

0

0

0

0

0

0

0

0
7

0

0

0

0

0

0

0

0

0

0

0
7

0

0

0

0

0

0

0

0

0

0

0
6

0

0

0

0

0

0

0

0

0

0

0
6

0

0

0

0

0

0

0

0

0

0

0
7

0

0

0

0

0

0

0

0

0

0

0
7

0

0

0

0

0

0

0

0

0

0

0
4

0
6

0
6

0
4

23.6389

0.453808
7

8.06896
5

0

0

0

0

0

0

0

0.0798828

0

0

5.57556
5

0

0

0

0

0

0

0

0

0

0

0
6

0

0

0

0

0

0

0

0

0

0

2.54361

0

0

0

0

0

0

0

0

0

0

3.57721
5

0

0

0

0

0

0

0

0

0

0

0.2981

0

0

0

0

0

0

0

0

0
6

3.04181

0
6

0
4

0

0

0

0

0

0

0
4

0
7

0

0

0

0

0

0

0

0

0

0

0

0

0

0

0

0

0

0

0

0

0

0

0

0

0

0

0
4

0
7

0
7

0
7

0
7

0
6

0
7

0

0

0

0

0

0

0

0

0

0

0

0
4

0
7

0
7

0
7

0

0

0

0

0
4

5.1449
7

2.00896

0
7

0

0

0

0

0

0

0

0

0

0

0
6

0

0

0

0

0

0

0

0

0

0

0

0

0

0

0

0

0

0

0

0

0

0
7

0

0

0

0

0

0

0

0

0

0
7

0

0

0
6

0

0
7

0
6

0

0

0

0

0

0

0

0

0

0.195996

0
6

0

0

0

0

0

0

0

2.59695

0

0

0
6

0

0

0

0

0

0

0

0

0

0

0.195996

0

0

0

0

0

0

0

0

0

0

0
7

0

0

0

0

0

0

0

0

0

0

0
7

0

0

0

0

0

0

0

0

0

0

0
7

0

0

0

0

0

0

0

0

0

0

0

0

0

0

0

0.146997

0

0

0

0

0

0
4

0
7

0
7

0
7

0
7

0
7

0

0

0

0

0

0
4

0
7

0
6

0

0
4

0
7

0

0

0
4

0
7

0

0

0

0

0
4

0
7

0

0

0

0
4

0

0

0

0
4

0

0

0

0

0

0

0
4

0.57933

0.57933

0

0

0
4

0

0

0

0

0
4

0

0

0
4

0

0

0
4

5.70132

4.39584

0

0

1.19824

0.107242

0

0

0

0

5.96744875736022e-16

0
4

0

0

0

0
4

0

0

0
4

0

0

0

0
4

0

0

0
4

0

0

0
4

0

0

0
4

0

0

0
4

0

0

0
4

0

0

0

0
4

0

0

0
4

0
3

0
3

0

0

0

0

0

0

0

0

0
4

0

0

0

0
4

0

0

0

0
4

0

0

0

0
4

0

0

0
4

0

0

0
4

0

0

0
4

0

0

0
4

0

0

0
4

0

0

0
4

0

0

0
4

0
7

0
7

0

0

0

0

0

0

0
4

0

0

0
4

0

0

0
4

0

0

0
4

0

0

0
4

0

0

0
4

0.319531

0.319531

0
4

0

0

0
4

0

0

0
4

0

0

0
4

0

0

0
4

0
7

0
7

0

0

0

0

0
4

0

0

0
4

0

0

0
4

0

0

0
4

0

0

0
4

0

0

0
4

0

0

0
4

0

0

0
4

0

0

0
4

0

0

0
4

0

0

0
4

0
7

0
7

0

0

0

0
4

0

0

0
4

0
7

0
7

0
7

0

0
4

0
7

0
7

0

0
4

0
3

0
3

0

0
4

2.38697950294409e-15
7

0
4

3.13594

3.13594
2

3.13594
2

0

0

0

0

0

0

0

0

0

0

0

0
4

0

0

0

0

0

0
4

0
2

0
2

0
4

0

0

0

0
4

0

0

0
4

0

0

0
4

0
4

1.59442

0

0
3

0

0

0

0

0

0

0

0

0

0

0
2

0

0

0

0

0

0

0

0

0

0

0
2

0

0

0

0

0

0

0

0

0

0

0
2

0

0

0

0

0

0

0
2

0
4

0.0761658

0.0761658

0

0
4

0

0

0

0
4

0

0

0

0
4

0

0

0

0
4

0

0

0
4

0

0

0
4

0.17772
3

0
3

0

0

0

0

0

0

0

0

0

0

0

0

0

0

0

0.0761658

0

0

0

0

0

0

0

0

0.101554

0
4

1.23329

1.23329

0

0

0

0
2

0
3

0

0

0

0

0

0

0
4

0.107242

0.107242
3

0

0

0

0

0

0

0

0

0

0

0
4

0

0

0

0

0
4

0
2

0

0

0

0

0

0
4

0
2

0

0

0

0

0

0
4

0

0

0

0
4

0

0

0
4

0
4

0.583938
2

0.583938
2

0.583938

0

0

0

0

0

0

0

0

0

0
4

0

0

0

0

0

0

0

0
4

0

0

0

0

0

0

0

0

0

0

0
4

0

0

0

0
4

0

0

0

0

0
4

0

0

0

0
4

0
4

0

0

0

0
4

0

0

0
4

0
4

0

0

0

0

0
4

0
4

0

0

0

0
4

0

0

0
4

0
4

0

0

0

0
4

0
4

0

0

0

0
4

0

0

0
4

0
4

0

0

0

0
4

0

0

0
4

0
4

0

0

0

0
4

0
4

0

0

0

0
4

0
4

0

0

0

0
4

0
4

0

0

0

0
4

0
4

0.0507772

0

0

0

0

0

0

0

0

0
4

0
3

0
2

0

0

0

0

0

0

0

0

0

0

0
4

0

0

0

0

0

0

0

0
4

0

0

0

0

0

0
4

0

0

0
4

0

0

0
4

0

0

0
4

0.0507772

0.0507772

0
4

0

0

0
4

0
4

0

0

0

0

0
4

0
4

0

0

0

0
4

0
4

0

0

0

0
4

0

0

0
4

0
4

0

0

0

0
4

0
4

0

0

0

0

0
4

0
4

0

0

0

0
4

0
4

0

0

0

0
4

0
4

0

0

0

0
4

0

0

0
4

0
4

0

0

0

0
4

0

0

0
4

0
4

0

0

0

0

0
4

0
4

0

0

0

0

0

0

0

0

0

0

0
4

0
7

0

0

0

0

0

0

0

0
4

0

0

0

0

0

0

0

0

0
4

0

0

0

0

0

0
4

0

0

0

0
4

0

0

0
4

0
4

0

0

0

0
4

0
4

0

0

0

0
4

0
4

0

0

0

0
4

0
4

0

0

0

0
4

0
4

0

0

0

0
4

0
4

0

0

0

0
4

0
4

0

0

0

0
4

0
4

0

0

0

0
4

0
4

0

0

0

0
4

0
4

0

0

0

0
4

0
4

0
2

0
1

0

0

0

0

0

0

0

0
4

0

0

0

0

0

0

0

0

0

0

0

0
4

0

0

0

0

0

0

0
4

0

0

0

0

0

0
4

0

0

0

0
4

0

0

0

0
4

0

0

0
4

0
4

0

0

0

0
4

0
4

0

0

0

0
4

0
4

0

0

0

0
4

0
4

0

0

0

0
4

0
4

0

0

0

0
4

0
4

0

0

0

0
4

0
4

0

0

0

0
4

0
4

0

0

0

0
4

0
4

0

0

0

0
4

0
4

0

0

0

0
4

0
4

3.27027

3.27027

1.82312
3

1.44715

0

0

0
4

0

0

0

0

0

0

0

0
4

0

0

0

0
4

0

0

0

0
4

0

0

0
4

0

0

0
4

0
4

0

0

0

0
4

0
4

0

0

0

0
4

0
4

0

0

0

0
4

0
4

0

0

0

0
4

0
4

0

0

0

0
4

0
4

0

0

0

0
4

0
4

0

0

0

0
4

0
4

0

0

0

0
4

0
4

0

0

0

0
4

0
4

0

0

0

0
4

0
4

0
7

0
7

0
7

0

0

0

0

0

0

0
4

0
7

0
7

0
4

0

0

0

0
4

0
4

0

0

0

0
4

0
4

0

0

0

0
4

0
4

0

0

0

0
4

0
4

0

0

0

0
4

0
4

0

0

0

0
4

0
4

0

0

0

0
4

0
4

0

0

0

0
4

0
4

0

0

0

0
4

0
4

0

0

0

0
4

0
4

0

0

0

0
4

0
4

41.1462
3

41.1462
3

40.9641
3

0

0.0533257

0.12874

0

0

0

0
4

0
4

0

0

0

0
4

0
4

0

0

0

0
4

0
4

0

0

0

0
4

0
4

0

0

0

0
4

0
4

0

0

0

0
4

0
4

0

0

0

0
4

0
4

0

0

0

0
4

0
4

0

0

0

0
4

0
4

0

0

0

0
4

0
4

0

0

0

0
4

0
4

0

0
6

0

0

0

0

0
4

0

0

0
4

0

0

0
4

0

0

0
4

0
7

0

0

0
4

0

0

0

0

0

0

0
4

0

0

0

0

0
4

0

0

0

0
4

0

0

0

0
4

0

0

0
4

0

0

0

0
4

0

0

0
4

0
4

0

0

0

0
4

0
4

0

0

0

0
4

0
4

0

0

0

0
4

0
4

0

0

0

0
4

0
4

0

0

0

0
4

0
4

0

0

0

0
4

0
4

0

0

0

0
4

0
4

0

0

0

0
4

0
4

0

0

0

0
4

0
4

0

0

0

0
4

0
4

0
7

0
7

0
7

0

0

0

0

0

0
4

0
7

0
6

0

0

0
4

0

0

0

0
4

0

0

0

0
4

0

0

0
4

0
4

0

0

0

0
4

0
4

0

0

0

0
4

0
4

0

0

0

0
4

0
4

0

0

0

0
4

0
4

0

0

0

0
4

0
4

0

0

0

0

0

0

0

0

0

0

0
4

0

0

0
4

0

0

0
4

0
4

0

0

0

0

0

0

0

0

0

0

0

0

0

0

0

0

0

0

0

0

0

0

0

0

0

0

0

0

0

0

0

0

0

0

0

0

0

0

0

0

0

0

0

0

0

0

0

0

0

0

0

0

0

0

0

0

0

0

0

0

0

0

0
4

0

0

0

0

0

0

0

0
4

0

0

0

0

0

0

0

0

0

0

0

0

0
4

0

0

0

0

0

0
4

0

0

0
4

0
4

1.54488

1.54488

0.917273

0.627608

0

0

0

0

0

0
4

0

0

0
4

0
4

3.86146

3.86146

3.59336

0.268106

0
4

0
4

1.78968
4

0
4

0

0

0

0

0

0
4

1.63233
4

1.41247

0.219855

0

8.32667268468867e-17
4

0
4

0.157353

0.0488567

0.108496

0

0

0
4

1.94289029309402e-16
4

0
4

0

0

0

0

0

0

0

0

0
4

0
4

0

0

0

0

0

0

0
4

0

0

0

0

0
4

0

0

0

0

0
4

0

0

0
4

0
4

0

0

0

0

0

0

0
4

0

0

0
4

0
4

0
3

0
3

0
3

0

0

0

0

0
4

0

0

0

0
4

0

0

0
4

0
4

0
8

0
8

0

0

0

0

0

0
4

0

0

0
4

0
4

0.627608
3

0.627608
3

0.627608
3

0
4

0
4

3.57979
3

0
2

0
2

0

0
4

3.57979
3

0

0

3.57979

0
4

0

0

0
4

0
4

0
1

0
1

0

0

0

0

0

0

0

0

0

0

0

0
2

0

0

0

0

0

0

0

0

0
4

0

0

0
4

0

0

0
4

0
1

0
2

0

0

0

0

0

0

0

0

0

0

0
1

0

0

0

0

0

0

0

0

0

0

0
4

0
1

0

0

0

0

0

0

0

0

0

0

0
4

0

0

0

0

0
4

0

0

0

0
4

0

0

0

0
4

0

0

0

0
4

0

0

0

0
4

0

0

0

0
4

0
4

0.309187
3

0
3

0
3

0

0

0

0

0
4

0.309187

0

0.309187

0

0

0
4

0
4

0.0507772

0

0

0

0

0

0
4

0

0

0

0

0

0

0
4

0

0

0
4

0

0

0

0
4

0.0507772

0

0.0507772

0
4

0

0

0
4

0
4

0

0

0

0

0

0

0
4

0

0

0

0

0

0

0
4

0
4

0

0

0

0

0

0

0
4

0
4

0
2

0

0

0

0

0

0

0
4

0

0

0
4

0

0

0
4

0
4

0.609327

0.609327

0.609327

0

0
4

0

0

0

0
4

0

0

0

0
4

0
4

0

0

0
2

0

0

0
4

0

0

0
4

0

0

0
4

0
4

0
7

0

0

0

0
4

0

0

0

0

0
4

0

0

0

0
4

0

0

0
4

0
4

0
2

0

0

0

0

0
4

0
3

0

0

0

0

0
4

0

0

0
4

0
4

0

0

0

0

0

0

0
4

0

0

0

0
4

0
4

0

0
7

0
7

0

0

0

0

0

0

0

0

0

0

0

0
4

0

0

0
4

0

0

0

0
4

0

0

0

0
4

0

0

0
4

0

0

0

0

0

0

0

0

0

0
4

0
1

0

0

0

0

0

0

0

0

0
4

0
6

0

0

0

0

0

0

0
4

0
6

0
6

0

0
4

0

0

0

0

0

0

0
4

0
7

0

0

0

0

0
4

0

0

0

0

0

0

0
4

0

0

0

0

0

0
4

0
4

0
8

0
8

0
8

0
4

0

0

0
4

0

0

0
4

0
4

0.0507772
3

0

0

0

0
4

0

0

0
4

0

0

0
4

0

0

0
4

0.0507772

0.0507772

0
4

0
4

0
7

0
7

0
7

0

0
4

0
4

0
7

0
7

0

0

0

0
4

0

0

0
4

0
4

0

0

0

0

0

0

0

0
4

0

0

0
4

0

0

0
4

0
4

0
2

0
2

0

0

0

0

0
4

0

0

0
4

0

0

0
4

0
4

1.85735
4

1.54817

0.213303

0.700341

0.154593

0.479931

0

0
4

0.309187

0.309187

0
4

1.11022302462516e-16
4

0
4

0

0

0

0

0

0

0
4

0
4

0
7

0
7

0
7

0

0

0
4

0
4

14.3516
4

13.9522
4

13.9522

0

0
4

0.399414

0.399414

0
4

0

0

0
4

0

0

0
4

5.55111512312578e-16
4

0
4

0
7

0
7

0
7

0
6

0
8

0

0

0

0

0

0

0
4

0
7

0
7

0

0

0
4

0
7

0
7

0

0

0

0

0
4

0
7

0
8

0

0

0

0

0

0

0
4

0

0

0

0

0
4

0

0

0

0

0
4

0

0

0

0
4

0

0

0
4

0
4

1.64144

0

0

0

0

0

0
4

1.64144

0

0

1.64144

0
4

0

0

0
4

0
4

0

0

0

0

0
4

0
4

0.533257

0.533257

0.533257

0
4

0
4

0

0

0

0

0

0

0
4

0
4

0
3

0
3

0

0

0

0
4

0

0

0
4

0
4

0

0

0

0

0
4

0

0

0
4

0

0

0
4

0
4

0

0

0

0

0

0

0
4

0
4

0

0

0

0

0
4

0

0

0

0
4

0
4

4.40044

4.40044

4.40044

0

0
4

0

0

0

0
4

0
4

0

0

0

0

0

0
4

0

0

0
4

0
4

38.8201
3

38.8201
3

29.1577
3

0.0798828

0

0

0

1.96739
3

3.7328

0

3.8823

0

0

0

0

0
4

0

0

0

0
4

0

0

0
4

0
4

0

0

0

0

0

0

0

0
4

0

0

0
4

0
4

0.29314

0.29314

0.219855

0

0.0732851

1.38777878078145e-17

0
4

0
4

0

0

0

0

0
4

0

0

0

0
4

0

0

0
4

0
4

0

0

0

0

0
4

0

0

0
4

0

0

0

0
4

0
4

0

0

0

0

0

0

0
4

0
4

0
8

0

0

0

0
4

0

0

0
4

0

0

0
4

0
4

0.751954

0.243329

0.243329

0
4

0.43534

0.43534

0
4

0.0732851

0.0732851

0
4

0
4

0

0

0

0

0

0
4

0

0

0
4

0
4

0

0

0

0

0
4

0
4

0

0

0

0

0
4

0

0

0

0
4

0

0

0
4

0
4

0.391992

0.391992

0

0

0

0

0

0

0

0

0

0.391992

0

0

0

0
4

0

0

0

0

0

0

0

0

0

0

0

0
4

0

0

0
4

0

0

0
4

0

0

0
4

0

0

0
4

0

0

0
4

0

0

0
4

0
4

0

0

0

0
4

0

0

0
4

0
4

0.17772

0.17772

0.17772

0
4

0

0

0
4

0
4

0

0

0

0

0
4

0
4

0

0

0

0
4

0

0

0

0
4

0

0

0
4

0
4

0

0

0

0
4

0

0

0

0
4

0
4

0
7

0

0

0
4

0

0

0

0
4

0
4

1.5117

1.5117

1.5117

0
4

0
4

0

0

0

0
4

0

0

0
4

0
4

0

0

0

0

0
4

0
4

0

0

0

0
4

0
4

0.097998

0.097998

0

0

0

0.097998

0

0

0

0

0

0

0

0
4

0

0

0
4

0

0

0
4

0
7

0
7

0

0

0

0
4

0
7

0

0

0

0

0

0
4

0

0

0

0

0

0
4

0

0

0

0
4

0

0

0
4

0

0

0
4

0

0

0
4

0

0

0
4

0
4

0

0

0

0
4

0
4

0

0

0

0

0

0
4

0
4

0

0

0

0
4

0
4

0

0

0

0

0
4

0
4

0

0

0

0

0
4

0

0

0
4

0
4

0

0

0

0

0
4

0

0

0
4

0
4

0

0

0

0

0
4

0

0

0
4

0
4

0

0

0

0

0
4

0
4

0

0

0

0

0

0
4

0
4

0

0

0

0
4

0
4

0

0

0

0

0

0

0

0

0

0

0

0

0

0

0

0

0

0

0

0

0

0

0

0

0

0

0

0

0

0
4

0

0

0

0
4

0
4

0

0

0

0
4

0
4

0

0

0

0

0

0
4

0
4

0

0

0

0

0
4

0

0

0
4

0
4

0

0

0

0

0
4

0

0

0
4

0
4

0

0

0

0

0

0
4

0
4

0

0

0

0

0
4

0
4

0

0

0

0

0
4

0
4

0

0

0

0
4

0

0

0
4

0
4

0

0

0

0
4

0

0

0
4

0

0

0
4

0
4

0

0

0

0

0
4

0

0

0
4

0
4

0
3

0
3

0

0

0

0

0

0

0
4

0
4

36.2103
3

36.2103
3

36.2103
3

0

0

0
4

0

0

0
4

0

0

0
4

0

0

0
4

0

0

0
4

0

0

0
4

0
4

4.25132

4.25132

0
7

0
7

0

0

0

0

0

0

0

0

0

0

0
8

0

0

0

0

0

0

0

0

0

0

0
3

0

0

0

0

0

0

0

0

0

0

0

0

0

0

0

0

0

0

0

0

0

0

0

0

0

0

0

0

0

0

0

0

0
7

0

0

0

0

0

0

0

0

0

0

0

0

0

0

0

0

0

0

0

0

0

0
7

0

0

0

0

0

0

0

0

0

0

0

0

0

0

0

0

0

0

0

0

0

0

0

0

0

0

0

0

1.70053
3

0
7

0

0

0
6

0

0
7

0
7

0

0

0

0
7

0

0
7

0

0

0

0

0

0

0

0

0
7

0

0

0

0

0

0

0

0

0

0

0
6

0

0

0

0

0

0

0

0

0

0

0
3

0

0

0

0

0

0

0

0

0

0

0

0

0

0

0

0

0

0

0

0

0

2.55079
3

0

0

0

0

0

0

0

0

0

0

0

0

0

0

0

0

0

0

0

0

0

0
4

0

0

0

0

0

0
4

0

0

0

0
4

0

0

0
4

0

0

0
4

0
4

2.66039
3

2.66039
4

0

2.66039

0
4

0

0

0
4

0

0

0
4

0

0

0
4

0

0

0
4

0

0

0
4

0

0

0
4

0
4

6.30606677987089e-14

0
4

215.323
3

201.701
3

10.7578

9.46202

0.19311

0

0.119824

0.0683282

0

0

0

0

0

0

0.153738

0.0854102

0

0.0798828

0.0854102

0.102492

0.151349

0.0683282

0.0341641

0

0.153738

1.47104550762833e-15

0
4

4.1594
5

1.91972
5

0.0533257
6

0.0533257

0

0

0

0.319954

0

0

0

1.81307

0

0

0

0
4

186.413
3

0.685065

9.80864
3

30.2826

1.23818
4

0

0.199707

0

0

0

0

0

0

0

0.0488567

1.06584
4

0

0

0

0

0

0.122142

0

0

0.0488567

0

14.062
3

0

0

0

0

0.0808509

0

0

0

0.399414

0

0.242553
3

0

0

0

0

0

0

0

0

0

0

2.17066
4

0

0

0

0

0

0

0

0

0

0

0

0.977135

0

0

0

0

0

0

0.154593

0

0

0.733986
3

0.121276

0

0

0

0

0

0.0808509

0

0.229344

0

0

0

0

0.309187

0

0

0.341997

0

0

0.229344

0.868263

8.37239
4

0

0

0

0

0

0

0

0

0

0

2.2121
4

0

0

0

0

0

0

0

0

0.119824

0

0

19.9038
4

0

0

0.183475

0

0

0

0

0

0

0

0.323404
3

0

0

0

0

0

0

0

0

0

0

0
4

0.202127

0

0.239648

0.390854

0

0.229344

0

0

0

0

6.43057

0

0

0

0

0

0

0

0

0

0

7.58724

0.119824

0

0

0.27959

0

0

0

0

0

0

1.70053
3

0

0

0

0

0

0.0798828

0

0.175085

0

0

0

0

0

0

0

0

1.57659
5

0
6

0

3.98792

1.00486
3

0.375348

0

0

0.44468
3

0.428969

5.82238

7.60085
4

0.40085

0

0

0
6

8.30781
4

0

0

0.360341
4

0

0

3.93067

0.646042

0

7.86336
4

0

11.6144

0

0

0.23189

0.391867

0

0

0

0

3.47305
3

4.61569

0.893134

0

1.25319

0

0

0

0

0

0

1.8805

0

0

0

0.27959

0

0

0

0

0.643454

0.154593

4.28886
3

0

0.14657

0

0

0

0

0

0

0

0.265526

0

0

0

0

0

0

0.341997

0

0

0

0.136656

0
4

0

0

0

0
4

0.27959

0.27959

0
4

0

0

0
4

0

0

0
4

0

0

0
4

0

0

0

0
4

0

0

0

0
4

0

0

0
4

0

0

0
4

0.0917376

0.0917376

0
4

0

0

0
4

0

0

0
4

0

0

0
4

1.62925228863742e-14
3

0
4

4.47963
3

4.47963
3

1.85335
3

0

0

0

0

0

0

0

0

0.262628

0

0

0

0

0

0

0

0

0

0

2.36365
4

0
3

0

0

0

0

0

0
4

0
3

0

0
2

0

0

0

0

0

0

0

0
4

0
4

0

0

0

0

0

0

0

0

0

0

0

0

0

0

0

0

0
4

0

0

0
4

0
4

1.51965
4

1.51965
4

1.51965
4

0
4

0

0

0
4

0
4

0

0

0

0

0

0

0

0
4

0

0

0
4

0
4

0.262628

0.262628

0.262628

0
4

0
4

0

0

0

0

0

0
4

0
4

0

0

0

0

0
4

0

0

0
4

0

0

0
4

0
4

0

0

0

0

0

0

0
4

0

0

0

0
4

0
4

0.275213

0.275213

0.183475

0.0917376

0
4

0
4

0

0

0

0

0

0
4

0

0

0
4

0

0

0
4

0
4

0

0

0

0
4

0

0

0
4

0
4

0

0

0

0

0
4

0
4

5.84401
3

5.84401
3

5.84401
3

0

0

0

0

0

0

0

0
4

0

0

0
4

0

0

0
4

0

0

0

0

0

0

0

0
4

0
2

0

0

0

0

0
4

0
3

0

0

0

0
4

0

0

0
4

0

0

0
4

0

0

0
4

0

0

0
4

0

0

0
4

0
4

0

0

0

0
4

0
4

0

0

0

0
4

0
4

0

0

0

0
4

0

0

0
4

0
4

0

0

0

0
4

0
4

0

0

0

0
4

0

0

0
4

0
4

0.0917376

0.0917376

0

0.0917376

0
4

0

0

0
4

0
4

0

0

0

0
4

0

0

0
4

0
4

0

0

0

0
4

0
4

0

0

0

0
4

0

0

0
4

0

0

0
4

0
4

0

0

0

0

0
4

0
4

0.175085
3

0.175085
3

0
3

0

0.175085

0

0

0

0
3

0
4

0

0

0

0

0

0

0
4

0

0

0
4

0

0

0
4

0

0

0

0
4

0
3

0

0

0

0

0

0

0
4

0

0

0
4

0

0

0

0
4

0

0

0

0
4

0

0

0

0
4

0

0

0
4

0

0

0
4

0
4

0

0

0

0
4

0

0

0
4

0

0

0
4

0
4

0

0

0

0
4

0
4

0

0

0

0
4

0
4

0

0

0

0
4

0
4

0

0

0

0

0
4

0
4

0

0

0

0

0
4

0
4

0

0

0

0
4

0
4

0

0

0

0
4

0
4

0

0

0

0
4

0
4

0

0

0

0
4

0
4

0
4

0
4

0
4

0
4

0

0

0

0

0

0

0
4

0
4

0
4

0

0

0

0

0

0
4

0

0

0

0

0
4

0

0

0

0
4

0
4

0

0

0

0
4

0
4

0

0

0

0
4

0
4

0

0

0

0
4

0
4

0

0

0

0
4

0
4

0

0

0

0
4

0
4

0

0

0

0
4

0
4

0

0

0

0
4

0
4

0

0

0

0

0

0

0

0

0

0

0

0

0

0

0

0

0
4

0

0

0

0

0

0

0

0

0

0

0

0

0

0

0
4

0

0

0

0

0

0
4

0

0

0

0
4

0
4

0

0

0

0

0

0

0

0

0

0
4

0

0

0

0

0

0

0

0

0
4

0

0

0

0

0

0

0
4

0
4

0.175085
4

0

0

0

0

0

0

0

0

0

0

0
4

0.175085

0.175085

0

0

0

0
4

0
4

0.798828

0.798828

0.439355

0.27959

0.0798828

0
4

0
4

0
4

0
4

0

0

0

0

0

0
4

0

0

0

0
4

0

0

0

0
4

0

0

0

0
4

0

0

0
4

0
4

2.00950367457153e-14
3

0
4

0.293994

0.293994

0.097998

0.097998

0

0

0

0

0

0

0

0

0

0

0
4

0.195996

0.195996

0

0

0

0
4

0

0

0

0

0

0
4

0

0

0

0
4

0
4

0
4

0
7

0
7

0
7

0
7

0

0

0
4

0
4

0
4

0

0

0

0

0
4

0
4

0
4

0.0798828

0.0798828

0.0798828

0

0.0798828

0
4

0
4

0
4

0.0649455

0.0649455

0.0649455

0

0.0649455

0
4

0
4

0
4

0

0

0

0

0
4

0
4

0
4

0

0

0

0

0
4

0
4

0
4

0

0

0

0

0
4

0
4

0
4

0

0

0

0

0
4

0
4

0
4

0

0

0

0

0
4

0
4

0
4

0

0

0

0

0
4

0
4

0
4

0

0

0

0

0

0
4

0
4

0
4

0
7

0
7

0
7

0
7

0

0

0
4

0
4

0
4

0

0

0

0

0

0
4

0
4

0
4

0

0

0

0

0
4

0
4

0
4

0

0

0

0

0
4

0
4

0
4

0

0

0

0

0
4

0
4

0
4

0

0

0

0

0
4

0
4

0
4

0

0

0

0

0
4

0
4

0
4

0

0

0

0

0
4

0
4

0
4

0

0

0

0

0
4

0

0

0
4

0
4

0
4

0

0

0

0

0
4

0
4

0
4

0

0

0

0

0
4

0
4

0
4

0
7

0
7

0
7

0
7

0
4

0

0

0
4

0
4

0
4

0

0

0

0

0
4

0

0

0
4

0
4

0
4

0

0

0

0

0
4

0
4

0
4

0

0

0

0

0
4

0

0

0
4

0
4

0
4

0

0

0

0

0
4

0

0

0
4

0
4

0
4

0

0

0

0

0
4

0
4

0
4

0

0

0

0

0

0
4

0
4

0
4

0

0

0

0

0
4

0
4

0
4

0

0

0

0

0
4

0
4

0
4

0

0

0

0

0
4

0
4

0
4

0

0

0

0

0
4

0
4

0
4

0.779345

0.129891

0.0649455

0.0649455

0

0

0
4

0.0649455

0

0.0649455

0
4

0
4

0.649455

0.649455

0.649455

0
4

0

0

0
4

0
4

0
4

0

0

0

0

0

0
4

0
4

0
4

0

0

0

0

0

0
4

0
4

0
4

0

0

0

0

0
4

0
4

0
4

0

0

0

0

0
4

0

0

0
4

0
4

0
4

0

0

0

0

0
4

0

0

0
4

0
4

0
4

0

0

0

0

0
4

0
4

0
4

0.097998

0.097998

0.097998

0.097998

0
4

0
4

0
4

0

0

0

0

0
4

0
4

0
4

0

0

0

0

0
4

0
4

0
4

0

0

0

0

0
4

0
4

0
4

0

0

0

0

0

0

0

0

0

0

0
4

0

0

0

0

0

0
4

0

0

0
4

0
4

0
4

0

0

0

0

0
4

0
4

0
4

0

0

0

0

0
4

0
4

0
4

0

0

0

0

0
4

0
4

0
4

0

0

0

0

0
4

0
4

0
4

0

0

0

0

0
4

0
4

0
4

0

0

0

0

0
4

0
4

0
4

2.58723

2.58723

2.58723

2.58723

0
4

0
4

0
4

0

0

0

0

0
4

0
4

0
4

0

0

0

0

0
4

0
4

0
4

0

0

0

0

0
4

0
4

0
4

0
7

0
7

0

0

0

0
4

0

0

0

0

0
4

0

0

0
4

0

0

0
4

0
4

0
6

0

0

0

0

0
4

0

0

0
4

0

0

0
4

0
4

0
4

0

0

0

0

0
4

0
4

0
4

0

0

0

0

0
4

0
4

0
4

0

0

0

0

0
4

0
4

0
4

0

0

0

0

0
4

0
4

0
4

0

0

0

0

0
4

0
4

0
4

0

0

0

0

0
4

0
4

0
4

0

0

0

0

0
4

0
4

0
4

0

0

0

0

0
4

0
4

0
4

0

0

0

0

0
4

0
4

0
4

0

0

0

0

0
4

0
4

0
4

0
3

0
3

0
3

0
3

0

0
4

0

0

0
4

0
4

0
4

0

0

0

0

0
4

0
4

0
4

0

0

0

0

0
4

0
4

0
4

0

0

0

0

0
4

0
4

0
4

0

0

0

0

0
4

0
4

0
4

0

0

0

0

0
4

0
4

0
4

0

0

0

0

0
4

0
4

0
4

0

0

0

0

0
4

0
4

0
4

0

0

0

0

0
4

0
4

0
4

0

0

0

0

0
4

0
4

0
4

0

0

0

0

0
4

0
4

0
4

10.6706
5

10.6706
5

10.6706
5

10.5634
5

0

0.107242

3.7470027081099e-16
5

0
4

0

0

0
4

0

0

0
4

0
4

0
4

0

0

0

0

0
4

0
4

0
4

0

0

0

0

0
4

0
4

0
4

0

0

0

0

0
4

0
4

0
4

0

0

0

0

0
4

0
4

0
4

0

0

0

0

0
4

0
4

0
4

0

0

0

0

0
4

0
4

0
4

0

0

0

0

0
4

0
4

0
4

0

0

0

0

0
4

0
4

0
4

0

0

0

0

0
4

0
4

0
4

0

0

0

0

0
4

0
4

0
4

0

0

0

0

0

0

0

0
4

0

0

0

0
4

0
4

0
4

0

0

0

0

0
4

0
4

0
4

0

0

0

0

0
4

0
4

0
4

0

0

0

0

0
4

0
4

0
4

0

0

0

0

0
4

0
4

0
4

0

0

0

0

0
4

0
4

0
4

0.0488567

0.0488567

0.0488567

0.0488567

0
4

0
4

0
4

0

0

0

0

0
4

0
4

0
4

0

0

0

0

0
4

0
4

0
4

0

0

0

0

0
4

0
4

0
4

0

0

0

0

0
4

0
4

0
4

0

0

0

0

0

0

0

0
4

0

0

0
4

0
4

0
4

0

0

0

0

0
4

0
4

0
4

0

0

0

0

0
4

0
4

0
4

0

0

0

0

0
4

0
4

0
4

0

0

0

0

0
4

0
4

0
4

0

0

0

0

0
4

0
4

0
4

0

0

0

0

0
4

0
4

0
4

0

0

0

0

0
4

0
4

0
4

0

0

0

0

0
4

0
4

0
4

0

0

0

0

0
4

0
4

0
4

0

0

0

0

0
4

0
4

0
4

0

0

0

0

0

0

0

0

0

0

0
4

0

0

0

0

0

0
4

0

0

0

0

0
4

0

0

0
4

0
4

0
4

0
7

0
7

0
7

0
7

0
4

0
4

0
4

0

0

0

0

0
4

0
4

0
4

0

0

0

0

0
4

0
4

0
4

0

0

0

0

0
4

0
4

0
4

0

0

0

0

0
4

0
4

0
4

0

0

0

0

0
4

0
4

0
4

0

0

0

0

0
4

0
4

0
4

0

0

0

0

0
4

0
4

0
4

0

0

0

0

0
4

0
4

0
4

0

0

0

0

0
4

0
4

0
4

0

0

0

0

0
4

0
4

0
4

12.5188

12.5188

12.5188

2.29523

3.72583

6.49771

1.77635683940025e-15

0
4

0
4

0
4

0

0

0

0

0
4

0
4

0
4

0

0

0

0

0
4

0
4

0
4

0

0

0

0

0
4

0
4

0
4

0

0

0

0

0
4

0
4

0
4

0

0

0

0

0
4

0
4

0
4

0

0

0

0

0
4

0
4

0
4

0

0

0

0

0
4

0
4

0
4

0

0

0

0

0
4

0
4

0
4

0

0

0

0

0
4

0
4

0
4

0.0798828

0.0798828

0.0798828

0.0798828

0
4

0
4

0
4

10.7086

10.7086

10.7086

4.92016

5.78842

0
4

0
4

0
4

0

0

0

0

0
4

0
4

0
4

0

0

0

0

0
4

0
4

0
4

0

0

0

0

0
4

0
4

0
4

0

0

0

0

0
4

0
4

0
4

0

0

0

0

0
4

0
4

0
4

0

0

0

0

0
4

0
4

0
4

0

0

0

0

0
4

0
4

0
4

0

0

0

0

0
4

0
4

0
4

0

0

0

0

0
4

0
4

0
4

0

0

0

0

0
4

0
4

0
4

0
7

0

0

0

0

0

0
4

0
4

0

0

0

0
4

0

0

0
4

0
4

0
4

0

0

0

0

0
4

0
4

0
4

0

0

0

0

0
4

0
4

0
4

0

0

0

0

0
4

0
4

0
4

0

0

0

0

0
4

0
4

0
4

0

0

0

0

0
4

0
4

0
4

0

0

0

0

0
4

0
4

0
4

0

0

0

0

0
4

0
4

0
4

0

0

0

0

0
4

0
4

0
4

0

0

0

0

0
4

0
4

0
4

0

0

0

0

0
4

0
4

0
4

0
6

0
6

0
6

0
6

0
4

0
4

0
4

0

0

0

0

0
4

0
4

0
4

0

0

0

0

0
4

0
4

0
4

0

0

0

0

0
4

0
4

0
4

0

0

0

0

0
4

0
4

0
4

0

0

0

0

0
4

0
4

0
4

0

0

0

0

0
4

0
4

0
4

0

0

0

0

0

0

0
4

0
4

0

0

0

0

0
4

0
4

0
4

0
3

0
3

0
3

0

0

0

0

0
4

0
4

0
4

0
6

0
6

0
6

0

0

0

0
4

0
4

0
4

14.6512
4

14.6512
4

14.0649
4

14.0649
4

0
4

0.586281

0.586281

0
4

2.22044604925031e-16
4

0
4

0
4

0
7

0
7

0
7

0
7

0
4

0
4

0
4

0
1

0
1

0
1

0
1

0

0

0

0

0
4

0

0

0
4

0
4

0
4

0
7

0
7

0
7

0
7

0
4

0
4

0
4

0
6

0
6

0
6

0
6

0

0
4

0
4

0
4

0

0

0

0

0

0

0

0
4

0
4

0
4

0

0

0
7

0
7

0

0
4

0

0

0
4

0
4

0
4

0

0

0

0

0

0

0
4

0

0

0

0

0
4

0
4

0

0

0

0
4

0

0

0
4

0
4

0
4

0
7

0
7

0
7

0
7

0

0
4

0
4

0
4

0

0

0

0

0

0
4

0
4

0
4

0

0

0

0

0

0
4

0

0

0

0
4

0
4

0
4

0

0

0

0

0

0

0
4

0
4

0
4

0

0

0

0

0

0
4

0

0

0

0
4

0

0

0
4

0

0

0
4

0
4

0
4

0
6

0
6

0
6

0
6

0

0
4

0
4

0
4

0
7

0
7

0
7

0

0

0

0

0
4

0
4

0
4

0
7

0
7

0
7

0
7

0

0
4

0
4

0
4

0

0

0

0

0

0
4

0

0

0
4

0
4

0
4

0

0

0

0

0

0
4

0
4

0
4

0

0

0

0

0

0

0
4

0

0

0
4

0

0

0
4

0
4

0
4

0

0

0

0

0

0

0

0
4

0
4

0
4

0

0

0

0

0
4

0
4

0
4

0

0

0

0

0

0
4

0
4

0
4

0
7

0
7

0
7

0
7

0
4

0
4

0
4

8.65723

8.65723

8.65723

0.23189

8.03885

0.23189

0.154593

1.4432899320127e-15

0
4

0
4

0
4

9.65181
5

4.71866
5

4.39694
5

4.39694
5

0

0
4

0

0

0
4

0.321727

0.321727

0

0
4

0

0

0
4

2.22044604925031e-16
5

0
4

4.93315

4.93315

4.82591

0

0.107242

3.7470027081099e-16

0
4

0
4

0
4

0

0

0

0

0

0

0

0
4

0
4

0
4

0
6

0
6

0
6

0
6

0
4

0
4

0
4

0

0

0

0

0

0
4

0

0

0
4

0
4

0
4

0.203109

0.203109

0.203109

0.0761658

0.126943

0

0
4

0
4

0
4

0

0

0

0

0
4

0

0

0

0
4

0
4

0
4

0

0

0

0

0

0
4

0

0

0

0
4

0
4

0
4

0

0

0

0

0

0

0

0
4

0
4

0
4

0.137606

0.137606

0.137606

0

0.137606

0
4

0

0

0
4

0
4

0
4

0

0

0

0

0
4

0

0

0
4

0

0

0
4

0

0

0
4

0
4

0
4

0

0

0

0

0

0

0
4

0
4

0
4

0
7

0
7

0
7

0
7

0

0

0
4

0
4

0
4

0

0

0

0

0

0

0
4

0
4

0
4

0

0

0

0

0

0
4

0
4

0
4

0
3

0
3

0

0

0
4

0

0

0
4

0
4

0
4

0

0

0

0

0
4

0
4

0
4

0

0

0

0

0
4

0
4

0
4

0

0

0

0

0
4

0
4

0
4

0

0

0

0

0

0
4

0
4

0
4

0

0

0

0

0
4

0
4

0
4

0

0

0

0

0

0
4

0
4

0
4

0

0

0

0

0

0
4

0
4

0
4

0

0

0

0

0

0

0

0

0

0

0

0

0
4

0

0

0

0

0

0

0
4

0

0

0

0
4

0

0

0

0
4

0
4

0
4

0

0

0

0

0

0

0
4

0
4

0
4

0

0

0

0

0

0
4

0
4

0
4

0

0

0

0

0
4

0
4

0
4

0

0

0

0

0

0

0
4

0
4

0
4

0

0

0

0

0
4

0
4

0
4

0

0

0

0

0
4

0
4

0
4

0

0

0

0

0

0
4

0

0

0

0
4

0
4

0
4

0

0

0

0

0
4

0

0

0
4

0
4

0

0

0

0
4

0
4

0
4

0

0

0

0

0

0
4

0
4

0
4

0

0

0

0

0
4

0
4

0
4

0
7

0
7

0
7

0
7

0

0

0

0

0
4

0
4

0
4

1.03913

1.03913

1.03913

1.03913

0

0
4

0

0

0
4

0
4

0
4

0

0

0

0

0
4

0
4

0
4

0

0

0

0

0
4

0
4

0
4

0

0

0

0

0
4

0
4

0
4

0.998535

0.998535

0.998535

0

0.998535

0
4

0
4

0
4

0

0

0

0

0
4

0
4

0
4

0

0

0

0

0

0
4

0
4

0
4

0

0

0

0

0

0

0
4

0
4

0
4

0

0

0

0

0

0
4

0

0

0
4

0
4

0
4

0

0

0

0

0
4

0

0

0
4

0
4

0
4

0
6

0
6

0

0

0

0

0

0

0
4

0

0

0

0

0

0
4

0

0

0
4

0
4

0
4

0

0

0

0

0
4

0
4

0
4

0

0

0

0

0
4

0
4

0
4

0

0

0

0

0
4

0

0

0
4

0
4

0
4

0.0798828

0.0798828

0.0798828

0

0.0798828

0

0
4

0
4

0
4

0.0761658

0

0

0

0
4

0
4

0.0761658

0.0761658

0.0761658

0
4

0
4

0
4

0

0

0

0

0
4

0
4

0
4

0

0

0

0

0

0
4

0

0

0
4

0
4

0
4

0

0

0

0

0
4

0
4

0
4

0

0

0

0

0

0
4

0
4

0
4

0

0

0

0

0
4

0
4

0
4

0
1

0

0

0

0
4

0
4

0

0

0

0
4

0
4

0

0

0

0
4

0
4

0

0

0

0

0
4

0
4

0

0

0

0
4

0
4

0

0

0

0
4

0
4

0

0

0

0
4

0
4

0

0

0

0
4

0

0

0
4

0
4

0

0

0

0
4

0
4

0

0

0

0

0
4

0
4

0

0

0

0
4

0
4

0
1

0
2

0

0

0

0

0

0

0

0

0

0
4

0

0

0
4

0
4

0
4

0
7

0

0

0

0

0
4

0
4

0

0

0

0
4

0
4

0

0

0

0
4

0
4

0

0

0

0
4

0
4

0

0

0

0
4

0
4

0

0

0

0
4

0
4

0

0

0

0
4

0
4

0
7

0
7

0
7

0

0
4

0
7

0
7

0
4

0

0

0
4

0

0

0
4

0

0

0
4

0

0

0
4

0

0

0
4

0

0

0
4

0

0

0
4

0
4

0
4

0
3

0
3

0
3

0

0

0

0

0

0

0

0

0

0

0

0
3

0

0

0

0

0

0

0

0

0

0

0
2

0

0

0

0

0
3

0
2

0
3

0
2

0
3

0
2

0
4

0

0

0
4

0

0

0
4

0
4

0

0

0

0

0

0
4

0
4

0

0

0

0

0
4

0
4

0

0

0

0
4

0
4

0

0

0

0
4

0
4

0

0

0

0

0
4

0
4

0

0

0

0
4

0

0

0
4

0
4

0

0

0

0
4

0

0

0
4

0
4

0

0

0

0
4

0
4

0

0

0

0
4

0
4

0
4

0
7

0

0

0

0
4

0
4

0

0

0

0
4

0
4

0

0

0

0
4

0
4

0

0

0

0
4

0
4

0

0

0

0
4

0
4

0

0

0

0
4

0
4

0

0

0

0
4

0
4

0

0

0

0
4

0
4

0

0

0

0
4

0
4

0

0

0

0
4

0
4

0

0

0

0
4

0
4

0
7

0
7

0
7

0
5

0

0

0

0

0

0

0

0

0

0

0
7

0

0

0

0

0

0

0

0

0

0

0
6

0

0

0

0

0

0

0

0

0

0

0

0

0

0
7

0

0

0

0

0
4

0

0

0
4

0

0

0
4

0

0

0
4

0
4

0
4

0

0
5

0
5

0

0

0

0

0
5

0
6

0

0

0

0

0

0

0
4

0

0

0
4

0
4

0
5

0
5

0
5

0

0

0

0

0
4

0

0

0
4

0
4

0

0

0

0
4

0
4

0

0

0

0
4

0
4

0

0

0

0
4

0
4

0

0
7

0

0

0

0

0

0

0

0

0

0

0

0

0

0

0

0

0

0

0

0

0

0

0
4

0

0

0

0

0
4

0

0

0
4

0

0

0
4

0

0

0
4

0
4

0
4

2.16992

0

0

0

0

0

0

0

0

0

0

0

0
4

0

0

0

0
4

0

0

0
4

0
4

0.325488
5

0.325488
5

0
5

0

0

0

0.0723307
7

0

0

0.253157

0

0

0

0

0
4

0
4

0

0

0

0

0
4

0

0

0
4

0
4

0

0

0

0
4

0
4

0

0

0

0
4

0
4

0

0

0

0
4

0
4

0

0

0

0
4

0
4

0

0

0

0
4

0
4

0

0

0

0

0
4

0
4

0

0

0

0

0
4

0
4

0

0

0

0
4

0
4

0

0

0

0
4

0
4

1.84443
5

1.84443
5

0
5

1.84443

0

0

0

0

0

0
4

0

0

0
4

0

0

0
4

0
4

0

0

0

0
4

0
4

0

0

0

0
4

0
4

0

0

0

0
4

0
4

0

0

0

0
4

0
4

0

0

0

0
4

0
4

0

0

0

0
4

0
4

0

0

0

0
4

0
4

0

0

0

0
4

0
4

0

0

0

0
4

0
4

0

0

0

0
4

0
4

0
7

0
7

0
7

0

0
7

0
7

0

0

0

0

0

0
4

0
4

0
5

0
5

0

0

0

0

0

0

0

0

0
4

0

0

0

0

0

0
4

0
4

0
5

0
5

0

0

0

0

0

0

0
4

0

0

0

0
4

0
4

0

0

0

0
4

0

0

0
4

0
4

0

0

0

0

0

0
4

0

0

0
4

0
4

0
7

0
7

0

0

0

0

0

0
4

0
4

0

0

0

0
4

0

0

0
4

0
4

0
4

9.98163

0

0

0

0

0

0

0

0

0

0

0
4

0

0

0
4

0
4

0

0

0

0

0

0
4

0
4

0

0

0

0

0
4

0
4

0

0

0

0

0
4

0

0

0
4

0
4

0

0

0

0

0
4

0
4

0

0

0

0
4

0

0

0
4

0
4

0

0

0

0
4

0
4

0

0

0

0
4

0
4

0

0

0

0
4

0
4

0

0

0

0
4

0

0

0
4

0
4

0

0

0

0
4

0
4

0

0

0

0

0

0
4

0
4

0

0

0

0
4

0
4

0

0

0

0
4

0
4

0

0

0

0
4

0
4

0

0

0

0
4

0
4

0

0

0

0
4

0
4

0

0

0

0
4

0
4

0

0

0

0
4

0
4

0

0

0

0
4

0
4

0

0

0

0
4

0
4

0

0

0

0
4

0
4

0

0

0

0

0
4

0

0

0

0
4

0
4

0

0

0

0
4

0
4

0

0

0

0
4

0
4

0

0

0

0
4

0
4

0

0

0

0
4

0
4

0

0

0

0
4

0
4

0

0

0

0
4

0
4

0

0

0

0

0
4

0
4

0

0

0

0

0

0
4

0

0

0
4

0
4

0

0

0

0

0
4

0

0

0
4

0

0

0
4

0
4

0

0

0

0
4

0

0

0

0
4

0
4

0

0

0

0

0
4

0
4

0

0

0

0

0
4

0

0

0

0
4

0
4

9.98163

9.98163

9.98163

0

0

0

0

0

0

0

0

0

0

0

0

0

0

0

0
3

0

0

0

0

0

0

0
4

0

0

0

0

0

0

0

0
4

0

0

0
4

0

0

0
4

0

0

0
4

0

0

0
4

0

0

0
4

0

0

0
4

0

0

0
4

0

0

0

0

0

0

0
4

0

0

0
4

0

0

0
4

0

0

0

0
4

0

0

0
4

0

0

0
4

0

0

0
4

0

0

0
4

0
4

0
4

0
5

0
6

0

0

0

0

0

0

0

0

0

0
4

0

0

0

0
4

0

0

0

0
4

0

0

0
4

0

0

0
4

0
4

0

0
5

0
6

0

0

0

0

0

0

0

0
4

0

0

0

0
4

0

0

0
4

0
4

0

0

0

0
4

0
4

0

0

0

0
4

0
4

0

0

0

0
4

0
4

0

0

0

0
4

0
4

0

0

0

0
4

0
4

0
5

0
5

0
6

0

0

0

0

0

0
4

0
4

0

0

0

0

0

0

0

0
4

0
4

0
5

0

0

0

0

0

0
4

0

0

0
4

0

0

0
4

0
4

0

0

0

0
4

0
4

0

0

0

0

0
4

0
4

0

0

0

0
4

0
4

0

0

0

0
4

0
4

0

0

0

0
4

0
4

0
4

3.9542
3

0

0

0

0

0
4

0
4

0

0

0

0
4

0
4

3.9542
3

3.9542

3.9542

0
4

0

0

0

0

0
4

0

0

0
4

0

0

0
4

0

0

0
4

0
4

0
4

0.675885

0

0

0
7

0

0

0

0

0

0

0

0

0

0
8

0
8

0
1

0

0

0

0

0

0
4

0

0

0

0

0
4

0

0

0

0
4

0
4

0

0

0

0

0
4

0
4

0

0

0

0

0

0
4

0

0

0
4

0
4

0

0

0

0

0
4

0

0

0

0
4

0
4

0

0

0

0
4

0
4

0

0

0

0
4

0
4

0

0

0

0

0
4

0

0

0
4

0
4

0

0

0

0
4

0
4

0

0

0

0

0
4

0
4

0

0

0

0
4

0
4

0

0

0

0
4

0

0

0
4

0
4

0

0
7

0

0

0

0

0

0

0

0

0

0

0

0
4

0
1

0
8

0

0

0
4

0

0

0
4

0

0

0
4

0
4

0

0

0

0
4

0
4

0

0

0

0

0
4

0
4

0

0

0

0
4

0
4

0

0

0

0
4

0
4

0

0

0

0
4

0
4

0

0

0

0
4

0
4

0

0

0

0
4

0
4

0

0

0

0
4

0
4

0

0

0

0
4

0
4

0

0

0

0
4

0
4

0

0

0

0

0

0

0
4

0

0

0

0
4

0

0

0
4

0

0

0
4

0
4

0

0

0

0
4

0
4

0

0

0

0
4

0
4

0

0

0

0
4

0
4

0

0

0

0
4

0
4

0

0

0

0
4

0
4

0

0

0

0
4

0
4

0

0

0

0
4

0
4

0

0

0

0
4

0
4

0

0

0

0
4

0
4

0

0

0

0
4

0
4

0

0

0

0

0

0

0
4

0

0

0

0
4

0
4

0

0

0

0

0
4

0

0

0

0

0

0
4

0

0

0

0
4

0
4

0.168971
3

0.120694
3

0

0.120694

0

0

0

0

0

0

0
4

0.0482775

0.0482775

0
4

0

0

0
4

0
4

0
3

0
3

0

0

0

0

0

0
4

0
4

0
6

0

0

0

0

0
4

0

0

0
4

0

0

0
4

0
4

0.120694

0.120694

0.120694

0

0

0
4

0
4

0.38622

0

0
7

0

0

0

0

0

0

0

0

0

0

0

0

0

0

0

0

0

0

0

0

0

0

0

0

0

0

0

0

0

0

0

0

0

0

0

0

0

0

0

0

0

0

0

0
7

0

0

0

0

0

0

0

0

0

0

0

0

0

0

0

0

0

0

0

0

0

0

0

0

0

0
4

0

0

0

0

0
4

0

0

0
4

0

0

0
4

0

0

0

0
4

0

0

0

0
4

0

0

0
4

0

0

0
4

0

0

0
4

0.38622

0.38622

0
4

0

0

0
4

0

0

0
4

0
6

0
6

0

0

0
4

0

0

0
4

0

0

0
4

0

0

0
4

0

0

0
4

0

0

0
4

0

0

0

0

0

0
4

0

0

0

0

0
4

0
3

0

0

0

0
4

0

0

0

0
4

0

0

0

0
4

0

0

0

0
4

0

0

0
4

0
4

0
4

0
7

0
6

0
6

0
6

0

0
4

0
4

0
6

0
6

0
6

0
4

0
4

0

0

0

0
4

0
4

0

0

0

0
4

0
4

0

0

0

0
4

0
4

0

0

0

0
4

0
4

0
7

0
7

0
7

0
4

0
4

0
4

0
4

12429.4

0

0

0

0

0

0

0
4

0
4

0
4

7763.98
3

0

0

0

0

0

0

0

0

0

0

0

0

0
2

0

0

0

0

0

0

0

0
4

0

0

0
4

0

0

0
4

0
4

7688.02
3

4.83422

0

0

0

0

0

0

0

0

0

0

0

0

0

0

0

0

0

0

0

0

0

0

0

0

2.30608

0

0

0

0.102492

0

0

0

0

0

0

0

0

0

0

0

0

0

0

0

0

0

0

0

0

0

0

0

0

0

0

0

0

0
6

0

0

0

0

0.0512461

0

0

0

0

0

0
7

0

0

0.0512461

0

0

0

0

0

0

0

0

0

0

0

0

0

0

0

0

0

0

0

0

0

0

0

0

0

0

0

0

0

0

0

0

0

0

0

0

0

0

0

0

0

0
5

0

0

0

0

0

0

0

0

0

0

0

0

0

0

0

0

0

0

0

0

0

0
2

0

0

0

0

0

0

0

0

0

0

0

0

0

0

0

0

0

0

0

0

0

0

0

0

0

0

0

0

0

0

0

0

0

0

0

0

0

0

0

0

0

0

0

0
6

0

0

0

0

0

0

0

0

0

0

0

0

0

0

0

0

0

0

0

0

0

0

0

0

0

0

0

0

0

0

0

0

0

0

0

0

0

0

0

0

0.0341641

0

0

2.28899

0

0

0

0

0

0

0

0

0

0

0

0

0

0

0

0

0

0

0

0

0

0

0
2

0

0

0

0

0

0

0

0

0

0

0

0

0

0

0

0

0

0

0

0

0

0

0

0

0

0

0

0

0

0

0

0

0

0

0

0

0

0

0

0

0

0

0

0

0

0

0

0

0

0

0

0

0

0

0

0

0

0

0

0

0

0

0

0
6

0

0

0
2

0

0

0
7

0

0

0

0

0

0

0

0

0

0

0

0

0

0

0

0

0

0

0

0

0

0

0

0

0

0

0

0

0

0

0

0

0

0

0

0

0

0

0

0

0

0

0

0

0

0

0

0

0

0

0

0

0

0

0

0

0
4

7351.6
4

281.854
3

4738.17
4

665.577
3

0
6

15.6633
4

0.0974182

0.433984

0

0

0

0

0

0.3572

0

0.536111

42.198
4

0

0

0

0

0

0

0

5.34089

0

3.06903

213.016
4

0

0

1.75085

0

0

2.1468

0.0723307

0

0

0

2.47021
4

0

0.275213

0.334851

0

0

0

0

0

0

0

2.35075
4

0

0

0

0

0

0

0

2.44309

0

0.262628

0
2

0

0

0

0

0.850263

0.154593

0.354366

0

0

0

0.38106
3

0

0

0

0

0

0

0

0

0

0

0.242553
4

2.52448

0

0

0

0

0

0

0

0

0

0

0

0

0

0

0

0.133314

1.92594

0

0

0

0
2

0

0

1.48823

0

0

0

0

0

0

0

15.0255
3

1.76277
4

0

0

0

0.137606

0.108496

0

0

0

0

0.0723307

0

0

0

0

0

0

0

0

0

0

0

3.32834

0

0

0

0

0.154593

0

0

0

0

0

3.12394
4

0

0

0

0

0.0723307

0

0

0

0

0

0.406975

0

0

1.01297

0

0

1.15945

1.26943

0

0

0

1.27648
3

0

0

0

0

0

0.875427

0

0

0.154593

0

0

0

0.0533257

1.75085

0

0.144661

0.275213

0

0.0488567

0

0

0

0

0.0732851

0.137606

0

0

0

0

0

0

0

0.175085
4

0

0

0.199707

0

0

0

0.350171

0.319954

0

0

1.13805
4

0.262628

0

0

0

0

0

0

0

0.175085

0.119824

88.7209
4

0.359473

0

0

0.434131

0

0

0

0

0.479931

0

0

0

0

0

0.253157

0

0

0

0

0

0

0.962969

0

0

0

0

0

0

0

0.0723307

0

0

0

0.0917376
4

0

0

0

0

0.0974182

0

0

0

0.159766

0

0
3

0

0.175085

0

0.0488567

0

0.0917376

0

0

0

0

0.561569

0

0

0

0

0

0

0

0

0

0

0

0.152332

0

0.108496

0

0

0.229344

0

0

0

0

0.525869
4

0

0

0

0

0

0

0

0.0977135

0.262628

0

0

0

0

0

0

0

0

0

6.36726

1.39795

0

0

0

0

0

0

0

0

1.28433

0

0

0

13.0675
4

0

0.180827

0

0

0

0

0

0

0

0

0

2.40016

0

0

0

0

0

2.20496

0

0

0

0

0.238308

0

0.0488567

0

0

0

0.146997

0

0

0

0

0
2

0

0

0

0

0

0

0

0

0

0

0

0.289323

0.578842

0.0488567

0.574862
4

0

0

0

0
3

16.1869
4

0

13.9633

0

28.378
4

0

0.330764
4

0

0

0

0.350171

231.419
4

0

8.91239

0.567163

837.664

0

0

0

34.3197

0.154593

0.180827

0.901418
4

0

0

0

0

0

0

0

0

0

0.850263

0
3

0.108496

0

0

0

0

0

6.34521

8.68263

0

0

11.8896
3

0

0

0

0

0.0533257

0.662042

0

2.51348

0

2.80958

6.97264468385583e-12
4

0
4

72.0842
3

3.03794
3

39.9969
3

21.7128
3

0.0798828
3

6.34829
2

0.679004
3

0
2

0
1

0

0

0

0

0

0

0

0

0

0

0

0

0

0

0

0

0

0

0

0

0

0

0

0

0

0.0917376

0

0
8

0.137606

0

0

0

0

0
4

6.22141

0
7

0
6

0

0

0

0

0

0

0

0.0724163

0

0

0
7

0

0

0

0

0

0

0

0

0

0

0
7

0

0

0

0

0

0

0

0

0

0

0
5

0

0

0

0

0

0

0

0

0

0

0
6

0

0

0

0

0

0

0

0

0

0

0
7

0.0965551

0

0

0

0

0

0

0

0

0

0

0

0

0

0

0

0

0

0

0

0

0

0.175085

0

0

0

0.107242

0

0.337943

0

0

0
8

0

0

0

0

0

0

0

0

0

0

0

0

1.30349

0

0

0

0

0

0

1.88282

0
3

0

0

0

0

0

0

0

0

0

0

0
7

0

0

0

0

0

0

0

0

0

2.10102

0
3

0

0

0

0

0

0

0

0

0

0

0
7

0.0482775

0

0

0

0

0

0

0

0

0

0
1

0

0.0482775

0

0

0.0482775

0

0

0

0

0

0

0

0

0

0

0

0

0

0

0

0

0
4

0
2

0

0

0

0

0

0

0

0

0

0

0

0

0

0

0

0

0

0

0

0

0

0

0

0

0

0

0

0

0

0

0

0

0

0

0

0

0

0

0

0

0

0

0

0

0

0

0

0

0

0

0

0

0

0

0

0

0

0

0

0

0

0

0

0

0

0

0

0

0

0

0

0

0

0

0

0

0

0

0

0

0

0

0

0

0

0

0
4

15.9161
3

11.4449
3

0.434131

0

0

0

0

0

0

0

0

0

0.359473
4

0

0

0

0

0.868263

0

0

0

0

0

0
4

0

0

0

0

0

0

2.08581
4

0.723552
3

0
4

0
3

0
4

0

0
4

0.998535
3

0.159766

0

0

0

0.439355
4

0

0.399414

0

0

0

0

0

0
4

0

0

0
4

0

0

0
4

0

0

0
4

0

0

0
4

0

0

0

0
4

0

0

0
4

0

0

0

0
4

0

0

0
4

0

0

0
4

0

0

0

0
4

0
3

0
4

0
3

0
3

0
3

0

0

0

0

0
4

2.45119

2.45119

0
4

0.453268

0.106651

0.346617

0
4

0

0

0
4

0.0798828

0

0.0798828

0
4

0

0

0
4

3.59473

3.59473

0
4

0

0

0

0
4

0

0

0
4

0.998535

0.998535

0
4

0

0

0
4

21.8457
3

20.2052
3

0

0

0.152213

0

0

0

1.48823

4.21884749357559e-15
3

0
4

0

0

0
4

0

0

0
4

0

0

0
4

0.144661

0.144661

0
4

0

0

0
4

0

0

0
4

0

0

0
4

0

0

0
4

0.0798828

0.0798828

0
4

0

0

0
4

0
2

0
2

0

0

0

0

0

0

0
4

0

0

0
4

0

0

0
4

1.1583

1.1583

0
4

0

0

0
4

0

0

0
4

0

0

0
4

1.39795

1.39795

0
4

0.0798828

0.0798828

0
4

0

0

0
4

0.0798828

0.0798828

0
4

0

0

0

0

0

0

0

0

0
4

0.133314

0.133314

0
4

0

0

0
4

0

0

0
4

0

0

0
4

0

0

0
4

0

0

0
4

0.262628

0.262628

0
4

0

0

0
4

0

0

0
4

0

0

0
4

3.76247
4

3.76247
4

0

0

0

0
4

0

0

0
4

0

0

0
4

0

0

0
4

0

0

0
4

0

0

0
4

0

0

0
4

0

0

0
4

0

0

0
4

0

0

0
4

0

0

0
4

2.45033

2.24171

0.159766

0.0488567

0

0
4

0

0

0
4

0

0

0
4

0

0

0
4

0

0

0
4

0

0

0
4

0.239648

0.239648

0
4

0

0

0
4

0

0

0
4

0.0799885

0.0799885

0
4

0.180827

0.180827

0
4

4.39642
3

4.39642
3

0

0

0

0
4

0

0

0
4

0

0

0
4

0

0

0
4

0

0

0
4

0

0

0
4

0

0

0
4

0

0

0
4

0

0

0
4

2.62628

2.62628

0
4

0

0

0
4

9.15309
4

7.26967
4

1.81847

0

0.0649455

0
4

0

0

0
4

0.0649455

0.0649455

0
4

0

0

0
4

0

0

0
4

0

0

0
4

0

0

0
4

0

0

0
4

0

0

0
4

0

0

0
4

0

0

0
4

12.9916

7.034

1.55171

2.3189

1.00486

1.08215

0
4

0

0

0
4

0

0

0
4

0

0

0
4

0

0

0
4

0

0

0
4

0

0

0
4

0

0

0
4

0

0

0
4

0

0

0
4

0

0

0
4

11.3649
3

10.3181
3

0

0

0

0

0

0

0

0

0.0723307

0

0.437713
4

0

0

0

0

0

0

0

0.464506
4

0
4

0.0723307
3

0

0

0

0

0
4

54.003

29.7776

23.8657

0.279801

0.0798828

3.31679128606766e-15

0
4

0

0

0
4

0

0

0
4

0

0

0
4

0

0

0
4

0

0

0
4

2.26745
3

2.26745
3

0

0

0
4

0.289421
4

0

0

0.289421

0

0

0
4

0
2

0

0

0

0

0

0

0

0
4

0.399414

0

0

0

0.399414

0

0

0

0

0
4

0

0

0

0
4

0.744039
3

0.0974182

0.386839

0.194836

0

0

0.0649455

0
4

0.30873
3

0

0.216992

0

0

0

0.0917376

0

0
4

6.73086

5.05593

1.67493

0
4

0
2

0

0

0

0

0

0
4

32.7572
3

32.7572
3

0

0

0

0

0

0

0

0

0

0

0
4

0

0

0
4

0

0

0

0

0

0
4

1.4131
3

0

1.22962

0.183475

0

0

2.77555756156289e-17
3

0
4

1.30239
4

0

0

0

0

1.30239

0
4

0

0

0
4

1.26227
4

0.561924
4

0

0

0.700341

0
4

1.8384

1.8384

0

0
4

25.8334

25.5704

0.154593

0.108496

0
4

5.1373
4

5.04557
4

0.0917376

0
4

0
5

0
5

0

0
4

0
2

0

0

0

0

0

0
4

2.30608
4

2.30608
4

0
4

0
5

0
5

0

0

0

0

0

0

0

0

0

0

0

0

0

0

0

0

0

0

0

0

0

0

0

0

0
4

0
4

0

0

0
4

0

0

0

0

0
4

0

0

0
4

0.759098

0.213091

0.439355

0.106651

0
4

0
4

0

0

0
4

0.183475

0

0.183475

0

0

0
4

0

0

0

0
4

0

0

0

0

0

0
4

0

0

0
4

0.434131

0.434131

0
4

0
6

0

0

0

0

0

0

0

0

0

0

0

0

0

0

0

0

0

0

0

0

0

0

0

0

0

0

0

0

0

0

0

0

0
4

0
2

0

0

0
4

0

0

0

0

0
4

0

0

0

0
4

0

0

0

0

0
4

0

0

0

0

0
4

0

0

0

0

0
4

0

0

0

0
4

0

0

0
4

0

0

0

0
4

1.13805

0.962969

0.175085

0

0
4

0
2

0
2

0
2

0

0

0

0

0

0

0
4

0

0

0
4

0.129891

0.129891

0

0
4

0

0

0
4

0

0

0

0
4

0

0

0
4

0

0

0
4

0

0

0
4

0

0

0

0

0

0
4

0.160864

0.160864

0

0
4

0

0

0
4

10.0844
3

1.81777
4

0

0.0917376

0

0

0

0

0.275213

0.0723307

0

0

7.45809

0

0

0

0

0

0

0

0

0.0798828

0

0

0

0

0

0.289421

0

0

2.16493489801906e-15
3

0
4

0.78433

0.78433

0
4

0

0

0
4

0

0

0

0
4

0

0

0

0
4

0

0

0

0
4

0

0

0

0
4

0

0

0

0
4

0

0

0

0
4

0

0

0

0
4

0

0

0

0
4

0
6

0
6

0

0

0

0

0

0

0

0

0

0

0

0
4

0

0

0

0
4

0

0

0

0
4

0

0

0
4

0.175085

0

0.175085

0
4

0

0

0
4

2.53668

2.53668

0
4

0

0

0

0
4

0

0

0

0
4

0

0

0
4

0

0

0
4

2.89421
4

2.60479
4

0
4

0.289421
4

0

0

0

0

0

0

0
4

0

0

0
4

0.159766

0.159766

0
4

0

0

0
4

0

0

0
4

0

0

0

0
4

0

0

0

0
4

0

0

0
4

0

0

0

0
4

0.259782

0.259782

0
4

0

0

0

0
4

1.06812891864649e-11
3

0
4

0
2

0
2

0
2

0

0

0

0

0

0

0

0

0

0

0

0

0

0

0

0

0

0

0

0

0

0
2

0

0

0

0

0

0

0

0

0

0

0

0

0

0

0

0

0

0

0

0

0

0

0

0

0

0

0

0

0

0

0

0

0

0

0

0

0

0

0

0

0

0

0

0

0

0

0

0

0

0

0

0

0
4

0
2

0

0

0

0

0

0

0

0

0

0

0

0
4

0

0

0

0

0

0

0

0

0
4

0

0

0

0

0

0
4

0

0

0
4

0
4

0

0

0
7

0

0

0

0

0

0
4

0

0

0
4

0

0

0
4

0
4

0

0

0

0
4

0

0

0
4

0
4

0

0

0

0
4

0

0

0
4

0
4

0

0

0

0
4

0

0

0
4

0
4

0

0

0

0

0
4

0
4

0

0

0

0
4

0

0

0
4

0
4

0

0

0

0
4

0
4

0

0

0

0
4

0
4

0

0

0

0
4

0
4

0

0

0

0
4

0

0

0
4

0
4

0.878711

0.878711

0.878711

0
4

0
4

0

0

0

0

0

0

0

0

0
4

0
4

0

0

0

0

0
4

0
4

0.55918

0.55918

0.55918

0
4

0

0

0
4

0
4

0

0

0

0

0
4

0
4

0

0

0

0
4

0
4

0

0

0

0

0
4

0
4

0

0

0

0
4

0
4

0.180827

0.180827

0.180827

0
4

0
4

0

0

0

0
4

0
4

0

0

0

0
4

0
4

0

0

0

0

0
4

0
4

0
7

0
7

0

0

0

0

0

0

0
4

0
4

0

0

0

0

0
4

0
4

0

0

0

0
4

0
4

0

0

0

0
4

0
4

0

0

0

0
4

0
4

0

0

0

0
4

0
4

0

0

0

0
4

0
4

0

0

0

0
4

0
4

0

0

0

0
4

0
4

0

0

0

0
4

0
4

0

0

0

0
4

0
4

0
2

0
2

0
2

0

0

0

0

0

0

0
4

0
4

0

0

0

0
4

0
4

0

0

0

0
4

0
4

0

0

0

0
4

0
4

0.0798828

0.0798828

0.0798828

0
4

0
4

0

0

0

0
4

0
4

0.798828

0.798828

0.798828

0
4

0
4

0

0

0

0
4

0
4

0

0

0

0
4

0
4

0

0

0

0
4

0
4

0

0

0

0
4

0
4

0.388385

0.241388
5

0

0

0.241388

0

0

0

0

0
4

0

0

0

0

0

0

0
4

0.146997

0.146997

0
4

0
4

0

0

0

0
4

0
4

0

0

0

0
4

0
4

0

0

0

0
4

0
4

0

0

0

0
4

0
4

0

0

0

0
4

0
4

0

0

0

0
4

0
4

0.0798828

0.0798828

0.0798828

0
4

0
4

0

0

0

0
4

0
4

0

0

0

0
4

0
4

0

0

0

0
4

0
4

0

0

0

0

0

0

0

0

0
4

0
4

0

0

0

0
4

0
4

0

0

0

0
4

0
4

0

0

0

0
4

0
4

0

0

0

0
4

0
4

0

0

0

0
4

0
4

0

0

0

0
4

0
4

0

0

0

0
4

0
4

0

0

0

0
4

0
4

0

0

0

0
4

0
4

0

0

0

0
4

0
4

0

0

0

0

0

0

0

0
4

0
4

0

0

0

0
4

0
4

0

0

0

0
4

0
4

0

0

0

0
4

0
4

0

0

0

0
4

0
4

0

0

0

0
4

0
4

0

0

0

0
4

0
4

0

0

0

0
4

0
4

0

0

0

0
4

0
4

0

0

0

0
4

0
4

0

0

0

0
4

0
4

0

0

0

0

0

0

0

0

0

0

0

0

0
4

0
4

0

0

0

0
4

0
4

0

0

0

0
4

0
4

0

0

0

0
4

0
4

0

0

0

0
4

0
4

0

0

0

0
4

0
4

0

0

0

0
4

0
4

0

0

0

0
4

0
4

0

0

0

0
4

0
4

0

0

0

0
4

0
4

0

0

0

0
4

0
4

0

0

0

0

0

0

0
4

0

0

0
4

0
4

0

0

0

0
4

0
4

0

0

0

0
4

0
4

0

0

0

0
4

0
4

0

0

0

0
4

0
4

0

0

0

0
4

0
4

0

0

0

0
4

0
4

0

0

0

0
4

0
4

0.199707

0.199707

0.199707

0
4

0
4

0

0

0

0
4

0
4

0

0

0

0
4

0
4

0
6

0
6

0
6

0

0

0

0

0

0
4

0
4

0

0

0

0
4

0
4

0.14657

0.14657

0.14657

0
4

0
4

0

0

0

0
4

0
4

0
2

0
2

0
2

0

0

0

0

0

0

0

0

0

0

0
2

0

0

0

0

0

0

0

0

0

0

0

0

0

0

0

0

0

0

0

0

0

0
2

0

0

0

0

0

0

0

0

0

0

0

0

0

0

0

0

0

0

0

0

0

0

0

0

0

0

0

0

0

0

0

0

0

0

0

0

0

0

0

0

0

0

0

0
2

0

0

0

0

0

0

0

0

0

0

0
4

0
2

0
2

0

0

0

0

0

0

0

0

0

0
4

0
2

0

0
2

0

0

0

0
4

0

0

0

0

0

0
4

0

0

0

0

0
4

0

0

0

0

0
4

0

0

0

0
4

0
4

0
2

0
2

0
2

0

0

0

0
4

0

0

0

0
4

0

0

0
4

0
4

0

0

0

0

0

0

0
4

0
4

0
5

0
5

0
5

0

0

0
4

0
4

0

0

0

0

0

0

0

0

0

0
4

0

0

0

0
4

0

0

0
4

0
4

2.76023

2.45105

1.48597

0.38106

0.272038

0.239648

0.0723307

0
4

0.309187

0.309187

0
4

0
4

0

0

0

0

0

0

0
4

0

0

0

0
4

0

0

0
4

0

0

0
4

0
4

0

0

0

0

0

0

0
4

0

0

0
4

0

0

0
4

0

0

0
4

0
4

0.375348
5

0.375348
5

0

0.214485

0

0.160864

0

0

0

0

2.77555756156289e-17
5

0
4

0
4

0
4

0

0

0

0

0
4

0

0

0
4

0

0

0
4

0
4

0
7

0
7

0
7

0

0

0
4

0
4

0
2

0
2

0
2

0

0

0

0

0

0

0

0

0
2

0

0

0

0

0

0

0

0
4

0
2

0
2

0

0

0

0
4

0

0

0

0

0

0

0

0

0

0

0
4

0

0

0

0
4

0
4

1.13805

1.13805

1.13805

0

0

0

0
4

0
4

0
6

0
6

0
6

0
4

0
4

0
2

0

0

0

0

0

0
4

0

0

0

0
4

0

0

0
4

0
4

0
7

0
7

0
7

0

0
4

0
4

0
7

0
7

0

0

0
4

0
4

0

0

0

0

0

0
4

0

0

0
4

0
4

0

0

0

0

0

0

0

0
4

0

0

0
4

0

0

0
4

0
4

0
6

0
6

0
6

0

0
4

0
4

0

0

0

0

0
4

0
4

0

0

0

0
4

0

0

0
4

0

0

0
4

0
4

0
2

0
2

0
2

0

0

0

0

0

0

0

0

0

0

0

0

0

0

0

0

0

0

0

0

0

0

0
4

0

0

0
4

0
2

0

0

0

0

0

0

0
4

0

0

0

0

0

0
4

0

0

0

0

0
4

0

0

0
4

0

0

0

0
4

0

0

0

0
4

0

0

0
4

0

0

0
4

0
4

0

0

0

0

0
4

0

0

0

0
4

0
4

0

0

0

0

0
4

0

0

0
4

0
4

0.773222

0.533257

0.213303

0.0799885

0.0799885

0.0799885

0.0799885

0
4

0.239966

0.106651

0.133314

0
4

0
4

0
6

0
6

0
6

0
4

0
4

0

0

0

0
4

0
4

0

0

0

0

0
4

0
4

0

0

0

0

0
4

0

0

0

0
4

0
4

0

0

0

0

0

0
4

0
4

0

0

0

0

0

0

0
4

0

0

0
4

0
4

0

0

0

0
4

0
4

59.2875

58.9142

52.7206

0.0799885

0.0533257

0.0533257

0.0798828

0.0799885

0.119824

0.0798828

3.78612

0.186534

0.359578

0.342511

0.239648

0.453268

0.0799885

0.199707

0
4

0.266628

0.266628

0
4

0.106651

0.0533257

0.0533257

0
4

1.1518563880486e-15

0
4

0.412819

0.229344

0.0917376

0

0.137606

0
4

0.0917376

0.0917376

0
4

0.0917376

0.0917376

0
4

0
4

0

0

0

0
4

0
4

0

0

0

0

0
4

0
4

0

0

0

0
4

0

0

0
4

0
4

1.83587

1.23675

0.92756

0.309187

1.11022302462516e-16

0
4

0.599121

0.599121

0
4

0
4

0

0

0

0

0
4

0
4

0

0

0

0

0

0
4

0

0

0
4

0

0

0
4

0
4

0

0

0

0

0
4

0

0

0
4

0

0

0
4

0

0

0
4

0
4

0

0

0

0

0
4

0
4

0

0

0

0
4

0
4

0
2

0
2

0
2

0
2

0

0

0

0

0

0

0

0
4

0

0

0

0
4

0
4

0

0

0

0

0
4

0

0

0
4

0

0

0
4

0

0

0
4

0
4

0
7

0

0

0

0
4

0

0

0
4

0
4

0

0

0

0
4

0
4

0

0

0

0
4

0

0

0

0
4

0

0

0
4

0
4

0

0

0

0

0
4

0
4

0

0

0

0

0
4

0

0

0
4

0
4

0

0

0

0

0

0
4

0
4

0

0

0

0
4

0

0

0
4

0
4

0

0

0

0
4

0
4

0

0

0

0

0
4

0
4

0
2

0
2

0
2

0

0

0

0

0

0
4

0
2

0

0

0

0

0

0

0

0
4

0
2

0

0

0

0

0

0
4

0

0

0

0
4

0

0

0

0
4

0

0

0
4

0

0

0
4

0

0

0
4

0

0

0
4

0
4

0

0

0

0
4

0
4

0

0

0

0
4

0

0

0
4

0
4

0

0

0

0

0

0
4

0
4

6.06747

6.06747

6.06747

0
4

0
4

0

0

0

0

0

0
4

0
4

0

0

0

0

0

0
4

0
4

0

0

0

0
4

0

0

0
4

0
4

0

0

0

0

0
4

0

0

0
4

0
4

0

0

0

0
4

0
4

0

0

0

0
4

0

0

0
4

0
4

0

0

0

0

0

0

0

0

0

0

0
4

0
4

0

0

0

0
4

0
4

0

0

0

0
4

0
4

0

0

0

0

0

0
4

0
4

0

0

0

0

0
4

0
4

0

0

0

0
4

0
4

0

0

0

0

0
4

0
4

0

0

0

0
4

0

0

0
4

0
4

0

0

0

0

0
4

0
4

0

0

0

0
4

0
4

0

0

0

0
4

0

0

0
4

0
4

0

0

0

0

0

0

0

0

0

0

0
4

0

0

0
4

0

0

0

0
4

0
4

0

0

0

0
4

0

0

0
4

0
4

0

0

0

0
4

0
4

0

0

0

0

0
4

0
4

0

0

0

0

0
4

0
4

0

0

0

0
4

0
4

0

0

0

0
4

0

0

0
4

0
4

0

0

0

0
4

0

0

0
4

0
4

0

0

0

0

0
4

0
4

0

0

0

0

0
4

0
4

0

0

0

0
4

0

0

0
4

0
4

0

0

0

0

0

0

0

0

0
4

0
1

0
1

0
4

0

0

0
4

0

0

0

0
4

0

0

0

0
4

0

0

0

0
4

0

0

0
4

0
4

2.65121258280487e-12
3

0
4

26.7033
4

26.7033
4

0.107242

0

0.107242

0
4

26.5961

26.5961

0
4

0

0

0
4

0

0

0
4

0
4

0

0

0

0
4

0
4

0

0

0

0

0
4

0
4

0

0

0

0
4

0
4

0

0

0

0
4

0
4

0
4

4563.64
5

320.117
4

320.117
4

9.71979
4

13.9237

0

0

0

0.0723307

0

0

0

0

0

0

0.133314

0

0

0

0

0.0723307

0

0

0

0

0.154593

0

0

0

0.108496

0.154593

0

0

0

0

0

0.108496

0

0

0

0

0.0723307

0

0

0

0

0

0

0.0533257

0

0.724189
4

0.759472

0.159977

0.159977

18.8118
4

18.7058

0

0

0.475963

0

0

0.133314

3.83353

0.397819

1.74301

0

2.40376
4

0

0

0.0723307

0.0799885

0

0

0.159977

0

1.08215

0

11.385
4

0

0.108496

1.23675

0

0

0

0

0.606022

0.275213

0.772967

97.1528
4

0.850263

0.0723307

0.0799885

0

0

0.0533257

0

0

0

0

0

0

0

0.618373

0

0

0

0.154593

0

0.309187

0

0.253559
4

0.154593

0

0

0

0

0.154593

0.0723307

0

0

0.23189

0.0723307
4

0

0.618373

0

0.0723307

0

0.154593

0

0

0

0

129.922
4

0

0

0

0.23189

0

0

0

0.0723307

0.154593

0

1.23956400699399e-13
4

0
4

0
4

4221.67
5

8.37429

1.21768

0
2

0

0.296835
4

0.239648

0.137606
4

0

0

0

0

0

0.392574

0

0
7

0.275213

0

0

0

0

0

0

0

0

0

3.68446
3

0

0

0

0

0

0

0

0

0

0

2.03853
4

0

0

0

0

0

0

0

0

0

0

0

0

0

0

0

0

0

0

0

0

0

0

0

0

0

0

0

0

0

0

0

0

0

0

0

0

0

0

0

0

0

0

0.0917376

0

0

0

0

0

0

0

0

0

0

0

0

0

0

0

1.02695629777827e-15

0
4

0

0

0

0

0

0
4

0

0

0
4

4212.89
5

1679.14
5

45.4629
3

652.254
3

1509.33
4

19.3193
5

33.3064
4

0

0.0798828

0

0

0

0

0

0

0

0

0

0

0

0

0

0

0.119824

0

0

0

0

0

0.0798828

0

0

0

0

0

0.27959

0

0

0

0

0

0

0

0

0

0

0

0

0

0

3.85188

0

0

0

0.0917376

0

0

0

0

0

0

0

0.0798828

0.716918
4

0.119824

0

0

0

0

0

0

0

0

0

0.0917376

0

0

0

0.0798828

0

0

0

0

0

0

0

0

0

0

0

0

0

0

0

0

0

0.538676

0

0

0

0.137606

0

0

0

0

0

0.262628

0.319531

0

0

0

0

0

0

0

0

0

0

1.53676
4

0

0

0

0

0

0

0

0.192155
3

0.159766

0.211562
4

0

0.327299
4

0

0

0.137606
4

0.154593

1.73764
4

0

0.0917376

0.479297
4

0.263464

0.0917376

0

0.424418

0.0649455
4

0

0

0

1.23846

0.4185

0.0917376

0

0.700341

0

0.137606

0.0798828

0.119824

0

0.0917376

0

0

0

0

0

0

0

0

0

0.0649455

0

0.0798828

0.0723307

0.0799885

0.202127

0

0

0.0799885

0

0

0.342616

6.55611
4

0

0.0798828

0

0.386483

0

0

0

0

0

0

0.978207
3

0

0

0

0

0.211562

0.0798828

0

0.411269

0

0

2.17847
4

0.0798828

0.17162

0

0.262628

0.199707

0

0

0.0917376

0

0.613245

1.64346
4

0.17162

0

0

0.458688

0.251503

0.229344

0

0

0

0.0649455

20.4855
4

0

0

0.679004

0.0798828

0

0

0

0.229344

0

0.137606

0

1.07559
4

0

0

0

0.183475

0

0

0.108496

0.137606

0

0

0

0

0

0

0

0.137606

0

0

0

1.06747

0

2.63764
4

0.27959

0

0

0

0

0

0

0

0

0.45202

0.0798828

0

0

0.119824

0.199707

0

0

0.119824

0

0

0

0.381557
4

0

0

0

0

0

0

0.253157

0

0

0.108496

2.83987
4

0

0

0.229344

0

0

0

0

0

0

0.0917376

0.446833
4

0.183475

0.133208

0

0

0

0.27959

0

0

0

0

0.607398
4

0

0

0

0

0.0798828

0

0

0.239648

0

0

0.524368
4

0

0

0

0

0

0.239648

0

0

0

0

1.19824

1.88222
4

0

0.119824

0

0

0

0

0

0

0

0

4.96963
4

0

0

0

0

0

0

0

0

0.0798828

0.239648

0

0

0.27959

0.0799885

0

0.175085

0

0

0

0

0.0917376

0

0

0

0

0

0

0

0.119824

0

0.119824

0

0.861154
4

0

0

0

0.0798828

1.23846

0

0

0.321081

0

0.412819

0.175085

0

0.137606

1.74301

0

0

0

0

0

0

0.355096

0

0

0

0

0

0.0917376

0

0

0.0723307

0.0798828

0

11.7124
4

0

0.23189

0

0

0.0798828

0

0

0.119824

0

0

0

0

0.159766

0

0

0

0

0

0.183475

0

0

0

0

0

0

0

0

0

0

0.0798828

0

0

26.2829
6

0.527574
3

0

0.0917376

0

0

0

0

0

0

0

0

0

0

0

0

0

0

0

0

0

0

0

3.68516
3

0

0

0

0

0

0

0

0

0

0

0.239754
4

0

0

0

0

0

0

0

0

0

0

0.654382

0

0

0

0

0

0.0798828

0

0

0

0

7.95354
4

0

0

0

0

0

0

0

0

0

0.0798828

0

0.0917376

0

0.199707

0

0

0

0

0

0

0

2.93886
4

0

0

0

0

0

0

0

0

1.8384

0

1.36549
4

0

0

0

0

0

0

0

0

0

0

0

0

0

0.137606

0

0.0649455

0

0

0

0

0.119824

0

0

0

0

0

0

0

0

0.0974182

0.875427

0.0798828

0

0

0

0

0

0

0

0

0

0

0

0.275213

0.47492
4

0

0.0798828

0

0

0

0

0.159766

0

0

0.0533257

0.199707
4

0

0

0.0649455

0

0

0

0.0798828

0

0

0

0

0

0

0

0

0

0

0

0

0

0

1.58447
4

0

0

0.0798828

0

0

0

0

0

0

0

0.592002
3

0

0

0

0

0

0

0

0

0

0.18664

0.538074
3

0

0

0

0.108496

0

0

0.137606

0

0

0

0

0

0

0

0

0

0

0

1.59766

0

0

1.17608
4

0

0

0

0.36695

0

0

0.239648

0

0

0

16.8589
4

0

0

0

0

0

0

0.0798828

0

0

0

0

0

0

0

0.0723307

0

0

0

0

0

0

0

0

0

0

0

0

0

0

0

0

0.0798828

0

0.555082
4

0.0723307

0

0

0

0

0.162364

0

0

0

0

0.0917376
4

0

0

0

0

0

0

0.0798828

0.119824

0

0

0.159766

0

0

0

0

0

0

0.119824

0

0

0.0798828

0

0

0

0

0

0

0

0

0

0

0

0.23189
4

0

0

0

0

0

0

0

0

0.137606

0

0

0

0.0723307

0

0

0

0

0.0798828

0

0.0798828

0

0

0

0

0

0

0

0

0

0

0

0

2.50043
4

0.881389
4

0

0.175085

0

0.0649455

0

0

0

0

0

0

0

0.0917376

0

0

0

0

0

0

0.0798828

0

0

0
4

0

0

0

0

0.0798828

0

0

0.0798828

0.0917376

0

0

0.0798828

0

0

0

0

0

0

0

0.23189

0

0.199707

0.0917376

0.119824

0

0

0

0

0

0

0

0

11.3118
3

0

0

0

0

0

0

0

0

0

0

0

0

0

0

0

0

0

0

0

0.0533257

0

0.650917
4

0

0

0

0

0

0.119824

0

0

0.0533257

0

0

0

0.0798828

0

0

0

0

0

0

0

0

0.497079
4

0

0

0.144661

0

0

0

0

0

0

0

79.5779
3

0

0

0

0

0

0

0

0

0

0

0

0

0

0

0

0

0

0

0.119824

0

0

0

0

0

0

0

0

0

0

0

0.319531

0

0

0

0

0

0

0

0

0.289421

0.0917376

0

0

0

0

0

0

0.0798828

0

0

0

0

0

0

0.119824

1.69715

0

0

0

0

0

0

0.119824

0

0

0

0

0

0

0

0

0

0

0

0

0

0

0.504557
4

0

0

0.0798828

0

0

0

0

0

0

0

0

0

0

0

0

0.0917376

0

0

0.137606

0

0

0

0

0

0

0

0

0

0

0

0

0

0

0

0

0

0

0

0

0

0

0.0533257

0

0

0.0798828

0

0

0

0

0

0

0.175085

0

0

0.159766

1.90938
4

0

0

0

0

0.0798828

0

0

0

0.119824

0

0.159766

0

0.0798828

0

0

0

0

0

0

0

0

0.343241
4

0

0

0

0

0

0

0

0

0

0.262628

0.229344

0

0

0

0

0

0.159766

0

0.0798828

0

0.0799885

0

0

0

0

0

0

0

0

0

0

0.199707

0.119824

0

0

0

0.119824

0

0.0798828

0

0

0

0

0

0

0

0

0

0

0

0

0

0

0

0.0798828

0

0

0

0

0.0917376

0

0

0

0

0

2.01560018675551e-11
5

0
4

0.175085

0.175085

0

0
4

0
4

0

0
4

0

0

0

0
4

0.0649455

0
5

0

0

0

0

0

0

0

0.0649455

0

0

0

0

0

0

0
4

0

0

0
4

0

0

0
4

0

0

0
4

0.0798828

0.0798828

0
4

0

0

0
4

0.0917376

0.0917376

0
4

0

0

0
4

0

0

0
4

1.1529249777098e-12
5

0
4

0

0

0

0
4

0
4

0

0

0

0
4

0
4

0

0

0

0
4

0
4

0

0

0

0
4

0
4

21.848
4

21.848
4

0

0

21.848
4

0
4

0

0

0

0
4

0

0

0
4

0

0

0
4

0
4

4.2632564145606e-13
5

0
4

0.290571
3

0

0

0

0

0

0

0
4

0
4

0.160864

0.160864

0.160864

0
4

0
4

0.0808509

0.0808509

0.0808509

0
4

0
4

0

0

0

0

0
4

0
4

0

0

0

0

0
4

0
4

0

0

0

0
4

0
4

0

0

0

0
4

0
4

0

0

0

0
4

0
4

0.0488567

0.0488567

0.0488567

0
4

0
4

0

0

0

0
4

0
4

0

0

0

0
4

0
4

0
4

5.20958

5.20958

5.20958

0

2.31537

0

0

0

0

0

0

2.89421

0

0

0
1

0

0

0

0

0

0

0

0

0

0

0

0

0

0

0

0

0

0

0

0

0

0

0

0

0

0

0

0

0

0

0

0

0

0

0
4

0
4

0

0

0

0
4

0
4

0
4

1.40068

1.40068

1.40068

0
7

0

0

0

0

0

0

0

0

0

0

0
6

0

0

0

0

0

0

0

0

0

0

0

0

0

0

0

0

0

0

0

0

0

0

0

0.612799

0

0

0

0.787884

0

0

0

0

0
6

0

0

0

0

0

0

0

0

0

0

0

0
4

0

0

0

0

0

0

0
4

0

0

0
4

0

0

0

0
4

0

0

0

0
4

0

0

0
4

0

0

0
4

0
4

0

0

0

0
4

0
4

0

0

0

0
4

0
4

0

0

0

0
4

0
4

0
4

0
7

0
7

0
7

0
7

0

0

0

0

0

0

0

0

0

0

0
6

0
7

0

0

0

0

0

0

0
4

0
6

0

0

0

0
4

0

0

0

0
4

0
4

0
4

0
7

0
7

0
7

0

0

0

0

0

0
4

0
4

0
4

0

0

0

0

0
4

0
4

0
4

0

0

0

0

0
4

0
4

0
4

0.599121

0.599121

0.599121

0.599121

0
4

0
4

0
4

0

0

0

0

0
4

0
4

0
4

0

0

0

0

0
4

0
4

0
4

0

0

0

0

0
4

0
4

0
4

0

0

0

0

0
4

0
4

0
4

0

0

0

0

0
4

0
4

0
4

0

0

0

0

0
4

0
4

0
4

0

0

0

0

0
4

0
4

0
4

0
7

0
7

0
7

0
7

0
7

0

0
4

0

0

0
4

0
4

0
4

0

0

0

0

0
4

0
4

0
4

0

0

0

0

0
4

0
4

0
4

0

0

0

0

0
4

0
4

0
4

0

0

0

0

0
4

0
4

0
4

0

0

0

0

0
4

0
4

0
4

0

0

0

0

0
4

0
4

0
4

0

0

0

0

0
4

0
4

0
4

0

0

0

0

0
4

0
4

0
4

0

0

0

0

0
4

0
4

0
4

0

0

0

0

0
4

0
4

0
4

0

0

0

0

0

0

0
4

0

0

0
4

0
4

0
4

0

0

0

0

0
4

0
4

0
4

0

0

0

0

0
4

0
4

0
4

0

0

0

0

0
4

0
4

0
4

0

0

0

0

0
4

0
4

0
4

0

0

0

0

0
4

0
4

0
4

0

0

0

0

0
4

0
4

0
4

0

0

0

0

0
4

0
4

0
4

0

0

0

0

0
4

0
4

0
4

0

0

0

0

0
4

0
4

0
4

0.525256

0.525256

0.525256

0.525256

0
4

0
4

0
4

0
7

0
7

0
7

0

0

0

0
4

0

0

0

0

0
4

0
4

0
4

0.0798828

0.0798828

0.0798828

0.0798828

0
4

0
4

0
4

0

0

0

0

0
4

0
4

0
4

0

0

0

0

0
4

0
4

0
4

0

0

0

0

0
4

0
4

0
4

0

0

0

0

0
4

0
4

0
4

0

0

0

0

0
4

0
4

0
4

0

0

0

0

0
4

0
4

0
4

0

0

0

0

0
4

0
4

0
4

0

0

0

0

0
4

0
4

0
4

0

0

0

0

0
4

0
4

0
4

0

0

0

0

0

0

0

0
4

0

0

0

0
4

0
4

0
4

0

0

0

0

0
4

0
4

0
4

0

0

0

0

0
4

0
4

0
4

0

0

0

0

0
4

0
4

0
4

0

0

0

0

0
4

0
4

0
4

0

0

0

0

0
4

0
4

0
4

0

0

0

0

0
4

0
4

0
4

0

0

0

0

0
4

0
4

0
4

0

0

0

0

0
4

0
4

0
4

0

0

0

0

0
4

0
4

0
4

0

0

0

0

0
4

0
4

0
4

31.3148

31.3148

31.3148

31.0467

0.107242

0.160864

4.44089209850063e-16

0
4

0
4

0
4

0

0

0

0

0
4

0
4

0
4

0

0

0

0

0
4

0
4

0
4

0

0

0

0

0
4

0
4

0
4

0

0

0

0

0
4

0
4

0
4

0

0

0

0

0
4

0
4

0
4

0

0

0

0

0
4

0
4

0
4

0

0

0

0

0
4

0
4

0
4

0

0

0

0

0
4

0
4

0
4

0

0

0

0

0
4

0
4

0
4

0

0

0

0

0
4

0
4

0
4

1.3068

1.3068

1.3068

0.967696

0.177406

0

0.161702

1.11022302462516e-16

0
4

0
4

0
4

0

0

0

0

0
4

0
4

0
4

0

0

0

0

0
4

0
4

0
4

0
7

0
7

0
7

0

0

0

0
4

0
4

0
4

0

0

0

0

0

0

0
4

0

0

0
4

0
4

0
4

0

0

0

0

0
4

0

0

0

0

0
4

0

0

0
4

0
4

0
4

0
7

0
7

0
7

0
7

0

0

0

0
4

0
4

0

0

0

0
4

0
4

0
4

0.108496

0.108496

0.108496

0

0.108496

0

0
4

0
4

0
4

0

0

0

0

0

0
4

0
4

0
4

0

0

0

0

0
4

0
4

0
4

2.54795

2.54795

2.54795

2.54795

0
4

0
4

0
4

0

0

0

0

0
4

0

0

0
4

0
4

0

0

0

0
4

0

0

0
4

0
4

0
4

1.44777

1.44777

1.44777

1.23329

0.214485

8.32667268468867e-17

0
4

0
4

0
4

0

0

0

0

0

0
4

0
4

0
4

0

0

0

0

0

0
4

0
4

0
4

0.18664

0.18664

0.18664

0.18664

0
4

0
4

0
4

0

0

0

0

0
4

0
4

0
4

0
2

0
2

0
2

0
2

0

0

0

0

0
4

0
2

0

0

0

0

0

0

0
4

0

0

0
4

0

0

0
4

0
4

0
4

0

0

0

0

0

0

0
4

0
4

0
4

0

0

0

0

0
4

0
4

0
4

0

0

0

0

0
4

0

0

0
4

0

0

0
4

0
4

0
4

0

0

0

0

0

0
4

0
4

0
4

0

0

0

0

0

0

0

0
4

0
4

0
4

0

0

0

0

0
4

0
4

0
4

0

0

0

0

0

0
4

0

0

0
4

0
4

0

0

0

0
4

0
4

0
4

0

0

0

0

0

0
4

0

0

0
4

0
4

0
4

0

0

0

0

0
4

0
4

0
4

0

0

0

0

0

0

0
4

0
4

0
4

0

0

0

0

0

0

0

0

0

0
4

0
4

0
4

0

0

0

0

0

0
4

0

0

0
4

0
4

0
4

0

0

0

0

0
4

0
4

0
4

0

0

0

0

0
4

0
4

0
4

0

0

0

0

0
4

0

0

0
4

0
4

0
4

0

0

0

0

0

0
4

0

0

0
4

0
4

0
4

0.787884

0.787884

0.787884

0

0.787884

0
4

0
4

0
4

1.57659

1.57659

1.57659

0.242553

1.33404

0
4

0
4

0
4

0

0

0

0

0
4

0
4

0
4

0

0

0

0

0

0
4

0
4

0
4

0.268106

0.268106

0.160864

0

0.160864

0
4

0.107242

0.107242

0
4

0
4

0
4

0
4

0
4

0
4

0

0

0

0

0

0

0
4

0

0

0
4

0
4

0
4

0.153274

0.0732851

0.0732851

0.0732851

0
4

0
4

0.0799885

0.0799885

0.0799885

0
4

0
4

1.38777878078145e-17

0
4

0

0

0

0

0
4

0

0

0
4

0

0

0
4

0
4

0
4

0

0

0

0

0

0
4

0
4

0
4

0

0

0

0

0

0
4

0
4

0
4

0

0

0

0

0

0
4

0
4

0
4

0

0

0

0

0
4

0

0

0
4

0
4

0
4

0

0

0

0

0
4

0

0

0
4

0
4

0
4

0

0

0

0

0
4

0
4

0
4

0

0

0

0

0
4

0

0

0
4

0
4

0
4

0

0

0

0

0
4

0

0

0
4

0
4

0
4

0
7

0
7

0
7

0

0

0

0

0

0

0

0

0
4

0

0

0

0
4

0
4

0
4

0.55918

0.55918

0.55918

0.55918

0
4

0
4

0
4

0.0341641

0.0341641

0.0341641

0.0341641

0
4

0
4

0
4

0

0

0

0

0

0
4

0
4

0
4

0

0

0

0

0
4

0

0

0
4

0
4

0
4

0

0

0

0

0
4

0
4

0
4

0

0

0

0

0
4

0

0

0
4

0
4

0
4

0

0

0

0

0
4

0

0

0
4

0
4

0
4

0

0

0

0

0

0
4

0
4

0
4

0.097998

0.097998

0.097998

0.097998

0
4

0

0

0
4

0
4

0
4

0

0

0

0

0

0
4

0
4

0
4

0.0723307

0

0

0

0

0

0

0

0

0
4

0

0

0
4

0
4

0.0723307

0.0723307

0.0723307

0

0
4

0
4

0
4

0.412819

0.412819

0.412819

0.412819

0
4

0
4

0
4

0

0

0

0

0
4

0

0

0
4

0
4

0
4

0

0

0

0

0
4

0
4

0
4

0

0

0

0

0

0
4

0
4

0
4

0

0

0

0

0
4

0
4

0
4

0.102492

0.102492

0.102492

0.102492

0
4

0

0

0
4

0
4

0
4

0

0

0

0

0
4

0
4

0
4

20.9123

20.9123

0

0

0
4

20.9123

20.9123

0
4

0
4

0
4

0.144661

0.144661

0.144661

0.144661

0
4

0

0

0
4

0
4

0
4

0

0

0

0

0

0
4

0
4

0
4

0
5

0
5

0
5

0
5

0

0

0
4

0

0

0

0

0

0
4

0
4

0
4

0

0

0

0

0

0
4

0
4

0
4

0

0

0

0

0

0
4

0
4

0
4

0

0

0

0

0

0
4

0
4

0
4

0

0

0

0

0
4

0
4

0
4

0

0

0

0

0
4

0

0

0
4

0
4

0
4

0

0

0

0

0
4

0
4

0
4

0

0

0

0

0
4

0
4

0
4

0

0

0

0

0
4

0
4

0
4

0

0

0

0

0
4

0

0

0
4

0
4

0
4

0

0

0

0

0
4

0
4

0
4

0
7

0
7

0
7

0
7

0

0
4

0

0

0

0
4

0

0

0
4

0
4

0

0

0

0
4

0
4

0
4

0

0

0

0

0

0
4

0
4

0
4

0

0

0

0

0
4

0
4

0
4

0

0

0

0

0
4

0
4

0
4

0

0

0

0

0
4

0
4

0
4

0

0

0

0

0
4

0
4

0
4

0

0

0

0

0
4

0
4

0
4

0

0

0

0

0
4

0
4

0
4

0

0

0

0

0
4

0
4

0
4

0.0798828

0.0798828

0.0798828

0.0798828

0
4

0
4

0
4

0

0

0

0

0
4

0
4

0
4

4.80865

4.80865

3.45064

0

0

0

0

0

0

0

0

0

0

0

0

0

0

0

0

0

0

0

0

2.27611

0

0.175085
4

0

0

0

0

0

0

0

0

0

0

0.175085

0

0

0

0.119824

0

0

0

0

0

0

0

0

0

0

0

0

0

0

0

0

0

0

0

0

0

0

0

0

0.146997

0

0

0

0

0

0

0

0

0

0

0

0

0

0.119824

0

0

0

0

0

0.437713

0

0

0

0

0
4

0

0

0

0

0

0
4

0

0

0
4

0

0

0
4

0

0

0
4

0

0

0
4

0

0

0
4

0

0

0

0

0
4

1.35801

0.998535

0.359473

5.55111512312578e-17

0
4

0

0

0

0

0
4

0

0

0

0

0
4

0

0

0

0
4

0

0

0
4

0

0

0
4

0

0

0
4

0
4

0

0

0

0

0

0

0
4

0
4

0

0

0

0

0

0
4

0

0

0
4

0
4

0

0

0

0

0
4

0
4

0

0

0

0
4

0
4

0

0

0

0
4

0
4

0
4

0

0

0

0

0
4

0
4

0
4

0
4

22.6022
3

15.4618
3

14.1599
3

1.4167

0

0

0

0

0

0

1.23329

0

0.107242

0

0.0761658

0
4

7.80056
3

7.80056
3

0

0

0
4

0

0

0
4

0.101554

0

0

0

0.0507772

0

0

0

0.0507772

0
4

2.40793

0.775882

1.17671

0.375348

0.0799885

1.38777878078145e-16

0
4

0

0

0
4

0

0

0
4

0

0

0
4

0

0

0
4

0

0

0

0
4

0

0

0
4

0

0

0

0
4

0

0

0

0
4

2.43311

2.43311

0
4

0

0

0

0
4

0

0

0
4

0

0

0
4

1.33226762955019e-15
3

0
4

0

0

0

0

0

0

0

0

0
4

0
4

0

0

0

0

0
4

0
4

1.30195

1.30195

1.30195

0
4

0
4

0

0

0

0
4

0
4

2.22044604925031e-16
3

0
4

0

0

0

0

0

0

0
4

0
4

0
4

0

0

0

0

0
4

0
4

0
4

0

0

0

0

0
4

0
4

0
4

0

0

0

0

0
4

0
4

0
4

0

0

0

0

0
4

0
4

0
4

0

0

0

0

0
4

0
4

0
4

0

0

0

0

0
4

0
4

0
4

5.73288

5.73288

5.73288

5.73288

0
4

0
4

0
4

0

0

0

0

0
4

0
4

0
4

0

0

0

0

0
4

0
4

0
4

0

0

0

0

0

0
4

0
4

0
4

0

0

0

0

0

0
4

0
4

0
4

1.32763

1.32763

1.32763

1.32763

0
4

0
4

0
4

0

0

0

0

0
4

0
4

0
4

0

0

0

0

0
4

0
4

0
4

0.0798828

0.0798828

0.0798828

0.0798828

0
4

0
4

0
4

0

0

0

0

0
4

0
4

0
4

5.96744875736022e-16
3

0
4

558.031

490.505
4

0

0

0

0

0

0

0

0

0

0

0

0

0

0

0

0

0

0

0

0

0

0

0

0

0

0

0

0

0

0

0

0

0

0

0

0
4

0

0

0

0

0
4

0

0

0

0
4

0

0

0

0
4

0
4

33.6675
4

9.4182

1.36428

2.71382

0.262628

5.07747

8.88178419700125e-16

0
4

23.3739

1.66331

16.2829

5.42764

0
4

0.175085

0.175085

0
4

0.700341

0.700341

0
4

0
4

0

0

0

0

0

0
4

0

0

0

0
4

0

0

0
4

0
4

0
6

0
6

0
6

0
4

0
4

0

0

0

0
4

0

0

0
4

0
4

2.31537

2.31537

2.31537

0

0
4

0

0

0

0
4

0

0

0
4

0
4

0

0

0

0

0
4

0
4

0.350171

0.350171

0.175085

0

0.175085

0
4

0
4

0

0

0

0

0
4

0

0

0
4

0
4

0

0

0

0
4

0
4

0

0

0

0
4

0

0

0
4

0
4

232.388
4

222.56
4

150.661

5.9529

0.437713

0.175085

2.01348

0.262628

0.262628

0.262628

0.437713

13.5074

29.3191

3.23908

8.84181

1.84657

4.20205

0.787884

0.350171

1.22124532708767e-15
4

0
4

6.68552

5.42764

0.612799

0.29491

0.175085

0.175085

1.16573417585641e-15

0
4

3.14266

2.40074

0.199707

0.0798828

0.0798828

0.262628

0.119824

0
4

0
4

0

0

0

0
4

0
4

0

0

0

0
4

0
4

0

0

0

0
4

0
4

0

0

0

0
4

0
4

0.160864

0.160864

0.160864

0
4

0
4

0.175085

0.175085

0.175085

0
4

0
4

0

0

0

0
4

0
4

4.02754
4

3.71836
4

2.97504

0

0

0

0.588725

0

0

0

0

0.154593

0
4

0

0

0
4

0

0

0

0
4

0.309187

0.309187

0
4

1.11022302462516e-16
4

0
4

7.68378
4

1.82398
4

0.772967

0.89642

0.154593

0

0
4

5.70521

5.0739

0.399414

0.23189

0
4

0.154593

0.154593

0
4

0

0

0
4

0
4

0.444014
4

0.444014
4

0

0
4

0

0.154593

0.289421

0

0

0

0

0
4

0
4

0

0

0

0

0

0

0

0

0
4

0

0

0

0

0
4

0

0

0

0

0

0
4

0

0

0
4

0

0

0
4

0
4

0
6

0
6

0
6

0

0

0
4

0
4

209.293

185.853

183.402

0.612799

0.262628

0.175085

0.350171

0.525256

0.262628

0.262628

0
4

22.3018

20.5509

1.75085

1.33226762955019e-15

0
4

0.700341

0

0.525256

0.175085

0
4

0.437713

0.437713

0
4

9.27036225562006e-15

0
4

0

0

0

0

0

0

0

0
4

0

0

0
4

0
4

0
4

3.50171
5

3.50171
5

0

0

0
4

3.50171
5

0
5

0

0

0

0

0

3.50171

0

0

0

0

0

0
4

0
4

0
4

8.0022
7

0
6

0
6

0

0

0
4

0

0

0
4

0
4

0

0

0

0
1

0

0

0

0

0

0

0

0

0

0

0

0

0

0

0

0

0

0

0

0

0

0

0

0

0

0

0

0

0

0

0

0

0

0

0

0

0

0

0

0

0

0

0

0

0

0

0

0

0

0

0

0

0

0

0

0

0

0

0

0

0

0

0

0

0

0

0
4

0

0

0

0

0

0

0

0

0

0

0

0
4

0

0

0

0

0

0

0

0

0

0

0

0

0

0

0

0

0

0

0

0

0

0

0

0

0

0

0

0

0

0

0

0

0

0

0

0

0

0

0

0

0

0

0

0

0

0

0

0

0

0

0

0

0

0

0

0

0

0

0

0

0

0

0

0

0

0

0

0

0

0

0

0

0

0

0
6

0

0

0

0

0

0

0

0

0

0

0
7

0

0

0

0

0

0

0

0

0

0

0
8

0

0

0

0

0

0

0

0

0

0

0

0

0

0

0

0

0

0

0

0

0

0

0

0

0

0

0

0

0

0

0

0

0

0

0

0

0

0

0

0

0

0

0

0
7

0

0

0

0

0

0

0

0

0

0

0

0

0

0

0

0

0

0

0

0

0

0
4

0
6

0

0

0

0

0

0

0
4

0

0

0
4

0
7

0

0

0
4

0

0

0

0

0
4

0

0

0

0

0
4

0

0

0

0

0
4

0

0

0

0
4

0

0

0
4

0

0

0

0
4

0

0

0

0
4

0

0

0

0

0

0

0

0

0

0

0

0

0
4

0

0

0
4

0

0

0

0
4

0

0

0
4

0

0

0

0
4

0

0

0
4

0

0

0
4

0

0

0
4

0

0

0
4

0

0

0
4

0

0

0
4

0

0

0

0

0

0

0

0

0
4

0

0

0
4

0

0

0
4

0

0

0
4

0

0

0
4

0

0

0
4

0

0

0
4

0

0

0
4

0

0

0
4

0

0

0
4

0

0

0
4

0

0
7

0

0

0

0
7

0

0

0

0

0

0

0
4

0

0

0
4

0

0

0
4

0

0

0
4

0

0

0
4

0

0

0
4

0

0

0
4

0

0

0
4

0

0

0
4

0

0

0
4

0

0

0
4

0

0

0

0

0

0

0

0
6

0

0

0

0

0

0

0

0
4

0

0

0
4

0

0

0
4

0

0

0
4

0

0

0

0

0

0

0

0

0

0

0
4

0

0
7

0

0

0

0

0

0
4

0

0

0

0

0

0

0
4

0

0

0

0

0

0

0

0

0
4

0
4

8.0022
7

0
6

0
6

0
7

0

0

0

0

0

0

0

0

0

0

0
6

0

0

0

0

0

0

0

0

0

0

0

0

0

0

0

0

0

0

0

0

0

0

0

0

0

0

0

0

0

0

0

0

0

0

0

0

0

0

0

0

0

0

0

0
5

0

0

0

0

0

0

0

0

0

0

0
6

0

0

0

0

0

0

0

0

0

0
6

0
6

0
4

0
7

0
7

0

0

0

0
4

4.17844
7

0.355441
7

3.61776
7

0

0

0

0

0

0

0

0

0

0

0

0

0

0

0.097998

0

0.107242

0

0

0

0

0

0

0

0

0

0

0

0

0

0

0

0

0

0

0

0

0

0

0

0

0

1.2490009027033e-16
7

0
4

0
7

0

0

0

0

0

0
4

0

0

0

0

0

0
4

0
7

0
7

0

0

0

0

0

0

0

0

0

0

0

0
7

0

0

0

0

0

0

0

0

0

0

0
6

0

0

0

0

0

0

0

0

0

0

0

0

0
4

0
6

0
6

0

0

0

0
4

0
6

0
6

0

0

0

0

0

0
4

0
6

0
7

0
6

0
7

0

0

0

0

0

0

0

0

0

0

0

0

0

0

0

0

0

0

0

0

0
7

0

0

0

0

0

0

0

0

0
6

0
7

0
6

0
7

0
7

0
6

0
4

0
7

0
7

0

0

0

0

0

0

0

0
4

0

0

0

0

0

0

0

0

0

0

0

0

0
4

0
6

0
6

0

0
4

0
6

0
6

0

0

0

0

0
4

0

0

0

0
4

0

0

0
4

0

0

0

0
4

0

0

0
4

0

0

0
4

0

0

0
4

0

0

0
4

0

0

0
4

0

0

0
4

0

0

0
4

0
7

0
7

0

0

0

0

0
4

0

0

0
4

0

0

0
4

0

0

0
4

0

0

0
4

0

0

0
4

0

0

0
4

0

0

0
4

0

0

0
4

0

0

0
4

0

0

0
4

0
7

0
7

0

0
4

0

0

0
4

0

0

0
4

0

0

0
4

0

0

0
4

0

0

0
4

0

0

0
4

0

0

0
4

0

0

0
4

0

0

0
4

0

0

0
4

0

0

0

0

0

0
4

0

0

0
4

0

0

0
4

0

0

0
4

0

0

0
4

0

0

0
4

0
7

0
7

0
4

3.82375

3.82375

0
4

0

0

0
4

0

0

0

0
4

0

0

0
4

8.88178419700125e-16
7

0
4

0
7

0
6

0

0
6

0

0

0

0
4

0

0

0

0
4

0
4

0
7

0

0

0
4

0
8

0
8

0

0
4

0

0

0

0
4

0

0

0
4

0

0

0
4

0

0

0
4

0

0

0
4

0

0

0
4

0

0

0
4

0

0

0
4

0
4

0

0

0

0

0
4

0

0

0

0

0

0

0
4

0

0

0
4

0

0

0
4

0

0

0
4

0

0

0
4

0
5

0
5

0

0
4

0
7

0

0

0
4

0

0

0
4

0

0

0

0
4

0

0

0

0
4

0

0

0

0
4

0

0

0
4

0

0

0
4

0
4

0
7

0
7

0

0

0

0
6

0

0
4

0
4

0

0

0

0
4

0
4

0

0

0

0
4

0
4

0

0

0

0
4

0
4

0

0

0

0
4

0
4

0

0

0

0

0

0
4

0
4

0

0

0

0
4

0
4

0

0

0

0

0
4

0
4

0

0

0

0
4

0
4

0

0

0

0
4

0
4

0

0

0

0
4

0
4

0

0

0

0
4

0
4

0

0

0

0
4

0
4

0
4

1.7695

0.697075

0.321727
7

0

0

0

0

0.321727

0

0

0

0

0

0

0
6

0

0

0

0

0

0

0

0

0

0

0

0
4

0

0

0
4

0

0
7

0

0

0

0
4

0

0

0

0
4

0

0

0
4

0.375348

0.375348

0
4

0

0

0
4

0

0

0
4

0
4

0

0

0

0

0

0

0

0

0
4

0
4

0

0

0

0
4

0
4

0

0

0

0
4

0
4

0

0

0

0
4

0
4

0

0

0

0

0
4

0
4

0

0

0

0
4

0

0

0
4

0
4

1.07242

1.07242

1.07242

0
4

0
4

0

0

0

0
4

0

0

0
4

0
4

0

0

0

0
4

0
4

0

0

0

0
4

0
4

0

0

0

0
4

0
4

0

0

0

0
4

0
4

0
4

0

0

0

0
6

0

0

0

0

0

0

0

0

0

0

0
6

0

0

0

0

0

0

0

0

0

0

0

0

0

0

0
4

0

0

0
4

0

0

0
4

0

0

0
4

0

0

0
4

0
4

0

0

0

0

0
4

0
4

0

0

0

0
4

0
4

0

0

0

0
4

0
4

0

0

0

0
4

0
4

0

0

0

0
4

0
4

0

0

0

0
4

0
4

0

0

0

0
4

0
4

0

0

0

0
4

0
4

0

0

0

0
4

0
4

0
4

0

0

0

0

0

0
4

0
4

0

0

0

0
4

0
1

0

0

0

0

0
4

0

0

0

0

0
4

0

0

0
4

0
4

0

0

0

0

0
4

0

0
1

0

0

0
4

0
4

0
4

37.8773
5

37.8773
5

14.9538

11.0829

0
7

0

0

0

0

0

0

0

3.87092

0

0

0

0

0

0

0
4

0
6

0
6

0
7

0
6

0

0

0

0

0

0
4

0

0

0
4

0

0

0
4

0

0

0
4

0

0

0
4

1.31807

1.31807

0
4

0

0

0
4

0
6

0
6

0

0
4

0

0

0

0

0

0

0
4

0

0

0

0

0
4

0

0

0

0

0
4

0

0

0

0
4

1.61696

1.61696

0
4

0

0

0
4

19.9885

19.9885

0
4

3.5527136788005e-15
5

0
4

0

0

0

0

0
4

0
4

0

0

0

0

0
4

0
4

0

0

0

0
4

0
4

0

0

0

0
4

0
4

0

0

0

0
4

0
4

0

0

0

0
4

0
4

0

0

0

0
4

0
4

0
4

0

0

0

0

0

0

0

0

0

0

0

0
4

0

0

0

0

0

0
4

0

0

0

0

0

0
4

0

0

0
4

0

0

0
4

0
4

0
4

0
3

0
3

0
3

0
3

0

0
4

0
4

0
4

0
6

0
6

0
6

0
6

0

0
4

0

0

0
4

0
4

0
4

0

0

0

0

0

0

0

0
4

0

0

0

0
4

0
4

0
4

0

0

0

0

0

0

0
4

0
4

0
4

0
4

0
4

0
4

0
4

0

0

0
4

0

0

0
4

0
4

0
4

0

0

0

0

0

0

0
4

0
4

0
4

0

0

0

0

0

0
4

0
4

0
4

0

0

0

0

0

0

0

0
4

0
4

0
4

0

0

0

0

0

0
4

0
4

0
4

0

0

0

0

0
4

0
4

0
4

0
6

0
6

0
6

0

0

0

0

0

0

0

0
4

0
4

0
4

0
6

0
6

0

0

0
4

0

0

0
4

0
4

0
4

0

0

0

0

0

0
4

0
4

0
4

0

0

0

0

0
4

0
4

0
4

0

0

0

0

0

0
4

0

0

0
4

0
4

0
4

0

0

0

0

0

0
4

0
4

0
4

0

0

0

0

0

0

0
4

0
4

0
4

0

0

0

0

0

0
4

0
4

0
4

0

0

0

0

0
4

0
4

0
4

0

0

0

0

0
4

0

0

0
4

0
4

0
4

0.439355

0.439355

0.439355

0.239648

0.119824

0.0798828

0
4

0
4

0
4

0
4

0
4

0
4

0

0

0

0

0

0

0

0
4

0
4

0

0

0

0
4

0
4

0
4

0.758887

0.758887

0.758887

0.519238

0.239648

0
4

0
4

0
4

0

0

0

0

0

0
4

0

0

0
4

0
4

0
4

0

0

0

0

0
4

0

0

0
4

0
4

0
4

0

0

0

0

0
4

0
4

0
4

0

0

0

0

0

0
4

0
4

0
4

0

0

0

0

0
4

0
4

0
4

0.671252

0.671252

0.321081

0

0.321081

0
4

0.350171

0.350171

0
4

5.55111512312578e-17

0
4

0
4

0

0

0

0

0
4

0
4

0
4

0

0

0

0

0
4

0
4

0
4

0

0

0

0

0
4

0
4

0
4

3.13598
4

3.13598
4

2.98139
4

0.578842

2.17066

0

0

0

0.23189

0

0

0
4

0

0

0

0

0

0
4

0.154593

0.154593

0
4

3.33066907387547e-16
4

0
4

0
4

0

0

0

0

0
4

0
4

0
4

0

0

0

0

0
4

0
4

0
4

0

0

0

0

0
4

0
4

0
4

0

0

0

0

0

0
4

0
4

0
4

0

0

0

0

0
4

0
4

0
4

0

0

0

0

0
4

0

0

0
4

0
4

0
4

0

0

0

0

0
4

0
4

0
4

0

0

0

0

0
4

0
4

0
4

0

0

0

0

0
4

0
4

0
4

0

0

0

0

0
4

0
4

0
4

11.1551
4

11.1551
4

7.36753
4

5.97619
4

0

1.23675

0.154593

0

0
4

3.78754

3.78754

0
4

0

0

0

0
4

0

0

0
4

1.33226762955019e-15
4

0
4

0
4

0

0

0

0

0
4

0
4

0
4

0

0

0

0

0
4

0
4

0
4

0

0

0

0

0
4

0
4

0
4

0

0

0

0

0
4

0
4

0
4

0

0

0

0

0
4

0
4

0
4

0

0

0

0

0
4

0
4

0
4

0

0

0

0

0
4

0
4

0
4

0

0

0

0

0
4

0
4

0
4

0

0

0

0

0
4

0
4

0
4

0

0

0

0

0
4

0
4

0
4

0

0

0

0

0

0

0

0

0
4

0

0

0

0

0

0
4

0

0

0
4

0

0

0

0
4

0

0

0
4

0
4

0
4

0

0

0

0

0
4

0
4

0
4

0

0

0

0

0
4

0
4

0
4

0

0

0

0

0
4

0
4

0
4

0

0

0

0

0
4

0
4

0
4

0

0

0

0

0
4

0
4

0
4

0.107242

0.107242

0.107242

0.107242

0
4

0
4

0
4

0

0

0

0

0
4

0
4

0
4

0

0

0

0

0
4

0
4

0
4

0

0

0

0

0
4

0
4

0
4

0

0

0

0

0
4

0
4

0
4

0
7

0
7

0
7

0
7

0

0

0

0

0

0

0
4

0
4

0
4

0

0

0

0

0
4

0
4

0
4

0

0

0

0

0
4

0
4

0
4

0

0

0

0

0
4

0
4

0
4

0

0

0

0

0
4

0
4

0
4

0

0

0

0

0
4

0
4

0
4

0

0

0

0

0
4

0
4

0
4

0

0

0

0

0
4

0
4

0
4

0

0

0

0

0
4

0
4

0
4

0

0

0

0

0
4

0
4

0
4

0

0

0

0

0
4

0
4

0
4

0
7

0
7

0
7

0
7

0

0

0
4

0
4

0
4

0

0

0

0

0
4

0
4

0
4

0

0

0

0

0
4

0
4

0
4

0

0

0

0

0
4

0
4

0
4

0

0

0

0

0
4

0
4

0
4

0

0

0

0

0
4

0
4

0
4

0

0

0

0

0
4

0
4

0
4

0

0

0

0

0
4

0
4

0
4

0

0

0

0

0
4

0
4

0
4

0

0

0

0

0
4

0
4

0
4

0

0

0

0

0
4

0
4

0
4

0

0

0

0

0

0

0

0

0
4

0
4

0
4

0

0

0

0

0
4

0
4

0
4

0

0

0

0

0
4

0
4

0
4

0

0

0

0

0
4

0
4

0
4

0

0

0

0

0
4

0
4

0
4

0.107242

0.107242

0.107242

0.107242
8

0

0
4

0

0

0
4

0
4

0

0

0

0
4

0
4

0

0

0

0
4

0
4

0
4

0
4

15401.4

2.0617
7

2.0617
7

0
7

0
7

0

0
4

0
7

0
7

0
7

0
7

0

0

0

0

0

0

0

0

0

0

0
7

0

0

0

0

0

0

0

0

0

0

0
7

0

0

0

0

0

0

0

0

0

0

0
7

0

0

0

0

0

0

0

0

0

0

0
7

0

0

0

0

0

0

0

0

0

0

0
6

0

0

0

0

0

0

0

0

0

0

0
6

0

0

0

0

0

0

0

0

0

0

0
7

0

0

0

0

0

0

0

0

0

0

0
7

0

0

0

0

0

0

0

0

0

0

0
7

0

0

0

0

0

0

0

0

0

0

0
7

0
6

0

0

0

0

0

0

0

0

0

0

0
7

0

0

0

0

0

0

0

0

0

0

0
7

0

0

0

0

0

0

0

0

0

0

0
7

0

0

0

0

0

0

0

0

0

0

0
7

0

0

0

0

0

0

0

0

0

0

0
7

0

0

0

0

0

0

0

0

0

0

0
7

0

0

0

0

0

0

0

0

0

0

0
6

0

0

0

0

0

0

0

0

0

0

0
7

0

0
7

0
7

0
6

0
6

0
7

0
7

0

0
7

0
8

0
7

0
7

0
7

0
7

0

0

0
7

0

0
7

0

0

0

0

0
8

0
6

0

0

0

0

0

0

0

0

0

0

0

0

0

0

0

0

0

0

0

0

0

0
6

0

0

0

0

0

0

0

0

0

0

0
6

0

0

0

0

0

0

0

0

0

0

0
7

0

0

0

0

0

0

0

0

0

0

0
4

0
7

0

0
7

0
7

0
7

0
7

0
7

0

0

0

0

0

0

0

0
4

0
7

0
7

0

0

0
4

0
7

0
7

0

0

0

0

0

0

0

0

0

0

0

0

0

0
4

0

0

0

0

0
4

2.0617
7

0
7

0
7

2.0617

0

0

0

0

0

0

0

0

0

0

0

0

0
4

0
7

0
7

0

0

0
4

0
8

0
8

0

0

0
4

0

0

0
4

0

0

0
4

0

0

0
4

0
4

0

0

0

0
4

0
4

0

0

0

0

0
4

0
4

0

0

0

0
4

0
4

0

0

0

0
4

0
4

0

0

0

0
4

0
4

0

0

0

0
4

0
4

0

0

0

0
4

0
4

0

0

0

0
4

0
4

0
4

9533.36

0

0

0

0

0

0

0

0

0

0

0

0

0
4

0

0

0
4

0

0

0
4

0

0

0
4

0

0

0
4

0

0

0
4

0

0

0
4

0

0

0
4

0

0

0
4

0

0

0
4

0

0

0
4

0

0

0

0

0

0

0

0

0
4

0

0

0
4

0

0

0
4

0

0

0
4

0

0

0

0

0

0

0

0
4

0

0

0

0

0
4

0

0

0

0
4

0

0

0

0

0

0
4

0

0

0
4

0

0

0

0
4

0

0

0
4

0
4

0

0

0

0
4

0
4

66.4035

2.47648
3

0.750489

1.56797

0

0.0507772

0

0.107242

9.71445146547012e-17
3

0
4

4.55934
3

0

4.39937

0

0

0

0.0533257

0

0

0

0

0

0

0

0

0

0

0.106651

0

0

0

0

0

0

0

0

0

0

0

0

0

0
4

0

0

0
4

39.677
3

39.502
3

0

0

0

0.175085

0

0

0

0

0

0

2.99760216648792e-15
3

0
4

6.54529

4.53181
5

0

0

0

0

0

0

0

0

0

0

1.75085

0

0

0

0

0

0

0
5

0.262628

0

0

0

0

9.99200722162641e-16

0
4

0

0

0

0

0

0

0
4

0

0

0
4

0

0

0

0

0

0
4

0

0

0

0

0

0

0

0
4

0

0

0

0

0

0
4

0

0

0

0

0

0
4

0

0

0

0

0

0
4

0.685986

0.587988

0.097998

0
4

0

0

0

0

0
4

0

0

0

0

0
4

2.96704
5

2.96704
5

0

0

0

0

0

0

0

0

0

0

0
4

0

0

0

0

0
4

0

0

0
4

0

0

0

0

0
4

0

0

0

0

0
4

0

0

0

0

0
4

0

0

0

0
4

0

0

0

0

0
4

0

0

0

0
4

0

0

0

0

0
4

0.121276

0.121276

0
4

0
5

0
5

0

0

0

0

0
4

0

0

0

0
4

0

0

0
4

0

0

0
4

0

0

0

0
4

0

0

0
4

0

0

0
4

0

0

0
4

0

0

0
4

0

0

0
4

0

0

0
4

0
3

0

0

0

0

0

0

0

0
4

0

0

0

0
4

0

0

0

0
4

0

0

0

0
4

0

0

0

0
4

0

0

0

0
4

0

0

0
4

0

0

0

0
4

0.213303

0.213303

0
4

1.22649

1.03985

0.18664

8.32667268468867e-17

0
4

0

0

0
4

0
5

0

0

0

0

0

0

0

0
4

0

0

0

0
4

0.159977

0.0533257

0.106651

1.38777878078145e-17

0
4

0

0

0
4

0

0

0
4

0

0

0
4

0

0

0
4

0.175085

0.175085

0
4

0

0

0
4

1.05051

1.05051

0
4

0

0

0
4

0

0

0

0

0

0

0

0

0
4

0

0

0
4

0

0

0
4

0

0

0
4

0.612799

0.612799

0
4

0

0

0
4

0

0

0
4

0

0

0
4

0

0

0
4

0

0

0
4

0

0

0
4

5.02093
5

4.94105
5

0.0798828

0
4

0

0

0
4

0

0

0
4

0

0

0
4

0.0507772

0.0507772

0
4

0

0

0
4

0

0

0
4

0

0

0
4

0

0

0
4

0

0

0
4

0.525256

0.525256

0
4

0.0732851

0

0

0

0

0

0.0732851

0
4

0

0

0
4

0.262628

0.262628

0
4

0

0

0
4

0

0

0
4

0

0

0
4

0

0

0
4

0

0

0
4

0

0

0
4

0

0

0
4

0

0

0
4

0

0

0

0

0

0

0
4

0

0

0
4

0
4

1265.75
3

2.33093

0.097998

0
3

0

0

0

0

0

0

0

0

0

0

0.723307

0

0

0

0

0.195996
5

0

0

0

0

0

0

0

0

0

0

0.180827

0

0

0

0

0

0

0

0

0

0

0
3

0

0

0

0

0

0.397819

0

0

0

0

0.244995

0

0

0

0

0

0

0

0

0

0

0
3

0

0

0

0

0

0

0

0

0

0

0.48999
6

0

0

0

0

0

0

0

0

0

0

0

0

0

0

0

0

0

0

0

0

0

0

0

0

0

0

0

0

0

0

0

0

1.11022302462516e-16

0
4

10.753

3.52902
6

0

0

0.304663

0

0

0

0.17772

0

0

0

0.304663

0

0

0

0

0

0

0.0761658

0

0

0

4.59326

0

0

0

0

0

0

0

0

0

0

0
7

0

0

0

0

0

0

0

0

0.126943

0

0

0

0

0

0

0

0

0

0

0

0

0
6

0

0

0

0

0

0

0

0

0

0

0
7

0

0

0.0761658

0

0

0

0

0

0

0

0.0507772

0

0

0

0

0

0

0

0

0

0

1.51367
3

0

6.66133814775094e-16

0
4

22.8739

0

22.7526

0

0.121276

0

0

0

0

0

0

0

0

0

0

0

0

0

0

0

0

0

0

0

0

0

8.18789480661053e-16

0
4

1162.64
3

160.843
4

6.15918
4

0

0

0.0488567

0

0

0

0

0

1.4471

0

0

0.725025
4

0

0.289421

0

0

0

0

0

0

11.8663

0

4.00472
4

0

0.439711

0

0

0

0

0

0

0.0488567

0

0

0

0.289421

0

0

0

0

0

0

0

0.0798828

3.57282
4

0

0

0

0.0488567

0

0

0

0

0

0

0

0

0

0.289421

0

0

0

0

0

0

0

3.68391

0

0

0.506315

0

0

0

0

0.434131

0

0

0

0

0

0

0

0

0.0917376

0

0

0

0

0

0

0

0

0.0799885

0

0

0

0

0

0

0

0.0798828

0

0

0

0

0

0

0

0

0

0

0

0

0

0

0

0

0

0

0

0

0

0

0

0

0

0.23189

0

0

0

0

0.0533257

0

0

0

0

0

0

0

0

0

0

0

0

0

0

0

0

0

2.7495

0

0.108496

0

0

0.46378

0

0.434131

0.0723307

0

0

0

0

0

0

0

0

0.630308

0

0

0

0

0

0

0

0.175085

0

0

15.6206

0

0

0

0

0

0

0.986525

0.0723307

0

0

0.606382

0

0

0

0

0

0

0

0

0

0

2.93378

0

0

0

2.17066

0

0

0

0

0

0

14.6158
4

0

0

0

0

0

0

0

0

0.0723307

0

0

0

0

0.175085

0

0

0

0

0

0

0

0

0.0799885

1.3598

0.0723307

0.0798828

0.350171

0

0

0

0.612799

0

0

10.1549
4

0

0

0

0

0

0

0

0

0

0

0

0

0

0

0

0

0

0.578842

0

0

0

0

0

0

0.0798828

0

0

0

0

0

0

0

2.38542

0

0

0

0.119824

0

0

0

0

0

0

0

0

0

2.62628

0

0

0

0

0.199707

0

0

0

0

0

0

0

0

0

1.15945

0

0

0

0

0

0

0

0

0

0

0

0

0

0

0

0

0

0

0

0

0

0.759472

0.519238

0

0

0

8.90374
4

0

0

0

0.386483

0

0.108496

0

0

0

0

0.229344
4

0

0

0

0

0.144661

0

0

0

0

0

0

0

0

0

0

0

0

0

0

0

0

0

0

0

0

0

0

0

0

0

0

1.01297

1.23381

0

0

0

0

0

0

0

0

0

0

0

0

0

0

0

0

0

0

0

0

0

0.437713

0

0

0

0

0

0

0

0

0

0

0

0

0

0

0

0

0

0

0

0.0723307

0.0488567

0

0

0

0

0

0.0798828

0

0

0

0

0

0

0.0917376

0

0

0

0

0

0

0

0.119824

0

0

0

0

0

0.578842

0

0

0

0

0

0

0

0.154593
4

0

0

0

0

0

0

0

0

0

0

0.434131
4

0

0

0

0

0

0

0

0

0

0

0

0

0

0

0.0723307

0

0

0.350171

0

0

0

0

0.0533257

0

0

0.439355

0.159766

0

0

0

0

0

0.219855

0

0

0

0

0

0

0

0

0

0

0

0

0

0

0

0.154593

0

0

0

0

0

0

0

0

0

0

0

0.289421

0

0

0

0

0

0

0

0

0

0

0

0

0

0

0.133314

0

0

0

0

0.289421

0

0

0

0.175085

0

0.426605

0

0.162364

0

0

0

0

0

0

0

0

0

0

0

0

0

0

0

0

0

0

0

0

0

0
7

0

0

0

0

0

0

0

0

0

0.319531

0

0

0

0

0

0

0

0

0

0

0

0

0

0.0917376

0

0

0

0

0

0

0

0

0

0

0

0

3.59948

0.0798828

0

0

0.525256

0

0

0.0723307

0

0

0

0

0

0

0

0

0

0.106651

0

0

0

0

0

1.8806

0

0

0

0

0

0

0

0.741925

3.11543

0

0

0

0

0

0

10.6913
3

0.121187

0

0

0

0

0

0

0

0.434082

0.183475

0

0.119824

0.254968

0

0

0.0798828

0

0

0

0

0

0

0

0

0.262628

0

4.11396

0

0

0

0

0

0

0

0

0

105.723

0.0649455

1.47783

0

0.871697

0

0

0

76.3774

0

0

0

0

0.23189

0

0

0.319954

0

5.64371

22.2755
4

0

0

0

0

0

0.262628

0

0

0

0.119824

2.08701
4

0

0

0

0.679004

0

0

0

0

0

0.289421

2.66133
3

0

1.3794

0

0

0

0

0

0

0

0

0

0

0

0

0.319531

0

0

0

0

0

0

0

0.309187

0

0

0

0

0

0

0.830565

0

0

3.97796
4

0.0723307

0

0

0

0

0.0799885

0

0

0

0

0
4

0

0

0

6.67072

0

0

0

0

0

0

290.713
4

1.01905
4

0.106651

0.175085

0.940299

3.96505

0

0

0.23189

0

0

0.810732

4.42264
4

0

0

0

0.27959

0

0

0

0

0.108496

0

0

0

0

0

0

0

0

1.29793

0

0

0

0

0

0

0

0.0488567

0

0.0977135

0

0

0

0

0

0.0723307

0

0

0

0

0

0

0

0

0

3.55573
4

0

0

1.40068

0

0.108496

0

0

0

0.700341

0

42.9969
4

0

0.262628

0

0

0

0

0.159977

0

0

0

2.12486
4

0

0

0

0

0

0

0.144661

0.133314

0

0

20.7892
4

0

0.471745

0

0

1.05051

0

0

0

0

0

0.747034
3

1.30648

0

0

0

0

0

0

0

0

0

0.772317
4

0

0

0

0

0.106651

0

0

0

0

0

0

1.50388
4

0

0

0.525256

0

0

0.35856

0

0

0

0.0917376

1.11965
4

0

0

0

0

0

0

0

0.0533257

0

0

0

0

0

0

0

0

0

0

0

0.437713

0

0.772967
4

0

0

0

0

0

0.0799885

0

0

0

0

0

0

0.0798828

0.868263

0

0

0

0

28.9684

0.175085

0

18.6505
3

0

0

0

0

0

0

0

0

0

0

0.359473
4

0.659566

0

0

0

0

0

0

0

0

0.216992

0

0

0

0

0

0.119824

0

0.733901

0

0.0732851

0.154593

0

0

0

0.289421

0

0

0.289421

0.0533257

0

0

0

0

3.00241
4

0

0

0

0

0

0

0

0

0.23189

0

2.2155
4

0

0

0.239648

0

0

0

0

0

0

0

24.965

0.122142

0

0

0

0

0

0

0.309187

0

0

0.314035
4

3.36338

0

0

0

0

0

0

0

0

0

0

0

0.0533257

0

0.199707

0

0

0.180827

0.0732851

0

0

0

0

0

0

0

0

0

0

0

0

0

0

0

0

0.119824

0

0

0.0732851

0

0

0

0

24.8831
4

0

0

0

0

0.519238

0

0

0

0.106651

0.0798828

0
7

0.108496

0

0.289323

0

0

0.144661

0

0

0.289421

0

0

0

0

0

0

0

0

0

0

0

0

3.21901
4

18.1248
4

0.27959

3.29105

0

0

0

0.0798828

0.239648

0

0

0.23189

0.144661
4

0.180827

0.0488567

0

0

0

0

0

0.216992

0

0

2.49614
4

0

0

0

0.180827

0.0917376

0

0.0732851

0

0

0

1.15729
4

0

0

0

0

0

0

0

0

0

0

0.561451
4

0

0

0

0

0.0723307

0

0

0

0

0

0
4

0

0

0

0.437713

0

0

0

0

0

0

10.8838

0

0.0798828

0

0

0.0723307

0

0.386483

0

0

0.0977135

4.15699
4

0

0

0

0.0798828

2.02595

0

0

0

0

0

3.38375
4

0

0

0

0

1.92594

0

0

0

0

0.871507

0

0

0

0

0

0

0

0

0

0

0

0

0.464506
4

0

0

0

0

0

0.289421

0

0

0.119824

0

1.39479
4

0

0.0723307

7.08841

0

0

0

0

0

0

0

0

0

0

0

0

0

0

0

0

0

0.159766

0

0

0.289421

0

0

0

0

0

0

0

0

10.6206

0

0

0.878711

0

0

0

0

0.437713

0

0

0

0

0

0

0

0.289421

0

0

0

0

0

0

0

0

0

0

0

0

0

0

0

0

0.694324
4

0.144661

0

0

0.289421

0

0

0.137606

0

0

0

1.03204

0

0

0

0.175085

0.199707

0

0.262628

0

0.723552

0.289421

8.96901

0

0

0

0

0

0

0

0

0

0

0

3.47835
4

0

0

0

0

0

0

0.289421

0

0

0

0

0

0

0

0.229344

0

0

0

0

0

0

0

0

0

0

0

0

0.180827

0

0

0

0

1.40458
4

0

0

0

0

0

0

0

0

0

0

0
4

0

0

0

0.119824

0

0

0

0

0

0

0
4

0

0

0

0

0

0.154593

0

0.108496

0

0

0

1.77782

0

0

0

0

0

0

0

0

0

0

0

0

0

0

0

0

0

0

0

0

0.215147
3

0.485106

0

0

0

0

0

0

0

0

0

19.0058
4

0

0

0

0

0

0

0

0.180827

0

0

0

0

0

0

0

0

0

0.122142

0

0

0.0798828

0

0.397917
4

0

0

0

0

0

0

0

0

0

0

0

0

0

0

0

0

0

0

0

0

0

0.0799885
4

0

0.325488

0.642163

0.199707

0

0

0

0

0

0

5.92947
4

0

0

0

0

1.01297

0

0

0

0

0

0.0723307
4

0

0

0

0

0.346617

0

0

0

0

0

0.205645
4

0

0

0

0

0

0

0

0.106651

0

0

0.289421

0

0

0

0

0

0.0798828

0

0

0

0

0

0.0723307

0

0.399414

0

0

0

0

0

0.0723307

0

0

0

0

0

0

0

0

0

0

0

0

0
4

0.119824

0

0

0

0

0

0

0

0

0

0.119824

0

0

0

0
4

10.0281
4

0
4

10.0281
4

0
4

0

0

0

0
4

0

0

0
4

3.31641
4

3.1031

0.213303

0

0

0

1.38777878078145e-16
4

0
4

1.57659

0.929786

0.565957

0.0808509

5.55111512312578e-17

0
4

0

0

0

0

0

0
4

0

0

0

0
4

0.437713

0

0.175085

0

0.262628

0

0

0
4

0.175085

0.175085

0

0

0

0
4

0

0

0
4

0

0

0

0
4

0.0533257

0.0533257

0

0

0
4

2.80632
3

0.226476
3

0

0

0

0

0

0

0

0

2.21811

0.133314

0

0.175085

0

0.0533257

0

0

7.63278329429795e-17
3

0
4

0.350171

0.350171

0
4

0

0

0

0

0
4

0

0

0
4

0

0

0
4

0

0

0

0
4

0.175085

0

0.175085

0

0
4

0

0

0

0
4

0

0

0

0

0
4

0

0

0

0
4

0

0

0
4

5.5359
3

3.15154
4

0

0

0

0

0

0

0

0

0.0533257

0.175085

0

2.15596

8.88178419700125e-16
3

0
4

0

0

0

0
4

0.15552

0.15552

0
4

0

0

0
4

0

0

0

0
4

0.0723307

0.0723307

0

0
4

0

0

0

0
4

0

0

0
4

0

0

0
4

0

0

0
4

0

0

0

0
4

2.30473
3

0.0799885

0

0.0533257

0

0.875427

0

0

0

0.0799885

0

0.0533257

0

0

0.962969

0.199707

4.44089209850063e-16
3

0
4

0

0

0
4

16.7116

16.7116

0
4

0

0

0
4

0

0

0

0
4

0

0

0

0
4

0.0533257

0.0533257

0

0
4

0.0808509

0.0808509

0

0
4

0

0

0

0
4

0

0

0

0
4

0.440991

0.440991

0
4

2.62628
3

2.45119

0

0

0

0.175085

0

0
4

0

0

0
4

0

0

0

0
4

0

0

0
4

0.175085

0.175085

0
4

0

0

0
4

0

0

0
4

0

0

0
4

0

0

0
4

0

0

0
4

0

0

0
4

18.2653
4

15.3841
4

0

2.80136

0

0

0

0.0798828

0

0

8.18789480661053e-16
4

0
4

0

0

0
4

0

0

0
4

0

0

0
4

0

0

0
4

0

0

0
4

0

0

0
4

0

0

0
4

0

0

0
4

0

0

0
4

0.0799885

0.0799885

0
4

0

0

0

0

0

0
4

0

0

0
4

0

0

0
4

0

0

0
4

0

0

0
4

0.289421

0.289421

0
4

0

0

0
4

0

0

0
4

0

0

0
4

0

0

0
4

0

0

0
4

0.241388

0

0.0482775

0

0.19311

0

0

0

0
4

0

0

0
4

0

0

0
4

0.159766

0.159766

0
4

0

0

0
4

0

0

0
4

0

0

0
4

0.723552

0.723552

0
4

0

0

0
4

0

0

0
4

0.0917376

0.0917376

0
4

0

0

0

0

0

0

0

0

0
4

0

0

0
4

0

0

0
4

0

0

0
4

0

0

0
4

0

0

0
4

0.107242

0.107242

0
4

0

0

0
4

0

0

0
4

0

0

0
4

4.343886361724e-13
3

0
4

0
4

0
4

0
4

0

0

0

0

0

0

0

0

0

0

0

0

0

0

0

0

0

0

0

0

0

0

0

0

0

0

0

0

0

0

0

0

0

0

0

0

0

0

0

0

0

0

0

0

0
4

0
4

0
4

0

0
4

0

0

0
4

0

0

0
4

0

0

0
4

0
4

291.512

10.9079

0
7

0
7

0

0

0

0

0

0

0

0

0

0

0
8

0

0

0

0

0

0

0

0

0

0

0
7

0

0

0

0

0

0

0

0

0

0

0

0

0

0

0

0

0

0

0

0

0

0
7

0

0

0

0

0

0

0

0
7

0

0
8

7.7943
3

0
7

0

0

0

0
7

0
8

0

0

0
7

0

0

0

0
7

0

0

0

0

0
7

0

0

0

0

0

0

0

0

0

0

0

0

0

0

0

0

0
8

0

0

0

0.0482775

0

0

0

0

0

0

3.06534
3

0

0

0

0

0

0

0

0

0

0

0
8

0

0

0

0

0

0

0

0

0

0

0
7

0

0

0

0

0

0

0

0

0

0

0
7

0

0

0

0

0

0

0

0

0

0

0
4

0.161702

0.0808509

0.0808509

0
4

0
2

0

0

0

0

0

0

0

0

0

0

0
4

157.336
4

0

44.3707
4

0.336816

0.541077

0

0

0.0649455

0

0

0

2.99852

0

0

0.325297

0.809545

0

0.279696

0

10.8873
4

0.434131

0.0761658

0.314359

0

0.309187

0

0

0.350171

0.234476

0.252307

56.9366
4

0.234476

0.578842

0.199813

0

0.108496

0

0

0

0

0

0.252307
3

1.22202

0.0482775

0

0

0

0

0

0

0.126943

0

3.3119
4

0

0.412819

0.0798828

0

0

0

1.62323

0.618373

5.64266

0.0488567

1.60346

0

0.0917376

0

0

0

0.0798828

0.0533257

0

0.23189

0.175085

2.96917

0.0533257

0

0.18664

0.0488567

0.0798828

0

1.73653

0.159766

0

0.154593

0
8

0

0

0

0.0482775

0.122142

0.0482775

0.23189

0

0

0.0798828

14.2968

0

0.119824

0.23189

0

0.119824

0.0732851

0

0.309187

0

0

2.77000644643977e-14
4

0
4

1.06256
5

0

0

0

0.0917376

0

0.175085

0

0

0

0

0

0.289421

0.506315

0

0

0

0

0

0
4

0.349795
6

0

0

0

0

0

0

0

0

0

0

0

0

0

0

0

0

0

0

0

0.242553

0

0

0

0

0.107242

0

0

0

0

0

0

0

0

0

0

0

0

0

0

0

0

0

0

0

0

0

0

0

0

0

0

0

0

0

0

0

1.38777878078145e-17
6

0
4

33.6448
5

10.5318
5

0

0.586582

0

0

0.18664

0

0.0533257

0.37328

0

0

0

0

1.11836

0

0

0

0.0533257

0

0.0533257

0.55992

0

0

0

0.0799885

0.0533257

0.0533257

1.31807

0

0

0

0

0.0533257

15.4111
5

0.0798828

0.0799885

0.0533257

0.0798828

0

0.0533257

0

0.0533257

0.0533257

1.03985

0.933199

0

0.533257

0

0.199707

4.10782519111308e-15
5

0
4

0

0

0

0

0

0

0

0

0

0

0

0

0

0

0

0

0

0

0

0

0
4

0

0

0

0

0

0

0

0

0
4

0

0

0
4

0.578645

0.578645

0
4

0.0808509

0.0808509

0
4

0

0

0
4

0

0

0
4

0.175085

0.175085

0
4

0

0

0
4

0

0

0
4

0

0

0
4

0

0

0
4

0
7

0
7

0

0

0

0

0
4

0

0

0
4

0.175085

0.175085

0
4

0

0

0
4

0.107242

0.107242

0
4

0

0

0
4

0

0

0
4

0

0

0
4

0

0

0
4

0

0

0
4

0

0

0
4

13.8714
4

13.4012

0.216992

0.253157

3.33066907387547e-16
4

0
4

0

0

0
4

0

0

0
4

0

0

0
4

0

0

0
4

0

0

0
4

0

0

0
4

0

0

0
4

0

0

0
4

0

0

0
4

0

0

0
4

0

0

0

0

0

0

0

0

0
4

0

0

0
4

0

0

0
4

0

0

0
4

0

0

0
4

0

0

0
4

0.0533257

0.0533257

0
4

0

0

0
4

0
7

0

0

0

0

0
4

28.186
4

27.5768

0.0723307

0.0723307

0.175085

0.289421

6.10622663543836e-16
4

0
4

0.136656
4

0

0

0

0.136656

0
4

0

0

0

0
4

0
7

0
7

0
4

0
7

0
7

0
4

34.2097
4

27.4163
4

3.24887
4

1.11936
4

0.651173

1.62935

0.144661

0
4

0.0533257

0.0533257

0

0
4

0
8

0

0

0
4

0
6

0

0

0

0
4

0

0

0

0

0

0
4

2.34468

2.34468

0
4

0

0

0

0

0
4

0.33542

0.33542

0
4

0

0

0

0

0

0
4

0

0

0

0

0
4

0

0

0

0
4

0
7

0
7

0

0

0
4

0.202127

0.202127

0
4

0.537065

0.154593

0.0732851

0.309187

0
4

0

0

0

0
4

1.22224

1.22224

0
4

0

0

0

0
4

0

0

0

0
4

0

0

0
4

0.517157

0.517157

0
4

0

0

0
4

1.22417

0.180827

0.868263

0.175085

0
4

0
7

0

0

0

0

0

0

0

0
4

0

0

0

0

0
4

0

0

0
4

0

0

0

0
4

0

0

0

0
4

1.25267

1.02332

0.229344

0
4

0

0

0

0

0
4

0

0

0

0
4

0

0

0
4

0

0

0

0
4

0

0

0

0
4

0

0

0

0

0

0

0

0

0

0

0

0

0
4

0

0

0

0
4

0

0

0

0
4

0

0

0
4

0

0

0

0
4

0

0

0
4

0.0533257

0.0533257

0
4

0

0

0

0
4

0

0

0

0
4

0

0

0

0
4

0

0

0
4

0
7

0
7

0

0

0

0
4

0

0

0

0
4

0.0798828

0.0798828

0
4

0

0

0

0
4

0

0

0
4

0

0

0
4

0

0

0
4

0

0

0

0
4

0

0

0

0
4

0

0

0
4

0.0808509

0.0808509

0
4

1.2043
3

0.46378

0.537065

0.20345

1.66533453693773e-16
3

0
4

0

0

0
4

0

0

0
4

0

0

0
4

0

0

0
4

0

0

0
4

0

0

0
4

0

0

0
4

0

0

0
4

0

0

0
4

0

0

0
4

1.25319

1.25319

0

0

0
4

0

0

0
4

0

0

0
4

0.0808509

0.0808509

0
4

0

0

0
4

0

0

0
4

0

0

0
4

0

0

0
4

0

0

0
4

0

0

0
4

0

0

0
4

0
7

0
7

0

0
4

0

0

0
4

0

0

0
4

0

0

0
4

0

0

0
4

0

0

0
4

0

0

0
4

0

0

0
4

0.0341641

0.0341641

0
4

0

0

0
4

0

0

0
4

0
4

1132.9

0.206687
7

0
7

0

0

0

0

0

0

0.0854102

0.121276

0
4

332.066

1.64144
7

0
7

0

0.46378
3

0
6

0

0

0

0

0

0.144833

0

83.4906
3

0

0

0

0

0

0

0

0

0

0

0

1.18302

0

0

0.614362

0

0

0.0798828

0

0

0

0
7

0

0

0.18664

0

0.168971

0

0

0

0.555455

0

0
7

0

0

0

0

0.218987

0

0

0

0

4.24467

0
6

0

0

0.525531

0

0

0

0

0

0.386483

0

0

0

0

0

0

0

0

0

0

0

0

52.1993

0

0.0732851

0

0

0.0482775

0

0

0

0

0

0

0

0

0

0

0

0

0

0

0

0

0.19311

0

0.154593

0

0.161702

0

0

0

0

0

0

25.2862
3

0
7

0

0

0

0

0

0.443842

0

0

0

0.0965551

0
7

0

0

0

0

0

0.0482775

0

0

0

0

0
2

0

0.471072

0

0

0

0

0.120694

0

0.0977135

0.202127

1.02847
3

0

0

0

0

0

0

0

0

0

0

37.6985
3

0

0.154593

0.0808509

0

0.879421

0

0

0.868995

0

0

0
7

0

0

0

0

0

0

0

0

0

0

0
7

0

0

0

0

0.309187

0

0

0

0

0

0.19311
3

0

0

0.404255

0

0

0

0

0

0

0

0
7

0

0

0

0

0

0.309187

0

0

0.0724163

0

0.282396
2

0

0.107242

0

0

0

0

0

0

0

0

2.38974
7

0

0

0

0

0

0

0

0

0

0

0

0.159766
6

0

0

0

0

0

0

0

0

0

0

0
2

0

0

0

0

0

0.241388

0

0

0

0

0.217828
3

0

0

0

0

0

0

0.0482775

0

0

0

0
7

0

0.106651

0

0

0

0

0

0

0

0

0
2

0

0

0

0

0.161702

0

0

0

0

0

0

0

0

0

0

0

0

0

0

0

0

0
5

0

0

0

0

0

0

0

0

0

0

1.82572
5

0

0

0

0

0

0.313804

0

0

0

0

1.13821
3

0

0

0

0.154593

0

0

0

0

0

0

58.0603
4

0
6

0

2.02127

0

0

0.202127

0.0808509

0

0

0

0

0.58893
3

0

0.0482775

0

0.23189

0

0.23189

0

0

0

0.107242

0

0.0482775

0

0

0

0

0

0

0

0

0

2.45455
3

0

0

0

0

0

0

0

0

0

0

0
7

0

0

0

0

0

0

0

0

0

0

20.0195
3

0

0.0965551

0

0

0

0

0

0

0

0

0.289665
3

0

0

0

0

0

0

0

0

0

0

0
7

0

0

0

0

0

0

0

0

0

0

0.743684
3

0

0

0

0

0

0

0

0

0

0

0
7

0.159766

0

0

0

0

0

0

0

0

0

0.482775
7

0
2

0.57933

0

0

0

0

0

0

0

0

0

0

0

0

0

0.161702

0

0

0

0

0

0

0

0

0

0

0

0

0

0

0

0

0

0

0

0

0

0

0

0

0

0

0

0

1.08279

0

0

0

0

0

0

0

0

0

0

0

0

0

0

0.121276

0

0

0.168971

0

0.0808509

0

0.0977135
3

0

0

0

0

0

0

0

0

0

0

0

0

0

0

0.0965551

0

0

0

0

0

0

0
5

0

0

0

0

0

3.69323

0

0

0

0

0.144833
3

0

0

0

0

0

0

0

0

0

0

0
7

0.0482775
3

0

0

0

0

0

0.144833

0

0

0

0

0
3

0

0

0

0

0

0

0

0

0

0

0.0488567
3

0

0

0

0

0

0

0.0488567

0

0

0

0
6

0

0

0

0

0

0

0

0

0

0

0
7

0

0

0

0

0

0.107242

0.241388

0

0

0

0
7

0

0

0

0

0

0

0

0

0

0

0
7

0

0

0

0

0

0

0

0

0

0

0
7

0

0

0

0

0

0

0

0

0.0724163

0

0
2

0

0

0

0

0

0.202127

0

0

0

0

2.55079
3

0

0

3.47835

0

0

0

0

0

0

0

0

0
2

0

0

0

0

0

0

0

0

0

0

0.492536
3

0

0

0

0

0

0

0

0.0965551

0

0

0.451111
3

0

0

0

0

0

0

0

0

0.154593

0

0

0

0

0

0

0

0

0

0.0808509

0

0

0

0

0

0

0

0

0

0

0

0

0

0
6

0

0

0

0

0

0

0

0

0

0

1.15945
3

0

0

0

0

0

0

0

0.107242

0

0

0
2

0

0

0

0

0

0

0

0

0

0

0
7

0

0

0.410359

0

0

0

0.107242

0

0

0

0

0

0

0

0

0

0

0

0

0.106651

0

2.55871
3

0

0

0

0

0

0

0

0.44468

0

0

0

0
7

0

0

0

0

0.309187

0

0

0

0

0

1.25319

0

0.363829

0

0

0

0.0808509

0

0.154593

0

0

0

0

0

0

0.482775

0

0

0

0

0

0
6

0

0
7

0

0
7

0

1.421

0

0.144833
3

0

0

0

0

0
6

0
7

0

0

0
4

5.78597

0

1.9

0

0

1.16788

0

0

0

0.939379

1.61702

0

0

0

0

0.161702

0
4

702.665

658.248

0
7

0.948366
4

0

0

1.42466

0.787884

0

0

0.289665

0

0

9.01045
4

0

0

0

0

0

0

0

0

0.254968

0

0
7

0

0

0.578842

0

0

0.0798828

0

0.227309

0

0

0
7

0

0

0

0

0.144661

0.506594

0

0

0

0.319531

26.1217
4

0.0974182

0

0

0

0.651747

0

0

0

0.289421

0

0
7

0

1.20694

0

0

0

0

0

0

0.0977135

0

0
4

0.159766

0

0

0.175085

0

0

0

0

0

0.199707

0

0.844291

0

0
7

0
4

0.787884
7

0.175085
7

0

0

0

0

0

0

0

0

0

0

0
6

0

0

0

0

0

0

0
7

0.612799

0
6

0
7

0
7

0

0

0
4

0.768001
4

0

0.46378

0.304221

0

5.55111512312578e-17
4

0
4

0

0

0
4

0

0

0
4

0

0

0
4

0

0

0
4

0

0

0
4

0

0

0
4

0.0723307

0.0723307

0
4

0

0

0
4

0

0

0
4

0.262628

0.262628

0
4

0.841683
4

0.361752
4

0.239966

0.239966

5.55111512312578e-17
4

0
4

0

0

0
4

0

0

0
4

0.180827

0.180827

0
4

0.0723307

0.0723307

0
4

0.119824

0.119824

0
4

0

0

0
4

0

0

0
4

0

0

0
4

0.119824

0.119824

0
4

0

0

0
4

0
7

0
7

0

0
4

0.0488567

0.0488567

0
4

0.0507772

0.0507772

0
4

0

0

0
4

0

0

0
4

0.119824

0.119824

0
4

0

0

0
4

0

0

0
4

0

0

0
4

0

0

0
4

0.0798828

0.0798828

0
4

1.99893

1.85236

0

0.0732851

0.0732851

0
4

0.0798828

0.0798828

0
4

0

0

0
4

0

0

0
4

0

0

0
4

0

0

0
4

0

0

0
4

0

0

0
4

0

0

0
4

0

0

0
4

0.0798828

0.0798828

0
4

0
3

0

0

0

0
4

0.0723307

0.0723307

0
4

0

0

0
4

0

0

0
4

0

0

0
4

0.0482775

0.0482775

0
4

0

0

0
4

0.0798828

0.0798828

0
4

0

0

0
4

0

0

0
4

0.0798828

0.0798828

0
4

0
2

0

0

0

0

0
4

0.614811

0.0723307

0.0723307

0.144661

0.0723307

0.253157

0
4

0
8

0

0

0
4

2.31158

1.40068

0

0.679004

0.23189

0
4

0
7

0
7

0
4

36.281
4

29.9301
4

0

0.152213

0

0.144661

0

0.386483

0.213303

0

0.23189

0

1.47457
4

0

0

0.154593

0

0

0

0

0

0

0

0.794355
4

0

1.15184
4

0.484272
4

0
4

0.106651

0.746873

0.309187

3.99680288865056e-15
4

0
4

0

0

0
4

0

0

0

0
4

0

0

0

0

0
4

0

0

0

0
4

0

0

0
4

0

0

0

0
4

2.64625

2.52643

0.119824

0
4

0

0

0

0

0
4

0

0

0
4

0.0723307

0

0

0.0723307

0
4

11.3307
4

1.54391
4

9.02339
4

0.0974182

0.434131

0

0

0

0.23189

1.38777878078145e-15
4

0
4

0

0

0

0

0
4

0

0

0

0
4

0

0

0
4

0

0

0

0

0
4

0

0

0

0
4

0

0

0

0

0
4

0.878711

0.159766

0.119824

0.599121

1.11022302462516e-16

0
4

0

0

0
4

0.375348

0.268106

0.107242

0
4

2.8209

2.8209

0

0
4

8.26787
5

0
7

0.199707
5

0.0798828

6.31074

0.0798828

1.59766

0

0

0
4

0.506914

0.120694

0.38622

0
4

0

0

0

0
4

0.214485

0

0.214485

0
4

0

0

0

0
4

2.91572

2.91572

0
4

1.07242

1.07242

0
4

0

0

0

0
4

0

0

0

0
4

0

0

0

0
4

0.811818

0.0974182

0.7144

0
4

1.13191
3

1.13191
3

0

0

0

0

0

0

0
4

0

0

0

0
4

0

0

0
4

0.578842

0.578842

0
4

0

0

0
4

0

0

0
4

0

0

0

0
4

0.440991

0.440991

0

0
4

0

0

0

0
4

0

0

0
4

0

0

0
4

5.58928
3

5.48204
3

0

0

0.107242

3.7470027081099e-16
3

0
4

0

0

0
4

0

0

0
4

0

0

0
4

0.0798828

0.0798828

0
4

0

0

0
4

0.0798828

0.0798828

0
4

0

0

0
4

0

0

0
4

0

0

0
4

0

0

0
4

0.509933
4

0.401437
4

0.108496

0
4

0

0

0
4

0

0

0
4

0

0

0
4

0

0

0
4

0.199707

0.199707

0
4

0

0

0
4

0

0

0
4

0

0

0
4

0

0

0
4

0

0

0
4

0.349994
3

0
4

0.251996

0

0.097998

2.77555756156289e-17
3

0
4

0

0

0
4

0

0

0
4

0

0

0
4

0

0

0
4

0

0

0
4

0

0

0
4

0

0

0
4

0.144661

0.144661

0
4

0

0

0
4

0

0

0
4

5.76887
4

5.09034

0.159766

0.229344

0

0.289421

0
4

0

0

0
4

0.14657

0.14657

0
4

0.0761658

0.0761658

0
4

0

0

0
4

0

0

0
4

0

0

0
4

0

0

0
4

0

0

0
4

0

0

0
4

0

0

0
4

0
4

290.1
3

263.664
3

234.296
3

0

0

0

0.612799

0

0

0

0

0

0

0

0

0

0

28.4924

0

0

0

0

0

0.262628

1.4432899320127e-15
3

0
4

5.8564
4

5.26768
4

0.588725

0

0
4

0.334851

0.334851

0
4

0.700341

0.700341

0
4

0

0

0

0
4

0.350171

0.175085

0.175085

0
4

0

0

0

0
4

0.262628

0.262628

0
4

0.660104

0.660104

0
4

0.0798828

0.0798828

0
4

0.0977135

0.0977135

0
4

0.175085

0.175085

0
4

3.37112

3.29783

0.0732851

2.08166817117217e-16

0
4

0.0799885

0.0799885

0
4

0

0

0
4

0.119824

0.119824

0
4

0

0

0
4

0

0

0
4

0

0

0
4

0

0

0
4

0

0

0
4

0

0

0
4

0.0799885

0.0799885

0
4

8.74717

8.74717

0
4

0.0732851

0.0732851

0
4

0

0

0
4

0.262628

0.262628

0
4

0

0

0
4

0

0

0
4

0

0

0
4

0

0

0
4

0.262628

0.262628

0
4

0

0

0
4

0

0

0
4

2.18857

1.57577

0.437713

0.175085

0
4

0.350171

0.350171

0
4

0

0

0
4

0.309187

0.309187

0
4

0.0761658

0.0761658

0
4

0

0

0

0

0
4

0.251313

0.251313

0
4

0.14657

0.14657

0
4

1.40044

1.40044

0
4

0.199707

0.119824

0.0798828

0
4

0
4

8.20404
2

8.20404
2

0
2

0
2

0

0

0

0

0

0

0

0

0

0

0

0

0

0

0

0

0

0

0

0

0

0

0.214485

0

0

0

0

4.28969

0

0

0

0

0

0

0

0

0

0

0

0

0

0

0

0

0

0.428969

0

0

3.16365

0

0

0

0

0

0

0

0

0

0

0

0

0

0

0

0

0

0

0

0

0

0

0

0

0

0

0

0

0

0

0

0

0

0

0

0

0

0.107242

0

0

0

0

0

0
2

0

0

0

0

0

0

0

0

0

0

0

0

0

0

0

0

0

0

0

0

0

0
4

0

0

0
4

0

0

0
4

0
4

35.1343

32.1803
7

3.07206
7

0
7

0

0

0

0

0

0

0

0

0

0

0

0

0

0

0

0

0

0.410359

0

0

0

0

0

0

0

0

0

0

0

0

0

0.436916

0
6

0

2.65549

0

0

0

0

0

0

0

0

0

0

0

0

0

0

0

0

0

0

0

0
7

0

0

0

0

0

0

0

0

0

0

0
6

0

0

0

0

0

0

0

0

0

0

0
7

0

0

0

0

0

0

0

0

0

0

0
7

0

0

0

0

0

0

0

0

0

0

0
7

0

0

0

0

0

0

0

0

0

0

4.53379
7

0
6

0

0

0

0

0

0

0

0

0

0

0
7

0

0

0

0

0

0

0

0

0

0

0
7

0

0

0

0

0

0

0

0

0

0

0
7

0

0

0

0

0

0

0

0.120694

0

0

0
7

0

0

0

0

0

0

0

0

0

0

0
6

0

0

0

0

0

0

0

0

0

0

0
7

0

0

0

0

0

0

0

0

0

0

0
7

0

0

0

0

0

0

0

0

0

0

0
6

0

0

0

0

0

0

0

0

0

0

0
7

0

0

0

0

0

0

0

0

0

0

0
7

0
5

0

0

0

0

0

0

0

0

0

0

0
7

0

0

0

0

0

0

0

0

0

0

0
7

0

0

0

0

0

0

0

0

0

0

0
7

0

0

0

0

0

0

0.0724163

0

0

0

0
7

0

0

0

0

0

0

0

0

0

0

0
7

0

0

0

0

0

0

0

0

0

0

0

0

0

0

0

0

0

0

0

0

0

0
7

0

0

0

0

0

0

0

0

0

0

0
6

0

0

0

0

0

0

0

0

0

0

0

0

0

0

0

0

0

0

0

0

0

0.0808509
6

0
6

0

0

0

0

0

0

0

0

0

0.844857

0

0

0

0

0

0

0

0

0

0

0

0
7

0

0

0

0

0

0

0

0

0

0

0
7

0

0

0

0

0

0

0

0.0482775

0

0

0

0

0

0

0

0

0.129891

0

0

0

0

0
6

0

0

0

0

0

0

0

0

0

0

0
6

0

0

0

0

0

0

0

0

0

0

0
7

0

0

0

0

0

0

0

0

0

0

0
7

0

0

0

0

0

0

0

0

0

0

0
7

0

0

0

0

0

0

0

0

0

0

0.262628
7

3.69323

0

0

0

0

0

0

0

0

0

0

0

0

0

0

0

0

0

0

0

0

0

0
7

0

0

0

0

0

0

0

0

0

0

0
6

0

0

0

0

0

0

0

0

0

0

0
7

0

0

0

0

0

0

0

0

0

0

0
7

0

0

0

0

0

0

0

0

0

0

0
6

0

0

0

0

0

0

2.31732

0

0

0

0

0

0

0

0

0

0

0

0

0

0

0
7

0

0

0

0

0

0

0

0

0

0

0
7

0

0

0

0

0

0

0

0

0

0

0.646808
7

0
7

0

0

0

0

0

0

0

0

0

0

0
7

0

0

0

0

0

0

0

0

0

0

0
7

0

0

0

0

0

0

0

0

0

0

0
6

0

0

0

0

0

0

0

0

0

0

0

0

0

0
6

0

0
8

0
5

0
7

0

0
6

0

0

0

0

0
7

0

0

0
7

0

0
7

1.54488

0

0

0
7

0

1.1233

0

0

0
7

0

0

0

0

10.1866

0

0

0

0

0

1.77635683940025e-15
7

0
4

0.685485
7

0.637208
7

0.0482775

0
4

2.26844
4

1.68971
4

0

0

0

0

0

0

0

0

0

0

0

0

0.506315

0.0724163

0

1.11022302462516e-16
4

0
4

0

0

0
4

0

0

0
4

0

0

0
4

0

0

0
4

0

0

0
4

0

0

0
4

0

0

0
4

0

0

0
4

0

0

0
4

0

0

0
4

0

0

0
4

0

0

0

0
4

0

0

0
4

0

0

0
4

0

0

0
4

0

0

0
4

0

0

0
4

0

0

0

0
4

0

0

0
4

0

0

0
4

0

0

0
4

0

0

0
4

1.46549439250521e-14

0
4

2363.51
4

2221.51
4

489.195
4

0
5

0.199707

0.309187

0.14657

0.160864

0.879421

0.0977135

0.266823

0

64.1759

0

4.60409
3

0.133314

0

2.31537

0.309187

0.469995

0.0488567

0

0

0.101554

0.0798828

130.976
4

0

0.888029

0.0488567

0.403496

2.45924

0.27959

0

0.0512461

0.122142

0.0488567

5.42027
4

0

0.122142

0

0

0

0.639908

1.38626

0.119824

0

0

0

0.723552

0.386483

0.280747

0

0.195427

0.472312

0

0

0

0.14657

0.838769

0.154593

17.7782

0.374792

0

0.509936

3.6045

0.867872

0

0

0.359473

2.42866
4

3.32376

0.27959

0

0.346617

0

0.386483

0.319531

0.154593

0.639062

0.0732851

0
5

0.199707

3.15154

0

0.122142

0.0798828

0.541077

3.4749

0.0488567

0.107242

0.262628

56.6693

0.0799885

5.499

0.23189

0

0.154593

0.23189

0.0533257

0

0

0.0488567

2.02048

0

0.170999

2.03701

0

1.15768

0.525256

0.578842

0

0.0732851

0

7.4018

23.6306
4

0

0.586281

0.195427

0

0.289421

0

0.0488567

0.723552

0

0.195427

3.25428

0.23189

0.868263

0

0

0.279275

0

0

0

1.90541

0.309187

0

0.0488567

0.154593

0

0.488567

0.154593

0.0799885

0

0

0

0.0533257

1.91265

0

0

0

0

0

0.0977135

0

0

0.0488567

2.21302

5.51191

0.154593

0.23189

0

0.309187

0.0799885

0.0488567

0

0

0

0.0488567

9.75208

0.154593

0.386483

0

0

0

0.0488567

0

0.0533257

0.0732851

0.69567

4.46233

1.47254

0.0488567

0.0732851

0.289421

0

0.541077

1.00486

0

0

0

0.708423

0.0732851

0

0.154593

0

0.0488567

0.0732851

0.0507772

0.154593

0

0

1.17866

0

0

0.23189

0

0.0798828

0.0732851

0.525256

0

0

0

23.9554
4

0.175085

0

0.0761658

0.0732851

0.0488567

0

0.154593

68.0574

0.175085

0.122142

21.1512

1.74817

0.170999

0

0.0488567

0.0798828

0

0.289421

0.154593

0.0488567

0

0.122142

0

0.159977

0.0488567

0.23189

0

0

0.154593

0.0761658

0

0

0

13.1979

0.154593

0

0

0

0.0488567

0.154593

0.0488567

0.386483

0.0761658

0.437713

0.366426

0

0.541077

0

0.0732851

0.0732851

0.144661

0

0.0488567

0.965181

0.0488567

3.77606

0.0488567

1.30239

0

0

0.0732851

2.2416

1.13805

0

0

0.175085

0
3

0

0.0507772

0.0798828

1.23329

0.0723307

0

0.0732851

0

0

0.0488567

0

0.122142

0.0798828

0.122142

0.0683282

0.14657

0.0977135

0

0

0.122142

0

2.30725

0.154593

0.0488567

0.618373

0.23189

0.0732851

0.386483

0

0

0.0798828

0

0

0.0488567

0.175085

0

0

0.154593

0.0799885

0

0.160864

0.772967

0.289421

1.03848

0

0.854993

0

0

0.262628

0.29314

0

0

0.618373

0

59.1322

3.83638

0

0

0.578842

0

1.01297

0

0.23189

0

0.289421

0.154593

0

0.350171

0.107242

0

0

0.0488567

0.213303

0.0488567

0.14657

0.609327

0.154593

7.34922

0.122142

0.289421

0

0.14657

0.0732851

0.106651

0.386483

0.108496

0.14657

0

1.57311

0

0

0

0.0488567

0

0

0

0

0

0

1.31807

0.0488567

0.0799885

0.0799885

0.0732851

0.0488567

0

0

0

0.0488567

0

0

0.152332

0.175085

0.868263

0

0.55992

0.175085

0

0

0.319531

0

3.90698
4

0.23189

0.850263

0.0732851

0

0.0732851

0

4.92016

0.0488567

0.119824

0.289421

0

0

0.0732851

0.154593

0.0732851

0.175085

0

1.31404

1.23675

0.0798828

0

0

0.868263

0.289421

0.0533257

1.14395
4

196.547
4

0

0.508625

0

0

0.341997

0.798828

0

0.330052

2.14572

1.00486

789.867
4

0.724963

0

11.4976

0

16.7176

0

1.19876

0.310351

2.31674

2.91572

0.121276
3

0.42616

1.25054

2.08701

0

0

4.83919

3.91248

1.6017

1.91719

0.0732851

10.3098

0.107242

1.74195

5.06205

0

5.13758

0.821823

0.244284

1.29824

4.90909

1.2856

0
5

2.02526

0

0

0

0

1.25796

0.126943

0

0

0.12874

0
4

51.1457
4

11.0234
4

0.439711

0.244284

0.0488567

0.0488567

0.23189

0.0732851

0.0732851

0.0732851

0.0732851

0.618373

27.9705

2.44505

3.21802

0.317569

3.02531

0.92756

0.170999

0.122142

6.80011602582908e-15
4

0
4

1.26371

1.00874

0.175085

0.0798828

0
4

0.450588

0.401732

0.0488567

5.55111512312578e-17

0
4

0.119824

0.119824

0

0
4

1.28398

0.119824

0.119824

0.262628

0.781708

0
4

0.170999

0.0977135

0.0732851

0
4

0

0

0
4

1.24404

1.19326

0.0507772

0
4

0

0

0

0

0
4

0

0

0

0
4

0.958594

0.718945

0.159766

0.0798828

1.38777878078145e-17

0
4

60.1501
4

31.8134
4

0.119824

0.0732851

26.1577
4

0.536876
4

0.195427

0.254968

0.334851

0.0732851

0.468419

0.122142

6.96664947952286e-15
4

0
4

0.760932

0.760932

0

0
4

0.701983

0.701983

0
4

0.350171

0.175085

0.175085

0
4

0

0

0
4

5.3085

5.3085

0
4

0.170999

0.0732851

0.0977135

0
4

0.193109

0.193109

0
4

0.239648

0.119824

0.119824

0
4

0

0

0

0
4

0.399414

0.399414

0
4

7.28799
3

5.13266
3

1.12279

0.39377

0

0.587988

0.0507772

0
4

0

0

0
4

0.107242

0.107242

0
4

0

0

0
4

0.0488567

0.0488567

0
4

0.0723307

0.0723307

0
4

0

0

0
4

0

0

0
4

0

0

0
4

0

0

0
4

0

0

0
4

5.82715
3

5.82715
3

0
4

0

0

0
4

0.195427

0.195427

0
4

0.561853

0.561853

0
4

0.289421

0.289421

0
4

0

0

0
4

0

0

0
4

0.0488567

0.0488567

0
4

0.350171

0.350171

0
4

0

0

0
4

0.0798828

0.0798828

0
4

1.39792

1.22692

0.170999

0

0
4

0.359473

0

0.27959

0.0798828

0
4

0.29314

0.219855

0.0732851

0

1.38777878078145e-17

0
4

0.170999

0.122142

0

0

0.0488567

0
4

0

0

0

0

0

0
4

3.93543531096441e-12
4

0
4

1194.15
4

20.0955
5

14.7428

1.57268
5

0.0533257
4

0

0

0

0

0

0

0

0

0

0

0
5

0

0

0

0

0

0

0

0

0

0

0
5

0

0

0

0

0

0

0

0

0

0

0

0

0

0

0.199707

0.106651

0

0

0

0

0.311485
5

0.541077

0

0

0

0

0.324559

0

0

0

0

1.03974
5

0

0

0

0

0

0.289421

0

0

0

0

0.0533257
6

0.0799885

0

0

0

0

0

0

0

0

0

0.175085
4

0

0

0

0

0

0

0

0

0

0.097998

0
5

0

0

0

0

0.146997

0

0

0

0

0

0

0

0

0

0

0

0

0

0

0.097998

0

0
5

0

0

0

0

0

0

0

0

0

0

0.262628

0

0

0

0

0

0

0

0

0

0

3.33066907387547e-16
5

0
4

1164.63
4

0
6

4.73646
3

265.639
4

102.689
5

0
5

0.097998
5

191.311
4

11.9675
4

74.4845
4

0.0917376
4

0

0

0

0

1.01297

0.289421

0.119824

0.0799885

0

0

2.15566
4

0

0

0

0

0

0.23189

0

0

0

0

0

0

0

0

0

0

0

0

0

0

0.154593

0.402329
4

0

0

0

0

0

0

0

0

0.437713

0

0

0

0

0

0

0

0

0

0

0

0

0.639592

0

0.199707

0

0

0

0

0

0

0

0

1.08215
4

0

0

0

0

0

0

0

0.23189

0

0

0

0

0.386483

0.119824

0

0

0

0

0

0

0.0341641

0.144661

0.154593

0

0.23189

0

0

0

0

0

0

0

0

0

0.0723307

0

0

0

0

0

0

0

0

135.234
4

0
7

0

0.0482775

0

0

0

0

0

0.102492

0

0

3.65556

0

0

0

0

0

0

0.0649455

0

0.217249

0

0

0

0

0

0

0

0

0

0

0

0

0

0

0

0

0

0

0

0

0.154593

0

0

0.213303

0

0

0

0

0

0.386483

0.119824

0

0

0

1.93009
4

0

0

0.0649455

0

0.0649455

0

0

0.154593

0

0

0

0

0

0

0

0

0

0

0

0.0917376

0

0.618373

0

0

0

0.106651

0

0

0

0

0

0

0.447885

1.01297

295.139
4

0

0

1.66395

0

0.23189

0

0

0.154593

0

0

38.6873
3

0

0.159766

0

0

0

0.881615

0.0798828

0

0

0

0

0

0

2.00971

0.224544

0

0

0.0723307

0.0799885

0

0

2.89774
4

0.434131

0

0

0

0.154593

0.599121

0

0

0

0

7.49619
4

0.144661

0.433984

0

0

0

0.718945

0.154593

0

0

0

0
4

0

0

0.485106

0

0

0.0649455

0

0.868263

0

0.0341641

0.226924
3

0

0

0

0

0

0.129891

6.51197

0

0

0

0
4

0.912532

0
8

0

0

0

0

0

0.0798828

0

0

0

0.0798828

0
8

0

0.199707

0

0

0

0.0723307

0

0

0.0341641

0

0

0

0

0

0

0

0.183475

0.154593

0

0

0

0

0.108496

0

0

0

0

0

2.77555756156289e-17

0
4

0

0

0

0

0

0

0

0

0
4

0

0

0
4

0

0

0
4

0.0965551

0.0965551

0
4

0

0

0
4

0

0

0
4

0

0

0
4

0

0

0
4

0

0

0
4

0.23189

0.23189

0
4

0

0

0
4

2.72903
4

2.3626
4

0.29314

0.0732851

0

3.19189119579733e-16
4

0
4

0

0

0
4

0

0

0
4

0

0

0
4

0

0

0
4

4.97316
4

4.78005
4

0.120694

0.0724163

0
4

0.387379
4

0.387379

0

0

0

0
4

0.0971343

0.0482775

0.0488567

0

0
4

0

0

0
4

0

0

0
4

0

0

0
4

0

0

0
4

4.16333634234434e-15
4

0
4

28.5517
3

19.273
4

18.3777
4

0

0

0.685265

0.209979

0

0

0

0

0

0

0
4

0

0

0

0

0

0
4

0.129128

0.129128

0
4

0.325488

0.325488

0
4

0

0

0
4

0

0

0
4

0.146997

0.146997

0
4

0
3

0
4

0

0

0

0

0

0

0

0
4

0

0
4

0

0

0

0
4

1.75996
3

1.75996
3

0
4

0
4

0
4

1.05053
4

0.7631
4

0.0482775

0.0512461

0

0

0

0.187902

5.55111512312578e-17
4

0
4

4.92043
3

0.265526

0

0

0

0

4.65491

0

0
4

0.699742
4

0.544222
4

0.0482775

0.107242

0

4.16333634234434e-17
4

0
4

0.0533257
4

0.0533257

0

0

0

0

0
4

0.19311

0.19311

0

0

0
4

0

0

0

0

0

0
4

0
4

3.56866

3.38901

2.2683

0

0

0.536212
7

0.214485

0

0.0482775

0

0

0

0.321727

3.33066907387547e-16

0
4

0.179659

0.179659

0

0

0

0
4

0

0

0

0

0

0

0
4

0

0

0

0
4

0

0

0
4

0
4

0
4

0
4

0

0

0

0

0
4

0

0

0

0
4

0
4

0

0

0

0

0

0

0
4

0

0

0

0
4

0
4

1.03848

0.159766

0.159766

0

0
4

0.27959

0

0.27959

0
4

0.599121

0.599121

0
4

0
4

0.586281

0.586281

0.341997

0.170999

0.0732851

0
4

0
4

11.4268
3

11.376
3

11.376
3

0

0
4

0.0507772

0.0507772

0
4

3.81639164714898e-16
3

0
4

4.9572
4

3.38143

3.1188

0.262628

1.11022302462516e-16

0
4

0

0

0
4

1.57577

1.57577

0
4

0
4

0.0798828

0

0

0

0

0

0
4

0.0798828

0.0798828

0
4

0
4

1.4471

0

0

0

0
4

0

0

0

0

0
4

1.4471

1.4471

0
4

0
4

0
6

0
6

0
6

0
4

0
4

0

0

0

0
4

0
4

1.07798
3

1.07798
3

1.07798
3

0

0

0
3

0

0

0

0

0

0

0

0
4

0

0

0

0

0

0
4

0

0

0

0

0
4

0
4

2.74394

2.74394

0.734985

0

2.00896

0
4

0

0

0
4

0
4

3.4847
4

3.4847

3.33011

0.154593

0

0
4

0

0

0
4

0

0

0
4

0
4

0

0

0

0

0
4

0
4

0.0533257
3

0

0

0
4

0.0533257

0.0533257

0

0
4

0

0

0
4

0

0

0
4

0

0

0
4

0

0

0
4

0
4

0

0

0

0

0
4

0
4

5.00736

4.2608

4.10083

0.159977

0
4

0.586582

0.18664

0.18664

0.213303

0
4

0.0533257

0.0533257

0
4

0.106651

0.106651

0
4

2.35922392732846e-16

0
4

0

0

0

0

0

0

0
4

0
4

0

0

0

0

0

0
4

0

0

0

0

0
4

0
4

0

0

0

0

0
4

0
4

0

0

0

0

0
4

0
4

14.1563
3

12.0871
3

4.90835
3

0

5.84205
4

0.321727

0.426605

0.159977

0.321727

0.106651

0

0

9.57567358739198e-16
3

0
4

0.159977

0.0533257

0.106651

0

1.38777878078145e-17

0
4

1.55009

1.22837

0.321727

0
4

0.179573

0.179573

0

0
4

0.179573

0.0723307

0.107242

0
4

1.0547118733939e-15
3

0
4

1.52821

1.32297

0.930981

0.391992

5.55111512312578e-17

0
4

0.20524

0.097998

0.107242

1.38777878078145e-17

0
4

0

0

0
4

0
4

0

0

0

0

0

0

0

0

0
4

0
4

0.0798828
4

0
4

0

0

0

0
4

0.0798828

0.0798828

0
4

0

0

0
4

0
4

0

0

0

0

0

0
4

0

0

0

0
4

0
4

0

0

0

0

0
4

0

0

0

0
4

0

0

0
4

0
4

0

0

0

0

0

0

0
4

0

0

0
4

0
4

0.133314

0.133314

0.0533257

0.0799885

0

0
4

0

0

0
4

0

0

0
4

0

0

0
4

0
4

0
4

0
4

0
4

0
4

0

0

0
4

0

0

0
4

0
4

0.172523
4

0.172523
4

0.121276

0

0.0512461

0
4

0
4

0.896703

0.383708

0.12874

0.254968

0
4

0.512996

0.195427

0.317569

0
4

1.11022302462516e-16

0
4

5.47725
3

2.35942
3

0
3

2.35942
3

0

0

0

0

0

0

0

0
4

0.0798828

0.0798828

0
4

0

0

0
4

0

0

0
4

0

0

0
4

0

0

0
4

0
4

0
4

0

0

0

0
4

3.03794
4

3.03794

0

0

0
4

0

0

0

0
4

0

0

0

0
4

0

0

0
4

0

0

0
4

0

0

0
4

0

0

0
4

4.44089209850063e-16
3

0
4

0
6

0
6

0
6

0
4

0
4

3.50171

0.437713

0.437713

0
4

2.71382

2.71382

0
4

0.350171

0.350171

0

0
4

0

0

0
4

2.22044604925031e-16

0
4

0

0

0

0

0
4

0
4

1.7001
3

1.59286
3

1.54208

0.0507772

0

0
4

0.107242

0.107242

0
4

0
4

0.720683

0.720683

0.0488567

0.14657

0.525256

0
4

0

0

0
4

0
4

0

0

0

0
4

0

0

0
4

0

0

0
4

0

0

0
4

0
4

0

0

0

0

0

0
4

0
4

0

0

0

0

0

0

0
4

0

0

0
4

0
4

0.268106

0

0

0
4

0.268106

0.268106

0
4

0

0

0
4

0
4

0

0

0

0

0

0

0
4

0
4

7.19204
5

7.19204
5

0.0533257
5

0

0

0

0

0

0

0.175085

0

0

0.199707

0.107242
5

0

6.36726
4

0

0

0

0

0.289421

0

4.44089209850063e-16
5

0
4

0
4

0.312483

0.312483

0.312483

0

0

0

0

0
4

0
4

0

0

0

0

0

0

0
4

0

0

0
4

0
4

0.0798828

0.0798828

0.0798828

0

0
4

0
4

0.0798828

0.0798828

0

0.0798828

0

0

0
4

0

0

0
4

0

0

0
4

0
4

0

0

0

0

0
4

0

0

0

0
4

0

0

0
4

0
4

0

0

0

0

0
4

0

0

0
4

0
4

0.519238

0.519238

0.199707

0

0.319531

0
4

0
4

6.79416

5.79563

5.6758

0.119824

0
4

0.998535

0.998535

0
4

0
4

0

0

0

0

0

0
4

0

0

0
4

0
4

0

0

0

0

0

0
4

0
4

0.759472
5

0.759472
5

0.470149
5

0

0

0.144661

0

0

0.144661

0

0

5.55111512312578e-17
5

0
4

0
4

0

0

0

0

0
4

0

0

0
4

0

0

0
4

0

0

0
4

0
4

0

0

0

0

0

0
4

0

0

0

0
4

0
4

0.951525

0.951525

0.561853

0.162364

0.227309

5.55111512312578e-17

0
4

0

0

0

0
4

0
4

0

0

0

0

0
4

0

0

0
4

0

0

0
4

0
4

0.575028

0.228411

0.175085

0.0533257

0
4

0.159977

0.159977

0
4

0.0799885

0.0799885

0
4

0.106651

0.106651

0
4

4.16333634234434e-17

0
4

0

0

0

0

0
4

0
4

0.23189
4

0

0

0

0
4

0.23189

0.23189

0
4

0
4

0

0

0

0
4

0
4

0.470248

0.470248

0.397917

0.0723307

0

0
4

0

0

0
4

0
4

0

0

0

0

0

0
4

0

0

0
4

0

0

0
4

0
4

247.878
4

244.326
4

199.718
4

0.175085

0.195427

0.386483

3.15318

1.05042

18.0101

1.36763

14.7662

5.1125

0.317569

0.0732851

3.87329057716101e-14
4

0
4

0.119824

0.119824

0
4

0.154593

0

0.154593

0
4

0.311485

0.311485

0
4

2.07641

2.07641

0
4

0.175085

0.175085

0
4

0.244284

0.244284

0
4

0.350171

0.350171

0
4

0.119824

0.119824

0
4

0

0

0
4

0
4

0
4

0

0

0
4

0

0

0
4

0
4

11.3563

11.1964

1.45699

9.73938

0
4

0.159977

0.159977

0
4

0
4

0.126943

0.126943

0.126943

0

0
4

0

0

0

0
4

0
4

0

0

0

0

0
4

0

0

0
4

0
4

1.04879

0.614811

0.0723307

0.144661

0.397819

0
4

0.433984

0.253157

0.180827

0
4

0
4

0

0

0

0
4

0
4

0

0

0

0

0

0
4

0

0

0

0
4

0
4

0

0

0

0

0
4

0

0

0

0
4

0

0

0
4

0
4

0

0

0

0

0

0
4

0

0

0

0
4

0
4

0

0

0

0

0

0
4

0

0

0
4

0

0

0
4

0
4

3.11322

1.15509

0.0533257
6

0.613245

0.328538

0.0533257

0

0.0533257

0.0533257

2.63677968348475e-16

0
4

0.624994

0.0917376
6

0

0.213303

0

0

0.0533257

0.0533257

0.213303

2.77555756156289e-17

0
4

1.22649

1.1465

0.0799885

2.77555756156289e-17

0
4

0.106651

0.0533257

0.0533257

0
4

0
4

0

0

0

0

0

0

0
4

0

0

0
4

0
4

0.126943

0.126943

0.126943

0

0

0
4

0

0

0
4

0

0

0
4

0
4

1.11836

0.878711

0.439355

0.239648

0.199707

0
4

0.239648

0.239648

0
4

0
4

1.74484

1.74484

1.74484

0
4

0
4

4.6715

0.101554

0.0507772

0.0507772

0
4

4.56995

4.56995

0
4

0

0

0
4

0
4

0.0723307

0

0

0

0
4

0

0

0

0
4

0.0723307

0.0723307

0
4

0
4

0

0

0

0
4

0
4

0

0

0

0

0

0
4

0
4

0.683994

0.683994

0.635138

0.0488567

0
4

0
4

0.101554

0.101554

0

0.0507772

0

0.0507772

0
4

0
4

0.17772

0.126943
6

0
6

0

0

0

0.126943

0

0

0

0
4

0.0507772
2

0

0

0

0.0507772

0

0

0
4

0
2

0

0

0

0

0
4

0

0

0

0

0
4

0

0

0

0
4

0

0

0

0
4

0

0

0
4

0
4

0

0

0

0

0

0

0
4

0
4

0

0

0

0

0
4

0

0

0

0
4

0
4

0.0723307

0.0723307

0.0723307

0
4

0
4

0

0

0

0

0
4

0

0

0
4

0
4

0

0

0

0

0
4

0

0

0
4

0
4

0

0

0

0

0
4

0
4

6.63124

6.63124

6.19092

0.359473

0.0808509

0
4

0
4

0.101554

0

0

0

0
4

0.101554

0.101554

0
4

0
4

0

0

0

0
4

0

0

0

0
4

0
4

0

0

0

0
4

0

0

0
4

0
4

13.2719
5

13.0968
5

13.0968
5

0

0

0
4

0

0

0
4

0.175085

0.175085

0
4

0
4

0

0

0

0

0

0
4

0

0

0
4

0
4

1.02599

1.02599

0.366426

0.659566

1.11022302462516e-16

0
4

0
4

0

0

0

0
4

0
4

0.454675

0.254968

0.175085

0.0798828

0
4

0.199707

0.199707

0
4

0
4

0

0

0

0
4

0

0

0

0
4

0
4

0.469995

0.29491

0.29491

0
4

0

0

0
4

0.175085

0.175085

0
4

0
4

0

0

0

0

0

0
4

0
4

7.29289

0.335027

0.237029

0.097998

2.77555756156289e-17

0
4

6.95786

6.95786

0
4

0
4

0

0

0

0

0

0
4

0

0

0
4

0
4

0

0

0

0

0

0

0
4

0
4

0

0

0

0

0

0

0

0

0

0

0

0

0

0

0

0

0

0

0

0

0

0

0

0

0

0

0

0

0

0

0

0

0

0

0

0

0

0
4

0

0

0

0

0

0

0

0

0

0

0
4

0
4

6.34575

6.34575

0.773222

0.106651

0.106651

0.0533257

0.0533257

0.0533257

0.719897

0.479931

1.59977

0.239966

1.8664

0.106651

0.0533257

0.133314

0
4

0
4

2.84194

2.84194

2.84194

0
4

0

0

0
4

0

0

0
4

0
4

6.58257

6.58257

0.23189

6.35068

0
4

0
4

0

0

0

0

0
4

0
4

0

0

0

0
4

0

0

0
4

0

0

0
4

0
4

1.15768

1.15768

1.15768

0
4

0

0

0
4

0
4

0

0

0

0
4

0

0

0
4

0
4

0.0977135

0.0977135

0.0977135

0

0
4

0
4

0

0

0

0

0
4

0

0

0
4

0
4

0

0

0

0

0
4

0

0

0
4

0
4

0.213303

0.159977

0.0533257

0.106651

1.38777878078145e-17

0
4

0.0533257

0.0533257

0
4

0
4

0

0

0

0

0

0

0

0

0

0

0

0

0

0

0

0

0

0

0

0

0

0
4

0

0

0

0

0

0

0

0
4

0

0

0

0
4

0
4

0.168971

0.168971

0.120694

0.0482775

0
4

0
4

0

0

0

0
4

0
4

0

0

0

0

0
4

0
4

0

0

0

0
4

0

0

0
4

0
4

0.386483

0

0

0

0
4

0.386483

0.386483

0
4

0
4

0.439355

0.439355

0.199707

0.159766

0.0798828

0
4

0
4

0

0

0

0
4

0

0

0
4

0
4

0

0

0

0
4

0

0

0
4

0
4

0

0

0

0

0
4

0

0

0
4

0
4

0

0

0

0

0
4

0
4

3.45446
3

3.45446
3

3.45446
3

0

0

0

0

0

0

0

0

0
4

0

0

0

0
4

0

0

0
4

0
4

0

0

0

0
4

0

0

0
4

0
4

3.47187

3.47187

0

3.47187

0
4

0
4

0

0

0

0
4

0

0

0
4

0

0

0
4

0
4

0.0808509

0.0808509

0.0808509

0

0
4

0

0

0
4

0
4

0

0

0

0

0
4

0

0

0
4

0
4

0

0

0

0

0
4

0

0

0
4

0
4

0.119824

0.119824

0.119824

0

0
4

0
4

0

0

0

0
4

0
4

0

0

0

0

0
4

0
4

0

0

0

0

0
4

0

0

0
4

0
4

0.343294
4

0.209979
4

0

0

0.209979

0

0

0

0

0

0
4

0.133314
4

0
4

0

0

0

0.133314

0
4

0

0

0

0

0

0
4

0

0

0

0
4

0

0

0
4

0
4

0

0

0

0
4

0

0

0
4

0
4

0

0

0

0
4

0

0

0
4

0

0

0
4

0
4

0.0482775

0.0482775

0.0482775

0

0
4

0
4

0

0

0

0

0
4

0
4

0

0

0

0
4

0
4

0

0

0

0
4

0

0

0
4

0
4

0

0

0

0
4

0
4

0

0

0

0

0
4

0
4

0.282978

0.282978

0

0.282978

0

0
4

0
4

0.700341

0.700341

0.437713

0.262628

5.55111512312578e-17

0
4

0
4

0.239648

0
5

0

0

0

0

0

0

0

0

0
4

0

0

0

0

0
4

0

0

0

0
4

0.239648

0.239648

0
4

0
4

0.772967

0.772967

0.386483

0.386483

0
4

0
4

0

0

0

0
4

0

0

0
4

0
4

0

0

0

0
4

0
4

0

0

0

0
4

0
4

0

0

0

0
4

0
4

0

0

0

0

0
4

0
4

0.121276

0

0

0
4

0.121276

0.121276

0
4

0
4

0

0

0

0

0
4

0
4

0.27959

0.27959

0.199707

0.0798828

0
4

0
4

0

0

0

0
4

0

0

0
4

0
4

0
2

0
2

0

0

0

0

0
2

0

0

0

0

0

0

0

0
4

0

0

0

0
4

0
4

0

0

0

0
4

0
4

0.170999

0.170999

0.0732851

0.0977135

0
4

0
4

0.0724163

0.0724163

0.0724163

0
4

0
4

0

0

0

0

0
4

0
4

0

0

0

0

0
4

0
4

0

0

0

0

0
4

0
4

0

0

0

0

0
4

0
4

0

0

0

0
4

0

0

0
4

0
4

0

0

0

0

0
4

0
4

1.23675

0.541077

0.541077

0
4

0.69567

0.69567

0
4

0
4

6.92811
3

6.14328
3

4.92999
3

0.425896

0.147801

0.202127

0

0.38622

0

0.0512461

3.19189119579733e-16
3

0
4

0.784832
3

0.0482775

0.0808509

0.409969

0.130719

0.0808509

0.0341641

0

1.04083408558608e-16
3

0
4

7.7715611723761e-16
3

0
4

0

0

0

0
4

0
4

0

0

0

0
4

0

0

0
4

0
4

0

0

0

0
4

0

0

0
4

0
4

0

0

0

0
4

0

0

0
4

0
4

0

0

0

0

0
4

0
4

0.102492

0.0341641

0.0341641

0
4

0.0683282

0.0683282

0
4

0
4

0

0

0

0
4

0
4

0

0

0

0

0
4

0
4

0

0

0

0

0
4

0
4

0

0

0

0
4

0

0

0
4

0
4

0
4

0
4

0
4

0

0

0

0

0

0

0

0
4

0

0

0

0

0

0

0
4

0

0

0

0

0
4

0

0

0

0
4

0

0

0

0
4

0

0

0

0
4

0

0

0
4

0
4

0

0

0

0
4

0

0

0
4

0
4

0

0

0

0

0
4

0
4

0.199707

0.199707

0.0798828

0.119824

0
4

0
4

0

0

0

0
4

0

0

0
4

0
4

0

0

0

0
4

0

0

0
4

0
4

3.0488

2.89421

2.89421

0
4

0.154593

0.154593

0
4

0
4

0

0

0

0

0
4

0
4

0

0

0

0

0
4

0
4

0.0507772

0

0

0
4

0.0507772

0.0507772

0
4

0
4

0

0

0

0
4

0
4

4.0059
3

4.0059
3

4.0059
3

0
2

0

0

0

0

0
4

0
3

0
3

0

0

0

0
4

0

0

0

0
4

0

0

0

0

0

0
4

0

0

0
4

0
4

0

0

0

0

0
4

0
4

0

0

0

0
4

0
4

0

0

0

0

0
4

0
4

0.0798828

0.0798828

0.0798828

0
4

0

0

0
4

0
4

0

0

0

0

0
4

0
4

1.49793

1.49793

0

1.49793

0
4

0
4

0

0

0

0
4

0
4

0

0

0

0

0
4

0
4

0

0

0

0

0
4

0
4

0

0

0

0

0
4

0
4

17.0671
4

17.0671
4

16.174
4

0.893134

0

0

0

0

0

0

2.44249065417534e-15
4

0
4

0

0

0
4

0

0

0
4

0
4

0.0798828

0.0798828

0.0798828

0
4

0
4

0

0

0

0
4

0

0

0
4

0
4

0

0

0

0

0
4

0
4

0

0

0

0
4

0
4

0.635138

0.586281

0.586281

0
4

0.0488567

0.0488567

0
4

5.55111512312578e-17

0
4

0

0

0

0

0
4

0
4

0

0

0

0
4

0

0

0
4

0
4

0

0

0

0

0
4

0
4

0.152332

0.152332

0.0761658

0.0761658

0
4

0
4

0

0

0

0
4

0

0

0
4

0
4

3.72029
2

3.24989
2

3.00489
2

0

0

0

0

0

0.244995
3

0

0

0

0

0

0

0
4

0.308699
2

0.146997
2

0

0

0

0

0

0

0

0

0

0

0.161702

0
2

0
3

0

0

0

0

0

0
4

0.161702

0.161702

0

0
4

0

0

0

0

0

0
4

0

0

0
4

0

0

0
4

4.9960036108132e-16
2

0
4

5.83439

5.83439

5.78611

0.0482775

0

0

0
4

0

0

0
4

0

0

0
4

0
4

0

0

0

0
4

0

0

0
4

0
4

0

0

0

0
4

0
4

0.399943

0.0533257

0.0533257

0
4

0.346617

0.346617

0
4

0
4

0

0

0

0
4

0
4

1.17234

1.09149

1.09149

0
4

0.0808509

0.0808509

0
4

0
4

0

0

0

0
4

0
4

0.12874

0.0798828

0.0798828

0
4

0.0488567

0.0488567

0
4

0
4

0

0

0

0
4

0
4

0

0

0

0
4

0
4

0

0

0

0
4

0
4

0
7

0
7

0
7

0
8

0

0

0

0

0

0

0

0
4

0

0

0

0

0

0
4

0

0

0

0
4

0
4

0.962969

0.962969

0.962969

0
4

0
4

0

0

0

0

0
4

0
4

0

0

0

0
4

0
4

0.119824

0.119824

0.119824

0
4

0
4

0

0

0

0
4

0
4

0

0

0

0
4

0
4

0

0

0

0
4

0
4

0

0

0

0
4

0
4

0

0

0

0
4

0
4

0

0

0

0
4

0
4

0.727658
2

0.242553
2

0
2

0
2

0

0.242553

0

0
4

0.485106

0.363829

0.121276

0
4

0

0

0
4

0

0

0
4

5.55111512312578e-17
2

0
4

0

0

0

0
4

0
4

0.0798828

0.0798828

0.0798828

0
4

0
4

0

0

0

0
4

0
4

0

0

0

0
4

0
4

0

0

0

0
4

0
4

0

0

0

0
4

0
4

0

0

0

0
4

0
4

0

0

0

0
4

0
4

0

0

0

0
4

0
4

0

0

0

0
4

0
4

0
4

0
4

0
4

0

0

0

0

0

0

0

0
4

0
4

0

0

0

0
4

0
4

0

0

0

0
4

0
4

0

0

0

0
4

0
4

0

0

0

0
4

0
4

0.23189

0.23189

0.23189

0
4

0
4

0

0

0

0
4

0
4

0

0

0

0
4

0
4

0

0

0

0
4

0
4

0

0

0

0
4

0
4

0

0

0

0
4

0
4

0

0

0

0

0

0

0

0

0

0
4

0

0

0
4

0
4

0

0

0

0
4

0
4

0

0

0

0
4

0
4

0.0799885

0.0799885

0.0799885

0
4

0
4

0

0

0

0
4

0
4

0

0

0

0
4

0
4

0

0

0

0
4

0
4

0

0

0

0
4

0
4

0

0

0

0
4

0
4

0

0

0

0
4

0
4

0.0488567

0.0488567

0.0488567

0
4

0
4

2.16307
3

2.16307
3

1.54601
3

0.617063
3

0

0

2.22044604925031e-16
3

0
4

0

0

0

0

0
4

0
4

0.0917376

0.0917376

0.0917376

0
4

0
4

0

0

0

0
4

0
4

0.0917376

0.0917376

0.0917376

0
4

0
4

0

0

0

0
4

0
4

0

0

0

0
4

0
4

0.0724163

0.0724163

0.0724163

0
4

0
4

0

0

0

0
4

0
4

0

0

0

0
4

0
4

0

0

0

0
4

0
4

0.175085

0.175085

0.175085

0
4

0
4

24.9538
4

9.06537
4

0.723194

0.435075

0.49053

0.257479

0.27531

2.07641

0.244284

4.48321

0.0798828

2.5951463200613e-15
4

0
4

1.52453

0.827698

0.519238

0.0488567

0.0798828

0.0488567

0
4

14.3639

14.3639

0
4

0
4

0

0

0

0
4

0
4

0

0

0

0
4

0
4

0

0

0

0
4

0
4

0

0

0

0
4

0
4

0

0

0

0
4

0
4

0

0

0

0
4

0
4

0

0

0

0
4

0
4

0

0

0

0
4

0
4

0.0799885

0.0799885

0.0799885

0
4

0
4

0.0798828

0.0798828

0.0798828

0
4

0
4

1.22498
3

1.22498
3

1.22498
3

0

0

0

0

0
4

0

0

0
4

0

0

0
4

0
4

0

0

0

0
4

0
4

0

0

0

0
4

0
4

0

0

0

0
4

0
4

0.144661

0.144661

0.144661

0
4

0
4

0

0

0

0
4

0
4

0

0

0

0
4

0
4

0.0488567

0.0488567

0.0488567

0
4

0
4

0

0

0

0
4

0
4

0

0

0

0
4

0
4

0.0798828

0.0798828

0.0798828

0
4

0
4

0

0

0

0

0

0

0

0
4

0

0

0
4

0
4

0

0

0

0
4

0
4

0

0

0

0
4

0
4

0

0

0

0
4

0
4

0

0

0

0
4

0
4

0

0

0

0
4

0
4

0

0

0

0
4

0
4

0

0

0

0
4

0
4

0

0

0

0
4

0
4

0

0

0

0
4

0
4

0.144661

0.144661

0.144661

0
4

0
4

6.55451

5.32908

4.43688

0.213091

0.0798828

0.27959

0.239648

0.0799885

0
4

0.559602

0.319954

0.119824

0.119824

0
4

0.479297

0.399414

0.0798828

0
4

0.106651

0.0533257

0.0533257

0
4

0.0798828

0.0798828

0
4

0
4

0

0

0

0
4

0
4

0.0507772

0.0507772

0.0507772

0
4

0
4

0.154593

0.154593

0.154593

0
4

0
4

0

0

0

0
4

0
4

0

0

0

0
4

0
4

0

0

0

0
4

0
4

0

0

0

0
4

0
4

0

0

0

0
4

0
4

0

0

0

0
4

0
4

0

0

0

0
4

0
4

0.17162
3

0
3

0
5

0

0

0

0

0

0

0

0

0

0

0

0

0

0

0

0

0

0

0
4

0

0

0

0
4

0

0

0

0

0
4

0

0

0

0

0

0
4

0

0

0

0

0
4

0

0

0

0
4

0

0

0

0
4

0

0

0

0
4

0

0

0

0
4

0

0

0

0
4

0

0

0
4

0

0

0

0

0

0

0

0

0

0

0

0
4

0

0

0
4

0.0798828

0.0798828

0
4

0
3

0

0

0

0

0

0

0

0

0

0

0

0

0
4

0
3

0

0

0

0

0

0

0
4

0.0917376
4

0.0917376

0

0

0

0

0

0

0

0

0
4

0

0

0

0

0

0
4

0
5

0

0

0

0

0

0
4

0
4

0

0

0

0

0

0
4

0

0

0

0

0

0
4

1.38777878078145e-17
3

0
4

0.54007
3

0.54007
3

0.202127
3

0

0

0.217249

0.120694

0

0

0
4

0

0

0

0

0
4

0

0

0

0
4

0
4

0

0

0

0
4

0
4

0

0

0

0
4

0
4

0

0

0

0
4

0
4

0

0

0

0
4

0
4

0.0488567

0.0488567

0.0488567

0
4

0
4

0.0512461

0.0512461

0.0512461

0
4

0
4

0.175085

0.175085

0.175085

0
4

0
4

0

0

0

0
4

0
4

0

0

0

0
4

0
4

0

0

0

0
4

0
4

0
4

0
4

0
4

0

0

0

0
4

0

0

0

0

0

0
4

0
4

0

0

0

0
4

0
4

0

0

0

0
4

0
4

1.13805

1.13805

1.13805

0
4

0
4

0.154593

0.154593

0.154593

0
4

0
4

0

0

0

0
4

0
4

0

0

0

0
4

0
4

0

0

0

0
4

0
4

0

0

0

0
4

0
4

0.217249

0.217249

0.217249

0
4

0
4

0

0

0

0
4

0
4

0.479297

0

0

0

0

0

0

0

0
4

0.479297

0.479297

0
4

0
4

0

0

0

0
4

0
4

0.160864

0.160864

0.160864

0
4

0
4

0

0

0

0
4

0
4

0

0

0

0
4

0
4

0

0

0

0
4

0
4

0

0

0

0
4

0
4

0

0

0

0
4

0
4

0

0

0

0
4

0
4

0

0

0

0
4

0
4

0

0

0

0
4

0
4

2.08391
4

1.77757

1.31807

0.219855

0.159766

0

0

0

0.0798828

9.71445146547012e-17

0
4

0

0

0

0

0

0
4

0.159766

0.159766

0

0
4

0.14657

0.14657

0
4

8.32667268468867e-17
4

0
4

0

0

0

0
4

0
4

0

0

0

0
4

0
4

0

0

0

0
4

0
4

0

0

0

0
4

0
4

0

0

0

0
4

0
4

0

0

0

0
4

0
4

0

0

0

0
4

0
4

0

0

0

0
4

0
4

0

0

0

0
4

0
4

0

0

0

0
4

0
4

2.47964
3

2.47964
3

0

2.34633

0

0.0533257

0

0.0799885

0

0
4

0

0

0

0

0
4

0

0

0
4

0
4

0.0798828

0.0798828

0.0798828

0
4

0
4

0

0

0

0
4

0
4

0

0

0

0
4

0
4

0

0

0

0
4

0
4

0

0

0

0
4

0
4

0

0

0

0
4

0
4

0

0

0

0
4

0
4

0

0

0

0
4

0
4

0

0

0

0
4

0
4

0

0

0

0
4

0
4

0
7

0
7

0
7

0
7

0
4

0
4

0

0

0

0
4

0
4

0

0

0

0
4

0
4

0

0

0

0
4

0
4

0

0

0

0
4

0
4

0

0

0

0
4

0
4

0

0

0

0
4

0
4

0

0

0

0
4

0
4

0.159766

0.159766

0.159766

0
4

0
4

0

0

0

0
4

0
4

0

0

0

0
4

0
4

0.17315
4

0.17315
4

0.119824
4

0

0

0.0533257

0

0

0

0
4

0

0

0

0

0
4

0

0

0

0
4

0
4

0

0

0

0
4

0
4

0

0

0

0
4

0
4

0

0

0

0
4

0
4

0

0

0

0
4

0
4

0

0

0

0
4

0
4

0

0

0

0
4

0
4

0

0

0

0
4

0
4

0

0

0

0
4

0
4

0.0917376

0.0917376

0.0917376

0
4

0
4

0

0

0

0
4

0
4

2.36351
4

2.00382
4

1.53208
4

0.319531

0.0798828

0.0723307

0

0

2.77555756156289e-17
4

0
4

0.199707
4

0

0

0.199707

0
4

0.159977

0.159977

0
4

0

0

0
4

0

0

0
4

2.22044604925031e-16
4

0
4

0.0798828

0.0798828

0.0798828

0
4

0
4

0

0

0

0
4

0
4

0

0

0

0
4

0
4

0.154593

0.154593

0.154593

0
4

0
4

0.106651

0.106651

0.106651

0
4

0
4

0

0

0

0
4

0
4

0

0

0

0
4

0
4

0.0482775

0.0482775

0.0482775

0
4

0
4

0

0

0

0
4

0
4

0

0

0

0
4

0
4

0

0

0

0

0

0

0

0

0

0

0
4

0

0

0

0
4

0

0

0
4

0

0

0
4

0

0

0
4

0
4

0

0

0

0
4

0
4

0

0

0

0
4

0
4

0.0799885

0.0799885

0.0799885

0
4

0
4

0

0

0

0
4

0
4

0

0

0

0
4

0
4

0.0798828

0.0798828

0.0798828

0
4

0
4

0

0

0

0
4

0
4

0

0

0

0
4

0
4

0

0

0

0
4

0
4

0.199707

0.199707

0.199707

0
4

0
4

0.758887

0.639062

0.639062

0
7

0

0

0
4

0

0

0

0
4

0.119824

0.119824

0
4

0
4

0

0

0

0
4

0
4

0

0

0

0
4

0
4

0

0

0

0
4

0
4

0

0

0

0
4

0
4

0

0

0

0
4

0
4

0

0

0

0
4

0
4

0

0

0

0
4

0
4

0

0

0

0
4

0
4

0

0

0

0
4

0
4

0

0

0

0
4

0
4

162.652
4

157.035
4

133.005

0.928278

0.0488567

0.29314

0.12874

0.268712

0.195427

0.830565

0.700341

0.0798828

0.341997

3.36078

0.219855

0.0798828

3.23908

0.488567

0.0732851

1.2256

0.170999

0.317569

0.154593

0.0732851

0.55918

0.119824

0.170999

0.0732851

0.537424

0.14657

0.0488567

0.0732851

0.0488567

0.962969

0.0488567

0.75728

0.0488567

0.390854

0.0732851

0.175085

0.122142

0.962969

0.14657

0.0732851

0.0732851

0.0488567

0.244284

0.175085

0.0732851

0

0.0488567

0.122142

0

0.0977135

0.683994

3.09242

0.610709

0
4

0.262628

0.262628

0
4

2.27593

1.49013

0.512996

0.175085

0.0488567

0.0488567

0
4

1.19699

0.806136

0.219855

0.170999

0
4

0.341997

0.122142

0.219855

0
4

0.488567

0.122142

0.122142

0.244284

0
4

0.170999

0.170999

0
4

0.806136

0

0.806136

0
4

0.0732851

0.0732851

0
4

0

0

0
4

0
4

3.33167
4

0.888246
4

0.268712

0.386483

0.0732851

0.0798828

0.0798828

0

1.94289029309402e-16
4

0
4

2.2414

0.951779

0.0798828

1.20974

0
4

0.122142

0.0488567

0.0732851

0
4

0.0798828

0

0.0798828

0
4

0
4

0

0

0

0
4

0
4

0.0798828

0.0798828

0.0798828

0
4

0
4

0

0

0

0
4

0
4

0.0533257

0.0533257

0.0533257

0
4

0
4

0

0

0

0
4

0
4

0.107242

0.107242

0.107242

0
4

0
4

0.0488567

0.0488567

0.0488567

0
4

0
4

0

0

0

0
4

0
4

0

0

0

0
4

0
4

0

0

0

0
4

0
4

0.785858
4

0.677362
4

0.437713

0.239648

0

0

0

0
4

0.108496
3

0

0

0

0.108496

0
4

0

0

0
4

0

0

0
4

2.77555756156289e-17
4

0
4

0

0

0

0
4

0
4

0.643454

0.643454

0.643454

0
4

0
4

26.3744

8.25309

7.75081

0.159766

0.0798828

0.262628

3.88578058618805e-16

0
4

17.2459

0.437713

16.8082

0
4

0.875427

0.875427

0
4

5.21804821573824e-15

0
4

0
4

0
4

0
4

0

0

0
4

0

0

0

0

0

0

0
4

0

0

0

0

0

0

0
4

0

0

0
4

0
4

1.02472
4

0.794475
4

0

0.161702

0.44468

0.0808509

0

0

0

0.107242

4.16333634234434e-17
4

0
4

0.230243

0.144833

0

0.0854102

1.38777878078145e-17

0
4

0

0

0
4

0
4

1.93036
3

0

0

0

0
4

0

0

0

0

0

0

0
4

0

0

0
4

0

0

0
4

1.93036

1.93036

0
4

0
4

0
2

0
2

0
2

0

0

0
4

0
4

0.625753
3

0.250405

0

0.0482775

0

0

0.202127

0
4

0

0

0

0

0

0
4

0

0

0

0

0
4

0.375348

0.375348

0
4

0

0

0
4

0

0

0
4

0
4

6.94588

6.48174

1.52669

2.27666

0.998535

1.55771

0

0.122142

1.38777878078145e-16

0
4

0.464139

0.170999

0.0732851

0

0.0977135

0.122142

4.16333634234434e-17

0
4

3.33066907387547e-16

0
4

0
3

0
4

0
4

0

0

0

0

0

0
4

0

0

0

0

0
4

0

0

0
4

0
4

0
3

0
3

0
4

0

0

0

0

0

0

0

0

0

0

0

0
4

0

0

0
4

0

0

0
4

0

0

0
4

0
3

0
5

0

0

0

0

0

0

0

0

0

0

0

0
4

0
4

0
4

0

0

0

0

0

0

0
4

0
3

0
7

0

0

0

0

0

0

0
4

0
5

0

0

0

0

0

0

0

0
4

0

0

0

0

0

0

0
4

0

0

0

0
4

0

0

0

0
4

0

0

0
4

0
4

0

0

0

0

0

0
4

0

0

0

0

0

0
4

0

0

0
4

0

0

0
4

0

0

0
4

0
4

3.19185

3.07202

0.91399

0.964767

0.685493

0.0761658

0.126943

0.101554

0.203109

1.38777878078145e-16

0
4

0.119824

0.119824

0
4

0
4

0

0

0

0

0

0

0

0

0

0
4

0

0

0

0

0

0
4

0
4

1.1462
4

1.1462
4

0

0

0

1.06632

0.0798828

0
4

0
4

0

0

0

0

0

0

0

0

0
4

0

0

0
4

0
4

0
7

0
7

0
7

0

0

0

0
4

0

0

0
4

0

0

0
4

0

0

0
4

0

0

0
4

0

0

0
4

0

0

0
4

0
4

10.6074
4

10.1734
4

0.798828

8.8667

0

0.108496

0.319531

0.0798828

0

2.08166817117217e-16
4

0
4

0.433984

0.433984

0

0
4

0
4

12.3409
3

12.3409
3

12.3409
3

0
4

0

0

0
4

0
4

4.75156
4

2.44284

1.75884

0.610709

0.0732851

0

9.71445146547012e-17

0
4

0.867446

0.199707

0.317569

0.350171

0
4

1.2947

0.14657

0

1.14813

0
4

0.14657

0.14657

0
4

0
4

3.23663

1.55909

0.586582

0.17315

0.213197

0.266628

0.239648

0.0798828

9.71445146547012e-17

0
4

1.67754

1.43789

0.239648

0
4

0
4

0.175085
7

0.175085
7

0
7

0

0

0

0

0

0

0

0
7

0.175085

0
7

0
7

0

0

0

0

0
4

0
7

0

0

0

0

0
4

0
4

14.8059
4

9.46983

0.504614

0.0732851

8.79421

0.0488567

0.0488567

0

9.99200722162641e-16

0
4

5.25617

0.154593

4.7151

0.386483

0
4

0.0798828

0.0798828

0
4

1.70696790036118e-15
4

0
4

0

0

0

0
4

0

0

0

0
4

0

0

0

0

0
4

0

0

0

0
4

0
4

0

0

0

0

0

0
4

0
4

14.0944
4

14.0944
4

14.0944
4

0
4

0
4

9.2371
4

4.43427

0.108496

4.03645

0.0723307

0.216992

3.33066907387547e-16

0
4

0.107242

0.107242

0
4

0.216992

0.216992

0
4

4.34131

0

4.34131

0
4

0.0723307

0.0723307

0
4

0.0649455

0.0649455

0
4

2.77555756156289e-17
4

0
4

0
2

0
2

0

0

0

0

0
4

0

0

0

0

0
4

0

0

0

0
4

0

0

0
4

0
4

0

0

0

0

0

0

0
4

0

0

0
4

0
4

0
7

0
7

0
7

0

0
4

0

0

0

0
4

0
4

0

0

0

0

0
4

0

0

0

0
4

0

0

0
4

0
4

0
4

0

0

0

0

0

0

0
4

0

0

0

0
4

0
4

3.15027
3

3.15027
3

0
2

3.15027
3

0
2

0

0

0

0

0

0
4

0

0

0

0

0

0

0
4

0

0

0

0

0

0
4

0

0

0

0

0
4

0

0

0
4

0

0

0
4

0

0

0
4

0
4

4.29263
4

1.91719

1.91719

0

0

0
4

0.958594

0.798828

0.0798828

0.0798828

8.32667268468867e-17

0
4

0.415282

0.415282

0
4

0.195427

0.195427

0
4

0.806136

0.806136

0
4

0
4

0.0798828
4

0.0798828

0

0

0

0.0798828

0

0
4

0

0

0

0

0

0
4

0

0

0
4

0

0

0
4

0

0

0
4

0
4

0

0

0

0

0

0

0

0

0

0
4

0

0

0

0

0
4

0
4

0.354459
3

0.202127
3

0.202127
3

0

0

0

0
4

0

0

0

0
4

0

0

0
4

0.152332

0.152332

0
4

0
4

0

0

0

0

0

0

0

0
4

0

0

0

0
4

0
4

0
1

0

0

0

0

0

0

0
4

0

0

0
4

0

0

0
4

0

0

0
4

0

0

0
4

0

0

0
4

0
4

0.240268
3

0.167852

0.119574

0.0482775

0

0

0

0
4

0.0724163
4

0

0

0

0

0.0724163

0
4

0
4

0.0798828

0

0

0

0
4

0.0798828

0

0.0798828

0
4

0

0

0

0

0
4

0
4

8.05392

8.05392

2.27611

4.20205

0.962969

0.437713

0

0.175085

2.77555756156289e-16

0
4

0
4

0

0

0

0

0

0

0

0
4

0

0

0
4

0

0

0

0
4

0

0

0
4

0

0

0
4

0

0

0
4

0
4

39.5869
3

12.4254
4

3.03078
4

0.0488567

0.0488567

0.479297

0.0798828

0.0798828

0.390854

0.373023

0.257479

5.41187

0.177596

0.582061

0.785633

0.439711

0.239648

4.27435864480685e-15
4

0
4

26.3488
3

18.769
4

0.0798828

0.399414

0

0.55918

0.0798828

0.636907
3

0.410647

2.68483

0.0798828

0.321849

0.75728

1.49013

0.0798828

0
4

0.812643
3

0.812643

0

0
4

0

0

0
4

0
4

0
2

0
2

0
2

0

0

0

0
4

0
4

0
7

0

0

0

0

0

0
4

0

0

0

0

0

0
4

0

0

0

0
4

0
4

0
4

0
4

0

0

0

0

0
4

0

0

0

0

0
4

0

0

0
4

0
4

0
7

0
7

0

0

0

0
4

0
4

0
4

0
4

0

0

0

0

0
4

0

0

0
4

0
4

4.01466
4

2.31414

0.980886

0.589933

0.309187

0.434131

0
4

1.39134

0.618373

0.618373

0.154593

1.11022302462516e-16

0
4

0

0

0
4

0.309187

0.309187

0
4

1.11022302462516e-16
4

0
4

4.86971
3

4.86971
3

3.85907

1.01064

0

0
4

0

0

0
4

0

0

0
4

0
4

0.282978
2

0.282978
2

0.161702

0

0

0.121276

0
4

0

0

0
4

0
4

7.15246
3

7.10121
3

7.10121
3

0

0
4

0.0512461

0

0

0.0512461

0
4

3.7470027081099e-16
3

0
4

0.325488
4

0.325488
4

0.325488
4

0

0

0
4

0
4

0.521351

0.337943

0.337943

0

0
4

0
6

0

0

0
4

0.183408

0.183408

0

0

0
4

0

0

0
4

0

0

0
4

0

0

0
4

5.55111512312578e-17

0
4

114.029
3

102.97
3

14.2983
3

0.175085

77.4189
3

2.53457

0
7

0

0

0

0.342993

0
7

0

0

0

0

0

0

0

0

0
6

0

0

0

0

0

0.27959

0

0

0

0

0
7

0

0

0

0

0

0

0

2.26383

0

0

0
7

0

0

0

0

0.434131

0

0

0

0

0

0
7

0

0

0

0

0

0

0

0

0

0

0
7

0.0533257

0

0

0

0

0

0

0

0

0

4.84959

0

0

0

0

0

0

0

0

0

0

0
7

0

0

0

0.319531

0

0

0

0

0

0

0
2

0

0

0

0

0

0

0

0

0

0

0
4

4.46468

4.46468
5

0

0
7

0
7

0

0

0

0

0

0

0
4

0

0

0
4

0

0

0
4

2.55625

2.55625

0
4

0

0

0
4

0

0

0
4

0

0

0
4

0

0

0
4

0

0

0
4

0

0

0
4

0.175085

0.175085

0
4

0.800923
4

0.366426
4

0.362081
4

0

0.0724163

0

0
4

0.437713

0.437713

0
4

0

0

0
4

0

0

0
4

0

0

0
4

0.27959

0.27959

0
4

0

0

0
4

0

0

0
4

0

0

0
4

0
7

0
7

0

0

0
4

0
7

0

0

0

0

0

0

0
4

2.29627

2.29627

0
4

0

0

0

0

0
4

0

0

0
4

0.0488567

0.0488567

0
4

0

0

0

0
4

0
4

23.2078

18.798

1.73308
3

0

0

0.119824
4

0

0
6

0

0

0

3.48538

0

2.09123

1.52074

0

0

0.0533257

0

0

0

0

0

0

0

0

0

0

0

0

0

0

0

0

0

1.34053

0

0

0

0

0

0

0

0

0

0

0

0

0

0.154593

0

0

0

0

0.434131

0

0.0533257

1.99971
4

0

0

0

0

0

0

0

0

0

0

0

0

0

2.31537

0

1.83455

0

0

0

0

0

0

0

0

0

0

0

0

0

1.66226

0
4

1.32095
3

1.32095
3

0
2

0

0

0

0

0

0

0
4

0.107242

0.107242

0

0
4

0

0

0

0
4

0

0

0

0
4

0

0

0
4

0

0

0

0
4

0

0

0

0
4

0

0

0

0
4

0

0

0
4

0

0

0

0
4

0

0

0
4

0

0

0

0
4

0

0

0
4

0

0

0
4

0

0

0
4

0

0

0
4

0

0

0
4

0

0

0
4

0

0

0
4

0

0

0
4

0

0

0
4

0

0

0
4

1.66226

0.482591

1.17967

0

0
4

0

0

0
4

0

0

0
4

0

0

0
4

0

0

0
4

0

0

0
4

0.386483

0.386483

0
4

0.557522
4

0.485106

0

0

0.0724163

5.55111512312578e-17
4

0
4

0
7

0
7

0
4

0.375348

0.214485

0

0.160864

2.77555756156289e-17

0
4

0

0

0

0

0

0
4

0

0

0

0

0
4

0

0

0
4

0
4

325.87

325.87

8.78711
6

95.3285
4

0.159766

0

0

0.525256

0

0.199707

0.303299

0

0

0.601422

22.467
3

0

0.0798828

0.0507772

0

0

0

0

0

0

0

0
7

0

0

0

0.183475

0

0

0

0

0

0

0
6

0

0

0.199707

0

0

0

0.145063

0

0

0.479297

0
2

0

0

0

0

0

0

0.439355

0.254968

0.0917376

0

1.29165
4

0

0.159766

0

0.186463

0

0.159766

0

0

0

0.239648

0
3

0

0

0

0

0

0

0

1.2256

0.700341

0

0.946989
4

0

0.175085

0

0

0

0

0

0

0

0.122142

0

0

0

0

0

0

0

0

0

0

0

0
3

0

0

0

0

0

0

0

0.0798828

0.159766

0

12.6215
6

0.364236
3

0

0

0

0

0

0.266823

0

0

0

0

2.27611

0.346617

0

0

0

0

0

0

0

0

0

0.904706
4

0.0798828

0

8.50752

0.746559

0

0

0

0

0

0.0533257

0
6

0

0

0.175085

0

0

0

0

0

0.199707

0

20.1876
4

0

0

0

0

0.0533257

0

0

0.0799885

0

0

2.11689
5

0

0

0

0

0

0

0.437713

0

0

0

0
7

0

0

0

0

0

0

0.0798828

0

0.0798828

0.119824

0

0

0

0

0

0

0

0

0

0

0

7.69833
4

0

0

0

0

0

0

0

0.0533257

0

0

0
7

0

0

0

0

0.718945

0

0.0798828

0

0

0

68.1807
4

0
3

0

0

0

0

0

0

0.159766

0

0

0.0798828

0

0

0

0

0

0

0

0

0

0

0

0.95107
4

0

0.0917376

0

0

0

0

0

0

0

0

0
6

0

0

0

0.0761658

0

0

0

0

0.159766

0

1.48687
4

0

0

0.159766

0

0

0

0.0482775

0

0

0

2.72672

0

0

0.0798828

0

0

0

0

0

0

0

0.164068
4

0

0

0

0

0

0.0799885

0

0

0

0

0

0

0.0798828

0

0

0

0

0

0

0

0

0

0

0

0

0.170999

0.0761658

0

0

0

0

0

0

0.0799885

0

0

0

0

0

0

0

0

3.99414

0
7

0.355193
3

0.27959

0

3.85298

3.93942

0

0.0798828

0

0.0488567

0.0917376

0

0.133208
3

0

0

0

0.199707

0

0

0

0

0

0

0

0.0917376

0

0

0.159766

0

0

0

0

0.27959

0

0
3

0

0

0

0

0

0

0

0.137606

0

0

0

0

0

0

0

0

0

0

0

0.119824

0

0.876929
4

0

0

0

0

0

0.0917376

0

0

0.0798828

1.14672

0
7

0

0

0.0533257

0

0

0

0

0

0

0

0

0

0

0

0

0

0

0

0

1.27812

0

0

0

0

0.159766

0.0798828

0

0

0.097998

0

0

0

0.310756
4

0

0

0

0

0

0

0.0798828

0

0

0

6.62298
4

0

0.239648

0.48999

0

0

0.0488567

0

0

0

0

0

0

0

1.07842

0.350171

0

0.195427

0

0

0

0

0.137606

0
2

0

0

0

0.878711

0

0.097998

0

0

0

0.0724163

0

0

0

0.199707

0.0798828

0.0798828

0

0.0488567

0

0

0.708423

0

0

0.199707

0

0.0761658

0

0.0488567

0

0

0

0

0

0.0533257

0

0.0533257

0

0

0

0.119824

0.639062

0.159766

0

0

0

0

0

0

0

0

0

0

0

0

0.912954
3

0

0

0

0

0

0

0.119824

0

0

0

3.23394

0

0

0.0798828

0

0

0.23189

0

0.0533257

0

0

0
6

0

0.239648

0

0

0

0.262628

0

0

0.159766

0

0

8.98682

0

0

0

0.097998

0

0

0.0798828

0

0

0.154593

0

0

0

0.17772

0

0.228498

0

0

0

0

0

0.159871

0

0

0

0

0

0

0

0

0

0.126943

0.37328
4

0

0

0

0

0.239648

0

0

0.519238

0

0

0.254968
4

0

0

0.0798828

0

0

0

0

0

0

0.0649455

0

0.0917376

0

0

0

0

0

0

0

0

0

0.346811
4

0

0.0798828

0

0

0

0

0.0533257

0

0

0

0

0.14657

0

0

0

0

0

0

0.0798828

0

0

0

0.399414

0.0799885

0

0

0

0

0

0

0

0

0

0

0

0

0

0

0

0

0

0

0

0
6

0

0

0

0

0

0

0

0.0799885

0.639062

0

0

0

2.3166

0

0

0

0

0

0

0

0

0.533257

0

0

0

0

0

0

0.0798828

0

0

0

0

0

0

0

0

0

0

0

0

0

0

0.101554

0

0

0

0

0

0

0

0

0

0.319531

0

0

0
6

0

0

0.312692

0

0.390639

0

0

2.62628

0

0.106651

0
6

0

0.27959

0

0

0.309227

0

0.119824

0

0

0

0
4

0

0

0

0
4

0

0

0
4

0

0

0

0
4

0

0

0
4

0

0

0
4

0

0

0
4

0

0

0
4

0

0

0
4

0

0

0
4

0
4

0.157017

0.0808509

0
7

0

0

0

0

0

0

0

0

0

0

0
7

0

0

0

0

0

0

0

0

0

0

0

0

0.0808509

0

0
4

0
7

0
7

0

0

0

0
6

0
6

0

0

0

0

0

0

0
4

0

0

0
4

0
7

0

0

0
4

0

0

0

0
4

0

0

0

0

0
4

0.0761658

0.0761658

0
4

0

0

0
4

0

0

0
4

0

0

0
4

0

0

0
4

1.38777878078145e-17

0
4

29.9493

0.680456
3

0
3

0

0

0

0

0

0

0

0

0

0

0

0

0

0.121276

0

0

0

0

0

0

0

0

0

0

0

0

0

0

0

0

0

0

0

0

0

0

0

0.55918

0

0

0
4

2.48808
7

2.48808
7

0

0

0

0

0

0

0
4

0
3

0

0

0

0

0
4

0.0808509

0.0808509

0
4

0

0

0
4

0

0

0
4

0.758887

0.758887

0
4

0

0

0
4

0

0

0
4

0

0

0
4

0
7

0
6

0

0
4

0
3

0

0

0

0

0

0
4

0

0

0

0

0

0
4

0

0

0

0
4

0
7

0
7

0
4

0

0

0

0

0
4

0

0

0
4

0
7

0

0

0
4

0

0

0

0
4

0
6

0
6

0

0

0

0

0

0

0

0
4

0

0

0

0
4

0

0

0

0
4

0

0

0

0

0

0
4

3.73659

3.73659

0
4

0

0

0

0

0

0
4

0

0

0

0
4

0

0

0

0
4

0

0

0

0
4

0

0

0
4

2.7959

2.7959

0

0

0
4

0
7

0
7

0

0

0

0
7

0

0

0

0

0

0

0

0
4

0

0

0
4

0

0

0

0
4

0

0

0

0

0
4

0

0

0

0
4

0

0

0

0
4

0

0

0
4

0

0

0
4

0

0

0
4

0

0

0
4

0.167852

0.119574

0.0482775

0
4

0
8

0
8

0

0

0

0

0

0

0

0

0
4

0

0

0
4

0

0

0

0
4

0

0

0

0
4

0

0

0

0
4

0

0

0

0
4

0

0

0
4

0

0

0
4

0

0

0
4

0

0

0

0
4

0

0

0
4

0
8

0

0

0

0

0

0

0

0

0
4

0

0

0
4

0

0

0
4

0

0

0

0
4

0

0

0
4

0

0

0
4

0

0

0

0
4

0

0

0

0
4

0.202127

0.202127

0
4

0

0

0
4

0

0

0
4

0.107242
3

0

0

0.107242

0

0

0

0
4

0

0

0
4

0

0

0
4

0.0533257

0.0533257

0
4

0

0

0
4

0

0

0
4

0

0

0
4

0

0

0
4

0

0

0
4

0

0

0
4

0

0

0
4

3.04104

2.20733

0.785434

0.0482775

1.11022302462516e-16

0
4

0

0

0
4

0

0

0
4

0

0

0
4

0

0

0
4

0

0

0
4

0

0

0
4

0

0

0
4

0

0

0
4

0

0

0
4

0

0

0
4

15.5944

1.16621

12.5172

0.244995

1.66597

1.99840144432528e-15

0
4

0

0

0
4

0

0

0
4

0

0

0
4

0

0

0
4

0

0

0
4

0

0

0
4

0

0

0
4

0

0

0
4

0

0

0
4

0

0

0
4

0

0

0

0

0

0
4

0

0

0
4

0.0808509

0.0808509

0
4

0

0

0
4

0

0

0
4

0

0

0
4

0

0

0
4

0

0

0
4

0.161702

0.161702

0
4

0

0

0
4

0

0

0
4

0
4

27.2977
3

25.0542
3

21.3997
3

0
2

0
2

0

0

0

0

0

0

0

0.0798828

0

0

0.195996
3

0

0

0

0

0

0

0

0

0

0

0.0723307
3

0

0

0

0

0

0

0

0.107242

0

0

0
2

0

0

0

0

0

0

0

0.0808509

0

0

0.346617

0

0

0

0

0

0

0

0

0

0

0
2

0

0

0

0

0

0

0

0

0

0

0.533257

0

0

0

0

0

0

0

0

0

0.0798828

0

0

0

0.0488567

0

0

0

0

0

0

0

0
2

0

0.0533257

0

0

0

0.107242

0

0

0

0

0

0

0

0

0

0

0.0732851

0

0

0

0

0

0

0

0

0

0

0

0

0

0

0

0

0

0

0

0

0

0

0

0

0

0

0

0

0

0

0

0

0

0

0

0

0

0

0

0

0

0

0

0

0

0

0

0

0

0

0

0.106651

0

0

0

0

0

0

0

0

0

0

0

0

0

0

0

0

0

0

0

0

0

0

0
2

0

0

0

0

0

0

0

0

0

0

0

0.434131

0

0

0

0

0

0

0

0

0

0.217249
3

0

0

0

0

0

0

0

0

0

0

0

0.144833

0

0

0

0

0

0

0

0

0

0.55992

0

0

0

0

0

0

0

0

0.27959

0

0
3

0

0

0

0

0

0

0

0

0

0

0

0

0

0

0

0

0

0

0.133314

0

0

3.30291349825984e-15
3

0
4

0

0

0

0

0

0
4

0

0

0

0

0
4

0.108496

0.108496

0
4

0

0

0

0

0
4

0

0

0

0

0
4

0

0

0

0
4

0

0

0
4

0

0

0

0
4

0

0

0

0
4

0

0

0

0
4

0

0

0
4

0.161702
3

0.0808509

0.0808509

0

0
4

0

0

0

0
4

0

0

0
4

0

0

0

0
4

0.0808509

0.0808509

0
4

0.106651

0.0533257

0.0533257

0
4

0.0723307

0.0723307

0
4

0.0507772

0.0507772

0
4

0

0

0
4

0

0

0
4

0

0

0
4

0
2

0

0

0

0
4

0

0

0
4

0

0

0
4

0

0

0
4

0.0732851

0.0732851

0
4

0

0

0
4

0

0

0
4

0

0

0
4

0

0

0
4

0.0799885

0.0799885

0
4

0

0

0
4

0.590272

0.590272

0
4

0

0

0
4

0

0

0
4

0

0

0
4

0.199707

0.199707

0
4

0.319531

0.319531

0
4

0

0

0
4

0.0799885

0.0799885

0
4

0

0

0
4

0

0

0
4

0

0

0
4

0

0

0

0
4

0

0

0
4

0

0

0
4

0

0

0
4

0

0

0
4

0.108496

0.108496

0
4

0.0808509

0.0808509

0
4

0

0

0
4

0

0

0
4

0

0

0
4

0.0798828

0.0798828

0
4

0.0507772

0.0507772

0
4

0

0

0
4

0

0

0
4

0

0

0

0

0
4

0

0

0

0

0
4

0

0

0

0

0
4

1.06165076729781e-15
3

0
4

0.39957
3

0
3

0
2

0

0

0

0

0

0

0

0

0

0

0
2

0

0

0

0

0

0

0

0

0

0

0
3

0

0

0

0

0

0

0

0

0

0

0

0

0

0

0

0

0

0

0

0

0

0

0

0
4

0.39957

0

0.323404

0

0.0761658

0

1.38777878078145e-17

0
4

0

0

0

0
4

0

0

0

0

0
4

0

0

0

0
4

0

0

0
4

0

0

0
4

0

0

0
4

0

0

0
4

0

0

0
4

0
4

75.9861
5

75.9861
5

75.9861
5

0
7

0

0

0

0

0

0

0

0

0

0

0

0

0

0

0

0

0

0

0

0

0

0

0

0

0

0

0

0

0
4

0

0

0
4

0

0

0
4

0

0

0
4

0

0

0
4

0

0

0
4

0

0

0
4

0

0

0
4

0
4

875.687
4

494.262
4

473.602
4

0
7

0

0

0

0

0

0

0

0

0

0

0

0

0

0

0

0

0

0

0

0

0

0

0

0

0

0

0

0

0

0

0

0

19.4372
4

0

0

0

0

0

0

0

0.186534

0

0

0
6

0

0

0

0

0

0

0

0

0

0

0
7

0

0

0

0.0533257

0

0

0

0

0.275213

0

0
7

0

0.0533257

0

0

0

0

0

0

0

0

0
7

0

0

0

0

0

0

0

0

0

0.0798828

0
7

0

0

0

0

0

0

0

0

0

0

0
5

0

0

0

0

0.0798828

0

0

0

0.122142

0.0533257

0
6

0

0

0.319531

0

0

0

0

0

0

0

0
4

0
6

0
6

0

0

0

0

0

0

0

0

0

0

0
6

0

0

0

0

0

0

0

0

0

0

0
6

0

0

0

0

0

0

0

0

0

0

0

0

0

0

0

0

0

0

0

0

0

0

0

0

0

0

0

0

0

0

0

0

0

0

0

0

0

0

0

0

0

0

0

0

0

0

0

0

0
4

2.9441
3

0

0

0

0

0

0.0808509

0

0

2.74197

0.121276

0
4

0

0

0
4

0

0

0

0
4

1.74301

1.74301

0
4

0

0

0

0
4

0

0

0

0
4

0

0

0
4

0

0

0

0
4

0

0

0

0
4

0

0

0

0
4

0.187902

0.187902

0
4

7.46778
4

2.54726
4

0.0798828
4

0

4.51701

0.23189

0

0.0917376

3.60822483003176e-16
4

0
4

0.0808509

0

0.0808509

0
4

0

0

0

0
4

0

0

0
4

0

0

0
4

0

0

0

0
4

0.0723307

0

0.0723307

0
4

0

0

0

0
4

0

0

0

0
4

0

0

0
4

0

0

0

0
4

1.33404
2

0

0.121276
3

1.21276

0

0

0

0

0

0
4

0

0

0
4

0

0

0

0
4

2.78268

2.78268

0
4

0

0

0
4

0

0

0

0
4

0.159766

0.0798828

0.0798828

0
4

0

0

0

0
4

0

0

0

0
4

0

0

0
4

0

0

0
4

0

0

0

0

0

0

0

0

0

0

0
4

0

0

0

0
4

0

0

0

0
4

0

0

0

0
4

0

0

0
4

0

0

0
4

0

0

0
4

0

0

0
4

0

0

0
4

0

0

0
4

0.154593

0.154593

0
4

0
5

0
5

0

0

0

0

0

0

0

0

0

0
4

0

0

0
4

0

0

0
4

0

0

0
4

0

0

0
4

0

0

0
4

0

0

0
4

0

0

0
4

0

0

0
4

0

0

0
4

0

0

0
4

0

0

0

0

0
4

0

0

0
4

0

0

0
4

0

0

0
4

0

0

0
4

0

0

0
4

0

0

0
4

0.133314

0.133314

0
4

0

0

0
4

0.0533257

0.0533257

0
4

0

0

0
4

0

0

0

0

0

0

0
4

0.428969

0.428969

0
4

0

0

0
4

0

0

0
4

0

0

0
4

0

0

0
4

0

0

0
4

0

0

0
4

0

0

0
4

0

0

0
4

0

0

0
4

0

0

0

0

0

0
4

0

0

0
4

0

0

0
4

0

0

0
4

0

0

0
4

0

0

0
4

0

0

0
4

0

0

0
4

0

0

0
4

0

0

0
4

0

0

0
4

1.48278
5

1.48278
5

0

0
4

0.0724163

0.0724163

0
4

0

0

0
4

0

0

0
4

0

0

0
4

0

0

0
4

0.154593

0.154593

0
4

0

0

0
4

0

0

0
4

0

0

0
4

0

0

0
4

0

0
5

0

0

0

0

0

0
4

0.0482775

0.0482775

0
4

0

0

0
4

0

0

0
4

0

0

0
4

0

0

0
4

0.0533257

0.0533257

0
4

0

0

0
4

0

0

0
4

0

0

0
4

0

0

0
4

79.2266
4

74.1841
4

0.108496

0

0

0.434131

0

0

0

0.541077

0

0

1.41647
4

0.434131

0.0799885

0

0

0

0

0

0.69567

0

0.129891

0.154593

0.157353

0.311879

0.0723307

0

0.506462

8.32667268468867e-15
4

0
4

0

0

0

0
4

0.289421

0.289421

0
4

0

0

0
4

0.119824

0.119824

0
4

0

0

0
4

0

0

0
4

0

0

0
4

0.23189

0.23189

0
4

0.154593

0.154593

0
4

0

0

0
4

2.94493

2.94493

0
4

0
6

0

0

0

0

0

0

0

0

0
4

0

0

0
4

0

0

0
4

0

0

0
4

0

0

0
4

0.199707

0.199707

0
4

0

0

0
4

0

0

0
4

0

0

0
4

0.154593

0.154593

0
4

0

0

0
4

0.929786
3

0.929786

0

0

0

0

0

0
4

0

0

0
4

0

0

0
4

0

0

0
4

0.0798828

0.0798828

0
4

0.0798828

0.0798828

0
4

0

0

0
4

0

0

0
4

0

0

0
4

0

0

0
4

0

0

0
4

0.315954
4

0

0.0533257

0

0

0

0

0.262628

0

0
4

0

0

0
4

0

0

0
4

0

0

0
4

0

0

0
4

0.321081

0.321081

0
4

0

0

0
4

0

0

0
4

0

0

0
4

0

0

0
4

0.175085

0.175085

0
4

4.17847
3

4.17847

0

0

0

0

0
4

0

0

0
4

0

0

0
4

0

0

0
4

0

0

0
4

0

0

0
4

0

0

0
4

0

0

0
4

0

0

0
4

0

0

0
4

12.1876
4

12.1876
4

0

0

0

0
4

0
2

0

0

0

0

0

0

0
4

0

0

0

0

0

0

0
4

0.832797
4

0.344895

0.144661

0

0.183475

0.159766

0

5.55111512312578e-17
4

0
4

0.0917376

0

0.0917376

0

0
4

26.0864
4

7.06982
4

0

1.31807

0.289421

0

0.0798828

0

0.0488567

0

0

0

3.12728
4

0

0

0

0

0.23189

0.734782
3

9.39121
4

0.69567

0.434131

2.3189

0.0798828

0.266628

1.4432899320127e-15
4

0
4

0.396913
4

0.305175
4

0

0.0917376

0

2.77555756156289e-17
4

0
4

1.09599
4

0.500653

0.351049

0

0

0

0.244284

5.55111512312578e-17
4

0
4

0

0

0

0

0

0

0

0
4

0.242553

0

0.0808509

0

0

0.0808509

0.0808509

0

0
4

0
7

0
7

0

0

0
4

17.0861
4

17.0128
4

0.0732851

2.08166817117217e-16
4

0
4

0

0

0

0

0
4

0
7

0
7

0
4

0.361752
4

0.361752

0

0

0
4

0

0

0

0

0

0
4

148.272
4

90.0721
4

0.154593

0

0

0

53.5052
4

1.12391
4

0.453268

0.0723307

0.0723307

2.74627

0

0.0723307

1.14769305170626e-14
4

0
4

0

0

0

0
4

0

0

0

0

0
4

0
6

0
6

0
4

0
5

0
5

0
4

0

0

0

0
4

0

0

0

0
4

0

0

0
4

0.17315

0

0.0533257

0

0.119824

0
4

0

0

0

0

0

0
4

0.0723307

0

0.0723307

0

0

0
4

21.923

21.8018

0.121276

0

0

0

0

0

0

0

0

8.18789480661053e-16

0
4

0

0

0

0

0
4

0

0

0

0
4

0.279801

0

0

0.159977

0.119824

0
4

0

0

0

0

0
4

0

0

0

0

0
4

0

0

0
4

0

0

0

0
4

0

0

0
4

0

0

0

0
4

0

0

0

0
4

14.7743

14.7743
3

0

0
2

0

0

0

0

0

0

0

0
4

0.225052

0.0533257

0.0799885

0.0917376

0
4

0.892791

0.772967

0.119824

5.55111512312578e-17

0
4

0

0

0
4

0

0

0

0

0

0
4

0

0

0

0
4

0.217249

0.0724163

0.0724163

0.0724163

0
4

0

0

0

0
4

0

0

0
4

1.92649

0

1.92649

0
4

1.48823

1.48823

0
4

7.48166
4

6.46844
4

0.246102
4

0.477702

0.289421

0

0
4

0

0

0
4

0

0

0

0

0
4

0

0

0

0

0
4

0

0

0

0
4

0

0

0
4

0

0

0

0
4

0.676555

0.676555

0

0
4

0

0

0
4

0

0

0

0
4

0

0

0

0

0
4

13.5574
4

13.3934
4

0

0.0723307

0

0

0

0

0.0917376

0
4

0

0

0

0

0
4

0

0

0

0

0
4

0

0

0

0
4

0

0

0

0

0
4

0

0

0
4

0

0

0

0
4

0

0

0

0
4

0

0

0

0
4

1.83455

1.83455

0
4

0.183475

0

0.183475

0
4

0
6

0
6

0
4

0

0

0

0
4

0

0

0
4

0.309187

0.309187

0
4

0

0

0
4

0

0

0

0
4

0

0

0

0
4

0

0

0
4

0

0

0

0
4

0

0

0

0
4

0

0

0
4

0
4

1.26967

1.26967

1.26967

0
6

0

0

0

0

0

0

0

0

0

0

0

0

0
4

0
6

0

0

0
4

0

0

0
4

0

0

0
4

0

0

0
4

0

0

0
4

0
4

56.6871
3

56.3924
3

16.7007
3

36.4933
4

0

0

0.687233

0

0

0

0.282978

0

0

0

0
4

0

0

0

0.0482775

0

0

0.144661

0.0965551

0

0.159977

0

0

0

0

0

0

0

0

0

0

0

0.219855
4

0

0.108496

0

0

0

0

0

0

0.0732851

1.15924

0.217831

0

0

0

2.83106871279415e-15
3

0
4

0
6

0
6

0

0

0

0
4

0

0

0
4

0

0

0
4

0

0

0
4

0

0

0
4

0.19311
4

0.19311
4

0
4

0

0

0

0
4

0

0

0

0
4

0

0

0
4

0

0

0
4

0

0

0

0
4

0.101554

0.101554

0
4

0

0

0
4

0
4

157.967

121.909

87.9452

0
7

0
6

0

0

0

0

0

0

0

0

0

0

0

0

0

13.3804

0

0

0

0

0

0

0

0
7

0

0

0

0

0

0

0

0

0

0

0.536212

0

0

0

0

0

0

0

0

0

0

0

0

0

0

0.0798828

0

0

0

0

0

0

0.662512

0

0

0

0

0

0

0

0

0

0.159766

0.482591
5

0

0

0

0

0

0

0

0

0

0

0
7

0
7

0
7

0

0

0

0

0.399414

0

0

0

0

0

0
6

0

0

0

0

0

0

0

0

0

0

0
7

0

0

0

0

0

0

0

0

0

0

5.7948

0

0

0

0

0.27959

0

0

0

0

0

2.07695
5

9.70211

0

0

0

0

0

0

0

0

0.0798828

0
7

0

0

0

0

0

0.0724163

0

0

0

0

0

0

0

0

0

0.159766

0

0

0

0

0

0

0

0

0

0

0

0

0

0

0.097998

0

0
4

0
7

0
7

0

0

0

0

0

0

0

0

0

0
7

0
8

0
7

0

0

0

0

0

0
4

0

0

0
4

0.0798828

0.0798828

0
4

0

0

0
4

0

0

0
4

0

0

0
4

0

0

0
4

5.07747

5.07747

0

0

0
4

2.67607

0.439355

0

0.639062

0.998535

0

0.27959

0

0.319531

3.33066907387547e-16

0
4

26.9604
4

26.601
4

0.27959

0

0.0798828

1.42941214420489e-15
4

0
4

0

0

0

0

0
4

0.375348

0

0.375348

0
4

0.808509

0.808509

0
4

0

0

0
4

0.0798828

0.0798828

0
4

1.79162240598885e-14

0
4

11.8189

11.739

2.42632

0

0

0

0

0.319531

0

0

0

0

0

0

0
6

0

0.159977

0

0

0

0

0

0

0

0

0
7

0

0

0.175085

0

0

0

0

0

0

0

0

0

0

0

0

0

0.106651

0

0

0

0

0

0

0

0

0

0

0

0

0

0

0

0
7

0

0

0

0

0

0

0

0

0

0

0.0533257

0

0.0799885

0

0

0

0

0

0

0

0

0

0

0

0

0

0

0

0

0

0

0

0

0

0

0

0

0

0

0

0

0

0

0
6

0

0

0

0

0
7

0

0
7

0

0

0
7

0
6

0

0
7

0

0
6

3.2262

0

0

0

0

0

0

0

0

0

0

0.426605

0

0

3.4084

0

1.19699

0

0

0

0

0

0
7

0

0

0

0

0

0

0

0

0

0

0
7

0

0

0

0

0

0

0

0

0

0

0
7

0

0

0

0

0

0

0

0

0.159977

0

0
7

0

0

0

0

0

0

0

0

0

0

0
6

0

0

0

0

0

0

0

0

0

0

0
4

0
6

0
6

0
4

0

0

0
4

0

0

0
4

0

0

0
4

0

0

0
4

0.0798828

0.0798828

0
4

0

0

0
4

0

0

0
4

8.18789480661053e-16

0
4

19.9184
3

19.9184
3

19.0748
3

0.843635
3

0

0

0

0

3.21964677141295e-15
3

0
4

0

0

0

0
4

0

0

0
4

0

0

0
4

0

0

0
4

0

0

0
4

0
4

67.8473

64.8143

32.6645

0
7

0

0

0

0

0

0

0

0

0

0

0
6

0

0

0

0

0

0

0

0.107242

0

0

0.20345
3

0

0

0

0

0

0

0

0

0

0

0.209141
3

0

0

0

0

0

2.99321

0

0

0

0

0.262762
3

0

0

0

0

0

0

0

0

0

0

0
7

0

0

0

0

0

0

0

0

0

0

0
7

0

0.214485

0

0

0

0

0

4.45056

0

0

0
7

0

0.0917376

0

0

0

0

0

0

0

0

0.120694
3

0

0

0

0

0

0

0

0

0

0

0
6

0

0

0

0

0

0

0

0

0

0

0
7

0

0

0

0

0

0.0917376

0.434498

0

0

0

0

0.0482775
3

0

0

0

0

0

0

0

0

0

0

0

0

0

0

0

0

0

0

0

0

0

0.20524
3

0

0

0

0

0

0.0724163

0

0

0

0

0

0

0

0

0

0

0.107242

0

0

0

0

0
7

0

0.137606

0

0

0

0

0.214485

0

0

0

0
7

0

0

0

0

0

0.538989

0

0

0

0

0

0

0

0

0

0

0

0

0

0

0

0
4

0

0

0

0

0.154593

0

0

0

0

0

0

0

1.51897

0

0

0

0

0

0

0

0

0
7

0
7

0

0

0

0

0

0

0

0

0

0

0.57532
3

0

0

0

0

0

0

0

0

0

0

0.479931

0

0.107242

0

0

0

0.265526

0

0

0

0

0
7

0

0

0

0

0

0

0

0

0

0

0.275213
3

0

0

0

0

0

0

0

0

0

0

0

0

0

0

0

0

0

0

0

0

0

0

0

0

0

0

0

0

0

0

0

0

0.0533257

0

0

0

0

0

0

0

0.244995

0

0

0

0

0

0

0

0

0

0

0

0

0

0

0

0

0

0

0

0

0

0

0

0

13.2909
4

0

0

0

0

0

0

0

0

0

0

0

0

0

0

0

0

0

0

0

0

0

0

0

0

0

0

0

0.0488567

0

0

0

0

0

0

0

0.0965551

0

0

0
7

0

0

0

0.632814

0

0.833276

0

0

0.675885

0

0
7

0

0

0

0

0

0

0

0

0

1.54593

0.22701
3

0

0

0

0

0

0

0

0

0.375348

0

0
7

0

0

0.097998

0

0

0

0

0.097998

0

0

0
7

0

0

0

0

0

0

0

0.0482775

0

0

0
4

1.54482

1.54482

0
4

0

0

0
4

0

0

0
4

0

0

0
4

0

0

0
4

1.48823

1.48823

0
4

0

0

0
4

0

0

0
4

0
4

209.068

27.3299
3

0
6

0
7

0

0

0.289421

1.34053

0

0

0

0

0

2.46373

0
6

0

0

0

0

0

0

0

0.239966

0.496591

0

0.180827
3

0.612799

0

0

0

0

0

0

0

0

0

0
6

0

0

0

0

0

0

0

0

0

0

0
2

0

0

0

0

0

0

0

0

0

0

0

0

0

0

0.723552

0

0.0488567

0

0

0

0

0.434131
4

0

0

0

0

0

0

0

0

0

0

0

0.234185

0

0

0

0

0

0

0.268106

0

0

0

0

0

0

0

0

0

0

0

0

0

0
7

0

0

0.107242

0

0

0

0

0

0

0.106651

0
2

0

0.268106

0

0.55918

0

0.133314

0.107242

0

0

0

0.0799885

0
6

0

0

0

0

0

4.45056

0

0

0

0

0
6

0

0

0

0

0

0

0

0

0

0

0
6

0

0

0

0

0

0

0

0

0

0

0

0

0

0

0

0

0

0

0

0

0

0
6

0

0

0

0

0

0

0

0

0

0

0

0

0

0

0

0

0

0

0

0

0

0
2

0

0

0.107242

0

0

0

0

0

0

0

0
6

0

0

0

0

0.107242

0

0

0.133314

0

0

0
7

0.107242

0

0

0

0

0

0

0

0

0

0
7

0

0

0

0

0

0

0.0507772

0

0

0

0

0.219855
4

0

0

0

0

0

0

0

0

0

0

0
7

0

0

0

0

0

0

0

0

0

0

0
5

0

0

0

0

0

0

0

0

0

0

0

0

0

0

0

0

0

0

0

0

0

0
7

0

0.0808509

0.37328

0

0

0

0

0

0

0

0

0

0

0

0

0

0

0

0

0.107242

0

0

0

0

0

0

0

0

0

0

0

0

0

0

0

0.0808509

0

0

0.107242

0

0

0

0

0

0

0

0

0

0

0

0

0

0

0

0.122142

0
7

0

0

0.214485

2.09123

0

0.0533257

0

0

0

0

0

0

0

0

0

0

0

0

0

0

0.0808509

0

0

0

0

0

0

0

0

0

0

0

0

0

0

0

0

0

0

0

0

0

0

0

0

0

0

0

0

0

0

0

0

0

0

0

0

0

0

0

0

0

0

0

0

0.289421

0

0

0

0

0

0

0

0

0

0

0

0

0

0

0

0

0

0

0

0

0

0

0

0

0

0

0

0

0

0

0

0

0

0

0

0

0

0

0

0

0

0

0

0
4

0
5

0

0

0

0

0

0

0

0

0

0

0

0

0.0533257

0

0

0

0

0

0

0

0

0

0

0

0

0

0

0

0

0

0

0

0

0

0

0

0.289421

0

0

0

0

0

0

0

0

0

0

0

0.0723307

0

0

0

0

0

0

0

0

0

0

0

0

0

0

0

0

0

0

0

0

0

0

0

0

0

0

0

0

0

0

0

0

0

0

0

0

0

0

0.195427

0

0

0

0

0

0

2.05796

0

0

0

0

0

0

0

0

0

0.453268

0

0

0

0

0
7

4.50418

0

0

0

0

0

0

0

0

0

0

0
7

0

0

0

0

0

0

0

0

0

0
7

0.0808509

0

0

0.795736
4

0.145063

0

0.299255
4

0
5

0

0

0

0

0

0

0

0

0

0

0
2

0

0

0

0.91156

0

0

0

0

0

0

0
6

0

0

0

0

0

0

0

0

0

0

7.54951656745106e-15
3

0
4

0

0

0

0

0

0

0

0

0

0

0

0

0

0
6

0

0

0

0

0

0

0

0

0

0

0
7

0

0

0

0

0

0

0

0

0

0

0

0

0

0

0

0

0

0

0

0

0

0

0

0

0

0

0

0

0

0

0

0

0

0

0

0

0

0

0

0

0

0

0

0
7

0

0

0

0

0

0

0

0

0

0

0

0

0

0

0

0

0
7

0
7

0

0

0

0

0

0
6

0

0

0

0

0
7

0

0

0

0

0

0

0

0

0

0

0

0

0

0

0

0

0

0

0

0

0

0
5

0

0

0

0

0

0

0

0

0

0

0
7

0

0

0

0

0

0

0

0

0

0

0

0

0

0

0

0

0

0

0

0

0

0

0

0

0

0

0

0

0

0

0

0

0

0

0

0

0

0

0

0

0

0

0

0
4

0
2

0
2

0

0

0

0
4

0

0

0

0
4

0

0

0

0
4

0

0

0
4

0

0

0

0
4

0

0

0
4

0

0

0
4

0

0

0

0
4

0

0

0

0
4

0

0

0

0
4

0

0

0
4

0
4

0
4

0

0

0

0

0

0

0

0

0

0
4

0

0

0
4

0

0

0

0
4

0

0

0

0
4

0

0

0
4

0

0

0

0
4

0.0533257

0.0533257

0
4

0.0917376

0.0917376

0
4

0

0

0
4

0

0

0
4

0

0

0
4

0
6

0
6

0

0

0

0

0

0

0

0

0
4

0

0

0
4

0

0

0
4

0

0

0
4

0

0

0
4

0

0

0
4

0

0

0
4

0

0

0
4

0

0

0
4

0

0

0
4

0

0

0
4

1.28699
3

0.925101
3

0.121276

0

0.0808509

0

0

0.159766

0

0

0

0
4

0

0

0
4

0

0

0
4

0

0

0
4

0

0

0
4

0

0

0
4

0

0

0
4

0

0

0
4

0

0

0
4

0

0

0
4

0

0

0
4

0
6

0
6

0

0

0

0

0

0
4

0

0

0
4

0

0

0
4

0

0

0
4

0

0

0
4

0

0

0
4

0

0

0
4

0

0

0
4

0

0

0
4

0

0

0
4

0

0

0
4

1.33812
3

1.33812
3

0

0

0

0

0

0
4

0

0

0
4

0

0

0
4

0

0

0
4

0.0799885

0.0799885

0
4

0

0

0
4

0

0

0
4

0

0

0
4

0

0

0
4

0

0

0
4

0

0

0
4

0

0

0

0

0
4

0

0

0
4

0

0

0
4

0

0

0
4

0

0

0
4

0

0

0
4

0

0

0
4

0

0

0
4

0

0

0
4

0

0

0
4

0

0

0
4

21.3412
3

19.4109
3

1.60864

0

0

0

0.107242

0

0

0.107242

0.107242

1.58206781009085e-15
3

0
4

0.121276

0.121276

0
4

0

0

0
4

0

0

0
4

0

0

0
4

0

0

0
4

0

0

0
4

0

0

0
4

0

0

0
4

0.0761658

0.0761658

0
4

0

0

0
4

0

0

0

0

0

0

0

0

0
4

0

0

0
4

0

0

0
4

0

0

0
4

0

0

0
4

0

0

0
4

0

0

0
4

0

0

0
4

0

0

0
4

0

0

0
4

0

0

0
4

0
5

0

0

0

0

0

0

0

0

0

0
4

0

0

0
4

0

0

0
4

0

0

0
4

0

0

0
4

0

0

0
4

0

0

0
4

0

0

0
4

0

0

0
4

0

0

0
4

38.4312

38.4312

0
4

0

0

0

0

0

0

0

0

0

0

0

0

0
7

0

0

0

0

0

0

0

0

0

0

0

0

0
4

10.0391
4

7.7473
4

2.1471

0

0.144661

0

0

2.77555756156289e-16
4

0
4

0.0798828

0.0798828

0
4

0

0

0
4

0

0

0
4

0.282978

0.282978

0
4

0.160864

0.160864

0
4

0

0

0
4

0

0

0
4

0

0

0
4

0

0

0
4

0

0

0
4

0

0

0

0

0

0

0
4

0

0

0
4

0.0798828

0.0798828

0
4

0

0

0
4

0

0

0
4

0.697075

0.697075

0
4

0

0

0
4

0
6

0

0

0

0

0

0
4

0.613245
4

0.133314

0

0.479931

0

0

0

0
4

2.22298
4

2.07641

0

0

0.14657

0

0
4

0

0

0

0

0

0

0

0
4

0
4

0

0

0

0

0
4

0

0

0

0

0

0

0
4

0.693234

0.639908

0.0533257

0

4.85722573273506e-17

0
4

0
6

0

0

0

0

0
4

21.3583
4

16.8553
4

0.723552

0.289421

0.289421

0

0

0

0

0

0

0

0

0

0

0

0

0

0

0

0

0

0

0

0

0

0

0

0

0

0

0

0

0

0.434131

0

0

0

0

0

0

0

0.161702

0

0

0

0

0

1.4471

0

0

0.289421

0

0

0

0

0.868263

0

0

0

0

0

0

0

0

5.44009282066327e-15
4

0
4

0
6

0
6

0
4

0
7

0

0

0
4

0

0

0

0

0

0
4

1.01297

1.01297

0

0

0
4

0

0

0

0

0
4

0

0

0

0

0

0
4

0

0

0

0
4

0

0

0

0
4

0

0

0

0
4

0.465384

0.212479

0.202127

0.0507772

2.08166817117217e-17

0
4

0

0
7

0

0

0

0

0

0

0

0

0

0

0
6

0

0

0

0

0

0

0

0

0

0

0

0

0

0

0
2

0

0

0

0
4

1.94965

1.50667

0.366816

0.0761658

0
4

0.107242

0

0

0.107242

0
4

0

0
7

0

0
4

0

0

0

0

0

0
4

0
7

0

0

0

0

0
4

0.578842

0

0.578842

0

0
4

12.0648

11.9575

0

0.107242

3.7470027081099e-16

0
4

0

0

0

0

0

0
4

0

0

0

0

0
4

0

0

0

0
4

0
6

0
6

0

0

0

0

0

0

0

0

0
4

0

0

0

0
4

44.1215

42.5457

1.57577

1.33226762955019e-15

0
4

0

0

0

0
4

0

0

0

0

0
4

0

0

0

0
4

0

0

0

0

0
4

3.22455

3.22455

0

0
4

0

0

0

0
4

0

0

0

0

0
4

1.08496

1.08496

0

0

0
4

0

0
6

0

0

0

0

0

0

0

0

0

0

0

0

0

0

0

0

0

0

0

0

0

0

0

0

0

0

0

0
4

0

0

0

0

0
4

0

0

0
4

0

0

0
4

1.37447

0

1.37447

0

0
4

0

0

0

0

0
4

0

0

0

0

0
4

0

0

0

0

0
4

0

0

0

0
4

0

0

0
4

0

0

0

0

0
4

1.04093
6

0
6

0

0

0

0

0
7

0

0

0

0

0

0

1.04093

0
4

0

0

0

0

0
4

1.83213

1.83213

0

0
4

0.108496

0.108496

0
4

0

0

0
4

0

0

0

0

0
4

6.42765

6.42765

0

0
4

0

0

0

0

0
4

0

0

0
4

0

0

0
4

0.121276

0

0.121276

0
4

6.2834

6.2834

0

0

0

0

0

0

0
4

0

0

0

0

0
4

0

0

0
4

0

0

0
4

0

0

0

0
4

0

0

0

0
4

0

0

0

0
4

0

0

0

0
4

0

0

0

0
4

0

0

0

0
4

0

0

0
4

0.759472
3

0.759472
3

0

0

0

0

0

0

0

0

0

0
4

0

0

0

0
4

0

0

0

0
4

0

0

0

0
4

0

0

0

0
4

0

0

0
4

0

0

0

0
4

0

0

0

0
4

0

0

0
4

0.133314

0.0799885

0.0533257

0
4

0

0

0

0
4

4.63795668537159e-14

0
4

1.86645365829463e-10

0
4

102.31
4

68.5157
4

59.6711
4

22.025
4

0

0

0

0

0.0799885

0

0

0

0

0.0798828

0

0

0

0

0

0.555192

0

0

0

0

0

0

0

0

0

0

0

0

0

0

0

0

0

0.108496

0

0

0

0

0

0

0.0533257

0

0.175085

0

0.175085
4

0

0

0

0

0

0

0

0

0

0.262628

0
4

0

0

0

0

0

0.437713

0

0

0

4.79931

0

0

0

0

0

0

0

0

0

0

0

0
4

0

0

0

0

0

0

0

0

0

0

0

0

0

0

0

0

0

0

0

0

0

0.23189
4

0

0

0

0

0

0

0

0.262628

26.3071
5

0

0

0

0

0.175085

0

0

0

0

0

0

0

0.437713

0

0

0

0

0

0

0

0

0

0

0.262628

0

0.787884

1.27812

0

0

0

0

0

0
6

0

0

0

0.437713

0

0

0

0

0

0

0

0

0

0

0

0

0

0

0

0

0

0

0

0

0

0

0

0

0

0

0.0798828

0

0
4

0

0

0

0

0

0

0.175085

0

0

0

0.216992
4

0

0.266628

0

0

0

0

0

0

0

0

0
4

1.03848
4

0

0

0

1.03848

0

0

0

0

0
4

1.66226

1.66226

0
4

5.50487

5.25173

0.119824

0.0799885

0.0533257

0
4

0.0798828

0

0

0

0.0798828

0
4

0

0

0

0
4

0

0

0

0
4

0.119824

0

0.119824

0
4

0

0

0
4

0.0798828

0.0798828

0
4

0.359473

0.359473

0
4

3.83582055007992e-14
4

0
4

12.9282

12.9282

6.79004

0.945738

0.359473

4.75303

0.0798828

3.48332473976143e-15

0
4

0
4

14.2591

14.2591

14.1393

0.119824

7.21644966006352e-16

0
4

0
4

0

0

0

0

0
4

0

0

0
4

0
4

0

0

0

0

0
4

0
4

0

0

0

0

0
4

0
4

0

0

0

0
4

0
4

1.05051

1.05051

1.05051

0
4

0
4

0

0

0

0
4

0
4

1.48823

0

0

0
4

1.48823

1.48823

0
4

0

0

0
4

0
4

0

0

0

0
4

0

0

0
4

0
4

0

0

0

0
4

0

0

0
4

0
4

0
4

0
4

0

0

0

0

0

0

0
4

0
4

0

0

0

0
4

0
4

0.315954

0.262628

0.262628

0
4

0.0533257

0.0533257

0
4

0
4

0

0

0

0
4

0
4

0

0

0

0
4

0
4

0

0

0

0

0
4

0
4

0.101554

0.101554

0.0507772

0.0507772

0
4

0
4

0

0

0

0
4

0
4

0

0

0

0
4

0
4

0

0

0

0
4

0
4

0

0

0

0
4

0
4

1.47783

1.07842

0.639062

0.239648

0.119824

0.0798828

0
4

0.0798828

0.0798828

0
4

0.119824

0.119824

0
4

0.199707

0.199707

0
4

0
4

0

0

0

0
4

0
4

0

0

0

0
4

0
4

0

0

0

0
4

0
4

0

0

0

0
4

0
4

0.0533257

0.0533257

0.0533257

0
4

0
4

0

0

0

0
4

0
4

0

0

0

0
4

0
4

0

0

0

0
4

0
4

0

0

0

0
4

0
4

0

0

0

0
4

0
4

0
6

0
6

0

0

0

0
4

0
4

0

0

0

0
4

0
4

0

0

0

0
4

0
4

0

0

0

0
4

0
4

0.962969

0.962969

0.962969

0
4

0
4

0

0

0

0
4

0
4

0.0798828

0.0798828

0.0798828

0
4

0
4

0

0

0

0
4

0
4

0.0798828

0.0798828

0.0798828

0
4

0
4

0

0

0

0
4

0
4

0

0

0

0
4

0
4

0

0

0

0

0

0

0
4

0
4

0

0

0

0
4

0
4

0

0

0

0
4

0
4

0

0

0

0
4

0
4

0.119824

0.119824

0.119824

0
4

0
4

0.437713

0.437713

0.437713

0
4

0
4

0

0

0

0
4

0
4

0

0

0

0
4

0
4

0

0

0

0
4

0
4

0.439355

0.439355

0.239648

0.0798828

0.119824

0
4

0
4

0

0

0

0
4

0
4

0

0

0

0

0
4

0

0

0
4

0
4

7.47735207085043e-14
4

0
4

110.702

32.1373
5

0
5

0
6

0
5

0
4

0

0

0

0

0

0

0
4

0

0

0
4

0

0

0
4

0.183475

0.183475

0
4

0.0917376

0.0917376

0
4

0

0

0
4

0

0

0
4

0

0

0
4

0

0

0
4

0

0

0
4

0

0

0
4

30.8237
4

30.259

0.0917376

0.289421

0

0.0917376

0.0917376

5.71764857681956e-15
4

0
4

0

0

0
4

0

0

0
4

0

0

0
4

1.03848

0

1.03848

0
4

0

0

0

0
4

0

0

0

0
4

0

0

0
4

0

0

0

0
4

0

0

0
4

0

0

0
4

0
4

70.7329

52.0458

0
7

0

0.275213

0

0

0

0

0

0

0

0

0

0.36695

0

0

0.0917376

0

0

0

0.133314

0

0

0

0
7

0

0

0

0

0

0

0

0.0649455

0.779769

0

0
7

0

0

0

0.129891

0

0

0

0

0

0

0
7

0

0.239966

0.106651

0

0.0917376

0.18664

0.0533257

0

0

0

0

0

0

0

0

0.0649455

0

0.133314

0.0533257

0.0533257

0

0
7

0

0

0

0

0

0

0

0

0.0649455

0

0

0

0

11.4679

0
6

0
6

0

0

1.60541

0

0

0
7

0
7

0

0.183475

0
8

0.20407

0.825638

0

0

0

0

0

0

0

0

0
7

0

0

0

0

0

0

4.99321

0

0

0

0
7

0

0

0

0.553073

0

0.278248

0

0.0917376

0

0

15.0811

0

0

0

0.19826

0

0

0.183475

0

0

0.183217

8.87552

0

0

0

0

0

0

0

0

0

0.0974182

3.86719

0

0

0

0

0

0

0

0

0.0974182

0

0
7

0.289421

0

0.0799885

0

0

0

0

0

0

0

9.63118473862323e-15

0
4

1.94024

0
7

0

0

0

0

0

0

0

0

0

0.487091

0
7

0

0

0

0

0

0

0

0

0

0

0
7

0

0

0

0

0

0

0

0

0

0

0
6

0.998535

0

0

0.454618

0

0

5.55111512312578e-17

0
4

2.58629

0
7

0

0

0
7

0
7

0

0

0

0

2.58629

0

0
7

0

0

0

0

0

0

0

0

0

0

0
8

0

0

0

0

0

0

0

0

0

0

0
8

0

0

0

0

0

0

0

0

0

0

0
7

0

0

0

0

0

0

0
7

0
7

0
7

0
7

0
4

0.875427

0

0

0

0.437713

0

0

0

0.437713

0

0

0

0

0

0

0

0

0

0

0

0
4

13.2053

0
7

8.77178

4.31367

0

0

0

0

0

0

0.119824

0

0

0

0

0

0

0

0

7.21644966006352e-16

0
4

0

0

0
4

0.0798828
7

0.0798828
7

0

0

0

0
4

0

0

0

0

0

0

0

0

0
4

0

0

0

0

0

0

0
4

0

0

0
4

0

0

0
4

0

0

0
4

0

0

0
4

0

0

0
4

0

0

0
4

0

0

0
4

8.18789480661053e-16

0
4

0

0

0

0

0

0

0

0

0

0

0
4

0

0

0

0

0
4

0
4

0

0

0

0

0
4

0

0

0
4

0
4

0

0

0

0
4

0
4

0

0

0

0

0
4

0

0

0
4

0
4

0

0

0

0

0

0
4

0
4

0.733901

0.0917376

0.0917376

0
4

0.642163

0.642163

0
4

0
4

0

0

0

0
4

0

0

0
4

0
4

0

0

0

0
4

0
4

0

0

0

0

0
4

0
4

0

0

0

0
4

0
4

0

0

0

0
4

0

0

0
4

0
4

0
7

0
7

0

0
7

0

0

0

0

0
4

0

0

0
4

0
4

0

0

0

0
4

0
4

0

0

0

0
4

0
4

0

0

0

0
4

0
4

0

0

0

0
4

0
4

0.649455

0.649455

0.649455

0
4

0
4

0

0

0

0
4

0
4

0

0

0

0
4

0
4

0

0

0

0
4

0
4

0

0

0

0
4

0
4

0

0

0

0
4

0
4

4.73282

4.73282

4.59521

0.137606

2.4980018054066e-16

0
4

0

0

0
4

0
4

0

0

0

0
4

0
4

0

0

0

0
4

0
4

1.71588

1.71588

1.50139

0.107242

0.107242

0
4

0

0

0
4

0
4

0

0

0

0
4

0
4

0
6

0

0

0

0

0
4

0

0

0
4

0

0

0
4

0
4

0

0

0

0

0

0
4

0

0

0

0
4

0
4

0

0

0

0

0
4

0
4

0

0

0

0

0
4

0
4

6.66133814775094e-15

0
4

2.26978

0

0

0

0

0

0

0

0

0

0

0

0

0

0
1

0

0

0

0

0

0

0

0
4

0

0

0

0
4

0

0

0

0
4

0

0

0
4

0

0

0
4

0

0

0
4

0

0

0
4

0
4

0

0

0

0
4

0
4

0.106651

0.106651

0.106651

0
4

0
4

0.106651

0.106651

0.0533257

0.0533257

0
4

0
4

0

0

0

0
4

0
4

0

0

0

0
4

0
4

0

0

0

0
4

0
4

0

0

0

0
4

0
4

2.05648

2.05648

2.05648

0
4

0
4

0

0

0

0
4

0
4

0

0

0

0
4

0
4

0
4

5.88083
5

5.88083
5

4.60271
5

4.60271
5

0

0

0

0

0

0

0

0

0
4

1.27812

0.519238

0.758887

0

0
4

0

0

0

0

0
4

0

0

0
4

0

0

0

0

0
4

0

0

0
4

0

0

0
4

4.44089209850063e-16
5

0
4

0
4

3.60285
4

3.60285
4

3.60285
4

1.67403
4

0.0798828

0.0917376

0

0.183475

0.515049
4

0.451965

0.251503

0.0917376

0

0.0917376

0.0799885

0.0917376

1.94289029309402e-16
4

0
4

0
4

0
4

0

0

0

0

0

0
4

0

0

0
4

0
4

0
4

0

0

0

0

0

0
4

0
4

0
4

0

0

0

0

0

0
4

0

0

0

0
4

0
4

0
4

0

0

0

0

0

0
4

0
4

0
4

0

0

0

0

0

0

0
4

0
4

0
4

0

0

0

0

0
4

0
4

0
4

0

0

0

0

0

0
4

0
4

0
4

0

0

0

0

0
4

0
4

0
4

0

0

0

0

0

0
4

0

0

0
4

0
4

0
4

0

0

0

0

0
4

0
4

0
4

0
2

0
2

0
2

0

0

0

0

0

0

0

0

0

0
4

0

0

0
4

0
4

0

0

0

0

0

0

0

0
4

0

0

0

0
4

0
4

0
4

0

0

0

0

0
4

0

0

0
4

0
4

0
4

0

0

0

0

0

0

0
4

0
4

0
4

0

0

0

0

0
4

0

0

0
4

0
4

0
4

0.0798828

0

0

0

0

0
4

0
4

0.0798828

0.0798828

0.0798828

0
4

0
4

0
4

0

0

0

0

0
4

0
4

0
4

0

0

0

0

0
4

0

0

0
4

0
4

0
4

0

0

0

0

0
4

0
4

0
4

0

0

0

0

0

0
4

0

0

0
4

0
4

0
4

0

0

0

0

0
4

0

0

0
4

0
4

0
4

0

0

0

0

0

0

0
4

0
4

0
4

0
7

0
7

0
7

0
7

0
7

0

0
4

0
7

0
7

0

0

0
4

0

0

0

0

0
4

0

0

0

0
4

0
4

0
4

0

0

0

0

0

0
4

0
4

0
4

0

0

0

0

0
4

0

0

0
4

0

0

0
4

0
4

0
4

1.46646

1.46646

0.586582

0.586582

0
4

0.879874

0.879874

0
4

0
4

0
4

0

0

0

0

0

0
4

0
4

0
4

0.183475

0.183475

0.183475

0.183475

0

0
4

0
4

0
4

0

0

0

0

0
4

0
4

0
4

0

0

0

0

0
4

0
4

0
4

0

0

0

0

0
4

0
4

0
4

0

0

0

0

0

0
4

0
4

0
4

0

0

0

0

0
4

0

0

0
4

0
4

0
4

5.67307

5.56642

5.56642

5.04808

0

0.133314

0

0.239966

0.0917376

0

0

0.0533257

0

0
4

0

0

0
4

0

0

0

0
4

0
4

0.106651

0.106651

0

0.106651

0
4

0
4

2.91433543964104e-16

0
4

0

0

0

0

0

0
4

0
4

0
4

0.199707

0.199707

0.119824

0.119824

0
4

0.0798828

0.0798828

0
4

0
4

0
4

0

0

0

0

0

0
4

0
4

0
4

0

0

0

0

0
4

0
4

0
4

0

0

0

0

0

0
4

0

0

0
4

0
4

0
4

0

0

0

0

0
4

0

0

0
4

0
4

0
4

0

0

0

0

0
4

0
4

0
4

0

0

0

0

0

0
4

0
4

0

0

0

0
4

0
4

0
4

0

0

0

0

0
4

0

0

0
4

0
4

0
4

0

0

0

0

0
4

0
4

0
4

0
2

0
2

0
2

0

0

0

0

0

0

0
4

0

0

0

0

0

0

0
4

0

0

0

0

0

0
4

0
4

0
4

0.119824

0.119824

0.119824

0.119824

0
4

0
4

0
4

0.23189

0.23189

0.23189

0.23189

0
4

0

0

0
4

0

0

0
4

0
4

0
4

0

0

0

0

0
4

0
4

0
4

0

0

0

0

0

0
4

0
4

0
4

0

0

0

0

0

0

0
4

0
4

0
4

0

0

0

0

0

0
4

0
4

0
4

0

0

0

0

0

0
4

0
4

0
4

0

0

0

0

0
4

0
4

0
4

0.18664

0.18664

0.106651

0.106651

0
4

0.0799885

0.0799885

0
4

0
4

0
4

0

0

0

0

0
4

0
4

0
4

0

0

0

0
6

0
7

0

0

0

0

0
4

0
6

0
6

0

0

0

0
4

0

0

0

0
4

0

0

0
4

0

0

0
4

0

0

0
4

0
4

0
4

0

0

0

0

0
4

0

0

0
4

0
4

0
4

0

0

0

0

0

0
4

0
4

0
4

0

0

0

0

0
4

0

0

0
4

0
4

0
4

0

0

0

0

0

0
4

0
4

0
4

0

0

0

0

0

0
4

0
4

0
4

0

0

0

0

0

0
4

0
4

0
4

0

0

0

0

0
4

0

0

0
4

0
4

0
4

0

0

0

0

0
4

0

0

0
4

0
4

0
4

0

0

0

0

0
4

0

0

0
4

0
4

0
4

0

0

0

0

0
4

0
4

0
4

0
7

0
7

0
7

0
7

0

0

0

0

0
4

0

0

0

0

0

0

0
4

0
4

0
4

0

0

0

0

0
4

0

0

0
4

0
4

0
4

0

0

0

0

0
4

0

0

0
4

0
4

0
4

0.159977

0.159977

0.159977

0.0799885

0.0799885

0
4

0
4

0
4

0

0

0

0

0

0
4

0
4

0
4

0

0

0

0

0

0
4

0
4

0
4

0

0

0

0

0
4

0
4

0
4

0

0

0

0

0
4

0
4

0
4

0.199707

0.199707

0.199707

0.199707

0
4

0

0

0
4

0
4

0
4

0

0

0

0

0
4

0

0

0
4

0
4

0
4

0

0

0

0

0
4

0
4

0
4

0
7

0
7

0
7

0
7

0

0

0

0

0

0
4

0

0

0

0

0
4

0

0

0
4

0
4

0
4

0

0

0

0

0
4

0

0

0
4

0
4

0
4

0

0

0

0

0
4

0
4

0
4

0

0

0

0

0
4

0

0

0
4

0
4

0
4

0

0

0

0

0

0
4

0
4

0
4

0

0

0

0

0
4

0
4

0
4

0.718945

0.718945

0.718945

0.639062

0.0798828

4.16333634234434e-17

0
4

0
4

0
4

0

0

0

0

0

0
4

0
4

0
4

0.126943

0.126943

0.126943

0.0761658

0.0507772

6.93889390390723e-18

0
4

0
4

0
4

0.097998

0.097998

0.097998

0.097998

0

0
4

0
4

0
4

0

0

0

0

0
4

0

0

0
4

0
4

0
4

0.228498
7

0.228498
7

0
7

0
7

0

0

0
4

0

0

0
4

0.228498

0.228498

0
4

0
4

0
4

0.159766

0.159766

0.159766

0.0798828

0.0798828

0
4

0
4

0
4

0

0

0

0

0
4

0
4

0
4

0

0

0

0

0
4

0
4

0
4

0.137606

0.137606

0.137606

0.137606

0
4

0

0

0
4

0
4

0
4

0

0

0

0

0
4

0
4

0
4

0

0

0

0

0

0
4

0
4

0
4

0.106651

0.106651

0

0

0
4

0.106651

0.106651

0
4

0
4

0
4

0

0

0

0

0
4

0
4

0
4

0

0

0

0

0
4

0
4

0
4

0

0

0

0

0
4

0
4

0
4

0
7

0
7

0
7

0
7

0
7

0

0

0

0
4

0
7

0
7

0

0

0
4

0
4

0
4

0

0

0

0

0
4

0

0

0
4

0
4

0
4

0

0

0

0

0

0
4

0
4

0
4

0

0

0

0

0
4

0
4

0
4

0

0

0

0

0
4

0

0

0
4

0
4

0
4

0

0

0

0

0
4

0
4

0
4

0

0

0

0

0

0
4

0
4

0
4

0

0

0

0

0
4

0

0

0
4

0
4

0
4

0

0

0

0

0
4

0
4

0
4

0

0

0

0

0
4

0

0

0
4

0
4

0
4

0.213303

0.213303

0.0799885

0.0799885

0
4

0.133314

0.133314

0
4

0
4

0
4

121.663
4

121.609
4

31.1955

22.0235

0.266628

0.133314

1.03985

0.159977

0.0533257

0.18664

0.0533257

0.213303

1.46646

0.933199

0.933199

1.46646

0.426605

1.27982

0.346617

0.213303

0
4

84.3612
4

75.8824

0.37328

0.399943

0.213303

0.18664

0.213303

0.133314

2.18635

3.27953

0.719897

0.399943

0.106651

0.106651

0.106651

0.0533257

1.92137972199191e-14
4

0
4

5.91915

4.77265

0.853211

0

0.0799885

0.106651

0.106651

7.21644966006352e-16

0
4

0.133314

0.0533257

0.0799885

0
4

1.80688797257744e-14
4

0
4

0.0533257

0.0533257

0.0533257

0
4

0
4

0
4

0

0

0

0

0

0

0

0

0

0

0
4

0

0

0

0

0
4

0
4

0
4

0

0

0

0

0
4

0
4

0
4

0

0

0

0

0

0
4

0
4

0
4

5.73746

5.73746

5.73746

5.73746

0
4

0
4

0
4

0

0

0

0

0

0
4

0
4

0
4

0

0

0

0

0

0
4

0
4

0
4

0.578842

0.578842

0.578842

0.578842

0
4

0
4

0
4

0

0

0

0

0
4

0

0

0
4

0
4

0
4

0

0

0

0

0
4

0
4

0
4

0.18664

0.18664

0.18664

0.0799885

0.106651

0
4

0
4

0
4

0

0

0

0

0

0
4

0
4

0
4

0
7

0
7

0
7

0
7

0
7

0

0
4

0

0

0

0
4

0
4

0

0

0

0
4

0
4

0
4

0

0

0

0

0
4

0
4

0
4

0

0

0

0

0
4

0
4

0
4

0

0

0

0

0
4

0
4

0
4

0

0

0

0

0
4

0
4

0
4

0

0

0

0

0
4

0
4

0
4

0

0

0

0

0
4

0
4

0
4

2.31537

2.31537

2.31537

2.31537

0
4

0
4

0
4

0.106651

0.106651

0.106651

0.106651

0
4

0
4

0
4

0

0

0

0

0
4

0
4

0
4

0

0

0

0

0
4

0
4

0
4

0

0

0

0
7

0

0

0
4

0

0

0
4

0

0

0
4

0

0

0
4

0
4

0
4

0

0

0

0

0
4

0
4

0
4

0

0

0

0

0
4

0
4

0
4

0

0

0

0

0
4

0
4

0
4

0

0

0

0

0
4

0
4

0
4

0

0

0

0

0
4

0
4

0
4

0

0

0

0

0
4

0
4

0
4

0

0

0

0

0
4

0
4

0
4

0

0

0

0

0
4

0
4

0
4

0

0

0

0

0
4

0
4

0
4

0

0

0

0

0
4

0
4

0
4

0
2

0
2

0
2

0
2

0

0

0

0

0

0
4

0
2

0

0

0

0
4

0

0

0
4

0
4

0
4

0

0

0

0

0
4

0
4

0
4

0

0

0

0

0
4

0
4

0
4

0

0

0

0

0
4

0
4

0
4

0

0

0

0

0
4

0
4

0
4

0

0

0

0

0
4

0
4

0
4

0

0

0

0

0
4

0
4

0
4

0

0

0

0

0
4

0
4

0
4

0.0798828

0.0798828

0.0798828

0.0798828

0
4

0
4

0
4

0

0

0

0

0
4

0
4

0
4

0

0

0

0

0
4

0
4

0
4

0
4

0
4

0
4

0

0

0

0

0

0

0

0

0

0
4

0
4

0
4

0
4

0
4

0

0

0

0
4

0
4

0
4

0

0

0

0

0
4

0
4

0
4

0

0

0

0

0
4

0
4

0
4

0

0

0

0

0
4

0
4

0
4

0

0

0

0

0
4

0
4

0
4

0

0

0

0

0
4

0
4

0
4

0

0

0

0

0
4

0
4

0
4

0

0

0

0

0
4

0
4

0
4

0

0

0

0

0
4

0
4

0
4

0

0

0

0

0
4

0
4

0
4

0

0

0

0

0
4

0
4

0
4

2.64007
5

2.64007
5

2.64007
5

2.64007
5

0

0

0
4

0
4

0
4

0

0

0

0

0
4

0
4

0
4

0

0

0

0

0
4

0
4

0
4

0

0

0

0

0
4

0
4

0
4

0.175085

0.175085

0.175085

0.175085

0
4

0
4

0
4

0

0

0

0

0
4

0
4

0
4

0

0

0

0

0
4

0
4

0
4

0

0

0

0

0
4

0
4

0
4

0

0

0

0

0
4

0
4

0
4

0

0

0

0

0
4

0
4

0
4

0.0533257

0.0533257

0.0533257

0.0533257

0
4

0
4

0
4

0
7

0
7

0
7

0
7

0

0
4

0

0

0
4

0
4

0

0

0

0

0

0
4

0
4

0
4

0

0

0

0

0
4

0
4

0
4

0

0

0

0

0
4

0
4

0
4

0

0

0

0

0
4

0
4

0
4

0

0

0

0

0
4

0
4

0
4

0

0

0

0

0
4

0
4

0
4

0

0

0

0

0
4

0
4

0
4

0.0533257

0.0533257

0.0533257

0.0533257

0
4

0
4

0
4

0

0

0

0

0
4

0
4

0
4

0

0

0

0

0
4

0
4

0
4

0.106651

0.106651

0.106651

0.106651

0
4

0
4

0
4

0.469079

0.469079

0.469079

0.371081

0

0

0

0.097998

0

0

0
4

0
4

0
4

0.137606

0.137606

0.137606

0.137606

0
4

0
4

0
4

0.0798828

0.0798828

0.0798828

0.0798828

0
4

0
4

0
4

0

0

0

0

0
4

0
4

0
4

0

0

0

0

0
4

0
4

0
4

0

0

0

0

0
4

0
4

0
4

0.144661

0.144661

0.144661

0.144661

0
4

0
4

0
4

0

0

0

0

0
4

0
4

0
4

0

0

0

0

0
4

0
4

0
4

0

0

0

0

0
4

0
4

0
4

0.289421

0.289421

0.289421

0.289421

0
4

0
4

0
4

9.06198

9.06198

9.06198

6.38092

0.697075

1.28691

0.589833

0.107242

0
4

0
4

0
4

0

0

0

0

0
4

0
4

0
4

0

0

0

0

0
4

0
4

0
4

0.0507772

0.0507772

0.0507772

0.0507772

0
4

0
4

0
4

0

0

0

0

0
4

0
4

0
4

0

0

0

0

0
4

0
4

0
4

0

0

0

0

0
4

0
4

0
4

0

0

0

0

0
4

0
4

0
4

0

0

0

0

0
4

0
4

0
4

0

0

0

0

0
4

0
4

0
4

0

0

0

0

0
4

0
4

0
4

0
4

0
4

0
4

0
4

0

0

0

0
4

0
4

0
4

0

0

0

0

0
4

0
4

0
4

0.133314

0.133314

0.133314

0.133314

0
4

0
4

0
4

0

0

0

0

0
4

0
4

0
4

0

0

0

0

0
4

0
4

0
4

0

0

0

0

0
4

0
4

0
4

0

0

0

0

0
4

0
4

0
4

0

0

0

0

0
4

0
4

0
4

0

0

0

0

0
4

0
4

0
4

0

0

0

0

0
4

0
4

0
4

2.27611

2.27611

2.27611

2.27611

0
4

0
4

0
4

0
4

0
4

0
4

0
4

0

0

0

0

0

0

0

0

0

0

0
4

0

0

0
4

0

0

0

0

0

0

0
4

0
4

0

0

0

0

0

0

0
4

0

0

0

0
4

0

0

0
4

0
4

0
4

0
5

0
5

0
5

0
5

0

0

0

0

0

0

0
4

0

0

0
4

0
4

0
4

0.0798828

0.0798828

0.0798828

0.0798828

0
4

0
4

0
4

0.0723307

0.0723307

0.0723307

0.0723307

0
4

0
4

0
4

0

0

0

0

0
4

0
4

0
4

0

0

0

0

0
4

0
4

0
4

0

0

0

0

0
4

0
4

0
4

0

0

0

0

0
4

0
4

0
4

0

0

0

0

0
4

0
4

0
4

0

0

0

0

0
4

0
4

0
4

0

0

0

0

0
4

0
4

0
4

0

0

0

0

0
4

0
4

0
4

0
4

0
4

0
4

0

0

0

0

0

0

0

0
4

0
4

0
4

0

0

0

0

0
4

0
4

0
4

0

0

0

0

0
4

0
4

0
4

0

0

0

0

0
4

0
4

0
4

0

0

0

0

0
4

0
4

0
4

0

0

0

0

0
4

0
4

0
4

0.106651

0.106651

0.106651

0.106651

0
4

0
4

0
4

0

0

0

0

0
4

0
4

0
4

0

0

0

0

0
4

0
4

0
4

0

0

0

0

0
4

0
4

0
4

0.350171

0.350171

0.350171

0.350171

0
4

0
4

0
4

0

0

0
6

0

0

0

0
4

0

0

0

0

0
4

0

0

0
4

0

0

0

0
4

0

0

0
4

0

0

0
4

0
4

0
4

0

0

0

0

0
4

0
4

0
4

0

0

0

0

0
4

0
4

0
4

0

0

0

0

0
4

0
4

0
4

0

0

0

0

0
4

0
4

0
4

0

0

0

0

0
4

0
4

0
4

0

0

0

0

0
4

0
4

0
4

0

0

0

0

0
4

0
4

0
4

0

0

0

0

0
4

0
4

0
4

0

0

0

0

0
4

0
4

0
4

0

0

0

0

0
4

0
4

0
4

0

0

0

0
7

0

0

0

0
4

0
4

0
4

0.107242

0.107242

0.107242

0.107242

0
4

0
4

0
4

0

0

0

0

0
4

0
4

0
4

0

0

0

0

0
4

0
4

0
4

0

0

0

0

0
4

0
4

0
4

0

0

0

0

0
4

0
4

0
4

0

0

0

0

0
4

0
4

0
4

0

0

0

0

0
4

0
4

0
4

0.119824

0.119824

0.119824

0.119824

0
4

0
4

0
4

0

0

0

0

0
4

0
4

0
4

0

0

0

0

0
4

0
4

0
4

0
3

0
3

0
4

0

0

0

0

0
4

0

0

0
4

0

0

0
4

0

0

0

0
4

0
4

0
4

0

0

0

0

0
4

0
4

0
4

0

0

0

0

0
4

0
4

0
4

0

0

0

0

0
4

0
4

0
4

0

0

0

0

0
4

0
4

0
4

0

0

0

0

0
4

0
4

0
4

0

0

0

0

0
4

0
4

0
4

0

0

0

0

0
4

0
4

0
4

0

0

0

0

0
4

0
4

0
4

0

0

0

0

0
4

0
4

0
4

0.160864

0.160864

0.160864

0.160864

0
4

0
4

0
4

0
7

0
7

0
7

0

0

0

0
4

0

0

0

0

0

0
4

0

0

0
4

0
4

0
4

0.183475

0.183475

0.183475

0.183475

0
4

0
4

0
4

0

0

0

0

0
4

0
4

0
4

0

0

0

0

0
4

0
4

0
4

0

0

0

0

0
4

0
4

0
4

0

0

0

0

0
4

0
4

0
4

0

0

0

0

0
4

0
4

0
4

0

0

0

0

0
4

0
4

0
4

0

0

0

0

0
4

0
4

0
4

0

0

0

0

0
4

0
4

0
4

0

0

0

0

0
4

0
4

0
4

0

0

0

0

0

0

0
4

0

0

0

0
4

0
4

0

0

0

0

0
4

0

0

0
4

0
4

0
4

0

0

0

0

0
4

0
4

0
4

0

0

0

0

0
4

0
4

0
4

0.195427

0.195427

0.195427

0.195427

0
4

0
4

0
4

0

0

0

0

0
4

0
4

0
4

0

0

0

0

0
4

0
4

0
4

0

0

0

0

0
4

0
4

0
4

0

0

0

0

0
4

0
4

0
4

0

0

0

0

0
4

0
4

0
4

0

0

0

0

0
4

0
4

0
4

0

0

0

0

0
4

0
4

0
4

0.962969
5

0.962969
5

0.962969
5

0.962969
5

0

0
4

0
4

0
4

0.107242

0.107242

0.107242

0.107242

0
4

0
4

0
4

0

0

0

0

0
4

0
4

0
4

0

0

0

0

0
4

0
4

0
4

0

0

0

0

0
4

0
4

0
4

0

0

0

0

0
4

0
4

0
4

0

0

0

0

0
4

0
4

0
4

0

0

0

0

0
4

0
4

0
4

0

0

0

0

0
4

0
4

0
4

0

0

0

0

0
4

0
4

0
4

0

0

0

0

0
4

0
4

0
4

0
7

0
7

0

0

0

0
4

0

0

0

0
4

0
4

0
4

0

0

0

0

0
4

0
4

0
4

0

0

0

0

0
4

0
4

0
4

0

0

0

0

0
4

0
4

0
4

0

0

0

0

0
4

0
4

0
4

0

0

0

0

0
4

0
4

0
4

0

0

0

0

0
4

0
4

0
4

0

0

0

0

0
4

0
4

0
4

0.0798828

0.0798828

0.0798828

0.0798828

0
4

0
4

0
4

0

0

0

0

0
4

0
4

0
4

0

0

0

0

0
4

0
4

0
4

1.35267
4

1.35267
4

0.473295
4

0.301675

0.0917376

0.0798828

0
4

0.667815
4

0.667815
4

0
4

0.211562

0.211562

0
4

0

0

0
4

8.32667268468867e-17
4

0
4

0
4

0

0

0

0

0
4

0
4

0
4

0

0

0

0

0
4

0
4

0
4

0.0798828

0.0798828

0.0798828

0.0798828

0
4

0
4

0
4

0

0

0

0

0
4

0
4

0
4

0

0

0

0

0
4

0
4

0
4

0

0

0

0

0
4

0
4

0
4

0

0

0

0

0
4

0
4

0
4

0.154593

0.154593

0.154593

0.154593

0
4

0
4

0
4

0.0482775

0.0482775

0.0482775

0.0482775

0
4

0
4

0
4

0

0

0

0

0
4

0
4

0
4

0.875427
7

0.875427
7

0.875427
7

0
7

0.875427

0
6

0

0

0

0
4

0
7

0
7

0
7

0
7

0
7

0

0

0

0
4

0
4

0
4

2.02213

2.02213

2.02213

1.39453

0.410359

0.217249

0

1.94289029309402e-16

0
4

0

0

0

0
4

0
4

0
4

0

0

0

0

0
4

0
4

0
4

0

0

0

0

0
4

0
4

0
4

0

0

0

0

0
4

0
4

0
4

0

0

0

0

0
4

0
4

0
4

0

0

0

0

0
4

0
4

0
4

0

0

0

0

0
4

0
4

0
4

0

0

0

0

0
4

0
4

0
4

0

0

0

0

0
4

0
4

0
4

0

0

0

0

0
4

0
4

0
4

0

0

0

0

0
4

0
4

0
4

0
7

0
7

0
7

0
6

0

0

0
4

0

0

0
4

0

0

0
4

0
4

0
4

0

0

0

0

0
4

0
4

0
4

0

0

0

0

0
4

0
4

0
4

0

0

0

0

0
4

0
4

0
4

0

0

0

0

0
4

0
4

0
4

0

0

0

0

0
4

0
4

0
4

0

0

0

0

0
4

0
4

0
4

0

0

0

0

0
4

0
4

0
4

0

0

0

0

0
4

0
4

0
4

0

0

0

0

0
4

0
4

0
4

0

0

0

0

0
4

0
4

0
4

0
7

0
7

0

0

0

0

0
4

0

0

0

0

0
4

0
4

0
4

0

0

0

0

0
4

0
4

0
4

0

0

0

0

0
4

0
4

0
4

0

0

0

0

0
4

0
4

0
4

0

0

0

0

0
4

0
4

0
4

0
6

0
6

0
6

0
6

0

0

0
4

0
4

0
4

46.1

45.8067

44.4736

0.55992

0.37328

42.8472

0.693234

1.33226762955019e-15

0
4

0.639908

0.506594

0.133314

2.77555756156289e-17

0
4

0.453268

0.453268

0
4

0.239966

0.106651

0.133314

0
4

3.88578058618805e-16

0
4

0.293291

0.293291

0.293291

0
4

0
4

1.38777878078145e-15

0
4

0
7

0
7

0
7

0
7

0

0
4

0

0

0
4

0

0

0
4

0
4

0
4

0
7

0
7

0
7

0
7

0
4

0
4

0
4

0

0

0

0

0

0

0

0

0
4

0

0

0
4

0
4

0
4

0
4

0
4

0

0

0

0

0
4

0

0

0
4

0
4

0
4

0.404255

0.404255

0.404255

0.404255

0

0
4

0
4

0
4

0
5

0
5

0
6

0
6

0

0

0

0

0

0

0

0

0

0

0

0

0

0

0

0

0
4

0
5

0
7

0
6

0

0

0
4

0

0

0

0

0
4

0
4

0
4

0

0

0

0

0

0
4

0

0

0

0
4

0
4

0
4

0.335062
4

0.335062
4

0.335062
4

0

0.106651

0.0533257

0.175085

0
4

0

0

0

0
4

0
4

0
4

20.6896

20.6896

20.6896

20.1305

0.119824

0.359473

0.0798828

1.1518563880486e-15

0
4

0
4

0
4

0
7

0
7

0
7

0

0

0
4

0
4

0
4

2.18371

2.18371

2.18371

0.18664

1.99707

0

0
4

0

0

0
4

0
4

0
4

0

0

0

0

0

0

0

0

0
4

0

0

0
4

0
4

0
4

0.262628

0.262628

0.262628

0

0.262628

0

0

0
4

0
4

0
4

0

0

0

0

0

0

0
4

0
4

0
4

0

0

0

0

0
4

0
4

0
4

0

0

0

0

0

0
4

0
4

0
4

0.321081

0.321081

0
5

0
6

0

0

0

0

0

0

0

0

0

0
4

0
7

0
7

0

0

0
4

0.321081

0

0

0

0.321081

0
4

0

0

0

0
4

0
4

0

0

0

0
4

0
4

0
4

0.23189

0.23189

0.23189

0.23189

0

0

0
4

0

0

0

0
4

0
4

0

0

0

0
4

0
4

0
4

0.384712
4

0.384712
4

0.384712
4

0.384712
4

0

0
4

0
4

0
4

0
7

0
7

0
7

0
7

0

0
4

0
4

0
4

0

0

0

0

0

0
4

0

0

0
4

0
4

0
4

0

0

0

0

0

0
4

0

0

0
4

0

0

0

0
4

0

0

0
4

0
4

0
4

0

0

0

0

0

0
4

0
4

0
4

0.57933
4

0.57933
4

0.57933

0.57933

0

0
4

0

0

0

0
4

0

0

0
4

0
4

0
4

0

0

0

0

0

0

0
4

0
4

0
4

6.64902
4

6.64902
4

0

0

0
4

6.64902

6.64902

0
4

0

0

0
4

0
4

0
4

0
5

0
5

0
5

0
5

0
4

0
4

0
4

103.524

103.524

7.9907

7.9907

0

0

0
4

0

0

0
4

0

0

0
4

94.1731
4

93.7199
4

0.159977

0.18664

0.0533257

0.0533257

0
4

1.11984

0.106651

0.319954

0.693234

0
4

0

0

0
4

0

0

0
4

0

0

0
4

0.0799885

0.0799885

0
4

0.159977

0.159977

0
4

0

0

0
4

4.91273688396632e-15

0
4

0
4

0.636987

0.636987

0.636987

0.48999

0.146997

5.55111512312578e-17

0
4

0
4

0
4

0

0

0

0

0

0

0
4

0

0

0

0
4

0
4

0
4

0

0

0

0

0
4

0
4

0
4

0

0

0

0

0

0
4

0

0

0

0
4

0
4

0
4

0

0

0

0

0

0
4

0
4

0
4

0

0

0

0

0

0

0

0

0
4

0

0

0
4

0
4

0
4

0

0

0

0

0

0

0
4

0
4

0
4

0

0

0

0

0
4

0

0

0

0
4

0
4

0
4

0
7

0
7

0

0

0
4

0

0

0
4

0
4

0
4

0

0

0

0

0

0
4

0

0

0

0
4

0
4

0
4

0

0

0

0

0

0

0

0

0

0

0

0

0

0

0
4

0

0

0

0

0

0

0

0

0
4

0

0

0

0

0

0

0

0

0

0
4

0
4

0
4

0

0

0

0

0

0

0
4

0
4

0
4

0.0799885
5

0.0799885
5

0.0799885
5

0

0.0799885

0
4

0
4

0
4

1.05051

1.05051

1.05051

1.05051

0
4

0
4

0
4

0

0

0

0

0
4

0
4

0
4

0

0

0

0

0

0
4

0
4

0
4

3.38093

3.38093

3.38093

3.38093

0
4

0
4

0
4

0.959862

0.959862

0.826548

0.18664

0.639908

0
4

0.133314

0.133314

0
4

2.77555756156289e-17

0
4

0
4

0

0

0

0

0

0

0
4

0
4

0
4

0

0

0

0

0

0
4

0
4

0
4

0

0

0

0

0

0
4

0

0

0

0
4

0
4

0
4

0
7

0
7

0
7

0
7

0
7

0
7

0

0

0

0

0

0

0
4

0

0

0

0

0
4

0

0

0
4

0
4

0
4

0

0

0

0

0

0

0
4

0
4

0
4

0

0

0

0

0

0

0
4

0
4

0
4

0

0

0

0

0

0
4

0
4

0
4

0

0

0

0

0
4

0
4

0
4

0

0

0

0

0

0
4

0
4

0
4

0

0

0

0

0
4

0

0

0
4

0
4

0
4

1.13805

1.13805

1.13805

1.13805

0
4

0
4

0
4

0

0

0

0

0

0
4

0
4

0
4

0

0

0

0

0

0
4

0

0

0
4

0
4

0

0

0

0
4

0
4

0
4

0

0

0

0

0

0

0
4

0
4

0
4

0.146997

0
4

0
4

0

0

0

0
4

0
4

0

0

0

0
4

0
4

0

0

0

0
4

0
4

0

0

0

0
4

0
4

0

0

0

0
4

0
4

0

0

0

0
4

0
4

0

0

0

0
4

0
4

0

0

0

0
4

0
4

0

0

0

0
4

0
4

0

0

0

0
4

0
4

0

0

0

0
4

0
4

0

0

0

0

0
4

0
4

0

0

0

0
4

0
4

0

0

0

0
4

0
4

0

0

0

0
4

0
4

0

0

0

0
4

0
4

0

0

0

0
4

0
4

0

0

0

0
4

0
4

0

0

0

0
4

0
4

0.146997

0.146997

0.146997

0
4

0
4

0

0

0

0
4

0
4

0

0

0

0
4

0
4

0

0

0

0
4

0
4

0

0

0

0
4

0
4

0

0

0

0

0

0
4

0
4

0

0

0

0

0
4

0
4

0

0

0

0
4

0
4

0

0

0

0
4

0

0

0
4

0
4

0

0

0

0
4

0
4

0

0

0

0
4

0
4

0
3

0
2

0
3

0
2

0

0

0

0

0

0

0

0

0

0

0
2

0

0

0

0

0

0

0

0

0

0

0

0

0

0

0

0

0

0

0

0

0

0

0

0

0

0
4

0

0

0

0

0

0

0

0

0

0

0
4

0

0

0
4

0

0

0

0
4

0

0

0
4

0

0

0

0
4

0

0

0
4

0

0

0
4

0

0

0
4

0

0

0
4

0

0

0
4

0

0

0
4

0

0

0

0

0

0

0

0
4

0

0

0
4

0

0

0
4

0

0

0
4

0

0

0
4

0

0

0
4

0

0

0
4

0

0

0
4

0

0

0
4

0

0

0
4

0

0

0
4

0

0

0

0

0
4

0

0

0
4

0

0

0
4

0

0

0
4

0

0

0

0
4

0

0

0

0
4

0

0

0

0

0
4

0

0

0

0

0
4

0

0

0
4

0

0

0

0
4

0
4

0
4

80.2949
3

0

0

0

0

0

0

0

0

0
4

0

0

0

0

0
4

0

0

0
4

0
4

0
6

0
6

0
6

0
4

0
4

0

0

0

0
4

0
4

0

0

0

0
4

0
4

0

0

0

0
4

0
4

0

0

0

0
4

0
4

0

0

0

0
4

0
4

0

0

0

0
4

0
4

0

0

0

0
4

0
4

0

0

0

0
4

0
4

0

0

0

0
4

0
4

0

0

0

0
4

0
4

0

0

0

0

0

0

0

0
4

0

0

0

0
4

0

0

0
4

0
4

0

0

0

0
4

0
4

0

0

0

0
4

0
4

0

0

0

0
4

0
4

0.0798828

0.0798828

0.0798828

0
4

0
4

0

0

0

0
4

0
4

0.0798828

0.0798828

0.0798828

0
4

0
4

0.0533257

0.0533257

0.0533257

0
4

0
4

0

0

0

0
4

0
4

0

0

0

0
4

0
4

0

0

0

0
4

0
4

0

0

0

0

0
4

0

0

0

0
4

0
4

0

0

0

0
4

0
4

0

0

0

0
4

0
4

0

0

0

0
4

0
4

0

0

0

0
4

0
4

0

0

0

0
4

0
4

0

0

0

0
4

0
4

0

0

0

0
4

0
4

0

0

0

0
4

0
4

0.0488567

0.0488567

0.0488567

0
4

0
4

0

0

0

0
4

0
4

0

0

0

0

0

0

0

0
4

0

0

0
4

0
4

0

0

0

0
4

0
4

0

0

0

0
4

0
4

0

0

0

0
4

0
4

0

0

0

0
4

0
4

0

0

0

0
4

0
4

0

0

0

0
4

0
4

0

0

0

0

0

0
4

0

0

0

0
4

0

0

0
4

0
4

0
3

0

0

0

0

0

0
4

0

0

0

0
4

0

0

0
4

0

0

0
4

0

0

0
4

0
4

0
2

0

0

0

0

0
4

0

0

0
4

0

0

0

0
4

0

0

0
4

0

0

0
4

0
4

0

0

0

0

0

0

0
4

0
4

0

0

0

0

0
4

0
4

0
6

0
6

0
6

0
4

0
4

62.7087
4

62.7087
4

62.7087
4

0
4

0

0

0
4

0

0

0
4

0

0

0
4

0
4

0
3

0

0

0

0

0

0
4

0

0

0
4

0

0

0
4

0
4

0
3

0

0

0

0

0
4

0

0

0
4

0

0

0
4

0

0

0
4

0

0

0
4

0
4

0

0

0

0

0
4

0

0

0
4

0
4

0

0

0

0

0
4

0
4

4.43762

4.43762

4.19263

0.244995

2.22044604925031e-16

0
4

0
4

0

0

0

0
4

0

0

0
4

0
4

0

0

0

0
4

0

0

0

0
4

0
4

0

0

0

0

0

0
4

0

0

0
4

0
4

1.48823
4

1.48823
4

0

0

0

0

1.48823

0

0
4

0
4

0
4

0

0

0

0

0

0
4

0

0

0

0
4

0
4

0.121276
3

0.121276
3

0.121276

0

0

0

0
4

0

0

0

0

0
4

0

0

0
4

0
4

0

0

0

0

0
4

0

0

0
4

0
4

0

0

0

0

0

0

0
4

0
4

0
6

0
6

0
6

0
4

0
4

0

0

0

0

0
4

0

0

0

0
4

0
4

0

0

0

0

0
4

0

0

0
4

0
4

0

0

0

0

0
4

0
4

0

0

0

0

0

0
4

0

0

0
4

0
4

0

0

0

0

0

0
4

0
4

0.18664

0

0

0

0
4

0.18664

0.18664

0
4

0
4

0

0

0

0

0
4

0
4

0
2

0

0

0

0

0
4

0

0

0

0

0
4

0

0

0
4

0

0

0
4

0

0

0
4

0
4

0

0

0

0

0
4

0

0

0
4

0
4

0

0

0

0
4

0
4

0

0

0

0
4

0
4

0

0

0

0
4

0
4

0

0

0

0
4

0
4

0

0

0

0

0
4

0
4

0

0

0

0
4

0

0

0
4

0
4

0

0

0

0

0
4

0

0

0
4

0
4

0

0

0

0
4

0
4

0

0

0

0
4

0
4

0

0

0

0

0

0

0

0
4

0
4

0

0

0

0
4

0

0

0
4

0
4

0

0

0

0

0
4

0

0

0
4

0
4

0

0

0

0

0
4

0
4

0

0

0

0

0
4

0
4

0

0

0

0

0
4

0
4

0

0

0

0
4

0

0

0
4

0
4

0

0

0

0

0
4

0
4

0

0

0

0
4

0

0

0
4

0
4

0

0

0

0
4

0

0

0
4

0
4

0

0

0

0
4

0
4

1.32495
4

0.500893

0.266417

0.0798828

0

0.154593

1.11022302462516e-16

0
4

0.648975

0.648975

0
4

0.175085

0.175085

0
4

0

0

0
4

0
4

0

0

0

0
4

0
4

0

0

0

0

0
4

0
4

0

0

0

0
4

0
4

0

0

0

0

0
4

0
4

0

0

0

0
4

0

0

0
4

0
4

0.0798828

0.0798828

0.0798828

0
4

0

0

0
4

0
4

0.0799885

0

0

0
4

0.0799885

0.0799885

0
4

0
4

0

0

0

0
4

0
4

0

0

0

0
4

0
4

0

0

0

0

0
4

0
4

0

0

0

0

0
4

0

0

0

0

0
4

0

0

0
4

0
4

0

0

0

0

0
4

0
4

0

0

0

0
4

0

0

0
4

0
4

0

0

0

0
4

0

0

0
4

0
4

0

0

0

0

0
4

0
4

0

0

0

0
4

0
4

0

0

0

0
4

0
4

0

0

0

0

0
4

0
4

0

0

0

0
4

0
4

0

0

0

0
4

0
4

0

0

0

0
4

0
4

0

0

0

0

0

0
4

0

0

0
4

0

0

0
4

0

0

0

0
4

0
4

0

0

0

0
4

0
4

0

0

0

0
4

0
4

0

0

0

0
4

0
4

0

0

0

0
4

0
4

2.0376

2.0376

2.0376

0
4

0
4

0

0

0

0
4

0
4

0

0

0

0
4

0
4

0

0

0

0
4

0
4

0

0

0

0
4

0
4

0

0

0

0
4

0
4

0.0798828
3

0

0

0

0
4

0.0798828

0.0798828

0

0

0
4

0

0

0
4

0

0

0
4

0
4

0

0

0

0
4

0
4

0

0

0

0
4

0
4

0

0

0

0
4

0
4

0

0

0

0
4

0
4

0

0

0

0
4

0
4

0

0

0

0
4

0
4

0

0

0

0
4

0
4

0

0

0

0
4

0
4

0

0

0

0
4

0
4

0

0

0

0
4

0
4

7.48809
3

6.63308
3

6.34366
3

0
3

0

0

0

0.289421

0

0

0

0

0

0

0

0

0

0

0

0

0

0

0

0

0

0

0

0

0

0

0

0

0

0

0

0

0

0

0

0

0

0

0

0

0

0

0

0

0

0

0

4.44089209850063e-16
3

0
4

0
7

0
7

0
4

0

0

0
4

0

0

0
4

0

0

0
4

0

0

0
4

0

0

0
4

0

0

0
4

0

0

0
4

0

0

0
4

0

0

0
4

0

0

0
4

0.506413

0.506413

0
4

0

0

0
4

0.0798828

0.0798828

0
4

0

0

0
4

0

0

0

0
4

0

0

0
4

0.268712

0.268712

0
4

0

0

0
4

0

0

0
4

0
4

1.4210854715202e-14
3

0
4

2896.18
4

0
7

0
7

0
7

0

0

0

0

0

0

0
4

0
4

0
7

0
7

0
7

0
4

0

0

0
4

0
4

0

0

0

0

0
4

0
4

0

0

0

0

0
4

0
4

0

0

0

0

0
4

0
4

0

0

0

0

0
4

0
4

0

0

0

0

0
4

0
4

0

0

0

0
4

0
4

0

0

0

0

0
4

0
4

0

0

0

0

0
4

0
4

0.599121

0.239648

0.239648

0
4

0.359473

0.359473

0
4

5.55111512312578e-17

0
4

0

0

0

0
4

0
4

0.415077
4

0.125656
4

0

0.0533257

0

0

0.0723307

0

0

0

1.38777878078145e-17
4

0
4

0.289421

0.289421

0

0
4

0

0

0

0
4

0

0

0
4

0

0

0
4

0
4

0

0

0

0
4

0

0

0
4

0
4

0

0

0

0
4

0

0

0
4

0
4

0

0

0

0
4

0

0

0
4

0
4

0

0

0

0
4

0

0

0
4

0
4

0

0

0

0

0
4

0
4

0.119824

0

0

0
4

0.119824

0.119824

0
4

0
4

1.55771

1.55771

1.55771

0
4

0
4

0

0

0

0

0
4

0
4

0.119824

0.119824

0.119824

0
4

0

0

0
4

0
4

0

0

0

0

0
4

0
4

0
7

0
7

0
7

0

0

0
4

0

0

0

0
4

0

0

0
4

0
4

0

0

0

0
4

0

0

0
4

0
4

0

0

0

0
4

0
4

0

0

0

0
4

0
4

0

0

0

0
4

0

0

0
4

0
4

0

0

0

0
4

0
4

0

0

0

0
4

0

0

0
4

0
4

0

0

0

0
4

0

0

0
4

0
4

0

0

0

0
4

0
4

0

0

0

0

0
4

0
4

0

0

0

0
4

0

0

0
4

0
4

0
4

0
4

0

0

0

0

0

0
4

0

0

0

0
4

0

0

0

0
4

0
4

0

0

0

0
4

0
4

0

0

0

0
4

0
4

0

0

0

0
4

0

0

0
4

0
4

0

0

0

0
4

0
4

0.254968

0.254968

0.254968

0
4

0
4

0

0

0

0
4

0
4

0.159766

0.0798828

0.0798828

0
4

0.0798828

0.0798828

0
4

0
4

0.27959

0.159766

0.159766

0
4

0.119824

0.119824

0
4

2.77555756156289e-17

0
4

0.154593

0.154593

0.154593

0
4

0

0

0
4

0
4

0

0

0

0
4

0

0

0
4

0
4

0
7

0
7

0
7

0

0
4

0

0

0
4

0
4

0.199707

0.0798828

0.0798828

0
4

0.119824

0.119824

0
4

0
4

2.59695

2.59695

2.59695

0
4

0
4

0

0

0

0
4

0

0

0
4

0
4

0

0

0

0
4

0

0

0
4

0
4

0

0

0

0
4

0
4

0

0

0

0
4

0
4

0

0

0

0
4

0
4

0.0798828

0.0798828

0.0798828

0
4

0
4

0

0

0

0
4

0
4

0

0

0

0
4

0
4

0
7

0
6

0

0

0

0

0

0

0
4

0

0

0
4

0

0

0
4

0

0

0
4

0
4

0

0

0

0
4

0
4

0

0

0

0
4

0
4

0

0

0

0
4

0
4

0

0

0

0
4

0
4

0.239648

0.239648

0.239648

0
4

0
4

0.137606

0.137606

0.137606

0
4

0
4

0

0

0

0
4

0
4

0.0798828

0.0798828

0.0798828

0
4

0
4

0

0

0

0
4

0
4

0.159766

0.159766

0.159766

0
4

0
4

0

0

0

0

0

0

0

0
4

0

0

0

0

0

0
4

0

0

0

0

0
4

0
4

0.0917376

0.0917376

0.0917376

0
4

0
4

0

0

0

0
4

0
4

0.119824

0.119824

0.119824

0
4

0
4

0

0

0

0
4

0
4

0.0507772

0.0507772

0.0507772

0
4

0
4

0

0

0

0
4

0
4

0

0

0

0
4

0
4

0

0

0

0
4

0
4

0.0533257

0.0533257

0.0533257

0
4

0
4

0

0

0

0
4

0
4

3.13515
5

3.04342

2.72388

0.239648

0.0798828

0
4

0

0

0

0
4

0

0

0
4

0.0917376

0.0917376

0
4

0

0

0
4

0

0

0
4

5.82867087928207e-16
5

0
4

0

0

0

0
4

0
4

0

0

0

0
4

0
4

0

0

0

0
4

0
4

0

0

0

0
4

0
4

0.175085

0.175085

0.175085

0
4

0
4

0

0

0

0
4

0
4

0

0

0

0
4

0
4

0

0

0

0
4

0
4

0

0

0

0
4

0
4

0

0

0

0
4

0
4

0
4

0
4

0
6

0

0

0

0
4

0

0

0

0

0
4

0
4

0.437713

0.437713

0.437713

0
4

0
4

0

0

0

0
4

0
4

0

0

0

0
4

0
4

0

0

0

0
4

0
4

0

0

0

0
4

0
4

0

0

0

0
4

0
4

0

0

0

0
4

0
4

0

0

0

0
4

0
4

0

0

0

0
4

0
4

0

0

0

0
4

0
4

2.56549
4

2.36578
4

1.4523

0

0.439355

0.159766

0.154593

0.159766

0
4

0.199707

0.119824

0.0798828

0
4

0
4

0

0

0

0
4

0
4

0.154593

0.154593

0.154593

0
4

0
4

0

0

0

0
4

0
4

0.0917376

0.0917376

0.0917376

0
4

0
4

0

0

0

0
4

0
4

0

0

0

0
4

0
4

0.0798828

0.0798828

0.0798828

0
4

0
4

0

0

0

0
4

0
4

0.119824

0.119824

0.119824

0
4

0
4

0

0

0

0
4

0
4

7.23137
4

6.44714
4

6.36726
4

0

0.0798828

0

0

0
4

0.297372

0

0.159766

0.137606

0
4

0

0

0

0
4

0.254968

0.0798828

0.175085

0
4

0.23189

0.23189

0

0
4

0

0

0
4

0

0

0
4

1.66533453693773e-16
4

0
4

0.195427
4

0.195427
4

0.195427
4

0

0

0
4

0

0

0

0
4

0

0

0
4

0
4

0

0

0

0
4

0
4

0

0

0

0
4

0
4

0

0

0

0
4

0
4

0

0

0

0
4

0
4

0.0488567

0.0488567

0.0488567

0
4

0
4

0

0

0

0
4

0
4

0

0

0

0
4

0
4

0

0

0

0
4

0
4

0

0

0

0
4

0
4

0

0

0

0
4

0
4

0

0

0

0

0

0
4

0

0

0

0

0
4

0

0

0

0
4

0
4

0

0

0

0
4

0
4

0

0

0

0
4

0
4

0.0732851

0.0732851

0.0732851

0
4

0
4

0

0

0

0
4

0
4

0

0

0

0
4

0
4

0

0

0

0
4

0
4

0.159766

0.159766

0.159766

0
4

0
4

0

0

0

0
4

0
4

0

0

0

0
4

0
4

0

0

0

0
4

0
4

4.39487

4.39487

3.94213

0.27959

0.119824

0.0533257

0

0

4.85722573273506e-17

0
4

0

0

0

0

0
4

0
4

0.107242

0.107242

0.107242

0
4

0
4

0

0

0

0
4

0
4

0

0

0

0
4

0
4

0.219855

0.219855

0.219855

0
4

0
4

0

0

0

0
4

0
4

0

0

0

0
4

0
4

0

0

0

0
4

0
4

0

0

0

0
4

0
4

0

0

0

0
4

0
4

0

0

0

0
4

0
4

0.519238
4

0.0798828

0

0

0.0798828

0

0
4

0.319531

0.239648

0

0.0798828

0
4

0.119824

0.119824

0
4

0

0

0
4

0
4

0

0

0

0
4

0
4

0

0

0

0
4

0
4

0

0

0

0
4

0
4

0

0

0

0
4

0
4

0.0533257

0.0533257

0.0533257

0
4

0
4

0

0

0

0
4

0
4

0

0

0

0
4

0
4

0

0

0

0
4

0
4

0

0

0

0
4

0
4

0

0

0

0
4

0
4

1.14513
4

1.0918

0.55918

0.0533257

0.479297

5.55111512312578e-17

0
4

0.0533257

0.0533257

0

0
4

0

0

0
4

0

0

0
4

4.85722573273506e-17
4

0
4

0.119824

0.119824

0.119824

0
4

0
4

0

0

0

0
4

0
4

0

0

0

0
4

0
4

0.0798828

0.0798828

0.0798828

0
4

0
4

0.106651

0.106651

0.106651

0
4

0
4

0

0

0

0
4

0
4

0

0

0

0
4

0
4

0

0

0

0
4

0
4

0

0

0

0
4

0
4

0

0

0

0
4

0
4

7.53647
4

7.4603
4

7.4603
4

0

0
4

0

0

0
4

0.0761658

0.0761658

0
4

0
4

0.175085

0.175085

0.175085

0
4

0
4

0

0

0

0
4

0
4

0

0

0

0
4

0
4

0

0

0

0
4

0
4

0

0

0

0
4

0
4

0

0

0

0
4

0
4

0

0

0

0
4

0
4

0

0

0

0
4

0
4

0

0

0

0
4

0
4

0.159766

0.159766

0.159766

0
4

0
4

0

0

0

0

0
4

0

0

0

0
4

0

0

0
4

0

0

0
4

0
4

0

0

0

0
4

0
4

0.175085

0.175085

0.175085

0
4

0
4

0.350171

0.350171

0.350171

0
4

0
4

0

0

0

0
4

0
4

0.0723307

0.0723307

0.0723307

0
4

0
4

0.386483

0.386483

0.154593

0

0.23189

0
4

0

0

0
4

0

0

0
4

0
4

0.348144
4

0.0798828

0

0.0798828

0
4

0.108496

0.108496

0

0
4

0

0

0
4

0.0798828

0.0798828

0
4

0.0798828

0.0798828

0
4

0
4

0.0799885

0

0

0

0

0

0

0

0
4

0

0

0
4

0.0799885

0.0799885

0
4

0

0

0
4

0
4

14.09
4

13.9065
4

13.1853
4

0.35856

0.0799885

0.0533257

0.137606

0

0.0917376

0
4

0.183475

0.0917376

0.0917376

0
4

0
4

0

0

0

0
4

0

0

0

0

0
4

0

0

0

0
4

0

0

0
4

0
4

0.175085
3

0.175085
3

0

0.175085

0

0

0

0
4

0
4

0
3

0

0

0

0

0
4

0

0

0

0
4

0

0

0
4

0
4

0.0482775

0

0

0

0
4

0

0

0

0
4

0

0

0
4

0

0

0
4

0.0482775

0.0482775

0
4

0

0

0
4

0
4

2.46008
4

2.46008
4

0

2.46008

0

0

0

0
4

0
4

0.667766

0.534558

0.159766

0.175085

0

0.119824

0.0798828

0
4

0.0798828

0.0798828

0
4

0.0533257

0.0533257

0
4

2.0122792321331e-16

0
4

0.159766
3

0.0798828

0

0.0798828

0
4

0

0

0

0
4

0

0

0
4

0.0798828

0.0798828

0
4

0
4

0

0

0

0

0

0
4

0
4

0

0

0

0
4

0

0

0
4

0

0

0
4

0

0

0
4

0

0

0
4

0

0

0
4

0
4

0
6

0

0

0
4

0

0

0
4

0
4

0
7

0
7

0
7

0
6

0

0

0

0

0

0
4

0
4

0

0

0

0

0

0
4

0

0

0
4

0

0

0
4

0
4

0

0

0

0

0

0
4

0
4

0
3

0
3

0
3

0
4

0

0

0
4

0

0

0
4

0
4

0.175085

0

0

0

0

0
4

0.175085

0.175085

0

0

0
4

0

0

0
4

0
4

0.262628
5

0

0

0
4

0

0

0
4

0.262628

0.262628

0
4

0
4

0

0

0

0

0
4

0
4

0

0

0

0

0
4

0

0

0

0

0
4

0
4

0

0

0

0

0
4

0

0

0
4

0

0

0
4

0
4

0.159766
4

0

0

0
4

0

0

0
4

0

0

0
4

0.159766

0.159766

0
4

0

0

0
4

0
4

0
4

0

0

0

0
4

0

0

0
4

0

0

0
4

0

0

0
4

0
4

0
4

0
4

0

0

0

0

0

0

0

0

0
4

0
4

0

0

0

0

0

0
4

0

0

0

0
4

0

0

0

0

0
4

0

0

0
4

0

0

0

0
4

0

0

0
4

0

0

0
4

0

0

0
4

0
4

0

0

0

0

0
4

0
4

0.359473

0.119824

0.119824

0
4

0.0798828

0.0798828

0
4

0.0798828

0.0798828

0
4

0.0798828

0.0798828

0
4

0

0

0
4

0
4

0.261245
4

0.261245

0.261245

0

0
4

0

0

0
4

0
4

0.757245

0.494617

0.175085

0.319531

0
4

0.262628

0.262628

0
4

0

0

0
4

0

0

0
4

0
4

0.359473

0.0798828

0.0798828

0

0
4

0.119824

0.119824

0
4

0.159766

0.159766

0
4

0

0

0
4

0
4

0.679004

0.679004

0.439355

0.239648

0
4

0
4

0

0

0

0

0

0
4

0

0

0
4

0
4

0

0

0

0

0
4

0

0

0
4

0
4

0.321727

0.321727

0

0.214485

0.107242

0

1.38777878078145e-17

0
4

0

0

0
4

0
4

0

0

0

0

0
4

0

0

0
4

0
4

6.72918
4

6.48404
4

6.40787
4

0

0

0.0761658

0

0
4

0.245137

0.245137

0

0

0
4

5.55111512312578e-17
4

0
4

0

0

0

0

0
4

0

0

0

0
4

0

0

0
4

0
4

6.35068

0.399414

0.159766

0.239648

0
4

5.95127

5.95127

0
4

0

0

0
4

0
4

0

0

0

0

0
4

0

0

0
4

0

0

0
4

0
4

0.46378

0.154593

0.154593

0

0
4

0.309187

0.154593

0.154593

0
4

0
4

0

0

0

0

0
4

0

0

0
4

0

0

0
4

0
4

0

0

0

0
4

0

0

0
4

0

0

0
4

0
4

0.439673

0.239966

0.239966

0
4

0

0

0
4

0.199707

0.199707

0
4

0
4

0

0

0

0

0
4

0
4

0.46378

0.46378

0.46378

0
4

0

0

0
4

0
4

0

0

0

0
4

0

0

0
4

0

0

0
4

0

0

0
4

0
4

0
6

0
7

0
7

0

0

0

0
4

0
6

0
6

0

0

0

0
4

0
4

0

0

0

0
4

0

0

0
4

0

0

0
4

0
4

0

0

0

0
4

0
4

0

0

0

0
4

0

0

0
4

0

0

0
4

0
4

0

0

0

0
4

0

0

0
4

0
4

0

0

0

0

0
4

0

0

0
4

0
4

0

0

0

0

0
4

0

0

0
4

0
4

0

0

0

0
4

0
4

0

0

0

0
4

0

0

0
4

0

0

0
4

0
4

0

0

0

0

0
4

0

0

0
4

0
4

0

0

0

0

0
4

0
4

0
7

0
7

0
7

0

0

0

0
4

0

0

0
4

0
4

0.0798828

0.0798828

0.0798828

0
4

0
4

0

0

0

0

0
4

0
4

1.07842

1.07842

1.07842

0
4

0

0

0
4

0
4

0

0

0

0
4

0

0

0
4

0
4

0

0

0

0
4

0

0

0
4

0

0

0
4

0
4

0.159766

0.159766

0.159766

0

0
4

0

0

0
4

0
4

0

0

0

0

0
4

0
4

0

0

0

0
4

0

0

0
4

0
4

0.159766

0.159766

0.0798828

0.0798828

0
4

0

0

0
4

0
4

0

0

0

0

0
4

0

0

0
4

0
4

0.838769
4

0.758887

0.359473

0.399414

0

0
4

0

0

0

0

0
4

0.0798828

0.0798828

0

0
4

0

0

0

0
4

0

0

0
4

0

0

0
4

0

0

0
4

4.16333634234434e-17
4

0
4

0

0

0

0

0
4

0

0

0
4

0
4

0

0

0

0
4

0

0

0
4

0
4

0.175085

0.175085

0.175085

0
4

0
4

0

0

0

0

0
4

0

0

0
4

0
4

0

0

0

0
4

0
4

0.372504

0.372504

0.321727

0

0.0507772

0
4

0
4

0.154593

0.154593

0.154593

0
4

0

0

0
4

0

0

0
4

0
4

0

0

0

0
4

0

0

0
4

0

0

0
4

0
4

0.309187

0.309187

0

0.309187

0
4

0

0

0
4

0
4

0

0

0

0

0
4

0
4

2819.5
4

2809.88
4

75.0707
4

2054.09
4

524.956
4

0
7

0.759472

0

0

0

0.806136

0

0

0

0

0

0.262628

2.15086

0

0

0

0

0.27959

0.233051

0.531845

0

0.0808509

0.175085

0
7

0.12874

0

0.154593

0

0.107242

0

0.0761658

0

0

0.0799885

0
6

0

0

0

0

0

0

0

0

0

0

0
7

0.159977

0.175085

0

0

0.107242

0

0

0

0.772967

0

0
7

0.154593

0

0

0

0

0

0

0

0

0

0
7

0

0

0

0

0

0.154593

0

0.23189

0

0

1.43789

0.119824

0

0

0

0

0

0

0

0

0

0
6

0

0

0

0

0

0

0

0

0

5.33347

0
7

0

0

0

0.289421

0

0

0

0

0.154593

0

0
7

1.08496
4

0.0488567

0

0

0

0

0

0

0.108496

0

0

0
7

0

0.175085

0

0

0

0

0

0

0

0

0
6

0

0.0798828

0

0

0

0

0

0

0

0

0
7

0

0

0

0

0

0

0

0

0

0.0533257

0
7

0

0

0

0

0

0

0

0

0

0

0
3

0

0.175085

0

0.199707

0

0

0

0

0

0

0
6

0

0

0

0

0

0

0

0

0

0

0

0

0

0

0

0

0

0.119824

0

2.97645

0

0
6

0

0

0

0

0

0

0

0

0

0

0
6

0.0798828

0.319531

0

0

0

0.55918

0.0798828

0

0.289421

0

0
7

0

0

0

0.0798828

0

0

0

0

0

0

0

0
6

0

0.154593

0

0

0

0.199707

0.386483

0

0

0

2.73437

0.289421

0

0

0

0

0

0

0

0

0

0
3

0

0

0

0

0.101554

0

0.0732851

0

0

0

14.8983
4

0

0

0

0

0

0

0

0.0488567

0

0

0

0

0

0

0

0.289421

0

0.0488567

0.119824

0

0

0
6

0

0

0

0

0

0

0

0

0

0

0
7

0

0

0

0

0

0

0

0

0

0

0
7

0.309187

0

0

0.119824

0

0

0

0.289421

0

0

0

0

0

0

0

0

0.119824

0

0

0

0

48.0891
4

0
7

0

0

0

0

0

0.0798828

0.525256

0

0.0798828

0

0

1.27812

0

1.0188

0

0.934538

0

0

0

0
7

0

0

0

0

0.226924

0

0

0.772967

0.772409

0.745178

0
3

0

0.341997

0.411563

0

0

0

0.519238

0

0.415282

1.19824

0
7

0

1.54593

0

0

0.338278

0

0

2.55625

0.0761658

0

0
7

0

0.531845

0

0

0.612799

0.137606

0

0

0

0.823126

49.9376
4

0

0

0

0

0

0

0

0

0

0.258862

5.26023669067399e-13
4

0
4

0
7

0
7

0

0

0

0
4

0.772967

0.772967

0

0

0
4

0.0798828

0.0798828

0
4

0

0

0
4

0

0

0
4

0

0

0
4

0

0

0
4

0

0

0

0

0

0
4

0

0

0

0
4

0

0

0

0
4

0

0

0

0
4

0

0

0

0

0
4

0

0

0

0
4

0

0

0
4

0.0488567

0.0488567

0

0
4

0

0

0
4

0

0

0

0

0

0
4

0

0

0

0
4

0

0

0
4

0

0

0
4

0

0

0

0
4

0

0

0

0
4

0

0

0
4

0.154593

0

0.154593

0
4

1.23818

1.23818

0

0
4

0

0

0

0
4

0

0

0

0
4

0
7

0

0

0

0
4

0

0

0

0
4

0

0

0
4

0.679004

0.0798828

0.599121

0
4

0

0

0
4

0

0

0
4

0

0

0
4

0

0

0
4

0.0798828

0.0798828

0
4

0

0

0
4

0

0

0
4

0
6

0
6

0
4

0.159766

0.159766

0
4

0

0

0
4

0

0

0
4

0

0

0
4

0

0

0
4

0

0

0
4

0.437713

0.437713

0
4

0

0

0
4

0

0

0
4

0

0

0
4

0
6

0
6

0
4

0.268106

0.268106

0
4

0

0

0
4

0

0

0
4

0

0

0
4

0

0

0
4

0

0

0
4

0.121276

0.121276

0
4

0

0

0
4

0

0

0
4

0.0798828

0.0798828

0
4

0.122142
4

0.122142
4

0
4

0.154593

0.154593

0
4

0

0

0
4

0.350171

0.350171

0
4

0

0

0
4

0

0

0
4

0.154593

0.154593

0
4

0

0

0
4

0

0

0
4

0

0

0
4

0

0

0
4

0

0

0

0

0
4

0

0

0
4

0

0

0
4

0.199707

0.199707

0
4

0

0

0
4

0

0

0
4

0

0

0
4

0

0

0
4

0

0

0
4

0

0

0
4

0

0

0
4

0

0

0

0

0
4

0

0

0
4

0

0

0
4

0

0

0
4

0.154593

0.154593

0
4

0

0

0
4

0

0

0
4

0

0

0
4

0.160864

0.160864

0
4

0.0917376

0.0917376

0
4

0

0

0
4

0

0

0

0
4

0.119824

0.119824

0
4

0

0

0
4

0

0

0
4

1.11836

1.11836

0
4

0

0

0
4

0

0

0
4

0

0

0
4

2.68106

2.68106

0
4

0.199707

0.199707

0
4

0

0

0
4

1.95288230031565e-12
4

0
4

0
4

2292.53
4

2269.78
4

0

0

0

0

0

0
4

2211.76
4

182.041
4

767.683
4

6.77425
3

273.195
4

101.972
4

690.098
4

141.084
4

47.8737
4

0.641684
3

0

0.119824

0.0798828

0.119824

0

0

0

0

0

0

0

0.0798828

8.32431346076135e-13
4

0
4

56.0709
3

18.0626
3

13.9365
3

18.0093
3

0.254019
4

0
4

0

0
4

0

0

0

0

0

0

0

0.273313
4

0

0

0

0

0

0

0

0

0

0

0
3

0

0

0

0

0

0

0

0

0

0

0.0798828
3

0

0

0

0

0

0

0

4.77545

0

0

0

0

0

0

0

0

0

0

0

0

0

0
3

0

0

0

0

0

0

0

0

0

0

0
4

0.154593

0

0

0

0.525256

0

0

0

0

0

0
4

0
4

0
4

0.787884
5

0.787884
5

0
4

0

0

0
4

0

0

0
4

0

0

0
4

0

0

0
4

0

0

0
4

0

0

0
4

0

0

0
4

0.0649455

0.0649455

0
4

0

0

0
4

0

0

0
4

0

0

0

0
4

0

0

0
4

0

0

0
4

0

0

0
4

0

0

0
4

0

0

0
4

0.0974182

0.0974182

0
4

0

0

0
4

0

0

0
4

0

0

0
4

0

0

0

0
4

0

0

0
4

0

0

0
4

0

0

0
4

0

0

0
4

0

0

0
4

0.998535

0.998535

0
4

0
4

1.68393
4

1.46439
4

0.386483

0.394242

0.159766

0

0.154593

0.289421

0

0.0798828

2.63677968348475e-16
4

0
4

0.0649455

0.0649455

0

0
4

0.154593

0.154593

0
4

0
4

0.26071
4

0.26071

0.180827

0.0798828

0

0
4

0

0

0
4

0
4

0.0917376
4

0

0

0
4

0.0917376

0.0917376

0
4

0

0

0
4

0

0

0
4

0
4

0

0

0

0
4

0

0

0

0
4

0

0

0
4

0
4

0.159766

0.159766

0.159766

0
4

0

0

0
4

0
4

0

0

0

0

0

0

0
4

0
4

0.965181

0.965181

0.965181

0
4

0

0

0
4

0

0

0
4

0
4

0

0

0

0
4

0

0

0
4

0

0

0
4

0
4

0.23189

0.23189

0.23189

0

0
4

0
4

0

0

0

0

0

0
4

0
4

0

0

0

0
4

0
4

2.3846
4

1.6464
4

1.41451
4

0.23189

0
4

0.738198

0.738198

0

0
4

0

0

0
4

0
4

0.0798828

0

0

0
4

0.0798828

0.0798828

0
4

0

0

0
4

0
4

0

0

0

0
4

0

0

0
4

0

0

0
4

0
4

0

0

0

0

0
4

0
4

0

0

0

0

0
4

0

0

0
4

0
4

0.199707

0.199707

0.199707

0

0
4

0
4

0.203109

0.203109

0

0.203109

0
4

0

0

0
4

0
4

0.23189

0.23189

0.23189

0
4

0
4

0

0

0

0
4

0
4

0.154593

0.154593

0

0.154593

0
4

0
4

0

0

0

0
4

0
4

0.527575
4

0.407751
4

0.154593
4

0.253157

0

0
4

0

0

0

0
4

0.119824

0.119824

0
4

0
4

0

0

0

0
4

0
4

0

0

0

0
4

0
4

0

0

0

0
4

0
4

0

0

0

0

0
4

0
4

0

0

0

0
4

0
4

0

0

0

0
4

0

0

0
4

0
4

0

0

0

0

0
4

0
4

0.145063

0.145063

0.0533257

0.0917376

0
4

0
4

0

0

0

0
4

0

0

0
4

0
4

0

0

0

0
4

0
4

0
5

0

0

0

0
4

0

0

0

0

0

0
4

0
4

0

0

0

0
4

0
4

0

0

0

0
4

0
4

11.1179

11.1179

11.1179

0
4

0
4

0

0

0

0
4

0
4

0.0798828

0.0798828

0.0798828

0
4

0
4

0

0

0

0
4

0
4

0

0

0

0
4

0
4

0

0

0

0
4

0
4

0

0

0

0
4

0
4

0.202127

0.202127

0.202127

0
4

0
4

0.410608
4

0.410608
4

0.361752
4

0.0488567

0

0
4

0

0

0
4

0

0

0
4

0
4

0.986525

0.986525

0.986525

0
4

0
4

0

0

0

0
4

0
4

0

0

0

0
4

0
4

0

0

0

0
4

0
4

0

0

0

0
4

0
4

0.0533257

0.0533257

0.0533257

0
4

0
4

0

0

0

0
4

0
4

0

0

0

0
4

0
4

0.119824

0.119824

0.119824

0
4

0
4

0

0

0

0
4

0
4

0
4

0
4

0

0

0

0

0
4

0
4

0

0

0

0
4

0
4

0

0

0

0
4

0
4

0

0

0

0
4

0
4

0.119824

0.119824

0.119824

0
4

0
4

0

0

0

0
4

0
4

0.0798828

0.0798828

0.0798828

0
4

0
4

0

0

0

0
4

0
4

0

0

0

0
4

0
4

0

0

0

0
4

0
4

0

0

0

0
4

0
4

0.23189

0.23189

0.23189

0

0
4

0

0

0
4

0

0

0
4

0

0

0
4

0
4

0

0

0

0
4

0
4

0.154593

0.154593

0.154593

0
4

0
4

0

0

0

0
4

0
4

0.0507772

0.0507772

0.0507772

0
4

0
4

0

0

0

0
4

0
4

0

0

0

0
4

0
4

0

0

0

0
4

0
4

0.0649455

0.0649455

0.0649455

0
4

0
4

0

0

0

0
4

0
4

0

0

0

0
4

0
4

0.599121

0.599121

0.319531

0

0.27959

0
4

0

0

0
4

0
4

0

0

0

0
4

0
4

0

0

0

0
4

0
4

0

0

0

0
4

0
4

0.160864

0.160864

0.160864

0
4

0
4

0

0

0

0
4

0
4

0

0

0

0
4

0
4

0

0

0

0
4

0
4

0

0

0

0
4

0
4

0.0732851

0.0732851

0.0732851

0
4

0
4

0

0

0

0
4

0
4

0.319531
4

0.319531
4

0.319531
4

0

0
4

0
4

0

0

0

0
4

0
4

0.506914

0.506914

0.506914

0
4

0
4

0

0

0

0
4

0
4

0

0

0

0
4

0
4

0

0

0

0
4

0
4

0

0

0

0
4

0
4

0

0

0

0
4

0
4

0

0

0

0
4

0
4

0.097998

0.097998

0.097998

0
4

0
4

1.06997743998249e-13
4

0
4

19.4944
5

0

0

0

0

0

0
4

0

0

0
4

0
4

0

0

0

0
4

0
4

0

0

0

0
4

0
4

0

0

0

0
4

0
4

0

0

0

0
4

0
4

0

0

0

0
4

0
4

0

0

0

0
4

0
4

0

0

0

0
4

0
4

0

0

0

0
4

0
4

0

0

0

0
4

0
4

0

0

0

0
4

0
4

9.98227
4

6.02807
4

0.0798828

5.499

0.289421

0.159766

0
4

3.63467

0

3.63467

0
4

0.319531

0.319531

0
4

0
4

0

0

0

0
4

0
4

0

0

0

0
4

0
4

0

0

0

0
4

0
4

0
6

0
6

0

0

0

0
4

0
4

0
7

0
7

0
7

0
4

0
4

0
6

0
6

0
6

0

0
4

0
4

7.23552

7.23552

7.23552

0
4

0

0

0
4

0
4

0

0

0

0
4

0
4

0

0

0

0

0
4

0
4

0.439355

0.439355

0.439355

0
4

0
4

1.8373

0
5

0

0

0

0

0

0

0

0
4

0
7

0

0

0
4

1.59766

1.59766

0
4

0

0

0
4

0

0

0
4

0

0

0
4

0

0

0
4

0

0

0

0
4

0.239648

0

0.239648

0
4

0

0

0
4

0

0

0
4

0

0

0
4

0

0

0
4

0

0

0
4

0

0

0
4

0
4

0
4

0
4

13.2047

12.6792

5.07318

4.41528
3

4.41528
3

0

0

0

0

0

0
4

0.657903
3

0.106651
3

0

0

0

0

0.146997

0

0

0

0
2

0.404255
3

0

0

0

0

0

0

0
4

0

0

0

0

0
4

0
7

0

0

0

0

0

0

0

0

0

0

0
6

0

0

0

0

0

0

0

0
4

0

0

0
4

0

0

0
4

0

0

0
4

4.44089209850063e-16

0
4

3.21638
3

2.77538

1.46906

1.30633

2.22044604925031e-16

0
4

0

0

0

0

0

0
4

0

0

0

0

0
4

0.440991

0.440991

0
4

0
4

0

0

0

0
4

0
4

0

0

0

0
4

0
4

0.097998

0.097998

0.097998

0
4

0
4

0

0

0

0
4

0

0

0
4

0
4

0.930981
3

0.930981
3

0.930981

0

0

0
4

0

0

0

0
4

0

0

0
4

0
4

0

0

0

0

0
4

0
4

0
2

0
2

0
2

0

0
4

0
4

0.405475

0.405475

0.371311

0.0341641

2.08166817117217e-17

0
4

0
4

2.95519

2.95519

2.95519

0
4

0
4

0

0

0

0
4

0
4

0

0

0

0
4

0
4

1.33226762955019e-15

0
4

0.525531
2

0.525531
2

0.282978
2

0.282978

0

0

0

0

0
4

0.242553
3

0.161702

0.0808509

0

0

0

0
4

0

0

0
4

0
4

0
4

0

0

0

0

0
4

0
4

0
4

0

0

0

0

0

0
4

0
4

0
4

0

0

0

0

0

0

0
4

0
4

0
4

0

0

0

0

0
4

0
4

0
4

0

0

0

0

0
4

0
4

0
4

0
4

379.939

365.625

135.814
3

135.814
3

38.2678

0

0.0341641

0

0

0

0

0

0

0.430455

0

0.632036
3

0

0.0683282

0

0.234589

0

0

0.0854102

0

0

0

1.19574
3

0.0512461

0

0

0

0

0

0.0341641

0

0

0

0.187902
3

0

0

0.102492

0

0

0

0.290395

0

0.0683282

0

0

0

0

0

0

0

0

0

0

0.0854102

0

66.567
4

0

0

0

27.4781
4

0

2.48689957516035e-14
3

0
4

0
4

0
3

0
3

0
1

0

0

0

0

0

0

0

0

0

0

0

0

0

0

0

0

0

0

0

0

0

0
4

0

0

0

0

0

0

0

0

0
4

0

0

0

0

0

0
4

0
4

72.4364
6

72.4364
6

0
6

1.40863

0

0

0

0

0
7

0

0

0.404255

0.334224

29.4145
5

0

0

0

0

0.121276

0.121276

0

0

0.121276

0

35.8917
5

0.242553

0.0808509

0

0

0.0808509

0

0

0.121276

0

0.0808509

0
6

0

0.121276

0.121276

0

3.77039

0
6

0
6

0
7

0
6

0
4

0

0

0
4

0
4

0
7

0
7

0
7

0
7

0
4

0
4

25.5576
3

7.33731
3

0

0

0

0

0

0

0

0

0

0

0

0

3.55441

0

0

0.533161

0

0

0

0

0

0

3.02124

0

0

0

0

0

0

0

0

0

0

0

0

0

0

0

0

0

0

0

0

0

0

0

0

0

0

0

0

0

0

0

0

0

0

0

0

0

0

0

0

0

0

0

0

0

0

0

0

0

0

0.17772

0

0

0

0

0

0

0

0

0

0

0

0.0507772

0

0

0

1.18655085756814e-15
3

0
4

4.50465

4.04714

0.0965551

0.288546

0.0724163

6.10622663543836e-16

0
4

0

0

0
4

0

0

0
4

6.74664

2.15571

3.35636

1.23457

0
4

6.93486

6.93486

0
4

0

0

0

0
4

0

0

0

0
4

0

0

0
4

0

0

0
4

0.0341641

0.0341641

0
4

0

0

0
4

0
4

62.052
3

6.90865
3

6.90865

0

0
4

49.7053
3

19.1455
4

9.86382

0

0

0.0482775

3.71537

0

1.27935

0

0

0

0
2

0.0808509

0

0.0808509

0

0.161702

0

0.0808509

0.242553

0.0808509

0

1.85957

0

0.202127

0

0

0

0

0

0

0

0.121276

9.05172
4

0

0

0.0808509

0

0

0

0

0

0

0

0

0

0

0

0.0808509

0

0

0.0808509

0.0482775

0

0.0808509

0.808509
4

0

0

1.30349

0.144833

0

0

0

0

1.06211

1.99840144432528e-15
3

0
4

0.820718

0.362081

0.458636

5.55111512312578e-17

0
4

0

0

0
4

0

0

0
4

2.5004

2.5004

0

0

0
4

0

0

0

0

0

0

0

0

0

0

0

0

0
4

0

0

0

0

0
4

2.11689

0.958594

0

0

0

0

0

0

0

0

0

1.1583

0

0

0

0

2.22044604925031e-16

0
4

0

0

0
4

0

0

0
4

4.44089209850063e-15
3

0
4

33.2874
5

0
6

0
6

0
4

33.2874
5

33.2874
5

0
4

0
7

0

0

0

0

0

0

0

0

0

0

0

0

0

0

0

0

0

0

0

0

0

0

0

0

0

0

0

0

0

0

0

0

0

0

0

0

0

0

0

0

0

0

0

0

0

0

0

0

0

0

0

0

0

0

0

0

0

0

0

0

0

0

0

0

0

0

0

0

0

0

0

0

0

0

0

0

0

0

0
4

0

0

0

0
4

0

0

0
4

0

0

0
4

0

0

0
4

0
4

8.56923
3

8.56923
3

8.56923
4

0

0

0

0

0

0

0

0

0

0
4

0
4

20.0491
3

19.7699
3

16.7801
3

0

0

0.0507772

0

0

2.58964
3

0.0507772

0

0.0917376

0.0798828

0

0.126943

0

4.57966997657877e-15
3

0
4

0

0

0
4

0.279275

0.279275

0
4

5.55111512312578e-17
3

0
4

0

0

0

0
4

0
4

0

0

0

0
4

0
4

0

0

0

0
4

0
4

0

0

0

0
4

0
4

0

0

0

0
4

0
4

0

0

0

0
4

0
4

0

0

0

0
4

0
4

0

0

0

0
4

0
4

0.217249

0.217249

0.217249

0
4

0
4

0

0

0

0
4

0
4

7.64197
3

7.64197
3

7.64197
3

0
4

0
4

0

0

0

0

0

0

0

0
4

0
4

0

0

0

0

0
4

0
4

0

0

0

0

0
4

0

0

0
4

0
4

0

0

0

0
4

0

0

0
4

0
4

0

0

0

0
4

0
4

0

0

0

0

0
4

0
4

0

0

0

0
4

0

0

0
4

0
4

0
4

13.8068
3

13.8068
3

13.2228
3

11.801
3

0.0761658
3

1.23846

0

0

0.107242

1.52655665885959e-16
3

0
4

0.0507772

0.0507772

0
4

0.533161

0.533161

0

0
4

0
4

0
4

0

0

0

0

0

0

0
4

0

0

0
4

0
4

0
4

0

0

0

0

0

0
4

0
4

0
4

0

0

0

0

0

0
4

0
4

0
4

0

0

0

0

0

0
4

0
4

0
4

0

0

0

0

0
4

0

0

0
4

0
4

0
4

0

0

0

0

0
4

0
4

0
4

0

0

0

0

0

0
4

0
4

0
4

0

0

0

0

0
4

0
4

0
4

0

0

0

0

0
4

0
4

0
4

0

0

0

0

0
4

0
4

0
4

0.507772
3

0.507772
3

0.507772
3

0.507772
3

0

0

0

0
4

0
4

0
4

0

0

0

0

0
4

0
4

0
4

0

0

0

0

0
4

0
4

0
4

0

0

0

0

0
4

0
4

0
4

0

0

0

0

0
4

0
4

0
4

0

0

0

0

0
4

0
4

0
4

0

0

0

0

0
4

0
4

0
4

0

0

0

0

0
4

0
4

0
4

0

0

0

0

0
4

0
4

0
4

0

0

0

0

0
4

0
4

0
4

0

0

0

0

0
4

0
4

0
4

0

0

0

0

0

0

0

0

0
4

0

0

0
4

0
4

0
4

0

0

0

0

0
4

0
4

0
4

0

0

0

0

0
4

0
4

0
4

0

0

0

0

0
4

0
4

0
4

0

0

0

0

0

0
4

0
4

0
4

0

0

0

0

0

0

0
4

0

0

0
4

0

0

0
4

0
4

0
4

0

0

0

0

0

0
4

0
4

0
4

0

0

0

0

0

0

0

0
4

0

0

0
4

0
4

0
4

0

0

0

0

0

0

0
4

0
4

0
4

0

0

0

0

0

0

0
4

0

0

0
4

0
4

0
4

6.07291994469961e-14

0
4

144.273
3

143.754
3

0
4

0
4

0
4

0
4

0
4

4.8019

4.8019

0.342993

0.146997

0.097998

0

0.146997

0.097998

0

0.195996

0

2.84194

0

0.293994

0.293994

0.097998

0.146997

0

0.097998

0
4

0

0

0

0

0

0

0

0

0

0

0

0

0

0

0
4

0

0

0

0
4

0
4

0

0

0

0
4

0
4

4.20205

2.18857

2.18857

0
4

2.01348

0

2.01348

0
4

0
4

3.57441

0
1

0
1

0
4

0

0

0
4

0.512461

0.512461

0
4

3.06195
4

2.47637

0.585585
4

0

0

0

0

0
4

0

0

0
4

0

0

0
4

0

0

0
4

0

0

0
4

4.44089209850063e-16

0
4

0
7

0
7

0
7

0
4

0
4

0.0649455
7

0.0649455
7

0.0649455
7

0

0

0

0
4

0
4

39.2571
4

39.2571
4

38.9479
4

0

0.154593

0.154593

0

0

0

0

0
4

0

0

0
4

0

0

0
4

0
4

1.39134
4

1.39134
4

1.00486

0.386483

0

0
4

0
4

0

0

0

0
4

0
4

0.319531

0.319531

0.319531

0
4

0
4

20.461
3

20.461
3

0

14.0594
3

0

6.07665
3

0

0

0

0

0

0.0854102

0

0

0

0

0

0

0

0

0.0533257

0

0

0

0

0

0

0

0

0

0

0

0

0.0649455

0

0

0

0

0

0

0.121276

0

0

0
4

0

0

0
4

0
4

69.6815
4

69.6815
4

38.5699
4

23.4699
4

3.04275
4

1.42097
3

3.02734

0.0533257

0

0.0974182

0

0
4

0
4

0

0

0

0

0
4

0
4

1.4210854715202e-14
3

0
4

0.239966

0.239966

0.239966

0.239966

0
4

0
4

0
4

0.27959

0.27959

0.27959

0.27959

0
4

0
4

0
4

3.61377594515488e-14
3

0
4

6023.93

129.214

30.6786

30.6786

30.6786

0
4

0
4

87.8652

87.8652
3

14.8543
3

0.107242

0.107242

0

0.107242

0

0

0

0

0

0

46.1055
3

0

0

0.107242

1.60864

0

0

0

0

10.2292
3

0.697075

1.23329

0

2.89554

9.81267

0

5.32907051820075e-15
3

0
4

0

0

0
4

0

0

0

0

0

0

0
4

0
4

10.6706

10.6706

10.6706

0

0

0

0

0

0

0

0

0

0

0

0

0

0

0

0

0

0

0

0

0
4

0

0

0

0

0

0

0
4

0
4

0

0

0

0
4

0
4

0

0

0

0
4

0
4

0
4

0
1

0

0

0

0

0

0

0

0

0

0

0

0

0

0

0
4

0

0

0

0

0

0

0
4

0

0

0
4

0

0

0
4

0
4

0

0

0

0
4

0
4

0

0

0

0
4

0
4

0

0

0

0

0

0

0

0

0
4

0

0

0
4

0

0

0
4

0
4

0
8

0
8

0

0

0

0
4

0

0

0
4

0
4

0

0

0

0

0

0
4

0

0

0
4

0
4

0

0

0

0
4

0

0

0
4

0
4

0

0

0

0

0

0
4

0

0

0
4

0
4

0

0

0

0

0

0
4

0
4

0

0

0

0
4

0

0

0
4

0
4

0

0

0

0
4

0
4

0
4

669.431
3

669.302
3

31.6722
3

7.6924

0

1.4471

1.36385

0.567604

0.584509

0

1.59182

0

0.0649455

0

2.52019

0

0

0

0

0

0

0.319531

0

0

0

0

0

0.0649455

0

2.02595

0

0

0

0

0

0.0649455

0

0

0

0

0

0

0

0

0

0

0

0

0

0

0

0

0

0

0

0

3.27089

0

0

10.0935

0
4

636.213
4

207.053
4

0.614877
4

0.0649455

0

0

0

0.227309

0.292255

2.72771

0

0

0

1.11846
4

0.974182

0

1.0716

0

0

0

0

0.0974182

0

0.162364

5.52057
4

0

0.199707

0

0.0798828

0

0

0

0

0

0

0.834141
4

0.194836

0

0

0

0.0649455

0.137606

0

0.119824

0

0

0.921893

0.0917376

0

0

0

0.0649455

0

0.519238

0

0.0649455

0

1.24504
4

0.0974182

0

0

0

0

0

0.0974182

0.106651

0

0.359473

0.0974182
4

9.77429

0.0798828

0

0

0

0

0

0

0.162364

0

0.7144
4

0

0

0

0.129891

0.0649455

0

0.0798828

0

0

0

15.9477
4

0

0

0.321081

0.0974182

0

0

0

0.159766

0

0

0.319047
4

0

0

1.31807

0.0917376

0.154593

0

0.0649455

0.479297

0

0.0798828

317.486
4

0.389673
4

0

0.239648

0

0

0.289421

0

0

0

0.119824

0.268106

0.162364
3

1.25637
4

0.194836
4

0
3

0.604317

0.988952
4

1.26644

0.159977

13.2013
4

0.0649455

1.23975
3

1.07842

0.921576

0.0649455

0.292255

0.948617

0

0.0649455

3.56628

4.05389
4

0.129891

0.0649455

0.0799885

0

0.259782

0.566974

0.0798828

0

0.162364

0.913762

5.05078
4

0

0

0.144661

0.159766

0.0723307

0

0

0

0.340158

0

13.0863
4

0

0

0

0

0

0.162364

0

0.199707

0

0

1.83899
4

0.249715

0.199707

0

0

0

0.159766

0.359473

0

0.0977135

0.63938

1.96399
4

0

0

0

0.0649455

0

0.0488567

0

0

0

0

1.01899
4

0

0

0.0798828

0

0

0.426605

3.06735

0

0.0799885

0.259782

0
4

0
2

0
2

0

0

0
4

0
2

0

0

0

0

0

0

0

0

0

0

0

0

0

0

0

0

0

0

0

0

0

0

0

0

0

0

0

0

0

0

0

0

0

0

0

0

0

0

0

0

0

0

0

0

0

0
4

0

0

0

0

0
4

0

0

0

0

0

0

0

0

0

0

0

0
4

0

0

0
4

0

0

0
4

1.41685

1.41685

0
4

0

0

0
4

0

0

0

0

0

0

0

0
4

0

0

0

0
4

0

0

0

0
4

0

0

0

0

0
4

0

0

0

0
4

0

0

0
4

0

0

0
4

0

0

0
4

1.11022302462516e-13
3

0
4

0.129891

0.0649455

0.0649455

0
4

0.0649455

0.0649455

0
4

0
4

0

0

0

0

0
4

0
4

0

0

0

0
4

0
4

0

0

0

0
4

0
4

0
4

93.1425
3

88.4796
3

77.4429
3

0
2

0

0

0

0.389673

0

1.11836

2.09123

0.844072

1.03848

0

0

2.43643

4.40991
3

0.909236

0

0.321727

0

0

0

0

7.39972

1.31807

0

7.85551

0

0

0

0

0

6.44287

0.342993

0

0

0

0

0.0798828

0

0

0

0

0

0

0

0

0

0

0

0.129891

0

0

0.857939

0

0

0

0

0

2.44995

0.160864

1.30239

0.097998

12.2256

0

0

0.596294

1.7695

0

0

8.57939

0

0

0.119824

0.107242

0

0

0

0

0

0

0.541077
4

0

0

0

0

0

0

0.0798828

0

0

0

10.0272

0.160864

0.160864

0

0.930981

0

0

0

0

0

0.146997

1.99285032920216e-14
3

0
4

3.43892
4

0.386483

3.05244
4

0

0
4

0

0

0
4

0

0

0
4

0

0

0
4

0

0

0

0
4

5.0008
4

5.0008
4

0
4

0

0

0
4

0

0

0

0
4

2.59695

2.59695

0
4

0

0

0
4

0

0

0
4

0

0

0
4

0
4

0

0

0

0

0

0

0
4

0

0

0
4

0
4

0

0

0

0
4

0

0

0
4

0
4

0

0

0

0

0

0
4

0
4

0

0

0

0
4

0
4

0

0

0

0
4

0
4

0

0

0

0
4

0
4

0

0

0

0
4

0
4

0

0

0

0
4

0
4

0

0

0

0
4

0
4

0

0

0

0
4

0
4

0

0

0

0
4

0
4

0

0

0

0

0
4

0

0

0
4

0
4

0

0

0

0
4

0
4

0.0798828

0.0798828

0.0798828

0
4

0
4

0.321727

0.321727

0.321727

0
4

0
4

0

0

0

0
4

0
4

0

0

0

0
4

0
4

0

0

0

0
4

0
4

0

0

0

0
4

0
4

0

0

0

0
4

0
4

0.106651

0.106651

0.106651

0
4

0
4

0.0533257

0.0533257

0.0533257

0
4

0
4

0

0

0

0

0

0

0
4

0

0

0
4

0
4

0.195996

0

0

0
4

0.195996

0.195996

0

0

0
4

0
4

0.832983

0.832983

0.734985

0.097998

0
4

0
4

0

0

0

0

0

0
4

0
4

0

0

0

0

0
4

0
4

3.07234

3.07234

3.07234

0
4

0
4

0

0

0

0
4

0
4

4.44089209850063e-15
3

0
4

0.812226

0
4

0
5

0

0

0

0

0

0

0

0

0

0

0

0
4

0

0

0

0
4

0

0

0
4

0
4

0.154593
7

0
8

0

0
8

0
4

0.154593
1

0
1

0

0

0.154593

0
4

0
1

0

0

0

0

0

0

0

0

0

0

0

0

0

0
4

0

0

0
4

0
7

0
7

0
7

0
7

0

0

0

0

0

0

0

0

0

0

0
7

0

0

0

0

0

0

0

0

0

0

0
7

0

0

0

0

0
7

0
7

0

0

0

0

0
4

0

0

0
7

0

0

0

0

0

0

0

0

0

0

0

0
4

0

0

0

0
4

0

0

0

0
4

0

0

0
4

0

0

0
4

0
4

0
7

0
7

0
7

0

0

0
4

0
7

0
6

0

0
4

0
4

0.55992

0
1

0

0

0

0

0

0

0

0

0

0

0

0

0

0

0

0

0

0

0

0

0

0

0

0

0

0

0

0

0

0

0

0

0

0

0

0

0

0

0

0

0

0

0

0

0

0

0

0

0

0

0

0

0

0

0

0

0

0

0
4

0

0

0

0

0

0

0

0

0

0

0

0

0
4

0

0

0

0

0

0

0

0

0

0

0
7

0

0
5

0

0

0

0

0
4

0

0

0
4

0.55992

0.55992

0
4

0
4

0

0

0

0

0

0

0
4

0

0

0
4

0
4

0

0

0

0
4

0
4

0

0

0

0
4

0
4

0

0

0

0
4

0
4

0

0

0

0
4

0
4

0

0

0

0

0
4

0

0

0
4

0
4

0

0

0

0

0

0
4

0
4

0

0

0

0

0
4

0

0

0
4

0
4

0

0

0

0
4

0
4

0

0

0

0
4

0
4

0

0

0

0

0
4

0
4

0

0

0

0
4

0
4

0.0977135

0.0977135

0.0977135

0
4

0
4

0
7

0
7

0
7

0
4

0
4

1.38777878078145e-17

0
4

4581.5
3

0
3

0

0

0
4

0
3

0
3

0

0

0

0
4

0
2

0
2

0
2

0
2

0

0

0

0

0

0
4

0

0

0

0
4

0

0

0

0
4

0

0

0

0
4

0

0

0
4

0

0

0
4

0

0

0
4

0

0

0
4

0
4

71.608
3

0
7

0
6

0

0
4

0
3

0
3

0
4

50.1131
3

49.3896
3

0.723552
2

2.1094237467878e-15
3

0
4

21.4949
3

0

14.2081
3

0

0

0

0

0

0.097998

0

4.34131

0

0

0

0

2.7495

0

0

0.097998

0

0

0

0

2.85882428840978e-15
3

0
4

0
3

0

0

0

0

0

0

0

0

0

0

0

0

0

0
7

0
7

0

0

0

0

0

0
4

0

0

0

0
4

0

0

0
4

0

0

0
4

0

0

0
4

0

0

0
4

3.5527136788005e-15
3

0
4

89.2001
3

4.53044

4.26218

0

0

0.0798828

0.0798828

0.108496

0
4

1.39795
5

0

0

0

0.119824

0

0

0

0

1.27812

0
4

3.61561

0

0
6

0

0

0
6

0

0

3.53573

0

0

0

0.0798828

0
4

77.6185
3

4.15565
3

39.2582
4

24.6903
4

0

0

0

0

0

7.9226
4

0

0

0

1.59182

0

0

0

1.28785870856518e-14
3

0
4

0

0

0

0
4

0

0

0

0
4

0

0

0
4

0

0

0
4

0

0

0
4

0

0

0
4

2.0376

2.0376

0

0

0
4

0

0

0

0
4

0

0

0
4

0

0

0
4

0

0

0
4

0

0

0
4

0

0

0
4

0

0

0
4

0
4

4209.36
4

0

0

0
4

3.90718

1.88124

2.02595

0
4

4201.98
4

2198.56
4

467.089
4

7.3724
4

1.01263

0.108496

0.259782

0

0

0

0.162364

0.194836

0

1.49375

6.67448
4

0

0.162364

0.129891

0.0723307

0.0649455

0.259782

0

0.3572

0.0649455

13.9308

96.0953
4

0

0

0.275213

0.129891

0.129891

0.229344

0.0799885

0.162364

0.0649455

0

2.66194
4

0

0

0.0649455

0.0974182

0.0798828

0

0

0.0533257

2.09793
4

2.39291

3.6079

2.79419
4

1.80685

5.78015

27.7222
4

4.71677

0.916952

10.179

11.3005

0

5.28832
4

0.722266
4

1.8636

0

5.18266

966.665
4

3.78969

0.573148

1.33138

0

1.23396

0.389673

0.679447

1.80623

0

3.65172

18.7829
4

6.00745

0.162364

0.259782

0

0.587988

0.313107

0.31441

1.12518

0.768084

0.649455

29.1494
4

5.0008

0

0.389673

0

4.15189

0.194836

0.324727

0.137606

178.697

0.336816

6.00825
4

0

0.194836

4.57949

1.07798

0

0.0917376

0.0533257

0.0974182

0.0533257

0

0.565957
3

0.0974182

0.137606

0.239648

0.0974182

0.54248

0

0.129891

0.795638

2.38518

56.0517

3.32884

0.0798828

0.0917376

0.292255

0.292255

0.649455

0.194836

0.0917376

0.584509

0

0

9.30875
4

0.0798828

0.0723307

0.183475

0.519564

0.262628

0.227309

0.412819

0.0974182

0

0.0723307

4.33744706818118e-12
4

0
4

0

0

0
4

0.881982

0.881982

0
4

0

0

0
4

0

0

0
4

0

0

0
4

0.389673

0.389673

0
4

0

0

0
4

0

0

0
4

0

0

0
4

0

0

0
4

0

0

0
4

0

0

0

0

0
4

0

0

0
4

0

0

0
4

0

0

0
4

0

0

0
4

0

0

0
4

0

0

0
4

0

0

0
4

0

0

0
4

0

0

0
4

0.0798828

0.0798828

0
4

0

0

0

0
4

0

0

0
4

0

0

0
4

0.0974182

0.0974182

0
4

0

0

0
4

0.144828

0.0798828

0.0649455

0
4

1.78888

1.78888

0
4

0

0

0
4

0.0917376

0.0917376

0
4

9.64034407857639e-13
4

0
4

120.247

0
6

0
6

0
6

0

0
6

0

0

0

0

0
4

4.60218
3

2.08754

0.267691

0

0

0

0

0

0

0

0

0

0

0.0965551

0

0

0

0

0

0

0

0

0

0

0

0

0

2.1504

0

0

0

0

0
4

9.60826
3

6.35379
3

0
2

0

0

0

0
2

0

2.71548

0

0

0

0.538989

0

0
4

0
2

0
2

0

0

0

0

0

0

0
2

0

0

0

0

0

0

0

0
4

27.6225

10.0938
3

0
7

4.39694
3

0

13.0337

0
2

0

0

0

0

0

0

0

0

0

0

0

0

0

0

0

0

0

0

0

0.097998

0

0

0

0

0

0

1.97064586870965e-15

0
4

19.1571

19.1571

0

0

0

0

0

0

0

0
4

0.391992
6

0
6

0

0

0

0

0

0

0

0

0

0

0

0

0

0

0

0

0

0

0

0

0

0

0

0

0

0

0

0

0

0

0

0

0

0.391992

0

0

0

0

0

0

0

0

0

0

0

0

0

0

0

0

0

0

0

0

0

0

0

0

0

0

0

0

0

0

0

0

0

0

0

0

0

0

0

0

0

0

0
6

0

0

0

0

0

0

0

0

0

0

0

0

0
4

11.5469
3

1.88062
3

0
2

0

0

0

0

0

0

0

0

0

0

0
3

0

0

0

2.2017

0

0

0

0

0

0

0
2

0

0

0

0

0

0

0

0

0

0

0

0

0

0

0

0

0

0

0

0

0

1.19434

0

0

0

0

0

0

0

0

0

0

0

0.144833

0

0

0

0

0

0

0

0

0

0

0

0

0.0917376

0

0
2

6.0337

8.88178419700125e-16
3

0
4

47.3178
3

0

0

7.23164
4

0

0

0.289421

0

0.0533257

0

0.578842

0

0

0

24.6118
4

0

0

0

0

0

0

13.5228
4

0

0

0.289421

0.740467

0

0

1.55431223447522e-15
3

0
4

0

0

0

0
4

0

0

0
4

0

0

0
4

0

0

0
4

0

0

0
4

0

0

0
4

0

0

0
4

0

0

0
4

0

0

0
4

0

0

0
4

0

0

0
4

0

0

0
4

0

0

0
4

0

0

0
4

0

0

0
4

0

0

0
4

0

0

0
4

0

0

0

0
4

0

0

0
4

0

0

0
4

0

0

0
4

0

0

0
4

0
4

1.46864
3

0

0

0
4

0.46378
3

0
2

0

0.46378

0

0

0
4

1.00486
3

1.00486
3

0

0

0

0

0

0

0

0

0

0
4

0

0

0
4

0

0

0
4

0

0

0
4

0

0

0
4

0

0

0
4

0

0

0

0
4

0

0

0
4

0

0

0
4

0

0

0
4

0

0

0
4

0

0

0
4

0
4

0

0

0

0

0
4

0
2

0

0

0

0
4

0

0

0
4

0

0

0
4

0

0

0
4

0
4

0
6

0
6

0
6

0

0

0

0
4

0
7

0
6

0
7

0

0

0

0

0

0

0

0
4

0
6

0

0

0

0

0

0

0

0

0
4

0
4

0

0

0

0
4

0
4

0

0

0

0
4

0

0

0
4

0
4

0

0

0

0

0

0
4

0

0

0
4

0
4

2.94098

2.94098

2.43643

0

0.504557

0
4

0

0

0
4

0

0

0
4

0
4

0.289421

0.289421

0

0.289421

0
4

0

0

0
4

0

0

0
4

0
4

0

0

0

0

0
4

0

0

0
4

0

0

0
4

0
4

0

0

0

0

0

0
4

0
4

0

0

0

0

0
4

0
4

0

0

0

0

0
4

0
4

0

0

0

0

0
4

0
4

2.2416
4

2.2416
4

2.2416
4

0

0

0
4

0

0

0
4

0
4

0

0

0

0

0
4

0
4

0

0

0

0
4

0
4

0

0

0

0

0
4

0
4

0

0

0

0
4

0
4

0

0

0

0

0
4

0
4

0

0

0

0
4

0
4

0

0

0

0
4

0
4

0

0

0

0

0
4

0
4

0

0

0

0

0
4

0
4

0.0341641

0.0341641

0.0341641

0
4

0
4

0.23189
3

0.23189
3

0.23189
3

0
4

0

0

0

0

0
4

0

0

0

0
4

0

0

0
4

0
4

0

0

0

0
4

0
4

0

0

0

0
4

0
4

0

0

0

0
4

0
4

0

0

0

0
4

0
4

0.239648

0.239648

0.239648

0
4

0
4

0

0

0

0
4

0
4

0

0

0

0
4

0
4

0

0

0

0
4

0
4

0

0

0

0
4

0
4

0

0

0

0
4

0
4

3.47835
3

3.47835
3

3.47835
3

0

0

0

0
4

0
4

0

0

0

0
4

0
4

0

0

0

0
4

0
4

0

0

0

0
4

0
4

0

0

0

0
4

0
4

0

0

0

0
4

0
4

0

0

0

0
4

0
4

0

0

0

0
4

0
4

0

0

0

0
4

0
4

0

0

0

0
4

0
4

0

0

0

0
4

0
4

0

0

0

0

0
4

0
4

0

0

0

0
4

0
4

0

0

0

0
4

0
4

0.504557

0.504557

0.504557

0
4

0
4

0.289421

0.289421

0.289421

0
4

0
4

0

0

0

0
4

0
4

0

0

0

0
4

0
4

0

0

0

0
4

0
4

0

0

0

0
4

0
4

0.292255

0.292255

0.292255

0
4

0
4

0

0

0

0
4

0
4

0

0

0

0

0

0

0
4

0
4

0

0

0

0
4

0
4

0
7

0
7

0

0

0
4

0
4

0

0

0

0
4

0
4

0
2

0
2

0

0

0
4

0
4

0

0

0
2

0
2

0

0

0
3

0
6

0

0

0

0
2

0

0
7

0

0
7

0

0

0

0

0

0

0

0

0

0

0
7

0

0

0

0

0

0

0

0

0

0

0

0

0

0

0

0

0

0

0

0

0

0
7

0

0

0

0

0

0

0

0

0

0

0

0

0

0

0

0

0

0

0

0

0

0
2

0

0

0

0

0
5

0
4

0
2

0
2

0

0

0

0

0

0
4

0

0

0

0
4

0

0

0
4

0

0

0
4

0
2

0
2

0

0

0
4

0

0

0

0

0
4

0
2

0

0

0

0
4

0

0

0
4

0

0

0
4

0

0

0

0
4

0

0

0
4

0

0

0

0
4

0
4

43.6144
4

43.6144
4

43.6144
4

0

0
4

0
7

0
7

0
7

0

0

0

0

0

0
4

0

0

0
4

0

0

0

0
4

0

0

0
4

0

0

0
4

0

0

0
4

0

0

0
4

0

0

0
4

0

0

0
4

0

0

0
4

0
4

0

0

0

0

0

0

0

0

0

0

0

0

0

0

0
4

0
3

0
3

0

0
4

0

0

0
4

0
4

1.86196

0.97998

0.97998

0
4

0

0

0
4

0

0

0
4

0.881982

0.881982

0
4

0

0

0
4

0

0

0
4

0

0

0
4

0
4

0

0

0

0

0

0

0

0

0

0

0

0

0

0

0

0

0

0
4

0

0

0
4

0

0

0
4

0

0

0
4

0

0

0
4

0
4

31.6904
3

28.2502
3

7.59391
3

0.107242
4

0.303238
3

0

0.107242
3

0.146997
4

0

0

0

0

0.097998

14.292
3

0

0

0

0

0

0

0

0

0

0

2.56644
3

0

0

0

0

0

0

0

0

0

0

0.440991
3

0

0

0

0

0

0

0

1.19909
3

0.48999
3

0.758096
3

0.146997
3

0

5.93969318174459e-15
3

0
4

0
2

0
2

0

0

0

0

0
4

0

0

0
4

0

0

0
4

0

0

0
4

0

0

0
4

0
2

0

0

0

0

0

0

0
4

3.34842

3.34842

0

0
4

0

0

0
4

0

0

0
4

0

0

0

0
4

0

0

0

0
4

0

0

0

0
4

0.0917376

0.0917376

0
4

0
4

1.91096
3

1.91096
3

0.881982
2

0

0

0

0

0

0

0

0

0

0

0
2

0

0

0

0

0

0

0

0

0

0

0
2

0

1.02898
2

0

0

0

0
4

0

0

0

0
4

0

0

0
4

0

0

0
4

0

0

0
4

0

0

0
4

0

0

0
4

0

0

0
4

0

0

0
4

0

0

0
4

0
4

0

0

0
2

0

0

0
4

0

0

0

0

0

0
4

0

0

0

0

0
4

0

0

0

0
4

0

0

0
4

0

0

0
4

0

0

0
4

0
4

0

0

0

0
4

0

0

0
4

0

0

0
4

0
4

4.19220214098459e-12
3

0
4

0
2

0
2

0

0

0

0

0

0

0
4

0
2

0
2

0
4

0

0

0
4

0

0

0
4

0

0

0
4

0
4

0

0

0

0
4

0
4

0
4

90.5281
4

0

0

0

0

0
4

0
4

0
2

0
2

0
2

0

0
2

0

0

0

0

0

0
4

0

0

0
4

0

0

0

0

0
4

0

0

0

0
4

0
4

0
4

0
4

0
7

0

0

0

0
7

0

0

0

0

0

0

0
4

0

0

0

0

0

0

0

0

0

0

0

0

0

0

0

0
4

0

0

0

0

0
4

0

0

0

0

0
4

0

0

0

0
4

0

0

0
4

0

0

0
4

0
4

0
7

0
7

0

0

0

0

0
4

0

0

0

0
4

0
4

0

0

0

0
4

0
4

0

0

0

0
4

0
4

0

0

0

0
4

0
4

0

0

0

0
4

0
4

0

0

0

0
4

0
4

0

0

0

0
4

0
4

0

0

0

0
4

0
4

0

0

0

0
4

0
4

0

0

0

0
4

0
4

0

0

0

0
4

0
4

5.63022
5

0
7

0
7

0
4

5.63022

5.63022

0
4

0

0

0
4

0
4

0

0

0

0
4

0
4

0.144661

0.144661

0.144661

0
4

0
4

0

0

0

0
4

0
4

0

0

0

0
4

0
4

0

0

0

0
4

0
4

0

0

0

0
4

0
4

0

0

0

0
4

0
4

0

0

0

0
4

0
4

0

0

0

0
4

0
4

0

0

0

0
4

0
4

0
7

0
7

0
7

0

0

0

0

0

0

0

0
4

0
4

0

0

0

0
4

0
4

0

0

0

0
4

0
4

0

0

0

0
4

0
4

0

0

0

0
4

0
4

0

0

0

0
4

0
4

0

0

0

0
4

0
4

0

0

0

0
4

0
4

0

0

0

0
4

0
4

0

0

0

0
4

0
4

0

0

0

0
4

0
4

0

0
7

0

0

0

0

0
4

0

0

0

0

0
4

0

0

0
4

0

0

0
4

0
4

0

0

0

0
4

0
4

0

0

0

0
4

0
4

0

0

0

0
4

0
4

0

0

0

0
4

0
4

0

0

0

0
4

0
4

0

0

0

0
4

0
4

0.0533257

0.0533257

0.0533257

0
4

0
4

0

0

0

0
4

0
4

0

0

0

0
4

0
4

0

0

0

0
4

0
4

0.107242

0

0

0

0

0

0
4

0

0

0

0
4

0

0

0

0
4

0.107242

0.107242

0
4

0

0

0
4

0

0

0
4

0

0

0
4

0
4

0

0

0

0
4

0
4

0

0

0

0
4

0
4

0.137606

0.137606

0.137606

0
4

0
4

0.27959

0.27959

0.27959

0
4

0
4

0

0

0

0
4

0
4

0

0

0

0
4

0
4

0

0

0

0
4

0
4

0

0

0

0
4

0
4

0

0

0

0
4

0
4

0

0

0

0
4

0
4

0

0

0

0

0

0
4

0
4

0

0

0

0
4

0
4

0

0

0

0
4

0
4

0
7

0
7

0
7

0

0
4

0

0

0
4

0

0

0
4

0
4

0.206297
5

0
7

0

0

0

0

0
4

0.206297

0.206297

0
4

0

0

0
4

0
4

0

0

0

0

0
4

0

0

0

0
4

0

0

0

0
4

0

0

0
4

0
4

5.70864

5.40346

4.70779

0.386483

0

0.309187

0
4

0.305175

0.0732851

0.23189

0
4

0
4

0

0

0

0

0

0

0

0

0

0

0

0

0

0

0

0

0

0

0

0

0
4

0

0

0

0

0

0

0
4

0
4

0.719897

0.719897

0.426605

0.293291

0
4

0
4

0

0

0

0

0

0

0
4

0

0

0
4

0
4

0.119824
4

0

0

0

0
4

0

0

0
4

0.119824

0.119824

0
4

0
4

0

0

0

0

0

0

0
4

0

0

0

0
4

0
4

0.0824416
3

0.0824416
3

0.0341641
3

0.0482775

0
4

0

0

0
4

0
4

0

0

0

0
4

0
4

0.107242
2

0

0

0
4

0.107242

0.107242

0
4

0

0

0
4

0
4

0

0

0

0

0
4

0

0

0
4

0
4

0
6

0

0

0

0

0
4

0

0

0

0
4

0
4

0

0

0

0

0

0
4

0
4

0.183475
4

0.0917376
4

0
4

0

0

0

0.0917376
4

0

0

0

0

0

0

0

0
4

0.0917376

0.0917376

0
4

0
4

0

0

0

0

0
4

0
4

0

0

0

0

0

0
4

0

0

0

0
4

0
4

0
5

0
5

0
5

0
4

0
4

0

0

0

0
4

0

0

0
4

0

0

0
4

0

0

0
4

0

0

0
4

0
4

0
2

0

0

0

0

0
4

0

0

0
4

0

0

0
4

0
4

0

0

0

0
4

0
4

0

0

0

0

0
4

0

0

0
4

0

0

0
4

0

0

0
4

0
4

0

0

0

0

0
4

0
4

6.91713

6.91713

0

0

6.91713

0
4

0
4

0

0

0

0
4

0
4

0
4

0

0

0

0

0

0

0

0

0

0
4

0

0

0

0

0

0
4

0

0

0

0

0
4

0

0

0
4

0

0

0
4

0
4

0

0

0

0
4

0
4

0

0

0

0

0
4

0

0

0
4

0
4

0

0

0

0

0
4

0
4

0

0

0

0

0
4

0
4

6.77236

0

0

0

0
4

6.77236

6.77236

0

0
4

0
4

0

0

0

0

0

0
4

0
4

0

0

0

0

0
4

0

0

0
4

0
4

0

0

0

0
4

0
4

0

0

0

0
4

0

0

0

0
4

0
4

0

0

0

0

0
4

0
4

0
6

0
6

0
6

0

0

0
4

0
4

0

0

0

0

0
4

0
4

0

0

0

0
4

0
4

0

0

0

0
4

0

0

0
4

0
4

0

0

0

0
4

0
4

0

0

0

0
4

0
4

0

0

0

0

0

0
4

0
4

0.750696

0.643454

0.643454

0
4

0.107242

0.107242

0
4

4.16333634234434e-17

0
4

0

0

0

0

0
4

0

0

0
4

0
4

0

0

0

0

0
4

0
4

0

0

0

0
4

0

0

0
4

0

0

0
4

0
4

1.29153
3

1.21068

0.296847

0.178849
3

0

0.734985

0
4

0.0808509

0

0.0808509

0
4

0

0

0
4

0

0

0
4

1.66533453693773e-16
3

0
4

0

0

0

0

0
4

0

0

0
4

0
4

0

0

0

0
4

0
4

0

0

0

0

0
4

0

0

0
4

0
4

0

0

0

0
4

0
4

0

0

0

0
4

0

0

0
4

0
4

0

0

0

0
4

0
4

0.87449

0.154593

0.154593

0
4

0.719897

0.719897

0
4

0
4

0

0

0

0
4

0

0

0
4

0
4

0

0

0

0
4

0

0

0
4

0
4

0.0649455

0.0649455

0.0649455

0
4

0
4

0
6

0
6

0
6

0

0
4

0
4

0

0

0

0
4

0
4

0

0

0

0

0
4

0
4

0

0

0

0
4

0

0

0
4

0
4

0.262628

0.262628

0.262628

0

0
4

0
4

0

0

0

0
4

0
4

0

0

0

0
4

0

0

0
4

0
4

0

0

0

0
4

0

0

0
4

0
4

0

0

0

0

0
4

0
4

0

0

0

0
4

0
4

0

0

0

0
4

0
4

0.132097
2

0.132097
2

0.0512461

0.0808509
2

0

0

0

0

0

0
4

0

0

0
4

0
4

0

0

0

0

0
4

0
4

0

0

0

0

0
4

0
4

0

0

0

0
4

0
4

0

0

0

0
4

0
4

0

0

0

0
4

0
4

0

0

0

0
4

0
4

0

0

0

0
4

0
4

1.48823

1.48823

1.48823

0
4

0
4

0

0

0

0
4

0
4

0

0

0

0
4

0
4

0

0

0
6

0

0

0

0

0
4

0

0

0

0

0
4

0

0

0
4

0

0

0
4

0
4

0

0

0

0
4

0
4

0

0

0

0
4

0
4

0

0

0

0
4

0
4

0

0

0

0
4

0
4

0

0

0

0
4

0
4

0

0

0

0
4

0
4

0

0

0

0
4

0
4

0

0

0

0
4

0
4

0

0

0

0
4

0
4

0

0

0

0
4

0
4

58.4935

49.3723

23.8058

11.0392

0.345337
2

9.8831
3

0
3

0

1.1583

0

0

0

0

0

0

0

0

0.275213
3

0.097998

0

0

0

0

0

0

0

0

0.0917376

0

0

0

0

0

0

0

0

0

0

0

0.321727

0

0

0

0

0

0

0

0

0

0

0.798828

0

0

0

0

1.55501

0

0
4

0

0

0

0
4

6.83049

6.83049

0
4

0

0

0

0
4

0

0

0
4

0

0

0
4

0

0

0

0
4

0

0

0
4

0

0

0
4

0

0

0
4

0

0

0

0
4

0

0

0
4

0

0

0
4

0

0

0
4

0

0

0
4

0

0

0
4

0

0

0
4

0

0

0
4

0.0533257

0.0533257

0
4

0

0

0
4

0

0

0
4

0

0

0
4

0

0

0
4

0

0

0

0

0

0

0
4

0

0

0
4

0

0

0
4

0

0

0
4

0.0488567

0.0488567

0
4

0

0

0
4

0

0

0
4

0

0

0
4

0

0

0
4

0

0

0
4

0

0

0
4

2.18857
3

2.18857
3

0
4

0

0

0
4

0

0

0
4

0

0

0
4

0

0

0
4

0

0

0
4

0

0

0
4

0

0

0
4

0

0

0

0
4

0

0

0
4

0

0

0

0
4

0

0

0

0
4

0

0

0

0

0
4

0
4

4.9737991503207e-14
4

0
4

0

0

0

0

0

0

0
4

0

0

0
4

0
4

0
4

115.823
3

13.6712
3

0.144833
3

0

0

0

0

0

0

0.0482775

0

0.0482775

0

0

0.0482775

0

0

0

0
4

8.59751
3

2.55871
3

0.313804
3

0

0

0
3

0

0

0
3

0

0

0

0.293994
3

0

0
2

0

0

0

0

0

0

0

0

0.440991
3

0

0

0

0

0

0

0.404255

0

0

0

0
3

0

0

0

0

0

0

0.0482775

0

0

0

2.72768
3

0

0

0

0

0

0

0

0

0

0

0.217249
3

0

0

0

0

0

0

0

0

0

0

0.293994
3

0

0

0

0

0

0

0

0

0

0

0.651747
3

0

0.646808

0

0

0

0

0

0

0

0

0
2

0

0

0

0

0

0

0

8.88178419700125e-16
3

0
4

1.34625
3

1.22498
3

0

0

0

0

0

0

0

0

0

0.121276

0
3

0

0

0

0

0

0

0

0

0

0
3

0
3

0
2

0

0

0

0

0
4

0
2

0
3

0

0

0

0

0

0

0
4

0

0

0

0
4

0

0

0
4

0

0

0
4

0

0

0
4

0

0

0

0
4

1.27269

1.27269

0
4

0

0

0
4

0

0

0
4

1.66226

1.66226

0
4

0

0

0
4

0
2

0
3

0

0

0

0

0

0
4

0

0

0
4

0.146997

0.146997

0
4

0

0

0
4

0

0

0
4

0

0

0
4

0

0

0
4

0

0

0
4

0

0

0
4

0

0

0
4

0

0

0
4

0

0

0
4

0

0

0
4

0

0

0
4

0

0

0
4

0

0

0
4

0.119824

0.119824

0
4

0

0

0
4

0

0

0
4

0.380829

0.380829

0

0
4

0

0

0

0
4

0

0

0

0

0

0

0

0

0
4

0

0

0

0
4

0

0

0

0
4

0

0

0

0

0
4

8.88178419700125e-16
3

0
4

74.6777
3

46.24
3

46.24
3

0

0

0

0

0

0

0

0

0
4

0
2

0

0

0

0

0

0

0

0

0

0

0
4

3.97345

3.97345

0

0

0
4

0

0

0

0
4

0

0

0

0
4

6.0169
2

0.0649455
3

0.321727
2

0
3

0

0

0

0

0

0

0

0

0

0

0

0

0

0

0

0

0

0

0

0

0

0.107242
2

0

0

0

0

0

0

0

0

0

0

0
2

0

0

0

0

0

0

0

0

0

0

0
3

0

0

0

0

0

0

0

0

0

0

0

0

0

0

0

0

0

0

0

0

0

0
2

0

0

0

0

0

0

0

0

0

0

0

0

0

0

0

0

0

0

0

0

0

0
2

0

0

0

0

0

0

0

0

0

0

0

0

0

0

0

0

0

0

0

0

0

3.8071
3

0

0

0

0

0

0

0

0

0

0

0

0
2

0

0

0

0

0

0
2

0
2

0

0

0

0

0

0

0

0

0
2

0

0

0

0

0
2

0.482591

0

0

0

0

0

0

0

0

0

1.12604
3

0

0

0

0

0

0

0

0

0

0

0
2

0

0

0

0

0

0

0

0

0

0

0.107242
3

0

0

0

0

0

0

0

0

0

0

0
2

0

0

0

0

0

0

0

0

0

0

0

0

0

0

0

0

0

0

0

0

0

0
4

14.2883
3

14.2883
3

0

0

0

0

0
4

0.742804
3

0

0

0.107242

0

0

0

0

0

0

0

0.229344

0

0.406218

0
4

0

0

0

0

0
4

0

0

0
4

0

0

0
4

0

0

0
4

0

0

0

0
4

0

0

0
4

0

0

0
4

3.41632

3.41632

0
4

0

0

0
4

0

0

0
4

0

0

0
4

0

0

0
4

0
4

1.22498

0

0

0

0

0

0

0

0

0

0
4

0

0

0

0

0

0

0

0

0

0

0

0

0

0

0

0

0

0

0

0

0

0

0

0

0

0

0

0

0

0

0

0

0

0

0

0

0

0

0

0

0

0

0

0

0

0

0

0

0

0

0

0

0

0

0

0

0

0

0

0

0

0

0

0

0

0

0

0

0

0

0

0

0

0

0

0

0

0

0

0

0

0

0

0

0

0
4

0

0
7

0

0

0
4

0

0

0
4

0

0

0
4

0

0

0
4

0

0

0
4

0

0

0
4

0

0

0
4

0

0

0
4

0

0

0
4

0

0

0
4

0

0

0
4

0

0

0
4

1.22498

0

1.22498

0

0
4

0
7

0
7

0
4

0

0

0

0
4

0

0

0
4

0

0

0

0

0
4

0

0

0
4

0

0

0
4

0
4

10.4822
3

6.45197
3

5.29189
3

0.0808509

0
3

0
2

0.097998
3

0.195996

0

0.097998

0.687233

0

0
4

0.521842
3

0.342993
3

0.0808509

0
2

0

0

0

0

0

0

0.097998

0
4

1.41023

1.2811

0.0808509

0.0482775

5.55111512312578e-17

0
4

0.342993
3

0

0

0.342993

0

0

0

0

0
4

0

0

0

0

0

0
4

1.29362

0

1.29362

0

0
4

0
2

0

0

0

0

0
4

0.265526

0

0.265526

0
4

0.195996

0.195996

0

0
4

3.02535774210355e-15
3

0
4

0

0

0

0
4

0

0

0

0
4

0
4

0

0

0

0

0
4

0

0

0
4

0
4

0

0

0

0
4

0
4

1.09171

1.09171

1.09171

0
4

0
4

2.0057

2.0057

0

2.0057

0
4

0
4

0

0

0

0
4

0
4

0

0

0

0
4

0
4

0

0

0

0
4

0
4

0

0

0

0

0
4

0
4

0

0

0

0
4

0
4

0

0

0

0

0

0

0

0

0

0

0

0

0

0

0

0

0

0

0

0

0

0

0

0

0
4

0

0

0

0

0
4

0

0

0
4

0
4

0

0

0

0
4

0
4

0.144661

0.144661

0.144661

0
4

0
4

0

0

0

0
4

0
4

0

0

0

0
4

0
4

0

0

0

0
4

0
4

0

0

0

0
4

0
4

0

0

0

0
4

0
4

0

0

0

0
4

0
4

0

0

0

0
4

0
4

0

0

0

0
4

0
4

0

0

0

0

0

0

0

0

0

0

0

0
4

0

0

0

0

0

0
4

0
4

0

0

0

0
4

0
4

0

0

0

0
4

0
4

0

0

0

0
4

0
4

0

0

0

0
4

0
4

12.1817
3

4.44125
3

0
3

0.097998

0

3.00922

1.33404

0

0

0

0

0
4

7.64385
3

7.64385
3

0

0
4

0

0

0
4

0.0965551

0.0965551

0
4

0

0

0
4

2.22044604925031e-16
3

0
4

0
3

0
3

0

0

0

0

0

0

0

0
4

0
3

0

0

0

0

0
4

0

0

0
4

0

0

0

0
4

0
4

0
2

0
2

0

0

0

0

0

0

0
4

0

0

0

0

0
4

0

0

0

0
4

0

0

0
4

0

0

0
4

0

0

0
4

0
4

0

0

0

0

0

0

0

0
4

0

0

0

0

0

0
4

0

0

0
4

0

0

0
4

0
4

0

0

0

0

0
4

0

0

0

0
4

0

0

0
4

0
4

0.342993

0.244995

0.244995

0
4

0.097998

0.097998

0
4

0
4

0

0

0

0
4

0
4

0
4

5.20314

0.107242

0

0

0

0

0
4

0

0

0
4

0

0

0
4

0.107242

0.107242

0
4

0
4

0

0

0

0
4

0

0

0
4

0

0

0

0

0

0

0
4

0

0

0

0

0

0

0

0

0
4

0
4

2.05796

0

0

0

0

0

0

0
4

0

0

0

0

0
4

0

0

0

0
4

0

0

0
4

2.05796

2.05796

0
4

0
4

0
5

0
5

0
5

0

0
4

0
4

0.097998

0

0

0

0
4

0
7

0
7

0
4

0
7

0
7

0
4

0
2

0
2

0

0

0

0

0

0

0

0

0

0

0

0

0

0

0
4

0

0

0

0

0

0
4

0

0

0
4

0

0

0
4

0

0

0

0

0
4

0

0

0
4

0

0

0

0

0
4

0

0

0

0

0
4

0

0

0
4

0

0

0

0
4

0

0

0
4

0
2

0
2

0

0

0

0

0

0

0
4

0

0

0

0
4

0

0

0
4

0

0

0

0
4

0

0

0
4

0

0

0
4

0

0

0

0
4

0

0

0

0
4

0

0

0

0
4

0

0

0
4

0

0

0
4

0
2

0
2

0

0

0
4

0

0

0
4

0

0

0
4

0

0

0
4

0

0

0
4

0

0

0
4

0

0

0
4

0

0

0
4

0

0

0
4

0.097998

0.097998

0
4

0

0

0
4

0
2

0

0

0

0

0

0

0

0

0
4

0

0

0
4

0

0

0
4

0

0

0
4

0

0

0
4

0

0

0
4

0

0

0
4

0

0

0
4

0

0

0
4

0

0

0
4

0

0

0
4

0
2

0

0

0

0

0

0

0
4

0

0

0

0
4

0

0

0

0

0
4

0
2

0
2

0
4

0

0

0

0

0

0

0
4

0
4

0
6

0
6

0
6

0
4

0

0

0
4

0
4

0

0

0

0
4

0
4

0

0

0

0
4

0
4

0

0

0

0
4

0
4

0

0

0

0
4

0
4

0
7

0
7

0
7

0
4

0
4

0.685986

0.685986

0.685986

0
4

0
4

1.95996

1.95996

1.12698

0.685986

0.146997

0
4

0
4

0.293994

0.293994

0

0.293994

0
4

0

0

0
4

0
4

0

0

0

0
4

0
4

0

0

0

0

0
4

0
4

0

0

0

0
4

0
4

0

0

0

0
4

0
4

0
4

0
6

0
6

0
7

0
7

0
6

0

0

0

0

0

0
6

0
6

0
7

0

0
7

0

0
6

0

0

0
6

0

0

0

0

0

0

0

0

0

0

0
7

0

0

0

0

0

0

0

0

0

0

0
6

0

0

0

0

0

0

0

0

0

0

0
6

0

0

0

0

0

0

0

0

0

0

0
6

0

0

0

0

0

0

0

0

0

0

0
6

0

0

0

0

0

0

0

0

0

0

0
7

0

0

0

0

0

0

0

0

0

0

0
6

0

0

0

0

0

0

0

0

0

0

0
4

0
6

0
6

0

0

0

0

0

0

0

0
4

0
6

0

0

0

0

0

0

0

0

0
4

0
7

0
7

0

0

0

0

0

0

0
4

0
6

0

0

0

0

0

0

0
4

0

0

0

0

0
4

0

0

0

0

0

0
4

0

0

0

0

0
4

0

0

0
4

0
4

0
4

0.321727

0.321727

0.321727

0

0

0

0

0.321727
5

0

0

0

0

0

0

0

0
4

0
2

0
2

0

0
4

0

0

0

0

0

0

0
4

0

0

0

0

0
4

0
4

0
7

0
7

0

0

0
4

0

0

0

0
4

0
4

0

0

0

0

0
4

0

0

0
4

0
4

0

0

0

0

0

0

0
4

0
4

0
4

0

0

0

0

0
4

0

0

0
4

0
4

0
4

0

0

0

0

0
4

0
4

0
4

0

0

0

0

0
4

0

0

0
4

0
4

0
4

0

0

0

0

0
4

0

0

0
4

0

0

0
4

0
4

0

0

0

0
4

0
4

0
4

0

0

0

0

0
4

0

0

0

0
4

0
4

0
4

0

0

0

0

0

0
4

0

0

0
4

0
4

0
4

0

0

0

0

0

0

0
4

0

0

0
4

0
4

0
4

0

0

0

0

0
4

0

0

0
4

0
4

0

0

0

0
4

0
4

0
4

0

0

0

0

0

0

0
4

0

0

0
4

0
4

0
4

0

0

0

0

0
4

0
4

0
4

0
6

0
6

0
6

0
6

0

0
4

0
6

0
7

0

0

0

0

0

0

0
4

0
6

0
7

0

0

0
4

0

0

0
4

0

0

0
4

0
4

0
4

1.82818

1.82818

1.82818

1.6531

0.175085

0
4

0
4

0
4

0

0

0

0

0
4

0
4

0
4

0

0

0

0

0
4

0
4

0
4

1.28691

1.28691

1.28691

1.28691

0
4

0

0

0
4

0
4

0
4

0

0

0

0

0

0
4

0

0

0
4

0
4

0
4

0

0

0

0

0
4

0
4

0
4

0

0

0

0

0
4

0

0

0
4

0
4

0

0

0

0
4

0
4

0
4

0

0

0

0

0

0

0
4

0
4

0
4

0

0

0

0

0
4

0

0

0
4

0
4

0
4

0

0

0

0

0

0
4

0
4

0
4

0
3

0
3

0
4

0
3

0

0

0

0

0

0

0

0

0

0

0
4

0
3

0

0

0

0

0
4

0

0

0
4

0
4

0
4

0

0

0

0

0
4

0
4

0
4

0

0

0

0

0
4

0
4

0
4

0

0

0

0

0
4

0

0

0
4

0

0

0
4

0
4

0
4

1.76396

1.76396

0

0

0
4

1.76396

1.76396

0
4

0
4

0
4

0

0

0

0

0

0
4

0
4

0
4

0

0

0

0

0
4

0

0

0
4

0
4

0
4

0

0

0

0

0
4

0
4

0
4

0

0

0

0

0
4

0
4

0
4

0.319531

0.319531

0.319531

0.319531

0
4

0

0

0
4

0
4

0
4

0

0

0

0

0
4

0
4

0
4

0.88275

0.129128

0.0482775

0
7

0

0

0.0482775

0

0

0

0

0

0
4

0

0

0

0

0
4

0

0

0

0

0
4

0.0808509

0.0808509

0
4

0

0

0
4

0
4

0.681291

0.265262

0

0.107242

0

0

0

0

0.0507772

0.107242

4.16333634234434e-17

0
4

0.308787

0.227936

0

0.0808509

0
4

0.107242

0

0.107242

0
4

0

0

0
4

0

0

0
4

4.16333634234434e-17

0
4

0

0

0

0
4

0

0

0
4

0
4

0.0723307

0.0723307

0.0723307

0
4

0
4

1.52655665885959e-16

0
4

0

0

0

0

0

0

0
4

0
4

0
4

0

0

0

0

0

0
4

0
4

0
4

0

0

0

0

0
4

0
4

0
4

0.175085

0.175085

0

0

0
4

0.175085

0.175085

0
4

0
4

0
4

0

0

0

0

0
4

0
4

0
4

1.49574

1.49574

1.49574

1.49574

0
4

0
4

0
4

0

0

0

0

0
4

0

0

0
4

0
4

0

0

0

0
4

0
4

0
4

0

0

0

0

0
4

0
4

0
4

0

0

0

0

0
4

0

0

0
4

0
4

0

0

0

0
4

0
4

0
4

0

0

0

0

0

0
4

0
4

0
4

17.0604

17.0604

2.53911

0.496603

2.04251

0

0

0

0
4

1.15729
5

1.15729
5

0

0

0
4

13.364

11.38

1.98398

0
4

0
4

0
4

0

0

0

0

0

0
4

0

0

0
4

0
4

0
4

1.22872

1.22872

1.22872

1.22872

0
4

0
4

0
4

0.3543

0.3543

0.199707

0.199707

0
4

0.154593

0.154593

0
4

0
4

0
4

0.144661

0.144661

0.144661

0

0

0.144661

0
4

0
4

0
4

0

0

0

0

0
4

0
4

0
4

0

0

0

0

0
4

0

0

0
4

0
4

0
4

0

0

0

0

0

0
4

0
4

0
4

0.350171

0.350171

0.350171

0.350171

0
4

0

0

0
4

0
4

0

0

0

0
4

0
4

0
4

0

0

0

0

0
4

0
4

0
4

0

0

0

0

0
4

0
4

0
4

1.21276

1.21276

1.09149

0.485106

0

0

0

0.606382

0
4

0.121276

0.121276

0

0

0

0
4

0
4

0
4

0

0

0

0

0

0
4

0

0

0
4

0
4

0
4

0.0974182

0.0974182

0.0974182

0.0974182

0
4

0

0

0
4

0
4

0

0

0

0
4

0
4

0
4

0

0

0

0

0
4

0

0

0
4

0
4

0
4

0

0

0

0

0
4

0

0

0
4

0
4

0
4

0

0

0

0

0

0
4

0
4

0
4

0.18664

0.18664

0.18664

0.106651

0.0799885

0
4

0
4

0
4

0

0

0

0

0

0
4

0
4

0
4

0

0

0

0

0
4

0
4

0
4

0

0

0

0

0
4

0

0

0
4

0
4

0
4

6.58525

6.58525

6.58525

6.58525

0
4

0
4

0
4

0
6

0
6

0
6

0
6

0

0

0

0

0
7

0
6

0

0

0

0

0

0

0
4

0

0

0
4

0
4

0
4

0

0

0

0

0
4

0

0

0
4

0
4

0
4

1.27927

1.27927

1.15945

1.15945

0
4

0.119824

0.119824

0
4

5.55111512312578e-17

0
4

0
4

0

0

0

0

0
4

0
4

0
4

0

0

0

0

0
4

0
4

0
4

0

0

0

0

0
4

0

0

0
4

0
4

0
4

0

0

0

0

0

0
4

0
4

0
4

0.107242

0.107242

0.107242

0

0.107242

0
4

0
4

0
4

0

0

0

0

0
4

0
4

0
4

0

0

0

0

0
4

0
4

0
4

0

0

0

0

0
4

0

0

0
4

0
4

0
4

0
7

0
7

0
7

0
7

0

0

0
4

0
4

0

0

0

0
4

0

0

0
4

0
4

0
4

0

0

0

0

0
4

0

0

0
4

0
4

0
4

0

0

0

0

0

0
4

0
4

0
4

0

0

0

0

0
4

0
4

0
4

0

0

0

0

0
4

0

0

0
4

0
4

0
4

0

0

0

0

0
4

0
4

0
4

0

0

0

0

0
4

0
4

0
4

0

0

0

0

0
4

0

0

0
4

0
4

0
4

0.614811

0.614811

0

0

0
4

0.614811

0.614811

0
4

0
4

0
4

0

0

0

0

0

0
4

0
4

0
4

3.66354

3.66354

3.66354

0.92756

2.73598

0
4

0
4

0
4

0

0

0

0

0

0

0

0

0

0

0
4

0

0

0
4

0
4

0
4

0.289421

0.289421

0.289421

0.289421

0

0
4

0
4

0
4

0.0649455

0.0649455

0.0649455

0.0649455

0
4

0
4

0
4

0

0

0

0

0
4

0

0

0
4

0
4

0
4

0.0974182

0.0974182

0

0

0
4

0.0974182

0.0974182

0
4

0
4

0
4

0.108496

0.108496

0

0

0
4

0.108496

0.108496

0
4

0
4

0
4

0

0

0

0

0
4

0

0

0
4

0
4

0
4

0

0

0

0

0
4

0

0

0
4

0
4

0
4

0

0

0

0

0

0
4

0
4

0
4

0

0

0

0

0
4

0
4

0
4

0

0

0

0

0

0
4

0
4

0
4

1.71755

1.59628

1.23329
5

0

0

0.857939

0

0.375348

0

0
4

0.241714

0.160864

0.0808509

0

0
4

0.121276
3

0.121276

0

0
4

0

0

0
4

0

0

0
4

1.52655665885959e-16

0
4

0.121276

0.121276

0

0

0.121276

0
4

0

0

0

0
4

0
4

0
4

2.95106

2.95106

2.95106

2.95106

0
4

0
4

0
4

0

0

0

0

0

0
4

0
4

0
4

0

0

0

0

0
4

0

0

0
4

0
4

0
4

0

0

0

0

0
4

0

0

0
4

0
4

0
4

0

0

0

0

0
4

0
4

0
4

0

0

0

0

0
4

0
4

0
4

1.75884

1.75884

1.75884

1.75884

0
4

0
4

0
4

0

0

0

0

0
4

0

0

0
4

0
4

0
4

0

0

0

0

0
4

0
4

0
4

0

0

0

0

0
4

0
4

0
4

12.7542

12.7542

12.7542

5.15821

0

0

0

0.409969

0

0

0.391992

0

0

0

3.00644

0

0

0

0

0

0

0

0

0

0

1.96443
3

1.09149

0

0

0

0

0

0

0

0

0.253373
3

0.222067
3

0.256231
3

0

0

0

0
4

0

0

0

0
4

0

0

0

0

0

0
4

0

0

0
4

0

0

0

0
4

0

0

0
4

0

0

0
4

0

0

0
4

0
4

0
4

0
7

0
7

0
7

0
7

0
7

0

0

0

0

0
4

0

0

0
4

0

0

0

0
4

0

0

0
4

0
4

0
4

0

0

0

0

0
4

0
4

0
4

0

0

0

0

0
4

0

0

0
4

0
4

0
4

0

0

0

0

0
4

0
4

0
4

0

0

0

0

0
4

0
4

0
4

0

0

0

0

0

0
4

0
4

0
4

0

0

0

0

0
4

0
4

0
4

0

0

0

0

0
4

0
4

0
4

0

0

0

0

0
4

0
4

0
4

0.27959

0.27959

0.27959

0.27959

0
4

0
4

0
4

0

0

0

0

0
4

0
4

0
4

1.06754

1.06754

0.268712
2

0
2

0.268712

0

0
4

0.798828
5

0.798828
5

0

0
4

0

0

0

0

0
4

0
4

0

0

0

0
4

0
4

0
4

0

0

0

0

0

0
4

0
4

0
4

0

0

0

0

0
4

0
4

0
4

0

0

0

0

0
4

0

0

0
4

0
4

0
4

0

0

0

0

0

0
4

0
4

0
4

0

0

0

0

0
4

0
4

0
4

0

0

0

0

0
4

0
4

0
4

0

0

0

0

0
4

0
4

0
4

0

0

0

0

0
4

0
4

0
4

0

0

0

0

0
4

0
4

0
4

0

0

0

0

0
4

0
4

0
4

0

0

0

0

0

0

0

0

0

0

0
4

0
4

0
4

0

0

0

0

0
4

0
4

0
4

0

0

0

0

0
4

0
4

0
4

0

0

0

0

0
4

0
4

0
4

0

0

0

0

0
4

0
4

0
4

0

0

0

0

0
4

0
4

0
4

0

0

0

0

0
4

0
4

0
4

0.119824

0.119824

0.119824

0.119824

0
4

0
4

0
4

1.44777

1.44777

1.44777

1.44777

0
4

0
4

0
4

0

0

0

0

0
4

0
4

0
4

0

0

0

0

0
4

0
4

0
4

0
6

0
6

0
6

0
6

0

0
4

0
6

0

0

0

0
4

0

0

0

0
4

0

0

0
4

0
4

0
4

0

0

0

0

0
4

0
4

0
4

0.612799

0.612799

0.612799

0.612799

0
4

0
4

0
4

0

0

0

0

0
4

0
4

0
4

0

0

0

0

0
4

0
4

0
4

0

0

0

0

0
4

0
4

0
4

0

0

0

0

0
4

0
4

0
4

0

0

0

0

0
4

0
4

0
4

0

0

0

0

0
4

0
4

0
4

0

0

0

0

0
4

0
4

0
4

0

0

0

0

0
4

0
4

0
4

0

0

0

0

0

0

0

0
4

0

0

0
4

0
4

0

0

0

0

0
4

0

0

0
4

0
4

0
4

0

0

0

0

0
4

0
4

0
4

0

0

0

0

0
4

0
4

0
4

0

0

0

0

0
4

0
4

0
4

0

0

0

0

0
4

0
4

0
4

0

0

0

0

0
4

0
4

0
4

0.107242

0.107242

0.107242

0.107242

0
4

0
4

0
4

0

0

0

0

0
4

0
4

0
4

0

0

0

0

0
4

0
4

0
4

0

0

0

0

0
4

0
4

0
4

0

0

0

0

0
4

0
4

0
4

0.214485

0.214485

0
2

0
2

0

0

0

0
4

0

0

0
4

0

0

0

0

0
4

0.214485

0.214485

0
4

0

0

0
4

0
4

0
4

0

0

0

0

0
4

0
4

0
4

0

0

0

0

0
4

0
4

0
4

0.106651

0.106651

0.106651

0.106651

0
4

0
4

0
4

0

0

0

0

0
4

0
4

0
4

0

0

0

0

0
4

0
4

0
4

0

0

0

0

0
4

0
4

0
4

0

0

0

0

0
4

0
4

0
4

0

0

0

0

0
4

0
4

0
4

0

0

0

0

0
4

0
4

0
4

0

0

0

0

0
4

0
4

0
4

0
7

0
7

0
7

0
7

0
4

0

0

0
4

0
4

0
4

0

0

0

0

0
4

0
4

0
4

0

0

0

0

0
4

0
4

0
4

0

0

0

0

0
4

0
4

0
4

0

0

0

0

0
4

0
4

0
4

0

0

0

0

0
4

0
4

0
4

0

0

0

0

0
4

0
4

0
4

0

0

0

0

0
4

0
4

0
4

0

0

0

0

0
4

0
4

0
4

0

0

0

0

0
4

0
4

0
4

0

0

0

0

0
4

0
4

0
4

0
7

0
7

0
7

0

0

0

0
4

0

0

0

0

0
4

0

0

0
4

0
4

0
4

0

0

0

0

0
4

0
4

0
4

0

0

0

0

0
4

0
4

0
4

0

0

0

0

0
4

0
4

0
4

0

0

0

0

0
4

0
4

0
4

0.0798828

0.0798828

0.0798828

0.0798828

0
4

0
4

0
4

0

0

0

0

0
4

0
4

0
4

0.14657

0.14657

0.14657

0.14657

0
4

0
4

0
4

0

0

0

0

0
4

0
4

0
4

0

0

0

0

0
4

0
4

0
4

0

0

0

0

0
4

0
4

0
4

0.219855

0.219855

0

0

0

0
4

0.219855

0.219855

0

0
4

0

0

0
4

0
4

0

0

0

0

0

0

0

0
4

0
4

0

0

0

0

0
4

0
4

0
4

0.437713

0.437713

0.437713

0.437713

0
4

0
4

0
4

1.57659

1.57659

1.57659

1.57659

0
4

0
4

0
4

0

0

0

0

0
4

0
4

0
4

0

0

0

0

0
4

0
4

0
4

0

0

0

0

0
4

0
4

0
4

0

0

0

0

0
4

0
4

0
4

0

0

0

0

0
4

0
4

0
4

0.0649455

0.0649455

0.0649455

0.0649455

0
4

0
4

0
4

0

0

0

0

0
4

0
4

0
4

0

0

0

0

0
4

0
4

0
4

0

0

0

0

0

0

0

0
4

0
4

0
4

0

0

0

0

0
4

0
4

0
4

0.289323

0.289323

0.289323

0.289323

0
4

0
4

0
4

0

0

0

0

0
4

0
4

0
4

0

0

0

0

0
4

0
4

0
4

0

0

0

0

0
4

0
4

0
4

0

0

0

0

0
4

0
4

0
4

0.108496

0.108496

0.108496

0.108496

0
4

0
4

0
4

0

0

0

0

0
4

0
4

0
4

0

0

0

0

0
4

0
4

0
4

0

0

0

0

0
4

0
4

0
4

0
7

0
7

0
7

0
7

0

0

0

0

0

0

0

0

0

0

0
7

0

0

0

0

0

0

0

0

0

0

0
6

0

0

0

0

0

0

0

0

0

0

0
7

0

0

0

0

0

0

0

0

0

0

0

0

0

0

0

0

0
4

0
6

0

0

0

0

0

0

0

0
4

0
4

0
6

0
6

0

0

0

0

0

0

0
4

0

0

0

0

0
4

0

0

0
4

0
4

0
4

0
4

0
4

0

0

0

0

0

0

0

0

0
4

0

0

0

0

0
4

0

0

0

0
4

0

0

0
4

0

0

0
4

0
4

0
4

0

0

0

0

0
4

0
4

0
4

0

0

0

0

0
4

0
4

0
4

0

0

0

0

0
4

0
4

0
4

0

0

0

0

0
4

0
4

0
4

0

0

0

0

0
4

0
4

0
4

0

0

0

0

0
4

0
4

0
4

0

0

0

0

0
4

0
4

0
4

0

0

0

0

0
4

0
4

0
4

0.0808509

0.0808509

0.0808509

0.0808509

0
4

0
4

0
4

0

0

0

0

0
4

0
4

0
4

0
4

0
4

0
4

0

0

0

0

0

0

0
4

0
4

0

0

0

0

0

0
4

0

0

0
4

0
4

0
4

0

0

0

0

0
4

0
4

0
4

0

0

0

0

0
4

0
4

0
4

0

0

0

0

0
4

0
4

0
4

0

0

0

0

0
4

0
4

0
4

0

0

0

0

0
4

0
4

0
4

0

0

0

0

0
4

0
4

0
4

0

0

0

0

0
4

0
4

0
4

0

0

0

0

0
4

0
4

0
4

0

0

0

0

0
4

0
4

0
4

0

0

0

0

0
4

0
4

0
4

0
6

0
7

0

0

0

0

0
4

0

0

0

0

0
4

0

0

0

0
4

0
4

0

0

0

0

0
4

0

0

0
4

0
4

0
4

0.0974182

0.0974182

0.0974182

0.0974182

0
4

0
4

0
4

0

0

0

0

0
4

0
4

0
4

0.159977

0.159977

0.159977

0.159977

0
4

0
4

0
4

0.0649455

0.0649455

0.0649455

0.0649455

0
4

0
4

0
4

0

0

0

0

0
4

0
4

0
4

0

0

0

0

0
4

0
4

0
4

0

0

0

0

0
4

0
4

0
4

0

0

0

0

0
4

0
4

0
4

0

0

0

0

0
4

0
4

0
4

0.119824

0.119824

0.119824

0.119824

0
4

0
4

0
4

0

0

0
6

0
6

0

0
4

0

0

0

0
4

0

0

0
4

0
4

0
4

0

0

0

0

0
4

0
4

0
4

0

0

0

0

0
4

0
4

0
4

0

0

0

0

0
4

0
4

0
4

0

0

0

0

0
4

0
4

0
4

0

0

0

0

0
4

0
4

0
4

0

0

0

0

0
4

0
4

0
4

0

0

0

0

0
4

0
4

0
4

0

0

0

0

0
4

0
4

0
4

0

0

0

0

0
4

0
4

0
4

0.0723307

0.0723307

0.0723307

0.0723307

0
4

0
4

0
4

0.501386

0.501386

0.107242
5

0.107242

0

0

0

0
4

0

0

0

0

0
4

0.394143

0.214485

0.107242

0.0724163

0
4

0

0

0
4

0

0

0
4

1.11022302462516e-16

0
4

0
4

0

0

0

0

0
4

0
4

0
4

0

0

0

0

0
4

0
4

0
4

0

0

0

0

0
4

0
4

0
4

0

0

0

0

0
4

0
4

0
4

0

0

0

0

0
4

0
4

0
4

0

0

0

0

0
4

0
4

0
4

0

0

0

0

0
4

0
4

0
4

0

0

0

0

0
4

0
4

0
4

0

0

0

0

0
4

0
4

0
4

0

0

0

0

0
4

0
4

0
4

5.47208
4

5.47208
4

5.47208
4

0.0799885

0

5.39209

0

0
4

0

0

0

0

0

0
4

0
4

0
4

0.0533257

0.0533257

0.0533257

0.0533257

0
4

0
4

0
4

0

0

0

0

0
4

0
4

0
4

0.0533257

0.0533257

0.0533257

0.0533257

0
4

0
4

0
4

0

0

0

0

0
4

0
4

0
4

0.119824

0.119824

0.119824

0.119824

0
4

0
4

0
4

0

0

0

0

0
4

0
4

0
4

0

0

0

0

0
4

0
4

0
4

0

0

0

0

0
4

0
4

0
4

0

0

0

0

0
4

0
4

0
4

0

0

0

0

0
4

0
4

0
4

0

0

0

0

0

0

0

0

0

0
4

0

0

0

0

0
4

0

0

0

0
4

0

0

0
4

0

0

0
4

0
4

0

0

0

0
4

0
4

0

0

0

0
4

0
4

0
4

0

0

0

0

0
4

0
4

0
4

0

0

0

0

0
4

0
4

0
4

0

0

0

0

0
4

0
4

0
4

0

0

0

0

0
4

0
4

0
4

0

0

0

0

0
4

0
4

0
4

0

0

0

0

0
4

0
4

0
4

0

0

0

0

0
4

0
4

0
4

0

0

0

0

0
4

0
4

0
4

0.773222

0.773222

0.773222

0.773222

0
4

0
4

0
4

0.0723307

0.0723307

0.0723307

0.0723307

0
4

0
4

0
4

0

0

0

0

0

0
4

0

0

0

0

0

0

0
4

0
4

0
4

0

0

0

0

0
4

0
4

0
4

0

0

0

0

0
4

0
4

0
4

0

0

0

0

0
4

0
4

0
4

0

0

0

0

0
4

0
4

0
4

0

0

0

0

0
4

0
4

0
4

0.0799885

0.0799885

0.0799885

0.0799885

0
4

0
4

0
4

0

0

0

0

0
4

0
4

0
4

0

0

0

0

0
4

0
4

0
4

0

0

0

0

0
4

0
4

0
4

0.0798828

0.0798828

0.0798828

0.0798828

0
4

0
4

0
4

1.45532

1.45532

1.45532

1.37447

0.0808509

0
4

0

0

0
4

0
4

0
4

0

0

0

0

0
4

0
4

0
4

0

0

0

0

0
4

0
4

0
4

0

0

0

0

0
4

0
4

0
4

0

0

0

0

0
4

0
4

0
4

0

0

0

0

0
4

0
4

0
4

0

0

0

0

0
4

0
4

0
4

0

0

0

0

0
4

0
4

0
4

0

0

0

0

0
4

0
4

0
4

0

0

0

0

0
4

0
4

0
4

0

0

0

0

0
4

0
4

0
4

0.146997

0.146997

0

0

0

0

0

0

0

0
4

0.146997

0.146997

0

0
4

0
4

0
4

0

0

0

0

0
4

0
4

0
4

0

0

0

0

0
4

0
4

0
4

0

0

0

0

0
4

0
4

0
4

0

0

0

0

0
4

0
4

0
4

0

0

0

0

0
4

0
4

0
4

0

0

0

0

0
4

0
4

0
4

0

0

0

0

0
4

0
4

0
4

0

0

0

0

0
4

0
4

0
4

0

0

0

0

0
4

0
4

0
4

0

0

0

0

0
4

0
4

0
4

0

0

0

0

0

0

0

0

0

0

0

0

0

0

0

0

0

0

0

0

0

0

0

0

0

0

0

0

0

0

0
4

0

0

0

0

0

0

0
4

0

0

0

0

0

0

0
4

0

0

0

0

0

0

0

0
4

0

0

0

0
4

0

0

0
4

0
4

0

0

0

0

0

0

0

0

0

0

0

0

0

0

0

0

0

0

0

0

0
4

0

0

0

0

0

0

0

0

0

0
4

0

0

0

0

0

0

0
4

0

0

0

0

0
4

0

0

0
4

0

0

0
4

0
4

0

0

0

0

0

0
4

0
4

0

0

0

0
4

0

0

0
4

0
4

0
4

0
6

0
6

0
6

0

0

0

0
4

0

0

0
4

0
4

0
4

0

0

0

0

0
4

0
4

0
4

0

0

0

0

0
4

0
4

0
4

0

0

0

0

0
4

0
4

0
4

0

0

0

0

0
4

0
4

0
4

0

0

0

0

0

0

0
4

0

0

0
4

0
4

0

0

0

0
4

0
4

0
4

0
6

0
6

0
6

0
6

0
4

0
4

0
4

0

0

0

0

0

0

0
4

0

0

0

0
4

0

0

0

0

0
4

0
4

0
4

0
7

0
7

0
7

0

0

0

0

0
4

0
4

0
4

0.897721
4

0.498307
4

0.311773

0.23189

0

0.0798828

0
4

0

0

0

0

0
4

0.106651

0.106651

0
4

0.0798828

0.0798828

0
4

0
4

0.399414

0.199707

0

0.199707

0
4

0

0

0
4

0.199707

0.199707

0
4

0
4

0
4

0

0

0

0

0

0
4

0

0

0
4

0
4

0

0

0

0
4

0
4

0
4

0.507772
3

0.507772
3

0.304663
3

0

0

0.304663

0
4

0.203109

0

0.0761658

0.0761658

0.0507772

6.93889390390723e-18

0
4

2.77555756156289e-17
3

0
4

0
4

0

0

0

0

0

0

0

0

0
4

0

0

0
4

0
4

0
4

0.589833
3

0.589833
3

0.589833
3

0

0.589833

0

0

0

0
4

0
4

0
4

0
7

0
7

0
7

0
7

0

0

0

0

0

0

0

0

0

0

0

0
4

0

0

0
4

0

0

0
4

0

0

0
6

0

0

0

0

0
4

0
6

0
6

0

0
4

0

0

0
4

0

0

0
4

0

0

0
4

0

0

0
4

0

0

0
4

0

0

0
4

0
4

0
4

2.37612

2.37612

2.37612

1.21843

1.15768

4.44089209850063e-16

0
4

0
4

0
4

1.18383
4

1.18383
4

0

0

0

0

0

0
4

1.18383

0.0799885

0.0533257

1.05051

0

0
4

0
4

0
4

0

0

0

0

0

0

0

0

0
4

0
4

0
4

2.66409
4

2.66409
4

2.66409
4

2.66409

0

0
4

0

0

0

0
4

0

0

0
4

0

0

0
4

0
4

0
4

0

0

0

0

0

0

0
4

0

0

0
4

0
4

0
4

4.08232
4

0
4

0
4

0

0

0

0
4

0
4

4.08232

4.08232

4.08232

0
4

0
4

0
4

0

0

0

0

0

0

0

0

0
4

0
4

0
4

0

0

0

0

0

0

0

0

0
4

0

0

0
4

0
4

0
4

0

0

0

0

0

0
4

0

0

0
4

0
4

0
4

1.22649

1.22649

1.22649

0.55992

0.426605

0.18664

0.0533257

1.87350135405495e-16

0
4

0
4

0
4

4.71528
5

4.63443
5

0.902316
6

0
6

0

0

0

0

0

0

0

0.097998

0

0

0

0

0

0

0

0.804318

0
4

0.50982

0.50982

0

0

0

0

0

0

0

0

0

0
4

3.22229
4

0.357647

0.697075

0.121276

0

1.65691

0.107242

0.160864

0.121276

0
4

0

0

0

0

0

0

0
4

0

0

0

0

0
4

0

0

0
4

0

0

0
4

0

0

0
4

4.44089209850063e-16
5

0
4

0
6

0

0

0

0

0

0
4

0

0

0

0

0

0
4

0

0

0

0

0

0
4

0

0

0
4

0
4

0.0808509

0

0

0
4

0.0808509

0.0808509

0
4

0
4

1.27675647831893e-15
5

0
4

0

0

0

0

0

0

0
4

0
4

0
4

0

0

0

0

0

0
4

0

0

0
4

0

0

0
4

0
4

0
4

0
6

0
6

0

0

0

0
4

0

0

0

0
4

0
4

0
4

0

0

0

0

0

0
4

0
4

0

0

0

0
4

0

0

0
4

0
4

0
4

0

0

0

0

0

0
4

0
4

0

0

0

0
4

0
4

0

0

0

0
4

0
4

0
4

0

0

0

0

0

0

0

0
4

0
4

0
4

0

0

0

0

0

0
4

0

0

0
4

0
4

0
4

0

0

0

0

0

0
4

0
4

0
4

0
3

0
3

0
3

0

0

0
4

0

0

0
4

0
4

0
4

0.262628

0.262628

0.262628

0

0

0.262628

0
4

0

0

0
4

0
4

0
4

8.71412

8.71412

4.45081

4.02376

0

0

0.119574
3

0.119574

0.0512461
3

0.0683282
3

0

0.0683282

0

0

7.21644966006352e-16

0
4

0

0

0
4

0
2

0

0

0

0

0
4

4.18245

0

4.18245

0

0
4

0

0

0
4

0

0

0
4

0

0

0

0
4

0

0

0

0
4

0.0808509

0.0808509

0
4

0

0

0
4

3.88578058618805e-16

0
4

0
4

0

0

0

0

0

0
4

0

0

0
4

0

0

0

0
4

0
4

0
4

0

0

0

0

0

0

0
4

0

0

0
4

0

0

0
4

0
4

0
4

0

0

0

0

0

0
4

0

0

0
4

0
4

0

0

0

0
4

0
4

0
4

0
6

0

0

0

0

0
4

0

0

0
4

0

0

0
4

0
4

0

0

0

0
4

0
4

0
4

0
6

0
6

0
7

0
7

0
4

0

0

0
4

0
4

0
4

0

0

0

0

0

0

0
4

0
4

0
4

0

0

0

0

0

0

0
4

0

0

0
4

0
4

0

0

0

0

0
4

0
4

0
4

0
7

0
7

0

0

0

0
4

0

0

0
4

0
4

0
4

1.07242

1.07242

1.07242

1.07242

0

0
4

0
4

0

0

0

0
4

0

0

0
4

0
4

0
4

0

0

0

0

0
4

0

0

0
4

0
4

0
4

0
7

0
6

0
7

0
6

0
7

0

0

0

0

0

0
4

0
6

0
6

0

0

0

0

0
4

0

0

0

0
4

0
4

0
7

0
7

0

0

0

0

0

0

0

0

0

0

0

0
4

0

0

0

0

0
4

0

0

0

0
4

0

0

0
4

0
4

0
4

0

0

0

0

0
4

0
4

0
4

0.202222

0.202222

0.0723307

0.0723307

0

0
4

0.129891

0

0.129891

0
4

0

0

0
4

2.77555756156289e-17

0
4

0
4

0

0

0

0

0

0
4

0
4

0
4

0

0

0

0

0

0
4

0

0

0
4

0
4

0
4

0

0

0

0

0
4

0

0

0
4

0
4

0
4

0

0

0

0

0

0
4

0

0

0
4

0
4

0

0

0

0
4

0
4

0

0

0

0
4

0
4

0
4

0

0

0

0

0
4

0

0

0
4

0
4

0
4

0

0

0

0

0

0

0
4

0

0

0
4

0
4

0
4

0

0

0

0

0
4

0

0

0
4

0

0

0
4

0
4

0
4

0

0

0

0

0

0

0
4

0
4

0
4

10.9655
4

5.97247
4

5.97247
4

5.43922
4

0.319954

0

0.0799885

0.133314

0

0

0
4

0

0

0
4

0
4

4.28711
4

0

0

0

0

0

0

0

0

0

0
4

4.23379

4.23379

0

0

0
4

0.0533257

0

0

0

0.0533257

0
4

0

0

0
4

0
4

0.18664

0.18664

0.18664

0

0

0
4

0

0

0
4

0

0

0
4

0
4

0.519238

0.519238

0.519238

0
4

0

0

0
4

0
4

0
4

0
6

0
6

0
6

0
6

0
4

0
4

0
4

0

0

0

0

0
4

0
4

0
4

0

0

0

0

0
4

0
4

0
4

0

0

0

0

0
4

0

0

0
4

0
4

0
4

0

0

0

0

0

0
4

0
4

0
4

0

0

0

0

0

0
4

0

0

0

0
4

0
4

0
4

0

0

0

0

0
4

0
4

0
4

0.519238

0.519238

0.519238

0.439355

0.0798828

4.16333634234434e-17

0
4

0
4

0
4

0.482591

0.482591

0.482591

0.482591

0

0

0
4

0
4

0
4

0

0

0

0

0
4

0
4

0
4

8.04475
3

8.04475
3

8.04475
3

1.901

0.0482775

0

0

0

1.17745
3

4.36283
3

0.555192

0

0

0

0

0

0
4

0

0

0
4

0

0

0
4

0

0

0
4

0
4

0

0

0

0

0

0
4

0
4

0

0

0

0
4

0
4

0
4

3.62461
3

0

0

0

0
4

0
4

0

0

0

0

0
4

0
4

0

0

0

0
4

0
4

0

0

0

0
4

0
4

0

0

0

0
4

0
4

3.62461
3

3.19012
3

0

0

0

0

0

0

0

0

0

0

0

0

0

0

0

0

0.0724163

0

0

0

0

0

0

0

0

0.651747

0

0

0

0

0

2.46595
3

0

0

0

0

0
4

0

0

0

0

0
4

0

0

0
4

0

0

0
4

0.434498

0.434498

0
4

0
4

0
4

4.87117
3

0

0

0

0
4

0
4

4.87117
3

4.77944
3

0.92756

0

3.85188

0

0

0

0
4

0

0

0
4

0.0917376

0.0917376

0
4

5.82867087928207e-16
3

0
4

0
4

42.0897
4

40.856
4

18.0263
4

5.82148

0

0

0.202127

0

0

0

0.685986
4

0.594402

0

1.25233

0.783984
4

8.53137
4

0

0.154593

7.7715611723761e-16
4

0
4

22.8297
3

0.798828

0

1.00486

0

0.133314

0

0.175085

0

20.7176

0

0

0

0

0

0

0

0
4

0

0

0
4

0

0

0
4

0

0

0
4

0
4

0.883587

0.883587

0.620959

0.262628

0
4

0
4

0

0

0

0
4

0
4

0

0

0

0
4

0
4

0.350171

0.350171

0.350171

0
4

0
4

0

0

0

0
4

0
4

0

0

0

0
4

0
4

0

0

0

0
4

0
4

0
4

7.16652

0

0

0

0

0
4

0
4

0

0

0

0

0
4

0
4

0

0

0

0
4

0
4

0

0

0

0

0
4

0
4

0

0

0

0
4

0
4

7.16652

4.16373

0

0

0

0

0

0.0507772

0

0.0507772

0

0

0

0

0

0

0

0

0

0.0507772

2.05648

1.95492

0

0

0

0

0

0
4

3.00279

3.00279

0
4

0

0

0
4

0

0

0
4

0
4

0
4

58.6079
3

58.6079
3

2.57382
2

2.57382
2

0

0

0
4

56.0341
3

4.07521

0.160864

0

0.804318

7.82869

0

0

0

18.8747

0

2.41295

21.663

0.107242

0.107242

0

0

7.49400541621981e-16
3

0
4

0

0

0
4

0

0

0
4

7.105427357601e-15
3

0
4

0

0

0

0

0
4

0
4

0

0

0

0
4

0
4

0

0

0

0
4

0
4

0

0

0

0
4

0
4

0

0

0

0
4

0
4

0
4

5.14229

0.0482775

0.0482775

0.0482775

0
4

0
4

5.09401

5.09401

0

0
3

0

5.09401

0

0

0

0

0

0

0
4

0

0

0
4

0
4

0
4

0
7

0
7

0
7

0
7

0
4

0
4

0
4

91.2796

91.1583

0

0

0

0

0
4

0

0

0
4

28.2724
3

15.3857
3

0
5

12.8867
3

0
4

62.2683
3

0

62.2683
3

0
4

0
4

0

0

0

0

0

0

0
4

0

0

0
4

0.617607

0.434131

0.183475

0
4

0

0

0
4

0

0

0
4

0

0

0
4

0
4

0.121276

0.121276

0.121276

0

0
4

0
4

0

0

0

0
4

0
4

0

0

0

0
4

0
4

0

0

0

0
4

0
4

0

0

0

0
4

0
4

4.37150315946155e-15

0
4

0
2

0
2

0
2

0

0

0

0

0

0
4

0
4

0
4

0

0

0

0

0
4

0
4

0

0

0

0

0
4

0
4

0

0

0

0
4

0

0

0
4

0
4

0

0

0

0

0
4

0
4

0

0

0

0
4

0
4

0

0

0

0
4

0
4

0

0

0

0

0
4

0

0

0
4

0

0

0
4

0

0

0
4

0

0

0
4

0

0

0
4

0

0

0
4

0

0

0
4

0
4

0
4

1.76072489921353e-11

0
4

2.13257
7

2.13257
7

2.13257
7

0.773542
6

0.266628

0
7

0.0724163

0

0

0

0

0

0

0

0

0

0

0

0

0

0

0

0

0

0

0

0

0.168971
7

0

0

0

0.0482775

0
6

0
6

0
6

0
6

0.217249

0

2.77555756156289e-17
6

0
4

0
7

0
7

0
7

0

0
4

1.35902
7

0

0
7

0
7

0.0965551
7

0
6

0.675885
7

0

0
6

0.0533257

0.479931

0.0533257

0

0

1.59594559789866e-16
7

0
4

0
4

0
4

0
4

0

0

0

0

0

0

0

0

0

0

0

0

0

0

0

0

0

0

0

0

0

0

0

0

0

0

0

0

0

0

0

0

0

0

0

0

0

0

0

0

0

0

0

0

0

0

0

0

0

0

0

0

0

0

0

0

0

0

0

0

0

0

0

0

0

0

0

0

0

0

0

0

0

0

0

0

0

0

0

0

0

0

0

0

0

0

0

0

0

0

0

0

0

0

0

0

0

0

0

0

0

0

0

0

0

0

0

0

0

0

0

0

0

0

0

0

0

0

0

0

0

0

0

0

0

0

0

0

0

0

0

0

0

0

0

0

0

0

0

0

0

0

0

0
4

0

0

0

0

0

0

0

0

0

0

0

0

0

0

0

0

0

0

0

0
4

0

0

0

0

0

0

0

0

0
4

0
4

0
4

0
4

0
7

0
7

0
7

0
7

0
7

0

0

0

0
4

0

0

0

0

0

0
4

0
4

0
4

0
4

0

0

0

0

0

0
4

0

0

0
4

0

0

0
4

0
4

0
4

0
4

0

0

0

0

0

0

0
4

0

0

0
4

0

0

0
4

0
4

0
4

0
4

0

0

0

0

0

0

0
4

0
4

0
4

0
4

10.4025

10.4025

10.4025

0

0

0

0
4

10.4025

10.4025

0
4

0
4

0
4

0
4

1.35177
3

1.35177
3

1.35177
3

1.35177
3

1.35177

0

0
4

0
4

0
4

0
4

0.129891

0.129891

0.129891

0.129891

0.129891

0

0
4

0
4

0
4

0
4

0

0

0

0

0

0

0
4

0
4

0
4

0
4

0

0

0

0

0

0

0
4

0
4

0
4

0
4

0

0

0

0

0

0
4

0
4

0
4

0
4

0.259782

0.259782

0.259782

0.259782

0.259782

0
4

0

0

0
4

0

0

0
4

0
4

0
4

0
4

4.94584
3

4.94584
3

4.94584
3

4.86595
3

4.4743
3

0.0798828

0.23189

0.0798828

0

0

0
4

0

0

0

0
4

0.0798828

0.0798828

0

0
4

0

0

0

0
4

0

0

0
4

0
4

0
4

0
4

0.724163

0.724163

0.724163

0.724163

0.675885

0.0482775

0
4

0
4

0
4

0
4

0

0

0

0

0

0

0
4

0
4

0
4

0
4

0

0

0

0

0

0
4

0
4

0
4

0
4

2.11689

2.11689

2.11689

2.11689

1.95713

0.159766

0
4

0
4

0
4

0
4

0

0

0

0

0

0

0
4

0
4

0
4

0
4

0.642163

0.642163

0.642163

0.642163

0.642163

0
4

0
4

0
4

0
4

0.773222

0.773222

0.773222

0.773222

0.773222

0
4

0
4

0
4

0
4

0.330052

0.330052

0.330052

0.0507772

0.0507772

0
4

0.0761658

0.0761658

0
4

0.0507772

0.0507772

0
4

0.152332

0.152332

0
4

2.77555756156289e-17

0
4

0
4

0
4

0

0

0

0

0

0
4

0
4

0
4

0
4

0

0

0

0

0

0

0
4

0
4

0
4

0
4

0
4

0
4

0
4

0
4

0
4

0

0

0

0
4

0
4

0
4

0
4

0
4

0
4

0
4

0

0

0
4

0

0

0

0

0

0

0
4

0
4

0
4

0
4

0

0

0

0

0

0
4

0
4

0
4

0
4

3.63294

3.63294

3.63294

0

0

0

0
4

3.63294

3.63294

0
4

0
4

0
4

0
4

0

0

0

0

0

0

0
4

0
4

0
4

0
4

0

0

0

0

0

0

0
4

0
4

0
4

0
4

0

0

0

0

0

0
4

0

0

0
4

0
4

0
4

0
4

7.48222

7.48222

7.48222

7.48222

7.48222

0
4

0
4

0
4

0
4

0.159977

0.159977

0.159977

0.159977

0.106651

0.0533257

6.93889390390723e-18

0
4

0
4

0
4

0
4

2.50638

2.50638

2.50638

2.50638

2.50638

0
4

0
4

0
4

0
4

0

0

0

0

0

0
4

0
4

0
4

0
4

0.550425

0.550425

0.550425

0.550425

0.550425

0

0
4

0
4

0
4

0
4

0
6

0
6

0
6

0
6

0
6

0

0

0

0

0

0

0

0
4

0

0

0

0
4

0
4

0
4

0
4

0.739006

0.739006

0.739006

0.570035

0.473479

0.0965551

0
4

0.168971

0.168971

0
4

5.55111512312578e-17

0
4

0
4

0
4

0

0

0

0

0

0

0

0
4

0

0

0
4

0
4

0
4

0
4

0

0

0

0

0

0

0
4

0
4

0
4

0
4

0.0649455

0.0649455

0.0649455

0.0649455

0.0649455

0

0

0
4

0

0

0
4

0
4

0
4

0
4

0

0

0

0

0

0
4

0
4

0
4

0
4

0

0

0

0

0

0
4

0
4

0
4

0
4

0

0

0

0

0

0

0
4

0
4

0
4

0
4

0.0723307

0.0723307

0.0723307

0.0723307

0

0

0.0723307

0
4

0
4

0
4

0
4

0

0

0

0

0

0

0
4

0

0

0
4

0
4

0
4

0
4

0

0

0

0

0

0
4

0
4

0
4

0
4

0

0

0

0

0

0

0

0

0

0

0

0

0
4

0

0

0

0

0

0

0

0
4

0

0

0

0

0
4

0
4

0
4

0

0

0

0

0

0

0
4

0

0

0

0
4

0
4

0
4

0

0

0

0

0
4

0
4

0
4

0
4

0

0

0

0

0

0
4

0

0

0
4

0

0

0
4

0
4

0
4

0
4

0

0

0

0

0

0

0
4

0
4

0
4

0

0

0

0

0
4

0
4

0
4

0
4

0

0

0

0

0

0
4

0

0

0
4

0
4

0
4

0
4

0

0

0

0

0

0

0
4

0
4

0
4

0
4

0

0

0

0

0

0

0
4

0
4

0
4

0
4

0

0

0

0

0

0
4

0

0

0
4

0
4

0
4

0
4

0

0

0

0

0

0
4

0
4

0
4

0
4

0

0

0

0

0

0
4

0
4

0
4

0
4

1.02898

1.02898

1.02898

1.02898

1.02898

0
4

0
4

0
4

0
4

0

0

0

0

0

0
4

0
4

0
4

0
4

4.03718
3

3.9573
3

3.9573
3

3.83748
3

3.06081
3

0.776669
3

0

0

0

0

0

0

1.11022302462516e-16
3

0
4

0.119824

0

0.119824

0
4

0
4

0
4

0.0798828

0.0798828

0.0798828

0.0798828

0
4

0
4

0
4

3.7470027081099e-16
3

0
4

0

0

0

0

0

0

0
4

0

0

0
4

0
4

0
4

0
4

0

0

0

0

0

0
4

0
4

0
4

0
4

0

0

0

0

0

0

0
4

0

0

0
4

0
4

0
4

0
4

0

0

0

0

0

0
4

0
4

0
4

0
4

0

0

0

0

0

0

0
4

0
4

0
4

0
4

0

0

0

0

0

0

0
4

0
4

0
4

0
4

0

0

0

0

0

0
4

0
4

0
4

0
4

0

0

0

0

0

0

0
4

0
4

0
4

0
4

0

0

0

0

0

0
4

0
4

0
4

0
4

0

0

0

0

0

0
4

0
4

0
4

0
4

0

0

0

0

0

0

0

0
4

0

0

0

0
4

0

0

0

0
4

0

0

0
4

0
4

0
4

0

0

0

0

0
4

0

0

0
4

0
4

0
4

0

0

0

0

0
4

0
4

0
4

0
4

0

0

0

0

0

0
4

0
4

0
4

0
4

0

0

0

0

0

0
4

0
4

0
4

0
4

0

0

0

0

0

0
4

0
4

0
4

0
4

0

0

0

0

0

0
4

0
4

0
4

0
4

0

0

0

0

0

0

0
4

0
4

0
4

0
4

0

0

0

0

0

0

0
4

0
4

0
4

0
4

0

0

0

0

0

0

0
4

0
4

0
4

0
4

0

0

0

0

0

0
4

0
4

0
4

0
4

0

0

0

0

0

0
4

0
4

0
4

0
4

0

0

0

0

0

0
4

0
4

0
4

0
4

0
7

0
7

0
7

0
7

0
7

0
7

0

0

0

0
4

0

0

0

0
4

0
4

0
4

0
4

0.159766

0.159766

0.159766

0.159766

0.159766

0
4

0
4

0
4

0
4

0

0

0

0

0

0
4

0
4

0
4

0
4

0.798828

0.798828

0.798828

0.798828

0.798828

0
4

0
4

0
4

0
4

0

0

0

0

0

0
4

0
4

0
4

0
4

1.40068

1.40068

1.40068

1.40068

1.40068

0
4

0
4

0
4

0
4

0

0

0

0

0

0
4

0
4

0
4

0
4

0

0

0

0

0

0
4

0
4

0
4

0
4

0

0

0

0

0

0

0
4

0
4

0
4

0
4

0

0

0

0

0

0
4

0
4

0
4

0
4

0

0

0

0

0

0
4

0
4

0
4

0
4

0.775257
4

0.775257
4

0.775257
4

0.553628
4

0.311276
4

0.0974182

0.144934

0

0

0

0

0
4

0.221628
4

0.0649455

0.0917376

0.0649455

1.38777878078145e-17
4

0
4

0
4

0
4

0
4

0

0

0

0

0

0
4

0
4

0
4

0
4

0

0

0

0

0

0

0
4

0
4

0
4

0
4

1.39633

1.39633

1.39633

1.39633

1.39633

0
4

0
4

0
4

0
4

0

0

0

0

0

0
4

0
4

0
4

0
4

0

0

0

0

0

0

0
4

0
4

0
4

0
4

0

0

0

0

0

0

0
4

0
4

0
4

0
4

2.11689

2.11689

2.11689

0.0798828

0.0798828

0
4

2.03701

2.03701

0
4

0
4

0
4

0
4

0

0

0

0

0

0
4

0
4

0
4

0
4

0.612799

0.612799

0.612799

0.612799

0.612799

0
4

0
4

0
4

0
4

1.40068

1.40068

1.40068

1.40068

1.40068

0
4

0
4

0
4

0
4

0

0

0

0

0
1

0

0

0

0

0
4

0

0

0
4

0

0

0
4

0
4

0
4

0

0

0

0

0
4

0
4

0
4

0
4

0

0

0

0

0

0
4

0
4

0
4

0
4

0

0

0

0

0

0

0
4

0
4

0
4

0
4

0

0

0

0

0

0

0
4

0
4

0
4

0
4

0

0

0

0

0

0
4

0
4

0
4

0
4

0

0

0

0

0

0
4

0
4

0
4

0
4

0

0

0

0

0

0

0
4

0
4

0
4

0
4

0.126943

0.126943

0.126943

0.0761658

0.0761658

0
4

0.0507772

0.0507772

0
4

6.93889390390723e-18

0
4

0
4

0
4

0

0

0

0

0

0

0
4

0
4

0
4

0
4

0

0

0

0

0

0

0
4

0
4

0
4

0
4

0

0

0

0

0

0
4

0
4

0
4

0
4

244.763
3

244.763
3

244.763
3

244.763
3

123.605
3

0
4

0
5

0
4

0
2

17.5969
4

0

0

0

0

0

36.1535
3

0

0

0

0

0.341997

0

0

0.0808509

0

0

28.8037
3

28.0707
3

1.09318

8.75729
3

0.259782
3

0

0
3

5.93969318174459e-15
3

0
4

0
4

0
4

0
4

7.20376
4

7.20376
4

7.20376
4

2.25892
3

2.25892
3

0
4

0.569246
3

0.569246
4

0

0
4

0.534501
4

0.454618

0

0.0798828

0
4

1.68761

0.129891

1.55771

0
4

0.608112

0.608112

0
4

1.23818

1.23818

0
4

0.307192

0.227309

0.0798828

1.38777878078145e-17

0
4

2.77555756156289e-16
4

0
4

0
4

0
4

0

0

0

0

0

0
4

0
4

0
4

0
4

0

0

0

0

0

0
4

0
4

0
4

0
4

0

0

0

0

0

0

0
4

0
4

0
4

0
4

0

0

0

0

0

0
4

0
4

0
4

0
4

0

0

0

0

0

0
4

0
4

0
4

0
4

0

0

0

0

0

0
4

0
4

0
4

0
4

0

0

0

0

0

0
4

0
4

0
4

0
4

0

0

0

0

0

0
4

0
4

0
4

0
4

0

0

0

0

0

0
4

0
4

0
4

0
4

0

0

0

0

0

0
4

0

0

0
4

0
4

0
4

0
4

0

0

0

0

0

0

0

0

0

0

0

0
4

0

0

0
4

0
4

0
4

0
4

0

0

0

0

0

0

0
4

0
4

0
4

0
4

0.144828

0.144828

0.144828

0.144828

0.144828

0
4

0
4

0
4

0
4

0

0

0

0

0

0
4

0
4

0
4

0
4

0

0

0

0

0

0

0
4

0
4

0
4

0
4

0

0

0

0

0

0
4

0
4

0
4

0
4

0

0

0

0

0

0
4

0
4

0
4

0
4

0

0

0

0

0

0
4

0
4

0
4

0
4

0

0

0

0

0

0

0
4

0
4

0
4

0
4

0

0

0

0

0

0
4

0

0

0
4

0
4

0
4

0
4

0

0

0

0

0

0
4

0

0

0
4

0
4

0
4

0
4

0.0977135
4

0.0977135
4

0.0977135
4

0.0977135
4

0
4

0

0

0.0488567

0

0

0.0488567

0

0
4

0
4

0
4

0
4

0

0

0

0

0

0
4

0
4

0
4

0
4

0

0

0

0

0

0

0
4

0
4

0
4

0
4

0

0

0

0

0

0
4

0
4

0
4

0
4

0.750696

0.750696

0.750696

0.750696

0.750696

0
4

0
4

0
4

0
4

0

0

0

0

0

0
4

0
4

0
4

0
4

0

0

0

0

0

0
4

0
4

0
4

0
4

0

0

0

0

0

0
4

0
4

0
4

0
4

0

0

0

0

0

0
4

0
4

0
4

0
4

0

0

0

0

0

0
4

0
4

0
4

0
4

0.0808509

0.0808509

0.0808509

0.0808509

0.0808509

0
4

0
4

0
4

0
4

0

0

0

0

0

0

0

0

0

0

0

0
4

0

0

0

0

0
4

0
4

0
4

0
4

0

0

0

0

0

0
4

0
4

0
4

0
4

0

0

0

0

0

0
4

0
4

0
4

0
4

0

0

0

0

0

0
4

0
4

0
4

0
4

0

0

0

0

0

0
4

0
4

0
4

0
4

0

0

0

0

0

0
4

0
4

0
4

0
4

0.428969

0.428969

0.428969

0.428969

0.428969

0
4

0
4

0
4

0
4

0

0

0

0

0

0
4

0
4

0
4

0
4

0

0

0

0

0

0
4

0
4

0
4

0
4

0

0

0

0

0

0
4

0
4

0
4

0
4

0

0

0

0

0

0
4

0
4

0
4

0
4

0
7

0
7

0
7

0
7

0
7

0
7

0

0
4

0
4

0
4

0
4

0

0

0

0

0

0
4

0
4

0
4

0
4

0.844857

0.844857

0.844857

0.844857

0.844857

0
4

0
4

0
4

0
4

0

0

0

0

0

0
4

0
4

0
4

0
4

0.097998

0.097998

0.097998

0.097998

0.097998

0
4

0
4

0
4

0
4

0

0

0

0

0

0
4

0
4

0
4

0
4

0

0

0

0

0

0
4

0
4

0
4

0
4

0

0

0

0

0

0
4

0
4

0
4

0
4

0

0

0

0

0

0
4

0
4

0
4

0
4

0.154593

0.154593

0.154593

0.154593

0.154593

0
4

0
4

0
4

0
4

0

0

0

0

0

0
4

0
4

0
4

0
4

0

0

0

0

0

0

0

0

0

0

0
4

0

0

0

0

0

0

0
4

0
4

0
4

0

0

0

0

0

0

0

0
4

0

0

0
4

0

0

0
4

0
4

0
4

0
4

0

0

0

0

0

0
4

0
4

0
4

0
4

0.538989

0.538989

0.538989

0.538989

0.538989

0
4

0
4

0
4

0
4

0

0

0

0

0

0
4

0
4

0
4

0
4

0

0

0

0

0

0
4

0
4

0
4

0
4

0

0

0

0

0

0
4

0
4

0
4

0
4

0

0

0

0

0

0
4

0
4

0
4

0
4

0

0

0

0

0

0
4

0
4

0
4

0
4

0

0

0

0

0

0
4

0
4

0
4

0
4

0

0

0

0

0

0
4

0
4

0
4

0
4

0

0

0

0

0

0
4

0
4

0
4

0
4

1.07233
3

1.07233
3

1.07233
3

1.07233
4

0.393328
4

0
3

0.679004

0
4

0

0

0

0
4

0
4

0
4

0
4

0

0

0

0

0

0
4

0
4

0
4

0
4

0

0

0

0

0

0
4

0
4

0
4

0
4

2.99586

2.99586

2.99586

2.99586

2.99586

0
4

0
4

0
4

0
4

0

0

0

0

0

0
4

0
4

0
4

0
4

0.0974182

0.0974182

0.0974182

0.0974182

0.0974182

0
4

0
4

0
4

0
4

0

0

0

0

0

0
4

0
4

0
4

0
4

0

0

0

0

0

0
4

0
4

0
4

0
4

0

0

0

0

0

0
4

0
4

0
4

0
4

0

0

0

0

0

0
4

0
4

0
4

0
4

0

0

0

0

0

0
4

0
4

0
4

0
4

0

0

0

0

0

0

0

0

0

0

0

0
4

0
4

0
4

0
4

0

0

0

0

0

0
4

0
4

0
4

0
4

0

0

0

0

0

0
4

0
4

0
4

0
4

0

0

0

0

0

0
4

0
4

0
4

0
4

0

0

0

0

0

0
4

0
4

0
4

0
4

0

0

0

0

0

0
4

0
4

0
4

0
4

0

0

0

0

0

0
4

0
4

0
4

0
4

0.244995

0.244995

0.244995

0.244995

0.244995

0
4

0
4

0
4

0
4

0

0

0

0

0

0
4

0
4

0
4

0
4

0

0

0

0

0

0
4

0
4

0
4

0
4

0

0

0

0

0

0
4

0
4

0
4

0
4

5.59011
4

4.239
4

4.239
4

1.00471
4

0.821238
4

0.183475

2.77555756156289e-17
4

0
4

0.333984

0.269039

0.0649455

0
4

0.364585

0.292255

0.0723307

0
4

0.194836

0.194836

0
4

2.34088

2.34088

0
4

0

0

0
4

0
4

0
4

1.28616

1.28616

1.28616

1.28616

0
4

0
4

0
4

0.0649455

0.0649455

0.0649455

0.0649455

0
4

0
4

0
4

0
4

0.92756

0.92756

0.92756

0.92756

0.92756

0
4

0
4

0
4

0
4

0

0

0

0

0

0
4

0
4

0
4

0
4

0

0

0

0

0

0
4

0
4

0
4

0
4

0

0

0

0

0

0
4

0
4

0
4

0
4

0

0

0

0

0

0
4

0
4

0
4

0
4

0

0

0

0

0

0
4

0
4

0
4

0
4

0

0

0

0

0

0
4

0
4

0
4

0
4

0

0

0

0

0

0
4

0
4

0
4

0
4

0

0

0

0

0

0
4

0
4

0
4

0
4

0

0

0

0

0

0
4

0
4

0
4

0
4

0.371422

0.371422

0.371422

0.371422

0.161702

0

0

0

0

0.160864

0

0

0

0

0

0.0488567

0
4

0

0

0

0
4

0
4

0
4

0
4

0

0

0

0

0

0
4

0
4

0
4

0
4

0

0

0

0

0

0
4

0
4

0
4

0
4

0

0

0

0

0

0
4

0
4

0
4

0
4

0

0

0

0

0

0
4

0
4

0
4

0
4

0.350171

0.350171

0.350171

0.350171

0.350171

0
4

0
4

0
4

0
4

0

0

0

0

0

0
4

0
4

0
4

0
4

0

0

0

0

0

0
4

0
4

0
4

0
4

0

0

0

0

0

0
4

0
4

0
4

0
4

0

0

0

0

0

0
4

0
4

0
4

0
4

0.107242

0.107242

0.107242

0.107242

0.107242

0
4

0
4

0
4

0
4

106.543
4

106.229
4

106.084
4

18.6258
4

4.48785
4

0.0974182

0.0649455

0

0.0974182

0

0

0

0

0

0.0798828

3.30305
4

0.199707

1.08215

0.0798828

0.0798828

0

0

0

0.23189

0

0

3.67233
4

0

0

0

0.154593

0

0

1.09251
4

0.524434
3

0.402012
4

2.55138

0.144828
3

0.27959

3.27515792264421e-15
4

0
4

85.025
4

82.1939
4

0

0.129891

1.61038
4

0.579313

0.23189

0.119824

0

0

0

0.159766

2.31203944878189e-14
4

0
4

2.19366
4

0.344535
4

1.36723
4

0.402012
4

0

0.0798828

0

0

0

0
4

0.239648

0.0798828

0

0

0.0798828

0.0798828

2.77555756156289e-17

0
4

2.37587727269783e-14
4

0
4

0.144828

0.0649455

0.0649455

0

0
4

0.0798828

0.0798828

0
4

0
4

1.15740750317173e-14
4

0
4

0.314359
4

0.314359
4

0.314359
4

0.234476
4

0.0798828

0
4

0

0

0

0
4

0

0

0
4

0
4

0
4

0

0

0

0

0
4

0

0

0
4

0
4

0
4

5.99520433297585e-15
4

0
4

0

0

0

0

0

0

0

0

0

0
4

0
4

0
4

0
4

1.1171

1.1171

1.1171

1.1171

1.1171

0
4

0
4

0
4

0
4

0

0

0

0

0

0
4

0
4

0
4

0
4

0

0

0

0

0

0
4

0
4

0
4

0
4

0

0

0

0

0

0
4

0
4

0
4

0
4

0

0

0

0

0

0
4

0
4

0
4

0
4

0

0

0

0

0

0
4

0
4

0
4

0
4

0

0

0

0

0

0
4

0
4

0
4

0
4

0

0

0

0

0

0
4

0
4

0
4

0
4

0

0

0

0

0

0
4

0
4

0
4

0
4

0

0

0

0

0

0
4

0
4

0
4

0
4

0
5

0
5

0
5

0
5

0

0

0

0

0

0

0

0

0
4

0

0

0
4

0
4

0
4

0
4

0

0

0

0

0

0
4

0
4

0
4

0
4

0

0

0

0

0

0
4

0
4

0
4

0
4

0

0

0

0

0

0
4

0
4

0
4

0
4

0.122142

0.122142

0.122142

0.122142

0.122142

0
4

0
4

0
4

0
4

0

0

0

0

0

0
4

0
4

0
4

0
4

0

0

0

0

0

0
4

0
4

0
4

0
4

0

0

0

0

0

0
4

0
4

0
4

0
4

0

0

0

0

0

0
4

0
4

0
4

0
4

0

0

0

0

0

0
4

0
4

0
4

0
4

0.525531

0.525531

0.525531

0.525531

0.525531

0
4

0
4

0
4

0
4

0
7

0
7

0
7

0
7

0
7

0
7

0

0

0

0

0
4

0
4

0
4

0
4

0

0

0

0

0

0
4

0
4

0
4

0
4

0

0

0

0

0

0
4

0
4

0
4

0
4

0

0

0

0

0

0
4

0
4

0
4

0
4

0

0

0

0

0

0
4

0
4

0
4

0
4

0

0

0

0

0

0
4

0
4

0
4

0
4

0.242553

0.242553

0.242553

0.242553

0.242553

0
4

0
4

0
4

0
4

0

0

0

0

0

0
4

0
4

0
4

0
4

0

0

0

0

0

0
4

0
4

0
4

0
4

0

0

0

0

0

0
4

0
4

0
4

0
4

0

0

0

0

0

0
4

0
4

0
4

0
4

0

0

0

0

0

0

0

0

0

0

0

0

0

0

0

0

0

0
4

0

0

0

0

0

0
4

0

0

0

0
4

0

0

0
4

0
4

0
4

0
4

0

0

0

0

0

0
4

0
4

0
4

0
4

0

0

0

0

0

0
4

0
4

0
4

0
4

5.1939

5.1939

5.1939

5.1939

5.1939

0
4

0
4

0
4

0
4

0

0

0

0

0

0
4

0
4

0
4

0
4

0

0

0

0

0

0
4

0
4

0
4

0
4

0

0

0

0

0

0
4

0
4

0
4

0
4

0

0

0

0

0

0
4

0
4

0
4

0
4

0

0

0

0

0

0
4

0
4

0
4

0
4

0

0

0

0

0

0
4

0
4

0
4

0
4

0

0

0

0

0

0
4

0
4

0
4

0
4

0
7

0
7

0
7

0
7

0
6

0

0

0

0

0

0

0

0
4

0
4

0
4

0
4

0

0

0

0

0

0
4

0
4

0
4

0
4

0

0

0

0

0

0
4

0
4

0
4

0
4

0

0

0

0

0

0
4

0
4

0
4

0
4

0.293994

0.293994

0.293994

0.293994

0.293994

0
4

0
4

0
4

0
4

0

0

0

0

0

0
4

0
4

0
4

0
4

0

0

0

0

0

0
4

0
4

0
4

0
4

0

0

0

0

0

0
4

0
4

0
4

0
4

0

0

0

0

0

0
4

0
4

0
4

0
4

0

0

0

0

0

0
4

0
4

0
4

0
4

0

0

0

0

0

0
4

0
4

0
4

0
4

4.47842

4.47842

4.47842

4.47842

4.47842

0

0

0

0

0

0

0
4

0

0

0

0
4

0

0

0
4

0
4

0
4

0
4

0

0

0

0

0

0
4

0
4

0
4

0
4

0

0

0

0

0

0
4

0
4

0
4

0
4

0

0

0

0

0

0

0

0

0

0

0

0
4

0
4

0
4

0
4

6.59779

6.50605

6.50605

6.50605

4.14571

2.36035

0
4

0
4

0
4

0.0917376

0.0917376

0.0917376

0.0917376

0
4

0

0

0
4

0
4

0
4

5.82867087928207e-16

0
4

7.22888

7.22888

7.22888

2.04288

0.7054

1.10045

0.237029

0
4

5.186

5.00828

0.17772

3.60822483003176e-16

0
4

0
4

0
4

0
4

0.121276

0.121276

0.121276

0.121276

0

0

0.121276

0
4

0
4

0
4

0
4

185.695
4

185.695
4

185.695
4

20.3795
4

8.42375
4

0.162364

0.129891

0.202552

0

0

0.0649455

0.0974182

0.0917376

0

0

3.84738
4

0

0

0.137606

0.0649455

0.0649455

0.0649455

0

0

0.0649455

0.0649455

2.06578
4

0.0649455

0

0.0917376

0.0649455

0.0798828

0.0649455

0.0649455

0

0

0

0.690291
4

0

0.0649455

0.175085

0

0

0

0.437083

0.603128
4

0.649138

1.49375

0.286574

2.94209101525666e-15
4

0
4

164.949
4

142.53
4

0.0799885

0.0798828

17.9314
4

1.44853

0.408136
4

0.0974182

1.91589

0.319531

0.137606

0

0
4

0.36695

0.137606

0.229344

0
4

5.71764857681956e-15
4

0
4

0
4

0
4

5.58022
3

5.58022
3

5.58022
3

5.12153
3

5.12153
3

0
4

0.458688

0.458688

0

0
4

2.77555756156289e-16
3

0
4

0
4

0
4

0.0798828

0.0798828

0.0798828

0.0798828

0.0798828

0

0

0

0

0
4

0
4

0
4

0
4

0

0

0

0

0

0

0
4

0
4

0
4

0

0

0

0

0
4

0
4

0
4

0
4

7.17331

7.17331

7.17331

7.17331

7.04

0.133314

0
4

0
4

0
4

0
4

9.63599
4

9.63599
4

9.63599
4

7.233
4

7.03817
4

0.194836

0
4

2.40298

2.40298

0
4

4.44089209850063e-16
4

0
4

0
4

0
4

1.06211

1.06211

1.06211

1.06211

1.06211

0
4

0
4

0
4

0
4

0.0798828
4

0.0798828
4

0

0

0

0

0

0

0
4

0
4

0.0798828

0.0798828

0.0798828

0
4

0
4

0
4

0

0

0

0

0

0
4

0

0

0
4

0
4

0
4

0
4

0.360925
4

0.360925
4

0.360925
4

0.360925
4

0.360925
4

0

0
4

0

0

0

0
4

0
4

0
4

0
4

0

0

0

0

0

0

0

0

0
4

0

0

0
4

0
4

0
4

0
4

0

0

0

0

0

0

0

0

0

0
4

0

0

0

0
4

0

0

0
4

0
4

0
4

0
4

32.4965
4

29.5439
4

29.5439
4

11.1394
4

0.975407
4

0.183475

0.257395

0.0649455

0.0649455

0.0649455

0.0649455

0

0.0649455

0

0.0917376

1.07996
4

0.0974182

0.119824

0.0974182

0.0649455

0.0649455

0

0.0723307

0.0649455

0.0649455

0.0974182

0.936789

2.97177
4

2.14202
4

0.689643

0.301488

0.360776

0.0799885

2.77555756156289e-17
4

0
4

11.8404
4

6.85351
4

2.22358
4

0.65717

0.359235

0.350576

0.552036

0.0649455

0.162364

0.616982

1.99840144432528e-15
4

0
4

6.40174
4

3.1527
4

2.36457
4

0.397388
4

0.0649455

0.324727

0.0974182

2.08166817117217e-16
4

0
4

0.0649455

0.0649455

0

0
4

0.0974182

0.0974182

0
4

0

0

0
4

0
4

0
4

2.95264
4

2.95264
4

2.34369
4

1.50586
4

0.729337

0

0.108496

1.38777878078145e-16
4

0
4

0.379606

0.282188

0.0974182

4.16333634234434e-17

0
4

0.229344

0.137606

0.0917376

0
4

0
4

0
4

0

0

0

0

0
4

0
4

0
4

0
4

0
5

0
5

0
5

0
5

0

0

0

0

0
4

0
4

0
4

0
4

0.0798828

0.0798828

0.0798828

0.0798828

0

0.0798828

0
4

0
4

0
4

0
4

0
5

0
5

0
5

0
5

0
4

0

0

0
4

0

0

0
4

0

0

0
4

0
4

0
4

0
4

0

0

0

0

0

0

0

0

0

0

0
4

0

0

0
4

0
4

0
4

0
4

0

0

0

0

0

0

0

0
4

0
4

0
4

0
4

0

0

0

0

0

0

0

0
4

0
4

0
4

0
4

0

0

0

0

0

0

0

0
4

0
4

0
4

0
4

0
3

0
3

0
3

0
3

0
3

0
4

0
4

0
4

0
4

0

0

0

0

0

0

0

0
4

0

0

0
4

0

0

0
4

0
4

0
4

0
4

0

0

0

0

0

0
4

0
4

0
4

0
4

37.9113
4

37.9113
4

37.9113
4

37.9113
4

37.7963
4

0

0

0

0

0.0808509

0

0

0.0341641

0
4

0
4

0
4

0
4

0

0

0

0

0

0

0
4

0
4

0
4

0
4

0

0

0

0

0

0

0

0

0

0
4

0
4

0
4

0

0

0

0

0

0
4

0

0

0
4

0
4

0
4

0
4

0
7

0
7

0
7

0
7

0

0

0

0

0
4

0
4

0
4

0
4

0
7

0
7

0
7

0
7

0
7

0
4

0
4

0
4

0
4

8.33765

8.33765

8.33765

0.279275

0.0507772

0.0761658

0.0507772

0.0507772

0.0507772

0
4

8.0076

7.90605

0.101554

5.68989300120393e-16

0
4

0.0507772

0.0507772

0
4

3.81639164714898e-16

0
4

0
4

0
4

0

0

0

0

0

0
4

0
4

0
4

0
4

0

0

0

0

0

0

0
4

0

0

0
4

0

0

0
4

0

0

0
4

0
4

0
4

0
4

7.3903

7.3903

7.3903

7.31031

7.25699

0.0533257

0
4

0.0799885

0.0799885

0
4

0
4

0
4

0
4

2.91063
4

2.91063
4

2.91063
4

2.91063
4

2.91063

0

0

0
4

0
4

0
4

0
4

0

0

0

0

0

0

0

0

0
4

0
4

0
4

0

0

0

0

0
4

0
4

0
4

0
4

230.753
4

230.753
4

230.753
4

204.3
4

18.0782
4

0.0799885

0.239648

0.137606

0

0.0974182

0

0.144661

0.0649455

164.979

0.601975

18.412

0.591894

0.183475

0.462003

0.0649455

0.162364

2.18991491607312e-14
4

0
4

0.546356

0.189156

0.162364

0.194836

0
4

24.4976
3

24.4976
3

0

0
4

0.819203

0.3572

0.389673

0.0723307

4.16333634234434e-17

0
4

0.159766

0.159766

0
4

0.332443

0.332443

0
4

0.0974182

0.0974182

0
4

0

0

0
4

0
4

0
4

0
4

2.25209

2.25209

2.25209

1.98398

1.55501

0.428969

0
4

0.268106

0.268106

0

0
4

2.22044604925031e-16

0
4

0
4

0
4

1.41064
4

1.41064
4

1.41064
4

1.41064
4

1.41064

0

0

0
4

0
4

0
4

0
4

28.2003
4

28.2003
4

28.2003
4

28.2003
4

27.9109
4

0.289421

1.33226762955019e-15
4

0
4

0
4

0
4

0
4

2.6449
4

2.6449
4

2.6449
4

2.57996
4

2.57996
4

0
4

0.0649455

0.0649455

0

0
4

9.71445146547012e-17
4

0
4

0
4

0
4

0

0

0

0

0

0

0

0
4

0
4

0
4

0
4

4.7529

4.7529

4.7529

4.16492

2.59695

1.56797

0
4

0.48999

0.293994

0.195996

2.77555756156289e-17

0
4

0.097998

0.097998

0
4

0
4

0
4

0
4

0

0

0

0

0

0
4

0

0

0

0
4

0

0

0

0
4

0

0

0
4

0
4

0
4

0
4

0

0

0

0

0

0

0

0

0
4

0

0

0
4

0
4

0
4

0
4

14.3362

14.0532

14.0532

9.46683

9.17658

0.0482775

0.0724163

0.121276

0.0482775

0
4

4.53809

4.53809

0
4

0.0482775

0.0482775

0
4

0
4

0
4

0.282978

0.282978

0.282978

0.282978

0
4

0
4

0
4

2.22044604925031e-16

0
4

0

0

0

0

0

0
4

0
4

0
4

0
4

124.271
4

124.271
4

124.271
4

121.293
4

119.283
4

1.28277
3

0.0974182

0.239648

0.162364

0.129891

0.0974182

1.6306400674182e-14
4

0
4

0.658906
4

0.217242

0.23189

0.0798828

0

0.129891

8.32667268468867e-17
4

0
4

1.47007

1.1106

0.359473

5.55111512312578e-17

0
4

0.449422

0.159766

0.159766

0

0.129891

0
4

0

0

0
4

0.399414

0.399414

0
4

0
4

0
4

0
4

0.552036

0.422145

0.422145

0.422145

0.422145

0
4

0

0

0
4

0

0

0
4

0
4

0
4

0.129891

0.129891

0.129891

0.0649455

0.0649455

0
4

0
4

0
4

2.77555756156289e-17

0
4

0

0

0

0

0

0
4

0

0

0
4

0
4

0
4

0

0

0

0

0
4

0
4

0
4

0
4

0
7

0
7

0
7

0
7

0
7

0
4

0
4

0
4

0
4

0

0

0

0

0

0

0

0
4

0

0

0
4

0
4

0
4

0
4

0

0

0

0

0

0

0

0
4

0
4

0
4

0
4

0

0

0

0

0

0

0

0
4

0
4

0
4

0
4

0

0

0

0

0

0

0
4

0
4

0
4

0
4

0

0

0

0

0

0
4

0
4

0
4

0
4

3.42517
3

3.42517
3

3.42517
3

0.958594
3

0

0

0.958594

0
4

2.46657

2.46657

0
4

0
4

0
4

0
4

0.416465

0.416465

0.416465

0.351519

0.351519

0
4

0.0649455

0.0649455

0
4

0
4

0
4

0
4

0

0

0

0

0

0

0

0

0

0

0

0

0

0

0

0
4

0

0

0
4

0

0

0
4

0
4

0
4

0
4

0.199707

0.199707

0.199707

0.119824

0

0.119824

0
4

0.0798828

0.0798828

0
4

0

0

0
4

0
4

0
4

0
4

0
2

0
2

0
2

0
2

0
2

0
4

0

0

0
4

0
4

0
4

0
4

0
7

0
7

0
7

0
7

0
7

0
4

0
4

0
4

0
4

0
7

0
7

0
7

0
7

0
7

0
4

0
4

0
4

0
4

0

0

0

0

0

0

0

0
4

0

0

0
4

0

0

0
4

0
4

0
4

0
4

0

0

0

0

0

0

0
4

0

0

0
4

0
4

0
4

0
4

0

0

0

0

0

0

0
4

0
4

0
4

0
4

0

0

0

0

0

0

0
4

0

0

0
4

0

0

0
4

0
4

0
4

0
4

0

0

0

0

0

0
4

0
4

0
4

0
4

5.43268

5.43268

5.43268

5.43268

5.14302

0.289665

0
4

0
4

0
4

0
4

1.23846

0

0

0

0

0
4

0
4

0
4

0

0

0

0

0
4

0
4

0
4

0

0

0

0

0
4

0
4

0
4

1.23846

1.23846
3

1.23846
3

1.23846
3

0

0
4

0

0

0
4

0

0

0

0
4

0
4

0

0

0

0
4

0
4

0

0

0

0
4

0
4

0

0

0

0
4

0
4

0

0

0

0

0
4

0

0

0
4

0

0

0
4

0
4

0

0

0

0

0
4

0
4

0

0

0

0

0
4

0
4

0

0

0

0
4

0
4

0

0

0

0
4

0

0

0
4

0

0

0
4

0
4

0

0

0

0
4

0
4

0

0

0

0

0
4

0
4

0

0

0

0
4

0
4

0
6

0
6

0
6

0

0
4

0
4

0
4

0
4

0

0

0

0

0

0

0
4

0
4

0
4

0

0

0

0

0

0

0
4

0
4

0
4

0

0

0

0

0
4

0

0

0
4

0
4

0
4

0

0

0

0

0
4

0
4

0
4

0

0

0

0

0
4

0
4

0
4

0

0

0

0

0
4

0
4

0
4

0

0

0

0

0
4

0
4

0
4

0

0

0

0

0
4

0
4

0
4

0

0

0

0

0

0
4

0
4

0

0

0

0

0
4

0
4

0

0

0

0
4

0
4

0

0

0

0
4

0
4

0

0

0

0
4

0
4

0

0

0

0

0
4

0

0

0
4

0

0

0
4

0

0

0
4

0
4

0
4

0
4

0

0

0

0

0

0
4

0
4

0
4

0
4

7.3461
3

0

0

0

0

0
4

0
4

0
4

0

0

0

0

0
4

0
4

0
4

7.3461
3

0

0

0

0
4

0
4

7.3461
3

7.3461
3

0
2

0

0

2.30571

0

0

0

0

0

0

0

0
2

0

0

3.8071

1.0188

0

0

0

0

0.107242

0

0
2

0

0

0

0

0

0

0

0

0

0

0
2

0

0

0

0

0

0

0

0

0

0

0
2

0

0

0

0

0

0

0.107242

0

0

0

0

0

0
2

0

3.05311331771918e-16
3

0
4

0
4

0
4

0
4

0

0

0

0

0

0
4

0
4

0
4

0
4

0
2

0

0

0

0

0
4

0
4

0
4

0

0

0

0

0
4

0
4

0
4

0
2

0
2

0
2

0
2

0

0

0

0

0

0

0
4

0
4

0
2

0
2

0
2

0

0
4

0

0

0
4

0
4

0

0

0

0
4

0
4

0
3

0
3

0

0

0

0

0

0

0

0

0

0

0

0

0

0

0

0
4

0
4

0
4

0
4

10.2146

0
7

0
7

0
7

0
7

0

0

0

0

0

0

0

0
4

0

0

0
4

0

0

0
4

0

0

0
4

0
4

0

0

0

0
4

0
4

0
4

0

0

0

0

0

0
4

0
4

0
4

0

0

0

0

0
4

0
4

0
4

0

0

0

0

0
4

0
4

0
4

0

0

0

0

0
4

0
4

0
4

0

0

0

0

0
4

0

0

0
4

0
4

0
4

0

0

0

0

0
4

0
4

0
4

0

0

0

0

0
4

0
4

0
4

0

0

0

0

0
4

0
4

0
4

0

0

0

0

0
4

0
4

0
4

0

0

0

0

0
4

0
4

0
4

0
7

0
7

0
7

0

0

0

0
4

0

0

0

0

0
4

0

0

0

0
4

0

0

0

0
4

0

0

0
4

0
4

0
4

0

0

0

0

0
4

0
4

0
4

0

0

0

0

0
4

0
4

0
4

0

0

0

0

0
4

0
4

0
4

0

0

0

0

0
4

0
4

0
4

0.0533257

0.0533257

0.0533257

0.0533257

0
4

0
4

0
4

0

0

0

0

0
4

0
4

0
4

0.0724163

0.0724163

0.0724163

0.0724163

0
4

0
4

0
4

0

0

0

0

0
4

0
4

0
4

0

0

0

0

0
4

0
4

0
4

0

0

0

0

0
4

0
4

0
4

0

0

0

0

0

0

0

0

0

0
4

0
4

0
4

0

0

0

0

0
4

0
4

0
4

0

0

0

0

0

0
4

0

0

0

0
4

0
4

0
4

0

0

0

0

0
4

0
4

0
4

0

0

0

0

0

0

0
4

0
4

0
4

0

0

0

0

0

0
4

0
4

0
4

0

0

0

0

0
4

0
4

0
4

0

0

0

0

0
4

0

0

0
4

0
4

0
4

10.0889

0

0

0

0
4

0
4

0.599121

0.599121

0.599121

0
4

0

0

0
4

0
4

0

0

0

0
4

0
4

0

0

0

0
4

0
4

0

0

0

0
4

0
4

0

0

0

0
4

0
4

0

0

0

0
4

0
4

0

0

0

0
4

0
4

0

0

0

0
4

0
4

0

0

0

0
4

0
4

0

0

0

0
4

0
4

0
7

0
7

0

0

0

0
4

0

0

0
4

0

0

0
4

0
4

0

0

0

0
4

0
4

0
7

0
7

0
7

0
4

0
4

0

0

0

0

0
4

0
4

0

0

0

0
4

0
4

0

0

0

0
4

0

0

0
4

0

0

0
4

0
4

0

0

0

0
4

0
4

0

0

0

0
4

0
4

0

0

0

0
4

0
4

9.48979

0.619704

0
6

0

0
7

0

0

0

0

0

0

0

0

0
5

0.239149

0

0.107242

0

0

0

0

0

0

0

0
7

0

0

0

0

0

0

0

0

0

0

0
6

0

0

0

0

0

0

0

0

0

0

0
7

0

0

0

0

0

0

0

0

0

0

0
5

0

0.273313

0

0

0

0

0

0

0

0

0
7

0

0

0

0

0

0

0

0

0

0

5.55111512312578e-17

0
4

0

0

0

0
4

8.81884

8.81884

0
4

0

0

0
4

0

0

0
4

0.0512461

0.0512461

0
4

0

0

0
4

0

0

0
4

0

0

0
4

0
4

0
4

0
4

40.2336

3.64989
3

3.36057
3

2.52877
3

0

1.08215
3

0

0

0

0

1.44661

0
4

0

0

0

0

0
4

0.831803

0

0.831803

0
4

0
4

0.289323

0.289323

0.289323

0

0

0
4

0

0

0
4

0
4

0

0

0

0

0
4

0

0

0
4

0
4

0

0

0

0
4

0

0

0
4

0
4

0
4

0

0

0

0

0
4

0
4

0
4

0.0798828

0.0798828

0.0798828

0

0.0798828

0

0
4

0
4

0
4

0

0

0

0

0
4

0
4

0
4

1.37197

1.37197

1.37197

1.27397

0.097998

0
4

0
4

0
4

1.7695

1.7695

1.7695

1.44777

0.321727

0
4

0
4

0
4

0

0

0

0

0
4

0
4

0
4

0

0

0

0

0

0
4

0
4

0
4

0

0

0

0

0
4

0
4

0
4

0

0

0

0

0

0
4

0
4

0
4

0

0

0

0

0

0
4

0
4

0
4

0
3

0
3

0
3

0
3

0

0

0

0

0

0

0
4

0

0

0
4

0

0

0
4

0

0

0
4

0
4

0
4

0

0

0

0

0
4

0
4

0
4

0

0

0

0

0
4

0
4

0
4

0

0

0

0

0
4

0

0

0
4

0
4

0
4

0

0

0

0

0

0
4

0
4

0
4

0

0

0

0

0
4

0
4

0
4

0

0

0

0

0
4

0

0

0
4

0
4

0
4

0

0

0

0

0
4

0
4

0
4

0.0798828

0.0798828

0.0798828

0.0798828

0
4

0
4

0
4

0.175085

0.175085

0.175085

0.175085

0
4

0
4

0
4

0

0

0

0

0
4

0
4

0
4

0
6

0
6

0
6

0
7

0

0

0

0

0

0
4

0
4

0
4

0

0

0

0

0
4

0
4

0
4

0

0

0

0

0
4

0
4

0
4

0

0

0

0

0
4

0
4

0
4

0

0

0

0

0
4

0
4

0
4

2.7996

2.7996

2.7996

2.7996

0
4

0
4

0
4

0

0

0

0

0
4

0
4

0
4

0

0

0

0

0
4

0
4

0
4

0

0

0

0

0
4

0
4

0
4

0

0

0

0

0
4

0
4

0
4

1.71497

1.71497

1.71497

1.71497

0
4

0
4

0
4

0
7

0
7

0
7

0
6

0

0

0

0

0
4

0

0

0
4

0
4

0
4

0

0

0

0

0
4

0
4

0
4

0.0798828

0.0798828

0.0798828

0.0798828

0
4

0
4

0
4

0

0

0

0

0
4

0
4

0
4

0

0

0

0

0
4

0
4

0
4

0

0

0

0

0
4

0
4

0
4

0

0

0

0

0
4

0
4

0
4

0

0

0

0

0
4

0
4

0
4

0

0

0

0

0
4

0
4

0
4

0

0

0

0

0
4

0
4

0
4

3.15537

3.15537

3.15537

0.639062

2.35654

0.159766

0
4

0
4

0
4

0

0

0

0

0

0
4

0
4

0
4

0

0

0

0

0

0

0
4

0
4

0

0

0

0
4

0
4

0
4

0
3

0
3

0
3

0

0

0

0
4

0
4

0

0

0

0
4

0
4

0
4

0.612799

0.612799

0.612799

0.612799

0
4

0

0

0
4

0

0

0
4

0
4

0
4

24.7447
3

0

0

0

0

0

0

0

0
4

0
4

0

0

0

0
4

0
4

0

0

0

0
4

0
4

0

0

0

0
4

0

0

0
4

0
4

0

0

0

0
4

0
4

0

0

0

0
4

0
4

0

0

0

0
4

0
4

0

0

0

0
4

0
4

0

0

0

0
4

0
4

0

0

0

0
4

0
4

0

0

0

0
4

0
4

0.525256

0.525256

0

0

0.525256

0
4

0
4

0

0

0

0
4

0
4

0.0533257

0.0533257

0.0533257

0
4

0
4

0

0

0

0
4

0
4

0.101554

0.101554

0.101554

0
4

0
4

0

0

0

0
4

0
4

0.0798828

0.0798828

0.0798828

0
4

0
4

0

0

0

0
4

0
4

0

0

0

0
4

0
4

0.266628

0.266628

0.266628

0
4

0
4

0.536212

0.536212

0.536212

0
4

0
4

7.52867

7.52867

0

7.52867

0
4

0

0

0
4

0
4

0

0

0

0
4

0
4

0

0

0

0
4

0
4

0

0

0

0
4

0
4

0.107242

0.107242

0.107242

0
4

0
4

2.05198

1.9787

1.92984

0.0488567

5.55111512312578e-17

0
4

0.0732851

0.0732851

0
4

2.08166817117217e-16

0
4

1.66331

0

0

0

0
4

1.66331

1.66331

0
4

0
4

0

0

0

0
4

0

0

0
4

0

0

0
4

0
4

0

0

0

0

0
4

0
4

0

0

0

0
4

0
4

0

0

0

0

0
4

0
4

11.8307
3

11.8307
3

1.75085
3

9.8305
3

0
7

0.195996

0

0.0533257

0

0

0

0

1.07552855510562e-15
3

0
4

0

0

0
4

0

0

0
4

0

0

0
4

0

0

0
4

0
4

0
4

0
4

9.18869

0
7

0
7

0
7

0
7

0

0
4

0

0

0

0

0
4

0
4

0
4

0

0

0

0

0
4

0
4

0
4

0

0

0

0

0
4

0
4

0
4

0

0

0

0

0
4

0
4

0
4

0

0

0

0

0
4

0
4

0
4

0.0798828

0.0798828

0.0798828

0.0798828

0
4

0
4

0
4

0

0

0

0

0
4

0
4

0
4

0

0
6

0
6

0
6

0

0

0

0

0

0
4

0
4

0

0

0

0
4

0
4

0
4

0
7

0
7

0
7

0
7

0

0

0
4

0
4

0
4

0

0

0

0

0
4

0

0

0
4

0
4

0
4

0.798828

0.798828

0.798828

0.798828

0
4

0

0

0
4

0
4

0
4

0

0

0

0

0
4

0

0

0
4

0
4

0
4

0

0

0

0

0

0
4

0
4

0
4

0

0

0

0

0

0
4

0
4

0
4

0

0

0

0

0
4

0
4

0
4

8.30997
3

0
7

0

0

0

0
4

0

0

0
4

0

0

0
4

0
4

0

0

0

0

0
4

0
4

0

0

0

0
4

0
4

0

0

0

0
4

0
4

0.700341

0.700341

0.700341

0
4

0
4

0

0

0

0
4

0
4

7.60963
3

7.60963
3

0

0

0

0.0808509

0

0

0.126943

0.0507772

0

0

0

0
7

0

0

0.334737

0

0

0

0

0

0

0

1.05204
5

0

0

0

0

0

0

0

0

0

0.0507772

0

0

0

0

0

0

0

0

0

0

0.160864

0
7

0

0

0

0

0

0

0

0

0

0

1.75181
3

0

0

0

0

0

0

0

0

0

0

0

0

0

0.0507772

0

0

0

0
7

3.95005

0
4

0

0

0

0
4

0

0

0
4

0

0

0
4

0
4

0
4

0
4

0

0

0

0

0

0

0
4

0
4

0
4

0
4

0
2

0

0

0

0

0

0

0
4

0
4

0
4

0

0

0

0

0
4

0
4

0
4

0

0

0

0

0

0

0

0
4

0

0

0
4

0
4

0
4

0

0

0

0

0
4

0
4

0
4

0

0

0

0

0
4

0
4

0
4

0

0

0

0

0
4

0
4

0
4

0

0

0

0

0
4

0
4

0
4

0

0

0

0

0
4

0
4

0
4

0

0

0

0

0
4

0
4

0
4

0

0

0

0

0
4

0
4

0
4

0
2

0

0

0

0

0
4

0

0

0
4

0
4

0

0

0

0

0
4

0
4

0

0

0

0
4

0
4

0

0

0

0
4

0
4

0

0

0

0
4

0
4

0

0

0

0
4

0
4

0

0

0

0
4

0
4

0

0

0

0
4

0
4

0

0

0

0
4

0
4

0
2

0
2

0

0
2

0
2

0
4

0

0

0

0
4

0

0

0
4

0

0

0
4

0

0

0
4

0

0

0
4

0

0

0
4

0

0

0
4

0

0

0
4

0

0

0
4

0

0

0
4

0

0

0
4

0
4

0
4

0
4

0
2

0
2

0
2

0
2

0

0

0

0

0

0

0

0

0

0

0

0
4

0
4

0
4

0
4

0

0

0

0

0

0
4

0
4

0
4

0
4

11.3086
2

0
2

0
2

0

0

0

0

0

0

0

0

0

0

0

0

0

0

0

0

0

0

0

0

0

0

0

0

0

0

0

0

0

0

0

0

0

0

0

0

0

0

0

0

0

0

0

0

0

0

0

0

0

0

0

0

0

0

0

0

0

0

0

0

0

0

0

0

0

0

0

0

0

0

0

0

0

0

0

0

0

0

0

0

0

0

0

0

0

0

0

0

0

0

0
4

0

0

0
4

0

0

0
4

0

0

0
4

0

0

0

0

0

0

0

0

0

0

0

0

0

0

0

0

0

0

0

0

0

0

0

0

0

0

0

0

0
4

0

0

0

0

0

0

0

0

0

0
4

0

0

0

0
4

0

0

0

0

0
4

0

0

0

0
4

0

0

0

0
4

0

0

0

0
4

0

0

0
4

0
4

0
4

1.93036
4

1.93036
4

1.93036
4

0

0

0

0

1.93036

0

0
4

0
4

0
4

0
7

0
7

0
7

0

0

0

0

0
4

0
4

0
4

0
4

0
4

0

0

0

0

0
4

0

0

0
4

0

0

0
4

0

0

0
4

0
4

0
4

0

0

0

0

0

0
4

0
4

0
4

0

0

0

0

0

0

0
4

0

0

0

0
4

0
4

0
4

0

0

0

0

0
4

0
4

0
4

0

0

0

0

0

0
4

0

0

0
4

0
4

0
4

0

0

0

0

0
4

0

0

0
4

0
4

0
4

0

0

0

0

0
4

0

0

0
4

0
4

0

0

0

0
4

0
4

0
4

0

0

0

0

0
4

0
4

0
4

8.84207
3

6.28112
3

0

0

0

0

0

0

0

0

0

0

0

0

0

0

0

0

0

0

0

0
4

6.28112
3

5.92888

0

0.107242

0.244995

6.10622663543836e-16
3

0
4

0

0

0

0
4

0

0

0
4

0

0

0
4

0

0

0
4

0
4

1.5516

0.244995
2

0
2

0

0.244995

0

0

0

0

0

0
4

1.30661

0.663155

0.643454

0

0
4

0

0

0

0

0

0

0
4

0

0

0
4

0

0

0
4

0

0

0
4

0

0

0
4

0

0

0
4

0

0

0
4

0
4

0.907797

0.746933

0.488072

0.160864

0.097998

0
4

0.160864

0.160864

0
4

5.55111512312578e-17

0
4

0.101554

0.101554

0.101554

0
4

0

0

0
4

0
4

0

0

0

0
4

0
4

0

0

0

0
4

0
4

0

0

0

0
4

0
4

0
4

0

0

0

0

0

0
4

0

0

0
4

0
4

0
4

0

0

0

0

0
4

0
4

0
4

0

0

0

0

0
4

0

0

0
4

0

0

0
4

0
4

0
4

0

0

0

0

0
4

0
4

0
4

0

0

0

0

0
4

0

0

0
4

0
4

0
4

0

0

0

0

0
4

0
4

0
4

0

0

0

0

0
4

0
4

0
4

0

0

0

0

0
4

0

0

0
4

0
4

0
4

0

0

0

0

0
4

0

0

0
4

0
4

0
4

0

0

0

0

0
4

0
4

0
4

0

0

0

0

0

0

0

0

0

0

0

0

0

0

0

0

0
4

0

0

0

0
4

0

0

0
4

0

0

0
4

0
4

0

0

0

0

0

0

0
4

0

0

0
4

0
4

0
4

0

0

0

0

0

0
4

0
4

0
4

0

0

0

0

0

0
4

0
4

0
4

0

0

0

0

0
4

0

0

0
4

0
4

0
4

0

0

0

0

0
4

0

0

0
4

0
4

0
4

0

0

0

0

0
4

0
4

0
4

0

0

0

0

0
4

0
4

0
4

0

0

0

0

0
4

0
4

0
4

0

0

0

0

0
4

0
4

0
4

0

0

0

0

0
4

0
4

0
4

0

0

0

0

0
4

0
4

0
4

0

0

0

0

0

0

0

0

0

0

0

0

0

0
4

0

0

0

0

0

0
4

0

0

0

0
4

0

0

0
4

0
4

0

0

0

0
4

0
4

0

0

0

0
4

0
4

0
4

0

0

0

0

0
4

0
4

0
4

0

0

0

0

0
4

0
4

0
4

0

0

0

0

0
4

0
4

0
4

0

0

0

0

0
4

0
4

0
4

0

0

0

0

0
4

0
4

0
4

0

0

0

0

0
4

0
4

0
4

0

0

0

0

0
4

0
4

0
4

0

0

0

0

0
4

0
4

0
4

0

0

0

0

0
4

0
4

0
4

0

0

0

0

0
4

0
4

0
4

0

0

0

0

0

0

0

0
4

0

0

0
4

0
4

0
4

0

0

0

0

0
4

0
4

0
4

0

0

0

0

0
4

0
4

0
4

0

0

0

0

0
4

0
4

0
4

0

0

0

0

0
4

0
4

0
4

0

0

0

0

0
4

0
4

0
4

0

0

0

0

0
4

0
4

0
4

0

0

0

0

0
4

0
4

0
4

0

0

0

0

0
4

0
4

0
4

0

0

0

0

0
4

0
4

0
4

0

0

0

0

0
4

0
4

0
4

0

0

0

0

0

0

0

0
4

0

0

0

0
4

0

0

0
4

0
4

0
4

0

0

0

0

0
4

0
4

0
4

0

0

0

0

0
4

0
4

0
4

0

0

0

0

0
4

0
4

0
4

0

0

0

0

0
4

0
4

0
4

0

0

0

0

0
4

0
4

0
4

0

0

0

0

0
4

0
4

0
4

0

0

0

0

0
4

0
4

0
4

0

0

0

0

0
4

0
4

0
4

0

0

0

0

0
4

0
4

0
4

0

0

0

0

0
4

0
4

0
4

0
2

0
2

0
2

0

0

0
4

0

0

0

0
4

0

0

0
4

0

0

0
4

0
4

0
4

0

0

0

0

0
4

0
4

0
4

0

0

0

0

0
4

0
4

0
4

0

0

0

0

0
4

0
4

0
4

0

0

0

0

0

0

0
4

0
4

0
4

0

0

0

0

0

0

0
4

0
4

0
4

0.536212

0

0

0

0

0

0

0

0

0
4

0

0

0

0
4

0

0

0

0
4

0

0

0
4

0

0

0
4

0

0

0
4

0
4

0

0

0

0

0
4

0

0

0

0
4

0

0

0
4

0
4

0

0

0

0

0
4

0
4

0

0

0

0
4

0
4

0

0

0

0
4

0

0

0
4

0
4

0

0

0

0
4

0
4

0

0

0

0
4

0
4

0

0

0

0
4

0
4

0

0

0

0
4

0
4

0

0

0

0
4

0
4

0

0

0

0
4

0
4

0
7

0
7

0
7

0

0

0

0
4

0
4

0

0

0

0
4

0
4

0

0

0

0
4

0
4

0

0

0

0
4

0
4

0

0

0

0
4

0
4

0

0

0

0
4

0
4

0

0

0

0
4

0
4

0

0

0

0
4

0
4

0

0

0

0
4

0
4

0

0

0

0
4

0
4

0

0

0

0
4

0
4

0
6

0

0

0

0

0
4

0

0

0

0
4

0
4

0

0

0

0
4

0
4

0.107242

0.107242

0.107242

0
4

0
4

0

0

0

0
4

0
4

0

0

0

0
4

0
4

0

0

0

0

0

0

0
4

0

0

0
4

0
4

0
7

0
7

0
7

0

0
4

0

0

0
4

0
4

0

0

0

0

0

0

0
4

0

0

0
4

0
4

0
8

0
8

0
8

0

0
4

0
4

0

0
6

0

0

0
4

0

0

0
4

0

0

0
4

0
4

0.428969
3

0.428969

0

0.107242

0.321727

0
4

0

0

0
4

0

0

0
4

0
4

0

0

0
7

0

0

0

0

0

0

0

0

0

0

0

0

0

0

0

0

0

0

0

0

0

0

0

0

0

0

0

0

0

0

0

0

0

0

0

0

0

0

0

0
4

0
7

0
7

0
4

0
4

0
4

1.88737914186277e-15
2

0
4

0.750696
7

0.643454

0.643454

0.643454

0.375348

0.160864

0.107242

0
4

0
4

0
4

0

0

0

0

0
4

0
4

0
4

0

0

0

0

0
4

0
4

0
4

0

0

0

0

0
4

0
4

0
4

0

0

0

0

0
4

0
4

0
4

0.107242
7

0

0

0

0

0

0
4

0
4

0

0

0

0
4

0
4

0

0

0

0
4

0
4

0

0

0

0
4

0
4

0

0

0

0
4

0
4

0.107242
7

0
7

0
7

0

0

0

0

0

0

0
4

0.107242

0.107242

0
4

0
4

0
4

4.16333634234434e-17
7

0
4

0

0

0

0

0

0

0
4

0

0

0
4

0

0

0
4

0

0

0
4

0
4

0
4

0
4

0

0

0

0

0

0
4

0
4

0
4

0

0
7

0
7

0
7

0
4

0
4

0

0

0

0
4

0
4

0

0

0

0
4

0
4

0

0

0

0

0
4

0

0

0
4

0
4

0
4

0
4

5.13403
3

0

0

0

0

0
4

0
4

0
4

0

0

0

0

0
4

0
4

0
4

5.13403
3

5.13403
3

5.13403
3

2.46595
3

0

0

0

0

0

0

0

0

0

0

2.50638
3

0

0.161702
3

0

0

0

0

0

0
4

0
4

0
4

0
4

0
7

0
7

0
7

0
7

0

0

0

0

0

0

0

0

0

0

0

0

0

0

0

0

0

0
6

0

0

0

0

0
4

0
4

0
4

0
4

631.104

123.578
4

114.469
4

112.215
4

80.6698
4

0.297372

0.17162

0.266823

0.779769

0.239648

0

0.0798828

0.0723307

0.0917376

0.119824

17.3623

0

0

0

0.175085

0.0917376

1.51208
4

2.3868
4

1.74939
4

3.45184

1.81658

0.400964

0.479297

4.59632332194815e-14
4

0
4

2.11623
3

1.56827
4

0.188379

0.27959

0

0.0799885

2.77555756156289e-17
3

0
4

0.137606

0.137606

0
4

0
4

8.82962
4

8.08542
4

1.87773

0.262628

0.479297

0.0798828

0

0.0798828

0.0533257

1.14672

0.119824

0.199707

0.213303

0.331196
4

0.0917376

0.0798828

0

0.0917376

0

0.565997
4

0.399414

1.17608

0.144661

0.0917376

0.27959

0.321081

0
4

0.36695

0.183475

0.183475

0
4

0.217489

0.217489

0
4

0.0798828

0.0798828

0
4

0.0798828

0.0798828

0
4

4.08006961549745e-15
4

0
4

0.27959

0.27959

0.27959

0
4

0

0

0
4

0
4

0

0

0

0
4

0
4

1.83741910575463e-14
4

0
4

0
5

0
5

0
5

0
5

0

0

0

0

0
4

0
4

0

0

0

0

0
4

0

0

0
4

0
4

0
4

0

0

0

0

0
4

0

0

0
4

0
4

0

0

0

0
4

0
4

0

0

0

0
4

0
4

0
4

6.50573

6.39908

6.29243

0

6.29243

0
4

0.106651

0.106651

0
4

2.91433543964104e-16

0
4

0.106651

0.106651

0.106651

0
4

0
4

2.91433543964104e-16

0
4

0

0

0

0

0
4

0

0

0
4

0
4

0
4

0

0

0

0

0

0
4

0

0

0
4

0
4

0
4

0

0

0

0

0

0
4

0

0

0
4

0
4

0
4

0

0

0

0

0

0
4

0
4

0
4

1.16788

1.16788

1.16788

1.1171

0.0507772

0
4

0
4

0
4

0.386483

0.386483

0

0

0

0
4

0.386483

0.386483

0
4

0

0

0
4

0
4

0
4

0

0

0

0

0
4

0
4

0
4

0

0

0

0

0

0
4

0
4

0
4

7.51892

7.51892

1.73308

0.55992

1.11984

0.0533257

2.70616862252382e-16

0
4

5.78584

5.38589

0.0799885

0.0533257

0.159977

0.106651

0
4

0
4

0
4

0

0

0

0

0

0
4

0

0

0

0
4

0
4

0
4

0

0

0

0

0

0
4

0

0

0
4

0
4

0
4

0

0

0

0

0

0

0
4

0
4

0
4

0

0

0

0

0

0
4

0
4

0
4

0

0

0

0

0
4

0

0

0
4

0
4

0
4

0

0

0

0

0
4

0

0

0
4

0
4

0
4

0

0

0

0

0
4

0
4

0
4

1.03848

1.03848

1.03848

0.878711

0.159766

0
4

0
4

0
4

0

0

0

0

0
4

0

0

0
4

0
4

0

0

0

0
4

0
4

0
4

0

0

0

0

0
4

0
4

0
4

0

0

0

0

0

0

0

0

0
4

0

0

0

0

0
4

0

0

0
4

0

0

0
4

0
4

0
4

0

0

0

0

0
4

0

0

0
4

0
4

0

0

0

0
4

0
4

0
4

0

0

0

0

0
4

0
4

0
4

0

0

0

0

0
4

0
4

0
4

0

0

0

0

0

0

0
4

0
4

0
4

0

0

0

0

0

0
4

0
4

0
4

1.39637

1.39637

1.39637

1.39637

0
4

0
4

0
4

0

0

0

0

0

0
4

0

0

0
4

0
4

0
4

0.838769

0.838769

0.838769

0.718945

0.119824

5.55111512312578e-17

0
4

0
4

0
4

0

0

0

0

0

0
4

0
4

0

0

0

0
4

0
4

0
4

0.0798828

0.0798828

0.0798828

0.0798828

0

0
4

0
4

0
4

1.76039
4

1.76039
4

1.44075
4

1.17813
4

0

0.262628

0
4

0

0

0
4

0.239648

0.239648

0
4

0

0

0
4

0

0

0
4

0.0799885

0.0799885

0
4

2.4980018054066e-16
4

0
4

0
4

0

0

0

0

0
4

0
4

0
4

0

0

0

0

0
4

0
4

0
4

0

0

0

0

0
4

0

0

0
4

0

0

0
4

0
4

0
4

0

0

0

0

0
4

0
4

0
4

0

0

0

0

0
4

0

0

0
4

0
4

0
4

0.129891

0.129891

0.129891

0

0.129891

0
4

0
4

0
4

0

0

0

0

0
4

0

0

0
4

0
4

0
4

0

0

0

0

0
4

0
4

0
4

0

0

0

0

0
4

0
4

0
4

0

0

0

0

0

0
4

0

0

0
4

0
4

0
4

0

0

0

0

0

0

0
4

0

0

0

0

0
4

0
4

0
4

0

0

0

0

0

0
4

0
4

0

0

0

0
4

0
4

0
4

0.394242

0.394242

0.394242

0.234476

0.159766

2.77555756156289e-17

0
4

0
4

0
4

0

0

0

0

0
4

0
4

0
4

0

0

0

0

0
4

0
4

0
4

0

0

0

0

0

0
4

0
4

0
4

7.78555

7.78555

7.73222

7.73222

0
4

0.0533257

0.0533257

0
4

0
4

0
4

0.316449

0.316449

0.316449

0.316449

0
4

0
4

0
4

0.997259

0.997259

0.263358

0.0798828

0.183475

2.77555756156289e-17

0
4

0.733901

0.733901

0
4

1.11022302462516e-16

0
4

0
4

0

0

0

0

0
4

0
4

0
4

0

0

0

0

0
4

0

0

0
4

0
4

0
4

1.34112
4

1.34112
4

1.34112
4

1.26124
4

0.0798828

0
4

0
4

0
4

0.289323

0.289323

0.289323

0.289323

0
4

0

0

0
4

0
4

0
4

2.10102

2.10102

2.10102

1.75085

0.350171

2.22044604925031e-16

0
4

0
4

0
4

0

0

0

0

0

0
4

0

0

0
4

0
4

0
4

0.877483

0.877483

0.779769

0.779769

0
4

0.0977135

0.0977135

0
4

1.38777878078145e-17

0
4

0
4

0

0

0

0

0

0
4

0
4

0
4

0

0

0

0

0
4

0

0

0
4

0
4

0
4

0.359473

0.359473

0.359473

0.359473

0
4

0
4

0
4

0

0

0

0

0
4

0

0

0
4

0
4

0
4

0

0

0

0

0
4

0

0

0
4

0
4

0
4

0

0

0

0

0
4

0

0

0
4

0
4

0
4

8.48266
3

8.48266
3

8.48266
3

4.25132
3

0

4.23135

0

0
4

0

0

0
4

0
4

0
4

0

0

0

0

0
4

0

0

0
4

0
4

0
4

0

0

0

0

0

0
4

0
4

0
4

4.19385

4.19385

4.19385

4.11396

0.0798828

0
4

0
4

0
4

0

0

0

0

0
4

0

0

0
4

0
4

0
4

0

0

0

0

0
4

0
4

0
4

0

0

0

0

0
4

0

0

0
4

0
4

0
4

0

0

0

0

0
4

0
4

0
4

0.0917376

0.0917376

0.0917376

0

0.0917376

0
4

0
4

0
4

0

0

0

0

0
4

0
4

0
4

0

0

0

0

0
4

0
4

0
4

0

0

0

0

0

0

0

0
4

0
4

0
4

2.18342

2.18342

2.18342

2.18342

0
4

0
4

0
4

0

0

0

0

0

0
4

0
4

0
4

0

0

0

0

0
4

0
4

0
4

0

0

0

0

0
4

0

0

0
4

0
4

0
4

0

0

0

0

0
4

0
4

0
4

0

0

0

0

0
4

0

0

0
4

0
4

0
4

0

0

0

0

0
4

0
4

0
4

0

0

0

0

0
4

0
4

0
4

0.319531

0.319531

0.0798828

0.0798828

0
4

0.239648

0.239648

0
4

0
4

0
4

0.958594

0.958594

0.958594

0.958594

0
4

0
4

0
4

0

0

0

0

0
4

0
4

0
4

0

0

0

0

0
4

0
4

0
4

0

0

0

0

0
4

0

0

0
4

0
4

0
4

0

0

0

0

0
4

0

0

0
4

0
4

0
4

0.239648

0.239648

0.239648

0.0798828

0.159766

2.77555756156289e-17

0
4

0
4

0
4

0

0

0

0

0
4

0

0

0
4

0
4

0
4

0

0

0

0

0
4

0

0

0
4

0
4

0
4

0

0

0

0

0
4

0
4

0
4

0

0

0

0

0
4

0

0

0
4

0
4

0
4

0

0

0

0

0
4

0
4

0
4

0.537424

0.537424

0.537424

0.0732851

0.464139

0
4

0
4

0
4

0
6

0
6

0
6

0
6

0

0
4

0

0

0
4

0

0

0
4

0
4

0
4

0

0

0

0

0
4

0
4

0
4

0

0

0

0

0
4

0
4

0
4

0

0

0

0

0

0
4

0
4

0
4

0.17315

0.17315

0.0533257

0.0533257

0
4

0.119824

0.119824

0
4

0
4

0
4

0.976464

0.976464

0.976464

0.687142

0.289323

0
4

0
4

0
4

0

0

0

0

0
4

0
4

0
4

0.92756

0.92756

0.92756

0.92756

0
4

0
4

0
4

0

0

0

0

0
4

0

0

0
4

0
4

0
4

0.168971

0.168971

0.168971

0

0.168971

0
4

0
4

0
4

0

0

0

0

0
4

0

0

0
4

0
4

0
4

130.355
4

130.355
4

127.122
4

124.986
4

0.229344

0.229344

0.119824

0.0917376

0.0798828

0.119824

0.0917376

0.251503

0

0.0649455

0.17162

0.239648

0.36695

0.0798828

0

3.09613445992341e-14
4

0
4

0.183475

0.183475

0
4

0

0

0
4

1.65901
4

0.76802

0

0.0917376

0

0.0798828

0.319954

0.399414

0
4

0.76474

0.192155

0.251503

0.321081

0

0
4

0.159766

0.159766

0
4

0.0917376

0.0917376

0
4

0.0533257

0.0533257

0
4

0

0

0
4

0.321081

0.321081

0
4

0

0

0
4

2.75335310107039e-14
4

0
4

0
4

0
4

0
4

0

0

0

0

0
4

0

0

0

0

0

0
4

0

0

0
4

0

0

0
4

0
4

0
4

0

0

0

0

0
4

0
4

0
4

0.107242

0.107242

0.107242

0.107242

0
4

0
4

0
4

0

0

0

0

0

0
4

0
4

0
4

0

0

0

0

0
4

0
4

0
4

0

0

0

0

0
4

0
4

0
4

0

0

0

0

0

0
4

0
4

0
4

0.289665

0.289665

0.289665

0.289665

0
4

0
4

0
4

0

0

0

0

0
4

0
4

0
4

0

0

0

0

0
4

0

0

0
4

0
4

0
4

0

0

0

0

0
4

0
4

0
4

24.8266

24.8266

24.8266

24.1295

0.482591

0.214485

1.9151347174784e-15

0
4

0
4

0
4

0

0

0

0

0
4

0

0

0
4

0
4

0
4

0

0

0

0

0
4

0

0

0
4

0
4

0
4

0

0

0

0

0

0
4

0
4

0
4

0

0

0

0

0
4

0

0

0
4

0
4

0
4

0

0

0

0

0
4

0
4

0
4

0

0

0

0

0
4

0

0

0
4

0
4

0
4

0

0

0

0

0
4

0

0

0
4

0
4

0
4

0

0

0

0

0
4

0

0

0
4

0
4

0
4

0

0

0

0

0
4

0
4

0
4

0

0

0

0

0
4

0
4

0
4

0
6

0
6

0

0

0

0
4

0

0

0

0
4

0
4

0
4

0

0

0

0

0
4

0
4

0
4

0.239966

0.239966

0.239966

0.239966

0
4

0
4

0
4

0

0

0

0

0
4

0
4

0
4

0

0

0

0

0
4

0
4

0
4

0

0

0

0

0
4

0
4

0
4

0

0

0

0

0
4

0
4

0
4

0

0

0

0

0
4

0
4

0
4

0

0

0

0

0
4

0
4

0
4

0.107242

0.107242

0.107242

0.107242

0
4

0
4

0
4

0

0

0

0

0
4

0
4

0
4

0

0

0

0

0

0

0
4

0
4

0
4

0

0

0

0

0
4

0
4

0
4

0

0

0

0

0
4

0
4

0
4

0.097998

0.097998

0.097998

0.097998

0
4

0
4

0
4

0

0

0

0

0
4

0
4

0
4

0.0533257

0.0533257

0.0533257

0.0533257

0
4

0
4

0
4

0

0

0

0

0
4

0
4

0
4

0

0

0

0

0
4

0
4

0
4

0

0

0

0

0
4

0
4

0
4

0

0

0

0

0
4

0
4

0
4

0

0

0

0

0
4

0
4

0
4

0

0

0

0

0

0

0
4

0
4

0
4

0.119824

0.119824

0.119824

0.119824

0
4

0
4

0
4

0

0

0

0

0
4

0
4

0
4

0

0

0

0

0
4

0
4

0
4

0

0

0

0

0
4

0
4

0
4

0

0

0

0

0
4

0
4

0
4

0

0

0

0

0
4

0
4

0
4

0

0

0

0

0
4

0
4

0
4

0

0

0

0

0
4

0
4

0
4

0

0

0

0

0
4

0
4

0
4

0

0

0

0

0
4

0
4

0
4

0

0

0

0

0

0

0
4

0
4

0
4

0

0

0

0

0
4

0
4

0
4

0.0533257

0.0533257

0.0533257

0.0533257

0
4

0
4

0
4

0

0

0

0

0
4

0
4

0
4

0

0

0

0

0
4

0
4

0
4

0

0

0

0

0
4

0
4

0
4

0

0

0

0

0
4

0
4

0
4

0

0

0

0

0
4

0
4

0
4

0

0

0

0

0
4

0
4

0
4

0

0

0

0

0
4

0
4

0
4

0.439355

0.439355

0.439355

0.439355

0
4

0
4

0
4

0

0

0

0

0

0

0

0

0
4

0
4

0
4

0

0

0

0

0
4

0
4

0
4

1.3456

1.3456

1.3456

1.3456

0
4

0
4

0
4

0

0

0

0

0
4

0
4

0
4

0

0

0

0

0
4

0
4

0
4

0

0

0

0

0
4

0
4

0
4

0.175085

0.175085

0.175085

0.175085

0
4

0
4

0
4

0

0

0

0

0
4

0
4

0
4

0

0

0

0

0
4

0
4

0
4

0

0

0

0

0
4

0
4

0
4

0

0

0

0

0
4

0
4

0
4

0

0

0

0

0

0
4

0
4

0
4

0

0

0

0

0
4

0
4

0
4

0

0

0

0

0
4

0
4

0
4

0

0

0

0

0
4

0
4

0
4

0

0

0

0

0
4

0
4

0
4

0

0

0

0

0
4

0
4

0
4

0

0

0

0

0
4

0
4

0
4

0

0

0

0

0
4

0
4

0
4

0

0

0

0

0
4

0
4

0
4

0

0

0

0

0
4

0
4

0
4

0

0

0

0

0
4

0
4

0
4

0

0

0

0

0

0

0
4

0
4

0
4

0

0

0

0

0
4

0
4

0
4

0

0

0

0

0
4

0
4

0
4

0

0

0

0

0
4

0
4

0
4

0

0

0

0

0
4

0
4

0
4

3.42993

3.42993

3.42993

3.42993

0
4

0
4

0
4

0

0

0

0

0
4

0
4

0
4

0

0

0

0

0
4

0
4

0
4

0

0

0

0

0
4

0
4

0
4

0

0

0

0

0
4

0
4

0
4

0

0

0

0

0
4

0
4

0
4

0
7

0
7

0
6

0
6

0
4

0

0

0
4

0
4

0

0

0

0
4

0
4

0
4

0

0

0

0

0
4

0
4

0
4

0

0

0

0

0
4

0
4

0
4

0.119824

0.119824

0.119824

0.119824

0
4

0
4

0
4

0

0

0

0

0
4

0
4

0
4

0

0

0

0

0
4

0
4

0
4

0

0

0

0

0
4

0
4

0
4

0

0

0

0

0
4

0
4

0
4

0

0

0

0

0
4

0
4

0
4

0.107242

0.107242

0.107242

0.107242

0
4

0
4

0
4

0

0

0

0

0
4

0
4

0
4

0
7

0
7

0
7

0
8

0

0

0

0

0

0
4

0
4

0
4

0.0798828
4

0.0798828
4

0

0

0

0
4

0

0

0
4

0.0798828

0.0798828

0
4

0

0

0
4

0

0

0
4

0

0

0
4

0

0

0
4

0
4

0
4

0

0

0

0

0
4

0
4

0
4

0

0

0

0

0
4

0
4

0
4

0

0

0

0

0
4

0
4

0
4

0

0

0

0

0
4

0
4

0
4

0

0

0

0

0
4

0
4

0
4

0

0

0

0

0
4

0
4

0
4

0

0

0

0

0
4

0
4

0
4

0

0

0

0

0
4

0
4

0
4

0

0

0

0

0
4

0
4

0
4

0

0

0

0

0
4

0
4

0
4

0
6

0
6

0
6

0
6

0

0
4

0
4

0
4

0

0

0

0

0
4

0
4

0
4

0.107242

0.107242

0.107242

0.107242

0
4

0
4

0
4

0.119824

0.119824

0.119824

0.119824

0
4

0
4

0
4

0

0

0

0

0
4

0
4

0
4

0

0

0

0

0
4

0
4

0
4

0

0

0

0

0
4

0
4

0
4

0

0

0

0

0
4

0
4

0
4

0

0

0

0

0
4

0
4

0
4

0

0

0

0

0
4

0
4

0
4

0.0798828

0.0798828

0.0798828

0.0798828

0
4

0
4

0
4

0.333021
4

0.333021
4

0.133314

0

0.133314

0
4

0

0

0

0
4

0.199707

0.199707

0
4

2.77555756156289e-17
4

0
4

0

0

0

0

0
4

0

0

0

0
4

0

0

0
4

0
4

0

0

0

0
4

0
4

0
4

2.53874

2.53874

2.53874

2.53874

0
4

0
4

0
4

0

0

0

0

0
4

0
4

0
4

0

0

0

0

0
4

0
4

0
4

0

0

0

0

0
4

0
4

0
4

0

0

0

0

0
4

0
4

0
4

0

0

0

0

0
4

0
4

0
4

0

0

0

0

0
4

0
4

0
4

0

0

0

0

0
4

0
4

0
4

0

0

0

0

0
4

0
4

0
4

0

0

0

0

0
4

0
4

0
4

0
6

0
6

0
6

0
6

0
4

0

0

0
4

0
4

0
4

0

0

0

0

0
4

0
4

0
4

0

0

0

0

0
4

0
4

0
4

0

0

0

0

0
4

0
4

0
4

0

0

0

0

0
4

0
4

0
4

0

0

0

0

0
4

0
4

0
4

0

0

0

0

0
4

0
4

0
4

0.350171

0.350171

0.350171

0.350171

0
4

0
4

0
4

0

0

0

0

0
4

0
4

0
4

0

0

0

0

0
4

0
4

0
4

0

0

0

0

0
4

0
4

0
4

14.6646
4

3.80377

0

0

0
4

1.39134

1.39134

0
4

0.154593

0.154593

0
4

0.23189

0.23189

0
4

2.02595

2.02595

0
4

0

0

0
4

0
4

10.0652

0.119824

0.119824

0
4

9.94541

9.94541

0
4

0
4

0.795638

0.795638

0.795638

0
4

0
4

0
4

0

0

0

0

0
4

0
4

0
4

0

0

0

0

0
4

0
4

0
4

0

0

0

0

0
4

0
4

0
4

0

0

0

0

0
4

0
4

0
4

0.0533257

0.0533257

0.0533257

0.0533257

0
4

0
4

0
4

0

0

0

0

0
4

0
4

0
4

0

0

0

0

0
4

0
4

0
4

0

0

0

0

0
4

0
4

0
4

0

0

0

0

0
4

0
4

0
4

0

0

0

0

0
4

0
4

0
4

0
6

0
6

0
6

0
6

0
4

0

0

0
4

0

0

0
4

0
4

0
4

0

0

0

0

0
4

0
4

0
4

0.0533257

0.0533257

0.0533257

0.0533257

0
4

0
4

0
4

0

0

0

0

0
4

0
4

0
4

0

0

0

0

0
4

0
4

0
4

0

0

0

0

0
4

0
4

0
4

0

0

0

0

0
4

0
4

0
4

0

0

0

0

0
4

0
4

0
4

0

0

0

0

0
4

0
4

0
4

0

0

0

0

0
4

0
4

0
4

0.0798828

0.0798828

0.0798828

0.0798828

0
4

0
4

0
4

2.38311
4

2.38311
4

2.38311
4

1.11095

0

0

0

0.309187

0

0.962969

0
4

0
4

0
4

0

0

0

0

0
4

0
4

0
4

0.0761658

0.0761658

0.0761658

0.0761658

0
4

0
4

0
4

0

0

0

0

0
4

0
4

0
4

0

0

0

0

0
4

0
4

0
4

0

0

0

0

0
4

0
4

0
4

0

0

0

0

0
4

0
4

0
4

0
[truncated: 167,432 more chars]
