# Supplementary material for: Fungal communities decline with urbanization—more in air than in soil
Source: ISME J. 2020 Aug 5;14(11):2806–15. doi: 10.1038/s41396-020-0732-1 (PMC7784924; doi:10.1038/s41396-020-0732-1)
Supplement: Supplementary file 2 — Supplemental data [file 41396_2020_732_MOESM2_ESM.zip › Krona_SoilUrbanCore.html]

Javascript must be enabled to view this page.

num
probth


38479

27411.3

428.884
3

428.884
3

428.884
3

428.884
3

428.884
3

0

0

0

0

0

0

0

0

0

0

0

0

0

0

0

0

0

0

0

0

0

0

0

0

0

0

0

0

0

0

0

0

0

0

0

0

0
4

0
4

0

0

0

0
4

0

0

0
4

0
4

0

0

0

0
4

0
4

0
4

0
4

0

0

0

0

0

0

0
4

0
4

0
4

0
4

0

0

0

0

0

0
4

0
4

0
4

0

0

0

0

0
4

0
4

0
4

0
4

5777.53

85.3778

84.4424

58.6294

2.94719
3

0.345215
2

0

0

0

0

0.115072

0

0.137103

0

0.0595838

0

0

4.56415

0.380801

0

0

0

0

0

0

0.0582163

0.054841

6.66668
3

0

0

0.184115

0

0

0

0

0.219364

0

0

0.438897

0

0

0.0397225

0

0.106147

0

0

0

0

0

0

0

0

0

0

0

0.0460286

0.0873245

0

0

0

0

0

0

0

0

0

0.192656

0

0

0

0

0
7

0.0582163

0

0

0

0

0.278058

0

0

0

0

0
7

0

0

0.174649

0.045462

0

0

0

0

0

0.105778

0
7

0

0

0

0.110524

0

0.054841

0

0

0

0

2.505

0.0582163

0.109682

0

0

0

0

0

0

0

0

1.75491

0

0

0

0

0

0

0

0

0

0

0

0

0

0

0

0

0

0

0.0530735

0

0.0530735

0

0
6

0

0.0582163

0.0598143

0

0

0

0.119629

0

0.054841

0

0

0

0

1.79129

0
6

0
7

2.83648

0

0

1.07286
1

0.054841

3.96533
2

0

0

0

0

0

0

0

0

1.19418
3

0

0

0

0.611272

0.0398762

0

0

0

0.44567

0

2.95843
2

2.05654

1.3915

0

0

0

3.11262

1.59966

0

1.10881

0.466149

3.36829
2

0.219716

0

0

0

0

0.0398762

0.341364

3.25404

0

0

0
7

0

0.655481

0

0

0

0

0

0

0

0

0
6

0.438728

0.431889

0

0

0

0

0

0

0

0

0
6

0

0.054841

0.378025

0.203757

0.045462

0

1.33483

0.815029

0

0.045462

1.0547118733939e-15

0
4

10.5808
3

8.62907
3

0.69043

1.26134

0

1.11022302462516e-15
3

0
4

0

0

0
4

0

0

0
4

0

0

0
4

0

0

0
4

0

0

0

0
4

0

0

0

0
4

0

0

0

0
4

0

0

0
4

0

0

0
4

0

0

0
4

0
6

0

0

0

0

0
4

5.65117

5.65117

0
4

1.01879

0

1.01879

0
4

0.126934

0.0846225

0.0423112

0
4

0

0

0

0
4

0

0

0
4

1.86627

1.86627

0

0
4

0

0

0

0
4

0.0846225

0.0846225

0
4

0.716162

0.716162

0
4

0

0

0
4

5.26474
2

2.49527

0

0

2.76947

0
4

0

0

0
4

0.145541

0.145541

0
4

0

0

0
4

0

0

0
4

0

0

0
4

0

0

0
4

0

0

0
4

0

0

0
4

0

0

0
4

0.0773585

0.0773585

0
4

0
6

0

0

0
4

0

0

0
4

0

0

0
4

0

0

0
4

0.0423112

0.0423112

0
4

0

0

0
4

0

0

0
4

0

0

0
4

0

0

0
4

0

0

0
4

0

0

0
4

0

0

0

0
4

0.069043

0.069043

0
4

0

0

0
4

0

0

0
4

0.169245

0.169245

0
4

0

0

0
4

0

0

0
4

0

0

0
4

0

0

0
4

0

0

0
4

0

0

0
4

0

0

0
4

0

0

0
4

0

0

0
4

0

0

0
4

0
7

0

0

0
4

0

0

0
4

0

0

0

0
4

2.38697950294409e-15

0
4

0
6

0
6

0
6

0
4

0
4

0.367379

0.206279

0.206279

0
4

0.1611

0.1611

0
4

0
4

0

0

0

0
4

0
4

0.383887

0.383887

0

0.383887

0
4

0
4

0

0

0

0
4

0
4

0

0

0

0
4

0
4

0

0

0

0
4

0
4

0

0

0

0
4

0
4

0

0

0

0
4

0
4

0

0

0

0
4

0
4

0

0

0

0
4

0
4

0
6

0
6

0
6

0
4

0

0

0
4

0

0

0
4

0
4

0

0

0

0
4

0
4

0

0

0

0
4

0
4

0

0

0

0
4

0
4

0

0

0

0
4

0
4

0

0

0

0
4

0
4

0

0

0

0
4

0
4

0

0

0

0
4

0
4

0.184115

0.184115

0.184115

0
4

0
4

0

0

0

0
4

0
4

0

0

0

0

0
4

0
4

0

0

0

0

0

0
4

0

0

0
4

0
4

0

0

0

0
4

0
4

0

0

0

0
4

0
4

0

0

0

0
4

0
4

0

0

0

0
4

0
4

0

0

0

0
4

0
4

2.94209101525666e-15

0
4

1690.57
7

4.77374

1.89203

1.16433

0.727704

0

0

0
4

2.47419

1.48452

0.989678

1.11022302462516e-16

0
4

0.145541

0.0582163

0.0873245

0
4

0.203757

0.145541

0.0582163

6.93889390390723e-18

0
4

0.0582163

0.0582163

0
4

8.67361737988404e-16

0
4

1577.31
7

1577.31
7

120.031
7

0
7

0
6

0

0

0.0663141

0

0

0

0

0

0

0

0

0
7

0

0

0

0

0

0

0

0

0

0

0
7

0

0

0

0

0

0

0

0

0

0

0.0460286

0

0

0

0

0

0

0

0

0

0

0
7

0

0

0

0

0

0

0

0

0

0

0

0

0

0

0

0

0

0

0

0

0

0
7

0

0

0

0

0

0

0

0

0

0

0.190435
8

0

0

0

0

0

0

0

0

0

0

0

0

0

0

0

0

0

0

0

0

0.326431

0

0

0

0

0

0

0

0

0

0

0

0
7

0
7

0

0

0

0

0

0

0.045462

0

0

0.115072

0.93266

0

0

0

0

0

0

0

0

0

0

0
6

0

0

0

0

0

0

0

0

0

0

0
7

0

0

0

0

0

0

0

0

0

0

0
7

0

0

0

0

0.0881189

0

0

0

0

0

0
8

0

0

0

0

0

0

0

0

0

0

0
8

0

0

0

0

0

0

0

0

0

0

0
7

0

0

0

0

0

0

0

0

0

0

0
7

0

0

0

0

0

0

0

0

0

0

0
6

0

0

0

0

0

0

0

0

0

0

0.139899
6

0.169577
1

0

0

0

0

0

0

0

0

0

0

0
7

0

0

0

0

0

0

0

0

0

0

0
7

0

0

0

0

0

0

0

0

0

0

20.5651
1

0

0

0

0

0

0

0

0

0

0

0
7

0

0

0

0

0

0

0

0

0

0

0
6

0

0

0

0

0

0

0

0

0

0

0
8

0

0

0

0

0

0

0

0

0

0

0
7

0

0

0

0

0

0

0

0

0

0

0

0

0

0

0

0

0

0

0

0

0

0
6

0

0

0

0

0

0

0

0

0

0

0.115072
7

0

0

0

0

0

0

0

0

0

0

0

0
6

0

0

0

0

0

0

0

0

0

0

0
1

0

0

0

0

0

0

0

0

0

0

0
7

0

0

0

0

0

0

0

0

0

0

0
7

0

0

0

0

0

0

0

0

0

0

0
7

0

0

0

0

0

0

0

0

0

0

0
7

0

0

0

0

0

0

0

0

0

0

0
5

0

0

0

0

0

0

0

0

0

0

0
7

0

0

0

0

0

0

0

0

0

0

0
6

0

0

0

0

0

0

0

0

0

1.10009

0
7

0
7

0

0

0

0

0

0

0

0

0

0

0
7

0

0

0

0
7

0
7

0.0498868
8

0
7

0
6

0
6

0
7

1.24485

0.06398
7

0
7

0
7

0
7

0
7

0.326431

0

0

0
7

0
7

0

0.0761741

0
7

0

0
7

0.0398762

0
7

0.069043
7

0
7

7.03933
3

0

0
6

0.0722459
7

0
7

0
7

0

0
7

0

0
7

0
7

0

0.409158
6

0
6

0.288862

0
8

0
6

0
7

0
6

0

0.630672

0

0

0
7

0

0
7

0
7

0
7

0.291954

0
7

0
6

0
7

0
2

0.14966

0

0
8

12.9021

0
7

0
6

0
6

0
8

0
7

0
7

0.045462
7

0
8

0
6

0.412072

0
6

1.42656

0

0

0
6

0
8

0
6

0
7

0
7

0
6

0
8

0
7

0
7

0
7

0
8

0

0
6

0
7

0.0332578

0
7

0
7

0.0460286
2

0.31595

0
7

0

0.304696

0
6

0.0761741
7

0
8

0
7

0

0

0
7

0
7

0.793649

0.252596
1

0.0920573
8

0
7

0.877794

0
7

0
7

0
6

0
7

0
7

0
7

0.139899
7

0
7

0

0
7

0.093266
6

0
7

0
6

0.139899

0

0
7

0
7

0.103018
7

0
6

0
7

0
7

0
7

0

0.0481639

0
6

0
8

0
6

0
7

0.260772
7

0
7

0
7

0
7

0
7

0
6

0
7

0
7

0
7

0
7

0
7

0.0481639
7

0.0761741

0
6

0
7

0
6

0
7

0
6

0
7

0
6

0
7

0
7

0
7

0
7

0
7

0
7

0

0
7

0
6

0
7

0

0
7

0
7

0.532236
7

0.126409
7

0
6

0.0481639

0
7

0
6

0
7

0

0
6

0
7

0
5

0
7

0
7

0
7

0
7

0
7

0
8

0
8

0.129757
7

0

0
7

1.48101
1

0.419697

0
7

0
7

0
7

0
6

0
7

0
7

0
5

0

0
7

0
6

0
6

0
7

0
7

0
8

0
6

0.0873245

0.329011
1

0
7

0
7

0
7

0

0
7

0
7

0.045462

0
6

0
7

0
7

0

0
7

0
7

0

0
6

0
7

0
7

0
7

0

0
6

0
7

0
7

0
7

0
7

0
7

0
7

0
6

0
7

0

0
7

0
7

0
7

0
7

0
6

0
7

0
7

1.63006
2

0

0
7

6.85912
1

0
7

0
7

0

0.10967

0
7

0
7

0
7

0

0
7

0
7

0

0
7

0
6

0
7

0
5

0
7

0
7

0
7

0
2

0.199381

0
7

0
7

0
7

0
7

0
5

0
6

0
7

0
7

0

0
8

0

0
7

0
7

0
7

0
7

0
7

0
6

0
7

0
7

0
7

0
7

0
7

0
7

0.0686787

0
7

0
7

0
7

0
7

0
8

0

0
7

0
7

0
7

0

0
7

0.0666674
7

0

0
7

0

0
6

0
2

0
7

0

0
7

0

0.22731

0
7

0
7

0
6

0
6

0
6

0
7

0

2.44509

0
7

0
7

0

0

0
7

0
7

0
7

0
5

0
7

0
7

0
2

0
7

0
7

0
7

0
6

0

0
7

0
6

0
7

0
6

0

0
7

0
7

0

0
7

0
7

0

0

0
2

0
7

0
7

0.0822527

0.0460286

0
6

0.512963

0.0914742
7

0
7

0
7

0
5

0
7

0
6

0
7

0

0
7

0
7

0
7

0
7

0
4

0
6

0
7

0
7

2.00273

2.30319
2

0
7

0

0
7

0
7

0.122138
6

0
6

4.67539

0
7

0
6

0
7

0
7

0
7

0
7

0
7

0
7

0.0787198
7

0.386427

0
6

0
7

0
7

0

0.123366

0
7

0
7

0.0352475

0
7

0
5

0.115072
7

0
6

0.0582163

0

0
6

0
7

0
6

1.16434
2

0
7

0
7

0

0
7

0.093266
4

0
8

0

1.10326

0
7

0
6

0
7

0
7

0
8

0
7

0
7

0
7

0
7

0
1

0
7

0.279134

0
6

0
6

0
7

0
7

0
8

0
7

0

0
7

0
7

0.114261

0.191923

0
7

0
7

0
6

0
6

0

0
7

0
8

0
8

0
6

0
7

0
7

0
7

0.054841
7

0
6

0
6

0
7

0.0582163

0

0

0

0
7

0.114261
7

0
7

0
7

0
7

0
7

0
7

0
7

0

0

0.0332578
7

0
7

0
6

0
8

0.0666674

0
7

0
6

0

0

0
7

0
7

0
7

0.494839

0
7

0
6

0
7

0
7

0
7

0.373064

0
7

0
6

0
7

0
6

0

0
7

0
1

0
7

0
6

0
7

0
6

0
7

0
7

0
7

0
8

0.0582163
7

0
6

0.054841
7

0
7

0
7

0
7

0

0
6

0.496531
7

0

0
7

0
7

0
7

0
7

0
7

0
7

0

0
7

0.0481639

0
7

0
6

0
6

0

0
7

0
7

0

0
6

0
7

0.131037

0

0
7

0
7

0
7

0.0681929
8

0
7

0.159505

0
6

0
7

0
5

0
7

0
6

0
7

0.069043
7

0
6

0
7

0
7

0
7

0
7

0
6

0
5

0
8

0
7

0
7

0
6

0
8

0
7

0
7

0
6

0
7

0
6

0
7

0.103018

0
7

0
7

0

0
7

0

0
7

0
7

0
7

0
6

0
7

0
7

0
7

0
7

0.0398762

0
7

0

0
7

0.837915

0
6

0

0.182918

0.565383

0
5

0.609393

0

0
6

0
6

0
1

0
6

0
7

0
8

0.495132

0.063149

0.979293

0
7

0
2

0.0681929

0

5.13219

0
7

0
7

0
7

0
7

0
7

0
6

0.535007

0
7

0
7

0.0704951

0.137088

0
6

0
7

0
6

0
6

0
7

0.606229

0
7

0

0
7

0
7

0

0
7

0

0
7

0
7

0
8

0

0

0.233165
7

0
6

0
7

0
7

0.317228

0
5

0
7

0
7

0
6

0
6

0
2

0
7

0.0397225
7

0.045462

0.115072
1

0
6

0
7

0
6

0
7

4.64275
2

0

0
7

0
7

0
7

0
7

0
8

0
7

0
7

0
7

0
6

0
7

0.45462
1

0.0582163

0
7

0
7

0
7

0

0

0
6

0
7

0

0
7

0
7

0
7

0
7

0
6

0
6

0.21934
6

0.0997735

0
7

0
7

0

0

0.45462
1

0
7

0.539161
7

0

0
6

0
8

0

0
8

0
1

0.186532

0
6

0
7

0
7

0.0460286
6

0
7

0

0
7

0.0460286

0
7

0
7

0

0

0.240375

0
7

0
7

0
7

0
7

0
7

0
7

0
6

0
8

0
8

0

0
7

0
6

0.269354
7

0

0

0.987032
1

0.093266

0
7

0
8

0
5

0
7

0
6

0
7

0.282692

0

0
7

0
6

0
7

0
7

0
7

0
7

0.12282

0
7

0
7

0.192656

0
7

0
5

0
7

0
7

0

0
7

0
7

0
7

0
7

0
6

0
6

0.164505
7

0.841046

0
7

0
7

0
7

1.53968
2

0
6

0
7

0
6

0
7

0
8

0
7

0
7

0
7

0
7

0
7

0

0
8

0
4

0

0
7

0
8

0
7

0
7

0
6

0
7

1.50024

0
6

0

0
6

0
7

0.0963278

0
7

0.208092

0.0548351

0
7

0
7

0

0

0
7

0
7

0
7

0
7

0.228522

0

0
7

0
7

0
6

0
7

0
7

0
7

0

0
7

0
7

0
7

0
6

0
5

0
6

0
6

0
6

0
5

0
7

0
6

0

0
7

0.318234

0
6

14.5493
2

0
6

0
8

0
7

0
7

0
6

0
7

0
7

0

0.093266

0.124338
7

0.0873245
7

0.0460286
6

0

0.168574
6

0

0
6

0.0873245

0

0
8

0
8

0
7

0
7

0.529803

0

0
7

0
6

0
7

0
6

0
7

0
6

0
7

0
6

0.266609
7

0
8

0
6

0
7

0
7

0
7

0
7

0
6

0

0
6

0
6

0.142887

0.0722459
1

0
5

0
6

0
7

0
6

0
6

0

0.0655183

0
6

0
2

0.114261

0
6

0
8

0

0
7

0
7

0
7

0
7

0
7

0
6

0
7

0.34732
6

0
7

0
6

0
7

0
8

0
7

0
6

0
5

0
7

0
5

0
7

0
7

0
7

0
7

0

0
7

0
7

0
7

0
5

0
7

0

0

0
6

0
7

0

0
6

0
6

0.0498868

0.553055
8

0.0681929

0

0
8

0
7

0
7

0
7

0
6

0
6

0
6

0
6

0
6

0
7

0
7

0
8

0.186532

0
4

0
7

0
5

0
7

0

0.0548351
1

0
6

0
6

0

0
7

0

0
7

0.638099

0
7

0
6

0
7

0
6

0
6

0
7

0
7

0

0
6

0.06398

0
7

0

0
6

0.0548351

0
7

0
7

0
7

0
7

0
8

0
7

0
6

0.0909239
7

0
8

0
7

0
8

0
6

0
6

0.0515724

0
6

0.253158
1

0
7

0
7

0.176262
7

0
6

0
7

0
7

0
7

0
6

0

0
6

0
7

0
7

0
8

78.4884
7

0
8

0
7

0
7

0
7

0
6

0
6

0
8

0
6

0
6

1.36357
1

0.233165
7

0

0

0
7

0
7

0
6

0
7

0
7

0
7

0

0
7

0
6

0
7

0
7

0
7

0
7

0

0
7

0

0
7

0
6

0
1

0
7

0
7

0
7

0
7

0
8

0
7

0

0
6

0
7

0
7

0
7

0
6

0
7

0
7

0

0
7

0
7

0
8

0
7

0
7

0
7

0
7

0.148107
5

0
7

0
7

0
2

0
7

0
6

0
7

0
7

0
6

0
7

0

676.638
7

0.349396
7

0
6

0
7

0
7

0
7

0
7

0.0761741
6

0
7

0
7

0
7

0
7

0
6

0
8

0
6

0
6

0
7

0
7

0

0.460286

0
7

0
5

0

0
7

0

0
7

0
7

0
6

0
8

0
7

0
7

0
4

0
7

0
6

0
7

0
7

0
7

0
7

0
1

0
7

0
6

0
6

0
7

0
6

0
7

0

0
7

0

0
6

0
8

0
7

0
6

0

0
7

0

0
7

0
8

0
7

0

0
7

0
7

0.0598143

0

0
8

0

0
6

0.0822527

0.0663141

0
7

0
7

0.505721

0
7

0
7

0
5

0
6

0
7

0.0481639
6

0

0
7

0.114261

0
7

0.0481639

0
7

0.186532

0

0
7

0
7

0
6

0
7

0
7

0

0
6

0

0
6

0
2

0
7

0
8

0
6

0
1

0
6

0
6

0
7

0
7

0.115072

0
8

0
7

0
7

0
6

0
7

0

0
7

0

0
7

0.545544

0
7

0
6

0

0
7

0
8

0
7

0
7

0
7

0
6

0
7

0.16944

0

0

0
6

0
7

0
7

0
7

0
6

1.33379

0
7

0

0
7

0
8

0
7

0
7

0
6

0
6

0

0.152348

0
6

0
7

0
7

0
7

0

0

0
5

0

0
7

0.0598143

0
7

0
6

0

0
7

0
7

2.87915
2

0
7

0.093266

0

0

0
7

0
7

0

0
7

0
7

0.093266
7

0
7

0
7

0
7

0.775446
1

0
7

0

0

0

0.093266

0
5

0.14179
7

0
7

0
7

0
1

0
6

0
7

0.113655
4

0

0
8

0
7

0
7

0
7

0

0
7

0
6

0.342783

0
7

0

0
6

0
2

0
6

0
8

0.093266
7

0

0
7

0
6

0.0595838
1

0

0
7

0
7

0
7

0

0
8

0.291082

0
7

0
7

0
8

0
7

0

0
8

0
7

0

0

0
8

0
7

0.203757

0

0
8

0
7

0
6

0
8

0
8

0
6

0
8

0

0
6

0.606229

0

0
7

0

0

0
7

0
6

0
7

0

0
6

0

0
7

0

0
6

0
7

0
7

0
6

0

0
7

0
7

0
8

0
7

0
7

0
7

0

0

0
7

0
6

0
6

0
6

0

0
6

0
5

0
6

0

0.0460286

0.0827448

0

0
7

0

0

0.591006
2

0
1

0

0
7

0
7

0
7

0
6

0

0
7

0

0.0582163

0
7

0
6

0
7

0
7

0

0.279798

0

1.22254

0
7

0
6

0
7

0
7

0

0

0

0.211485

0
7

0

0

0
7

0
7

0
7

0
6

0
7

0
6

0.342783
7

0
5

0
8

0

0

0.0548351

0
7

0.0548351

0

0

0

0
7

0
6

0
7

0
5

0
7

0
6

0
6

0
7

0
7

0
7

0
6

0.190435
7

0
7

0
7

0
7

0

0

0
7

0
7

0
5

0
7

0
7

0
7

0.0582163

0

0
8

0

0
7

0

0
7

0
7

0
6

0

0
7

0.24082

0
6

0.0460286

0
7

0
8

0
7

0.261974

0
7

0.593082

0
7

0

0.324393

0
6

0
7

0
6

0

0

0

0
1

0

0

0
7

0.0722459
7

0

0
6

0.045462
8

0
6

0
6

0
7

0

0

0
8

0
6

0
7

0
1

0
7

0.465424
1

0

0.0515724

0
8

0
7

0
7

0
6

0
7

0
7

0
7

0

0
7

0
6

0
7

0

0
7

0
6

0.0761741

0
6

0
6

0
6

0
4

0.674295

0
6

0
7

0
6

0

0
7

0
7

0
8

0
6

1.08271

0
7

0

0

0
6

0
7

0.0761741
1

0
7

0

0
6

0
6

0
7

0

0
6

0
7

0
7

0
7

0
7

0
7

0
8

0
7

0.063149
7

0.0761741

0

0.190435

0
6

0
6

0

0
6

0
1

0.164505

0

0
7

0
7

0

0
6

0

0
7

0

0
7

0
8

0

0
6

0

0
7

0
7

0
6

0

0
7

0
7

0
7

0
6

0
6

0
7

0

0.145541
7

0

0

0

0

0

0

0

0

0

0

0.291082
7

0

0

0

0

0

2.495

0

0

0

0

0.0398762
7

0

0.0909239

0

0

0

0

0

0

0

0

0.326431

0

0

0

0

0

0

0

0

0

0.154717

0
6

0

0

0

0.044445

0

0

0

0

0

0

0.348728

0

0

0

0

0

0

0

0

0

0

0.0761741
7

0

0

0

0

0

0

0

0

0

0

0
7

0
6

0

0

0

0

0

0

0

0

0

0

0
7

0

0

0.35189

0

0

0

0.228522

0

0

0

0
7

0

0.533219

0

0

0

0

0

0.0920573

0

0

0
7

0.0481639

0

0

0

0.113655

0

0

0

0

0

0.0722459
7

0

0.438728

0

0

0

0

0

0

0

0.457045

0

0

0

0

0

0.24082

0

0

0

0

0

0.23646
7

0

0

0

0

0

0

0

0

0

0

0
7

0.186532

0

0

0

0

0

0

0

0

0

0
7

0

0

0

0

0

0

0

0

0

0

0
6

0

0

0

0

0

0

0

0.114261

0

0

0
7

0.157853
6

0

0

0

0

0

0.168574

0

0

0

0.0528713

0
7

0

0.0681929

0

0

0

0

0

0

1.44562

0

0.0997735
7

0.044445

0

0

0

0

0

0

0.349298

0

0

0
7

0

0

0

0

0

0

0

0

0.186532

0

0

0

0

0

0

0

0

0

0

0

0

0
7

0

0

0

0

0

0

0

0.233165

0

1.8166

0
7

0

0

0

0

0

0.0963278

0

3.52209

0

0

0.0822527
7

0

0

0

0

0

0

0

0

0

0

0.850036

0

0

0

0

0

0

0

0

0

0

0
7

0

0

0

0

0

0

0.345215

0

0

0

0
7

2.7727

0

0

0

0

0

0

0

0

0

0

0
7

0

0

0

0

0

0

0.233165

0

0

0

0
6

0

0

0.0681929

0

0.258167

0.0548351

0

0

0

0

3.11457

0

0

0

0

0

0

0

0

0

0

0.164505

0

0

0

0

0

0

0

0

0

0

2.91673

0

0

0

0

0

0

0

0

0

0

0.137103
7

0

0

0

0

0

0

0

0

0

0

0
7

0

0.136386

0

0

0

0

0

0

0

0

0
7

0

0

0.136386

0

0

0

0

0

0

0

0
6

0

0

0

0

0

0

0

0

0

0

0.636468
7

0
7

0

0

0

0

0

0

0

0

0

0

0.093266
7

0

0

0

0

0

0

0

0

0

0

0.0761741
7

0

0

0

0

0

0

0

0

0

0

1.56793

0

0

0

0

0

0

0

0

0

0

0
7

0

0

0

0

0

0

0

0

0

0

0.17365

0

0

0

0

0.054841

0

0

0

0.203757

0

0

0

0

0

0

0

0

0

0

0.181848

0

0
7

0

0

0

0

0

0

0

0

0

0

0
7

0.466098

0

0

0

0

0

0

0

0

0

0
7

0

0

0

0.045462

0

0

0

0

0

0

0
7

0.276172
7

0

0

0

0

0

0

0.438728

0

0

0

0
6

0

0

0

0

0

0

0

0

0

0

0
7

0

0

0

0

0

0

0

0.0761741

0

0

0.233165

0

0

0

0.0761741

0

0

0

0

0.0909239

0

0
7

0

0

0

0

0

0

0

0.0332578

0

0

0

0

0

0

0

0

0

0

0

0

0

0.105778
7

0

0

0

0

0

0

0

0

0

0

0

0

0

0

0

3.0493

0

0

0

0

0

0
7

0

0

0

0

0

0

0

0

0

0

0
7

0

0

0.772853

0

0

0

0

0

0

0

86.4905

0.305411
7

0
7

0

0

0

0

0

0

0

0

0

0

0
7

2.05185

0

0

0

0

0

0

0

0

0

0
6

0

0

0

0

0

0

0

0

0

0

0.114261
7

0

0

0

0

0

0

0

0

0

0

0
7

0

0

0

0

0

0

0

0

0

0

0
7

0

0

0

0

0

0

0

0

0

0

0
7

0

0

0

0

0

0

0

0

0

0

0
7

0

0

0

0

0

0

0

0

0

0

0

0

0

0

0

9.75807

0

0

0

0

0

0.0515724
7

0

0.795584

0

0

0

0

0

0

0

0

0
7

0
7

0

0

0

0

0

0

0

0

0

0

0.412579
6

0

0.0997735

0

0

0

0

0

0

0

0

0
7

0

0

0

0

0

0

0

0

0

0

0
7

0

0

0.0761741

0

0

0

0

0

0.10967

0

0
7

0

0

0

0

0

0

0

0

0

0

0
7

0

0

0

0

0

0

0

0

0

0

0
7

0

0

0

0

0

0

0

0

0

0

0
7

1.02593

0

0

0.160534

0

0

0

5.91006

0

0

0
7

0

0

1.23616

0

0

0

0

0

0

0

0
7

0.411263

0

0

0

0

0

0

0

0

0

5.16094

0.159967

0

0

0

0

0

0

0

0

0

0

0
7

0

0

0

0

0

0

0

0

0

0

0
7

0

0

0

0

0

0

0

0

6.81758

0

0.114261
7

0

0

0

0

0

0

0

0

0

0

0.162425

0

0

0

0

0

0

0

0

0

0

0.0665157

0

0

0

0

0

0

0

0

0

0

0
7

0

0

0

0

0

0

0

0

0

0

0
7

0

0

0

0

0

0

0

0

0

0

0
4

0

0

0

0

0

0

0

0

0

0

0.10967
7

0

0.553055

0

0

0

0

0

0

0

0

0.0722459
6

0
7

0

0

0

0

0

0

0

0

0

0

0
5

0.093266

0

0

0

0

0

0

0

0

0

0
7

0

0

0

0

0

0

0

0

0

0

0.28158
7

0

0

0

0

0

0

0

0

0

0

0
7

0

0

0

0

0

0

0

0

0

0

0.0920573

0

0.0481639

0

0

0

0

0

0

0

0

0
7

0

0

0

0

0

0

0

0

0

0

0.12041
7

0

0

0

0

0

0

0

0

0

0

0.0460286
7

0

0

0

0

0

0

0

0

0

0

0
7

0

0

0

0

0

0

0

0

0

0

1.16157

0
7

0

0.745463

0

0

1.02593

0

0

0

0

0

0.0873245
7

0

0

0

0

0

0

0

0

0

0

0
7

0

0

0

0

0

0

0

0

0

0

0
6

0

0

0

0

0

0.0909239

0

0

0

0

0.304696
7

0

0

0

0

0

0

0

0

0

0

0
6

0

0

0

0

0

0

0

0

0

0

0.0797524
7

0

0

0

0

0

0

0

0

0

0

0
7

0

0

0.103145

0

0

0

0

0

0

0

0.0481639
7

0

0

0

0

0

0

0

0

0

0

0
7

0

0

0

0

0

0

0

0

0

0

0.19194
7

0.33522

0

0.12041

0

0

0

0

0

0

0

0

0
7

0

0

0

0

0

0

0

0.207129

0

0

0
5

0

0

0

0

0

0

0

0

0

0

0
6

0

0

0

0

0

0

0

0

0

0

0
7

0

0

0

0

0

0

0

0

0

0

0.0481639
7

0

0

0

0

0

0

0

0.186532

0

0

0
7

0

0

0

0

0

0

0

0

0

0

3.87454

0

0

0

0

0

0

0

0

0

0

0
7

0

0

0

0

0

0.0909239

0

0

0

0

0

0

0

0

0.0548351

0

0

0

0.0460286

0

0

0.383144
7

0
7

0

0

0

0

0

0

0

0

0

0

0
7

0

0

0

0

0

0

0

0

0

0

11.0452
2

0

0

0

0

0

0

0.688897

0

0

0

0

0

0

0

0

0

0

0

0

0

0

0
7

0.299036

0

0

0

0

0

0

0

0.0548351

0

0
7

0

0

0

0

0

0

0

0

0

0

0
7

0

0

0

0

0

0

0

0

0.0722459

0

0
7

0

0

0

0

0

0

0

0

0

0

0.669488

0

0

0

0

0

0

0

0

0

0

0

0

0

0

0

0

0

0

0

0

0

0.202378
7

0
7

0

0

0

0

0

0

0

0

0

0

0
7

0

0

0.0681929

0

0

0

0

0

0

0

0

0

0

0

0

0

0

0

0

0

0

0
7

0

0

0

0

0

0.418957

0

0

0

0

0.0582163
7

0

0

0

0

0

0

0

0.361006

0

0

0
7

0

0

0

0

0

0

0

0

0

0

0
5

0.553885

0

0

0

0

0

0

0

0

0

0
6

0

0

0

0

0

0

0.0481639

0

0

0

0
7

0

0

0

0

0

0

0

0

0

0

0.0827448
7

0

0

0

0

0

0

0

0

0

0

1.1107
7

0
6

0

0

0

0

0

0

0

0

0

0

0
6

0

0

0.0460286

0

0

0

0

0

0

0

0
7

0

0

0

0

0

0

0

0

0

0

0.0773585
7

0

0

0

0

0

0

0

0

0

0

0

0

0

0

0

0.232805

0

0

0

0

0

0.412072

0

0

0

0

0.0881189

0

0

0

0

0

0
6

0

0

0

0

0

0

0

0

0

0

0
7

0

0

0.228522

0

0.326431

0

0

0

0

0

0
7

0

0

0

0

0

0

0

0

0

0

0.0909239

0

0

0

0

0

0

0

0

0

0

0.113655
7

0
7

0

0

0

0

0

0

0

0

0

0

0
7

0

0

0

0

0

0

0

0

0

0

0

0

0

0

0

0

0

0

0

0

0

0
7

0

0

0

0

0

0

0

0

0

0

1.55709

0

0

0

0

0

0

0

0

0

0

0
7

0

0

0

0

0

0

0

0

0

0

0.598372

0.973179

0

0

0

0

0

0

0

0

0

0.0761741
8

0.190435

0

0

0

0

0

0

0

0

0

0
7

0

0

0

0

0

0

2.21761

0.093266

0

0

0
6

0

0

0

0

0

0

0

0

0

0

59.0542

4.27691

0.093266
7

0

0

0

0

0.500082

0

0

0

0

0

0.0920573
7

0.114261

0

0

0

0

0

0

0

0

0

3.34376

0.266063

0

0

0

0

0

0

0

0

0

0.327084

0

0

0

0

0

0

0

0

0

0

0

0

0

0

0

0

0

0

0

0

0

0
6

0

0

0

0

0

0

0

0

0

0

0.0582163
7

0

0

0

0

0

0.0548351

0

0

0

0

0

0

0

0

0

0

0

0

0

0

0

0
7

0

0

0

0

0

0

0.0515724

0

0

0

0

0

0

0

0

0

0

0

0

0

0

0.0598143
7

0
6

0

0

0

0

0

0

0

0

0

0.279798

0
7

0

0

0

0

0

0

0

0

0

0

3.72832

0

0

1.42689

0

0

0

0

0

0

0

0
7

0

0

0

0

0

0

0

0

0

0

0
7

0

0

0

0

0

0

0

0

0

0

0
7

0

0

0

0

0

0

0

0

0

0

0
7

0

0

0

0

0

0

0

0

0

0

0
6

0

0

0

0

0

0

0

0

0

0

0
7

0

0

0

0

0

0

0

0

0

0

0
6

0

0

0

0

0.0761741

0

0

0

0

0

1.75182

7.99521

0

0

0

0

0

0

0

0

0

0

0.093266
7

0

0

0

0

0

0

0

0

0

0

0.617334

0

0

0

0

0

0

0

0

0

0

0
7

0

0

0

0

0

0

0

0

0

0

0

0

0

0

0

0

0

0

0

0

0

0
7

0

0

0

0.137088

0

0

0

0

0

0

0
7

0

0

0

0

0

0

0

0

0.093266

0

1.80085

0

0

0

0

0

0

0

0

0

0.093266

0.0963278
6

0

0

0

0

0

0

0

0

0

0

0
7

0

0

0

0

0

0

0

0

0

0

0.685513
7

0
7

0

1.95486

0

0

0

0

0

0

0

0.0460286

0
7

0

0

0

0

0

0

0

0

0

0

0

0

0

0

0

0

0

0

0

0

0

1.2041
1

0

0

0

0

0

0

0

0

0

0

0.054841

0

0

0

0

0

0

0

0

0

0

0
7

0

0

0

0

0

0

0

0

0

0

0
7

0

0

0

0

0

0

0

0

0

0

0

0

0

0

0

0

0

0

0

0.045462

0

0.159505
7

0

0

0

0

0

0

0

0

0

0

0
7

0

0

0

0.0761741

0

0

0

0

0

0

0.330175
7

0
7

0

0

0

0

0

0

0

0

0

0

0
7

0

0

0

0

0

0

0

0

0

0

0
7

0

0

0

0

0

0

0

0

0

0

0.0996905
7

0

0

0

0

0.115072

0

0

0

0

0

0
7

0

0

0

0

0

0

0

0

0

0

0
6

0

0

0

0

0

0.591006

0

0

0.304696

0

0
6

0

0

0

0

0

0

0

0

0

0

0

0

0

0

0

0

0

0

0

0

0

0

0

0

0

0

0

0

0

0

0

0

0
7

0

0

0

0

0

0.0582163

2.13338

0.31595

0

0

0
7

0
7

0

0

0

0

0

0

0.093266

0

0

0

0
7

0.295503

0

0

0

0

0

0

0

0

0

0
7

0

0

0

0

0

0

0

0

0

0

0
7

0

0

0

0

0

0.0648786

0

0

0

0

0

0

0.0655183

0

0

0

0

0

0

0

0

0
7

0

0

0

0

0

0

0

0

0

0

0

0

0

0

0

0

0

0

0

0

0

0.0822527
7

0

0

0

0

0

0

0

0

0

0

0
7

0

0

0

0

0

0

0

0

0

0

5.50149

0

0

0

0

0

0

0

0

0

0

0.045462
7

0
7

0

0

0

0

0

0

0

0

0

0

0

0

0

0

0

0

0

0

0

0

0

0
7

0

0

0

0

0

0

0

0

0

0

0
7

0

0

0

0.0909239

0

0

0

0

0

0

0
7

0

0

0

0

0.266609

0

0

0

0

0

0
7

0

0

0

0

0

0

0

0

0

0

0

0

0

0

0

0

0

0

0

0

0

0.358886

0

0

0

0

0

0

0

0

0

0

0.0909239
6

0

0

0

0

0

0

0

0

0

0

0
7

0

0

0

0

0

0

0

0

0

0

5.0179

0.093266

0

0

0

0

0

0

0

0

0

0

0

0.045462

0

0.304696

0

0

0

0

0

0

0

0
7

0

0

0

0

0

0

0

0.0332578

0

0

0.0598143
7

0

0

0

0

0

0

0

0

0

0

0
7

0

0

0

0

0

0

0

0

0

0

0.139088

0

0

0

0

0

0

0

0

0

0

0
7

0

0

0

0

0

0

0

0

0

0

0
7

0

0

0

0

0

0

0

0

0

0

0

0

0

0

0

0

0

0

0

0

0

0

0

0

0

0

0

0

0

0

0

0

0
7

0
7

0

0

0

0

0

0

0.493569

0

0.0460286

0

0
7

0

0

0

0

0

0

0

0

0

0

0.356428
7

0

0

0

0

0

0

0

0

0

0

0

0

0

0

0

0

0

0

0

0

0

0.0460286

0

0

0

0

0

0

0

0

0

0

0
7

0

0

0

0

0

0

0

0

0

0

0

0

0

0

0

0

0

0

0

0

0

0
5

0

0

0

0.10967

0

0

0

0

0

0

0
7

0

0

0

0

0.0761741

0

0.0481639

0

0

0

0
7

0

0

0

0

0

0

0

0

0

0

0.25889
7

0
7

0

0

0

0

0

0

0

0

0

0

0
7

0

0

0

0

0

0

0

0

0

0

0
7

0

0

0

0

0

0

0

0

0

0

0
7

0

0

0

0

0

0

0.692334

0

0

0

0.0460286
6

0

0

0

0

0

0

0

0

0

0

0
7

0

3.35166

0

0.494839

0

0

0

0

0

0

0
7

0

0

0

0

0

0

0

0

0

0

0
6

0

0

0

0

0

0

0

0

0.398762

0

0.0686787

0

0

0

0

0

0

0

0

0

0

0
7

0

0

0

0

0

0

0

0

0

0

18.0451

1.09958
7

0
7

0

0

0

0

0

0

0

0

0

0

0
7

0

0

0

0

0

0.915114

0

0

0

0

0.155557
8

0

0

0

0

0

0

0

0

0

0

0
6

0

0

0

0.0997735

0

0

0

0

0

0

0
6

0

0

0.0460286

0.045462

0

0

0

0

0

0

0
7

0

0

0

0

0

0

0

0

0

0

0.152348

0

0

0

0

0

0

0

0

0

0

0

0

0

0

0

0

0

0

0

0

0

0
7

0

0

0

0

0

0

0

0

0

0

0
7

0

0

0

0

0

0

0

0

0

0

0
7

0
7

0

0

0.22731

0

0

0

0

0

0

0.0398762

0
7

0

0

0

0

0

0

0

0

0

0

0
7

0

0

0

0

0

0

0

0

0

0

0.139899

0

0

0.139899

0

0

0

0

0

0

0

0
7

0

0

0

0

0

0

0

0

0

0

0
7

0

0

0

0

0

0

0

0

0

0

0

0

0

0

0

0

0

0

0

0

0

0.366927

0

0

0

0

0

0

0

0

0

0

8.61602

0

0

0

0

0.109682

0

0

0

0

0

0
6

0

0

0

0

0

0

0

0

0

0

5.49494

0.069043
7

0

0

0

0

0

0

0

0

0

0

0
7

0

0

0

0

0

0

0

0

0

0

0
7

0

0

0

0

0

0

0

0

0

0

0
7

0

0

0

0

0

0

0

0

0

0

0.0332578
7

0

0

0.0332578

0

0

0

0.313065

0

0

0

0

0

0

0

0

0

0

0

0

0

0

0.0761741
7

0

0

0

0

0

0

0

0

0

0

0.727391

0

0

0

0

0

0

0

0

0

0

0
7

0

0

0

0

0.0398762

0

1.72731

0

0

0

0
7

0

0

0

0

0

0

0

0

0

0

5.12721

0

0

0

0

5.92239

0

0

0

0

0

0

0

0

0.045462

0

0

0.0665157

0

0

0

0

0

0
7

0

0

0

0

0.0761741

0

0

0.0582163

0

0

0.0761741
7

0

1.16582

0

0

0

0

0

0

0

0

0
7

0

0

0

0

0

0

0

0

0

0

0.139899

0

0

0

0

0

0

0

0

0

0

0

0

0

0

0

0

0

0

0

0

0

0
7

0

0

0

0

0

0

0

0

0

0

0.939196

0

0

0

0

0

0

0

0

0

0

0
7

0

0

0

0

0

0

0

0

0.0873245

0

0.395527
7

0.0761741
7

0

0

0

0

0

0

0

0

0

0

0
7

0

0

0

0

0

0

0

0

0

0

0.137103
6

0

0

0

0

0

0

0

0

0

0

0
7

0

0

0

0

0

0

0

0

0

0

0.09597
7

0

0

0

0

0

0

0

0

0

0

0
6

0

0

0

0

0.0332578

0

0

0

0

0

0
6

0

0

0

0

0

0

0

0

0

0

0
5

0

0

0

0

0

0

0

0

0

0

0
7

0

0

0

0

0

0

0

0

0

0

0
7

0

0

0

0

0

0

0

0

0

0

0.411308
7

0
7

0

0

0

0

0

0

0

0

0

0

0
7

0

0

0

0

0

0

0

0

0

0

0
7

0

0

0

0

0

0

0

0

0

0

0
8

0

0

0

0

0

0

0

0

0

0

0
7

0

0

0

0

0

0

0

0

0

0

0
7

0

0

0

0

0

0

0

0

0.139899

0

0
7

0

0

0

0

0

0

0

0

0

0

20.9744
2

0.0704951

0

0

0

0

0

0

0

0

0

0.093266

0

0

0

0

0

0

0

0

0

0

0
7

0

0

0

0

0

0

0

0

0

0

0
7

0
7

0

0

0

0

0

0

0

0

0

0

0

0

0

0

0

0

0

0

0

0

0

0
7

0

0

0

0

0

0

0

0

0

0

0
7

0

0

0

0

0

0

0

0

0

0

0.069043
7

0

0

0

0

0

0

0

0

0

0

0
6

0

0

0

0

0

0

0

0

0

0

0
7

0

0

0

0

0

0

0

0

0

0

0

0

0

0

0

0

0

0

0

0.0973179

0

0
7

0

0

0

0

0

0

0

0

0

0

0
6

0

0

0

0

0

0.093266

0

0

0

0

0
7

0.0761741
7

1.20637

0

0

0

0

0

0

0

0

0

0
7

0

0

0

0

0

0

0

0

0

0

0
6

0

0

0

0

0

0

0

0

0

0

0

0

0

0

0

0

0

0

0

0

0

0
6

0

0

0

0

0

0

0

0

0

0

0.358886
6

0

0

0

0

0

0

0

0

0

0

0
7

0

0

0

0.186532

0

0

0.782487

0

0

0

0
8

0

0

0

0

0

0

0

0

0

0.0761741

0
7

0

0

0

0

0

0

0.045462

0

0

0

0

0

0

0

0

0

0

0

0

0

0

0.52133

0
7

0

0

0

0

0

0

0

0

0

0

2.61921

0

0

0

0

0

0

0

0

0

0

0.152348

0

0

0

0

0

0

0

0

0

0

0
6

0

0

0

0

0

0

0

0

0

0

0
6

0

0

0

0

0

0

0

0

0

0

0
6

0

0

0

0

0

0

0

0

0

0

0
6

0

0

0

0

0

0

0

0

0

0

0.0398762

0

0

0

0

0

0

0

0

0

0

0
8

0

0

0

0

0

0

0

0

0

0

0
7

0

0

0

0

0

0

0

0

0

0

2.95733

0
6

0

0

0

0

0

0

0

0

0

0

0.045462
7

0

0

0

0

0

0

0

0

0

0

0
7

0.0582163

0

0

0

0

0

0

0

0

0

0
6

0

0

0

0

0

0

0

0

0

0

0
7

0

0

0

0

0

0

0

0

0

0

0
7

0

0

0

0

0

0

0

0

0

0

0
7

0

0

0

0

0

0

0

0

0

0

0
7

0

0

0

0

0

0

0

0

0

0

0

0

0

0.186532

0

0

0

0

0

0

0

0
6

0

0

0

0

0

0

0

0

0

0

22.6477
7

0.435502

0
7

0.0822527

0

0

0

0

0

0

0

0

0

0
7

0

0

0

0

0

0

0

0

0

0

0
6

0

0

0

0

0

0

0

0

0

0

0

0

0

0

0

0

0

0

0

0

0

0
6

0

0

0

0

0

0

0

0

0

0

0
7

0

0

0

0

0

0

0

0

0

0

0
7

0

0

0

0

0

0

0

0

0

0

1.42571
8

0

0

0

0

0

0

0

0

0

0

0
7

0

0

0

0

0

0

0

0

2.19175

0

0
7

0

0

0

0

0

0

0

0

0

0

0.30473

0
5

0

0

0

0

0

0

0

0

0

0

0
7

0

0

0

0

0

0

0

0

0

0

0
7

0

0

0

0

0

0

0

0

0

0

0
7

0

0

0

0

0

0

0

0.184115

0

0

0
7

0

0

0

0

0

0

0

0

0

0

0.136386
6

0

0

0

0

0.389272

0

0

0

0.194636

0

0

0

0

0

0

0

0

0

0

0

1.21246

0
7

0

0

0

0

0

0

0

0

0

0

0
7

0

0

0

0

0

0

0

0

0

0

0.0681929

0

0

0

0

0

0

0

0

0

0

0
7

0
6

0

0

0

0

0

0

0

0

0

0

0.0598143
7

0

0

0

0

0

0

0

0

0

0

0
7

0

0

0

0

0

0

0

0

0

0

0
7

0

0

0

0

0

0

0

0

0

0

0.0797524
7

0

0

0

0

0

0

0

0

0

0

0
7

0

0

0

0.414258

0

0

0

0

0

0

0
7

0

0

0

0

0

0

0

0

0

0

0
7

0

0

0

0

0

0

0

0

0

0

0

0

0

0

0

0

0

0

0

0

0

0.0634669

0

0

0

0

0

0

0

0

0

0

0
7

0
6

0

0

0

0

0

0

0

0

0

0

0
8

0

0

0

0

0

0

0

0

0

0

0.669488

0.139899

0

0

0

0

0

0

0

0

0

0
6

0

0

0

0

0

0

0

0

0

0

0
6

0

0

0

0

0

0

0

0

0

0

0

0

0

0

0

0

0

0

0

0

0

0

0

0

0

0

0

0

0

0

0

0

0.0460286
7

0

0

0

0

0

0

0

0

0

0

0.114261
7

0

0

0

0

0

0

0

0

0

0

0
6

0

0

0

0

0

0

0

0

0

0

3.32958

0
7

0

0

0

0

0

0

0

0

0

0

0

0

0

0

0

0

0

0

0

0

0

0
7

0

0

0

0

0

0

0

0

0

0

0
7

0

0

0

0

0

0

0

0

0

0

0
7

0

0

0

0

0

0

0

0

0

0

0
7

0

0

0

0

0

0

0

0

0

0

0
7

0

0

0.0332578

0

0

0

0

0

0

0

0
7

0

0

0

0

0

0

0

0

0

0

0
7

0

0

0

0

0

0

0

0

0

0

0
7

0

0

0

0

0

0

0

0

0

0

1.54196
7

0
7

0

0

0

0

0

0

0

0

0

0

0
7

0

0

0

0

0

0

0

0

0

0

0
6

0

0

0

0

0

0

0

0

0

0

0

0

0

0

0

0

0

0

0

0

0

0
7

0

0

0

0

0

0

0

0

0

0

0
7

0

0

0

0

0

0

0

0

0

0

0.0722459

0

0

0

0

0

0

0

0.0655183

0

0

0
7

0

0

0

0

0

0

0

0

0

0

0
7

0

0

0

0.154717

0

0

0

0

0

0

0

0

0

0

0

0

0

0

0

0

0

0
7

0
7

0

0

0

0

0

0

0

0

0.45415

0

0
7

0

0

0

0

0

0

0

0

0

0

0
7

0

0

0

0

0

0

0.0460286

0

0

0

2.97079
1

0

0

0

0

0

0

0

0

0

0

0
6

0

0

0

0

0

0

0

0

0

0

0
7

0

0

0

0

0

0

0

0

0

0

0
6

0

0

0

0

0

0

0

0

0

0

0
7

0

0

0

0

0

0

0

0

0

0

0
6

0

0

0

0

0

0

0

0

0

0

0
7

0

0

0

0

0

0

0

0

0

0

0
7

0
7

0

0

0

0

0

0

0

0

0

0

0

0

0

0

0

0

0

0

0

0

0

0
7

0

0

0

0

0

0

0

0

0

0

0
7

0

0

0

0

0

0

0

0.207129

0

0

0
7

0

0

0

0

0

0

0

0

0

0

0
7

0

0

0

0

0

0

0

0

0

0

0
6

0

0.0997735

0

0

0

0

0

0

0

0

0
7

0

0

0

0

0

0

0

0

0

0

0
7

0

0.0761741

0

0

0

0

0

0

0

0

0.46633

0

0

0

0

0

0

0

0

0

0

0.207527
7

0
6

0

0

0

0

0

0

0

0

0

0

0

0

0

0

0

0

0

0

0

0

0

0

0

0

0

0

0

0

0

0

0

0

0
7

0

0

0

0

0

0

0

0

0

0

0
7

0

0

0

0

0

0

0

0

0

0

0

0

0

0

0

0

0.093266

0

0

0

0

0

0

0

0

0

0

0

0

0

0

0

0
7

0

0

0

0

0

0

0

0

0

0

0
6

0

0

0

0

0

0

0

0

0

0

0
7

0

0

0

0

0

0

0

0

0

0

2.97315

0
7

0

0

0

0

0

0

0

0

0

0

0

0

0

0

0

0

0

0

0

0

0

0.218474

0

0

0

0

0

0

0

0

0

0

0
7

0

0

0

0

0

0

0

0

0.304696

0

0
7

0

0

0

0

0

0

0

0

0

0

0
7

0

0

0

0

0

0

0

0

0

0

0
8

0

0

0

0

0

0

0

0

0

0

0
7

0

0

0

0

0

0

0

0

0

0

0.069043
7

0

0

0

0

0

0

0.777586

0

0

0

0

0

0

0

0

0

0

0

0

0

0

13.9869

0.069043
7

0.093266
7

0

0

0

0

0

0

0

0

0

0

0
7

0

0

0

0

0

0

0

0

0.0398762

0

0
7

0

0

0

0

0

0

0

0

0

0

0
7

0

0

0

4.15034

0

0

0

0

0

0

0
7

0

0

0

0

0

0

0

0

0

0

0.188385

0

0

0

0

0

0

0

0

0

0

0
7

0

0

0

0

0

0

0

0

0

0

0

0

0

0

0

0

0

0

0

0

0

0
7

0

0

0

0

0

0

0

0

0

0

0.250041
1

0

0

0

0

0

0

0

0

0

0

0
6

0.152348

21.6334

0

0

0

0

0

0

0

0

0

0.304974

0

0

0

0

0

0

0.0481639

0

0

0

0

0

0

0

0

0

0

0

0

0

0

0
7

0

0

0

0

0

0

0

0

0

0

0.0481639

0

0.233165

0.115072

0.228522

0

0

0

0

0

0

0
7

0

0

0

0

0

0

0

0

0

0

0.0460286

0

0

0.0761741

0

0

0

0

0

0

0

0
7

0

0

0

0

0

0

0

0

0

0

0
7

0

0

0

0

0

0

0

0

0.259515

0

0
5

0

0

0

0

1.1332

0

0

0

0

0

0
7

0.963278

0

0

0

0

0

0

0

0

0

0

0
7

0

0

0

0

0

0

0

0

0

0

0
7

0

0

0

0

0

0

0

0

0

0

0
2

0

0

0

0.261974

0

0

0

0

0

0

0.0515724

0

0

0

0

0

0

0

0

0

0

0
7

0

0

0

0

0

0

0

0.842869

0

0

0
6

0

0

0

0.139899

0

0

0

0

0

0

0.431889

0

0.0704951

0

0

0

0

0

0

0

0

0
6

0

0

0

0

0

0

0.0831446

0

0

0

0
7

0

0

0

0

0

0

0

0

0

0

0.342783
7

0.334852

0

0

0

0

0

0

0

0

0

0

0

0

0

1.0967

0

1.97923

0

0

0

0

0

0
7

0

0

0

0

0

0

0

0

0

0

0
7

0

0

0

0

0

0

0

0

0

0

0
7

0

0

0

0

0

0

0

0

0

0

0
7

0

0

0

0

0

0

0

0

0

0

0.291082
6

0

0

0

0

0

0

0

0

0

0

0
7

0

0

0

0

0

0

0

0

0

0

0.0797524

0

0

0

0

0

0

0

0

0

0.0634669

0.644452

0

0

0

0

0

0

0

0

0

0

0
7

0.069043

0

0

0

0

0

0

0

0

0

0

0
7

0

0.38087

0

0

0

0

0

0

0

0

0
6

0

0

0

0

0

0

0

0

0

0

0

0

0

0

0

0

0

0

0

0

0

0
6

0

0

0

0

0

0

0

0

0

0

0
7

0

0

0

0.113655

0

0

0

0

0

0

0
7

0

0

0

0

0

0

0

0

0

0

0
7

0

0

0

0

0

0

0

0

0

0

0
7

0

0

0

0

0

0

0

0

0

0

0
7

0

0

0

0

0

0

0

0

0

0

0
6

0

0

0

0

0

0

0

0

0

0

0

0
6

0

0

0

0

0

0

0

0

0

0

0
6

0

0

0

0

0

0

0

0

0

0

0
5

0

0

0

0

0

0

0

0

0

0

0

0

0

0

0

0

0

0

0

0

0

0.0686787
7

0

0

0

0

0

0

0

0

0

0

0

0

0

0

0

0

0

0

0

0

0

0
6

0

0

0

0

0

0

0

0

0

0

0.0515724

0

0

0

0.400005

0

0

0

0

0

0

0
7

0

0

0

0

0

0

0

0

0

0

0
6

0
7

0

0

0

0.0582163

0

0

0

0

0

0.0797524

0
7

0

0

0

0

0

0

0

0

0

0

0
7

0

0

0

0

0

0

0

0

0

0

0
6

0

0

0

0

0

0

0

0

0

0

0
6

0

0

0

0

0

0

0

0

0

0

0
7

0

0

0

0

0

0

0

0

0

0

0.1611

0

0

0

0

0

0

0

0

0

0

0
7

0

0

0

0

0

0

0

0

0.093266

0

0
7

0

0

0

0

0

0

0

0

0

0

0
8

0

0

0

0

0

0

0

0

0

0

0.334813
7

0
7

0

0

0

0

0

0

0

0

0

0

0
7

0

0

0

0

0

0

0

0

0

0

0.0681929
7

0

0

0

0

0

0

0

0

0

0

0
6

0

0

0

0

0

0

0

0

0

0

0
7

0

0

0

0

0

0

0

0

0

0

0
7

0

0

0

0

0

0

0

0

0

0

0
7

0

0

0

0

0

0

0

0

0

0

0
7

0

0

0

0

0

0

0.0548351

0

0

0

0
7

0

0

0

0

0

0

0

0

0

0

0
7

0

0

0

0

0

0

0

0

0

0

0
7

0
7

0

0

0

0

0

0

0

0

0

0

0
7

0

0

0

0

0

0

0

0

0

0

0
7

0

0

0

0

0

0

0

0

0

0

0
7

0

0

0

0

0

0

0

0

0

0

0.0761741
7

0

0

0

0

0

0

0

0

0

0

0
7

0

0

0

0

0

0

0

0

0

0

0
6

0

0

0

0

0

0

0

0

0

0

0
7

0.158614

0

0

0

0

0

0

0

0

0

0
7

0

0

0

0

0

0

0

0

0

0

0
7

0

0

0

0

0

0

0

0

0

0

0
7

0
7

0

0

0

0

0

0

0

0

0

0

0
6

0

0

0

0

0

0

0

0

0

0

27.0994
2

0

0

0

0

0

0

0

0

0

0

0
6

0

0

0

0

0

0

0

0

0

0

0.0761741
7

0

0

0

0

0

0

0

0

0

0

0
7

0

0

0

0

0

0

0

0

0

0

0
8

0

0

0

0

0

0

0

0

0

0

0
7

0

0

0

0

0

0

0

0

0

0

2.62225

0

0

0

0

0

0

0

0

0

0

0
7

0

0

0

0

0

0

0

0

0

0

1.48877
7

0
7

0
7

0

0

0

0

0

0

0

0

0

0

0
7

0

0

0

0

0

0

0

0

0

0

0
7

0

0

0

0

0

0

0

0

0

0

0

0

0

0

0

0

0

0

0

0

0

0.093266

0

0

0

0

0

0

0

0

0

0

0.113655

0.464199

0

0

0

0

0

0

0

0

0

0
7

0

0

0

0

0

0

0

0

0

0

0.0761741

0

0

0

0

0

0

0

0

0

0

0.0761741
6

0

0

0

0

0

0

0

0

0

0

0.139899

0

0

0

0

0

0

0

0

0

0

0.121636
7

0
7

0

0

0

0

0

0

0

0

0

0

0
7

0

0

0

0

0

0

0

0

0

0

0
7

0

0

0

0

0

0

0

0

0

0

0
5

0

0

0

0

0

0

0

0

0

0

0.483301

0

0

0

0

0

0

0

0

0

0

0.045462
7

0

0

0

0

0

0

0

0

0

0

0.233165

0

0

0

0

0

0

0

0

0

0

0
7

0

0

0

0

0

0

0

0

0

0

0
7

0

0

0

0

0

0

0

0

0

0

0
7

0

0

0

0

0

0

0

0

0

0

0.139295
7

0
7

0

0

0

0

0

0

0

0

0

0

0
7

0

0

0

0

0

0

0

0

0

0

0
7

0

0

0

0

0

0

0.054841

0

0

0

0
7

0

0

0

0

0

0

0

0

0

0

0.045462

0

0

0

0

0

0

0

0

0

0

0.0761741
6

0

0

0

0

0

0

0

0

0

0

0
7

0

0

0

0

0

0

0

0

0

0

0

0

0

0

0

0

0

0

0

0

0

0
7

0

0.0648786

0

0

0

0

0

0

0

0

0
7

0

0

0

0

0

0

0

0

0

0

0
7

0
7

0

0

0

0

0

0

0

0

0

0

0

0

0

0

0

0

0

0

0

0

0

0
6

0

0

0

0

0

0

0

0

0

0

0
7

0

0

0

0

0

0

0

0

0

0

0
7

0

0

0

0

0

0

0

0

0

0

0
7

0

0

0

0

0

0

0

0

0

0

0
4

0

0

0

0

0

0

0

0

0

0

0.063149

0

0

0

0

0

0

0

0

0

0.264902

0
7

0

0

0

0

0

0

0

0

0

0

0
7

0

0

0

0

0

0

0

0

0

0

0.10967
6

0
7

0

0

0

0

0

0

0

0

0

0

0
7

0

0

0

0

0

0

0

0

0

0

0.0481639
7

0

0

0

0

0

0

0

0

0

0

0
7

0

0

0.0761741

0

0

0.093266

0

0

0

0

0
7

0

0

0.512963

0

0

0

0

0

0

0

0
7

0

0

0

0

0

0

0

0

0

0

0
7

0

0

0

0

0

0

0

0

0

0

0

0

0

0

0

0.12041

0

0

0.0722459

0

0

0
7

0

0

0

0

0

0

0

0

0

0

0
7

0

0

0

0

0.0761741

0

0

0

0

0

0.43958
7

0
6

0

0

0

0

0

0

0

0

0

0

0

0

0

0

0

0

0

0

0

0

0

0
7

0

0

0

0

0

0

0

0

0

0

0
7

0

0

0

0

0

0

0

0

0

0

0
7

0

0

0.438365

0

0

0

0

0

0

0

0.114261
7

0

0

0

0

0

0

0

0

0

0.180503

0.0761741

0

0

0

0

0

0.0548351

0

0

0

0

0
7

0

0

0

0

0

0

0

0

0

0

0
6

0

0

0

0

0

0

0

0

0.199381

0

0
7

0

0

0

0

0

0

0

0

0

0

0.054841
7

0

0

0

0

0.0352475

0

0

0

0

0

0

0
7

0

0

0

0

0

0

0

0

0

0

0.219364

0

0

0

0

0

0

0

0

0

0

0
6

0

0

0

0

0

0

0

0

0

0

0
7

0

0

0

0

0

0

0

0

0

0

0

0

0

0

0

0

0

0

0

0

0

0
7

0

0

0

0

0

0

0

0

0

0

0
7

0

0.044445

0

0

0

0

0

0.279134

0

0

0

0

0

0

0

0

0

0

0

0

0

0

0

0

0

0

0

0

0

0

0

0

0
7

0

0

0

0

0

0

0

0

0

0

0

0
7

0

0

0

0

0

0

0

0

0

0

0
7

0

0

0

0

0

0

0

0

0

0

0
6

0

0

0

0

0.0761741

0

0

0

0

0

0
6

0

0

0

0

0

0

0

0

0

0

0
5

0

0

0

0.0761741

0

0

0

0

0

0

0
6

0

0

0

0

0

0

0

0

0

0

0
7

0

0

0

0

0

0

0

0

0

0

0.045462

0

0

0

0

0

0

0

0

0

0

0

0

0

0

0

0

0

0

0

0

0

0.194069
7

0
7

0

0

0

0

0

0

0

0

0

0

0.1611
7

0

0

0

0

0

0

0

0

0

0

0
7

0

0

0

0

0

0.373064

0

0

0

0.045462

0
7

0

0.415723

0

0

0

0

0

0

0

0

0.0460286
7

0

0

0

0

0

0

0

0

0

0

0
7

0

0

0

0

0

0

0

0

0

0

0.329493

0

0

0

0

0

0

0

0

0

0

0
7

0

0

0

0

0

0

0

0

0

0

0
7

0

0

0

0

0

0

0

0

0

0.16549

0

0

0

0

0

0

0

0

0

0

0

0
7

0

0

0

0

0

0

0

0

0

0

0

6.61762
2

0

0

0

0

0

0

0

0

0

0

0
7

0

0

0

0

0

0

0

0

0

0

0
7

0

0

0

0

0

0

0

0

0

0

0
6

0

0

0

0

0

0

0

0

0

0

0.318483

0

0

0

0

0

0

0

0

0

0

0
7

0

0

0

0

0

0

0

0

0

0

0
7

0

0

0

0

0

0

0

0

0

0

0

0

0

0

0

0

0

0

0

0

0

0
7

0

0

0

0

0

0

0

0

0

0

0
7

0
7

0

0

0

0

0.114261

0

0

0.309466

0

0

0

0.190435

0

0

0

0

0

0

0

0

0

0

0
6

0

0

0

0

0

0

0

0

0

0

0
7

0

0

0

0

0

0

0

0

0

0

0
7

0

0.194636

0

0

0

0

0

0

0

0

0
7

0

0

0

0

0

0

0

0

0

0.266063

0.0982775

0

0

0

0

0

0

0

0

0

0

0.48789
6

0

0

0

0

0

0

0

0

0

0

0
8

0

0

0

0

0

0

0

0

0

0

0
7

0

0

0

0

0

0.571306

0

0

0

0

0

0.0582163

0

0

0

0

0

0

0

0

0

0

0
7

0

0

0

0

0

0

0

0

0

0

0
7

0

0

0

0

0

0

0

0

0

0

0
7

0

0

0

0

0

0

0

0

0

0

0
7

0

0

0

0

0

0

0

0

0

0.138086

0
6

0

0

0

0

0

0

0

0

0

0

0
7

0

0

0

0

0

0

0

0

0

0

0
7

0

0

0

0

0.116433

0

0

0

0

0.0548351

0
6

0

0

0

0

0

0

0

0

0

0

0
7

0

0

0

0

0.287361

0

0

0

0

0

0
7

0
7

0

0.0704951

0

0

0

0

0

0

0

0

0

0

0

0

0

0

0

0

0

0

0

0
7

0

0

0

0

0

0.0398762

0

0

0

0

0
7

0

0

0

0

0

0

0

0.0498868

0

0

0.0761741

0

0

0

0

0

0

0

0

0

0

0
7

0

0

0

0

0

0

0

0

0

0

0
7

0.093266

0

0

0

0

0

0

0

0

0

0
7

0

0

0

0

0

0

0

0

0

0

0
6

0

0

0

0

0

0

0

0

0

0

0
7

0

0

0

0

0

0

0

0

0

0

0
7

0
7

0

0

0

0

0

0

0

0

0

0

0
7

0

0

0

0

0

0

0

0

0

0

0
7

0

0

0

0

0

0

0

0

0

0

0

0

0

0

0

0

0

0

0

0

0

0
7

0

0

0

0

0

0

0

0

0

0

0
6

0

0

0

0

0

0

0

0

0

0

0
7

0

0

0

0

0

0

0

0

0

0.093266

14.7792
2

0

0

0

0

0

0

0

0

0

0

0
7

0

0

0

0

0

0

0

0

0

0

0

0

0

0

0.0515724

0

0

0

0

0

0

0
7

0.0460286
7

0

0

0

0

0

0

0

0

0

0

0
7

0

0

0

0

0

0

0

0

0

0

0
7

0

0

0

0

0.0398762

0

0

0

0

0

0
8

0

0

0

0

0

0

0

0

0

0

0

0

0

0

0

0

0

0

0

0

0

0

0

0

0

0

0

0

0

0

0

0

0.113655

0

0

0

0

0

0

0

0

0

0

0
7

0

0

0

0

0

0

0

0

0

0

0
7

0

0

0

0

0.0773585

0

0

0

0

0

0
7

0

0

0

0

0

0

0

0

0

0

0.114261
7

0
7

0

0

0

0

0

0

0

0

0

0

0
6

0

0

0

0

0

0.114261

0

0

0

0

0
7

0

0

0

0

0

0

0

0

0

0

0
7

0

0

0

0

0

0

0

0

0

0

0

0

0

0

0

0

0

0

0

0

0

0
7

0

0

0

0

0

0

0

0

0

0

0.0442094
7

0

0

0

0

0

0

0

0

0

0

0
6

0

0

0

0

0

0

0

0

0

0

0
7

0

0

0

0

0

0

0

0

0

0

0
7

0

0

0

0

0

0

0

0

0

0

0
7

0
7

0

0

0

0

0.0773585

0

0

0

0

0

1.13077

0

0

0

0

0

0

0

0

0

0

0

0

0

0

0

0

0

0

0

0

0

0
6

0

0

0

0

0

0

0

0

0

0

0
7

0

0

0

0

0

0

0

0

0

0

0

0.979293

0

0

0

0

0

0

0

0

0

0
7

0

0

0

0

0

0

0

0

0

0

0

0

0

0

0

0

0

0

0

0

0

0
7

0

0

0

0

0

0

0

0

0

0

0
7

0

0

0

0

0

0

0

0

0

0

0.045462
7

0
7

0

0

0

0

0

0

0

0

0

0

0
7

0

0

0

0

0

0

0

0

0

0

0
7

0

0

0

0.0761741

0

0

0

0

0

0

0
7

0

0

0

0

0

0

0

0

0

0

0.0582163
7

0

0

0

0

0

0

0

0

0

0

0
8

0

0

0

0

0

0

0

0

0

0

0
7

0

0

0

0

0

0

0

0

0

0

0
7

0

0

0

0

0

0

0

0

0

0

0
6

0

0

0

0

0

0

0

0

0

0

0
7

0

0

0

0

0

0

0

0

0

0

0
7

0
8

0

0

0

0

0

0

0

0

0

0

0
7

0

0

0

0

0

0

0

0

0

0

0
7

0

0

0

0

0

0

0

0

0

0

0

0

0

0

0

0

0

0

0

0

0

0
6

0

0

0

0

0

0

0

0

0

0

0
7

0

0

0

0

0

0

0

0

0

0

0
5

0

0

0

0

0

0

0

0

0

0

0
1

0

0

0.093266

0

0

0

0

0

0

0

0
7

0

0

0

0

0

0

0

0

0

0

0
6

0

0

0

0

0

0

0

0

0

0

0.1611
7

0
7

0

0

0

0

0

0

0

0

0

0

0
5

0

0

0

0

0

0

0

0

0

0

0
7

0

0

0

0

0

0

0

0

0

0

0
7

0

0

0

0

0

0

0

0

0

0

0.378406
8

0

0

0

0

0

0

0

0

0

0

0.365836

0

0

0

0

0

0

0

0

0

0.114261

0
7

0

0

0

0

0

0

0

0

0

0

0.0481639
7

0

0

0

0

0

0

0

0

0

0

0
6

0

0

0

0

0

0

0

0

0

0

0
7

0

0

0

0

0

0

0

0

0

0

3.10159536942578e-12
7

0
4

0
5

0

0

0

0
4

0
7

0
7

0
4

0
7

0

0

0
4

0

0

0
4

0

0

0
4

0

0

0

0
4

0

0

0
4

0

0

0
4

0

0

0
4

0
4

0
7

0
7

0
7

0
7

0
7

0

0
4

0

0

0
4

0
4

12.2247

0.476997
7

0
7

0
7

0.233165

0

0

0

0

0

0

0

0

0

0
7

0

0

0

0

0

0

0

0

0

0

0
6

0

0

0

0

0

0

0

0

0

0

0

0

0

0

0

0

0

0

0

0

0

0
7

0
6

0

0
7

0
6

0

0
7

0
7

0

0
7

0.243832

0

0

0

0

0

0

0
7

0

0

0

0

0

0

0

0

0

0

0
6

0

0

0

0

0

0

0

0

0

0

0
6

0

0

0

0

0

0

0

0

0

0

0
7

0

0

0

0

0

0

0

0

0

0

0
7

0

0

0

0

0

0

0

0

0

0

0
6

0

0

0

0

0

0

0

0

0

0

0
6

0

0

0

0

0

0

0

0

0

0

2.77555756156289e-17
7

0
4

0

0

0

0

0

0

0
4

0

0

0
4

1.33941

1.33941

0

0
7

0

0
4

0

0

0

0

0

0

0

0
4

0.326431
7

0
7

0

0
6

0
6

0

0

0
5

0

0

0

0

0

0

0

0

0

0

0

0

0

0

0

0
6

0

0

0

0

0

0

0

0

0

0

0
7

0

0

0

0

0

0

0

0

0

0

0
7

0

0

0

0

0

0

0

0

0

0

0
7

0

0

0

0

0

0

0

0

0

0

0
6

0

0.326431

0

0

0

0

0

0

0

0

0
7

0

0

0

0

0

0

0

0

0

0
4

0
6

0
6

0

0

0

0

0

0

0

0

0

0

0

0

0
6

0

0

0

0

0

0

0
4

7.85151
6

0
6

0
7

0

0

0

0

0

0

0

0

0

0

0
6

0

0

0

0

0

0

0

0

0

0

1.31956

0

0

0

0

0

0

0

0

0

0

0
7

0

0

0

0

0

0

0

0

0

0

0
6

0

0

0

0

0

0

0

0

0

0

2.29202

0

0

0

0

0

0

0

0

0

0

0
7

0

0

0

0

0

0

0

0

0

0

0
6

0

0

0

0

0

0

0

0

0

0

0
6

0

0

0

0

0

0

0

0

0

0

0
7

0

0

0

0

0

0

0

0

0

0

0
6

0
6

0

0

0

0

0

0

0

0

0

0

0

0

0

0

0

0

0

0

0

0

0

0
6

0

0

0

0

0

0

0

0

0

0

0
7

0

0

0

0

0

0

0

0

0

0

0
6

0

0

0

0

0

0

0
7

0
6

0
6

0
7

0
6

0
6

0
6

0
6

0

0
6

0

0
6

0

0
6

0
6

0
7

0
6

0

0
7

0
6

0
6

0
7

0
6

0

0

0

0

2.04819

0

2.19175

0
7

0

0

0
6

0

0

0

0

0
6

0

0

0

0

0

0

0

0

0

0

0
6

0

0

0

0

0

0

0

0

0

0

0
7

0

0

0

0

0

0

0

0

0

0

0
7

0

0

0

0

0

0

0

0

0

0

0
4

0

0

0

0
5

0

0

0

0

0
4

0

0

0
4

0

0

0

0

0
4

0

0

0
4

0

0

0
4

0

0

0

0
4

0

0

0
4

0

0

0
4

0

0

0
4

0

0

0
4

0

0

0
4

0

0

0

0
4

0

0

0
4

0

0

0
4

0

0

0
4

0

0

0
4

0

0

0
4

0

0

0
4

0

0

0
4

0

0

0

0

0
4

0.243832

0.243832

0
4

0

0

0

0

0

0

0
4

0

0

0

0

0
4

1.98652

1.98652

0
4

0

0

0

0
4

0

0

0
4

8.88178419700125e-16

0
4

27.5124

0
7

0
7

0
4

1.25129
5

1.14555
5

0
5

0

0.105743

0

0

0

0

0

0
4

1.44731

1.44731

0

0

0

0

0

0
4

0
7

0
7

0
7

0

0
4

2.82845

2.63338

0.0975327

0.0975327

0

0

0

0

0
4

3.03115
5

3.03115
5

0

0

0
4

0
7

0
7

0

0

0
4

0
5

0
5

0

0

0

0

0

0

0
4

0
6

0
6

0

0

0
4

0

0

0

0

0
4

0.0528713
4

0
4

0

0

0

0

0

0.0528713

0
4

2.75177

1.34313

0.196555

0.131037

0

0.0655183

1.01553

0
4

2.81844

2.13287
5

0

0

0

0

0.114261

0.571306
6

0

0

0

0

0

0

0

2.22044604925031e-16

0
4

0.252596
3

0.063149
4

0.189447

0
4

0

0

0

0
4

1.10452

0.914089

0.114261

0.0761741

2.77555756156289e-17

0
4

0
7

0

0

0
4

0.0352475
5

0

0

0.0352475

0

0

0

0

0

0
4

1.44731

1.44731

0
4

0

0

0

0

0

0

0

0
4

0
7

0
7

0
4

0
6

0
6

0

0
4

0

0

0
4

0
5

0
5

0

0

0

0

0

0

0

0

0

0

0
5

0

0

0

0

0

0

0

0

0

0
4

0
4

0

0

0
4

0

0

0

0

0

0

0
4

0
7

0

0

0

0

0
4

0

0

0

0
4

0

0

0

0
4

0

0

0
4

0

0

0

0
4

0

0

0

0

0
4

0.0975327

0.0975327

0
4

0

0

0

0
4

0
7

0
7

0

0

0
7

0
7

0
7

0
6

0
7

0

0

0

0
4

2.88281

2.88281

0
4

0.0761741

0.0761741

0

0
4

1.29496

1.29496

0
4

0.0352475

0.0352475

0
4

0

0

0

0

0
4

0

0

0
4

0

0

0
4

0

0

0

0

0
4

0.0352475

0

0.0352475

0
4

0

0

0

0

0
4

2.46281
5

2.27337
5

0.189447
3

0
5

0

0

0

0

0
4

0

0

0

0
4

0

0

0
4

0

0

0

0
4

0

0

0
4

0

0

0

0
4

0

0

0
4

0

0

0
4

0

0

0

0
4

0

0

0
4

0

0

0

0
4

0.190435

0.190435

0
6

0

0

0

0

0

0

0
4

0

0

0
4

0

0

0
4

0.0761741

0.0761741

0
4

0

0

0
4

0

0

0
4

0.063149

0.063149

0
4

0

0

0
4

0

0

0
4

0

0

0
4

0

0

0
4

3.08505

3.08505

0

0

0

0

0

0

0

0
4

0

0

0
4

0

0

0
4

0

0

0
4

0

0

0
4

0

0

0
4

0

0

0
4

0

0

0
4

0

0

0
4

0

0

0
4

0

0

0
4

0

0

0

0

0

0

0

0

0

0

0

0

0
4

0

0

0
4

0

0

0
4

0

0

0
4

0.126298

0.126298

0
4

0

0

0
4

0

0

0
4

0.0655183

0.0655183

0
4

0

0

0
4

0

0

0
4

0

0

0
4

0
5

0

0

0

0

0

0
4

0

0

0
4

0

0

0
4

0

0

0
4

0
4

2.30505

2.30505

2.03232

0.0442094

0.228522

0

0

0

2.77555756156289e-17

0
4

0
4

0

0

0

0
4

0
4

0.264902

0.264902

0.264902

0
4

0
4

0.176238

0.176238

0.14099

0.0352475

0
4

0
4

0

0

0

0

0

0
4

0
4

0

0

0

0
4

0
4

0.390131

0.390131

0

0.390131

0
4

0
4

0.0827448

0.0827448

0.0827448

0

0
4

0
4

0

0

0

0
4

0
4

0

0

0

0
4

0
4

0

0

0

0

0
4

0
4

0.72026
3

0.72026
3

0.0332578

0.0722459

0

0.093266

0

0

0

0

0.12796

0

0

0.184857

0

0

0

0.0332578

0

0

0.079445

0

0

0

0
5

0

0

0

0

0

0
3

0.09597
3

0

0

0

0

6.93889390390723e-17
3

0
4

0

0

0
4

0
4

0

0

0

0

0
4

0
4

0.511089

0.352475

0.352475

0
4

0.158614

0.158614

0
4

2.77555756156289e-17

0
4

0.11996

0

0

0
4

0.11996

0.11996

0
4

0
4

0

0

0

0
4

0
4

0

0

0

0
4

0
4

0

0

0

0
4

0
4

0

0

0

0
4

0
4

0

0

0

0
4

0
4

0

0

0

0
4

0
4

0

0

0

0
4

0
4

0
3

0
3

0

0

0

0

0
4

0

0

0
4

0

0

0
4

0
4

0

0

0

0
4

0
4

0

0

0

0
4

0
4

0

0

0

0
4

0
4

0

0

0

0
4

0
4

0

0

0

0
4

0
4

0

0

0

0
4

0
4

0

0

0

0
4

0
4

0

0

0

0
4

0
4

0

0

0

0
4

0
4

0

0

0

0
4

0
4

0
6

0
6

0
6

0

0

0

0

0
4

0
4

0

0

0

0
4

0
4

0

0

0

0
4

0
4

0

0

0

0
4

0
4

0

0

0

0
4

0
4

0

0

0

0
4

0
4

0

0

0

0
4

0
4

0

0

0

0
4

0
4

3.51118

3.51118

3.51118

0
4

0
4

0

0

0

0

0

0

0
4

0
4

0
5

0

0

0
4

0

0

0
4

0

0

0
4

0
4

0

0

0

0

0
4

0
4

0

0

0

0

0
4

0
4

9.18024

6.56879

6.51592

0

0

0

0

0

0

0.0528713

8.74300631892311e-16

0
4

0
6

0
6

0

0

0

0

0

0

0

0
4

0

0

0
4

0

0

0
4

0

0

0
4

0
5

0

0

0

0

0

0

0

0

0

0
4

0
7

0
6

0

0

0
4

0
7

0
7

0

0
4

2.61145

0

2.61145

0
4

0

0

0
4

0

0

0
4

0

0

0
4

0

0

0
4

8.88178419700125e-16

0
4

7.1603

7.1603

0.639786

0
5

0
7

0

0

0
5

0

0

0
5

0

0

0

0

0

0

0

0

0

0

0.387723

0

0

0
6

3.24277

0

0

0

0

0

0

0

0

0

0
5

0

0

0

0

0

0

0

0

0

0

0
6

0

0

0

0

0

0

0

0

0

0

0.105743

0

0

0

0

2.09695

0

0

0

0

0.211485

0.246733

0

0

0

0

0

0

0

0

0

0

0
7

0

0

0.0352475

0

0

0.193861

0

0

0

1.11022302462516e-15

0
4

0

0
7

0

0
4

0

0

0

0
4

0

0

0
4

0

0

0
4

0

0

0
4

0

0

0
4

0

0

0
4

0

0

0
4

0

0

0
4

0
5

0
6

0

0

0

0
4

0
6

0

0

0

0

0
4

0
5

0
5

0
4

0
7

0
7

0

0

0
4

0
7

0
7

0
4

0

0

0

0
4

0

0

0

0
4

0

0

0
4

0
4

6.66852

6.66852

0
5

0

0

0

0

0

0

0

0

0

3.26431

0
5

0

0.093266

0

0

0

0.093266

0

0

0

0

0

0

0

0.093266

0
6

2.79798

0

0

0.326431

0

0
4

0

0

0
4

0

0

0
4

0

0

0
4

0

0

0
4

0
4

0
5

0
5

0
5

0
5

0
5

0
6

0
5

0

0

0

0

0

0

0

0

0

0

0

0

0

0

0

0

0

0
6

0

0

0

0

0

0

0

0

0

0

0
5

0

0

0

0

0

0

0

0

0

0

0
5

0

0

0

0

0

0

0

0

0

0

0
5

0

0

0

0

0

0

0

0

0

0

0
6

0

0

0

0

0

0

0
4

0
6

0
4

0

0

0

0
4

0

0

0

0
4

0

0

0
4

0

0

0
4

0
4

33.9712

31.9383

24.2031

0
5

0

0

0

0

0

0

0

0

0

0

0
5

0

0

0

0

0.146299

0

0

0

0.373064

0

3.11941

0

0

0.195065

3.90131

0

0
7

0

0

1.33226762955019e-15

0
4

0
5

0
5

0
4

0

0

0
4

0

0

0

0
4

0

0

0

0

0
4

0

0

0
4

0

0

0
4

0

0

0
4

0

0

0

0
4

0

0

0
4

0

0

0
4

0

0

0
4

0.606229

0

0.606229

0
4

0

0

0
4

0

0

0
4

0.792761

0.792761

0
4

0

0

0
4

0

0

0
4

0

0

0
4

0

0

0
4

0

0

0
4

0

0

0
4

0

0

0
4

0

0

0

0

0
4

0

0

0
4

0

0

0
4

0

0

0
4

0

0

0
4

0

0

0
4

0

0

0
4

0

0

0
4

0

0

0

0

0

0
4

0

0

0

0

0

0
4

0

0

0
4

0.633962

0.633962

0
4

0

0

0
4

0

0

0

0
4

0
4

0
5

0
7

0
7

0
7

0

0
4

0
4

0
4

0

0

0
4

0

0

0
4

0

0

0
4

0

0

0
4

0

0

0
4

0

0

0

0
4

0
4

0
4

0

0

0
4

0

0

0

0

0
4

0

0

0

0

0
4

0

0

0
4

0

0

0
4

0

0

0
4

0

0

0
4

0
4

0

0

0

0
5

0

0

0

0

0

0

0

0

0

0

0

0

0

0

0

0

0

0

0

0

0

0

0

0

0

0

0

0

0

0

0

0
7

0

0
7

0

0

0

0

0
4

0
4

0
4

0

0

0

0

0

0
4

0

0

0

0

0
4

0

0

0
4

0

0

0
4

0

0

0
4

0

0

0
4

0
4

3.68977
4

1.40335
4

0
5

0

0

0

0

0

0

0

0

0

0

0.563961

0.839394

0

0

0

0

1.11022302462516e-16
4

0
4

2.28642

2.28642

0
4

0

0

0
4

0

0

0
4

0
4

0
4

224.286

83.114

3.91874

3.91874
5

0

0

0

0

0

0

0

0

0

0

0
4

7.00839

7.00839

0
7

0

0

0

0

0

0

0

0

0

0

0
7

0

0

0
7

0

0

0

0

0

0

0
4

63.0326

26.9955
7

0
7

0.163796

0

0

0

0

0

0

0

0

0

0
7

0

0

0

0

0

0

0

0

0

0

0
7

0

0

0

0

0

0

0

0

0

0

0
8

0

0

0

0

0

0

0

0

0

0

0
7

0

0

0

0

0

0

0

0

0

0.252596

0
7

0

0

0

0

0

0

0

0

0

0

0.0460286
1

0

0

0

0

0

0

0

0

0

0

0

0

0

0

0

0

0

0

0

0

0

0
8

0

0

0

0

0

0

0

0

0

0.131037

0.0655183

0

0

0

0

0

0

0

0.0655183

0

0.0460286

0.891583

0

0

0

0

0

0

0

0

0

0

0

0
8

0

0

0

0

0

0

0

0

0

0

0
7

0

0

0

0

0

0

0

0

0

0

0
7

0

0

0.229314

0

0

0

0

0

0

0

0
7

0

0

0

0

0.0873245

0

0

0

0

0
7

0

0

1.02802
7

0
7

0

0

0

0

0

0

0.477351

0.496737

0

18.6607

0

0

0

0

0

0.0548351

0

0

0

0.69464

0.220206
7

0

0

0

0

0

0

0

0

0

0

8.6913
1

0

0

0

0.356428

0

0

0

0

0

0

2.24781
1

0

0

0

0

0

0

0

0

0.785921

0

0
8

0

0

0

0

0.0982775

0

0

0

0

0

0
8

0.18059

0

0

0

0

0

0

0

0

0.0655183

0
4

0
7

0
7

0

0
4

0
7

0
7

0

0

0

0

0

0

0

0

0

0
4

0
6

0
6

0
6

0

0

0

0
4

0
7

0
7

0
6

0

0

0
4

0
7

0

0

0

0

0

0

0

0

0

0

0
4

0

0

0

0

0

0

0
4

0.114505
1

0.114505
1

0
4

0
6

0
6

0

0

0
4

0.0963278

0

0

0.0963278

0
4

0

0

0

0
4

0
5

0
7

0

0
6

0

0

0

0

0

0

0

0

0

0

0

0

0

0

0

0

0

0

0

0
6

0

0

0

0
6

0
5

0
6

0
6

0
6

0
4

0
6

0
6

0
4

0

0

0

0
4

0

0

0

0
4

0

0

0

0
4

0

0

0

0
4

0

0

0

0
4

0

0

0

0
4

0

0

0
4

0

0

0
4

0

0

0
4

7.61089

0.46727
1

0

0

0

0.666841

0.632116

0.317287

0.0548351

0
7

5.47255
2

0
8

0
6

8.88178419700125e-16

0
4

0

0

0
4

0

0

0
4

0

0

0
4

0

0

0
4

0

0

0
4

0

0

0
4

0

0

0
4

0

0

0
4

0

0

0
4

0

0

0
4

0

0
5

0

0

0
7

0
7

0

0

0

0

0

0
4

0.0975327

0.0975327

0
4

0

0

0
4

0

0

0
4

0.0460286

0.0460286

0
4

0

0

0
4

0

0

0
4

0

0

0
4

0

0

0
4

0

0

0
4

0

0

0
4

1.10167

0.778189
1

0.323477
1

0

0

0

0

0

0

1.66533453693773e-16

0
4

0

0

0
4

0
7

0
7

0
8

0

0

0

0

0
4

0
6

0
6

0
7

0

0

0

0

0

0
4

0.0873245
8

0.0873245
8

0
8

0

0

0

0

0

0
4

0

0

0

0

0

0

0

0
4

1.9151347174784e-15

0
4

105.3

104.843

102.454

0

0

0

0

2.30332

0

0

0

0

0

0

0

0

0.0460286

0

0.0397225

0

0

0

0

0

0

0

0

0

0
4

0
8

0
8

0

0

0

0

0

0

0

0

0

0

0
8

0

0

0

0

0

0

0

0

0

0

0
8

0

0

0

0

0

0

0

0

0
4

0

0

0
4

0.391243

0.391243

0
4

0.0655183

0.0655183

0
4

0

0

0
4

0

0

0
4

0

0

0
4

0

0

0
4

0

0

0
4

0

0

0
4

0

0

0
4

2.01366701091388e-14

0
4

21.5813

17.9818

3.15913

0
5

0

0

0

0

0

0

0

0

0

0

0

0

0

0

0

0

0

0.0973179

0

0

0

0
6

0.221022

0

0

0

0

0

0

0

0

0

0
7

0

0

0

0

0

0

0

0

0

0

0

0.229314

0

0

0

0

0

0

0

0

0

0.352475

0

0

0
6

0
7

0

0
6

1.96713

0
7

0

0

0
7

0

0

0.69043

0

0

0.932197

3.52163
7

0

0.0920573

0

0

0

0

0

0

0

0

4.53231

0

0

0

0

0

0

0

0

0

0.0773585

0
6

0

0

0

0

0

0

0

0

0

0

0
7

0

0

0

0

0

0.0548351

0

0.0548351

0

0

1.47225
1

0

0

0

0

0

0

0

0

0

0

0.527559

0

0

0

0

0

0

0

0

0

0

0
7

0

0

0

0

0

0

0

0

0

0

7.7715611723761e-16

0
4

0

0

0

0

0
4

0

0

0

0

0
4

0

0

0

0
4

0

0

0
4

0

0

0
4

0

0

0
4

0

0

0
4

0

0

0
4

3.48517

2.7911

0

0

0

0

0

0

0

0

0

0

0
6

0

0

0

0

0

0

0

0

0

0

0.228522
6

0

0

0

0

0

0

0

0

0

0

0
6

0

0

0

0.0973179

0

0

0

0

0

0

0

0

0

0

0

0

0

0.368229

2.22044604925031e-16

0
4

0
5

0
6

0

0

0

0

0

0

0

0

0

0
4

0.114261

0
6

0

0

0.114261

0

0

0

0

0

0

0

0
4

0
7

0
7

0

0

0

0
4

0
6

0

0

0

0

0

0
4

0
7

0

0

0

0

0
4

0

0

0

0

0

0
4

0

0

0

0

0

0

0
4

4.5102810375397e-15

0
4

0

0

0

0
4

0

0

0
4

0
4

0

0

0

0
4

0

0

0
4

0
4

0.0352475

0.0352475

0.0352475

0

0
4

0
4

0

0

0

0

0

0
4

0
4

0

0

0

0

0
4

0
4

0

0

0

0

0
4

0
4

0.438897

0.438897

0.341364

0.0975327

0
4

0
4

0.054841

0.054841

0.054841

0

0
4

0

0

0
4

0
4

0

0

0

0
4

0
4

0

0

0

0
4

0
4

1.95409
7

0.0515724
7

0
7

0

0

0

0

0

0

0.0515724
7

0
7

0
7

0

0

0

0

0

0
4

0
6

0
6

0
7

0

0

0

0

0

0

0
4

0.0352475

0.0352475

0

0
4

1.86727

1.86727

0

0
4

0
6

0

0

0

0
4

0

0

0

0
4

0

0

0
4

0

0

0
4

0

0

0
4

0
4

0

0

0

0
4

0

0

0
4

0
4

0

0

0

0
4

0

0

0
4

0
4

0

0

0

0

0
4

0
4

0

0

0

0
4

0
4

0

0

0

0
4

0
4

0

0

0

0
4

0
4

0

0

0

0
4

0
4

0

0

0

0
4

0
4

0

0

0

0
4

0
4

0

0

0

0
4

0
4

0.063149
7

0.063149
7

0.063149
7

0

0

0

0

0
4

0
7

0
7

0

0

0

0

0

0
4

0
6

0

0

0

0
4

0

0

0
4

0
4

0

0

0

0
4

0
4

0

0

0

0
4

0
4

0

0

0

0
4

0
4

0

0

0

0
4

0
4

0

0

0

0
4

0
4

0.093266

0.093266

0.093266

0
4

0
4

0
6

0
6

0
6

0

0

0

0

0

0
4

0
7

0
7

0

0

0
4

0
7

0
6

0

0
4

0

0

0

0

0
4

0

0

0
4

0

0

0
4

0
4

0
6

0
6

0
6

0

0

0

0

0
4

0
4

4.3768
2

4.3768
2

4.26804
2

0

0.069043

0

0

0.0397225

0

0

7.35522753814166e-16
2

0
4

0
4

0
6

0
6

0

0

0

0

0

0
4

0

0

0

0
4

0
4

0

0

0

0
4

0
4

0

0

0

0

0

0

0

0
4

0
4

7.2747

7.2747

3.20675

0
7

0
7

0

0

0
7

0

0.0975327

0

0

0

0
7

0

0

0

0

0

0

0

0

0

0

3.77048

0

0

0

0

0

0

0

0.0799732

0

0

0

0

0

0

0

0

0

0

0

0

0

0
7

0

0

0

0

0

0

0

0

0

0

0

0

0

0

0

0

0

0

0

0

0

0
8

0

0

0

0

0

0

0

0

0

0

0

0

0

0

0

0

0

0

0

0

0.11996

0

0

0

0
4

0

0

0
4

0

0

0
4

0

0

0
4

0
4

6.21724893790088e-15

0
4

2.49494

0
7

0

0

0

0
4

0

0

0

0
4

0

0

0
4

0

0

0
4

0

0

0
4

0

0

0
4

0

0

0
4

0

0

0
4

0
4

0
7

0
7

0

0

0

0

0

0

0

0

0

0

0

0

0

0

0

0

0

0

0

0

0

0

0

0

0

0

0

0

0

0

0

0

0

0

0

0

0

0

0

0

0

0

0

0

0

0

0

0

0

0

0

0

0

0

0

0

0

0

0

0

0

0

0

0

0

0

0

0

0

0

0

0

0

0

0
4

0

0

0
4

0
4

0
6

0
6

0
6

0

0
4

0
4

0

0

0

0
4

0
4

0

0

0

0
4

0
4

0
7

0
7

0
7

0
4

0
4

0

0

0

0
4

0
4

0

0

0

0
4

0
4

0

0

0

0
4

0
4

0

0

0

0
4

0
4

0

0

0

0
4

0
4

0

0

0

0
4

0
4

0

0

0

0
4

0
4

2.49494

2.49494

0.493466

0
7

0

0

0

1.02218

0

0

0

0

0

0

0
6

0.979293

0

0

0

0

0

0

0

0

0

0

0

0

0

0
7

0

0
7

0

0

0

0
4

0

0

0

0

0
4

0

0

0
4

0

0

0
4

0

0

0
4

0

0

0

0
4

0

0

0
4

0

0

0
4

0

0

0
4

0
4

0
4

120.454
4

120.454
4

120.454
4

116.949
4

1.44731

0

0

0

0

0

0

0

0

0

0

1.752

0

0

0

0

0

0

0

0

0

0

0
7

0

0

0

0

0

0

0

0

0

0.115072

0

0

0

0

0

0

0

0

0

0

0

0

0

0.115072

0

0

0

0

0

0

0.0761741

0
6

0

0

0

5.12090370108353e-15
4

0
4

0

0

0
4

0

0

0
4

0

0

0
4

0

0

0
4

0

0

0
4

0

0

0
4

0

0

0
4

0

0

0
4

0

0

0
4

0

0

0
4

0

0

0
4

0

0

0
4

0

0

0
4

0
4

0

0

0

0
4

0
4

0

0

0

0

0
4

0
4

0

0

0

0
4

0
4

0

0

0

0
4

0
4

0

0

0

0
4

0
4

0

0

0

0
4

0
4

0

0

0

0
4

0
4

0

0

0

0
4

0
4

0

0

0

0
4

0
4

0

0

0

0
4

0
4

0

0

0

0
4

0
4

0

0

0

0

0
4

0
4

0

0

0

0
4

0
4

0

0

0

0
4

0
4

0

0

0

0
4

0
4

0

0

0

0
4

0
4

0

0

0

0
4

0
4

0

0

0

0
4

0
4

0

0

0

0
4

0
4

0

0

0

0
4

0
4

0

0

0

0

0
4

0
4

0

0

0

0
4

0
4

0

0

0

0

0
4

0
4

0

0

0

0
4

0
4

0

0

0

0
4

0
4

0
4

3182.11

0
1

0

0

0

0

0

0

0
4

0

0

0
4

0

0

0
4

0

0

0
4

0

0

0
4

0

0

0
4

0

0

0
4

0

0

0
4

0

0

0
4

0

0

0
4

0
4

69.5275

44.5952

10.3808

2.91495
2

0.297855
3

0

0.545544

0.26502

0

0.148089

0.156816

0

0

13.5808

0.155711

0.118308

0

0

6.46702

0

0

0

0

0

0
4

0

0.054841

0

0.1611

0.054841

0

0

0

0

0

2.79254

0

0.0460286

0

0

0

0

0

0

0.253158

0

0

0

0

0

0

0

0

0

0.114261

0

0

0
4

0

0.115072

0

0

0

0

0

0.0423112

0

0

0.21663
3

0.0423112

0

0

0

0

0

0

0.0846225

0

0

2.55429

0

0

0.0423112

0.0822615

0.0423112

0

0

0

0

0

2.59455
3

0

0

0

0.228522

0

0

0.0423112

0

0

0
4

5.55027
3

0.483688
3

4.30484

0.228522

0

0.533219

1.11022302462516e-16
3

0
4

0

0

0
4

0

0

0
4

0

0

0
4

0

0

0
4

0

0

0
4

0

0

0
4

0.114261

0.114261

0
4

0

0

0
4

0

0

0
4

3.33669

3.33669

0
4

15.9311
3

15.9311
3

0
4

0

0

0

0
4

0

0

0

0
4

0

0

0
4

0

0

0

0
4

0

0

0
4

0

0

0
4

0

0

0
4

1.95399252334028e-14

0
4

122.048
1

20.3549
1

0
1

0

0

0

0

0

0

0

0

0

0

1.63796
1

0

0

0

0

0

0

0

0

0

0

0

0

0

0

0

0

0

0

0

0

0.12041

0

0

0

0

0

0

0

0

0

0

0

0

0

0

0

0

0

0

0.0982775

0

0

0.114261

1.24485

0

0

0

0

0

0

0

0

0

0

17.1392

0

0
4

91.0266
2

91.0266
2

0

0
4

0

0

0

0

0

0
4

0.667415

0.667415

0
4

0.159117

0.159117

0
4

0

0

0
4

0

0

0
4

0.093266

0.093266

0
4

0

0

0
4

0.152348

0.152348

0
4

0

0

0
4

0.105778

0.105778

0
4

0

0

0
4

0

0

0

0

0
4

0.0666674

0.0666674

0
4

3.53686

3.53686

0
4

2.62801

2.62801

0
4

3.16122

3.08505

0.0761741

1.80411241501588e-16

0
4

0.09597

0.09597

0
4

0

0

0
4

0

0

0
4

0

0

0
4

1.39610545346613e-14
1

0
4

356.714

26.0232

15.0759
2

0

0.181769

0

0.212186

0.12041

0.0548351

0.0971464

0

0

0

0
6

0.0634669

0

0

0

0.0548351

0

0

0.0548351

0

0

1.0474
3

0

0

0

0

0

0

0

0.274176

0

0

2.94068
2

0

0

0

0.0634669

0

0

0.0481639

0

0

0

4.08774
3

0

0

0

0

0

0.0481639

0

0.0548351

0

0.0548351

0
6

0

0

0

0

0

0

0

1.13681
2

0

0.351531
2

0
4

265.102

242.479

0.0761741
7

2.78384

0

0

0

0

0

0

0

0

0

0.139899

0
7

0

0

0

0

0

0

1.71392

0

0

0

0

0.57582

0

0.114261

0

0

0

0

0

0

0

0
7

0

0

0

0

0

0

0

0

0

0

0.292598

0.240375

0.190435

0.114261

0

0

0.06398

0

0

0.0761741

0

0
7

0

0

0

0

0

0

0.137357

0

0

0

0
6

0

0

0

0

0

0

0

0

0

0

0
7

0.0761741

0

0

0.152348

0

0

0

0

5.90349

0

0
7

0

0

0.0761741

0

0

0

0

0

0

0

0
7

0

0.0761741

0

0

0

0

0.146299

0

0

0

1.22399
7

0
7

0.190435

0

0

0

0

0

0.0761741

0

0

0

0
7

0

0

0

0

0

0

0.152348

0

0

0

0
7

0

0

0

0

0

0

0.0761741

0

0

0

0
7

0

0

0

0

0

0

0.09597

0.0761741

0

0.105743

0
6

0.152348

0

0

0

0.373064

0

0

0

0

0

0
2

0

0.0761741

0

0.38087

0

0

0

0.480751

0

0

0
7

0

0

0.152348

0

0

0

0

0

0

0

0.0704951

0

0

0

0

0

0

0

0

0

0.0761741

0
7

0

0

0

0

0

0.0761741

0

0

0

0

0
6

0

0

0

0

0

0

0

0

0

0

0
7

0
6

0

0

0.093266

0

0

0

0.114261

0

0

0

0
7

0

0

0

0

0

0

0

0

0

0

0

0

0

0

0

0

0

0

0

0.114261

0

0
7

0

0

0

0.3199

0

0

0

0

0

0

0
7

0.131037

0

0

0

0

0

0

0

0

0

0
8

0.09597

0

0

0

0

0

0

0

0

0

0.327592
1

0

0

0

0

0.09597

0

0

0

0

0

0
7

0

0

0

0

0

0

0

0

0

0

0

0

0

0

0

0

0.266609

0

0

0

0

0
7

0.0761741

0

0.0460286

0

0

0

0

0

0
7

0

0
7

0

0
7

0
7

0
6

0
8

0

0.186532

0

0.0799732
7

0
7

0
7

0

0
6

0

0

0

0

0

0

0
7

0

0

0

0

0

0

0

0

0

0

0
7

0

0

0

0.15995

0

0

0

0

0

0

1.82818

0

0

0

0

0

0

0

1.80435

0.0975327

0

0

0

0

0

0

0

0

0

0

0

0

0
4

2.89854
7

2.79213
7

0.0515724

0.0548351

0
4

59.8784

5.0031

5.87576

0

0

0

0

0

0

0

0

0.266609

6.09361

0
6

1.53889

0.266609

0.0761741

0

0.0761741
4

0.216328

12.7972

0.174649

0

0

0

0

0

0.609393

0

4.06273
2

0

0.152348

0.723654

0

0.152348

0

0

0.0761741

0

0

4.19423
3

0

0

0

0

0

0.06398

0

0.0761741

0

0

0.06398
6

0

0

0

0.0761741

0

0.12796

0

0

0

0

0.190435
1

0

0

0

0

0

0.114261

0.64748

0.06398

0

0

0.0761741

0

0

0

0

0

0.262073

0

0

0

0

15.6622
3

0

0

0

0.0975327

0

0

0

0

0

0

0
4

0

0

0

0

0

0

0

0

0

0

4.38538094726937e-15

0
4

0

0

0
7

0

0
4

0

0

0
4

0

0

0

0
4

0.0582163

0.0582163

0
4

0.228522

0.228522

0
4

0

0

0
4

0

0

0
4

0

0

0
4

0

0

0
4

0

0

0
4

0

0

0
4

0.815557

0.240808
1

0

0

0.0761741

0

0.326431

0.172144

0

0

0

0

0

0
4

0

0

0
4

0

0

0
4

0.0530735

0.0530735

0
4

0.0352475

0.0352475

0
4

0.114261
7

0.114261
7

0

0

0

0

0

0
4

0.614035

0.114261
1

0.245614

0.114261

0

0.139899

0

0

0
4

0

0

0

0

0

0

0
4

0.371514

0

0

0

0.371514

0
4

0
1

0

0

0

0
4

0.521024

0

0.152348

0.06398

0.304696

5.55111512312578e-17

0
4

0

0

0

0

0
4

0
4

25.637

10.0127

9.21989

0

0

0.792761

0

0

0

6.66133814775094e-16

0
4

0.0398762
1

0.0398762
2

0
1

0

0

0
4

0

0

0
4

0

0

0
4

0

0

0
4

0

0

0

0
4

0

0

0

0
4

0.727704

0.727704

0
4

0

0

0

0
4

0.233165

0.233165

0
4

0

0

0
4

0

0

0
4

1.36005

1.36005

0

0
4

0

0

0

0
4

0

0

0

0
4

0

0

0
4

0

0

0
4

0

0

0
4

0

0

0
4

0.093266

0.093266

0
4

0

0

0
4

0.069043

0.069043

0
4

0

0

0
4

0
2

0
2

0

0
4

0

0

0
4

0

0

0
4

0.0761741

0.0761741

0
4

0

0

0
4

0

0

0
4

0

0

0
4

0

0

0
4

0.114261

0.114261

0
4

0

0

0
4

0.093266

0.093266

0
4

7.10011
2

7.10011
2

0
4

0

0

0
4

0

0

0
4

0

0

0
4

0

0

0
4

0

0

0
4

0

0

0
4

0

0

0
4

0

0

0
4

1.4956

1.4956

0
4

0.228522

0.114261

0.114261

0
4

3.99332

3.99332

0
4

0

0

0

0
4

0
4

74.3641
3

63.9959

63.9959

0

0

0

0
4

0

0

0

0

0
4

0

0

0
4

8.83619

8.83619

0

0
4

0

0

0

0

0
4

0.0634669

0.0634669

0
4

0

0

0

0
4

0.0722459

0.0722459

0
4

1.39627

1.39627

0
4

0

0

0
4

0

0

0
4

0
4

85.7911

64.749

0
8

0

0

0

0

0.0665157

0.0332578

0

0

0

0.0761741

0.152348

0

0.0460286

0

0

0.0761741

0

0

0

0

0.0761741

0

0

0

7.57932

0

0

1.16582

0.399094

0

0

0

0

0

0

0.38087

0

0.199547

3.96105

0

1.44562

0

4.63947

0

0

0

0

0

0

0

0

0

0

0

0.528519

0.486303

0.415723

0

0

0

0

0.761741

0

0

0

0.914089

0

0

0.152348

0.0332578

0

0

0

0

0

11.6864

0

0

0

0

0.0761741

0.0460286

0.0761741

0

0

0

0

0.0997735

0.069043

0.0332578

0

0

0

0

0

0.0761741

0

3.76479

0.0460286

0

0.0595838

0.114261

0.114261

0

0

0

0

0.069043

19.9153

0

0

0.0761741

4.72279

0

0

0.114261

0

0

0

9.57567358739198e-16

0
4

0

0

0

0

0

0

0

0

0
4

0

0

0
4

0

0

0

0

0

0
4

0

0

0
4

0

0

0

0
4

0.342783

0.342783

0
4

0

0

0

0

0
4

0

0

0

0

0
4

0

0

0

0

0
4

0

0

0

0
4

0

0

0
4

0

0

0

0

0

0
4

0.299321

0.0332578

0.232805

0.0332578

0
4

0.217665

0.217665

0
4

0

0

0
4

0

0

0

0

0
4

5.02194

0.0332578

4.90553

0.0831446

5.55111512312578e-17

0
4

0

0

0
4

0

0

0
4

0

0

0

0
4

0

0

0
4

0

0

0

0
4

11.5838

10.9364

0.64748

1.11022302462516e-16

0
4

0

0

0
4

0

0

0
4

0

0

0
4

0.0352475

0.0352475

0
4

0

0

0
4

0.166289

0.166289

0
4

0

0

0
4

0

0

0
4

0.228522

0.228522

0
4

0

0

0
4

1.16402

0.914591

0.0831446

0.0498868

0.0332578

0.0332578

0.0498868

4.85722573273506e-17

0
4

0

0

0
4

0

0

0
4

0

0

0
4

0

0

0
4

0

0

0
4

0.0595838

0.0595838

0
4

0.0663141

0.0663141

0
4

1.3926

1.3926

0
4

0

0

0
4

0

0

0
4

0
7

0
7

0

0
4

0

0

0
4

0

0

0
4

0

0

0
4

0

0

0
4

0

0

0
4

0.0831446

0.0831446

0
4

0

0

0
4

0

0

0
4

0

0

0
4

0

0

0

0

0

0

0
4

0.38087

0.38087

0
4

0

0

0

0

0
4

0

0

0

0
4

0
4

14.646

9.94072

9.1028

0

0

0

0

0

0.837915

0

0

0

0

0

0

0

0

0

0

0

0

0

0

0

0

0

0

0

0

0

0

0

0

0
4

0.190435

0

0

0.114261

0.0761741

0

0
4

0

0

0
4

0.0761741

0.0761741

0

0
4

0.38087

0.38087

0
4

0

0

0
4

4.0578

4.0578

0
4

0

0

0
4

0
4

51.0601

25.7799

25.7799

0

0
4

0

0

0

0

0

0

0

0

0

0

0
4

0

0

0
4

0

0

0

0
4

0

0

0
4

0

0

0
4

0

0

0

0
4

0

0

0
4

0

0

0

0
4

0

0

0
4

0

0

0
4

0.553055

0.553055

0
4

0

0

0

0

0

0
4

0

0

0
4

0

0

0
4

0

0

0
4

0

0

0
4

0

0

0
4

0

0

0
4

0

0

0
4

0

0

0
4

0

0

0

0

0

0
4

0

0

0

0
4

3.64903

3.64903

0

0
4

0

0

0

0
4

0

0

0

0
4

21.0781
2

20.9382
2

0.139899

0

4.9960036108132e-16
2

0
4

0

0

0

0
4

0
4

224.731

223.536

222.825

0.430902

0.140154

0

0.0761741

0

0

0

0.06398

0
4

0.38087

0.304696

0.0761741

1.38777878078145e-17

0
4

0.685567

0.571306

0.114261

0
4

0

0

0
4

0

0

0
4

0.12796

0.12796

0
4

6.07569550226117e-14

0
4

7.95169

2.36177

0

0

0.928786

0

1.37991

0

0

0

0.0530735

0

0
4

5.58992

5.58992

0
4

0

0

0

0
4

8.88178419700125e-16

0
4

79.2122

0
7

0

0

0

0

0

0

0

0

0

0

0

0

0

0

0

0

0

0

0

0

0

0

0

0

0

0

0

0

0

0

0

0

0
4

4.49226

0
7

0

0

0

0

0

0

0.09597

0

0

0

0
7

0

0.754728

0

0
7

0

2.84711

0

0

0.15889

0.63556

0
4

10.5123

4.68471
7

0
7

0

0

0

0

0

0

0

0

0

0

0
7

0

0

0

0

0.571306

0

0

0

0

0

0
6

0

0

0

0

0

0

0

0

0.191944

0

0

0

0

0

0

0

0

0

0

0

0.109682

1.37103

0.0796102

0

0

0

0

0

0

0

0

0

0

0

0

0

0

0

0

0

0

0

0

3.50401

0

0

0

0

8.88178419700125e-16

0
4

20.4936

18.8969

0
7

0

0

0

0

0.35556

0

0

0

0

0

0

0
7

0

0

0

0

0

0

0

0

0

0

0
7

0

0

0

0

0

0

0

0

0

0

0
7

0

0

0

0

0

0

0

0

0

0

0
6

0

0

0

0

0

0

0

0

0

0

0

0

0

0

0

0

0

0

0

0

0

0

0.333337

0

0

0

0

0

0

0

0
7

0

0

0
7

0

0

0

0

0

0

0

0

0

0

0

0

0

0

0

0

0

0

0

0

0

0
5

0

0

0

0

0

0

0

0

0

0

0
7

0.831594

0

0

0

0

0

0

0

0

0

0
7

0

0

0

0

0

0

0

0

0

0

0

0

0

0

0

0

0

0

0

0

0

0
7

0

0

0

0

0

0

0

0

0

0

0

0

0

0

0

0

0.0761741

0

0

0

0

0
4

22.9539
2

5.14182
2

0

3.35166

0.230143

0.0920573

0.0460286

0

0

0.1611

0.0460286

0

8.74174
2

0.0515724

0.0515724

0.0460286

0.253158

0.917153

0

0

0

0

0

0

0

0.0920573

0

0

0

0.128931

0

0

0

0

0.678411
2

0

0

0

0

0

0

0

0

0

0

0

0

0

0

0

0

0

0.0398762

0

0

0

1.19674

0

0

0.115072

0.0761741

0

0

0

0

0.414258

0.777586

0

0.304696

0
4

5.04591

5.04591

0
4

0
7

0
7

0

0

0
4

0

0

0

0

0

0

0

0

0

0

0

0

0

0

0

0

0

0

0
4

3.98926

2.65029

0

0

0

0

0

0

0

0

0

0

0

0

0

1.33898

0

0

0

0

0

0

0
4

0.401287
3

0.136386

0.192656

0.0722459

0

0
4

0
6

0
6

0

0
4

0

0

0

0

0

0
4

0

0

0

0

0

0

0
4

0

0

0

0
4

0

0

0

0
4

0.555562

0.555562

0

0

0
4

0

0

0

0

0
4

0

0

0

0

0
4

1.40922

1.40922

0
4

6.2027

6.2027

0

0

0

0

0

0
7

0

0

0

0

0

0

0

0
4

0.0666674

0.0666674

0
4

0

0

0

0
4

0

0

0
4

0

0

0
4

0

0

0

0

0
4

0

0

0

0

0
4

0

0

0

0
4

0

0

0

0
4

0

0

0
4

0

0

0

0
4

0
7

0
7

0
7

0

0

0

0

0

0
4

0

0

0

0
4

0

0

0

0
4

0

0

0

0
4

0

0

0
4

0

0

0

0
4

0

0

0

0
4

0

0

0
4

0

0

0
4

0

0

0
4

0

0

0
4

0
7

0
7

0

0
4

0

0

0
4

0

0

0
4

0

0

0
4

0

0

0
4

0

0

0
4

0

0

0
4

0

0

0
4

0

0

0
4

0.044445

0.044445

0
4

0

0

0
4

1.40002
7

1.40002

0

0

0

0
4

1.59966

1.59966

0
4

0

0

0
4

0

0

0
4

0

0

0
4

0

0

0
4

0.045462

0.045462

0
4

0

0

0
4

0

0

0
4

0

0

0
4

0

0

0
4

0
7

0
7

0
4

0

0

0
4

0

0

0
4

0

0

0
4

0

0

0
4

0

0

0
4

0

0

0
4

0

0

0
4

0
7

0

0

0

0
4

0
5

0

0

0

0

0
4

0
7

0
7

0

0

0
4

0
4

26.2493

3.42042

0.244447

0

3.17598

0

0
4

0

0

0

0

0

0

0

0
4

0

0

0
4

0

0

0
4

0.093266

0.093266

0
4

0

0

0
4

0

0

0
4

0.093266

0.093266

0
4

20.1254

16.3481

0.279798

0.233165

0.139899

0.093266

0.326431

2.70471

0
4

1.95859

0.373064

0.186532

0.279798

0.419697

0.326431

0.373064

0
4

0

0

0

0

0

0
4

0.46633

0.186532

0.279798

0
4

0

0

0

0
4

0

0

0

0
4

0.0920573

0.0460286

0.0460286

0
4

0

0

0
4

0
4

48.6626
7

45.4237
7

0
7

1.01263
7

0
7

0

0.069043

0

0

0

0

0

0

0

0

0
7

0

0

0

0

0

0

0

0

0

0

0
7

0

0

0

0

0

0

0

0

0

0

0
7

0

0

0

0

0

0

0

0

0

0

0
7

0

0

0

0

0

0

0

0

0

0

0

0

0.054841

0

0

0

0

0

0

0

0

0
7

0

0

0

0

0

0

0

0

0

0

0
7

0

0

0

0

0

0

0

0

0

0

0
7

0

0

0

0

0

0

0

0

0

0

0.0822615

0

0

0

0

0

0

0

0

0

0

0
7

0
8

0

0

0

0

0.207129

0

0

0

0

0

0
7

0

0

0

0

0

0

0

0

0

0

0
8

0

0

0

0

0

0

0

0

0

0

0
7

0

0

0

0

0

0

0

0

0

0

0
7

0

0

0

0

0

0

0

0

0

0

0
7

0

0

0

0

0

0

0

0

0

0

0
7

0

0

0

0

0

0

0

0

0

0

0
7

0

0

0

0

0

0

0

0

0

0

0
7

0

0

0

0

0

0

0

0

0

0

0
7

0

0

0

0

0

0

0

0

0

0

0
7

0
7

0

0

0

0

0

0

0

0

0

0

0
7

0

0

0

0

0

0

0

0

0

0

0
7

0

0

0

0

0

0

0

0

0

0

0
7

0

0

0

0

0

0

0

0

0

0

0
7

0

0

0

0

0

0

0

0

0

0

0
6

0

0

0

0

0

0

0

0

0

0

0
7

0

0

0

0

0

0

0

0

0

0

0
7

0

0

0

0

0

0

0

0

0

0

0
7

0

0

0

0

0

0

0

0

0

0

0
7

0

0

0

0

0

0

0

0

0

0.0761741

1.65703
7

0
7

0

0

0

0

0

0

0

0

0

0

0
7

0

0

0

0

0

0

0

0

0

0

0
7

0

0

0

0

0

0

0

0

0

0

0
7

0

0

0

0

0

0

0

0

0

0

0
6

0

0

0

0

0

0

0

0

0

0

0
7

0

0

0

0

0

0

0

0

0

0

0

0

0

0

0

0

0

0

0

0

0

0
7

0

0

0

0

0

0

0

0

0

0

0
7

0

0

0

0

0

0

0

0

0

0

0
7

0

0

0

0

0

0

0

0

0

0

0
7

0
7

0

0

0

0

0

0

0

0

0

0

0
7

0

0

0

0

0

0

0

0

0

0

0
8

0

0

0

0

0

0

0

0

0

0

0
7

0

0

0

0

0

0

0

0

0

0

0
6

0

0

0

0

0

0

0

0

0

0

0
6

0

0

0

0

0

0

0

0

0

0

0

0

0

0

0

0

0

0

0

0

0

0
7

0

0

0

0

0

0

0

0

0

0

0
8

0

0

0

0

0

0

0

0

0

0

0

0

0

0

0

0

0

0

0

0

0

0
7

0
7

0

0

0

0

0

0

0

0

0

0

0
7

0

0

0

0

0

0

0

0

0

0

0
7

0

0

0

0

0

0

0

0

0

0

0
7

0

0

0

0

0

0

0

0

0

0

0
7

0

0

0

0

0

0

0

0

0

0

0
7

0

0

0

0

0

0

0

0

0

0

0
7

0

0

0

0

0

0

0

0

0

0

0
7

0

0

0

0

0

0

0

0

0

0

0
6

0

0

0

0

0

0

0

0

0

0

0
7

0

0

0

0

0

0

0

0

0

0

0
7

0
7

0

0

0

0

0

0

0

0

0

0

0
7

0

0

0

0

0

0

0

0

0

0

0
6

0

0

0

0

0

0

0

0

0

0

0
6

0

0

0

0

0

0

0

0

0

0

0

0

0

0

0

0

0

0

0

0

0

0
7

0

0

0

0

0

0

0

0

0

0.069043

0

0

0

0

0

0

0

0

0

0

0

0
7

0

0

0

0

0

0

0

0

0

0

0
7

0

0

0

0

0

0

0

0

0

0

0
7

0

0

0

0

0

0

0

0

0

0

0
7

0
6

0

0

0

0

0

0

0

0

0

0

0
8

0

0

0

0

0

0

0

0

0

0

0
7

0

0

0

0

0

0

0

0

0

0

0
7

0

0

0

0

0

0

0

0

0

0

0
6

0

0

0

0

0

0

0

0

0

0

0
7

0

0

0

0

0

0

0

0

0

0

0
7

0

0

0

0

0

0

0

0

0

0

0
7

0

0

0

0

0

0

0

0

0

0

0
7

0

0

0

0

0

0

0

0

0

0

0
8

0

0

0

0

0

0

0

0

0

0

0
7

0
7

0

0

0

0

0

0

0

0

0

0

0
7

0

0

0

0

0

0

0

0

0

0

0
8

0

0

0

0

0

0

0

0

0

0

0
7

0

0

0

0

0

0

0

0

0

0

0
7

0

0

0

0

0

0

0

0

0

0

0
7

0

0

0

0

0

0

0

0

0

0

0
6

0

0

0

0

0

0

0

0

0

0

0
7

0

0

0

0

0

0

0

0

0

0

0
7

0

0

0

0

0

0

0

0

0

0

0

0

0

0

0

0

0

0

0

0

0

0
7

0
6

0

0

0

0

0

0

0

0

0

0

0
7

0

0

0

0

0

0

0

0

0

0

0
6

0

0

0

0

0

0

0

0

0

0

0
7

0

0

0

0

0

0

0

0

0

0

0
7

0

0

0

0

0

0

0

0

0

0

0
7

0

0

0

0

0

0

0

0

0

0

0

0

0

0

0

0

0

0

0

0

0

0
7

0

0

0

0

0.06398

0

0

0

0

0

0
7

0

0

0

0

0

0

0

0

0

0

0
7

0

0

0

0

0

0

0

0

0

0

17.077
7

0
7

0
7

0

0

0

0

0

0

0

0

0

0

0
6

0

0

0

0

0

0

0

0

0

0

0.054841

0

0

0

0

0

0

0

0

0

0

0
7

0

0

0

0

0

0

0

0

0

0

0
7

0

0

0

0

0

0

0

0

0

0

0
7

0

0

0

0

0

0

0

0

0

0

0
7

0

0

0

0

0

0

0

0

0

0

0
8

0

0

0

0

0

0

0

0

0

0

0
7

0

0

0

0

0

0

0

0

0

0

0
7

0

0

0

0

0

0

0

0

0

0

0
7

0
7

0

0

0

0

0

0

0

0

0

0

0
7

0

0

0

0

0

0

0

0

0

0

0
7

0

0

0

0

0

0

0

0

0

0

0
7

0

0

0

0

0

0

0

0

0

0

0
7

0

0

0

0

0

0

0

0

0

0

0
6

0

0

0

0

0

0

0

0

0

0

0.06398
6

0

0

0

0

0

0

0

0

0

0

0
6

0

0

0

0

0

0

0

0

0

0

0

0

0

0

0

0

0

0

0

0

0

0
7

0

0

0

0

0

0

0

0

0

0

0
7

0
7

0

0

0

0

0

0

0

0

0

0

0
7

0

0

0

0

0

0

0

0

0

0

0
6

0

0

0

0

0

0

0

0

0

0

0
7

0

0

0

0

0

0

0

0

0

0

0
7

0

0

0

0

0

0

0

0

0

0

0
7

0

0

0

0

0

0

0

0

0

0

0
7

0

0

0

0

0

0

0

0

0

0

0
7

0

0

0

0

0

0

0

0

0

0

0
7

0

0

0

0

0

0

0

0

0

0

0
8

0

0

0

0

0

0

0

0

0

0

0
7

0
6

0

0

0

0

0

0

0

0

0

0

0

0

0

0

0

0

0

0

0

0

0

0
8

0

0

0

0

0

0

0

0

0

0

0
7

0

0

0

0

0

0

0

0

0

0

0
8

0

0

0

0

0

0

0

0

0

0

0.159723

0

0

0

0

0

0

0

0

0

0

0
6

0

0

0

0

0

0

0

0

0

0

0
7

0

0

0

0

0

0

0

0

0

0

0
7

0

0

0

0

0

0

0

0

0

0

0
6

0

0

0

0

0

0

0

0

0

0

0
7

0

0

0

0

0

0

0

0

0

0

0

0
7

0

0

0

0

0

0

0

0

0

0

0
6

0

0

0

0

0

0

0

0

0

0

0
7

0

0

0

0

0

0

0

0

0

0

0
7

0

0

0

0

0

0

0

0

0

0

0

0

0

0

0

0

0

0

0

0

0

0
7

0

0

0

0

0

0

0

0

0

0

0
7

0

0

0

0

0

0

0

0

0

0

0
8

0

0

0

0

0

0

0

0

0

0

0
7

0

0

0

0

0

0

0

0

0

0

0
7

0

0

0

0

0

0

0

0

0

0

0

0
7

0

0

0

0

0

0

0

0

0

0

0
7

0

0

0

0

0

0

0

0

0

0

0

0

0

0

0

0

0

0

0

0

0

0
7

0

0

0

0

0

0

0

0

0

0

0

0

0

0

0

0

0

0

0

0

0

0
6

0

0

0

0

0

0

0

0

0

0.09597

0
7

0

0

0

0

0

0

0

0

0

0

0

0

0

0

0

0

0

0

0

0

0

0
6

0

0

0

0

0

0

0

0

0

0

0
7

0

0

0

0

0

0

0

0

0

0

0

0
7

0

0

0

0

0

0

0

0

0

0

0
8

0

0

0

0

0

0

0

0

0

0

0
7

0

0

0

0

0

0

0

0

0

0

0
7

0

0

0

0

0

0

0

0

0

0

0
6

0

0

0

0

0

0

0

0

0

0

0
7

0

0

0

0

0

0

0

0

0

0

0
7

0

0

0

0

0

0

0

0

0

0

0

0

0

0

0

0

0

0

0

0

0

0

0

0

0

0

0

0

0

0

0

0

0.457045

0
7

0

0

0

0

0

0

0

0

0

0

0
7

0

0

0

0

0

0

0

0

0

0

0
7

0

0

0

0

0

0

0

0

0

0

0
6

0

0

0

0

0

0

0

0

0

0

0

0

0

0

0

0

0

0

0

0

0

0
7

0

0

0

0

0

0

0

0

0

0

0

0

0

0.06398

0

0

0

0

0

0

0

0

0

0

0

0

0

0

0

0

0

0

0

0

0

0

0

0

0

0

0

0

0

0

0

0

0

0

0

0

0

0

0

0

0
7

0

0
7

0

0
7

0
7

0
7

0
7

0
8

0

0
7

0
7

0
7

0
6

0
7

0

0
7

0
7

0
7

0
6

0
6

0
8

0
7

0
6

0

0

0

0
6

0
7

0

0

0

0
8

0

0
7

0

0
7

0

0

0
7

0

0

0
8

0
7

0
7

0
7

0
6

0
6

0

0

0
6

0
7

0
7

0

0

0
8

0
7

0
7

0
6

0
7

0
7

0
7

0.0761741

0

0

0

0

0
7

0
8

0
7

0
6

0

0

0

0

0

0

0
7

0
7

0

0

0
6

0

0

0

0.054841

0

0

0

0
7

0
6

0

0
7

0
7

0

0

0

0

0

0

0.121636
5

0

0

0

0

0

0

0

0

0

0

0
7

0

0

0

0

0

0

0

0

0

0

0
7

0

0

0

0

0

0

0

0

0

0

17.768
7

0
7

0

0

0

0

0

0

0

0

0

0

0
7

0

0

0

0

0

0

0

0

0

0

0
7

0

0

0

0

0

0

0

0

0

0

0
7

0

0

0

0

0

0

0

0.0822615

0

0

0
7

0

0

0

0

0

0

0

0

0

0.1611

0.0460286
7

0

0

0

0

0

0

0

0

0

0

0
6

0

0

0

0

0

0

0

0

0

0

0
7

0

0

0

0

0

0

0

0

0

0

0
7

0

0

0

0

0

0

0

0

0

0

0
7

0

0

0

0

0

0

0

0

0

0

0
7

0
7

0

0

0

0

0

0

0

0

0

0

2.8791
7

0

0

0

0

0

0

0

0

0

0

0
7

0

0

0

0

0

0

0

0

0

0

0
7

0

0

0

0

0

0

0

0

0

0

0
7

0

0

0

0

0

0

0

0

0

0

0
7

0

0

0

0

0

0

0

0

0

0

0
7

0

0

0

0

0

0

0

0

0

0

0
7

0

0

0

0

0

0

0

0

0

0

0
7

0

0

0

0

0

0

0

0

0

0

0
7

0

0

0

0

0

0

0

0

0

0

0
7

0
7

0

0

0

0

0

0

0

0

0

0

0
7

0

0

0

0

0

0

0

0

0

0

0
7

0

0

0

0

0

0

0

0

0

0

0
7

0

0

0

0

0

0

0

0

0

0

0
7

0

0

0

0

0

0

0

0

0

0

0
7

0

0

0

0

0

0

0

0

0

0

0.177875

0

0

0

0

0

0

0

0

0

0

0.06398
7

0

0

0

0

0

0

0

0

0

0

0
8

0

0

0

0

0

0

0

0

0

0

0

0

0

0

0

0

0

0

0

0

0

0
7

0

0

0

0

0

0

0

0

0

0

0

0.100303

0

0

0

0

0

0

0

0

0

0

0
7

0

0

0

0

0

0

0

0

0

0

0
7

0

0

0

0

0

0

0

0

0

0

0
7

0

0

0

0

0

0

0

0

0

0

0
7

0

0

0

0

0

0

0

0

0

0

0
7

0

0

0

0

0

0

0

0

0

0

0
7

0

0

0

0

0

0

0

0

0

0

0.0761741
7

0

0

0

0

0

0

0

0

0

0

0

0

0.152348

0

0

0

0

0

0

0

0

0
7

0
7

0

0

0

0

0

0

0

0

0

0

0
7

0

0

0

0

0

0

0

0

0

0

0
7

0

0

0

0

0

0

0

0

0

0

0
7

0

0

0

0

0

0

0

0

0

0

0
6

0

0

0

0

0

0

0

0

0

0

0
8

0

0

0

0

0

0

0

0

0

0

0
7

0

0

0

0

0

0

0

0

0

0

0
7

0

0

0

0

0

0

0

0

0

0

0
7

0

0

0

0

0

0

0

0

0

0

0
7

0

0

0

0

0

0

0

0

0

0

2.39925
7

0

0

0

0

0

0

0

0

0

0

0

0
7

0

0

0

0

0

0

0

0

0

0

0
8

0

0

0

0

0

0

0

0

0

0

0
7

0

0

0

0

0

0

0

0

0

0

0
7

0

0

0

0

0

0

0

0

0

0

0
7

0

0

0

0

0

0

0

0

0

0

0
6

0

0

0

0

0

0

0

0

0

0

0
6

0

0

0

0

0

0

0

0

0

0

0
7

0

0

0

0

0

0

0

0

0

0

0
7

0

0

0

0

0

0

0

0

0

0

3.5527136788005e-15
7

0
4

0
6

0
6

0
4

0

0
7

0
5

0

0

0

0

0

0

0

0

0

0

0
5

0

0

0

0

0

0

0

0

0

0

0
6

0

0

0

0

0

0

0

0

0

0

0

0

0

0

0

0

0

0

0

0

0

0
5

0

0

0

0

0

0

0

0

0

0

0

0

0

0

0

0

0

0

0

0
6

0

0
6

0
4

0

0

0

0

0

0

0
4

0.609393
7

0

0

0

0

0

0

0

0

0

0

0

0

0

0

0

0

0.609393

0

0

0

0

0

0

0

0

0

0
4

0
7

0
7

0
7

0
7

0
6

0
7

0

0

0

0

0

0

0

0

0

0

0

0
4

0
7

0
7

0
7

0

0

0

0

0
4

0.268114
7

0

0
7

0

0

0

0

0

0

0

0

0

0

0
6

0

0

0

0

0

0

0

0

0

0

0

0

0

0

0

0

0

0

0

0

0

0
7

0

0

0

0

0

0

0

0

0

0
7

0

0

0
6

0

0
7

0
6

0

0

0

0

0

0

0

0

0

0

0
6

0

0

0

0

0

0

0

0

0

0

0
6

0

0

0

0

0

0

0

0

0

0

0.12796

0

0

0

0

0

0

0

0

0

0

0
7

0

0

0

0

0

0

0

0

0

0

0
7

0

0

0

0

0

0

0

0

0

0

0
7

0

0

0.06398

0

0

0

0

0

0

0

0

0

0.0761741

0

0

0

0

0

0

0

0

2.77555756156289e-17
7

0
4

0
7

0
7

0
7

0
7

0
7

0

0

0

0

0

0
4

0
7

0
6

0

0
4

0
7

0

0

0
4

0
7

0

0

0

0

0
4

0
7

0

0

0

0
4

0

0

0

0
4

0

0

0

0

0

0

0
4

0.723654

0.64748

0.0761741

0

0
4

0

0

0

0

0
4

0

0

0
4

0

0

0
4

1.21879

1.21879

0

0

0

0

0

0

0

0

0
4

0

0

0

0
4

0

0

0
4

0

0

0

0
4

0.304696

0.304696

0
4

0

0

0
4

0

0

0
4

0

0

0
4

0

0

0
4

0

0

0

0
4

0

0

0
4

0
3

0
3

0

0

0

0

0

0

0

0

0
4

0

0

0

0
4

0

0

0

0
4

0

0

0

0
4

0

0

0
4

0

0

0
4

0

0

0
4

0

0

0
4

0

0

0
4

0

0

0
4

0

0

0
4

0
7

0
7

0

0

0

0

0

0

0
4

0

0

0
4

0

0

0
4

0

0

0
4

0

0

0
4

0

0

0
4

0

0

0
4

0

0

0
4

0

0

0
4

0.114261

0.114261

0
4

0

0

0
4

0
7

0
7

0

0

0

0

0
4

0

0

0
4

0

0

0
4

0

0

0
4

0

0

0
4

0

0

0
4

0

0

0
4

0

0

0
4

0

0

0
4

0

0

0
4

0

0

0
4

0
7

0
7

0

0

0

0
4

0

0

0
4

0
7

0
7

0
7

0

0
4

0
7

0
7

0

0
4

0
3

0
3

0

0
4

7.23032744787133e-15
7

0
4

292.176

253.424
2

241.883
2

8.95624

0.137103

0

0

2.16787

0

0

0

0

0.279798

0

0
4

34.6356

31.7072

1.29972

1.55246

0.0761741

2.62290189567693e-15

0
4

2.6957
2

2.6957
2

0
4

1.37511

1.11919

0.25592

0
4

0

0

0
4

0.0460286

0.0460286

0
4

9.00043928275807e-14

0
4

133.668

46.096

12.0893
3

0.21256

0.38087

0.295628

0.609393

0

0.190435

0.0761741

0.0663141

0

0.0761741

2.64436
2

2.9327

0.219319

1.25997

4.26575

0

0.0530735

0

0

0.0582163

0

6.80356
2

0.190435

0.64748

0.06398

0

0.266609

0

0

0.0761741

0.228522

0.113655

7.94826
2

0.152348

0

1.72251

0.38087

1.13985

0.672174

0.259322
2

0
4

0

0

0

0
4

0.36115

0.132628

0.228522

0
4

0.145541

0.0582163

0.0873245

0
4

0.92656

0.829028

0.0975327

5.55111512312578e-17

0
4

0.0761741

0.0761741

0
4

0.0761741

0.0761741

0
4

28.1629
3

13.5617
3

0

0.0975327

0.291082

0

0.0761741

0

0

0.0982775

0.76776

0.243832

2.97823

1.21916

0.0773585

0

0.0761741

0

0.0799732

0.152348

4.92358

0.396074

0.494694

1.8214

0.3199

0.487663

0

0
4

38.2044

9.30287

0.09597

0.0761741

0.0873245

13.6943
2

11.7835
3

2.56618

0.304696

0

0.114261

0.114261

0.0648786

0
4

2.59238

0.682603
3

0.0761741

0.131037

0.451249

0.526411

0.155784

0.0530735

0

0.229314

0.0582163

0.228522

2.22044604925031e-16

0
4

0

0

0

0

0
4

12.6413
2

5.03494

0.5555

0.110524

0.198942

6.74141

8.88178419700125e-16
2

0
4

0.98573
2

0.304696

0.228522

0.120383

0.266609

0.0655183

0
4

3.40006

3.19463

0.205422

2.4980018054066e-16

0
4

0

0

0
4

7.14983627858601e-14

0
4

42.6379
2

32.7785
2

2.51374

1.81869

20.8717

5.99604

0.914089

0.266609

0.054841

0.0761741

0.190435

0.0761741

5.50948175970234e-15
2

0
4

3.8087

2.70418

0.495132

0.342783

0.114261

0.0761741

0.0761741

4.71844785465692e-16

0
4

4.26575

0.152348

0.0761741

2.81844

0.0761741

0.418957

0.0761741

0.228522

0.152348

0.266609

6.10622663543836e-16

0
4

0.457045

0.342783

0.114261

1.38777878078145e-17

0
4

0.457045

0.0761741

0.304696

0.0761741

1.38777878078145e-17

0
4

0.870911

0.495132

0.37578

0
4

1.57651669496772e-14
2

0
4

0

0

0

0
4

0

0

0
4

0
4

1.752

1.752

0.0761741

1.67583

0
4

0
4

0

0

0

0
4

0

0

0
4

0
4

0

0

0

0
4

0
4

0

0

0

0
4

0

0

0
4

0
4

0

0

0

0
4

0

0

0
4

0
4

0

0

0

0
4

0
4

0

0

0

0
4

0
4

0.204134

0.204134

0.204134

0
4

0
4

0

0

0

0
4

0
4

64.0224

58.7625

54.7373

0.306486

2.0146

0.0663141

0.114261

1.37113

0.152348

5.74540415243519e-15

0
4

1.37434
3

0.49979
2

0.0397225

0.0761741

0.364511

0.0397225

0.218662

0

0.0761741

0

0

0.0595838

0
4

2.54477

0.360405

1.68684

0.142488

0.196558

0.114261

0.0442094

5.27355936696949e-16

0
4

1.10452

0.152348

0.152348

0.685567

0.114261

1.80411241501588e-16

0
4

0.0397225

0.0397225

0
4

0.0761741

0.0761741

0
4

0.0442094

0.0442094

0
4

0

0

0
4

0.0761741

0.0761741

0
4

5.78703751585863e-15

0
4

0

0

0

0

0
4

0
4

0

0

0

0
4

0
4

0

0

0

0
4

0

0

0
4

0
4

0

0

0

0
4

0
4

0

0

0

0

0
4

0
4

0

0

0

0
4

0
4

0

0

0

0
4

0
4

0.530735

0.477661

0.477661

0
4

0.0530735

0.0530735

0
4

0
4

0

0

0

0
4

0

0

0
4

0
4

0

0

0

0

0
4

0
4

213.881

120.012

118.603

0

0

0

0

0.190435

1.14261

0.0761741

0
4

1.73622
7

0.952176

0.403171

0

0.38087

0

0

0

0
4

54.5787

48.9038

0.38087

4.18957

0.190435

0.609393

0.304696

0

1.0547118733939e-15

0
4

37.4776

37.4776

0

0

0

0
4

0.0761741

0.0761741

0

0
4

0

0

0
4

0
4

0

0

0

0
4

0
4

0

0

0

0
4

0
4

0

0

0

0
4

0
4

0.0761741

0.0761741

0.0761741

0
4

0
4

0

0

0

0
4

0
4

0

0

0

0
4

0
4

0

0

0

0
4

0
4

0

0

0

0
4

0
4

0

0

0

0
4

0
4

0.0909239

0.0909239

0.0909239

0
4

0
4

30.6247
2

12.4666
1

10.7193

0.261974

0

0

0

0.304696

1.1807

0
4

3.07728

1.98892

0.64748

0.11605

0

0

0.190435

0

0.0761741

0.0582163

0

6.03683769639929e-16

0
4

0.902646

0.0582163

0.239673

0.279931

0.266609

0.0582163

0
4

12.6912

12.6912

0

0

0

0
4

1.23616

1.23616

0

0
4

0.192607

0.0761741

0.116433

0
4

0.0582163

0.0582163

0
4

4.53109771925142e-15
2

0
4

0.139899

0.139899

0.139899

0
4

0
4

0

0

0

0
4

0
4

0

0

0

0
4

0
4

0

0

0

0
4

0
4

0

0

0

0
4

0
4

0.418957

0.418957

0.418957

0
4

0
4

0

0

0

0
4

0
4

0.0582163

0.0582163

0.0582163

0
4

0
4

0

0

0

0
4

0
4

0

0

0

0
4

0
4

33.0123

6.96847

5.28301
3

0

1.68546

0

0
4

24.1013

22.9587

0.152348

0.266609

0.418957

0.152348

0.152348

0
4

1.67583

0.152348

1.52348

0
4

0.190435

0.114261

0.0761741

0
4

0

0

0
4

0.0761741

0.0761741

0
4

1.98452365651747e-15

0
4

0

0

0

0
4

0
4

0

0

0

0
4

0
4

0

0

0

0
4

0
4

0

0

0

0
4

0
4

0.054841

0.054841

0.054841

0
4

0
4

0

0

0

0
4

0
4

0

0

0

0
4

0
4

0

0

0

0
4

0
4

0

0

0

0
4

0
4

1.79009

1.79009

1.79009

0
4

0
4

0
7

0
7

0
7

0

0

0

0

0

0

0
4

0
7

0
7

0
4

0

0

0

0
4

0
4

0

0

0

0
4

0
4

0.139899

0.139899

0.139899

0
4

0
4

0

0

0

0
4

0
4

0

0

0

0
4

0
4

0.09597

0.09597

0.09597

0
4

0
4

0.0975327

0.0975327

0.0975327

0
4

0
4

0

0

0

0
4

0
4

0

0

0

0
4

0
4

0.0873245

0.0873245

0.0873245

0
4

0
4

0

0

0

0
4

0
4

1.91936
3

1.91936
3

1.83938
3

0

0.0799732

0

0

0

0

1.11022302462516e-16
3

0
4

0
4

0

0

0

0
4

0
4

0

0

0

0
4

0
4

0

0

0

0
4

0
4

0

0

0

0
4

0
4

0

0

0

0
4

0
4

0

0

0

0
4

0
4

0

0

0

0
4

0
4

0

0

0

0
4

0
4

0

0

0

0
4

0
4

0

0

0

0
4

0
4

0

0
6

0

0

0

0

0
4

0

0

0
4

0

0

0
4

0

0

0
4

0
7

0

0

0
4

0

0

0

0

0

0

0
4

0

0

0

0

0
4

0

0

0

0
4

0

0

0

0
4

0

0

0
4

0

0

0

0
4

0

0

0
4

0
4

0

0

0

0
4

0
4

0

0

0

0
4

0
4

0

0

0

0
4

0
4

0

0

0

0
4

0
4

0.152348

0.152348

0.152348

0
4

0
4

0

0

0

0
4

0
4

0

0

0

0
4

0
4

0

0

0

0
4

0
4

0

0

0

0
4

0
4

0

0

0

0
4

0
4

0
7

0
7

0
7

0

0

0

0

0

0
4

0
7

0
6

0

0

0
4

0

0

0

0
4

0

0

0

0
4

0

0

0
4

0
4

0.0418477

0.0418477

0.0418477

0
4

0
4

0

0

0

0
4

0
4

0

0

0

0
4

0
4

0

0

0

0
4

0
4

0

0

0

0
4

0
4

6.8564

6.58979

3.65636

0.326055

2.07417

0.190435

0

0.0761741

0.152348

0.114261

2.08166817117217e-16

0
4

0.190435

0.190435

0
4

0.0761741

0.0761741

0
4

0
4

168.84

167.05

107.482

0

0

0

0

0.266609

0.952176

0.342783

0.190435

1.29496

0

3.04696

0.114261

0.114261

0.266609

0.799828

0.114261

0.0761741

0.495132

0

0

0.114261

0

0

0.418957

0.114261

0.228522

0

0

0.38087

0.0761741

0

0.418957

1.86627

0

39.4582

0

0.190435

2.13287

0.152348

0

0

0.114261

0

0

0.533219

0.0761741

0.0761741

0.152348

0.0761741

0.0761741

0

0

0.0761741

0.114261

0

1.71392

0.495132

0.837915

1.59966

6.19504447740837e-14

0
4

0.457045

0

0

0.114261

0

0.342783

0

0
4

0.990263

0

0

0.0761741

0

0

0.0761741

0.0761741

0.114261

0

0.0761741

0.571306

2.22044604925031e-16

0
4

0.342783

0

0

0.342783

0

0
4

0

0

0
4

8.04911692853238e-15

0
4

0

0

0

0

0

0

0

0

0

0
4

0

0

0
4

0
4

4.0055

4.0055

3.74353

0.261974

5.55111512312578e-17

0
4

0
4

0
4

0
4

0

0

0

0

0

0
4

0
4

0

0

0

0
4

0

0

0

0

0

0
4

0
4

1.29959

1.29959

0

0

1.10452

0.195065

0

0

5.55111512312578e-17

0
4

0
4

134.252

3.09023

1.05547

1.05547

0.886027

0.093266

1.80411241501588e-16

0
4

95.3472

87.3436

7.91039

0.093266

6.50868248186498e-15

0
4

35.1146

35.0214

0

0.093266

2.95596880306448e-15

0
4

0.699495

0.699495

0
4

0
4

0

0

0

0

0

0

0
4

0

0

0
4

0
4

37.0307
3

36.4213
3

15.3459
3

19.9708

0.0761741

0.952176

0.0761741

4.95437024738976e-15
3

0
4

0.418957

0.266609

0.152348

2.77555756156289e-17

0
4

0.190435

0.190435

0
4

0
4

0
8

0
8

0

0

0

0

0

0
4

0

0

0
4

0
4

4.41012
3

4.41012
3

4.41012
3

0
4

0
4

13.9182
3

13.557
2

13.557
2

0

0
4

0.361168
3

0.297701

0.0634669

0

0
4

0

0

0
4

0
4

58.3386
1

44.1187
1

12.8643

0.0873245

0

0.571306

0

0.0582163

0.349298

0.342783

0.228522

0

0.0582163

27.0567
2

0.114261

0.450237

0.488324

0.853408

0.286739

0.0761741

0

0.232865

8.99280649946377e-15
1

0
4

0

0

0
4

0.0761741

0.0761741

0
4

7.85943
1

2.5899
2

0.799828

0

0

0.0582163

0

0.266609

0

0.116433

0

0.116433

0.512797
1

0

0.291082

0.0873245

0.203757

1.4263

0.64748

0.436623

0.219319

0

0.0873245

0
4

5.90349
1

2.01861

0.228522

2.17096

0.837915

0

0

0.64748

0

0

0

0
4

0.38087

0.38087

0

0

0
4

0

0

0

0
4

0

0

0

0
4

0

0

0

0
4

0

0

0

0
4

0

0

0

0
4

1.96509475358653e-14
1

0
4

5.16703
3

5.07106
3

3.67207
3

0.139899

0.139899

1.02593

0.093266

9.57567358739198e-16
3

0
4

0.09597

0

0

0

0.09597

0
4

1.94289029309402e-16
3

0
4

15.6458

13.5891

6.27635

0.0761741

0.114261

7.12228

0
4

1.56157

0.0761741

1.02835

0.190435

0.114261

0.152348

2.77555756156289e-17

0
4

0.228522

0.228522

0
4

0.190435

0.0761741

0.114261

0
4

0.0761741

0.0761741

0

0
4

0

0

0
4

6.52256026967279e-16

0
4

12.548

2.95002

1.39361

0.246785

1.11919

0.190435

2.22044604925031e-16

0
4

9.59793

0.685567

0.266609

0.0761741

0.0761741

8.49341

1.77635683940025e-15

0
4

1.77635683940025e-15

0
4

0

0

0

0

0

0

0
4

0
4

45.4284
2

42.5938

5.0588

29.1366

5.44645

1.54273

1.40922

0
4

2.30391

2.30391

0
4

0.530735

0.530735

0
4

3.33066907387547e-15
2

0
4

17.1011

15.2729

11.8832

3.38975

4.44089209850063e-16

0
4

1.06644

0.342783

0.723654

0
4

0.761741

0.609393

0.152348

0
4

0
4

2.57571

2.1644

2.0049
2

0.119629

0.0398762

0
4

0.329046

0.329046

0
4

0.0822615

0.0822615

0
4

1.2490009027033e-16

0
4

0
7

0

0

0

0
4

0

0

0

0

0
4

0

0

0

0
4

0

0

0
4

0
4

5.90811
2

4.53601

4.28613

0.249881

0

0
4

1.3721
3

1.14261

0.174649

0.054841

0

3.46944695195361e-17
3

0
4

0

0

0
4

0
4

0

0

0

0

0

0

0
4

0

0

0

0
4

0
4

1.31655

0
7

0
7

0

0

0

0

0

0

0

0

0

0

0

0
4

0

0

0
4

0

0

0

0
4

0

0

0

0
4

0

0

0
4

0

0

0

0

0

0

0

0

0

0
4

1.31655
1

1.22923

0

0

0

0

0

0

0.0873245

0
4

0
6

0

0

0

0

0

0

0
4

0
6

0
6

0

0
4

0

0

0

0

0

0

0
4

0
7

0

0

0

0

0
4

0

0

0

0

0

0

0
4

0

0

0

0

0

0
4

0
4

0
8

0
8

0
8

0
4

0

0

0
4

0

0

0
4

0
4

1.55328
3

0.0873245

0

0.0873245

0
4

0.131546

0.131546

0
4

1.27957

1.27957

0
4

0.054841

0.054841

0
4

0

0

0
4

0
4

0
7

0
7

0
7

0

0
4

0
4

0
7

0
7

0

0

0

0
4

0

0

0
4

0
4

0.990263

0.342783

0

0.0761741

0.114261

0

0.152348

0
4

0.190435

0.190435

0
4

0.457045

0.457045

0
4

5.55111512312578e-17

0
4

0.765974
2

0.43423
2

0.21934

0.0822615

0.132628

0

0
4

0.331744

0.331744

0
4

0

0

0
4

0
4

0
4

0

0

0

0

0

0

0
4

0

0

0
4

0
4

3.34259

3.34259

1.43858

0.232712

1.26934

0.401957

0
4

0
4

0
7

0
7

0
7

0

0

0
4

0
4

0
4

0
4

0

0

0
4

0

0

0
4

0

0

0
4

0

0

0
4

0
4

0.0761741
7

0
7

0
7

0
6

0
8

0

0

0

0

0

0

0
4

0
7

0
7

0

0

0
4

0
7

0
7

0

0

0

0

0
4

0.0761741
7

0
8

0

0

0

0.0761741

0

0

0
4

0

0

0

0

0
4

0

0

0

0

0
4

0

0

0

0
4

0

0

0
4

0
4

0

0

0

0

0

0

0
4

0

0

0

0

0
4

0

0

0
4

0
4

0

0

0

0

0
4

0
4

0

0

0

0
4

0
4

1.32178

1.32178

0.722575

0.0352475

0.0704951

0.493466

5.55111512312578e-17

0
4

0
4

1.24861
3

1.24861
3

0.818979

0.332094

0.0975327

0
4

0

0

0
4

0
4

0.0761741

0.0761741

0.0761741

0

0
4

0

0

0
4

0

0

0
4

0
4

0

0

0

0

0

0

0
4

0
4

0.376065

0.172144

0.172144

0

0
4

0.203921

0.152348

0.0515724

0
4

0
4

4.34901

1.8166

1.6544

0.162197

0
4

2.53241

2.47419

0.0582163

0
4

4.44089209850063e-16

0
4

0

0

0

0

0

0
4

0

0

0
4

0
4

1.7994
3

1.7994
3

0
3

0

0

0

0

1.59946
3

0.199933

0

0

0

0

0

0

8.32667268468867e-17
3

0
4

0

0

0

0
4

0

0

0
4

0
4

0

0

0

0

0

0

0

0
4

0

0

0
4

0
4

0

0

0

0

0

0
4

0
4

0.266609

0.266609

0.190435

0.0761741

1.38777878078145e-17

0
4

0

0

0

0
4

0

0

0
4

0
4

1.65705

0.63577

0.0761741

0.559596

0
4

0.792761

0.792761

0
4

0.228522

0.228522

0

0
4

2.77555756156289e-17

0
4

0

0

0

0

0

0

0
4

0
4

0
8

0

0

0

0
4

0

0

0
4

0

0

0
4

0
4

0

0

0

0
4

0

0

0
4

0

0

0
4

0
4

0.0975327

0

0

0

0

0
4

0.0975327

0.0975327

0
4

0
4

1.3003

1.3003

1.24723

0.0530735

6.24500451351651e-17

0
4

0
4

3.85238

0.105778

0.0423112

0.0634669

0
4

3.49068

3.42721

0.0634669

9.71445146547012e-17

0
4

0.25592

0.25592

0
4

1.66533453693773e-16

0
4

7.6555

4.14994

0.761741

0

0

0

0

0

0.114261

0

0

3.19777

0

0.0761741

0

0
4

3.14615

1.68175

0.0761741

0.571306

0.152348

0.207527

0.266609

0

0

0.190435

0

2.77555756156289e-16

0
4

0.279798

0.279798

0
4

0

0

0
4

0

0

0
4

0

0

0
4

0

0

0
4

0.0796102

0.0796102

0
4

0
4

0

0

0

0
4

0

0

0
4

0
4

0

0

0

0
4

0

0

0
4

0
4

0

0

0

0

0
4

0
4

0.0761741

0

0

0
4

0

0

0

0
4

0.0761741

0.0761741

0
4

0
4

0.799535

0.799535

0.799535

0
4

0

0

0

0
4

0
4

0
7

0

0

0
4

0

0

0

0
4

0
4

0

0

0

0
4

0
4

0

0

0

0
4

0

0

0
4

0
4

0

0

0

0

0
4

0
4

0

0

0

0
4

0
4

25.0517

23.6795

8.16959

0

0

14.868

0.510804

0

0.131037

0

0

0

0

8.60422844084496e-16

0
4

0

0

0
4

0.152348

0.152348

0
4

0
7

0
7

0

0

0

0
4

0.136386
7

0.136386

0

0

0

0

0
4

1.08353

0.931181

0.152348

0

0

0
4

0

0

0

0
4

0

0

0
4

0

0

0
4

0

0

0
4

0

0

0
4

0
4

0.434779

0.434779

0.434779

0
4

0
4

0.426559

0.426559

0.266609

0.06398

0.09597

1.38777878078145e-17

0
4

0
4

0.973194

0.973194

0.973194

0
4

0
4

0

0

0

0

0
4

0
4

7.3261

3.99914

0.0761741

3.92297

0
4

3.32696

3.32696

0
4

0
4

0.207189

0.131015

0.054841

0.0761741

0
4

0.0761741

0.0761741

0
4

1.38777878078145e-17

0
4

0.158614

0.105743

0.0528713

0.0528713

0
4

0.0528713

0.0528713

0
4

1.38777878078145e-17

0
4

0

0

0

0

0
4

0
4

0.322287

0.322287

0.0722459

0.113655

0.136386

0
4

0
4

0

0

0

0
4

0
4

102.867

101.939

16.9038

0.474136

1.72542

0.139899

0.419697

0.326431

0.886027

0.139899

0.233165

0.139899

0.093266

2.68762

0.093266

2.09848

0.186532

0.233165

0.139899

0.152348

0.0761741

0.139899

38.0908

1.86068

0.886027

29.8195

0.309339

1.07256

2.61145

6.61692922676593e-14

0
4

0.928017

0.788118

0.139899

5.55111512312578e-17

0
4

0
4

0

0

0

0
4

0
4

0

0

0

0

0

0
4

0
4

0.0681929

0.0681929

0.0681929

0

0
4

0

0

0
4

0
4

0

0

0

0

0
4

0

0

0
4

0
4

0

0

0

0

0

0
4

0
4

0

0

0

0

0
4

0
4

0

0

0

0

0
4

0
4

0

0

0

0
4

0

0

0
4

0
4

0.0761741

0

0

0
4

0

0

0
4

0.0761741

0.0761741

0
4

0
4

0

0

0

0

0
4

0

0

0
4

0
4

6.31571
3

6.31571
3

6.0491

0

0

0

0.266609

0

5.55111512312578e-17
3

0
4

0
4

185.061
3

184.322
3

98.7323
3

85.5134

0.0761741

0
4

0.0761741

0.0761741

0
4

0.495132

0.495132

0
4

0

0

0
4

0.0761741

0.0761741

0
4

0.0920573

0.0920573

0
4

1.04777297948999e-14
3

0
4

279.57

275.53

0
7

0
7

0

0

0

0

0

0

0.09597

0

0

0

0
8

0

0

0

0

0

0

0

0

0

0

1.48348
3

0

0

0

0

0

0

0

0

0

0

0

0

0

0

0

0

0

0

0

0

0

0

0

0.57582

0

0

0

0

0

0

0

0

0
7

0

0

0

0

0

0

0

0

0

0

0

0

0

0

0

0

0

0

0

0.09597

0

0
7

0

0

0

0.186532

0.22393

0

0

0

0

0.28791

0

0

0

0

0

0

0.093266

0

0

0

0

0

0

0

0

0

0

0

123.396
3

0
7

0

0

0
6

0

0
7

0
7

0

0.06398

0

0
7

0

0
7

0

0

0

0

0

0

0

0

0
7

0

0

0

0

0

0

0

0

0.390411

0

0
6

0

0

0

0

0

0

0

0

0

2.14512

10.7348
3

0

0

0

0

0

0

0

0

0

0

0

0

0

0

0

0

0

0

0

2.32529

0

130.773
3

0

0

0

0

0

0

2.65808

0

0

0

0

0

0

0

0

0

0

0

0

0

0

8.88178419700125e-15

0
4

3.67614

0.157246

3.5189

0

0

0
4

0.22393

0

0.22393

0
4

0.139899

0.139899

0
4

0

0

0
4

0
4

2.74085
3

0.0397225
4

0.0397225

0

0
4

2.66141

2.66141

0
4

0

0

0
4

0

0

0
4

0

0

0
4

0.0397225

0.0397225

0
4

0

0

0
4

8.32667268468867e-17
3

0
4

0
4

231.919
3

164.112
3

3.29081

2.92229

0.159946

0

0

0

0

0

0

0

0.0460286

0

0

0.0460286

0

0

0

0

0

0

0.0761741

0

0.0403499

5.41233724504764e-16

0
4

2.20731
5

2.20731
5

0
6

0

0

0

0

0

0

0

0

0

0

0

0

0
4

158.614
3

1.97525

10.4943
3

29.8603

0
4

0

0

0

0

0

0

0

0

0

0

0
4

0

0

0

0.38087

0

0

0

0

0

0.0761741

37.0076
3

0

0

0

0.0920573

0

0

0

0

0

0

1.41332
3

0.0873245

0

0

0

0

0

0

0

0

0

0
4

0.146299

0

0

0

0

0

0.114261

0

0

0.0397225

0.318441

0

0

0

0

0

0

0

0

0

0

0
3

0

0

0

0

0

0

0

0

0

0

1.82724

0

0

0

0

0

0

0

0.195065

0

0

0
4

0

0

0

0

0

0

0

0

0

0

0
4

0

0

0

0.15995

0

0

0

0

0

0

0

0
4

0

0

0

0

0

0

0.0799732

0

0

0

0
3

0

0

0

0

0

0

0.06398

0.0975327

0

0

0
4

0

0

0

0

0

0

0

0.341364

0

0

0

0.266609

0

0

0

0.0403499

0

0

0

0.079445

0

8.88266

0

0

0

0

0

0

0

0

0

0

1.31669
3

0

0

0.975327

0

0

0

0

0

0

0

0

0

0

0

0

0

0
5

0.0595838
6

0

1.00512

1.18595
3

0

0

0.0799732

0.228522
3

0

0

0
4

1.34694

2.48652

0.460286

0
6

0
4

0

0

0
4

0.401

0

0

0.506315

0

0
4

0

0

0

0

0

0

0

0

0

0

27.1921
3

0

0.719759

0

0

0.119168

0

0

0

0

0.551642

13.4235

0

0

0

0

0

0.198613

0

0

0

0.0799732

4.33884
3

0.959679

0

0.0799732

0.106147

0

0.297919

0

0.53643

0

0

4.46849

0

0

1.31037

0.139029

0

0

0

0

0

0

1.16573417585641e-14
3

0
4

0

0

0

0
4

0

0

0
4

0

0

0
4

0

0

0
4

0

0

0
4

0

0

0

0
4

0

0

0

0
4

0

0

0
4

0

0

0
4

0

0

0
4

0

0

0
4

0

0

0
4

0

0

0
4

0
4

26.168
3

18.3853
3

12.9135
3

0

0.282502

0.15995

0.968935

0

0.469034

0

0

0

0.438897

0

0

0

0

0

0

0.292598

0

0

0
4

1.22528
3

0.963913

0.445156

0

0.225493

0

0
4

7.57568
3

0.329046

7.10953
2

0

0

0

0.137103

0

0

0

3.60822483003176e-16
3

0
4

0.206975
4

0.0975327

0

0

0.045462

0

0

0

0

0

0

0

0.06398

0

0

0

0

0
4

0

0

0
4

0
4

0
4

0
4

0
4

0
4

0

0

0
4

0
4

0.86373

0.86373

0.57582

0

0.28791

0

0

0
4

0

0

0
4

0
4

0

0

0

0
4

0
4

2.46084

2.46084

2.29631

0.0822615

0.0822615

2.77555756156289e-17

0
4

0
4

0

0

0

0

0
4

0

0

0
4

0

0

0
4

0
4

0

0

0

0

0

0

0
4

0

0

0

0
4

0
4

0

0

0

0

0
4

0
4

0

0

0

0

0

0
4

0

0

0
4

0

0

0
4

0
4

0

0

0

0
4

0

0

0
4

0
4

0.133915

0.133915

0.0941926

0.0397225

0
4

0
4

30.8367
3

30.5081
3

29.6093
3

0.578945

0

0.12796

0.09597

0.09597

0

0

0
4

0

0

0
4

0

0

0
4

0

0

0

0

0

0

0

0
4

0.209745
2

0.06398

0.0909239

0.054841

0

2.08166817117217e-17
2

0
4

0.054841
3

0

0.054841

0

0
4

0

0

0
4

0

0

0
4

0

0

0
4

0

0

0
4

0.06398

0.06398

0
4

0
4

0

0

0

0
4

0
4

0

0

0

0
4

0
4

0.582163

0.291082

0.291082

0
4

0.291082

0.291082

0
4

0
4

0

0

0

0
4

0
4

0.243832

0.243832

0.243832

0
4

0

0

0
4

0
4

0

0

0

0

0
4

0

0

0
4

0
4

0

0

0

0
4

0

0

0
4

0
4

0

0

0

0
4

0
4

0

0

0

0
4

0

0

0
4

0

0

0
4

0
4

0

0

0

0

0
4

0
4

1.096
3

1.096
3

0.0761741
3

0

0

0

0

0.054841

0.681626
3

0
4

0.228522

0

0

0

0

0.054841

0
4

0

0

0
4

0

0

0
4

0

0

0

0
4

0
3

0

0

0

0

0

0

0
4

0

0

0
4

0

0

0

0
4

0

0

0

0
4

0

0

0

0
4

0

0

0
4

0

0

0
4

0
4

0

0

0

0
4

0

0

0
4

0

0

0
4

0
4

0

0

0

0
4

0
4

0

0

0

0
4

0
4

0

0

0

0
4

0
4

0

0

0

0

0
4

0
4

0

0

0

0

0
4

0
4

0.0548351

0.0548351

0.0548351

0
4

0
4

0

0

0

0
4

0
4

0

0

0

0
4

0
4

0

0

0

0
4

0
4

0
4

0
4

0
4

0
4

0

0

0

0

0

0

0
4

0
4

0
4

0

0

0

0

0

0
4

0

0

0

0

0
4

0

0

0

0
4

0
4

0

0

0

0
4

0
4

0

0

0

0
4

0
4

0.06398

0.06398

0.06398

0
4

0
4

0

0

0

0
4

0
4

0

0

0

0
4

0
4

0

0

0

0
4

0
4

0

0

0

0
4

0
4

4.78656

0.973202

0.198613

0.0397225

0.0397225

0

0.079445

0

0.278058

0.15889

0

0.0993063

0.079445

0

0

0

1.2490009027033e-16

0
4

3.73392

1.62862

0.0595838

0

1.19168

0

0

0.754728

0

0

0

0

0.0993063

0

1.94289029309402e-16

0
4

0.079445

0

0.079445

0

0

0
4

0

0

0

0
4

7.07767178198537e-16

0
4

0.516393

0.47667

0.0993063

0.079445

0.0397225

0.0595838

0.0397225

0.079445

0.079445

0
4

0

0

0

0

0

0

0

0

0
4

0.0397225

0

0

0.0397225

0

0

0
4

0
4

0
4

0

0

0

0

0

0

0

0

0

0

0
4

0

0

0

0

0

0
4

0
4

0

0

0

0

0

0
4

0
4

0
4

0
4

0

0

0

0

0

0
4

0

0

0

0
4

0

0

0

0
4

0

0

0

0
4

0

0

0
4

0
4

0
4

0

0

0

0

0

0

0

0

0

0

0

0

0

0

0
4

0

0

0

0

0

0
4

0

0

0

0

0

0
4

0

0

0

0
4

0
4

0
4

0
7

0
7

0
7

0
7

0

0

0
4

0
4

0
4

0

0

0

0

0
4

0
4

0
4

0

0

0

0

0

0
4

0
4

0
4

0

0

0

0

0

0
4

0
4

0
4

0

0

0

0

0
4

0
4

0
4

0.47985

0.47985

0.47985

0.47985

0
4

0
4

0
4

0

0

0

0

0
4

0
4

0
4

0

0

0

0

0
4

0
4

0
4

0

0

0

0

0
4

0
4

0
4

0

0

0

0

0
4

0
4

0
4

0

0

0

0

0

0
4

0
4

0
4

0
7

0
7

0
7

0
7

0

0

0
4

0
4

0
4

0.115072

0.115072

0.115072

0.069043

0.0460286

6.93889390390723e-18

0
4

0
4

0
4

0

0

0

0

0
4

0
4

0
4

0

0

0

0

0
4

0
4

0
4

0

0

0

0

0
4

0
4

0
4

0

0

0

0

0
4

0
4

0
4

0

0

0

0

0
4

0
4

0
4

0

0

0

0

0
4

0
4

0
4

0

0

0

0

0
4

0

0

0
4

0
4

0
4

0.568341

0.568341

0.568341

0.568341

0
4

0
4

0
4

0

0

0

0

0
4

0
4

0
4

0
7

0
7

0
7

0
7

0
4

0

0

0
4

0
4

0
4

0

0

0

0

0
4

0

0

0
4

0
4

0
4

0

0

0

0

0
4

0
4

0
4

1.83451

1.83451

1.83451

1.83451

0
4

0

0

0
4

0
4

0
4

0.188315

0.188315

0.125543

0.125543

0
4

0.0627716

0.0627716

0
4

0
4

0
4

0

0

0

0

0
4

0
4

0
4

0

0

0

0

0

0
4

0
4

0
4

0

0

0

0

0
4

0
4

0
4

0

0

0

0

0
4

0
4

0
4

0

0

0

0

0
4

0
4

0
4

0

0

0

0

0
4

0
4

0
4

0

0

0

0

0

0

0
4

0

0

0

0
4

0
4

0

0

0

0
4

0

0

0
4

0
4

0
4

0.191923

0.191923

0.191923

0.0548351

0.137088

0
4

0
4

0
4

0

0

0

0

0

0
4

0
4

0
4

0

0

0

0

0
4

0
4

0
4

0

0

0

0

0
4

0

0

0
4

0
4

0
4

0

0

0

0

0
4

0

0

0
4

0
4

0
4

0.0761741

0.0761741

0.0761741

0.0761741

0
4

0
4

0
4

0

0

0

0

0
4

0
4

0
4

0

0

0

0

0
4

0
4

0
4

0

0

0

0

0
4

0
4

0
4

0

0

0

0

0
4

0
4

0
4

0

0

0

0

0

0

0

0

0

0

0
4

0

0

0

0

0

0
4

0

0

0
4

0
4

0
4

0

0

0

0

0
4

0
4

0
4

0

0

0

0

0
4

0
4

0
4

0

0

0

0

0
4

0
4

0
4

0

0

0

0

0
4

0
4

0
4

0

0

0

0

0
4

0
4

0
4

0

0

0

0

0
4

0
4

0
4

0

0

0

0

0
4

0
4

0
4

0

0

0

0

0
4

0
4

0
4

0.0963278

0.0963278

0.0963278

0.0963278

0
4

0
4

0
4

0

0

0

0

0
4

0
4

0
4

0
7

0
7

0

0

0

0
4

0

0

0

0

0
4

0

0

0
4

0

0

0
4

0
4

0
6

0

0

0

0

0
4

0

0

0
4

0

0

0
4

0
4

0
4

0

0

0

0

0
4

0
4

0
4

0

0

0

0

0
4

0
4

0
4

0

0

0

0

0
4

0
4

0
4

2.63338

2.63338

2.63338

2.63338

0
4

0
4

0
4

0

0

0

0

0
4

0
4

0
4

0

0

0

0

0
4

0
4

0
4

0

0

0

0

0
4

0
4

0
4

0.0761741

0.0761741

0.0761741

0.0761741

0
4

0
4

0
4

0

0

0

0

0
4

0
4

0
4

0

0

0

0

0
4

0
4

0
4

20.4161
3

20.4161
3

20.2049
3

20.2049
3

0

0
4

0.211225

0.211225

0
4

2.72004641033163e-15
3

0
4

0
4

0

0

0

0

0
4

0
4

0
4

0

0

0

0

0
4

0
4

0
4

0

0

0

0

0
4

0
4

0
4

0

0

0

0

0
4

0
4

0
4

1.11965

1.11965

1.11965

1.11965

0
4

0
4

0
4

0

0

0

0

0
4

0
4

0
4

0

0

0

0

0
4

0
4

0
4

0

0

0

0

0
4

0
4

0
4

0

0

0

0

0
4

0
4

0
4

0

0

0

0

0
4

0
4

0
4

0
5

0
5

0
5

0
5

0

0

0
4

0

0

0
4

0

0

0
4

0
4

0
4

0.0975327

0.0975327

0.0975327

0.0975327

0
4

0
4

0
4

0.163796

0.163796

0.163796

0.163796

0
4

0
4

0
4

0

0

0

0

0
4

0
4

0
4

0.093266

0.093266

0.093266

0.093266

0
4

0
4

0
4

0

0

0

0

0
4

0
4

0
4

0.54383

0.54383

0.54383

0.54383

0
4

0
4

0
4

0.12796

0.12796

0.12796

0.12796

0
4

0
4

0
4

0.585196

0.585196

0.585196

0.585196

0
4

0
4

0
4

0

0

0

0

0
4

0
4

0
4

0

0

0

0

0
4

0
4

0
4

0

0

0

0

0

0

0

0
4

0

0

0

0
4

0
4

0
4

0

0

0

0

0
4

0
4

0
4

0

0

0

0

0
4

0
4

0
4

0

0

0

0

0
4

0
4

0
4

0

0

0

0

0
4

0
4

0
4

0

0

0

0

0
4

0
4

0
4

0

0

0

0

0
4

0
4

0
4

0

0

0

0

0
4

0
4

0
4

0

0

0

0

0
4

0
4

0
4

0

0

0

0

0
4

0
4

0
4

0

0

0

0

0
4

0
4

0
4

0

0

0

0

0

0

0

0
4

0

0

0
4

0
4

0
4

0

0

0

0

0
4

0
4

0
4

0

0

0

0

0
4

0
4

0
4

0

0

0

0

0
4

0
4

0
4

0

0

0

0

0
4

0
4

0
4

0

0

0

0

0
4

0
4

0
4

0

0

0

0

0
4

0
4

0
4

0

0

0

0

0
4

0
4

0
4

0.0460286

0.0460286

0.0460286

0.0460286

0
4

0
4

0
4

0

0

0

0

0
4

0
4

0
4

0

0

0

0

0
4

0
4

0
4

0

0

0

0

0

0

0

0

0

0

0
4

0

0

0

0

0

0
4

0

0

0

0

0
4

0

0

0
4

0
4

0
4

0
7

0
7

0
7

0
7

0
4

0
4

0
4

0

0

0

0

0
4

0
4

0
4

0

0

0

0

0
4

0
4

0
4

0

0

0

0

0
4

0
4

0
4

0

0

0

0

0
4

0
4

0
4

0

0

0

0

0
4

0
4

0
4

0

0

0

0

0
4

0
4

0
4

0

0

0

0

0
4

0
4

0
4

0.53643

0.53643

0.53643

0.53643

0
4

0
4

0
4

0

0

0

0

0
4

0
4

0
4

0

0

0

0

0
4

0
4

0
4

0

0

0

0

0

0

0
4

0
4

0
4

0

0

0

0

0
4

0
4

0
4

0

0

0

0

0
4

0
4

0
4

0

0

0

0

0
4

0
4

0
4

0

0

0

0

0
4

0
4

0
4

0

0

0

0

0
4

0
4

0
4

0

0

0

0

0
4

0
4

0
4

0

0

0

0

0
4

0
4

0
4

0

0

0

0

0
4

0
4

0
4

0

0

0

0

0
4

0
4

0
4

0

0

0

0

0
4

0
4

0
4

0

0

0

0

0

0
4

0
4

0
4

0

0

0

0

0
4

0
4

0
4

1.45541

1.45541

1.45541

1.45541

0
4

0
4

0
4

0

0

0

0

0
4

0
4

0
4

0

0

0

0

0
4

0
4

0
4

0

0

0

0

0
4

0
4

0
4

0

0

0

0

0
4

0
4

0
4

0

0

0

0

0
4

0
4

0
4

0.0773585

0.0773585

0.0773585

0.0773585

0
4

0
4

0
4

0.06398

0.06398

0.06398

0.06398

0
4

0
4

0
4

0

0

0

0

0
4

0
4

0
4

0
7

0

0

0

0

0

0
4

0
4

0

0

0

0
4

0

0

0
4

0
4

0
4

0

0

0

0

0
4

0
4

0
4

0.27201

0.27201

0.27201

0.27201

0
4

0
4

0
4

0

0

0

0

0
4

0
4

0
4

0

0

0

0

0
4

0
4

0
4

0.232865

0.232865

0.232865

0.232865

0
4

0
4

0
4

0

0

0

0

0
4

0
4

0
4

2.29314

2.29314

2.29314

2.29314

0
4

0
4

0
4

0

0

0

0

0
4

0
4

0
4

0

0

0

0

0
4

0
4

0
4

0

0

0

0

0
4

0
4

0
4

0
6

0
6

0
6

0
6

0
4

0
4

0
4

0.0655183

0.0655183

0.0655183

0.0655183

0
4

0
4

0
4

0.0582163

0.0582163

0.0582163

0.0582163

0
4

0
4

0
4

0

0

0

0

0
4

0
4

0
4

0

0

0

0

0
4

0
4

0
4

0

0

0

0

0
4

0
4

0
4

0

0

0

0

0
4

0
4

0
4

0.884627

0

0

0

0

0

0
4

0
4

0.884627

0.884627

0.815584

0.069043

0
4

0
4

0
4

1.35785
3

1.35785
3

1.35785
3

0.920573

0.437272

0

0

5.55111512312578e-17
3

0
4

0
4

0
4

0
6

0
6

0
6

0

0

0

0
4

0
4

0
4

0
4

0
4

0
4

0
4

0
4

0

0

0
4

0
4

0
4

0
7

0
7

0
7

0
7

0
4

0
4

0
4

2.95901
1

2.95901
1

2.95901
1

2.95901
1

0

0

0

0

0
4

0

0

0
4

0
4

0
4

0
7

0
7

0
7

0
7

0
4

0
4

0
4

0.292598
6

0.292598
6

0.292598
6

0
6

0.292598

0
4

0
4

0
4

1.18056

1.18056

1.18056

0.970591

0.100297

0.0548351

0.0548351

0
4

0
4

0
4

0

0

0
7

0
7

0

0
4

0

0

0
4

0
4

0
4

0

0

0

0

0

0

0
4

0

0

0

0

0
4

0
4

0

0

0

0
4

0

0

0
4

0
4

0
4

0
7

0
7

0
7

0
7

0

0
4

0
4

0
4

0

0

0

0

0

0
4

0
4

0
4

0.639786

0.639786

0.639786

0.559813

0.0799732

0
4

0

0

0

0
4

0
4

0
4

0

0

0

0

0

0

0
4

0
4

0
4

0

0

0

0

0

0
4

0

0

0

0
4

0

0

0
4

0

0

0
4

0
4

0
4

0
6

0
6

0
6

0
6

0

0
4

0
4

0
4

0
7

0
7

0
7

0

0

0

0

0
4

0
4

0
4

0
7

0
7

0
7

0
7

0

0
4

0
4

0
4

0

0

0

0

0

0
4

0

0

0
4

0
4

0
4

0

0

0

0

0

0
4

0
4

0
4

0

0

0

0

0

0

0
4

0

0

0
4

0

0

0
4

0
4

0
4

0

0

0

0

0

0

0

0
4

0
4

0
4

0

0

0

0

0
4

0
4

0
4

0

0

0

0

0

0
4

0
4

0
4

0
7

0
7

0
7

0
7

0
4

0
4

0
4

0

0

0

0

0

0

0

0
4

0
4

0
4

0
5

0
5

0
5

0
5

0

0
4

0

0

0
4

0

0

0

0
4

0

0

0
4

0
4

0

0

0

0

0

0
4

0
4

0
4

0.352475

0.352475

0.352475

0.0352475

0.0352475

0.246733

0.0352475

0
4

0
4

0
4

0
6

0
6

0
6

0
6

0
4

0
4

0
4

0

0

0

0

0

0
4

0

0

0
4

0
4

0
4

0.238831

0.238831

0.238831

0.132684

0.106147

0

0
4

0
4

0
4

0

0

0

0

0
4

0

0

0

0
4

0
4

0
4

0

0

0

0

0

0
4

0

0

0

0
4

0
4

0
4

2.4165

2.4165

2.4165

0.644401

0.253158

0.0460286

1.47292

0
4

0
4

0
4

0.438897

0.438897

0

0

0

0
4

0.438897

0.438897

0
4

0
4

0
4

1.74649

1.74649

1.74649

1.74649

0
4

0

0

0
4

0

0

0
4

0

0

0
4

0
4

0
4

0

0

0

0

0

0

0
4

0
4

0
4

0
7

0
7

0
7

0
7

0

0

0
4

0
4

0
4

0.518371

0.518371

0.518371

0.186532

0.19194

0.139899

0
4

0
4

0
4

0

0

0

0

0

0
4

0
4

0
4

0.492781
3

0.492781
3

0.492781

0.492781

0
4

0

0

0
4

0
4

0
4

0

0

0

0

0
4

0
4

0
4

0

0

0

0

0
4

0
4

0
4

0

0

0

0

0
4

0
4

0
4

0

0

0

0

0

0
4

0
4

0
4

0

0

0

0

0
4

0
4

0
4

0

0

0

0

0

0
4

0
4

0
4

0

0

0

0

0

0
4

0
4

0
4

25.2401

25.2401

0.9597

0.06398

0.09597

0.06398

0.28791

0.12796

0.06398

0.06398

0.09597

0.09597

5.55111512312578e-17

0
4

23.7686

21.4333

0.47985

0.06398

1.2796

0.51184

0
4

0.38388

0.28791

0.09597

0
4

0.12796

0.06398

0.06398

0
4

0
4

0
4

0

0

0

0

0

0

0
4

0
4

0
4

0

0

0

0

0

0
4

0
4

0
4

0

0

0

0

0
4

0
4

0
4

0

0

0

0

0

0

0
4

0
4

0
4

0.361229

0.361229

0.361229

0.361229

0
4

0
4

0
4

0

0

0

0

0
4

0
4

0
4

0.60781

0.60781

0.44786

0.25592

0.19194

0
4

0.15995

0.09597

0.06398

1.38777878078145e-17

0
4

0
4

0
4

1.38219

0.114261

0

0

0
4

0.114261

0.114261

0
4

0
4

1.26792

1.26792

1.26792

0
4

0
4

0
4

0

0

0

0

0

0
4

0
4

0
4

0

0

0

0

0
4

0
4

0
4

0
7

0
7

0
7

0
7

0

0

0

0

0
4

0
4

0
4

0

0

0

0

0

0
4

0

0

0
4

0
4

0
4

0

0

0

0

0
4

0
4

0
4

0

0

0

0

0
4

0
4

0
4

0

0

0

0

0
4

0
4

0
4

0

0

0

0

0

0
4

0
4

0
4

0

0

0

0

0
4

0
4

0
4

0

0

0

0

0

0
4

0
4

0
4

0

0

0

0

0

0

0
4

0
4

0
4

0

0

0

0

0

0
4

0

0

0
4

0
4

0
4

0.139899

0.139899

0

0

0
4

0.139899

0.139899

0
4

0
4

0
4

0
6

0
6

0

0

0

0

0

0

0
4

0

0

0

0

0

0
4

0

0

0
4

0
4

0
4

0

0

0

0

0
4

0
4

0
4

0

0

0

0

0
4

0
4

0
4

0

0

0

0

0
4

0

0

0
4

0
4

0
4

0

0

0

0

0

0

0
4

0
4

0
4

0.661772

0.661772

0.661772

0.661772

0
4

0
4

0

0

0

0
4

0
4

0
4

0

0

0

0

0
4

0
4

0
4

0.448981

0.448981

0

0

0

0
4

0.448981

0.448981

0
4

0
4

0
4

0

0

0

0

0
4

0
4

0
4

0

0

0

0

0

0
4

0
4

0
4

0

0

0

0

0
4

0
4

0
4

2.8634
1

0

0

0

0
4

0
4

0

0

0

0
4

0
4

0

0

0

0
4

0
4

0

0

0

0

0
4

0
4

0

0

0

0
4

0
4

0.137103

0.137103

0.137103

0
4

0
4

0.344978

0.344978

0.344978

0
4

0
4

0.0481639

0.0481639

0.0481639

0
4

0

0

0
4

0
4

0

0

0

0
4

0
4

0

0

0

0

0
4

0
4

0

0

0

0
4

0
4

2.33315
1

2.33315
2

0.722459

1.22818

0.054841

0.0481639

0.0481639

0

0.168574

0

0.0627716

2.77555756156289e-17
2

0
4

0

0

0
4

0
4

0
4

0.0582163
7

0

0

0

0

0
4

0
4

0

0

0

0
4

0
4

0.0582163

0.0582163

0.0582163

0
4

0
4

0

0

0

0
4

0
4

0

0

0

0
4

0
4

0

0

0

0
4

0
4

0

0

0

0
4

0
4

0
7

0
7

0
7

0

0
4

0
7

0
7

0
4

0

0

0
4

0

0

0
4

0

0

0
4

0

0

0
4

0

0

0
4

0

0

0
4

0

0

0
4

0
4

0
4

31.3263
3

31.2803
3

31.2803
3

13.3366

0.214547

0

0.0481639

0.054841

0.0548351

0.0423112

0.054841

0

0

0.239257

14.5849
3

0.054841

0

0

0.0515724

0

0

0

0

0.0598143

0

0.703221
2

0.0681929

0

0

0.0423112

0.219452
3

0.558205
2

0.181666
3

0.261786
2

0.111387
3

0.337495
2

0
4

0

0

0
4

0

0

0
4

0
4

0

0

0

0

0

0
4

0
4

0

0

0

0

0
4

0
4

0

0

0

0
4

0
4

0

0

0

0
4

0
4

0

0

0

0

0
4

0
4

0

0

0

0
4

0

0

0
4

0
4

0

0

0

0
4

0

0

0
4

0
4

0.0460286

0.0460286

0.0460286

0
4

0
4

0

0

0

0
4

0
4

0
4

4.28512
7

0

0

0

0
4

0
4

0

0

0

0
4

0
4

0

0

0

0
4

0
4

0

0

0

0
4

0
4

0

0

0

0
4

0
4

0

0

0

0
4

0
4

0

0

0

0
4

0
4

0

0

0

0
4

0
4

0

0

0

0
4

0
4

0

0

0

0
4

0
4

0

0

0

0
4

0
4

4.28512
7

4.28512
7

3.7219
7

0
5

0

0

0

0

0

0

0.299604

0

0

0

0
7

0

0

0

0

0

0

0

0

0

0

0
6

0

0

0

0

0

0.157873

0

0

0

0

0

0

0.105743

0
7

0

0

0

0

0
4

0

0

0
4

0

0

0
4

0

0

0
4

0
4

0
4

2.04819

0.877794
5

0.877794
5

0.780261

0

0

0

0
5

0
6

0

0

0.0975327

0

0

0

0
4

0

0

0
4

0
4

1.17039
5

1.17039
5

1.17039
5

0

0

0

0

0
4

0

0

0
4

0
4

0

0

0

0
4

0
4

0

0

0

0
4

0
4

0

0

0

0
4

0
4

0

0
7

0

0

0

0

0

0

0

0

0

0

0

0

0

0

0

0

0

0

0

0

0

0

0
4

0

0

0

0

0
4

0

0

0
4

0

0

0
4

0

0

0
4

0
4

0
4

2.14512

0.886027

0.886027

0

0.326431

0

0

0.559596

0

0

0

0

0
4

0

0

0

0
4

0

0

0
4

0
4

0
5

0
5

0
5

0

0

0

0
7

0

0

0

0

0

0

0

0
4

0
4

0

0

0

0

0
4

0

0

0
4

0
4

0

0

0

0
4

0
4

0

0

0

0
4

0
4

0

0

0

0
4

0
4

0

0

0

0
4

0
4

0

0

0

0
4

0
4

0

0

0

0

0
4

0
4

0

0

0

0

0
4

0
4

0

0

0

0
4

0
4

0

0

0

0
4

0
4

0
5

0
5

0
5

0

0

0

0

0

0

0
4

0

0

0
4

0

0

0
4

0
4

0

0

0

0
4

0
4

0

0

0

0
4

0
4

0

0

0

0
4

0
4

0

0

0

0
4

0
4

0

0

0

0
4

0
4

0

0

0

0
4

0
4

0

0

0

0
4

0
4

0

0

0

0
4

0
4

0

0

0

0
4

0
4

0

0

0

0
4

0
4

0
7

0
7

0
7

0

0
7

0
7

0

0

0

0

0

0
4

0
4

0.233165
5

0.233165
5

0

0

0.233165

0

0

0

0

0

0
4

0

0

0

0

0

0
4

0
4

0
5

0
5

0

0

0

0

0

0

0
4

0

0

0

0
4

0
4

0

0

0

0
4

0

0

0
4

0
4

0

0

0

0

0

0
4

0

0

0
4

0
4

0
7

0
7

0

0

0

0

0

0
4

0
4

1.02593

0.886027

0.886027

0
4

0.139899

0.139899

0
4

0
4

0
4

88.1184

13.1557

13.1075

8.52319

1.07106

0.238118

2.02288

0.0963278

1.01144

0.0963278

0.0481639

0
4

0.0481639

0.0481639

0
4

0
4

0

0

0

0

0

0
4

0
4

0

0

0

0

0
4

0
4

2.01113

1.89072

1.81848

0.0722459

6.93889390390723e-17

0
4

0.12041

0.12041

0
4

0
4

0

0

0

0

0
4

0
4

0

0

0

0
4

0

0

0
4

0
4

0

0

0

0
4

0
4

0.0663141

0.0663141

0.0663141

0
4

0
4

0

0

0

0
4

0
4

0

0

0

0
4

0

0

0
4

0
4

0

0

0

0
4

0
4

0

0

0

0

0

0
4

0
4

0

0

0

0
4

0
4

0

0

0

0
4

0
4

0

0

0

0
4

0
4

0.0704951

0.0704951

0.0704951

0
4

0
4

0

0

0

0
4

0
4

0

0

0

0
4

0
4

0

0

0

0
4

0
4

0

0

0

0
4

0
4

0.079445

0.079445

0.079445

0
4

0
4

0

0

0

0
4

0
4

0.972607

0.839979

0.243152

0.596827

0
4

0.132628

0.132628

0

0
4

0
4

0

0

0

0
4

0
4

0

0

0

0
4

0
4

0

0

0

0
4

0
4

0

0

0

0
4

0
4

0

0

0

0
4

0
4

0

0

0

0
4

0
4

0

0

0

0

0
4

0
4

10.7619

10.7165

0.0963278

10.572

0.0481639

0
4

0.045462

0.045462

0
4

8.32667268468867e-16

0
4

0

0

0

0

0
4

0

0

0
4

0

0

0
4

0
4

0

0

0

0
4

0

0

0

0
4

0
4

0

0

0

0

0
4

0
4

0.259498

0.117708

0.0722459

0.045462

0
4

0.14179

0.045462

0.0963278

1.38777878078145e-17

0
4

0
4

60.7413

54.2754

41.5113

0.902353

0

0

0.0397225

0

0.0397225

0.0397225

0.0352475

0

0

0.261974

8.69923

0.0397225

0

0.0595838

1.28076
3

1.22703

0

0

0

0.0993063

0.0397225

1.79301018476963e-14

0
4

0

0

0

0

0

0

0

0
4

0

0

0
4

0

0

0
4

0

0

0
4

0

0

0
4

0

0

0
4

5.99826

5.99826

0
4

0

0

0
4

0.0975327

0

0

0.0975327

0

0

0
4

0

0

0
4

0

0

0
4

0

0

0

0
4

0.370099

0.370099

0
4

0

0

0
4

0

0

0
4

0

0

0
4

0
4

0
4

0
5

0
6

0

0

0

0

0

0

0

0

0

0
4

0

0

0

0
4

0

0

0

0
4

0

0

0
4

0

0

0
4

0
4

0

0
5

0
6

0

0

0

0

0

0

0

0
4

0

0

0

0
4

0

0

0
4

0
4

0

0

0

0
4

0
4

0

0

0

0
4

0
4

0

0

0

0
4

0
4

0

0

0

0
4

0
4

0

0

0

0
4

0
4

0
5

0
5

0
6

0

0

0

0

0

0
4

0
4

0

0

0

0

0

0

0

0
4

0
4

0
5

0

0

0

0

0

0
4

0

0

0
4

0

0

0
4

0
4

0

0

0

0
4

0
4

0

0

0

0

0
4

0
4

0

0

0

0
4

0
4

0

0

0

0
4

0
4

0

0

0

0
4

0
4

0
4

1.69787
3

0.115072

0.115072

0.0460286

0.069043

1.38777878078145e-17

0
4

0
4

0

0

0

0
4

0
4

1.5828
3

0.0481639

0.0481639

0
4

1.39441

1.33483

0

0.0595838

0
4

0

0

0
4

0.140221

0.140221

0
4

0

0

0
4

2.77555756156289e-17
3

0
4

0
4

28.9531

2.48708

2.48708

1.46299
7

0

0

0

0

0

0

0

0

0

0
8

0
8

1.02409
1

0

0

0

0

0

0
4

0

0

0

0

0
4

0

0

0

0
4

0
4

0

0

0

0

0
4

0
4

0

0

0

0

0

0
4

0

0

0
4

0
4

0.294833

0.131037

0.0655183

0.0655183

0
4

0.163796

0.0655183

0.0982775

1.38777878078145e-17

0
4

2.77555756156289e-17

0
4

0

0

0

0
4

0
4

0.397225

0.397225

0.397225

0
4

0
4

0.15889

0.0993063

0.0595838

0.0397225

0
4

0.0595838

0.0595838

0
4

0
4

0

0

0

0
4

0
4

0.266609

0.266609

0.114261

0.152348

0
4

0
4

1.12163

1.12163

1.12163

0
4

0
4

0

0

0

0
4

0

0

0
4

0
4

2.73091

0.146299
7

0

0

0

0

0.146299

0

0

0

0

0

0

0
4

2.58462
1

0
8

2.58462

0

0
4

0

0

0
4

0

0

0
4

0
4

0

0

0

0
4

0
4

2.2759

2.2759

2.22106

0.054841

0
4

0
4

0

0

0

0
4

0
4

0

0

0

0
4

0
4

0

0

0

0
4

0
4

0

0

0

0
4

0
4

0

0

0

0
4

0
4

0

0

0

0
4

0
4

0

0

0

0
4

0
4

0

0

0

0
4

0
4

0

0

0

0

0

0

0
4

0

0

0

0
4

0

0

0
4

0

0

0
4

0
4

0

0

0

0
4

0
4

0

0

0

0
4

0
4

0

0

0

0
4

0
4

0

0

0

0
4

0
4

0.152348

0.152348

0.152348

0
4

0
4

0

0

0

0
4

0
4

0

0

0

0
4

0
4

0

0

0

0
4

0
4

0

0

0

0
4

0
4

0

0

0

0
4

0
4

0

0

0

0

0

0

0
4

0

0

0

0
4

0
4

2.2759

2.19364

2.11138

0.0822615

0
4

0.0822615

0

0

0.0822615

0

0
4

0

0

0

0
4

0
4

0.739147
3

0.604757
3

0.201586

0.0582163

0

0

0.0582163

0.0582163

0.114261

0.114261

1.11022302462516e-16
3

0
4

0.0582163

0.0582163

0
4

0.0761741

0.0761741

0
4

0
4

0
3

0
3

0

0

0

0

0

0
4

0
4

0
6

0

0

0

0

0
4

0

0

0
4

0

0

0
4

0
4

4.4181

4.4181

4.18957

0.152348

0.0761741

0
4

0
4

11.6346

2.50982

0
7

0

0

0

0

0

0

0

0

0

1.752

0

0

0

0

0

0

0

0

0

0

0

0

0

0

0

0

0

0

0

0

0

0

0

0

0

0

0

0

0

0

0

0

0

0
7

0

0

0

0

0

0

0

0.685567

0

0

0

0

0

0

0

0

0.0722459

0

0

0

0

0

0

0

0

0
4

0

0

0

0

0
4

0

0

0
4

0.114261

0.114261

0
4

0

0

0

0
4

0

0

0

0
4

0.0582163

0.0582163

0
4

0

0

0
4

3.11212

3.11212

0
4

0

0

0
4

2.04819

2.04819

0
4

0.114261

0.114261

0
4

0
6

0
6

0

0

0
4

0

0

0
4

0

0

0
4

0

0

0
4

0

0

0
4

0

0

0
4

0

0

0

0

0

0
4

2.78453

2.68625

0.0982775

0

0
4

0.555519
3

0.116433

0.304696

0.13439

0
4

0

0

0

0
4

0

0

0

0
4

0.337641

0.238335

0.0993063

2.77555756156289e-17

0
4

0

0

0
4

0
4

0
4

1.95486
7

0
6

0
6

0
6

0

0
4

0
4

0
6

0
6

0
6

0
4

0
4

0

0

0

0
4

0
4

0

0

0

0
4

0
4

0

0

0

0
4

0
4

0

0

0

0
4

0
4

1.95486
7

1.95486
7

1.95486
7

0
4

0
4

0
4

0
4

6081.55

0

0

0

0

0

0

0
4

0
4

0
4

5314.67
3

91.919

91.8047

77.2351

2.08396

0.191944

0.054841

0.0993063

0

0

0

0.373064

0

2.0057
2

2.52269

5.54129

0.82434

0.44786

0.424588

0

0

2.15938378289593e-14

0
4

0

0

0
4

0.114261

0.114261

0
4

5.39845945723982e-15

0
4

4042.46
3

388.567

1.41743

13.0952

0.330589

0

0

0.38087

0.780261

0

0

0

0.266609

0

0.28239

1.40922

0

5.52554

0

0

2.14707

0.780261

0

7.70508

0

0

0

0

0

0

0

0

0

0.457045

0

0

0.06398

2.73091

0

2.33165

0

0

0.210565

0

1.20153

2.53932

0.481413

0.291904

1.51568

1.80175

0

0

4.64662

0.349298

0

0

4.79897

0

0.0663141

0
6

0

0

0

0

0

0

0

0.0582163

0

0.397885

0
7

0

0.063149

0

0

0.266609

0

0.723654

2.91109

0.114261

0

0.839394

0

0

0

0.152348

0

0.114261

0.0761741

0

0

0

0.287968

0

0.114261

0

0

0

0.0761741

0

0.114261

0

0.378406

1.95065

0

0

0

0

0

0

0

0

0.0460286

0

2.80851

0
5

0

0

0.146299

0

0

0.06398

0

0.0761741

0.0761741

0

30.9167

0.0761741

0

0

0

0

0

0

0.06398

0

0

113.467
2

0

0

0.0975327

0

0.09597

0.093266

0

0.063149

0

0

0

1.25687

0

0

0

0

0

0

0.093266

0

0

0.457045

0

0.358886

0

0

0

0

0

0

0

0

0.0761741

0

0

0

0

0

0.610345

0

0

0

0.0442094

0.0975327
6

0

0.06398

0

0.0761741

0.114261

0.093266

0

0.152348

0

0

0

0.0761741

0

0.0761741

0.0761741

0.0873245

0

0

0

0

0.0873245

0.266609

0

0.266609

0

0.0761741

0

0

0.25592

0

0.0761741

0

2.51398

0

0

0

0

0.152348

0

0

0

0

0

0

0.093266

0.114261

0

0.0761741

0

0

0

0.228522

0

0

0

2.94704

0

0

0

0

0

0

0

0

0

0

12.442
2

0.114261

0

0

0.238831

0.190435

0.114261

0

0

0

0

7.43028

0.152348

0

0.152348

0

0

0

0.114261

0

0

0

0

0

0.190435

0

0.279798

0.06398

0.0530735

0.0761741

0

0.0761741

0

2.14707

0

0.0761741

0.0582163

0

0.114261

0

1.39719

0

0.0530735

0

11.6165

0

0.799828

0

0.0598143

0.533219

0.3199

0

0

0

0

0.517229

0

0

7.6555

4.26256

2.62318

5.42341

0

0

0
6

3.00888

0

0.29326
2

1.47747

0.489082

0
7

3.75501

3.93057

0.487663

0

0.597265

3.35464

0

5.2941

1.55583

0

5.52578

0

2.24325

0

29.0754

1.99942

0

6.30042

0

0

0.0975327

0

0

4.14832

1.19983

0.38087

0

0

2.07482

0.349298

0.266609

0.38087

0

10.512

3.53221

1.09101

0.38087

0

2.92713

0

0

0

0.886027

0

1.25687

2.46148

0

0.884087

0

0

0.288331

0

0.621637

0.553055

0.238831

0

0
4

1463.93
4

469.125
3

104.817
4

434.851
3

0
6

1.39953
4

0

0

0

0

0.685567

0.459822

0.332541

0

0

0

0
4

0.774946

0

0

0

0

0

0

0

0.357694

0

0
4

0

0

0

0

0

0

0

1.40922

0

0.0996905

0
4

0

0

0

0

0

0

0.131534

0

0.163838

14.7335

0
4

0.116433

0

0

0

0.10619

0

0.342783

0

0

0

271.39
2

0

0

0

0

0

0

0

0

0

0

4.39853
3

0

16.3367

0

2.17203

0.221047

0

0.571306

0

0

0.229314

0
4

0

0.304696

0.654762

0

0

0

0

0

0

0

0

0

0

0

0.271239

0

0

0

0.110524

0.0761741

0

25.8591
2

0.152348

0

0

0.0873245

0

0

0.0582163

0

0

0

7.63744
3

0
4

0

0

0

0

0

0

2.24714

0

0.0582163

0

4.06845

0

0

0.0442094

0

0

0

0

0.0582163

0

0

0

0.574722

0

0

0.0442094

0

0

0

0

0

0.228522

0
4

0

0

0

0

0

0

0.0598143

0.0663141

0

0

0

0.0665157

0

0

0.198942

41.1721

0

0

0.0442094

0

0

0.35988
3

0.152348

0.0884188

0

0

0

0

0

0.0884188

0

0.0582163

1.71392

0

0

0

0.0655183

0

0

0

0

0

0

0

0.152348

0

0

0

0.64748

0.418957

0

0.131037

0

0

0
4

0.0442094

0

0

0

0

0

0

0

4.46672

0.327592

0
4

0

0

0

0

0

0

0

0

0

0

0
4

0

0

0

0

0.163796

0

0

0

0

0.0582163

0

1.07474

0

0

0

0

0

0

0.0663141

0

0.0442094

0

0

0

0

0

0

0

0.0530735

0

0

0.0582163

0.0442094

0.367682
4

0

0

0.0634669

0

0

3.04448

0

0

0

0

0
3

0

0

0

0

0

0

0.116433

0.0442094

0

0

0

0.221047

0

0.0442094

0

0

0

0

0.0663141

0

0

0

0

0

0

0.0975327

0

0

0

0

0.0442094

0.0582163

0
4

0

0

0.0947236

0.114261

0.0761741

0

0

0

0

0

0

0.190435

0

0

0

0

0.0582163

0.110524

0

0

0

2.97079

0

0

0

0

0

0.243832

0

0.0398762

0

0

0.0530735
4

0.887909

0

0.0442094

0.0442094

0

0

0

0

0

0.0663141

0.292598

0

0

0

0

0

0

0

0

0

0

0

0

0

0

1.02835

0

0.0442094

0

0

0

0

0.0442094

5.25743
2

0

0

0

1.04446

0

0.152348

0

0.228522

0

0.0761741

2.6541

0

0

0

0
4

0.759746

0

2.09995

0
3

1.87937
4

0

0

0.275434

0
4

1.18679

0
4

0

0

0.221047

0

0
4

0

0

0

0

0.223594

2.42095

0

0

0

0

0
4

6.79667

0

1.21879

0.940216

1.752

0

0.448003

0

0

0

2.55621
3

0

0

0

0

0

0

0

0

0

0

0.11996
3

0

0.319326

0.0663141

0.464199

0

0

0

0

0.551523

0

0
4

1175.27
3

2.02022
3

131.092
3

547.141
3

69.4817
3

394.15
2

20.2364
3

4.64895
2

0.933479
1

0.0582163

0.14099

0

0.0442094

0.0663141

0.0975327

1.37465

0.0582163

0

0.0442094

1.62862

0

0

0.0397225

0.0403499

0

0.0975327

0

0

0.0996905

0.0442094

0

0

0.0873245

0

0

0.0655183

0
8

0.10619

0.397225

1.07505

0

0

0
4

26.3528

0
7

0
6

0

0

0

0

0

0

0

0

0

0

0
7

0

0.06398

0

0

0.06398

0

0

0

0

0

0
7

0

0

0

0

0

0

0

0

0

0.0761741

0
5

0

0

0

0

0

0.0761741

0

0

0

0

0
6

0

0

0

0.152348

0

0

0

0

0

0.523947

0
7

0

0

0

0

0

0

0

0

0

0

0

0

0

0

0

0

0

0

0

0

0.228522

4.15149

0

0

0

0

0

0

0.0773585

0.254415

0

0
8

0

0.0761741

0

0

0

0

0

0

0

0

0

0

1.05723

0

0

0

0

0

0

1.28931

5.52547
3

0.0761741

0

0

0

0

0

0

0.955323

0

0.440258

0
7

0

0.0761741

0

0

0

0

0

0

0

0

11.01
3

0

0.06398

0

0

0

0

0

0

0

0

0
7

0

0

0

0

0

0

0

0

0

0

0
1

0

0

0

0

0

0

0

0

0

0

0.114261

0

0

0

0

0

0

0

0

0

0

1.38777878078145e-15

0
4

228.701
2

26.8667

6.29325

3.61653

22.6691

0.361181

18.1411

2.33038

8.78742

0

0.568218

0.443444

7.75111

0

0.76776

0.494839

2.38955

0

0.35189

10.7939

0.556115

0.243832

0.460089

17.0189

0.190799

0.195065

0.243832

0.28791

0

0

0

0.157246

0.3199

0.0975327

2.24325

0.233165

0.09597

0.093266

0.0582163

0.06398

0.0582163

0.146299

0.195065

0.06398

0.0582163

44.0049

0.15995

0

0.06398

0.292598

0.195065

0.146299

0.195065

4.4865

0.438897

0.146299

2.74399

0.093266

0.233165

0.174649

0.89572

0.146299

0

0.093266

0.139899

0.093266

0.06398

11.265

0

0.715005

0.0582163

0.119168

0.292598

0.6398

0.0761741

0.139899

0.0873245

0.09597

0.611272

0.146299

0.146299

0.0975327

0.093266

0.0582163

0.0975327

0.698596

22.7489

0
4

87.7865
3

84.1016
3

0

0

0

0

0

0

0

0.146299

0

0

0.585196
4

0

0

0

0

0

0.243832

0

0

0.0975327

0

0.390131
4

0

0

0

0

0

0

0.417538
4

0.487663
3

0
4

1.17039
3

0.146299
4

0

0
4

3.80377
3

0.53643

0

0

0

0
4

0.146299

0.975327

0

2.14572

0

0

0

0
4

0

0

0
4

0

0

0
4

4.0517

4.0517

0
4

0

0

0
4

0.133335

0.044445

0.0888899

0
4

0.264902

0.264902

0
4

0

0

0

0
4

0

0

0
4

0.0799732

0.0799732

0
4

0

0

0

0
4

1.53916
3

0
4

0.222473
3

0.292598
3

0.292598
3

0.146299

0.195065

0.390131

0

2.77555756156289e-16
3

0
4

0

0

0
4

0

0

0

0
4

0.266609

0.266609

0
4

0

0

0

0
4

0

0

0
4

0

0

0
4

0.37259

0.284171

0.0884188

0
4

0

0

0
4

0

0

0
4

0

0

0
4

1.35954
3

0
3

0

1.35954

0

0

0

0

0

0
4

0

0

0
4

0.326431

0.326431

0
4

0.0352475

0.0352475

0
4

0

0

0
4

0

0

0
4

0

0

0
4

0.477661

0.477661

0
4

0.596827

0.596827

0
4

0

0

0
4

0.114261

0.114261

0
4

72.7839
2

4.26304
2

7.82933

4.74944

43.3567

5.21773

7.10239

0.265256

0
4

0

0

0
4

0

0

0
4

0

0

0
4

0

0

0
4

0

0

0
4

0

0

0
4

0

0

0
4

0

0

0
4

0

0

0
4

0

0

0
4

12.7025

11.0475

1.02097

0.177845

0.309466

0.0582163

0.0442094

0.0442094

0
4

0

0

0
4

0.0761741

0.0761741

0
4

1.33305

1.33305

0
4

0.0975327

0.0975327

0
4

0.360351

0.360351

0
4

2.69164

2.69164

0
4

0

0

0
4

0

0

0
4

0

0

0
4

2.13287

2.13287

0
4

1.70682
4

1.70682
4

0

0

0

0
4

2.43832

2.43832

0
4

0.228522

0.228522

0
4

0

0

0
4

0.342783

0.342783

0
4

1.40922

1.40922

0
4

0.0761741

0.0761741

0
4

0.0761741

0.0761741

0
4

0.0761741

0.0761741

0
4

0.294833

0.294833

0
4

0

0

0
4

0

0

0

0

0

0
4

0

0

0
4

0.196555

0.196555

0
4

0

0

0
4

1.16582

1.16582

0
4

0.0655183

0.0655183

0
4

0

0

0
4

0.589665

0.589665

0
4

0

0

0
4

0

0

0
4

0

0

0
4

0.799732
3

0.799732
3

0

0

0

0
4

0.19194

0.19194

0
4

0.0761741

0.0761741

0
4

0.720702

0.720702

0
4

2.04579

2.04579

0
4

0

0

0
4

0

0

0
4

0.139029

0.139029

0
4

0

0

0
4

0

0

0
4

0.0797524

0.0797524

0
4

0
4

0
4

0

0

0

0
4

0

0

0
4

0

0

0
4

0

0

0
4

0.0975327

0.0975327

0
4

0.0761741

0.0761741

0
4

0

0

0
4

0.0666674

0.0666674

0
4

0

0

0
4

0

0

0
4

0

0

0
4

0

0

0

0

0

0

0
4

2.12935

2.12935

0
4

0

0

0
4

0

0

0
4

0.0530735

0.0530735

0
4

0.994712

0.994712

0
4

0

0

0
4

0

0

0
4

0

0

0
4

0

0

0
4

0.0975327

0.0975327

0
4

6.21005
3

3.90131
3

0

0

0.243832

0.341364

0.0975327

0

0

0

0

0

0.845756
4

0

0

0.0975327

0

0

0

0

0.0975327
4

0
4

0.341364
3

0

0

0.243832

0

0
4

0

0

0

0

0

0
4

0

0

0
4

0.342783

0.342783

0
4

0

0

0
4

0

0

0
4

0.262073

0.262073

0
4

1.59946
3

1.59946
3

0

0

0
4

0
4

0

0

0

0

0

0
4

65.2392
2

51.6309

6.16997

1.027

2.12205

0.262073

3.78406

0.243152

2.52575738102223e-15
2

0
4

0

0

0

0

0

0

0

0

0

0
4

0

0

0

0
4

0
3

0

0

0

0

0

0

0
4

0
3

0

0

0

0

0

0

0

0
4

0

0

0

0
4

0.941859
2

0.672987

0.0761741

0.0761741

0.0403499

0.0761741

0
4

114.134
3

113.159
3

0

0

0

0

0.243832

0

0

0

0

0

0
4

0.438897

0

0
4

0.292598

0

0

0

0

4.71844785465692e-15
3

0
4

0.159946
3

0

0

0.0799732

0.0799732

0

0
4

0.342783
4

0.342783

0

0

0

0

0
4

0

0

0
4

0
4

0
4

0

0

0

0
4

0

0

0

0
4

0

0

0

0

0
4

0
4

0
4

0

0
4

0
5

0
5

0

0
4

12.4291
2

0.442474

0.0442094

0.378406

11.4985

0.0655183

5.41233724504764e-16
2

0
4

0
4

0
4

0
4

0
5

0
5

0

0

0

0

0

0

0

0

0

0

0

0

0

0

0

0

0

0

0

0

0

0

0

0

0
4

0
4

0

0

0
4

0

0

0

0

0
4

0

0

0
4

0

0

0

0

0
4

0
4

0

0

0
4

0

0

0

0

0

0
4

0.330334

0.216073

0.114261

1.38777878078145e-17

0
4

0.631309

0.186696

0.299072

0.0582163

0.0873245

0
4

18.4957

18.4957

0
4

0

0

0
4

0
6

0

0

0

0

0

0

0

0

0

0

0

0

0

0

0

0

0

0

0

0

0

0

0

0

0

0

0

0

0

0

0

0

0
4

0.778693
2

0

0.778693

0
4

5.40836

3.65636

1.67583

0.0761741

1.80411241501588e-16

0
4

0

0

0

0
4

0

0

0

0

0
4

0

0

0

0

0
4

0.401775

0.212539

0.093266

0.09597

4.16333634234434e-17

0
4

0

0

0

0
4

0.0971464

0.0971464

0
4

0.195065

0

0.195065

0
4

0

0

0

0

0
4

304.095
2

273.241
2

28.8502
2

0.783099

0.712254

0.304696

0.12796

0.0761741

0

0
4

1.40922

1.40922

0
4

0

0

0

0
4

0

0

0
4

0

0

0

0
4

0

0

0
4

0

0

0
4

0

0

0
4

1.83789

1.50813

0.114261

0.152348

0.063149

4.16333634234434e-17

0
4

0

0

0

0
4

0

0

0
4

0.599799
3

0.11996
4

0

0

0

0

0

0

0

0

0

0

0

0

0

0

0

0

0

0

0

0

0

0.479839

0

0

0

0

0

0

0
4

0

0

0
4

0

0

0
4

0

0

0

0
4

5.02167

4.66132

0.360351

0
4

0

0

0

0
4

0

0

0

0
4

1.24442

1.13016

0.114261

6.93889390390723e-17

0
4

0

0

0

0
4

0

0

0

0
4

0.255954

0.190435

0.0655183

0
4

0
6

0
6

0

0

0

0

0

0

0

0

0

0

0

0
4

4.59591

4.5517

0.0442094

1.38777878078145e-17

0
4

0

0

0

0
4

0

0

0
4

0

0

0

0
4

0

0

0
4

0

0

0
4

0.107358

0.063149

0.0442094

0
4

0

0

0

0
4

0.132628

0.132628

0
4

0.552344

0.552344

0
4

1.46299
4

1.31669
4

0.146299
4

0
4

0

0

0

0

0

0

0
4

0.131832

0.131832

0
4

0

0

0
4

0

0

0
4

0

0

0
4

0

0

0

0
4

0

0

0

0
4

0.196555

0.196555

0
4

0.374585

0.294833

0.0797524

0
4

0

0

0
4

0

0

0

0
4

0
4

260.721
2

253.833
2

94.8291
2

1.53653

2.78864

8.05414

2.45083

2.34977

4.63104

3.34508

2.45518

0.321668

0.721184

30.4574

0.220251

0.327592

0.327592

0.197848

0.163796

0.12453

0.156494

0.982775

0.590261

0.109728

36.9884
2

0.556906

0.0655183

0.294833

0.0982775

0.0982775

0.0582163

0.0655183

0.0982775

0.174649

0.0582163

7.92632

0.0442094

0.0663141

0.0655183

3.63107

0.0655183

0.0582163

0.163796

0.0797524

0.0982775

0.063149

7.64718

0.0655183

0.556906

0.145541

0.0582163

0.989678

6.48632

4.88112

0.0982775

0.0655183

0.0884188

2.05653

0.0982775

0.0398762

0.294833

0.327592

0.0655183

0.198942

0.0655183

0.0663141

0.950016

0.146299

3.09156

0.163796

7.69841

0.0442094

0.116433

0.0442094

0.0947236

7.24322

1.98298

5.75095526755831e-14
2

0
4

5.59582
2

2.71901

0.163796

0.0655183

0.356797

1.41072

0.0840856

0.191816

0.414816

0.0655183

0.0582163

0.0655183

0
4

1.01316

0.418567

0.123735

0.0582163

0.0982775

0.0982775

0.157873

0.0582163

3.46944695195361e-17

0
4

0.212949

0.0442094

0.0442094

0.0582163

0.0663141

1.38777878078145e-17

0
4

0.0663141

0.0663141

0
4

4.3534620353114e-14
2

0
4

0.975327

0.975327

0
7

0.731495

0.146299

0.0975327

0

0

0
4

0

0

0
4

0

0

0
4

0
4

0

0

0

0
4

0

0

0
4

0
4

0.240764

0.196555

0.196555

0
4

0.0442094

0.0442094

0
4

1.38777878078145e-17

0
4

0.0884188

0.0442094

0.0442094

0
4

0.0442094

0.0442094

0
4

0
4

0

0

0

0

0
4

0
4

0.0975327

0.0975327

0.0975327

0
4

0

0

0
4

0
4

0.0881189

0.0881189

0.0881189

0
4

0
4

1.71392

1.71392

1.71392

0
4

0
4

0.685567

0.685567

0.685567

0
4

0
4

4.89351

4.76088

4.76088

0
4

0.132628

0.132628

0
4

0
4

0

0

0

0
4

0
4

2.09695

2.09695

1.85312

0.243832

0

0

0

0

5.55111512312578e-17

0
4

0
4

0

0

0

0

0
4

0
4

0

0

0

0
4

0

0

0
4

0
4

0

0

0

0

0
4

0
4

0

0

0

0
4

0
4

0

0

0

0

0
4

0
4

3.98431

3.98431

3.98431

0
4

0
4

0

0

0

0
4

0
4

0.176042

0.176042

0.176042

0
4

0
4

0

0

0

0
4

0
4

0

0

0

0

0
4

0
4

0
7

0
7

0

0

0

0

0

0

0
4

0
4

0

0

0

0

0
4

0
4

0

0

0

0
4

0
4

0

0

0

0
4

0
4

0.0975327

0.0975327

0.0975327

0
4

0
4

0.0993063

0.0993063

0.0993063

0
4

0
4

0

0

0

0
4

0
4

0

0

0

0
4

0
4

0.0582163

0.0582163

0.0582163

0
4

0
4

0.233165

0.233165

0.233165

0
4

0
4

0.0398762

0.0398762

0.0398762

0
4

0
4

6.6008
2

6.6008
2

2.44857
2

1.71287

0.487663

1.02835

0

0.390131

0.533219

0
4

0
4

0.114261

0.114261

0.114261

0
4

0
4

0

0

0

0
4

0
4

0

0

0

0
4

0
4

0

0

0

0
4

0
4

0

0

0

0
4

0
4

0

0

0

0
4

0
4

0

0

0

0
4

0
4

0.0761741

0.0761741

0.0761741

0
4

0
4

0

0

0

0
4

0
4

0

0

0

0
4

0
4

0

0
5

0

0

0

0

0

0

0

0
4

0

0

0

0

0

0

0
4

0

0

0
4

0
4

0

0

0

0
4

0
4

0

0

0

0
4

0
4

0

0

0

0
4

0
4

0

0

0

0
4

0
4

0

0

0

0
4

0
4

0.0442094

0.0442094

0.0442094

0
4

0
4

0

0

0

0
4

0
4

0

0

0

0
4

0
4

0

0

0

0
4

0
4

0.0796102

0.0796102

0.0796102

0
4

0
4

15.9585

15.9585

12.7972

2.28522

0.342783

0.342783

0.0761741

0.114261

0
4

0
4

0

0

0

0
4

0
4

0

0

0

0
4

0
4

0

0

0

0
4

0
4

0

0

0

0
4

0
4

0

0

0

0
4

0
4

0

0

0

0
4

0
4

0

0

0

0
4

0
4

0.0442094

0.0442094

0.0442094

0
4

0
4

0.0663141

0.0663141

0.0663141

0
4

0
4

0.0655183

0.0655183

0.0655183

0
4

0
4

3.13028

3.13028

1.97387

0.757648

0.398762

0

0

1.66533453693773e-16

0
4

0
4

0

0

0

0
4

0
4

0.15922

0.15922

0.15922

0
4

0
4

0

0

0

0
4

0
4

0.0442094

0.0442094

0.0442094

0
4

0
4

0

0

0

0
4

0
4

0

0

0

0
4

0
4

0

0

0

0
4

0
4

0

0

0

0
4

0
4

0.0761741

0.0761741

0.0761741

0
4

0
4

0

0

0

0
4

0
4

4.03991

4.03991

0.761741

0.152348

0.272697

1.56157

0.457045

0.190435

0.249881

0.203757

0.114261

0.0761741

0
4

0
4

0.0761741

0.0761741

0.0761741

0
4

0
4

0

0

0

0
4

0
4

0

0

0

0
4

0
4

0.190435

0.190435

0.190435

0
4

0
4

0.46633

0.46633

0.46633

0
4

0
4

0.0761741

0.0761741

0.0761741

0
4

0
4

0.0799732

0.0799732

0.0799732

0
4

0
4

0

0

0

0
4

0
4

0

0

0

0
4

0
4

0

0

0

0
4

0
4

4.17533

4.10982

3.86965

0.116433

0.0655183

0.0582163

0
4

0.0655183

0.0655183

0
4

0
4

0.0982775

0.0982775

0.0982775

0
4

0
4

0.0761741

0.0761741

0.0761741

0
4

0
4

0

0

0

0
4

0
4

0

0

0

0
4

0
4

0

0

0

0
4

0
4

0

0

0

0
4

0
4

0

0

0

0
4

0
4

0

0

0

0
4

0
4

0.093266

0.093266

0.093266

0
4

0
4

0.0398762

0.0398762

0.0398762

0
4

0
4

0
6

0
6

0
6

0

0

0

0

0

0
4

0
4

0.114261

0.114261

0.114261

0
4

0
4

0

0

0

0
4

0
4

0.0761741

0.0761741

0.0761741

0
4

0
4

190.87
2

165.756
2

72.3805
2

1.27168

4.2992

1.43756

0.884498

0.198942

0.163796

0.31048

0.131037

3.24316

0.218361

19.9036
2

0.174503

0.198942

0.196555

0.0398762

0.552618

0.243152

0.131037

0.131037

0.229314

0.0398762

4.34372

0.0655183

0.0884188

7.3053

0.131037

0.0884188

0.137103

0.0655183

0.37578

1.06644

0.0442094

6.98971
2

0.294833

0.0582163

0.163796

3.98782

0.198942

0.131037

0.442094

0.884498

0.0982775

0.0442094

2.45193

0.0982775

0.0655183

0.063149

0.131037

0.0655183

0.110524

0.262073

0.163796

0.0655183

0.174649

7.51503

0.132628

0.0398762

0.0982775

0.906293

0.262073

0.327592

0.0655183

0.196555

0.0982775

0.265256

1.60093

0.196555

0.131037

0.203757

0.0655183

0.219364

0.75156

0.126298

5.60182

0.116433

0.508408

7.71885
2

0.0442094

0.0982775

0.0655183

0.132628

0.233165

0

0.0797524

0.0655183

0.0442094

0.808026

1.75748304798162e-13
2

0
4

17.4194
2

12.0031
2

0.163796

1.92055

0.929735

1.02588

0.622424

0.131534

0.252215

0.0655183

0.304696

0
4

2.11595
2

0.881943

0.530083
2

0.458628

0.131037

0.114261

0
4

3.90443

1.60379

0.844137

1.34007

0.116433

0
4

0.349298

0.145541

0.116433

0.0873245

0
4

0.418761

0.189447

0.131037

0.0982775

0
4

0.906293

0.0663141

0.839979

0
4

0
4

3.17873
2

2.7545
2

1.37361
2

0.897499

0.190799

0.292598

1.66533453693773e-16
2

0
4

0.13632

0.09597

0.0403499

0
4

0.28791

0.28791

0
4

0
4

0

0

0

0

0

0

0
4

0
4

0
5

0
5

0
5

0

0

0
4

0
4

0

0

0

0

0

0

0

0

0

0
4

0

0

0

0
4

0

0

0
4

0
4

0

0

0

0

0

0

0

0
4

0

0

0
4

0
4

1.73111

0.846421

0.47541

0.180575

0.114261

0.0761741

0
4

0.589855

0.247072

0.342783

0
4

0.228522

0.228522

0
4

0.0663141

0.0663141

0
4

1.52655665885959e-16

0
4

2.66609

2.09479

1.40922

0.304696

0.304696

0.0761741

2.91433543964104e-16

0
4

0.266609

0.266609

0
4

0.0761741

0.0761741

0
4

0.228522

0.228522

0
4

2.4980018054066e-16

0
4

0.0423112
5

0.0423112
5

0

0

0

0

0.0423112

0

0

0

0
4

0
4

0
4

0

0

0

0

0
4

0

0

0
4

0

0

0
4

0
4

0
7

0
7

0
7

0

0

0
4

0
4

169.055
2

157.274
2

97.1669
2

0.0761741

0.0761741

0.533219

0.152348

0.457045

0.457045

0.0598143

0.152348

52.7269
2

1.14261

0.495132

0.228522

0.469219

0.190435

2.6996

0.190435

6.52811138479592e-14
2

0
4

5.31408
2

3.74908
2

1.20011

0.288713

0.0761741

6.93889390390723e-17
2

0
4

6.27473

2.47566

2.47589

0.142488

0.190435

0.457045

0.0761741

0.0761741

0.190435

0.190435

1.11022302462516e-16

0
4

0.192607

0.116433

0.0761741

0
4

0
4

0

0

0

0

0

0

0
4

0
4

0
6

0
6

0
6

0
4

0
4

11.8797
2

3.84857

3.55759

0.138627

0.0761741

0.0761741

1.38777878078145e-16

0
4

7.96559

7.96559

0

0
4

0.0655183

0.0655183

0
4

5.41233724504764e-16
2

0
4

0
7

0
7

0
7

0

0
4

0
4

0
7

0
7

0

0

0
4

0
4

2.04819

2.04819

2.04819

0

0

0
4

0

0

0
4

0
4

0

0

0

0

0

0

0

0
4

0

0

0
4

0

0

0
4

0
4

0
6

0
6

0
6

0

0
4

0
4

3.22023

3.22023

1.06723

2.153

0
4

0
4

1.30416

0

0

0
4

1.19842

1.19842

0
4

0.105743

0.105743

0
4

0
4

209.823
2

168.1
2

107.357
2

8.28807

0.272927

1.60115

0.556906

0.28388

0.327592

0.262073

0.0655183

0.291082

0.0655183

12.7829

0.524147

0.0947236

0.0655183

0.0982775

1.37531

13.0066

15.4082

1.20114

0.436623

3.60351

0.131037

7.74380559676047e-15
2

0
4

0.0655183

0.0655183

0
4

14.8565
2

13.0292

0.673751

0.284675

0.549419

0.122963

0.196555

0
4

1.20341

0.503335

0.20869

0.131037

0.360351

0
4

23.4381

0.363423

0.110524

22.9642

0
4

0.550101

0.550101

0
4

1.2339

0.64423

0.589665

1.11022302462516e-16

0
4

0.193443

0.129463

0.06398

1.38777878078145e-17

0
4

0.0655183

0.0655183

0
4

0.116433

0.116433

0
4

6.77374822899424e-14
2

0
4

0

0

0

0

0
4

0

0

0

0
4

0
4

1.87395

1.82974

1.63356

0.196185

5.55111512312578e-17

0
4

0.0442094

0.0442094

0
4

1.38777878078145e-17

0
4

0

0

0

0

0

0

0

0
4

0

0

0

0
4

0
4

0
6

0
6

0
6

0
4

0
4

0

0

0

0
4

0
4

0

0

0

0

0
4

0
4

0

0

0

0

0
4

0

0

0

0
4

0
4

0

0

0

0

0

0
4

0
4

0

0

0

0

0

0

0
4

0

0

0
4

0
4

4.88228

4.88228

4.88228

0
4

0
4

0

0

0

0

0

0

0

0

0

0

0

0

0

0

0

0

0

0

0
4

0

0

0
4

0

0

0

0
4

0
4

0

0

0

0

0

0
4

0

0

0
4

0

0

0
4

0
4

0

0

0

0
4

0
4

4.72892

4.72892

4.68471

0.0442094

9.0205620750794e-16

0
4

0
4

0

0

0

0
4

0

0

0
4

0
4

0

0

0

0

0
4

0

0

0
4

0
4

1.46668

1.46668

0.466672

1.00001

0
4

0
4

1.23102

0.783844

0.114261

0.0982775

0.571306

1.11022302462516e-16

0
4

0.152348

0.152348

0
4

0.294833

0.294833

0
4

0
4

0.387785

0.131037

0.0655183

0.0655183

0
4

0.114261

0.114261

0
4

0.0442094

0.0442094

0
4

0.0982775

0.0982775

0
4

0
4

0

0

0

0

0
4

0
4

6.26056

6.26056

6.26056

0
4

0
4

22.1237
2

19.0403
2

7.36518
2

6.37559
2

2.17093

0.38087

1.34313

0.542202

0.0655183

0.720702

0.0761741

0
4

3.08338

1.21608

1.8673

0
4

0
4

0.180413

0.06398

0.06398

0

0
4

0.0582163

0.0582163

0
4

0

0

0
4

0.0582163

0.0582163

0
4

0
4

0
7

0

0

0

0
4

0

0

0
4

0
4

2.14572

2.14572

2.14572

0
4

0
4

1.1807

0.266609

0.266609

0
4

0.723654

0.304696

0.418957

0
4

0.190435

0.190435

0
4

0
4

0

0

0

0

0
4

0
4

0.457045

0.342783

0.266609

0.0761741

1.38777878078145e-17

0
4

0.114261

0.114261

0
4

1.38777878078145e-17

0
4

0.152348

0.152348

0

0.152348

0

0
4

0
4

1.67392

1.63405

1.63405

0
4

0.0398762

0.0398762

0
4

0
4

0

0

0

0
4

0
4

0

0

0

0

0
4

0
4

102
2

47.2396
2

8.35864
2

1.05235

25.4169

0.84367

0.528126

11.0398

1.77635683940025e-15
2

0
4

51.3326
2

0.389592

0.990334

1.3433

0.8406

47.468

0.221022

0.0797524

0
4

1.97706
2

1.20281

0.220056

0.0398762

0.116433

0.397885

2.77555756156289e-16
2

0
4

0.39311

0.327592

0.0655183

0
4

0.182747

0.0663141

0.116433

0
4

0.536393

0.536393

0
4

0.203757

0.203757

0
4

0.0761741

0.0761741

0
4

0.0582163

0.0582163

0
4

3.1412372702988e-14
2

0
4

0

0

0

0
4

0
4

1.4099

0

0

0
4

1.4099

1.4099

0
4

0
4

0

0

0

0

0

0
4

0
4

0

0

0

0
4

0
4

1.12121

1.12121

0.95669

0

0.164523

0
4

0
4

0

0

0

0

0

0
4

0
4

5.33328

5.235

5.235

0
4

0.0982775

0.0982775

0
4

0
4

0

0

0

0

0
4

0

0

0
4

0
4

0

0

0

0
4

0
4

0

0

0

0
4

0

0

0
4

0
4

18.8435

18.8435

16.7986

0.449567

0.445832

0.265256

0.508408

0.110524

0.221047

0.0442094

0
4

0
4

0

0

0

0
4

0
4

0.185417

0.185417

0.185417

0
4

0
4

0.0996905

0.0996905

0.0598143

0

0.0398762

6.93889390390723e-18

0
4

0
4

0

0

0

0

0
4

0
4

0

0

0

0
4

0
4

0

0

0

0

0
4

0
4

0

0

0

0
4

0

0

0
4

0
4

0

0

0

0

0
4

0
4

0.397885

0.397885

0.397885

0
4

0
4

1.09299

0.120383

0.120383

0
4

0.972607

0.972607

0
4

0
4

0

0

0

0

0

0

0

0

0

0

0
4

0

0

0
4

0

0

0

0
4

0
4

14.9682

14.892

14.892

0
4

0.0761741

0.0761741

0
4

0
4

0

0

0

0
4

0
4

2.37828

2.37828

0.139899

2.23838

0
4

0
4

0.0761741

0.0761741

0.0761741

0

0
4

0
4

0

0

0

0
4

0
4

0

0

0

0
4

0

0

0
4

0
4

0.171871

0.0582163

0.0582163

0
4

0.113655

0.113655

0
4

0
4

0

0

0

0

0
4

0
4

0.106147

0.106147

0.0530735

0.0530735

0
4

0
4

0.152348

0.0761741

0.0761741

0
4

0.0761741

0.0761741

0
4

0
4

75.0401

60.3456

60.1003

0

0.0761741

0.054841

0

0.114261

0
4

0.495132
1

0.495132
1

0
4

0.495132

0.495132

0
4

13.0568

12.9425

0.114261

6.93889390390723e-17

0
4

0.64748

0.533219

0.114261

0
4

0

0

0

0
4

0

0

0
4

1.4432899320127e-14

0
4

0
4

2.15643
4

0.09597
4

0

0

0

0
4

0

0

0
4

0

0

0
4

0.09597

0.09597

0
4

0
4

2.01861

2.01861

2.01861

0
4

0
4

0.0418477

0.0418477

0

0.0418477

0
4

0
4

0

0

0

0
4

0
4

0

0

0

0
4

0
4

0
4

486.737
5

0.0397225
4

0.0397225
4

0
4

0

0

0

0

0

0

0

0

0

0

0

0

0

0

0

0

0

0

0

0

0

0

0

0

0

0

0

0

0

0

0

0

0

0

0

0

0

0

0

0

0

0

0

0

0

0

0
4

0

0

0

0
4

0

0

0

0

0

0

0

0

0

0

0

0
4

0.0397225

0

0

0

0

0

0

0

0

0

0
4

0

0

0

0

0

0

0

0

0

0

0
4

0

0

0

0

0

0

0

0

0

0

0

0

0

0

0

0

0

0

0

0

0

0
4

0

0

0

0

0

0

0

0

0

0

0
4

0

0

0

0

0

0

0

0

0

0

0
4

0

0

0

0

0

0

0

0

0

0

0
4

0
4

485.246
5

12.8306

0.931399

0.159946
2

0

0
4

0

0
4

0

0.0799732

0

3.15894

1.12522

0

0

0
7

0

0

0

0

0.0530735

0

0

0

1.59946

2.14948

0
3

0

0

0

1.01445

0

0.279906

0

0

0

0

0
4

0

0

0

0

0

0

0

0

0

0

0

0

0

0

0

0

0

0

0.0530735

0

0

0

0

0

0

0

0.093266

0

0

0

0

0

0

0

0

0

0

0

0

0.0799732

0

0

0

0

0

0.11996

0

0

0

0

0

0

0

0.0530735

1.87937

0

0

0

0
4

0

0

0

0

0

0
4

0

0

0
4

462.861
5

72.1747
5

22.6324
3

333.719
3

24.0732
4

5.16682
5

0.291954
4

0

0

0.0773585

0

0

0

0

0

0

0

0

0

0

0

0

0

0

0

0

0

0

0

0

0

0

0

0

0

0

0

0

0

0.0799732

0

0

0

0

0

0

0

0

0

0

0

0

0

0

0

0

0

0

0

0

0

0

0

0
4

0

0

0

0

0

0

0

0

0

0

0

0

0

0

0

0

0

0

0

0

0

0

0

0

0

0

0

0

0

0

0

0

0

0

0

0

0

0

0

0

0

0

0

0

0

0

0

0

0

0

0

0

0

0

0
4

0

0

0

0

0

0

0

0
3

0

0
4

0

0
4

0

0

0
4

0

0.195276
4

0

0

0
4

0

0

0

0

0
4

0

0

0

0

0

0

0

0

0

0

0

0

0

0

0

0

0

0

0

0

0

0

0

0

0

0

0

0

0

0

0

0

0

0

0

0.128667
4

0

0

0

0

0

0

0

0

0

0

0.0982775
3

0.0648786

0

0

0

0

0

0

0

0

0

0
4

0

0

0

0

0

0

0

0

0

0

0
4

0

0

0

0

0

0

0

0

0

0

0
4

0

0

0

0

0

0

0

0

0

0

0

0.0648786
4

0

0

0

0

0.11996

0

0

0.227075

0

0

0

0

0

0

0

0

0

0

0

0

0

0
4

0

0

0

0

0

0

0

0

0

0

0

0

0

0

0

0

0

0

0

0

0

0
4

0

0

0

0

0

0

0

0

0

0

0
4

0

0

0

0

0

0

0

0

0

0

0.0655183
4

0

0

0

0

0

0

0

0

0

0

0
4

0

0

0

0

0

0

0

0

0

0

0
4

0

0

0

0

0

0

0

0

0

0

0

0
4

0

0

0

0

0

0

0

0

0

0

0
4

0

0

0

0

0

0

0

0

0

0

0

0

0

0

0

0

0

0

0

0

0

0

0

0

0

0

0

0

0

0

0

0

0
4

0

0

0

0

0

0

0

0

0

0

0

0

0

0

0

0

0

0

0

0

0

0

0

0

0

0

0

0

0

0

0

0

0
4

0

0

0

0

0

0

0

0

0

0

0

0

0

0

0

0

0

0

0

0

0

0

0

0

0

0

0

0

0

0

0

0

0
6

0
3

0

0

0

0

0

0

0

0

0

0

0

0

0

0

0

0

0

0

0

0

0

0.0973179
3

0

0

0

0

0

0

0

0.0722459

0

0

0
4

0

0

0

0

0

0

0

0

0

0

0

0

0

0

0

0

0

0

0

0

0

0
4

0

0

0

0

0

0

0

0

0

0

0

0

0

0

0

0

0

0

1.72767

0

0

0
4

0

0

0

0

0

0

0

0

0

0

0
4

0

0

0

0

0

0

0

0

0

0

0

0

0

0

0

0

0

0

0

0

0

0

0

0

0

0

0

0

0

0

0

0

0

0

0

0

0

0

0

0

0

0

0

0

0
4

0

0

0

0

0

0

0

0

0

0

0
4

0

0

0

0

0

0

0

0

0

0

0

0

0

0.0648786

0

0

0

0

0

0

0

0
4

0

0

0

0

0

0

0

0

0

0

0
3

0

0

0

0

0

0

0

0

0

0

0
3

0

0

0

0

0

0

0

0

0

0

0

0

0

0

0

0

0

0

0

0

0

0
4

0

0

0

0

0

0

0

0

0

0

0.161427
4

0

0

0

0

0

0

0

0

0

0

0

0

0

0

0

0

0

0

0

0

0

0

0

0

0

0

0

0

0

0

0

0

0

0
4

0

0

0

0

0

0

0

0

0

0

0
4

0

0

0

0

0

0

0

0

0

0

0

0

0

0

0

0

0

0

0

0

0

0

0

0

0

0

0

0

0

0

0

0

0
4

0

0

0

0

0

0

0

0

0

0

0

0

0

0

0

0

0

0

0

0

0

0

0

0

0

0

0

0

0

0

0

0

0.063149
4

0
4

0

0

0

0

0

0

0

0

0

0

0

0

0

0

0

0

0

0

0

0

0

0
4

0

0

0

0

0

0

0

0

0

0

0

0

0

0

0

0

0

0

0

0

0

0

0

0

0

0

0

0

0

0

0

0

0
3

0

0

0

0

0

0.09597

0

0

0

0

0

0

0

0

0

0

0

0

0

0

0

0
4

0

0

0

0

0

0

0

0

0

0

0

0

0

0

0

0

0

0

0

0

0

0.0686787
4

0

0

0

0

0

0

0

0

0

0

0.750725
3

0

0.0548351

0

0

0

0

0

0

0

0.0423112

0

0

0

0

0

0

0

0

0

0

0

0

0

0

0

0

0

0

0

0

0

0

0

0

0

0

0

0

0

0

0

0

0

0

0

0

0.19194

0

0

0

0

0

0

0

0

0

0

0

0

0

0

0

0

0

0

0

0

0

0

0

0

0

0

0

0

0

0

0
4

0

0

0

0

0

0

0

0

0

0

0

0

0

0

0

0

0

0

0

0

0

0

0

0

0

0

0

0

0.288984

0

0

0

0

0

0

0

0

0

0

0

0

0

0

0

0

0

0

0

0

0

0

0

0

0

0

0
4

0

0

0

0

0

0

0

0

0

0

0

0

0

0

0

0

0

0

0

0

0

0
4

0

0

0

0

0

0

0

0

0

0

0

0

0

0

0

0

0

0

0

0

0

0

0

0

0

0

0

0

0

0

0

0

0

0

0

0

0

0

0

0

0

0

0

0

0

0

0

0

0

0

0

0

0

0

0

0

0

0

0

0

0

0

0

0

0

0
4

4.0949

4.0949

0

0
4

2.75358
4

0

2.59886
4

0.154717

0

0

0
4

2.70674

0
5

0

0

0

0.180503

0

1.10881

1.41743

0

0

0

0

0

0

0

0
4

0

0

0
4

0

0

0
4

0

0

0
4

0

0

0
4

0

0

0
4

0

0

0
4

0

0

0
4

0

0

0
4

4.13002965160558e-14
5

0
4

0

0

0

0
4

0
4

0

0

0

0
4

0
4

0

0

0

0
4

0
4

0

0

0

0
4

0
4

1.4506
4

1.31956
4

0

0

1.31956
4

0
4

0

0

0

0
4

0.131037

0.131037

0
4

0

0

0
4

0
4

0
4

5.61444
3

2.30741

2.30741

0.36827

1.57087

0.325959

0.0423112

0
4

0
4

0

0

0

0
4

0
4

0

0

0

0
4

0
4

2.7021

2.7021

2.61748

0.0846225

5.55111512312578e-17

0
4

0
4

0.281651

0.281651

0.230078

0.0515724

0
4

0
4

0

0

0

0
4

0
4

0

0

0

0
4

0
4

0

0

0

0
4

0
4

0

0

0

0
4

0
4

0.079445

0.079445

0.079445

0
4

0
4

0.243832

0.243832

0.243832

0
4

0
4

2.77555756156289e-16
3

0
4

0

0

0

0

0

0

0

0

0

0

0

0

0

0

0
1

0

0

0

0

0

0

0

0

0

0

0

0

0

0

0

0

0

0

0

0

0

0

0

0

0

0

0

0

0

0

0

0

0

0

0
4

0
4

0

0

0

0
4

0
4

0
4

123.584

117.326

86.5836

0
7

5.05442

4.24267

0.806001

0

16.5841

0

0.79975

1.24761

0

1.31669

0
6

0

22.53

0.44786

0.243832

0

0.243832

0.15995

5.95014

0.390131

0

14.6787

0.51184

0.15995

0

0.09597

0.09597

0.06398

0.0975327

0.06398

0

0.06398

7.32028

0.09597

0

0.0975327

0.06398

0.146299

0

0.06398

0

0

0

0
6

0.0975327

0.06398

0.06398

0.19194

0.12796

0.12796

0.0975327

1.59054

0

0

0.585196

5.45119505090952e-14

0
4

12.0922

9.9169

0.25592

0.92771

0.09597

0.89572

4.44089209850063e-16

0
4

0

0

0
4

18.2343

18.0104

0.22393

0
4

0.28791

0.06398

0.22393

2.77555756156289e-17

0
4

0.12796

0.12796

0
4

0

0

0
4

4.69069227904129e-15

0
4

6.25765

6.25765

6.25765

0
4

0
4

0

0

0

0
4

0
4

0

0

0

0
4

0
4

1.33226762955019e-14

0
4

0
7

0
7

0
7

0
7

0

0

0

0

0

0

0

0

0

0

0
6

0
7

0

0

0

0

0

0

0
4

0
6

0

0

0

0
4

0

0

0

0
4

0
4

0
4

0
7

0
7

0
7

0

0

0

0

0

0
4

0
4

0
4

0.0582163

0.0582163

0.0582163

0.0582163

0
4

0
4

0
4

0

0

0

0

0
4

0
4

0
4

0

0

0

0

0
4

0
4

0
4

0

0

0

0

0
4

0
4

0
4

1.03663

1.03663

1.03663

1.03663

0
4

0
4

0
4

0

0

0

0

0
4

0
4

0
4

0

0

0

0

0
4

0
4

0
4

0.0947236

0.0947236

0.0947236

0.0947236

0
4

0
4

0
4

0

0

0

0

0
4

0
4

0
4

0

0

0

0

0
4

0
4

0
4

0
7

0
7

0
7

0
7

0
7

0

0
4

0

0

0
4

0
4

0
4

0

0

0

0

0
4

0
4

0
4

0

0

0

0

0
4

0
4

0
4

0.0761741

0.0761741

0.0761741

0.0761741

0
4

0
4

0
4

0

0

0

0

0
4

0
4

0
4

0

0

0

0

0
4

0
4

0
4

0.093266

0.093266

0.093266

0.093266

0
4

0
4

0
4

0.0481639

0.0481639

0.0481639

0.0481639

0
4

0
4

0
4

0

0

0

0

0
4

0
4

0
4

0

0

0

0

0
4

0
4

0
4

0

0

0

0

0
4

0
4

0
4

0

0

0

0

0

0

0
4

0

0

0
4

0
4

0
4

0

0

0

0

0
4

0
4

0
4

0

0

0

0

0
4

0
4

0
4

0

0

0

0

0
4

0
4

0
4

0

0

0

0

0
4

0
4

0
4

0

0

0

0

0
4

0
4

0
4

0

0

0

0

0
4

0
4

0
4

0

0

0

0

0
4

0
4

0
4

0

0

0

0

0
4

0
4

0
4

0.09597

0.09597

0.09597

0.09597

0
4

0
4

0
4

0

0

0

0

0
4

0
4

0
4

0
7

0
7

0
7

0

0

0

0
4

0

0

0

0

0
4

0
4

0
4

0

0

0

0

0
4

0
4

0
4

0

0

0

0

0
4

0
4

0
4

0

0

0

0

0
4

0
4

0
4

0

0

0

0

0
4

0
4

0
4

0.0686787

0.0686787

0.0686787

0.0686787

0
4

0
4

0
4

0

0

0

0

0
4

0
4

0
4

0

0

0

0

0
4

0
4

0
4

0

0

0

0

0
4

0
4

0
4

0.0442094

0.0442094

0.0442094

0.0442094

0
4

0
4

0
4

0.0442094

0.0442094

0.0442094

0.0442094

0
4

0
4

0
4

0.0423112

0.0423112

0.0423112

0

0.0423112

0

0

0
4

0

0

0

0
4

0
4

0
4

0

0

0

0

0
4

0
4

0
4

0

0

0

0

0
4

0
4

0
4

0

0

0

0

0
4

0
4

0
4

0

0

0

0

0
4

0
4

0
4

0

0

0

0

0
4

0
4

0
4

0

0

0

0

0
4

0
4

0
4

0

0

0

0

0
4

0
4

0
4

0

0

0

0

0
4

0
4

0
4

0

0

0

0

0
4

0
4

0
4

0

0

0

0

0
4

0
4

0
4

0

0

0

0

0

0

0
4

0
4

0
4

0

0

0

0

0
4

0
4

0
4

0

0

0

0

0
4

0
4

0
4

0.0799732

0.0799732

0.0799732

0.0799732

0
4

0
4

0
4

0.0761741

0.0761741

0.0761741

0.0761741

0
4

0
4

0
4

0

0

0

0

0
4

0
4

0
4

0.0761741

0.0761741

0.0761741

0.0761741

0
4

0
4

0
4

0.0332578

0.0332578

0.0332578

0.0332578

0
4

0
4

0
4

0

0

0

0

0
4

0
4

0
4

0

0

0

0

0
4

0
4

0
4

0

0

0

0

0
4

0
4

0
4

0

0

0

0

0

0

0

0
4

0
4

0
4

0

0

0

0

0
4

0
4

0
4

0.0442094

0.0442094

0.0442094

0.0442094

0
4

0
4

0
4

0
7

0
7

0
7

0

0

0

0
4

0
4

0
4

2.24325

2.24325

2.24325

1.85312

0.146299

0.243832

2.77555756156289e-17

0
4

0

0

0
4

0
4

0
4

0

0

0

0

0
4

0

0

0

0

0
4

0

0

0
4

0
4

0
4

0.233165
7

0
7

0
7

0
7

0

0

0

0
4

0
4

0.233165

0.233165

0.233165

0
4

0
4

0
4

0

0

0

0

0

0

0
4

0
4

0
4

21.6794

21.6794

21.6794

21.5861

0.093266

0
4

0
4

0
4

0

0

0

0

0
4

0
4

0
4

0

0

0

0

0
4

0
4

0
4

0

0

0

0

0
4

0

0

0
4

0
4

0

0

0

0
4

0

0

0
4

0
4

0
4

0

0

0

0

0

0
4

0
4

0
4

0

0

0

0

0

0
4

0
4

0
4

0

0

0

0

0

0
4

0
4

0
4

0

0

0

0

0
4

0
4

0
4

0

0

0

0

0
4

0
4

0
4

42.219
2

42.219
2

36.3764
2

26.3865
2

5.00053

0.0761741

4.83705

0.0761741

5.06539254985228e-15
2

0
4

5.27131
2

2.24714

1.48057

1.25687

0

0.0582163

0.228522

0
4

0.38087

0.38087

0
4

0.190435

0.190435

0
4

1.25455201782643e-14
2

0
4

0
4

0

0

0

0

0

0

0
4

0
4

0
4

0

0

0

0

0
4

0
4

0
4

0.26291

0.26291

0.145857

0.145857

0
4

0.0530735

0.0530735

0
4

0.06398

0.06398

0
4

0
4

0
4

0

0

0

0

0

0
4

0
4

0
4

0

0

0

0

0

0

0

0
4

0
4

0
4

0

0

0

0

0
4

0
4

0
4

0

0

0

0

0

0
4

0

0

0
4

0
4

0

0

0

0
4

0
4

0
4

0

0

0

0

0

0
4

0

0

0
4

0
4

0
4

3.91276

3.91276

3.91276

3.91276

0
4

0
4

0
4

0.192071

0.192071

0.192071

0.0761741

0.0761741

0.0397225

0
4

0
4

0
4

20.263

20.263

20.263

9.749

7.20311

0.559596

0.652862

0.093266

2.00522

0
4

0
4

0
4

0.168574

0.168574

0

0

0

0
4

0.168574

0.168574

0
4

0
4

0
4

0

0

0

0

0
4

0
4

0
4

0

0

0

0

0
4

0
4

0
4

0.396074

0.396074

0.3199

0.3199

0
4

0.0761741

0.0761741

0
4

1.38777878078145e-17

0
4

0
4

0.0975327

0.0975327

0.0975327

0

0.0975327

0
4

0

0

0
4

0
4

0
4

0

0

0

0

0

0
4

0
4

0
4

0

0

0

0

0

0
4

0
4

0
4

0.12796

0.12796

0.12796

0.12796

0
4

0
4

0
4

0

0

0

0

0

0
4

0
4

0
4

0

0

0

0

0

0
4

0

0

0
4

0
4

0
4

0
4

0
4

0
4

0

0

0

0

0

0

0
4

0

0

0
4

0
4

0
4

0

0

0

0

0
4

0
4

0

0

0

0
4

0
4

0
4

0

0

0

0

0
4

0

0

0
4

0

0

0
4

0
4

0
4

2.24714

2.24714

2.24714

2.17096

0.0761741

1.80411241501588e-16

0
4

0
4

0
4

0

0

0

0

0

0
4

0
4

0
4

0

0

0

0

0

0
4

0
4

0
4

0

0

0

0

0
4

0

0

0
4

0
4

0
4

0.38087

0.38087

0.114261

0.114261

0
4

0.266609

0.266609

0
4

0
4

0
4

0.326431

0.326431

0.326431

0.326431

0
4

0
4

0
4

0

0

0

0

0
4

0

0

0
4

0
4

0
4

0

0

0

0

0
4

0

0

0
4

0
4

0
4

0
7

0
7

0
7

0

0

0

0

0

0

0

0

0
4

0

0

0

0
4

0
4

0
4

0

0

0

0

0
4

0
4

0
4

0

0

0

0

0
4

0
4

0
4

0.195065

0.195065

0.195065

0.0975327

0.0975327

0
4

0
4

0
4

0

0

0

0

0
4

0

0

0
4

0
4

0
4

0

0

0

0

0
4

0
4

0
4

0

0

0

0

0
4

0

0

0
4

0
4

0
4

0

0

0

0

0
4

0

0

0
4

0
4

0
4

0

0

0

0

0

0
4

0
4

0
4

0

0

0

0

0
4

0

0

0
4

0
4

0
4

0

0

0

0

0

0
4

0
4

0
4

0.0761741

0.0761741

0.0761741

0

0.0761741

0

0

0

0

0
4

0

0

0
4

0
4

0

0

0

0

0
4

0
4

0
4

0

0

0

0

0
4

0
4

0
4

0

0

0

0

0
4

0

0

0
4

0
4

0
4

0.318234

0.318234

0.318234

0.318234

0
4

0
4

0
4

0

0

0

0

0

0
4

0
4

0
4

0.233165

0.233165

0.233165

0.233165

0
4

0
4

0
4

0

0

0

0

0
4

0

0

0
4

0
4

0
4

0

0

0

0

0
4

0
4

0
4

0

0

0

0

0
4

0

0

0
4

0
4

0
4

0

0

0

0

0
4

0

0

0
4

0
4

0
4

0

0

0

0

0

0
4

0
4

0
4

0
5

0
5

0
5

0
5

0

0

0
4

0

0

0

0

0

0
4

0
4

0
4

0.0761741

0.0761741

0.0761741

0.0761741

0

0
4

0
4

0
4

0.373064

0.373064

0.373064

0.279798

0.093266

1.38777878078145e-17

0
4

0
4

0
4

0.331839

0.331839

0.331839

0.139899

0.19194

0
4

0
4

0
4

0

0

0

0

0
4

0
4

0
4

0.19194

0.19194

0.12796

0.12796

0
4

0.06398

0.06398

0
4

1.38777878078145e-17

0
4

0
4

0

0

0

0

0
4

0
4

0
4

0

0

0

0

0
4

0
4

0
4

0

0

0

0

0
4

0
4

0
4

0.792761

0.792761

0.279798

0.279798

0
4

0.512963

0.512963

0
4

0
4

0
4

0

0

0

0

0
4

0
4

0
4

0
7

0
7

0
7

0
7

0

0
4

0

0

0

0
4

0

0

0
4

0
4

0

0

0

0
4

0
4

0
4

0.105778

0.105778

0.105778

0.105778

0

0
4

0
4

0
4

0

0

0

0

0
4

0
4

0
4

0

0

0

0

0
4

0
4

0
4

0

0

0

0

0
4

0
4

0
4

0

0

0

0

0
4

0
4

0
4

0

0

0

0

0
4

0
4

0
4

0

0

0

0

0
4

0
4

0
4

0

0

0

0

0
4

0
4

0
4

0

0

0

0

0
4

0
4

0
4

0

0

0

0

0
4

0
4

0
4

49.2551

48.5695

34.4581

1.80435

0

0

0

1.14555

0

0

0

0

0

0.146299

6.53469

0

4.84968

2.09479

0

0

0

0

0

0

0.0704951

0
4

0.243832

0

0

0

0

0

1.94856

0

0.228522

0

0

0

0

0

0

0

0

0

0.0761741

0.0352475

0.53643

0

0

0.114261

0

0.159505

0

0

0

0

0

0.0398762

3.04696

0.0761741

0

0

0

0

0

0

0

0

0.0528713

9.9792

0

0

0

0

1.19842

0

0

0

0

0

0

0.0761741

0

0

0

0

0

0

0

0

5.50948175970234e-15

0
4

0

0

0

0

0

0
4

0

0

0
4

0.19194

0.19194

0
4

0

0

0
4

0

0

0
4

0

0

0
4

13.0214

11.5361

1.37113

0.114261

6.93889390390723e-17

0
4

0

0

0

0
4

0.821931

0.669583

0.0761741

0.0761741

0
4

0

0

0

0

0
4

0

0

0

0
4

0

0

0
4

0.0761741

0.0761741

0
4

0

0

0
4

1.35030875370035e-14

0
4

0

0

0

0

0

0

0
4

0
4

0

0

0

0

0

0
4

0

0

0
4

0
4

0.685567

0.685567

0.457045

0.228522

0
4

0
4

0

0

0

0
4

0
4

0

0

0

0
4

0
4

9.99200722162641e-16

0
4

0.0761741

0.0761741

0.0761741

0.0761741

0
4

0
4

0
4

1.42437450723065e-12

0
4

116.442
3

109.835
3

106.807
3

21.7127

8.55714

1.4795

1.71942

9.31688

0.319893

0

0

0.23992

0

0.0799732

0

2.80331313717852e-15

0
4

81.3071
3

81.1872
3

0.11996

0

6.12010442324618e-15
3

0
4

0

0

0
4

0

0

0

0

0

0

0

0

0

0
4

0

0

0

0

0

0
4

0

0

0
4

0

0

0
4

0

0

0
4

0

0

0
4

0

0

0

0
4

0

0

0
4

0

0

0

0
4

1.3182

1.08804

0.230163

5.55111512312578e-17

0
4

0

0

0
4

0

0

0

0
4

0

0

0
4

2.46902

2.46902

0
4

7.99360577730113e-15
3

0
4

1.45891

1.45891

0.154733

1.10524

0.0884188

0.0442094

0

0.0663141

5.55111512312578e-17

0
4

0
4

1.56943

1.56943

1.56943

0

0
4

0
4

0

0

0

0
4

0
4

0

0

0

0
4

0
4

0
4

0.44786

0.44786

0.44786

0.38388

0

0.06398

0
4

0
4

0
4

0

0

0

0

0
4

0
4

0
4

2.00002

2.00002

2.00002

2.00002

0
4

0
4

0
4

2.0793

2.0793

2.0793

2.0793

0
4

0
4

0
4

0.35988

0.35988

0.35988

0.35988

0
4

0
4

0
4

0

0

0

0

0
4

0
4

0
4

1.71942

1.71942

1.71942

1.71942

0
4

0
4

0
4

0

0

0

0

0
4

0
4

0
4

0

0

0

0

0
4

0
4

0
4

0

0

0

0

0
4

0
4

0
4

0

0

0

0

0

0
4

0
4

0
4

0

0

0

0

0

0
4

0
4

0
4

0

0

0

0

0
4

0
4

0
4

0

0

0

0

0
4

0
4

0
4

0

0

0

0

0
4

0
4

0
4

0

0

0

0

0
4

0
4

0
4

0

0

0

0

0
4

0
4

0
4

3.99680288865056e-15
3

0
4

150.631

2.24833
4

0

0

0

0

0

0

0

0

0

0

0

0

0

0

0

0

0

0

0

0

0

0

0

0

0

0

0

0

0

0

0

0

0

0

0

0
4

0

0

0

0

0
4

0

0

0

0
4

0

0

0

0
4

0
4

0
4

0

0

0

0

0

0
4

0

0

0

0

0
4

0

0

0
4

0

0

0
4

0
4

0

0

0

0

0

0
4

0

0

0

0
4

0

0

0
4

0
4

0
6

0
6

0
6

0
4

0
4

0.35556

0

0

0
4

0.35556

0.35556

0
4

0
4

0

0

0

0

0
4

0

0

0

0
4

0

0

0
4

0
4

1.60002

1.60002

1.55557

0.044445

8.32667268468867e-17

0
4

0
4

0

0

0

0

0

0
4

0
4

0

0

0

0

0
4

0

0

0
4

0
4

0

0

0

0
4

0
4

0.0888899

0.044445

0.044445

0
4

0.044445

0.044445

0
4

0
4

0
4

0
4

0

0

0

0

0

0

0

0

0

0

0

0

0

0

0

0

0

0
4

0

0

0

0

0

0

0
4

0

0

0

0

0

0

0

0
4

0
4

0

0

0

0
4

0
4

0.079445

0.079445

0.079445

0
4

0
4

0

0

0

0
4

0
4

0

0

0

0
4

0
4

0

0

0

0
4

0
4

0

0

0

0
4

0
4

0

0

0

0
4

0
4

0
4

0
4

0

0

0

0

0

0

0

0

0

0

0
4

0

0

0
4

0

0

0

0
4

0

0

0
4

0
4

0.044445
4

0
4

0

0

0

0

0
4

0

0

0

0

0
4

0

0

0
4

0.044445

0.044445

0
4

0
4

0
4

0
4

0

0
4

0

0

0

0

0

0

0

0
4

0
4

0

0

0

0

0

0

0

0

0
4

0

0

0

0

0
4

0

0

0

0

0

0
4

0

0

0
4

0

0

0
4

0
4

0
6

0
6

0
6

0

0

0
4

0
4

0

0

0

0

0

0

0

0

0

0

0
4

0

0

0

0
4

0

0

0

0

0
4

0

0

0
4

0
4

0.0799732

0.0799732

0.0799732

0

0

0

0

0
4

0

0

0
4

0
4

0
4

1.51949
5

1.51949
5

0

0

0
4

1.51949
5

0
5

1.51949

0

0

0

0

0

0

0

0

0

0

0
4

0
4

0
4

79.9768
7

0
6

0
6

0

0

0
4

0

0

0
4

0
4

48.1633

4.84654

4.3707

0.158614
1

0.0881189

0

0

0

0

0

0.123366

0

0

0

0

0

0

0

0

0

0

0

0

0

0

0

0

0

0

0

0

0

0

0

0

0

0

0

0

0

0

0

0

0

0

0

0

0

0

0

0

0

0

0

0

0

0

0

0

0

0

0.105743

0

0

0

0

0

0

0

0

0
4

0

0

0

0

0

0

0

0

0

0

0

0
4

13.5731

4.87549

0

0

0

0

0

0

0

0.0881189

0

0

0

0

0

0

0

0

0

0

0

0

0

0

0.0352475

0

0

0

0

0

0

0

0

0

0

0.845941

0

0

0

0

0

0

0

0

0

0

0

0

0

0

0

0

0

0

0

0

0

0

0

0

0

0

0

0

0

0

0

0

0.0881189

0

0

0

0

0

0
6

0.14099

2.53585

0

0

0.123366

0

0

0

0

0

0
7

0

0.53643

0

0

0.317228

0

0

0

0

0

0.0352475
8

0

0

0

0

0

0

0

0

0

0

0.28198

0

0

0

0

0

0

0

0.528713

0

0

2.58462

0

0

0

0

0

0

0

0

0

0

0.0528713

0.0352475

0

0

0

0

0

0

0

0

0

0
7

0.0352475

0

0

0

0

0

0

0

0

0

0.0975327

0

0

0.0352475

0

0

0.299604

0

0

0

0

3.33066907387547e-16

0
4

0
6

0

0

0

0

0

0

0
4

2.82845

2.82845

0
4

0
7

0

0

0
4

0

0

0

0

0
4

0.292598

0.292598

0

0

0
4

0

0

0

0

0
4

0

0

0

0
4

0

0

0
4

0

0

0

0
4

0

0

0

0
4

20.598

20.5098

0

0

0

0.0881189

0

0

0

0

0

0

0
4

0

0

0
4

0

0

0

0
4

0

0

0
4

0

0

0

0
4

0

0

0
4

0

0

0
4

0

0

0
4

0

0

0
4

0

0

0
4

0

0

0
4

1.65204

0.791372

0.616832

0.243832

0

0

0

0

5.55111512312578e-17

0
4

0

0

0
4

0

0

0
4

0

0

0
4

0

0

0
4

0

0

0
4

0

0

0
4

0

0

0
4

0

0

0
4

0

0

0
4

0

0

0
4

0.370099

0
7

0

0

0.229109

0
7

0.0704951

0

0.0352475

0

0

0.0352475

5.55111512312578e-17

0
4

0

0

0
4

0

0

0
4

0.123366

0.123366

0
4

0

0

0
4

0

0

0
4

0

0

0
4

0

0

0
4

0

0

0
4

0

0

0
4

0

0

0
4

1.46398

0.881189

0

0

0

0

0.195065

0.0528713
6

0.264357

0.0704951

0

0

0

0

0

1.66533453693773e-16

0
4

0

0

0
4

0

0

0
4

0

0

0
4

1.23366

0.599208

0

0.158614

0.405347

0

0

0.0704951

0

0

0
4

0.829028

0
7

0.829028

0

0

0

0

0
4

0.246733

0.158614

0

0

0.0881189

0

0
4

0.105743

0

0.0352475

0.0352475

0

0.0352475

0

0

0
4

3.08086889333481e-15

0
4

10.0077
7

0
6

0
6

0
7

0

0

0

0

0

0

0

0

0

0

0
6

0

0

0

0

0

0

0

0

0

0

0

0

0

0

0

0

0

0

0

0

0

0

0

0

0

0

0

0

0

0

0

0

0

0

0

0

0

0

0

0

0

0

0

0
5

0

0

0

0

0

0

0

0

0

0

0
6

0

0

0

0

0

0

0

0

0

0
6

0
6

0
4

0
7

0
7

0

0

0

0
4

1.15961
7

1.15961
7

0
7

0

0

0

0

0

0

0

0

0

0

0

0

0

0

0

0

0

0

0

0

0

0

0

0

0

0

0

0

0

0

0

0

0

0

0

0

0

0

0

0

0

0

0
4

0
7

0

0

0

0

0

0
4

0

0

0

0

0

0
4

4.24733
7

4.17683
7

0

0

0

0

0.0704951

0

0

0

0

0

0

0
7

0

0

0

0

0

0

0

0

0

0

0
6

0

0

0

0

0

0

0

0

0

0

0

0

2.77555756156289e-16
7

0
4

0
6

0
6

0

0

0

0
4

0
6

0
6

0

0

0

0

0

0
4

0
6

0
7

0
6

0
7

0

0

0

0

0

0

0

0

0

0

0

0

0

0

0

0

0

0

0

0

0
7

0

0

0

0

0

0

0

0

0
6

0
7

0
6

0
7

0
7

0
6

0
4

0
7

0
7

0

0

0

0

0

0

0

0
4

0

0

0

0

0

0

0

0

0

0

0

0

0
4

0
6

0
6

0

0
4

0
6

0
6

0

0

0

0

0
4

2.68215

2.53585

0.146299

0
4

0

0

0
4

0

0

0

0
4

0.780261

0.780261

0
4

0

0

0
4

0

0

0
4

0

0

0
4

0

0

0
4

0

0

0
4

0

0

0
4

0
7

0
7

0

0

0

0

0
4

0

0

0
4

0

0

0
4

0

0

0
4

0.114261

0.114261

0
4

0

0

0
4

0

0

0
4

0

0

0
4

1.02409

1.02409

0
4

0

0

0
4

0

0

0
4

0
7

0
7

0

0
4

0

0

0
4

0

0

0
4

0

0

0
4

0

0

0
4

0

0

0
4

0

0

0
4

0

0

0
4

0

0

0
4

0

0

0
4

0

0

0
4

0

0

0

0

0

0
4

0

0

0
4

0

0

0
4

0

0

0
4

0

0

0
4

0

0

0
4

0
7

0
7

0
4

0

0

0
4

0

0

0
4

0

0

0

0
4

0

0

0
4

0
4

0
7

0
6

0

0
6

0

0

0

0
4

0

0

0

0
4

0
4

0.0975327
7

0

0

0
4

0.0975327
8

0.0975327
8

0

0
4

0

0

0

0
4

0

0

0
4

0

0

0
4

0

0

0
4

0

0

0
4

0

0

0
4

0

0

0
4

0

0

0
4

0
4

21.2207

20.6977

17.2414

3.45639

0
4

0.487663

0.487663

0

0

0

0

0
4

0

0

0
4

0

0

0
4

0

0

0
4

0

0

0
4

0
5

0
5

0

0
4

0
7

0

0

0
4

0.0352475

0.0352475

0
4

0

0

0

0
4

0

0

0

0
4

0

0

0

0
4

0

0

0
4

0

0

0
4

0
4

0
7

0
7

0

0

0

0
6

0

0
4

0
4

0

0

0

0
4

0
4

0

0

0

0
4

0
4

0

0

0

0
4

0
4

0

0

0

0
4

0
4

0

0

0

0

0

0
4

0
4

0.292598

0.292598

0.292598

0
4

0
4

0

0

0

0

0
4

0
4

0.195065

0.195065

0.195065

0
4

0
4

0

0

0

0
4

0
4

0

0

0

0
4

0
4

0

0

0

0
4

0
4

0

0

0

0
4

0
4

2.42028619368284e-14
7

0
4

7.36372

6.43716

0
7

0

0

0

0

0

0

0

0

0

0

0

0
6

0

0

0

0

0

0

0

0

0

0

0

0
4

0

0

0
4

6.43716

0
7

6.43716

0

0

0
4

0

0

0

0
4

0

0

0
4

0

0

0
4

0

0

0
4

0

0

0
4

0
4

0

0

0

0

0

0

0

0

0
4

0
4

0

0

0

0
4

0
4

0

0

0

0
4

0
4

0

0

0

0
4

0
4

0.92656

0.92656

0.829028

0.0975327

5.55111512312578e-17

0
4

0
4

0

0

0

0
4

0

0

0
4

0
4

0

0

0

0
4

0
4

0

0

0

0
4

0

0

0
4

0
4

0

0

0

0
4

0
4

0

0

0

0
4

0
4

0

0

0

0
4

0
4

0

0

0

0
4

0
4

0
4

11.543

10.1287

10.1287

0
6

0

0

0

4.61439

0

0

0

0.159946

0

0

0
6

0

0

0

0

0

0

0

5.11447

0

0

0.23992

0

0

0

6.93889390390723e-16

0
4

0

0

0
4

0

0

0
4

0

0

0
4

0

0

0
4

0
4

0

0

0

0

0
4

0
4

1.41422

1.41422

1.41422

0
4

0
4

0

0

0

0
4

0
4

0

0

0

0
4

0
4

0

0

0

0
4

0
4

0

0

0

0
4

0
4

0

0

0

0
4

0
4

0

0

0

0
4

0
4

0

0

0

0
4

0
4

0
4

6.87744

0

0

0

0

0
4

0
4

0

0

0

0
4

0
1

0

0

0

0

0
4

0

0

0

0

0
4

0

0

0
4

0
4

6.87744

0

0

0

0
4

6.87744

6.87744
1

0

0

0
4

0
4

0
4

1.88173
5

1.88173
5

1.80175

1.80175

0
7

0

0

0

0

0

0

0

0

0

0

0

0

0

0

0
4

0
6

0
6

0
7

0
6

0

0

0

0

0

0
4

0

0

0
4

0

0

0
4

0

0

0
4

0

0

0
4

0

0

0
4

0

0

0
4

0
6

0
6

0

0
4

0

0

0

0

0

0

0
4

0

0

0

0

0
4

0.0799732

0.0799732

0

0

0
4

0

0

0

0
4

0

0

0
4

0

0

0
4

0

0

0
4

1.11022302462516e-16
5

0
4

0

0

0

0

0
4

0
4

0

0

0

0

0
4

0
4

0

0

0

0
4

0
4

0

0

0

0
4

0
4

0

0

0

0
4

0
4

0

0

0

0
4

0
4

0

0

0

0
4

0
4

0
4

0

0

0

0

0

0

0

0

0

0

0

0
4

0

0

0

0

0

0
4

0

0

0

0

0

0
4

0

0

0
4

0

0

0
4

0
4

0
4

35.342
3

35.342
3

35.342
3

35.1088
3

0.233165

3.85802501057242e-15
3

0
4

0
4

0
4

0
6

0
6

0
6

0
6

0

0
4

0

0

0
4

0
4

0
4

0

0

0

0

0

0

0

0
4

0

0

0

0
4

0
4

0
4

0

0

0

0

0

0

0
4

0
4

0
4

0
4

0
4

0
4

0
4

0

0

0
4

0

0

0
4

0
4

0
4

0

0

0

0

0

0

0
4

0
4

0
4

0

0

0

0

0

0
4

0
4

0
4

0

0

0

0

0

0

0

0
4

0
4

0
4

0

0

0

0

0

0
4

0
4

0
4

0

0

0

0

0
4

0
4

0
4

0
6

0
6

0
6

0

0

0

0

0

0

0

0
4

0
4

0
4

0
6

0
6

0

0

0
4

0

0

0
4

0
4

0
4

0.201717

0.201717

0.201717

0.201717

0

0
4

0
4

0
4

0

0

0

0

0
4

0
4

0
4

0

0

0

0

0

0
4

0

0

0
4

0
4

0
4

0

0

0

0

0

0
4

0
4

0
4

0

0

0

0

0

0

0
4

0
4

0
4

0

0

0

0

0

0
4

0
4

0
4

0

0

0

0

0
4

0
4

0
4

0

0

0

0

0
4

0

0

0
4

0
4

0
4

0

0

0

0

0

0

0
4

0
4

0
4

1.23959
4

1.23959
4

1.11963
4

0

1.11963

0

0

0

0

0

0
4

0.11996
4

0

0.11996

0

0
4

0
4

0
4

0

0

0

0

0

0
4

0
4

0
4

0

0

0

0

0

0
4

0

0

0
4

0
4

0
4

0

0

0

0

0
4

0

0

0
4

0
4

0
4

0

0

0

0

0
4

0
4

0
4

0

0

0

0

0

0
4

0
4

0
4

0

0

0

0

0
4

0
4

0
4

0

0

0

0

0

0
4

0

0

0
4

0
4

0
4

0

0

0

0

0
4

0
4

0
4

0

0

0

0

0
4

0
4

0
4

0

0

0

0

0
4

0
4

0
4

0
4

0
4

0
4

0

0

0

0

0

0

0

0

0
4

0

0

0

0

0

0
4

0

0

0
4

0
4

0
4

0

0

0

0

0
4

0
4

0
4

0

0

0

0

0
4

0
4

0
4

0

0

0

0

0
4

0
4

0
4

0

0

0

0

0

0
4

0
4

0
4

0

0

0

0

0
4

0
4

0
4

0

0

0

0

0
4

0

0

0
4

0
4

0
4

0

0

0

0

0
4

0
4

0
4

0

0

0

0

0
4

0
4

0
4

0

0

0

0

0
4

0
4

0
4

0

0

0

0

0
4

0
4

0
4

1.91113
4

1.91113
4

0
4

0
4

0

0

0

0

0
4

0

0

0
4

0

0

0

0
4

1.91113

1.91113

0
4

0
4

0
4

0

0

0

0

0
4

0
4

0
4

0

0

0

0

0
4

0
4

0
4

0

0

0

0

0
4

0
4

0
4

0

0

0

0

0
4

0
4

0
4

0

0

0

0

0
4

0
4

0
4

0

0

0

0

0
4

0
4

0
4

0

0

0

0

0
4

0
4

0
4

0

0

0

0

0
4

0
4

0
4

0

0

0

0

0
4

0
4

0
4

0

0

0

0

0
4

0
4

0
4

0

0

0

0

0

0

0

0

0
4

0

0

0

0

0

0
4

0

0

0
4

0

0

0

0
4

0

0

0
4

0
4

0
4

0

0

0

0

0
4

0
4

0
4

0

0

0

0

0
4

0
4

0
4

0

0

0

0

0
4

0
4

0
4

0.0481639

0.0481639

0.0481639

0.0481639

0
4

0
4

0
4

0

0

0

0

0
4

0
4

0
4

0

0

0

0

0
4

0
4

0
4

0

0

0

0

0
4

0
4

0
4

0

0

0

0

0
4

0
4

0
4

0

0

0

0

0
4

0
4

0
4

0

0

0

0

0
4

0
4

0
4

0
7

0
7

0
7

0
7

0

0

0

0

0

0

0
4

0
4

0
4

0.25592

0.25592

0.25592

0.25592

0
4

0
4

0
4

0

0

0

0

0
4

0
4

0
4

0

0

0

0

0
4

0
4

0
4

0

0

0

0

0
4

0
4

0
4

0

0

0

0

0
4

0
4

0
4

0

0

0

0

0
4

0
4

0
4

0

0

0

0

0
4

0
4

0
4

0

0

0

0

0
4

0
4

0
4

0

0

0

0

0
4

0
4

0
4

0.163796

0.163796

0.163796

0.163796

0
4

0
4

0
4

0
7

0
7

0
7

0
7

0

0

0
4

0
4

0
4

0.0582163

0.0582163

0.0582163

0.0582163

0
4

0
4

0
4

0

0

0

0

0
4

0
4

0
4

0

0

0

0

0
4

0
4

0
4

0

0

0

0

0
4

0
4

0
4

0

0

0

0

0
4

0
4

0
4

0

0

0

0

0
4

0
4

0
4

0

0

0

0

0
4

0
4

0
4

0

0

0

0

0
4

0
4

0
4

0

0

0

0

0
4

0
4

0
4

0

0

0

0

0
4

0
4

0
4

0

0

0

0

0

0

0

0

0
4

0
4

0
4

0

0

0

0

0
4

0
4

0
4

0

0

0

0

0
4

0
4

0
4

0

0

0

0

0
4

0
4

0
4

0

0

0

0

0
4

0
4

0
4

0

0

0

0
8

0

0
4

0

0

0
4

0
4

0

0

0

0
4

0
4

0

0

0

0
4

0
4

0
4

0
4

6703.39

4.68943
7

4.68943
7

0
7

0
7

0

0
4

0
7

0
7

0
7

0
7

0

0

0

0

0

0

0

0

0

0

0
7

0

0

0

0

0

0

0

0

0

0

0
7

0

0

0

0

0

0

0

0

0

0

0
7

0

0

0

0

0

0

0

0

0

0

0
7

0

0

0

0

0

0

0

0

0

0

0
6

0

0

0

0

0

0

0

0

0

0

0
6

0

0

0

0

0

0

0

0

0

0

0
7

0

0

0

0

0

0

0

0

0

0

0
7

0

0

0

0

0

0

0

0

0

0

0
7

0

0

0

0

0

0

0

0

0

0

0
7

0
6

0

0

0

0

0

0

0

0

0

0

0
7

0

0

0

0

0

0

0

0

0

0

0
7

0

0

0

0

0

0

0

0

0

0

0
7

0

0

0

0

0

0

0

0

0

0

0
7

0

0

0

0

0

0

0

0

0

0

0
7

0

0

0

0

0

0

0

0

0

0

0
7

0

0

0

0

0

0

0

0

0

0

0
6

0

0

0

0

0

0

0

0

0

0

0
7

0

0
7

0
7

0
6

0
6

0
7

0
7

0

0
7

0
8

0
7

0
7

0
7

0
7

0

0

0
7

0

0
7

0

0

0

0

0
8

0
6

0

0

0

0

0

0

0

0

0

0

0

0

0

0

0

0

0

0

0

0

0

0
6

0

0

0

0

0

0

0

0

0

0

0
6

0

0

0

0

0

0

0

0

0

0

0
7

0

0

0

0

0

0

0

0

0

0

0
4

2.61618
7

0

0
7

0
7

0
7

0
7

0
7

0.797524

0.163796

1.2561

0.0398762

0

0.31901

0.0398762

5.13478148889135e-16
7

0
4

0
7

0
7

0

0

0
4

0
7

0
7

0

0

0

0

0

0

0

0

0

0

0

0

0

0
4

0

0

0

0

0
4

2.07325
7

0
7

2.07325
7

0

0

0

0

0

0

0

0

0

0

0

0

0

0
4

0
7

0
7

0

0

0
4

0
8

0
8

0

0

0
4

0

0

0
4

0

0

0
4

0

0

0
4

0
4

0

0

0

0
4

0
4

0

0

0

0

0
4

0
4

0

0

0

0
4

0
4

0

0

0

0
4

0
4

0

0

0

0
4

0
4

0

0

0

0
4

0
4

0

0

0

0
4

0
4

0

0

0

0
4

0
4

0
4

5671.52

4.52271

1.45243

0.599916

0

0.69464

0.157873

0

0

0

0

0

0

0
4

1.39683

1.39683

0
4

0

0

0
4

0

0

0
4

0

0

0
4

0

0

0
4

0

0

0
4

0

0

0
4

0

0

0
4

0

0

0
4

0

0

0
4

0

0

0

0

0

0

0

0

0
4

0

0

0
4

0

0

0
4

0

0

0
4

0

0

0

0

0

0

0

0
4

1.67345

1.67345

0

0

0
4

0

0

0

0
4

0

0

0

0

0

0
4

0

0

0
4

0

0

0

0
4

0

0

0
4

4.44089209850063e-16

0
4

0

0

0

0
4

0
4

149.974

2.75734
3

0.713665

1.94636

0.0973179

0

0

0

8.32667268468867e-17
3

0
4

25.0007
3

9.83956

0

0

0.139899

0

0

0

0.279798

0.093266

0.093266

0.093266

0

0

0.186532

0.093266

0.19194

0

0.279798

12.4976

0

0

0.139899

0

0

0

0

0

0

1.07256

0

0
4

0

0

0
4

104.561
3

104.561
3

0

0

0

0

0

0

0

0

0

0

0
4

0

0
5

0

0

0

0

0

0

0

0

0

0

0

0

0

0

0

0

0

0
5

0

0

0

0

0

0
4

0.737308

0.193478

0.22393

0.09597

0.12796

0.09597

0
4

0

0

0
4

0

0

0

0

0

0
4

0

0

0

0

0

0

0

0
4

2.71772

0.162257

1.94848

0.063149

0.54383

1.11022302462516e-16

0
4

0.457045

0.152348

0.114261

0.0761741

0.114261

0
4

0

0

0

0

0

0
4

0

0

0

0
4

0

0

0

0

0
4

0

0

0

0

0
4

0
5

0
5

0

0

0

0

0

0

0

0

0

0

0
4

0

0

0

0

0
4

0

0

0
4

0.868637

0.356797

0.3199

0.19194

0
4

0.852512

0.0947236

0.757789

0

0
4

2.01861

0

0

2.01861

0
4

0

0

0

0
4

0.152348

0

0.152348

0

0
4

0

0

0

0
4

0

0

0

0

0
4

0.354428

0.354428

0
4

0
5

0
5

0

0

0

0

0
4

0

0

0

0
4

0

0

0
4

0

0

0
4

0.126298

0.063149

0.063149

0
4

0

0

0
4

0

0

0
4

0

0

0
4

0

0

0
4

0

0

0
4

0

0

0
4

4.3708
3

1.02253

0.196555

0.11996

1.63774

0.0655183

0.0655183

1.26298

0
4

0.126298

0.063149

0.063149

0
4

0

0

0

0
4

0

0

0

0
4

0

0

0

0
4

0

0

0

0
4

0

0

0
4

0

0

0

0
4

0

0

0
4

0

0

0

0
4

0

0

0
4

0
5

0

0

0

0

0

0

0

0
4

0

0

0

0
4

0

0

0

0
4

0

0

0
4

0

0

0
4

0

0

0
4

0

0

0
4

0

0

0
4

0

0

0
4

0

0

0
4

0

0

0
4

2.58992

1.90435

0.38087

0

0.152348

0

0.0761741

0.0761741

4.44089209850063e-16

0
4

0

0

0
4

0

0

0
4

0

0

0
4

0

0

0
4

0

0

0
4

0

0

0
4

0

0

0
4

0

0

0
4

0

0

0
4

0

0

0
4

0
5

0
5

0

0
4

0

0

0
4

0.063149

0.063149

0
4

0

0

0
4

0

0

0
4

0

0

0
4

0

0

0
4

0

0

0
4

0

0

0
4

0

0

0
4

0

0

0
4

0

0

0

0

0

0

0

0
4

0

0

0
4

0

0

0
4

0.329046

0.329046

0
4

0

0

0
4

0

0

0
4

0

0

0
4

0.063149

0.063149

0
4

0

0

0
4

0

0

0
4

0

0

0
4

1.82818

0.799828

0.723654

0.152348

0.152348

0

5.55111512312578e-17

0
4

0

0

0
4

8.28226376370367e-14

0
4

678.609
3

10.5873

0.194636

0
3

0

0

0

0.0648786

0

0

0

0

0.191923

0

0

0

0

1.56157

0

0.843422
5

0.228522

0.0761741

0

0

0

0

0

0

0

0

0

0

0

0

0

0

0

0

0

0

0

1.3983
3

0

0

0

0

0

0

0

0

0

0

0.0648786

0

0

0

0

0

0

0

0

0.228522

0

4.10396
3

0

0

0

0

0

0

0.266609

0

0

0

0
6

0

0

0

0

0

0

0.114261

0

0

0

1.13538

0

0

0.114261

0

0

0

0

0

0

0

0

0

0

0

0

0

0

0

0

0

0

0
4

0.34216

0
6

0

0

0

0

0

0

0

0

0

0

0

0

0

0

0

0

0

0

0

0

0

0

0

0

0

0

0

0

0

0

0

0

0
7

0

0

0

0

0

0

0

0

0

0

0

0

0

0

0

0

0

0

0

0

0

0
6

0

0

0

0

0.15995

0

0

0

0

0

0
7

0

0

0

0

0

0

0

0.0423112

0

0

0

0

0

0

0

0

0

0

0

0

0

0
3

0.139899

0
4

98.2425

0

95.6672

0

0

0

0

0

0

0

0

0

0

0

0

0

0

0

0

0

2.57535

0

0

0

0

0

0
4

518.916
3

2.999
4

0.0799732
4

0

0

0

0

0

0

0

0

0

0.11996

0

0
4

0

0

0

0

0

0.0634669

0

0

0

0

0
4

0

0

0

0

0

0.179443

0

0.92656

0

0

0

0

0

0

0

0

0

0

0

0

0

0
4

0

0

0

0

0

0

0

0

0

0

0

0

0

0

0

0

0

0

0

0

0.0655183

0

0

0

0

0

0

0

0

0

0

0

0.0920573

0

0

0

0

0

0

0

0

0

0

0

0

0

0

0

0

0

0

0

0

0

11.8914

0

0

0

0

0

0

0

0.0975327

0

0

0

0

0.297919

0

0

0

0.279906

0.279906

0

0

0

0

0

0

0

0

0

0

0

0

0

0

0

0

0

0

0

0

0

0.276172

0

0

0

0

0

0

0.146299

0

0

0

0

0

0

0

0

0

0

0

0

0

0.146299

0

0

0

0

0

0

0

0

0

0

0

0

0

0

0

0

0

0

0

0.137103

0

0

0

0

0

0

0

5.40707

0

0

0

0

0

0

0.0799732

0

0

0

0

0

0.0975327

0

0

0

0

0

0

0

0

0
4

0

0

0

0

0

0

0

0

0

0

0

0

0

0

0

0

0

0

0

0

0

0

0

0

0

0

0

0

0

0.069043

0

0.069043

0

0
4

0

0.0799732

0

0

0

0

0

0

0

0

0

0

0

0.115072

0

0

0

0

0

0

0

0

0

0

0

0

0.0975327

0

0

0

0

0

0

0

0

0

0

0

0

0

0

0.195065

0

0

0

0

0

0

0

0

0

0

0

0

1.51176

0

0

0

0

0

0

0

0.146299

0

0

0

0

0.0975327

0

0

0

0

0

0

0

0

0

0

0

0

0.0975327

0

0

0

0

0.0975327

0

0

0
4

0

0

0

0

0.0975327

0

0

0

0

0

0
4

0

0

0

0

0

0

0

0

0

0

0

0

0

0

0

0.11996

0

0.199933

0

0

0

0

0

0

0

0

0

0

0

0

0

0

0

0

0

0.0595838

0

0

0

0

0

0

0

0

0

0

0

0.190435

0

0

0

0

0

0

0

0

0.0761741

0

0.0975327

0

0.0460286

0.195065

0

0.0975327

0.131037

0

0

0

0

0

0

0

0.0634669

0

0

0

0.673363

0

0

0

0

0

0

0.195065

0

0.0423112

0

0

0

0

0

0

0

0

0

0

0

0

0

0

0

0

0

0

0

0

0

0

0

0

0
4

0

0

0

0

0

0

0

0

0.195065

0

0
4

0

0.079445

0

0.119168

0

0

0

0

0

0

0

0

0.731495

0

0

0

0

0

0

0

0

0

0

0

0

0

0

0

0

0

0

0

1.07964

0

0

0.0975327

0

0

0

0

0

0

0

0

0

0

0

0

0

0.243832

0

0

0

0

0

0

0

0

0

0

0

1.1996

0

0

0

0

0

0

0

0

0

0

0

0

0.0799732

0

0

0

0.11996

0

0

0

0

0

0

0

0

0.398492

0

0

0

0.1611

0

0

0

0.159946

0

0

0

1.24485

0

0

0

0

0.0598143

0

0

0

0

0

0
7

0

0

0

0

0

0

0

0

0

0

0

0

0

0

0

0

0

0

0

0.0634669

0

0

0.207129

0

0

0

0

0

0

0

0

0

0.069043

0

0

0

0

0

0.0460286

0.0920573

0

0

0

0

0.0975327

0

0

0

0

0

0

0

0

0.139899

0

0

0

0

0

0

0

0

0.258196

0

0

0

0.138086

0

0

0

0

0

0

0

0

4.63845
3

0.11996

0

1.01553

1.21916

0

0

0

0

0

0

0

0

0

0.200823

0

0

0

0

0

190.237

0

0

144.3

0

0

0

0

0

0

0

0

0

0

7.51496

0

0

0

0

0

0

0

0

0

0

0

0

3.60726

0

0

0

0

0

0

0

0

8.47716
4

0

0

0

0

0

0

0

0

0

0

0
4

0.185057

0

0

0

0

0

0

0

0

0

0.479839
3

0.184115

0

0.567759

7.26618

0

0

0

0

0

4.53527

0

0.13178

0.0665157

0

0

0

0

0

0

0

0

0

0

0

0

0.276172

0.23992

0

0

0

0

0.0332578

0
4

0

0

0

0

0

0

0

0

0.414258

0

0
4

0.341364

0

0

0

0

0

0

0

0

0

19.605
4

0.279906
4

0

0

0

0

0

0

0

0

0

0

0
4

0

0.0460286

0

0

0

0

0

0

0

0

12.5815

0

0

0.069043

0.0920573

0

0

0

0

0

0

0.0920573

0.0460286

0

0

0.0799732

0

0

0

0

0

0

0

0

0

0.0460286

0

0

0

0

0

0

0

0
4

0

0

0

0

0

0

0

1.02409

0

0

0
4

0

0

0

0

0.53643

0

0

0

0.184115

0

0.0799732
4

0

0

0

0

0.163796

0

0

0

0.195065

0

0
4

0.877794

0

0

0

0

0

0

0

0

0

0.959679
3

0

0

0

0

0

0

0.146892

0

0

0.682427

11.6761
4

11.4113

0

0

0

0

0

0

0.0920573

0

0.152843

0

0
4

1.03965

0

0

0

0

0

0.0460286

0

0

0

0.159946
4

0

0

0

0.138086

0

0

0

0

0

0.115072

0

0

0

5.80319

0

0

0

0

0.1611

0

0

0
4

0

0

0

0

0

0

0

0

0

0

0

0

0

0

0

0.877794

0.118308

0

0

0

0

0
3

0

0

0

0

0

0

0

0

0

0

0
4

0

0

0

0.131037

0

0

0

0

0.11996

0

3.0329

0

0.0920573

0

0

0

0

0

0

0.0799732

0

0

0

0

0

0

0

0

0

0

0

0.67698

0

0
4

0

0

0

0

0

0

0

0

0

0

0.0799732
4

0

0

0

0

0

0

0.054841

0

0

0

0

0

0

0

0

0

0

0

0

0

0

0.11996
4

0

0

0

0

0

0

0

0

0

0.146299

0

0.877794

0

0

0

0

0

0

0

0

0

2.04819

0

0

0

0.243832

0

0

0

0

0

0

0

0

0

0

0

0

0

0

0

0

0

0
4

0

0

0.319893

0.11996

0

0

0

0

0

0

0
7

0

0

0

0

0

0

0

0

0

0

0

0.0975327

0

0.0975327

0

0

0.0822615

0

0

0

0

0.0799732
4

0
4

0

0

0

0

0

0

0

0

0

0

0
4

0

0

0

0

0

0.169245

0.390131

0

0

0

0
4

0

0

0

0

0

0

0

0

0

0

0
4

0

0

0

0

0

0

0

0

0

0

0
4

0

0

0

0

0

0

0

0

0

0

0
4

0

0

0.390131

0

0

0

0.146299

0

0

0

0

0

0

0

0

0

0

0

0

0

0

0
4

0

0.483301

0

0

0

0

0

0

0

0

0.0799732
4

0

0

0

0

0

0

0.146299

0

0

0

0

0

0

0

0

0

0

0

0

0

0

0

0
4

0

0

0

0

0

0

0

0

0

0.53643

0
4

0

0

0

0

0

0

0

0

0

0

0

0

0

0

0.0423112

0

0

0

0.341364

0

0

0

0

0

0

0

0

0

0

0

0

0

0

0

0.243832

0

0

0

0

0.0460286

0

0

0.253158

0

0

0

0

0

0

0

0

0

0

0

0

0

0

0

0

0

0

0

0

0

0.919692

0
4

0

0

0

0

0

0

0

0

0

0

0

0.243832

0

0

0

0

0

0

0

0

0

0

0

0

0

0

0.195065

0

1.27957

0

0.0846225

0.243832

0

0
4

0

0

0

0

0

0

0

0

0

0

0

0

0

0

0

0

0

0

0

0

0

4.10188

0

0

0

0

0

0

0

0

0

0

0
4

0

0

0

0

0

0

0

0

0

0

0
4

0

0

0

0

0.0460286

0

0

0

0

0

0
4

0

0

0

0

0

0

0

0

0

0

1.26579

0

0

0

0

0

0

0

0

0

0.243832

0

0

0

0

0

0

0

0

0

0

0.0975327

0.0799732
3

0

0

0

0

0

0

0.0920573

0

0

0

0
4

0

0

0

0

0.682729

0

0

0

0

0.0460286

0

0

0

0

0

0

0

0

0

0

0

0

0
4

0

0

0

0

0

0

0

0.054841

0

0

0.277115

0

0

0

0

0

0

0

0

0

0

0
4

0

0

0

0

0

0

0

0

0

0

0
4

0

0

0

0

0

0

0

0

0

0

0
4

0

0

0

0.0975327

0

0

0.069043

0.0423112

0

0

0
4

0

0.341364

0

0

0

0

0

0

0

0

0

0

0

0

0

0

0

0

0

0

0

0

0

0

0

0

0

0

0

0

0

0

15.405

0

0

0

0

0

0

0

0

0

0

0
4

10.6826

10.6826

0

0

0

0

0

0

0

0

0

0

0

0

0
4

1.7366
4

0
4

1.7366
4

0
4

0

0

0

0
4

0

0

0
4

0.852512
4

0

0.505192

0.221022

0.126298

0

2.77555756156289e-17
4

0
4

0

0

0

0

0
4

0

0

0

0

0

0
4

0.754728

0.754728

0

0
4

0.189447

0

0

0.189447

0

0

0

0
4

0.663065

0.063149

0.378894

0.126298

0.0947236

0
4

2.24179

2.24179

0
4

0.448981

0.448981

0

0
4

0.284171

0.0947236

0.189447

0

0
4

7.32529
3

0.663065
3

0

0

0.0947236

0.063149

0

0

0

0

0.221022

0.284171

0.063149

5.93601

0

0

0

0

8.88178419700125e-16
3

0
4

0

0

0
4

0

0

0

0

0
4

0

0

0
4

0

0

0
4

0

0

0

0
4

0.126298

0.126298

0

0

0
4

0

0

0

0
4

0.126298

0.063149

0

0.063149

0
4

0

0

0

0
4

0

0

0
4

13.4823
3

4.79933
4

0

0

0

2.21022

0

6.37805

0

0.0947236

0

0

0

0

2.91433543964104e-16
3

0
4

0

0

0

0
4

0

0

0
4

0

0

0
4

0

0

0

0
4

0

0

0

0
4

0

0

0

0
4

0

0

0
4

0.163402

0.163402

0
4

0

0

0
4

0

0

0

0
4

5.46239
3

4.98877

0

0

0.063149

0

0

0.063149

0.221022

0.126298

0

0

0

0

0

0

0
4

0

0

0
4

0

0

0
4

0

0

0
4

0

0

0

0
4

0

0

0

0
4

0.063149

0

0.063149

0
4

0

0

0

0
4

0

0

0

0
4

0

0

0

0
4

0

0

0
4

0
3

0

0

0

0

0

0

0
4

0.230143

0.230143

0
4

0

0

0

0
4

0

0

0
4

0

0

0
4

0

0

0
4

0

0

0
4

0.154733

0.154733

0
4

0.0947236

0.0947236

0
4

0

0

0
4

0

0

0
4

2.14707
4

0
4

0

0

0

0

0

0

0

2.14707

0
4

0

0

0
4

0

0

0
4

0

0

0
4

0

0

0
4

0.606229

0.606229

0
4

0

0

0
4

1.67345

1.67345

0
4

0.221022

0.221022

0
4

0

0

0
4

0

0

0
4

0.063149

0

0

0

0.063149

0
4

0

0

0
4

0

0

0
4

0

0

0
4

0

0

0
4

0

0

0
4

0

0

0
4

0

0

0
4

0

0

0
4

0

0

0
4

0

0

0
4

0

0

0

0

0

0

0

0

0
4

0

0

0
4

0.0947236

0.0947236

0
4

0

0

0
4

0

0

0
4

0

0

0
4

0

0

0
4

0

0

0
4

0

0

0
4

0.063149

0.063149

0
4

0

0

0
4

0.378894

0

0

0

0.315745

0.063149

0

0

1.38777878078145e-17

0
4

0.0761741

0.0761741

0
4

0

0

0
4

0.114261

0.114261

0
4

0

0

0
4

0

0

0
4

0

0

0
4

0

0

0
4

0

0

0
4

0

0

0
4

0
4

0
4

0
4

0
4

0

0

0

0

0

0

0

0

0

0

0

0

0

0

0

0

0

0

0

0

0

0

0

0

0

0

0

0

0

0

0

0

0

0

0

0

0

0

0

0

0

0

0

0

0
4

0
4

0
4

0

0
4

0

0

0
4

0

0

0
4

0

0

0
4

0
4

82.5548

48.2488

0
7

0
7

0

0

0

0

0

0

0

0

0

0

0.06398
8

0

0.174649

0

0

0

0

0

0

0

0

0
7

0

0.06398

0

0

0

0

0

0.06398

0

0

0

0

0

0

0

0

0

0

0

0.0332578

0

0.06398
7

0

0

0

0

0

0

0

0
7

0

0
8

6.14033
3

0
7

1.72746

0

0

0
7

0
8

0

1.34617

0
7

0

0

0

0
7

0

0

0

0

0
7

0

0

0

0

0

2.11134

0

0

0

0

0

0

0

0

0

0

0
8

0

0.103018

0

0.0582163

0

0

0

0

0.0761741

0.195065

34.474
3

0.134197

0.0792865

0

0

0

0

0

0

0

0

0
8

0

0

0.214978

0.566884

0

0

0

0

0

0

0
7

0.265256

0

0

0

0

0

0

0

0

0.0873245

0
7

0

0

0

0.145541

0

0

0

0

0.0598143

0

0
4

0

0

0

0
4

4.11792
2

2.15736

0.0681929

1.07513

0.187767

0.197871

0.0975327

0.0975327

0.128931

0.044445

0.063149

2.35922392732846e-16
2

0
4

1.4936
4

0

0
4

0

0

0

0

0

0

0

0

0.759746

0

0

0.0799732

0

0

0

0

0
4

0

0

0

0

0

0

0

0

0

0

0
4

0

0

0

0

0.0332578

0

0

0

0

0

0.35988
3

0

0

0

0

0

0

0

0.221022

0

0

0
4

0

0

0

0

0

0

0

0

0

0

0

0.0397225

0

0

0

0

0

0

0

0

0

0

0

0

0

0

0

0

0

0

0

0

0
8

0

0

0

0

0

0

0

0

0

0

0

0

0

0

0

0

0

0

0

0

0

2.77555756156289e-17
4

0
4

0
5

0

0

0

0

0

0

0

0

0

0

0

0

0

0

0

0

0

0

0
4

7.95698
6

2.57753

0.166576

0

0

0

0

0

0

0

0

0.0761741

0

0

1.24277

0

0

0

0

2.74205

0

0

0.0498868

0

0

0

0

0.326431

0

0

0

0

0

0

0

0

0

0.09597

0

0

0

0

0.292598

0.146299

0

0.164523

0

0

0

0.0761741

0

0

0

0

0

0

0

2.08166817117217e-16
6

0
4

0.06398
5

0
5

0

0

0

0

0

0

0

0

0

0

0

0

0

0

0

0

0

0

0

0

0.06398

0

0

0

0

0

0

0

0

0

0

0

0
5

0

0

0

0

0

0

0

0

0

0

0

0

0

0

0

0
4

0

0

0

0

0

0

0

0

0

0

0

0

0

0

0

0

0

0

0

0

0
4

0

0

0

0

0

0

0

0

0
4

0

0

0
4

0

0

0
4

0

0

0
4

0.0582163

0.0582163

0
4

0

0

0
4

0

0

0
4

1.02835

1.02835

0
4

0.0598143

0.0598143

0
4

0

0

0
4

0

0

0
4

0
7

0
7

0

0

0

0

0
4

0.0582163

0.0582163

0
4

0

0

0
4

0.0761741

0.0761741

0
4

0

0

0
4

0

0

0
4

0

0

0
4

0

0

0
4

0

0

0
4

0.0655183

0.0655183

0
4

0

0

0
4

0
4

0

0

0

0
4

0

0

0
4

0

0

0
4

0

0

0
4

0

0

0
4

0.063149

0.063149

0
4

0

0

0
4

0

0

0
4

0.0481639

0.0481639

0
4

0.0761741

0.0761741

0
4

0

0

0
4

0

0

0

0

0

0

0

0

0
4

0

0

0
4

0

0

0
4

0

0

0
4

0

0

0
4

0

0

0
4

0

0

0
4

0

0

0
4

0
7

0

0

0

0

0
4

0
4

0

0

0

0

0

0
4

0
4

0

0

0

0

0
4

0

0

0

0
4

0
7

0
7

0
4

0
7

0
7

0
4

0
4

0
4

0
4

0
4

0

0

0

0
4

0

0

0

0
4

0
8

0

0

0
4

0
6

0

0

0

0
4

0.127087

0

0.054841

0.0722459

0

0
4

0

0

0
4

0.199933

0.199933

0

0

0
4

0

0

0
4

0

0

0

0

0

0
4

0

0

0

0

0
4

2.01153

2.01153

0

0
4

0
7

0
7

0

0

0
4

0

0

0
4

0

0

0

0

0
4

2.91902

2.91902

0

0
4

0

0

0
4

0

0

0

0
4

0

0

0

0
4

0

0

0
4

0.291954

0.291954

0
4

0

0

0
4

0

0

0

0

0
4

0
7

0

0

0

0

0

0

0

0
4

0.288984

0.0722459

0.12041

0.0963278

1.38777878078145e-17

0
4

0

0

0
4

0

0

0

0
4

0

0

0

0
4

0

0

0

0
4

0

0

0

0

0
4

0

0

0

0
4

0

0

0
4

0

0

0

0
4

0.199333

0.054841

0.144492

0
4

0

0

0

0

0

0

0

0

0

0

0

0

0
4

0

0

0

0
4

0

0

0

0
4

0

0

0
4

0

0

0

0
4

0

0

0
4

0.0460286

0.0460286

0
4

0

0

0

0
4

0

0

0

0
4

0

0

0

0
4

0

0

0
4

0
7

0
7

0

0

0

0
4

0

0

0

0
4

0

0

0
4

0

0

0

0
4

0

0

0
4

0

0

0
4

0

0

0
4

0

0

0

0
4

0.063149

0

0.063149

0
4

0

0

0
4

0

0

0
4

0.0799732
3

0

0.0799732

0

0
4

0

0

0
4

0

0

0
4

0

0

0
4

0

0

0
4

0

0

0
4

0

0

0
4

0

0

0
4

0

0

0
4

0

0

0
4

0.11996

0.11996

0
4

0

0

0

0

0
4

0.418957

0.418957

0
4

0

0

0
4

0

0

0
4

0

0

0
4

0

0

0
4

0

0

0
4

2.35776

2.35776

0
4

9.83856

9.83856

0
4

0

0

0
4

0

0

0
4

0
7

0
7

0

0
4

0.0975327

0.0975327

0
4

0

0

0
4

0

0

0
4

0.0799732

0.0799732

0
4

0

0

0
4

0

0

0
4

0

0

0
4

0

0

0
4

0

0

0
4

0

0

0
4

1.02695629777827e-14

0
4

482.152

0.0873245
7

0
7

0

0

0

0.0873245

0

0

0

0

0
4

440.95

0
7

0
7

0.0548351

1.05868
3

0
6

0

0.169245

0.219761

0

0

0.183109

0.0846225

92.2029
3

0

0

0.126298

0

0

0

0

0.37844

0.0797524

0

0

0

0

0

0.145541

0

0.191944

0

9.92603

0

0.11996

0
7

0.0634669

0

0

0

0.279134

0.0799732

0

0

0

0

0
7

0

0

0.528925

0.0423112

0.0982775

0

0

0.279534

0.159946

0.133335

0
6

0

0

0.244447

0.519826

0

0.159946

0.169245

0

0

0

0

0

0.0799732

0

0

0

0

0

0

0

0

25.755

0.307335

0

0

0

0.11996

0

29.1873

0

0

0

17.0783

0.192656

0

0.283405

33.9087

5.77967

0.0423112

0

0

0.137054

0

0

0

0.11996

0.138154

0.044445

0.23992

0

0

0.125333

0

0

36.6848
3

0.0666674
7

0

0

1.17908

0

0.252596

0

0.0332578

0

0

0

0
7

0

0.457045

0

0

0

0

0

0.0498868

0

0.0498868

11.8361
2

0.199547

0

0

0

0

2.0793

0

0.229314

0

0.133335

1.73931
3

0.199933

0.199933

0.53334

0

0.137103

0.0943317

0

0

0.378406

0

1.56854
3

0

0

0

0

0

0

0

0

0

0

0
7

0.296179

0

0.140773

0

0

0.362952

0

1.0705

0

0

0
7

0.458628

0

0.133335

0

0

0

0

0

0

0.2428

0.716327
3

0

0

0

0

0

0.0846225

0.131037

0

0

0.126298

0
7

0

0

0

1.15154

0

0

0

0

0

0

12.8224
2

0

0

0

0

0.0982775

0

0

0

0

0.753461

0
7

0

2.95901

0

0

0

0

0

0

0

0

0

0
6

0

0

0

0.0665157

0

0

0

0

0

0

16.3277
2

0

0

0

0

0

0

0

0

0

0

1.18624
3

0

0.0722459

0

0

0

0

0

0

0

0

0
7

0.0722459

0

0.399094

0

0

0

0

0.294833

0

0

0.900218
2

0

0

0

0.144492

0

0.063149

0

0.519826

0

0

1.73305

0

0

0

0.0722459

0

0.0963278

0.0481639

0.0332578

0

0

0
5

0

0.0481639

0

0

0

0

0

0

0

0

0
5

0.0481639

0

0.044445

0.221022

0

0

0.0982775

0

0

0

0.600271
3

0

0

0.963278

0

0

0

0

0

0.0799732

0

20.9467
4

0
6

0

0

0.126298

1.27957

0

0

0

0

0.0722459

0

0.853869
3

0

0

0

0

0

0

0.11996

0

0

0

3.90128

0

0

0

0

0.0655183

0

0

0

0

0

1.1119
3

0.192656

0

0

0.0761741

0

0

0.0481639

0.0598143

0

0

0
7

0

0

0.0947236

0

0

1.48452

0.0722459

0

0.144492

0

3.03115
3

0

0

0

0.0481639

0.0873245

0

0

0.0481639

0

0

12.8949
3

0

0

0

0

0

0

0

0.0799732

0

0

0
7

0.192656

0.159946

0

0.0722459

0

0

0

0.12041

0

0

0.839431
3

0

0.063149

0

0

0.0498868

0

0

0

0

0

0
7

0

0.0722459

0.282692

0

0

0

0

0.611272

0

0

0
7

1.92516
2

0

0

0

0.168574

0

0

0

0

0

0.12041

6.76366

0

0

0

0

0

0

0

0

0.111112

0

0

0

0

0

0.0666674

0

0

0

0.0481639

0

0

1.37038

0.23992

0

0

0

0.221022

0

0.0799732

0

0

0

0.422227

0

0

0

0

0

0.0481639

0

0.0481639

0.0873245

0

2.63338

0

0

0.116433

0

0

0

0

0

0

0

4.55847
3

0

0

0

0

0

0

0.0481639

0.111112

0

0

0

0

0

0

0

0.133031

0

0

0.133335

0

0

0
5

0

0

0.222225

0

0.0963278

0

0

0.552618

0

0

0.319223
3

0

0

0.0481639

0

0.044445

0

0

0

0

0.0398762

0
7

0.520961
3

0

0

0.0655183

0

0

0

0.111112

0

0.139567

0

0.797925
3

0

0

0

0

0

0

0.884498

0

0

0

0.199933
3

0

0

0

0

0

0

0

0

0

0.0582163

0.0831446
6

0.0481639

0

0.157873

0

0

0

0

0

0.0973179

0

0
7

0

0

0

0.505721

0.157873

0

0

0

0.0722459

0

0
7

0

0.0498868

0

0

0.144492

0.144492

0

0

0

0.0481639

0
7

0

0

0

0.851738

0

0.0722459

0

0.0873245

0

0.0797524

0
7

0

0

0

0

0

1.99933

0

0

0

0

0.339466
2

0

0

0

0.439853

0

0

0

0.0722459

0.0799732

0.0888899

0.88353
3

0.291082

0

0

0

0

0

0

0

0

0.0799732

6.97226

2.01103
2

0

0.519826

0

0.262073

0

0

0.063149

0.0982775

0.044445

0

0.309582
3

0.0722459

0

0

0

0

0

0

0

0

0

0.41878
3

0.044445

0.144492

0

0

0

0.266063

0

0.0963278

0

0

2.4667

0

0

0

0.0963278

0

0.14966

0.0598143

0

0.163796

0

0

0

0

0.0822615

0.319893

0.0722459

0

0

0

0

0.0761741

0
6

0.0799732

0

0

0

0

0

0

0

0

0

0.312284
3

0

0.39311

0

0

0

0.0722459

0

0

0.133335

0.216738

0.246367
2

0

0

0

0

0

0.319893

0

0.0398762

0.0332578

0

0
7

0

0.14966

0

0

0

0

0

0

0.182918

0.0665157

0

0

0.264902

0

0

0

0

0.116433

0

0

0.0799732

10.1442
3

0

0

0.111112

0.12041

0

0

0

0

0

0

0

0
7

0.063149

0.0666674

0

0

0

0

0

0

0.179443

0.0722459

0.133335

0

0

0

0

0

0

0

0

0

0

2.51916

0

0

0

0

0

0

1.53335

0

0

0
6

0.599799

0
7

0.0634669

0
7

0

2.24325

0

0.328741
3

0

0

0

0.458576

0
6

0
7

0

0.0398762

1.18641207969006e-13

0
4

0

0

0

0

0

0

0

0

0

0

0

0

0

0

0

0

0
4

24.2315

23.1718

0
7

0
4

0

0

0

0

0

0

0

0

0

0
4

0

0

0

0

0

0

0

0

0

0

0
7

0

0

0

0

0.0397225

0

0

0

0.0799732

0

0
7

0

0

0

0

0

0

0.0397225

0

0

0

0
4

0

0

0

0

0

0

0

0

0

0

0
7

0

0

0

0

0

0

0

0.378406

0

0.243832

0
4

0

0

0

0

0

0

0.218474

0

0

0

0

0

0.0595838

0
7

0
4

0
7

0
7

0

0

0

0

0

0

0

0

0

0

0
6

0

0

0

0

0

0

0
7

0

0
6

0
7

0
7

0

0

0
4

0
4

0

0

0

0

0
4

0

0

0
4

0

0

0
4

0

0

0
4

0

0

0
4

0

0

0
4

0

0

0
4

0

0

0
4

0

0

0
4

0.0332578

0.0332578

0
4

0

0

0
4

0
4

0
4

0

0

0
4

0

0

0
4

0

0

0
4

0

0

0
4

0

0

0
4

0

0

0
4

0

0

0
4

0

0

0
4

0

0

0
4

0

0

0
4

0

0

0
4

0
7

0
7

0

0
4

0

0

0
4

0

0

0
4

0

0

0
4

0

0

0
4

0

0

0
4

0

0

0
4

0

0

0
4

0

0

0
4

0.0975327

0.0975327

0
4

0

0

0
4

0

0

0

0

0

0
4

0

0

0
4

0

0

0
4

0

0

0
4

0

0

0
4

0

0

0
4

0

0

0
4

0

0

0
4

0

0

0
4

0

0

0
4

0

0

0
4

0
3

0

0

0

0
4

0

0

0
4

0.0582163

0.0582163

0
4

0

0

0
4

0

0

0
4

0

0

0
4

0.0665157

0.0665157

0
4

0

0

0
4

0

0

0
4

0.054841

0.054841

0
4

0

0

0
4

4.07224
2

0

0.0846225

3.98762

0

0
4

0

0

0

0

0

0

0
4

0
8

0

0

0
4

0

0

0

0

0

0
4

0
7

0
7

0
4

0
4

0
4

0

0

0

0

0

0

0

0

0

0

0
4

0

0

0

0

0

0

0

0

0

0

0
4

0

0
4

0
4

0
4

0

0

0

0
4

0

0

0
4

0

0

0

0
4

0

0

0

0

0
4

0.158436

0.158436

0

0
4

0

0

0
4

0.788158

0.719115

0.069043

0
4

0

0

0

0
4

0.663363

0.228522

0.0920573

0.342783

0
4

0

0

0
4

0

0

0

0

0
4

0.599799
4

0
4

0.599799
4

0

0

0

0

0

0

0
4

0

0

0

0

0
4

0.448981

0.448981

0

0
4

0.123735

0.123735

0
4

0

0

0

0

0
4

0

0

0

0
4

0

0

0

0

0
4

0

0

0

0

0
4

0

0

0
4

0

0

0

0
4

0

0

0

0
4

0
5

0
7

0
5

0

0

0

0

0

0

0
4

0

0

0

0
4

0

0

0

0
4

0

0

0

0
4

0.16029

0.114261

0.0460286

6.93889390390723e-18

0
4

0

0

0
4

0.162197

0.162197

0
4

0.0397225

0.0397225

0

0
4

0.190435

0.114261

0.0761741

0
4

1.26935

1.17181

0.0975327

0
4

0

0

0

0
4

4.71764
3

2.35579
3

0.439087

0.063149

0.418957

0.0975327

0

1.34313

0
4

0

0

0

0
4

0.103018

0.103018

0
4

0

0

0
4

0

0

0
4

0

0

0
4

0

0

0

0
4

0

0

0

0
4

0

0

0

0
4

0.299072

0.299072

0
4

0

0

0
4

1.35316
3

0.159505
3

1.19365

0

0

0
4

0

0

0
4

0

0

0
4

0

0

0
4

0

0

0
4

0

0

0
4

0

0

0
4

0

0

0
4

0

0

0
4

0

0

0
4

0

0

0
4

0
4

0
4

0

0
4

0

0

0
4

0

0

0
4

0

0

0
4

0

0

0
4

0

0

0
4

0

0

0
4

0

0

0
4

0

0

0
4

0

0

0
4

0

0

0
4

1.2314
3

0
4

0

1.2314

0

0
4

0

0

0
4

0

0

0
4

0

0

0
4

0

0

0
4

0

0

0
4

0

0

0
4

0

0

0
4

0

0

0
4

0.0442094

0.0442094

0
4

0.0655183

0.0655183

0
4

0
4

0

0

0

0

0

0
4

0

0

0
4

0

0

0
4

0

0

0
4

0

0

0
4

0.0822615

0.0822615

0
4

0

0

0
4

0

0

0
4

0

0

0
4

0

0

0
4

0

0

0
4

0
4

112.205
3

111.262
3

106.564
3

0

0.06398

0

0

0.0530735

0.21934

3.07936

0.0548351

0

0

0

0

0

0

0

0.0530735

0

1.17373

0

0

0

1.48769885299771e-14
3

0
4

0
4

0
4

0

0

0
4

0

0

0
4

0

0

0
4

0

0

0

0
4

0

0

0

0
4

0

0

0

0
4

0

0

0
4

0

0

0
4

0

0

0
4

0

0

0
4

0

0

0
4

0

0

0

0
4

0

0

0
4

0.06398

0.06398

0
4

0

0

0
4

0

0

0
4

0

0

0
4

0

0

0
4

0

0

0
4

0

0

0
4

0

0

0
4

0

0

0
4

0

0

0
4

0

0

0
4

0

0

0
4

0

0

0
4

0

0

0
4

0.759746

0.759746

0
4

0

0

0
4

0

0

0
4

0

0

0
4

0

0

0
4

0

0

0
4

0

0

0

0

0
4

0

0

0
4

0

0

0
4

0

0

0
4

0

0

0
4

0

0

0

0

0
4

0

0

0
4

0

0

0
4

0

0

0
4

0.11996

0.11996

0

0
4

9.56179579958416e-15
3

0
4

8.51267
2

8.51267
2

3.26366
2

0.287725
2

0

0

0

0

0

0

0

0.229314

0

0

0

0

0.196555

0

0

0

0

0

0

0

0

0.0655183

0

0.0947236

0

0

0

0

0

0

0

0

1.38928

0

0

0

0

0

0

0

0

0

0

0

0

0

0

0

0

0

0.196555

0

0

0

0

0

0

0

0

0

0

0

0

0

0

0

0

0

0

0

0.126298

0

0

0

0

0

0

0

0

0

0

0

0

0

0

0

0

0

0

0

0

0

2.62073
2

0

0

0

0

0

0

0

0

0

0

0.0423112

0

0

0

0

0

0

0

0

0

0

0
4

0

0

0
4

0

0

0
4

0
4

54.6054

50.1942
7

0
7

0
7

0

0

0

0

0

0

0

0

0

0

0

0

0

0

0

0

0

0

0

0

0

0

0

0

0

0

0

0

0

0

0

0

0
6

0

0

0

0

0

0

0

0

0

0

0

0

0

0

0

0

0

0

0

0

0

0
7

0

0

0

0

0

0

0

0

0

0

0
6

0

0

0

0

0

0

0

0

0

0

0
7

0

0

0

0

0.276172

0

0

0

0

0

0
7

0

0

0

0

0

0

0

0

0

0

0
7

0

0

0

0

0

0.138086

0

0

0

0

25.8911
7

0
6

0

0

0

0

0

0

0

0

0

0

0
7

0

0

0

0

0

0

0

0

0

0

0
7

0

0

0

0

0

0

0

0

0

0

0
7

0

0.230143

0

0

0

0

0

0

0

0

0
7

0

0

0

0

0

0

0

0

0

0

0
6

0

0

0

0

0

0

0

0

0

0

0
7

0

0

0

0

0

0

0

0

0

0

0
7

0

0

0

0.184115

0

0

0

0

0

0

0
6

0

0

0

0

0

0

0

0

0

0

0
7

0

0

0

0

0

0

0

0

0

0

0
7

0
5

0

0

0

0

0

0.069043

0

0

0

0

0
7

0

0

0

0

0

0

0

0

0

0

0
7

0

0

0

0

0

0

0

0

0.0460286

0.0460286

0
7

0

0

0

0

0

0

0

0

0

0

0
7

0

0

0

0

0

0

0.0460286

0

0

0

0
7

0

0

0

0

0

0

0

0

0

0

19.9074

0

0

0

0

0

0

0

0.0460286

0

0

0
7

0

0

0

0

0

0

0

0

0

0

0
6

0

0

0

0

0

0

0

0

0

0.138086

0

0

0

0

0

0

0.0920573

0

0

0

0

0
6

0
6

0

0

0

0

0

0

0

0

0

0

0.575358

0

0

0

0

0.1611

0

0

0

0

0

0
7

0

0

0

1.1277

0

0

0

0

0

0

0
7

0

0

0

0

0

0

0

0

0

0

0

0

0

0

0

0

0

0

0

0

0

0
6

0

0

0

0

0

0

0

0

0

0

0
6

0.069043

0

0

0

0

0

0

0

0

0

0
7

0

0

0.0920573

0

0

0

0

0

0

0

0
7

0

0

0

0

0

0

0

0

0

0

0
7

0

0

0

0

0

0

0

0

0

0

0
7

0

0.069043

0

0

0

0

0

0

0

0

0

0

0

0

0

0

0

0

0

0

0

0

0
7

0

0.207129

0

0

0

0

0

0

0

0

0
6

0

0

0

0

0

0

0

0

0

0

0
7

0

0

0

0

0.0460286

0

0

0

0

0.322201

0
7

0

0

0

0

0

0

0.0460286

0

0

0

0
6

0

0

0

0

0

0

0

0

0

0

0

0

0

0

0

0

0

0

0

0

0

0
7

0

0.0460286

0

0

0

0

0

0

0

0

0
7

0

0

0

0

0

0

0

0

0

0

0
7

0
7

0

0

0

0

0

0

0

0

0

0

0
7

0

0

0

0

0

0

0

0

0

0

0
7

0

0

0

0

0

0

0

0

0

0

0
6

0

0

0.069043

0

0.0460286

0

0

0

0

0

0

0

0

0
6

0

0
8

0
5

0
7

0

0
6

0

0

0

0

0
7

0

0

0
7

0

0
7

0

0

0

0
7

0

0

0

0

0
7

0

0

0.207129

0

0

0

0

0

0

0

0
4

0.253158
7

0.253158
7

0

0
4

3.82038
4

3.17598
4

0

0

0

0

0

0

0

0.644401

0

0

0

0

0

0

0

0
4

0

0

0
4

0

0

0
4

0

0

0
4

0

0

0
4

0

0

0
4

0

0

0
4

0

0

0
4

0

0

0
4

0

0

0
4

0

0

0
4

0

0

0
4

0

0

0

0
4

0

0

0
4

0

0

0
4

0

0

0
4

0

0

0
4

0

0

0
4

0

0

0

0
4

0.337641

0.337641

0
4

0

0

0
4

0

0

0
4

0

0

0
4

0
4

67.6753
4

56.4724
4

0
4

0
5

0

0

0

0

0

0

0

0

0

0

10.3249
3

0

0

0

0

0

0

0.182918

0

0.0332578

0

8.27723
4

0

0

0

0

0

0

0

0

0

0

0
4

0

0

0

0

0

0

0

0

0.199933

0

0

0

0

0

0

0

0

0

0

0

0

2.87904

0

0

0

0

0

0

0

0

0

0

0.0947236
4

0

0

0

0

0

0

0

0

0

0

0
5

0

0

0.23992

0

0

0

0

0

0

0

0

0

0

0

0

0

0

0

0

0.12796

0

0

0

0

0

0

0

0

0

0

0

0

0

0.11996
4

0.0799732

0

0

0

0

0

0

0

0

0

0

0

0

0

0

0

0

0

0

0

0

0

0

0

0

0

0

0

0

0

0

0

0

0

0

0

0

0

0

0

0.0799732

0

0

0.0799732

0

0

0

0

0

0

0

0

0.0332578

0

0

0

0

0

0

0

0

0

0

0

0

0

0

0

0

0

0

0

0

0

0.0332578

0

0

0

0

0

0

0

0

0

0

0

0

0

0

0

0

0

0

0

0

0

0

0

0.279906
4

0

0

0

0

0

0

0

0

0

0

0

0

0

0

0

0

0

0

0

0

0

0

0

0

0

0

0

0

0

0

0

0.11996

0

0

0

0.159946

0

0

0

0

0

0

0

0

0

0

0

0

0

0

0

0

0

0

0

0

0

0

0

0

0

0

0

0.23992

0

0

0.221022
3

0

0

0

0

0

0.0442094

0

0

0

0

0

0

0

0

0

0

0

0

0.06398

0

0

0

0

0

0

0

0

0

0

0

0

0

0

0

0

0

0.0975327

0

0

0

0

0

0

1.63945

0

0

0

0

0

0

0

0

0

0

0

3.95867

0

0

0

0

0

0

0

0

0

0

0

0

0

0

0

0

0

0

0

0

0

4.55847

0

0

0

0

0

0

0

0

0

0

0

0

0

0.0799732

0

0

0

0

0.063149

0.063149

0

2.71909

0

0

0

0

0

0

0

0

0

0

0

0

0

0

0

0

0

0

0

0

0

0
4

0

0

0

0.0498868

0

0

0

0

0

0

0

0

0

0

0

0

0

0

0

0

0

0

0

0

0

0
4

0
4

1.07964

0

0

0

0

0

0

0

0

0

0
4

0

0

2.69389

0

0

0

0

0.282692

0

0

7.86206
3

0

0

0

0

0.279906

0

0

0

0

0

2.09524

0

0

0

2.51916

0

0

0

0

0

0

0
5

0

2.19926

0

0

0

0

0.31595

0

0

0

1.2712053631958e-14
4

0
4

0
4

0
4

0

0

0

0

0

0

0

0

0

0

0

0

0

0

0

0

0

0

0
4

0

0

0

0

0
4

0

0

0

0
4

0

0

0

0
4

0

0

0

0

0

0
4

0

0

0

0
4

0

0

0
4

0

0

0

0
4

0

0

0

0

0
4

0.839719

0.839719

0

0
4

0

0

0

0

0
4

1.7994
4

0.679772
4

0

0

0
4

0.0799732
4

0.11996

0

0

0.919692

0

0

1.11022302462516e-16
4

0
4

0

0

0

0
4

0

0

0
4

0

0

0

0
4

0

0

0
4

0

0

0
4

0

0

0

0
4

0

0

0
4

0

0

0

0
4

0

0

0

0
4

0

0

0
4

2.86776
3

2.00607
3

0.778544

0

0.0831446

0

0

0
4

0

0

0
4

0

0

0
4

0

0

0
4

0

0

0
4

0

0

0
4

0

0

0
4

0

0

0
4

0

0

0
4

0

0

0
4

0

0

0
4

1.29757
3

1.29757
3

0
4

0

0

0
4

0

0

0
4

0

0

0
4

0

0

0
4

0

0

0
4

0

0

0
4

0

0

0
4

0

0

0
4

0

0

0
4

0

0

0
4

0.11996

0

0

0.11996

0
4

4.27857

4.27857

0

0

0
4

0

0

0

0

0
4

0

0

0

0

0

0
4

0

0

0

0

0

0
4

0
4

117.981
4

12.7115
5

7.00588

2.97475
5

0
4

0

0

0

0

0

0

0

0

0

0

0
5

0

0

0

0

0

0

0

0

0

0

0
5

0

0

0

0

0

0

0

0

0

0

0

0

0

0

0

0

0

0

0

0

0.780261
5

0

0

0

0

0

0

0

0

0

0

1.80435
5

0

0

0

0

0

0

0

0

0

0

0
6

0

0

0

0

0

0

0

0

0

0

0.146299
4

0

0

0

0

0

0

0

0

0

0

0
5

0

0

0

0

0

0

0

0

0

0

0

0

0

0

0

0

0

0

0

0

0

0
5

0

0

0

0

0

0

0

0

0

0

0

0

0

0

0

0

0

0

0

0

0

1.30451205393456e-15
5

0
4

105.2
4

0
6

15.2067
3

11.2336
4

0
5

1.8955
5

0
5

0
4

1.04205
4

7.35772
4

0
4

0

0

0

0

0

0

0

0

0

0

0
4

0

0

0

1.77205

0

0

0

0

0

0

0

0

0

0

0

0

0

0.195065

0

0

0

0.0975327
4

0.292598

0

0

0

0

0

0

0

0

0

0

0

0

0

0

0

0

0

0

0

0

0

0

0

0

0.0548351

0

0

0

0

0

0

0
4

0

0

0

0

0.633962

0

0

0

0

0

0

0

0

0

0

0

0

0

0

0

0

0.0975327

0

0

0

0

0

0

0

0

0

0

0

0

0

0

0

0

0

0

0

0

0

0.146299
4

0
7

0

0

0

0

0

0

0

0

0

0

0

0

0

0

0

0

0

0

0

0

0

0

0

0

0

0

0

0

0

0

0

0

0

0

0

0

0

0

0

0

0

0

0

0

0.682729

0

0

0

0

0

0

0

0

0

0
4

0

0

0

0

0

0

0

0

0

0

0

0

0

0

0

0

0

0

0

0

0

0

0

0

0

0

0

0

0

0

0

0

0

0

37.3981
4

0.0799732

0

0

0

0

0

0

0

0

0

18.6955
3

0

0

0

0

0

0

0

4.14514

0

0

2.06987

0.233165

0

0

0

0

0

0

0

0

0

0
4

0

0

0

0

0

0

0.0530735

0

0

0

0
4

0

0

0

0

0

0

0

0

0

0

0.15922
4

0

0

0

0

0

0

0

0

0

0

0.292598
3

0

0

0

0

0

0

0

0

1.36546

0

0
4

0

0
8

0

0

0

0

0

0

0

0

0

0

0
8

0

0

0

0

0

0

0

0

0

0

0

0

0

0

0

0

0

0

0

0

0

0

0

0

0

0

0

0

0
4

0

0

0

0

0

0

0

0

0
4

0

0

0
4

0

0

0
4

0

0

0
4

0

0

0
4

0

0

0
4

0

0

0
4

0

0

0
4

0

0

0
4

0

0

0
4

0

0

0
4

0
4

0
4

0

0

0

0
4

0

0

0
4

0

0

0
4

0

0

0
4

0

0

0
4

0
4

0
4

0

0

0
4

0
4

0

0

0

0

0
4

0

0

0

0

0
4

0

0

0
4

0.069043

0.069043

0
4

0

0

0
4

0

0

0
4

0
4

53.6792
3

0.0332578
4

0
4

0

0

0

0

0

0.0332578

0

0

0

0

0
4

0

0

0

0

0

0
4

0

0

0
4

0

0

0
4

0.0332578

0.0332578

0
4

0

0

0
4

0

0

0
4

49.9709
3

0
4

0

0

0

0

0

49.4943

0

0.0993063
4

0

0.377364
4

0

0

0

0
4

0
3

0
3

0
4

0
4

0
4

0
4

0
4

0

0

0

0

0

0

0
4

3.64173
3

3.60848

0

0

0

0.0332578

0

0

0
4

0
4

0
4

0

0

0

0
4

0
4

0

0

0

0

0

0
4

0

0

0

0

0
4

0

0

0

0

0

0
4

4.44089209850063e-16
3

0
4

1.04762

1.04762

0

0

0

0
7

0

0

0

0

1.04762

0

0

0
4

0

0

0

0

0

0
4

0

0

0

0

0

0

0
4

0

0

0

0
4

0

0

0
4

0
4

0
4

0
4

0

0

0

0

0
4

0

0

0

0
4

0
4

2.2614

1.11779

0.670326

0.092796

0.0530735

0.301593

5.55111512312578e-17

0
4

1.14361

0.904779

0.238831

0
4

4.44089209850063e-16

0
4

0

0

0

0

0
4

0

0

0

0
4

0

0

0
4

0
4

0

0

0

0

0

0
4

0
4

5.27046
3

5.2372
3

5.13989
3

0.0973179

9.71445146547012e-16
3

0
4

0.0332578

0.0332578

0
4

0
4

0
4

0

0

0

0
4

0

0

0
4

0

0

0
4

0
4

0

0

0

0

0

0

0
4

0

0

0
4

0
4

0

0

0

0

0
4

0

0

0

0

0
4

0

0

0
4

0
4

0
6

0
6

0
6

0
4

0
4

0

0

0

0
4

0
4

34.0099
3

30.358
3

22.7282
3

0

0

2.32771
3

1.37015

0.320192

2.63965

0.622424

0

0.266609

0.0831446

1.77635683940025e-15
3

0
4

3.02351

1.26792

0.682729

0.975327

0.0975327

0
4

0.628344

0.189447

0.438897

0

5.55111512312578e-17

0
4

0
4

0.232712

0.0634669

0

0.0634669

0

0
4

0.169245

0.169245

0
4

0
4

0
4

0

0

0

0

0
4

0

0

0
4

0

0

0
4

0
4

0

0

0

0

0
4

0
4

0.297919
3

0.139029

0.139029

0
4

0

0

0

0
4

0

0

0
4

0

0

0
4

0

0

0
4

0.15889

0.15889

0
4

0
4

0

0

0

0

0
4

0
4

0

0

0

0

0
4

0

0

0

0

0
4

0

0

0
4

0

0

0
4

0
4

0

0

0

0

0

0

0
4

0
4

1.73496

0

0

0

0

0
4

1.73496

0.0398762

0.618081

1.077

0
4

0
4

0

0

0

0

0
4

0
4

36.6176

36.6176

36.2746

0.342975

0
4

0
4

0
3

0
3

0
3

0

0
4

0

0

0

0

0

0

0

0
4

0

0

0

0

0
4

0

0

0

0
4

0

0

0

0
4

0

0

0

0
4

0
4

0

0

0

0

0
4

0

0

0

0
4

0

0

0
4

0
4

0.464024

0.464024

0.0806999

0.0403499

0.0403499

0.0403499

0.0403499

0.221925

0
4

0
4

0
4

0
4

0

0

0

0
4

0

0

0
4

0

0

0
4

0
4

0.164523

0.164523

0.164523

0

0

0
4

0

0

0

0
4

0
4

0

0

0

0

0
4

0

0

0

0
4

0

0

0
4

0
4

4.08735

4.02247

3.76296

0.0973179

0.0648786

0.0973179

2.4980018054066e-16

0
4

0.0648786

0.0648786

0
4

8.32667268468867e-17

0
4

0

0

0

0

0

0
4

0

0

0
4

0

0

0
4

0

0

0
4

0
4

0
4

0
4

0
4

0
4

0

0

0
4

0

0

0
4

0
4

0
4

0
4

0

0

0

0
4

0
4

0

0

0

0

0
4

0

0

0

0
4

0
4

44.4857
3

6.51093
3

6.27614
3

0
3

0

0

0.0397225

0.195065

0

0

0

0
4

0

0

0
4

0

0

0
4

0

0

0
4

0.0595838

0.0595838

0
4

0

0

0
4

0
4

0
4

0

0

0

0
4

0
4

0

0

0

0
4

37.8158

37.7563

0.0595838

1.23512311489549e-15

0
4

0

0

0

0
4

0.0993063

0.0993063

0
4

0

0

0
4

0

0

0
4

0

0

0
4

0
4

0
6

0
6

0
6

0
4

0
4

0

0

0

0
4

0

0

0
4

0

0

0

0
4

0

0

0
4

0
4

0

0

0

0

0
4

0
4

0.859793
3

0.859793
3

0.626988

0

0.232805

0
4

0

0

0
4

0
4

0.159946

0

0

0

0

0
4

0.159946

0.159946

0
4

0
4

1.86292

0

0

0
4

0

0

0
4

0

0

0
4

1.86292

1.86292

0
4

0
4

0

0

0

0

0

0
4

0
4

0

0

0

0

0

0

0
4

0

0

0
4

0
4

1.54589

1.48101

1.48101

0
4

0

0

0
4

0.0648786

0.0648786

0
4

8.32667268468867e-17

0
4

0

0

0

0

0

0

0
4

0
4

0.327592
5

0.327592
5

0.196555
5

0

0

0

0

0

0

0

0

0

0

0.131037
5

0

0
4

0

0

0

0

0

0

0
4

0
4

0.421711

0.421711

0

0

0

0.291954

0.129757

2.77555756156289e-17

0
4

0
4

0.34732

0.34732

0

0.126298

0.126298

0.0947236

1.38777878078145e-17

0
4

0

0

0
4

0
4

0.79007

0.79007

0.731854

0.0582163

0
4

0
4

0

0

0

0

0

0

0
4

0

0

0
4

0

0

0
4

0
4

0.0799732

0.0799732

0.0799732

0

0
4

0

0

0

0
4

0

0

0
4

0
4

0

0

0

0

0
4

0

0

0
4

0
4

0

0

0

0

0

0
4

0
4

0

0

0

0

0
4

0

0

0
4

0
4

0

0

0

0

0

0
4

0

0

0
4

0
4

0

0

0

0

0

0
4

0
4

0
5

0
5

0
5

0

0

0

0

0

0

0

0

0
4

0
4

0

0

0

0

0
4

0

0

0
4

0

0

0
4

0

0

0
4

0
4

0.490874

0.326368

0.246758

0.0796102

0

2.77555756156289e-17

0
4

0.164505

0.0822527

0.0822527

0
4

0
4

0.199933

0

0

0

0

0
4

0.199933

0.199933

0

0
4

0
4

0

0

0

0

0
4

0

0

0
4

0

0

0
4

0
4

0

0

0

0

0
4

0

0

0
4

0

0

0
4

0

0

0
4

0
4

0

0

0

0

0
4

0
4

0
4

0

0

0

0
4

0

0

0
4

0
4

0

0

0

0
4

0
4

0

0

0

0

0

0
4

0

0

0
4

0
4

0

0

0

0

0

0
4

0

0

0
4

0

0

0
4

0
4

0
4

0
4

0
4

0

0

0

0

0

0

0

0

0

0

0

0
4

0

0

0
4

0

0

0

0
4

0

0

0
4

0

0

0
4

0

0

0
4

0

0

0
4

0

0

0
4

0

0

0
4

0

0

0
4

0
4

0
4

0

0

0
4

0

0

0
4

0
4

0

0

0

0

0
4

0

0

0
4

0
4

0

0

0

0

0
4

0

0

0

0
4

0
4

1.4795

1.39953

0.11996

1.27957

0
4

0.0799732

0.0799732

0
4

0
4

0

0

0

0

0

0
4

0

0

0

0
4

0
4

0

0

0

0
4

0
4

0.410469

0.252596

0.063149

0.126298

0.063149

0
4

0.157873

0.063149

0.0947236

0
4

2.77555756156289e-17

0
4

0

0

0

0

0
4

0

0

0

0
4

0

0

0
4

0
4

0

0

0

0

0

0
4

0

0

0

0
4

0
4

0.0655183

0

0

0

0

0
4

0.0655183

0.0655183

0
4

0

0

0
4

0
4

0

0

0
6

0

0

0

0

0

0

0
4

0

0
6

0

0

0

0

0

0

0

0
4

0

0

0

0
4

0

0

0

0
4

0
4

0

0

0

0

0

0

0
4

0

0

0
4

0
4

0.104024

0

0

0

0

0
4

0.0442094

0.0442094

0
4

0.0598143

0.0598143

0
4

6.93889390390723e-18

0
4

0

0

0

0

0

0
4

0

0

0
4

0
4

0

0

0

0
4

0
4

0

0

0

0

0
4

0

0

0
4

0

0

0
4

0
4

0

0

0

0

0
4

0

0

0

0
4

0

0

0
4

0
4

0

0

0

0
4

0
4

0

0

0

0

0

0
4

0
4

0

0

0

0

0
4

0
4

0.0997735

0.0997735

0.0332578

0

0.0665157

0

0
4

0
4

5.43928

2.36652
6

0
6

0

0.850944

0

0

0.757789

0

0.757789

0
4

1.02001
2

0.464151

0.105743

0.243832

0

0.154717

0.0515724

2.77555756156289e-17
2

0
4

1.64262
2

1.14637

0.180503

0

0.315745

0
4

0.157873

0.157873

0

0

0
4

0.0975327

0

0.0975327

0
4

0

0

0

0
4

0.154717

0.154717

0
4

7.7715611723761e-16

0
4

0

0

0

0

0

0

0
4

0
4

1.39029

0.0595838

0

0.0595838

0
4

1.3307

0.63556

0.695144

0
4

0
4

0

0

0

0
4

0
4

0

0

0

0

0
4

0

0

0
4

0
4

0

0

0

0

0
4

0

0

0
4

0
4

0

0

0

0

0
4

0
4

0

0

0

0

0

0
4

0
4

0.999665

0.999665

0.999665

0

0
4

0

0

0
4

0
4

0.0799732

0.0799732

0.0799732

0
4

0

0

0

0
4

0
4

0

0

0

0
4

0

0

0
4

0
4

0
5

0
5

0
5

0

0

0
4

0

0

0
4

0

0

0
4

0
4

0.192102

0.139029

0.0397225

0.0595838

0.0397225

1.38777878078145e-17

0
4

0.0530735

0.0530735

0
4

6.93889390390723e-18

0
4

0

0

0

0

0
4

0
4

0.78622

0.78622

0.78622

0
4

0
4

0

0

0

0

0
4

0

0

0
4

0
4

0

0

0

0
4

0

0

0

0
4

0
4

0

0

0

0
4

0

0

0
4

0

0

0
4

0
4

0

0

0

0

0

0
4

0
4

0

0

0

0

0
4

0

0

0
4

0
4

0

0

0

0

0

0
4

0

0

0
4

0
4

0.0799732

0.0799732

0

0

0.0799732

0

0
4

0
4

120.061

113.827

3.61132

0.20175

1.02892

0.544724

0.0806999

1.43242

0.342975

0.100875

0.1614

0.0806999

0.0605249

67.9695

0.0806999

0.0403499

0.0605249

0.0605249

0.0403499

0.786824

0.0403499

3.3894

0.0605249

0.12105

0.383325

0.0403499

0.0605249

0.0806999

0.12105

0.141225

0.0403499

0.423674

24.8354

1.0491

0.221925

0.484199

5.64899

0
4

6.23407

0.181575

0.645599

0.20175

4.92269

0.12105

0.0403499

0.0403499

0.0403499

0.0403499

1.55431223447522e-15

0
4

2.66453525910038e-14

0
4

0

0

0

0

0

0

0

0

0

0

0

0

0

0

0

0

0
4

0
4

0.359646

0

0

0
4

0.0423112

0.0423112

0
4

0.317334

0.317334

0
4

0
4

0

0

0

0

0
4

0
4

0

0

0

0

0
4

0
4

0

0

0

0
4

0

0

0
4

0

0

0
4

0
4

0

0

0

0
4

0

0

0
4

0
4

0

0

0

0
4

0

0

0
4

0
4

0

0

0

0

0
4

0
4

0.332831

0.239565

0.146299

0.093266

0
4

0.093266

0.093266

0
4

1.38777878078145e-17

0
4

0

0

0

0

0
4

0

0

0
4

0
4

0

0

0

0

0
4

0

0

0
4

0
4

8.96987

7.87277

2.77583

0.0530735

0.0530735

0.0530735

0.12796

0.12796

0.06398

0.212294

0.106147

0.106147

0.15922

2.62318

0.419864

0.3199

0.22393

0.06398

0.22393

0.106147

0.0530735

0
4

0.83174

0

0.19194

0.19194

0.19194

0.25592

0

0
4

0.265367

0.212294

0.0530735

6.93889390390723e-18

0
4

7.21644966006352e-16

0
4

0

0

0

0

0
4

0
4

0

0

0

0
4

0
4

1.80471

1.80471

1.74649

0.0582163

0
4

0
4

0

0

0

0
4

0

0

0
4

0
4

0

0

0

0

0
4

0

0

0
4

0
4

0

0

0

0

0

0
4

0
4

0

0

0

0
4

0

0

0
4

0
4

0

0

0

0
4

0

0

0
4

0
4

0

0

0

0

0
4

0

0

0
4

0
4

0

0

0

0

0
4

0
4

62.2196
3

62.0321
3

60.8139
3

0.464409

0.242221

0.25592

0

0.114261

0

0.09597

0.045462

0
4

0.118815

0.06398

0.0548351

0
4

0.0686787

0.0686787

0
4

2.19269047363468e-15
3

0
4

3.0666

2.32012

2.32012

0
4

0.746474

0.746474

0
4

1.11022302462516e-16

0
4

0

0

0

0

0
4

0
4

0.928049

0.262275

0.262275

0
4

0.585074

0.585074

0
4

0.0806999

0.0806999

0
4

1.52655665885959e-16

0
4

0

0

0

0

0
4

0

0

0
4

0
4

0

0

0

0

0
4

0

0

0
4

0
4

0

0

0

0

0
4

0

0

0
4

0
4

0

0

0

0

0
4

0
4

0

0

0

0
4

0
4

0

0

0

0

0
4

0
4

0

0

0

0

0
4

0

0

0
4

0
4

0
4

0
4

0

0

0

0

0

0

0

0

0
4

0
4

0
4

0

0

0

0

0
4

0

0

0

0

0

0
4

0

0

0

0
4

0

0

0
4

0
4

0

0

0

0
4

0

0

0
4

0
4

2.0121

1.92896

1.92896

0
4

0.0498868

0.0498868

0
4

0.0332578

0.0332578

0
4

4.85722573273506e-17

0
4

0

0

0

0

0
4

0
4

0

0

0

0

0
4

0
4

0

0

0

0
4

0
4

0

0

0

0
4

0

0

0
4

0
4

0.389272

0.389272

0.389272

0
4

0
4

0

0

0

0

0
4

0
4

0

0

0

0

0

0
4

0
4

0

0

0

0

0
4

0
4

0.23992

0.159946
5

0.159946

0

0

0

0

0

0

0

0
4

0.0799732

0

0.0799732

0

0
4

0

0

0

0
4

0

0

0
4

0
4

0

0

0

0

0
4

0
4

0

0

0

0
4

0

0

0
4

0
4

0

0

0

0
4

0
4

0

0

0

0
4

0
4

0

0

0

0
4

0
4

0

0

0

0

0
4

0
4

0.0975327

0.0975327

0.0975327

0
4

0

0

0
4

0
4

0.655566

0.655566

0.559596

0.09597

0
4

0
4

0

0

0

0

0
4

0
4

0

0

0

0
4

0

0

0
4

0
4

3.14316
2

3.08334
2

1.39489

0

0

0

0.585812
2

0.164505

0.119714

0.778544

0

0.0398762

0

0

6.93889390390723e-17
2

0
4

0.0598143

0.0598143

0

0
4

2.70616862252382e-16
2

0
4

0.342783

0.342783

0.342783

0
4

0
4

0

0

0

0

0
4

0
4

0

0

0

0
4

0
4

0

0

0

0

0
4

0
4

0

0

0

0

0
4

0
4

0

0

0

0

0
4

0
4

0

0

0

0

0
4

0
4

0

0

0

0
4

0

0

0
4

0
4

0

0

0

0

0
4

0
4

0

0

0

0
4

0

0

0
4

0
4

1.44446
3

1.35557
3

1.22224
3

0.0666674

0

0

0

0

0.0666674

0

2.77555756156289e-17
3

0
4

0.0888899
3

0.044445

0

0

0

0

0

0.044445

0
4

1.66533453693773e-16
3

0
4

0

0

0

0
4

0
4

0

0

0

0
4

0

0

0
4

0
4

0

0

0

0
4

0

0

0
4

0
4

0

0

0

0
4

0

0

0
4

0
4

0

0

0

0

0
4

0
4

0

0

0

0
4

0

0

0
4

0
4

0

0

0

0
4

0
4

0.138086

0.138086

0.0920573

0.0460286

0
4

0
4

0

0

0

0

0
4

0
4

0.122203

0.0460286

0.0460286

0
4

0.0761741

0.0761741

0
4

1.38777878078145e-17

0
4

0.0423112
4

0
4

0
4

0

0

0

0

0

0

0

0
4

0

0

0

0

0

0

0
4

0

0

0

0

0
4

0

0

0

0
4

0

0

0

0
4

0

0

0

0
4

0.0423112

0.0423112

0
4

0
4

0.079445

0.079445

0.079445

0
4

0

0

0
4

0
4

0

0

0

0

0
4

0
4

0

0

0

0

0
4

0
4

0

0

0

0
4

0

0

0
4

0
4

0

0

0

0
4

0

0

0
4

0
4

0

0

0

0
4

0

0

0
4

0
4

0.512963

0.512963

0.419697

0.093266

0
4

0
4

0

0

0

0

0
4

0
4

0

0

0

0
4

0

0

0
4

0
4

0

0

0

0
4

0
4

31.3245
3

21.9107
3

9.90582
3

7.65549
2

0.842869

0

3.44833

0.0582163

4.23272528138341e-16
3

0
4

5.5233
3

3.97597
3

1.54733

0

0

0
4

0

0

0

0
4

1.52522

0

0

1.52522

0

0
4

2.3652

2.3652

0
4

2.66453525910038e-15
3

0
4

0

0

0

0

0
4

0
4

0.0873245

0.0873245

0.0873245

0
4

0
4

0.079445

0.079445

0.0397225

0.0397225

0
4

0
4

0

0

0

0
4

0

0

0
4

0
4

0

0

0

0

0
4

0
4

0.26667

0.26667

0.26667

0

0
4

0
4

0

0

0

0
4

0
4

0

0

0

0

0
4

0
4

0

0

0

0

0
4

0
4

0

0

0

0

0
4

0
4

4.93879
4

4.93879
4

0.232805
4

4.67273

0

0

0

0

0

0.0332578

0
4

0

0

0
4

0

0

0
4

0
4

0.399866

0.399866

0.399866

0
4

0
4

0

0

0

0
4

0

0

0
4

0
4

0

0

0

0

0
4

0
4

0

0

0

0
4

0
4

0

0

0

0
4

0

0

0
4

0
4

2.23831

2.23831

0

2.23831

0
4

0
4

0.0460286

0

0

0
4

0.0460286

0.0460286

0
4

0
4

0

0

0

0

0
4

0
4

0

0

0

0

0
4

0
4

0

0

0

0
4

0

0

0
4

0
4

67.3461
2

55.6126
2

48.9037
2

0.171697

0.15995

0.045462

0

2.96392

0.141339
3

0.901879

0.833368

0.450937

0.149105

0.825721

0.0655183

0
4

9.83541
2

2.0644
2

0.06398

0.09597

0.229314

0.09597

0.09597

0

0

0

0.28791

0.266609

1.49501

1.82617
2

0.498882
3

1.16932

0.337964

0.688576

0.395431

0.22393

0
4

1.21447

1.21447

0

0
4

0.532984

0.06398

0.309054

0.06398

0.09597

0
4

0.0975327

0.0975327

0
4

0.0530735

0.0530735

0
4

0
4

0.0665157

0.0332578

0

0

0.0332578

0

0
4

0.0332578

0.0332578

0
4

0

0

0
4

0
4

0.0940962

0.0498868

0.0498868

0
4

0.0442094

0.0442094

0
4

6.93889390390723e-18

0
4

0.093266

0.093266

0.093266

0
4

0
4

0

0

0

0
4

0

0

0
4

0
4

0

0

0

0
4

0
4

0

0

0

0
4

0

0

0
4

0
4

0

0

0

0
4

0
4

0

0

0

0
4

0

0

0
4

0
4

0

0

0

0
4

0
4

0

0

0

0
4

0
4

0

0

0

0
4

0
4

0
7

0
7

0
7

0
8

0

0

0

0

0

0

0

0
4

0

0

0

0

0

0
4

0

0

0

0
4

0
4

0

0

0

0
4

0
4

0

0

0

0

0
4

0
4

0.0822615

0.0822615

0.0822615

0
4

0
4

0

0

0

0
4

0
4

0.06398

0.06398

0.06398

0
4

0
4

0

0

0

0
4

0
4

0

0

0

0
4

0
4

0

0

0

0
4

0
4

0

0

0

0
4

0
4

0

0

0

0
4

0
4

4.46726
2

4.29261
2

2.78674
2

0.479674
2

0.793329

0

0.232865

0
4

0.174649

0.174649

0

0
4

0

0

0
4

0

0

0
4

5.55111512312578e-17
2

0
4

1.06147

1.06147

1.06147

0
4

0
4

0

0

0

0
4

0
4

0

0

0

0
4

0
4

0

0

0

0
4

0
4

0

0

0

0
4

0
4

0

0

0

0
4

0
4

0

0

0

0
4

0
4

0

0

0

0
4

0
4

0

0

0

0
4

0
4

0

0

0

0
4

0
4

0
4

0
4

0
4

0

0

0

0

0

0

0

0
4

0
4

0

0

0

0
4

0
4

0

0

0

0
4

0
4

0

0

0

0
4

0
4

0

0

0

0
4

0
4

0

0

0

0
4

0
4

0

0

0

0
4

0
4

0

0

0

0
4

0
4

0

0

0

0
4

0
4

0

0

0

0
4

0
4

0

0

0

0
4

0
4

0

0

0

0

0

0

0

0

0

0
4

0

0

0
4

0
4

0

0

0

0
4

0
4

0

0

0

0
4

0
4

0

0

0

0
4

0
4

0

0

0

0
4

0
4

0.877794

0.877794

0.877794

0
4

0
4

0.326431

0.326431

0.326431

0
4

0
4

0

0

0

0
4

0
4

0

0

0

0
4

0
4

0.0582163

0.0582163

0.0582163

0
4

0
4

0

0

0

0
4

0
4

4.42043
3

4.42043
3

1.484
3

2.71541
3

0.221022

0

0
4

0

0

0

0

0
4

0
4

0

0

0

0
4

0
4

0

0

0

0
4

0
4

0

0

0

0
4

0
4

0

0

0

0
4

0
4

0

0

0

0
4

0
4

0

0

0

0
4

0
4

0.093266

0.093266

0.093266

0
4

0
4

0

0

0

0
4

0
4

0

0

0

0
4

0
4

0

0

0

0
4

0
4

0
4

0
4

0

0

0

0

0

0

0

0

0

0
4

0

0

0

0

0

0

0
4

0

0

0
4

0
4

0

0

0

0
4

0
4

0

0

0

0
4

0
4

0

0

0

0
4

0
4

0

0

0

0
4

0
4

0

0

0

0
4

0
4

0.0423112

0.0423112

0.0423112

0
4

0
4

0

0

0

0
4

0
4

0

0

0

0
4

0
4

0

0

0

0
4

0
4

0

0

0

0
4

0
4

41.7041
3

41.467
3

41.2473
3

0.154137

0

0.0655183

0

0
4

0.0975327

0.0975327

0
4

0.139567

0.139567

0
4

0
4

0

0

0

0
4

0
4

0

0

0

0
4

0
4

0

0

0

0
4

0
4

0

0

0

0
4

0
4

0

0

0

0
4

0
4

0.06398

0.06398

0.06398

0
4

0
4

0

0

0

0
4

0
4

0

0

0

0
4

0
4

0.0515724

0.0515724

0.0515724

0
4

0
4

0

0

0

0
4

0
4

2.38988

2.3259

2.26038

0.0655183

0

0

0

9.71445146547012e-17

0
4

0.06398

0.06398

0
4

0
4

0

0

0

0
4

0
4

0

0

0

0
4

0
4

0

0

0

0
4

0
4

0

0

0

0
4

0
4

0

0

0

0
4

0
4

0

0

0

0
4

0
4

0

0

0

0
4

0
4

0.0648786

0.0648786

0.0648786

0
4

0
4

0

0

0

0
4

0
4

0

0

0

0
4

0
4

0

0

0

0

0

0

0

0

0
4

0

0

0

0

0
4

0

0

0

0
4

0

0

0

0
4

0

0

0
4

0
4

0

0

0

0
4

0
4

0

0

0

0
4

0
4

0

0

0

0
4

0
4

0.06398

0.06398

0.06398

0
4

0
4

0

0

0

0
4

0
4

0

0

0

0
4

0
4

0

0

0

0
4

0
4

0

0

0

0
4

0
4

0

0

0

0
4

0
4

0

0

0

0
4

0
4

24.8682
3

16.3279
3

0
5

0.218474

0.119168

0

0

0

0.0595838

0.0397225

0

0

0.438897

11.0826

0.774589

1.86696

0.218474

0.0397225

0

1.46973

0

0
4

0

0

0

0
4

0

0

0

0

0
4

0.0595838

0.0595838

0

0

0

0
4

0

0

0

0

0
4

0

0

0

0
4

0

0

0

0
4

0

0

0

0
4

0

0

0

0
4

0.31778

0.278058

0.0397225

0
4

0

0

0
4

1.82724

0.456809

0.0397225

0.297919

0.079445

0.15889

0.63556

0.0397225

0.0397225

0

0.079445

2.08166817117217e-16

0
4

0

0

0
4

0

0

0
4

1.35057
3

0.814312

0.0595838

0

0

0.139029

0.0397225

0.079445

0.0397225

0.0397225

0.0397225

0.0993063

0

4.9960036108132e-16
3

0
4

0.754728
3

0.337641

0.31778

0

0

0.0595838

0.0397225

0
4

0.31778
4

0

0

0

0

0

0.079445

0

0

0.238335

0
4

0.575977

0

0.516393

0.0595838

0

1.38777878078145e-17

0
4

0
5

0

0

0

0

0

0
4

0
4

0

0

0

0

0

0
4

3.33669

0

3.25725

0.079445

0

0
4

0
4

1.9443
3

0.271166
3

0.116433
3

0

0

0

0

0.0884188

0.0663141

1.38777878078145e-17
3

0
4

1.67314

0.419989

0.0873245

1.16582

2.22044604925031e-16

0
4

0

0

0

0
4

0
4

0

0

0

0
4

0
4

0

0

0

0
4

0
4

2.65517

2.65517

2.65517

0
4

0
4

0

0

0

0
4

0
4

0

0

0

0
4

0
4

0

0

0

0
4

0
4

0

0

0

0
4

0
4

0.356467

0.356467

0.356467

0
4

0
4

0

0

0

0
4

0
4

0.0761741

0.0761741

0.0761741

0
4

0
4

0
4

0
4

0
4

0

0

0

0
4

0

0

0

0

0

0
4

0
4

0

0

0

0
4

0
4

0

0

0

0
4

0
4

0

0

0

0
4

0
4

0

0

0

0
4

0
4

0

0

0

0
4

0
4

0

0

0

0
4

0
4

0

0

0

0
4

0
4

0

0

0

0
4

0
4

0

0

0

0
4

0
4

0

0

0

0
4

0
4

0

0

0

0

0

0

0

0

0
4

0

0

0
4

0
4

0.0460286

0.0460286

0.0460286

0
4

0
4

0

0

0

0
4

0
4

0

0

0

0
4

0
4

0

0

0

0
4

0
4

0

0

0

0
4

0
4

0

0

0

0
4

0
4

0

0

0

0
4

0
4

0

0

0

0
4

0
4

0.15995

0.15995

0.15995

0
4

0
4

0

0

0

0
4

0
4

0
4

0

0

0

0

0

0

0

0

0
4

0

0

0

0

0

0
4

0

0

0

0
4

0

0

0
4

0
4

0.157873

0.157873

0.157873

0
4

0
4

0

0

0

0
4

0
4

0

0

0

0
4

0
4

0.0975327

0.0975327

0.0975327

0
4

0
4

0.114261

0.114261

0.114261

0
4

0
4

0.0530735

0.0530735

0.0530735

0
4

0
4

0

0

0

0
4

0
4

0

0

0

0
4

0
4

0

0

0

0
4

0
4

0.0582163

0.0582163

0.0582163

0
4

0
4

1.81756
3

0.138086
3

0

0

0.138086

0

0

0

0

0
4

1.67948

1.58742

0.0920573

0

2.77555756156289e-17

0
4

0

0

0
4

0
4

0

0

0

0
4

0
4

0.12796

0.12796

0.12796

0
4

0
4

0

0

0

0
4

0
4

0

0

0

0
4

0
4

0

0

0

0
4

0
4

0

0

0

0
4

0
4

0

0

0

0
4

0
4

0

0

0

0
4

0
4

0

0

0

0
4

0
4

0.116433

0.116433

0.116433

0
4

0
4

0
7

0
7

0
7

0
7

0
4

0
4

0

0

0

0
4

0
4

0

0

0

0
4

0
4

0

0

0

0
4

0
4

0.06398

0.06398

0.06398

0
4

0
4

0

0

0

0
4

0
4

0

0

0

0
4

0
4

0

0

0

0
4

0
4

0

0

0

0
4

0
4

0

0

0

0
4

0
4

0

0

0

0
4

0
4

0
4

0
4

0
4

0

0

0

0

0

0

0
4

0

0

0

0

0
4

0

0

0

0
4

0
4

0

0

0

0
4

0
4

0

0

0

0
4

0
4

0

0

0

0
4

0
4

0

0

0

0
4

0
4

0

0

0

0
4

0
4

0

0

0

0
4

0
4

0

0

0

0
4

0
4

0

0

0

0
4

0
4

0

0

0

0
4

0
4

0

0

0

0
4

0
4

0
4

0
4

0
4

0

0

0

0

0

0
4

0
4

0

0

0

0
4

0

0

0
4

0

0

0
4

0

0

0
4

0
4

0

0

0

0
4

0
4

0

0

0

0
4

0
4

0

0

0

0
4

0
4

0

0

0

0
4

0
4

0

0

0

0
4

0
4

0

0

0

0
4

0
4

0

0

0

0
4

0
4

0

0

0

0
4

0
4

0.0799732

0.0799732

0.0799732

0
4

0
4

0

0

0

0
4

0
4

60.815

31.843

30.6296

0.238831

0.19194

0.266097

0.3199

0.06398

0.0796102

0.0530735

0
4

27.7993

0.44786

27.3514

0
4

0.0530735

0.0530735

0
4

0.3199

0.3199

0
4

0.79975

0.79975

0
4

9.10382880192628e-15

0
4

0

0

0

0
4

0
4

0

0

0

0
4

0
4

0

0

0

0
4

0
4

0

0

0

0
4

0
4

0.0884188

0.0884188

0.0884188

0
4

0
4

0

0

0

0
4

0
4

0

0

0

0
4

0
4

0

0

0

0
4

0
4

0

0

0

0
4

0
4

0

0

0

0
4

0
4

0

0

0

0
7

0

0

0
4

0

0

0

0
4

0

0

0
4

0
4

0

0

0

0
4

0
4

0

0

0

0
4

0
4

0.457045

0.457045

0.457045

0
4

0
4

0

0

0

0
4

0
4

0

0

0

0
4

0
4

0

0

0

0
4

0
4

0

0

0

0
4

0
4

0

0

0

0
4

0
4

0

0

0

0
4

0
4

0

0

0

0
4

0
4

0
4

0
4

0

0

0

0

0

0

0

0

0

0

0

0

0

0

0

0

0

0

0

0

0

0

0

0

0

0

0

0

0

0

0

0

0

0

0

0

0

0

0

0

0

0

0

0

0

0

0

0

0

0

0

0

0

0

0

0
4

0

0

0
4

0

0

0

0

0

0

0
4

0

0

0

0

0
4

0

0

0

0
4

0

0

0

0

0
4

0

0

0
4

0

0

0

0
4

0

0

0
4

0

0

0
4

0
4

0
4

0
4

0

0

0

0

0

0

0
4

0

0

0

0

0
4

0

0

0

0
4

0

0

0

0
4

0
4

0

0

0

0
4

0
4

0

0

0

0
4

0
4

0

0

0

0
4

0
4

0

0

0

0
4

0
4

0

0

0

0
4

0
4

0

0

0

0
4

0
4

0

0

0

0
4

0
4

0

0

0

0
4

0
4

0

0

0

0
4

0
4

0

0

0

0
4

0
4

0.23992
4

0
4

0

0

0

0

0

0
4

0.23992
3

0

0.23992

0

0

0
4

0

0

0
4

0

0

0
4

0
4

0.145541

0.145541

0.145541

0
4

0
4

0

0

0

0
4

0
4

0

0

0

0

0

0

0
4

0

0

0

0
4

0

0

0
4

0
4

0
4

0
4

0
4

0

0

0
4

0

0

0

0

0

0

0
4

0

0

0

0

0

0

0
4

0

0

0
4

0
4

0
4

0
4

0

0

0

0

0

0

0

0

0
4

0

0

0

0

0
4

0

0

0
4

0
4

9.06764
3

0

0

0

0
4

9.06764

7.29554

0.1611

0.230143

0

1.38086

8.88178419700125e-16

0
4

0

0

0
4

0

0

0
4

0

0

0
4

0
4

14.8156
2

14.8156
2

14.8156
2

0

0

0
4

0
4

0.748302
3

0.216176

0.0997735

0.0332578

0

0.0831446

0

0
4

0.182918

0.0332578

0.116402

0

0.0332578

0
4

0.116402

0.0498868

0

0.0665157

0
4

0

0

0
4

0.232805

0.232805

0
4

0

0

0
4

0
4

0

0

0

0

0

0

0

0

0
4

0

0

0

0

0

0

0
4

0
4

0.615699
3

0
4

0
4

0

0

0

0

0

0
4

0.615699

0.0595838

0

0.556115

0
4

0

0

0
4

0
4

6.60119
3

0.595838
3

0
4

0.0397225

0

0

0.417086

0.139029

0

0

0

0

0

0

5.55111512312578e-17
3

0
4

0

0

0
4

0

0

0
4

0

0

0
4

1.25888
3

0.292598
5

0

0

0.0975327

0.673685

0

0

0

0.195065

0

0

0

2.77555756156289e-16
3

0
4

0
4

0
4

0

0

0

0

0

0

0
4

4.24267
3

0
7

4.24267

0

0

0

0

0

0
4

0.225744
5

0

0

0.079445

0

0

0

0.146299

2.77555756156289e-17
5

0
4

0.238335

0

0.0595838

0.119168

0

0.0595838

0
4

0

0

0

0
4

0.0397225

0.0397225

0

0
4

0

0

0
4

0
4

0.41587

0

0

0

0

0
4

0

0

0

0

0

0
4

0

0

0
4

0

0

0
4

0.41587

0.41587

0
4

0
4

0

0

0

0

0

0

0

0

0

0
4

0

0

0
4

0
4

0

0

0

0

0

0

0

0

0

0
4

0

0

0

0

0

0
4

0
4

0
4

0
4

0

0

0

0

0

0
4

0
4

43.7289

43.6649

22.5195

20.4096

0.22393

0.15995

0.25592

0.09597

0
4

0.06398

0.06398

0
4

0
4

0
7

0
7

0
7

0

0

0

0
4

0

0

0
4

0

0

0
4

0

0

0
4

0

0

0
4

0

0

0
4

0

0

0
4

0
4

0
4

0
4

0

0

0

0

0

0

0

0
4

0

0

0

0
4

0
4

0
3

0
3

0
3

0
4

0

0

0
4

0
4

0
4

0

0

0

0

0

0
4

0

0

0

0

0
4

0

0

0

0

0
4

0

0

0
4

0
4

0

0

0

0

0

0

0

0

0
4

0

0

0

0
4

0
4

0
7

0
7

0
7

0

0

0

0

0

0

0

0
7

0

0
7

0
7

0

0

0

0

0
4

0
7

0

0

0

0

0
4

0
4

0
4

0

0

0

0

0

0

0

0
4

0

0

0

0

0
4

0

0

0
4

0
4

24.795

19.6504

19.6504

0
4

0.685949

0.625424

0.0605249

3.46944695195361e-17

0
4

2.84467

0.564899

1.02892

1.25085

4.44089209850063e-16

0
4

1.614

0.403499

1.2105

0
4

3.5527136788005e-15

0
4

4.36591

4.36591

3.0938

1.22608

0.0460286

7.63278329429795e-17

0
4

0
4

0
4

0
4

0
4

0
4

0
4

0
4

0

0

0

0

0

0
4

0

0

0
4

0

0

0
4

0

0

0

0
4

0

0

0
4

0

0

0
4

0
4

1.84716
2

0.884612
2

0.195567

0.406353

0.0332578

0.249434

0
4

0.467418

0.299186

0.0761741

0.0920573

0
4

0.304696

0.304696

0

0
4

0.190435

0.190435

0
4

1.11022302462516e-16
2

0
4

0

0

0

0

0

0

0
4

0

0

0
4

0
4

0
7

0
7

0
7

0

0
4

0

0

0

0
4

0
4

1.74604

1.1474

0.964478

0.182918

0
4

0.415723

0.282692

0.133031

0
4

0.182918

0.182918

0
4

0
4

0
4

0

0

0

0

0

0

0
4

0

0

0

0
4

0
4

31.0587
3

20.0316
3

7.26198
2

3.81317
3

3.0713
2

3.53718

0.897576

1.30987

0.0582163

0.0822615

0
4

10.8282

7.0735

0.331571

0.132628

0

3.29046

1.77635683940025e-15

0
4

0.198942

0

0.198942

0

0

0
4

0

0

0

0

0
4

0

0

0
4

0

0

0
4

0

0

0
4

1.58206781009085e-15
3

0
4

0
4

0

0

0

0

0
4

0

0

0

0

0
4

0

0

0
4

0

0

0
4

0

0

0
4

0
4

0
4

0

0

0

0

0

0

0
4

0

0

0

0

0

0
4

0

0

0
4

0

0

0
4

0

0

0
4

0
4

0

0

0

0

0

0

0

0

0

0
4

0

0

0

0

0
4

0
4

3.10992
3

0.911785
3

0.768849
3

0.0423112

0.0551632

0.045462

0
4

0.315745

0.221022

0.0947236

1.38777878078145e-17

0
4

1.88239

1.88239

0
4

0

0

0
4

0
4

1.6544

0.551468

0

0.259515

0.162197

0

0.129757

0
4

1.10294

1.10294

0

0
4

2.22044604925031e-16

0
4

0.621387
1

0.621387

0.115072

0.253158

0.115072

0.069043

0.069043

0
4

0

0

0
4

0

0

0
4

0

0

0
4

0

0

0
4

0

0

0
4

0
4

0.282692
3

0.249434

0.0831446

0

0.0831446

0.0498868

0.0332578

0
4

0.0332578
4

0

0.0332578

0

0

0

0
4

2.77555756156289e-17
3

0
4

0

0

0

0

0
4

0

0

0

0
4

0

0

0

0

0
4

0
4

0

0

0

0

0

0

0

0

0
4

0
4

0.0975327

0

0

0

0

0

0

0
4

0

0

0
4

0

0

0

0
4

0

0

0
4

0.0975327

0.0975327

0
4

0

0

0
4

0
4

4.99833
3

0
4

0
4

0

0

0

0

0

0

0

0

0

0

0

0

0

0

0
4

4.75841
3

0.0799732
4

0

0

0

0

0

3.83872
3

0.839719

0

0

0

0

0

0

3.33066907387547e-16
3

0
4

0.23992
3

0.23992

0

0
4

0

0

0
4

0
4

7.30153
2

7.30153
2

7.30153
2

0

0

0

0
4

0
4

0
7

0

0

0

0

0

0
4

0

0

0

0

0

0
4

0

0

0

0
4

0
4

0
4

0
4

0

0

0

0

0
4

0

0

0

0

0
4

0

0

0
4

0
4

0
7

0
7

0

0

0

0
4

0
4

0
4

0
4

0

0

0

0

0
4

0

0

0
4

0
4

0
4

0

0

0

0

0

0
4

0

0

0

0

0
4

0

0

0
4

0

0

0
4

0
4

1.47731
3

1.25629
3

0.969307

0

0.28698

0
4

0

0

0
4

0.221022

0.221022

0
4

0
4

0.908617
2

0.908617
2

0.60083

0.146299

0.161488

0

2.77555756156289e-17
2

0
4

0

0

0
4

0
4

1.60002
3

1.60002
3

1.55557
3

0.044445

8.32667268468867e-17
3

0
4

0

0

0

0

0
4

0
4

0
4

0
4

0
4

0

0

0
4

0
4

1.01845

0.38087

0.38087

0

0
4

0
6

0

0

0
4

0.637581

0.438638

0.132628

0.0663141

6.93889390390723e-17

0
4

0

0

0
4

0

0

0
4

0

0

0
4

0
4

718.683
3

717.246
3

261.258
3

54.8631

355.943
3

4.79275

0
7

1.05629

0.218474

0

0

0
7

0

0

0

0

0.093266

0

3.02835

0.576943

0
6

0.15922

0

0

0

0.186532

0

0.0993063

0.0831446

4.88276

0.0530735

0
7

0

1.13209

0

0

0

0.0397225

0

0.216176

0.079445

0

0
7

0

0

0

0.3199

0

0

0

0.06398

0

0.093266

0
7

0

0.146299

0

0

0

0

0

0.975327

0

0

0
7

0

0

0

0

0.0796102

0

0.0993063

0

0.133335

0.106147

0

0

0

0

0

0

0

0

0.0873245

0

0

0
7

0

0

0

0

0

0.207129

0

0

0.0796102

0

25.3426
2

0

0

0

0.0975327

0.337641

0.12041

0

0.195065

0

0

1.18571819029967e-13
3

0
4

0

0
5

0

0
7

0
7

0

0

0

0

0

0

0
4

0

0

0
4

0.15889

0.15889

0
4

0

0

0
4

0

0

0
4

0.196555

0.196555

0
4

0.0761741

0.0761741

0
4

0

0

0
4

0

0

0
4

0

0

0
4

0

0

0
4

0
4

0
4

0
4

0

0

0

0
4

0

0

0
4

0

0

0
4

0.093266

0.093266

0
4

0.633962

0.633962

0
4

0

0

0
4

0

0

0
4

0

0

0
4

0.079445

0.079445

0
4

0
7

0
7

0

0

0
4

0
7

0

0

0

0

0

0

0
4

0

0

0
4

0.15889

0.0595838

0.0397225

0.0595838

0
4

0

0

0
4

0

0

0
4

0.0397225

0

0.0397225

0
4

2.69811950559529e-13
3

0
4

149.431

96.8334

0
3

1.59966

3.38975

0.0799732
4

11.9593

0
6

21.9381

1.10452

0

6.45445

0

16.1898

0

0

0

0

4.55067

0.979293

0.58962

0.46633

1.33305

0.063149

2.09848

0.266609

0

0.304696

0

0.093266

0.228522

0.152348

0

0

0.190435

9.51663

0.0761741

0

0

0

0.139899

0.114261

0

0

0.114261

0

0

0

0.186532

0

0

0.139899

0

0.0498868

0

0.186532

0

0
4

0

0

0

0.152348

0

0.114261

0.139899

0

0.0398762

1.98053

3.21768

0.114261

0.228522

0

0

0

0

0.152348

0.0761741

0.228522

0

1.10452

0

0

0

0.418957

0.190435

0

0

4.11893

0
4

23.1317
3

12.6716
3

3.90573
2

2.447

0.0873245

3.83806

0.0655183

0.116433

0

4.57966997657877e-16
3

0
4

3.46704

2.27075

1.19629

0
4

0.829028

0.341364

0.487663

0
4

0.785921

0.785921

0

0
4

0.876002

0.876002

0
4

0

0

0

0
4

3.05997

0.38087

2.6791

0
4

0

0

0

0
4

13.7113

13.7113

0
4

0

0

0

0
4

1.33305

1.33305

0
4

1.33288

1.33288

0

0
4

0

0

0
4

0

0

0
4

0.0442094

0.0442094

0
4

0

0

0
4

0

0

0
4

0

0

0
4

0

0

0
4

0

0

0
4

0

0

0
4

0

0

0
4

2.63667

2.5605

0

0.0761741

0
4

0

0

0
4

0

0

0
4

0

0

0
4

0

0

0
4

1.10452

1.10452

0
4

0

0

0
4

0
4

0

0

0

0

0
4

0
7

0
7

0
4

0.285701

0.239673

0.0460286

0

0
4

0

0

0

0

0

0
4

0

0

0

0

0
4

0

0

0
4

2.8643754035329e-14

0
4

150.523

150.408

0
6

0.799732
4

0

0

0

0

0

0

0

0

0.106544

0

52.5521
3

0.973159

0

0

0

0.969307

0.392452

0

0.0761741

0

0.186183

0
7

0

0

0.922386

0.792761

0

0

0.326431

0

0

0

0
6

0.138086

0

0

0

0

0

0

0

0

0

21.3619
2

0

0.322201

0

0

0

0

0

0

0

0

0.0799732
4

0

0

0.139899

0

0

0

0.665794

0.0920573

0.373064

0

6.33088
3

0

0

0

0

0.438481

0

0

0

0

0.0460286

0
4

0

0

0

2.18636

0

0

0

0

1.21246

0

0

0

0.943587

0.0460286

0

0

0

0

1.47292

0

2.65808

1.22446
3

0.0460286

0

0

0

0.652862

0

0.189447

0

0

0.897559

0
6

0
3

0

0

0.093266

0.253158

0

0

0.115072

0

0

0.46633

0

0

0

0.069043

0

0.115072

0

0

0

0

0

0
4

0

0

0

0

0

0

8.2074

0.069043

0.0460286

0.114261

0
6

0

0

0

0

0

0

0

0

0

0.139899

0
4

0

0

0.233165

0

0

0

0

0

0

0

0
5

0

0.186532

0.233165

0

0.0799732

0

0

0

0

0

0
7

0

0

0.979293

0.0423112

0

0

0

0

0

0

0

0

0

0

0

0.069043

0

0

0.0352475

0

0

0
4

0

0

0

0

0.093266

0

0

0

0

0

0
7

0

0

0

0

0

0

0

0

0

0.093266

0.559813
4

6.41848
3

0

0

0

0

0

0

0

0

0.186532

0

1.54196

0

0

0

0

0.0460286

1.24818

0

2.52721

0.115072

0

0
4

0

0

0

0

0

0

0

0

0

0

0
6

0

0.069043

0.0460286

0

0

0

0

0

0

0

0
4

0

0

0

0

0

0

0

0

0

0

0

0.093266

0

0

0.190435

0

0

0

0

0

0

0
4

0

0.0761741

0

0

0

0

2.62069

0

0.233165

0

0

0

0

0

0

0.990263

0.0460286

0

0

0

0.138086

0

0

0

0

0

0

0

0

0.0332578

0

0

0

0

0

0

0

0

0

0

0

0.0799732

0

0
7

0
3

0

0

0

0

0

0

0

0

0

0

0
3

0

0

0

0

0

0.25592

0

0

0.0460286

0

0

0

0

0

0

0

0

0

0

0

0

0.179052
3

0

0

0

0

0.093266

0

0

0

0

0.559596

0

0

0

0

0

0

0

0

0

0

0

0
4

0.06398

0

0

0

0

0

0

0

0

0

0
7

0

0

0

0

0.1611

0

0

0

0

0

0

0

0

0

0

0

0

0

0

0

0

0

0

0.0634669

0

0

0.186532

0

0

0

0

0.279798

0
4

0

0

0.115072

0

0.0332578

0

0

0.0460286

0.157873

0.093266

0
4

0

0

0

0

0

0

0.0423112

0

0

0

0

0

0

0

0

0

0

0

0

0

0

0

0.938583
2

0

0

0.279798

0

1.16582

0

0

0

0

0

0

0

0

0

0

0

0

0

0

0

0

0

0

0

0

0

0

0

0.0460286

0

0

0

0

0

0

0

0

0

0

0

0

0

0.0352475

0

0

0.828516

0

0.093266

0.0460286

0

0

0.345215

0

0.186532

0.11996
3

0

0.419697

0

0

0.0423112

0.279798

0

0

0

0

0

0

0

0

0

0

0

0

0

0.190435

0

0
6

0

0

0

0

0.054841

0

0

0

0

0.616832

0

0

0

0

0.0460286

0

0

0.233165

0

0

0

0

0

0

0

0

0

0

0.06398

0

0

0

0.06398

0.230143

0

0.1611

0

0.093266

0

0

0

0

0

0

0
4

0

0

0

0

0

0

0

0

0

0.253158

0
4

0

0

0

0.559596

0.746128

0.0460286

0

0.069043

0

0

0.237274

0

0

0.83174

0

0

0

0.414258

0

0.317334

0

0
4

0

0

0.139899

0

0

0.0920573

0

0

0

0.0460286

1.35785

0

0.1611

0

0

0

0

0

0

0

0

0

0

0

0.230143

0

0

0

0

0.114261

0

0

0.439853

0.0352475

0

0

0

0.069043

0

0

0.0352475

0

0.552344

0
6

0.178803

0.44786

0

0

0

1.74475

0.093266

0

0

0

0

0

0

0

0.093266

0

0

0

0

0

0

0

0.46633

0

0.0460286

0

0

0

0

0

0

0

0.0423112

0

0

0

0

0

0

0

0

0

0

0

0

0

0

0

0

0

0

0

0

0

0.115072

0

0
6

0

0

0

0

0

0

0

0

0.179052

0

0
6

0

0

0.28791

0

0

0

0

2.39966

0

0

0
4

0

0

0

0
4

0.115072

0.115072

0
4

0

0

0

0
4

0

0

0
4

0

0

0
4

0

0

0
4

0

0

0
4

0

0

0
4

0

0

0
4

0
4

4.68202

4.56143

0
7

0.169245

0.0796102

0

0

0

0

0.132684

0

0

0

0
7

0.09597

0

0.0423112

0

0

0

0

0

0.952003

2.24043

0

0.663419

0

0.185757

0
4

0
7

0
7

0

0

0

0
6

0
6

0

0

0

0

0

0

0
4

0

0

0
4

0
7

0

0

0
4

0

0

0

0
4

0

0

0

0

0
4

0.0332578

0.0332578

0
4

0.0873245

0.0873245

0
4

0

0

0
4

0

0

0
4

0

0

0
4

2.22044604925031e-16

0
4

105.134

82.6307
3

76.4181
3

0.735206

0

0

0.275023

0

0

0.232712

0

0

0.0846225

0.0423112

0.0598143

0

0

0.0648786

0.190401

0.0582163

0

0

0

0

0

0

0

0

0

0.359646

0

0

0.571202

0

0

0.0846225

0.190401

0

0.785638

1.94632

0

0.401957

0.129636

0
4

0
7

0
7

0

0

0

0

0

0

0
4

0.83174
3

0.83174

0

0

0

0
4

0

0

0
4

0

0

0
4

0

0

0
4

0

0

0
4

0

0

0
4

0

0

0
4

0

0

0
4

0
7

0
6

0

0
4

3.07482
3

0.429533

1.52266

0.50454

0.618081

0

2.22044604925031e-16
3

0
4

0

0

0

0

0

0
4

4.45355

2.21222

2.24133

0
4

0
7

0
7

0
4

6.84667

6.78115

0.0655183

0

0
4

0

0

0
4

0
7

0

0

0
4

0

0

0

0
4

0
6

0
6

0

0

0

0

0

0

0

0
4

0

0

0

0
4

0

0

0

0
4

0

0

0

0

0

0
4

0

0

0
4

0

0

0

0

0

0
4

0

0

0

0
4

0

0

0

0
4

0

0

0

0
4

0

0

0
4

0

0

0

0

0
4

0
7

0
7

0

0

0

0
7

0

0

0

0

0

0

0

0
4

0

0

0
4

0.0423112

0

0.0423112

0
4

0

0

0

0

0
4

0

0

0

0
4

0.418957

0.304696

0.114261

1.38777878078145e-17

0
4

0

0

0
4

0

0

0
4

0

0

0
4

0

0

0
4

0

0

0

0
4

0
8

0
8

0

0

0

0

0

0

0

0

0
4

0

0

0
4

0.1611

0.1611

0

0
4

0

0

0

0
4

0

0

0

0
4

0

0

0

0
4

0.139567

0.139567

0
4

0

0

0
4

0

0

0
4

0

0

0

0
4

0.712933

0.712933

0
4

0
8

0

0

0

0

0

0

0

0

0
4

0

0

0
4

0

0

0
4

0.390131

0.243832

0.146299

2.77555756156289e-17

0
4

0

0

0
4

0

0

0
4

0.195065

0.0975327

0.0975327

0
4

0

0

0

0
4

0

0

0
4

0.0423112

0.0423112

0
4

0

0

0
4

2.62639
3

0.303363

0.240395

0.154538

0.192719

0.221754

1.51362

0
4

0

0

0
4

0.99169

0.99169

0
4

0

0

0
4

0.0423112

0.0423112

0
4

0.169245

0.169245

0
4

0

0

0
4

0

0

0
4

0

0

0
4

0.79975

0.79975

0
4

0

0

0
4

0

0

0

0

0
4

0

0

0
4

0

0

0
4

0.279798

0.279798

0
4

0

0

0
4

0

0

0
4

0

0

0
4

0

0

0
4

0

0

0
4

0

0

0
4

0

0

0
4

0

0

0

0

0

0
4

0

0

0
4

0

0

0
4

0

0

0
4

0.0398762

0.0398762

0
4

0

0

0
4

0

0

0
4

0

0

0
4

0

0

0
4

0.0582163

0.0582163

0
4

0.186532

0.186532

0
4

0

0

0

0

0

0
4

0

0

0
4

0

0

0
4

0

0

0
4

0

0

0
4

0

0

0
4

0

0

0
4

0

0

0
4

0

0

0
4

0

0

0
4

0

0

0
4

2.31759056390501e-14

0
4

289.012
3

279.701
3

247.982
3

0.747808
2

0.677432
2

0

0.0582163

0.0530735

0

0

0.292598

0

0

0

0

0.608218
3

0

0.371514

0

0

0

0.206289

0

0.15922

0.0598143

0.341364

0
3

0.0398762

0.195065

0

0

0.0548351

0

0.093266

0

0

0.0996905

0.246716
2

0.0884188

0

0

0

0

0.063149

0

0

0

0

0

0.0397225

0.243152

0

0

0

1.08801

0

0

0

0

0.197007
2

0

0.0884188

0.106147

0

0

0

0.119168

0

0

0

0

0

0.106147

0.0442094

0

0

0.138086

0.292598

0

0

0

0.290132

0.0796102

0.0846225

0

0.110524

0.0975327

0

0.159505

0.0548351

0.0975327

0

0.152188
2

0.291904

0

0.0663141

0

0

0

0

0.152348

0

0

0.309466

0

0

0

0.12796

0

0

0.054841

0

0

0

0

2.15021

0

0

0.0582163

0

0.063149

0.0822615

0.0398762

0

0

0.0548351

0

0.458576

1.90435

0.063149

0

0

0

0

0.063149

0

0

0.129757

0

0.0761741

0.137103

0.0663141

0.301593

0.15922

0

0.309466

3.46241

0

0

0.0598143

0

0.0398762

0

0.186532

0.0947236

0.10967

0.0884188

0

0

0.154532

0

0

0

0.054841

0

0

0

0

0

0

0

0.06398

0

0

0

0

0.0460286

0

0

0

0

0

0.386412

0

0.51759
2

0.123962

0.119629

0.130397

0

0.159505

0.246758

0

0.110524

0.127201

0.289144

1.87466

0

0.17447

0.41587

0

0

0.164511

0.15922

0

0

1.36245

0.147854
3

0

0.179443

0

0.334801

0.393327

0

0

0.158436

0.09597

0

0.563582

0

0

0.0663141

0

0.0996905

0.0648786

0.0648786

0

0.0731341

0.344978

0

0.17068

0

0.260644

0.489297

0.110524

0

0.0442094

0

0

0.0663141

0.544226
3

0.213023

0

0

0

0.0884188

0.0884188

0

0

0

0.0797524

0.0797524

0

0.419864

0

0

0.287361

0.114261

0

0

0

0.185757

1.11244347067441e-13
3

0
4

0.582606

0.23342

0.15995

0.09597

0.093266

0
4

0.107594

0.063149

0.044445

0

0
4

0

0

0
4

0

0

0

0

0
4

0

0

0

0

0
4

0.200551

0.200551

0

0
4

0.0397225

0.0397225

0
4

0.553055

0.436623

0.116433

2.77555756156289e-17

0
4

0.352475

0.0881189

0.264357

0
4

0.465731

0.174649

0.291082

0
4

2.20491

2.20491

0
4

0.363745
3

0.112606

0.093266

0.157873

0
4

0.626147

0.571306

0.054841

0
4

0

0

0
4

0

0

0

0
4

0.169245

0.169245

0
4

0

0

0

0
4

0

0

0
4

0

0

0
4

0

0

0
4

0.119168

0.119168

0
4

0

0

0
4

1.13092
2

1.03039

0.0423112

0.0582163

2.28983498828939e-16
2

0
4

0

0

0
4

0

0

0
4

0.116433

0.116433

0
4

0

0

0
4

0.09597

0.09597

0
4

0.38388

0.38388

0
4

0

0

0
4

0

0

0
4

0

0

0
4

0.291904

0.291904

0
4

0

0

0
4

0.0761741

0.0761741

0
4

0

0

0
4

0

0

0
4

0

0

0
4

0

0

0
4

0.232865

0.232865

0
4

0

0

0
4

0

0

0
4

0

0

0
4

0

0

0
4

0.261974

0.174649

0.0873245

0
4

0.0582163

0.0582163

0
4

0.063149

0.063149

0
4

0.0582163

0.0582163

0
4

0

0

0
4

0

0

0
4

0

0

0
4

0

0

0
4

0.0352475

0.0352475

0
4

0

0

0
4

0

0

0
4

0.322051

0.322051

0
4

0

0

0
4

0.0398762

0.0398762

0
4

0

0

0

0

0
4

0

0

0

0

0
4

0.359537

0.131015

0.0761741

0.152348

0
4

0
4

31.433
3

29.9228
3

8.73776
2

0.0481639

0.207129

0.118275

0

0.406763

0

0.70378

0.12041

0

0.0963278

14.0543
2

0.0460286

0

0

0.06398

0

0

0.24082

0

0.12041

0.15995

1.33593
3

0

0

0

0.0460286

0

0

0

0

0

0.0963278

0.347267

0

0.144492

0.0722459

0

0.0963278

0

0.06398

0.329891

0.572998

0.713477

0.250104

0.729658

0
4

0.377437

0.093266

0

0.221022

0

0.063149

1.38777878078145e-17

0
4

0.279527

0.116357

0.16317

0
4

0.853205

0.327691

0.477351

0.0481639

0
4

0

0

0

0
4

0

0

0
4

0

0

0
4

0

0

0
4

0

0

0
4

0

0

0
4

2.77555756156289e-15
3

0
4

3.02556
5

2.95652
5

2.75908
5

0
7

0

0

0

0.155132

0

0

0

0

0

0

0

0

0

0

0

0

0

0

0

0

0

0

0

0

0

0

0.0423112

0

1.66533453693773e-16
5

0
4

0.069043

0.069043

0
4

0

0

0
4

0

0

0
4

0

0

0
4

0

0

0
4

0

0

0
4

0

0

0
4

0
4

203.551
4

31.7805
4

28.6615
4

0
7

0

0

0

0

0

0

0.0799732

0

0

0

0

0

0

0

0

0

0

0

0

0

0

0

0

0

0

0

0

0

0

0

0

0

2.95901
4

0

0

0

0

0

0

0

0

0

0

0
6

0

0

0

0

0

0

0

0

0

0

0
7

0

0

0

0

0

0

0.0799732

0

0

0

0
7

0

0

0

0

0

0

0

0

0

0

0
7

0

0

0

0

0

0

0

0

0

0

0
7

0

0

0

0

0

0

0

0

0

0

0
5

0

0

0

0

0

0

0

0

0

0

0
6

0

0

0

0

0

0

0

0

0

0

2.33146835171283e-15
4

0
4

0
6

0
6

0

0

0

0

0

0

0

0

0

0

0
6

0

0

0

0

0

0

0

0

0

0

0
6

0

0

0

0

0

0

0

0

0

0

0

0

0

0

0

0

0

0

0

0

0

0

0

0

0

0

0

0

0

0

0

0

0

0

0

0

0

0

0

0

0

0

0

0

0

0

0

0

0
4

2.88953
3

0.726214

0.0947236

0.928302

0.979293

0

0.161

0

0

0

0

5.55111512312578e-17
3

0
4

0

0

0
4

0

0

0

0
4

0

0

0
4

3.01698

0.0515724

2.96541

0
4

0

0

0

0
4

0.11996

0.11996

0
4

0

0

0

0
4

0

0

0

0
4

0

0

0

0
4

0

0

0
4

0
4

0
4

0
4

0

0

0

0

0

0
4

0

0

0

0
4

0

0

0

0
4

0

0

0
4

0

0

0
4

0

0

0

0
4

0

0

0

0
4

0

0

0

0
4

0

0

0

0
4

0

0

0
4

0

0

0

0
4

40.5034
2

32.5218

0.309051
3

4.57831

0.69464

0.315745

0.0947236

1.89447

0.0947236

0
4

0

0

0
4

0

0

0

0
4

0

0

0
4

0

0

0
4

0

0

0

0
4

0

0

0

0
4

0

0

0

0
4

0

0

0

0
4

0

0

0
4

0

0

0
4

0

0

0

0

0

0

0

0

0

0

0
4

0

0

0

0
4

0

0

0

0
4

0

0

0

0
4

0

0

0
4

0

0

0
4

1.55948

1.55948

0
4

0.0947236

0.0947236

0
4

0.145541

0.145541

0
4

0

0

0
4

0

0

0
4

0
5

0
5

0

0

0

0

0

0

0

0

0

0
4

0

0

0
4

0

0

0
4

0

0

0
4

0

0

0
4

0

0

0
4

0

0

0
4

0

0

0
4

0

0

0
4

0

0

0
4

0

0

0
4

0

0

0

0

0
4

0

0

0
4

0

0

0
4

0

0

0
4

0

0

0
4

0.0515724

0.0515724

0
4

0

0

0
4

0

0

0
4

0

0

0
4

0

0

0
4

0

0

0
4

0

0

0

0

0

0

0
4

0

0

0
4

0

0

0
4

0.0515724

0.0515724

0
4

0

0

0
4

0

0

0
4

0

0

0
4

0.0481639

0.0481639

0
4

0

0

0
4

0

0

0
4

0

0

0
4

0

0

0

0

0

0
4

0.184115

0.184115

0
4

0

0

0
4

0

0

0
4

0

0

0
4

0

0

0
4

0

0

0
4

0

0

0
4

0

0

0
4

0.0595838

0.0595838

0
4

0

0

0
4

0
5

0
5

0

0
4

0

0

0
4

0

0

0
4

0

0

0
4

0

0

0
4

0

0

0
4

0

0

0
4

0

0

0
4

0

0

0
4

0

0

0
4

0

0

0
4

0

0
5

0

0

0

0

0

0
4

0

0

0
4

0

0

0
4

0

0

0
4

0

0

0
4

0

0

0
4

0

0

0
4

0

0

0
4

0

0

0
4

0

0

0
4

0

0

0
4

0
4

0
4

0

0

0

0

0

0

0

0

0

0

0
4

0

0

0

0

0

0

0

0

0

0

0

0

0

0

0

0

0
4

0

0

0

0
4

0

0

0
4

0

0

0
4

0

0

0
4

0

0

0
4

0

0

0
4

0

0

0
4

0

0

0
4

0

0

0
4

0

0

0
4

0

0

0
4

0
6

0

0

0

0

0

0

0

0

0
4

0

0

0
4

0

0

0
4

0

0

0
4

0

0

0
4

0

0

0
4

0

0

0
4

0

0

0
4

0

0

0
4

0

0

0
4

0

0

0
4

1.15156
3

0.190799

0

0.780261

0.180503

0

0

5.55111512312578e-17
3

0
4

0.399866

0.399866

0
4

0

0

0
4

0

0

0
4

0

0

0
4

0

0

0
4

0

0

0
4

0

0

0
4

0

0

0
4

0

0

0
4

0

0

0
4

0.439853
4

0.439853

0

0

0

0

0

0

0

0
4

0

0

0
4

0

0

0
4

0

0

0
4

0

0

0
4

0

0

0
4

0

0

0
4

0

0

0
4

0

0

0
4

0

0

0
4

0

0

0
4

0.23992
3

0

0

0

0.23992

0

0
4

0

0

0
4

0

0

0
4

0

0

0
4

0

0

0
4

0

0

0
4

0

0

0
4

0

0

0
4

0

0

0
4

0

0

0
4

0
4

0
4

0

0

0

0
4

4.93291
2

4.47176

0.221022

0

0.0822615

0.063149

0.0947236

2.77555756156289e-17
2

0
4

0

0

0

0

0

0

0
4

0
4

0

0

0

0

0

0

0
4

2.75912

2.65598

0

0.103145

1.2490009027033e-16

0
4

0.159946
4

0.159946
4

0

0

0

0

0

0

0

0

0

0

0
4

0

0

0

0

0

0
3

0
4

0

0

0

0

0

0
4

0
4

0
4

0

0

0

0
4

0
4

0

0

0

0

0

0

0
4

0

0

0

0

0

0

0

0
4

0.628335

0.0515724

0.103145

0.157873

0.252596

0

0

0.063149

0
4

0
7

0
7

0

0

0
4

0
4

0
4

0

0
4

0

0

0

0

0
4

0
7

0
7

0
4

0
4

0

0

0

0
4

0

0

0

0

0

0
4

0.799732
4

0.799732
4

0

0

0

0

0
4

0
4

0

0

0

0

0

0

0
4

0

0

0

0
4

0

0

0

0

0
4

0
6

0
6

0
4

0
5

0
5

0
4

17.6815

17.5883

0.093266

2.95596880306448e-15

0
4

0

0

0

0
4

0

0

0
4

0.0799732

0

0

0.0799732

0

0
4

0

0

0

0

0

0
4

0

0

0

0

0

0
4

76.6533

68.5158

0

7.8533

0.284171

0

0

0

0

0

0

2.38697950294409e-15

0
4

0

0

0

0

0
4

0

0

0

0
4

0

0

0

0

0

0
4

0

0

0

0

0
4

0

0

0

0

0
4

0

0

0
4

0

0

0

0
4

0

0

0
4

0

0

0

0
4

0

0

0

0
4

16.3839

11.1285
3

0

2.93571
2

0.214036

0

0.975327

0

0.0975327

0.98736

0.045462

0
4

0

0

0

0

0
4

0

0

0

0
4

0

0

0
4

0

0

0

0

0

0
4

0

0

0

0
4

0.0332578

0.0332578

0

0

0
4

0

0

0

0
4

0

0

0
4

0

0

0

0
4

0

0

0
4

0
4

0
4

0
4

0

0

0

0
4

0.479839

0.479839

0
4

0

0

0

0

0
4

0

0

0

0

0
4

0

0

0

0
4

0

0

0
4

0

0

0

0
4

0

0

0

0
4

0

0

0
4

0

0

0

0
4

0.0799732

0

0.0799732

0

0
4

0
4

0
4

0

0

0

0

0

0

0

0
4

0

0

0

0

0
4

0

0

0

0

0
4

0

0

0

0
4

0.0975327

0

0

0.0975327

0
4

0

0

0
4

0

0

0

0
4

0

0

0

0
4

0

0

0

0
4

0

0

0
4

0

0

0

0
4

0
6

0
6

0
4

0.045462

0.045462

0

0
4

0

0

0
4

0

0

0
4

0

0

0
4

0

0

0

0
4

0

0

0

0
4

0

0

0
4

0

0

0

0
4

0

0

0

0
4

0

0

0
4

9.14268660778816e-14
4

0
4

2.07328

2.07328

1.72205

0
6

0.0530735

0

0.0481639

0

0.114655

0

0

0

0.0822615

0

0.0530735

0

0
4

0
6

0

0

0
4

0

0

0
4

0

0

0
4

0

0

0
4

0

0

0
4

0
4

32.0802
3

29.3477
3

24.464
3

0
4

0.0460286

0

0

0

0.276172

0

0

0

0

0.079445

0
4

0.139029

0.0595838

0

0

0

0

0

0

0.138086

0

0

0.337641

0.1611

0

0

0.0460286

0

0.0993063

0.258196

0.0397225

0

0
4

0.253158

0

0.299186

0

0

0.0397225

0.069043

0

0

0

0

1.15195

1.39029

0

0
4

0
6

0
6

0

0

0

0
4

0.138086

0.138086

0
4

0.126298

0.126298

0
4

0.529803

0.529803

0
4

0.0993063

0.0993063

0
4

0
4

0
4

0
4

1.38086

1.17373

0.207129

0
4

0

0

0

0
4

0

0

0
4

0.458076

0.458076

0
4

0

0

0

0
4

0

0

0
4

0

0

0
4

0
4

81.4306

81.1874

69.8733

0
7

0
6

0.438728

0

0

0.176838

0

0.0822615

0

0

0.190401

0

0

0

0

0

0

0

0

0

0.06398

0

0

0
7

0

0

0.38388

0

0.530735

0.186532

0.849176

0

0

0

0

0

0

0.0442094

0

0

0

0

0

1.13185

0

0

0.06398

0

0

0

0.139899

0

0.19194

0

0

0

0

0

0

0

0

0.246785

0

0

0

0

0

0
5

0

0

0

0

0

0

0

0.06398

0

0.22393

0
7

0
7

0
7

0

0

0.290033

0

0

0

0.15922

0

0

0

0
6

0

0.09597

0

0

0

0

0

0

0.169245

0

0
7

0

0

0.0530735

0

0

0

0

0

0

0

0

0

0

0

0

0

0

0

0

0

0

0
5

0

0.3199

0

0

0

0

0.093266

0

0.093266

0

0
7

0

0

0

0

0

0

0

0

0

0

0

0

0

0

0

0

0

0.190435

0

0.0796102

0.0530735

3.91516

0

0

0

0

0

0

0

0

0

0.792761

0
4

0
7

0
7

0

0

0

0

0

0

0

0

0

0
7

0
8

0
7

0

0

0

0

0

0
4

0

0

0
4

0

0

0
4

0

0

0
4

0.243152

0.243152

0
4

0

0

0
4

0

0

0
4

0

0

0

0

0
4

0

0

0

0

0

0

0

0

0

0
4

0
4

0
4

0

0

0

0
4

0

0

0

0

0
4

0

0

0

0
4

0

0

0
4

0

0

0
4

0

0

0
4

3.05311331771918e-16

0
4

0.0548351

0.0548351

0

0

0

0

0

0

0

0

0

0

0

0

0
6

0

0

0

0

0

0

0

0

0

0

0
7

0

0

0

0

0

0

0

0

0

0

0

0

0

0

0

0

0

0

0

0

0

0

0

0

0

0

0

0

0

0

0

0

0
7

0

0

0

0

0

0

0

0

0

0

0

0

0

0

0

0

0

0

0

0

0

0

0

0

0

0

0

0

0

0

0

0

0

0

0

0

0

0

0

0

0

0

0

0
6

0

0

0

0

0
7

0

0
7

0

0

0
7

0
6

0

0
7

0

0
6

0

0

0

0

0

0

0

0

0

0

0

0

0

0

0

0

0

0

0

0

0

0

0
7

0

0

0

0.0548351

0

0

0

0

0

0

0
7

0

0

0

0

0

0

0

0

0

0

0
7

0

0

0

0

0

0

0

0

0

0

0
7

0

0

0

0

0

0

0

0

0

0

0
6

0

0

0

0

0

0

0

0

0

0

0
4

0
6

0
6

0
4

0

0

0
4

0

0

0
4

0

0

0
4

0

0

0
4

0

0

0
4

0

0

0
4

0

0

0
4

0
4

1074.54
3

1074.23
3

1047.75
3

26.3606
3

0

0.0595838

0.06398

0

3.98570065840431e-14
3

0
4

0

0

0

0
4

0.15995

0.15995

0
4

0.093266

0.093266

0
4

0

0

0
4

0.0530735

0.0530735

0
4

0
4

100.743

100.743

49.7042

0
7

0.146489

0

0

0

0.0498868

0

0

0

0.35988

0

0
6

0.0799732

0

0.11996

0

0

0

0

0

0.045462

0.519826

0.0888899
3

0

0

0

0

0

0

0

0

0

0.045462

0.359494
3

0.166289

0

0.0332578

0

0

0

0

0.159946

0.045462

0

0.241135
3

0

0

0.044445

0.35988

0

0

0

0

0.159946

0.14966

0
7

0

0

0

0

0

0

0

0.239257

0

0

0
7

0

0

0

0

1.50312

0

0.288892

0

0

0

0
7

0

0

0.11996

0

0

0

0

0

0

0

0.302768
3

0

0

0

0

0

0

0

0

0.841046

0

0
6

0.365836

0.0888899

0

0

0

0

0

0

0

0

0.578205
7

0

0

0

0

0

0

0

0

0.0799732

0

0

0.354052
3

0

0

0

0

0

0

0

0.35988

0

0

0

0

0

0

0

0.0398762

0

0

0

0

0

1.28491
3

0

0

0

0.0398762

0

0

0

0

0

0.0997735

0.562702

0

0

0

0

0.0498868

0

0

0

0

0

0
7

0.0665157

0

0

0

0

0

0

0.0799732

0

0

0
7

0

0.0799732

0

0

0.559813

0

0

0.199933

0

0

0.133031

0

0

0

0

0.174649

0.0997735

0

0

0

0

0
4

0

0

0

0.596827

0

0.159946

0

0.415723

0

0

0

1.4795

0

17.2742

0.0665157

0

0

0

0

0

0

0
7

0
7

0

0

0

0

0.199547

0

0

0.0332578

0

0

0.0940962
3

0.11996

0

0.116402

0

0.119629

0

0

0

0

0

0

0

0

0.0799732

0

0

0

0.14966

0

0

0

0
7

0

0

0

0.11996

0

0

0.0831446

0.279906

0

0

0.197317
3

0

0

0

0

0.166289

0.0873245

0

0.0663141

0

0

0

0

0

0

0.0996905

0

0

0.0665157

7.64931

0

0.35988

0

0

0

0

0

0

0

0

0.332578

0

0.200002

0

0

0

0

0

0

0

0

0

0

0

0

0.0884188

0

0

0

0

0

0

0

0

0

0

0

0

0.0332578

0.0332578

0.439853

0

0

0.548754

0

0

0
4

0

0.044445

0

0

0.0598143

0.0799732

0

0

0

0

0

0

0

0

0

0

0

0.559813

0.0873245

0.0663141

0

0

0

0.0498868

0

0

0

0

0

0.0797524

0

0

0

0

0.288892

0.71303

0.127354

0.12759

0
7

0

0

0

0

0

0

0

0

0.209833

1.2889

0
7

0

0

0

0

0.365635

0.11996

0.0799732

0

0.0498868

0

0.335158
3

0

0.767825

0

0

0

0

0

0

0.216176

0

0
7

0

0

0.46561

0

0.0665157

0

0.498868

0.0332578

0.045462

0

0
7

0

0.0831446

0.319893

0

0

0.0665157

0.0799732

0.0332578

0.104259

0

3.00315328161105e-14

0
4

0

0

0
4

0

0

0
4

0

0

0
4

0

0

0
4

0

0

0
4

0

0

0
4

0

0

0
4

0

0

0
4

0
4

136.578

30.997
3

0
6

0
7

0

0

0

0

0

0

0

0

0

0

0
6

0.470233

0

0.52099

0

0

0

0

0

0

0

0
3

0

0

0

0

0

0

0

0

0

0.10967

0
6

0

0

0

0

0

0

0

0

0

0

8.32541
2

0

0

0.329046

0

0

0

0.207129

0

0

0.0761741

0

0

0

0

0

0

0

0.627224

0.319893

0

0

0
4

0

0

0

0

0

0

0

0

0

0

0.22393

0

0

0.287517

0

0

0

0

0

0

0

0

0

0

0.06398

0

0

0

0

0

0

0

0
7

0

0

0

0

0

0

0

0

0

0

5.60453
2

0

0

0

0

0.152348

0

0

0

0

0

0

0
6

0

0

0

0

0

0

0

0

0

0

0
6

0

1.10452

0

0

0

0

0

0

0

0

0
6

0

0

0

0

0

0

0

0.243832

0.146299

0

0

0

0

0

0

0.063149

0

0.0515724

0

0

0

0
6

0

0

0

0

0

0

0

0

0

0.114261

0

0

0

0.0975327

0

0

0

0

0

0

0

0.454462
2

0

0

0

0

0

0

0

0.054841

0.0761741

0

0
6

0

0

0

0.38087

0

0

0

0

0

0

0
7

0

0

0

0

0

0

0

0

0.054841

0

0
7

0

0

0

0.246785

0.146299

0

0

0

0

0

0

0
4

0

0

0

0

0

0

0

0

0

0

0
7

0

0

0

0

0

0

0

0.536254

0

0

0
5

0

0

0

0

0

0

0

0

0

0

0

0

0

0.09597

0

0

0

0

0

0

0

0
7

0

0

0

0

0

0

0

0

0

0

0

0

0

0.06398

0.110326

0

0

0

0

0

0

0

0

0

0

0

0

0

0

0

0

0

0

0

0.0423112

0

0

0

0

0

0

0

0

0

0

0

0

0

0

0

0

0

0.06398

0

0

0
7

0

0

0

0

0

0

0

0

0

0

0

0

0

0

0

0

0

0

0

0

0

0.495132

1.24761

0

0

0

0

0

0

0

0

0

0

0

0

0

0

0

0

0

0

0

0

0

0

0

0

0

0

0

0

0

0

0

0

0

0

0

0

0

0

0

0

0

0.114261

0

0.633962

0

0.0460286

0

0

0.109682

0

0

0.169245

0

0

0

0

0

0

0

0

0

0

0

0

0.862225

0

0

0

0

0

0

0

0

0

0.0663141

0.152348

0

0

0

0

0

0

0

0

0

0.329046

0.309326
4

0
5

0

0

0

0

0

0.266609

0

0

0.0761741

0

0.158436

0

0

0

0

0

0

0

0

0

0

0

0

0

0

0.19194

0

0

0

0

0

0

0.174462

0

0

0

0

0

0

0

0

0

0

0

0

0

0

0

0

0

0.06398

0

0.0822615

0

0

0

0

0

0

0

0.063149

0

0

0

0

0

0

0

0

0

0

0

0

0

0

0

0

0

0

0

0

0

0

0

0

0

0

0

0

0

0

0

0

0

0

0

0

0

0

0

0

0

0

0

0

0

0

0

0

0
7

0.06398

0

0

0

0.146299

0

0

0

0

0.89572

0.12796

0
7

0

0

0

0

0

0

0

0

0

0
7

0

0

0

0
4

0

0

0
4

0
5

0

0

0

0

0

0.411308

0

0.439853

0.221022

0

0.460054
2

0

0.771065

0

0

0

0

0

0

0.381234

0

0
6

0

0

0

0

0

0

0

0

0

0

4.44089209850063e-15
3

0
4

19.6381

6.96283

0

0

0

0

0

0

0

0

0

0

0

0
6

0

0

0

0

0

0

0

0

0

0

0
7

0

0

0

0

0

0

0

0

0

0

0

0

0

0

0

0.114261

0.0761741

0

0

0

0

0

0

0

0

0

0

0

0

0

0

0

0

0

0

0

0

0

0

0

0

0

0

0
7

0

0.139899

0

0

0

0

0

0

0

0

0

0

0

0

0

0

0
7

0
7

0

0

0

0

0

0
6

0

0

0

0

0
7

0

0

0

0

0

0

0

0

0

0

0

0

1.56157

0

0

0

0

0

0

0

0

0.152348
5

0

0

0

0

0

0

0

0

0

0

0
7

0

0

0

0

0

0

0

0

0

0.152348

9.96358

0

0.438897

0

0

0

0

0

0

0

0

0

0

0

0

0

0

0

0

0

0

0

0

0

0

0

0

0.0761741

0

0

0

0

0

0
4

1.69745
2

0.710314
2

0.987138

0

0

0
4

0

0

0

0
4

0

0

0

0
4

0

0

0
4

0

0

0

0
4

0

0

0
4

0

0

0
4

0.223831

0.126298

0.0975327

0
4

8.17131

8.17131

0

0
4

0.228522

0.0761741

0.152348

0
4

0

0

0
4

0
4

0
4

0

0

0

0

0

0

0

0

0

0
4

0

0

0
4

0

0

0

0
4

0

0

0

0
4

0

0

0
4

0

0

0

0
4

0

0

0
4

0

0

0
4

0

0

0
4

0.0666674

0.0666674

0
4

0

0

0
4

0
6

0
6

0

0

0

0

0

0

0

0

0
4

0

0

0
4

0

0

0
4

0

0

0
4

0

0

0
4

0

0

0
4

0

0

0
4

0

0

0
4

0

0

0
4

0

0

0
4

0

0

0
4

4.034
3

0.220182
3

0

1.17165

0.63149

1.32613

0.473618

0

0.063149

0.0846225

0.063149

0
4

0

0

0
4

0

0

0
4

0.0397225

0.0397225

0
4

0

0

0
4

0

0

0
4

0

0

0
4

0

0

0
4

0

0

0
4

0

0

0
4

0

0

0
4

0
6

0
6

0

0

0

0

0

0
4

0

0

0
4

0

0

0
4

0

0

0
4

0

0

0
4

0

0

0
4

0

0

0
4

0

0

0
4

0.0761741

0.0761741

0
4

0

0

0
4

0

0

0
4

0
3

0
3

0

0

0

0

0

0
4

0

0

0
4

0

0

0
4

0

0

0
4

0

0

0
4

0

0

0
4

0

0

0
4

0

0

0
4

0

0

0
4

0

0

0
4

0

0

0
4

6.90037

5.5445

1.21033

0.145541

3.60822483003176e-16

0
4

0

0

0
4

0

0

0
4

0

0

0
4

0

0

0
4

0

0

0
4

0

0

0
4

0

0

0
4

0

0

0
4

0

0

0
4

0.0920573

0.0920573

0
4

7.99464
3

7.15071
3

0

0.0761741

0

0.51184

0

0

0.25592

0

0

0
4

0

0

0
4

0

0

0
4

0

0

0
4

0

0

0
4

0

0

0
4

0

0

0
4

0

0

0
4

0

0

0
4

0

0

0
4

0

0

0
4

1.23241

1.09225

0

0.06398

0

0.0761741

0

0

0
4

0

0

0
4

0

0

0
4

0

0

0
4

0

0

0
4

0

0

0
4

0

0

0
4

0

0

0
4

0

0

0
4

0

0

0
4

0

0

0
4

0
5

0

0

0

0

0

0

0

0

0

0
4

0

0

0
4

0

0

0
4

0

0

0
4

0.131037

0.131037

0
4

0.0873245

0.0873245

0
4

0

0

0
4

0.87673

0.87673

0
4

0

0

0
4

0

0

0
4

0

0

0
4

22.2099

13.4691

0

0

0

0

0.0761741

1.99942

0

0

0

0

0
7

0

0

0

0

0

0

6.66523

0

0

0

0

0

1.77635683940025e-15

0
4

0
4

0
4

0

0

0

0

0

0
4

0

0

0
4

0

0

0
4

0

0

0
4

0

0

0
4

0

0

0
4

0

0

0
4

0

0

0
4

0

0

0
4

0

0

0
4

0

0

0
4

0

0

0

0

0

0

0
4

0

0

0
4

0

0

0
4

0

0

0
4

0

0

0
4

0

0

0
4

0

0

0
4

0
6

0

0

0

0

0

0
4

0
4

0

0

0

0

0

0

0
4

0
4

0

0

0

0

0

0
4

0

0

0

0

0

0

0

0
4

0
4

0

0

0

0

0
4

0

0

0

0

0

0

0
4

0

0

0

0

0
4

0
6

0

0

0

0

0
4

0
4

0
4

0

0

0

0

0

0

0

0

0

0

0

0

0

0

0

0

0

0

0

0

0

0

0

0

0

0

0

0

0

0

0

0

0

0

0

0

0

0

0

0

0

0

0

0

0

0

0

0

0

0

0

0

0

0

0

0

0

0

0

0

0

0

0

0
4

0
6

0
6

0
4

0
7

0

0

0
4

0

0

0

0

0

0
4

0

0

0

0

0
4

0

0

0

0

0
4

0

0

0

0

0

0
4

0

0

0

0
4

1.26134

1.26134

0

0
4

0

0

0

0
4

0.222197

0.222197

0

0

0
4

1.17908

0
7

0

0

0

0

0

0

0

0

0

0

0
6

0

0

0

0

0

0

0

0

0

0

0

0

0

0

1.17908
2

0

0

0

0
4

0

0

0

0

0
4

0.799828

0.38087

0.418957

0

0
4

0

0
7

0

0
4

0

0

0

0

0

0
4

0
7

0

0

0

0

0
4

0

0

0

0

0
4

0

0

0

0

0
4

0

0

0

0

0

0
4

0

0

0

0

0
4

0

0

0

0
4

0
6

0
6

0

0

0

0

0

0

0

0

0
4

1.67944

1.67944

0

0
4

0

0

0

0
4

0.159117

0

0.159117

0
4

0

0

0

0

0
4

0

0

0

0
4

0

0

0

0

0
4

0

0

0

0
4

1.56052

1.46299

0.0975327

0
4

5.347

0

0

5.347

0
4

0

0

0

0

0
4

6.11102

0
6

0

0

0

0

0

0

0

0

0

0

0

1.06644

0

0

0

0

0

0.190435

0

0

0

4.68471

0

0

0.16944

0

0

2.77555756156289e-17

0
4

1.41277

1.29633

0.0582163

0.0582163

0
4

0.747799

0.747799

0
4

7.01507

7.01507

0
4

0.113655

0

0

0.113655

0
4

1.31455

0.430467

0.284171

0.599916

1.11022302462516e-16

0
4

0

0

0

0

0
4

0

0

0

0

0
4

0

0

0

0
4

0

0

0
4

0

0

0

0

0
4

0
6

0
6

0

0

0

0

0
7

0

0

0

0

0

0

0

0
4

0

0

0

0

0
4

0

0

0

0
4

0

0

0
4

0

0

0
4

0

0

0

0

0
4

0

0

0

0
4

0

0

0

0

0
4

0

0

0
4

0

0

0
4

0

0

0

0
4

1.80975

1.80975

0

0

0

0

0

0

0
4

0.0515724

0

0

0.0515724

0
4

0

0

0
4

0

0

0
4

0

0

0

0
4

0.114261

0.114261

0

0
4

0

0

0

0
4

0.442043

0.157873

0.284171

5.55111512312578e-17

0
4

0

0

0

0
4

0.271239

0.195065

0.0761741

1.38777878078145e-17

0
4

0

0

0
4

0
3

0
3

0

0

0

0

0

0

0

0

0

0
4

0

0

0

0
4

0.109682

0.054841

0.054841

0
4

0.06398

0

0.06398

0
4

0

0

0

0
4

0

0

0
4

0.0947236

0.0947236

0

0
4

0

0

0

0
4

0

0

0
4

0

0

0

0
4

0

0

0

0
4

1.04916075827077e-13

0
4

0
4

9.67834
4

8.33483
4

6.09558
4

2.4712
4

0

0

0

0

0

0

0

0

0

0

0

0

0

0

0

0

0

0

0

0

0

0

0

0

0

0

0

0

0

0

0

0

0.129757

0

0

0

0

0

0

0

0

0

0

0

0
4

0

0

0

0

0

0

0

0

0

0

0
4

0.15995

0

0

0

0

0

0

0

0

0

0

0

0

0

0

0

0

0

0

0

0

0
4

0

0

0

0

0

0

0

0

0

0.06398

0

0

0

0

0

0

0

0

0

0

0

0
4

0

0

0

0

0

0

0

0

3.07101
5

0

0

0

0

0

0

0

0

0

0

0

0

0

0

0

0

0

0

0

0

0

0

0

0

0

0

0

0

0

0

0

0

0
6

0

0

0

0

0

0

0

0

0

0

0

0

0

0

0

0

0

0

0

0

0

0

0

0

0

0

0

0

0

0

0

0

0.06398
4

0

0

0

0

0

0.09597

0

0

0

0

0
4

0

0

0

0

0

0

0

0.0397225

0

0

2.77555756156289e-16
4

0
4

0.919692
4

0

0.919692

0

0

0

0

0

0

0
4

0

0

0
4

0

0

0

0

0

0
4

0

0

0

0

0

0
4

0

0

0

0
4

1.31956

1.31956

0

0
4

0

0

0

0
4

0

0

0
4

0

0

0
4

0

0

0
4

0
4

0

0

0

0

0

0

0

0
4

0
4

0

0

0

0

0
4

0
4

0

0

0

0

0
4

0

0

0
4

0
4

0.126298

0.126298

0

0.126298

0
4

0
4

0

0

0

0

0
4

0
4

0

0

0

0
4

0
4

0

0

0

0
4

0
4

0.736458

0.736458

0.736458

0
4

0
4

0

0

0

0
4

0

0

0
4

0

0

0
4

0
4

0

0

0

0
4

0

0

0
4

0
4

0

0

0

0
4

0

0

0
4

0
4

0
4

0
4

0

0

0

0

0

0

0
4

0
4

0

0

0

0
4

0
4

0

0

0

0
4

0

0

0
4

0
4

0

0

0

0
4

0
4

0

0

0

0
4

0
4

0

0

0

0

0
4

0
4

0

0

0

0

0
4

0
4

0

0

0

0
4

0
4

0

0

0

0
4

0
4

0

0

0

0
4

0
4

0

0

0

0
4

0
4

0

0

0

0

0

0

0
4

0

0

0
4

0

0

0
4

0

0

0
4

0
4

0

0

0

0
4

0
4

0

0

0

0
4

0
4

0

0

0

0
4

0
4

0

0

0

0
4

0
4

0

0

0

0
4

0
4

0.0761741

0.0761741

0.0761741

0
4

0
4

0

0

0

0
4

0
4

0

0

0

0
4

0
4

0

0

0

0
4

0
4

0

0

0

0
4

0
4

0
6

0
6

0

0

0

0
4

0
4

0

0

0

0
4

0
4

0

0

0

0
4

0
4

0.06398

0.06398

0.06398

0
4

0
4

0

0

0

0
4

0
4

0

0

0

0
4

0
4

0

0

0

0
4

0
4

0

0

0

0
4

0
4

0

0

0

0
4

0
4

0

0

0

0
4

0
4

0

0

0

0
4

0
4

0

0

0

0

0

0

0
4

0
4

0.195065

0.195065

0.195065

0
4

0
4

0

0

0

0
4

0
4

0.145541

0.145541

0.145541

0
4

0
4

0

0

0

0
4

0
4

0

0

0

0
4

0
4

0

0

0

0
4

0
4

0

0

0

0
4

0
4

0

0

0

0
4

0
4

0

0

0

0

0

0
4

0
4

0

0

0

0
4

0
4

0

0

0

0

0
4

0

0

0
4

0
4

0
4

45.6274

0
5

0
5

0
6

0
5

0
4

0

0

0

0

0

0

0
4

0

0

0
4

0

0

0
4

0

0

0
4

0

0

0
4

0

0

0
4

0

0

0
4

0

0

0
4

0

0

0
4

0

0

0
4

0

0

0
4

0
4

0

0

0

0

0

0

0
4

0

0

0
4

0

0

0
4

0

0

0
4

0

0

0

0
4

0

0

0

0
4

0

0

0

0
4

0

0

0
4

0

0

0

0
4

0

0

0
4

0

0

0
4

0
4

45.6274

0

0
7

0

0

0

0

0

0

0

0

0

0

0

0

0

0

0

0

0

0

0

0

0

0

0
7

0

0

0

0

0

0

0

0

0

0

0
7

0

0

0

0

0

0

0

0

0

0

0
7

0

0

0

0

0

0

0

0

0

0

0

0

0

0

0

0

0

0

0

0

0

0
7

0

0

0

0

0

0

0

0

0

0

0

0

0

0

0
6

0
6

0

0

0

0

0

0
7

0
7

0

0

0
8

0

0

0

0

0

0

0

0

0

0

0
7

0

0

0

0

0

0

0

0

0

0

0
7

0

0

0

0

0

0

0

0

0

0

0

0

0

0

0

0

0

0

0

0

0

0

0

0

0

0

0

0

0

0

0

0

0

0

0

0

0

0

0

0

0

0

0

0
7

0

0

0

0

0

0

0

0

0

0

0
4

0

0
7

0

0

0

0

0

0

0

0

0

0

0
7

0

0

0

0

0

0

0

0

0

0

0
7

0

0

0

0

0

0

0

0

0

0

0
6

0

0

0

0

0

0

0
4

10.1556

0
7

0

0

0
7

0
7

0

0

0

0

10.1556

0

0
7

0

0

0

0

0

0

0

0

0

0

0
8

0

0

0

0

0

0

0

0

0

0

0
8

0

0

0

0

0

0

0

0

0

0

0
7

0

0

0

0

0

0

0
7

0
7

0
7

0
7

0
4

0

0

0

0

0

0

0

0

0

0

0

0

0

0

0

0

0

0

0

0

0
4

33.3512

0
7

31.9517

1.39953

0

0

0

0

0

0

0

0

0

0

0

0

0

0

0

0
4

1.72941

1.72941

0
4

0.391243
7

0
7

0

0.391243

0

0
4

0

0

0

0

0

0

0

0

0
4

0

0

0

0

0

0

0
4

0

0

0
4

0

0

0
4

0

0

0
4

0

0

0
4

0

0

0
4

0

0

0
4

0

0

0
4

3.60822483003176e-15

0
4

0

0

0

0

0

0

0

0

0

0

0
4

0

0

0

0

0
4

0
4

0

0

0

0

0
4

0

0

0
4

0
4

0

0

0

0
4

0
4

0

0

0

0

0
4

0

0

0
4

0
4

0

0

0

0

0

0
4

0
4

0

0

0

0
4

0

0

0
4

0
4

0

0

0

0
4

0

0

0
4

0
4

0

0

0

0
4

0
4

0

0

0

0

0
4

0
4

0

0

0

0
4

0
4

0

0

0

0
4

0

0

0
4

0
4

0
7

0
7

0

0
7

0

0

0

0

0
4

0

0

0
4

0
4

0

0

0

0
4

0
4

0

0

0

0
4

0
4

0

0

0

0
4

0
4

0

0

0

0
4

0
4

0

0

0

0
4

0
4

0

0

0

0
4

0
4

0

0

0

0
4

0
4

0

0

0

0
4

0
4

0

0

0

0
4

0
4

0

0

0

0
4

0
4

0

0

0

0

0
4

0

0

0
4

0
4

0

0

0

0
4

0
4

0

0

0

0
4

0
4

0

0

0

0

0

0
4

0

0

0
4

0
4

0

0

0

0
4

0
4

0
6

0

0

0

0

0
4

0

0

0
4

0

0

0
4

0
4

0

0

0

0

0

0
4

0

0

0

0
4

0
4

0

0

0

0

0
4

0
4

0

0

0

0

0
4

0
4

0
4

21.4593

21.3283

21.3283

8.06136

0

0.114261

0.0655183

0

0

0

0.0655183

0.0761741

0

0

1.55148
1

0.953414

2.68625

0.0423112

4.49427

3.10988

0

0.10783

0
4

0

0

0

0
4

0

0

0

0
4

0

0

0
4

0

0

0
4

0

0

0
4

0

0

0
4

0
4

0

0

0

0
4

0
4

0

0

0

0
4

0
4

0

0

0

0

0
4

0
4

0.0655183

0.0655183

0.0655183

0
4

0
4

0

0

0

0
4

0
4

0

0

0

0
4

0
4

0

0

0

0
4

0
4

0

0

0

0
4

0
4

0

0

0

0
4

0
4

0.0655183

0.0655183

0.0655183

0
4

0
4

0
4

1.96846
5

1.96846
5

1.40864
5

1.40864
5

0

0

0

0

0

0

0

0

0
4

0

0

0

0

0
4

0.559813

0.479839

0.0799732

0

0
4

0

0

0
4

0

0

0

0

0
4

0

0

0
4

0

0

0
4

0
4

0
4

0
4

0
4

0
4

0
4

0

0

0

0

0
4

0

0

0

0

0

0

0

0
4

0
4

0
4

0

0

0

0

0

0
4

0

0

0
4

0
4

0
4

0

0

0

0

0

0
4

0
4

0
4

0

0

0

0

0

0
4

0

0

0

0
4

0
4

0
4

0.233165

0.233165

0.233165

0.093266

0.139899

0
4

0
4

0
4

0

0

0

0

0

0

0
4

0
4

0
4

0

0

0

0

0
4

0
4

0
4

0

0

0

0

0

0
4

0
4

0
4

0

0

0

0

0
4

0
4

0
4

0

0

0

0

0

0
4

0

0

0
4

0
4

0
4

0

0

0

0

0
4

0
4

0
4

17.9215
2

13.6703
2

12.98
2

6.29973

5.02677

0.715719

0.28791

0.06398

0.15995

0.138086

0.15995

0.12796

0
4

0.69026

0.69026

0
4

9.99200722162641e-16
2

0
4

4.25122

3.99218

0.438897

1.60929

0.83174

0.53643

0.57582

1.11022302462516e-16

0
4

0.259045

0.06398

0.195065

0
4

0
4

0
4

0

0

0

0

0
4

0

0

0
4

0
4

0
4

0

0

0

0

0

0

0
4

0
4

0
4

0

0

0

0

0
4

0

0

0
4

0
4

0
4

0

0

0

0

0

0
4

0
4

0

0

0

0
4

0
4

0
4

0

0

0

0

0
4

0
4

0
4

0.063149

0.063149

0

0

0
4

0.063149

0.063149

0
4

0
4

0
4

0

0

0

0

0
4

0
4

0
4

0

0

0

0

0

0
4

0

0

0
4

0
4

0
4

0.225744

0.225744

0.186022

0.186022

0
4

0.0397225

0.0397225

0
4

0
4

0
4

0.119168

0.119168

0.119168

0.0397225

0.0397225

0.0397225

0
4

0
4

0
4

0
7

0
7

0
7

0
7

0
7

0

0
4

0
7

0
7

0

0

0
4

0

0

0

0

0
4

0

0

0

0
4

0
4

0
4

0

0

0

0

0

0
4

0
4

0
4

0

0

0

0

0
4

0

0

0
4

0

0

0
4

0
4

0
4

0

0

0

0

0
4

0

0

0
4

0
4

0
4

0

0

0

0

0

0
4

0
4

0
4

0

0

0

0

0

0
4

0
4

0
4

0

0

0

0

0
4

0
4

0
4

2.56415

2.56415

2.56415

2.56415

0
4

0
4

0
4

0

0

0

0

0
4

0
4

0
4

0

0

0

0

0

0
4

0
4

0
4

0

0

0

0

0
4

0

0

0
4

0
4

0
4

0

0

0

0

0

0

0

0

0

0

0

0

0

0
4

0

0

0
4

0

0

0

0
4

0
4

0

0

0

0

0
4

0
4

0
4

0.157116

0.157116

0.157116

0.157116

0

0
4

0
4

0
4

0

0

0

0

0
4

0

0

0
4

0
4

0
4

0.154073

0.154073

0.154073

0.154073

0

0
4

0
4

0
4

0

0

0

0

0
4

0
4

0
4

0.186532

0.186532

0.093266

0.093266

0

0
4

0.093266

0.093266

0
4

0
4

0
4

0

0

0

0

0
4

0

0

0
4

0
4

0
4

7.16036

7.16036

7.16036

7.16036

0
4

0
4

0
4

0

0

0

0

0

0
4

0
4

0

0

0

0
4

0
4

0
4

0

0

0

0

0
4

0

0

0
4

0
4

0
4

0

0

0

0

0
4

0
4

0
4

5.86194
2

5.86194
2

4.31554
2

0.176838

3.91243

0.15995

0

0

0.0663141

0
4

0.795769

0.0663141

0.729455

0

0

0

0
4

0.750636

0.50564

0.191923

0.0530735

0

0
4

3.33066907387547e-16
2

0
4

0
4

0

0

0

0

0
4

0
4

0
4

0

0

0

0

0
4

0

0

0
4

0

0

0
4

0
4

0
4

0.830159

0.830159

0.830159

0.830159

0
4

0
4

0
4

0

0

0

0

0

0
4

0
4

0
4

0

0

0

0

0

0

0
4

0
4

0
4

0.417969

0.417969

0.417969

0.228522

0.189447

0
4

0
4

0
4

2.41598

2.41598

2.41598

2.41598

0

0
4

0
4

0
4

0

0

0

0

0
4

0
4

0
4

0

0

0

0

0
4

0

0

0
4

0
4

0
4

0

0

0

0

0
4

0
4

0
4

2.12935

2.12935

1.99831

0
6

0
7

0

1.99831

0

0

0
4

0
6

0
6

0

0

0

0
4

0

0

0

0
4

0.0655183

0.0655183

0
4

0

0

0
4

0.0655183

0.0655183

0
4

1.94289029309402e-16

0
4

0
4

0

0

0

0

0
4

0

0

0
4

0
4

0
4

0

0

0

0

0

0
4

0
4

0
4

0

0

0

0

0
4

0

0

0
4

0
4

0
4

0

0

0

0

0

0
4

0
4

0
4

0

0

0

0

0

0
4

0
4

0
4

0

0

0

0

0

0
4

0
4

0
4

0

0

0

0

0
4

0

0

0
4

0
4

0
4

0

0

0

0

0
4

0

0

0
4

0
4

0
4

0.126934

0.126934

0

0

0
4

0.126934

0.126934

0
4

0
4

0
4

0

0

0

0

0
4

0
4

0
4

0
7

0
7

0
7

0
7

0

0

0

0

0
4

0

0

0

0

0

0

0
4

0
4

0
4

0

0

0

0

0
4

0

0

0
4

0
4

0
4

0

0

0

0

0
4

0

0

0
4

0
4

0
4

0

0

0

0

0

0
4

0
4

0
4

0

0

0

0

0

0
4

0
4

0
4

0

0

0

0

0

0
4

0
4

0
4

3.37369

3.37369

3.37369

3.37369

0
4

0
4

0
4

0

0

0

0

0
4

0
4

0
4

0

0

0

0

0
4

0

0

0
4

0
4

0
4

0

0

0

0

0
4

0

0

0
4

0
4

0
4

0

0

0

0

0
4

0
4

0
4

0
7

0
7

0
7

0
7

0

0

0

0

0

0
4

0

0

0

0

0
4

0

0

0
4

0
4

0
4

0

0

0

0

0
4

0

0

0
4

0
4

0
4

3.30881

3.30881

3.30881

3.30881

0
4

0
4

0
4

0

0

0

0

0
4

0

0

0
4

0
4

0
4

0

0

0

0

0

0
4

0
4

0
4

0

0

0

0

0
4

0
4

0
4

0

0

0

0

0

0
4

0
4

0
4

0.195065

0.195065

0.195065

0.0975327

0.0975327

0
4

0
4

0
4

0

0

0

0

0

0
4

0
4

0
4

0

0

0

0

0

0
4

0
4

0
4

0

0

0

0

0
4

0

0

0
4

0
4

0
4

0
7

0
7

0
7

0
7

0

0

0
4

0

0

0
4

0

0

0
4

0
4

0
4

0

0

0

0

0

0
4

0
4

0
4

0

0

0

0

0
4

0
4

0
4

0.0481639

0.0481639

0.0481639

0.0481639

0
4

0
4

0
4

0

0

0

0

0
4

0

0

0
4

0
4

0
4

0

0

0

0

0
4

0
4

0
4

0

0

0

0

0

0
4

0
4

0
4

0

0

0

0

0
4

0

0

0
4

0
4

0
4

0

0

0

0

0
4

0
4

0
4

0

0

0

0

0
4

0
4

0
4

0

0

0

0

0
4

0
4

0
4

0
7

0
7

0
7

0
7

0
7

0

0

0

0
4

0
7

0
7

0

0

0
4

0
4

0
4

0

0

0

0

0
4

0

0

0
4

0
4

0
4

0.12796

0.12796

0.12796

0.06398

0.06398

0
4

0
4

0
4

0

0

0

0

0
4

0
4

0
4

0

0

0

0

0
4

0

0

0
4

0
4

0
4

0

0

0

0

0
4

0
4

0
4

0.0397225

0.0397225

0.0397225

0.0397225

0

0
4

0
4

0
4

0.304696

0.304696

0.228522

0.228522

0
4

0.0761741

0.0761741

0
4

0
4

0
4

0

0

0

0

0
4

0
4

0
4

0.559596

0.559596

0

0

0
4

0.559596

0.559596

0
4

0
4

0
4

0

0

0

0

0
4

0

0

0
4

0
4

0
4

0
4

0
4

0

0

0

0

0

0

0

0

0

0

0

0

0

0

0

0

0

0

0
4

0
4

0

0

0

0

0

0

0

0

0

0

0

0

0

0

0

0
4

0

0

0

0

0

0

0

0
4

0

0

0

0
4

0
4

0

0

0

0
4

0
4

0
4

0

0

0

0

0

0

0

0

0

0

0
4

0

0

0

0

0
4

0
4

0
4

0.152348

0.152348

0.152348

0.152348

0
4

0
4

0
4

4.33167

4.33167

4.33167

4.13973

0.19194

0
4

0
4

0
4

0

0

0

0

0
4

0
4

0
4

0.118022

0.118022

0.118022

0.0418477

0.0761741

0
4

0
4

0
4

0

0

0

0

0

0
4

0
4

0
4

0

0

0

0

0
4

0
4

0
4

0

0

0

0

0
4

0

0

0
4

0
4

0
4

0

0

0

0

0
4

0
4

0
4

0

0

0

0

0

0
4

0
4

0
4

0

0

0

0

0

0
4

0
4

0
4

0
7

0
7

0
7

0
7

0
7

0

0
4

0

0

0

0
4

0
4

0

0

0

0
4

0
4

0
4

0

0

0

0

0
4

0
4

0
4

0

0

0

0

0
4

0
4

0
4

0

0

0

0

0
4

0
4

0
4

0

0

0

0

0
4

0
4

0
4

0

0

0

0

0
4

0
4

0
4

0

0

0

0

0
4

0
4

0
4

0

0

0

0

0
4

0
4

0
4

0

0

0

0

0
4

0
4

0
4

0

0

0

0

0
4

0
4

0
4

1.1996

1.1996

1.1996

1.1996

0
4

0
4

0
4

0

0

0

0
7

0

0

0
4

0

0

0
4

0

0

0
4

0

0

0
4

0
4

0
4

0

0

0

0

0
4

0
4

0
4

0

0

0

0

0
4

0
4

0
4

0.0481639

0.0481639

0.0481639

0.0481639

0
4

0
4

0
4

0

0

0

0

0
4

0
4

0
4

0

0

0

0

0
4

0
4

0
4

0

0

0

0

0
4

0
4

0
4

0

0

0

0

0
4

0
4

0
4

2.65349

2.65349

2.65349

2.65349

0
4

0
4

0
4

0

0

0

0

0
4

0
4

0
4

1.02409

1.02409

1.02409

1.02409

0
4

0
4

0
4

56.1404
2

56.1404
2

34.2948
2

28.4944
2

3.38065

0.06398

0.0761741

0.093266

2.18636

1.24344978758018e-14
2

0
4

13.9747
2

1.06103

12.9137

0

0
4

7.87087

7.87087

0
4

0
4

0
4

0.0655183

0.0655183

0.0655183

0.0655183

0
4

0
4

0
4

0

0

0

0

0
4

0
4

0
4

0.164523

0.164523

0.164523

0.164523

0
4

0
4

0
4

0

0

0

0

0
4

0
4

0
4

0

0

0

0

0
4

0
4

0
4

0

0

0

0

0
4

0
4

0
4

0

0

0

0

0
4

0
4

0
4

0

0

0

0

0
4

0
4

0
4

0

0

0

0

0
4

0
4

0
4

0.0515724

0.0515724

0.0515724

0.0515724

0
4

0
4

0
4

0
4

0
4

0
4

0

0

0

0

0

0

0

0

0

0
4

0
4

0
4

0
4

0
4

0

0

0

0
4

0
4

0
4

0

0

0

0

0
4

0
4

0
4

0

0

0

0

0
4

0
4

0
4

0

0

0

0

0
4

0
4

0
4

0

0

0

0

0
4

0
4

0
4

0

0

0

0

0
4

0
4

0
4

0

0

0

0

0
4

0
4

0
4

0

0

0

0

0
4

0
4

0
4

0

0

0

0

0
4

0
4

0
4

0

0

0

0

0
4

0
4

0
4

0

0

0

0

0
4

0
4

0
4

0
5

0
5

0
5

0
5

0

0

0
4

0
4

0
4

0

0

0

0

0
4

0
4

0
4

0

0

0

0

0
4

0
4

0
4

0

0

0

0

0
4

0
4

0
4

0

0

0

0

0
4

0
4

0
4

0

0

0

0

0
4

0
4

0
4

0

0

0

0

0
4

0
4

0
4

0

0

0

0

0
4

0
4

0
4

0

0

0

0

0
4

0
4

0
4

0

0

0

0

0
4

0
4

0
4

0

0

0

0

0
4

0
4

0
4

0
7

0
7

0
7

0
7

0

0
4

0

0

0
4

0
4

0

0

0

0

0

0
4

0
4

0
4

0

0

0

0

0
4

0
4

0
4

0

0

0

0

0
4

0
4

0
4

0

0

0

0

0
4

0
4

0
4

0.06398

0.06398

0.06398

0.06398

0
4

0
4

0
4

0.877794

0.877794

0.877794

0.877794

0
4

0
4

0
4

0

0

0

0

0
4

0
4

0
4

0

0

0

0

0
4

0
4

0
4

0

0

0

0

0
4

0
4

0
4

0

0

0

0

0
4

0
4

0
4

0

0

0

0

0
4

0
4

0
4

0

0

0

0

0

0

0

0

0

0

0
4

0
4

0
4

0

0

0

0

0
4

0
4

0
4

0

0

0

0

0
4

0
4

0
4

0

0

0

0

0
4

0
4

0
4

0

0

0

0

0
4

0
4

0
4

0

0

0

0

0
4

0
4

0
4

0

0

0

0

0
4

0
4

0
4

0

0

0

0

0
4

0
4

0
4

0

0

0

0

0
4

0
4

0
4

0.291082

0.291082

0.291082

0.291082

0
4

0
4

0
4

0

0

0

0

0
4

0
4

0
4

0

0

0

0

0

0

0

0

0
4

0
4

0
4

0

0

0

0

0
4

0
4

0
4

0.0655183

0.0655183

0.0655183

0.0655183

0
4

0
4

0
4

0

0

0

0

0
4

0
4

0
4

7.82664

7.82664

7.82664

7.82664

0
4

0
4

0
4

0

0

0

0

0
4

0
4

0
4

0

0

0

0

0
4

0
4

0
4

0.0920573

0.0920573

0.0920573

0.0920573

0
4

0
4

0
4

0

0

0

0

0
4

0
4

0
4

0.0397225

0.0397225

0.0397225

0.0397225

0
4

0
4

0
4

0.0993063

0.0993063

0.0993063

0.0993063

0
4

0
4

0
4

0
4

0
4

0
4

0
4

0

0

0

0
4

0
4

0
4

0

0

0

0

0
4

0
4

0
4

0

0

0

0

0
4

0
4

0
4

0.044445

0.044445

0.044445

0.044445

0
4

0
4

0
4

0

0

0

0

0
4

0
4

0
4

0

0

0

0

0
4

0
4

0
4

0.304696

0.304696

0.304696

0.304696

0
4

0
4

0
4

0.0530735

0.0530735

0.0530735

0.0530735

0
4

0
4

0
4

0

0

0

0

0
4

0
4

0
4

0

0

0

0

0
4

0
4

0
4

0

0

0

0

0
4

0
4

0
4

0
4

0
4

0
4

0
4

0

0

0

0

0

0

0

0

0

0

0
4

0

0

0
4

0

0

0

0

0

0

0
4

0
4

0

0

0

0

0

0

0
4

0

0

0

0
4

0

0

0
4

0
4

0
4

0
5

0
5

0
5

0
5

0

0

0

0

0

0

0
4

0

0

0
4

0
4

0
4

0

0

0

0

0
4

0
4

0
4

0

0

0

0

0
4

0
4

0
4

0

0

0

0

0
4

0
4

0
4

0

0

0

0

0
4

0
4

0
4

0

0

0

0

0
4

0
4

0
4

0

0

0

0

0
4

0
4

0
4

0

0

0

0

0
4

0
4

0
4

0.06398

0.06398

0.06398

0.06398

0
4

0
4

0
4

0

0

0

0

0
4

0
4

0
4

0

0

0

0

0
4

0
4

0
4

0
4

0
4

0
4

0

0

0

0

0

0

0

0
4

0
4

0
4

0

0

0

0

0
4

0
4

0
4

0

0

0

0

0
4

0
4

0
4

0.46633

0.46633

0.46633

0.46633

0
4

0
4

0
4

0

0

0

0

0
4

0
4

0
4

0

0

0

0

0
4

0
4

0
4

0

0

0

0

0
4

0
4

0
4

0.079445

0.079445

0.079445

0.079445

0
4

0
4

0
4

0

0

0

0

0
4

0
4

0
4

0

0

0

0

0
4

0
4

0
4

0

0

0

0

0
4

0
4

0
4

0

0

0
6

0

0

0

0
4

0

0

0

0

0
4

0

0

0
4

0

0

0

0
4

0

0

0
4

0

0

0
4

0
4

0
4

0

0

0

0

0
4

0
4

0
4

0

0

0

0

0
4

0
4

0
4

0

0

0

0

0
4

0
4

0
4

0

0

0

0

0
4

0
4

0
4

0

0

0

0

0
4

0
4

0
4

0

0

0

0

0
4

0
4

0
4

0

0

0

0

0
4

0
4

0
4

0

0

0

0

0
4

0
4

0
4

0

0

0

0

0
4

0
4

0
4

0.110326

0.110326

0.110326

0.110326

0
4

0
4

0
4

2.3259

2.3259

2.3259

0
7

0

0

2.3259

0
4

0
4

0
4

0

0

0

0

0
4

0
4

0
4

0.0655183

0.0655183

0.0655183

0.0655183

0
4

0
4

0
4

0

0

0

0

0
4

0
4

0
4

0

0

0

0

0
4

0
4

0
4

0

0

0

0

0
4

0
4

0
4

0

0

0

0

0
4

0
4

0
4

0.15889

0.15889

0.15889

0.15889

0
4

0
4

0
4

0

0

0

0

0
4

0
4

0
4

0

0

0

0

0
4

0
4

0
4

0

0

0

0

0
4

0
4

0
4

15.2639
3

15.2639
3

0
4

0

0

0

0

0
4

15.2639

15.2639

0
4

0

0

0
4

0

0

0

0
4

0
4

0
4

0

0

0

0

0
4

0
4

0
4

0

0

0

0

0
4

0
4

0
4

0

0

0

0

0
4

0
4

0
4

0

0

0

0

0
4

0
4

0
4

0

0

0

0

0
4

0
4

0
4

0.06398

0.06398

0.06398

0.06398

0
4

0
4

0
4

0

0

0

0

0
4

0
4

0
4

0

0

0

0

0
4

0
4

0
4

0

0

0

0

0
4

0
4

0
4

0

0

0

0

0
4

0
4

0
4

0
7

0
7

0
7

0

0

0

0
4

0

0

0

0

0

0
4

0

0

0
4

0
4

0
4

0

0

0

0

0
4

0
4

0
4

0

0

0

0

0
4

0
4

0
4

0

0

0

0

0
4

0
4

0
4

0

0

0

0

0
4

0
4

0
4

0

0

0

0

0
4

0
4

0
4

0

0

0

0

0
4

0
4

0
4

0

0

0

0

0
4

0
4

0
4

0

0

0

0

0
4

0
4

0
4

0

0

0

0

0
4

0
4

0
4

0

0

0

0

0
4

0
4

0
4

0

0

0

0

0

0

0
4

0

0

0

0
4

0
4

0

0

0

0

0
4

0

0

0
4

0
4

0
4

0

0

0

0

0
4

0
4

0
4

0

0

0

0

0
4

0
4

0
4

0

0

0

0

0
4

0
4

0
4

0

0

0

0

0
4

0
4

0
4

0

0

0

0

0
4

0
4

0
4

0

0

0

0

0
4

0
4

0
4

0

0

0

0

0
4

0
4

0
4

0

0

0

0

0
4

0
4

0
4

0

0

0

0

0
4

0
4

0
4

0

0

0

0

0
4

0
4

0
4

0
5

0
5

0
5

0
5

0

0
4

0
4

0
4

0

0

0

0

0
4

0
4

0
4

0

0

0

0

0
4

0
4

0
4

0

0

0

0

0
4

0
4

0
4

0

0

0

0

0
4

0
4

0
4

0

0

0

0

0
4

0
4

0
4

0

0

0

0

0
4

0
4

0
4

0.06398

0.06398

0.06398

0.06398

0
4

0
4

0
4

0

0

0

0

0
4

0
4

0
4

0

0

0

0

0
4

0
4

0
4

0.0975327

0.0975327

0.0975327

0.0975327

0
4

0
4

0
4

0
7

0
7

0

0

0

0
4

0

0

0

0
4

0
4

0
4

0

0

0

0

0
4

0
4

0
4

0

0

0

0

0
4

0
4

0
4

0

0

0

0

0
4

0
4

0
4

0.06398

0.06398

0.06398

0.06398

0
4

0
4

0
4

0

0

0

0

0
4

0
4

0
4

0.64038

0.64038

0.64038

0.64038

0
4

0
4

0
4

0

0

0

0

0
4

0
4

0
4

0

0

0

0

0
4

0
4

0
4

0

0

0

0

0
4

0
4

0
4

0

0

0

0

0
4

0
4

0
4

0
4

0
4

0
4

0

0

0

0
4

0
4

0
4

0
4

0

0

0
4

0

0

0
4

0
4

0
4

0

0

0

0

0
4

0
4

0
4

0

0

0

0

0
4

0
4

0
4

0

0

0

0

0
4

0
4

0
4

0

0

0

0

0
4

0
4

0
4

0

0

0

0

0
4

0
4

0
4

0

0

0

0

0
4

0
4

0
4

0

0

0

0

0
4

0
4

0
4

0

0

0

0

0
4

0
4

0
4

0

0

0

0

0
4

0
4

0
4

0

0

0

0

0
4

0
4

0
4

0
7

0
7

0
7

0
7

0

0
6

0

0

0

0
4

0
7

0
7

0
7

0
7

0
7

0

0

0

0
4

0
4

0
4

1.86323

1.86323

1.55542

1.01159

0

0

0.54383

1.11022302462516e-16

0
4

0.307812

0.243832

0.06398

0
4

0
4

0
4

0

0

0

0

0
4

0
4

0
4

0

0

0

0

0
4

0
4

0
4

0

0

0

0

0
4

0
4

0
4

0

0

0

0

0
4

0
4

0
4

0.847349

0.847349

0.847349

0.847349

0
4

0
4

0
4

0

0

0

0

0
4

0
4

0
4

0

0

0

0

0
4

0
4

0
4

0.093266

0.093266

0.093266

0.093266

0
4

0
4

0
4

0

0

0

0

0
4

0
4

0
4

0

0

0

0

0
4

0
4

0
4

0
7

0
7

0
7

0
6

0

0

0
4

0

0

0
4

0

0

0
4

0
4

0
4

0

0

0

0

0
4

0
4

0
4

0

0

0

0

0
4

0
4

0
4

0.0634669

0.0634669

0.0634669

0.0634669

0
4

0
4

0
4

0

0

0

0

0
4

0
4

0
4

0

0

0

0

0
4

0
4

0
4

0

0

0

0

0
4

0
4

0
4

0

0

0

0

0
4

0
4

0
4

0

0

0

0

0
4

0
4

0
4

0

0

0

0

0
4

0
4

0
4

0

0

0

0

0
4

0
4

0
4

0
7

0
7

0

0

0

0

0
4

0

0

0

0

0
4

0
4

0
4

0

0

0

0

0
4

0
4

0
4

0

0

0

0

0
4

0
4

0
4

0

0

0

0

0
4

0
4

0
4

0

0

0

0

0
4

0
4

0
4

0
6

0
6

0
6

0
6

0

0

0
4

0
4

0
4

0

0

0

0

0

0

0

0
4

0

0

0

0
4

0

0

0
4

0

0

0

0
4

0
4

0

0

0

0
4

0
4

0
4

0
7

0
7

0
7

0
7

0

0
4

0

0

0
4

0

0

0
4

0
4

0
4

0
7

0
7

0
7

0
7

0
4

0
4

0
4

7.64056

7.64056

6.66523

5.33219

0.495132

0.457045

0.304696

0.0761741

0
4

0.975327

0.975327

0
4

3.33066907387547e-16

0
4

0
4

0
4

0
4

0

0

0

0

0
4

0

0

0
4

0
4

0
4

9.1684

9.1684

9.1684

6.99308

2.17532

4.44089209850063e-16

0
4

0
4

0
4

0
5

0
5

0
6

0
6

0

0

0

0

0

0

0

0

0

0

0

0

0

0

0

0

0
4

0
5

0
7

0
6

0

0

0
4

0

0

0

0

0
4

0
4

0
4

7.2888

7.2888

6.88345

2.68955

4.19391

0
4

0.405344

0.259045

0.146299

2.77555756156289e-17

0
4

1.11022302462516e-15

0
4

0
4

0
4

0
4

0
4

0

0

0

0

0
4

0

0

0

0
4

0
4

0
4

0

0

0

0

0

0

0

0
4

0
4

0
4

0
7

0
7

0
7

0

0

0
4

0
4

0
4

2.93246

2.93246

2.93246

2.93246

0

0

0
4

0

0

0
4

0
4

0
4

0

0

0

0

0

0

0

0

0
4

0

0

0
4

0
4

0
4

0.184115

0.184115

0.184115

0

0

0

0.184115

0
4

0
4

0
4

0

0

0

0

0

0

0
4

0
4

0
4

2.7249

2.7249

2.7249

2.7249

0
4

0
4

0
4

0

0

0

0

0

0
4

0
4

0
4

0

0

0
5

0
6

0

0

0

0

0

0

0

0

0

0
4

0
7

0
7

0

0

0
4

0

0

0

0

0

0
4

0

0

0

0
4

0
4

0

0

0

0
4

0
4

0
4

0

0

0

0

0

0

0
4

0

0

0

0
4

0
4

0

0

0

0
4

0
4

0
4

0
4

0
4

0
4

0
4

0

0
4

0
4

0
4

0
7

0
7

0
7

0
7

0

0
4

0
4

0
4

0

0

0

0

0

0
4

0

0

0
4

0
4

0
4

0

0

0

0

0

0
4

0

0

0
4

0

0

0

0
4

0

0

0
4

0
4

0
4

1.14944

1.14944

1.14944

1.10524

0.0442094

1.38777878078145e-17

0
4

0
4

0
4

0
4

0
4

0

0

0

0
4

0

0

0

0
4

0

0

0
4

0
4

0
4

0

0

0

0

0

0

0
4

0
4

0
4

0.063149
4

0.063149
4

0

0

0
4

0

0

0
4

0.063149

0.063149

0
4

0
4

0
4

0
5

0
5

0
5

0
5

0
4

0
4

0
4

0

0

0

0

0

0

0
4

0

0

0
4

0

0

0
4

0
4

0
4

0

0

0

0

0
4

0

0

0

0

0
4

0

0

0
4

0

0

0
4

0

0

0
4

0

0

0
4

0

0

0
4

0

0

0
4

0
4

0
4

0

0

0

0

0

0
4

0
4

0
4

0

0

0

0

0

0

0
4

0

0

0

0
4

0
4

0
4

0

0

0

0

0
4

0
4

0
4

0

0

0

0

0

0
4

0

0

0

0
4

0
4

0
4

0

0

0

0

0

0
4

0
4

0
4

0

0

0

0

0

0

0

0

0
4

0

0

0
4

0
4

0
4

0.06398

0.06398

0.06398

0

0

0.06398

0
4

0
4

0
4

0

0

0

0

0
4

0

0

0

0
4

0
4

0
4

0
7

0
7

0

0

0
4

0

0

0
4

0
4

0
4

0

0

0

0

0

0
4

0

0

0

0
4

0
4

0
4

0

0

0

0

0

0

0

0

0

0

0

0

0

0

0
4

0

0

0

0

0

0

0

0

0
4

0

0

0

0

0

0

0

0

0

0
4

0
4

0
4

0

0

0

0

0

0

0
4

0
4

0
4

0
5

0
5

0
5

0

0

0
4

0
4

0
4

0

0

0

0

0
4

0
4

0
4

0

0

0

0

0
4

0
4

0
4

0

0

0

0

0

0
4

0
4

0
4

0

0

0

0

0
4

0
4

0
4

0

0

0

0

0

0
4

0

0

0
4

0
4

0
4

0

0

0

0

0

0

0
4

0
4

0
4

0

0

0

0

0

0
4

0
4

0
4

0

0

0

0

0

0
4

0

0

0

0
4

0
4

0
4

0
7

0
7

0
7

0
7

0
7

0
7

0

0

0

0

0

0

0
4

0

0

0

0

0
4

0

0

0
4

0
4

0
4

0

0

0

0

0

0

0
4

0
4

0
4

2.8871

2.8871

2.8871

2.62758

0.129757

0.129757

0
4

0
4

0
4

0

0

0

0

0

0
4

0
4

0
4

2.0943

2.0943

2.0943

2.0943

0
4

0
4

0
4

0

0

0

0

0

0
4

0
4

0
4

0.679772

0.679772

0.679772

0.679772

0
4

0

0

0
4

0
4

0
4

0

0

0

0

0
4

0
4

0
4

0

0

0

0

0

0
4

0
4

0
4

0.551643

0.487663

0.341364

0.195065

0.146299

2.77555756156289e-17

0
4

0.146299

0.146299

0
4

0
4

0.06398

0.06398

0.06398

0
4

0
4

0
4

0

0

0

0

0

0

0
4

0
4

0
4

47.5313

0
4

0
4

0

0

0

0
4

0
4

0

0

0

0
4

0
4

0

0

0

0
4

0
4

0.069043

0.069043

0.069043

0
4

0
4

0

0

0

0
4

0
4

0

0

0

0
4

0
4

0

0

0

0
4

0
4

0.079445

0.079445

0.079445

0
4

0
4

0

0

0

0
4

0
4

0

0

0

0
4

0
4

0

0

0

0
4

0
4

0

0

0

0

0
4

0
4

0

0

0

0
4

0
4

0

0

0

0
4

0
4

0

0

0

0
4

0
4

0

0

0

0
4

0
4

0

0

0

0
4

0
4

0

0

0

0
4

0
4

0

0

0

0
4

0
4

0

0

0

0
4

0
4

0

0

0

0
4

0
4

0

0

0

0
4

0
4

0

0

0

0
4

0
4

0

0

0

0
4

0
4

0

0

0

0

0

0
4

0
4

0

0

0

0

0
4

0
4

0

0

0

0
4

0
4

0.326431

0.233165

0.233165

0
4

0.093266

0.093266

0
4

0
4

0

0

0

0
4

0
4

0

0

0

0
4

0
4

47.0563
3

46.5685
2

0.770922
3

17.7517
2

0.29863

0.126934

0

0

0.0423112

0

0

0.232712

0

0

21.7751
2

0.0963278

0.0722459

0.574722

0.0722459

0.0722459

0.0423112

0

0

0.0722459

0

2.77475

0

0.190401

0

0.148089

0

0

0.137357

0

0

0.179669

0.111631

0.105778

0.879757

0.0403499

0
4

0

0

0

0

0

0

0

0

0

0

0
4

0

0

0
4

0

0

0

0
4

0

0

0
4

0

0

0

0
4

0

0

0
4

0

0

0
4

0

0

0
4

0

0

0
4

0

0

0
4

0

0

0
4

0

0

0

0

0

0

0

0
4

0

0

0
4

0.0982775

0.0982775

0
4

0

0

0
4

0

0

0
4

0.0605249

0.0605249

0
4

0

0

0
4

0

0

0
4

0

0

0
4

0

0

0
4

0

0

0
4

0

0

0

0

0
4

0

0

0
4

0

0

0
4

0

0

0
4

0

0

0

0
4

0

0

0

0
4

0

0

0

0

0
4

0

0

0

0

0
4

0.329046

0.329046

0
4

0

0

0

0
4

0
4

7.105427357601e-15

0
4

191.767
3

7.55658

6.44933

1.81869

3.29946

0.25592

0.655566

0.233165

0.186532

2.4980018054066e-16

0
4

0.979293

0.279798

0.559596

0.139899

5.55111512312578e-17

0
4

0.12796

0.12796

0
4

5.82867087928207e-16

0
4

0
6

0
6

0
6

0
4

0
4

0

0

0

0
4

0
4

0

0

0

0
4

0
4

0

0

0

0
4

0
4

0

0

0

0
4

0
4

0

0

0

0
4

0
4

0

0

0

0
4

0
4

0

0

0

0
4

0
4

0

0

0

0
4

0
4

0

0

0

0
4

0
4

0

0

0

0
4

0
4

0

0

0

0

0

0

0

0
4

0

0

0

0
4

0

0

0
4

0
4

0

0

0

0
4

0
4

0

0

0

0
4

0
4

0

0

0

0
4

0
4

0

0

0

0
4

0
4

0

0

0

0
4

0
4

0

0

0

0
4

0
4

0

0

0

0
4

0
4

0

0

0

0
4

0
4

0

0

0

0
4

0
4

0

0

0

0
4

0
4

34.2672

0.0332578

0

0.0332578

0
4

34.234

1.02409

33.2099

0
4

0
4

0.0423112

0.0423112

0.0423112

0
4

0
4

0

0

0

0
4

0
4

0

0

0

0
4

0
4

0

0

0

0
4

0
4

1.72749

1.72749

1.72749

0
4

0
4

0.06398

0.06398

0.06398

0
4

0
4

0

0

0

0
4

0
4

0.06398

0.06398

0.06398

0
4

0
4

0

0

0

0
4

0
4

0

0

0

0
4

0
4

9.11619

9.02292

4.07982

2.00522

1.16582

1.44562

0.326431

7.7715611723761e-16

0
4

0.093266

0.093266

0
4

0
4

0.093266

0.093266

0.093266

0
4

0
4

1.85312

1.85312

1.85312

0
4

0
4

0

0

0

0
4

0
4

0

0

0

0
4

0
4

0

0

0

0
4

0
4

0.0530735

0.0530735

0.0530735

0
4

0
4

55.3172

55.02

54.5976

0.282502

0.139899

1.94289029309402e-15

0
4

0.203879

0.06398

0.139899

0
4

0.093266

0.093266

0
4

3.01147995429574e-15

0
4

1.55694
3

0.630693

0.15995

0.06398

0.06398

0.342783

5.55111512312578e-17

0
4

0

0

0

0
4

0.318441

0.318441

0
4

0

0

0
4

0.60781

0.60781

0
4

0
4

0.913037
2

0.149043

0.0530735

0

0.09597

0
4

0.286198

0.286198

0
4

0.140154

0.0761741

0.06398

0
4

0.297919

0.297919

0
4

0.0397225

0.0397225

0
4

0
4

0

0

0

0

0

0

0
4

0
4

0

0

0

0

0
4

0
4

0
6

0
6

0
6

0
4

0
4

0
4

0
4

0
4

0
4

0

0

0
4

0

0

0
4

0

0

0
4

0
4

0.449601
3

0.384083

0

0.129757

0.0648786

0.189447

2.77555756156289e-17

0
4

0.0655183

0.0655183

0
4

0

0

0
4

0
4

5.31946
3

4.92401

0.0947236

0.342783

4.4865

0
4

0

0

0
4

0.0975327

0.0975327

0
4

0.15889

0.15889

0
4

0.139029

0.139029

0
4

3.88578058618805e-16
3

0
4

0

0

0

0

0
4

0

0

0
4

0
4

0

0

0

0

0
4

0
4

0

0

0

0

0
4

0
4

0.745878

0.682729

0.682729

0
4

0.063149

0.063149

0
4

1.38777878078145e-17

0
4

0

0

0

0
4

0

0

0

0
4

0
4

0

0

0

0

0

0
4

0

0

0
4

0
4

0
4

0
4

0

0

0

0

0

0

0
4

0
4

0
4

0

0

0

0

0

0
4

0

0

0

0
4

0
4

4.46265
3

2.06299
3

0

0.0796102

1.88741

0.09597

0
4

2.39966

2.24179

0.063149

0.0947236

0
4

0

0

0
4

0
4

0.475454

0.315504

0.315504

0

0
4

0.15995

0.15995

0
4

2.77555756156289e-17

0
4

0.129757

0.129757

0.0648786

0

0

0.0648786

0
4

0
4

0
6

0
6

0
6

0
4

0
4

0.297919

0

0

0

0
4

0.297919

0

0.297919

0
4

0
4

0.196839

0.196839

0.137255

0.0595838

0
4

0

0

0
4

0
4

0

0

0

0

0
4

0
4

0.254415

0.254415

0.0761741

0.114261

0.06398

0
4

0

0

0
4

0
4

0.238335

0.238335

0.0993063

0.0397225

0.0993063

0
4

0
4

0

0

0

0

0
4

0

0

0
4

0
4

0

0

0

0

0
4

0
4

1.29174
2

0.401624

0.291954

0.10967

0

2.77555756156289e-17

0
4

0

0

0

0

0
4

0.179571

0.179571

0
4

0.548351

0.548351

0
4

0.162197

0.162197

0
4

8.32667268468867e-17
2

0
4

0

0

0

0

0
4

0

0

0
4

0
4

3.27602

3.27602

3.27602

0
4

0
4

0

0

0

0
4

0
4

0

0

0

0
4

0
4

0

0

0

0
4

0
4

5.62525

5.62525

5.40423

0.221022

0
4

0
4

0

0

0

0
4

0

0

0
4

0
4

0

0

0

0

0
4

0

0

0
4

0
4

0

0

0

0
4

0
4

0

0

0

0
4

0
4

0

0

0

0

0

0

0

0
4

0
4

0

0

0

0
4

0

0

0
4

0
4

0.167682

0.103702

0.06398

0.0397225

0
4

0.06398

0.06398

0
4

0
4

0

0

0

0

0
4

0
4

0

0

0

0

0
4

0
4

0

0

0

0

0
4

0
4

0.0722459

0

0

0
4

0.0722459

0.0722459

0
4

0
4

0

0

0

0

0
4

0
4

0.149043

0.09597

0.09597

0
4

0.0530735

0.0530735

0
4

6.93889390390723e-18

0
4

0.117053

0.06398

0.06398

0
4

0.0530735

0.0530735

0
4

0
4

0

0

0

0
4

0
4

0
4

0

0

0

0

0

0
4

0

0

0
4

0

0

0
4

0

0

0
4

0
4

0.495132

0.495132

0.495132

0
4

0
4

0.132684

0.132684

0.0796102

0.0530735

6.93889390390723e-18

0
4

0
4

0

0

0

0
4

0
4

0

0

0

0

0
4

0
4

0

0

0

0
4

0

0

0
4

0
4

0

0

0

0
4

0

0

0
4

0
4

0

0

0

0
4

0

0

0
4

0
4

0

0

0

0
4

0
4

0

0

0

0
4

0
4

0

0

0

0

0
4

0
4

0.47667

0

0

0

0
4

0.417086

0.278058

0.0993063

0.0397225

0
4

0.0595838

0.0595838

0
4

1.38777878078145e-17

0
4

0.603251

0.603251

0.164523

0.438728

5.55111512312578e-17

0
4

0
4

1.91065

0.106147

0.106147

0
4

1.8045

1.8045

0
4

0
4

0

0

0

0
4

0

0

0
4

0
4

0.0460286

0.0460286

0

0.0460286

0
4

0
4

0

0

0

0
4

0
4

0.21256

0.21256

0.21256

0
4

0
4

0

0

0

0

0
4

0
4

0

0

0

0
4

0
4

0

0

0

0
4

0
4

0

0

0

0
4

0
4

0

0

0

0

0

0
4

0

0

0
4

0

0

0
4

0

0

0

0
4

0
4

0

0

0

0
4

0
4

0

0

0

0
4

0
4

0

0

0

0
4

0
4

0

0

0

0
4

0
4

0

0

0

0
4

0
4

0

0

0

0
4

0
4

0

0

0

0
4

0
4

0.06398

0.06398

0.06398

0
4

0
4

0

0

0

0
4

0
4

0

0

0

0
4

0
4

0.490193
3

0.255405

0.255405

0

0
4

0.234788

0.195065

0.0397225

0

0
4

0

0

0
4

0

0

0
4

0
4

0.284171

0.284171

0.284171

0
4

0
4

0.093266

0.093266

0.093266

0
4

0
4

0

0

0

0
4

0
4

0.09597

0.09597

0.09597

0
4

0
4

0

0

0

0
4

0
4

0

0

0

0
4

0
4

0

0

0

0
4

0
4

0.093266

0.093266

0.093266

0
4

0
4

0

0

0

0
4

0
4

0

0

0

0
4

0
4

50.8753
3

50.0527
3

10.0173
3

1.71079
3

0.975327

0

0

0

0

1.78059

0

0

1.24723

0

22.4006

0.344978

0

0

0

0.146299

0

0

0

0

0.0530735

6.32623

0.186532

0.09597

0

0.093266

0

0

0

0.105778

0

0

0

0

0

0

0

0

0

0

0

0

3.90131

0

0.667462

0

0

2.88657986402541e-15
3

0
4

0
7

0
7

0
4

0

0

0
4

0

0

0
4

0

0

0
4

0

0

0
4

0

0

0
4

0

0

0
4

0.822615

0.822615

0
4

0

0

0
4

0

0

0
4

0

0

0
4

0

0

0
4

0

0

0
4

0

0

0
4

0

0

0
4

0

0

0

0
4

0

0

0
4

0

0

0
4

0

0

0
4

0

0

0
4

0
4

0
4

219.253
4

0
7

0
7

0
7

0

0

0

0

0

0

0
4

0
4

0
7

0
7

0
7

0
4

0

0

0
4

0
4

0

0

0

0

0
4

0
4

0.19194

0.19194

0.06398

0.12796

2.77555756156289e-17

0
4

0
4

0

0

0

0

0
4

0
4

0

0

0

0

0
4

0
4

0

0

0

0

0
4

0
4

0

0

0

0
4

0
4

0

0

0

0

0
4

0
4

0

0

0

0

0
4

0
4

0

0

0

0
4

0

0

0
4

0
4

0

0

0

0
4

0
4

0
4

0
4

0

0

0

0

0

0

0

0

0
4

0

0

0

0
4

0

0

0

0
4

0

0

0
4

0

0

0
4

0
4

0

0

0

0
4

0

0

0
4

0
4

0

0

0

0
4

0

0

0
4

0
4

0

0

0

0
4

0

0

0
4

0
4

0

0

0

0
4

0

0

0
4

0
4

0

0

0

0

0
4

0
4

0

0

0

0
4

0

0

0
4

0
4

0

0

0

0
4

0
4

0

0

0

0

0
4

0
4

0

0

0

0
4

0

0

0
4

0
4

0

0

0

0

0
4

0
4

0
7

0
7

0
7

0

0

0
4

0

0

0

0
4

0

0

0
4

0
4

0

0

0

0
4

0

0

0
4

0
4

0

0

0

0
4

0
4

0

0

0

0
4

0
4

0.079445

0.0397225

0.0397225

0
4

0.0397225

0.0397225

0
4

0
4

0

0

0

0
4

0
4

0

0

0

0
4

0

0

0
4

0
4

0

0

0

0
4

0

0

0
4

0
4

0

0

0

0
4

0
4

0

0

0

0

0
4

0
4

0

0

0

0
4

0

0

0
4

0
4

0
4

0
4

0

0

0

0

0

0
4

0

0

0

0
4

0

0

0

0
4

0
4

0

0

0

0
4

0
4

0

0

0

0
4

0
4

0.254674

0.15995

0.15995

0
4

0.0947236

0.0947236

0
4

0
4

0

0

0

0
4

0
4

0

0

0

0
4

0
4

0

0

0

0
4

0
4

0

0

0

0
4

0

0

0
4

0
4

0

0

0

0
4

0

0

0
4

0
4

0

0

0

0
4

0

0

0
4

0
4

0

0

0

0
4

0

0

0
4

0
4

0
7

0
7

0
7

0

0
4

0

0

0
4

0
4

0

0

0

0
4

0

0

0
4

0
4

0

0

0

0
4

0
4

0

0

0

0
4

0

0

0
4

0
4

0.326431

0.326431

0.326431

0
4

0

0

0
4

0
4

0

0

0

0
4

0
4

0

0

0

0
4

0
4

0

0

0

0
4

0
4

0

0

0

0
4

0
4

0

0

0

0
4

0
4

0

0

0

0
4

0
4

0
7

0
6

0

0

0

0

0

0

0
4

0

0

0
4

0

0

0
4

0

0

0
4

0
4

0

0

0

0
4

0
4

0

0

0

0
4

0
4

0

0

0

0
4

0
4

0

0

0

0
4

0
4

0

0

0

0
4

0
4

0

0

0

0
4

0
4

0

0

0

0
4

0
4

0

0

0

0
4

0
4

0

0

0

0
4

0
4

0

0

0

0
4

0
4

0

0

0

0

0

0

0

0
4

0

0

0

0

0

0
4

0

0

0

0

0
4

0
4

0

0

0

0
4

0
4

0

0

0

0
4

0
4

0

0

0

0
4

0
4

0

0

0

0
4

0
4

0

0

0

0
4

0
4

6.25176

6.25176

6.25176

0
4

0
4

0

0

0

0
4

0
4

0

0

0

0
4

0
4

0

0

0

0
4

0
4

0

0

0

0
4

0
4

0
5

0

0

0

0

0
4

0

0

0

0
4

0

0

0
4

0

0

0
4

0

0

0
4

0

0

0
4

0
4

0

0

0

0
4

0
4

0

0

0

0
4

0
4

0

0

0

0
4

0
4

0

0

0

0
4

0
4

0

0

0

0
4

0
4

0

0

0

0
4

0
4

0

0

0

0
4

0
4

0

0

0

0
4

0
4

0

0

0

0
4

0
4

0

0

0

0
4

0
4

0
4

0
4

0
6

0

0

0

0
4

0

0

0

0

0
4

0
4

0

0

0

0
4

0
4

0

0

0

0
4

0
4

0

0

0

0
4

0
4

0

0

0

0
4

0
4

0.15922

0.15922

0.15922

0
4

0
4

0

0

0

0
4

0
4

0

0

0

0
4

0
4

0

0

0

0
4

0
4

0

0

0

0
4

0
4

0

0

0

0
4

0
4

0
4

0
4

0

0

0

0

0

0

0
4

0

0

0

0
4

0
4

0.0530735

0.0530735

0.0530735

0
4

0
4

0

0

0

0
4

0
4

0

0

0

0
4

0
4

0

0

0

0
4

0
4

0

0

0

0
4

0
4

0

0

0

0
4

0
4

0

0

0

0
4

0
4

0

0

0

0
4

0
4

0

0

0

0
4

0
4

0

0

0

0
4

0
4

0.159946
4

0.159946
4

0.159946
4

0

0

0

0

0
4

0

0

0

0

0
4

0

0

0

0
4

0

0

0

0
4

0

0

0

0
4

0

0

0
4

0

0

0
4

0
4

0
4

0
4

0
4

0

0

0
4

0

0

0

0
4

0

0

0
4

0
4

0

0

0

0
4

0
4

0

0

0

0
4

0
4

0.0947236

0.0947236

0.0947236

0
4

0
4

0.0332578

0.0332578

0.0332578

0
4

0
4

0

0

0

0
4

0
4

0

0

0

0
4

0
4

0

0

0

0
4

0
4

0

0

0

0
4

0
4

0

0

0

0
4

0
4

0

0

0

0
4

0
4

9.03874

8.36195

8.24551

0.0582163

0.0582163

0
4

0.553055

0.145541

0.0582163

0.349298

0
4

0.123735

0.0655183

0.0582163

0
4

1.63757896132211e-15

0
4

0

0

0

0
4

0
4

0

0

0

0
4

0
4

0

0

0

0
4

0
4

0

0

0

0
4

0
4

0

0

0

0
4

0
4

0.685567

0.685567

0.685567

0
4

0
4

0

0

0

0
4

0
4

0

0

0

0
4

0
4

0.093266

0.093266

0.093266

0
4

0
4

0

0

0

0
4

0
4

0

0

0

0

0

0

0

0

0
4

0

0

0

0

0
4

0
4

0

0

0

0
4

0
4

0

0

0

0
4

0
4

0.06398

0.06398

0.06398

0
4

0
4

0

0

0

0
4

0
4

0

0

0

0
4

0
4

0

0

0

0
4

0
4

0

0

0

0
4

0
4

0

0

0

0
4

0
4

0

0

0

0
4

0
4

0

0

0

0
4

0
4

0.06398
4

0

0

0

0

0

0
4

0

0

0

0

0
4

0

0

0
4

0.06398

0.06398

0
4

0
4

0.139899

0.139899

0.139899

0
4

0
4

0

0

0

0
4

0
4

0

0

0

0
4

0
4

0

0

0

0
4

0
4

0

0

0

0
4

0
4

0

0

0

0
4

0
4

0

0

0

0
4

0
4

0

0

0

0
4

0
4

0

0

0

0
4

0
4

0

0

0

0
4

0
4

0
4

0

0

0

0

0
4

0

0

0

0
4

0

0

0
4

0

0

0
4

0
4

0

0

0

0
4

0
4

0

0

0

0
4

0
4

0

0

0

0
4

0
4

0

0

0

0
4

0
4

0

0

0

0
4

0
4

0

0

0

0
4

0
4

0

0

0

0
4

0
4

0.063149

0.063149

0.063149

0
4

0
4

0

0

0

0
4

0
4

0

0

0

0
4

0
4

0
4

0
4

0
4

0

0
4

0

0

0
4

0

0

0
4

0
4

0

0

0

0
4

0
4

0

0

0

0
4

0
4

0

0

0

0
4

0
4

0.06398

0.06398

0.06398

0
4

0
4

0.09597

0.09597

0.09597

0
4

0
4

0

0

0

0
4

0
4

0

0

0

0
4

0
4

0

0

0

0
4

0
4

0

0

0

0
4

0
4

0

0

0

0
4

0
4

0

0

0

0

0
4

0

0

0

0
4

0

0

0
4

0

0

0
4

0
4

0

0

0

0
4

0
4

0

0

0

0
4

0
4

0

0

0

0
4

0
4

0

0

0

0
4

0
4

0

0

0

0
4

0
4

0

0

0

0

0

0
4

0

0

0
4

0

0

0
4

0
4

0
4

0

0

0

0
4

0

0

0

0
4

0

0

0
4

0

0

0
4

0

0

0
4

0
4

0

0

0

0

0

0

0

0

0
4

0

0

0
4

0

0

0
4

0

0

0
4

0
4

0
4

0
4

0
4

0

0

0

0

0

0

0
4

0

0

0

0
4

0
4

0

0

0

0
4

0

0

0

0

0
4

0

0

0

0
4

0

0

0
4

0
4

2.25193
3

2.25193
3

1.99933

0

0.252596

0

0

0
4

0
4

0.57544
3

0.31952

0.279798

0.0397225

0

0
4

0.12796

0.06398

0.06398

0
4

0.12796

0.12796

0
4

0
4

0.815681

0.145541

0.0582163

0.0873245

0
4

0.0332578

0

0.0332578

0
4

0.636882

0.636882

0
4

0

0

0
4

0

0

0
4

0

0

0
4

0
4

0
4

0
4

0

0

0

0

0

0
4

0
4

0

0

0

0

0

0

0

0
4

0

0

0
4

0

0

0
4

0
4

0.859445
3

0.763475

0.763475

0

0
4

0

0

0

0
4

0.09597

0.09597

0
4

0

0

0
4

0
4

0

0

0

0

0

0
4

0
4

0.564766

0.326431

0.326431

0
4

0

0

0
4

0.0595838

0.0595838

0
4

0

0

0
4

0.178751

0.178751

0
4

0

0

0
4

0
4

0
6

0

0

0
4

0

0

0
4

0
4

0
7

0
7

0
7

0
6

0

0

0

0

0

0
4

0
4

0

0

0

0

0

0
4

0

0

0
4

0

0

0
4

0
4

0

0

0

0

0

0
4

0
4

2.24432
3

0
3

0
3

0
4

2.24432

2.24432

0
4

0

0

0
4

0
4

0

0

0

0

0

0
4

0

0

0

0

0
4

0

0

0
4

0
4

0
5

0

0

0
4

0

0

0
4

0

0

0
4

0
4

0

0

0

0

0
4

0
4

0

0

0

0

0
4

0

0

0

0

0
4

0
4

0

0

0

0

0
4

0

0

0
4

0

0

0
4

0
4

0
4

0

0

0
4

0

0

0
4

0

0

0
4

0

0

0
4

0

0

0
4

0
4

0
4

0

0

0

0
4

0

0

0
4

0

0

0
4

0

0

0
4

0
4

0.74359
4

0.251139
4

0.0947236

0

0

0

0

0

0.063149

0.093266

0
4

0.266197
4

0

0.139899

0

0

0.126298

2.77555756156289e-17
4

0
4

0.093266

0

0.093266

0
4

0.132988

0

0.0397225

0.093266

0
4

0

0

0
4

0

0

0

0
4

0

0

0
4

0

0

0
4

0

0

0
4

0
4

0

0

0

0

0
4

0
4

0

0

0

0
4

0

0

0
4

0

0

0
4

0

0

0
4

0

0

0
4

0
4

0
4

0

0

0

0
4

0

0

0
4

0
4

0

0

0

0

0
4

0

0

0
4

0

0

0
4

0

0

0
4

0
4

0

0

0

0

0
4

0

0

0
4

0

0

0
4

0

0

0
4

0
4

0

0

0

0

0
4

0
4

0.0975327

0.0975327

0

0

0.0975327

0
4

0

0

0
4

0
4

0

0

0

0

0
4

0

0

0
4

0
4

0

0

0

0

0

0

0
4

0

0

0
4

0
4

0

0

0

0

0
4

0

0

0
4

0
4

0
4

0
4

0
4

0

0

0

0

0
4

0

0

0

0

0
4

0
4

0.545774

0.156415

0.063149

0.093266

0
4

0.331143

0.232865

0.0982775

2.77555756156289e-17

0
4

0.0582163

0.0582163

0
4

0
4

0

0

0

0

0
4

0

0

0
4

0

0

0
4

0
4

0

0

0

0

0
4

0

0

0
4

0

0

0
4

0
4

0

0

0

0

0
4

0

0

0

0
4

0
4

0.132988

0

0

0

0
4

0.0397225

0.0397225

0
4

0.093266

0.093266

0
4

0
4

0

0

0

0
4

0

0

0
4

0

0

0
4

0
4

0

0

0

0
4

0

0

0
4

0

0

0
4

0
4

0

0

0

0

0
4

0
4

0

0

0

0
4

0

0

0
4

0
4

0

0

0

0
4

0

0

0
4

0

0

0
4

0

0

0
4

0
4

0
6

0
7

0
7

0

0

0

0
4

0
6

0
6

0

0

0

0
4

0
4

0

0

0

0
4

0

0

0
4

0

0

0
4

0
4

0

0

0

0
4

0
4

0

0

0

0
4

0

0

0
4

0

0

0
4

0
4

0

0

0

0
4

0

0

0
4

0
4

0

0

0

0

0
4

0

0

0
4

0
4

0.716842

0.576943

0.512963

0.06398

0
4

0.139899

0.139899

0
4

5.55111512312578e-17

0
4

0

0

0

0
4

0
4

0.063149

0.063149

0.063149

0
4

0

0

0
4

0

0

0
4

0
4

0

0

0

0

0
4

0

0

0
4

0
4

0

0

0

0

0
4

0
4

0
7

0
7

0
7

0

0

0

0
4

0

0

0
4

0
4

0

0

0

0
4

0
4

0.326431

0.326431

0.186532

0.139899

0
4

0
4

0

0

0

0
4

0

0

0
4

0
4

0

0

0

0
4

0

0

0
4

0
4

0.373064

0.233165

0.233165

0
4

0.139899

0.139899

0
4

0

0

0
4

2.77555756156289e-17

0
4

0

0

0

0

0
4

0

0

0
4

0
4

0

0

0

0

0
4

0
4

0

0

0

0
4

0

0

0
4

0
4

0

0

0

0

0
4

0

0

0
4

0
4

0

0

0

0

0
4

0

0

0
4

0
4

0.234788
4

0

0

0

0

0
4

0

0

0

0

0
4

0

0

0

0
4

0.195065

0.0975327

0.0975327

0
4

0

0

0
4

0

0

0
4

0.0397225

0.0397225

0
4

0
4

0

0

0

0

0
4

0

0

0
4

0
4

0

0

0

0
4

0

0

0
4

0
4

0

0

0

0
4

0
4

0

0

0

0

0
4

0

0

0
4

0
4

0

0

0

0
4

0
4

0

0

0

0

0

0
4

0
4

0

0

0

0
4

0

0

0
4

0

0

0
4

0
4

1.08368

1.02409

1.02409

0
4

0.0595838

0.0595838

0
4

0

0

0
4

0
4

0

0

0

0

0
4

0

0

0
4

0
4

0

0

0

0

0
4

0
4

189.711
4

179.6
4

150.656
4

13.8848
4

8.42457
4

0
7

0

0

0

0

0

0

0

0

0

0

0

0

0.063149

0

0

0

0

0

0

0

0

0

0
7

0

0

0

0

0

0

0

0

0

0

0
6

0

0

0

0.159946

0

0

0

0

0

0

0
7

0

0

0

0

0

0

0

0

0

0

0
7

0

0

0

0

0

0.126298

0

0

0

0

0
7

0

0

0

0

0.284171

0

0

0

0

0

0

0

0

0

0

0

0

0

0

0

0

0
6

0

0

0

0

0

0

0

0.093266

0

0

0
7

0

0

0

0

0

0

0

0

0

0

0
7

0
4

0

0.0975327

0

0

0

0

0

0

0

0

0
7

0

0

0

0

0.093266

0.0595838

0.09597

0.0655183

0

0

0
6

0

0

0

0

0

0

0

0

0

0

0
7

0

0

0

0

0

0

0

0

0

0

0
7

0

0.063149

0

0

0

0

0

0

0

0

0.719759
3

0

0

0

0

0

0

0

0

0

0

0
6

0

0

0

0

0

0

0

0

0

0

0

0

0

0

0

0

0

0

0

0

0

0
6

0

0

0

0

0

0

0

0

0.063149

0

0
6

0

0

0

0

0

0

0

0

0

0

0
7

0

0

0

0

0

0

0

0

0

0

0

0
6

0.232865

0

0

0.294833

0

0

0

0

0

0

0

0

0

0

0

0

0

0

0

0

0

0.206271
3

0

0

0

0

0

0

0

0

0

0

0
4

0

0.157873

0

0

0

0

0

0

0

0

0.183109

0

0.0655183

0

0

0

0

0

0

0

0

0
6

0

0

0

0

0

0

0

0

0

0.252596

0
7

0

0

0

0

0

0

0

0

0

0.063149

0
7

0

0

0

0

0.063149

0

0

0

0

0

0.334643

0

0

0

0

0

0

0

0

0

0

0
4

0
7

0

0

0.0947236

0

0

0

0

0

0

0

0

0

0

0

0

0

0

0.568341

0

0
7

0

0

0.183109

0

0

0

0

0

0

0

1.31748
3

0

0

0

0

0

0

0

0

0

0

0
7

0.34732

0

0

0

0

0

0

0

0

0

0
7

0

0

0.284171

0

0

0

0

0

0

0

0
4

0

0

0

0

0

0

0

0

0

0

8.21009926710303e-14
4

0
4

0
7

0
7

0

0

0

0
4

0

0

0

0

0
4

0

0

0
4

0

0

0
4

0

0

0
4

0.0582163

0.0582163

0
4

0.0947236

0.0947236

0
4

0

0

0

0

0

0
4

0.218474

0

0.218474

0
4

0

0

0

0
4

0

0

0

0
4

0

0

0

0

0
4

0

0

0

0
4

0

0

0
4

0

0

0

0
4

0

0

0
4

0

0

0

0

0

0
4

0

0

0

0
4

0

0

0
4

0

0

0
4

0

0

0

0
4

0.0498868

0

0.0498868

0
4

0

0

0
4

0

0

0

0
4

0

0

0

0
4

0

0

0

0
4

0

0

0

0
4

0
7

0

0

0

0
4

0

0

0

0
4

0

0

0
4

0

0

0

0
4

0

0

0
4

0

0

0
4

0

0

0
4

0

0

0
4

0

0

0
4

0.063149

0.063149

0
4

0

0

0
4

0
6

0
6

0
4

0

0

0
4

0

0

0
4

0.0397225

0.0397225

0
4

0

0

0
4

0

0

0
4

0

0

0
4

0

0

0
4

0.0397225

0.0397225

0
4

0

0

0
4

0

0

0
4

0
6

0
6

0
4

0

0

0
4

0

0

0
4

0

0

0
4

0

0

0
4

0

0

0
4

0

0

0
4

0

0

0
4

0

0

0
4

0

0

0
4

0

0

0
4

0
4

0
4

0
4

0

0

0
4

0.0582163

0.0582163

0
4

0

0

0
4

0

0

0
4

0

0

0
4

0

0

0
4

0

0

0
4

0

0

0
4

0

0

0
4

0

0

0
4

0

0

0

0

0
4

0

0

0
4

0

0

0
4

0

0

0
4

0

0

0
4

0

0

0
4

0

0

0
4

0

0

0
4

0

0

0
4

0

0

0
4

0

0

0
4

0

0

0

0

0
4

0

0

0
4

8.41226

8.41226

0
4

0

0

0
4

0

0

0
4

0

0

0
4

0

0

0
4

0

0

0
4

0

0

0
4

0

0

0
4

0

0

0
4

0.873245

0.815029

0.0582163

0
4

0

0

0
4

0

0

0
4

0.203757

0.203757

0
4

0

0

0
4

0

0

0
4

0

0

0
4

0

0

0
4

0

0

0
4

0

0

0
4

0

0

0
4

5.88973314563646e-14
4

0
4

5.6843418860808e-14
4

0
4

303.872
4

295.83
4

23.8753

2.82222

8.79105

11.7755

0.48659

2.55351295663786e-15

0
4

60.89
4

2.0793
4

1.77901
4

1.484
3

27.5367
4

2.33018
4

12.2359
4

0
4

9.31688
4

4.08815
3

0

0

0

0

0

0

0.0398762

0

0

0

0

0

0
4

211.025
3

119.463
3

55.3162
3

30.4771
3

0.669633
4

0
4

0

0.0442094
4

0.36315

0

0.0442094

0

0

0

0

0.559813
4

0

0

0.12105

0

0

0

0.100875

0

0

0

0.0799732
3

0

0

0

0

0

0

0

0

0

0

0.0442094
3

0.0806999

0

0

0

0

0

0

0

0

0

1.71487

0

0

0

0

0

0

0

0

0.0799732

0

1.52371
3

0

0

0.1614

0

0

0

0

0

0

0

0
4

0

0

0

0.11996

0

0.0605249

0

0

0

0

0
4

0
4

0
4

0
5

0
5

0
4

0

0

0
4

0

0

0
4

0

0

0
4

0

0

0
4

0

0

0
4

0.0403499

0.0403499

0
4

0

0

0
4

0

0

0
4

0

0

0
4

0

0

0
4

0

0

0

0
4

0

0

0
4

0

0

0
4

0

0

0
4

0

0

0
4

0

0

0
4

0

0

0
4

0

0

0
4

0

0

0
4

0

0

0
4

0

0

0

0
4

0

0

0
4

0

0

0
4

0

0

0
4

0

0

0
4

0

0

0
4

0

0

0
4

1.72362124573056e-14
4

0
4

0
4

0
4

0

0

0

0

0

0

0

0

0
4

0

0

0

0
4

0

0

0
4

0
4

0
4

0

0

0

0

0
4

0

0

0
4

0
4

0
4

0

0

0
4

0

0

0
4

0

0

0
4

0

0

0
4

0
4

0

0

0

0
4

0

0

0

0
4

0

0

0
4

0
4

0

0

0

0
4

0

0

0
4

0
4

0

0

0

0

0

0

0
4

0
4

0

0

0

0
4

0

0

0
4

0

0

0
4

0
4

0

0

0

0
4

0

0

0
4

0

0

0
4

0
4

0

0

0

0

0
4

0
4

0

0

0

0

0

0
4

0
4

0

0

0

0
4

0
4

0
4

0
4

0
4

0

0
4

0

0

0

0
4

0

0

0
4

0
4

0

0

0

0
4

0

0

0
4

0

0

0
4

0
4

0

0

0

0
4

0

0

0
4

0

0

0
4

0
4

0

0

0

0

0
4

0
4

0

0

0

0

0
4

0

0

0
4

0
4

0

0

0

0

0
4

0
4

0

0

0

0

0
4

0

0

0
4

0
4

0

0

0

0
4

0
4

0

0

0

0
4

0
4

0

0

0

0

0
4

0
4

0

0

0

0
4

0
4

0
4

0
4

0
4

0

0

0
4

0

0

0

0
4

0

0

0
4

0
4

0

0

0

0
4

0
4

0

0

0

0
4

0
4

0

0

0

0
4

0
4

0

0

0

0

0
4

0
4

0

0

0

0
4

0
4

0

0

0

0
4

0

0

0
4

0
4

0

0

0

0

0
4

0
4

0

0

0

0

0
4

0
4

0

0

0

0
4

0

0

0
4

0
4

0

0

0

0
4

0
4

0
5

0

0

0

0
4

0

0

0

0

0

0
4

0
4

0

0

0

0
4

0
4

0

0

0

0
4

0
4

0

0

0

0
4

0
4

0

0

0

0
4

0
4

0

0

0

0
4

0
4

0

0

0

0
4

0
4

0

0

0

0
4

0
4

0

0

0

0
4

0
4

0

0

0

0
4

0
4

0

0

0

0
4

0
4

0
4

0
4

0
4

0

0

0
4

0

0

0
4

0

0

0
4

0
4

0

0

0

0
4

0
4

0

0

0

0
4

0
4

0.0975327

0.0975327

0.0975327

0
4

0
4

0

0

0

0
4

0
4

0

0

0

0
4

0
4

0

0

0

0
4

0
4

0

0

0

0
4

0
4

0.146299

0.146299

0.146299

0
4

0
4

0

0

0

0
4

0
4

0

0

0

0
4

0
4

0
4

0
4

0

0

0

0

0
4

0
4

0

0

0

0
4

0
4

0.0655183

0.0655183

0.0655183

0
4

0
4

0

0

0

0
4

0
4

0

0

0

0
4

0
4

0

0

0

0
4

0
4

0

0

0

0
4

0
4

0

0

0

0
4

0
4

0

0

0

0
4

0
4

0.0423112

0.0423112

0.0423112

0
4

0
4

0

0

0

0
4

0
4

0

0

0

0

0
4

0

0

0
4

0

0

0
4

0

0

0
4

0
4

0

0

0

0
4

0
4

0

0

0

0
4

0
4

0.0975327

0.0975327

0.0975327

0
4

0
4

0

0

0

0
4

0
4

6.81758

6.81758

6.81758

0
4

0
4

0.361229

0.361229

0.361229

0
4

0
4

0

0

0

0
4

0
4

0

0

0

0
4

0
4

0

0

0

0
4

0
4

0

0

0

0
4

0
4

0

0

0

0

0

0
4

0

0

0
4

0
4

0.105778

0.105778

0.105778

0
4

0
4

0

0

0

0
4

0
4

0

0

0

0
4

0
4

0

0

0

0
4

0
4

0

0

0

0
4

0
4

0

0

0

0
4

0
4

0

0

0

0
4

0
4

0

0

0

0
4

0
4

0

0

0

0
4

0
4

0.243832

0.243832

0.243832

0
4

0
4

0
4

0
4

0
4

0

0
4

0
4

0

0

0

0
4

0
4

0

0

0

0
4

0
4

0.06398

0.06398

0.06398

0
4

0
4

0

0

0

0
4

0
4

0

0

0

0
4

0
4

0

0

0

0
4

0
4

0

0

0

0
4

0
4

0

0

0

0
4

0
4

0

0

0

0
4

0
4

6.09234884763055e-15
4

0
4

2.02376
5

0

0

0

0

0

0
4

0

0

0
4

0
4

0

0

0

0
4

0
4

1.02409

1.02409

1.02409

0
4

0
4

0.519826

0.519826

0.519826

0
4

0
4

0

0

0

0
4

0
4

0

0

0

0
4

0
4

0

0

0

0
4

0
4

0

0

0

0
4

0
4

0

0

0

0
4

0
4

0

0

0

0
4

0
4

0

0

0

0
4

0
4

0
4

0
4

0

0

0

0

0
4

0

0

0

0
4

0

0

0
4

0
4

0

0

0

0
4

0
4

0

0

0

0
4

0
4

0

0

0

0
4

0
4

0
6

0
6

0

0

0

0
4

0
4

0
7

0
7

0
7

0
4

0
4

0
6

0
6

0
6

0

0
4

0
4

0

0

0

0
4

0

0

0
4

0
4

0

0

0

0
4

0
4

0

0

0

0

0
4

0
4

0

0

0

0
4

0
4

0.479839

0.0799732
5

0

0

0.0799732

0

0

0

0

0
4

0
7

0

0

0
4

0

0

0
4

0

0

0
4

0

0

0
4

0.399866

0.399866

0
4

0

0

0
4

0

0

0

0
4

0

0

0

0
4

0

0

0
4

0

0

0
4

0

0

0
4

0

0

0
4

0

0

0
4

0

0

0
4

0
4

3.88578058618805e-16
5

0
4

1.00381924994508e-11

0
4

65.5719

57.743

42.5008

22.7176
3

16.7681
3

5.41306

0.341364

0

0

0.195065

5.55111512312578e-17
3

0
4

19.3779
3

0.999665
3

0

0

0

0

0

0.054841

0

0

7.13697
2

5.93032
3

3.62828

1.28239

0.0655183

0

0

0.279906

1.0547118733939e-15
3

0
4

0

0

0

0

0
4

0
7

0

0

0

0

0

0

0

0

0

0

0
6

0

0

0

0

0

0

0

0
4

0

0

0
4

0.405347

0.405347

0
4

0

0

0
4

0
4

2.6791
3

2.6791

2.6791

0

0
4

0

0

0

0

0

0
4

0

0

0

0

0
4

0

0

0
4

0
4

0

0

0

0
4

0
4

0

0

0

0
4

0
4

0

0

0

0
4

0
4

4.69055

4.20891

4.20891

0
4

0.481639

0.481639

0
4

1.11022302462516e-16

0
4

2.71909
3

2.71909
3

2.71909

0

0

0
4

0

0

0

0
4

0

0

0
4

0
4

0

0

0

0

0
4

0
4

5.037
2

5.037
2

4.48312
2

0.553885

1.11022302462516e-16
2

0
4

0
4

0

0

0

0

0
4

0
4

0

0

0

0
4

0
4

0

0

0

0
4

0
4

0.116433

0.116433

0.116433

0
4

0
4

0
4

5.69272
2

5.69272
2

5.17886
2

2.65808

0.809601

1.61791

0

0.093266

0
4

0.448347
3

0.192656

0.0481639

0.114261

0.093266

0

2.77555756156289e-17
3

0
4

0.0655183

0.0655183

0
4

4.85722573273506e-16
2

0
4

0
4

0.847801

0.847801

0.847801

0.847801

0
4

0
4

0
4

0.872838

0.872838

0.872838

0.546407

0.326431

0
4

0
4

0
4

0.0982775

0.0982775

0.0982775

0

0

0.0982775

0
4

0
4

0
4

0

0

0

0

0
4

0
4

0
4

0.317228

0.317228

0.317228

0.317228

0
4

0
4

0
4

0
4

480.09

429.588

48.0216
3

48.0216
3

0.518391

4.64146

0.0873245

3.24906

2.06036

0.128931

7.71

0

1.23774

0

0.0582163

1.93225
3

0

0

0

0

0.0515724

0

0

0.128931

0

0.371514

7.04747
3

0

0.291082

0

0

0

0.232865

0

0

0.093266

0

3.23779
3

1.23774

0

0

0.0873245

0.0515724

0

0

0.116433

0

0

3.24906

0

0

0

0

0

0

0.0582163

4.32676

0

0

0.598143
4

1.47751

0.232865

0

2.09848
4

1.40932

1.77635683940025e-15
3

0
4

0
4

2.35921
3

2.35921
3

0
1

0

0

0

0

0

0

0

0

0

0

0.0799732

0

0

0

0

0

0

0

0

0

0

0.639786
4

0

0

0

0

0

0

0

0

1.27957
4

0

0

0

0

0.35988

0
4

0
4

8.65142
6

8.65142
6

0
6

0.568341

0

0

0

0

0
7

0

0

0

0

3.66264
5

0

0

0

0

0.063149

0

0

0

0

0

4.35728
5

0

0

0

0

0

0

0

0

0

0

0
6

0

0

0

0

0

0
6

0
6

0
7

0
6

8.88178419700125e-16
6

0
4

0

0

0
4

0
4

0
7

0
7

0
7

0
7

0
4

0
4

138.014
3

138.014
3

24.07

0.727704

1.70502

1.13668

0.915661

0.0873245

0

1.6103

0

0

0.746104

2.27044

0

0

0

0

1.077

32.5218

0

0.0873245

0

0.551468

0

0.757789

0.157873

0

0.34732

0.189447

0

0.189447

0.126298

0.96057

0.599916

0.97881

0.884087

26.3732

1.19983

0.0947236

0.063149

0.291904

0.157873

0.063149

0

1.6103

2.39966

0

0.0973179

0.063149

0.356832

0

0.0947236

0

0

1.3577

0

0

0.284171

0.0648786

0

0

0.129757

0.129757

1.07353

0.129757

0.45415

0.947236

3.88367

0

0.063149

0.0947236

0.315745

0.0947236

0.063149

0

0.0947236

0

0.0648786

21.4075

0.126298

0.162197

0.063149

0.145541

0.063149

0.0648786

0.0947236

0

0.129757

0

0.947236

0
4

0

0

0

0

0

0
4

0

0

0
4

0

0

0
4

0

0

0

0

0
4

0

0

0
4

0

0

0

0
4

0

0

0

0
4

0

0

0
4

0

0

0
4

0

0

0
4

0

0

0
4

0
4

85.1112
3

1.9464
3

1.9464

0

0
4

50.7573
3

0
4

0

0

0

0.154733

0

1.0889

1.61764

2.08543

0.133047

0

13.0524
2

0

0

0.422227

0.39311

0

0

0

0

0

0.11996

0.0442094

0

0

0

3.27484

0.0666674

0

0.0655183

0.159946

0.199933

0

1.93206
4

0

0.265367

0

0.0799732

0

0

0.132684

0

0.119168

4.8156

8.17911

0

0

0.139029

0

0.11996

0

0

0

0

0

0
4

0.044445

0

0

0

0

6.43785

4.16041

1.45313

0

0
4

0

0

0

0
4

11.6946

11.6946

0
4

0

0

0
4

2.10611

0

1.84121

0.264902

0
4

5.39165

1.37991

2.12294

0.0530735

0.398051

0.15922

0.873895

0.0530735

0.0595838

0.132684

0.0530735

0.106147

7.7715611723761e-16

0
4

3.61216

0.994211

2.06342

0.554537

2.22044604925031e-16

0
4

9.07188

9.07188

0

0

0

0

0

0

0

0

0

0

0

0

0

0

0
4

0.0397225

0.0397225

0
4

0.491388

0.491388

0
4

2.51465515077598e-14
3

0
4

17.0638
5

0
6

0
6

0
4

8.957
5

8.957
5

0
4

7.19962
7

0

2.85005

0

0

0

0

0

0

0

0

0

0

0

0

0

0

0

0

0

0

0

0

0

0

0

0

0

0

0

0

0

0

0

0

0

2.78453

0

0

0

0

0

0

0

0

0

0

0

0

0.0655183

0

0

0

0

0.219319

0

0

0

0

0.06398

0

1.21622

0

0

0

0

0

0

0

0

0

0

0

0

0

0

0

0

0

0
4

0.779183

0.0655183

0.713665

0
4

0

0

0
4

0.06398

0.06398

0
4

0.06398

0.06398

0
4

2.19269047363468e-15
5

0
4

76.8906
3

76.8906
3

37.7387
4

21.7233

14.6441

0.341364

0.0947236

0.0947236

1.99831

0.0975327

0.063149

0.0947236

0
4

0
4

19.3985
3

19.3189
3

16.3445
3

0.0796102

0

0

0

0.139899

0.0972378
3

0.132684

2.06987

0.0530735

0.221022

0.0530735

0

0.12796

2.19269047363468e-15
3

0
4

0.0796102

0.0796102

0
4

0

0

0
4

0
4

0

0

0

0
4

0
4

0.863777

0.863777

0.863777

0
4

0
4

0.179443

0.179443

0.179443

0
4

0
4

0

0

0

0
4

0
4

0

0

0

0
4

0
4

0.06398

0.06398

0.06398

0
4

0
4

0

0

0

0
4

0
4

0.652862

0.652862

0.652862

0
4

0
4

0

0

0

0
4

0
4

0

0

0

0
4

0
4

17.5222
3

17.5222
3

17.5222
3

0
4

0
4

7.42177

7.42177

1.62862

1.16547

0.119168

4.42906

0.079445

2.63677968348475e-16

0
4

0
4

0

0

0

0

0
4

0
4

0.41587

0.28791

0.12796

0.15995

0
4

0.12796

0.12796

0
4

2.77555756156289e-17

0
4

6.84586

6.74989

6.74989

0
4

0.09597

0.09597

0
4

1.94289029309402e-16

0
4

0

0

0

0
4

0
4

0.11129

0.11129

0.0530735

0.0582163

0
4

0
4

0

0

0

0
4

0

0

0
4

0
4

0
4

6.8409
3

6.8409
3

5.35484
3

4.68088
3

0.0981365
3

0.19194

0.3199

0.06398

0

0
4

0.132684

0.132684

0
4

1.35337

0

1.35337

0
4

8.88178419700125e-16
3

0
4

0
4

0.54383

0.54383

0.47985

0.06398

0.06398

0.35189

5.55111512312578e-17

0
4

0.06398

0.06398

0
4

0
4

0
4

0

0

0

0

0

0
4

0
4

0
4

0

0

0

0

0

0
4

0
4

0
4

0

0

0

0

0

0
4

0
4

0
4

0.111112

0.111112

0.044445

0.044445

0
4

0.0666674

0.0666674

0
4

0
4

0
4

1.18002

1.18002

1.18002

1.18002

0
4

0
4

0
4

2.09513

2.09513

2.09513

0.866951

1.22818

0
4

0
4

0
4

0

0

0

0

0
4

0
4

0
4

0.0397225

0.0397225

0.0397225

0.0397225

0
4

0
4

0
4

0

0

0

0

0
4

0
4

0
4

2.01153
3

2.01153
3

2.01153
3

1.55421
3

0.374166

0.0831446

0

0
4

0
4

0
4

0.0666674

0.0666674

0.0666674

0.0666674

0
4

0
4

0
4

0

0

0

0

0
4

0
4

0
4

0.054841

0.054841

0.054841

0.054841

0
4

0
4

0
4

0

0

0

0

0
4

0
4

0
4

0

0

0

0

0
4

0
4

0
4

0.0761741

0.0761741

0.0761741

0.0761741

0
4

0
4

0
4

0.0598143

0.0598143

0.0598143

0.0598143

0
4

0
4

0
4

0

0

0

0

0
4

0
4

0
4

0

0

0

0

0
4

0
4

0
4

34.3253

34.3253

34.3253

34.3253

0
4

0
4

0
4

2.8791

2.8791

2.39925

0.12796

1.56751

0.3199

0.12796

0.25592

5.55111512312578e-17

0
4

0.47985

0.47985

0
4

1.11022302462516e-16

0
4

0
4

0

0

0

0

0
4

0
4

0
4

0

0

0

0

0
4

0
4

0
4

0

0

0

0

0
4

0
4

0
4

0

0

0

0

0

0
4

0
4

0
4

0

0

0

0

0

0

0
4

0

0

0
4

0

0

0
4

0
4

0
4

0

0

0

0

0

0
4

0
4

0
4

0

0

0

0

0

0

0

0
4

0

0

0
4

0
4

0
4

0

0

0

0

0

0

0
4

0
4

0
4

0.218474

0.218474

0.178751

0.079445

0.0595838

0.0397225

0
4

0.0397225

0.0397225

0
4

0
4

0
4

1.45550238528358e-13

0
4

23.2765
3

23.2765
3

0.0799732
4

0.0799732
4

0.0799732
4

0
4

0
4

0

0

0

0

0

0

0

0

0

0

0

0

0

0

0

0

0

0

0

0
4

0

0

0

0

0

0

0

0

0

0

0

0

0

0

0
4

0

0

0

0
4

0
4

4.61988

4.61988

4.61988

0
4

0
4

0

0

0

0
4

0

0

0

0
4

0
4

5.39436

5.39436
1

5.39436
1

0
4

0

0

0
4

0

0

0
4

0
4

0

0
4

0

0

0

0

0
4

0

0

0
4

0

0

0
4

0

0

0
4

0

0

0
4

0
4

0
7

0
7

0
7

0
4

0
4

0
7

0
7

0
7

0

0

0

0
4

0
4

3.31889
4

3.31889
4

3.31889
4

0

0

0

0

0

0

0

0
4

0

0

0
4

0

0

0
4

0
4

0
4

0
4

0

0

0

0
4

0
4

0.717121

0.717121

0.717121

0
4

0
4

0

0

0

0
4

0
4

0.882611
3

0.882611
3

0

0
3

0

0
3

0

0

0

0

0

0

0

0

0

0

0

0

0

0

0

0.882611

0

0

0

0

0

0

0

0

0

0

0

0

0

0

0

0

0

0

0

0

0

0
4

0

0

0
4

0
4

8.20848
4

8.20848
4

0
4

0
4

0
4

8.20848
3

0

0

0

0

0

0
4

0
4

0.0551632

0.0551632

0

0.0551632

0
4

0
4

0
4

0

0

0

0

0
4

0
4

0
4

0

0

0

0

0
4

0
4

0
4

0
4

4420.75

113.367

58.0136

58.0136

58.0136

0
4

0
4

32.6946

28.6145
3

2.94279
3

0.115072

0

0.157576

0.0460286

0.0460286

0.299186

0

0

0.0982775

0

14.0744
3

0

0.0920573

0

0

0.0460286

0.131037

0.982775

0.0920573

9.02452
3

0.0460286

0.190435

0

0.1611

0

0.069043

8.81239525796218e-15
3

0
4

0

0

0
4

4.08016

0

2.02962

0.0627716

0.0418477

1.94592

2.22044604925031e-16

0
4

0
4

21.0656

7.61032

5.39077

1.03892

0

0

0

0

0

0

0.0423112

0.0442094

0

0

0.0442094

0.0442094

0

0.265256

0

0

0.74043

0

0

0
4

13.4553

12.8107

0.644581

0

0

0

6.66133814775094e-16

0
4

1.77635683940025e-15

0
4

1.59294

1.59294

1.59294

0
4

0
4

0

0

0

0
4

0
4

2.66453525910038e-15

0
4

0.41587
1

0

0

0

0

0

0

0

0

0

0

0

0

0

0

0
4

0

0

0

0

0

0

0
4

0

0

0
4

0

0

0
4

0
4

0

0

0

0
4

0
4

0.41587

0.41587

0.41587

0
4

0
4

0

0

0

0

0

0

0

0

0
4

0

0

0
4

0

0

0
4

0
4

0
8

0
8

0

0

0

0
4

0

0

0
4

0
4

0

0

0

0

0

0
4

0

0

0
4

0
4

0

0

0

0
4

0

0

0
4

0
4

0

0

0

0

0

0
4

0

0

0
4

0
4

0

0

0

0

0

0
4

0
4

0

0

0

0
4

0

0

0
4

0
4

0

0

0

0
4

0
4

0
4

397.677
3

396.755
3

18.5301
3

0

0

0

0

0

0

0

0

0

0

2.47342

0

0.19194

0

2.97507

0

0

0

0

0

0

0

3.3976

0

0

0

0

0.0993063

0

0

0

0

0

1.96983

0

0

0

0

0.079445

0

0

0

0

0

0

0

0.09597

0.079445

0

0

0

0.06398

0.0397225

0

7.06436

0

0

0
4

30.9292
4

3.71876
4

0
4

0

0

0

0

0

0

0

0

0

0

0
4

0

0

0

0

0

0

0

0

0

0

0
4

0

0

0

0

0

0

0

0.11996

0.0799732

0

0
4

0

0

0

0

0

0

0

0

0.119168

0.319893

0

0

0

0

0

0

0

0

0

0

0

0.0799732
4

0

0

0

0

0

0

0

0

0

0

0
4

0

0

0

0

0

0

0

0

0

0

0.159946
4

0

0

0.159946

0

0

0

0

0

0

0

0
4

0

0

0

0

0

0

0.23992

0

0

0

0
4

0

0

0

0

0

0

0

0

0

0

15.2749
4

0
4

0

0

0

0

0

0

0

0

0

0

0.11996
3

0
4

0
4

0.279906
3

0

0
4

0

0

8.99699
4

0

0
3

0

0

0

0.0799732

0

0.2739

0

0

0.11996
4

0

0

0

0

0

0

0

0

0

0

0
4

0

0

0

0

0

0

0

0

0

0

0.23992
4

0

0

0

0

0

0

0.199933

0

0

0

0.159946
4

0

0

0

0

0

0

0

0

0

0

0
4

0

0.0663141

0

0

0

0

0

0

0

0

0
4

0

0

0.11996

0

0

0

0

0

0

0

0
4

148.148
2

94.0966
2

0.70378

53.3481

0
4

75.7047
2

4.57572

12.873

0

0.13439

0.156309

0.174649

0.259802

0.0761741

0.0873245

0.0582163

5.29769

13.1956

0.407514

0.0582163

0.116433

0.0582163

0

0.174649

0.232865

0.638019

1.10611

0.0582163

8.58673

0.0582163

0.0873245

0.0873245

0.0582163

0.152348

0

0.195065

0.174649

0.0873245

0.0582163

1.83845

0.494839

0.0582163

0.0582163

0.174649

0.873245

0.145541

17.7755

0.214525

3.0891

0.837915

0.859923

2.1094237467878e-15
2

0
4

0

0

0

0

0
4

84.3924

70.349

0.06398

6.79897

0.587759

4.8032

0.093266

0.46633

0.233165

0.06398

0.93266

1.35447209004269e-14

0
4

0

0

0
4

0

0

0
4

0

0

0
4

0

0

0
4

24.209

1.59251

21.4333

0.93266

0.093266

0.093266

0.06398

0
4

0.449569

0.310375

0.139194

0
4

12.6452

12.6055

0.0397225

0
4

1.43001

0

0

1.43001

0
4

0.200002

0.200002

0

0
4

0.116433

0.116433

0
4

0

0

0
4

0

0

0
4

8.47377723545151e-14
3

0
4

0

0

0

0
4

0

0

0
4

0
4

0

0

0

0

0
4

0
4

0.863777

0.863777

0.863777

0
4

0
4

0.0582163

0.0582163

0.0582163

0
4

0
4

0
4

53.6592
3

38.0621
3

35.7764
3

8.93137
2

0

1.29098

0.0722459

0

0.706687

0

0

0

0

0.357503

0

0.864704

14.5702
3

0

0

0

0.284171

1.91113

0

0.433475

0

0

0.229577

0

0

0

0.279134

0

0.109496

0

0

0

0

0.0481639

0

0

0

0.410469

0

0

0

0.0481639

0

0

0.274205

0.881333

0

0

0

0

0

0

0.0332578

0

0.0722459

0.12041

1.33335

0

0

0

0

0.0963278

0

0

0

0

0

0

0

0

0

0

0.410469

0.0397225

0

0

0.663065

0.0975327

0
4

0

0.0822527

0

0

0

0

0

0.893757

0

0

0

0

0

0

0

0

0.0498868

0

0.054841

0.126298

0

1.02695629777827e-15
3

0
4

0
4

0

0
4

0

0
4

0

0

0
4

0

0

0
4

0

0

0
4

0

0

0

0
4

0
4

0
4

0
4

0

0

0
4

0.216738

0.0722459

0.144492

0
4

0

0

0
4

0.0481639

0.0481639

0
4

2.02077

2.02077

0
4

0

0

0
4

0
4

0

0

0

0

0

0

0
4

0

0

0
4

0
4

0

0

0

0
4

0

0

0
4

0
4

0

0

0

0

0

0
4

0
4

3.53516

3.53516

3.53516

0
4

0
4

0

0

0

0
4

0
4

0

0

0

0
4

0
4

0

0

0

0
4

0
4

0

0

0

0
4

0
4

0.045462

0.045462

0.045462

0
4

0
4

0

0

0

0
4

0
4

0

0

0

0
4

0
4

0

0

0

0

0
4

0

0

0
4

0
4

0

0

0

0
4

0
4

0

0

0

0
4

0
4

0

0

0

0
4

0
4

0

0

0

0
4

0
4

0.06398

0.06398

0.06398

0
4

0
4

4.09393

4.09393

4.09393

0
4

0
4

0

0

0

0
4

0
4

0.093266

0.093266

0.093266

0
4

0
4

0

0

0

0
4

0
4

0

0

0

0
4

0
4

1.48891

0

0

0

0

0

0
4

1.48891

1.48891

0
4

0
4

1.05889

0.904779

0.904779

0
4

0.154115

0

0.044445

0.10967

0
4

0
4

0

0

0

0

0
4

0
4

0

0

0

0

0

0
4

0
4

3.85311

3.85311

3.78087

0.0722459

0
4

0
4

1.36438

1.36438

1.36438

0
4

0
4

0

0

0

0
4

0
4

9.99200722162641e-15
3

0
4

44.6569

0
4

0
5

0

0

0

0

0

0

0

0

0

0

0

0
4

0

0

0

0
4

0

0

0
4

0
4

12.7536
7

0
8

0

0
8

0
4

5.43082
1

5.43082
1

0

0

0

0
4

2.23925
1

0

0

0

0

0

0

0

0

2.23925

0

0

0

0

0
4

0

0

0
4

0
7

0
7

0
7

0
7

0

0

0

0

0

0

0

0

0

0

0
7

0

0

0

0

0

0

0

0

0

0

0
7

0

0

0

0

0
7

0
7

0

0

0

0

0
4

5.0835

0.063149

0
7

0.284171

0

0

0

0

4.73618

0

0

0

0

0

8.88178419700125e-16

0
4

0

0

0

0
4

0

0

0

0
4

0

0

0
4

0

0

0
4

0
4

0
7

0
7

0
7

0

0

0
4

0
7

0
6

0

0
4

0
4

31.2513

12.181
1

0

0

0

0

0

0

0

0

0

0

0.0863786

0

0.685949

0

0

8.53384

0

0

0

0

0

0

0

0

0

0

0

0

0

0

0

0

0

0

0

0

0

0

0

1.33483

0

0

0

0

0

0

0

0

0

0

0

0

0

0

0

1.54002

0

0

0
4

10.2559

8.72037

1.40756

0

0.12796

0

0

0

0

0

0

0

0
4

8.81442

3.58018

0.342783

0

0.09597

0

0

0

0

3.48691

0.38087
7

0.06398

0
5

0.86373

0

0

0

0
4

0

0

0
4

0

0

0
4

0
4

0.41587

0.25592

0.25592

0

0

0

0
4

0.15995

0.15995

0
4

2.77555756156289e-17

0
4

0

0

0

0
4

0
4

0

0

0

0
4

0
4

0

0

0

0
4

0
4

0

0

0

0
4

0
4

0.0761741

0.0761741

0

0.0761741

0
4

0

0

0
4

0
4

0

0

0

0

0

0
4

0
4

0

0

0

0

0
4

0

0

0
4

0
4

0.159946

0.159946

0.159946

0
4

0
4

0

0

0

0
4

0
4

0

0

0

0

0
4

0
4

0

0

0

0
4

0
4

0

0

0

0
4

0
4

0
7

0
7

0
7

0
4

0
4

1.85407245112401e-14

0
4

1780.86
3

50.1734
3

0.139029

0.139029

0
4

22.439
3

21.2651
3

1.17393

0

0

0
4

27.1928
2

19.2209
2

6.89399
2

0.419172
2

0

0

0.658696

0

0

8.88178419700125e-16
2

0
4

0.136386

0.0681929

0.0681929

0
4

0.100303

0.100303

0

0
4

0.12041

0.12041

0

0
4

0

0

0
4

0

0

0
4

0.045462

0.045462

0
4

0

0

0
4

0
4

98.7014
3

0
7

0
6

0

0
4

1.54273
3

1.54273
3

0
4

63.8603
3

20.6798
3

43.1805
2

7.105427357601e-15
3

0
4

31.3149
3

0

18.4572
3

0.272772

0.0548351

0.0398762

0.045462

0.093266

0

0.045462

0

0.093266

0.0481639

4.51982

0.0398762

0

2.54209

0.191944

0

4.77996

0

0.045462

0.045462

0
4

1.57849
3

0.559813

0

0

0

0

0

0

0

0

0

0

0

0

0
7

0
7

0

0.639786

0

0.378894

0

0
4

0

0

0

0
4

0.174649

0.174649

0
4

0.137103

0.137103

0
4

0

0

0
4

0.093266

0.093266

0
4

0
4

283.602
3

9.35998

9.35998

0

0

0

0

0

0
4

0
5

0

0

0

0

0

0

0

0

0

0
4

32.6729

32.6729

0
6

0

0

0
6

0

0

0

0

0

0

0

0
4

234.715
3

230.543
3

3.95121
4

0
4

0

0

0

0

0.221022

0
4

0

0

0

0

0

0

0

0
4

0

0

0

0
4

0

0

0

0
4

0.633962

0.633962

0
4

0.0582163

0.0582163

0
4

0.772853

0.772853

0
4

0

0

0
4

0.232865

0

0.232865

0

0
4

0

0

0

0
4

0

0

0
4

2.18311

2.18311

0
4

2.97212

2.97212

0
4

0

0

0
4

0

0

0
4

0

0

0
4

1.55431223447522e-14
3

0
4

52.1414
4

0

0

0
4

2.89124

2.89124

0

0
4

43.0282
4

36.0404
4

0.782155
4

0
4

0

0

0

0

0

0

0

0

0

0

0
4

0

0

0

0

0

0

0

0

0

0

0
4

0

0

0

0

0

0

0

0

0

0

0
4

0

0

0

0

0

0.0681929

0

0

0.0398762
4

0

0

0
4

0

0

0
4

0

0

0

0

0.639786

0
4

0
4

0

0

0

1.03497
4

0

0

0

0.844137

0

0

0

0

0

0

0
4

0

0

0

0

0

0

0

0

0

0

0
4

0

0

0

0.0799732

0

0

0

0

0

0

0
4

0.0598143

0

0

0

0

0

0

0

0

0

3.43885
3

0

0

0

0

0

0

0

0

0

0

0

0

0

0

0

0

0

0

0

0

0

0
4

0

0

0

0

0

0

0

0

0

0

1.77635683940025e-15
4

0
4

0

0

0
4

0

0

0
4

0

0

0
4

0

0

0
4

0

0

0
4

0

0

0
4

0.114261

0.114261

0
4

0

0

0
4

0

0

0
4

0

0

0
4

0

0

0
4

4.27857

4.19859

0

0.0799732

0
4

0

0

0
4

0

0

0
4

1.10611

1.10611

0
4

0

0

0
4

0

0

0
4

0

0

0
4

0

0

0
4

0.0595838

0.0595838

0
4

0

0

0
4

0

0

0
4

0

0

0

0
4

0

0

0
4

0

0

0
4

0

0

0
4

0.663419

0.663419

0
4

0

0

0

0
4

0

0

0
4

0

0

0
4

0

0

0
4

0
4

895.099

0
6

0
6

0
6

0

0
6

0

0

0

0

0
4

41.5327
3

1.13209

0

0.282502

0

0.12796

0.418957

0.093266

0

3.10303

0.15995

0.06398

1.55399

0

0.09597

0.12796

0.09597

0.0397225

0

0.19194

0.114261

0.09597

0.0397225

0

0.09597

0.09597

11.3245

1.25126

2.65517

17.9887

0

0.38388

0
4

157.896
3

143.689
3

9.95289
2

0.0595838

0.0761741

0.0761741

1.20327
2

2.04819

0

0.740746

0

0

0.0498868

0

0
4

51.6055
2

33.2719
2

0.447112

0.0846225

0.109682

0

0.0352475

1.25909

11.1943
2

3.56993

0.321747

0.822639

0.148107

0.144492

0

0.196686

1.41553435639707e-15
2

0
4

76.2745

34.6294
3

0.063149
7

0
3

0

11.8138

25.1841
2

0

0

0

0.228522

0

1.09751

1.06644

0.0515724

0

0

0.815711

0

0

0

0

0.152348

0

0.06398

0

0

0

0

1.02835

0

0.0796102

0

0
4

403.421

403.269

0

0

0.152348

0

0

0

0

6.18949336228525e-15

0
4

0.354708
6

0
6

0

0

0

0

0

0

0

0

0

0

0

0.190435

0

0

0

0

0

0

0

0

0

0

0

0

0

0

0

0

0

0

0

0

0

0

0

0

0

0

0

0

0

0.054841

0

0

0

0

0

0

0

0

0

0

0

0

0

0

0

0

0.0761741

0

0

0

0

0

0

0

0

0

0

0

0

0

0

0

0

0

0
6

0

0

0

0

0

0.0332578

0

0

0

0

0

0

1.38777878078145e-17
6

0
4

139.327
3

30.5039
3

30.0979
2

1.74736

0

0.837915

0.493569

0.152348

40.0676

0.51184

0

0.131015

0.46633

1.32506
3

0.145765

0

0.603251

0

0.0481639

0

0

0.272697

0.366398

0.319387

8.35757
2

0

0

0.190435

1.52348

0

0.0530735

0.0481639

0.0975327

0.114261

0.28791

1.38789

0

0.313065

1.00016

0.0761741

0.152348

0.0481639

0

0.0975327

0

0.054841

0.166289

0

1.30572

0.0761741

0.0909239

0

0.0481639

0.0332578

0

0

0

4.14557

0

0

0

0.06398

0.093266

0

0

0

0

0

3.6054

0.796102

0.0761741

0

0.73577

2.04717
2

4.14985

0
4

14.0261
3

12.7949

0.09597

0
4

0

0

0

0

0

0

0

0

0

0

0
4

0

0

0

0

0

0

0
4

0

0

0

0

0

1.13522

4.44089209850063e-16
3

0
4

0.135873

0.0551632

0.0807095

0
4

0.133031

0.133031

0
4

0

0

0
4

0

0

0
4

0

0

0
4

0.0975327

0.0975327

0
4

0.0761741

0.0761741

0
4

0

0

0
4

0.0582163

0.0582163

0
4

0

0

0
4

0

0

0
4

0

0

0
4

0

0

0
4

0

0

0
4

0.341364

0.341364

0
4

9.22328

9.22328

0
4

0.457557

0.457557

0
4

0.0423112

0

0.0423112

0
4

0

0

0
4

0

0

0
4

0

0

0
4

0.09597

0.09597

0
4

0
4

29.6986
3

0.493569

0.493569

0
4

10.7072
3

9.57373
2

0.815029

0

0

0.318441

1.11022302462516e-16
3

0
4

9.90543
3

8.08652
3

0.69464

0

0.726214

0

0

0.185757

0.212294

0

0

8.88178419700125e-16
3

0
4

3.00107

3.00107

0
4

0

0

0
4

0

0

0
4

5.59134

5.59134

0
4

0

0

0
4

0

0

0

0
4

0

0

0
4

0

0

0
4

0

0

0
4

0

0

0
4

0

0

0
4

3.5527136788005e-15
3

0
4

2.01929

0.844137

0.844137

0

0
4

0.601745
2

0.556283

0

0.045462

0
4

0

0

0
4

0.398762

0.398762

0
4

0.174649

0.174649

0
4

0
4

0
6

0
6

0
6

0

0

0

0
4

0
7

0
6

0
7

0

0

0

0

0

0

0

0
4

0
6

0

0

0

0

0

0

0

0

0
4

0
4

6.68225

6.68225

6.68225

0
4

0
4

0

0

0

0
4

0

0

0
4

0
4

0

0

0

0

0

0
4

0

0

0
4

0
4

0.19194

0.12796

0

0.12796

0

0
4

0.06398

0.06398

0
4

0

0

0
4

1.38777878078145e-17

0
4

0

0

0

0

0
4

0

0

0
4

0

0

0
4

0
4

0

0

0

0

0
4

0

0

0
4

0

0

0
4

0
4

0.324989

0.324989

0.0936259

0.0722459

0.159117

0
4

0
4

0.15987

0.15987

0.15987

0

0
4

0
4

0

0

0

0

0
4

0
4

0.666382

0.666382

0.602402

0.06398

0
4

0
4

2.3992
4

2.3992
4

2.23925
4

0

0.159946

0
4

0

0

0
4

0
4

0

0

0

0

0
4

0
4

0

0

0

0
4

0
4

0

0

0

0

0
4

0
4

0

0

0

0
4

0
4

0

0

0

0

0
4

0
4

0

0

0

0
4

0
4

0

0

0

0
4

0
4

0.98186

0.98186

0.0530735

0.928786

0
4

0
4

0

0

0

0

0
4

0
4

0

0

0

0
4

0
4

0.159946
3

0.159946
3

0.159946
3

0
4

0

0

0

0

0
4

0

0

0

0
4

0

0

0
4

0
4

0.09597

0.09597

0.09597

0
4

0
4

0.0418477

0.0418477

0.0418477

0
4

0
4

0

0

0

0
4

0
4

0

0

0

0
4

0
4

0

0

0

0
4

0
4

0.0873245

0.0873245

0.0873245

0
4

0
4

0

0

0

0
4

0
4

0

0

0

0
4

0
4

2.02912

2.02912

2.02912

0
4

0
4

0

0

0

0
4

0
4

2.75908
3

2.75908
3

2.75908
3

0

0

0

0
4

0
4

0.09597

0.09597

0.09597

0
4

0
4

0

0

0

0
4

0
4

0

0

0

0
4

0
4

0.204579

0.204579

0.204579

0
4

0
4

0

0

0

0
4

0
4

0

0

0

0
4

0
4

0

0

0

0
4

0
4

0

0

0

0
4

0
4

0

0

0

0
4

0
4

0

0

0

0
4

0
4

5.81006

5.81006

5.53586

0.274205

0
4

0
4

0

0

0

0
4

0
4

0

0

0

0
4

0
4

0

0

0

0
4

0
4

0

0

0

0
4

0
4

0.818315

0.818315

0.818315

0
4

0
4

0.09597

0.09597

0.09597

0
4

0
4

0

0

0

0
4

0
4

0.12796

0.12796

0.12796

0
4

0
4

0

0

0

0
4

0
4

0

0

0

0
4

0
4

1.21887

1.21887

0.384535

0.397225

0.258362

0.178751

0
4

0
4

0

0

0

0
4

0
4

0
7

0
7

0

0

0
4

0
4

0

0

0

0
4

0
4

7.37895
2

7.37895
2

0.893028

6.48592

8.88178419700125e-16
2

0
4

0
4

194.633

95.9476

11.9396
2

28.9192
2

2.65399

2.76555

1.31155
3

0
6

0

0

0

0.316398
2

0.605703

0
7

0

0
7

0

0.717772

0.232712

0

0

0.166048

0

0

0

0.159505

0
7

0

0.0722459

0.131037

0.261974

3.55341

0

0

0.045462

23.2232

0

4.36186

0

0

0

0

0.045462

1.57511

0.63556

0

2.13671

0.0423112

0
7

0.0515724

0.0655183

0.045462

0.0423112

0.25592

0

0.0442094

0

0

0.0423112

0

0

0

0

0

0.045462

0

0.0681929

0.079445

1.19415

0.0655183

2.99141
2

0

0

0.349298

4.73446

0
5

3.01980662698043e-14

0
4

30.3124
2

29.9487
2

0.0909239

0.159117

0

0.045462

0.0681929

7.17481629664007e-15
2

0
4

0.454855

0.409393

0.045462

0
4

0

0

0
4

0.796102

0.796102

0
4

63.6418
2

49.3887
2

14.0845

0.168574

0
4

0.434591

0.340965

0.0481639

0.045462

6.93889390390723e-18

0
4

0.331099
2

0.211334

0.0515724

0.0681929

4.16333634234434e-17
2

0
4

0.577349

0.577349

0
4

1.66007

1.66007

0
4

0

0

0

0
4

0

0

0
4

0.477351

0.477351

0

0
4

0
4

0.291082
4

0
4

0
4

0

0
4

0
7

0
7

0
7

0

0

0

0

0

0
4

0

0

0
4

0

0

0

0
4

0

0

0
4

0

0

0
4

0

0

0
4

0

0

0
4

0

0

0
4

0.291082

0.291082

0
4

0

0

0
4

0
4

3.20249

2.75902

0

0

0

0.357503

0.767692

1.17895

0.137088

0.31778

0

0

0

0

3.33066907387547e-16

0
4

0.315745
3

0.315745
3

0

0
4

0.127723

0.127723

0
4

0
4

0.512963

0

0

0
4

0.512963

0.512963

0
4

0

0

0
4

0

0

0
4

0

0

0
4

0

0

0
4

0

0

0
4

0
4

0.310721

0

0

0

0

0

0

0

0

0

0

0

0

0

0

0

0

0
4

0.219797

0.219797

0
4

0.045462

0.045462

0
4

0.045462

0.045462

0
4

0

0

0
4

2.77555756156289e-17

0
4

86.4308
3

74.1046
3

18.3667
3

0
4

0.175943
3

1.49474

0.0398762
3

0.0938836
4

1.35783

0.191427

0.0515724

0.135846

0.0799732

32.7761
3

0.06398

0.299849

0.0773585

0.0634669

0

0

0.146296

0.22393

0

0

1.19545
3

0.06398

0

0

0.0397225

0

0

0.06398

0

0

0

2.83946
3

0

0

0

0.09597

0.0975327

0

0

1.38018
3

1.33553
3

1.61869
3

3.41762
3

6.31765

2.39808173319034e-14
3

0
4

8.64681
2

7.56903
2

0.292406

0.692575

0.0530735

0.0397225

0
4

0.0582163

0.0582163

0
4

1.2796

1.2796

0
4

0.0975327

0.0975327

0
4

0.276172

0.276172

0
4

0.665339
2

0.202195

0.194364

0.0775588

0.0909239

0.0548351

0.045462

0
4

0

0

0

0
4

0

0

0
4

0.219433

0.219433

0
4

0.0704951

0.0352475

0.0352475

0
4

0.823414

0.636882

0.186532

5.55111512312578e-17

0
4

0.189236

0.09597

0.093266

0
4

0

0

0
4

0
4

40.8917
3

38.9055
3

11.2685
2

0.164523

2.01679

0

0.0796102

0

0.0530735

0

0.0530735

0.0530735

0.0530735

2.79699
2

0.0551632

0.33849

0

0.0530735

0.0796102

0.0530735

0

0

0

2.50452

2.99009
2

1.3003

12.3654
2

0

0.146611

2.48044

4.44089209850063e-16
3

0
4

0.164523

0.164523

0

0
4

0

0

0
4

0

0

0
4

0.611272

0.611272

0
4

0.09597

0.09597

0
4

0.54383

0.54383

0
4

0

0

0
4

0.411308

0.411308

0
4

0.15922

0.15922

0
4

5.05151476204446e-15
3

0
4

9.41724

8.54139

0.0398762
2

8.27299

0.228522

0
4

0

0

0

0

0

0
4

0.498453

0.279134

0.179443

0.0398762

1.38777878078145e-17

0
4

0.377404

0.377404

0

0
4

0

0

0
4

0

0

0
4

0

0

0
4

0
4

1.40756

1.40756

1.40756

0
4

0

0

0
4

0

0

0
4

0
4

3.11972669919669e-13
3

0
4

34.9682
2

34.9682
2

0

0

0

0

0

0

0
4

34.9682
2

34.9682
2

0
4

0

0

0
4

0

0

0
4

0

0

0
4

0
4

0

0

0

0
4

0
4

0
4

224.456
4

0

0

0

0

0
4

0
4

20.516
2

9.1397
2

3.125
2

0.895797

4.63239
2

0.32654

0.0515724

0

0.0397225

0.0686787

0
4

10.9556

10.9556

0
4

0.272662

0.0846225

0.105778

0.0822615

0
4

0.148089

0

0.148089

0
4

0
4

0.0551632
4

0
4

0
7

0

0

0

0
7

0

0

0

0

0

0

0
4

0.0551632

0.0551632

0

0

0

0

0

0

0

0

0

0

0

0

0

0
4

0

0

0

0

0
4

0

0

0

0

0
4

0

0

0

0
4

0

0

0
4

0

0

0
4

0
4

0
7

0
7

0

0

0

0

0
4

0

0

0

0
4

0
4

0

0

0

0
4

0
4

0

0

0

0
4

0
4

0.0515724

0.0515724

0.0515724

0
4

0
4

0

0

0

0
4

0
4

0

0

0

0
4

0
4

0

0

0

0
4

0
4

0

0

0

0
4

0
4

0

0

0

0
4

0
4

2.23206

2.23206

2.23206

0
4

0
4

0

0

0

0
4

0
4

0
5

0
7

0
7

0
4

0

0

0
4

0

0

0
4

0
4

0

0

0

0
4

0
4

0

0

0

0
4

0
4

0.0582163

0.0582163

0.0582163

0
4

0
4

0

0

0

0
4

0
4

0

0

0

0
4

0
4

0

0

0

0
4

0
4

0

0

0

0
4

0
4

0

0

0

0
4

0
4

0

0

0

0
4

0
4

0

0

0

0
4

0
4

0
7

0
7

0
7

0

0

0

0

0

0

0

0
4

0
4

0

0

0

0
4

0
4

0

0

0

0
4

0
4

0

0

0

0
4

0
4

0

0

0

0
4

0
4

0

0

0

0
4

0
4

0

0

0

0
4

0
4

0

0

0

0
4

0
4

0

0

0

0
4

0
4

0

0

0

0
4

0
4

0

0

0

0
4

0
4

0

0
7

0

0

0

0

0
4

0

0

0

0

0
4

0

0

0
4

0

0

0
4

0
4

0

0

0

0
4

0
4

0

0

0

0
4

0
4

0

0

0

0
4

0
4

0.054841

0.054841

0.054841

0
4

0
4

0

0

0

0
4

0
4

0

0

0

0
4

0
4

0

0

0

0
4

0
4

0.0397225

0.0397225

0.0397225

0
4

0
4

0.152348

0.152348

0.152348

0
4

0
4

0

0

0

0
4

0
4

0

0

0

0

0

0

0
4

0

0

0

0
4

0

0

0

0
4

0

0

0
4

0

0

0
4

0

0

0
4

0

0

0
4

0
4

0.0799732

0.0799732

0.0799732

0
4

0
4

0.243832

0.243832

0.243832

0
4

0
4

0

0

0

0
4

0
4

0

0

0

0
4

0
4

0

0

0

0
4

0
4

0

0

0

0
4

0
4

0

0

0

0
4

0
4

0

0

0

0
4

0
4

0.0761741

0.0761741

0.0761741

0
4

0
4

0

0

0

0
4

0
4

0

0

0

0

0

0
4

0
4

0

0

0

0
4

0
4

0

0

0

0
4

0
4

0
7

0
7

0
7

0

0
4

0

0

0
4

0

0

0
4

0
4

0
5

0
7

0

0

0

0

0
4

0

0

0
4

0

0

0
4

0
4

0

0

0

0

0
4

0

0

0

0
4

0

0

0

0
4

0

0

0
4

0
4

0

0

0

0

0

0

0
4

0

0

0

0
4

0
4

0

0

0

0

0

0

0

0

0

0

0

0

0

0

0

0

0

0

0

0

0
4

0

0

0

0

0

0

0
4

0
4

0

0

0

0

0
4

0
4

0

0

0

0

0

0

0
4

0

0

0
4

0
4

0
4

0

0

0

0
4

0

0

0
4

0

0

0
4

0
4

0

0

0

0

0

0

0
4

0

0

0

0
4

0
4

0.0498868
3

0.0498868
3

0.0498868
3

0

0
4

0

0

0
4

0
4

0.279906

0.279906

0.279906

0
4

0
4

0.188031
2

0.188031

0.188031

0
4

0

0

0
4

0

0

0
4

0
4

14.7202

14.6804

14.5811

0.0993063

0
4

0.0397225

0.0397225

0
4

0
4

0.0947236
6

0

0

0

0

0
4

0.0947236

0.0947236

0

0
4

0
4

0

0

0

0

0

0
4

0
4

0
4

0
4

0
4

0

0

0

0
4

0

0

0

0

0

0

0

0
4

0

0

0
4

0
4

0

0

0

0

0
4

0
4

0.0551632

0

0

0

0

0
4

0.0551632

0

0.0551632

0
4

0
4

0
5

0
5

0
5

0
4

0
4

0

0

0

0
4

0

0

0
4

0

0

0
4

0

0

0
4

0

0

0
4

0
4

0.0822527
2

0

0

0

0

0
4

0

0

0
4

0.0822527

0.0822527

0
4

0
4

2.16338

2.16338

2.16338

0
4

0
4

0.19194

0

0

0

0
4

0.12796

0.12796

0
4

0.06398

0.06398

0
4

0

0

0
4

1.38777878078145e-17

0
4

0.0873245

0.0873245

0

0.0873245

0
4

0
4

0

0

0

0

0

0
4

0
4

0

0

0

0
4

0
4

0.0648786
4

0.0648786

0

0.0648786

0

0

0

0

0

0

0
4

0

0

0

0

0

0
4

0

0

0

0

0
4

0

0

0
4

0

0

0
4

0
4

0

0

0

0
4

0
4

0

0

0

0

0
4

0

0

0
4

0
4

0

0

0

0

0
4

0
4

0

0

0

0

0
4

0
4

0

0

0

0

0
4

0

0

0

0
4

0
4

0

0

0

0

0

0
4

0
4

0

0

0

0

0
4

0

0

0
4

0
4

0.854984

0.854984

0.854984

0
4

0
4

0

0

0

0
4

0

0

0

0
4

0
4

3.28091

3.28091

0.897559

2.38335

0
4

0
4

0.92771
6

0.92771
6

0.86373
6

0.06398

0

0
4

0
4

0

0

0

0

0
4

0
4

0

0

0

0
4

0
4

0

0

0

0
4

0

0

0
4

0
4

1.16762

1.16762

1.16762

0
4

0
4

19.2486

19.2486

19.2486

0
4

0
4

0

0

0

0

0

0
4

0
4

0

0

0

0
4

0

0

0
4

0
4

0

0

0

0

0
4

0

0

0
4

0
4

0

0

0

0

0
4

0
4

0

0

0

0
4

0

0

0
4

0

0

0
4

0
4

0.266609
3

0.266609

0.114261

0
3

0

0.152348

0
4

0

0

0

0
4

0

0

0
4

0

0

0
4

0
4

0

0

0

0

0
4

0

0

0
4

0
4

0

0

0

0
4

0
4

0

0

0

0

0
4

0

0

0
4

0
4

0

0

0

0
4

0
4

0

0

0

0
4

0

0

0
4

0
4

0

0

0

0
4

0
4

0

0

0

0
4

0

0

0
4

0
4

0

0

0

0
4

0

0

0
4

0
4

0

0

0

0
4

0

0

0
4

0
4

0

0

0

0
4

0
4

4.92646
6

4.92646
6

4.92646
6

0

0
4

0
4

0

0

0

0
4

0
4

0

0

0

0

0
4

0
4

0

0

0

0
4

0

0

0
4

0
4

0

0

0

0

0
4

0
4

0

0

0

0
4

0
4

0

0

0

0
4

0

0

0
4

0
4

3.80377

3.80377

3.80377

0
4

0

0

0
4

0
4

0

0

0

0

0
4

0
4

0

0

0

0
4

0
4

0

0

0

0
4

0
4

0.601876
2

0.601876
2

0.518731

0.0498868
2

0.0332578

0

0

0

0

0
4

0

0

0
4

0
4

10.837

10.837

0.0920573

10.7449

0
4

0
4

0

0

0

0

0
4

0
4

0

0

0

0
4

0
4

0

0

0

0
4

0
4

0

0

0

0
4

0
4

0

0

0

0
4

0
4

0

0

0

0
4

0
4

0

0

0

0
4

0
4

0

0

0

0
4

0
4

0

0

0

0
4

0
4

0

0

0
6

0

0

0

0

0
4

0

0

0

0

0
4

0

0

0
4

0

0

0
4

0
4

0

0

0

0
4

0
4

0

0

0

0
4

0
4

0.0873245

0.0873245

0.0873245

0
4

0
4

0.0822615

0.0822615

0.0822615

0
4

0
4

0

0

0

0
4

0
4

0.178751

0.178751

0.178751

0
4

0
4

0

0

0

0
4

0
4

0

0

0

0
4

0
4

0

0

0

0
4

0
4

0

0

0

0
4

0
4

136.654

125.492

1.95486

16.5793

73.484
2

3.57081
3

0
3

1.29189

0

0

0

0

0

2.56481

0

0

0

0
3

0

0

0

0

0

0

0

0.195065

0

0

0

0

0

0

0

0

0

0.0975327

0

0

0

0

0

0

0

0

0.0975327

0.25592

0

9.89957

0.146299

0

0

0

0

0

0

0

15.3548

0
4

0

0

0

0
4

0

0

0
4

0

0

0

0
4

0

0

0
4

0

0

0
4

0

0

0

0
4

0.914089

0.914089

0
4

0

0

0
4

0

0

0
4

0

0

0

0
4

0.0530735

0.0530735

0
4

3.80377

3.80377

0
4

0

0

0
4

0

0

0
4

0.0722459

0.0722459

0
4

0

0

0
4

0

0

0
4

0

0

0
4

0

0

0
4

0.0397225

0.0397225

0
4

0

0

0
4

0

0

0
4

0.447275

0.138281

0.12796

0.0530735

0.06398

0.06398

0
4

0

0

0
4

0

0

0
4

0

0

0
4

0

0

0
4

0

0

0
4

0

0

0
4

0

0

0
4

0

0

0
4

0.132684

0.132684

0
4

0.243832

0.243832

0
4

0
3

0
3

0
4

0

0

0
4

0.0397225

0.0397225

0
4

0.0515724

0.0515724

0
4

0

0

0
4

0

0

0
4

0.495132

0.495132

0
4

0

0

0
4

4.38897

4.38897

0

0
4

0

0

0
4

0

0

0

0
4

0.479839

0.479839

0

0
4

0

0

0

0

0
4

0
4

0
4

0

0

0

0

0

0

0
4

0

0

0
4

0
4

0
4

997.643
3

250.53
3

2.67883
3

0.194636

0.063149

0.0634669

0

0.0796102

0

0

0

0.116338

0

2.10856

0

0.0530735

0

0

0
4

131.285
3

13.1272
3

1.4414
3

1.49221

0.274715

0.408865
3

6.01517

1.95734

0.448023
3

9.40898

7.00523

0.274715

5.81452
3

3.51897

0.234113
2

0

0.550046

0.557272

0

0

2.13377

0.244617

0.0909239

10.2752
3

0.210647

0.368229

0.0686787

0.093266

0.137357

0.063149

0

0

0.103018

0.0530735

17.03
3

0.189236

0

0.09597

0

0

0

0

1.03493

0

0.530735

20.213
3

0

0.093266

0.0666674

1.38928

0

1.45974

0.265367

0

0

0

0.238176
3

0.371514

0

0

0.09597

0

0.15995

0

0

0.06398

0

5.73006
3

0.0655183

0.852512

0.109682

0.0530735

0.15922

0

0

0.200002

0

0

4.59562
3

1.72793

0

1.3003

0

0

0

0

0.0873245

0

0

5.53288
2

0.0423112

0.232712

0.06398

0

0.86373

0

0

5.35127497869325e-14
3

0
4

82.3094
3

63.2618
3

0.093266

0.239605

0.0530735

0.045462

0.0799732

0.0530735

0.113655

0

0.0655183

0

11.0138
3

0.244447

0.15995

0.06398

0.0655183

0.0423112

0

0.045462

0.0403499

0.06398

3.27706
3

0.414171
3

2.07602
2

0.161513

0.139899

0.347677

0.147803

3.45279360658424e-14
3

0
4

9.99962
2

7.85035
3

0.591416

1.10469

0.178146

0.115072

0.06398

0.09597

7.07767178198537e-16
2

0
4

2.75559

2.75559

0

0
4

0.371514

0.371514

0
4

0.875713

0.875713

0
4

0

0

0
4

0

0

0

0
4

0

0

0
4

0

0

0
4

0

0

0
4

0

0

0
4

0

0

0
4

11.9584
2

8.17046
3

3.19808

0.0460286

0.41587

0.06398

0.06398

0
4

0

0

0
4

0

0

0
4

0

0

0
4

0

0

0
4

0

0

0
4

0.0846225

0.0846225

0
4

0

0

0
4

0.309434

0.309434

0
4

0

0

0
4

0

0

0
4

0

0

0
4

0

0

0
4

3.22327

3.22327

0
4

0

0

0
4

0

0

0
4

0

0

0
4

0

0

0
4

0.698135

0.698135

0
4

0

0

0

0
4

3.40004

2.26669

1.13335

0
4

0

0

0

0

0

0

0

0

0
4

0

0

0

0
4

0

0

0

0
4

0.580463

0.263129

0.0846225

0.232712

2.77555756156289e-17

0
4

0
4

512.403
3

232.138
3

220.648
3

10.8425

0.436368

0.0846225

0

0

0.126934

0

0

1.55431223447522e-15
3

0
4

19.0445
2

3.08275

0.0423112

1.50536

3.62839

6.91164

1.12125

0.157132

0

2.53585

0.0598143

2.49106291150269e-15
2

0
4

1.55614

1.43647

0.0773585

0.0423112

5.55111512312578e-17

0
4

0

0

0

0
4

0

0

0

0
4

158.927
2

4.17897
3

24.3985
2

0.906574
3

0

0.132628

0.12041

0

0.131037

0

0

0.044445

0

0

0.246785

0

0.0442094

0.0582163

0.480751

0

0

0

0

0

0

1.75606
2

0

0

0

0

0.0963278

0

0.0442094

0.0681929

0.0423112

0.0582163

7.93148
2

0.232865

0

0

0

0.666674

0

0

0

0

0.190435

0.306501
3

2.23831

0.0686787

0

0

0.0634669

0.0648786

0.0442094

0.0582163

0

0

1.66068

0

0

0

0.67698

0

0.0681929

0

0.054841

0.67698

0

0.665471
2

1.89203

0.340965

0.229314

0.545544

0.0663141

0

0.755465

0

0

0.045462

6.19703

0.0663141

0

0

0.198942

0

0

0.0634669

0

0.0460286

0

0.0582163
2

0

0

0.0846225

0

0.0722459

0

0.0884188

0

0.0460286

0.0722459

1.78416

0.0648786

0

0.0655183

0.0873245

0

0.32019

0

0.486579

0

0.216738

37.4553
3

2.34396

0

0

0

0

0

0

0

0.0634669

0

0.0655183

11.9319
2

0.0873245

0.054841

1.10239

0.126409

0.0963278

0.356322
2

1.0966
2

2.338

1.46517

0.877277

0

0.302908

0.491388

0.652951

0

0.213406
2

0

0.262

0.152843

0.0663141

1.90822
2

0.216738

5.64373

0

0.142487

0.291585

0.0481639

0

0.136386

1.68891

0.045462

3.42856
3

0.237659

0.169245

4.07225

0

0.494839

0.054841

0.103678

0.251086

2.34501

0.226753

0.31903
2

0.169245

0.113655

2.97148

0

0.191003

0.253867

0.129636

0.113655

0

0

1.18431
3

0.109682

0

0

0

0.0822615

0.453798

0

0.054841

0.221047

0

0.937772
2

2.51651

0.0686787

0.129757

1.15593

0.186696

0.493569

0.044445

0.129757

0

0

0.0598143

0

0.123735

0.158471

0

0

0.229314

0.103005

0.298592

0.133711

0.311115

0
4

92.8381
3

88.1511
3

1.29728

2.69918

0.0627716

0.627716

0
4

1.75778
3

0

0

0

0

0.045462

0.127087

1.15164

0

0.295503

0.0920573

0

0.0460286

0

0
4

0.465424

0.465424

0

0

0
4

0.409393

0.409393

0
4

0.417086

0.417086

0
4

0

0

0
4

2.38456

0.417086

1.96747

0
4

0

0

0
4

0.371514

0.371514

0
4

0

0

0
4

0

0

0
4

0

0

0
4

1.91065

1.91065

0
4

0.181848

0.181848

0
4

1.78052017574259e-13
3

0
4

31.7306

0

0

0

0

0

0

0

0

0

0
4

15.7299

6.4748

0

0

0

0

0

0

0

0

0

0

0

0

0

0

0

0

0

0

0

0

0

0.228522

0

0

0

0

0

0

0

0

0

0

8.11254

0

0

0

0

0

0

0

0

0

0

0.114261

0

0

0

0

0

0

0

0

0

0

0

0

0

0

0

0

0

0

0

0

0

0.533219

0

0

0

0

0

0

0

0

0

0

0

0

0

0

0

0

0.266609

1.16573417585641e-15

0
4

1.01292

0
7

1.01292

0

0
4

6.06865

6.06865

0
4

0

0

0
4

0

0

0
4

0.044445

0.044445

0
4

0.164523

0.164523

0
4

0.275023

0.275023

0
4

0

0

0
4

0

0

0
4

0

0

0
4

0

0

0
4

7.21889

7.21889

0
4

0

0

0

0

0
4

0
7

0
7

0
4

0.174649

0.174649

0

0
4

0

0

0
4

0.139029

0.0397225

0.0993063

0

0
4

0.0846225

0.0846225

0
4

0.817874

0.817874

0
4

4.44089209850063e-16

0
4

28.0793
3

20.89
3

11.0759
3

0

0.132948
3

8.30509
2

0.650517
3

0.13965

0.541721

0

0

0.0442094

0
4

1.63257
3

0.634823
3

0

0.129586
2

0.177845

0.170297

0.26412

0.0873245

0

0.168574

0

0
4

0.873245

0.873245

0

0

0
4

1.83883
3

1.34518

0.247536

0.0797524

0

0.0598143

0.0398762

0.0666674

0
4

1.12813

0.433475

0.0926089

0.602049

0

1.11022302462516e-16

0
4

0

0

0

0

0
4

0.3407
2

0

0.292536

0

0.0481639

6.93889390390723e-18
2

0
4

1.27604

1.27604

0

0
4

0.0997735

0.0997735

0

0
4

0
4

0

0

0

0
4

0

0

0

0
4

0
4

0.0722459

0.0722459

0.0722459

0

0
4

0

0

0
4

0
4

0

0

0

0
4

0
4

0

0

0

0
4

0
4

0

0

0

0

0
4

0
4

0

0

0

0
4

0
4

0.11996

0.11996

0.11996

0
4

0
4

0

0

0

0
4

0
4

0

0

0

0

0
4

0
4

0.0722459

0.0722459

0.0722459

0
4

0
4

45.9327

45.8163

37.0256

0.0873245

0

0

0

0

0.0582163

0

0

0

0

0.553055

0

1.86292

0.174649

0.436623

3.81317

0.0873245

1.30987

0.174649

0.116433

0.116433

0
4

0.0582163

0

0.0582163

0

0
4

0.0582163

0.0582163

0
4

0
4

0

0

0

0
4

0
4

0

0

0

0
4

0
4

0

0

0

0
4

0
4

0

0

0

0
4

0
4

0

0

0

0
4

0
4

0

0

0

0
4

0
4

0.0481639

0.0481639

0.0481639

0
4

0
4

0

0

0

0
4

0
4

0

0

0

0
4

0
4

0

0

0

0
4

0
4

36.6674

30.8261

21.558

2.63237

0.575831

4.4147

1.09682

0.219364

0.137103

0.109682

0.0822615

0
4

5.84128

3.37272

0.357179

0.877456

1.23392

0
4

0
4

0.045462

0.045462

0.045462

0
4

0
4

0

0

0

0
4

0
4

0

0

0

0
4

0
4

0.199933

0.199933

0.199933

0
4

0
4

29.6931
3

28.6229
3

16.8908
3

6.09986

2.98616

0.989678

0.168574

0.0722459

0

0.0398762

1.37573

4.44089209850063e-16
3

0
4

0.931461
3

0.931461
3

0

0
4

0.105504

0.105504

0
4

0

0

0
4

0.0332578

0.0332578

0
4

0
4

3.19122
3

2.71279
3

2.13338

0.0886544

0.216426

0.159505

0.0666674

0

0.0481639

0
4

0.338862
3

0.0666674

0.139567

0

0.132628

0
4

0.139567

0.139567

0
4

0

0

0

0
4

0
4

4.21185
2

1.89875
2

1.57756

0.0880401

0.0886544

0.0481639

0.0963278

0

6.93889390390723e-17
2

0
4

2.16022

0

0.31901

1.84121

2.22044604925031e-16

0
4

0.11301

0.0332578

0.0797524

0
4

0

0

0
4

0.0398762

0.0398762

0
4

0

0

0
4

4.02455846426619e-16
2

0
4

15.4206

0.756812

0.0873245

0.407514

0.145541

0

0.116433

5.55111512312578e-17

0
4

4.71552

0.0873245

0.0582163

4.56998

0

0
4

9.83856

9.83856

0
4

0.109682

0.109682

0
4

0
4

39.0811

38.6972

38.5327

0.164523

0
4

0.164523

0.109682

0.054841

0
4

0.219364

0.219364

0
4

2.08166817117217e-15

0
4

0.144492

0.0963278

0.0963278

0
4

0.0481639

0.0481639

0
4

0
4

0

0

0

0
4

0
4

0
4

287.102

3.45311

3.45311

2.83068

0.556906

0.0655183

0
4

0

0

0
4

0

0

0
4

0

0

0
4

0
4

2.32331

2.32331

2.32331

0
4

0

0

0
4

0

0

0

0

0

0

0
4

0

0

0

0

0

0

0

0

0
4

0
4

7.44417

0.189447

0

0

0

0

0.189447

0
4

0

0

0

0

0
4

6.53402

6.47087

0.063149

0
4

0.720702

0.720702

0
4

0

0

0
4

0
4

0
5

0
5

0
5

0

0
4

0
4

259.245

8.4609

8.4609

0

0
4

0
7

0
7

0
4

0
7

0
7

0
4

98.6501
2

90.785
2

0.0993063

0.119168

1.82724

0.0397225

0.0982775

0.238335

3.13808

0.0993063

0.557191

0.079445

0.734867

0.218474

0.15889

0.456809

0
4

0.403313

0.144963

0.079445

0.139183

0.0397225

0
4

4.76651

4.76651

0
4

2.04333

2.04333

0
4

0

0

0

0

0
4

0

0

0
4

0.695144

0.536254

0.0397225

0.119168

0
4

7.73627

7.57247

0.0982775

0.0655183

0
4

0.06398

0.06398

0
4

1.5628

1.5628

0

0
4

0

0

0
4

2.55796
2

1.89456
2

0.045462

0.197861

0.35189

0

0

0.0681929

0
4

0.238335

0.198613

0.0397225

0
4

0

0

0
4

0.079445

0.0397225

0.0397225

0
4

3.50523

3.50523

0
4

0.258196

0.258196

0
4

0.119168

0.079445

0.0397225

0
4

4.36948

4.32975

0.0397225

0
4

5.18238

0.06398

5.1184

0
4

0

0

0
4

0

0

0
4

1.85791
2

1.04187
2

0.438681

0.377364

0
4

0.09597

0.09597

0
4

0

0

0
4

0.0655183

0.0655183

0
4

0.0397225

0.0397225

0
4

0

0

0
4

0.0397225

0.0397225

0
4

0

0

0
4

0

0

0
4

0

0

0
4

0

0

0
4

89.005
2

1.23496

85.3271

0.516393

0.337641

0.198613

0.218474

0.47667

0.695144

0
4

0

0

0
4

0

0

0
4

0

0

0
4

0.0822527

0.0822527

0
4

0

0

0
4

0

0

0
4

0

0

0
4

0.360351

0.360351

0
4

0

0

0
4

2.22909

2.22909

0
4

10.5653
2

5.05169

5.17595

0.0397225

0.139029

0.0595838

0.0993063

0
4

7.08108

6.85177

0.229314

3.60822483003176e-16

0
4

3.34144

2.98108

0.262073

0.0982775

0
4

2.84424
2

2.84424
2

0
4

0.945348

0.423021

0.125102

0.0397225

0.0397225

0.31778

0
4

1.07802655691103e-13

0
4

1.37049
6

1.37049
6

1.37049
6

0
4

0

0

0
4

0
4

0

0

0

0
4

0
4

0

0

0

0
4

0
4

0

0

0

0
4

0
4

0

0

0

0
4

0
4

0
7

0
7

0
7

0
4

0
4

0

0

0

0
4

0
4

0

0

0

0

0

0
4

0
4

0

0

0

0

0
4

0

0

0
4

0
4

13.1372

13.1372

13.1372

0
4

0
4

0.128667

0.128667

0.063149

0.0655183

0
4

0
4

0

0

0

0
4

0
4

0

0

0

0
4

0
4

2.32036612146658e-14

0
4

0
6

0
6

0
7

0
7

0
6

0

0

0

0

0

0
6

0
6

0
7

0

0
7

0

0
6

0

0

0
6

0

0

0

0

0

0

0

0

0

0

0
7

0

0

0

0

0

0

0

0

0

0

0
6

0

0

0

0

0

0

0

0

0

0

0
6

0

0

0

0

0

0

0

0

0

0

0
6

0

0

0

0

0

0

0

0

0

0

0
6

0

0

0

0

0

0

0

0

0

0

0
7

0

0

0

0

0

0

0

0

0

0

0
6

0

0

0

0

0

0

0

0

0

0

0
4

0
6

0
6

0

0

0

0

0

0

0

0
4

0
6

0

0

0

0

0

0

0

0

0
4

0
7

0
7

0

0

0

0

0

0

0
4

0
6

0

0

0

0

0

0

0
4

0

0

0

0

0
4

0

0

0

0

0

0
4

0

0

0

0

0
4

0

0

0
4

0
4

0
4

1.92893

1.92893

0.105778

0.0634669

0

0

0.0423112

0
5

0

0

0

0

0

0

0

0
4

1.82315
2

1.82315
2

0

0
4

0

0

0

0

0

0

0
4

0

0

0

0

0
4

0
4

0
7

0
7

0

0

0
4

0

0

0

0
4

0
4

0

0

0

0

0
4

0

0

0
4

0
4

0

0

0

0

0

0

0
4

0
4

0
4

0

0

0

0

0
4

0

0

0
4

0
4

0
4

0

0

0

0

0
4

0
4

0
4

0.28791

0.28791

0.28791

0.28791

0
4

0

0

0
4

0
4

0
4

0

0

0

0

0
4

0

0

0
4

0

0

0
4

0
4

0

0

0

0
4

0
4

0
4

0

0

0

0

0
4

0

0

0

0
4

0
4

0
4

0

0

0

0

0

0
4

0

0

0
4

0
4

0
4

4.6743

4.6743

0.139029

0.0397225

0.0595838

0.0397225

1.38777878078145e-17

0
4

4.53527

4.53527

0
4

0
4

0
4

0

0

0

0

0
4

0

0

0
4

0
4

0

0

0

0
4

0
4

0
4

0

0

0

0

0

0

0
4

0

0

0
4

0
4

0
4

0

0

0

0

0
4

0
4

0
4

0
6

0
6

0
6

0
6

0

0
4

0
6

0
7

0

0

0

0

0

0

0
4

0
6

0
7

0

0

0
4

0

0

0
4

0

0

0
4

0
4

0
4

0

0

0

0

0

0
4

0
4

0
4

0.720783

0.720783

0.720783

0.720783

0
4

0
4

0
4

0

0

0

0

0
4

0
4

0
4

0

0

0

0

0
4

0

0

0
4

0
4

0
4

0

0

0

0

0

0
4

0

0

0
4

0
4

0
4

0

0

0

0

0
4

0
4

0
4

0.15889

0.0993063

0

0

0
4

0.0993063

0.0993063

0
4

0
4

0.0595838

0.0595838

0.0595838

0
4

0
4

0
4

0

0

0

0

0

0

0
4

0
4

0
4

0

0

0

0

0
4

0

0

0
4

0
4

0
4

0.0418477

0.0418477

0.0418477

0

0.0418477

0
4

0
4

0
4

0
3

0
3

0
4

0
3

0

0

0

0

0

0

0

0

0

0

0
4

0
3

0

0

0

0

0
4

0

0

0
4

0
4

0
4

0

0

0

0

0
4

0
4

0
4

0

0

0

0

0
4

0
4

0
4

0

0

0

0

0
4

0

0

0
4

0

0

0
4

0
4

0
4

0

0

0

0

0
4

0

0

0
4

0
4

0
4

1.54571

1.54571

1.54571

1.3184

0.22731

0
4

0
4

0
4

0

0

0

0

0
4

0

0

0
4

0
4

0
4

0

0

0

0

0
4

0
4

0
4

0

0

0

0

0
4

0
4

0
4

0

0

0

0

0
4

0

0

0
4

0
4

0
4

0

0

0

0

0
4

0
4

0
4

0.159946

0

0

0
7

0

0

0

0

0

0

0

0

0
4

0

0

0

0

0
4

0

0

0

0

0
4

0

0

0
4

0

0

0
4

0
4

0.159946

0.159946

0

0.159946

0

0

0

0

0

0

0
4

0

0

0

0

0
4

0

0

0

0
4

0

0

0
4

0

0

0
4

0
4

0

0

0

0
4

0

0

0
4

0
4

0

0

0

0
4

0
4

0
4

0

0

0

0

0

0

0
4

0
4

0
4

0

0

0

0

0

0
4

0
4

0
4

0

0

0

0

0
4

0
4

0
4

0

0

0

0

0
4

0

0

0
4

0
4

0
4

0.0548351

0.0548351

0.0548351

0.0548351

0
4

0
4

0
4

0

0

0

0

0
4

0
4

0
4

0

0

0

0

0
4

0

0

0
4

0
4

0

0

0

0
4

0
4

0
4

0

0

0

0

0
4

0
4

0
4

0

0

0

0

0
4

0

0

0
4

0
4

0

0

0

0
4

0
4

0
4

0

0

0

0

0

0
4

0
4

0
4

0.799732

0.799732

0.799732

0

0

0.799732

0

0

0
4

0
5

0
5

0

0

0
4

0

0

0

0
4

0
4

0
4

0

0

0

0

0

0
4

0

0

0
4

0
4

0
4

0

0

0

0

0
4

0
4

0
4

0

0

0

0

0
4

0

0

0
4

0
4

0
4

0

0

0

0

0

0

0
4

0
4

0
4

0.0799732

0.0799732

0.0799732

0.0799732

0
4

0
4

0
4

0

0

0

0

0
4

0

0

0
4

0
4

0
4

0

0

0

0

0

0
4

0
4

0
4

0

0

0

0

0
4

0

0

0
4

0
4

0

0

0

0
4

0
4

0
4

0

0

0

0

0
4

0
4

0
4

0.432352

0.432352

0.432352

0.432352

0
4

0
4

0
4

1.31592

1.31592

1.05672

0.877277

0.179443

0

0

0

0
4

0.259195

0.259195

0

0

0

0
4

0
4

0
4

0

0

0

0

0

0
4

0

0

0
4

0
4

0
4

0

0

0

0

0
4

0

0

0
4

0
4

0

0

0

0
4

0
4

0
4

0

0

0

0

0
4

0

0

0
4

0
4

0
4

0

0

0

0

0
4

0

0

0
4

0
4

0
4

0.079445

0.079445

0.079445

0.0397225

0.0397225

0
4

0
4

0
4

0

0

0

0

0

0
4

0
4

0
4

0

0

0

0

0

0
4

0
4

0
4

0

0

0

0

0
4

0
4

0
4

0

0

0

0

0
4

0

0

0
4

0
4

0
4

0

0

0

0

0
4

0
4

0
4

0
6

0
6

0
6

0
6

0

0

0

0

0
7

0
6

0

0

0

0

0

0

0
4

0

0

0
4

0
4

0
4

0

0

0

0

0
4

0

0

0
4

0
4

0
4

0

0

0

0

0
4

0

0

0
4

0
4

0
4

0

0

0

0

0
4

0
4

0
4

0

0

0

0

0
4

0
4

0
4

0

0

0

0

0
4

0

0

0
4

0
4

0
4

0

0

0

0

0

0
4

0
4

0
4

0

0

0

0

0

0
4

0
4

0
4

0

0

0

0

0
4

0
4

0
4

0

0

0

0

0
4

0
4

0
4

0

0

0

0

0
4

0

0

0
4

0
4

0
4

0
7

0
7

0
7

0
7

0

0

0
4

0
4

0

0

0

0
4

0

0

0
4

0
4

0
4

0

0

0

0

0
4

0

0

0
4

0
4

0
4

0

0

0

0

0

0
4

0
4

0
4

0

0

0

0

0
4

0
4

0
4

0

0

0

0

0
4

0

0

0
4

0
4

0
4

0

0

0

0

0
4

0
4

0
4

0.0481639

0.0481639

0.0481639

0.0481639

0
4

0
4

0
4

0

0

0

0

0
4

0

0

0
4

0
4

0
4

0

0

0

0

0
4

0

0

0
4

0
4

0
4

0

0

0

0

0

0
4

0
4

0
4

0

0

0

0

0

0
4

0
4

0
4

0

0

0

0

0

0

0

0

0

0

0
4

0

0

0
4

0
4

0
4

0

0

0

0

0

0
4

0
4

0
4

0

0

0

0

0
4

0
4

0
4

0

0

0

0

0
4

0

0

0
4

0
4

0
4

0

0

0

0

0
4

0

0

0
4

0
4

0
4

0

0

0

0

0
4

0

0

0
4

0
4

0
4

0

0

0

0

0
4

0

0

0
4

0
4

0
4

0

0

0

0

0
4

0

0

0
4

0
4

0
4

0

0

0

0

0

0
4

0
4

0
4

0

0

0

0

0
4

0
4

0
4

0

0

0

0

0

0
4

0
4

0
4

0.0398762

0.0398762

0
5

0

0

0

0

0

0

0
4

0

0

0

0

0
4

0.0398762
3

0.0398762

0

0
4

0

0

0
4

0

0

0
4

0
4

0

0

0

0

0

0
4

0

0

0

0
4

0
4

0
4

0.598641

0.598641

0.598641

0.598641

0
4

0
4

0
4

0

0

0

0

0

0
4

0
4

0
4

0.06398

0.06398

0.06398

0.06398

0
4

0

0

0
4

0
4

0
4

0

0

0

0

0
4

0

0

0
4

0
4

0
4

0

0

0

0

0
4

0
4

0
4

0

0

0

0

0
4

0
4

0
4

0

0

0

0

0
4

0
4

0
4

0.044445

0.044445

0.044445

0.044445

0
4

0

0

0
4

0
4

0
4

0

0

0

0

0
4

0
4

0
4

0

0

0

0

0
4

0
4

0
4

10.9728

10.9728

10.8966

3.6841

0

0.0665157

0

0

0

0

0

0

0

0

4.65083

0

0

0

0

0

0

0

0

0

0

0.728764
3

0

0

0.761741

0

0.0722459

0

0.0963278

0

0

0.166289
3

0.174649
3

0
3

0.0761741

0

0.418957

0
4

0

0

0

0
4

0

0

0

0

0

0
4

0

0

0
4

0

0

0

0
4

0

0

0
4

0.0761741

0.0761741

0
4

0

0

0
4

0
4

0
4

0
7

0
7

0
7

0
7

0
7

0

0

0

0

0
4

0

0

0
4

0

0

0

0
4

0

0

0
4

0
4

0
4

0

0

0

0

0
4

0
4

0
4

0

0

0

0

0
4

0

0

0
4

0
4

0
4

0

0

0

0

0
4

0
4

0
4

0.67179

0.67179

0.67179

0.67179

0
4

0
4

0
4

0.15889

0.15889

0.15889

0.119168

0.0397225

0
4

0
4

0
4

0

0

0

0

0
4

0
4

0
4

0

0

0

0

0
4

0
4

0
4

0

0

0

0

0
4

0
4

0
4

0

0

0

0

0
4

0
4

0
4

0

0

0

0

0
4

0
4

0
4

0.971135

0.857481

0.211556
2

0
2

0.211556

0

0
4

0
5

0
5

0

0
4

0.645924

0.59776

0

0.0481639

6.93889390390723e-18

0
4

0
4

0.113655

0.113655

0.113655

0
4

0
4

2.77555756156289e-17

0
4

0

0

0

0

0

0
4

0
4

0
4

0

0

0

0

0
4

0
4

0
4

0

0

0

0

0
4

0

0

0
4

0
4

0
4

0.0403499

0.0403499

0.0403499

0.0403499

0

0
4

0
4

0
4

0

0

0

0

0
4

0
4

0
4

0

0

0

0

0
4

0
4

0
4

0

0

0

0

0
4

0
4

0
4

0

0

0

0

0
4

0
4

0
4

0

0

0

0

0
4

0
4

0
4

0

0

0

0

0
4

0
4

0
4

0

0

0

0

0

0

0

0

0

0

0
4

0
4

0
4

0

0

0

0

0
4

0
4

0
4

1.11965

1.11965

1.11965

1.11965

0
4

0
4

0
4

0

0

0

0

0
4

0
4

0
4

0

0

0

0

0
4

0
4

0
4

0.069043

0.069043

0.069043

0.069043

0
4

0
4

0
4

0

0

0

0

0
4

0
4

0
4

0

0

0

0

0
4

0
4

0
4

0

0

0

0

0
4

0
4

0
4

0.0595838

0.0595838

0.0595838

0.0595838

0
4

0
4

0
4

0

0

0

0

0
4

0
4

0
4

0
6

0
6

0
6

0
6

0

0
4

0
6

0

0

0

0
4

0

0

0

0
4

0

0

0
4

0
4

0
4

0.045462

0.045462

0.045462

0.045462

0
4

0
4

0
4

0

0

0

0

0
4

0
4

0
4

0

0

0

0

0
4

0
4

0
4

0

0

0

0

0
4

0
4

0
4

0

0

0

0

0
4

0
4

0
4

0

0

0

0

0
4

0
4

0
4

0

0

0

0

0
4

0
4

0
4

0

0

0

0

0
4

0
4

0
4

0

0

0

0

0
4

0
4

0
4

0

0

0

0

0
4

0
4

0
4

13.1371

8.6558

8.60977

8.31689

0.1611

0.0397225

0.0920573

0
4

0.0460286

0.0460286

0
4

7.63278329429795e-17

0
4

4.4813

1.31149

0.436948

0.874544

0
4

3.16981

3.16981

0
4

0
4

0
4

0

0

0

0

0
4

0
4

0
4

0

0

0

0

0
4

0
4

0
4

0.0460286

0.0460286

0.0460286

0.0460286

0
4

0
4

0
4

0

0

0

0

0
4

0
4

0
4

0

0

0

0

0
4

0
4

0
4

0

0

0

0

0
4

0
4

0
4

0

0

0

0

0
4

0
4

0
4

0

0

0

0

0
4

0
4

0
4

0

0

0

0

0
4

0
4

0
4

0

0

0

0

0
4

0
4

0
4

0.50224

0.50224

0.39257
2

0.215448
2

0.0634669

0.113655

0

0
4

0.0548351

0.0548351

0
4

0

0

0

0

0
4

0

0

0
4

0.0548351

0.0548351

0
4

0
4

0
4

0

0

0

0

0
4

0
4

0
4

0

0

0

0

0
4

0
4

0
4

0

0

0

0

0
4

0
4

0
4

0

0

0

0

0
4

0
4

0
4

0.194636

0.194636

0.194636

0.194636

0
4

0
4

0
4

0

0

0

0

0
4

0
4

0
4

0

0

0

0

0
4

0
4

0
4

0

0

0

0

0
4

0
4

0
4

0

0

0

0

0
4

0
4

0
4

0

0

0

0

0
4

0
4

0
4

0
7

0
7

0
7

0
7

0
4

0

0

0
4

0
4

0
4

0

0

0

0

0
4

0
4

0
4

0

0

0

0

0
4

0
4

0
4

0

0

0

0

0
4

0
4

0
4

0

0

0

0

0
4

0
4

0
4

0.0648786

0.0648786

0.0648786

0.0648786

0
4

0
4

0
4

0

0

0

0

0
4

0
4

0
4

0

0

0

0

0
4

0
4

0
4

0

0

0

0

0
4

0
4

0
4

0

0

0

0

0
4

0
4

0
4

0

0

0

0

0
4

0
4

0
4

0
7

0
7

0
7

0

0

0

0
4

0

0

0

0

0
4

0

0

0
4

0
4

0
4

0.577967

0.577967

0.577967

0.577967

0
4

0
4

0
4

0

0

0

0

0
4

0
4

0
4

0

0

0

0

0
4

0
4

0
4

0.0397225

0.0397225

0.0397225

0.0397225

0
4

0
4

0
4

0

0

0

0

0
4

0
4

0
4

0

0

0

0

0
4

0
4

0
4

0

0

0

0

0
4

0
4

0
4

0

0

0

0

0
4

0
4

0
4

0

0

0

0

0
4

0
4

0
4

0

0

0

0

0
4

0
4

0
4

2.43946

0.161819

0

0

0

0
4

0.0681929

0

0.0681929

0
4

0.0936259

0.0936259

0
4

0
4

1.69245

1.69245

1.69245

0

0

0

0

0
4

0
4

0.585196

0.585196

0.585196

0

0
4

0
4

0
4

0

0

0

0

0
4

0
4

0
4

0

0

0

0

0
4

0
4

0
4

0.0648786

0.0648786

0.0648786

0.0648786

0
4

0
4

0
4

0.139899

0.139899

0.139899

0.139899

0
4

0
4

0
4

0

0

0

0

0
4

0
4

0
4

0

0

0

0

0
4

0
4

0
4

0

0

0

0

0
4

0
4

0
4

0

0

0

0

0
4

0
4

0
4

0

0

0

0

0
4

0
4

0
4

0

0

0

0

0
4

0
4

0
4

3.32696

3.32696

3.32696

3.32696

0

0

0

0
4

0
4

0
4

0

0

0

0

0
4

0
4

0
4

0

0

0

0

0
4

0
4

0
4

0

0

0

0

0
4

0
4

0
4

0.0481639

0.0481639

0.0481639

0.0481639

0
4

0
4

0
4

0

0

0

0

0
4

0
4

0
4

0

0

0

0

0
4

0
4

0
4

0

0

0

0

0
4

0
4

0
4

0

0

0

0

0
4

0
4

0
4

0

0

0

0

0
4

0
4

0
4

0

0

0

0

0
4

0
4

0
4

4.37387
7

4.37387
7

4.37387
7

3.18903
7

0

0

0

0

0

0

0

0

0

0

0.774378
7

0

0

0

0

0

0

0

0

0

0.252596

0
6

0

0

0

0

0

0

0

0

0

0

0.063149
7

0

0

0

0

0

0

0

0

0

0

0

0

0

0.0947236

0

0

1.38777878078145e-16
7

0
4

0
6

0

0

0

0

0

0

0

0
4

0
4

0
6

0
6

0

0

0

0

0

0

0
4

0

0

0

0

0
4

0

0

0
4

0
4

0
4

0
4

0
4

0

0

0

0

0

0

0

0

0
4

0

0

0

0

0
4

0

0

0

0
4

0

0

0
4

0

0

0
4

0
4

0
4

0

0

0

0

0
4

0
4

0
4

0

0

0

0

0
4

0
4

0
4

0

0

0

0

0
4

0
4

0
4

0

0

0

0

0
4

0
4

0
4

0

0

0

0

0
4

0
4

0
4

0

0

0

0

0
4

0
4

0
4

0

0

0

0

0
4

0
4

0
4

0

0

0

0

0
4

0
4

0
4

0

0

0

0

0
4

0
4

0
4

0

0

0

0

0
4

0
4

0
4

0
4

0
4

0
4

0

0

0

0

0

0

0
4

0
4

0

0

0

0

0

0
4

0

0

0
4

0
4

0
4

0

0

0

0

0
4

0
4

0
4

0

0

0

0

0
4

0
4

0
4

0

0

0

0

0
4

0
4

0
4

0

0

0

0

0
4

0
4

0
4

0

0

0

0

0
4

0
4

0
4

0

0

0

0

0
4

0
4

0
4

0

0

0

0

0
4

0
4

0
4

0

0

0

0

0
4

0
4

0
4

0.06398

0.06398

0.06398

0.06398

0
4

0
4

0
4

0

0

0

0

0
4

0
4

0
4

0
6

0
7

0

0

0

0

0
4

0

0

0

0

0
4

0

0

0

0
4

0
4

0

0

0

0

0
4

0

0

0
4

0
4

0
4

0

0

0

0

0
4

0
4

0
4

0

0

0

0

0
4

0
4

0
4

0

0

0

0

0
4

0
4

0
4

0

0

0

0

0
4

0
4

0
4

0.195065

0.195065

0.195065

0.195065

0
4

0
4

0
4

0

0

0

0

0
4

0
4

0
4

0

0

0

0

0
4

0
4

0
4

0

0

0

0

0
4

0
4

0
4

0

0

0

0

0
4

0
4

0
4

0

0

0

0

0
4

0
4

0
4

0.719674

0.719674

0
6

0
6

0

0
4

0.475842

0

0.475842

0
4

0.243832

0.243832

0
4

0
4

0
4

0

0

0

0

0
4

0
4

0
4

0

0

0

0

0
4

0
4

0
4

0

0

0

0

0
4

0
4

0
4

0.06398

0.06398

0.06398

0.06398

0
4

0
4

0
4

0

0

0

0

0
4

0
4

0
4

0

0

0

0

0
4

0
4

0
4

0

0

0

0

0
4

0
4

0
4

0

0

0

0

0
4

0
4

0
4

0

0

0

0

0
4

0
4

0
4

0

0

0

0

0
4

0
4

0
4

0

0

0
5

0

0

0

0

0
4

0

0

0

0

0
4

0

0

0

0

0
4

0

0

0
4

0

0

0
4

0
4

0
4

0

0

0

0

0
4

0
4

0
4

0.0595838

0.0595838

0.0595838

0.0595838

0
4

0
4

0
4

0

0

0

0

0
4

0
4

0
4

0

0

0

0

0
4

0
4

0
4

0

0

0

0

0
4

0
4

0
4

0

0

0

0

0
4

0
4

0
4

0

0

0

0

0
4

0
4

0
4

0

0

0

0

0
4

0
4

0
4

0

0

0

0

0
4

0
4

0
4

0.06398

0.06398

0.06398

0.06398

0
4

0
4

0
4

0
4

0
4

0
4

0

0

0

0

0
4

0

0

0

0

0

0
4

0
4

0
4

0

0

0

0

0
4

0
4

0
4

0.487663

0.487663

0.487663

0.487663

0
4

0
4

0
4

0

0

0

0

0
4

0
4

0
4

0

0

0

0

0
4

0
4

0
4

0

0

0

0

0
4

0
4

0
4

0

0

0

0

0
4

0
4

0
4

0

0

0

0

0
4

0
4

0
4

0

0

0

0

0
4

0
4

0
4

0

0

0

0

0
4

0
4

0
4

0.203757

0.203757

0.203757

0.203757

0
4

0
4

0
4

0

0

0

0

0

0

0

0

0

0
4

0

0

0

0

0
4

0

0

0

0
4

0

0

0
4

0

0

0
4

0
4

0

0

0

0
4

0
4

0

0

0

0
4

0
4

0
4

0

0

0

0

0
4

0
4

0
4

0

0

0

0

0
4

0
4

0
4

0

0

0

0

0
4

0
4

0
4

0

0

0

0

0
4

0
4

0
4

0

0

0

0

0
4

0
4

0
4

0

0

0

0

0
4

0
4

0
4

0

0

0

0

0
4

0
4

0
4

0.0761741

0.0761741

0.0761741

0.0761741

0
4

0
4

0
4

0

0

0

0

0
4

0
4

0
4

0

0

0

0

0
4

0
4

0
4

5.60158

5.60158

4.406

4.12972

0.276284

5.55111512312578e-16

0
4

1.19557

0.742313

0.0975327

0.178751

0.0975327

0.079445

1.2490009027033e-16

0
4

0
4

0
4

0

0

0

0

0
4

0
4

0
4

0

0

0

0

0
4

0
4

0
4

0

0

0

0

0
4

0
4

0
4

0

0

0

0

0
4

0
4

0
4

0

0

0

0

0
4

0
4

0
4

0

0

0

0

0
4

0
4

0
4

0

0

0

0

0
4

0
4

0
4

0

0

0

0

0
4

0
4

0
4

0

0

0

0

0
4

0
4

0
4

0

0

0

0

0
4

0
4

0
4

1.37573

1.37573

1.33585

1.33585

0

0
4

0.0398762

0.0398762

0
4

0
4

0
4

0

0

0

0

0
4

0
4

0
4

0

0

0

0

0
4

0
4

0
4

0

0

0

0

0
4

0
4

0
4

0

0

0

0

0
4

0
4

0
4

0

0

0

0

0
4

0
4

0
4

0

0

0

0

0
4

0
4

0
4

0

0

0

0

0
4

0
4

0
4

0.103145

0.103145

0.103145

0.103145

0
4

0
4

0
4

0

0

0

0

0
4

0
4

0
4

0

0

0

0

0
4

0
4

0
4

0

0

0

0

0

0

0

0

0

0
4

0

0

0

0
4

0
4

0
4

0

0

0

0

0
4

0
4

0
4

0

0

0

0

0
4

0
4

0
4

0

0

0

0

0
4

0
4

0
4

0

0

0

0

0
4

0
4

0
4

0

0

0

0

0
4

0
4

0
4

3.39556

3.39556

3.39556

3.39556

0
4

0
4

0
4

0

0

0

0

0
4

0
4

0
4

0

0

0

0

0
4

0
4

0
4

0

0

0

0

0
4

0
4

0
4

0

0

0

0

0
4

0
4

0
4

0

0

0

0

0

0

0

0

0

0

0

0

0

0

0

0

0

0

0

0

0

0

0

0

0

0

0

0

0

0

0
4

0

0

0

0

0

0

0
4

0

0

0

0

0

0

0
4

0

0

0

0

0

0

0

0
4

0

0

0

0
4

0

0

0
4

0
4

0

0

0

0

0

0

0

0

0

0

0

0

0

0

0

0

0

0

0

0

0
4

0

0

0

0

0

0

0

0

0

0
4

0

0

0

0

0

0

0
4

0

0

0

0

0
4

0

0

0
4

0

0

0
4

0
4

0

0

0

0

0

0
4

0
4

0

0

0

0
4

0

0

0
4

0
4

0
4

0
6

0
6

0
6

0

0

0

0
4

0

0

0
4

0
4

0
4

0.06398

0.06398

0.06398

0.06398

0
4

0
4

0
4

0

0

0

0

0
4

0
4

0
4

0

0

0

0

0
4

0
4

0
4

0

0

0

0

0
4

0
4

0
4

0

0

0

0

0

0

0
4

0

0

0
4

0
4

0

0

0

0
4

0
4

0
4

0
6

0
6

0
6

0
6

0
4

0
4

0
4

0

0

0

0

0

0

0
4

0

0

0

0
4

0

0

0

0

0
4

0
4

0
4

0
7

0
7

0
7

0

0

0

0

0
4

0
4

0
4

0.189447
4

0
4

0

0

0

0

0
4

0

0

0

0

0
4

0

0

0
4

0

0

0
4

0
4

0.189447

0.189447

0.189447

0

0
4

0

0

0
4

0

0

0
4

0
4

0
4

0

0

0

0

0

0
4

0

0

0
4

0
4

0

0

0

0
4

0
4

0
4

1.21916
3

1.21916
3

1.21916
3

0

1.21916

0

0
4

0

0

0

0

0

0
4

0
4

0
4

0

0

0

0

0

0

0

0

0
4

0

0

0
4

0
4

0
4

0.406669
3

0.406669
3

0.406669
3

0.406669

0

0

0

0

0
4

0
4

0
4

5.26351
7

5.26351
7

4.74332
7

4.66714
7

0

0

0.0761741

0

0

0

0

0

0

0

0

0
4

0

0

0
4

0

0

0
4

0.063149

0.063149

0
6

0

0

0

0

0
4

0
6

0
6

0

0
4

0

0

0
4

0

0

0
4

0.457045

0.457045

0
4

0

0

0
4

0

0

0
4

0

0

0
4

0
4

0
4

0

0

0

0

0

0
4

0
4

0
4

0.126298
4

0.126298
4

0.126298

0

0

0

0.126298

0
4

0

0

0

0

0

0
4

0
4

0
4

0

0

0

0

0

0

0

0

0
4

0
4

0
4

0
4

0
4

0
4

0

0

0
4

0

0

0

0
4

0

0

0
4

0

0

0
4

0
4

0
4

0

0

0

0

0

0

0
4

0

0

0
4

0
4

0
4

0
4

0
4

0
4

0

0

0

0
4

0
4

0

0

0

0
4

0
4

0
4

8.36613

8.36613

8.36613

7.82362

0.054841

0.292598

0.0975327

0.0975327

2.05391259555654e-15

0
4

0
4

0
4

0

0

0

0

0

0

0

0

0
4

0

0

0
4

0
4

0
4

0

0

0

0

0

0
4

0

0

0
4

0
4

0
4

0

0

0

0

0

0

0

0
4

0
4

0
4

0
5

0
5

0
6

0
6

0

0

0

0

0

0

0

0

0

0

0

0

0

0

0

0

0
4

0

0

0

0

0

0

0

0

0

0

0

0
4

0
4

0

0

0

0

0

0

0

0

0
4

0

0

0

0

0

0

0
4

0

0

0

0

0
4

0

0

0
4

0

0

0
4

0

0

0
4

0
4

0
6

0

0

0

0

0

0
4

0

0

0

0

0

0
4

0

0

0

0

0

0
4

0

0

0
4

0
4

0

0

0

0
4

0

0

0
4

0
4

0
4

0.657958

0.657958

0.657958

0.657958

0

0

0
4

0
4

0
4

0

0

0

0

0

0
4

0

0

0
4

0

0

0
4

0
4

0
4

0
6

0
6

0

0

0

0
4

0

0

0

0
4

0
4

0
4

0

0

0

0

0

0
4

0
4

0

0

0

0
4

0

0

0
4

0
4

0
4

0.195065

0

0

0

0

0
4

0
4

0.195065

0.195065

0.195065

0
4

0
4

0

0

0

0
4

0
4

0
4

0

0

0

0

0

0

0

0
4

0
4

0
4

0

0

0

0

0

0
4

0

0

0
4

0
4

0
4

0

0

0

0

0

0
4

0
4

0
4

0
3

0
3

0
3

0

0

0
4

0

0

0
4

0
4

0
4

0

0

0

0

0

0

0
4

0

0

0
4

0
4

0
4

4.73351

4.73351

4.73351

4.52506

0

0

0
3

0.0398762

0.0481639
3

0.0722459
3

0.0481639

0

0

0

4.64905891561784e-16

0
4

0

0

0
4

0
2

0

0

0

0

0
4

0

0

0

0

0
4

0

0

0
4

0

0

0
4

0

0

0

0
4

0

0

0

0
4

0

0

0
4

0

0

0
4

0
4

0
4

0

0

0

0

0

0
4

0

0

0
4

0

0

0

0
4

0
4

0
4

0

0

0

0

0

0

0
4

0

0

0
4

0

0

0
4

0
4

0
4

0.438897

0.438897

0.0975327

0

0.0975327

0
4

0.341364

0.341364

0
4

0
4

0

0

0

0
4

0
4

0
4

0
6

0

0

0

0

0
4

0

0

0
4

0

0

0
4

0
4

0

0

0

0
4

0
4

0
4

0
6

0
6

0
7

0
7

0
4

0

0

0
4

0
4

0
4

0

0

0

0

0

0

0
4

0
4

0
4

0

0

0

0

0

0

0
4

0

0

0
4

0
4

0

0

0

0

0
4

0
4

0
4

0
7

0
7

0

0

0

0
4

0

0

0
4

0
4

0
4

0

0

0

0

0

0
4

0
4

0

0

0

0
4

0

0

0
4

0
4

0
4

0

0

0

0

0
4

0

0

0
4

0
4

0
4

0
7

0
6

0
7

0
6

0
7

0

0

0

0

0

0
4

0
6

0
6

0

0

0

0

0
4

0

0

0

0
4

0
4

0
7

0
7

0

0

0

0

0

0

0

0

0

0

0

0
4

0

0

0

0

0
4

0

0

0

0
4

0

0

0
4

0
4

0
4

0

0

0

0

0
4

0
4

0
4

0

0

0

0

0

0
4

0

0

0

0
4

0

0

0
4

0
4

0
4

0

0

0

0

0

0
4

0
4

0
4

0

0

0

0

0

0
4

0

0

0
4

0
4

0
4

0

0

0

0

0
4

0

0

0
4

0
4

0
4

0

0

0

0

0

0
4

0

0

0
4

0
4

0

0

0

0
4

0
4

0

0

0

0
4

0
4

0
4

0

0

0

0

0
4

0

0

0
4

0
4

0
4

0.337641

0.337641

0.15889

0.079445

0.0397225

0.0397225

0
4

0.178751

0.178751

0
4

0
4

0
4

0

0

0

0

0
4

0

0

0
4

0

0

0
4

0
4

0
4

0

0

0

0

0

0

0
4

0
4

0
4

0
4

0
4

0
4

0
4

0

0

0

0

0

0

0
4

0

0

0
4

0
4

0
4

0

0

0

0

0

0

0

0

0

0
4

0

0

0

0

0
4

0

0

0

0

0

0
4

0

0

0
4

0
4

0

0

0

0

0

0
4

0

0

0
4

0

0

0
4

0
4

0

0

0

0
4

0

0

0
4

0
4

0
4

0
6

0
6

0
6

0
6

0
4

0
4

0
4

0

0

0

0

0
4

0
4

0
4

0

0

0

0

0
4

0
4

0
4

0

0

0

0

0
4

0

0

0
4

0
4

0
4

0

0

0

0

0

0
4

0
4

0
4

0

0

0

0

0

0
4

0

0

0

0
4

0
4

0
4

1.29567

1.29567

1.29567

1.29567

0
4

0
4

0
4

0

0

0

0

0

0
4

0
4

0
4

0

0

0

0

0

0

0
4

0
4

0
4

0

0

0

0

0
4

0
4

0
4

4.23858
3

4.23858
3

4.23858
3

1.7994

0

0

0

0

0.159946
3

2.27924
3

0

0

0

0

0

0

0
4

0

0

0
4

0

0

0
4

0

0

0
4

0
4

0

0

0

0

0

0
4

0
4

0

0

0

0
4

0
4

0
4

77.6691
3

3.95007

3.95007

3.95007

0
4

0
4

0.519826

0.519826

0

0.519826

0
4

0
4

1.91406

1.91406

1.91406

0
4

0
4

0

0

0

0
4

0
4

0

0

0

0
4

0
4

71.2852
3

68.6061
3

3.7933

0.349298

0.174649

0

0.0722459

25.3241

0

0.203757

0

0.0797524

0.241511

21.8899

0.0873245

0.145541

0.063149

0.203757

0

0

0.063149

0.0582163

0.0873245

0.06398

0.948752

1.47416

0.0582163

0

0

0.145541

0

0.0481639

1.42313

2.16502
3

1.83381

3.96676

2.00212

1.63945

0
4

0.06398

0.06398

0

0

0
4

2.6151

2.6151

0
4

0

0

0
4

0

0

0
4

0
4

1.4210854715202e-14
3

0
4

3.8239
3

0

0

0

0
4

0
4

3.8239
3

3.8239
3

0

0.186532

0

0

3.63737

0

0
4

0

0

0
4

0

0

0
4

0
4

0
4

3.50472
4

2.04931
4

0
4

0

0

0

0

0

0

0

0
4

0

0

0

0
4

0
4

0

0

0
4

1.98264
3

0

0

0

0

0

0

0

0

0

0

0.340965

0

0.431889

1.16433

0.045462

0

0
4

0.0666674

0.0666674

0
4

0

0

0
4

0

0

0
4

0
4

0

0

0

0

0
4

0
4

0

0

0

0
4

0
4

0

0

0

0
4

0
4

0

0

0

0
4

0
4

1.45541

1.45541

1.45541

0
4

0
4

0

0

0

0
4

0
4

0

0

0

0
4

0
4

0
4

4.63009

1.69547

1.69547

0.67179

1.02368

0
4

0
4

0

0

0

0

0
4

0
4

1.00705

1.00705

1.00705

0
4

0
4

0.109088

0.109088

0.0648786

0.0442094

0
4

0
4

0

0

0

0
4

0
4

1.81848

0

0

0

0

0

0

0

0

0

0

0

0

0

0

0

0

0

0

0

0

0

0

0

0

0

0

0
4

0

0

0
4

0

0

0
4

1.81848

1.81848

0
4

0
4

8.88178419700125e-16

0
4

184.344
3

183.575
3

183.117
2

183.117
2

0

0

0
4

0.0655183
3

0

0

0

0

0

0

0.0655183

0

0

0

0

0

0

0

0

0

0
4

0

0

0
4

0.39311

0.39311

0
4

1.26565424807268e-14
3

0
4

0

0

0

0

0
4

0
4

0

0

0

0
4

0
4

0.0481639

0.0481639

0.0481639

0
4

0
4

0.720702

0.720702

0.720702

0
4

0
4

0

0

0

0
4

0
4

0
4

39.9097

0

0

0

0
4

0
4

39.9097

39.6768

1.67583

34.3767
3

0

3.51115

0.054841

0.0582163

0

0

0

0

1.29063426612674e-15

0
4

0.232865

0.232865

0
4

3.27515792264421e-15

0
4

0
4

0
7

0
7

0
7

0
7

0
4

0
4

0
4

58.5535

57.9015

9.61369

9.48269

0.0761741

0.0548351

0
4

0

0

0
4

19.2239
3

11.6907
3

0
5

7.53325
3

1.77635683940025e-15
3

0
4

27.9459
3

0

27.293
3

0
4

0
4

0

0.233165

0

0.419697

0

0

0
4

0.767692

0.767692

0
4

0

0

0

0
4

0.0548351

0.0548351

0
4

0

0

0
4

0.295503

0.295503

0
4

0
4

0

0

0

0

0
4

0
4

0.575769

0.575769

0.575769

0
4

0
4

0.0761741

0.0761741

0.0761741

0
4

0
4

0

0

0

0
4

0
4

0

0

0

0
4

0
4

9.57567358739198e-16

0
4

3.50321
2

3.50321
2

3.50321
2

1.69835

0

0

1.72489

0.0799732

1.11022302462516e-16
2

0
4

0
4

0
4

17.9213

0.699495

0.699495

0.699495

0
4

0
4

0.522813

0.522813

0.477351

0.045462

0
4

0
4

0.772853

0.772853

0.772853

0
4

0

0

0
4

0
4

0.772853

0.772853

0.772853

0

0
4

0
4

0

0

0

0
4

0
4

0.0442094

0.0442094

0.0442094

0
4

0
4

15.1091

15.1091

0

15.1091

0
4

0

0

0
4

0

0

0
4

0

0

0
4

0

0

0
4

0

0

0
4

0

0

0
4

0

0

0
4

0
4

0
4

0
4

8.52822
7

8.52822
7

8.52822
7

0
6

0

0
7

0

0

0

0

0

0

0

0

0

0

0

0

0

0

0

0

0

0

0

0

0

0
7

0

0

0

0

0
6

0
6

0
6

0
6

0

0

0
4

0
7

0
7

0
7

0

0
4

8.52822
7

3.84679

0
7

0
7

1.69547
7

0
6

2.98596
7

0

0
6

0

0

0

0

0

0
4

0
4

0
4

0
4

0

0

0

0

0

0

0

0

0

0

0

0

0

0

0

0

0

0

0

0

0

0

0

0

0

0

0

0

0

0

0

0

0

0

0

0

0

0

0

0

0

0

0

0

0

0

0

0

0

0

0

0

0

0

0

0

0

0

0

0

0

0

0

0

0

0

0

0

0

0

0

0

0

0

0

0

0

0

0

0

0

0

0

0

0

0

0

0

0

0

0

0

0

0

0

0

0

0

0

0

0

0

0

0

0

0

0

0

0

0

0

0

0

0

0

0

0

0

0

0

0

0

0

0

0

0

0

0

0

0

0

0

0

0

0

0

0

0

0

0

0

0

0

0
4

0

0

0

0

0

0

0

0

0

0

0

0

0

0

0

0

0

0

0

0
4

0

0

0

0

0

0

0

0

0
4

0
4

0
4

0
4

0
7

0
7

0
7

0
7

0
7

0

0

0

0
4

0

0

0

0

0

0
4

0
4

0
4

0
4

0

0

0

0

0

0
4

0

0

0
4

0

0

0
4

0
4

0
4

0
4

0.559813

0.559813

0.559813

0.439853

0.35988

0.0799732

0
4

0.11996

0.11996

0
4

0

0

0
4

1.38777878078145e-17

0
4

0
4

0
4

0

0

0

0

0

0

0
4

0
4

0
4

0
4

29.3646

29.3646

29.3646

29.3646

8.1

21.2646

0
4

0

0

0
4

0
4

0
4

0
4

5.01552
3

5.01552
3

5.01552
3

5.01552
3

2.25994

2.75558

0
4

0
4

0
4

0
4

0

0

0

0

0

0

0
4

0
4

0
4

0
4

0.105743

0.105743

0.105743

0.105743

0

0.105743

0
4

0
4

0
4

0
4

0

0

0

0

0

0

0
4

0
4

0
4

0
4

0

0

0

0

0

0
4

0
4

0
4

0
4

0

0

0

0

0

0
4

0

0

0
4

0

0

0
4

0
4

0
4

0
4

0
3

0
3

0
3

0
3

0
3

0

0

0

0

0

0
4

0

0

0

0
4

0

0

0

0
4

0

0

0

0
4

0

0

0
4

0
4

0
4

0
4

0

0

0

0

0

0

0
4

0
4

0
4

0
4

2.14572

2.14572

2.14572

2.14572

2.04819

0.0975327

0
4

0
4

0
4

0
4

0.299186

0.299186

0.299186

0.299186

0.299186

0
4

0
4

0
4

0
4

0

0

0

0

0

0

0
4

0
4

0
4

0
4

0

0

0

0

0

0

0
4

0
4

0
4

0
4

0.154717

0.154717

0.154717

0.154717

0.154717

0
4

0
4

0
4

0
4

0

0

0

0

0

0
4

0
4

0
4

0
4

0

0

0

0

0

0
4

0

0

0
4

0

0

0
4

0

0

0
4

0
4

0
4

0
4

0

0

0

0

0

0
4

0
4

0
4

0
4

0.104619

0.104619

0.104619

0.104619

0.104619

0

0
4

0
4

0
4

0
4

0
4

0
4

0
4

0
4

0
4

0

0

0

0
4

0
4

0
4

0
4

0
4

0
4

0
4

0

0

0
4

0

0

0

0

0

0

0
4

0
4

0
4

0
4

0

0

0

0

0

0
4

0
4

0
4

0
4

0

0

0

0

0

0

0
4

0

0

0
4

0
4

0
4

0
4

0

0

0

0

0

0

0
4

0
4

0
4

0
4

0

0

0

0

0

0

0
4

0
4

0
4

0
4

0

0

0

0

0

0
4

0

0

0
4

0
4

0
4

0
4

0

0

0

0

0

0
4

0
4

0
4

0
4

0

0

0

0

0

0

0
4

0
4

0
4

0
4

0

0

0

0

0

0
4

0
4

0
4

0
4

0

0

0

0

0

0
4

0
4

0
4

0
4

0.180503

0.180503

0.180503

0.180503

0

0.180503

0
4

0
4

0
4

0
4

0
6

0
6

0
6

0
6

0
6

0

0

0

0

0

0

0

0
4

0

0

0

0
4

0
4

0
4

0
4

0

0

0

0

0

0

0
4

0

0

0
4

0
4

0
4

0
4

0

0

0

0

0

0

0

0
4

0

0

0
4

0
4

0
4

0
4

0

0

0

0

0

0

0
4

0
4

0
4

0
4

0

0

0

0

0

0

0

0
4

0

0

0
4

0
4

0
4

0
4

0

0

0

0

0

0
4

0
4

0
4

0
4

0

0

0

0

0

0
4

0
4

0
4

0
4

0

0

0

0

0

0

0
4

0
4

0
4

0
4

0

0

0

0

0

0

0

0
4

0
4

0
4

0
4

0

0

0

0

0

0

0
4

0

0

0
4

0
4

0
4

0
4

0

0

0

0

0

0
4

0
4

0
4

0
4

40.3153

21.289

21.289

16.3286

6.18892

4.37585

2.1841

0.28791

0.792761

0.06398

2.01537

0.419697

6.66133814775094e-16

0
4

4.43013

0.746128

0.093266

2.05185

0.839394

0.233165

0.46633

0
4

0.53031

0.233165

0.06398

0.233165

8.32667268468867e-17

0
4

5.44009282066327e-15

0
4

0
4

18.933

18.933

14.6894

13.85

0.46633

0.373064

1.38777878078145e-15

0
4

4.2436

1.53889

2.70471

0
4

1.77635683940025e-15

0
4

0
4

0.093266

0.093266

0.093266

0.093266

0
4

0
4

0
4

1.00613961606655e-14

0
4

0.343778

0.343778

0.343778

0.093266

0.093266

0
4

0.06398

0.06398

0
4

0.186532

0.186532

0
4

0
4

0
4

0
4

0

0

0

0

0

0

0
4

0
4

0
4

0

0

0

0

0
4

0
4

0
4

0
4

6.48896

6.48896

6.48896

6.48896

6.48896

0
4

0

0

0
4

0
4

0
4

0
4

0

0

0

0

0

0

0
4

0
4

0
4

0
4

0

0

0

0

0

0

0
4

0
4

0
4

0
4

0

0

0

0

0

0
4

0

0

0
4

0
4

0
4

0
4

0

0

0

0

0

0
4

0
4

0
4

0
4

0.530513

0.530513

0.530513

0.530513

0.530513

0
4

0
4

0
4

0
4

0

0

0

0

0

0
4

0
4

0
4

0
4

0

0

0

0

0

0
4

0
4

0
4

0
4

3.07897
3

3.07897
3

3.07897
3

3.07897
3

2.31922
3

0.559813
3

0

0

0.0799732

0

0.11996

0

1.38777878078145e-17
3

0
4

0

0

0

0
4

0
4

0
4

0

0

0

0

0
4

0
4

0
4

0
4

0

0

0

0

0

0

0
4

0

0

0
4

0
4

0
4

0
4

0

0

0

0

0

0
4

0
4

0
4

0
4

0

0

0

0

0

0

0
4

0

0

0
4

0
4

0
4

0
4

0

0

0

0

0

0
4

0
4

0
4

0
4

0

0

0

0

0

0

0
4

0
4

0
4

0
4

0

0

0

0

0

0

0
4

0
4

0
4

0
4

0

0

0

0

0

0
4

0
4

0
4

0
4

0

0

0

0

0

0

0
4

0
4

0
4

0
4

0

0

0

0

0

0
4

0
4

0
4

0
4

0

0

0

0

0

0
4

0
4

0
4

0
4

0

0

0

0

0

0

0

0
4

0

0

0

0
4

0

0

0

0
4

0

0

0
4

0
4

0
4

0

0

0

0

0
4

0

0

0
4

0
4

0
4

0

0

0

0

0
4

0
4

0
4

0
4

0

0

0

0

0

0
4

0
4

0
4

0
4

0

0

0

0

0

0
4

0
4

0
4

0
4

0

0

0

0

0

0
4

0
4

0
4

0
4

1.16433

1.16433

1.16433

1.16433

1.16433

0
4

0
4

0
4

0
4

5.88787

5.88787

5.88787

5.88787

0.609524

5.27835

0
4

0
4

0
4

0
4

0

0

0

0

0

0

0
4

0
4

0
4

0
4

0

0

0

0

0

0

0
4

0
4

0
4

0
4

0.794705

0.794705

0.794705

0.794705

0.794705

0
4

0
4

0
4

0
4

0

0

0

0

0

0
4

0
4

0
4

0
4

0

0

0

0

0

0
4

0
4

0
4

0
4

0
7

0
7

0
7

0
7

0
7

0
7

0

0

0

0
4

0

0

0

0
4

0
4

0
4

0
4

0

0

0

0

0

0
4

0
4

0
4

0
4

0

0

0

0

0

0
4

0
4

0
4

0
4

0

0

0

0

0

0
4

0
4

0
4

0
4

0

0

0

0

0

0
4

0
4

0
4

0
4

0

0

0

0

0

0
4

0
4

0
4

0
4

0

0

0

0

0

0
4

0
4

0
4

0
4

0

0

0

0

0

0
4

0
4

0
4

0
4

0.15889

0.15889

0.15889

0.15889

0.0397225

0.119168

0
4

0
4

0
4

0
4

0

0

0

0

0

0
4

0
4

0
4

0
4

0.189447

0.189447

0.189447

0.189447

0.189447

0
4

0
4

0
4

0
4

0
4

0
4

0
4

0
4

0
4

0

0

0

0

0

0

0
4

0
4

0

0

0

0
4

0
4

0
4

0
4

2.13338

2.13338

2.13338

2.13338

2.13338

0
4

0
4

0
4

0
4

0

0

0

0

0

0

0
4

0
4

0
4

0
4

0

0

0

0

0

0
4

0
4

0
4

0
4

0

0

0

0

0

0
4

0
4

0
4

0
4

0

0

0

0

0

0

0
4

0
4

0
4

0
4

0

0

0

0

0

0

0
4

0
4

0
4

0
4

0

0

0

0

0

0
4

0

0

0
4

0
4

0
4

0
4

0

0

0

0

0

0
4

0
4

0
4

0
4

0

0

0

0

0

0
4

0
4

0
4

0
4

0

0

0

0

0

0
4

0
4

0
4

0
4

0.278765

0.278765

0.278765

0.278765

0.150805
1

0.12796

0

0

0

0
4

0

0

0
4

0

0

0
4

0
4

0
4

0

0

0

0

0
4

0
4

0
4

0
4

0

0

0

0

0

0
4

0
4

0
4

0
4

0

0

0

0

0

0

0
4

0
4

0
4

0
4

0

0

0

0

0

0

0
4

0
4

0
4

0
4

0

0

0

0

0

0
4

0
4

0
4

0
4

1.72749

1.72749

1.72749

1.72749

1.72749

0
4

0
4

0
4

0
4

0

0

0

0

0

0

0
4

0
4

0
4

0
4

0

0

0

0

0

0
4

0

0

0
4

0
4

0
4

0
4

0

0

0

0

0

0

0
4

0
4

0
4

0
4

0.0442094

0.0442094

0.0442094

0.0442094

0.0442094

0

0
4

0
4

0
4

0
4

0.312071

0.312071

0.312071

0.312071

0.312071

0
4

0
4

0
4

0
4

122.59
3

122.59
3

122.59
3

122.59
3

67.8837
3

0.304696
4

0
5

0.0634669
4

0.0655183
2

0
4

0

0

0.126934

0

0.261974

32.1773
3

0

0

0

0

0

0

0

0

0

0.583908

9.29581
3

5.81381
3

0.0648786

2.2677
3

0.206289
3

0.137088

3.33747
3

0
4

0
4

0
4

0
4

0.759746
4

0.759746
4

0.759746
4

0.35988
3

0.35988
3

0
4

0.399866
3

0
4

0.399866

0
4

0
4

0

0

0

0
4

0

0

0

0
4

0

0

0
4

0

0

0
4

0

0

0

0
4

5.55111512312578e-17
4

0
4

0
4

0
4

0

0

0

0

0

0
4

0
4

0
4

0
4

0

0

0

0

0

0
4

0
4

0
4

0
4

0.473618

0.473618

0.473618

0.473618

0.063149

0.410469

0
4

0
4

0
4

0
4

0.773585

0.773585

0.773585

0.773585

0.773585

0
4

0
4

0
4

0
4

0

0

0

0

0

0
4

0
4

0
4

0
4

0.877794

0.877794

0.877794

0.877794

0.877794

0
4

0
4

0
4

0
4

4.87663

4.87663

4.87663

4.87663

4.87663

0
4

0
4

0
4

0
4

0

0

0

0

0

0
4

0
4

0
4

0
4

0

0

0

0

0

0
4

0
4

0
4

0
4

0

0

0

0

0

0
4

0

0

0
4

0
4

0
4

0
4

0

0

0

0

0

0

0

0

0

0

0

0
4

0

0

0
4

0
4

0
4

0
4

0.0442094

0.0442094

0.0442094

0.0442094

0

0.0442094

0
4

0
4

0
4

0
4

0

0

0

0

0

0
4

0
4

0
4

0
4

0

0

0

0

0

0
4

0
4

0
4

0
4

0

0

0

0

0

0

0
4

0
4

0
4

0
4

0

0

0

0

0

0
4

0
4

0
4

0
4

0

0

0

0

0

0
4

0
4

0
4

0
4

0.360351

0.360351

0.360351

0.360351

0.360351

0
4

0
4

0
4

0
4

0

0

0

0

0

0

0
4

0
4

0
4

0
4

0

0

0

0

0

0
4

0

0

0
4

0
4

0
4

0
4

0

0

0

0

0

0
4

0

0

0
4

0
4

0
4

0
4

0
4

0
4

0
4

0
4

0
4

0

0

0

0

0

0

0

0
4

0
4

0
4

0
4

0

0

0

0

0

0
4

0
4

0
4

0
4

2.51916

2.51916

2.51916

2.51916

2.51916

0

0
4

0
4

0
4

0
4

0

0

0

0

0

0
4

0
4

0
4

0
4

0

0

0

0

0

0
4

0
4

0
4

0
4

0.11996

0.11996

0.11996

0.11996

0.11996

0
4

0
4

0
4

0
4

0

0

0

0

0

0
4

0
4

0
4

0
4

0

0

0

0

0

0
4

0
4

0
4

0
4

0.093266

0.093266

0.093266

0.093266

0.093266

0
4

0
4

0
4

0
4

0

0

0

0

0

0
4

0
4

0
4

0
4

0

0

0

0

0

0
4

0
4

0
4

0
4

11.2075

11.2075

11.2075

8.59404

5.6996

1.634

0.818979

0.153937

0.163796

0.0582163

0.0655183

1.92901250528621e-15

0
4

2.61346

1.21962

0.869894

0.523947

0
4

0
4

0
4

0
4

0.641036

0.641036

0.641036

0.641036

0.641036

0
4

0
4

0
4

0
4

0

0

0

0

0

0
4

0
4

0
4

0
4

0

0

0

0

0

0
4

0
4

0
4

0
4

0

0

0

0

0

0
4

0
4

0
4

0
4

3.20518

3.20518

3.20518

3.20518

3.20518

0
4

0
4

0
4

0
4

0

0

0

0

0

0
4

0
4

0
4

0
4

0

0

0

0

0

0
4

0
4

0
4

0
4

0.904877

0.904877

0.904877

0.904877

0.904877

0
4

0
4

0
4

0
4

0

0

0

0

0

0
4

0
4

0
4

0
4

0

0

0

0

0

0
4

0
4

0
4

0
4

0
7

0
7

0
7

0
7

0
7

0
7

0

0
4

0
4

0
4

0
4

0

0

0

0

0

0
4

0
4

0
4

0
4

0

0

0

0

0

0
4

0
4

0
4

0
4

0

0

0

0

0

0
4

0
4

0
4

0
4

0

0

0

0

0

0
4

0
4

0
4

0
4

0

0

0

0

0

0
4

0
4

0
4

0
4

0

0

0

0

0

0
4

0
4

0
4

0
4

0

0

0

0

0

0
4

0
4

0
4

0
4

0

0

0

0

0

0
4

0
4

0
4

0
4

0

0

0

0

0

0
4

0
4

0
4

0
4

0

0

0

0

0

0
4

0
4

0
4

0
4

0

0

0

0

0

0

0

0

0

0

0
4

0

0

0

0

0

0

0
4

0
4

0
4

0

0

0

0

0

0

0

0
4

0

0

0
4

0

0

0
4

0
4

0
4

0
4

0.465731

0.465731

0.465731

0.465731

0.465731

0
4

0
4

0
4

0
4

0

0

0

0

0

0
4

0
4

0
4

0
4

0

0

0

0

0

0
4

0
4

0
4

0
4

2.97475

2.97475

2.97475

2.97475

2.97475

0
4

0
4

0
4

0
4

0

0

0

0

0

0
4

0
4

0
4

0
4

0

0

0

0

0

0
4

0
4

0
4

0
4

0

0

0

0

0

0
4

0
4

0
4

0
4

0

0

0

0

0

0
4

0
4

0
4

0
4

0

0

0

0

0

0
4

0
4

0
4

0
4

0

0

0

0

0

0
4

0
4

0
4

0
4

0.304427
3

0.304427
3

0.304427
3

0.199672
4

0.0397225
4

0.06398
3

0.09597

1.38777878078145e-17
4

0
4

0.104755

0.0648786

0.0398762

0
4

0
4

0
4

0
4

0

0

0

0

0

0
4

0
4

0
4

0
4

0

0

0

0

0

0
4

0
4

0
4

0
4

0

0

0

0

0

0
4

0
4

0
4

0
4

0

0

0

0

0

0
4

0
4

0
4

0
4

0

0

0

0

0

0
4

0
4

0
4

0
4

0

0

0

0

0

0
4

0
4

0
4

0
4

0

0

0

0

0

0
4

0
4

0
4

0
4

0

0

0

0

0

0
4

0
4

0
4

0
4

0.114261

0.114261

0.114261

0.114261

0.114261

0
4

0
4

0
4

0
4

0

0

0

0

0

0
4

0
4

0
4

0
4

0

0

0

0

0

0

0

0

0

0

0

0
4

0
4

0
4

0
4

0

0

0

0

0

0
4

0
4

0
4

0
4

0

0

0

0

0

0
4

0
4

0
4

0
4

0

0

0

0

0

0
4

0
4

0
4

0
4

0

0

0

0

0

0
4

0
4

0
4

0
4

0

0

0

0

0

0
4

0
4

0
4

0
4

0

0

0

0

0

0
4

0
4

0
4

0
4

0

0

0

0

0

0
4

0
4

0
4

0
4

0

0

0

0

0

0
4

0
4

0
4

0
4

0

0

0

0

0

0
4

0
4

0
4

0
4

0

0

0

0

0

0
4

0
4

0
4

0
4

0
4

0
4

0
4

0
4

0
4

0

0
4

0

0

0

0
4

0

0

0

0
4

0

0

0
4

0

0

0
4

0

0

0
4

0
4

0
4

0

0

0

0

0
4

0
4

0
4

0

0

0

0

0
4

0
4

0
4

0
4

0

0

0

0

0

0
4

0
4

0
4

0
4

0

0

0

0

0

0
4

0
4

0
4

0
4

0

0

0

0

0

0
4

0
4

0
4

0
4

0

0

0

0

0

0
4

0
4

0
4

0
4

0

0

0

0

0

0
4

0
4

0
4

0
4

0.216738

0.216738

0.216738

0.216738

0.216738

0
4

0
4

0
4

0
4

0

0

0

0

0

0
4

0
4

0
4

0
4

0

0

0

0

0

0
4

0
4

0
4

0
4

0

0

0

0

0

0
4

0
4

0
4

0
4

0

0

0

0

0

0
4

0
4

0
4

0
4

0.239008

0.239008

0.239008

0.175859

0.110341

0

0

0

0

0.0655183

0

0

0

0

0

0

1.38777878078145e-17

0
4

0.063149

0

0.063149

0
4

1.38777878078145e-17

0
4

0
4

0
4

1.13538

1.13538

1.13538

1.13538

1.13538

0
4

0
4

0
4

0
4

0

0

0

0

0

0
4

0
4

0
4

0
4

0

0

0

0

0

0
4

0
4

0
4

0
4

0

0

0

0

0

0
4

0
4

0
4

0
4

0

0

0

0

0

0
4

0
4

0
4

0
4

0

0

0

0

0

0
4

0
4

0
4

0
4

0.0909239

0.0909239

0.0909239

0.0909239

0.0909239

0
4

0
4

0
4

0
4

0

0

0

0

0

0
4

0
4

0
4

0
4

0

0

0

0

0

0
4

0
4

0
4

0
4

0

0

0

0

0

0
4

0
4

0
4

0
4

16.4345
4

16.4345
4

16.4345
4

1.7994
4

0.23992
4

0

0

0.0799732

0

0

0

0.0799732

0.159946

0

0

0.319893
4

0

0

0

0

0

0

0

0

0

0

0.0799732
4

0.11996

0

0.35988

0

0

0

0.0799732
4

0.0799732
3

0
4

0

0.199933
3

0

1.94289029309402e-16
4

0
4

14.4352
4

14.3552
4

0

0

0
4

0

0.0799732

0

0

0

0

0

5.55111512312578e-16
4

0
4

0.0799732
4

0
4

0
4

0
4

0.0799732

0

0

0

0

0
4

0.11996

0

0.11996

0

0

0

0
4

0
4

0

0

0

0

0
4

0

0

0
4

0
4

0
4

0
4

0
4

0
4

0
4

0

0
4

0

0

0

0
4

0

0

0
4

0
4

0
4

0

0

0

0

0
4

0

0

0
4

0
4

0
4

0
4

0

0

0

0

0

0

0

0

0

0
4

0
4

0
4

0
4

0

0

0

0

0

0
4

0
4

0
4

0
4

0.0799732

0.0799732

0.0799732

0.0799732

0.0799732

0
4

0
4

0
4

0
4

0

0

0

0

0

0
4

0
4

0
4

0
4

0

0

0

0

0

0
4

0
4

0
4

0
4

0

0

0

0

0

0
4

0
4

0
4

0
4

0

0

0

0

0

0
4

0
4

0
4

0
4

0

0

0

0

0

0
4

0
4

0
4

0
4

0

0

0

0

0

0
4

0
4

0
4

0
4

0

0

0

0

0

0
4

0
4

0
4

0
4

0

0

0

0

0

0
4

0
4

0
4

0
4

0
5

0
5

0
5

0
5

0

0

0

0

0

0

0

0

0
4

0

0

0
4

0
4

0
4

0
4

0

0

0

0

0

0
4

0
4

0
4

0
4

0.773585

0.773585

0.773585

0.773585

0.773585

0
4

0
4

0
4

0
4

0

0

0

0

0

0
4

0
4

0
4

0
4

0

0

0

0

0

0
4

0
4

0
4

0
4

0

0

0

0

0

0
4

0
4

0
4

0
4

0

0

0

0

0

0
4

0
4

0
4

0
4

0.0460286

0.0460286

0.0460286

0.0460286

0.0460286

0
4

0
4

0
4

0
4

0

0

0

0

0

0
4

0
4

0
4

0
4

0

0

0

0

0

0
4

0
4

0
4

0
4

0

0

0

0

0

0
4

0
4

0
4

0
4

0
7

0
7

0
7

0
7

0
7

0
7

0

0

0

0

0
4

0
4

0
4

0
4

0

0

0

0

0

0
4

0
4

0
4

0
4

1.28076

1.28076

1.28076

1.28076

1.28076

0
4

0
4

0
4

0
4

0

0

0

0

0

0
4

0
4

0
4

0
4

0

0

0

0

0

0
4

0
4

0
4

0
4

0

0

0

0

0

0
4

0
4

0
4

0
4

0

0

0

0

0

0
4

0
4

0
4

0
4

0

0

0

0

0

0
4

0
4

0
4

0
4

0

0

0

0

0

0
4

0
4

0
4

0
4

0

0

0

0

0

0
4

0
4

0
4

0
4

0

0

0

0

0

0
4

0
4

0
4

0
4

102.142

102.142

102.142

96.0449

91.4769

0.0397225

0.198613

0.119168

0.417086

0.655422

0.834173

0.47667

0.0397225

0.0397225

0.0595838

0.655422

1.03279

0
4

4.60781

1.96626

0.238335

1.76765

0.63556

0
4

1.09237

0.754728

0.337641

0
4

0.397225

0.397225

0
4

0
4

0
4

0
4

0

0

0

0

0

0
4

0
4

0
4

0
4

0

0

0

0

0

0
4

0
4

0
4

0
4

0

0

0

0

0

0
4

0
4

0
4

0
4

0

0

0

0

0

0
4

0
4

0
4

0
4

0.054841

0.054841

0.054841

0.054841

0.054841

0
4

0
4

0
4

0
4

0

0

0

0

0

0
4

0
4

0
4

0
4

0

0

0

0

0

0
4

0
4

0
4

0
4

0

0

0

0

0

0
4

0
4

0
4

0
4

0

0

0

0

0

0
4

0
4

0
4

0
4

0

0

0

0

0

0
4

0
4

0
4

0
4

0
7

0
7

0
7

0
7

0
6

0

0

0

0

0

0

0

0
4

0
4

0
4

0
4

0

0

0

0

0

0
4

0
4

0
4

0
4

0.243832

0.243832

0.243832

0.243832

0.243832

0
4

0
4

0
4

0
4

0

0

0

0

0

0
4

0
4

0
4

0
4

0

0

0

0

0

0
4

0
4

0
4

0
4

0

0

0

0

0

0
4

0
4

0
4

0
4

0

0

0

0

0

0
4

0
4

0
4

0
4

0

0

0

0

0

0
4

0
4

0
4

0
4

0

0

0

0

0

0
4

0
4

0
4

0
4

0

0

0

0

0

0
4

0
4

0
4

0
4

0

0

0

0

0

0
4

0
4

0
4

0
4

1.59966

1.59966

1.59966

1.59966

1.59966

0

0

0

0

0

0

0
4

0

0

0

0
4

0

0

0
4

0
4

0
4

0
4

0

0

0

0

0

0
4

0
4

0
4

0
4

0

0

0

0

0

0
4

0
4

0
4

0
4

0

0

0

0

0

0

0

0

0

0

0

0
4

0
4

0
4

0
4

0

0

0

0

0

0

0
4

0
4

0
4

0

0

0

0

0
4

0

0

0
4

0
4

0
4

0
4

0

0

0

0

0

0

0

0
4

0

0

0

0
4

0
4

0
4

0
4

2.1339

2.1339

2.1339

2.1339

2.1339

0

0

0
4

0
4

0
4

0
4

7.11762
4

7.11762
4

7.11762
4

0.479839
4

0.199933
4

0

0

0

0

0

0

0

0

0

0

0.0799732
4

0

0.11996

0

0

0

0

0

0

0

0

0
4

0

0

0

0

0

0

0

0

0

0

0.0799732
4

0

0

0

0

0

0

0

0
4

0

0

0

1.38777878078145e-17
4

0
4

6.63778
4

5.51815
4

0

0

0.799732
4

0

0
4

0

0

0.159946

0.159946

0

0
4

0

0

0

0
4

0
4

0
4

0
4

3.75874
3

3.75874
3

3.75874
3

3.75874
3

3.75874
3

0
4

0

0

0

0
4

0
4

0
4

0
4

0.19194

0.19194

0.19194

0.19194

0

0

0.19194

0

0

0
4

0
4

0
4

0
4

3.46827

3.46827

3.46827

3.46827

3.36526

0.103018

1.11022302462516e-16

0
4

0
4

0
4

0

0

0

0

0
4

0
4

0
4

0
4

0

0

0

0

0

0

0
4

0
4

0
4

0
4

0
4

0
4

0
4

0
4

0
4

0

0
4

0

0

0
4

0
4

0
4

0
4

0.139029

0.139029

0.139029

0.139029

0.139029

0
4

0
4

0
4

0
4

0.11996
4

0.11996
4

0.11996

0.11996

0.11996

0

0

0

0
4

0
4

0

0

0

0
4

0
4

0
4

0

0

0

0

0

0
4

0

0

0
4

0
4

0
4

0
4

0
4

0
4

0
4

0
4

0
4

0

0
4

0

0

0

0
4

0
4

0
4

0
4

0

0

0

0

0

0

0

0

0
4

0

0

0
4

0
4

0
4

0
4

11.4445

11.4445

11.4445

11.1897

8.98924

0.131534

0.931461

0.154733

0.982775

7.7715611723761e-16

0
4

0.156494

0.0982775

0.0582163

6.93889390390723e-18

0
4

0.0982775

0.0982775

0
4

0
4

0
4

0
4

0.479839
4

0.35988
4

0.35988
4

0.11996
4

0
4

0.11996

0

0

0

0

0

0

0

0

0

0
4

0

0

0

0

0

0

0

0

0

0

0

0
4

0
4

0

0

0

0

0
4

0.23992
4

0.159946
4

0.0799732
4

0

0

0

0

0

0

0

0
4

0
4

0
4

0
4

0
4

0

0

0

0
4

0

0

0

0
4

0

0

0
4

0

0

0
4

0
4

0
4

0.11996
4

0.11996
4

0.11996
4

0
4

0

0.11996

0

0
4

0

0

0

0
4

0

0

0

0
4

0
4

0
4

0

0

0

0

0
4

0
4

0
4

1.38777878078145e-17
4

0
4

0
5

0
5

0
5

0
5

0

0

0

0

0
4

0
4

0
4

0
4

0.0481639

0.0481639

0.0481639

0.0481639

0.0481639

0

0
4

0
4

0
4

0
4

0
5

0
5

0
5

0
5

0
4

0

0

0
4

0

0

0
4

0

0

0
4

0
4

0
4

0
4

0

0

0

0

0

0

0

0

0

0

0
4

0

0

0
4

0
4

0
4

0
4

1.77315

1.77315

1.77315

1.77315

1.5365

0.170338

0.0663141

9.71445146547012e-17

0
4

0
4

0
4

0
4

0

0

0

0

0

0

0

0
4

0
4

0
4

0
4

0

0

0

0

0

0

0

0
4

0
4

0
4

0
4

0
3

0
3

0
3

0
3

0
3

0
4

0
4

0
4

0
4

0

0

0

0

0

0

0

0
4

0

0

0
4

0

0

0
4

0
4

0
4

0
4

0

0

0

0

0

0
4

0
4

0
4

0
4

3.19893
4

3.19893
4

3.19893
4

3.19893
4

3.19893
4

0

0

0

0

0

0

0

0

0
4

0
4

0
4

0
4

2.07784

2.07784

2.07784

2.07784

2.07784

0

0
4

0
4

0
4

0
4

10.1833

0.839394

0.839394

0.839394

0.186532

0.186532

0.093266

0.186532

0.186532

1.11022302462516e-16

0
4

0
4

0
4

9.34394

9.34394

9.25068

9.1867

0.06398

0
4

0.093266

0.093266

0
4

0
4

0
4

0
4

0
7

0
7

0
7

0
7

0

0

0

0

0
4

0
4

0
4

0
4

0
7

0
7

0
7

0
7

0
7

0
4

0
4

0
4

0
4

0

0

0

0

0

0

0

0

0

0
4

0

0

0

0
4

0

0

0
4

0
4

0
4

0
4

0

0

0

0

0

0
4

0
4

0
4

0
4

0

0

0

0

0

0

0
4

0

0

0
4

0

0

0
4

0

0

0
4

0
4

0
4

0
4

0

0

0

0

0

0

0
4

0

0

0
4

0
4

0
4

0
4

0
4

0
4

0
4

0
4

0

0

0

0
4

0
4

0
4

0
4

0

0

0

0

0

0

0

0

0
4

0
4

0
4

0

0

0

0

0
4

0
4

0
4

0
4

8.0773
4

8.0773
4

8.0773
4

0.0799732
4

0.0799732
4

0

0

0

0

0

0

0

0

0

0

0

0

0

0

0

0

0
4

0

0

0

0

0
4

7.99732
3

7.99732
3

0

0
4

0

0

0

0

0
4

0

0

0
4

0

0

0
4

0

0

0
4

0

0

0
4

8.88178419700125e-16
4

0
4

0
4

0
4

0

0

0

0

0

0

0
4

0

0

0

0
4

0
4

0
4

0
4

0
4

0
4

0
4

0
4

0

0

0

0
4

0
4

0
4

0
4

0
4

0
4

0
4

0
4

0
4

0

0
4

0
4

0
4

0
4

0
4

0
4

0
4

0
4

0
4

0
4

0

0

0

0
4

0
4

0
4

0
4

0

0

0

0

0

0

0

0
4

0
4

0
4

0
4

0

0

0

0

0

0

0
4

0

0

0

0
4

0

0

0
4

0
4

0
4

0
4

1.40169

1.40169

1.40169

0.559596

0.559596

0
4

0.512963

0.139899

0.373064

0
4

0.233165

0.139899

0.093266

1.38777878078145e-17

0
4

0.09597

0.09597

0
4

8.32667268468867e-17

0
4

0
4

0
4

0

0

0

0

0

0

0

0

0
4

0

0

0
4

0
4

0
4

0
4

0

0

0

0

0

0

0

0

0

0
4

0

0

0
4

0

0

0
4

0
4

0
4

0

0

0

0

0
4

0
4

0
4

0
4

0

0

0

0

0

0
4

0
4

0
4

0
4

18.4338
4

18.4338
4

18.4338
4

18.2739
4

16.6344
4

1.51949
3

0

0

0.11996

0

0

0
4

0.0799732
4

0.0799732

0

0

0

0

0
4

0

0

0

0
4

0

0

0

0

0

0
4

0.0799732

0.0799732

0
4

0

0

0
4

1.11022302462516e-15
4

0
4

0
4

0
4

0

0

0

0

0

0
4

0

0

0
4

0

0

0
4

0
4

0
4

0

0

0

0

0

0
4

0
4

0
4

0
4

14.0488

5.56157

5.56157

5.28177

5.28177

0
4

0.279798

0.279798

0
4

2.22044604925031e-16

0
4

0
4

8.4872

8.4872

8.4872

8.4872

0
4

0
4

0
4

0
4

0
7

0
7

0
7

0
7

0
7

0
4

0
4

0
4

0
4

0

0

0

0

0

0

0

0
4

0

0

0
4

0
4

0
4

0
4

0

0

0

0

0

0

0

0
4

0
4

0
4

0
4

0

0

0

0

0

0

0

0
4

0
4

0
4

0
4

3.33092

3.33092

3.33092

3.33092

3.33092

0

0
4

0
4

0
4

0
4

2.58701

2.58701

2.58701

2.58701

2.58701

0
4

0
4

0
4

0
4

1.67944
3

1.67944
3

1.67944
3

1.67944
3

0

1.67944

0

0
4

0

0

0
4

0
4

0
4

0
4

0

0

0

0

0

0
4

0

0

0
4

0
4

0
4

0
4

92.8395

92.8395

92.8395

92.7201

86.3049

0.079445

0.139029

1.3307

2.78058

0.834173

0.715005

0.397225

0.0397225

0.0397225

0.0595838

0
4

0.0796102

0.0796102

0
4

0.0397225

0.0397225

0
4

0
4

0
4

0
4

0.279906

0.279906

0.279906

0

0

0

0
4

0

0

0
4

0.279906

0.279906

0
4

0
4

0
4

0
4

2.54229
2

2.54229
2

2.54229
2

2.49683
2

2.49683
2

0
4

0.045462

0.045462

0
4

0
4

0
4

0
4

0
7

0
7

0
7

0
7

0
7

0
4

0
4

0
4

0
4

0
7

0
7

0
7

0
7

0
7

0
4

0
4

0
4

0
4

0

0

0

0

0

0

0

0
4

0

0

0
4

0

0

0
4

0
4

0
4

0
4

6.40782

6.40782

6.40782

6.33164

6.27857

0.0530735

2.84494650060196e-16

0
4

0.0761741

0.0761741

0
4

0
4

0
4

0
4

0

0

0

0

0

0

0
4

0
4

0
4

0
4

0

0

0

0

0

0

0
4

0

0

0
4

0

0

0
4

0
4

0
4

0
4

0

0

0

0

0

0
4

0
4

0
4

0
4

0

0

0

0

0

0

0
4

0
4

0
4

0
4

5.67186

0.0423112

0.0423112

0.0423112

0.0423112

0
4

0
4

0
4

0.0423112

0.0423112

0.0423112

0.0423112

0
4

0
4

0
4

0.0423112

0.0423112

0.0423112

0.0423112

0
4

0
4

0
4

5.54493

0
3

0
3

0
3

0

0
4

0

0

0
4

0

0

0

0
4

0
4

0

0

0

0
4

0
4

0.126934

0.126934

0.126934

0
4

0
4

0.195065

0.195065

0.195065

0
4

0
4

1.36546

1.02409

0.92656

0.0975327

5.55111512312578e-17

0
4

0.0975327

0.0975327

0
4

0.243832

0.243832

0
4

0
4

0

0

0

0

0
4

0
4

0

0

0

0

0
4

0
4

0

0

0

0
4

0
4

1.26792

1.07286

1.07286

0
4

0.0975327

0.0975327

0
4

0.0975327

0.0975327

0
4

0
4

0.248617

0.248617

0.248617

0
4

0
4

0

0

0

0

0
4

0
4

0.0975327

0.0975327

0.0975327

0
4

0
4

2.2434
6

2.2434
6

2.2434
6

0

0
4

0
4

0
4

0
4

0.387721

0

0

0

0

0

0
4

0
4

0
4

0

0
[truncated: 202,630 more chars]
