# Supplementary material for: Fungal communities decline with urbanization—more in air than in soil
Source: ISME J. 2020 Aug 5;14(11):2806–15. doi: 10.1038/s41396-020-0732-1 (PMC7784924; doi:10.1038/s41396-020-0732-1)
Supplement: Supplementary file 2 — Supplemental data [file 41396_2020_732_MOESM2_ESM.zip › Krona_SoilUrbanEdge.html]

Javascript must be enabled to view this page.

num
probth


30343

18928.6

828.859
3

828.859
3

828.859
3

828.859
3

828.665
3

0

0

0

0

0

0

0

0

0.116713

0

0

0

0

0

0

0

0.077809

0

0

0

0

0

0

0

0

0

0

0

0

0

0

0

0

0

0

0

1.92762472650543e-14
3

0
4

0
4

0

0

0

0
4

0

0

0
4

0
4

0

0

0

0
4

0
4

0
4

0
4

0

0

0

0

0

0

0
4

0
4

0
4

0
4

0.0367698

0

0

0

0

0
4

0
4

0
4

0.0367698

0.0367698

0.0367698

0.0367698

0
4

0
4

0
4

0
4

6062.64

128.291

127.932

82.8838

5.91406
3

2.44718
2

0

0

0

0

0.077809

0

0

0.322802

0.220619

0.451923

0

0

0

0

0

0

0.258242

0

0

0

0

8.69114
3

0

0

0

0

0

0

0

0

0

0

0

0

0

0

0

0

0.658285

0

0

0

0.0484412

0

0

0

0

0

0

0

0

0.193681

0

0

0

0.14617

0

0.129121

0

0

0

0

0.219428

0.129121

0

0
7

0

0

0

0.0877713

0

0

0

0

0

0

0
7

0

0

0

0

0

0

0

0

0

0

0
7

0

0

0

0

0

0

0

0

0

0

0.516483

0

0

0

0

0

0

0

0

0

0

15.4299

0

0

0

0

0

0

0.129121

0

0

0.0950773

0

0

0

0.0877713

0

0

0

0.438857

0

0

0

0

0
6

0.263314

0

0

0

0

0

0

0

0

0.131657

0

0

0

0

0
6

0
7

0

0

0

0
1

0.193681

9.48858
2

0.404696

0

0

0

1.93339

0

0

0

3.47761
3

0

0

0

0

0.570464

0

0

0

0.0551547

4.19643

7.57479
2

0

5.03571

0

0

0

0

0

0

0

0.774725

4.51923
2

0.0367698

0

0

0

0

0.192506

0

0

3.4217

0

0
7

0

0

0

0

0

0

0

0

0

1.14103

0
6

0

0.497354

0

0

0

1.93097

0

0

0

0

0
6

0

0.129121

0

0

0.0950773

0

0

0

0

0.12677

0
4

3.64565
3

3.64565
3

0

0

0

0
4

0

0

0
4

0

0

0
4

0

0

0
4

0

0

0
4

0

0

0

0
4

0

0

0

0
4

0

0

0

0
4

0

0

0
4

0

0

0
4

0

0

0
4

0
6

0

0

0

0

0
4

0

0

0
4

0

0

0

0
4

0

0

0

0
4

0

0

0

0
4

0

0

0
4

0

0

0

0
4

0

0

0

0
4

0.858752

0.858752

0
4

0

0

0
4

0

0

0
4

39.059
2

38.6071

0

0.451923

0

0
4

0

0

0
4

0

0

0
4

0

0

0
4

0

0

0
4

0

0

0
4

0

0

0
4

0

0

0
4

0

0

0
4

0

0

0
4

0

0

0
4

0
6

0

0

0
4

0

0

0
4

0

0

0
4

0

0

0
4

0

0

0
4

0

0

0
4

0

0

0
4

0

0

0
4

0

0

0
4

0

0

0
4

0

0

0
4

0

0

0

0
4

0

0

0
4

0

0

0
4

0

0

0
4

0

0

0
4

0

0

0
4

0

0

0
4

0

0

0
4

0

0

0
4

0

0

0
4

0

0

0
4

0

0

0
4

0

0

0
4

0

0

0
4

0

0

0
4

0
7

0

0

0
4

0

0

0
4

1.48489

0.451923

1.03297

0
4

1.68753899743024e-14

0
4

0
6

0
6

0
6

0
4

0
4

0

0

0

0
4

0

0

0
4

0
4

0

0

0

0
4

0
4

0.0367698

0.0367698

0.0367698

0

0
4

0
4

0

0

0

0
4

0
4

0

0

0

0
4

0
4

0

0

0

0
4

0
4

0

0

0

0
4

0
4

0

0

0

0
4

0
4

0

0

0

0
4

0
4

0

0

0

0
4

0
4

0
6

0
6

0
6

0
4

0

0

0
4

0

0

0
4

0
4

0.0633849

0.0633849

0.0633849

0
4

0
4

0

0

0

0
4

0
4

0

0

0

0
4

0
4

0

0

0

0
4

0
4

0

0

0

0
4

0
4

0

0

0

0
4

0
4

0

0

0

0
4

0
4

0

0

0

0
4

0
4

0.258242

0.258242

0.258242

0
4

0
4

0

0

0

0

0
4

0
4

0

0

0

0

0

0
4

0

0

0
4

0
4

0

0

0

0
4

0
4

0

0

0

0
4

0
4

0

0

0

0
4

0
4

0

0

0

0
4

0
4

0

0

0

0
4

0
4

0
4

1874.6
7

0

0

0

0

0

0

0
4

0

0

0

0
4

0

0

0

0
4

0

0

0

0
4

0

0

0
4

0
4

1838.74
7

1838.62
7

135.064
7

0
7

0
6

0.219428

0

0

0

0

0

0

0

0

0

0

0
7

0

0

0

0

0

0

0

0

0

0

0
7

0

0

0

0

0

0

0

0

0

0

0.0548139

0

0

0

0

0

0

0

0

0

0

0
7

0

0

0

0

0

0

0

0

0

0

0

0

0

0

0

0

0

0

0

0

0

0
7

0

0

0

0

0

0

0

0

0

0

0
8

0

0

0

0

0

0

0

0

0

0

0

0

0

0

0

0

0

0

0

0

0

1.55293

0

0

0

0

0

0

0

0

0

0

0
7

0
7

0

0

0

0

0

0

0

0

0

0

0

0

0

0

0

0

0

0

0

0

0

0
6

0

0

0

0

0

0

0

0

0

0

0
7

0

0

0

0

0

0

0

0

0

0

0
7

0

0

0

0

0

0

0

0

0

0

0
8

0

0

0

0

0

0

0

0

0

0.0868184

0
8

0.0877713

0

0

0

0

0

0

0

0

0

0
7

0

0

0

0

0

0

0

0

0

0

0
7

0

0

0

0

0

0

0

0

0

0

0
6

0

0

0

0

0

0

0

0

0

0

0
6

0.121103
1

0

0

0

0

0

0

0

0

0

0

0
7

0

0

0

0

0

0

0

0

0

0

0
7

0.0633849

0

0

0

0

0

0

0

0

0

0
1

0

0

0

0

0

0

0

0

0

0

0
7

0

0

0

0

0

0

0

0

0

0

0
6

0

0

0

0

0

0

0

0

0

0

0
8

0

0

0

0

0

0

0

0

0

0

0
7

0

0

0

0

0

0

0

0

0

0

0

0

0

0

0

0

0

0

0

0

0

0
6

0

0

0

0

0

0

0

0

0

0

0
7

0.0877713

0

0

0.175543

0

0

0

0

0

0

0

0
6

0

0

0

0

0

0

0

0

0

0

0.190155
1

0

0

0

0

0

0

0

0

0

0

0
7

0

0

0

0

0

0

0

0

0

0

0
7

0

0

0

0

0

0.0877713

0

0

0

0

0
7

0

0

0

0

0

0

0

0

0

0

0
7

0

0

0

0

0

0

0

0

0

0

0
5

0

0

0

0

0

0

0

0

0

0

0
7

0

0

0

0

0

0

0

0

0

0

0
6

0

0

0

0

0

0

0

0

0

0

0
7

0
7

0

0

0

0

0

0

0

0

0

0

0
7

0

0

0

0
7

0
7

0
8

0
7

0
6

0
6

0
7

0

0.346253
7

0
7

0
7

0
7

0
7

0

0.0877713

0

0
7

0
7

0

0.339082

0
7

0.0633849

0
7

0

0
7

0
7

0
7

122.967
3

0.0367698

0
6

0
7

0
7

0
7

0.0950773

0
7

0.131657

0
7

0
7

0.0633849

0
6

0
6

0.40624

0
8

0.0551547
6

0
7

0
6

0

0

0.175543

2.90522

0
7

0.131657

0.263314
7

0
7

0
7

0.0950773

0
7

0
6

0
7

13.5692
2

0

0.282813

0
8

15.3474

0
7

0
6

0
6

0
8

0
7

0
7

0
7

0
8

0
6

0

0
6

0.0735396

0.219428

0.175543

0
6

0
8

0
6

0
7

0
7

0
6

0
8

0
7

0
7

0.0367698
7

0
8

0

0
6

0
7

0

0
7

0
7

3.86083
2

0

0
7

0

0

0
6

0
7

0
8

0
7

0

0.0726619

0
7

0
7

1.22323

0
1

0
8

0
7

0.175543

0
7

0
7

0
6

0
7

0
7

0
7

0.0950773
7

0
7

0.131657

0
7

0
6

0
7

0
6

0

0.0877713

0
7

0
7

0.0726619
7

0
6

0
7

0
7

0
7

0.855696

0.482742

0
6

0
8

0.0950773
6

0
7

0
7

0
7

0
7

0
7

0.0367698
7

0
6

0
7

0
7

0
7

0
7

0
7

0
7

0

0
6

0.0367698
7

0
6

0
7

0
6

0
7

0
6

0
7

0
7

0
7

0
7

0
7

0
7

0

0
7

0
6

0
7

0

0
7

0
7

1.87707
7

0.184804
7

0
6

0

0
7

0
6

0
7

0.221765

0
6

0
7

0
5

0
7

0
7

0
7

0
7

0
7

0
8

0
8

0
7

0

0
7

0
1

0.0877713

0
7

0
7

0
7

0
6

0
7

0
7

0
5

0

0
7

0
6

0
6

0
7

0
7

0.0726619
8

0
6

0

0
1

0
7

0
7

0
7

0

0
7

0.183849
7

0

0.0633849
6

0
7

0
7

0.131657

0
7

0
7

0.219428

0
6

0
7

0
7

0
7

0

0
6

0
7

0
7

0
7

0
7

0
7

0
7

0
6

0.0367698
7

0.12677

0
7

0
7

0
7

0
7

0
6

0
7

0
7

0.0968825
2

0

0
7

0
1

0
7

0
7

0.175543

0.263314

0
7

0
7

0
7

0

0
7

0
7

0.482742

0
7

0
6

0
7

0
5

0
7

0
7

0
7

3.06691
2

0.158462

0
7

0
7

0
7

0
7

0
5

0
6

0
7

0
7

0.0633849

0
8

0.0633849

0
7

0
7

0
7

0
7

0
7

0
6

0
7

0
7

0
7

0
7

0
7

0
7

2.76232

0
7

0
7

0
7

0
7

0.0877713
8

0.263314

0
7

0
7

0
7

0.0484412

0
7

0.0950773
7

0

0
7

0

0
6

3.1081
2

0
7

0.965484

0
7

1.10309

0.0633849

0
7

0
7

0.0633849
6

0
6

0
6

0
7

0.219428

0

0
7

0
7

0

0

0
7

0
7

0
7

0
5

0
7

0
7

8.8649
2

0
7

0
7

0
7

0
6

0.0877713

0
7

0
6

0
7

0
6

0

0.0633849
7

0
7

0.158462

0
7

0
7

0

0.0877713

6.94064
2

0
7

0
7

0.0633849

0

0
6

0

0
7

0
7

0
7

0
5

0
7

0
6

0
7

0

0
7

0
7

0
7

0
7

0
4

0
6

0
7

0
7

0

0
2

0
7

0

0
7

0
7

0
6

0
6

0

0
7

0
6

0
7

0
7

0
7

0
7

0
7

0
7

0.521771
7

0.0739218

0
6

0
7

0
7

0.0877713

0

0
7

0
7

0

0
7

0
5

0
7

0
6

0

0

0
6

0
7

0
6

0
2

0
7

0
7

0

0.219428
7

0
4

0.219428
8

0

0

0
7

0
6

0
7

0
7

0
8

0
7

0
7

0
7

0
7

0.351085
1

0
7

0

0
6

0
6

0
7

0
7

0
8

0.219428
7

0

0
7

0
7

0

0

0
7

0
7

0
6

0
6

0.131657

0
7

0
8

0
8

0
6

0
7

0
7

0
7

0
7

0
6

0
6

0
7

0

0.299078

0

0.175543

0
7

0
7

0
7

0
7

0
7

0
7

0
7

0
7

0.193681

1.92737

0
7

0
7

0
6

0
8

0

0
7

0
6

0

0

0
7

0
7

0
7

0

0
7

0
6

0
7

0
7

0
7

0

0
7

0
6

0
7

0
6

0.219428

0
7

0.0877713
1

0
7

0
6

0
7

0
6

0
7

0
7

0
7

0
8

0
7

0
6

0
7

0
7

0
7

0
7

0.0877713

0
6

0
7

0

0
7

0
7

0
7

0
7

0
7

0
7

0.0950773

0
7

0

0
7

0
6

0
6

0

0
7

0
7

0.351085

0
6

0
7

0

1.09753

0
7

0
7

0
7

0
8

0
7

0

0
6

0
7

0
5

0
7

0
6

0.0484412
7

0
7

0
6

0
7

0
7

0
7

0
7

0
6

0
5

0
8

0
7

0
7

0
6

0
8

0
7

0
7

0
6

0
7

0
6

0
7

0

0
7

0.282813
7

0

0
7

0

0
7

0
7

0
7

0
6

0
7

0
7

0
7

0.0367698
7

0

0
7

0

0
7

0

0
6

1.13837

0

0

0
5

0.0877713

0

0
6

0
6

0.131657
1

0
6

0
7

0
8

0

0

0

0
7

13.6484
2

0

0.3072

0

0
7

0
7

0
7

0
7

0
7

0
6

0.859377

0.136362
7

0
7

0

0

0
6

0
7

0
6

0
6

0
7

0

0
7

2.01874

0
7

0
7

0.681808

0
7

0.0877713

0
7

0
7

0
8

0.480492

0.175543

0
7

0
6

0
7

0
7

0

0
5

0
7

0
7

0
6

0
6

45.4217
2

0
7

0
7

0

0
1

0
6

0
7

0
6

0
7

0
2

0.183849

0
7

0.0877713
7

0
7

0
7

0
8

0
7

0
7

0
7

0
6

0
7

0.0633849
1

0.0877713

0
7

0
7

0
7

0.0367698

0

0
6

0
7

0

0
7

0
7

0
7

0
7

0
6

0
6

0
6

0

0
7

0
7

0.3072

1.95893

0
1

0
7

0
7

0.131657

0
6

0
8

0.394971

0
8

0.65982
1

0

0
6

0
7

0
7

0.122499
6

0
7

0

0
7

0

0
7

0
7

0.110883

0

0

0
7

0
7

0
7

0
7

0
7

0
7

0
6

0
8

0
8

0

0
7

0
6

0.343969
7

0

0.131657

0
1

0

0
7

0
8

0
5

0
7

0
6

0
7

0.761796

1.44823

0
7

0
6

0
7

0
7

0
7

0
7

0

0
7

0
7

0

0
7

0
5

0
7

0
7

0.131657

0
7

0
7

0
7

0
7

0
6

0
6

0
7

0

0
7

0
7

0
7

0
2

0
6

0
7

0
6

0
7

0
8

0
7

0
7

0
7

0
7

0
7

0.0633849

0
8

0
4

0.0877713

0
7

0
8

0.3072
7

0
7

0
6

0
7

0

0
6

0.0694547

0
6

0
7

0

0
7

0.482742

0

0
7

0
7

0

0

0
7

0
7

0
7

0
7

0

3.02811

0
7

0
7

0
6

0
7

0
7

0
7

0.0968825

0
7

0
7

0
7

0
6

0
5

0
6

0
6

0
6

0
5

0
7

0
6

0.0633849

0
7

0

0
6

0
2

0
6

0
8

0
7

0
7

0
6

0
7

0
7

0.0877713

0

0.263314
7

0.3072
7

0
6

0.0877713

0
6

0.0877713

0
6

0

0.0877713

0
8

0
8

0
7

0
7

0

0

0
7

0
6

0
7

0
6

0
7

0
6

0
7

0
6

0
7

0
8

0
6

0
7

0
7

0
7

0
7

0
6

0

0
6

0
6

0.281453

0
1

0
5

0
6

0
7

0
6

0
6

0.0877713

0

0
6

5.81295
2

14.568

0
6

0
8

0

0
7

0
7

0
7

0
7

0
7

0
6

0
7

0.0877713
6

0
7

0
6

0
7

0
8

0
7

0
6

0
5

0
7

0
5

0
7

0
7

0
7

0
7

0.121103

0
7

0
7

0
7

0
5

0
7

0.0735396

0

0.204319
6

0
7

0

0
6

0
6

0.0367698

0
8

0

0

0
8

0
7

0
7

0
7

0
6

0
6

0
6

0.0551547
6

0
6

0
7

0
7

0
8

0

0
4

0
7

0
5

0
7

0.322802

0.0877713
1

0
6

0
6

0

0.0877713
7

0.0633849

0
7

0.0950773

0
7

0
6

0
7

0
6

0
6

0
7

0
7

0.0877713

0
6

0

0
7

0.0877713

0
6

0.0877713

0
7

0
7

0
7

0
7

0
8

0
7

0
6

0.0484412
7

0.0484412
8

0
7

0
8

0
6

0
6

0

0
6

0
1

0
7

0
7

0
7

0
6

0
7

0
7

0
7

0
6

0

0
6

0
7

0
7

0
8

69.2777
7

0
8

0
7

0
7

0
7

0
6

0
6

0
8

0
6

0
6

0
1

0
7

0.628335

0

0
7

0
7

0
6

0
7

0
7

0
7

0

0
7

0
6

0
7

0
7

0
7

0
7

0.0877713

0
7

0

0
7

0
6

0.219428
1

0
7

0
7

0
7

0
7

0
8

0
7

0.138909

0
6

0
7

0
7

0
7

0
6

0
7

0
7

0

0
7

0
7

0
8

0
7

0
7

0
7

0
7

0.131657
5

0
7

0
7

1.40434
2

0
7

0
6

0
7

0
7

0
6

0
7

0

702.526
7

1.54526
7

0
6

0
7

0
7

0
7

0
7

0
6

0
7

0
7

0
7

0
7

0
6

0.0347274
8

0
6

0
6

0
7

0
7

0

0

0
7

0
5

1.08471

0
7

0.519512

0
7

0
7

0
6

0
8

0
7

0
7

0
4

0
7

0
6

0
7

0
7

0
7

0
7

2.11609
1

0
7

0
6

0
6

0
7

0
6

0
7

0

0
7

0

0
6

0
8

0
7

0
6

0

0
7

0.0551547

0
7

0
8

0
7

0.131657

0
7

0
7

0

0

0
8

0

0
6

0

0.219428

0
7

0
7

0

0
7

0
7

0
5

0
6

0
7

0
6

0

0
7

2.04542

0
7

0

0
7

0

0

0
7

0
7

0
6

0
7

0
7

0.38753

0
6

0

0
6

2.64551
2

0
7

0
8

0
6

13.824
1

0
6

0
6

0.0950773
7

0
7

0

0
8

0
7

0
7

0
6

0
7

0

0
7

0

0
7

0.131657

0
7

0
6

0

0
7

0
8

0
7

0
7

0
7

0
6

0
7

0.0877713

0

0.0877713

0
6

0
7

0
7

0
7

0
6

0

0
7

0

0
7

0
8

0
7

0
7

0
6

0
6

0

0

0.0950773
6

0
7

0
7

0
7

0.219428

0

0
5

0

0
7

0

0
7

0
6

0

0
7

0
7

0.220619
2

0
7

0

0

0

0
7

0
7

0

0
7

0
7

0
7

0
7

0
7

0
7

0
1

0
7

0

0

0

0

0
5

0
7

0
7

0
7

0.182713
1

0
6

0
7

0
4

0.129121

0
8

0
7

0
7

0
7

0

0
7

0
6

0

0
7

0

0
6

0.726619
2

0
6

0
8

0
7

0

0
7

0
6

0
1

0

0
7

0
7

0
7

0

0
8

0.327424

0
7

0
7

0
8

0
7

0

0
8

0
7

0

0

0
8

0
7

0

0

0
8

0
7

0
6

0
8

0
8

0
6

0
8

0.0877713

0
6

0

0

0
7

0

0

0
7

0
6

0
7

0

0
6

0.0633849

0.351085
7

0

0
6

0
7

0
7

0
6

0

0
7

0
7

0
8

0
7

0
7

0
7

0

0

0
7

0
6

0
6

0
6

0

0.0551547
6

0
5

0
6

0

0

0.0633849

0

0
7

0

0

0
2

0.136362
1

0

0
7

0
7

0
7

0
6

0

0
7

0

0

0
7

0
6

0
7

0.332648
7

0

0

0

0

0
7

0
6

0
7

0
7

0

0

2.26018

0

0
7

0.131657

0

0
7

0
7

0
7

0
6

0
7

0
6

0
7

0
5

0
8

0

0

0.0877713

0
7

0

0.0877713

0

0.0367698

0
7

0
6

0
7

0
5

0
7

0
6

0
6

0
7

0
7

0
7

0
6

0.131657
7

0
7

0
7

0
7

0

0

0
7

0
7

0
5

0
7

0
7

0
7

0.0551547

0

0
8

0

0
7

0

0
7

0
7

0
6

0

0
7

0

0
6

0

0
7

0
8

0
7

0

0
7

0

0
7

0

0

0
6

0
7

0
6

0

0

0

0.266427
1

0

0

0
7

0
7

0

0
6

0
8

0
6

0
6

0
7

0

0

0
8

0
6

0
7

2.94034
1

0
7

0
1

0

0

0
8

0
7

0
7

0
6

0
7

0
7

0
7

0

0
7

0
6

0
7

0

0
7

0
6

0

0
6

0
6

0
6

0
4

0

0
6

0
7

0
6

0

0
7

0
7

0
8

0.0551547
6

0

0
7

0

0.650929

0
6

0
7

0
1

0
7

0

0
6

0
6

0
7

0

0
6

0
7

0
7

0
7

0
7

0
7

0
8

0
7

0
7

0.175543

0.263314

0

0
6

0
6

0

0
6

0.52091
1

0

0

0
7

0.0367698
7

0

0
6

0

0
7

0

0
7

0
8

0

0
6

0

0
7

0
7

0
6

0

0
7

0
7

0
7

0
6

0
6

0
7

0

0
7

0

0

0.133937

0

0

0

0

0

0

0

0.435894
7

0

0

0

0

0

0

0

0

0

0

0
7

0

0

0

0

0

0

0

0

0

0

0

0

0

0

0

0

0

0

0

0

0

0
6

0

0

0

0

0

0

0

0

0

0

0

0

0

0

0

0

0

0

0

0

0

0
7

0

0

0

0

0

0

0

0

0

0

1.14103
7

0
6

0.121103

0

0

0

0

0

0

0

0.886679

0.0633849

0.147844
7

0

0

0

0

0

0

0.131657

0

0

0

0.0367698
7

0

0

0

0

0

0

0

0

0

0

0
7

0

0

0

0

0

0

0

0

0

0

0
7

0

0

0

0

0

0

0.0877713

0

0

0

0.794662

0

0

0

0

0

0

0

0

0

0

0
7

0

0

0

6.23248

0

0

0

0

0

0

0
7

0

0

0

0

0

0

0

0

0

0

0.0551547
7

0

0

0

0

0

0

0

0

0

0

0.110883
6

0

0

0

0

0

0

0.131657

0.0633849

0

0

0.572932
7

0.0739218
6

0

0

0

0

0

0

0

0

0

0

0.0877713
7

0

0

0

0

0

0

0

0

0

0

0
7

0

0

0

0

0

0

0

0

0

0

0.0633849
7

0

0

0

0

0.110309

0

0

0

0

0

0.3072

0.0633849

0

0

0

0

0

0

0

0

0

0.111826
7

0

0

0

0

0

0

0

0.0950773

0

0

0.184804
7

0

0

0

0

0

0

0

0

0

0

0.351085
7

0

0

0

0

0.175543

0

0

0

0

0

0

0

0

0

0

0

0

0

0

0

0

0
7

0

0

0

0

0

0

0

0

0

0

0.0877713
7

0

0

0

0

0

0

0

0

0

0.0877713

0

0
7

0

0

0

0

0

0

0

0

0

0

0
6

0

0

0

0

0

0

0

0

0

0

0

0

0

0

0

0

0

0

0.443694

0

0

0.131657

0

0

0

0

0.0484412

0

0

0

0

0

0

0

0

0

0

0

0

0

0

0

0.12677

0
7

0

0

0

0

0

0

0

0

0

0

0
7

0

0

0

0

0

0

0

0

0

0

0
7

0

0

0.150679

0

0

0.131657

0

0

0.181815

0

0
6

0

0

0

0

0

0

0.570514

0

0

0

0.175543
7

0
7

0

0

0

0

0

0

0

0

0.387362

0

0
7

0

0

0

0

0

0

0.131657

0

0

0

0.314506
7

0

0

0

0

0

0

0

0

0

0

0

0

0

0

0

0

0.351085

0

0.175543

0

0

0
7

0

0

0

0

0

0

0

0

0

0

0

0

0

0

0

0

0

0

0

0

0

0.175543

0

0

0

0

0

0

0

0

0

0.077809

0.0367698
7

0

0

0

0

0

0

0

0

0

0

0.12677
7

0

0

0

0

0

0

0

0

0

0

0
7

0.658285

0

0

0

0

0

0

0

0

0

1.49211
7

0
7

0

0.3072

0

0.110883

0

0

0

0

0

0

0
6

0

0

0

0

0

0

0

0.244997

0

0.0484412

0
7

0

0

0

0

0

0

0

0

0

0

0.438857

0

0

0

0

0

0

0

0

0

0

0.131657
7

0

0

0

0

0

0

0

0

0

0

0

0

0

0

0

0

0

0

0

0

0

0
7

0

0

0

0

0

0

0

0

0

0

1.01416

0

0

0.0735396

0

0

0

0

0

0

0

0
7

0

0

0

0

0

0

0

0

0

0

0
7

0

0

0

0

0

0

0

0

0

0

107.494

0
7

0
7

0

0

0

0

0

0

0

0

0

0

0
7

0

0

0

0

0

0

0

0

0

0

0
6

0

0

0

0

0

0

0.0877713

0

0

0

0
7

0

0

0

0

0

0

0

0

0

0

0
7

0

0

0

0

0

0

0

0

0

0

0
7

0

0

0

0

0

0

0

0

0

0

0
7

0

0

0

0

0

0

0

0

0

0

0
7

0

0

0

0

0

0

0.0877713

0

0

0

0.133937

0

0

0

0

0

0

0

0.0367698

0

0

0.369609
7

0

0

0

0

0

0

0

0

0

0

0
7

0.0950773
7

0

0

0

0

0

0

0

0

0

0

0.363849
6

0

0

0

0

0

0

0

0

0

0

0
7

0

0

0

0

0

0

0

0

0

0.104182

0
7

0

0

0

0.0877713

0

0

0

0

0

0

0
7

0

0

0

0

0

0

0

0

0

0

0
7

0

0

0

0

0

0

0

0

0

0

0
7

0

0

0

0

0

0

0

0

0

0

0
7

0

0

0

0

0

0

0

0

0

0

0
7

0

0

0

0

0

0

0

0

0

0

0
7

0

0

0

0

0.0950773

0

0

0

0

0

5.39173

0

0

0

0

0

0

0

0

0

0

0

0
7

0

0

0

0

0

0

0

0

0

0

0
7

0

0

0

0

0

0

0

0

0

0

0
7

0

0

0

0

0

0

0

0

0

0

0

0

0

0

0

0

0

0

0

0

0.0633849

0.481264

0

0

0

0

0

0

0

0

0

0

0
7

0

0

0

0

0

0

0

0

0

0

0
7

0

0

0

0

0

0

0

0

0

0

0
4

0

0

0

0

0

0

0

0

0

0

0
7

0

0

0.175543

0

0

0

0

0

0

4.74331

0
6

0
7

0

0

0

0

0

0

0

0

0

0

0
5

0

0

0

0

0

0

4.98945

0

0

0

0.0735396
7

0

0

0.263314

0

0

0

0

0

0

0

0
7

0

0

0

0

0

0

0

0

0

0

0.131657
7

0

0

0

0

0

0

0

0

0

0

0

0

0

0

0

0.0367698

0

0

0

0

0

0
7

0

0

0

0

0

7.09657

0

0

0

0

0
7

0

0

0

0

0

0

0

0

0

0

0
7

0.0367698

0

0

0

0

0

0

0

0

1.42616

0
7

0

0

0

0

0

0

0

0

0

0

0

0
7

0

0

0

0

0

0

0

0

0

0

0
7

0

0

0

0

0

0

0

0

0

0.0367698

0.219428
7

0

0

0

0

0

0

0

0

0

0

0
6

0

0

0

0

0

0

0

0

0

0

0.0551547
7

0

0

0

0

0

0

0

0

0

0

0.131657
6

0

0

0

0

0

0

0

0

0

0

0
7

0

0

0

0

0

0

0

0

0

0

0
7

0

0

0

0

0

0

0

0

0

0

0
7

0

0

0

0

0

0

0

0

0

0

0
7

0

0

0

0

0

0

0

0

0

0

0.282128
7

0.533162

0

0

0

0

0

0

0

0

5.79291

0

0.0877713
7

0

0

0

0

0

0

0

0

0

0

0
5

0

0

0

0

0

0

0

0

0

0

0
6

0

0

0

0

0

0

0

0

0

0

0
7

0

0

0

0

0

0

0.175543

0

0

0

0
7

0

0

0

0

0

0

0

0

0

0

0
7

0

0

0

0

0

0

0

0

0

0

0

0

0

0

0.0968825

0

0

0

0

0

0

0.0694547
7

0

0

0

0

0

0

0

0

0

0

0

0

0

0

0

0

0

0

0

0

0

0
7

0
7

0.175543

0

0

0

0.581044

0

0

0

0

0

0
7

0

0

0

0

0

0

0

0

0

0

0.219428
2

0

0

0

0

0

0

0

0

0

0

0.0950773

0

0

0

0

0.0367698

0

0

0.131657

0

0

0
7

0

0

0

0

0

0

0

0

0

0

0
7

0

0

0

0

0

0

0

0

0

0

0
7

0

0.351085

0

0

0

0

0.0877713

1.25667

0

0

0.131657
7

0

0

0

0

0

0

0

0

0

0

0

0

0

0

0

0

0

0

0

0

0

0.622068

0

0

0

0

0

0

0

0

0

0

0.526628
7

0
7

0

0

0

0

0

0

0

0

0

0

0
7

0

0

0

0

0

0

0

0

0

0

0.219428

0

0

0

0

0

0

0

0

0

0

0
7

0

0

0

0

0

0

0

0

0

0

0
7

0

0

0

0

0.147844

0

0

0

0.183849

0.0877713

0
7

0

0

0

0

0

0

0

0

0

0

0.131657
5

0

0

0

0.0877713

0

0

0

0

0

0

0
6

0

0

0

0

0

0

0.175543

0

0.3072

3.88089

0
7

0

0

0

0

0

0

0

0

0

0

0
7

0

0

0

0

0

0

0

0

0

0

0.0730852
7

0
6

0

0

0

0

0

1.22665

0

0

0

0

0
6

0

0

0

0

0

0

0

0

0

0

0
7

0

0

0

0

0

0

0

0

0

0

0
7

0

0

0

0.0551547

0.0877713

0

0

0

0

0

0.255345

0

0

0

0.330863

0

0

0

0

0

0

0

0

0

0

0

0

0

0

0

0

0

0
6

0

0

0

0

0

0

0

0

0

0

0
7

0

0

0

0

0

0

0

0

0

0

0
7

0

0

0

0

0

0

0

0

0

0

0.0484412

0

0

0

0

0

0

0

0

0

0

1.72018
7

0
7

0

0

0

0

0

0.0877713

0

0

0

0

0.131657
7

0

0

0

0

0

0

0

0

0

0

0

0

0.131657

0

0

0

0

0

0

0

0

0.0347274
7

0

0

0

0

0

0

0

0

0

0

0

0

0

0

0

0

0

0

0

0

0

0
7

0

0

0

0

0

0.0633849

0

0

0

0

1.79931

0

0

0

0

0

0

0

0

0

0

0
8

0.0877713

0

0

0

0

0

0

0

0

0

0
7

0

0

0

0

0

0.0367698

0

0

0

0

0
6

0

0

0

0

0

0.131657

0

0

0

0

160.49

3.51883

0
7

0

0

0

0.316924

0

0

0

0

0

0

0
7

0

0

0

0

0

0

0

0

0

0

0.131657

0

0

0

0

0

0

0

0

0

0

0

0

0

0

0

0

0

0

0.131657

0

0

0.168427

0

0

0

0

0

0.175543

0

0

0

0

0
6

0

0

0

0

0

0.965484

0

0

0.0633849

0

0
7

0

0

0

0

0.175543

0

0

0

0.219428

0

0.131657

0

0

0

0

0

0

0

0

0

0

0
7

0

0

0

0

0

0

0

0

0

0

14.7649

0

0

0

0

0

0

0

0

0

0

0
7

0
6

0

0

0

0

0

0

0

0

0

0

0
7

0

0

0

0

0

0

0

0

0

0

0

0

0

0

0

0

0

0

0

0

0

0
7

0

0

0

0

0

0

0

0

0

0

0
7

0

0

0

0

0

0

0

0

0

0.0950773

0
7

0

0

0

0

0

0

0

0

0

0

0
7

0

0

0

0.175543

0

0

0

0

0

0

0
6

0

0

0

0

0.0877713

0

0

0

0

0

0
7

0

0

0

0

0

0

0

0

0

0

0
6

0

0

0

0

0

0

0

0

0

0

3.09788

0

0

0

0

0

0

0

0

0

0

0

0
7

0

0

0

0

0

0

0

0

0

0

0.0950773

0

0

0

0

0

0

0

0

0

0

0
7

0

0

0

0

0

0

0

0

0

0

0

0

0

0

0

0

0

0

0

0

0

0
7

0

0

0

0

0

0

0

0

0

0.0877713

0.0502263
7

0

0

0

0.131657

0

0

0

0

0

0

0

0

0

0

0

0

0

0

0

0

0

0.0877713
6

0

0

0

0

0

0

0

0

0

0

0.0694547
7

0

0

0

0

0

0

0

0

0

0

0
7

0
7

0

0

0

0

0

0

0.0367698

0

0

0

0.0877713
7

0

0

0

0

0

0

0

0

0.103596

0

0.219428

0

0

0

0

0

0

0.217986

0

0

0

0.526628
1

0

0

0

0

0

0

0

0

0

0

0.131657

0

0

0

0

0

0

0

0

0

0

0
7

0

0

0

0

0

0

0

0

0

0

0
7

0

0

0

0

0

0

0

0

0

0

0.0950773

0

0

0

0

0

0

0

0

0

0

0
7

0

0

0

0

0

0

0

0

0

0

0
7

0

0

0

0

0

0

0.0633849

0

0

0

0.658285
7

0
7

0

0

0

0

0

0

0

0

0

0

0
7

0

0

0

0

0

0

0

0

0

0

0
7

0

0

0

0

0

0

0

0

0

0

0.0877713
7

0.190155

0

0

0

0.824003

0

0.0909077

0

0

0

0
7

0

0

0

0

0

0

0

0

0

0

0
6

0

0

0

0

0

0

0

0

0

0

0
6

0

0

0

0

0

0

0

0

0

0

0.190155

0

0

0

0

0

0

0

0

0

0

0.131657

0

0

0

0

0

0

0

0

0

0

0.318177
7

0

0

0

0

0

0

0

0

0

0

0
7

0
7

0

0

0

0

0

0

0

0

0

0

0
7

0

0

0

0

0

0

0

0

0

0

0
7

0

0

0

0

0

0

0

0

0

0

0
7

0

0

0

0

0

0

0

0

0

0

0.175543

0

0

0

0

0.25354

0

0

0

0

0

0
7

0

0

0

0

0

0

0

0

0

0

0.614399

0

0

0.219428

0

0

0

0

0

0

0

0
7

0

0

0

0

0

0

0

0

0

0

0
7

0

0

0

0

0

0

0

0

0

0

0.389045

0

0

0

0

0

0

0

0

0

0

0.219428
7

0
7

0

0

0

0

0

0

0

0

0

0

0.570514

0

0

0

0

0

0

0

0

0

0

0
7

0

0

0.0367698

0

0

0

0

0

0

0

0
7

0

0

0

0

0

0

0

0

0

0

0
7

0

0

0

0

0

0

0

0

0

0

0
7

0

0

0

0

0

0

0.052091

0

0

0

0

0.0950773

0

0

0

0

0

0

0

0

0

0

0

0

0

0

0

0

0

0

0

0

0
6

0

0

0

0

0

0

0

0

0

0

0
7

0

0

0

0

0

0

0

0

0

0

3.77298

0.137307

0

0

0

0

0

0

0

0

0

0

0

0

0

0

0

0

0

0

0

0

0

0
7

0

0

0

0

0

0

0

0

0

0

0
7

0

0

0

0

0

0

0

0

0

0

0
7

0

0

0.0968825

0

0

0

0

0

0

0

0

0

0

0

0

0

0

0

0

0

0

0.295183
7

0

0

0

0

0

0

0

0

0

0

0
7

0

0

0

0

0

0

0

0

0

0

0.175543

0

0

0

0

0

0

0.0551547

0

0

0

0

0

0

0

0

0

0.0347274

0

0

0

0

0
7

0
7

0

0

0

0

0

0

0

0

0

0

0
7

0

0

0

0

0

0

0

0

0

0

0
7

0

0

0

0

0

0

0

0

0

0

3.33531

0

0

0

0

0

0

0

0

0

0

0.0633849

0

0

0

0

0

0

0

0

0

0

0
7

0

0

0

0

0.0877713

0

0

0

0

0

0.217986

0

0

0

0

0

0

0

0.138508

0

0

0
5

0.121546

0

0

0

0

0

0

0

0

0

0.158462
7

0

0

0

0

0

0

0.158462

0

0

0

0
7

0

0

0

0

0

0

0

0

0

0

0.317541
7

0
7

0

0

0

0

0.131657

0

0

0

0

0

0.131657
7

0

0

0

0

0

0

0

0

0

0

0
7

0

0.175543

0

0

0

0

0

0

0

0

0
7

0

0

0

0

0

0

0

0

0

0

0.0367698
6

0

0

0

0

0

0

0

0

0

0

0
7

0

0

0

0

0

0

0

0

0

0

0
7

0

0.0877713

0

0

0

0

0

0

0

0

0
6

0

0

0

0

0

0

3.29142

0

0

0

0.290309

0

0

0

0

0

0

0

0

0

0

0.0334842
7

0

0

0

0

0

0

0

0

0

0

32.6029

0
7

0.0877713
7

0

0

0

0

0

0

0

0

0

0

0.0735396
7

0

0

0

0

0

0

0

0

0

0

0.0633849
8

0

0

0

0

0

0

0

0

0

0

0
6

0

0

0

0

0

0

0.242206

0

0

0.175543

0
6

0

0

0

0

0

0

0

0

0

0

0
7

0

0

0

1.40434

0

0

7.17041

0

0

0

1.2288

0

0

0.242206

0

0

0

0

0

0

0

7.81165

0

0

0

0

0

0

0

0

0

0

0
7

0

0

0

0

0

0.0735396

0

0

0

0

0
7

0

0

0

0

0

0

0

0

0

0

0
7

0
7

0

0

0

0

0

0

0

0

0

0

0
7

0

0

0

0

0

0

0

0

0

0

0
7

0

0

0

0

0

0

0

0

0

0

0.0950773

0

0

0

0

0

0

0.131657

0

0

0

0
7

0

0

0

0

0

0

0

0

0

0

0
7

0

0

0

0

0

0

0

0

0

0

0

0

0

0

0

0

0

0

0

0.382001

0

0.131657

0

0

0

0

0

0

0

0

0

0.077809

0

0

0

0

0

0

0

0

0

0

0

0
6

0

0

0

0

0

0

0

0

0

0

3.69041

0
7

0

0

0

0

0

0

0

0

0

0

0
7

0

0

0

0

0

0

0

0

0

0

0.0877713
7

0

0

0

0

0

0

0

0

0

0

0
7

0

0

0

0

0

0

0

0

0

0

0
7

0

0

0

0

0

0

0.147844

0

0

0

0.0919245

0

0

0

0

0

0

0

0

0

0

0
7

0

0

0

0

0

0

0.394971

0

0

0

0

0

0

0.0877713

0.0877713

0

0

0

0

0

0

0.131657
7

0

0

0

0

0

0

0

0

0

0

0
7

0

0

0

0

0

0

0

0

0

0

4.68043

0

0

0

0

0

0

0

0

0

0

0

0.0551547

0.183849

0

0.263314

0

0

0

0

0

0

0

0
7

0

0

0

0

0

0

0

0

0

0

0
7

0

0

0

0

0

0

0

0

0

0

0
7

0

0

0

0

0

0

0

0

0

0

0

0

0

0

0

0

0

0

0

0

0

0

0

0

0

0

0

0

0

0

0

0

0
7

0

0

0

0

0

0.0877713

0

0

0

0

0

0

0

0

0

0

0

0

0

0

0

0
7

0

0

0

0

0

0

0.131657

0

0

0

0.171482
7

0
7

0

0

0

0

0

0

0

0

0

0

0
7

0

0

0

0

0

0

0

0

0

0

0
6

0

0

0

0

0

0

0

0

0

0

0
7

0

0

0

0

0

0

0

0

0

0

0
7

0

0

0

0

0

0

0

0

0

0

0.351085
6

0

0

0

0

0

0

0

0

0

0

0
6

0

0

0

0

0

0

0

0

0

0

0.0633849
5

0

0

0

0

0

0

0

1.54543

0

0

0
7

0

0

0

0

0

0

0

6.45604

0

0.0877713

0
7

0

0

0

0

0

0

0

0

0

0

0
7

0
7

0

0

0

0

0

0

0

0

0

0

0
7

0

0

0

0

0

0

0

0

0

0

0
7

0

0

0

0

0

0

0

0

0

0

0
8

0

0

0

0

0

0

0

0

0

0

0
7

0

0

0

0

0

0

0

0

0

0

0
7

0

0

0

0

0

0

0

0

0

0

0.0367698
7

0

0

0

0

0

0

0

0

0

0

0
2

0

0

0

0

0

0

0

0

0

0

0.131657

0

0

0

0

0

0

0

0

0

0

0
7

0

0

0

0

0

0

0

0

0

0

0
7

0
7

0

0

0

0

0

0

0

0

0

0

0.131657

0

0

0

0

0

0

0

0

0

0

0
7

0

0

0

0

0

0

0

0

0

0

0
7

0

0

0

0

0

0

0

0

0

0

0
7

0.0726619

0

0

0

0

0

0

0

0

0

0
6

0

0

0

0

0.219428

0

0

0

0

0

0
7

0

0

0

0

0

0

0

0

0

0

0.175543

0

0

0

0

0

0

0

0

0

0

0
7

0

0

0

0

0

0

0

0

0

0

0
6

0

0

0

0

0

0

0

0

0.0484412

0

0
7

0
7

0

0

0

0

0

0

0

0

0

0

0
7

0

0

0

0

0

0

0

0

0

0

0.12677
6

0

0

0

0

0

0

0

0

0

0

1.88708

0

0

0

0

0

0

0

0

0

0

0
6

0

0

0

0

0

0

0

0

0

0

0.110309
6

0

0

0

0

0

0

0

0

0

0

0
7

0

0

0

0

0

0

0

0

0

0

0.0633849
8

0

0

0

0

0

0.263314

0

0

0

0

0
7

0

0

0

0

0

0

0

0

0

0

0.3072

0

0

0

0

0

0

0

0

0

0

0.146553

0
7

0

0

0

0

0

0

0

0

0

0

0.158462

0

0

0

0

0

0

0

0

0

0

0.3072

0

0

0

0

0

0

0

0

0

0

0
6

0

0

0

0

0

0

0

0

0

0

0
6

0

0

0

0

0

0

0

0

0

0

0
6

0

0

0

0

0

0

0

0

0

0

0
6

0

0

0

0

0

0

0

0

0

0

0.219428

0

0

0

0

0

0

0

0

0

0

0
8

0

0

0

0

0

0

0

0

0

0

0
7

0

0

0

0

0

0

0

0

0

0

2.74852

0
6

0

0

0

0

0

0

0

0

0

0

0
7

0

0

0

0

0

0

0

0

0

0

0
7

0

0

0

0

0

0

0

0

0

0

0
6

0

0

0

0

0

0

0

0

0

0

0
7

0

0

0

0

0

0

0

0

0

0

0
7

0

0

0

0

0

0

0

0

0

0

0
7

0

0

0

0

0

0

0

0

0

0

0
7

0

0

0

0

0

0

0

0

0

0

0.0877713

0

0

0

0

0

0

0

0

0

0

0
6

0

0

0

0

0

0

0

0

0

0

21.7037
7

1.01294

0
7

0

0

0

0

0

0

0

0

0

0

0
7

0

0

0

0

0

0

0

0

0

0

0
6

0

0

0

0

0

0

0

0

0

0

0.284004

0

0

0

0

0

0

0

0

0

0

0
6

0

0

0

0

0

0

0

0

0

0

0
7

0

0

0

0

0

0

0

0

0

0

0
7

0

0

0

0

0

0

0

0.0735396

0

0

0
8

0

0

0

0

0

0

0

0

0

0

0
7

0

0

0

0

0

0

0

0

0

0

0
7

0

0

0

0

0

0

0

0

0

0

0.600762

0
5

0

0

0

0

0

0

0

0

0

0

0
7

0

0

0

0

0

0

0

0

0

0

0
7

0

0

0

0

0

0

0

0

0

0

0.0484412
7

0

0

0

0

0

0

0

0

0

0

0
7

0

0

0

0.0633849

0

0

0

0

0

0

0
6

0

0

0

0

0

0

0

0

0

0

0.629011

0

0

0

0

0

0

0

0

0

0

0.128694
7

0

0

0

0

0

0

0

0

0

0

0
7

0

0

0

0

0

0

0

0

0

0

0.0367698

0

0

0

0

0

0

0

0

0

0

0
7

0
6

0

0

0

0

0

0

0

0

0

0

0
7

0

0

0

0

0

0

0

0

0

0

0
7

0

0

0

0

0

0

0

0

0

0

0
7

0

0

0

0

0

0

0

0

0

0

0
7

0

0

0

0

0

0

0

0

0

0

0
7

0

0

0

0

0.0551547

0.131657

0

0

0

0

0
7

0

0

0

0

0

0

0

0

0

0

0.175543
7

0

0

0

0

0

0

0

0

0

0

0

0.0909077

0

0

0

0

0

0

0

0

0

0.3072

0

0

0

0

0

0

0

0

0

0

0.688112
7

0
6

0

0

0

0

0

0.147079

0

0

0

0

0
8

0

0

0

0

0

0

0

0

0

0

0

0

0

0

0

0

0

0.052091

0

0

0

0
6

0

0

0.0877713

0

0

0

0

0

0

0

0
6

0

0

0

0

0

0

0

0

0

0

0.263314

0

0

0

0

0

0

0

0

0

0

0.175543

0

0

0

0

0

0

0

0

0

0

0
7

0

0

0

0

0

0

0

0

0

0

0
7

0

0

0

0

0

0

0

0

0

0

0
6

0

0

0

0

0

0

0

0

0

0

2.64065

0
7

0

0

0

0

0

0

0

0

0

0

0.0633849

0

0

0

0

0

0

0

0

0

0

0
7

0

0

0

0

0

0

0

0

0

0

0
7

0

0

0

0

0

0

0

0

0

0

0
7

0

0

0

0

0

0

0

0

0

0

0
7

0

0

0

0

0

0

0

0

0

0

0
7

0

0

0

0

0

0

0

0

0

0

0
7

0

0

0

0

0

0

0

0

0

0

0.0551547
7

0

0

0

0

0

0

0

0

0

0

0
7

0.614399

0

0

0

0

0

0

0

0

0

0.326699
7

0
7

0

0

0

0

0.110309

0

0

0

0

0

0
7

0

0

0

0

0

0

0

0

0

0

0
6

0

0

0

0

0

0

0

0

0

0

0.3072

0

0

0

0

0

0

0

0

0

0

0
7

0

0

0.131657

0

0

0

0

0

0

0

0
7

0

0

0

0

0

0

0

0

0

0

0

0

0

0

0

0

0

0

0

0

0

0
7

0

0

0

0

0

0

0

0

0

0

0
7

0

0

0

0

0

0

0

0

0

0

0

0

0

0

0

0

0

0

0

0

0

0
7

0
7

0

0

0

0

0

0

0

0

0

0

0
7

0

0

0

0

0

0

0

0

0

0

0
7

0

0

0

0

0

0

0

0

0

0

0
1

0

0

0

0

0

0

0

0

0

0

0
6

0

0

0

0

0

0

0

0

0

0

0
7

2.06263

0

0

0

0

0

0

0

0

0

0
6

0.746056

0

0

0

0

0

0

0

0

0

0
7

0

0

0.0633849

0

0

0

0

0

0

0

0
6

0

0

0

0

0

0

0

0

0

0

0
7

0

0

0

0

0

0

0

0

0

0

0.0633849
7

0
7

0

0

0

0

0

0

0

0

0

0

0.110883

0

0

0

0

0

0

0

0

0

0

0
7

0

0

0

0

0

0

0

0

0

0

0
7

0

0

0

0

0

0

0

0

0

0

0
7

0

0

0

0

0

0

0

0

0

0

0
7

0

0

0

0

0

0

0

0

0

0

0
6

0

0

0

0

0.0950773

0

0

0

0

0

0
7

0

0

0

0

0

0

0

0

0

0

0
7

0

0

0

0

0

0

0

0

0

0

0

0

0

0

0

0

0

0

0

0

0

0.3072
7

0
6

0

0

0

0

0

0

0

0

0

0

0.544663

0

0

0

0

0

0

0

0

0

0

0

0

0

0

0

0

0

0

0

0

0

0
7

0

0

0

0

0

0

0

0

0

0

0
7

0.193765

0

0

0

0

0

0

0

0

0.0877713

0.219428

0

0

0

0

0

0

0

0

0

0

0.431083

0

0.131657

0

0

0

0

0

0

0

0

0
7

0

0

0

0

0

0

0

0

0

0

0
6

0

0

0

0

0

0.3072

0.219428

0

0

0

0
7

0

0

0

0

0

0

0

0

0

0

2.88679

0
7

0

0

0

0

0

0

0

0

0

0

0.147844

0

0

0.0877713

0

0

0

0

0

0

0

0.131657

0

0

0

0

0

0

0

0

0

0

0
7

0.0877713

0

0

0

0

0

0

0

0

0

0
7

0

0

0

0

0

0.242206

0.0739218

0

0

0

0
7

0

0

0

0

0

0

0

0

0

0

0
8

0

0

0

0

0

0

0

0

0

0

0
7

0

0

0

0

0

0

0

0

0.131657

0

0
7

0

0

0

0

0

0

0

0

0

0

0.295687

0

0

0

0

0

0

0

0

0

0

30.8629

0
7

0
7

0

0

0

0

0

0

0

0

0

0

0
7

0

0

0

0.3072

0

0

0

0

0

0

0
7

0

0

0

0

0

0

0

0

0

0

0
7

0

0

0

0

0

0

0

0

0

0

0
7

0

0

0

0

0

0

0

0

0

0

0.147844

0

0

0

0

0

0

0

0

0

0

0
7

0

0

0

0

0

0

0

0

0

0

0.0633849

0

0

0

0

0

0

0

0

0

0

0
7

0

0

0

0

0

0

0

0

0

0

0
1

0

0

0

0

0

0

0

0

0

0

0.147079
6

0

0

0

0

0

0

0

0

0

0

0

0

0

0

0

0

0

0

0

0

0

0

0

0

0

0

0

0

0

0

0

0

0

0
7

0

0

0

0

0

0

0

0

0

0

0

0

0

0

0

0

0

0

0

0.263314

0

0.0877713
7

0

0

0

0

0

0

0

0

0

0

0.052091

0

0

0

0

0

0

0

0

0

0

0
7

0

0

0

0

0

0

0

0

0

0

0
7

0

0.110309

0

0

0

0

0

0

0

0

0
5

0

0

0

0

0

0

0

0

0

0

0
7

0

0

0

0

0

0

0

0

0

0

0

0
7

0

0

0

0

0

0

0

0

0

0

0
7

0

0

0

0

0

0

0

0

0

0

4.94108
2

0

0

0

0

0

0

0

0

0

0

0.077809

0

0

0

0

0

0

0

0

0

0

0
7

0

0

0

0

0

0

0

0

0

0

0
6

0

0

0

0

0

0

0

0

0

0

0

0

0

0

0

0

0

0

0

0

0

0
6

0

0

0

0

0

0

0

0

0

0

0
7

0

0

0

0

0

0

0

0

0

0

0.0968825
7

0

0

0

0

0

0

0

0

0

0

0

0.219428

0

0

0

0

0

0

0

0

0

0

0
7

0

0

0

0

0

0

0

0

0

0

0
7

0

0

0

0

0

0

0

0

0

0

0
7

0

0

0

0

0

0

0

0

0

0

0
7

0

0

0

0

0

0

0

0

0

0

0
6

0

0

0

0

0

0

0

0

0

0

0
7

0

0

0

0

0

0

0

0

0

0

0

0

0

0

0

0

0

0

0

0.322802

0

0

0

0

0

0

0

0

0

0

0

0

0
7

0.0367698

0

0

0

0

0

0

0

0

0

0

0
7

0

0

0

0

0

0

0

0

0

0

0
6

0

0

0

0

0

0

0

0

0

0

0

0

0

0

0

0

0

0

0

0

0

0
6

0

0

0

0

0

0

0

0

0

0

0
7

0

0

0

0

0

0

0

0

0

0

0
7

0

0

0

0

0

0

0

0

0

0

0
7

0

0

0

0

0

0

0

0

0

0

0
7

0

0

0

0

0

0

0

0

0

0

0
7

0

0

0

0

0

0

0

0

0

0

0
6

0

0

0

0

0

0

0

0

0

0

0

0
6

0

0

0

0

0

0

0

0

0.175543

0

0
6

0

0

0

0

0

0

0

0

0

0

0
5

0

0

0

0

0

0

0

0

0

0

0.131657

0

0

0

0

0

0

0

0

0

0

0
7

0

0

0

0

0

0

0

0

0

0

0

0

0

0

0

0

0

0

0

0

0

0
6

0

0

0

0

0

0

0

0

0

0

0

0

0

0

0

0

0

0

0

0

0

0
7

0

0

0

0

0

0

0

0

0

0

0
6

0
7

0

0

0

0

0

0

0

0

0

0

0
7

0

0

0

0

0

0.0919245

0

0

0

0

0
7

0

0

0

0

0

0

0

0

0

0

0
6

0

0

0

0

0

0

0

0

0

0

0
6

0

0

0

0

0

0

0

0

0

0

0
7

0

0

0

0

0

0

0

0

0

0

0

0

0

1.27268

0

0

0

0

0

0

0

0
7

0

0

0

0

0

0

0

0

0

0

0
7

0

0

0

0

0

0

0

0

0

0

0
8

0

0

0

0

0

0

0

0

0

0

0.0837105
7

0
7

0

0

0

0

0

0

0

0

0

0

0
7

0

0

0

0

0

0

0

0

0

0

0
7

0

0

0

0

0

0

0

0

0

0

0
6

0

0

0

0

0

0

0

0

0

0

0
7

0

0

0

0

0

0

0

0

0

0

0
7

0

0

0

0

0

0

0

0

0

0

0
7

0

0

0

0

0

0

0

0

0

0

0
7

0

0

0

0

0

0

0

0

0

0

0
7

0

0

0

0

0

0

0

0

0

0

0
7

0

0

0

0

0

0

0

0

0

0

0
7

0
7

0

0

0

0

0

0

0

0

0

0

0
7

0

0

0

0

0

0

0

0

0

0

0
7

0

0

0

0

0

0

0

0

0

0.921599

0
7

0

0

0

0

0

0

0

0

0

0

0
7

0

0

0

0

0

0

0

0

0

0

0
7

0

0

0

0

0

0

0

0

0

0

0
6

0

0

0

0

0

0

0

0

0

0

0
7

0

0

0

0

0

0

0

0

0

0

0
7

0

0

0

0

0

0

0

0

0

0

0
7

0

0

0

0

0

0

0

0

0

0

0.438857
7

0
7

0

0

0

0

0

0

0

0

0

0

0
6

0

0

0

0

0

0

0

0

0

0

0
2

0

0

0

0

0

0

0

0

0

0

0
6

0

0

0

0

0

0

0

0

0

0

0
7

0

0

0

0

0

0

0

0

0

0

0
7

0

0

0

0

0

0

0

0

0

0

0
8

0

0

0

0

0

0

0

0

0

0

0
7

0

0

0

0

0

0

0

0

0

0

0

0

0

0

0

0

0

0

0

0

0

0
7

0

0

0

0

0

0

0

0

0

0

2.83055
7

0
7

0
7

0

0

0

0

0

0

0

0

0

0

0
7

0

0

0

0

0

0

0

0

0

0

0
7

0

0

0

0

0

0

0

0

0

0

0.158462

0

0

0

0

0

0

0

0

0

0

0

0

0

0

0

0

0

0

0

0

0

0

0

0

0

0

0

0

0

0

0

0

0
7

0

0

0

0

0

0

0

0

0

0

0.25354

0

0

0

0

0

0

0

0

0

0

0
6

0

0

0

0

0.0877713

0

0

0

0

0

0

0

0

0

0

0

0

0

0

0

0

0.0968825
7

0
7

0

0

0

0

0.23439

0

0

0

0

0

0
7

0

0

0

0

0

0

0

0

0

0

0
7

0

0

0

0

0

0

0

0

0

0

0
5

0

0

0

0

0

0

0.0739218

0

0

0

0

0

0

0

0

0

0

0

0

0

0.0877713

0
7

0

0

0

0

0

0

0

0

0

0

0.0877713

0

0

0

0

0

0

0

0

0

0

0
7

0

0

0.175543

0

0

0

0

0

0

0

0
7

0

0

0

0

0

0

0

0

0

0.0877713

0
7

0

0

0

0

0

0

0

0

0

0

0.847478
7

0
7

0

0

0

0

0

0

0

0

0

0

0
7

0

0

0

0

0

0

0

0

0

0

0
7

0

0

0

0

0

0

0

0

0

0

0
7

0

0

0

0

0

0

0

0

0

0

0.077809

0.0909077

0

0

0

0

0

0

0.0551547

0

0

0
6

0

0

0

0

0

0

0

0

0

0

0
7

0

0

0

0

0

0

0.363309

0

0

0

1.4977

0

0

0

0

0

0

0

0

0

0

0
7

0

0

0

0

0

0

0

0

0

0

0
7

0

0

0

0

0

0

0

0

0

0

0
7

0
7

0

0

0

0

0

0

0

0

0

0

0.614399

0

0

0

0

0

0

0

0

0

0

0
6

0

0

0

0

0

0

0

0

0

0

0
7

0

0

0

0

0

0

0

0

0

0

0
7

0

0

0

0

0

0

0

0

0

0

0
7

0

0

0

0

0

0

0

0

0

0

0
4

0

0

0

0

0

0

0

0

0

0

0

0

0.0484412

0

0

0

0

0

0

0

0

0
7

0

0

0

0

0

0

0

0

0

0

0
7

0

0

0

0

0

0

0

0

0

0

0
6

0
7

0

0

0

0

0

0

0

0

0

0

0
7

0

0

0

0

0

0

0

0

0

0

0
7

0

0

0

0

0

0

0

0

0

0

0
7

0

0

0

0

0

0

0

0

0

0

0
7

0

0

0

0

0

0

0

0

0

0

0
7

0

0

0

0

0

0

0

0

0

0

0
7

0

0

0

0

0

0

0

0

0

0

0

0

0

0

0

0

0

0

0

0

0

0
7

0

0

0

0

0

0

0

0

0

0

0
7

0

0

0

14.1665

0

0

0

0

0

0

0
7

0
6

0

0

0

0

0

0

0

0

0

0

0.0633849

0

0

0

0

0

0

0

0

0

0

0
7

0

0

0

0

0

0

0

0

0

0

0.131657
7

0

0

0

0

0

0

0

0

0

0

0
7

0

0

0

0

0

0

0

0

0

0

0.0367698
7

0

0

0

0

0

0

0

0

0

0

0

0

0

0

0

0

0

0

0

0

0

0.0633849
7

0

0

0

0

0

0

0

0

0

0

0
6

0

0

0

0

0

0

0

0

0

0

0
7

0

0

0

0

0

0

0

0

0

0

0
7

0.158462

0

0

0

0

0

0

0

0

0

0

0
7

0

0

0

0

0

0

0

0

0

0.131657

0

0

0

0

0

0

0

0

0

0

0

0
6

0

0

0

0

0

0

0

0

0.0551547

1.82851

0
7

0

0

0

0

0

0

0

0

0

0

0.0968825

0

0

0

0

0

0

0

0

0

0

0
7

0

0

0

0

0

0

0

0

0

0

0
7

0

0

0

0

0

0

0

0

0

0

0.3072

0

0

0

0

0

0

0

0

0

0

0.0367698

0

0

0

0

0

0.950773

0

0

0

0

0
7

0.0877713

0

0

0

0

5.74902

0

0

0

0

0

0
7

0

0

0

0

0

0

0

0

0

0

0
7

0

0

0

0

0

0

0

0

0

0

0
6

0

0

0

0

0

0

0

0

0

0

0
6

0

0

0

0

0

0

0

0

0

0

0
5

0

0

0

0

0

0

0

0

0

0

0
6

0

0

0

0

0

0

0

0

0

0

0
7

0

0

0

0

0

0

0

0

0

0

0

0

0

0

0

0

0

0

0

0

0

0

0

0

0

0

0

0

0

0

0

0

0
7

0
7

0

0

0

0

0

0

0

0

0

0

0
7

0

0

0

0

0

0

0

0

0

0

0
7

0

0

0

0

0

0

0

0

0

0

0
7

0

0

0

0

0

0

0

0

0

0

0
7

0

0

0

0

0

0

0

0

0

0

0
7

0

0

0

0

0

0

0

0

0

0

0.443694

0

0.0551547

0

0

0

0

0

0

0

0

0
7

0

0

0

0.404468

0

0

0

0

0

0

0
7

0

0

0

0

0

0

0

0

0

0

0.131657

0

0

0.0877713

0

0

0

0

0

0

0

0.256266
7

0

0

0

0

0

0

0

0

0

0

0

0
2

0

0

0

0

0

0

0

0

0

0

0
7

0

0

0

0

0

0

0

0

0

0

0
7

0

0

0

0

0

0

0

0

0

0

0
6

0

0

0

0

0

0

0

0

0

0

0

0

0

0

0

0

0

0

0

0

0.175543

0
7

0

0

0

0

0

0

0

0

0

0

0
7

0

0

0

0

0

0

0

0

0

0

0

0

0

0

0

0

0

0

0

0

0

0
7

0

0

0

0

0.147079

0

0

0

0

0

0
7

0
7

0

0

0

0

0

0

0

0

0

0

0

0

0

0

0

0

0

0

0

0

0

0

0
6

0

0

0

0

0

0

0

0

0

0

0
7

0

0

0

0

0

0

0

0

0

0

0
7

0

0

0

0

0

0

0

0

0

0

0
7

0

0

0

0

0

0

0

0

0

0

0.0877713

0

0

0

0

0

0

0

0

0

0

0.351085
6

0

0

0

0

0

0

0

0

0

0

0
8

0

0

0

0

0

0

0

0

0

0

0
7

0

0

0

0

0

0

0

0

0

0

1.26506

0.161311

0

0.526628

0

0

0

0

0

0

0

0.0367698

0
7

0

0

0

0

0

0

0

0

0

0

0.181815
7

0

0

0

0

0

0

0

0

0

0

0
7

0

0

0

0

0

0

0

0

0

0

0
7

0

0

0

0

0

0

0

0

0

0

0
6

0

0

0

0

0

0

0

0

0

0

0
7

0

0

0

0

0

0

0

0

0

0

0
7

0

0

0

0

0

0

0

0.0877713

0

0

0
6

0

0

0

0

0

0

0

0

0

0

0
7

0

0

0

0

0

0

0

0

0

0

0.0950773
7

0
7

0

0

0

0

0

0

0

0

0

0

0

0

0

0

0

0

0

0

0

0

0

0
7

0

0

0

0

0

0

0

0

0

0

0
7

0

0

0

0

0

0

0

0

0

0

0

0

0

0

0

0

0

0

0

0

0

0
7

0

0

0

0

0

0

0

0

0

0

0
7

0

0

0

0.0484412

0

0

0

0

0

0

0
7

0

0

0

0

0

0

0

0

0

0

0
6

0

0

0

0

0

0

0

0

1.40434

0

0
7

0

0

0

0

0

0

0

0

0

0

0.0633849
7

0
7

0

0

0

0

0

0

0

0

0

0

0
7

0

0

0

0

0

0.0877713

0

0

0

0

0
7

0

0

0

0

0.792311

0

0

0

0

0

0.405321

0

0

0

0

0

0

0

0

0

0

0
7

0

0

0.175543

0

0

0

0

0

0

0

0
6

0

0

0

0

0

0

0

0

0

0

0
7

0

0

0

0

0

0

0

0

0

0

0
2

0

0

0

0

0

0

0

0

0

0

0
7

0

0

0

0

0

0

0

0

0

0

0

0

0

0

0

0

0

0

0

0

0

0
7

0.0877713
7

0

0

0

0

0

0

0

0

0

0

0
7

0

0

0

0

0.0367698

0

0

0

0

0

0
7

0

0

0

0

0

0

0

0

0

0

0
8

0

0

0

0

0

0

0

0

0

0

0.219428

0

0

0

0

0

0

0

0.0633849

0

0

0.0877713

0

0

0

0

0

0

0

0

0

0

0

0

0

0

0

0

0

0

0

0

0

0
7

0

0

0

0

0

0

0

0

0

0

0
7

0

0

0

0

0

0

0

0

0

0

0
7

0

0

0

0.833827

0.3072

0

0

0

0

0

0
7

0
7

0

0

0

0

0.131657

0

0

0

0

0

0
6

0

0

0

0

0

0

0

0

0

0

0
7

0

0

0

0

0

0

0

0

0

0

0
7

0

0

0

0

0

0

0

0

0

0

0

0

0

0

0

0

0

0

0

0

0

0
7

0

0

0

0

0

0

0

0

0

0

0
7

0

0

0

0

0

0

0

0

0

0

0
6

0

0

0

0

0

0

0

0

0

0

0
7

0

0

0

0

0

0

0

0

0

0

0
7

0

0

0

0

0

0

0

0

0

0

0
7

0
7

0

0

0

0

0

0.0877713

0

0

0

0

0

0

0

0

0

0

0

0

0

0

0

0.0633849

0

0

0

0

0

0

0

0

0

0

0
6

0

0

0

0

0

0

0

0

0

0

0
7

0

0

0

0

0

0

0

0

0

0

0

0

0

0

0

0

0

0

0

0

0

0
7

0.0367698

0

0

0

0

0

0

0

0

0

0

0

0

0

0

0

0

0

0

0

0

0
7

0

0

0

0

0

0

0

0

0

0

0
7

0

0

0

0

0

0

0

0

0

0

0
7

0
7

0

0

0

0

0

0

0

0

0

0

0
7

0

0

0

0

0

0

0

0

0

0

0
7

0

0

0

0

0

0

0

0

0

0

0
7

0

0

0

0

0

0

0

0

0

0

0
7

0

0

0

0

0

0

0

0

0

0

0.692542
8

0

0

0

0

0

0

0

0

0

0

0
7

0

0

0

0

0

0

0

0

0

0

0
7

0

0

0

0

0

0

0

0

0

0

0
6

0

0

0

0

0

0

0

0

0.128694

0

0
7

0

0

0

0

0

0

0

0

0

0

0
7

0
8

0

0

0

0

0

0

1.04794

0

0

0

0
7

0

0

0

0

0

0

0

0

0

0

0
7

0

0

0

0

0

0

0

0

0

0

0

0

0

0

0

0

0

0

0

0

0

0.0484412
6

0

0

0

0

0

0

0

0

0

0

0
7

0.193681

0

0

0

0

0

0

0

0

0

0
5

0

0

0

0

0

0

0

0

0

0

0.220619
1

0

0

0

0

0

0

0

0

0

0

0
7

0

0

0

0.0633849

0

0

0

0

0

0

0
6

0

0

0

0

0

0

0

0

0

0

0
7

0
7

0

0

0

0

0

0

0

0

0

0

0
5

0

0

0

0

0

0

0

0

0

0

0
7

0

0

0

0

0

0

0

0

0

0

0
7

0

0

0

0

0

0

0

0

0

0

0
8

0

0

0

0

0

0

0

0

0

0

0

0

0

0

0

0

0

0

0

0

0

0
7

0

0

0

0

0

0

0

0

0

0

0.175543
7

0

0

0

0

0

0

0

0

0

0

0
6

0

0

0

0

0

0

0

0

0

0

0
7

0

0

0

0

0

0

0

0

0

0

0
4

0
5

0

0

0

0
4

0
7

0
7

0
4

0
7

0

0

0
4

0

0

0
4

0

0

0
4

0

0

0

0
4

0.129121

0.129121

0
4

0

0

0
4

0

0

0
4

7.89646126264643e-14
7

0
4

0
7

0
7

0
7

0
7

0
7

0

0
4

0

0

0
4

0
4

10.1638

0
7

0
7

0
7

0

0

0

0

0

0

0

0

0

0

0
7

0

0

0

0

0

0

0

0

0

0

0
6

0

0

0

0

0

0

0

0

0

0

0

0

0

0

0

0

0

0

0

0

0

0
7

0
6

0

0
7

0
6

0

0
7

0
7

0

0
7

0

0

0

0

0

0

0

0
7

0

0

0

0

0

0

0

0

0

0

0
6

0

0

0

0

0

0

0

0

0

0

0
6

0

0

0

0

0

0

0

0

0

0

0
7

0

0

0

0

0

0

0

0

0

0

0
7

0

0

0

0

0

0

0

0

0

0

0
6

0

0

0

0

0

0

0

0

0

0

0
6

0

0

0

0

0

0

0

0

0

0

0
4

0

0

0

0

0

0

0
4

3.67994

3.67994

0
4

0

0

0

0
7

0

0
4

0.129121

0

0.129121

0

0

0

0

0
4

0
7

0
7

0

0
6

0
6

0

0

0
5

0

0

0

0

0

0

0

0

0

0

0

0

0

0

0

0
6

0

0

0

0

0

0

0

0

0

0

0
7

0

0

0

0

0

0

0

0

0

0

0
7

0

0

0

0

0

0

0

0

0

0

0
7

0

0

0

0

0

0

0

0

0

0

0
6

0

0

0

0

0

0

0

0

0

0

0
7

0

0

0

0

0

0

0

0

0

0
4

0
6

0
6

0

0

0

0

0

0

0

0

0

0

0

0

0
6

0

0

0

0

0

0

0
4

3.3242
6

0
6

0
7

0

0

0

0

0

0

0

0

0

0

0
6

0

0

0

0

0

0

0

0

0

0

3.3242

0

0

0

0

0

0

0

0

0

0

0
7

0

0

0

0

0

0

0

0

0

0

0
6

0

0

0

0

0

0

0

0

0

0

0

0

0

0

0

0

0

0

0

0

0

0
7

0

0

0

0

0

0

0

0

0

0

0
6

0

0

0

0

0

0

0

0

0

0

0
6

0

0

0

0

0

0

0

0

0

0

0
7

0

0

0

0

0

0

0

0

0

0

0
6

0
6

0

0

0

0

0

0

0

0

0

0

0

0

0

0

0

0

0

0

0

0

0

0
6

0

0

0

0

0

0

0

0

0

0

0
7

0

0

0

0

0

0

0

0

0

0

0
6

0

0

0

0

0

0

0
7

0
6

0
6

0
7

0
6

0
6

0
6

0
6

0

0
6

0

0
6

0

0
6

0
6

0
7

0
6

0

0
7

0
6

0
6

0
7

0
6

0

0

0

0

0

0

0

0
7

0

0

0
6

0

0

0

0

0
6

0

0

0

0

0

0

0

0

0

0

0
6

0

0

0

0

0

0

0

0

0

0

0
7

0

0

0

0

0

0

0

0

0

0

0
7

0

0

0

0

0

0

0

0

0

0

0
4

0

0

0

0
5

0

0

0

0

0
4

0

0

0
4

0

0

0

0

0
4

0

0

0
4

0

0

0
4

0

0

0

0
4

0

0

0
4

0

0

0
4

0

0

0
4

0

0

0
4

0

0

0
4

0

0

0

0
4

0

0

0
4

0

0

0
4

0

0

0
4

0

0

0
4

0

0

0
4

0

0

0
4

0

0

0
4

0

0

0

0

0
4

0

0

0
4

0

0

0

0

0

0

0
4

0

0

0

0

0
4

3.0305

3.0305

0
4

0

0

0

0
4

0

0

0
4

4.44089209850063e-16

0
4

9.4399

0
7

0
7

0
4

0
5

0
5

0
5

0

0

0

0

0

0

0

0
4

0

0

0

0

0

0

0

0
4

0
7

0
7

0
7

0

0
4

0

0

0

0

0

0

0

0

0
4

0
5

0
5

0

0

0
4

0
7

0
7

0

0

0
4

0
5

0
5

0

0

0

0

0

0

0
4

0
6

0
6

0

0

0
4

1.93681

1.93681

0

0

0
4

0
4

0
4

0

0

0

0

0

0

0
4

2.64698

2.13049

0.387362

0

0.129121

0

0

2.77555756156289e-17

0
4

0

0
5

0

0

0

0

0

0
6

0

0

0

0

0

0

0

0
4

0
3

0
4

0

0
4

0

0

0

0
4

0

0

0

0

0
4

0
7

0

0

0
4

0
5

0

0

0

0

0

0

0

0

0
4

0

0

0
4

0

0

0

0

0

0

0

0
4

0
7

0
7

0
4

0
6

0
6

0

0
4

0.207762

0.207762

0
4

0
5

0
5

0

0

0

0

0

0

0

0

0

0

0
5

0

0

0

0

0

0

0

0

0

0
4

0
4

0

0

0
4

0

0

0

0

0

0

0
4

0
7

0

0

0

0

0
4

0

0

0

0
4

0

0

0

0
4

0

0

0
4

0

0

0

0
4

0

0

0

0

0
4

0

0

0
4

0

0

0

0
4

0
7

0
7

0

0

0
7

0
7

0
7

0
6

0
7

0

0

0

0
4

3.16346

3.16346

0
4

0

0

0

0
4

0

0

0
4

0

0

0
4

0

0

0

0

0
4

0

0

0
4

0

0

0
4

0

0

0

0

0
4

0

0

0

0
4

0

0

0

0

0
4

0
5

0
5

0
3

0
5

0

0

0

0

0
4

0

0

0

0
4

0

0

0
4

0

0

0

0
4

0

0

0
4

0

0

0

0
4

0

0

0
4

0

0

0
4

0

0

0

0
4

0

0

0
4

0

0

0

0
4

0

0

0
6

0

0

0

0

0

0

0
4

0

0

0
4

0

0

0
4

0

0

0
4

0

0

0
4

0

0

0
4

0

0

0
4

0

0

0
4

0

0

0
4

0

0

0
4

0

0

0
4

0

0

0

0

0

0

0

0

0

0
4

0

0

0
4

0

0

0
4

0

0

0
4

0

0

0
4

0

0

0
4

0

0

0
4

0

0

0
4

0

0

0
4

0

0

0
4

0

0

0
4

1.48489

1.48489

0

0

0

0

0

0

0

0

0

0

0
4

0

0

0
4

0

0

0
4

0

0

0
4

0

0

0
4

0

0

0
4

0

0

0
4

0

0

0
4

0

0

0
4

0

0

0
4

0

0

0
4

0
5

0

0

0

0

0

0
4

0

0

0
4

0

0

0
4

0

0

0
4

0
4

4.15275

4.15275

3.93213

0

0.220619

0

0

0

0
4

0
4

0.0347274

0.0347274

0.0347274

0
4

0
4

0.077809

0.077809

0.077809

0
4

0
4

0

0

0

0

0
4

0
4

0

0

0

0

0

0
4

0
4

0

0

0

0
4

0
4

0

0

0

0

0
4

0
4

0

0

0

0

0
4

0
4

0

0

0

0
4

0
4

0

0

0

0
4

0
4

0

0

0

0

0
4

0
4

0.507857
3

0.507857
3

0.230451

0

0

0

0

0

0

0

0

0

0

0.077809

0

0

0

0

0

0

0

0

0

0

0
5

0

0

0

0

0

0.111826
3

0.0877713
3

0

0

0

0

0
4

0

0

0
4

0
4

0

0

0

0

0
4

0
4

0

0

0

0
4

0

0

0
4

0
4

0

0

0

0
4

0

0

0
4

0
4

0

0

0

0
4

0
4

0

0

0

0
4

0
4

0

0

0

0
4

0
4

0

0

0

0
4

0
4

0

0

0

0
4

0
4

0

0

0

0
4

0
4

0

0

0

0
4

0
4

0.808209
3

0.774725
3

0

0.774725

0

0

0
4

0

0

0
4

0.0334842

0.0334842

0
4

6.93889390390723e-18
3

0
4

0

0

0

0
4

0
4

0

0

0

0
4

0
4

0

0

0

0
4

0
4

0

0

0

0
4

0
4

0

0

0

0
4

0
4

0

0

0

0
4

0
4

0

0

0

0
4

0
4

0

0

0

0
4

0
4

0

0

0

0
4

0
4

0

0

0

0
4

0
4

0
6

0
6

0
6

0

0

0

0

0
4

0
4

0

0

0

0
4

0
4

0

0

0

0
4

0
4

0

0

0

0
4

0
4

0

0

0

0
4

0
4

0

0

0

0
4

0
4

2.25961

2.25961

2.25961

0
4

0
4

0

0

0

0
4

0
4

0

0

0

0
4

0
4

1.33763

1.33763

0.581044

0.304663

0.193681

0.258242

1.66533453693773e-16

0
4

0
4

0
5

0

0

0
4

0

0

0
4

0

0

0
4

0
4

0

0

0

0

0
4

0
4

0

0

0

0

0
4

0
4

0.138508

0.138508

0.138508

0

0

0

0

0

0

0

0
4

0
6

0
6

0

0

0

0

0

0

0

0
4

0

0

0
4

0

0

0
4

0

0

0
4

0
5

0

0

0

0

0

0

0

0

0

0
4

0
7

0
6

0

0

0
4

0
7

0
7

0

0
4

0

0

0

0
4

0

0

0
4

0

0

0
4

0

0

0
4

0

0

0
4

0
4

1.38508

0

0

0
5

0
7

0

0

0
5

0

0

0
5

0

0

0

0

0

0

0

0

0

0

0

0

0

0
6

0

0

0

0

0

0

0

0

0

0

0
5

0

0

0

0

0

0

0

0

0

0

0
6

0

0

0

0

0

0

0

0

0

0

0

0

0

0

0

0

0

0

0

0

0

0

0

0

0

0

0

0

0

0

0

0

0
7

0

0

0

0

0

0

0

0

0

0
4

1.38508

0
7

1.38508

0
4

0

0

0

0
4

0

0

0
4

0

0

0
4

0

0

0
4

0

0

0
4

0

0

0
4

0

0

0
4

0

0

0
4

0
5

0
6

0

0

0

0
4

0
6

0

0

0

0

0
4

0
5

0
5

0
4

0
7

0
7

0

0

0
4

0
7

0
7

0
4

0

0

0

0
4

0

0

0

0
4

0

0

0
4

0
4

1.24657

0

0
5

0

0

0

0

0

0

0

0

0

0

0
5

0

0

0

0

0

0

0

0

0

0

0

0

0

0

0
6

0

0

0

0

0

0
4

1.24657

1.24657

0
4

0

0

0
4

0

0

0
4

0

0

0
4

0
4

0
5

0
5

0
5

0
5

0
5

0
6

0
5

0

0

0

0

0

0

0

0

0

0

0

0

0

0

0

0

0

0
6

0

0

0

0

0

0

0

0

0

0

0
5

0

0

0

0

0

0

0

0

0

0

0
5

0

0

0

0

0

0

0

0

0

0

0
5

0

0

0

0

0

0

0

0

0

0

0
6

0

0

0

0

0

0

0
4

0
6

0
4

0

0

0

0
4

0

0

0

0
4

0

0

0
4

0

0

0
4

0
4

0

0

0

0
5

0

0

0

0

0

0

0

0

0

0

0
5

0

0

0

0

0

0

0

0

0

0

0

0

0

0

0

0

0
7

0

0

0
4

0
5

0
5

0
4

0

0

0
4

0

0

0

0
4

0

0

0

0

0
4

0

0

0
4

0

0

0
4

0

0

0
4

0

0

0

0
4

0

0

0
4

0

0

0
4

0

0

0
4

0

0

0

0
4

0

0

0
4

0

0

0
4

0

0

0
4

0

0

0
4

0

0

0
4

0

0

0
4

0

0

0
4

0

0

0
4

0

0

0
4

0

0

0
4

0

0

0

0

0
4

0

0

0
4

0

0

0
4

0

0

0
4

0

0

0
4

0

0

0
4

0

0

0
4

0

0

0
4

0

0

0

0

0

0
4

0

0

0

0

0

0
4

0

0

0
4

0

0

0
4

0

0

0
4

0

0

0

0
4

0
4

0
5

0
7

0
7

0
7

0

0
4

0
4

0
4

0

0

0
4

0

0

0
4

0

0

0
4

0

0

0
4

0

0

0
4

0

0

0

0
4

0
4

0
4

0

0

0
4

0

0

0

0

0
4

0

0

0

0

0
4

0

0

0
4

0

0

0
4

0

0

0
4

0

0

0
4

0
4

4.15037

4.15037

0

0
5

0

0

0.0877713

0.131657

0

0

0

0

0

0

0

0

0

0

0

0

0

0

0

0

0

0

0

0

0

0

0

0

0

0

0

0
7

0

0
7

1.56266

2.36828

0

0

0
4

0
4

0
4

0

0

0

0

0

0
4

0

0

0

0

0
4

0

0

0
4

0

0

0
4

0

0

0
4

0

0

0
4

0
4

0.155618
4

0.155618
4

0.155618
5

0

0

0

0

0

0

0

0

0

0

0

0

0

0

0

0

0
4

0

0

0
4

0

0

0
4

0

0

0
4

0
4

0
4

88.3176

13.5872

2.70091

0
5

2.70091

0

0

0

0

0

0

0

0

0

0
4

5.35025

5.35025

0
7

0

0

0

0

0

0

0

0

0

0

0
7

0

0

0
7

0

0

0

0

0

0

0
4

2.4533

1.61401
7

0
7

0

0

0

0

0

0

0

0

0

0

0
7

0

0

0

0

0

0

0

0

0

0

0
7

0

0

0

0

0

0

0

0

0

0

0
8

0

0

0

0

0

0

0

0

0

0

0
7

0

0

0

0

0

0

0

0

0

0

0
7

0

0

0

0

0

0

0

0

0

0

0.129121
1

0

0

0

0

0

0

0

0

0

0

0

0

0

0

0

0

0

0

0

0

0

0
8

0

0

0

0

0

0

0

0

0

0

0

0

0

0

0

0

0

0

0

0

0

0.516483

0

0

0

0

0

0

0

0

0

0

0

0
8

0

0

0

0

0

0

0

0

0

0

0
7

0

0

0

0

0

0

0

0

0

0

0
7

0

0

0

0

0

0

0

0

0

0

0
7

0

0

0

0

0

0

0

0

0

0
7

0

0

0.193681
7

0
7

0

0

0

0

0

0

0

0

0

0

0

0

0

0

0

0

0

0

0

0

0
7

0

0

0

0

0

0

0

0

0

0

0
1

0

0

0

0

0

0

0

0

0

0

0
1

0

0

0

0

0

0

0

0

0

0

0
8

0

0

0

0

0

0

0

0

0

0

0
8

0

0

0

0

0

0

0

0

0

0

0
4

0
7

0
7

0

0
4

0
7

0
7

0

0

0

0

0

0

0

0

0

0
4

0
6

0
6

0
6

0

0

0

0
4

0
7

0
7

0
6

0

0

0
4

0
7

0

0

0

0

0

0

0

0

0

0

0
4

0

0

0

0

0

0

0
4

0
1

0
1

0
4

0
6

0
6

0

0

0
4

0

0

0

0

0
4

0

0

0

0
4

0
5

0
7

0

0
6

0

0

0

0

0

0

0

0

0

0

0

0

0

0

0

0

0

0

0

0
6

0

0

0

0
6

0
5

0
6

0
6

0
6

0
4

0
6

0
6

0
4

0

0

0

0
4

0

0

0

0
4

0.387362

0.258242

0.129121

0
4

0

0

0

0
4

0

0

0

0
4

0

0

0

0
4

0

0

0
4

0

0

0
4

0

0

0
4

2.64698

0
1

0

0

0

0

0

0

0

0
7

2.64698
2

0
8

0
6

0
4

0.0484412

0.0484412

0
4

0

0

0
4

0

0

0
4

0

0

0
4

0

0

0
4

0

0

0
4

0

0

0
4

0

0

0
4

0

0

0
4

0

0

0
4

0

0
5

0

0

0
7

0
7

0

0

0

0

0

0
4

0

0

0
4

0

0

0
4

0

0

0
4

0

0

0
4

0

0

0
4

0

0

0
4

0

0

0
4

0

0

0
4

0

0

0
4

0

0

0
4

0

0
1

0
1

0

0

0

0

0

0

0
4

0

0

0
4

0
7

0
7

0
8

0

0

0

0

0
4

0
6

0
6

0
7

0

0

0

0

0

0
4

0
8

0
8

0
8

0

0

0

0

0

0
4

0

0

0

0

0

0

0

0
4

0
4

38.0235

36.4121

0.193681

1.93681

0

0.322802

0

0

0

0

0

0

0

0.193681

0

0.129121

0

0

0

0

0

0

0

0

1.67857

16.9794

3.93818

11.0398

8.88178419700125e-15

0
4

0
8

0
8

0

0

0

0

0

0

0

0

0

0

0
8

0

0

0

0

0

0

0

0

0

0

0
8

0

0

0

0

0

0

0

0

0
4

0

0

0
4

0.268062

0.268062

0
4

0

0

0
4

1.22665

1.22665

0
4

0.116713

0.116713

0
4

0

0

0
4

0

0

0
4

0

0

0
4

0

0

0
4

0

0

0
4

9.71445146547012e-16

0
4

3.03434

1.67857

0

0
5

0

0

0

0

0

0

0

0

0

0.129121

0.387362

0

0

0

0

0

0

0

0

0

0

0
6

0

0

0

0

0

0

0

0

0.129121

0

0
7

0

0

0

0

0

0

0

0

0

0

0

0

0

0

0

0

0

0

0

0

0

0

0

0

0
6

0
7

0

0
6

0

0
7

0

0

0
7

0

0

0.387362

0.516483

0

0

0
7

0

0.129121

0

0

0

0

0

0

0

0

0

0

0

0

0

0

0

0

0

0

0

0
6

0

0

0

0

0

0

0

0

0

0

0
7

0

0

0

0

0

0

0

0

0

0

0
1

0

0

0

0

0

0

0

0

0

0

0

0

0

0

0

0

0

0

0

0

0

0
7

0

0

0

0

0

0

0

0

0

0

2.77555756156289e-17

0
4

0

0

0

0

0
4

0

0

0

0

0
4

0

0

0

0
4

0

0

0
4

0

0

0
4

0

0

0
4

0

0

0
4

0

0

0
4

1.22665

0

0

0

0

0

0

0

0

0

0

0

0
6

0

0

0

0

0

0

0

0

0

0

0
6

0

0

0

0

0

0

0

0

0

0

0
6

0

0

0

0

0

0

0

0

0

0

0

0

0

0

0

0

0

1.22665

0
4

0
5

0
6

0

0

0

0

0

0

0

0

0

0
4

0.129121

0
6

0

0

0

0

0

0

0

0

0

0.129121

0
4

0
7

0
7

0

0

0

0
4

0
6

0

0

0

0

0

0
4

0
7

0

0

0

0

0
4

0

0

0

0

0

0
4

0

0

0

0

0

0

0
4

0
4

0

0

0

0
4

0

0

0
4

0
4

0

0

0

0
4

0

0

0
4

0
4

0

0

0

0

0
4

0
4

4.21439

4.21439

0.0484412

4.11751

0.0484412

0
4

0
4

0

0

0

0

0
4

0
4

0.968406

0.968406

0.774725

0.193681

0
4

0
4

0

0

0

0

0
4

0
4

2.84066

2.71154

0

2.71154

0
4

0.129121

0.129121

0
4

0
4

0

0

0

0
4

0
4

0

0

0

0
4

0
4

0.129121
7

0.129121
7

0
7

0

0

0

0

0

0.129121

0
7

0
7

0
7

0

0

0

0

0

0
4

0
6

0
6

0
7

0

0

0

0

0

0

0
4

0

0

0

0
4

0

0

0

0
4

0
6

0

0

0

0
4

0

0

0

0
4

0

0

0
4

0

0

0
4

0

0

0
4

0
4

0

0

0

0
4

0

0

0
4

0
4

0

0

0

0
4

0

0

0
4

0
4

0

0

0

0

0
4

0
4

3.16346

3.16346

3.16346

0
4

0
4

0

0

0

0
4

0
4

0

0

0

0
4

0
4

0

0

0

0
4

0
4

0

0

0

0
4

0
4

0

0

0

0
4

0
4

0

0

0

0
4

0
4

0
7

0
7

0
7

0

0

0

0

0
4

0
7

0
7

0

0

0

0

0

0
4

0
6

0

0

0

0
4

0

0

0
4

0
4

0.129121

0.129121

0.129121

0
4

0
4

0

0

0

0
4

0
4

0

0

0

0
4

0
4

0

0

0

0
4

0
4

0

0

0

0
4

0
4

0

0

0

0
4

0
4

0
6

0
6

0
6

0

0

0

0

0

0
4

0
7

0
7

0

0

0
4

0
7

0
6

0

0
4

0

0

0

0

0
4

0

0

0
4

0

0

0
4

0
4

0
6

0
6

0
6

0

0

0

0

0
4

0
4

22.2273
2

22.2273
2

17.292
2

0.645604

0

4.13187

0.0726619

0

0.0367698

0.0484412

1.3392065234541e-15
2

0
4

0
4

0
6

0
6

0

0

0

0

0

0
4

0

0

0

0
4

0
4

0

0

0

0
4

0
4

0

0

0

0

0

0

0

0
4

0
4

0

0

0

0
7

0
7

0

0

0
7

0

0

0

0

0

0
7

0

0

0

0

0

0

0

0

0

0

0

0

0

0

0

0

0

0

0

0

0

0

0

0

0

0

0

0

0

0

0

0

0
7

0

0

0

0

0

0

0

0

0

0

0

0

0

0

0

0

0

0

0

0

0

0
8

0

0

0

0

0

0

0

0

0

0

0

0

0

0

0

0

0

0

0

0

0

0

0

0

0
4

0

0

0
4

0

0

0
4

0

0

0
4

0
4

1.06581410364015e-14

0
4

1.22665

0
7

0

0

0

0
4

0

0

0

0
4

0

0

0
4

0

0

0
4

0

0

0
4

0

0

0
4

0

0

0
4

0

0

0
4

0
4

0
7

0
7

0

0

0

0

0

0

0

0

0

0

0

0

0

0

0

0

0

0

0

0

0

0

0

0

0

0

0

0

0

0

0

0

0

0

0

0

0

0

0

0

0

0

0

0

0

0

0

0

0

0

0

0

0

0

0

0

0

0

0

0

0

0

0

0

0

0

0

0

0

0

0

0

0

0

0
4

0

0

0
4

0
4

0
6

0
6

0
6

0

0
4

0
4

0

0

0

0
4

0
4

0

0

0

0
4

0
4

0
7

0
7

0
7

0
4

0
4

0

0

0

0
4

0
4

0

0

0

0
4

0
4

0.129121

0.129121

0.129121

0
4

0
4

0

0

0

0
4

0
4

0

0

0

0
4

0
4

0

0

0

0
4

0
4

0

0

0

0
4

0
4

1.09753

1.09753

0

0
7

0

0

0

0

0

0

0

0

0

0

0
6

0

0

0

0

0

1.09753

0

0

0

0

0

0

0

0

0
7

0

0
7

0

0

0

0
4

0

0

0

0

0
4

0

0

0
4

0

0

0
4

0

0

0
4

0

0

0

0
4

0

0

0
4

0

0

0
4

0

0

0
4

0
4

0
4

32.3189
4

31.4151
4

31.2214
4

25.992
4

0

0

0

0

0

0

0

0

0

0

0

0

0

0

0

0

0

0

0

0

0

0

0
7

0

0

0

0

0

0

0

0

0

0

0

0

0

3.03434

0

0

0

0

0

0

0

0

0

0

0

0.193681

0

0

0

0

0

0
6

0

2.00137

0

2.22044604925031e-15
4

0
4

0

0

0
4

0

0

0
4

0

0

0
4

0

0

0
4

0

0

0
4

0

0

0
4

0

0

0
4

0

0

0
4

0.193681

0.193681

0
4

0

0

0
4

0

0

0
4

0

0

0
4

0

0

0
4

1.22124532708767e-15
4

0
4

0

0

0

0
4

0
4

0

0

0

0

0
4

0
4

0

0

0

0
4

0
4

0

0

0

0
4

0
4

0

0

0

0
4

0
4

0

0

0

0
4

0
4

0

0

0

0
4

0
4

0

0

0

0
4

0
4

0

0

0

0
4

0
4

0

0

0

0
4

0
4

0

0

0

0
4

0
4

0

0

0

0

0
4

0
4

0

0

0

0
4

0
4

0

0

0

0
4

0
4

0

0

0

0
4

0
4

0.903846

0.903846

0.903846

0
4

0
4

0

0

0

0
4

0
4

0

0

0

0
4

0
4

0

0

0

0
4

0
4

0

0

0

0
4

0
4

0

0

0

0

0
4

0
4

0

0

0

0
4

0
4

0

0

0

0

0
4

0
4

0

0

0

0
4

0
4

0

0

0

0
4

0
4

3.66373598126302e-15
4

0
4

2718.81

14.2182
1

4.22981

3.47649

0.0633849

0.25354

0.348617

0.0877713

1.38777878078145e-16

0
4

0.0633849

0.0633849

0
4

0

0

0
4

6.524

6.524

0
4

1.51403

1.51403

0
4

0

0

0
4

1.71139

1.71139

0
4

0.0877713

0.0877713

0
4

0

0

0
4

0.0877713

0.0877713

0
4

4.71844785465692e-15
1

0
4

44.0988

39.9661

11.8078

0.352985
2

0.0950773
3

0.309618

0.0633849

0

0

0.0633849

0

0

0

15.1362

0

0

0

0

1.32275

0

0

0

0.0633849

0

0
4

0

0.475387

0

0.0633849

0

0

0

0

0

0

0.238927

0

0

0

0.077809

0

0

0

0

0

0

0

0.0633849

0

0.285232

0.0735396

0

0

0

0

0

0

0
4

0.0877713

0

4.3573

0

0

0

0.0633849

0

0

0

0.322002
3

0

0

0

0.0633849

0

0

0.0633849

0

0

0

1.42616

0

0

0

0

0

0

0

0.0551547

0

0

2.84003
3

0

0.0367698

0

0

0.0950773

0.0633849

0

0

0

8.53483950180589e-15

0
4

0.386083
3

0.257389
3

0

0.0367698

0.0919245

0

2.77555756156289e-17
3

0
4

0

0

0
4

0

0

0
4

0.0367698

0.0367698

0
4

0

0

0
4

0

0

0
4

0

0

0
4

0

0

0
4

0

0

0
4

0

0

0
4

0

0

0
4

3.2909
3

3.2909
3

0
4

0.155618

0.155618

0

0
4

0

0

0

0
4

0

0

0
4

0

0

0

0
4

0.131657

0.131657

0
4

0.131657

0.131657

0
4

0

0

0
4

0
4

118.972
1

55.6298
1

26.0987
1

0

0.183849

0.0367698

0.147079

0

0

0.239004

0

0

0

25.4301
1

0

0.116713

0

0

0

0.0735396

0

0

0

0

0.459623

0.183849

0.0735396

0.275774

0

0.275774

0

0

0

0

0

0

0

0

0

0

1.16713

0.0367698

0

0

0

0

0.0551547

0.147079

0.0367698

0.0919245

0.110309

0

0

0

0

0

0

0

0

0

0

0

0

0

0

0

0

0.353583

0

0.0367698

5.62050406216485e-16
1

0
4

63.3424
2

63.3424
2

0

0
4

0

0

0

0

0

0
4

0

0

0
4

0

0

0
4

0

0

0
4

0

0

0
4

0

0

0
4

0

0

0
4

0

0

0
4

0

0

0
4

0

0

0
4

0

0

0
4

0

0

0

0

0
4

0

0

0
4

0

0

0
4

0

0

0
4

0

0

0

0
4

0

0

0
4

0

0

0
4

0

0

0
4

0

0

0
4

1.4210854715202e-14
1

0
4

284.329

30.9963

15.7688
2

0

0.0367698

0

0

0.131657

0

0.0367698

0

0

0

0
6

0.110309

0.526628

0

0.110692

0

0.131657

0

0.0877713

0

0

4.18128
3

0

0

0.0551547

0

0

0

0.0367698

0

0

0

1.95767
2

0

0

0

0

0

0

0

0.165464

0

0

6.94109
3

0

0

0

0

0.0551547

0

0

0

0

0

0
6

0.0367698

0.0919245

0

0

0

0

0

0.239768
2

0

0.294158
2

1.11022302462516e-14

0
4

249.908

232.309

0.263314
7

3.58465

0

0

0

0

0

0

0

0

0.314506

0

0
7

0

0

0

0

0

0

1.54945

0

0

0

0.900304

0

0

0

0

0

0

0

0

0

0

0
7

0

0

0

0

0

0

0

0

0

0

0

0

0

0

0

0

0

0

0

0

0

0
7

0

0

0

0

0

0

0

0

0

0

0
6

0

0

0

0

0

0.0484412

0

0

0

0

0
7

0

0

0

0

0

0

0

0

0

0

0
7

0

0

0

0

0

0

0

0.131657

0

0

0
7

0

0

0

0

0

0

0

0

0

0

0.340531
7

0
7

0

0

0

0

0

0

0

0

0

0

0
7

0

0

0

0

0

0

0

0

0

0

0
7

0

0

0

0

0

0

0

0

0

0

0
7

0

0

0

0

0

0.0484412

0

0

0

0

0
6

0

0

0.290647

0

0

0

0

0

0

0

2.67702
2

0

0

0

0

0

0

0

0

0

0

0
7

0

0

0

0

0

0

0

0

0

0

0

0

0

0

0

0

0

0

0

0

0

0
7

0

0

0

0

0

0

0

0

0

0

0
6

0

0

0

0

0

0

0

0

0

0

0
7

0
6

0

0

0

0

0

0

0

0

0

0

0
7

0

0

0

0

0

0

0

0

0

0

0.193681

0

0.0968825

1.84077

0

0

0

0

0.0484412

0

0.219428

0
7

0

0

0

0

0

0

0

0

0

0

0
7

0

0

0

0

0

0

0

0

0

0

0
8

0

0

0

0

0

0

0

0

0

0

0
1

0

0

0

0

0

0

0

0

0

0

0
7

0

0

0

0

0

0

0

0

0

0

0.0739218

0

0

0

0

0

0

0

0

0

0

0
7

0

0

0

0.0968825

0

0

0

0

0.0633849
7

0

0
7

0

0
7

0
7

0
6

0
8

0

0.0484412

0

0
7

0
7

0
7

0

0
6

0

0

0

0

0

0

0.131657
7

0

0

0

0

0

0

0

0

0

2.35464

0
7

0

0

0

0

0

0

0

0

0

0

0

0

0

0

0

1.00937

0

0

0

0

0

1.27268

0

0

0

0

0

0

0

0

0

0

0
4

1.62628
7

1.62628
7

0

0

0
4

0.826447

0.438857

0

0

0.175543

0

0

0

0

0

0

0

0

0
6

0

0

0

0

0
4

0

0

0

0

0

0

0

0

0

0

0
2

0

0

0

0

0

0

0

0

0

0

0.0735396
3

0

0

0

0

0

0

0

0

0

0

0
6

0

0

0

0

0

0

0

0

0

0

0.138508
1

0

0

0

0

0

0

0

0

0

0

0

0

0

0

0

0

0

0

0

0

0

0
3

0

0

0

0

0

0

0

0

0

0

0
4

0

0

0

0

0

0

0

0

0

0

0
4

0.131657

0.131657

0
7

0

0
4

0

0

0
4

0

0

0

0
4

0

0

0
4

0

0

0
4

0

0

0
4

0

0

0
4

0

0

0
4

0

0

0
4

0

0

0
4

0

0

0
4

0.0367698

0.0367698
1

0

0

0

0

0

0

0

0

0

0

0

0
4

0

0

0
4

0

0

0
4

0

0

0
4

0

0

0
4

0
7

0
7

0

0

0

0

0

0
4

0.277017

0.277017
1

0

0

0

0

0

0

0
4

0.0877713

0

0.0877713

0

0

0

0
4

0.175543

0.175543

0

0

0

0
4

0.263314
1

0.263314

0

0

0
4

0

0

0

0

0

0
4

0

0

0

0

0
4

0
4

673.403

642.808

642.713

0

0

0

0.0950773

0

0

0
4

1.91939
1

1.65294
2

0.26645
1

0

0

1.11022302462516e-16
1

0
4

2.85232

2.85232

0
4

3.60578

3.60578

0
4

0

0

0
4

0

0

0

0
4

0.316924

0.221847

0.0950773

0
4

0

0

0
4

0.0877713

0

0.0877713

0
4

0

0

0
4

0

0

0
4

0

0

0
4

1.09635

0.967231

0.129121

0
4

0

0

0

0
4

0

0

0

0
4

0

0

0
4

0

0

0
4

0

0

0
4

0

0

0
4

0

0

0
4

0

0

0
4

0

0

0
4

2.58242

2.58242

0
4

2.51363
2

1.46037
2

1.05326

2.22044604925031e-16
2

0
4

0

0

0
4

0

0

0
4

0

0

0
4

0

0

0
4

0

0

0
4

0

0

0
4

0

0

0
4

0

0

0
4

0

0

0
4

0

0

0
4

15.2938
2

15.2938
2

0
4

0

0

0
4

0

0

0
4

0

0

0
4

0

0

0
4

0

0

0
4

0.0877713

0.0877713

0
4

0.0877713

0.0877713

0
4

0

0

0
4

0

0

0
4

0

0

0

0
4

0

0

0
4

0.151156

0.151156

0

0
4

3.75532938079459e-14

0
4

67.0384
3

63.45

60.0491

3.40096

0

0

0
4

2.28186

1.07754

0.25354

0.950773

0
4

0

0

0
4

1.08471

0

1.08471

0
4

0

0

0

0

0
4

0

0

0
4

0.158462

0.0950773

0.0633849

0
4

0

0

0
4

0

0

0
4

0

0

0
4

0.0633849

0.0633849

0
4

0
4

46.9351

39.1607

0
8

0

0

0

0

0

0

0

0

0

0

0

3.4625

0

0

0

0

0

0

0

0

0

0

0

0

0

0

0

0

0

0

0

10.9774

0

0

0

0

0

0

3.7445

0.077809

0

0

0

0.40657

0.233427

0

0

0

0

15.08

0

0

0

0

0

0

0

0

0.258726

0

0

0

2.66118

0

0

0

0

0

0

0

0.628335

0

0

0

0

0

0

0.110883

0.184804

0

0

0

0

0

0

0

0

0

0

0

0

0

0.077809

0

0.147844

0

0

0

0

0.258726

0

0

0

0

0

0

0.517453

0

0

0.184804

0.147844

0

0

0

0

0

0

0

0

0
4

0

0

0

0

0

0

0

0

0
4

0

0

0
4

0

0

0

0

0

0
4

0

0

0
4

0

0

0

0
4

0

0

0
4

0

0

0

0

0
4

0

0

0

0

0
4

0

0

0

0

0
4

0

0

0

0
4

0

0

0
4

0

0

0

0

0

0
4

0

0

0

0

0
4

0

0

0
4

0

0

0
4

0

0

0

0

0
4

0

0

0

0

0
4

4.16278

4.16278

0
4

0

0

0
4

0

0

0

0
4

0

0

0
4

0

0

0

0
4

0.322802

0.322802

0

0
4

0

0

0
4

0

0

0
4

0

0

0
4

0

0

0
4

0

0

0
4

0

0

0
4

0

0

0
4

0

0

0
4

0

0

0
4

0

0

0
4

0

0

0

0

0

0

0

0
4

0

0

0
4

0

0

0
4

0.129121

0.129121

0
4

0

0

0
4

0

0

0
4

0

0

0
4

0

0

0
4

0

0

0
4

0

0

0
4

0

0

0
4

0
7

0
7

0

0
4

0

0

0
4

0

0

0
4

3.15977

3.15977

0
4

0

0

0
4

0

0

0
4

0

0

0
4

0

0

0
4

0

0

0
4

0

0

0
4

0

0

0

0

0

0

0
4

0

0

0
4

0

0

0

0

0
4

0

0

0

0
4

1.06581410364015e-14

0
4

1.44343

1.44343

0

0

0

0

0

0

0

0

0.14617

0

0

0

0

0

0

0.712581

0

0.0730852

0

0.310612

0

0

0

0

0

0

0

0

0

0

0.200984

1.66533453693773e-16

0
4

0

0

0

0

0

0

0
4

0

0

0
4

0

0

0

0
4

0

0

0
4

0

0

0
4

0

0

0
4

0

0

0
4

0
4

32.1459

0.0877713

0.0877713

0

0
4

0

0

0

0

0

0

0

0

0

0

0
4

11.0924

11.0924

0
4

0

0

0

0
4

0

0

0
4

0.294158

0.294158

0
4

0

0

0

0
4

0

0

0
4

0

0

0

0
4

0.0633849

0.0633849

0
4

0

0

0
4

0

0

0
4

11.5994

9.34927

2.0917

0.0950773

0.0633849

0
4

0

0

0
4

0

0

0
4

0

0

0
4

0

0

0
4

0

0

0
4

0

0

0
4

0

0

0
4

0

0

0
4

0

0

0

0

0

0
4

0

0

0

0
4

9.00883

8.68603

0.322802

0
4

0

0

0

0
4

0

0

0

0
4

0
2

0
2

0

0

0
4

0

0

0

0
4

0
4

622.251

622.251

622.173

0

0.077809

0

0

0

0

0

0

5.33878496966622e-14

0
4

0

0

0

0
4

0

0

0

0
4

0

0

0
4

0

0

0
4

0

0

0
4

0
4

0.0877713

0

0

0

0

0

0

0

0

0

0

0

0
4

0

0

0
4

0.0877713

0

0.0877713

0
4

0
4

114.973

0
7

0

0

0

0

0

0

0

0

0

0

0

0

0

0

0

0

0

0

0

0

0

0

0

0

0

0

0

0

0

0

0

0

0
4

9.11521

0
7

0.0367698

0.0739218

0

0

0.147844

0

0

0

0.0739218

0

0
7

0

0

0

0
7

2.11828

0

4.44682

2.21765

0

0

8.88178419700125e-16

0
4

4.00378

0.0739218
7

0
7

0

0

0

0

0

0

0

0

0

0

0
7

0

0

0

0

0

0

0

0

0

0

0
6

0

0

0

0

0

0

0

0

0

0

0

0

0

0

0

0

0

0

0

0

0

3.86648

0

0

0

0

0

0

0

0.0633849

0

0

0

0

0

0

0

0

0

0

0

0

0

0

0

0

0

0

4.9960036108132e-16

0
4

14.3939

14.2354

0
7

0

0

0

0

0

0

0

0

0

0

0

0
7

0

0

0

0

0

0

0

0

0

0

0
7

0

0

0

0

0

0

0

0

0

0

0
7

0

0

0

0

0

0

0

0

0.0633849

0

0
6

0

0

0

0

0

0

0

0

0

0

0

0

0

0

0

0

0

0

0

0

0

0

0

0

0

0

0

0

0

0

0
7

0

0

0
7

0

0

0

0

0

0

0

0

0

0

0

0

0

0

0

0

0

0

0

0

0

0
5

0

0

0

0

0

0

0

0

0

0

0
7

0

0

0

0

0

0

0

0

0

0

0
7

0

0

0

0

0

0

0

0

0

0

0

0.0950773

0

0

0

0

0

0

0

0

0

0
7

0

0

0

0

0

0

0

0

0

0

0

0

0

0

0

0

0

0

0

0

0

5.41233724504764e-16

0
4

58.6919
2

11.7558
2

3.81805

0

0.35014

0.349695

0.169734

0.516549

0

0.147079

0.0735396

0.422853

23.7826
2

0

0.0919245

0.0367698

0.0877713

2.10677

0.294158

0.0367698

0.183849

0

0.0551547

0.746056

0

0

0.0367698

0

0.40657

0

0

0

0.0633849

0.0919245

1.08057
2

0.175543

0.0877713

0.0735396

0

0

0.0735396

0.438857

0.128694

0

0.110883

1.39812

0.0919245

0.116713

0

0.0877713

0.110309

0.0367698

0

0.0739218

0.351085

0.131657

4.31197

0.0633849

0

0

0

0.0919245

0.0367698

0.0367698

0.0367698

0

1.62628

1.36545

0.931096

1.75415237890775e-14
2

0
4

0

0

0
4

0
7

0
7

0

0

0
4

2.63314

0

0

0

0

0

0

0

0

0

0

0

0

0

0

2.63314

0

0

0
4

15.4815

3.48617

0

0

0

0

0.0950773

0

0

0

0

0

0

0

0

0.507079

0

0

0.158462

0

11.2347

0

0
4

0.590013
3

0.0633849

0

0.219428

0.3072

1.11022302462516e-16
3

0
4

0
6

0
6

0

0
4

0

0

0

0

0

0
4

0

0

0

0

0

0

0
4

0

0

0

0
4

0

0

0

0
4

0

0

0

0

0
4

0

0

0

0

0
4

0.175543

0

0.175543

0

0
4

0

0

0
4

9.56144

9.32251

0

0

0

0

0

0
7

0

0.238927

0

0

0

0

0

0
4

0

0

0
4

0

0

0

0
4

0

0

0
4

0

0

0
4

0

0

0

0

0
4

0

0

0

0

0
4

0

0

0

0
4

0

0

0

0
4

0.326699

0.326699

0
4

0

0

0

0
4

0
7

0
7

0
7

0

0

0

0

0

0
4

0

0

0

0
4

0

0

0

0
4

0

0

0

0
4

0

0

0
4

0

0

0

0
4

0

0

0

0
4

0

0

0
4

0

0

0
4

0

0

0
4

0

0

0
4

0
7

0
7

0

0
4

0

0

0
4

0

0

0
4

0

0

0
4

0

0

0
4

0

0

0
4

0

0

0
4

0

0

0
4

0

0

0
4

0

0

0
4

0

0

0
4

0
7

0

0

0

0

0
4

0

0

0
4

0

0

0
4

0

0

0
4

0

0

0
4

0

0

0
4

0

0

0
4

0

0

0
4

0

0

0
4

0

0

0
4

0

0

0
4

0
7

0
7

0
4

0

0

0
4

0

0

0
4

0

0

0
4

0

0

0
4

0

0

0
4

0

0

0
4

0

0

0
4

0
7

0

0

0

0
4

0
5

0

0

0

0

0
4

0
7

0
7

0

0

0
4

0
4

18.4736

12.7923

12.2793

0.190155

0

0.322802

3.88578058618805e-16

0
4

2.38874

2.00137

0

0

0.387362

0

0

1.11022302462516e-16

0
4

0.193681

0.193681

0
4

0

0

0
4

0

0

0
4

0

0

0
4

3.0989

3.0989

0
4

0

0

0
4

0

0

0

0

0

0

0

0

0
4

0

0

0

0

0

0

0

0
4

0

0

0

0

0

0
4

0

0

0

0
4

0

0

0

0
4

0

0

0

0
4

0

0

0

0
4

0

0

0
4

0
4

59.8856
7

18.5537
7

2.32594
7

1.25333
7

0
7

0

0

0

0

0

0

0

0

0

0

0
7

0

0

0

0

0

0

0

0

0

0

0
7

0

0

0

0

0

0

0

0

0

0

0
7

0

0

0

0

0

0

0

0

0

0

0
7

0

0

0

0

0

0

0

0

0

0

0

0

0

0

0

0

0

0

0

0

0

0
7

0

0

0

0

0

0

0

0

0

0

0
7

0

0

0

0

0

0

0

0

0

0

0
7

0

0

0

0

0

0

0

0

0

0

0

0

0

0

0

0

0

0

0

0

0

0
7

0
8

0

0

0.0367698

0

0

0

0

0

0

0

0
7

0

0

0

0

0

0

0

0

0

0

0
8

0

0

0

0

0

0

0

0

0

0

0
7

0

0

0

0

0

0

0

0

0

0

0
7

0

0

0

0

0

0

0

0

0

0

0
7

0

0

0

0

0

0

0

0

0

0

0
7

0

0

0

0

0

0

0

0

0

0

0
7

0

0

0

0

0

0

0

0

0

0

0
7

0

0

0

0

0

0

0

0

0

0

0
7

0

0

0

0

0

0

0

0

0

0

0.263314
7

0
7

0

0

0

0

0

0

0

0

0

0

0
7

0

0

0

0

0

0

0

0

0

0

0
7

0

0

0

0

0

0

0

0

0

0

0
7

0

0

0

0

0

0

0

0

0

0

0
7

0

0

0

0

0

0

0

0

0

0

0
6

0

0

0

0

0

0

0

0

0

0

0
7

0

0

0

0

0

0

0

0

0

0

0
7

0

0

0

0

0

0

0

0

0

0

0
7

0

0

0

0

0

0

0

0

0

0

0
7

0

0

0

0

0

0

0

0

0

0

2.50911
7

0
7

0

0

0

0

0

0

0

0

0

0

0
7

0

0

0

0

0

0

0

0

0

0

0
7

0

0

0

0

0

0

0

0

0

0

0
7

0

0

0

0

0

0

0

0

0

0

0
6

0

0

0

0

0

0

0

0

0

0

0
7

0

0

0

0

0

0

0

0

0

0

0

0

0

0

0

0

0

0

0

0

0

0
7

0

0

0

0

0

0

0

0

0

0

0
7

0

0

0

0

0

0

0

0

0

0

0
7

0

0

0

0

0

0

0

0

0

0

0
7

0
7

0

0

0

0

0

0

0

0

0

0

0
7

0

0

0

0

0

0

0

0

0

0

0
8

0

0

0

0

0

0

0

0

0

0

0
7

0

0

0

0

0

0

0

0

0

0

0
6

0

0

0

0

0

0

0

0

0

0

0
6

0

0

0

0

0

0

0

0

0

0

0

0

0.165464

0

0

0

0

0

0

0

0

0
7

0

0

0

0

0

0

0

0

0

0

0
8

0

0

0

0

0

0

0

0

0

0

0

0

0

0

0

0

0

0

0

0

0

0
7

0
7

0

0

0

0

0

0

0

0

0

0

0
7

0

0

0

0

0

0

0

0

0

0

0
7

0

0

0

0

0

0

0

0

0

0

0
7

0

0

0

0

0

0

0

0

0

0

0
7

0

0

0

0

0

0

0

0

0

0

0
7

0

0

0

0

0

0

0

0

0

0

0
7

0

0

0

0

0

0

0

0

0

0

0
7

0

0

0

0

0

0

0

0

0

0

0
6

0

0

0

0

0

0

0

0

0

0

0
7

0

0

0

0

0

0

0

0

0

0

0
7

0
7

0

0

0

0

0

0

0

0

0

0

0
7

0

0

0

0

0

0

0

0

0

0

0
6

0

0

0

0

0

0

0

0

0

0

0
6

0

0

0

0

0

0

0

0

0

0

0.348617

0

0

0

0

0

0

0

0

0

0

0
7

0

0

0

0

0

0

0

0

0

0

0

0

0

0

0

0

0

0

0

0

0

0
7

0

0

0

0

0

0

0

0

0

0

0
7

0

0

0

0

0

0

0

0

0

0

0
7

0

0

0

0

0

0

0

0

0

0

0
7

0
6

0

0

0

0

0

0

0

0

0

0

0
8

0

0

0

0

0

0

0

0

0

0

0
7

0

0

0

0

0

0

0

0

0

0

0
7

0

0

0

0

0

0

0

0

0

0

0
6

0

0

0

0

0

0

0

0

0

0

0
7

0

0

0

0

0

0

0

0

0

0

0
7

0

0

0

0

0

0

0

0

0

0

0
7

0

0

0

0

0

0

0

0

0

0

0
7

0

0

0

0

0

0

0

0

0

0

0
8

0

0

0

0

0

0

0

0

0

0

0
7

0
7

0

0

0

0

0

0

0

0

0

0

0
7

0

0

0

0

0

0

0

0

0

0

0
8

0

0

0

0

0

0

0

0

0

0

0
7

0

0

0

0

0

0

0

0

0

0

0
7

0

0

0

0

0

0

0

0

0

0

0
7

0

0

0

0

0

0

0

0

0

0

0
6

0

0

0

0

0

0

0

0

0

0

0
7

0

0

0

0

0

0

0

0

0

0

0
7

0

0

0

0

0

0

0

0

0

0

0

0

0

0

0

0

0

0

0

0

0

0
7

0
6

0

0

0

0

0

0

0

0

0

0

0
7

0

0

0

0

0

0

0

0

0

0

0
6

0

0

0

0

0

0

0

0

0

0

0
7

0

0

0

0

0

0

0

0

0

0

0
7

0

0

0

0

0

0

0

0

0

0

0
7

0

0

0

0

0

0

0

0

0

0

0

0

0

0

0

0

0

0

0

0

0

0
7

0

0

0

0

0

0

0

0

0

0

0
7

0

0

0

0

0

0

0

0

0

0

0
7

0

0

0

0

0

0

0

0

0

0

3.51564
7

0
7

0
7

0

0

0

0

0

0

0

0

0

0

0
6

0

0

0

0

0

0

0

0

0

0

0

0

0

0

0

0

0

0

0

0

0

0
7

0

0

0

0

0

0

0

0

0

0

0
7

0

0

0

0

0

0

0

0

0

0

0
7

0

0

0

0

0

0

0

0

0

0

0
7

0

0

0

0

0

0

0

0

0

0

0
8

0

0

0

0

0

0

0

0

0

0

0
7

0

0

0

0

0

0

0

0

0

0

0
7

0

0

0

0

0

0

0

0

0

0

0
7

0
7

0

0

0

0

0

0

0

0

0

0

0
7

0

0

0

0

0

0

0

0

0

0

0
7

0

0

0

0

0

0

0

0

0

0

0
7

0

0

0

0

0

0

0

0

0

0

0
7

0

0

0

0

0

0

0

0

0

0

0
6

0

0

0

0

0

0

0

0

0

0

0
6

0

0

0

0

0

0

0

0

0

0

0
6

0

0

0

0

0

0

0

0

0

0

0

0

0

0

0

0

0

0

0

0

0

0
7

0

0

0

0

0

0

0

0

0

0

0.110309
7

0
7

0

0

0

0

0

0

0

0

0

0

0
7

0

0

0

0

0

0

0

0

0

0

0
6

0

0

0

0

0

0

0

0

0

0

0
7

0

0

0

0

0

0

0

0

0

0

0
7

0

0

0

0

0

0

0

0

0

0

0
7

0

0

0

0

0

0

0

0

0

0

0
7

0

0

0

0

0

0

0

0

0

0

0
7

0

0

0

0

0

0

0

0

0

0

0
7

0

0

0

0

0

0

0

0

0

0

0
8

0

0

0

0

0

0

0

0

0

0

0
7

0
6

0

0

0

0

0

0

0

0

0

0

0

0

0

0

0

0

0

0

0

0

0

0
8

0

0

0

0

0

0

0

0

0

0

0
7

0

0

0

0

0

0

0

0

0

0

0
8

0

0

0

0

0

0

0

0

0

0

0

0

0

0

0

0

0

0

0

0

0

0
6

0

0

0

0

0

0

0

0

0

0

0
7

0

0

0

0

0

0

0

0

0

0

0
7

0

0

0

0

0

0

0

0

0

0

0
6

0

0

0

0

0

0

0

0

0

0

0
7

0

0

0

0

0

0

0

0

0

0

0

0
7

0

0

0

0

0

0

0

0

0

0

0
6

0

0

0

0

0

0

0

0

0

0

0
7

0

0

0

0

0

0

0

0

0

0

0
7

0

0

0

0

0

0

0

0

0

0

0

0

0

0

0

0

0

0

0

0

0

0
7

0

0

0

0

0

0

0

0

0

0

0
7

0

0

0

0

0

0

0

0

0

0

0
8

0

0

0

0

0

0

0

0

0

0

0
7

0

0

0

0

0

0

0

0

0

0

0
7

0

0

0

0

0

0

0

0

0

0

0

0
7

0

0

0

0

0

0

0

0

0

0

0
7

0

0

0

0

0

0

0

0

0

0

0

0

0

0

0

0

0

0

0

0

0

0
7

0

0

0

0

0

0

0

0

0

0

0

0

0

0

0

0

0

0

0

0

0

0
6

0

0

0

0

0

0

0

0

0

0

0
7

0

0

0

0

0

0

0

0

0

0

0

0

0

0

0

0

0

0

0

0

0

0
6

0

0

0

0

0

0

0

0

0

0

0
7

0

0

0

0

0

0

0

0

0

0

0

0
7

0

0

0

0

0

0

0

0

0

0

0
8

0

0

0

0

0

0

0

0

0

0

0
7

0

0

0

0

0

0

0

0

0

0

0
7

0

0

0

0

0

0

0

0

0

0

0
6

0

0

0

0

0

0

0

0

0

0

0
7

0

0

0

0

0

0

0

0

0

0

0
7

0

0

0

0

0

0

0

0

0

0

0

0

0

0

0

0

0

0

0

0

0

0

0

0

0

0

0

0

0

0

0

0

0

0
7

0

0

0

0

0

0

0

0

0

0

0
7

0

0

0

0

0

0

0

0

0

0

0
7

0

0

0

0

0

0

0

0

0

0

0
6

0

0

0

0

0

0

0

0

0

0

0

0

0

0

0

0

0

0

0

0

0

0
7

0

0

0

0

0

0

0

0

0

0

0

0

0

0

0

0

0

0

0

0

0

0

0

0

0

0

0

0

0

0

0

0

0

0

0

0

0

0

0

0

0

0

0

0

0

0

0

0

0

0

0

0

0

0

0
7

0

0
7

0

0
7

0
7

0
7

0
7

0
8

0

0
7

0
7

0
7

0
6

0
7

0

0
7

0
7

0
7

0
6

0
6

0
8

0
7

0
6

0

0

0

0
6

0
7

0

0

0

0
8

0

0
7

0

0
7

0

0

0
7

0

0

0
8

0
7

0
7

0
7

0
6

0
6

0

0

0
6

0
7

0
7

0

0

0
8

0
7

0
7

0
6

0
7

0
7

0
7

0

0

0

0

0

0
7

0
8

0
7

0
6

0

0

0

0

0

0

0
7

0.0367698
7

0

0

0
6

0

0

0

0

0

0

0

0
7

0
6

0

0
7

0
7

0

0

0

0

0

0

0
5

0

0

0

0

0

0

0

0

0

0

0
7

0

0

0

0

0

0

0

0

0

0

0
7

0

0

0

0

0

0

0

0

0

0

4.2885
7

0
7

0

0

0

0

0

0

0

0

0

0

0
7

0

0

0

0

0

0

0

0

0

0

0
7

0

0

0

0

0

0

0

0

0

0

0
7

0

0

0

0

0

0

0

0

0

0

2.85257
7

0

0

0

0

0

0

0

0

0

0

0
7

0

0

0

0

0

0

0

0

0

0

0
6

0

0

0

0

0

0

0

0

0

0

0
7

0

0

0

0

0

0

0

0

0

0

0
7

0

0

0

0

0

0

0

0

0

0

0
7

0

0

0

0

0

0

0

0

0

0

0
7

0
7

0

0

0

0

0

0

0

0

0

0

0
7

0

0

0

0

0

0

0

0

0

0

0
7

0

0

0

0

0

0

0

0

0

0

0
7

0

0

0

0

0

0

0

0

0

0

0
7

0

0

0

0

0

0

0

0

0

0

0
7

0

0

0

0

0

0

0

0

0

0

0
7

0

0

0

0

0

0

0

0

0

0

0
7

0

0

0

0

0

0

0

0

0

0

0
7

0

0

0

0

0

0

0

0

0

0

0
7

0

0

0

0

0

0.175543

0

0

0

0

0
7

0
7

0

0

0

0

0

0

0

0

0

0

0
7

0

0

0

0

0

0

0

0

0

0

0
7

0

0

0

0

0

0

0

0

0

0

0
7

0

0

0

0.0735396

0

0

0

0

0

0

0
7

0

0

0

0

0

0.183849

0

0

0

0

0
7

0

0

0

0

0

0

0

0

0

0

0

0

0

0

0

0

0

0

0

0

0

0
7

0

0

0

0

0

0

0

0

0

0

0
8

0

0.0633849

0

0

0

0

0

0

0

0

0

0

0

0

0

0

0

0

0

0

0

0
7

0

0

0

0

0

0

0

0

0

0

0

0

0

0

0

0

0

0

0

0

0

0

0
7

0

0

0

0

0

0

0

0

0

0

0
7

0

0

0

0

0

0

0

0

0

0

0
7

0

0

0

0

0

0

0

0

0

0

0
7

0

0

0

0

0

0

0

0

0

0

0
7

0

0

0

0

0

0

0

0

0

0

0
7

0

0

0

0

0

0

0

0

0

0

0
7

0

0

0

0

0

0

0

0

0

0

0

0

0.0877713

0

0

0

0

0

0

0

0

0
7

0
7

0

0

0

0

0

0

0

0

0

0

0
7

0

0

0

0

0

0

0

0

0

0

0
7

0

0

0

0

0

0

0

0

0

0

0
7

0

0

0

0

0

0

0

0

0

0

0.263314
6

0

0

0

0

0

0

0

0

0

0

0
8

0

0

0

0

0

0

0

0

0

0

0
7

0

0

0

0

0

0

0

0

0

0

0
7

0

0

0

0

0

0

0

0

0

0

0
7

0

0

0

0

0

0

0

0

0

0

0
7

0

0

0

0

0

0

0

0

0

0

0
7

0

0

0

0

0

0

0

0

0

0

0

0
7

0

0

0

0

0

0

0

0

0

0

0
8

0

0

0

0

0

0

0

0

0

0

0
7

0

0

0

0

0

0

0

0

0

0

0
7

0

0

0

0

0

0

0

0

0

0

0
7

0

0

0

0

0

0

0

0

0

0

0
6

0

0

0

0

0

0

0

0

0

0

0
6

0

0

0

0

0

0

0

0

0

0

0
7

0

0

0

0

0

0

0

0

0

0

0
7

0

0

0

0

0

0

0

0

0

0

3.05311331771918e-15
7

0
4

0
6

0
6

0
4

18.4643

0
7

0
5

0

0

0

0

0

0

0

0

0

0

0
5

0

0

0

0

0

0

0

0

0

0

0
6

0

0.322802

0

0

0.129121

0

0

0

0

0

3.93818

0.129121

0

0

0

0

0

0

0

0

0

0
5

0

0

0

0

0

0

0

0

0

0

4.19643

0.193681

0

0

0

0

0

0

0

0
6

9.55494

0
6

0
4

0

0

0

0

0

0

0
4

0
7

0

0

0

0

0

0

0

0

0

0

0

0

0

0

0

0

0

0

0

0

0

0

0

0

0

0

0
4

0
7

0
7

0
7

0
7

0
6

0
7

0

0

0

0

0

0

0

0

0

0

0

0
4

0
7

0
7

0
7

0

0

0

0

0
4

0.131657
7

0

0
7

0

0

0

0

0

0

0

0

0

0

0
6

0

0

0

0

0

0

0

0

0

0

0

0

0

0

0

0

0

0

0

0

0

0
7

0

0

0

0

0

0

0

0

0

0
7

0

0

0.131657
6

0

0
7

0
6

0

0

0

0

0

0

0

0

0

0

0
6

0

0

0

0

0

0

0

0

0

0

0
6

0

0

0

0

0

0

0

0

0

0

0

0

0

0

0

0

0

0

0

0

0

0
7

0

0

0

0

0

0

0

0

0

0

0
7

0

0

0

0

0

0

0

0

0

0

0
7

0

0

0

0

0

0

0

0

0

0

0

0

0

0

0

0

0

0

0

0

0

0
4

0
7

0
7

0
7

0
7

0
7

0

0

0

0

0

0
4

0
7

0
6

0

0
4

0
7

0

0

0
4

0
7

0

0

0

0

0
4

0
7

0

0

0

0
4

0

0

0

0
4

0

0

0

0

0

0

0
4

0.0919245

0.0551547

0

0.0367698

0
4

0

0

0

0

0
4

2.89645

2.89645

0
4

0

0

0
4

0

0

0

0

0

0

0

0

0

0

0
4

0

0

0

0
4

0

0

0
4

0

0

0

0
4

0

0

0
4

0

0

0
4

0

0

0
4

0

0

0
4

0

0

0
4

0

0

0

0
4

0

0

0
4

12.7822
3

10.5158
3

0

0

0.272331

1.67289

0.0877713

0

0.077809

0.155618

8.60422844084496e-16
3

0
4

0

0

0

0
4

0

0

0

0
4

0

0

0

0
4

0

0

0
4

0

0

0
4

0

0

0
4

0.0877713

0.0877713

0
4

0

0

0
4

0

0

0
4

0

0

0
4

0
7

0
7

0

0

0

0

0

0

0
4

0

0

0
4

0

0

0
4

0

0

0
4

0

0

0
4

0

0

0
4

0

0

0
4

0

0

0
4

0

0

0
4

0

0

0
4

0

0

0
4

0
7

0
7

0

0

0

0

0
4

0

0

0
4

0

0

0
4

0

0

0
4

0.0877713

0.0877713

0
4

0

0

0
4

0

0

0
4

0

0

0
4

0

0

0
4

0

0

0
4

0

0

0
4

0
7

0
7

0

0

0

0
4

0

0

0
4

0
7

0
7

0
7

0

0
4

0
7

0
7

0

0
4

6.78974
3

6.78974
3

0

0
4

1.24344978758018e-14
7

0
4

115.796

100.957
2

95.3669
2

0

0

2.71154

2.4533

0

0

0.147844

0.0739218

0.129121

0

0.0739218

1.26287869051112e-15
2

0
4

2.63314

2.63314

0

0

0

0
4

12.0771
2

12.0771
2

0
4

0

0

0

0
4

0.129121

0.129121

0
4

0

0

0
4

0
4

16.2146

7.49895

0
3

0.77506

0

0

0

0

0

0

0

0

0

2.13935
2

0

0

0

0

0.0484412

0

0

0

0

0.0968825

1.96972
2

0

0

0

0

0

0

0.193681

0

0

0

2.22738
2

0

0

0

0

0

0

0.0484412
2

0
4

0.193681

0

0.193681

0
4

0

0

0

0
4

0

0

0

0
4

0

0

0

0
4

0

0

0
4

0

0

0
4

6.8434
3

6.64972
3

0

0

0

0

0

0

0

0

0

0

0

0

0

0

0

0

0

0

0

0

0

0

0

0

0.193681

3.33066907387547e-16
3

0
4

0

0

0

0

0

0
2

0
3

0

0

0

0

0

0

0
4

1.29121

0
3

0

0

0

0

0

0

1.29121

0

0

0

0
4

0

0

0

0

0
4

0
2

0

0

0

0

0

0
4

0.387362
2

0.387362

0

0

0

0

0
4

0

0

0

0
4

0

0

0
4

0
4

0
2

0
2

0

0

0

0

0

0

0

0

0

0

0
4

0

0

0

0

0

0

0

0
4

0

0

0

0

0

0

0

0

0

0

0
4

0

0

0

0
4

0

0

0

0

0
4

0

0

0

0
4

0
4

0

0

0

0
4

0

0

0
4

0
4

0

0

0

0

0
4

0
4

0

0

0

0
4

0

0

0
4

0
4

0

0

0

0
4

0
4

0

0

0

0
4

0

0

0
4

0
4

0

0

0

0
4

0

0

0
4

0
4

0

0

0

0
4

0
4

1.00937

1.00937

1.00937

0
4

0
4

0

0

0

0
4

0
4

0

0

0

0
4

0
4

0.314868

0.145324

0.145324

0

0

0

0

0

0

0
4

0.169544
3

0.0726619
2

0

0

0

0

0

0.0484412

0

0

0.0484412

0

0
4

0

0

0

0

0

0

0

0
4

0

0

0

0

0

0
4

0

0

0
4

0

0

0
4

0

0

0
4

0

0

0
4

0

0

0
4

0
4

0

0

0

0

0
4

0
4

0

0

0

0
4

0
4

0

0

0

0
4

0

0

0
4

0
4

0

0

0

0
4

0
4

0

0

0

0

0
4

0
4

0

0

0

0
4

0
4

0

0

0

0
4

0
4

0

0

0

0
4

0

0

0
4

0
4

0.110309

0.0735396

0.0735396

0
4

0.0367698

0.0367698

0
4

6.93889390390723e-18

0
4

0

0

0

0

0
4

0
4

32.6948

0

0

0

0

0

0

0

0

0

0
4

0.263314
7

0.263314

0

0

0

0

0

0

0
4

0

0

0

0

0

0

0

0

0
4

32.4315

31.9926

0.175543

0.131657

0.131657

2.60902410786912e-15

0
4

0

0

0

0
4

0

0

0
4

0
4

0.0735396

0.0735396

0.0735396

0
4

0
4

0

0

0

0
4

0
4

0

0

0

0
4

0
4

0

0

0

0
4

0
4

0

0

0

0
4

0
4

0.0877713

0.0877713

0.0877713

0
4

0
4

0

0

0

0
4

0
4

0

0

0

0
4

0
4

0

0

0

0
4

0
4

0

0

0

0
4

0
4

54.5938
2

2.32594
1

0

0.526628

0

0.131657

1.66765

0

0

0
4

0.0877713

0

0

0

0.0877713

0

0

0

0

0

0

0
4

0.0877713

0.0877713

0

0

0

0

0
4

51.9606

51.3901

0.131657

0.351085

0.0877713

6.68909372336657e-15

0
4

0.131657

0

0.131657

0
4

0

0

0

0
4

0

0

0
4

0
4

0

0

0

0
4

0
4

0

0

0

0
4

0
4

0.0950773

0.0950773

0.0950773

0
4

0
4

0

0

0

0
4

0
4

0

0

0

0
4

0
4

0

0

0

0
4

0
4

0

0

0

0
4

0
4

0

0

0

0
4

0
4

0

0

0

0
4

0
4

0

0

0

0
4

0
4

16.4972

10.1702

8.29798
3

1.87225

0

0

0
4

6.1978

6.06868

0.129121

0

0

0

0

0
4

0

0

0

0
4

0.129121

0.129121

0

0
4

0

0

0
4

0

0

0
4

3.46944695195361e-15

0
4

0

0

0

0
4

0
4

0.110309

0.110309

0.110309

0
4

0
4

0.0633849

0.0633849

0.0633849

0
4

0
4

0

0

0

0
4

0
4

0

0

0

0
4

0
4

0

0

0

0
4

0
4

0

0

0

0
4

0
4

0.0877713

0.0877713

0.0877713

0
4

0
4

0

0

0

0
4

0
4

0

0

0

0
4

0
4

0
7

0
7

0
7

0

0

0

0

0

0

0
4

0
7

0
7

0
4

0

0

0

0
4

0
4

0

0

0

0
4

0
4

0

0

0

0
4

0
4

0.0909077

0.0909077

0.0909077

0
4

0
4

0.332648

0.332648

0.332648

0
4

0
4

0

0

0

0
4

0
4

0

0

0

0
4

0
4

0

0

0

0
4

0
4

0

0

0

0
4

0
4

0

0

0

0
4

0
4

0

0

0

0
4

0
4

23.9619
3

23.9619
3

23.7542
3

0.207762

0

0

0

0

0

1.16573417585641e-15
3

0
4

0
4

0

0

0

0
4

0
4

0

0

0

0
4

0
4

0

0

0

0
4

0
4

0

0

0

0
4

0
4

0

0

0

0
4

0
4

0

0

0

0
4

0
4

0

0

0

0
4

0
4

0.484779

0.484779

0.484779

0
4

0
4

0

0

0

0
4

0
4

0

0

0

0
4

0
4

17.5543

0
6

0

0

0

0

0
4

0

0

0
4

0

0

0
4

0

0

0
4

0
7

0

0

0
4

17.5543

17.2471

0.219428

0

0.0877713

0

0
4

0

0

0

0

0
4

0

0

0

0
4

0

0

0

0
4

0

0

0
4

0

0

0

0
4

0

0

0
4

0
4

0

0

0

0
4

0
4

0.0877713

0.0877713

0.0877713

0
4

0
4

0

0

0

0
4

0
4

0.0367698

0.0367698

0.0367698

0
4

0
4

0

0

0

0
4

0
4

0

0

0

0
4

0
4

0

0

0

0
4

0
4

0

0

0

0
4

0
4

0.221765

0.221765

0.221765

0
4

0
4

0

0

0

0
4

0
4

0
7

0
7

0
7

0

0

0

0

0

0
4

0
7

0
6

0

0

0
4

0

0

0

0
4

0

0

0

0
4

0

0

0
4

0
4

0

0

0

0
4

0
4

0

0

0

0
4

0
4

0

0

0

0
4

0
4

0

0

0

0
4

0
4

0

0

0

0
4

0
4

1.61401

1.61401

0.710164

0.129121

0

0.387362

0.387362

0

0

0

0
4

0

0

0
4

0

0

0
4

0
4

0.175543

0

0

0

0

0

0

0

0

0

0

0

0

0

0

0

0

0

0

0

0

0

0

0

0

0

0

0

0

0

0

0

0

0

0

0

0

0

0

0

0

0

0

0

0

0

0

0

0

0

0

0

0

0

0

0

0

0

0

0

0

0

0
4

0

0

0

0

0

0

0

0
4

0

0

0

0

0

0

0

0

0

0

0

0

0
4

0

0

0

0

0

0
4

0.175543

0.175543

0
4

0
4

11.536

10.9339

4.97571

0.919081

0.190155

0.0633849

4.37356

0.190155

0.221847

0
4

0.602156

0.602156

0
4

0
4

0

0

0

0

0
4

0
4

0
4

0
4

0

0

0

0

0

0
4

0
4

0

0

0

0
4

0

0

0

0

0

0
4

0
4

2.68441

2.68441

2.41208

0.155618

0

0

0.116713

0

2.77555756156289e-16

0
4

0
4

0

0

0

0

0

0

0
4

0

0

0

0

0
4

0

0

0

0

0
4

0

0

0
4

0
4

0

0

0

0

0

0

0
4

0

0

0
4

0
4

6.26236
3

6.26236
3

6.26236
3

0

0

0

0

0
4

0

0

0

0
4

0

0

0
4

0
4

0
8

0
8

0

0

0

0

0

0
4

0

0

0
4

0
4

2.11426
3

2.11426
3

2.11426
3

0
4

0
4

15.2805
3

12.229
2

12.1922
2

0.0367698

0
4

3.05147
3

0.0367698

3.0147

0

0
4

0

0

0
4

0
4

80.8374
1

1.05326
1

0.0877713

0

0

0

0.0877713

0

0

0

0

0.131657

0

0.131657
2

0

0

0.0877713

0

0

0

0.526628

0

0
4

0

0

0
4

0

0

0
4

77.2388
1

59.1579
2

0

0

0

0

3.59862

0

0

0

6.80228

0

0.3072
1

0.70217

0

0

1.00937

0.131657

1.36046

2.72091

1.14103

0.3072

0

0
4

0.833827
1

0.833827

0

0

0

0

0

0

0

0

0

0
4

0.614399

0.0877713

0.526628

0

0
4

0

0

0

0
4

0.526628

0.526628

0

0
4

0.0877713

0

0.0877713

0
4

0.175543

0.0877713

0.0877713

0
4

0.3072

0.175543

0.131657

2.77555756156289e-17

0
4

4.05231403988182e-15
1

0
4

0
3

0
3

0
3

0

0

0

0

0
4

0

0

0

0

0

0
4

0
4

0

0

0

0

0

0

0
4

0

0

0

0

0

0

0
4

0

0

0
4

0

0

0

0
4

0

0

0

0
4

0

0

0
4

0
4

0

0

0

0

0

0

0
4

0

0

0

0

0

0

0
4

0
4

0

0

0

0

0

0

0
4

0
4

0.129121
2

0

0

0

0

0

0

0
4

0.129121

0.129121

0
4

0

0

0
4

0
4

0

0

0

0

0
4

0

0

0

0
4

0

0

0

0
4

0
4

6.61376

6.61376

0.311236
2

6.0302

0.272331

0
4

0

0

0
4

0

0

0
4

0
4

0.0877713
7

0

0

0

0
4

0

0

0

0

0
4

0

0

0

0
4

0.0877713

0.0877713

0
4

0
4

0.0726619
2

0

0

0

0

0
4

0
3

0

0

0

0

0
4

0.0726619

0.0726619

0
4

0
4

0

0

0

0

0

0

0
4

0

0

0

0
4

0
4

5.76343

0
7

0
7

0

0

0

0

0

0

0

0

0

0

0

0
4

0

0

0
4

0

0

0

0
4

0

0

0

0
4

0

0

0
4

2.31355

0

0

0

0

1.99662

0.316924

0

0

0
4

3.31823
1

0.833827

0

0

0.195042

2.01874

0.175543

0.0950773

0

0
4

0
6

0

0

0

0

0

0

0
4

0
6

0
6

0

0
4

0

0

0

0

0

0

0
4

0
7

0

0

0

0

0
4

0.131657

0

0

0.131657

0

0

0
4

0

0

0

0

0

0
4

1.13797860024079e-15

0
4

0
8

0
8

0
8

0
4

0

0

0
4

0

0

0
4

0
4

0.0484412
3

0

0

0

0
4

0.0484412

0.0484412

0
4

0

0

0
4

0

0

0
4

0

0

0
4

0
4

0
7

0
7

0
7

0

0
4

0
4

0
7

0
7

0

0

0

0
4

0

0

0
4

0
4

0

0

0

0

0

0

0

0
4

0

0

0
4

0

0

0
4

0
4

1.37716
2

0.33131
2

0.0739218

0.0551547

0

0.202234

0
4

1.04585

1.04585

0
4

0

0

0
4

0
4

0
4

0

0

0

0

0

0

0
4

0

0

0
4

0
4

0

0

0

0

0

0

0
4

0
4

0
7

0
7

0
7

0

0

0
4

0
4

1.24657
4

0.138508
4

0

0.138508

0
4

0

0

0
4

0

0

0
4

1.10807

1.10807

0
4

0
4

0
7

0
7

0
7

0
6

0
8

0

0

0

0

0

0

0
4

0
7

0
7

0

0

0
4

0
7

0
7

0

0

0

0

0
4

0
7

0
8

0

0

0

0

0

0

0
4

0

0

0

0

0
4

0

0

0

0

0
4

0

0

0

0
4

0

0

0
4

0
4

12.4234

0.507079

0.0633849

0.25354

0.0950773

0.0950773

0
4

11.853

11.7579

0.0950773

0

0
4

0.0633849

0.0633849

0
4

0
4

0

0

0

0

0
4

0
4

0

0

0

0
4

0
4

0

0

0

0

0

0

0
4

0
4

0
3

0
3

0

0

0

0
4

0

0

0
4

0
4

0.877713

0.614399

0.526628

0.0877713

2.77555756156289e-17

0
4

0

0

0
4

0.263314

0.263314

0
4

5.55111512312578e-17

0
4

0

0

0

0

0

0

0
4

0
4

0.387362

0.258242

0.129121

0.129121

0
4

0.129121

0.129121

0

0
4

0
4

0

0

0

0

0
4

0

0

0

0
4

0
4

1.40434

1.31657

0.921599

0.263314

0.131657

8.32667268468867e-17

0
4

0.0877713

0.0877713

0
4

0
4

23.6849
3

23.2001
3

22.1613
3

0

0

0

0

0.900304
3

0

0

0

0

0

0.138508

0

3.33066907387547e-16
3

0
4

0.207762

0.207762

0

0
4

0.277017

0.277017

0
4

2.38697950294409e-15
3

0
4

0

0

0

0

0

0

0

0
4

0

0

0
4

0
4

0

0

0

0

0

0
4

0
4

0

0

0

0

0
4

0

0

0

0
4

0

0

0
4

0
4

0

0

0

0

0
4

0

0

0
4

0

0

0

0
4

0
4

0

0

0

0

0

0

0
4

0
4

0
8

0

0

0

0
4

0

0

0
4

0

0

0
4

0
4

0

0

0

0
4

0

0

0
4

0

0

0
4

0
4

0

0

0

0

0

0
4

0

0

0
4

0
4

0

0

0

0

0
4

0
4

0

0

0

0

0
4

0

0

0

0
4

0

0

0
4

0
4

1.68228

1.31459

1.03881

0

0

0

0

0

0

0.275774

0

0

0

0

0

5.55111512312578e-17

0
4

0

0

0

0

0

0

0

0

0

0

0

0
4

0.367698

0.367698

0
4

0

0

0
4

0

0

0
4

0

0

0
4

0

0

0
4

0

0

0
4

0
4

0

0

0

0
4

0

0

0
4

0
4

0

0

0

0
4

0

0

0
4

0
4

0

0

0

0

0
4

0
4

0

0

0

0
4

0

0

0

0
4

0

0

0
4

0
4

0.0877713

0

0

0
4

0.0877713

0.0877713

0

0
4

0
4

0
7

0

0

0
4

0

0

0

0
4

0
4

0

0

0

0
4

0
4

0

0

0

0
4

0

0

0
4

0
4

0

0

0

0

0
4

0
4

0

0

0

0
4

0
4

1.04379

0.956015

0.956015

0

0

0

0

0

0

0

0

0

0

0
4

0

0

0
4

0

0

0
4

0
7

0
7

0

0

0

0
4

0
7

0

0

0

0

0

0
4

0

0

0

0

0

0
4

0

0

0

0
4

0

0

0
4

0.0877713

0.0877713

0
4

0

0

0
4

0

0

0
4

2.77555756156289e-17

0
4

0

0

0

0
4

0
4

0

0

0

0

0

0
4

0
4

0

0

0

0
4

0
4

0

0

0

0

0
4

0
4

0

0

0

0

0
4

0

0

0
4

0
4

0

0

0

0

0
4

0

0

0
4

0
4

0

0

0

0

0
4

0

0

0
4

0
4

0.0633849

0.0633849

0

0.0633849

0
4

0
4

0

0

0

0

0

0
4

0
4

0

0

0

0
4

0
4

0

0

0

0

0

0

0

0

0

0

0

0

0

0

0

0

0

0

0

0

0

0

0

0

0

0

0

0

0

0
4

0

0

0

0
4

0
4

0

0

0

0
4

0
4

0

0

0

0

0

0
4

0
4

0

0

0

0

0
4

0

0

0
4

0
4

0

0

0

0

0
4

0

0

0
4

0
4

0

0

0

0

0

0
4

0
4

0

0

0

0

0
4

0
4

0

0

0

0

0
4

0
4

0

0

0

0
4

0

0

0
4

0
4

0.25354

0

0

0
4

0.25354

0.25354

0
4

0

0

0
4

0
4

0

0

0

0

0
4

0

0

0
4

0
4

5.02668
3

5.02668
3

0

4.76796

0.184804

0.0739218

0

0

0
4

0
4

145.118
3

145.118
3

139.179
3

5.93956

0

0
4

0

0

0
4

0

0

0
4

0

0

0
4

0

0

0
4

0

0

0
4

0
4

12.1383

12.1383

0
7

0
7

0

0.0633849

0

0.0633849

0

0

0

0

0

0

0
8

0

0

0

0

0.533162

0

0

0

0

0

0
3

0

0

0

0

0

0

0

0

0

0.0950773

0

0

0

0

0

0

0

0

0

0

0

0

0

0

0

0

0

0.0877713

0

0

0

0

0
7

0

0

0

0

0

0

0

0

0

3.77417

2.50148

0

0

0.0633849

0

0

0

0

0

0

0

0
7

0

0

0

0

0

0

0

0

0

0

0

0

0

0

0

0

0

0

0

0

0

0

0

0

0

0

0

0

0
3

0
7

0

0

0
6

0

0
7

0
7

0

0

0

0
7

0

0
7

0.3072

0

0

0

2.76223

0

0

0

0
7

0

0

0

0

0

0

0

0

0

0

0
6

0

0

0

0

0

0

0

0

0

0

0
3

0

0

0

0

0

0

0

0

0

0

1.79931

0

0

0

0

0

0

0

0

0

0

0
3

0

0

0

0

0

0

0

0

0

0

0

0

0

0

0.0877713

0

0

0

0

0

0

3.02535774210355e-15

0
4

0

0

0

0

0

0
4

0

0

0

0
4

0

0

0
4

0

0

0
4

0
4

0
3

0
4

0

0

0
4

0

0

0
4

0

0

0
4

0

0

0
4

0

0

0
4

0

0

0
4

0

0

0
4

0
4

0
4

217.584
3

196.802
3

22.6056

21.6107

0

0

0

0

0

0

0.129121

0

0

0.155618

0

0.322802

0

0

0.129121

0.258242

0

0

0

0

0

8.32667268468867e-16

0
4

0
5

0
5

0
6

0

0

0

0

0

0

0

0

0

0

0

0

0
4

174.196
3

0

3.85643
3

78.5534

0
4

0

0

0.774725

0

0

0

0.581044

0

0

0

0
4

0

0

0

0

0

0

0.129121

0

0

0

5.352
3

0.077809

0

1.03491

0

0

0.129121

0

0

0

0

1.09753
3

0

0

0

0

0

0

0

0

0

0

0
4

0

0

0

0

0

0.129121

0

0.516483

0

0

0

0

0

0

0

0

0

0

0

0

0

0.693508
3

0

0

0

0

0

0.129121

0

0

0

0

0

2.06593

0

0

0.484779

0

0

0

0

0

0

0
4

0

0

0

0

0

0

0.387362

0

0

0

0
4

0

0

0

0

0

0

0

0

0

0

0

0
4

0

0

0

0

0

0.387362

0

1.22665

0

0

0.451923
3

0

0

0

0

0

0

0

0

0

0

0
4

0

0

0

0

0

0

0

0

0.129121

0.193681

0

0

0

0

0

0

0

0

0

0

0.387362

0

0

0.387362

0

0

0

0

0

0

0

0

0
3

0

0

0

0

0

0

0

0

0

0

0.129121

0

0

0

0

0

0
5

0
6

6.64972

2.19505

0
3

37.1868

0

0.138508

1.48489
3

0

0

0
4

0

0

1.03297

0
6

0
4

0

0

0
4

0

0

0

0

0

0
4

0.129121

0

1.35577

0

0

0

0

2.06593

0

0

1.93912
3

0

0

6.1978

0

0

0

0

0

0.451923

0

0

0

0

0

0

0

0

0.387362

0

0

0

12.0728
3

0

0

0

0

0

0

0

0.138508

0

0

1.35577

0

0

0.129121

0

0

0

0

0

0

0

0
4

0

0

0

0
4

0

0

0
4

0

0

0
4

0

0

0
4

0

0

0
4

0

0

0

0
4

0

0

0

0
4

0

0

0
4

0

0

0
4

0

0

0
4

0

0

0
4

0

0

0
4

0

0

0
4

0
4

7.701
3

7.31731
3

6.53164
3

0

0

0

0

0

0

0

0

0

0

0

0

0

0

0

0

0

0

0

0
4

0
3

0

0

0

0.584682

0.200984

0
4

0.164442
3

0

0.164442
2

0

0

0

0

0

0

0

0
4

0.219256
4

0.127899

0

0

0

0

0

0

0

0.0365426

0.0548139

0

0

0

0

0

0

0
4

0

0

0
4

3.33066907387547e-16
3

0
4

0
4

0
4

0
4

0
4

0

0

0
4

0
4

1.64037

1.60383

0.735872

0.535306

0

0.184804

0.147844

0
4

0.0365426

0.0365426

0
4

0
4

0

0

0

0
4

0
4

0

0

0

0

0

0
4

0
4

0

0

0

0

0
4

0

0

0
4

0

0

0
4

0
4

0

0

0

0

0

0

0
4

0

0

0

0
4

0
4

0

0

0

0

0
4

0
4

0

0

0

0

0

0
4

0

0

0
4

0

0

0
4

0
4

0

0

0

0
4

0

0

0
4

0
4

0.0367698

0.0367698

0.0367698

0

0
4

0
4

9.63985
3

9.12826
3

9.12826
3

0

0

0

0

0

0

0

0
4

0

0

0
4

0

0

0
4

0.255798

0.109628

0.109628

0.0365426

0

0

0

0
4

0.14617
2

0.109628

0.0365426

0

0

6.93889390390723e-18
2

0
4

0.109628
3

0.0548139

0.0548139

0

0
4

0

0

0
4

0

0

0
4

0

0

0
4

0

0

0
4

0

0

0
4

4.34374758384592e-15
3

0
4

0

0

0

0
4

0
4

0

0

0

0
4

0
4

0

0

0

0
4

0

0

0
4

0
4

0

0

0

0
4

0
4

0

0

0

0
4

0

0

0
4

0
4

0

0

0

0

0
4

0

0

0
4

0
4

0

0

0

0
4

0

0

0
4

0
4

0

0

0

0
4

0
4

0

0

0

0
4

0

0

0
4

0

0

0
4

0
4

0

0

0

0

0
4

0
4

1.27983
3

0.274906
3

0.274906
3

0

0

0

0

0

0
3

0
4

0

0

0

0

0

0

0
4

0

0

0
4

0

0

0
4

0.730852

0.730852

0

0
4

0.164442
3

0

0.164442

0

0

0

0

0
4

0.109628

0.109628

0
4

0

0

0

0
4

0

0

0

0
4

0

0

0

0
4

0

0

0
4

0

0

0
4

0
4

0

0

0

0
4

0

0

0
4

0

0

0
4

0
4

0

0

0

0
4

0
4

0

0

0

0
4

0
4

0

0

0

0
4

0
4

0

0

0

0

0
4

0
4

0

0

0

0

0
4

0
4

0

0

0

0
4

0
4

0

0

0

0
4

0
4

0

0

0

0
4

0
4

0

0

0

0
4

0
4

0
4

0
4

0
4

0
4

0

0

0

0

0

0

0
4

0
4

0
4

0

0

0

0

0

0
4

0

0

0

0

0
4

0

0

0

0
4

0
4

0

0

0

0
4

0
4

0

0

0

0
4

0
4

0

0

0

0
4

0
4

0

0

0

0
4

0
4

0

0

0

0
4

0
4

0

0

0

0
4

0
4

0

0

0

0
4

0
4

0

0

0

0

0

0

0

0

0

0

0

0

0

0

0

0

0
4

0

0

0

0

0

0

0

0

0

0

0

0

0

0

0
4

0

0

0

0

0

0
4

0

0

0

0
4

0
4

0

0

0

0

0

0

0

0

0

0
4

0

0

0

0

0

0

0

0

0
4

0

0

0

0

0

0

0
4

0
4

0
4

0

0

0

0

0

0

0

0

0

0

0
4

0

0

0

0

0

0
4

0
4

0.484779

0.484779

0.277017

0.207762

0

0
4

0
4

0
4

0
4

0

0

0

0

0

0
4

0

0

0

0
4

0

0

0

0
4

0

0

0

0
4

0

0

0
4

0
4

0
4

600.776

600.776

594.901

583.151

0.258242

0.387362

2.51786

1.16209

0.581044

1.35577

0.581044

2.84066

0.516483

1.54945

2.25153229393982e-13

0
4

2.4533

0.774725

1.22665

0.258242

0.193681

0
4

2.84066

1.35577

0.129121

0.322802

1.03297

0
4

0.581044

0.129121

0.451923

0
4

0
4

0
4

0
7

0
7

0
7

0
7

0

0

0
4

0
4

0
4

0

0

0

0

0
4

0
4

0
4

0

0

0

0

0

0
4

0
4

0
4

0

0

0

0

0

0
4

0
4

0
4

0

0

0

0

0
4

0
4

0
4

0

0

0

0

0
4

0
4

0
4

0

0

0

0

0
4

0
4

0
4

0

0

0

0

0
4

0
4

0
4

0

0

0

0

0
4

0
4

0
4

0

0

0

0

0
4

0
4

0
4

0

0

0

0

0

0
4

0
4

0
4

0
7

0
7

0
7

0
7

0

0

0
4

0
4

0
4

0

0

0

0

0

0
4

0
4

0
4

0

0

0

0

0
4

0
4

0
4

0

0

0

0

0
4

0
4

0
4

0

0

0

0

0
4

0
4

0
4

0

0

0

0

0
4

0
4

0
4

0

0

0

0

0
4

0
4

0
4

0

0

0

0

0
4

0
4

0
4

0

0

0

0

0
4

0

0

0
4

0
4

0
4

0

0

0

0

0
4

0
4

0
4

0

0

0

0

0
4

0
4

0
4

0
7

0
7

0
7

0
7

0
4

0

0

0
4

0
4

0
4

0

0

0

0

0
4

0

0

0
4

0
4

0
4

0

0

0

0

0
4

0
4

0
4

0

0

0

0

0
4

0

0

0
4

0
4

0
4

0

0

0

0

0
4

0

0

0
4

0
4

0
4

0

0

0

0

0
4

0
4

0
4

0

0

0

0

0

0
4

0
4

0
4

0

0

0

0

0
4

0
4

0
4

0

0

0

0

0
4

0
4

0
4

0

0

0

0

0
4

0
4

0
4

0

0

0

0

0
4

0
4

0
4

0

0

0

0

0

0

0
4

0

0

0

0
4

0
4

0

0

0

0
4

0

0

0
4

0
4

0
4

0

0

0

0

0

0
4

0
4

0
4

0

0

0

0

0

0
4

0
4

0
4

0

0

0

0

0
4

0
4

0
4

0

0

0

0

0
4

0

0

0
4

0
4

0
4

0.387362

0.387362

0.129121

0.129121

0
4

0.258242

0.258242

0
4

0
4

0
4

0

0

0

0

0
4

0
4

0
4

0

0

0

0

0
4

0
4

0
4

0

0

0

0

0
4

0
4

0
4

0

0

0

0

0
4

0
4

0
4

0

0

0

0

0
4

0
4

0
4

0

0

0

0

0

0

0

0

0

0

0
4

0

0

0

0

0

0
4

0

0

0
4

0
4

0
4

0

0

0

0

0
4

0
4

0
4

0

0

0

0

0
4

0
4

0
4

0

0

0

0

0
4

0
4

0
4

0

0

0

0

0
4

0
4

0
4

0

0

0

0

0
4

0
4

0
4

0

0

0

0

0
4

0
4

0
4

0

0

0

0

0
4

0
4

0
4

0.129121

0.129121

0.129121

0.129121

0
4

0
4

0
4

0

0

0

0

0
4

0
4

0
4

0

0

0

0

0
4

0
4

0
4

0
7

0
7

0

0

0

0
4

0

0

0

0

0
4

0

0

0
4

0

0

0
4

0
4

0
6

0

0

0

0

0
4

0

0

0
4

0

0

0
4

0
4

0
4

0

0

0

0

0
4

0
4

0
4

0

0

0

0

0
4

0
4

0
4

0

0

0

0

0
4

0
4

0
4

0

0

0

0

0
4

0
4

0
4

0

0

0

0

0
4

0
4

0
4

0

0

0

0

0
4

0
4

0
4

0

0

0

0

0
4

0
4

0
4

0

0

0

0

0
4

0
4

0
4

0

0

0

0

0
4

0
4

0
4

0

0

0

0

0
4

0
4

0
4

28.6553
3

28.6553
3

28.4899
3

28.412
3

0.077809

3.6498581934552e-15
3

0
4

0.165464

0.165464

0
4

1.11022302462516e-16
3

0
4

0
4

0.0877713

0.0877713

0.0877713

0.0877713

0
4

0
4

0
4

0.138508

0.138508

0.138508

0.138508

0
4

0
4

0
4

0.0633849

0.0633849

0.0633849

0.0633849

0
4

0
4

0
4

0

0

0

0

0
4

0
4

0
4

0

0

0

0

0
4

0
4

0
4

0

0

0

0

0
4

0
4

0
4

0

0

0

0

0
4

0
4

0
4

0

0

0

0

0
4

0
4

0
4

0

0

0

0

0
4

0
4

0
4

0.158462

0.158462

0.158462

0.158462

0
4

0
4

0
4

0
5

0
5

0
5

0
5

0

0

0
4

0

0

0
4

0

0

0
4

0
4

0
4

0

0

0

0

0
4

0
4

0
4

0

0

0

0

0
4

0
4

0
4

0

0

0

0

0
4

0
4

0
4

0

0

0

0

0
4

0
4

0
4

0

0

0

0

0
4

0
4

0
4

0

0

0

0

0
4

0
4

0
4

0

0

0

0

0
4

0
4

0
4

0

0

0

0

0
4

0
4

0
4

0

0

0

0

0
4

0
4

0
4

0

0

0

0

0
4

0
4

0
4

0

0

0

0

0

0

0

0
4

0

0

0

0
4

0
4

0
4

0

0

0

0

0
4

0
4

0
4

0

0

0

0

0
4

0
4

0
4

0

0

0

0

0
4

0
4

0
4

0

0

0

0

0
4

0
4

0
4

0

0

0

0

0
4

0
4

0
4

0

0

0

0

0
4

0
4

0
4

0

0

0

0

0
4

0
4

0
4

0

0

0

0

0
4

0
4

0
4

0

0

0

0

0
4

0
4

0
4

0

0

0

0

0
4

0
4

0
4

0

0

0

0

0

0

0

0
4

0

0

0
4

0
4

0
4

2.90522

2.90522

2.90522

2.90522

0
4

0
4

0
4

0

0

0

0

0
4

0
4

0
4

0

0

0

0

0
4

0
4

0
4

0

0

0

0

0
4

0
4

0
4

0

0

0

0

0
4

0
4

0
4

0

0

0

0

0
4

0
4

0
4

0

0

0

0

0
4

0
4

0
4

0

0

0

0

0
4

0
4

0
4

0

0

0

0

0
4

0
4

0
4

0

0

0

0

0
4

0
4

0
4

0

0

0

0

0

0

0

0

0

0

0
4

0

0

0

0

0

0
4

0

0

0

0

0
4

0

0

0
4

0
4

0
4

0
7

0
7

0
7

0
7

0
4

0
4

0
4

0

0

0

0

0
4

0
4

0
4

0

0

0

0

0
4

0
4

0
4

0

0

0

0

0
4

0
4

0
4

0

0

0

0

0
4

0
4

0
4

0

0

0

0

0
4

0
4

0
4

0

0

0

0

0
4

0
4

0
4

0

0

0

0

0
4

0
4

0
4

0

0

0

0

0
4

0
4

0
4

0

0

0

0

0
4

0
4

0
4

0

0

0

0

0
4

0
4

0
4

0

0

0

0

0

0

0
4

0
4

0
4

0

0

0

0

0
4

0
4

0
4

0

0

0

0

0
4

0
4

0
4

0

0

0

0

0
4

0
4

0
4

0

0

0

0

0
4

0
4

0
4

0

0

0

0

0
4

0
4

0
4

0

0

0

0

0
4

0
4

0
4

0

0

0

0

0
4

0
4

0
4

0

0

0

0

0
4

0
4

0
4

0

0

0

0

0
4

0
4

0
4

0

0

0

0

0
4

0
4

0
4

0

0

0

0

0

0
4

0
4

0
4

0

0

0

0

0
4

0
4

0
4

0

0

0

0

0
4

0
4

0
4

0

0

0

0

0
4

0
4

0
4

0.507079

0.507079

0.507079

0.507079

0
4

0
4

0
4

0

0

0

0

0
4

0
4

0
4

0

0

0

0

0
4

0
4

0
4

0

0

0

0

0
4

0
4

0
4

0

0

0

0

0
4

0
4

0
4

0

0

0

0

0
4

0
4

0
4

0

0

0

0

0
4

0
4

0
4

0
7

0

0

0

0

0

0
4

0
4

0

0

0

0
4

0

0

0
4

0
4

0
4

0

0

0

0

0
4

0
4

0
4

0

0

0

0

0
4

0
4

0
4

0

0

0

0

0
4

0
4

0
4

0

0

0

0

0
4

0
4

0
4

0

0

0

0

0
4

0
4

0
4

0

0

0

0

0
4

0
4

0
4

0

0

0

0

0
4

0
4

0
4

0

0

0

0

0
4

0
4

0
4

0

0

0

0

0
4

0
4

0
4

0

0

0

0

0
4

0
4

0
4

0
6

0
6

0
6

0
6

0
4

0
4

0
4

0

0

0

0

0
4

0
4

0
4

0

0

0

0

0
4

0
4

0
4

0

0

0

0

0
4

0
4

0
4

0

0

0

0

0
4

0
4

0
4

0

0

0

0

0
4

0
4

0
4

0

0

0

0

0
4

0
4

0
4

3.56043

3.56043

3.56043

2.54317

0.38753

0.629736

1.11022302462516e-16

0
4

0
4

0

0

0

0

0
4

0
4

0
4

0.516483
3

0.516483
3

0.516483
3

0.516483

0

0

0

0
4

0
4

0
4

0
6

0
6

0
6

0

0

0

0
4

0
4

0
4

0
4

0
4

0
4

0
4

0
4

0

0

0
4

0
4

0
4

0
7

0
7

0
7

0
7

0
4

0
4

0
4

1.96381
1

1.96381
1

1.96381
1

1.33924
1

0.295687

0

0

0.328884

2.22044604925031e-16
1

0
4

0

0

0
4

0
4

0
4

0
7

0
7

0
7

0
7

0
4

0
4

0
4

0
6

0
6

0
6

0
6

0

0
4

0
4

0
4

0

0

0

0

0

0

0

0
4

0
4

0
4

0

0

0
7

0
7

0

0
4

0

0

0
4

0
4

0
4

0

0

0

0

0

0

0
4

0

0

0

0

0
4

0
4

0

0

0

0
4

0

0

0
4

0
4

0
4

0
7

0
7

0
7

0
7

0

0
4

0
4

0
4

0

0

0

0

0

0
4

0
4

0
4

0

0

0

0

0

0
4

0

0

0

0
4

0
4

0
4

0

0

0

0

0

0

0
4

0
4

0
4

0

0

0

0

0

0
4

0

0

0

0
4

0

0

0
4

0

0

0
4

0
4

0
4

0
6

0
6

0
6

0
6

0

0
4

0
4

0
4

0
7

0
7

0
7

0

0

0

0

0
4

0
4

0
4

0
7

0
7

0
7

0
7

0

0
4

0
4

0
4

0

0

0

0

0

0
4

0

0

0
4

0
4

0
4

0

0

0

0

0

0
4

0
4

0
4

0

0

0

0

0

0

0
4

0

0

0
4

0

0

0
4

0
4

0
4

0

0

0

0

0

0

0

0
4

0
4

0
4

0

0

0

0

0
4

0
4

0
4

2.36829

2.36829

2.36829

2.31348

0.0548139

0
4

0
4

0
4

0
7

0
7

0
7

0
7

0
4

0
4

0
4

0

0

0

0

0

0

0

0
4

0
4

0
4

0
5

0
5

0
5

0
5

0

0
4

0

0

0
4

0

0

0

0
4

0

0

0
4

0
4

0

0

0

0

0

0
4

0
4

0
4

0

0

0

0

0

0

0

0
4

0
4

0
4

0
6

0
6

0
6

0
6

0
4

0
4

0
4

0

0

0

0

0

0
4

0

0

0
4

0
4

0
4

0

0

0

0

0

0

0
4

0
4

0
4

0

0

0

0

0
4

0

0

0

0
4

0
4

0
4

0

0

0

0

0

0
4

0

0

0

0
4

0
4

0
4

0

0

0

0

0

0

0

0
4

0
4

0
4

0

0

0

0

0

0
4

0

0

0
4

0
4

0
4

0

0

0

0

0
4

0

0

0
4

0

0

0
4

0

0

0
4

0
4

0
4

0

0

0

0

0

0

0
4

0
4

0
4

0
7

0
7

0
7

0
7

0

0

0
4

0
4

0
4

0

0

0

0

0

0

0
4

0
4

0
4

0

0

0

0

0

0
4

0
4

0
4

0
3

0
3

0

0

0
4

0

0

0
4

0
4

0
4

0

0

0

0

0
4

0
4

0
4

0

0

0

0

0
4

0
4

0
4

0

0

0

0

0
4

0
4

0
4

0

0

0

0

0

0
4

0
4

0
4

0

0

0

0

0
4

0
4

0
4

0

0

0

0

0

0
4

0
4

0
4

0

0

0

0

0

0
4

0
4

0
4

1.35577

1.35577

0.387362

0.129121

0.129121

0.129121

0

0

0

0

0

0

0
4

0.968406

0.129121

0.839285

0

0

0

0
4

0

0

0

0
4

0

0

0

0
4

0
4

0
4

0

0

0

0

0

0

0
4

0
4

0
4

0

0

0

0

0

0
4

0
4

0
4

0

0

0

0

0
4

0
4

0
4

0

0

0

0

0

0

0
4

0
4

0
4

0.272331

0.272331

0.272331

0.272331

0
4

0
4

0
4

0

0

0

0

0
4

0
4

0
4

0

0

0

0

0

0
4

0

0

0

0
4

0
4

0
4

0

0

0

0

0
4

0

0

0
4

0
4

0

0

0

0
4

0
4

0
4

0

0

0

0

0

0
4

0
4

0
4

0

0

0

0

0
4

0
4

0
4

0
7

0
7

0
7

0
7

0

0

0

0

0
4

0
4

0
4

0

0

0

0

0

0
4

0

0

0
4

0
4

0
4

0

0

0

0

0
4

0
4

0
4

0

0

0

0

0
4

0
4

0
4

0

0

0

0

0
4

0
4

0
4

0

0

0

0

0

0
4

0
4

0
4

0

0

0

0

0
4

0
4

0
4

0

0

0

0

0

0
4

0
4

0
4

0

0

0

0

0

0

0
4

0
4

0
4

0

0

0

0

0

0
4

0

0

0
4

0
4

0
4

0

0

0

0

0
4

0

0

0
4

0
4

0
4

0
6

0
6

0

0

0

0

0

0

0
4

0

0

0

0

0

0
4

0

0

0
4

0
4

0
4

0

0

0

0

0
4

0
4

0
4

0

0

0

0

0
4

0
4

0
4

0

0

0

0

0
4

0

0

0
4

0
4

0
4

0

0

0

0

0

0

0
4

0
4

0
4

0

0

0

0

0
4

0
4

0

0

0

0
4

0
4

0
4

0

0

0

0

0
4

0
4

0
4

0.310612

0.310612

0.310612

0.0548139

0.255798

0
4

0

0

0
4

0
4

0
4

0

0

0

0

0
4

0
4

0
4

0

0

0

0

0

0
4

0
4

0
4

0

0

0

0

0
4

0
4

0
4

3.40912
1

1.36876

1.36876

1.36876

0
4

0
4

0

0

0

0
4

0
4

0

0

0

0
4

0
4

0

0

0

0

0
4

0
4

0

0

0

0
4

0
4

1.69435

1.69435

1.69435

0
4

0
4

0

0

0

0
4

0
4

0

0

0

0
4

0

0

0
4

0
4

0

0

0

0
4

0
4

0

0

0

0

0
4

0
4

0.258242

0.258242

0.258242

0
4

0
4

0.0877713
1

0.0877713
2

0

0

0.0877713

0

0

0

0

0

0

0
4

0

0

0
4

0
4

0
4

0
7

0

0

0

0

0
4

0
4

0

0

0

0
4

0
4

0

0

0

0
4

0
4

0

0

0

0
4

0
4

0

0

0

0
4

0
4

0

0

0

0
4

0
4

0

0

0

0
4

0
4

0
7

0
7

0
7

0

0
4

0
7

0
7

0
4

0

0

0
4

0

0

0
4

0

0

0
4

0

0

0
4

0

0

0
4

0

0

0
4

0

0

0
4

0
4

0
4

82.2507
3

82.2507
3

82.2507
3

35.6837

0

0.355811

0.290119

0.124541

0.190155

0.0551547

0

0.151349

0

0

37.7463
3

0

1.32275

0.077809

0

0.155618

0.190155

0.077809

0.0950773

0

0

1.7244
2

0

0.0877713

0.155618

0

1.36872
3

0.966628
2

0.16354
3

0.302505
2

0.373814
3

0.591347
2

1.22124532708767e-14
3

0
4

0

0

0
4

0

0

0
4

0
4

0

0

0

0

0

0
4

0
4

0

0

0

0

0
4

0
4

0

0

0

0
4

0
4

0

0

0

0
4

0
4

0

0

0

0

0
4

0
4

0

0

0

0
4

0

0

0
4

0
4

0

0

0

0
4

0

0

0
4

0
4

0

0

0

0
4

0
4

0

0

0

0
4

0
4

0
4

0
7

0

0

0

0
4

0
4

0

0

0

0
4

0
4

0

0

0

0
4

0
4

0

0

0

0
4

0
4

0

0

0

0
4

0
4

0

0

0

0
4

0
4

0

0

0

0
4

0
4

0

0

0

0
4

0
4

0

0

0

0
4

0
4

0

0

0

0
4

0
4

0

0

0

0
4

0
4

0
7

0
7

0
7

0
5

0

0

0

0

0

0

0

0

0

0

0
7

0

0

0

0

0

0

0

0

0

0

0
6

0

0

0

0

0

0

0

0

0

0

0

0

0

0
7

0

0

0

0

0
4

0

0

0
4

0

0

0
4

0

0

0
4

0
4

0
4

0

0
5

0
5

0

0

0

0

0
5

0
6

0

0

0

0

0

0

0
4

0

0

0
4

0
4

0
5

0
5

0
5

0

0

0

0

0
4

0

0

0
4

0
4

0

0

0

0
4

0
4

0

0

0

0
4

0
4

0

0

0

0
4

0
4

0

0
7

0

0

0

0

0

0

0

0

0

0

0

0

0

0

0

0

0

0

0

0

0

0

0
4

0

0

0

0

0
4

0

0

0
4

0

0

0
4

0

0

0
4

0
4

0
4

0

0

0

0

0

0

0

0

0

0

0

0

0
4

0

0

0

0
4

0

0

0
4

0
4

0
5

0
5

0
5

0

0

0

0
7

0

0

0

0

0

0

0

0
4

0
4

0

0

0

0

0
4

0

0

0
4

0
4

0

0

0

0
4

0
4

0

0

0

0
4

0
4

0

0

0

0
4

0
4

0

0

0

0
4

0
4

0

0

0

0
4

0
4

0

0

0

0

0
4

0
4

0

0

0

0

0
4

0
4

0

0

0

0
4

0
4

0

0

0

0
4

0
4

0
5

0
5

0
5

0

0

0

0

0

0

0
4

0

0

0
4

0

0

0
4

0
4

0

0

0

0
4

0
4

0

0

0

0
4

0
4

0

0

0

0
4

0
4

0

0

0

0
4

0
4

0

0

0

0
4

0
4

0

0

0

0
4

0
4

0

0

0

0
4

0
4

0

0

0

0
4

0
4

0

0

0

0
4

0
4

0

0

0

0
4

0
4

0
7

0
7

0
7

0

0
7

0
7

0

0

0

0

0

0
4

0
4

0
5

0
5

0

0

0

0

0

0

0

0

0
4

0

0

0

0

0

0
4

0
4

0
5

0
5

0

0

0

0

0

0

0
4

0

0

0

0
4

0
4

0

0

0

0
4

0

0

0
4

0
4

0

0

0

0

0

0
4

0

0

0
4

0
4

0
7

0
7

0

0

0

0

0

0
4

0
4

0

0

0

0
4

0

0

0
4

0
4

0
4

190.08

0

0

0

0

0

0

0

0

0

0

0
4

0

0

0
4

0
4

0

0

0

0

0

0
4

0
4

0.909077

0.909077

0.363631

0.545446

0
4

0
4

0

0

0

0

0
4

0

0

0
4

0
4

0

0

0

0

0
4

0
4

0

0

0

0
4

0

0

0
4

0
4

0

0

0

0
4

0
4

0

0

0

0
4

0
4

0

0

0

0
4

0
4

0

0

0

0
4

0

0

0
4

0
4

0

0

0

0
4

0
4

0

0

0

0

0

0
4

0
4

0

0

0

0
4

0
4

0

0

0

0
4

0
4

0.0739218

0.0739218

0.0739218

0
4

0
4

0

0

0

0
4

0
4

0

0

0

0
4

0
4

0

0

0

0
4

0
4

0

0

0

0
4

0
4

1.03491

1.03491

1.03491

0
4

0
4

0

0

0

0
4

0
4

0

0

0

0
4

0
4

0

0

0

0

0
4

0

0

0

0
4

0
4

0

0

0

0
4

0
4

0.0367698

0.0367698

0.0367698

0
4

0
4

4.7272

4.7272

4.7272

0
4

0
4

0

0

0

0
4

0
4

0

0

0

0
4

0
4

0

0

0

0
4

0
4

0

0

0

0

0
4

0
4

0

0

0

0

0

0
4

0

0

0
4

0
4

0

0

0

0

0
4

0

0

0
4

0

0

0
4

0
4

0

0

0

0
4

0

0

0

0
4

0
4

0

0

0

0

0
4

0
4

0

0

0

0

0
4

0

0

0

0
4

0
4

183.298

180.253

179.934

0

0

0

0

0

0

0

0

0

0

0

0

0

0.318177

0

0
3

0

0

0

0

0

0

0
4

0

0

0

0

0

0

0

0
4

0

0

0
4

0

0

0
4

0

0

0
4

0

0

0
4

0

0

0
4

0

0

0
4

0

0

0
4

0

0

0

0

0

0

0
4

3.04541

3.04541

0
4

0

0

0
4

0

0

0

0
4

0

0

0
4

0

0

0
4

0

0

0
4

0

0

0
4

3.01980662698043e-14

0
4

0
4

0
5

0
6

0

0

0

0

0

0

0

0

0

0
4

0

0

0

0
4

0

0

0

0
4

0

0

0
4

0

0

0
4

0
4

0

0
5

0
6

0

0

0

0

0

0

0

0
4

0

0

0

0
4

0

0

0
4

0
4

0

0

0

0
4

0
4

0

0

0

0
4

0
4

0

0

0

0
4

0
4

0

0

0

0
4

0
4

0

0

0

0
4

0
4

0
5

0
5

0
6

0

0

0

0

0

0
4

0
4

0

0

0

0

0

0

0

0
4

0
4

0
5

0

0

0

0

0

0
4

0

0

0
4

0

0

0
4

0
4

0

0

0

0
4

0
4

0

0

0

0

0
4

0
4

0

0

0

0
4

0
4

0

0

0

0
4

0
4

0

0

0

0
4

0
4

0
4

2.156
3

0.132964

0.132964

0.132964

0

0
4

0
4

0

0

0

0
4

0
4

2.02303
3

0

0

0
4

2.02303

0

2.02303

0

0
4

0

0

0
4

0

0

0
4

0

0

0
4

0
4

0
4

79.4406

0

0

0
7

0

0

0

0

0

0

0

0

0

0
8

0
8

0
1

0

0

0

0

0

0
4

0

0

0

0

0
4

0

0

0

0
4

0
4

0

0

0

0

0
4

0
4

5.48553

5.36882

5.13539

0.155618

0.077809

2.91433543964104e-16

0
4

0.116713

0.116713

0
4

0
4

0

0

0

0

0
4

0

0

0

0
4

0
4

0

0

0

0
4

0
4

0

0

0

0
4

0
4

0

0

0

0

0
4

0

0

0
4

0
4

0

0

0

0
4

0
4

0

0

0

0

0
4

0
4

0

0

0

0
4

0
4

0

0

0

0
4

0

0

0
4

0
4

0

0
7

0

0

0

0

0

0

0

0

0

0

0

0
4

0
1

0
8

0

0

0
4

0

0

0
4

0

0

0
4

0
4

0

0

0

0
4

0
4

0

0

0

0

0
4

0
4

0

0

0

0
4

0
4

0

0

0

0
4

0
4

0

0

0

0
4

0
4

0

0

0

0
4

0
4

0

0

0

0
4

0
4

0

0

0

0
4

0
4

0

0

0

0
4

0
4

0

0

0

0
4

0
4

0

0

0

0

0

0

0
4

0

0

0

0
4

0

0

0
4

0

0

0
4

0
4

0

0

0

0
4

0
4

0

0

0

0
4

0
4

0

0

0

0
4

0
4

0.0877713

0.0877713

0.0877713

0
4

0
4

0

0

0

0
4

0
4

0

0

0

0
4

0
4

0

0

0

0
4

0
4

0

0

0

0
4

0
4

0

0

0

0
4

0
4

0

0

0

0
4

0
4

0

0

0

0

0

0

0
4

0

0

0

0
4

0
4

0

0

0

0

0
4

0

0

0

0

0

0
4

0

0

0

0
4

0
4

0.294158
3

0.239004
3

0.147079

0.0551547

0.0367698

0

0

0

0

0

0
4

0.0551547

0.0551547

0
4

0

0

0
4

0
4

3.51918
3

3.51918
3

1.57396

1.94522

0

0

0

0
4

0
4

0
6

0

0

0

0

0
4

0

0

0
4

0

0

0
4

0
4

0.220619

0.220619

0.220619

0

0

0
4

0
4

69.8333

60.3067

0
7

0

0

0

0

0

0

0

0

0.433784

0

23.7517

0

0

0

0.0950773

0.219428

0

0

0

0.131657

0

0

0.0633849

0

0

0

0

0

0

0.0877713

0

0.0877713

0

0

0

0

0

0

0

0.175543

0

0.0877713

0.129121

0
7

0

0

0.263314

0

0

0

0.0877713

0

0.0633849

0

30.6093

0

0

0.129121

0.0877713

3.80309

0

0

0

0

0

0

0

0

0

0
4

8.82992

7.74285

0.71171

0.375354

3.88578058618805e-16

0
4

0

0

0
4

0.0367698

0.0367698

0
4

0.12677

0.0633849

0.0633849

0
4

0

0

0

0
4

0

0

0
4

0

0

0
4

0

0

0
4

0

0

0
4

0

0

0
4

0

0

0
4

0
6

0
6

0

0

0
4

0

0

0
4

0

0

0
4

0

0

0
4

0

0

0
4

0

0

0
4

0

0

0

0

0

0
4

0

0

0

0

0
4

0.533162
3

0.533162

0

0

0
4

0

0

0

0
4

0

0

0

0
4

0

0

0

0
4

0

0

0
4

6.10622663543836e-15

0
4

0
4

0
7

0
6

0
6

0
6

0

0
4

0
4

0
6

0
6

0
6

0
4

0
4

0

0

0

0
4

0
4

0

0

0

0
4

0
4

0

0

0

0
4

0
4

0

0

0

0
4

0
4

0
7

0
7

0
7

0
4

0
4

0
4

0
4

2112.28

4.05663

4.05663

4.05663

1.42616

0.443694

2.18678

4.44089209850063e-16

0
4

0
4

0
4

894.173
3

9.83039

9.83039

0.3072

3.59862

0

0

0

0.0877713

0

0.0877713

0

0

1.44823
2

4.12525

0

0

0

0

0.175543

0

0
4

0

0

0
4

0

0

0
4

0
4

858.122
3

30.1497

0

0

0

0

0

0

0

0

0

0

0

0

0

0

0

0

0

0

0

0

0

0

0

0

0

0

0

0

0

0

24.0165

0

0

0

0

0

0

0

0

0

0

0

0

0

0

0

0

0

0

0

0

0

0

0

0

0

0

0
6

0

0

0

0

0

0

0

0

0

0

0
7

0

0

0

0

0

0

0

0

0

0

0

0

0

0

0

0

0

0

0

0

0

0

0

0

0

0

0

0

0

0

0

0

0

0

0

0

0

0

0

0

0

0

0

0

0
5

0

0

0

0

0

0

0

0

0

0

0

0

0

0

0

0

0

0

0

0

0

0
2

0

0

0

0

0

0

0

0

0

0

0

0

0

2.7761

0

0

0

0

0

0

0

0

0

0

0

0

0

0

0

0

0

0

0

0

0

0

0.193681

0

0

0

0

0

0

0
6

0

0

0

0

0

0

0

0

0

0

0

0

0

0

0

0

0

0

0

0

0

0

0

0

0

0

0

0

0

0

0

0

0

0

0

0

0

0

0

0

0

0

0

0

0

0

0

0

0

0.451923

0

0

0

0

0

0.581044

0

0

0

0

0

0

0

0

0

0

1.87225
2

0

0

0

0

0

0

0

0

0

0

0

0

0

0

0

0

0

0

0

0

0

0

0

0

0

0

0

0

0

0

0

0

0

0

0

0

0

0

0

0

0

0

0

0

0

0

0

0

0

0

0

0

0

0

0

0

0

0

0

0

0

0

0

0
6

0

0

0.258242
2

0

0

0
7

0

0

0

0

0

0

0

0

0

0

0

0

0

0

0

0

0

0

0

0

0

0

0

0

0

0

0

0

0

0

0

0

0

0

0

0

0

0

0

0

0

0

0

0

0

0

0

0

0

0

0

0

0

0

0

0

1.16573417585641e-15

0
4

354.263
4

81.0708
3

56.5881
4

113.309
3

0
6

0
4

0

0

0

0

0

0

0

0

0

0

0
4

0

0

0

0

0

0

0

0.693508

0

0

0
4

0

0

0

0

0

0

0

0

0

0

0
4

0

0

0

0

0

0

0

0

0

0

0
4

0

0

0

0

0

0

0

0

0

2.63166

0
2

0

0

0

0

0

0

0

0

0

0

4.57077
3

0

0

0

0

0

0

0

0

0

0

0.207762
4

0

0

0

0

0

0

0

0

0

0

0

0

0

0

0

0

0

0

0

0

0

0
2

0

0

0

0

0

0.207762

0

0

0

0

6.57914
3

0
4

0

0

0

0

0

0

0

0

0

0

0

0

0

0

0

0

0

0

0

0

0

0

0

0

0

0

0

0

0

0

0

0

0
4

0

0

0

0

0

0

0

0

0

0

0

0

0

0

0

0

0

0

0

0

0

0.900304
3

0

0

0

0

0

0

0

0

0

0

0

0

0

0

0

0

0

0

0

0

0

0

0

0

0

0

0

0

0

0

0

0

0
4

0

0

0

3.57921

0

0

0

0

0

0

0
4

0

0

0.761796

0

0

0

0

0

0

0

6.16362
4

0

0

0

0

0

1.17732

0

0

0

0

0

0

0

0

0

0

0

0

0

0

0

0

12.4883

0

0

0

0

0

0

0

0

0

0

0
4

0

0

0

0

0

0

0

0

0

0

0.138508
3

0

0

0

0

0

0

0

0

0

0

0

0

1.35577

0

0

0

0

0

0

0

0

0

0

0

0

0

0

0

0

0

0

0

0
4

0

0

0

0

0

0

0

0

0

0

0

0

0

0

0

0

0

0

0

0

0

0

0

0

0

0

0

0

0

0

0

0

0
4

0

0

0

0

0

0

0

7.35989

0

0

0

0

0

0.138508

0

0

0

0

0

0

0

0

0

0

0

0

0

0

0

0

0

0

0

0
2

0

0

0

0

0

0

0

0

0

0

0

0

0

0

0
4

0

0

0

0.692542
3

6.50989
4

0

0

0

0
4

0

0
4

0

0

0

0

0
4

4.50152

0

0

0

0.129121

0

0

0.0347274

0

0

0.900304
4

0

0

0

0

0

16.9794

0

0

0

0

0.761796
3

0

0

0

0

0

0

0

0.077809

0

0.207762

6.23287
3

0

0

0

17.3135

0

0

0

0

0

0

0
4

138.996
3

0
3

41.9874
3

61.3826
3

0.0633849
3

31.7158
2

3.84658
3

0
2

0
1

0

0

0

0

0

0

0

0

0

0

0

0

0

0

0

0

0

0

0

0

0

0

0

0

0

0

0

0
8

0

0

0

0

0

2.26485497023532e-14
3

0
4

20.5271

0
7

0
6

0

0

0

0

0

0

0

0

0

0

0
7

0

0

0

0

0

0

0.147079

0

0

0

0
7

0

0

0

0

0.110309

0.0913565

0

0

0

0

0
5

0

0

0

0

0

0

0

0

0

0

0
6

0

0

0

0

0

0

0.0367698

0

0

0

0
7

0

0

0

0

0

0

0

0.0365426

0

0

0

0

0

0.165464

0

0

0

0

0

0

0

0

0

0

0

0

0

0

0

0.0913565

5.62578

0
8

0.0919245

0

0

0

0

0

0

0.147079

0.0735396

3.64021

0.0367698

0

0

0.0551547

0

0.110309

0.588317

0

0

0

0
3

0.0730852

0

0

0.0365426

0

0

0

0

0

0

0.0367698
7

0.193681

0

0

0

0

0.69431

0

0

0

0

4.0745
3

0

0

0

0.25354

0.365426

0.0548139

0

0

0

0.129121

0
7

0

0

0

0

0

0.0919245

0.110309

0

0

0

0.257389
1

0

0

0.0735396

0

0

0.0730852

0

0

0.200984

0

2.53971

0

0

0

0

0

0

0

0.0367698

0.0365426

0.147079

0
4

8.71565
2

0

0

0

0

0.129121

0

0

4.32555

0

0

0

3.7445

0

0

0

0

0

0

0

0

0

0

0

0

0

0

0

0

0

0

0

0

0

0

0

0

0

0

0

0

0

0

0

0

0

0

0

0

0

0

0

0

0

0

0

0

0

0

0

0

0

0

0

0

0

0

0

0

0

0

0

0

0

0

0

0

0

0.516483

0

0

0

0

0

0

0

0

0
4

0
3

0
3

0

0

0

0

0

0

0

0

0

0

0
4

0

0

0

0

0

0

0

0

0

0

0
4

0

0

0

0

0

0

0
4

0
3

0
4

0
3

0
4

0

0
4

0
3

0

0

0

0

0
4

0

0

0

0

0

0

0

0
4

0

0

0
4

0

0

0
4

0

0

0
4

0

0

0
4

0

0

0

0
4

0

0

0
4

0

0

0

0
4

0

0

0
4

0

0

0
4

0

0

0

0
4

0
3

0
4

0
3

0
3

0
3

0

0

0

0

0
4

0

0

0
4

0

0

0

0
4

0

0

0
4

0

0

0

0
4

0

0

0
4

0

0

0
4

0

0

0

0
4

0

0

0
4

0

0

0
4

0

0

0
4

79.6423
3

79.1575
3

0

0.138508

0

0.207762

0.138508

0

0

0
4

0

0

0
4

0

0

0
4

0

0

0
4

0

0

0
4

0

0

0
4

0

0

0
4

0

0

0
4

0

0

0
4

0

0

0
4

0

0

0
4

0
2

0
2

0

0

0

0

0

0

0
4

0

0

0
4

0

0

0
4

0

0

0
4

0

0

0
4

0

0

0
4

0

0

0
4

0

0

0
4

0

0

0
4

0

0

0
4

0

0

0
4

0

0

0

0

0

0

0

0

0
4

0

0

0
4

0

0

0
4

0

0

0
4

0

0

0
4

0

0

0
4

0

0

0
4

0

0

0
4

0

0

0
4

0

0

0
4

0

0

0
4

0
4

0
4

0

0

0

0
4

0

0

0
4

0

0

0
4

0

0

0
4

0

0

0
4

0

0

0
4

0

0

0
4

0

0

0
4

0

0

0
4

0

0

0
4

0

0

0
4

0

0

0

0

0

0
4

0

0

0
4

0

0

0
4

0

0

0
4

0

0

0
4

0

0

0
4

0

0

0
4

0

0

0
4

0

0

0
4

0

0

0
4

0

0

0
4

1.03881
3

0.761796
3

0.277017

0

0

0
4

0

0

0
4

0

0

0
4

0

0

0
4

0

0

0
4

0

0

0
4

0

0

0
4

0

0

0
4

0

0

0
4

0

0

0
4

0

0

0
4

0.415525
4

0.415525
4

0

0

0

0
4

0

0

0
4

0

0

0
4

0

0

0
4

0

0

0
4

0

0

0
4

0

0

0
4

0

0

0
4

0

0

0
4

0

0

0
4

0

0

0
4

0

0

0

0

0

0

0
4

0

0

0
4

0

0

0
4

0

0

0
4

0

0

0
4

0

0

0
4

0

0

0
4

0

0

0
4

0

0

0
4

0

0

0
4

0

0

0
4

0
3

0
3

0

0

0

0

0

0

0

0

0

0

0
4

0

0

0

0

0

0

0

0
4

0
4

0
3

0

0

0

0

0
4

0

0

0

0

0

0
4

0

0

0
4

0

0

0
4

0

0

0
4

0

0

0
4

0

0

0
4

0
3

0
3

0

0

0
4

0
4

0

0

0

0

0

0
4

0
2

0

0

0

0

0

0

0

0
4

0

0

0

0

0

0

0

0

0

0
4

0

0

0

0
4

0.415525
3

0.138508

0

0

0.277017

0

0

0
4

1.03881
3

0.277017

0.207762

0.415525

0.138508

0

0

0

0
4

0

0

0

0
4

0
2

0

0

0

0

0

0
4

0
3

0
3

0

0

0

0

0

0

0

0

0

0

0
4

0

0

0
4

0

0

0

0

0

0
4

0.277017
3

0.138508

0

0

0

0.138508

0
4

0
4

0

0

0

0

0

0
4

0

0

0
4

0
4

0
4

0

0

0

0
4

0

0

0

0
4

0

0

0

0

0
4

0
4

0
4

0

0
4

0
5

0
5

0

0
4

0
2

0

0

0

0

0

0
4

0
4

0
4

0
4

0
5

0
5

0

0

0

0

0

0

0

0

0

0

0

0

0

0

0

0

0

0

0

0

0

0

0

0

0
4

0
4

0

0

0
4

0

0

0

0

0
4

0

0

0
4

0

0

0

0

0
4

0
4

0

0

0
4

0

0

0

0

0

0
4

0.258242

0.258242

0

0
4

0

0

0

0

0

0
4

0

0

0
4

0

0

0
4

0
6

0

0

0

0

0

0

0

0

0

0

0

0

0

0

0

0

0

0

0

0

0

0

0

0

0

0

0

0

0

0

0

0

0
4

0.451923
2

0.451923

0

0
4

0

0

0

0

0
4

0

0

0

0
4

0

0

0

0

0
4

0

0

0

0

0
4

0

0

0

0

0
4

0

0

0

0
4

0.193681

0.193681

0
4

0

0

0

0
4

0

0

0

0

0
4

200.21
2

190.784
2

9.2967
2

0

0

0

0

0

0.129121

6.13398221105399e-15
2

0
4

0

0

0
4

0

0

0

0
4

0

0

0
4

0

0

0

0
4

0

0

0
4

0

0

0
4

0

0

0
4

0

0

0

0

0

0
4

0.129121

0

0.129121

0
4

0

0

0
4

20.9148
3

0
4

0

0

0

0

0

0

0

0

0

0

0

0

0

0

3.25495

0

0

0

0

0

0

3.73972

13.9201

0

0

0

0

0

1.77635683940025e-15
3

0
4

0

0

0
4

0

0

0
4

0

0

0

0
4

0

0

0

0
4

0

0

0

0
4

0

0

0

0
4

0

0

0

0
4

0.484779

0

0.484779

0
4

0

0

0

0
4

0

0

0

0
4

0
6

0
6

0

0

0

0

0

0

0

0

0

0

0

0
4

0

0

0

0
4

0

0

0

0
4

0

0

0
4

0

0

0

0
4

0

0

0
4

0

0

0
4

0

0

0

0
4

0

0

0

0
4

0

0

0
4

0

0

0
4

0
4

0
4

0
4

0
4

0

0

0

0

0

0

0
4

0

0

0
4

0

0

0
4

0

0

0
4

0

0

0
4

0

0

0

0
4

0

0

0

0
4

0

0

0
4

0

0

0

0
4

0

0

0
4

0

0

0

0
4

0
4

0
2

0
2

0
2

0

0

0

0

0

0

0

0

0

0

0

0

0

0

0

0

0

0

0

0

0

0
2

0

0

0

0

0

0

0

0

0

0

0

0

0

0

0

0

0

0

0

0

0

0

0

0

0

0

0

0

0

0

0

0

0

0

0

0

0

0

0

0

0

0

0

0

0

0

0

0

0

0

0

0

0
4

0
2

0

0

0

0

0

0

0

0

0

0

0

0
4

0

0

0

0

0

0

0

0

0
4

0

0

0

0

0

0
4

0

0

0
4

0
4

0

0

0
7

0

0

0

0

0

0
4

0

0

0
4

0

0

0
4

0
4

0

0

0

0
4

0

0

0
4

0
4

0

0

0

0
4

0

0

0
4

0
4

0

0

0

0
4

0

0

0
4

0
4

0

0

0

0

0
4

0
4

0

0

0

0
4

0

0

0
4

0
4

0

0

0

0
4

0
4

0

0

0

0
4

0
4

0

0

0

0
4

0
4

0

0

0

0
4

0

0

0
4

0
4

0

0

0

0
4

0
4

0

0

0

0

0

0

0

0

0
4

0
4

0.628335

0.628335

0.554413

0.0739218

1.38777878078145e-17

0
4

0
4

0

0

0

0
4

0

0

0
4

0
4

0

0

0

0

0
4

0
4

0

0

0

0
4

0
4

0

0

0

0

0
4

0
4

0

0

0

0
4

0
4

0

0

0

0
4

0
4

0

0

0

0
4

0
4

0.968406

0.968406

0.968406

0
4

0
4

0

0

0

0

0
4

0
4

0
7

0
7

0

0

0

0

0

0

0
4

0
4

0

0

0

0

0
4

0
4

0

0

0

0
4

0
4

0

0

0

0
4

0
4

0

0

0

0
4

0
4

0

0

0

0
4

0
4

0

0

0

0
4

0
4

0

0

0

0
4

0
4

0

0

0

0
4

0
4

0

0

0

0
4

0
4

0

0

0

0
4

0
4

18.7871
2

18.7871
2

18.3997
2

0

0.258242

0

0.129121

0

0

2.41473507855972e-15
2

0
4

0
4

0

0

0

0
4

0
4

0

0

0

0
4

0
4

0

0

0

0
4

0
4

0

0

0

0
4

0
4

0

0

0

0
4

0
4

0

0

0

0
4

0
4

0

0

0

0
4

0
4

0

0

0

0
4

0
4

0

0

0

0
4

0
4

0

0

0

0
4

0
4

0.193681

0
5

0

0

0

0

0

0

0

0
4

0.193681

0.193681

0

0

0

0

0
4

0

0

0
4

0
4

0

0

0

0
4

0
4

0

0

0

0
4

0
4

0

0

0

0
4

0
4

0

0

0

0
4

0
4

0

0

0

0
4

0
4

0

0

0

0
4

0
4

0

0

0

0
4

0
4

0

0

0

0
4

0
4

0

0

0

0
4

0
4

0

0

0

0
4

0
4

0

0

0

0

0

0

0

0

0
4

0
4

0

0

0

0
4

0
4

0

0

0

0
4

0
4

0

0

0

0
4

0
4

0

0

0

0
4

0
4

0

0

0

0
4

0
4

0

0

0

0
4

0
4

0

0

0

0
4

0
4

0

0

0

0
4

0
4

0

0

0

0
4

0
4

0

0

0

0
4

0
4

0

0

0

0

0

0

0

0
4

0
4

0

0

0

0
4

0
4

0

0

0

0
4

0
4

0.0739218

0.0739218

0.0739218

0
4

0
4

0

0

0

0
4

0
4

0

0

0

0
4

0
4

0

0

0

0
4

0
4

0

0

0

0
4

0
4

0

0

0

0
4

0
4

0

0

0

0
4

0
4

0

0

0

0
4

0
4

0

0

0

0

0

0

0

0

0

0

0

0

0
4

0
4

0

0

0

0
4

0
4

0

0

0

0
4

0
4

0

0

0

0
4

0
4

0

0

0

0
4

0
4

0

0

0

0
4

0
4

0

0

0

0
4

0
4

0

0

0

0
4

0
4

0

0

0

0
4

0
4

0

0

0

0
4

0
4

0

0

0

0
4

0
4

0

0

0

0

0

0

0
4

0

0

0
4

0
4

0

0

0

0
4

0
4

0

0

0

0
4

0
4

0

0

0

0
4

0
4

0

0

0

0
4

0
4

0

0

0

0
4

0
4

0

0

0

0
4

0
4

1.29121

1.29121

1.29121

0
4

0
4

0

0

0

0
4

0
4

0

0

0

0
4

0
4

0

0

0

0
4

0
4

0
6

0
6

0
6

0

0

0

0

0

0
4

0
4

0

0

0

0
4

0
4

0

0

0

0
4

0
4

0

0

0

0
4

0
4

0
2

0
2

0
2

0

0

0

0

0

0

0

0

0

0

0
2

0

0

0

0

0

0

0

0

0

0

0

0

0

0

0

0

0

0

0

0

0

0
2

0

0

0

0

0

0

0

0

0

0

0

0

0

0

0

0

0

0

0

0

0

0

0

0

0

0

0

0

0

0

0

0

0

0

0

0

0

0

0

0

0

0

0

0
2

0

0

0

0

0

0

0

0

0

0

0
4

0
2

0
2

0

0

0

0

0

0

0

0

0

0
4

0
2

0

0
2

0

0

0

0
4

0

0

0

0

0

0
4

0

0

0

0

0
4

0

0

0

0

0
4

0

0

0

0
4

0
4

2.71154
2

2.71154
2

1.16209
2

1.42033

0.129121

0

0
4

0

0

0

0
4

0

0

0
4

0
4

0

0

0

0

0

0

0
4

0
4

0
5

0
5

0
5

0

0

0
4

0
4

0

0

0

0

0

0

0

0

0

0
4

0

0

0

0
4

0

0

0
4

0
4

0

0

0

0

0

0

0

0
4

0

0

0
4

0
4

0

0

0

0

0

0

0
4

0

0

0

0
4

0

0

0
4

0

0

0
4

0
4

0

0

0

0

0

0

0
4

0

0

0
4

0

0

0
4

0

0

0
4

0
4

0
5

0
5

0

0

0

0

0

0

0

0

0
4

0
4

0
4

0

0

0

0

0
4

0

0

0
4

0

0

0
4

0
4

0
7

0
7

0
7

0

0

0
4

0
4

0
2

0
2

0
2

0

0

0

0

0

0

0

0

0
2

0

0

0

0

0

0

0

0
4

0
2

0
2

0

0

0

0
4

0

0

0

0

0

0

0

0

0

0

0
4

0

0

0

0
4

0
4

0

0

0

0

0

0

0
4

0
4

0
6

0
6

0
6

0
4

0
4

0
2

0

0

0

0

0

0
4

0

0

0

0
4

0

0

0
4

0
4

0
7

0
7

0
7

0

0
4

0
4

0
7

0
7

0

0

0
4

0
4

0

0

0

0

0

0
4

0

0

0
4

0
4

0

0

0

0

0

0

0

0
4

0

0

0
4

0

0

0
4

0
4

0
6

0
6

0
6

0

0
4

0
4

0

0

0

0

0
4

0
4

0

0

0

0
4

0

0

0
4

0

0

0
4

0
4

0
2

0
2

0
2

0

0

0

0

0

0

0

0

0

0

0

0

0

0

0

0

0

0

0

0

0

0

0
4

0

0

0
4

0
2

0

0

0

0

0

0

0
4

0

0

0

0

0

0
4

0

0

0

0

0
4

0

0

0
4

0

0

0

0
4

0

0

0

0
4

0

0

0
4

0

0

0
4

0
4

0

0

0

0

0
4

0

0

0

0
4

0
4

0

0

0

0

0
4

0

0

0
4

0
4

0

0

0

0

0

0

0

0
4

0

0

0

0
4

0
4

0
6

0
6

0
6

0
4

0
4

0

0

0

0
4

0
4

0

0

0

0

0
4

0
4

0

0

0

0

0
4

0

0

0

0
4

0
4

0

0

0

0

0

0
4

0
4

0

0

0

0

0

0

0
4

0

0

0
4

0
4

0

0

0

0
4

0
4

0

0

0

0

0

0

0

0

0

0

0

0

0

0

0

0

0

0

0
4

0

0

0
4

0

0

0

0
4

0
4

0

0

0

0

0

0
4

0

0

0
4

0

0

0
4

0
4

0

0

0

0
4

0
4

0

0

0

0

0
4

0
4

0

0

0

0
4

0

0

0
4

0
4

0

0

0

0

0
4

0

0

0
4

0
4

0

0

0

0

0
4

0
4

0

0

0

0

0

0
4

0

0

0
4

0

0

0
4

0
4

0

0

0

0

0
4

0

0

0
4

0

0

0
4

0

0

0
4

0
4

0

0

0

0

0
4

0
4

0

0

0

0
4

0
4

1.42033
2

1.42033
2

1.42033
2

0
2

0

0

0

0

0

0

0

0
4

0

0

0

0
4

0
4

0

0

0

0

0
4

0

0

0
4

0

0

0
4

0

0

0
4

0
4

0
7

0

0

0

0
4

0

0

0
4

0
4

0

0

0

0
4

0
4

0

0

0

0
4

0

0

0

0
4

0

0

0
4

0
4

0

0

0

0

0
4

0
4

0

0

0

0

0
4

0

0

0
4

0
4

0.0730852

0.0730852

0

0

0.0730852

0
4

0
4

0

0

0

0
4

0

0

0
4

0
4

0

0

0

0
4

0
4

0

0

0

0

0
4

0
4

0
2

0
2

0
2

0

0

0

0

0

0
4

0
2

0

0

0

0

0

0

0

0
4

0
2

0

0

0

0

0

0
4

0

0

0

0
4

0

0

0

0
4

0

0

0
4

0

0

0
4

0

0

0
4

0

0

0
4

0
4

0

0

0

0
4

0
4

0

0

0

0
4

0

0

0
4

0
4

0

0

0

0

0

0
4

0
4

0

0

0

0
4

0
4

0

0

0

0

0

0
4

0
4

0

0

0

0

0

0
4

0
4

0

0

0

0
4

0

0

0
4

0
4

0

0

0

0

0
4

0

0

0
4

0
4

0

0

0

0
4

0
4

0

0

0

0
4

0

0

0
4

0
4

0

0

0

0

0

0

0

0

0

0

0
4

0
4

0

0

0

0
4

0
4

0

0

0

0
4

0
4

0

0

0

0

0

0
4

0
4

0

0

0

0

0
4

0
4

0

0

0

0
4

0
4

0

0

0

0

0
4

0
4

0

0

0

0
4

0

0

0
4

0
4

0

0

0

0

0
4

0
4

0

0

0

0
4

0
4

0

0

0

0
4

0

0

0
4

0
4

0

0

0

0

0

0

0

0

0

0

0
4

0

0

0
4

0

0

0

0
4

0
4

0

0

0

0
4

0

0

0
4

0
4

0

0

0

0
4

0
4

0

0

0

0

0
4

0
4

0.0730852

0.0730852

0

0.0730852

0
4

0
4

0

0

0

0
4

0
4

0

0

0

0
4

0

0

0
4

0
4

0

0

0

0
4

0

0

0
4

0
4

0

0

0

0

0
4

0
4

0

0

0

0

0
4

0
4

0

0

0

0
4

0

0

0
4

0
4

0

0

0

0

0

0

0

0

0
4

0
1

0
1

0
4

0

0

0
4

0

0

0

0
4

0

0

0

0
4

0

0

0

0
4

0

0

0
4

0
4

1.33809630042947e-13
3

0
4

0.0347274
4

0.0347274
4

0

0

0

0
4

0.0347274

0.0347274

0
4

0

0

0
4

0

0

0
4

0
4

0

0

0

0
4

0
4

0

0

0

0

0
4

0
4

0

0

0

0
4

0
4

0

0

0

0
4

0
4

0
4

1192.25
5

0.136362
4

0.136362
4

0
4

0

0

0

0

0

0

0

0

0

0

0

0

0

0

0

0

0

0

0

0

0

0

0

0

0

0

0

0

0

0

0

0

0

0

0

0

0

0

0

0

0

0

0

0

0

0

0
4

0

0

0

0
4

0

0

0

0

0

0

0

0

0

0

0

0
4

0

0

0

0

0

0

0

0

0

0

0.136362
4

0

0

0

0

0

0

0

0

0

0

0
4

0

0

0

0

0

0

0

0

0

0

0

0

0

0

0

0

0

0

0

0

0

0
4

0

0

0

0

0

0

0

0

0

0

0
4

0

0

0

0

0

0

0

0

0

0

0
4

0

0

0

0

0

0

0

0

0

0

0
4

0
4

1189
5

119.851

1.93912

116.208
2

0

0
4

0

0.116713
4

0

0

0

0

0

0

0

0
7

0

0

0

0

0.0633849

0

0

0

0

0

1.38508
3

0

0

0

0

0

0

0

0

0

0

0
4

0

0

0

0

0

0

0

0

0

0

0

0

0

0

0

0

0

0

0

0

0

0

0

0

0

0

0

0

0

0

0

0

0

0

0

0

0

0

0

0

0

0

0

0

0

0

0

0

0

0

0

0

0

0

0.138508

0

0

0

0
4

6.97782

0

2.80868

4.16914

0

0
4

0

0

0
4

1059.6
5

488.195
5

1.31583
3

286.963
3

86.5251
4

0
5

1.59285
4

0

0

0

0

0

0

0

0

0

0

0

0

0

0

0

0

0

0

0

0

0

0

0

0

0

0

0

0

0

0

0

0

0

0

0

0

0

0

0

0

0

0

0

0

0

0

0

0

0

0

0

0

0

0

0

0

0
4

0

0

0

0

0

0

0

0

0

0

0

0

0

0

0

0

0

0

0

0

0

0

0

0

0

0

0

0

0

0

0

0

0.623287

0

0

0

0

0

0

0

0

0

0

0

0

0

0

0

0

0

0

0

0

0

0.138508
4

0

0

0

0

0

0

0

0.138508
3

0

0
4

0

0
4

0

0

0
4

0

0.415525
4

0

0

0.138508
4

0

0

0

0

0
4

0

0

0

0

0

0

0

0

0

0

0

0

0.138508

0

0

0

0

0

0

0

0

0

0

0

0

0

0

0

0

0

0

0

0

0

0

0
4

0

0

0

0

0.207762

0.692542

0

0

0

0

0.83105
3

0

0

0

0

0

0

0

0

0

0

0.277017
4

0

0

0

0

0

0

0

0

0

0

0.077809
4

0

0

0

0.138508

0

0.138508

0

0

0

0

2.08618
4

0

0

0

0

0

0

0

0

0

0

0

0.415525
4

0.277017

0

0

0

0.138508

0

0

0

0

0

0

0

0

0

0

0

0

0

0

0

0

0
4

0

0

0

0

0

0

0

0

0

0

0

0

0

0

0

0

0

0

0

0

0

0.277017
4

0

0

0

0

0

0

0

0

0

0

1.45434
4

0

0

0

0

0

0

0

0

0

0

0.277017
4

0.207762

0

0

0

0

0

0

0

0

0

0.415525
4

0

0

0

0

0

0

0

0

0

0.138508

0
4

0

0

0

0

0

0

0

0

0

0

0

0
4

0

0

0

0

0

0

0

0

0

0

0.138508
4

0

0

0

0

0

0

0

0

0

0

0

0

0

0

0

0

0

0

0

0

0

0

0

0

0

0

0

0

0

0

0

0

0
4

0

0

0

0

0

0

0

0

0

0

0

0

0

0

0

0

0

0

0

0

0

0

0

0

0

0

0

0.138508

0

0

0

0

0
4

0

0

0

0

0

0.346271

0

0

0

0

0

0

0

0

0

0

0

0.207762

0

0

0

0

0

0

0

0

0

0

0

0

0

0

0
6

1.38508
3

0

0

0

0

0

0

0

0

0

0

0

0

0

0

0

0

0

0

0

0

0

0.770351
3

0

0

0

0

0

0

0

0

0

0

0
4

0

0

0

0.138508

0

0

0

0

0

0

0

0

0

0

0

0

0

0

0

0

0

0
4

0

0

0

0

0

0

0

0

0

0

0

0

0

0

0

0

0

0

0

0

0

0.415525
4

0

0

0

0

0

0

0

0

0

0

0
4

0

0

0

0

0

0

0

0

0

0

0

0

0

0

0

0

0

0.138508

0

0

0

0

0

0

0

0

0

0

0

0

0

0

0

0

0

0

0

0

0

0

0

0

0

0

0
4

0.0347274

0

0

0

0

0

0

0

0

0

0
4

0

0

0

0

0

0

0

0

0

0

0

0

0

0

0

0

0

0

0

0

0

0
4

0

0

0

0

0

0

0

0

0

0

0.692542
3

0

0.761796

0

0

0

0

0

0

0

0

0.138508
3

0

0

0

0

0

0

0

0

0

0

0

0

0

0

0

0

0

0

0

0

0

0
4

0

0

0

0

0

0

0

0

0

0

2.28539
4

0

0

0

0

0

0

0

0

0

0

0

0

0

0

0

0

0

0.277017

0

0

0

0.138508

0

0

0

0

0

0

0

0

0

0

0

0
4

0

0

0

0

0

0

0

0

0

0

0.138508
4

0

0

0

0

0

0

0

0

0

0.207762

0

0

0

0

0

0

0

0

0

0

0

0

0.138508

0

0

0

0

0

0

0

0

0

0
4

0

0

0

0

0

0.207762

0

0

0

0

0

0

0

0

0

0

0

0

0

0

0

0

0

0

0

0

0

0

0.138508

0

0

0

0.138508
4

0
4

0

0

0

0

0

0

0

0

0

0

0

0

0

0

0

0

0

0

0

0

0

0
4

0

0

0

0

0

0

0

0

0

0

0

0

0

0

0

0

0

0

0

0

0

0

0

0

0

0

0

0

0

0

0

0

10.4574
3

0

0

0

0

0

0

0

0

0

0

0

0

0

0

0

0

0

0

0

0

0

0
4

0

0

0

0

0

0

0

0

0

0

0

0

0

0

0

0

0

0

0

0

0

0
4

0

0

0

0

0

0

0

0

0

0

164.963
3

0

0

0

0

0

0

0

0

0

0

0

0

0

0

0

0

0

0

0

0

0

0

0

0

0

0

0

0

0

0.207762

0

0

0

0

0

0

0

0

0

0

0

0

0

0

0

0

0

0

0

0

0

0

0

0

0

0

0

0

0

0

0

0

0

0

0

0

0

0

0

0

0

0

0

0

0

0

0

0
4

0

0

0

0

0

0

0

0

0

0

0

0

0

0

0

0

0.346271

0

0

0

0

0

0

0

0

0

0

0

0

0

0

0

0

0

0

0

0

0

0

0

0

0

0

0

0

0

0

0

0

0

0.277017

0

0

0

0

0
4

0

0

0

0

0

0

0

0

0

0

0

0

0

0

0

0

0

0

0

0

0

0
4

0

0

0

0

0

0

0

0

0

0

0

0

0

0

0

0

0

0

0

0

0

0

0

0

0

0

0

0

0

0

0

0

0

0

0.138508

0

0

0

0

0

0

0

0

0

0

0

0

0

0

0

0

0

0

0

0

0

0

0

0

0

0

0

0

0

0

0
4

0

0

0

0
4

2.37317
4

0

2.37317
4

0

0

0

0
4

0

0
5

0

0

0

0

0

0

0

0

0

0

0

0

0

0

0
4

0

0

0
4

0

0

0
4

0

0

0
4

0

0

0
4

0.193681

0.193681

0
4

0

0

0
4

0

0

0
4

0

0

0
4

0
4

0

0

0

0
4

0
4

0

0

0

0
4

0
4

0

0

0

0
4

0
4

0

0

0

0
4

0
4

3.11236
4

2.14688
4

0

0

2.14688
4

0
4

0.965484

0.746056

0.219428

0
4

0

0

0
4

0

0

0
4

0
4

7.19424519957101e-14
5

0
4

1.66324
3

0.776179

0.776179

0.776179

0

0

0

0
4

0
4

0

0

0

0
4

0
4

0

0

0

0
4

0
4

0.887062

0.887062

0.887062

0

0
4

0
4

0

0

0

0

0
4

0
4

0

0

0

0
4

0
4

0

0

0

0
4

0
4

0

0

0

0
4

0
4

0

0

0

0
4

0
4

0

0

0

0
4

0
4

0

0

0

0
4

0
4

0
4

5.16483

5.16483

5.16483

0

0

0

0

0

0

0

0

0

0

0.129121

2.32417
1

0

0

0

0

0

0

0

0

0

0

0

0

0

0

0

0

0

0

0

0

0

0

0

0

0

0

0

0

0

2.71154

0

0

0

0

0
4

0
4

0

0

0

0
4

0
4

0
4

0

0

0

0
7

0

0

0

0

0

0

0

0

0

0

0
6

0

0

0

0

0

0

0

0

0

0

0

0

0

0

0

0

0

0

0

0

0

0

0

0

0

0

0

0

0

0

0

0

0
6

0

0

0

0

0

0

0

0

0

0

0

0
4

0

0

0

0

0

0

0
4

0

0

0
4

0

0

0

0
4

0

0

0

0
4

0

0

0
4

0

0

0
4

0
4

0

0

0

0
4

0
4

0

0

0

0
4

0
4

0

0

0

0
4

0
4

0
4

0
7

0
7

0
7

0
7

0

0

0

0

0

0

0

0

0

0

0
6

0
7

0

0

0

0

0

0

0
4

0
6

0

0

0

0
4

0

0

0

0
4

0
4

0
4

0
7

0
7

0
7

0

0

0

0

0

0
4

0
4

0
4

0

0

0

0

0
4

0
4

0
4

0

0

0

0

0
4

0
4

0
4

0

0

0

0

0
4

0
4

0
4

0

0

0

0

0
4

0
4

0
4

0

0

0

0

0
4

0
4

0
4

0

0

0

0

0
4

0
4

0
4

0

0

0

0

0
4

0
4

0
4

0

0

0

0

0
4

0
4

0
4

0

0

0

0

0
4

0
4

0
4

0.129121

0.129121

0.129121

0.129121

0
4

0
4

0
4

0
7

0
7

0
7

0
7

0
7

0

0
4

0

0

0
4

0
4

0
4

0

0

0

0

0
4

0
4

0
4

0

0

0

0

0
4

0
4

0
4

0

0

0

0

0
4

0
4

0
4

0

0

0

0

0
4

0
4

0
4

0

0

0

0

0
4

0
4

0
4

0

0

0

0

0
4

0
4

0
4

0

0

0

0

0
4

0
4

0
4

0

0

0

0

0
4

0
4

0
4

0

0

0

0

0
4

0
4

0
4

0

0

0

0

0
4

0
4

0
4

0

0

0

0

0

0

0
4

0

0

0
4

0
4

0
4

0

0

0

0

0
4

0
4

0
4

0.0669684

0.0669684

0.0669684

0.0669684

0
4

0
4

0
4

0

0

0

0

0
4

0
4

0
4

0

0

0

0

0
4

0
4

0
4

0

0

0

0

0
4

0
4

0
4

0

0

0

0

0
4

0
4

0
4

0

0

0

0

0
4

0
4

0
4

0

0

0

0

0
4

0
4

0
4

0

0

0

0

0
4

0
4

0
4

0

0

0

0

0
4

0
4

0
4

0
7

0
7

0
7

0

0

0

0
4

0

0

0

0

0
4

0
4

0
4

0

0

0

0

0
4

0
4

0
4

1.3896

1.3896

1.3896

1.3896

0
4

0
4

0
4

0

0

0

0

0
4

0
4

0
4

0

0

0

0

0
4

0
4

0
4

0

0

0

0

0
4

0
4

0
4

0

0

0

0

0
4

0
4

0
4

0

0

0

0

0
4

0
4

0
4

0

0

0

0

0
4

0
4

0
4

0

0

0

0

0
4

0
4

0
4

0

0

0

0

0
4

0
4

0
4

0

0

0

0

0

0

0

0
4

0

0

0

0
4

0
4

0
4

0.516483

0.516483

0.516483

0.516483

0
4

0
4

0
4

0.0877713

0.0877713

0.0877713

0.0877713

0
4

0
4

0
4

0

0

0

0

0
4

0
4

0
4

0

0

0

0

0
4

0
4

0
4

0

0

0

0

0
4

0
4

0
4

0

0

0

0

0
4

0
4

0
4

0

0

0

0

0
4

0
4

0
4

0

0

0

0

0
4

0
4

0
4

0

0

0

0

0
4

0
4

0
4

0

0

0

0

0
4

0
4

0
4

0

0

0

0

0

0

0
4

0
4

0
4

0

0

0

0

0
4

0
4

0
4

0

0

0

0

0
4

0
4

0
4

0

0

0

0

0
4

0
4

0
4

0

0

0

0

0
4

0
4

0
4

0

0

0

0

0
4

0
4

0
4

0

0

0

0

0
4

0
4

0
4

0

0

0

0

0
4

0
4

0
4

0

0

0

0

0
4

0
4

0
4

0

0

0

0

0
4

0
4

0
4

0

0

0

0

0
4

0
4

0
4

0

0

0

0

0

0

0

0
4

0
4

0
4

0

0

0

0

0
4

0
4

0
4

0

0

0

0

0
4

0
4

0
4

0
7

0
7

0
7

0

0

0

0
4

0
4

0
4

0

0

0

0

0

0

0
4

0

0

0
4

0
4

0
4

0

0

0

0

0
4

0

0

0

0

0
4

0

0

0
4

0
4

0
4

0
7

0
7

0
7

0
7

0

0

0

0
4

0
4

0

0

0

0
4

0
4

0
4

0

0

0

0

0

0

0
4

0
4

0
4

0

0

0

0

0

0
4

0
4

0
4

0

0

0

0

0
4

0
4

0
4

0

0

0

0

0
4

0
4

0
4

0

0

0

0

0
4

0

0

0
4

0
4

0

0

0

0
4

0

0

0
4

0
4

0
4

0

0

0

0

0

0
4

0
4

0
4

0

0

0

0

0

0
4

0
4

0
4

0

0

0

0

0

0
4

0
4

0
4

0

0

0

0

0
4

0
4

0
4

0

0

0

0

0
4

0
4

0
4

9.74862
2

9.74862
2

0
2

0
2

0

0

0

0

0
4

9.74862
2

3.0989

0

0

6.64972

0

0

0
4

0

0

0
4

0

0

0
4

0
4

0
4

0

0

0

0

0

0

0
4

0
4

0
4

0

0

0

0

0
4

0
4

0
4

0

0

0

0

0
4

0

0

0
4

0

0

0
4

0
4

0
4

0

0

0

0

0

0
4

0
4

0
4

0

0

0

0

0

0

0

0
4

0
4

0
4

0

0

0

0

0
4

0
4

0
4

0

0

0

0

0

0
4

0

0

0
4

0
4

0

0

0

0
4

0
4

0
4

1.35577

1.35577

1.35577

0.193681

1.16209

0
4

0

0

0
4

0
4

0
4

0

0

0

0

0
4

0
4

0
4

0

0

0

0

0

0

0
4

0
4

0
4

0

0

0

0

0

0

0

0

0

0
4

0
4

0
4

0

0

0

0

0

0
4

0

0

0
4

0
4

0
4

0

0

0

0

0
4

0
4

0
4

0

0

0

0

0
4

0
4

0
4

0

0

0

0

0
4

0

0

0
4

0
4

0
4

0

0

0

0

0

0
4

0

0

0
4

0
4

0
4

0

0

0

0

0

0
4

0
4

0
4

0

0

0

0

0

0
4

0
4

0
4

0

0

0

0

0
4

0
4

0
4

0

0

0

0

0

0
4

0
4

0
4

0.0633849

0.0633849

0.0633849

0.0633849

0

0
4

0

0

0
4

0
4

0
4

0
4

0
4

0
4

0

0

0

0

0

0

0
4

0

0

0
4

0
4

0
4

0

0

0

0

0
4

0
4

0

0

0

0
4

0
4

0
4

0

0

0

0

0
4

0

0

0
4

0

0

0
4

0
4

0
4

0

0

0

0

0

0
4

0
4

0
4

0

0

0

0

0

0
4

0
4

0
4

0

0

0

0

0

0
4

0
4

0
4

0.175543

0.175543

0

0

0
4

0.175543

0.175543

0
4

0
4

0
4

0

0

0

0

0
4

0

0

0
4

0
4

0
4

0

0

0

0

0
4

0
4

0
4

0

0

0

0

0
4

0

0

0
4

0
4

0
4

0

0

0

0

0
4

0

0

0
4

0
4

0
4

0
7

0
7

0
7

0

0

0

0

0

0

0

0

0
4

0

0

0

0
4

0
4

0
4

0

0

0

0

0
4

0
4

0
4

0

0

0

0

0
4

0
4

0
4

0

0

0

0

0

0
4

0
4

0
4

0

0

0

0

0
4

0

0

0
4

0
4

0
4

0

0

0

0

0
4

0
4

0
4

0

0

0

0

0
4

0

0

0
4

0
4

0
4

0

0

0

0

0
4

0

0

0
4

0
4

0
4

0

0

0

0

0

0
4

0
4

0
4

0.184804

0.184804

0

0

0
4

0.184804

0.184804

0
4

0
4

0
4

0

0

0

0

0

0
4

0
4

0
4

0.378486

0.378486

0.378486

0.184804

0

0

0

0

0.193681

0
4

0

0

0
4

0
4

0

0

0

0

0
4

0
4

0
4

0

0

0

0

0
4

0
4

0
4

0

0

0

0

0
4

0

0

0
4

0
4

0
4

0

0

0

0

0
4

0
4

0
4

0

0

0

0

0

0
4

0
4

0
4

0

0

0

0

0
4

0
4

0
4

0

0

0

0

0
4

0

0

0
4

0
4

0
4

0

0

0

0

0
4

0
4

0
4

0

0

0

0

0
4

0

0

0
4

0
4

0
4

0

0

0

0

0
4

0

0

0
4

0
4

0
4

0

0

0

0

0

0
4

0
4

0
4

0
5

0
5

0
5

0
5

0

0

0
4

0

0

0

0

0

0
4

0
4

0
4

0.710164

0.710164

0.710164

0

0.710164

0
4

0
4

0
4

0

0

0

0

0

0
4

0
4

0
4

0

0

0

0

0

0
4

0
4

0
4

0

0

0

0

0
4

0
4

0
4

0

0

0

0

0
4

0

0

0
4

0
4

0
4

0

0

0

0

0
4

0
4

0
4

0

0

0

0

0
4

0
4

0
4

0

0

0

0

0
4

0
4

0
4

0

0

0

0

0
4

0

0

0
4

0
4

0
4

0

0

0

0

0
4

0
4

0
4

0
7

0
7

0
7

0
7

0

0
4

0

0

0

0
4

0

0

0
4

0
4

0

0

0

0
4

0
4

0
4

0

0

0

0

0

0
4

0
4

0
4

0

0

0

0

0
4

0
4

0
4

0

0

0

0

0
4

0
4

0
4

0

0

0

0

0
4

0
4

0
4

0

0

0

0

0
4

0
4

0
4

0

0

0

0

0
4

0
4

0
4

0.129121

0.129121

0.129121

0.129121

0
4

0
4

0
4

0

0

0

0

0
4

0
4

0
4

0

0

0

0

0
4

0
4

0
4

0

0

0

0

0
4

0
4

0
4

0

0

0

0

0

0

0

0

0

0

0

0

0

0

0

0

0

0

0

0

0

0

0

0

0

0
4

0

0

0

0

0

0

0

0

0

0

0

0

0

0

0

0

0

0

0

0

0

0

0

0

0

0

0

0

0

0

0

0

0

0

0

0

0

0

0

0

0

0

0

0

0

0

0

0

0

0

0

0

0

0

0

0

0

0

0

0

0

0

0

0

0
4

0

0

0

0

0

0
4

0

0

0
4

0

0

0
4

0

0

0
4

0

0

0
4

0

0

0
4

0

0

0

0

0
4

0

0

0

0
4

0

0

0

0

0
4

0

0

0

0

0
4

0

0

0

0
4

0

0

0
4

0

0

0
4

0

0

0
4

0
4

0

0

0

0

0

0

0
4

0
4

0

0

0

0

0

0
4

0

0

0
4

0
4

0

0

0

0

0
4

0
4

0

0

0

0
4

0
4

0

0

0

0
4

0
4

0
4

0

0

0

0

0
4

0
4

0
4

1.90764071206218e-13

0
4

9.2967
3

9.2967
3

6.13324
3

0

0

0

0

0

0

0

0

0

0

0

0

0
4

0
3

0
3

0

0

0
4

0

0

0
4

0

0

0

0

0

0

0

0

0

0
4

0

0

0

0

0

0
4

0

0

0
4

6.13324

6.13324

0
4

0

0

0
4

0

0

0
4

0

0

0

0
4

0

0

0
4

0

0

0

0
4

0

0

0

0
4

0

0

0
4

0

0

0

0
4

0

0

0
4

0

0

0
4

0
4

0

0

0

0

0

0

0

0

0
4

0
4

0

0

0

0

0
4

0
4

3.16346

3.16346

3.16346

0
4

0
4

0

0

0

0
4

0
4

8.88178419700125e-16
3

0
4

0

0

0

0

0

0

0
4

0
4

0
4

0

0

0

0

0
4

0
4

0
4

0

0

0

0

0
4

0
4

0
4

0

0

0

0

0
4

0
4

0
4

0

0

0

0

0
4

0
4

0
4

0

0

0

0

0
4

0
4

0
4

0

0

0

0

0
4

0
4

0
4

0

0

0

0

0
4

0
4

0
4

0

0

0

0

0
4

0
4

0
4

0

0

0

0

0
4

0
4

0
4

0

0

0

0

0

0
4

0
4

0
4

0

0

0

0

0

0
4

0
4

0
4

0

0

0

0

0
4

0
4

0
4

0

0

0

0

0
4

0
4

0
4

0

0

0

0

0
4

0
4

0
4

0

0

0

0

0
4

0
4

0
4

0

0

0

0

0
4

0
4

0
4

0
4

18.2034

0.155618
4

0

0

0

0

0

0

0

0

0

0

0

0

0

0

0

0

0

0

0

0

0

0

0

0

0

0

0

0

0

0

0

0

0

0

0

0
4

0

0

0

0

0
4

0

0

0

0
4

0

0

0

0
4

0
4

0
4

0

0

0

0

0

0
4

0

0

0

0

0
4

0

0

0
4

0

0

0
4

0
4

0

0

0

0

0

0
4

0

0

0

0
4

0

0

0
4

0
4

0
6

0
6

0
6

0
4

0
4

0

0

0

0
4

0

0

0
4

0
4

0

0

0

0

0
4

0

0

0

0
4

0

0

0
4

0
4

0

0

0

0

0
4

0
4

0

0

0

0

0

0
4

0
4

0

0

0

0

0
4

0

0

0
4

0
4

0

0

0

0
4

0
4

0

0

0

0
4

0

0

0
4

0
4

0
4

0
4

0

0

0

0

0

0

0

0

0

0

0

0

0

0

0

0

0

0
4

0

0

0

0

0

0

0
4

0

0

0

0

0

0

0

0
4

0
4

0

0

0

0
4

0
4

0.155618

0.155618

0.155618

0
4

0
4

0

0

0

0
4

0
4

0

0

0

0
4

0
4

0

0

0

0
4

0
4

0

0

0

0
4

0
4

0

0

0

0
4

0
4

0
4

0
4

0

0

0

0

0

0

0

0

0

0

0
4

0

0

0
4

0

0

0

0
4

0

0

0
4

0
4

0
4

0
4

0

0

0

0

0
4

0

0

0

0

0
4

0

0

0
4

0

0

0
4

0
4

0
4

0
4

0

0
4

0

0

0

0

0

0

0

0
4

0
4

0

0

0

0

0

0

0

0

0
4

0

0

0

0

0
4

0

0

0

0

0

0
4

0

0

0
4

0

0

0
4

0
4

0
6

0
6

0
6

0

0

0
4

0
4

0

0

0

0

0

0

0

0

0

0

0
4

0

0

0

0
4

0

0

0

0

0
4

0

0

0
4

0
4

0

0

0

0

0

0

0

0
4

0

0

0
4

0
4

0
4

0
5

0
5

0

0

0
4

0
5

0
5

0

0

0

0

0

0

0

0

0

0

0

0
4

0
4

0
4

4.26685
7

0
6

0
6

0

0

0
4

0

0

0
4

0
4

3.82686

0

0

0
1

0

0

0

0

0

0

0

0

0

0

0

0

0

0

0

0

0

0

0

0

0

0

0

0

0

0

0

0

0

0

0

0

0

0

0

0

0

0

0

0

0

0

0

0

0

0

0

0

0

0

0

0

0

0

0

0

0

0

0

0

0

0

0

0

0

0

0
4

3.82686

0

0.0484412

0.847722

0

1.01727

0

1.81655

0.0484412

0.0484412

0

0
4

0

0

0

0

0

0

0

0

0

0

0

0

0

0

0

0

0

0

0

0

0

0

0

0

0

0

0

0

0

0

0

0

0

0

0

0

0

0

0

0

0

0

0

0

0

0

0

0

0

0

0

0

0

0

0

0

0

0

0

0

0

0

0

0

0

0

0

0

0

0

0

0

0

0

0
6

0

0

0

0

0

0

0

0

0

0

0
7

0

0

0

0

0

0

0

0

0

0

0
8

0

0

0

0

0

0

0

0

0

0

0

0

0

0

0

0

0

0

0

0

0

0

0

0

0

0

0

0

0

0

0

0

0

0

0

0

0

0

0

0

0

0

0

0
7

0

0

0

0

0

0

0

0

0

0

0

0

0

0

0

0

0

0

0

0

0

0
4

0
6

0

0

0

0

0

0

0
4

0

0

0
4

0
7

0

0

0
4

0

0

0

0

0
4

0

0

0

0

0
4

0

0

0

0

0
4

0

0

0

0
4

0

0

0
4

0

0

0

0
4

0

0

0

0
4

0

0

0

0

0

0

0

0

0

0

0

0

0
4

0

0

0
4

0

0

0

0
4

0

0

0
4

0

0

0

0
4

0

0

0
4

0

0

0
4

0

0

0
4

0

0

0
4

0

0

0
4

0

0

0
4

0

0

0

0

0

0

0

0

0
4

0

0

0
4

0

0

0
4

0

0

0
4

0

0

0
4

0

0

0
4

0

0

0
4

0

0

0
4

0

0

0
4

0

0

0
4

0

0

0
4

0

0
7

0

0

0

0
7

0

0

0

0

0

0

0
4

0

0

0
4

0

0

0
4

0

0

0
4

0

0

0
4

0

0

0
4

0

0

0
4

0

0

0
4

0

0

0
4

0

0

0
4

0

0

0
4

0

0

0

0

0

0

0

0
6

0

0

0

0

0

0

0

0
4

0

0

0
4

0

0

0
4

0

0

0
4

0

0

0

0

0

0

0

0

0

0

0
4

0

0
7

0

0

0

0

0

0
4

0

0

0

0

0

0

0
4

0

0

0

0

0

0

0

0

0
4

0
4

0.322802
7

0
6

0
6

0
7

0

0

0

0

0

0

0

0

0

0

0
6

0

0

0

0

0

0

0

0

0

0

0

0

0

0

0

0

0

0

0

0

0

0

0

0

0

0

0

0

0

0

0

0

0

0

0

0

0

0

0

0

0

0

0

0
5

0

0

0

0

0

0

0

0

0

0

0
6

0

0

0

0

0

0

0

0

0

0
6

0
6

0
4

0
7

0
7

0

0

0

0
4

0.193681
7

0.193681
7

0
7

0

0

0

0

0

0

0

0

0

0

0

0

0

0

0

0

0

0

0

0

0

0

0

0

0

0

0

0

0

0

0

0

0

0

0

0

0

0

0

0

0

0

0
4

0
7

0

0

0

0

0

0
4

0

0

0

0

0

0
4

0
7

0
7

0

0

0

0

0

0

0

0

0

0

0

0
7

0

0

0

0

0

0

0

0

0

0

0
6

0

0

0

0

0

0

0

0

0

0

0

0

0
4

0
6

0
6

0

0

0

0
4

0
6

0
6

0

0

0

0

0

0
4

0
6

0
7

0
6

0
7

0

0

0

0

0

0

0

0

0

0

0

0

0

0

0

0

0

0

0

0

0
7

0

0

0

0

0

0

0

0

0
6

0
7

0
6

0
7

0
7

0
6

0
4

0
7

0
7

0

0

0

0

0

0

0

0
4

0

0

0

0

0

0

0

0

0

0

0

0

0
4

0
6

0
6

0

0
4

0
6

0
6

0

0

0

0

0
4

0

0

0

0
4

0

0

0
4

0

0

0

0
4

0

0

0
4

0

0

0
4

0

0

0
4

0

0

0
4

0

0

0
4

0

0

0
4

0

0

0
4

0
7

0
7

0

0

0

0

0
4

0

0

0
4

0

0

0
4

0

0

0
4

0

0

0
4

0

0

0
4

0

0

0
4

0

0

0
4

0

0

0
4

0

0

0
4

0

0

0
4

0.129121
7

0.129121
7

0

0
4

0

0

0
4

0

0

0
4

0

0

0
4

0

0

0
4

0

0

0
4

0

0

0
4

0

0

0
4

0

0

0
4

0

0

0
4

0

0

0
4

0

0

0

0

0

0
4

0

0

0
4

0

0

0
4

0

0

0
4

0

0

0
4

0

0

0
4

0
7

0
7

0
4

0

0

0
4

0

0

0
4

0

0

0

0
4

0

0

0
4

2.77555756156289e-17
7

0
4

0
7

0
6

0

0
6

0

0

0

0
4

0

0

0

0
4

0
4

0
7

0

0

0
4

0
8

0
8

0

0
4

0

0

0

0
4

0

0

0
4

0

0

0
4

0

0

0
4

0

0

0
4

0

0

0
4

0

0

0
4

0

0

0
4

0
4

0

0

0

0

0
4

0

0

0

0

0

0

0
4

0

0

0
4

0

0

0
4

0

0

0
4

0

0

0
4

0
5

0
5

0

0
4

0
7

0

0

0
4

0

0

0
4

0

0

0

0
4

0

0

0

0
4

0

0

0

0
4

0

0

0
4

0

0

0
4

0
4

0
7

0
7

0

0

0

0
6

0

0
4

0
4

0

0

0

0
4

0
4

0

0

0

0
4

0
4

0

0

0

0
4

0
4

0

0

0

0
4

0
4

0.117195

0.117195

0.0334842

0.0502263

0.0334842

0
4

0
4

0

0

0

0
4

0
4

0

0

0

0

0
4

0
4

0

0

0

0
4

0
4

0

0

0

0
4

0
4

0

0

0

0
4

0
4

0

0

0

0
4

0
4

0

0

0

0
4

0
4

0
4

0

0

0
7

0

0

0

0

0

0

0

0

0

0

0

0
6

0

0

0

0

0

0

0

0

0

0

0

0
4

0

0

0
4

0

0
7

0

0

0

0
4

0

0

0

0
4

0

0

0
4

0

0

0
4

0

0

0
4

0

0

0
4

0
4

0

0

0

0

0

0

0

0

0
4

0
4

0

0

0

0
4

0
4

0

0

0

0
4

0
4

0

0

0

0
4

0
4

0

0

0

0

0
4

0
4

0

0

0

0
4

0

0

0
4

0
4

0

0

0

0
4

0
4

0

0

0

0
4

0

0

0
4

0
4

0

0

0

0
4

0
4

0

0

0

0
4

0
4

0

0

0

0
4

0
4

0

0

0

0
4

0
4

0
4

3.90582

3.51085

3.3792

0
6

0

0

0

0

0

0

1.31657

0

0

0

0
6

0

0

0

0

2.06263

0

0

0

0

0

0

0

0

0

0
4

0

0

0
4

0

0

0
4

0

0

0
4

0.131657

0.131657

0
4

0
4

0

0

0

0

0
4

0
4

0

0

0

0
4

0
4

0

0

0

0
4

0
4

0.394971

0.394971

0.394971

0
4

0
4

0

0

0

0
4

0
4

0

0

0

0
4

0
4

0

0

0

0
4

0
4

0

0

0

0
4

0
4

0

0

0

0
4

0
4

0
4

5.69159

0

0

0

0

0
4

0
4

5.11055

0

0

0
4

5.11055
1

0

0

5.11055

0

0
4

0

0

0

0

0
4

0

0

0
4

0
4

0.581044

0

0

0

0
4

0.581044

0
1

0

0.581044

0
4

0
4

0
4

0
5

0
5

0

0

0
7

0

0

0

0

0

0

0

0

0

0

0

0

0

0

0
4

0
6

0
6

0
7

0
6

0

0

0

0

0

0
4

0

0

0
4

0

0

0
4

0

0

0
4

0

0

0
4

0

0

0
4

0

0

0
4

0
6

0
6

0

0
4

0

0

0

0

0

0

0
4

0

0

0

0

0
4

0

0

0

0

0
4

0

0

0

0
4

0

0

0
4

0

0

0
4

0

0

0
4

0
4

0

0

0

0

0
4

0
4

0

0

0

0

0
4

0
4

0

0

0

0
4

0
4

0

0

0

0
4

0
4

0

0

0

0
4

0
4

0

0

0

0
4

0
4

0

0

0

0
4

0
4

0
4

0

0

0

0

0

0

0

0

0

0

0

0
4

0

0

0

0

0

0
4

0

0

0

0

0

0
4

0

0

0
4

0

0

0
4

0
4

0
4

0
3

0
3

0
3

0
3

0

0
4

0
4

0
4

0
6

0
6

0
6

0
6

0

0
4

0

0

0
4

0
4

0
4

0

0

0

0

0

0

0

0
4

0

0

0

0
4

0
4

0
4

0

0

0

0

0

0

0
4

0
4

0
4

0.138508
4

0.138508
4

0.138508
4

0.138508
4

0

0

0
4

0

0

0
4

0
4

0
4

0

0

0

0

0

0

0
4

0
4

0
4

0

0

0

0

0

0
4

0
4

0
4

0

0

0

0

0

0

0

0
4

0
4

0
4

0

0

0

0

0

0
4

0
4

0
4

0

0

0

0

0
4

0
4

0
4

0
6

0
6

0
6

0

0

0

0

0

0

0

0
4

0
4

0
4

0
6

0
6

0

0

0
4

0

0

0
4

0
4

0
4

0.581044

0.581044

0.581044

0.581044

0

0
4

0
4

0
4

0

0

0

0

0
4

0
4

0
4

0

0

0

0

0

0
4

0

0

0
4

0
4

0
4

0

0

0

0

0

0
4

0
4

0
4

0

0

0

0

0

0

0
4

0
4

0
4

0

0

0

0

0

0
4

0
4

0
4

0

0

0

0

0
4

0
4

0
4

0

0

0

0

0
4

0

0

0
4

0
4

0
4

0

0

0

0

0

0

0
4

0
4

0
4

0
4

0
4

0
4

0

0

0

0

0

0

0

0
4

0
4

0

0

0

0
4

0
4

0
4

0

0

0

0

0

0
4

0
4

0
4

0

0

0

0

0

0
4

0

0

0
4

0
4

0
4

0

0

0

0

0
4

0

0

0
4

0
4

0
4

0

0

0

0

0
4

0
4

0
4

0

0

0

0

0

0
4

0
4

0
4

0

0

0

0

0
4

0
4

0
4

0.277017

0.277017

0.277017

0.277017

0

0
4

0

0

0
4

0
4

0
4

0

0

0

0

0
4

0
4

0
4

0.322802

0.322802

0.322802

0.322802

0
4

0
4

0
4

0

0

0

0

0
4

0
4

0
4

0
4

0
4

0
4

0

0

0

0

0

0

0

0

0
4

0

0

0

0

0

0
4

0

0

0
4

0
4

0
4

0

0

0

0

0
4

0
4

0
4

2.38874

2.38874

2.38874

2.38874

0
4

0
4

0
4

0

0

0

0

0
4

0
4

0
4

0

0

0

0

0

0
4

0
4

0
4

0

0

0

0

0
4

0
4

0
4

0.322802

0.322802

0.322802

0.322802

0
4

0

0

0
4

0
4

0
4

0.0694547

0.0694547

0.0694547

0.0694547

0
4

0
4

0
4

0

0

0

0

0
4

0
4

0
4

0

0

0

0

0
4

0
4

0
4

0.0347274

0.0347274

0.0347274

0.0347274

0
4

0
4

0
4

0
4

0
4

0
4

0
4

0

0

0

0

0
4

0

0

0
4

0

0

0

0
4

0

0

0
4

0
4

0
4

0

0

0

0

0
4

0
4

0
4

0

0

0

0

0
4

0
4

0
4

0

0

0

0

0
4

0
4

0
4

0

0

0

0

0
4

0
4

0
4

0

0

0

0

0
4

0
4

0
4

0

0

0

0

0
4

0
4

0
4

0

0

0

0

0
4

0
4

0
4

0

0

0

0

0
4

0
4

0
4

0

0

0

0

0
4

0
4

0
4

0

0

0

0

0
4

0
4

0
4

0

0

0

0

0

0

0

0

0
4

0

0

0

0

0

0
4

0

0

0
4

0

0

0

0
4

0

0

0
4

0
4

0
4

0

0

0

0

0
4

0
4

0
4

0

0

0

0

0
4

0
4

0
4

0

0

0

0

0
4

0
4

0
4

0

0

0

0

0
4

0
4

0
4

0

0

0

0

0
4

0
4

0
4

0

0

0

0

0
4

0
4

0
4

0

0

0

0

0
4

0
4

0
4

0

0

0

0

0
4

0
4

0
4

0

0

0

0

0
4

0
4

0
4

0

0

0

0

0
4

0
4

0
4

0
7

0
7

0
7

0
7

0

0

0

0

0

0

0
4

0
4

0
4

0

0

0

0

0
4

0
4

0
4

0

0

0

0

0
4

0
4

0
4

0

0

0

0

0
4

0
4

0
4

0

0

0

0

0
4

0
4

0
4

0

0

0

0

0
4

0
4

0
4

0

0

0

0

0
4

0
4

0
4

0

0

0

0

0
4

0
4

0
4

0

0

0

0

0
4

0
4

0
4

0

0

0

0

0
4

0
4

0
4

0

0

0

0

0
4

0
4

0
4

0
7

0
7

0
7

0
7

0

0

0
4

0
4

0
4

0

0

0

0

0
4

0
4

0
4

0.0484412

0.0484412

0.0484412

0.0484412

0
4

0
4

0
4

0

0

0

0

0
4

0
4

0
4

0

0

0

0

0
4

0
4

0
4

0

0

0

0

0
4

0
4

0
4

0

0

0

0

0
4

0
4

0
4

0

0

0

0

0
4

0
4

0
4

0

0

0

0

0
4

0
4

0
4

0

0

0

0

0
4

0
4

0
4

0

0

0

0

0
4

0
4

0
4

0

0

0

0

0

0

0

0

0
4

0
4

0
4

0

0

0

0

0
4

0
4

0
4

0

0

0

0

0
4

0
4

0
4

0

0

0

0

0
4

0
4

0
4

0

0

0

0

0
4

0
4

0
4

0

0

0

0
8

0

0
4

0

0

0
4

0
4

0

0

0

0
4

0
4

0

0

0

0
4

0
4

0
4

8.81239525796218e-16

0
4

4734.46

0
7

0
7

0
7

0
7

0

0
4

0
7

0
7

0
7

0
7

0

0

0

0

0

0

0

0

0

0

0
7

0

0

0

0

0

0

0

0

0

0

0
7

0

0

0

0

0

0

0

0

0

0

0
7

0

0

0

0

0

0

0

0

0

0

0
7

0

0

0

0

0

0

0

0

0

0

0
6

0

0

0

0

0

0

0

0

0

0

0
6

0

0

0

0

0

0

0

0

0

0

0
7

0

0

0

0

0

0

0

0

0

0

0
7

0

0

0

0

0

0

0

0

0

0

0
7

0

0

0

0

0

0

0

0

0

0

0
7

0
6

0

0

0

0

0

0

0

0

0

0

0
7

0

0

0

0

0

0

0

0

0

0

0
7

0

0

0

0

0

0

0

0

0

0

0
7

0

0

0

0

0

0

0

0

0

0

0
7

0

0

0

0

0

0

0

0

0

0

0
7

0

0

0

0

0

0

0

0

0

0

0
7

0

0

0

0

0

0

0

0

0

0

0
6

0

0

0

0

0

0

0

0

0

0

0
7

0

0
7

0
7

0
6

0
6

0
7

0
7

0

0
7

0
8

0
7

0
7

0
7

0
7

0

0

0
7

0

0
7

0

0

0

0

0
8

0
6

0

0

0

0

0

0

0

0

0

0

0

0

0

0

0

0

0

0

0

0

0

0
6

0

0

0

0

0

0

0

0

0

0

0
6

0

0

0

0

0

0

0

0

0

0

0
7

0

0

0

0

0

0

0

0

0

0

0
4

0
7

0

0
7

0
7

0
7

0
7

0
7

0

0

0

0

0

0

0

0
4

0
7

0
7

0

0

0
4

0
7

0
7

0

0

0

0

0

0

0

0

0

0

0

0

0

0
4

0

0

0

0

0
4

0
7

0
7

0
7

0

0

0

0

0

0

0

0

0

0

0

0

0

0
4

0
7

0
7

0

0

0
4

0
8

0
8

0

0

0
4

0

0

0
4

0

0

0
4

0

0

0
4

0
4

0

0

0

0
4

0
4

0

0

0

0

0
4

0
4

0

0

0

0
4

0
4

0

0

0

0
4

0
4

0

0

0

0
4

0
4

0

0

0

0
4

0
4

0

0

0

0
4

0
4

0

0

0

0
4

0
4

0
4

3675.07

4.2245

0

0

0

0

0

0

0

0

0

0

0

0
4

0

0

0
4

0

0

0
4

0

0

0
4

0

0

0
4

0

0

0
4

2.35464

2.35464

0
4

0

0

0
4

0

0

0
4

0

0

0
4

0

0

0
4

0

0

0

0

0

0

0

0

0
4

0

0

0
4

0

0

0
4

0

0

0
4

0

0

0

0

0

0

0

0
4

0

0

0

0

0
4

0

0

0

0
4

0

0

0

0

0

0
4

1.86986

1.86986

0
4

0

0

0

0
4

0

0

0
4

4.44089209850063e-16

0
4

0

0

0

0
4

0
4

0.516483

0
3

0

0

0

0

0

0

0
4

0
3

0

0

0

0

0

0

0

0

0

0

0

0

0

0

0

0

0

0

0

0

0

0

0

0

0

0

0

0

0

0

0
4

0

0

0
4

0.258242
3

0.129121
3

0

0

0

0

0

0

0

0

0.129121

0

0
4

0

0
5

0

0

0

0

0

0

0

0

0

0

0

0

0

0

0

0

0

0
5

0

0

0

0

0

0
4

0

0

0

0

0

0

0
4

0

0

0
4

0

0

0

0

0

0
4

0

0

0

0

0

0

0

0
4

0

0

0

0

0

0
4

0

0

0

0

0

0
4

0

0

0

0

0

0
4

0

0

0

0
4

0

0

0

0

0
4

0

0

0

0

0
4

0
5

0
5

0

0

0

0

0

0

0

0

0

0

0
4

0

0

0

0

0
4

0

0

0
4

0

0

0

0

0
4

0

0

0

0

0
4

0

0

0

0

0
4

0

0

0

0
4

0

0

0

0

0
4

0

0

0

0
4

0

0

0

0

0
4

0

0

0
4

0
5

0
5

0

0

0

0

0
4

0

0

0

0
4

0

0

0
4

0

0

0
4

0

0

0

0
4

0

0

0
4

0

0

0
4

0

0

0
4

0

0

0
4

0

0

0
4

0

0

0
4

0
3

0

0

0

0

0

0

0

0
4

0

0

0

0
4

0

0

0

0
4

0

0

0

0
4

0

0

0

0
4

0

0

0

0
4

0

0

0
4

0

0

0

0
4

0

0

0
4

0

0

0

0
4

0

0

0
4

0
5

0

0

0

0

0

0

0

0
4

0

0

0

0
4

0

0

0

0
4

0

0

0
4

0.129121

0.129121

0
4

0

0

0
4

0

0

0
4

0

0

0
4

0.129121

0.129121

0
4

0

0

0
4

0

0

0
4

0

0

0

0

0

0

0

0

0
4

0

0

0
4

0

0

0
4

0

0

0
4

0

0

0
4

0

0

0
4

0

0

0
4

0

0

0
4

0

0

0
4

0

0

0
4

0

0

0
4

0
5

0
5

0

0
4

0

0

0
4

0

0

0
4

0

0

0
4

0

0

0
4

0

0

0
4

0

0

0
4

0

0

0
4

0

0

0
4

0

0

0
4

0

0

0
4

0

0

0

0

0

0

0

0
4

0

0

0
4

0

0

0
4

0

0

0
4

0

0

0
4

0

0

0
4

0

0

0
4

0

0

0
4

0

0

0
4

0

0

0
4

0

0

0
4

0

0

0

0

0

0

0
4

0

0

0
4

0
4

529.174
3

30.5785

0.0919245

1.46061
3

0.184804

0

0

0

0.0484412

0

2.09503

0

0.287275

0

0

0

0.238596

0

0

0.0735396
5

0

0

0.208709

0

0

0

0

0

0.195025

0

0

0

0

0.443531

0.0877713

1.18491

0

0

0

0

0

5.82531
3

0

0.0877713

0.394971

0

0.219428

0

0

0

0

0

0

0

0

0.0739218

0

0

0

0

0

0

0

13.5136
3

0.322802

0.0726619

0

0

0

0

0

0

0

0

1.02955
6

0

0

0

0

0

0

0

0

0

0

0

0

0

0

0

0

0

1.66765

0

0.38753

0

0

0

0

0

0

0.272331

0

0

0

0.110883

0

7.61890550649014e-15

0
4

32.4138

0
6

0

0

0

0

0

0

0

0

0

0

0

0

0

0

0

0

0

0

0

3.15126

0

0

0

0

0

0

0

0

0

0

0

0

0
7

0

0

0

0

0.077809

0.116713

0

0

0

0

0

0

0

0

0

0.129121

0

0

0

0

0

0
6

0

0

0

0

0

0

0

0

0

0

0
7

0

0

0

0

0

0

0

0

0

0

0

0

0

0

0

0.129121

0.484779

0

0

0

0

28.325
3

0

0
4

6.25512

0

5.9781

0

0

0

0

0

0

0

0

0

0

0

0

0

0

0

0.277017

0

0

0

0

0

0

0

3.33066907387547e-16

0
4

455.264
3

17.5213
4

0.346271
4

0

0

0

0

0

0

0.623287

0

0

0

0

0
4

0

0

0

0

0

0

0

0

0

0

0
4

0

0

0

0

0

0

0

0

0

0

0

0

0

0

0.155618

0

0

0

0

0

0

0
4

0

0.155618

0

0

0

0

0

0

0

0

0

0

0

0

0

0

0

0

0

0

0

0

0

0

0

0

0

0

0

0

0

0

14.5892

0

0

0

0

0

0

0

0

0

0

0

0

0

0

0

0

0

0.116713

0

0

1.47837

101.774

0

0

0

6.78691

0

0

0

0

0

0

0

0

0

0

0

0

0

0

0.077809

0

0

0

2.5624

0

0

0

0

0

0

0.138508

0

0

0.116713

0

0

0

0

0

0

0

0

0

0

0

0

0

0

0.116713

0

0

0

0

0

0

0

0

0

0

0

0

0

0

0

0

0

0

1.31583

0

0

0

0

0

0

0

0

0

0

0

0

0

0

0

0

0

0

0

0

0

0

0

0

0

0

0

0

0

0

0.077809

0

0

0

0

0

0

0

0

0

0

0

0

0
4

0

0

0

0

0

0

0

0

0

0

0

0

0

0

0

0

0

0

0

0

0

0

0

0

0

0

0

0

0

0

0

0

0

0.277017
4

0

0

0

0

0.387362

0

0

0

0

0

0

0

0

0

0

0

0

0

0

0.129121

0

0

0

0

0

0

0

0

0

0

0

0

0.346271

0.116713

0

0

0

0

0

0

0

0

0

0

0

0

0

0

0

0

0

0

0

0

0

0

0

0

0

0

0

0

0

0

0

0

0

0

0

0

0

0

0.077809

0

0

0

0

0

0

0

0

0

0

0

0

0

0

0

0
4

0.466854

0

0.129121

0

0

0

0

0

0

0

0
4

0

0

0

0

0

0

0

0

0

0

0

0

0

0

0

0

0

0

0.077809

0.155618

0

0

0

0

0

0

0

1.16713

0

0

0

0

0

0

0

0

0

0

0

0

0

0

0

0

0

0

0

0

0

0

0

0

0.155618

0

0

0

0

0

0

0

0

0

0

0

0

0

0

0

0

0

0.207762

0

0

0

0

0

0

0

0

0

0

0

0

0

0

0

0

0

0

0

0

0

0.116713

0

0

0

0

0

0

0

0

0

0

0

0

0

0

0

0

0.077809

0
4

0

0

0

0

0

0.277017

0

0

0

0

0
4

0

0

0

0

0

0

0

0

0

0

0

0

0

3.67047

0

0

0

0

0

0

0.116713

0

0

0

0

0

0

0.138508

0

0

0

0

0

0

0

0

0

0

0.155618

0

0

0

0

0

0

0

0

0

0

0

0

0

0.077809

0

0

0

0

0

0

0

0

0

0

0

0

0

0

0

0

0

0

0

0

0

0

0

0

0

0

0

0

0

0

0

0

0

0

73.8407

0

0

0

0

0

0

0.077809

0

0

0

0

0

0

0

0

0

0

0

0

0.155618

0

0

0
7

0

0

0

0

0

0

0

0

0

0

0

0

0

0

0

0

0

0

0

0

0

0

0

0

0

0

0

0

0

0

0

0

3.22907

0

0

0

0

0

0

0

0

0

0

0

0

0

0

0

0.155618

0

0

0

0

0

0

1.42033

0

0

0

0

0

0

0

0

0

0

5.44663

0

0

0

0

0

0

0

0

0
3

0

0

0

0

0

0

0

0

0.138508

0

0

0

0

0.583567

0.138508

0.207762

0

0

0

0

0

0

0

0

0

0

0

0

0

0

0

0

0

22.1887

0

0

0

0

0

0

0

0

0

0

0

0

10.4588

0

0

0

0

0

0

0

0

1.67857
4

0

0

0

0

0

0

0

0

0.207762

0

0
4

0

0.583567

0

0

0

0

0

0

0

0

0.761796
3

2.80112

0

0

0

0

0

0

0

0

0

0

0.35014

0

0

0

0.484779

0

0

1.24657

0

0

0

0

0.233427

0

0.155618

0

0

0

0

0

0

0
4

0

0

0

0

0

0

0

0

1.16209

0

0
4

0

0

0

0

0

0

0

0

0

0

49.309
4

0
4

0

0

0

0

0

0

0

0

0.207762

0

0
4

0

0.077809

0

0

0

0

0.505758

0

0

0

0

0

0

0.466854

0.322802

0

0

0

0

0

5.91348

2.06194

7.15843

0

0

0

0

0

0.207762

0

0

0

0

0

0

0.233427

0

0

0

0

0

0

0

0
4

0

0

0

37.4781

0

0

0

0

0

0

0
4

0

0

0

0

0

0

0

0

0

0

0.138508
4

0

0

0

0

0

0

0

0.346271

0

0

0
4

0

0

0

0

0

0

0

0

0

0

0
3

0

0

0

0

0

0

0

0

0.277017

0

7.20243
4

0

0

0

0

0

0

0

0.389045

0

0

0

0
4

0

0

0

0

0

0

0.389045

0

0

0

0
4

0

0

0

0

0

0

0

0

0

0

0

0.207762

0

0

0

0

0

3.94749

0.77809

0

0

0
4

0

0

0

0

1.16713

0

0

0

0

0

0

0

0

0

2.28539

0

0

0

0

0

0

5.54033
3

0

0

0

4.12388

0

0

0

0

0

0

0
4

0

0

0

0

0

0

0.389045

0

0

0

8.78022

0

0.233427

0

0

0

0

0

0

0

0

0

0

0

0

0.583567

0

0

0

0

0

0

0

0
4

0

0

0

0

0

0

0

0

0

0

0
4

0

0

0

0

0

0

0

0

0

0

0

0

0

0.233427

0

0.077809

0

0

0

0

0

0
4

0

0

0

0

0

0

0

0

0

0

0

0

0

1.80061

0

0

0

0

0

0

0

0

0

0.077809

0

0

0

0

0

0

0

0

0

0

0

0

0

0

0

0

0

0

0

0
4

0

0.138508

0

0

0

0

0

0

0

0

0
7

0

0

0

0

0

0

0

0

0

0

0

0

0

0

0

0

0

0

0.466854

0

0

0.277017
4

0.484779
4

0

0

0

0

0

0

0

0

0

0

0
4

0

0

0

0

0

0

0

0

0

0

0.277017
4

0.427949

0

0

0

0

0

0

0

0

0

0
4

0

0

0

0

0

0

0

0.116713

0

0

0.207762
4

0

0

0

0

0

0

0

0

0

0

0
4

0

0

0

0

0

0

0

0

0

0

0

0

0

0

0

0

0

0

0

0

0

0
4

0

0

0

0

0

0

0

0

0.277017

0

0
4

0

0

0

0

0

0

0

0

0

0

0

0

0.077809

0

0

0

0

0

0

0

0

0

0
4

0

0

0

0

0

0

0

0

0

0

0
4

0

0

0

0

0

0

0

0

0.194522

0

0

0

0

0

0

0

0

0

0

0

0

0

0

0

0

0

0

0

0

0

0.077809

0

4.64003

0

0

0

0

0

0

0

0

0

0

4.90197

0

0

0

0

0

0

0

1.03881

0

0

0

0

0

0

0

0

0

0

0

0

0

0
4

0

0

0.505758

0

0

0

0

0

0

0

0

0

0

0

0

0

0

0

0

0

0

0

0

0

0

0

0

0

0

0

0

0

0

0
4

0

0

0

0

0

0.077809

0

0

0

0

0

0

0

0.077809

0

0

0

0

1.24494

0

0

0

0

0

0

0

0

0

0

0

0

0

0
4

0

0

0

0

0

0

0

0

0

0

0
4

0

0

0

0

0

0

0

0

0

0

0
4

0

0

0

0

0

0

0

0

0

0

9.41489

0

0

0

0

0

0

0

0

0

0

0

0

0

0

0

0

0

0

0

0

0

0.207762
3

0

0

0

0

0

0

0

0

0

0

0
4

0

0

0

0

0

0

0

0

0

0

0

0

0

0

0

0

0

0

0

0

0

0

0
4

0

0.116713

0

0

1.52359

0

0

0

0

0

0.933708

0

0

0.311236

0

0

0

0

0

0

0

0
4

0

0

0

0

0

0

0

0

0

0.207762

0
4

0

0

0

0

0

0

0

0

0

0

0
4

0

0

0

0

0

0

0

0

0

0

0
4

0

0

0

0

0

0

0

0

0

0

0

0

0

0

0

0

0

0

0

0.077809

0

0

0

0

0

0

0

0

0.0633849

0

0

0

0

0

0

0

0

0

0

0

0

0

0

0
4

0

0

0

0

0

0

0

0

0

0

0

0

0

0

0
4

0
4

0
4

0
4

0
4

0

0

0

0
4

0

0

0
4

0
4

0

0

0

0

0

0
4

0

0

0

0

0
4

0

0

0

0

0

0
4

0

0

0

0
4

0

0

0

0

0

0

0

0
4

0

0

0

0

0

0
4

0

0

0
4

0

0

0

0
4

0

0

0

0

0
4

0
3

0
3

0

0

0

0

0

0

0

0

0

0

0

0

0

0

0

0

0
4

0

0

0
4

0

0

0

0

0
4

0

0

0
4

0

0

0
4

0

0

0

0
4

0

0

0

0

0
4

0

0

0

0
4

0

0

0

0

0
4

0

0

0

0
4

0

0

0
4

1.45434
3

1.45434
4

0

0

0

0

0

0

0

0

0

0

0

0

0
4

0

0

0

0
4

0

0

0
4

0

0

0
4

0

0

0

0
4

0

0

0

0
4

0

0

0

0
4

0

0

0
4

0

0

0
4

0.332648

0.332648

0
4

0

0

0

0
4

0
3

0

0

0

0

0

0

0

0

0

0

0

0

0

0

0

0
4

0

0

0
4

0

0

0
4

0

0

0
4

0

0

0

0
4

0

0

0

0
4

0

0

0

0
4

0

0

0

0
4

0

0

0

0
4

0

0

0

0
4

0

0

0
4

1.17732
3

0.692542

0.484779

0

0

0

0

5.55111512312578e-17
3

0
4

0.544663

0.544663

0
4

0

0

0

0
4

0

0

0
4

0

0

0
4

0

0

0
4

0

0

0
4

0

0

0
4

0

0

0
4

0

0

0
4

0

0

0
4

0
4

0
4

0

0

0

0

0

0

0

0

0
4

0

0

0
4

0

0

0
4

0

0

0
4

0

0

0
4

0

0

0
4

0

0

0
4

0

0

0
4

0

0

0
4

0

0

0
4

0

0

0
4

0

0

0

0

0

0
4

0

0

0
4

0.129121

0.129121

0
4

0

0

0
4

0

0

0
4

0

0

0
4

0

0

0
4

0

0

0
4

0.443531

0.443531

0
4

0

0

0
4

0

0

0
4

0.581044

0.581044

0

0

0

0

0

0

0
4

0

0

0
4

0

0

0
4

0

0

0
4

0

0

0
4

0

0

0
4

0

0

0
4

0

0

0
4

0

0

0
4

0

0

0
4

0

0

0
4

0

0

0

0

0

0

0

0

0
4

0

0

0
4

0

0

0
4

0

0

0
4

0

0

0
4

0

0

0
4

0

0

0
4

0

0

0
4

0

0

0
4

0

0

0
4

8.03801469828613e-14
3

0
4

0.193681
4

0
4

0
4

0

0

0

0

0

0

0

0

0

0

0

0

0

0

0

0

0

0

0

0

0

0

0

0

0

0

0

0

0

0

0

0

0

0

0

0

0

0

0

0

0

0

0

0

0
4

0
4

0
4

0

0
4

0.193681

0.193681

0
4

0

0

0
4

0

0

0
4

0
4

17.5947

11.801

0
7

0
7

0.0502263

0

0

0

0

0

0

0

0

0

0
8

0

0

0

0.3181

0

0

0

0

0

0

0
7

0

0

0

0

0

0

0

0

0

0

0

0

0

0

0

0

0.0334842

0

0

0

0

0
7

0

0

0

0

0

0

0

0
7

0

0
8

2.4533
3

0
7

0

0

0

0
7

0
8

0

0

0
7

0

0

0

0
7

0

0

0

0

0
7

0

0

0

0

0

0

0

0

0

0

0

0

0

0

0

0

0
8

0

0

0

0

0

0

0

0

0

0

8.90913
3

0

0

0

0

0

0

0

0

0

0

0
8

0

0

0

0

0

0

0

0

0

0

0
7

0

0

0

0

0

0

0

0.0367698

0

0

0
7

0

0

0

0

0

0

0

0

0

0

0
4

0

0

0

0
4

0
2

0

0

0

0

0

0

0

0

0

0

0
4

1.79004
4

0

0
4

0

0

0

0

0

0

0

0

0

0

0

0

0

0

0

0

0
4

0

0

0

0

0

0

0

0

0

0

0
4

0

0

0

0

0

0

0

0

0

0

0.554033
3

0

0

0

0

0

0

0

0

0

0

0
4

0

0

0

0

0

0

0

0

0

0

0

0

0

0.0950773

0

0

0

0

0

0

0

0

0

0

0

0

0

0

0

0

1.14093

0

0
8

0

0

0

0

0

0

0

0

0

0

0

0

0

0

0

0

0

0

0

0

0

0
4

0.207762
5

0

0

0

0

0

0

0

0

0.207762

0

0

0

0

0

0

0

0

0

0
4

0
6

0

0

0

0

0

0

0

0

0

0

0

0

0

0

0

0

0

0

0

0

0

0

0

0

0

0

0

0

0

0

0

0

0

0

0

0

0

0

0

0

0

0

0

0

0

0

0

0

0

0

0

0

0

0

0

0

0
4

0
5

0
5

0

0

0

0

0

0

0

0

0

0

0

0

0

0

0

0

0

0

0

0

0

0

0

0

0

0

0

0

0

0

0

0

0
5

0

0

0

0

0

0

0

0

0

0

0

0

0

0

0

0
4

0

0

0

0

0

0

0

0

0

0

0

0

0

0

0

0

0

0

0

0

0
4

0

0

0

0

0

0

0

0

0
4

0

0

0
4

0

0

0
4

0

0

0
4

0

0

0
4

0

0

0
4

0

0

0
4

0

0

0
4

0

0

0
4

0

0

0
4

0

0

0
4

0
7

0
7

0

0

0

0

0
4

0

0

0
4

0

0

0
4

0

0

0
4

0

0

0
4

0

0

0
4

0

0

0
4

0

0

0
4

0

0

0
4

0

0

0
4

0

0

0
4

0
4

0

0

0

0
4

0

0

0
4

0

0

0
4

0

0

0
4

0

0

0
4

0

0

0
4

0

0

0
4

0

0

0
4

0

0

0
4

0

0

0
4

0

0

0
4

0

0

0

0

0

0

0

0

0
4

0

0

0
4

0

0

0
4

0

0

0
4

0

0

0
4

0

0

0
4

0

0

0
4

0

0

0
4

0
7

0

0

0

0

0
4

0
4

0

0

0

0

0

0
4

0
4

0

0

0

0

0
4

0

0

0

0
4

0
7

0
7

0
4

0
7

0
7

0
4

0
4

0
4

0
4

0
4

0

0

0

0
4

0

0

0

0
4

0
8

0

0

0
4

0
6

0

0

0

0
4

0

0

0

0

0

0
4

0

0

0
4

0

0

0

0

0
4

0

0

0
4

0

0

0

0

0

0
4

0

0

0

0

0
4

0

0

0

0
4

0
7

0
7

0

0

0
4

0

0

0
4

0

0

0

0

0
4

1.15521

0

1.15521

0
4

0

0

0
4

0

0

0

0
4

0

0

0

0
4

0

0

0
4

0

0

0
4

0

0

0
4

0

0

0

0

0
4

0
7

0

0

0

0

0

0

0

0
4

0

0

0

0

0
4

0

0

0
4

0

0

0

0
4

0

0

0

0
4

0

0

0

0
4

0

0

0

0

0
4

0

0

0

0
4

0

0

0
4

0

0

0

0
4

0

0

0

0
4

0

0

0

0

0

0

0

0

0

0

0

0

0
4

0

0

0

0
4

0

0

0

0
4

0

0

0
4

0

0

0

0
4

0

0

0
4

0

0

0
4

0

0

0

0
4

0.443531

0.332648

0.110883

1.38777878078145e-17

0
4

0

0

0

0
4

0

0

0
4

0
7

0
7

0

0

0

0
4

1.01596

0.0968825

0.919081

0
4

0

0

0
4

0

0

0

0
4

0

0

0
4

0

0

0
4

0

0

0
4

0

0

0

0
4

0

0

0

0
4

0

0

0
4

0

0

0
4

0.138508
3

0.138508

0

0

0
4

0

0

0
4

0

0

0
4

0

0

0
4

0.894803

0.894803

0
4

0

0

0
4

0

0

0
4

0

0

0
4

0

0

0
4

0

0

0
4

0

0

0
4

0

0

0

0

0
4

0

0

0
4

0

0

0
4

0

0

0
4

0

0

0
4

0

0

0
4

0

0

0
4

0

0

0
4

0

0

0
4

0

0

0
4

0

0

0
4

0
7

0
7

0

0
4

0

0

0
4

0

0

0
4

0

0

0
4

0

0

0
4

0

0

0
4

0

0

0
4

0.147844

0.147844

0
4

0

0

0
4

0

0

0
4

0

0

0
4

2.3037127760972e-15

0
4

355.454

0
7

0
7

0

0

0

0

0

0

0

0

0
4

328.538

4.26099
7

0
7

0.835628

0
3

0
6

13.9201

1.25823

0

13.3412

0

0

0.227269

0
3

0

0

0

0

0

2.72091

0

0.409085

0.710164

1.71154

0

0

0

0

0

0.387362

3.61538

0

0

0

0

0
7

0.295011

0

0

0

0

0

0

0

0

0

0
7

0

0

0

0.645604

0

1.87081

0

0

0

0.322802

0
6

0

0

0

0

0

0

0.887355

13.2535

0

0

0

0

0.138508

0.322802

0

0

0

0

0

0

0

8.02427

0

0

0

0

0

0

0

0

0

0

1.38702

0

0

0

0

0

0.0877713

0

0

0

0

0.258242

1.75037

0

0

0

0

0

0

0

0.387362

0

0.623287
3

0
7

0

0

0

0

0

0

0

0

0

0

0
7

0

0

0

0

0.184276

0

0

0

0

0

58.328
2

0

0

0

0

0.70217

0.692542

0

0

0

0

0
3

0

0

0

0

0

0

0

0

0

0

6.39148
3

0

0

0.277017

0

0

0

0

0

0

0

0
7

3.4217

33.7481

0

0

0

0

0

0

0

0

0
7

0

0

0

0

0

0

0

0

0

0

0
3

0

0

0

0

0

0

0

0.3072

0

0

0
7

0

0

0

0

0

0

0

0

0

0

13.5727
2

0

0

0

0

0

0

0

0

0

0

5.35851
7

0

0

0

0

0

0

0

0

0

0

0

0
6

0

0

0

0

0

0

0

0

0

0

77.2165
2

0

0

0

0.0877713

0

0

0

0

0.129121

0

0.260778
3

0

0

0

0.0909077

0

0

0

0

0

0

0
7

0

0

0

0

0

0

0

0

0.129121

0

4.68879
2

0

0

0.0367698

0

0

0

0

0

0.394971

0

6.64972

0

0.263314

0

0

0

0

0

0

0

0

0
5

0

0

0

0

0

0

0

0

0

0.129121

0
5

0

0

0

0

0

0

0

0

0

0

0
3

0

0

0

0

0

0

0

0

0

0

0
4

0
6

0

0

0

0

0

0

0

0.129121

0

0

0.33219
3

0

0

0

0

0

0

0

0

0

0

0

0

2.64698

0

0

0

0

0

0

0

0

0
3

0

0

0.387362

0

0

0

0

0

0

0

0
7

0

0

0

0

0

0

0

0

0

0.129121

0
3

0

0

0

0

0

0

0

0

0

0

2.83942
3

0

0

0

0

0

0

0

0

0

0.0551547

0
7

0

0

0.623287

0

0

0

0

0

0

0

0
3

0

0

0

0

0

0

0

0

0

0

0
7

0

0

0

0

0.129121

0

0

0

0

0

0.258242
7

1.66272
2

0

0

0

0

0

0

0

0

0

0

0

0

0.129121

0

0

0

0

0

0

0

0

0

0.219428

0

0

0

0.193681

0

0

0

0

0

1.35577

0

0.710164

0

0

0

0

0

0

0.351085

0

0.886223

0

0

0

0

0

0

0.0877713

0

0

0

0

0

0

0

0

0

0.258242

0

0

0

0

0
3

0

0

0

0

0.077809

0

0

0

0

0

0

0

0

0

0

0

0

0

0

0

0.129121

0
5

0

0

0

0

0

0

0

0

0

0

0.0909077
3

0

0

0

0

0

0

0

0

0

0

0
7

0
3

0

0

0

0

0

0

0

0

0

0.482742

0.375497
3

0

0

0

0

0

0

0

0

0

0

1.52359
3

0

0

0

0

0

0

0

0

0

0

0
6

0

0

0

0

0

0

0

0

0

0

0
7

0

0

0

0

0

0

0

0.129121

0

0

0
7

0

0

0

0

0

0

0.175543

0

0

0

0
7

0

0

0

0

0

0

0.0367698

0

0

0

0
7

0

0

0

0

0

0

0

0

0

0

0.369224
2

0

0

0

0

0

0

0

0

0

0

0
3

0

0.258242

0

0

0

0

0

0

0

0

15.6143

3.35714
2

0

0

0

0

0

0

0

0

0

0

0
3

0

0

0

0

0

0

0

0

0

0

0
3

0

0

0

0.882475

0.136362

0

0.138508

0

0

0

0

0

0

0

0

0

0

0

0

0

0

0

0

0.129121

0

0

0

0

0

0

0

0

0
6

0

0

0.774725

0

0

0

0

0

0

0

0
3

0

0

0

0

0

0

0

0

0

0

0.544766
2

0

0

0

0

0

0

0

0

0

0

0
7

0

0

0

0

0

0

0

0

0

0

0

0

0

0

0

0.193681

0

0

0

0

0

0
3

0

0

0

0

0

0

0

0

0

0

0

0
7

0

0

0.0551547

0

0

0

0

0

0

0

0

0

0

0

0

0

0

0

0

0

0

0

0

0

0

0

0

0

0

0

0

0
6

0

0
7

2.96574

0
7

0

0

0

0
3

0

0

0

0

0
6

0
7

0

0

1.32338584535319e-13

0
4

0

0

0

0

0

0

0

0

0

0

0

0

0

0

0

0

0
4

16.6952

16.6952

0
7

0
4

0

0

0

0

0

0

0

0

0

0
4

0

0

0

0

0

0

0

0

0

0

0
7

0

0

0

0

0

0

0

0

0

0

0
7

0

0

0

0

0

0

0

0

0

0

0
4

0

0

0

0

0

0

0

0

0

0

0
7

0

0

0

0

0

0

0

0

0

0

0
4

0

0

0

0

0

0

0

0

0

0

0

0

0

0
7

0
4

0
7

0
7

0

0

0

0

0

0

0

0

0

0

0
6

0

0

0

0

0

0

0
7

0

0
6

0
7

0
7

0

0

0
4

0
4

0

0

0

0

0
4

0

0

0
4

0

0

0
4

0

0

0
4

0

0

0
4

0

0

0
4

0

0

0
4

0

0

0
4

0

0

0
4

0

0

0
4

0

0

0
4

0
4

0
4

0

0

0
4

0

0

0
4

0

0

0
4

0

0

0
4

0

0

0
4

0

0

0
4

0.516483

0.516483

0
4

0

0

0
4

0

0

0
4

0

0

0
4

0

0

0
4

0
7

0
7

0

0
4

0

0

0
4

0

0

0
4

0

0

0
4

0

0

0
4

0

0

0
4

0

0

0
4

0

0

0
4

0

0

0
4

0

0

0
4

0

0

0
4

0.277017

0

0.277017

0

0

0
4

0

0

0
4

0

0

0
4

0

0

0
4

0

0

0
4

0

0

0
4

0

0

0
4

0

0

0
4

0.0669684

0.0669684

0
4

0

0

0
4

0

0

0
4

4.9863
3

0

0

4.9863

0
4

0

0

0
4

0

0

0
4

0

0

0
4

0

0

0
4

0

0

0
4

0

0

0
4

0

0

0
4

0

0

0
4

0

0

0
4

0

0

0
4

1.0849
2

0.538771

0.394971

0.0633849

0.0877713

0
4

0

0

0

0

0

0

0
4

0
8

0

0

0
4

0

0

0

0

0

0
4

0
7

0
7

0
4

0
4

0
4

0

0

0

0

0

0

0

0

0

0

0
4

0

0

0

0

0

0

0

0

0

0

0
4

0

0
4

0
4

0
4

0

0

0

0
4

0

0

0
4

0

0

0

0
4

0

0

0

0

0
4

0

0

0

0
4

0

0

0
4

0

0

0

0
4

0

0

0

0
4

0

0

0

0

0
4

0

0

0
4

0

0

0

0

0
4

0
4

0
4

0
4

0

0

0

0

0

0

0
4

0.129121

0

0.129121

0

0
4

0

0

0

0
4

0

0

0
4

0

0

0

0

0
4

0

0

0

0
4

0.451923

0

0

0.451923

0
4

0

0

0

0

0
4

0

0

0
4

0

0

0

0
4

0

0

0

0
4

0
5

0
7

0
5

0

0

0

0

0

0

0
4

0

0

0

0
4

0

0

0

0
4

0

0

0

0
4

0

0

0

0
4

0

0

0
4

0

0

0
4

0

0

0

0
4

0

0

0

0
4

0

0

0

0
4

0

0

0

0
4

0.233427
3

0
3

0

0

0

0

0.233427

0

0
4

0

0

0

0
4

0

0

0
4

0

0

0
4

0

0

0
4

0

0

0
4

0

0

0

0
4

0.12677

0

0.12677

0
4

0

0

0

0
4

0

0

0
4

0

0

0
4

2.00137
3

2.00137
3

0

0

0

0
4

0

0

0
4

0

0

0
4

0

0

0
4

0

0

0
4

0

0

0
4

0

0

0
4

0

0

0
4

0

0

0
4

0

0

0
4

0

0

0
4

0
4

0
4

0

0
4

0

0

0
4

0

0

0
4

0

0

0
4

0

0

0
4

0

0

0
4

0

0

0
4

0

0

0
4

0

0

0
4

0

0

0
4

0

0

0
4

0
3

0
4

0

0

0

0
4

0

0

0
4

0

0

0
4

0

0

0
4

0

0

0
4

0

0

0
4

0

0

0
4

0

0

0
4

0

0

0
4

0

0

0
4

0

0

0
4

0.346271
4

0

0.346271

0

0

0

0
4

0

0

0
4

0

0

0
4

0

0

0
4

0

0

0
4

0

0

0
4

0

0

0
4

0

0

0
4

0

0

0
4

0

0

0
4

0

0

0
4

2.13107309576799e-13

0
4

23.5353
3

23.4062
3

23.3347
3

0

0

0

0

0

0

0

0

0

0.0347274

0.0367698

0

0

0

0

0

0

0

0

0

0

1.4432899320127e-15
3

0
4

0
4

0
4

0

0

0
4

0

0

0
4

0

0

0
4

0

0

0

0
4

0

0

0

0
4

0

0

0

0
4

0

0

0
4

0

0

0
4

0

0

0
4

0

0

0
4

0

0

0
4

0

0

0

0
4

0

0

0
4

0

0

0
4

0

0

0
4

0

0

0
4

0

0

0
4

0

0

0
4

0

0

0
4

0

0

0
4

0

0

0
4

0

0

0
4

0

0

0
4

0

0

0
4

0

0

0
4

0

0

0
4

0

0

0
4

0

0

0
4

0

0

0
4

0

0

0
4

0

0

0
4

0

0

0
4

0

0

0
4

0

0

0

0

0
4

0

0

0
4

0.129121

0.129121

0
4

0

0

0
4

0

0

0
4

0

0

0

0

0
4

0

0

0
4

0

0

0
4

0

0

0
4

0

0

0

0
4

8.04911692853238e-16
3

0
4

362.619
2

362.49
2

14.0052
2

25.3596
2

0.221765

0

0.25354

0.129121

1.29363

2.71154

0.131657

0

0.0950773

0.258242

0

0.0877713

0

0

0.387362

0

0.258242

0.193681

0

58.927

1.67857

0.701288

0

1.01872

5.16483

0

53.1978

0

0.491801

0.454459

2.77863

0.396724

0.131657

0.687538

0.429296

0

0.322802

59.8954

0.575569

0.525845

0.451923

0.470646

0

0.387362

0.322802

0

0.475134

0

0

0.346013

0

0.193681

0.0739218

0

0.258242

0.0739218

0

0.387362

0.258726

8.26373

0.193681

0.322802

0

0.110883

0

0

0

0.129121

0

0.129121

0.629311

0

0.322802

0.129121

0

0.147844

0

0.387362

0

0.258242

0.129121

8.57562

6.91169

13.5577

0

0

0

0.0739218

0.129121

0.129121

0.129121

0.903846

76.2516
2

0.258242

0.131657

0.184804

0.219428

0.258242

0.710164

0

0

0.129121

0.221765

5.03571

0.0633849

0.184804

0.193681

0.129121

0

0

0.158462

0.0739218

0

0.258242

0
4

0.129121

0.129121

0
4

0

0

0
4

2.21211937656562e-14
2

0
4

0

0
7

0
7

0
7

0

0

0

0

0

0

0

0

0

0

0

0

0

0

0

0

0

0

0

0

0

0

0

0

0

0

0

0

0

0

0

0

0
6

0

0

0

0

0

0

0

0

0

0

0

0

0

0

0

0

0

0

0

0

0

0
7

0

0

0

0

0

0

0

0

0

0

0
6

0

0

0

0

0

0

0

0

0

0

0
7

0

0

0

0

0

0

0

0

0

0

0
7

0

0

0

0

0

0

0

0

0

0

0
7

0

0

0

0

0

0

0

0

0

0

0
7

0
6

0

0

0

0

0

0

0

0

0

0

0
7

0

0

0

0

0

0

0

0

0

0

0
7

0

0

0

0

0

0

0

0

0

0

0
7

0

0

0

0

0

0

0

0

0

0

0
7

0

0

0

0

0

0

0

0

0

0

0
6

0

0

0

0

0

0

0

0

0

0

0
7

0

0

0

0

0

0

0

0

0

0

0
7

0

0

0

0

0

0

0

0

0

0

0
6

0

0

0

0

0

0

0

0

0

0

0
7

0

0

0

0

0

0

0

0

0

0

0
7

0
5

0

0

0

0

0

0

0

0

0

0

0
7

0

0

0

0

0

0

0

0

0

0

0
7

0

0

0

0

0

0

0

0

0

0

0
7

0

0

0

0

0

0

0

0

0

0

0
7

0

0

0

0

0

0

0

0

0

0

0
7

0

0

0

0

0

0

0

0

0

0

0

0

0

0

0

0

0

0

0

0

0

0
7

0

0

0

0

0

0

0

0

0

0

0
6

0

0

0

0

0

0

0

0

0

0

0

0

0

0

0

0

0

0

0

0

0

0
6

0
6

0

0

0

0

0

0

0

0

0

0

0

0

0

0

0

0

0

0

0

0

0

0
7

0

0

0

0

0

0

0

0

0

0

0
7

0

0

0

0

0

0

0

0

0

0

0

0

0

0

0

0

0

0

0

0

0

0
6

0

0

0

0

0

0

0

0

0

0

0
6

0

0

0

0

0

0

0

0

0

0

0
7

0

0

0

0

0

0

0

0

0

0

0
7

0

0

0

0

0

0

0

0

0

0

0
7

0

0

0

0

0

0

0

0

0

0

0
7

0

0

0

0

0

0

0

0

0

0

0

0

0

0

0

0

0

0

0

0

0

0

0
7

0

0

0

0

0

0

0

0

0

0

0
6

0

0

0

0

0

0

0

0

0

0

0
7

0

0

0

0

0

0

0

0

0

0

0
7

0

0

0

0

0

0

0

0

0

0

0
6

0

0

0

0

0

0

0

0

0

0

0

0

0

0

0

0

0

0

0

0

0

0
7

0

0

0

0

0

0

0

0

0

0

0
7

0

0

0

0

0

0

0

0

0

0

0
7

0
7

0

0

0

0

0

0

0

0

0

0

0
7

0

0

0

0

0

0

0

0

0

0

0
7

0

0

0

0

0

0

0

0

0

0

0
6

0

0

0

0

0

0

0

0

0

0

0

0

0

0
6

0

0
8

0
5

0
7

0

0
6

0

0

0

0

0
7

0

0

0
7

0

0
7

0

0

0

0
7

0

0

0

0

0
7

0

0

0

0

0

0

0

0

0

0

0
4

0
7

0
7

0

0
4

0
4

0
4

0

0

0

0

0

0

0

0

0

0

0

0

0

0

0

0
4

0

0

0
4

0

0

0
4

0

0

0
4

0

0

0
4

0

0

0
4

0

0

0
4

0

0

0
4

0

0

0
4

0

0

0
4

0

0

0
4

0

0

0
4

0

0

0

0
4

0

0

0
4

0

0

0
4

0

0

0
4

0

0

0
4

0

0

0
4

0

0

0

0
4

0

0

0
4

0

0

0
4

0

0

0
4

0

0

0
4

0
4

62.6967
4

62.5676
4

0
4

0
5

0

0

0

0

0

0

0

0

0

0

2.99565
3

0

0

0

0

0

0

0

0

0

0.129121

0
4

0.387362

0

0

0

0

0

0

0

0

0

0
4

0

0

0

0

0

0

0

0

0

0

0

0

0

0

0

0

0

0

0

0

0

0

0

0

0

0.233427

0

0

0

1.32275

0

0

0.583567
4

0

0

0

0

0

0

0

0

0.129121

0

0
5

0

0

0

0

0

0

0

0

0

0

0

0

0

0

0

0

0

0

0

0

0

0

0

0

0

0

0

0

0

0

0

0

0

0
4

0

0

0

0

0

0

0

0

0

0

0

0

0

0

0

0

0

0

0

0

0

2.40198

0

0

0

0

0

0

0

0

0

0

0

0

0

0

0

0

0

0

0

0

0

0

0

0

0

0

0

0

0

0

0

0

0

0

0

0

0

0

0

0

0

0

0

0

0

0

0

0

0

0

0

0

0

0

0

0

0

0

0

0

0

0

0

0

0

0

0

0.322802

0

0

0

0

0

0

0.258242

0

0
4

0

0

0

0

0

0

0

0

0

0

0

0

0

0.193681

0

0

0

0

0

0

0

0

0

0

0

0

0

0

0

0

0

0

0

0

0

0

0

0.129121

0

0

0

0

0

0

0

0.129121

0

0

0

0

0

0

0

0

0

0

0

0

0

1.61401

0

0

0

0

0.129121

0

0.077809
3

0

0

0

0

0

0

0

0

0

0

12.8418

0

0

0

0

0

0

0

0

0

0

0

0

0

0

0

0

0

0

0

0

0

0

0

0

0.387362

0

0

0

0

0

0

0

0

0

0

0

0

0

0

0

0

0

0

0

0

0

0

0

0

0

0

0

0

0

0

0

0

0

0

0

0

0

0

0

0

0

0

0

0

0

0

0

0

0

0

0

0

1.59285

0

0

0

0

0

0

0

0

0

0

0

0

0

0

0

0

0

0

0

0

0

0

0

0

0

0

0

0

0

0

0

0

0
4

0

0

0

0

0

0.129121

0

0

0

0

7.61813

0

0

0

0

0

0

0

0

0

0

0

0

0

0

0
4

0
4

0

0

0

0

0

10.0069

0

0

0

0

0.0347274
4

0

8.65109

0

0

0

0

0

0

0

0

8.78379
3

0

0

0

0

0

0

0

0

0

0

0

0

0

0

0

0

0

0

0

0

0

0
5

0

0

0

0

0

0

0

1.48489

0

0

0
4

0
4

0
4

0

0

0

0

0

0

0

0

0

0

0

0

0

0

0

0

0

0

0
4

0

0

0

0

0
4

0

0

0

0
4

0

0

0

0
4

0

0

0

0

0

0
4

0

0

0

0
4

0

0

0
4

0

0

0

0
4

0

0

0

0

0
4

0

0

0

0
4

0

0

0

0

0
4

0
4

0
4

0

0

0
4

0
4

0

0

0

0

0

0

0
4

0

0

0

0
4

0

0

0
4

0

0

0

0
4

0

0

0
4

0

0

0
4

0

0

0

0
4

0

0

0
4

0

0

0

0
4

0

0

0

0
4

0

0

0
4

0
3

0
3

0

0

0

0

0

0
4

0

0

0
4

0

0

0
4

0

0

0
4

0

0

0
4

0

0

0
4

0.129121

0.129121

0
4

0

0

0
4

0

0

0
4

0

0

0
4

0

0

0
4

0
3

0
3

0
4

0

0

0
4

0

0

0
4

0

0

0
4

0

0

0
4

0

0

0
4

0

0

0
4

0

0

0
4

0

0

0
4

0

0

0
4

0

0

0
4

0

0

0

0

0
4

0

0

0

0

0
4

0

0

0

0

0
4

0

0

0

0

0

0
4

0

0

0

0

0

0
4

0
4

198.786
4

4.83371
5

4.55669

0
5

0
4

0

0

0

0

0

0

0

0

0

0

0
5

0

0

0

0

0

0

0

0

0

0

0
5

0

0

0

0

0

0

0

0

0

0

0

0

0

0

0

0

0

0

0

0

0
5

0.277017

0

0

0

0

0

0

0

0

0

0
5

0

0

0

0

0

0

0

0

0

0

0
6

0

0

0

0

0

0

0

0

0

0

0
4

0

0

0

0

0

0

0

0

0

0

0
5

0

0

0

0

0

0

0

0

0

0

0

0

0

0

0

0

0

0

0

0

0

0
5

0

0

0

0

0

0

0

0

0

0

0

0

0

0

0

0

0

0

0

0

0

3.33066907387547e-16
5

0
4

181.583
4

0
6

72.0635
3

31.5128
4

0
5

0
5

0.258242
5

0
4

0.267629
4

12.535
4

0
4

0

0

0

0

0

0

0

0

0

0

0
4

0

0

0

0

0

0

0

0

0

0.129121

0

0

0

0

0

0

0

0

0

0

0

0
4

0

0

0.116713

0

1.24494

0

0

0

0

0

0

0

0

0

0

0

0

0

0

0

0

0

0.839285

0

0

0

0

0

0

0

0.129121

0

0
4

0

0

0

0

0

0

0

0

0

0

0

0

0

0

0

0.311236

0

0

0

0

0

0

0

0

0

0

0

0

0

0

0

0

3.55082

0

0

0

0

0

0

0

0

0

0

6.50989
4

0
7

0

0

0

0

0

0

0

0

0

0

0

0

0

0

0

0

0

0

0

0

0

0

0

0

0

0

0

0

0

0

0

0

0

0

0

0

0

0

0

0

0

0

0

0

0

0

0

0

0

0

0

0

1.35577

0

0.129121
4

0

0

0

0

0

0

0

0

0

0

0

0

0

0

0

0

0

0

0

0

0

0

0

1.48489

0

0

0

0

0

0

0

0

0

0

27.9213
4

0

0

0

0

0

0

3.16346

0.207762

0

0

0.415525
3

0

0

0

0

0

0

0

0

0

0

0

0

0

0

0

0

0

0

0

0

0

0
4

0

16.2747

0

0

0

0

0

0

0

0

0
4

0

0

0

0

0

0

0

0

0

0

0
4

0

1.16209

0

0

0

0

0

0

0

0

0
3

0

0

0

0

0

0

0

0

0

0

0
4

0

0
8

0

0

0

0

0

0

0

0

0

0

0
8

0

0

0

0

0

0

0

0

0

0

0

0

0

0

0

0

0

0

0

0

0

0

0

0

0

0

0

0

0
4

11.1069

7.61813

0.454459

1.48489

0.516483

0.516483

0.258242

0.258242

7.7715611723761e-16

0
4

0

0

0
4

0

0

0
4

0

0

0
4

0

0

0
4

0.138508

0.138508

0
4

0.129121

0.129121

0
4

0

0

0
4

0.129121

0.129121

0
4

0

0

0
4

0

0

0
4

0
4

0
4

0

0

0

0
4

0.710164

0.710164

0
4

0

0

0
4

0

0

0
4

0

0

0
4

0
4

0
4

0

0

0
4

0
4

0

0

0

0

0
4

0

0

0

0

0
4

0

0

0
4

0.155618

0.155618

0
4

0

0

0
4

0

0

0
4

0
4

4.71291
3

0
4

0
4

0

0

0

0

0

0

0

0

0

0

0
4

0

0

0

0

0

0
4

0

0

0
4

0

0

0
4

0

0

0
4

0

0

0
4

0

0

0
4

0.774725
3

0
4

0.774725

0

0

0

0

0

0

0
4

0

0
4

0

0

0

0
4

3.93818
3

3.93818
3

0
4

0
4

0
4

0
4

0
4

0

0

0

0

0

0

0
4

0
3

0

0

0

0

0

0

0

0
4

0
4

0
4

0

0

0

0
4

0
4

0

0

0

0

0

0
4

0

0

0

0

0
4

0

0

0

0

0

0
4

0
4

0

0

0

0

0

0
7

0

0

0

0

0

0

0

0
4

0

0

0

0

0

0
4

0

0

0

0

0

0

0
4

0

0

0

0
4

0

0

0
4

0
4

0
4

0
4

0

0

0

0

0
4

0

0

0

0
4

0
4

0

0

0

0

0

0

0
4

0

0

0

0
4

0
4

0

0

0

0

0
4

0

0

0

0
4

0

0

0
4

0
4

0

0

0

0

0

0
4

0
4

0
3

0
3

0
3

0

0
4

0

0

0
4

0
4

0
4

0

0

0

0
4

0

0

0
4

0

0

0
4

0
4

0

0

0

0

0

0

0
4

0

0

0
4

0
4

0

0

0

0

0
4

0

0

0

0

0
4

0

0

0
4

0
4

0
6

0
6

0
6

0
4

0
4

0

0

0

0
4

0
4

0.972612
3

0.972612
3

0.972612
3

0

0

0
3

0

0

0

0

0

0

0

0
4

0

0

0

0

0

0
4

0

0

0

0

0
4

0
4

0

0

0

0

0

0
4

0

0

0
4

0
4

0
4

0

0

0

0

0
4

0

0

0
4

0

0

0
4

0
4

0

0

0

0

0
4

0
4

0
3

0

0

0
4

0

0

0

0
4

0

0

0
4

0

0

0
4

0

0

0
4

0

0

0
4

0
4

0

0

0

0

0
4

0
4

0

0

0

0

0
4

0

0

0

0

0
4

0

0

0
4

0

0

0
4

0
4

0

0

0

0

0

0

0
4

0
4

0

0

0

0

0

0
4

0

0

0

0

0
4

0
4

0

0

0

0

0
4

0
4

0

0

0

0

0
4

0
4

6.48066
3

5.93602
3

4.39011
3

0.129121

0.761796
4

0.516483

0.138508

0

0

0

0

0

0
4

0.415525

0

0

0.415525

0
4

0

0

0

0
4

0.129121

0

0.129121

0
4

0

0

0

0
4

3.60822483003176e-16
3

0
4

0

0

0

0

0
4

0

0

0

0
4

0

0

0
4

0
4

0

0

0

0

0

0

0

0

0
4

0
4

0
4

0
4

0

0

0

0
4

0

0

0
4

0

0

0
4

0
4

2.03285

0.147844

0

0.0739218

0.0739218

0
4

1.88501

1.81108

0.0739218

1.38777878078145e-17

0
4

0
4

0

0

0

0

0
4

0

0

0

0
4

0

0

0
4

0
4

0

0

0

0

0

0

0
4

0

0

0
4

0
4

0.645604

0.322802

0.129121

0

0.193681

2.77555756156289e-17

0
4

0

0

0
4

0

0

0
4

0.322802

0.322802

0
4

0
4

0
4

0
4

0
4

0
4

0

0

0
4

0

0

0
4

0
4

0
4

0
4

0

0

0

0
4

0
4

0

0

0

0

0
4

0

0

0

0
4

0
4

12.0082
3

12.0082
3

5.74588
3

6.26236
3

0

0

0

0

0

0

0

0
4

0

0

0
4

0

0

0
4

0

0

0
4

0

0

0
4

0

0

0
4

0
4

0
4

0

0

0

0
4

0
4

0

0

0

0
4

0

0

0

0
4

0

0

0

0
4

0

0

0
4

0

0

0
4

0

0

0
4

0

0

0
4

0
4

0
6

0
6

0
6

0
4

0
4

0

0

0

0
4

0

0

0
4

0

0

0

0
4

0

0

0
4

0
4

0

0

0

0

0
4

0
4

0
3

0
3

0

0

0

0
4

0

0

0
4

0
4

0

0

0

0

0

0
4

0

0

0
4

0
4

0

0

0

0
4

0

0

0
4

0

0

0
4

0

0

0
4

0
4

0

0

0

0

0

0
4

0
4

0

0

0

0

0

0

0
4

0

0

0
4

0
4

0

0

0

0
4

0

0

0
4

0

0

0
4

0
4

0

0

0

0

0

0

0
4

0
4

0
5

0
5

0
5

0

0

0

0

0

0

0

0

0

0

0
5

0

0
4

0

0

0

0

0

0

0
4

0
4

0

0

0

0

0

0

0

0
4

0
4

0.077809

0.077809

0.077809

0

0

0

0
4

0

0

0
4

0
4

0

0

0

0

0
4

0
4

0

0

0

0

0

0

0
4

0

0

0
4

0

0

0
4

0
4

0

0

0

0

0
4

0

0

0

0
4

0

0

0
4

0
4

54.0076

54.0076

53.8967

0.110883

1.51267887105178e-15

0
4

0

0

0
4

0
4

0

0

0

0

0

0
4

0
4

0

0

0

0

0
4

0

0

0
4

0
4

0

0

0

0

0

0
4

0

0

0
4

0
4

1.28385

1.28385

0.972612

0.155618

0.155618

0
4

0
4

0
5

0
5

0
5

0

0

0

0

0

0

0

0

0
4

0
4

0

0

0

0

0
4

0

0

0
4

0

0

0
4

0

0

0
4

0
4

0.0347274

0.0347274

0

0

0.0347274

0
4

0

0

0

0
4

0
4

0

0

0

0

0

0
4

0

0

0

0
4

0
4

0

0

0

0

0
4

0

0

0
4

0

0

0
4

0
4

0

0

0

0

0
4

0

0

0
4

0

0

0
4

0

0

0
4

0
4

0

0

0

0

0
4

0
4

0
4

0

0

0

0
4

0

0

0
4

0
4

0

0

0

0
4

0
4

0

0

0

0

0

0
4

0

0

0
4

0
4

0

0

0

0

0

0
4

0

0

0
4

0

0

0
4

0
4

0
4

0
4

0
4

0

0

0

0

0

0

0

0

0

0

0

0
4

0

0

0
4

0

0

0

0
4

0

0

0
4

0

0

0
4

0

0

0
4

0

0

0
4

0

0

0
4

0

0

0
4

0

0

0
4

0
4

0
4

0

0

0
4

0

0

0
4

0
4

0

0

0

0

0
4

0

0

0
4

0
4

0

0

0

0

0
4

0

0

0

0
4

0
4

0

0

0

0

0
4

0

0

0
4

0
4

0

0

0

0

0

0
4

0

0

0

0
4

0
4

0

0

0

0
4

0
4

0

0

0

0

0

0
4

0

0

0

0
4

0
4

0

0

0

0

0
4

0

0

0

0
4

0

0

0
4

0
4

0.193681

0.193681

0.193681

0

0

0
4

0

0

0

0
4

0
4

0

0

0

0

0

0
4

0

0

0
4

0

0

0
4

0
4

2.90522

0.193681

0
6

0

0

0

0.193681

0

0

0
4

2.71154

0
6

1.54945

0.710164

0.451923

0

0

0

0

0
4

0

0

0

0
4

0

0

0

0
4

0
4

0

0

0

0

0

0

0
4

0

0

0
4

0
4

0.322802

0.322802

0

0.193681

0.129121

2.77555756156289e-17

0
4

0

0

0
4

0

0

0
4

0
4

0

0

0

0

0

0
4

0

0

0
4

0
4

0

0

0

0
4

0
4

0.193681

0

0

0

0
4

0

0

0
4

0.193681

0.193681

0
4

0
4

0

0

0

0

0
4

0

0

0

0
4

0

0

0
4

0
4

0

0

0

0
4

0
4

0

0

0

0

0

0
4

0
4

0

0

0

0

0
4

0
4

0

0

0

0

0

0

0
4

0
4

0.201957

0
6

0
6

0

0

0

0

0

0

0

0
4

0
2

0

0

0

0

0

0

0
4

0.0739218
2

0

0

0.0739218

0

0
4

0.077809

0

0.077809

0

0
4

0.0502263

0.0502263

0

0
4

0

0

0

0
4

0

0

0
4

0
4

0

0

0

0

0

0

0
4

0
4

0

0

0

0

0
4

0

0

0

0
4

0
4

0

0

0

0
4

0
4

0

0

0

0

0
4

0

0

0
4

0
4

0

0

0

0

0
4

0

0

0
4

0
4

0

0

0

0

0
4

0
4

0

0

0

0

0

0
4

0
4

0

0

0

0

0
4

0

0

0
4

0
4

0.138508

0.138508

0.138508

0
4

0

0

0

0
4

0
4

0

0

0

0
4

0

0

0
4

0
4

0
5

0
5

0
5

0

0

0
4

0

0

0
4

0

0

0
4

0
4

0

0

0

0

0

0
4

0

0

0
4

0
4

0

0

0

0

0
4

0
4

0

0

0

0
4

0
4

0

0

0

0

0
4

0

0

0
4

0
4

0

0

0

0
4

0

0

0

0
4

0
4

0

0

0

0
4

0

0

0
4

0

0

0
4

0
4

0

0

0

0

0

0
4

0
4

0

0

0

0

0
4

0

0

0
4

0
4

0

0

0

0

0

0
4

0

0

0
4

0
4

0

0

0

0

0

0

0
4

0
4

0

0

0

0

0

0

0

0

0

0

0

0

0

0

0

0

0

0

0

0

0

0

0

0

0

0

0

0

0

0

0

0

0

0

0

0

0

0
4

0

0

0

0

0

0

0

0

0

0

0
4

0
4

0

0

0

0

0

0

0

0

0

0

0

0

0

0

0

0

0
4

0
4

0

0

0

0
4

0

0

0
4

0

0

0
4

0
4

0

0

0

0

0
4

0
4

0

0

0

0

0
4

0
4

0

0

0

0
4

0

0

0
4

0

0

0
4

0
4

0

0

0

0
4

0

0

0
4

0
4

0

0

0

0
4

0

0

0
4

0
4

0

0

0

0

0
4

0
4

0

0

0

0

0
4

0

0

0
4

0
4

0

0

0

0

0
4

0

0

0
4

0
4

0

0

0

0

0
4

0

0

0
4

0
4

0.427949

0

0

0

0

0

0

0

0

0

0

0

0

0

0

0

0

0

0

0

0

0
4

0.427949

0.311236

0

0

0

0

0.116713

2.77555756156289e-17

0
4

0

0

0

0
4

0
4

0

0

0

0

0
4

0
4

0

0

0

0
4

0
4

0

0

0

0

0
4

0
4

0

0

0

0
4

0

0

0
4

0
4

0

0

0

0

0
4

0

0

0
4

0
4

0

0

0

0

0

0
4

0
4

0

0

0

0
4

0

0

0
4

0
4

0

0

0

0
4

0

0

0
4

0
4

0.427949

0

0

0

0
4

0.427949

0.427949

0
4

0
4

0

0

0

0

0
4

0
4

7.2612
3

7.2612
3

6.59982
3

0.194522

0

0

0.272331

0

0.194522

0

0

1.13797860024079e-15
3

0
4

0

0

0

0
4

0

0

0
4

0
4

0

0

0

0
4

0

0

0
4

0
4

1.09753

1.09753

1.09753

0

0
4

0
4

0

0

0

0
4

0

0

0
4

0

0

0
4

0
4

0.216892

0.0877713

0

0.0877713

0
4

0.129121

0.129121

0
4

0
4

2.38874

2.06593

1.93681

0.129121

0
4

0.322802

0.322802

0
4

1.11022302462516e-16

0
4

0

0

0

0

0
4

0

0

0
4

0
4

0

0

0

0

0
4

0
4

0

0

0

0
4

0
4

0

0

0

0

0
4

0
4

0

0

0

0

0
4

0

0

0
4

0
4

0
4

0
4

0

0

0

0

0

0

0

0

0
4

0
4

0
4

0

0

0

0

0
4

0

0

0

0

0

0
4

0

0

0

0
4

0

0

0
4

0
4

0

0

0

0
4

0

0

0
4

0
4

0

0

0

0
4

0

0

0
4

0

0

0
4

0
4

0.322802

0.322802

0.129121

0.193681

2.77555756156289e-17

0
4

0
4

0

0

0

0

0
4

0
4

0

0

0

0
4

0
4

0

0

0

0
4

0

0

0
4

0
4

0

0

0

0
4

0
4

0

0

0

0

0
4

0
4

0

0

0

0

0

0
4

0
4

0

0

0

0

0
4

0
4

0

0
5

0

0

0

0

0

0

0

0

0
4

0

0

0

0

0
4

0

0

0

0
4

0

0

0
4

0
4

0

0

0

0

0
4

0
4

0

0

0

0
4

0

0

0
4

0
4

0.258242

0.258242

0.258242

0
4

0
4

0

0

0

0
4

0
4

0

0

0

0
4

0
4

0

0

0

0

0
4

0
4

0

0

0

0
4

0

0

0
4

0
4

0

0

0

0

0
4

0
4

0

0

0

0

0
4

0
4

0

0

0

0
4

0

0

0
4

0
4

24.507
2

19.2313
2

15.363

0.0739218

0.0633849

0.129121

2.01148
2

0.88139

0.193681

0.129121

0.0633849

0

0.129121

0.193681

3.30291349825984e-15
2

0
4

5.27572

5.08203

0.193681

3.33066907387547e-16

0
4

4.44089209850063e-15
2

0
4

0

0

0

0
4

0
4

0

0

0

0

0
4

0
4

0

0

0

0
4

0
4

0

0

0

0

0
4

0
4

0

0

0

0

0
4

0
4

0.0837105

0.0837105

0.0334842

0.0502263

0
4

0
4

0

0

0

0

0
4

0
4

0

0

0

0
4

0

0

0
4

0
4

0

0

0

0

0
4

0
4

0

0

0

0
4

0

0

0
4

0
4

0
3

0
3

0
3

0

0

0

0

0

0

0

0
4

0
3

0

0

0

0

0

0

0

0
4

0
4

0

0

0

0
4

0
4

0

0

0

0
4

0

0

0
4

0
4

0

0

0

0
4

0

0

0
4

0
4

0

0

0

0
4

0

0

0
4

0
4

0

0

0

0

0
4

0
4

0

0

0

0
4

0

0

0
4

0
4

0.258242

0.258242

0.258242

0
4

0
4

0

0

0

0

0
4

0
4

0

0

0

0

0
4

0
4

0

0

0

0
4

0

0

0
4

0
4

0
4

0
4

0
4

0

0

0

0

0

0

0

0
4

0

0

0

0

0

0

0
4

0

0

0

0

0
4

0

0

0

0
4

0

0

0

0
4

0

0

0

0
4

0

0

0
4

0
4

0

0

0

0
4

0

0

0
4

0
4

0

0

0

0

0
4

0
4

0

0

0

0

0
4

0
4

0

0

0

0
4

0

0

0
4

0
4

0

0

0

0
4

0

0

0
4

0
4

0

0

0

0
4

0

0

0
4

0
4

0

0

0

0

0
4

0
4

0

0

0

0

0
4

0
4

0

0

0

0
4

0

0

0
4

0
4

0

0

0

0
4

0
4

49.793
3

11.0561
3

5.40963
3

0
2

1.51463

4.13187

0

0

0
4

4.30147
3

1.93
3

0

1.74313

0.628335

0
4

6.45604

0

6.45604

0
4

27.9794

14.4147

13.4907

0

0.0739218

1.56819002228303e-15

0
4

0

0

0
4

0
4

0

0

0

0

0
4

0
4

0.077809

0.077809

0.077809

0
4

0
4

0

0

0

0

0
4

0
4

0

0

0

0
4

0

0

0
4

0
4

0

0

0

0

0
4

0
4

0

0

0

0

0
4

0
4

0

0

0

0
4

0
4

0

0

0

0

0
4

0
4

0

0

0

0

0
4

0
4

0

0

0

0

0
4

0
4

0
4

0
4

0
4

0

0

0

0

0

0

0

0
4

0

0

0
4

0

0

0
4

0
4

0

0

0

0
4

0
4

0

0

0

0
4

0

0

0
4

0
4

0

0

0

0

0
4

0
4

0

0

0

0
4

0
4

0

0

0

0
4

0

0

0
4

0
4

0

0

0

0

0
4

0
4

0

0

0

0
4

0

0

0
4

0
4

0

0

0

0

0
4

0
4

0

0

0

0

0
4

0
4

0

0

0

0
4

0

0

0
4

0
4

31.68
2

27.4822
2

26.7165
2

0

0

0

0.138508

0.35014

0.277017
3

0

0

0

0

0

0

3.05311331771918e-15
2

0
4

3.84767
2

0.933708
2

0.194522

0

0

0

0

0.505758

0.077809

0.077809

0

0

0.311236

0.700281
2

0.622472
3

0.346271

0.077809

0

0

0

0
4

0.35014

0

0.35014

0
4

0

0

0

0

0

0
4

0

0

0
4

0

0

0
4

6.16173778666962e-15
2

0
4

1.09753

1.09753

1.09753

0

0

0

0
4

0

0

0
4

0

0

0
4

0
4

0

0

0

0
4

0

0

0
4

0
4

0

0

0

0
4

0
4

0

0

0

0
4

0

0

0
4

0
4

0

0

0

0
4

0
4

0

0

0

0
4

0

0

0
4

0
4

0.40657

0.40657

0.40657

0
4

0
4

0

0

0

0
4

0

0

0
4

0
4

0

0

0

0
4

0
4

0

0

0

0
4

0
4

0

0

0

0
4

0
4

0
7

0
7

0
7

0
8

0

0

0

0

0

0

0

0
4

0

0

0

0

0

0
4

0

0

0

0
4

0
4

0

0

0

0
4

0
4

0

0

0

0

0
4

0
4

0

0

0

0
4

0
4

0

0

0

0
4

0
4

0

0

0

0
4

0
4

0

0

0

0
4

0
4

0

0

0

0
4

0
4

0

0

0

0
4

0
4

0

0

0

0
4

0
4

0

0

0

0
4

0
4

7.1422
2

6.84652
2

5.7559
2

1.09062
2

0

0

0

4.44089209850063e-16
2

0
4

0

0

0

0
4

0

0

0
4

0.295687

0.295687

0
4

0
4

0

0

0

0
4

0
4

0

0

0

0
4

0
4

0

0

0

0
4

0
4

0

0

0

0
4

0
4

0

0

0

0
4

0
4

0

0

0

0
4

0
4

0

0

0

0
4

0
4

0

0

0

0
4

0
4

0

0

0

0
4

0
4

0

0

0

0
4

0
4

0
4

0
4

0
4

0

0

0

0

0

0

0

0
4

0
4

0

0

0

0
4

0
4

0

0

0

0
4

0
4

0

0

0

0
4

0
4

0

0

0

0
4

0
4

0

0

0

0
4

0
4

0

0

0

0
4

0
4

0

0

0

0
4

0
4

0

0

0

0
4

0
4

0

0

0

0
4

0
4

0

0

0

0
4

0
4

0

0

0

0

0

0

0

0

0

0
4

0

0

0
4

0
4

0

0

0

0
4

0
4

0

0

0

0
4

0
4

0

0

0

0
4

0
4

0.129121

0.129121

0.129121

0
4

0
4

0

0

0

0
4

0
4

0

0

0

0
4

0
4

0.116713

0.116713

0.116713

0
4

0
4

0

0

0

0
4

0
4

0

0

0

0
4

0
4

0

0

0

0
4

0
4

0.867465
3

0.711847
3

0.311236
3

0.077809
3

0

0.322802

5.55111512312578e-17
3

0
4

0.155618

0.077809

0

0.077809

0
4

0
4

0

0

0

0
4

0
4

0

0

0

0
4

0
4

0

0

0

0
4

0
4

0.0739218

0.0739218

0.0739218

0
4

0
4

0

0

0

0
4

0
4

0

0

0

0
4

0
4

0

0

0

0
4

0
4

0

0

0

0
4

0
4

0

0

0

0
4

0
4

0

0

0

0
4

0
4

0
4

0
4

0

0

0

0

0

0

0

0

0

0
4

0

0

0

0

0

0

0
4

0

0

0
4

0
4

0

0

0

0
4

0
4

0

0

0

0
4

0
4

0.0950773

0.0950773

0.0950773

0
4

0
4

0

0

0

0
4

0
4

0.0950773

0.0950773

0.0950773

0
4

0
4

0

0

0

0
4

0
4

0

0

0

0
4

0
4

0

0

0

0
4

0
4

0

0

0

0
4

0
4

0

0

0

0
4

0
4

1.63399
3

1.63399
3

1.63399
3

0

0

0

0

0
4

0

0

0
4

0

0

0
4

0
4

0

0

0

0
4

0
4

0.138508

0.138508

0.138508

0
4

0
4

0

0

0

0
4

0
4

0

0

0

0
4

0
4

0

0

0

0
4

0
4

0

0

0

0
4

0
4

0

0

0

0
4

0
4

0

0

0

0
4

0
4

0

0

0

0
4

0
4

0

0

0

0
4

0
4

0

0

0

0

0

0

0

0
4

0

0

0
4

0
4

0

0

0

0
4

0
4

0

0

0

0
4

0
4

0

0

0

0
4

0
4

0

0

0

0
4

0
4

0

0

0

0
4

0
4

0

0

0

0
4

0
4

0

0

0

0
4

0
4

0

0

0

0
4

0
4

0

0

0

0
4

0
4

0

0

0

0
4

0
4

0

0

0

0

0

0

0

0

0
4

0

0

0

0

0
4

0

0

0

0
4

0

0

0

0
4

0

0

0
4

0
4

0

0

0

0
4

0
4

0

0

0

0
4

0
4

0

0

0

0
4

0
4

0

0

0

0
4

0
4

0

0

0

0
4

0
4

0

0

0

0
4

0
4

0

0

0

0
4

0
4

0

0

0

0
4

0
4

0

0

0

0
4

0
4

0

0

0

0
4

0
4

0.258242
3

0.258242
3

0
5

0

0

0

0

0

0

0

0

0

0

0

0

0

0

0

0

0

0.258242

0
4

0

0

0

0
4

0

0

0

0

0
4

0

0

0

0

0

0
4

0

0

0

0

0
4

0

0

0

0
4

0

0

0

0
4

0

0

0

0
4

0

0

0

0
4

0

0

0

0
4

0

0

0
4

0

0

0

0

0

0

0

0

0

0

0

0
4

0

0

0
4

0

0

0
4

0
3

0

0

0

0

0

0

0

0

0

0

0

0

0
4

0
3

0

0

0

0

0

0

0
4

0
4

0

0

0

0

0

0

0

0

0

0
4

0

0

0

0

0

0
4

0
5

0

0

0

0

0

0
4

0
4

0

0

0

0

0

0
4

0

0

0

0

0

0
4

0
4

7.50857
3

7.50857
3

0.661376
3

6.84719

0

0

0

0

0

0
4

0

0

0

0

0
4

0

0

0

0
4

0
4

0

0

0

0
4

0
4

0

0

0

0
4

0
4

0

0

0

0
4

0
4

0

0

0

0
4

0
4

0

0

0

0
4

0
4

0

0

0

0
4

0
4

0

0

0

0
4

0
4

0

0

0

0
4

0
4

0

0

0

0
4

0
4

0

0

0

0
4

0
4

0
4

0
4

0
4

0

0

0

0
4

0

0

0

0

0

0
4

0
4

0

0

0

0
4

0
4

0

0

0

0
4

0
4

0

0

0

0
4

0
4

0

0

0

0
4

0
4

0

0

0

0
4

0
4

0.877713

0.877713

0.877713

0
4

0
4

0

0

0

0
4

0
4

0

0

0

0
4

0
4

0

0

0

0
4

0
4

0

0

0

0
4

0
4

0

0

0

0

0

0

0

0

0
4

0

0

0
4

0
4

0

0

0

0
4

0
4

0

0

0

0
4

0
4

0

0

0

0
4

0
4

0

0

0

0
4

0
4

0.267874

0.267874

0.267874

0
4

0
4

0

0

0

0
4

0
4

0

0

0

0
4

0
4

0

0

0

0
4

0
4

0

0

0

0
4

0
4

0

0

0

0
4

0
4

0
4

0

0

0

0

0

0

0

0

0
4

0

0

0

0

0

0
4

0

0

0

0
4

0

0

0
4

0
4

0

0

0

0
4

0
4

0

0

0

0
4

0
4

0

0

0

0
4

0
4

0

0

0

0
4

0
4

0

0

0

0
4

0
4

0

0

0

0
4

0
4

0

0

0

0
4

0
4

0

0

0

0
4

0
4

0

0

0

0
4

0
4

0

0

0

0
4

0
4

0.903846
3

0.193681
3

0

0

0

0

0

0

0.193681

0
4

0.516483

0.387362

0

0.129121

2.77555756156289e-17

0
4

0.193681

0.193681

0
4

0
4

0

0

0

0
4

0
4

0

0

0

0
4

0
4

0

0

0

0
4

0
4

0

0

0

0
4

0
4

0.0739218

0.0739218

0.0739218

0
4

0
4

0

0

0

0
4

0
4

0

0

0

0
4

0
4

0

0

0

0
4

0
4

0

0

0

0
4

0
4

0

0

0

0
4

0
4

0
7

0
7

0
7

0
7

0
4

0
4

0

0

0

0
4

0
4

0

0

0

0
4

0
4

0

0

0

0
4

0
4

0

0

0

0
4

0
4

0

0

0

0
4

0
4

0

0

0

0
4

0
4

0

0

0

0
4

0
4

0

0

0

0
4

0
4

0

0

0

0
4

0
4

0

0

0

0
4

0
4

0
4

0
4

0
4

0

0

0

0

0

0

0
4

0

0

0

0

0
4

0

0

0

0
4

0
4

0

0

0

0
4

0
4

0

0

0

0
4

0
4

0

0

0

0
4

0
4

0

0

0

0
4

0
4

0

0

0

0
4

0
4

0

0

0

0
4

0
4

0

0

0

0
4

0
4

0

0

0

0
4

0
4

0

0

0

0
4

0
4

0

0

0

0
4

0
4

0
4

0
4

0
4

0

0

0

0

0

0
4

0
4

0

0

0

0
4

0

0

0
4

0

0

0
4

0

0

0
4

0
4

0

0

0

0
4

0
4

0

0

0

0
4

0
4

0

0

0

0
4

0
4

0

0

0

0
4

0
4

0

0

0

0
4

0
4

0

0

0

0
4

0
4

0

0

0

0
4

0
4

0

0

0

0
4

0
4

0

0

0

0
4

0
4

0

0

0

0
4

0
4

11.4176

0

0

0

0

0

0

0

0

0

0
4

11.4176

11.4176

0

0
4

0

0

0
4

0

0

0
4

0

0

0
4

0
4

0

0

0

0
4

0
4

0

0

0

0
4

0
4

0

0

0

0
4

0
4

0

0

0

0
4

0
4

0

0

0

0
4

0
4

0

0

0

0
4

0
4

0

0

0

0
4

0
4

0

0

0

0
4

0
4

0

0

0

0
4

0
4

0

0

0

0
4

0
4

0

0

0

0
7

0

0

0
4

0

0

0

0
4

0

0

0
4

0
4

0

0

0

0
4

0
4

0

0

0

0
4

0
4

0

0

0

0
4

0
4

0

0

0

0
4

0
4

0

0

0

0
4

0
4

0

0

0

0
4

0
4

0

0

0

0
4

0
4

0

0

0

0
4

0
4

0

0

0

0
4

0
4

0

0

0

0
4

0
4

0
4

0
4

0

0

0

0

0

0

0

0

0

0

0

0

0

0

0

0

0

0

0

0

0

0

0

0

0

0

0

0

0

0

0

0

0

0

0

0

0

0

0

0

0

0

0

0

0

0

0

0

0

0

0

0

0

0

0

0
4

0

0

0
4

0

0

0

0

0

0

0
4

0

0

0

0

0
4

0

0

0

0
4

0

0

0

0

0
4

0

0

0
4

0

0

0

0
4

0

0

0
4

0

0

0
4

0
4

0
4

0
4

0

0

0

0

0

0

0
4

0

0

0

0

0
4

0

0

0

0
4

0

0

0

0
4

0
4

0

0

0

0
4

0
4

0

0

0

0
4

0
4

0

0

0

0
4

0
4

0

0

0

0
4

0
4

0

0

0

0
4

0
4

0

0

0

0
4

0
4

0

0

0

0
4

0
4

0

0

0

0
4

0
4

0

0

0

0
4

0
4

0

0

0

0
4

0
4

0
4

0
4

0

0

0

0

0

0
4

0
3

0

0

0

0

0
4

0

0

0
4

0

0

0
4

0
4

0

0

0

0
4

0
4

0

0

0

0
4

0
4

0

0

0

0

0

0

0
4

0

0

0

0
4

0

0

0
4

0
4

0
4

0
4

0
4

0

0

0
4

0

0

0

0

0

0

0
4

0

0

0

0

0

0

0
4

0

0

0
4

0
4

0
4

0
4

0

0

0

0

0

0

0

0

0
4

0

0

0

0

0
4

0

0

0
4

0
4

0.077809
3

0.077809

0.077809

0

0
4

0

0

0

0

0

0

0
4

0

0

0
4

0

0

0
4

0

0

0
4

0
4

20.6988
2

20.6988
2

20.5477
2

0.0633849

0.0877713

0
4

0
4

0
3

0

0

0

0

0

0

0
4

0

0

0

0

0

0
4

0

0

0

0

0
4

0

0

0
4

0

0

0
4

0

0

0
4

0
4

0

0

0

0

0

0

0

0

0
4

0

0

0

0

0

0

0
4

0
4

0
3

0
4

0
4

0

0

0

0

0

0
4

0

0

0

0

0
4

0

0

0
4

0
4

3.93818
3

0
3

0
4

0

0

0

0

0

0

0

0

0

0

0

0
4

0

0

0
4

0

0

0
4

0

0

0
4

0.193681
3

0
5

0.193681

0

0

0

0

0

0

0

0

0

0

0
4

0.129121
4

0
4

0

0

0

0

0

0.129121

0
4

3.61538
3

0
7

3.61538

0

0

0

0

0

0
4

0
5

0

0

0

0

0

0

0

0
4

0

0

0

0

0

0

0
4

0

0

0

0
4

0

0

0

0
4

0

0

0
4

4.44089209850063e-16
3

0
4

0

0

0

0

0

0
4

0

0

0

0

0

0
4

0

0

0
4

0

0

0
4

0

0

0
4

0
4

0

0

0

0

0

0

0

0

0

0
4

0

0

0
4

0
4

0

0

0

0

0

0

0

0

0

0
4

0

0

0

0

0

0
4

0
4

0
4

0
4

0

0

0

0

0

0
4

0
4

0

0

0

0

0

0

0

0

0
4

0

0

0
4

0
4

0
7

0
7

0
7

0

0

0

0
4

0

0

0
4

0

0

0
4

0

0

0
4

0

0

0
4

0

0

0
4

0

0

0
4

0
4

0
4

0
4

0

0

0

0

0

0

0

0
4

0

0

0

0
4

0
4

5.35851
3

5.35851
3

5.35851
3

0
4

0

0

0
4

0
4

0
4

0

0

0

0

0

0
4

0

0

0

0

0
4

0

0

0

0

0
4

0

0

0
4

0
4

0

0

0

0

0

0

0

0

0
4

0

0

0

0
4

0
4

0
7

0
7

0
7

0

0

0

0

0

0

0

0
7

0

0
7

0
7

0

0

0

0

0
4

0
7

0

0

0

0

0
4

0
4

0
4

0

0

0

0

0

0

0

0
4

0

0

0

0

0
4

0

0

0
4

0
4

0

0

0

0
4

0

0

0

0
4

0

0

0

0

0
4

0

0

0

0
4

0
4

0

0

0

0

0

0
4

0
4

0
4

0
4

0
4

0
4

0
4

0
4

0

0

0

0

0

0
4

0

0

0
4

0

0

0
4

0

0

0

0
4

0

0

0
4

0

0

0
4

0
4

0.110309
2

0.110309
2

0

0.110309

0

0

0
4

0

0

0

0

0
4

0

0

0

0
4

0

0

0
4

0
4

0

0

0

0

0

0

0
4

0

0

0
4

0
4

0
7

0
7

0
7

0

0
4

0

0

0

0
4

0
4

0

0

0

0

0
4

0

0

0

0
4

0

0

0
4

0
4

0
4

0

0

0

0

0

0

0
4

0

0

0

0
4

0
4

19.631
3

15.1733
3

1.7909
2

11.7179
3

1.15747
2

0.348617

0.158462

0

0

0

4.9960036108132e-16
3

0
4

3.0989

2.4533

0.193681

0.258242

0.193681

0

0
4

0.693508

0

0.693508

0

0

0
4

0.591374

0.369609

0.221765

0

2.77555756156289e-17

0
4

0

0

0
4

0

0

0
4

0.0739218

0.0739218

0
4

0
4

0
4

0

0

0

0

0
4

0

0

0

0

0
4

0

0

0
4

0

0

0
4

0

0

0
4

0
4

0
4

0

0

0

0

0

0

0
4

0

0

0

0

0

0
4

0

0

0
4

0

0

0
4

0

0

0
4

0
4

0

0

0

0

0

0

0

0

0

0
4

0

0

0

0

0
4

0
4

0
3

0
3

0
3

0

0

0

0
4

0

0

0

0
4

0

0

0
4

0

0

0
4

0
4

0

0

0

0

0

0

0

0
4

0

0

0

0
4

0
4

0.16302
1

0

0

0

0

0

0

0
4

0

0

0
4

0

0

0
4

0.0367698

0.0367698

0
4

0.077809

0.077809

0
4

0.0484412

0.0484412

0
4

0
4

0
3

0

0

0

0

0

0

0
4

0
4

0

0

0

0

0

0
4

0
4

0

0

0

0

0
4

0

0

0

0
4

0

0

0

0

0
4

0
4

0

0

0

0

0

0

0

0

0
4

0
4

0

0

0

0

0

0

0

0
4

0

0

0
4

0

0

0

0
4

0

0

0
4

0

0

0
4

0

0

0
4

0
4

0
3

0
4

0
4

0

0

0

0

0

0

0

0

0

0

0

0

0

0

0
4

0
3

0
4

0

0

0

0

0

0
3

0

0

0

0

0

0

0

0
4

0
3

0

0

0
4

0

0

0
4

0
4

53.1069
2

53.1069
2

52.4613
2

0.387362

0.129121

0.129121

3.05311331771918e-15
2

0
4

0
4

0
7

0

0

0

0

0

0
4

0

0

0

0

0

0
4

0

0

0

0
4

0
4

0
4

0
4

0

0

0

0

0
4

0

0

0

0

0
4

0

0

0
4

0
4

0
7

0
7

0

0

0

0
4

0
4

0
4

0
4

0

0

0

0

0
4

0

0

0
4

0
4

0
4

0

0

0

0

0

0
4

0

0

0

0

0
4

0

0

0
4

0

0

0
4

0
4

0
3

0
3

0

0

0

0
4

0

0

0
4

0

0

0
4

0
4

3.81264
2

3.73483
2

0

3.73483

0

0

0
4

0.077809

0.077809

0
4

9.71445146547012e-17
2

0
4

0
3

0
3

0
3

0

0
4

0

0

0

0

0
4

0
4

0
4

0
4

0
4

0

0

0
4

0
4

0.258242

0.258242

0.258242

0

0
4

0
6

0

0

0
4

0

0

0

0

0
4

0

0

0
4

0

0

0
4

0

0

0
4

0
4

129.041
3

129.041
3

5.93956
3

0.0484412

121.143
3

0

0.158462
7

0

0

0

0

0
7

0

0

0

0

0.129121

0

0

0

0
6

0

0

0

0

0

0

0

0

0

0

0
7

0

0

0

0

0

0

0

0

0

0

0
7

0.129121

0

0

0

0

0

0

0

0

0

0
7

0

0

0

0

0

0

0

0

0

0

0.0730852
7

0

0

0.129121

0

0

0

0

0

0

0

0

0

0

0

0

0

0

0

0

0

0

0
7

0

0.129121

0

0

0

0

0

0.258242

0

0

0
2

0

0

0

0

0

0

0

0

0

0.903846

0
4

0

0
5

0

0
7

0
7

0

0

0

0

0

0

0
4

0

0

0
4

0

0

0
4

0

0

0
4

0

0

0
4

0

0

0
4

0

0

0
4

0

0

0
4

0

0

0
4

0

0

0
4

0

0

0
4

0
4

0
4

0
4

0

0

0

0
4

0

0

0
4

0

0

0
4

0

0

0
4

0

0

0
4

0

0

0
4

0

0

0
4

0

0

0
4

0

0

0
4

0
7

0
7

0

0

0
4

0
7

0

0

0

0

0

0

0
4

0

0

0
4

0

0

0

0

0
4

0

0

0
4

0

0

0
4

0

0

0

0
4

0
4

6.63422

5.46779

0.623287
3

0

0

0
4

0

0
6

0

0

0

0

0

1.32371

0

0

0

0

0

0

0

0

0

0

0

0

0

0

0

0

0

0

0

0

0

1.01117

0

0

0

0

0

0

0

0

0

0

0

0

0

0

0

0

1.67857

0

0

0

0

0.83105
4

0

0

0

0

0

0

0

0

0

0

0

0

0

0

0

0

0

0

0

0

0

0

0

0

0

0

0

0

0

0

0
4

0.622472
3

0.622472
3

0
2

0

0

0

0

0

0

0
4

0

0

0

0
4

0

0

0

0
4

0

0

0

0
4

0

0

0
4

0.452037

0.418553

0.0334842

6.93889390390723e-18

0
4

0

0

0

0
4

0

0

0

0
4

0

0

0
4

0

0

0

0
4

0

0

0
4

0.0919245

0.0919245

0

0
4

0

0

0
4

0

0

0
4

0

0

0
4

0

0

0
4

0

0

0
4

0

0

0
4

0

0

0
4

0

0

0
4

0

0

0
4

0

0

0
4

0

0

0

0

0
4

0

0

0
4

0

0

0
4

0

0

0
4

0

0

0
4

0

0

0
4

0

0

0
4

0
4

0

0

0

0

0
4

0
7

0
7

0
4

0

0

0

0

0
4

0

0

0

0

0

0
4

0

0

0

0

0
4

0

0

0
4

0
4

177.023

176.764

0
6

0
4

0

0

1.47837

0

0

0

0

0

0

0

12.7452
3

0

0

0

0

0

0.077809

0

0.116713

0

0

0
7

0

0

0.816994

0

0

0

0.116713

0

0

0

0
6

0.077809

0

0

0

0

0

0

0

0

0

15.901
2

0

14.1223

0

0.158462

0

0

0

0

0

0

0
4

0

0

0

0

0

0

0

0

0

0

0
3

0

0

0

0.332648

0

0

0

0

0

0.311236

0
4

0

0

0

0

0

0

0

0

0

0

44.4564

0

0

0.077809

0

0

0

0

0

0

0

0.077809
3

4.66854

0

0

0

0

0

0

0

0

0

0
6

0.277017
3

0

0

0.155618

0

0

0

0

0

0

0

0

0

0

0.077809

0

0

0

0

0

0.20693

0

0.415525
4

0

0

0

0

0

0

0

0.155618

0.116713

0

0
6

0

1.25667

0

0

0

0

0

0

0

0

0
4

0

0

0

0

0

0

0

0

0

0

0
5

0

0

0

0.155618

0

0

0

0

0

0

0
7

0.110883

0

0

0

0

0

0

0

0

0

0

0

0

0

0

0

0

0

0

0

0

0
4

0

0

0

0

0

0

0

0

0

0.077809

0
7

0

0

0

0

0

0

0

0

0

0

6.30213
4

36.0256
3

0

0

0

0

0

0

0

0

0

0

0.272331

0

0.194522

0

0

0

0

0

0

0

0.155618

0
4

0

0

0

0

0

0

0

0

0

0

0
6

0

0

0

0

0

0

0

0

0

0

0
4

0

0

0

0

0

0

0

0

0

0

0

0

0

0

0

0

0

0

0

0

0

0
4

0.077809

0

0

0

0

0

0

0

0

0

0

0

0

0

0

0

0

0

0

0

0

0

0

0

0

0

0

0

0

0

0

0

0

0

0

0

0

0

0.311236

0

0

0

0

0
7

0.138508
3

0

0

0

0

0

0

0.155618

0

0

0

0.427949
3

0

0

0

0

0

0

0

0

0

0

0

0

0.628335

0

0

0

0

0

0

0

0

0.311236
3

0

0

0

0

0

0.443531

0

0

0

0

0

0

0.077809

0

0

0

0

0.184804

0

0

0

0
4

0

0

0

0

0

0

0

0

0

0

0
7

0

0

0

0

0

0

0

0.700281

0

0

0

0

0

0

0

0

0

0

0

0

0

0

0

0

0

0

0

0

0

0

0

0

0
4

0

0

0

0

0

0.077809

0

0

0

0

0
4

0

0

0

0

0

0

0

0

0.129121

0

0

0.316924

0

0

0

0

0

0

0

0

0

0

0.077809
2

0

0

0

0

0

0

0

0

0

0

0

0

0.0739218

0

0

0

0.077809

0

0

0

0

0

0

0

0

0

0

0

0

0

0

0

3.36303

0

0

0

0

0.233427

0

0

0

0

0

0.443694

0

0

2.29536

0

0

0

0.0739218

0

0

0

0
3

0

0

0

0.110883

0

0

0

0

0

0

0

0.193681

0

0

0

0

0

0

0

0

0

0
6

0

0

0.155618

0

0

0

0

0

0

0

0

0

10.1541

0

0

0

0

0

0

0

0

0

0

0

0

0

0

0

0

0

0

0

0

0

0

0

0

0

0.077809

0

0

0.35014

0

0

0
4

0

0

0

0

0

0

0

0

0

0

0
4

0

0

0

0

0

0

0

0

0

0

0.466854

0

0

0

0

0

0.258726

0

0

0

0

0
4

0

0

0

0

0

0

0

0

0

0

9.88174

0

0

0

0

0

0

0.077809

0

0

0

0

0

0

0

0

0

0

0

0

0

0

0

0

0

0

0

0

0.0739218

0

0

0

0

0
6

0.25354

0

0

0

0

0

0

0

0

0

0

0

0

0

0

0

0

0

0

0

0

0

0.505758

0

0

0

0

0

0

0

0

0

0

0

0

0.077809

0

0

0

0

0

0

0

0

0

0

0

0

0.147844

0

0

0

0.348617

0

0.116713

1.05042

0
6

0

0

0

0

0.077809

0

0

0

0.116713

0.155618

0
6

0

0

0

0

0

0

0

0

0

0

0
4

0

0

0

0
4

0

0

0
4

0

0

0

0
4

0

0

0
4

0.258726

0.258726

0
4

0

0

0
4

0

0

0
4

0

0

0
4

0

0

0
4

0
4

0.997944

0.369609

0
7

0

0

0

0

0

0

0

0

0

0

0
7

0

0.184804

0

0.184804

0

0

0

0

0

0

0

0

0

0

0
4

0
7

0
7

0

0

0

0
6

0
6

0

0

0

0

0

0

0
4

0

0

0
4

0
7

0

0

0
4

0

0

0

0
4

0

0

0

0

0
4

0

0

0
4

0

0

0
4

0

0

0
4

0.628335

0.628335

0
4

0

0

0
4

0
4

29.362

24.6803
3

23.7267
3

0

0

0

0

0

0

0

0

0

0

0

0

0

0

0

0

0

0

0

0.0877713

0.129121

0

0

0.155618

0

0

0

0

0.581044

0

0

0

0

0

0

0

0

0

0

0

0
4

0
7

0
7

0

0

0

0

0

0

0
4

2.864
3

2.59251

0.193681

0

0.077809

4.30211422042248e-16
3

0
4

0

0

0
4

0

0

0
4

0

0

0
4

0

0

0
4

0

0

0
4

0

0

0
4

0

0

0
4

0
7

0
6

0

0
4

0
3

0

0

0

0

0

0
4

0

0

0

0

0

0
4

0

0

0

0
4

0
7

0
7

0
4

0

0

0

0

0
4

0

0

0
4

0
7

0

0

0
4

0

0

0

0
4

0
6

0
6

0

0

0

0

0

0

0

0
4

0

0

0

0
4

0

0

0

0
4

0

0

0

0

0

0
4

0

0

0
4

0

0

0

0

0

0
4

0

0

0

0
4

0

0

0

0
4

0

0

0

0
4

0

0

0
4

0

0

0

0

0
4

1.6621
7

0
7

0

0

0

0
7

0

0

0

0

0

0

1.6621

0
4

0

0

0
4

0

0

0

0
4

0

0

0

0

0
4

0

0

0

0
4

0

0

0

0
4

0

0

0
4

0

0

0
4

0

0

0
4

0

0

0
4

0

0

0

0
4

0
8

0
8

0

0

0

0

0

0

0

0

0
4

0

0

0
4

0

0

0

0
4

0

0

0

0
4

0

0

0

0
4

0

0

0

0
4

0

0

0
4

0

0

0
4

0

0

0
4

0

0

0

0
4

0

0

0
4

0
8

0

0

0

0

0

0

0

0

0
4

0

0

0
4

0

0

0
4

0

0

0

0
4

0

0

0
4

0

0

0
4

0

0

0

0
4

0

0

0

0
4

0

0

0
4

0

0

0
4

0

0

0
4

0
3

0

0

0

0

0

0

0
4

0

0

0
4

0

0

0
4

0

0

0
4

0

0

0
4

0

0

0
4

0.077809

0.077809

0
4

0

0

0
4

0

0

0
4

0

0

0
4

0

0

0
4

0

0

0

0

0
4

0

0

0
4

0

0

0
4

0

0

0
4

0

0

0
4

0

0

0
4

0

0

0
4

0

0

0
4

0

0

0
4

0

0

0
4

0

0

0
4

0

0

0

0

0

0
4

0

0

0
4

0

0

0
4

0

0

0
4

0

0

0
4

0

0

0
4

0

0

0
4

0

0

0
4

0

0

0
4

0

0

0
4

0

0

0
4

0

0

0

0

0

0
4

0.077809

0.077809

0
4

0

0

0
4

0

0

0
4

0

0

0
4

0

0

0
4

0

0

0
4

0

0

0
4

0

0

0
4

0

0

0
4

0

0

0
4

2.85882428840978e-15

0
4

135.124
3

132.9
3

102.579
3

0.589681
2

0
2

0

0

0

0

0

0

0.0739218

0

0

0

0
3

0

0

0

0

0.258242

0

0

0

0

0

0.129121
3

0

0

0.221765

0.369609

0

0

0

0

0

0

0.246234
2

0

0

0

0

0

0

0

0

0

0

0

0

0

0

0.147844

0.184804

0

0

0

0

0.0739218

0.203751
2

0

0

0

0

0

0

0

0

0

0

0

0

0

0

0.40657

0

0

0

0

0.40657

0

0

0

0

0

0

0

0

0

0

0

0

0.111826
2

0

0

0

0

0.0739218

0

0.0739218

0

0

0.077809

0.279501

0.129121

0

0

0

0.0739218

0

0

0

0

0

0

0

0.110883

0

0

0

0

0

0

0

0

0

0

0

0

0

0

0

0.0739218

0

0

0.110883

0

0.077809

0.131657

0

0

0

0

0

0

0

0

0

0.369609

0

0.0502263

0

0

0

0

0

0

0

0

0.446163

0

0

0

0

0

0

0.147844

0

0

0

1.84804

0

0

0

0

0

0

0.517453

0

0.0739218

0.743105

0

0.0633849

1.73984

0.145324
2

0.0633849

0.195232

0

4.43531

0.0484412

0.433685

0.665296

8.78022

0.0726619

0

0

0

0.12677

0

0

0.184804

0

0

0

0

0

0.226734
3

0

0

1.40451

0

0

0

0.443531

0

0.077809

0

0

0

0

0.0739218

0.248189

0

0.0950773

0.0633849

0

0

0

0

0

0

0

0

0.0739218

0

0.110883

0

0

0.184804

0
3

0

0

0.295687

0

0.147844

0

0.295687

0.387362

0

0

0.340387

0

0

0.0633849

0

0

0

0

0

0

0

3.04756220259605e-14
3

0
4

0

0

0

0

0

0
4

0.0837105

0

0

0.0837105

0
4

0

0

0
4

0

0

0

0

0
4

2.03285

0.332648

0.184804

1.5154

0
4

0

0

0

0
4

0

0

0
4

0

0

0

0
4

0

0

0

0
4

0

0

0

0
4

0

0

0
4

0
3

0

0

0

0
4

0

0

0

0
4

0

0

0
4

0

0

0

0
4

0

0

0
4

0

0

0

0
4

0

0

0
4

0

0

0
4

0

0

0
4

0

0

0
4

0

0

0
4

0
2

0

0

0

0
4

0

0

0
4

0

0

0
4

0

0

0
4

0

0

0
4

0

0

0
4

0

0

0
4

0.0739218

0.0739218

0
4

0

0

0
4

0

0

0
4

0

0

0
4

0

0

0
4

0

0

0
4

0

0

0
4

0

0

0
4

0

0

0
4

0

0

0
4

0

0

0
4

0

0

0
4

0

0

0
4

0

0

0
4

0

0

0
4

0

0

0

0
4

0

0

0
4

0

0

0
4

0

0

0
4

0

0

0
4

0

0

0
4

0

0

0
4

0.0334842

0.0334842

0
4

0

0

0
4

0

0

0
4

0

0

0
4

0

0

0
4

0

0

0
4

0

0

0
4

0

0

0

0

0
4

0

0

0

0

0
4

0

0

0

0

0
4

2.6999236180103e-14
3

0
4

8.30199
3

8.23861
3

3.33822
2

0

0

0

0

0

0

0.077809

0

0

0

4.2959
2

0

0

0

0

0

0

0

0

0

0

0.258242
3

0

0.0739218

0

0

0

0.194522

0

0

0

0

0

0

0

0

0

0

0

0

0

0

0

0

0

0
4

0

0

0

0

0

0

0
4

0

0

0

0
4

0

0

0

0

0
4

0

0

0

0
4

0

0

0
4

0.0633849

0.0633849

0
4

0

0

0
4

0

0

0
4

0

0

0
4

9.43689570931383e-16
3

0
4

3.12475
5

3.12475
5

0.313925
5

0
7

0

0

0

0

0

0

0

2.38874

0

0.0367698

0

0.0367698

0

0

0

0

0.0877713

0

0

0

0

0

0

0

0.131657

0

0.129121

0

5.55111512312578e-17
5

0
4

0

0

0
4

0

0

0
4

0

0

0
4

0

0

0
4

0

0

0
4

0

0

0
4

0

0

0
4

0
4

43.1716
4

26.6153
4

25.6498
4

0
7

0

0

0

0

0

0

0

0

0

0

0

0

0

0

0

0

0

0

0

0

0

0.965484

0

0

0

0

0

0

0

0

0

0

0
4

0

0

0

0

0

0

0

0

0

0

0
6

0

0

0

0

0

0

0

0

0

0

0
7

0

0

0

0

0

0

0

0

0

0

0
7

0

0

0

0

0

0

0

0

0

0

0
7

0

0

0

0

0

0

0

0

0

0

0
7

0

0

0

0

0

0

0

0

0

0

0
5

0

0

0

0

0

0

0

0

0

0

0
6

0

0

0

0

0

0

0

0

0

0

1.99840144432528e-15
4

0
4

0
6

0
6

0

0

0

0

0

0

0

0

0

0

0
6

0

0

0

0

0

0

0

0

0

0

0
6

0

0

0

0

0

0

0

0

0

0

0

0

0

0

0

0

0

0

0

0

0

0

0

0

0

0

0

0

0

0

0

0

0

0

0

0

0

0

0

0

0

0

0

0

0

0

0

0

0
4

2.7518
3

0

0

0

0.883142

0.551547

0

0.655735

0.661376

0

0

1.11022302462516e-16
3

0
4

0

0

0
4

0

0

0

0
4

0

0

0
4

0

0

0

0
4

0

0

0

0
4

0

0

0
4

0

0

0

0
4

0

0

0

0
4

0

0

0

0
4

0

0

0
4

0
4

0
4

0
4

0

0

0

0

0

0
4

0

0

0

0
4

0

0

0

0
4

0

0

0
4

0

0

0
4

0

0

0

0
4

0

0

0

0
4

0

0

0

0
4

0

0

0

0
4

0

0

0
4

0

0

0

0
4

0.466854
2

0

0.466854
3

0

0

0

0

0

0

0
4

0

0

0
4

0

0

0

0
4

0

0

0
4

0

0

0
4

0

0

0

0
4

0

0

0

0
4

0

0

0

0
4

0

0

0

0
4

0

0

0
4

0

0

0
4

0

0

0

0

0

0

0

0

0

0

0
4

0

0

0

0
4

0

0

0

0
4

0

0

0

0
4

0

0

0
4

0

0

0
4

0

0

0
4

0

0

0
4

0

0

0
4

0.0877713

0.0877713

0
4

0

0

0
4

0
5

0
5

0

0

0

0

0

0

0

0

0

0
4

0

0

0
4

0

0

0
4

0.138508

0.138508

0
4

0

0

0
4

0

0

0
4

0

0

0
4

0

0

0
4

0

0

0
4

0

0

0
4

0

0

0
4

0

0

0

0

0
4

0

0

0
4

0

0

0
4

0

0

0
4

0

0

0
4

0

0

0
4

0

0

0
4

0

0

0
4

0

0

0
4

0

0

0
4

0

0

0
4

0

0

0

0

0

0

0
4

0

0

0
4

0

0

0
4

0

0

0
4

0

0

0
4

0

0

0
4

0

0

0
4

0

0

0
4

0

0

0
4

0

0

0
4

0

0

0
4

0

0

0

0

0

0
4

0

0

0
4

0

0

0
4

0

0

0
4

0

0

0
4

0

0

0
4

0

0

0
4

0

0

0
4

0

0

0
4

0

0

0
4

0

0

0
4

0
5

0
5

0

0
4

0

0

0
4

0

0

0
4

0

0

0
4

0

0

0
4

0

0

0
4

0

0

0
4

0

0

0
4

0

0

0
4

0

0

0
4

0

0

0
4

0

0
5

0

0

0

0

0

0
4

0

0

0
4

0

0

0
4

0

0

0
4

0

0

0
4

0

0

0
4

0

0

0
4

0

0

0
4

0

0

0
4

0

0

0
4

0

0

0
4

0
4

0
4

0

0

0

0

0

0

0

0

0

0

0
4

0

0

0

0

0

0

0

0

0

0

0

0

0

0

0

0

0
4

0

0

0

0
4

0

0

0
4

0

0

0
4

0

0

0
4

0

0

0
4

0

0

0
4

0

0

0
4

0

0

0
4

0

0

0
4

0

0

0
4

0

0

0
4

0
6

0

0

0

0

0

0

0

0

0
4

0

0

0
4

0

0

0
4

0

0

0
4

0

0

0
4

0

0

0
4

0

0

0
4

0

0

0
4

0

0

0
4

0

0

0
4

0

0

0
4

0.40698
3

0

0.295687

0

0

0.077809

0.0334842

0
4

0

0

0
4

0

0

0
4

0

0

0
4

0

0

0
4

0

0

0
4

0

0

0
4

0

0

0
4

0

0

0
4

0

0

0
4

0

0

0
4

0
4

0

0

0

0

0

0

0

0

0
4

0

0

0
4

0

0

0
4

0

0

0
4

0

0

0
4

0

0

0
4

0

0

0
4

0

0

0
4

0

0

0
4

0

0

0
4

0

0

0
4

1.80061
3

0

0.484779

1.31583

0

0

0
4

0

0

0
4

0

0

0
4

0

0

0
4

0

0

0
4

0

0

0
4

0

0

0
4

0

0

0
4

0

0

0
4

0

0

0
4

0
4

0
4

0

0

0

0
4

0
2

0

0

0

0

0

0

0
4

0

0

0

0

0

0

0
4

0
4

0

0

0

0

0

0

0
4

1.48489

1.48489

0

0

0
4

0.484779
4

0.277017
4

0

0

0

0

0

0

0

0

0

0

0
4

0

0

0

0

0

0.207762
3

0
4

0

0

0

0

0

0
4

0
4

0
4

0

0

0

0
4

0
4

0

0

0

0

0

0

0
4

0

0

0

0

0

0

0

0
4

0.0739218

0.0739218

0

0

0

0

0

0

0
4

0
7

0
7

0

0

0
4

0
4

0
4

0

0
4

0

0

0

0

0
4

0
7

0
7

0
4

0
4

0

0

0

0
4

0

0

0

0

0

0
4

0
4

0
4

0

0

0

0

0
4

0
4

0

0

0

0

0

0

0
4

0

0

0

0
4

0

0

0

0

0
4

0
6

0
6

0
4

0
5

0
5

0
4

0

0

0

0
4

0

0

0

0
4

0

0

0
4

0

0

0

0

0

0
4

0

0

0

0

0

0
4

0

0

0

0

0

0
4

4.27458

4.08977

0

0

0

0

0.110883

0

0.0739218

0

0

0
4

0

0

0

0

0
4

0

0

0

0
4

0

0

0

0

0

0
4

0

0

0

0

0
4

0

0

0

0

0
4

0

0

0
4

0

0

0

0
4

0

0

0
4

0

0

0

0
4

0

0

0

0
4

0.0735396

0
3

0

0
2

0

0

0

0.0735396

0

0

0

0
4

0

0

0

0

0
4

0

0

0

0
4

0

0

0
4

0

0

0

0

0

0
4

0

0

0

0
4

0

0

0

0

0
4

0

0

0

0
4

0

0

0
4

1.67857

1.67857

0

0
4

0

0

0
4

0
4

0
4

0
4

0

0

0

0
4

0

0

0
4

0

0

0

0

0
4

0

0

0

0

0
4

0.346271

0.346271

0

0
4

0

0

0
4

0

0

0

0
4

0

0

0

0
4

0

0

0
4

0

0

0

0
4

0

0

0

0

0
4

2.14688
4

0
4

0

0

2.14688

0

0

0

0

0
4

0

0

0

0

0
4

0

0

0

0

0
4

0

0

0

0
4

0.211228

0.147844

0.0633849

0

0
4

0

0

0
4

0

0

0

0
4

0

0

0

0
4

0

0

0

0
4

0

0

0
4

0

0

0

0
4

0
6

0
6

0
4

0.129121

0

0.129121

0
4

0

0

0
4

0

0

0
4

0

0

0
4

0

0

0

0
4

0

0

0

0
4

0

0

0
4

0

0

0

0
4

0

0

0

0
4

0

0

0
4

0
4

2.10087

2.10087

2.0641

0
6

0

0.0367698

0

0

0

0

0

0

0

0

0

0

6.93889390390723e-18

0
4

0
6

0

0

0
4

0

0

0
4

0

0

0
4

0

0

0
4

0

0

0
4

0
4

22.4071
3

21.0844
3

20.399
3

0
4

0.077809

0

0.129121

0

0

0

0.322802

0

0

0

0
4

0

0

0

0

0

0

0

0

0

0

0

0

0

0

0

0

0

0

0

0

0

0
4

0

0

0

0

0

0

0

0

0

0

0

0.155618

0

0

2.08166817117217e-15
3

0
4

0
6

0
6

0

0

0

0
4

0

0

0
4

0

0

0
4

0

0

0
4

0

0

0
4

0
4

0
4

0
4

1.32275

1.32275

0

0
4

0

0

0

0
4

0

0

0
4

0

0

0
4

0

0

0

0
4

0

0

0
4

0

0

0
4

1.11022302462516e-15
3

0
4

31.2588

31.2588

20.0658

0
7

0
6

0

0

0

0

0

0

0

0

0

0

2.12548

0

0

0

0

0.158462

0

0

0

0

0

0
7

0

0

0

0

0

0

0

0

0

0

0

0

0

0

0

0

0

0

0

0

0

0

0

0

0

0

0

0

0

0

0

0

0

0

0

0

0

0

0

0

0

0

0

0
5

0

0

0

0.110883

0

0

0

0

0

1.84077

0
7

0
7

0
7

0

0

0

0

0

0

0

0

0

0

0
6

0

0

0

0

3.27411

0

0

0

0.526628

0

0
7

0

0

0

0

0.411751

0

0

0

0

0

0

0

0

0

0

0

0

0

0

0

0

0
5

0

0

0

0

0

0

0

0

0

0

0
7

0

0

0

0

0

0

0

0

0

0

0

0

0

0

0

0

0

0

0

0

0

0.129121

0

0

0

0

0

0

0

2.61583

0

0

2.66453525910038e-15

0
4

0
7

0
7

0

0

0

0

0

0

0

0

0

0
7

0
8

0
7

0

0

0

0

0

0
4

0

0

0
4

0

0

0
4

0

0

0
4

0

0

0
4

0

0

0
4

0

0

0
4

0

0

0

0

0
4

0

0

0

0

0

0

0

0

0

0
4

0
4

0
4

0

0

0

0
4

0

0

0

0

0
4

0

0

0

0
4

0

0

0
4

0

0

0
4

0

0

0
4

0
4

0

0

0

0

0

0

0

0

0

0

0

0

0

0

0
6

0

0

0

0

0

0

0

0

0

0

0
7

0

0

0

0

0

0

0

0

0

0

0

0

0

0

0

0

0

0

0

0

0

0

0

0

0

0

0

0

0

0

0

0

0
7

0

0

0

0

0

0

0

0

0

0

0

0

0

0

0

0

0

0

0

0

0

0

0

0

0

0

0

0

0

0

0

0

0

0

0

0

0

0

0

0

0

0

0

0
6

0

0

0

0

0
7

0

0
7

0

0

0
7

0
6

0

0
7

0

0
6

0

0

0

0

0

0

0

0

0

0

0

0

0

0

0

0

0

0

0

0

0

0

0
7

0

0

0

0

0

0

0

0

0

0

0
7

0

0

0

0

0

0

0

0

0

0

0
7

0

0

0

0

0

0

0

0

0

0

0
7

0

0

0

0

0

0

0

0

0

0

0
6

0

0

0

0

0

0

0

0

0

0

0
4

0
6

0
6

0
4

0

0

0
4

0

0

0
4

0

0

0
4

0

0

0
4

0

0

0
4

0

0

0
4

0

0

0
4

0
4

497.075
3

497.075
3

486.719
3

10.3561
3

0

0

0

0

0
4

0

0

0

0
4

0

0

0
4

0

0

0
4

0

0

0
4

0

0

0
4

0
4

8.52106

8.52106

4.75049

0
7

0

0

0

0

0

0

0

0

0

0

0
6

0

0

0

0

0

0

0

0

0

0

0
3

0

0

0

0

0

0

0

0

0

0

0
3

0

0

0

0

0

0

0

0

0

0

0
3

0

0

0

0

0

0

0

0

0

0

0
7

0

0.193681

0

0

0

0

0

0

0

0

0
7

0

0

0

0

0

0

0

0

0

0

0
7

0

0

0

0

0

0

0

0

0

0

0
3

0

0

0

0

0

0

0

0

0

0

0
6

0

0

0

0

0

0

0

0

1.04026

0

0
7

0

0

0

0

0

0

0

0

0

0

0

0
3

0

0

0

0

0

0

0

0

0

0

0

0

0

0

0

0

0

0

0

0

0

0
3

0

0

0.138508

0

0

0

0

0

0

0

0

0

0

0

0

0

0

0

0

0

0

0
7

0

0

0

0

0

0

0

0

0

0

0
7

0

0

0

0

0

0

0

0

0

0

0

0

0

0

0

0

0

0

0

0

0

0
4

0

0

0

0

0

0

0

0

0

0

0

0

0

0

0

0

0

0

0

0

0

0
7

0
7

0

0

0

0

0

0

0

0

0

0

0
3

0

0

0

0

0

0

0

0

0

0

0

0

0

0

0

0

0

0

0

0

0

0
7

0

0

0

0

0

0

0

0

0

0

0
3

1.67857

0

0

0

0

0

0

0

0

0

0

0

0

0

0

0

0

0

0

0

0

0

0

0.193681

0

0

0

0

0

0

0

0

0

0

0

0

0

0

0

0

0

0

0

0

0

0

0

0

0.258242

0

0

0

0

0

0

0

0

0

0

0

0

0

0

0

0

0.138508
4

0

0

0

0

0

0

0

0

0

0

0

0

0

0

0

0

0

0

0

0

0

0

0

0

0

0

0

0

0

0

0

0

0

0

0

0

0

0

0
7

0

0

0

0

0

0

0

0

0

0

0
7

0

0

0

0

0

0

0

0

0

0

0
3

0

0

0

0

0

0

0

0

0

0

0
7

0

0

0

0

0

0

0.129121

0

0

0

0
7

0

0

0

0

0

0

0

0

0

0

1.08246744900953e-15

0
4

0

0

0
4

0

0

0
4

0

0

0
4

0

0

0
4

0

0

0
4

0

0

0
4

0

0

0
4

0

0

0
4

0
4

680.858

602.782
3

0
6

0
7

0

0

0

0

0

0

0

0

0

0

0
6

0

0

0

0

0

1.10883

0

0

0

0

1.52359
3

0

0

0.332648

0

0

0

0

0

0

0.394971

0
6

0

0

0

0

0

0.258242

0

0

0

0

295.555
2

0

0

0

1.64371

0

0

0

0.295687

0

0

2.32854

0

0.14617

0

0

0

0

0

0

0

0

0.207762
4

1.25583

0

0

0

0

0

0

0.517453

0

0

2.59717

0

0.138508

0

0

0

0

0

0

0

0

0

0

0

0.109628

0

2.49381

0

0

0

0

0

0
7

0

2.95687

0.175543

2.67019

0

0.346271

0

0

0

0

248.517
2

0

0

0

0

0

0

0

0.184804

0

0

0.129121

0
6

0

0

0

0

0

0

0.184804

0

1.36713

0

0
6

0

0.184804

0

0

0

0.0365426

0

0

0

0.665296

0
6

0.221765

0

0

0

0

0

0

0

0

0

0

0

0

0

0

0

0

0

0

0

0

0
6

0

0

0

0.0877713

0

0

0

0

0

0

1.73716

0

0

0

0

0

0

0

0

0

0

1.52078
2

0.0739218

0

0

0

0

0

0

0

0

0

0
6

0

0

0

0

0

0

0.0739218

0

0

0.110883

0
7

0

0

0

0

0

0

0

0.0739218

0

0.110883

0
7

0.077809

0.0739218

0

0

0

0.221765

0

0

0.0739218

0

0

0
4

0

0.0739218

0

0

0.110883

0

0

0

0

0

0
7

0

0

0

0

0.184804

0

0

0

0

0

0
5

0

0.887062

0

0

0

0

0

0

0

0

0

0

0

0

0

0

0

0

0

0

0

0
7

0

0

0

0

0

0

0

0

0

0

0

0

0.138508

0

0

0

0

0

0

0

0

0.131657

0

0

0

1.07187

0

0

0

0

0

0

0

0

0

0

0

0

0

0

0

0

0

1.33059

0

0

0

0

0

1.7002

0

0

0

0

0

0
7

0

0

0

0

0

0

0

0

0

0

0

0

0

0

0

0

0

0

0

0

0

0

0

0

0

0

0

0

0

0

0

0

0.924022

0

0

0

0

0

0

0

0.110883

0

0

0

0.0365426

0

0

0

0.110883

0

0

0

0

0

0

0

0

0

0

0.295687

0

0

0

0

0

0

0

0.0739218

0

0

0.665296

0

0

0

0

0

0

0

0

0

0

0

0.221765

0

0

0

0

0

0

0.110883

0

0

0

0

0

0

0

0

0.702257

0

0

0

0

0

0

0

0

0

0

0
4

0
5

0.0739218

0

0

0

0

0

0

0

0

0.81314

2.23467

0

0

0

0

0

0

0

0

0

0

0

0

0.0739218

0

0

0

0

0

0

0

0

0

0

0

0.0739218

0

0

0

0

0

0

0.129121

0

0

0

0.0739218

0

0

0

0

0

0

0.147844

0

0

0

0

0

0

0

0

0

0

0

0.554413

0

0

0

0

0

0

0

0

0

0.110883

0

0.138508

0

0

0

0

0

0

0

0

0

0

0

0

0

0

0.0739218

0

0

0

0

0

0

0

0

0

0

0

0

0

0

0.0739218

0

0
7

0.207762

0

0

0

0

0

0

0

0

0

0

0
7

0

0

0

1.14579

0.0739218

0

0

0

0

0
7

0

0

0

0
4

0

0

0
4

0
5

0

0

0

0

0

0

0

0

0

0

11.8267
2

0

0

0

0

0

0.258308

0

2.06981

0

0.665296

0
6

0

0

0

0.295687

0

0

0

0

0

0

6.62636612247525e-13
3

0
4

2.51297

0

0

0

0

0

0

0

0

0

0

0

0

0
6

0

0

0

0

0

0

0

0

0

0

0
7

0

0

0

0

0

0

0

0

0

0

0

0

0

0

0

0

0

0

0

0

0

0

0

0

0

0

0

0

0

0

0

0

0

0

0

0

0

0

0

0

0

0

0

0
7

0

0

0

0

0.258242

0

0

0

0

0

0

0

0

0

0

0

0
7

0
7

0

0

0

0

0

0
6

0

0

0

0

0
7

0

0

0

0

0

0

0

0

0

0

0

1.74313

0

0

0

0

0

0

0

0

0

0
5

0

0

0

0

0

0

0

0

0

0

0
7

0

0

0

0

0

0

0

0

0

0

0.401969

0

0

0

0

0

0

0

0.109628

0

0

0

0

0

0

0

0

0

0

0

0

0

0

0

0

0

0

0

0

0

0

0

0

0
4

14.8906
2

14.3362
2

0

0.443531

0.110883

4.57966997657877e-16
2

0
4

0

0

0

0
4

0

0

0

0
4

0

0

0
4

0

0

0

0
4

0

0

0
4

0

0

0
4

0

0

0

0
4

0

0

0

0
4

0

0

0

0
4

0

0

0
4

0
4

0
4

0

0

0

0

0

0

0

0

0

0
4

0

0

0
4

0

0

0

0
4

0

0

0

0
4

0

0

0
4

0

0

0

0
4

0

0

0
4

0

0

0
4

0

0

0
4

0

0

0
4

0

0

0
4

0
6

0
6

0

0

0

0

0

0

0

0

0
4

0

0

0
4

0

0

0
4

0

0

0
4

0

0

0
4

0

0

0
4

0

0

0
4

0

0

0
4

0

0

0
4

0

0

0
4

0

0

0
4

0.110883
3

0.110883
3

0

0

0

0

0

0

0

0

0

0
4

0

0

0
4

0

0

0
4

0

0

0
4

0

0

0
4

0

0

0
4

0

0

0
4

0

0

0
4

0

0

0
4

0

0

0
4

0

0

0
4

0
6

0
6

0

0

0

0

0

0
4

0

0

0
4

0

0

0
4

0

0

0
4

0

0

0
4

0

0

0
4

0

0

0
4

0

0

0
4

0

0

0
4

0

0

0
4

0

0

0
4

19.3912
3

19.2527
3

0

0

0

0.138508

0

1.11022302462516e-16
3

0
4

0

0

0
4

0

0

0
4

0.147844

0.147844

0
4

0

0

0
4

0

0

0
4

0

0

0
4

0

0

0
4

0

0

0
4

0

0

0
4

0

0

0
4

2.58925

2.32594

0.175543

0.0877713

0
4

0.295687

0.295687

0
4

0

0

0
4

0

0

0
4

2.19505

2.19505

0
4

0

0

0
4

0

0

0
4

0

0

0
4

0

0

0
4

0

0

0
4

0

0

0
4

2.49315
3

2.35464
3

0

0

0.138508

0

0

0

0

0

0

1.11022302462516e-16
3

0
4

0

0

0
4

0

0

0
4

0.0739218

0.0739218

0
4

0.0739218

0.0739218

0
4

0

0

0
4

0

0

0
4

0

0

0
4

0

0

0
4

0

0

0
4

0

0

0
4

0

0

0

0

0

0

0

0

0
4

0

0

0
4

0

0

0
4

0

0

0
4

0

0

0
4

0

0

0
4

0

0

0
4

0

0

0
4

0

0

0
4

0

0

0
4

0

0

0
4

0
5

0

0

0

0

0

0

0

0

0

0
4

0

0

0
4

0

0

0
4

0

0

0
4

0

0

0
4

0

0

0
4

0

0

0
4

0

0

0
4

0

0

0
4

0

0

0
4

0

0

0
4

3.35956

2.64698

0

0

0

0

0

0

0

0

0

0

0
7

0

0

0

0

0

0

0.712581

0

0

0

0

0

2.22044604925031e-16

0
4

0
4

0
4

0

0

0

0

0

0
4

0

0

0
4

0

0

0
4

0

0

0
4

0

0

0
4

0

0

0
4

0

0

0
4

0

0

0
4

0

0

0
4

0

0

0
4

0

0

0
4

0

0

0

0

0

0

0
4

0

0

0
4

0

0

0
4

0.0739218

0.0739218

0
4

0

0

0
4

0

0

0
4

0

0

0
4

0
6

0

0

0

0

0

0
4

0
4

0

0

0

0

0

0

0
4

0
4

0

0

0

0

0

0
4

0

0

0

0

0

0

0

0
4

0.168717
4

0.077809

0.0909077

0

0

0
4

0

0

0

0

0

0

0
4

0.903846

0.903846

0

0

0
4

0
6

0

0

0

0

0
4

1.31583
4

0
4

0

0

0

0

0

0

0

0

0

0

0

0

0

0

0

0

0

0

0

0

0

0

0

0

0

0

0

0

0

0

0

0

0.415525

0

0

0

0

0

0

0

0

0

0

0

0

0

0

0

0

0

0

0

0

0

0

0

0

0

0

0

0

0

0.900304

0
4

0
6

0
6

0
4

0
7

0

0

0
4

0

0

0

0

0

0
4

0

0

0

0

0
4

0

0

0

0

0
4

0

0

0

0

0

0
4

0

0

0

0
4

0

0

0

0
4

0

0

0

0
4

0

0

0

0

0
4

3.11588

0
7

0

0

0

0

0

0

0

0

0

0

0
6

0

0

0

0

0

0

0

0

0

0

0.0877713

0

0

0

3.02811
2

0

0

0

0
4

0

0

0

0

0
4

0

0

0

0

0
4

0

0
7

0

0
4

0

0

0

0

0

0
4

0
7

0

0

0

0

0
4

0

0

0

0

0
4

0

0

0

0

0
4

1.38508

0

0

0

1.38508

0
4

0

0

0

0

0
4

0

0

0

0
4

0
6

0
6

0

0

0

0

0

0

0

0

0
4

0

0

0

0
4

0

0

0

0
4

0.107406

0.107406

0

0
4

0

0

0

0

0
4

0

0

0

0
4

0.110883

0

0

0.110883

0
4

0

0

0

0
4

0

0

0

0
4

0

0

0

0

0
4

0.258242

0

0

0.258242

0
4

0

0
6

0

0

0

0

0

0

0

0

0

0

0

0

0

0

0

0

0

0

0

0

0

0

0

0

0

0

0

0
4

0.0877713

0.0877713

0

0

0
4

0

0

0
4

3.99359

3.99359

0
4

0.329171

0.329171

0

0

0
4

0

0

0

0

0
4

0

0

0

0

0
4

0

0

0

0

0
4

0.645604

0.387362

0.258242

0
4

0

0

0
4

0

0

0

0

0
4

0
6

0
6

0

0

0

0

0
7

0

0

0

0

0

0

0

0
4

0.710164

0.258242

0.451923

0

0
4

0

0

0

0
4

0

0

0
4

0

0

0
4

0

0

0

0

0
4

0.129121

0

0.129121

0
4

0

0

0

0

0
4

0

0

0
4

0

0

0
4

0

0

0

0
4

4.46873

4.46873

0

0

0

0

0

0

0
4

0.129077

0.0739218

0.0551547

0

6.93889390390723e-18

0
4

0

0

0
4

0

0

0
4

0

0

0

0
4

0.443531

0.295687

0.147844

2.77555756156289e-17

0
4

0

0

0

0
4

0

0

0

0
4

0

0

0

0
4

0

0

0

0
4

0

0

0
4

11.5654
3

11.2884
3

0

0

0

0

0

0

0.277017

0

0

0
4

0

0

0

0
4

0

0

0

0
4

0

0

0

0
4

0

0

0

0
4

0

0

0
4

0

0

0

0
4

0

0

0

0
4

0

0

0
4

0

0

0

0
4

0

0

0

0
4

1.54543045027822e-13

0
4

2.03499439521693e-11

0
4

6.91735
4

6.64972
4

6.64972
4

0.258242
4

0

0

0

0

0

0

0

0

0

0

0

0

0

0

0

0

0

0

0

0

0

0

0

0

0

0

0

0

0

0

0

0

0

0

0

0

0

0

0

0

0

0

0

0

0
4

0

0

0

0

0

0

0

0

0

0

0
4

0

0

0

0

0

0

0

0

0

0

0

0

0

0

0

0

0

0

0

0

0

0
4

0

0

0

0

0

0

0

0

0

0

0

0

0

0

0

0

0

0

0

0

0

0
4

0

0

0

0

0

0

0

0

3.4217
5

0

0

0

0

0

0

0

0

0

0

2.58242

0

0

0

0

0

0

0

0

0

0

0.387362

0

0

0

0

0

0

0

0

0

0

0
6

0

0

0

0

0

0

0

0

0

0

0

0

0

0

0

0

0

0

0

0

0

0

0

0

0

0

0

0

0

0

0

0

0
4

0

0

0

0

0

0

0

0

0

0

0
4

0

0

0

0

0

0

0

0

0

0

0
4

0
4

0

0

0

0

0

0

0

0

0
4

0

0

0
4

0

0

0

0

0

0
4

0

0

0

0

0

0
4

0

0

0

0
4

0

0

0

0
4

0

0

0

0
4

0

0

0
4

0

0

0
4

0

0

0
4

0
4

0

0

0

0

0

0

0

0
4

0
4

0

0

0

0

0
4

0
4

0

0

0

0

0
4

0

0

0
4

0
4

0

0

0

0

0
4

0
4

0

0

0

0

0
4

0
4

0

0

0

0
4

0
4

0

0

0

0
4

0
4

0

0

0

0
4

0
4

0

0

0

0
4

0

0

0
4

0

0

0
4

0
4

0

0

0

0
4

0

0

0
4

0
4

0

0

0

0
4

0

0

0
4

0
4

0.138508
4

0.138508
4

0

0

0

0

0.138508

0

0
4

0
4

0

0

0

0
4

0
4

0

0

0

0
4

0

0

0
4

0
4

0

0

0

0
4

0
4

0

0

0

0
4

0
4

0

0

0

0

0
4

0
4

0

0

0

0

0
4

0
4

0

0

0

0
4

0
4

0

0

0

0
4

0
4

0

0

0

0
4

0
4

0

0

0

0
4

0
4

0

0

0

0

0

0

0
4

0

0

0
4

0

0

0
4

0

0

0
4

0
4

0

0

0

0
4

0
4

0

0

0

0
4

0
4

0

0

0

0
4

0
4

0

0

0

0
4

0
4

0

0

0

0
4

0
4

0

0

0

0
4

0
4

0

0

0

0
4

0
4

0

0

0

0
4

0
4

0

0

0

0
4

0
4

0

0

0

0
4

0
4

0
6

0
6

0

0

0

0
4

0
4

0

0

0

0
4

0
4

0

0

0

0
4

0
4

0

0

0

0
4

0
4

0

0

0

0
4

0
4

0

0

0

0
4

0
4

0

0

0

0
4

0
4

0

0

0

0
4

0
4

0

0

0

0
4

0
4

0.129121

0.129121

0.129121

0
4

0
4

0

0

0

0
4

0
4

0

0

0

0

0

0

0
4

0
4

0

0

0

0
4

0
4

0

0

0

0
4

0
4

0

0

0

0
4

0
4

0

0

0

0
4

0
4

0

0

0

0
4

0
4

0

0

0

0
4

0
4

0

0

0

0
4

0
4

0

0

0

0
4

0
4

0

0

0

0

0

0
4

0
4

0

0

0

0
4

0
4

0

0

0

0

0
4

0

0

0
4

0
4

2.77555756156289e-17
4

0
4

52.0324

0
5

0
5

0
6

0
5

0
4

0

0

0

0

0

0

0
4

0

0

0
4

0

0

0
4

0

0

0
4

0

0

0
4

0

0

0
4

0

0

0
4

0

0

0
4

0

0

0
4

0

0

0
4

0

0

0
4

0
4

0

0

0

0

0

0

0
4

0

0

0
4

0

0

0
4

0

0

0
4

0

0

0

0
4

0

0

0

0
4

0

0

0

0
4

0

0

0
4

0

0

0

0
4

0

0

0
4

0

0

0
4

0
4

51.4794

0

0
7

0

0

0

0

0

0

0

0

0

0

0

0

0

0

0

0

0

0

0

0

0

0

0
7

0

0

0

0

0

0

0

0

0

0

0
7

0

0

0

0

0

0

0

0

0

0

0
7

0

0

0

0

0

0

0

0

0

0

0

0

0

0

0

0

0

0

0

0

0

0
7

0

0

0

0

0

0

0

0

0

0

0

0

0

0

0
6

0
6

0

0

0

0

0

0
7

0
7

0

0

0
8

0

0

0

0

0

0

0

0

0

0

0
7

0

0

0

0

0

0

0

0

0

0

0
7

0

0

0

0

0

0

0

0

0

0

0

0

0

0

0

0

0

0

0

0

0

0

0

0

0

0

0

0

0

0

0

0

0

0

0

0

0

0

0

0

0

0

0

0
7

0

0

0

0

0

0

0

0

0

0

0
4

0

0
7

0

0

0

0

0

0

0

0

0

0

0
7

0

0

0

0

0

0

0

0

0

0

0
7

0

0

0

0

0

0

0

0

0

0

0
6

0

0

0

0

0

0

0
4

0

0
7

0

0

0
7

0
7

0

0

0

0

0

0

0
7

0

0

0

0

0

0

0

0

0

0

0
8

0

0

0

0

0

0

0

0

0

0

0
8

0

0

0

0

0

0

0

0

0

0

0
7

0

0

0

0

0

0

0
7

0
7

0
7

0
7

0
4

0

0

0

0

0

0

0

0

0

0

0

0

0

0

0

0

0

0

0

0

0
4

51.4794

0
7

51.4794

0

0

0

0

0

0

0

0

0

0

0

0

0

0

0

0

0
4

0

0

0
4

0
7

0
7

0

0

0

0
4

0

0

0

0

0

0

0

0

0
4

0

0

0

0

0

0

0
4

0

0

0
4

0

0

0
4

0

0

0
4

0

0

0
4

0

0

0
4

0

0

0
4

0

0

0
4

0
4

0

0

0

0

0

0

0

0

0

0

0
4

0

0

0

0

0
4

0
4

0

0

0

0

0
4

0

0

0
4

0
4

0

0

0

0
4

0
4

0

0

0

0

0
4

0

0

0
4

0
4

0.516483

0.516483

0.129121

0.258242

0.129121

0
4

0
4

0

0

0

0
4

0

0

0
4

0
4

0

0

0

0
4

0

0

0
4

0
4

0

0

0

0
4

0
4

0

0

0

0

0
4

0
4

0

0

0

0
4

0
4

0

0

0

0
4

0

0

0
4

0
4

0
7

0
7

0

0
7

0

0

0

0

0
4

0

0

0
4

0
4

0

0

0

0
4

0
4

0

0

0

0
4

0
4

0

0

0

0
4

0
4

0

0

0

0
4

0
4

0

0

0

0
4

0
4

0

0

0

0
4

0
4

0

0

0

0
4

0
4

0

0

0

0
4

0
4

0

0

0

0
4

0
4

0

0

0

0
4

0
4

0

0

0

0

0
4

0

0

0
4

0
4

0

0

0

0
4

0
4

0.0365426

0.0365426

0.0365426

0
4

0
4

0

0

0

0

0

0
4

0

0

0
4

0
4

0

0

0

0
4

0
4

0
6

0

0

0

0

0
4

0

0

0
4

0

0

0
4

0
4

0

0

0

0

0

0
4

0

0

0

0
4

0
4

0

0

0

0

0
4

0
4

0

0

0

0

0
4

0
4

0
4

3.1498

3.072

3.072

1.27268

0

0

0

0

0

0

0

0

0

0

0.175543
1

0

1.536

0

0

0

0.0877713

0

0
4

0

0

0

0
4

0

0

0

0
4

0

0

0
4

0

0

0
4

0

0

0
4

0

0

0
4

0
4

0

0

0

0
4

0
4

0

0

0

0
4

0
4

0

0

0

0

0
4

0
4

0

0

0

0
4

0
4

0.077809

0.077809

0.077809

0
4

0
4

0

0

0

0
4

0
4

0

0

0

0
4

0
4

0

0

0

0
4

0
4

0

0

0

0
4

0
4

0

0

0

0
4

0
4

9.71445146547012e-17

0
4

0
5

0
5

0
5

0
5

0

0

0

0

0

0

0

0

0
4

0

0

0

0

0
4

0

0

0

0

0
4

0

0

0
4

0

0

0

0

0
4

0

0

0
4

0

0

0
4

0
4

0
4

0.194522
4

0.194522
4

0.194522
4

0
4

0

0

0

0

0
4

0.077809

0.116713

0

0

0

0

0

1.38777878078145e-17
4

0
4

0
4

0
4

0

0

0

0

0

0
4

0

0

0
4

0
4

0
4

0

0

0

0

0

0
4

0
4

0
4

0

0

0

0

0

0
4

0

0

0

0
4

0
4

0
4

1.03297

1.03297

1.03297

1.03297

0

0
4

0
4

0
4

0

0

0

0

0

0

0
4

0
4

0
4

0

0

0

0

0
4

0
4

0
4

0

0

0

0

0

0
4

0
4

0
4

0

0

0

0

0
4

0
4

0
4

0

0

0

0

0

0
4

0

0

0
4

0
4

0
4

0

0

0

0

0
4

0
4

0
4

0.0739218
2

0.0739218
2

0.0739218
2

0

0

0

0

0.0739218

0

0

0

0

0
4

0

0

0
4

0
4

0

0

0

0

0

0

0

0
4

0

0

0

0
4

0
4

0
4

0

0

0

0

0
4

0

0

0
4

0
4

0
4

0

0

0

0

0

0

0
4

0
4

0
4

0

0

0

0

0
4

0

0

0
4

0
4

0
4

0

0

0

0

0

0
4

0
4

0

0

0

0
4

0
4

0
4

0

0

0

0

0
4

0
4

0
4

0

0

0

0

0
4

0

0

0
4

0
4

0
4

0

0

0

0

0
4

0
4

0
4

0

0

0

0

0

0
4

0

0

0
4

0
4

0
4

0

0

0

0

0
4

0

0

0
4

0
4

0
4

0

0

0

0

0

0

0
4

0
4

0
4

0
7

0
7

0
7

0
7

0
7

0

0
4

0
7

0
7

0

0

0
4

0

0

0

0

0
4

0

0

0

0
4

0
4

0
4

0

0

0

0

0

0
4

0
4

0
4

0

0

0

0

0
4

0

0

0
4

0

0

0
4

0
4

0
4

0

0

0

0

0
4

0

0

0
4

0
4

0
4

0

0

0

0

0

0
4

0
4

0
4

0

0

0

0

0

0
4

0
4

0
4

0

0

0

0

0
4

0
4

0
4

0

0

0

0

0
4

0
4

0
4

0

0

0

0

0
4

0
4

0
4

0

0

0

0

0

0
4

0
4

0
4

0.263314

0.263314

0

0

0
4

0.263314

0.263314

0
4

0
4

0
4

28.1483

28.1483

28.1483

25.695

0

1.42033

0.451923

0

0

0.322802

0

0

0.258242

2.16493489801906e-15

0
4

0

0

0
4

0

0

0

0
4

0
4

0

0

0

0

0
4

0
4

0
4

0

0

0

0

0

0
4

0
4

0
4

0

0

0

0

0
4

0

0

0
4

0
4

0
4

0

0

0

0

0

0
4

0
4

0
4

0

0

0

0

0
4

0
4

0
4

0.129121

0.129121

0.129121

0

0.129121

0
4

0

0

0
4

0
4

0
4

0

0

0

0

0
4

0

0

0
4

0
4

0
4

1.48954

1.48954

1.48954

1.48954

0
4

0
4

0
4

0

0

0

0

0

0
4

0
4

0

0

0

0
4

0
4

0
4

0

0

0

0

0
4

0

0

0
4

0
4

0
4

0

0

0

0

0
4

0
4

0
4

8.95239
2

8.95239
2

2.24887
2

2.09325

0

0

0.077809

0.077809

0

1.94289029309402e-16
2

0
4

6.62571

0.696301

1.81834

3.88618

0.077809

0.147079

0
4

0.077809

0

0

0

0.077809

0
4

1.87350135405495e-15
2

0
4

0
4

0

0

0

0

0
4

0
4

0
4

0

0

0

0

0
4

0

0

0
4

0

0

0
4

0
4

0
4

0

0

0

0

0
4

0
4

0
4

0

0

0

0

0

0
4

0
4

0
4

0

0

0

0

0

0

0
4

0
4

0
4

0

0

0

0

0

0
4

0
4

0
4

0.263314

0.263314

0.263314

0.263314

0

0
4

0
4

0
4

0

0

0

0

0
4

0
4

0
4

0

0

0

0

0
4

0

0

0
4

0
4

0
4

0

0

0

0

0
4

0
4

0
4

0

0

0

0
6

0
7

0

0

0

0

0
4

0
6

0
6

0

0

0

0
4

0

0

0

0
4

0

0

0
4

0

0

0
4

0

0

0
4

0
4

0
4

0

0

0

0

0
4

0

0

0
4

0
4

0
4

0

0

0

0

0

0
4

0
4

0
4

0

0

0

0

0
4

0

0

0
4

0
4

0
4

0

0

0

0

0

0
4

0
4

0
4

0

0

0

0

0

0
4

0
4

0
4

0

0

0

0

0

0
4

0
4

0
4

0

0

0

0

0
4

0

0

0
4

0
4

0
4

0

0

0

0

0
4

0

0

0
4

0
4

0
4

0

0

0

0

0
4

0

0

0
4

0
4

0
4

0

0

0

0

0
4

0
4

0
4

0
7

0
7

0
7

0
7

0

0

0

0

0
4

0

0

0

0

0

0

0
4

0
4

0
4

0

0

0

0

0
4

0

0

0
4

0
4

0
4

0

0

0

0

0
4

0

0

0
4

0
4

0
4

0

0

0

0

0

0
4

0
4

0
4

0

0

0

0

0

0
4

0
4

0
4

0

0

0

0

0

0
4

0
4

0
4

0

0

0

0

0
4

0
4

0
4

0

0

0

0

0
4

0
4

0
4

0

0

0

0

0
4

0

0

0
4

0
4

0
4

0

0

0

0

0
4

0

0

0
4

0
4

0
4

0

0

0

0

0
4

0
4

0
4

0
7

0
7

0
7

0
7

0

0

0

0

0

0
4

0

0

0

0

0
4

0

0

0
4

0
4

0
4

0

0

0

0

0
4

0

0

0
4

0
4

0
4

0

0

0

0

0
4

0
4

0
4

0

0

0

0

0
4

0

0

0
4

0
4

0
4

0

0

0

0

0

0
4

0
4

0
4

0

0

0

0

0
4

0
4

0
4

0

0

0

0

0

0
4

0
4

0
4

0

0

0

0

0

0
4

0
4

0
4

0

0

0

0

0

0
4

0
4

0
4

1.74313

1.74313

1.74313

0

1.74313

0
4

0
4

0
4

0

0

0

0

0
4

0

0

0
4

0
4

0
4

0
7

0
7

0
7

0
7

0

0

0
4

0

0

0
4

0

0

0
4

0
4

0
4

0

0

0

0

0

0
4

0
4

0
4

0

0

0

0

0
4

0
4

0
4

0.0551547

0.0551547

0.0551547

0.0551547

0
4

0
4

0
4

0

0

0

0

0
4

0

0

0
4

0
4

0
4

0

0

0

0

0
4

0
4

0
4

0

0

0

0

0

0
4

0
4

0
4

0

0

0

0

0
4

0

0

0
4

0
4

0
4

0

0

0

0

0
4

0
4

0
4

0

0

0

0

0
4

0
4

0
4

0

0

0

0

0
4

0
4

0
4

0
7

0
7

0
7

0
7

0
7

0

0

0

0
4

0
7

0
7

0

0

0
4

0
4

0
4

0

0

0

0

0
4

0

0

0
4

0
4

0
4

0

0

0

0

0

0
4

0
4

0
4

0

0

0

0

0
4

0
4

0
4

0.628335

0.628335

0.628335

0.628335

0
4

0

0

0
4

0
4

0
4

0

0

0

0

0
4

0
4

0
4

0.389045

0.389045

0.389045

0

0.389045

0
4

0
4

0
4

0

0

0

0

0
4

0

0

0
4

0
4

0
4

0

0

0

0

0
4

0
4

0
4

0

0

0

0

0
4

0

0

0
4

0
4

0
4

0

0

0

0

0
4

0

0

0
4

0
4

0
4

0
4

0
4

0

0

0

0

0

0

0

0

0

0

0

0

0

0

0

0

0

0

0
4

0
4

0

0

0

0

0

0

0

0

0

0

0

0

0

0

0

0
4

0

0

0

0

0

0

0

0
4

0

0

0

0
4

0
4

0

0

0

0
4

0
4

0
4

0

0

0

0

0

0

0

0

0

0

0
4

0

0

0

0

0
4

0
4

0
4

0

0

0

0

0
4

0
4

0
4

0

0

0

0

0

0
4

0
4

0
4

0

0

0

0

0
4

0
4

0
4

0

0

0

0

0

0
4

0
4

0
4

0

0

0

0

0

0
4

0
4

0
4

0

0

0

0

0
4

0
4

0
4

0

0

0

0

0
4

0

0

0
4

0
4

0
4

0

0

0

0

0
4

0
4

0
4

0

0

0

0

0

0
4

0
4

0
4

0

0

0

0

0

0
4

0
4

0
4

0
7

0
7

0
7

0
7

0
7

0

0
4

0

0

0

0
4

0
4

0

0

0

0
4

0
4

0
4

0

0

0

0

0
4

0
4

0
4

0

0

0

0

0
4

0
4

0
4

0

0

0

0

0
4

0
4

0
4

0

0

0

0

0
4

0
4

0
4

0

0

0

0

0
4

0
4

0
4

0

0

0

0

0
4

0
4

0
4

0

0

0

0

0
4

0
4

0
4

0

0

0

0

0
4

0
4

0
4

0

0

0

0

0
4

0
4

0
4

0

0

0

0

0
4

0
4

0
4

0

0

0

0
7

0

0

0
4

0

0

0
4

0

0

0
4

0

0

0
4

0
4

0
4

0

0

0

0

0
4

0
4

0
4

0

0

0

0

0
4

0
4

0
4

0

0

0

0

0
4

0
4

0
4

0

0

0

0

0
4

0
4

0
4

0

0

0

0

0
4

0
4

0
4

0

0

0

0

0
4

0
4

0
4

0

0

0

0

0
4

0
4

0
4

0

0

0

0

0
4

0
4

0
4

0

0

0

0

0
4

0
4

0
4

0

0

0

0

0
4

0
4

0
4

9.38531
2

9.38531
2

0.295687
2

0
2

0

0.295687

0

0

0

0
4

9.08962
2

9.08962

0

0

0
4

0

0

0
4

0
4

0
4

0

0

0

0

0
4

0
4

0
4

0

0

0

0

0
4

0
4

0
4

0

0

0

0

0
4

0
4

0
4

0

0

0

0

0
4

0
4

0
4

0.129121

0.129121

0.129121

0.129121

0
4

0
4

0
4

0

0

0

0

0
4

0
4

0
4

0

0

0

0

0
4

0
4

0
4

0

0

0

0

0
4

0
4

0
4

0

0

0

0

0
4

0
4

0
4

0

0

0

0

0
4

0
4

0
4

0
4

0
4

0
4

0

0

0

0

0

0

0

0

0

0
4

0
4

0
4

0
4

0
4

0

0

0

0
4

0
4

0
4

0.155618

0.155618

0.155618

0.155618

0
4

0
4

0
4

0

0

0

0

0
4

0
4

0
4

0

0

0

0

0
4

0
4

0
4

0

0

0

0

0
4

0
4

0
4

0

0

0

0

0
4

0
4

0
4

0

0

0

0

0
4

0
4

0
4

0

0

0

0

0
4

0
4

0
4

0

0

0

0

0
4

0
4

0
4

0

0

0

0

0
4

0
4

0
4

0

0

0

0

0
4

0
4

0
4

0
5

0
5

0
5

0
5

0

0

0
4

0
4

0
4

0

0

0

0

0
4

0
4

0
4

0

0

0

0

0
4

0
4

0
4

0

0

0

0

0
4

0
4

0
4

0

0

0

0

0
4

0
4

0
4

0

0

0

0

0
4

0
4

0
4

0

0

0

0

0
4

0
4

0
4

0

0

0

0

0
4

0
4

0
4

0

0

0

0

0
4

0
4

0
4

0

0

0

0

0
4

0
4

0
4

0

0

0

0

0
4

0
4

0
4

0
7

0
7

0
7

0
7

0

0
4

0

0

0
4

0
4

0

0

0

0

0

0
4

0
4

0
4

0

0

0

0

0
4

0
4

0
4

0

0

0

0

0
4

0
4

0
4

0

0

0

0

0
4

0
4

0
4

0

0

0

0

0
4

0
4

0
4

0

0

0

0

0
4

0
4

0
4

0

0

0

0

0
4

0
4

0
4

0

0

0

0

0
4

0
4

0
4

0

0

0

0

0
4

0
4

0
4

0

0

0

0

0
4

0
4

0
4

0

0

0

0

0
4

0
4

0
4

42.8593

42.8593

42.8593

40.7702

1.31442

0.322802

0.129121

0

0.193681

0.129121

0
4

0
4

0
4

0

0

0

0

0
4

0
4

0
4

0

0

0

0

0
4

0
4

0
4

0.12677

0.12677

0.12677

0.12677

0
4

0
4

0
4

0

0

0

0

0
4

0
4

0
4

0

0

0

0

0
4

0
4

0
4

0

0

0

0

0
4

0
4

0
4

0

0

0

0

0
4

0
4

0
4

0

0

0

0

0
4

0
4

0
4

0

0

0

0

0
4

0
4

0
4

0

0

0

0

0
4

0
4

0
4

0

0

0

0

0

0

0

0

0
4

0
4

0
4

0

0

0

0

0
4

0
4

0
4

0

0

0

0

0
4

0
4

0
4

0

0

0

0

0
4

0
4

0
4

0

0

0

0

0
4

0
4

0
4

0

0

0

0

0
4

0
4

0
4

0

0

0

0

0
4

0
4

0
4

0

0

0

0

0
4

0
4

0
4

0

0

0

0

0
4

0
4

0
4

0

0

0

0

0
4

0
4

0
4

0

0

0

0

0
4

0
4

0
4

0
4

0
4

0
4

0
4

0

0

0

0
4

0
4

0
4

0

0

0

0

0
4

0
4

0
4

0

0

0

0

0
4

0
4

0
4

0

0

0

0

0
4

0
4

0
4

0

0

0

0

0
4

0
4

0
4

0

0

0

0

0
4

0
4

0
4

0

0

0

0

0
4

0
4

0
4

0

0

0

0

0
4

0
4

0
4

0.0877713

0.0877713

0.0877713

0.0877713

0
4

0
4

0
4

0

0

0

0

0
4

0
4

0
4

0

0

0

0

0
4

0
4

0
4

0
4

0
4

0
4

0
4

0

0

0

0

0

0

0

0

0

0

0
4

0

0

0
4

0

0

0

0

0

0

0
4

0
4

0

0

0

0

0

0

0
4

0

0

0

0
4

0

0

0
4

0
4

0
4

0
5

0
5

0
5

0
5

0

0

0

0

0

0

0
4

0

0

0
4

0
4

0
4

0

0

0

0

0
4

0
4

0
4

0

0

0

0

0
4

0
4

0
4

0

0

0

0

0
4

0
4

0
4

0.207762

0.207762

0.207762

0.207762

0
4

0
4

0
4

0

0

0

0

0
4

0
4

0
4

0

0

0

0

0
4

0
4

0
4

0

0

0

0

0
4

0
4

0
4

0

0

0

0

0
4

0
4

0
4

0

0

0

0

0
4

0
4

0
4

0

0

0

0

0
4

0
4

0
4

0
4

0
4

0
4

0

0

0

0

0

0

0

0
4

0
4

0
4

0

0

0

0

0
4

0
4

0
4

0

0

0

0

0
4

0
4

0
4

0

0

0

0

0
4

0
4

0
4

0

0

0

0

0
4

0
4

0
4

0.184804

0.184804

0.184804

0.184804

0
4

0
4

0
4

0

0

0

0

0
4

0
4

0
4

0

0

0

0

0
4

0
4

0
4

0

0

0

0

0
4

0
4

0
4

0

0

0

0

0
4

0
4

0
4

0

0

0

0

0
4

0
4

0
4

0

0

0
6

0

0

0

0
4

0

0

0

0

0
4

0

0

0
4

0

0

0

0
4

0

0

0
4

0

0

0
4

0
4

0
4

0

0

0

0

0
4

0
4

0
4

0

0

0

0

0
4

0
4

0
4

0

0

0

0

0
4

0
4

0
4

0

0

0

0

0
4

0
4

0
4

0

0

0

0

0
4

0
4

0
4

0

0

0

0

0
4

0
4

0
4

0

0

0

0

0
4

0
4

0
4

0

0

0

0

0
4

0
4

0
4

0

0

0

0

0
4

0
4

0
4

0

0

0

0

0
4

0
4

0
4

0

0

0

0
7

0

0

0

0
4

0
4

0
4

0

0

0

0

0
4

0
4

0
4

0

0

0

0

0
4

0
4

0
4

0

0

0

0

0
4

0
4

0
4

0

0

0

0

0
4

0
4

0
4

0

0

0

0

0
4

0
4

0
4

0

0

0

0

0
4

0
4

0
4

0

0

0

0

0
4

0
4

0
4

0

0

0

0

0
4

0
4

0
4

0

0

0

0

0
4

0
4

0
4

0

0

0

0

0
4

0
4

0
4

1.78961
3

1.78961
3

0
4

0

0

0

0

0
4

1.78961

1.78961

0
4

0

0

0
4

0

0

0

0
4

0
4

0
4

0

0

0

0

0
4

0
4

0
4

0

0

0

0

0
4

0
4

0
4

0

0

0

0

0
4

0
4

0
4

0

0

0

0

0
4

0
4

0
4

0

0

0

0

0
4

0
4

0
4

0

0

0

0

0
4

0
4

0
4

0

0

0

0

0
4

0
4

0
4

0

0

0

0

0
4

0
4

0
4

0

0

0

0

0
4

0
4

0
4

0

0

0

0

0
4

0
4

0
4

0
7

0
7

0
7

0

0

0

0
4

0

0

0

0

0

0
4

0

0

0
4

0
4

0
4

0

0

0

0

0
4

0
4

0
4

0

0

0

0

0
4

0
4

0
4

0

0

0

0

0
4

0
4

0
4

0

0

0

0

0
4

0
4

0
4

0

0

0

0

0
4

0
4

0
4

0

0

0

0

0
4

0
4

0
4

0

0

0

0

0
4

0
4

0
4

0.40657

0.40657

0.40657

0.40657

0
4

0
4

0
4

0

0

0

0

0
4

0
4

0
4

0

0

0

0

0
4

0
4

0
4

0

0

0

0

0

0

0
4

0

0

0

0
4

0
4

0

0

0

0

0
4

0

0

0
4

0
4

0
4

0

0

0

0

0
4

0
4

0
4

0

0

0

0

0
4

0
4

0
4

0

0

0

0

0
4

0
4

0
4

0

0

0

0

0
4

0
4

0
4

0

0

0

0

0
4

0
4

0
4

0

0

0

0

0
4

0
4

0
4

0

0

0

0

0
4

0
4

0
4

0

0

0

0

0
4

0
4

0
4

0

0

0

0

0
4

0
4

0
4

0

0

0

0

0
4

0
4

0
4

0
5

0
5

0
5

0
5

0

0
4

0
4

0
4

0

0

0

0

0
4

0
4

0
4

0

0

0

0

0
4

0
4

0
4

0

0

0

0

0
4

0
4

0
4

0

0

0

0

0
4

0
4

0
4

0

0

0

0

0
4

0
4

0
4

0

0

0

0

0
4

0
4

0
4

0

0

0

0

0
4

0
4

0
4

0

0

0

0

0
4

0
4

0
4

0

0

0

0

0
4

0
4

0
4

0

0

0

0

0
4

0
4

0
4

0
7

0
7

0

0

0

0
4

0

0

0

0
4

0
4

0
4

0

0

0

0

0
4

0
4

0
4

0

0

0

0

0
4

0
4

0
4

0

0

0

0

0
4

0
4

0
4

0

0

0

0

0
4

0
4

0
4

0

0

0

0

0
4

0
4

0
4

0

0

0

0

0
4

0
4

0
4

0

0

0

0

0
4

0
4

0
4

0

0

0

0

0
4

0
4

0
4

0.322802

0.322802

0.322802

0.322802

0
4

0
4

0
4

0

0

0

0

0
4

0
4

0
4

0
4

0
4

0
4

0

0

0

0
4

0
4

0
4

0
4

0

0

0
4

0

0

0
4

0
4

0
4

0.0739218

0.0739218

0.0739218

0.0739218

0
4

0
4

0
4

0

0

0

0

0
4

0
4

0
4

0

0

0

0

0
4

0
4

0
4

0.193681

0.193681

0.193681

0.193681

0
4

0
4

0
4

0

0

0

0

0
4

0
4

0
4

0

0

0

0

0
4

0
4

0
4

0

0

0

0

0
4

0
4

0
4

0

0

0

0

0
4

0
4

0
4

0

0

0

0

0
4

0
4

0
4

0

0

0

0

0
4

0
4

0
4

0
7

0
7

0
7

0
7

0

0
6

0

0

0

0
4

0
7

0
7

0
7

0
7

0
7

0

0

0

0
4

0
4

0
4

0

0

0

0

0

0

0

0
4

0

0

0

0
4

0
4

0
4

0

0

0

0

0
4

0
4

0
4

0

0

0

0

0
4

0
4

0
4

0

0

0

0

0
4

0
4

0
4

0

0

0

0

0
4

0
4

0
4

0

0

0

0

0
4

0
4

0
4

0

0

0

0

0
4

0
4

0
4

0

0

0

0

0
4

0
4

0
4

0

0

0

0

0
4

0
4

0
4

0

0

0

0

0
4

0
4

0
4

0

0

0

0

0
4

0
4

0
4

0
7

0
7

0
7

0
6

0

0

0
4

0

0

0
4

0

0

0
4

0
4

0
4

0

0

0

0

0
4

0
4

0
4

0

0

0

0

0
4

0
4

0
4

0

0

0

0

0
4

0
4

0
4

0

0

0

0

0
4

0
4

0
4

0

0

0

0

0
4

0
4

0
4

0.129121

0.129121

0.129121

0.129121

0
4

0
4

0
4

0.0739218

0.0739218

0.0739218

0.0739218

0
4

0
4

0
4

0

0

0

0

0
4

0
4

0
4

0

0

0

0

0
4

0
4

0
4

0

0

0

0

0
4

0
4

0
4

0
7

0
7

0

0

0

0

0
4

0

0

0

0

0
4

0
4

0
4

0

0

0

0

0
4

0
4

0
4

0

0

0

0

0
4

0
4

0
4

0

0

0

0

0
4

0
4

0
4

0

0

0

0

0
4

0
4

0
4

0
6

0
6

0
6

0
6

0

0

0
4

0
4

0
4

0

0

0

0

0

0

0

0
4

0

0

0

0
4

0

0

0
4

0

0

0

0
4

0
4

0

0

0

0
4

0
4

0
4

0
7

0
7

0
7

0
7

0

0
4

0

0

0
4

0

0

0
4

0
4

0
4

0
7

0
7

0
7

0
7

0
4

0
4

0
4

0

0

0

0

0

0

0

0

0
4

0

0

0
4

0
4

0
4

0
4

0
4

0

0

0

0

0
4

0

0

0
4

0
4

0
4

0

0

0

0

0

0
4

0
4

0
4

0.258242
5

0.258242
5

0.258242
6

0
6

0

0

0

0

0

0

0

0

0

0

0.258242

0

0

0

0

0

0
4

0
5

0
7

0
6

0

0

0
4

0

0

0

0

0
4

0
4

0
4

0

0

0

0

0

0
4

0

0

0

0
4

0
4

0
4

0
4

0
4

0
4

0

0

0

0

0
4

0

0

0

0
4

0
4

0
4

0

0

0

0

0

0

0

0
4

0
4

0
4

0
7

0
7

0
7

0

0

0
4

0
4

0
4

0

0

0

0

0

0

0
4

0

0

0
4

0
4

0
4

0

0

0

0

0

0

0

0

0
4

0

0

0
4

0
4

0
4

0

0

0

0

0

0

0

0
4

0
4

0
4

0

0

0

0

0

0

0
4

0
4

0
4

0

0

0

0

0
4

0
4

0
4

0

0

0

0

0

0
4

0
4

0
4

22.0921

22.0921

0.900304
5

0
6

0

0

0

0.900304

0

0

0

0

0

0
4

0
7

0
7

0

0

0
4

21.1918

19.5989

0.83105

0.761796

0

2.22044604925031e-16

0
4

0

0

0

0
4

0
4

0

0

0

0
4

0
4

0
4

0

0

0

0

0

0

0
4

0

0

0

0
4

0
4

0

0

0

0
4

0
4

0
4

0
4

0
4

0
4

0
4

0

0
4

0
4

0
4

0
7

0
7

0
7

0
7

0

0
4

0
4

0
4

0

0

0

0

0

0
4

0

0

0
4

0
4

0
4

0

0

0

0

0

0
4

0

0

0
4

0

0

0

0
4

0

0

0
4

0
4

0
4

0

0

0

0

0

0
4

0
4

0
4

0.155618
4

0.155618
4

0

0

0

0
4

0

0

0

0
4

0.155618

0.155618

0
4

0
4

0
4

0

0

0

0

0

0

0
4

0
4

0
4

0
4

0
4

0

0

0
4

0

0

0
4

0

0

0
4

0
4

0
4

0
5

0
5

0
5

0
5

0
4

0
4

0
4

30.3434

30.3434

30.3434

30.3434

0

0

0
4

0

0

0
4

0

0

0
4

0
4

0
4

0

0

0

0

0
4

0

0

0

0

0
4

0

0

0
4

0

0

0
4

0

0

0
4

0

0

0
4

0

0

0
4

0

0

0
4

0
4

0
4

0

0

0

0

0

0
4

0
4

0
4

1.24657

1.24657

0.900304

0.277017

0.484779

0.138508

5.55111512312578e-17

0
4

0.346271

0.138508

0.207762

0
4

5.55111512312578e-17

0
4

0
4

0

0

0

0

0
4

0
4

0
4

0

0

0

0

0

0
4

0

0

0

0
4

0
4

0
4

0

0

0

0

0

0
4

0
4

0
4

3.11644

3.11644

2.35464

0.346271

0.346271

0.138508

1.38508

0.138508

3.33066907387547e-16

0
4

0.761796

0.761796

0
4

1.11022302462516e-16

0
4

0
4

0

0

0

0

0

0

0
4

0
4

0
4

0

0

0

0

0
4

0

0

0

0
4

0
4

0
4

0
7

0
7

0

0

0
4

0

0

0
4

0
4

0
4

0

0

0

0

0

0
4

0

0

0

0
4

0
4

0
4

0

0

0

0

0

0

0

0

0

0

0

0

0

0

0
4

0

0

0

0

0

0

0

0

0
4

0

0

0

0

0

0

0

0

0

0
4

0
4

0
4

0

0

0

0

0

0

0
4

0
4

0
4

0
5

0
5

0
5

0

0

0
4

0
4

0
4

0

0

0

0

0
4

0
4

0
4

0

0

0

0

0
4

0
4

0
4

0

0

0

0

0

0
4

0
4

0
4

0

0

0

0

0
4

0
4

0
4

0

0

0

0

0

0
4

0

0

0
4

0
4

0
4

0

0

0

0

0

0

0
4

0
4

0
4

0

0

0

0

0

0
4

0
4

0
4

0

0

0

0

0

0
4

0

0

0

0
4

0
4

0
4

0
7

0
7

0
7

0
7

0
7

0
7

0

0

0

0

0

0

0
4

0

0

0

0

0
4

0

0

0
4

0
4

0
4

0

0

0

0

0

0

0
4

0
4

0
4

0

0

0

0

0

0

0
4

0
4

0
4

0

0

0

0

0

0
4

0
4

0
4

0

0

0

0

0
4

0
4

0
4

0

0

0

0

0

0
4

0
4

0
4

0

0

0

0

0
4

0

0

0
4

0
4

0
4

0

0

0

0

0
4

0
4

0
4

0

0

0

0

0

0
4

0
4

0
4

0

0

0

0

0

0
4

0

0

0
4

0
4

0

0

0

0
4

0
4

0
4

0

0

0

0

0

0

0
4

0
4

0
4

32.4336

0
4

0
4

0

0

0

0
4

0
4

0

0

0

0
4

0
4

0

0

0

0
4

0
4

0

0

0

0
4

0
4

0

0

0

0
4

0
4

0

0

0

0
4

0
4

0

0

0

0
4

0
4

0

0

0

0
4

0
4

0

0

0

0
4

0
4

0

0

0

0
4

0
4

0

0

0

0
4

0
4

0

0

0

0

0
4

0
4

0

0

0

0
4

0
4

0

0

0

0
4

0
4

0

0

0

0
4

0
4

0

0

0

0
4

0
4

0

0

0

0
4

0
4

0

0

0

0
4

0
4

0

0

0

0
4

0
4

0

0

0

0
4

0
4

0

0

0

0
4

0
4

0

0

0

0
4

0
4

0

0

0

0
4

0
4

0

0

0

0
4

0
4

0

0

0

0

0

0
4

0
4

0

0

0

0

0
4

0
4

0

0

0

0
4

0
4

0

0

0

0
4

0

0

0
4

0
4

0

0

0

0
4

0
4

0

0

0

0
4

0
4

32.4336
3

31.0485
2

0.547963
3

11.9939
2

0.169544

0.0968825

0.314868

0

0.0968825

0.207762

0

0

0

0

15.7689
2

0

0

0

0

0

0

0

0

0

0.0726619

0.467783

0.0484412

0

0.339089

0

0

0

0

0.0484412

0

0.184654

0.157873

0.532854

0

0

0
4

0

0

0

0

0

0

0

0

0

0

0
4

0

0

0
4

0

0

0

0
4

0

0

0
4

0

0

0

0
4

0

0

0
4

0

0

0
4

0

0

0
4

1.24657

1.24657

0
4

0

0

0
4

0

0

0
4

0

0

0

0

0

0

0

0
4

0

0

0
4

0

0

0
4

0

0

0
4

0

0

0
4

0

0

0
4

0

0

0
4

0

0

0
4

0

0

0
4

0

0

0
4

0

0

0
4

0

0

0

0

0
4

0

0

0
4

0

0

0
4

0

0

0
4

0

0

0

0
4

0

0

0

0
4

0

0

0

0

0
4

0.138508

0

0.138508

0

0
4

0

0

0
4

0

0

0

0
4

0
4

0
4

60.7077
3

0

0

0

0

0

0

0

0

0
4

0

0

0

0

0
4

0

0

0
4

0
4

0
6

0
6

0
6

0
4

0
4

0

0

0

0
4

0
4

0

0

0

0
4

0
4

0

0

0

0
4

0
4

0

0

0

0
4

0
4

0.131657

0.131657

0.131657

0
4

0
4

0.129121

0.129121

0.129121

0
4

0
4

0

0

0

0
4

0
4

0

0

0

0
4

0
4

0

0

0

0
4

0
4

0

0

0

0
4

0
4

0

0

0

0

0

0

0

0
4

0

0

0

0
4

0

0

0
4

0
4

0

0

0

0
4

0
4

0

0

0

0
4

0
4

0

0

0

0
4

0
4

0

0

0

0
4

0
4

0

0

0

0
4

0
4

0

0

0

0
4

0
4

0

0

0

0
4

0
4

0

0

0

0
4

0
4

0

0

0

0
4

0
4

0.129121

0.129121

0.129121

0
4

0
4

0

0

0

0

0
4

0

0

0

0
4

0
4

0

0

0

0
4

0
4

0

0

0

0
4

0
4

0

0

0

0
4

0
4

0

0

0

0
4

0
4

0

0

0

0
4

0
4

0

0

0

0
4

0
4

0

0

0

0
4

0
4

0

0

0

0
4

0
4

0

0

0

0
4

0
4

0.129121

0.129121

0.129121

0
4

0
4

0

0

0

0

0

0

0

0
4

0

0

0
4

0
4

0

0

0

0
4

0
4

0

0

0

0
4

0
4

0

0

0

0
4

0
4

0

0

0

0
4

0
4

0

0

0

0
4

0
4

0

0

0

0
4

0
4

0

0

0

0

0

0
4

0

0

0

0
4

0

0

0
4

0
4

1.54945
3

0

0

0

0

0

0
4

1.54945

0.193681

1.35577

2.22044604925031e-16

0
4

0

0

0
4

0

0

0
4

0

0

0
4

0
4

0.129121
2

0.129121

0

0.129121

0

0
4

0

0

0
4

0

0

0

0
4

0

0

0
4

0

0

0
4

0
4

0

0

0

0

0

0

0
4

0
4

0

0

0

0

0
4

0
4

0
6

0
6

0
6

0
4

0
4

0.554033
4

0.554033
4

0.554033
4

0
4

0

0

0
4

0

0

0
4

0

0

0
4

0
4

0
3

0

0

0

0

0

0
4

0

0

0
4

0

0

0
4

0
4

0
3

0

0

0

0

0
4

0

0

0
4

0

0

0
4

0

0

0
4

0

0

0
4

0
4

0

0

0

0

0
4

0

0

0
4

0
4

0

0

0

0

0
4

0
4

0

0

0

0

0
4

0
4

0

0

0

0
4

0

0

0
4

0
4

0

0

0

0
4

0

0

0

0
4

0
4

0

0

0

0

0

0
4

0

0

0
4

0
4

0
4

0
4

0

0

0

0

0

0

0
4

0
4

0
4

0

0

0

0

0

0
4

0

0

0

0
4

0
4

0.401452
3

0.401452
3

0.272331

0.129121

0

0

0
4

0

0

0

0

0
4

0

0

0
4

0
4

0.129121

0.129121

0

0.129121

0
4

0

0

0
4

0
4

0.311574

0.311574

0.174268

0.0633849

0.0739218

0

1.38777878078145e-17

0
4

0
4

0
6

0
6

0
6

0
4

0
4

0

0

0

0

0
4

0

0

0

0
4

0
4

0

0

0

0

0
4

0

0

0
4

0
4

0

0

0

0

0
4

0
4

0

0

0

0

0

0
4

0

0

0
4

0
4

0

0

0

0

0

0
4

0
4

0

0

0

0

0
4

0

0

0
4

0
4

3.16346

3.16346

3.03434

0.129121

0
4

0
4

2.03107
2

0.793932

0.387362

0.147844

0.258726

5.55111512312578e-17

0
4

0.899144

0.385011

0.12677

0.387362

0
4

0.337998

0.337998

0
4

0

0

0
4

0

0

0
4

1.11022302462516e-16
2

0
4

0

0

0

0

0
4

0

0

0
4

0
4

0

0

0

0
4

0
4

0

0

0

0
4

0
4

0

0

0

0
4

0
4

0

0

0

0
4

0
4

0

0

0

0

0
4

0
4

0

0

0

0
4

0

0

0
4

0
4

0

0

0

0

0
4

0

0

0
4

0
4

0.117195

0.117195

0.117195

0
4

0
4

0

0

0

0
4

0
4

0

0

0

0

0

0

0

0
4

0
4

0

0

0

0
4

0

0

0
4

0
4

0

0

0

0

0
4

0

0

0
4

0
4

0

0

0

0

0
4

0
4

0

0

0

0

0
4

0
4

0

0

0

0

0
4

0
4

0.184804

0.184804

0.184804

0
4

0

0

0
4

0
4

0

0

0

0

0
4

0
4

0

0

0

0
4

0

0

0
4

0
4

0

0

0

0
4

0

0

0
4

0
4

0.839285

0.839285

0.839285

0
4

0
4

0
4

0

0

0

0

0

0
4

0

0

0
4

0

0

0
4

0

0

0
4

0
4

0

0

0

0
4

0
4

0

0

0

0

0
4

0
4

0

0

0

0
4

0
4

0

0

0

0

0
4

0
4

0

0

0

0
4

0

0

0
4

0
4

0

0

0

0
4

0

0

0
4

0
4

0

0

0

0
4

0

0

0
4

0
4

0.387362

0.387362

0.387362

0
4

0
4

0

0

0

0
4

0
4

0.0837105

0.0837105

0.0502263

0.0334842

0
4

0
4

0

0

0

0

0
4

0

0

0

0

0
4

0

0

0
4

0
4

0

0

0

0

0
4

0
4

0

0

0

0
4

0

0

0
4

0
4

0

0

0

0
4

0

0

0
4

0
4

0.116713

0.116713

0.116713

0

0
4

0
4

0

0

0

0
4

0
4

0

0

0

0
4

0
4

0

0

0

0

0
4

0
4

0

0

0

0
4

0
4

0

0

0

0
4

0
4

0

0

0

0
4

0
4

0

0

0

0

0

0
4

0

0

0
4

0

0

0
4

0

0

0

0
4

0
4

0

0

0

0
4

0
4

0

0

0

0
4

0
4

0

0

0

0
4

0
4

0

0

0

0
4

0
4

0

0

0

0
4

0
4

0.516483

0.516483

0.516483

0
4

0
4

0

0

0

0
4

0
4

0

0

0

0
4

0
4

0

0

0

0
4

0
4

0

0

0

0
4

0
4

0
3

0

0

0

0
4

0

0

0

0

0
4

0

0

0
4

0

0

0
4

0
4

0

0

0

0
4

0
4

0

0

0

0
4

0
4

0

0

0

0
4

0
4

0

0

0

0
4

0
4

0

0

0

0
4

0
4

0

0

0

0
4

0
4

0

0

0

0
4

0
4

0

0

0

0
4

0
4

0

0

0

0
4

0
4

0

0

0

0
4

0
4

49.6738
3

45.0452
3

30.2854
3

0
3

0

0

0.258242

0

0

0

0

0.129121

0

0

0

0

0

0

0.129121

0

0

0

0

0

0

0

0

0

1.22665

0

0

0.077809

0

0

0

0

8.00549

0

0

0.0739218

0

0.258242

0.193681

0

0

0.184804

0

4.22272

0

0

0

0
4

0
7

0
7

0
4

0

0

0
4

0

0

0
4

0

0

0
4

0

0

0
4

0

0

0
4

0

0

0
4

0

0

0
4

0.129121

0.129121

0
4

0

0

0
4

0

0

0
4

0

0

0
4

0.207762

0.207762

0
4

0

0

0
4

4.03103

4.03103

0
4

0.260778

0.129121

0.131657

0
4

0

0

0
4

0

0

0
4

0

0

0
4

0

0

0
4

1.0547118733939e-15
3

0
4

0
4

200.971
4

0
7

0
7

0
7

0

0

0

0

0

0

0
4

0
4

0
7

0
7

0
7

0
4

0

0

0
4

0
4

0

0

0

0

0
4

0
4

0

0

0

0

0
4

0
4

0

0

0

0

0
4

0
4

0

0

0

0

0
4

0
4

0

0

0

0

0
4

0
4

0

0

0

0
4

0
4

0

0

0

0

0
4

0
4

0

0

0

0

0
4

0
4

0

0

0

0
4

0

0

0
4

0
4

0

0

0

0
4

0
4

0
4

0
4

0

0

0

0

0

0

0

0

0
4

0

0

0

0
4

0

0

0

0
4

0

0

0
4

0

0

0
4

0
4

0

0

0

0
4

0

0

0
4

0
4

0

0

0

0
4

0

0

0
4

0
4

0

0

0

0
4

0

0

0
4

0
4

0

0

0

0
4

0

0

0
4

0
4

0

0

0

0

0
4

0
4

0

0

0

0
4

0

0

0
4

0
4

0

0

0

0
4

0
4

0

0

0

0

0
4

0
4

0

0

0

0
4

0

0

0
4

0
4

0

0

0

0

0
4

0
4

0
7

0
7

0
7

0

0

0
4

0

0

0

0
4

0

0

0
4

0
4

0

0

0

0
4

0

0

0
4

0
4

0

0

0

0
4

0
4

0

0

0

0
4

0
4

0

0

0

0
4

0

0

0
4

0
4

0

0

0

0
4

0
4

0

0

0

0
4

0

0

0
4

0
4

0

0

0

0
4

0

0

0
4

0
4

0

0

0

0
4

0
4

0

0

0

0

0
4

0
4

0

0

0

0
4

0

0

0
4

0
4

0
4

0
4

0

0

0

0

0

0
4

0

0

0

0
4

0

0

0

0
4

0
4

0

0

0

0
4

0
4

0

0

0

0
4

0
4

0

0

0

0
4

0

0

0
4

0
4

0

0

0

0
4

0
4

0

0

0

0
4

0
4

0

0

0

0
4

0
4

0

0

0

0
4

0

0

0
4

0
4

0

0

0

0
4

0

0

0
4

0
4

0

0

0

0
4

0

0

0
4

0
4

0

0

0

0
4

0

0

0
4

0
4

0
7

0
7

0
7

0

0
4

0

0

0
4

0
4

0

0

0

0
4

0

0

0
4

0
4

0.0950773

0.0950773

0.0950773

0
4

0
4

0

0

0

0
4

0

0

0
4

0
4

0

0

0

0
4

0

0

0
4

0
4

0

0

0

0
4

0
4

0

0

0

0
4

0
4

0

0

0

0
4

0
4

0

0

0

0
4

0
4

0

0

0

0
4

0
4

0

0

0

0
4

0
4

0
7

0
6

0

0

0

0

0

0

0
4

0

0

0
4

0

0

0
4

0

0

0
4

0
4

0

0

0

0
4

0
4

0.129121

0.129121

0.129121

0
4

0
4

0

0

0

0
4

0
4

0

0

0

0
4

0
4

0

0

0

0
4

0
4

0

0

0

0
4

0
4

0

0

0

0
4

0
4

0

0

0

0
4

0
4

0

0

0

0
4

0
4

0

0

0

0
4

0
4

3.80906

1.42033

0.387362

0.387362

0.258242

0.129121

0.258242

1.66533453693773e-16

0
4

0.516483

0.129121

0.129121

0.129121

0.129121

0
4

1.87225

1.22665

0.516483

0.129121

0
4

0
4

0

0

0

0
4

0
4

0

0

0

0
4

0
4

0

0

0

0
4

0
4

0

0

0

0
4

0
4

0

0

0

0
4

0
4

0

0

0

0
4

0
4

0

0

0

0
4

0
4

0

0

0

0
4

0
4

0

0

0

0
4

0
4

0

0

0

0
4

0
4

0
5

0

0

0

0

0
4

0

0

0

0
4

0

0

0
4

0

0

0
4

0

0

0
4

0

0

0
4

0
4

0

0

0

0
4

0
4

0

0

0

0
4

0
4

0

0

0

0
4

0
4

0

0

0

0
4

0
4

0

0

0

0
4

0
4

0

0

0

0
4

0
4

0

0

0

0
4

0
4

0

0

0

0
4

0
4

0

0

0

0
4

0
4

0

0

0

0
4

0
4

0
4

0
4

0
6

0

0

0

0
4

0

0

0

0

0
4

0
4

0

0

0

0
4

0
4

0

0

0

0
4

0
4

0

0

0

0
4

0
4

0

0

0

0
4

0
4

0

0

0

0
4

0
4

0

0

0

0
4

0
4

0

0

0

0
4

0
4

0

0

0

0
4

0
4

0

0

0

0
4

0
4

0

0

0

0
4

0
4

0
4

0
4

0

0

0

0

0

0

0
4

0

0

0

0
4

0
4

0

0

0

0
4

0
4

0

0

0

0
4

0
4

0

0

0

0
4

0
4

0

0

0

0
4

0
4

0

0

0

0
4

0
4

0

0

0

0
4

0
4

0

0

0

0
4

0
4

0

0

0

0
4

0
4

0

0

0

0
4

0
4

0

0

0

0
4

0
4

0
4

0
4

0
4

0

0

0

0

0
4

0

0

0

0

0
4

0

0

0

0
4

0

0

0

0
4

0

0

0

0
4

0

0

0
4

0

0

0
4

0
4

0
4

0
4

0
4

0

0

0
4

0

0

0

0
4

0

0

0
4

0
4

0

0

0

0
4

0
4

0

0

0

0
4

0
4

0

0

0

0
4

0
4

0

0

0

0
4

0
4

0

0

0

0
4

0
4

0

0

0

0
4

0
4

0

0

0

0
4

0
4

0

0

0

0
4

0
4

0

0

0

0
4

0
4

0

0

0

0
4

0
4

0

0

0

0

0

0
4

0

0

0

0

0
4

0

0

0

0
4

0
4

0

0

0

0
4

0
4

0

0

0

0
4

0
4

0

0

0

0
4

0
4

0

0

0

0
4

0
4

0

0

0

0
4

0
4

0

0

0

0
4

0
4

0

0

0

0
4

0
4

0

0

0

0
4

0
4

0

0

0

0
4

0
4

0

0

0

0
4

0
4

0

0

0

0

0

0

0

0

0
4

0

0

0

0

0
4

0
4

0

0

0

0
4

0
4

0

0

0

0
4

0
4

0

0

0

0
4

0
4

0

0

0

0
4

0
4

0

0

0

0
4

0
4

0

0

0

0
4

0
4

0

0

0

0
4

0
4

0

0

0

0
4

0
4

0

0

0

0
4

0
4

0

0

0

0
4

0
4

0
4

0

0

0

0

0

0
4

0

0

0

0

0
4

0

0

0
4

0

0

0
4

0
4

0

0

0

0
4

0
4

0

0

0

0
4

0
4

0

0

0

0
4

0
4

0

0

0

0
4

0
4

0

0

0

0
4

0
4

0

0

0

0
4

0
4

0

0

0

0
4

0
4

0

0

0

0
4

0
4

0

0

0

0
4

0
4

0

0

0

0
4

0
4

0
4

0

0

0

0

0
4

0

0

0

0
4

0

0

0
4

0

0

0
4

0
4

0

0

0

0
4

0
4

0

0

0

0
4

0
4

0

0

0

0
4

0
4

0

0

0

0
4

0
4

0

0

0

0
4

0
4

0

0

0

0
4

0
4

0

0

0

0
4

0
4

0

0

0

0
4

0
4

0

0

0

0
4

0
4

0

0

0

0
4

0
4

0
4

0
4

0
4

0

0
4

0

0

0
4

0

0

0
4

0
4

0

0

0

0
4

0
4

0

0

0

0
4

0
4

0

0

0

0
4

0
4

0

0

0

0
4

0
4

0

0

0

0
4

0
4

0

0

0

0
4

0
4

0

0

0

0
4

0
4

0

0

0

0
4

0
4

0

0

0

0
4

0
4

0

0

0

0
4

0
4

0

0

0

0

0
4

0

0

0

0
4

0

0

0
4

0

0

0
4

0
4

0

0

0

0
4

0
4

0

0

0

0
4

0
4

0

0

0

0
4

0
4

0

0

0

0
4

0
4

0

0

0

0
4

0
4

0

0

0

0

0

0
4

0

0

0
4

0

0

0
4

0
4

0
4

0

0

0

0
4

0

0

0

0
4

0

0

0
4

0

0

0
4

0

0

0
4

0
4

0

0

0

0

0

0

0

0

0
4

0

0

0
4

0

0

0
4

0

0

0
4

0
4

0
4

0
4

0
4

0

0

0

0

0

0

0
4

0

0

0

0
4

0
4

0

0

0

0
4

0

0

0

0

0
4

0

0

0

0
4

0

0

0
4

0
4

0
3

0
3

0

0

0

0

0

0
4

0
4

0
3

0

0

0

0

0
4

0

0

0

0
4

0

0

0
4

0
4

0

0

0

0

0
4

0

0

0

0
4

0

0

0
4

0

0

0
4

0

0

0
4

0

0

0
4

0
4

0
4

0
4

0

0

0

0

0

0
4

0
4

0

0

0

0

0

0

0

0
4

0

0

0
4

0

0

0
4

0
4

0
3

0

0

0

0
4

0

0

0

0
4

0

0

0
4

0

0

0
4

0
4

0

0

0

0

0

0
4

0
4

0

0

0

0
4

0

0

0
4

0

0

0
4

0

0

0
4

0

0

0
4

0

0

0
4

0
4

0
6

0

0

0
4

0

0

0
4

0
4

0
7

0
7

0
7

0
6

0

0

0

0

0

0
4

0
4

0

0

0

0

0

0
4

0

0

0
4

0

0

0
4

0
4

0

0

0

0

0

0
4

0
4

0.207762
3

0.207762
3

0.207762
3

0
4

0

0

0
4

0

0

0
4

0
4

0

0

0

0

0

0
4

0

0

0

0

0
4

0

0

0
4

0
4

0
5

0

0

0
4

0

0

0
4

0

0

0
4

0
4

0

0

0

0

0
4

0
4

0

0

0

0

0
4

0

0

0

0

0
4

0
4

0

0

0

0

0
4

0

0

0
4

0

0

0
4

0
4

0
4

0

0

0
4

0

0

0
4

0

0

0
4

0

0

0
4

0

0

0
4

0
4

0
4

0

0

0

0
4

0

0

0
4

0

0

0
4

0

0

0
4

0
4

0.710164
4

0
4

0

0

0

0

0

0

0

0

0
4

0
4

0

0

0

0

0

0
4

0

0

0

0
4

0

0

0

0

0
4

0.710164

0.710164

0
4

0

0

0

0
4

0

0

0
4

0

0

0
4

0

0

0
4

0
4

0

0

0

0

0
4

0
4

0

0

0

0
4

0

0

0
4

0

0

0
4

0

0

0
4

0

0

0
4

0
4

0
4

0

0

0

0
4

0

0

0
4

0
4

0

0

0

0

0
4

0

0

0
4

0

0

0
4

0

0

0
4

0
4

0

0

0

0

0
4

0

0

0
4

0

0

0
4

0

0

0
4

0
4

0

0

0

0

0
4

0
4

0

0

0

0

0

0
4

0

0

0
4

0
4

0

0

0

0

0
4

0

0

0
4

0
4

0

0

0

0

0

0

0
4

0

0

0
4

0
4

0

0

0

0

0
4

0

0

0
4

0
4

0
4

0
4

0
4

0

0

0

0

0
4

0

0

0

0

0
4

0
4

0

0

0

0

0
4

0

0

0

0
4

0

0

0
4

0
4

0

0

0

0

0
4

0

0

0
4

0

0

0
4

0
4

0

0

0

0

0
4

0

0

0
4

0

0

0
4

0
4

0

0

0

0

0
4

0

0

0

0
4

0
4

0

0

0

0

0
4

0

0

0
4

0

0

0
4

0
4

0

0

0

0
4

0

0

0
4

0

0

0
4

0
4

0

0

0

0
4

0

0

0
4

0

0

0
4

0
4

0

0

0

0

0
4

0
4

0

0

0

0
4

0

0

0
4

0
4

0

0

0

0
4

0

0

0
4

0

0

0
4

0

0

0
4

0
4

0
6

0
7

0
7

0

0

0

0
4

0
6

0
6

0

0

0

0
4

0
4

0

0

0

0
4

0

0

0
4

0

0

0
4

0
4

0

0

0

0
4

0
4

0

0

0

0
4

0

0

0
4

0

0

0
4

0
4

0

0

0

0
4

0

0

0
4

0
4

0

0

0

0

0
4

0

0

0
4

0
4

0

0

0

0

0
4

0

0

0
4

0
4

0

0

0

0
4

0
4

0

0

0

0
4

0

0

0
4

0

0

0
4

0
4

0

0

0

0

0
4

0

0

0
4

0
4

0

0

0

0

0
4

0
4

0
7

0
7

0
7

0

0

0

0
4

0

0

0
4

0
4

0

0

0

0
4

0
4

0

0

0

0

0
4

0
4

0

0

0

0
4

0

0

0
4

0
4

0

0

0

0
4

0

0

0
4

0
4

0

0

0

0
4

0

0

0
4

0

0

0
4

0
4

0

0

0

0

0
4

0

0

0
4

0
4

0

0

0

0

0
4

0
4

0

0

0

0
4

0

0

0
4

0
4

0

0

0

0

0
4

0

0

0
4

0
4

0

0

0

0

0
4

0

0

0
4

0
4

0
4

0

0

0

0

0
4

0

0

0

0

0
4

0

0

0

0
4

0

0

0

0
4

0

0

0
4

0

0

0
4

0

0

0
4

0
4

0

0

0

0

0
4

0

0

0
4

0
4

0

0

0

0
4

0

0

0
4

0
4

0

0

0

0
4

0
4

0

0

0

0

0
4

0

0

0
4

0
4

0

0

0

0
4

0
4

0

0

0

0

0

0
4

0
4

0

0

0

0
4

0

0

0
4

0

0

0
4

0
4

0

0

0

0
4

0

0

0
4

0

0

0
4

0
4

0

0

0

0

0
4

0

0

0
4

0
4

0

0

0

0

0
4

0
4

196.02
4

188.134
4

105.061
4

37.6944
4

16.9794
4

0
7

0

0

0

0

0

0

0

0

0

0

0

0

0

0

0

0

0

0

0

0

0

0

0
7

0

0

0

0

0

0

0

0

0

0

0
6

0

0

0

0

0

0

0

0

0

0.387362

0
7

0

0

0

0

0

0

0

0

0

0

0
7

0

0

0

0

0.129121

0

0

0

0

0

0
7

0

0

0

0

0

0

0

0

0

0

0

0

0

0

0

0

0

0

0

0

0

0
6

0

0

0.129121

0

0

0

0

0

0.138508

0

0
7

0

0

0

0

0

0

0

0

0

0

0
7

0
4

0

0

0

0

0

0.193681

0

0

0

0

0
7

0

0

0

0

0

0

0

0

0

0

0
6

0

0

0

0

0

0

0

0

0

0

0
7

0

0

0

0

0

0

0

0

0

0

0
7

0

0

0

0

0

0

0

0

0

0

0.415525
3

8.90934

0

0

0

0

0

0

0

0

0

0
6

0

0

0

0

0

1.35577

0

0

0

0

14.5261

0

0

0

0

0.387362

0

0

0

0

0

0
6

0

0

0

0

0

0

0

0

0

0

0
6

0

0

0

0

0

0

0

0

0

0

0
7

0

0

0

0

0

0

0

0

0

0

0

0
6

0

0

0

0

0

0

0

0

0

0

0

0

0

0

0

0

0

0

0

0

0

0.207762
3

0

0

0

0

0

0

0

0

0

0

0
4

0

0

0

0

0

0

0

0

0

0

0

0

0

0

0

0

0

0

0

0

0

0
6

0

0

0

0

0

0

0

0

0

0

0
7

0

0

0

0.415525

0

0

0

0

0

0

0
7

0

0

0

0

0

0

0

0

0

0.258242

0

0

0

0

0

0

0

0

0

0

0

0
4

0
7

0

0

0

0

0

0

0

0

0

0

0

0

0

0

0

0.129121

0

0

0

0
7

0

0

0

0

0

0

0

0

0

0

0.623287
3

0

0

0

0

0

0

0

0

0

0

0
7

0

0

0

0

0

0

0

0

0.193681

0

0
7

0

0

0

0

0

0

0

0

0

0

0
4

0

0

0

0

0

0

0

0

0

0

0
4

0
7

0
7

0

0

0

0
4

0

0

0

0

0
4

0

0

0
4

0

0

0
4

0

0

0
4

0

0

0
4

0

0

0
4

0

0

0

0

0

0
4

0

0

0

0
4

0

0

0

0
4

0

0

0

0
4

0

0

0

0

0
4

0

0

0

0
4

0

0

0
4

0

0

0

0
4

0

0

0
4

0

0

0

0

0

0
4

0

0

0

0
4

0

0

0
4

0

0

0
4

0

0

0

0
4

0

0

0

0
4

0

0

0
4

0

0

0

0
4

0

0

0

0
4

0

0

0

0
4

0

0

0

0
4

0
7

0

0

0

0
4

0.645604

0.645604

0

0
4

0

0

0
4

0

0

0

0
4

0

0

0
4

0

0

0
4

0

0

0
4

0

0

0
4

0

0

0
4

0

0

0
4

0

0

0
4

0
6

0
6

0
4

0

0

0
4

0

0

0
4

0

0

0
4

0

0

0
4

0

0

0
4

0.138508

0.138508

0
4

0

0

0
4

0

0

0
4

0

0

0
4

0

0

0
4

0
6

0
6

0
4

0

0

0
4

0

0

0
4

0

0

0
4

0

0

0
4

0

0

0
4

0

0

0
4

0

0

0
4

0

0

0
4

0.193681

0.193681

0
4

0

0

0
4

0
4

0
4

0
4

0

0

0
4

0

0

0
4

0

0

0
4

0

0

0
4

0

0

0
4

0

0

0
4

0

0

0
4

0

0

0
4

0

0

0
4

0

0

0
4

0

0

0

0

0
4

0

0

0
4

0

0

0
4

0

0

0
4

0

0

0
4

0

0

0
4

0

0

0
4

0

0

0
4

0

0

0
4

0

0

0
4

0

0

0
4

0.387362

0

0

0.387362

0
4

0

0

0
4

0

0

0
4

0

0

0
4

0

0

0
4

0

0

0
4

0

0

0
4

0

0

0
4

0

0

0
4

0

0

0
4

0

0

0
4

0

0

0

0
4

0

0

0
4

0

0

0
4

0

0

0
4

0

0

0
4

0

0

0
4

0

0

0
4

0

0

0
4

0

0

0
4

0

0

0
4

6.5206

6.5206

0
4

0
4

2.8421709430404e-14
4

0
4

546.48
4

545.646
4

0

0

0

0

0

0
4

150.394
4

39.5059
4

51.1896
4

34.8935
3

1.24657
4

4.96124
4

0.153483
4

0
4

15.7985
4

2.64551
3

0

0

0

0

0

0

0

0

0

0

0

0

0
4

311.186
3

131.514
3

149.403
3

26.7369
3

0.484779
4

0
4

0.138508

0
4

0

0

0.138508

0

0

0

0

0
4

0

0

0

0

0

0

0

0

0

0

1.80061
3

0

0

0

0.138508

0

0

0

0

0

0

0.554033
3

0

0

0

0

0

0

0

0

0

0.138508

0

0

0

0

0

0

0

0

0

0

0

0
3

0

0

0

0

0

0

0

0

0

0

0
4

0

0

0

0

0

0

0

0

0

0

0.138508
4

0
4

1.37334588146132e-13
3

0
4

0
5

0
5

0
4

0

0

0
4

0

0

0
4

0

0

0
4

0

0

0
4

0

0

0
4

0

0

0
4

0

0

0
4

0

0

0
4

0

0

0
4

0

0

0
4

0

0

0

0
4

0

0

0
4

0

0

0
4

1.42033

1.42033

0
4

0.330928

0.330928

0
4

0

0

0
4

0

0

0
4

0

0

0
4

0

0

0
4

0

0

0
4

0

0

0

0
4

0

0

0
4

0

0

0
4

0

0

0
4

0

0

0
4

82.3145

82.3145

0
4

0

0

0
4

0
4

0
4

0
4

0

0

0

0

0

0

0

0

0
4

0

0

0

0
4

0

0

0
4

0
4

0
4

0

0

0

0

0
4

0

0

0
4

0
4

0
4

0

0

0
4

0

0

0
4

0

0

0
4

0

0

0
4

0
4

0

0

0

0
4

0

0

0

0
4

0

0

0
4

0
4

0

0

0

0
4

0

0

0
4

0
4

0.581044

0.581044

0.129121

0.129121

0.193681

0.129121

0
4

0
4

0

0

0

0
4

0

0

0
4

0

0

0
4

0
4

0

0

0

0
4

0

0

0
4

0

0

0
4

0
4

0

0

0

0

0
4

0
4

0

0

0

0

0

0
4

0
4

0

0

0

0
4

0
4

0
4

0
4

0
4

0

0
4

0

0

0

0
4

0

0

0
4

0
4

0

0

0

0
4

0

0

0
4

0

0

0
4

0
4

0

0

0

0
4

0

0

0
4

0

0

0
4

0
4

0

0

0

0

0
4

0
4

0

0

0

0

0
4

0

0

0
4

0
4

0

0

0

0

0
4

0
4

0

0

0

0

0
4

0

0

0
4

0
4

0

0

0

0
4

0
4

0

0

0

0
4

0
4

0

0

0

0

0
4

0
4

0

0

0

0
4

0
4

0
4

0
4

0
4

0

0

0
4

0

0

0

0
4

0

0

0
4

0
4

0

0

0

0
4

0
4

0

0

0

0
4

0
4

0

0

0

0
4

0
4

0

0

0

0

0
4

0
4

0

0

0

0
4

0
4

0

0

0

0
4

0

0

0
4

0
4

0

0

0

0

0
4

0
4

0

0

0

0

0
4

0
4

0

0

0

0
4

0

0

0
4

0
4

0

0

0

0
4

0
4

0
5

0

0

0

0
4

0

0

0

0

0

0
4

0
4

0

0

0

0
4

0
4

0

0

0

0
4

0
4

0

0

0

0
4

0
4

0

0

0

0
4

0
4

0

0

0

0
4

0
4

0

0

0

0
4

0
4

0

0

0

0
4

0
4

0

0

0

0
4

0
4

0

0

0

0
4

0
4

0

0

0

0
4

0
4

0
4

0
4

0
4

0

0

0
4

0

0

0
4

0

0

0
4

0
4

0

0

0

0
4

0
4

0.25354

0.25354

0.25354

0
4

0
4

0

0

0

0
4

0
4

0

0

0

0
4

0
4

0

0

0

0
4

0
4

0

0

0

0
4

0
4

0

0

0

0
4

0
4

0

0

0

0
4

0
4

0

0

0

0
4

0
4

0

0

0

0
4

0
4

0
4

0
4

0

0

0

0

0
4

0
4

0

0

0

0
4

0
4

0

0

0

0
4

0
4

0

0

0

0
4

0
4

0

0

0

0
4

0
4

0

0

0

0
4

0
4

0

0

0

0
4

0
4

0

0

0

0
4

0
4

0

0

0

0
4

0
4

0

0

0

0
4

0
4

0

0

0

0
4

0
4

0

0

0

0

0
4

0

0

0
4

0

0

0
4

0

0

0
4

0
4

0

0

0

0
4

0
4

0

0

0

0
4

0
4

0

0

0

0
4

0
4

0

0

0

0
4

0
4

0

0

0

0
4

0
4

0

0

0

0
4

0
4

0

0

0

0
4

0
4

0

0

0

0
4

0
4

0

0

0

0
4

0
4

0

0

0

0
4

0
4

0

0

0

0

0

0
4

0

0

0
4

0
4

0

0

0

0
4

0
4

0

0

0

0
4

0
4

0

0

0

0
4

0
4

0

0

0

0
4

0
4

0

0

0

0
4

0
4

0

0

0

0
4

0
4

0

0

0

0
4

0
4

0

0

0

0
4

0
4

0

0

0

0
4

0
4

0

0

0

0
4

0
4

0
4

0
4

0
4

0

0
4

0
4

0

0

0

0
4

0
4

0

0

0

0
4

0
4

0

0

0

0
4

0
4

0

0

0

0
4

0
4

0

0

0

0
4

0
4

0

0

0

0
4

0
4

0

0

0

0
4

0
4

0

0

0

0
4

0
4

0

0

0

0
4

0
4

0
4

0
5

0

0

0

0

0

0
4

0

0

0
4

0
4

0

0

0

0
4

0
4

0

0

0

0
4

0
4

0

0

0

0
4

0
4

0

0

0

0
4

0
4

0

0

0

0
4

0
4

0

0

0

0
4

0
4

0

0

0

0
4

0
4

0

0

0

0
4

0
4

0

0

0

0
4

0
4

0

0

0

0
4

0
4

0
4

0
4

0

0

0

0

0
4

0

0

0

0
4

0

0

0
4

0
4

0

0

0

0
4

0
4

0

0

0

0
4

0
4

0

0

0

0
4

0
4

0
6

0
6

0

0

0

0
4

0
4

0
7

0
7

0
7

0
4

0
4

0
6

0
6

0
6

0

0
4

0
4

0

0

0

0
4

0

0

0
4

0
4

0

0

0

0
4

0
4

0

0

0

0

0
4

0
4

0

0

0

0
4

0
4

0

0
5

0

0

0

0

0

0

0

0
4

0
7

0

0

0
4

0

0

0
4

0

0

0
4

0

0

0
4

0

0

0
4

0

0

0
4

0

0

0

0
4

0

0

0

0
4

0

0

0
4

0

0

0
4

0

0

0
4

0

0

0
4

0

0

0
4

0

0

0
4

0
4

0
4

6.3664629124105e-12

0
4

25.919

18.4253

17.0464

7.22203
3

7.22203
3

0

0

0

0

0

0
4

9.8244
3

0.969558
3

1.29121

1.87225

0

0

0

0

0

0

3.85577
2

0
3

0

0

0

1.83561

0

0

8.88178419700125e-16
3

0
4

0

0

0

0

0
4

0
7

0

0

0

0

0

0

0

0

0

0

0
6

0

0

0

0

0

0

0

0
4

0

0

0
4

0

0

0
4

0

0

0
4

1.77635683940025e-15

0
4

0
3

0

0

0

0
4

0

0

0

0

0

0
4

0

0

0

0

0
4

0

0

0
4

0
4

0

0

0

0
4

0
4

0

0

0

0
4

0
4

0

0

0

0
4

0
4

0

0

0

0
4

0

0

0
4

0
4

0
3

0
3

0

0

0

0
4

0

0

0

0
4

0

0

0
4

0
4

0.68945

0.68945

0.588998

0.100453

0
4

0
4

0.68945
2

0.68945
2

0.68945
2

0

0
4

0
4

0

0

0

0

0
4

0
4

0

0

0

0
4

0
4

0

0

0

0
4

0
4

0

0

0

0
4

0
4

0
4

1.94696
2

1.94696
2

1.63976
2

0

0.47767

0.581044

0.581044

0

0
4

0.3072
3

0

0

0

0

0.3072

0
4

0

0

0
4

0
4

0
4

0

0

0

0

0
4

0
4

0
4

4.51377

4.51377

4.51377

4.51377

0

0
4

0
4

0
4

0.387362

0.387362

0.387362

0.193681

0.193681

0

0
4

0
4

0
4

0.645604

0.645604

0.645604

0.645604

0
4

0
4

0
4

0

0

0

0

0
4

0
4

0
4

0
4

161.941

141.071

15.3417
3

15.3417
3

0

0

1.2677

0

0

0

0

0

0

0

0.0739218

3.09953
3

0

0

1.23601

0

0

0

0

0

0.110883

0.0633849

7.17699
3

0

0

0.0633849

0

0

0

0

0

0

0.12677

0.40657
3

0

0.0633849

0

0

0

0

0

0

0

0.258726

0

0

0

0

0.12677

0.25354

0

0

0

0

0

0.507079
4

0

0

0

0.507079
4

0

1.33226762955019e-15
3

0
4

0
4

15.0061
3

15.0061
3

6.39424
1

0.184804

0

0

0

0

0

0

0

0.0739218

0

0

0

0.0739218

0

0

0.110883

0

0

0.110883

0

0

0
4

0

0.0739218

0

0

0

0

0

0

0
4

0

7.83571

0

0.147844

0

8.60422844084496e-16
3

0
4

0
4

0.077809
6

0.077809
6

0
6

0

0

0

0

0

0
7

0

0

0

0

0
5

0

0

0

0

0

0

0.077809

0

0

0

0
5

0

0

0

0

0

0

0

0

0

0

0
6

0

0

0

0

0

0
6

0
6

0
7

0
6

0
4

0

0

0
4

0
4

0
7

0
7

0
7

0
7

0
4

0
4

26.5674
3

26.5674
3

0

7.21711

0

0

0

0.285232

0

0

0

0

0

10.9714

0

0

0.285232

0

5.76802

0

0

0.190155

0

0

0

0

0

0

0

0

0

0

0

0.380309

0

0

0

0

0

0

0

0

0

0

0.12677

0

0

0.0633849

0

0

0

0.0633849

0

0

0

0

0.0633849

0.867775

0

0

0.0950773

0

0

0

0

0

0

0

0

0.0633849

0

0

0

0

0

0

0

0.12677

0

0

0

0

0

0

0

0

0

0

0

0

0

6.30051566474776e-15
3

0
4

0

0

0

0

0

0
4

0

0

0
4

0

0

0
4

0

0

0

0

0
4

0

0

0
4

0

0

0

0
4

0

0

0

0
4

0

0

0
4

0

0

0
4

0

0

0
4

0

0

0
4

0
4

63.8155
3

0.077809
3

0

0.077809

0
4

13.7169
3

0
4

0

0

0

0

0

0

0

0

0.129121

0.581295

6.42723
2

0

0

0

0

0

0

0

0

0

0.129121

0

0

0

0.121103

0

0

0

0

0

0

0

0
4

0

0

0

0

0

0

0

0.116713

0

0

5.11436

0

0.129121

0

0

0

0

0

0

0

0

0
4

0

0.968825

0

0

0

0

0

0

0

1.11022302462516e-15
3

0
4

0

0

0

0
4

0

0

0
4

0

0

0
4

0

0

0

0

0
4

0

0

0

0

0

0

0

0

0

0

0

0

0
4

4.12388

1.78961

2.33427

0

0
4

45.8969

18.4349

17.963

0

0

0.554413

0

0

0

0.369609

8.35316

0

0.147844

0.0739218

0

0

8.64586180426841e-15

0
4

0

0

0
4

0

0

0
4

0
4

13.8217
5

0
6

0
6

0
4

8.10274
5

8.10274
5

0
4

0
7

0

0

0

0

0

0

0

0

0

0

0

0

0

0

0

0

0

0

0

0

0

0

0

0

0

0

0

0

0

0

0

0

0

0

0

0

0

0

0

0

0

0

0

0

0

0

0

0

0

0

0

0

0

0

0

0

0

0

0

0

0

0

0

0

0

0

0

0

0

0

0

0

0

0

0

0

0

0

0
4

0

0

0

0
4

5.71896

5.71896

0
4

0

0

0
4

0

0

0
4

0
4

0
3

0
3

0
4

0

0

0

0

0

0

0

0

0

0
4

0
4

3.615
3

3.615
3

1.74014
3

0

0

0

0

0

0.685538
3

0.942717

0.194522

0

0

0.052091

0

0

5.27355936696949e-16
3

0
4

0

0

0
4

0

0

0
4

0
4

0

0

0

0
4

0
4

0

0

0

0
4

0
4

0

0

0

0
4

0
4

0.077809

0.077809

0.077809

0
4

0
4

0.0633849

0.0633849

0.0633849

0
4

0
4

0

0

0

0
4

0
4

0

0

0

0
4

0
4

0

0

0

0
4

0
4

0

0

0

0
4

0
4

0

0

0

0
4

0
4

0
3

0
3

0
3

0
4

0
4

0

0

0

0

0

0

0

0
4

0
4

2.68441

2.68441

2.6066

0.077809

9.71445146547012e-17

0
4

0
4

0

0

0

0

0
4

0

0

0
4

0
4

0

0

0

0
4

0

0

0
4

0
4

0

0

0

0
4

0
4

0

0

0

0

0
4

0
4

0

0

0

0
4

0

0

0
4

0
4

0
4

2.05727
3

2.05727
3

2.02254
3

1.97045
3

0.052091
3

0

0

0

0

1.11022302462516e-16
3

0
4

0.0347274

0.0347274

0
4

0

0

0

0
4

1.04083408558608e-16
3

0
4

0
4

0

0

0

0

0

0

0
4

0

0

0
4

0
4

0
4

2.69386

2.69386

2.69386

2.5037

0.190155

0
4

0
4

0
4

3.45448

3.45448

3.45448

3.39109

0.0633849

5.55111512312578e-17

0
4

0
4

0
4

0

0

0

0

0

0
4

0
4

0
4

0

0

0

0

0
4

0

0

0
4

0
4

0
4

0

0

0

0

0
4

0
4

0
4

0

0

0

0

0

0
4

0
4

0
4

0

0

0

0

0
4

0
4

0
4

0

0

0

0

0
4

0
4

0
4

0

0

0

0

0
4

0
4

0
4

0.975233
3

0.975233
3

0.975233
3

0.975233
3

0

0

0

0
4

0
4

0
4

0

0

0

0

0
4

0
4

0
4

0.933708

0.933708

0.933708

0.933708

0
4

0
4

0
4

0

0

0

0

0
4

0
4

0
4

0

0

0

0

0
4

0
4

0
4

0.207762

0.207762

0.207762

0.207762

0
4

0
4

0
4

0

0

0

0

0
4

0
4

0
4

0

0

0

0

0
4

0
4

0
4

0

0

0

0

0
4

0
4

0
4

0.077809

0.077809

0.077809

0.077809

0
4

0
4

0
4

0

0

0

0

0
4

0
4

0
4

0.129121

0.129121

0.129121

0.129121

0

0

0

0

0
4

0

0

0
4

0
4

0
4

2.80112

2.80112

2.80112

2.80112

0
4

0
4

0
4

0

0

0

0

0
4

0
4

0
4

0

0

0

0

0
4

0
4

0
4

4.63756

4.63756

4.63756

4.43666

0.200905

1.11022302462516e-16

0
4

0
4

0
4

0

0

0

0

0

0

0
4

0

0

0
4

0

0

0
4

0
4

0
4

2.5677

2.5677

2.5677

2.48989

0.077809

9.71445146547012e-17

0
4

0
4

0
4

0.334842

0.334842

0.217647

0.0669684

0.0669684

0.0502263

0.0334842

1.38777878078145e-17

0
4

0.117195

0.117195

0
4

2.77555756156289e-17

0
4

0
4

0

0

0

0

0

0

0
4

0
4

0
4

0

0

0

0

0

0

0
4

0

0

0
4

0
4

0
4

0
4

63.8214
3

63.8214
3

0
4

0
4

0
4

0
4

0
4

0

0

0

0

0

0

0

0

0

0

0

0

0

0

0

0

0

0

0

0
4

0

0

0

0

0

0

0

0

0

0

0

0

0

0

0
4

0

0

0

0
4

0
4

0

0

0

0
4

0
4

0

0

0

0
4

0

0

0

0
4

0
4

1.02955

0.0367698
1

0.0367698
1

0
4

0

0

0
4

0

0

0
4

0.992785
4

0

0
4

0

0

0.992785

0

0
4

0

0

0
4

0

0

0
4

0

0

0
4

0

0

0
4

0
4

0
7

0
7

0
7

0
4

0
4

0
7

0
7

0
7

0

0

0

0
4

0
4

2.37317
4

2.29536
4

2.29536
4

0

0

0

0

0

0

0

0
4

0

0

0
4

0.077809

0.077809

0
4

9.71445146547012e-17
4

0
4

0.272331
4

0.272331
4

0.272331

0

0

0
4

0
4

0

0

0

0
4

0
4

0

0

0

0
4

0
4

55.011
3

55.011
3

0

26.1049
3

0

28.4003
3

0

0

0

0

0

0.155618

0

0

0

0.116713

0

0

0

0

0

0

0

0

0

0

0

0

0

0

0.155618

0

0

0

0

0.077809

0

0

0

0

0

0

0

0
4

0

0

0
4

0
4

5.13539
4

5.13539
4

0
4

0
4

0
4

5.13539
3

0

0

0

0

0

0
4

0
4

0

0

0

0

0
4

0
4

0
4

0

0

0

0

0
4

0
4

0
4

0

0

0

0

0
4

0
4

0
4

0
4

2722.87

40.6608

10.2708

10.2708

10.2708

0
4

0
4

5.35851

5.35851
3

1.80769
3

0.193681

0

0

0

0

0

0

0

0

0

2.84066
3

0

0

0

0

0

0

0

0

0.516483
3

0

0

0

0

0

0

8.88178419700125e-16
3

0
4

0

0

0
4

0

0

0

0

0

0

0
4

0
4

25.0315

10.6498

3.55082

4.9963

0.343267

0

0

0

0.151156

0

0

0

0

0

0

0

0

0

0

1.25713

0

0

0.351085

3.33066907387547e-16

0
4

14.3818

13.8007

0.451923

0

0.129121

0

8.60422844084496e-16

0
4

0
4

0

0

0

0
4

0
4

0

0

0

0
4

0
4

0
4

2.46358
1

1.12148

1.12148

0

0

0

0

1.06632

0

0

0

0

0

0.0551547

0

0
4

0

0

0

0

0

0

0
4

0

0

0
4

0

0

0
4

0
4

0

0

0

0
4

0
4

0

0

0

0
4

0
4

0

0

0

0

0

0

0

0

0
4

0

0

0
4

0

0

0
4

0
4

0
8

0
8

0

0

0

0
4

0

0

0
4

0
4

0

0

0

0

0

0
4

0

0

0
4

0
4

0

0

0

0
4

0

0

0
4

0
4

1.3421

1.28694

1.2134

0.0735396

0

0
4

0.0551547

0.0551547

0
4

0
4

0

0

0

0

0

0
4

0
4

0

0

0

0
4

0

0

0
4

0
4

0

0

0

0
4

0
4

2.22044604925031e-16
1

0
4

21.7097
3

21.3869
3

0.933708
3

0

0

0

0

0

0

0

0

0

0

0

0

0

0

0

0

0

0

0

0

0

0

0.389045

0

0

0

0

0

0

0

0

0

0

0

0

0

0

0

0

0

0

0

0

0

0

0

0

0

0

0

0

0

0

0

0.544663

0

0

0
4

18.3524
4

3.46271
4

0
4

0

0

0

0

0

0

0

0

0

0

0
4

0

0

0

0

0

0

0

0

0

0

0
4

0

0

0

0

0

0

0

0

0

0

0
4

0

0

0

0

0

0

0

0

0

0

0

0

0

0

0

0

0

0

0

0

0

0
4

0

0

0

0

0

0

0

0

0

0

0
4

0

0

0

0

0

0

0

0

0

0

0
4

0

0

0

0

0

0

0

0

0

0

0
4

0

0

0

0

0

0

0

0

0

0

0
4

0

0

0

0

0

0

0

0

0

0

9.41857
4

0
4

0

0

0

0

0

0

0

0

0

0

0
3

0
4

0
4

0
3

0

0
4

0

0

4.70928
4

0

0.277017
3

0

0

0

0

0

0

0

0

0.484779
4

0

0

0

0

0

0

0

0

0

0

0
4

0

0

0

0

0

0

0

0

0

0

0
4

0

0

0

0

0

0

0

0

0

0

0
4

0

0

0

0

0

0

0

0

0

0

0
4

0

0

0

0

0

0

0

0

0

0

0
4

0

0

0

0

0

0

0

0

0

0

0
4

0
2

0
2

0

0

0
4

2.10084
2

0.816994

0

0

0

0

0

0

0

0

0

0

0

0

0

0

0

0

0

0

0

0

0

0

0

0

0

0

0

0.077809

0

0

0

0

0.077809

0

0

0

0

0

0

1.12823

0

0

0

0

0
4

0

0

0

0

0
4

0

0

0

0

0

0

0

0

0

0

0

0
4

0

0

0
4

0

0

0
4

0

0

0
4

0

0

0
4

0

0

0

0

0

0

0

0
4

0

0

0

0
4

0

0

0

0
4

0

0

0

0

0
4

0

0

0

0
4

0

0

0
4

0

0

0
4

0

0

0
4

8.88178419700125e-16
3

0
4

0

0

0

0
4

0

0

0
4

0
4

0.322802

0.322802

0.193681

0.129121

2.77555756156289e-17

0
4

0
4

0

0

0

0
4

0
4

0

0

0

0
4

0
4

0
4

62.1164
3

21.8558
3

21.8558
3

2.24908
2

0

0

0

0

0

0

0

0

0

0

0.770137

0

7.00842
3

0

0

0

0

0

0

0.839285

0

0

0

0

0

0.516483

0.129121

0

0

0

0

0

0.0502263

0

0

0

0

0

0

0.820363

0

0

0.0739218

0.0739218

0

0

0

0

0

0.131657

0

0

0

0.0334842

0

0

3.55082

0

0

0

0

0

0

0

0

0.0877713

0

0

0

0

0

0

0

0

0.0334842

3.4217

0

0

0
4

0

0

0

1.29121

0

0

0

0

0.774725

0

0

0

0

0

0

0

0

0

0

0

0

0
4

0
4

0

0
4

0

0
4

0

0

0
4

0

0

0
4

0

0

0
4

0

0

0

0
4

0
4

0
4

0
4

0

0

0
4

0

0

0

0
4

0

0

0
4

0

0

0
4

0

0

0
4

0

0

0
4

0
4

0

0

0

0

0

0

0
4

0

0

0
4

0
4

0

0

0

0
4

0

0

0
4

0
4

19.82

19.82

0.129121

0.451923

19.239

0
4

0
4

0

0

0

0
4

0
4

0

0

0

0
4

0
4

0.557074

0.557074

0.557074

0
4

0
4

0

0

0

0
4

0
4

0

0

0

0
4

0
4

0

0

0

0
4

0
4

0

0

0

0
4

0
4

0

0

0

0
4

0
4

18.8516

18.658

18.0769

0.581044

0
4

0.193681

0.193681

0
4

1.22124532708767e-15

0
4

0.0633849

0.0633849

0.0633849

0
4

0
4

0

0

0

0
4

0
4

0

0

0

0
4

0
4

0

0

0

0
4

0
4

0

0

0

0
4

0
4

0

0

0

0
4

0
4

0.968406

0.968406

0.968406

0
4

0
4

0

0

0

0
4

0
4

0

0

0

0
4

0
4

0

0

0

0
4

0
4

0

0

0

0

0

0

0
4

0

0

0
4

0
4

0

0

0

0
4

0

0

0

0

0
4

0
4

0

0

0

0

0
4

0
4

0

0

0

0

0

0
4

0
4

0

0

0

0

0
4

0
4

0

0

0

0
4

0
4

0

0

0

0
4

0
4

4.88498130835069e-15
3

0
4

18.0403

0
4

0
5

0

0

0

0

0

0

0

0

0

0

0

0
4

0

0

0

0
4

0

0

0
4

0
4

13.2275
7

0
8

0

0
8

0
4

0
1

0
1

0

0

0

0
4

13.2275
1

0

0

0

0

0

0

0

0

13.2275

0

0

0

0

0
4

0

0

0
4

0
7

0
7

0
7

0
7

0

0

0

0

0

0

0

0

0

0

0
7

0

0

0

0

0

0

0

0

0

0

0
7

0

0

0

0

0
7

0
7

0

0

0

0

0
4

0

0

0
7

0

0

0

0

0

0

0

0

0

0

0

0
4

0

0

0

0
4

0

0

0

0
4

0

0

0
4

0

0

0
4

0
4

0
7

0
7

0
7

0

0

0
4

0
7

0
6

0

0
4

0
4

0

0
1

0

0

0

0

0

0

0

0

0

0

0

0

0

0

0

0

0

0

0

0

0

0

0

0

0

0

0

0

0

0

0

0

0

0

0

0

0

0

0

0

0

0

0

0

0

0

0

0

0

0

0

0

0

0

0

0

0

0

0
4

0

0

0

0

0

0

0

0

0

0

0

0

0
4

0

0

0

0

0

0

0

0

0

0

0
7

0

0
5

0

0

0

0

0
4

0

0

0
4

0

0

0
4

0
4

0

0

0

0

0

0

0
4

0

0

0
4

0
4

0

0

0

0
4

0
4

0

0

0

0
4

0
4

0

0

0

0
4

0
4

0

0

0

0
4

0
4

0

0

0

0

0
4

0

0

0
4

0
4

0

0

0

0

0

0
4

0
4

0

0

0

0

0
4

0

0

0
4

0
4

4.29376

4.29376

4.29376

0
4

0
4

0

0

0

0
4

0
4

0

0

0

0

0
4

0
4

0.519005

0.519005

0.519005

0
4

0
4

0

0

0

0
4

0
4

0
7

0
7

0
7

0
4

0
4

0
4

1264.63
3

43.2265
3

0

0

0
4

29.1553
3

28.9615
3

0

0.193765

0

0
4

13.0635
2

9.03793
2

1.50168
2

2.22942
2

0

0

0

0.294427

0

0
4

0.0484412

0.0484412

0

0
4

0

0

0

0
4

0.314868

0.145324

0.169544

0
4

0

0

0
4

0.0633849

0.0633849

0
4

0

0

0
4

0.581044

0.581044

0
4

5.99520433297585e-15
3

0
4

123.312
3

0
7

0
6

0

0
4

0
3

0
3

0
4

29.0139
3

28.3117
3

0.70217
2

0
4

18.9493
3

3.56607

15.1499
3

0

0

0

0

0

0

0

0

0

0

0

0

0

0

0

0.077809

0

0.155618

0

0

3.05311331771918e-16
3

0
4

75.3485
3

35.3196

0

0

0.138508

0.138508

0.484779

0.554033

0.138508

0.207762

0

0.277017

0

0.138508

0
7

0
7

0

37.9513

0

0

0

0
4

0

0

0

0
4

0

0

0
4

0

0

0
4

0

0

0
4

0

0

0
4

0
4

26.8926
3

17.5694

17.5694

0

0

0

0

0

0
4

0
5

0

0

0

0

0

0

0

0

0

0
4

0

0

0
6

0

0

0
6

0

0

0

0

0

0

0

0
4

4.56411
3

0.877713
3

3.68639
4

0
4

0

0

0

0

0

0
4

0

0

0

0

0

0

0

0
4

0

0

0

0
4

0.736653

0.418553

0.3181

0
4

0

0

0
4

0

0

0
4

0

0

0
4

0.219428

0.219428

0
4

2.02166

1.76342

0

0.258242

0
4

1.29121

0.645604

0.645604

0
4

0

0

0
4

0

0

0
4

0

0

0
4

0.0837105

0.0837105

0
4

0.322802

0.322802

0
4

0.0837105

0.0837105

0
4

0
4

128.428
4

0

0

0
4

0

0

0

0
4

128.299
4

106
4

12.535
4

0
4

0

0

0

0

0

0.0877713

0

0

0

0

0
4

0

0

0

0

0

0

0

0

0

0

0
4

2.13141

0

0

0

0

0

0

0

0

0

0
4

0

0.155618

0

0

0

0

0

0

0
4

0

0

0
4

0

0

0
4

0

0

0

0

0

0
4

0
4

0

0

0

6.85616
4

0

0

0

0

0

0

0

0

0

0

0
4

0

0

0

0

0

0

0

0

0

0

0
4

0

0

0

0

0

0

0

0

0

0

0
4

0.532854

0

0

0

0

0

0

0

0

0

0
3

0

0

0

0

0

0

0

0

0

0

0

0

0

0

0

0

0

0

0

0

0

0
4

0

0

0

0

0

0

0

0

0

0

1.64313007644523e-14
4

0
4

0

0

0
4

0

0

0
4

0

0

0
4

0

0

0
4

0

0

0
4

0

0

0
4

0

0

0
4

0

0

0
4

0

0

0
4

0

0

0
4

0

0

0
4

0

0

0

0

0
4

0

0

0
4

0

0

0
4

0

0

0
4

0

0

0
4

0

0

0
4

0

0

0
4

0

0

0
4

0

0

0
4

0

0

0
4

0

0

0
4

0

0

0

0
4

0.129121

0.129121

0
4

0

0

0
4

0

0

0
4

0

0

0
4

0

0

0

0
4

0

0

0
4

0

0

0
4

0

0

0
4

0
4

757.355

0
6

0
6

0
6

0

0
6

0

0

0

0

0
4

0
3

0

0

0

0

0

0

0

0

0

0

0

0

0

0

0

0

0

0

0

0

0

0

0

0

0

0

0

0

0

0

0

0
4

17.7359
3

14.2279
3

0.661376
2

0

0

0

0.35014
2

2.45965

0

0

0

0.0367698

0

0

0
4

11.5487
2

5.76379
2

0

0

0

0.0739218

0

0

4.9371
2

0.739185

0

0

0.0347274

0

0

0

0
4

482.825

188.095
3

5.49591
7

5.99151
3

6.77807

247.964

23.2905
2

0

0

0

0

0.0877713

0

0

0

0

0.0950773

2.19505

0

0.351085

2.38874

0

0

0

0

0

0.0919245

0

0

0

0

0

0

6.45317133063372e-14

0
4

220.922

220.533

0.233427

0

0

0

0.077809

0.077809

0

0
4

0.263314
6

0
6

0

0

0

0

0

0

0

0

0

0

0

0

0

0

0

0

0

0

0

0

0

0

0

0

0

0.263314

0

0

0

0

0

0

0

0

0

0

0

0

0

0

0

0

0

0

0

0

0

0

0

0

0

0

0

0

0

0

0

0

0

0

0

0

0

0

0

0

0

0

0

0

0

0

0

0

0

0

0
6

0

0

0

0

0

0

0

0

0

0

0

0

0
4

14.0249
3

5.32002
3

0.855899
2

0

0.179696

0

0

2.13975

0

0

0

0

0

0.208364
3

0

0.0735396

0.052091

1.04585

0.0551547

0

0.475387

0

0

0

0
2

0

0.0347274

0

0

0.0919245

0

0

0

0

0

0

0

0

0

0

0

0

0.614399

0

0.175543

0

0

0

0

0

0

0

0

0

0

0

0

0

0

0

0

0

0

0.0367698

0

0

0

0

2.66581

0

0

0

0

0
2

0

0
4

7.10948
3

0

6.53896

0
4

0

0

0

0.351085

0

0

0

0

0.0877713

0.131657

0
4

0

0

0

0

0

0

0
4

0

0

0

0

0

0

4.9960036108132e-16
3

0
4

0.0726619

0.0726619

0

0
4

0

0

0
4

0

0

0
4

0.570514

0.570514

0
4

0

0

0
4

0

0

0
4

0

0

0
4

0

0

0
4

0

0

0
4

0

0

0
4

0

0

0
4

1.31657

1.31657

0
4

0

0

0
4

0

0

0
4

0

0

0
4

0

0

0
4

0

0

0
4

0

0

0

0
4

0

0

0
4

0.965484

0.965484

0
4

0

0

0
4

0

0

0
4

0
4

14.8027
3

0

0

0
4

0.658285
3

0
2

0

0

0.658285

0

0
4

10.9504
3

0.0730852
3

0

1.93912

0

3.11588

0

0

0

4.78354

1.03881

0
4

0

0

0
4

0

0

0
4

0

0

0
4

0

0

0
4

0.692542

0.692542

0
4

0

0

0

0
4

2.50148

2.50148

0
4

0

0

0
4

0

0

0
4

0

0

0
4

0

0

0
4

2.66453525910038e-15
3

0
4

1.99589

0

0

0

0
4

1.99589
2

1.99589

0

0

0
4

0

0

0
4

0

0

0
4

0

0

0
4

0
4

0
6

0
6

0
6

0

0

0

0
4

0
7

0
6

0
7

0

0

0

0

0

0

0

0
4

0
6

0

0

0

0

0

0

0

0

0
4

0
4

3.93381

3.93381

3.93381

0
4

0
4

0.207762

0.207762

0.207762

0
4

0

0

0
4

0
4

0

0

0

0

0

0
4

0

0

0
4

0
4

0

0

0

0

0

0
4

0

0

0
4

0

0

0
4

0
4

0.138508

0

0

0

0
4

0.138508

0.138508

0
4

0

0

0
4

0
4

0

0

0

0

0
4

0

0

0
4

0

0

0
4

0
4

0

0

0

0

0

0
4

0
4

0

0

0

0

0
4

0
4

0

0

0

0

0
4

0
4

0

0

0

0

0
4

0
4

0.12677
4

0.12677
4

0.12677
4

0

0

0
4

0

0

0
4

0
4

2.95492

2.95492

2.95492

0

0
4

0
4

3.03855

3.03855

3.03855

0
4

0
4

0.322802

0.322802

0.193681

0.129121

2.77555756156289e-17

0
4

0
4

0

0

0

0
4

0
4

0

0

0

0

0
4

0
4

0

0

0

0
4

0
4

0

0

0

0
4

0
4

0

0

0

0

0
4

0
4

0

0

0

0

0
4

0
4

0

0

0

0
4

0
4

0.887388
3

0.887388
3

0.887388
3

0
4

0

0

0

0

0
4

0

0

0

0
4

0

0

0
4

0
4

0

0

0

0
4

0
4

0

0

0

0
4

0
4

0

0

0

0
4

0
4

0

0

0

0
4

0
4

0

0

0

0
4

0
4

0

0

0

0
4

0
4

0

0

0

0
4

0
4

0.077809

0.077809

0.077809

0
4

0
4

0

0

0

0
4

0
4

0

0

0

0
4

0
4

1.23601
3

1.23601
3

1.23601
3

0

0

0

0
4

0
4

0

0

0

0
4

0
4

0

0

0

0
4

0
4

0

0

0

0
4

0
4

0

0

0

0
4

0
4

0

0

0

0
4

0
4

0.0877713

0.0877713

0.0877713

0
4

0
4

0

0

0

0
4

0
4

0.193681

0.193681

0.193681

0
4

0
4

0

0

0

0
4

0
4

0

0

0

0
4

0
4

3.32693

3.32693

3.32693

0

0
4

0
4

0

0

0

0
4

0
4

0

0

0

0
4

0
4

0

0

0

0
4

0
4

0

0

0

0
4

0
4

0

0

0

0
4

0
4

0

0

0

0
4

0
4

0

0

0

0
4

0
4

0

0

0

0
4

0
4

0

0

0

0
4

0
4

0

0

0

0
4

0
4

0

0

0

0

0

0

0
4

0
4

0

0

0

0
4

0
4

0
7

0
7

0

0

0
4

0
4

0

0

0

0
4

0
4

29.2312
2

29.2312
2

0.193681

29.0375

0
4

0
4

45.3596

14.429

2.58455
2

3.36038
2

1.27104

0.460192

0.0367698
3

0
6

0

0

0.792311

0.0968825
2

0

0
7

0

0
7

1.14093

0

0

0

0

0

0

2.37317

0.290647

0

0
7

0

0

0

0

0

0.0726619

0

0.0726619

0

0

1.52881

0

0

0

0

0

0

0

0

0

0

0
7

0

0

0

0

0

0

0

0

0.0367698

0

0

0

0

0

0

0

0

0

0

0

0

0
2

0.311236

0

0

0

0
5

0
4

14.3386
2

14.2902
2

0.0484412

0

0

0

0

0
4

0

0

0

0
4

0.110883

0.110883

0
4

0

0

0
4

11.937
2

11.937
2

0

0

0
4

0.121103

0.121103

0

0

0
4

0.0726619
2

0

0

0.0726619

0
4

0

0

0
4

0

0

0
4

0.438857

0.0877713

0.351085

0
4

2.02234

2.02234

0
4

1.88921

0

1.88921

0
4

1.33226762955019e-15

0
4

7.67423
4

1.80061
4

1.80061
4

0

0
4

0
7

0
7

0
7

0

0

0

0

0

0
4

0

0

0
4

0

0

0

0
4

0

0

0
4

4.51085

4.51085

0
4

0

0

0
4

0

0

0
4

1.36277

1.36277

0
4

0

0

0
4

0

0

0
4

4.44089209850063e-16
4

0
4

3.04933

0

0

0

0

0

0

0

0

0

0

0

0

0

0
4

3.04933
3

3.04933
3

0

0
4

0

0

0
4

0
4

0

0

0

0
4

0

0

0
4

0

0

0
4

0

0

0
4

0

0

0
4

0

0

0
4

0

0

0
4

0
4

1.21358

0.862496

0

0

0

0

0

0.0877713

0

0

0

0

0

0

0

0.774725

0

0
4

0

0

0
4

0

0

0
4

0

0

0
4

0.351085

0.351085

0
4

1.11022302462516e-16

0
4

35.1188
3

32.8874
3

7.41945
3

0
4

0.175543
3

0

0
3

0
4

0

0

0.0739218

0

0

16.8364
3

0

0.311236

0.0633849

0

0

0.0877713

0

0

0

0.077809

2.16169
3

0

0.351085

0.131657

0

0

0

0

0

0

0

0.838351
3

0

0.077809

0.0877713

0

0

0

0

0.27062
3

0.385009
3

0.077809
3

3.46007
3

0

1.06581410364015e-14
3

0
4

1.4669
2

1.31964
2

0.147264

0

0

0

2.22044604925031e-16
2

0
4

0

0

0
4

0

0

0
4

0

0

0
4

0

0

0
4

0.225728
2

0.052091

0

0.0694547

0.0347274

0.0694547

0

0
4

0.538771

0

0.538771

0
4

0

0

0
4

0

0

0
4

0

0

0

0
4

0

0

0

0
4

0

0

0

0
4

0

0

0
4

0
4

6.51195
3

6.43929
3

0
2

0

0

0.665541

0

0

0

0

0

0

0

2.1229
2

0

0

0.0367698

0

0

0

0

0

0.0367698

0.478008

2.49259
2

0

0
2

0

0

0.606702

5.55111512312578e-16
3

0
4

0

0

0

0
4

0

0

0
4

0

0

0
4

0

0

0
4

0

0

0
4

0

0

0
4

0.0726619

0.0726619

0
4

0

0

0
4

0

0

0
4

3.33066907387547e-16
3

0
4

23.9308

14.866

3.80025
2

11.0658

0

0
4

8.94803

7.93652

0.389045

0.505758

0.116713

0
4

0

0

0

0

0
4

0

0

0

0
4

0

0

0
4

0.116713

0.116713

0
4

0

0

0
4

3.19189119579733e-15

0
4

0

0

0

0
4

0

0

0
4

0

0

0
4

0
4

4.9737991503207e-14
3

0
4

99.4289
2

99.4289
2

0

0

0

0

0

0

0
4

99.1702
2

99.1702
2

0
4

0

0

0
4

0.258726

0.258726

0
4

0

0

0
4

0
4

0

0

0

0
4

0
4

0
4

87.7426
4

0

0

0

0

0
4

0
4

2.96931
2

2.58322
2

0.937558
2

0.24523

1.31673
2

0

0.0334842

0.0502263

0

0

6.93889390390723e-17
2

0
4

0

0

0
4

0

0

0

0

0
4

0.386083

0.386083

0

0
4

0
4

0
4

0
4

0
7

0

0

0

0
7

0

0

0

0

0

0

0
4

0

0

0

0

0

0

0

0

0

0

0

0

0

0

0

0
4

0

0

0

0

0
4

0

0

0

0

0
4

0

0

0

0
4

0

0

0
4

0

0

0
4

0
4

0
7

0
7

0

0

0

0

0
4

0

0

0

0
4

0
4

0

0

0

0
4

0
4

0

0

0

0
4

0
4

0

0

0

0
4

0
4

0

0

0

0
4

0
4

0

0

0

0
4

0
4

0.0739218

0.0739218

0.0739218

0
4

0
4

0

0

0

0
4

0
4

0

0

0

0
4

0
4

0

0

0

0
4

0
4

0

0

0

0
4

0
4

0
5

0
7

0
7

0
4

0

0

0
4

0

0

0
4

0
4

0

0

0

0
4

0
4

0

0

0

0
4

0
4

0

0

0

0
4

0
4

0

0

0

0
4

0
4

0

0

0

0
4

0
4

0

0

0

0
4

0
4

0

0

0

0
4

0
4

0

0

0

0
4

0
4

0

0

0

0
4

0
4

0

0

0

0
4

0
4

0
7

0
7

0
7

0

0

0

0

0

0

0

0
4

0
4

0

0

0

0
4

0
4

0

0

0

0
4

0
4

0

0

0

0
4

0
4

0

0

0

0
4

0
4

0

0

0

0
4

0
4

0

0

0

0
4

0
4

0

0

0

0
4

0
4

0.138508

0.138508

0.138508

0
4

0
4

0.138508

0.138508

0.138508

0
4

0
4

0.129121

0.129121

0.129121

0
4

0
4

0.387362

0
7

0

0

0

0

0
4

0.258242

0

0.258242

0

0
4

0

0

0
4

0.129121

0.129121

0
4

0
4

0

0

0

0
4

0
4

0

0

0

0
4

0
4

0

0

0

0
4

0
4

0

0

0

0
4

0
4

0

0

0

0
4

0
4

0

0

0

0
4

0
4

0

0

0

0
4

0
4

0

0

0

0
4

0
4

0

0

0

0
4

0
4

2.21847

2.21847

2.21847

0
4

0
4

1.54945

0

0

0

0

0

0
4

0

0

0

0
4

0

0

0

0
4

0

0

0
4

0

0

0
4

0

0

0
4

1.54945

1.54945

0
4

0
4

0

0

0

0
4

0
4

0

0

0

0
4

0
4

0

0

0

0
4

0
4

0

0

0

0
4

0
4

0

0

0

0
4

0
4

0

0

0

0
4

0
4

2.58925

2.58925

2.58925

0
4

0
4

0

0

0

0
4

0
4

0

0

0

0
4

0
4

0

0

0

0
4

0
4

0

0

0

0

0

0
4

0
4

0

0

0

0
4

0
4

0

0

0

0
4

0
4

0
7

0
7

0
7

0

0
4

0

0

0
4

0

0

0
4

0
4

0
5

0
7

0

0

0

0

0
4

0

0

0
4

0

0

0
4

0
4

0

0

0

0

0
4

0

0

0

0
4

0

0

0

0
4

0

0

0
4

0
4

0

0

0

0

0

0

0
4

0

0

0

0
4

0
4

0

0

0

0

0

0

0

0

0

0

0

0

0

0

0

0

0

0

0

0

0
4

0

0

0

0

0

0

0
4

0
4

0

0

0

0

0
4

0
4

0.0877713

0.0877713

0

0.0877713

0

0

0
4

0

0

0
4

0
4

0
4

0

0

0

0
4

0

0

0
4

0

0

0
4

0
4

0

0

0

0

0

0

0
4

0

0

0

0
4

0
4

0.60921
3

0.545825
3

0.545825
3

0

0
4

0.0633849

0.0633849

0
4

5.55111512312578e-17
3

0
4

0

0

0

0
4

0
4

27.2677
2

0.475134

0.475134

0
4

0

0

0
4

26.7926

26.7926

0
4

0
4

0

0

0

0

0
4

0

0

0
4

0
4

0.221765
6

0

0

0

0

0
4

0.221765

0

0.221765

0
4

0
4

0

0

0

0

0

0
4

0
4

2.21613
4

2.21613
4

1.52359
4

0

0

0

0
4

0

0

0

0.692542

0

0

0

1.11022302462516e-16
4

0
4

0

0

0
4

0
4

0

0

0

0

0
4

0
4

0

0

0

0

0

0
4

0

0

0

0
4

0
4

0
5

0
5

0
5

0
4

0
4

0

0

0

0
4

0

0

0
4

0

0

0
4

0

0

0
4

0

0

0
4

0
4

1.72499
2

1.72499

1.54945

0.175543

0

0
4

0

0

0
4

0

0

0
4

0
4

0

0

0

0
4

0
4

0.443531

0.443531

0.0739218

0.369609

0
4

0

0

0
4

0

0

0
4

0

0

0
4

0
4

0

0

0

0

0
4

0
4

0

0

0

0

0

0
4

0
4

0

0

0

0
4

0
4

0
4

0

0

0

0

0

0

0

0

0

0
4

0

0

0

0

0

0
4

0

0

0

0

0
4

0

0

0
4

0

0

0
4

0
4

0

0

0

0
4

0
4

0

0

0

0

0
4

0

0

0
4

0
4

0

0

0

0

0
4

0
4

0

0

0

0

0
4

0
4

0

0

0

0

0
4

0

0

0

0
4

0
4

0

0

0

0

0

0
4

0
4

0

0

0

0

0
4

0

0

0
4

0
4

0

0

0

0
4

0
4

0.129121

0

0

0
4

0.129121

0

0.129121

0
4

0
4

0

0

0

0

0
4

0
4

0
6

0
6

0
6

0

0

0
4

0
4

0

0

0

0

0
4

0
4

0

0

0

0
4

0
4

0

0

0

0
4

0

0

0
4

0
4

0

0

0

0
4

0
4

0

0

0

0
4

0
4

0

0

0

0

0

0
4

0
4

0

0

0

0
4

0

0

0
4

0
4

0

0

0

0

0
4

0

0

0
4

0
4

0

0

0

0

0
4

0
4

0

0

0

0
4

0

0

0
4

0

0

0
4

0
4

1.57541
3

0.994368

0.274842

0.258242
3

0.461284

0

5.55111512312578e-17

0
4

0.129121

0.129121

0

0
4

0.129121

0.129121

0
4

0.322802

0.322802

0
4

1.11022302462516e-16
3

0
4

0

0

0

0

0
4

0

0

0
4

0
4

0

0

0

0
4

0
4

0

0

0

0

0
4

0

0

0
4

0
4

0

0

0

0
4

0
4

0

0

0

0
4

0

0

0
4

0
4

0

0

0

0
4

0
4

0

0

0

0
4

0

0

0
4

0
4

0

0

0

0
4

0

0

0
4

0
4

0

0

0

0
4

0

0

0
4

0
4

0

0

0

0
4

0
4

0
6

0
6

0
6

0

0
4

0
4

0

0

0

0
4

0
4

0

0

0

0

0
4

0
4

0

0

0

0
4

0

0

0
4

0
4

0

0

0

0

0
4

0
4

0

0

0

0
4

0
4

0

0

0

0
4

0

0

0
4

0
4

0

0

0

0
4

0

0

0
4

0
4

0

0

0

0

0
4

0
4

0

0

0

0
4

0
4

0

0

0

0
4

0
4

5.52335
2

5.52335
2

0

5.02848
2

0

0.236629

0.129121

0

0.129121

4.9960036108132e-16
2

0
4

0

0

0
4

0
4

0

0

0

0

0
4

0
4

0

0

0

0

0
4

0
4

0

0

0

0
4

0
4

0

0

0

0
4

0
4

0

0

0

0
4

0
4

0

0

0

0
4

0
4

0

0

0

0
4

0
4

0

0

0

0
4

0
4

0

0

0

0
4

0
4

0

0

0

0
4

0
4

0.216892

0.0877713

0
6

0

0

0.0877713

0

0
4

0

0

0

0

0
4

0.129121

0.129121

0
4

0

0

0
4

0
4

0

0

0

0
4

0
4

0

0

0

0
4

0
4

0

0

0

0
4

0
4

0

0

0

0
4

0
4

0.0739218

0.0739218

0.0739218

0
4

0
4

0

0

0

0
4

0
4

0

0

0

0
4

0
4

0

0

0

0
4

0
4

0

0

0

0
4

0
4

0.322802

0.322802

0.322802

0
4

0
4

37.1361

34.2054

6.61318

3.54459

5.60752
2

0
3

3.3242
3

0

0

0

0

0

0

0

0

0

0

15.0282
3

0

0

0.0877713

0

0

0

0

0

0

0

0

0

0

0

0

0

0

0

0

0

0

0

0

0

0

0

0

0

0

0

0

0

0

0

0

0

0

0

0

3.60822483003176e-16

0
4

0

0

0

0
4

0

0

0
4

0

0

0

0
4

0

0

0
4

0

0

0
4

0

0

0

0
4

0

0

0
4

0

0

0
4

0

0

0
4

0.0877713

0.0877713

0

0
4

0

0

0
4

0

0

0
4

0

0

0
4

0

0

0
4

0

0

0
4

0

0

0
4

0

0

0
4

0

0

0
4

0

0

0
4

0

0

0
4

0

0

0
4

0

0

0
4

0

0

0

0

0

0

0
4

0

0

0
4

0

0

0
4

0

0

0
4

0

0

0
4

0

0

0
4

0

0

0
4

0

0

0
4

0

0

0
4

0

0

0
4

0

0

0
4

2.07762
3

2.07762
3

0
4

0

0

0
4

0

0

0
4

0

0

0
4

0

0

0
4

0.710164

0.710164

0
4

0

0

0
4

0

0

0
4

0.0551547

0

0.0551547

0
4

0

0

0
4

0

0

0

0
4

0

0

0

0
4

0

0

0

0

0
4

0
4

4.2632564145606e-14
4

0
4

0

0

0

0

0

0

0
4

0

0

0
4

0
4

0
4

649.669
3

113.81
3

0.6362
3

0.200905

0

0

0

0

0

0

0

0

0

0.435295

0

0

0

0

0
4

63.1623
3

11.0497
3

1.73135
3

0.267874

0.460192

0.67374
3

0

7.50839

0
3

9.76091

1.4048

0.605515

3.87637
3

0

0.0909077
2

0

0

0

1.92379

0

0.526628

0

0.0739218

2.72938
3

1.80061

0.0334842

0

0

0.193765

0

0

0

0.0726619

0

0
3

0

0

0

0

0.0726619

0.0334842

0

0

0

0

12.3758
3

0

0

0

0

0

0

0

0

0.145324

0.0726619

0.277017
3

0

0

0

0

0

0

0

0.0726619

0

0.207762

0
3

0

0

0

0

0

2.0258

0

0

0

0

2.39249
3

0

0

0

0

0.0726619

0

0

0

0

0.0484412

0.412002
2

0

0

0

0.0484412

0

0.121103

0

0
4

13.4002
3

12.4044
3

0

0

0

0

0

0

0

0

0

0

0.332163
3

0

0

0

0

0

0

0

0

0

0
3

0.534438
3

0
2

0.129121

0

0

0

0
4

0
2

0
3

0

0

0

0

0

0

0
4

0

0

0

0
4

0

0

0
4

0

0

0
4

0

0

0
4

0.221847

0.12677

0.0950773

1.38777878078145e-17

0
4

0

0

0
4

0

0

0
4

0

0

0
4

0

0

0
4

0

0

0
4

0
2

0
3

0

0

0

0

0

0
4

0

0

0
4

0

0

0
4

0

0

0
4

0

0

0
4

0

0

0
4

0

0

0
4

0

0

0
4

0

0

0
4

0.0877713

0.0877713

0
4

0

0

0
4

25.1638

25.1638

0
4

0.0877713

0.0877713

0
4

0

0

0
4

0

0

0
4

0

0

0
4

0

0

0
4

0

0

0
4

0

0

0
4

0.470062

0

0.470062

0
4

0

0

0

0
4

6.99129

6.35744

0.0633849

0.0633849

0.221847

0.0633849

0.0950773

0.12677

3.05311331771918e-16

0
4

3.5883

3.45918

0.129121

0
4

0

0

0

0
4

0

0

0

0

0
4

9.76996261670138e-15
3

0
4

458.488
3

121.432
3

119.937
3

0.633849

0.0633849

0.538771

0.158462

0

0

0.0367698

0.0633849

8.60422844084496e-15
3

0
4

0.808936
2

0.275774

0

0.0551547

0

0.478008

0

0

0

0

0

5.55111512312578e-17
2

0
4

0.792311

0.697234

0.0950773

0

4.16333634234434e-17

0
4

0

0

0

0
4

2.59227

0

2.59227

0
4

253.82
2

1.47844
3

5.37033
2

0.27062
3

0.077809

0

0

0.295687

0

0.0877713

0.0633849

0

0.175543

0.190155

2.49023

0.116713

0

0

0

2.31355

0.0334842

0.0739218

0.0334842

0.077809

0.0633849

0.25354
2

0.0367698

0.0739218

0.110883

0

0

0.412002

0

0

0

0

1.42616
2

0

0.129121

0.0739218

0.147844

0

0

0.0334842

0

0.175543

0

1.42868
3

0

0

0.116713

0.116713

0

0

0

0

0.0633849

0

0.40657

0.0633849

0

0.190155

0

0.0502263

0

0

0

0

0.155618

0.221847
2

0

0

0

0

0

0.110883

0

0.0633849

0.0877713

0

7.40442

0

0.116713

0.0367698

0

0.077809

0.258726

0

1.88501

0

0

1.85554
2

0

0.0633849

0

0.184804

0

0

0

0.0909077

0

0

0

0

0.443694

0

0

0.193765

0

0.0950773

0

0

0

13.897
3

0

0

0

0.258726

0.193681

1.45452

0.0633849

0.0669684

0

0.0950773

0

65.5245
2

0

4.37281

0

0.25715

1.39058

0.232384
2

30.7969
2

0

0.0633849

22.2894

0

1.62628

0.728926

0

0.427949

0.0633849
2

0.239004

0

0.184804

0.175543

23.3716
2

0

0.412002

0.433453

0.182849

0

5.0391

0.341123

1.07754

0

0.161109

3.44731
3

0.0633849

0

0

5.52199

0.746056

0.235615

0

0

0

0

3.10084
2

0

0

2.68441

1.22725

0.0633849

0

0.0633849

0

0.427344

0.235615

0.670728
3

0.147844

0

0.0633849

0.517453

0.184804

0

0

0.184804

0

0.309618

19.0472
2

0

0

0.116713

0

0

0

0.147844

0

0

0.158462

6.70653

0

0

0

0.147844

0.311236

0

0

0

0

0

7.49400541621981e-15
2

0
4

76.6729
3

76.6729
3

0

0

0

0

0
4

1.50839
3

0.150679

0

0

0

0

0.0877713

0

1.26994

0

0

0

0

0

0
4

0

0

0

0

0
4

0

0

0
4

0

0

0
4

0.258726

0.258726

0
4

0.0739218

0.0739218

0

0
4

0.412241

0.412241

0
4

0

0

0
4

0

0

0
4

0

0

0
4

0.116713

0.116713

0
4

0

0

0
4

0

0

0
4

7.90756349289268e-14
3

0
4

4.49111

0

0

0

0

0

0

0

0

0

0
4

0

0

0

0

0

0

0

0

0

0

0

0

0

0

0

0

0

0

0

0

0

0

0

0

0

0

0

0

0

0

0

0

0

0

0

0

0

0

0

0

0

0

0

0

0

0

0

0

0

0

0

0

0

0

0

0

0

0

0

0

0

0

0

0

0

0

0

0

0

0

0

0

0

0

0

0

0

0

0

0

0

0

0

0

0

0
4

0

0
7

0

0

0
4

0

0

0
4

0.739218

0.739218

0
4

0

0

0
4

0

0

0
4

0

0

0
4

0

0

0
4

0.554413

0.554413

0
4

0.0633849

0.0633849

0
4

0

0

0
4

0

0

0
4

0

0

0
4

0.982466

0.380309

0.538771

0.0633849

0
4

0
7

0
7

0
4

0.351085

0.263314

0.0877713

0
4

0

0

0
4

0

0

0

0

0
4

0.0633849

0.0633849

0
4

1.73716

1.73716

0
4

0
4

18.6784
3

15.057
3

11.7648
3

0

3.25538
3

0
2

0.0367698
3

0

0

0

0

0

6.93889390390723e-18
3

0
4

3.11989
3

0.553253
3

0

2.32721
2

0.20266

0.0367698

0

0

0

0

0

0
4

0

0

0

0

0
4

0
3

0

0

0

0

0

0

0

0
4

0

0

0

0

0

0
4

0

0

0

0

0
4

0.501515
2

0.423706

0

0.077809

0

4.16333634234434e-17
2

0
4

0

0

0

0
4

0

0

0

0
4

7.99360577730113e-15
3

0
4

0

0

0

0
4

0

0

0

0
4

0
4

0

0

0

0

0
4

0

0

0
4

0
4

0

0

0

0
4

0
4

0

0

0

0
4

0
4

0

0

0

0

0
4

0
4

0

0

0

0
4

0
4

0

0

0

0
4

0
4

0

0

0

0
4

0
4

0

0

0

0

0
4

0
4

0

0

0

0
4

0
4

29.7106

29.535

18.8708

0

0.70217

0.175543

0

0.175543

0

0.131657

1.36046

0.0877713

0.263314

1.36046

0.131657

0

0

2.98422

1.44823

0.658285

0.394971

0.789942

0

0

0
4

0.0877713

0.0877713

0

0

0
4

0.0877713

0.0877713

0
4

0
4

0

0

0

0
4

0
4

0

0

0

0
4

0
4

0

0

0

0
4

0
4

0

0

0

0
4

0
4

0.131657

0.131657

0.131657

0
4

0
4

0

0

0

0
4

0
4

0

0

0

0
4

0
4

1.17262

1.17262

1.17262

0
4

0
4

0.131657

0.131657

0.131657

0
4

0
4

0

0

0

0
4

0
4

0

0

0

0

0

0

0

0

0

0

0

0
4

0

0

0

0

0

0
4

0
4

0

0

0

0
4

0
4

0

0

0

0
4

0
4

0.0502263

0.0502263

0.0502263

0
4

0
4

0

0

0

0
4

0
4

21.2163
3

8.97798
3

6.06868
3

0.147079

2.76222

0

0

0

0

0

0

0
4

12.2384
3

12.2384
3

0

0
4

0

0

0
4

0

0

0
4

0

0

0
4

1.77635683940025e-15
3

0
4

0
3

0
3

0

0

0

0

0

0

0

0
4

0
3

0

0

0

0

0
4

0

0

0
4

0

0

0

0
4

0
4

0
2

0
2

0

0

0

0

0

0

0
4

0

0

0

0

0
4

0

0

0

0
4

0

0

0
4

0

0

0
4

0

0

0
4

0
4

0.263314

0.175543

0.175543

0

0

0

0

0
4

0.0877713

0

0

0

0.0877713

0
4

0

0

0
4

0

0

0
4

2.77555756156289e-17

0
4

0

0

0

0

0
4

0

0

0

0
4

0

0

0
4

0
4

0

0

0

0
4

0

0

0
4

0
4

1.5259

1.5259

1.5259

0
4

0
4

3.98125976630581e-13
3

0
4

97.7685

0

0

0

0

0

0
4

0

0

0
4

0

0

0
4

0

0

0
4

0
4

0

0

0

0
4

0

0

0
4

0

0

0

0

0

0

0
4

0

0

0

0

0

0

0

0

0
4

0
4

0

0

0

0

0

0

0

0
4

0

0

0

0

0
4

0

0

0

0
4

0

0

0
4

0

0

0
4

0
4

0
5

0
5

0
5

0

0
4

0
4

97.7685

20.8176

20.8176

0

0
4

0
7

0
7

0
4

0
7

0
7

0
4

0
2

0
2

0

0

0

0

0

0

0

0

0

0

0

0

0

0

0
4

0

0

0

0

0

0
4

0

0

0
4

0

0

0
4

0.658853

0.336051

0.129121

0.193681

0
4

0

0

0
4

0

0

0

0

0
4

0

0

0

0

0
4

0.581044

0.581044

0
4

0.0633849

0

0.0633849

0
4

0

0

0
4

31.6365
2

26.1678
2

2.69261

0.710164

1.93681

0

0.129121

0

1.2490009027033e-15
2

0
4

0

0

0

0
4

0

0

0
4

0

0

0

0
4

0

0

0
4

0

0

0
4

0

0

0

0
4

0

0

0

0
4

0

0

0

0
4

0

0

0
4

0

0

0
4

8.8354
2

8.8354
2

0

0

0
4

0

0

0
4

0

0

0
4

0

0

0
4

0

0

0
4

0

0

0
4

0

0

0
4

0.129121

0.129121

0
4

0

0

0
4

0

0

0
4

0

0

0
4

0
2

0

0

0

0

0

0

0

0

0
4

0

0

0
4

0

0

0
4

0

0

0
4

0

0

0
4

0.129121

0.129121

0
4

0

0

0
4

0

0

0
4

0

0

0
4

0

0

0
4

0

0

0
4

0
2

0

0

0

0

0

0

0
4

0

0

0

0
4

0

0

0

0

0
4

34.9175
2

34.9175
2

0
4

0

0

0

0

0

0

0
4

0
4

0
6

0
6

0
6

0
4

0

0

0
4

0
4

0

0

0

0
4

0
4

0

0

0

0
4

0
4

0

0

0

0
4

0
4

0

0

0

0
4

0
4

0
7

0
7

0
7

0
4

0
4

0

0

0

0
4

0
4

0

0

0

0

0

0
4

0
4

0

0

0

0

0
4

0

0

0
4

0
4

0

0

0

0
4

0
4

0

0

0

0

0
4

0
4

0

0

0

0
4

0
4

0

0

0

0
4

0
4

0
4

0
6

0
6

0
7

0
7

0
6

0

0

0

0

0

0
6

0
6

0
7

0

0
7

0

0
6

0

0

0
6

0

0

0

0

0

0

0

0

0

0

0
7

0

0

0

0

0

0

0

0

0

0

0
6

0

0

0

0

0

0

0

0

0

0

0
6

0

0

0

0

0

0

0

0

0

0

0
6

0

0

0

0

0

0

0

0

0

0

0
6

0

0

0

0

0

0

0

0

0

0

0
7

0

0

0

0

0

0

0

0

0

0

0
6

0

0

0

0

0

0

0

0

0

0

0
4

0
6

0
6

0

0

0

0

0

0

0

0
4

0
6

0

0

0

0

0

0

0

0

0
4

0
7

0
7

0

0

0

0

0

0

0
4

0
6

0

0

0

0

0

0

0
4

0

0

0

0

0
4

0

0

0

0

0

0
4

0

0

0

0

0
4

0

0

0
4

0
4

0
4

20.3729

20.3729

18.5757

13.9662

0

0.839285

0

0
5

0

0

0.47767

0

0

0

3.29258

0
4

0.43632
2

0.3072
2

0.129121

2.77555756156289e-17
2

0
4

1.36084

0

1.23172

0

0

0.129121

0
4

0

0

0

0

0
4

2.44249065417534e-15

0
4

0
7

0
7

0

0

0
4

0

0

0

0
4

0
4

0

0

0

0

0
4

0

0

0
4

0
4

0

0

0

0

0

0

0
4

0
4

0
4

0

0

0

0

0
4

0

0

0
4

0
4

0
4

0

0

0

0

0
4

0
4

0
4

0

0

0

0

0
4

0

0

0
4

0
4

0
4

0

0

0

0

0
4

0

0

0
4

0

0

0
4

0
4

0

0

0

0
4

0
4

0
4

0

0

0

0

0
4

0

0

0

0
4

0
4

0
4

0

0

0

0

0

0
4

0

0

0
4

0
4

0
4

0

0

0

0

0

0

0
4

0

0

0
4

0
4

0
4

0

0

0

0

0
4

0

0

0
4

0
4

0

0

0

0
4

0
4

0
4

1.97557

1.97557

0.133937

0.0334842

0.0334842

0.0669684

0
4

1.84163

1.84163

0
4

0
4

0
4

0

0

0

0

0
4

0
4

0
4

0
6

0
6

0
6

0
6

0

0
4

0
6

0
7

0

0

0

0

0

0

0
4

0
6

0
7

0

0

0
4

0

0

0
4

0

0

0
4

0
4

0
4

0

0

0

0

0

0
4

0
4

0
4

0

0

0

0

0
4

0
4

0
4

0

0

0

0

0
4

0
4

0
4

0

0

0

0

0
4

0

0

0
4

0
4

0
4

8.57493

8.57493

0.776179

0.221765

0.554413

0
4

7.79875

7.79875

0
4

0
4

0
4

0

0

0

0

0
4

0
4

0
4

0

0

0

0

0
4

0

0

0
4

0
4

0

0

0

0
4

0
4

0
4

0

0

0

0

0

0

0
4

0
4

0
4

0

0

0

0

0
4

0

0

0
4

0
4

0
4

0

0

0

0

0

0
4

0
4

0
4

8.58752
3

8.58752
3

4.50152
4

4.36301
3

0

0

0

0

0

0.138508

0

0

0

0

1.11022302462516e-16
4

0
4

4.086
3

0

4.086

0

0

0
4

0

0

0
4

0
4

0
4

0

0

0

0

0
4

0
4

0
4

0

0

0

0

0
4

0
4

0
4

0

0

0

0

0
4

0

0

0
4

0

0

0
4

0
4

0
4

3.59686

3.59686

3.59686

3.59686

0
4

0

0

0
4

0
4

0
4

0

0

0

0

0

0
4

0
4

0
4

0

0

0

0

0
4

0

0

0
4

0
4

0
4

0

0

0

0

0
4

0
4

0
4

0

0

0

0

0
4

0
4

0
4

0

0

0

0

0
4

0

0

0
4

0
4

0
4

0

0

0

0

0
4

0
4

0
4

0.710164

0.451923

0.322802

0
7

0

0

0

0

0

0

0.322802

0

0
4

0

0

0

0

0
4

0

0

0

0

0
4

0

0

0
4

0.129121

0.129121

0
4

2.77555756156289e-17

0
4

0.258242

0.258242

0.129121

0

0

0

0.129121

0

0

0

0
4

0

0

0

0

0
4

0

0

0

0
4

0

0

0
4

0

0

0
4

0
4

0

0

0

0
4

0

0

0
4

0
4

0

0

0

0
4

0
4

5.55111512312578e-17

0
4

0

0

0

0

0

0

0
4

0
4

0
4

0

0

0

0

0

0
4

0
4

0
4

0

0

0

0

0
4

0
4

0
4

0

0

0

0

0
4

0

0

0
4

0
4

0
4

0.193681

0.193681

0.193681

0.193681

0
4

0
4

0
4

0

0

0

0

0
4

0
4

0
4

0

0

0

0

0
4

0

0

0
4

0
4

0

0

0

0
4

0
4

0
4

0

0

0

0

0
4

0
4

0
4

0

0

0

0

0
4

0

0

0
4

0
4

0

0

0

0
4

0
4

0
4

0

0

0

0

0

0
4

0
4

0
4

1.74313

1.74313

1.74313

1.74313

0

0

0

0

0
4

0
5

0
5

0

0

0
4

0

0

0

0
4

0
4

0
4

0

0

0

0

0

0
4

0

0

0
4

0
4

0
4

0

0

0

0

0
4

0
4

0
4

0

0

0

0

0
4

0

0

0
4

0
4

0
4

0

0

0

0

0

0

0
4

0
4

0
4

0

0

0

0

0
4

0
4

0
4

4.43543

4.43543

4.36151

4.36151

0
4

0.0739218

0.0739218

0
4

0
4

0
4

0

0

0

0

0

0
4

0
4

0
4

0

0

0

0

0
4

0

0

0
4

0
4

0

0

0

0
4

0
4

0
4

0

0

0

0

0
4

0
4

0
4

0

0

0

0

0
4

0
4

0
4

0.193681

0.193681

0.193681

0.193681

0

0

0

0

0
4

0

0

0

0

0

0
4

0
4

0
4

0

0

0

0

0

0
4

0

0

0
4

0
4

0
4

0

0

0

0

0
4

0

0

0
4

0
4

0

0

0

0
4

0
4

0
4

0

0

0

0

0
4

0

0

0
4

0
4

0
4

0

0

0

0

0
4

0

0

0
4

0
4

0
4

0

0

0

0

0

0
4

0
4

0
4

0

0

0

0

0

0
4

0
4

0
4

0

0

0

0

0

0
4

0
4

0
4

0

0

0

0

0
4

0
4

0
4

0

0

0

0

0
4

0

0

0
4

0
4

0
4

0

0

0

0

0
4

0
4

0
4

0
6

0
6

0
6

0
6

0

0

0

0

0
7

0
6

0

0

0

0

0

0

0
4

0

0

0
4

0
4

0
4

0.221765

0.221765

0

0

0
4

0.221765

0.221765

0
4

0
4

0
4

0

0

0

0

0
4

0

0

0
4

0
4

0
4

0

0

0

0

0
4

0
4

0
4

0

0

0

0

0
4

0
4

0
4

0.517453

0.517453

0.110883

0.110883

0
4

0.40657

0.40657

0
4

0
4

0
4

0

0

0

0

0

0
4

0
4

0
4

0

0

0

0

0

0
4

0
4

0
4

0

0

0

0

0
4

0
4

0
4

0

0

0

0

0
4

0
4

0
4

0

0

0

0

0
4

0

0

0
4

0
4

0
4

0
7

0
7

0
7

0
7

0

0

0
4

0
4

0

0

0

0
4

0

0

0
4

0
4

0
4

0

0

0

0

0
4

0

0

0
4

0
4

0
4

0

0

0

0

0

0
4

0
4

0
4

0

0

0

0

0
4

0
4

0
4

0

0

0

0

0
4

0

0

0
4

0
4

0
4

0

0

0

0

0
4

0
4

0
4

0

0

0

0

0
4

0
4

0
4

0

0

0

0

0
4

0

0

0
4

0
4

0
4

0

0

0

0

0
4

0

0

0
4

0
4

0
4

0

0

0

0

0

0
4

0
4

0
4

0

0

0

0

0

0
4

0
4

0
4

0

0

0

0

0

0

0

0

0

0

0
4

0

0

0
4

0
4

0
4

0

0

0

0

0

0
4

0
4

0
4

0

0

0

0

0
4

0
4

0
4

0.443531

0.443531

0.295687

0.295687

0
4

0.147844

0.147844

0
4

2.77555756156289e-17

0
4

0
4

0

0

0

0

0
4

0

0

0
4

0
4

0
4

0

0

0

0

0
4

0

0

0
4

0
4

0
4

0.147844

0.147844

0.0739218

0.0739218

0
4

0.0739218

0.0739218

0
4

0
4

0
4

0

0

0

0

0
4

0

0

0
4

0
4

0
4

0

0

0

0

0

0
4

0
4

0
4

0

0

0

0

0
4

0
4

0
4

0

0

0

0

0

0
4

0
4

0
4

0.774725

0.774725

0
5

0

0

0

0

0

0

0
4

0

0

0

0

0
4

0.774725
3

0.774725

0

0
4

0

0

0
4

0

0

0
4

0
4

0

0

0

0

0

0
4

0

0

0

0
4

0
4

0
4

0

0

0

0

0
4

0
4

0
4

0

0

0

0

0

0
4

0
4

0
4

0

0

0

0

0
4

0

0

0
4

0
4

0
4

0

0

0

0

0
4

0

0

0
4

0
4

0
4

5.64209

5.64209

5.64209

5.64209

0
4

0
4

0
4

0

0

0

0

0
4

0
4

0
4

0

0

0

0

0
4

0
4

0
4

0

0

0

0

0
4

0

0

0
4

0
4

0
4

0

0

0

0

0
4

0
4

0
4

0

0

0

0

0
4

0
4

0
4

45.162

45.162

45.1254

32.2062

0.507079

0.0365426

0.190155

0

0

0.255891

0

0

0

0.316924

3.80803

0.0633849

0

0

0

0

0

0

0.0633849

0.0950773

0.129121

2.80687
3

0

0

0

0

0

0.0950773

0

0.12677

0

2.24433
3

0.337998
3

1.75484
3

0

0.0877713

0

3.69149155687865e-15

0
4

0

0

0

0
4

0.0365426

0

0

0

0.0365426

0
4

0

0

0
4

0

0

0

0
4

0

0

0
4

0

0

0
4

0

0

0
4

0
4

0
4

0
7

0
7

0
7

0
7

0
7

0

0

0

0

0
4

0

0

0
4

0

0

0

0
4

0

0

0
4

0
4

0
4

0

0

0

0

0
4

0
4

0
4

0

0

0

0

0
4

0

0

0
4

0
4

0
4

0

0

0

0

0
4

0
4

0
4

0

0

0

0

0
4

0
4

0
4

0

0

0

0

0

0
4

0
4

0
4

0

0

0

0

0
4

0
4

0
4

0

0

0

0

0
4

0
4

0
4

0

0

0

0

0
4

0
4

0
4

0

0

0

0

0
4

0
4

0
4

0

0

0

0

0
4

0
4

0
4

7.20564

7.20564

7.20564
2

6.24466
2

0

0.960983

1.11022302462516e-16
2

0
4

0
5

0
5

0

0
4

0

0

0

0

0
4

0
4

0

0

0

0
4

0
4

0
4

0

0

0

0

0

0
4

0
4

0
4

0

0

0

0

0
4

0
4

0
4

0

0

0

0

0
4

0

0

0
4

0
4

0
4

0

0

0

0

0

0
4

0
4

0
4

0

0

0

0

0
4

0
4

0
4

0.184804

0.184804

0.184804

0.184804

0
4

0
4

0
4

0

0

0

0

0
4

0
4

0
4

0

0

0

0

0
4

0
4

0
4

0

0

0

0

0
4

0
4

0
4

0

0

0

0

0
4

0
4

0
4

0

0

0

0

0

0

0

0

0

0

0
4

0
4

0
4

0

0

0

0

0
4

0
4

0
4

0

0

0

0

0
4

0
4

0
4

0

0

0

0

0
4

0
4

0
4

0

0

0

0

0
4

0
4

0
4

0

0

0

0

0
4

0
4

0
4

0

0

0

0

0
4

0
4

0
4

0

0

0

0

0
4

0
4

0
4

0

0

0

0

0
4

0
4

0
4

0

0

0

0

0
4

0
4

0
4

0

0

0

0

0
4

0
4

0
4

0
6

0
6

0
6

0
6

0

0
4

0
6

0

0

0

0
4

0

0

0

0
4

0

0

0
4

0
4

0
4

0

0

0

0

0
4

0
4

0
4

0

0

0

0

0
4

0
4

0
4

0

0

0

0

0
4

0
4

0
4

0

0

0

0

0
4

0
4

0
4

0

0

0

0

0
4

0
4

0
4

0

0

0

0

0
4

0
4

0
4

0

0

0

0

0
4

0
4

0
4

0

0

0

0

0
4

0
4

0
4

0

0

0

0

0
4

0
4

0
4

0

0

0

0

0
4

0
4

0
4

0

0

0

0

0

0

0

0
4

0

0

0
4

0
4

0

0

0

0

0
4

0

0

0
4

0
4

0
4

0

0

0

0

0
4

0
4

0
4

0

0

0

0

0
4

0
4

0
4

0

0

0

0

0
4

0
4

0
4

0

0

0

0

0
4

0
4

0
4

0

0

0

0

0
4

0
4

0
4

0

0

0

0

0
4

0
4

0
4

0

0

0

0

0
4

0
4

0
4

0

0

0

0

0
4

0
4

0
4

0

0

0

0

0
4

0
4

0
4

0

0

0

0

0
4

0
4

0
4

2.11489

2.11489

2.02712
2

1.10006
2

0.475134

0.451923

0

0
4

0.0877713

0.0877713

0
4

0

0

0

0

0
4

0

0

0
4

0

0

0
4

3.60822483003176e-16

0
4

0
4

0

0

0

0

0
4

0
4

0
4

0

0

0

0

0
4

0
4

0
4

0

0

0

0

0
4

0
4

0
4

0.0877713

0.0877713

0.0877713

0.0877713

0
4

0
4

0
4

0

0

0

0

0
4

0
4

0
4

0

0

0

0

0
4

0
4

0
4

0

0

0

0

0
4

0
4

0
4

0

0

0

0

0
4

0
4

0
4

0

0

0

0

0
4

0
4

0
4

0

0

0

0

0
4

0
4

0
4

0
7

0
7

0
7

0
7

0
4

0

0

0
4

0
4

0
4

0

0

0

0

0
4

0
4

0
4

0

0

0

0

0
4

0
4

0
4

0

0

0

0

0
4

0
4

0
4

0

0

0

0

0
4

0
4

0
4

0

0

0

0

0
4

0
4

0
4

0

0

0

0

0
4

0
4

0
4

0

0

0

0

0
4

0
4

0
4

0

0

0

0

0
4

0
4

0
4

0

0

0

0

0
4

0
4

0
4

0

0

0

0

0
4

0
4

0
4

0
7

0
7

0
7

0

0

0

0
4

0

0

0

0

0
4

0

0

0
4

0
4

0
4

0

0

0

0

0
4

0
4

0
4

0

0

0

0

0
4

0
4

0
4

0

0

0

0

0
4

0
4

0
4

0

0

0

0

0
4

0
4

0
4

0

0

0

0

0
4

0
4

0
4

0

0

0

0

0
4

0
4

0
4

0

0

0

0

0
4

0
4

0
4

0

0

0

0

0
4

0
4

0
4

0

0

0

0

0
4

0
4

0
4

0

0

0

0

0
4

0
4

0
4

4.1564

1.81108

0

0

0

0
4

1.81108

1.81108

0

0
4

0

0

0
4

0
4

2.34532

2.34532

0

0

0.903846

1.44147

0

0
4

0
4

0

0

0

0

0
4

0
4

4.44089209850063e-16

0
4

0

0

0

0

0
4

0
4

0
4

0

0

0

0

0
4

0
4

0
4

0

0

0

0

0
4

0
4

0
4

0

0

0

0

0
4

0
4

0
4

0

0

0

0

0
4

0
4

0
4

0

0

0

0

0
4

0
4

0
4

0

0

0

0

0
4

0
4

0
4

0

0

0

0

0
4

0
4

0
4

0

0

0

0

0
4

0
4

0
4

0

0

0

0

0
4

0
4

0
4

0

0

0

0

0

0

0

0
4

0
4

0
4

0

0

0

0

0
4

0
4

0
4

0

0

0

0

0
4

0
4

0
4

0

0

0

0

0
4

0
4

0
4

0

0

0

0

0
4

0
4

0
4

0

0

0

0

0
4

0
4

0
4

0

0

0

0

0
4

0
4

0
4

0

0

0

0

0
4

0
4

0
4

0

0

0

0

0
4

0
4

0
4

0

0

0

0

0
4

0
4

0
4

0

0

0

0

0
4

0
4

0
4

0
7

0
7

0
7

0
7

0

0

0

0

0

0

0

0

0

0

0
7

0

0

0

0

0

0

0

0

0

0

0
6

0

0

0

0

0

0

0

0

0

0

0
7

0

0

0

0

0

0

0

0

0

0

0

0

0

0

0

0

0
4

0
6

0

0

0

0

0

0

0

0
4

0
4

0
6

0
6

0

0

0

0

0

0

0
4

0

0

0

0

0
4

0

0

0
4

0
4

0
4

0
4

0
4

0

0

0

0

0

0

0

0

0
4

0

0

0

0

0
4

0

0

0

0
4

0

0

0
4

0

0

0
4

0
4

0
4

6.09855

6.09855

6.09855

6.09855

0
4

0
4

0
4

0

0

0

0

0
4

0
4

0
4

0

0

0

0

0
4

0
4

0
4

0

0

0

0

0
4

0
4

0
4

0

0

0

0

0
4

0
4

0
4

0

0

0

0

0
4

0
4

0
4

0

0

0

0

0
4

0
4

0
4

0

0

0

0

0
4

0
4

0
4

0

0

0

0

0
4

0
4

0
4

0

0

0

0

0
4

0
4

0
4

0
4

0
4

0
4

0

0

0

0

0

0

0
4

0
4

0

0

0

0

0

0
4

0

0

0
4

0
4

0
4

0

0

0

0

0
4

0
4

0
4

0

0

0

0

0
4

0
4

0
4

0

0

0

0

0
4

0
4

0
4

0.184804

0.184804

0.184804

0.184804

0
4

0
4

0
4

0

0

0

0

0
4

0
4

0
4

0

0

0

0

0
4

0
4

0
4

0

0

0

0

0
4

0
4

0
4

0

0

0

0

0
4

0
4

0
4

0

0

0

0

0
4

0
4

0
4

0

0

0

0

0
4

0
4

0
4

0
6

0
7

0

0

0

0

0
4

0

0

0

0

0
4

0

0

0

0
4

0
4

0

0

0

0

0
4

0

0

0
4

0
4

0
4

0

0

0

0

0
4

0
4

0
4

0

0

0

0

0
4

0
4

0
4

0

0

0

0

0
4

0
4

0
4

0

0

0

0

0
4

0
4

0
4

0

0

0

0

0
4

0
4

0
4

0

0

0

0

0
4

0
4

0
4

0

0

0

0

0
4

0
4

0
4

0

0

0

0

0
4

0
4

0
4

0.184804

0.184804

0.184804

0.184804

0
4

0
4

0
4

0

0

0

0

0
4

0
4

0
4

0

0

0
6

0
6

0

0
4

0

0

0

0
4

0

0

0
4

0
4

0
4

0.077809

0.077809

0.077809

0.077809

0
4

0
4

0
4

0

0

0

0

0
4

0
4

0
4

0

0

0

0

0
4

0
4

0
4

0

0

0

0

0
4

0
4

0
4

0

0

0

0

0
4

0
4

0
4

0

0

0

0

0
4

0
4

0
4

0

0

0

0

0
4

0
4

0
4

0

0

0

0

0
4

0
4

0
4

0

0

0

0

0
4

0
4

0
4

0

0

0

0

0
4

0
4

0
4

0

0

0
5

0

0

0

0

0
4

0

0

0

0

0
4

0

0

0

0

0
4

0

0

0
4

0

0

0
4

0
4

0
4

0

0

0

0

0
4

0
4

0
4

0

0

0

0

0
4

0
4

0
4

0

0

0

0

0
4

0
4

0
4

0

0

0

0

0
4

0
4

0
4

0

0

0

0

0
4

0
4

0
4

0.710164

0.710164

0.710164

0.710164

0
4

0
4

0
4

0

0

0

0

0
4

0
4

0
4

0

0

0

0

0
4

0
4

0
4

0

0

0

0

0
4

0
4

0
4

0

0

0

0

0
4

0
4

0
4

0
4

0
4

0
4

0

0

0

0

0
4

0

0

0

0

0

0
4

0
4

0
4

0

0

0

0

0
4

0
4

0
4

0

0

0

0

0
4

0
4

0
4

0

0

0

0

0
4

0
4

0
4

0

0

0

0

0
4

0
4

0
4

0

0

0

0

0
4

0
4

0
4

0

0

0

0

0
4

0
4

0
4

0

0

0

0

0
4

0
4

0
4

0

0

0

0

0
4

0
4

0
4

0

0

0

0

0
4

0
4

0
4

0

0

0

0

0
4

0
4

0
4

0

0

0

0

0

0

0

0

0

0
4

0

0

0

0

0
4

0

0

0

0
4

0

0

0
4

0

0

0
4

0
4

0

0

0

0
4

0
4

0

0

0

0
4

0
4

0
4

0

0

0

0

0
4

0
4

0
4

0

0

0

0

0
4

0
4

0
4

0

0

0

0

0
4

0
4

0
4

0

0

0

0

0
4

0
4

0
4

0

0

0

0

0
4

0
4

0
4

0

0

0

0

0
4

0
4

0
4

0

0

0

0

0
4

0
4

0
4

0

0

0

0

0
4

0
4

0
4

0

0

0

0

0
4

0
4

0
4

0

0

0

0

0
4

0
4

0
4

0

0

0

0

0

0
4

0

0

0

0

0

0

0
4

0
4

0
4

0

0

0

0

0
4

0
4

0
4

0

0

0

0

0
4

0
4

0
4

0

0

0

0

0
4

0
4

0
4

0

0

0

0

0
4

0
4

0
4

0

0

0

0

0
4

0
4

0
4

0

0

0

0

0
4

0
4

0
4

0

0

0

0

0
4

0
4

0
4

0

0

0

0

0
4

0
4

0
4

0

0

0

0

0
4

0
4

0
4

0

0

0

0

0
4

0
4

0
4

0.322802

0.322802

0.322802

0.322802

0

0
4

0

0

0
4

0
4

0
4

0

0

0

0

0
4

0
4

0
4

0

0

0

0

0
4

0
4

0
4

0

0

0

0

0
4

0
4

0
4

0

0

0

0

0
4

0
4

0
4

0

0

0

0

0
4

0
4

0
4

0

0

0

0

0
4

0
4

0
4

0

0

0

0

0
4

0
4

0
4

0

0

0

0

0
4

0
4

0
4

0

0

0

0

0
4

0
4

0
4

0.138508

0.138508

0.138508

0.138508

0
4

0
4

0
4

2.4533

2.4533

1.74313

0.516483

0.258242

0.387362

0.129121

0.193681

0.258242

0
4

0.710164

0.581044

0.129121

2.77555756156289e-17

0
4

1.11022302462516e-16

0
4

0
4

0

0

0

0

0
4

0
4

0
4

0

0

0

0

0
4

0
4

0
4

0

0

0

0

0
4

0
4

0
4

0

0

0

0

0
4

0
4

0
4

0

0

0

0

0
4

0
4

0
4

0

0

0

0

0
4

0
4

0
4

0

0

0

0

0
4

0
4

0
4

0

0

0

0

0
4

0
4

0
4

0

0

0

0

0
4

0
4

0
4

0

0

0

0

0
4

0
4

0
4

0

0

0

0

0

0

0

0

0

0

0

0

0

0

0

0

0

0

0

0

0

0

0

0

0

0

0

0

0

0

0
4

0

0

0

0

0

0

0
4

0

0

0

0

0

0

0
4

0

0

0

0

0

0

0

0
4

0

0

0

0
4

0

0

0
4

0
4

0

0

0

0

0

0

0

0

0

0

0

0

0

0

0

0

0

0

0

0

0
4

0

0

0

0

0

0

0

0

0

0
4

0

0

0

0

0

0

0
4

0

0

0

0

0
4

0

0

0
4

0

0

0
4

0
4

0

0

0

0

0

0
4

0
4

0

0

0

0
4

0

0

0
4

0
4

0
4

0
6

0
6

0
6

0

0

0

0
4

0

0

0
4

0
4

0
4

0

0

0

0

0
4

0
4

0
4

0

0

0

0

0
4

0
4

0
4

0

0

0

0

0
4

0
4

0
4

0

0

0

0

0
4

0
4

0
4

0

0

0

0

0

0

0
4

0

0

0
4

0
4

0

0

0

0
4

0
4

0
4

0
6

0
6

0
6

0
6

0
4

0
4

0
4

0

0

0

0

0

0

0
4

0

0

0

0
4

0

0

0

0

0
4

0
4

0
4

0
7

0
7

0
7

0

0

0

0

0
4

0
4

0
4

0
4

0
4

0

0

0

0

0
4

0

0

0

0

0
4

0

0

0
4

0

0

0
4

0
4

0

0

0

0

0
4

0

0

0
4

0

0

0
4

0
4

0
4

0

0

0

0

0

0
4

0

0

0
4

0
4

0

0

0

0
4

0
4

0
4

0
3

0
3

0
3

0

0

0

0
4

0

0

0

0

0

0
4

0
4

0
4

0

0

0

0

0

0

0

0

0
4

0

0

0
4

0
4

0
4

4.02596
3

4.02596
3

4.02596
3

0.0877713

1.42033

0.258242

0.581044

1.67857

4.44089209850063e-16
3

0
4

0
4

0
4

0
7

0
7

0
7

0
7

0

0

0

0

0

0

0

0

0

0

0

0
4

0

0

0
4

0

0

0
4

0

0

0
6

0

0

0

0

0
4

0
6

0
6

0

0
4

0

0

0
4

0

0

0
4

0

0

0
4

0

0

0
4

0

0

0
4

0

0

0
4

0
4

0
4

0

0

0

0

0

0
4

0
4

0
4

0
4

0
4

0

0

0

0

0

0
4

0

0

0

0

0

0
4

0
4

0
4

0

0

0

0

0

0

0

0

0
4

0
4

0
4

0
4

0
4

0
4

0

0

0
4

0

0

0

0
4

0

0

0
4

0

0

0
4

0
4

0
4

0

0

0

0

0

0

0
4

0

0

0
4

0
4

0
4

0
4

0
4

0
4

0

0

0

0
4

0
4

0

0

0

0
4

0
4

0
4

0

0

0

0

0

0

0

0

0
4

0
4

0
4

0

0

0

0

0

0

0

0

0
4

0

0

0
4

0
4

0
4

0

0

0

0

0

0
4

0

0

0
4

0
4

0
4

0

0

0

0

0

0

0

0
4

0
4

0
4

0
5

0
5

0
6

0
6

0

0

0

0

0

0

0

0

0

0

0

0

0

0

0

0

0
4

0

0

0

0

0

0

0

0

0

0

0

0
4

0
4

0

0

0

0

0

0

0

0

0
4

0

0

0

0

0

0

0
4

0

0

0

0

0
4

0

0

0
4

0

0

0
4

0

0

0
4

0
4

0
6

0

0

0

0

0

0
4

0

0

0

0

0

0
4

0

0

0

0

0

0
4

0

0

0
4

0
4

0

0

0

0
4

0

0

0
4

0
4

0
4

1.40434

1.40434

1.40434

0.219428

0.570514

0.614399

0
4

0
4

0
4

0

0

0

0

0

0
4

0

0

0
4

0

0

0
4

0
4

0
4

0
6

0
6

0

0

0

0
4

0

0

0

0
4

0
4

0
4

0

0

0

0

0

0
4

0
4

0

0

0

0
4

0

0

0
4

0
4

0
4

0

0

0

0

0

0
4

0
4

0

0

0

0
4

0
4

0

0

0

0
4

0
4

0
4

0

0

0

0

0

0

0

0
4

0
4

0
4

0

0

0

0

0

0
4

0

0

0
4

0
4

0
4

0

0

0

0

0

0
4

0
4

0
4

0.554033
3

0.554033
3

0.554033
3

0.554033

0

0
4

0

0

0
4

0
4

0
4

0

0

0

0

0

0

0
4

0

0

0
4

0
4

0
4

6.13255

6.13255

5.74871

5.04178

0

0

0.158462
3

0.13162

0.25354
3

0.12677
3

0.0365426

0

0

0

0
4

0

0

0
4

0.383836
2

0.193681

0.190155

0

0

0
4

0

0

0

0

0
4

0

0

0
4

0

0

0
4

0

0

0

0
4

0

0

0

0
4

0

0

0
4

0

0

0
4

6.10622663543836e-16

0
4

0
4

0

0

0

0

0

0
4

0

0

0
4

0

0

0

0
4

0
4

0
4

0

0

0

0

0

0

0
4

0

0

0
4

0

0

0
4

0
4

0
4

0

0

0

0

0

0
4

0

0

0
4

0
4

0

0

0

0
4

0
4

0
4

0
6

0

0

0

0

0
4

0

0

0
4

0

0

0
4

0
4

0

0

0

0
4

0
4

0
4

0
6

0
6

0
7

0
7

0
4

0

0

0
4

0
4

0
4

0

0

0

0

0

0

0
4

0
4

0
4

0

0

0

0

0

0

0
4

0

0

0
4

0
4

0

0

0

0

0
4

0
4

0
4

0
7

0
7

0

0

0

0
4

0

0

0
4

0
4

0
4

0

0

0

0

0

0
4

0
4

0

0

0

0
4

0

0

0
4

0
4

0
4

2.4239

2.4239

2.4239

2.4239

0
4

0

0

0
4

0
4

0
4

0
7

0
6

0
7

0
6

0
7

0

0

0

0

0

0
4

0
6

0
6

0

0

0

0

0
4

0

0

0

0
4

0
4

0
7

0
7

0

0

0

0

0

0

0

0

0

0

0

0
4

0

0

0

0

0
4

0

0

0

0
4

0

0

0
4

0
4

0
4

0

0

0

0

0
4

0
4

0
4

0

0

0

0

0

0
4

0

0

0

0
4

0

0

0
4

0
4

0
4

0

0

0

0

0

0
4

0
4

0
4

0.569232

0.569232

0.284616

0.251132

0.0334842

6.93889390390723e-18

0
4

0.284616

0.284616

0
4

5.55111512312578e-17

0
4

0
4

0.591374

0.591374

0.517453

0.517453

0
4

0.0739218

0.0739218

0
4

1.38777878078145e-17

0
4

0
4

0

0

0

0

0

0
4

0

0

0
4

0
4

0

0

0

0
4

0
4

0

0

0

0
4

0
4

0
4

0

0

0

0

0
4

0

0

0
4

0
4

0
4

0

0

0

0

0

0

0
4

0

0

0
4

0
4

0
4

0

0

0

0

0
4

0

0

0
4

0

0

0
4

0
4

0
4

0

0

0

0

0

0

0
4

0
4

0
4

0
4

0
4

0
4

0
4

0

0

0

0

0

0

0
4

0

0

0
4

0
4

0
4

0

0

0

0

0

0

0

0

0

0
4

0

0

0

0

0
4

0

0

0

0

0

0
4

0

0

0
4

0
4

0

0

0

0

0

0
4

0

0

0
4

0

0

0
4

0
4

0

0

0

0
4

0

0

0
4

0
4

0
4

0
6

0
6

0
6

0
6

0
4

0
4

0
4

0

0

0

0

0
4

0
4

0
4

0

0

0

0

0
4

0
4

0
4

0

0

0

0

0
4

0

0

0
4

0
4

0
4

0

0

0

0

0

0
4

0
4

0
4

0

0

0

0

0

0
4

0

0

0

0
4

0
4

0
4

0

0

0

0

0
4

0
4

0
4

0

0

0

0

0

0
4

0
4

0
4

0.539694

0.539694

0.539694

0

0.451923

0.0877713

0
4

0
4

0
4

0

0

0

0

0
4

0
4

0
4

23.2001
3

23.2001
3

23.2001
3

8.37975

0

0

0

1.45434

1.17732
3

11.7732
3

0

0.138508

0.138508

0

0.138508

0

3.33066907387547e-16
3

0
4

0

0

0
4

0

0

0
4

0

0

0
4

0
4

0

0

0

0

0

0
4

0
4

0

0

0

0
4

0
4

0
4

11.3049
3

0

0

0

0
4

0
4

6.57914

6.57914

6.57914

0

0
4

0
4

0

0

0

0
4

0
4

0

0

0

0
4

0
4

0

0

0

0
4

0
4

4.72576
3

2.03855
3

0

0

0

0.554033

0.3072

0

0

0

0

0

0

0

0

0

0

0

0

0.346271

0

0

0

0

0

0

0

0

0.138508

0

0

0

0

0
3

0

0

0

0.692542

0
4

0

0

0

0

0
4

0.263314

0.263314

0
4

2.4239

2.4239

0
4

0

0

0
4

0
4

8.88178419700125e-16
3

0
4

0
3

0

0

0

0
4

0
4

0
3

0
3

0

0

0

0

0

0

0
4

0

0

0
4

0

0

0
4

0
4

0
4

10.6551
4

10.2211
4

0
4

0

0

0

0

0

0

0

0
4

0

0

0

0
4

0
4

0

0

0
4

9.87001
3

0

0

0

0

0

0

0

0

0

6.40731

0

3.46271

0

0

0

0

4.44089209850063e-16
3

0
4

0

0

0
4

0

0

0
4

0.351085

0.351085

0
4

0
4

0

0

0

0

0
4

0
4

0.346271

0.346271

0.346271

0
4

0
4

0

0

0

0
4

0
4

0

0

0

0
4

0
4

0

0

0

0
4

0
4

0

0

0

0
4

0
4

0.0877713

0.0877713

0.0877713

0
4

0
4

0
4

6.70257

0

0

0

0

0
4

0
4

0

0

0

0

0
4

0
4

0.3072

0.3072

0.3072

0
4

0
4

0

0

0

0

0
4

0
4

1.62628

1.62628

1.62628

0
4

0
4

4.76909

4.44629

0

0

0

0

0

0

0

0

0

0

0

0.774725

0

0

0

0.387362

0

0

2.36514

0

0

0.129121

0.789942

0

0

0
4

0.322802

0.322802

0
4

0

0

0
4

0

0

0
4

1.11022302462516e-16

0
4

0
4

66.5308
3

66.3991
3

26.3979
2

26.0907
2

0

0.3072

6.66133814775094e-16
2

0
4

40.0012
3

1.44111

0

0.175543

0

0

0.0877713

0

0

6.58285

1.34506

0

0.263314

0.0877713

0.175543

29.7545

0.0877713

0
4

0

0

0
4

0

0

0
4

1.4210854715202e-14
3

0
4

0

0

0

0

0
4

0
4

0

0

0

0
4

0
4

0

0

0

0
4

0
4

0

0

0

0
4

0
4

0.131657

0.131657

0.131657

0
4

0
4

0
4

54.0253

0

0

0

0
4

0
4

54.0253

54.0253

15.8886

37.7855
3

0

0

0

0

0

0

0.0877713

0.263314

1.83186799063151e-15

0
4

0

0

0
4

0
4

0
4

0
7

0
7

0
7

0
7

0
4

0
4

0
4

60.7173

59.7299

7.70798

7.70798

0

0

0
4

4.52022

4.52022

0
4

14.2227
3

12.1846
3

0
5

2.03802
3

0
4

31.743
3

0

31.743
3

0
4

0
4

0

0

0

0

0

0

0
4

1.44823

1.44823

0
4

0

0

0

0
4

0

0

0
4

0.0877713

0.0877713

0
4

0

0

0
4

1.76803016671556e-14

0
4

0

0

0

0

0
4

0
4

0.987439

0.987439

0.987439

0
4

0
4

0

0

0

0
4

0
4

0

0

0

0
4

0
4

0

0

0

0
4

0
4

1.66533453693773e-15

0
4

1.31657
2

1.31657
2

1.31657
2

0.482742

0

0

0.833827

0

1.11022302462516e-16
2

0
4

0
4

0
4

0.726619

0

0

0

0
4

0
4

0.678177

0.678177

0.678177

0

0
4

0
4

0

0

0

0
4

0

0

0
4

0
4

0.0484412

0.0484412

0

0.0484412

0
4

0
4

0

0

0

0
4

0
4

0

0

0

0
4

0
4

0

0

0

0

0
4

0

0

0
4

0

0

0
4

0

0

0
4

0

0

0
4

0

0

0
4

0

0

0
4

0

0

0
4

0
4

0
4

7.95996601965498e-12

0
4

4.04607
7

4.04607
7

4.04607
7

0
6

0

0
7

0

0

0

0

0

0

0

0

0

0

0

0

0

0

0

0

0

0

0

0

0

0
7

0

0

0

0

0
6

0
6

0
6

0
6

0

0

0
4

0
7

0
7

0
7

0

0
4

4.04607
7

0

0
7

3.73483
7

0
7

0
6

0
7

0

0
6

0.116713

0.194522

0

0

0

0
4

0
4

0
4

0
4

0

0

0

0

0

0

0

0

0

0

0

0

0

0

0

0

0

0

0

0

0

0

0

0

0

0

0

0

0

0

0

0

0

0

0

0

0

0

0

0

0

0

0

0

0

0

0

0

0

0

0

0

0

0

0

0

0

0

0

0

0

0

0

0

0

0

0

0

0

0

0

0

0

0

0

0

0

0

0

0

0

0

0

0

0

0

0

0

0

0

0

0

0

0

0

0

0

0

0

0

0

0

0

0

0

0

0

0

0

0

0

0

0

0

0

0

0

0

0

0

0

0

0

0

0

0

0

0

0

0

0

0

0

0

0

0

0

0

0

0

0

0

0

0
4

0

0

0

0

0

0

0

0

0

0

0

0

0

0

0

0

0

0

0

0
4

0

0

0

0

0

0

0

0

0
4

0
4

0
4

0
4

0
7

0
7

0
7

0
7

0
7

0

0

0

0
4

0

0

0

0

0

0
4

0
4

0
4

0
4

0

0

0

0

0

0
4

0

0

0
4

0

0

0
4

0
4

0
4

0
4

0

0

0

0

0

0

0
4

0

0

0
4

0

0

0
4

0
4

0
4

0
4

0

0

0

0

0

0

0
4

0
4

0
4

0
4

0

0

0

0

0

0

0
4

0

0

0
4

0
4

0
4

0
4

0
3

0
3

0
3

0
3

0

0

0
4

0
4

0
4

0
4

0

0

0

0

0

0

0
4

0
4

0
4

0
4

0.933708

0.933708

0.933708

0.933708

0.933708

0

0
4

0
4

0
4

0
4

0

0

0

0

0

0

0
4

0
4

0
4

0
4

0

0

0

0

0

0
4

0
4

0
4

0
4

0

0

0

0

0

0
4

0

0

0
4

0

0

0
4

0
4

0
4

0
4

44.1842
3

44.1842
3

44.1842
3

13.0198
3

3.25495
3

6.92542

2.35464

0.346271

0.138508

0

0
4

28.1864

3.11644

25.07

0
4

0.415525

0.138508

0.277017

0
4

2.5624

2.5624

0

0
4

0

0

0
4

0
4

0
4

0
4

0

0

0

0

0

0

0
4

0
4

0
4

0
4

0

0

0

0

0

0

0
4

0
4

0
4

0
4

3.61538

3.61538

3.61538

3.61538

3.61538

0
4

0
4

0
4

0
4

0

0

0

0

0

0

0
4

0
4

0
4

0
4

0

0

0

0

0

0

0
4

0
4

0
4

0
4

0

0

0

0

0

0
4

0
4

0
4

0
4

0

0

0

0

0

0
4

0
4

0
4

0
4

0

0

0

0

0

0
4

0

0

0
4

0

0

0
4

0

0

0
4

0
4

0
4

0
4

0

0

0

0

0

0
4

0
4

0
4

0
4

0

0

0

0

0

0

0
4

0
4

0
4

0
4

0.969558
4

0.969558
4

0.969558
4

0.969558
4

0.969558
4

0

0

0

0
4

0
4

0
4

0
4

0
4

0
4

0
4

0

0

0
4

0

0

0

0

0

0

0
4

0
4

0
4

0
4

0

0

0

0

0

0
4

0
4

0
4

0
4

0

0

0

0

0

0

0
4

0

0

0
4

0
4

0
4

0
4

0

0

0

0

0

0

0
4

0
4

0
4

0
4

0

0

0

0

0

0

0
4

0
4

0
4

0
4

0.0502263

0.0502263

0.0502263

0.0502263

0.0502263

0
4

0

0

0
4

0
4

0
4

0
4

0

0

0

0

0

0
4

0
4

0
4

0
4

0

0

0

0

0

0

0
4

0
4

0
4

0
4

0

0

0

0

0

0
4

0
4

0
4

0
4

0

0

0

0

0

0
4

0
4

0
4

0
4

0

0

0

0

0

0

0
4

0
4

0
4

0
4

0
6

0
6

0
6

0
6

0
6

0

0

0

0

0

0

0

0
4

0

0

0

0
4

0
4

0
4

0
4

0

0

0

0

0

0

0
4

0

0

0
4

0
4

0
4

0
4

0

0

0

0

0

0

0

0
4

0

0

0
4

0
4

0
4

0
4

0

0

0

0

0

0

0
4

0
4

0
4

0
4

0

0

0

0

0

0

0

0
4

0

0

0
4

0
4

0
4

0
4

0

0

0

0

0

0
4

0
4

0
4

0
4

0

0

0

0

0

0
4

0
4

0
4

0
4

0

0

0

0

0

0

0
4

0
4

0
4

0
4

0

0

0

0

0

0

0

0
4

0
4

0
4

0
4

0

0

0

0

0

0

0
4

0

0

0
4

0
4

0
4

0
4

0

0

0

0

0

0
4

0
4

0
4

0
4

0

0

0

0

0

0

0

0

0

0

0

0

0
4

0

0

0

0

0

0

0

0
4

0

0

0

0

0
4

0
4

0
4

0

0

0

0

0

0

0
4

0

0

0

0
4

0
4

0
4

0

0

0

0

0
4

0
4

0
4

0
4

0

0

0

0

0

0
4

0

0

0
4

0

0

0
4

0
4

0
4

0
4

0

0

0

0

0

0

0
4

0
4

0
4

0

0

0

0

0
4

0
4

0
4

0
4

1.05042

1.05042

1.05042

0

0

0
4

1.05042

1.05042

0
4

0
4

0
4

0
4

0

0

0

0

0

0

0
4

0
4

0
4

0
4

0

0

0

0

0

0

0
4

0
4

0
4

0
4

0

0

0

0

0

0
4

0

0

0
4

0
4

0
4

0
4

0

0

0

0

0

0
4

0
4

0
4

0
4

0

0

0

0

0

0
4

0
4

0
4

0
4

3.99178

3.99178

3.99178

3.99178

3.99178

0
4

0
4

0
4

0
4

0

0

0

0

0

0
4

0
4

0
4

0
4

1.6621
3

1.6621
3

1.6621
3

1.6621
3

1.17732
3

0.207762
3

0

0.138508

0

0.138508

0

0

5.55111512312578e-17
3

0
4

0

0

0

0
4

0
4

0
4

0

0

0

0

0
4

0
4

0
4

0
4

0

0

0

0

0

0

0
4

0

0

0
4

0
4

0
4

0
4

0

0

0

0

0

0
4

0
4

0
4

0
4

0

0

0

0

0

0

0
4

0

0

0
4

0
4

0
4

0
4

0

0

0

0

0

0
4

0
4

0
4

0
4

0

0

0

0

0

0

0
4

0
4

0
4

0
4

0

0

0

0

0

0

0
4

0
4

0
4

0
4

0

0

0

0

0

0
4

0
4

0
4

0
4

0

0

0

0

0

0

0
4

0
4

0
4

0
4

0

0

0

0

0

0
4

0
4

0
4

0
4

0

0

0

0

0

0
4

0
4

0
4

0
4

0

0

0

0

0

0

0

0
4

0

0

0

0
4

0

0

0

0
4

0

0

0
4

0
4

0
4

0

0

0

0

0
4

0

0

0
4

0
4

0
4

0

0

0

0

0
4

0
4

0
4

0
4

0

0

0

0

0

0
4

0
4

0
4

0
4

0

0

0

0

0

0
4

0
4

0
4

0
4

0

0

0

0

0

0
4

0
4

0
4

0
4

0

0

0

0

0

0
4

0
4

0
4

0
4

0

0

0

0

0

0

0
4

0
4

0
4

0
4

0

0

0

0

0

0

0
4

0
4

0
4

0
4

0

0

0

0

0

0

0
4

0
4

0
4

0
4

0

0

0

0

0

0
4

0
4

0
4

0
4

0.681808

0.681808

0.681808

0.681808

0.681808

0
4

0
4

0
4

0
4

3.65702

3.65702

3.65702

3.65702

3.65702

0
4

0
4

0
4

0
4

0
7

0
7

0
7

0
7

0
7

0
7

0

0

0

0
4

0

0

0

0
4

0
4

0
4

0
4

0

0

0

0

0

0
4

0
4

0
4

0
4

0

0

0

0

0

0
4

0
4

0
4

0
4

0

0

0

0

0

0
4

0
4

0
4

0
4

0

0

0

0

0

0
4

0
4

0
4

0
4

0

0

0

0

0

0
4

0
4

0
4

0
4

0

0

0

0

0

0
4

0
4

0
4

0
4

0

0

0

0

0

0
4

0
4

0
4

0
4

0

0

0

0

0

0

0
4

0
4

0
4

0
4

0

0

0

0

0

0
4

0
4

0
4

0
4

0

0

0

0

0

0
4

0
4

0
4

0
4

0
4

0
4

0
4

0
4

0
4

0

0

0

0

0

0

0
4

0
4

0

0

0

0
4

0
4

0
4

0
4

15.6882

15.6882

15.6882

15.6882

15.6882

0
4

0
4

0
4

0
4

0

0

0

0

0

0

0
4

0
4

0
4

0
4

0

0

0

0

0

0
4

0
4

0
4

0
4

0

0

0

0

0

0
4

0
4

0
4

0
4

0

0

0

0

0

0

0
4

0
4

0
4

0
4

0

0

0

0

0

0

0
4

0
4

0
4

0
4

0

0

0

0

0

0
4

0

0

0
4

0
4

0
4

0
4

1.78334

1.78334

1.78334

1.78334

1.78334

0
4

0
4

0
4

0
4

0

0

0

0

0

0
4

0
4

0
4

0
4

0

0

0

0

0

0
4

0
4

0
4

0
4

0

0

0

0

0
1

0

0

0

0

0
4

0

0

0
4

0

0

0
4

0
4

0
4

0

0

0

0

0
4

0
4

0
4

0
4

0

0

0

0

0

0
4

0
4

0
4

0
4

0.614399

0.614399

0.614399

0.614399

0.614399

0

0
4

0
4

0
4

0
4

0

0

0

0

0

0

0
4

0
4

0
4

0
4

0

0

0

0

0

0
4

0
4

0
4

0
4

2.58925

2.58925

2.58925

2.58925

2.58925

0
4

0
4

0
4

0
4

0

0

0

0

0

0

0
4

0
4

0
4

0
4

0

0

0

0

0

0
4

0

0

0
4

0
4

0
4

0
4

0

0

0

0

0

0

0
4

0
4

0
4

0
4

0

0

0

0

0

0

0
4

0
4

0
4

0
4

0

0

0

0

0

0
4

0
4

0
4

0
4

56.3546
3

56.3546
3

56.3546
3

56.3546
3

19.4127
3

0
4

0
5

0
4

1.2134
2

0
4

0

0

0

0

0

4.04218
3

0

0

0

0

0

0

0

0

0

0

0.369224
3

22.7731
3

0

0
3

8.54401
3

0

0
3

3.5527136788005e-15
3

0
4

0
4

0
4

0
4

0
4

0
4

0
4

0
3

0
3

0
4

0
3

0
4

0

0
4

0
4

0

0

0

0
4

0

0

0

0
4

0

0

0
4

0

0

0
4

0

0

0

0
4

0
4

0
4

0
4

0

0

0

0

0

0
4

0
4

0
4

0
4

0

0

0

0

0

0
4

0
4

0
4

0
4

0

0

0

0

0

0

0
4

0
4

0
4

0
4

0

0

0

0

0

0
4

0
4

0
4

0
4

0

0

0

0

0

0
4

0
4

0
4

0
4

0

0

0

0

0

0
4

0
4

0
4

0
4

0

0

0

0

0

0
4

0
4

0
4

0
4

0

0

0

0

0

0
4

0
4

0
4

0
4

0

0

0

0

0

0
4

0
4

0
4

0
4

0

0

0

0

0

0
4

0

0

0
4

0
4

0
4

0
4

0

0

0

0

0

0

0

0

0

0

0

0
4

0

0

0
4

0
4

0
4

0
4

0

0

0

0

0

0

0
4

0
4

0
4

0
4

0

0

0

0

0

0
4

0
4

0
4

0
4

0

0

0

0

0

0
4

0
4

0
4

0
4

0

0

0

0

0

0

0
4

0
4

0
4

0
4

0.583567

0.583567

0.583567

0.583567

0.583567

0
4

0
4

0
4

0
4

0

0

0

0

0

0
4

0
4

0
4

0
4

0

0

0

0

0

0
4

0
4

0
4

0
4

0

0

0

0

0

0

0
4

0
4

0
4

0
4

0

0

0

0

0

0
4

0

0

0
4

0
4

0
4

0
4

0

0

0

0

0

0
4

0

0

0
4

0
4

0
4

0
4

0
4

0
4

0
4

0
4

0
4

0

0

0

0

0

0

0

0
4

0
4

0
4

0
4

0

0

0

0

0

0
4

0
4

0
4

0
4

0

0

0

0

0

0

0
4

0
4

0
4

0
4

0

0

0

0

0

0
4

0
4

0
4

0
4

0

0

0

0

0

0
4

0
4

0
4

0
4

0

0

0

0

0

0
4

0
4

0
4

0
4

0

0

0

0

0

0
4

0
4

0
4

0
4

0

0

0

0

0

0
4

0
4

0
4

0
4

0

0

0

0

0

0
4

0
4

0
4

0
4

0

0

0

0

0

0
4

0
4

0
4

0
4

0

0

0

0

0

0
4

0
4

0
4

0
4

0

0

0

0

0

0

0

0

0

0

0

0
4

0

0

0

0

0
4

0
4

0
4

0
4

0

0

0

0

0

0
4

0
4

0
4

0
4

0

0

0

0

0

0
4

0
4

0
4

0
4

0

0

0

0

0

0
4

0
4

0
4

0
4

0

0

0

0

0

0
4

0
4

0
4

0
4

0

0

0

0

0

0
4

0
4

0
4

0
4

0

0

0

0

0

0
4

0
4

0
4

0
4

0

0

0

0

0

0
4

0
4

0
4

0
4

0

0

0

0

0

0
4

0
4

0
4

0
4

0

0

0

0

0

0
4

0
4

0
4

0
4

0.919081

0.919081

0.919081

0.919081

0.919081

0
4

0
4

0
4

0
4

0
7

0
7

0
7

0
7

0
7

0
7

0

0
4

0
4

0
4

0
4

0

0

0

0

0

0
4

0
4

0
4

0
4

0

0

0

0

0

0
4

0
4

0
4

0
4

0

0

0

0

0

0
4

0
4

0
4

0
4

0

0

0

0

0

0
4

0
4

0
4

0
4

0

0

0

0

0

0
4

0
4

0
4

0
4

0.190155

0.190155

0.190155

0.190155

0.190155

0
4

0
4

0
4

0
4

0

0

0

0

0

0
4

0
4

0
4

0
4

0

0

0

0

0

0
4

0
4

0
4

0
4

0

0

0

0

0

0
4

0
4

0
4

0
4

0

0

0

0

0

0
4

0
4

0
4

0
4

0

0

0

0

0

0

0

0

0

0

0
4

0

0

0

0

0

0

0
4

0
4

0
4

0

0

0

0

0

0

0

0
4

0

0

0
4

0

0

0
4

0
4

0
4

0
4

0

0

0

0

0

0
4

0
4

0
4

0
4

0

0

0

0

0

0
4

0
4

0
4

0
4

0

0

0

0

0

0
4

0
4

0
4

0
4

0

0

0

0

0

0
4

0
4

0
4

0
4

0

0

0

0

0

0
4

0
4

0
4

0
4

0

0

0

0

0

0
4

0
4

0
4

0
4

0

0

0

0

0

0
4

0
4

0
4

0
4

0

0

0

0

0

0
4

0
4

0
4

0
4

0

0

0

0

0

0
4

0
4

0
4

0
4

0

0

0

0

0

0
4

0
4

0
4

0
4

0.663618
3

0.663618
3

0.663618
3

0.600233
4

0.0739218
4

0.526311
3

0

0
4

0.0633849

0.0633849

0

0
4

0
4

0
4

0
4

0

0

0

0

0

0
4

0
4

0
4

0
4

0

0

0

0

0

0
4

0
4

0
4

0
4

0

0

0

0

0

0
4

0
4

0
4

0
4

0.0633849

0.0633849

0.0633849

0.0633849

0.0633849

0
4

0
4

0
4

0
4

0

0

0

0

0

0
4

0
4

0
4

0
4

0

0

0

0

0

0
4

0
4

0
4

0
4

0

0

0

0

0

0
4

0
4

0
4

0
4

0

0

0

0

0

0
4

0
4

0
4

0
4

0

0

0

0

0

0
4

0
4

0
4

0
4

0

0

0

0

0

0
4

0
4

0
4

0
4

0

0

0

0

0

0

0

0

0

0

0

0
4

0
4

0
4

0
4

0

0

0

0

0

0
4

0
4

0
4

0
4

0

0

0

0

0

0
4

0
4

0
4

0
4

0

0

0

0

0

0
4

0
4

0
4

0
4

0

0

0

0

0

0
4

0
4

0
4

0
4

0.81817

0.81817

0.81817

0.81817

0.81817

0
4

0
4

0
4

0
4

0

0

0

0

0

0
4

0
4

0
4

0
4

0

0

0

0

0

0
4

0
4

0
4

0
4

0.334842

0.334842

0.334842

0.334842

0.334842

0
4

0
4

0
4

0
4

0

0

0

0

0

0
4

0
4

0
4

0
4

0

0

0

0

0

0
4

0
4

0
4

0
4

0
4

0
4

0
4

0
4

0
4

0

0
4

0

0

0

0
4

0

0

0

0
4

0

0

0
4

0

0

0
4

0

0

0
4

0
4

0
4

0

0

0

0

0
4

0
4

0
4

0

0

0

0

0
4

0
4

0
4

0
4

0

0

0

0

0

0
4

0
4

0
4

0
4

0

0

0

0

0

0
4

0
4

0
4

0
4

0

0

0

0

0

0
4

0
4

0
4

0
4

0

0

0

0

0

0
4

0
4

0
4

0
4

0

0

0

0

0

0
4

0
4

0
4

0
4

0

0

0

0

0

0
4

0
4

0
4

0
4

0

0

0

0

0

0
4

0
4

0
4

0
4

0

0

0

0

0

0
4

0
4

0
4

0
4

0

0

0

0

0

0
4

0
4

0
4

0
4

0

0

0

0

0

0
4

0
4

0
4

0
4

0

0

0

0

0

0

0

0

0

0

0

0

0

0

0

0

0
4

0

0

0

0
4

0
4

0
4

0
4

0

0

0

0

0

0
4

0
4

0
4

0
4

0

0

0

0

0

0
4

0
4

0
4

0
4

0

0

0

0

0

0
4

0
4

0
4

0
4

0

0

0

0

0

0
4

0
4

0
4

0
4

0

0

0

0

0

0
4

0
4

0
4

0
4

0

0

0

0

0

0
4

0
4

0
4

0
4

0

0

0

0

0

0
4

0
4

0
4

0
4

0

0

0

0

0

0
4

0
4

0
4

0
4

0

0

0

0

0

0
4

0
4

0
4

0
4

0

0

0

0

0

0
4

0
4

0
4

0
4

0
4

0
4

0
4

0
4

0
4

0

0

0

0

0

0

0

0

0

0

0
4

0

0

0

0

0

0

0

0

0

0

0
4

0

0

0

0

0

0

0
4

0
3

0
4

0

0
3

0

0
4

0
4

0
4

0

0

0
4

0

0

0

0

0

0

0

0
4

0
4

0
4

0
4

0
4

0

0

0

0

0

0
4

0

0

0

0

0

0

0
4

0
4

0

0

0

0

0
4

0

0

0
4

0
4

0
4

0
4

0
4

0
4

0
4

0

0
4

0

0

0

0
4

0

0

0
4

0
4

0
4

0

0

0

0

0
4

0

0

0
4

0
4

0
4

0
4

0

0

0

0

0

0

0

0

0

0
4

0
4

0
4

0
4

0

0

0

0

0

0
4

0
4

0
4

0
4

0

0

0

0

0

0
4

0
4

0
4

0
4

2.25646

2.25646

2.25646

2.25646

2.25646

0
4

0
4

0
4

0
4

0

0

0

0

0

0
4

0
4

0
4

0
4

0

0

0

0

0

0
4

0
4

0
4

0
4

0

0

0

0

0

0
4

0
4

0
4

0
4

0

0

0

0

0

0
4

0
4

0
4

0
4

0

0

0

0

0

0
4

0
4

0
4

0
4

0

0

0

0

0

0
4

0
4

0
4

0
4

0

0

0

0

0

0
4

0
4

0
4

0
4

0
5

0
5

0
5

0
5

0

0

0

0

0

0

0

0

0
4

0

0

0
4

0
4

0
4

0
4

0

0

0

0

0

0
4

0
4

0
4

0
4

0

0

0

0

0

0
4

0
4

0
4

0
4

0

0

0

0

0

0
4

0
4

0
4

0
4

0

0

0

0

0

0
4

0
4

0
4

0
4

0

0

0

0

0

0
4

0
4

0
4

0
4

0

0

0

0

0

0
4

0
4

0
4

0
4

0

0

0

0

0

0
4

0
4

0
4

0
4

0

0

0

0

0

0
4

0
4

0
4

0
4

0

0

0

0

0

0
4

0
4

0
4

0
4

0

0

0

0

0

0
4

0
4

0
4

0
4

0
7

0
7

0
7

0
7

0
7

0
7

0

0

0

0

0
4

0
4

0
4

0
4

0

0

0

0

0

0
4

0
4

0
4

0
4

0

0

0

0

0

0
4

0
4

0
4

0
4

0

0

0

0

0

0
4

0
4

0
4

0
4

0

0

0

0

0

0
4

0
4

0
4

0
4

0

0

0

0

0

0
4

0
4

0
4

0
4

0

0

0

0

0

0
4

0
4

0
4

0
4

0

0

0

0

0

0
4

0
4

0
4

0
4

0

0

0

0

0

0
4

0
4

0
4

0
4

0

0

0

0

0

0
4

0
4

0
4

0
4

0

0

0

0

0

0
4

0
4

0
4

0
4

0

0

0

0

0

0

0

0

0

0

0

0

0

0

0

0

0

0
4

0

0

0

0

0

0
4

0

0

0

0
4

0

0

0
4

0
4

0
4

0
4

0

0

0

0

0

0
4

0
4

0
4

0
4

0

0

0

0

0

0
4

0
4

0
4

0
4

0

0

0

0

0

0
4

0
4

0
4

0
4

0

0

0

0

0

0
4

0
4

0
4

0
4

0

0

0

0

0

0
4

0
4

0
4

0
4

0

0

0

0

0

0
4

0
4

0
4

0
4

0

0

0

0

0

0
4

0
4

0
4

0
4

0

0

0

0

0

0
4

0
4

0
4

0
4

0

0

0

0

0

0
4

0
4

0
4

0
4

0

0

0

0

0

0
4

0
4

0
4

0
4

0
7

0
7

0
7

0
7

0
6

0

0

0

0

0

0

0

0
4

0
4

0
4

0
4

0

0

0

0

0

0
4

0
4

0
4

0
4

0

0

0

0

0

0
4

0
4

0
4

0
4

1.61401

1.61401

1.61401

1.61401

1.61401

0
4

0
4

0
4

0
4

0

0

0

0

0

0
4

0
4

0
4

0
4

0

0

0

0

0

0
4

0
4

0
4

0
4

0

0

0

0

0

0
4

0
4

0
4

0
4

0

0

0

0

0

0
4

0
4

0
4

0
4

0

0

0

0

0

0
4

0
4

0
4

0
4

0

0

0

0

0

0
4

0
4

0
4

0
4

0

0

0

0

0

0
4

0
4

0
4

0
4

0

0

0

0

0

0

0

0

0

0

0

0
4

0

0

0

0
4

0

0

0
4

0
4

0
4

0
4

0

0

0

0

0

0
4

0
4

0
4

0
4

0

0

0

0

0

0
4

0
4

0
4

0
4

0

0

0

0

0

0

0

0

0

0

0

0
4

0
4

0
4

0
4

0

0

0

0

0

0

0
4

0
4

0
4

0

0

0

0

0
4

0

0

0
4

0
4

0
4

0
4

0

0

0

0

0

0

0

0
4

0

0

0

0
4

0
4

0
4

0
4

0

0

0

0

0

0

0

0
4

0
4

0
4

0
4

1.03881
4

1.03881
4

1.03881
4

0
4

0
4

0

0

0

0

0

0

0

0

0

0

0
4

0

0

0

0

0

0

0

0

0

0

0
4

0

0

0

0

0

0

0

0

0

0

0
4

0

0

0

0

0

0

0

0
4

0

0

0

0
4

1.03881
4

1.03881
4

0

0

0
4

0

0
4

0

0

0

0

0

0
4

0

0

0

0
4

0
4

0
4

0
4

1.86986
3

1.86986
3

1.86986
3

1.86986
3

1.86986
3

0
4

0

0

0

0
4

0
4

0
4

0
4

0

0

0

0

0

0

0

0

0

0
4

0
4

0
4

0
4

0

0

0

0

0

0

0
4

0
4

0
4

0

0

0

0

0
4

0
4

0
4

0
4

0

0

0

0

0

0

0
4

0
4

0
4

0
4

0
4

0
4

0
4

0
4

0
4

0

0
4

0

0

0
4

0
4

0
4

0
4

0

0

0

0

0

0
4

0
4

0
4

0
4

0
4

0
4

0

0

0

0

0

0

0
4

0
4

0

0

0

0
4

0
4

0
4

0

0

0

0

0

0
4

0

0

0
4

0
4

0
4

0
4

0
4

0
4

0
4

0
4

0
4

0

0
4

0

0

0

0
4

0
4

0
4

0
4

0

0

0

0

0

0

0

0

0
4

0

0

0
4

0
4

0
4

0
4

0

0

0

0

0

0

0

0

0

0
4

0

0

0

0
4

0

0

0
4

0
4

0
4

0
4

0
4

0
4

0
4

0
4

0
4

0

0

0

0

0

0

0

0

0

0

0
4

0

0

0

0

0

0

0

0

0

0

0

0
4

0
4

0

0

0

0

0
4

0
4

0
4

0
4

0

0

0

0

0

0

0

0
4

0
4

0
4

0
4

0
4

0

0

0

0
4

0

0

0

0
4

0

0

0
4

0

0

0
4

0
4

0
4

0
4

0
4

0
4

0
4

0

0

0

0
4

0

0

0

0
4

0

0

0

0
4

0
4

0
4

0

0

0

0

0
4

0
4

0
4

0
4

0
5

0
5

0
5

0
5

0

0

0

0

0
4

0
4

0
4

0
4

0

0

0

0

0

0

0
4

0
4

0
4

0
4

0
5

0
5

0
5

0
5

0
4

0

0

0
4

0

0

0
4

0

0

0
4

0
4

0
4

0
4

0

0

0

0

0

0

0

0

0

0

0
4

0

0

0
4

0
4

0
4

0
4

0

0

0

0

0

0

0

0
4

0
4

0
4

0
4

0

0

0

0

0

0

0

0
4

0
4

0
4

0
4

0

0

0

0

0

0

0

0
4

0
4

0
4

0
4

3.11644
3

3.11644
3

3.11644
3

3.11644
3

3.11644
3

0
4

0
4

0
4

0
4

0

0

0

0

0

0

0

0
4

0

0

0
4

0

0

0
4

0
4

0
4

0
4

0

0

0

0

0

0
4

0
4

0
4

0
4

3.81264
4

3.81264
4

3.81264
4

3.81264
4

3.81264
4

0

0

0

0

0

0

0

0

0
4

0
4

0
4

0
4

0

0

0

0

0

0

0
4

0
4

0
4

0
4

0

0

0

0

0

0

0

0

0

0
4

0
4

0
4

0

0

0

0

0

0
4

0

0

0
4

0
4

0
4

0
4

0
7

0
7

0
7

0
7

0

0

0

0

0
4

0
4

0
4

0
4

0
7

0
7

0
7

0
7

0
7

0
4

0
4

0
4

0
4

0

0

0

0

0

0

0

0

0

0
4

0

0

0

0
4

0

0

0
4

0
4

0
4

0
4

0

0

0

0

0

0
4

0
4

0
4

0
4

43.5609

43.5609

43.5609

39.7519

39.7519

0

0
4

3.3242

3.3242

0
4

0.138508

0.138508

0
4

0.346271

0.346271

0
4

5.55111512312578e-17

0
4

0
4

0
4

0

0

0

0

0

0

0
4

0

0

0
4

0
4

0
4

0
4

0.311236
4

0.311236
4

0.311236
4

0.311236
4

0.311236

0

0

0
4

0
4

0
4

0
4

0

0

0

0

0

0

0

0

0
4

0
4

0
4

0

0

0

0

0
4

0
4

0
4

0
4

1.52359
4

1.52359
4

1.52359
4

0
4

0
4

0

0

0

0

0

0

0

0

0

0

0

0

0

0

0

0

0
4

0

0

0

0

0
4

1.52359
3

1.52359
3

0

0
4

0

0

0

0

0
4

0

0

0
4

0

0

0
4

0

0

0
4

0

0

0
4

0
4

0
4

0
4

0

0

0

0

0

0

0
4

0

0

0

0
4

0
4

0
4

0
4

0
4

0
4

0
4

0
4

0

0

0

0
4

0
4

0
4

0
4

0
4

0
4

0
4

0
4

0
4

0

0
4

0
4

0
4

0
4

0
4

0
4

0
4

0
4

0
4

0
4

0

0

0

0
4

0
4

0
4

0
4

0

0

0

0

0

0

0

0
4

0
4

0
4

0
4

0

0

0

0

0

0

0
4

0

0

0

0
4

0

0

0
4

0
4

0
4

0
4

0

0

0

0

0

0
4

0

0

0

0
4

0

0

0

0
4

0

0

0
4

0
4

0
4

0
4

0

0

0

0

0

0

0

0

0
4

0

0

0
4

0
4

0
4

0
4

0

0

0

0

0

0

0

0

0

0
4

0

0

0
4

0

0

0
4

0
4

0
4

0

0

0

0

0
4

0
4

0
4

0
4

0

0

0

0

0

0
4

0
4

0
4

0
4

0
4

0
4

0
4

0
4

0
4

0
3

0

0

0

0

0

0
4

0
4

0

0

0

0

0

0
4

0

0

0

0
4

0

0

0

0

0

0
4

0

0

0
4

0

0

0
4

0
4

0
4

0
4

0

0

0

0

0

0
4

0

0

0
4

0

0

0
4

0
4

0
4

0

0

0

0

0

0
4

0
4

0
4

0
4

0

0

0

0

0

0
4

0

0

0
4

0
4

0
4

0

0

0

0

0
4

0
4

0
4

0
4

0
7

0
7

0
7

0
7

0
7

0
4

0
4

0
4

0
4

0

0

0

0

0

0

0

0
4

0

0

0
4

0
4

0
4

0
4

1.80582

1.80582

1.80582

1.80582

1.80582

0

0

0
4

0
4

0
4

0
4

0

0

0

0

0

0

0

0
4

0
4

0
4

0
4

0

0

0

0

0

0

0
4

0
4

0
4

0
4

0

0

0

0

0

0
4

0
4

0
4

0
4

0
3

0
3

0
3

0
3

0

0

0

0
4

0

0

0
4

0
4

0
4

0
4

0

0

0

0

0

0
4

0

0

0
4

0
4

0
4

0
4

0

0

0

0

0

0

0

0

0

0

0

0

0

0

0

0
4

0

0

0
4

0

0

0
4

0
4

0
4

0
4

0

0

0

0

0

0

0
4

0

0

0
4

0

0

0
4

0
4

0
4

0
4

0.496856
2

0.496856
2

0.496856
2

0.496856
2

0.496856
2

0
4

0

0

0
4

0
4

0
4

0
4

0
7

0
7

0
7

0
7

0
7

0
4

0
4

0
4

0
4

0
7

0
7

0
7

0
7

0
7

0
4

0
4

0
4

0
4

0

0

0

0

0

0

0

0
4

0

0

0
4

0

0

0
4

0
4

0
4

0
4

0

0

0

0

0

0

0
4

0

0

0
4

0
4

0
4

0
4

0.698626

0.698626

0.698626

0.698626

0.643472

0.0551547

2.08166817117217e-17

0
4

0
4

0
4

0
4

0

0

0

0

0

0

0
4

0

0

0
4

0

0

0
4

0
4

0
4

0
4

0

0

0

0

0

0
4

0
4

0
4

0
4

0

0

0

0

0

0

0
4

0
4

0
4

0
4

5.12481

0

0

0

0

0
4

0
4

0
4

0

0

0

0

0
4

0
4

0
4

0

0

0

0

0
4

0
4

0
4

5.12481

5.12481
3

5.12481
3

5.12481
3

0

0
4

0

0

0
4

0

0

0

0
4

0
4

0

0

0

0
4

0
4

0

0

0

0
4

0
4

0

0

0

0
4

0
4

0

0

0

0

0
4

0

0

0
4

0

0

0
4

0
4

0

0

0

0

0
4

0
4

0

0

0

0

0
4

0
4

0

0

0

0
4

0
4

0

0

0

0
4

0

0

0
4

0

0

0
4

0
4

0

0

0

0
4

0
4

0

0

0

0

0
4

0
4

0

0

0

0
4

0
4

0
6

0
6

0
6

0

0
4

0
4

0
4

0
4

0.0877713

0

0

0

0

0

0
4

0
4

0
4

0

0

0

0

0

0

0
4

0
4

0
4

0.0877713

0.0877713

0

0

0
4

0.0877713

0.0877713

0
4

0
4

0
4

0

0

0

0

0
4

0
4

0
4

0

0

0

0

0
4

0
4

0
4

0

0

0

0

0
4

0
4

0
4

0

0

0

0

0
4

0
4

0
4

0

0

0

0

0
4

0
4

0
4

0

0

0

0

0

0
4

0
4

0

0

0

0

0
4

0
4

0

0

0

0
4

0
4

0

0

0

0
4

0
4

0

0

0

0
4

0
4

0

0

0

0

0
4

0

0

0
4

0

0

0
4

0

0

0
4

0
4

0
4

0
4

0

0

0

0

0

0
4

0
4

0
4

0
4

91.2592
3

0

0

0

0

0
4

0
4

0
4

0

0

0

0

0
4

0
4

0
4

91.2592
3

0

0

0

0
4

0
4

91.2592
3

91.2592
3

10.7826
2

1.07825

0

0.735396

0.0548139

0.118199

0.0548139

0

0.0365426

0

0

21.8727
2

1.09753

0.238749

0.864091

0

0

0.193681

0.0913565

0.0367698

0

0

45.4559
2

0

0.0367698

0

0

0.129121

0

0

0

0.0365426

0.129121

1.08705
2

0

0.774725

0

0

0

0

0

0

0

0

1.17479
2

0

0.0365426

0

0.129121

0

0

0

0.0633849

0

0

4.27079

0

0.679872
2

0

3.10862446895044e-15
3

0
4

0
4

0
4

0
4

0

0

0

0

0

0
4

0
4

0
4

0
4

2.02337
2

0.636354

0.636354

0.636354

0.636354

0
4

0
4

0
4

0

0

0

0

0
4

0
4

0
4

1.38702
2

0
2

0
2

0
2

0

0

0

0

0

0

0
4

0
4

0
2

0
2

0
2

0

0
4

0

0

0
4

0
4

0

0

0

0
4

0
4

1.38702
3

1.38702
3

0

0

0

0

0

0

0

0

0

0

1.38702

0

0

0

0

0
4

0
4

0
4

2.22044604925031e-16
2

0
4

6.02346

0
7

0
7

0
7

0
7

0

0

0

0

0

0

0

0
4

0

0

0
4

0

0

0
4

0

0

0
4

0
4

0

0

0

0
4

0
4

0
4

0

0

0

0

0

0
4

0
4

0
4

0

0

0

0

0
4

0
4

0
4

0

0

0

0

0
4

0
4

0
4

0

0

0

0

0
4

0
4

0
4

0

0

0

0

0
4

0

0

0
4

0
4

0
4

0

0

0

0

0
4

0
4

0
4

0

0

0

0

0
4

0
4

0
4

0

0

0

0

0
4

0
4

0
4

0.129121

0.129121

0.129121

0.129121

0
4

0
4

0
4

0

0

0

0

0
4

0
4

0
4

0
7

0
7

0
7

0

0

0

0
4

0

0

0

0

0
4

0

0

0

0
4

0

0

0

0
4

0

0

0
4

0
4

0
4

0

0

0

0

0
4

0
4

0
4

0

0

0

0

0
4

0
4

0
4

0

0

0

0

0
4

0
4

0
4

0

0

0

0

0
4

0
4

0
4

0

0

0

0

0
4

0
4

0
4

0

0

0

0

0
4

0
4

0
4

0

0

0

0

0
4

0
4

0
4

0

0

0

0

0
4

0
4

0
4

0

0

0

0

0
4

0
4

0
4

0

0

0

0

0
4

0
4

0
4

0

0

0

0

0

0

0

0

0

0
4

0
4

0
4

0

0

0

0

0
4

0
4

0
4

0

0

0

0

0

0
4

0

0

0

0
4

0
4

0
4

0.263314

0.263314

0.263314

0.263314

0
4

0
4

0
4

0

0

0

0

0

0

0
4

0
4

0
4

0

0

0

0

0

0
4

0
4

0
4

0

0

0

0

0
4

0
4

0
4

0

0

0

0

0
4

0

0

0
4

0
4

0
4

5.63102

0

0

0

0
4

0
4

0

0

0

0
4

0

0

0
4

0
4

0

0

0

0
4

0
4

0

0

0

0
4

0
4

0

0

0

0
4

0
4

0

0

0

0
4

0
4

0

0

0

0
4

0
4

0.903846

0.903846

0.903846

0
4

0
4

0

0

0

0
4

0
4

0

0

0

0
4

0
4

0

0

0

0
4

0
4

0
7

0
7

0

0

0

0
4

0

0

0
4

0

0

0
4

0
4

0

0

0

0
4

0
4

0
7

0
7

0
7

0
4

0
4

4.26099

4.26099

4.06731

0.193681

3.33066907387547e-16

0
4

0
4

0

0

0

0
4

0
4

0

0

0

0
4

0

0

0
4

0

0

0
4

0
4

0

0

0

0
4

0
4

0

0

0

0
4

0
4

0

0

0

0
4

0
4

0.46619

0.46619

0
6

0

0
7

0

0

0

0

0

0

0

0

0
5

0

0

0.175543

0

0

0

0

0

0

0

0
7

0

0

0

0

0

0

0

0

0

0

0
6

0

0

0

0

0

0

0

0

0

0

0
7

0

0

0

0

0

0

0

0

0

0

0
5

0.217986

0

0

0

0

0

0

0

0

0

0
7

0

0

0

0

0

0

0

0

0

0.0726619

0
4

0

0

0

0
4

0

0

0
4

0

0

0
4

0

0

0
4

0

0

0
4

0

0

0
4

0

0

0
4

0

0

0
4

0
4

1.11022302462516e-15

0
4

0
4

48.2897

3.85217
3

3.54094
3

3.07493
3

2.01462

0.129121
3

0.931184

0

0

0

0

0
4

0.349299

0.349299

0

0

0
4

0.116713

0.116713

0

0
4

1.94289029309402e-16
3

0
4

0

0

0

0

0

0
4

0

0

0
4

0
4

0.194522

0

0

0

0
4

0.194522

0.194522

0
4

0
4

0.116713

0

0

0
4

0.116713

0.116713

0
4

0
4

2.77555756156289e-16
3

0
4

23.0704

23.0704

23.0704

23.0704

0
4

0
4

0
4

0

0

0

0

0

0

0
4

0
4

0
4

0

0

0

0

0
4

0
4

0
4

0

0

0

0

0

0
4

0
4

0
4

0

0

0

0

0

0
4

0
4

0
4

0

0

0

0

0
4

0
4

0
4

0

0

0

0

0

0
4

0
4

0
4

0

0

0

0

0
4

0
4

0
4

0

0

0

0

0

0
4

0
4

0
4

0

0

0

0

0

0
4

0
4

0
4

0.544663
3

0.544663
3

0.544663
3

0.194522
3

0

0.077809

0.116713

0.155618

0

0

5.55111512312578e-17
3

0
4

0

0

0
4

0

0

0
4

0

0

0
4

0
4

0
4

0

0

0

0

0
4

0
4

0
4

0

0

0

0

0
4

0
4

0
4

0

0

0

0

0
4

0

0

0
4

0
4

0
4

0

0

0

0

0

0
4

0
4

0
4

0

0

0

0

0
4

0
4

0
4

0

0

0

0

0
4

0

0

0
4

0
4

0
4

0

0

0

0

0
4

0
4

0
4

0

0

0

0

0
4

0
4

0
4

0

0

0

0

0
4

0
4

0
4

0

0

0

0

0
4

0
4

0
4

0
6

0
6

0
6

0
7

0

0

0

0

0

0
4

0
4

0
4

0

0

0

0

0
4

0
4

0
4

0

0

0

0

0
4

0
4

0
4

0

0

0

0

0
4

0
4

0
4

0.0347274

0.0347274

0.0347274

0.0347274

0
4

0
4

0
4

0

0

0

0

0
4

0
4

0
4

0

0

0

0

0
4

0
4

0
4

0

0

0

0

0
4

0
4

0
4

0.194522

0.194522

0.194522

0.194522

0
4

0
4

0
4

0

0

0

0

0
4

0
4

0
4

0

0

0

0

0
4

0
4

0
4

0
7

0
7

0
7

0
6

0

0

0

0

0
4

0

0

0
4

0
4

0
4

0

0

0

0

0
4

0
4

0
4

0

0

0

0

0
4

0
4

0
4

0

0

0

0

0
4

0
4

0
4

0

0

0

0

0
4

0
4

0
4

0

0

0

0

0
4

0
4

0
4

0

0

0

0

0
4

0
4

0
4

0

0

0

0

0
4

0
4

0
4

0

0

0

0

0
4

0
4

0
4

0

0

0

0

0
4

0
4

0
4

0

0

0

0

0

0

0
4

0
4

0
4

0

0

0

0

0

0
4

0
4

0
4

0

0

0

0

0

0

0
4

0
4

0

0

0

0
4

0
4

0
4

8.9657
3

8.9657
3

8.9657
3

7.87637

1.08933

0

8.88178419700125e-16
3

0
4

0
4

0

0

0

0
4

0
4

0
4

0

0

0

0

0
4

0

0

0
4

0

0

0
4

0
4

0
4

11.6276
3

0

0

0

0

0

0

0

0
4

0
4

0

0

0

0
4

0
4

0

0

0

0
4

0
4

0.322802

0.129121

0.129121

0
4

0.193681

0.193681

0
4

2.77555756156289e-17

0
4

0

0

0

0
4

0
4

0

0

0

0
4

0
4

0

0

0

0
4

0
4

0

0

0

0
4

0
4

0

0

0

0
4

0
4

0

0

0

0
4

0
4

0

0

0

0
4

0
4

0

0

0

0

0

0
4

0
4

0

0

0

0
4

0
4

0

0

0

0
4

0
4

0

0

0

0
4

0
4

0

0

0

0
4

0
4

0

0

0

0
4

0
4

0

0

0

0
4

0
4

0

0

0

0
4

0
4

0

0

0

0
4

0
4

0

0

0

0
4

0
4

0

0

0

0
4

0
4

0

0

0

0

0
4

0

0

0
4

0
4

0

0

0

0
4

0
4

0

0

0

0
4

0
4

0

0

0

0
4

0
4

0

0

0

0
4

0
4

0

0

0

0

0
4

0

0

0
4

0
4

0

0

0

0

0
4

0

0

0
4

0
4

0.272331

0.272331

0.272331

0
4

0

0

0
4

0

0

0
4

0
4

0

0

0

0

0
4

0
4

0

0

0

0
4

0
4

0

0

0

0

0
4

0
4

11.0324
3

11.0324
3

10.8846
3

0.147844
3

0
7

0

0

0

0

0

0

0

0
4

0

0

0
4

0

0

0
4

0

0

0
4

0

0

0
4

0
4

0
4

0
4

7.45995

0
7

0
7

0
7

0
7

0

0
4

0

0

0

0

0
4

0
4

0
4

0

0

0

0

0
4

0
4

0
4

0

0

0

0

0
4

0
4

0
4

0

0

0

0

0
4

0
4

0
4

0

0

0

0

0
4

0
4

0
4

0

0

0

0

0
4

0
4

0
4

0

0

0

0

0
4

0
4

0
4

0.169544

0
6

0
6

0
6

0

0

0

0

0

0
4

0
4

0.169544

0.169544

0.169544

0
4

0
4

0
4

0
7

0
7

0
7

0
7

0

0

0
4

0
4

0
4

0

0

0

0

0
4

0

0

0
4

0
4

0
4

0

0

0

0

0
4

0

0

0
4

0
4

0
4

0

0

0

0

0
4

0

0

0
4

0
4

0
4

0

0

0

0

0

0
4

0
4

0
4

0

0

0

0

0

0
4

0
4

0
4

0

0

0

0

0
4

0
4

0
4

7.29041
3

0
7

0

0

0

0
4

0

0

0
4

0

0

0
4

0
4

0

0

0

0

0
4

0
4

4.02062

4.02062

4.02062

0
4

0
4

0

0

0

0
4

0
4

0

0

0

0
4

0
4

0

0

0

0
4

0
4

3.26978
3

3.19712
3

0.968825

0.0484412

0

0

0

0

0

0

0

1.74388

0

0
7

0.0484412

0.0484412

0

0

0

0

0

0

0

0

0
5

0.0484412

0

0

0

0

0

0

0

0

0

0

0

0

0

0.0484412

0

0

0

0

0

0

0
7

0

0

0

0

0

0

0

0

0

0

0
3

0

0.0968825

0

0

0

0

0

0

0

0

0.145324

0

0

0

0

0

0

0
7

0

5.55111512312578e-16
3

0
4

0

0

0

0
4

0.0726619

0.0726619

0
4

0

0

0
4

0
4

0
4

0
4

0

0

0

0

0

0

0
4

0
4

0
4

0
4

208.467
2

0

0

0

0

0

0

0
4

0
4

0
4

0

0

0

0

0
4

0
4

0
4

0

0

0

0

0

0

0

0
4

0

0

0
4

0
4

0
4

0

0

0

0

0
4

0
4

0
4

0

0

0

0

0
4

0
4

0
4

0

0

0

0

0
4

0
4

0
4

0

0

0

0

0
4

0
4

0
4

0

0

0

0

0
4

0
4

0
4

0

0

0

0

0
4

0
4

0
4

0

0

0

0

0
4

0
4

0
4

208.467
2

0

0

0

0

0
4

0

0

0
4

0
4

0

0

0

0

0
4

0
4

0

0

0

0
4

0
4

0

0

0

0
4

0
4

0

0

0

0
4

0
4

0

0

0

0
4

0
4

0

0

0

0
4

0
4

0

0

0

0
4

0
4

0

0

0

0
4

0
4

208.467
2

208.467
2

0

208.467
2

0
2

0
4

0

0

0

0
4

0

0

0
4

0

0

0
4

0

0

0
4

0

0

0
4

0

0

0
4

0

0

0
4

0

0

0
4

0

0

0
4

0

0

0
4

0

0

0
4

0
4

0
4

0
4

6.57781
2

6.57781
2

6.57781
2

6.57781
2

1.97485

0

0

0

2.63314

0.855696

0

0

0.982466

0

0.131657

3.60822483003176e-16
2

0
4

0
4

0
4

0
4

0

0

0

0

0

0
4

0
4

0
4

0
4

16.6187
2

0
2

0
2

0

0

0

0

0

0

0

0

0

0

0

0

0

0

0

0

0

0

0

0

0

0

0

0

0

0

0

0

0

0

0

0

0

0

0

0

0

0

0

0

0

0

0

0

0

0

0

0

0

0

0

0

0

0

0

0

0

0

0

0

0

0

0

0

0

0

0

0

0

0

0

0

0

0

0

0

0

0

0

0

0

0

0

0

0

0

0

0

0

0

0
4

0

0

0
4

0

0

0
4

0

0

0
4

0

0

0

0

0

0

0

0

0

0

0

0

0

0

0

0

0

0

0

0

0

0

0

0

0

0

0

0

0
4

0

0

0

0

0

0

0

0

0

0
4

0

0

0

0
4

0

0

0

0

0
4

0

0

0

0
4

0

0

0

0
4

0

0

0

0
4

0

0

0
4

0
4

0
4

0
4

0
4

0
4

0

0

0

0

0

0

0
4

0
4

0
4

0
7

0
7

0
7

0

0

0

0

0
4

0
4

0
4

0
4

0
4

0

0

0

0

0
4

0

0

0
4

0

0

0
4

0

0

0
4

0
4

0
4

0.570464

0.570464

0.570464

0.443694

0.12677

0
4

0
4

0
4

0

0

0

0

0

0

0
4

0

0

0

0
4

0
4

0
4

0

0

0

0

0
4

0
4

0
4

0

0

0

0

0

0
4

0

0

0
4

0
4

0
4

0.131657

0.131657

0.131657

0.131657

0
4

0

0

0
4

0
4

0
4

0

0

0

0

0
4

0

0

0
4

0
4

0

0

0

0
4

0
4

0
4

0.238927

0.238927

0.238927

0.238927

0
4

0
4

0
4

7.88377
3

1.63399
3

0

0

0

0

0

0

0

0

0

0

0

0

0

0

0

0

0

0

0

0
4

1.63399
3

1.63399

0

0

0

0
4

0

0

0

0
4

0

0

0
4

0

0

0
4

0

0

0
4

0
4

6.24978

2.48146
2

2.48146
2

0

0

0

0

0

0

0

0
4

0

0

0

0

0
4

3.23263

0

0

0

3.23263

0

0
4

0

0

0
4

0

0

0
4

0.129121

0.129121

0
4

0

0

0
4

0

0

0
4

0.40657

0.40657

0
4

0
4

0

0

0

0

0

0
4

0

0

0
4

0
4

0

0

0

0
4

0

0

0
4

0
4

0

0

0

0
4

0
4

0

0

0

0
4

0
4

0

0

0

0
4

0
4

0
4

0

0

0

0

0

0
4

0

0

0
4

0
4

0
4

0

0

0

0

0
4

0
4

0
4

0

0

0

0

0
4

0

0

0
4

0

0

0
4

0
4

0
4

0

0

0

0

0
4

0
4

0
4

0

0

0

0

0
4

0

0

0
4

0
4

0
4

0

0

0

0

0
4

0
4

0
4

0

0

0

0

0
4

0
4

0
4

0

0

0

0

0
4

0

0

0
4

0
4

0
4

0

0

0

0

0
4

0

0

0
4

0
4

0
4

0

0

0

0

0
4

0
4

0
4

0

0

0

0

0

0

0

0

0

0

0

0

0

0

0

0

0
4

0

0

0

0
4

0

0

0
4

0

0

0
4

0
4

0

0

0

0

0

0

0
4

0

0

0
4

0
4

0
4

0

0

0

0

0

0
4

0
4

0
4

0

0

0

0

0

0
4

0
4

0
4

0

0

0

0

0
4

0

0

0
4

0
4

0
4

0

0

0

0

0
4

0

0

0
4

0
4

0
4

0

0

0

0

0
4

0
4

0
4

0

0

0

0

0
4

0
4

0
4

0

0

0

0

0
4

0
4

0
4

0

0

0

0

0
4

0
4

0
4

0

0

0

0

0
4

0
4

0
4

0.0950773

0.0950773

0.0950773

0.0950773

0
4

0
4

0
4

0

0

0

0

0

0

0

0

0

0

0

0

0

0
4

0

0

0

0

0

0
4

0

0

0

0
4

0

0

0
4

0
4

0

0

0

0
4

0
4

0

0

0

0
4

0
4

0
4

0

0

0

0

0
4

0
4

0
4

0

0

0

0

0
4

0
4

0
4

0

0

0

0

0
4

0
4

0
4

0

0

0

0

0
4

0
4

0
4

0

0

0

0

0
4

0
4

0
4

0

0

0

0

0
4

0
4

0
4

0

0

0

0

0
4

0
4

0
4

0

0

0

0

0
4

0
4

0
4

0

0

0

0

0
4

0
4

0
4

0

0

0

0

0
4

0
4

0
4

1.70887

1.70887

1.70887

1.70887

0

0

0

0
4

0

0

0
4

0
4

0
4

0

0

0

0

0
4

0
4

0
4

0

0

0

0

0
4

0
4

0
4

0

0

0

0

0
4

0
4

0
4

0

0

0

0

0
4

0
4

0
4

0

0

0

0

0
4

0
4

0
4

0

0

0

0

0
4

0
4

0
4

0

0

0

0

0
4

0
4

0
4

0

0

0

0

0
4

0
4

0
4

0

0

0

0

0
4

0
4

0
4

0

0

0

0

0
4

0
4

0
4

0

0

0

0

0

0

0

0
4

0

0

0

0
4

0

0

0
4

0
4

0
4

0

0

0

0

0
4

0
4

0
4

0

0

0

0

0
4

0
4

0
4

0

0

0

0

0
4

0
4

0
4

0

0

0

0

0
4

0
4

0
4

0

0

0

0

0
4

0
4

0
4

0

0

0

0

0
4

0
4

0
4

0

0

0

0

0
4

0
4

0
4

0

0

0

0

0
4

0
4

0
4

0

0

0

0

0
4

0
4

0
4

0

0

0

0

0
4

0
4

0
4

4.56964
2

4.56964
2

4.56964
2

4.56964

0

0
4

0

0

0

0
4

0

0

0
4

0

0

0
4

0
4

0
4

0

0

0

0

0
4

0
4

0
4

0

0

0

0

0
4

0
4

0
4

0

0

0

0

0
4

0
4

0
4

0

0

0

0

0

0

0
4

0
4

0
4

0

0

0

0

0

0

0
4

0
4

0
4

1.42033

0

0

0

0

0

0

0

0

0
4

0

0

0

0
4

0

0

0

0
4

0

0

0
4

0

0

0
4

0

0

0
4

0
4

0

0

0

0

0
4

0

0

0

0
4

0

0

0
4

0
4

0

0

0

0

0
4

0
4

0

0

0

0
4

0
4

0

0

0

0
4

0

0

0
4

0
4

0

0

0

0
4

0
4

0

0

0

0
4

0
4

0

0

0

0
4

0
4

0

0

0

0
4

0
4

0

0

0

0
4

0
4

0

0

0

0
4

0
4

0
7

0
7

0
7

0

0

0

0
4

0
4

0

0

0

0
4

0
4

0

0

0

0
4

0
4

0

0

0

0
4

0
4

0

0

0

0
4

0
4

0

0

0

0
4

0
4

0

0

0

0
4

0
4

0

0

0

0
4

0
4

0

0

0

0
4

0
4

0

0

0

0
4

0
4

0

0

0

0
4

0
4

0
6

0

0

0

0

0
4

0

0

0

0
4

0
4

0

0

0

0
4

0
4

0

0

0

0
4

0
4

0

0

0

0
4

0
4

0

0

0

0
4

0
4

0

0

0

0

0

0

0
4

0

0

0
4

0
4

0
7

0
7

0
7

0

0
4

0

0

0
4

0
4

0

0

0

0

0

0

0
4

0

0

0
4

0
4

0
8

0
8

0
8

0

0
4

0
4

0

0
6

0

0

0
4

0

0

0
4

0

0

0
4

0
4

1.42033
3

1.42033

1.42033

0

0

0
4

0

0

0
4

0

0

0
4

0
4

0

0

0
7

0

0

0

0

0

0

0

0

0

0

0

0

0

0

0

0

0

0

0

0

0

0

0

0

0

0

0

0

0

0

0

0

0

0

0

0

0

0

0

0
4

0
7

0
7

0
4

0
4

0
4

3.99680288865056e-15
2

0
4

0
7

0

0

0

0

0

0

0
4

0
4

0
4

0

0

0

0

0
4

0
4

0
4

0

0

0

0

0
4

0
4

0
4

0

0

0

0

0
4

0
4

0
4

0

0

0

0

0
4

0
4

0
4

0
7

0

0

0

0

0

0
4

0
4

0

0

0

0
4

0
4

0

0

0

0
4

0
4

0

0

0

0
4

0
4

0

0

0

0
4

0
4

0
7

0
7

0
7

0

0

0

0

0

0

0
4

0

0

0
4

0
4

0
4

0
4

1.94208

1.94208

1.94208

0.937558

0.87059

0.0669684

2.77555756156289e-17

0
4

0

0

0
4

0

0

0
4

1.00453

1.00453

0
4

2.22044604925031e-16

0
4

0
4

0
4

0

0

0

0

0

0
4

0
4

0
4

0

0
7

0
7

0
7

0
4

0
4

0

0

0

0
4

0
4

0

0

0

0
4

0
4

0

0

0

0

0
4

0

0

0
4

0
4

0
4

0
4

62.4299
3

0

0

0

0

0
4

0
4

0
4

0

0

0

0

0
4

0
4

0
4

62.4299
3

62.4299
3

62.4299
3

20.0783
3

0

1.74313

0

0

0

0.129121

5.10027

0

0.129121

0.258242

24.4684
3

5.03571

0.581044
3

0

4.32555

0

0.322802

0.258242

0
4

0
4

0
4

0
4

0
7

0
7

0
7

0
7

0

0

0

0

0

0

0

0

0

0

0

0

0

0

0

0

0

0
6

0

0

0

0

0
4

0
4

0
4

0
4

76.4297

5.76102
4

5.2561
4

4.99786
4

4.64772
4

0

0

0

0

0

0

0

0

0

0

0

0.194522

0

0

0

0

0
4

0
4

0.155618
4

0

0

0

0

0
4

0.258242
3

0
4

0

0

0.258242

0

0
4

0

0

0
4

0
4

0.310395
4

0.310395
4

0

0

0

0

0

0

0

0

0

0

0

0
4

0

0

0

0

0

0
4

0

0

0

0.310395

0

0

0
4

0

0

0

0
4

0

0

0
4

0

0

0
4

0

0

0
4

0
4

0.194522

0

0

0
4

0.194522

0.194522

0
4

0
4

0

0

0

0
4

0
4

0
4

0
5

0
5

0
5

0
5

0

0

0

0

0
4

0
4

0

0

0

0

0
4

0

0

0
4

0
4

0
4

0.233427

0.233427

0

0

0
4

0.233427

0.233427

0
4

0
4

0

0

0

0
4

0
4

0

0

0

0
4

0
4

0
4

0

0

0

0

0

0
4

0

0

0
4

0
4

0

0

0

0
4

0
4

0
4

0

0

0

0

0
4

0

0

0
4

0
4

0
4

0

0

0

0

0

0
4

0

0

0
4

0
4

0
4

0

0

0

0

0

0
4

0

0

0
4

0
4

0
4

0

0

0

0

0

0
4

0
4

0
4

0

0

0

0

0

0
4

0
4

0
4

0

0

0

0

0

0
4

0

0

0
4

0

0

0
4

0
4

0
4

0

0

0

0

0
4

0
4

0
4

1.88708

1.88708

1.88708

1.49211

0.394971

0
4

0
4

0
4

0

0

0

0

0

0

0
4

0

0

0

0

0

0

0
4

0
4

0
4

0

0

0

0

0

0
4

0

0

0

0
4

0
4

0
4

0

0

0

0

0

0
4

0

0

0
4

0
4

0
4

0

0

0

0

0

0

0
4

0
4

0
4

0

0

0

0

0

0
4

0
4

0
4

0

0

0

0

0
4

0

0

0
4

0
4

0
4

0

0

0

0

0
4

0

0

0
4

0
4

0
4

0.138909

0.138909

0.138909

0.138909

0
4

0
4

0
4

0

0

0

0

0

0
4

0
4

0
4

0

0

0

0

0
4

0

0

0
4

0
4

0

0

0

0
4

0
4

0
4

0

0

0

0

0
4

0
4

0
4

0

0

0

0

0

0

0

0

0
4

0

0

0

0

0
4

0

0

0
4

0

0

0
4

0
4

0
4

0.077809

0

0

0

0
4

0

0

0
4

0
4

0.077809

0.077809

0.077809

0
4

0
4

0
4

0

0

0

0

0
4

0
4

0
4

0

0

0

0

0
4

0
4

0
4

0

0

0

0

0

0

0
4

0
4

0
4

0

0

0

0

0

0
4

0
4

0
4

0

0

0

0

0
4

0
4

0
4

0

0

0

0

0

0
4

0

0

0
4

0
4

0
4

0

0

0

0

0

0
4

0
4

0
4

0

0

0

0

0

0
4

0
4

0

0

0

0
4

0
4

0
4

0

0

0

0

0

0
4

0
4

0
4

0
4

0
4

0
4

0
4

0

0

0
4

0

0

0
4

0

0

0
4

0

0

0
4

0

0

0
4

0

0

0
4

0
4

0
4

0.0347274

0.0347274

0.0347274

0.0347274

0
4

0
4

0
4

0

0

0

0

0
4

0
4

0
4

0

0

0

0

0
4

0

0

0
4

0

0

0
4

0
4

0
4

0

0

0

0

0
4

0
4

0
4

0

0

0

0

0
4

0

0

0
4

0
4

0
4

0

0

0

0

0

0
4

0
4

0
4

0

0

0

0

0
4

0

0

0
4

0
4

0
4

0

0

0

0

0
4

0
4

0
4

0

0

0

0

0
4

0
4

0
4

0

0

0

0

0

0
4

0

0

0
4

0
4

0
4

0

0

0

0

0

0

0
4

0

0

0

0

0
4

0
4

0
4

0

0

0

0

0

0
4

0
4

0

0

0

0
4

0
4

0
4

0

0

0

0

0

0
4

0
4

0
4

0

0

0

0

0
4

0
4

0
4

0

0

0

0

0
4

0
4

0
4

0

0

0

0

0

0
4

0
4

0
4

0

0

0

0

0
4

0

0

0
4

0
4

0
4

0

0

0

0

0
4

0
4

0
4

0

0

0

0

0

0
4

0

0

0
4

0
4

0
4

0

0

0

0

0
4

0
4

0
4

0

0

0

0

0
4

0

0

0
4

0
4

0
4

0
4

0
4

0
4

0
4

0

0
4

0
4

0
4

0

0

0

0

0
4

0

0

0
4

0
4

0
4

0

0

0

0

0

0
4

0
4

0
4

0.258242

0.258242

0.258242

0

0.258242

0
4

0

0

0
4

0
4

0
4

0

0

0

0

0
4

0

0

0
4

0
4

0
4

0

0

0

0

0

0
4

0
4

0
4

0

0

0

0

0
4

0

0

0
4

0
4

0
4

0

0

0

0

0
4

0
4

0
4

0

0

0

0

0
4

0

0

0
4

0
4

0
4

0

0

0

0

0
4

0

0

0
4

0
4

0
4

0

0

0

0

0
4

0

0

0
4

0
4

0
4

3.78614
3

3.78614
3

3.63053
3

0
3

3.63053

0

0

0
4

0.155618

0.155618

0
4

1.94289029309402e-16
3

0
4

0
4

0

0

0

0

0
4

0

0

0
4

0
4

0
4

0

0

0

0

0

0
4

0
4

0
4

0

0

0

0

0

0
4

0
4

0
4

0

0

0

0

0
4

0

0

0
4

0
4

0
4

0

0

0

0

0
4

0
4

0
4

0

0

0

0

0
4

0

0

0
4

0
4

0
4

0

0

0

0

0
4

0
4

0
4

0

0

0

0

0

0
4

0
4

0
4

0

0

0

0

0
4

0
4

0
4

0

0

0

0

0
4

0
4

0
4

0

0

0

0

0

0

0

0
4

0
4

0
4

0

0

0

0

0
4

0
4

0
4

0

0

0

0

0

0
4

0
4

0
4

0

0

0

0

0
4

0
4

0
4

0

0

0

0

0
4

0

0

0
4

0
4

0
4

0

0

0

0

0
4

0
4

0
4

0

0

0

0

0
4

0

0

0
4

0
4

0
4

0

0

0

0

0
4

0
4

0
4

0

0

0

0

0
4

0
4

0
4

0

0

0

0

0
4

0

0

0
4

0
4

0
4

0

0

0

0

0
4

0
4

0
4

4.19643

4.19643

4.19643

4.19643

0
4

0
4

0
4

0

0

0

0

0
4

0
4

0
4

0.0484412

0.0484412

0.0484412

0.0484412

0
4

0

0

0
4

0
4

0
4

0

0

0

0

0
4

0

0

0
4

0
4

0
4

0

0

0

0

0

0
4

0
4

0
4

0

0

0

0

0
4

0

0

0
4

0
4

0
4

0

0

0

0

0
4

0

0

0
4

0
4

0
4

0

0

0

0

0
4

0
4

0
4

0

0

0

0

0
4

0

0

0
4

0
4

0
4

0

0

0

0

0
4

0
4

0
4

0

0

0

0

0

0
4

0
4

0
4

0
6

0
6

0
6

0
6

0

0
4

0

0

0
4

0

0

0
4

0
4

0
4

0

0

0

0

0
4

0
4

0
4

0

0

0

0

0
4

0
4

0
4

0

0

0

0

0

0
4

0
4

0
4

0

0

0

0

0
4

0

0

0
4

0
4

0
4

0

0

0

0

0

0
4

0
4

0
4

0

0

0

0

0
4

0
4

0
4

0

0

0

0

0
4

0
4

0
4

0

0

0

0

0
4

0

0

0
4

0
4

0
4

0.774725

0.774725

0.774725

0.774725

0

0
4

0
4

0
4

0

0

0

0

0
4

0

0

0
4

0
4

0
4

5.99445
4

5.99445
4

5.99445
4

5.99445
4

0

0

0

0

0

0

0

0

0

0

0

0

0

0

0

0
4

0

0

0
4

0

0

0
4

0
4

0

0

0

0

0

0

0

0
4

0

0

0

0

0

0
4

0

0

0
4

0

0

0
4

0

0

0
4

0

0

0
4

0

0

0
4

0

0

0
4

0
4

0
4

0
4

0
4

0

0

0

0

0
4

0

0

0

0

0

0
4

0

0

0
4

0

0

0
4

0
4

0
4

0

0

0

0

0
4

0
4

0
4

0

0

0

0

0
4

0
4

0
4

0

0

0

0

0

0
4

0
4

0
4

0

0

0

0

0
4

0
4

0
4

0

0

0

0

0
4

0
4

0
4

0

0

0

0

0

0
4

0
4

0
4

0.116713

0.116713

0.116713

0.116713

0
4

0
4

0
4

0

0

0

0

0
4

0
4

0
4

0

0

0

0

0
4

0

0

0
4

0
4

0
4

0

0

0

0

0
4

0
4

0
4

0

0

0

0

0

0

0
4

0
4

0
4

0

0

0

0

0
4

0

0

0
4

0
4

0
4

0

0

0

0

0
4

0

0

0
4

0
4

0
4

0

0

0

0

0

0
4

0
4

0
4

0

0

0

0

0
4

0

0

0
4

0
4

0
4

0

0

0

0

0
4

0
4

0
4

0

0

0

0

0
4

0

0

0
4

0
4

0
4

0

0

0

0

0
4

0

0

0
4

0
4

0
4

0

0

0

0

0
4

0

0

0
4

0
4

0
4

0

0

0

0

0
4

0
4

0
4

0

0

0

0

0
4

0
4

0
4

0
6

0
6

0

0

0

0
4

0

0

0

0
4

0
4

0
4

0

0

0

0

0
4

0
4

0
4

0

0

0

0

0
4

0
4

0
4

0

0

0

0

0
4

0
4

0
4

0

0

0

0

0
4

0
4

0
4

0

0

0

0

0
4

0
4

0
4

0

0

0

0

0
4

0
4

0
4

0

0

0

0

0
4

0
4

0
4

0

0

0

0

0
4

0
4

0
4

0

0

0

0

0
4

0
4

0
4

0

0

0

0

0
4

0
4

0
4

0

0

0

0

0

0

0
4

0
4

0
4

0

0

0

0

0
4

0
4

0
4

0

0

0

0

0
4

0
4

0
4

0

0

0

0

0
4

0
4

0
4

0

0

0

0

0
4

0
4

0
4

0

0

0

0

0
4

0
4

0
4

0

0

0

0

0
4

0
4

0
4

0

0

0

0

0
4

0
4

0
4

0

0

0

0

0
4

0
4

0
4

0

0

0

0

0
4

0
4

0
4

0

0

0

0

0
4

0
4

0
4

0

0

0

0

0

0

0
4

0
4

0
4

0

0

0

0

0
4

0
4

0
4

0

0

0

0

0
4

0
4

0
4

0

0

0

0

0
4

0
4

0
4

0

0

0

0

0
4

0
4

0
4

0

0

0

0

0
4

0
4

0
4

0

0

0

0

0
4

0
4

0
4

0

0

0

0

0
4

0
4

0
4

0

0

0

0

0
4

0
4

0
4

0

0

0

0

0
4

0
4

0
4

0

0

0

0

0
4

0
4

0
4

0

0

0

0

0

0

0
4

0
4

0
4

0

0

0

0

0
4

0
4

0
4

0

0

0

0

0
4

0
4

0
4

0

0

0

0

0
4

0
4

0
4

0

0

0

0

0
4

0
4

0
4

0

0

0

0

0
4

0
4

0
4

0

0

0

0

0
4

0
4

0
4

0.692542

0.692542

0.692542

0.692542

0
4

0
4

0
4

0

0

0

0

0
4

0
4

0
4

0

0

0

0

0
4

0
4

0
4

0

0

0

0

0
4

0
4

0
4

0

0

0

0

0

0

0

0

0
4

0
4

0
4

0

0

0

0

0
4

0
4

0
4

0

0

0

0

0
4

0
4

0
4

0

0

0

0

0
4

0
4

0
4

0

0

0

0

0
4

0
4

0
4

0

0

0

0

0
4

0
4

0
4

0

0

0

0

0
4

0
4

0
4

0

0

0

0

0
4

0
4

0
4

0

0

0

0

0
4

0
4

0
4

0.129121

0.129121

0.129121

0.129121

0
4

0
4

0
4

0

0

0

0

0
4

0
4

0
4

0

0

0

0

0

0
4

0
4

0
4

0

0

0

0

0
4

0
4

0
4

0

0

0

0

0
4

0
4

0
4

0

0

0

0

0
4

0
4

0
4

0

0

0

0

0
4

0
4

0
4

0

0

0

0

0
4

0
4

0
4

0

0

0

0

0
4

0
4

0
4

0

0

0

0

0
4

0
4

0
4

0

0

0

0

0
4

0
4

0
4

0

0

0

0

0
4

0
4

0
4

0

0

0

0

0
4

0
4

0
4

4.88842

4.88842

4.88842

4.18814

0.700281

0

0
4

0
4

0
4

0

0

0

0

0
4

0
4

0
4

0

0

0

0

0
4

0
4

0
4

0

0

0

0

0
4

0
4

0
4

0

0

0

0

0
4

0
4

0
4

0

0

0

0

0
4

0
4

0
4

0

0

0

0

0
4

0
4

0
4

0

0

0

0

0
4

0
4

0
4

0

0

0

0

0
4

0
4

0
4

0.155618

0.155618

0.155618

0.155618

0
4

0
4

0
4

0.129121

0.129121

0.129121

0.129121

0
4

0
4

0
4

0
7

0
7

0
6

0
6

0
4

0

0

0
4

0
4

0

0

0

0
4

0
4

0
4

0

0

0

0

0
4

0
4

0
4

0

0

0

0

0
4

0
4

0
4

0

0

0

0

0
4

0
4

0
4

0

0

0

0

0
4

0
4

0
4

0

0

0

0

0
4

0
4

0
4

0

0

0

0

0
4

0
4

0
4

0.394971

0.394971

0.394971

0.394971

0
4

0
4

0
4

0.077809

0.077809

0.077809

0.077809

0
4

0
4

0
4

0

0

0

0

0
4

0
4

0
4

0

0

0

0

0
4

0
4

0
4

0
7

0
7

0
7

0
8

0

0

0

0

0

0
4

0
4

0
4

0.415525
4

0.415525
4

0.277017

0

0.277017

0
4

0

0

0
4

0

0

0
4

0

0

0
4

0

0

0
4

0.138508

0.138508

0
4

0

0

0
4

0
4

0
4

0

0

0

0

0
4

0
4

0
4

0

0

0

0

0
4

0
4

0
4

0

0

0

0

0
4

0
4

0
4

0

0

0

0

0
4

0
4

0
4

0.052091

0.052091

0.052091

0.052091

0
4

0
4

0
4

0

0

0

0

0
4

0
4

0
4

0

0

0

0

0
4

0
4

0
4

0

0

0

0

0
4

0
4

0
4

0

0

0

0

0
4

0
4

0
4

0

0

0

0

0
4

0
4

0
4

0
6

0
6

0
6

0
6

0

0
4

0
4

0
4

0

0

0

0

0
4

0
4

0
4

0

0

0

0

0
4

0
4

0
4

0

0

0

0

0
4

0
4

0
4

0

0

0

0

0
4

0
4

0
4

0

0

0

0

0
4

0
4

0
4

0

0

0

0

0
4

0
4

0
4

0

0

0

0

0
4

0
4

0
4

0

0

0

0

0
4

0
4

0
4

0

0

0

0

0
4

0
4

0
4

0

0

0

0

0
4

0
4

0
4

0
4

0
4

0

0

0

0
4

0

0

0

0
4

0

0

0
4

0
4

0

0

0

0

0
4

0

0

0

0
4

0

0

0
4

0
4

0

0

0

0
4

0
4

0
4

0

0

0

0

0
4

0
4

0
4

0

0

0

0

0
4

0
4

0
4

0

0

0

0

0
4

0
4

0
4

0

0

0

0

0
4

0
4

0
4

0

0

0

0

0
4

0
4

0
4

0

0

0

0

0
4

0
4

0
4

0

0

0

0

0
4

0
4

0
4

0

0

0

0

0
4

0
4

0
4

0

0

0

0

0
4

0
4

0
4

0

0

0

0

0
4

0
4

0
4

0
6

0
6

0
6

0
6

0
4

0

0

0
4

0
4

0
4

0

0

0

0

0
4

0
4

0
4

0

0

0

0

0
4

0
4

0
4

0

0

0

0

0
4

0
4

0
4

0

0

0

0

0
4

0
4

0
4

0

0

0

0

0
4

0
4

0
4

0

0

0

0

0
4

0
4

0
4

0

0

0

0

0
4

0
4

0
4

0

0

0

0

0
4

0
4

0
4

0

0

0

0

0
4

0
4

0
4

0

0

0

0

0
4

0
4

0
4

0
4

0

0

0

0
4

0

0

0
4

0

0

0
4

0

0

0
4

0

0

0
4

0

0

0
4

0
4

0

0

0

0
4

0

0

0
4

0
4

0

0

0

0
4

0
4

0
4

0

0

0

0

0
4

0
4

0
4

0

0

0

0

0
4

0
4

0
4

0

0

0

0

0
4

0
4

0
4

0

0

0

0

0
4

0
4

0
4

0

0

0

0

0
4

0
4

0
4

0

0

0

0

0
4

0
4

0
4

0

0

0

0

0
4

0
4

0
4

0

0

0

0

0
4

0
4

0
4

0

0

0

0

0
4

0
4

0
4

0

0

0

0

0
4

0
4

0
4

0
6

0
6

0
6

0
6

0
4

0

0

0
4

0

0

0
4

0
4

0
4

0

0

0

0

0
4

0
4

0
4

0

0

0

0

0
4

0
4

0
4

0

0

0

0

0
4

0
4

0
4

0

0

0

0

0
4

0
4

0
4

0

0

0

0

0
4

0
4

0
4

0

0

0

0

0
4

0
4

0
4

0

0

0

0

0
4

0
4

0
4

0

0

0

0

0
4

0
4

0
4

0

0

0

0

0
4

0
4

0
4

0

0

0

0

0
4

0
4

0
4

0
4

0
4

0
4

0

0

0

0

0

0

0

0
4

0
4

0
4

0

0

0

0

0
4

0
4

0
4

0

0

0

0

0
4

0
4

0
4

0.138508

0.138508

0.138508

0.138508

0
4

0
4

0
4

0

0

0

0

0
4

0
4

0
4

0

0

0

0

0
4

0
4

0
4

0

0

0

0

0
4

0
4

0
4

0

0

0

0

0
4

0
4

0
4

0

0

0

0

0
4

0
4

0
4

0

0

0

0

0
4

0
4

0
4

0

0

0

0

0
4

0
4

0
4

0
7

0
7

0
7

0
7

0
4

0
4

0
4

0

0

0

0

0
4

0
4

0
4

0

0

0

0

0
4

0
4

0
4

0

0

0

0

0
4

0
4

0
4

0

0

0

0

0
4

0
4

0
4

0

0

0

0

0
4

0
4

0
4

0

0

0

0

0
4

0
4

0
4

0

0

0

0

0
4

0
4

0
4

0

0

0

0

0
4

0
4

0
4

0

0

0

0

0
4

0
4

0
4

0

0

0

0

0
4

0
4

0
4

0
4

0
4

0
4

0
4

0

0

0
4

0
4

0
4

0

0

0

0

0
4

0
4

0
4

0

0

0

0

0
4

0
4

0
4

0

0

0

0

0
4

0
4

0
4

0

0

0

0

0
4

0
4

0
4

0

0

0

0

0
4

0
4

0
4

0

0

0

0

0
4

0
4

0
4

0

0

0

0

0
4

0
4

0
4

0

0

0

0

0
4

0
4

0
4

0

0

0

0

0
4

0
4

0
4

0

0

0

0

0
4

0
4

0
4

2.70091
3

2.70091
3

2.70091
3

2.70091
3

0
4

0
4

0
4

0

0

0

0

0
4

0
4

0
4

0

0

0

0

0
4

0
4

0
4

0

0

0

0

0
4

0
4

0
4

0

0

0

0

0
4

0
4

0
4

0

0

0

0

0
4

0
4

0
4

0

0

0

0

0
4

0
4

0
4

0

0

0

0

0
4

0
4

0
4

0

0

0

0

0
4

0
4

0
4

0

0

0

0

0
4

0
4

0
4

0

0

0

0

0
4

0
4

0
4

0
5

0
5

0
5

0

0

0

0

0

0

0

0
4

0
4

0

0

0

0

0
4

0
4

0
4

0

0

0

0

0

0

0
4

0

0

0

0
4

0

0

0

0
4

0
4

0
4

0

0

0

0

0

0

0

0
4

0
4

0
4

0

0

0

0

0
4

0
4

0
4

0

0

0

0

0
4

0
4

0
4

0

0

0

0

0
4

0
4

0
4

0

0

0

0

0
4

0
4

0
4

0

0

0

0

0
4

0
4

0
4

0

0

0

0

0
4

0
4

0
4

0

0

0

0

0
4

0
4

0
4

0

0

0

0

0
4

0
4

0
4

0

0

0

0

0
4

0
4

0
4

0

0

0

0

0
4

0
4

0
4

0

0

0

0

0
4

0
4

0
4

0

0

0

0

0
4

0
4

0
4

0

0

0

0

0
4

0
4

0
4

0

0

0

0

0
4

0
4

0
4

0

0

0

0

0
4

0
4

0
4

0

0

0

0

0
4

0
4

0
4

0

0

0

0

0
4

0
4

0
4

0

0

0

0

0
4

0
4

0
4

0

0

0

0

0
4

0
4

0
4

0

0

0

0

0
4

0
4

0
4

0

0

0

0

0
4

0
4

0
4

0
4

0
4

0

0

0

0
4

0

0

0
4

0

0

0
4

0

0

0
4

0

0

0
4

0

0

0
4

0

0

0
4

0
4

0
4

0

0

0

0

0
4

0
4

0
4

0

0

0

0

0
4

0
4

0
4

0

0

0

0

0
4

0
4

0
4

0

0

0

0

0
4

0
4

0
4

0

0

0

0

0
4

0
4

0
4

0

0

0

0

0
4

0
4

0
4

0

0

0

0

0
4

0
4

0
4

0

0

0

0

0
4

0
4

0
4

0

0

0

0

0
4

0
4

0
4

0

0

0

0

0
4

0
4

0
4

0
4

0
4

0
4

0
4

0

0
4

0

0

0
4

0
4

0
4

0

0

0

0

0
4

0
4

0
4

0

0

0

0

0
4

0
4

0
4

0

0

0

0

0
4

0
4

0
4

0

0

0

0

0
4

0
4

0
4

0

0

0

0

0
4

0
4

0
4

0

0

0

0

0
4

0
4

0
4

0

0

0

0

0
4

0
4

0
4

0

0

0

0

0
4

0
4

0
4

0

0

0

0

0
4

0
4

0
4

0

0

0

0

0
4

0
4

0
4

0

0

0

0

0

0
4

0
4

0
4

0

0

0

0

0
4

0
4

0
4

0

0

0

0

0
4

0
4

0
4

0

0

0

0

0
4

0
4

0
4

0
4

0
4

0
4

0
4

0
4

0
4

0
4

0.0950773
3

0.0950773

0.0950773

0.0950773

0

0
4

0

0

0

0
4

0

0

0
4

0
4

0

0

0

0

0
4

0

0

0
4

0
4

0
4

0
4

0

0

0

0
4

0

0

0
4

0

0

0
4

0
4

0

0

0

0
4

0
4

0
4

0

0

0

0

0

0

0
4

0

0

0
4

0
4

0
4

0

0

0

0

0
4

0

0

0

0
4

0
4

0

0

0

0
4

0
4

0
4

0
7

0

0

0

0

0

0

0

0

0

0
4

0

0

0
4

0
4

0

0

0

0
4

0
4

0
4

0

0

0

0

0

0

0

0
4

0
4

0

0

0

0
4

0
4

0
4

0
7

0
7

0
7

0
7

0

0
4

0

0

0
4

0
4

0
4

0

0

0

0

0

0

0
4

0

0

0
4

0
4

0
4

0

0

0

0

0

0
4

0
4

0
4

0

0

0

0

0

0

0
4

0

0

0

0

0
4

0
4

0
4

1.45324

1.45324

0

0

0

0

0
4

1.45324

1.45324

0
4

0
4

0
4

0

0

0

0

0

0

0
4

0

0

0
4

0

0

0
4

0
4

0
4

0

0

0

0

0

0
4

0
4

0
4

0
6

0
6

0
6

0

0

0
4

0
4

0
4

0

0

0

0

0

0

0
4

0
4

0
4

0
4

0
4

0
4

0
5

0

0

0

0
4

0

0

0
4

0

0

0
4

0
4

0
4

0

0

0

0

0

0
4

0
4

0
4

0
6

0
6

0
6

0
6

0
4

0
4

0
4

0

0

0

0

0

0
4

0

0

0
4

0

0

0
4

0
4

0

0

0

0
4

0
4

0
4

0

0

0

0

0
4

0

0

0
4

0
4

0

0

0

0
4

0
4

0

0

0

0
4

0
4

0
4

0

0

0

0

0

0
4

0
4

0
4

0

0

0

0

0

0
4

0
4

0
4

0
7

0
7

0

0

0

0

0
4

0

0

0
4

0

0

0
4

0
4

0
4

0

0

0

0

0

0
4

0

0

0
4

0
4

0
4

0

0

0

0

0

0

0
4

0

0

0
4

0
4

0
4

0

0

0

0

0

0
4

0

0

0
4

0

0

0
4

0
4

0
4

1.98413
3

1.98413
3

1.98413
3

0.194522

1.63399

0

0.155618

0

0
4

0

0

0

0
4

0
4

0
4

0

0

0

0

0

0
4

0

0

0

0
4

0
4

0
4

0

0

0

0

0

0
4

0

0

0
4

0

0

0
4

0
4

0
4

0
7

0
7

0
7

0
7

0

0
4

0
4

0
4

0
7

0
7

0
7

0
7

0
4

0
4

0
4

0

0

0

0

0
4

0
4

0

0

0

0
4

0

0

0
4

0
4

0
4

0

0

0

0

0
4

0
4

0
4

0
4

0
4

0

0

0
4

0

0

0
4

0
4

0
4

0

0

0

0

0

0

0
4

0

0

0
4

0

0

0
4

0
4

0
4

1.03881
3

1.03881
3

0

0

0

0
4

1.03881

1.03881

0
4

0
4

0
4

0

0

0

0

0
4

0
4

0

0

0

0
4

0

0

0
4

0
4

0
4

0

0

0

0

0

0
4

0
4

0
4

0
6

0
6

0
6

0
6

0
4

0
4

0
4

0

0

0

0

0
4

0

0

0

0
4

0

0

0
4

0
4

0
4

0

0

0

0

0
4

0
4

0

0

0

0
4

0

0

0
4

0
4

0
4

0

0

0

0

0

0
4

0

0

0
4

0
4

0
4

0

0

0

0

0

0
4

0
4

0
4

0
6

0
6

0
6

0
6

0
4

0
4

0
4

0

0

0

0

0

0

0
4

0
4

0
4

0

0

0

0

0
4

0

0

0
4

0

0

0
4

0
4

0
4

0

0

0

0

0

0
4

0

0

0
4

0
4

0
4

0

0

0

0

0

0
4

0
4

0
4

2.49315
3

2.49315
3

2.49315
3

1.59285
3

0.900304
3

0

0

0

0
4

0
4

0
4

0

0

0

0

0

0
4

0

0

0
4

0
4

0
4

0

0

0

0

0

0

0

0
4

0
4

0
4

0

0

0

0

0

0
4

0

0

0
4

0
4

0

0

0

0
4

0
4

0
4

0

0

0

0

0
4

0
4

0
4

0

0

0

0

0

0
4

0
4

0
4

0

0

0

0

0

0

0
4

0
4

0
4

0

0

0

0

0
4

0

0

0
4

0
4

0
4

0

0

0

0

0
4

0
4

0
4

0

0

0

0

0

0
4

0

0

0
4

0
4

0
4

0

0

0

0

0
4

0
4

0
4

36.2825

15.5736

4.70744

0

0

0

0

0

0

0

0

0

0

0

0

0

0

0

0

0

0

0

0

0

0

0

0

0

0

0

0

0

0

0

0

0

0

0

0

0

0

0

0

0

0

0

2.87893

0

0

0

0

0

0

0

0

0

0

0

1.82851

0

0

0
4

9.12304

7.54307

0.348169

0
5

0

0

0

0

0

0

0

0

0

0

0

0

0

0

0

0

0

0

0

0

0

0

0

0

0

0

0

0

0

0

0

0

0

0

0

0

0

0

0

0

0

0

0

0

0

0

0

0

0

0

0

0

0

0

0

0

0

0

0

0

0

0

0

0

0

0

0

0

0

0

0

0

0

0

0

0

0

0

0

0

0

0

0

0

0

0

0

0

0

0.272331

0

0

0

0

0

0

0

0

0

0

0

0

0

0

0

0

0

0

0

0

0

0

0

0

0

0

0

0

0

0

0

0

0

0

0

0

0

0

0

0

0

0

0
5

0

0

0

0

0

0

0

0

0

0

0
7

0

0

0

0

0

0

0.885548

0

0

0

0

0

0

0

0

0

0

0

0

0

0

0
7

0

0

0

0

0

0

0

0

0

0

0

0

0

0

0

0

0

0

0

0

0

0

0

0

0

0

0

0

0

0

0

0

0

0

0.0739218

0

0

0

0

0

0

0

0

0
4

0
7

0
7

0
8

0

0

0

0

0

0

0

0
4

0

0

0
4

0

0

0
4

0

0

0
4

0

0

0

0
4

0

0

0
4

0

0

0
4

0

0

0
4

0

0

0

0
4

0

0

0
4

0

0

0
4

0
7

0
7

0

0
4

0

0

0
4

0

0

0

0
4

0

0

0
4

0

0

0

0
4

0

0

0

0
4

0

0

0

0
4

0

0

0

0
4

0

0

0

0
4

0

0

0
4

0

0

0

0
4

0.711911

0.208364

0.382001

0.121546

0

4.16333634234434e-17

0
4

0

0

0
4

0.427949

0.427949

0
4

0

0

0
4

0

0

0
4

0

0

0
4

0

0

0
4

0

0

0
4

0

0

0
4

0

0

0
4

0

0

0
4

0

0

0

0
4

0

0

0
4

0

0

0
4

0.0347274

0.0347274

0
4

0

0

0
4

0

0

0
4

0

0

0
4

0

0

0
4

0

0

0
4

0.516483

0.516483

0
4

0

0

0
4

0.052091

0.052091

0

0

0

0
4

0

0

0
4

0

0

0
4

0

0

0
4

0

0

0
4

0

0

0
4

0

0

0
4

0

0

0
4

0

0

0
4

0

0

0
4

0

0

0
4

0

0

0

0
4

0

0

0
4

0

0

0
4

0

0

0
4

0

0

0
4

0

0

0
4

0

0

0
4

0

0

0
4

0

0

0
4

0

0

0
4

0

0

0
4

0

0

0
4

0

0

0
4

0

0

0
4

0

0

0
4

0

0

0
4

0

0

0
4

0

0

0
4

0

0

0

0
4

0

0

0

0
4

0
4

0

0

0

0

0

0

0

0

0

0

0

0

0

0

0

0

0

0

0
4

0

0

0

0

0

0

0

0

0
4

0

0

0
4

0
4

0
6

0
6

0
7

0

0
4

0

0

0
4

0

0

0
4

0
4

0

0

0

0
4

0
4

0

0

0

0
4

0
4

0

0

0

0
4

0
4

0

0

0

0
4

0
4

0

0

0

0
4

0
4

0

0

0

0
4

0
4

0

0

0

0
4

0
4

0

0

0

0
4

0
4

0

0

0

0
4

0
4

0

0

0

0
4

0
4

7.35989
3

7.35989
3

7.35989

0

0
4

0

0

0

0
4

0
4

0

0

0

0
4

0
4

0

0

0

0
4

0
4

0

0

0

0
4

0
4

0

0

0

0
4

0
4

0

0

0

0
4

0
4

0

0

0

0
4

0
4

0

0

0

0
4

0
4

0

0

0

0
4

0
4

0

0

0

0
4

0
4

0

0

0

0
4

0
4

0
7

0
7

0
7

0

0
4

0

0

0
4

0
4

0

0

0

0
4

0
4

0

0

0

0
4

0
4

0

0

0

0
4

0
4

0

0

0

0
4

0
4

0

0

0

0
4

0
4

0

0

0

0
4

0
4

0

0

0

0
4

0
4

0

0

0

0
4

0
4

0

0

0

0
4

0
4

0

0

0

0
4

0
4

2.74813

2.74813

2.74813

0

0
4

0
4

0

0

0

0
4

0
4

0

0

0

0
4

0
4

0

0

0

0
4

0
4

0

0

0

0
4

0
4

0

0

0

0
4

0
4

0

0

0

0
4

0
4

0

0

0

0
4

0
4

0

0

0

0
4

0
4

0

0

0

0
4

0
4

0

0

0

0
4

0
4

0
4

0
4

0
4

0
4

0
4

0

0

0

0
4

0
4

0

0

0

0
4

0
4

0

0

0

0
4

0
4

0

0

0

0
4

0
4

0

0

0

0
4

0
4

0

0

0

0
4

0
4

0

0

0

0
4

0
4

0

0

0

0
4

0
4

0

0

0

0
4

0
4

0

0

0

0
4

0
4

0

0

0

0
4

0
4

0

0

0

0
4

0
4

0

0

0

0
4

0
4

0

0

0

0
4

0
4

0

0

0

0
4

0
4

0

0

0

0
4

0
4

0

0

0

0
4

0
4

0

0

0

0
4

0
4

0

0

0

0
4

0
4

0

0

0

0
4

0
4

0

0

0

0
4

0
4

0
4

0

0

0

0
4

0

0

0
4

0

0

0
4

0
4

0

0

0

0
4

0
4

0

0

0

0
4

0
4

0.427949

0.427949

0.427949

0
4

0
4

0

0

0

0
4

0
4

0

0

0

0
4

0
4

0

0

0

0
4

0
4

0

0

0

0
4

0
4

0

0

0

0
4

0
4

0

0

0

0
4

0
4

0

0

0

0
4

0
4

0

0

0

0

0

0
4

0

0

0
4

0

0

0
4

0

0

0
4

0
4

0

0

0

0
4

0
4

0

0

0

0
4

0
4

0

0

0

0
4

0
4

0

0

0

0
4

0
4

0

0

0

0
4

0
4

0

0

0

0
4

0
4

0

0

0

0
4

0
4

0

0

0

0
4

0
4

0

0

0

0
4

0
4

0

0

0

0
4

0
4

0
7

0
7

0
7

0
4

0
4

0

0

0

0
4

0
4

0

0

0

0
4

0
4

0

0

0

0
4

0
4

0

0

0

0
4

0
4

0

0

0

0
4

0
4

0

0

0

0
4

0
4

0

0

0

0
4

0
4

0

0

0

0
4

0
4

0

0

0

0
4

0
4

0

0

0

0
4

0
4

0

0

0

0

0

0
4

0
4

0

0

0

0
4

0
4

0

0

0

0
4

0
4

0

0

0

0
4

0
4

0

0

0

0
4

0
4

0.138508

0.138508

0.138508

0
4

0
4

0

0

0

0
4

0
4

0

0

0

0
4

0
4

0

0

0

0
4

0
4

0

0

0

0
4

0
4

0

0

0

0
4

0
4

0
4

0
4

0
4

0

0

0

0

0

0

0
4

0

0

0
4

0
4

0
6

0
6

0
6

0
4

0
4

0

0

0

0
4

0
4

0

0

0

0
4

0
4

0

0

0

0
4

0
4

0

0

0

0
4

0
4

0

0

0

0
4

0
4

0

0

0

0
4

0
4

0

0

0

0
4

0
4

0

0

0

0
4

0
4

0

0

0

0
4

0
4

0

0

0

0
4

0
4

0

0

0

0

0

0
4

0
4

0

0

0

0
4

0
4

0

0

0

0
4

0
4

0

0

0

0
4

0
4

0

0

0

0
4

0
4

0

0

0

0
4

0
4

0

0

0

0
4

0
4

0

0

0

0
4

0
4

0

0

0

0
4

0
4

0

0

0

0
4

0
4

0

0

0

0
4

0
4

0
3

0

0

0

0
4

0

0

0
4

0

0

0
4

0

0

0
4

0

0

0
4

0
4

0

0

0

0
4

0
4

0.129121

0.129121

0.129121

0
4

0
4

0

0

0

0
4

0
4

0

0

0

0
4

0
4

0

0

0

0
4

0
4

0
3

0
3

0
3

0
4

0
4

0

0

0

0

0
4

0
4

0

0

0

0

0

0

0
4

0
4

0

0

0

0

0
4

0

0

0
4

0

0

0
4

0
4

0
7

0

0

0
4

0

0

0
4

0
4

0
3

0

0

0

0
4

0

0

0
4

0

0

0
4

0

0

0
4

0
4

0

0

0

0

0

0

0

0
4

0

0

0

0
4

0
4

0

0

0

0

0

0
4

0

0

0

0
4

0
4

0

0

0

0
4

0

0

0
4

0

0

0
4

0

0

0
4

0
4

0

0

0

0

0
4

0
4

0

0

0

0
4

0

0

0
4

0
4

0

0

0

0

0

0
4

0
4

0

0

0

0

0

0
4

0
4

0

0

0

0

0

0
4

0
4

0

0

0

0

0
4

0

0

0
4

0
4

0

0

0

0
4

0
4

0

0

0

0
4

0
4

0
7

0
7

0
7

0

0

0
4

0

0

0
4

0
4

0

0

0

0

0
4

0
4

0

0

0

0
4

0
4

0

0

0

0
4

0
4

3.6545

3.6545

3.6545

0
4

0

0

0
4

0
4

0

0

0

0
4

0

0

0
4

0

0

0
4

0
4

0

0

0

0
4

0
4

0

0

0

0
4

0

0

0
4

0

0

0
4

0
4

0

0

0

0
4

0

0

0
4

0
4

0

0

0

0
4

0

0

0
4

0
4

0

0

0

0
4

0
4

5.93956
3

5.93956

0.710164

4.58379

0.645604

0

3.33066907387547e-16

0
4

0

0

0
4

0
4

0

0

0

0

0
4

0
4

0

0

0

0

0
4

0

0

0
4

0
4

0

0

0

0

0
4

0
4

0

0

0

0

0
4

0

0

0
4

0
4

0

0

0

0
4

0

0

0
4

0
4

0

0

0

0
4

0
4

0

0

0

0

0
4

0

0

0
4

0
4

0

0

0

0
4

0
4

0

0

0

0
4

0

0

0
4

0
4

0

0

0

0
4

0

0

0
4

0
4

0
7

0
7

0
7

0
4

0
4

0

0

0

0
4

0
4

0

0

0

0
4

0

0

0
4

0
4

0

0

0

0
4

0
4

0

0

0

0
4

0

0

0
4

0
4

0

0

0

0
4

0
4

0

0

0

0
4

0
4

0

0

0

0
4

0
4

0

0

0

0

0
4

0
4

0

0

0

0
4

0
4

0

0

0

0
4

0
4

0

0

0

0

0

0

0
4

0
4

0

0

0

0
4

0
4

0

0

0

0
4

0

0

0
4

0
4

0

0

0

0

0
4

0
4

0

0

0

0
4

0
4

0

0

0

0
4

0

0

0
4

0
4

0

0

0

0

0
4

0
4

0.311236

0.311236

0.311236

0
4

0
4

0

0

0

0
4

0
4

0

0

0

0
4

0

0

0
4

0
4

0

0

0

0
4

0

0

0
4

0
4

0
4

0
4

0
4

0

0

0
4

0
4

0

0

0

0
4

0
4

0

0

0

0
4

0

0

0
4

0
4

0

0

0

0

0
4

0
4

0

0

0

0
4

0

0

0
4

0
4

0

0

0

0

0
4

0
4

0

0

0

0
4

0
4

0

0

0

0
4

0
4

0

0

0

0
4

0
4

0

0

0

0
4

0
4

0

0

0

0
4

0

0

0
4

0
4

0
6

0
6

0
6

0
4

0
4

0

0

0

0
4

0
4

0

0

0

0
4

0

0

0
4

0
4

0

0

0

0

0
4

0
4

0

0

0

0
4

0
4

0

0

0

0
4

0

0

0
4

0
4

0

0

0

0

0
4

0
4

0

0

0

0

0
4

0
4

0

0

0

0

0
4

0
4

0

0

0

0

0
4

0
4

0

0

0

0
4

0
4

9.27036225562006e-15

0
4

0
4

3.7445
4

3.7445
4

3.7445
4

3.7445
4

3.7445
4

0

0

0

0

0

0

0

0

0

0

0
4

0
4

0
4

0
4

0.9744

0

0

0

0

0
4

0

0

0
4

0
4

0
4

0

0

0

0

0
4

0
4

0
4

0.9744

0.9744

0.9744

0.9744

0

0

0

0
4

0
4

0
4

0
4

0

0

0

0

0

0
4

0
4

0
4

0

0

0

0

0
4

0
4

0
4

0
4

0

0

0

0

0

0
4

0
4

0
4

0
4

3.04195
3

3.04195
3

3.04195
3

3.04195
3

0

0

0.645604

0.387362

0.175543

0.0877713

0.387362

0

1.22665

0.131657

0

2.4980018054066e-16
3

0
4

0
4

0
4

0
4

0

0

0

0

0

0

0
4

0

0

0
4

0

0

0

0
4

0
4

0
4

0
4

20.1891
3

0
4

0
4

0
4

0
4

0

0

0
4

0
4

0
4

0

0

0

0

0
4

0
4

0
4

0

0

0

0

0
4

0
4

0
4

0

0

0

0

0
4

0
4

0
4

0

0

0

0

0
4

0
4

0
4

0

0

0

0

0
4

0
4

0
4

0

0

0

0

0
4

0
4

0
4

0
4

0
4

0
4

0
4

0
4

0
4

0
4

0

0

0

0

0

0
4

0
4

0
4

0

0

0

0

0

0

0

0
4

0
4

0
4

0

0

0

0

0

0
4

0
4

0
4

0

0

0

0

0

0
4

0
4

0
4

0

0

0

0

0

0
4

0
4

0
4

0

0

0

0

0
4

0
4

0
4

0

0

0

0

0
4

0
4

0
4

20.1891
3

0

0

0

0

0
4

0
4

0

0

0

0
4

0
4

0

0

0

0
4

0
4

0

0

0

0
4

0
4

20.1891
3

20.1891
3

20.1891
3

0

0

0

0

0

0

0

0

0
4

0

0

0
4

0

0

0
4

0
4

0
4

0
4

0.814003
5

0

0

0

0

0
4

0
4

0
4

0

0

0

0

0
4

0
4

0
4

0

0

0

0

0
4

0
4

0
4

0

0

0

0

0
4

0
4

0
4

0

0

0

0

0
4

0
4

0
4

0

0

0

0

0
4

0
4

0
4

0

0

0

0

0
4

0
4

0
4

0

0

0

0

0
4

0
4

0
4

0

0

0

0

0
4

0
4

0
4

0

0

0

0

0
4

0
4

0
4

0

0

0

0

0
4

0
4

0
4

0.193681
2

0.193681
2

0

0

0

0
4

0.193681

0

0.193681

0
4

0
4

0
4

0.221765

0.221765

0.221765

0.221765

0
4

0
4

0
4

0

0

0

0

0
4

0
4

0
4

0

0

0

0

0
4

0
4

0
4

0

0

0

0

0
4

0
4

0
4

0

0

0

0

0
4

0
4

0
4

0

0

0

0

0
4

0
4

0
4

0

0

0

0

0
4

0
4

0
4

0

0

0

0

0
4

0
4

0
4

0

0

0

0

0
4

0
4

0
4

0.3072

0.3072

0.3072

0.3072

0
4

0
4

0
4

0

0

0

0

0

0
4

0
4

0
4

0

0

0

0

0
4

0
4

0
4

0

0

0

0

0
4

0
4

0
4

0

0

0

0

0
4

0
4

0
4

0

0

0

0

0
4

0
4

0
4

0

0

0

0

0
4

0
4

0
4

0

0

0

0

0
4

0

0

0
4

0
4

0
4

0

0

0

0

0
4

0

0

0
4

0
4

0
4

0

0

0

0

0
4

0

0

0
4

0
4

0
4

0

0

0

0

0
4

0
4

0
4

0

0

0

0

0
4

0
4

0
4

0

0

0

0

0
4

0
4

0
4

0.0913565
5

0
7

0
7

0

0

0

0

0
4

0

0

0
4

0
4

0

0

0

0
4

0
4

0

0

0

0
4

0
4

0

0

0

0
4

0
4

0

0

0

0
4

0
4

0

0

0

0

0
4

0
4

0

0

0

0
4

0
4

0.0913565

0.0913565

0.0913565

0

0
4

0
4

0

0

0

0
4

0

0

0
4

0
4

0

0

0

0
4

0
4

0

0

0

0
4

0
4

0

0

0

0
4

0
4

0

0

0

0
4

0
4

0
5

0
5

0

0

0

0

0

0

0

0

0

0

0

0

0

0

0

0

0

0

0

0

0

0

0

0

0

0

0

0

0

0

0

0

0

0
5

0

0

0

0

0

0

0

0

0

0

0

0

0

0

0

0

0

0

0
4

0

0

0

0

0
4

0

0

0
4

0

0

0
4

0

0

0
4

0

0

0
4

0
4

0
4

2.77555756156289e-17
5

0
4

42.5025

0
7

0
7

0
7

0
7

0

0

0

0
4

0
6

0
6

0

0

0

0

0

0
4

0

0

0
4

0
4

0
4

0.385196

0.385196

0.25354

0.25354

0
4

0.131657

0.131657

0
4

0
4

0
4

0.077809

0.077809

0.077809

0

0.077809

0
4

0

0

0
4

0
4

0
4

0

0

0

0

0
4

0

0

0
4

0
4

0
4

0

0

0

0

0
4

0
4

0
4

0

0

0

0

0
4

0

0

0
4

0
4

0
4

0

0

0

0

0
4

0
4

0
4

0

0

0

0

0

0
4

0
4

0
4

0

0

0

0

0

0
4

0
4

0
4

0

0

0

0

0
4

0
4

0
4

0

0

0

0

0
4

0
4

0
4

0

0

0

0

0

0
4

0
4

0
4

0

0

0

0

0
4

0
4

0
4

0

0

0

0

0
4

0
4

0
4

0

0

0

0

0
4

0
4

0
4

0

0

0

0

0
4

0
4

0
4

0

0

0

0

0
4

0
4

0
4

0

0

0

0

0
4

0
4

0
4

0

0

0

0

0
4

0
4

0
4

0

0

0

0

0
4

0
4

0
4

0

0

0

0

0
4

0
4

0
4

0

0

0

0

0
4

0
4

0
4

0
7

0
8

0
8

0
8

0
4

0
4

0

0

0

0
4

0
4

0

0

0

0
4

0
4

0
4

0

0

0

0

0
4

0
4

0
4

0
5

0
5

0
5

0

0

0
4

0
4

0
4

0

0

0

0

0

0

0

0
4

0
4

0
4

0

0

0

0

0

0
4

0
4

0
4

0

0

0

0

0

0

0

0
4

0
4

0
4

0

0

0

0

0
4

0

0

0
4

0
4

0

0

0

0
4

0
4

0
4

0

0

0

0

0
4

0

0

0
4

0
4

0

0

0

0
4

0
4

0
4

42.0395

4.19643

4.19643

4.19643

0

0

0

0

0

0

0

0

0

0

0

0

0

0

0

0

0
4

0

0

0
4

0
4

0

0

0

0

0
4

0

0

0
4

0
4

0

0

0

0

0
4

0
4

0.285232

0.285232

0.285232

0
4

0
4

0

0

0

0
4

0
4

0

0

0

0
4

0

0

0
4

0
4

0

0

0

0
4

0

0

0
4

0
4

0

0

0

0

0
4

0
4

0

0

0

0
4

0
4

0

0

0

0
4

0
4

0

0

0

0
4

0
4

1.52359

1.52359

1.52359

0

0

0
4

0

0

0

0

0

0
4

0

0

0
4

0
4

0

0

0

0
4

0
4

0

0

0

0
4

0
4

0

0

0

0
4

0
4

0

0

0

0
4

0
4

0

0

0

0

0

0

0

0
4

0

0

0

0
4

0

0

0

0
4

0
4

0

0

0

0

0

0

0

0
4

0

0

0
4

0
4

1.86986
4

1.86986

1.86986

0

0

0
4

0

0

0
4

0

0

0
4

0
4

0.412002
3

0

0

0

0

0

0

0

0
4

0.412002

0.412002

0

0
4

0

0

0
4

0

0

0
4

0
4

1.2288

1.2288

0.921599

0.219428

0.0877713

0

0
4

0
4

5.29395

5.29395

5.29395

0

0
4

0

0

0

0
4

0
4

0

0

0

0

0
4

0
4

27.2296

23.7898

0
6

0

0

0

0

0

0

0.282813

0

0

0.475054

0.0548139

9.57219
2

0

0

0

0

0

0

0

0

0

0

12.5656
2

0

0

0

0

0

0

0

0.0877713

0

0

0

0

0

0.219428

0

0

0

0

0

0

0

0

0.0633849

0

0

0

0.0633849

0

0

0

0

0

0.278664
2

0

0

0

0

0.0633849

0

0

0.0633849

0

0

0

0

0

0

0

0

0

0

0

0

0

0

0

0

0

0

0
4

3.29258

3.29258

0
7

0

0

0

0

0

0

0

0
4

0

0

0
4

0

0

0
4

0

0

0
4

0

0

0
4

0

0

0
4

0

0

0
4

0

0

0
4

0

0

0
4

0

0

0
4

0.052091

0.052091

0
4

0

0

0
4

0

0

0
4

0

0

0
4

0.0950773

0.0950773

0
4

0

0

0
4

0

0

0
4

0

0

0
4

0

0

0
4

0
4

0
4

0
4

1026.51
3

0

0

0
7

0
7

0

0

0

0

0
4

0

0

0

0
4

0

0

0
4

0

0

0
4

0

0

0
4

0

0

0
4

0

0

0
4

0

0

0
4

0
4

0
4

0

0

0

0

0

0

0
4

0

0

0
4

0

0

0
4

0
4

0
4

0
7

0
7

0
7

0
7

0
4

0
4

0
4

0

0

0

0

0

0

0

0
4

0
4

0
4

0

0

0

0

0
4

0

0

0
4

0

0

0
4

0

0

0
4

0
4

0
4

0
7

0
7

0

0

0

0

0
4

0

0

0

0
4

0
4

0
4

0

0

0

0

0
4

0

0

0
4

0
4

0

0

0

0

0
4

0
4

0
4

0

0

0

0

0

0
4

0
4

0

0

0

0
4

0

0

0
4

0
4

0
4

0

0

0

0

0
4

0
4

0
4

0

0

0

0

0
4

0
4

0

0

0

0
4

0

0

0
4

0
4

0
4

0

0

0

0

0
4

0
4

0
4

0
7

0
7

0
7

0

0

0

0

0

0

0
4

0

0

0

0
4

0

0

0

0
4

0

0

0
4

0
4

0

0

0

0
4

0
4

0
4

0.0739218

0.0739218

0.0739218

0.0739218

0
4

0

0

0
4

0
4

0
4

0

0

0

0

0

0
4

0
4

0
4

0

0

0

0

0
4

0

0

0
4

0
4

0
4

0

0

0

0

0

0
4

0
4

0
4

0

0

0

0

0

0
4

0
4

0
4

0

0

0

0

0
4

0

0

0
4

0
4

0
4

0

0

0

0

0
4

0
4

0
4

0

0

0

0

0
4

0
4

0
4

0

0

0

0

0
4

0
4

0
4

0

0

0

0

0
4

0
4

0
4

19.7555

19.7555

19.7555

10.136

3.03434

0.581044

5.48763

0.258242

0.129121

0.129121

2.33146835171283e-15

0
4

0
4

0
4

0

0

0

0

0
4

0

0

0
4

0
4

0
4

0

0

0

0

0

0
4

0
4

0
4

0

0

0

0

0
4

0
4

0
4

0

0

0

0

0
4

0
4

0
4

0

0

0

0

0

0
4

0
4

0
4

0

0

0

0

0
4

0
4

0
4

0

0

0

0

0
4

0

0

0
4

0
4

0
4

0

0

0

0

0

0
4

0
4

0
4

0

0

0

0

0
4

0
4

0
4

0

0

0

0

0
4

0
4

0
4

0

0

0

0

0

0

0

0

0
4

0

0

0
4

0
4

0
4

0

0

0

0

0

0
4

0
4

0
4

0

0

0

0

0

0
4

0
4

0
4

0

0

0

0

0
4

0

0

0
4

0
4

0
4

0

0

0

0

0
4

0
4

0
4

0

0

0

0

0
4

0
4

0
4

0

0

0

0

0
4

0
4

0
4

0

0

0

0

0
4

0
4

0
4

0

0

0

0

0
4

0
4

0
4

0

0

0

0

0
4

0
4

0
4

0

0

0

0

0
4

0
4

0
4

0
6

0
6

0
6

0

0

0

0
4

0
4

0
4

0

0

0

0

0
4

0
4

0
4

0

0

0

0

0
4

0
4

0
4

0

0

0

0

0
4

0
4

0
4

0

0

0

0

0
4

0
4

0
4

0

0

0

0

0
4

0
4

0
4

0

0

0

0

0
4

0
4

0
4

0

0

0

0

0
4

0
4

0
4

0

0

0

0

0
4

0
4

0
4

0

0

0

0

0
4

0
4

0
4

0

0

0

0

0
4

0
4

0
4

0

0

0

0

0

0
4

0

0

0
4

0
4

0
4

0

0

0

0

0
4

0
4

0
4

0

0

0

0

0
4

0
4

0
4

0

0

0

0

0
4

0
4

0
4

0

0

0

0

0
4

0
4

0
4

0

0

0

0

0
4

0
4

0
4

0

0

0

0

0
4

0
4

0
4

0

0

0

0

0
4

0
4

0
4

0

0

0

0

0
4

0
4

0
4

0

0

0

0

0
4

0
4

0
4

0.0739218

0.0739218

0.0739218

0.0739218

0
4

0
4

0
4

657.935
2

657.935
2

657.935
2

657.935
2

0
4

0
4

0
4

0

0

0

0

0
4

0
4

0
4

0

0

0

0

0
4

0
4

0
4

0

0

0

0

0
4

0
4

0
4

0.129121

0.129121

0.129121

0.129121

0
4

0
4

0
4

0

0

0

0

0
4

0
4

0
4

0

0

0

0

0
4

0
4

0
4

0

0

0

0

0
4

0
4

0
4

0

0

0

0

0
4

0
4

0
4

0

0

0

0

0
4

0
4

0
4

0

0

0

0

0
4

0
4

0
4

0

0

0

0

0
4

0
4

0
4

0

0

0

0

0
4

0
4

0
4

0

0

0

0

0
4

0
4

0
4

0

0

0

0

0
4

0
4

0
4

0

0

0

0

0
4

0
4

0
4

0

0

0

0

0
4

0
4

0
4

0

0

0

0

0
4

0
4

0
4

0

0

0

0

0
4

0
4

0
4

0

0

0

0

0
4

0
4

0
4

0

0

0

0

0
4

0
4

0
4

0

0

0

0

0
4

0
4

0
4

0
7

0
7

0
7

0
7

0

0
4

0
4

0
4

0

0

0

0

0
4

0
4

0
4

0

0

0

0

0
4

0
4

0
4

0

0

0

0

0
4

0
4

0
4

0

0

0

0

0
4

0
4

0
4

0

0

0

0

0
4

0
4

0
4

0

0

0

0

0
4

0
4

0
4

0

0

0

0

0
4

0
4

0
4

0

0

0

0

0
4

0
4

0
4

0

0

0

0

0
4

0
4

0
4

348.542
3

101.992

101.862

13.2349

2.71154

0.710164

0.516483

0.129121

0.129121

0.193681

0.645604

0.258242

0.839285

0.258242

53.0041

0.258242

0.645604

0.903846

0.451923

0.516483

0.839285

0.903846

5.53836

4.26099

4.32555

3.16346

1.61401

4.51923

1.29121

0
4

0.129121

0.129121

0
4

0
4

0

0

0

0

0
4

0

0

0
4

0
4

0

0

0

0

0
4

0
4

0

0

0

0
4

0

0

0
4

0
4

0

0

0

0
4

0
4

0

0

0

0
4

0
4

0

0

0

0
4

0
4

0

0

0

0
4

0

0

0
4

0
4

0

0

0

0

0
4

0
4

0

0

0

0
4

0
4

0

0

0

0
4

0

0

0
4

0
4

0
6

0
6

0
6

0

0
4

0
4

0

0

0

0
4

0
4

0

0

0

0
4

0
4

0

0

0

0
4

0
4

0

0

0

0
4

0
4

0

0

0

0
4

0
4

0

0

0

0
4

0
4

0

0

0

0
4

0
4

0

0

0

0
4

0
4

0

0

0

0
4

0
4

0

0

0

0
4

0
4

0
7

0

0

0
4

0

0

0

0
4

0
4

0

0

0

0
4

0
4

0

0

0

0
4

0
4

0

0

0

0
4

0
4

0

0

0

0
4

0
4

0

0

0

0
4

0
4

0

0

0

0
4

0
4

0

0

0

0
4

0
4

0

0

0

0
4

0
4

0

0

0

0
4

0
4

0

0

0

0
4

0
4

0
7

0

0

0
4

0

0

0
4

0

0

0
4

0

0

0
4

0
4

0

0

0

0
4

0
4

0

0

0

0
4

0
4

0

0

0

0
4

0
4

0

0

0

0
4

0
4

0

0

0

0
4

0
4

0

0

0

0
4

0
4

0

0

0

0
4

0
4

0

0

0

0
4

0
4

0

0

0

0
4

0
4

0

0

0

0
4

0
4

0

0

0

0

0

0

0
4

0
4

0

0

0

0
4

0
4

0

0

0

0
4

0
4

0

0

0

0
4

0
4

0

0

0

0
4

0
4

0

0

0

0
4

0
4

0

0

0

0
4

0
4

0

0

0

0
4

0
4

0

0

0

0
4

0
4

0

0

0

0
4

0
4

0

0

0

0
4

0
4

0
7

0
7

0

0

0
4

0
4

0

0

0

0
4

0
4

0

0

0

0
4

0
4

0

0

0

0
4

0
4

238.735

238.735

238.735

0
4

0
4

0

0

0

0
4

0
4

0

0

0

0
4

0

0

0
4

0
4

7.8156

7.8156
3

6.84719
3

0

0

0

0

0.968406

0

0

0

0

0

0

0

0

0

0

0

0

0

0

0

0

0

0

4.44089209850063e-16
3

0
4

0
6

0
6

0
4

0

0

0

0
4

0

0

0
4

0

0

0
4

0

0

0
4

0

0

0
4

0

0

0
4

0

0

0
4

0

0

0
4

0
4

3.5527136788005e-15
3

0
4

0
4

0

0

0

0

0

0

0

0

0
4

0

0

0
4

0

0

0
4

0

0

0
4

0
4

0
4

0
4

350.195

1.68072

1.68072

0
7

0

0

0

0

0

0

0

0

0

0
4

1.68072

1.09714

0.451923

0

0.131657

8.32667268468867e-17

0
4

0
4

0
4

4.25691

4.25691

4.25691

4.25691

0
4

0
4

0
4

0

0

0

0

0

0

0
4

0

0

0
4

0
4

0
4

0

0

0

0

0

0
4

0

0

0
4

0

0

0
4

0
4

0
4

0

0

0

0

0
4

0

0

0
4

0
4

0
4

0

0

0

0

0
4

0
4

0
4

0

0

0

0

0

0
4

0
4

0
4

0

0

0

0

0

0
4

0
4

0
4

0

0

0

0

0
4

0
4

0
4

0.424808

0.424808

0.424808

0.295687

0.129121

0
4

0
4

0
4

0

0

0

0

0
4

0

0

0
4

0
4

0

0

0

0
4

0
4

0
4

2.80868

2.80868

2.80868

0

2.80868

0
4

0
4

0
4

0

0

0

0

0
4

0
4

0
4

0

0

0

0

0
4

0

0

0
4

0
4

0
4

0

0

0

0

0

0
4

0
4

0
4

0

0

0

0

0

0
4

0
4

0
4

0

0

0

0

0
4

0
4

0
4

0

0

0

0

0

0
4

0
4

0
4

0

0

0

0

0
4

0

0

0
4

0
4

0
4

0

0

0

0

0
4

0
4

0
4

0

0

0

0

0
4

0
4

0
4

0

0

0

0

0
4

0
4

0
4

0

0

0

0

0
4

0

0

0
4

0
4

0
4

0

0

0

0

0
4

0
4

0
4

0

0

0

0

0
4

0
4

0
4

0

0

0

0

0
4

0
4

0
4

0.129121

0.129121

0.129121

0.129121

0
4

0
4

0
4

0

0

0

0

0
4

0
4

0
4

0

0

0

0

0
4

0
4

0
4

0

0

0

0

0
4

0
4

0
4

0

0

0

0

0
4

0
4

0
4

0

0

0

0

0
4

0
4

0
4

0

0

0

0

0
4

0
4

0
4

0

0

0

0

0
4

0

0

0
4

0
4

0
4

0

0

0

0

0
4

0
4

0
4

0

0

0

0

0
4

0
4

0
4

0

0

0

0

0
4

0
4

0
4

0

0

0

0

0
4

0
4

0
4

0

0

0

0

0
4

0
4

0
4

0

0

0

0

0
4

0
4

0
4

0

0

0

0

0
4

0
4

0
4

0

0

0

0

0
4

0
4

0
4

0

0

0

0

0
4

0
4

0
4

0

0

0

0

0
4

0
4

0
4

0

0

0

0

0

0

0
4

0

0

0
4

0
4

0
4

0

0

0

0

0
4

0
4

0
4

0

0

0

0

0
4

0
4

0
4

0

0

0

0

0
4

0
4

0
4

0

0

0

0

0
4

0
4

0
4

0

0

0

0

0
4

0
4

0
4

0

0

0

0

0
4

0
4

0
4

0

0

0

0

0
4

0
4

0
4

0

0

0

0

0
4

0
4

0
4

0

0

0

0

0
4

0
4

0
4

0

0

0

0

0
4

0
4

0
4

0

0

0

0

0
4

0
4

0
4

0

0

0

0

0
4

0
4

0
4

0

0

0

0

0
4

0
4

0
4

0

0

0

0

0
4

0
4

0
4

0

0

0

0

0
4

0
4

0
4

0

0

0

0

0
4

0
4

0
4

0

0

0

0

0
4

0
4

0
4

0

0

0

0

0
4

0
4

0
4

0

0

0

0

0
4

0
4

0
4

0

0

0

0

0

0
4

0

0

0
4

0

0

0
4

0
4

0
4

0

0

0

0

0

0

0

0
4

0
4

0
4

0

0

0

0

0

0
4

0

0

0
4

0

0

0
4

0
4

0
4

340.895

1.03297
5

0
6

0
6

0

0

0

0

0

0

0

0

0

0

0
6

0

0

0

0

0

0

0

0

0

0

0
6

0

0
6

0

0
6

0

0

0
4

0

0

0

0
4

0

0

0

0
4

0.129121

0.129121

0

0
4

0

0

0
4

0

0

0
4

0

0

0
4

0.645604

0.516483

0

0

0

0
7

0

0

0.129121

0

0

0

0

2.77555756156289e-17

0
4

0
6

0
6

0

0

0

0

0
7

0

0

0

0

0

0

0

0
4

0

0

0

0

0

0

0
4

0

0

0

0

0

0

0

0

0

0
4

0

0

0

0
4

0

0

0

0
4

0.258242

0

0

0

0

0

0.258242

0
4

0

0

0

0

0
4

5.55111512312578e-17
5

0
4

3.17864

0

0

0

0

0
4

0

0

0

0

0

0

0
4

3.17864

2.95687

0.0739218

0.147844

0
4

0

0

0

0
4

0

0

0

0
4

0
4

0
4

0
4

0

0

0

0

0

0
4

0

0

0

0

0

0
4

0

0

0
4

0
4

0

0

0

0

0

0

0

0
4

0

0

0

0
4

0

0

0
4

0

0

0
4

0
4

0
5

0
5

0

0

0

0

0

0

0

0
4

0

0

0
4

0
4

0

0

0
6

0

0

0

0

0

0
4

0
4

0

0

0

0

0

0

0

0

0
4

0

0

0

0
4

0
4

0
4

0
4

0

0

0

0

0

0

0
4

0

0

0
4

0
4

0
5

0

0

0
4

0

0

0

0
4

0

0

0
4

0

0

0
4

0
4

0

0

0

0

0
4

0
4

0

0

0

0

0
4

0

0

0
4

0

0

0
4

0

0

0
4

0
4

70.8306

37.7678

0
5

0

0

0

0

0

0

0

0

0

0

5.10027

0

0

0

0

0

0

0
6

0
6

32.6676
2

0

0

0

0

0
4

0

0

0
4

0

0

0
4

0

0
[truncated: 130,810 more chars]
